# Detection and Alignment of 3D Domain Swapping Proteins Using Angle-Distance Image-Based Secondary Structural Matching Techniques

**DOI:** 10.1371/journal.pone.0013361

**Published:** 2010-10-14

**Authors:** Chia-Han Chu, Wei-Cheng Lo, Hsin-Wei Wang, Yen-Chu Hsu, Jenn-Kang Hwang, Ping-Chiang Lyu, Tun-Wen Pai, Chuan Yi Tang

**Affiliations:** 1 Department of Computer Science, National Tsing Hua University, Hsinchu, Taiwan, Republic of China; 2 Institute of Bioinformatics and Structural Biology, National Tsing Hua University, Hsinchu, Taiwan, Republic of China; 3 Institute of Bioinformatics and Systems Biology, National Chiao Tung University, Hsinchu, Taiwan, Republic of China; 4 Department of Computer Science and Engineering, National Taiwan Ocean University, Keelung, Taiwan, Republic of China; 5 Department of Computer Science and Information Engineering, Providence University, Taichung, Taiwan, Republic of China; Griffith University, Australia

## Abstract

This work presents a novel detection method for three-dimensional domain swapping (DS), a mechanism for forming protein quaternary structures that can be visualized as if monomers had “opened” their “closed” structures and exchanged the opened portion to form intertwined oligomers. Since the first report of DS in the mid 1990s, an increasing number of identified cases has led to the postulation that DS might occur in a protein with an unconstrained terminus under appropriate conditions. DS may play important roles in the molecular evolution and functional regulation of proteins and the formation of depositions in Alzheimer's and prion diseases. Moreover, it is promising for designing auto-assembling biomaterials. Despite the increasing interest in DS, related bioinformatics methods are rarely available. Owing to a dramatic conformational difference between the monomeric/closed and oligomeric/open forms, conventional structural comparison methods are inadequate for detecting DS. Hence, there is also a lack of comprehensive datasets for studying DS. Based on angle-distance (A-D) image transformations of secondary structural elements (SSEs), specific patterns within A-D images can be recognized and classified for structural similarities. In this work, a matching algorithm to extract corresponding SSE pairs from A-D images and a novel DS score have been designed and demonstrated to be applicable to the detection of DS relationships. The Matthews correlation coefficient (MCC) and sensitivity of the proposed DS-detecting method were higher than 0.81 even when the sequence identities of the proteins examined were lower than 10%. On average, the alignment percentage and root-mean-square distance (RMSD) computed by the proposed method were 90% and 1.8Å for a set of 1,211 DS-related pairs of proteins. The performances of structural alignments remain high and stable for DS-related homologs with less than 10% sequence identities. In addition, the quality of its hinge loop determination is comparable to that of manual inspection. This method has been implemented as a web-based tool, which requires two protein structures as the input and then the type and/or existence of DS relationships between the input structures are determined according to the A-D image-based structural alignments and the DS score. The proposed method is expected to trigger large-scale studies of this interesting structural phenomenon and facilitate related applications.

## Introduction

Involved in the formation of quaternary structures from monomers, three-dimensional (3D) domain swapping refers to two or more identical proteins exchanging equivalent parts of their structures to form intertwined oligomers, inclusive of dimers [1], [2], [3]. The term “3D domain swapping” was first created in 1994 to describe the dimeric structure of diphtheria toxin [4], [5]. Subsequently, this led to the discovery of a considerable number of other domain-swapped proteins, such as some ribonucleases [6], [7], [8], cysteine proteinase inhibitors [9], [10], [11], SH2 and SH3 domains [12], [13], L-histidinol dehydrogenase [14], glyoxalase I [15], nitric oxide synthase [16], suppressor of cyclin dependent kinase [17], [18], and prion proteins [19]. Related studies posited that 3D domain swapping may occur in any protein with an unconstrained terminus under appropriate conditions [1], [2], [20], implying that it plays important roles in protein molecular evolution, functional regulation and the formation of protein conformational/deposition diseases, such as amyloid and prion diseases [9], [19], [21]. Furthermore, bioengineers have been applying 3D domain swapping to the design of artificial biopolymers [20], [22].

A subunit of a 3D domain-swapped oligomer appears to have two conformational states, a monomeric closed-form and an oligomeric open-form. 3D domain swapping (abbreviated as DS) has been accordingly classified into three types [2]. First, in bona fide cases, both the closed monomer and domain-swapped oligomer of a protein exist stably. Second, although capable of forming intertwined and apparently domain-swapped oligomers, some proteins cannot exist as closed monomers. Quasi-domain-swapped cases refer to the domain-swapped proteins that have structural homologs known to be closed monomers. Third, DS candidates refer to the opposite situation in which no closed homolog is found for these oligomeric proteins.

DS can originate from environmental changes such as variations in pH values and protein concentrations [1], [23]. Additionally, two evolutionary mechanisms have been proposed for DS [1], [3]. First, as the hinge loop, the loop connecting the swapped domain to the protein body (main domain), of a closed monomer becomes shorter by residue deletion during evolution, the closed conformation might no longer remain stable because it is difficult for the domain to be swapped to reach the protein body, thus exposing the residues normally buried in the domain-domain contact interface. The domain-swapped form is then energetically favored [24], [25]. Second, changes or mutations in the hinge loop and/or the contact interface of domains might destabilize the closed monomer due to steric or electrostatic effects and subsequently promote swapping conditions [26], [27].

Although DS is an interesting and important structural phenomenon, related bioinformatics resources are rarely available. Previous studies have shown that 3D domain-swapping homologs may share minor sequence similarity [28] (see also a summary of DS cases in [2]). Therefore, sequence-based alignment tools may be inadequately sensitive to detect evolutionarily distantly related DS cases. Moreover, conventional structural comparison algorithms are insufficiently flexible to detect global similarities between proteins related by DS [29]. When aligning a “closed” monomer and its domain-swapped “open” homolog (referred to hereinafter as a “DS_CO_ pair”), several methods, such as FAST [30] and TM-align [31], tend to output one local alignment restricted only to the protein bodies or the swapped domains. Although other methods, such as DALI [32] and CE [33], can simultaneously make several alignments with statistical Z-scores, most of them are local/partial alignments. Even if a global similarity were detected, a low Z-score and a large root-mean-square distance (RMSD) of the structural superposition would likely occur. Failing to visually inspect the superimposed structures would make it extremely difficult to identify the DS relationships. Although capable of detecting the structural similarity of 3D domain-swapping proteins, structural comparison methods with a more flexible nature, such as Flexible structure AlignmenT by Chaining Aligned fragment pairs allowing Twists (FATCAT) [29] and Structural similarity search Aided by Ramachandran Sequential Transformation (SARST) [34] provide no information to help users distinguish the domain-swapped homologs from the common structural homologs in the hit list. DS is sometimes considered as a unique domain motion [35], [36]. However, tested with the known cases described by Eisenberg *et al.*, the well-known domain motion detection method DynDom [35] failed to identify most of those DS relationships (see Table S1 for details).

Perhaps because of the unavailability of suitable detection and analytical methods, currently the datasets for DS_CO_ pairs, including the largest literature-based dataset by [2] (33 pairs) and the predicted dataset with experimental verifications by [37] (7 pairs), are very small. This situation has greatly limited the scale and depth of DS-related researches. As a result, there is still much uncertainty about how frequently DS occurs in Nature, which proposed mechanism plays the major role in the evolution of DS or whether there is any undiscovered mechanism for DS [23]. There are also few solid data available about the sequence compositions and structural properties of 3D domain-swapped proteins and their hinge loops, which shall be very valuable for researchers who are looking for treatments for protein deposition diseases or interested in creating protein-based fibril materials or oligomerized enzymes. We believe that, in this post-genomic era, when protein structure data increase rapidly, it is very possible that plenty of information can be extracted to reveal the natural prevalence and evolutionary mechanism of DS as well as to accelerate the medical and bioengineering applications of DS. A suitable detection and analytical bioinformatics method shall be the key to these possibilities. Motivated by the importance of DS and the insufficiency of related bioinformatics developments, this work aims to design a DS-specific identification and structural comparison method.

**Figure 1:**
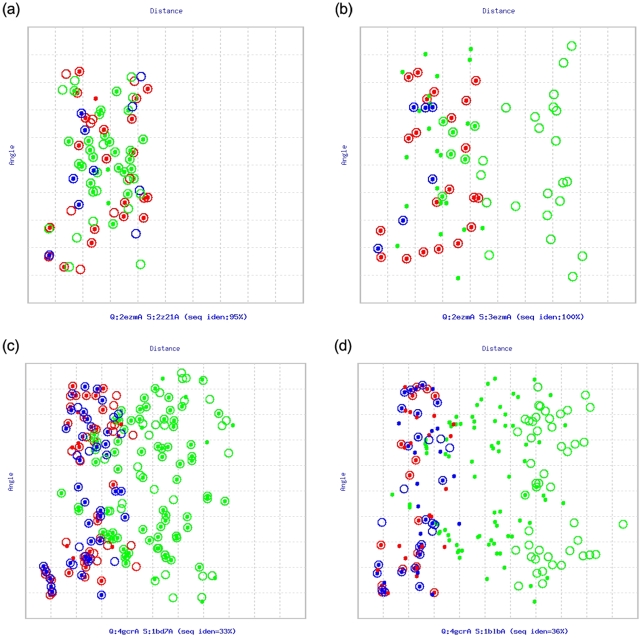
Matched angle-distance (A-D) images. (**a**) Two open-form cyanovirin-N molecules (PDB entries: 2ezmA and 2z21A). (**b**) 3D domain-swapping cyanovirin-N proteins (2ezmA and 3ezmA), a “bona fide” case [2]. (**c**) γB-crystallin from bovine (4gcrA) and βB2-crystallin from rat (1bd7A), common structural homologs with closed conformations. (**d**) γB-crystallin from bovine (4gcrA) and an iron-dependent regulator from *Mycobacterium tuberculosis* (1b1bA), an example of “quasi-domain swapping” [2]. Two secondary structural elements (SSEs), which have been transformed into vectors, in a protein structure form an SSE pair. In these images, the angle of SSE pairs is plotted on the y-axis, and the Euclidean distance of geometric centers of the SSEs is plotted on the x-axis. Both axes have been normalized. The dots and circles represent SSE pairs from the query and subject proteins, respectively. If two SSE pairs from different proteins can be matched (see MATERIALS AND METHODS), they are drawn as a concentric pair of dot and circle. Every protein shown here can be divided into two parts, *i.e.*, a main domain and a swapped domain, within which the SSE pairs are painted red and blue, respectively, while the SSE pairs formed between these (inter-domain SSE pairs) are painted green. Clearly, 3D domain-swapping homologs ((b) and (d)) have a different pattern from common structural homologs ((a) and (c)) in the matched A-D images, where the data points of inter-domain SSE pairs of the open- and closed-form homologs are distributed separately and cannot be well-matched.

We previously developed a protein structural comparison technique based on angle-distance (A-D) image transformation [38], which has been shown to detect structural similarities between evolutionarily distantly related proteins and to identify structurally similar proteins with different connectivities of secondary structural elements (SSEs) [38]. The A-D image is first constructed based on protein secondary structural information, and is then separated into three different sub-images focusing on various types of SSEs. Structural similarities can subsequently be identified using modified cross-correlation approaches to recognize specific patterns in the corresponding sub-images of the query and target structures. Our current work finds that homologous proteins with and without DS relationships reflect significantly different patterns in the matched A-D images through SSE matching algorithms (see Fig. 1). This finding confirms the feasibility of applying A-D images to develop automated DS detection procedures, which appear to be highly promising for DS-related researches.

### Overview of the Proposed Method

Each point in an A-D image records the angular difference between representative vectors and the distance between the centers of two SSEs in a protein [38]. In this work, as two structures are transformed into corresponding A-D images, the pairings of points from the two images are then analyzed and scored using a pair graph. Following an optimization procedure, the equivalence of SSEs between the two proteins is extracted based on the scores of the identified pairs. These processes, the pair graph analysis and extraction of SSE equivalence, are referred to as “matching”. Notably, SSEs in the protein bodies and swapped domains of a DS_CO_ pair can be matched simultaneously because the matching process does not depend on structural superposition. This feature markedly differs from that of most conventional structural alignment methods, which can only align one region at a time. Exploiting this significant difference allows us to compare the results of the SSE matching with those of a typical protein structural alignment and thus locate the boundary between the protein body and the swapped domain, *i.e.*, the approximate location of the hinge loop. Next, superimposing the two structures based on their protein bodies enables us to measure and normalize the conformational difference between these two structures with respect to the swapped domains into the DS score defined here. Two proteins with a DS score higher than a specific cutoff trained on known data are identified as a DS_CO_ pair. Finally, the superpositions of the protein bodies and swapped domains can be output to the users simultaneously, separately or in a fused form. Meanwhile, the location and range of the hinge loop is refined according to the results of structure superpositions based on an improved version of the hinge loop determination algorithm by Eisenberg *et al.* [1], who created the term 3D domain swapping.

Given the lack of a DS database, we collected many DS cases either reported in the literature or annotated in the Protein Data Bank (PDB) [39] to train and test our DS-scoring system. Additionally, a significantly larger number of DS cases were retrieved and identified manually from the PDB and Protein Quaternary Structure database [40] to perform more detailed assessments and experiments, including those on the quality and stability of binary classifications, the database independence of the discriminatory model, the performances for various DS types and sequence identities, and the quality of domain-swapped alignments and hinge loop determinations. The results revealed the uniqueness of the proposed method. In all experiments, most MCC (Matthews correlation coefficient), sensitivity and specificity values were considerably greater than 0.80, even when the sequence identities of the examined proteins were lower than 10%. On average, the alignment percentage and root-mean-square distance (RMSD) computed by the proposed method were 90% and 1.8Å for a set of 1,211 DS-related pairs of proteins, which is the largest DS dataset available. In addition, the range of hinge loops determined by the proposed method corresponded well to the results of manual inspections. To our knowledge, this work presents for the first time a detection and alignment method specifically developed for 3D domain swapping. The unique evaluation system and developmental processes of the proposed method and some unusual properties of DS for structure/sequence comparison methods that are observed for the first time are described in detail in this report, which concluded with some future perspectives on the post-genomic researches and applications of DS that may be enabled or facilitated by computational methods with high performances.

## Results

### Feasibility of Conventional Protein Structure Comparison Methods for Detecting the 3D Domain Swapping Phenomenon

Although it has been noticed that conventional protein structural comparison (PSC) methods may not be adequate for detecting DS relationships among proteins [29], extensive evaluations of the DS detection abilities of PSC methods have not been performed, probably due to the limited DS data and no standard evaluation mechanism for DS detection. This experiment involves Dataset L, which consists of literature-derived DS cases and their DS-related homologs, common structural homologs, and structurally non-homologous proteins (see MATERIALS AND METHODS for the preparation procedure). More specifically, the structural alignment ratio, that is, the percentage of structurally aligned/equivalent residues (see Experimental Parameters Subsection), calculated by FAST [30] was used to gradually filter out common global structural homologs, which are not DS cases in general (see Figure S1 for more information about the compositions of the filtered dataset at various structural alignment ratio cutoffs). The classification qualities of various PSC methods based on the structural diversity (S-div) [41], a general structural similarity measure, were therefore monitored over the increasing ratio of indistinguishable cases, *i.e.*, partial structural homologs and DS-related homologs, remaining in the test set by calculating the average MCC, sensitivity and specificity over the five-fold cross-validation. As an extensively adopted measure in machine learning for evaluating the quality of binary classifications, the MCC ranges from −1 (inverse prediction) to 0 (random prediction) to +1 (perfect prediction) and is generally considered to be a balanced measure even if the size of classes varies remarkably.

**Figure 2:**
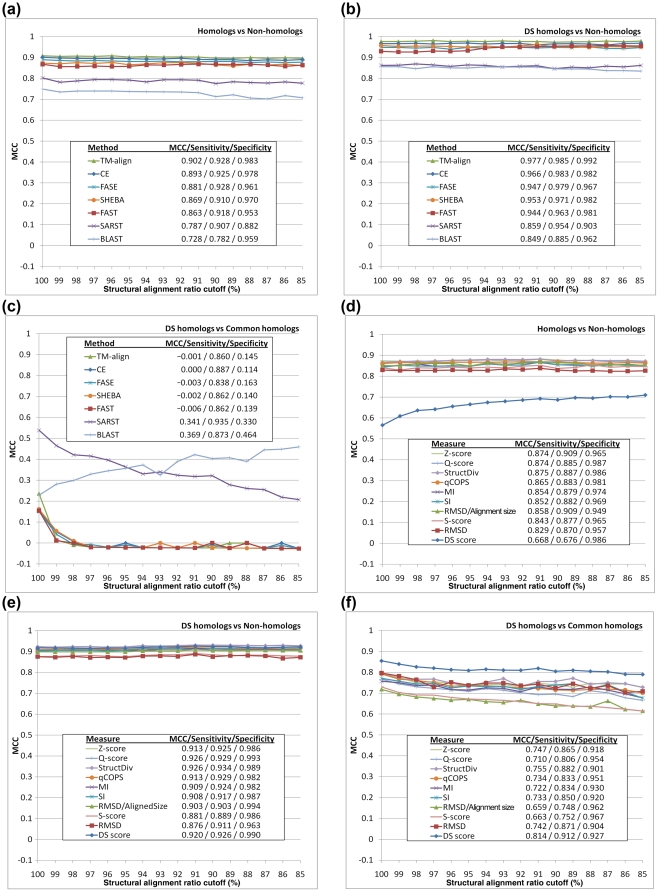
Performance of various alignment methods and similarity measures in identifying common homologs and/or DS-related homologs. The binary classification performance of several conventional alignment methods in distinguishing homologous from non-homologous structures is shown in (**a**), while their performances in distinguishing DS homologs from non-homologs and common homologs are shown in (**b**) and (**c**), respectively. The results of the binary classification tests for several similarity measures to distinguish homologs from non-homologs are summarized in (**d**). The results of distinguishing DS from non-homologs and common homologs by those measures are shown in (**e**) and (**f**), respectively. In these experiments, which involve Dataset L, a number of known DS-related homologous pairs (L_ds_), common homologous pairs (L_ch_) and non-homologous pairs (L_nh_) of protein structures were used as positive or negative data for different purposes. In (a) and (d) both L_ds_ and L_ch_ were used as positive data, and L_nh_ served as the negative data, in (b) and (e) L_ds_ was the positive and L_nh_ was the negative data, whereas in (c) and (f) L_ds_ and L_ch_ were respectively viewed as the positive and negative data. The x-axes indicate that proteins pairs with globally superimposable structures are gradually filtered out as the alignment ratio cutoff decreases; meanwhile, the average MCC obtained by five-fold cross-validations is plotted on the y-axes. TM-align [31], CE [33] and FAST [30] are order-dependent structural alignment methods. FASE [58] and SHEBA [59] can perform order-independent alignments. SARST acquires a more flexible nature than conventional methods by using a structure linear-encoding methodology [34]. BLAST [42] is a widely-used sequence alignment method. The MCCs of various alignment methods shown in (a)–(c) were determined based on a structural similarity measure known as structural diversity (S-div) [41] except those for BLAST, which were based on a normalized sequence similarity score (refer to Table S2). See the RESULTS and DISCUSSION Sections for explanations of these results. The structural similarity measures assessed in (d)–(f) include the Q-score [43], S-div, qCOPS [56], [60], MI [61], SI [61], S-score [44], RMSD, RMSD over the alignment size and the Z-score of TM-align.

According to Fig. 2 and Table S2, all of the tested structural comparison methods could well discriminate homologs, both common ones and DS-related ones, from non-homologs (Fig. 2a). They could also well distinguish DS-related homologs from non-homologs (Fig. 2b). Their MCC values were generally greater than 0.80. However, their abilities to distinguish domain-swapped homologs from common homologs were low. The MCC values were all lower than 0.54, and they declined dramatically as the cutoff of the structural alignment ratio became lower. When the structural alignment ratio cutoff was lower than 98%, the MCC values of most methods approached zero (Fig. 2c). Besides, their specificity values were mostly less than 0.20 (Table S2). Interestingly, those methods with good performance for separating homologs from non-homologs (MCC>0.86) were much weaker at distinguishing DS from common homologs (MCC<0.24) than those methods with relatively poor performance (Fig. 2c). The widely-used protein sequence comparison method BLAST [42] was also employed here for comparison, and its DS-detecting performance was unusually better than many structure-based alignment methods. See the DISCUSSION Section for explanations of these observations.

### Performance of the Proposed A-D Image Based DS-detecting Method Combined with Conventional Structural Measures

We supposed that the weakness of most conventional PSC methods at detecting DS relationship lies in the fact that they can only identify the structural similarities of a part, *i.e.*, the main domains or swapped domains, of DS-related proteins (see DISCUSSION for supporting information). Based on this supposition, a delicate A-D image-based PSC procedure was designed to align the structures, identify possible hinge loops and determine the global structural similarities between a DS_CO_ pair (see MATERIALS AND METHODS). The output of this procedure includes a “virtual structure alignment” produced by allowing the two domains of a DS_CO_ pair to be independently rotated and translated and thus superimposed simultaneously. The alignment size and RMSD based on this virtual alignment are termed the “virtual alignment size” and “virtual RMSD (vRMSD)”, respectively. Moreover, most well-defined protein structural similarity measures, *e.g.*, the Q-score [43], S-score [44] and S-div [41], can be computed based on this alignment as well and are hence termed the “virtual (v)” structural similarity measures, such as the vQ-score, vS-score and vS-div. These flexible virtual structural similarity measures are more feasible for describing the structural similarity of DS-related proteins than their conventional rigid versions (see Experimental Parameters Subsection for more information).

This experiment attempts to evaluate this DS-detecting procedure and examine the capabilities of various virtual structural similarity measures as suitable discriminators for 3D domain-swapping proteins and common homologous proteins. As shown in Fig. 2d and Table S2, all tested virtual measures adequately discriminated homologous from non-homologous proteins. The obtained MCC values ranged stably between 0.82 and 0.89, with average sensitivities ≥0.87. When only DS-related homologs were used as the positive data while non-homologous proteins were the negative data, their classification performances were good as well (all MCCs = 0.90
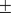
0.03 and all average sensitivities ≥0.89; see Fig. 2e). However, these measures were not as effective in separating DS-related homologs from common homologs, as evidenced by the relatively low MCC and sensitivity for this part (MCCs<0.80 and average sensitivities≤0.88), which declined as the alignment ratio cutoff became lower (Fig. 2f). Because the DS detection power of the S-div calculated by many PSC methods was verified, and most of the MCC values were lower than 0.24 (Fig. 2c), the high MCC values (all MCCs>0.72) achieved by vS-div shown in Fig. 2f confirmed the feasibility of the proposed DS detection procedure.

### Definition and Evaluations of a Novel DS Score

**Figure 3:**
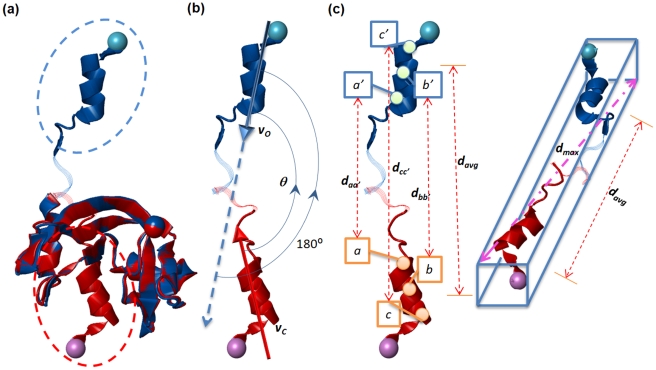
Measurement and normalization of the angular difference and displacement of swapped domains. (**a**) Structure superposition of a bona-fide DS_CO_ pair, the monomeric (red; PDB entry 5rsaA) and dimeric (blue; PDB entry 1a2wA) forms of ribonuclease A from bovine. This superposition was performed with a typical structural alignment algorithm that treats protein structures as rigid bodies. In this case, the main domains of two proteins were superimposed well, but the swapped domains (in dotted ellipses) were not superimposed or aligned because of the great difference in the orientation and position. The hinge loops (shown as thin strands) also could not be aligned because of the different conformations. (**b**) Computation of the normalized angular difference (*γ_θ_*). Using vector transformation techniques, the swapped domains can be represented as two vectors, ***v_c_*** and ***v_o_***. The angle *θ* between ***v_c_*** and ***v_o_*** can be determined based on the law of cosines. The *γ_θ_* of two swapped domains is thus normalized as *θ*/180°. (**c**) Computation of the normalized average residue displacement (*γ_d_*). In the process of hinge loop detection, the equivalent residues of two swapped domains, *e.g.*, *a* and *a′*, were determined (see MATERIALS AND METHODS). The *γ_d_* was calculated by dividing the average Euclidean distance of all equivalent residue pairs (*d_avg_*) by *d_max_*_,_, which is the length of the diagonal of the virtual box defined by the boundary of two swapped domains.

**Figure 4:**
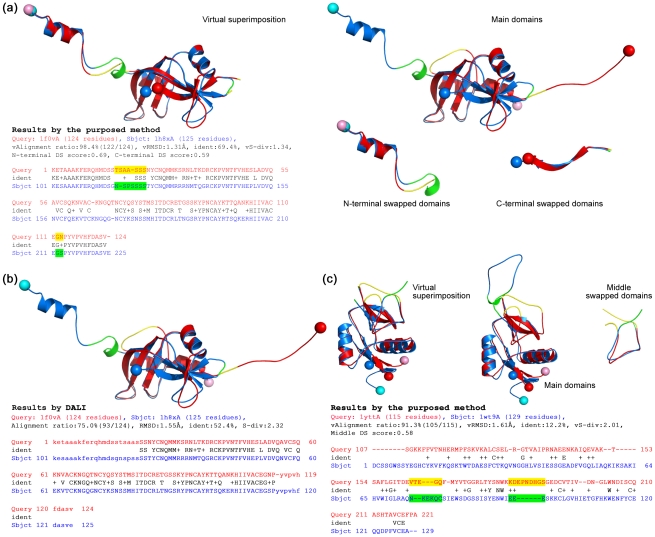
3D domain-swapping homologs with special swapped domains. Given that the proposed method can align two DS-related proteins along the full length, it is more capable of detecting the global structural and sequence similarities of a DS_CO_ pair than conventional PSC methods. (**a**) A pair of ribonucleases (RNases) exhibiting DS phenomena at both termini. The RNase A from *Bos taurus* (PDB entry 1f0vA) and the RNase from human pancreas (PDB entry 1h8xA) are both domain-swapped dimers; however, their swapped domains are respectively located at the C- and N-terminus. Therefore, when they are superimposed in a conventional manner (the upper right region), both termini appear swapped and unaligned, even if the individual terminal domains are structurally similar (superpositions are shown in the lower right region). The virtual superposition made by the proposed method (left) revealed the actual structural similarity between these RNases, which share a sequence identity of 69% calculated based on the structure-based sequence alignment. In this figure, hinge loops determined by our method are highlighted in the alignment text. (**b**) Structural alignment of the same RNases performed by DALI [32]. The alignment size and sequence identity computed by DALI are clearly smaller than those by the proposed method. (**c**) Structural alignment of the snake venom protein Aa-X-bp-I (PDB entry 1yttA) and a subtilisin fragment of mannose binding protein A from *Rattus norvegicus* (SUB-MPB-A; PDB entry 1wt9A), a quasi-domain swapping case. Aa-X-bp-I is a domain-swapped dimer, while SUB-MPB-A possesses a closed conformation. Interestingly, their swapped domains are located in the middle of the structures. The virtual superposition of the whole proteins is shown on the left side followed by superpositions of the main and swapped domains to the right. Comparing these proteins structures by DALI, the alignment ratio, RMSD and sequence identity were 78%, 2.4 Å and 10%, respectively. All of these values are worse than those calculated by the proposed method.

Despite the feasibility of the A-D image-based PSC method to detect DS, appropriate scoring functions must be identified or designed to assist the determination of the likelihood that the proteins under examination are related by DS. Because conventional protein (virtual) structural similarity measures are relatively weak at distinguishing DS-related proteins from other structural homolog types, we hypothesized that a practical scoring system for DS should be defined in a more complex manner in which the different properties of the DS and non-DS homologs revealed by the A-D image-based PSC method could be fully exploited. We observed that the A-D image transformation and matching allowed us to match SSEs from corresponding domains of a DS_CO_ pair (Fig. 1 and the MATERIALS AND METHODS). However, in an optimized structural superposition using conventional alignment algorithms, SSE pairs from the swapped domains were still orientationally and spatially different/distant (examples can be found in Fig. 3 and Fig. 4), implying a large product of the angle and distance (angle
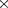
distance, or A-D product) between the matched SSEs. Therefore, we postulate that, in a profile of the A-D product (or “A·D profile” for simplicity) of a DS_CO_ pair, a high-valued region can be considered as a swapped domain, and the transition zone between a low-valued and a high-valued region represents the location of a hinge loop (see MATERIALS AND METHODS). The existence of a candidate hinge loop can be described by a binary function; in addition, other DS-specific structural properties can be analyzed and quantified. A DS score is defined here by integrating several such properties with the following formulas:
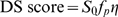
(1.1)

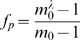
(1.2)

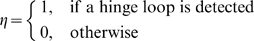
(1.3)

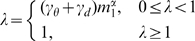
(1.4)

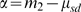
(1.5)

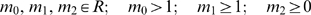
(1.6)where *m*_0_, *m*_1_, and *m*_2_ are parameters that can be trained by a dataset with both known DS and non-DS protein pairs. *S*_0_ can be any normalized structural similarity measure ranging from 0 (low similarity) to 1 (high similarity); *f_p_* represents an exponential penalty function that reduces *S*_0_ for common structural homologs while maintaining the high value of *S*_0_ for domain-swapped homologs; *η* denotes the binary function determined by whether a suspected hinge loop was detected or not. Function *f_p_* consists of three DS-specific factors, *i.e.*, the angular difference factor *γ_θ_*, displacement factor *γ_d_* and minimal structural diversity *μ_sd_* for the swapped domains, the last of which is defined as,
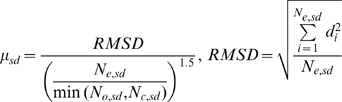
(2)where *N*_e,sd_ represents the number of equivalent residues between two swapped domains, while *N*_o,sd_ and *N*_c,sd_ refer to the size of the swapped domain of the open oligomer and closed monomer, respectively. A greater similarity between the two domains implies a smaller *μ_sd_*. Two “identical domains” result in a zero *μ_sd_*.

The *S*_0_ value selected in this work was the vQ-score. As long as a candidate hinge loop is identified, two proteins can be aligned by the main domains in general (see MATERIALS AND METHODS). In this case, the conformational difference in a DS_CO_ pair can be viewed as a large swinging movement of the swapped domain, which can be further described as an angular motion resulting in the displacement of all residues in the swapped domain. The angular difference (*γ_θ_*) and average displacement of residues (*γ_d_*) of the swapped domains are thus, respectively, determined and normalized within a range of 0 to 1. Fig. 3 provides further details.

The DS score has a theoretical minimum value of zero and maximum value of one. According to these algorithms, two proteins with a low structural similarity possess a low DS score owing to their low vQ-score. Interestingly, a pair of common structural homologs with a high structural similarity also has a low DS score as well because its *γ_θ_* and *γ_d_* cannot be large, in addition to the fact that *η* can be zero when no hinge loop is detected. By using this scoring scheme, only proteins with solid DS relationships yield a high DS score, which greatly facilitates the development of the DS detection method. Indeed, a repeat of the experiment stated in the previous subsection demonstrated that the DS score performed better than most conventional structural similarity measures at separating DS-related homologs from non-homologs (Fig. 2e) and clearly outperformed all conventional measures for distinguishing DS-related from common structural homologs (Fig. 2f). The MCCs were generally >0.80, while the sensitivity and specificity values were all >0.89 (Table S2). As expected, the DS score is relatively weak at binarily classifying homologs and non-homologs (Fig. 2d), for which the MCC was 0.57 at a 100% alignment ratio cutoff and gradually increased to 0.71 as the cutoff decreased to 85%. This unique increase in the performance of the DS score also reveals its specific nature for detecting 3D domain swapping relationships. See DISCUSSION for explanations.

### Evaluations of the Proposed Method Using Literature-derived and Manually Identified 3D Domain Swapping Cases

With a novel DS score defined based on the structural properties of DS, this study developed a complete A-D image-based DS detection method (refer to the MATERIALS AND METHODS). To more extensively evaluate this method and perform larger scale experiments in the following context than supported by the current literature-derived data, Dataset M was manually established utilizing the Protein Quaternary Structure database [40]. Similar to Dataset L, Dataset M consists of a number of DS-related homologs, common structural homologs and non-homologs (see MATERIALS AND METHODS). Because the proposed method is a DS detection method, the following experiments were all performed based on the condition that only DS homologs were treated as positive data, whereas common homologs and non-homologs were both regarded as negative data.

The dependency of the proposed method on the collected datasets is evaluated here. The parameters *m*_0_, *m*_1_, and *m*_2_ required in the formulas for the DS score and the discriminatory cutoff for the DS score were first determined by taking Dataset M as the training set, and the proposed method was evaluated by Dataset L. This procedure was then repeated by switching the roles of these two datasets. Receiver operating characteristic (ROC) analyses indicate that, regardless of whether trained or tested by literature-derived DS cases or manually identified DS candidates, the effectiveness of the proposed method remained high. The area under the ROC curve (AUC) in each experiment was greater than 0.95. Moreover, the MCC, sensitivity and specificity values all exceeded 0.80 (see Table S3). As Dataset M is involved in this experiment, in addition to verifying the performance of the proposed method with a larger range of data, the above results demonstrate that a classification made by this method generally corresponds to that by manual examination, which involves a considerable amount of manual labor and time.

The stability of the proposed discriminatory model related to the DS score was tested by *k*-fold cross-validations, where *k* ranged from 3 to 10. According to Figure S2, the classification quality of the discriminatory model remained high and stable in both datasets, even though Dataset L was much smaller than Dataset M. Combining these performance data obtained by both the inter-dataset (Table S3) and intra-dataset (Figure S2) training and testing, the feasibility and robustness of the proposed DS detection methods was confirmed again. In this report, all experiments described hereafter were performed with a discriminatory model trained using all data from Datasets L and M.

### Quality of Structural Alignments

Because the proposed method is the first DS-specific detection and alignment approach, the quality of its structural alignments may serve as a standard for the evaluation of related future works. There are 1,211 DS_CO_ pairs in total in Datasets L and M, among which 1,093 pairs could be successfully identified by the proposed method. The results of the structural alignment, superposition and hinge loop determination for the successful cases are listed in detail in Table S4. In addition, the (structure-based) sequence alignments of these DS_CO_ pairs calculated by the proposed and several other PSC methods are listed in Table S5. It is notable that our method correctly distinguished main domains from swapped domains for almost all of these cases, such that the primary alignment of every DS_CO_ pair was generally based on the main domains. The average (structural) alignment ratio and RMSD of the main domains and swapped domains and the average virtual alignment ratio and vRMSD of whole proteins were calculated and are listed in Table 1, where the alignment ratios were calculated according to Formula **3**.
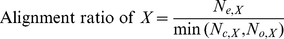
(3)where *X* can be the main domain, swapped domain or the whole protein; *N_e_* denotes the number of equivalent residue pairs, *i.e.*, the alignment size; *N_c_* and *N_o_* refer to the number of residues in the closed-form and open-form homologs, respectively.

**Table 1:** Average alignment size and RMSD of all available DS_CO_ pairs calculated by the proposed method.

Region	Average size (residues)	Alignment size (residues)	Alignment ratio (%)	RMSD (Å)
Main domains	113.3	105.8	93.4	1.717
Swapped domains	22.7	19.8	86.9	1.880
Whole proteins^1^	139.4	125.6	90.1	1.793

1 The alignment size/ratio and RMSD between whole proteins were calculated as the average virtual alignment size/ratio and average vRMSD. See the text for the definition of the virtual measures.

On average, 90% of the residues of a DS_CO_ pair could be aligned with a vRMSD of 1.8 Å. The average alignment ratio calculated by the proposed method for main domains were 7% smaller than that for swapped domains. Detailed analyses revealed that the alignment ratios calculated by the proposed method for the two domains of DS-related proteins differed more as the sequence identity decreased, although the differences were not very large (<9%; see Figure S3). The average running time for a pairwise comparison by the web-based Java implementation of the proposed method was 5.3 seconds with a 2.66GHz processor.

### Detecting Various 3D Domain Swapping Types

There are three types of DS regarding the location of the swapped domain, *i.e.*, N-terminal-, C-terminal- and middle-domain swapping [2]. This work evaluated the performance of the proposed method for these three DS types by separating all of the DS_CO_ pairs from Datasets L and M into three groups. Table 2 lists the classification sensitivities of the proposed method for these groups. Because the sensitivities were all >0.88, the proposed method performed satisfactorily for all reported DS types.

**Table 2:** Sensitivity for the detection of various DS types.

DS type (swapped domain)	Total No. (pairs)	No. of true positive predictions (pairs)	Sensitivity
N-terminal	433	384	0.887
C-terminal	676	614	0.908
Middle	102	91	0.892

### Effects of Sequence Identity on the Performance of the Proposed Method

As is well known, protein structural homologs may have low amino acid sequence identities [45]. This phenomenon is also observed in 3D domain-swapping proteins [2], [28]. This work evaluated how sequence identity affects the performance of the proposed method by classifying all protein pairs from Datasets L and M into ten groups with decreasing levels of sequence identity and then examining the quality of the binary classification and structural alignment of the proposed method. According to Table 3, the MCC, sensitivity and specificity varied slightly and still remain high as the sequence identity reduced. Even when the identities were lower than 10%, the values of MCC, sensitivity and specificity remained at the level of 0.83, 0.81 and 0.98 on average for DS detection. The alignment ratio and RMSD also varied with the sequence identity; however, because the variations were moderate, and it is reasonable that evolutionarily more distantly related proteins develop more structural differences, these results might just reflect the nature of the aligned proteins and should not be considered as a decrease in performance. Although it appeared that the decreasing sequence identity had minor influences on the performance of the proposed method, we did observe that the alignment qualities of this method became a little unbalanced as the identity decreased. As shown in Figure S3, the average difference of the alignment ratios calculated by this method for the two domains in a DS_CO_ pair with <10% sequence identity was 9%. It is not clear yet whether this unbalance revealed a natural property of DS or whether it stood for a decrease of alignment quality; nevertheless, this extent of unbalance brought little effect on the DS detection power of the proposed method for proteins with low sequence identities, as demonstrated in Table 3. Notably, in accordance with Table 3, ∼40% of the DS_CO_ pairs identified in this study shared less than 20% sequence identities. The DS relationships of these low-identity DS_CO_ pairs would normally escape detection by conventional structure/sequence comparison methods (see Figure S3). More importantly, by referring to Table S4, ∼25% of these low-identity DS_CO_ pairs involved hypothetical proteins with unknown or putative functions. These facts imply that the proposed method can be applied to suggest possible functions for functionally-unknown or hypothetical proteins, which are increasing rapidly because of many high throughput structural genomics efforts.

**Table 3:** Performance of DS-detection over various sequence identities.

Identity (%)	No. of DS cases (pairs)	No. of non-DS cases (pairs)	MCC	Sensitivity	Specificity	Alignment ratio^1^ (%)	RMSD^1^ (Å)
0–10	242	2478	0.825	0.810	0.988	85.824	2.339
10–20	253	1284	0.864	0.893	0.976	88.237	2.178
20–30	216	456	0.902	0.944	0.963	90.983	1.955
30–40	126	190	0.901	0.929	0.968	92.788	1.613
40–50	67	64	0.783	0.806	0.969	96.164	1.532
50–60	14	56	0.863	0.857	0.982	97.366	1.294
60–70	38	42	0.836	0.816	1.000	98.989	1.127
70–80	52	26	0.972	0.981	1.000	98.038	1.199
80–90	69	40	1.000	1.000	1.000	94.391	0.957
90–100	134	195	0.987	0.993	0.995	99.400	0.825

1 The alignment ratio and RMSD listed here are the average virtual alignment ratio and average vRMSD. Only DS cases with true positive predictions were included in the calculation of these two values.

### Identification of Hinge Loops

The hinge loop is the region linking the main domain and the swapped domain, and it is barely aligned in the structural alignment of a DS_CO_ pair [1]. Eisenberg *et al.* proposed that, after the approximate location of a hinge loop is assigned as the segment not superimposable between the open and closed homologs, its full length can be determined by extending both ends to include residues with large phi (*ϕ*), psi (*ψ*) differences until two consecutive residues have a torsion angular difference smaller than a cutoff *θ*_0_ (see Formula **10**), which was empirically set as 20° for bona fide and 30° for quasi domain swapping in their report [1].

While attempting to develop a fully automated DS-detecting procedure, this work designed a novel method for identifying the location and range of hinge loops by improving Eisenberg's method [1], [2] with a procedure dependent on the information extracted from the A-D image and A-D image-based structural alignments (see MATERIALS AND METHODS). Table 4 compares the hinge loops identified by the improved method with those reported in [2]. The automated determinations correlated well with those semi-manual identifications. The average difference in length between the hinge loops determined by the improved and original methods was small (1.4 residues), and the centers of the determined hinge loops only differed by 0.8 residue on average. However, the original method tended to lengthen the hinge loop identifications. For circumstances in which the two methods did not correlate, only five were determined longer by the improved method than by the original one; meanwhile, the original method determined longer hinge loops than the proposed method in 19 cases. For a detailed comparison between the two methods, a larger-scaled experiment than the above one was performed by computing the locations of the hinge loops of all DS_CO_ pairs available in Datasets L and M. According to Table S4, the improved version of Eisenberg's method determined the ranges of the hinge loops more strictly than the original method in general. The hinge loops determined by the improved method were shorter than those calculated by the original formula (Formula **10**) by 51% and 35% on average when *θ*_0_ was set as 20° and 30°, respectively. A close examination of the determined hinge loops revealed that by using either 20° or 30° as the cutoff, Formula **10** is likely to over-extend the boundary of hinge loops. Calculated hinge loops with obviously excessive or insufficient ranges judged by manual verifications are highlighted in this table. See the DISCUSSION Section for more information.

**Table 4:** Comparison of manually examined hinge loops and hinge loops identified by the proposed method.

Closed form	Open form	Hinge loops examined by [2]		Hinge loops identified by the proposed method		Length difference of hinge loops^1^ (residues)	Shift of the centers of hinge loops^2^ (residues)
		Range*_e_* *	Length*_e_*	Range*_i_*	Length*_i_*		
1msbA	***1ixxA***	72-75	4	72-80	9	−5	2.5
2ezmA	***3ezmA***	50-53	4	49-54	6	−2	0
1hz5A	***1jmlA***	52-55	4	52-56	5	−1	0.5
1wwwX	***1wwbX***	299-301	3	297-300	4	−1	1.5
1orcA	***5croA***	55-55	1	55-56	2	−1	0.5
***1orcA***	5croA	55-56	2	55-56	2	0	0
***1sncA***	1sndA	112-120	9	112-120	9	0	0
***1mupA***	1obpA	126-130	5	126-130	5	0	0
***1fynA***	1aojA	112-118	7	112-118	7	0	0
1sncA	***1sndA***	112-120	9	112-120	9	0	0
1mupA	***1obpA***	121-124	4	121-124	4	0	0
1fynA	***1aojA***	34-49	16	34-49	16	0	0
1brnL	***1yvsA***	37-41	5	37-41	5	0	0
5rsaA	***1bsrA***	15-22	8	15-22	8	0	0
1wwwX	***1wwaX***	297-299	3	296-298	3	0	1
1dksA	***1cksA***	60-65	6	60-65	6	0	0
1msbA	***1ixxA***	93-98	6	95-100	6	0	2
1griA	***1fyrA***	121-123	3	121-123	3	0	0
1nloC	***1aojA***	34-39	6	34-39	6	0	0
1mdtA	***1ddtA***	379-387	9	379-386	8	1	0.5
1qmpA	***1dz3A***	103-109	7	106-111	6	1	2.5
1wwwX	***1wwcA***	317-319	3	316-317	2	1	1.5
1k3sA	***1k3eA***	33-36	4	34-36	3	1	0.5
1eydA	***1sndA***	112-120	9	113-120	8	1	0.5
1pv3A	***1k04A***	943-948	6	943-947	5	1	0.5
1qd0A	***1sjvA***	95-100	6	94-98	5	1	1.5
1cunA	***2spcA***	72-75	4	72-73	2	2	1
***1gmfA***	1hulA	87-99	13	87-97	11	2	1
1qlxA	***1i4mA***	188-198	11	189-197	9	2	0
5rsaA	***1f0vA***	112-115	4	112-113	2	2	1
5rsaA	***1js0A***	112-115	4	112-113	2	2	1
1cewI	***1g96A***	55-59	5	57-59	3	2	1
5rsaA	***1a2wA***	15-22	8	18-22	5	3	1.5
1a5pA	***1a2wA***	15-22	8	18-22	5	3	1.5
1gmfA	***1hulA***	82-89	8	82-85	4	4	2
1hngA	***1cdcA***	44-50	7	44-46	3	4	2
4icbA	***1ht9A***	38-47	10	41-45	5	5	0.5
4gcrA	***1blbA***	79-87	9	86-87	2	7	3.5
				**Unsigned average**		**1.4**	**0.8**

* According to [2], this range was determined for the protein indicated by the bold italic text.

1 The length difference of the hinge loops was calculated as Length*_e_* – Length*_i_*.

2 This shift was calculated as the distance between the center of Range*_e_* and the center of Range*_i_*.

### Implementation and Illustrative Examples of Structural Alignments

The proposed DS-detecting method has been implemented as a web-based tool [http://ADiDoS.cs.nthu.edu.tw/]. The basic output on the web interface includes graphical and interactive Jmol [46] objects for the superpositions of the input protein structures, a table listing the detailed results of the structural alignments, and the DS score. If a DS relationship is identified, superpositions of the main and swapped domains and a virtual superposition of the whole proteins are generated. Additionally, the determined range of hinge loops and some novel structural measures defined in this study, such as the virtual alignment size, vRMSD and vS-div, are also provided. The alignment results of two DS_CO_ pairs identified in this work performed using the web interface are shown in Fig. 4. In the case of ribonucleases, our method precisely detected their overall structure and sequence similarities whereas DALI [47] only aligned them partially. As for the Aa-X-bp-I, a snake venom protein from *Agkistrodon acutus*, and SUB-MPB-A, a subtilisin fragment of mannose binding protein A from the rat, their DS relationships were well identified by the proposed method even though their overall sequence identity is only 12%.

## Discussion

### Difficulties in DS-detection for Conventional Alignment Approaches

The fact that conventional protein structural comparison (PSC) methods are weak at specifically identifying domain-swapped homologs implies that detecting DS relationships is a very different problem from detecting common structural similarities between proteins. As shown in Fig. 2c, at the 100% alignment ratio cutoff, which actually means that no protein was filtered out from the testing set, the best MCC value achieved was only 0.54 by SARST [34]. Because a lower alignment ratio cutoff means a more thorough exclusion of proteins with global similarities, the dramatic decline of the MCCs of the tested methods suggests that they are much less able to distinguish between DS homologs and other homolog types remaining in the testing dataset, which might be “partial homologs” (proteins with only local structural similarities) or “low-similarity homologs” (proteins with similar overall topologies but large displacements of the corresponding residues/SSEs). Difficulties in detecting DS relationships for conventional structural alignment approaches originate in the nature of the algorithms. A common goal of PSC is to determine a possible largest set of equivalent residues accompanying the possible smallest RMSD of a superposition. This can be visualized as if one structure were translated and rotated with respect to the other to make as many residues aligned and as close as possible. Under this circumstance, the protein structure is treated as a rigid body that always moves as a whole. However, the two objectives, a large number of aligned residues and a small RMSD, cannot be achieved simultaneously in a DS_CO_ pair due to a significant orientational difference between the swapped domains. A situation in which a method highly prioritizes RMSD implies its feasibility in detecting local structural similarities, but it is subsequently less applicable to the detection of DS relationships. Among the PSC methods assessed in the experiments of Fig. 2c that treated protein structures as rigid bodies, TM-align performed best. An experiment based on all identified DS cases listed in Table S4 and Table S5 was performed to determine the simultaneous alignment quality of TM-align on both domains of DS_CO_ pairs. Figure S3 clearly verifies that for most DS_CO_ pairs TM-align could only align one domain and thus could not detect their global structural similarities. On the other hand, despite the availability of several more flexible PSC methods that do not completely treat protein structures as rigid bodies and may detect the global structural similarity of 3D domain-swapping proteins by determining more aligned residues, their scoring systems prevent them from distinguishing between DS-related homologs and common structural homologs. Take SARST for example. It is a PSC method working based on a structural linear encoding methodology [34]. By transforming protein local backbone conformations into a conformational alphabet [34], it converts geometric structural comparison problems into string comparison problems that can be solved by conventional sequence alignment algorithms. As a hybrid, SARST was almost as precise as conventional PSC methods like CE in structural similarity searches while at the same time it possessed some properties of sequence alignment methods such as the high running speed [34]. Just like a sequence alignment algorithm, as SARST aligns structural strings, some minor local differences will result in mismatches and/or gaps but will not terminate the alignment as long as the score reduction effects of those local differences can be compensated by the score increasing effects of nearby string similarities. Besides, SARST does not consider the RMSD of structure superposition during its alignment but focuses completely on local backbone conformational similarities. As a result, when SARST deals with DS_CO_ pairs, in many cases the hinge loop will only cause some gaps but not prevent it from simultaneously aligning the main and swapped domains (see Figure S3 and Table S5 for experimental results demonstrating this property). These algorithmic features of SARST were the reasons that it could detect the overall structural similarity of many DS-related proteins (Fig. 2c). For a DS_CO_ pair and a pair of common homologs, when SARST reports similar alignment ratios for both cases, usually the RMSD of the former will be much larger than that of the latter, resulting in very different structural diversity (S-div) [41] values. In many DS cases, the RMSD values reported by SARST can be larger than 12 Å (∼28% in Table S5), a very extreme value that most conventional PSC methods barely report. For instance, the highest RMSD reported by TM-align in Table S5 was only 4.1 Å. Although this extreme difference in RMSD and S-div values has made SARST more capable of detecting DS relationships than most conventional PSC methods, it is not extreme enough to efficiently distinguish between DS-related and common homologs. As compared with SARST, the DS scoring system of the proposed method gives DS-related and common homologs much more different scores. A bona fide DS_CO_ pair usually has a DS score around 1 while a pair of common global homologs possesses a DS score close to 0 (note that the range of DS score is between 0 and 1 by definition).

Although it is normally considered that structure-based alignment methods are better than sequence-based ones at detecting protein structural similarities [45], very interestingly, in the case of DS-detection, the widely-used sequence alignment method BLAST outperformed most PSC methods, especially when the cutoff of structural alignment ratio (calculated by FAST [30]) was lower than 95%, forming an MCC curve with very different tendency from those of the PSC methods in Fig. 2c. This novel discovery also resulted from the nature of the alignment method. Sequence alignment does not consider any 3D structural information and is thus not affected by the conformational difference between closed and open homologs in the comparison processes. Similar to the situation shown by SARST, in many cases hinge loops only cause gaps but do not terminate the alignment. Provided that there are sequence similarities detectable by BLAST both in the main and swapped domains, a global alignment can be made and scored. As shown in Table S5 and Figure S3, for those DS-related proteins with global sequence identities lower than 20%, in most cases BLAST aligned them with only one domain. As the sequence identity increased, more and more protein pairs were aligned with two domains. At sequence identities ≥20%, the alignment ratio calculated by BLAST for the two domains of domain-swapped proteins differed less than 30%; at sequence identities ≥50%, the difference reduced to <10%. Although BLAST is capable of making global alignment for many DS_CO_ pairs, unfortunately it makes no difference to BLAST whether a high alignment score is achieved by a DS_CO_ pair or by a globally superimposable pair of common homologs. In the experiment of Fig. 2c, at a high structural alignment ratio cutoff, only those DS_CO_ pairs with small swapped domains and common homologous pairs with a small number of non-superimposable residues were eliminated. Thus, there were still many highly globally superimposable homologs, which were not distinguishable by BLAST from the DS_CO_ pairs, remaining in the testing set. As a result, the MCC of BLAST was only ∼0.3. However, as the alignment ratio cutoff decreased, the number of common global homologs in the testing set decreased much more rapidly than that of DS_CO_ pairs (refer to Figure S1) and the remaining DS_CO_ pairs maintained high scores that became relatively higher and higher than the scores of the partial and low-similarity homologs remaining in the testing set. Subsequently, BLAST registered higher MCC values at lower structural alignment ratio cutoffs than at the high cutoffs, and it achieved the highest MCC of 0.46 at the 85% cutoff; nevertheless, it was still inadequate to serve as an accurate DS-detecting method.

### Crucial Factors for the DS-detecting Ability of the Proposed Method

The DS-detecting ability of the proposed method has three critical factors. (1) The A-D image-based approach functions through SSE matching rather than structure superposition. This approach recognizes protein structural similarities without considering RMSD. As long as the relative geometric relationship of the corresponding SSEs is retained, a novel global structural relationship like 3D domain swapping can be detected. (2) The A·D profile generated by combining the results of the structural comparisons from the A-D image-based SSE matching and a conventional structural alignment greatly facilitates efforts to locate hinge loops. Possibly the origin [24], [25] and also the consequence [26], [27] of 3D domain swapping, the hinge loop is the most obvious feature of DS, implying that detecting its existence and location can assist the identification of DS relationships. Obviously, the main/large domain and candidate swapped/small domain can be distinguished after an approximate determination of the location of hinge loop. Next, superimposing protein structures by their main/large domains allowed us to examine conformational differences between two proteins caused by the swapping phenomena on a solid basis. (3) Carefully designed to integrate DS-specific structural properties, the DS score serves as a highly effective final indicator of a DS relationship (Fig. 2f, the blue curve). This score has a very different property from conventional structural similarity measures: no matter how similar two protein structures are, they cannot have a high DS score unless they are in different conformational states. The specific nature of the DS score is well revealed by the unique ascending MCC curve in Fig. 2d. Figure S1 shows that, as the alignment ratio cutoff decreased, the relative amount of DS-related homologs remaining in the testing set increased, while that of the common homologs decreased. Because the DS score is specifically designed for detecting DS, its MCC for distinguishing homologs (both DS and common homologs) from non-homologs is supposed to increase as the alignment ratio cutoff declines. Moreover, by integrating the three DS-specific factors, which describe the angular difference (*γ_θ_*), spatial displacement (*γ_d_*) and structural similarity (*μ_sd_*) of the swapped domains (see Formulas **1.1** to **1.6**), the DS score has a theoretical minimum value of zero (no DS relationship) and maximum value of one (definite DS relationship). In addition to simplifying the development of an automated procedure, this normalized score also offers users an easy way to recognize the DS relationship between proteins.

### Precision of Hinge Loop Determinations

Both the current two evolutionary mechanisms proposed for DS involve the hinge loop. Deletions may shorten the hinge loop and turn a closed monomer into an open oligomer [24], [25]; besides, mutations in the hinge loop and/or the contact interface of domains may promote swapping conditions [26], [27]. However, which proposed mechanism plays the major role or whether there is any undiscovered mechanism for DS remain uncertain. Examinations of the lengths and amino acid compositions of hinge loops may help reveal detailed evolutionary mechanisms of DS; additionally, the results of such examinations can provide important information for protein engineering studies utilizing 3D domain swapping. Precise examinations of the hinge loops depend on precise determinations of their positions and ranges. It is conceptually clear to define a hinge loop as the non-superimposable region linking the protein body with the swapped domain [1]. However, the numerous factors that can greatly complicate the implementation of this concept include the limitations of conventional PSC methods, effects of sequence identity and lack of robust ways to identify the boundary of a hinge loop. Consequently, manual labor is usually an indispensable factor in determining the location and range of hinge loops.

First, regardless of whether a conventional PSC algorithm is used, including the one utilized by the proposed method, superimposing the main and swapped domains simultaneously such that the non-superimposable portion of a DS_CO_ pair can be easily identified is very improbable. The proposed method bypasses this difficulty by using the A·D profile along with a morphological smoothing technique (see MATERIALS AND METHODS), which allows the preliminary identification of the hinge loop to be fully automated because the approximate location of a hinge loop can be simply recognized by a sudden decline or increase within the profile.

After the approximate location of a hinge loop is identified, the boundary must be determined. Eisenberg *et al.* suggested extending the hinge loop at both ends until two consecutive residues have *ϕ*, *ψ* differences lower than a cutoff (*θ*_0_). According to their algorithm, a higher *θ*_0_ results in a more restricted extension, allowing us to infer that they reasonably set a higher cutoff for quasi-DS_CO_ pairs (*θ*_0_ = 30°), which are evolutionarily more distant than bona fide cases (*θ*_0_ = 20°). Such an extension step is assumed here to be an excellent design and is thus applied as the fundamental hinge loop determination procedure in our DS-detecting system. However, as the discrimination of bona fide and quasi-domain swapping is somewhat empirical as well, manual inspections may be unavoidable to handle ambiguous cases. Although Eisenberg's algorithm was shown to be feasible based on a dataset of 33 DS_CO_ pairs, according to the large-scale test results shown in the Table S4, by setting *θ*_0_ as either 20° or 30°, the algorithm tended to over-extend the hinge loop of DS_CO_ pairs, especially for those with low sequence identities. This work also attempts to simplify the requirement for manual examinations and increase the precision of hinge loop identification by, first simply unifying the cutoff for bona fide and quasi-DS cases as 25° multiplied by *n_hl_*, the number of hinge loops detected in the same swapped domain (see Formula **11**). Then, the extension is restricted by a distance constraint of aligned residues in the front of the extending region. Given that the estimated range of hinge loops agrees well with the semi-manually verified ones reported in [2] (Table 4), and the over extension is well prevented (as also shown in the Table S4), we conclude that the proposed procedure is a fully automated, precise, and generally applicable method for hinge loop detection.

### Sensitivities to Middle-domain Swapping Cases

The experiment for detecting the three types of DS, N-terminal-, C-terminal-, and middle-domain swapping, discloses the robustness of the proposed method. Although the sensitivities for these three DS types seem evenly high (Table 2), the detection of middle domain swapping is actually more difficult than the other types. It is noteworthy that, as mentioned in the above subsection, a parameter *n_hl_* was introduced to help define the boundary condition (*θ*_0_) of a hinge loop. Because a middle-swapped domain has two hinge loops, its *θ*_0_ is twice as large as that of N-terminal- or C-terminal-swapped domains, resulting in a situation in which a higher restriction is imposed upon middle-DS than upon other DS types. Without the parameter *n_hl_*, the hinge loop(s) of a candidate middle-swapped domain may be determined to be invalid because of its over-extension (see MATERIALS AND METHODS), and thus the candidacy of the swapped domain is incorrectly rejected. Actually, according to our preliminary tests, the sensitivity to middle-DS of the proposed method without *n_hl_* was obviously lower. Two phenomena may explain the difficulty in detecting middle-DS.

First, a higher complexity of the “swapping movement” may hinder the calculation of the orientational difference (estimated by factor *γ_θ_*) between the swapped domains. The swapping of domains of an N-terminal- or C-terminal-DS_CO_ pair can be visualized as a swinging movement, during which the single hinge loop might bend and slightly twist, but the overall conformation of the swapped domain is preserved. Differently, the swapping movement of a middle-DS_CO_ pair could lead to some torsion of the swapped domain, especially when the extents or directions of bending of the two hinge loops differed. In this case, the way we transform the swapped domains into representative vectors (refer to Fig. 3) may not be adequate. To resolve the problem, we plan to design a multiple vector transformation technique to more precisely estimate the orientational difference between swapped domains.

Second, structural dissimilarities between small equivalent swapped domains can blur the boundary of hinge loops. Many of the swapped domains of known middle-DS_CO_ pairs are small and/or have few regular SSEs (see Table S6), meaning that they are either prone to be affected by the complicated type of swinging movement, or they are structurally quite flexible. Therefore, the structure of these swapped domains, including the hinge loops, may tend to be variable. It was observed that the structures of hinge loops of some small pairs of middle-swapped domains and/or the middle-swapped domains themselves were very varied that and thus boundaries of hinge loops were greatly over-extended when the parameter *n_hl_* was not applied. In some cases, they were so over-extended that the two determined hinge loops overlapped and hence overwhelmed the candidate swapped domain. No matter how visually apparent the middle-DS relationship between two proteins is, when a candidate middle-swapped domain is overwhelmed, or when the boundary of its hinge loop(s) cannot be determined, its candidacy is inevitably rejected based on our methodology. This is why *n_hl_* was introduced into Formula **11** to make the extension step more restricted in the determination process of hinge loops for middle-DS cases.

### Conclusions

We have designed the first specific detection method for 3D domain swapping. This pairwise comparison method does not work as an *ab initio* predictor for DS but determines the existence and type of DS relationship between two given protein structures. The A-D image-based algorithm proposed and the DS score defined here achieved a satisfactory performance, *i.e.*, an average MCC>0.80 in every experiment (Fig. 2, Figure S2 and Table S3). In addition, the robustness of this DS-detecting method have been evidenced by (1) the high true positive prediction rates for all three types of DS (Table 2), (2) the high sensitivity, specificity and structure alignment quality at low sequence identities (Table 3 and Figure S3), and, (3) the high precision of hinge loop determination (Table 4 and Table S4). With good performances, this method can greatly reduce the requirement for manual examinations for the identification of DS relationships among proteins, making it possible to develop automated procedures. As revealed by the fact that the structural similarities of DS homologs were prone to be underestimated by conventional PSC methods (Fig. 2 and Figure S3), the proposed method may also serve as a functional assignment system for novel hypothetical proteins which escape typical sequence and structural similarity searches/detections. Through several forms of structural alignments, the proposed method can present the actual structural similarities of main domains, swapped domains and the overall structures of DS_CO_ pairs, helping users study the structural and functional relationships among DS-related proteins. Changes in the hinge loops may profoundly impact the formation of 3D domain swapping [24], [25]. Thoroughly elucidating the hinge loops may help scientists to clarify the evolutionary mechanisms of DS, and the proposed method may be applicable to this field because of its well-developed function for determining the location of hinge loops. At present, several DS-related research fields appear to advance slowly or pause at theoretical stages. There is still much uncertainty about the natural prevalence of DS, the dominance of possible mechanisms for DS and how/why Nature achieves evolutionary diversities and functional regulations of proteins by using 3D domain swapping. Studying DS can deepen our knowledge about the structural dynamics and folding of proteins. Understanding the mechanisms of DS may help find new treatments for several protein conformational diseases like Alzheimer's disease and bovine spongiform encephalopathy [9], [19], [21]. Biotechnological applications of DS, such as the production of auto-assembling biomaterials and artificial biopolymers [20], [22], also require enough background knowledge. A key to solving those uncertainties and facilitating those medical and bioengineering applications shall be a comprehensive DS database. By manually screening only a small fraction of PDB, over a thousand DS cases had been identified in this study. As the number of protein structures is increasing at an unprecedented rate in this post-genomic era, we believe that the proposed method can greatly contribute to retrieving a much larger amount of relevant DS-related data than before from protein structural databases and thus move related fields forward.

## Materials and Methods

The experiments were performed using a Linux computer with a 2.66GHz Intel processor and 4 GB RAM. The source of protein structure files was a snapshot of the protein data bank (PDB) from August 2008. Specifically, the 90% sequence identity non-redundant subset of this PDB snapshot (abbreviated as nrPDB-90) was used. Programs were written in the Java, Asp.net and PHP languages. The structures shown in the figures were rendered using PyMol [48], Jmol [46], or Java OpenGL [49].

### Preparation of Experimental Datasets

This work established two main datasets for the 3D domain swapping, one based on literature-derived information (Dataset L) and the other based on manual inspections (Dataset M).

#### Dataset L

The “bona fide” and “quasi-domain swapping” DS_CO_ pairs summarized in [2], the domain-swapped dimers listed in [37] and a number of PDB entries with 3D domain swapping annotations located by keyword searches were collected into a primary dataset consisting of 263 proteins. Additional relevant data was retrieved by using each protein in this primary dataset as a query to search nrPDB-90 for DS-related homologs, common structural homologs and non-homologous structures following the procedure below:

1. For each protein *Q*, a rapid protein structural similarity search service, *i*SARST, was applied to search against nrPDB-90 for its structural neighbors with an E-value cutoff of 10 [50].
2. Protein *Q* and any protein *S* retrieved by *i*SARST in the hit list were defined as a neighboring structural pair (NS pair).
3. DaliLite v.3 [47] was utilized to perform structure superposition and to compute the Z-score of every NS pair.
4. According to the suggestion of [47], NS pairs with a Z-score ≥2 were provisionally considered as homologous structural pairs, which were classified into ten groups with decreasing sequence identities: 100–90%, 90–80%, 80–70%, etc.
5. Each NS pair belonging to the ten groups was then carefully examined by manual inspection. A DS relationship was identified when structural complementarities were observed. For instance, assume that *Q* is an open-form dimer; when *S* was found to have a similar structure to the known closed monomer of *Q*, *Q* and *S* would be considered a DS_CO_ pair.
6. After a DS_CO_ pair was identified, homologs of the closed and open forms were paired and examined to identify additional DS_CO_ pairs.
7. While all identified DS_CO_ pairs were selected into a dataset L_ds_, those homologous structural pairs without DS relationships were randomly selected into another dataset L_ch_, where the subscript ch refers to “common homologs”.
8. NS pairs with a Z-score <2 were generally considered as non-homologous (nh) structures, from which some were randomly selected into dataset L_nh_.
9. Finally, Dataset L consisted of datasets L_ds_ (737 pairs), L_ch_ (499 pairs) and L_nh_ (720 pairs). Dataset S1 provides a full list.

#### Dataset M

Due to the small number of experimentally and theoretically identified DS cases available at present, this work constructed Dataset M for a more detailed development and evaluation of the proposed method by screening nrPDB-90 for candidate cases of 3D domain swapping. According to the definition of DS, regardless of whether in the “bona fide”, “quasi” or “candidate” categories, an important prerequisite is the existence of homo-oligomers. The Protein Quaternary Structure (PQS) database maintained by the European Bioinformatics Institute contains predicted and experimentally-confirmed oligomeric proteins for PDB entries determined by X-ray crystallography [40]. The PQS team also established and implemented rules for distinguishing true biological oligomers from non-specific quaternary structures resulting from crystal packing. In this work, a 90% sequence identity subset of the PQS downloaded in August 2008 (nrPQS-90) was used. Because oligomers do not always form through 3D domain swapping, non-DS oligomers must be filtered out by manual inspection before utilizing the PQS. Considering the huge amount of data in nrPQS-90, only biological homodimers were examined. In accordance with the descriptions of DS in previous studies, dimers with obviously intertwined structures were collected into a preliminary dataset (472 polypeptides; all different from the primary dataset of Dataset L), based on which three datasets, M_ds_ (474 pairs), M_ch_ (1,803 pairs), and M_nh_ (1,809 pairs), were generated by following the same procedure as used for Dataset L. See the Dataset S2 for a full list of Dataset M.

### A-D Image-based Protein Secondary Structural Matching

**Figure 5:**
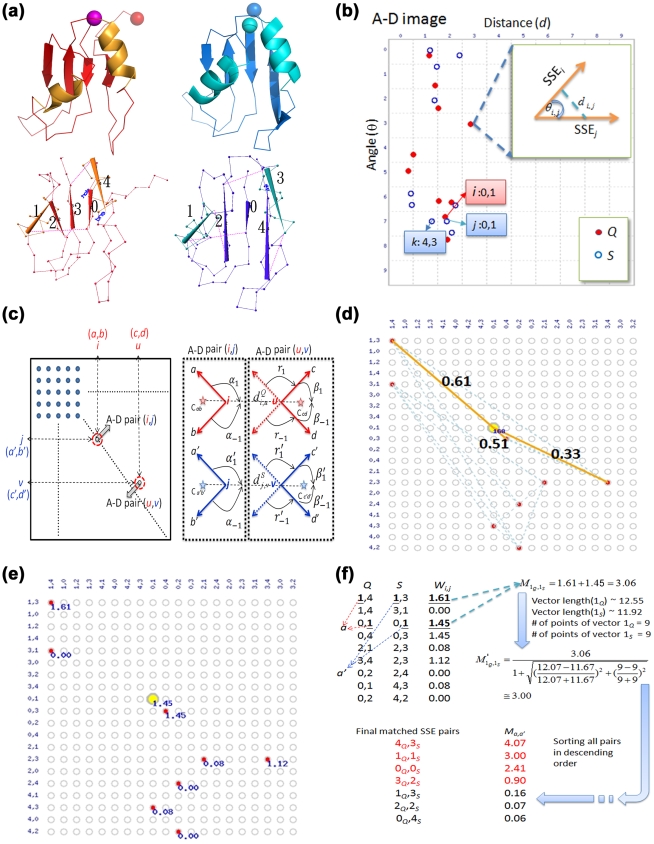
A-D image-based SSE matching. (**a**) RNA binding domains 2u2fA (red; *Q*) and 1no8A (blue; *S*) and their corresponding vectorized SSEs. (**b**) After transforming these structures into A-D images, in which each point comprised two SSEs [38], the A-D images were virtually superimposed and compared to find probable inter-image pairings between A-D points from the two images. Take the point *i* from *Q* for instance; it could be paired with its nearby points *j* and *k* from *S*. (**c**) Each probable pair of points, such as (*i*, *j*), was allowed to form a vertex in the pair-graph. Then, a delicate scoring scheme was applied to determine the geometric similarity between vertices. As illustrated here, many distances and angles of the component SSEs of two vertices were incorporated in this scheme (see the main text for details). (**d**) An edge (yellow line) was formed between two vertices sharing a positive score. (**e**) A weight was thus assigned to a vertex by summing the scores of the edges associated with the vertex. For example, the weight of the central yellow vertex was 1.45, *i.e.*, 0.61+0.51+0.33. (**f**) In the last stage, for every SSE pair (*a*, *a′*), where *a* is from *Q* and *a′* from *S*, a matching score 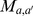 was assigned to it as the summation of all weights of the vertices associated with both *a* and *a′*. In this example, because *a* = 1*_Q_* and *a′* = 1*_S_*, the scores of the rows possessing both 1*_Q_* and 1*_S_* were summed to yield 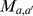 = 3.06. 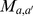 was then refined by a weighting function to become 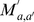. After sorting all SSE pairs according to 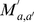 in a descending order, the first SSE pair was treated as the first matched SSE pair to identify the successive matched SSE pairs as described in the main text.

A-D image is a novel alignment-free PSC technique based on the angle-distance image transformation of SSEs [38]. The A-D image-based approach was initially based on comparing corresponding sub-images between two protein structures by using modified cross-correlation algorithms to identify the similarity of various patterns. This algorithm is effective at classifying protein structures at the “fold” level [38]. This work extends this technique to detect and align DS-related proteins through the development of a second version in which an SSE-matching algorithm is introduced. This SSE matching attempts to determine equivalent SSEs between two proteins. To achieve this, the equivalence of points (where each point represents a pair of SSEs in a protein) between the two A-D images is computed first. This task can be performed by utilizing a pair graph represented as *G*(*V*,*E*), in which vertices (*V*) denote possible pairings between points from the two A-D images, and edges (*E*) denote the compatibility between such pairings. Pair graphs were applied in a residue-based protein structural alignment method FAST, in which two residues from two proteins can be paired because of their similar local backbone conformation [30]. In this work, two points, *i.e.*, two pairs of SSEs, from two proteins can be paired due to their similar geometric relationships. The SSE matching algorithm is illustrated in Fig. 5 and is divided into several stages, which are explained below.

#### Construction of A-D images and determination of allowed vertices in the pair graph

The two proteins under examination, *Q* and *S*, are transformed into two A-D images. If *Q* and *S* have *E_Q_* and *E_S_* SSEs, respectively, there are 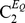 and 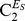 points on the respective A-D images *I^Q^* and *I^S^*. If all combinations of these points are allowed to form vertices in the pair graph, there are 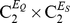 vertices. To reduce the computational cost, each A-D image is first dissected into 100 (10
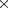
10) blocks, and then each point in block 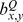 in *I^Q^* is allowed to be paired only with the points residing within 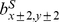, *i.e.*, block 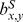 and its nearest 24 blocks in *I^S^*. Next, a geometric similarity score is assigned to each resulting pair of points *i* in *I^Q^* and *j* in *I^S^* according to the following formula:
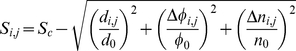
(4)where *d_i,j_* represents the Euclidean distance between *i* and *j* in the virtually superimposed A-D images (Fig. 5b), 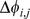 denotes the angular difference between the dihedral angle formed by the two SSEs constituting point *i* and the one formed by the two SSEs constituting *j*, and 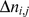 refers to the difference in length between the respective polypeptide chains connecting the component SSEs of *i* and *j*. The parameters 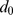, 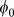, and 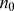 are scaling constants used to limit the three terms to similar ranges. The *S_c_* is a threshold to remove pairs of points with low geometric similarities; in this work, it was set to 3. Finally, only pairs with positive *S_i,j_* are allowed to form vertices. Based on this strategy, typically 70% of pairs are purged from the pair graph without substantially affecting the outcome of SSE matching.

#### Scoring scheme for edge computation

Let *a* and *b* denote the two SSEs constituting point *i*, and *a′* and *b′* denote those constituting *j*. Similarly, let *c* and *d* represent the component SSEs of point *u*, while *c′* and *d′* represent those of *v*. An edge connecting two vertices *p*(*i*,*j*) and *q*(*u*,*v*) in the pair graph cannot be assigned under the following conditions: (1) it is redundant with an existing edge, for instance, *p*((*a*,*b*),(*a′*,*b′*)) and *q*((*c*,*d*),(*c′*,*d′*)) are redundant with *p*((*a*,*b*),(*a′*,*b′*)) and *q*((*d*,*c*),(*d′*,*c′*)); and (2) *p* and *q* are contradictory, *e.g.*, *p*((*a*,*b*),(*c* = *a′*,*d* = *b′*)) and *q*((*a*,*b*),(*c* = *a′*,*d* = *e′*)). Because *d* cannot be equivalent to *b′* and *e′* simultaneously, the edge between *p* and *q* leads to a contradictory outcome. Except for the above “bad edges”, each possible edge between two vertices *p* and *q* in pair graph is assigned a weight calculated as follows:
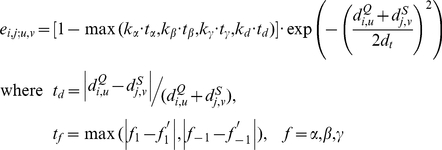
(5)


As shown in Fig. 5c, 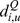 denotes the distance between the center point of *i* (calculated as the geometric center of SSEs *a* and *b* represented as *C_ab_*) and the center of *u* in protein *Q*, while 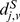 represents the distance between the centers of *j* and *v* in protein *S*. The exponential decay envelope and the distance term *t_d_* are adopted from the elastic score defined by Holm and Sander [32]. The other terms *t_α_*, *t_β_* and *t_γ_* are used to assess the similarity in the SSE directionality by using six angles, *α*_1_, *β*_1_, *γ*_1_, *α*_−1_, *β*_−1_, and *γ*_−1_. The threshold *d_t_* and other scaling factors, *k_α_*, *k_β_*, *k_γ_*, and *k_d_*, were empirically determined as 25 Å, 10 Å, 4/π (rad), 4/π (rad), and 3/π (rad), respectively.

#### Deducing the equivalence of SSEs from the equivalence of A-D points

A high positive weight of an edge suggests a high likelihood that the two pairs of A-D points are both equivalent pairs. A situation in which all positive weights of edges associated with vertex *p*(*i*,*j*) are summed up according to Formula **6** and assigned to *p* as its total weight *W_i,j_* suggests that *W_i,j_* is high if *p* is contained in the optimal set of equivalent pairs of A-D points. However, this work does not attempt to determine this optimal set. As long as the relative probability of equivalence of each pair of A-D points is approximately figured out, the equivalence of SSEs between the two proteins can be determined efficiently through an empirical voting process.
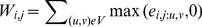
(6)


The equivalence of SSEs is extracted from the equivalence of A-D points, which is estimated by *W_i,j_*. Any A-D point *i* has two component SSEs *a* and *b*, with each possibly appearing in more than one A-D point. If SSE *a* of protein *Q* is matched with SSE *a′* of protein *S*, *a* and *a′* are supposed to co-exist in one or more vertices. A weighted matching score (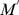) for SSEs *a* and *a′* is defined by summing up all weights of vertices associated with both *a* and *a′* and then being divided by a weighting function of the size similarity between SSEs *a* and *a′*.
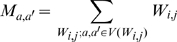
(7.1)

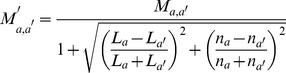
(7.2)where *L_x_* denotes the length of the representative vector of an SSE (*x* = *a* or *a′*), and *n_x_* represents the number of residues that the SSE contains. The summation is a voting procedure in essence. A higher 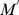 score implies a pair of SSEs with a higher priority to be selected as a matched pair at the final stage.

#### SSE matching

SSE pairs with a zero 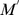 score are neglected in the descending order list, and the top-ranked SSE pair possessing the highest score is used as a seed to identify any SSE pair satisfying the following criteria during traversed checking: (1) a new matched pair must maintain the sequential order of SSEs that the existing matched pairs have already defined; and (2) an existing SSE cannot be repeatedly selected. The entire process ends after the last SSE pair is examined.

The first criterion guarantees a sequential or order-dependent matching because 3D domain swapping itself does not affect the sequential order of SSEs in a protein structure. Without this restriction, the algorithm can perform order-independent SSE matching and, thus, detect non-sequential structural similarities that can be observed in proteins with circular or crumbled permutations [51], [52], [53], [54].

### Locating Candidate Hinge Loops by the Profile of the A-D Product

The fact that matching processes do not depend on structure superposition allows us to identify the equivalence of the SSEs of a DS_CO_ pair throughout the main domains and swapped domains. Conversely, most conventional structural alignment methods, which are superposition-dependent, only report the equivalence of SSEs (and residues) for one of the two domains. Comparing the results of SSE matching and those of a superposition-dependent protein structural alignment reveals that the boundary between the region well aligned by both methods, and the region only aligned by matching can be recognized as the approximate location of a hinge loop. The profile of the A-D product is thus designed for quantifying and analyzing the differences between the results of the two methods.

#### Superposition-dependent protein structural alignment

To develop a well-integrated system, a superposition-dependent structural alignment method based on SSE matching has been designed and utilized here. However, this design is not required because most conventional structural alignment algorithms can actually be applied in this step.

The equivalent SSEs of two proteins determined by SSE matching are used as the seed anchors for the two protein structures. Using dynamic programming, the equivalent residues for each pair of seed anchors are determined in a manner such that the total number of equivalent residue pairs is as large as possible. These equivalent residues are then initially superimposed, and the RMSD is calculated by a classical singular value decomposition method developed by Kabsch [55]. Based on the initial superposition, equivalent residues with a distance exceeding a cutoff are eliminated; then, the remaining equivalent residues are in turn used as the new anchors for determining a new set of equivalent residues based on which the two structures are superimposed, and a new RMSD is computed as well. This process is iteratively performed until the RMSD stabilizes. Because the distance of equivalent residues is restricted, the main domains and swapped domains are unlikely to be aligned simultaneously. Additionally, because the dynamic programming focuses on a large number of equivalent residues, this method tends to align two proteins by a larger domain, which is normally the main domain in a DS_CO_ pair.

#### Profile of the A-D product (A·D profile)

Following the above structural alignment, a pair of aligned SSEs should have a similar position and orientation in the superposition. An SSE is represented by a transformed vector in the N- to C-terminus direction and its centroid point. The angular difference between two SSE vectors (A) and the distance between their centroids (D) can describe the similarities and differences between the aligned and matched-only SSEs. A measure called angle-distance product (A-D product or *P_ad_*) is thus formulated as follows:
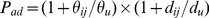
(8)where *θ_u_* and *d_u_* are set as 180° and 25 Å, respectively, as scaling factors, and *θ_ij_* and *d_ij_* denote the A and D factors of a pair of aligned/matched SSEs *i* and *j*, respectively.

The profile of *P_ad_* (or A·D profile) is extremely useful for rapidly locating possible hinge loop(s) in a DS_CO_ pair. Aligned SSE pairs normally possess low *P_ad_*, while matched-only SSEs possess high *P_ad_* values. Consequently, a low-valued region in the A·D profile is normally formed by two well-aligned domains (the candidate main domains). A high-valued region reflects similar and matched substructures with different orientations (the candidate swapped domains), while the transition zone in between can be considered as a candidate hinge loop. In other words, a hinge loop can be recognized as a sudden increase or decrease in the A·D profile. According to the example in Figure S4, the main domains of these two DS-related crystallins (PDB entries 4gcrA and 1blbA) form a low-valued region in the N-terminal region of the profile, and the swapped domains result in a high-valued region towards the C-terminus. Therefore, the hinge loops are assumed to appear somewhere in the transition zone, which lies between SSE No. 6 and No. 7 for 4gcrA and SSE No. 4 and No. 5 for 1blbA.

#### Identification of significant transitions in the A·D profile

It is not necessary that every increase or decrease corresponds to a hinge loop. A short peak or valley is normally attributed to a local dissimilarity between two structures. In this study, an attempt is made to identify a transition (a significant increase or decrease) in an A·D profile by applying a morphological smoothing algorithm, which is commonly used in image processing to remove isolated dark and bright spots, to reduce the noise of the A·D profile (see Figure S4). After morphological smoothing operations, the difference in the *P_ad_* between every two adjacent points is transformed into a sequence, the elements of which are subjected to a t-test to identify the most significant difference. For instance, the smoothened A·D profile of the above-mentioned crystallins consists of ten points: {*p*_1_, *p*_2_, *p*_3_, *p*_4_, *p*_5_, *p*_6_, *p*_7_, *p*_8_, *p*_9_, *p*_10_} = {1.10, 1.10, 1.10, 1.10, 4.50, 4.50, 4.50, 4.37, 4.37, 4.37}. Thus, the sequence of differences contains nine elements: {Abs(*d*_2-1_), Abs(*d*_3-2_), Abs(*d*_4-3_), Abs(*d*_5-4_), Abs(*d*_6-5_), Abs(*d*_7-6_), Abs(*d*_8-7_), Abs(*d*_9-8_), Abs(*d*_10-9_)} = {0.00, 0.00, 0.00, 3.40, 0.00, 0.00, 0.13, 0.00, 0.00}. A t-test is then performed at the 80% confidence interval on this sequence, with a t-value of 0.92 subsequently obtained. Because Abs(*d*_5-4_) = 3.40 was the only significant (>0.92) difference, a candidate for the hinge loop was identified between the SSE pairs constituting *p*_4_ and *p*_5_.

#### Identification of the 3D domain swapping type

Because swapped domains of a DS_CO_ pair typically result in a high-valued region in an A·D profile, when a high-valued region is on the N-/C-terminal side or in the middle of an A·D profile, the two proteins are identified as N-/C-domain-swapped or middle-domain-swapped cases, respectively.

#### Determination of the approximate opening point of a hinge loop

The “opening point” of hinge loops is defined here as the boundary between two well-superimposable main domains and the non-superimposable regions that cover the swapped domains. After a candidate hinge loop in a protein has been located between two consecutive SSEs by analyzing the A·D profile, its opening point can be determined as follows.

Let *SSE_N_* and *SSE_C_* denote these two consecutive SSEs, where *N* and *C* specify that the SSE is close to the N- or C-terminus. (1) In an N-domain-swapped case, the opening point of the hinge loop is determined as the first residue of *SSE_C_*. (2) In a C-domain-swapped case, the opening point is determined as the last residue of *SSE_N_*. (3) For a middle-domain-swapped protein, the opening points of its N- and C-terminal hinge loops are determined in the same manner as in (2) and (1), respectively.

### Refinement of the Location and Range of Hinge Loops

In theory, the above procedures can identify the approximate opening point of a candidate hinge loop for DS cases with large swapped domains possessing at least two SSEs. However, the term “domain” swapping is sometimes not well defined because the swapped “domain” may only be a small structural fragment with a few or even no regular SSEs. A situation in which the swapped domain is small, or its SSE(s) is distorted almost into a loop form, implies no significant increase or decrease in the A·D profile. Therefore, whether a small swapped “domain” exists in a flat region of an A·D profile must be further analyzed (see Figure S4c for an example). The feasibility of a candidate hinge loop is evaluated by using a refinement procedure described below, and its actual range will be identified as well.

#### Assigning small candidate swapped domains

When no significant peak or valley appears in the N-terminal low-valued region of an A·D profile, the N-terminal region before the first aligned SSE pair is temporarily considered as a swapped domain. Additionally, the first residue of this SSE pair is considered as the opening point of the hinge loop. A similar situation in which the C-terminal region of an A·D profile has low values means that the C-terminal region after the last aligned SSE pair is treated as a swapped domain.

Theoretically, middle-domain swapping requires a swapped domain with more than three SSEs to form a high-valued region recognizable in a smoothened A·D profile. Unfortunately, a large proportion (62%) of the known middle-swapped domains contains only one or even no SSE. The main feature of middle-DS is that both the N-terminal region prior to and the C-terminal region after the swapped domains of two proteins can be well-superimposed, leaving the swapped domains bifurcated. An unaligned fragment located in the flat region of an A·D profile is thus considered as a candidate swapped domain. The opening point of each hinge loop of the candidate middle-swapped domain is determined approximately as the first unaligned residue in the bifurcation region of the two superimposed protein structures.

#### Refining the location of opening points of candidate hinge loops

After the above procedures, each candidate hinge loop is determined with an approximate opening point. A refining procedure is then applied to identify its precise location through the following steps,

1. Starting from the initially assigned opening point (*o*) and moving a probe (*p*) along the backbone of a protein (*Q*), two directions are applied including towards the peak (the well-superimposed region) and towards the bifurcation of the structural superposition. The principle of the scanning processes is peak first and bifurcation last (PF and BL).
2. At the PF stage, the stop condition works when more than five consecutive residues are aligned with the other protein (*S*).

At the BL stage, starting from the stop point identified in step (2), the number of unaligned residues is counted by *N_u_* as *p* moves towards the bifurcation. When *N_u_* surpasses a specific cutoff *T_N_*, which was set to be 4 in this report, the search is terminated, and the new opening point (*o′*) is thus determined. If *p* has reached the terminus, and *N_u_* still does not exceed *T_N_*, the feasibility of this over-extended candidate hinge loop is rejected.

#### Validating the feasibility of candidate hinge loops and swapped domains

The feasibility of a candidate hinge loop is quickly rejected if this criterion is not satisfied:
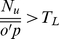
(9)where *T_L_* is set to be 0.5.

In addition, by cutting at *o′*, the non-superimposed fragments of both proteins, which should contain the swapped domains in a DS case, are subjected to a typical superposition-dependent structural alignment. Because swapped domains are supposed to resemble each other, a situation in which either their alignment ratio is too small (<50% of the smaller fragment) or the RMSD is too large (≥4 Å) suggests that these two fragments will not receive approval as a pair of swapped domains, subsequently leading to the rejection of the feasibility of this candidate hinge loop. The feasibilities of a candidate hinge loop and the swapped domain that it links are both validated if the candidate hinge loop is not rejected by these criteria.

#### Structural alignment of the determined main and swapped domains

Once a hinge loop is validated, the protein structure can be cut into a main/large domain and a swapped/small domain at *o′*. Next, the main domains and swapped domains of the two proteins are, respectively, subjected to the above-mentioned typical structural alignment to obtain two superpositions and two sets of equivalent residues, ultimately forming the basis for determining the range of the hinge loop and the computation of the DS score.

#### Determining the range of hinge loops

This step is a modified version of the hinge loop determination method of Eisenberg *et al* [1]. Based on the superpositions of the main domains and swapped domains, a pair of determined hinge loops can be extended at both ends until two consecutive aligned residue pairs satisfy the following criterion:
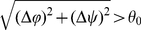
(10)where *ϕ* and *ψ* refer to the torsion angles of the protein backbone conformation. In their work, the cutoff *θ*_0_ was empirically set as 20° and 30° for bona fide and quasi domain swapping cases, respectively.

To avoid the manual determination of the category of 3D domain swapping, the setting of *θ*_0_ was modified in this study as follows:
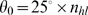
(11)where *n_hl_* stands for the number of hinge loops identified for a swapped domain. For an N- or C-terminal-swapped domain, *n_hl_* = 1, while a middle-swapped domain has *n_hl_* = 2.

In addition, there are two extra conditions that will terminate the extension: (1) Each of the last three pairs of aligned (equivalent) residues included has a Cα-Cα distance ≤2.6 Å. (2) The total number of residues remaining in the candidate swapped domain ≤10.

### Calculation of the DS Score

The RESULTS Section provides the definition and formulas of the DS score. A minimal structural diversity factor (*μ_sd_*) was defined to determine the structural similarity of the swapped domains. Interestingly, as implied by the angle-distance (A-D) image technique, this work designed two A-D factors to determine the conformational difference of a candidate DS_CO_ pair with respect to the swapped domains and hinge loops, *i.e.*, the angular difference factor *γ_θ_* and displacement factor *γ_d_*.

Following the identification of the hinge loops and swapped domains as stated above, these are transformed into representative vectors using two methods. The first is a conventional regression model for solving the minimum distance problems. This is the same method that we utilized to vectorize SSEs, except that here we use a whole swapped domain, excluding its hinge loop(s), as an input. See the Supporting Information of [38] for detailed algorithm. The steps of the second method are described here: (1) Assume that the position of a residue can be represented by the coordinate of its Cα atom. (2) Compute the geometric center (*C_0_*) of the central residue and the terminal residue of the swapped domain. (3) Make a vector pointing from the refined opening point (*o′*) of the hinge loop to *C_0_*.

If the angular differences between the representative vectors generated by these two methods are *θ_x_* and *θ_y_*, then the angular difference factor *γ_θ_* is calculated as 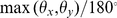. The displacement factor *γ_d_* is calculated as the normalized average displacement of equivalent residues in the swapped domains as the two proteins are superimposed by their main domains (Fig. 3).

In the proposed model of the DS score, three parameters, *m*_0_, *m*_1_, and *m*_2_, are assumed to be obtained during the dataset training. A heuristic and exhaustive range-searching strategy (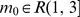, 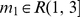, 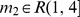) was implemented to adjust and evaluate the settings of these parameters based on the MCC values.

### The Choices of Positive and Negative Data for Binary Classification Experiments

In this work, the choices of positive and negative data were made in order to suitably demonstrate the uniqueness of the proposed method and the properties of various conventional methods and structural similarity measures. By using both DS and common homologs as the positive data and non-homologs as the negative data, such as the experiments described in Fig. 2a and Fig. 2d, the abilities of various methods and measures to serve as a general detector for all types of structural homologs were compared. Since the DS score designed here was specific for DS-related homologs, its performance was expected not as good as other virtual structural similarity measures (Fig. 2d). To test whether the accessed methods and measures could well distinguish DS-related homologs from non-homologs, DS homologs and non-homologs were respectively considered as the positive and negative data (Fig. 2b and Fig. 2e). Now that DS-related homologs are still a kind of homologs, in expectation all methods and measures should perform well. When using DS and common homologs respectively as the positive and negative data (Fig. 2c and Fig. 2f), the DS-specific detection ability was empathized and hence the uniqueness of the proposed DS-detecting method would be clearly revealed. For Table 3 Figure S2, and Table S3, only DS homologs were the positive data while common homologs and non-homologs were all defined as the negative data because in those experiments we were not comparing the proposed method with other approaches but only specifically testing its DS-detecting power.

### Experimental Parameters

#### Alignment ratio

In this work, the first evaluation mechanism of DS-detection ability for PSC methods is proposed. Since common global structural homologs usually possess a large structural alignment size, *i.e.*, the number of structurally aligned/equivalent residues, they can be easily distinguished from DS-related homologs, which are not so easily distinguishable from common homologs with only partial or low structural similarities. By gradually filtering out homologs with large alignment sizes from a structural dataset, the remaining data will be more and more challenging for PSC methods to classify DS and common homologs. Therefore, the (structural) alignment size is a very important parameter in this evaluation mechanism. To rule out the effects of various protein sizes, in implementation the alignment size was normalized by the protein size with Formula **3** and was thus called the alignment ratio (unit: percentage). In the experiments of Fig. 2, the alignment ratios of all protein pairs were uniformly calculated by FAST [30]. In this way, all alignment methods and similarity measures were assessed on an equal basis, no matter at which alignment ratio cutoff point.

#### Virtual structural similarity measures

The only difference between the virtual structural similarity measures proposed in this study, *e.g.*, vRMSD, vQ-score and vS-div, and their conventional versions, *e.g.*, RMSD, Q-score and S-Div, is that the calculation of virtual measures depends on the virtual structural superposition of proteins. In the proposed methodology, a virtual superposition of a DS_CO_ pair is done by flexibly allowing the main and swapped domains to be independently transformed in the Cartesian coordinate system; hence, the superpositions of main and swapped domains are optimal at the same time in the virtual superposition. Conventional PSC methods are more rigid than the proposed method. They usually align (superimpose) only one domain for a given DS_CO_ pair, or separately align the two domains and output two sets of similarity values. In either case, the structural similarity of the DS_CO_ pair as a whole is poorly described and cannot be directly conceived. It is supposed that the virtual structural similarity measures and the virtual structural superposition are currently the best ways to describe and visualize the structural similarities of DS-related proteins.

The original rigid versions of the virtual structural similarity measures utilized in the evaluation tests (Fig. 2) were all commonly used measures. Many of them have been reviewed and compared in [56] (see Fig. 2 for more references). Among these measures, RMSD and alignment size are the most widely used ones while others are complex measures calculated based on them and the sizes of the compared proteins. The structural diversity (S-div) defined as the RMSD divided by the normalized alignment size (see [41] and Formula **2**) is conceptionally the simplest one and the Q-score [43] is a measure with a clearly defined range (0 to 1); these two measures also showed high performances in our experiments and were thus extensively used in this study.

#### BLAST and SARST alignment parameters

BLAST [42] is the most widely used protein sequence alignment search method. SARST [34] is an extremely rapid protein structural alignment search method which describes protein structures as one-dimensional structural strings and recruits BLAST as its alignment engine. Since SARST works through BLAST, these methods have the same parameter sets. In order to make as long alignments as possible, especially for those proteins with very low sequence identities, we disabled the filter for low complexity regions, set the word size to be 2 and chose the highest E-value cutoff available for both tools. After trying BLOSUM45, 62 and 80 substitution matrices [57], BLOSUM62 was found to exert the best performance in Fig. 2c and was thus used in all other experiments. As for SARST, the standard scoring matrix for its Ramachandran codes, SARSTSM20 [34], was utilized. Gap penalties, inclusive of gap opening penalty (GOP) and gap extension penalty (GEP), for these tools were determined by trials-and-errors and the optimal conditions found for BLAST were GOP = 6 and GEP = 2 while the optimal settings for SARST were GOP = 9 and GEP = 1.

### Nomenclatural Acts

The electronic version of this document does not represent a published work according to the International Code of Zoological Nomenclature (ICZN), and hence the nomenclatural acts contained in the electronic version are not available under that Code from the electronic edition. Therefore, a separate edition of this document was produced by a method that assures numerous identical and durable copies, and those copies were simultaneously obtainable (from the publication date noted on the first page of this article) for the purpose of providing a public and permanent scientific record, in accordance with Article 8.1 of the Code. The separate print-only edition is available on request from PLoS by sending a request to PLoS ONE, 185 Berry Street, Suite 3100, San Francisco, CA 94107, USA along with a check for $10 (to cover printing and postage) payable to “Public Library of Science”.

In addition, this published work and the nomenclatural acts it contains have been registered in ZooBank, the proposed online registration system for the ICZN. The ZooBank LSIDs (Life Science Identifiers) can be resolved and the associated information viewed through any standard web browser by appending the LSID to the prefix “http://zoobank.org/”. The LSID for this publication is: (to be determined by PLoS ONE)

## Supporting Information

Figure S1 The number of DS-related homologs, common homologs and non-homologs remaining in the test set from the experiments presented in Fig. 2 as the alignment ratio cutoff decreases. The alignment cutoff applied in this study is designed to remove globally-superimposeable homologous protein pairs from the testing datasets. Since many common homologous pairs are globally-superimposeable, as this cutoff lowers, the amount of common homologs decreases much more rapidly than the amount of DS-related homologs, which are only partially-superimposeable, decreases. Meanwhile, the amount of non-homologous pairs remains nearly unchanged. Interestingly, relative to the amount of all homologs, including DS-related and common ones, the amount of DS-related homologs remaining in the dataset increases as the alignment ratio cutoff becomes lower within the tested range.
(0.46 MB PDF)

Figure S2 Stability evaluations of the discriminatory model of the proposed method by *k*-fold cross-validations. The stability of the discriminatory model applied in the proposed DS-scoring scheme was evaluated based on two datasets. **(a)** Evaluations based on Dataset L. **(b)** Evaluations based on Dataset M.
(1.34 MB PDF)

Figure S3 Performances of several protein structure/sequence comparison methods for the detection of global structural similarities between DS-related homologs with various sequence identities. An experiment that determines the simultaneous alignment qualities of the hinge loops, main domains and swapped domains for several protein structure/sequence comparison methods.
(0.53 MB PDF)

Figure S4 Examples of the A⋅D profile and related hinge loop detection procedure. **(a)** Crystallins with PDB identifiers 4gcrA and 1blbA, a quasi-domain swapping case [2]. **(b)** Crystallins with PDB identifiers 4gcrA and 2a5mA, a pair of common global homologs. **(c)** Acetyltransferases with PDB identifiers 1s60A and 1b6bA, a pair of quasi-domain swapping homologs with a small C-terminal-swapped “domain”.
(1.56 MB PDF)

Table S1 DS-detecting performance of DynDom assessed based on Eisenberg's DS dataset. Among the 39 query proteins, 12 are detected to posses hinge loops by DynDom [35]. The locations and ranges of hinge loops determined by DynDom are compared to those reported by Eisenberg *et al.* in [2].
(0.11 MB PDF)

Table S2 Sensitivity and specificity of various alignment methods and structural similarity measures for the identification of common structural homologs and/or DS-related homologs. Sensitivity and specificity values of all alignment methods were determined based on S-div [41], except those of BLAST, which were determined based on a normalized sequence similarity score calculated according to the Formula **8** in [34].
(0.07 MB XLS)

Table S3 Results of inter-dataset training and testing of the proposed method for the identification of DS-related homologs. Only DS-related homologs were used as positive data in this experiment, in which common homologs and non-homologs were both regarded as negative data. Performance measures listed in this table include AUC, MCC, sensitivity and specificity.
(0.06 MB PDF)

Table S4 Results of the structural alignments and hinge loop determinations for DS_CO_ pairs in Datasets L and M. The 1,093 DS_CO_ pairs successfully identified by the proposed method are listed here each with detailed information of the ranges of hinge loops determined by Eisenberg's and our methods, several structural similarity measures as well as the DS score defined in this work, and the virtual superimposition computed by our method. Structural superimpositions shown in this table were drawn using Jmol.
(9.67 MB PDF)

Table S5 Structure-based sequence alignments for DS_CO_ pairs in Datasets L and M performed by several protein structural comparison methods. The structure-based sequence alignments performed by TM-align [31], SARST [34] and the proposed DS-detecting method as well as the sequence alignments performed by BLAST [42] for the 1,093 DS_CO_ pairs shown in Table S4 are listed here.
(9.84 MB PDF)

Table S6 Number of SSEs in the swapped domains. Here an SSE means an α-helix or a β-strand. The number of SSEs that a swapped domain contains roughly reflects the size of the domain. The ranges of SSEs were extracted from the PDB files according to the HELIX and SHEET records.
(0.07 MB PDF)

Dataset S1 PDB entry list for the Dataset L. A list of the PDB entries of the protein pairs constituting sub-datasets L_ds_, L_ch_ and L_nh_.
(0.13 MB XLS)

Dataset S2 PDB entry list for the Dataset M. A list of the PDB entries of the protein pairs constituting sub-datasets M_ds_, M_ch_ and M_nh_.
(0.24 MB XLS)
