# Supplementary material for: Detection and Alignment of 3D Domain Swapping Proteins Using Angle-Distance Image-Based Secondary Structural Matching Techniques
Source: PLoS One. 2010 Oct 14;5(10):e13361. doi: 10.1371/journal.pone.0013361 (PMC2955075; doi:10.1371/journal.pone.0013361)
Supplement: Figure S1 — The number of DS-related homologs, common homologs and non-homologs remaining in the test set from the experiments presented in Fig. 2 as the alignment ratio cutoff decreases. The alignment cutoff applied in this study is designed to remove globally-superimposeable homologous protein pairs from the testing datasets. Since many common homologous pairs are globally-superimposeable, as this cutoff lowers, the amount of common homologs decreases much more rapidly than the amount of DS-related homologs, which are only partially-superimposeable, decreases. Meanwhile, the amount of non-homologous pairs remains nearly unchanged. Interestingly, relative to the amount of all homologs, including DS-related and common ones, the amount of DS-related homologs remaining in the dataset increases as the alignment ratio cutoff becomes lower within the tested range. (0.46 MB PDF) [file pone.0013361.s001.pdf]

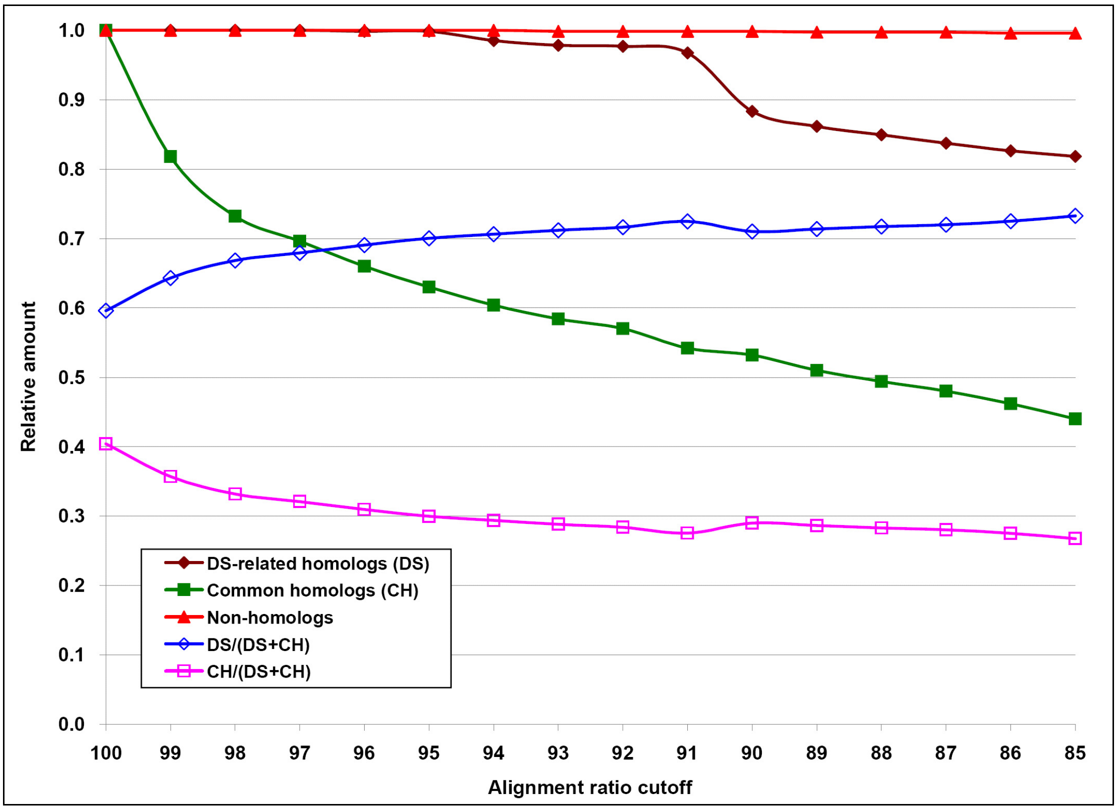

Figure S1. The number of DS-related homologs, common homologs and non-homologs remaining in the test set from the experiments presented in Fig. 2 as the alignment ratio cutoff decreases. The alignment cutoff (x-axis) applied in this study is designed to remove globally-superimposeable homologous protein pairs from the testing datasets. Since many common homologous pairs are globally-superimposeable, as this cutoff lowers, the amount of common homologs (the green cure) decreases much more rapidly than the amount of DS-related homologs, which are only partially-superimposeable, decreases (see the brown cure). Meanwhile, the amount of non-homologous pairs (the orange curve) remains nearly unchanged. Interestingly, relative to the amount of all homologs, including DS-related and common ones, the amount of DS-related homologs remaining in the dataset increases as the alignment ratio cutoff becomes lower within the tested range (see the blue curve). Alignment ratios were calculated by FAST [30]. Raw data of this figure are listed in the following table.

Alignment ratio cutoff (%)	DS-related homologs (DS) (pairs)	Common homologs (CH) (pairs)	Non-homologs (NH) (pairs)	DS (%)	CH (%)	NH (%)
100	737	500	721	1.000	1.000	1.000
99	737	409	721	1.000	0.818	1.000
98	737	366	721	1.000	0.732	1.000
97	737	348	721	1.000	0.696	1.000
96	736	330	721	0.999	0.660	1.000
95	736	315	721	0.999	0.630	1.000
94	726	302	721	0.985	0.604	1.000
93	721	292	720	0.978	0.584	0.999
92	720	285	720	0.977	0.570	0.999
91	713	271	720	0.967	0.542	0.999
90	651	266	720	0.883	0.532	0.999
89	635	255	719	0.862	0.510	0.997
88	626	247	719	0.849	0.494	0.997
87	617	240	719	0.837	0.480	0.997
86	609	231	718	0.826	0.462	0.996
85	603	220	718	0.818	0.440	0.996
