# Supplementary material for: Detection and Alignment of 3D Domain Swapping Proteins Using Angle-Distance Image-Based Secondary Structural Matching Techniques
Source: PLoS One. 2010 Oct 14;5(10):e13361. doi: 10.1371/journal.pone.0013361 (PMC2955075; doi:10.1371/journal.pone.0013361)
Supplement: Figure S2 — Stability evaluations of the discriminatory model of the proposed method by k-fold cross-validations. The stability of the discriminatory model applied in the proposed DS-scoring scheme was evaluated based on two datasets. (a) Evaluations based on Dataset L. (b) Evaluations based on Dataset M. (1.34 MB PDF) [file pone.0013361.s002.pdf]

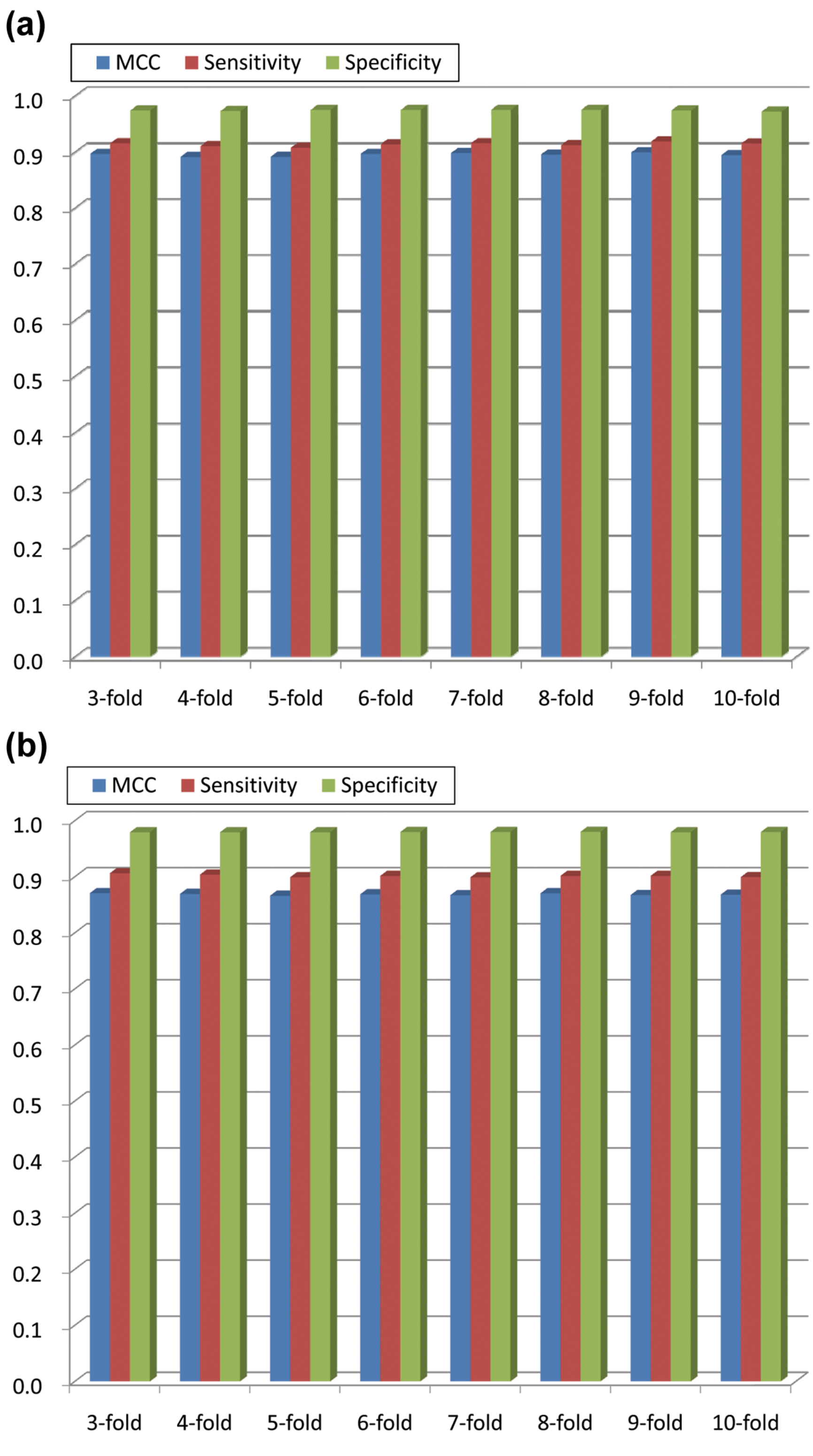

Figure S2. Stability evaluations of the discriminatory model of the proposed method by *k*-fold cross-validations. The stability of the discriminatory model applied in the proposed DS-scoring scheme was evaluated based on two datasets. **(a)** Evaluations based on Dataset L. **(b)** Evaluations based on Dataset M. In both experiments, only the DS-related homologs were treated as the positive data, while common homologs and non-homologs were used as the negative data.
