# Supplementary material for: Detection and Alignment of 3D Domain Swapping Proteins Using Angle-Distance Image-Based Secondary Structural Matching Techniques
Source: PLoS One. 2010 Oct 14;5(10):e13361. doi: 10.1371/journal.pone.0013361 (PMC2955075; doi:10.1371/journal.pone.0013361)
Supplement: Figure S3 — Performances of several protein structure/sequence comparison methods for the detection of global structural similarities between DS-related homologs with various sequence identities. An experiment that determines the simultaneous alignment qualities of the hinge loops, main domains and swapped domains for several protein structure/sequence comparison methods. (0.53 MB PDF) [file pone.0013361.s003.pdf]

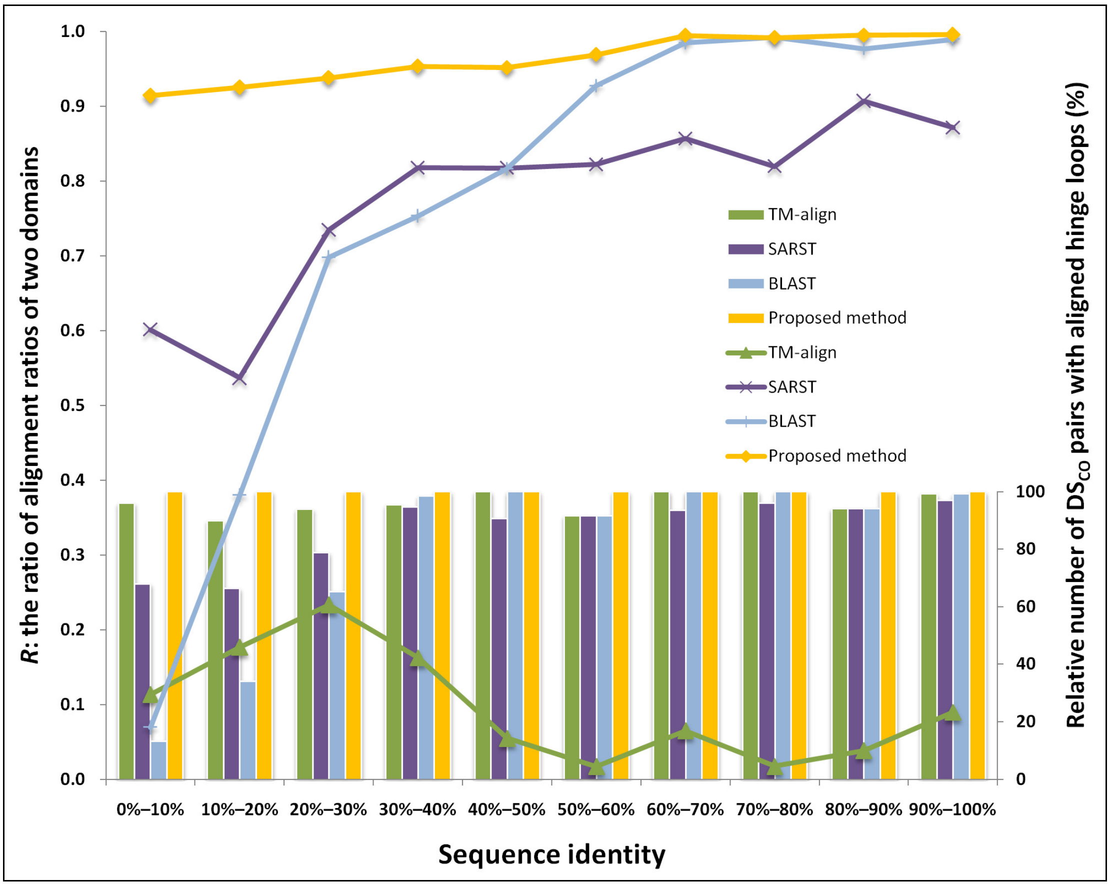

Figure S3. Performances of several protein structure/sequence comparison methods for the detection of global structural similarities between DS-related homologs with various sequence identities. Since a pair of DS-related homologs has at least one pair of hinge loops, which separates the main domains and swapped domains, if a method can detect the global structural/sequence similarities of the pair of DS-related homologs, it shall at least be able to simultaneously align some part of the main domains and some part of the swapped domains. And, if the alignment is correct, equivalent hinge loops of the query and subject proteins shall be positioned close to each other. This experiment utilized the structure-based sequence alignments performed by TM-align [31], SARST [34] and the proposed structural comparison method, and the sequence alignments performed by BLAST [42] for the 1,093 DS$_{CO}$ pairs listed in Table S5. For every DS$_{CO}$ pair, we first examined whether the equivalent hinge loops were closely positioned (two equivalent hinge loops were regarded as closely positioned when their alignment size ≥ 1) by various methods. Next, for each method we took the cases that it correctly positioned the equivalent hinge loops and calculated the alignment ratios, r_x and r_y, of the two domains flanking the hinge loop. Finally, the ratio (R) of r_x and r_y was computed according to the formula $R=\min(r_x,r_y)/\max(r_x,r_y)$ to determine the relative quality of alignments on both domains. If a method simultaneously and evenly detects the structural/ sequence similarities of both domains, R is close to 1. The bar chart reveals that, TM-align and the proposed method performed almost equally well at detecting the location of equivalent hinge loops and the performances remained high (>90% correct detections) even at <10% sequence identities. As the sequence identity decreased, SARST became less capable of correctly aligning equivalent hinge loops while BLAST performed even worse. When the sequence identities were lower than 20%, SARST and BLAST could only correctly align less than 66% and 34% equivalent hinge loops of all the DS$_{CO}$ pairs, respectively. The line chart shows that, among the DS$_{CO}$ pairs with properly aligned hinge loops and ≥20% sequence identities, the alignment ratios calculated by BLAST for the two domains were rather

balanced (R>0.70). SARST could align the two domains linked by hinge loops in a more balanced manner than BLAST at sequence identities <20%, although the average R values ranged only between 0.54 and 0.60. Even if TM-align could properly align most equivalent hinge loops, at every identity level it produced very low R values, which meant that its alignments were generally concentrated on one domain only. The performance of the proposed method was very impressive. The balance of the alignment qualities for both domains of DS_{CO} pairs was violated only slightly by the decrease of sequence identities. Even at <10% sequence identities, the R value was still 0.91, meaning that this method could detect the global structural similarities of DS-related proteins with very low sequence homology. The sequence identity values used in this experiment were obtained using BLAST. FASTA had also been utilized [http://fasta.bioch.virginia.edu/fasta_www2/fasta_list2.shtml] and the experimental results were very similar.
