# Supplementary material for: Detection and Alignment of 3D Domain Swapping Proteins Using Angle-Distance Image-Based Secondary Structural Matching Techniques
Source: PLoS One. 2010 Oct 14;5(10):e13361. doi: 10.1371/journal.pone.0013361 (PMC2955075; doi:10.1371/journal.pone.0013361)
Supplement: Figure S4 — Examples of the A⋅D profile and related hinge loop detection procedure. (a) Crystallins with PDB identifiers 4gcrA and 1blbA, a quasi-domain swapping case [2]. (b) Crystallins with PDB identifiers 4gcrA and 2a5mA, a pair of common global homologs. (c) Acetyltransferases with PDB identifiers 1s60A and 1b6bA, a pair of quasi-domain swapping homologs with a small C-terminal-swapped “domain”. (1.56 MB PDF) [file pone.0013361.s004.pdf]

(a)

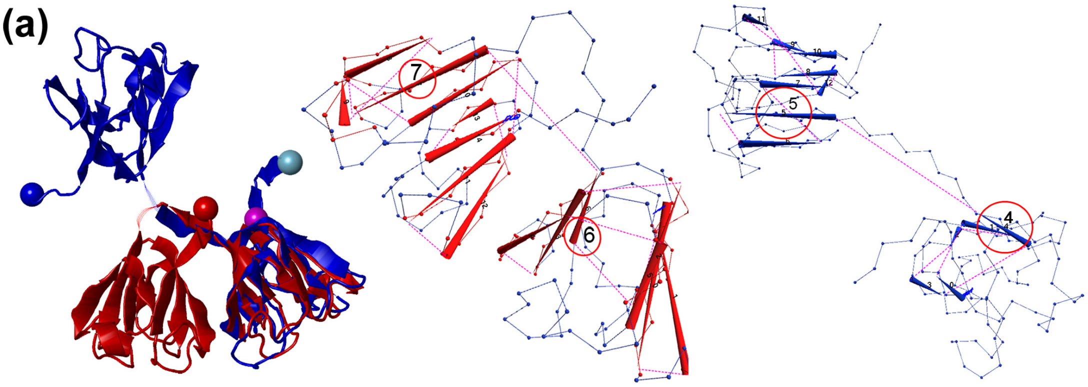

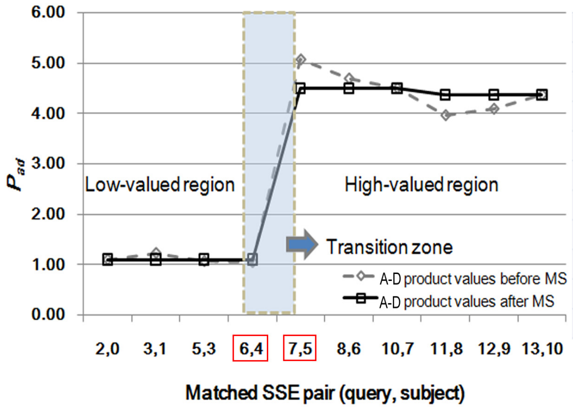

SSE pairs	Before MS	After MS
2,0	1.10	1.10
3,1	1.23	1.10
5,3	1.07	1.10
6,4	1.05	1.10
7,5	5.07	4.50
8,6	4.69	4.50
10,7	4.50	4.50
11,8	3.97	4.37
12,9	4.09	4.37
13,10	4.37	4.37

(b)

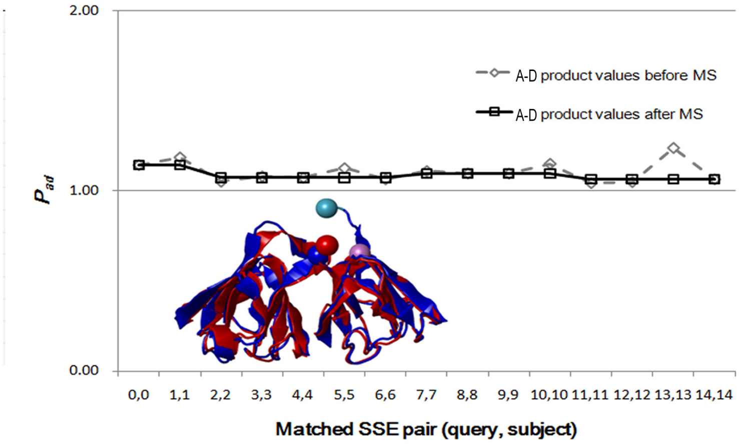

SSE pairs	Before MS	After MS
0,0	1.14	1.14
1,1	1.19	1.14
2,2	1.05	1.07
3,3	1.08	1.07
4,4	1.07	1.07
5,5	1.13	1.07
6,6	1.07	1.07
7,7	1.11	1.10
8,8	1.10	1.10
9,9	1.10	1.10
10,10	1.15	1.10
11,11	1.04	1.07
12,12	1.05	1.07
13,13	1.24	1.07
14,14	1.07	1.07

(c)

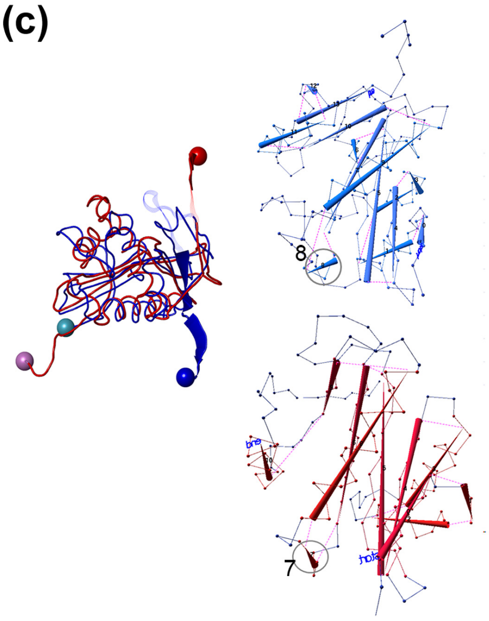

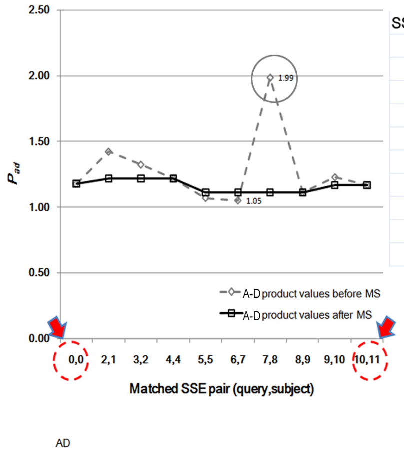

SSE pairs	Before MS	After MS
0,0	1.18	1.18
2,1	1.42	1.22
3,2	1.32	1.22
4,4	1.22	1.22
5,5	1.07	1.11
6,7	1.05	1.11
7,8	1.99	1.11
8,9	1.11	1.11
9,10	1.23	1.17
10,11	1.17	1.17

Figure S4. Examples of the A·D profile and related hinge loop detection procedure. (a) Crystallins with PDB identifiers 4gcr and 1blb, a quasi-domain swapping case [2]. The superimposed and SSE-vectorized structures shown in the upper region reveal that the hinge loop of 4gcr lies between its SSE No. 6 and No. 7 and the one of 1blb lies between its SSE No. 4 and No. 5 (Note that in this report SSEs are numbered from 0). A·D profiles of these crystallins is demonstrated by the plot on the lower left, in which P_{ad} values are plotted on the y-axis and the matched SSEs are indicated along the x-axis. The gray dotted line represents the raw A·D profile of these proteins while the black line represents the A·D profile obtained after applying morphological smoothing (MS). It is clear that the transition zone between the low- and high-valued regions of an A·D profile well reveal the approximate location of hinge loops. The table on the lower right side lists the matched SSEs and P_{ad} values shown in the A·D profiles. The red boxes indicate the boundary SSEs of the transition zone, which are exactly the SSEs No. 6 and 7 of 4gcr and No. 4 and 5 of 1blb. **(b)** Crystallins with PDB identifiers 4gcr and 2a5m, a pair of common global homologs. As shown by the superimposed structures, these proteins should not possess any swapped domain or hinge loop. As a result, no high-valued region is observed in the A·D profile. Although there are small fluctuations in the raw A·D profile, the noise caused by these fluctuations are well suppressed by MS, which prevents false positive detections of hinge loops. **(c)** Acetyltransferases with PDB identifiers 1s60 and 1b6b, a pair of quasi-domain swapping homologs with a small C-terminal swapping "domain". Since the two matched SSEs encircled by the gray circles in the vectorized structures have very different directions, they form a peak in the raw A·D profile. However, because these two SSEs can be aligned by a conventional superimposition-dependent alignment algorithm and they do not have obvious displacements and structural complementary properties like most swapped domains do, this peak is a false signal of swapped domain. The MS procedure correctly wipes out this false signal. After MS, no matter whether there is any candidate hinge loop identified, approximate opening points of candidate hinge loops will be assigned to the most terminal residues of an aligned pair of SSEs existing in the low-valued region of the A·D profile, as indicated by the red arrows. In this example, because the whole A·D profile is flat and low-valued, both the N- and C-terminal aligned SSEs are assigned with approximate opening points of candidate hinge loops. Finally, a refinement process is carried out to determine the precise positions of these opening points and whether the terminal regions after these points are valid swapped domains. A situation in which no precise opening point or valid swapped domain can be identified for a candidate hinge loop rejects the existence of this candidate hinge loop. For these acetyltransferases, only the C-terminal regions are identified with valid hinge loops and swapped domains, even if the swapped "domains" are so small that the one of 1s60 contains no regular SSE while the one of 1b6b possesses only one β-strand. Manual inspections of the superimposed structures have well verified these results.
