# Supplementary material for: Detection and Alignment of 3D Domain Swapping Proteins Using Angle-Distance Image-Based Secondary Structural Matching Techniques
Source: PLoS One. 2010 Oct 14;5(10):e13361. doi: 10.1371/journal.pone.0013361 (PMC2955075; doi:10.1371/journal.pone.0013361)
Supplement: Table S1 — DS-detecting performance of DynDom assessed based on Eisenberg's DS dataset. Among the 39 query proteins, 12 are detected to posses hinge loops by DynDom [35]. The locations and ranges of hinge loops determined by DynDom are compared to those reported by Eisenberg et al. in [2]. (0.11 MB PDF) [file pone.0013361.s005.pdf]

Table S1. DS-detecting performance of DynDom assessed based on Eisenberg's DS dataset

Closed form	Open form	Hinge loops examined by [2]		Hinge loops identified by DynDom [35]		Length difference of hinge loops[1] (residues)	Shift of the centers of hinge loops[1] (residues)
		Range$_e$	Length$_e$	Range$_i$	Length$_i$		
1brnL	***1yvsA***	37-41	5	37-44	7	-2	1.5
1griA	***1fyrA***	121-123	3	120-123	3	0	0.5
1griA	1fyrA	121-123	3	120-123	3	0	0.5
1mdtA	***1ddtA***	379-387	9	379-388	9	0	0.5
1hngA	***1cdcA***	44-50	7	43-50	7	0	0.5
1cunA	***2spcA***	72-75	4	76-79	3	1	4
2ezmA	***3ezmA***	50-53	4	49-50	1	3	2
1cewI	***1g96A***	55-59	5	55-57	2	3	1
1pv3A	***1k04A***	943-948	6	933-934	1	5	12
4icbA	***1ht9A***	38-47	10	42-47	5	5	2
5rsaA	***1bsrA***	15-22	8	22-23	1	7	4
1qlxA	***1i4mA***	188-198	11	203-207	4	7	12
1orcA	5croA	55-56	2			N/A*	
1sncA	1sndA	112-120	9			N/A*	
1mupA	1obpA	126-130	5			N/A*	
1fynA	1aojA	112-118	7			N/A*	
1gmfA	1hulA	87-99	13			N/A*	
1msbA	***1ixxA***	72-75	4			N/A*	
1hz5A	***1jmlA***	52-55	4			N/A*	
1wwwX	***1wwbX***	299-301	3			N/A*	
1orcA	***5croA***	55-55	1			N/A*	
1sncA	***1sndA***	112-120	9			N/A*	
1mupA	***1obpA***	121-124	4			N/A*	
1fynA	***1aojA***	34-49	16			N/A*	
1wwwX	***1wwaX***	297-299	3			N/A*	
1dksA	***1cksA***	60-65	6			N/A*	
1msbA	***1ixxA***	93-98	6			N/A*	
1nloC	***1aojA***	34-39	6			N/A*	
1qmpA	***1dz3A***	103-109	7			N/A*	
1wwwX	***1wwcA***	317-319	3			N/A*	
1k3sA	***1k3eA***	33-36	4			N/A*	
1eydA	***1sndA***	112-120	9			N/A*	
1qd0A	***1sjvA***	95-100	6			N/A*	
1gmfA	***1hulA***	82-89	8			N/A*	
5rsaA	***1f0vA***	112-115	4			N/A*	
5rsaA	***1js0A***	112-115	4			N/A*	
5rsaA	***1a2wA***	15-22	8			N/A*	
1a5pA	***1a2wA***	15-22	8			N/A*	
4gcrA	***1blbA***	79-87	9			N/A*	
				**Unsigned average**		**2.8**	**3.4**

*Not available because DynDom cannot identify any hinge loop.

[1]See Table 4 for how to calculate these values.
