# Supplementary material for: Detection and Alignment of 3D Domain Swapping Proteins Using Angle-Distance Image-Based Secondary Structural Matching Techniques
Source: PLoS One. 2010 Oct 14;5(10):e13361. doi: 10.1371/journal.pone.0013361 (PMC2955075; doi:10.1371/journal.pone.0013361)
Supplement: Table S3 — Results of inter-dataset training and testing of the proposed method for the identification of DS-related homologs. Only DS-related homologs were used as positive data in this experiment, in which common homologs and non-homologs were both regarded as negative data. Performance measures listed in this table include AUC, MCC, sensitivity and specificity. (0.06 MB PDF) [file pone.0013361.s007.pdf]

Table S3. Results of inter-dataset training and testing of the proposed method for the identification of DS-related homologs

Training set	Testing set	AUC	MCC	Sensitivity	Specificity
Dataset M	Dataset L	0.958	0.901	0.896	0.989
Dataset L	Dataset M	0.969	0.801	0.937	0.954

Only DS-related homologs were used as positive data in this experiment, in which common homologs and non-homologs were both regarded as negative data.
