# Supplementary material for: Detection and Alignment of 3D Domain Swapping Proteins Using Angle-Distance Image-Based Secondary Structural Matching Techniques
Source: PLoS One. 2010 Oct 14;5(10):e13361. doi: 10.1371/journal.pone.0013361 (PMC2955075; doi:10.1371/journal.pone.0013361)
Supplement: Table S4 — Results of the structural alignments and hinge loop determinations for DSCO pairs in Datasets L and M. The 1,093 DSCO pairs successfully identified by the proposed method are listed here each with detailed information of the ranges of hinge loops determined by Eisenberg's and our methods, several structural similarity measures as well as the DS score defined in this work, and the virtual superimposition computed by our method. Structural superimpositions shown in this table were drawn using Jmol. (9.67 MB PDF) [file pone.0013361.s008.pdf]

Table S4. Results of the structural alignments and hinge loop determinations for DS_{CO} pairs in Datasets L and M

The 1,093 DS_{CO} pairs successfully identified by the proposed method are listed here each with detailed information of the ranges of hinge loops determined by Eisenberg's and our methods, several structural similarity measures as well as the DS score defined in this work, and the virtual superimposition computed by our method. Structural superimpositions shown in this table were drawn using Jmol. The cells of determined hinge loops highlighted with yellow or green background colors mean that the calculated ranges for these hinge loops are obviously insufficient or over-extended, respectively. These highlighted parts were judged with manual inspections by the authors and their colleagues. The authors suggest that these parts should be used only for reference at the current stage where there is still no universal rule for verifying the range of a predicted or calculated hinge loop.

The authors would like to thank Chi-Ching Lee, Che-Wei Chang, Zong-Han Tsai and Yi-Chou Lin, the authors' colleagues at National Tsing Hua University, Shih-Chung Yen at National Chiao Tung University and Yu-Jie Lin at National Cheng Kung University, for their effort in preparing this table.

No.	PDB entry (size)	Hinge loop (size) Eisenberg's algorithm; θc=20°	Hinge loop (size) Eisenberg's algorithm; θc=30°	Hinge loop (size) The proposed method	DS type	DS score	vRMSD	vAli.Ratio	Identity	Superimposed structures
1	1brnL (108)	37-44 (8)	37-41 (5)	37-41 (5)	N	0.92	0.47	99.07% (107/108)	99.07% (107/108)	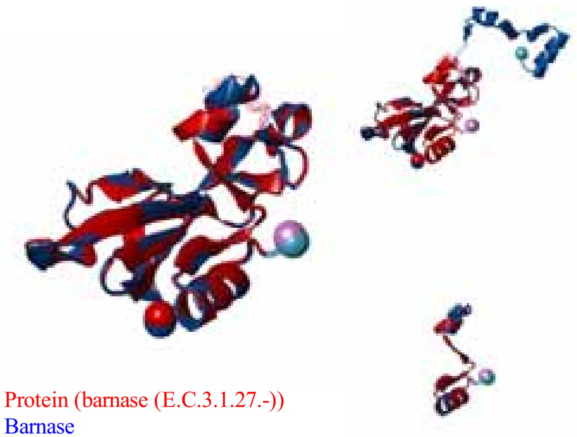 Protein (barnase (E.C.3.1.27.-)) Barnase
	1yvsA (108)	37-44 (8)	37-41 (5)	37-41 (5)						
2	4icbA (76)	39-45 (7)	39-45 (7)	41-45 (5)	C	0.86	0.97	97.37% (74/76)	96.05% (73/76)	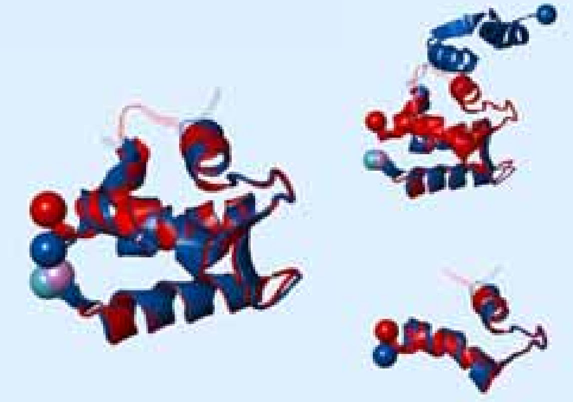 Calbindin D9K Calbindin D9K
	1ht9A (76)	39-45 (7)	39-45 (7)	41-45 (5)						

3	1hngA (175)	44-50 (7)	44-46 (3)	44-46 (3)	C	0.49	1.01	100.00% (96/96)	97.92% (94/96)	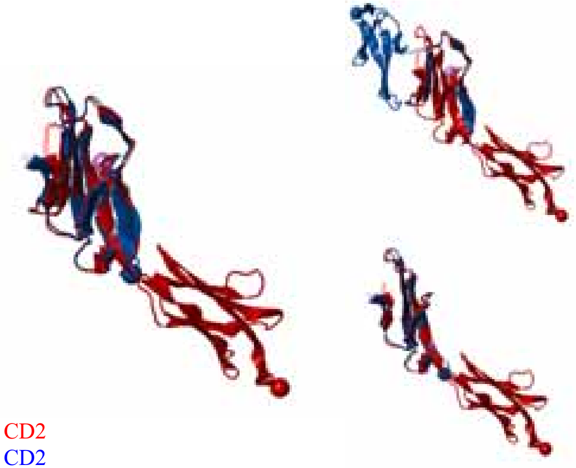 CD2 CD2
	1cdcA (96)	44-50 (7)	44-46 (3)	44-46 (3)						
4	1orcA (64)	54-60 (7)	55-60 (6)	55-56C (2)	C	0.76	1.24	96.72% (59/61)	93.44% (57/61)	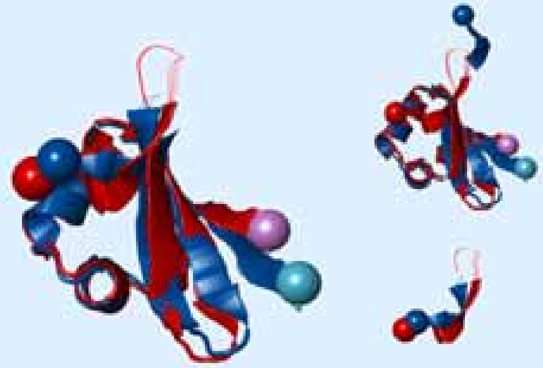 CRO repressor insertion mutant K56-[dgevk] CRO repressor protein
	5croA (61)	54-60 (7)	55-60 (6)	55-56C (2)						
5	2ezmA (101)	49-55 (7)	49-54 (6)	49-54 (6)	C	0.93	0.82	100.00% (101/101)	100.00% (101/101)	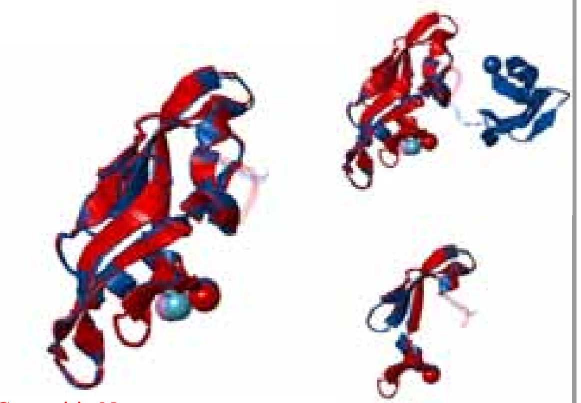 Cyanovirin-N Protein (cyanovirin-N)
	3ezmA (101)	49-55 (7)	49-54 (6)	49-54 (6)						

6	1mdtA (523)	377-386 (10)	379-386 (8)	379-386 (8)	C	0.91	0.84	98.85% (517/523)	98.85% (517/523)	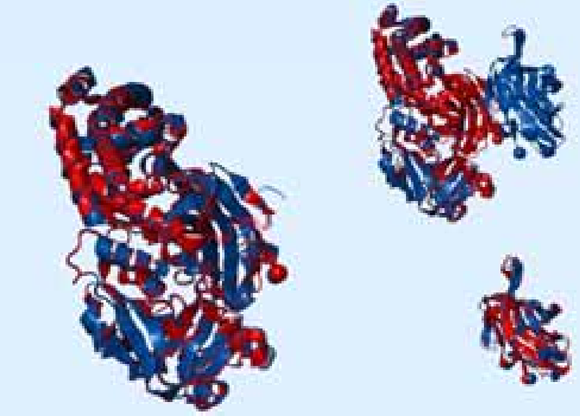 Diphtheria toxin
	1ddtA (523)	377-386 (10)	379-386 (8)	379-386 (8)						Diphtheria toxin
7	1qlxA (104)	188-198 (11)	188-191 (4)	189-197 (9)	C	0.69	1.56	95.19% (99/104)	92.31% (96/104)	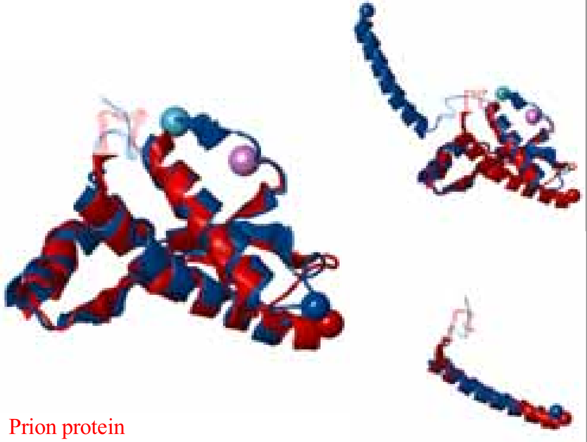 Prion protein
	1i4mA (108)	188-198 (11)	188-194 (7)	189-197 (9)						Major prion protein
8	1hz5A (72)	51-56 (6)	52-56 (5)	52-56 (5)	C	0.74	1.58	97.22% (70/72)	88.89% (64/72)	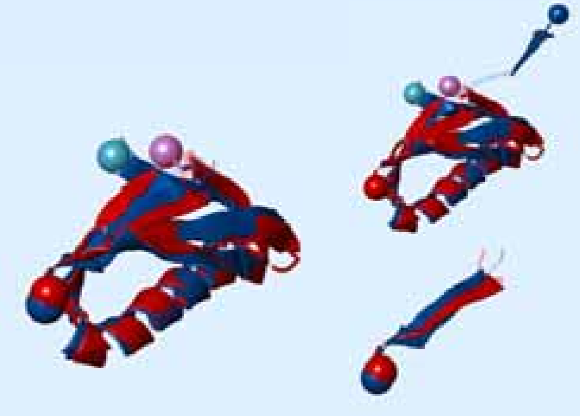 Protein L
	1jmlA (72)	51-56 (6)	52-56 (5)	52-56 (5)						Protein L

9	5rsaA (124)	15-22 (8)	16-22 (7)	18-22 (5)	N	0.97	0.54	100.00% (124/124)	100.00% (124/124)	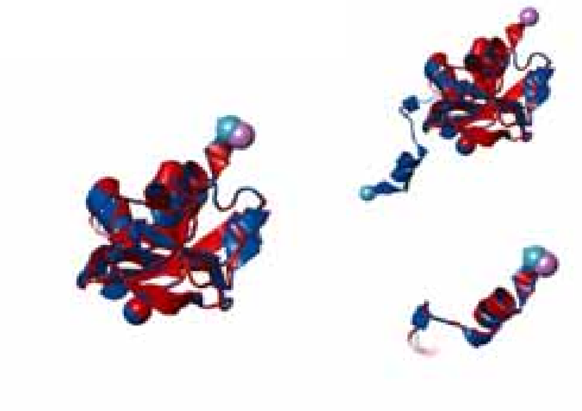 Ribonuclease A<br>Ribonuclease A
	1a2wA (124)	15-22 (8)	16-22 (7)	18-22 (5)						
10	5rsaA (124)	111-113 (3)	112-113 (2)	112-113 (2)	C	0.98	0.43	100.00% (124/124)	100.00% (124/124)	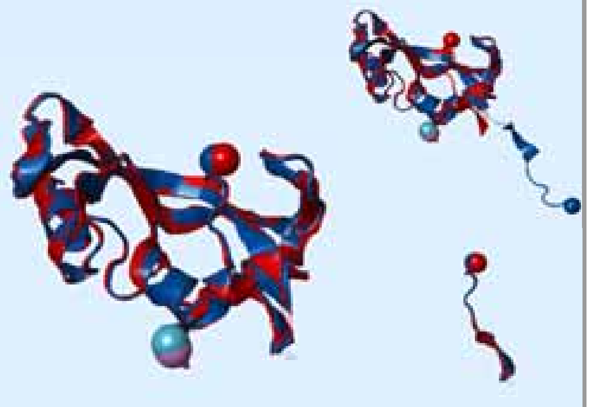 Ribonuclease A<br>Ribonuclease A
	1f0vA (124)	111-113 (3)	112-113 (2)	112-113 (2)						
11	5rsaA (124)	112-115 (4)	112-113 (2)	112-113 (2)	C	0.78	0.57	100.00% (124/124)	100.00% (124/124)	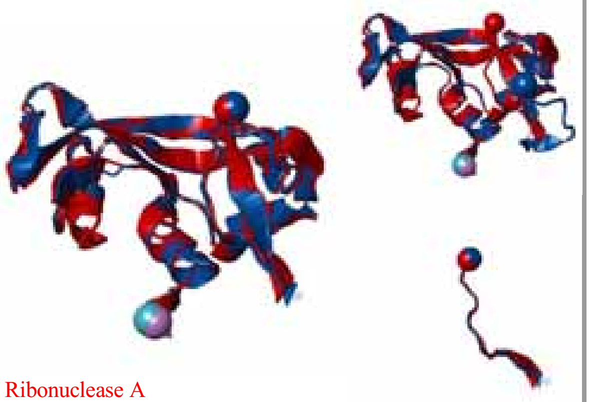 Ribonuclease A<br>Ribonuclease A
	1js0A (124)	112-115 (4)	112-113 (2)	112-113 (2)						

12	5rsaA (124)	15-22 (8)	15-22 (8)	15-22 (8)	N	0.57	0.91	100.00% (124/124)	81.45% (101/124)	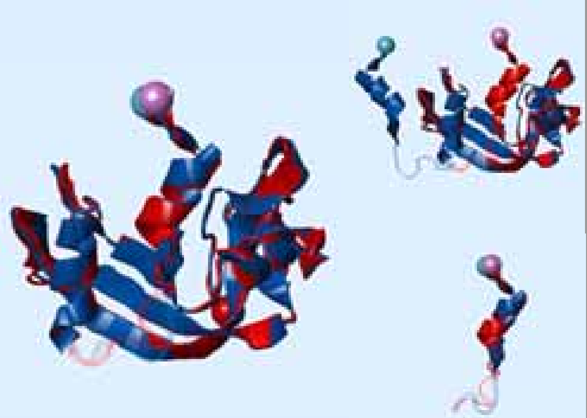 Ribonuclease A<br>Bovine seminal ribonuclease
	1bsrA (124)	15-22 (8)	15-22 (8)	15-22 (8)						
13	1qmpA (126)	100-112 (13)	102-108 (7)	107-111 (5)	C	0.79	1.33	98.37% (121/123)	89.43% (110/123)	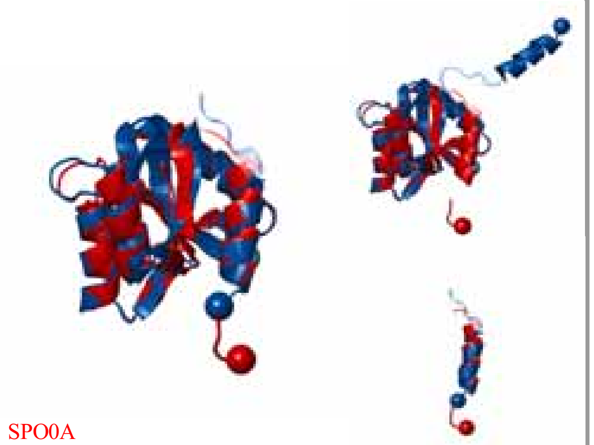 SPO0A<br>Stage 0 sporulation protein a
	1dz3A (123)	100-112 (13)	101-108 (8)	107-111 (5)						
14	1sncA (135)	112-120 (9)	112-120 (9)	112-120 (9)	C	0.87	0.93	100.00% (129/129)	99.22% (128/129)	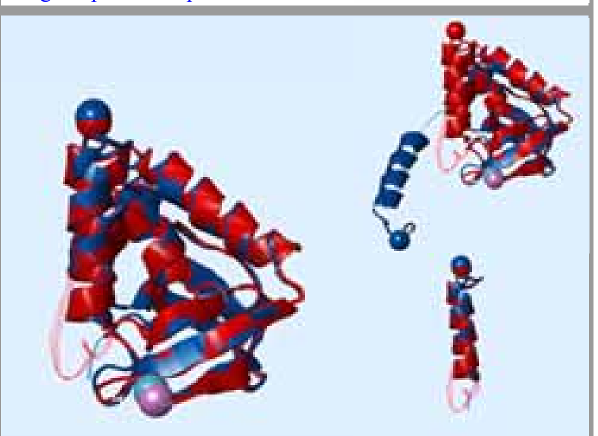 Thermonuclease precursor<br>Staphylococcal nuclease dimer
	1sndA (129)	112-120 (9)	112-120 (9)	112-120 (9)						

15	1wwwX (101)	296-299 (4)	296-299 (4)	296-298 (3)	N	0.92	0.68	100.00% (101/101)	100.00% (101/101)	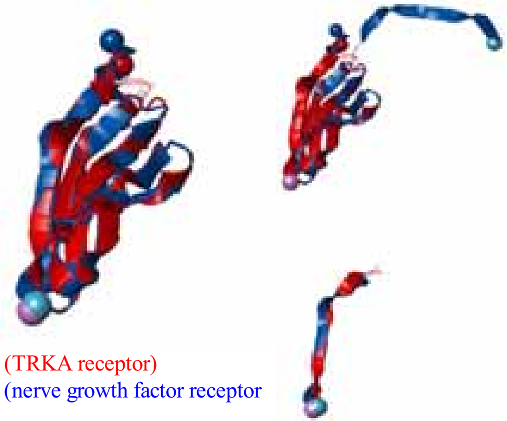 Protein (TRKA receptor) Protein (nerve growth factor receptor TRKA)
	1wwaX (105)	296-299 (4)	296-299 (4)	296-298 (3)						
16	1wwwX (101)	287-299 (13)	294-299 (6)	295-298 (4)	N	0.83	1.10	98.02% (99/101)	45.54% (46/101)	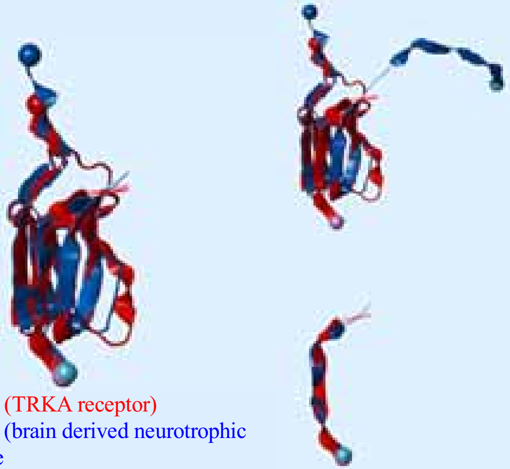 Protein (TRKA receptor) Protein (brain derived neurotrophic factor Re
	1wwbX (103)	288-301 (14)	296-301 (6)	295-298 (4)						
17	1wwwX (101)	295-300 (6)	295-300 (6)	296-297 (2)	N	0.62	1.13	96.04% (97/101)	41.58% (42/101)	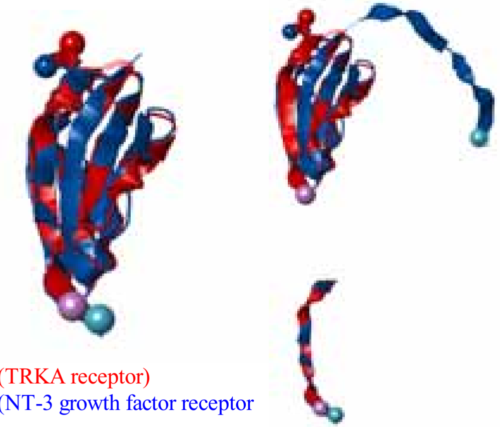 Protein (TRKA receptor) Protein (NT-3 growth factor receptor TRKC)
	1wwcA (105)	315-320 (6)	315-320 (6)	296-297 (2)						

18	1dksA (76)	60-65 (6)	60-65 (6)	60-65 (6)	C	0.78	1.37	98.65% (73/74)	83.78% (62/74)	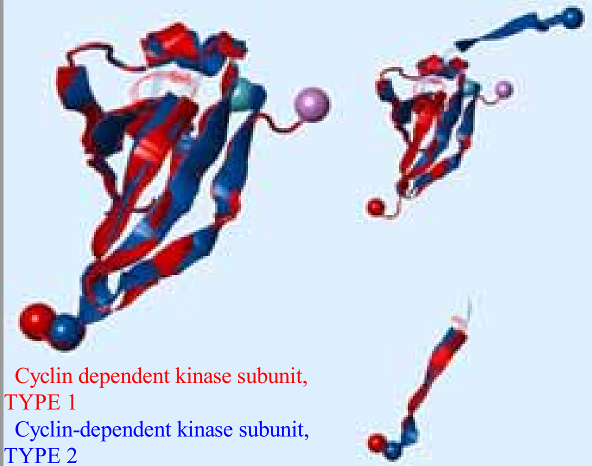 Cyclin dependent kinase subunit, TYPE 1<br>Cyclin-dependent kinase subunit, TYPE 2
	1cksA (74)	60-65 (6)	60-65 (6)	60-65 (6)						
19	4gcrA (174)	44-96 (53)	82-92 (11)	86-87 (2)	N	0.77	1.30	98.28% (171/174)	36.78% (64/174)	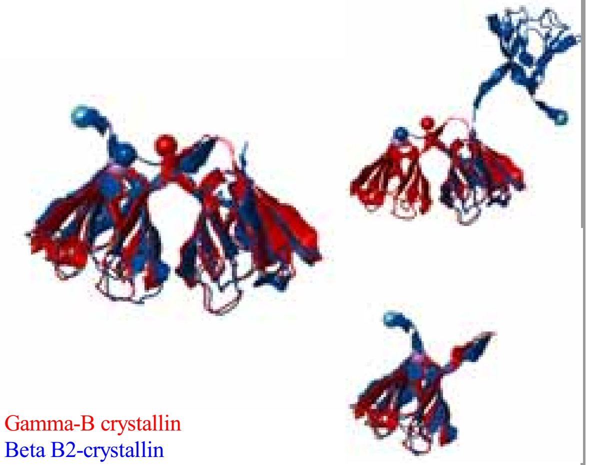 Gamma-B crystallin<br>Beta B2-crystallin
	1blbA (185)	44-96 (53)	82-92 (11)	86-87 (2)						
20	1cewI (108)	53-60 (8)	54-57 (4)	55-57 (3)	N	0.55	1.45	96.30% (104/108)	39.81% (43/108)	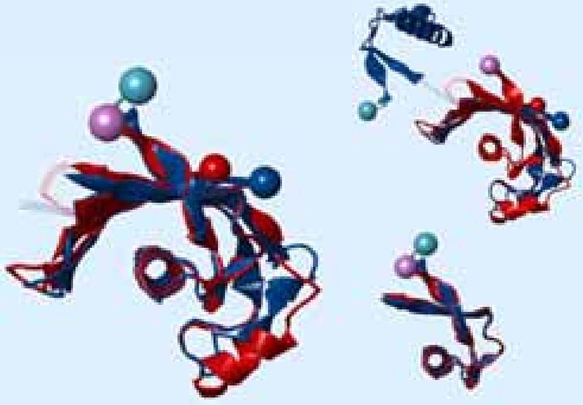 Cystatin<br>Cystatin C
	1g96A (111)	55-62 (8)	56-59 (4)	55-57 (3)						

21	2gmfA (121)	85-101 (17)	87-101 (15)	87-97 (11)	C	0.43	2.26	87.04% (94/108)	10.19% (11/108)	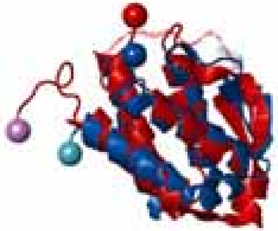 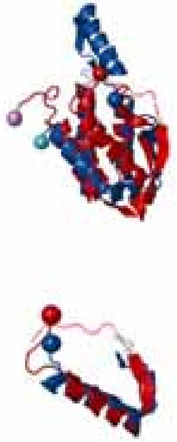 Granulocyte-macrophage colony-stimulating FAC Interleukin-5
	1hulA (108)	80-91 (12)	82-91 (10)	87-97 (11)						
22	1msbA (115)	150-170 (21), 173-194 (22)	158-168 (11), 182-194 (13)	161-168 (8), 182-193 (12)	M	0.57	1.64	91.30% (105/115)	13.04% (15/115)	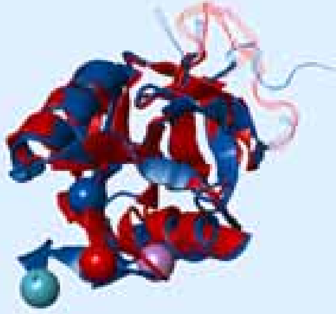 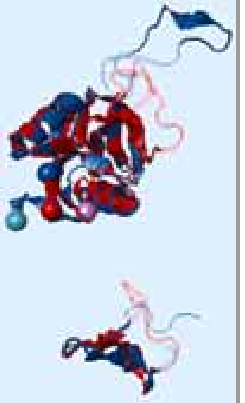 Mannose-binding protein-a Coagulation factors IX/X-binding protein
	1ixxA (129)	54-83 (30), 86-101 (16)	69-80 (12), 95-101 (7)	161-168 (8), 182-193 (12)						
23	1mupA (157)	125-134 (10)	126-130 (5)	126-130 (5)	C	0.60	1.92	92.36% (145/157)	24.84% (39/157)	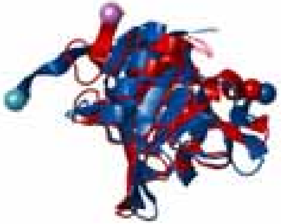 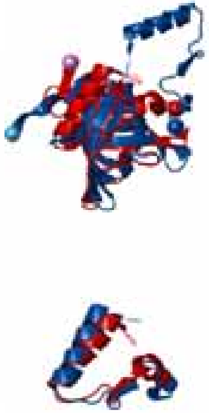 Major urinary protein Odorant-binding protein
	1obpA (158)	120-128 (9)	121-124 (4)	126-130 (5)						

24	1f0vA (124)	15-23 (9)	15-23 (9)	17-23 (7)	N	0.69	1.14	89.52% (111/124)	60.48% (75/124)	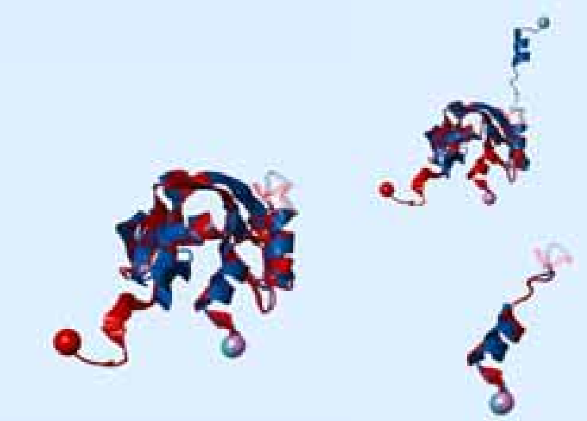 Ribonuclease A<br>Ribonuclease 1
	1h8xA (125)	115-123 (9)	115-123 (9)	17-23 (7)						
25	1f0vA (124)	111-113 (3)	111-113 (3)	112-113 (2)	C	0.59	1.20	83.06% (103/124)	57.26% (71/124)	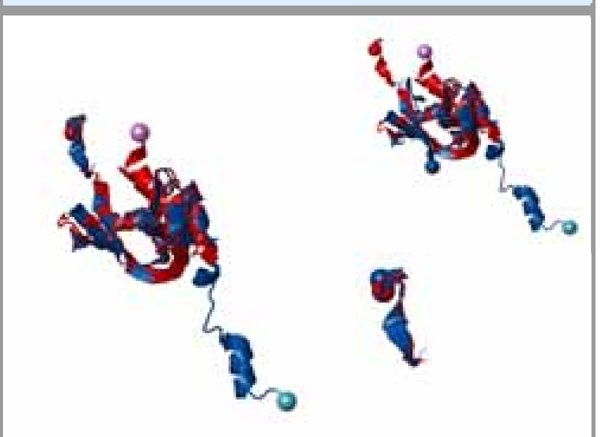 Ribonuclease A<br>Ribonuclease 1
	1h8xA (125)	211-213 (3)	211-213 (3)	112-113 (2)						
26	1griA (211)	121-126 (6)	121-124 (4)	121-123 (3)	C	0.28	1.35	100.00% (95/95)	100.00% (95/95)	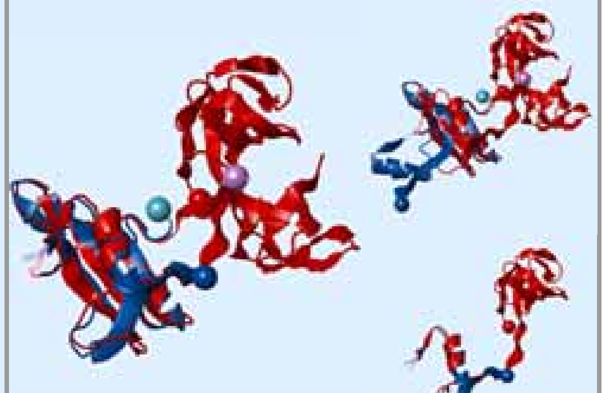 Growth factor bound protein 2<br>Growth factor receptor-bound protein 2
	1fyrA (95)	121-126 (6)	121-124 (4)	121-123 (3)						

27	1fynA (62)	112-132 (21)	112-119 (8)	112-118 (7)	C	0.69	1.10	90.00% (54/60)	26.67% (16/60)	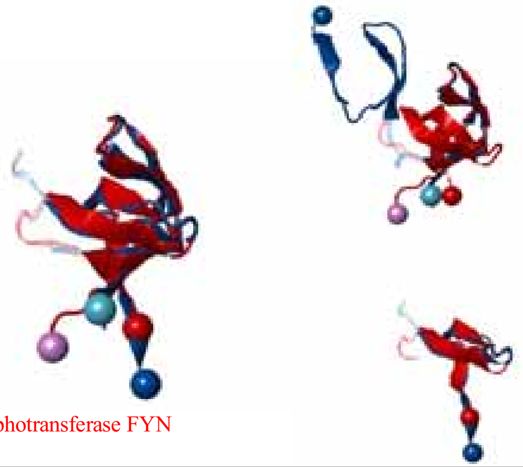 Phosphotransferase FYN EPS8
	1aojA (60)	34-52 (19)	34-40 (7)	112-118 (7)						
28	1k3sA (109)	12-40 (29)	27-32 (6)	27-28 (2)	N	0.56	1.75	98.17% (107/109)	12.84% (14/109)	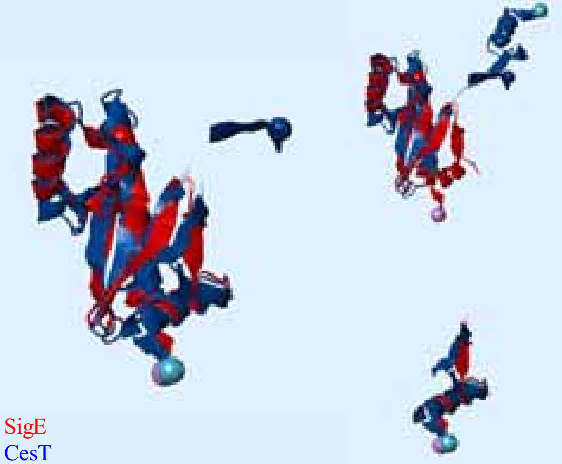 SigE CesT
	1k3eA (141)	16-49 (34)	34-40 (7)	27-28 (2)						
29	1cunA (213)	182-186 (5)	182-186 (5)	182-186 (5)	C	0.33	2.01	98.13% (105/107)	15.89% (17/107)	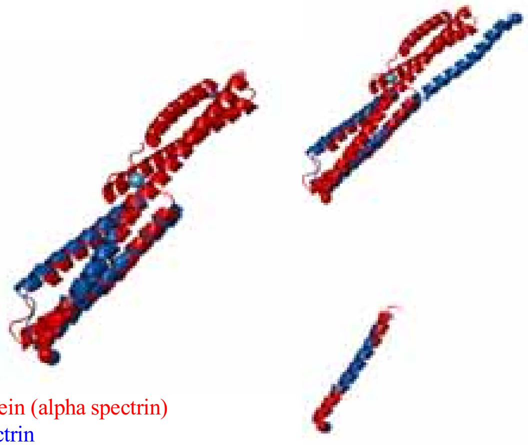 Protein (alpha spectrin) Spectrin
	2spcA (107)	72-73 (2)	72-73 (2)	182-186 (5)						

30	1wu3I (161)	96-114 (19)	96-103 (8)	96-104 (9)	C	0.38	2.80	86.75% (131/151)	9.93% (15/151)	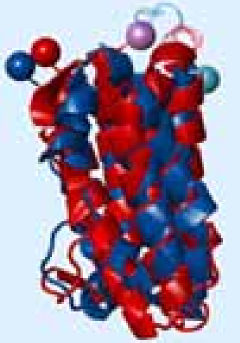 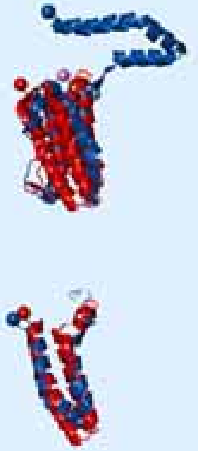 Interferon beta Interleukin-10
	1ilkA (151)	107-118 (12)	107-112 (6)	96-104 (9)						
31	1vjqA (73)	57-72 (16)	58-72 (15)	67-68 (2)	C	0.42	2.55	98.63% (72/73)	9.59% (7/73)	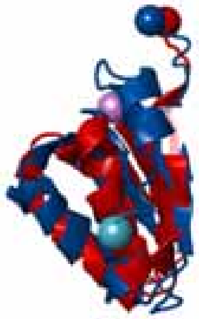 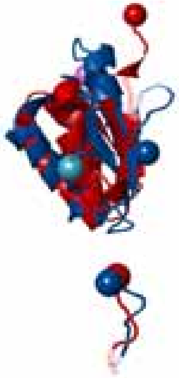 Designed protein Mesoderm development candidate 2
	2i9sA (97)	78-96 (19)	79-96 (18)	67-68 (2)						
32	2d8dA (80)	19-20 (2)	19-20 (2)	19-20 (2)	N	0.40	2.28	90.00% (72/80)	11.25% (9/80)	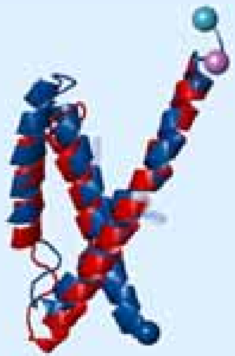 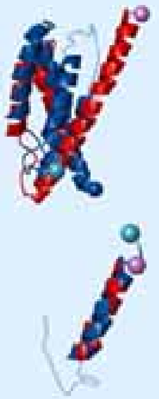 Phospho-2-dehydro-3-deoxyheptonate aldolase/c Chorismate mutase
	2gtvX (104)	19-32 (14)	19-32 (14)	19-20 (2)						

33	2d8dA (80)	22-24 (3)	22-24 (3)	23-24 (2)	N	0.49	1.73	93.75% (75/80)	11.25% (9/80)	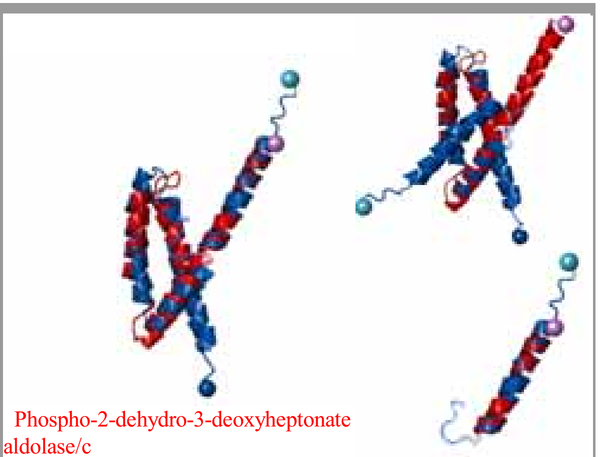<br>Phospho-2-dehydro-3-deoxyheptonate aldolase/c<br>Circadian clock protein KaiA homolog
	1q6aA (107)	27-36 (10)	27-36 (10)	23-24 (2)						
34	2c5jA (82)	61-72 (12)	62-65 (4)	63-64 (2)	C	0.78	0.89	98.78% (81/82)	62.20% (51/82)	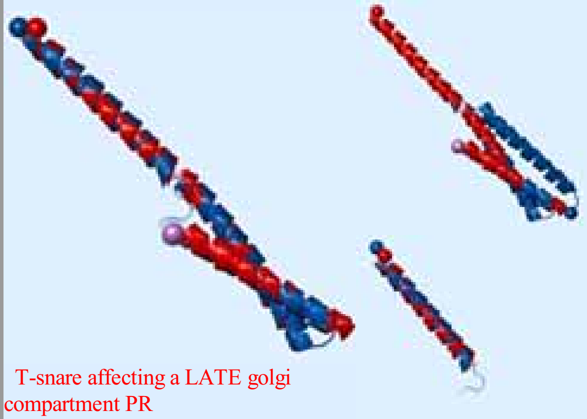<br>T-snare affecting a LATE golgi compartment PR<br>T-snare affecting a LATE golgi compartment PR
	2c5iT (94)	61-77 (17)	62-70 (9)	63-64 (2)						
35	2c5jA (82)	63-64 (2)	63-64 (2)	63-64 (2)	C	0.58	1.74	100.00% (82/82)	15.85% (13/82)	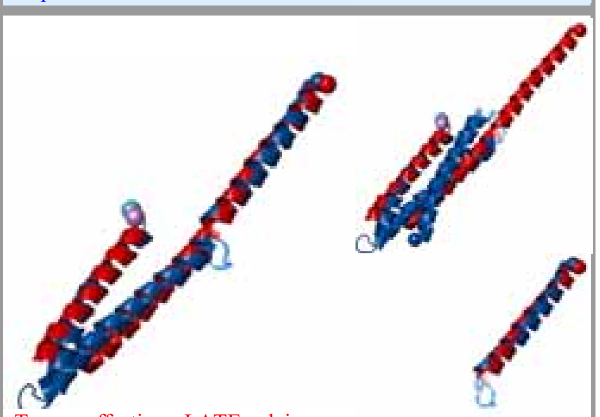<br>T-snare affecting a LATE golgi compartment PR<br>Syntaxin 6
	1lvfA (106)	72-81 (10)	72-81 (10)	63-64 (2)						

36	2c5jA (82)	70-72 (3)	70-72 (3)	70-72 (3)	C	0.41	1.75	89.02% (73/82)	7.32% (6/82)	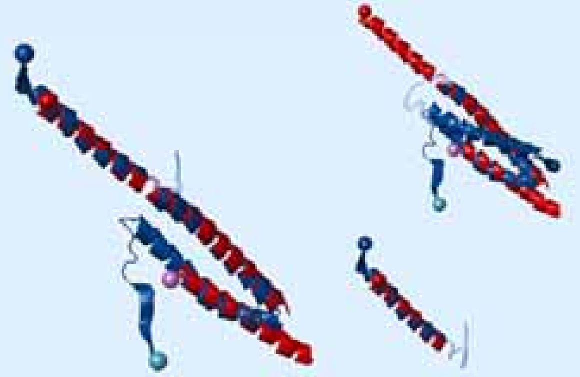 T-snare affecting a LATE golgi compartment PR
	2jmhA (117)	78-91 (14)	78-91 (14)	70-72 (3)						Mite allergen Blo t 5
37	2c5jA (82)	67-71 (5)	67-69 (3)	68-69 (2)	C	0.48	1.70	92.68% (76/82)	4.88% (4/82)	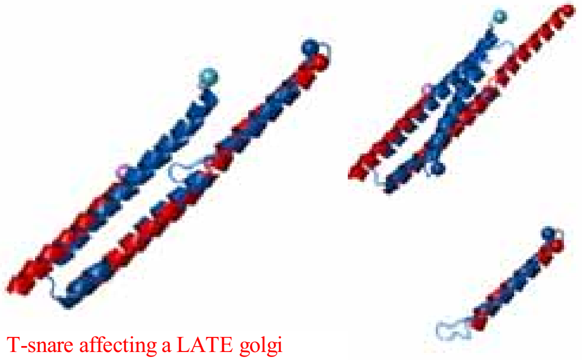 T-snare affecting a LATE golgi compartment PR
	1hx1B (112)	222-236 (15)	222-234 (13)	68-69 (2)						BAG-family molecular chaperone regulator-1
38	1wz3A (84)	49-65 (17)	49-62 (14)	59-61 (3)	C	0.53	1.60	97.62% (82/84)	17.86% (15/84)	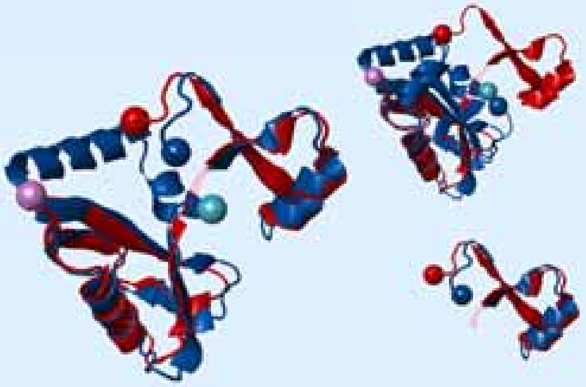 Autophagy 12b
	1gnuA (117)	69-86 (18)	69-83 (15)	59-61 (3)						Gabarap

39	1wz3A (84)	50-63 (14)	50-62 (13)	59-62 (4)	C	0.53	1.46	95.24% (80/84)	17.86% (15/84)	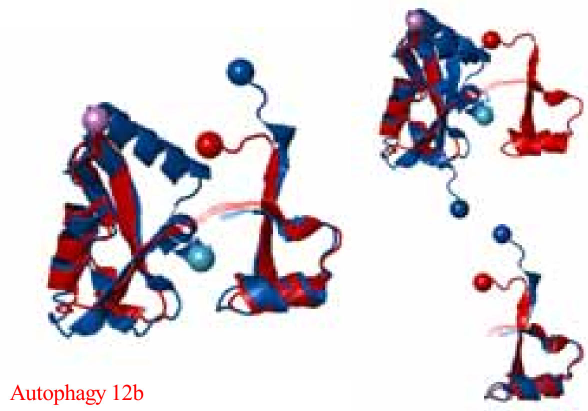 Autophagy 12b<br>Golgi-associated atpase enhancer OF 16 KD
	1eo6A (116)	70-84 (15)	70-83 (14)	59-62 (4)						
40	1wz3A (84)	49-61 (13)	51-61 (11)	59-61 (3)	C	0.56	1.35	94.05% (79/84)	21.43% (18/84)	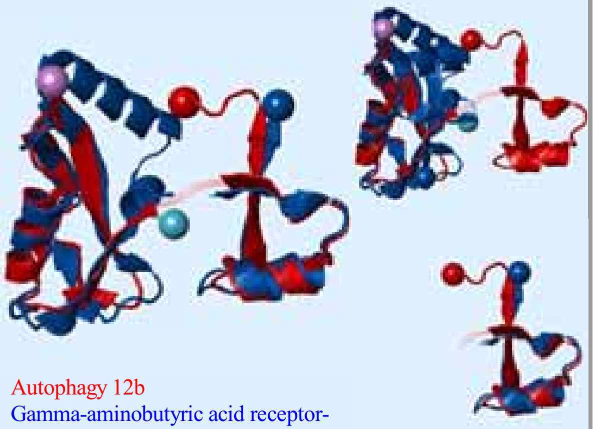 Autophagy 12b<br>Gamma-aminobutyric acid receptor-associated p
	2r2qA (110)	68-82 (15)	70-82 (13)	59-61 (3)						
41	1wz3A (84)	49-67 (19)	52-67 (16)	57-67 (11)	C	0.45	2.31	89.29% (75/84)	10.71% (9/84)	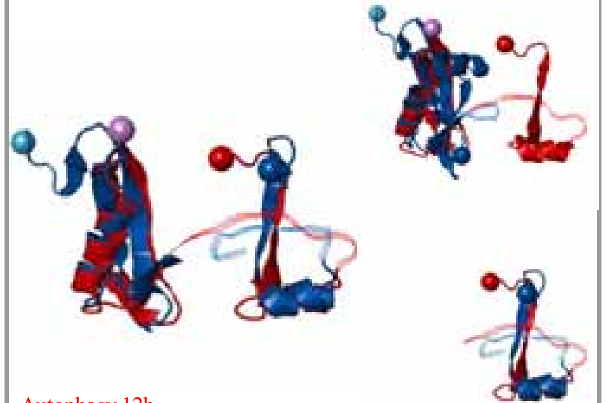 Autophagy 12b<br>Hypothetical protein ZK652.3
	1l7yA (94)	50-70 (21)	53-70 (18)	57-67 (11)						

42	1wz3A<br>(84)	51-64 (14)	52-64 (13)	58-62 (5)	C	0.25	2.33	89.29%<br>(75/84)	5.95%<br>(5/84)	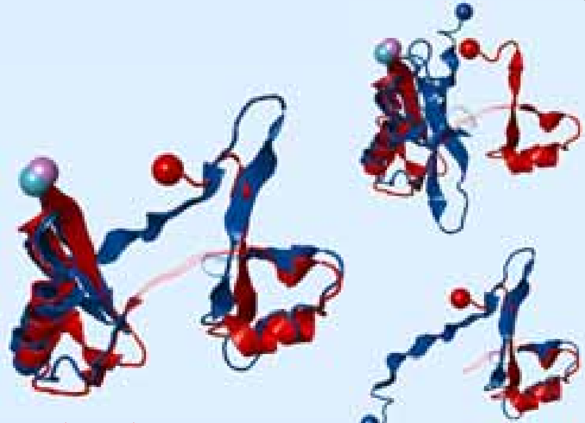<br>Autophagy 12b<br>Elongin B
	1lm8B<br>(106)	37-51 (15)	38-51 (14)	58-62 (5)						
43	1wz3A<br>(84)	51-65 (15)	51-65 (15)	59-61 (3)	C	0.54	2.03	90.12%<br>(73/81)	6.17%<br>(5/81)	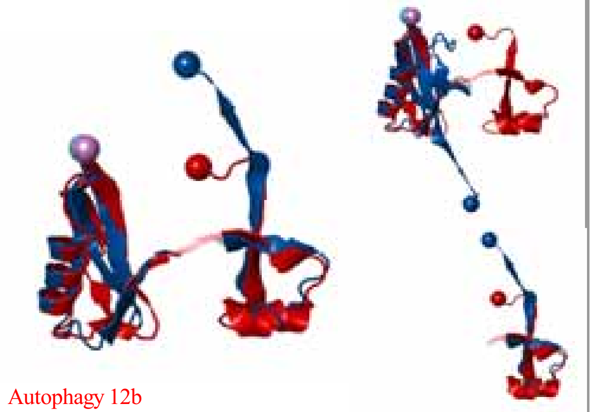<br>Autophagy 12b<br>Small ubiquitin-related modifier 3 precursor
	2io1B<br>(81)	52-67 (16)	52-67 (16)	59-61 (3)						
44	1wz3A<br>(84)	48-69 (22)	48-62 (15)	59-62 (4)	C	0.49	2.24	88.89%<br>(72/81)	6.17%<br>(5/81)	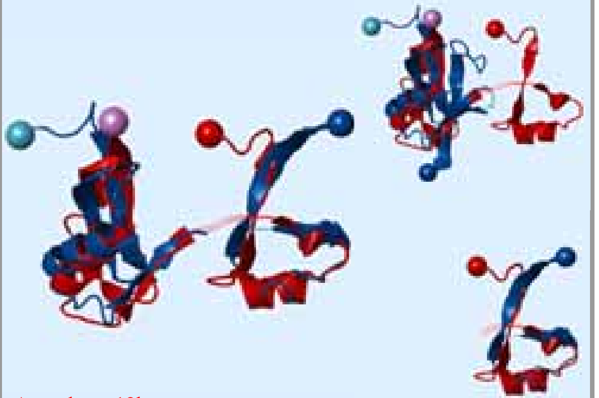<br>Autophagy 12b<br>Ubiquitin-like protein 4A
	2dziA<br>(81)	41-62 (22)	41-55 (15)	59-62 (4)						

45	1wz3A (84)	54-65 (12)	54-65 (12)	59-61 (3)	C	0.41	2.20	86.90% (73/84)	1.19% (1/84)	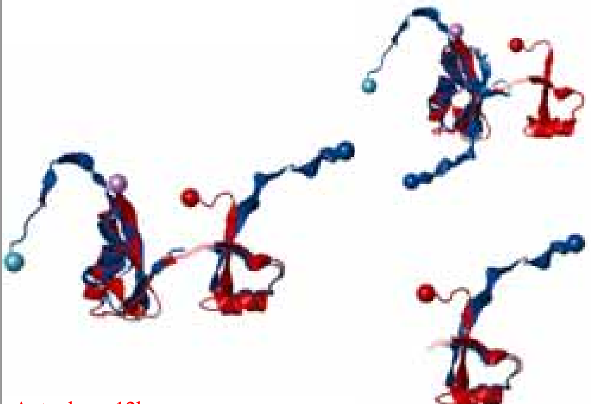 Autophagy 12b<br>NEDD8 ultimate buster-1
	1wjuA (100)	60-70 (11)	60-70 (11)	59-61 (3)						
46	1wz3A (84)	52-70 (19)	52-61 (10)	58-61 (4)	C	0.32	2.15	84.52% (71/84)	9.52% (8/84)	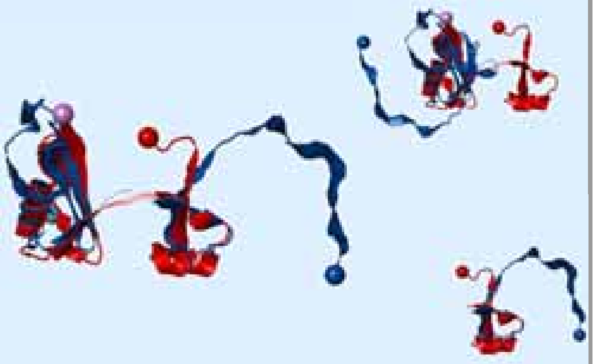 Autophagy 12b<br>Ubiquilin 3
	1wx7A (106)	53-72 (20)	53-63 (11)	58-61 (4)						
47	1wz3A (84)	49-64 (16)	56-64 (9)	59-61 (3)	C	0.41	1.93	83.33% (70/84)	4.76% (4/84)	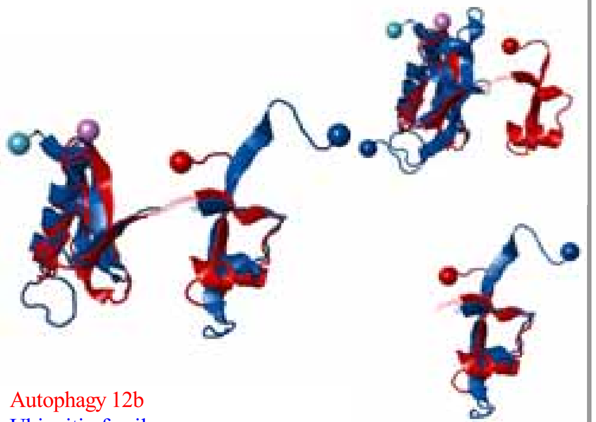 Autophagy 12b<br>Ubiquitin family
	1se9A (101)	42-65 (24)	58-65 (8)	59-61 (3)						

48	1wz3A (84)	49-70 (22)	49-70 (22)	58-66 (9)	C	0.32	2.41	88.10% (74/84)	9.52% (8/84)	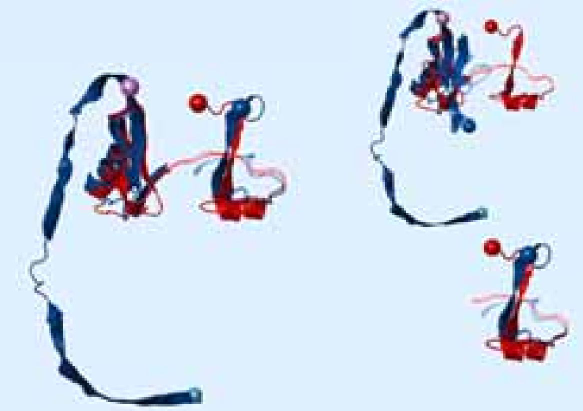 Autophagy 12b KIAA0794 protein
	1wj4A (124)	77-103 (27)	77-103 (27)	58-66 (9)						
49	1wz3A (84)	51-66 (16)	56-62 (7)	59-62 (4)	C	0.44	2.34	85.71% (72/84)	5.95% (5/84)	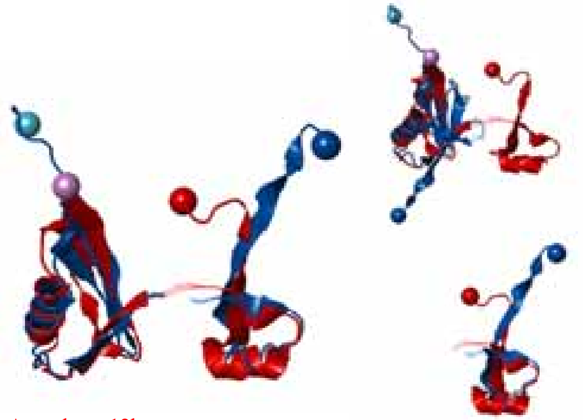 Autophagy 12b Ubiquilin 3
	1yqbA (88)	56-72 (17)	63-68 (6)	59-62 (4)						
50	1wz3A (84)	50-62 (13)	57-62 (6)	59-62 (4)	C	0.55	1.94	91.14% (72/79)	10.13% (8/79)	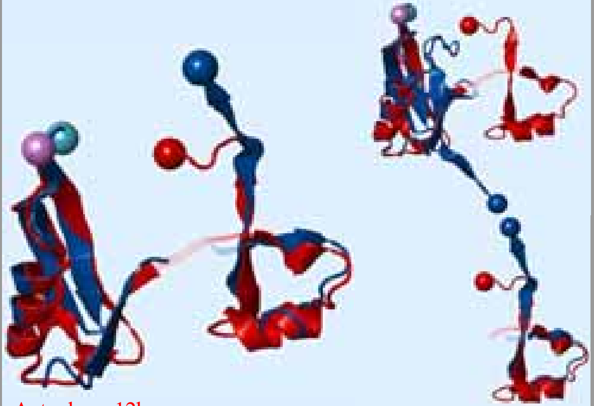 Autophagy 12b Ubitqutin-LIKE protein SMT3
	1euvB (79)	57-70 (14)	66-70 (5)	59-62 (4)						

51	1wz3A (84)	43-61 (19)	50-61 (12)	59-61 (3)	C	0.45	2.31	85.71% (72/84)	3.57% (3/84)	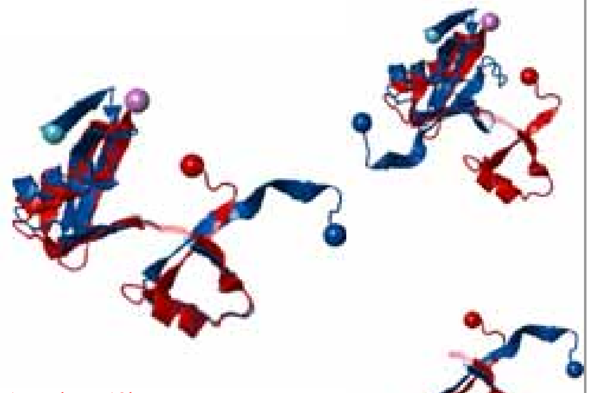 Autophagy 12b<br>59 kDa 2'-5'-oligoadenylate synthetase like p
	1wh3A (87)	35-54 (20)	42-54 (13)	59-61 (3)						
52	1wz3A (84)	48-68 (21)	49-61 (13)	58-61 (4)	C	0.49	2.41	95.89% (70/73)	10.96% (8/73)	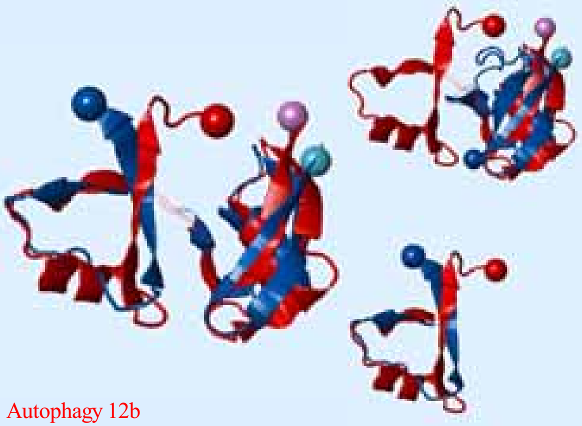 Autophagy 12b<br>Protein YNR032c-a
	1m94A (73)	34-55 (22)	35-48 (14)	58-61 (4)						
53	1wz3A (84)	47-69 (23)	57-65 (9)	57-61 (5)	C	0.44	2.30	85.71% (72/84)	11.90% (10/84)	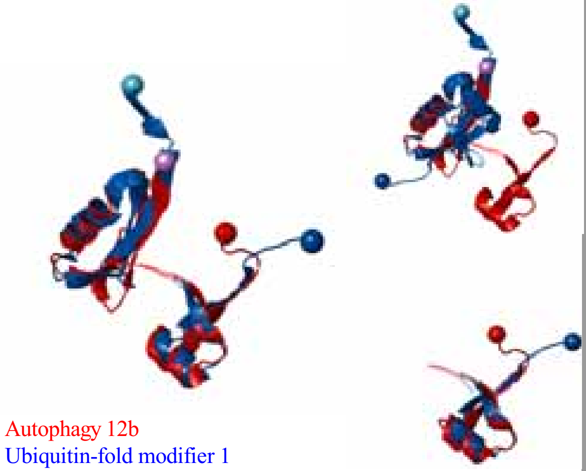 Autophagy 12b<br>Ubiquitin-fold modifier 1
	1wxsA (88)	39-62 (24)	50-58 (9)	57-61 (5)						

54	1wz3A (84)	50-65 (16)	50-65 (16)	59-61 (3)	C	0.43	2.34	85.19% (69/81)	9.88% (8/81)	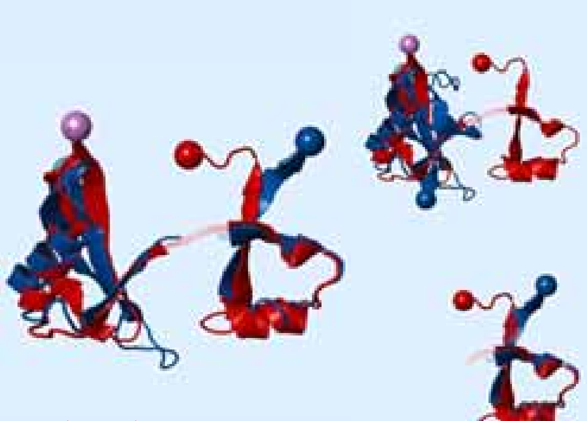 Autophagy 12b<br>Ubiquitin
	2gbjA (81)	43-59 (17)	43-59 (17)	59-61 (3)						
55	1wz3A (84)	52-69 (18)	56-65 (10)	59-63 (5)	C	0.36	2.09	84.52% (71/84)	5.95% (5/84)	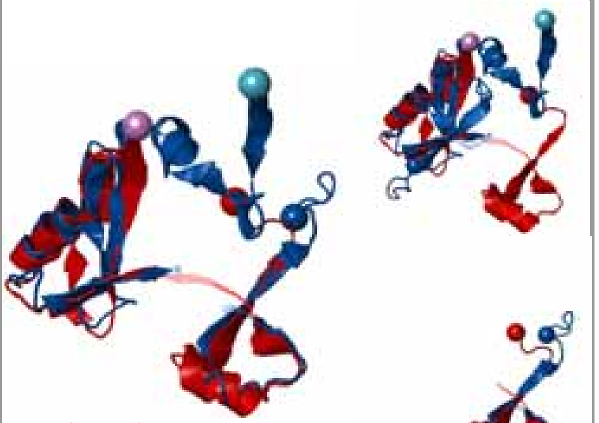 Autophagy 12b<br>Splicing factor, putative
	1we6A (111)	68-86 (19)	74-82 (9)	59-63 (5)						
56	1wz3A (84)	47-93 (47)	48-93 (46)	59-62 (4)	C	0.35	2.31	85.71% (72/84)	9.52% (8/84)	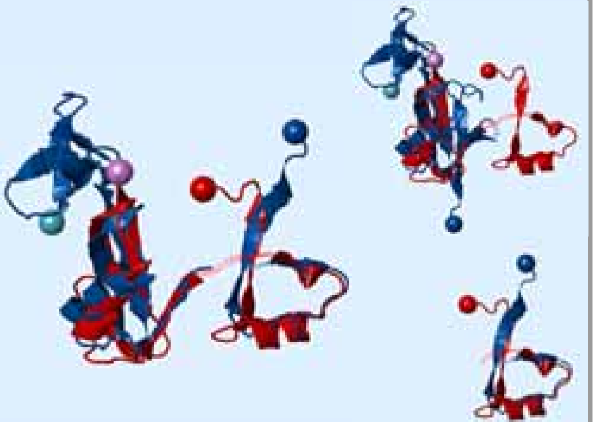 Autophagy 12b<br>Ubiquitin
	2ojrA (110)	132-175 (44)	134-175 (42)	59-62 (4)						

57	1wz3A (84)	49-69 (21)	57-68 (12)	58-65 (8)	C	0.52	2.08	90.48% (76/84)	8.33% (7/84)	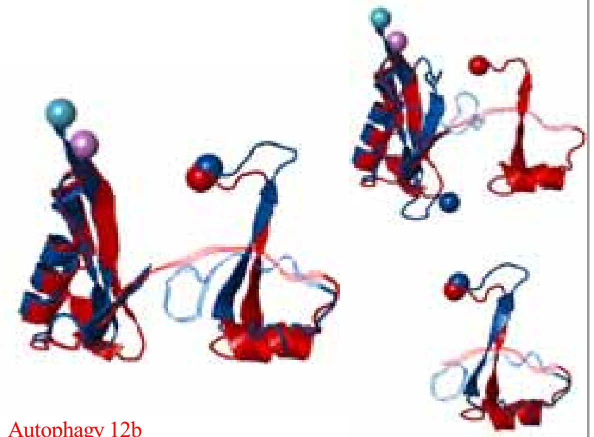<br>Autophagy 12b<br>Tubulin folding cofactor B
	1t0yA (90)	39-67 (29)	49-66 (18)	58-65 (8)						
58	1wz3A (84)	50-65 (16)	50-65 (16)	59-61 (3)	C	0.46	2.25	85.37% (70/82)	8.54% (7/82)	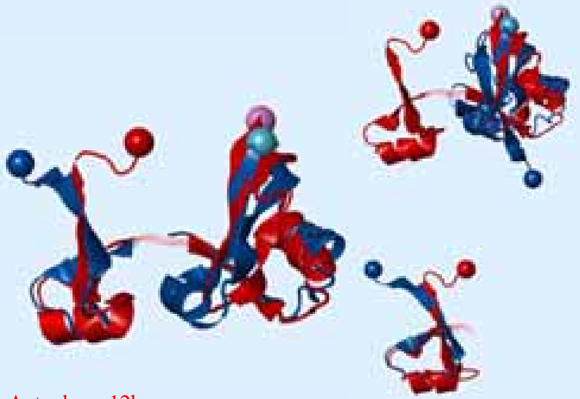<br>Autophagy 12b<br>Ubiquitin
	2gbkA (82)	42-58 (17)	42-58 (17)	59-61 (3)						
59	1wz3A (84)	49-69 (21)	49-66 (18)	58-62 (5)	C	0.50	2.19	91.03% (71/78)	6.41% (5/78)	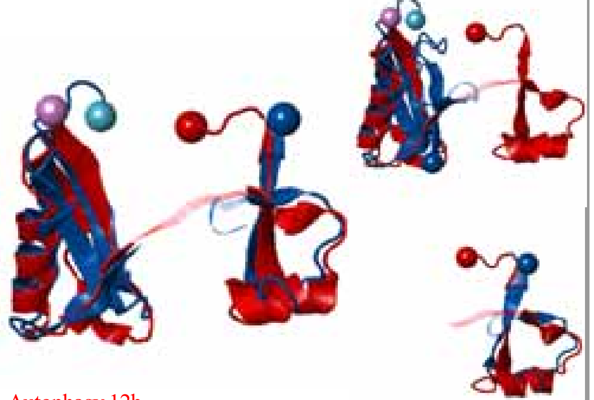<br>Autophagy 12b<br>Nfatc2-interacting protein
	2jxxA (78)	60-81 (22)	60-78 (19)	58-62 (5)						

60	1wz3A (84)	49-69 (21)	57-62 (6)	59-61 (3)	C	0.52	2.22	97.18% (69/71)	8.45% (6/71)	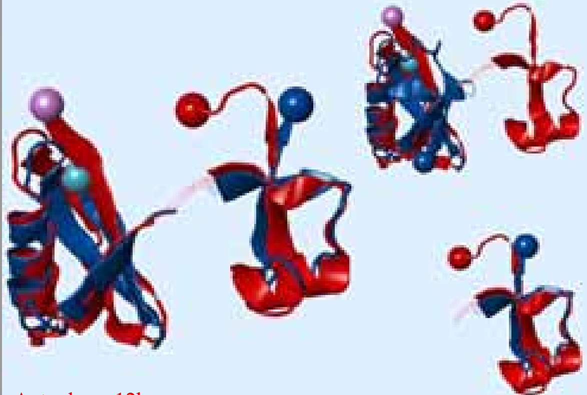
	1sifA (71)	34-55 (22)	44-48 (5)	59-61 (3)						
61	1wz3A (84)	47-69 (23)	51-62 (12)	59-62 (4)	C	0.48	2.55	96.05% (73/76)	7.89% (6/76)	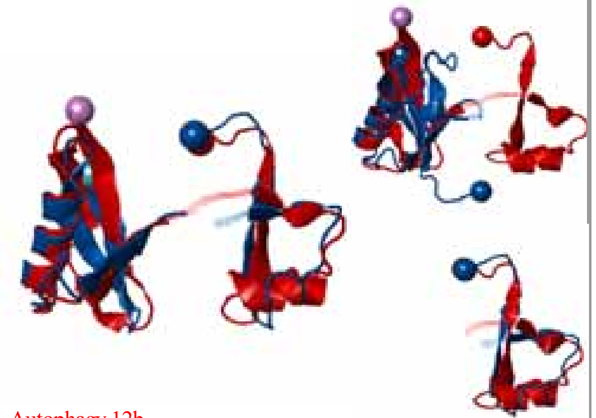
	1ud7A (76)	32-55 (24)	36-48 (13)	59-62 (4)						
62	1wz3A (84)	45-65 (21)	47-65 (19)	58-64 (7)	C	0.38	2.19	83.33% (70/84)	2.38% (2/84)	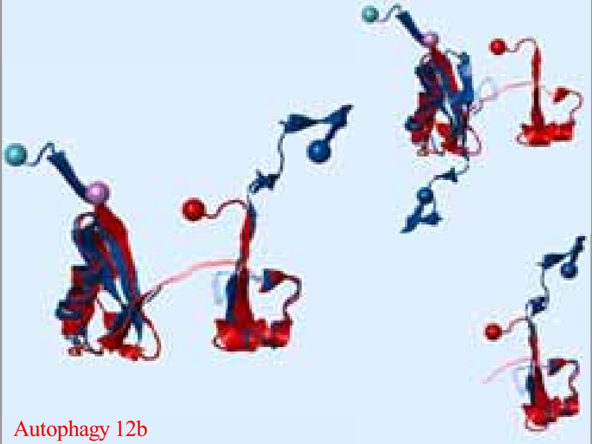
	2dzmA (100)	38-60 (23)	40-60 (21)	58-64 (7)						

63	1wz3A<br>(84)	48-93 (46)	49-61 (13)	59-61 (3)	C	0.41	2.51	84.52%<br>(71/84)	8.33%<br>(7/84)	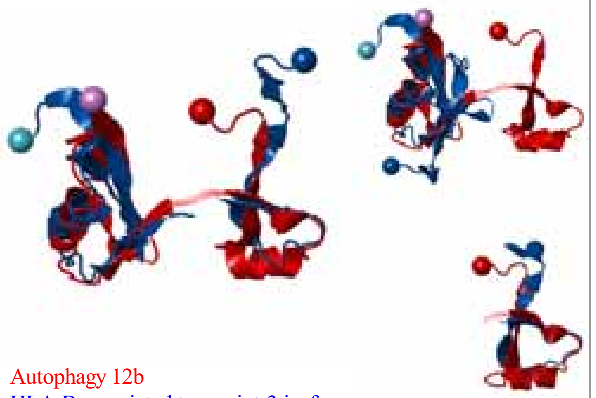<br>Autophagy 12b<br>HLA-B associated transcript-3 isoform b
	1wx9A<br>(86)	40-86 (47)	41-54 (14)	59-61 (3)						
64	1wz3A<br>(84)	42-73 (32)	60-71 (12)	60-63 (4)	C	0.42	2.19	88.10%<br>(74/84)	7.14%<br>(6/84)	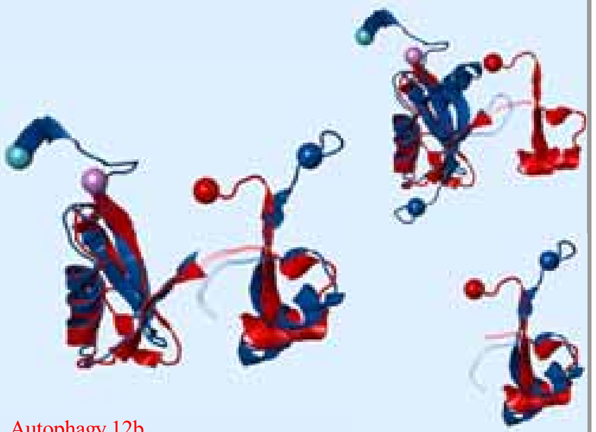<br>Autophagy 12b<br>KIAA0049 protein
	1wj6A<br>(101)	42-77 (36)	60-75 (16)	60-63 (4)						
65	1wz3A<br>(84)	47-89 (43)	51-61 (11)	58-61 (4)	C	0.53	2.21	97.26%<br>(71/73)	8.22%<br>(6/73)	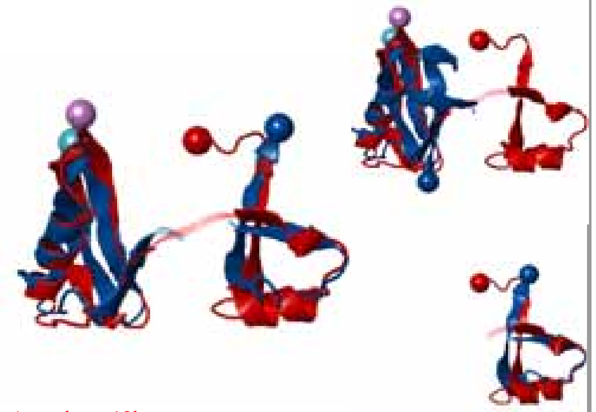<br>Autophagy 12b<br>Ubiquitin-like 5
	1p0rA<br>(73)	33-72 (40)	37-48 (12)	58-61 (4)						

66	1wz3A (84)	51-69 (19)	56-69 (14)	59-62 (4)	C	0.51	2.33	94.81% (73/77)	7.79% (6/77)	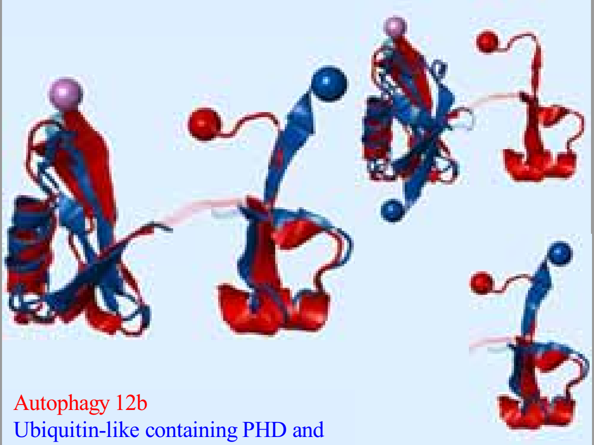 Autophagy 12b Ubiquitin-like containing PHD and RING finger
	2fazA (77)	38-57 (20)	45-57 (13)	59-62 (4)						
67	1wz3A (84)	48-69 (22)	60-69 (10)	60-62 (3)	C	0.35	2.89	90.48% (76/84)	11.90% (10/84)	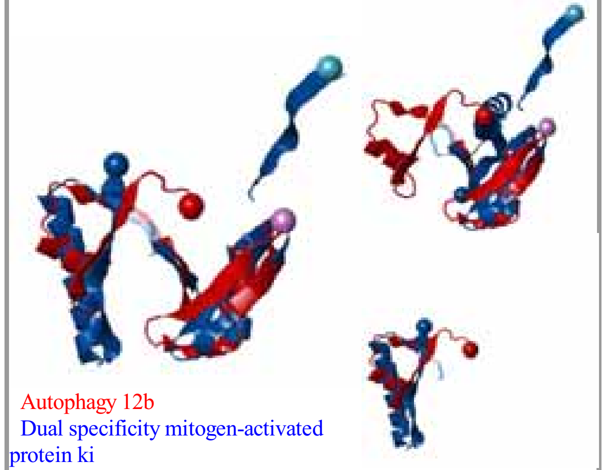 Autophagy 12b Dual specificity mitogen-activated protein ki
	2nptA (102)	51-77 (27)	64-77 (14)	60-62 (3)						
68	1wz3A (84)	46-90 (45)	48-69 (22)	51-64 (14)	C	0.35	2.67	83.33% (70/84)	10.71% (9/84)	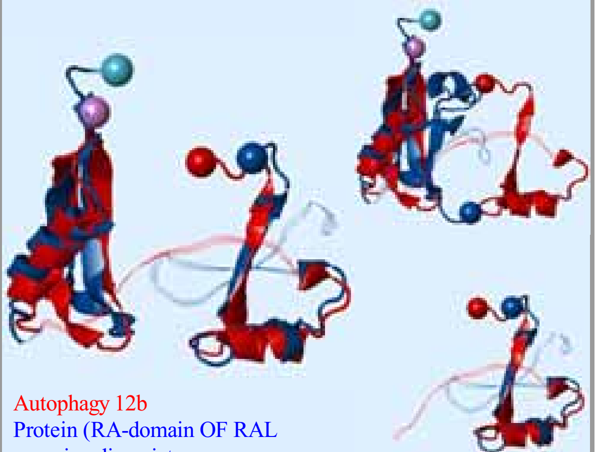 Autophagy 12b Protein (RA-domain OF RAL guanosine dissociat
	1raxA (93)	62-114 (53)	66-94 (29)	51-64 (14)						

69	1wz3A (84)	49-72 (24)	49-69 (21)	60-63 (4)	C	0.37	2.44	86.90% (73/84)	4.76% (4/84)	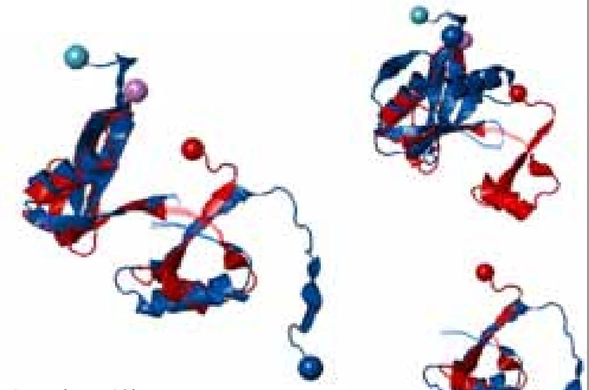 Autophagy 12b Mitogen-activated protein kinase kinase kinas
	2cu1A (103)	41-66 (26)	41-63 (23)	60-63 (4)						
70	1wz3A (84)	49-71 (23)	49-71 (23)	59-62 (4)	C	0.49	2.22	89.87% (71/79)	5.06% (4/79)	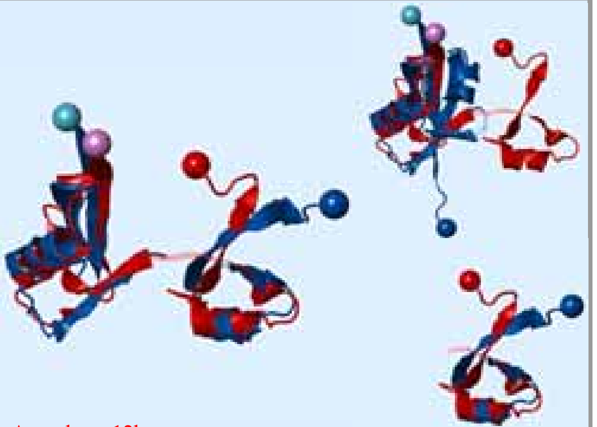 Autophagy 12b Ubiquitin-like protein SMT3A
	1u4aA (79)	50-71 (22)	50-71 (22)	59-62 (4)						
71	1wz3A (84)	51-62 (12)	51-62 (12)	59-62 (4)	C	0.47	2.38	92.11% (70/76)	9.21% (7/76)	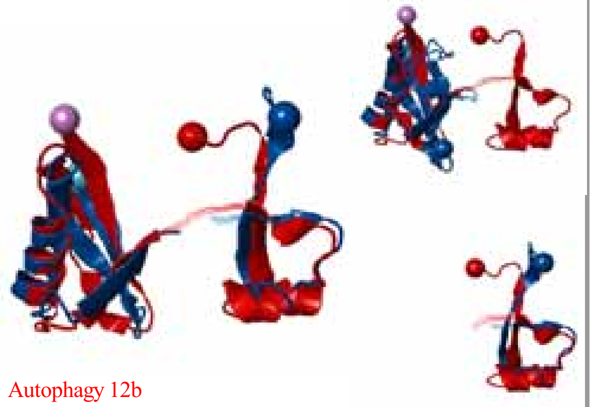 Autophagy 12b Protein (1D8 ubiquitin)
	1c3tA (76)	36-48 (13)	36-48 (13)	59-62 (4)						

72	1wz3A (84)	48-65 (18)	50-61 (12)	58-61 (4)	C	0.48	2.40	93.42% (71/76)	9.21% (7/76)	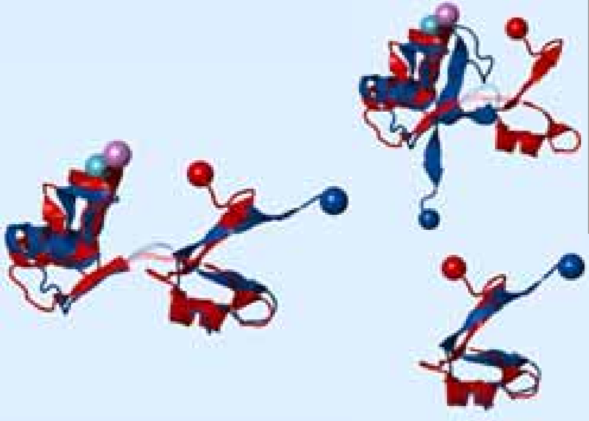<br>Autophagy 12b<br>Ubiquitin
	2jwzA (76)	33-51 (19)	35-47 (13)	58-61 (4)						
73	1wz3A (84)	48-69 (22)	48-69 (22)	59-69 (11)	C	0.30	2.93	90.48% (76/84)	8.33% (7/84)	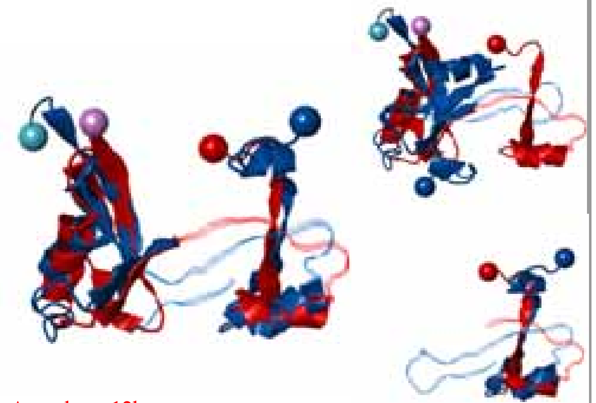<br>Autophagy 12b<br>Phospholipase C, epsilon 1
	2byfA (116)	42-87 (46)	42-87 (46)	59-69 (11)						
74	1wz3A (84)	51-69 (19)	57-69 (13)	59-63 (5)	C	0.41	2.63	89.29% (75/84)	8.33% (7/84)	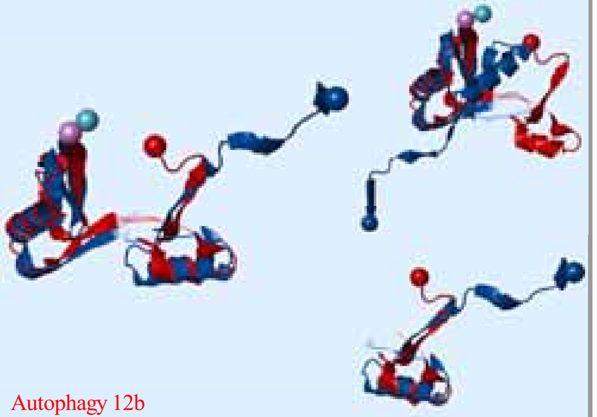<br>Autophagy 12b<br>Mitogen-activated protein kinase<br>kinase kinas
	2jrhA (93)	37-58 (22)	43-58 (16)	59-63 (5)						

75	1wz3A (84)	48-59 (12)	48-59 (12)	48-59 (12)	C	0.33	2.54	76.54% (62/81)	11.11% (9/81)	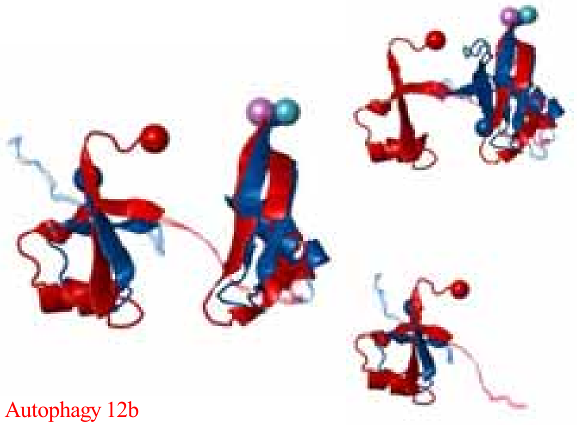<br>Autophagy 12b<br>YUKD protein
	2bpsA (81)	38-56 (19)	38-56 (19)	48-59 (12)						
76	1wz3A (84)	48-69 (22)	48-64 (17)	58-64 (7)	C	0.43	2.62	88.10% (74/84)	8.33% (7/84)	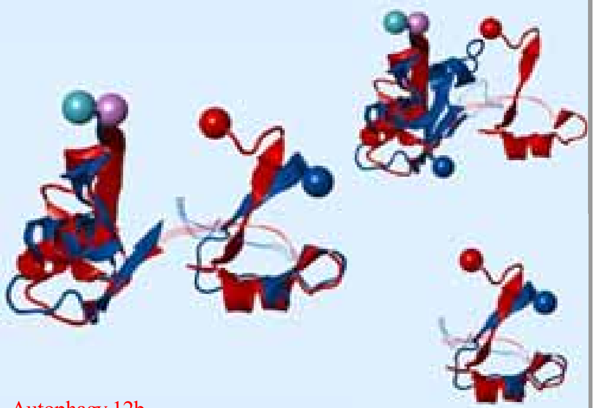<br>Autophagy 12b<br>Ralgds
	1lfdA (87)	54-82 (29)	54-77 (24)	58-64 (7)						
77	1wz3A (84)	48-69 (22)	59-69 (11)	59-63 (5)	C	0.47	2.51	90.24% (74/82)	10.98% (9/82)	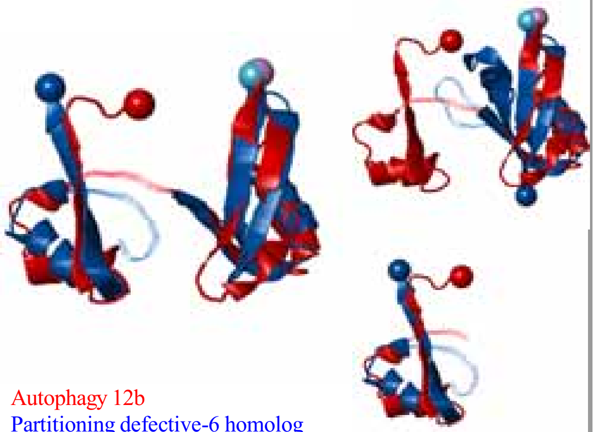<br>Autophagy 12b<br>Partitioning defective-6 homolog alpha
	1wmhB (82)	51-76 (26)	62-76 (15)	59-63 (5)						

78	1wz3A (84)	50-68 (19)	50-68 (19)	58-66 (9)	C	0.43	2.55	90.79% (69/76)	11.84% (9/76)	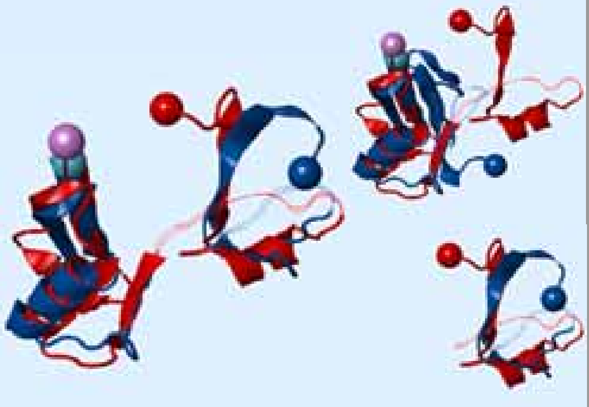<br>Autophagy 12b<br>TUG long isoform
	2al3A (76)	44-63 (20)	44-63 (20)	58-66 (9)						
79	1wz3A (84)	34-93 (60)	50-66 (17)	58-63 (6)	C	0.39	2.43	85.71% (72/84)	9.52% (8/84)	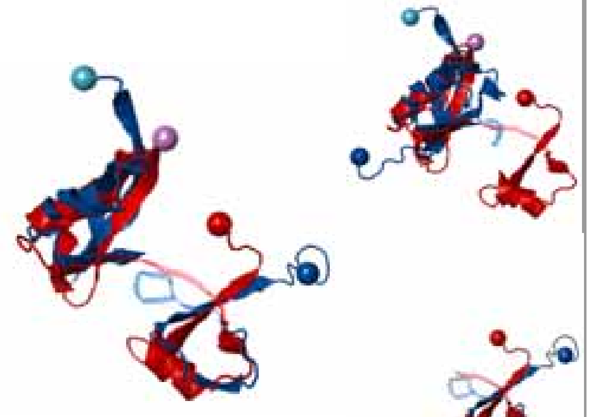<br>Autophagy 12b<br>Cytoskeleton-associated protein 1
	1v6eA (95)	27-95 (69)	43-67 (25)	58-63 (6)						
80	1wz3A (84)	48-65 (18)	48-59 (12)	58-59 (2)	C	0.37	2.71	86.90% (73/84)	9.52% (8/84)	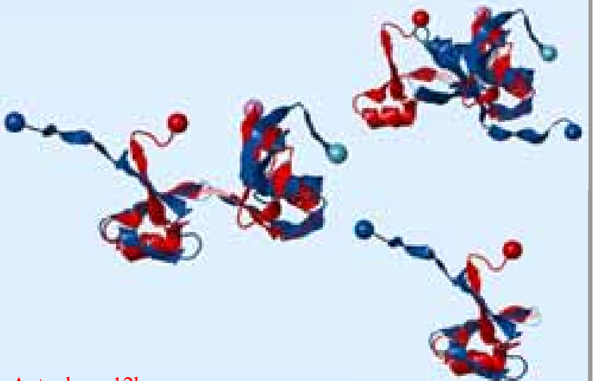<br>Autophagy 12b<br>Protein C12orf2
	2cs4A (95)	41-61 (21)	41-55 (15)	58-59 (2)						

81	1wz3A (84)	46-69 (24)	48-69 (22)	59-62 (4)	C	0.42	2.54	86.90% (73/84)	8.33% (7/84)	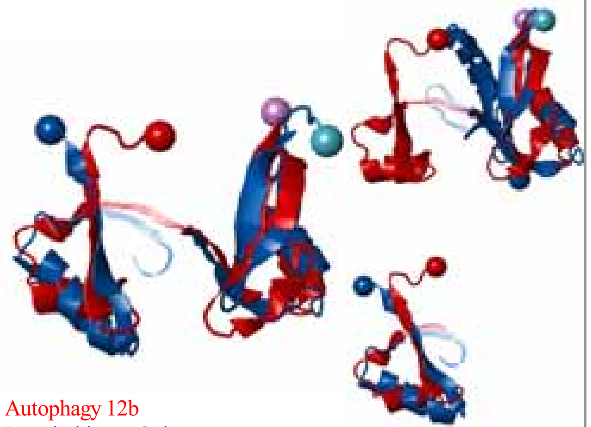 Autophagy 12b<br>Protein kinase C, iota type
	1vd2A (89)	46-76 (31)	50-76 (27)	59-62 (4)						
82	1zvnA (99)	1-23 (23)	1-7 (7)	7-7 (1)	N	0.40	1.64	95.96% (95/99)	28.28% (28/99)	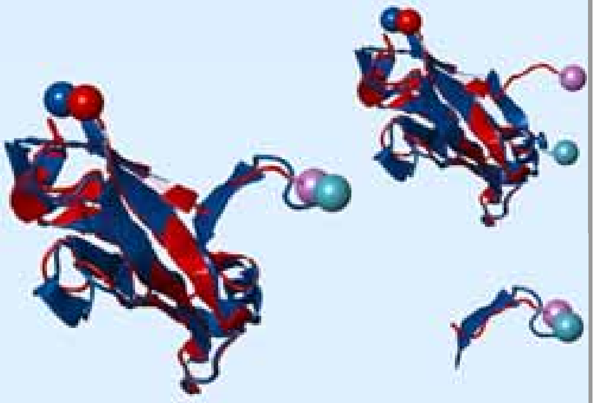 MN-cadherin<br>Epithelial-cadherin
	2omwB (105)	-1-23 (25)	-1-7 (9)	7-7 (1)						
83	2oyaA (102)	426-431 (6)	426-431 (6)	429-430 (2)	N	0.67	0.45	100.00% (98/98)	97.96% (96/98)	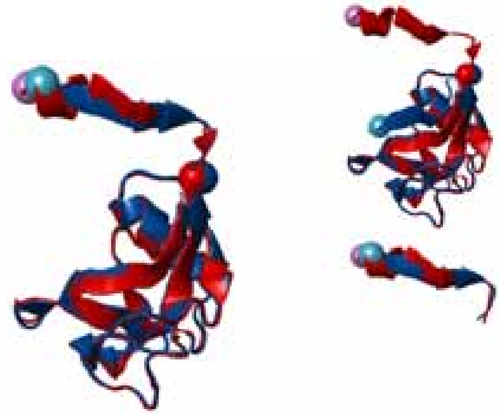 Macrophage receptor marco<br>Macrophage receptor marco
	2oy3A (98)	426-431 (6)	426-431 (6)	429-430 (2)						

84	2oyaA (102)	428-431 (4)	428-431 (4)	428-430 (3)	N	0.50	1.18	99.02% (101/102)	47.06% (48/102)	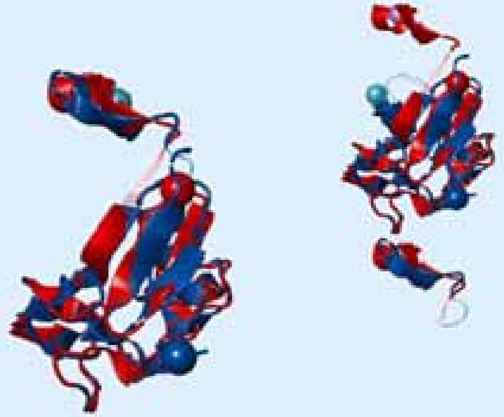 Macrophage receptor marco<br>MAC-2 binding protein
	1by2A (112)	11-16 (6)	11-16 (6)	428-430 (3)						
85	1v9yA (103)	28-34 (7)	28-29 (2)	29-29 (1)	N	0.26	2.15	96.12% (99/103)	15.53% (16/103)	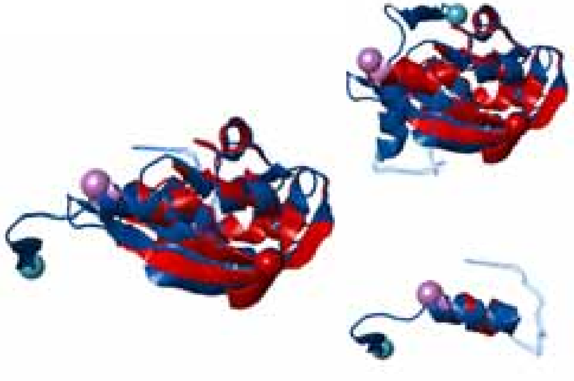 Heme pas sensor protein<br>Vivid PAS protein VVD
	2pd7A (149)	56-74 (19)	56-69 (14)	29-29 (1)						
86	1v9yA (103)	25-27 (3)	25-27 (3)	26-26 (1)	N	0.54	1.93	96.12% (99/103)	14.56% (15/103)	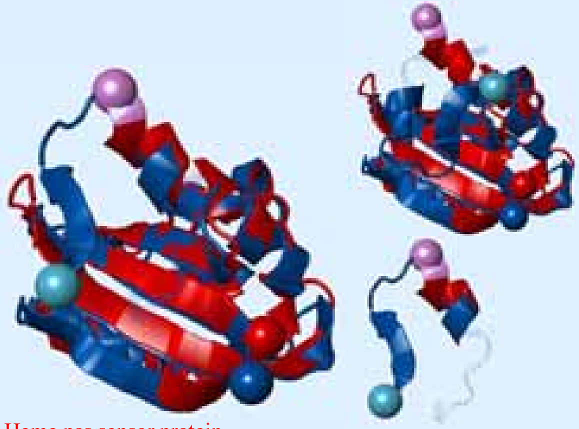 Heme pas sensor protein<br>Photoactive yellow protein
	1f98A (125)	14-24 (11)	14-24 (11)	26-26 (1)						

87	1v9yA (103)	25-27 (3)	25-26 (2)	25-26 (2)	N	0.50	2.12	93.20% (96/103)	16.50% (17/103)	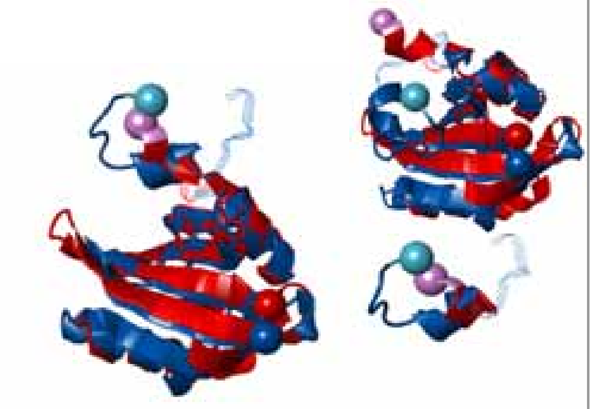<br>Heme pas sensor protein<br>Photoactive yellow protein
	1d7eA (119)	15-24 (10)	15-23 (9)	25-26 (2)						
88	1lgqA (112)	85-94 (10)	85-94 (10)	85-88 (4)	C	0.57	1.75	89.29% (100/112)	24.11% (27/112)	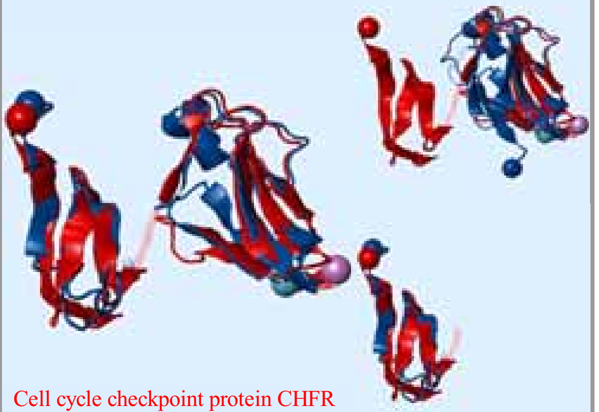<br>Cell cycle checkpoint protein CHFR<br>Serine/threonine-protein kinase CHK2
	1gxcA (116)	171-180 (10)	171-180 (10)	85-88 (4)						
89	1lgqA (112)	64-103 (40)	77-101 (25)	85-88 (4)	C	0.41	2.14	92.86% (104/112)	16.96% (19/112)	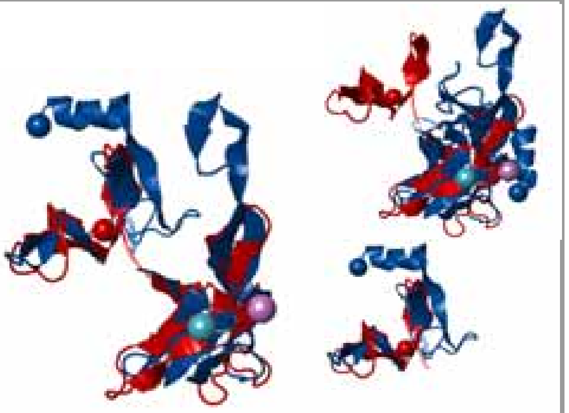<br>Cell cycle checkpoint protein CHFR<br>Protein (protein kinase SPK1)
	1dmzA (158)	626-678 (53)	652-676 (25)	85-88 (4)						

90	1lgqA (112)	84-102 (19)	85-97 (13)	85-88 (4)	C	0.46	1.89	91.07% (102/112)	24.11% (27/112)	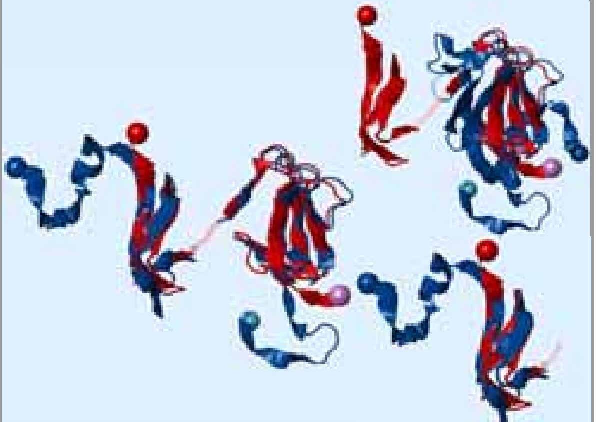<br>Cell cycle checkpoint protein CHFR<br>Ubiquitin ligase protein RNF8
	2cswA (145)	87-105 (19)	88-100 (13)	85-88 (4)						
91	1lgqA (112)	64-100 (37)	85-95 (11)	85-87 (3)	C	0.57	1.98	96.00% (96/100)	28.00% (28/100)	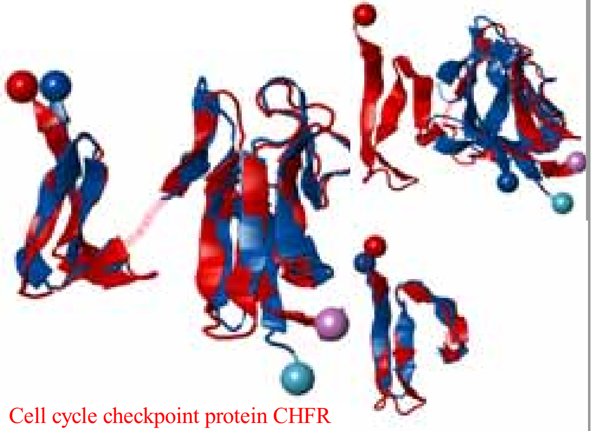<br>Cell cycle checkpoint protein CHFR<br>Antigen Ki-67
	1r21A (100)	52-85 (34)	72-80 (9)	85-87 (3)						
92	1lgqA (112)	74-116 (43)	85-101 (17)	85-88 (4)	C	0.53	1.70	85.71% (96/112)	21.43% (24/112)	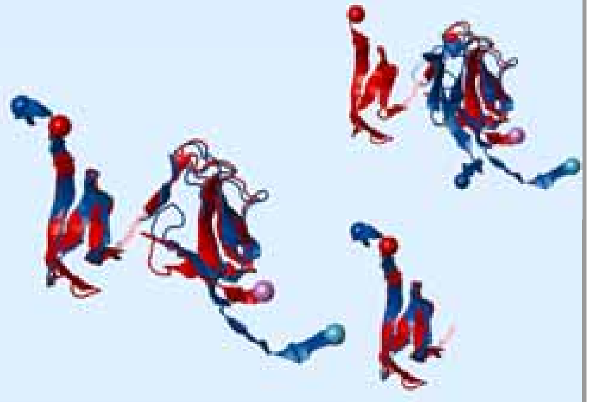<br>Cell cycle checkpoint protein CHFR<br>Expressed protein
	1uhtA (118)	69-105 (37)	81-97 (17)	85-88 (4)						

93	1lgqA (112)<br>2jpeA (132)	75-123 (49)<br>86-129 (44)	76-87 (12)<br>87-99 (13)	84-87 (4)<br>84-87 (4)	C	0.44	2.27	90.18% (101/112)	17.86% (20/112)	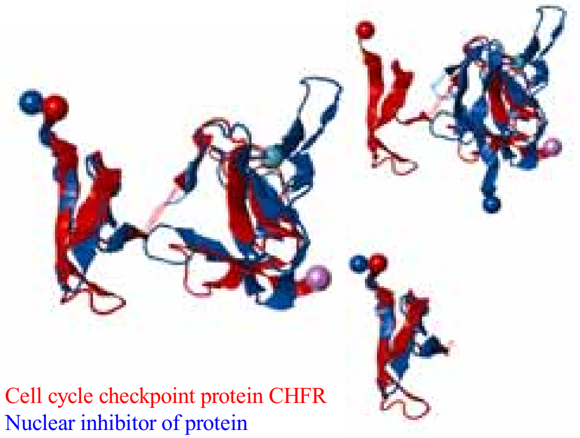<br>Cell cycle checkpoint protein CHFR<br>Nuclear inhibitor of protein phosphatase 1
94	1lgqA (112)<br>1wlnA (120)	63-95 (33)<br>64-93 (30)	84-95 (12)<br>84-93 (10)	85-88 (4)<br>85-88 (4)	C	0.52	1.97	89.29% (100/112)	14.29% (16/112)	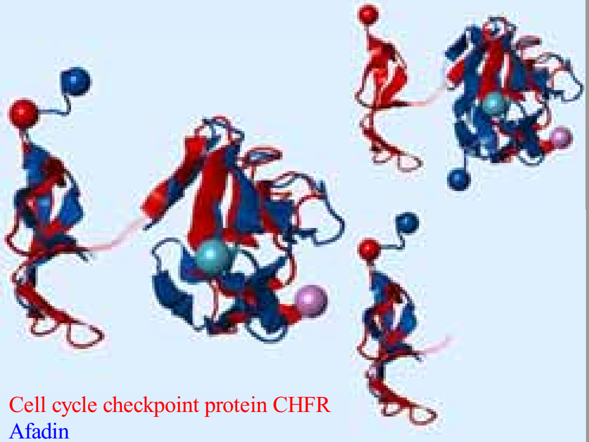<br>Cell cycle checkpoint protein CHFR<br>Afadin
95	1lgqA (112)<br>2g1lA (103)	77-97 (21)<br>563-581 (19)	85-97 (13)<br>571-581 (11)	85-87 (3)<br>85-87 (3)	C	0.56	1.75	90.29% (93/103)	17.48% (18/103)	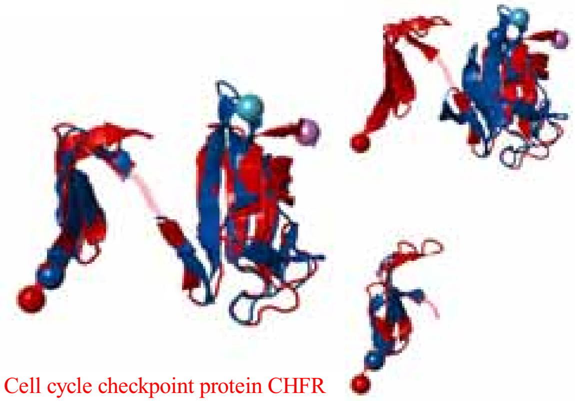<br>Cell cycle checkpoint protein CHFR<br>Kinesin-like protein KIF1C

96	1lgqA (112)	83-95 (13)	85-95 (11)	85-87 (3)	C	0.49	2.02	91.96% (103/112)	24.11% (27/112)	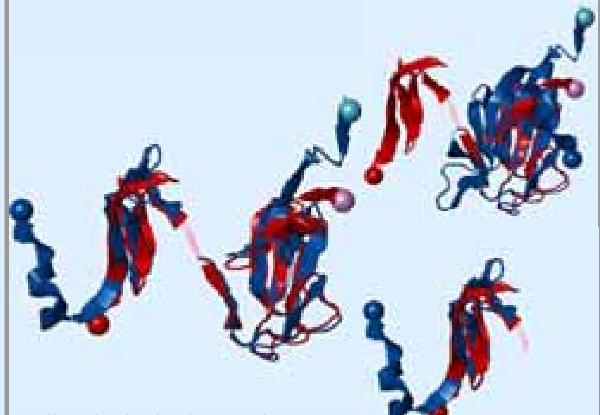 Cell cycle checkpoint protein CHFR<br>E3 ubiquitin-protein ligase RNF8
	2pieA (132)	86-98 (13)	88-98 (11)	85-87 (3)						
97	1lgqA (112)	15-123 (109)	85-99 (15)	85-88 (4)	C	0.46	1.94	87.50% (98/112)	18.75% (21/112)	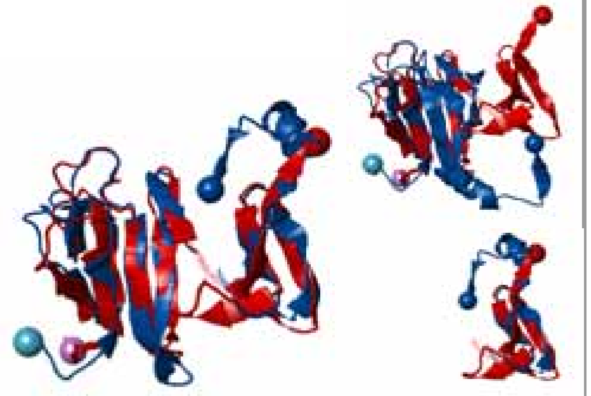 Cell cycle checkpoint protein CHFR<br>Kinesin-like protein KIF1B
	2eh0A (130)	7-114 (108)	85-97 (13)	85-88 (4)						
98	1lgqA (112)	84-98 (15)	84-98 (15)	84-87 (4)	C	0.45	2.48	89.52% (94/105)	16.19% (17/105)	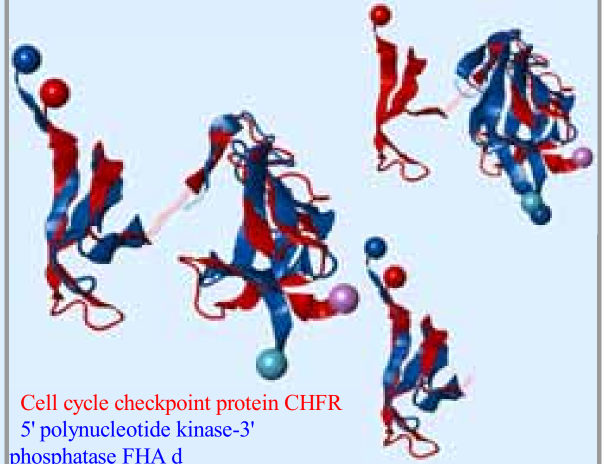 Cell cycle checkpoint protein CHFR<br>5' polynucleotide kinase-3' phosphatase FHA d
	1yj5C (105)	74-88 (15)	74-88 (15)	84-87 (4)						

99	1lgqA (112)	15-99 (85)	78-98 (21)	84-87 (4)	C	0.40	2.29	82.14% (92/112)	15.18% (17/112)	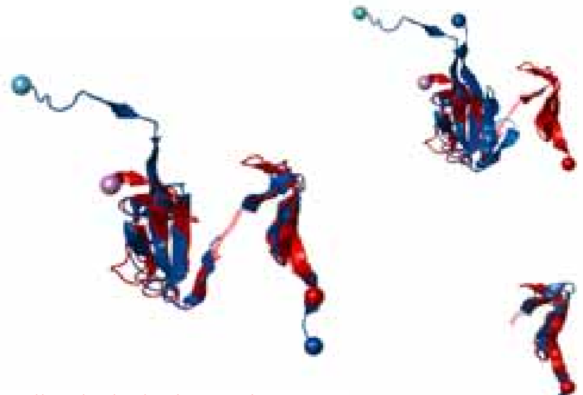 Cell cycle checkpoint protein CHFR<br>Polynucleotide kinase 3'-phosphatase
	1ujxA (119)	15-96 (82)	75-95 (21)	84-87 (4)						
100	1lgqA (112)	76-116 (41)	85-97 (13)	85-89 (5)	C	0.36	2.41	92.86% (104/112)	24.11% (27/112)	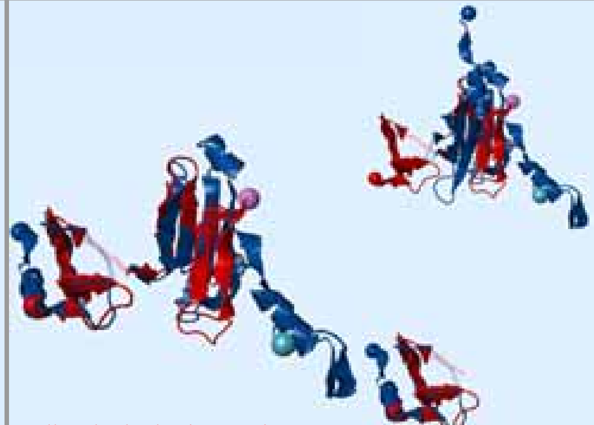 Cell cycle checkpoint protein CHFR<br>Protien kinase SPK1
	1g3gA (164)	103-141 (39)	112-124 (13)	85-89 (5)						
101	1lgqA (112)	80-98 (19)	85-96 (12)	84-88 (5)	C	0.46	2.37	91.00% (91/100)	18.00% (18/100)	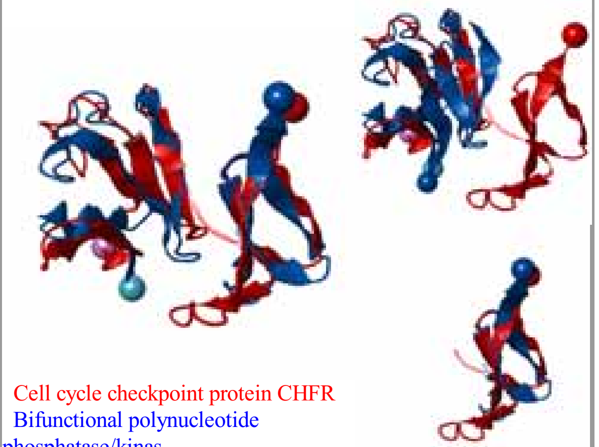 Cell cycle checkpoint protein CHFR<br>Bifunctional polynucleotide phosphatase/kinas
	2brfA (100)	70-88 (19)	75-86 (12)	84-88 (5)						

102	2pfsA (125)	41-63 (23), 68-69 (2)	42-62 (21), 68-69 (2)	42-57 (16), 68-69 (2)	M	0.41	1.71	95.20% (119/125)	27.20% (34/125)	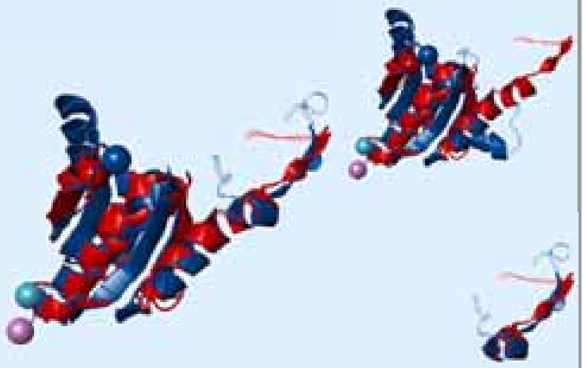 Universal stress protein<br>Universal stress protein a
	1jmvA (140)	39-52 (14), 57-64 (8)	40-51 (12), 57-64 (8)	42-57 (16), 68-69 (2)						
103	2qntA (125)	47-75 (29)	47-75 (29)	47-70 (24)	C	0.41	2.24	81.60% (102/125)	12.00% (15/125)	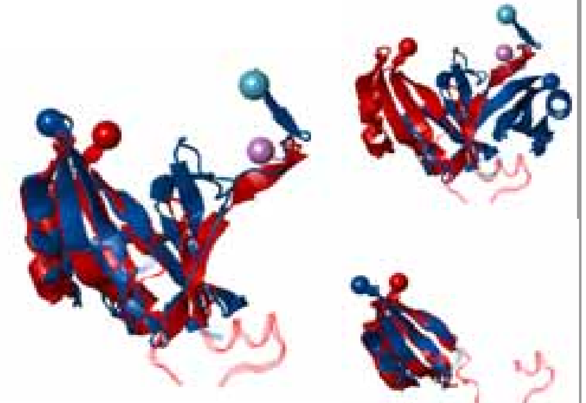 Uncharacterized protein Atu1872<br>Uncharacterized protein
	2r6uA (130)	65-76 (12)	65-76 (12)	47-70 (24)						
104	2qntA (125)	22-72 (51)	38-72 (35)	63-71 (9)	N	0.26	2.23	87.20% (109/125)	11.20% (14/125)	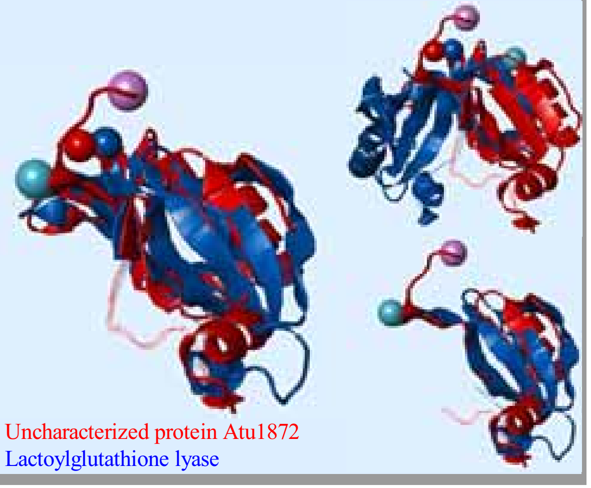 Uncharacterized protein Atu1872<br>Lactoylglutathione lyase
	2qh0A (129)	24-78 (55)	46-78 (33)	63-71 (9)						

105	2rbbA (129)	61-106 (46)	76-105 (30)	85-104 (20)	N	0.45	2.08	85.27% (110/129)	14.73% (19/129)	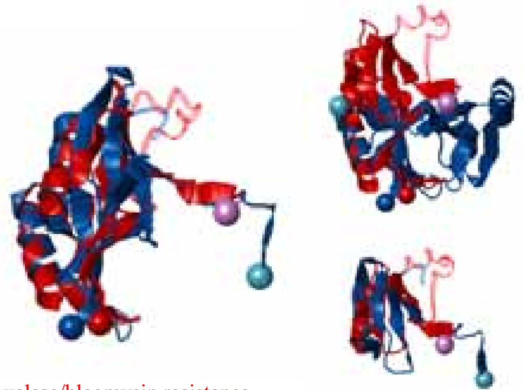 Glyoxalase/bleomycin resistance protein/dioxy Uncharacterized protein
	2r6uA (130)	30-73 (44)	44-72 (29)	85-104 (20)						
106	2rbbA (129)	55-115 (61)	72-104 (33)	85-104 (20)	C	0.51	2.05	86.82% (112/129)	10.08% (13/129)	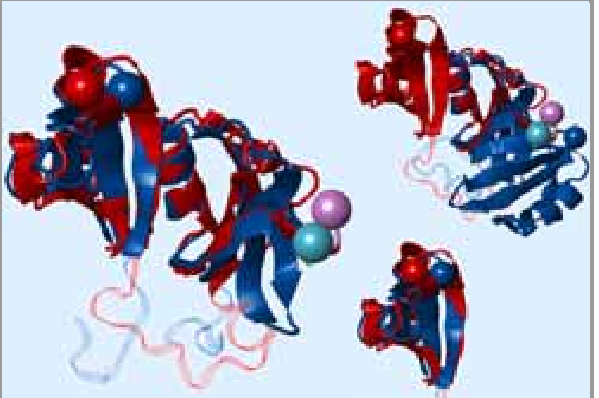 Glyoxalase/bleomycin resistance protein/dioxy Lactoylglutathione lyase
	2qh0A (129)	24-84 (61)	45-76 (32)	85-104 (20)						
107	2p7oA (127)	30-69 (40)	59-69 (11)	59-67 (9)	C	0.42	2.07	79.53% (101/127)	13.39% (17/127)	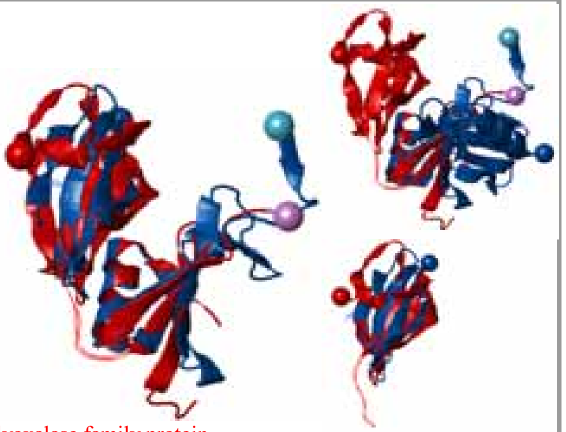 Glyoxalase family protein Uncharacterized protein
	2r6uA (130)	30-72 (43)	65-72 (8)	59-67 (9)						

108	2p7oA (127)	24-70 (47)	55-68 (14)	58-68 (11)	N	0.39	2.06	83.46% (106/127)	11.81% (15/127)	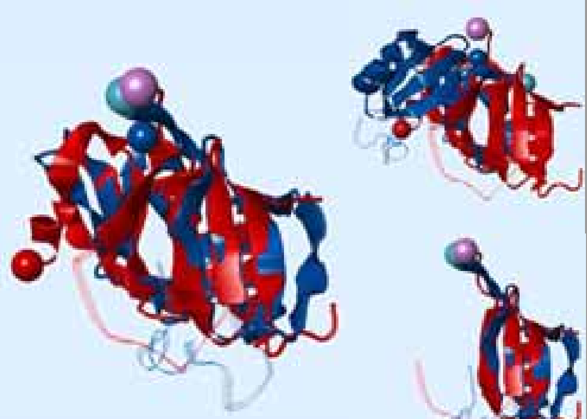 Glyoxalase family protein<br>Lactoylglutathione lyase
	2qh0A (129)	24-78 (55)	54-76 (23)	58-68 (11)						
109	2f5gA (130)	109-116 (8)	109-116 (8)	109-116 (8)	C	0.75	1.46	96.15% (125/130)	34.62% (45/130)	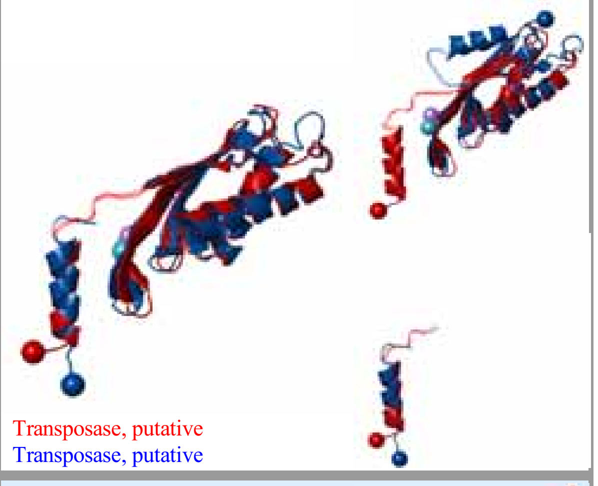 Transposase, putative<br>Transposase, putative
	2fyxA (130)	111-115 (5)	111-115 (5)	109-116 (8)						
110	2axwA (134)	110-132 (23)	110-132 (23)	117-122 (6)	C	0.51	2.54	96.27% (129/134)	79.85% (107/134)	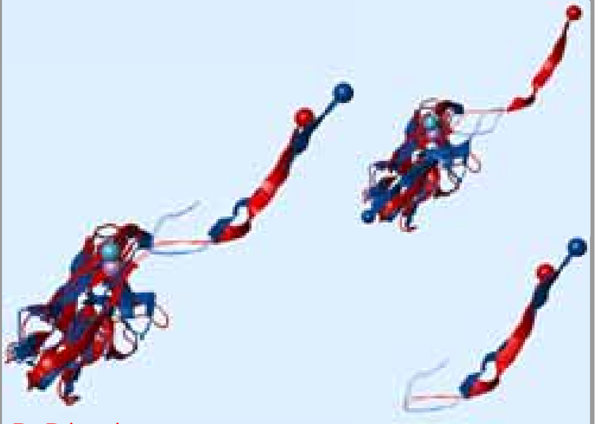 DraD invasin<br>Protein afaD
	2fvnA (142)	111-138 (28)	111-138 (28)	117-122 (6)						

111	2pa7A (135)	12-32 (21)	15-32 (18)	30-32 (3)	N	0.30	2.26	83.70% (113/135)	11.11% (15/135)	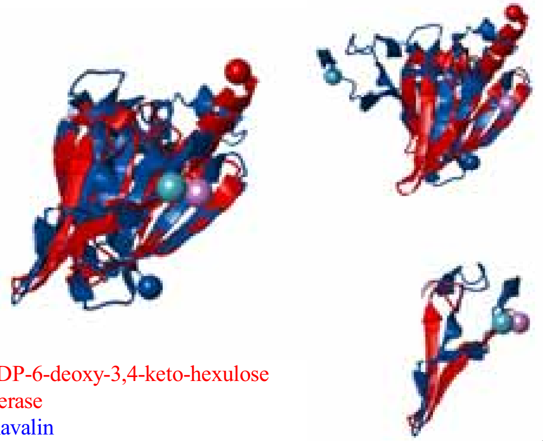 DTDP-6-deoxy-3,4-keto-hexulose isomerase Canavalin
	1cauA (181)	59-86 (28)	64-86 (23)	30-32 (3)						
112	2pa7A (135)	2-57 (56)	2-48 (47)	22-32 (11)	N	0.40	2.17	79.26% (107/135)	8.89% (12/135)	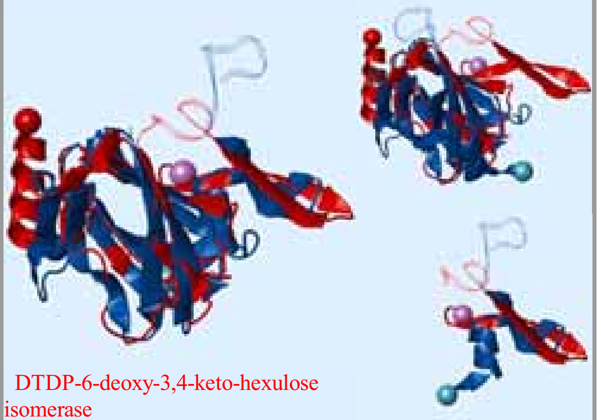 DTDP-6-deoxy-3,4-keto-hexulose isomerase Hypothetical protein Atu3615
	1znpA (140)	4-64 (61)	4-55 (52)	22-32 (11)						
113	2pa7A (135)	3-32 (30)	20-32 (13)	30-32 (3)	N	0.38	2.39	88.15% (119/135)	11.85% (16/135)	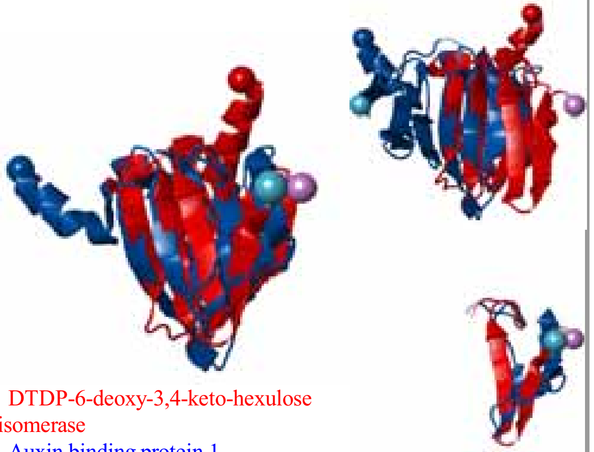 DTDP-6-deoxy-3,4-keto-hexulose isomerase Auxin binding protein 1
	1lr5A (160)	8-41 (34)	29-41 (13)	30-32 (3)						

114	1s60A (152)	130-144 (15)	135-138 (4)	137-137 (1)	C	0.49	2.15	91.45% (139/152)	10.53% (16/152)	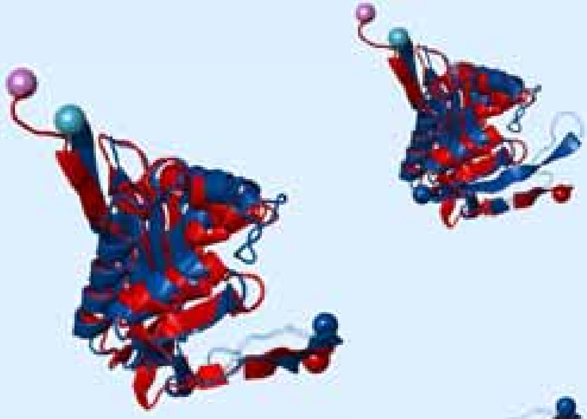 Aminoglycoside 6'-N-acetyltransferase<br>Acetyltransferase, GNAT family
	2i79A (171)	142-166 (25)	147-160 (14)	137-137 (1)						
115	1s60A (152)	129-139 (11)	131-139 (9)	138-138 (1)	C	0.50	2.37	91.45% (139/152)	8.55% (13/152)	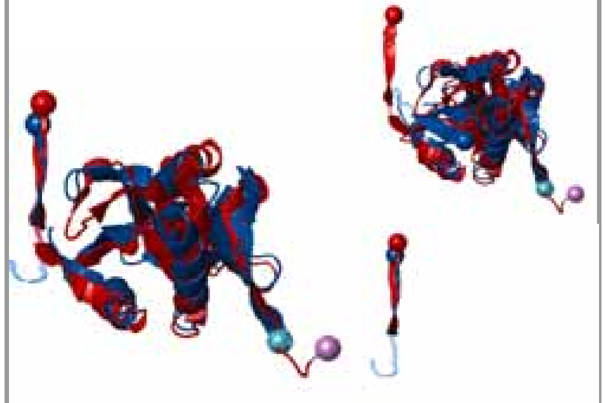 Aminoglycoside 6'-N-acetyltransferase<br>Hypothetical protein PH1933
	1wwzA (157)	138-152 (15)	139-152 (14)	138-138 (1)						
116	1s60A (152)	137-144 (8)	137-144 (8)	137-138 (2)	C	0.46	1.97	86.75% (131/151)	14.57% (22/151)	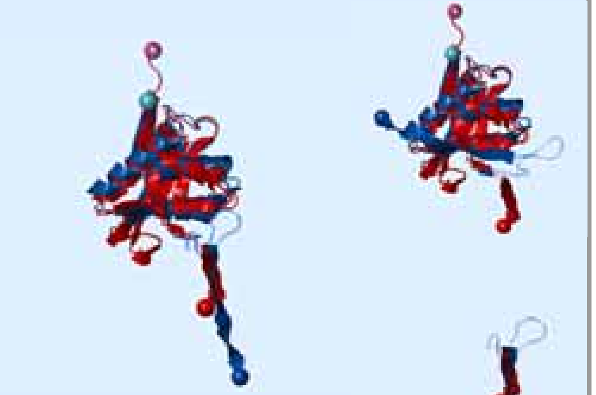 Aminoglycoside 6'-N-acetyltransferase<br>Modification OF 30S ribosomal subunit protein
	2cnmA (151)	125-144 (20)	125-144 (20)	137-138 (2)						

117	1s60A (152)	127-144 (18)	139-144 (6)	139-139 (1)	C	0.55	1.87	93.42% (142/152)	17.11% (26/152)	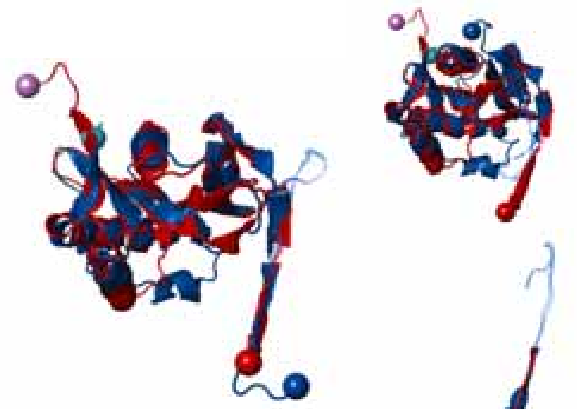 Aminoglycoside 6'-N-acetyltransferase Ttk003001606
	1wk4A (174)	140-171 (32)	152-171 (20)	139-139 (1)						
118	1s60A (152)	136-144 (9)	136-140 (5)	138-138 (1)	C	0.50	1.96	89.47% (136/152)	15.13% (23/152)	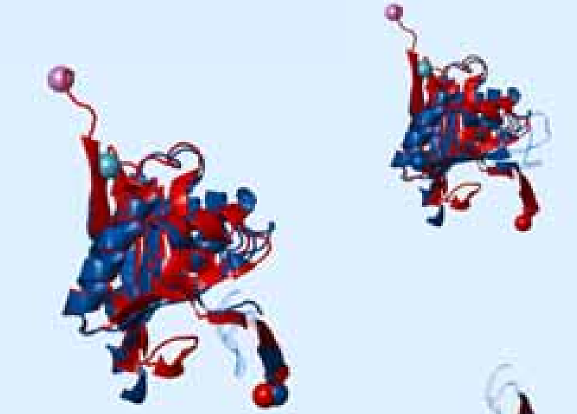 Aminoglycoside 6'-N-acetyltransferase Acetyltransferase
	1gheA (170)	150-172 (23)	150-168 (19)	138-138 (1)						
119	1s60A (152)	128-144 (17)	139-140 (2)	139-139 (1)	C	0.40	2.27	88.16% (134/152)	13.16% (20/152)	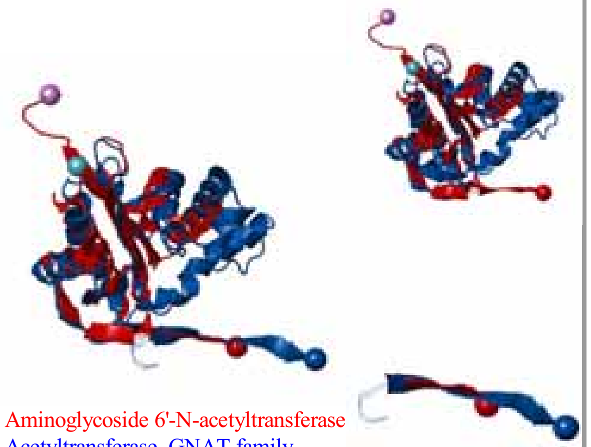 Aminoglycoside 6'-N-acetyltransferase Acetyltransferase, GNAT family
	1u6mA (189)	165-185 (21)	176-181 (6)	139-139 (1)						

120	1s60A (152)	135-144 (10)	137-143 (7)	139-139 (1)	C	0.52	2.18	90.79% (138/152)	15.13% (23/152)	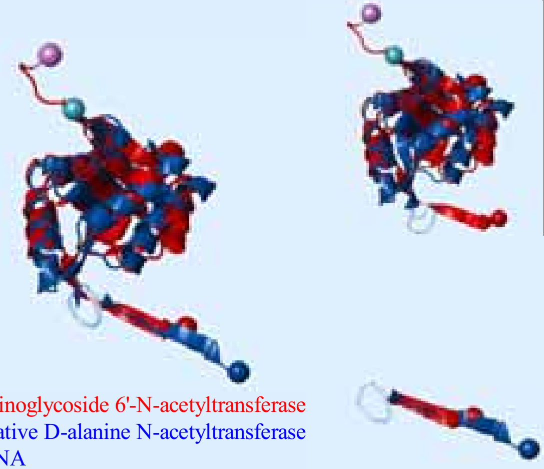 Aminoglycoside 6'-N-acetyltransferase Putative D-alanine N-acetyltransferase of GNA
	2r7hA (157)	155-171 (17)	157-170 (14)	139-139 (1)						
121	1s60A (152)	129-141 (13)	136-141 (6)	138-138 (1)	C	0.52	2.04	90.13% (137/152)	17.11% (26/152)	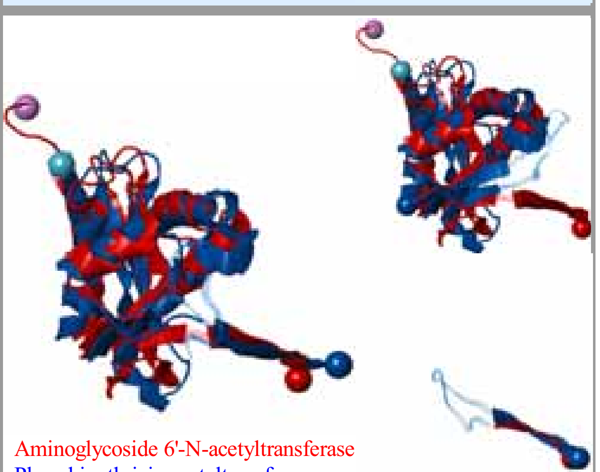 Aminoglycoside 6'-N-acetyltransferase Phosphinothricin acetyltransferase
	1yr0A (163)	137-161 (25)	144-161 (18)	138-138 (1)						
122	1s60A (152)	129-144 (16)	131-139 (9)	138-138 (1)	C	0.45	2.25	84.87% (129/152)	13.16% (20/152)	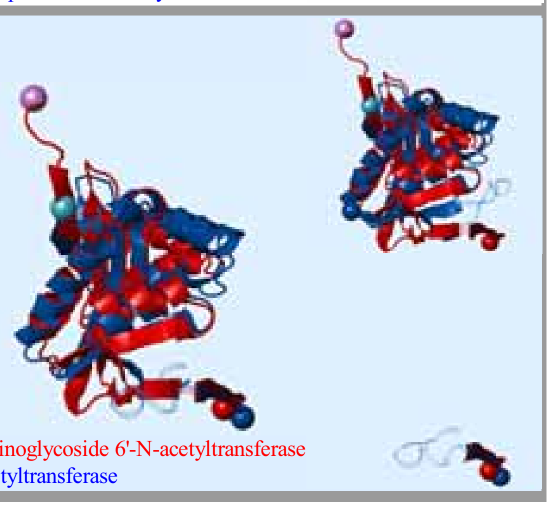 Aminoglycoside 6'-N-acetyltransferase Acetyltransferase
	2fiaA (157)	129-155 (27)	130-150 (21)	138-138 (1)						

123	1s60A (152)	129-144 (16)	129-144 (16)	135-135 (1)	C	0.46	2.12	85.53% (130/152)	7.89% (12/152)	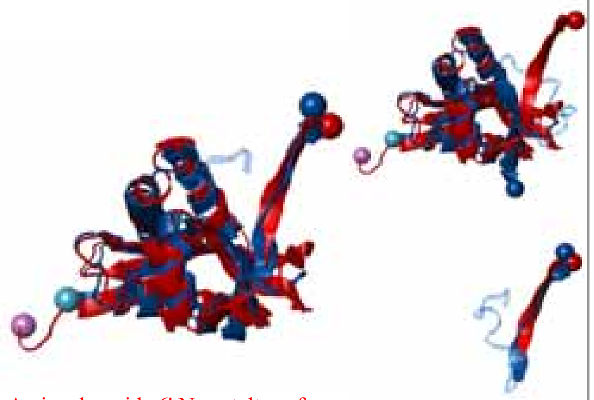 Aminoglycoside 6'-N-acetyltransferase<br>P300/CBP associating factor
	1cm0B (161)	619-651 (33)	619-651 (33)	135-135 (1)						
124	1s60A (152)	131-142 (12)	131-142 (12)	136-136 (1)	C	0.47	2.07	84.21% (128/152)	19.74% (30/152)	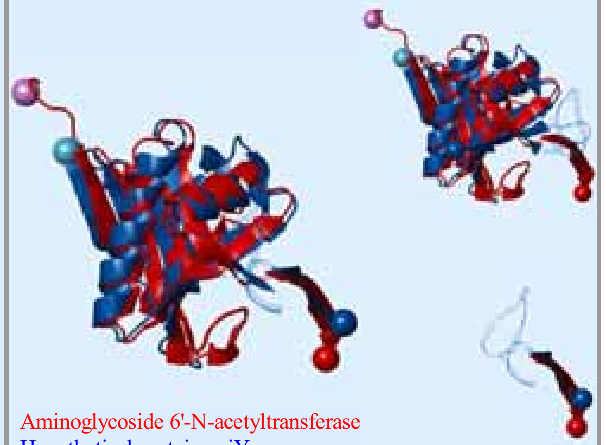 Aminoglycoside 6'-N-acetyltransferase<br>Hypothetical protein yqjY
	1mk4A (157)	123-155 (33)	123-155 (33)	136-136 (1)						
125	1s60A (152)	131-144 (14)	133-144 (12)	135-135 (1)	C	0.44	2.26	86.18% (131/152)	7.24% (11/152)	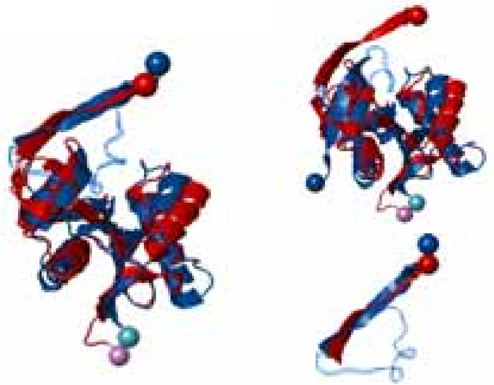 Aminoglycoside 6'-N-acetyltransferase<br>General control of amino acid synthesis prote
	1z4rA (163)	626-656 (31)	628-656 (29)	135-135 (1)						

126	1s60A (152)	137-144 (8)	137-144 (8)	139-139 (1)	C	0.39	2.23	89.47% (136/152)	13.16% (20/152)	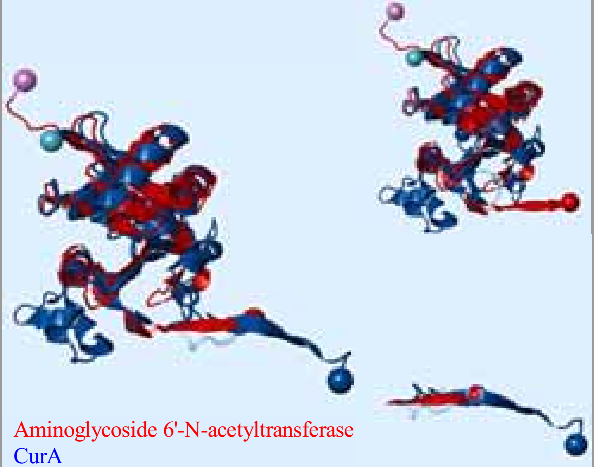
	2reeA (199)	399-415 (17)	399-415 (17)	139-139 (1)						
127	1s60A (152)	-5-12 (18)	-5-2 (8)	0-0 (1)	N	0.43	2.24	88.82% (135/152)	11.18% (17/152)	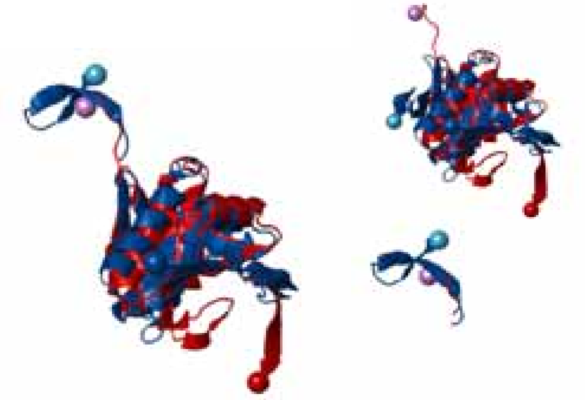
	1s7fA (181)	-4-22 (27)	-4-12 (17)	0-0 (1)						
128	1s60A (152)	135-144 (10)	136-144 (9)	139-139 (1)	C	0.42	2.21	88.16% (134/152)	10.53% (16/152)	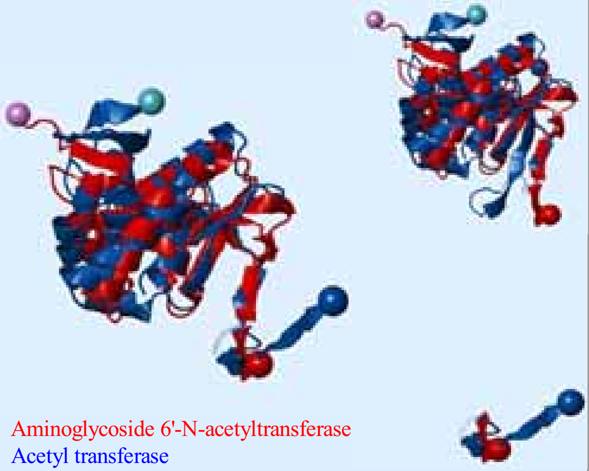
	1s7fA (181)	153-169 (17)	154-169 (16)	139-139 (1)						

129	1s60A (152)	129-144 (16)	129-144 (16)	135-136 (2)	C	0.43	2.27	85.53% (130/152)	8.55% (13/152)	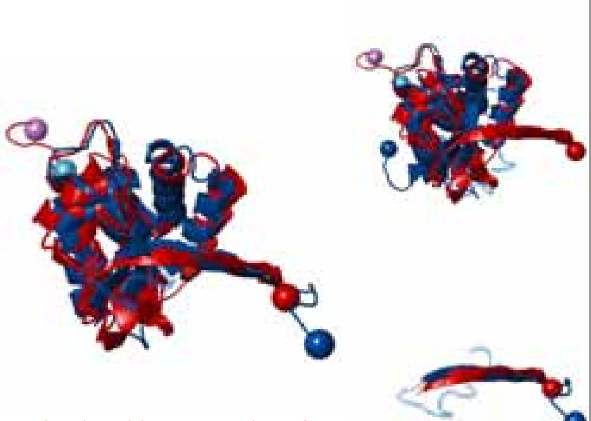 Aminoglycoside 6'-N-acetyltransferase TGCN5 histone acetyl transferase
	1m1dA (163)	171-203 (33)	171-203 (33)	135-136 (2)						
130	1s60A (152)	131-139 (9)	135-139 (5)	138-138 (1)	C	0.41	2.02	89.47% (136/152)	15.13% (23/152)	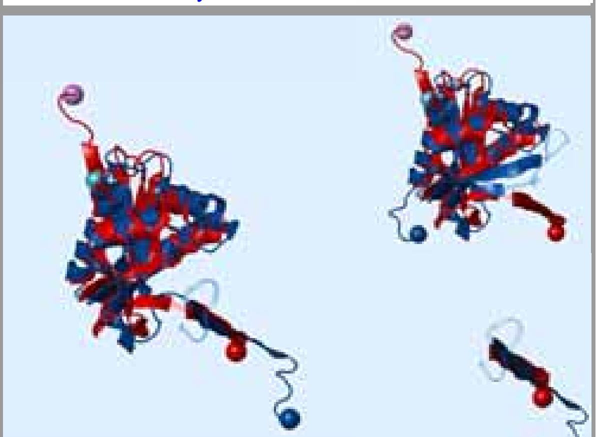 Aminoglycoside 6'-N-acetyltransferase Conserved hypothetical protein
	1yvoA (169)	138-156 (19)	142-156 (15)	138-138 (1)						
131	1s60A (152)	131-143 (13)	138-140 (3)	139-139 (1)	C	0.51	2.13	88.16% (134/152)	10.53% (16/152)	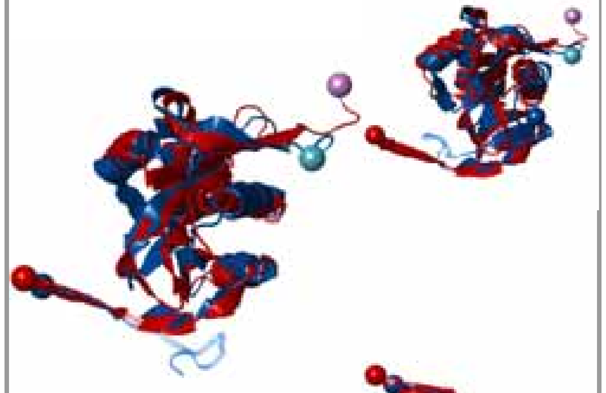 Aminoglycoside 6'-N-acetyltransferase Human MAK3 homolog
	2ob0A (154)	128-153 (26)	135-150 (16)	139-139 (1)						

132	1s60A (152)	131-144 (14)	137-141 (5)	137-137 (1)	C	0.47	2.35	90.13% (137/152)	14.47% (22/152)	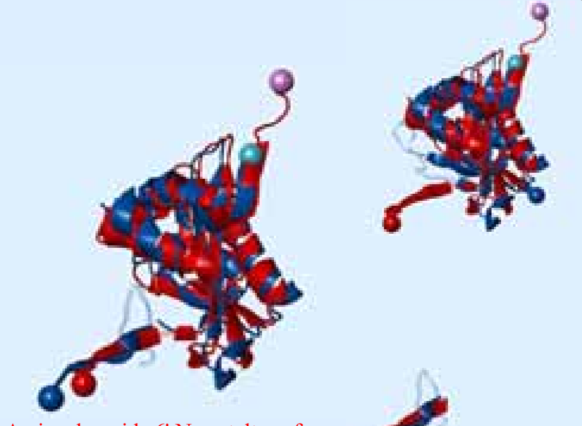 Aminoglycoside 6'-N-acetyltransferase<br>Acetyltransferase PA1377
	2vi7A (163)	142-167 (26)	148-164 (17)	137-137 (1)						
133	1s60A (152)	137-140 (4)	137-140 (4)	138-138 (1)	C	0.50	2.02	89.29% (125/140)	12.86% (18/140)	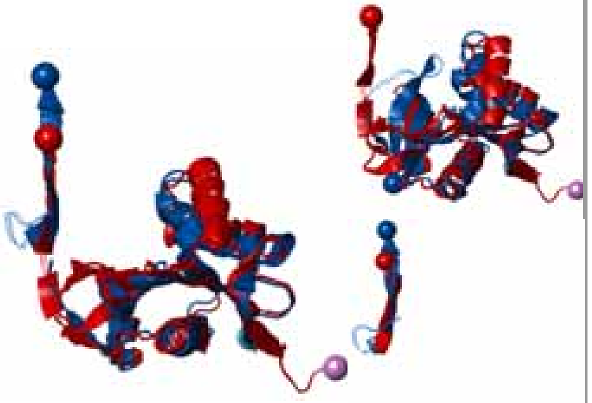 Aminoglycoside 6'-N-acetyltransferase<br>Similar to hypothetical proteins
	1q2yA (140)	124-133 (10)	124-133 (10)	138-138 (1)						
134	1s60A (152)	135-144 (10)	139-144 (6)	139-140 (2)	C	0.37	2.74	94.08% (143/152)	12.50% (19/152)	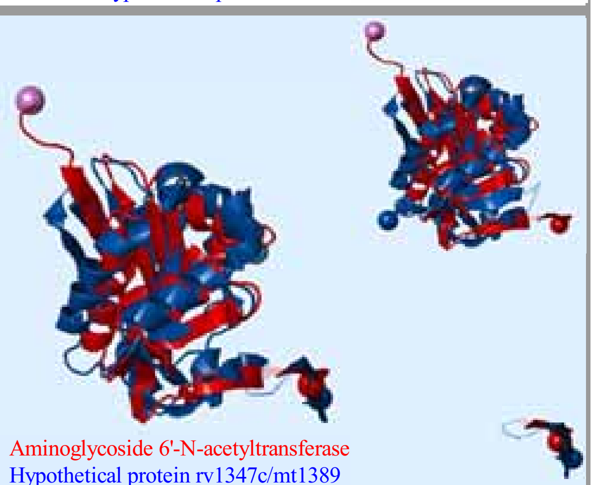 Aminoglycoside 6'-N-acetyltransferase<br>Hypothetical protein rv1347c/mt1389
	1yk3A (198)	188-201 (14)	192-201 (10)	139-140 (2)						

135	1s60A (152)	133-144 (12)	133-137 (5)	135-135 (1)	C	0.41	2.40	85.53% (130/152)	7.24% (11/152)	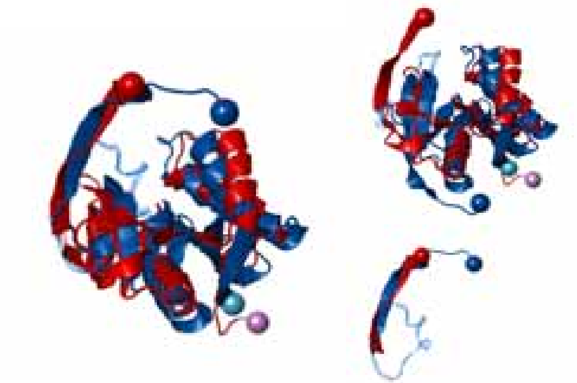<br>Aminoglycoside 6'-N-acetyltransferase<br>Protein (transcriptional activator GCN5)
	1yghA (164)	227-257 (31)	227-249 (23)	135-135 (1)						
136	1s60A (152)	131-141 (11)	135-141 (7)	137-137 (1)	C	0.52	2.21	91.72% (133/145)	15.17% (22/145)	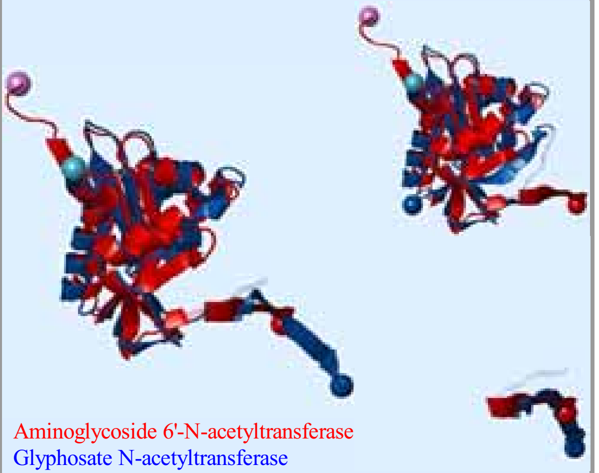<br>Aminoglycoside 6'-N-acetyltransferase<br>Glyphosate N-acetyltransferase
	2bswA (145)	122-137 (16)	126-137 (12)	137-137 (1)						
137	1s60A (152)	135-140 (6)	136-138 (3)	137-137 (1)	C	0.45	2.30	90.13% (137/152)	15.79% (24/152)	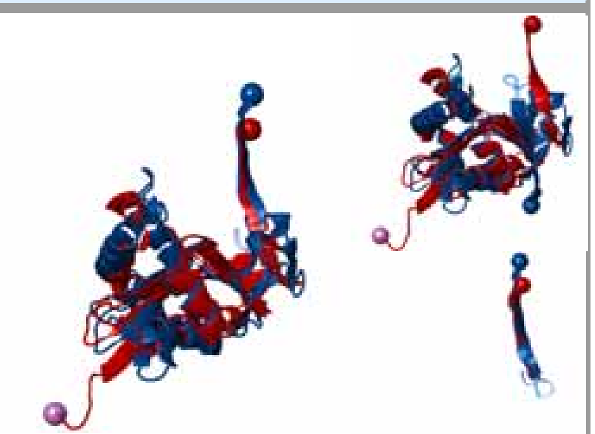<br>Aminoglycoside 6'-N-acetyltransferase<br>Acetyltransferase, GNAT family
	2pc1A (173)	149-160 (12)	150-158 (9)	137-137 (1)						

138	1s60A (152)	134-137 (4)	134-137 (4)	134-136 (3)	C	0.51	2.08	90.13% (137/152)	14.47% (22/152)	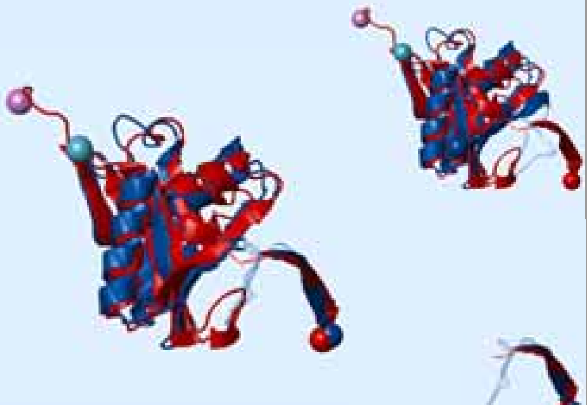 Aminoglycoside 6'-N-acetyltransferase Acetyltransferase, GNAT family
	2q7bA (164)	137-153 (17)	137-153 (17)	134-136 (3)						
139	1s60A (152)	134-144 (11)	134-144 (11)	134-135 (2)	C	0.47	1.88	82.89% (126/152)	14.47% (22/152)	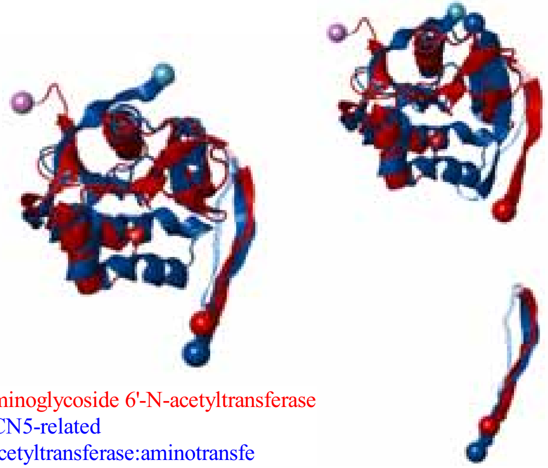 Aminoglycoside 6'-N-acetyltransferase GCN5-related N-acetyltransferase:aminotransfe
	2fiwA (160)	135-154 (20)	135-154 (20)	134-135 (2)						
140	1s60A (152)	129-143 (15)	134-139 (6)	136-137 (2)	C	0.51	2.06	94.53% (121/128)	15.63% (20/128)	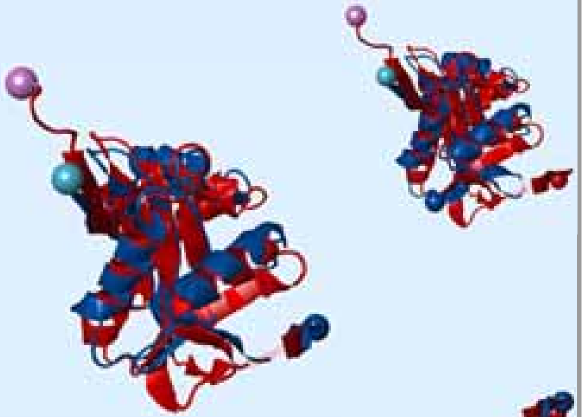 Aminoglycoside 6'-N-acetyltransferase Uncharacterized protein yhhK
	2k5tA (128)	114-127 (14)	119-123 (5)	136-137 (2)						

141	1s60A (152)	130-144 (15)	130-144 (15)	138-138 (1)	C	0.49	2.08	86.84% (132/152)	13.82% (21/152)	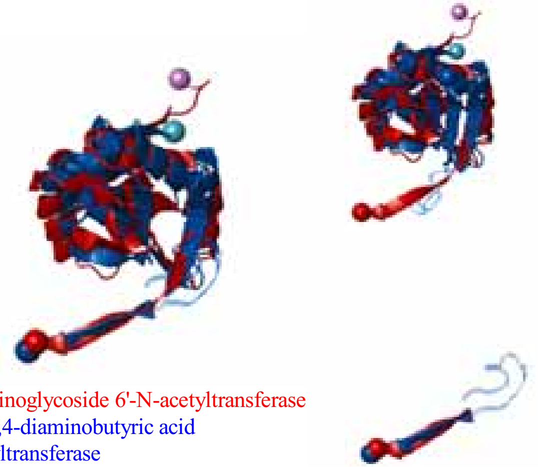 Aminoglycoside 6'-N-acetyltransferase L-2,4-diaminobutyric acid acetyltransferase
	3d3sA (159)	124-158 (35)	124-158 (35)	138-138 (1)						
142	1s60A (152)	131-141 (11)	138-141 (4)	138-138 (1)	C	0.39	2.74	90.13% (137/152)	14.47% (22/152)	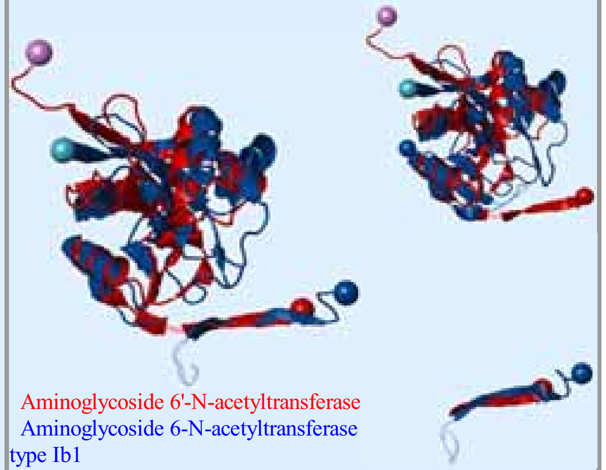 Aminoglycoside 6'-N-acetyltransferase Aminoglycoside 6-N-acetyltransferase type Ib1
	2pr8A (173)	158-174 (17)	165-174 (10)	138-138 (1)						
143	1s60A (152)	-1-30 (32)	15-30 (16)	15-30 (16)	N	0.43	2.48	86.39% (127/147)	12.24% (18/147)	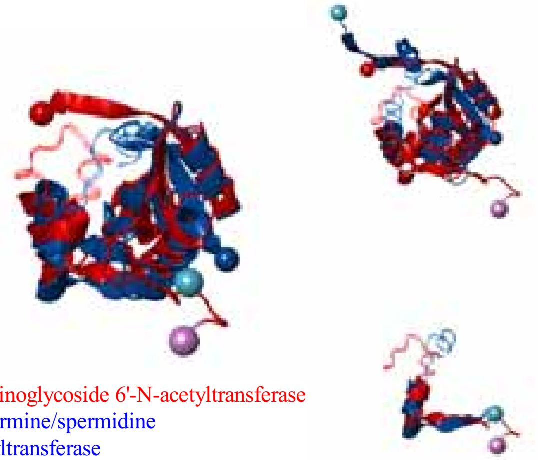 Aminoglycoside 6'-N-acetyltransferase Spermine/spermidine acetyltransferase
	2fl4A (147)	1-32 (32)	18-32 (15)	15-30 (16)						

144	1s60A (152)	130-142 (13)	131-142 (12)	136-136 (1)	C	0.34	2.36	75.51% (111/147)	11.56% (17/147)	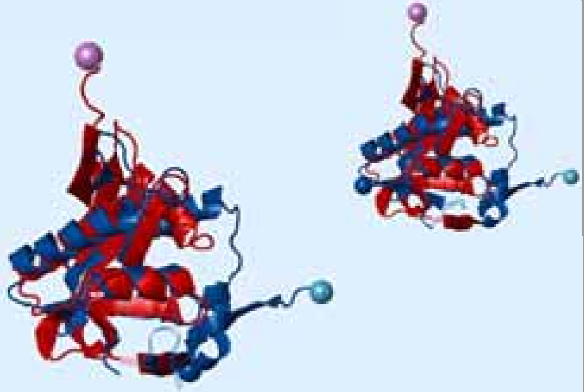 Aminoglycoside 6'-N-acetyltransferase Spermine/spermidine acetyltransferase 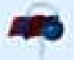
	2fl4A (147)	125-145 (21)	126-145 (20)	136-136 (1)						
145	1s60A (152)	121-144 (24)	136-144 (9)	139-141 (3)	C	0.36	2.57	82.89% (126/152)	12.50% (19/152)	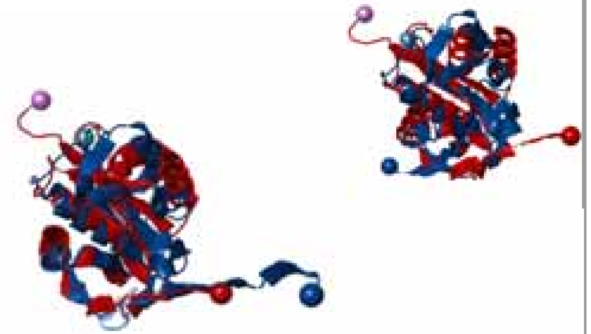 Aminoglycoside 6'-N-acetyltransferase Protein (arylalkylamine N-acetyltransferase) 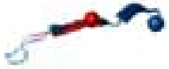
	1b6bA (168)	163-192 (30)	177-192 (16)	139-141 (3)						
146	1s60A (152)	133-139 (7)	135-139 (5)	135-137 (3)	C	0.42	2.38	82.89% (126/152)	8.55% (13/152)	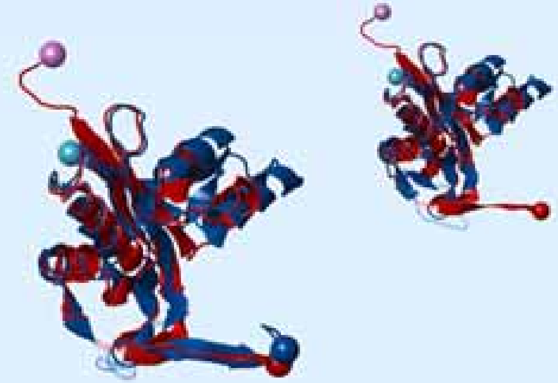 Aminoglycoside 6'-N-acetyltransferase Uncharacterized N-acetyltransferase ylbP 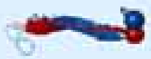
	2pr1A (152)	129-143 (15)	131-143 (13)	135-137 (3)						

147	1s60A (152)	104-142 (39)	129-139 (11)	138-138 (1)	C	0.42	2.44	84.56% (126/149)	10.74% (16/149)	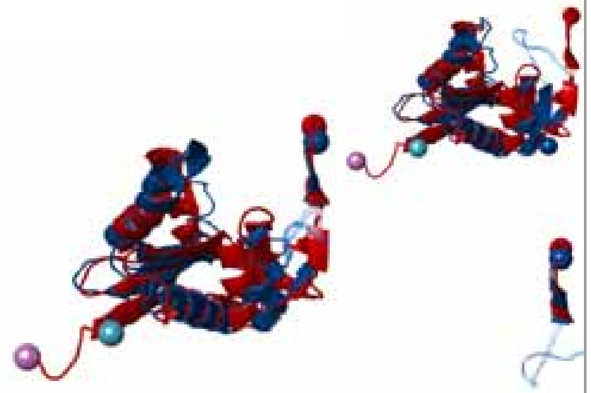 Aminoglycoside 6'-N-acetyltransferase Hypothetical protein PA0115
	1xebA (149)	104-149 (46)	127-146 (20)	138-138 (1)						
148	1s60A (152)	-5-12 (18)	-5-12 (18)	1-1 (1)	N	0.42	2.60	91.45% (139/152)	8.55% (13/152)	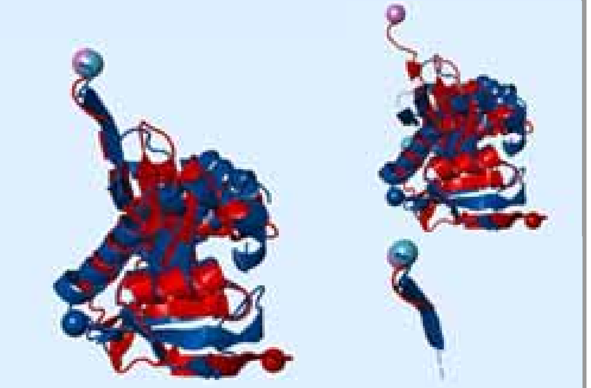 Aminoglycoside 6'-N-acetyltransferase Ribosomal-protein-serine acetyltransferase, p
	2fckA (174)	2-23 (22)	2-23 (22)	1-1 (1)						
149	1s60A (152)	135-144 (10)	135-144 (10)	140-140 (1)	C	0.40	2.58	89.47% (136/152)	9.21% (14/152)	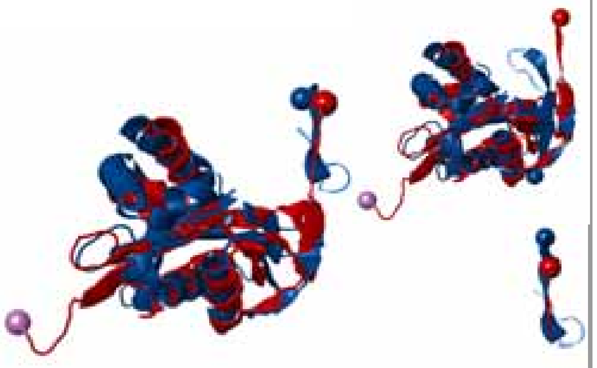 Aminoglycoside 6'-N-acetyltransferase Ribosomal-protein-serine acetyltransferase, p
	2fckA (174)	155-170 (16)	155-170 (16)	140-140 (1)						

150	1tiyA (157)	110-141 (32)	112-129 (18)	114-130 (17)	C	0.58	1.49	92.99% (146/157)	26.11% (41/157)	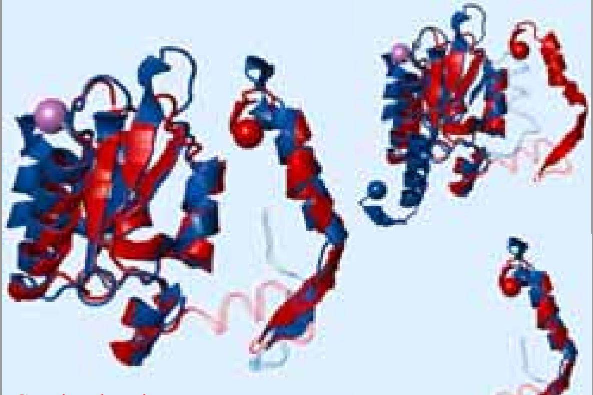 Guanine deaminase<br>Cytidine and deoxycytidylate deaminase zinc-b
	2g84A (189)	139-171 (33)	141-158 (18)	114-130 (17)						
151	1tiyA (157)	101-142 (42)	101-141 (41)	104-135 (32)	C	0.56	1.72	87.01% (134/154)	29.22% (45/154)	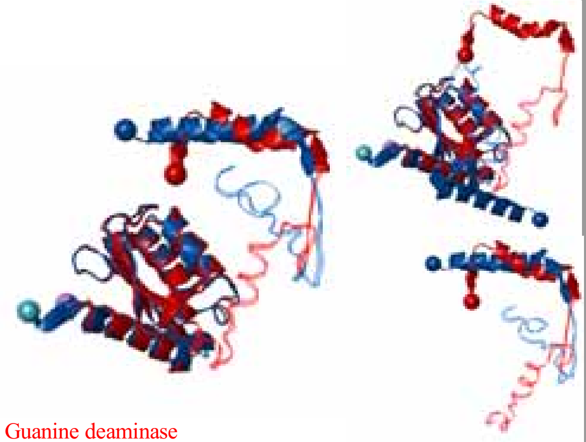 Guanine deaminase<br>TRNA adenosine deaminase TadA
	1wwrA (154)	100-134 (35)	100-133 (34)	104-135 (32)						
152	1tiyA (157)	104-146 (43)	104-133 (30)	104-131 (28)	C	0.58	1.59	88.08% (133/151)	29.14% (44/151)	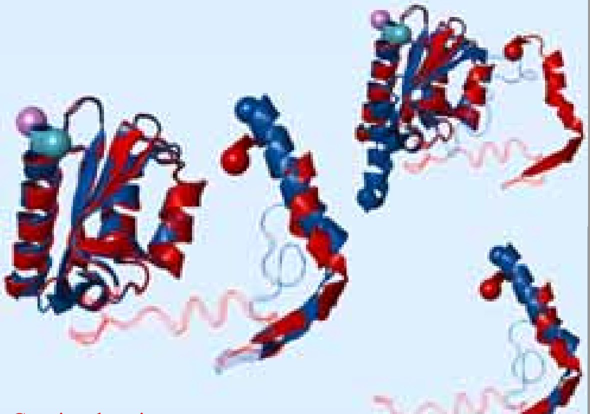 Guanine deaminase<br>TRNA adenosine deaminase
	2b3jA (151)	104-140 (37)	104-127 (24)	104-131 (28)						

153	1tiyA (157)	93-141 (49)	104-141 (38)	104-141 (38)	C	0.47	1.92	84.08% (132/157)	24.20% (38/157)	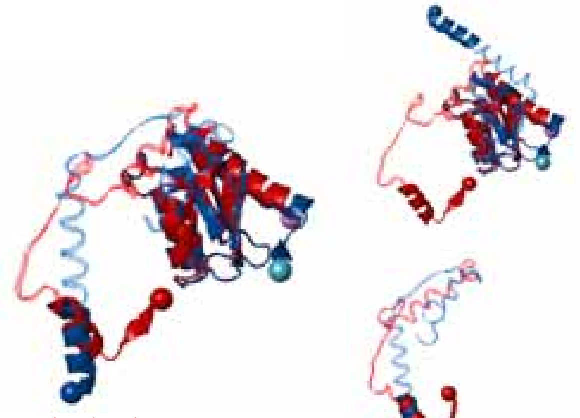 Guanine deaminase<br>TRNA-specific adenosine deaminase
	2nx8A (168)	105-161 (57)	116-161 (46)	104-141 (38)						
154	1tiyA (157)	103-143 (41)	104-130 (27)	105-133 (29)	C	0.58	1.62	86.54% (135/156)	25.64% (40/156)	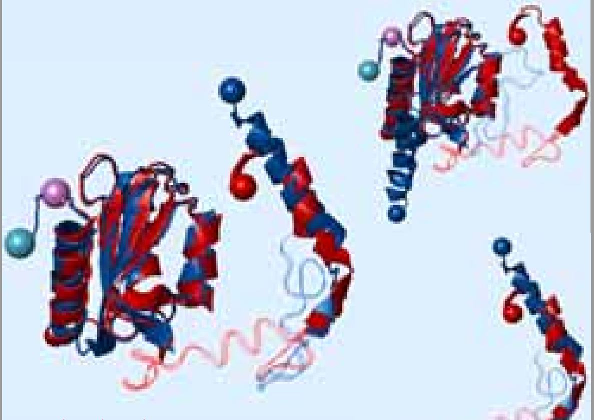 Guanine deaminase<br>TRNA-specific adenosine deaminase
	1z3aA (156)	118-152 (35)	119-139 (21)	105-133 (29)						
155	2p1jA (164)	491-501 (11)	492-501 (10)	494-501 (8)	C	0.68	1.49	95.12% (156/164)	29.27% (48/164)	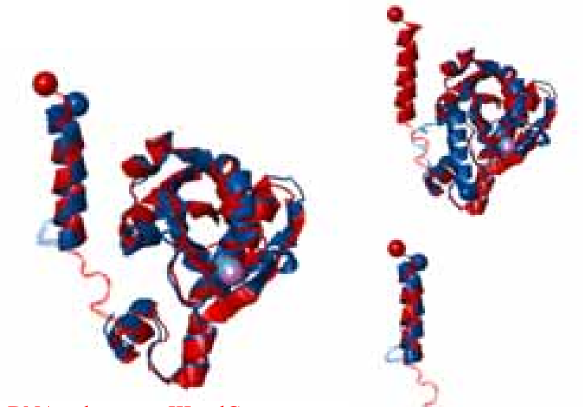 DNA polymerase III polC-type<br>DNA polymerase III, epsilon chain
	1j53A (174)	151-163 (13)	152-163 (12)	494-501 (8)						

156	2p1jA (164)	492-501 (10)	492-501 (10)	493-501 (9)	C	0.45	2.06	94.51% (155/164)	25.00% (41/164)	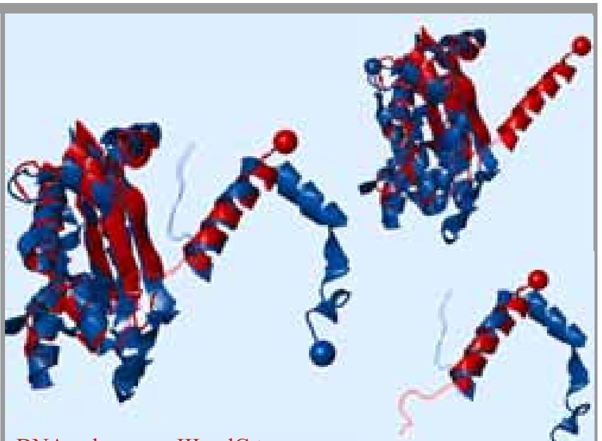 DNA polymerase III polC-type
	2iocB (220)	186-196 (11)	186-196 (11)	493-501 (9)						Three prime repair exonuclease 1
157	2p1jA (164)	491-501 (11)	493-501 (9)	494-501 (8)	C	0.38	2.14	93.29% (153/164)	18.90% (31/164)	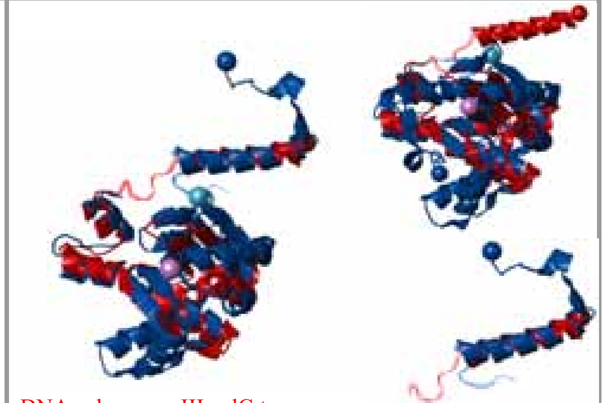 DNA polymerase III polC-type
	2d5rA (252)	215-226 (12)	217-226 (10)	494-501 (8)						CCR4-NOT transcription complex subunit 7
158	2p1jA (164)	492-501 (10)	494-501 (8)	494-501 (8)	C	0.36	2.23	92.68% (152/164)	17.68% (29/164)	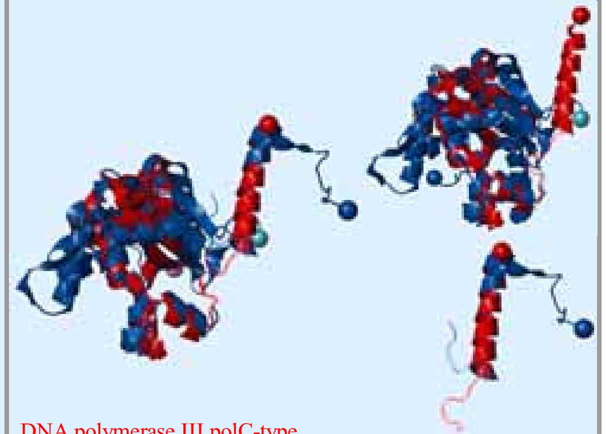 DNA polymerase III polC-type
	2p51A (255)	226-236 (11)	228-236 (9)	494-501 (8)						Spcc18.06c protein

159	2p1jA (164)	491-504 (14)	491-501 (11)	492-501 (10)	C	0.51	2.14	89.02% (146/164)	19.51% (32/164)	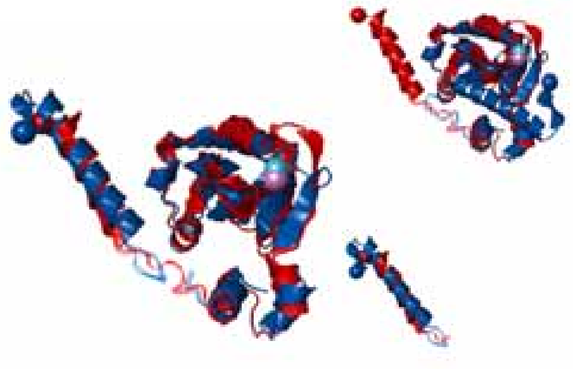 DNA polymerase III polC-type<br>Interferon stimulated gene 20kDa
	1wljA (168)	136-153 (18)	136-150 (15)	492-501 (10)						
160	2p1jA (164)	477-501 (25)	477-501 (25)	477-501 (25)	C	0.42	2.09	82.93% (136/164)	15.85% (26/164)	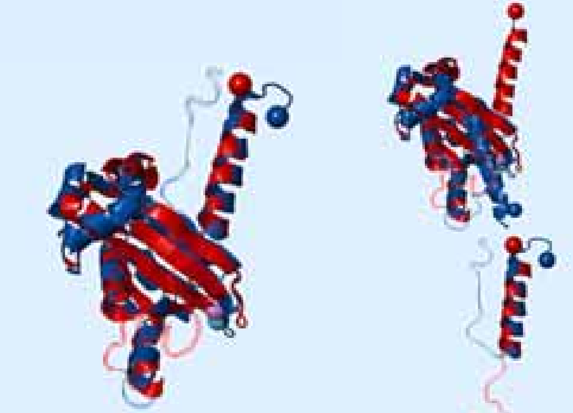 DNA polymerase III polC-type<br>Oligoribonuclease
	2gbzA (179)	144-162 (19)	144-162 (19)	477-501 (25)						
161	2jevA (169)	147-162 (16)	147-156 (10)	149-152 (4)	C	0.39	2.82	89.35% (151/169)	10.65% (18/169)	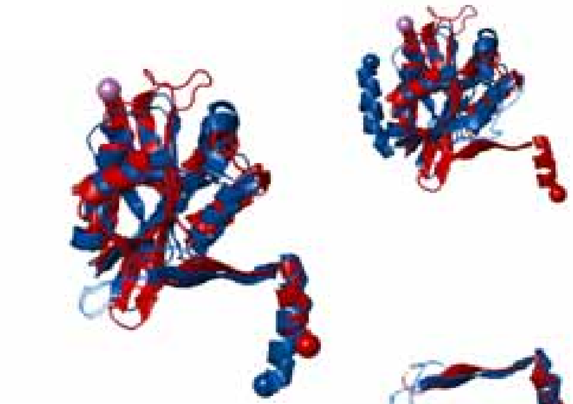 Diamine acetyltransferase 1<br>Hypothetical protein PA3270
	1yreA (183)	154-180 (27)	154-174 (21)	149-152 (4)						

162	1vguA (214)	125-150 (26), 153-171 (19)	125-141 (17), 153-166 (14)	138-141 (4), 156-160 (5)	M	0.52	2.12	93.93% (201/214)	14.49% (31/214)	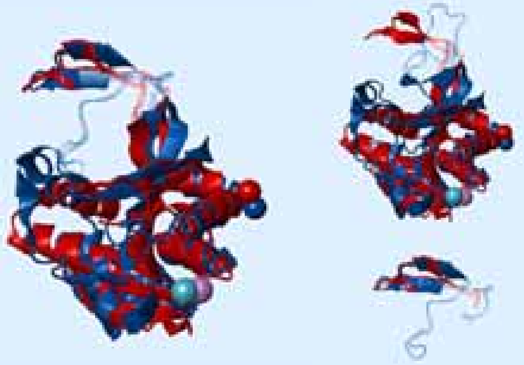 2-C-methyl-D-erythritol 4-phosphate cytidylyl 3-deoxy-manno-octulosonate cytidylyltransfera
	1gq9A (241)	115-149 (35), 152-180 (29)	115-141 (27), 152-175 (24)	138-141 (4), 156-160 (5)						
163	1vguA (214)	125-139 (15), 142-173 (32)	137-139 (3), 142-161 (20)	137-138 (2), 159-161 (3)	M	0.42	2.29	95.79% (205/214)	13.55% (29/214)	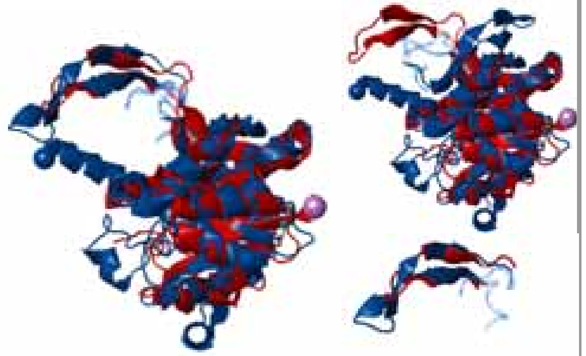 2-C-methyl-D-erythritol 4-phosphate cytidylyl UTP-glucose-1-phosphate uridylyltransferase
	2pa4A (294)	159-179 (21), 182-223 (42)	171-179 (9), 182-212 (31)	137-138 (2), 159-161 (3)						
164	1vguA (214)	125-141 (17), 144-169 (26)	139-141 (3), 145-160 (16)	139-140 (2), 156-157 (2)	M	0.32	2.45	92.06% (197/214)	19.16% (41/214)	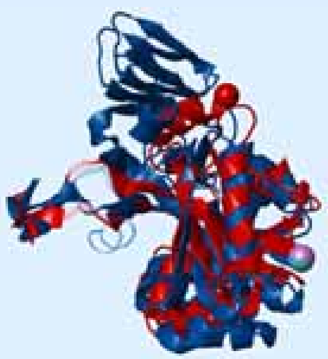 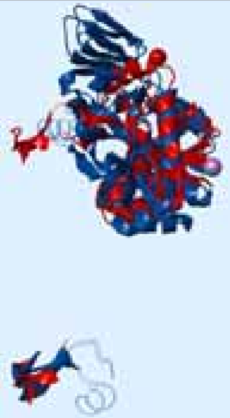 2-C-methyl-D-erythritol 4-phosphate cytidylyl Putative mannose-1-phosphate guanylyl transfe
	2cu2A (335)	127-150 (24), 153-192 (40)	143-150 (8), 154-183 (30)	139-140 (2), 156-157 (2)						

165	1vguA (214)	125-139 (15), 144-166 (23)	125-138 (14), 145-161 (17)	137-138 (2), 157-161 (5)	M	0.40	2.37	93.93% (201/214)	11.21% (24/214)	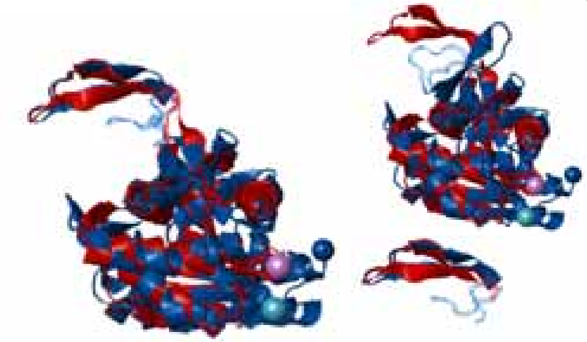 2-C-methyl-D-erythritol 4-phosphate cytidylyl Glucose-1-phosphate thymidylyltransferase
	1h5rA (290)	128-146 (19), 151-176 (26)	128-145 (18), 152-171 (20)	137-138 (2), 157-161 (5)						
166	1vguA (214)	124-139 (16), 144-166 (23)	124-139 (16), 144-166 (23)	137-138 (2), 157-161 (5)	M	0.40	2.40	94.39% (202/214)	11.21% (24/214)	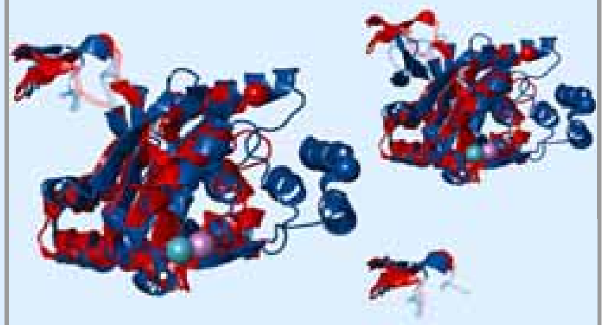 2-C-methyl-D-erythritol 4-phosphate cytidylyl Glucose-1-phosphate thymidylyltransferase
	1iimA (289)	127-146 (20), 151-176 (26)	127-146 (20), 151-176 (26)	137-138 (2), 157-161 (5)						
167	1vguA (214)	122-140 (19), 153-166 (14)	122-140 (19), 153-161 (9)	139-140 (2), 156-160 (5)	M	0.39	2.38	93.93% (201/214)	14.49% (31/214)	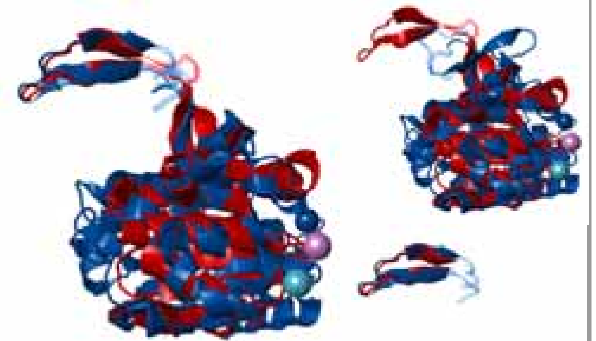 2-C-methyl-D-erythritol 4-phosphate cytidylyl Glucose-1-phosphate thymidylyltransferase
	1lvwA (295)	125-144 (20), 156-173 (18)	125-144 (20), 156-168 (13)	139-140 (2), 156-160 (5)						

168	1vguA (214)	122-139 (18), 144-166 (23)	122-139 (18), 145-161 (17)	137-138 (2), 157-161 (5)	M	0.40	2.32	93.46% (200/214)	11.21% (24/214)	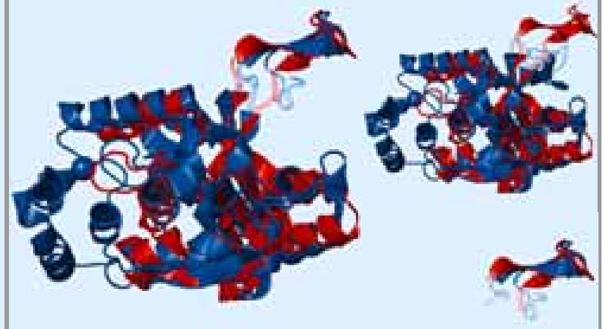 2-C-methyl-D-erythritol 4-phosphate cytidylyl Glucose-1-phosphate thymidylyltransferase
	1fxoA (292)	127-145 (19), 150-175 (26)	127-145 (19), 151-170 (20)	137-138 (2), 157-161 (5)						
169	2c49A (299)	7-35 (29), 24-40 (17)	17-25 (9), 35-39 (5)	22-25 (4), 35-36 (2)	M	0.50	2.11	92.98% (278/299)	20.07% (60/299)	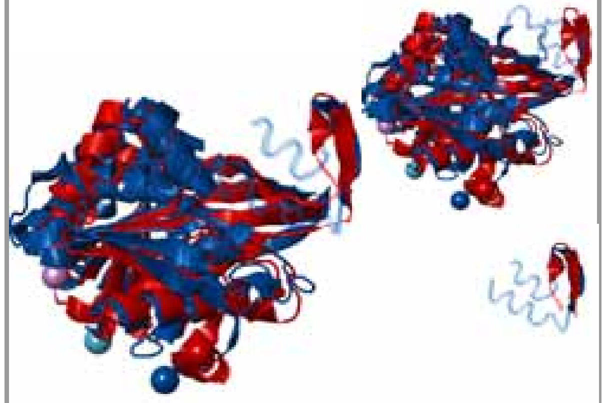 Sugar kinase MJ0406 Protein (adenosine kinase)
	1bx4A (342)	5-42 (38), 31-61 (31)	18-32 (15), 42-60 (19)	22-25 (4), 35-36 (2)						
170	1h1cA (329)	7-57 (51)	7-57 (51)	41-54 (14)	N	0.26	2.67	93.01% (306/329)	12.16% (40/329)	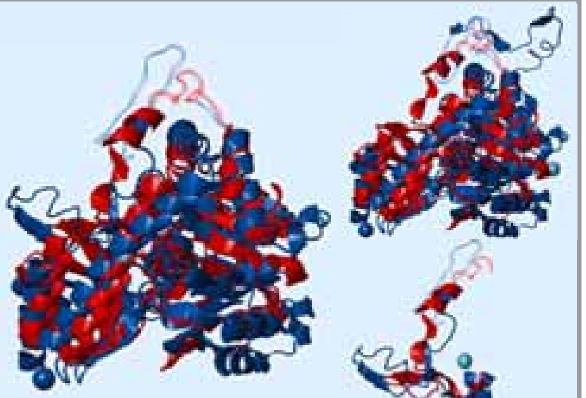 Histidinol-phosphate aminotransferase Kynurenine/alpha-aminoadipate aminotransferas
	2qlrA (425)	6-79 (74)	6-79 (74)	41-54 (14)						

171	1a5pA (124)	15-22 (8)	18-22 (5)	18-22 (5)	N	0.95	0.68	100.00% (124/124)	98.39% (122/124)	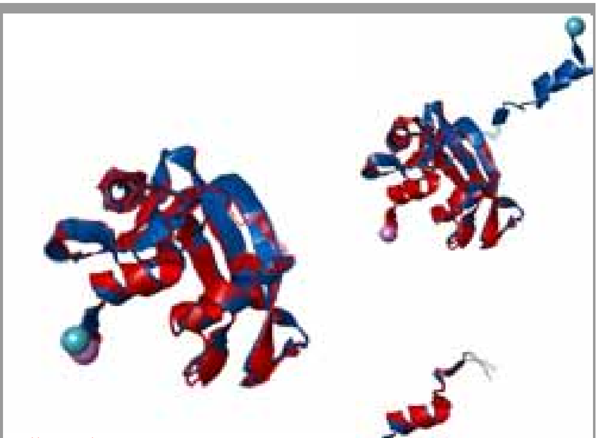 Ribonuclease A<br>Ribonuclease A
	1a2wA (124)	15-22 (8)	18-22 (5)	18-22 (5)						
172	1nloC (56)	28-56 (29)	29-42 (14)	35-41 (7)	C	0.69	1.23	92.86% (52/56)	25.00% (14/56)	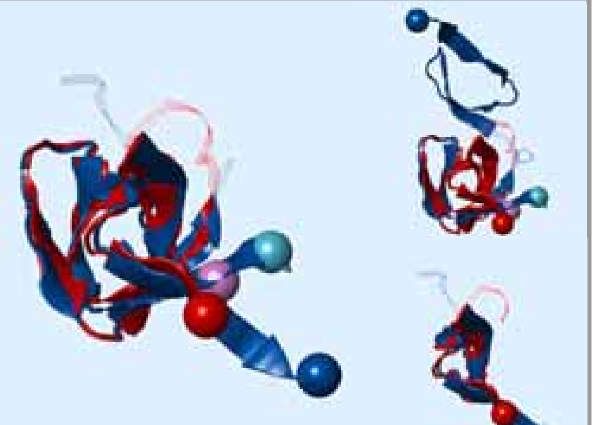 C-SRC<br>EPS8
	1aojA (60)	27-53 (27)	28-40 (13)	35-41 (7)						
173	1eydA (136)	112-120 (9)	112-120 (9)	113-120 (8)	C	0.92	0.51	100.00% (129/129)	100.00% (129/129)	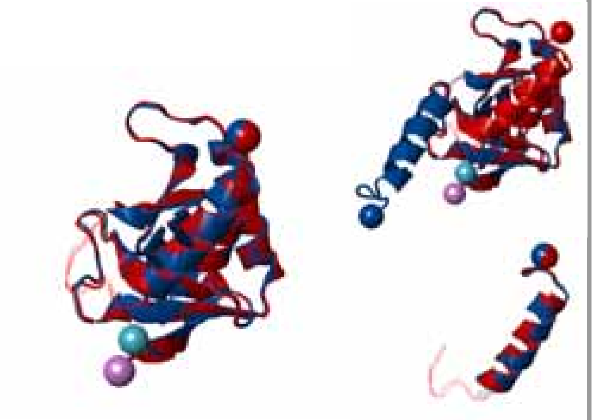 Staphylococcal nuclease<br>Staphylococcal nuclease dimer
	1sndA (129)	112-120 (9)	112-120 (9)	113-120 (8)						

174	1qd0A (128)	99-116 (18)	101-116 (16)	102-116 (15)	C	0.64	1.49	100.00% (102/102)	70.59% (72/102)	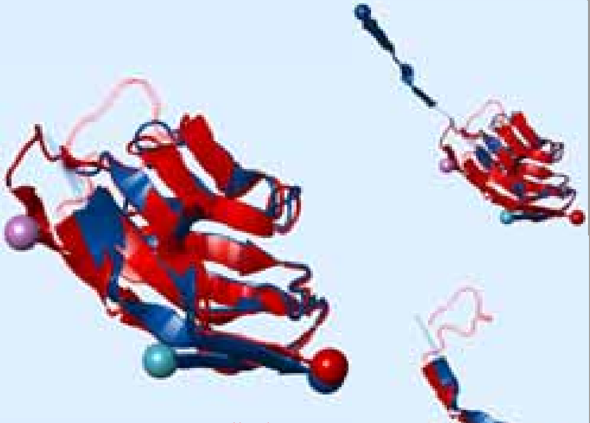 VHH-R2 ANTI-RR6 antibody<br>R9
	1sjvA (102)	91-98 (8)	93-98 (6)	102-116 (15)						
175	1pv3A (146)	941-951 (11)	941-951 (11)	944-948 (5)	N	0.42	2.02	91.55% (130/142)	83.80% (119/142)	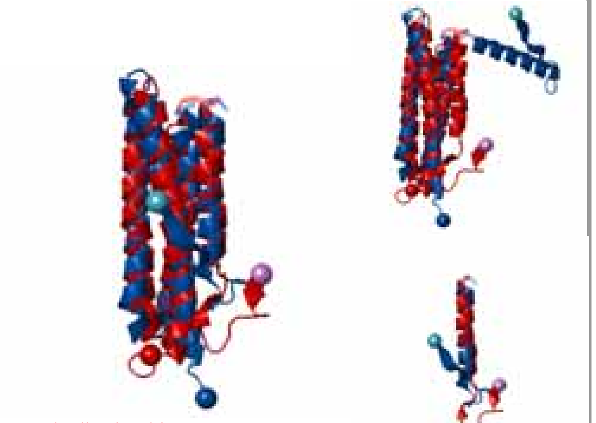 Focal adhesion kinase 1<br>Focal adhesion kinase 1
	1k04A (142)	940-950 (11)	940-950 (11)	944-948 (5)						
176	1b6bA (168)	50-90 (41)	50-82 (33)	50-80 (31)	N	0.38	2.03	81.43% (114/140)	15.71% (22/140)	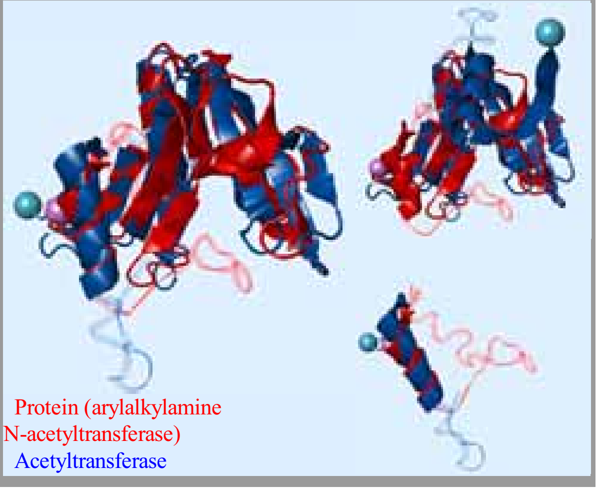 Protein (arylalkylamine N-acetyltransferase)<br>Acetyltransferase
	1y9wA (140)	23-51 (29)	23-42 (20)	50-80 (31)						

177	1bsrA (124)	12-27 (16)	12-22 (11)	15-22 (8)	N	0.50	1.55	91.06% (112/123)	31.71% (39/123)	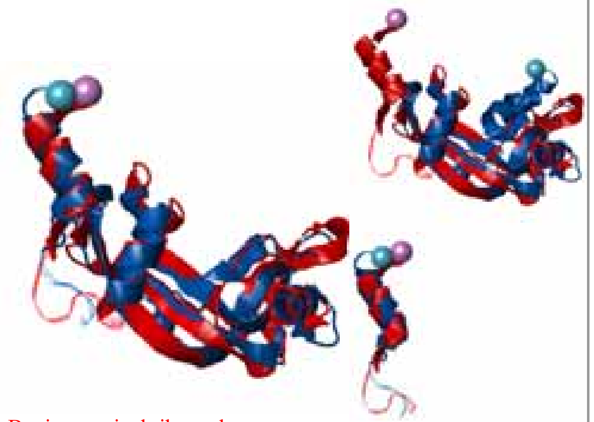 Bovine seminal ribonuclease<br>Angiogenin
	1awzA (123)	13-27 (15)	13-22 (10)	15-22 (8)						
178	1bsrA (124)	15-23 (9)	15-23 (9)	15-23 (9)	N	0.54	1.35	99.16% (118/119)	71.43% (85/119)	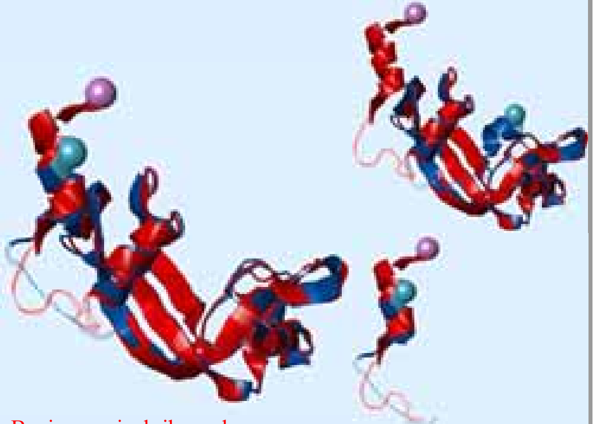 Bovine seminal ribonuclease<br>Ribonuclease 1
	1e21A (119)	15-23 (9)	15-23 (9)	15-23 (9)						
179	1bsrA (124)	9-24 (16)	9-24 (16)	13-24 (12)	N	0.57	1.89	89.52% (111/124)	33.06% (41/124)	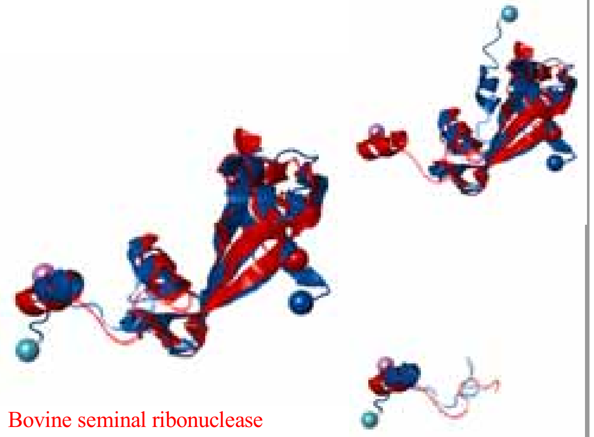 Bovine seminal ribonuclease<br>Angiogenin
	1gioA (125)	11-25 (15)	11-25 (15)	13-24 (12)						

180	1bsrA (124)	14-52 (39)	14-28 (15)	14-26 (13)	N	0.32	2.06	92.52% (99/107)	24.30% (26/107)	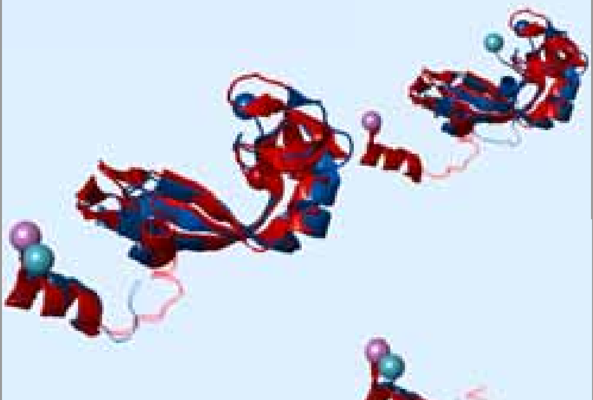<br>Bovine seminal ribonuclease<br>RC-rnase4
	1kvzA (107)	13-43 (31)	13-22 (10)	14-26 (13)						
181	1bsrA (124)	11-25 (15)	14-25 (12)	14-25 (12)	N	0.38	1.79	93.33% (98/105)	23.81% (25/105)	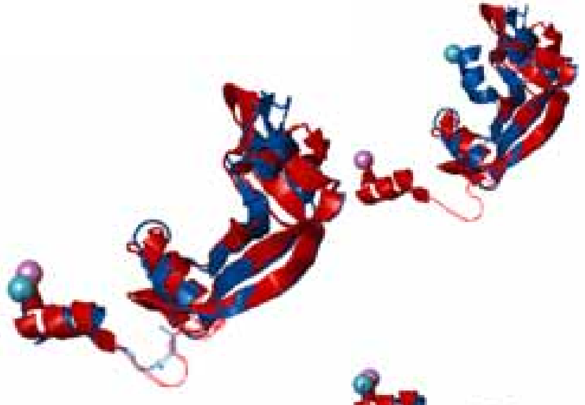<br>Bovine seminal ribonuclease<br>P-30 protein
	1pu3A (105)	10-19 (10)	13-19 (7)	14-25 (12)						
182	1bsrA (124)	15-25 (11)	15-24 (10)	15-23 (9)	N	0.49	1.19	95.83% (115/120)	42.50% (51/120)	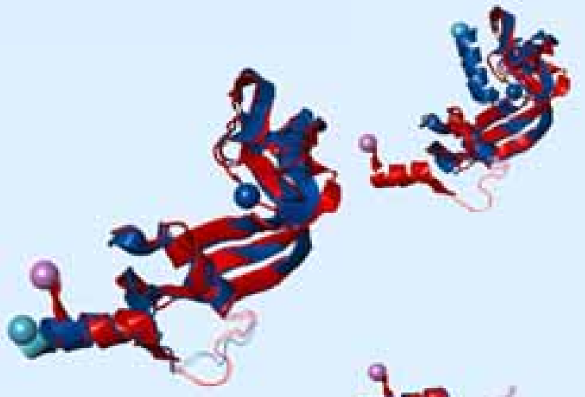<br>Bovine seminal ribonuclease<br>Protein (ribonuclease 4)
	1rnfA (120)	15-24 (10)	15-23 (9)	15-23 (9)						

183	1bsrA (124)	12-24 (13)	12-24 (13)	15-24 (10)	N	0.55	1.40	100.00% (124/124)	81.45% (101/124)	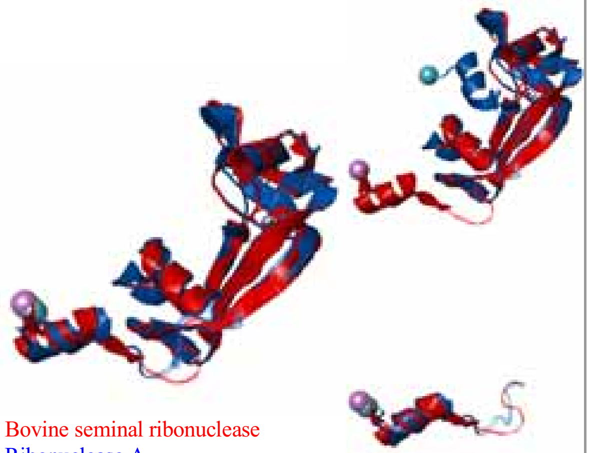 Bovine seminal ribonuclease<br>Ribonuclease A
	2aasA (124)	12-24 (13)	12-24 (13)	15-24 (10)						
184	1bsrA (124)	8-25 (18)	8-25 (18)	13-25 (13)	N	0.37	1.85	91.13% (113/124)	35.48% (44/124)	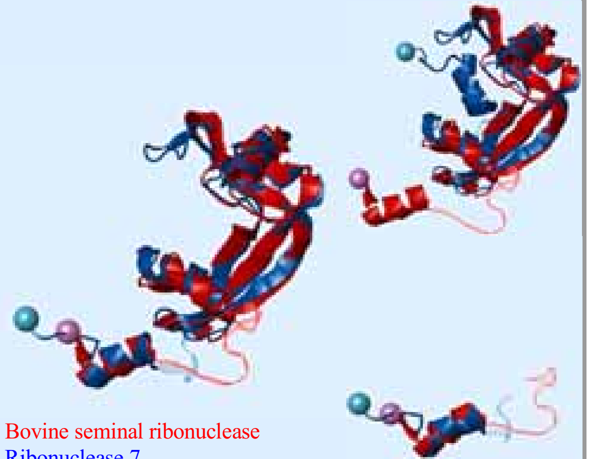 Bovine seminal ribonuclease<br>Ribonuclease 7
	2hkyA (129)	11-22 (12)	11-22 (12)	13-25 (13)						
185	1bsrA (124)	10-32 (23)	10-24 (15)	14-24 (11)	N	0.53	1.40	98.39% (122/124)	70.16% (87/124)	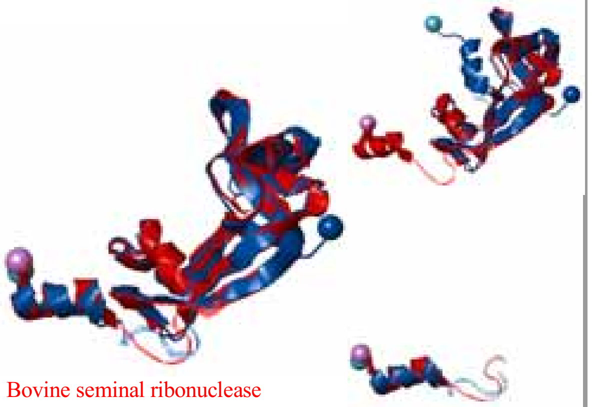 Bovine seminal ribonuclease<br>Pancreatic ribonuclease
	2k11A (127)	10-32 (23)	10-24 (15)	14-24 (11)						

186	1bsrA (124)	14-25 (12)	14-25 (12)	14-25 (12)	N	0.46	1.61	90.00% (99/110)	26.36% (29/110)	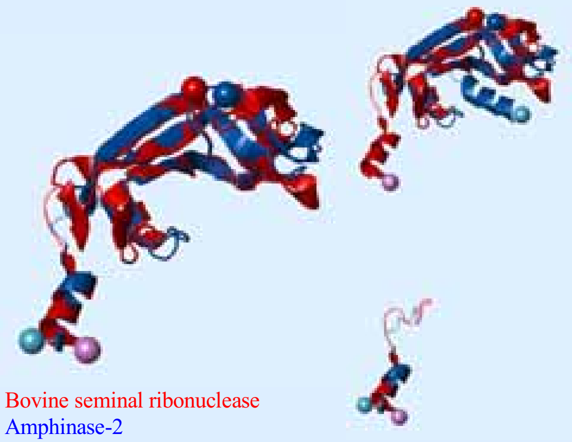 Bovine seminal ribonuclease Amphinase-2
	2p7sA (110)	17-25 (9)	17-25 (9)	14-25 (12)						
187	1cauA (181)	45-107 (63)	45-86 (42)	81-86 (6)	N	0.26	2.39	69.61% (126/181)	10.50% (19/181)	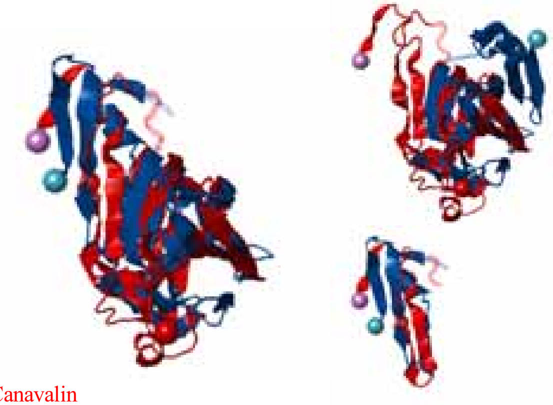 Canavalin DTDP-4-dehydrorhamnose 3,5-epimerase
	1dzrA (183)	4-72 (69)	4-47 (44)	81-86 (6)						
188	1cauA (181)	145-224 (80)	162-224 (63)	176-202 (27)	C	0.32	2.10	69.61% (126/181)	6.63% (12/181)	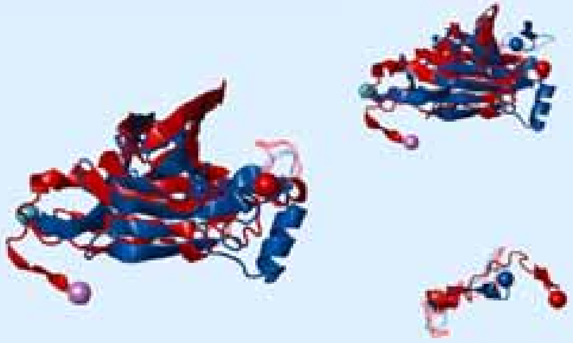 Canavalin Phosphoglucose isomerase
	1j3rB (185)	136-187 (52)	152-187 (36)	176-202 (27)						

189	1cauA (181)	177-194 (18)	177-194 (18)	177-181 (5)	C	0.39	2.44	86.16% (137/159)	11.95% (19/159)	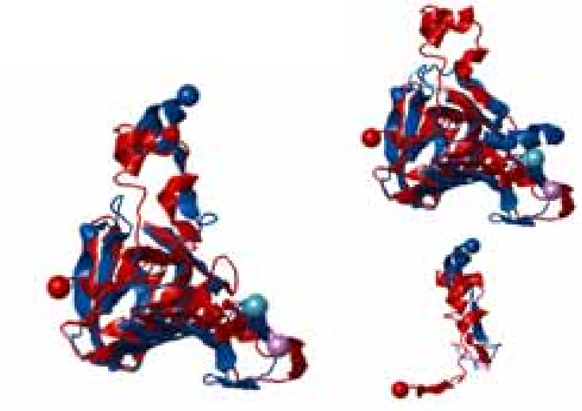 Canavalin<br>Auxin binding protein 1
	1lr5B (159)	133-149 (17)	133-149 (17)	177-181 (5)						
190	1cauA (181)	131-211 (81)	162-211 (50)	176-185 (10)	C	0.31	2.40	72.93% (132/181)	5.52% (10/181)	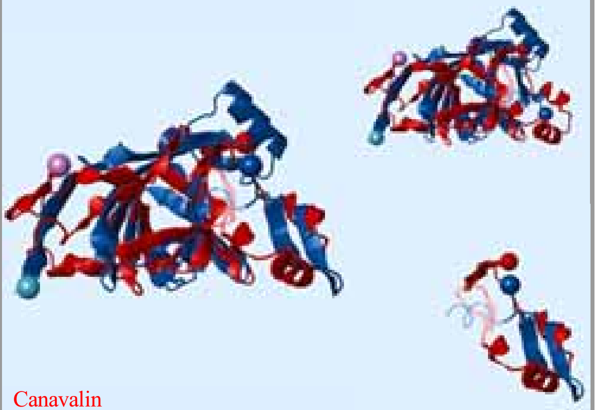 Canavalin<br>Glucose-6-phosphate isomerase
	1x7nA (189)	121-186 (66)	151-186 (36)	176-185 (10)						
191	1cauA (181)	45-86 (42)	45-86 (42)	76-86 (11)	N	0.31	2.24	70.17% (127/181)	9.94% (18/181)	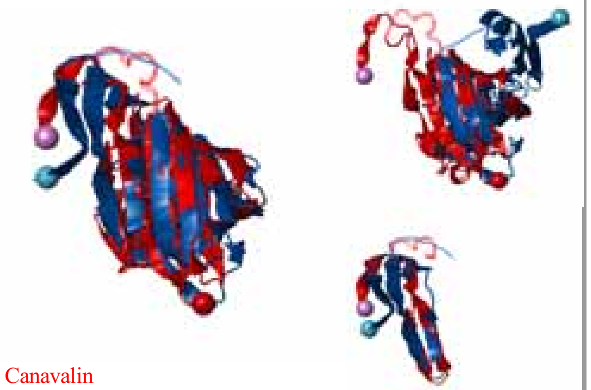 Canavalin<br>DTDP-4-dehydrorhamnose<br>3,5-epimerase
	2ixkA (184)	6-49 (44)	6-49 (44)	76-86 (11)						

192	1cauA (181)	50-101 (52)	50-86 (37)	74-86 (13)	N	0.28	2.32	84.33% (113/134)	11.19% (15/134)	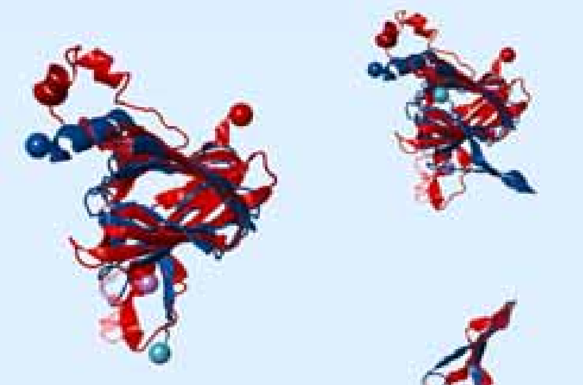 Canavalin DTDP-6-deoxy-3,4-keto-hexulose isomerase
	2pakB (134)	3-48 (46)	3-32 (30)	74-86 (13)						
193	1cauA (181)	44-101 (58)	44-86 (43)	84-86 (3)	N	0.30	2.14	82.96% (112/135)	9.63% (13/135)	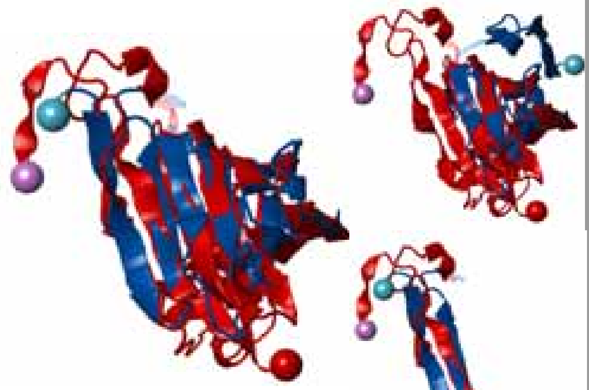 Canavalin DTDP-6-deoxy-3,4-keto-hexulose isomerase
	2pamA (135)	2-48 (47)	2-32 (31)	84-86 (3)						
194	1cm0B (161)	623-640 (18), 642-652 (11)	623-630 (8), 642-645 (4)	625-630 (6), 642-644 (3)	M	0.43	2.43	88.20% (142/161)	6.83% (11/161)	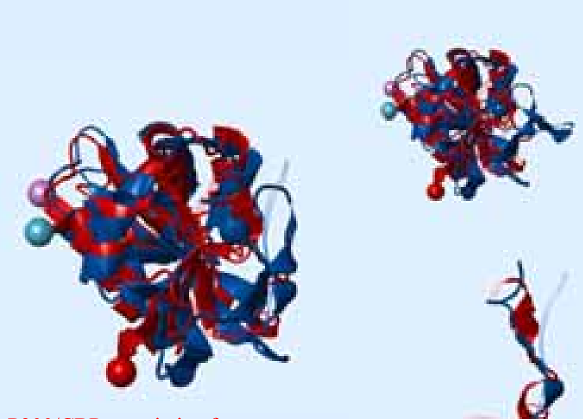 P300/CBP associating factor Aminoglycoside 2'-N-acetyltransferase
	1m44A (177)	132-150 (19), 154-181 (28)	132-142 (11), 154-156 (3)	625-630 (6), 642-644 (3)						

195	1cm0B (161)	623-651 (29)	623-651 (29)	625-642 (18)	C	0.48	2.14	89.66% (130/145)	8.97% (13/145)	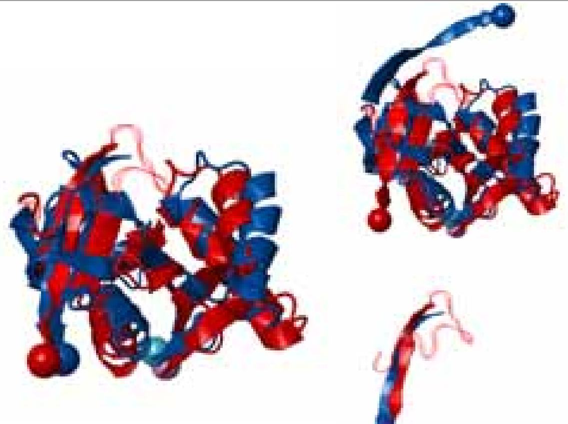 P300/CBP associating factor<br>Aminoglycoside 6'-N-acetyltransferase
	1s3zB (145)	133-144 (12)	133-144 (12)	625-642 (18)						
196	1cm0B (161)	623-651 (29)	623-651 (29)	625-642 (18)	C	0.45	2.15	84.97% (130/153)	7.84% (12/153)	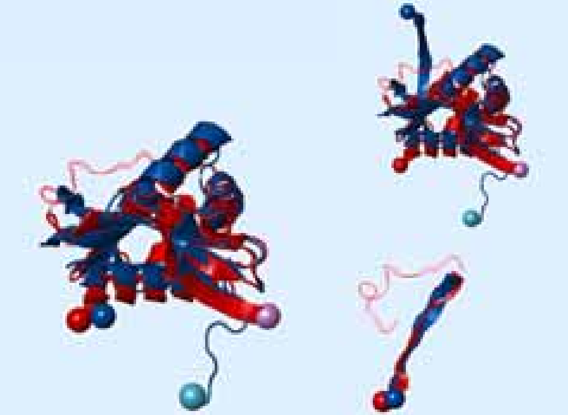 P300/CBP associating factor<br>Aminoglycoside 6'-N-acetyltransferase
	1s5kA (153)	133-144 (12)	133-144 (12)	625-642 (18)						
197	1cm0B (161)	623-651 (29)	624-651 (28)	625-642 (18)	C	0.47	2.10	88.89% (128/144)	8.33% (12/144)	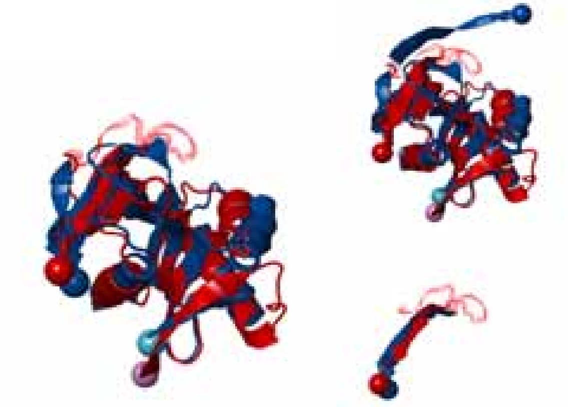 P300/CBP associating factor<br>Aminoglycoside 6'-N-acetyltransferase
	2vbqB (144)	133-144 (12)	134-144 (11)	625-642 (18)						

198	1d7eA (119)	5-43 (39)	5-40 (36)	17-28 (12)	N	0.39	2.12	85.09% (97/114)	4.39% (5/114)	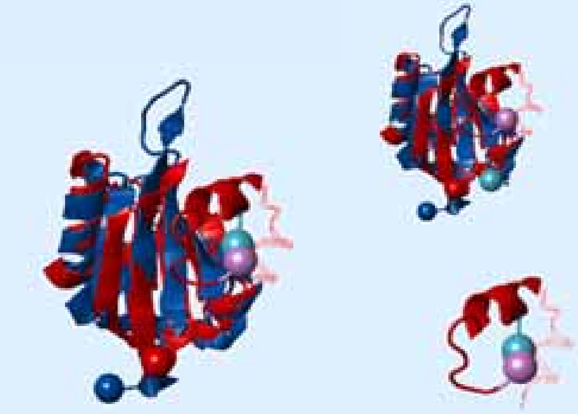 Photoactive yellow protein<br>Endothelial PAS domain protein 1
	1p97A (114)	2-22 (21)	2-19 (18)	17-28 (12)						
199	1d7eA (119)	6-31 (26)	6-23 (18)	18-23 (6)	N	0.53	2.24	90.76% (108/119)	13.45% (16/119)	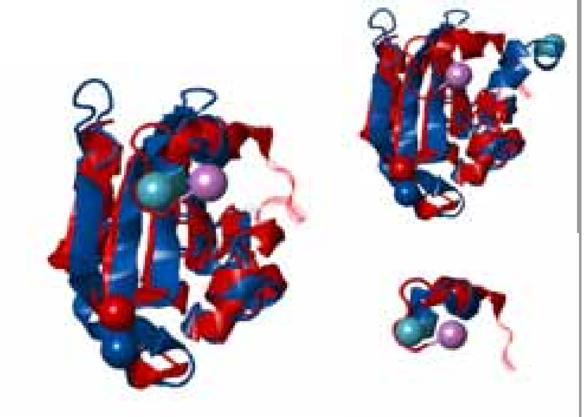 Photoactive yellow protein<br>Hypothetical protein yddU
	1s67L (119)	17-34 (18)	17-26 (10)	18-23 (6)						
200	1d7eA (119)	15-22 (8)	15-22 (8)	16-22 (7)	N	0.52	2.16	90.43% (104/115)	14.78% (17/115)	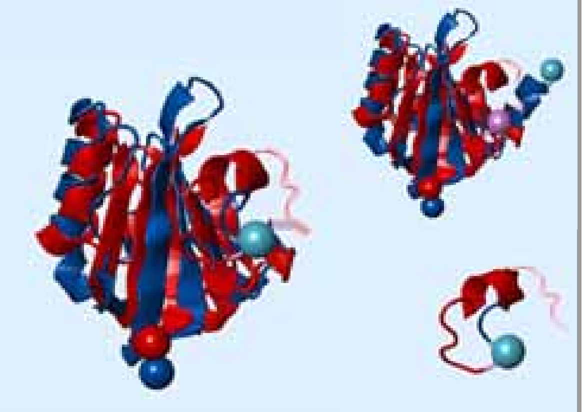 Photoactive yellow protein<br>Hypothetical protein yddU
	1s67U (115)	24-25 (2)	24-25 (2)	16-22 (7)						

201	1d7eA (119)	15-24 (10)	15-22 (8)	20-22 (3)	N	0.46	2.10	91.15% (103/113)	14.16% (16/113)	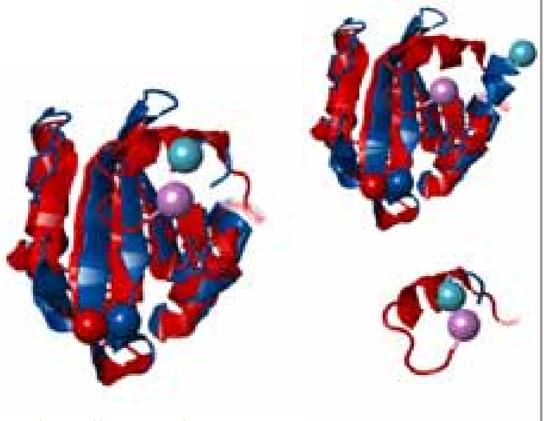 Photoactive yellow protein<br>Heme pas sensor protein
	1v9zA (113)	21-27 (7)	21-25 (5)	20-22 (3)						
202	1d7eA (119)	15-24 (10)	17-22 (6)	19-22 (4)	N	0.52	2.10	91.23% (104/114)	14.04% (16/114)	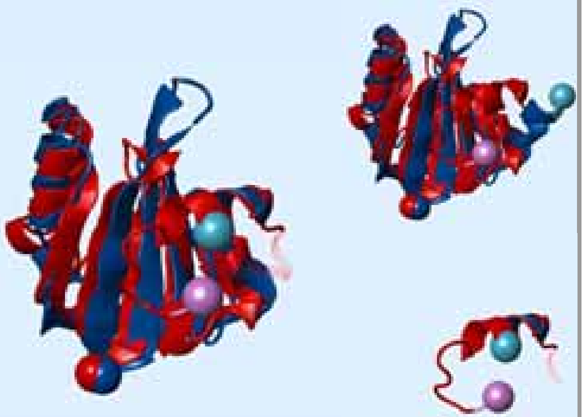 Photoactive yellow protein<br>Heme pas sensor protein
	1vb6A (114)	21-27 (7)	23-25 (3)	19-22 (4)						
203	1d7eA (119)	15-22 (8)	16-22 (7)	17-22 (6)	N	0.54	1.85	86.55% (103/119)	15.13% (18/119)	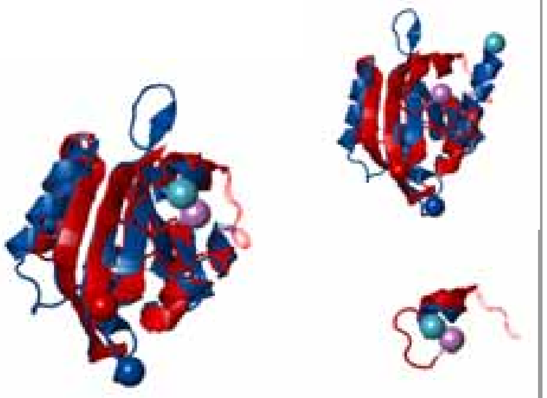 Photoactive yellow protein<br>Nitrogen fixation regulatory protein
	2gj3A (119)	28-30 (3)	29-30 (2)	17-22 (6)						

204	1d7eA (119)	9-22 (14)	17-22 (6)	18-22 (5)	N	0.56	1.88	88.24% (105/119)	16.81% (20/119)	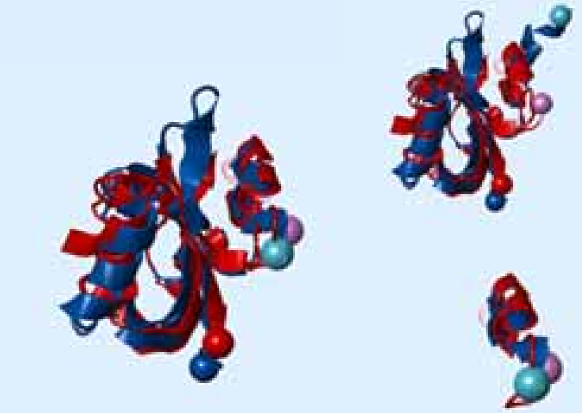 Photoactive yellow protein<br>Nitrogen fixation regulatory protein
	2gj3B (119)	22-30 (9)	29-30 (2)	18-22 (5)						
205	1dgmA (346)	27-53 (27),<br>36-64 (29)	27-53 (27),<br>36-63 (28)	27-38 (12),<br>53-63 (11)	M	0.46	2.34	92.64% (277/299)	16.39% (49/299)	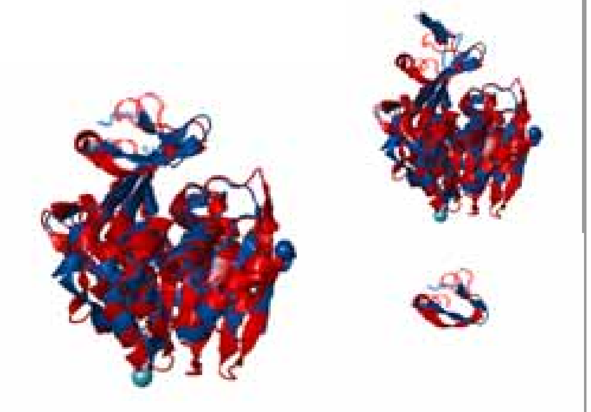 Adenosine kinase<br>Sugar kinase MJ0406
	2c4eA (299)	20-36 (17),<br>22-38 (17)	20-36 (17),<br>22-37 (16)	27-38 (12),<br>53-63 (11)						
206	1dmzA (158)	628-721 (94)	697-721 (25)	697-721 (25)	C	0.32	2.28	72.15% (114/158)	13.29% (21/158)	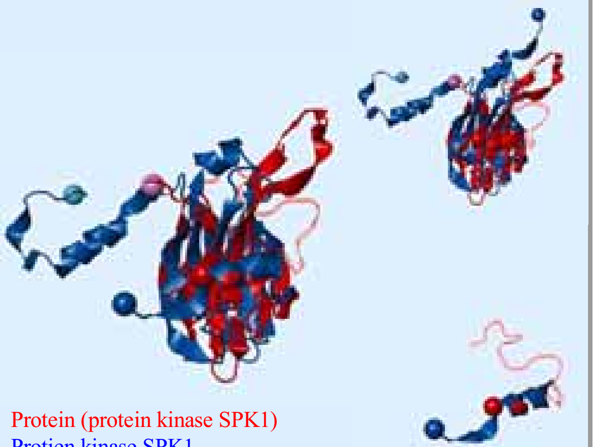 Protein (protein kinase SPK1)<br>Protien kinase SPK1
	1g3gA (164)	94-149 (56)	148-149 (2)	697-721 (25)						

207	1dmzA (158)	670-730 (61)	687-730 (44)	696-714 (19)	C	0.29	2.52	84.14% (122/145)	13.79% (20/145)	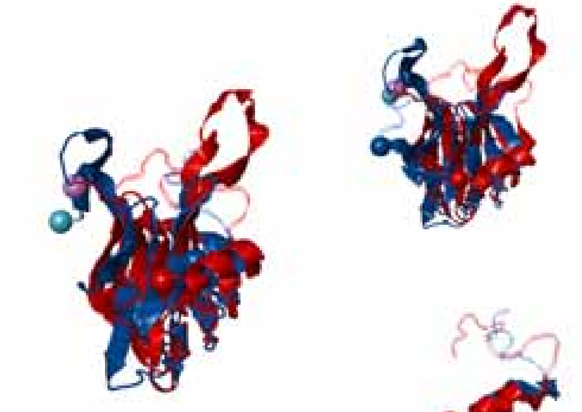 Protein (protein kinase SPK1) Ubiquitin ligase protein RNF8
	2cswA (145)	98-145 (48)	115-145 (31)	696-714 (19)						
208	1dmzA (158)	687-721 (35)	697-721 (25)	697-721 (25)	C	0.41	1.81	76.16% (115/151)	13.91% (21/151)	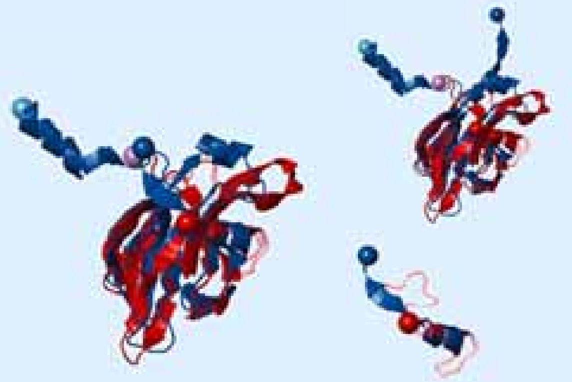 Protein (protein kinase SPK1) Serine/threonine-protein kinase RAD53
	2jqiA (151)	138-149 (12)	148-149 (2)	697-721 (25)						
209	1dmzA (158)	679-730 (52)	695-730 (36)	697-701 (5)	C	0.28	2.34	80.14% (113/141)	17.02% (24/141)	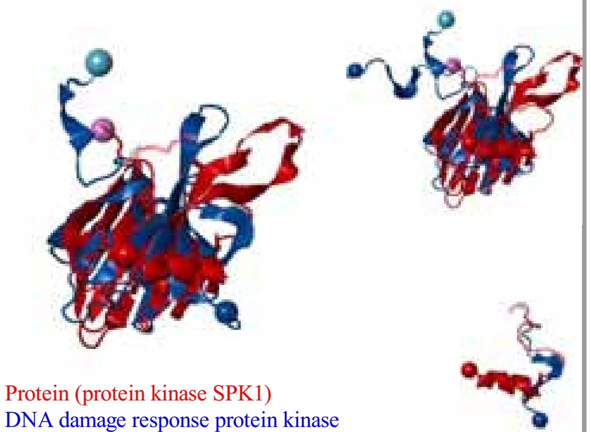 Protein (protein kinase SPK1) DNA damage response protein kinase DUN1
	2jqjA (141)	126-159 (34)	136-159 (24)	697-701 (5)						

210	1dz3A (123)	97-112 (16)	98-112 (15)	101-112 (12)	C	0.69	1.71	97.48% (116/119)	29.41% (35/119)	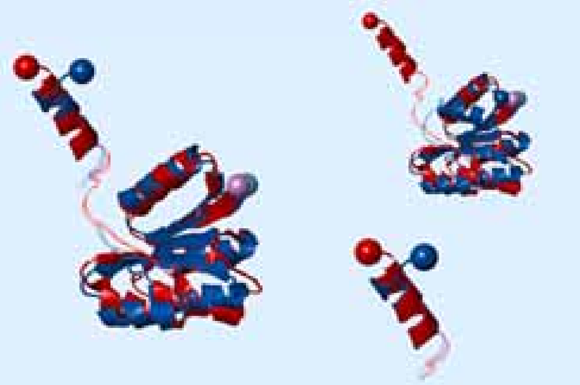<br>Stage 0 sporulation protein a<br>Sporulation response regulatory protein
	1natA (119)	95-109 (15)	96-109 (14)	101-112 (12)						
211	1dz3A (123)	100-113 (14)	100-112 (13)	101-112 (12)	C	0.70	1.67	96.67% (116/120)	29.17% (35/120)	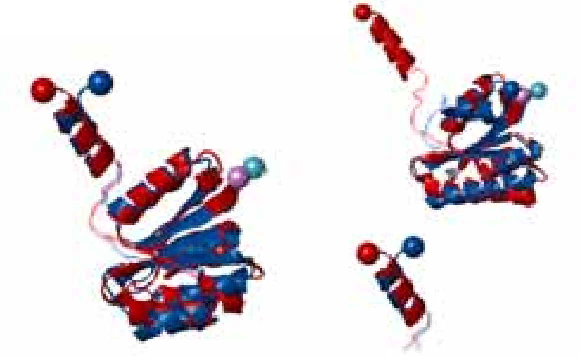<br>Stage 0 sporulation protein a<br>Sporulation initiation phosphotransferase F
	1peyC (120)	98-110 (13)	98-109 (12)	101-112 (12)						
212	1dz3A (123)	96-112 (17)	98-112 (15)	101-109 (9)	C	0.51	2.43	92.68% (114/123)	26.83% (33/123)	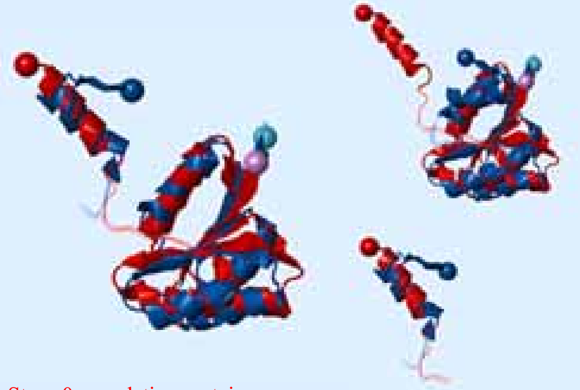<br>Stage 0 sporulation protein a<br>Sporulation initiation phosphotransferase F
	1puxA (124)	94-109 (16)	96-109 (14)	101-109 (9)						

213	1dz3A (123)	105-112 (8)	105-112 (8)	105-111 (7)	C	0.26	2.50	87.80% (108/123)	9.76% (12/123)	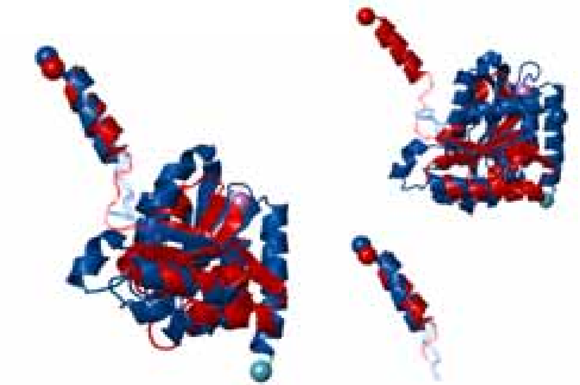 Stage 0 sporulation protein a Probable hydrogenase nickel incorporation pro
	2hf9A (211)	198-208 (11)	198-208 (11)	105-111 (7)						
214	1dz3A (123)	105-110 (6)	105-110 (6)	105-109 (5)	C	0.26	2.59	87.80% (108/123)	10.57% (13/123)	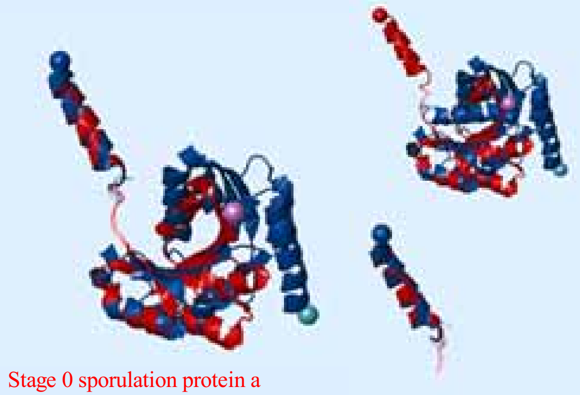 Stage 0 sporulation protein a Probable hydrogenase nickel incorporation pro
	2hf9B (209)	198-203 (6)	198-203 (6)	105-109 (5)						
215	1dz3A (123)	98-112 (15)	99-112 (14)	104-111 (8)	C	0.51	2.40	93.28% (111/119)	12.61% (15/119)	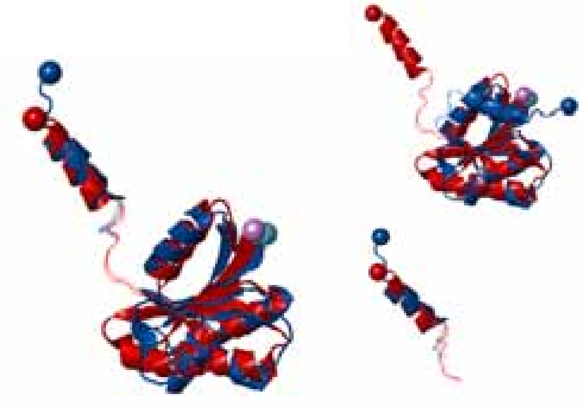 Stage 0 sporulation protein a TWO-component sensor kinase
	2j48A (119)	720-731 (12)	721-731 (11)	104-111 (8)						

216	1dz3A (123)	95-112 (18)	107-112 (6)	104-108 (5)	C	0.64	1.98	98.37% (121/123)	22.76% (28/123)	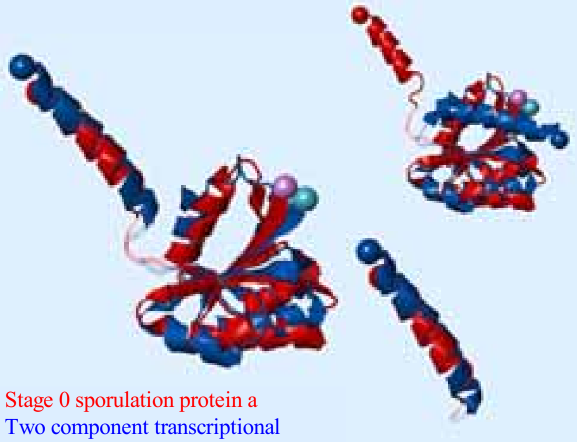 Stage 0 sporulation protein a<br>Two component transcriptional regulator, AraC
	3cu5B (129)	95-115 (21)	107-115 (9)	104-108 (5)						
217	1dz3A (123)	105-112 (8)	105-112 (8)	106-112 (7)	C	0.68	1.74	97.56% (120/123)	21.14% (26/123)	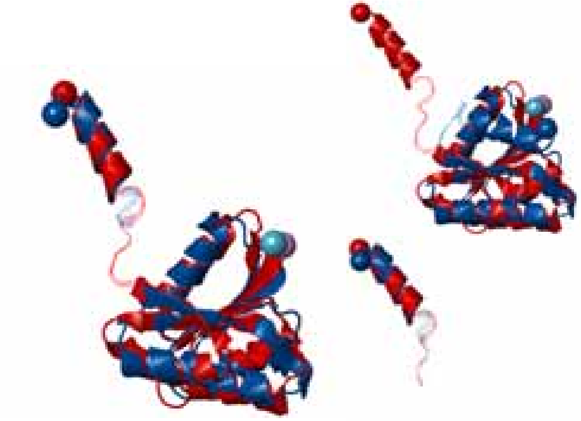 Stage 0 sporulation protein a<br>CHEY
	6chyA (128)	109-115 (7)	109-115 (7)	106-112 (7)						
218	1f0vA (124)	111-113 (3)	112-113 (2)	112-113 (2)	C	0.97	0.52	100.00% (124/124)	98.39% (122/124)	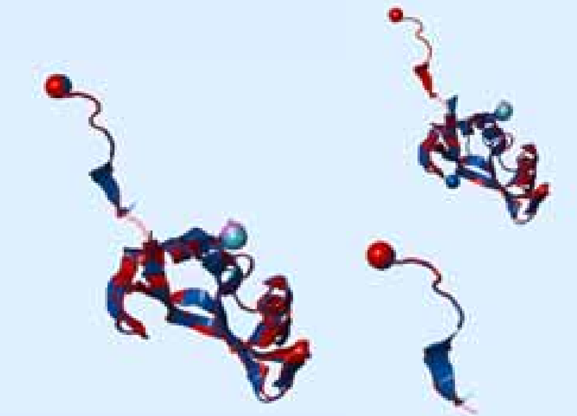 Ribonuclease A<br>Ribonuclease A
	1a5pA (124)	111-113 (3)	112-113 (2)	112-113 (2)						

219	1f0vA (124)	111-116 (6)	112-113 (2)	112-113 (2)	C	0.98	0.45	100.00% (124/124)	99.19% (123/124)	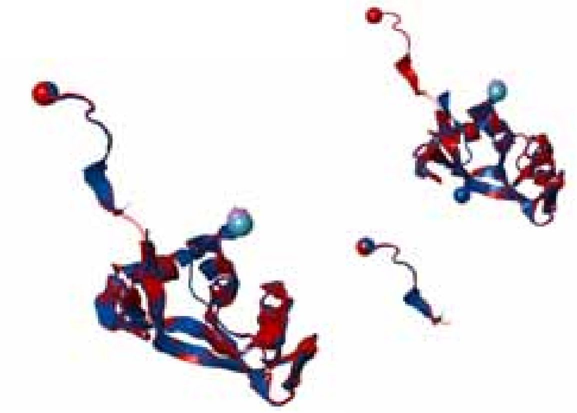 Ribonuclease A<br>Ribonuclease A
	1a5qA (124)	111-116 (6)	112-113 (2)	112-113 (2)						
220	1f0vA (124)	112-123 (12)	112-123 (12)	112-120 (9)	C	0.65	1.75	93.50% (115/123)	27.64% (34/123)	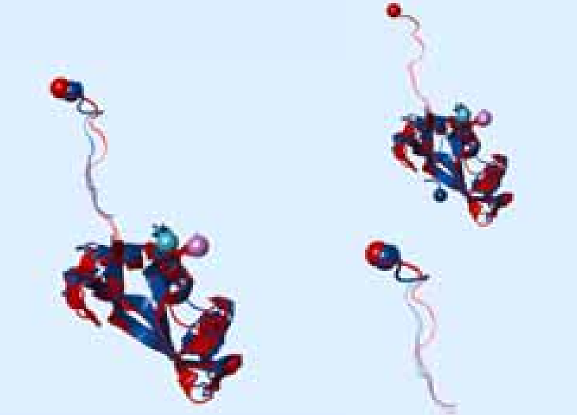 Ribonuclease A<br>Angiogenin
	1awzA (123)	109-122 (14)	109-122 (14)	112-120 (9)						
221	1f0vA (124)	111-113 (3)	111-113 (3)	112-113 (2)	C	0.97	0.56	100.00% (124/124)	94.35% (117/124)	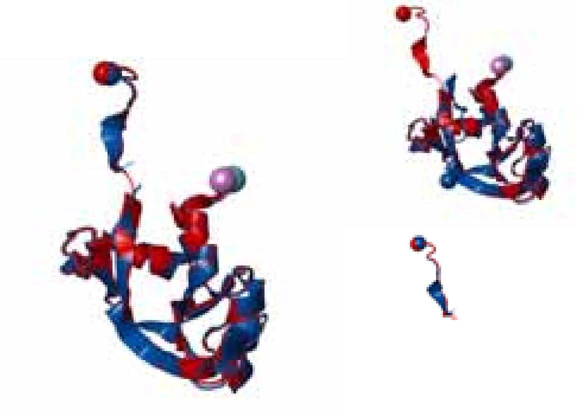 Ribonuclease A<br>Ribonuclease
	1b6vA (124)	111-113 (3)	111-113 (3)	112-113 (2)						

222	1f0vA (124)	111-113 (3)	111-113 (3)	112-113 (2)	C	0.95	0.66	100.00% (124/124)	99.19% (123/124)	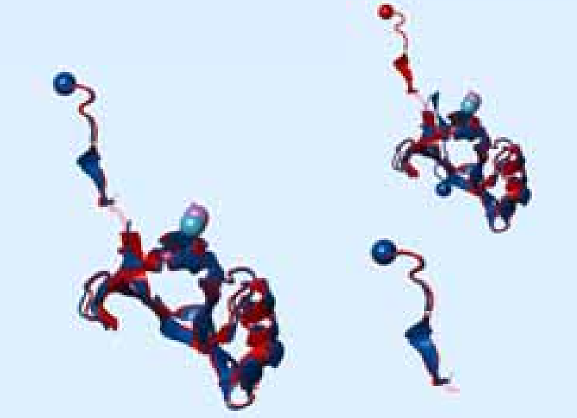 Ribonuclease A<br>Protein (ribonuclease A)
	1c8wA (124)	111-113 (3)	111-113 (3)	112-113 (2)						
223	1f0vA (124)	111-113 (3)	111-113 (3)	112-113 (2)	C	0.97	0.56	100.00% (124/124)	99.19% (123/124)	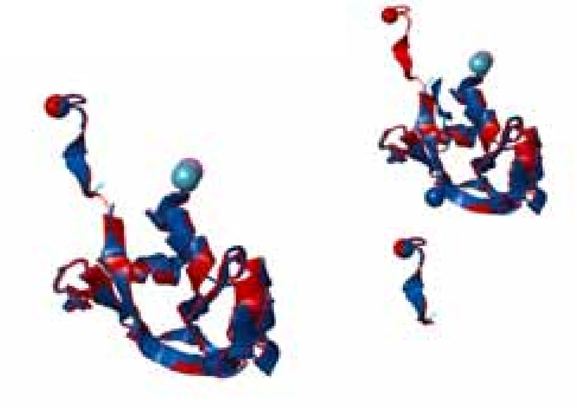 Ribonuclease A<br>Ribonuclease A
	1c9vA (124)	111-113 (3)	111-113 (3)	112-113 (2)						
224	1f0vA (124)	111-113 (3)	111-113 (3)	112-113 (2)	C	0.97	0.48	100.00% (124/124)	99.19% (123/124)	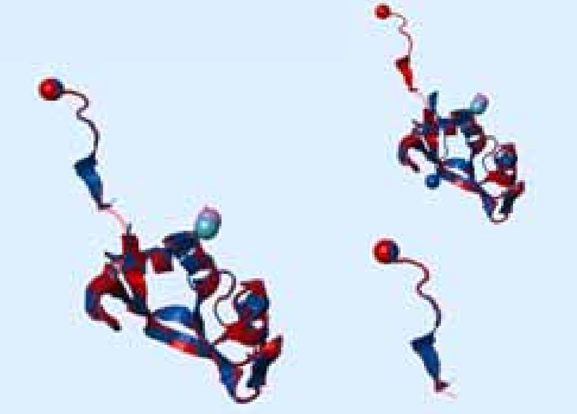 Ribonuclease A<br>Ribonuclease A
	1c9xA (124)	111-113 (3)	111-113 (3)	112-113 (2)						

225	1f0vA (124)	111-113 (3)	111-113 (3)	112-113 (2)	C	0.80	1.21	98.32% (117/119)	68.91% (82/119)	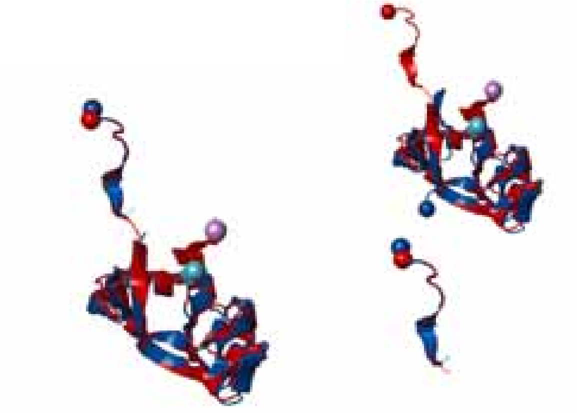
	1e21A (119)	111-113 (3)	111-113 (3)	112-113 (2)						Ribonuclease A<br>Ribonuclease 1
226	1f0vA (124)	111-113 (3)	111-113 (3)	112-113 (2)	C	0.97	0.56	100.00% (124/124)	99.19% (123/124)	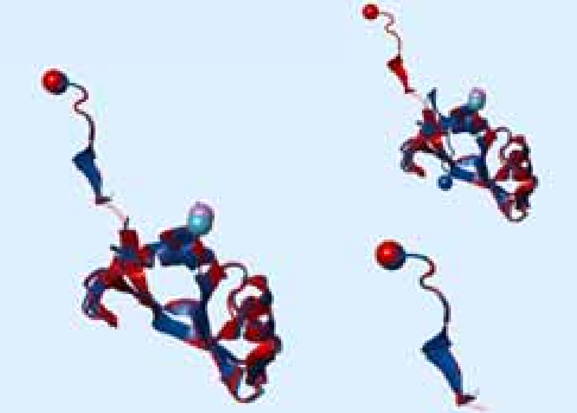
	1eicA (124)	111-113 (3)	111-113 (3)	112-113 (2)						Ribonuclease A<br>Ribonuclease A
227	1f0vA (124)	111-113 (3)	112-113 (2)	112-113 (2)	C	0.96	0.61	100.00% (124/124)	99.19% (123/124)	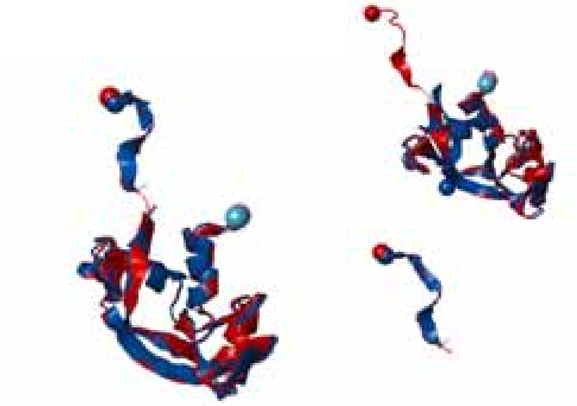
	1eidA (124)	111-113 (3)	112-113 (2)	112-113 (2)						Ribonuclease A<br>Ribonuclease A

228	1f0vA (124)	111-113 (3)	111-113 (3)	112-113 (2)	C	0.97	0.56	100.00% (124/124)	99.19% (123/124)	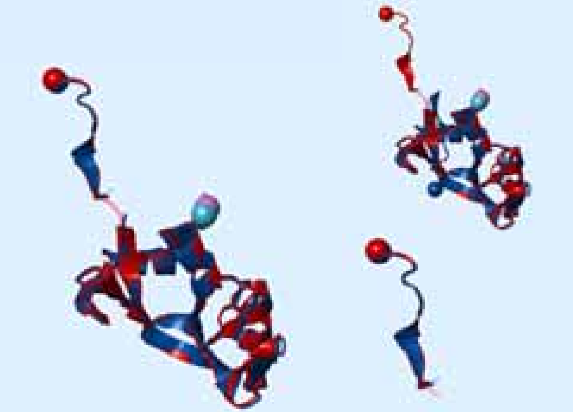 Ribonuclease A<br>Ribonuclease A
	1eieA (124)	111-113 (3)	111-113 (3)	112-113 (2)						
229	1f0vA (124)	112-123 (12)	112-115 (4)	112-115 (4)	C	0.56	1.95	89.52% (111/124)	33.06% (41/124)	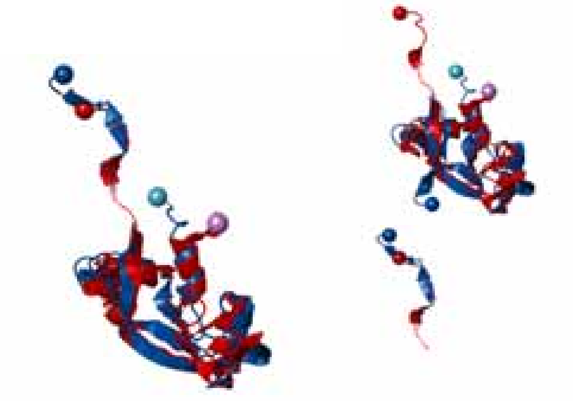 Ribonuclease A<br>Angiogenin
	1gioA (125)	110-120 (11)	110-111 (2)	112-115 (4)						
230	1f0vA (124)	111-116 (6)	111-116 (6)	112-115 (4)	C	0.74	1.40	95.12% (117/123)	41.46% (51/123)	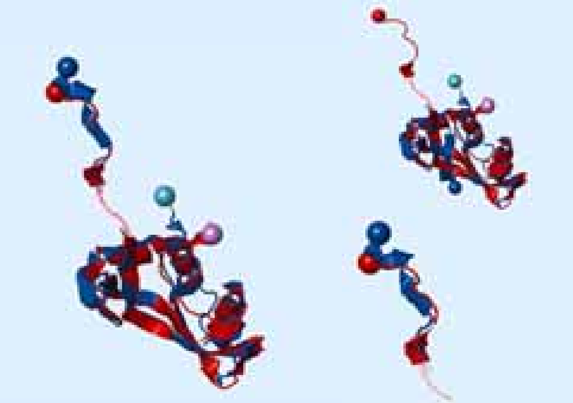 Ribonuclease A<br>Angiogenin
	1gv7A (123)	110-113 (4)	110-113 (4)	112-115 (4)						

231	1f0vA (124)	111-113 (3)	111-113 (3)	112-113 (2)	C	0.97	0.48	100.00% (124/124)	99.19% (123/124)	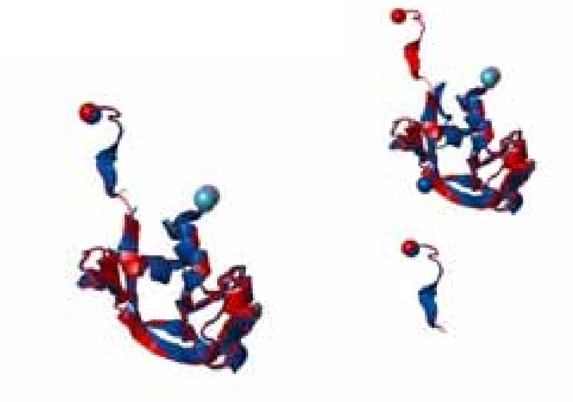 Ribonuclease A<br>Ribonuclease A
	1izpA (124)	111-113 (3)	111-113 (3)	112-113 (2)						
232	1f0vA (124)	111-113 (3)	111-113 (3)	112-113 (2)	C	0.97	0.52	100.00% (124/124)	99.19% (123/124)	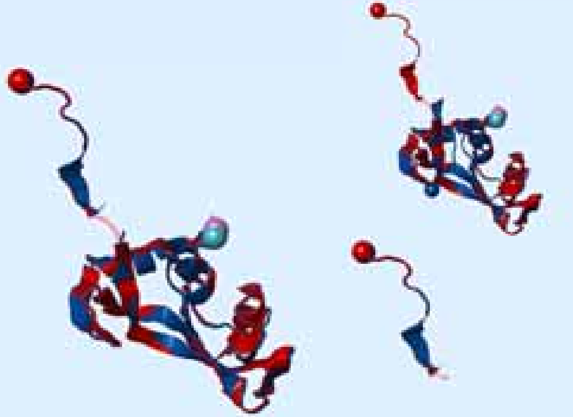 Ribonuclease A<br>Ribonuclease A
	1izqA (124)	111-113 (3)	111-113 (3)	112-113 (2)						
233	1f0vA (124)	111-113 (3)	111-113 (3)	112-113 (2)	C	0.91	0.96	100.00% (124/124)	99.19% (123/124)	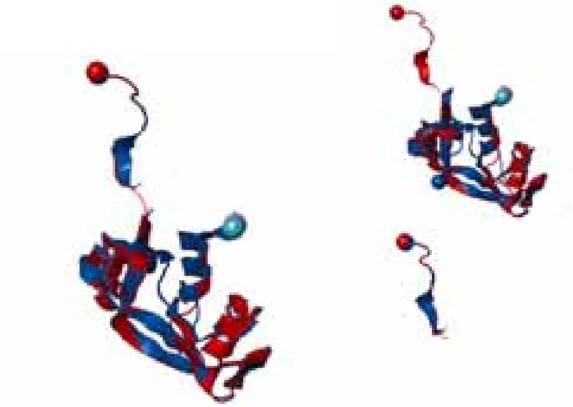 Ribonuclease A<br>Ribonuclease A
	1izrA (124)	111-113 (3)	111-113 (3)	112-113 (2)						

234	1f0vA (124)	111-113 (3)	112-113 (2)	112-113 (2)	C	0.77	0.71	100.00% (101/101)	100.00% (101/101)	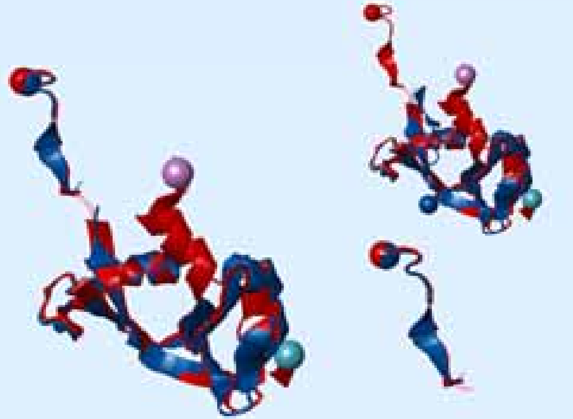 Ribonuclease A<br>Ribonuclease pancreatic
	1j80B (101)	111-113 (3)	112-113 (2)	112-113 (2)						
235	1f0vA (124)	110-116 (7)	111-116 (6)	111-116 (6)	C	0.91	0.73	98.39% (122/124)	97.58% (121/124)	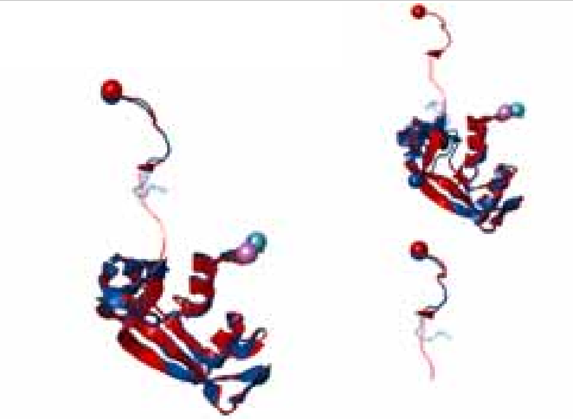 Ribonuclease A<br>Pancreatic ribonuclease a
	1kh8A (125)	110-116 (7)	111-116 (6)	111-116 (6)						
236	1f0vA (124)	104-123 (20)	112-119 (8)	112-116 (5)	C	0.49	1.91	89.72% (96/107)	23.36% (25/107)	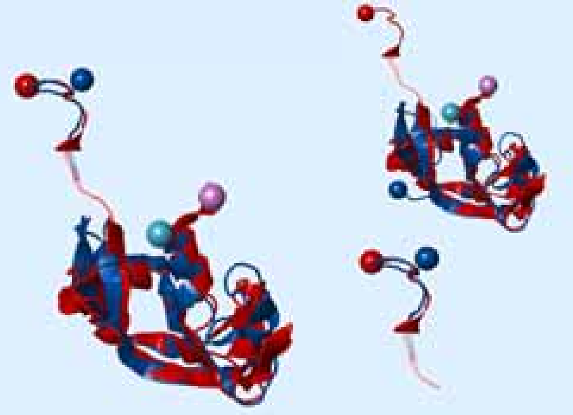 Ribonuclease A<br>RC-rnase4
	1kvzA (107)	86-103 (18)	94-99 (6)	112-116 (5)						

237	1f0vA (124)	105-119 (15)	109-116 (8)	112-115 (4)	C	0.55	1.68	92.38% (97/105)	24.76% (26/105)	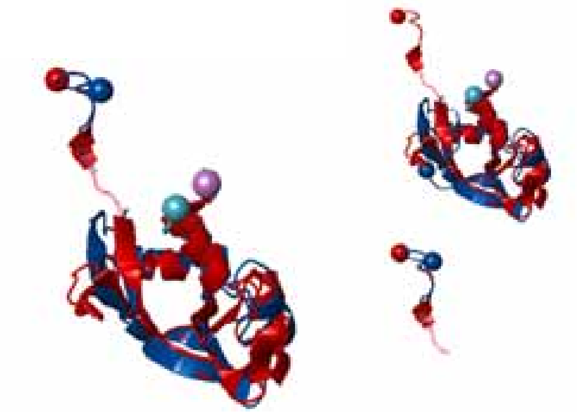 Ribonuclease A<br>P-30 protein
	1pu3A (105)	86-98 (13)	90-95 (6)	112-115 (4)						
238	1f0vA (124)	107-114 (8)	108-114 (7)	113-114 (2)	C	0.67	1.90	96.77% (120/124)	77.42% (96/124)	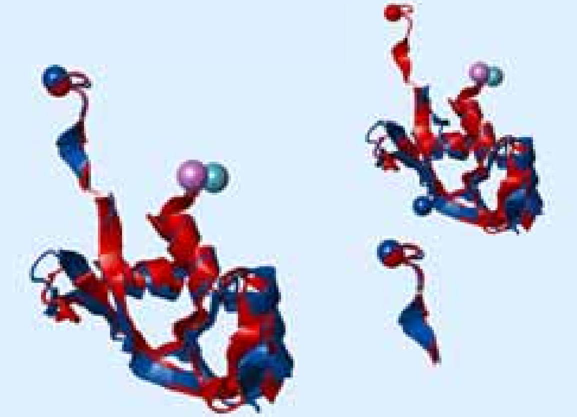 Ribonuclease A<br>Ribonuclease
	1qwqA (124)	107-114 (8)	108-114 (7)	113-114 (2)						
239	1f0vA (124)	111-113 (3)	112-113 (2)	112-113 (2)	C	0.97	0.47	100.00% (123/123)	100.00% (123/123)	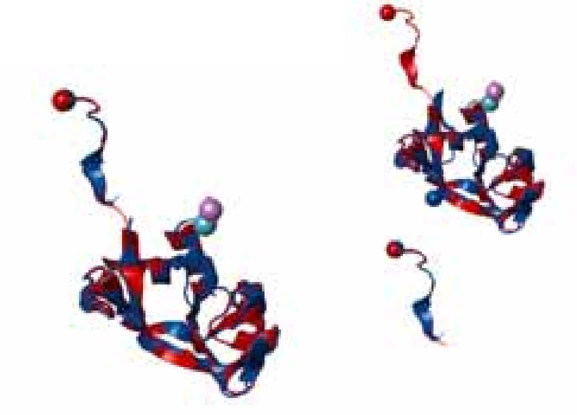 Ribonuclease A<br>Ribonuclease A
	1rasA (123)	111-113 (3)	112-113 (2)	112-113 (2)						

240	1f0vA (124)	112-115 (4)	112-115 (4)	112-113 (2)	C	0.79	1.20	97.50% (117/120)	43.33% (52/120)	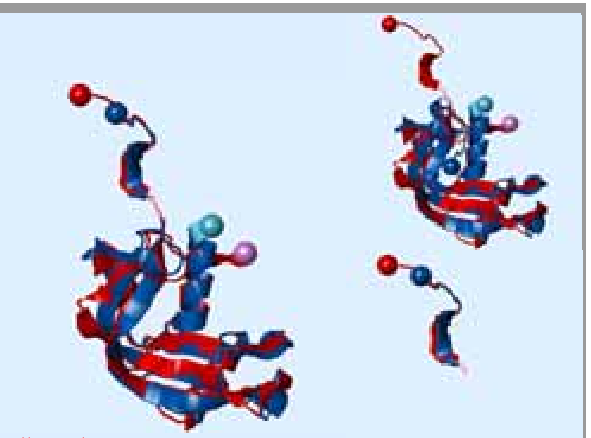<br>Ribonuclease A<br>Protein (ribonuclease 4)
	1rnfA (120)	109-112 (4)	109-112 (4)	112-113 (2)						
241	1f0vA (124)	108-116 (9)	111-114 (4)	112-114 (3)	C	0.89	1.04	100.00% (124/124)	66.94% (83/124)	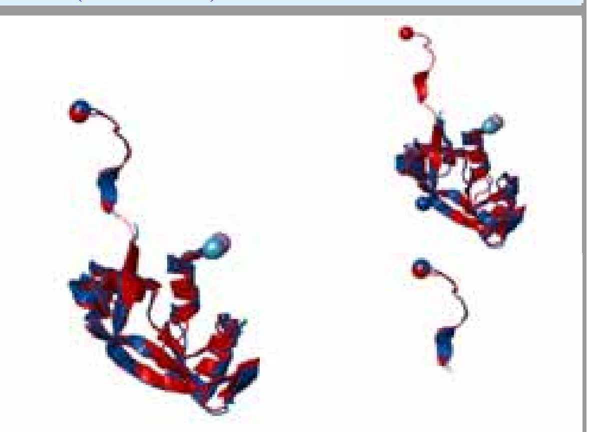<br>Ribonuclease A<br>Protein (ribonuclease)
	1rraA (124)	108-116 (9)	111-114 (4)	112-114 (3)						
242	1f0vA (124)	111-113 (3)	111-113 (3)	112-113 (2)	C	0.94	0.74	100.00% (124/124)	99.19% (123/124)	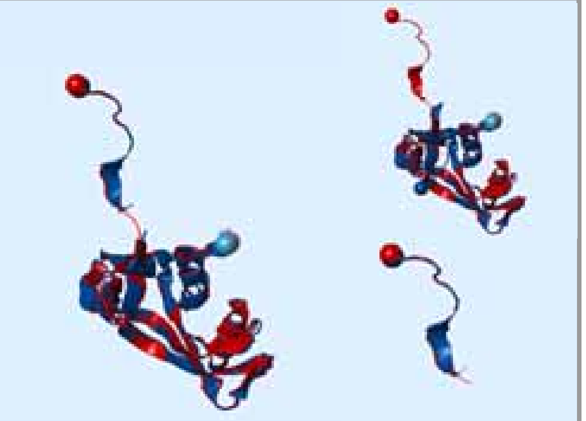<br>Ribonuclease A<br>Ribonuclease pancreatic
	1ymnA (124)	111-113 (3)	111-113 (3)	112-113 (2)						

243	1f0vA (124)	111-113 (3)	111-113 (3)	112-113 (2)	C	0.97	0.52	100.00% (124/124)	99.19% (123/124)	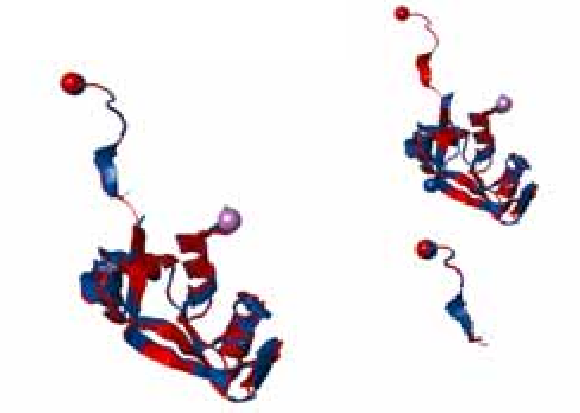 Ribonuclease A
	1ymrA (124)	111-113 (3)	111-113 (3)	112-113 (2)						Ribonuclease pancreatic
244	1f0vA (124)	111-113 (3)	111-113 (3)	112-113 (2)	C	0.97	0.55	100.00% (124/124)	99.19% (123/124)	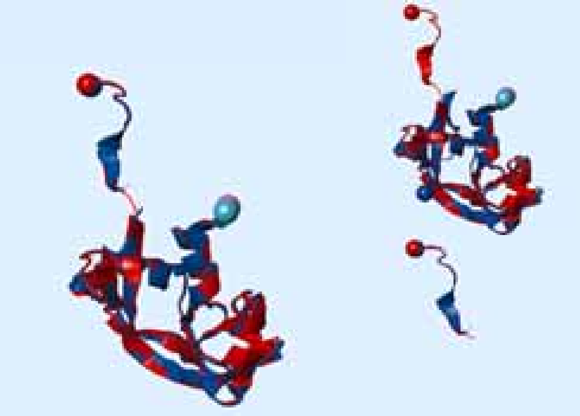 Ribonuclease A
	1ymwA (124)	111-113 (3)	111-113 (3)	112-113 (2)						Ribonuclease pancreatic
245	1f0vA (124)	104-116 (13)	110-115 (6)	112-115 (4)	C	0.54	1.78	93.27% (97/104)	26.92% (28/104)	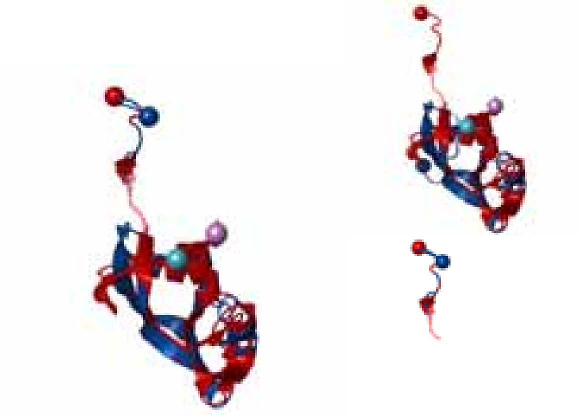 Ribonuclease A
	1yv4A (104)	84-94 (11)	90-93 (4)	112-115 (4)						P-30 protein

246	1f0vA (124)	107-113 (7)	111-113 (3)	112-113 (2)	C	0.88	1.13	100.00% (124/124)	100.00% (124/124)	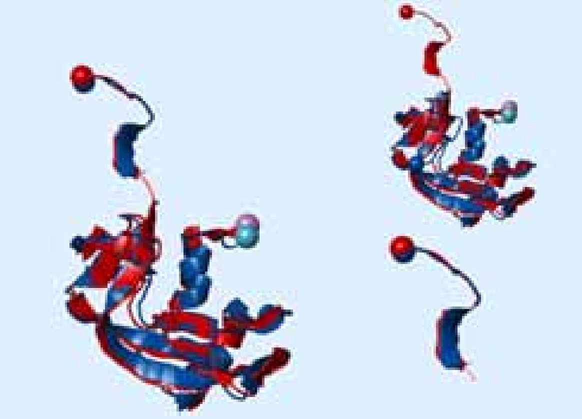<br>Ribonuclease A<br>Ribonuclease A
	2aasA (124)	107-113 (7)	111-113 (3)	112-113 (2)						
247	1f0vA (124)	111-113 (3)	111-113 (3)	112-113 (2)	C	0.85	1.13	100.00% (124/124)	70.16% (87/124)	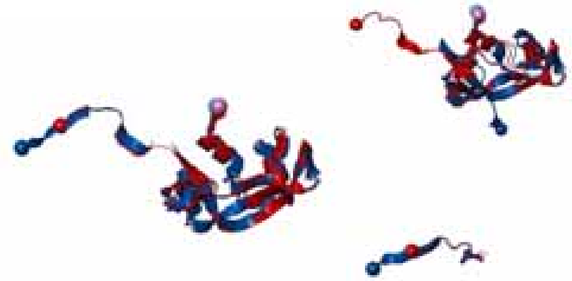<br>Ribonuclease A<br>Ribonuclease
	2e0jA (128)	111-113 (3)	111-113 (3)	112-113 (2)						
248	1f0vA (124)	111-113 (3)	111-113 (3)	112-113 (2)	C	0.90	0.86	100.00% (124/124)	69.35% (86/124)	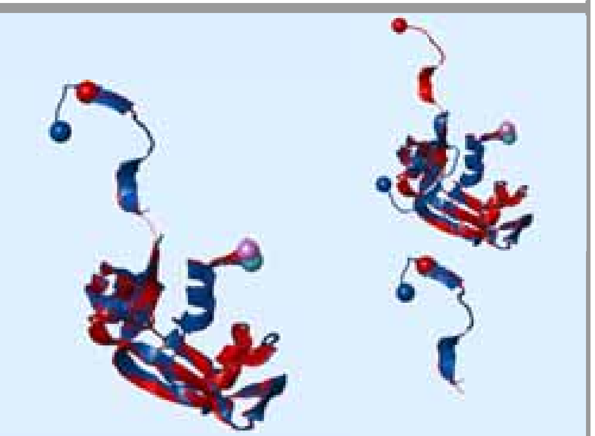<br>Ribonuclease A<br>Ribonuclease
	2e0lA (128)	111-113 (3)	111-113 (3)	112-113 (2)						

249	1f0vA (124)	111-113 (3)	111-113 (3)	112-113 (2)	C	0.90	0.85	100.00% (124/124)	69.35% (86/124)	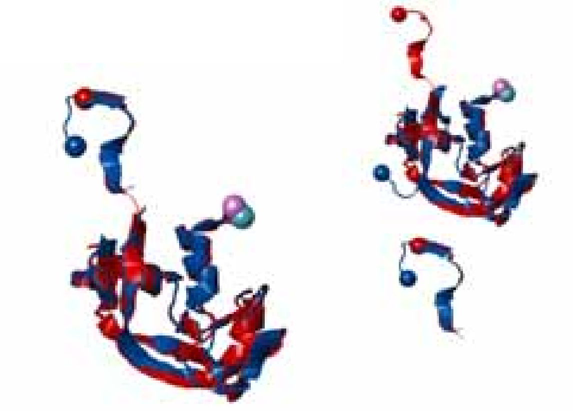 Ribonuclease A<br>Ribonuclease
	2e0mA (128)	111-113 (3)	111-113 (3)	112-113 (2)						
250	1f0vA (124)	112-113 (2)	112-113 (2)	112-113 (2)	C	0.89	0.92	99.19% (123/124)	68.55% (85/124)	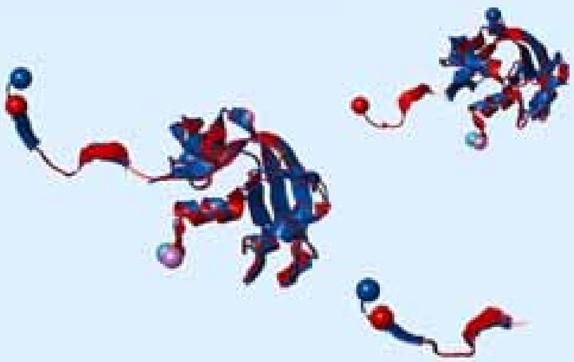 Ribonuclease A<br>Ribonuclease
	2e0oA (126)	112-113 (2)	112-113 (2)	112-113 (2)						
251	1f0vA (124)	112-113 (2)	112-113 (2)	112-113 (2)	C	0.89	1.01	100.00% (124/124)	69.35% (86/124)	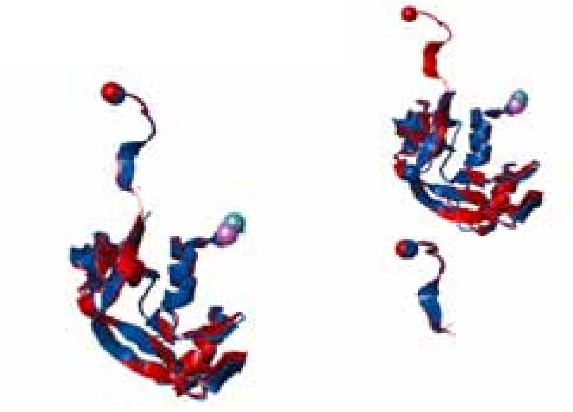 Ribonuclease A<br>Ribonuclease
	2e0oB (125)	112-113 (2)	112-113 (2)	112-113 (2)						

252	1f0vA (124)	105-123 (19)	112-123 (12)	112-116 (5)	C	0.58	1.80	90.32% (112/124)	41.13% (51/124)	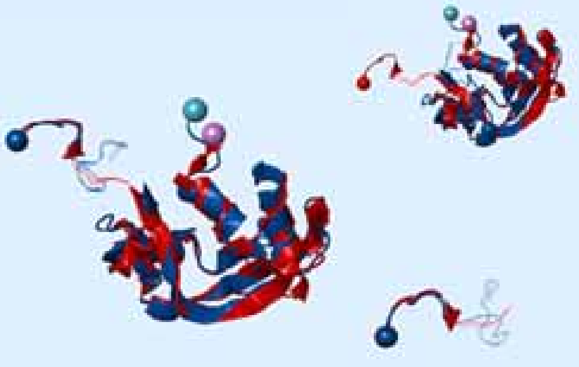 Ribonuclease A<br>Ribonuclease 7
	2hkyA (129)	101-127 (27)	108-127 (20)	112-116 (5)						
253	1f0vA (124)	110-116 (7)	112-115 (4)	112-115 (4)	C	0.54	1.75	93.27% (97/104)	27.88% (29/104)	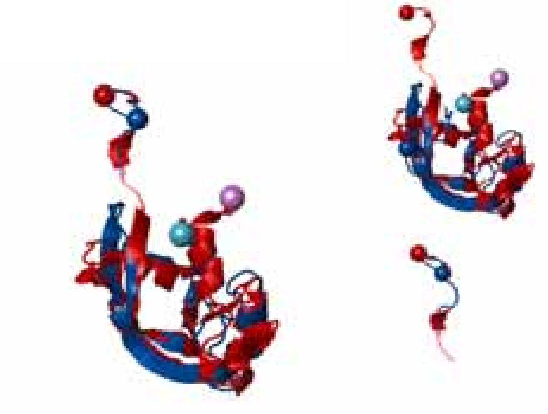 Ribonuclease A<br>P-30 protein
	2i5sX (104)	90-94 (5)	92-93 (2)	112-115 (4)						
254	1f0vA (124)	109-114 (6)	109-114 (6)	112-113 (2)	C	0.76	1.56	99.19% (123/124)	69.35% (86/124)	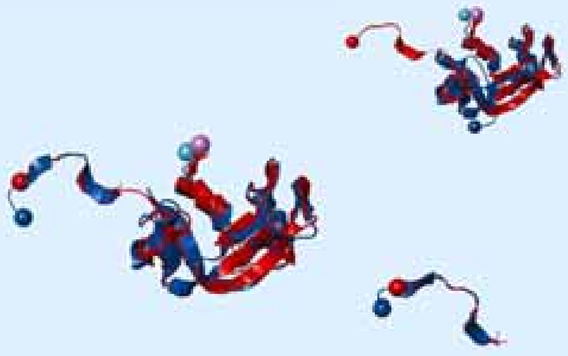 Ribonuclease A<br>Pancreatic ribonuclease
	2k11A (127)	109-114 (6)	109-114 (6)	112-113 (2)						

255	1f0vA (124)	111-113 (3)	112-113 (2)	112-113 (2)	C	0.97	0.52	100.00% (124/124)	99.19% (123/124)	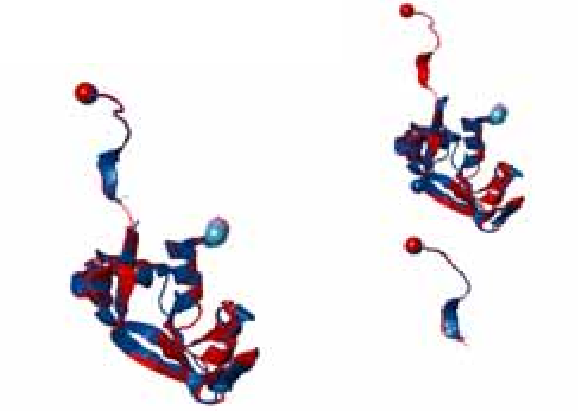 Ribonuclease A<br>Ribonuclease pancreatic
	2nuiA (124)	111-113 (3)	112-113 (2)	112-113 (2)						
256	1f0vA (124)	111-113 (3)	112-113 (2)	112-113 (2)	C	0.95	0.65	100.00% (124/124)	98.39% (122/124)	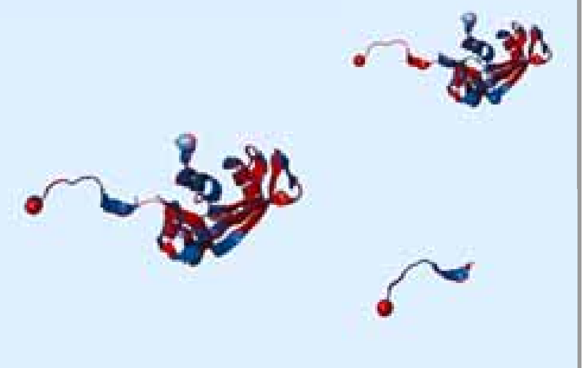 Ribonuclease A<br>Ribonuclease pancreatic
	2op2A (124)	111-113 (3)	112-113 (2)	112-113 (2)						
257	1f0vA (124)	110-116 (7)	111-116 (6)	111-115 (5)	C	0.55	1.63	89.91% (98/109)	26.61% (29/109)	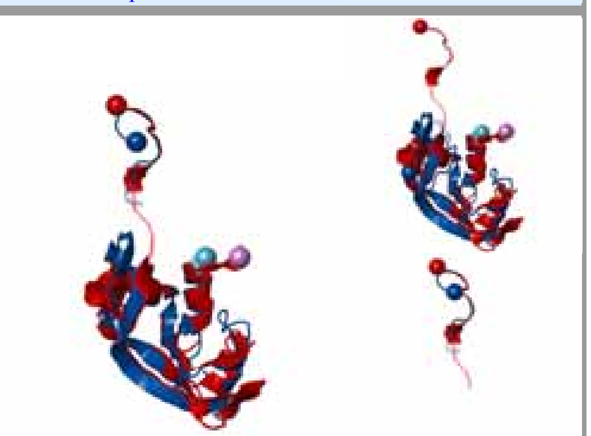 Ribonuclease A<br>Recombinant amphinase-2
	2p6zB (109)	100-104 (5)	101-104 (4)	111-115 (5)						

258	1f0vA (124)	111-115 (5)	111-113 (3)	112-113 (2)	C	0.90	0.81	99.19% (123/124)	69.35% (86/124)	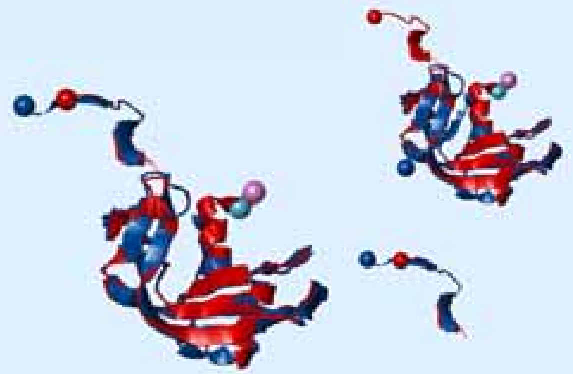 Ribonuclease A<br>Ribonuclease pancreatic
	2q4gX (126)	111-115 (5)	111-113 (3)	112-113 (2)						
259	1f0vA (124)	111-113 (3)	112-113 (2)	112-113 (2)	C	0.97	0.49	100.00% (124/124)	99.19% (123/124)	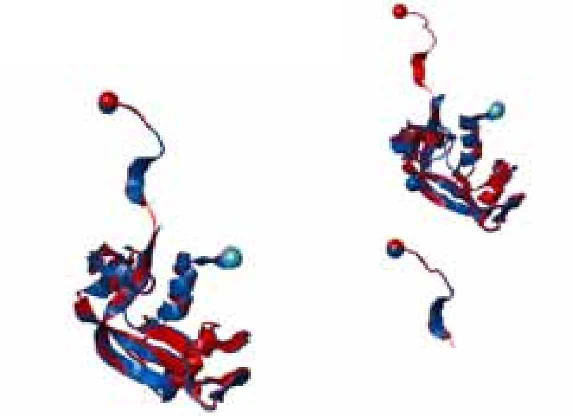 Ribonuclease A<br>Ribonuclease pancreatic
	3dh6A (124)	111-113 (3)	112-113 (2)	112-113 (2)						
260	1f0vA (124)	111-113 (3)	111-113 (3)	112-113 (2)	C	0.97	0.50	100.00% (124/124)	99.19% (123/124)	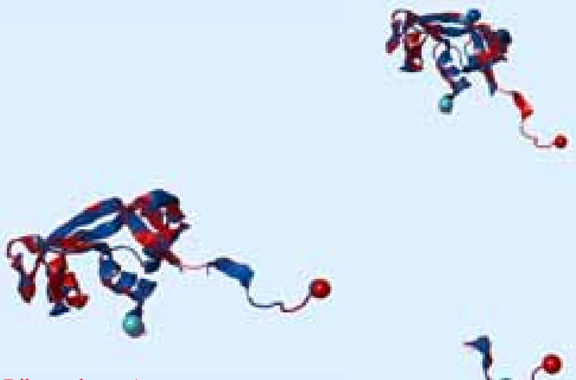 Ribonuclease A<br>Ribonuclease pancreatic
	3di7A (124)	111-113 (3)	111-113 (3)	112-113 (2)						

261	1f0vA (124)	111-113 (3)	112-113 (2)	112-113 (2)	C	0.98	0.47	100.00% (124/124)	99.19% (123/124)	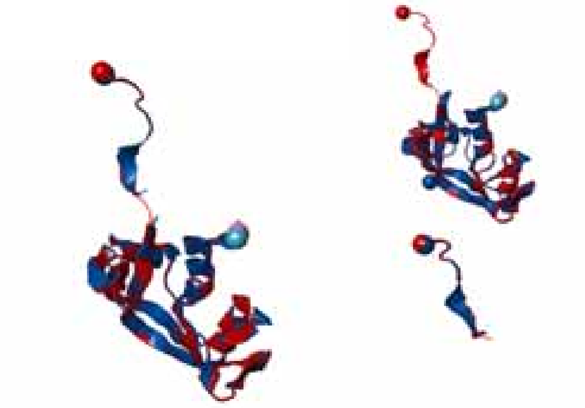 Ribonuclease A<br>Ribonuclease pancreatic
	3di8A (124)	111-113 (3)	112-113 (2)	112-113 (2)						
262	1f0vA (124)	111-113 (3)	111-113 (3)	112-113 (2)	C	0.97	0.55	100.00% (124/124)	99.19% (123/124)	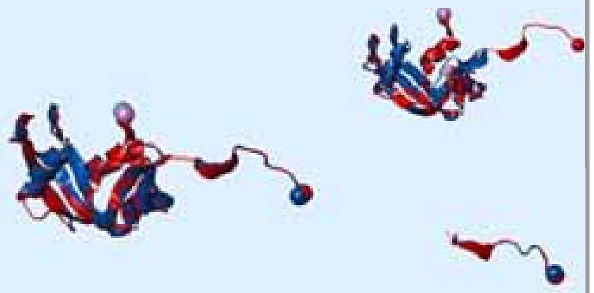 Ribonuclease A<br>Ribonuclease pancreatic
	3di9A (124)	111-113 (3)	111-113 (3)	112-113 (2)						
263	1f0vA (124)	111-113 (3)	112-113 (2)	112-113 (2)	C	0.97	0.51	100.00% (124/124)	99.19% (123/124)	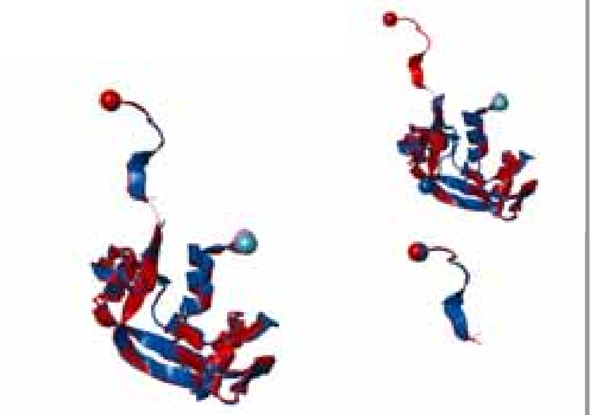 Ribonuclease A<br>Ribonuclease pancreatic
	3dibA (124)	111-113 (3)	112-113 (2)	112-113 (2)						

264	1f0vA (124)	111-113 (3)	111-113 (3)	112-113 (2)	C	0.97	0.48	100.00% (124/124)	99.19% (123/124)	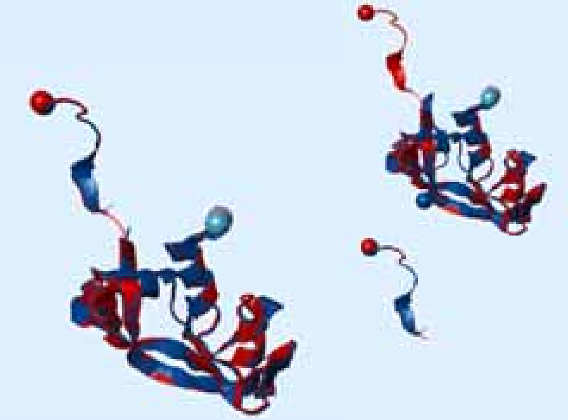 Ribonuclease A<br>Ribonuclease pancreatic
	3dicA (124)	111-113 (3)	111-113 (3)	112-113 (2)						
265	1f0vA (124)	111-113 (3)	112-113 (2)	112-113 (2)	C	0.98	0.46	100.00% (124/124)	99.19% (123/124)	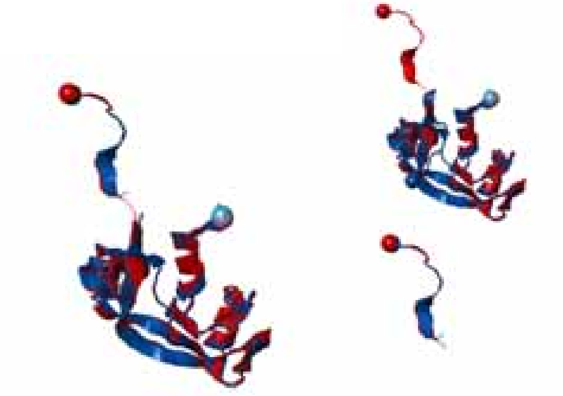 Ribonuclease A<br>Ribonuclease A
	3rsdA (124)	111-113 (3)	112-113 (2)	112-113 (2)						
266	1f0vA (124)	111-113 (3)	111-113 (3)	112-113 (2)	C	0.98	0.48	100.00% (124/124)	97.58% (121/124)	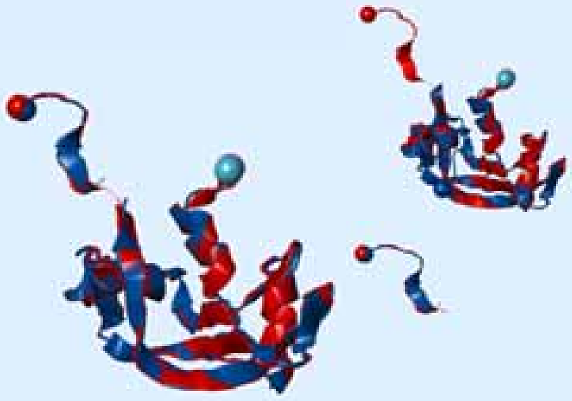 Ribonuclease A<br>Ribonuclease A
	3rskA (124)	111-113 (3)	111-113 (3)	112-113 (2)						

267	1f0vA (124)	111-113 (3)	111-113 (3)	112-113 (2)	C	0.96	0.58	100.00% (124/124)	99.19% (123/124)	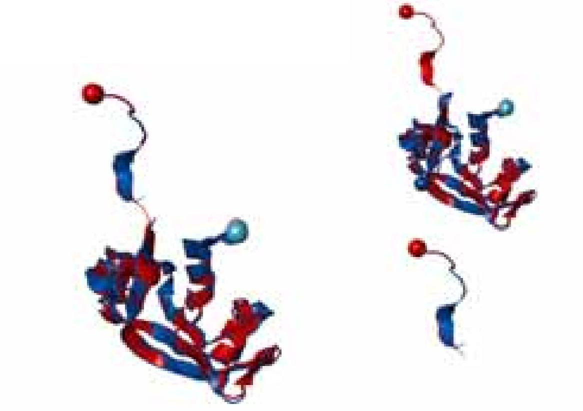 Ribonuclease A<br>Ribonuclease A
	3rspA (124)	111-113 (3)	111-113 (3)	112-113 (2)						
268	1f0vA (124)	111-113 (3)	111-113 (3)	112-113 (2)	C	0.96	0.59	100.00% (124/124)	99.19% (123/124)	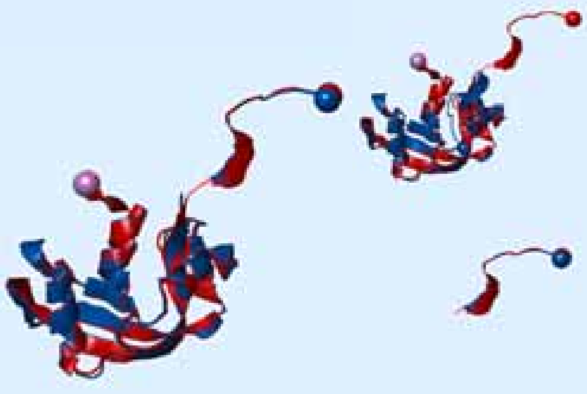 Ribonuclease A<br>Ribonuclease A
	4rsdA (124)	111-113 (3)	111-113 (3)	112-113 (2)						
269	1f98A (125)	10-31 (22)	10-23 (14)	19-23 (5)	N	0.54	2.10	91.60% (109/119)	13.45% (16/119)	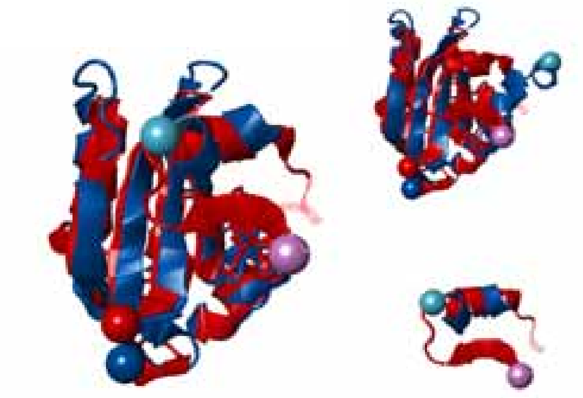 Photoactive yellow protein<br>Hypothetical protein yddU
	1s67L (119)	17-34 (18)	17-26 (10)	19-23 (5)						

270	1f98A (125)	22-28 (7)	22-23 (2)	22-23 (2)	N	0.37	2.23	93.04% (107/115)	13.91% (16/115)	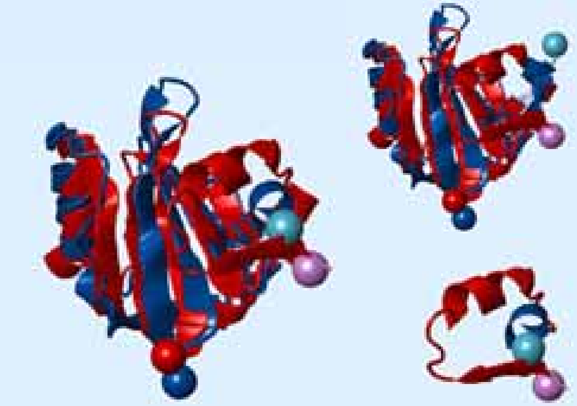 Photoactive yellow protein<br>Hypothetical protein yddU
	1s67U (115)	25-31 (7)	25-26 (2)	22-23 (2)						
271	1f98A (125)	10-24 (15)	14-24 (11)	15-23 (9)	N	0.55	2.00	93.81% (106/113)	14.16% (16/113)	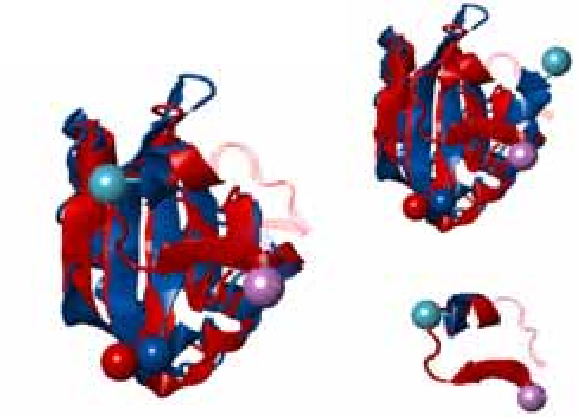 Photoactive yellow protein<br>Heme pas sensor protein
	1v9zA (113)	21-27 (7)	25-27 (3)	15-23 (9)						
272	1f98A (125)	14-25 (12)	14-24 (11)	15-23 (9)	N	0.55	2.04	93.86% (107/114)	14.04% (16/114)	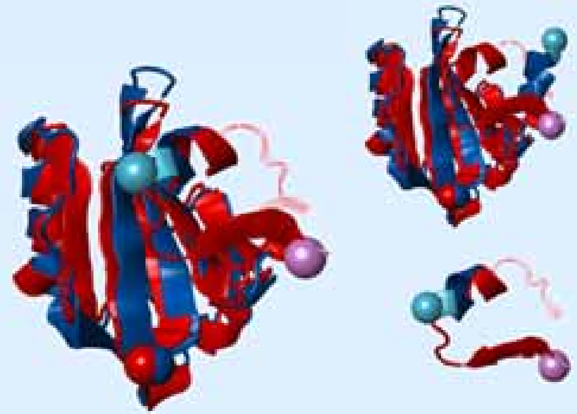 Photoactive yellow protein<br>Heme pas sensor protein
	1vb6A (114)	25-28 (4)	25-27 (3)	15-23 (9)						

273	1fyrA (95)	98-127 (30)	114-123 (10)	120-123 (4)	C	0.41	1.64	93.68% (89/95)	26.32% (25/95)	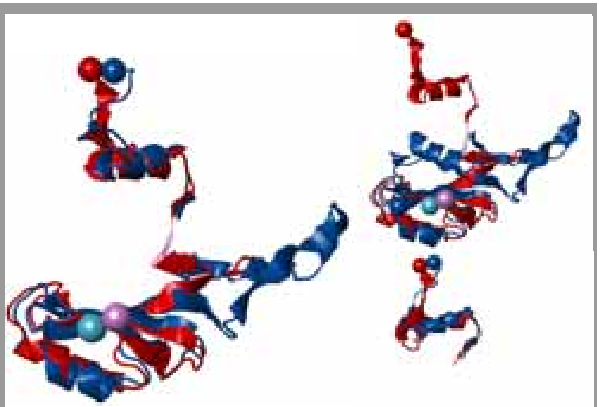 Growth factor receptor-bound protein 2 Crk
	1ju5A (109)	50-96 (47)	83-92 (10)	120-123 (4)						
274	1gq9A (241)	182-198 (17), 196-213 (18)	185-198 (14), 196-213 (18)	130-140 (11), 159-168 (10)	M	0.41	2.30	88.16% (201/228)	17.54% (40/228)	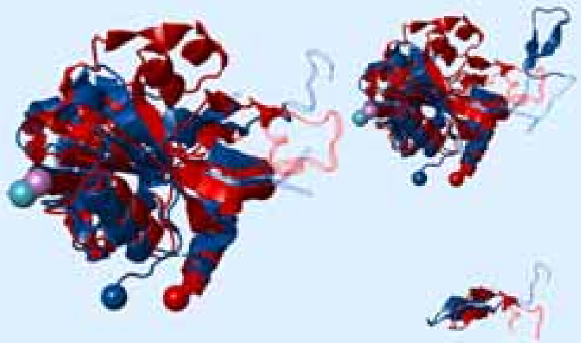 3-deoxy-manno-octulosonate cytidylyltransfera Cytidine monophospho-N-acetylneuraminic acid
	1qwjA (228)	222-232 (11), 230-234 (5)	225-232 (8), 230-234 (5)	130-140 (11), 159-168 (10)						
275	1h8xA (125)	112-126 (15)	112-124 (13)	116-124 (9)	N	0.63	1.65	91.06% (112/123)	33.33% (41/123)	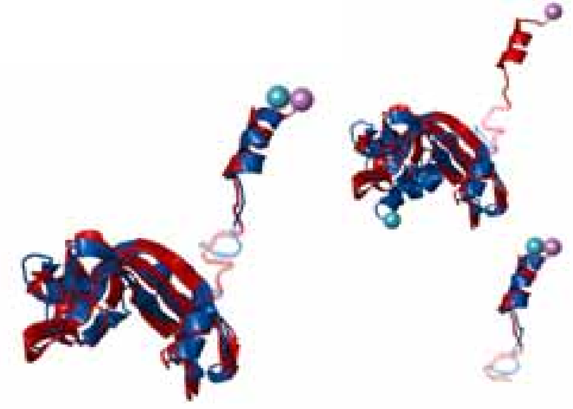 Ribonuclease 1 Angiogenin
	1awzA (123)	13-26 (14)	13-24 (12)	116-124 (9)						

276	1h8xA (125)	117-123 (7)	117-123 (7)	118-123 (6)	N	0.86	0.78	98.32% (117/119)	94.12% (112/119)	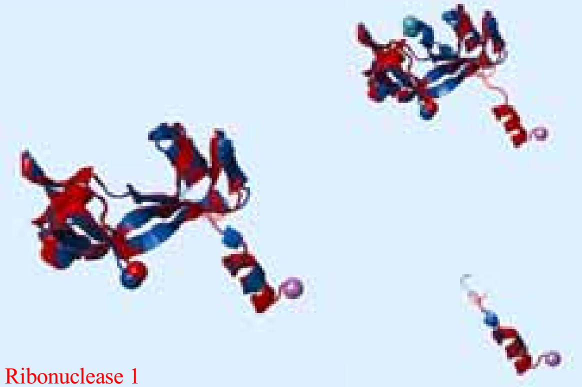 Ribonuclease 1<br>Ribonuclease 1
	1e21A (119)	17-23 (7)	17-23 (7)	118-123 (6)						
277	1h8xA (125)	109-124 (16)	112-124 (13)	115-124 (10)	N	0.58	1.90	90.40% (113/125)	30.40% (38/125)	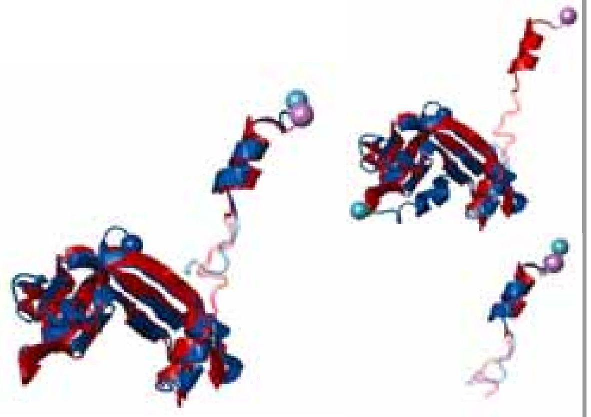 Ribonuclease 1<br>Angiogenin
	1gioA (125)	11-25 (15)	14-25 (12)	115-124 (10)						
278	1h8xA (125)	111-128 (18)	111-127 (17)	116-124 (9)	N	0.63	1.54	90.24% (111/123)	32.52% (40/123)	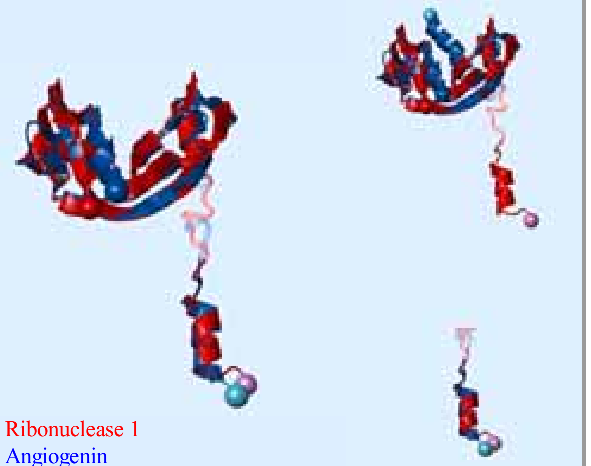 Ribonuclease 1<br>Angiogenin
	1k58A (123)	12-28 (17)	12-27 (16)	116-124 (9)						

279	1h8xA (125)	114-152 (39)	114-128 (15)	114-126 (13)	N	0.51	1.94	91.59% (98/107)	23.36% (25/107)	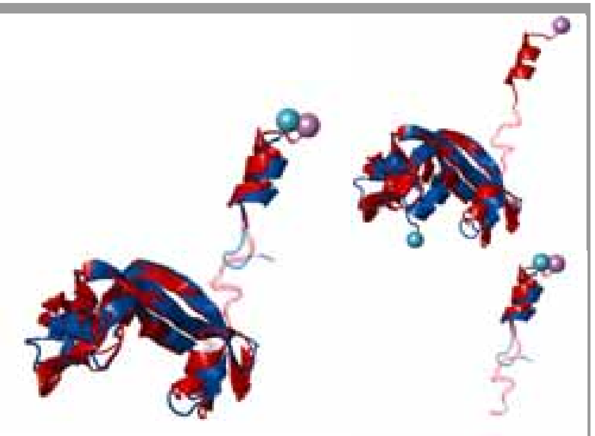 Ribonuclease 1<br>RC-rnase4
	1kvzA (107)	13-43 (31)	13-22 (10)	114-126 (13)						
280	1h8xA (125)	114-127 (14)	114-125 (12)	118-125 (8)	N	0.56	1.65	93.27% (97/104)	24.04% (25/104)	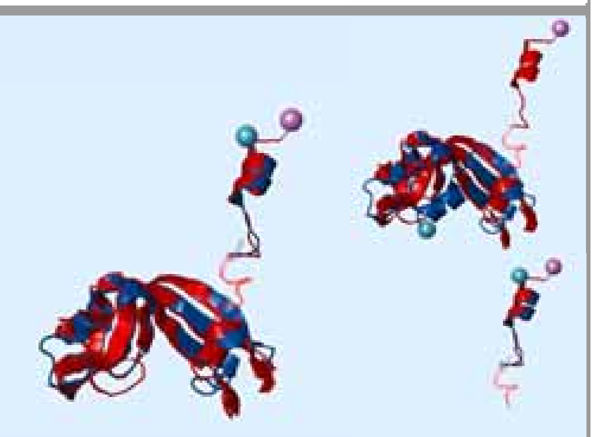 Ribonuclease 1<br>P-30 protein
	1oncA (104)	12-20 (9)	12-18 (7)	118-125 (8)						
281	1h8xA (125)	112-124 (13)	112-124 (13)	121-124 (4)	N	0.68	1.68	95.16% (118/124)	70.97% (88/124)	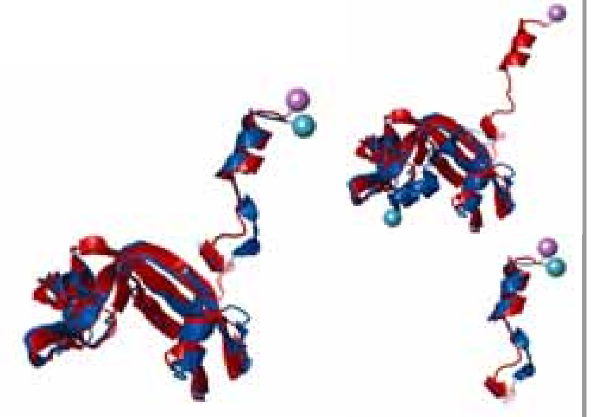 Ribonuclease 1<br>Ribonuclease
	1qwqA (124)	12-24 (13)	12-24 (13)	121-124 (4)						

282	1h8xA (125)	112-127 (16)	114-125 (12)	118-125 (8)	N	0.56	1.67	95.10% (97/102)	24.51% (25/102)	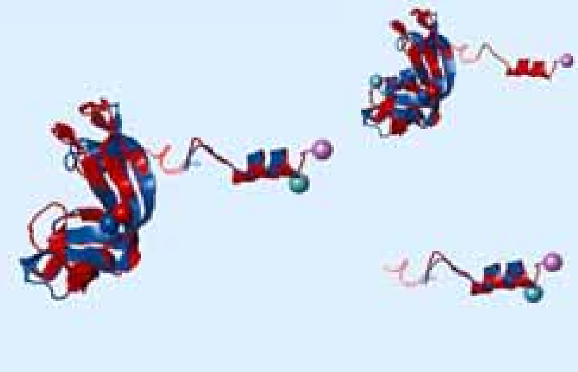 Ribonuclease 1<br>P-30 protein
	1yv7A (102)	10-20 (11)	12-18 (7)	118-125 (8)						
283	1h8xA (125)	112-125 (14)	112-125 (14)	119-125 (7)	N	0.78	1.32	96.77% (120/124)	70.16% (87/124)	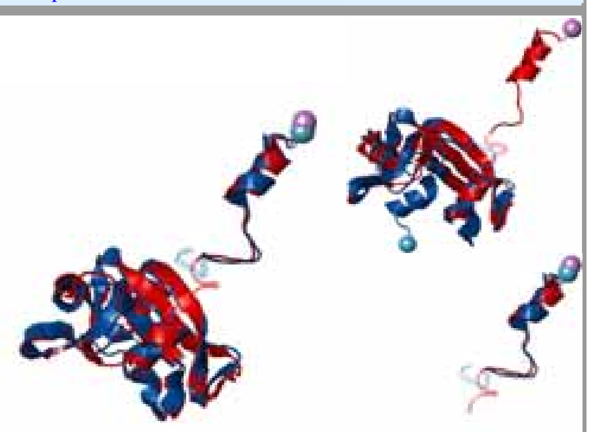 Ribonuclease 1<br>Ribonuclease A
	2aasA (124)	12-25 (14)	12-25 (14)	119-125 (7)						
284	1h8xA (125)	108-125 (18)	108-125 (18)	113-125 (13)	N	0.57	1.78	88.80% (111/125)	33.60% (42/125)	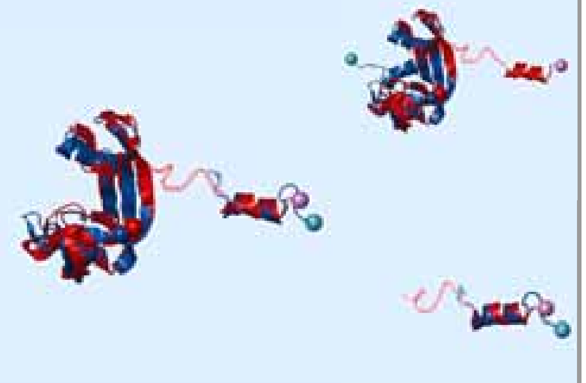 Ribonuclease 1<br>Ribonuclease 7
	2hkyA (129)	11-22 (12)	11-22 (12)	113-125 (13)						

285	1h8xA (125)	110-130 (21)	110-124 (15)	115-123 (9)	N	0.73	1.58	97.60% (122/125)	89.60% (112/125)	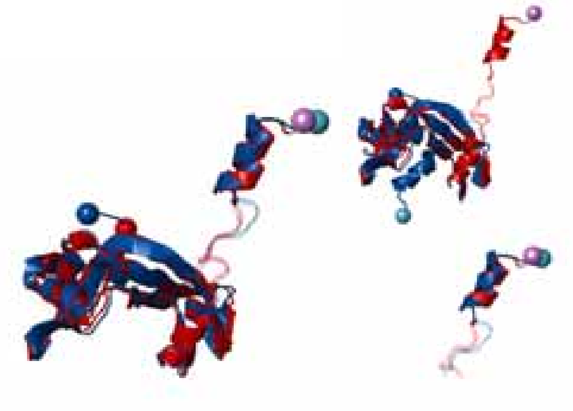 Ribonuclease 1<br>Pancreatic ribonuclease
	2k11A (127)	10-30 (21)	10-24 (15)	115-123 (9)						
286	1jmlA (72)	51-56 (6)	52-56 (5)	52-56 (5)	C	0.74	1.58	97.22% (70/72)	87.50% (63/72)	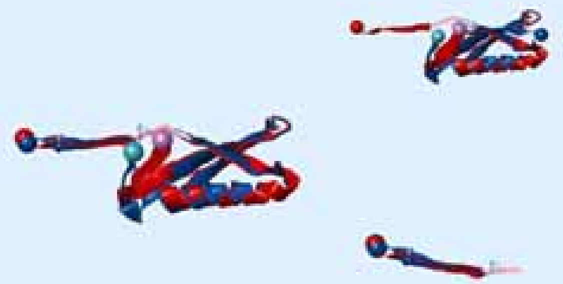 Protein L<br>Protein L
	1k52A (72)	51-56 (6)	52-56 (5)	52-56 (5)						
287	1jmlA (72)	51-56 (6)	52-56 (5)	52-56 (5)	C	0.74	1.56	97.22% (70/72)	87.50% (63/72)	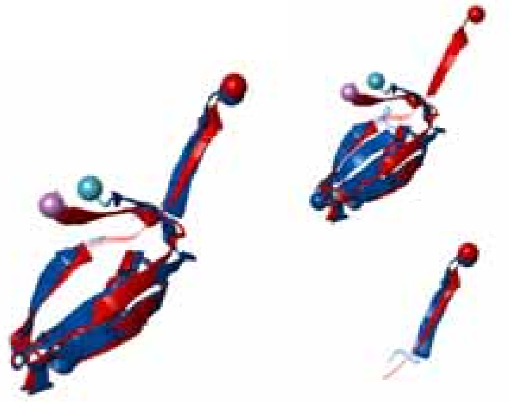 Protein L<br>Protein L
	1k53A (72)	51-56 (6)	52-56 (5)	52-56 (5)						

288	1jmlA (72)	35-63 (29)	35-63 (29)	51-56 (6)	C	0.73	1.40	98.48% (65/66)	54.55% (36/66)	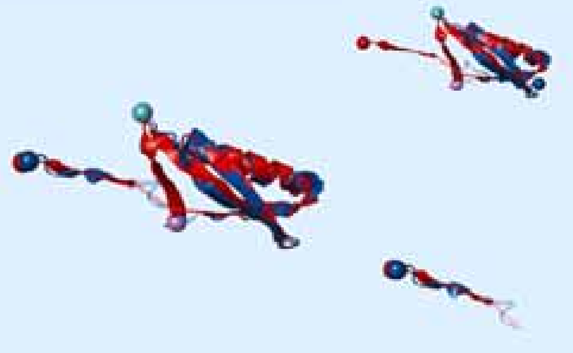 Protein L<br>Protein L
	1xcqL (66)	52-81 (30)	52-81 (30)	51-56 (6)						
289	1jmlA (72)	37-58 (22)	37-58 (22)	51-58 (8)	C	0.73	1.28	100.00% (62/62)	66.13% (41/62)	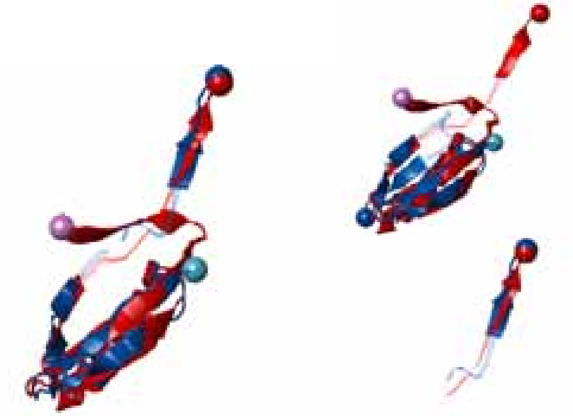 Protein L<br>Protein L
	1xcqM (62)	54-75 (22)	54-75 (22)	51-58 (8)						
290	1jmlA (72)	38-59 (22)	38-58 (21)	51-56 (6)	C	0.71	1.28	96.88% (62/64)	65.63% (42/64)	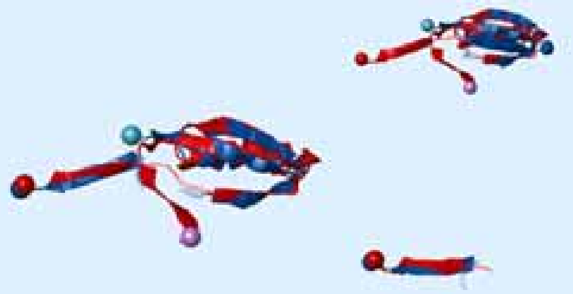 Protein L<br>Protein L
	1xcqN (64)	55-76 (22)	55-75 (21)	51-56 (6)						

291	1jmlA (72)	49-63 (15)	52-57 (6)	52-55 (4)	C	0.69	1.17	92.54% (62/67)	55.22% (37/67)	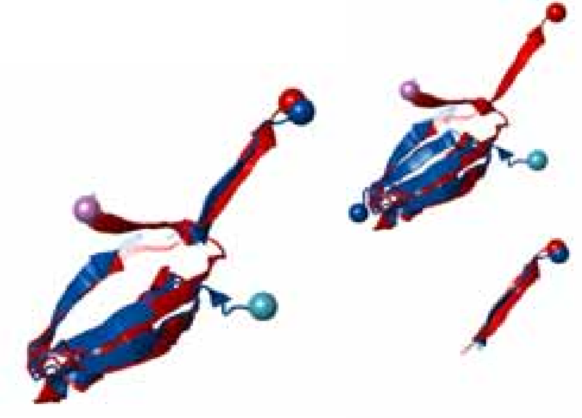 Protein L Protein L
	1xf5M (67)	66-81 (16)	69-75 (7)	52-55 (4)						
292	1js0A (124)	112-113 (2)	112-113 (2)	112-113 (2)	C	0.81	0.61	100.00% (124/124)	98.39% (122/124)	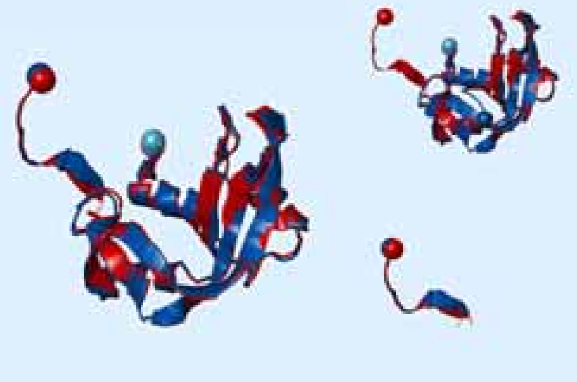 Ribonuclease A Ribonuclease A
	1a5pA (124)	112-113 (2)	112-113 (2)	112-113 (2)						
293	1js0A (124)	112-113 (2)	112-113 (2)	112-113 (2)	C	0.79	0.60	100.00% (124/124)	99.19% (123/124)	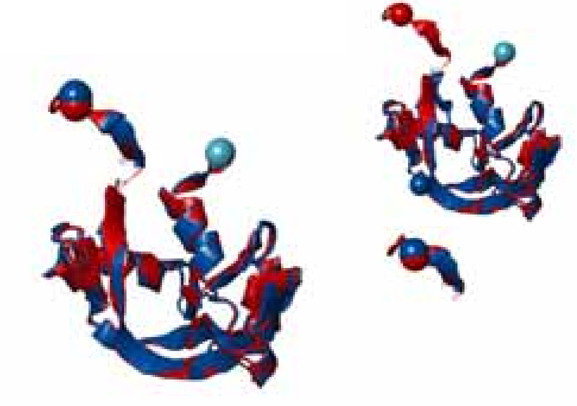 Ribonuclease A Ribonuclease A
	1a5qA (124)	112-113 (2)	112-113 (2)	112-113 (2)						

294	1js0A (124)	107-123 (17)	111-123 (13)	112-115 (4)	C	0.59	1.74	93.50% (115/123)	27.64% (34/123)	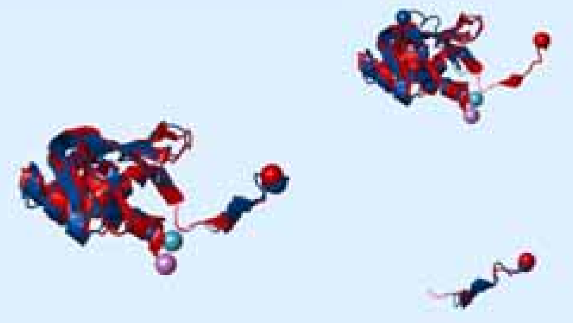<br>Ribonuclease A<br>Angiogenin
	1awzA (123)	104-122 (19)	108-122 (15)	112-115 (4)						
295	1js0A (124)	111-115 (5)	112-113 (2)	112-113 (2)	C	0.80	0.68	100.00% (124/124)	94.35% (117/124)	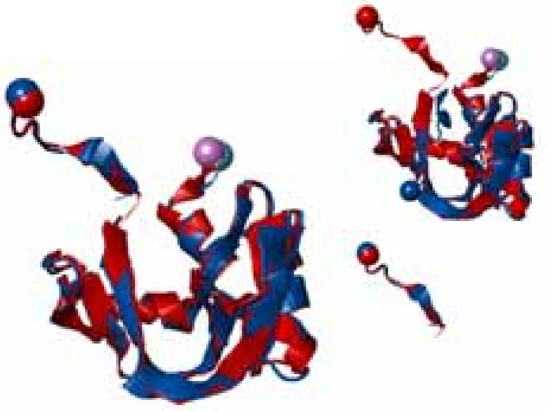<br>Ribonuclease A<br>Ribonuclease
	1b6vA (124)	111-115 (5)	112-113 (2)	112-113 (2)						
296	1js0A (124)	112-113 (2)	112-113 (2)	112-113 (2)	C	0.75	0.76	100.00% (124/124)	99.19% (123/124)	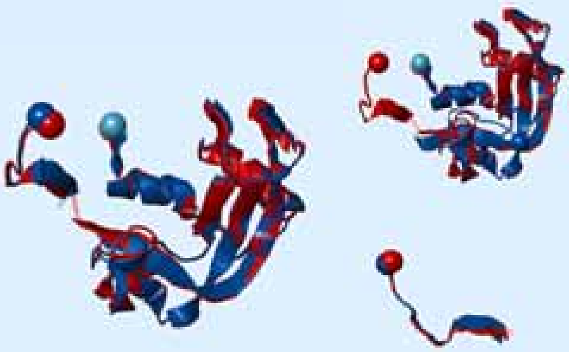<br>Ribonuclease A<br>Protein (ribonuclease A)
	1c8wA (124)	112-113 (2)	112-113 (2)	112-113 (2)						

297	1js0A (124)	112-115 (4)	112-113 (2)	112-113 (2)	C	0.78	0.66	100.00% (124/124)	99.19% (123/124)	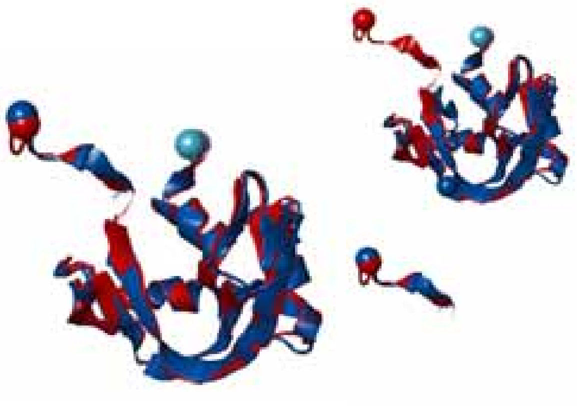
	1c9vA (124)	112-115 (4)	112-113 (2)	112-113 (2)						Ribonuclease A<br>Ribonuclease A
298	1js0A (124)	112-113 (2)	112-113 (2)	112-113 (2)	C	0.79	0.58	100.00% (124/124)	99.19% (123/124)	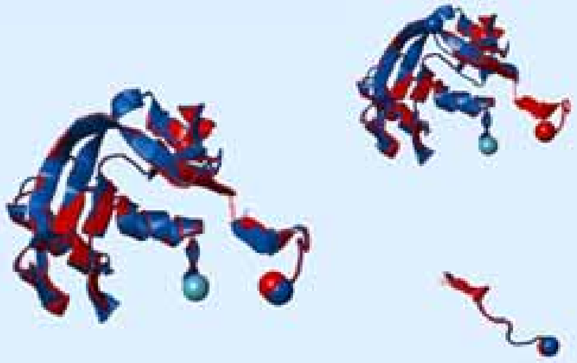
	1c9xA (124)	112-113 (2)	112-113 (2)	112-113 (2)						Ribonuclease A<br>Ribonuclease A
299	1js0A (124)	112-115 (4)	112-113 (2)	112-114 (3)	C	0.71	1.29	98.32% (117/119)	68.91% (82/119)	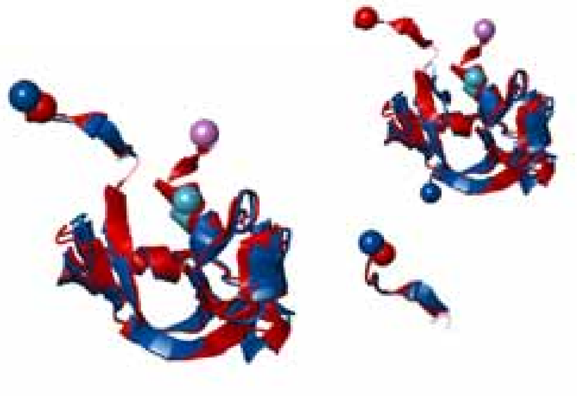
	1e21A (119)	112-115 (4)	112-113 (2)	112-114 (3)						Ribonuclease A<br>Ribonuclease 1

300	1js0A (124)	112-115 (4)	112-113 (2)	112-113 (2)	C	0.75	0.76	100.00% (124/124)	99.19% (123/124)	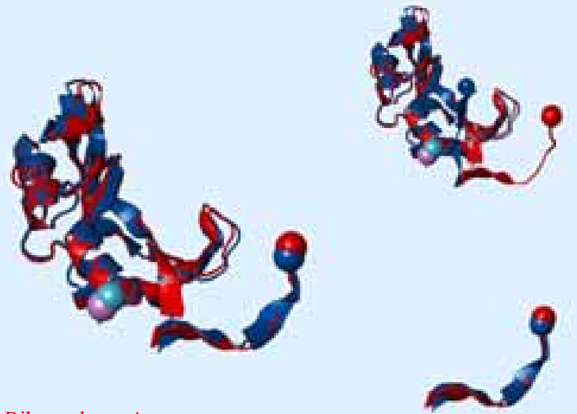 Ribonuclease A
	1eidA (124)	112-115 (4)	112-113 (2)	112-113 (2)						Ribonuclease A
301	1js0A (124)	112-115 (4)	112-113 (2)	112-113 (2)	C	0.74	0.73	100.00% (124/124)	99.19% (123/124)	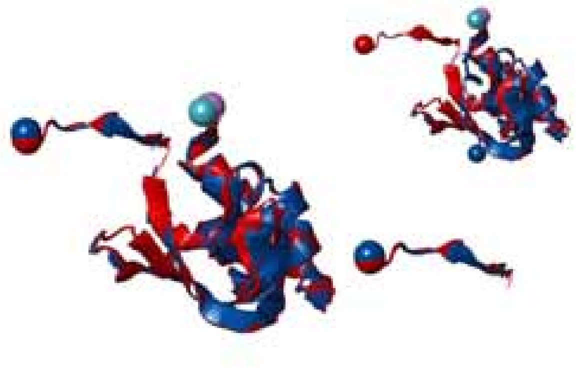 Ribonuclease A
	1eieA (124)	112-115 (4)	112-113 (2)	112-113 (2)						Ribonuclease A
302	1js0A (124)	107-123 (17)	112-115 (4)	112-115 (4)	C	0.45	1.92	88.71% (110/124)	32.26% (40/124)	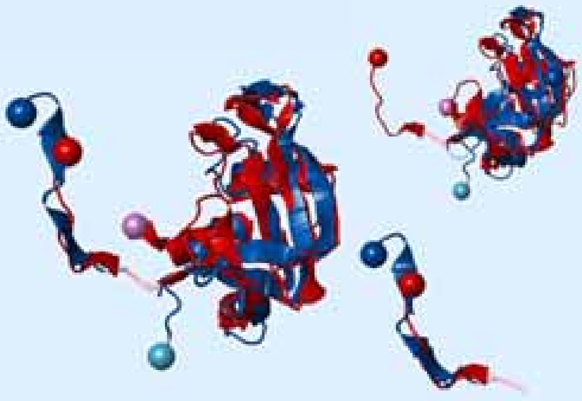 Ribonuclease A
	1gioA (125)	105-120 (16)	110-111 (2)	112-115 (4)						Angiogenin

303	1js0A (124)	111-116 (6)	111-115 (5)	111-115 (5)	C	0.57	1.41	95.12% (117/123)	41.46% (51/123)	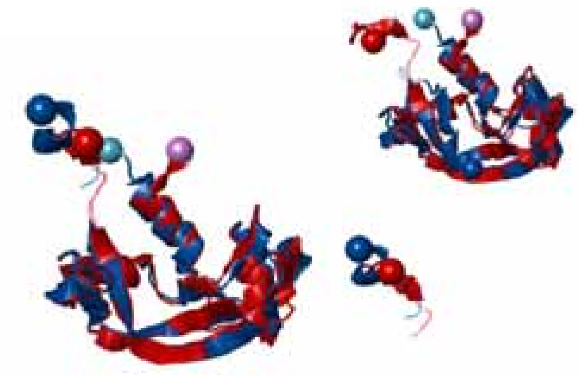<br>Ribonuclease A<br>Angiogenin
	1gv7A (123)	110-113 (4)	110-112 (3)	111-115 (5)						
304	1js0A (124)	112-115 (4)	112-113 (2)	112-113 (2)	C	0.80	0.61	100.00% (124/124)	99.19% (123/124)	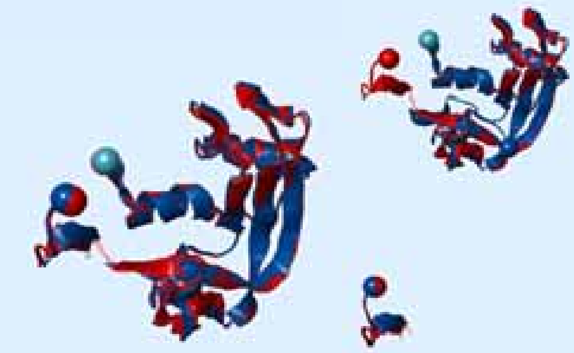<br>Ribonuclease A<br>Ribonuclease A
	1izpA (124)	112-115 (4)	112-113 (2)	112-113 (2)						
305	1js0A (124)	112-115 (4)	112-113 (2)	112-113 (2)	C	0.77	0.66	100.00% (124/124)	99.19% (123/124)	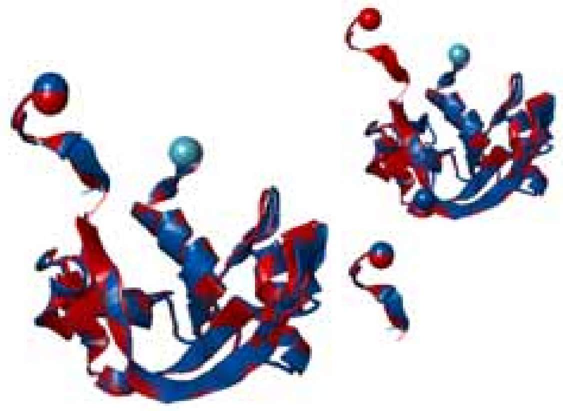<br>Ribonuclease A<br>Ribonuclease A
	1izqA (124)	112-115 (4)	112-113 (2)	112-113 (2)						

306	1js0A (124)	112-115 (4)	112-113 (2)	112-113 (2)	C	0.75	1.05	100.00% (124/124)	99.19% (123/124)	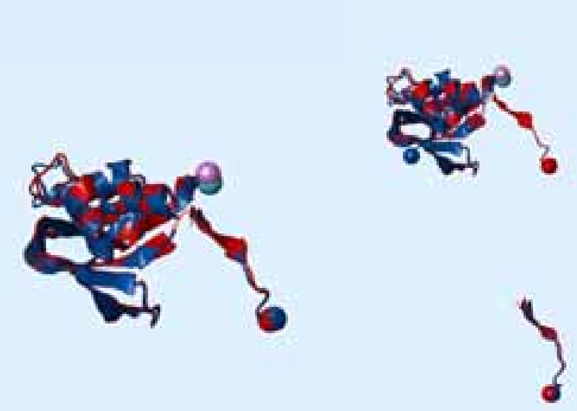 Ribonuclease A<br>Ribonuclease A
	1izrA (124)	112-115 (4)	112-113 (2)	112-113 (2)						
307	1js0A (124)	112-113 (2)	112-113 (2)	112-113 (2)	C	0.65	0.70	100.00% (101/101)	100.00% (101/101)	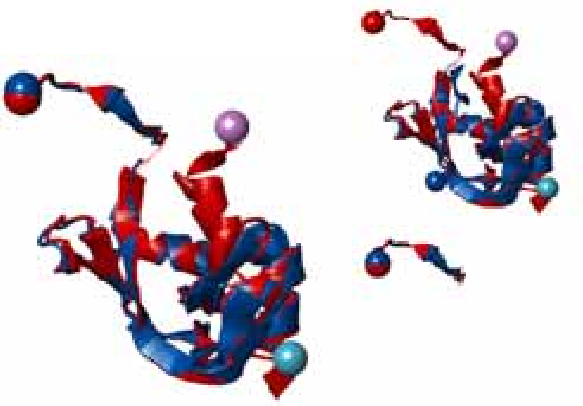 Ribonuclease A<br>Ribonuclease pancreatic
	1j80B (101)	112-113 (2)	112-113 (2)	112-113 (2)						
308	1js0A (124)	111-116 (6)	111-115 (5)	112-115 (4)	C	0.54	1.56	95.76% (113/118)	31.36% (37/118)	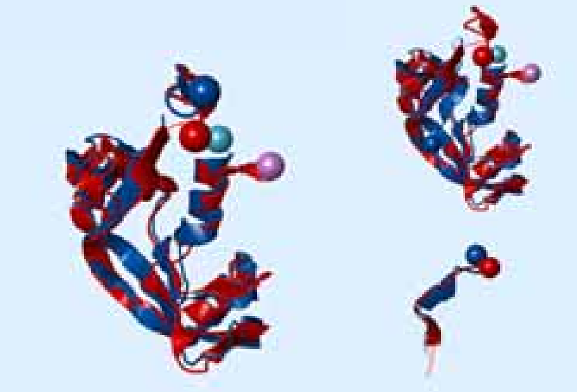 Ribonuclease A<br>Angiogenin
	1k5aA (118)	108-111 (4)	108-110 (3)	112-115 (4)						

309	1js0A (124)	110-116 (7)	110-116 (7)	111-116 (6)	C	0.92	0.66	98.39% (122/124)	98.39% (122/124)	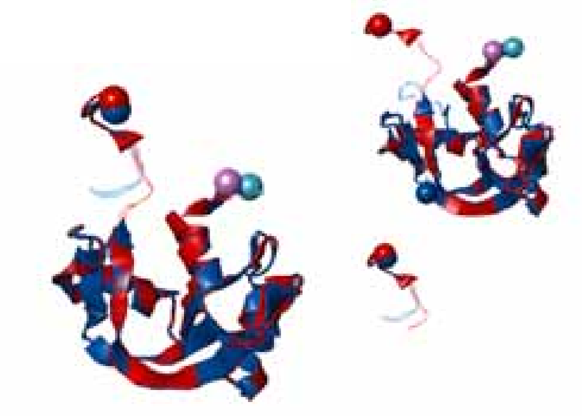 Ribonuclease A<br>Pancreatic ribonuclease a
	1kh8A (125)	110-116 (7)	110-116 (7)	111-116 (6)						
310	1js0A (124)	112-119 (8)	112-119 (8)	112-116 (5)	C	0.41	2.08	91.59% (98/107)	26.17% (28/107)	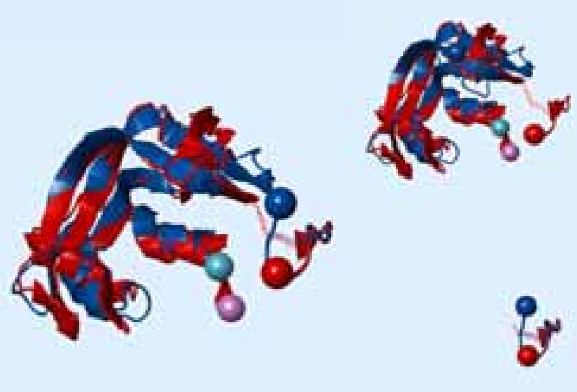 Ribonuclease A<br>RC-rnase4
	1kvzA (107)	94-99 (6)	94-99 (6)	112-116 (5)						
311	1js0A (124)	106-116 (11)	110-116 (7)	111-115 (5)	C	0.34	2.06	91.51% (97/106)	28.30% (30/106)	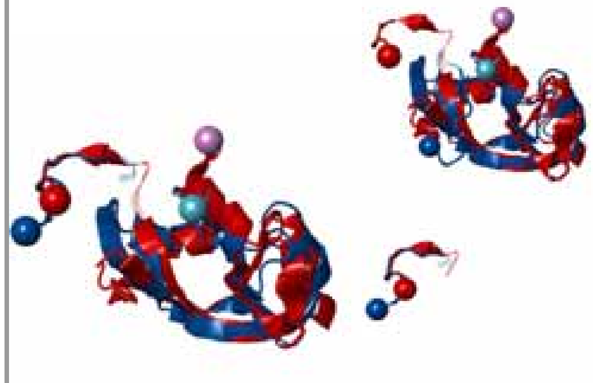 Ribonuclease A<br>RC-rnase2 ribonuclease
	1m58A (106)	87-95 (9)	91-95 (5)	111-115 (5)						

312	1js0A (124)	111-116 (6)	111-115 (5)	112-115 (4)	C	0.41	1.84	92.38% (97/105)	23.81% (25/105)	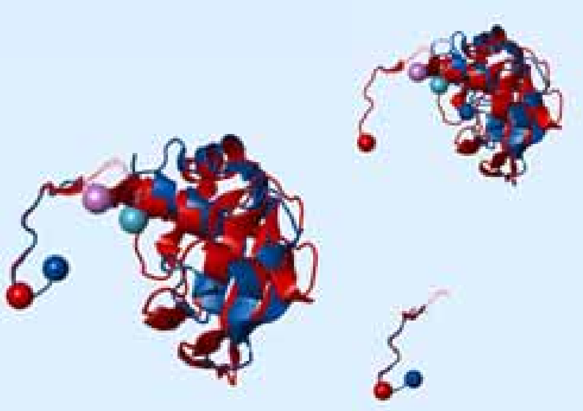 Ribonuclease A<br>RC-rnase6 ribonuclease
	1oj8A (105)	91-94 (4)	91-93 (3)	112-115 (4)						
313	1js0A (124)	105-123 (19)	106-118 (13)	111-115 (5)	C	0.44	1.76	92.38% (97/105)	26.67% (28/105)	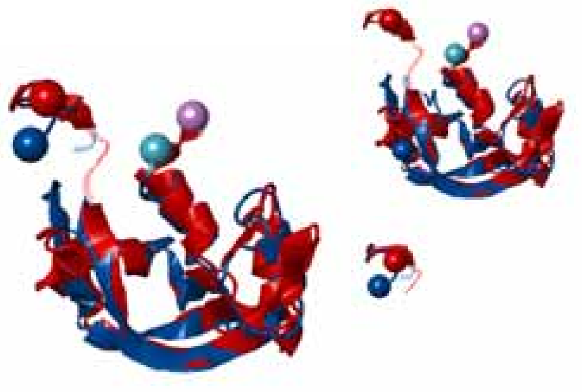 Ribonuclease A<br>P-30 protein
	1pu3A (105)	86-102 (17)	87-97 (11)	111-115 (5)						
314	1js0A (124)	107-113 (7)	108-113 (6)	111-113 (3)	C	0.55	1.92	95.97% (119/124)	75.81% (94/124)	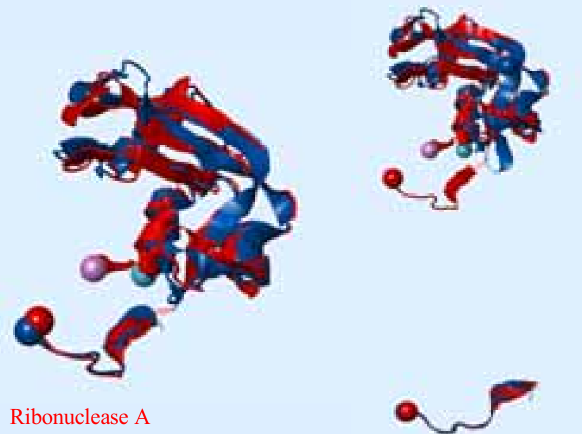 Ribonuclease A<br>Ribonuclease
	1qwqA (124)	107-113 (7)	108-113 (6)	111-113 (3)						

315	1js0A (124)	112-115 (4)	112-115 (4)	112-113 (2)	C	0.79	0.54	100.00% (123/123)	100.00% (123/123)	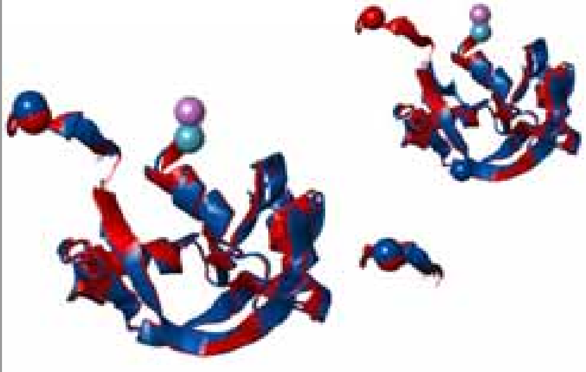<br>Ribonuclease A<br>Ribonuclease A
	1rasA (123)	112-115 (4)	112-115 (4)	112-113 (2)						
316	1js0A (124)	112-113 (2)	112-113 (2)	112-113 (2)	C	0.55	1.31	97.50% (117/120)	43.33% (52/120)	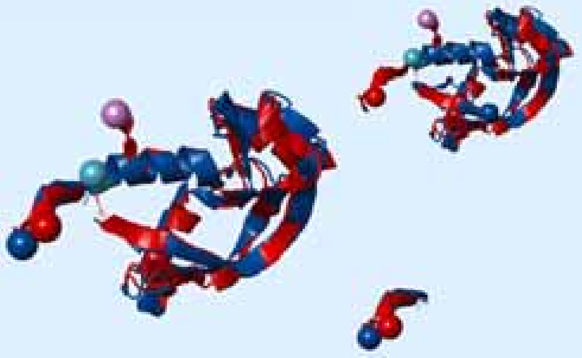<br>Ribonuclease A<br>Protein (ribonuclease 4)
	1rnfA (120)	109-110 (2)	109-110 (2)	112-113 (2)						
317	1js0A (124)	107-115 (9)	112-115 (4)	112-115 (4)	C	0.76	1.07	100.00% (124/124)	66.94% (83/124)	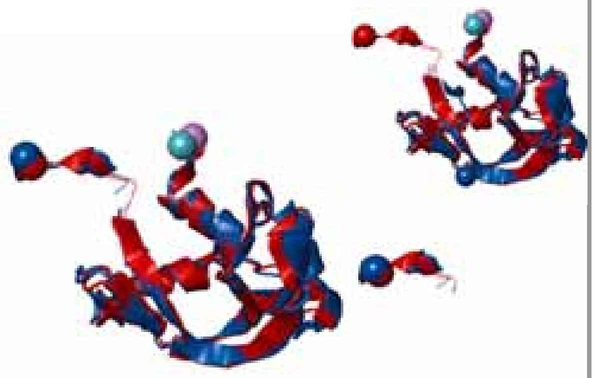<br>Ribonuclease A<br>Protein (ribonuclease)
	1rraA (124)	107-115 (9)	112-115 (4)	112-115 (4)						

318	1js0A<br>(124)	112-115 (4)	112-113 (2)	112-113 (2)	C	0.77	0.88	100.00%<br>(124/124)	99.19%<br>(123/124)	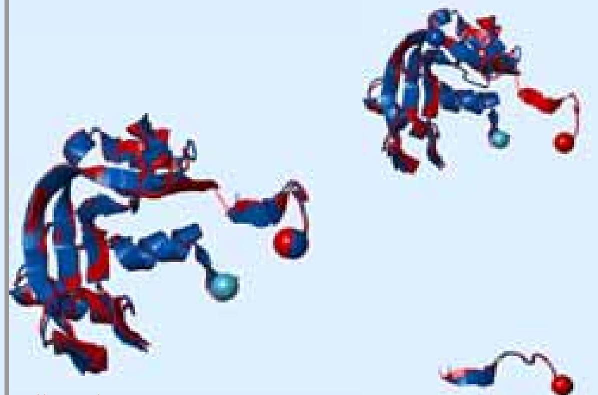<br>Ribonuclease A<br>Ribonuclease pancreatic
	1ymnA<br>(124)	112-115 (4)	112-113 (2)	112-113 (2)						
319	1js0A<br>(124)	111-115 (5)	112-113 (2)	112-113 (2)	C	0.79	0.61	100.00%<br>(124/124)	99.19%<br>(123/124)	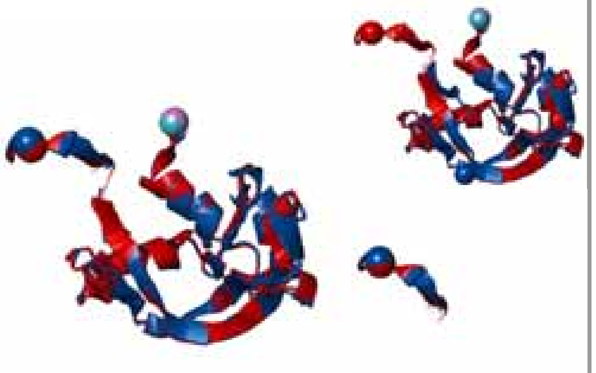<br>Ribonuclease A<br>Ribonuclease pancreatic
	1ymrA<br>(124)	111-115 (5)	112-113 (2)	112-113 (2)						
320	1js0A<br>(124)	111-115 (5)	112-113 (2)	112-113 (2)	C	0.80	0.67	100.00%<br>(124/124)	99.19%<br>(123/124)	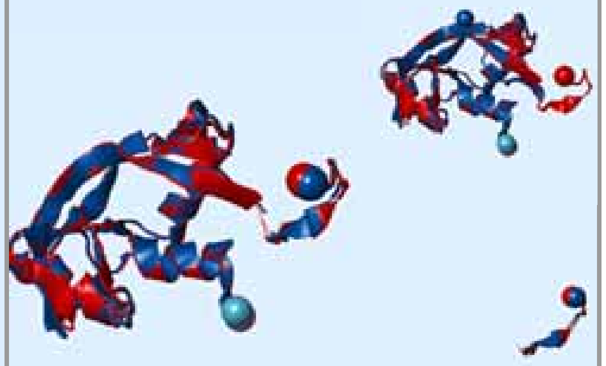<br>Ribonuclease A<br>Ribonuclease pancreatic
	1ymwA<br>(124)	111-115 (5)	112-113 (2)	112-113 (2)						

321	1js0A (124)	110-116 (7)	110-115 (6)	111-115 (5)	C	0.44	1.83	95.10% (97/102)	28.43% (29/102)	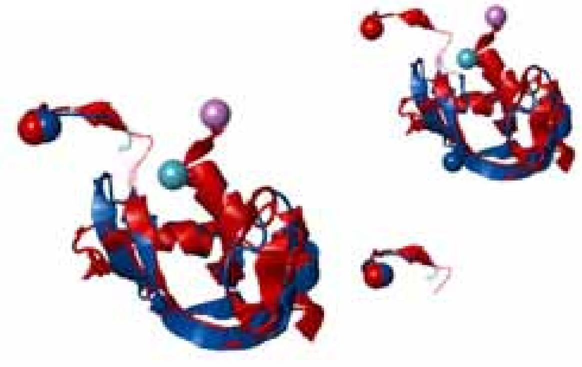 Ribonuclease A<br>P-30 protein
	1yv7A (102)	90-94 (5)	90-93 (4)	111-115 (5)						
322	1js0A (124)	107-123 (17)	111-113 (3)	112-113 (2)	C	0.67	1.27	100.00% (124/124)	100.00% (124/124)	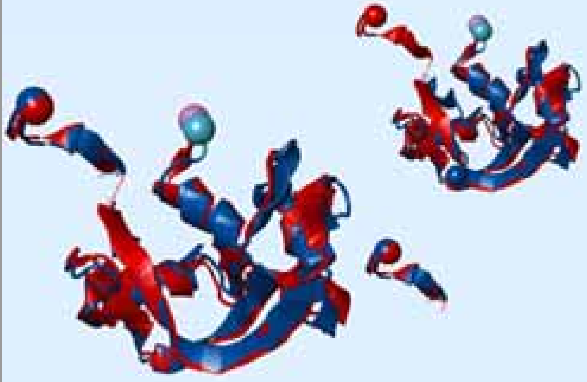 Ribonuclease A<br>Ribonuclease A
	2aasA (124)	107-123 (17)	111-113 (3)	112-113 (2)						
323	1js0A (124)	112-113 (2)	112-113 (2)	112-113 (2)	C	0.65	1.08	99.19% (123/124)	68.55% (85/124)	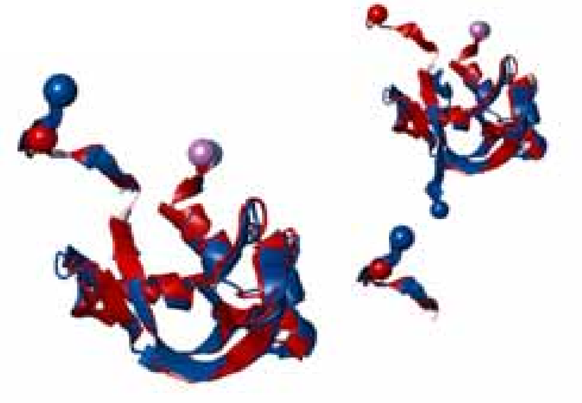 Ribonuclease A<br>Ribonuclease
	2e0jA (128)	112-113 (2)	112-113 (2)	112-113 (2)						

324	1js0A (124)	112-113 (2)	112-113 (2)	112-113 (2)	C	0.78	0.88	100.00% (124/124)	69.35% (86/124)	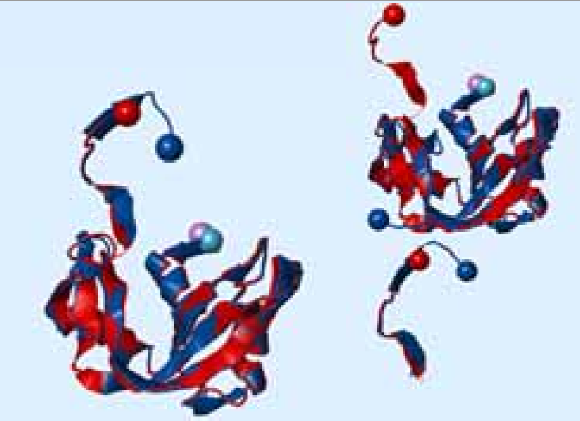 Ribonuclease A<br>Ribonuclease
	2e0lA (128)	112-113 (2)	112-113 (2)	112-113 (2)						
325	1js0A (124)	112-113 (2)	112-113 (2)	112-113 (2)	C	0.74	0.88	100.00% (124/124)	69.35% (86/124)	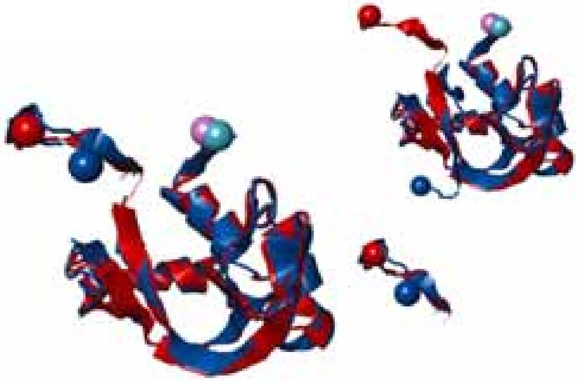 Ribonuclease A<br>Ribonuclease
	2e0mA (128)	112-113 (2)	112-113 (2)	112-113 (2)						
326	1js0A (124)	112-113 (2)	112-113 (2)	112-113 (2)	C	0.70	1.02	99.19% (123/124)	68.55% (85/124)	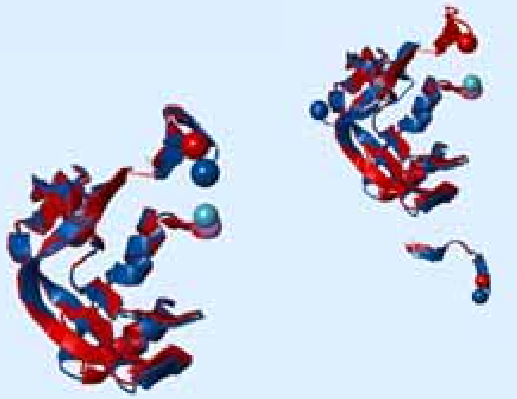 Ribonuclease A<br>Ribonuclease
	2e0oA (126)	112-113 (2)	112-113 (2)	112-113 (2)						

327	1js0A (124)	112-115 (4)	112-113 (2)	112-113 (2)	C	0.70	1.12	100.00% (124/124)	69.35% (86/124)	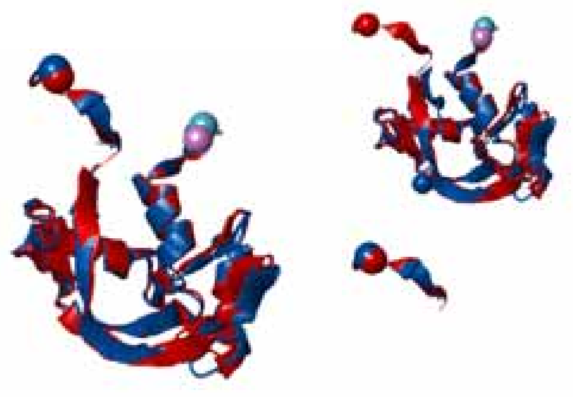 Ribonuclease A<br>Ribonuclease
	2e0oB (125)	112-115 (4)	112-113 (2)	112-113 (2)						
328	1js0A (124)	112-119 (8)	113-119 (7)	112-116 (5)	C	0.51	1.86	90.32% (112/124)	41.94% (52/124)	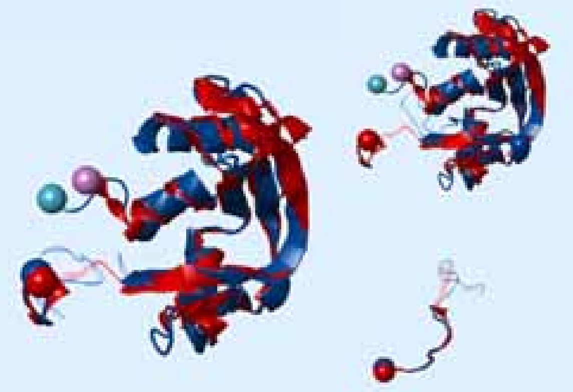 Ribonuclease A<br>Ribonuclease 7
	2hkyA (129)	108-123 (16)	109-123 (15)	112-116 (5)						
329	1js0A (124)	109-123 (15)	112-115 (4)	112-115 (4)	C	0.68	1.63	99.19% (123/124)	69.35% (86/124)	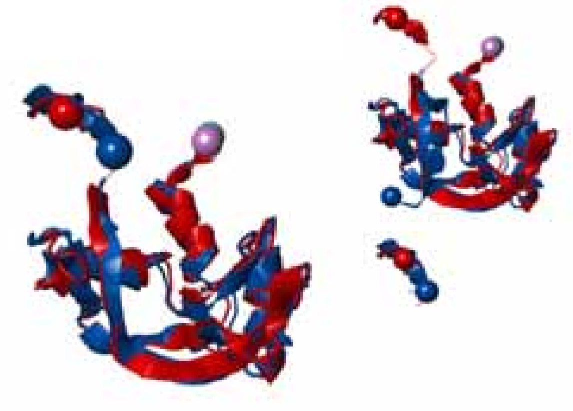 Ribonuclease A<br>Pancreatic ribonuclease
	2k11A (127)	109-123 (15)	112-115 (4)	112-115 (4)						

330	1js0A (124)	112-113 (2)	112-113 (2)	112-113 (2)	C	0.79	0.59	100.00% (124/124)	99.19% (123/124)	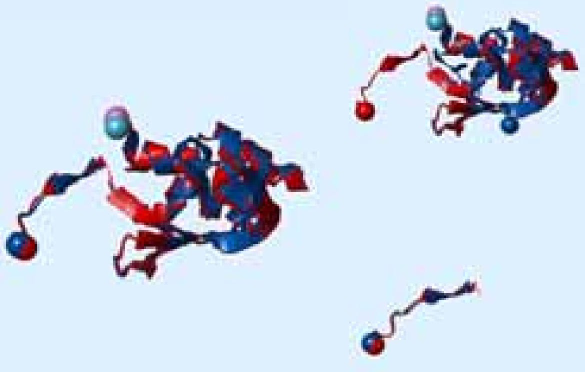 Ribonuclease A<br>Ribonuclease pancreatic
	2nuiA (124)	112-113 (2)	112-113 (2)	112-113 (2)						
331	1js0A (124)	112-113 (2)	112-113 (2)	112-113 (2)	C	0.76	0.79	100.00% (124/124)	98.39% (122/124)	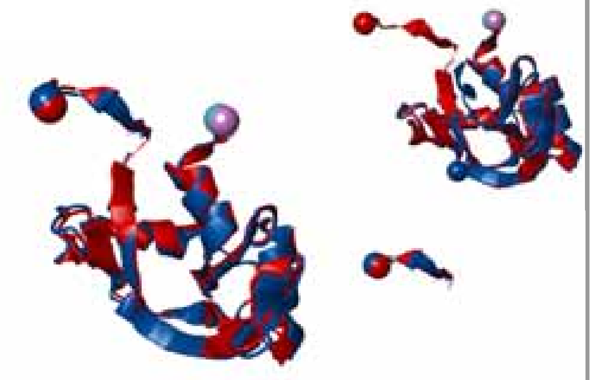 Ribonuclease A<br>Ribonuclease pancreatic
	2op2A (124)	112-113 (2)	112-113 (2)	112-113 (2)						
332	1js0A (124)	112-119 (8)	112-113 (2)	112-113 (2)	C	0.74	0.84	99.19% (123/124)	69.35% (86/124)	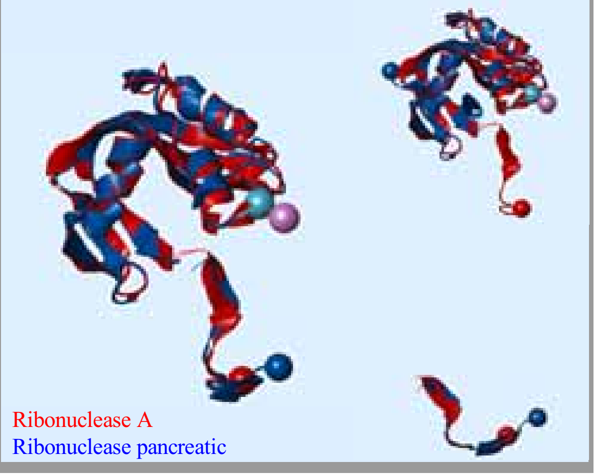 Ribonuclease A<br>Ribonuclease pancreatic
	2q4gX (126)	112-119 (8)	112-113 (2)	112-113 (2)						

333	1js0A (124)	112-113 (2)	112-113 (2)	112-113 (2)	C	0.78	0.62	100.00% (124/124)	99.19% (123/124)	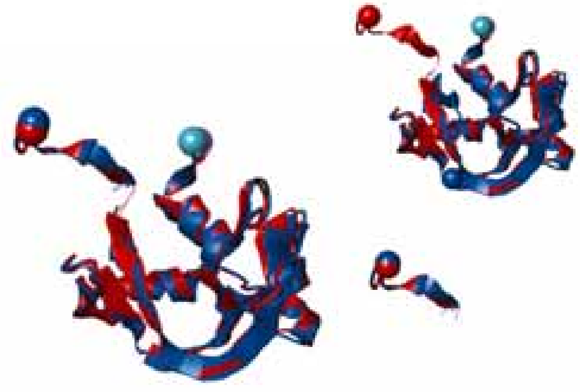 Ribonuclease A<br>Ribonuclease pancreatic
	3dh6A (124)	112-113 (2)	112-113 (2)	112-113 (2)						
334	1js0A (124)	112-115 (4)	112-113 (2)	112-113 (2)	C	0.76	0.64	100.00% (124/124)	99.19% (123/124)	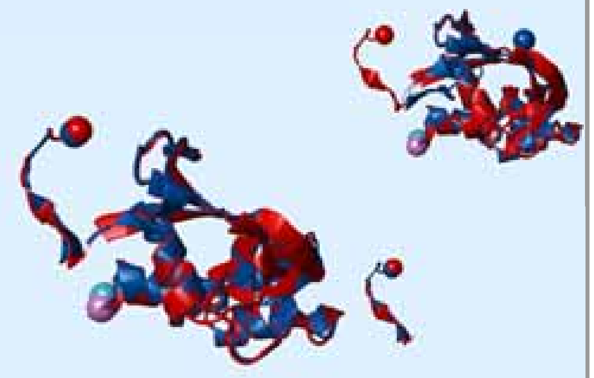 Ribonuclease A<br>Ribonuclease pancreatic
	3di7A (124)	112-115 (4)	112-113 (2)	112-113 (2)						
335	1js0A (124)	112-115 (4)	112-113 (2)	112-113 (2)	C	0.77	0.59	100.00% (124/124)	99.19% (123/124)	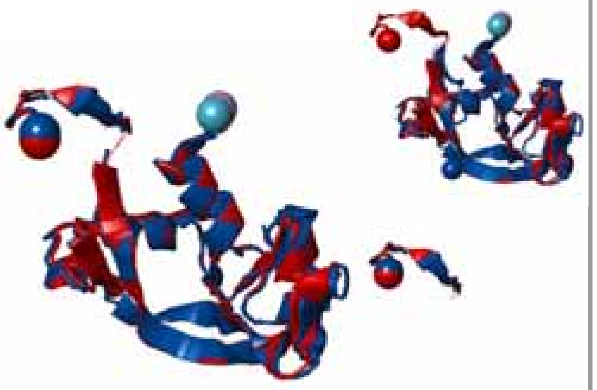 Ribonuclease A<br>Ribonuclease pancreatic
	3di8A (124)	112-115 (4)	112-113 (2)	112-113 (2)						

336	1js0A (124)	112-115 (4)	112-113 (2)	112-113 (2)	C	0.76	0.71	100.00% (124/124)	99.19% (123/124)	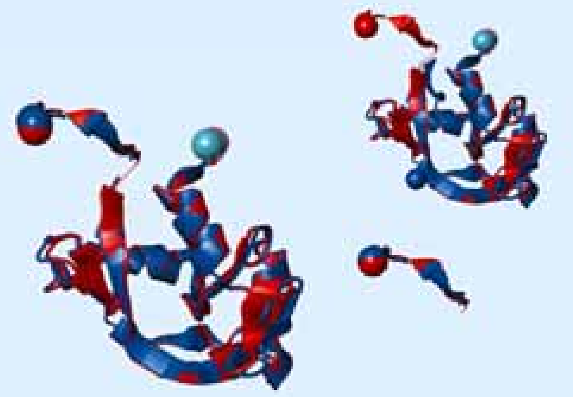 Ribonuclease A<br>Ribonuclease pancreatic
	3di9A (124)	112-115 (4)	112-113 (2)	112-113 (2)						
337	1js0A (124)	112-115 (4)	112-113 (2)	112-113 (2)	C	0.79	0.63	100.00% (124/124)	99.19% (123/124)	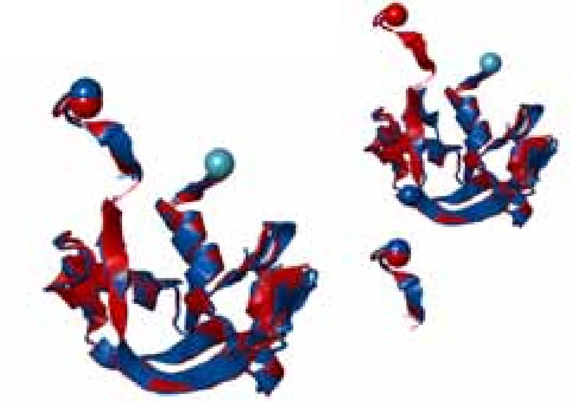 Ribonuclease A<br>Ribonuclease pancreatic
	3dibA (124)	112-115 (4)	112-113 (2)	112-113 (2)						
338	1js0A (124)	112-115 (4)	112-113 (2)	112-113 (2)	C	0.76	0.61	100.00% (124/124)	99.19% (123/124)	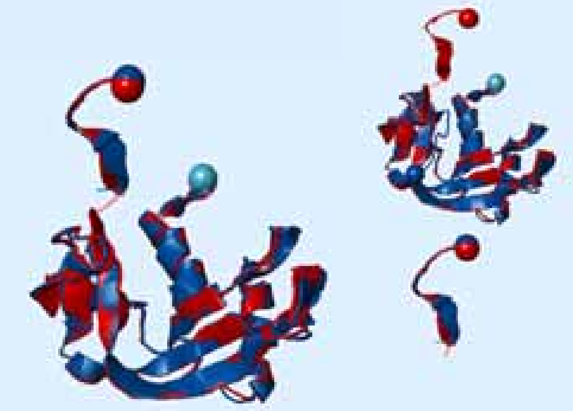 Ribonuclease A<br>Ribonuclease pancreatic
	3dicA (124)	112-115 (4)	112-113 (2)	112-113 (2)						

339	1js0A (124)	112-115 (4)	112-113 (2)	112-113 (2)	C	0.78	0.62	100.00% (124/124)	99.19% (123/124)	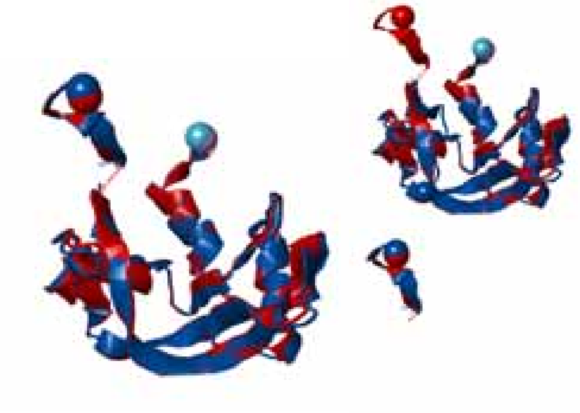 Ribonuclease A<br>Ribonuclease A
	3rsdA (124)	112-115 (4)	112-113 (2)	112-113 (2)						
340	1js0A (124)	112-113 (2)	112-113 (2)	112-113 (2)	C	0.80	0.53	100.00% (124/124)	97.58% (121/124)	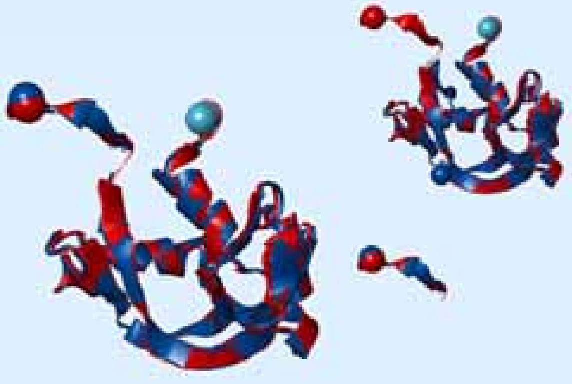 Ribonuclease A<br>Ribonuclease A
	3rskA (124)	112-113 (2)	112-113 (2)	112-113 (2)						
341	1js0A (124)	112-115 (4)	112-113 (2)	112-113 (2)	C	0.77	0.69	100.00% (124/124)	99.19% (123/124)	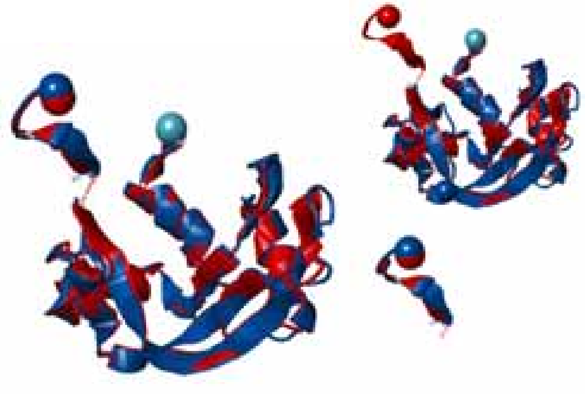 Ribonuclease A<br>Ribonuclease A
	3rspA (124)	112-115 (4)	112-113 (2)	112-113 (2)						

342	1js0A (124)	112-113 (2)	112-113 (2)	112-113 (2)	C	0.76	0.72	100.00% (124/124)	99.19% (123/124)	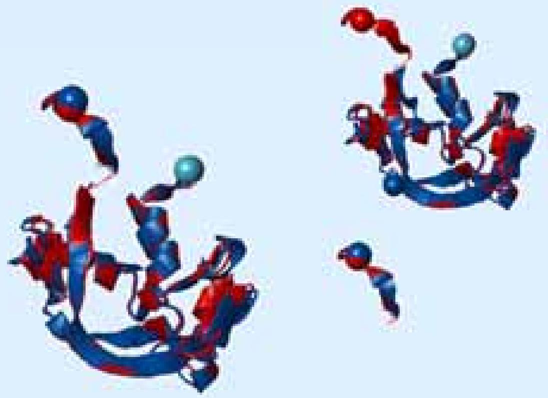<br>Ribonuclease A<br>Ribonuclease A
	4rsdA (124)	112-113 (2)	112-113 (2)	112-113 (2)						
343	1lgqA (112)	14-123 (110)	65-93 (29)	85-87 (3)	C	0.38	1.87	92.86% (104/112)	19.64% (22/112)	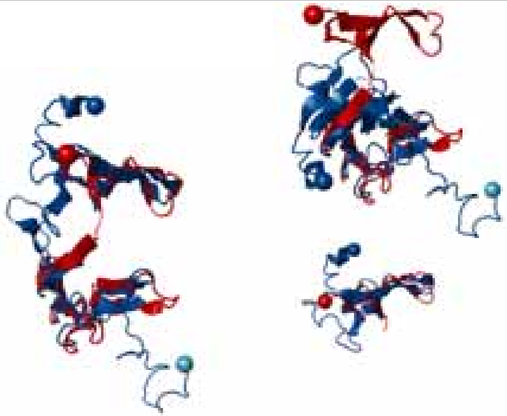<br>Cell cycle checkpoint protein CHFR<br>Protein kinase SPK1
	1qu5A (182)	575-698 (124)	627-668 (42)	85-87 (3)						
344	1lr5A (160)	122-159 (38)	133-139 (7)	133-136 (4)	C	0.37	2.32	87.50% (140/160)	10.63% (17/160)	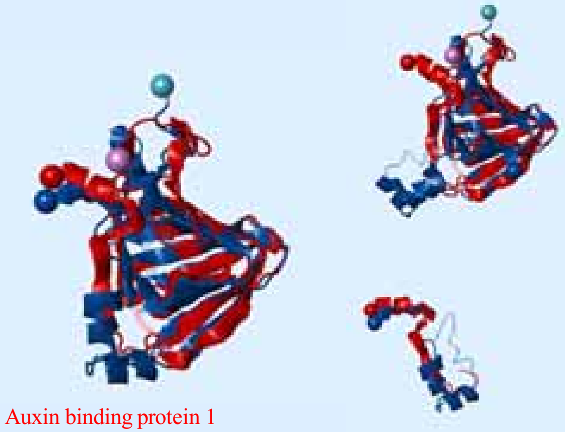<br>Auxin binding protein 1<br>Canavalin
	1dgwA (178)	162-222 (61)	177-203 (27)	133-136 (4)						

345	1lr5A (160)	11-41 (31)	12-41 (30)	37-41 (5)	N	0.34	2.39	80.63% (129/160)	8.13% (13/160)	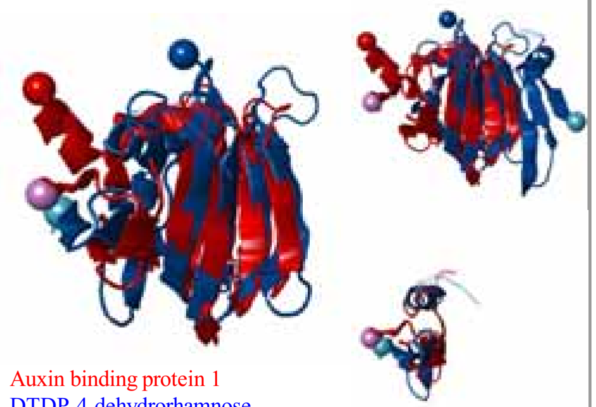 Auxin binding protein 1 DTDP-4-dehydrorhamnose 3,5-epimerase
	1dzrA (183)	14-47 (34)	15-47 (33)	37-41 (5)						
346	1lr5A (160)	35-41 (7)	36-41 (6)	36-41 (6)	N	0.35	2.30	80.00% (128/160)	8.13% (13/160)	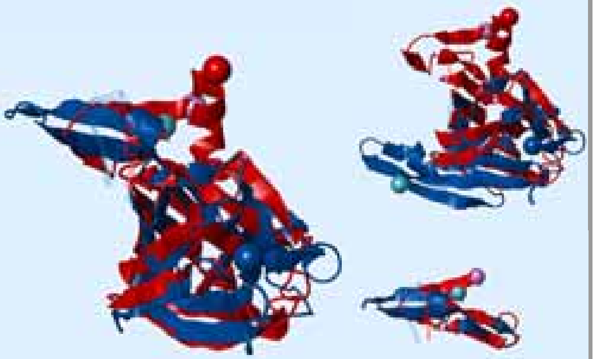 Auxin binding protein 1 DTDP-6-deoxy-D-XYLO-4-hexulose 3,5-epimerase
	1ep0A (183)	37-48 (12)	38-48 (11)	36-41 (6)						
347	1lr5A (160)	35-41 (7)	36-40 (5)	36-40 (5)	N	0.30	2.45	80.63% (129/160)	4.38% (7/160)	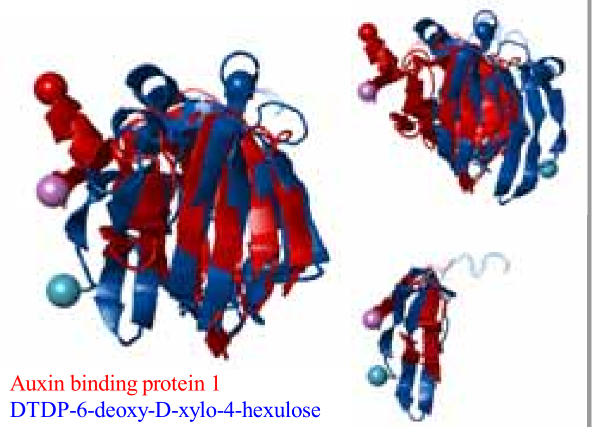 Auxin binding protein 1 DTDP-6-deoxy-D-xylo-4-hexulose 3,5-epimerase
	1nxmA (194)	48-60 (13)	49-59 (11)	36-40 (5)						

348	1lr5A<br>(160)	5-44 (40)	29-41 (13)	36-41 (6)	N	0.32	2.43	82.50%<br>(132/160)	9.38%<br>(15/160)	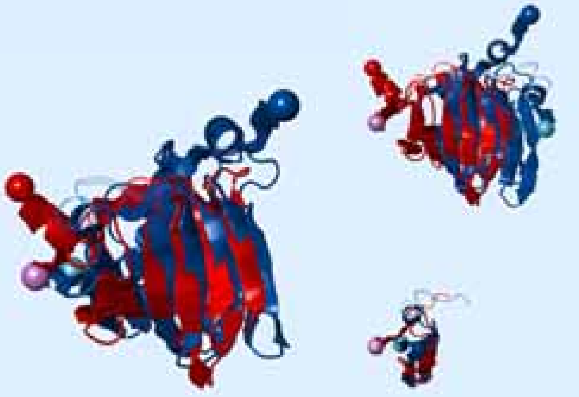<br>Auxin binding protein 1<br>PCZA361.16
	1wa4A<br>(205)	3-50 (48)	28-47 (20)	36-41 (6)						
349	1lr5A<br>(160)	36-41 (6)	36-40 (5)	36-40 (5)	N	0.32	2.53	81.88%<br>(131/160)	5.63%<br>(9/160)	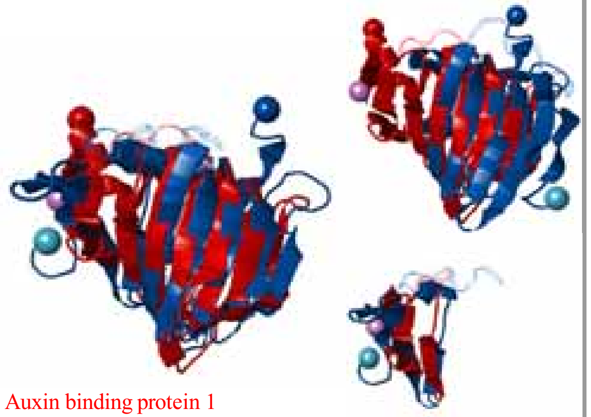<br>Auxin binding protein 1<br>DTDP-4-dehydrorhamnose<br>3,5-epimerase
	2ixlA<br>(196)	46-60 (15)	46-59 (14)	36-40 (5)						
350	1lr5A<br>(160)	36-41 (6)	36-40 (5)	36-40 (5)	N	0.32	2.58	82.50%<br>(132/160)	5.63%<br>(9/160)	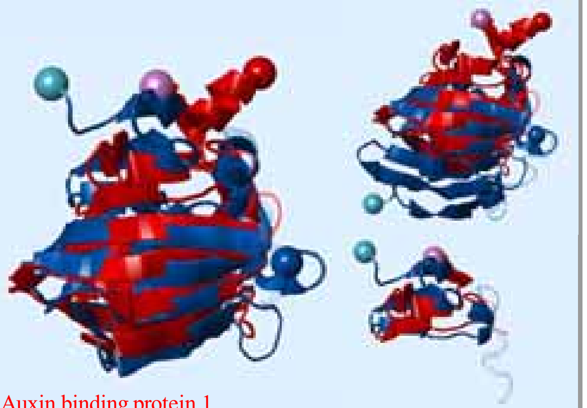<br>Auxin binding protein 1<br>DTDP-4-dehydrorhamnose<br>3,5-epimerase
	2ixlB<br>(195)	46-60 (15)	46-59 (14)	36-40 (5)						

351	1lr5A (160)	36-41 (6)	36-40 (5)	36-40 (5)	N	0.29	2.81	82.50% (132/160)	3.75% (6/160)	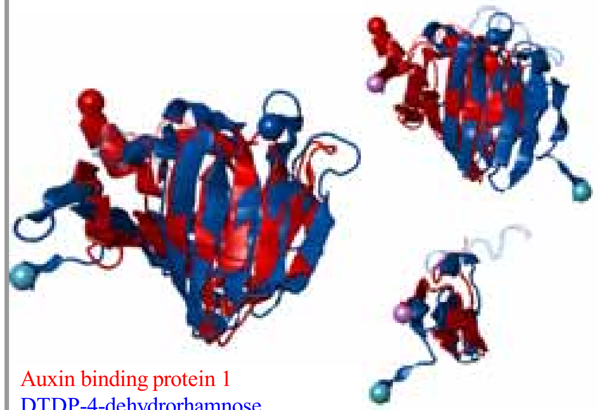<br>Auxin binding protein 1<br>DTDP-4-dehydrorhamnose 3,5-epimerase
	2ixlC (197)	46-60 (15)	46-59 (14)	36-40 (5)						
352	1lr5A (160)	1-41 (41)	29-41 (13)	39-41 (3)	N	0.32	2.38	88.06% (118/134)	11.94% (16/134)	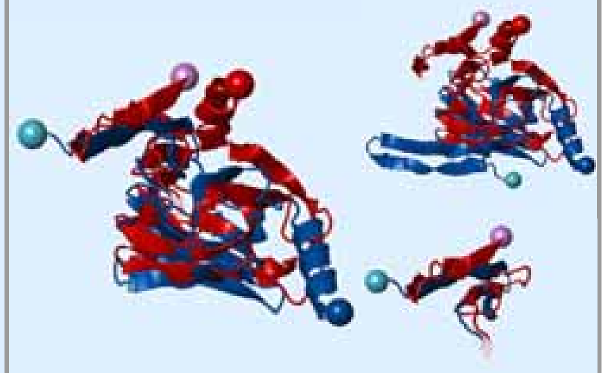<br>Auxin binding protein 1<br>DTDP-6-deoxy-3,4-keto-hexulose isomerase
	2pa7B (134)	2-32 (31)	20-32 (13)	39-41 (3)						
353	1lr5A (160)	122-153 (32)	132-153 (22)	133-153 (21)	C	0.27	2.04	70.15% (94/134)	11.94% (16/134)	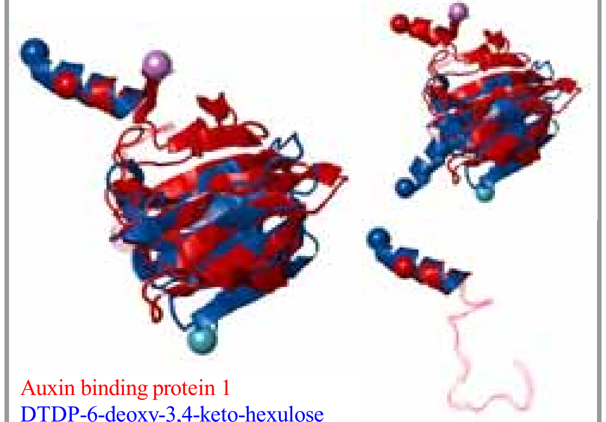<br>Auxin binding protein 1<br>DTDP-6-deoxy-3,4-keto-hexulose isomerase
	2pa7B (134)	109-123 (15)	121-123 (3)	133-153 (21)						

354	1lr5A (160)	18-63 (46)	29-63 (35)	39-41 (3)	N	0.38	2.39	87.50% (119/136)	11.03% (15/136)	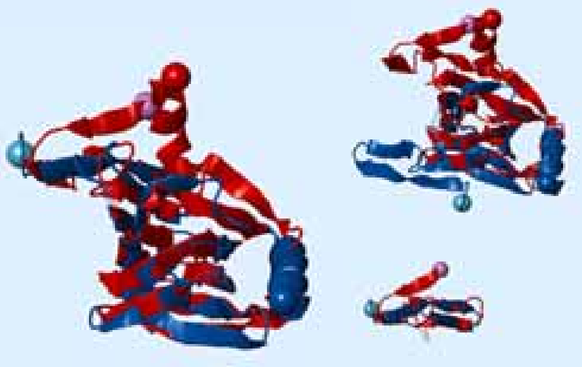 Auxin binding protein 1<br>DTDP-6-deoxy-3,4-keto-hexulose isomerase
	2paeA (136)	11-56 (46)	20-56 (37)	39-41 (3)						
355	1lr5A (160)	109-154 (46)	128-154 (27)	133-153 (21)	C	0.26	2.02	69.12% (94/136)	11.03% (15/136)	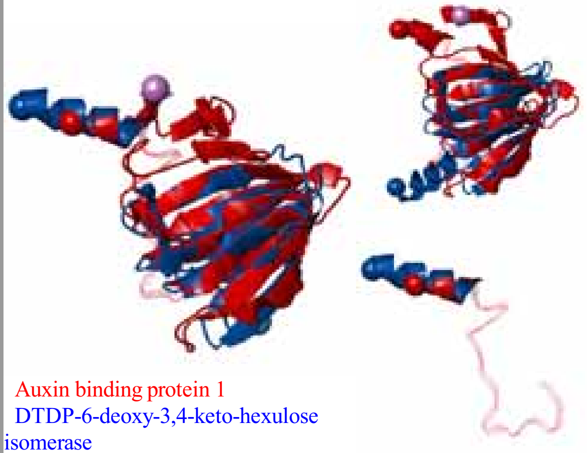 Auxin binding protein 1<br>DTDP-6-deoxy-3,4-keto-hexulose isomerase
	2paeA (136)	98-124 (27)	115-124 (10)	133-153 (21)						
356	1lr5A (160)	18-41 (24)	18-41 (24)	39-41 (3)	N	0.32	2.41	88.06% (118/134)	11.19% (15/134)	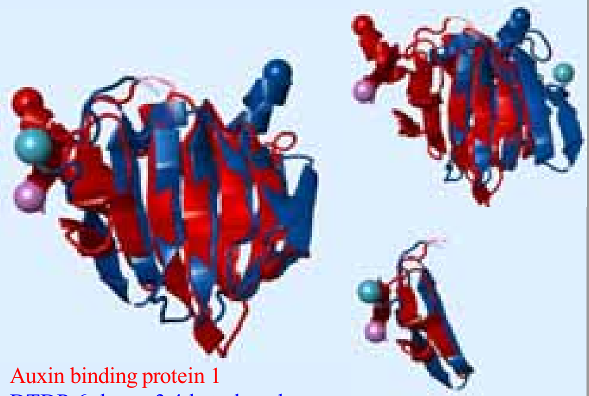 Auxin binding protein 1<br>DTDP-6-deoxy-3,4-keto-hexulose isomerase
	2paeB (134)	11-32 (22)	11-32 (22)	39-41 (3)						

357	1lr5A (160)	124-153 (30)	132-153 (22)	133-153 (21)	C	0.27	2.04	70.15% (94/134)	11.19% (15/134)	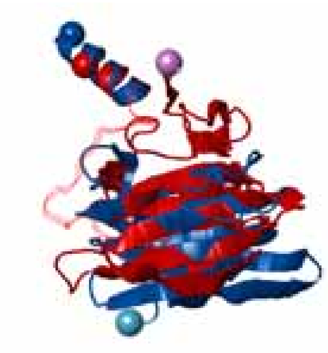 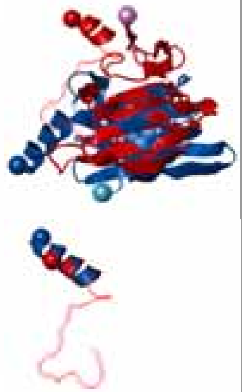 Auxin binding protein 1 DTDP-6-deoxy-3,4-keto-hexulose isomerase
	2paeB (134)	111-123 (13)	121-123 (3)	133-153 (21)						
358	1lr5A (160)	29-41 (13)	29-41 (13)	39-41 (3)	N	0.38	2.33	87.41% (118/135)	11.11% (15/135)	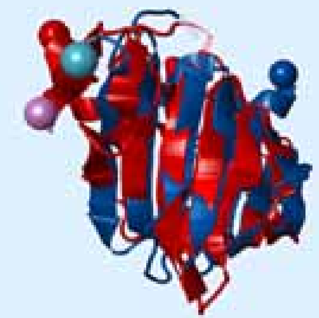 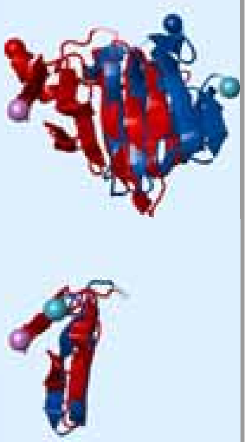 Auxin binding protein 1 DTDP-6-deoxy-3,4-keto-hexulose isomerase
	2pakA (135)	20-32 (13)	20-32 (13)	39-41 (3)						
359	1lr5A (160)	109-153 (45)	124-153 (30)	133-153 (21)	C	0.26	2.02	69.63% (94/135)	11.11% (15/135)	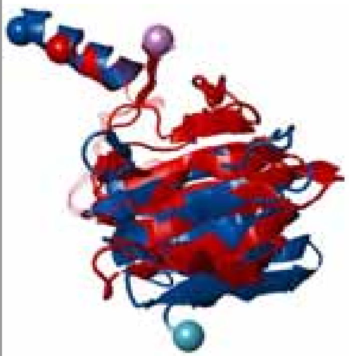 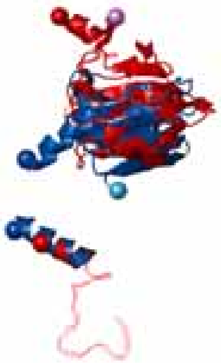 Auxin binding protein 1 DTDP-6-deoxy-3,4-keto-hexulose isomerase
	2pakA (135)	98-123 (26)	111-123 (13)	133-153 (21)						

360	1lr5A (160)	1-41 (41)	29-41 (13)	39-41 (3)	N	0.32	2.33	87.31% (117/134)	11.19% (15/134)	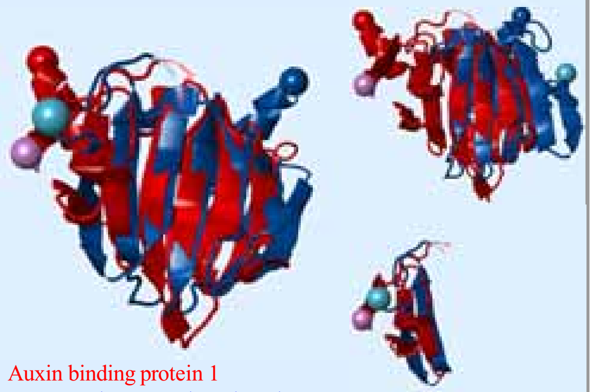<br>Auxin binding protein 1<br>DTDP-6-deoxy-3,4-keto-hexulose isomerase
	2pakB (134)	2-32 (31)	20-32 (13)	39-41 (3)						
361	1lr5A (160)	132-153 (22)	132-153 (22)	133-153 (21)	C	0.27	2.04	70.15% (94/134)	11.19% (15/134)	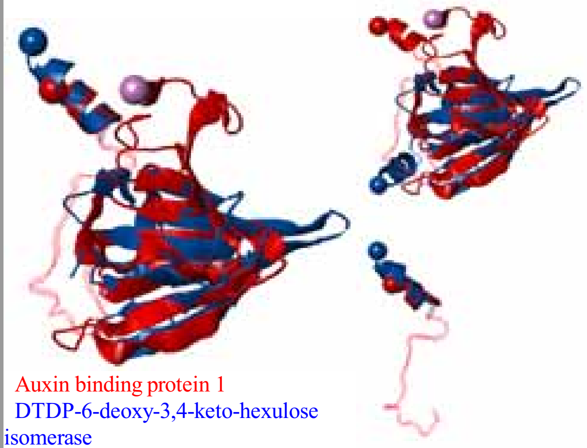<br>Auxin binding protein 1<br>DTDP-6-deoxy-3,4-keto-hexulose isomerase
	2pakB (134)	121-123 (3)	121-123 (3)	133-153 (21)						
362	1lr5A (160)	18-56 (39)	18-41 (24)	39-41 (3)	N	0.37	2.41	88.15% (119/135)	10.37% (14/135)	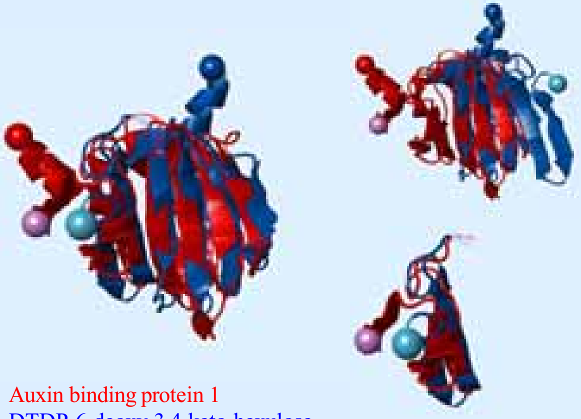<br>Auxin binding protein 1<br>DTDP-6-deoxy-3,4-keto-hexulose isomerase
	2pamA (135)	11-48 (38)	11-32 (22)	39-41 (3)						

363	1lr5A (160)	120-153 (34)	128-153 (26)	133-153 (21)	C	0.27	2.01	69.63% (94/135)	10.37% (14/135)	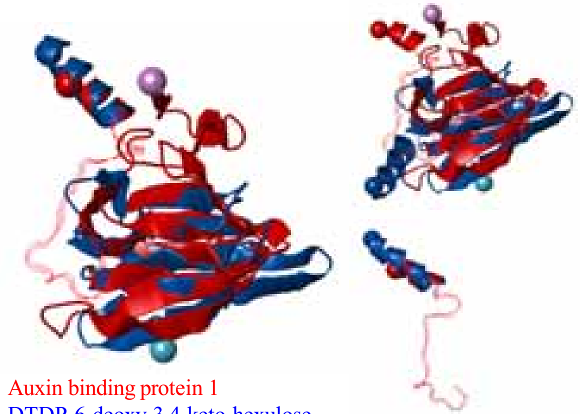 Auxin binding protein 1<br>DTDP-6-deoxy-3,4-keto-hexulose isomerase
	2pamA (135)	107-123 (17)	115-123 (9)	133-153 (21)						
364	1lr5A (160)	1-41 (41)	29-41 (13)	39-41 (3)	N	0.32	2.30	87.31% (117/134)	10.45% (14/134)	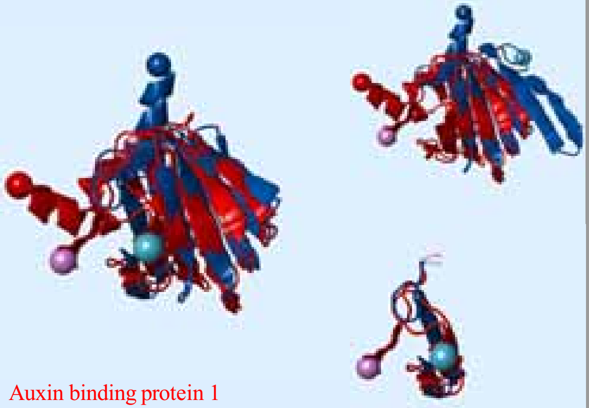 Auxin binding protein 1<br>DTDP-6-deoxy-3,4-keto-hexulose isomerase
	2pamB (134)	2-32 (31)	20-32 (13)	39-41 (3)						
365	1lr5A (160)	132-153 (22)	132-153 (22)	133-153 (21)	C	0.28	2.00	70.15% (94/134)	10.45% (14/134)	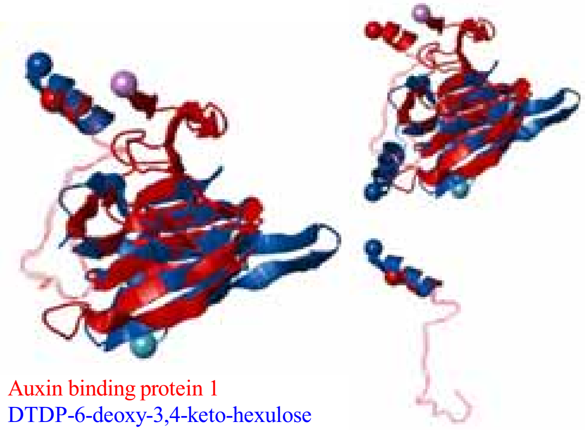 Auxin binding protein 1<br>DTDP-6-deoxy-3,4-keto-hexulose isomerase
	2pamB (134)	121-123 (3)	121-123 (3)	133-153 (21)						

366	1m1dA (163)	176-197 (22)	176-195 (20)	177-194 (18)	C	0.38	2.03	75.16% (121/161)	11.18% (18/161)	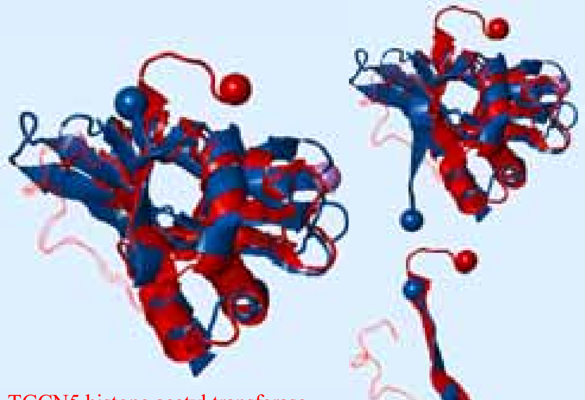
	1i1dD (161)	150-154 (5)	150-152 (3)	177-194 (18)						TGCN5 histone acetyl transferase<br>Glucosamine-phosphate N-acetyltransferase
367	1m1dA (163)	178-192 (15), 179-199 (21)	178-192 (15), 179-195 (17)	178-181 (4), 191-195 (5)	M	0.28	2.36	89.81% (141/157)	14.01% (22/157)	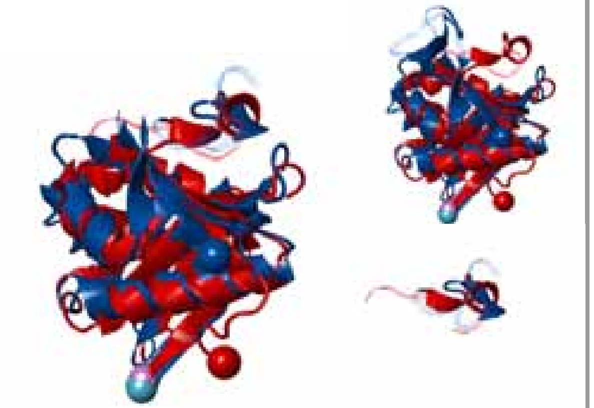
	1mk4A (157)	128-146 (19), 133-153 (21)	128-146 (19), 133-149 (17)	178-181 (4), 191-195 (5)						TGCN5 histone acetyl transferase<br>Hypothetical protein yqjY
368	1m1dA (163)	168-194 (27)	171-193 (23)	173-193 (21)	C	0.43	2.29	86.00% (129/150)	13.33% (20/150)	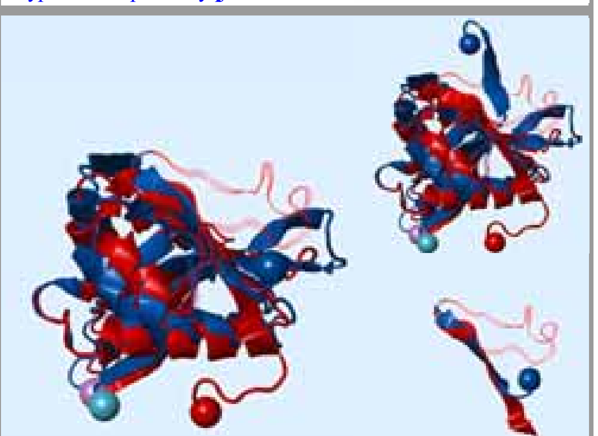
	1qsmA (150)	138-147 (10)	141-146 (6)	173-193 (21)						TGCN5 histone acetyl transferase<br>HPA2 histone acetyltransferase

369	1m1dA (163)	170-205 (36)	170-197 (28)	173-197 (25)	C	0.44	2.22	86.58% (129/149)	13.42% (20/149)	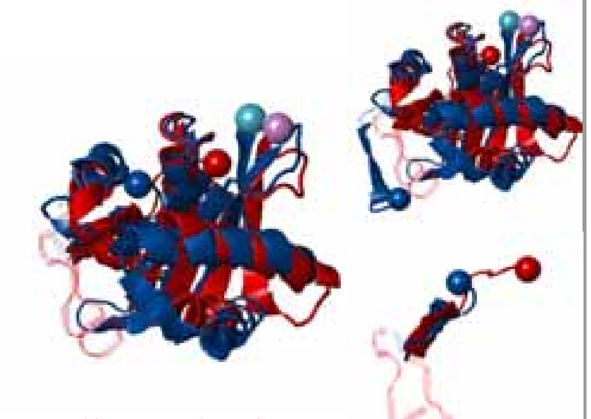 TGCN5 histone acetyl transferase<br>HPA2 histone acetyltransferase
	1qsmB (149)	140-155 (16)	140-147 (8)	173-197 (25)						
370	1m1dA (163)	168-193 (26)	171-193 (23)	173-193 (21)	C	0.43	2.25	84.87% (129/152)	13.16% (20/152)	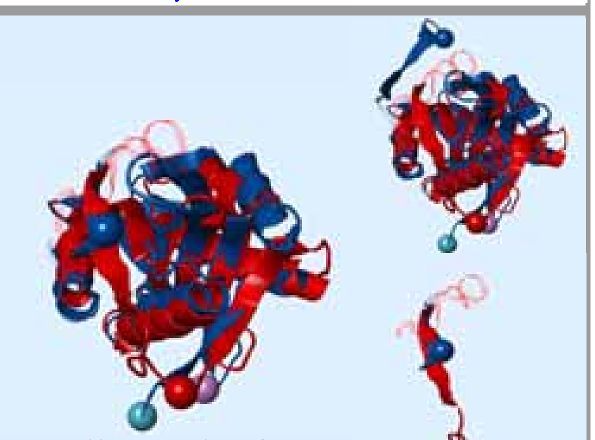 TGCN5 histone acetyl transferase<br>HPA2 histone acetyltransferase
	1qsmD (152)	138-146 (9)	141-146 (6)	173-193 (21)						
371	1m1dA (163)	175-202 (28)	176-202 (27)	177-195 (19)	C	0.47	2.20	89.66% (130/145)	9.66% (14/145)	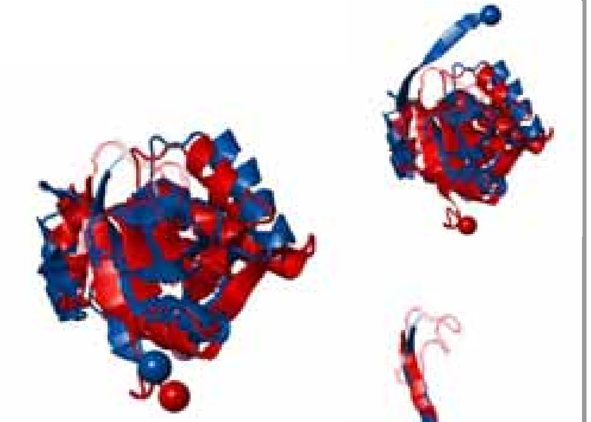 TGCN5 histone acetyl transferase<br>Aminoglycoside 6'-N-acetyltransferase
	1s3zB (145)	133-143 (11)	134-143 (10)	177-195 (19)						

372	1m1dA (163)	175-203 (29)	176-202 (27)	177-195 (19)	C	0.44	2.16	84.31% (129/153)	8.50% (13/153)	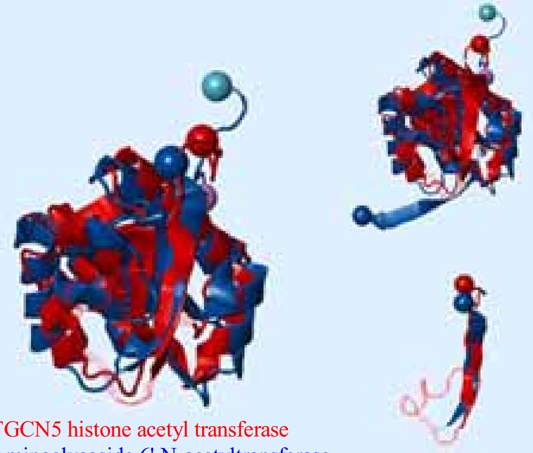 TGCN5 histone acetyl transferase<br>Aminoglycoside 6'-N-acetyltransferase
	1s5kA (153)	133-144 (12)	134-143 (10)	177-195 (19)						
373	1m1dA (163)	175-197 (23)	176-194 (19)	176-193 (18)	C	0.39	2.46	83.23% (129/155)	9.68% (15/155)	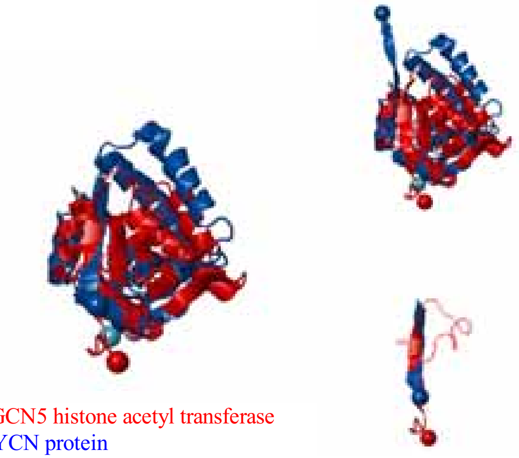 TGCN5 histone acetyl transferase<br>YYCN protein
	1ufhA (155)	146-151 (6)	147-148 (2)	176-193 (18)						
374	1m1dA (163)	176-197 (22)	176-197 (22)	176-193 (18)	C	0.44	2.08	83.12% (128/154)	9.09% (14/154)	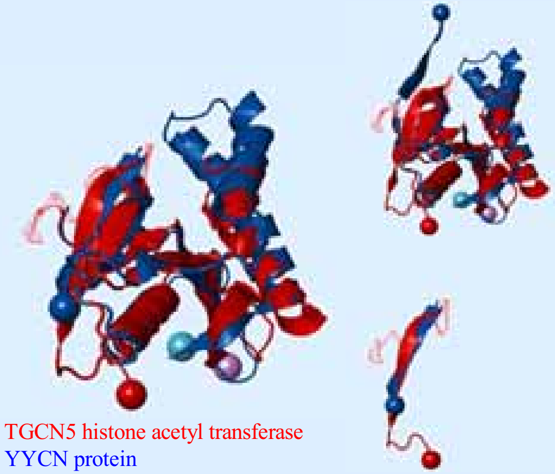 TGCN5 histone acetyl transferase<br>YYCN protein
	1ufhB (154)	147-151 (5)	147-151 (5)	176-193 (18)						

375	1m1dA (163)	175-198 (24)	176-198 (23)	176-193 (18)	C	0.45	2.10	85.91% (128/149)	12.08% (18/149)	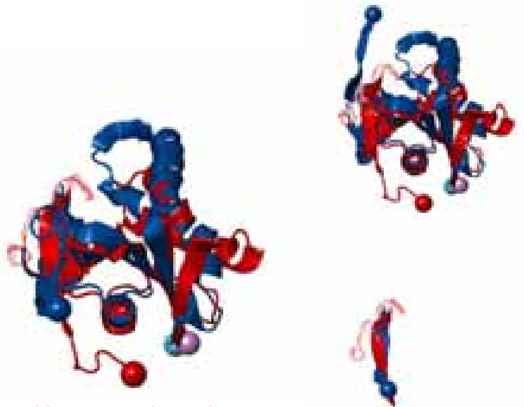 TGCN5 histone acetyl transferase<br>Putative acetyl transferase
	1vkcA (149)	140-146 (7)	141-146 (6)	176-193 (18)						
376	1m1dA (163)	176-200 (25)	176-197 (22)	177-194 (18)	C	0.38	1.92	73.62% (120/163)	10.43% (17/163)	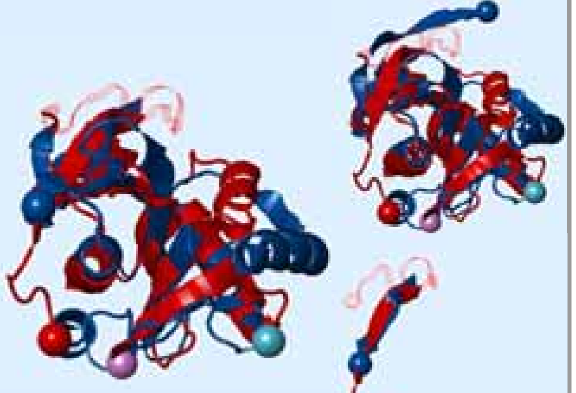 TGCN5 histone acetyl transferase<br>Glucosamine 6-phosphate acetyltransferase
	2vxkA (165)	181-188 (8)	181-185 (5)	177-194 (18)						
377	1mk4A (157)	125-148 (24)	125-148 (24)	127-146 (20)	C	0.51	2.01	89.66% (130/145)	20.69% (30/145)	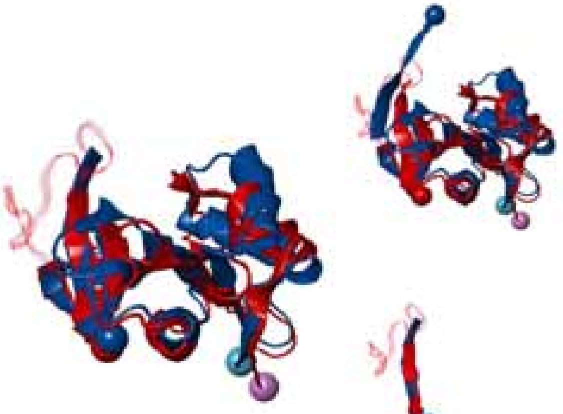 Hypothetical protein yqjY<br>Aminoglycoside 6'-N-acetyltransferase
	1s3zB (145)	133-137 (5)	133-137 (5)	127-146 (20)						

378	1mk4A (157)	125-155 (31)	125-155 (31)	128-149 (22)	C	0.47	2.04	83.66% (128/153)	19.61% (30/153)	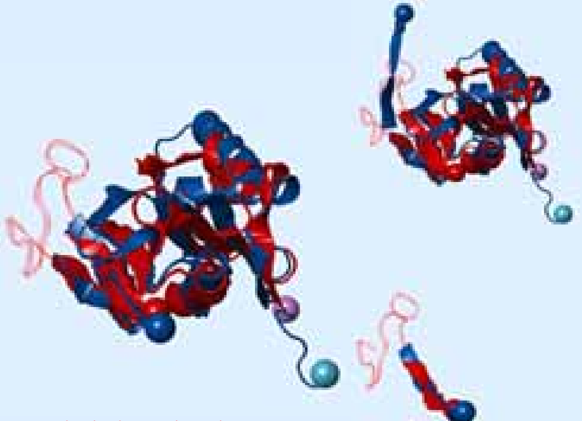 Hypothetical protein yqjY<br>Aminoglycoside 6'-N-acetyltransferase
	1s5kA (153)	133-142 (10)	133-142 (10)	128-149 (22)						
379	1mk4A (157)	125-153 (29)	125-150 (26)	127-148 (22)	C	0.37	2.56	80.65% (125/155)	9.68% (15/155)	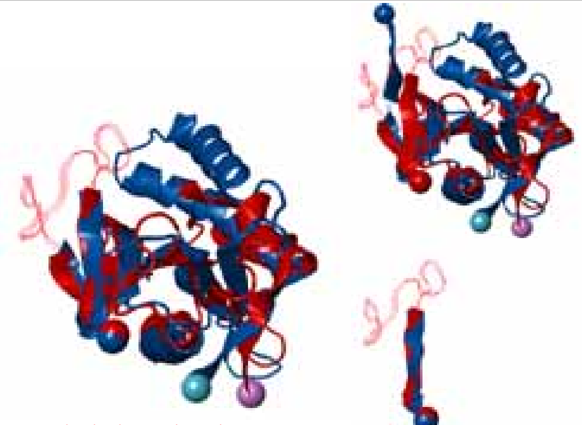 Hypothetical protein yqjY<br>YYCN protein
	1ufhA (155)	146-153 (8)	146-150 (5)	127-148 (22)						
380	1mk4A (157)	125-153 (29)	125-150 (26)	127-148 (22)	C	0.39	2.51	82.47% (127/154)	10.39% (16/154)	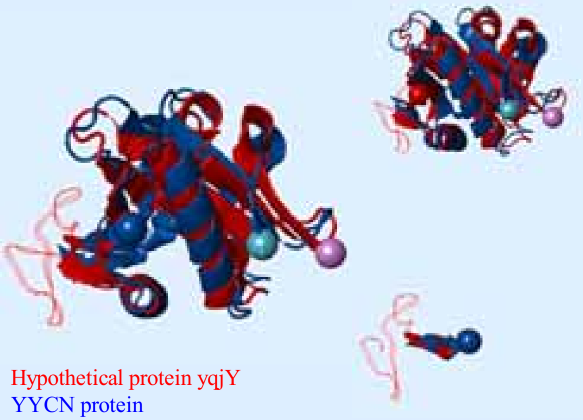 Hypothetical protein yqjY<br>YYCN protein
	1ufhB (154)	146-153 (8)	146-150 (5)	127-148 (22)						

381	1mk4A (157)	122-154 (33)	125-149 (25)	127-147 (21)	C	0.47	2.26	87.33% (131/150)	18.67% (28/150)	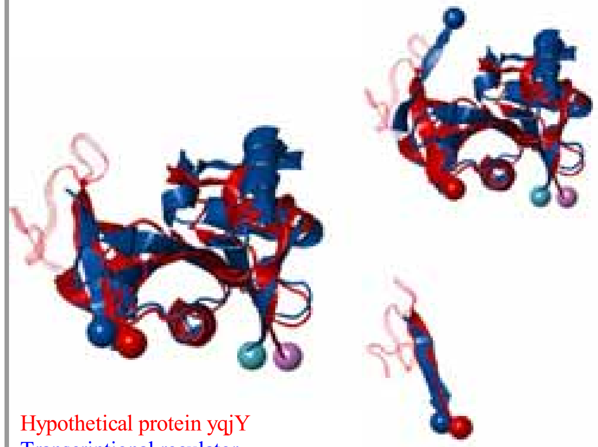 Hypothetical protein yqjY<br>Transcriptional regulator
	1z4eA (150)	140-152 (13)	143-147 (5)	127-147 (21)						
382	1mk4A (157)	125-155 (31)	125-150 (26)	125-151 (27)	C	0.47	2.25	93.85% (122/130)	13.85% (18/130)	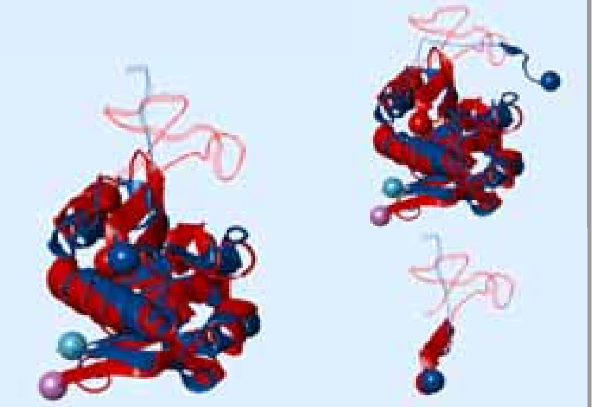 Hypothetical protein yqjY<br>Hypothetical protein TTHA1254
	2d4pA (130)	116-129 (14)	116-124 (9)	125-151 (27)						
383	1mk4A (157)	125-156 (32)	125-156 (32)	127-135 (9)	C	0.35	2.50	87.26% (137/157)	13.38% (21/157)	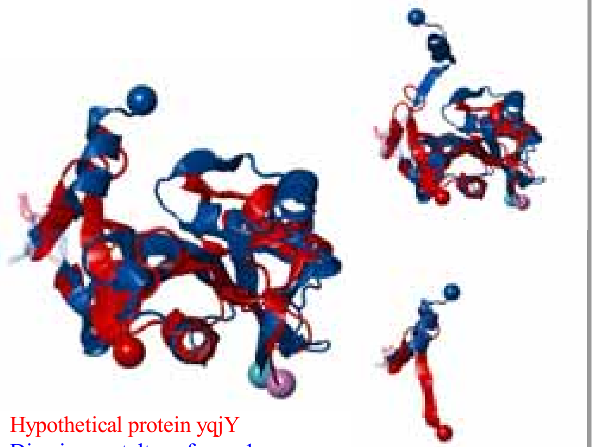 Hypothetical protein yqjY<br>Diamine acetyltransferase 1
	2g3tA (169)	146-171 (26)	146-171 (26)	127-135 (9)						

384	1mk4A (157)	125-154 (30)	125-149 (25)	127-149 (23)	C	0.40	2.23	82.17% (129/157)	13.38% (21/157)	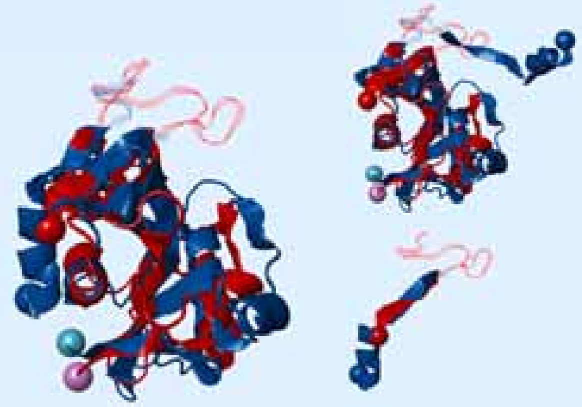 Hypothetical protein yqjY<br>Diamine acetyltransferase 1
	2jevA (169)	146-159 (14)	146-154 (9)	127-149 (23)						
385	1mk4A (157)	125-155 (31)	125-155 (31)	128-149 (22)	C	0.47	2.16	87.07% (128/147)	20.41% (30/147)	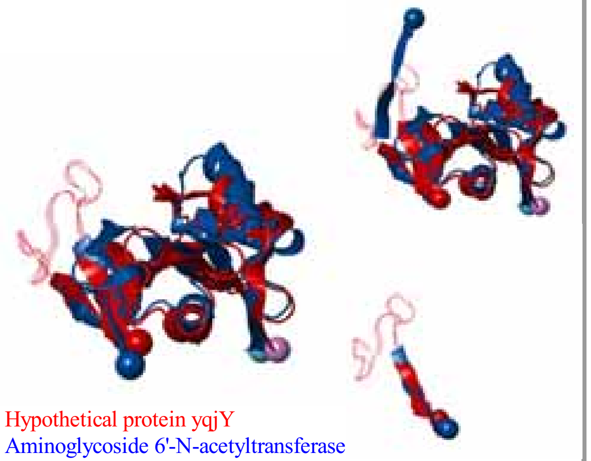 Hypothetical protein yqjY<br>Aminoglycoside 6'-N-acetyltransferase
	2vbqA (147)	133-142 (10)	133-142 (10)	128-149 (22)						
386	1mk4A (157)	125-155 (31)	125-155 (31)	128-149 (22)	C	0.48	2.09	88.19% (127/144)	19.44% (28/144)	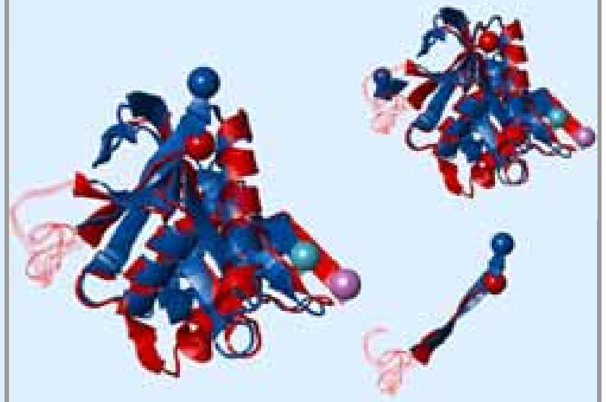 Hypothetical protein yqjY<br>Aminoglycoside 6'-N-acetyltransferase
	2vbqB (144)	133-142 (10)	133-142 (10)	128-149 (22)						

387	1obpA (158)	93-126 (34)	101-126 (26)	121-126 (6)	C	0.41	2.77	88.61% (140/158)	14.56% (23/158)	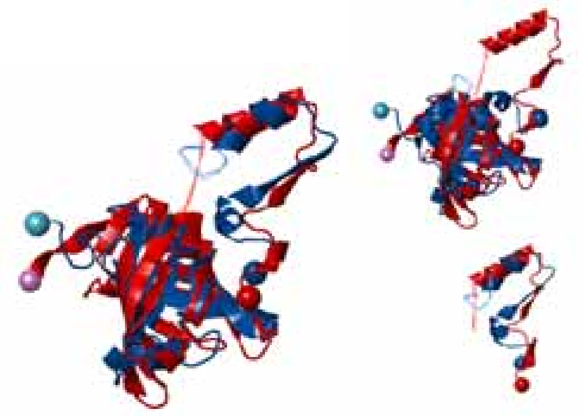 Odorant-binding protein<br>Beta-lactoglobulin a
	1cj5A (162)	96-130 (35)	105-130 (26)	121-126 (6)						
388	1obpA (158)	119-129 (11)	121-126 (6)	121-126 (6)	C	0.53	2.11	89.87% (142/158)	15.19% (24/158)	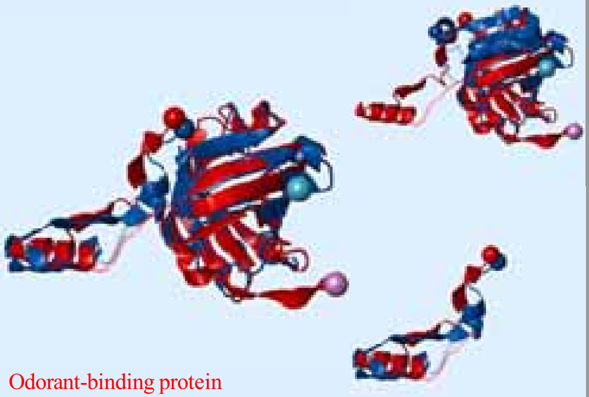 Odorant-binding protein<br>Beta-lactoglobulin
	1dv9A (162)	122-133 (12)	124-130 (7)	121-126 (6)						
389	1obpA (158)	3-12 (10)	3-10 (8)	10-10 (1)	N	0.88	0.81	97.45% (153/157)	91.72% (144/157)	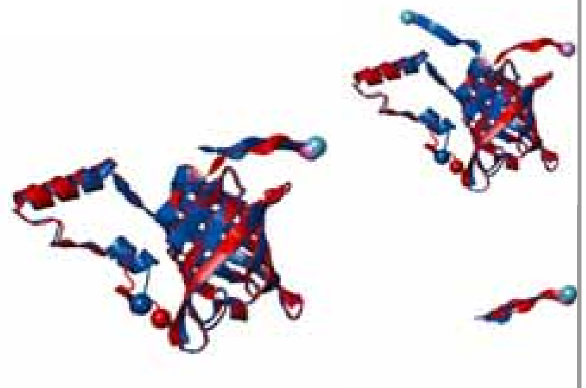 Odorant-binding protein<br>Odorant binding protein
	1pboA (157)	2-12 (11)	2-10 (9)	10-10 (1)						

390	1obpA (158)	121-129 (9)	121-129 (9)	121-129 (9)	C	0.37	2.38	83.54% (132/158)	7.59% (12/158)	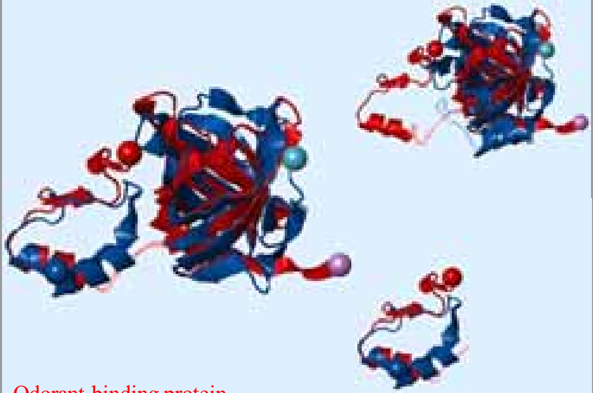 Odorant-binding protein<br>Nitrophorin 4
	1sy2A (184)	139-146 (8)	139-146 (8)	121-129 (9)						
391	1obpA (158)	107-123 (17)	121-123 (3)	121-123 (3)	C	0.33	2.55	81.65% (129/158)	6.96% (11/158)	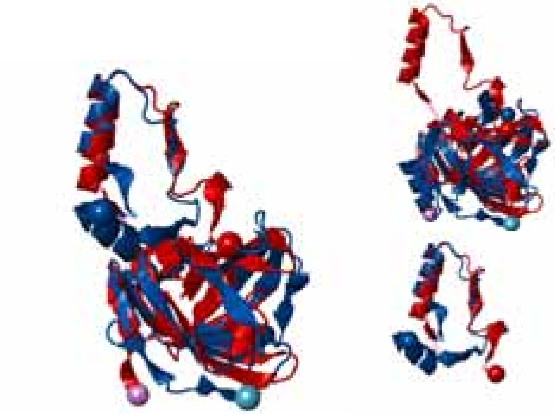 Odorant-binding protein<br>Nitrophorin 1
	1u17A (185)	126-143 (18)	140-143 (4)	121-123 (3)						
392	1q2yA (140)	122-136 (15)	122-133 (12)	125-130 (6)	C	0.53	2.02	89.29% (125/140)	12.14% (17/140)	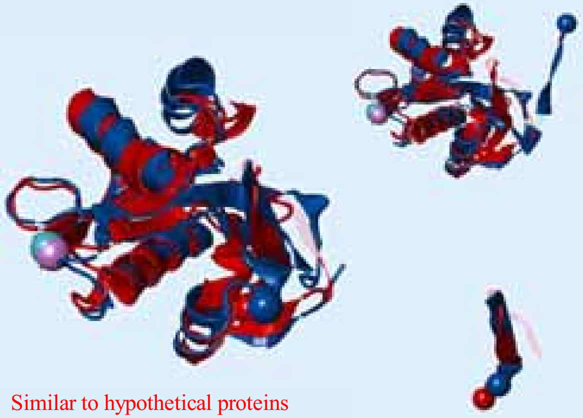 Similar to hypothetical proteins<br>Aminoglycoside 6'-N-acetyltransferase
	1s3zB (145)	135-144 (10)	135-141 (7)	125-130 (6)						

393	1q2yA (140)	122-139 (18)	122-139 (18)	122-132 (11)	C	0.35	2.20	83.57% (117/140)	15.71% (22/140)	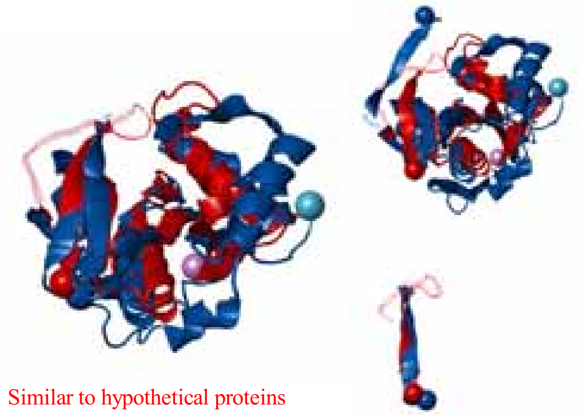<br>Similar to hypothetical proteins<br>Glucosamine 6-phosphate N-acetyltransferase
	2o28A (182)	173-181 (9)	173-181 (9)	122-132 (11)						
394	1q2yA (140)	122-133 (12)	122-133 (12)	125-131 (7)	C	0.52	2.02	88.57% (124/140)	12.14% (17/140)	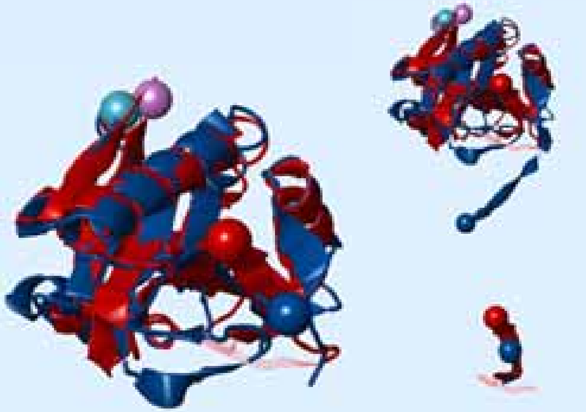<br>Similar to hypothetical proteins<br>Aminoglycoside 6'-N-acetyltransferase
	2vbqB (144)	135-140 (6)	135-140 (6)	125-131 (7)						
395	1q2yA (140)	122-139 (18)	122-137 (16)	123-135 (13)	C	0.40	2.00	82.14% (115/140)	18.57% (26/140)	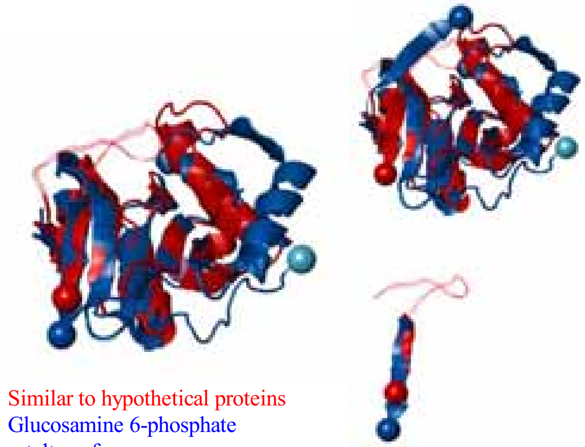<br>Similar to hypothetical proteins<br>Glucosamine 6-phosphate acetyltransferase
	2vxkA (165)	182-187 (6)	182-185 (4)	123-135 (13)						

396	1s60A (152)	129-144 (16)	135-139 (5)	138-138 (1)	C	0.50	1.73	88.82% (135/152)	13.82% (21/152)	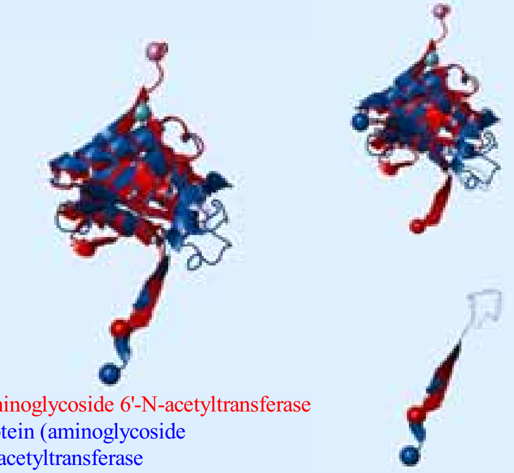 Aminoglycoside 6'-N-acetyltransferase
	1b87A (181)	149-176 (28)	155-170 (16)	138-138 (1)						Protein (aminoglycoside N6'-acetyltransferase
397	1s60A (152)	110-145 (36)	110-145 (36)	115-136 (22)	C	0.43	2.14	88.24% (120/136)	8.82% (12/136)	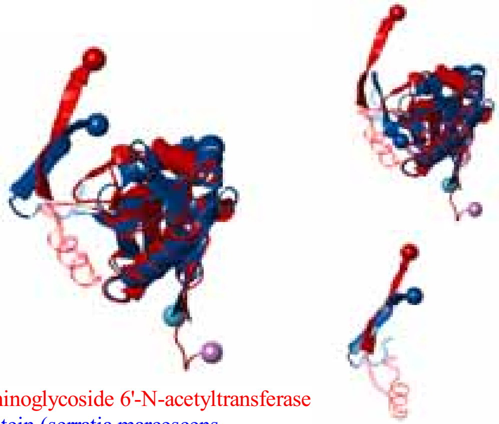 Aminoglycoside 6'-N-acetyltransferase
	1bo4B (136)	140-160 (21)	140-160 (21)	115-136 (22)						Protein (serratia marcescens aminoglycoside-3
398	1s60A (152)	134-136 (3)	134-136 (3)	134-136 (3)	C	0.46	2.25	88.16% (134/152)	13.16% (20/152)	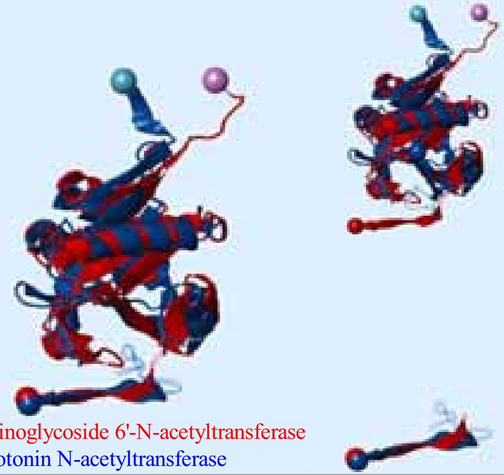 Aminoglycoside 6'-N-acetyltransferase
	1kuxA (166)	175-186 (12)	175-186 (12)	134-136 (3)						Serotonin N-acetyltransferase

399	1s60A (152)	128-141 (14)	129-141 (13)	140-140 (1)	C	0.44	2.29	87.50% (133/152)	11.84% (18/152)	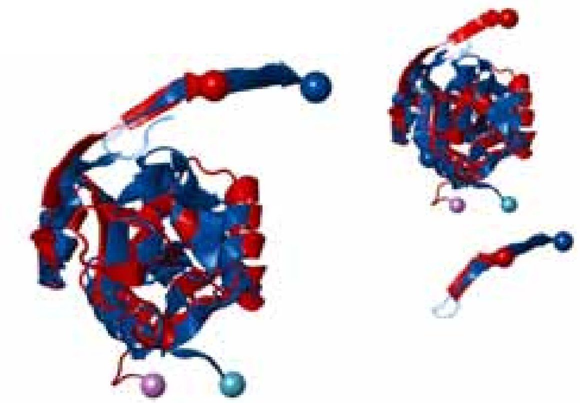 Aminoglycoside 6'-N-acetyltransferase<br>Serotonin N-acetyltransferase
	1l0cA (166)	169-187 (19)	170-187 (18)	140-140 (1)						
400	1s60A (152)	129-141 (13)	135-141 (7)	139-139 (1)	C	0.50	1.76	88.82% (135/152)	13.82% (21/152)	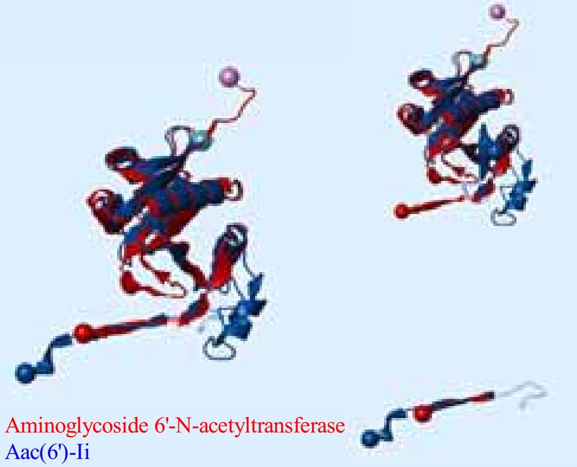 Aminoglycoside 6'-N-acetyltransferase<br>Aac(6')-Ii
	1n71A (180)	149-170 (22)	155-170 (16)	139-139 (1)						
401	1s60A (152)	129-144 (16)	135-143 (9)	139-139 (1)	C	0.44	1.84	88.82% (135/152)	13.82% (21/152)	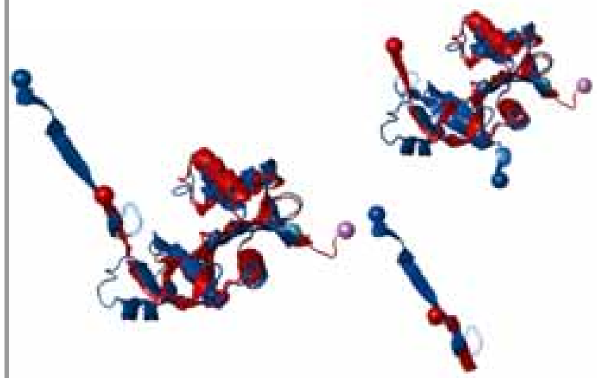 Aminoglycoside 6'-N-acetyltransferase<br>Aac(6')-Ii
	1n71B (179)	149-168 (20)	155-167 (13)	139-139 (1)						

402	1s60A (152)	126-144 (19)	131-138 (8)	135-136 (2)	C	0.41	2.40	84.87% (129/152)	7.89% (12/152)	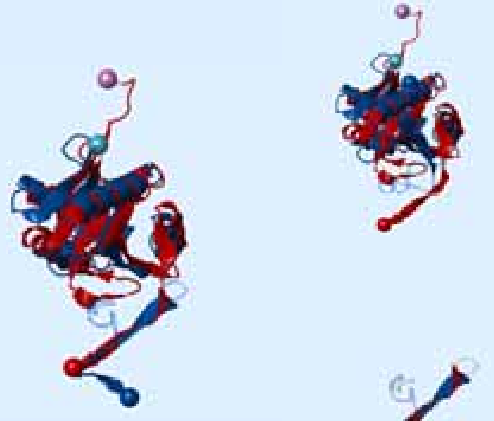 Aminoglycoside 6'-N-acetyltransferase<br>Histone acetyltransferase GCN5
	1q2dA (161)	168-203 (36)	173-197 (25)	135-136 (2)						
403	1s60A (152)	135-143 (9)	135-140 (6)	139-139 (1)	C	0.45	2.18	90.13% (137/152)	11.84% (18/152)	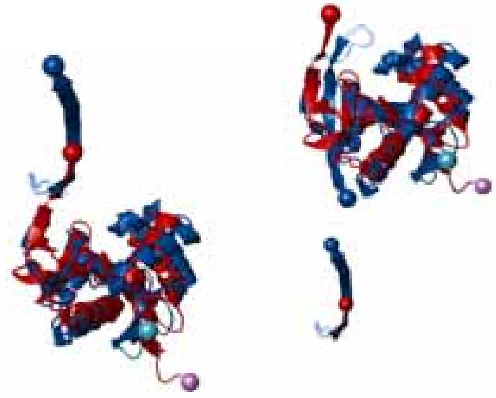 Aminoglycoside 6'-N-acetyltransferase<br>Acetyl transferase
	1s7nA (177)	153-169 (17)	153-166 (14)	139-139 (1)						
404	1s60A (152)	135-144 (10)	135-143 (9)	139-140 (2)	C	0.46	2.19	90.13% (137/152)	11.84% (18/152)	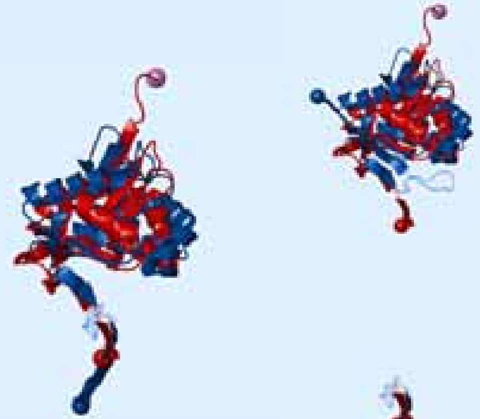 Aminoglycoside 6'-N-acetyltransferase<br>Acetyl transferase
	1s7nD (177)	153-172 (20)	153-171 (19)	139-140 (2)						

405	1s60A (152)	131-140 (10)	131-139 (9)	138-138 (1)	C	0.50	2.36	91.45% (139/152)	11.18% (17/152)	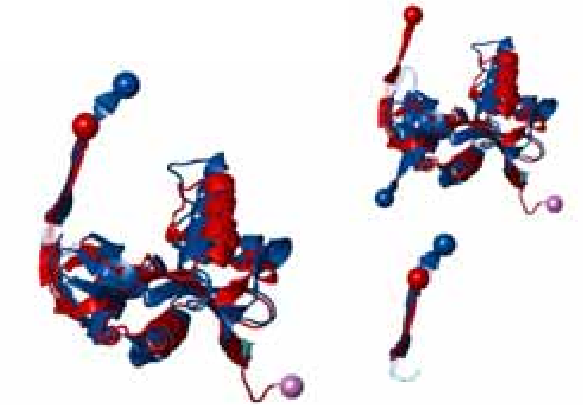<br>Aminoglycoside 6'-N-acetyltransferase<br>Hypothetical protein PH1933
	1wwzB (157)	140-152 (13)	140-151 (12)	138-138 (1)						
406	1s60A (152)	18-42 (25)	20-42 (23)	20-42 (23)	N	0.42	1.95	80.71% (113/140)	11.43% (16/140)	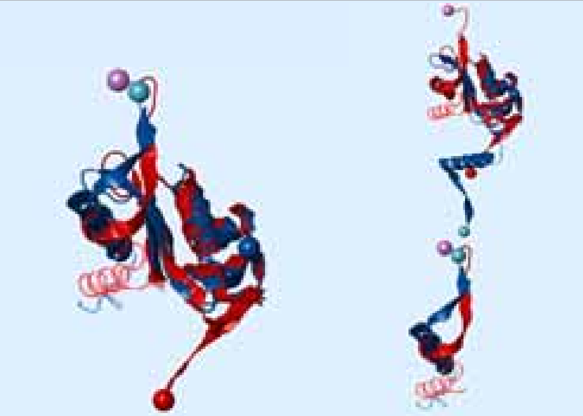<br>Aminoglycoside 6'-N-acetyltransferase<br>Acetyltransferase
	1y9wA (140)	25-38 (14)	27-38 (12)	20-42 (23)						
407	1s60A (152)	135-141 (7)	135-140 (6)	137-137 (1)	C	0.33	1.50	66.43% (93/140)	12.86% (18/140)	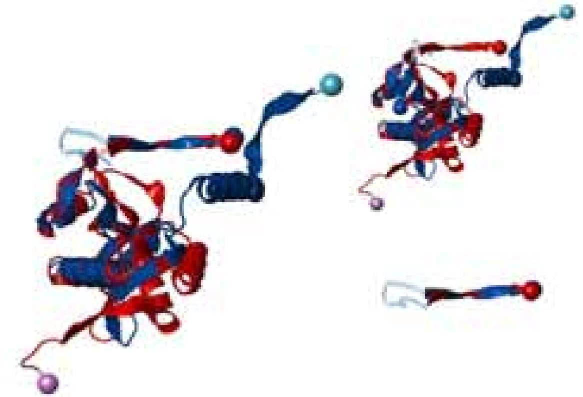<br>Aminoglycoside 6'-N-acetyltransferase<br>Acetyltransferase
	1y9wA (140)	121-136 (16)	121-135 (15)	137-137 (1)						

408	1s60A (152)	124-144 (21)	134-140 (7)	135-136 (2)	C	0.38	2.48	80.13% (121/151)	11.92% (18/151)	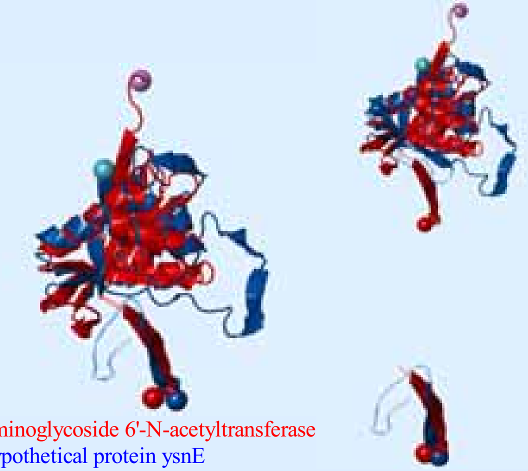 Aminoglycoside 6'-N-acetyltransferase<br>Hypothetical protein ysnE
	1yx0A (151)	121-150 (30)	131-146 (16)	135-136 (2)						
409	1s60A (152)	137-144 (8)	137-141 (5)	138-138 (1)	C	0.48	1.91	86.09% (130/151)	14.57% (22/151)	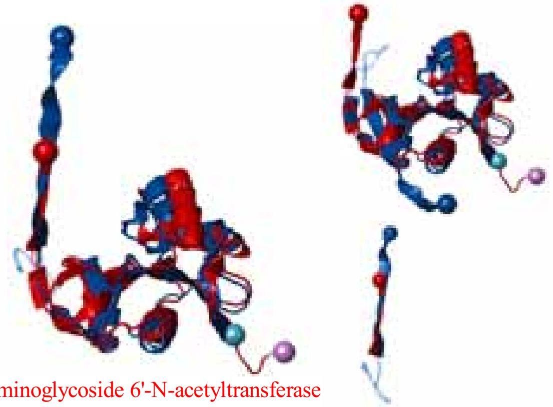 Aminoglycoside 6'-N-acetyltransferase<br>Modification OF 30S ribosomal subunit protein
	2cntA (151)	125-144 (20)	125-141 (17)	138-138 (1)						
410	1s60A (152)	131-144 (14)	135-139 (5)	138-138 (1)	C	0.43	2.06	90.13% (137/152)	17.76% (27/152)	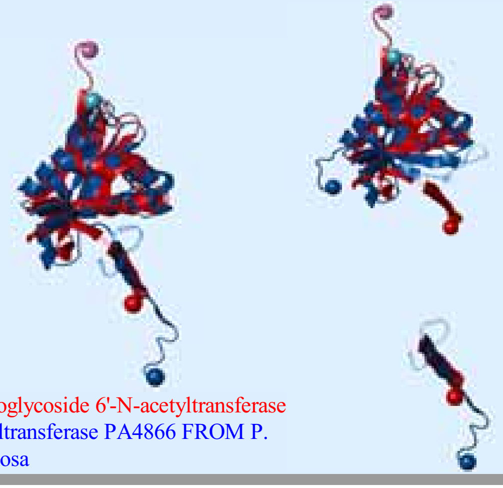 Aminoglycoside 6'-N-acetyltransferase<br>Acetyltransferase PA4866 FROM P. aeruginosa
	2j8mA (171)	138-161 (24)	142-156 (15)	138-138 (1)						

411	1s60A (152)	129-139 (11)	135-139 (5)	138-138 (1)	C	0.40	2.11	90.13% (137/152)	15.79% (24/152)	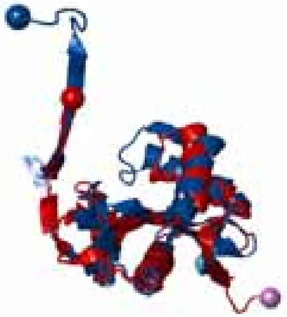 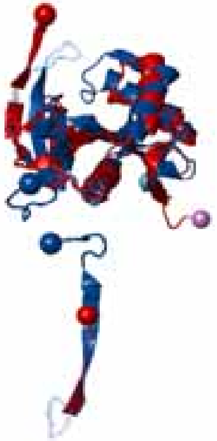 Aminoglycoside 6'-N-acetyltransferase<br>Acetyltransferase PA4866 FROM P. aeruginosa
	2j8mB (170)	136-156 (21)	142-156 (15)	138-138 (1)						
412	1s60A (152)	129-144 (16)	135-141 (7)	137-137 (1)	C	0.45	2.05	89.47% (136/152)	16.45% (25/152)	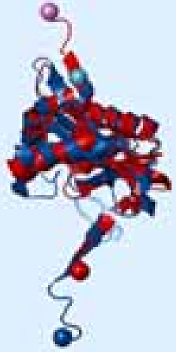 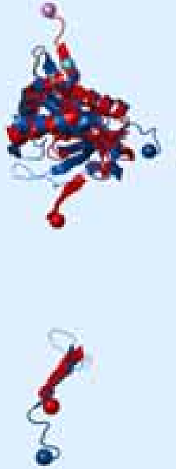 Aminoglycoside 6'-N-acetyltransferase<br>Acetyltransferase PA4866 FROM P. aeruginosa
	2j8nA (169)	136-163 (28)	142-160 (19)	137-137 (1)						
413	1s60A (152)	131-141 (11)	134-141 (8)	137-137 (1)	C	0.52	2.19	91.72% (133/145)	15.17% (22/145)	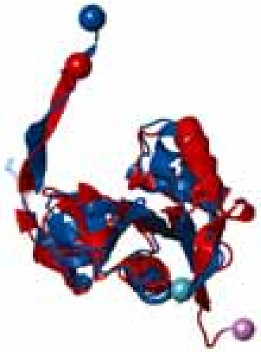 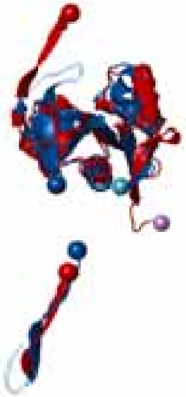 Aminoglycoside 6'-N-acetyltransferase<br>Glyphosate N-acetyltransferase
	2jdcA (145)	122-140 (19)	125-140 (16)	137-137 (1)						

414	1s60A (152)	-6-15 (22)	-6-15 (22)	2-2 (1)	N	0.34	2.43	84.21% (128/152)	10.53% (16/152)	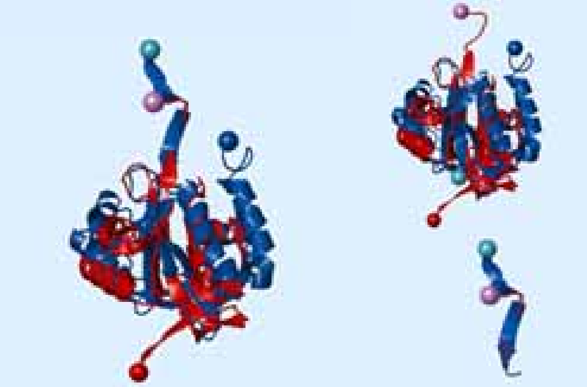<br>Aminoglycoside 6'-N-acetyltransferase<br>Putative uncharacterized protein TTHA1799
	2z0zA (194)	1-23 (23)	1-23 (23)	2-2 (1)						
415	1s60A (152)	105-144 (40)	131-143 (13)	131-138 (8)	C	0.35	2.37	78.95% (120/152)	7.89% (12/152)	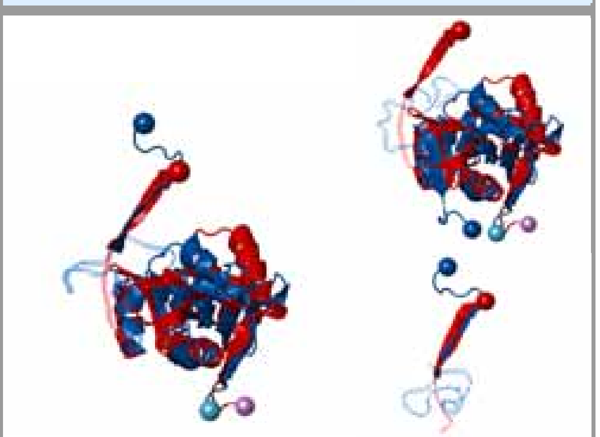<br>Aminoglycoside 6'-N-acetyltransferase<br>Histone acetyltransferase GCN5
	5gcnA (166)	106-159 (54)	129-158 (30)	131-138 (8)						
416	1s7fA (181)	148-174 (27)	154-174 (21)	154-166 (13)	C	0.34	2.84	83.83% (140/167)	10.18% (17/167)	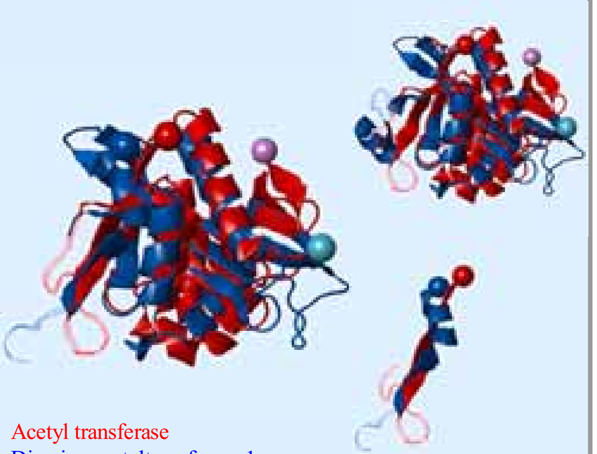<br>Acetyl transferase<br>Diamine acetyltransferase 1
	2f5iA (167)	143-168 (26)	149-168 (20)	154-166 (13)						

417	1s7fA (181)	-7-16 (24)	6-12 (7)	7-10 (4)	N	0.39	2.61	86.14% (143/166)	10.24% (17/166)	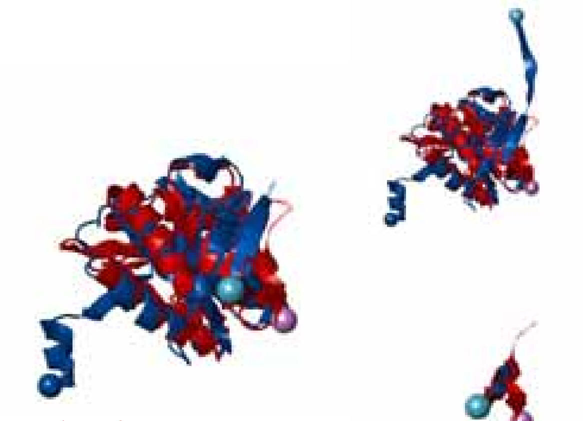 Acetyl transferase<br>Probable N-acetyltransferase
	2fe7B (166)	-7-8 (16)	1-4 (4)	7-10 (4)						
418	1s7fA (181)	148-175 (28)	153-171 (19)	153-168 (16)	C	0.38	2.52	83.73% (139/166)	10.84% (18/166)	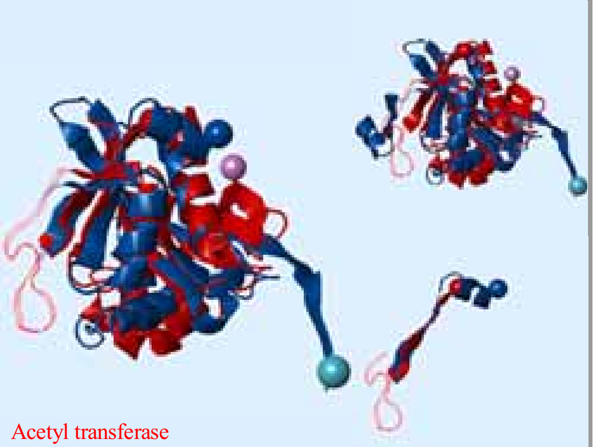 Acetyl transferase<br>Probable N-acetyltransferase
	2fe7B (166)	135-152 (18)	140-145 (6)	153-168 (16)						
419	1s7fA (181)	148-175 (28)	154-175 (22)	154-165 (12)	C	0.35	2.70	82.84% (140/169)	11.24% (19/169)	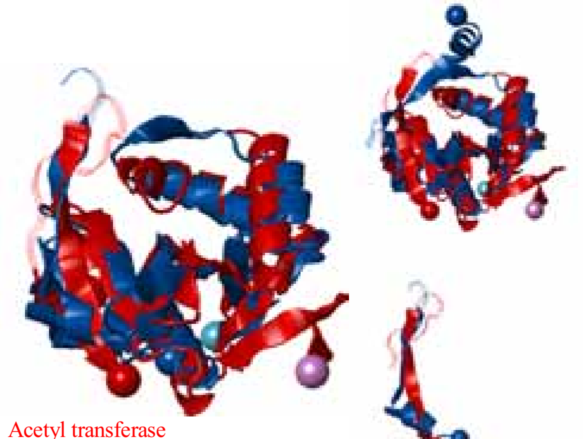 Acetyl transferase<br>Diamine acetyltransferase 1
	2g3tA (169)	143-169 (27)	149-169 (21)	154-165 (12)						

420	1s7fA (181)	148-175 (28)	152-175 (24)	154-167 (14)	C	0.34	2.88	84.02% (142/169)	11.24% (19/169)	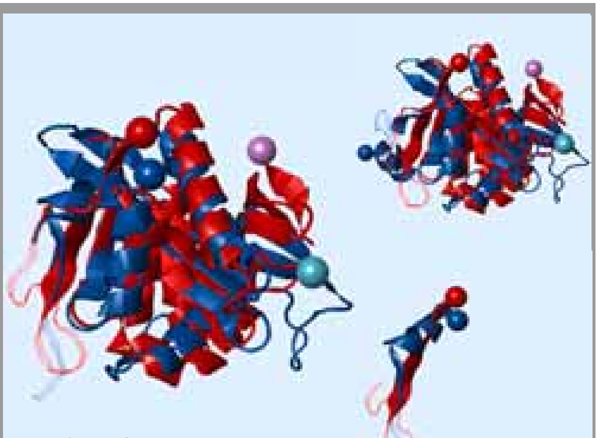<br>Acetyl transferase<br>Diamine acetyltransferase 1
	2jevA (169)	143-169 (27)	147-169 (23)	154-167 (14)						
421	1sjvA (102)	90-98 (9)	93-98 (6)	93-98 (6)	C	0.64	1.65	100.00% (102/102)	62.75% (64/102)	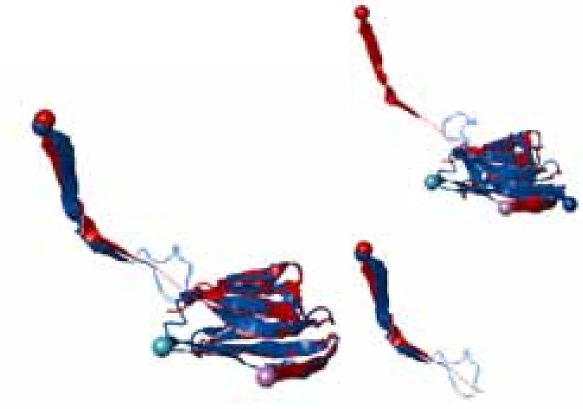<br>R9<br>IGM MEZ immunoglobulin
	1dqlH (123)	95-111 (17)	98-111 (14)	93-98 (6)						
422	1sjvA (102)	93-103 (11)	94-103 (10)	95-99 (5)	C	0.65	1.34	98.04% (100/102)	69.61% (71/102)	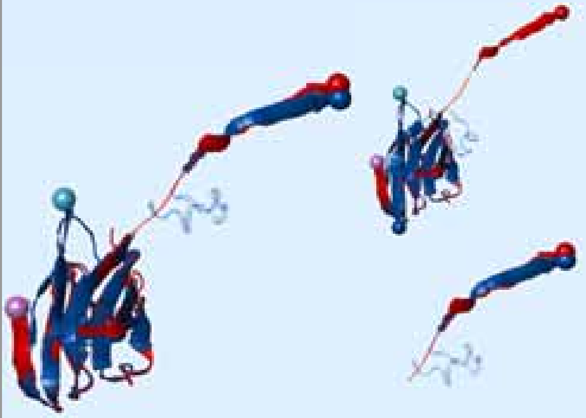<br>R9<br>Antibody heavy chain
	1f2xK (126)	896-919 (24)	897-919 (23)	95-99 (5)						

423	1sjvA (102)	93-103 (11)	94-98 (5)	94-98 (5)	C	0.65	1.43	99.02% (101/102)	69.61% (71/102)	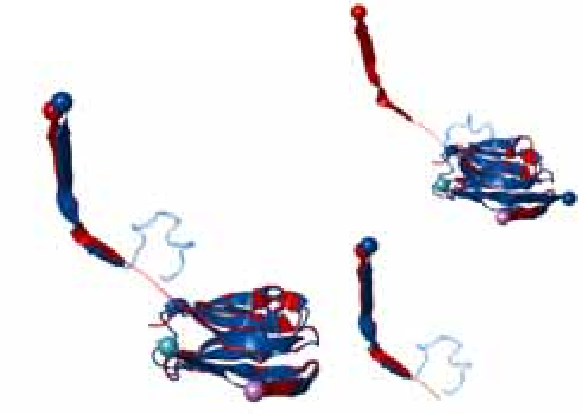<br>R9<br>Antibody heavy chain
	1f2xL (126)	1096-1119 (24)	1097-1114 (18)	94-98 (5)						
424	1sjvA (102)	91-98 (8)	94-98 (5)	94-98 (5)	C	0.62	1.50	99.02% (101/102)	67.65% (69/102)	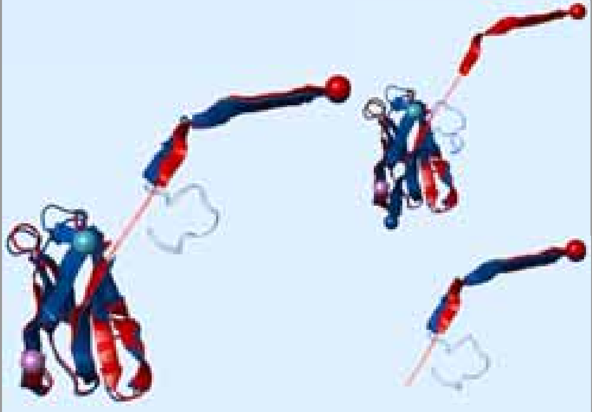<br>R9<br>Antibody VHH LAMA domain
	1i3vA (129)	101-117 (17)	104-117 (14)	94-98 (5)						
425	1sjvA (102)	93-99 (7)	93-99 (7)	93-99 (7)	C	0.64	1.63	98.04% (100/102)	57.84% (59/102)	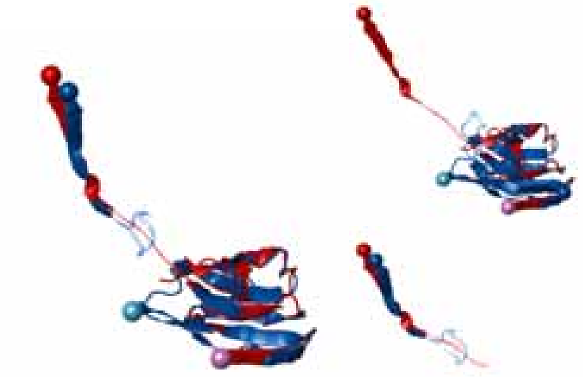<br>R9<br>Epidermal growth factor receptor antibody MR1
	1i8kB (119)	398-409 (12)	398-409 (12)	93-99 (7)						

426	1sjvA (102)	90-104 (15)	93-103 (11)	93-98 (6)	C	0.65	1.71	98.04% (100/102)	40.20% (41/102)	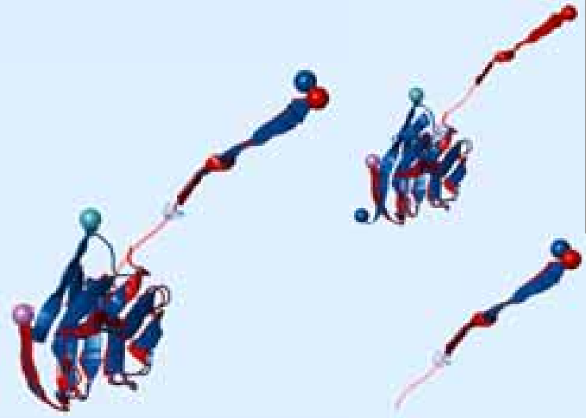 R9 IGG1 FAB chain H
	1ic4H (114)	94-107 (14)	97-106 (10)	93-98 (6)						
427	1sjvA (102)	90-104 (15)	93-98 (6)	93-98 (6)	C	0.66	1.66	98.04% (100/102)	40.20% (41/102)	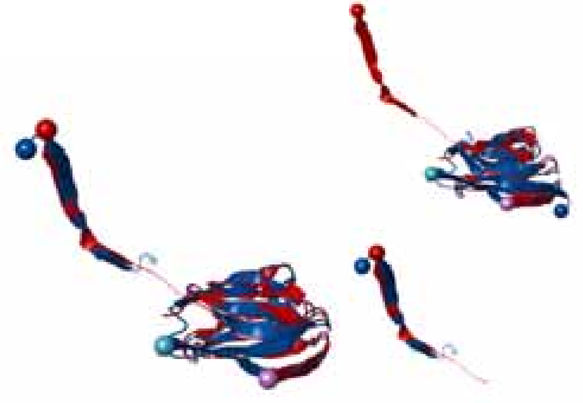 R9 IGG1 FAB chain H
	1ic5H (114)	94-107 (14)	97-101 (5)	93-98 (6)						
428	1sjvA (102)	90-99 (10)	93-98 (6)	93-99 (7)	C	0.66	1.63	98.04% (100/102)	39.22% (40/102)	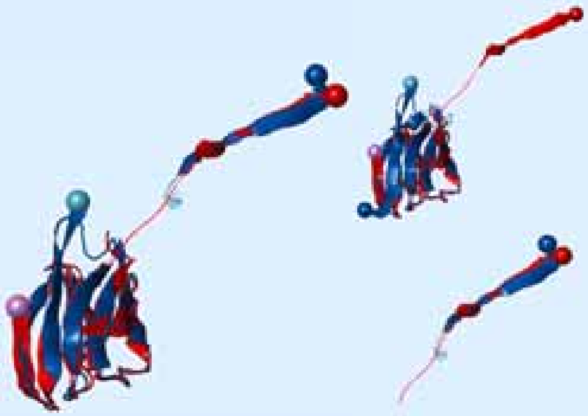 R9 IGG1 FAB chain H
	1ic7H (114)	94-102 (9)	97-101 (5)	93-99 (7)						

429	1sjvA (102)	93-98 (6)	93-98 (6)	94-98 (5)	C	0.65	1.65	98.04% (100/102)	41.18% (42/102)	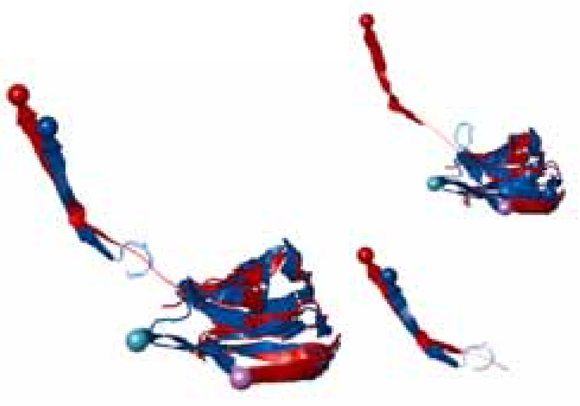 R9 IGG1-kappa D11.15 FV (heavy chain)
	1jhlH (116)	98-106 (9)	98-106 (9)	94-98 (5)						
430	1sjvA (102)	94-101 (8)	94-99 (6)	94-99 (6)	C	0.63	1.34	99.02% (101/102)	72.55% (74/102)	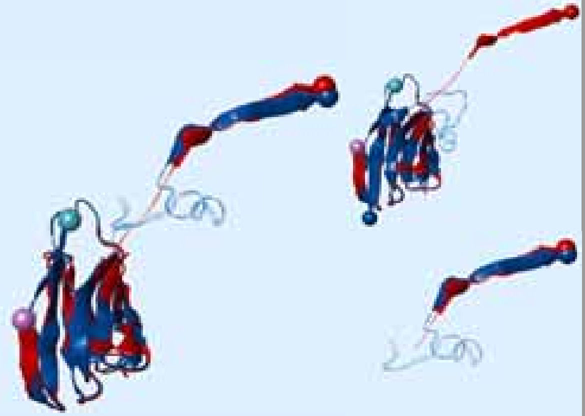 R9 Vh single-domain antibody
	1jtoA (132)	99-124 (26)	99-122 (24)	94-99 (6)						
431	1sjvA (102)	94-98 (5)	94-98 (5)	94-98 (5)	C	0.65	1.13	99.02% (101/102)	72.55% (74/102)	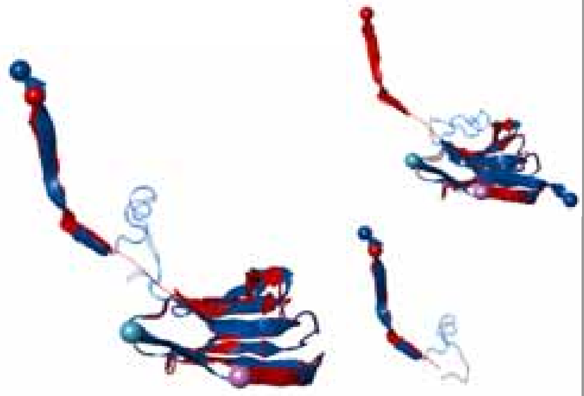 R9 Single-domain antibody
	1jtpA (135)	99-121 (23)	99-121 (23)	94-98 (5)						

432	1sjvA (102)	94-99 (6)	94-98 (5)	94-98 (5)	C	0.66	1.13	99.02% (101/102)	73.53% (75/102)	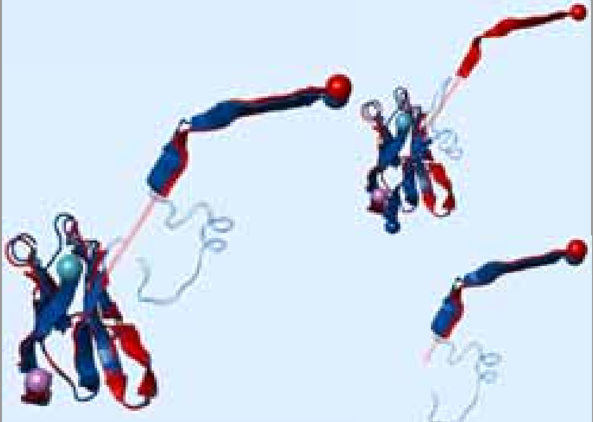 R9 Single-domain antibody
	1jtpB (133)	99-122 (24)	99-121 (23)	94-98 (5)						
433	1sjvA (102)	93-103 (11)	93-98 (6)	94-99 (6)	C	0.67	1.43	98.04% (100/102)	66.67% (68/102)	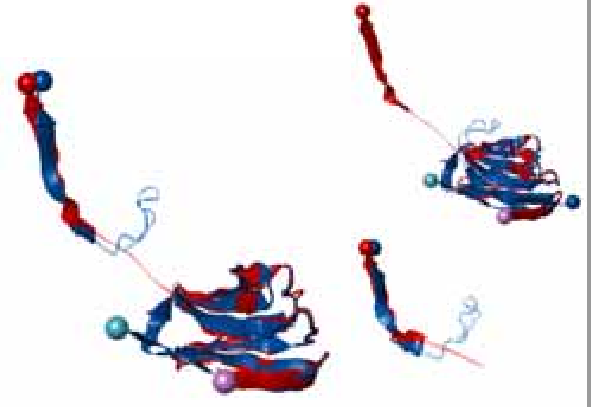 R9 Antibody VHH fragment cabamd9
	1kxqE (120)	95-113 (19)	95-108 (14)	94-99 (6)						
434	1sjvA (102)	94-98 (5)	95-98 (4)	96-98 (3)	C	0.70	1.12	99.02% (101/102)	68.63% (70/102)	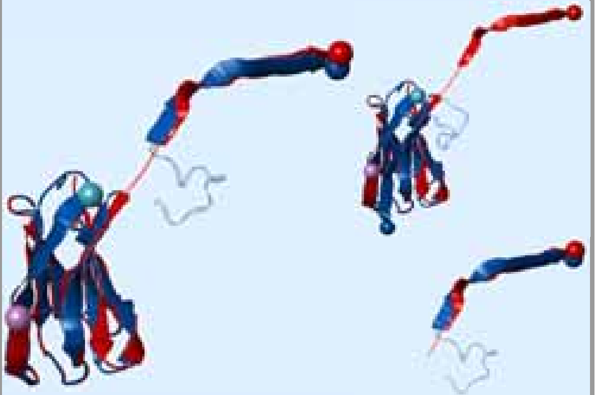 R9 Immunoglobulin heavy chain variable region
	1mvfA (126)	99-114 (16)	100-114 (15)	96-98 (3)						

435	1sjvA (102)	93-103 (11)	93-99 (7)	94-99 (6)	C	0.67	1.55	100.00% (102/102)	44.12% (45/102)	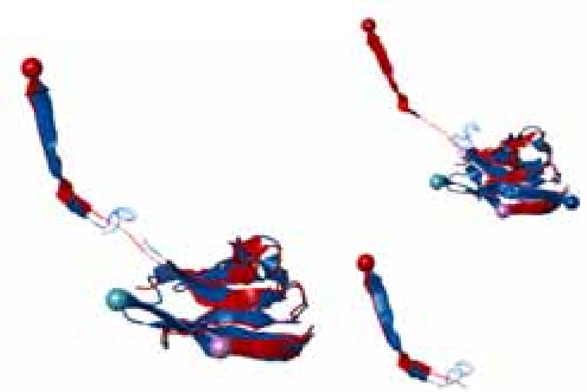 R9 Immunoglobulin heavy chain variable region
	1n4xH (120)	398-413 (16)	398-409 (12)	94-99 (6)						
436	1sjvA (102)	93-106 (14)	93-105 (13)	94-105 (12)	C	0.65	1.72	100.00% (102/102)	35.29% (36/102)	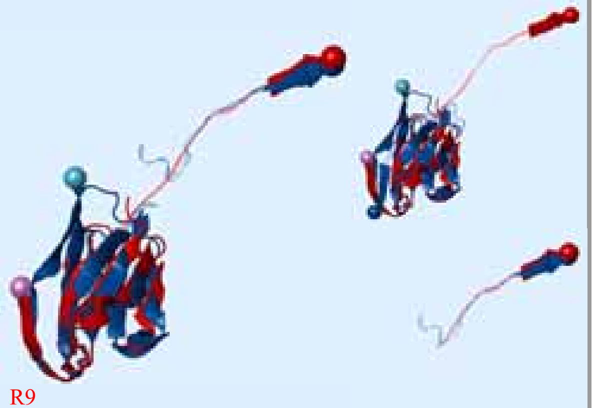 R9 Immunoglobulin heavy chain variable region
	1n4xI (119)	398-415 (18)	398-414 (17)	94-105 (12)						
437	1sjvA (102)	87-109 (23)	91-98 (8)	94-100 (7)	C	0.63	1.69	100.00% (102/102)	41.18% (42/102)	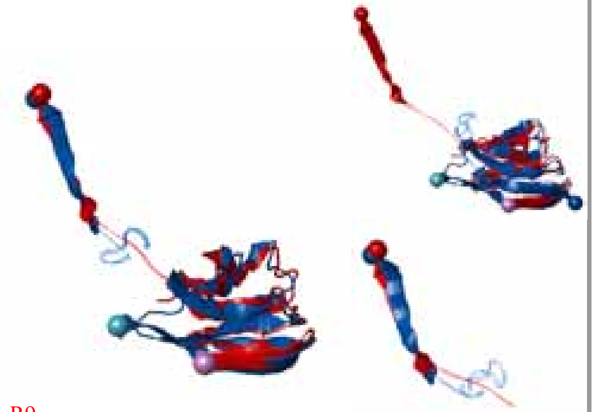 R9 Single chain antibody
	1nmcH (122)	88-112 (25)	92-101 (10)	94-100 (7)						

438	1sjvA (102)	93-109 (17)	93-103 (11)	94-98 (5)	C	0.28	1.89	94.12% (96/102)	26.47% (27/102)	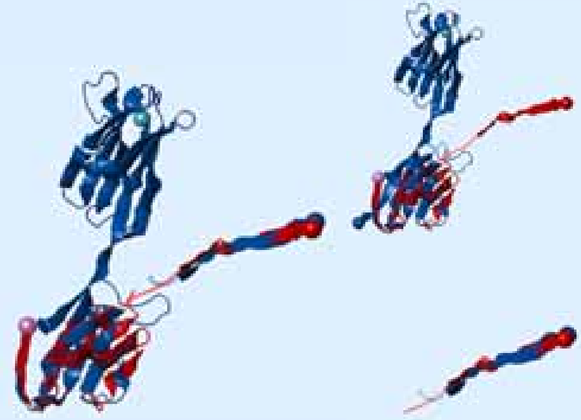 R9 Single-chain antibody fragment
	1nqbA (232)	215-232 (18)	215-226 (12)	94-98 (5)						
439	1sjvA (102)	93-104 (12)	94-103 (10)	94-98 (5)	C	0.66	1.35	98.04% (100/102)	67.65% (69/102)	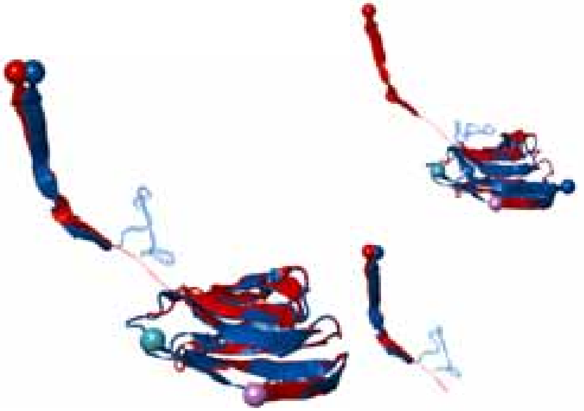 R9 Camelid antibody heavy chain
	1ri8A (124)	97-119 (23)	98-118 (21)	94-98 (5)						
440	1sjvA (102)	93-103 (11)	93-103 (11)	93-103 (11)	C	0.66	1.54	97.06% (99/102)	62.75% (64/102)	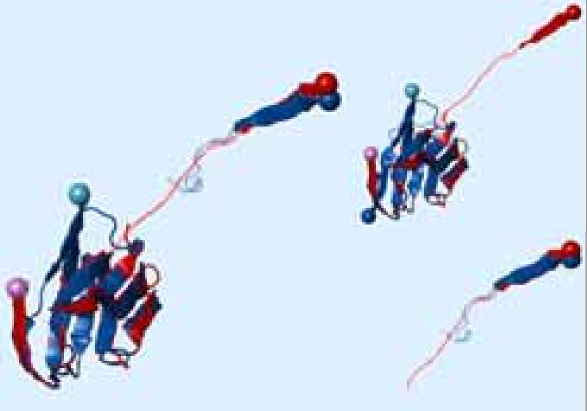 R9 M12-variable heavy domain
	1t2jA (116)	94-106 (13)	94-106 (13)	93-103 (11)						

441	1sjvA (102)	90-104 (15)	93-98 (6)	93-98 (6)	C	0.66	1.56	97.06% (99/102)	41.18% (42/102)	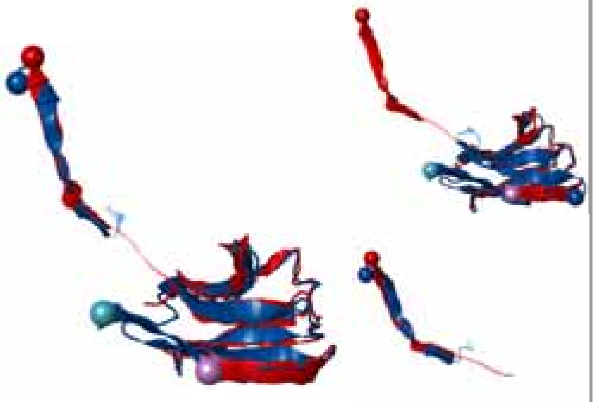 R9 Ig vh,anti-lysozyme
	1uacH (114)	94-107 (14)	97-101 (5)	93-98 (6)						
442	1sjvA (102)	91-109 (19)	91-108 (18)	95-102 (8)	C	0.66	1.71	99.02% (101/102)	38.24% (39/102)	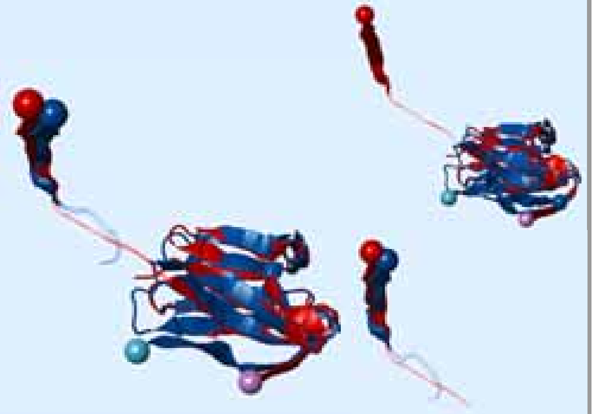 R9 ANTI EGFR antibody FV region
	1wt5A (115)	96-114 (19)	96-113 (18)	95-102 (8)						
443	1sjvA (102)	94-106 (13)	94-106 (13)	94-99 (6)	C	0.62	1.45	99.02% (101/102)	61.76% (63/102)	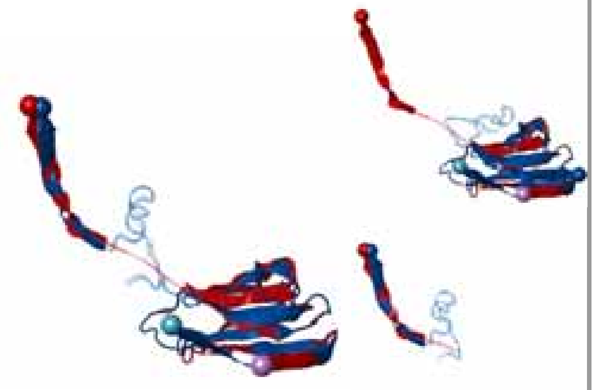 R9 Heavy chain antibody
	1xfpA (131)	99-128 (30)	99-128 (30)	94-99 (6)						

444	1sjvA (102)	94-104 (11)	94-98 (5)	94-98 (5)	C	0.62	1.43	99.02% (101/102)	69.61% (71/102)	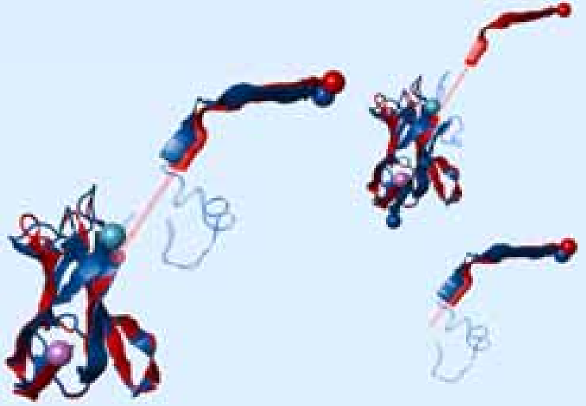 R9 Antibody cabbcii-10:lys3
	1zmyA (132)	99-127 (29)	99-121 (23)	94-98 (5)						
445	1sjvA (102)	94-98 (5)	94-98 (5)	94-98 (5)	C	0.65	1.54	98.04% (100/102)	69.61% (71/102)	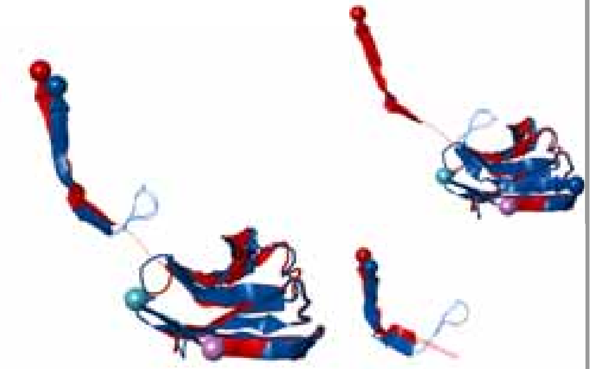 R9 Immunoglobulin heavy chain antibody variable
	1zv5A (119)	99-109 (11)	99-109 (11)	94-98 (5)						
446	1sjvA (102)	93-98 (6)	95-98 (4)	95-98 (4)	C	0.68	1.22	98.04% (100/102)	64.71% (66/102)	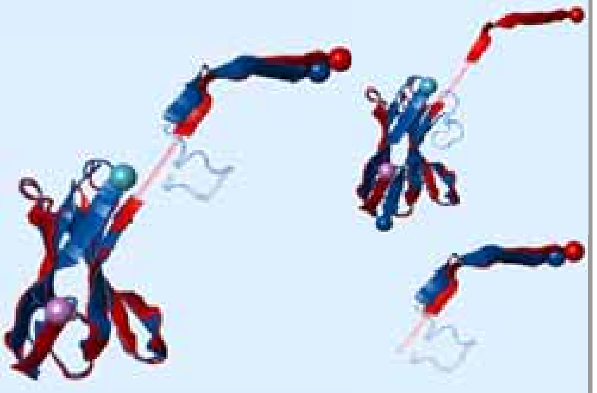 R9 Immunoglobulin heavy chain antibody variable
	1zvhA (124)	98-113 (16)	100-113 (14)	95-98 (4)						

447	1sjvA (102)	95-107 (13)	95-107 (13)	95-105 (11)	C	0.64	1.41	98.04% (100/102)	52.94% (54/102)	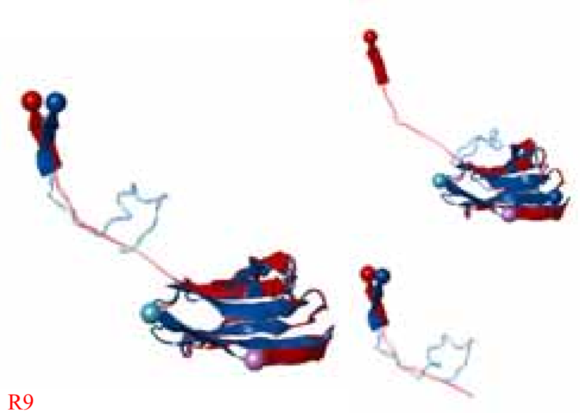 R9 Immunoglobulin heavy chain antibody variable
	1zvyA (125)	100-122 (23)	100-122 (23)	95-105 (11)						
448	1sjvA (102)	95-103 (9)	95-103 (9)	95-99 (5)	C	0.66	1.47	99.02% (101/102)	64.71% (66/102)	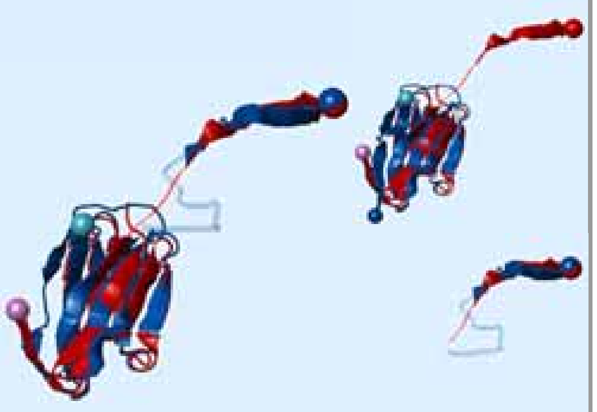 R9 Llama immunoglobulin
	2bseD (122)	100-116 (17)	100-116 (17)	95-99 (5)						
449	1sjvA (102)	91-99 (9)	93-98 (6)	93-98 (6)	C	0.66	1.60	97.06% (99/102)	39.22% (40/102)	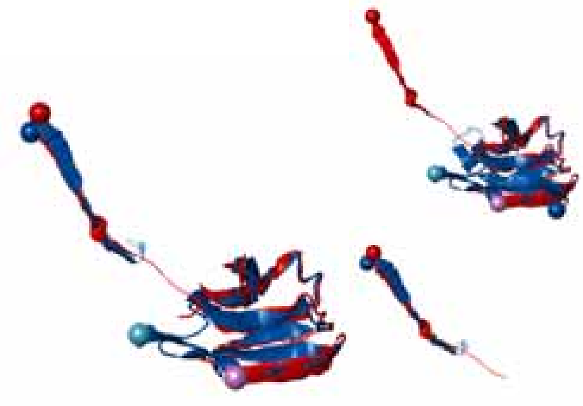 R9 Ig vh,anti-lysozyme
	2dqcH (114)	95-102 (8)	97-101 (5)	93-98 (6)						

450	1sjvA (102)	91-104 (14)	93-103 (11)	93-98 (6)	C	0.66	1.56	97.06% (99/102)	40.20% (41/102)	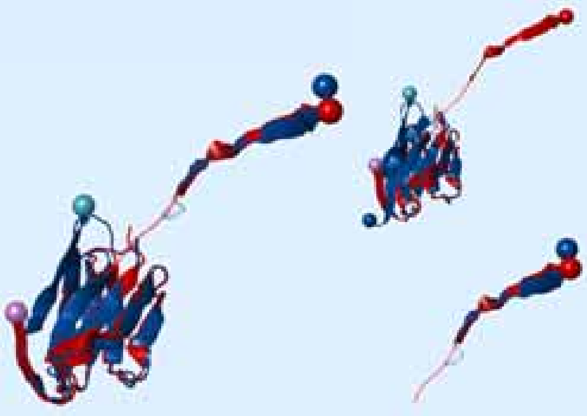 R9 Ig vh,anti-lysozyme
	2dqdH (114)	95-107 (13)	97-106 (10)	93-98 (6)						
451	1sjvA (102)	91-104 (14)	93-98 (6)	93-98 (6)	C	0.66	1.61	97.06% (99/102)	38.24% (39/102)	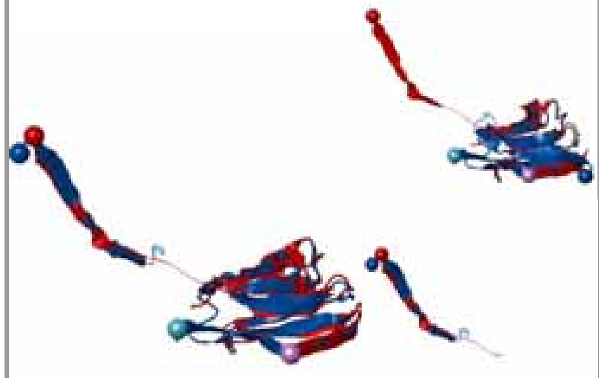 R9 Ig vh,anti-lysozyme
	2dqeH (114)	95-107 (13)	97-101 (5)	93-98 (6)						
452	1sjvA (102)	90-98 (9)	91-98 (8)	93-98 (6)	C	0.66	1.56	97.06% (99/102)	40.20% (41/102)	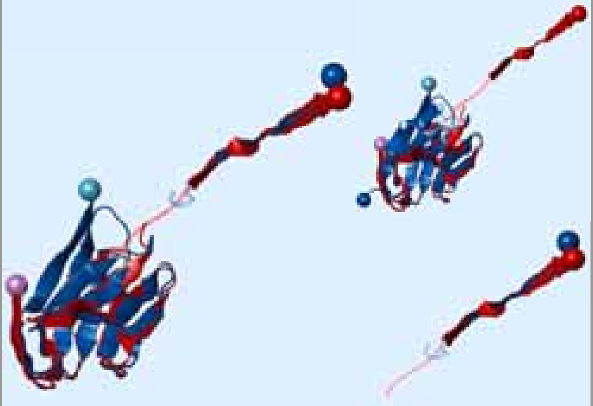 R9 Ig vh,anti-lysozyme
	2dqfB (114)	94-101 (8)	95-101 (7)	93-98 (6)						

453	1sjvA (102)	91-104 (14)	93-103 (11)	93-99 (7)	C	0.65	1.69	98.04% (100/102)	41.18% (42/102)	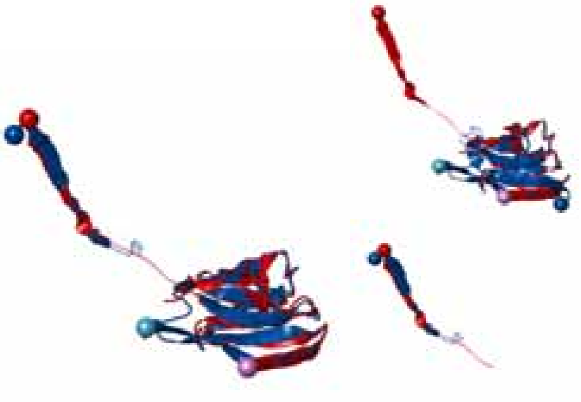 R9 Ig vh,anti-lysozyme
	2dqgH (114)	95-107 (13)	97-106 (10)	93-99 (7)						
454	1sjvA (102)	91-103 (13)	91-98 (8)	93-98 (6)	C	0.65	1.62	97.06% (99/102)	38.24% (39/102)	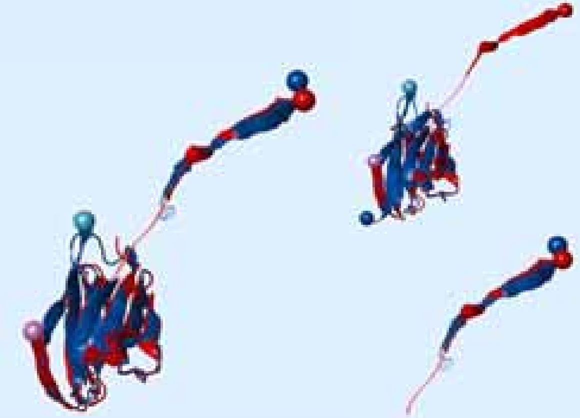 R9 Ig vh,anti-lysozyme
	2dqhH (114)	95-106 (12)	95-101 (7)	93-98 (6)						
455	1sjvA (102)	91-99 (9)	93-98 (6)	93-98 (6)	C	0.66	1.60	97.06% (99/102)	38.24% (39/102)	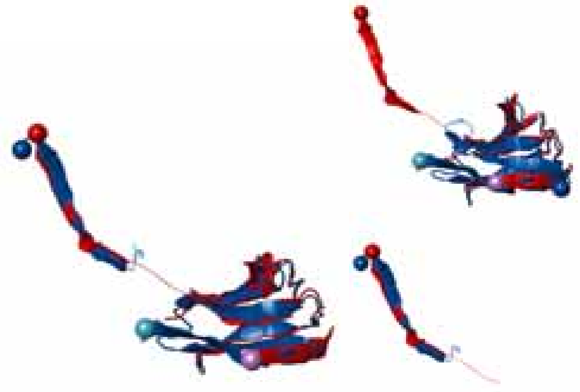 R9 Ig vh,anti-lysozyme
	2dqjH (114)	95-102 (8)	97-101 (5)	93-98 (6)						

456	1sjvA (102)	90-99 (10)	93-98 (6)	93-98 (6)	C	0.66	1.71	98.04% (100/102)	41.18% (42/102)	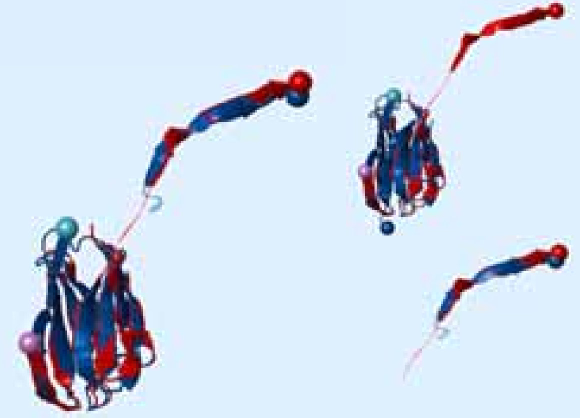 R9 ANTI-lysozyme antibody FV region
	2yssB (113)	94-102 (9)	97-101 (5)	93-98 (6)						
457	1sjvA (102)	91-103 (13)	93-99 (7)	93-103 (11)	C	0.61	1.61	99.02% (101/102)	45.10% (46/102)	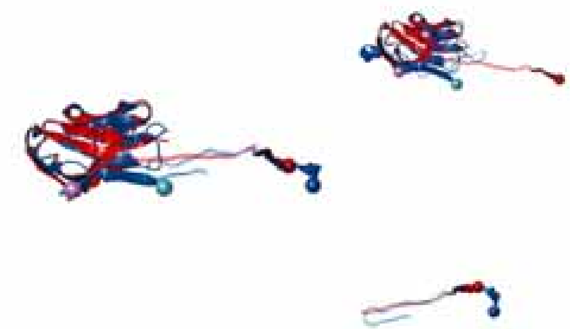 R9 Heavy chain (VH) OF FV-fragment
	3cx5J (127)	96-115 (20)	98-111 (14)	93-103 (11)						
458	1sndA (129)	104-124 (21)	108-121 (14)	112-121 (10)	C	0.83	1.07	100.00% (129/129)	99.22% (128/129)	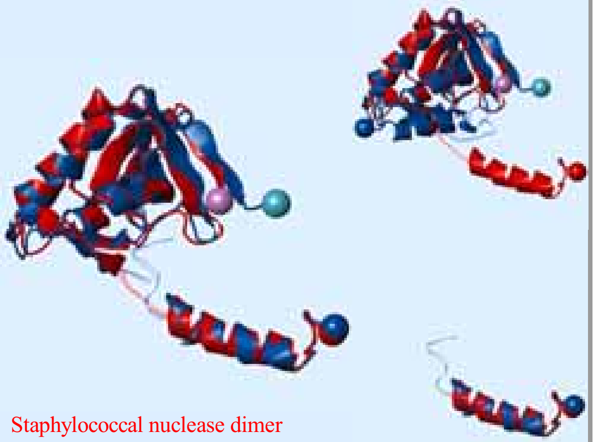 Staphylococcal nuclease dimer Staphylococcal nuclease
	1nsnS (138)	104-124 (21)	108-121 (14)	112-121 (10)						

459	1tiyA (157)	101-157 (57)	104-157 (54)	104-119 (16)	C	0.25	1.53	91.54% (119/130)	30.00% (39/130)	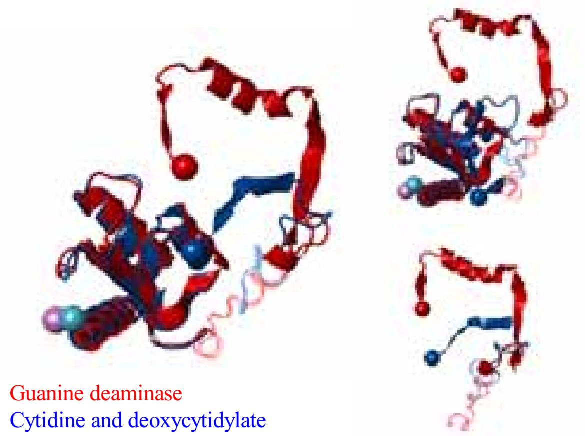<br>Guanine deaminase<br>Cytidine and deoxycytidylate deaminase
	2a8nA (130)	101-131 (31)	104-131 (28)	104-119 (16)						
460	1tiyA (157)	104-143 (40)	104-133 (30)	104-133 (30)	C	0.58	1.71	89.33% (134/150)	29.33% (44/150)	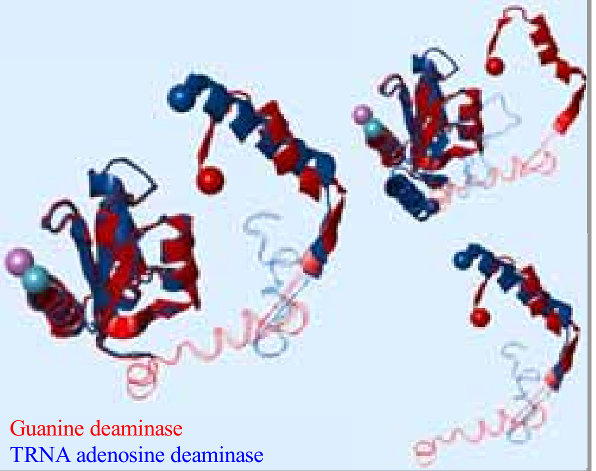<br>Guanine deaminase<br>TRNA adenosine deaminase
	2b3jD (150)	104-137 (34)	104-127 (24)	104-133 (30)						
461	1tiyA (157)	110-141 (32)	110-141 (32)	114-141 (28)	C	0.57	1.59	91.72% (144/157)	27.39% (43/157)	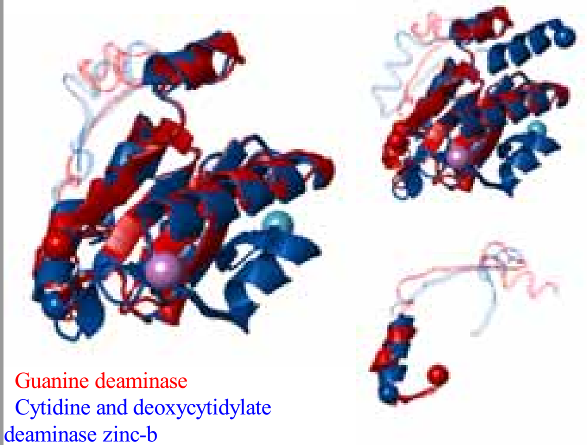<br>Guanine deaminase<br>Cytidine and deoxycytidylate deaminase zinc-b
	2g84B (182)	139-168 (30)	139-168 (30)	114-141 (28)						

462	1tiyA (157)	87-114 (28)	94-114 (21)	103-114 (12)	C	0.33	1.97	78.32% (112/143)	18.88% (27/143)	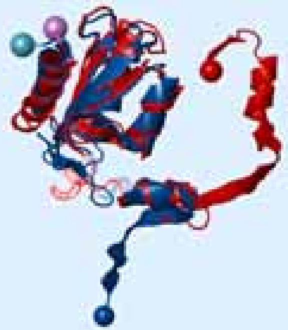 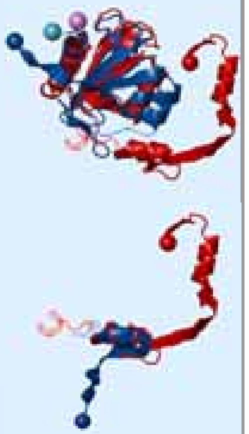 Guanine deaminase<br>Deoxycytidylate deaminase
	2hvvA (143)	103-124 (22)	110-124 (15)	103-114 (12)						
463	1u6mA (189)	173-189 (17)	176-189 (14)	176-185 (10)	C	0.37	2.42	87.92% (131/149)	20.81% (31/149)	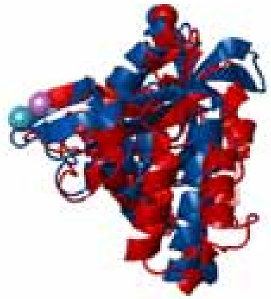 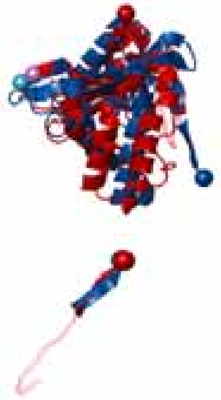 Acetyltransferase, GNAT family<br>Putative acetyl transferase
	1vkcA (149)	142-149 (8)	145-149 (5)	176-185 (10)						
464	1u6mA (189)	170-185 (16)	170-184 (15)	176-180 (5)	C	0.35	2.53	93.08% (121/130)	16.15% (21/130)	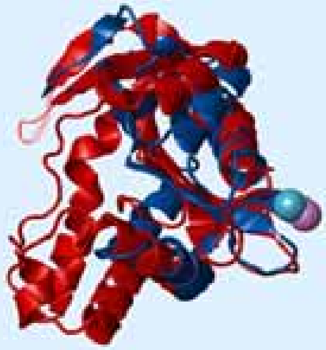 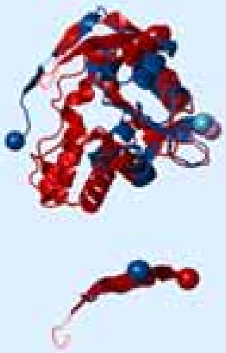 Acetyltransferase, GNAT family<br>Hypothetical protein TTHA1254
	2d4pA (130)	116-129 (14)	116-128 (13)	176-180 (5)						

465	1u6mA (189)	170-183 (14)	170-183 (14)	170-180 (11)	C	0.40	2.04	82.04% (137/167)	21.56% (36/167)	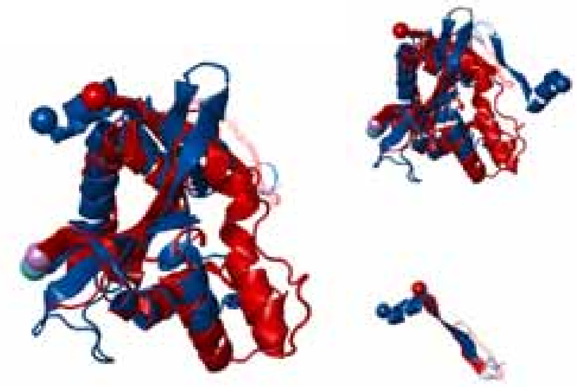 Acetyltransferase, GNAT family<br>Diamine acetyltransferase 1
	2f5iA (167)	146-155 (10)	146-155 (10)	170-180 (11)						
466	1u6mA (189)	171-183 (13)	171-183 (13)	171-180 (10)	C	0.42	2.00	82.14% (138/168)	22.02% (37/168)	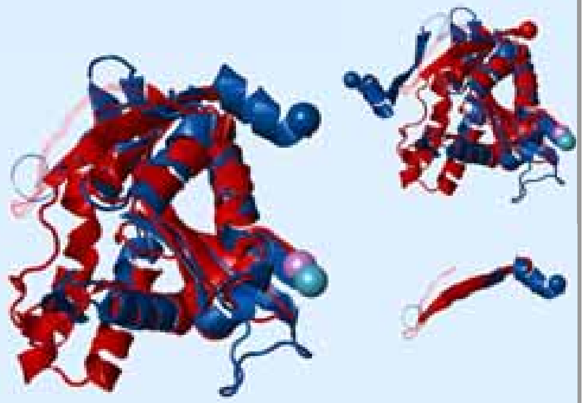 Acetyltransferase, GNAT family<br>Diamine acetyltransferase 1
	2g3tB (168)	147-155 (9)	147-155 (9)	171-180 (10)						
467	1u6mA (189)	169-183 (15)	169-183 (15)	169-180 (12)	C	0.39	2.07	80.47% (136/169)	20.71% (35/169)	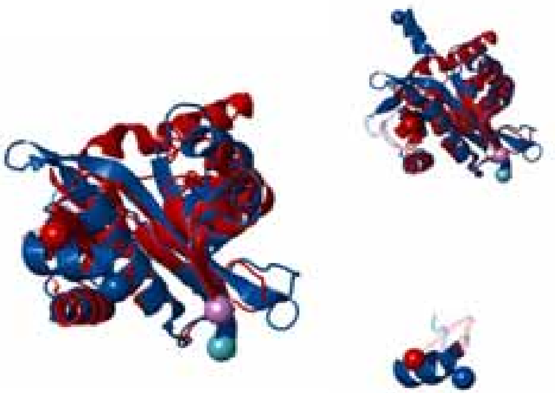 Acetyltransferase, GNAT family<br>Diamine acetyltransferase 1
	2jevA (169)	145-155 (11)	145-155 (11)	169-180 (12)						

468	1v9yA (103)	21-27 (7)	23-26 (4)	26-26 (1)	N	0.49	1.95	96.12% (99/103)	15.53% (16/103)	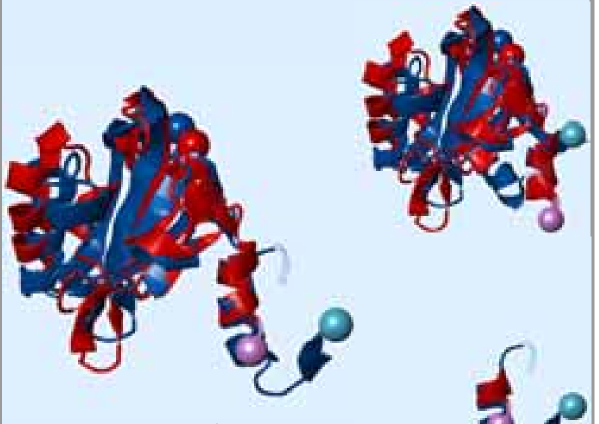 Heme pas sensor protein<br>Photoactive yellow protein
	1f9iA (125)	15-24 (10)	17-23 (7)	26-26 (1)						
469	1v9yA (103)	21-27 (7)	25-27 (3)	26-26 (1)	N	0.53	2.05	96.12% (99/103)	15.53% (16/103)	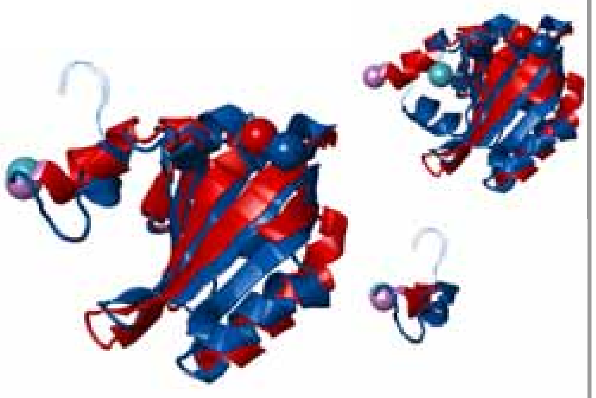 Heme pas sensor protein<br>Photoactive yellow protein
	1gsvA (122)	5-24 (20)	16-24 (9)	26-26 (1)						
470	1v9yA (103)	25-27 (3)	25-27 (3)	25-26 (2)	N	0.53	2.04	96.12% (99/103)	15.53% (16/103)	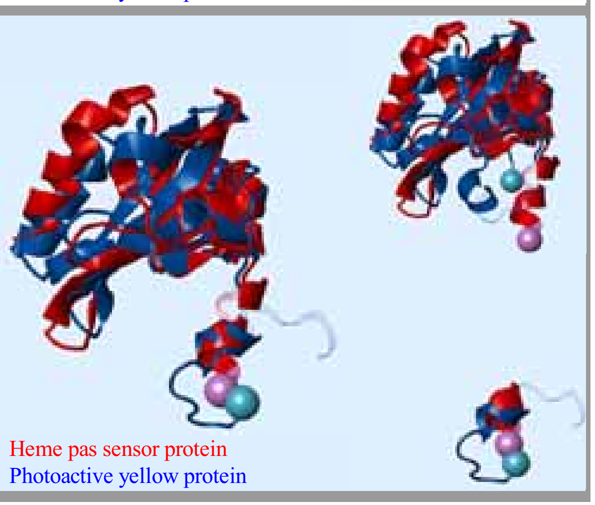 Heme pas sensor protein<br>Photoactive yellow protein
	1gswA (122)	15-24 (10)	15-24 (10)	25-26 (2)						

471	1v9yA (103)	25-28 (4)	25-27 (3)	26-26 (1)	N	0.53	2.06	96.12% (99/103)	13.59% (14/103)	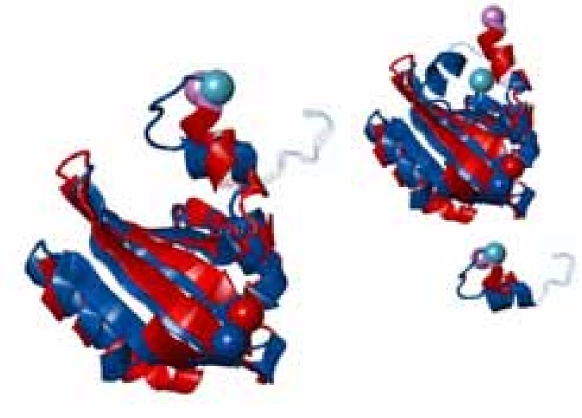 Heme pas sensor protein<br>Photoactive yellow protein
	1gsxA (122)	15-25 (11)	15-24 (10)	26-26 (1)						
472	1v9yA (103)	23-27 (5)	24-25 (2)	25-25 (1)	N	0.44	1.92	95.15% (98/103)	13.59% (14/103)	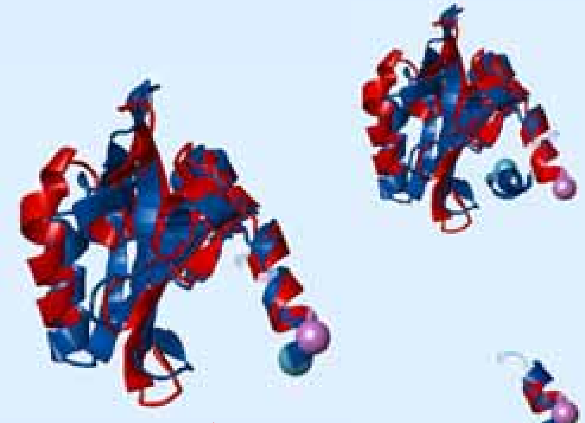 Heme pas sensor protein<br>PPR
	1mzuB (114)	16-24 (9)	17-22 (6)	25-25 (1)						
473	1v9yA (103)	21-28 (8)	21-26 (6)	26-26 (1)	N	0.51	2.03	96.12% (99/103)	14.56% (15/103)	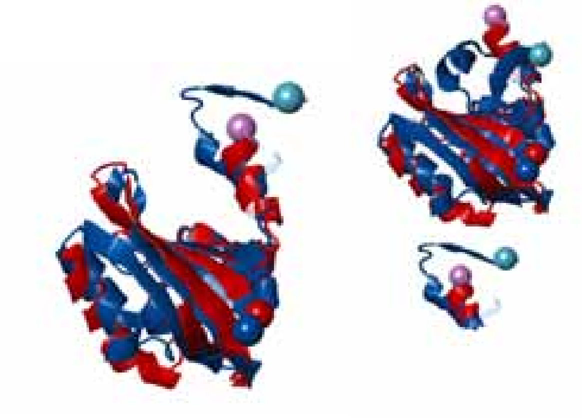 Heme pas sensor protein<br>Photoactive yellow protein
	1ot6A (125)	5-25 (21)	5-23 (19)	26-26 (1)						

474	1v9yA (103)	21-27 (7)	21-25 (5)	25-25 (1)	N	0.39	2.05	96.12% (99/103)	14.56% (15/103)	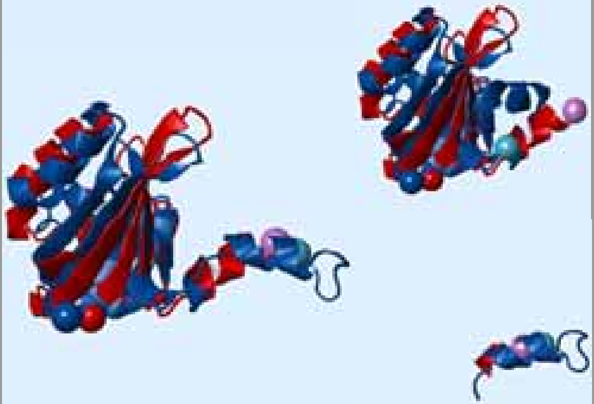 Heme pas sensor protein<br>Photoactive yellow protein
	1uguA (122)	15-24 (10)	15-22 (8)	25-25 (1)						
475	1v9yA (103)	25-27 (3)	25-26 (2)	26-26 (1)	N	0.53	1.99	96.12% (99/103)	15.53% (16/103)	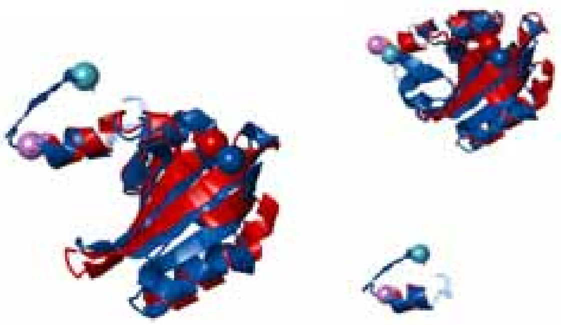 Heme pas sensor protein<br>Photoactive yellow protein
	2d01A (124)	15-24 (10)	15-23 (9)	26-26 (1)						
476	1v9yA (103)	24-27 (4)	24-25 (2)	24-25 (2)	N	0.51	2.14	96.12% (99/103)	15.53% (16/103)	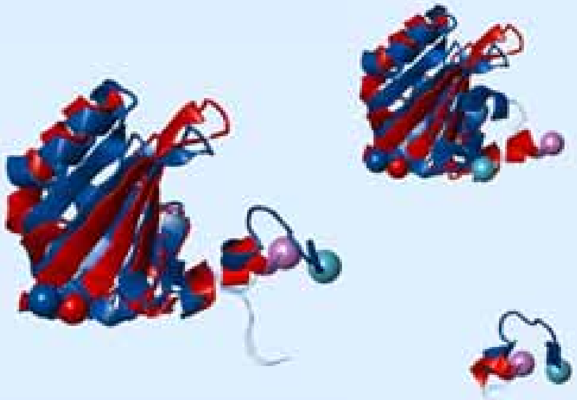 Heme pas sensor protein<br>Photoactive yellow protein
	2qj7A (123)	14-24 (11)	14-22 (9)	24-25 (2)						

477	1v9yA (103)	21-31 (11)	25-31 (7)	26-26 (1)	N	0.48	2.29	96.12% (99/103)	15.53% (16/103)	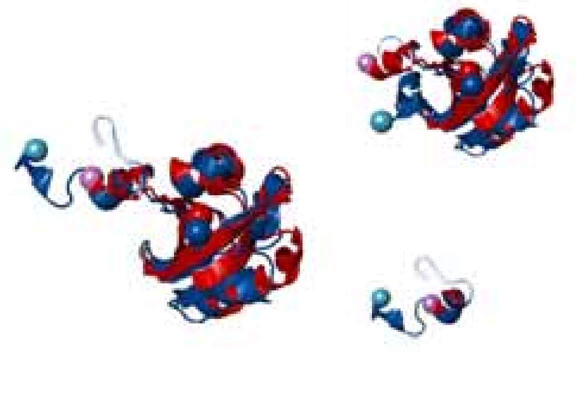 Heme pas sensor protein<br>Photoactive yellow protein
	3phyA (125)	10-28 (19)	15-28 (14)	26-26 (1)						
478	1wk4A (174)	149-162 (14)	149-162 (14)	152-161 (10)	C	0.58	1.85	97.93% (142/145)	16.55% (24/145)	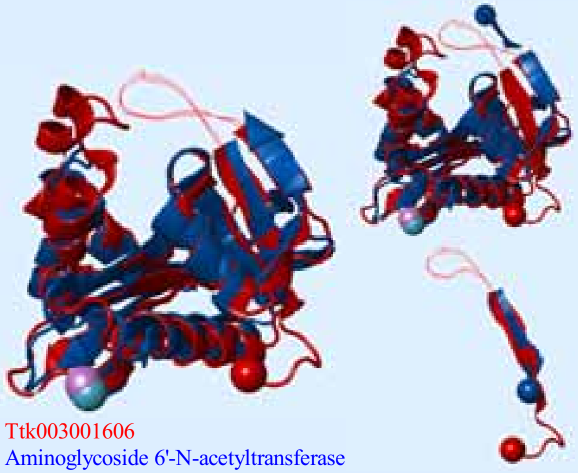 Ttk003001606<br>Aminoglycoside 6'-N-acetyltransferase
	1s3zB (145)	136-140 (5)	136-140 (5)	152-161 (10)						
479	1wk4A (174)	139-155 (17)	149-155 (7)	150-151 (2)	C	0.35	2.96	88.39% (137/155)	10.32% (16/155)	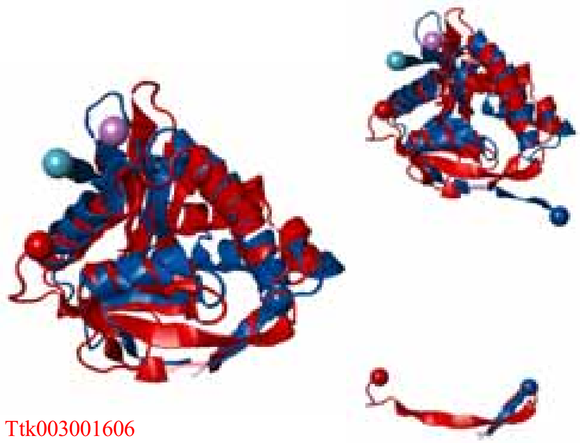 Ttk003001606<br>YYCN protein
	1ufhA (155)	139-155 (17)	149-155 (7)	150-151 (2)						

480	1wk4A (174)	139-165 (27)	140-165 (26)	152-160 (9)	C	0.43	2.25	94.62% (123/130)	16.92% (22/130)	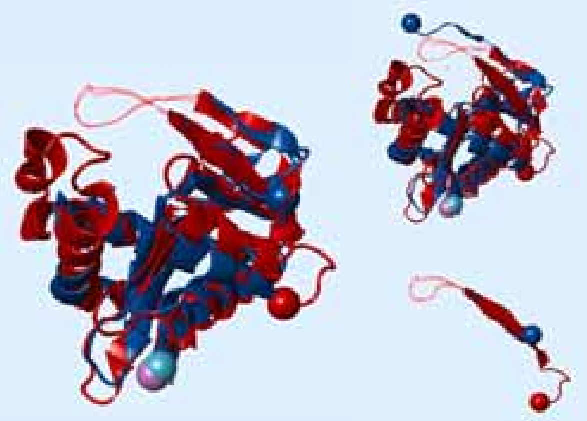 Ttk003001606 Hypothetical protein TTHA1254
	2d4pA (130)	109-129 (21)	110-129 (20)	152-160 (9)						
481	1wk4A (174)	140-173 (34)	145-162 (18)	148-161 (14)	C	0.52	2.17	91.02% (152/167)	14.97% (25/167)	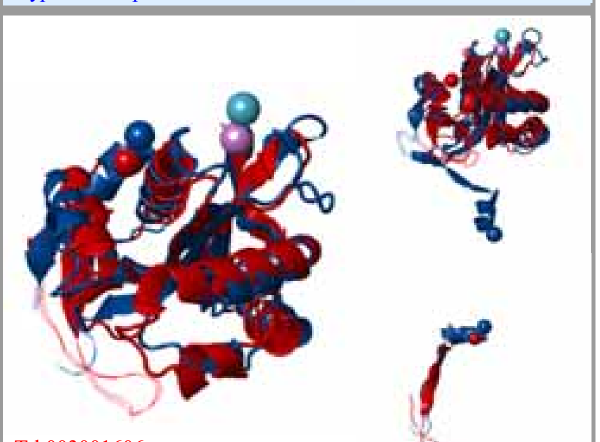 Ttk003001606 Diamine acetyltransferase 1
	2f5iA (167)	140-166 (27)	145-154 (10)	148-161 (14)						
482	1wk4A (174)	144-173 (30)	145-161 (17)	148-161 (14)	C	0.50	2.13	89.16% (148/166)	24.10% (40/166)	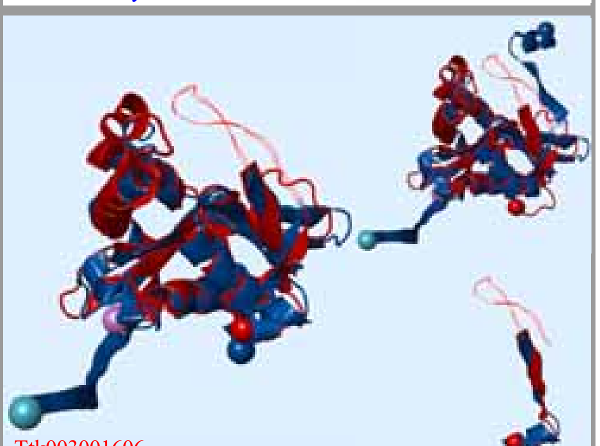 Ttk003001606 Probable N-acetyltransferase
	2fe7B (166)	136-155 (20)	137-142 (6)	148-161 (14)						

483	1wk4A (174)	139-173 (35)	145-173 (29)	148-163 (16)	C	0.51	2.30	91.12% (154/169)	13.61% (23/169)	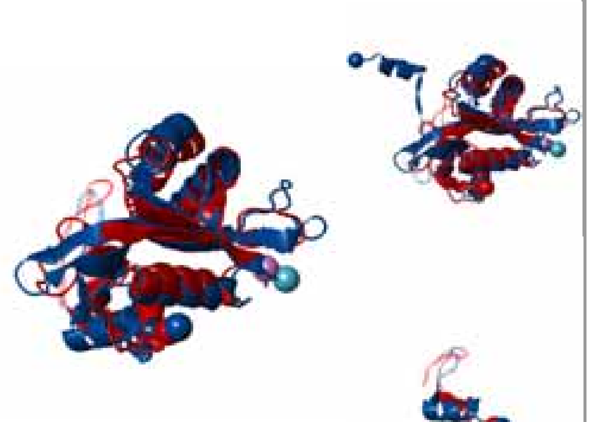 Ttk003001606 Diamine acetyltransferase 1
	2g3tA (169)	139-170 (32)	145-170 (26)	148-163 (16)						
484	1wk4A (174)	144-173 (30)	145-164 (20)	148-161 (14)	C	0.51	2.22	90.48% (152/168)	13.69% (23/168)	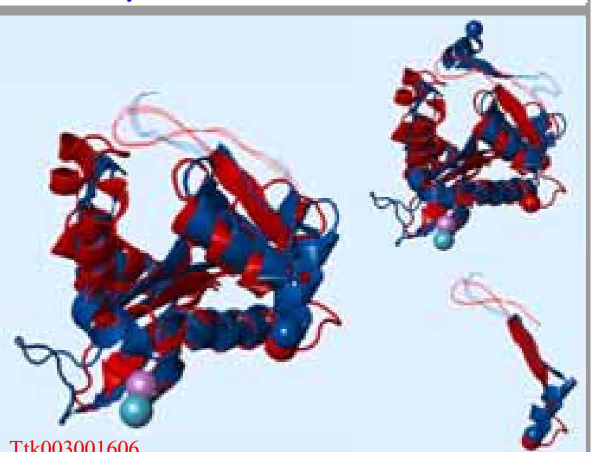 Ttk003001606 Diamine acetyltransferase 1
	2g3tB (168)	144-169 (26)	145-158 (14)	148-161 (14)						
485	1wk4A (174)	144-173 (30)	145-173 (29)	148-160 (13)	C	0.51	2.21	89.94% (152/169)	14.79% (25/169)	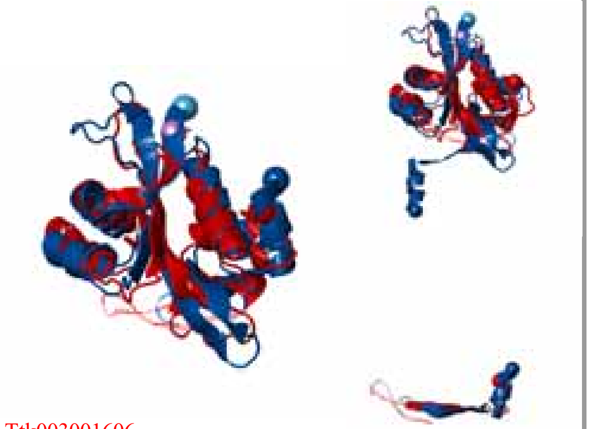 Ttk003001606 Diamine acetyltransferase 1
	2jevA (169)	144-166 (23)	145-166 (22)	148-160 (13)						

486	1wk4A (174)	149-173 (25)	149-173 (25)	152-164 (13)	C	0.54	2.01	96.60% (142/147)	17.01% (25/147)	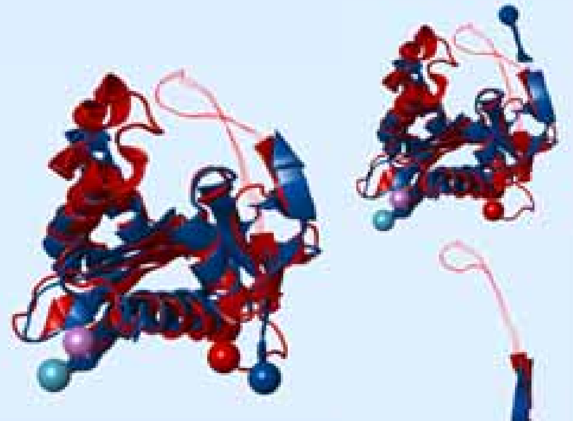 Ttk003001606<br>Aminoglycoside 6'-N-acetyltransferase
	2vbqA (147)	136-144 (9)	136-144 (9)	152-164 (13)						
487	1wk4A (174)	149-165 (17)	149-161 (13)	152-160 (9)	C	0.57	1.86	97.92% (141/144)	16.67% (24/144)	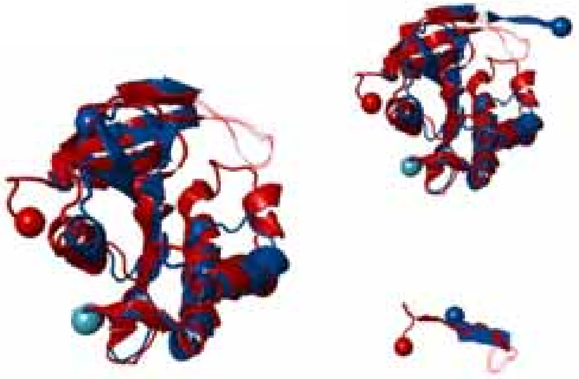 Ttk003001606<br>Aminoglycoside 6'-N-acetyltransferase
	2vbqB (144)	136-144 (9)	136-140 (5)	152-160 (9)						
488	1wwaX (105)	282-315 (34)	295-299 (5)	295-299 (5)	N	0.49	1.94	85.71% (90/105)	18.10% (19/105)	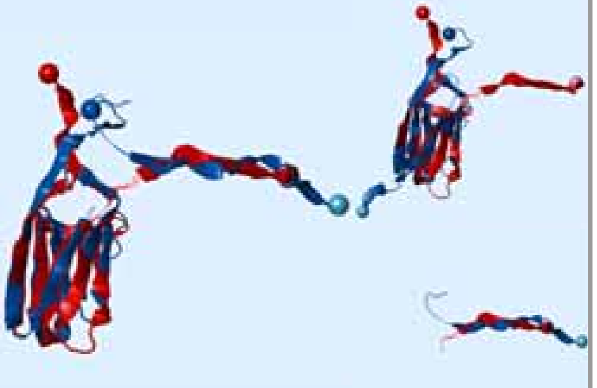 Protein (nerve growth factor receptor TRKA)<br>Palladin
	2dm2A (110)	1-42 (42)	19-26 (8)	295-299 (5)						

489	1wwaX (105)	286-298 (13)	286-298 (13)	295-298 (4)	N	0.45	2.18	87.62% (92/105)	9.52% (10/105)	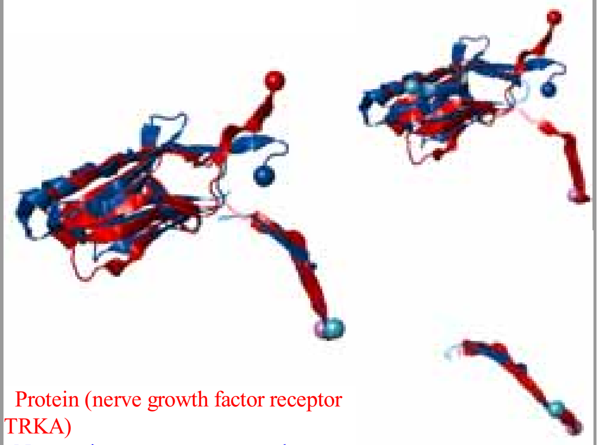 Protein (nerve growth factor receptor TRKA) New antigen receptor ancestral
	2i26O (117)	3-17 (15)	3-17 (15)	295-298 (4)						
490	1wwaX (105)	284-299 (16)	291-299 (9)	294-299 (6)	N	0.44	2.23	85.71% (90/105)	8.57% (9/105)	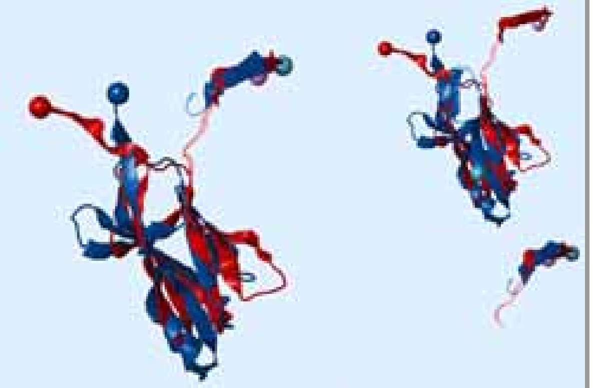 Protein (nerve growth factor receptor TRKA) New antigen receptor ancestral
	2i26P (114)	3-18 (16)	11-18 (8)	294-299 (6)						
491	1wwbX (103)	286-305 (20)	286-301 (16)	297-299 (3)	N	0.26	2.15	81.55% (84/103)	10.68% (11/103)	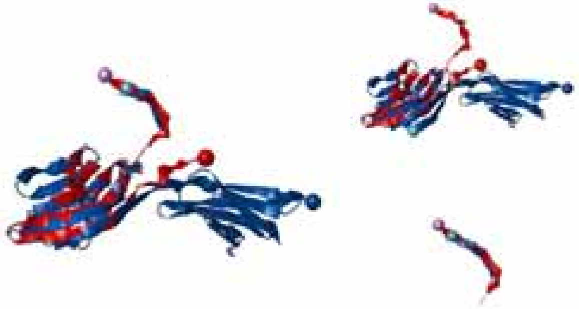 Protein (brain derived neurotrophic factor Re CD2
	1hngA (175)	3-18 (16)	3-14 (12)	297-299 (3)						

492	1wwbX (103)	298-310 (13)	298-299 (2)	298-299 (2)	N	0.50	2.04	87.38% (90/103)	13.59% (14/103)	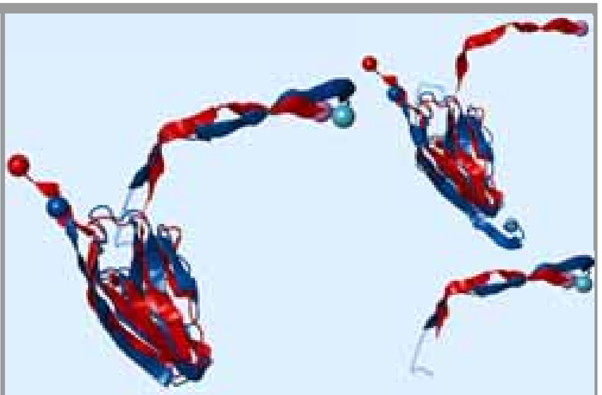 Protein (brain derived neurotrophic factor Re<br>Neural cell adhesion molecule
	1ie5A (107)	23-40 (18)	23-29 (7)	298-299 (2)						
493	1wwbX (103)	285-353 (69)	286-315 (30)	297-301 (5)	N	0.43	2.06	85.44% (88/103)	9.71% (10/103)	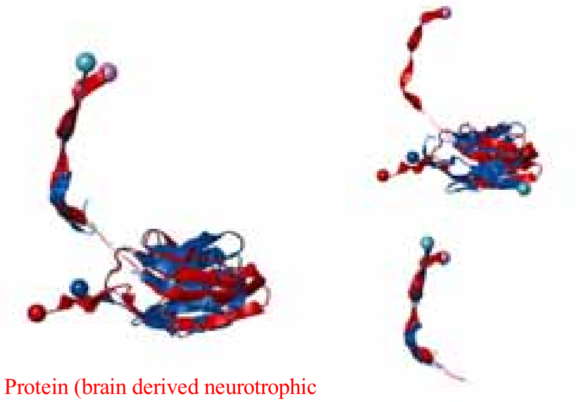 Protein (brain derived neurotrophic factor Re<br>Antibody VHH fragment cabamd9
	1kxqE (120)	2-85 (84)	3-31 (29)	297-301 (5)						
494	1wwbX (103)	285-300 (16)	293-300 (8)	293-300 (8)	N	0.41	2.29	84.47% (87/103)	7.77% (8/103)	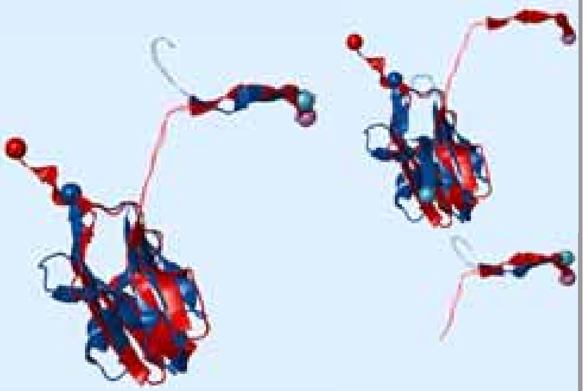 Protein (brain derived neurotrophic factor Re<br>Novel antigen receptor
	1sq2N (112)	3-17 (15)	11-17 (7)	293-300 (8)						

495	1wwbX (103)	284-311 (28)	285-306 (22)	294-300 (7)	N	0.53	2.25	94.17% (97/103)	13.59% (14/103)	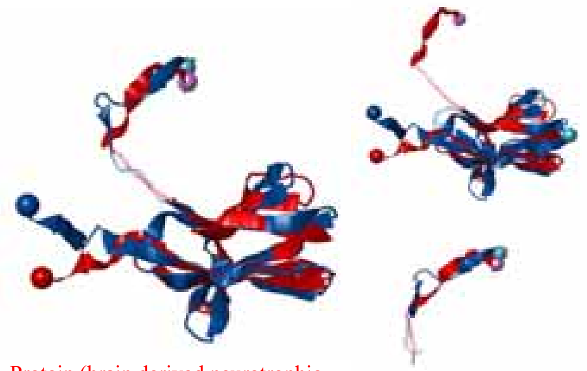 Protein (brain derived neurotrophic factor Re<br>B and T lymphocyte attenuator
	2aw2A (110)	35-65 (31)	36-59 (24)	294-300 (7)						
496	1wwbX (103)	284-345 (62)	285-312 (28)	297-300 (4)	N	0.54	2.14	92.23% (95/103)	13.59% (14/103)	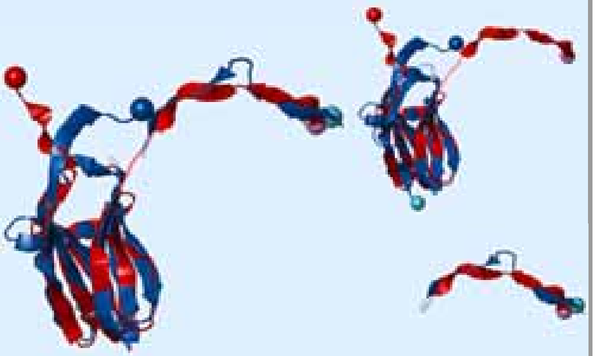 Protein (brain derived neurotrophic factor Re<br>B and T lymphocyte attenuator
	2aw2X (107)	35-99 (65)	36-66 (31)	297-300 (4)						
497	1wwbX (103)	285-345 (61)	285-315 (31)	297-301 (5)	N	0.29	2.42	94.17% (97/103)	6.80% (7/103)	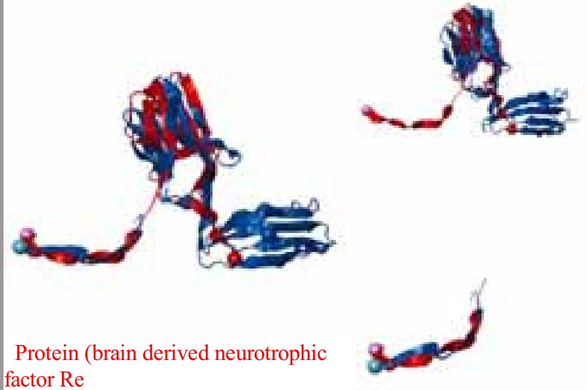 Protein (brain derived neurotrophic factor Re<br>INKT-TCR
	2cdeA (192)	3-74 (72)	3-33 (31)	297-301 (5)						

498	1wwbX (103)	287-317 (31)	287-301 (15)	298-301 (4)	N	0.48	2.21	90.29% (93/103)	6.80% (7/103)	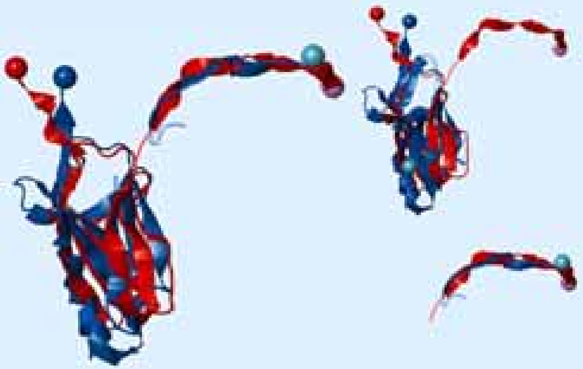 Protein (brain derived neurotrophic factor Re
	2i26P (114)	3-36 (34)	3-18 (16)	298-301 (4)						New antigen receptor ancestral
499	1wwbX (103)	283-299 (17)	283-299 (17)	294-299 (6)	N	0.45	2.24	88.35% (91/103)	10.68% (11/103)	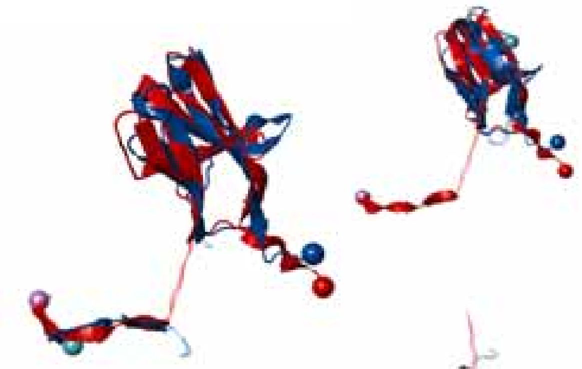 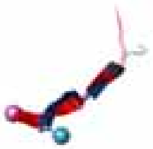 Protein (brain derived neurotrophic factor Re
	2rheA (114)	1-16 (16)	1-16 (16)	294-299 (6)						Bence-jones protein RHE (light chain)
500	1wwbX (103)	286-316 (31)	286-301 (16)	294-301 (8)	N	0.47	2.15	87.38% (90/103)	8.74% (9/103)	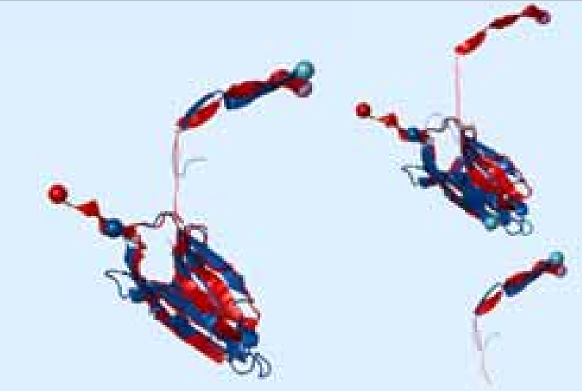 Protein (brain derived neurotrophic factor Re
	2ywzA (111)	3-35 (33)	3-18 (16)	294-301 (8)						New antigen receptor variable domain

501	1wwbX (103)	283-300 (18)	283-300 (18)	298-300 (3)	N	0.53	2.00	88.89% (88/99)	16.16% (16/99)	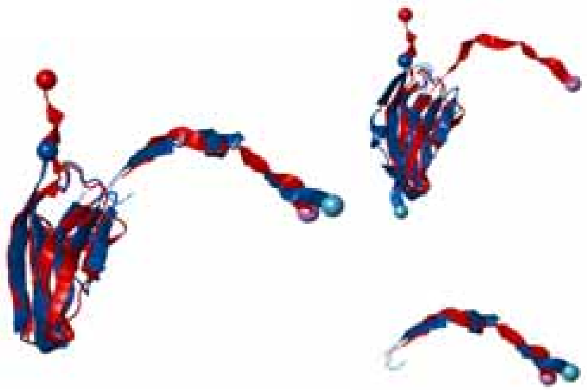 Protein (brain derived neurotrophic factor Re Fibroblast growth factor receptor 2
	3darA (99)	151-174 (24)	151-174 (24)	298-300 (3)						
502	1wwcA (105)	310-363 (54)	310-333 (24)	311-318 (8)	N	0.34	2.31	84.76% (89/105)	5.71% (6/105)	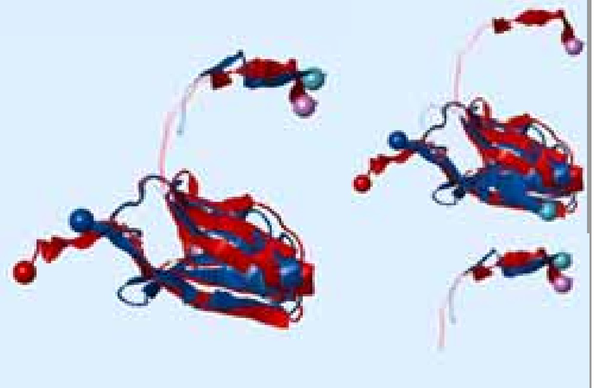 Protein (NT-3 growth factor receptor TRKC) B1-8 FV (light chain)
	1a6wL (109)	10-75 (66)	10-35 (26)	311-318 (8)						
503	1wwcA (105)	303-331 (29)	316-331 (16)	316-318 (3)	N	0.26	2.45	91.43% (96/105)	4.76% (5/105)	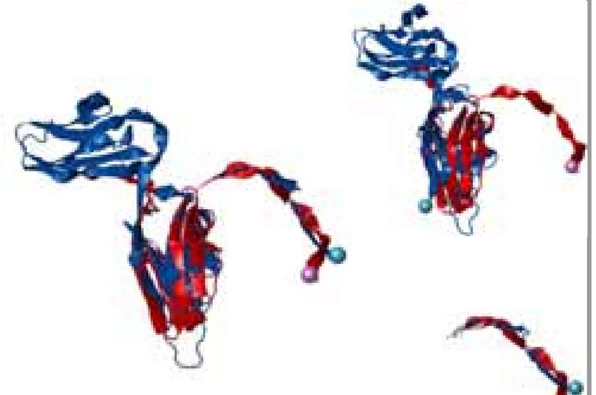 Protein (NT-3 growth factor receptor TRKC) Gamma-delta T-cell receptor
	1hxmA (206)	2-33 (32)	16-33 (18)	316-318 (3)						

504	1wwcA (105)	305-318 (14)	305-318 (14)	316-318 (3)	N	0.43	2.11	84.76% (89/105)	10.48% (11/105)	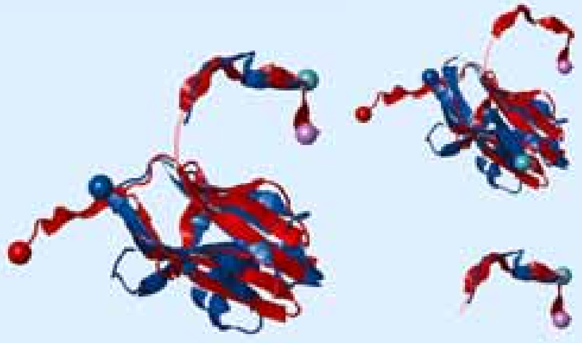 Protein (NT-3 growth factor receptor TRKC) Novel antigen receptor
	1sq2N (112)	3-17 (15)	3-17 (15)	316-318 (3)						
505	1wwcA (105)	301-329 (29)	304-324 (21)	312-319 (8)	N	0.37	2.18	86.67% (91/105)	13.33% (14/105)	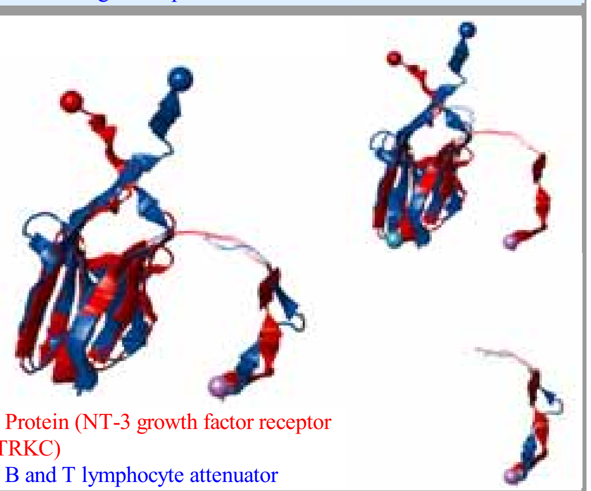 Protein (NT-3 growth factor receptor TRKC) B and T lymphocyte attenuator
	2aw2A (110)	35-65 (31)	38-59 (22)	312-319 (8)						
506	1wwcA (105)	301-335 (35)	309-318 (10)	315-318 (4)	N	0.37	1.98	85.44% (88/103)	9.71% (10/103)	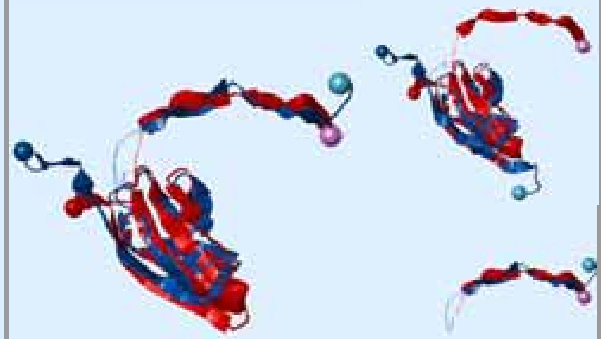 Protein (NT-3 growth factor receptor TRKC) KIAA1556 protein
	2dkuA (103)	5-41 (37)	13-25 (13)	315-318 (4)						

507	1wwcA (105)	310-318 (9)	310-318 (9)	312-318 (7)	N	0.37	2.31	86.67% (91/105)	10.48% (11/105)	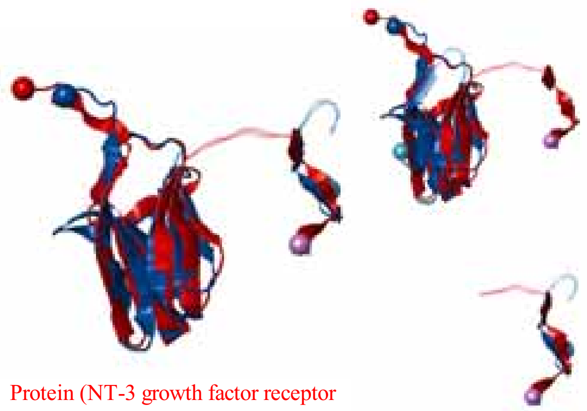 Protein (NT-3 growth factor receptor TRKC) Bence-jones protein RHE (light chain)
	2rheA (114)	10-17 (8)	10-17 (8)	312-318 (7)						
508	1wwcA (105)	309-330 (22)	309-319 (11)	318-319 (2)	N	0.46	2.02	86.00% (86/100)	12.00% (12/100)	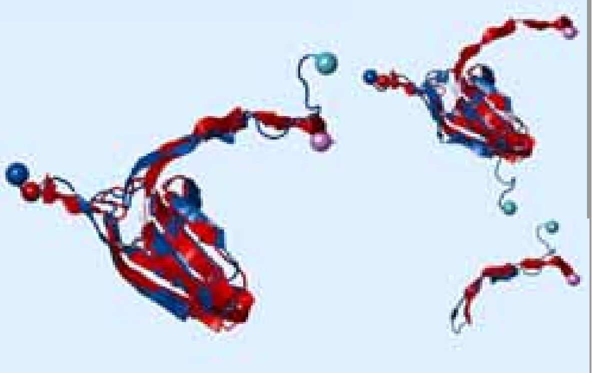 Protein (NT-3 growth factor receptor TRKC) Myosin-binding protein C, slow-type
	2yuvA (100)	12-36 (25)	12-26 (15)	318-319 (2)						
509	1wwcA (105)	304-320 (17)	304-318 (15)	316-318 (3)	N	0.46	1.94	86.67% (91/105)	8.57% (9/105)	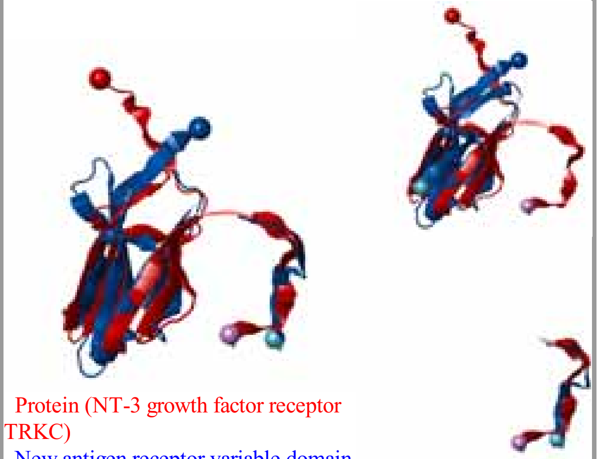 Protein (NT-3 growth factor receptor TRKC) New antigen receptor variable domain
	2ywyA (113)	2-19 (18)	2-17 (16)	316-318 (3)						

510	1wwcA (105)	304-333 (30)	304-318 (15)	316-318 (3)	N	0.47	1.93	86.67% (91/105)	8.57% (9/105)	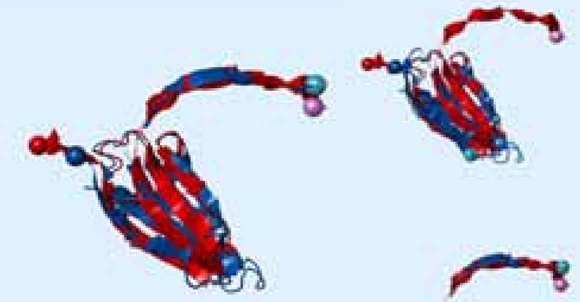 Protein (NT-3 growth factor receptor TRKC)
	2ywzA (111)	2-34 (33)	2-17 (16)	316-318 (3)						New antigen receptor variable domain
511	1wwrA (154)	100-131 (32)	100-130 (31)	104-124 (21)	C	0.58	1.56	86.36% (133/154)	29.87% (46/154)	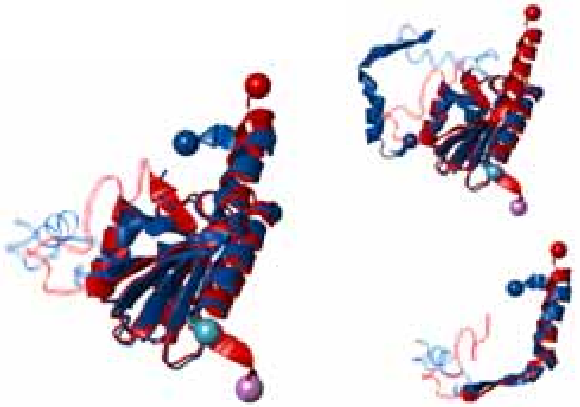 TRNA adenosine deaminase TadA
	1wkqB (155)	101-136 (36)	101-135 (35)	104-124 (21)						Guanine deaminase
512	1wwrA (154)	90-135 (46)	99-118 (20)	90-116 (27)	C	0.32	2.80	80.28% (114/142)	14.08% (20/142)	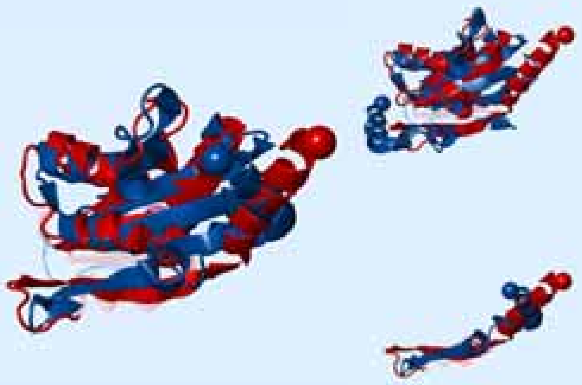 TRNA adenosine deaminase TadA
	3b8fB (142)	97-128 (32)	106-109 (4)	90-116 (27)						Putative blasticidin S deaminase

513	1wwrA (154)	90-135 (46)	99-133 (35)	97-116 (20)	C	0.31	2.88	81.43% (114/140)	14.29% (20/140)	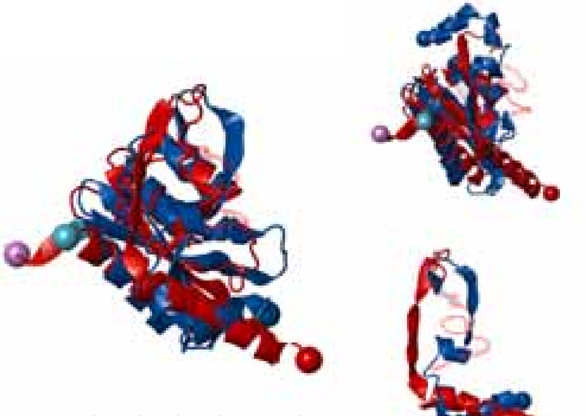 TRNA adenosine deaminase TadA
	3b8fC (140)	97-128 (32)	106-126 (21)	97-116 (20)						Putative blasticidin S deaminase
514	1wwzA (157)	138-150 (13)	138-149 (12)	146-147 (2)	C	0.46	2.54	91.33% (137/150)	18.00% (27/150)	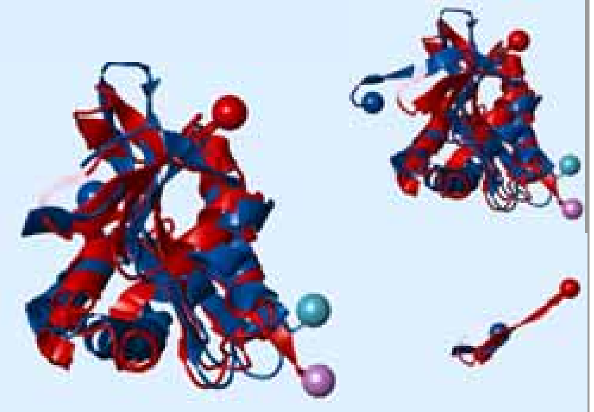 Hypothetical protein PH1933
	1qsmA (150)	141-148 (8)	141-147 (7)	146-147 (2)						HPA2 histone acetyltransferase
515	1wwzA (157)	135-152 (18)	138-152 (15)	146-147 (2)	C	0.46	2.74	93.96% (140/149)	19.46% (29/149)	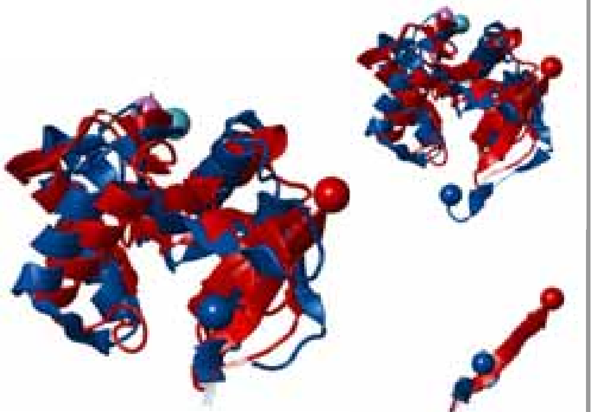 Hypothetical protein PH1933
	1qsmB (149)	138-155 (18)	141-155 (15)	146-147 (2)						HPA2 histone acetyltransferase

516	1wwzA (157)	138-149 (12)	140-149 (10)	140-148 (9)	C	0.47	2.68	93.42% (142/152)	19.74% (30/152)	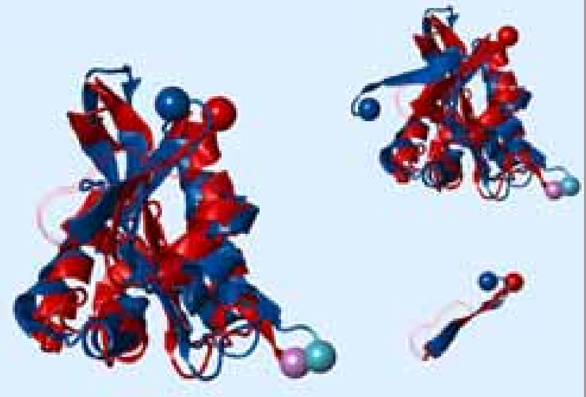 Hypothetical protein PH1933<br>HPA2 histone acetyltransferase
	1qsmD (152)	141-147 (7)	143-147 (5)	140-148 (9)						
517	1wwzA (157)	144-150 (7)	144-150 (7)	145-148 (4)	C	0.52	2.44	96.55% (140/145)	12.41% (18/145)	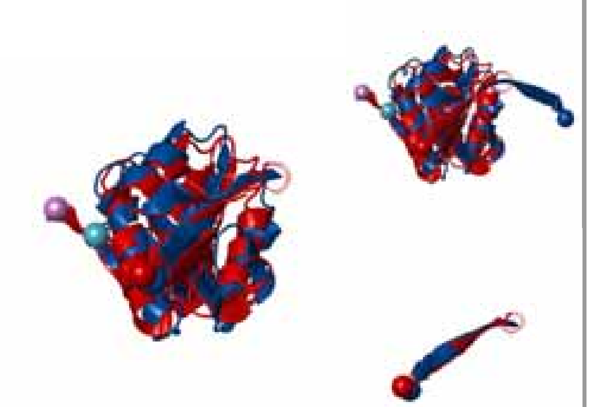 Hypothetical protein PH1933<br>Aminoglycoside 6'-N-acetyltransferase
	1s3zB (145)	135-138 (4)	135-138 (4)	145-148 (4)						
518	1wwzA (157)	143-153 (11)	143-149 (7)	143-149 (7)	C	0.49	2.32	90.67% (136/150)	17.33% (26/150)	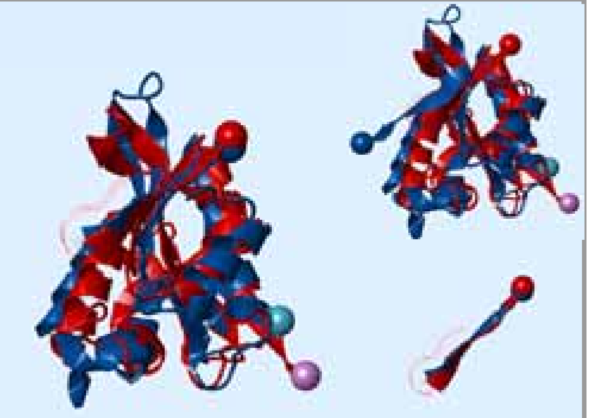 Hypothetical protein PH1933<br>Transcriptional regulator
	1z4eA (150)	144-149 (6)	144-145 (2)	143-149 (7)						

519	1wwzA (157)	143-150 (8)	143-150 (8)	144-149 (6)	C	0.46	2.31	89.17% (140/157)	15.29% (24/157)	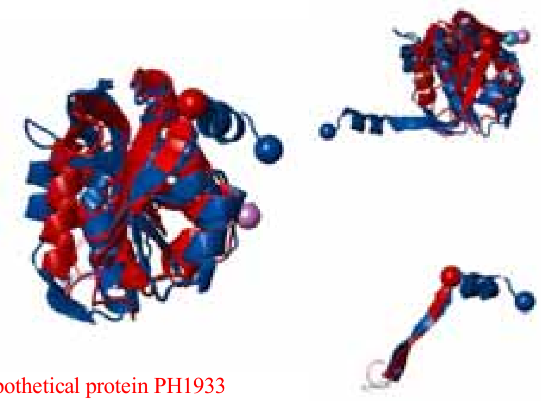 Hypothetical protein PH1933<br>Diamine acetyltransferase 1
	2g3tA (169)	147-154 (8)	147-154 (8)	144-149 (6)						
520	1wx7A (106)	2-20 (19)	2-19 (18)	12-16 (5)	N	0.47	1.93	96.88% (93/96)	35.42% (34/96)	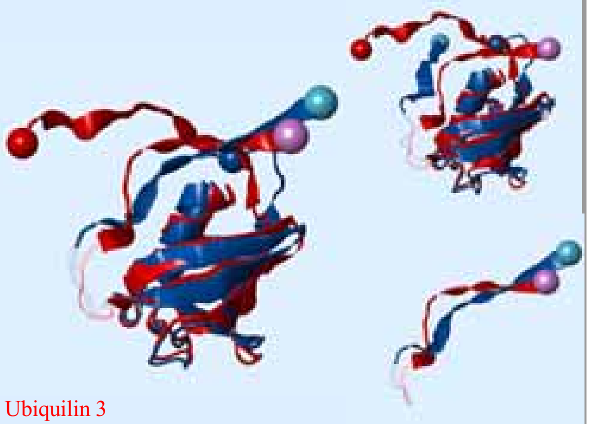 Ubiquilin 3<br>Riken cDNA 4931431F19
	1wx8A (96)	3-20 (18)	3-19 (17)	12-16 (5)						
521	1yghA (164)	228-254 (27)	228-253 (26)	229-247 (19)	C	0.32	2.43	75.78% (122/161)	11.18% (18/161)	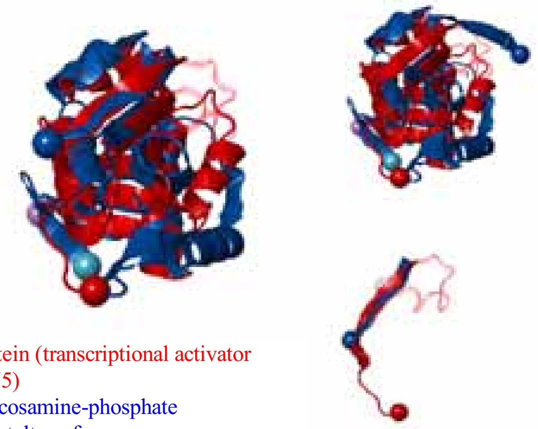 Protein (transcriptional activator GCN5)<br>Glucosamine-phosphate N-acetyltransferase
	1i1dD (161)	150-158 (9)	150-157 (8)	229-247 (19)						

522	1yghA (164)	225-257 (33)	228-249 (22)	229-247 (19)	C	0.44	2.33	89.66% (130/145)	8.28% (12/145)	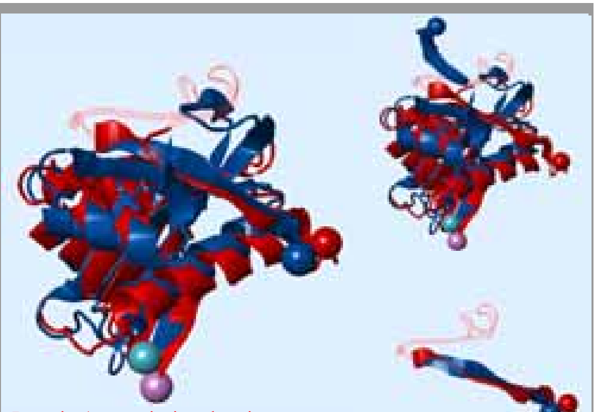 Protein (transcriptional activator GCN5) Aminoglycoside 6'-N-acetyltransferase
	1s3zB (145)	131-144 (14)	134-137 (4)	229-247 (19)						
523	1yghA (164)	227-257 (31)	228-249 (22)	229-247 (19)	C	0.42	2.32	84.31% (129/153)	7.19% (11/153)	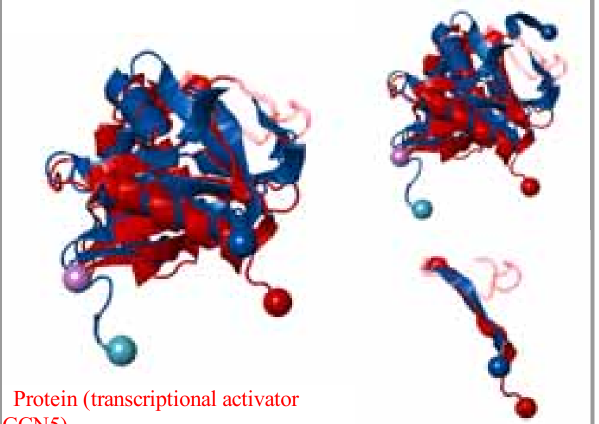 Protein (transcriptional activator GCN5) Aminoglycoside 6'-N-acetyltransferase
	1s5kA (153)	133-144 (12)	134-137 (4)	229-247 (19)						
524	1yghA (164)	228-253 (26)	228-249 (22)	228-244 (17)	C	0.39	2.41	82.58% (128/155)	10.97% (17/155)	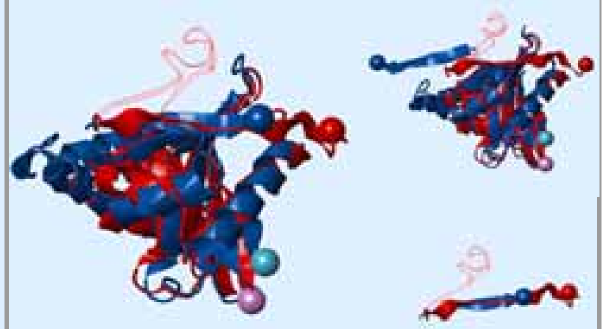 Protein (transcriptional activator GCN5) YYCN protein
	1ufhA (155)	147-155 (9)	147-151 (5)	228-244 (17)						

525	1yghA (164)	228-253 (26)	228-249 (22)	228-244 (17)	C	0.42	2.25	83.77% (129/154)	11.04% (17/154)	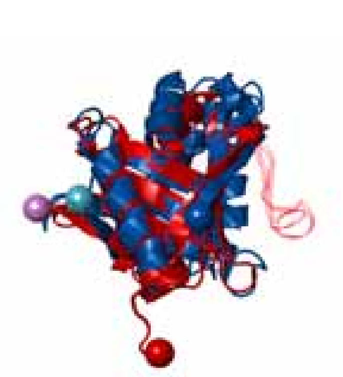 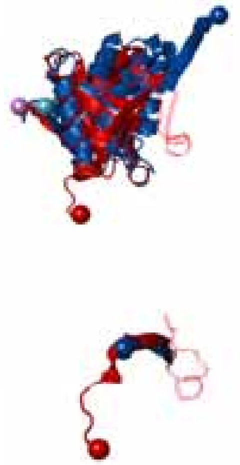 Protein (transcriptional activator GCN5) YYCN protein
	1ufhB (154)	147-155 (9)	147-151 (5)	228-244 (17)						
526	1yghA (164)	224-251 (28)	224-250 (27)	230-248 (19)	C	0.38	2.58	91.54% (119/130)	7.69% (10/130)	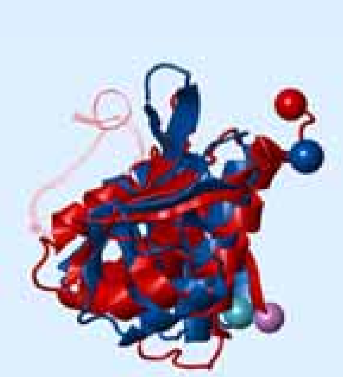 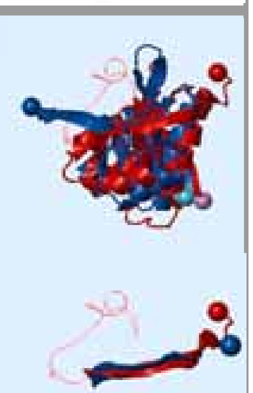 Protein (transcriptional activator GCN5) Hypothetical protein TTHA1254
	2d4pA (130)	113-123 (11)	113-122 (10)	230-248 (19)						
527	1yghA (164)	226-248 (23)	228-248 (21)	228-248 (21)	C	0.41	2.36	82.93% (136/164)	9.15% (15/164)	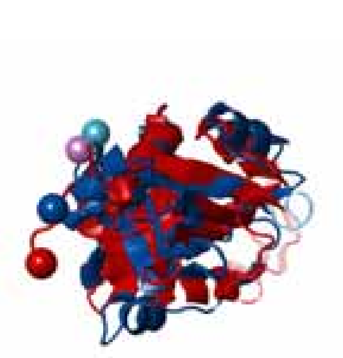 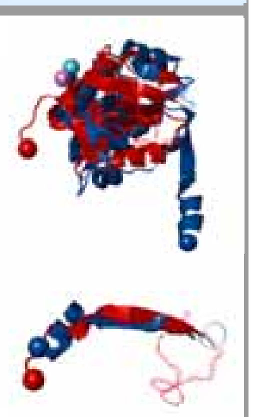 Protein (transcriptional activator GCN5) Diamine acetyltransferase 1
	2f5iA (167)	145-153 (9)	147-153 (7)	228-248 (21)						

528	1yghA (164)	225-259 (35)	228-243 (16)	228-250 (23)	C	0.39	2.48	83.54% (137/164)	10.37% (17/164)	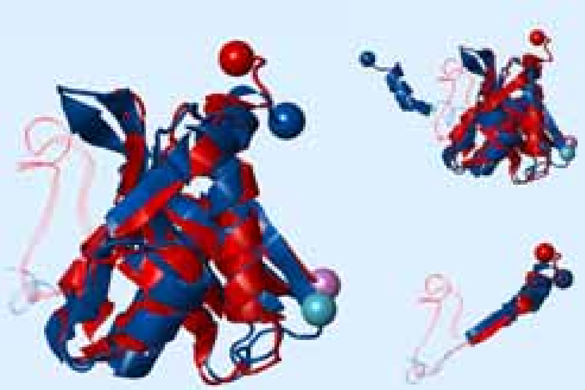 Protein (transcriptional activator GCN5) Diamine acetyltransferase 1
	2g3tA (169)	144-170 (27)	147-150 (4)	228-250 (23)						
529	1yghA (164)	221-248 (28)	228-248 (21)	228-248 (21)	C	0.41	2.35	82.93% (136/164)	9.15% (15/164)	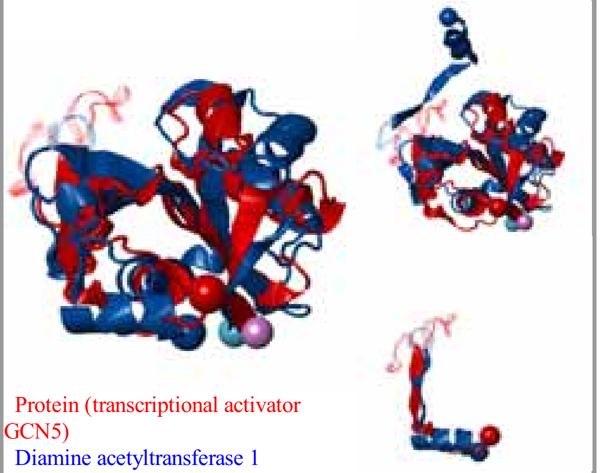 Protein (transcriptional activator GCN5) Diamine acetyltransferase 1
	2g3tB (168)	140-153 (14)	147-153 (7)	228-248 (21)						
530	1yghA (164)	228-254 (27)	228-250 (23)	229-246 (18)	C	0.30	2.43	75.00% (123/164)	10.37% (17/164)	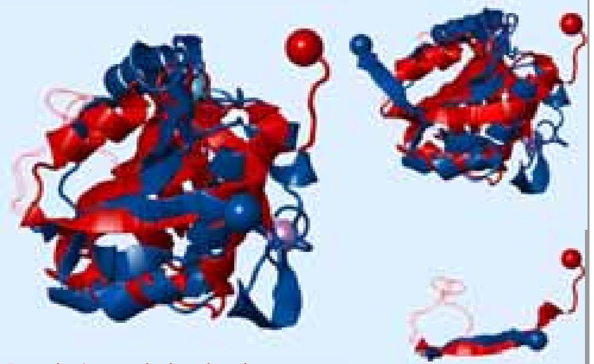 Protein (transcriptional activator GCN5) Glucosamine 6-phosphate N-acetyltransferase
	2o28B (180)	172-181 (10)	172-177 (6)	229-246 (18)						

531	1yghA (164)	227-257 (31)	228-249 (22)	229-247 (19)	C	0.43	2.37	89.58% (129/144)	8.33% (12/144)	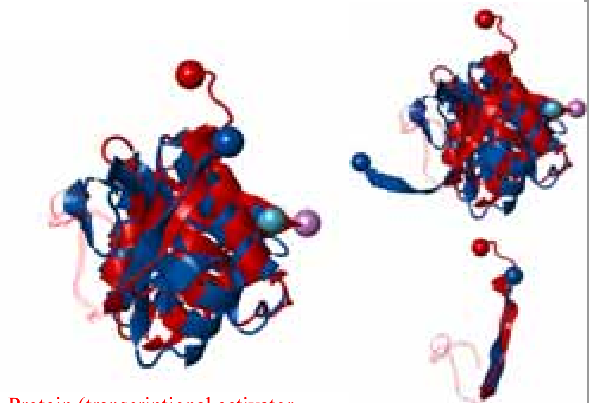 Protein (transcriptional activator GCN5)<br>Aminoglycoside 6'-N-acetyltransferase
	2vbqB (144)	133-144 (12)	134-137 (4)	229-247 (19)						
532	1yghA (164)	228-254 (27)	228-250 (23)	229-247 (19)	C	0.33	2.28	73.78% (121/164)	10.37% (17/164)	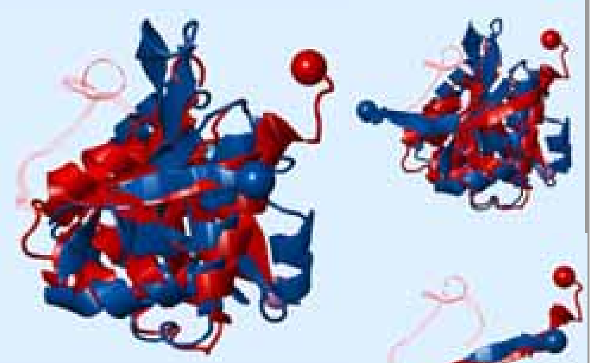 Protein (transcriptional activator GCN5)<br>Glucosamine 6-phosphate acetyltransferase
	2vxkA (165)	181-189 (9)	181-185 (5)	229-247 (19)						
533	1yk3A (198)	186-198 (13)	188-198 (11)	192-193 (2)	C	0.40	2.61	97.93% (142/145)	12.41% (18/145)	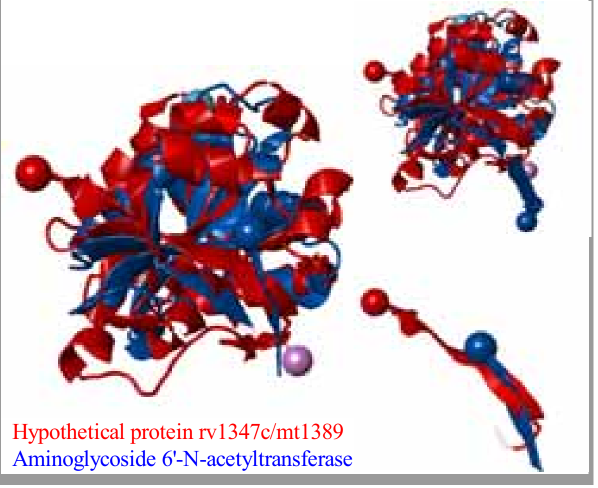 Hypothetical protein rv1347c/mt1389<br>Aminoglycoside 6'-N-acetyltransferase
	1s3zB (145)	133-144 (12)	135-144 (10)	192-193 (2)						

534	1yk3A (198)	183-201 (19)	192-201 (10)	192-197 (6)	C	0.40	2.58	94.77% (145/153)	13.07% (20/153)	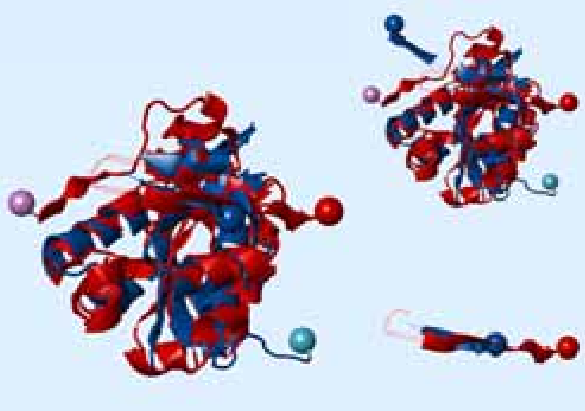 Hypothetical protein rv1347c/mt1389<br>Aminoglycoside 6'-N-acetyltransferase
	1s5kA (153)	130-144 (15)	139-144 (6)	192-197 (6)						
535	1yk3A (198)	186-198 (13)	192-198 (7)	192-193 (2)	C	0.40	2.60	97.92% (141/144)	13.19% (19/144)	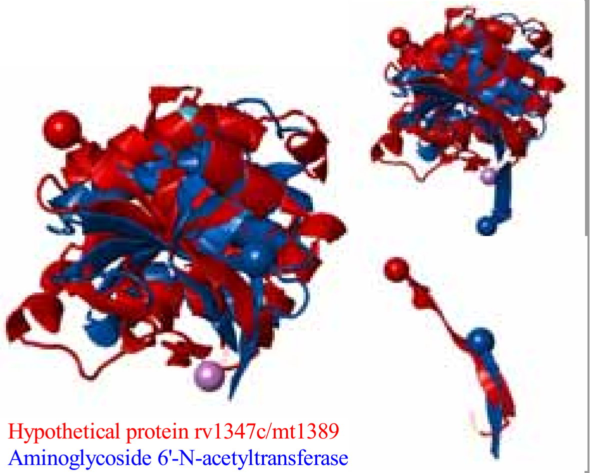 Hypothetical protein rv1347c/mt1389<br>Aminoglycoside 6'-N-acetyltransferase
	2vbqB (144)	133-144 (12)	139-144 (6)	192-193 (2)						
536	1yr0A (163)	143-160 (18)	144-160 (17)	146-159 (14)	C	0.54	2.04	94.48% (137/145)	16.55% (24/145)	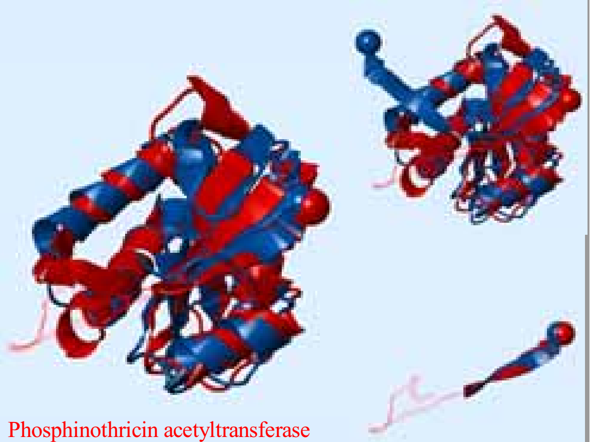 Phosphinothricin acetyltransferase<br>Aminoglycoside 6'-N-acetyltransferase
	1s3zB (145)	135-139 (5)	136-139 (4)	146-159 (14)						

537	1yr0A (163)	144-160 (17)	144-160 (17)	146-159 (14)	C	0.52	2.02	89.54% (137/153)	16.99% (26/153)	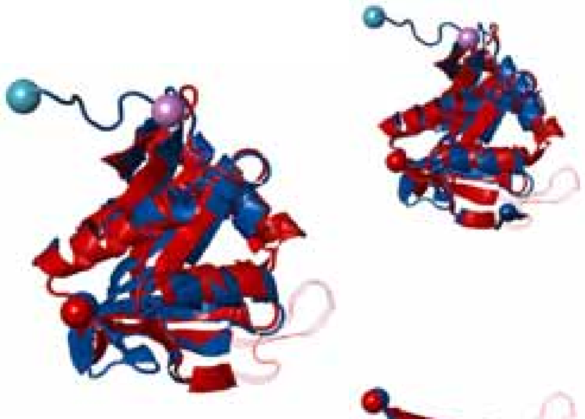<br>Phosphinothricin acetyltransferase<br>Aminoglycoside 6'-N-acetyltransferase
	1s5kA (153)	136-139 (4)	136-139 (4)	146-159 (14)						
538	1yr0A (163)	142-159 (18)	142-159 (18)	143-158 (16)	C	0.52	2.05	90.67% (136/150)	19.33% (29/150)	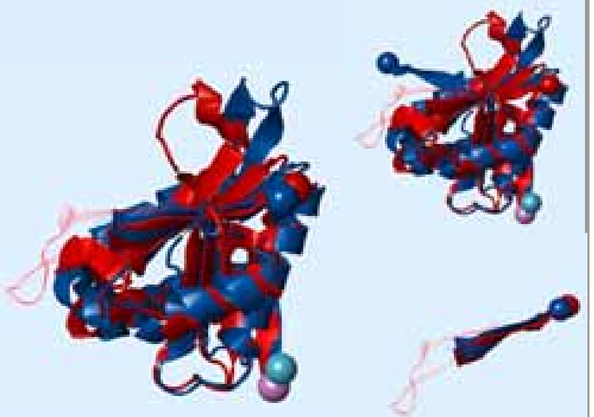<br>Phosphinothricin acetyltransferase<br>Transcriptional regulator
	1z4eA (150)	144-147 (4)	144-147 (4)	143-158 (16)						
539	1yr0A (163)	142-165 (24)	142-155 (14)	144-155 (12)	C	0.50	2.22	88.96% (145/163)	16.56% (27/163)	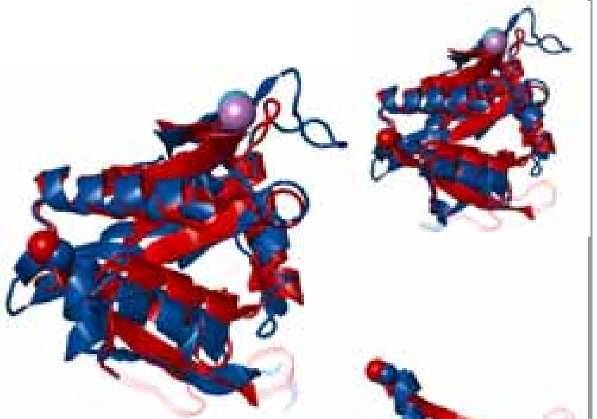<br>Phosphinothricin acetyltransferase<br>Diamine acetyltransferase 1
	2f5iA (167)	147-168 (22)	147-153 (7)	144-155 (12)						

540	1yr0A (163)	142-165 (24)	142-165 (24)	144-157 (14)	C	0.49	2.21	88.34% (144/163)	16.56% (27/163)	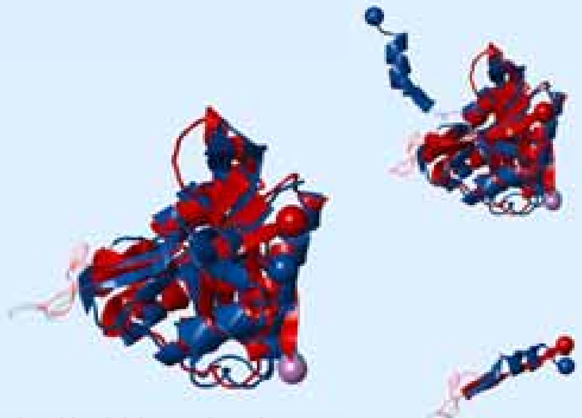 Phosphinothricin acetyltransferase<br>Diamine acetyltransferase 1
	2g3tA (169)	147-169 (23)	147-169 (23)	144-157 (14)						
541	1yr0A (163)	142-155 (14)	142-155 (14)	144-155 (12)	C	0.51	2.11	88.34% (144/163)	15.95% (26/163)	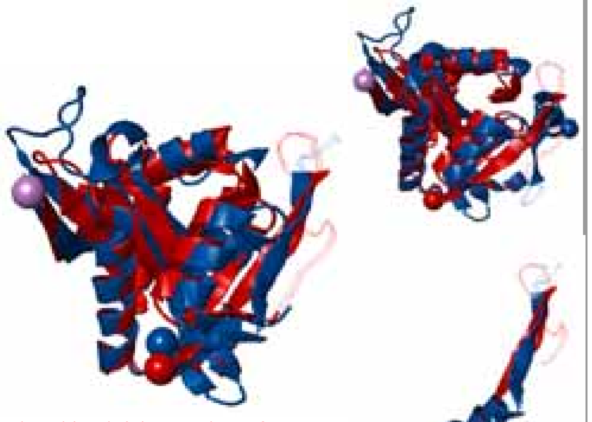 Phosphinothricin acetyltransferase<br>Diamine acetyltransferase 1
	2g3tB (168)	147-153 (7)	147-153 (7)	144-155 (12)						
542	1yr0A (163)	144-160 (17)	144-160 (17)	146-159 (14)	C	0.53	2.13	93.88% (138/147)	17.01% (25/147)	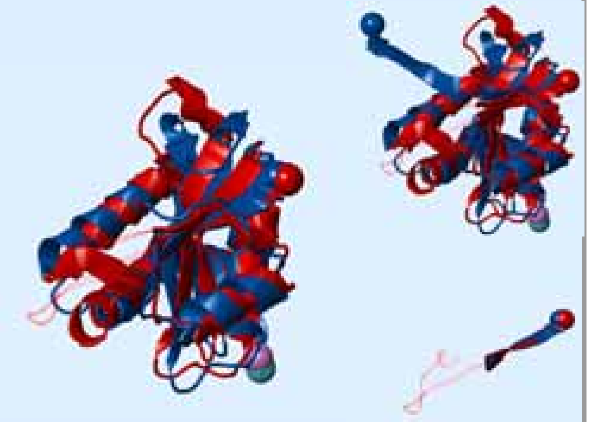 Phosphinothricin acetyltransferase<br>Aminoglycoside 6'-N-acetyltransferase
	2vbqA (147)	136-139 (4)	136-139 (4)	146-159 (14)						

543	1yr0A (163)	143-153 (11)	143-153 (11)	145-146 (2)	C	0.38	2.32	82.21% (134/163)	14.72% (24/163)	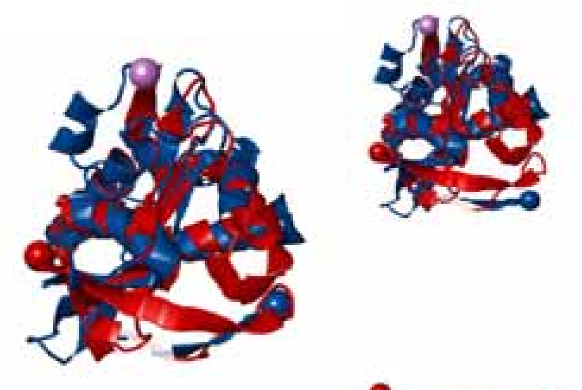 Phosphinothricin acetyltransferase Glucosamine 6-phosphate acetyltransferase
	2vxkA (165)	182-189 (8)	182-189 (8)	145-146 (2)						
544	1yvoA (169)	141-156 (16)	141-156 (16)	143-154 (12)	C	0.45	1.92	90.67% (136/150)	18.67% (28/150)	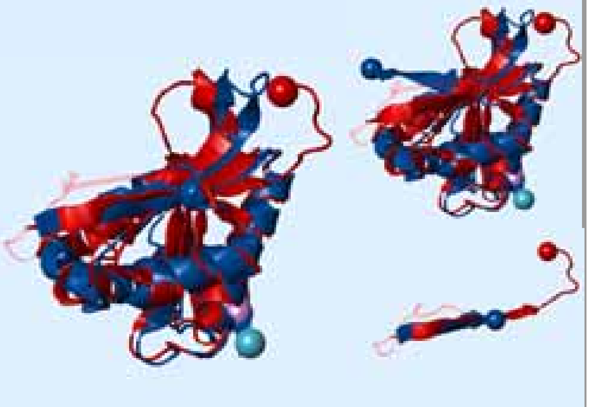 Conserved hypothetical protein Transcriptional regulator
	1z4eA (150)	144-148 (5)	144-148 (5)	143-154 (12)						
545	1yvoA (169)	141-169 (29)	141-157 (17)	142-157 (16)	C	0.47	2.35	88.02% (147/167)	17.96% (30/167)	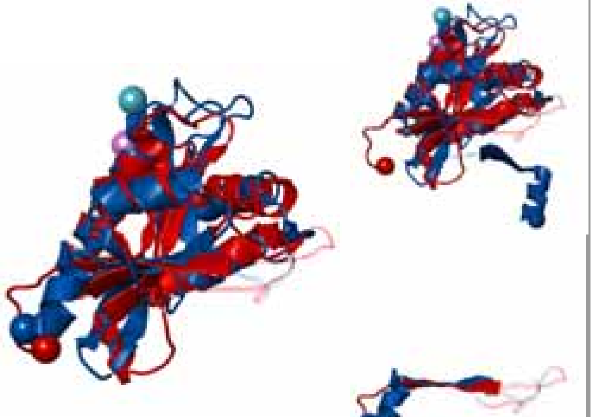 Conserved hypothetical protein Diamine acetyltransferase 1
	2f5iA (167)	147-168 (22)	147-154 (8)	142-157 (16)						

546	1yvoA (169)	142-156 (15)	142-156 (15)	142-156 (15)	C	0.47	2.38	87.95% (146/166)	18.07% (30/166)	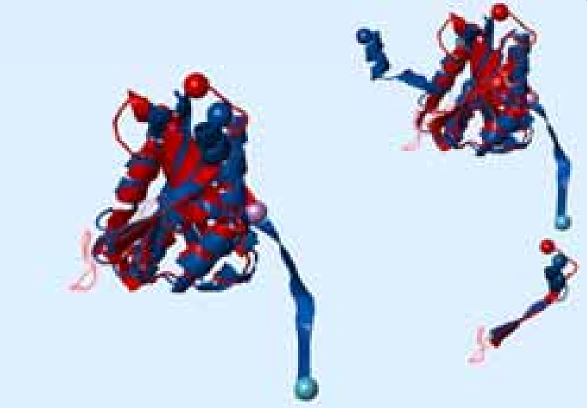<br>Conserved hypothetical protein<br>Probable N-acetyltransferase
	2fe7B (166)	140-142 (3)	140-142 (3)	142-156 (15)						
547	1yvoA (169)	141-171 (31)	141-157 (17)	142-147 (6)	C	0.49	2.34	88.69% (149/168)	18.45% (31/168)	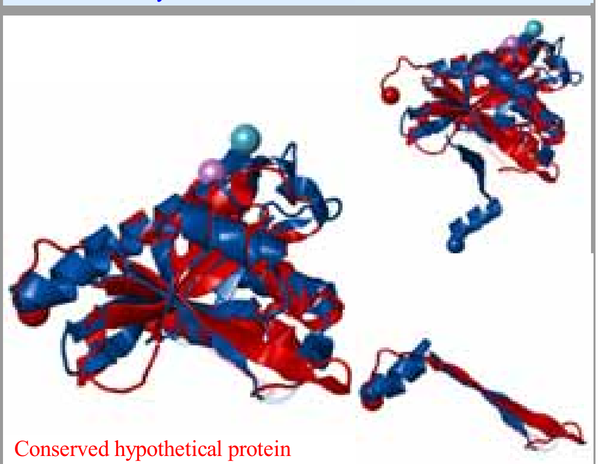<br>Conserved hypothetical protein<br>Diamine acetyltransferase 1
	2g3tB (168)	147-169 (23)	147-154 (8)	142-147 (6)						
548	1yvoA (169)	141-171 (31)	141-171 (31)	142-158 (17)	C	0.48	2.30	86.98% (147/169)	17.75% (30/169)	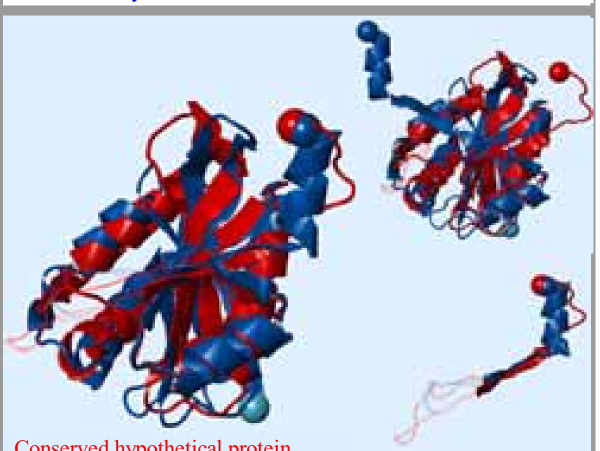<br>Conserved hypothetical protein<br>Diamine acetyltransferase 1
	2jevA (169)	147-169 (23)	147-169 (23)	142-158 (17)						

549	1yvsA (108)	45-52 (8)	45-52 (8)	46-52 (7)	N	0.39	2.48	86.32% (82/95)	22.11% (21/95)	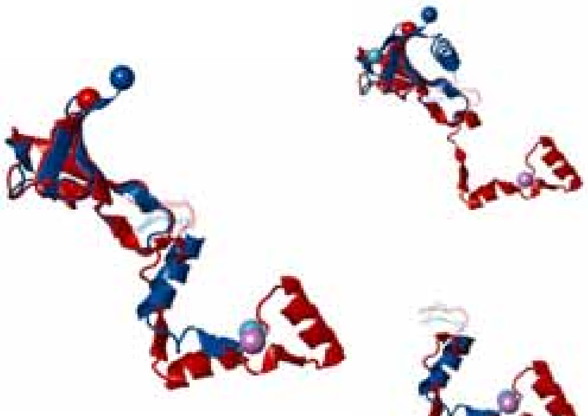 Barnase<br>Guanyl-specific ribonuclease SA
	1boxA (95)	23-33 (11)	23-33 (11)	46-52 (7)						
550	1yvsA (108)	45-109 (65)	45-58 (14)	45-54 (10)	N	0.37	2.42	83.33% (80/96)	20.83% (20/96)	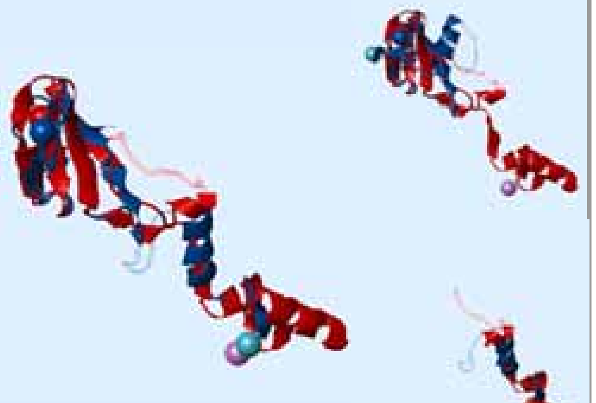 Barnase<br>Ribonuclease SA
	1c54A (96)	24-92 (69)	24-39 (16)	45-54 (10)						
551	1yvsA (108)	45-52 (8)	46-52 (7)	45-52 (8)	N	0.41	2.16	83.33% (80/96)	20.83% (20/96)	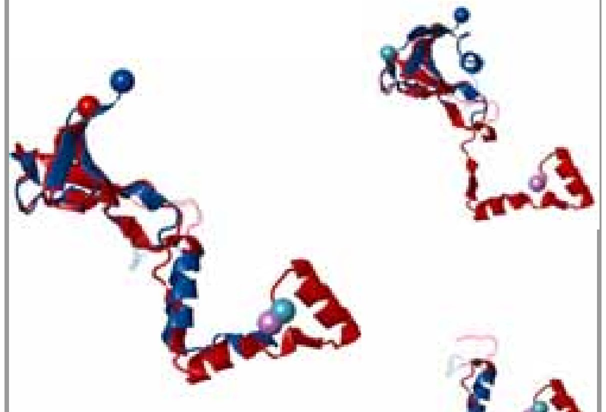 Barnase<br>Ribonuclease SA
	1gmpA (96)	24-33 (10)	25-33 (9)	45-52 (8)						

552	1yvsA (108)	26-52 (27)	28-52 (25)	26-52 (27)	N	0.40	2.33	85.42% (82/96)	22.92% (22/96)	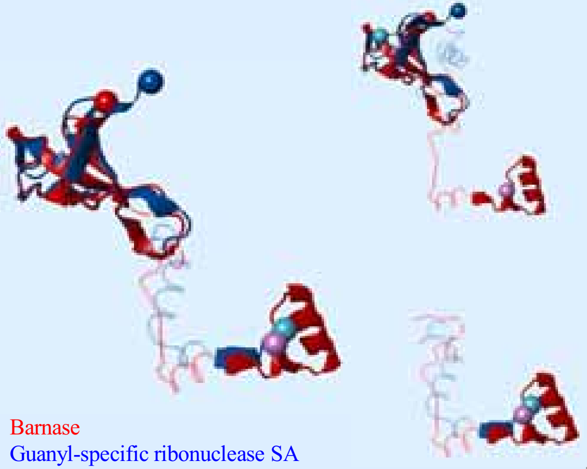
	1i70A (96)	7-33 (27)	9-33 (25)	26-52 (27)						
553	1yvsA (108)	45-52 (8)	45-52 (8)	46-52 (7)	N	0.40	2.27	84.38% (81/96)	19.79% (19/96)	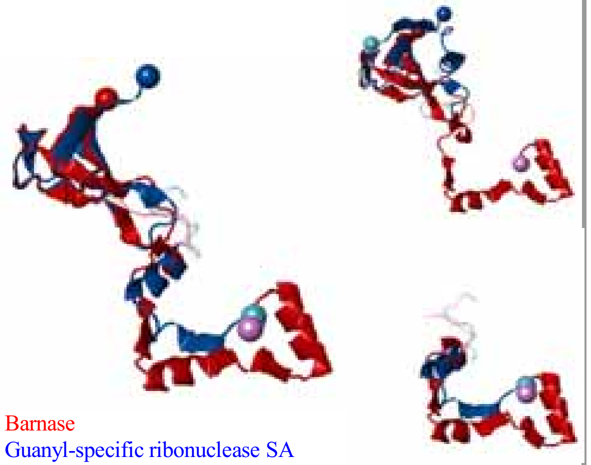
	1i8vA (96)	20-33 (14)	20-33 (14)	46-52 (7)						
554	1yvsA (108)	34-54 (21)	34-53 (20)	34-54 (21)	N	0.26	2.84	75.96% (79/104)	11.54% (12/104)	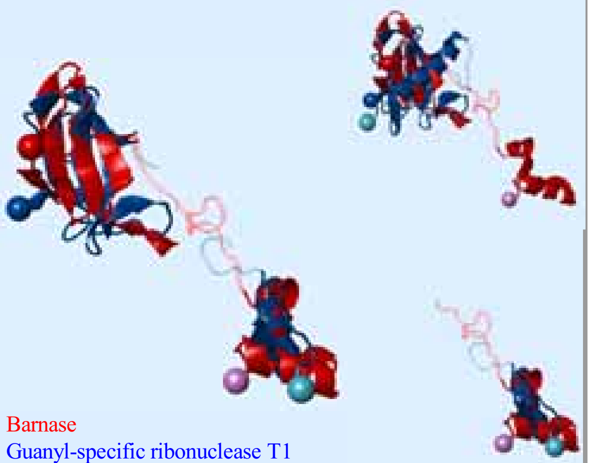
	1lovA (104)	30-40 (11)	30-39 (10)	34-54 (21)						

555	1yvsA (108)	45-52 (8)	45-52 (8)	46-52 (7)	N	0.41	2.43	86.60% (84/97)	22.68% (22/97)	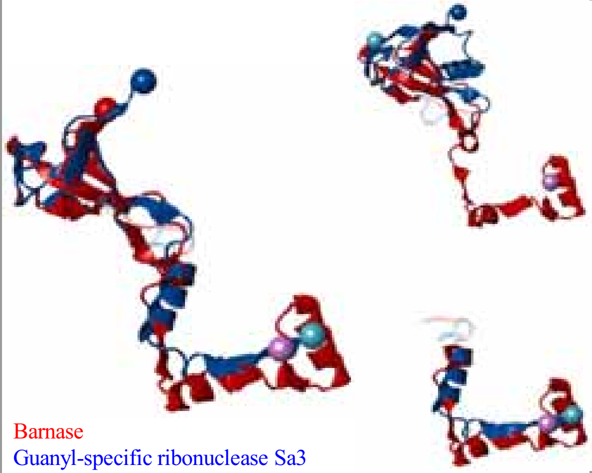 Barnase<br>Guanyl-specific ribonuclease Sa3
	1mgrA (97)	26-36 (11)	26-36 (11)	46-52 (7)						
556	1yvsA (108)	45-52 (8)	45-52 (8)	46-52 (7)	N	0.40	2.43	84.85% (84/99)	22.22% (22/99)	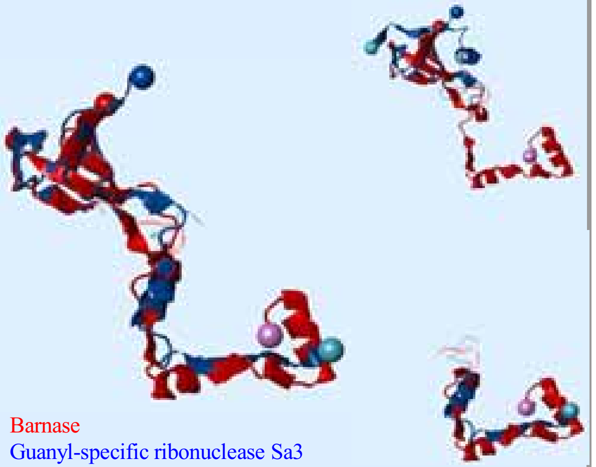 Barnase<br>Guanyl-specific ribonuclease Sa3
	1mgwA (99)	26-36 (11)	26-36 (11)	46-52 (7)						
557	1yvsA (108)	26-52 (27)	45-52 (8)	45-52 (8)	N	0.42	2.19	85.42% (82/96)	20.83% (20/96)	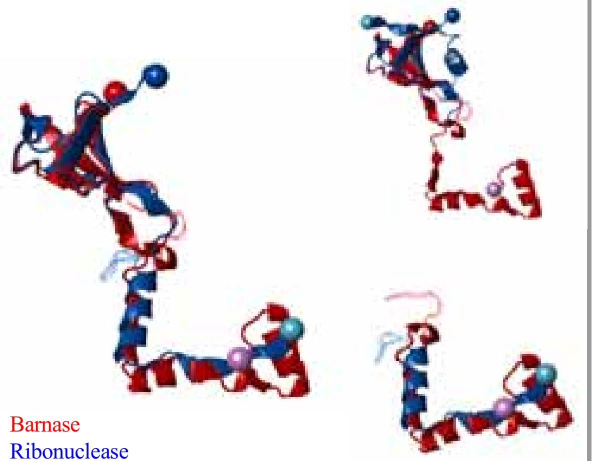 Barnase<br>Ribonuclease
	1pylA (96)	9-35 (27)	26-35 (10)	45-52 (8)						

558	1yvsA (108)	45-52 (8)	45-52 (8)	46-52 (7)	N	0.40	2.53	87.50% (84/96)	22.92% (22/96)	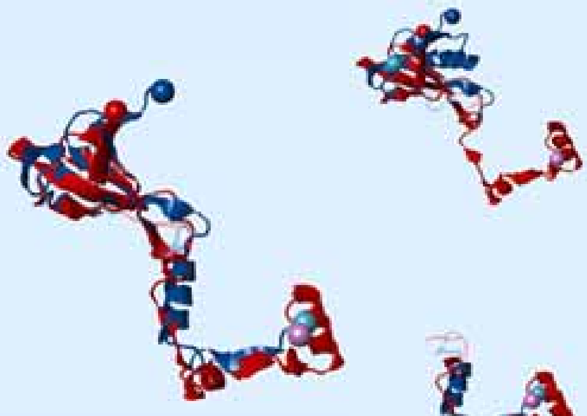 Barnase<br>Guanyl-specific ribonuclease Sa
	1t2iA (96)	23-33 (11)	23-33 (11)	46-52 (7)						
559	1yvsA (108)	45-52 (8)	46-52 (7)	45-52 (8)	N	0.40	2.27	84.38% (81/96)	21.88% (21/96)	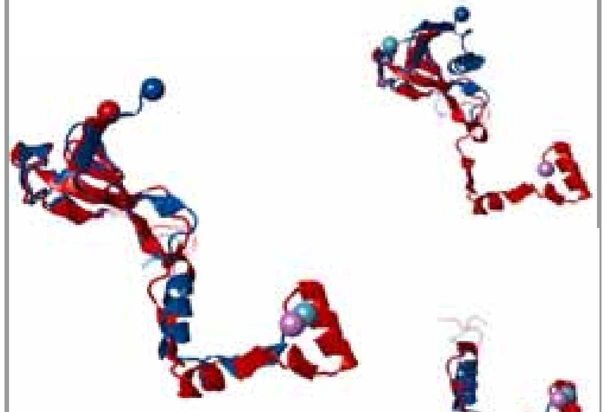 Barnase<br>Guanyl-specific ribonuclease Sa
	1uciA (96)	24-33 (10)	25-33 (9)	45-52 (8)						
560	1yvsA (108)	45-52 (8)	46-52 (7)	46-52 (7)	N	0.41	2.13	83.33% (80/96)	20.83% (20/96)	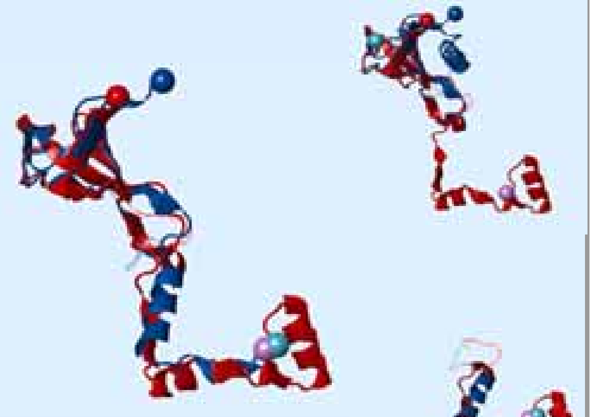 Barnase<br>Guanyl-specific ribonuclease Sa
	1ucjA (96)	24-33 (10)	25-33 (9)	46-52 (7)						

561	1yvsA (108)	45-52 (8)	45-52 (8)	46-52 (7)	N	0.41	2.36	86.46% (83/96)	21.88% (21/96)	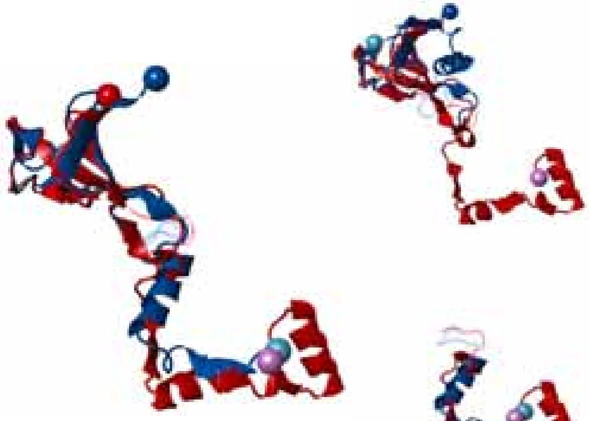 Barnase<br>Guanyl-specific ribonuclease Sa
	1uckA (96)	23-33 (11)	23-33 (11)	46-52 (7)						
562	1yvsA (108)	5-54 (50)	46-52 (7)	30-52 (23)	N	0.39	2.37	84.38% (81/96)	21.88% (21/96)	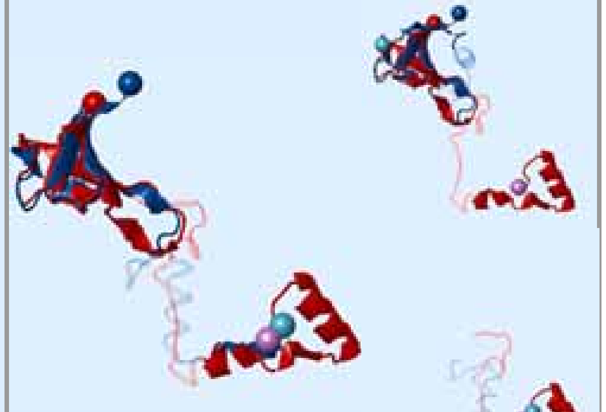 Barnase<br>Guanyl-specific ribonuclease Sa
	1ynvX (96)	2-35 (34)	25-33 (9)	30-52 (23)						
563	1z3aA (156)	114-150 (37)	118-150 (33)	120-142 (23)	C	0.56	1.70	86.45% (134/155)	26.45% (41/155)	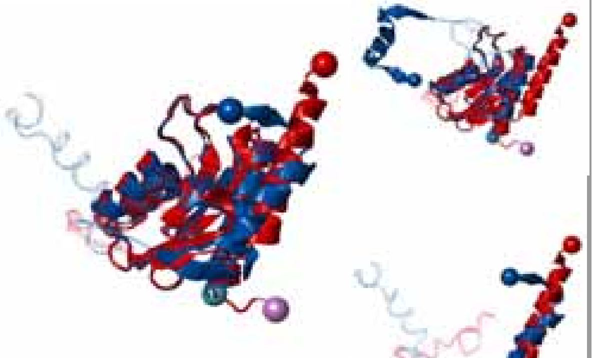 TRNA-specific adenosine deaminase<br>Guanine deaminase
	1wkqB (155)	99-141 (43)	103-141 (39)	120-142 (23)						

564	1z4rA (163)	617-658 (42)	626-647 (22)	626-646 (21)	C	0.44	2.19	84.87% (129/152)	14.47% (22/152)	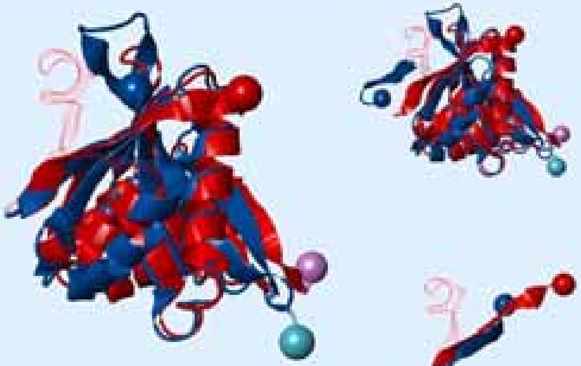 General control of amino acid synthesis prote<br>HPA2 histone acetyltransferase
	1qsmD (152)	132-156 (25)	143-147 (5)	626-646 (21)						
565	1znpA (140)	4-56 (53)	26-40 (15)	26-40 (15)	N	0.34	2.22	82.86% (116/140)	10.71% (15/140)	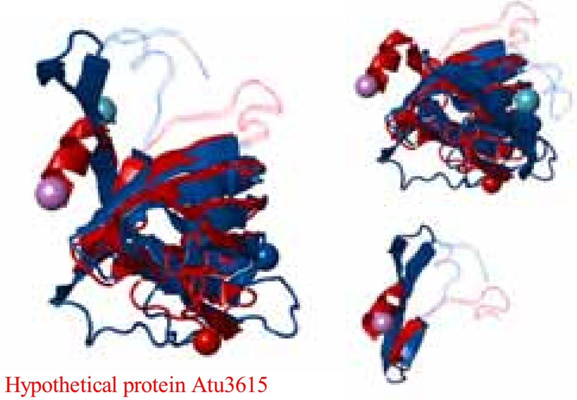 Hypothetical protein Atu3615<br>DTDP-6-deoxy-D-XYLO-4-hexulose 3,5-epimerase
	1ep0A (183)	3-64 (62)	31-48 (18)	26-40 (15)						
566	1znpA (140)	4-44 (41)	4-44 (41)	36-40 (5)	N	0.30	2.23	84.29% (118/140)	8.57% (12/140)	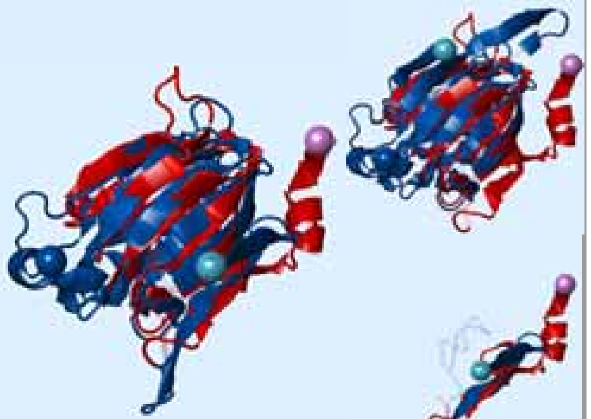 Hypothetical protein Atu3615<br>RFBC
	1pm7A (199)	1-50 (50)	1-50 (50)	36-40 (5)						

567	1znpA (140)	4-44 (41)	4-44 (41)	28-40 (13)	N	0.31	2.21	84.29% (118/140)	8.57% (12/140)	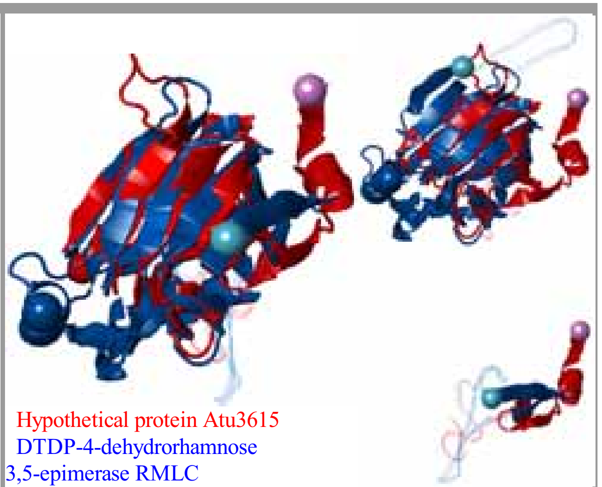 Hypothetical protein Atu3615 DTDP-4-dehydrorhamnose 3,5-epimerase RMLC
	2ixcA (198)	1-50 (50)	1-50 (50)	28-40 (13)						
568	1znpA (140)	4-44 (41)	4-40 (37)	36-40 (5)	N	0.29	2.24	83.57% (117/140)	6.43% (9/140)	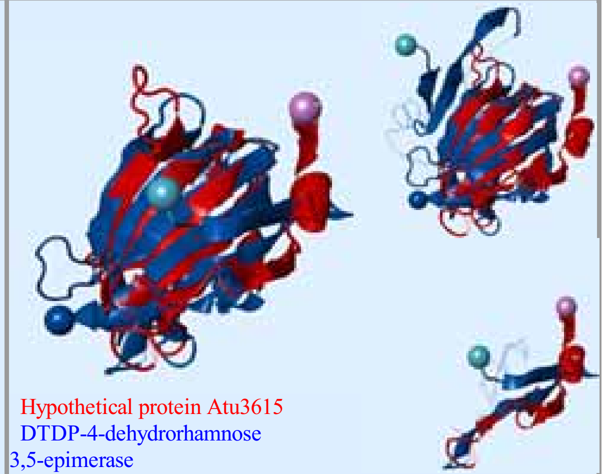 Hypothetical protein Atu3615 DTDP-4-dehydrorhamnose 3,5-epimerase
	2ixhA (184)	1-53 (53)	1-49 (49)	36-40 (5)						
569	1znpA (140)	4-55 (52)	4-55 (52)	26-40 (15)	N	0.39	2.23	79.85% (107/134)	7.46% (10/134)	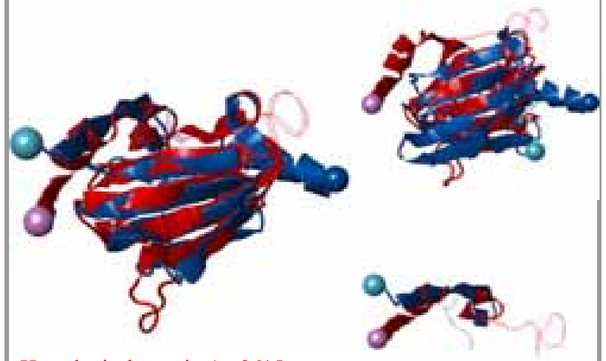 Hypothetical protein Atu3615 DTDP-6-deoxy-3,4-keto-hexulose isomerase
	2paeB (134)	2-48 (47)	2-48 (47)	26-40 (15)						

570	1zvnA (99)	1-70 (70)	1-6 (6)	4-5 (2)	N	0.28	3.18	79.80% (79/99)	7.07% (7/99)	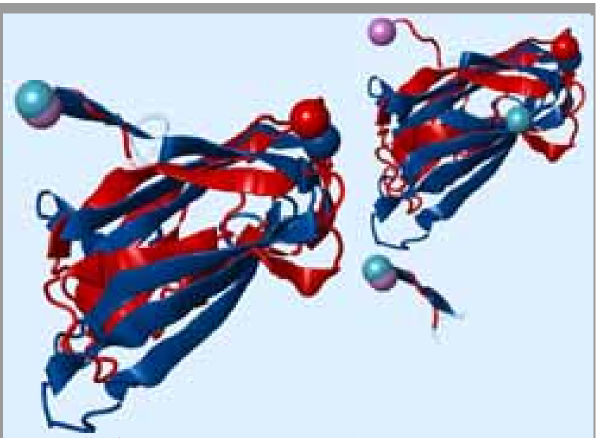 MN-cadherin<br>Chagasin
	2h7wA (108)	4-82 (79)	4-12 (9)	4-5 (2)						
571	2b3jA (151)	93-135 (43)	103-135 (33)	105-135 (31)	C	0.56	1.84	90.07% (136/151)	29.80% (45/151)	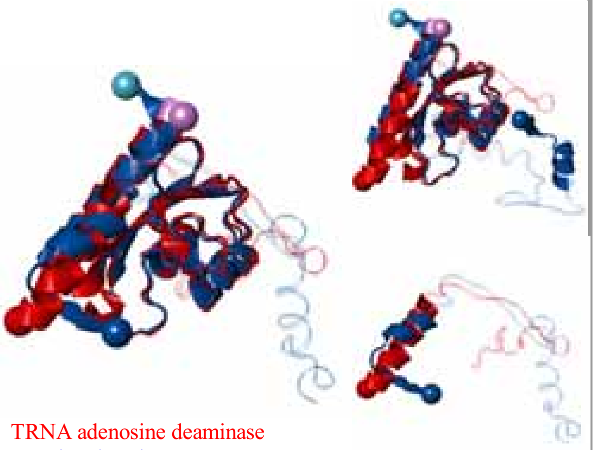 TRNA adenosine deaminase<br>Guanine deaminase
	1wkqA (158)	93-141 (49)	103-141 (39)	105-135 (31)						
572	2bswA (145)	125-141 (17)	126-141 (16)	127-138 (12)	C	0.57	1.95	91.03% (132/145)	13.79% (20/145)	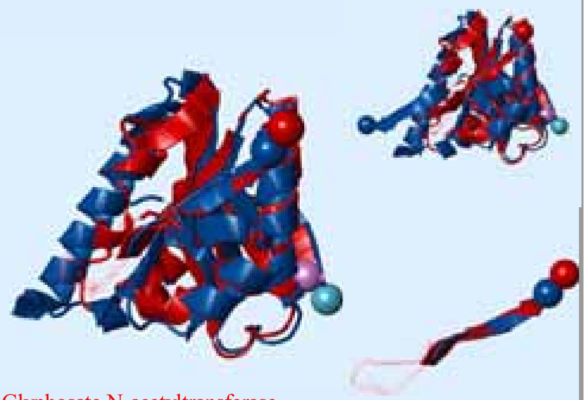 Glyphosate N-acetyltransferase<br>Putative acetyl transferase
	1vkcA (149)	141-146 (6)	142-146 (5)	127-138 (12)						

573	2bswA (145)	125-143 (19)	126-140 (15)	128-136 (9)	C	0.55	2.05	90.34% (131/145)	14.48% (21/145)	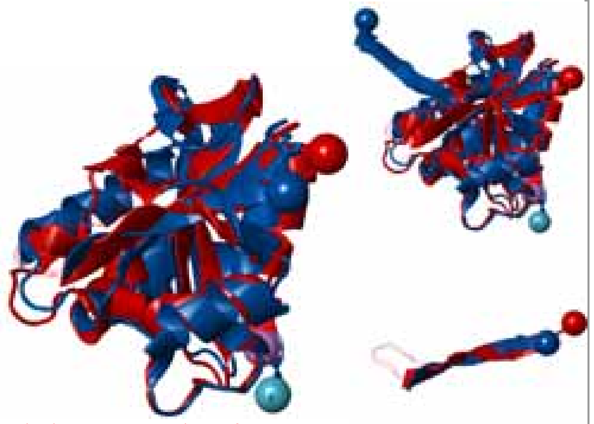<br>Glyphosate N-acetyltransferase<br>Aminoglycoside 6'-N-acetyltransferase
	2vbqA (147)	134-144 (11)	135-141 (7)	128-136 (9)						
574	2bswA (145)	125-142 (18)	126-142 (17)	128-136 (9)	C	0.55	2.15	91.67% (132/144)	15.28% (22/144)	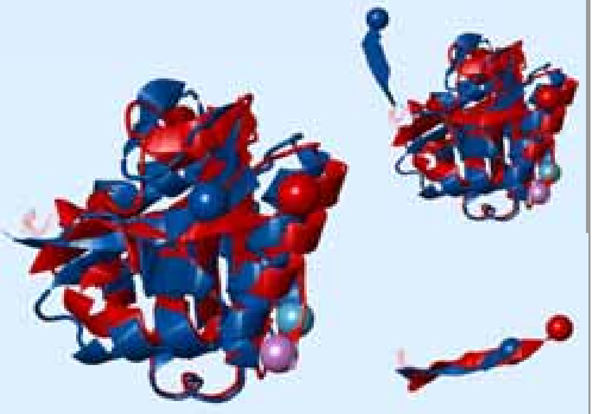<br>Glyphosate N-acetyltransferase<br>Aminoglycoside 6'-N-acetyltransferase
	2vbqB (144)	134-144 (11)	135-144 (10)	128-136 (9)						
575	2c49A (299)	3-40 (38),<br>18-42 (25)	11-23 (13),<br>26-42 (17)	17-18 (2),<br>37-42 (6)	M	0.32	2.31	85.28% (255/299)	17.39% (52/299)	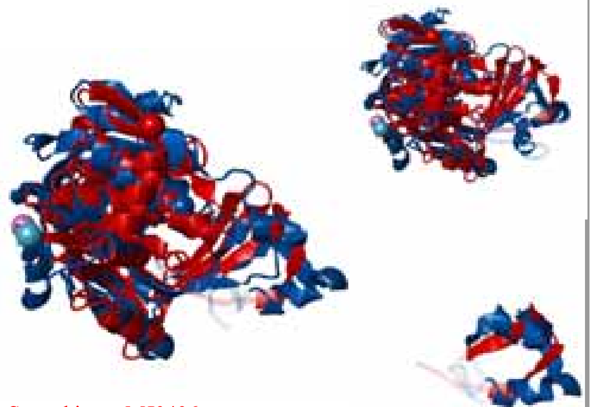<br>Sugar kinase MJ0406<br>Adenosine kinase
	2i6bB (326)	3-57 (55),<br>21-63 (43)	12-29 (18),<br>35-63 (29)	17-18 (2),<br>37-42 (6)						

576	2cnmA (151)	123-143 (21)	126-143 (18)	126-137 (12)	C	0.45	1.88	90.34% (131/145)	14.48% (21/145)	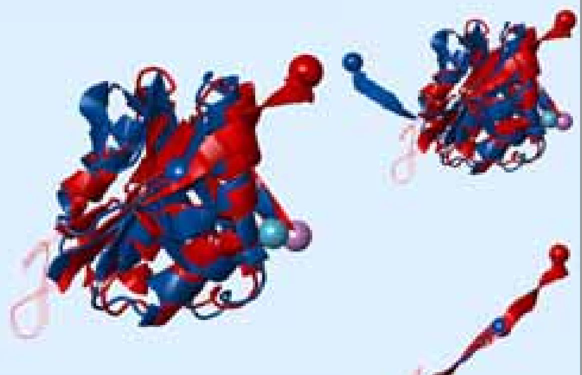 Modification OF 30S ribosomal subunit protein<br>Aminoglycoside 6'-N-acetyltransferase
	1s3zB (145)	135-144 (10)	138-144 (7)	126-137 (12)						
577	2cnmA (151)	118-135 (18)	121-135 (15)	126-134 (9)	C	0.45	2.00	89.12% (131/147)	13.61% (20/147)	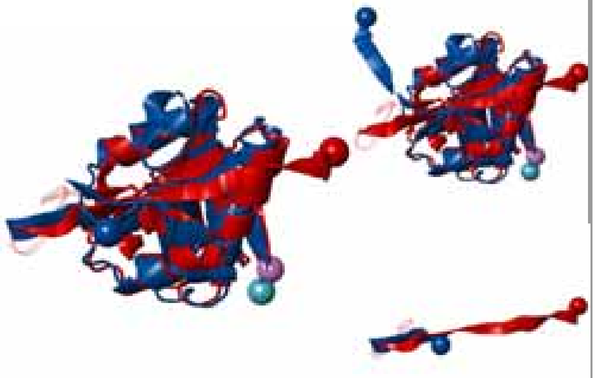 Modification OF 30S ribosomal subunit protein<br>Aminoglycoside 6'-N-acetyltransferase
	2vbqA (147)	130-140 (11)	134-140 (7)	126-134 (9)						
578	2cswA (145)	104-145 (42)	108-145 (38)	126-135 (10)	C	0.27	2.22	82.76% (120/145)	13.79% (20/145)	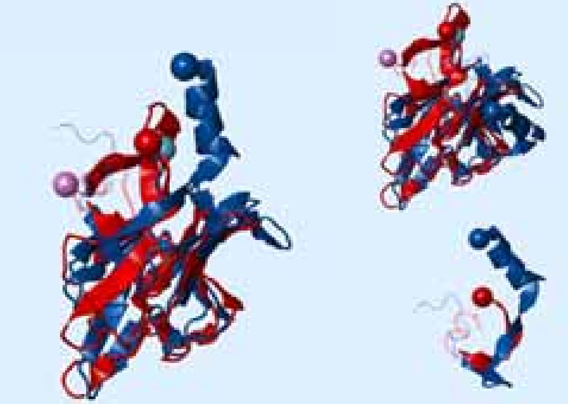 Ubiquitin ligase protein RNF8<br>Protein kinase SPK1
	1j4kA (158)	676-730 (55)	680-730 (51)	126-135 (10)						

579	2cu2A (335)	142-168 (27), 149-184 (36)	143-149 (7), 152-182 (31)	143-149 (7), 167-181 (15)	M	0.35	2.39	92.76% (205/221)	18.55% (41/221)	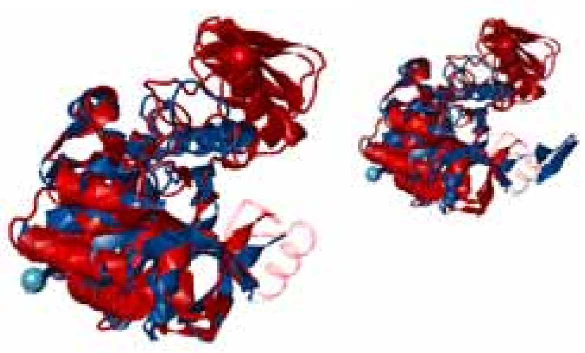 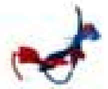 Putative mannose-1-phosphate guanylyl transfe 2-C-methyl-D-erythritol 4-phosphate cytidylyl
	1vpaA (221)	133-149 (17), 135-153 (19)	134-135 (2), 138-151 (14)	143-149 (7), 167-181 (15)						
580	2cu2A (335)	142-168 (27), 149-183 (35)	142-149 (8), 152-181 (30)	143-149 (7), 168-181 (14)	M	0.34	2.44	92.73% (204/220)	16.82% (37/220)	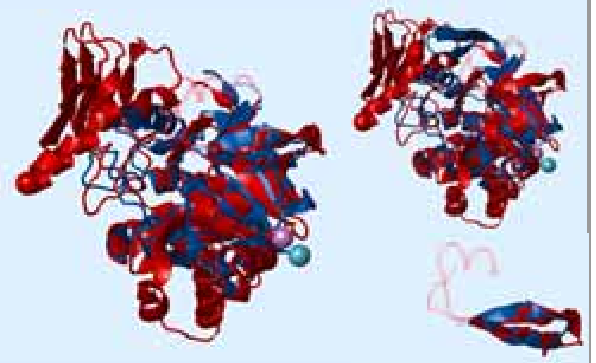 Putative mannose-1-phosphate guanylyl transfe 2-C-methyl-D-erythritol 4-phosphate cytidylyl
	1vpaB (220)	133-148 (16), 135-152 (18)	133-135 (3), 138-150 (13)	143-149 (7), 168-181 (14)						
581	2e3dA (286)	159-198 (40), 178-224 (47)	170-178 (9), 181-212 (32)	173-178 (6), 197-211 (15)	M	0.43	2.47	96.00% (216/225)	15.11% (34/225)	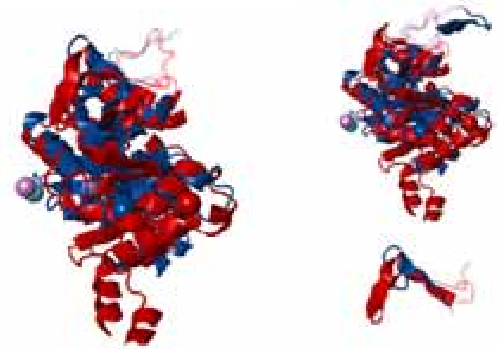 UTP--glucose-1-phosphate uridylyltransferase 4-diphosphocytidyl-2-C-methylerythritol synth
	1iniA (225)	124-153 (30), 140-174 (35)	135-140 (6), 143-161 (19)	173-178 (6), 197-211 (15)						

582	2e3dA (286)	159-200 (42), 177-224 (48)	172-200 (29), 177-212 (36)	172-177 (6), 199-211 (13)	M	0.44	2.35	95.93% (212/221)	14.48% (32/221)	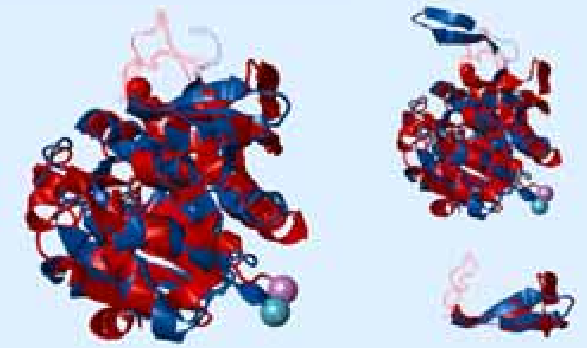<br>UTP--glucose-1-phosphate uridylyltransferase<br>2-C-methyl-D-erythritol 4-phosphate cytidylyl
	1vpaA (221)	120-148 (29), 133-166 (34)	132-148 (17), 133-153 (21)	172-177 (6), 199-211 (13)						
583	2f5gA (130)	81-121 (41)	81-119 (39)	109-119 (11)	C	0.27	2.62	72.31% (94/130)	5.38% (7/130)	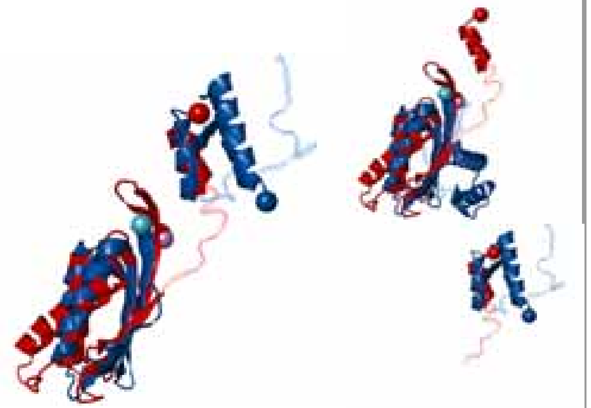<br>Transposase, putative<br>Hypothetical protein Tll0078
	1x0pE (141)	4067-4104 (38)	4067-4102 (36)	109-119 (11)						
584	2fckA (174)	154-174 (21)	154-174 (21)	159-170 (12)	C	0.34	3.06	84.62% (143/169)	11.24% (19/169)	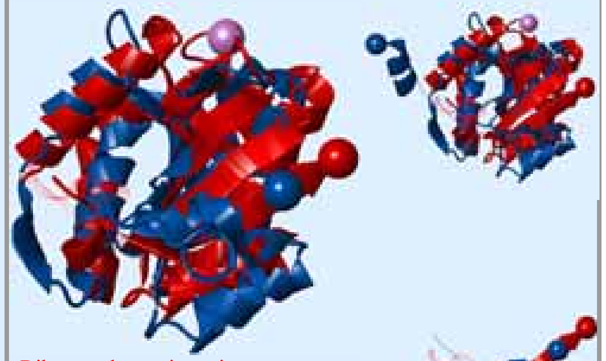<br>Ribosomal-protein-serine acetyltransferase, p<br>Diamine acetyltransferase 1
	2g3tA (169)	147-169 (23)	147-169 (23)	159-170 (12)						

585	2fckA (174)	151-173 (23)	151-173 (23)	160-169 (10)	C	0.29	3.04	83.43% (141/169)	9.47% (16/169)	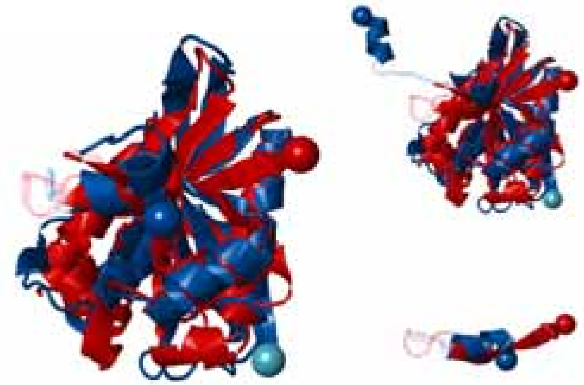 Ribosomal-protein-serine acetyltransferase, p Diamine acetyltransferase 1
	2jevA (169)	143-169 (27)	143-169 (27)	160-169 (10)						
586	2fiaA (157)	136-149 (14)	137-149 (13)	137-147 (11)	C	0.43	2.43	85.06% (131/154)	14.94% (23/154)	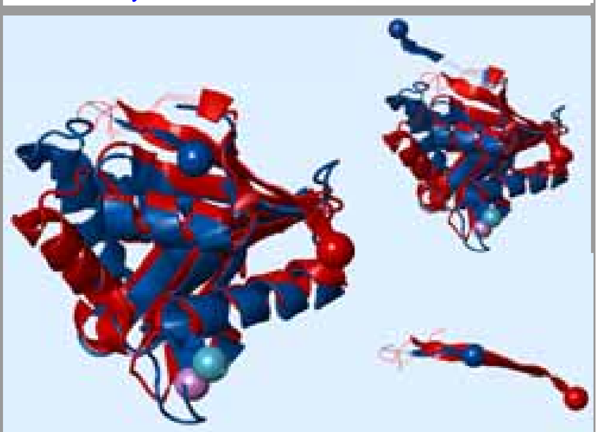 Acetyltransferase YYCN protein
	1ufhB (154)	149-154 (6)	150-154 (5)	137-147 (11)						
587	2fl4A (147)	27-37 (11)	27-37 (11)	27-37 (11)	N	0.45	2.33	86.39% (127/147)	12.24% (18/147)	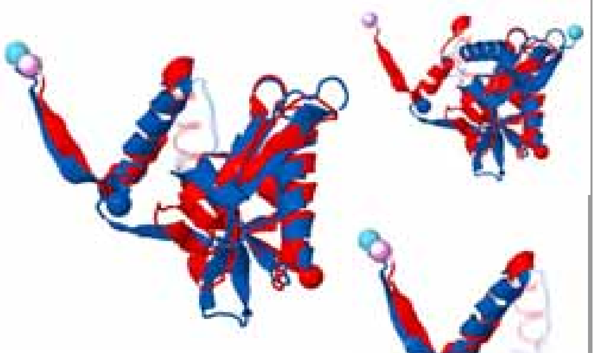 Spermine/spermidine acetyltransferase HPA2 histone acetyltransferase
	1qsmD (152)	33-43 (11)	33-43 (11)	27-37 (11)						

588	2fl4A (147)	119-137 (19)	126-137 (12)	133-134 (2)	C	0.34	2.29	74.15% (109/147)	9.52% (14/147)	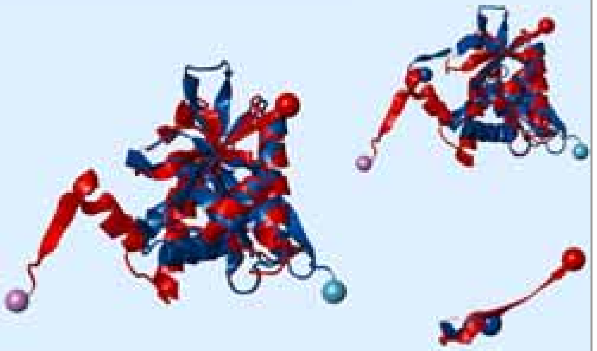 Spermine/spermidine acetyltransferase<br>HPA2 histone acetyltransferase
	1qsmD (152)	136-152 (17)	143-152 (10)	133-134 (2)						
589	2fl4A (147)	35-48 (14)	38-47 (10)	35-44 (10)	N	0.42	2.47	85.71% (126/147)	14.97% (22/147)	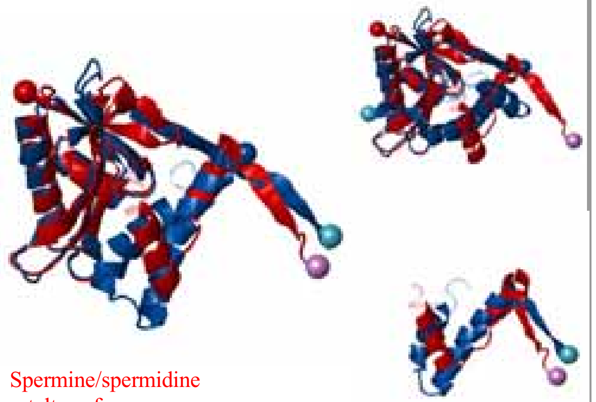 Spermine/spermidine acetyltransferase<br>YYCN protein
	1ufhA (155)	48-63 (16)	50-62 (13)	35-44 (10)						
590	2fl4A (147)	130-139 (10)	130-139 (10)	130-137 (8)	C	0.36	1.80	71.43% (105/147)	14.97% (22/147)	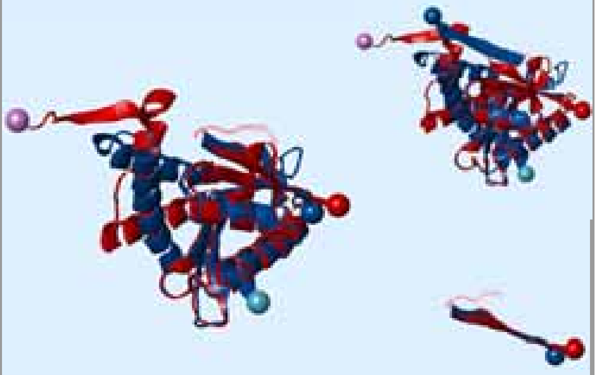 Spermine/spermidine acetyltransferase<br>YYCN protein
	1ufhA (155)	148-150 (3)	148-150 (3)	130-137 (8)						

591	2fl4A (147)	35-48 (14)	38-48 (11)	35-42 (8)	N	0.41	2.56	86.39% (127/147)	14.29% (21/147)	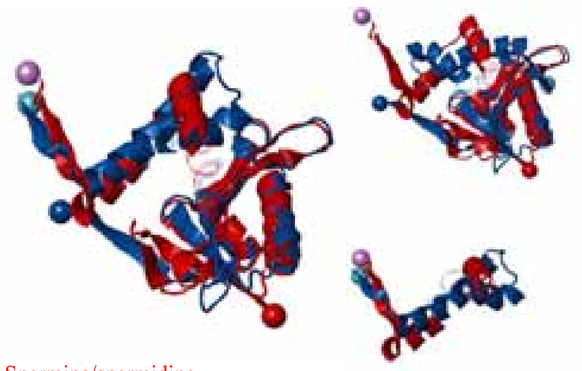 Spermine/spermidine acetyltransferase YYCN protein
	1ufhB (154)	48-63 (16)	50-63 (14)	35-42 (8)						
592	2fl4A (147)	129-145 (17)	129-140 (12)	129-138 (10)	C	0.34	2.14	73.47% (108/147)	13.61% (20/147)	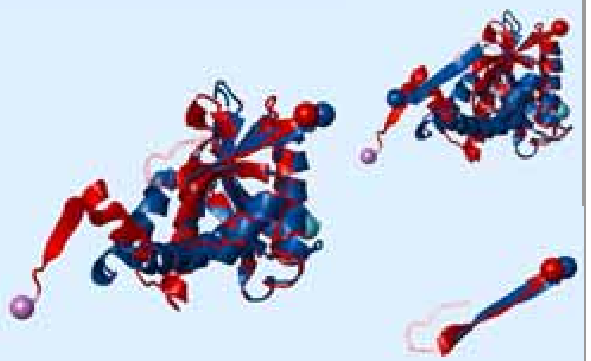 Spermine/spermidine acetyltransferase YYCN protein
	1ufhB (154)	147-154 (8)	147-149 (3)	129-138 (10)						
593	2fl4A (147)	18-37 (20)	18-37 (20)	26-37 (12)	N	0.39	2.56	82.31% (121/147)	11.56% (17/147)	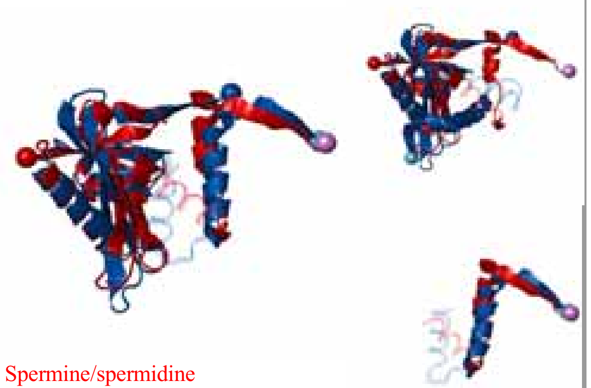 Spermine/spermidine acetyltransferase Putative acetyl transferase
	1vkcA (149)	17-45 (29)	17-45 (29)	26-37 (12)						

594	2fl4A (147)	129-141 (13)	129-140 (12)	129-136 (8)	C	0.34	2.38	74.83% (110/147)	12.93% (19/147)	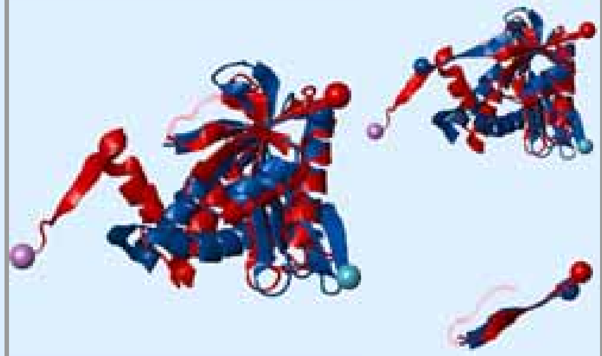 Spermine/spermidine acetyltransferase<br>Putative acetyl transferase
	1vkcA (149)	141-146 (6)	141-145 (5)	129-136 (8)						
595	2fl4A (147)	29-36 (8)	29-36 (8)	29-36 (8)	N	0.43	2.49	86.39% (127/147)	14.29% (21/147)	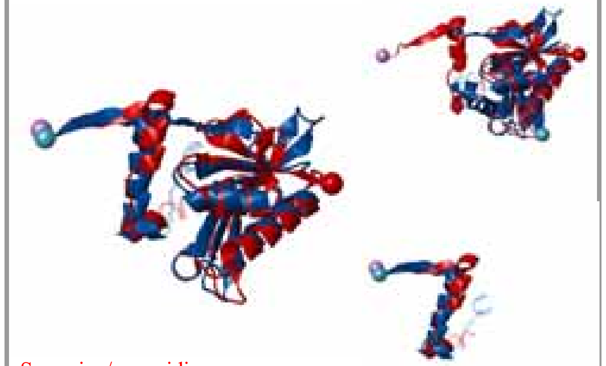 Spermine/spermidine acetyltransferase<br>Transcriptional regulator
	1z4eA (150)	30-46 (17)	30-46 (17)	29-36 (8)						
596	2fl4A (147)	128-144 (17)	129-140 (12)	131-138 (8)	C	0.37	2.37	78.23% (115/147)	12.93% (19/147)	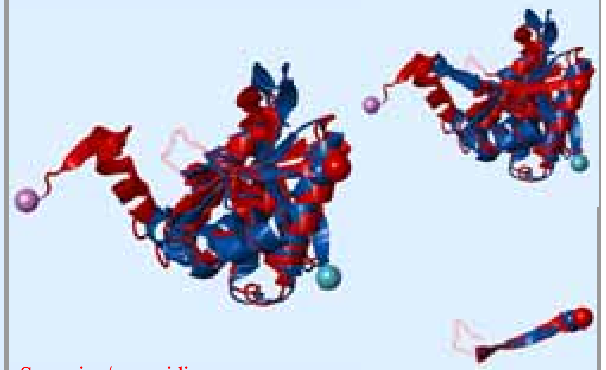 Spermine/spermidine acetyltransferase<br>Transcriptional regulator
	1z4eA (150)	143-152 (10)	144-148 (5)	131-138 (8)						

597	2fyxA (130)	112-117 (6)	112-117 (6)	112-117 (6)	C	0.76	1.51	97.69% (127/130)	33.08% (43/130)	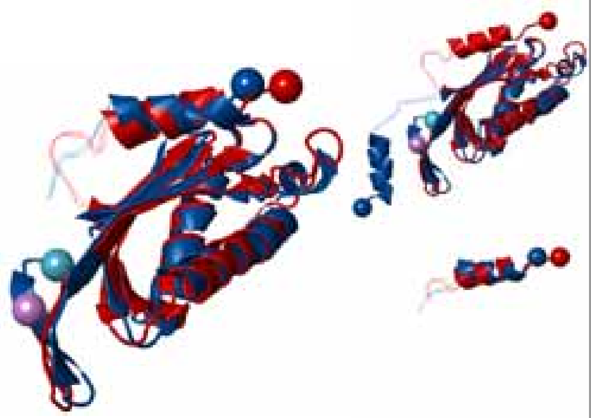 Transposase, putative<br>ISHp608 transposase
	2a6mA (130)	116-121 (6)	116-121 (6)	112-117 (6)						
598	2fyxA (130)	111-116 (6)	111-116 (6)	111-116 (6)	C	0.73	1.68	97.69% (127/130)	40.00% (52/130)	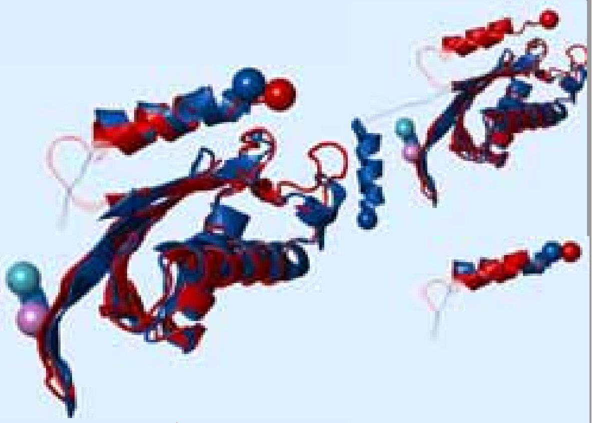 Transposase, putative<br>136aa long hypothetical transposase
	2ec2A (130)	112-117 (6)	112-117 (6)	111-116 (6)						
599	2fyxA (130)	111-119 (9)	112-119 (8)	112-118 (7)	C	0.78	1.42	99.21% (125/126)	34.13% (43/126)	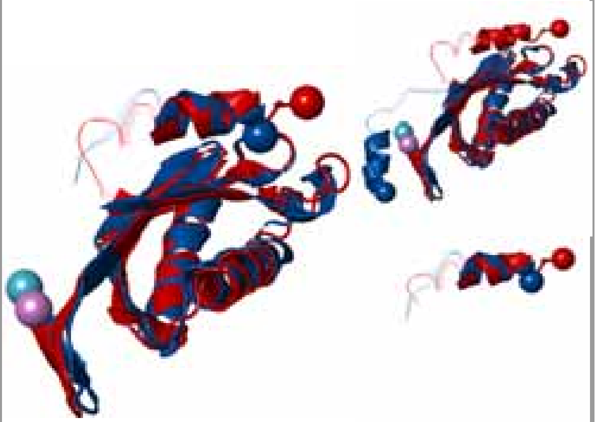 Transposase, putative<br>Transposase ORFA
	2vhgA (126)	115-123 (9)	116-123 (8)	112-118 (7)						

600	2fyxA (130)	112-115 (4)	112-115 (4)	112-115 (4)	C	0.64	1.54	96.92% (126/130)	30.00% (39/130)	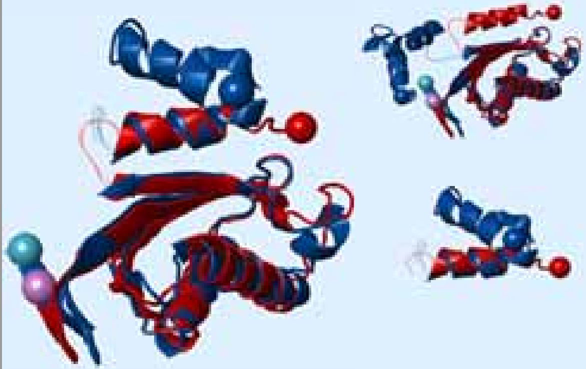<br>Transposase, putative<br>Transposase ORFA
	2vihA (151)	116-120 (5)	116-120 (5)	112-115 (4)						
601	2fyxA (130)	112-116 (5)	112-116 (5)	112-116 (5)	C	0.74	1.51	96.90% (125/129)	33.33% (43/129)	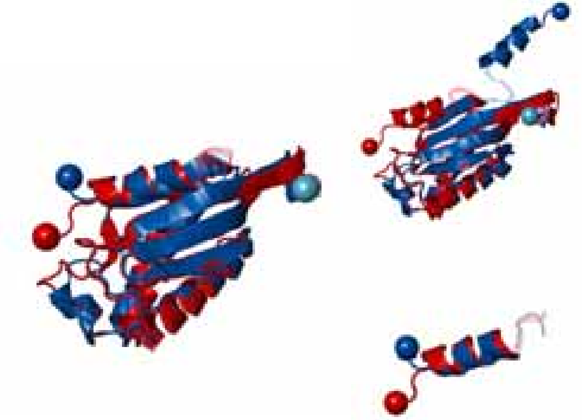<br>Transposase, putative<br>Transposase ORFA
	2vihB (129)	116-120 (5)	116-120 (5)	112-116 (5)						
602	2fyxA (130)	112-116 (5)	112-116 (5)	112-116 (5)	C	0.72	1.58	97.60% (122/125)	33.60% (42/125)	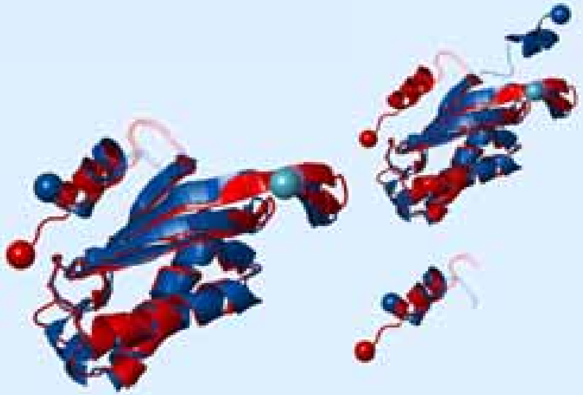<br>Transposase, putative<br>Transposase ORFA
	2vjvA (125)	116-120 (5)	116-120 (5)	112-116 (5)						

603	2g84A (189)	142-158 (17)	142-150 (9)	142-150 (9)	C	0.57	1.44	91.77% (145/158)	27.22% (43/158)	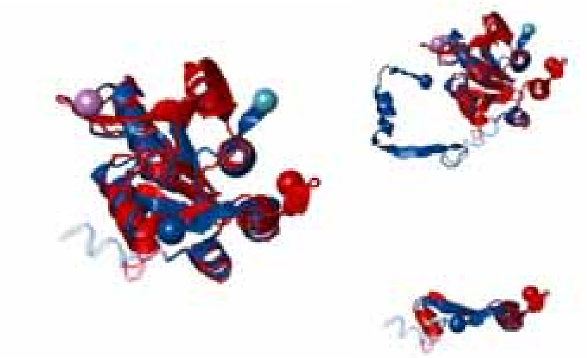 Cytidine and deoxycytidylate deaminase zinc-b<br>Guanine deaminase
	1wkqA (158)	113-129 (17)	113-124 (12)	142-150 (9)						
604	2gbzA (179)	135-162 (28)	139-162 (24)	139-162 (24)	C	0.40	2.07	78.36% (134/171)	12.87% (22/171)	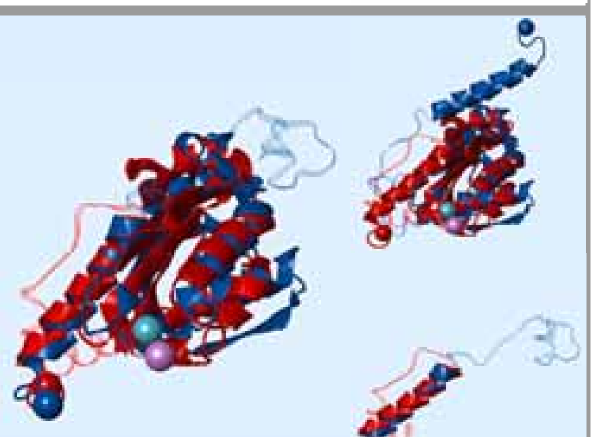 Oligoribonuclease<br>DNA polymerase III polC-type
	2p1jB (171)	468-503 (36)	472-503 (32)	139-162 (24)						
605	2gtvX (104)	19-33 (15)	20-31 (12)	21-32 (12)	N	0.75	1.20	100.00% (91/91)	31.87% (29/91)	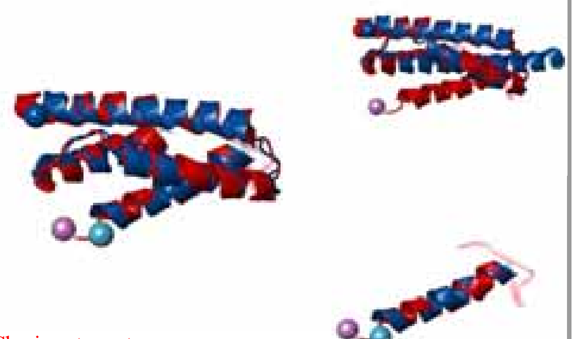 Chorismate mutase<br>ENDO-oxabicyclic transition state analogue
	1ecmA (91)	21-27 (7)	22-25 (4)	21-32 (12)						

606	2gtvX (104)	19-32 (14)	20-32 (13)	21-32 (12)	N	0.71	1.47	97.89% (93/95)	27.37% (26/95)	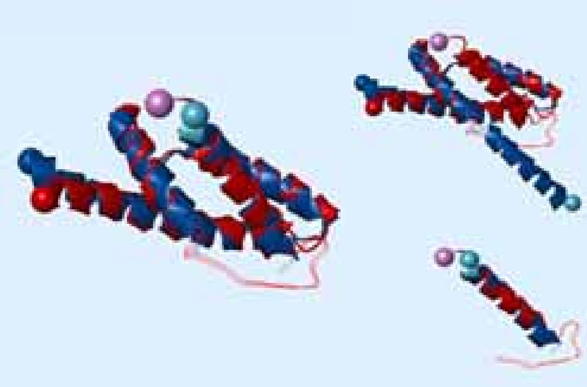 Chorismate mutase<br>ENDO-oxabicyclic transition state analogue
	1ecmB (95)	22-26 (5)	23-26 (4)	21-32 (12)						
607	2gtvX (104)	19-33 (15)	20-33 (14)	21-32 (12)	N	0.58	1.69	89.00% (89/100)	16.00% (16/100)	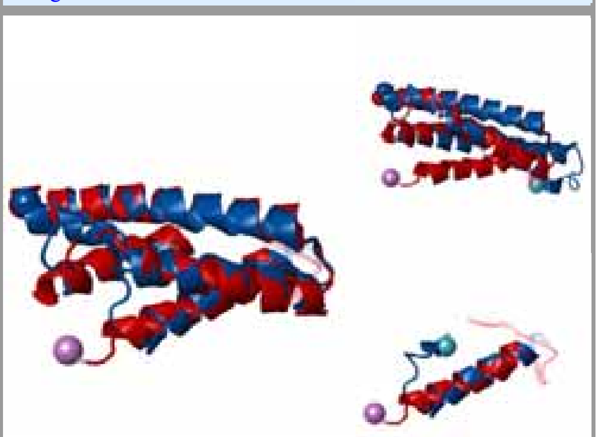 Chorismate mutase<br>Salicylate biosynthesis protein pchB
	2h9dD (100)	23-30 (8)	24-30 (7)	21-32 (12)						
608	2i79A (171)	147-166 (20)	147-166 (20)	147-159 (13)	C	0.46	2.32	86.75% (144/166)	19.28% (32/166)	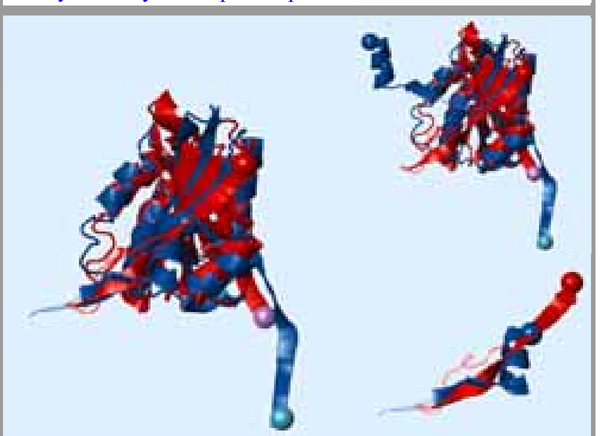 Acetyltransferase, GNAT family<br>Probable N-acetyltransferase
	2fe7B (166)	140-157 (18)	140-157 (18)	147-159 (13)						

609	2i9sA (97)	78-96 (19)	78-96 (19)	91-91 (1)	C	0.41	2.43	82.47% (80/97)	6.19% (6/97)	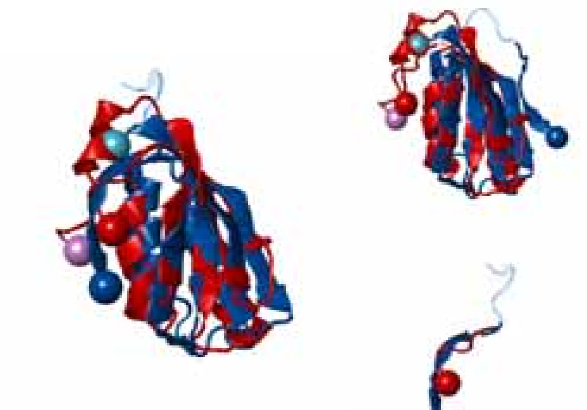
	2acyA (98)	65-96 (32)	65-96 (32)	91-91 (1)						Mesoderm development candidate 2<br>Acylphosphatase
610	2jevA (169)	146-161 (16)	147-158 (12)	148-152 (5)	C	0.45	2.31	87.90% (138/157)	15.92% (25/157)	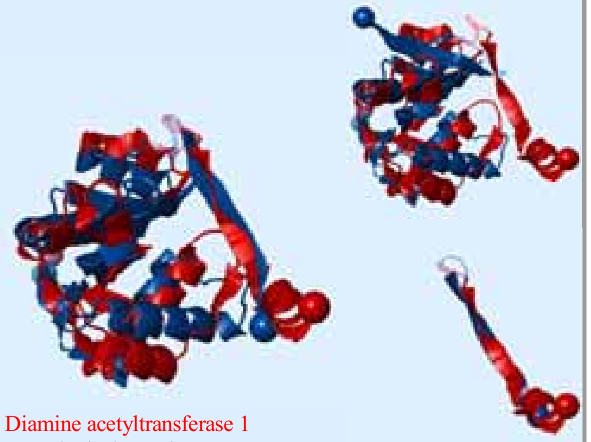
	1wwzB (157)	142-158 (17)	143-155 (13)	148-152 (5)						Diamine acetyltransferase 1<br>Hypothetical protein PH1933
611	2jevA (169)	147-169 (23)	147-154 (8)	148-155 (8)	C	0.48	2.29	87.57% (148/169)	18.34% (31/169)	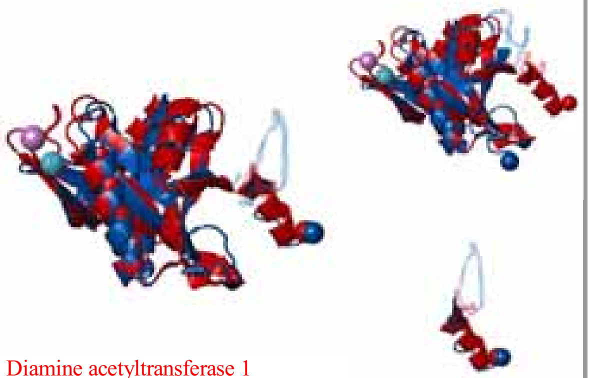
	2j8mB (170)	141-171 (31)	141-157 (17)	148-155 (8)						Diamine acetyltransferase 1<br>Acetyltransferase PA4866 FROM P. aeruginosa

612	2jevA (169)	147-169 (23)	147-156 (10)	148-151 (4)	C	0.48	2.25	86.98% (147/169)	18.34% (31/169)	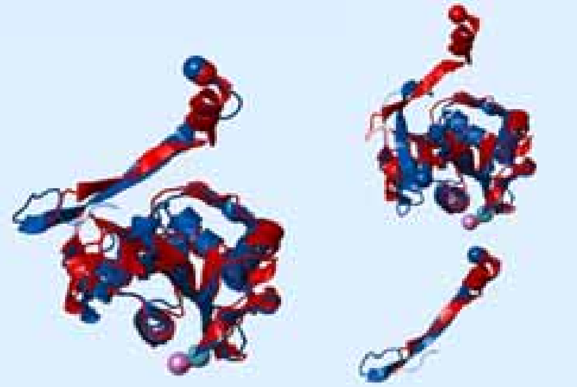 Diamine acetyltransferase 1 Acetyltransferase PA4866 FROM P. aeruginosa
	2j8nA (169)	141-171 (31)	141-159 (19)	148-151 (4)						
613	2ob0A (154)	131-153 (23)	132-150 (19)	136-149 (14)	C	0.56	1.87	91.03% (132/145)	11.03% (16/145)	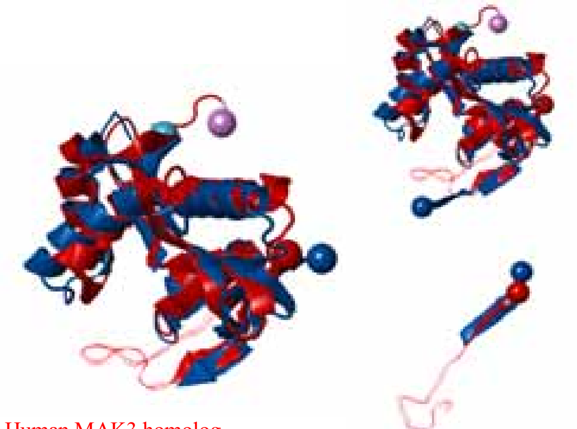 Human MAK3 homolog Aminoglycoside 6'-N-acetyltransferase
	1s3zB (145)	134-143 (10)	135-140 (6)	136-149 (14)						
614	2ob0A (154)	132-152 (21)	135-152 (18)	135-147 (13)	C	0.54	2.00	88.89% (136/153)	10.46% (16/153)	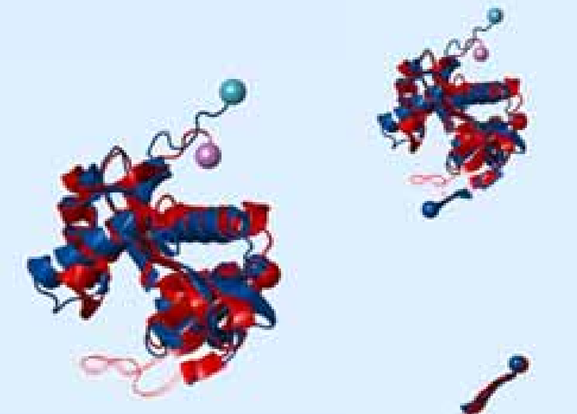 Human MAK3 homolog Aminoglycoside 6'-N-acetyltransferase
	1s5kA (153)	135-143 (9)	138-143 (6)	135-147 (13)						

615	2ob0A (154)	131-153 (23)	134-148 (15)	134-147 (14)	C	0.54	2.06	91.16% (134/147)	11.56% (17/147)	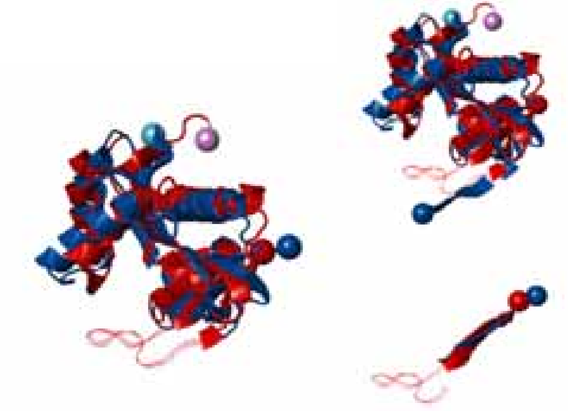<br>Human MAK3 homolog<br>Aminoglycoside 6'-N-acetyltransferase
	2vbqA (147)	134-143 (10)	137-138 (2)	134-147 (14)						
616	2pa4A (294)	155-179 (25), 182-224 (43)	171-179 (9), 185-217 (33)	171-178 (8), 204-211 (8)	M	0.45	2.27	96.00% (216/225)	15.11% (34/225)	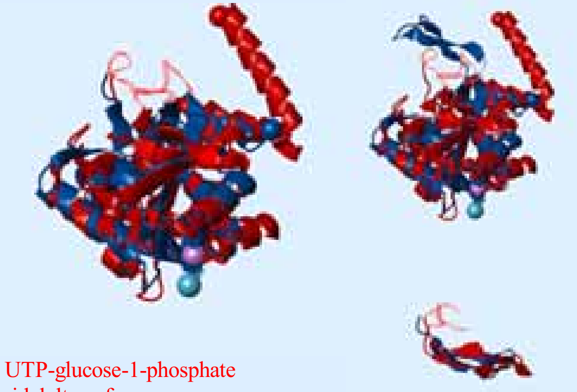<br>UTP-glucose-1-phosphate uridylyltransferase<br>4-diphosphocytidyl-2-C-methylerythritol synth
	1i52A (225)	121-139 (19), 142-174 (33)	137-139 (3), 145-166 (22)	171-178 (8), 204-211 (8)						
617	2pa7A (135)	19-55 (37)	19-48 (30)	23-32 (10)	N	0.31	2.42	83.70% (113/135)	15.56% (21/135)	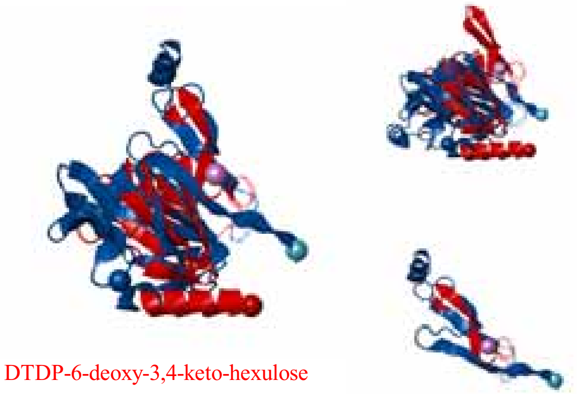<br>DTDP-6-deoxy-3,4-keto-hexulose isomerase<br>Phosphoglucose isomerase
	1j3qA (187)	56-97 (42)	56-88 (33)	23-32 (10)						

618	2pa7A (135)	2-55 (54)	19-55 (37)	23-32 (10)	N	0.32	2.37	83.70% (113/135)	15.56% (21/135)	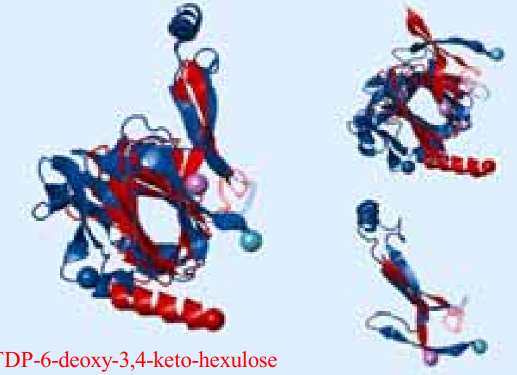 DTDP-6-deoxy-3,4-keto-hexulose isomerase Phosphoglucose isomerase
	1j3rB (185)	3-97 (95)	56-97 (42)	23-32 (10)						
619	2pa7A (135)	2-55 (54)	21-55 (35)	23-32 (10)	N	0.31	2.43	83.70% (113/135)	14.07% (19/135)	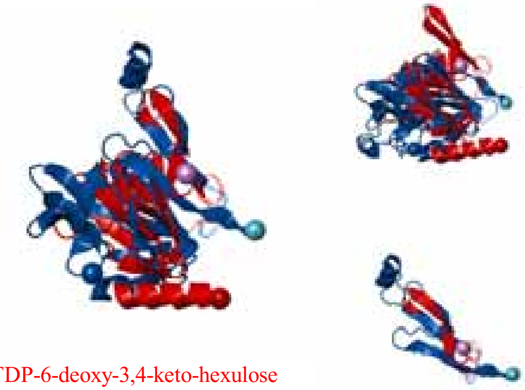 DTDP-6-deoxy-3,4-keto-hexulose isomerase Glucose-6-phosphate isomerase
	1qxrA (187)	1-96 (96)	57-96 (40)	23-32 (10)						
620	2pa7A (135)	109-129 (21)	112-125 (14)	122-123 (2)	C	0.27	1.88	70.37% (95/135)	10.37% (14/135)	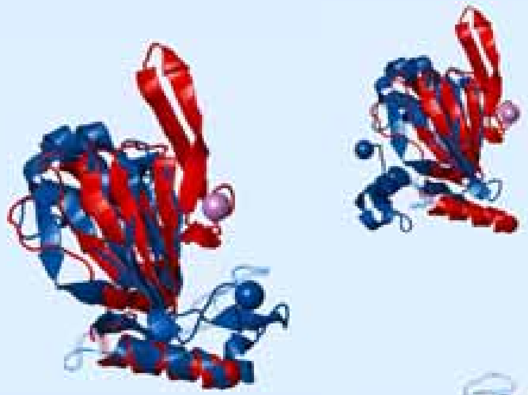 DTDP-6-deoxy-3,4-keto-hexulose isomerase 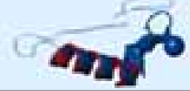 3-hydroxyanthranilate 3,4-dioxygenase
	1zvfA (176)	110-150 (41)	112-146 (35)	122-123 (2)						

621	2pa7A (135)	16-65 (50)	21-33 (13)	21-32 (12)	N	0.25	2.52	85.93% (116/135)	13.33% (18/135)	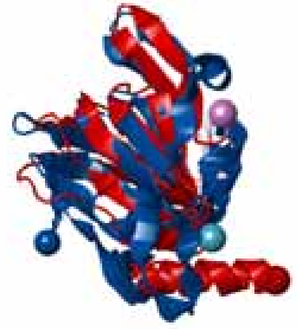 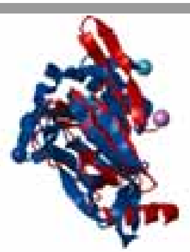 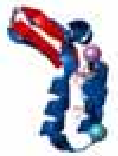 DTDP-6-deoxy-3,4-keto-hexulose isomerase<br>Cysteine dioxygenase type I
	2b5hA (186)	57-102 (46)	62-71 (10)	21-32 (12)						
622	2pa7A (135)	19-55 (37)	23-55 (33)	19-32 (14)	N	0.30	2.49	83.70% (113/135)	13.33% (18/135)	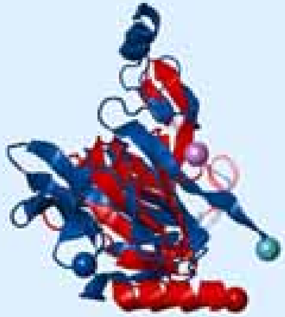 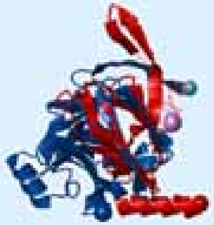 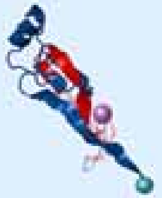 DTDP-6-deoxy-3,4-keto-hexulose isomerase<br>Glucose-6-phosphate isomerase
	2gc1A (188)	55-96 (42)	59-96 (38)	19-32 (14)						
623	2pc1A (173)	149-159 (11)	149-158 (10)	151-157 (7)	C	0.48	2.23	94.48% (137/145)	15.86% (23/145)	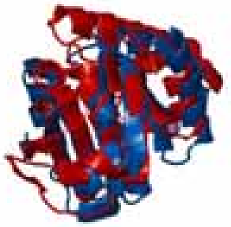 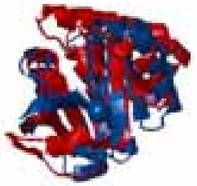 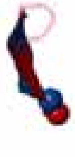 Acetyltransferase, GNAT family<br>Aminoglycoside 6'-N-acetyltransferase
	1s3zB (145)	135-139 (5)	135-138 (4)	151-157 (7)						

624	2pc1A (173)	148-162 (15)	153-162 (10)	153-157 (5)	C	0.42	2.11	91.54% (119/130)	8.46% (11/130)	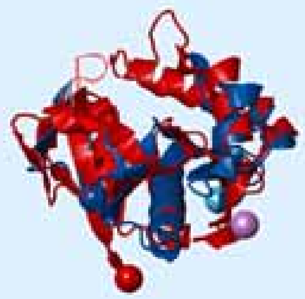 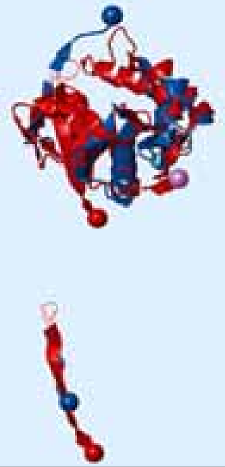 Acetyltransferase, GNAT family<br>Hypothetical protein TTHA1254
	2d4pA (130)	117-129 (13)	124-129 (6)	153-157 (5)						
625	2pc1A (173)	150-162 (13)	153-162 (10)	152-158 (7)	C	0.45	2.39	93.20% (137/147)	14.97% (22/147)	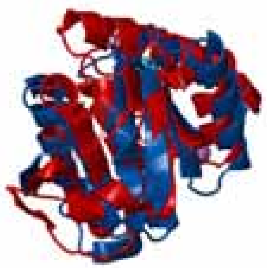 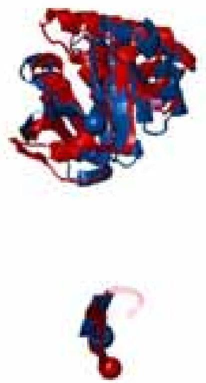 Acetyltransferase, GNAT family<br>Aminoglycoside 6'-N-acetyltransferase
	2vbqA (147)	136-144 (9)	140-144 (5)	152-158 (7)						
626	2pd7A (149)	58-74 (17)	58-73 (16)	58-71 (14)	N	0.48	1.52	95.80% (114/119)	23.53% (28/119)	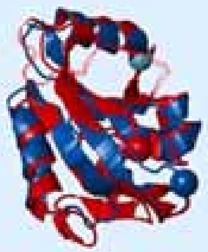 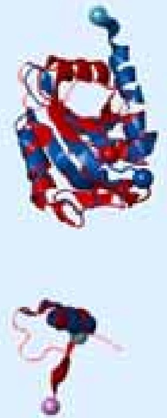 Vivid PAS protein VVD<br>Nitrogen fixation regulatory protein
	2gj3B (119)	35-39 (5)	35-38 (4)	58-71 (14)						

627	2pd7A (149)	54-74 (21)	54-72 (19)	54-72 (19)	N	0.40	1.57	95.04% (115/121)	30.58% (37/121)	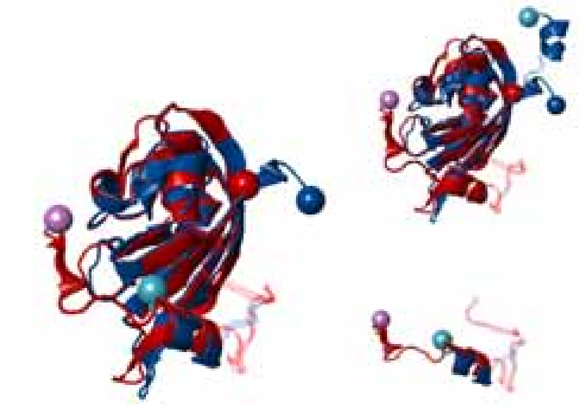 Vivid PAS protein VVD<br>Phototropin-1
	2z6cA (121)	192-200 (9)	192-198 (7)	54-72 (19)						
628	2pieA (132)	125-128 (4)	125-128 (4)	126-128 (3)	C	0.37	2.01	84.85% (112/132)	15.15% (20/132)	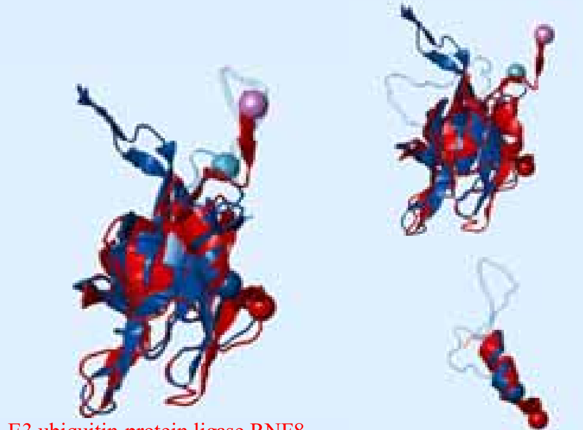 E3 ubiquitin-protein ligase RNF8<br>Protein kinase SPK1
	1j4kA (158)	695-722 (28)	695-722 (28)	126-128 (3)						
629	2pr1A (152)	105-152 (48)	122-146 (25)	133-144 (12)	C	0.40	2.24	79.33% (119/150)	9.33% (14/150)	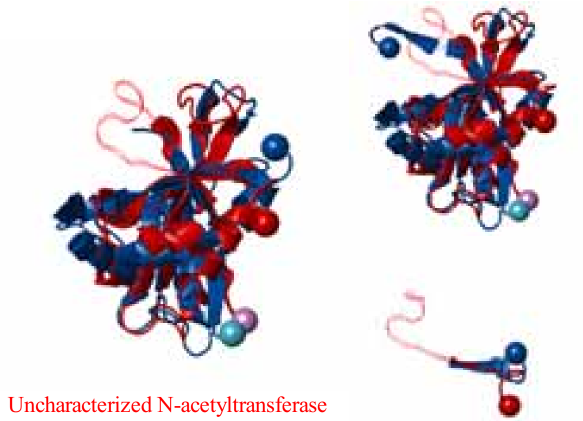 Uncharacterized N-acetyltransferase ylbP<br>HPA2 histone acetyltransferase
	1qsmA (150)	113-156 (44)	138-151 (14)	133-144 (12)						

630	2pr1A (152)	118-149 (32)	124-143 (20)	132-142 (11)	C	0.40	2.41	81.88% (122/149)	10.07% (15/149)	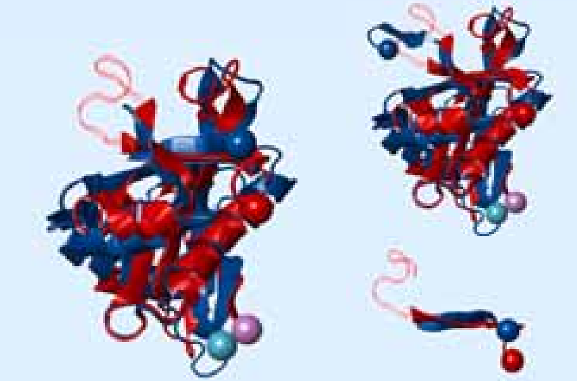 Uncharacterized N-acetyltransferase ylbP HPA2 histone acetyltransferase
	1qsmB (149)	132-155 (24)	140-149 (10)	132-142 (11)						
631	2pr1A (152)	105-143 (39)	126-143 (18)	133-142 (10)	C	0.40	2.18	78.29% (119/152)	9.21% (14/152)	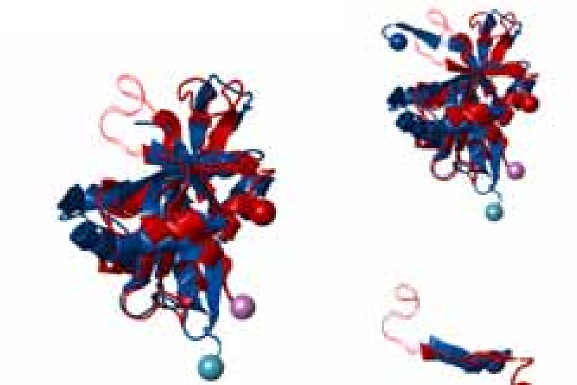 Uncharacterized N-acetyltransferase ylbP HPA2 histone acetyltransferase
	1qsmD (152)	113-150 (38)	142-150 (9)	133-142 (10)						
632	2pr1A (152)	131-143 (13)	131-143 (13)	132-140 (9)	C	0.45	2.28	86.21% (125/145)	8.97% (13/145)	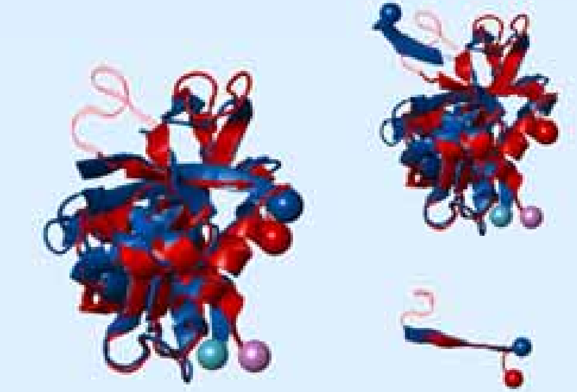 Uncharacterized N-acetyltransferase ylbP Aminoglycoside 6'-N-acetyltransferase
	1s3zB (145)	135-139 (5)	135-139 (5)	132-140 (9)						

633	2pr1A (152)	131-143 (13)	131-143 (13)	132-140 (9)	C	0.43	2.28	82.24% (125/152)	8.55% (13/152)	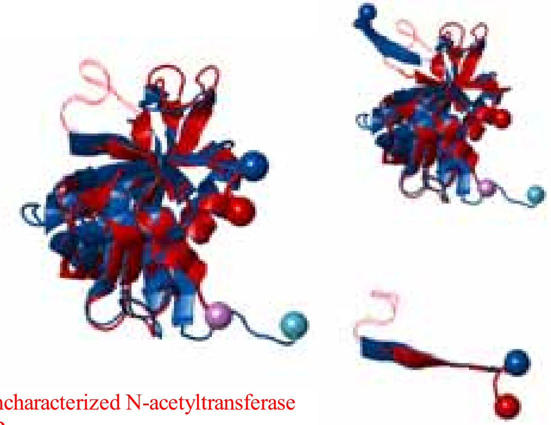 Uncharacterized N-acetyltransferase ylbP<br>Aminoglycoside 6'-N-acetyltransferase
	1s5kA (153)	135-139 (5)	135-139 (5)	132-140 (9)						
634	2pr1A (152)	131-143 (13)	131-143 (13)	131-141 (11)	C	0.33	2.42	74.34% (113/152)	5.26% (8/152)	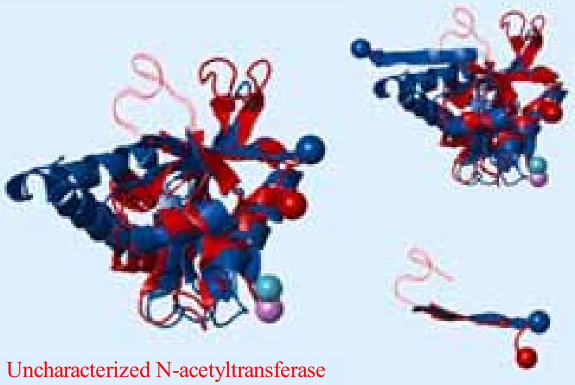 Uncharacterized N-acetyltransferase ylbP<br>YYCN protein
	1ufhA (155)	148-150 (3)	148-150 (3)	131-141 (11)						
635	2pr1A (152)	130-143 (14)	130-143 (14)	131-140 (10)	C	0.44	1.87	78.67% (118/150)	11.33% (17/150)	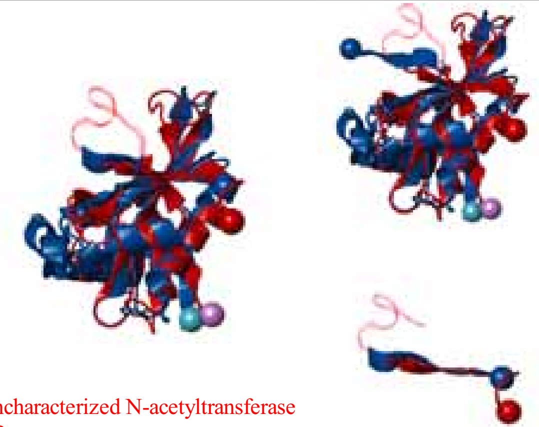 Uncharacterized N-acetyltransferase ylbP<br>Transcriptional regulator
	1z4eA (150)	144-148 (5)	144-148 (5)	131-140 (10)						

636	2pr1A (152)	125-151 (27)	130-143 (14)	132-142 (11)	C	0.39	2.47	84.87% (129/152)	10.53% (16/152)	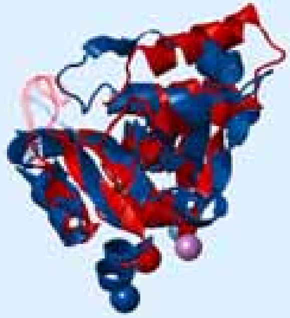 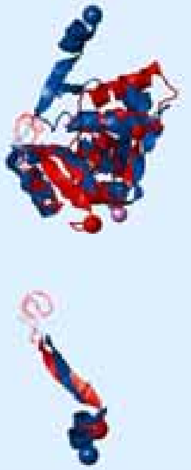 Uncharacterized N-acetyltransferase ylbP<br>Diamine acetyltransferase 1
	2f5iA (167)	142-164 (23)	147-156 (10)	132-142 (11)						
637	2pr1A (152)	125-151 (27)	125-142 (18)	132-142 (11)	C	0.37	2.63	85.53% (130/152)	10.53% (16/152)	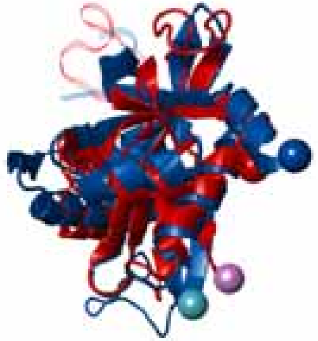 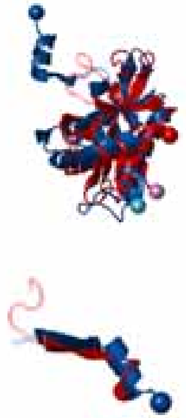 Uncharacterized N-acetyltransferase ylbP<br>Diamine acetyltransferase 1
	2g3tA (169)	142-168 (27)	142-155 (14)	132-142 (11)						
638	2pr1A (152)	125-151 (27)	130-142 (13)	131-142 (12)	C	0.38	2.56	85.53% (130/152)	9.87% (15/152)	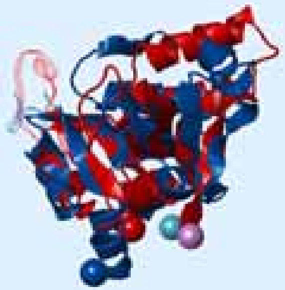 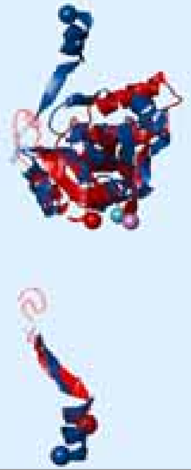 Uncharacterized N-acetyltransferase ylbP<br>Diamine acetyltransferase 1
	2jevA (169)	142-164 (23)	147-155 (9)	131-142 (12)						

639	2pr1A (152)	132-149 (18)	132-143 (12)	133-141 (9)	C	0.35	2.32	81.58% (124/152)	8.55% (13/152)	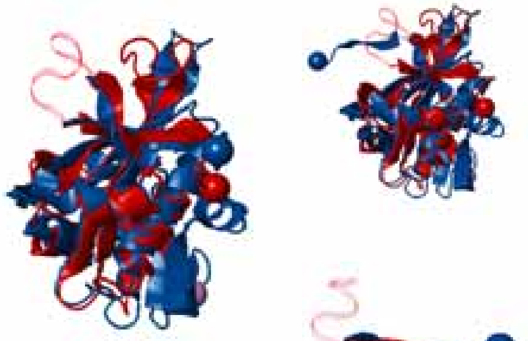 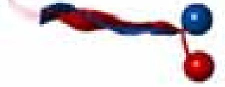 Uncharacterized N-acetyltransferase ylbP Glucosamine 6-phosphate N-acetyltransferase
	2o28A (182)	174-183 (10)	174-177 (4)	133-141 (9)						
640	2pr1A (152)	132-148 (17)	132-143 (12)	133-142 (10)	C	0.34	2.36	80.26% (122/152)	8.55% (13/152)	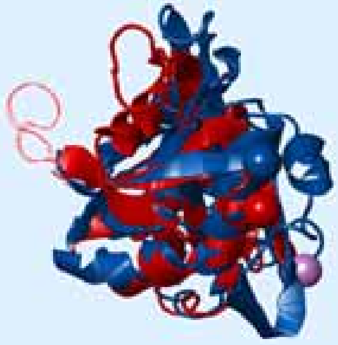 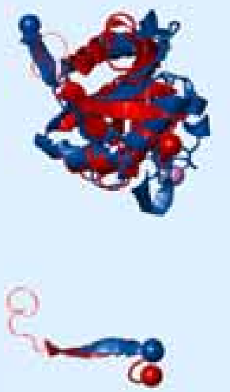 Uncharacterized N-acetyltransferase ylbP Glucosamine 6-phosphate N-acetyltransferase
	2o28B (180)	174-181 (8)	174-176 (3)	133-142 (10)						
641	2pr1A (152)	131-143 (13)	131-143 (13)	132-140 (9)	C	0.45	2.27	86.11% (124/144)	9.03% (13/144)	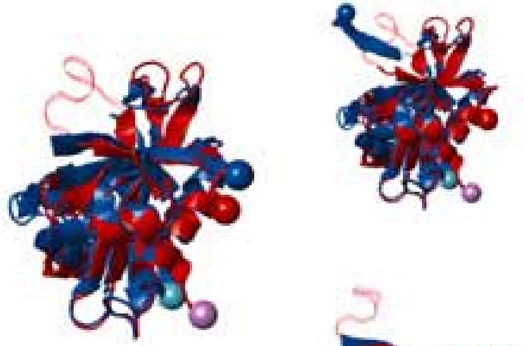 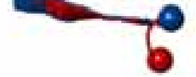 Uncharacterized N-acetyltransferase ylbP Aminoglycoside 6'-N-acetyltransferase
	2vbqB (144)	135-139 (5)	135-139 (5)	132-140 (9)						

642	2pr1A (152)	131-143 (13)	131-143 (13)	131-140 (10)	C	0.36	2.34	79.61% (121/152)	10.53% (16/152)	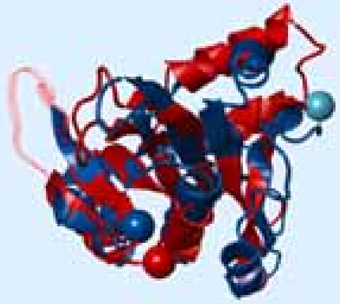 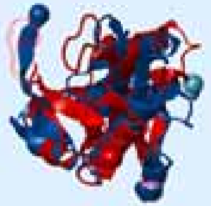 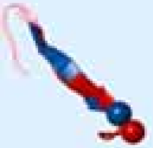 Uncharacterized N-acetyltransferase ylbP<br>Glucosamine 6-phosphate acetyltransferase
	2vxkA (165)	182-185 (4)	182-185 (4)	131-140 (10)						
643	2pr8A (173)	161-180 (20)	161-180 (20)	161-169 (9)	C	0.35	2.56	82.58% (128/155)	12.26% (19/155)	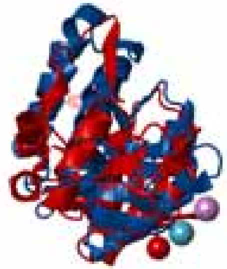 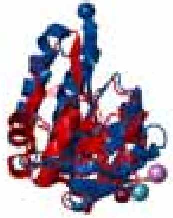 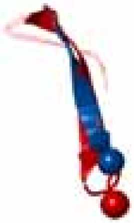 Aminoglycoside 6-N-acetyltransferase type Ib1<br>YYCN protein
	1ufhA (155)	147-155 (9)	147-155 (9)	161-169 (9)						
644	2pr8A (173)	154-155 (2)	154-155 (2)	154-155 (2)	C	0.36	3.09	92.62% (138/149)	12.75% (19/149)	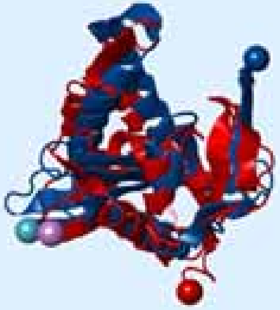 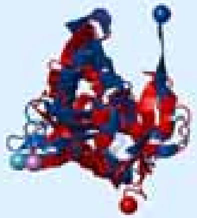 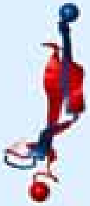 Aminoglycoside 6-N-acetyltransferase type Ib1<br>Putative acetyl transferase
	1vkcA (149)	134-135 (2)	134-135 (2)	154-155 (2)						

645	2pr8A (173)	162-182 (21)	162-182 (21)	164-172 (9)	C	0.41	2.78	94.56% (139/147)	14.29% (21/147)	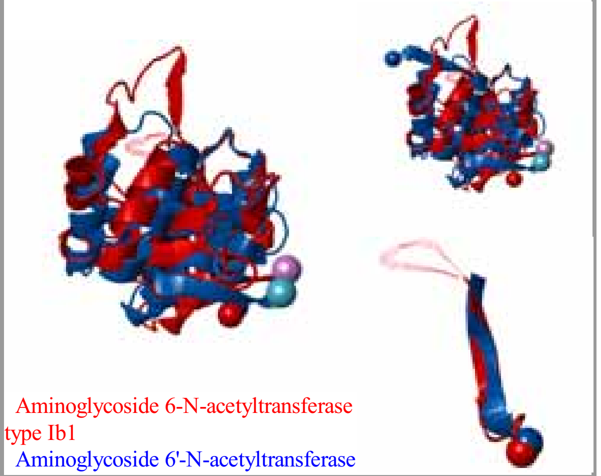
	2vbqA (147)	135-144 (10)	135-144 (10)	164-172 (9)						
646	2qlrA (425)	35-76 (42)	35-76 (42)	41-72 (32)	N	0.42	2.63	89.39% (354/396)	13.38% (53/396)	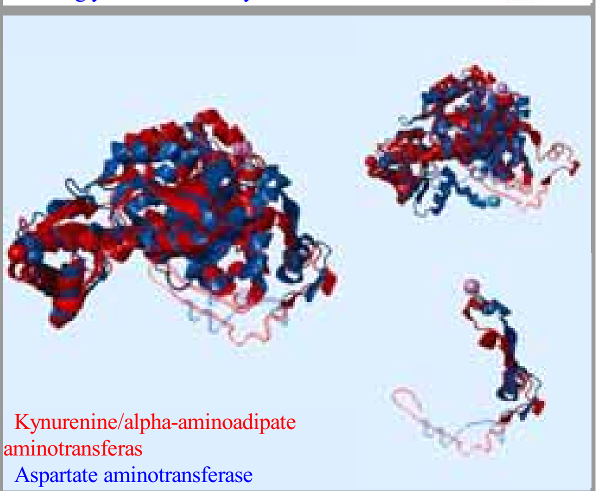
	1ahxA (396)	34-72 (39)	34-72 (39)	41-72 (32)						
647	2qlrA (425)	35-75 (41)	37-75 (39)	49-72 (24)	N	0.36	2.71	89.90% (356/396)	13.89% (55/396)	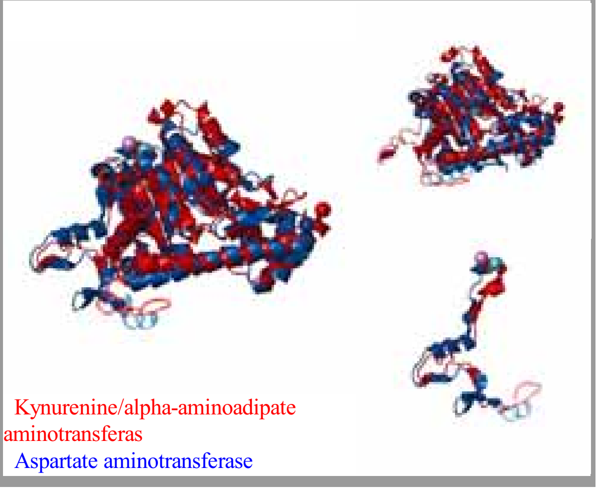
	1aiaA (396)	34-71 (38)	36-71 (36)	49-72 (24)						

648	2qlrA (425)	3-76 (74)	16-76 (61)	51-72 (22)	N	0.33	2.72	89.65% (355/396)	12.63% (50/396)	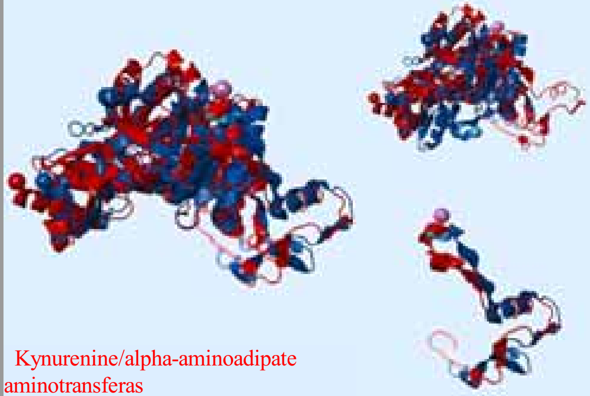<br>Kynurenine/alpha-aminoadipate aminotransferas<br>Aspartate aminotransferase
	1arhA (396)	6-72 (67)	13-72 (60)	51-72 (22)						
649	2qlrA (425)	3-76 (74)	3-76 (74)	49-72 (24)	N	0.34	2.61	89.14% (353/396)	12.88% (51/396)	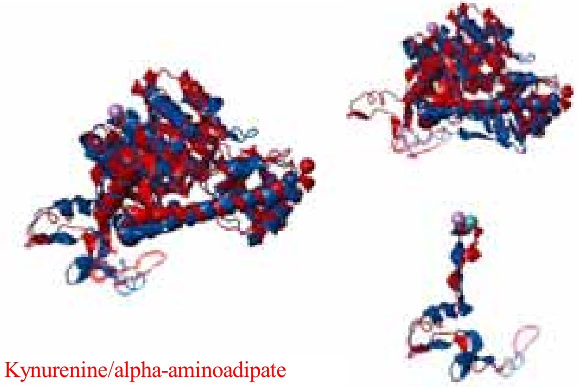<br>Kynurenine/alpha-aminoadipate aminotransferas<br>Aspartate aminotransferase
	1ariA (396)	6-72 (67)	6-72 (67)	49-72 (24)						
650	2qlrA (425)	35-75 (41)	38-75 (38)	43-72 (30)	N	0.42	2.67	89.39% (354/396)	13.38% (53/396)	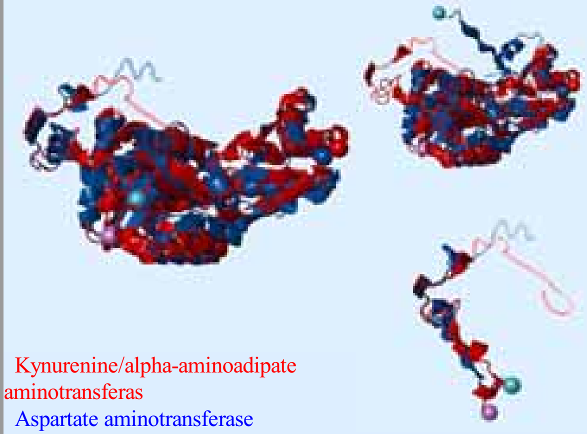<br>Kynurenine/alpha-aminoadipate aminotransferas<br>Aspartate aminotransferase
	1arsA (396)	34-71 (38)	37-71 (35)	43-72 (30)						

651	2qlrA (425)	33-76 (44)	33-72 (40)	49-72 (24)	N	0.36	2.78	90.40% (358/396)	12.88% (51/396)	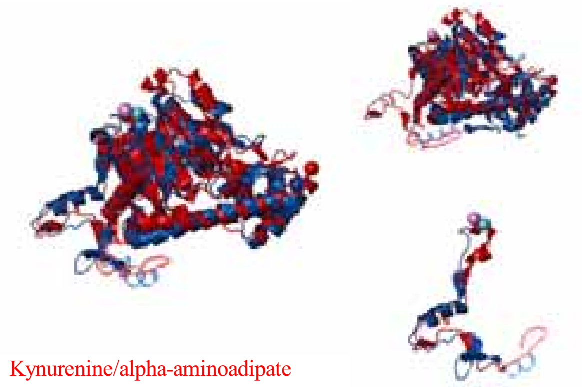 Kynurenine/alpha-aminoadipate aminotransferas Aspartate aminotransferase
	1asfA (396)	39-79 (41)	39-75 (37)	49-72 (24)						
652	2qlrA (425)	15-72 (58)	16-72 (57)	17-71 (55)	N	0.53	2.10	93.98% (359/382)	20.68% (79/382)	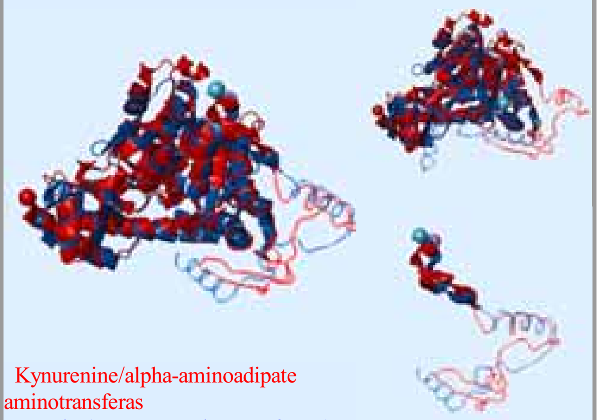 Kynurenine/alpha-aminoadipate aminotransferas Protein (aspartate aminotransferase)
	1b5oA (382)	12-62 (51)	13-62 (50)	17-71 (55)						
653	2qlrA (425)	15-72 (58)	16-72 (57)	20-72 (53)	N	0.48	2.00	94.50% (361/382)	20.42% (78/382)	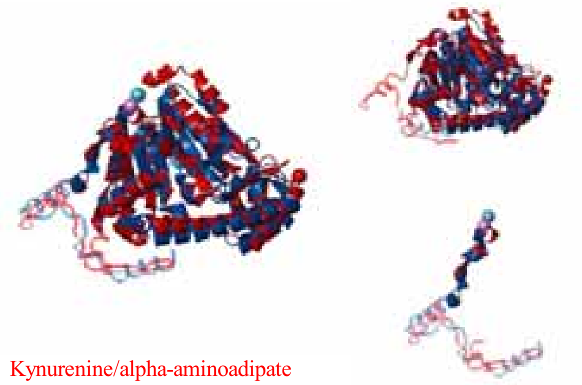 Kynurenine/alpha-aminoadipate aminotransferas Aspartate aminotransferase
	1bkgA (382)	12-62 (51)	13-62 (50)	20-72 (53)						

654	2qlrA (425)	35-79 (45)	37-75 (39)	43-72 (30)	N	0.41	2.66	89.14% (353/396)	12.63% (50/396)	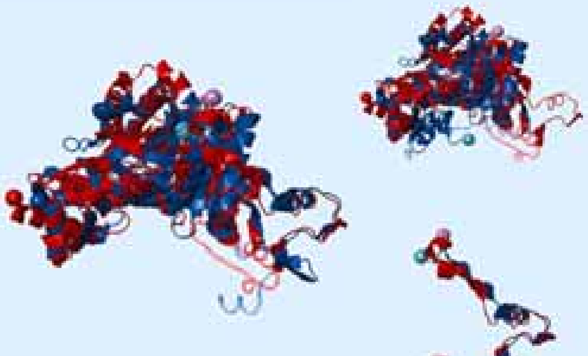 Kynurenine/alpha-aminoadipate aminotransferas Aspartate aminotransferase
	1g4vA (396)	34-75 (42)	36-71 (36)	43-72 (30)						
655	2qlrA (425)	3-80 (78)	36-75 (40)	43-73 (31)	N	0.41	2.68	88.89% (352/396)	13.13% (52/396)	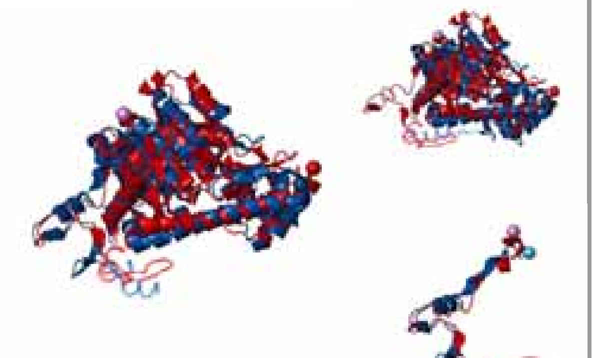 Kynurenine/alpha-aminoadipate aminotransferas Aspartate aminotransferase
	1g4xA (396)	5-76 (72)	35-71 (37)	43-73 (31)						
656	2qlrA (425)	1-79 (79)	36-75 (40)	51-73 (23)	N	0.35	2.77	88.38% (350/396)	13.89% (55/396)	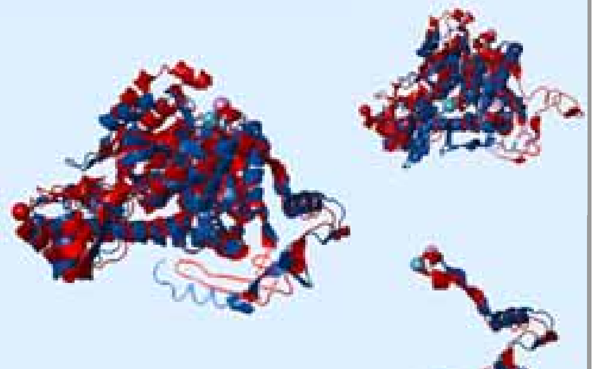 Kynurenine/alpha-aminoadipate aminotransferas Aspartate aminotransferase
	1g7wA (396)	5-75 (71)	35-71 (37)	51-73 (23)						

657	2qlrA (425)	3-76 (74)	37-75 (39)	43-72 (30)	N	0.41	2.71	89.39% (354/396)	12.88% (51/396)	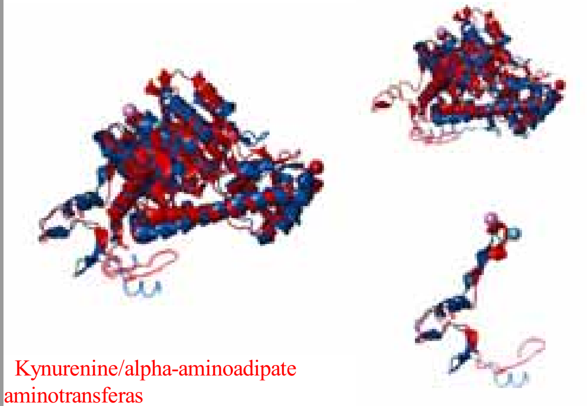 Kynurenine/alpha-aminoadipate aminotransferas Aspartate aminotransferase
	1g7xA (396)	5-72 (68)	36-71 (36)	43-72 (30)						
658	2qlrA (425)	2-75 (74)	14-72 (59)	22-71 (50)	N	0.47	2.06	95.03% (363/382)	19.90% (76/382)	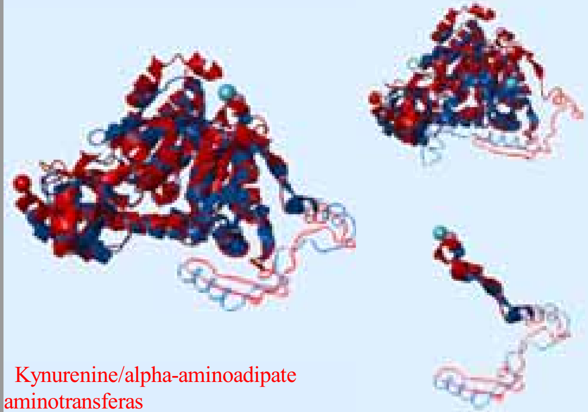 Kynurenine/alpha-aminoadipate aminotransferas Aspartate aminotransferase
	1gc3A (382)	2-65 (64)	11-62 (52)	22-71 (50)						
659	2qlrA (425)	15-72 (58)	44-71 (28)	49-71 (23)	N	0.41	2.21	93.56% (363/388)	20.62% (80/388)	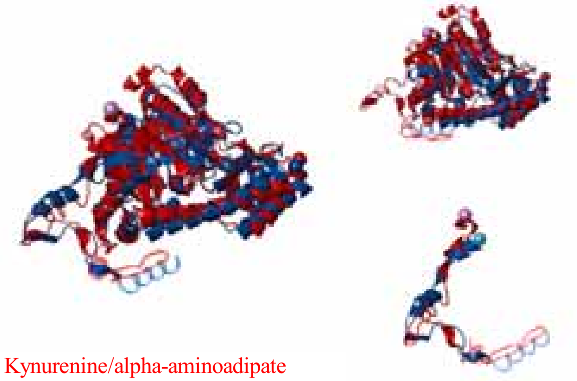 Kynurenine/alpha-aminoadipate aminotransferas Aspartate aminotransferase
	1gdeA (388)	11-57 (47)	38-56 (19)	49-71 (23)						

660	2qlrA (425)	10-73 (64)	43-73 (31)	54-73 (20)	N	0.30	2.67	94.35% (334/354)	13.84% (49/354)	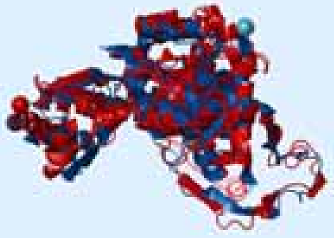 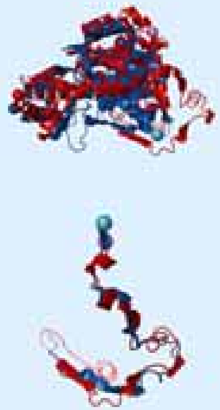 Kynurenine/alpha-aminoadipate aminotransferas Histidinol phosphate aminotransferase
	1ijiA (354)	13-54 (42)	40-54 (15)	54-73 (20)						
661	2qlrA (425)	35-76 (42)	37-75 (39)	47-72 (26)	N	0.41	2.68	88.89% (352/396)	13.38% (53/396)	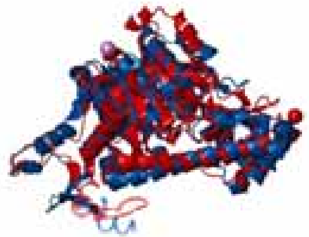 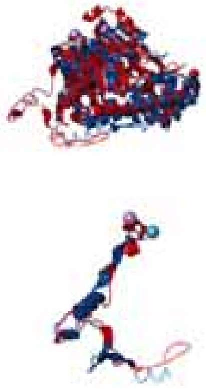 Kynurenine/alpha-aminoadipate aminotransferas Aspartate aminotransferase
	1ix6A (396)	34-72 (39)	36-71 (36)	47-72 (26)						
662	2qlrA (425)	3-76 (74)	3-75 (73)	53-73 (21)	N	0.33	2.78	88.38% (350/396)	13.13% (52/396)	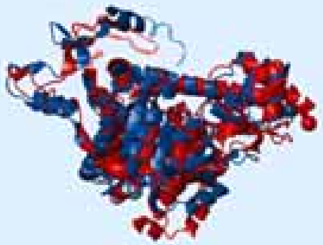 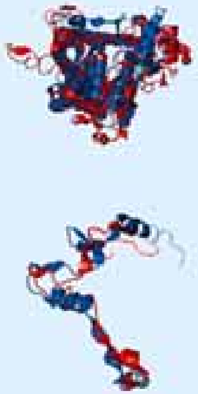 Kynurenine/alpha-aminoadipate aminotransferas Aspartate aminotransferase
	1ix8A (396)	6-72 (67)	6-71 (66)	53-73 (21)						

663	2qlrA (425)	3-72 (70)	14-72 (59)	53-71 (19)	N	0.46	2.07	94.33% (366/388)	16.49% (64/388)	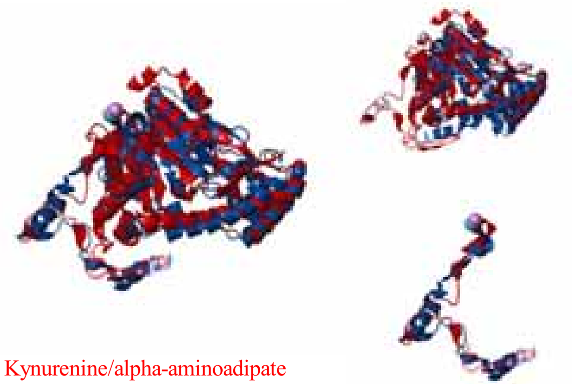 Kynurenine/alpha-aminoadipate aminotransferas Aspartate aminotransferase
	1j32A (388)	2-61 (60)	10-61 (52)	53-71 (19)						
664	2qlrA (425)	38-76 (39)	38-72 (35)	47-72 (26)	N	0.47	2.07	92.00% (345/375)	18.67% (70/375)	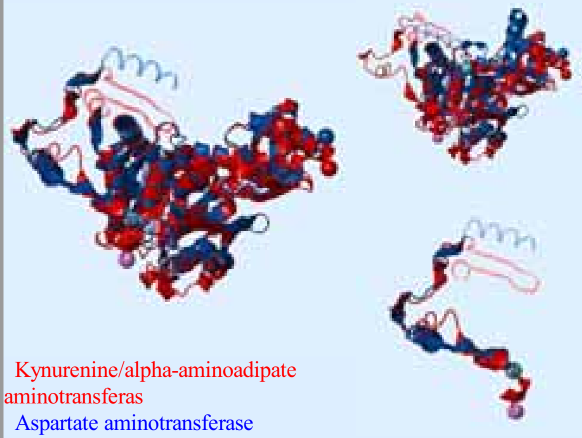 Kynurenine/alpha-aminoadipate aminotransferas Aspartate aminotransferase
	1o4sA (375)	36-64 (29)	36-60 (25)	47-72 (26)						
665	2qlrA (425)	3-76 (74)	3-76 (74)	43-72 (30)	N	0.42	2.66	89.65% (355/396)	12.63% (50/396)	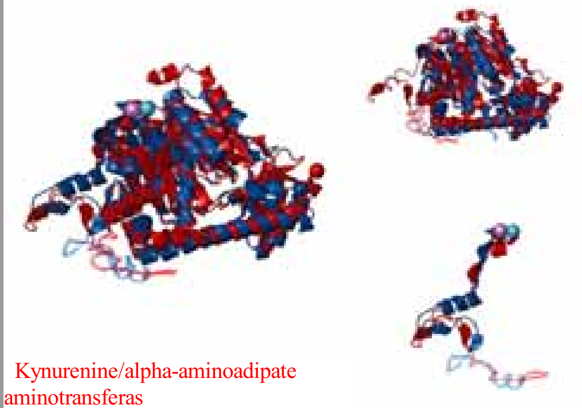 Kynurenine/alpha-aminoadipate aminotransferas Aspartate aminotransferase
	1qirA (396)	6-72 (67)	6-72 (67)	43-72 (30)						

666	2qlrA (425)	35-76 (42)	35-75 (41)	43-72 (30)	N	0.42	2.64	88.89% (352/396)	12.88% (51/396)	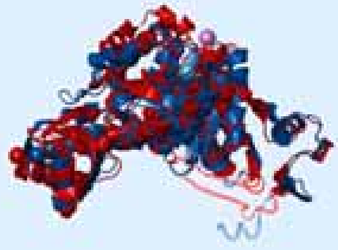 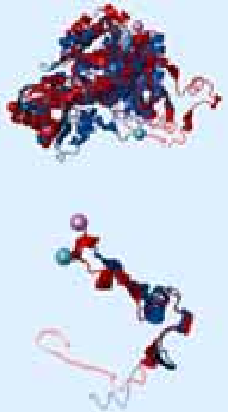 Kynurenine/alpha-aminoadipate aminotransferas
	1spaA (396)	34-72 (39)	34-71 (38)	43-72 (30)						Aspartate aminotransferase
667	2qlrA (425)	4-69 (66)	4-69 (66)	49-67 (19)	N	0.26	2.72	93.13% (312/335)	11.64% (39/335)	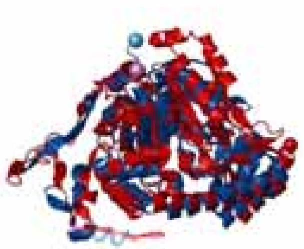 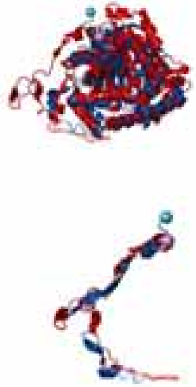 Kynurenine/alpha-aminoadipate aminotransferas
	1uu1C (335)	1-48 (48)	1-48 (48)	49-67 (19)						Histidinol-phosphate aminotransferase
668	2qlrA (425)	1-79 (79)	42-69 (28)	51-67 (17)	N	0.31	2.58	93.15% (299/321)	12.77% (41/321)	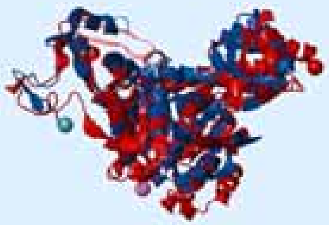 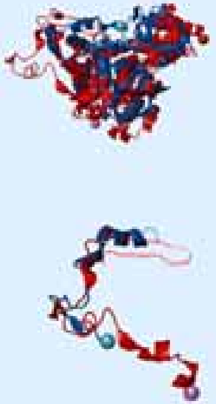 Kynurenine/alpha-aminoadipate aminotransferas
	1uu2A (321)	14-57 (44)	30-48 (19)	51-67 (17)						Histidinol-phosphate aminotransferase

669	2qlrA (425)	14-75 (62)	17-71 (55)	39-71 (33)	N	0.52	2.15	95.10% (349/367)	20.44% (75/367)	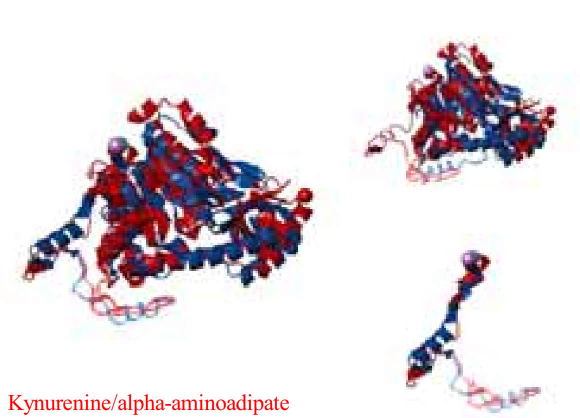 Kynurenine/alpha-aminoadipate aminotransferas Glutamine aminotransferase
	1v2fB (367)	10-58 (49)	13-54 (42)	39-71 (33)						
670	2qlrA (425)	47-80 (34)	47-71 (25)	47-71 (25)	N	0.60	1.69	94.04% (379/403)	29.28% (118/403)	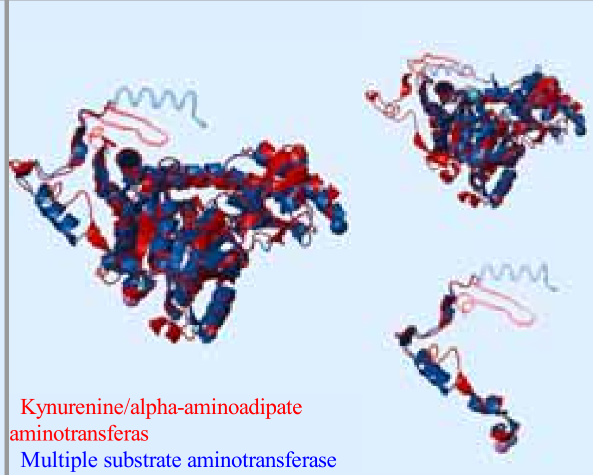 Kynurenine/alpha-aminoadipate aminotransferas Multiple substrate aminotransferase
	1wstA (403)	57-85 (29)	57-76 (20)	47-71 (25)						
671	2qlrA (425)	47-72 (26)	47-71 (25)	47-71 (25)	N	0.59	1.74	94.54% (381/403)	29.28% (118/403)	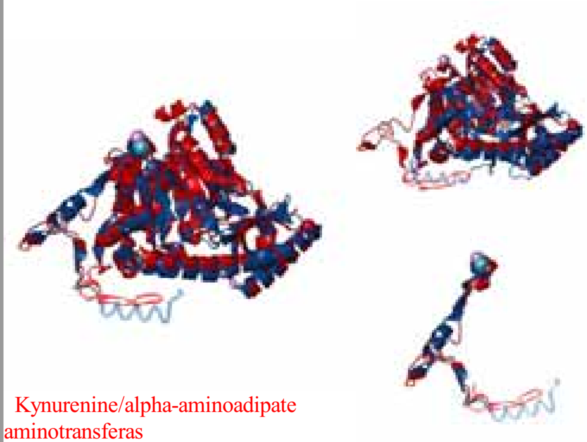 Kynurenine/alpha-aminoadipate aminotransferas Aminotransferase II homologue
	1x0mA (403)	72-92 (21)	72-91 (20)	47-71 (25)						

672	2qlrA (425)	2-76 (75)	2-75 (74)	36-68 (33)	N	0.42	2.59	87.14% (359/412)	10.44% (43/412)	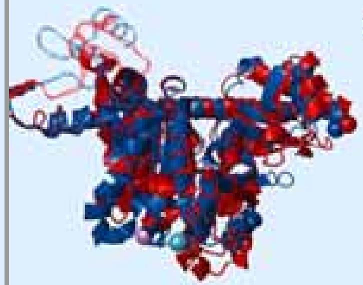 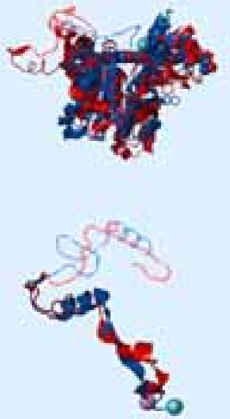 Kynurenine/alpha-aminoadipate aminotransferas Aspartate aminotransferase
	1yaaA (412)	4-72 (69)	4-71 (68)	36-68 (33)						
673	2qlrA (425)	3-80 (78)	6-76 (71)	18-72 (55)	N	0.41	2.70	89.65% (355/396)	12.63% (50/396)	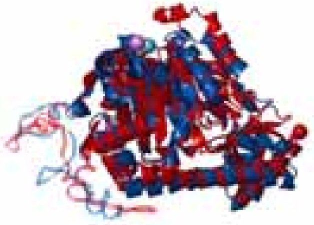 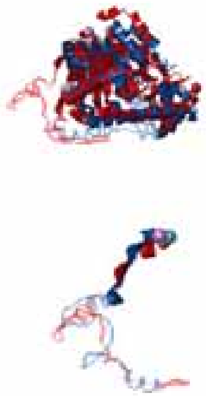 Kynurenine/alpha-aminoadipate aminotransferas Aspartate aminotransferase
	1yooA (396)	6-76 (71)	9-72 (64)	18-72 (55)						
674	2qlrA (425)	3-76 (74)	3-75 (73)	49-72 (24)	N	0.35	2.67	89.65% (355/396)	12.63% (50/396)	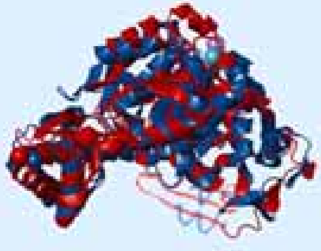 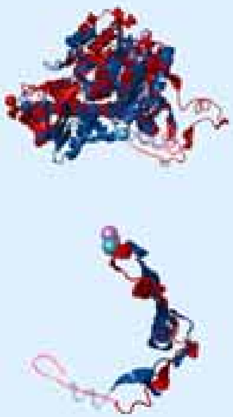 Kynurenine/alpha-aminoadipate aminotransferas Aspartate aminotransferase
	2d5yA (396)	6-72 (67)	6-71 (66)	49-72 (24)						

675	2qlrA (425)	35-76 (42)	48-75 (28)	48-72 (25)	N	0.39	2.61	88.38% (350/396)	14.14% (56/396)	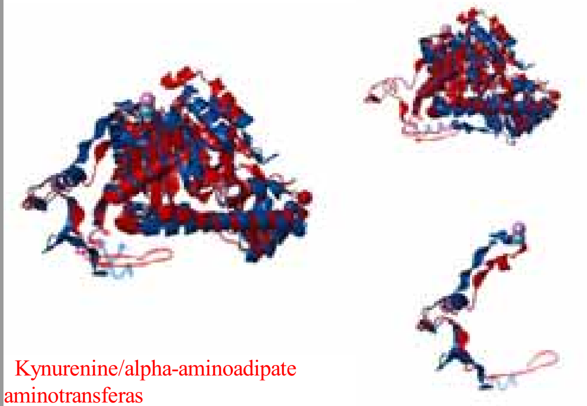 Kynurenine/alpha-aminoadipate aminotransferas Aspartate aminotransferase
	2d61A (396)	34-72 (39)	52-71 (20)	48-72 (25)						
676	2qlrA (425)	3-76 (74)	35-75 (41)	41-72 (32)	N	0.41	2.70	89.65% (355/396)	13.89% (55/396)	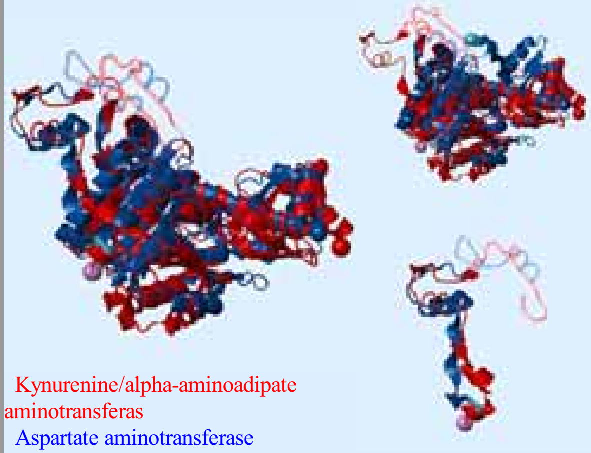 Kynurenine/alpha-aminoadipate aminotransferas Aspartate aminotransferase
	2d64A (396)	6-72 (67)	34-71 (38)	41-72 (32)						
677	2qlrA (425)	3-76 (74)	3-75 (73)	43-72 (30)	N	0.42	2.68	89.65% (355/396)	12.63% (50/396)	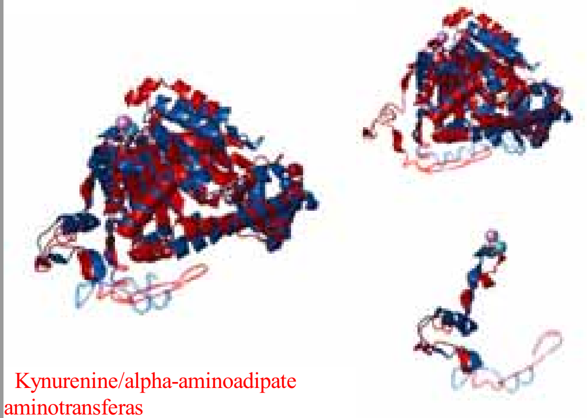 Kynurenine/alpha-aminoadipate aminotransferas Aspartate aminotransferase
	2d66A (396)	6-72 (67)	6-71 (66)	43-72 (30)						

678	2qlrA (425)	1-83 (83)	1-68 (68)	35-68 (34)	N	0.50	2.13	93.82% (349/372)	17.47% (65/372)	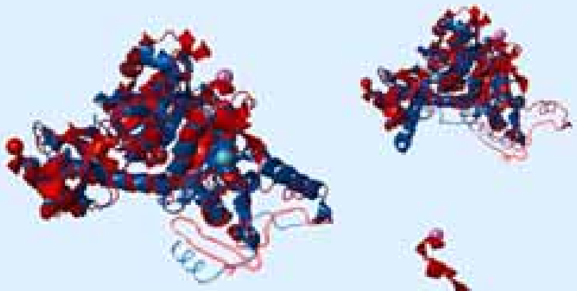 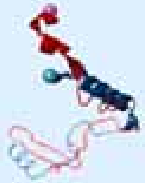 Kynurenine/alpha-aminoadipate aminotransferas Probable N-succinyldiaminopimelate aminotrans
	2douA (372)	4-68 (65)	4-53 (50)	35-68 (34)						
679	2qlrA (425)	17-83 (67)	17-68 (52)	44-68 (25)	N	0.51	2.18	94.84% (349/368)	17.66% (65/368)	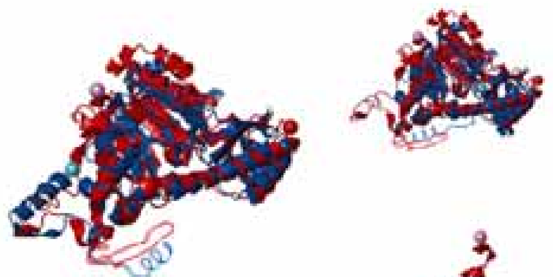 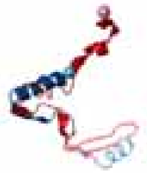 Kynurenine/alpha-aminoadipate aminotransferas Probable N-succinyldiaminopimelate aminotrans
	2douB (368)	9-68 (60)	9-53 (45)	44-68 (25)						
680	2qlrA (425)	47-69 (23)	47-64 (18)	47-64 (18)	N	0.56	1.82	96.94% (380/392)	27.30% (107/392)	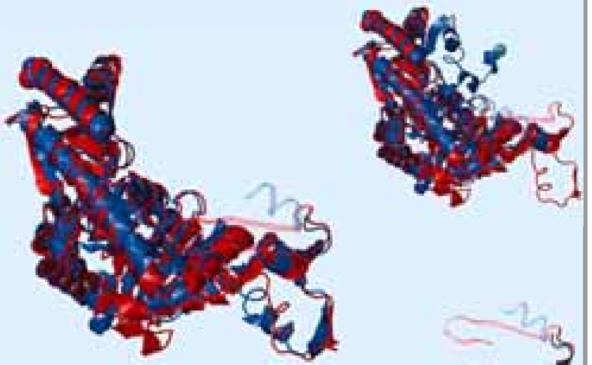 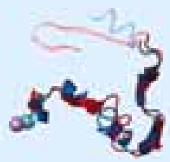 Kynurenine/alpha-aminoadipate aminotransferas Alpha-aminodipate aminotransferase
	2egyA (392)	48-65 (18)	48-60 (13)	47-64 (18)						

681	2qlrA (425)	47-70 (24)	47-70 (24)	48-67 (20)	N	0.52	1.78	96.19% (379/394)	26.65% (105/394)	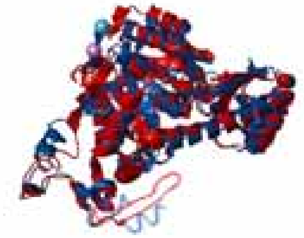 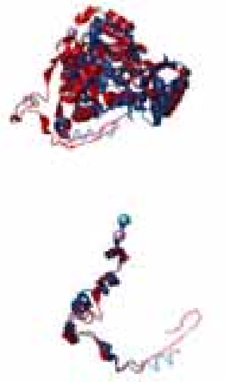<br>Kynurenine/alpha-aminoadipate aminotransferas<br>Alpha-aminodipate aminotransferase
	2egyB (394)	48-66 (19)	48-66 (19)	48-67 (20)						
682	2qlrA (425)	47-67 (21)	47-67 (21)	48-67 (20)	N	0.53	1.78	96.72% (383/396)	27.27% (108/396)	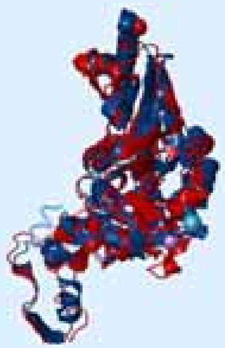 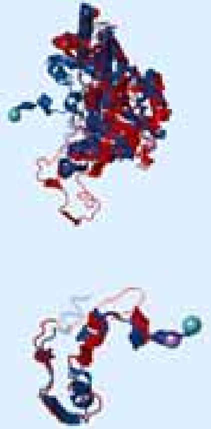<br>Kynurenine/alpha-aminoadipate aminotransferas<br>Alpha-aminodipate aminotransferase
	2egyC (396)	48-63 (16)	48-63 (16)	48-67 (20)						
683	2qlrA (425)	44-70 (27)	44-68 (25)	45-67 (23)	N	0.52	1.67	96.16% (376/391)	27.37% (107/391)	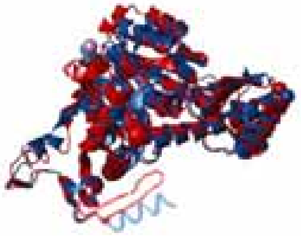 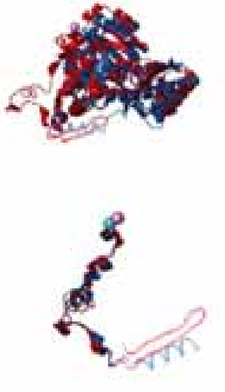<br>Kynurenine/alpha-aminoadipate aminotransferas<br>Alpha-aminodipate aminotransferase
	2egyD (391)	45-66 (22)	45-64 (20)	45-67 (23)						

684	2qlrA (425)	13-68 (56)	13-68 (56)	49-67 (19)	N	0.26	2.69	93.13% (312/335)	11.64% (39/335)	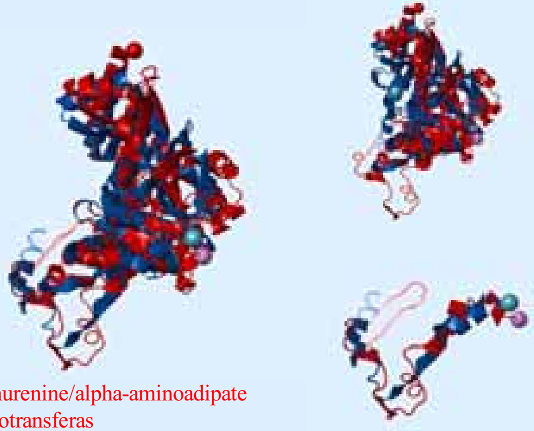 Kynurenine/alpha-aminoadipate aminotransferas Histidinol-phosphate aminotransferase
	2f8jA (335)	9-47 (39)	9-47 (39)	49-67 (19)						
685	2qlrA (425)	47-70 (24)	47-67 (21)	47-67 (21)	N	0.54	1.90	96.70% (381/394)	25.63% (101/394)	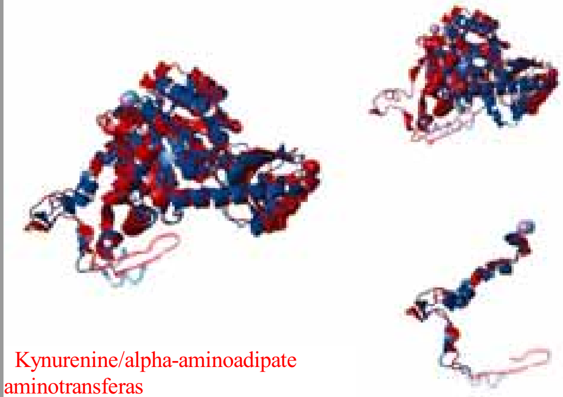 Kynurenine/alpha-aminoadipate aminotransferas Alpha-aminodipate aminotransferase
	2z1yB (394)	48-66 (19)	48-63 (16)	47-67 (21)						
686	2qlrA (425)	44-72 (29)	47-69 (23)	46-69 (24)	N	0.56	2.04	95.06% (385/405)	28.15% (114/405)	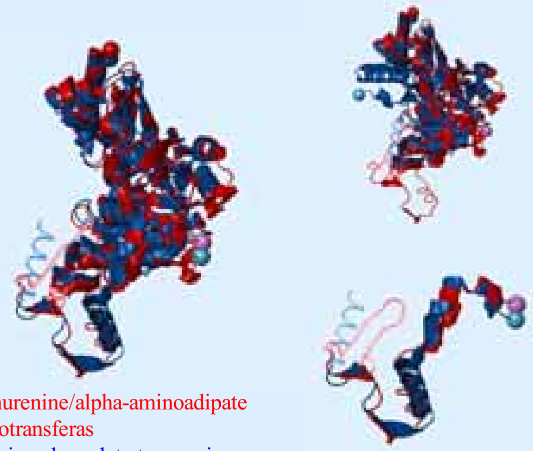 Kynurenine/alpha-aminoadipate aminotransferas Alanine glyoxylate transaminase
	2zc0A (405)	47-69 (23)	50-66 (17)	46-69 (24)						

687	2qlrA (425)	4-76 (73)	4-75 (72)	49-72 (24)	N	0.35	2.72	89.14% (353/396)	12.37% (49/396)	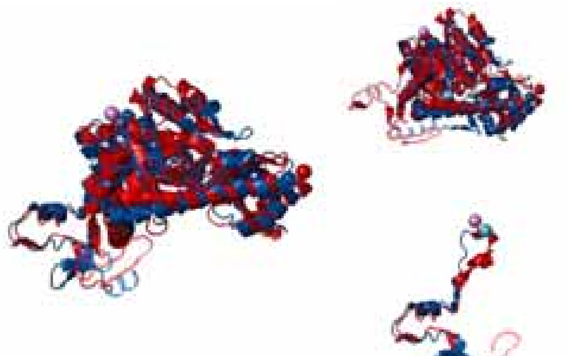 Kynurenine/alpha-aminoadipate aminotransferas Aspartate aminotransferase
	3aatA (396)	7-72 (66)	7-71 (65)	49-72 (24)						
688	2qlrA (425)	6-74 (69)	36-74 (39)	36-72 (37)	N	0.40	2.78	89.17% (354/397)	12.34% (49/397)	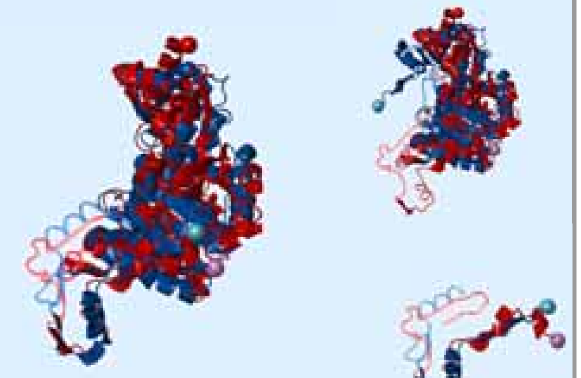 Kynurenine/alpha-aminoadipate aminotransferas Tyrosine aminotransferase
	3tatA (397)	6-70 (65)	35-70 (36)	36-72 (37)						
689	2qlrA (425)	2-75 (74)	41-75 (35)	51-72 (22)	N	0.33	2.79	86.53% (347/401)	14.46% (58/401)	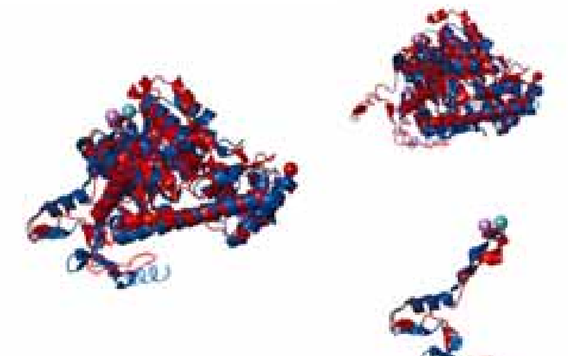 Kynurenine/alpha-aminoadipate aminotransferas Aspartate aminotransferase
	7aatA (401)	4-71 (68)	38-71 (34)	51-72 (22)						

690	2r7hA (157)	158-172 (15)	158-172 (15)	158-168 (11)	C	0.55	2.17	95.17% (138/145)	18.62% (27/145)	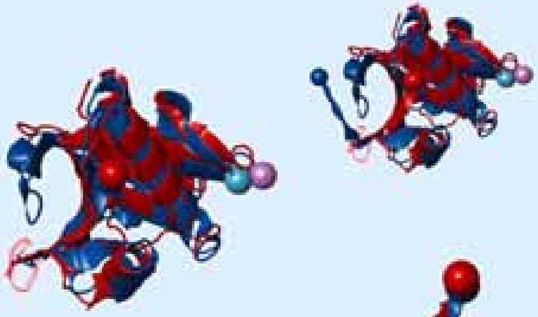 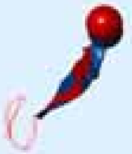 Putative D-alanine N-acetyltransferase of GNA<br>Aminoglycoside 6'-N-acetyltransferase
	1s3zB (145)	138-142 (5)	138-142 (5)	158-168 (11)						
691	2r7hA (157)	157-169 (13)	158-169 (12)	159-160 (2)	C	0.52	2.20	90.20% (138/153)	15.69% (24/153)	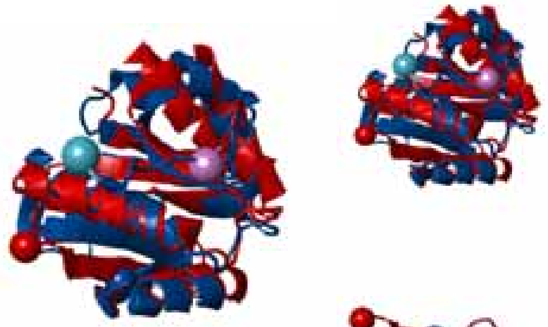 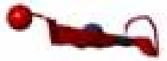 Putative D-alanine N-acetyltransferase of GNA<br>Aminoglycoside 6'-N-acetyltransferase
	1s5kA (153)	137-144 (8)	138-144 (7)	159-160 (2)						
692	2r7hA (157)	154-173 (20)	157-173 (17)	157-168 (12)	C	0.39	2.52	81.94% (127/155)	12.26% (19/155)	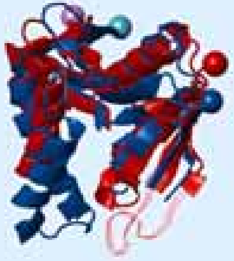 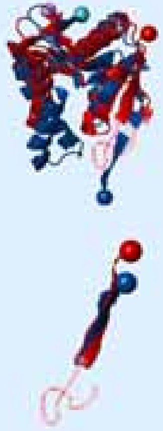 Putative D-alanine N-acetyltransferase of GNA<br>YYCN protein
	1ufhA (155)	147-155 (9)	150-155 (6)	157-168 (12)						

693	2r7hA (157)	154-173 (20)	154-173 (20)	154-166 (13)	C	0.43	2.45	85.06% (131/154)	12.99% (20/154)	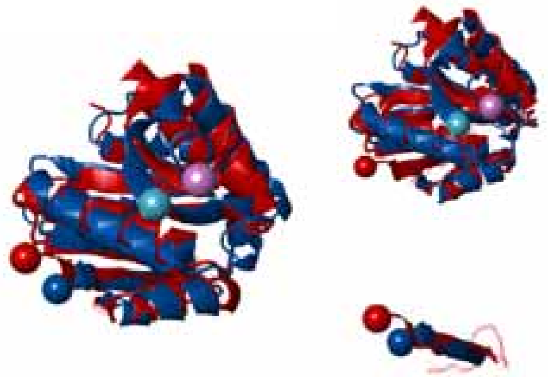 Putative D-alanine N-acetyltransferase of GNA YYCN protein
	1ufhB (154)	147-155 (9)	147-155 (9)	154-166 (13)						
694	2r7hA (157)	154-167 (14)	154-167 (14)	154-167 (14)	C	0.50	2.19	90.45% (142/157)	15.92% (25/157)	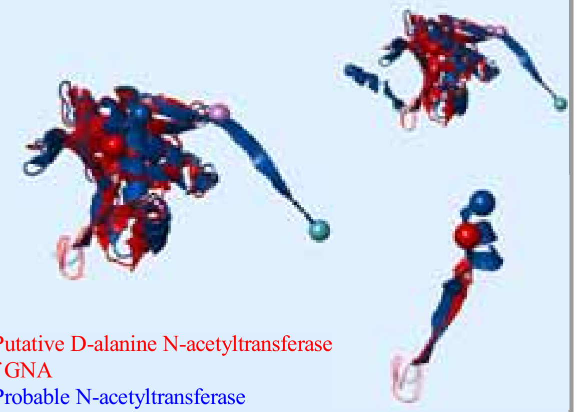 Putative D-alanine N-acetyltransferase of GNA Probable N-acetyltransferase
	2fe7B (166)	139-142 (4)	139-142 (4)	154-167 (14)						
695	2r7hA (157)	154-169 (16)	158-169 (12)	159-160 (2)	C	0.52	2.27	93.88% (138/147)	16.33% (24/147)	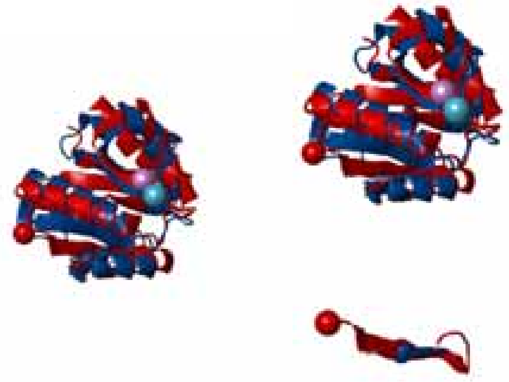 Putative D-alanine N-acetyltransferase of GNA Aminoglycoside 6'-N-acetyltransferase
	2vbqA (147)	134-144 (11)	138-144 (7)	159-160 (2)						

696	2r7hA (157)	154-173 (20)	158-168 (11)	158-167 (10)	C	0.54	2.20	95.14% (137/144)	16.67% (24/144)	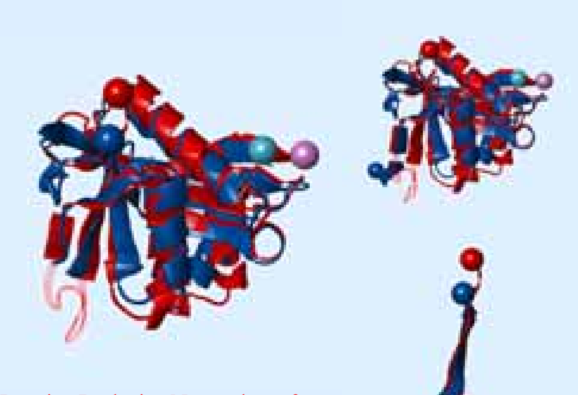 Putative D-alanine N-acetyltransferase of GNA<br>Aminoglycoside 6'-N-acetyltransferase
	2vbqB (144)	134-144 (11)	138-139 (2)	158-167 (10)						
697	2rbbA (129)	72-105 (34)	72-104 (33)	84-104 (21)	C	0.44	1.86	82.95% (107/129)	12.40% (16/129)	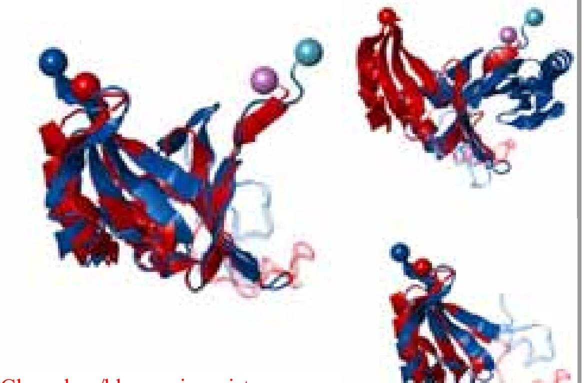 Glyoxalase/bleomycin resistance protein/dioxy<br>Methylmalonyl-CoA epimerase
	1jc5D (145)	50-91 (42)	50-90 (41)	84-104 (21)						
698	2vi7A (163)	140-170 (31)	142-170 (29)	147-158 (12)	C	0.45	2.40	88.82% (135/152)	17.11% (26/152)	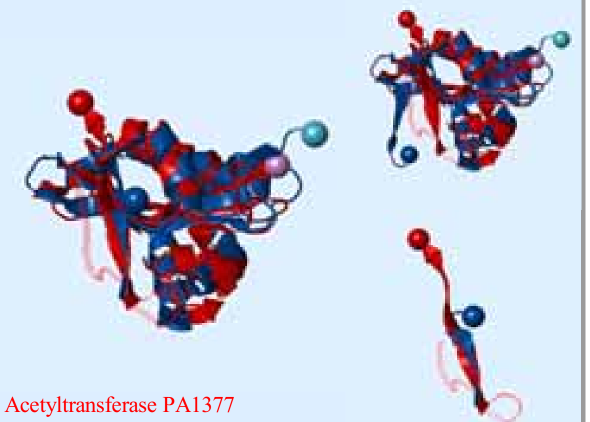 Acetyltransferase PA1377<br>HPA2 histone acetyltransferase
	1qsmD (152)	141-156 (16)	143-156 (14)	147-158 (12)						

699	2vi7A (163)	146-166 (21)	148-166 (19)	148-159 (12)	C	0.46	2.27	89.54% (137/153)	15.03% (23/153)	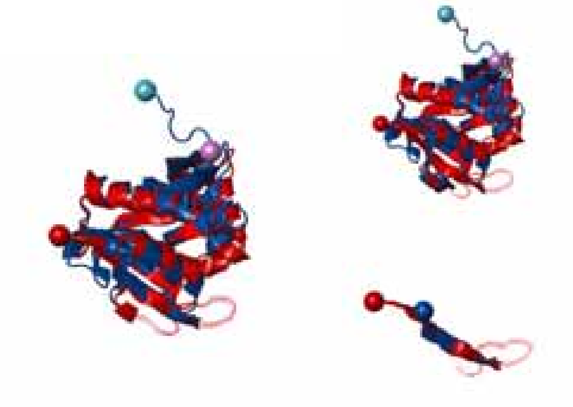<br>Acetyltransferase PA1377<br>Aminoglycoside 6'-N-acetyltransferase
	1s5kA (153)	135-144 (10)	137-144 (8)	148-159 (12)						
700	2vi7A (163)	146-170 (25)	146-170 (25)	146-158 (13)	C	0.45	2.29	84.66% (138/163)	20.86% (34/163)	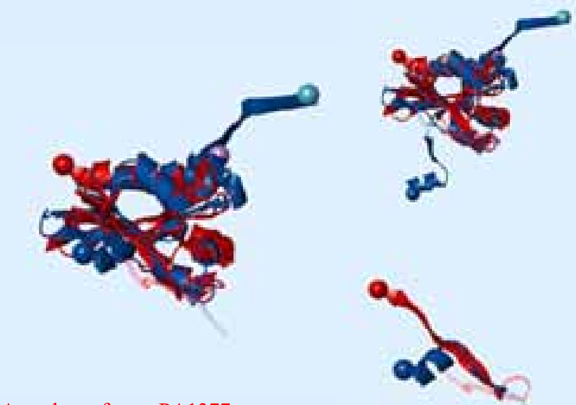<br>Acetyltransferase PA1377<br>Probable N-acetyltransferase
	2fe7B (166)	140-158 (19)	140-158 (19)	146-158 (13)						
701	2vi7A (163)	145-170 (26)	145-158 (14)	147-158 (12)	C	0.44	2.51	87.73% (143/163)	11.66% (19/163)	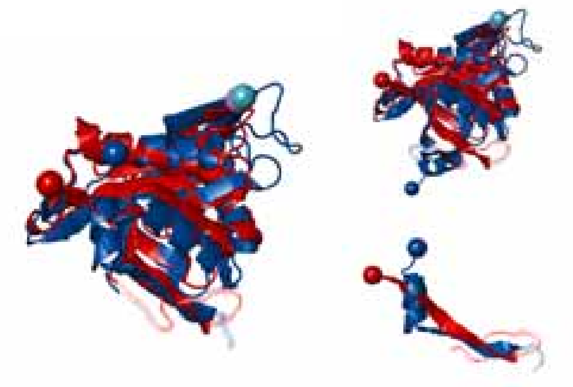<br>Acetyltransferase PA1377<br>Diamine acetyltransferase 1
	2g3tA (169)	147-171 (25)	147-153 (7)	147-158 (12)						

702	2vi7A (163)	147-166 (20)	147-166 (20)	147-160 (14)	C	0.48	1.85	82.21% (134/163)	19.02% (31/163)	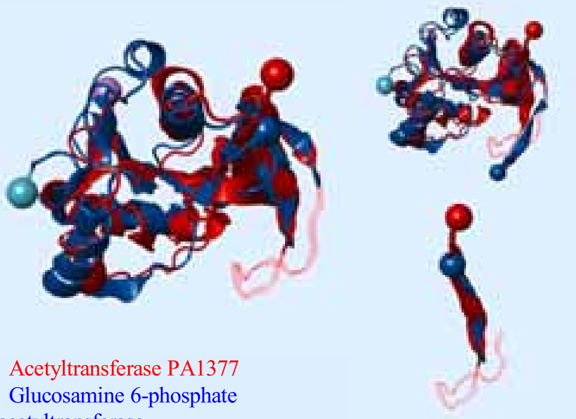 Acetyltransferase PA1377 Glucosamine 6-phosphate acetyltransferase
	2vxkA (165)	183-189 (7)	183-189 (7)	147-160 (14)						
703	5croA (61)	54-60 (7)	54-60 (7)	55-56 (2)	C	0.86	0.93	100.00% (61/61)	91.80% (56/61)	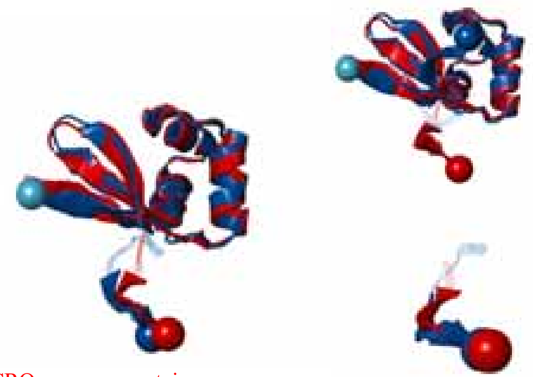 CRO repressor protein Lambda CRO repressor
	1d1mB (65)	54-64 (11)	54-64 (11)	55-56 (2)						
704	5croA (61)	42-60 (19)	52-60 (9)	54-54 (1)	C	0.58	2.03	95.08% (58/61)	19.67% (12/61)	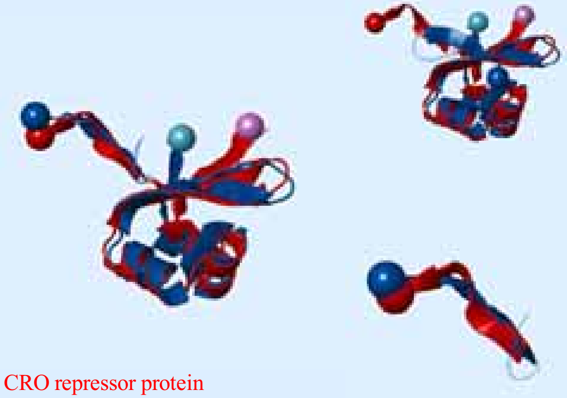 CRO repressor protein SN4m
	2cw1A (65)	42-64 (23)	52-64 (13)	54-54 (1)						

705	5croA (61)	44-60 (17)	45-60 (16)	55-55 (1)	C	0.69	1.49	100.00% (61/61)	93.44% (57/61)	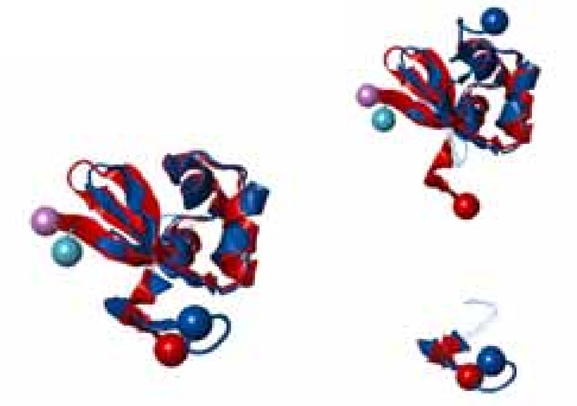<br>CRO repressor protein<br>CRO repressor
	2orcA (71)	44-61 (18)	45-61 (17)	55-55 (1)						
706	1gheA (170)	132-151 (20)	145-151 (7)	149-150 (2)	C	0.50	2.04	90.13% (137/152)	13.16% (20/152)	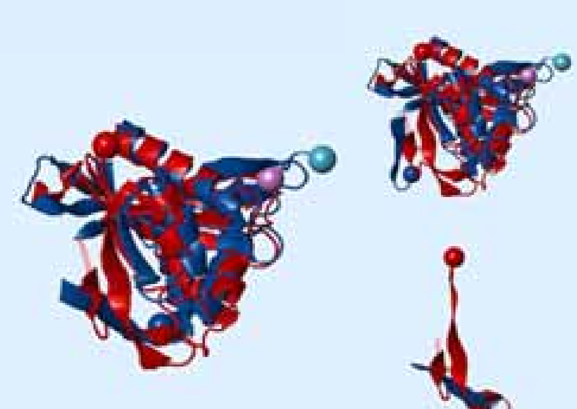<br>Acetyltransferase<br>HPA2 histone acetyltransferase
	1qsmD (152)	129-148 (20)	143-148 (6)	149-150 (2)						
707	1k53A (72)	40-58 (19)	40-58 (19)	52-57 (6)	C	0.40	2.36	83.33% (60/72)	8.33% (6/72)	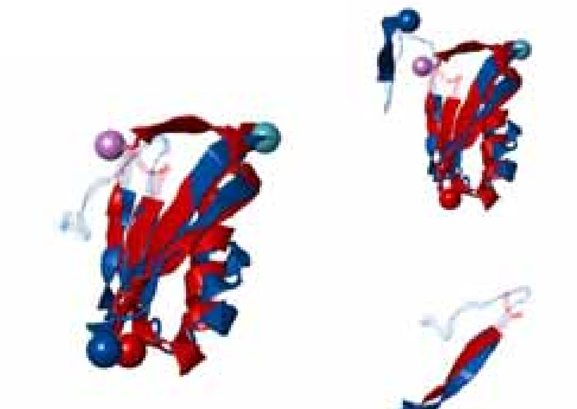<br>Protein L<br>Hypothetical protein
	2hj1A (77)	50-80 (31)	50-80 (31)	52-57 (6)						

708	2c5jA (82)	28-41 (14)	32-41 (10)	32-41 (10)	N	0.32	1.64	93.90% (77/82)	3.66% (3/82)	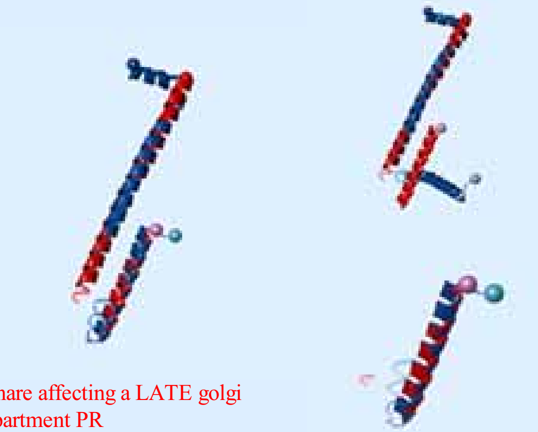 T-snare affecting a LATE golgi compartment PR
	1mi7R (103)	27-44 (18)	31-44 (14)	32-41 (10)						Trp operon repressor
709	1pucA (101)	86-90 (5)	86-88 (3)	86-88 (3)	C	0.72	0.73	95.05% (96/101)	71.29% (72/101)	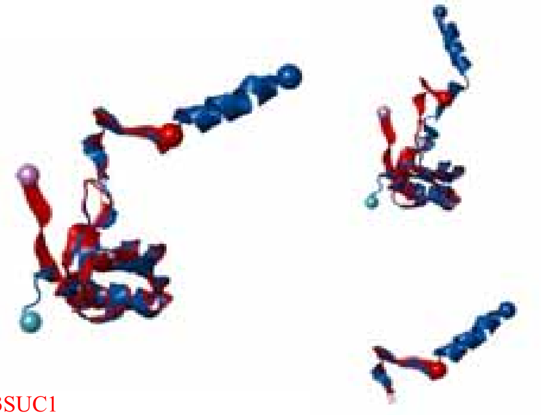 P13SUC1
	1qb3A (113)	89-93 (5)	89-91 (3)	86-88 (3)						Cyclin-dependent kinases regulatory subunit
710	3bcoA (124)	16-22 (7)	16-22 (7)	16-22 (7)	N	0.64	0.33	100.00% (119/119)	100.00% (119/119)	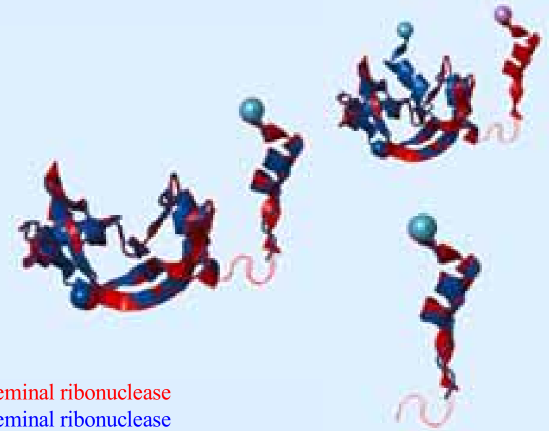 Seminal ribonuclease
	3bcmA (119)	16-22 (7)	16-22 (7)	16-22 (7)						Seminal ribonuclease

711	3bcoA (124)	15-22 (8)	15-22 (8)	15-22 (8)	N	0.43	0.93	91.13% (113/124)	74.19% (92/124)	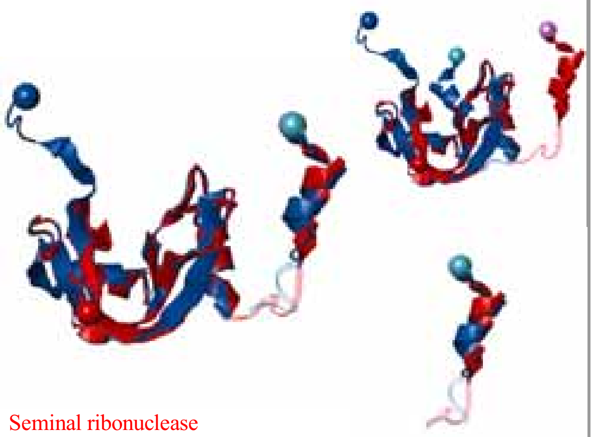 Seminal ribonuclease<br>Ribonuclease A
	1js0A (124)	15-22 (8)	15-22 (8)	15-22 (8)						
712	3bcoA (124)	111-115 (5)	112-113 (2)	112-113 (2)	C	0.55	0.83	83.87% (104/124)	69.35% (86/124)	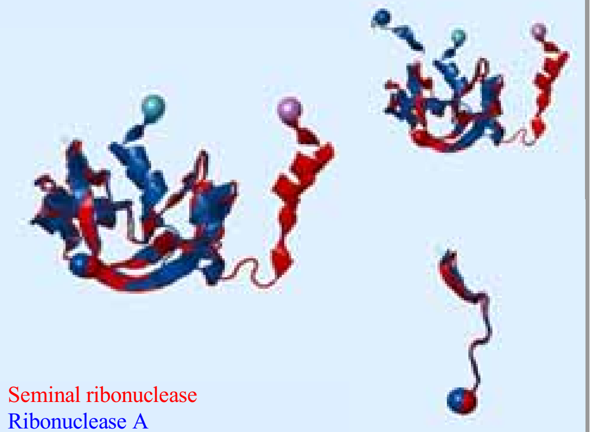 Seminal ribonuclease<br>Ribonuclease A
	1js0A (124)	111-115 (5)	112-113 (2)	112-113 (2)						
713	3bcoA (124)	16-22 (7)	16-22 (7)	16-22 (7)	N	0.62	0.55	100.00% (122/122)	96.72% (118/122)	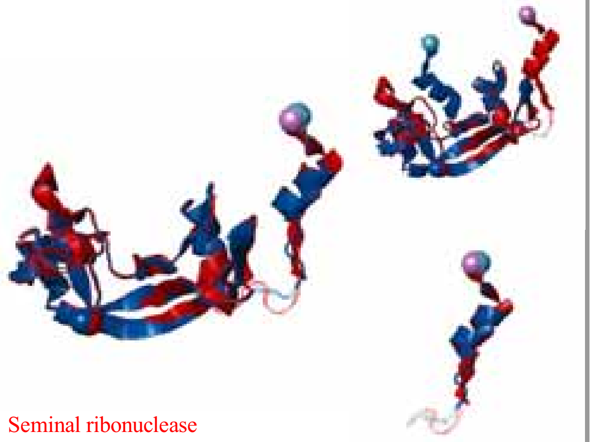 Seminal ribonuclease<br>Ribonuclease, seminal
	1r3mB (122)	16-22 (7)	16-22 (7)	16-22 (7)						

714	3bcoA (124)	15-22 (8)	15-22 (8)	15-22 (8)	N	0.90	1.01	100.00% (124/124)	82.26% (102/124)	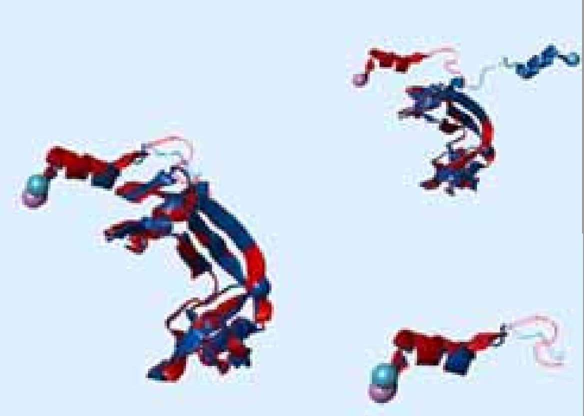 Seminal ribonuclease<br>Ribonuclease A
	1a2wA (124)	15-22 (8)	15-22 (8)	15-22 (8)						
715	3bcoA (124)	15-24 (10)	15-23 (9)	15-23 (9)	N	0.86	1.09	99.19% (123/124)	74.19% (92/124)	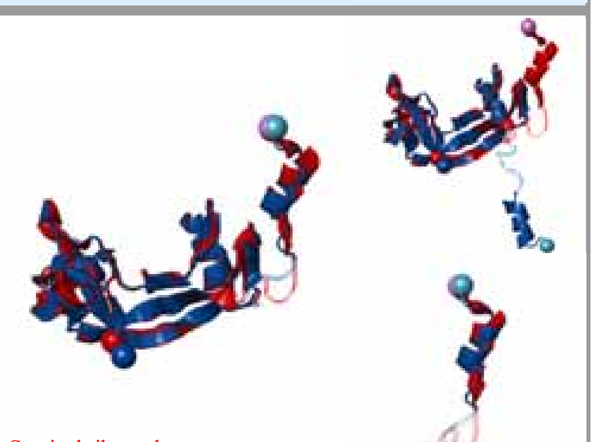 Seminal ribonuclease<br>Ribonuclease 1
	1h8xA (125)	115-124 (10)	115-123 (9)	15-23 (9)						
716	1a2wA (124)	15-23 (9)	15-22 (8)	15-22 (8)	N	0.91	0.86	99.19% (123/124)	80.65% (100/124)	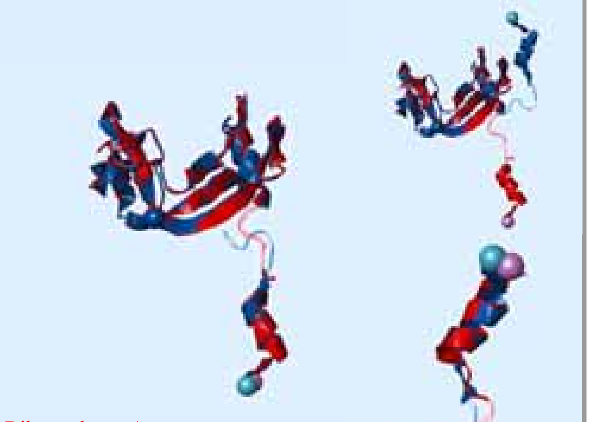 Ribonuclease A<br>Protein (ribonuclease, seminal)
	11baA (124)	15-23 (9)	15-22 (8)	15-22 (8)						

717	3b8iA (283)	254-271 (18)	254-271 (18)	254-264 (11)	C	0.62	1.91	94.70% (268/283)	26.15% (74/283)	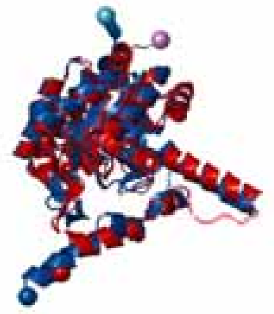 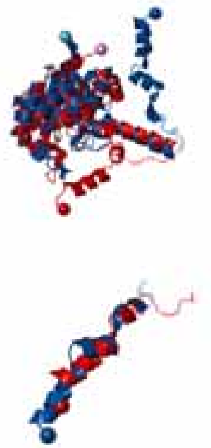 PA4872 oxaloacetate decarboxylase<br>2-methylisocitrate lyase
	1oqfA (290)	255-268 (14)	255-268 (14)	254-264 (11)						
718	3b8iA (283)	254-271 (18)	254-263 (10)	254-264 (11)	C	0.65	1.63	94.10% (255/271)	25.83% (70/271)	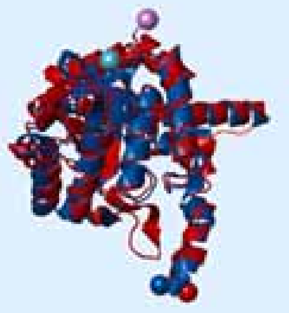 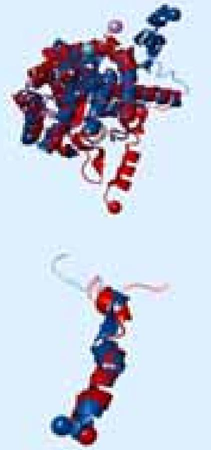 PA4872 oxaloacetate decarboxylase<br>Probable methylisocitrate lyase
	1o5qA (271)	256-269 (14)	256-263 (8)	254-264 (11)						
719	3b8iA (283)	250-265 (16)	254-264 (11)	254-264 (11)	C	0.59	1.95	91.87% (260/283)	23.67% (67/283)	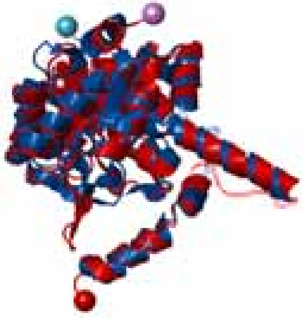 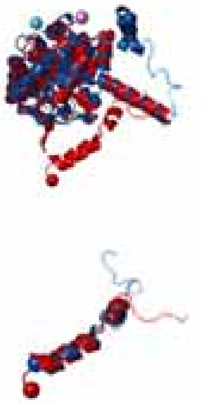 PA4872 oxaloacetate decarboxylase<br>Petal death protein
	1zlpA (284)	273-293 (21)	277-292 (16)	254-264 (11)						

720	3b8iA (283)	246-265 (20)	246-265 (20)	246-265 (20)	C	0.39	2.12	92.58% (262/283)	19.43% (55/283)	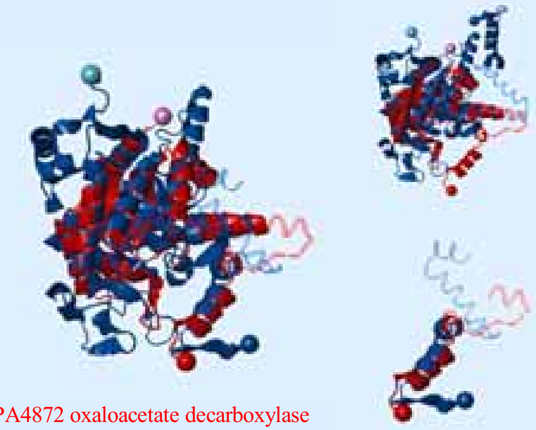 PA4872 oxaloacetate decarboxylase<br>Isocitrate lyase
	1f61A (418)	359-392 (34)	359-392 (34)	246-265 (20)						
721	3b8iA (283)	245-266 (22)	245-265 (21)	245-263 (19)	C	0.37	2.05	90.81% (257/283)	19.08% (54/283)	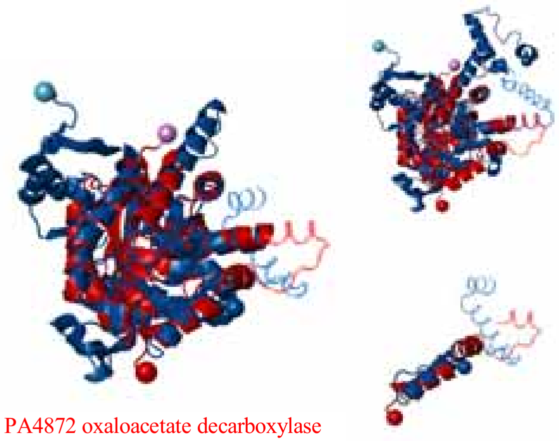 PA4872 oxaloacetate decarboxylase<br>Isocitrate lyase
	1f8mA (427)	358-393 (36)	358-392 (35)	245-263 (19)						
722	3b8iA (283)	242-265 (24)	242-265 (24)	242-265 (24)	C	0.38	2.03	90.81% (257/283)	19.43% (55/283)	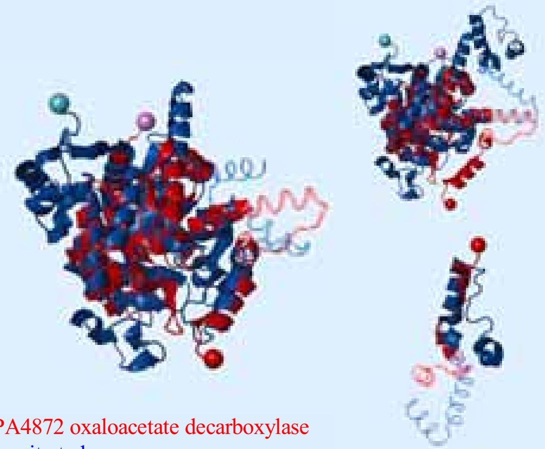 PA4872 oxaloacetate decarboxylase<br>Isocitrate lyase
	1f8iA (427)	355-392 (38)	355-392 (38)	242-265 (24)						

723	1k51A (72)	39-59 (21)	39-57 (19)	54-54 (1)	C	0.41	2.30	83.33% (60/72)	4.17% (3/72)	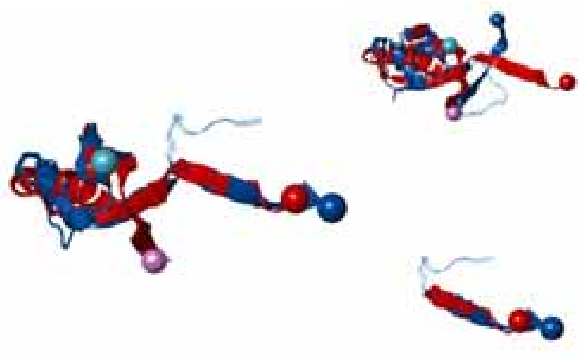 Protein L<br>Hypothetical protein
	2hj1A (77)	49-80 (32)	49-78 (30)	54-54 (1)						
724	1k52A (72)	39-63 (25)	40-57 (18)	52-54 (3)	C	0.41	2.21	81.94% (59/72)	6.94% (5/72)	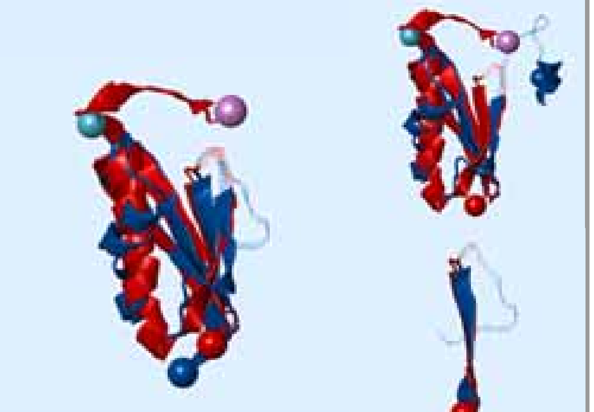 Protein L<br>Hypothetical protein
	2hj1A (77)	49-84 (36)	50-78 (29)	52-54 (3)						
725	1mo1A (87)	10-15 (6)	10-15 (6)	10-15 (6)	N	0.28	1.57	59.77% (52/87)	24.14% (21/87)	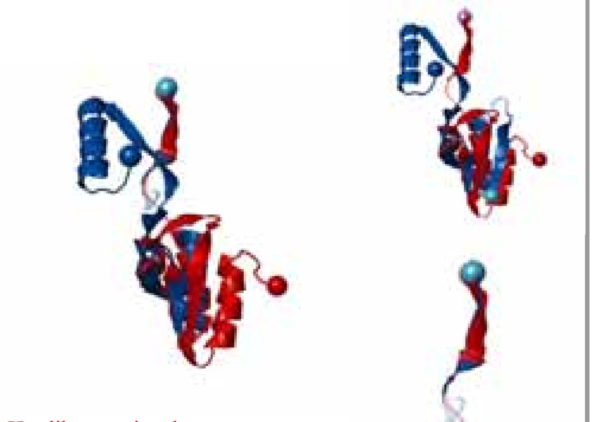 Hpr-like protein crh<br>Phosphocarrier protein HPr
	1y50A (87)	10-15 (6)	10-15 (6)	10-15 (6)						

726	1mo1A (87)	51-56 (6)	52-56 (5)	52-56 (5)	C	0.50	1.63	80.46% (70/87)	31.03% (27/87)	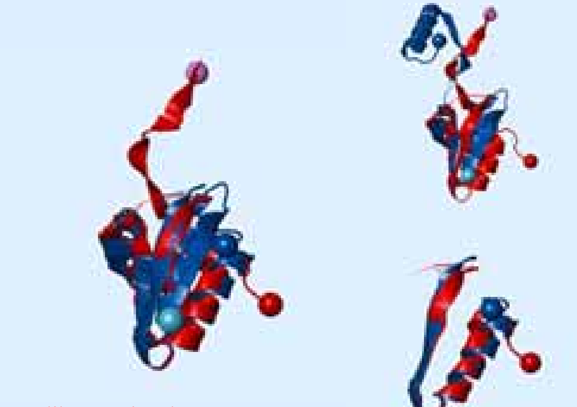 Hpr-like protein crh<br>Phosphocarrier protein HPr
	1y50A (87)	51-56 (6)	52-56 (5)	52-56 (5)						
727	3bcmA (119)	16-24 (9)	16-22 (7)	16-22 (7)	N	0.62	0.39	100.00% (119/119)	98.32% (117/119)	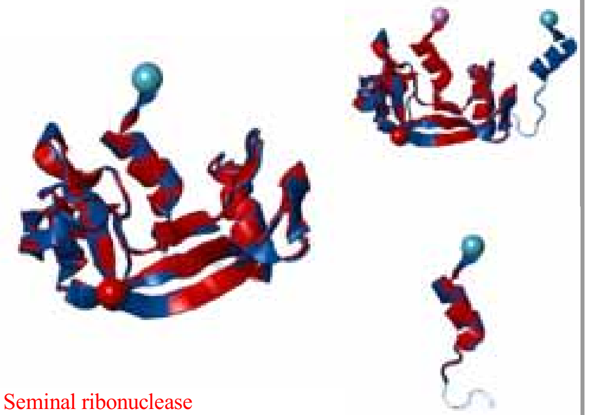 Seminal ribonuclease<br>Ribonuclease, seminal
	1r5cA (124)	16-24 (9)	16-22 (7)	16-22 (7)						
728	3bcmA (119)	112-113 (2)	112-113 (2)	112-113 (2)	C	0.74	0.59	100.00% (119/119)	83.19% (99/119)	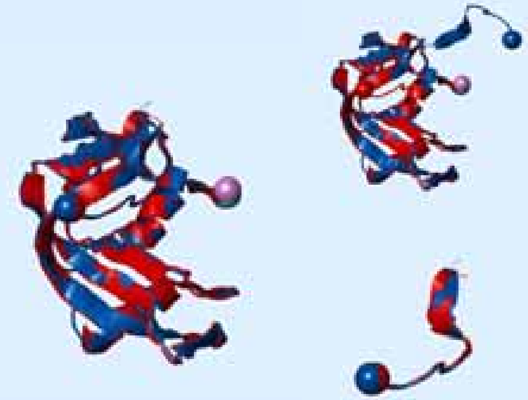 Seminal ribonuclease<br>Ribonuclease A
	1js0A (124)	112-113 (2)	112-113 (2)	112-113 (2)						

729	3bcmA (119)	16-24 (9)	16-22 (7)	16-22 (7)	N	0.59	0.42	100.00% (119/119)	98.32% (117/119)	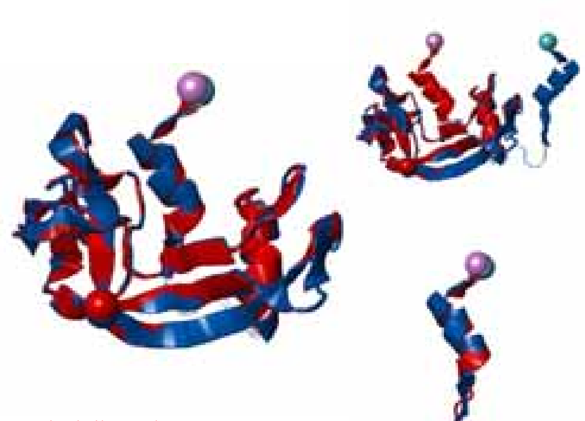<br>Seminal ribonuclease<br>Protein (bovine seminal ribonuclease)
	11bgA (124)	16-24 (9)	16-22 (7)	16-22 (7)						
730	3bcmA (119)	15-22 (8)	16-22 (7)	16-22 (7)	N	0.87	0.41	100.00% (119/119)	96.64% (115/119)	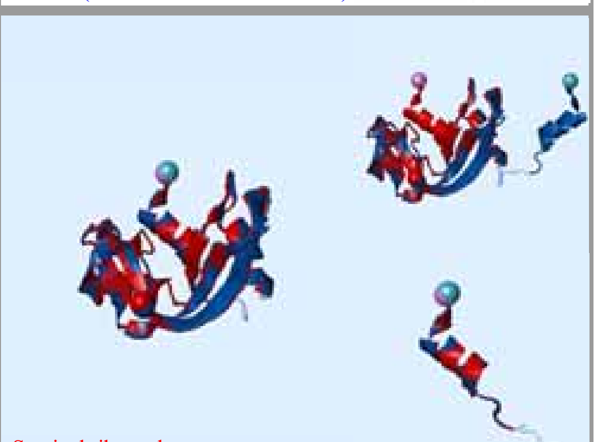<br>Seminal ribonuclease<br>Ribonuclease, seminal
	1tq9A (124)	15-22 (8)	16-22 (7)	16-22 (7)						
731	3bcmA (119)	16-24 (9)	16-22 (7)	16-22 (7)	N	0.58	0.44	100.00% (119/119)	98.32% (117/119)	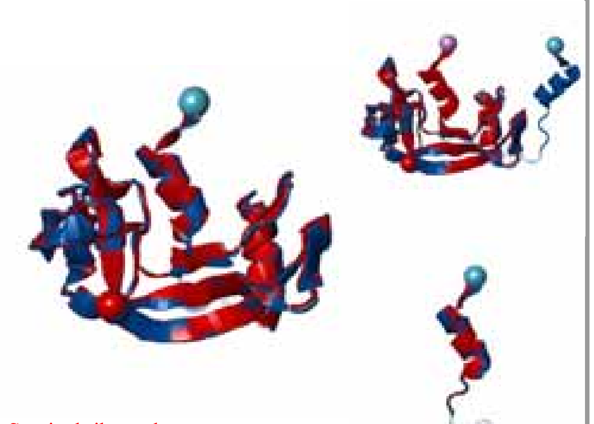<br>Seminal ribonuclease<br>Ribonuclease, seminal
	1r5dA (124)	16-24 (9)	16-22 (7)	16-22 (7)						

732	3bcmA (119)	15-22 (8)	15-22 (8)	15-22 (8)	N	0.93	0.58	100.00% (119/119)	83.19% (99/119)	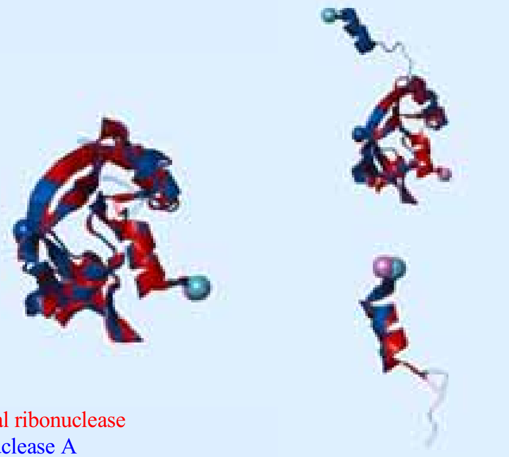 Seminal ribonuclease<br>Ribonuclease A
	1a2wA (124)	15-22 (8)	15-22 (8)	15-22 (8)						
733	3bcmA (119)	16-24 (9)	16-22 (7)	16-22 (7)	N	0.59	0.36	100.00% (119/119)	98.32% (117/119)	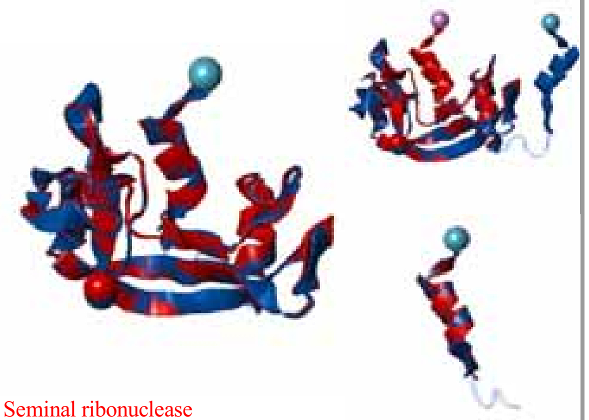 Seminal ribonuclease<br>Protein (ribonuclease, seminal)
	11baA (124)	16-24 (9)	16-22 (7)	16-22 (7)						
734	3bcmA (119)	15-23 (9)	15-23 (9)	15-23 (9)	N	0.85	1.04	100.00% (119/119)	75.63% (90/119)	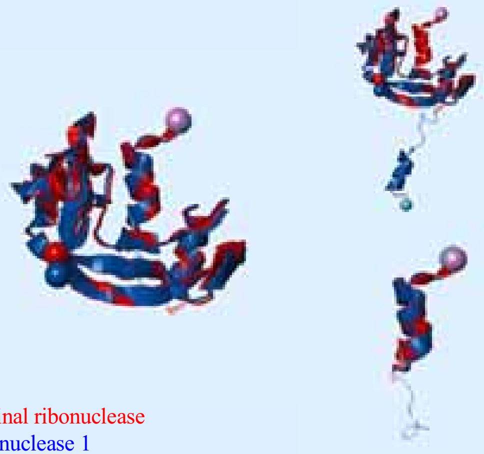 Seminal ribonuclease<br>Ribonuclease 1
	1h8xA (125)	115-123 (9)	115-123 (9)	15-23 (9)						

735	1tq9A (124)	16-22 (7)	16-22 (7)	18-22 (5)	N	0.81	0.62	100.00% (122/122)	98.36% (120/122)	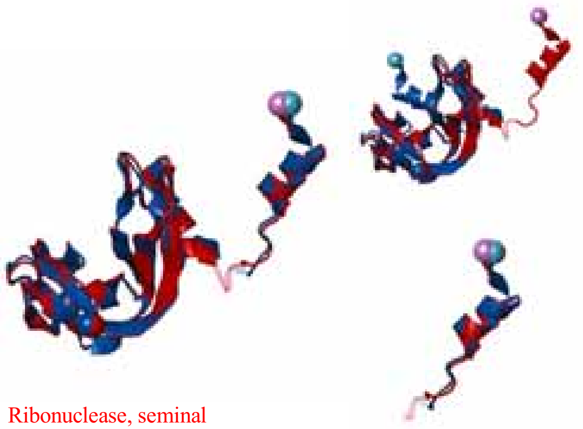
	1r3mB (122)	16-22 (7)	16-22 (7)	18-22 (5)						
736	1k50A (63)	50-56 (7)	50-56 (7)	53-56 (4)	C	0.76	1.15	100.00% (63/63)	96.83% (61/63)	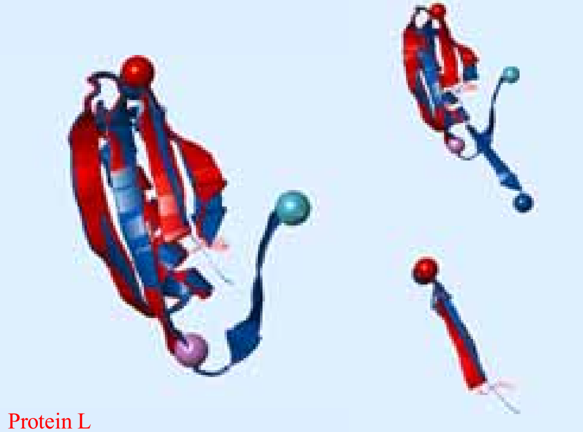
	1k51A (72)	50-56 (7)	50-56 (7)	53-56 (4)						
737	1k50A (63)	50-56 (7)	50-56 (7)	52-56 (5)	C	0.76	1.19	100.00% (63/63)	93.65% (59/63)	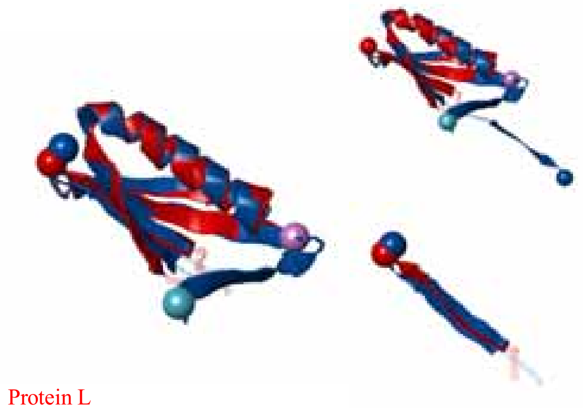
	1jmlA (72)	50-56 (7)	50-56 (7)	52-56 (5)						

738	1jmlA (72)	39-59 (21)	40-57 (18)	54-54 (1)	C	0.40	2.36	83.33% (60/72)	4.17% (3/72)	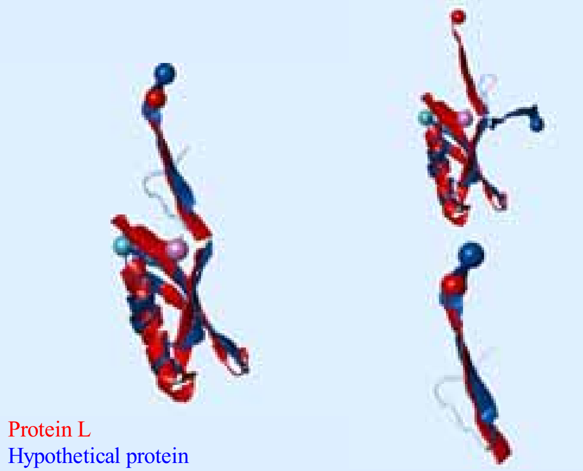 Protein L<br>Hypothetical protein
	2hj1A (77)	49-80 (32)	50-78 (29)	54-54 (1)						
739	1mu4A (86)	10-15 (6)	10-15 (6)	10-15 (6)	N	0.28	1.55	60.47% (52/86)	26.74% (23/86)	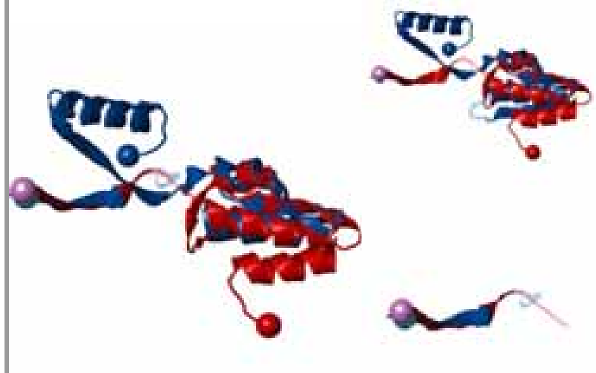 HPr-like protein crh<br>Phosphocarrier protein HPr
	1y50A (87)	10-15 (6)	10-15 (6)	10-15 (6)						
740	1mu4A (86)	51-56 (6)	52-56 (5)	53-55 (3)	C	0.50	1.75	82.56% (71/86)	34.88% (30/86)	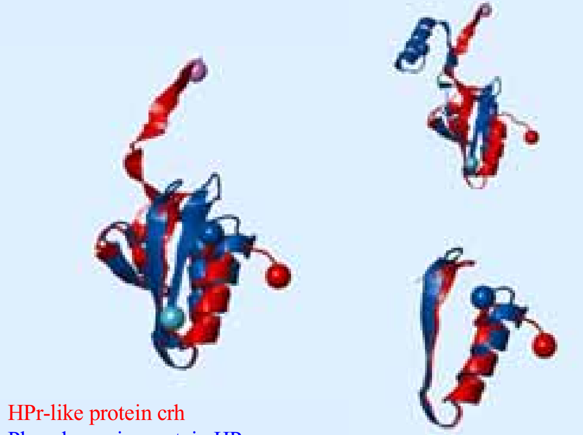 HPr-like protein crh<br>Phosphocarrier protein HPr
	1y50A (87)	51-56 (6)	52-56 (5)	53-55 (3)						

741	2ck2B (96)	2-68 (67)	2-21 (20)	14-19 (6)	N	0.31	2.90	81.25% (78/96)	9.38% (9/96)	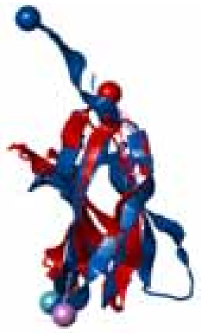 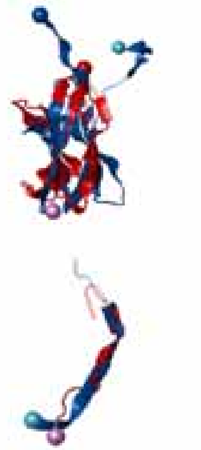<br>Human fibronectin<br>Protein (nerve growth factor receptor TRKA)
	1wwaX (105)	284-357 (74)	284-302 (19)	14-19 (6)						
742	1f0vA (124)	15-25 (11)	15-25 (11)	16-22 (7)	N	0.74	1.04	91.13% (113/124)	74.19% (92/124)	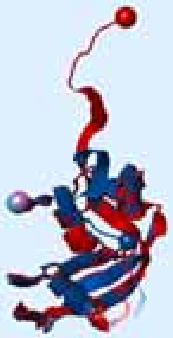 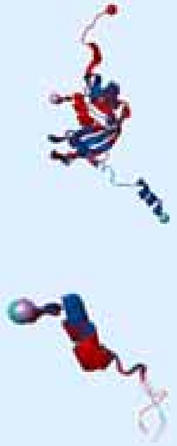<br>Ribonuclease A<br>Seminal ribonuclease
	3bcpA (124)	15-25 (11)	15-25 (11)	16-22 (7)						
743	1f0vA (124)	109-113 (5)	111-113 (3)	112-113 (2)	C	0.65	0.83	83.87% (104/124)	69.35% (86/124)	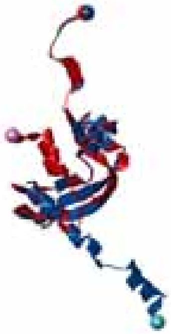 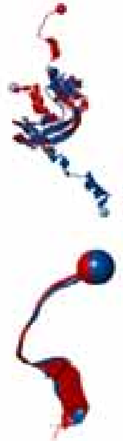<br>Ribonuclease A<br>Seminal ribonuclease
	3bcpA (124)	109-113 (5)	111-113 (3)	112-113 (2)						

744	1f0vA (124)	15-22 (8)	15-22 (8)	15-22 (8)	N	0.44	0.91	91.13% (113/124)	74.19% (92/124)	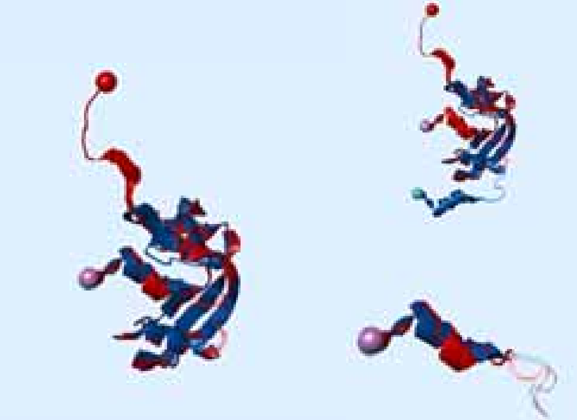 Ribonuclease A<br>Seminal ribonuclease
	3bcoA (124)	15-22 (8)	15-22 (8)	15-22 (8)						
745	1f0vA (124)	111-113 (3)	111-113 (3)	112-113 (2)	C	0.65	0.83	83.87% (104/124)	69.35% (86/124)	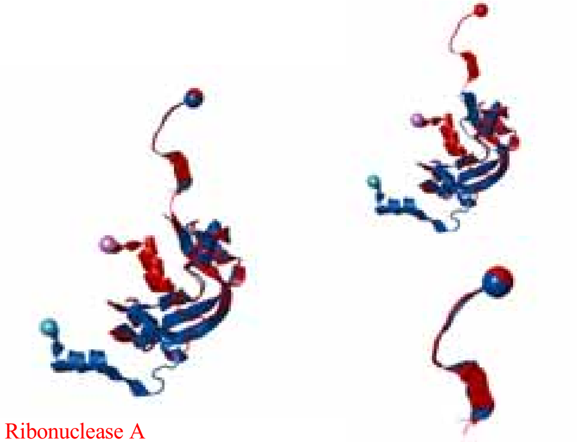 Ribonuclease A<br>Seminal ribonuclease
	3bcoA (124)	111-113 (3)	111-113 (3)	112-113 (2)						
746	1f0vA (124)	109-113 (5)	111-113 (3)	112-113 (2)	C	0.92	0.59	100.00% (119/119)	83.19% (99/119)	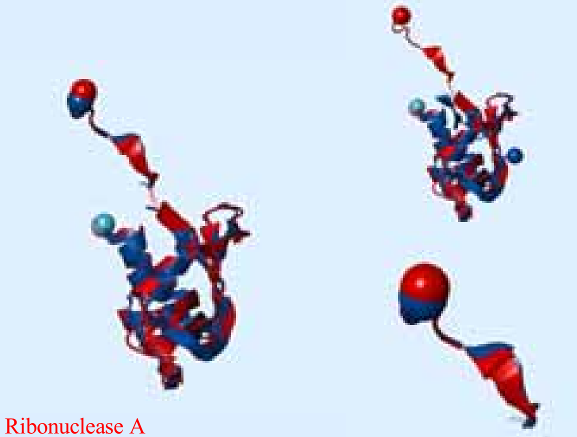 Ribonuclease A<br>Seminal ribonuclease
	3bcmA (119)	109-113 (5)	111-113 (3)	112-113 (2)						

747	1f0vA (124)	15-22 (8)	15-22 (8)	15-22 (8)	N	0.42	0.84	90.32% (112/124)	72.58% (90/124)	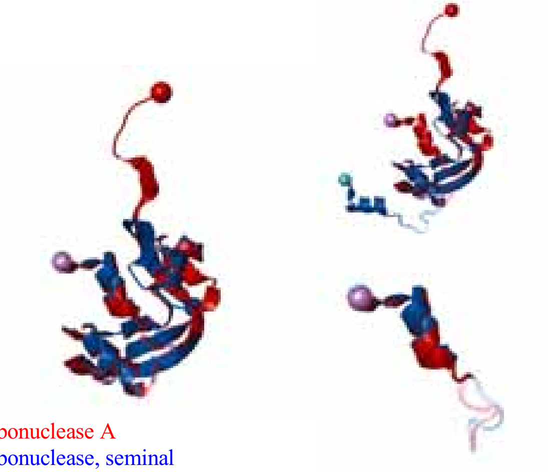 Ribonuclease A<br>Ribonuclease, seminal
	1r5cA (124)	15-22 (8)	15-22 (8)	15-22 (8)						
748	1f0vA (124)	111-113 (3)	111-113 (3)	112-113 (2)	C	0.66	0.65	83.06% (103/124)	68.55% (85/124)	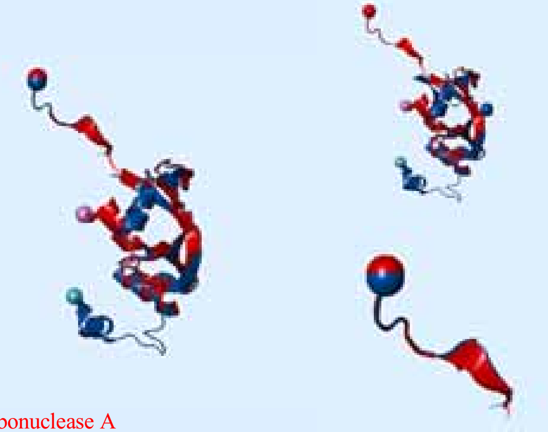 Ribonuclease A<br>Ribonuclease, seminal
	1r5cA (124)	111-113 (3)	111-113 (3)	112-113 (2)						
749	1f0vA (124)	15-22 (8)	15-22 (8)	15-22 (8)	N	0.44	0.93	91.13% (113/124)	73.39% (91/124)	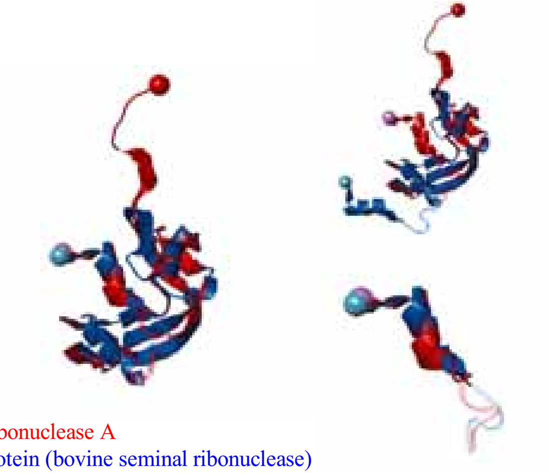 Ribonuclease A<br>Protein (bovine seminal ribonuclease)
	11bgA (124)	15-22 (8)	15-22 (8)	15-22 (8)						

750	1f0vA (124)	111-115 (5)	112-114 (3)	113-114 (2)	C	0.65	0.82	83.87% (104/124)	69.35% (86/124)	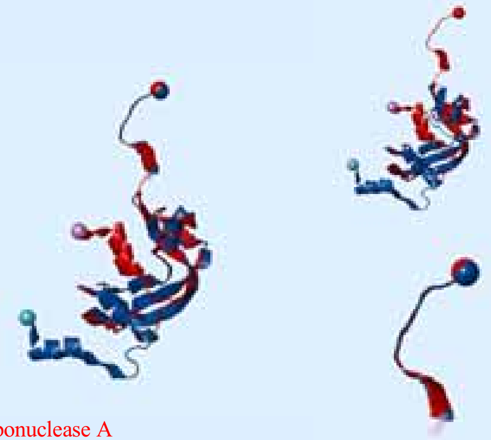 Ribonuclease A<br>Protein (bovine seminal ribonuclease)
	11bgA (124)	111-115 (5)	112-114 (3)	113-114 (2)						
751	1f0vA (124)	15-22 (8)	15-22 (8)	15-22 (8)	N	0.69	0.93	91.13% (113/124)	73.39% (91/124)	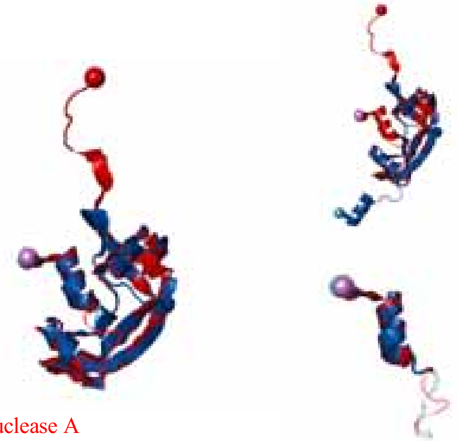 Ribonuclease A<br>Ribonuclease, seminal
	1tq9A (124)	15-22 (8)	15-22 (8)	15-22 (8)						
752	1f0vA (124)	111-115 (5)	111-113 (3)	112-113 (2)	C	0.67	0.66	83.87% (104/124)	69.35% (86/124)	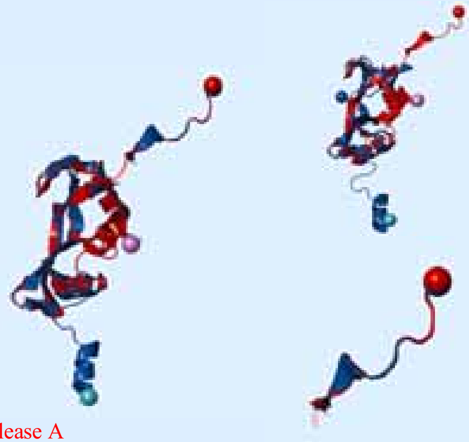 Ribonuclease A<br>Ribonuclease, seminal
	1tq9A (124)	111-115 (5)	111-113 (3)	112-113 (2)						

753	1f0vA (124)	111-113 (3)	111-113 (3)	112-113 (2)	C	0.92	0.79	100.00% (122/122)	81.15% (99/122)	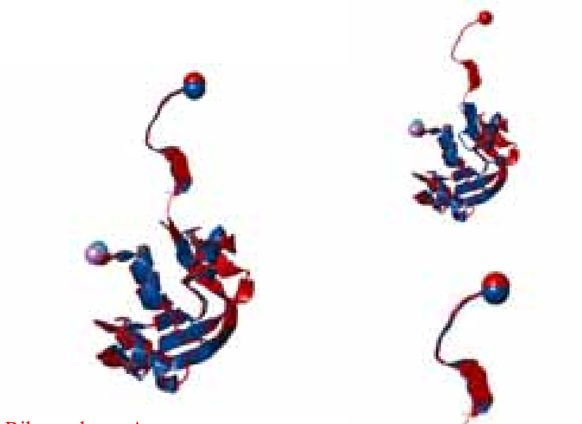 Ribonuclease A<br>Ribonuclease, seminal
	1r3mB (122)	111-113 (3)	111-113 (3)	112-113 (2)						
754	1f0vA (124)	15-22 (8)	15-22 (8)	15-22 (8)	N	0.42	0.84	90.32% (112/124)	72.58% (90/124)	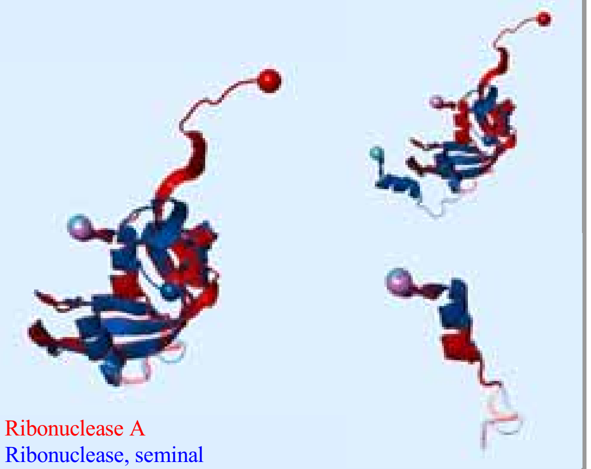 Ribonuclease A<br>Ribonuclease, seminal
	1r5dA (124)	15-22 (8)	15-22 (8)	15-22 (8)						
755	1f0vA (124)	111-113 (3)	111-113 (3)	112-113 (2)	C	0.66	0.66	83.06% (103/124)	68.55% (85/124)	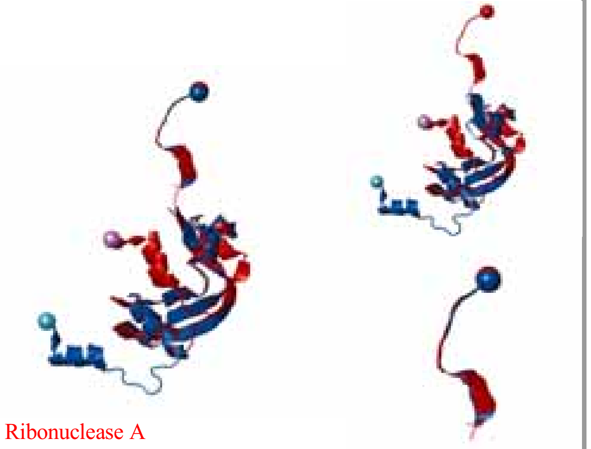 Ribonuclease A<br>Ribonuclease, seminal
	1r5dA (124)	111-113 (3)	111-113 (3)	112-113 (2)						

756	1f0vA (124)	15-22 (8)	15-22 (8)	19-22 (4)	N	0.40	0.75	90.32% (112/124)	72.58% (90/124)	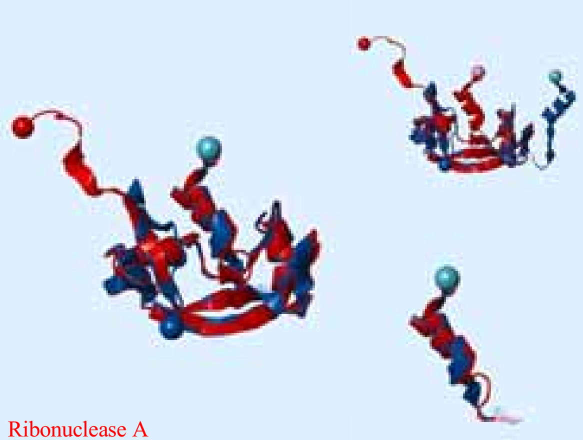
	1lbaA (124)	15-22 (8)	15-22 (8)	19-22 (4)						
757	1f0vA (124)	111-115 (5)	111-113 (3)	112-113 (2)	C	0.67	0.55	83.06% (103/124)	68.55% (85/124)	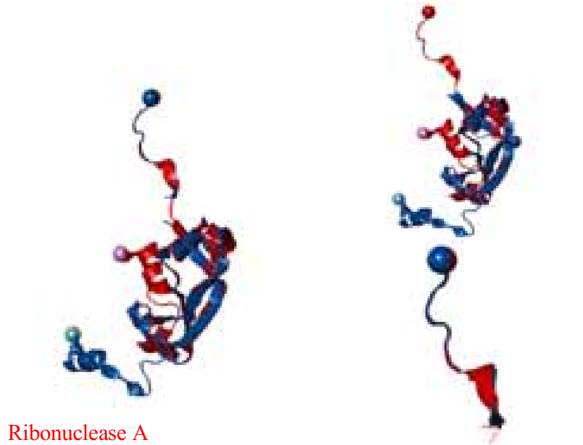
	1lbaA (124)	111-115 (5)	111-113 (3)	112-113 (2)						
758	1r5cA (124)	15-22 (8)	15-22 (8)	15-22 (8)	N	0.41	0.88	90.32% (112/124)	72.58% (90/124)	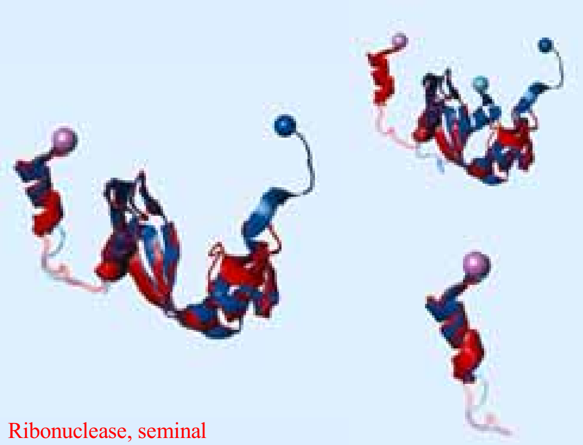
	1js0A (124)	15-22 (8)	15-22 (8)	15-22 (8)						

759	1r5cA (124)	111-115 (5)	112-113 (2)	112-113 (2)	C	0.54	0.67	83.06% (103/124)	68.55% (85/124)	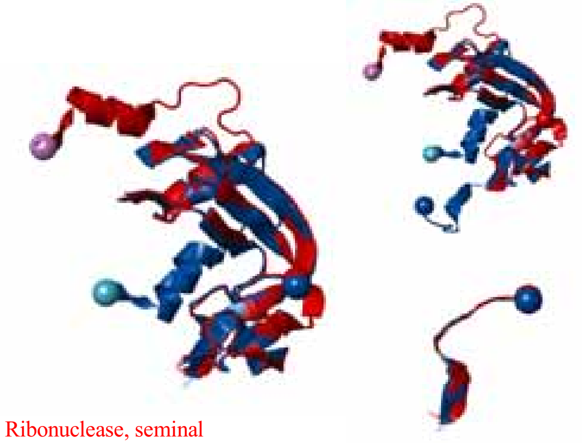 Ribonuclease, seminal Ribonuclease A
	1js0A (124)	111-115 (5)	112-113 (2)	112-113 (2)						
760	1r5cA (124)	15-23 (9)	16-22 (7)	16-22 (7)	N	0.59	0.51	100.00% (122/122)	98.36% (120/122)	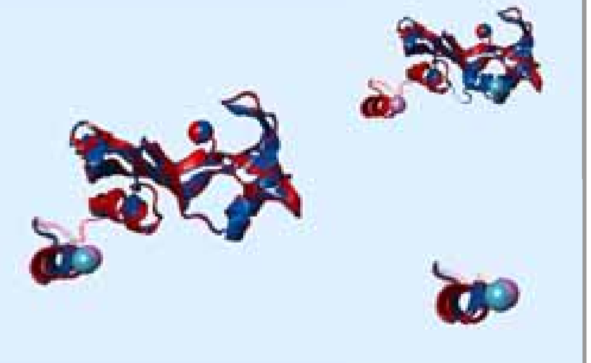 Ribonuclease, seminal Ribonuclease, seminal
	1r3mB (122)	15-23 (9)	16-22 (7)	16-22 (7)						
761	1r5cA (124)	15-22 (8)	15-22 (8)	15-22 (8)	N	0.90	0.92	99.19% (123/124)	80.65% (100/124)	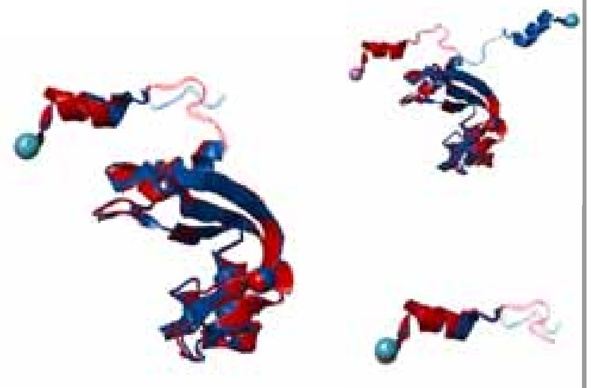 Ribonuclease, seminal Ribonuclease A
	1a2wA (124)	15-22 (8)	15-22 (8)	15-22 (8)						

762	1r5cA (124)	15-24 (10)	15-23 (9)	15-23 (9)	N	0.87	1.00	98.39% (122/124)	71.77% (89/124)	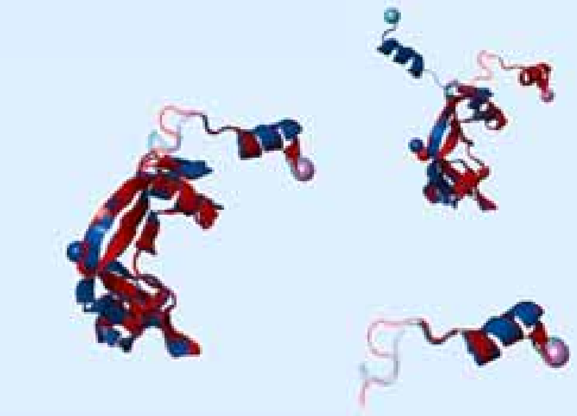 Ribonuclease, seminal
	1h8xA (125)	115-124 (10)	115-123 (9)	15-23 (9)						Ribonuclease 1
763	1r3mB (122)	15-22 (8)	16-22 (7)	16-22 (7)	N	0.61	0.53	100.00% (122/122)	99.18% (121/122)	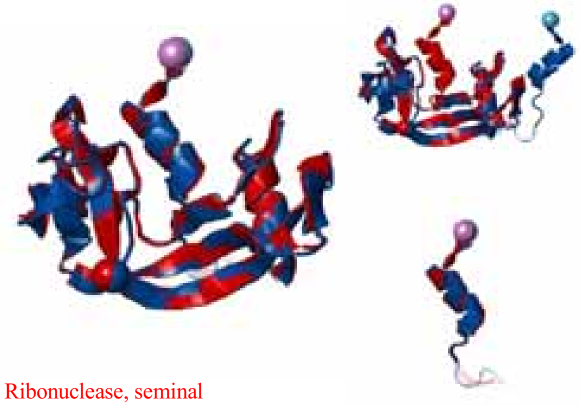 Ribonuclease, seminal
	1r5dA (124)	15-22 (8)	16-22 (7)	16-22 (7)						Ribonuclease, seminal
764	1r3mB (122)	15-23 (9)	15-22 (8)	19-22 (4)	N	0.93	0.76	100.00% (122/122)	81.15% (99/122)	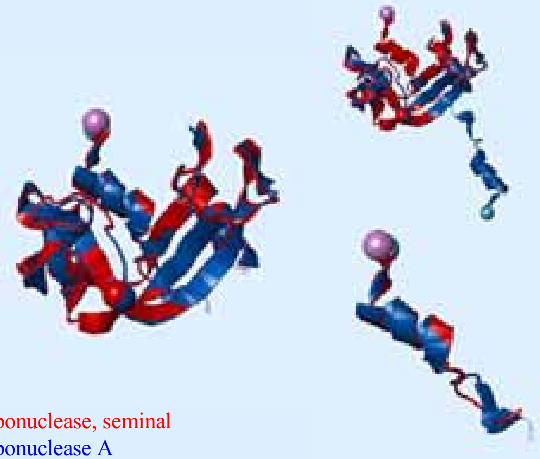 Ribonuclease, seminal
	1a2wA (124)	15-23 (9)	15-22 (8)	19-22 (4)						Ribonuclease A

765	1r3mB (122)	15-22 (8)	15-22 (8)	16-22 (7)	N	0.56	0.61	100.00% (122/122)	99.18% (121/122)	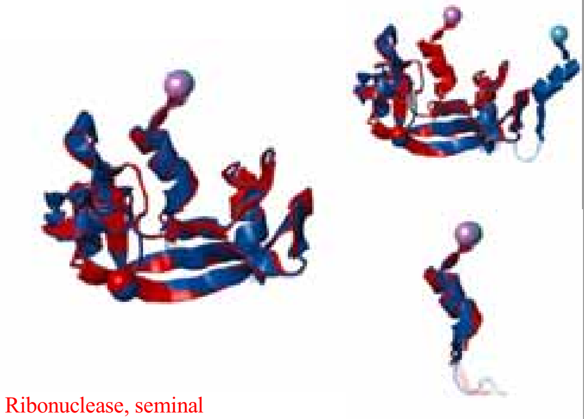 Ribonuclease, seminal<br>Protein (ribonuclease, seminal)
	11baA (124)	15-22 (8)	15-22 (8)	16-22 (7)						
766	1r3mB (122)	15-23 (9)	15-23 (9)	15-23 (9)	N	0.86	0.92	98.36% (120/122)	73.77% (90/122)	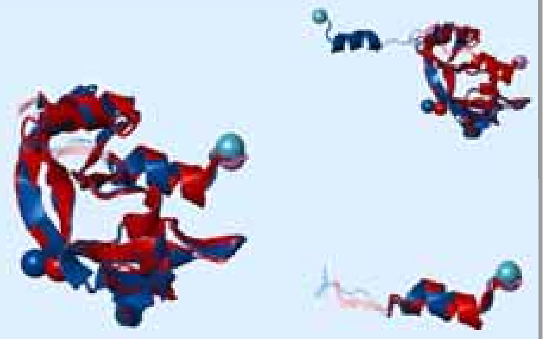 Ribonuclease, seminal<br>Ribonuclease 1
	1h8xA (125)	115-123 (9)	115-123 (9)	15-23 (9)						
767	1dz3A (123)	105-114 (10)	105-110 (6)	105-109 (5)	C	0.27	2.52	78.86% (97/123)	8.94% (11/123)	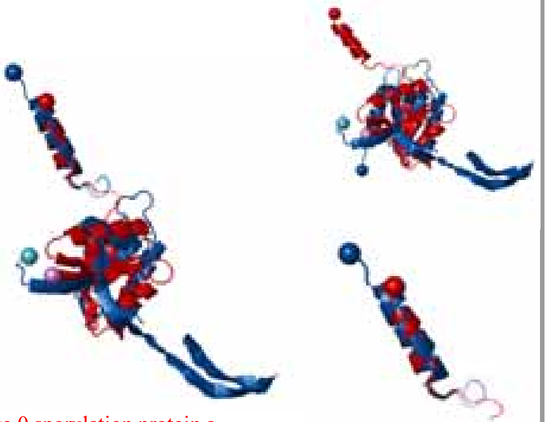 Stage 0 sporulation protein a<br>Ras-related protein Rab-27B
	2ieyA (164)	162-171 (10)	162-167 (6)	105-109 (5)						

768	1q10A (56)	36-55 (20)	37-50 (14)	38-41 (4)	C	0.54	2.28	98.21% (55/56)	5.36% (3/56)	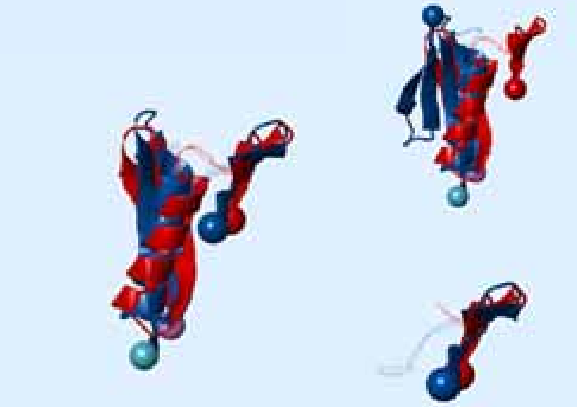 Immunoglobulin G binding protein G<br>Protein L
	1k50A (63)	39-61 (23)	40-56 (17)	38-41 (4)						
769	1q10A (56)	36-55 (20)	37-54 (18)	38-41 (4)	C	0.49	2.18	98.21% (55/56)	5.36% (3/56)	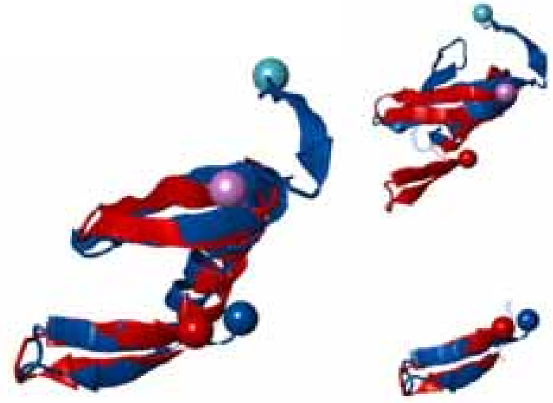 Immunoglobulin G binding protein G<br>Protein L
	1k53A (72)	39-61 (23)	40-60 (21)	38-41 (4)						
770	1q10A (56)	36-55 (20)	37-43 (7)	38-42 (5)	C	0.50	2.15	98.21% (55/56)	16.07% (9/56)	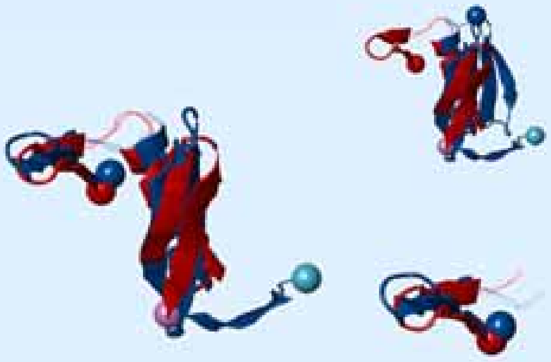 Immunoglobulin G binding protein G<br>Protein L
	1k52A (72)	39-63 (25)	40-47 (8)	38-42 (5)						

771	1q10A (56)	33-43 (11)	33-42 (10)	34-43 (10)	C	0.29	2.91	87.50% (49/56)	1.79% (1/56)	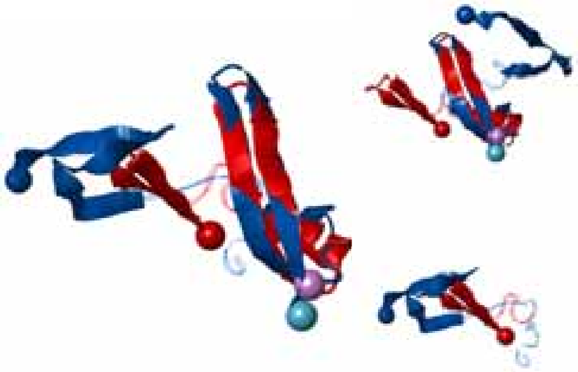<br>Immunoglobulin G binding protein G<br>Hypothetical protein
	2hj1A (77)	46-63 (18)	46-62 (17)	34-43 (10)						
772	2rlzA (85)	3-16 (14)	3-16 (14)	11-14 (4)	N	0.27	1.92	62.35% (53/85)	27.06% (23/85)	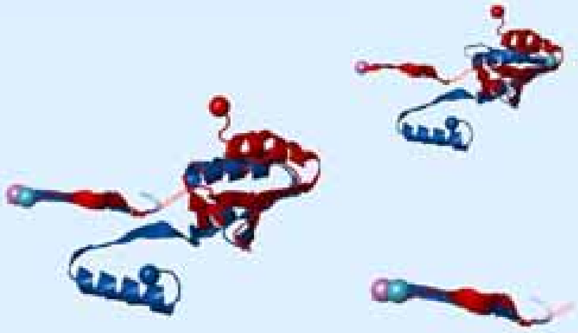<br>HPr-like protein crh<br>Phosphocarrier protein HPr
	1y50A (87)	3-16 (14)	3-16 (14)	11-14 (4)						
773	2rlzA (85)	50-56 (7)	51-56 (6)	52-55 (4)	C	0.50	2.00	85.88% (73/85)	35.29% (30/85)	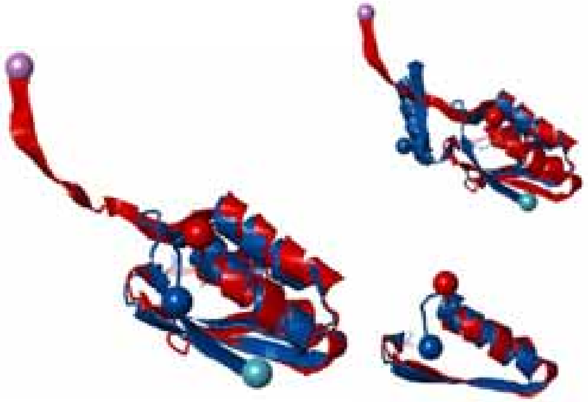<br>HPr-like protein crh<br>Phosphocarrier protein HPr
	1y50A (87)	50-56 (7)	51-56 (6)	52-55 (4)						

774	1wwaX (105)	378-381 (4)	378-381 (4)	379-379 (1)	C	0.90	0.88	100.00% (105/105)	91.43% (96/105)	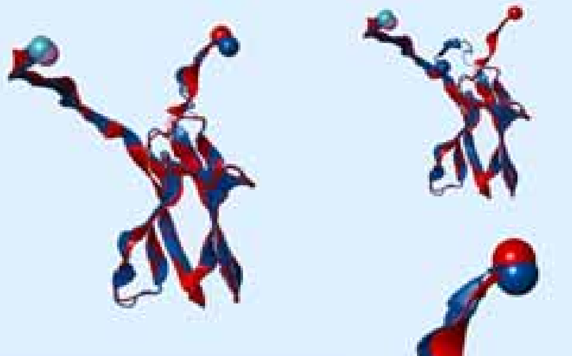 Protein (nerve growth factor receptor TRKA) HIGH affinity nerve growth factor receptor
	1he7A (107)	378-383 (6)	378-383 (6)	379-379 (1)						
775	1wwcA (105)	393-399 (7)	395-399 (5)	396-396 (1)	C	0.56	1.51	84.76% (89/105)	36.19% (38/105)	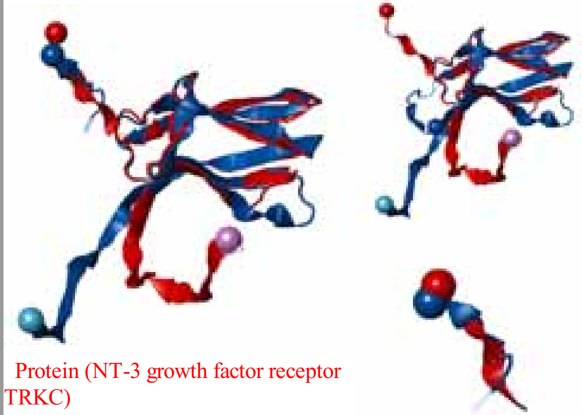 Protein (NT-3 growth factor receptor TRKC) HIGH affinity nerve growth factor receptor
	1he7A (107)	376-383 (8)	378-383 (6)	396-396 (1)						
776	3bcpA (124)	15-25 (11)	15-25 (11)	15-22 (8)	N	0.90	0.97	100.00% (124/124)	98.39% (122/124)	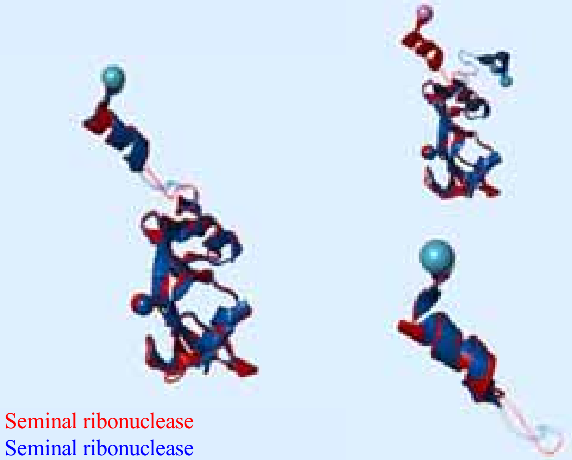 Seminal ribonuclease Seminal ribonuclease
	3bcoA (124)	15-25 (11)	15-25 (11)	15-22 (8)						

777	3bcpA (124)	15-25 (11)	15-24 (10)	15-22 (8)	N	0.91	0.72	100.00% (119/119)	98.32% (117/119)	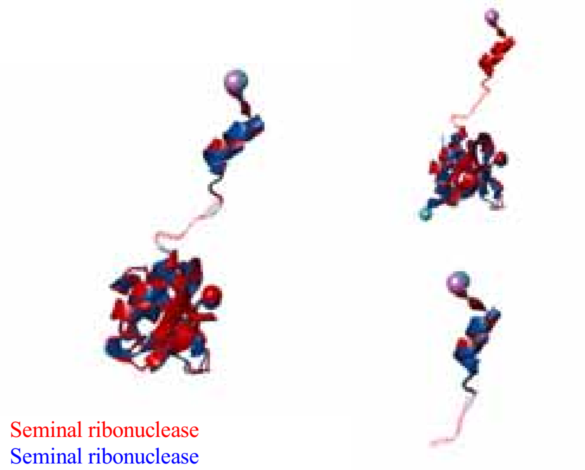
	3bcmA (119)	15-25 (11)	15-24 (10)	15-22 (8)						
778	3bcpA (124)	15-25 (11)	15-25 (11)	16-22 (7)	N	0.73	1.03	90.32% (112/124)	74.19% (92/124)	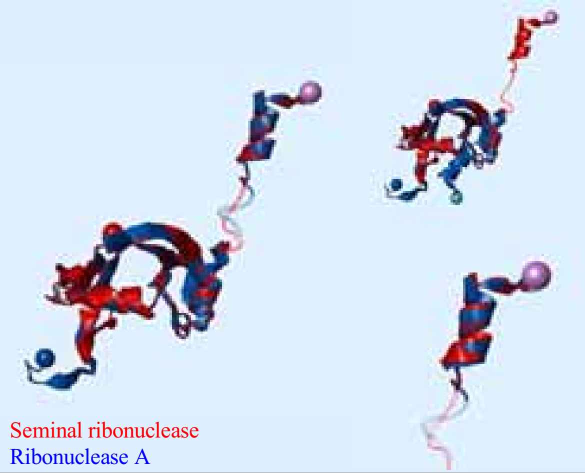
	1js0A (124)	15-25 (11)	15-25 (11)	16-22 (7)						
779	3bcpA (124)	108-116 (9)	112-113 (2)	112-113 (2)	C	0.54	0.91	83.87% (104/124)	69.35% (86/124)	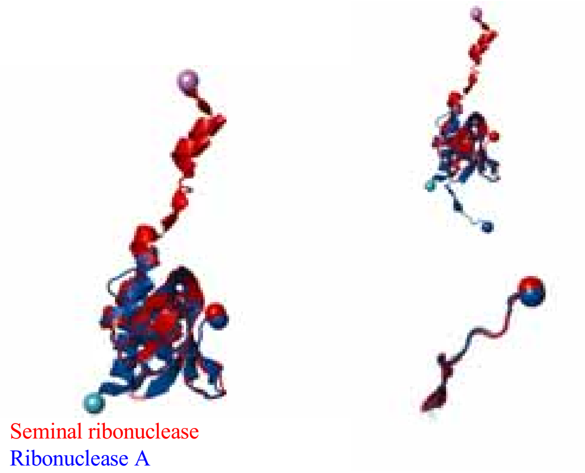
	1js0A (124)	108-116 (9)	112-113 (2)	112-113 (2)						

780	3bcpA (124)	15-25 (11)	15-24 (10)	16-22 (7)	N	0.91	0.85	100.00% (122/122)	95.90% (117/122)	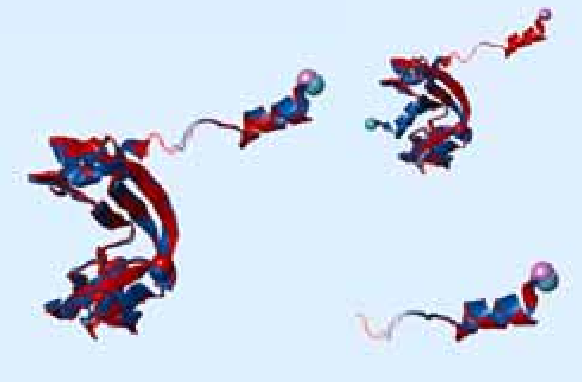 Seminal ribonuclease<br>Ribonuclease, seminal
	1r3mB (122)	15-25 (11)	15-24 (10)	16-22 (7)						
781	1js0A (124)	15-22 (8)	15-22 (8)	15-22 (8)	N	0.43	0.78	90.32% (112/124)	72.58% (90/124)	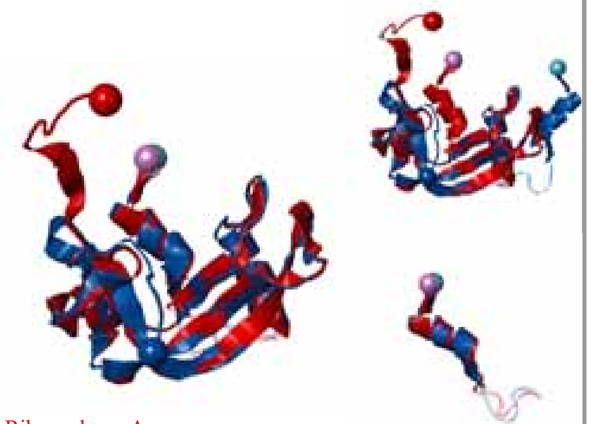 Ribonuclease A<br>Protein (bovine seminal ribonuclease)
	11bgA (124)	15-22 (8)	15-22 (8)	15-22 (8)						
782	1js0A (124)	112-115 (4)	112-113 (2)	112-113 (2)	C	0.56	0.74	83.87% (104/124)	69.35% (86/124)	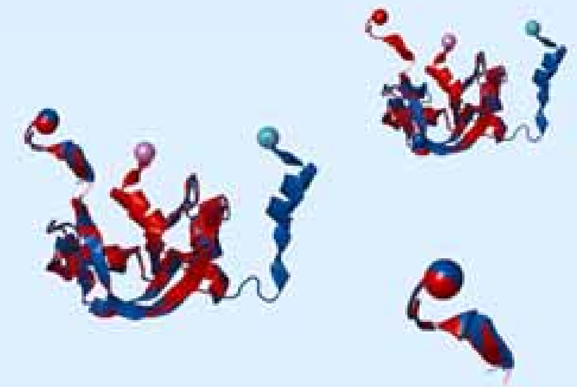 Ribonuclease A<br>Protein (bovine seminal ribonuclease)
	11bgA (124)	112-115 (4)	112-113 (2)	112-113 (2)						

783	1js0A (124)	15-22 (8)	15-22 (8)	15-22 (8)	N	0.73	0.78	90.32% (112/124)	72.58% (90/124)	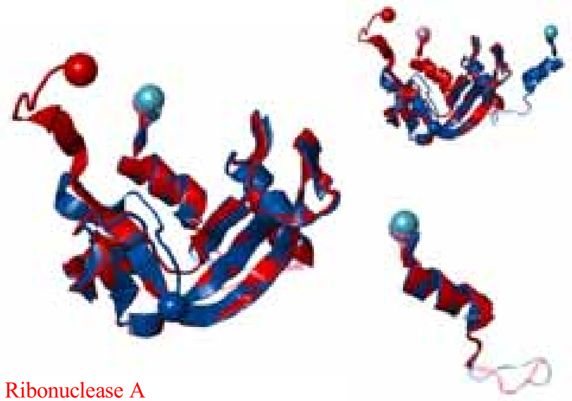
	1tq9A (124)	15-22 (8)	15-22 (8)	15-22 (8)						Ribonuclease A<br>Ribonuclease, seminal
784	1js0A (124)	112-113 (2)	112-113 (2)	112-113 (2)	C	0.57	0.66	83.87% (104/124)	69.35% (86/124)	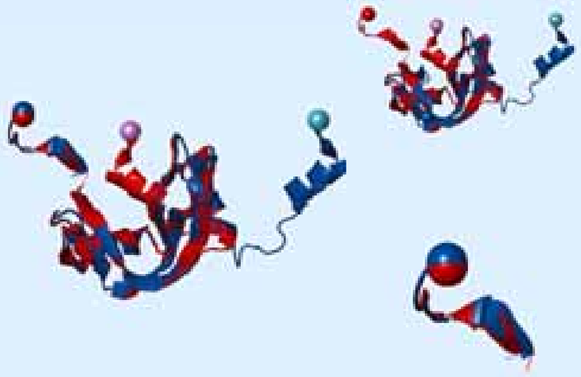
	1tq9A (124)	112-113 (2)	112-113 (2)	112-113 (2)						Ribonuclease A<br>Ribonuclease, seminal
785	1js0A (124)	111-113 (3)	112-113 (2)	112-113 (2)	C	0.76	0.79	100.00% (122/122)	81.15% (99/122)	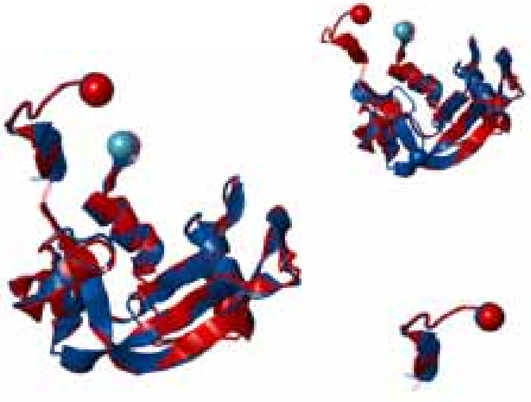
	1r3mB (122)	111-113 (3)	112-113 (2)	112-113 (2)						Ribonuclease A<br>Ribonuclease, seminal

786	1js0A (124)	15-22 (8)	15-22 (8)	15-22 (8)	N	0.44	0.91	90.32% (112/124)	72.58% (90/124)	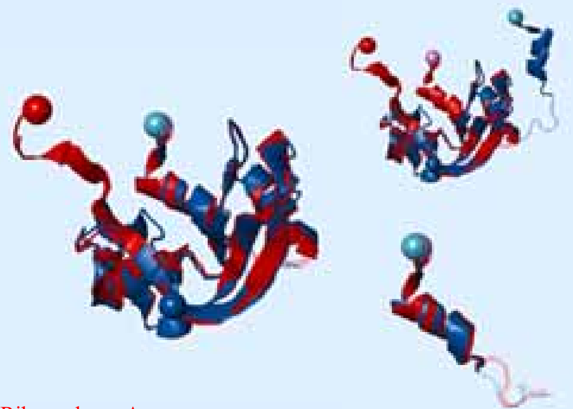 Ribonuclease A<br>Ribonuclease, seminal
	1r5dA (124)	15-22 (8)	15-22 (8)	15-22 (8)						
787	1js0A (124)	112-113 (2)	112-113 (2)	112-113 (2)	C	0.53	0.70	83.06% (103/124)	68.55% (85/124)	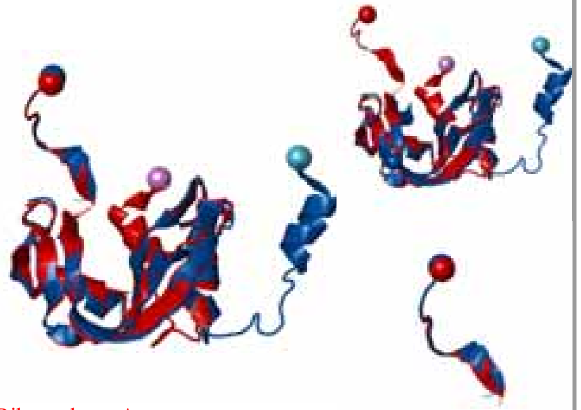 Ribonuclease A<br>Ribonuclease, seminal
	1r5dA (124)	112-113 (2)	112-113 (2)	112-113 (2)						
788	1js0A (124)	15-22 (8)	15-22 (8)	19-22 (4)	N	0.41	0.76	90.32% (112/124)	72.58% (90/124)	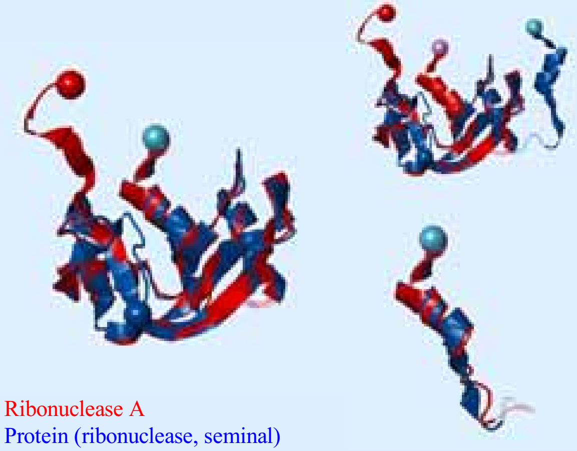 Ribonuclease A<br>Protein (ribonuclease, seminal)
	11baA (124)	15-22 (8)	15-22 (8)	19-22 (4)						

789	1js0A (124)	111-115 (5)	112-113 (2)	112-113 (2)	C	0.57	0.50	83.06% (103/124)	68.55% (85/124)	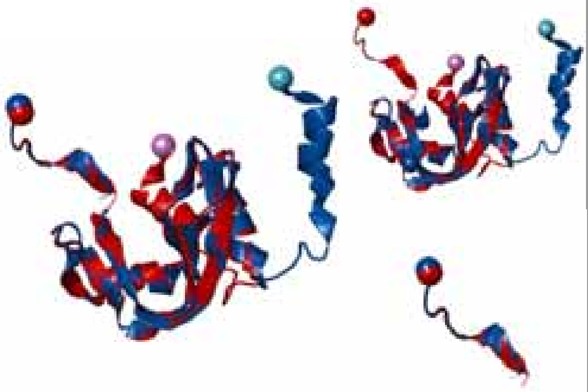<br>Ribonuclease A<br>Protein (ribonuclease, seminal)
	11baA (124)	111-115 (5)	112-113 (2)	112-113 (2)						
790	1r5dA (124)	15-22 (8)	15-22 (8)	15-22 (8)	N	0.90	0.92	99.19% (123/124)	80.65% (100/124)	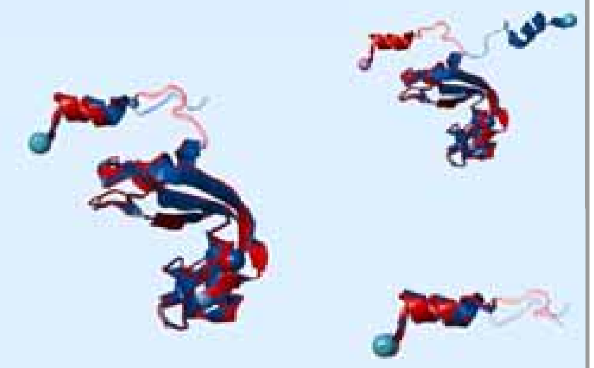<br>Ribonuclease, seminal<br>Ribonuclease A
	1a2wA (124)	15-22 (8)	15-22 (8)	15-22 (8)						
791	1r5dA (124)	15-24 (10)	15-23 (9)	15-23 (9)	N	0.86	1.01	98.39% (122/124)	71.77% (89/124)	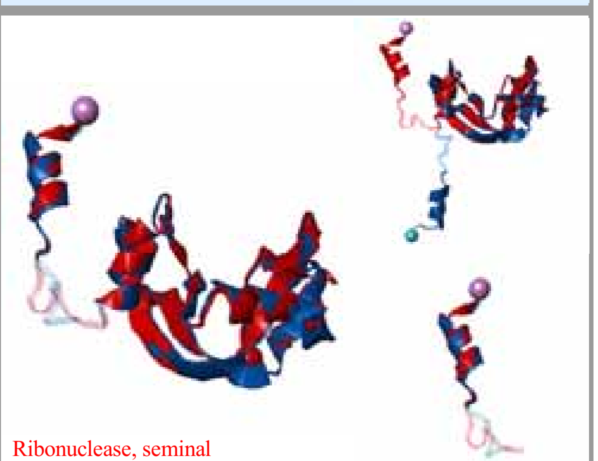<br>Ribonuclease, seminal<br>Ribonuclease 1
	1h8xA (125)	115-124 (10)	115-123 (9)	15-23 (9)						

792	1g85A (159)	121-123 (3)	121-123 (3)	121-123 (3)	C	0.76	0.88	97.32% (145/149)	92.62% (138/149)	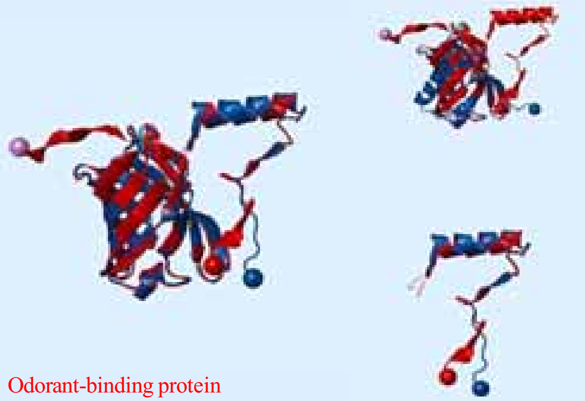 Odorant-binding protein
	2hlvA (149)	121-123 (3)	121-123 (3)	121-123 (3)						Odorant-binding protein
793	1oy0A (248)	217-254 (38)	220-254 (35)	230-254 (25)	C	0.30	2.26	83.87% (208/248)	15.32% (38/248)	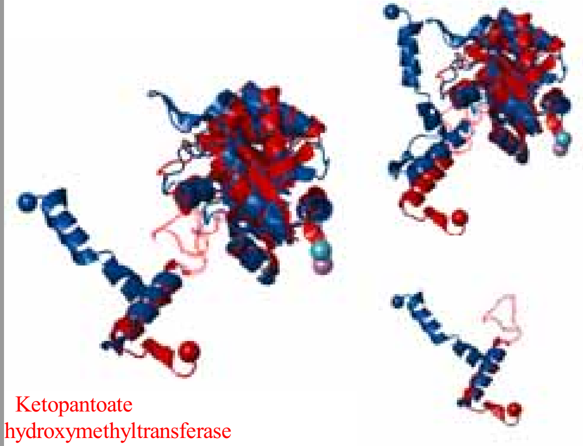 Ketopantoate hydroxymethyltransferase
	3b8iA (283)	207-237 (31)	210-237 (28)	230-254 (25)						PA4872 oxaloacetate decarboxylase
794	1dktB (71)	60-74 (15)	60-65 (6)	60-65 (6)	C	0.79	1.18	100.00% (71/71)	84.51% (60/71)	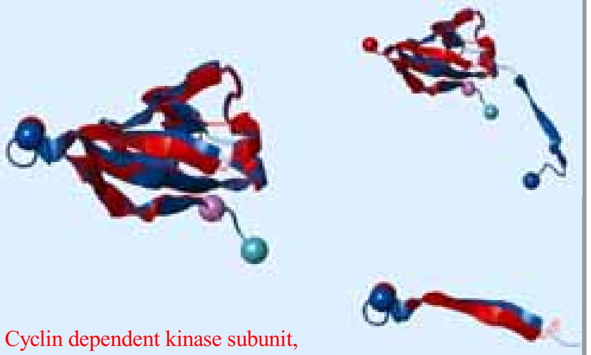 Cyclin dependent kinase subunit, TYPE 1
	1cksB (78)	60-74 (15)	60-65 (6)	60-65 (6)						Cyclin-dependent kinase subunit, TYPE 2

795	1dktB (71)	56-65 (10)	56-63 (8)	57-63 (7)	C	0.50	1.31	95.77% (68/71)	53.52% (38/71)	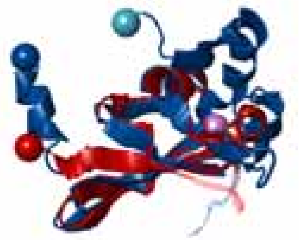 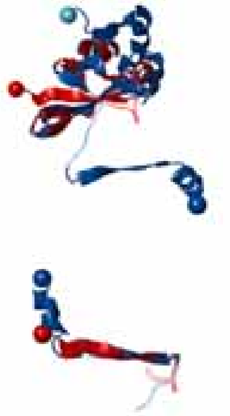 Cyclin dependent kinase subunit, TYPE 1
	1qb3C (109)	487-496 (10)	487-494 (8)	57-63 (7)						Cyclin-dependent kinases regulatory subunit
796	1buhB (70)	59-65 (7)	59-65 (7)	60-65 (6)	C	0.79	1.11	100.00% (70/70)	84.29% (59/70)	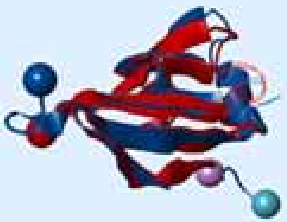 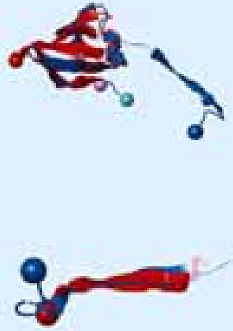 Protein (ckshs1 human)
	1cksB (78)	59-65 (7)	59-65 (7)	60-65 (6)						Cyclin-dependent kinase subunit, TYPE 2
797	1buhB (70)	56-64 (9)	57-64 (8)	57-63 (7)	C	0.49	1.59	98.57% (69/70)	55.71% (39/70)	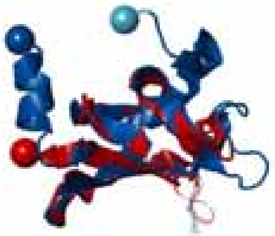 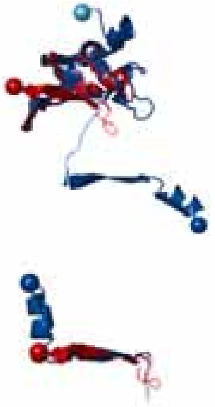 Protein (ckshs1 human)
	1qb3C (109)	487-495 (9)	488-495 (8)	57-63 (7)						Cyclin-dependent kinases regulatory subunit

798	1dktA (72)	60-70 (11)	60-66 (7)	61-65 (5)	C	0.82	1.09	100.00% (72/72)	83.33% (60/72)	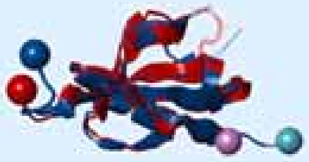 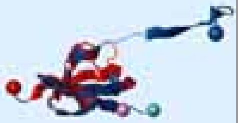 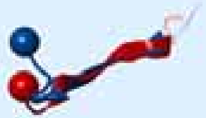 Cyclin dependent kinase subunit, TYPE 1 Cyclin-dependent kinase subunit, TYPE 2
	1cksB (78)	60-70 (11)	60-66 (7)	61-65 (5)						
799	1dktA (72)	56-64 (9)	57-63 (7)	57-63 (7)	C	0.47	1.58	95.83% (69/72)	54.17% (39/72)	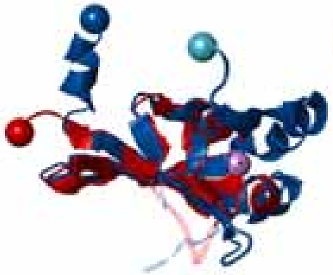 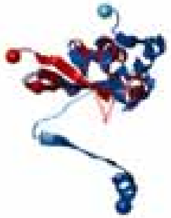 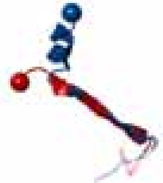 Cyclin dependent kinase subunit, TYPE 1 Cyclin-dependent kinases regulatory subunit
	1qb3C (109)	487-495 (9)	488-494 (7)	57-63 (7)						
800	2astC (69)	3060-3065 (6)	3060-3065 (6)	3060-3065 (6)	C	0.79	1.01	100.00% (69/69)	84.06% (58/69)	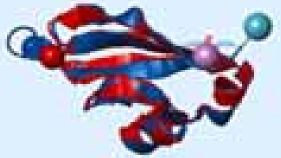 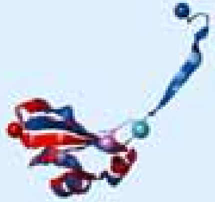 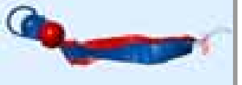 Cyclin-dependent kinases regulatory subunit 1 Cyclin-dependent kinase subunit, TYPE 2
	1cksB (78)	60-65 (6)	60-65 (6)	3060-3065 (6)						

801	2astC<br>(69)	3056-3063 (8)	3057-3063 (7)	3057-3063 (7)	C	0.48	1.62	98.55%<br>(68/69)	56.52%<br>(39/69)	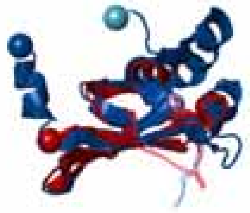 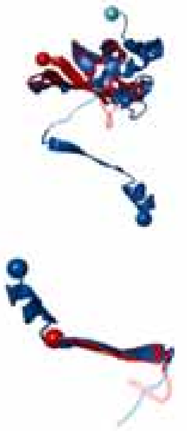<br>Cyclin-dependent kinases regulatory subunit 1<br>Cyclin-dependent kinases regulatory subunit
	1qb3C<br>(109)	487-494 (8)	488-494 (7)	3057-3063 (7)						
802	2hdxA<br>(108)	587-593 (7)	587-593 (7)	588-593 (6)	C	0.57	2.00	94.90%<br>(93/98)	23.47%<br>(23/98)	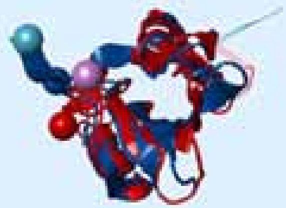 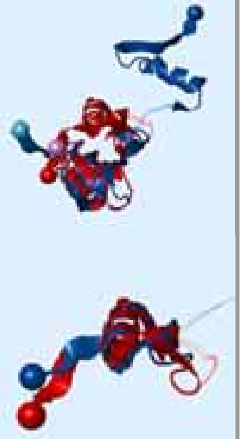<br>SH2-B PH domain containing signaling mediator<br>Growth receptor binding protein 2
	2h46E<br>(98)	118-124 (7)	118-124 (7)	588-593 (6)						
803	2hdxA<br>(108)	568-593 (26)	579-593 (15)	588-593 (6)	C	0.61	1.82	96.88%<br>(93/96)	21.88%<br>(21/96)	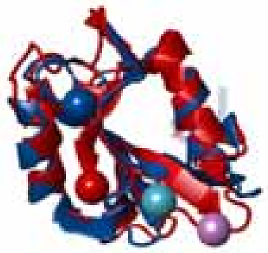 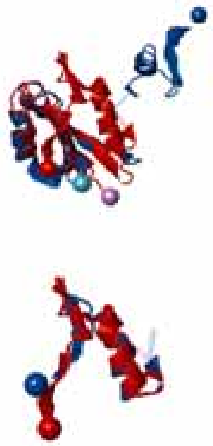<br>SH2-B PH domain containing signaling mediator<br>Growth factor receptor-bound protein 2
	2h5kB<br>(96)	99-124 (26)	110-124 (15)	588-593 (6)						

804	2hdxA (108)	551-596 (46)	588-593 (6)	588-593 (6)	C	0.45	1.83	96.77% (90/93)	22.58% (21/93)	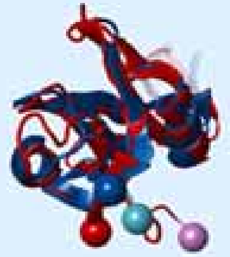 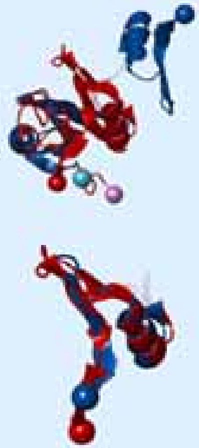 SH2-B PH domain containing signaling mediator Growth factor receptor-bound protein 2
	2aobB (93)	82-127 (46)	119-124 (6)	588-593 (6)						
805	2hdxA (108)	556-593 (38)	588-593 (6)	588-593 (6)	C	0.48	1.68	95.83% (92/96)	21.88% (21/96)	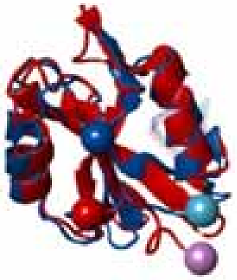 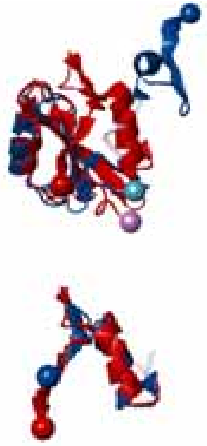 SH2-B PH domain containing signaling mediator Growth factor receptor-bound protein 2
	2aobA (96)	87-124 (38)	119-124 (6)	588-593 (6)						
806	2hdxA (108)	560-593 (34)	588-593 (6)	588-593 (6)	C	0.48	1.62	96.77% (90/93)	22.58% (21/93)	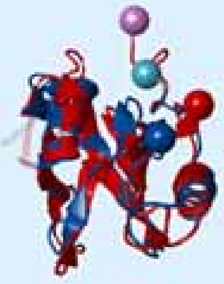 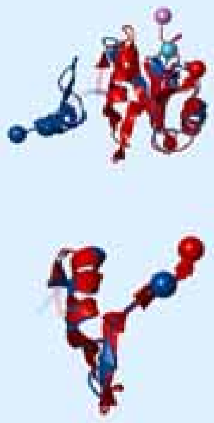 SH2-B PH domain containing signaling mediator Growth factor receptor-bound protein 2
	2aoaA (93)	91-124 (34)	119-124 (6)	588-593 (6)						

807	1p13A (102)	223-232 (10)	223-232 (10)	229-232 (4)	C	0.63	1.74	96.94% (95/98)	29.59% (29/98)	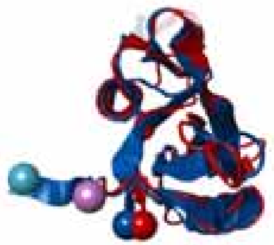 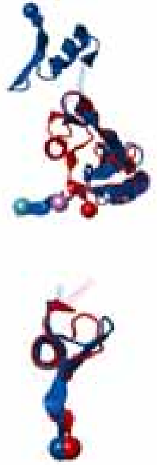 Proto-oncogene tyrosine-protein kinase Src<br>Growth receptor binding protein 2
	2h46E (98)	116-124 (9)	116-124 (9)	229-232 (4)						
808	1p13A (102)	219-232 (14)	223-232 (10)	228-232 (5)	C	0.66	1.65	97.92% (94/96)	29.17% (28/96)	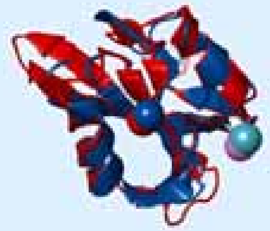 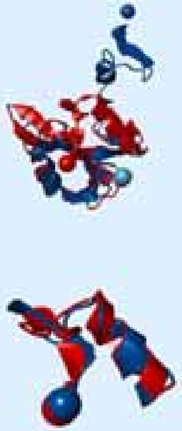 Proto-oncogene tyrosine-protein kinase Src<br>Growth factor receptor-bound protein 2
	2h5kB (96)	112-124 (13)	116-124 (9)	228-232 (5)						
809	1p13A (102)	228-232 (5)	229-232 (4)	229-232 (4)	C	0.66	1.28	97.85% (91/93)	32.26% (30/93)	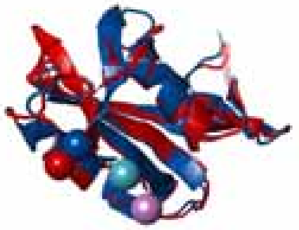 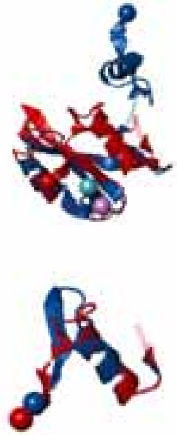 Proto-oncogene tyrosine-protein kinase Src<br>Growth factor receptor-bound protein 2
	2aobB (93)	121-124 (4)	122-124 (3)	229-232 (4)						

810	1p13A (102)	228-232 (5)	228-232 (5)	229-232 (4)	C	0.58	1.79	97.92% (94/96)	30.21% (29/96)	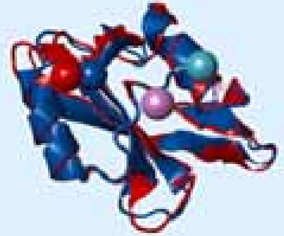 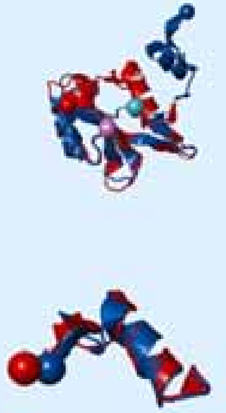 Proto-oncogene tyrosine-protein kinase Src<br>Growth factor receptor-bound protein 2
	2aobA (96)	121-124 (4)	121-124 (4)	229-232 (4)						
811	1p13A (102)	227-232 (6)	228-232 (5)	229-232 (4)	C	0.58	1.68	98.92% (92/93)	31.18% (29/93)	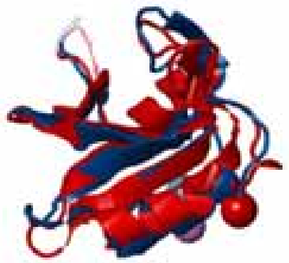 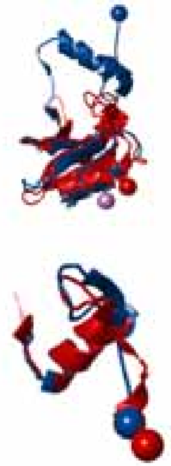 Proto-oncogene tyrosine-protein kinase Src<br>Growth factor receptor-bound protein 2
	2aoaA (93)	120-124 (5)	121-124 (4)	229-232 (4)						
812	1rjaA (100)	45-74 (30)	56-73 (18)	66-70 (5)	C	0.60	1.90	92.86% (91/98)	38.78% (38/98)	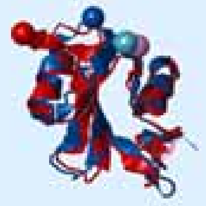 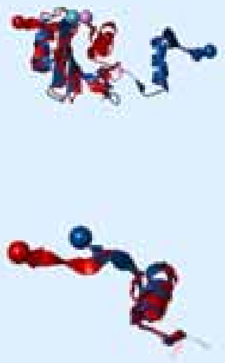 Tyrosine-protein kinase 6<br>Growth receptor binding protein 2
	2h46E (98)	100-128 (29)	111-127 (17)	66-70 (5)						

813	1rjaA (100)	56-76 (21)	56-69 (14)	66-70 (5)	C	0.62	1.90	94.79% (91/96)	39.58% (38/96)	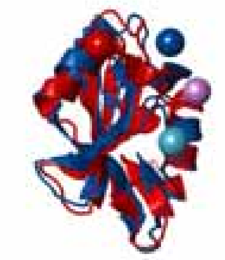 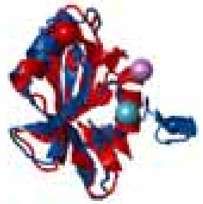 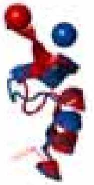 Tyrosine-protein kinase 6<br>Growth factor receptor-bound protein 2
	2h5kB (96)	111-130 (20)	111-123 (13)	66-70 (5)						
814	1rjaA (100)	45-75 (31)	57-70 (14)	67-70 (4)	C	0.42	1.84	94.62% (88/93)	39.78% (37/93)	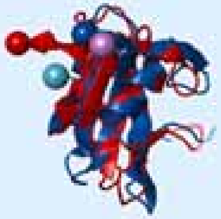 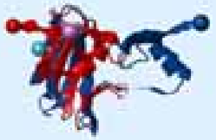 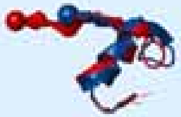 Tyrosine-protein kinase 6<br>Growth factor receptor-bound protein 2
	2aobB (93)	100-129 (30)	112-124 (13)	67-70 (4)						
815	1rjaA (100)	45-75 (31)	57-70 (14)	66-70 (5)	C	0.43	1.62	92.71% (89/96)	38.54% (37/96)	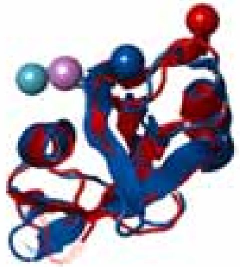 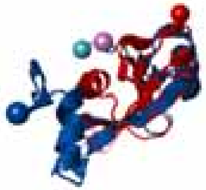 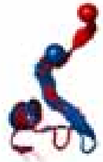 Tyrosine-protein kinase 6<br>Growth factor receptor-bound protein 2
	2aobA (96)	100-129 (30)	112-124 (13)	66-70 (5)						

816	1rjaA (100)	45-72 (28)	57-72 (16)	66-70 (5)	C	0.43	1.72	95.70% (89/93)	38.71% (36/93)	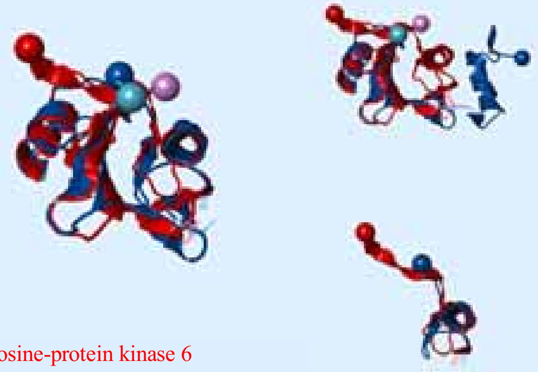 Tyrosine-protein kinase 6
	2aoaA (93)	100-126 (27)	112-126 (15)	66-70 (5)						Growth factor receptor-bound protein 2
817	1r1pB (100)	120-122 (3)	120-122 (3)	120-122 (3)	C	0.83	0.97	96.94% (95/98)	52.04% (51/98)	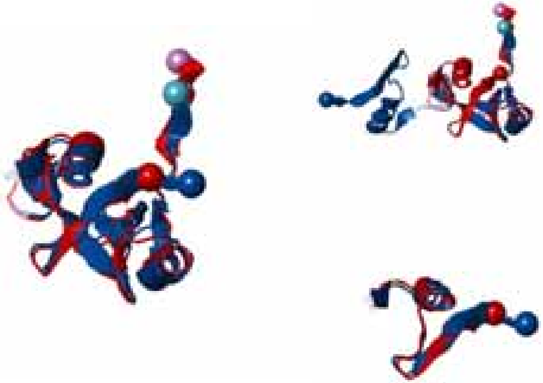 GRB2-related adaptor protein 2
	2h46E (98)	121-123 (3)	121-123 (3)	120-122 (3)						Growth receptor binding protein 2
818	1r1pB (100)	117-125 (9)	118-122 (5)	120-122 (3)	C	0.84	0.79	96.88% (93/96)	53.13% (51/96)	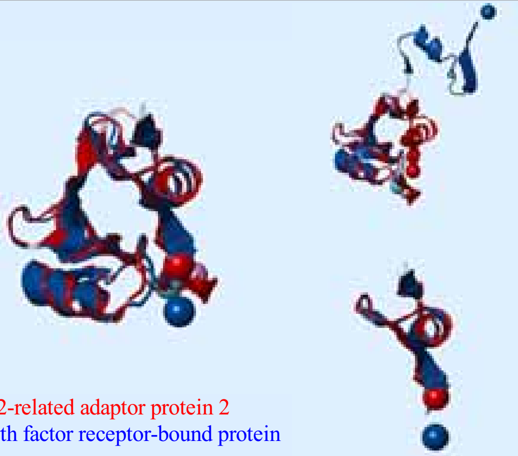 GRB2-related adaptor protein 2
	2h5kB (96)	118-126 (9)	119-123 (5)	120-122 (3)						Growth factor receptor-bound protein 2

819	1r1pB (100)	120-123 (4)	120-123 (4)	120-122 (3)	C	0.75	1.22	96.77% (90/93)	53.76% (50/93)	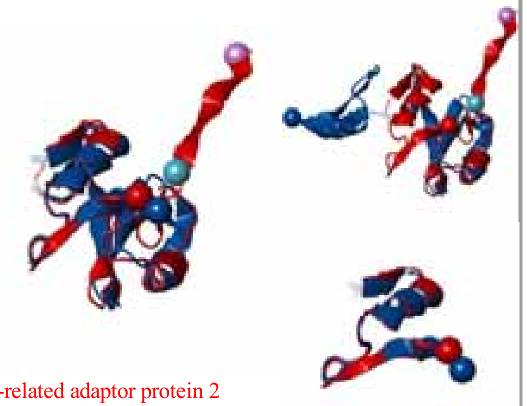 GRB2-related adaptor protein 2 Growth factor receptor-bound protein 2
	2aobB (93)	121-124 (4)	121-124 (4)	120-122 (3)						
820	1r1pB (100)	119-125 (7)	120-123 (4)	120-122 (3)	C	0.82	1.05	97.92% (94/96)	53.13% (51/96)	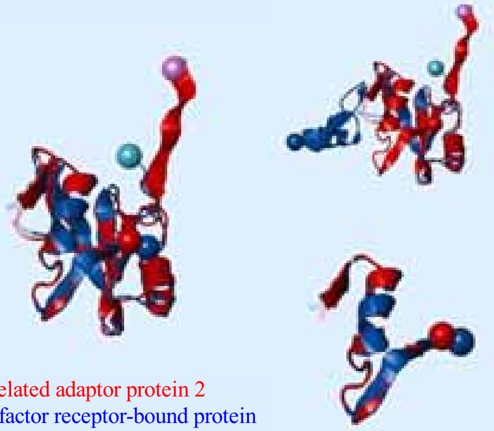 GRB2-related adaptor protein 2 Growth factor receptor-bound protein 2
	2aobA (96)	120-126 (7)	121-124 (4)	120-122 (3)						
821	1r1pB (100)	120-123 (4)	120-123 (4)	120-122 (3)	C	0.82	0.98	98.92% (92/93)	55.91% (52/93)	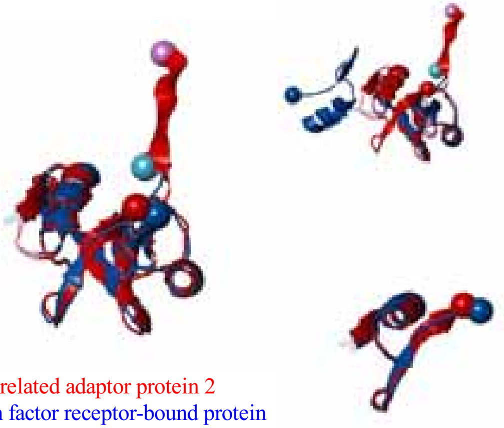 GRB2-related adaptor protein 2 Growth factor receptor-bound protein 2
	2aoaA (93)	121-124 (4)	121-124 (4)	120-122 (3)						

822	1fhsA (112)	61-76 (16)	61-74 (14)	70-73 (4)	C	0.45	1.73	97.92% (94/96)	90.63% (87/96)	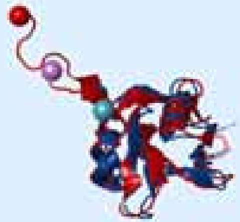 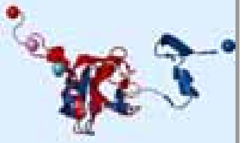 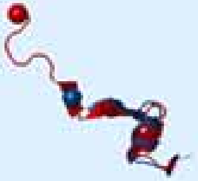 Growth factor receptor bound protein-2 Growth factor receptor-bound protein 2
	2h5kB (96)	112-127 (16)	112-125 (14)	70-73 (4)						
823	1fhsA (112)	57-76 (20)	58-74 (17)	69-72 (4)	C	0.29	1.50	98.92% (92/93)	95.70% (89/93)	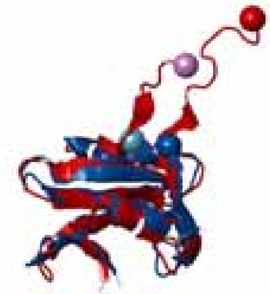 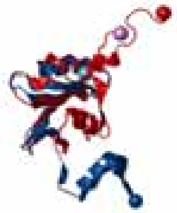 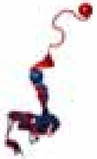 Growth factor receptor bound protein-2 Growth factor receptor-bound protein 2
	2aobB (93)	108-127 (20)	109-125 (17)	69-72 (4)						
824	1fhsA (112)	56-76 (21)	61-74 (14)	70-72 (3)	C	0.27	1.70	96.88% (93/96)	91.67% (88/96)	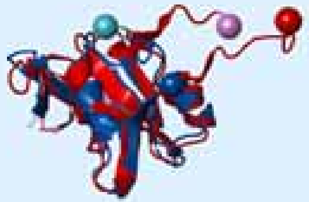 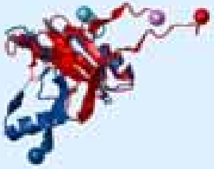 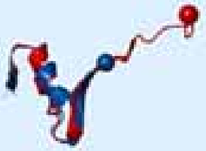 Growth factor receptor bound protein-2 Growth factor receptor-bound protein 2
	2aobA (96)	107-127 (21)	112-125 (14)	70-72 (3)						

825	1fhsA (112)	61-80 (20)	61-76 (16)	69-73 (5)	C	0.26	1.57	97.85% (91/93)	92.47% (86/93)	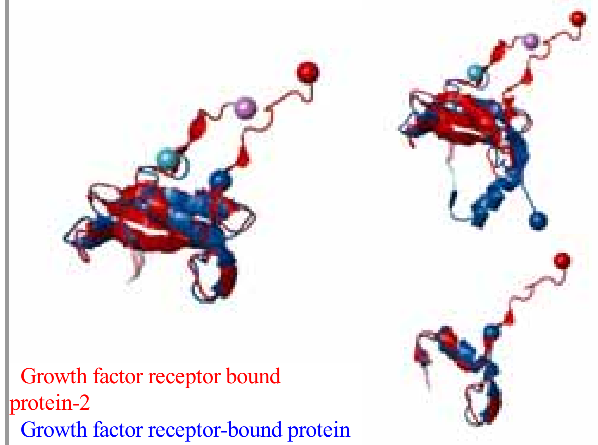 Growth factor receptor bound protein-2
	2aoaA (93)	112-131 (20)	112-127 (16)	69-73 (5)						Growth factor receptor-bound protein 2
826	2jzjA (116)	35-55 (21)	50-55 (6)	50-55 (6)	C	0.54	1.27	99.01% (100/101)	25.74% (26/101)	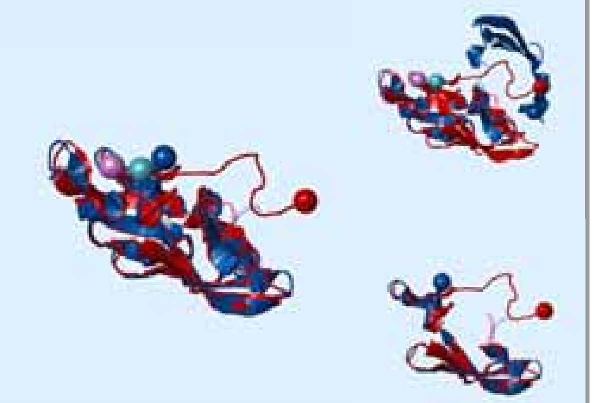 Cyanovirin-N homolog
	1l5eA (101)	34-54 (21)	49-54 (6)	50-55 (6)						Cyanovirin-N
827	2z21A (102)	49-54 (6)	49-54 (6)	50-54 (5)	N	0.91	0.79	99.01% (100/101)	94.06% (95/101)	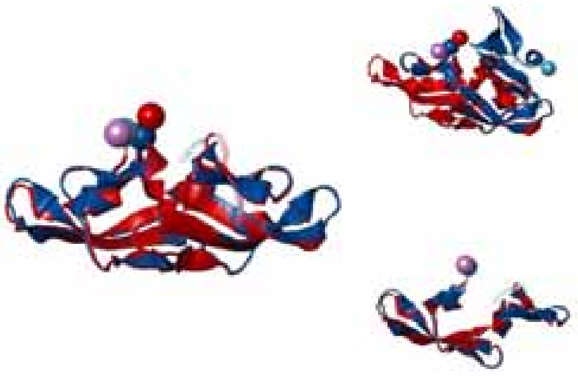 Cyanovirin-N
	1l5eA (101)	49-54 (6)	49-54 (6)	50-54 (5)						Cyanovirin-N

828	2z21A (102)	49-53 (5)	49-53 (5)	50-53 (4)	N	0.89	0.71	99.01% (100/101)	94.06% (95/101)	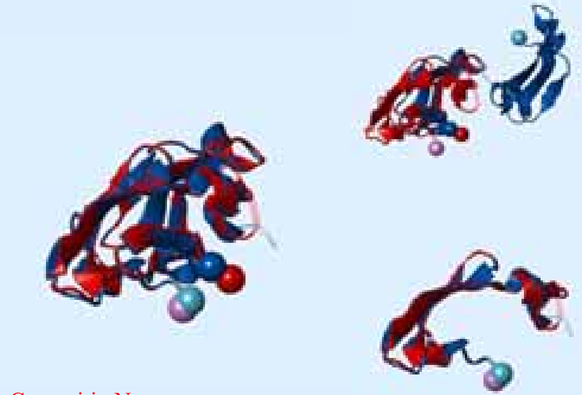 Cyanovirin-N Cyanovirin-N
	1lomA (101)	49-53 (5)	49-53 (5)	50-53 (4)						
829	2rdkA (101)	49-54 (6)	49-54 (6)	50-54 (5)	N	0.90	0.79	98.02% (99/101)	93.07% (94/101)	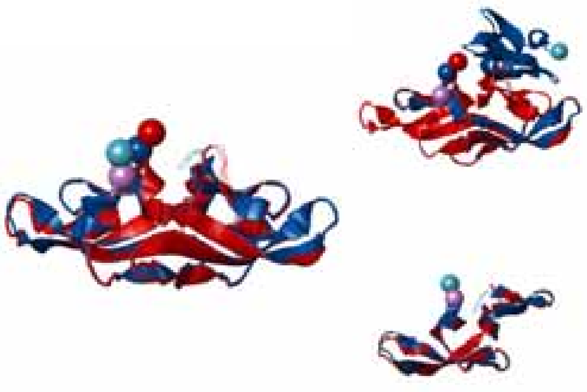 Cyanovirin-N Cyanovirin-N
	1l5eA (101)	49-54 (6)	49-54 (6)	50-54 (5)						
830	2rdkA (101)	49-53 (5)	49-53 (5)	50-53 (4)	N	0.83	0.69	98.02% (99/101)	93.07% (94/101)	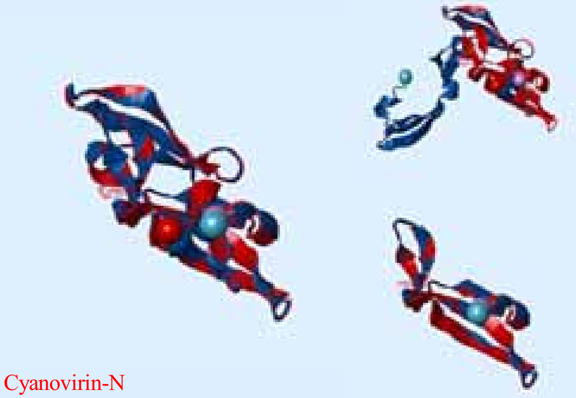 Cyanovirin-N Cyanovirin-N
	1lomA (101)	49-53 (5)	49-53 (5)	50-53 (4)						

831	2z21B (102)	49-56 (8)	49-54 (6)	50-54 (5)	N	0.90	0.74	98.02% (99/101)	93.07% (94/101)	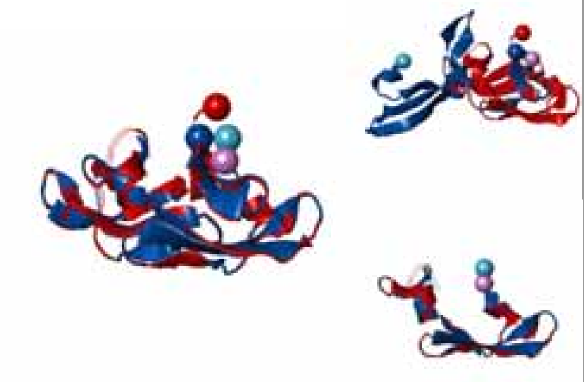 Cyanovirin-N<br>Cyanovirin-N
	1l5eA (101)	49-56 (8)	49-54 (6)	50-54 (5)						
832	2z21B (102)	49-53 (5)	49-53 (5)	50-53 (4)	N	0.87	0.65	98.02% (99/101)	93.07% (94/101)	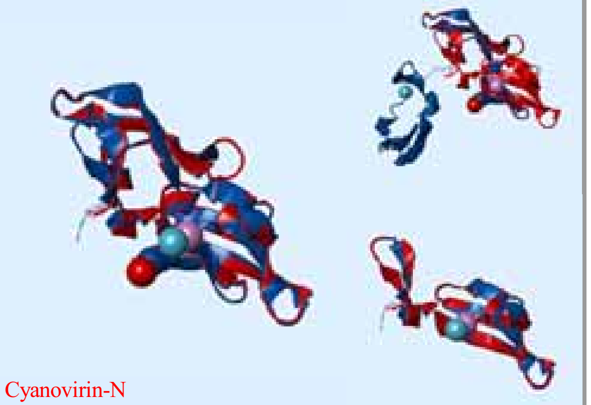 Cyanovirin-N<br>Cyanovirin-N
	1lomA (101)	49-53 (5)	49-53 (5)	50-53 (4)						
833	2jzkA (103)	48-70 (23)	49-55 (7)	51-55 (5)	N	0.71	1.43	94.06% (95/101)	25.74% (26/101)	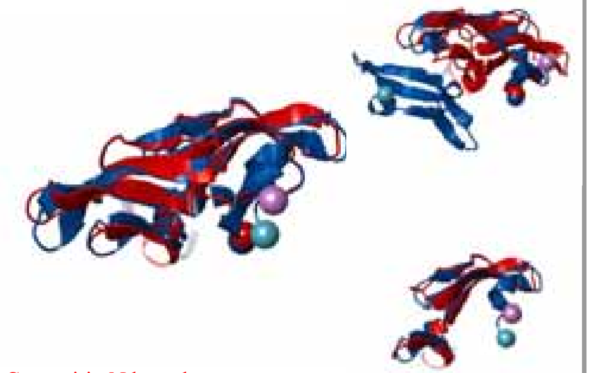 Cyanovirin-N homolog<br>Cyanovirin-N
	1l5eA (101)	47-68 (22)	48-54 (7)	51-55 (5)						

834	2rdkB (99)	49-57 (9)	49-54 (6)	50-54 (5)	N	0.91	0.73	98.99% (98/99)	93.94% (93/99)	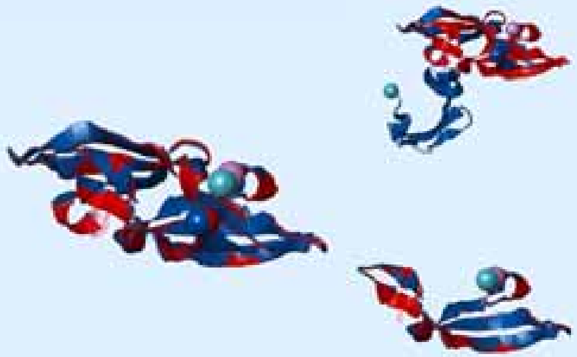 Cyanovirin-N
	1l5eA (101)	49-57 (9)	49-54 (6)	50-54 (5)						Cyanovirin-N
835	2rdkB (99)	49-53 (5)	49-53 (5)	50-53 (4)	N	0.90	0.64	98.99% (98/99)	93.94% (93/99)	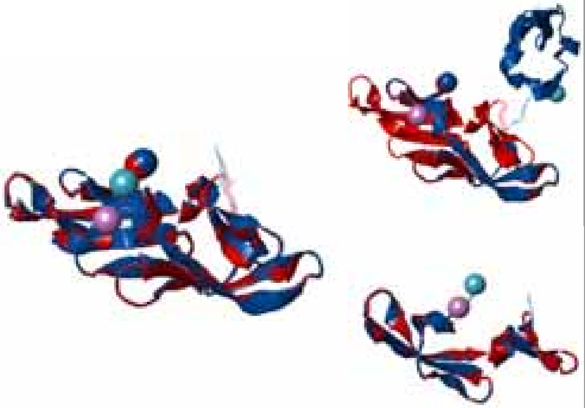 Cyanovirin-N
	1lomA (101)	49-53 (5)	49-53 (5)	50-53 (4)						Cyanovirin-N
836	2pysB (103)	49-57 (9)	49-54 (6)	50-54 (5)	N	0.89	0.73	98.02% (99/101)	93.07% (94/101)	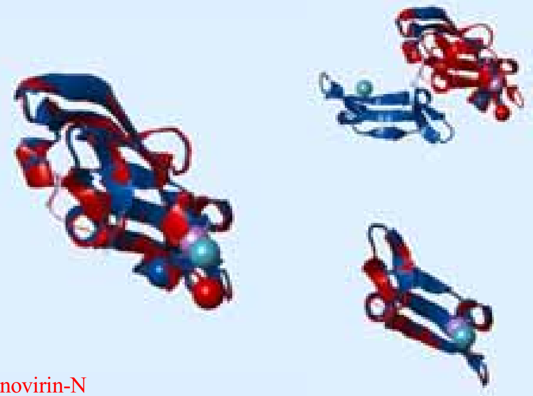 Cyanovirin-N
	1l5eA (101)	49-57 (9)	49-54 (6)	50-54 (5)						Cyanovirin-N

837	2pysB (103)	49-53 (5)	49-53 (5)	50-53 (4)	N	0.86	0.63	98.02% (99/101)	93.07% (94/101)	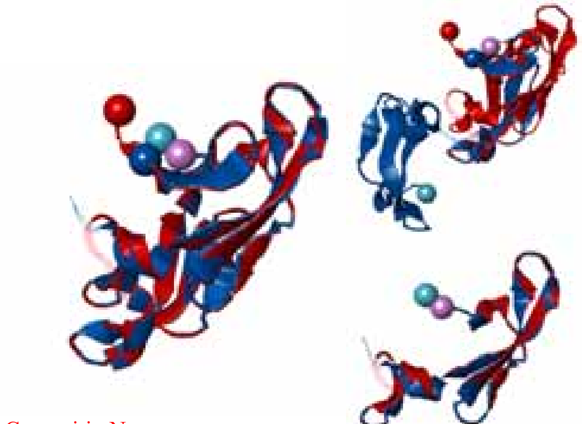
	1lomA (101)	49-53 (5)	49-53 (5)	50-53 (4)						Cyanovirin-N<br>Cyanovirin-N
838	2vq8A (128)	21-25 (5)	24-25 (2)	23-25 (3)	N	0.56	1.71	87.20% (109/125)	25.60% (32/125)	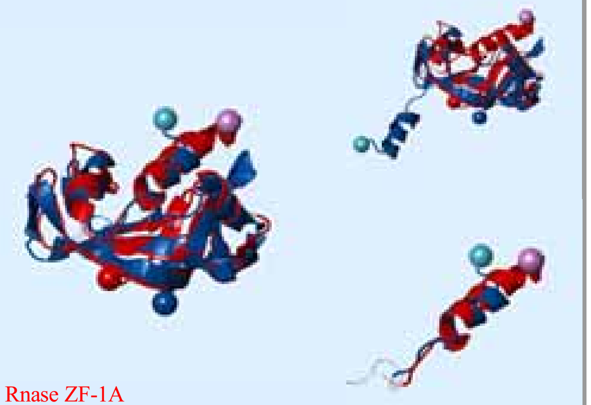
	1h8xA (125)	117-125 (9)	120-125 (6)	23-25 (3)						Rnase ZF-1A<br>Ribonuclease 1
839	1pu3A (105)	8-19 (12)	13-19 (7)	16-19 (4)	N	0.56	1.58	92.38% (97/105)	21.90% (23/105)	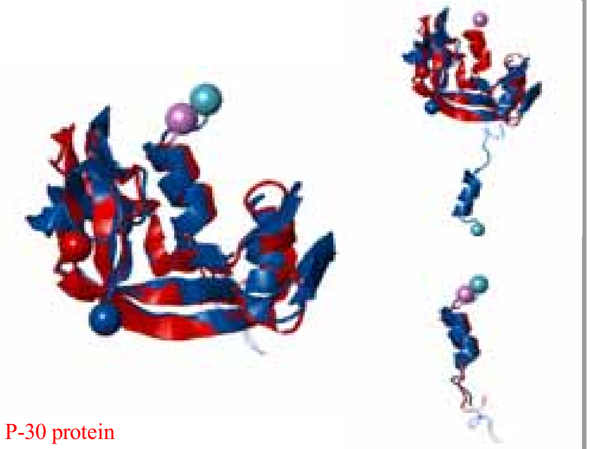
	1h8xA (125)	109-125 (17)	114-125 (12)	16-19 (4)						P-30 protein<br>Ribonuclease 1

840	1un4A (119)	14-24 (11)	15-24 (10)	17-24 (8)	N	0.65	1.48	92.44% (110/119)	34.45% (41/119)	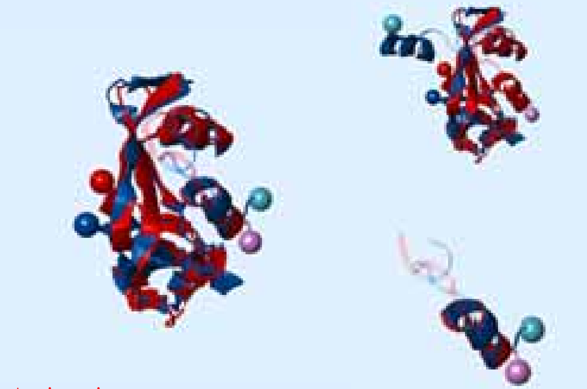 Angiogenin<br>Ribonuclease 1
	1h8xA (125)	113-124 (12)	114-124 (11)	17-24 (8)						
841	1rnfA (120)	16-24 (9)	16-23 (8)	16-23 (8)	N	0.73	1.37	95.83% (115/120)	41.67% (50/120)	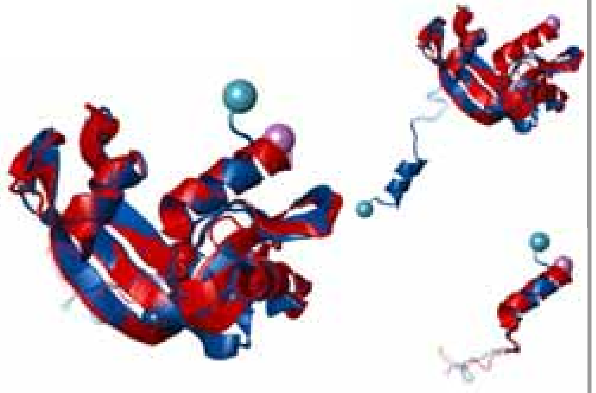 Protein (ribonuclease 4)<br>Ribonuclease 1
	1h8xA (125)	116-125 (10)	116-124 (9)	16-23 (8)						
842	2j4tA (119)	12-24 (13)	16-23 (8)	16-23 (8)	N	0.68	1.56	94.96% (113/119)	36.13% (43/119)	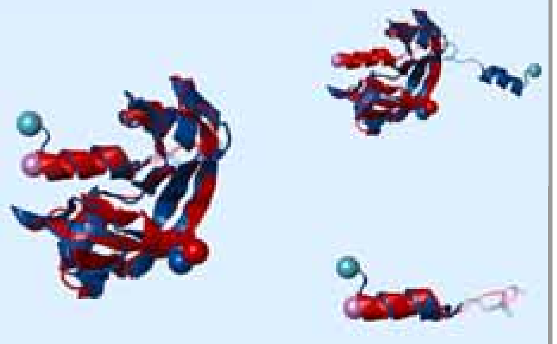 Angiogenin-4<br>Ribonuclease 1
	1h8xA (125)	112-125 (14)	116-124 (9)	16-23 (8)						

843	1b1eA (123)	13-24 (12)	17-24 (8)	17-24 (8)	N	0.63	1.63	91.06% (112/123)	32.52% (40/123)	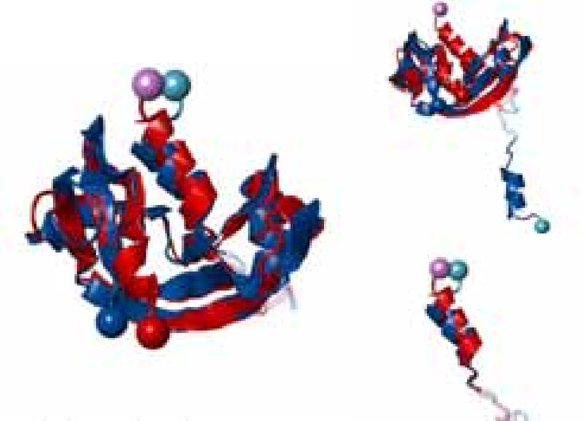 Hydrolase angiogenin<br>Ribonuclease 1
	1h8xA (125)	112-124 (13)	116-124 (9)	17-24 (8)						
844	2hkyA (129)	11-22 (12)	11-22 (12)	16-22 (7)	N	0.57	1.78	88.80% (111/125)	33.60% (42/125)	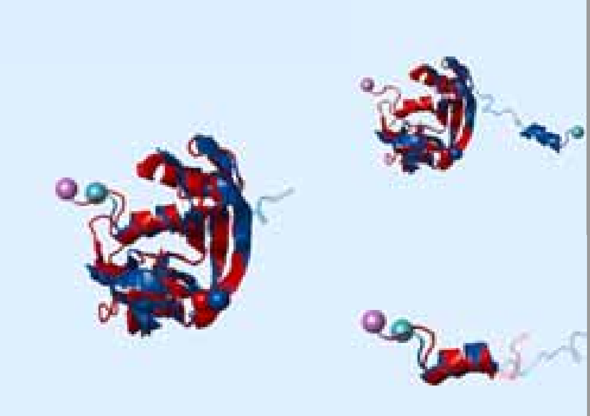 Ribonuclease 7<br>Ribonuclease 1
	1h8xA (125)	108-125 (18)	108-125 (18)	16-22 (7)						
845	1m58A (106)	12-19 (8)	12-19 (8)	15-19 (5)	N	0.50	2.07	93.40% (99/106)	23.58% (25/106)	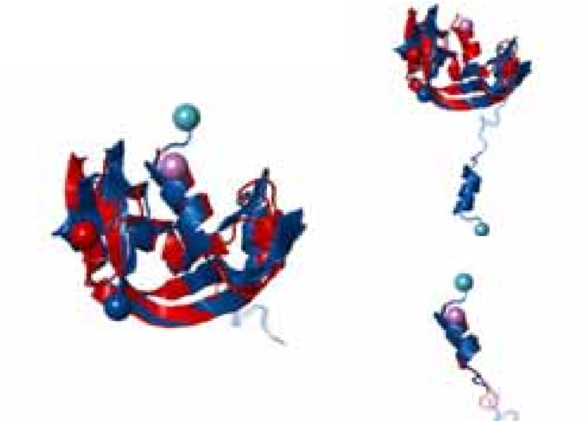 RC-rnase2 ribonuclease<br>Ribonuclease 1
	1h8xA (125)	113-125 (13)	113-125 (13)	15-19 (5)						

846	2e0oB (125)	16-24 (9)	16-24 (9)	17-24 (8)	N	0.85	0.95	96.80% (121/125)	86.40% (108/125)	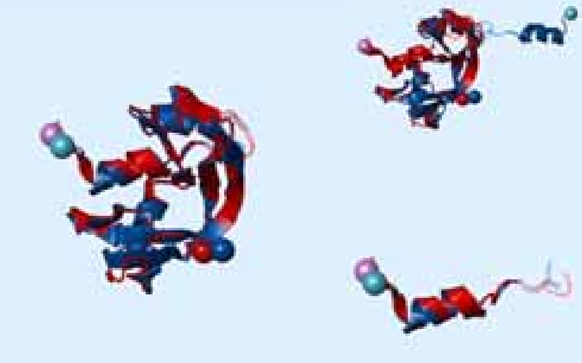 Ribonuclease<br>Ribonuclease 1
	1h8xA (125)	116-124 (9)	116-124 (9)	17-24 (8)						
847	1ymnA (124)	15-24 (10)	15-23 (9)	17-23 (7)	N	0.84	1.13	98.39% (122/124)	69.35% (86/124)	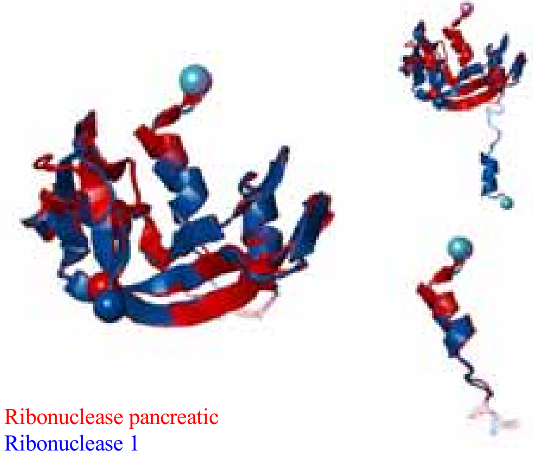 Ribonuclease pancreatic<br>Ribonuclease 1
	1h8xA (125)	115-124 (10)	115-123 (9)	17-23 (7)						
848	1a5pA (124)	15-24 (10)	15-23 (9)	17-23 (7)	N	0.85	1.05	98.39% (122/124)	68.55% (85/124)	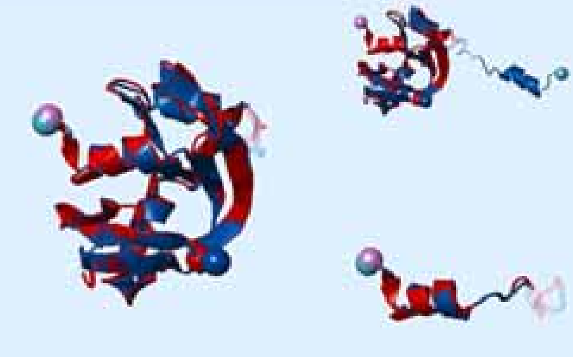 Ribonuclease A<br>Ribonuclease 1
	1h8xA (125)	115-124 (10)	115-123 (9)	17-23 (7)						

849	2bwlA (118)	13-24 (12)	15-24 (10)	17-24 (8)	N	0.68	1.56	95.76% (113/118)	37.29% (44/118)	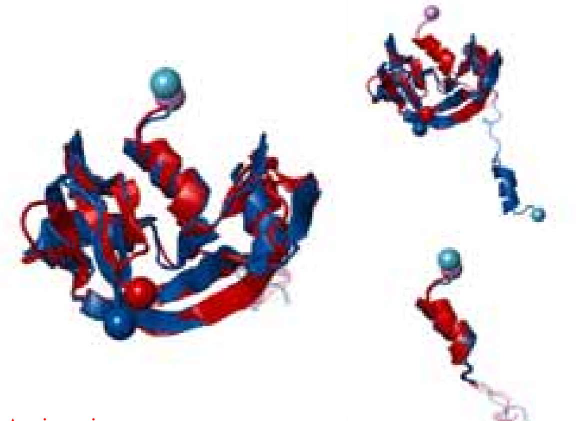 Angiogenin<br>Ribonuclease 1
	1h8xA (125)	112-124 (13)	114-124 (11)	17-24 (8)						
850	1gv7A (123)	12-24 (13)	17-24 (8)	17-24 (8)	N	0.68	1.67	95.12% (117/123)	40.65% (50/123)	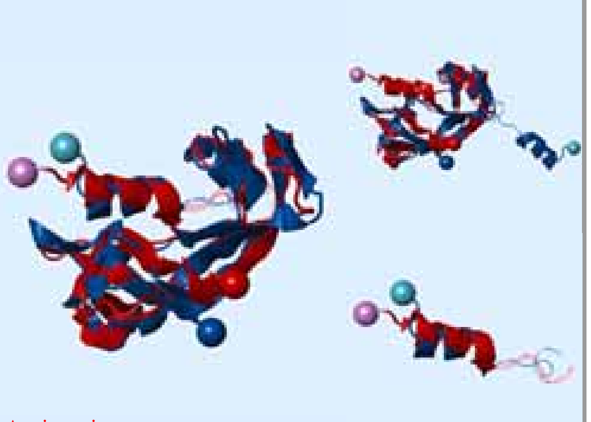 Angiogenin<br>Ribonuclease 1
	1h8xA (125)	111-124 (14)	116-124 (9)	17-24 (8)						
851	1rraA (124)	16-23 (8)	17-23 (7)	17-23 (7)	N	0.84	1.14	98.39% (122/124)	62.10% (77/124)	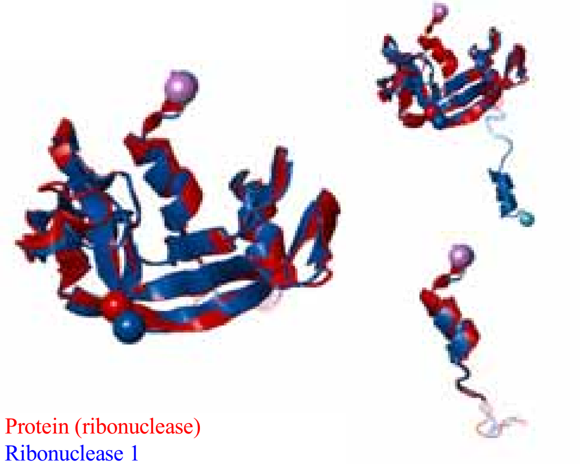 Protein (ribonuclease)<br>Ribonuclease 1
	1h8xA (125)	116-123 (8)	117-123 (7)	17-23 (7)						

852	2q4gX (126)	15-25 (11)	15-24 (10)	17-23 (7)	N	0.83	1.11	97.60% (122/125)	89.60% (112/125)	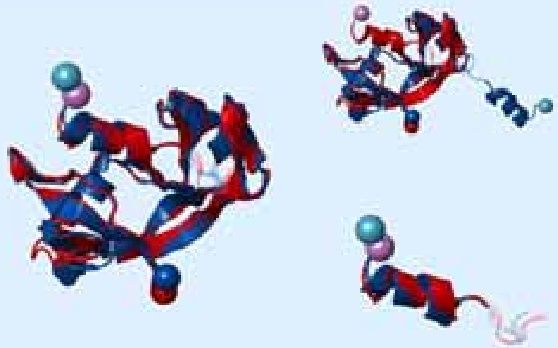 Ribonuclease pancreatic<br>Ribonuclease 1
	1h8xA (125)	115-125 (11)	115-124 (10)	17-23 (7)						
853	2k11A (127)	10-30 (21)	10-24 (15)	15-23 (9)	N	0.73	1.58	97.60% (122/125)	89.60% (112/125)	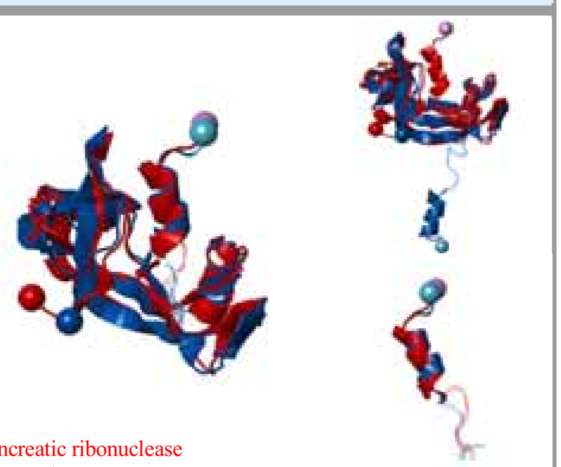 Pancreatic ribonuclease<br>Ribonuclease 1
	1h8xA (125)	110-130 (21)	110-124 (15)	15-23 (9)						
854	2aasA (124)	12-25 (14)	12-25 (14)	20-25 (6)	N	0.78	1.32	96.77% (120/124)	70.16% (87/124)	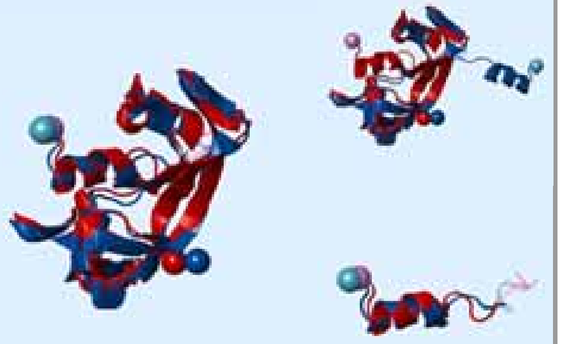 Ribonuclease A<br>Ribonuclease 1
	1h8xA (125)	112-125 (14)	112-125 (14)	20-25 (6)						

855	2nuiA (124)	15-24 (10)	15-23 (9)	16-23 (8)	N	0.86	1.04	98.39% (122/124)	68.55% (85/124)	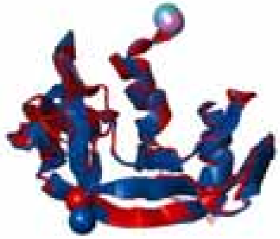 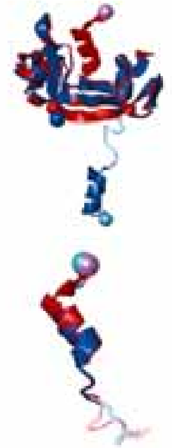 Ribonuclease pancreatic
	1h8xA (125)	115-124 (10)	115-123 (9)	16-23 (8)						Ribonuclease 1
856	1qwqA (124)	12-24 (13)	12-24 (13)	19-24 (6)	N	0.68	1.68	95.16% (118/124)	70.97% (88/124)	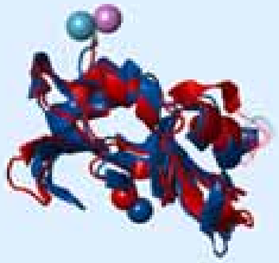 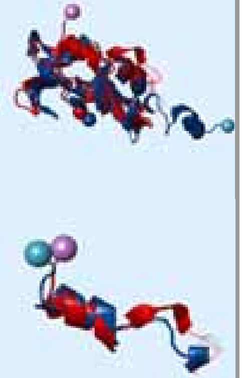 Ribonuclease
	1h8xA (125)	112-124 (13)	112-124 (13)	19-24 (6)						Ribonuclease 1
857	1izpA (124)	15-23 (9)	15-23 (9)	20-23 (4)	N	0.86	0.95	97.58% (121/124)	68.55% (85/124)	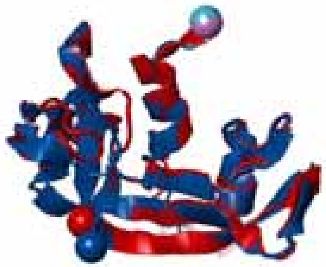 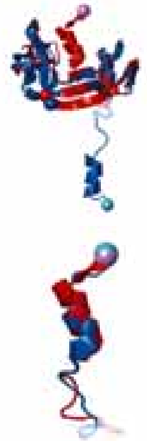 Ribonuclease A
	1h8xA (125)	115-123 (9)	115-123 (9)	20-23 (4)						Ribonuclease 1

858	2vq8A (128)	17-25 (9)	19-25 (7)	19-25 (7)	N	0.39	1.67	87.10% (108/124)	22.58% (28/124)	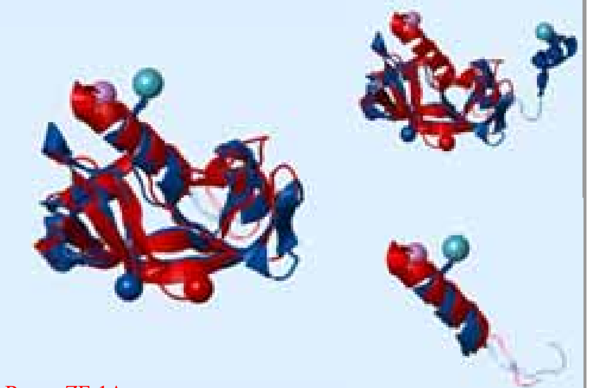 Rnase ZF-1A<br>Protein (bovine seminal ribonuclease)
	11bgA (124)	13-25 (13)	15-25 (11)	19-25 (7)						
859	2vq8A (128)	19-25 (7)	19-25 (7)	23-25 (3)	N	0.34	1.69	87.90% (109/124)	23.39% (29/124)	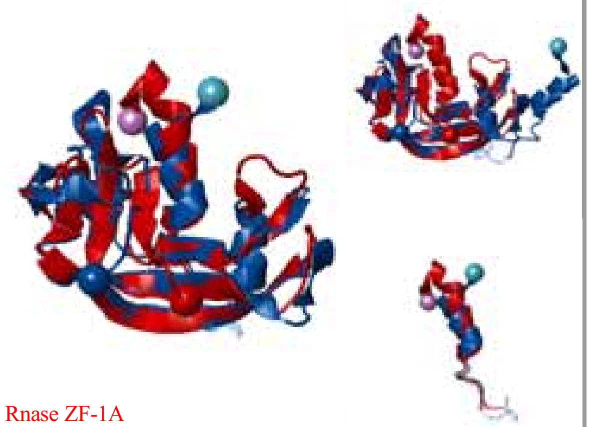 Rnase ZF-1A<br>Seminal ribonuclease
	1y92B (124)	15-25 (11)	15-25 (11)	23-25 (3)						
860	2vq8A (128)	19-25 (7)	19-25 (7)	19-25 (7)	N	0.38	1.78	87.90% (109/124)	22.58% (28/124)	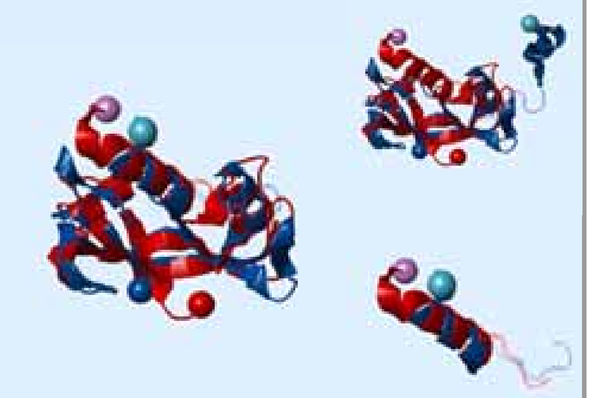 Rnase ZF-1A<br>Seminal ribonuclease
	3bcoA (124)	15-25 (11)	15-25 (11)	19-25 (7)						

861	2vq8A (128)	17-25 (9)	19-25 (7)	19-25 (7)	N	0.36	1.76	87.90% (109/124)	22.58% (28/124)	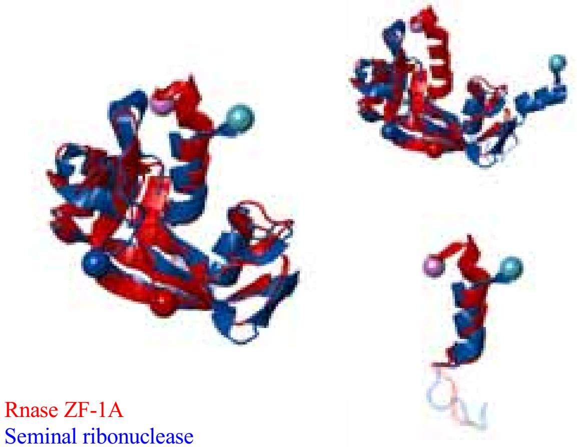 Rnase ZF-1A<br>Seminal ribonuclease
	1y94A (124)	13-25 (13)	15-25 (11)	19-25 (7)						
862	1kvzA (107)	13-42 (30)	13-22 (10)	14-20 (7)	N	0.35	2.11	92.52% (99/107)	24.30% (26/107)	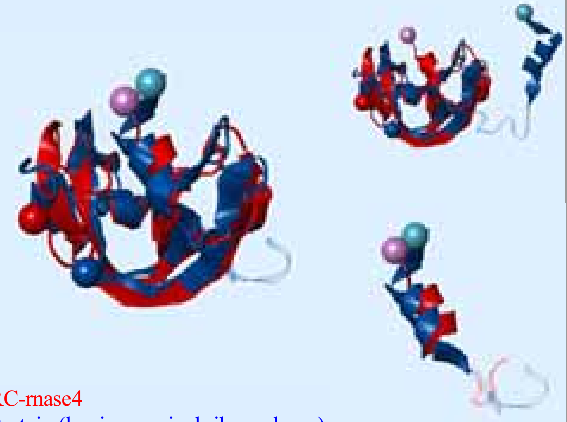 RC-rnase4<br>Protein (bovine seminal ribonuclease)
	11bgA (124)	14-51 (38)	14-28 (15)	14-20 (7)						
863	1kvzA (107)	9-42 (34)	11-22 (12)	13-20 (8)	N	0.30	2.04	91.59% (98/107)	23.36% (25/107)	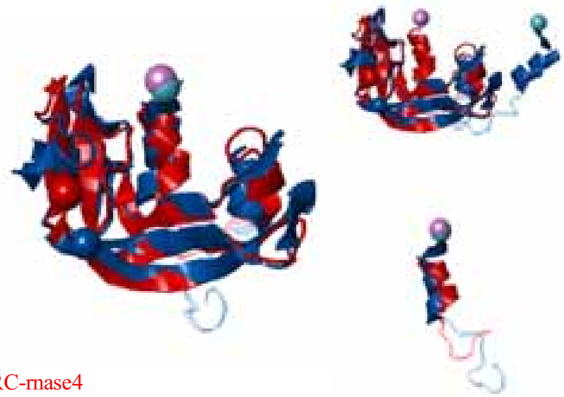 RC-rnase4<br>Seminal ribonuclease
	1y92B (124)	9-51 (43)	11-28 (18)	13-20 (8)						

864	1kvzA (107)	13-43 (31)	13-22 (10)	14-20 (7)	N	0.37	2.10	92.52% (99/107)	24.30% (26/107)	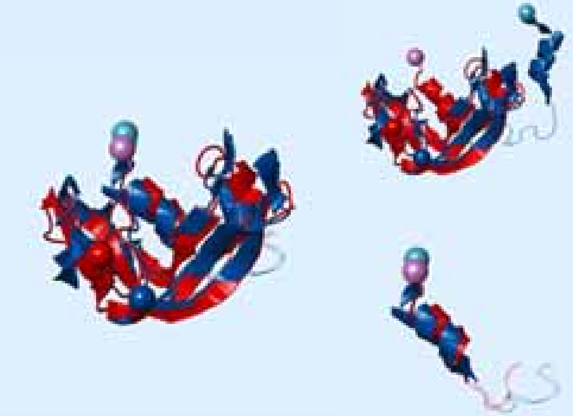 RC-rnase4 Seminal ribonuclease
	3bcoA (124)	14-52 (39)	14-28 (15)	14-20 (7)						
865	1kvzA (107)	13-42 (30)	13-20 (8)	14-20 (7)	N	0.35	2.15	93.46% (100/107)	24.30% (26/107)	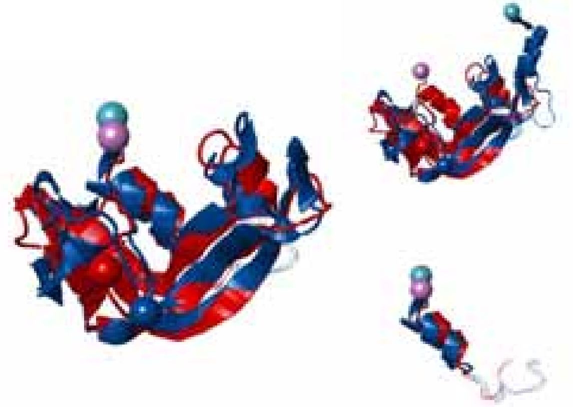 RC-rnase4 Seminal ribonuclease
	1y94A (124)	14-51 (38)	14-26 (13)	14-20 (7)						
866	1pu3A (105)	12-19 (8)	13-19 (7)	13-19 (7)	N	0.37	1.82	93.33% (98/105)	23.81% (25/105)	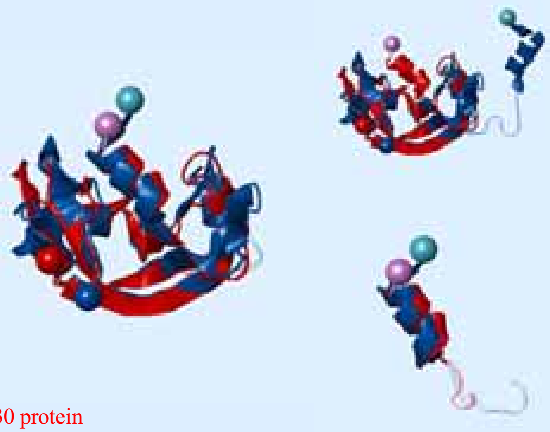 P-30 protein Protein (bovine seminal ribonuclease)
	11bgA (124)	13-25 (13)	14-25 (12)	13-19 (7)						

867	1pu3A (105)	8-19 (12)	13-19 (7)	13-19 (7)	N	0.35	1.77	93.33% (98/105)	23.81% (25/105)	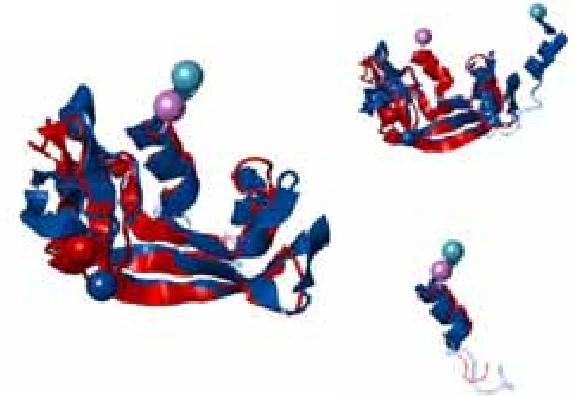 P-30 protein<br>Seminal ribonuclease
	1y92B (124)	9-25 (17)	14-25 (12)	13-19 (7)						
868	1pu3A (105)	8-21 (14)	13-19 (7)	13-19 (7)	N	0.36	1.68	92.38% (97/105)	23.81% (25/105)	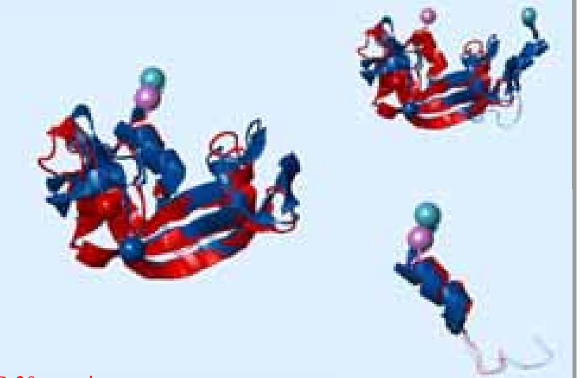 P-30 protein<br>Seminal ribonuclease
	3bcoA (124)	9-27 (19)	14-25 (12)	13-19 (7)						
869	1pu3A (105)	8-21 (14)	13-19 (7)	13-19 (7)	N	0.36	1.63	92.38% (97/105)	23.81% (25/105)	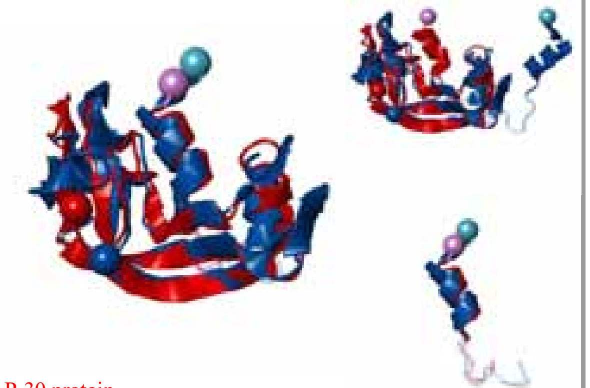 P-30 protein<br>Seminal ribonuclease
	1y94A (124)	9-27 (19)	14-25 (12)	13-19 (7)						

870	1un4A (119)	14-24 (11)	15-24 (10)	16-22 (7)	N	0.52	1.37	93.28% (111/119)	32.77% (39/119)	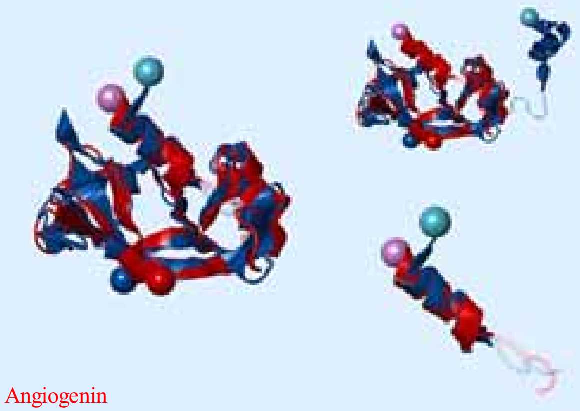 Angiogenin<br>Protein (bovine seminal ribonuclease)
	11bgA (124)	13-24 (12)	14-24 (11)	16-22 (7)						
871	1un4A (119)	15-24 (10)	16-24 (9)	19-22 (4)	N	0.50	1.35	93.28% (111/119)	30.25% (36/119)	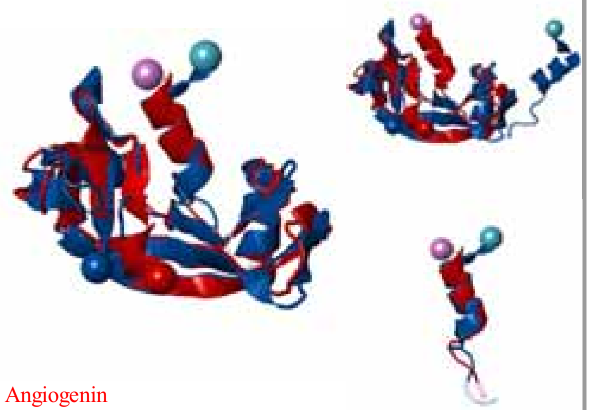 Angiogenin<br>Seminal ribonuclease
	1y92B (124)	14-24 (11)	15-24 (10)	19-22 (4)						
872	1un4A (119)	15-24 (10)	15-24 (10)	16-22 (7)	N	0.49	1.50	94.12% (112/119)	31.93% (38/119)	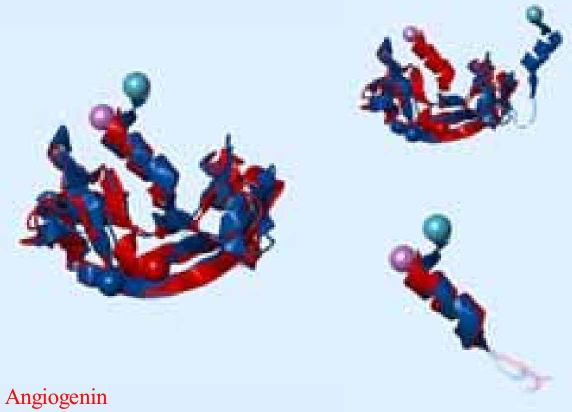 Angiogenin<br>Seminal ribonuclease
	3bcoA (124)	14-24 (11)	14-24 (11)	16-22 (7)						

873	1un4A (119)	14-24 (11)	15-24 (10)	16-22 (7)	N	0.48	1.47	93.28% (111/119)	31.09% (37/119)	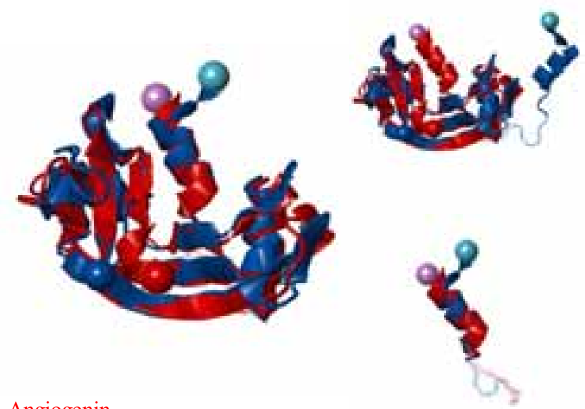 Angiogenin<br>Seminal ribonuclease
	1y94A (124)	13-24 (12)	14-24 (11)	16-22 (7)						
874	1rnfA (120)	15-24 (10)	15-23 (9)	18-22 (5)	N	0.51	1.41	97.50% (117/120)	43.33% (52/120)	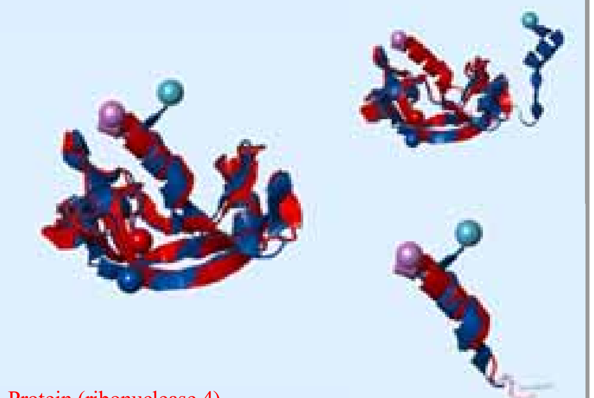 Protein (ribonuclease 4)<br>Protein (bovine seminal ribonuclease)
	11bgA (124)	15-25 (11)	15-24 (10)	18-22 (5)						
875	1rnfA (120)	15-24 (10)	17-23 (7)	18-23 (6)	N	0.56	1.20	96.67% (116/120)	42.50% (51/120)	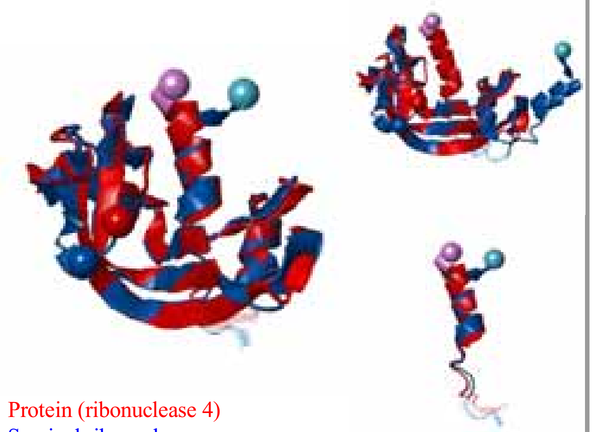 Protein (ribonuclease 4)<br>Seminal ribonuclease
	1y92B (124)	15-25 (11)	17-24 (8)	18-23 (6)						

876	1rnfA (120)	15-24 (10)	15-23 (9)	15-22 (8)	N	0.51	1.40	97.50% (117/120)	41.67% (50/120)	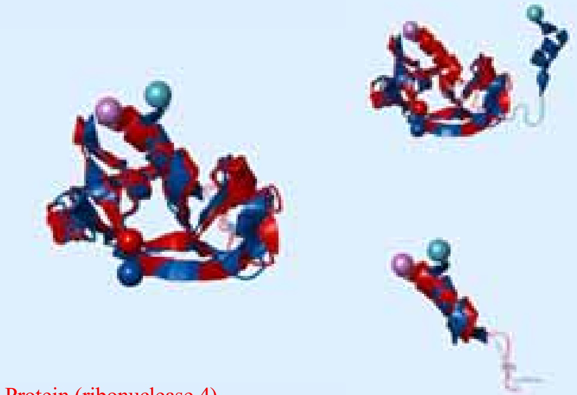
	3bcoA (124)	15-25 (11)	15-24 (10)	15-22 (8)						Protein (ribonuclease 4)<br>Seminal ribonuclease
877	1rnfA (120)	15-24 (10)	15-23 (9)	18-22 (5)	N	0.46	1.41	97.50% (117/120)	42.50% (51/120)	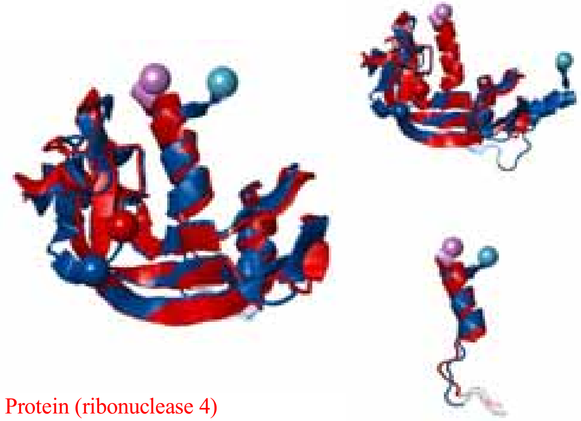
	1y94A (124)	15-25 (11)	15-24 (10)	18-22 (5)						Protein (ribonuclease 4)<br>Seminal ribonuclease
878	2j4tA (119)	12-24 (13)	15-23 (9)	15-21 (7)	N	0.52	1.29	94.12% (112/119)	34.45% (41/119)	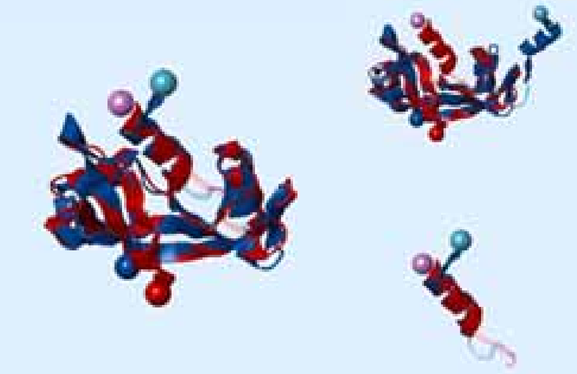
	11bgA (124)	12-25 (14)	15-24 (10)	15-21 (7)						Angiogenin-4<br>Protein (bovine seminal ribonuclease)

879	2j4tA (119)	13-24 (12)	15-23 (9)	18-21 (4)	N	0.51	1.40	94.96% (113/119)	33.61% (40/119)	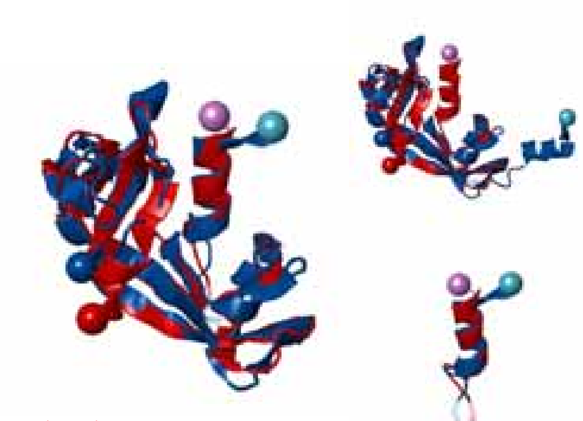 Angiogenin-4<br>Seminal ribonuclease
	1y92B (124)	13-25 (13)	15-24 (10)	18-21 (4)						
880	2j4tA (119)	12-24 (13)	15-23 (9)	15-21 (7)	N	0.50	1.43	94.96% (113/119)	33.61% (40/119)	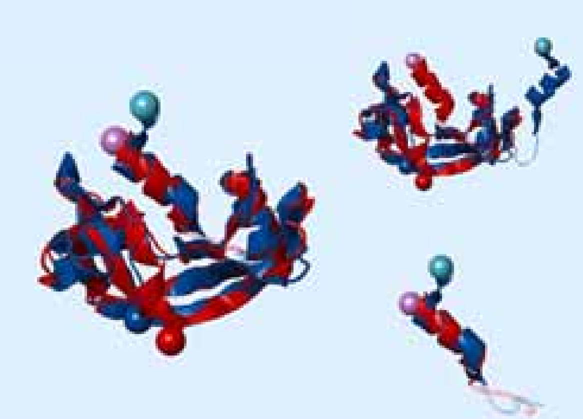 Angiogenin-4<br>Seminal ribonuclease
	3bcoA (124)	12-25 (14)	15-24 (10)	15-21 (7)						
881	2j4tA (119)	12-23 (12)	14-23 (10)	15-21 (7)	N	0.49	1.34	94.12% (112/119)	33.61% (40/119)	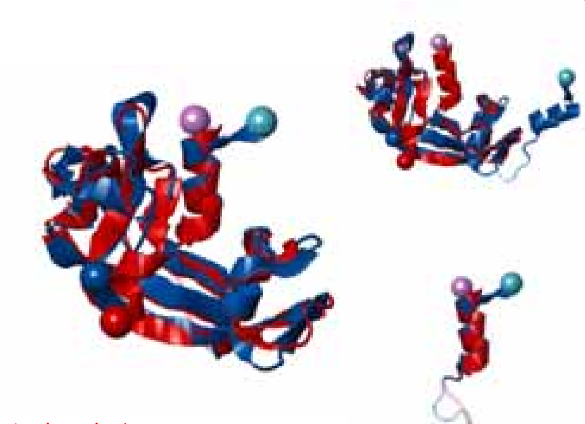 Angiogenin-4<br>Seminal ribonuclease
	1y94A (124)	12-24 (13)	14-24 (11)	15-21 (7)						

882	1b1eA (123)	14-24 (11)	15-24 (10)	16-22 (7)	N	0.48	1.34	90.24% (111/123)	30.89% (38/123)	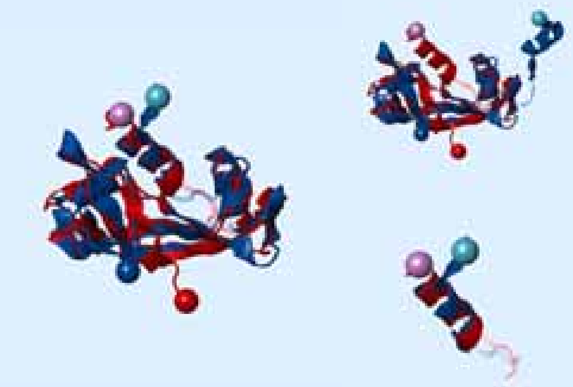 Hydrolase angiogenin<br>Protein (bovine seminal ribonuclease)
	11bgA (124)	13-24 (12)	14-24 (11)	16-22 (7)						
883	1b1eA (123)	14-24 (11)	16-24 (9)	19-22 (4)	N	0.48	1.46	91.06% (112/123)	28.46% (35/123)	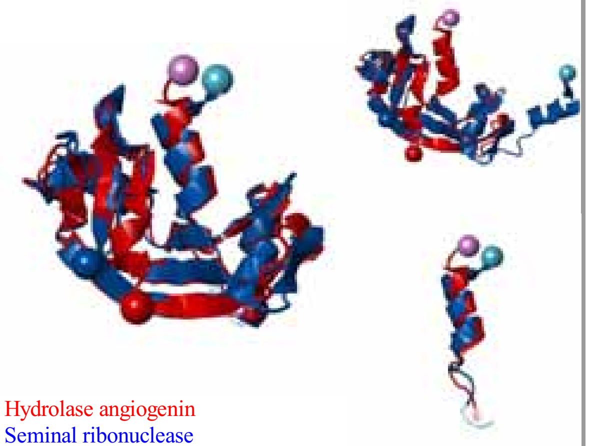 Hydrolase angiogenin<br>Seminal ribonuclease
	1y92B (124)	13-24 (12)	15-24 (10)	19-22 (4)						
884	1b1eA (123)	14-25 (12)	15-24 (10)	16-22 (7)	N	0.45	1.39	90.24% (111/123)	28.46% (35/123)	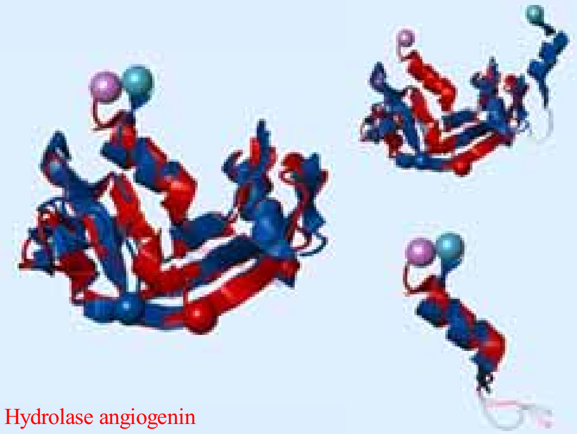 Hydrolase angiogenin<br>Seminal ribonuclease
	3bcoA (124)	13-25 (13)	14-24 (11)	16-22 (7)						

885	1b1eA (123)	13-24 (12)	15-24 (10)	16-23 (8)	N	0.44	1.37	89.43% (110/123)	28.46% (35/123)	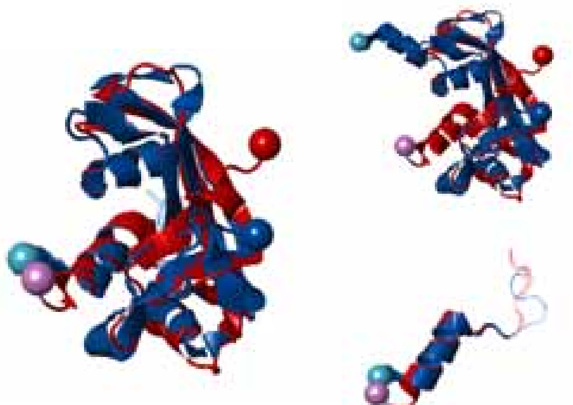 Hydrolase angiogenin Seminal ribonuclease
	1y94A (124)	12-24 (13)	14-24 (11)	16-23 (8)						
886	2hkyA (129)	11-22 (12)	11-22 (12)	16-22 (7)	N	0.33	1.91	91.13% (113/124)	35.48% (44/124)	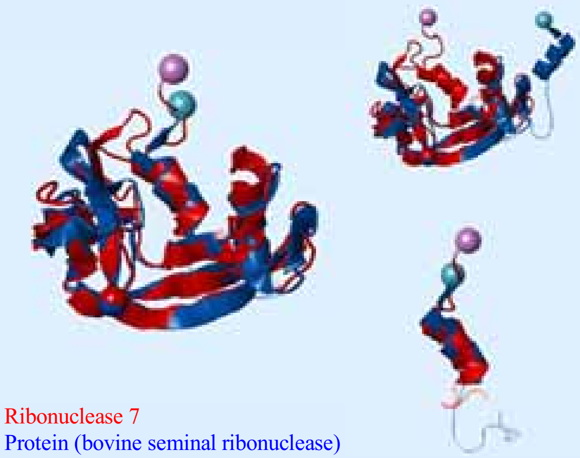 Ribonuclease 7 Protein (bovine seminal ribonuclease)
	11bgA (124)	8-25 (18)	8-25 (18)	16-22 (7)						
887	2hkyA (129)	11-22 (12)	15-22 (8)	17-22 (6)	N	0.31	1.68	91.13% (113/124)	35.48% (44/124)	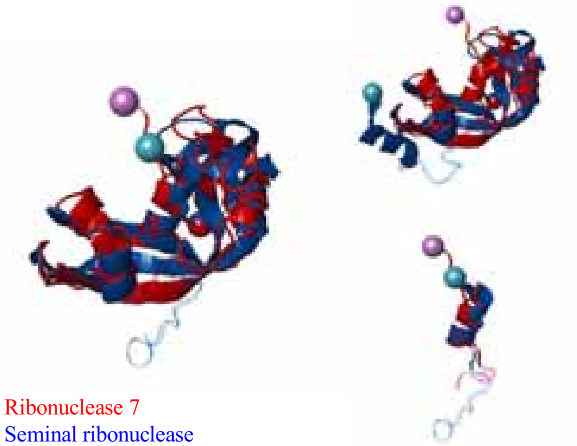 Ribonuclease 7 Seminal ribonuclease
	1y92B (124)	8-25 (18)	12-25 (14)	17-22 (6)						

888	2hkyA (129)	11-22 (12)	11-22 (12)	17-22 (6)	N	0.28	1.86	91.13% (113/124)	35.48% (44/124)	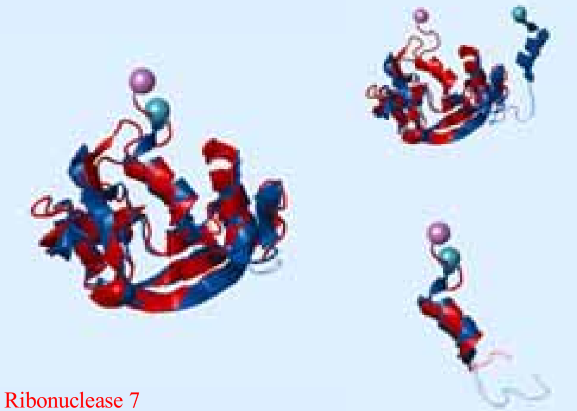 Ribonuclease 7<br>Seminal ribonuclease
	3bcoA (124)	8-25 (18)	8-25 (18)	17-22 (6)						
889	2hkyA (129)	11-22 (12)	11-22 (12)	16-22 (7)	N	0.31	1.82	91.13% (113/124)	36.29% (45/124)	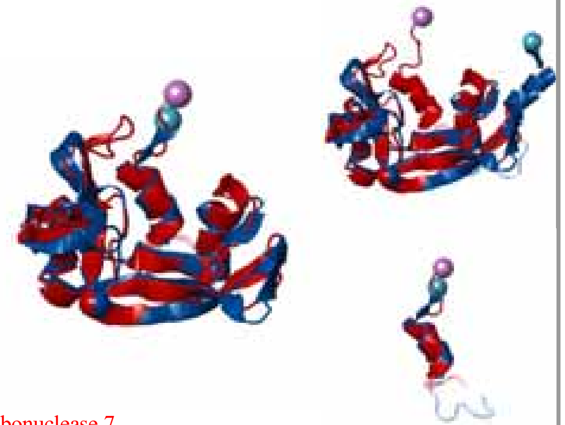 Ribonuclease 7<br>Seminal ribonuclease
	1y94A (124)	8-25 (18)	8-25 (18)	16-22 (7)						
890	1m58A (106)	12-19 (8)	12-19 (8)	17-19 (3)	N	0.37	1.97	91.51% (97/106)	23.58% (25/106)	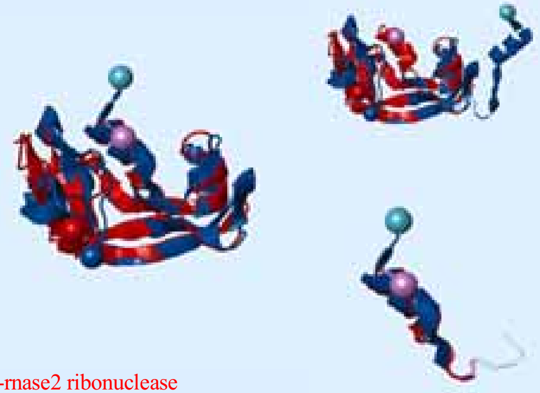 RC-rnase2 ribonuclease<br>Protein (bovine seminal ribonuclease)
	11bgA (124)	13-25 (13)	13-25 (13)	17-19 (3)						

891	1m58A (106)	12-19 (8)	12-19 (8)	13-19 (7)	N	0.37	2.06	93.40% (99/106)	23.58% (25/106)	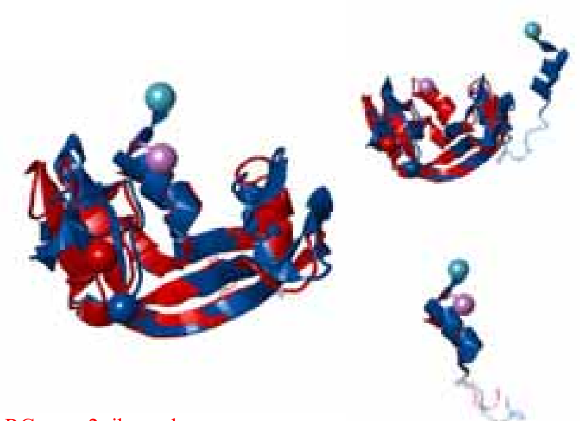 RC-rnase2 ribonuclease<br>Seminal ribonuclease
	1y92B (124)	13-25 (13)	13-25 (13)	13-19 (7)						
892	1m58A (106)	12-19 (8)	12-19 (8)	18-19 (2)	N	0.34	2.08	92.45% (98/106)	23.58% (25/106)	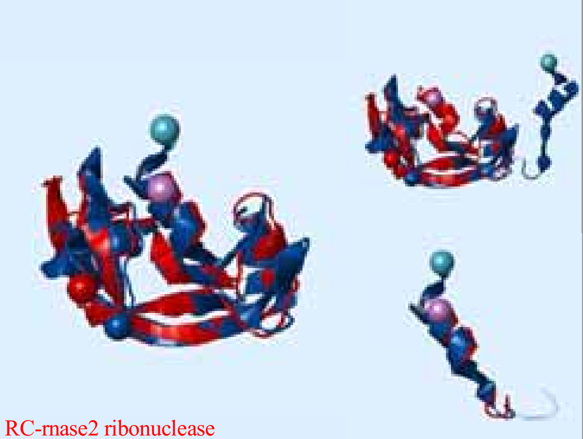 RC-rnase2 ribonuclease<br>Seminal ribonuclease
	3bcoA (124)	13-25 (13)	13-25 (13)	18-19 (2)						
893	1m58A (106)	12-19 (8)	12-19 (8)	13-19 (7)	N	0.36	2.02	92.45% (98/106)	23.58% (25/106)	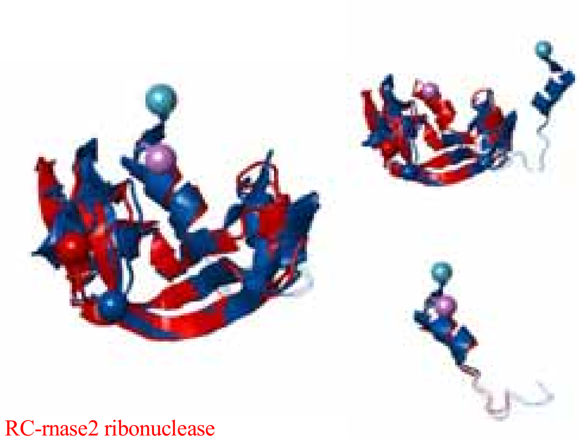 RC-rnase2 ribonuclease<br>Seminal ribonuclease
	1y94A (124)	13-25 (13)	13-25 (13)	13-19 (7)						

894	2e0oB (125)	15-24 (10)	15-24 (10)	17-23 (7)	N	0.57	1.01	99.19% (123/124)	69.35% (86/124)	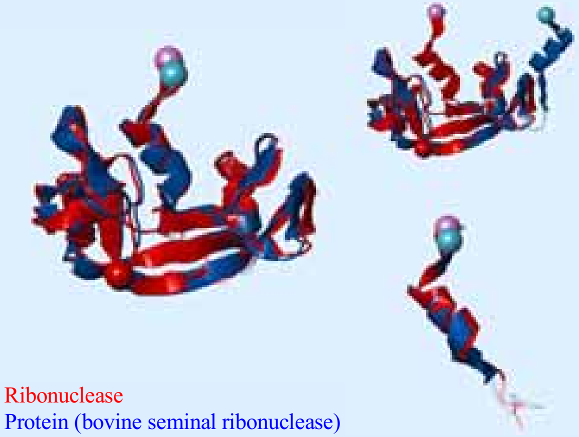 Ribonuclease<br>Protein (bovine seminal ribonuclease)
	1lbgA (124)	15-24 (10)	15-24 (10)	17-23 (7)						
895	2e0oB (125)	15-24 (10)	15-24 (10)	18-23 (6)	N	0.58	0.89	100.00% (124/124)	70.16% (87/124)	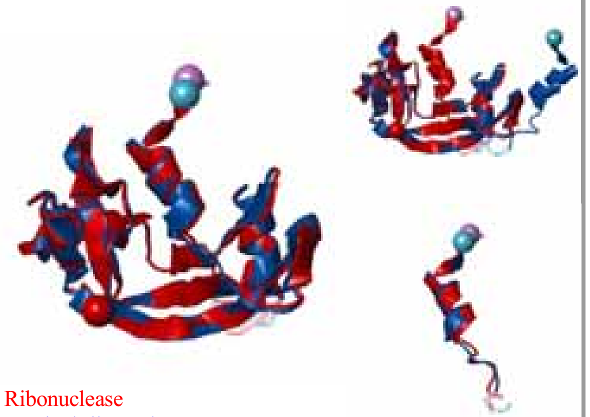 Ribonuclease<br>Seminal ribonuclease
	1y92B (124)	15-24 (10)	15-24 (10)	18-23 (6)						
896	2e0oB (125)	15-24 (10)	15-24 (10)	18-23 (6)	N	0.51	0.93	99.19% (123/124)	69.35% (86/124)	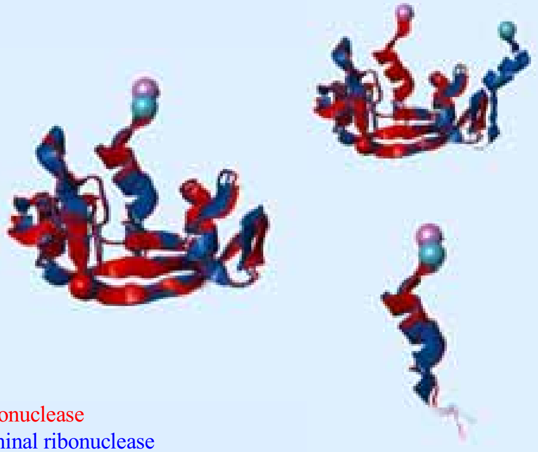 Ribonuclease<br>Seminal ribonuclease
	3bcoA (124)	15-24 (10)	15-24 (10)	18-23 (6)						

897	2e0oB (125)	15-25 (11)	20-24 (5)	15-23 (9)	N	0.49	0.98	99.19% (123/124)	68.55% (85/124)	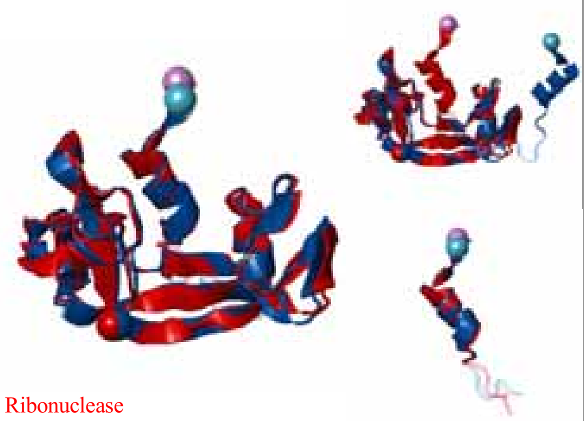<br>Ribonuclease<br>Seminal ribonuclease
	1y94A (124)	15-25 (11)	20-24 (5)	15-23 (9)						
898	1ymnA (124)	15-22 (8)	15-22 (8)	15-22 (8)	N	0.55	1.10	100.00% (124/124)	80.65% (100/124)	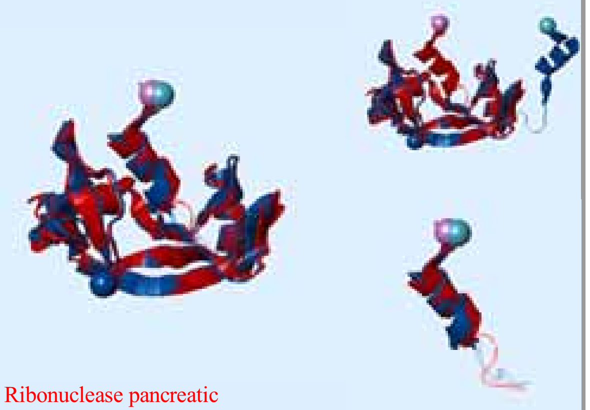<br>Ribonuclease pancreatic<br>Protein (bovine seminal ribonuclease)
	11bgA (124)	15-22 (8)	15-22 (8)	15-22 (8)						
899	1ymnA (124)	15-23 (9)	15-23 (9)	17-22 (6)	N	0.65	0.99	100.00% (124/124)	80.65% (100/124)	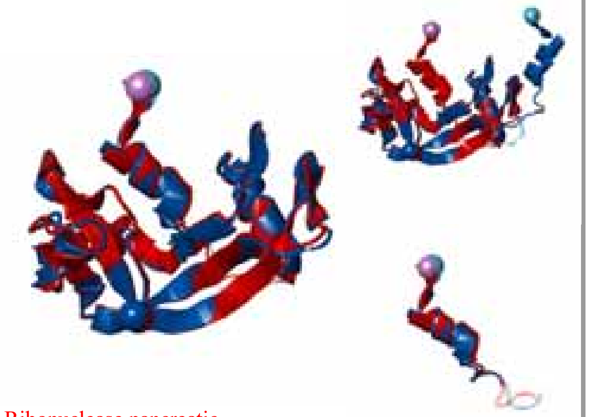<br>Ribonuclease pancreatic<br>Seminal ribonuclease
	1y92B (124)	15-23 (9)	15-23 (9)	17-22 (6)						

900	1ymnA (124)	15-22 (8)	15-22 (8)	15-22 (8)	N	0.55	1.09	100.00% (124/124)	81.45% (101/124)	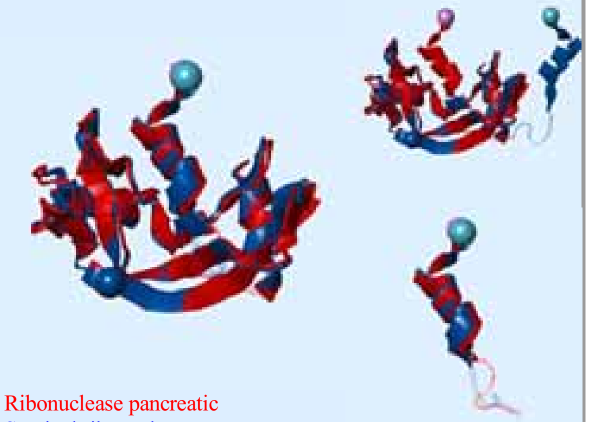 Ribonuclease pancreatic<br>Seminal ribonuclease
	3bcoA (124)	15-22 (8)	15-22 (8)	15-22 (8)						
901	1ymnA (124)	15-22 (8)	15-22 (8)	15-22 (8)	N	0.51	1.15	100.00% (124/124)	83.06% (103/124)	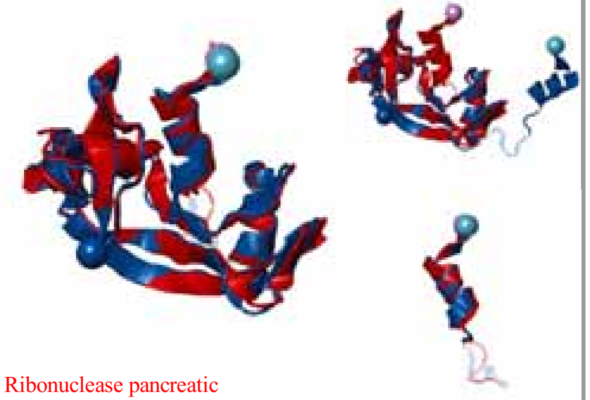 Ribonuclease pancreatic<br>Seminal ribonuclease
	1y94A (124)	15-22 (8)	15-22 (8)	15-22 (8)						
902	1a5pA (124)	15-22 (8)	15-22 (8)	15-22 (8)	N	0.55	0.91	100.00% (124/124)	79.84% (99/124)	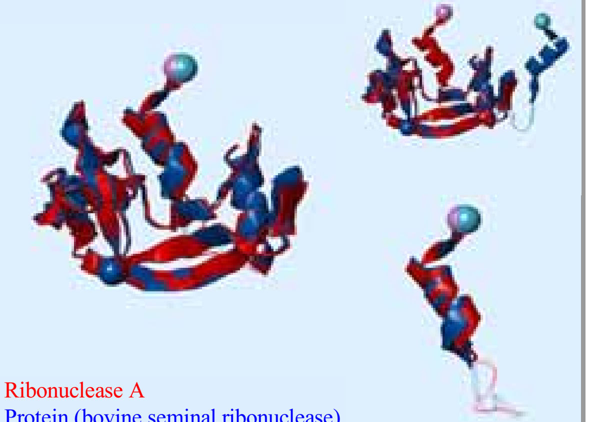 Ribonuclease A<br>Protein (bovine seminal ribonuclease)
	11bgA (124)	15-22 (8)	15-22 (8)	15-22 (8)						

903	1a5pA (124)	15-23 (9)	21-23 (3)	17-22 (6)	N	0.65	0.89	100.00% (124/124)	79.84% (99/124)	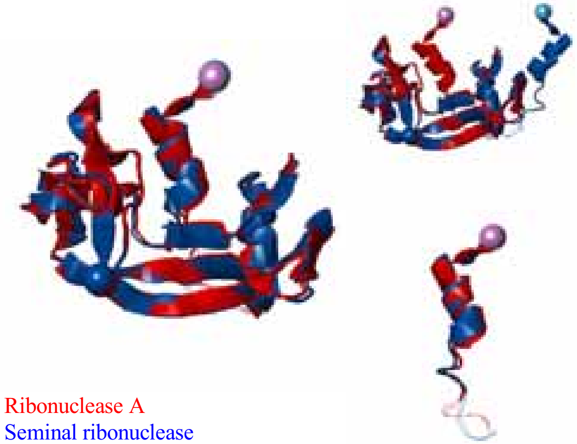 Ribonuclease A<br>Seminal ribonuclease
	1y92B (124)	15-23 (9)	21-23 (3)	17-22 (6)						
904	1a5pA (124)	15-22 (8)	15-22 (8)	15-22 (8)	N	0.57	0.89	100.00% (124/124)	80.65% (100/124)	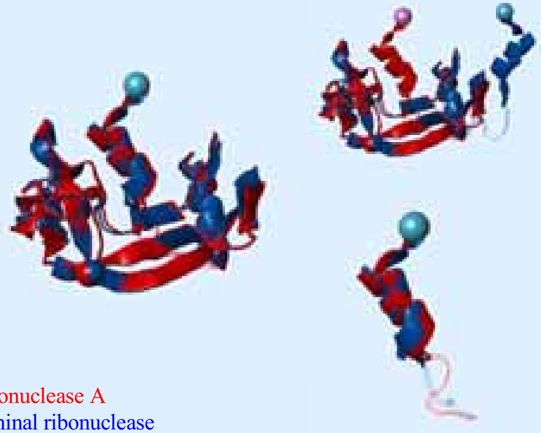 Ribonuclease A<br>Seminal ribonuclease
	3bcoA (124)	15-22 (8)	15-22 (8)	15-22 (8)						
905	1a5pA (124)	15-22 (8)	15-22 (8)	15-22 (8)	N	0.55	0.97	100.00% (124/124)	82.26% (102/124)	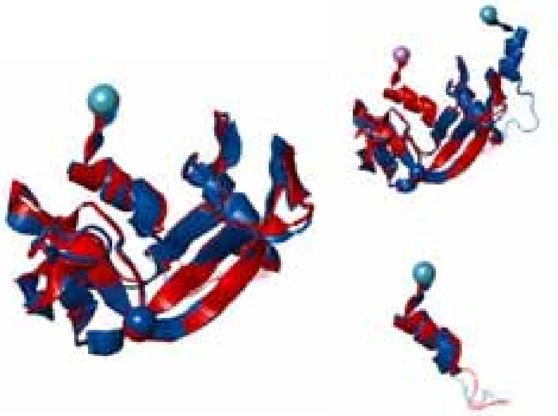 Ribonuclease A<br>Seminal ribonuclease
	1y94A (124)	15-22 (8)	15-22 (8)	15-22 (8)						

906	1z5fA (105)	1-32 (32)	10-18 (9)	17-18 (2)	N	0.30	2.32	91.43% (96/105)	21.90% (23/105)	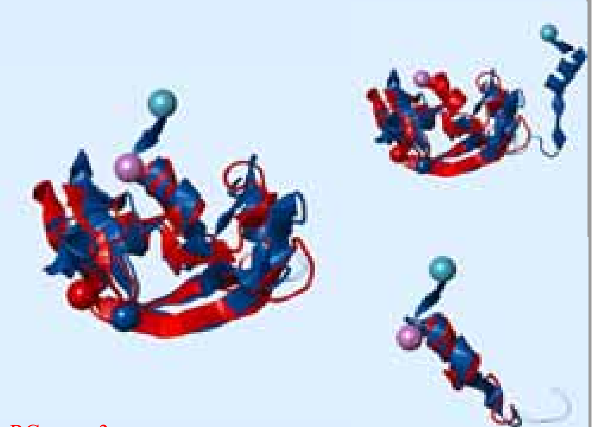 RC-rnase 3<br>Protein (bovine seminal ribonuclease)
	11bgA (124)	1-42 (42)	12-25 (14)	17-18 (2)						
907	1z5fA (105)	2-32 (31)	11-18 (8)	11-18 (8)	N	0.39	2.27	91.43% (96/105)	20.95% (22/105)	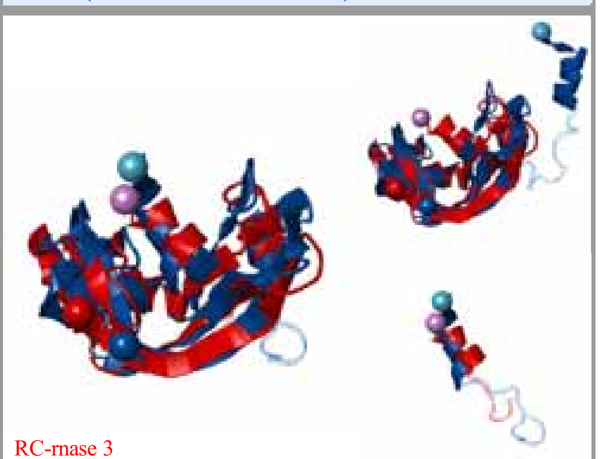 RC-rnase 3<br>Seminal ribonuclease
	1y92B (124)	3-42 (40)	12-25 (14)	11-18 (8)						
908	1z5fA (105)	2-32 (31)	11-18 (8)	12-18 (7)	N	0.34	2.47	92.38% (97/105)	20.00% (21/105)	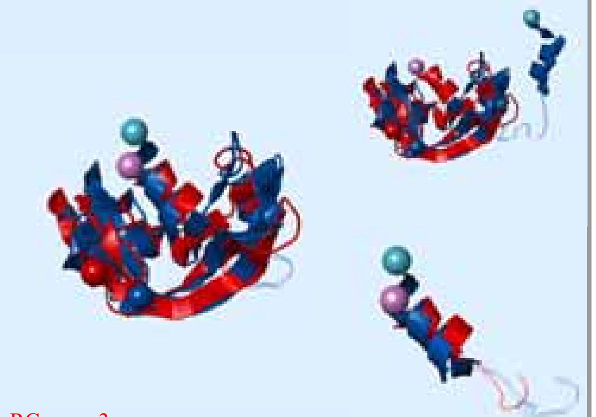 RC-rnase 3<br>Seminal ribonuclease
	3bcoA (124)	3-42 (40)	12-25 (14)	12-18 (7)						

909	2bwlA (118)	15-24 (10)	15-24 (10)	16-23 (8)	N	0.42	1.46	95.76% (113/118)	35.59% (42/118)	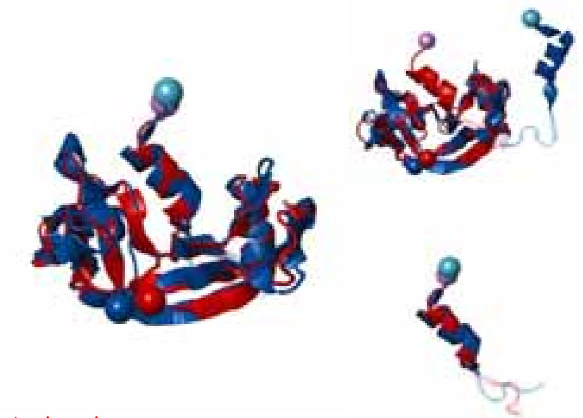 Angiogenin<br>Protein (bovine seminal ribonuclease)
	11bgA (124)	14-24 (11)	14-24 (11)	16-23 (8)						
910	2bwlA (118)	15-24 (10)	17-24 (8)	18-24 (7)	N	0.45	1.59	96.61% (114/118)	34.75% (41/118)	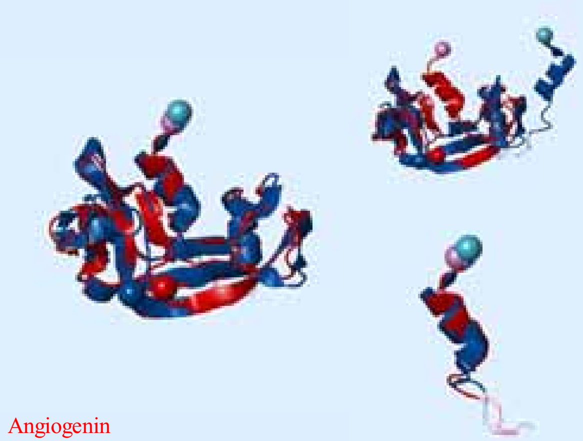 Angiogenin<br>Seminal ribonuclease
	1y92B (124)	14-24 (11)	16-24 (9)	18-24 (7)						
911	2bwlA (118)	15-24 (10)	15-24 (10)	16-23 (8)	N	0.39	1.54	95.76% (113/118)	35.59% (42/118)	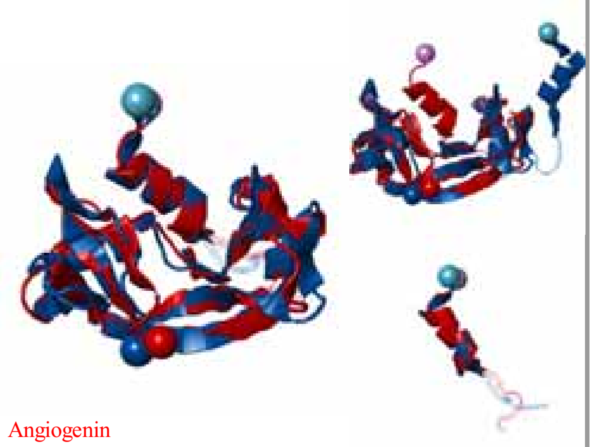 Angiogenin<br>Seminal ribonuclease
	3bcoA (124)	14-24 (11)	14-24 (11)	16-23 (8)						

912	2bwlA (118)	15-24 (10)	15-24 (10)	16-23 (8)	N	0.45	1.42	94.92% (112/118)	33.90% (40/118)	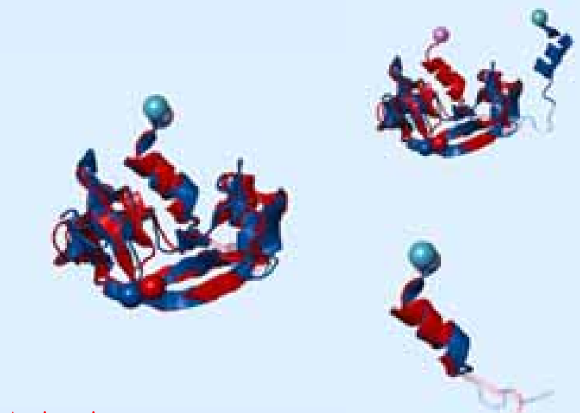 Angiogenin<br>Seminal ribonuclease
	1y94A (124)	14-24 (11)	14-24 (11)	16-23 (8)						
913	1gv7A (123)	12-24 (13)	16-24 (9)	16-22 (7)	N	0.46	1.42	95.12% (117/123)	40.65% (50/123)	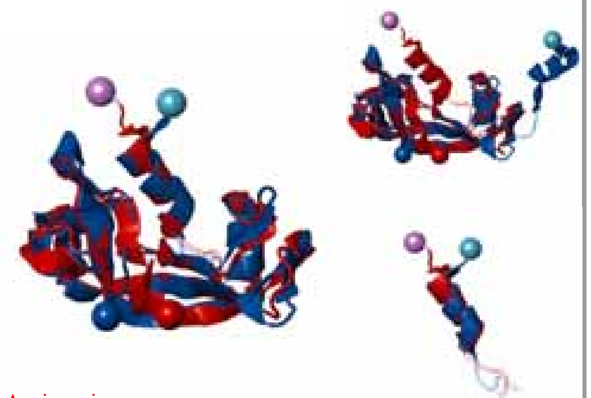 Angiogenin<br>Protein (bovine seminal ribonuclease)
	11bgA (124)	11-24 (14)	15-24 (10)	16-22 (7)						
914	1gv7A (123)	12-24 (13)	16-24 (9)	18-24 (7)	N	0.52	1.28	93.50% (115/123)	39.02% (48/123)	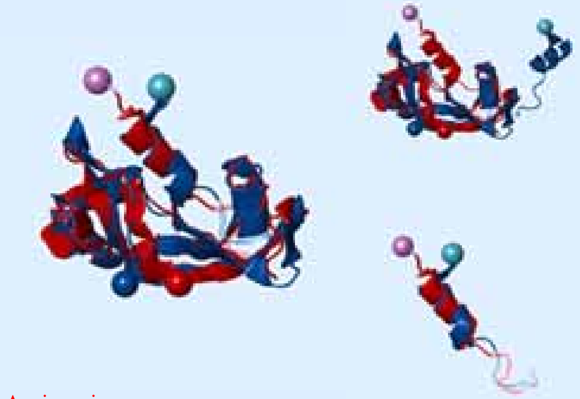 Angiogenin<br>Seminal ribonuclease
	1y92B (124)	11-24 (14)	15-24 (10)	18-24 (7)						

915	1gv7A (123)	11-25 (15)	16-24 (9)	16-23 (8)	N	0.47	1.36	93.50% (115/123)	39.84% (49/123)	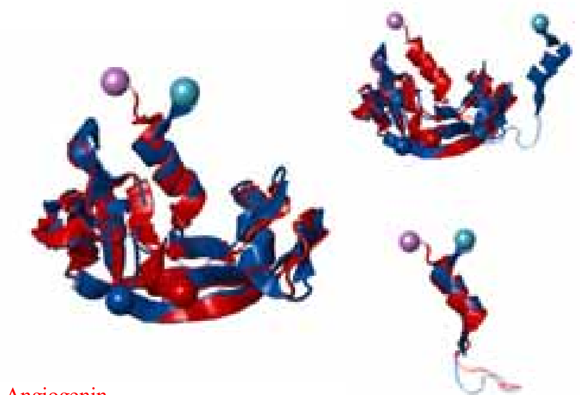 Angiogenin<br>Seminal ribonuclease
	3bcoA (124)	10-25 (16)	15-24 (10)	16-23 (8)						
916	1gv7A (123)	11-24 (14)	15-24 (10)	16-23 (8)	N	0.47	1.36	93.50% (115/123)	39.84% (49/123)	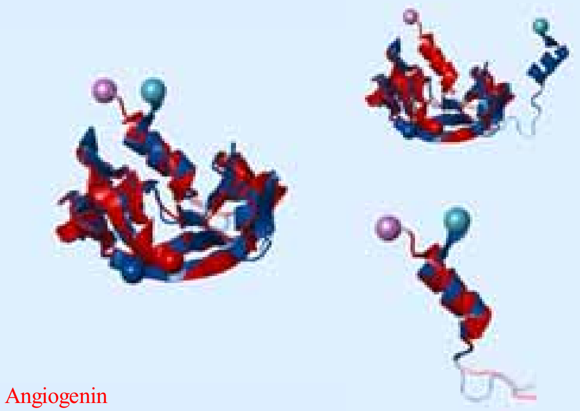 Angiogenin<br>Seminal ribonuclease
	1y94A (124)	10-24 (15)	14-24 (11)	16-23 (8)						
917	1rraA (124)	15-24 (10)	15-22 (8)	15-22 (8)	N	0.54	1.18	100.00% (124/124)	66.13% (82/124)	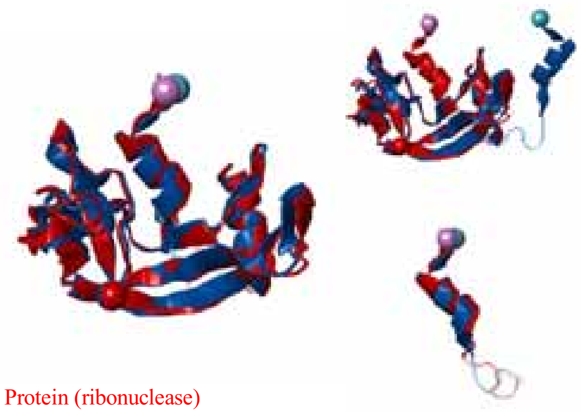 Protein (ribonuclease)<br>Protein (bovine seminal ribonuclease)
	1lbgA (124)	15-24 (10)	15-22 (8)	15-22 (8)						

918	1rraA (124)	20-23 (4)	20-23 (4)	21-22 (2)	N	0.55	0.76	100.00% (124/124)	65.32% (81/124)	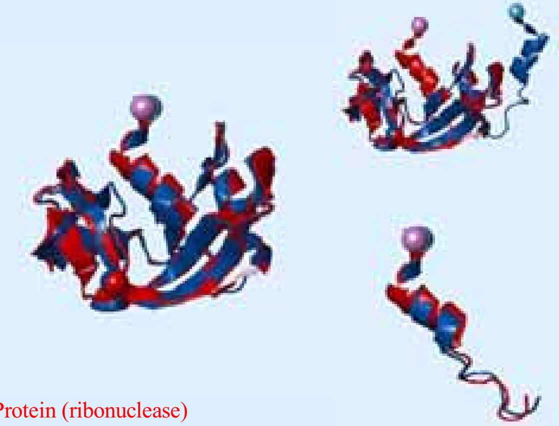 Protein (ribonuclease) Seminal ribonuclease
	1y92B (124)	20-23 (4)	20-23 (4)	21-22 (2)						
919	1rraA (124)	15-22 (8)	15-22 (8)	15-22 (8)	N	0.54	1.13	100.00% (124/124)	66.13% (82/124)	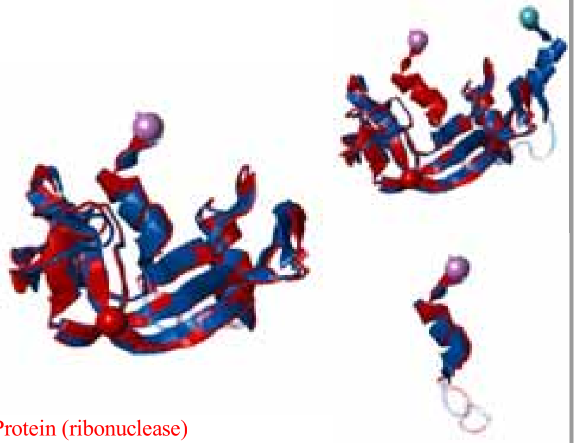 Protein (ribonuclease) Seminal ribonuclease
	3bcoA (124)	15-22 (8)	15-22 (8)	15-22 (8)						
920	1rraA (124)	15-22 (8)	15-22 (8)	15-22 (8)	N	0.53	1.16	100.00% (124/124)	65.32% (81/124)	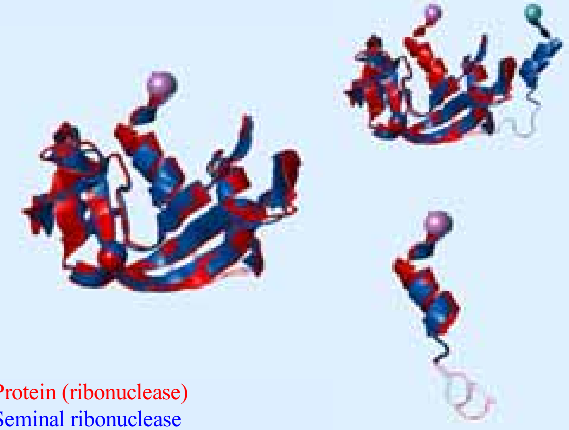 Protein (ribonuclease) Seminal ribonuclease
	1y94A (124)	15-22 (8)	15-22 (8)	15-22 (8)						

921	2q4gX (126)	15-25 (11)	15-22 (8)	15-22 (8)	N	0.52	0.85	98.39% (122/124)	69.35% (86/124)	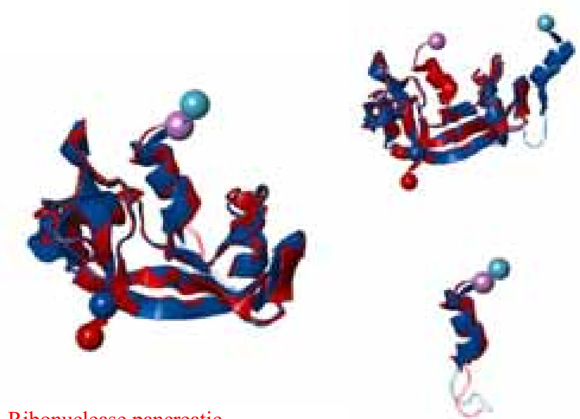<br>Ribonuclease pancreatic<br>Protein (bovine seminal ribonuclease)
	11bgA (124)	15-25 (11)	15-22 (8)	15-22 (8)						
922	2q4gX (126)	14-25 (12)	19-24 (6)	20-22 (3)	N	0.59	0.75	99.19% (123/124)	70.16% (87/124)	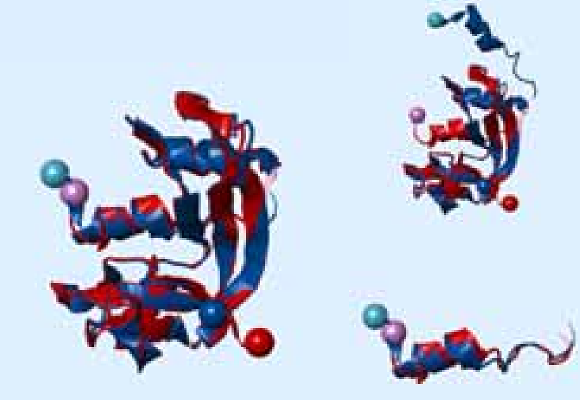<br>Ribonuclease pancreatic<br>Seminal ribonuclease
	1y92B (124)	14-25 (12)	19-24 (6)	20-22 (3)						
923	2q4gX (126)	15-24 (10)	15-22 (8)	15-22 (8)	N	0.53	0.95	99.19% (123/124)	70.97% (88/124)	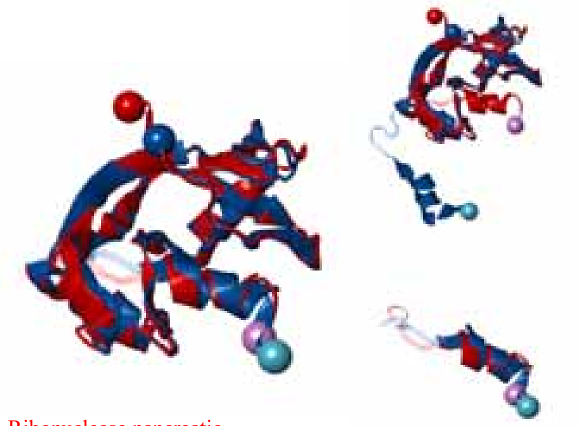<br>Ribonuclease pancreatic<br>Seminal ribonuclease
	3bcoA (124)	15-24 (10)	15-22 (8)	15-22 (8)						

924	2q4gX (126)	15-25 (11)	15-22 (8)	15-22 (8)	N	0.49	1.03	99.19% (123/124)	69.35% (86/124)	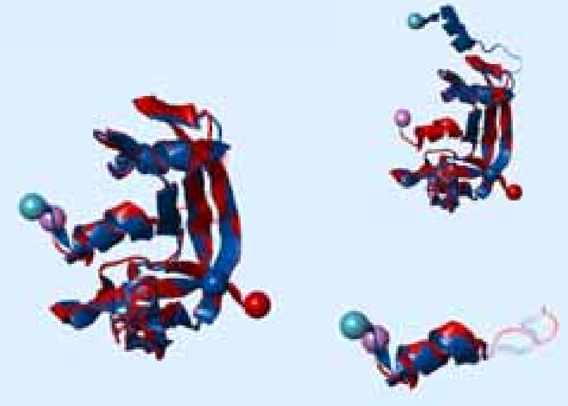<br>Ribonuclease pancreatic<br>Seminal ribonuclease
	1y94A (124)	15-25 (11)	15-22 (8)	15-22 (8)						
925	2k11A (127)	10-32 (23)	10-24 (15)	14-24 (11)	N	0.53	1.31	97.58% (121/124)	67.74% (84/124)	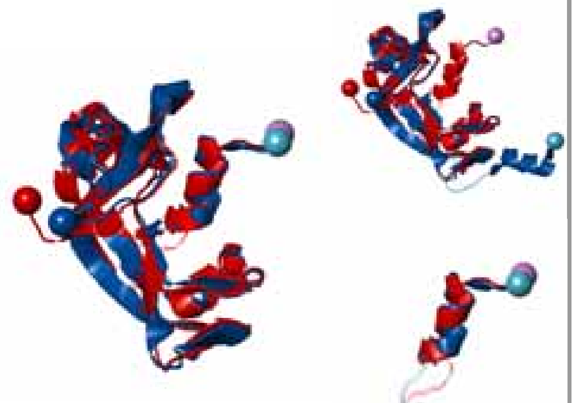<br>Pancreatic ribonuclease<br>Protein (bovine seminal ribonuclease)
	1lbgA (124)	10-32 (23)	10-24 (15)	14-24 (11)						
926	2k11A (127)	10-32 (23)	19-24 (6)	19-23 (5)	N	0.45	1.43	100.00% (124/124)	70.97% (88/124)	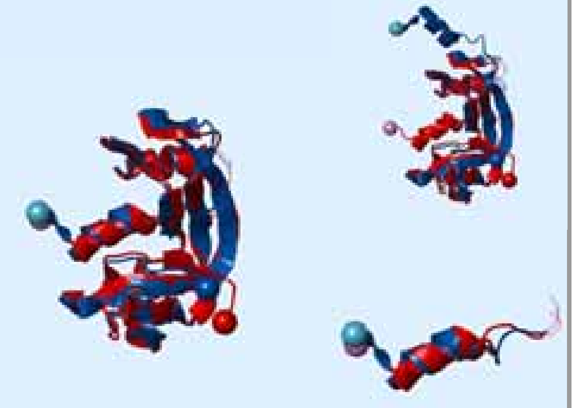<br>Pancreatic ribonuclease<br>Seminal ribonuclease
	1y92B (124)	10-32 (23)	19-24 (6)	19-23 (5)						

927	2k11A (127)	10-24 (15)	10-24 (15)	14-24 (11)	N	0.48	1.31	97.58% (121/124)	68.55% (85/124)	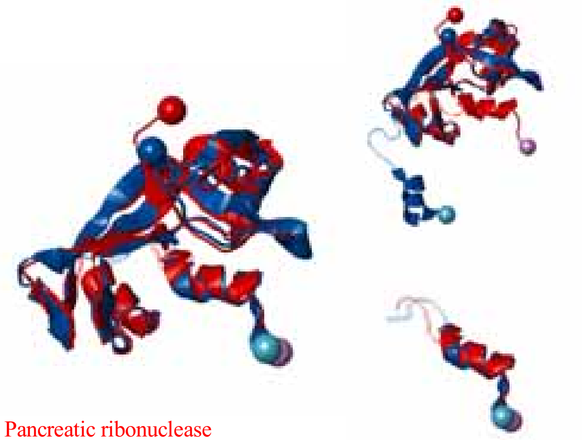 Pancreatic ribonuclease
	3bcoA (124)	10-24 (15)	10-24 (15)	14-24 (11)						Seminal ribonuclease
928	2k11A (127)	10-32 (23)	10-24 (15)	14-23 (10)	N	0.48	1.33	97.58% (121/124)	67.74% (84/124)	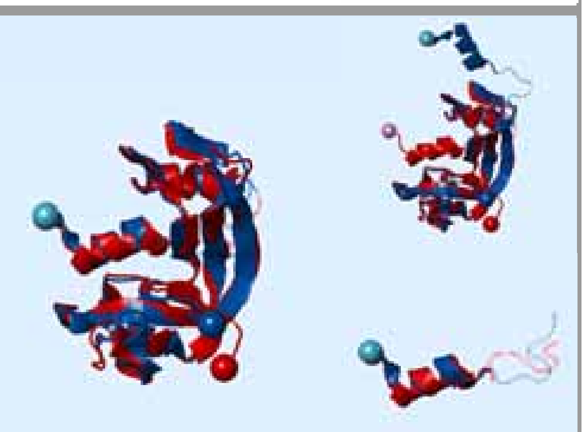 Pancreatic ribonuclease
	1y94A (124)	10-32 (23)	10-24 (15)	14-23 (10)						Seminal ribonuclease
929	2aasA (124)	11-24 (14)	12-24 (13)	15-24 (10)	N	0.49	1.42	100.00% (124/124)	81.45% (101/124)	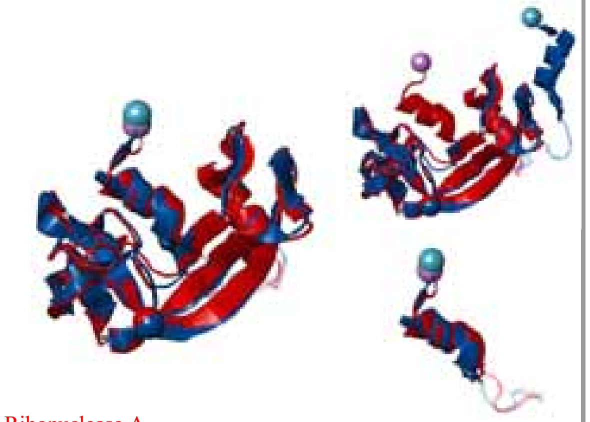 Ribonuclease A
	11bgA (124)	11-24 (14)	12-24 (13)	15-24 (10)						Protein (bovine seminal ribonuclease)

930	2aasA (124)	10-24 (15)	21-24 (4)	17-24 (8)	N	0.61	1.38	100.00% (124/124)	81.45% (101/124)	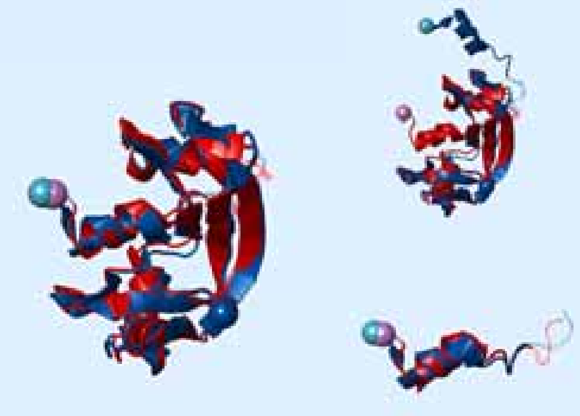 Ribonuclease A<br>Seminal ribonuclease
	1y92B (124)	10-24 (15)	21-24 (4)	17-24 (8)						
931	2aasA (124)	12-24 (13)	12-24 (13)	19-24 (6)	N	0.51	1.41	100.00% (124/124)	82.26% (102/124)	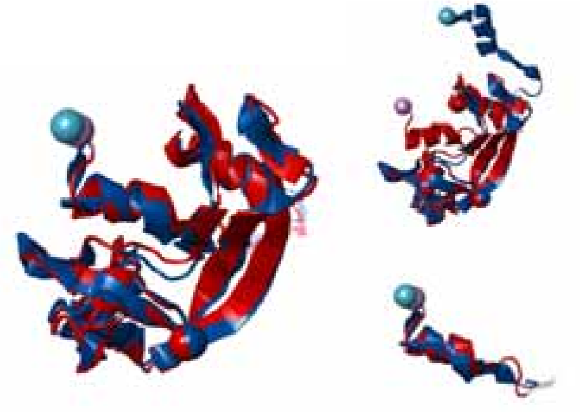 Ribonuclease A<br>Seminal ribonuclease
	3bcoA (124)	12-24 (13)	12-24 (13)	19-24 (6)						
932	2aasA (124)	12-24 (13)	12-24 (13)	15-24 (10)	N	0.52	1.47	100.00% (124/124)	83.87% (104/124)	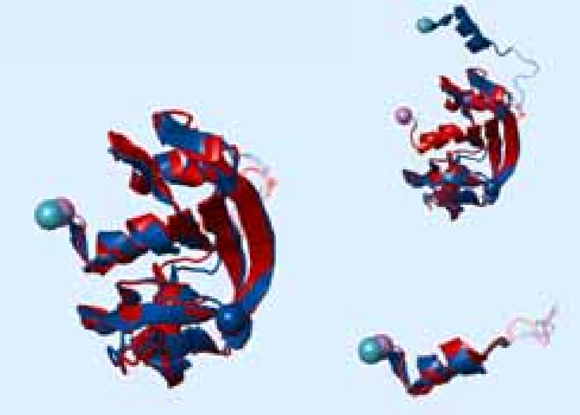 Ribonuclease A<br>Seminal ribonuclease
	1y94A (124)	12-24 (13)	12-24 (13)	15-24 (10)						

933	2nuiA (124)	15-22 (8)	15-22 (8)	15-22 (8)	N	0.60	0.84	100.00% (124/124)	80.65% (100/124)	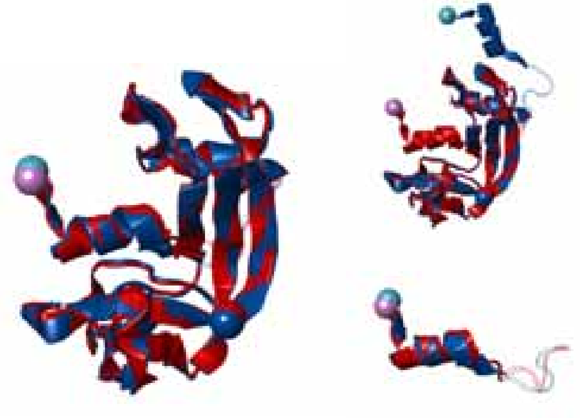 Ribonuclease pancreatic<br>Protein (bovine seminal ribonuclease)
	11bgA (124)	15-22 (8)	15-22 (8)	15-22 (8)						
934	2nuiA (124)	15-23 (9)	15-23 (9)	17-22 (6)	N	0.66	0.87	100.00% (124/124)	80.65% (100/124)	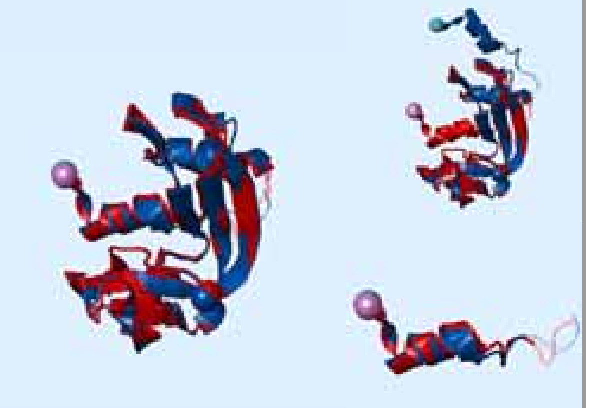 Ribonuclease pancreatic<br>Seminal ribonuclease
	1y92B (124)	15-23 (9)	15-23 (9)	17-22 (6)						
935	2nuiA (124)	15-22 (8)	15-22 (8)	15-22 (8)	N	0.58	0.83	100.00% (124/124)	81.45% (101/124)	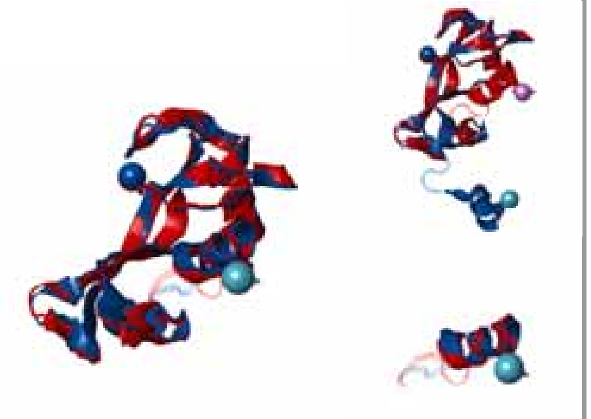 Ribonuclease pancreatic<br>Seminal ribonuclease
	3bcoA (124)	15-22 (8)	15-22 (8)	15-22 (8)						

936	2nuiA (124)	15-22 (8)	15-22 (8)	15-22 (8)	N	0.56	0.89	100.00% (124/124)	83.06% (103/124)	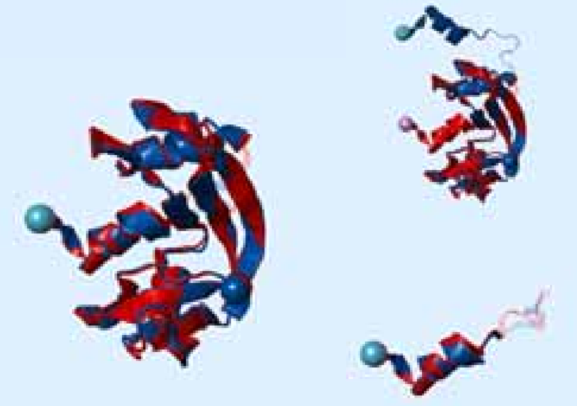 Ribonuclease pancreatic<br>Seminal ribonuclease
	1y94A (124)	15-22 (8)	15-22 (8)	15-22 (8)						
937	1qwqA (124)	12-29 (18)	12-24 (13)	13-24 (12)	N	0.65	1.79	97.58% (121/124)	92.74% (115/124)	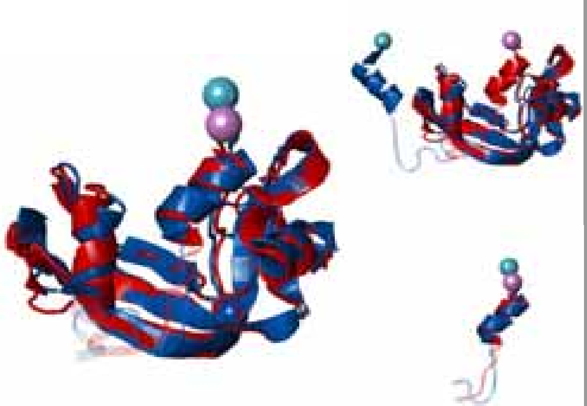 Ribonuclease<br>Protein (bovine seminal ribonuclease)
	11bgA (124)	12-29 (18)	12-24 (13)	13-24 (12)						
938	1qwqA (124)	12-26 (15)	12-24 (13)	19-24 (6)	N	0.54	1.59	99.19% (123/124)	98.39% (122/124)	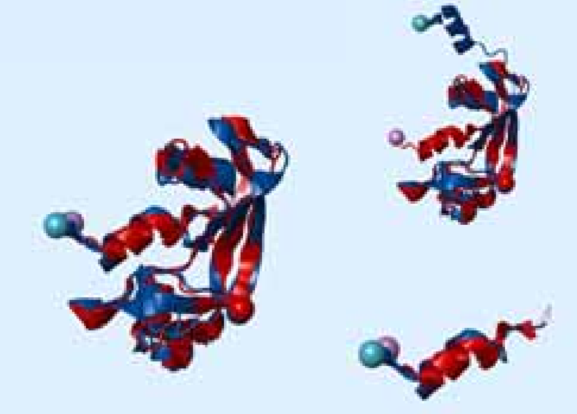 Ribonuclease<br>Seminal ribonuclease
	1y92B (124)	12-26 (15)	12-24 (13)	19-24 (6)						

939	1qwqA (124)	12-29 (18)	12-24 (13)	13-24 (12)	N	0.62	1.78	97.58% (121/124)	92.74% (115/124)	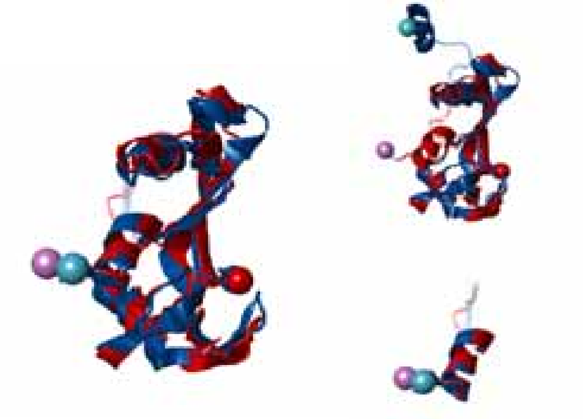 Ribonuclease<br>Seminal ribonuclease
	3bcoA (124)	12-29 (18)	12-24 (13)	13-24 (12)						
940	1qwqA (124)	12-24 (13)	12-24 (13)	13-24 (12)	N	0.63	1.79	98.39% (122/124)	95.97% (119/124)	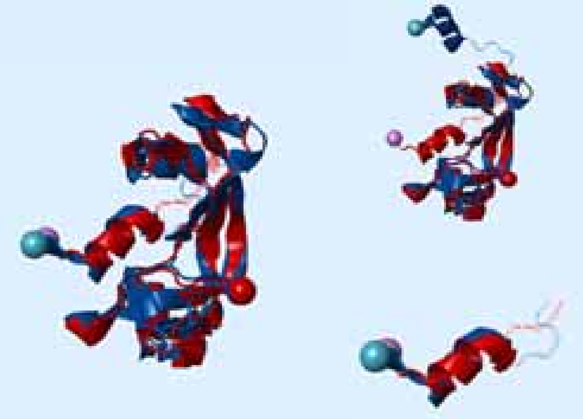 Ribonuclease<br>Seminal ribonuclease
	1y94A (124)	12-24 (13)	12-24 (13)	13-24 (12)						
941	1izpA (124)	15-22 (8)	15-22 (8)	15-22 (8)	N	0.57	0.89	100.00% (124/124)	80.65% (100/124)	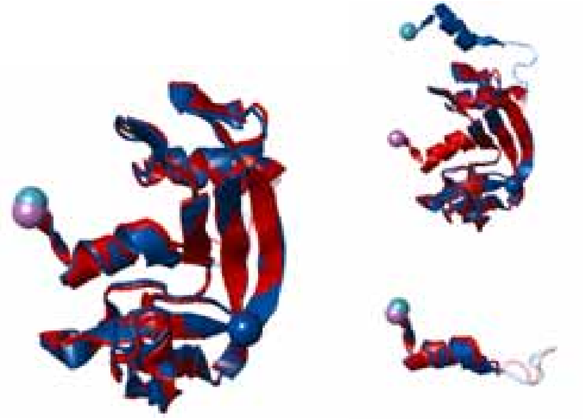 Ribonuclease A<br>Protein (bovine seminal ribonuclease)
	11bgA (124)	15-22 (8)	15-22 (8)	15-22 (8)						

942	1izpA (124)	15-23 (9)	15-22 (8)	17-22 (6)	N	0.65	0.86	100.00% (124/124)	80.65% (100/124)	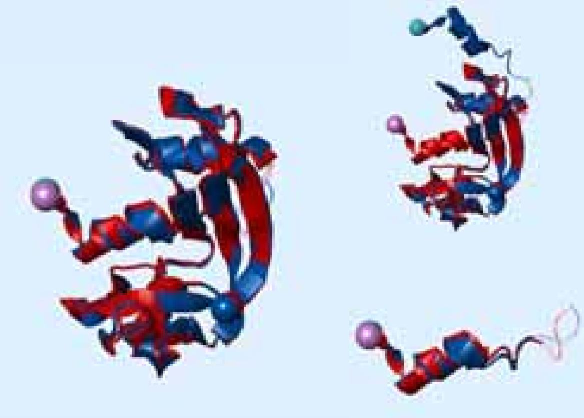 Ribonuclease A<br>Seminal ribonuclease
	1y92B (124)	15-23 (9)	15-22 (8)	17-22 (6)						
943	1izpA (124)	15-22 (8)	15-22 (8)	15-22 (8)	N	0.58	0.85	100.00% (124/124)	81.45% (101/124)	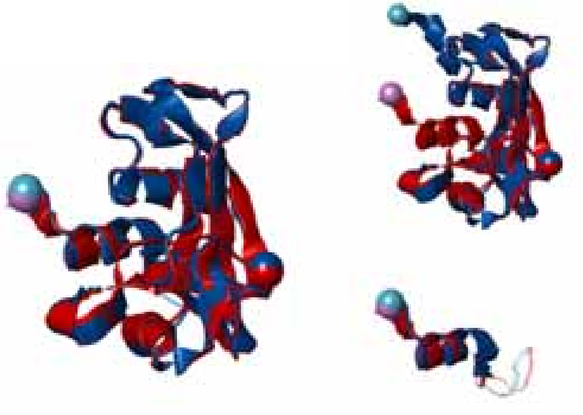 Ribonuclease A<br>Seminal ribonuclease
	3bcoA (124)	15-22 (8)	15-22 (8)	15-22 (8)						
944	1izpA (124)	15-22 (8)	15-22 (8)	15-22 (8)	N	0.53	0.95	100.00% (124/124)	83.06% (103/124)	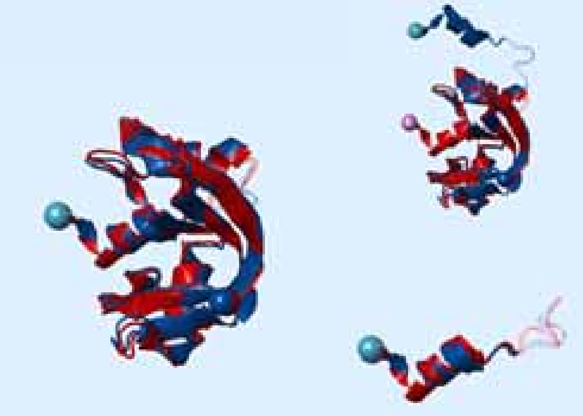 Ribonuclease A<br>Seminal ribonuclease
	1y94A (124)	15-22 (8)	15-22 (8)	15-22 (8)						

945	2v1qB (58)	27-38 (12)	30-38 (9)	30-37 (8)	C	0.72	1.12	91.38% (53/58)	31.03% (18/58)	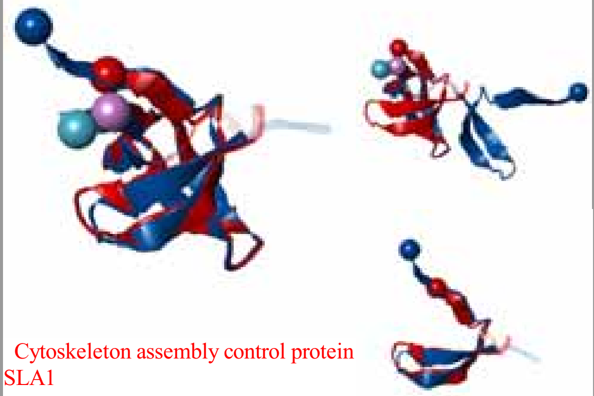<br>Cytoskeleton assembly control protein SLA1<br>Epidermal growth factor receptor kinase subst
	1i07A (59)	31-40 (10)	34-40 (7)	30-37 (8)						
946	1sshA (60)	31-42 (12)	31-39 (9)	31-39 (9)	C	0.65	1.41	89.83% (53/59)	27.12% (16/59)	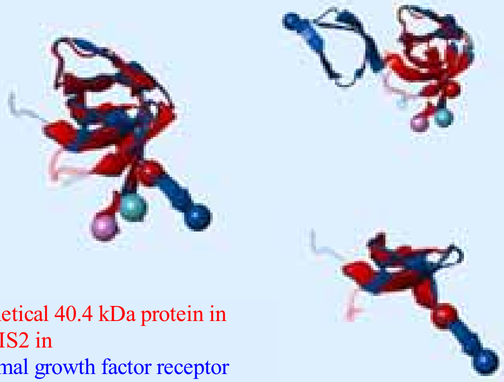<br>Hypothetical 40.4 kDa protein in PES4-HIS2 in<br>Epidermal growth factor receptor kinase subst
	1i07A (59)	34-42 (9)	34-39 (6)	31-39 (9)						
947	1h3hA (60)	46-53 (8)	46-53 (8)	50-53 (4)	C	0.69	1.48	93.22% (55/59)	30.51% (18/59)	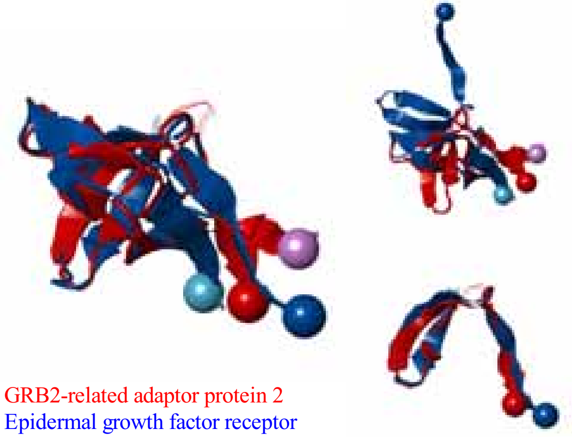<br>GRB2-related adaptor protein 2<br>Epidermal growth factor receptor kinase subst
	1i07A (59)	34-40 (7)	34-40 (7)	50-53 (4)						

948	2semB (59)	284-302 (19)	284-291 (8)	284-291 (8)	C	0.75	1.35	94.92% (56/59)	22.03% (13/59)	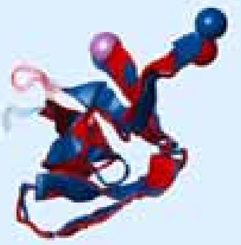 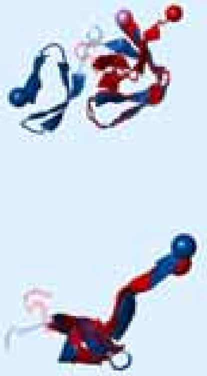 Protein (SEX muscle abnormal protein 5)<br>Epidermal growth factor receptor kinase subst
	1i07A (59)	34-52 (19)	34-40 (7)	284-291 (8)						
949	1jo8A (58)	25-35 (11)	26-34 (9)	26-34 (9)	C	0.70	1.41	93.10% (54/58)	22.41% (13/58)	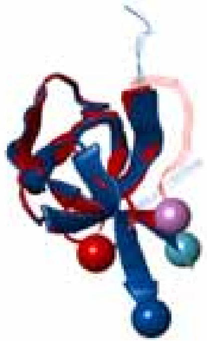 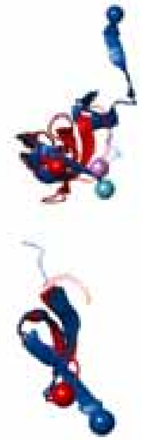 Actin binding protein<br>Epidermal growth factor receptor kinase subst
	1i07A (59)	31-40 (10)	32-39 (8)	26-34 (9)						
950	4hckA (61)	90-137 (48)	108-114 (7)	110-114 (5)	N	0.72	1.52	96.61% (57/59)	27.12% (16/59)	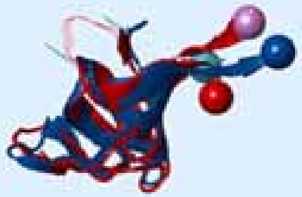 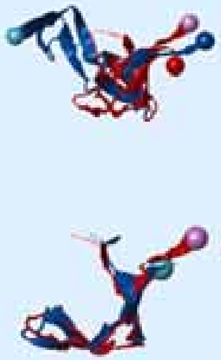 Hematopoietic cell kinase<br>Epidermal growth factor receptor kinase subst
	1i07A (59)	16-62 (47)	34-40 (7)	110-114 (5)						

951	1rn7A (112)	57-65 (9)	58-63 (6)	58-61 (4)	N	0.56	0.92	99.09% (109/110)	52.73% (58/110)	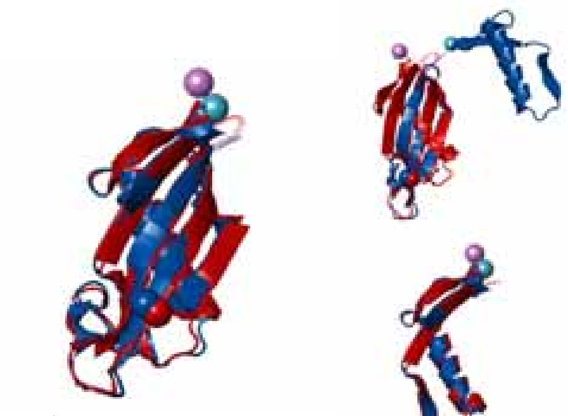 Cystatin D<br>Cystatin C
	1r4cA (110)	56-63 (8)	57-61 (5)	58-61 (4)						
952	1yvbI (111)	48-64 (17)	53-59 (7)	55-57 (3)	C	0.82	1.24	98.18% (108/110)	44.55% (49/110)	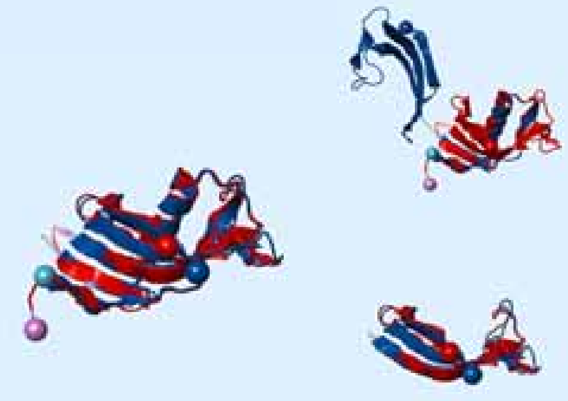 Cystatin<br>Cystatin C
	1r4cA (110)	50-66 (17)	55-61 (7)	55-57 (3)						
953	1yupE (154)	120-129 (10)	124-129 (6)	124-129 (6)	C	0.56	1.94	90.91% (140/154)	16.23% (25/154)	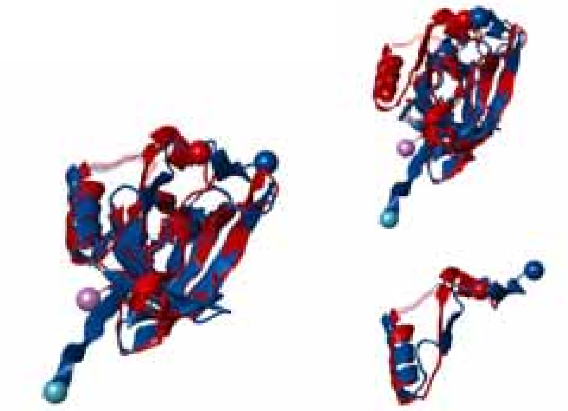 Beta-lactoglobulin<br>Odorant-binding protein
	1g85A (159)	117-125 (9)	121-125 (5)	124-129 (6)						

954	1yupE (154)	120-129 (10)	124-129 (6)	124-128 (5)	C	0.59	1.89	91.39% (138/151)	16.56% (25/151)	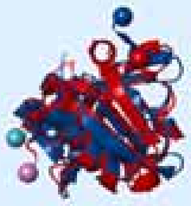 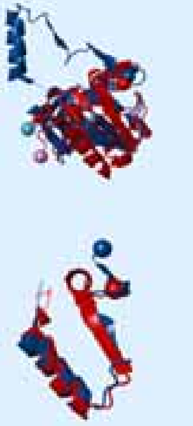 Beta-lactoglobulin<br>Odorant binding protein
	1pboB (151)	117-125 (9)	121-125 (5)	124-128 (5)						
955	1yupE (154)	124-129 (6)	124-129 (6)	124-129 (6)	C	0.56	1.94	90.91% (140/154)	16.23% (25/154)	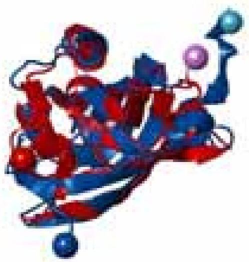 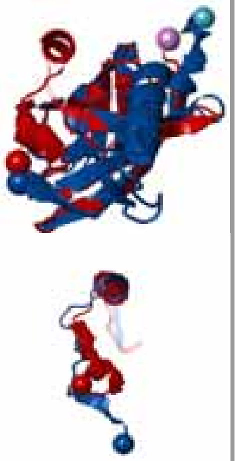 Beta-lactoglobulin<br>Odorant-binding protein
	1gt3A (159)	121-125 (5)	121-125 (5)	124-129 (6)						
956	1yupE (154)	121-129 (9)	124-129 (6)	124-129 (6)	C	0.56	2.03	91.56% (141/154)	16.23% (25/154)	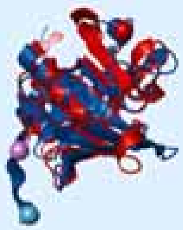 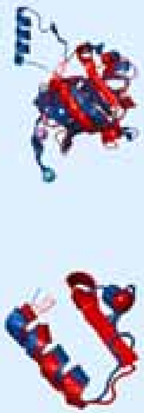 Beta-lactoglobulin<br>Odorant-binding protein
	1gt1A (158)	118-125 (8)	121-125 (5)	124-129 (6)						

957	1qwdA (167)	142-147 (6)	143-147 (5)	143-147 (5)	C	0.35	2.34	77.36% (123/159)	8.18% (13/159)	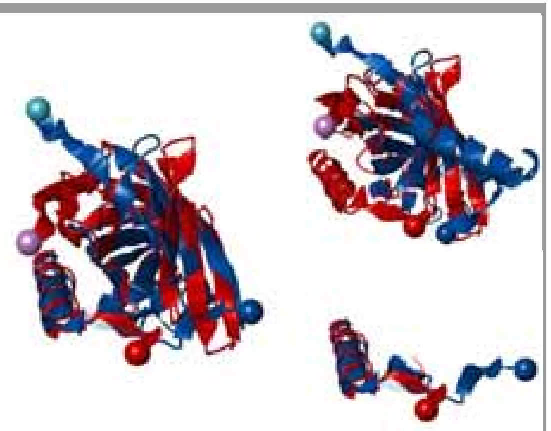<br>Outer membrane lipoprotein blc<br>Odorant-binding protein
	1g85A (159)	120-124 (5)	121-124 (4)	143-147 (5)						
958	1qwdA (167)	142-147 (6)	143-147 (5)	143-147 (5)	C	0.36	2.33	77.36% (123/159)	8.18% (13/159)	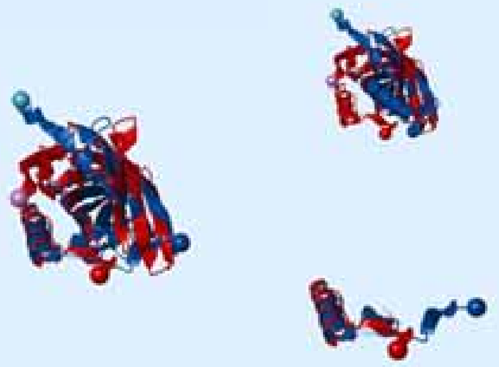<br>Outer membrane lipoprotein blc<br>Odorant-binding protein
	1gt3A (159)	120-124 (5)	121-124 (4)	143-147 (5)						
959	1qwdA (167)	142-147 (6)	143-147 (5)	143-147 (5)	C	0.36	2.40	78.48% (124/158)	8.23% (13/158)	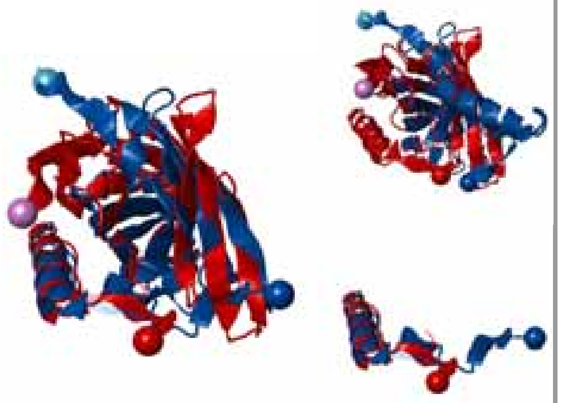<br>Outer membrane lipoprotein blc<br>Odorant-binding protein
	1gt1A (158)	120-124 (5)	121-124 (4)	143-147 (5)						

960	2q39B (151)	306-329 (24)	324-329 (6)	324-329 (6)	C	0.59	1.99	92.05% (139/151)	15.89% (24/151)	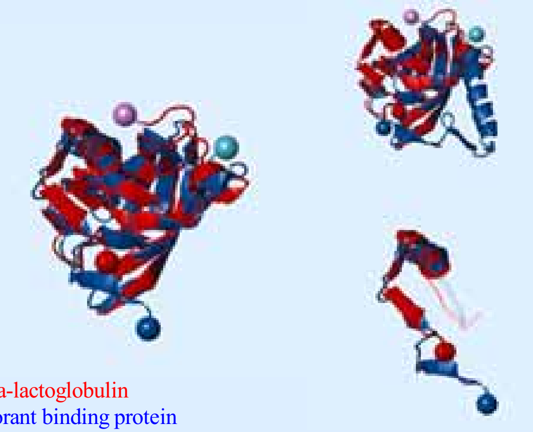 Beta-lactoglobulin<br>Odorant binding protein
	1pboB (151)	102-125 (24)	121-125 (5)	324-329 (6)						
961	1bj7A (150)	113-123 (11)	119-123 (5)	119-123 (5)	C	0.67	1.80	98.00% (147/150)	26.67% (40/150)	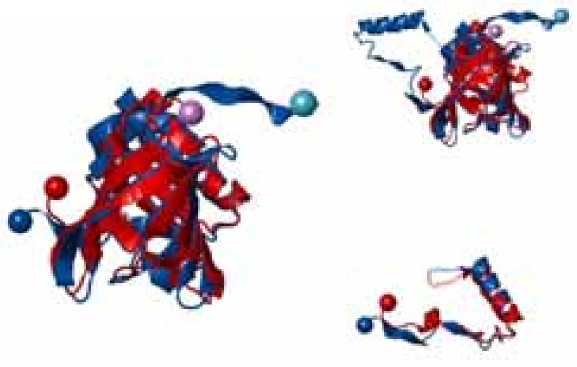 D 2<br>Odorant-binding protein
	1g85A (159)	115-124 (10)	121-124 (4)	119-123 (5)						
962	1bj7A (150)	113-124 (12)	119-122 (4)	119-123 (5)	C	0.70	1.81	98.00% (147/150)	26.67% (40/150)	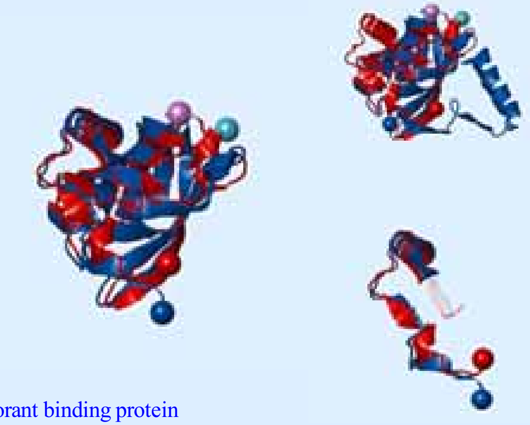 D 2<br>Odorant binding protein
	1pboB (151)	115-125 (11)	121-123 (3)	119-123 (5)						

963	1bj7A<br>(150)	115-124 (10)	119-123 (5)	119-123 (5)	C	0.67	1.79	98.00%<br>(147/150)	27.33%<br>(41/150)	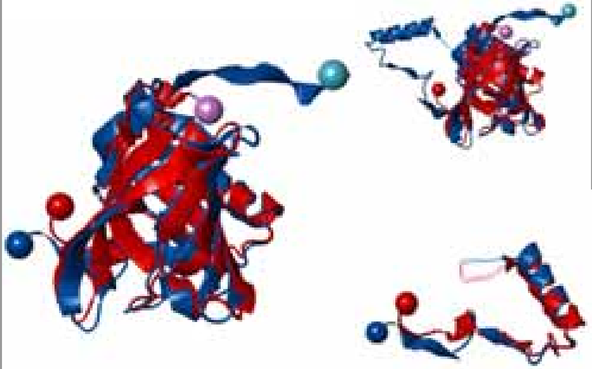<br>D 2<br>Odorant-binding protein
	1gt3A<br>(159)	117-125 (9)	121-124 (4)	119-123 (5)						
964	1bj7A<br>(150)	113-124 (12)	119-123 (5)	119-123 (5)	C	0.67	1.80	98.00%<br>(147/150)	27.33%<br>(41/150)	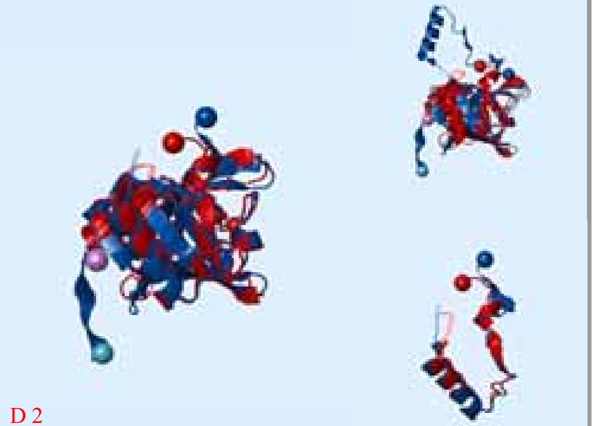<br>D 2<br>Odorant-binding protein
	1gt1A<br>(158)	115-125 (11)	121-124 (4)	119-123 (5)						
965	1dzkA<br>(148)	117-125 (9)	119-124 (6)	119-123 (5)	C	0.74	1.41	98.65%<br>(146/148)	42.57%<br>(63/148)	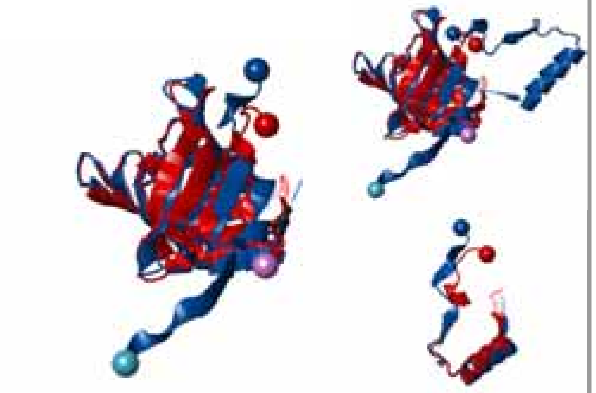<br>Odorant-binding protein<br>Odorant-binding protein
	1g85A<br>(159)	118-125 (8)	120-124 (5)	119-123 (5)						

966	1dzkA (148)	119-125 (7)	119-124 (6)	119-123 (5)	C	0.78	1.43	98.65% (146/148)	43.24% (64/148)	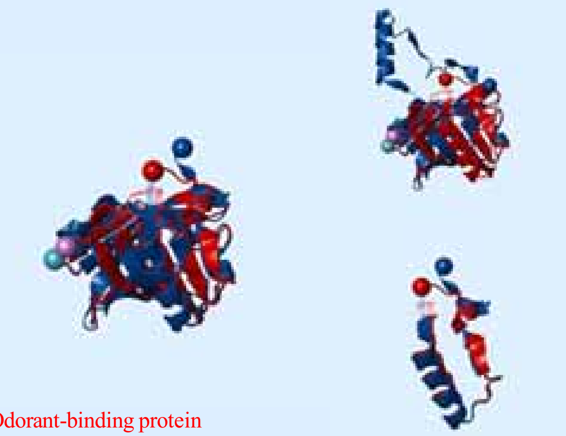 Odorant-binding protein<br>Odorant binding protein
	1pboB (151)	120-125 (6)	120-124 (5)	119-123 (5)						
967	1dzkA (148)	117-125 (9)	119-124 (6)	119-123 (5)	C	0.74	1.40	98.65% (146/148)	41.89% (62/148)	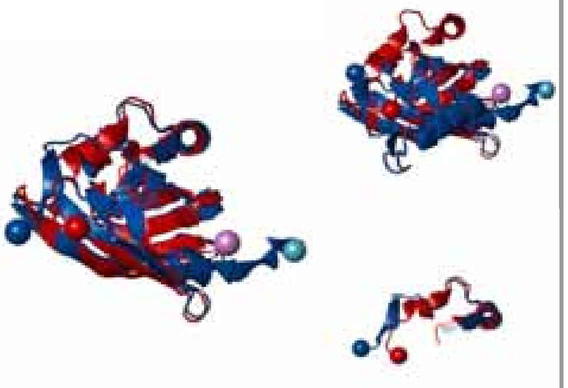 Odorant-binding protein<br>Odorant-binding protein
	1gt3A (159)	118-125 (8)	120-124 (5)	119-123 (5)						
968	1dzkA (148)	119-125 (7)	119-124 (6)	119-123 (5)	C	0.74	1.42	98.65% (146/148)	41.89% (62/148)	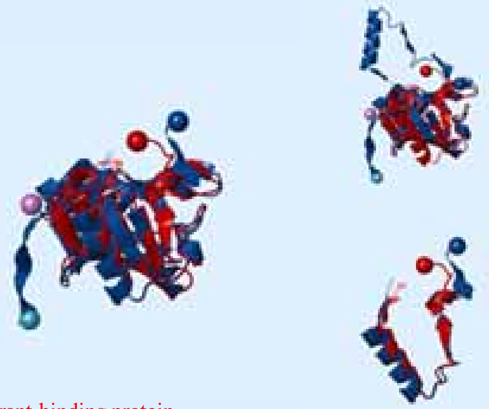 Odorant-binding protein<br>Odorant-binding protein
	1gt1A (158)	120-125 (6)	120-124 (5)	119-123 (5)						

969	1ew3A (159)	24-32 (9)	24-32 (9)	30-30 (1)	N	0.30	1.83	75.47% (120/159)	16.35% (26/159)	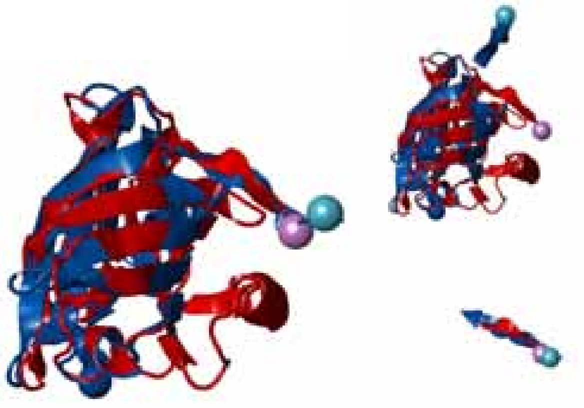<br>Allergen EQU C 1<br>Odorant-binding protein
	1g85A (159)	3-12 (10)	3-12 (10)	30-30 (1)						
970	1ew3A (159)	140-145 (6)	141-145 (5)	141-145 (5)	C	0.62	1.89	93.08% (148/159)	22.01% (35/159)	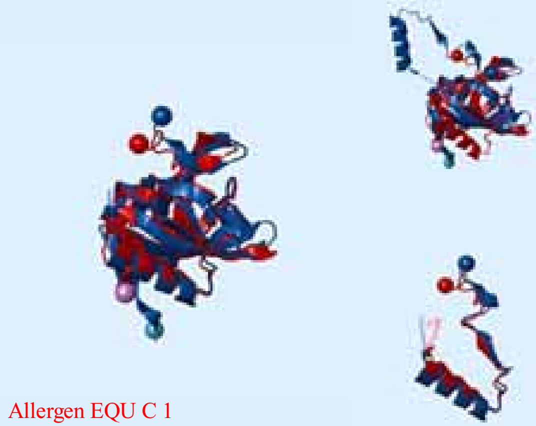<br>Allergen EQU C 1<br>Odorant-binding protein
	1g85A (159)	120-124 (5)	121-124 (4)	141-145 (5)						
971	1ew3A (159)	140-146 (7)	141-145 (5)	141-145 (5)	C	0.65	1.91	98.01% (148/151)	23.18% (35/151)	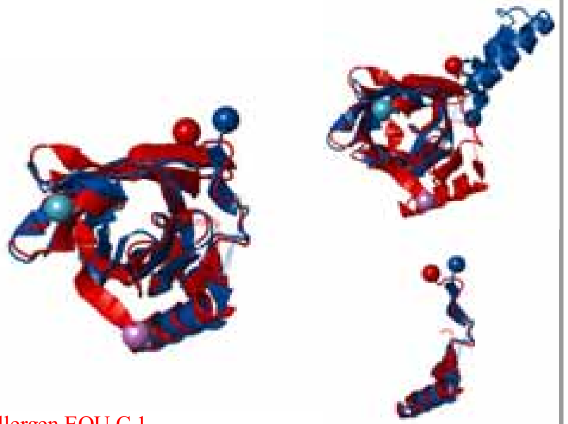<br>Allergen EQU C 1<br>Odorant binding protein
	1pboB (151)	120-125 (6)	121-124 (4)	141-145 (5)						

972	1ew3A (159)	24-35 (12)	24-32 (9)	30-30 (1)	N	0.30	1.83	75.47% (120/159)	16.98% (27/159)	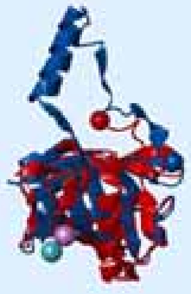 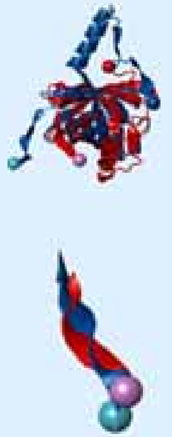 Allergen EQU C 1<br>Odorant-binding protein
	1gt3A (159)	3-15 (13)	3-12 (10)	30-30 (1)						
973	1ew3A (159)	141-145 (5)	141-145 (5)	141-145 (5)	C	0.62	1.89	93.08% (148/159)	22.64% (36/159)	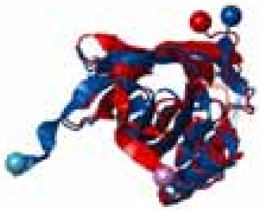 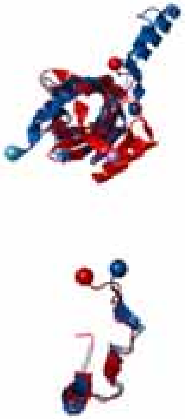 Allergen EQU C 1<br>Odorant-binding protein
	1gt3A (159)	121-124 (4)	121-124 (4)	141-145 (5)						
974	1ew3A (159)	24-32 (9)	29-32 (4)	30-30 (1)	N	0.31	1.83	75.95% (120/158)	17.09% (27/158)	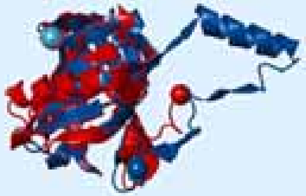 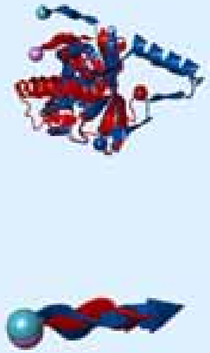 Allergen EQU C 1<br>Odorant-binding protein
	1gt1A (158)	3-12 (10)	8-12 (5)	30-30 (1)						

975	1ew3A (159)	140-145 (6)	141-145 (5)	141-145 (5)	C	0.63	1.89	93.67% (148/158)	22.78% (36/158)	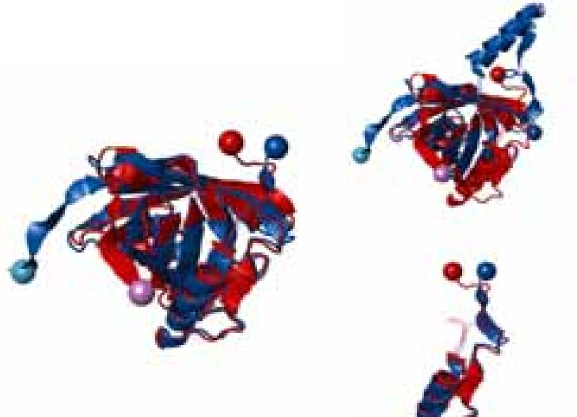 Allergen EQU C 1<br>Odorant-binding protein
	1gt1A (158)	120-124 (5)	121-124 (4)	141-145 (5)						
976	2a2uA (158)	122-129 (8)	122-129 (8)	122-129 (8)	C	0.70	1.47	95.36% (144/151)	26.49% (40/151)	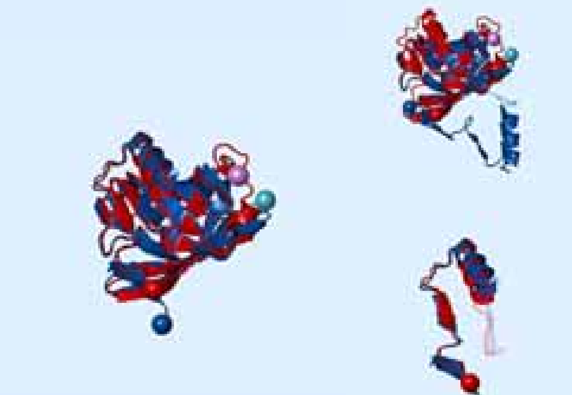 Protein (alpha-2U-globulin)<br>Odorant binding protein
	1pboB (151)	121-127 (7)	121-127 (7)	122-129 (8)						
977	1hcfX (101)	298-301 (4)	298-300 (3)	298-300 (3)	N	0.73	1.29	96.04% (97/101)	45.54% (46/101)	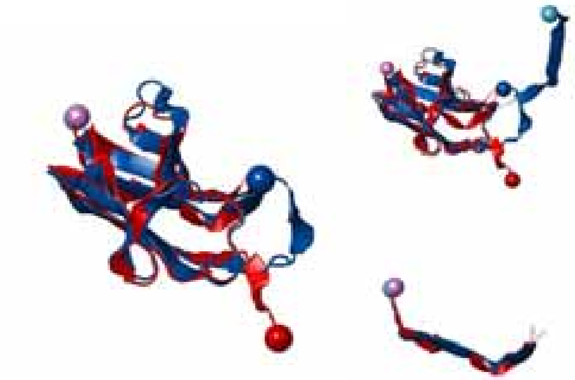 Bdnf/nt-3 growth factors receptor<br>HIGH affinity nerve growth factor receptor
	1he7A (107)	296-299 (4)	296-298 (3)	298-300 (3)						

978	1fhgA (102)	50-65 (16)	50-58 (9)	50-57 (8)	N	0.56	1.99	92.16% (94/102)	14.71% (15/102)	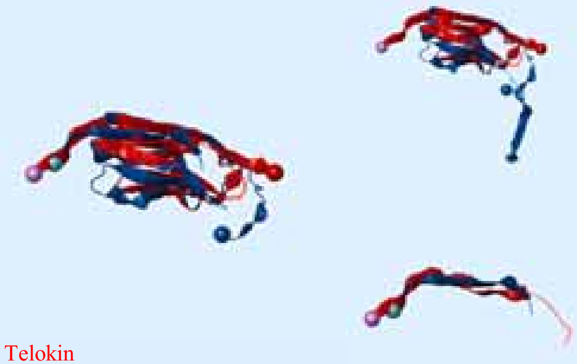 Telokin<br>HIGH affinity nerve growth factor receptor
	1he7A (107)	295-305 (11)	295-298 (4)	50-57 (8)						
979	2dm3A (110)	7-44 (38)	7-31 (25)	21-25 (5)	N	0.55	1.90	88.79% (95/107)	18.69% (20/107)	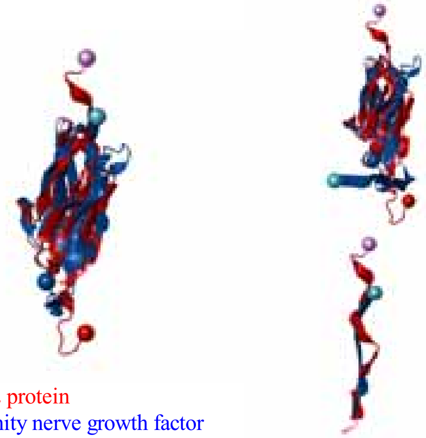 KIAA0992 protein<br>HIGH affinity nerve growth factor receptor
	1he7A (107)	283-316 (34)	283-303 (21)	21-25 (5)						
980	1g1cA (98)	5-28 (24)	5-21 (17)	20-21 (2)	N	0.63	1.75	95.92% (94/98)	17.35% (17/98)	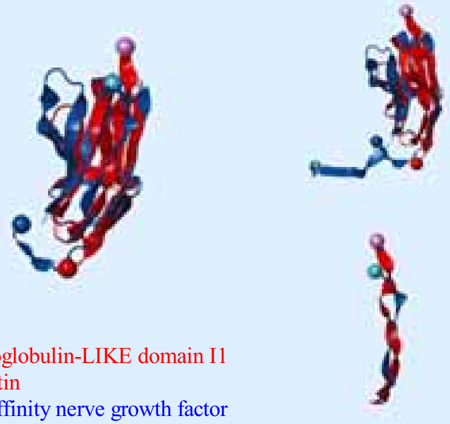 Immunoglobulin-LIKE domain I1 FROM titin<br>HIGH affinity nerve growth factor receptor
	1he7A (107)	283-304 (22)	283-297 (15)	20-21 (2)						

981	1u2hA (96)	19-40 (22)	19-36 (18)	34-35 (2)	N	0.67	1.46	95.83% (92/96)	19.79% (19/96)	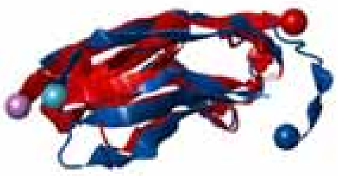 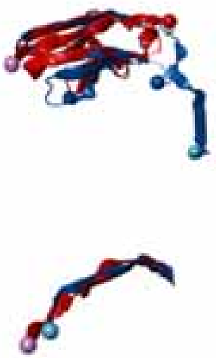 Aortic preferentially expressed protein 1 HIGH affinity nerve growth factor receptor
	1he7A (107)	283-302 (20)	283-298 (16)	34-35 (2)						
982	2a5mA (177)	86-93 (8)	86-92 (7)	89-92 (4)	C	0.77	1.42	98.31% (174/177)	33.90% (60/177)	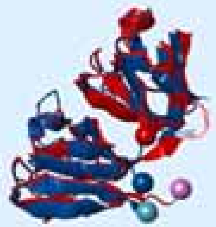 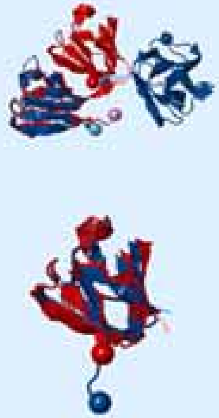 Gamma crystallin S Beta crystallin B2
	1ytqA (181)	100-106 (7)	100-105 (6)	89-92 (4)						
983	2a5mA (177)	83-93 (11)	84-93 (10)	89-93 (5)	C	0.77	1.43	98.31% (174/177)	33.90% (60/177)	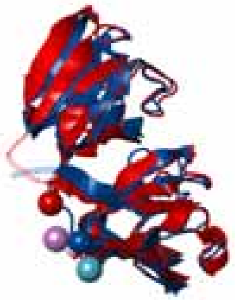 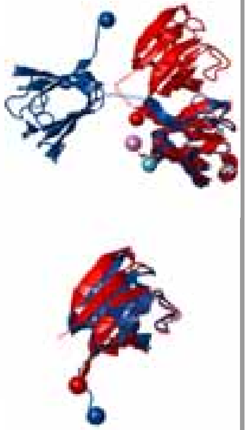 Gamma crystallin S Beta B2-crystallin
	2bb2A (181)	79-88 (10)	80-88 (9)	89-93 (5)						

984	1a45A (173)	79-91 (13)	82-89 (8)	82-88 (7)	C	0.74	1.51	98.27% (170/173)	35.26% (61/173)	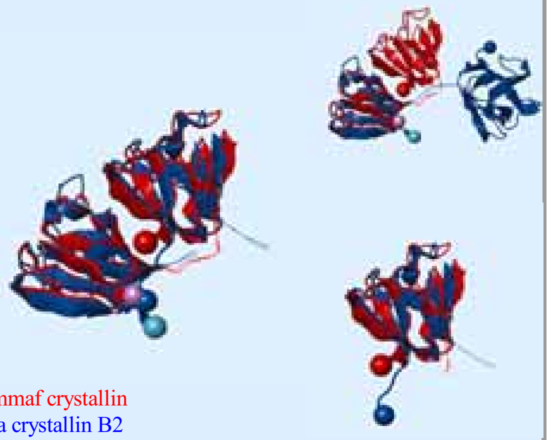 Gammaf crystallin<br>Beta crystallin B2
	1ytqA (181)	97-109 (13)	100-107 (8)	82-88 (7)						
985	2v2uA (173)	79-89 (11)	79-87 (9)	84-87 (4)	N	0.80	1.22	98.84% (171/173)	35.84% (62/173)	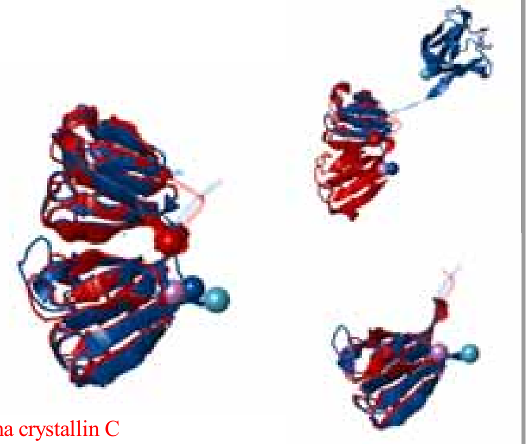 Gamma crystallin C<br>Beta B2-crystallin
	2bb2A (181)	79-90 (12)	79-88 (10)	84-87 (4)						
986	1ammA (174)	79-90 (12)	79-87 (9)	84-87 (4)	C	0.80	1.23	98.85% (172/174)	36.78% (64/174)	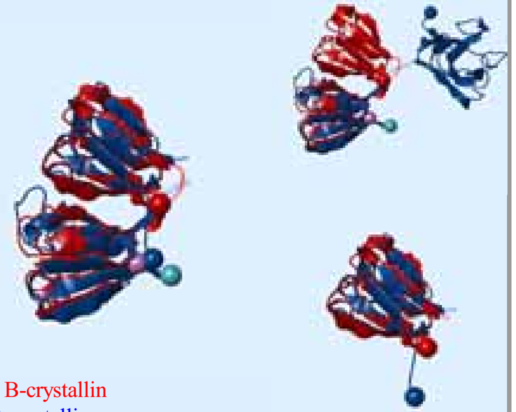 Gamma B-crystallin<br>Beta B2-crystallin
	2bb2A (181)	79-90 (12)	79-87 (9)	84-87 (4)						

987	1zwmA (177)	86-93 (8)	86-92 (7)	89-92 (4)	C	0.77	1.45	98.31% (174/177)	33.90% (60/177)	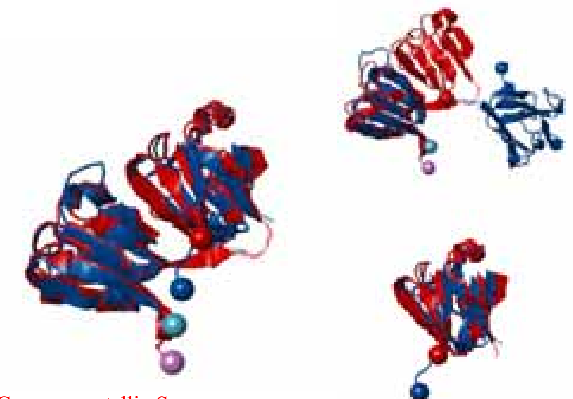 Gamma crystallin S<br>Beta crystallin B2
	1ytqA (181)	100-106 (7)	100-105 (6)	89-92 (4)						
988	2jdfA (175)	79-91 (13)	82-89 (8)	82-87 (6)	C	0.79	1.30	98.29% (172/175)	34.86% (61/175)	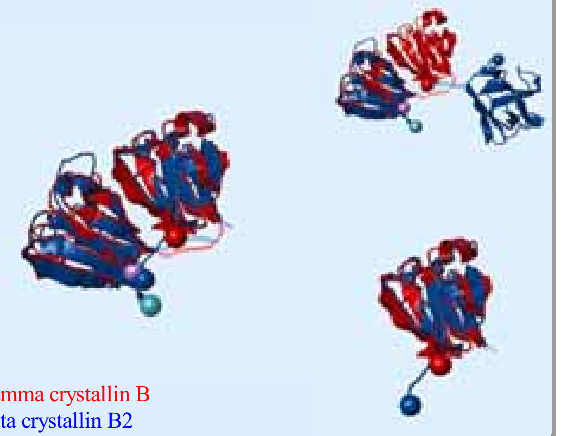 Gamma crystallin B<br>Beta crystallin B2
	1ytqA (181)	97-109 (13)	100-107 (8)	82-87 (6)						
989	2jdfA (175)	79-90 (12)	80-88 (9)	82-87 (6)	C	0.78	1.31	98.29% (172/175)	34.86% (61/175)	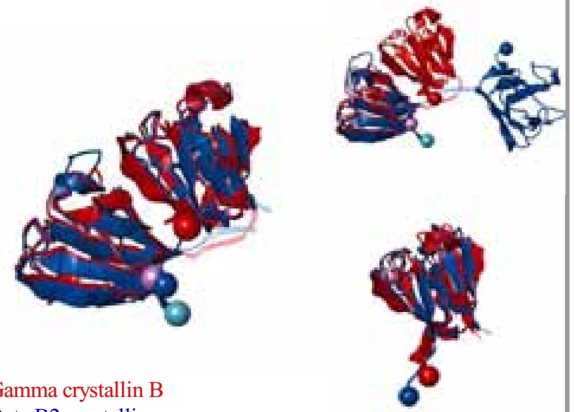 Gamma crystallin B<br>Beta B2-crystallin
	2bb2A (181)	79-90 (12)	80-88 (9)	82-87 (6)						

990	1h4aX (173)	77-89 (13)	79-88 (10)	82-88 (7)	N	0.80	1.23	98.84% (171/173)	34.68% (60/173)	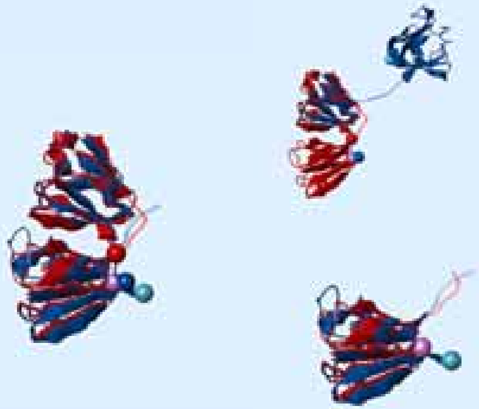 Gamma crystallin D<br>Beta B2-crystallin
	2bb2A (181)	77-89 (13)	79-88 (10)	82-88 (7)						
991	2jdgA (171)	80-90 (11)	82-90 (9)	82-88 (7)	C	0.80	1.29	100.00% (171/171)	34.50% (59/171)	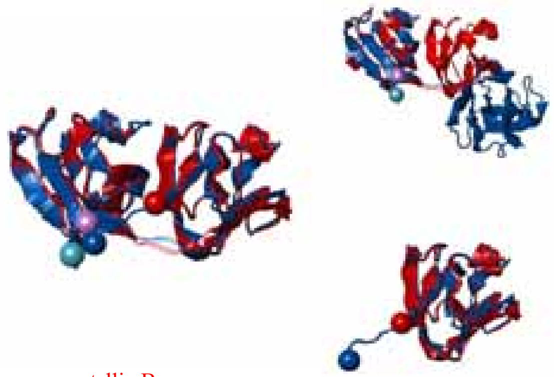 Gamma crystallin B<br>Beta crystallin B2
	1ytqA (181)	98-108 (11)	100-108 (9)	82-88 (7)						
992	1ez3B (124)	104-114 (11)	104-114 (11)	104-114 (11)	C	0.41	1.59	85.39% (76/89)	12.36% (11/89)	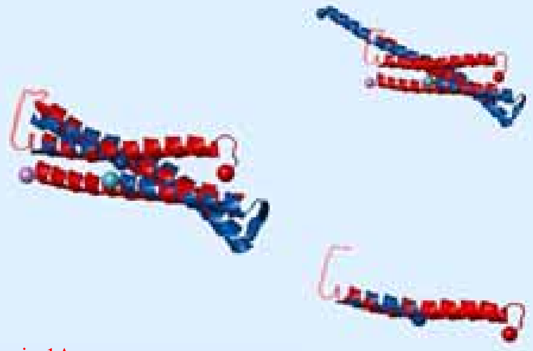 Syntaxin-1A<br>T-snare affecting a LATE golgi compartment PR
	2c5jB (89)	77-78 (2)	77-78 (2)	104-114 (11)						

993	1lvfA (106)	70-76 (7)	70-76 (7)	70-76 (7)	C	0.59	1.90	98.88% (88/89)	22.47% (20/89)	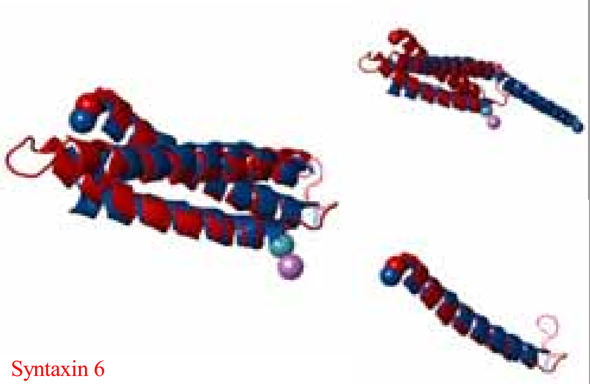 Syntaxin 6<br>T-snare affecting a LATE golgi compartment PR
	2c5jB (89)	61-62 (2)	61-62 (2)	70-76 (7)						
994	2iggA (64)	40-64 (25)	41-55 (15)	51-55 (5)	C	0.51	2.10	92.19% (59/64)	7.81% (5/64)	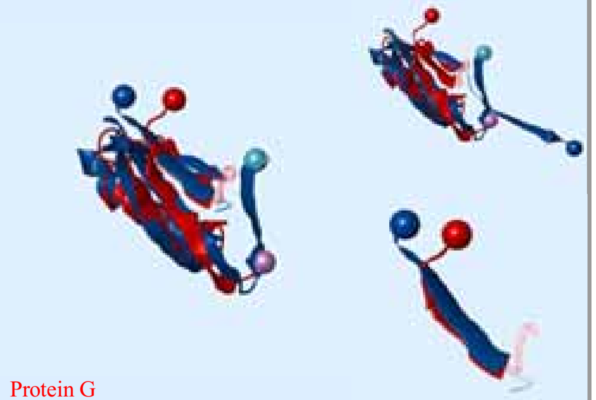 Protein G<br>Protein L
	1k51A (72)	38-64 (27)	39-55 (17)	51-55 (5)						
995	2igdA (61)	49-60 (12)	49-60 (12)	52-52 (1)	C	0.53	2.02	95.08% (58/61)	14.75% (9/61)	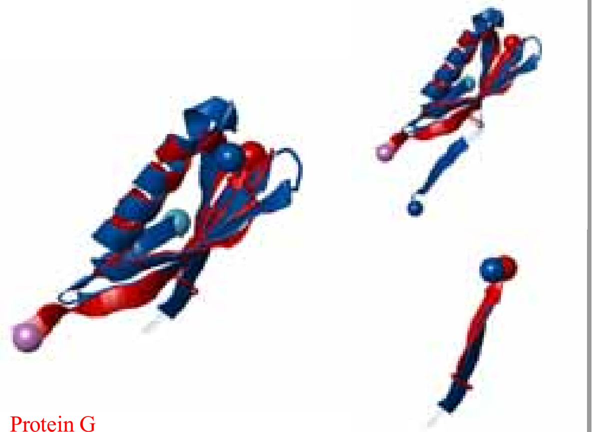 Protein G<br>Protein L
	1k51A (72)	50-63 (14)	50-63 (14)	52-52 (1)						

996	1mhhE (63)	868-877 (10)	868-874 (7)	869-874 (6)	C	0.77	0.93	98.41% (62/63)	58.73% (37/63)	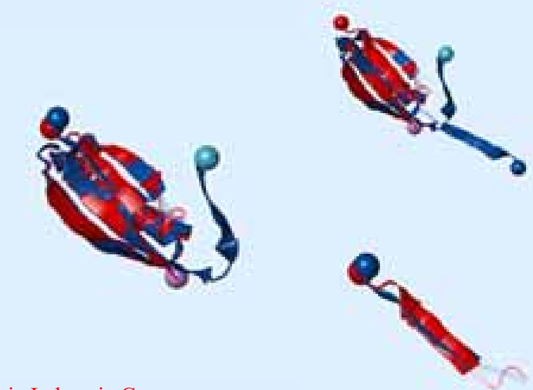 Protein L domain C<br>Protein L
	1k51A (72)	51-59 (9)	51-56 (6)	869-874 (6)						
997	1kh0A (65)	45-58 (14)	50-58 (9)	55-57 (3)	C	0.78	0.86	96.92% (63/65)	78.46% (51/65)	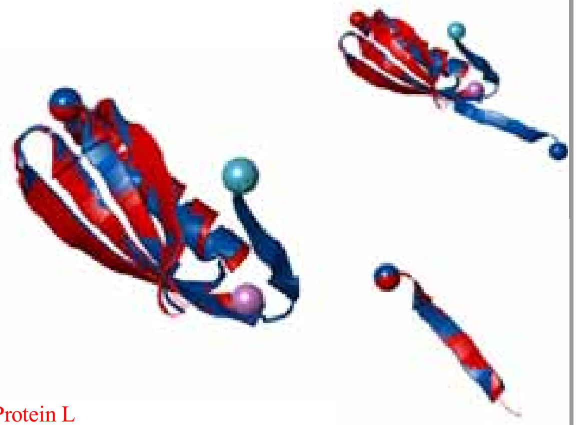 Protein L<br>Protein L
	1k51A (72)	45-56 (12)	50-56 (7)	55-57 (3)						
998	1hz6B (63)	49-57 (9)	50-56 (7)	53-57 (5)	C	0.74	1.29	100.00% (63/63)	98.41% (62/63)	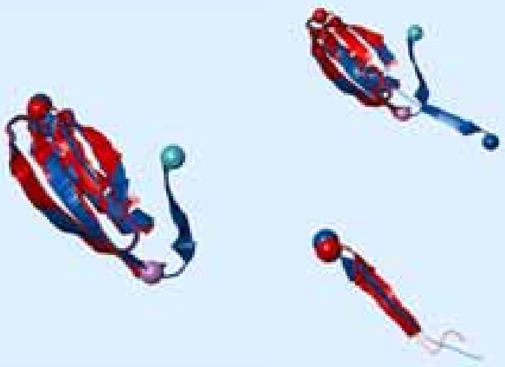 Protein L<br>Protein L
	1k51A (72)	49-57 (9)	50-56 (7)	53-57 (5)						

999	1le4A (139)	39-56 (18)	39-56 (18)	41-56 (16)	N	0.27	2.31	82.84% (111/134)	11.94% (16/134)	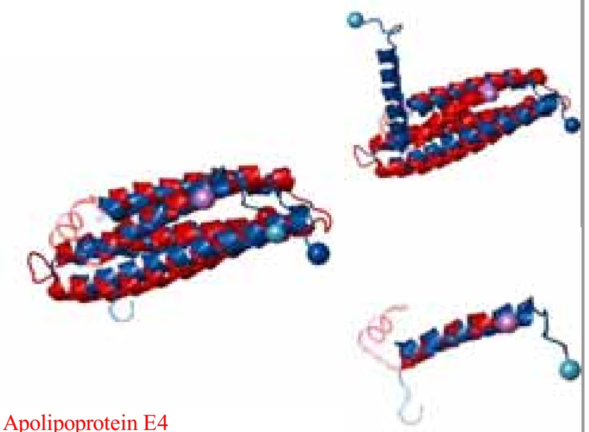 Apolipoprotein E4<br>Focal adhesion kinase 1
	1ow6A (134)	941-950 (10)	941-950 (10)	41-56 (16)						
1000	1k40A (126)	942-950 (9)	942-950 (9)	943-944 (2)	N	0.64	1.05	100.00% (126/126)	96.83% (122/126)	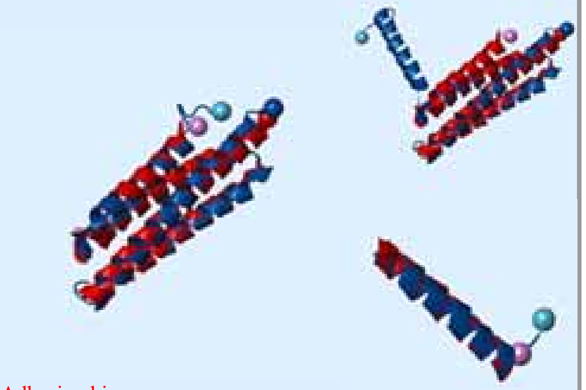 Adhesion kinase<br>Focal adhesion kinase 1
	1ow6A (134)	942-950 (9)	942-950 (9)	943-944 (2)						
1001	1e87A (117)	133-154 (22), 161-183 (23)	150-153 (4), 161-182 (22)	150-153 (4), 168-173 (6)	M	0.67	1.65	95.73% (112/117)	21.37% (25/117)	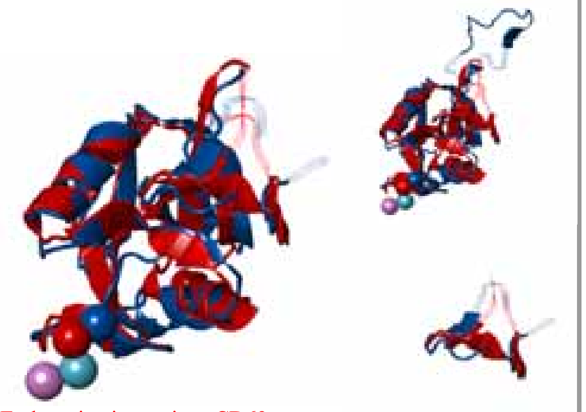 Early activation antigen CD69<br>Echicetin B-chain
	1oz7B (123)	52-78 (27), 85-108 (24)	73-77 (5), 85-107 (23)	150-153 (4), 168-173 (6)						

1002	1e87A (117)	145-155 (11), 162-172 (11)	145-152 (8), 162-172 (11)	146-149 (4), 168-172 (5)	M	0.65	1.67	94.87% (111/117)	22.22% (26/117)	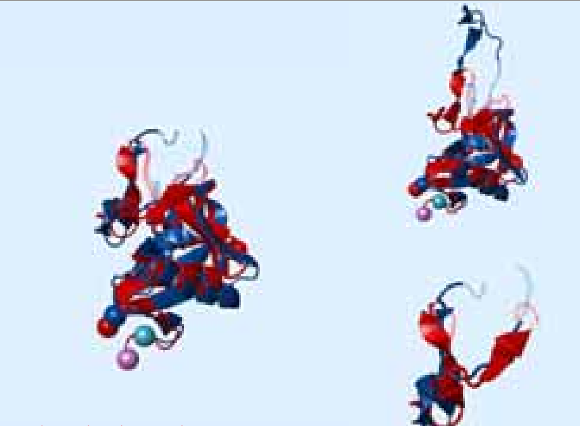 Early activation antigen CD69<br>EMS16 B chain
	1ukmB (124)	67-78 (12), 85-94 (10)	67-75 (9), 85-94 (10)	146-149 (4), 168-172 (5)						
1003	1e87A (117)	147-153 (7), 162-171 (10)	147-153 (7), 162-171 (10)	149-153 (5), 169-171 (3)	M	0.65	1.60	96.58% (113/117)	24.79% (29/117)	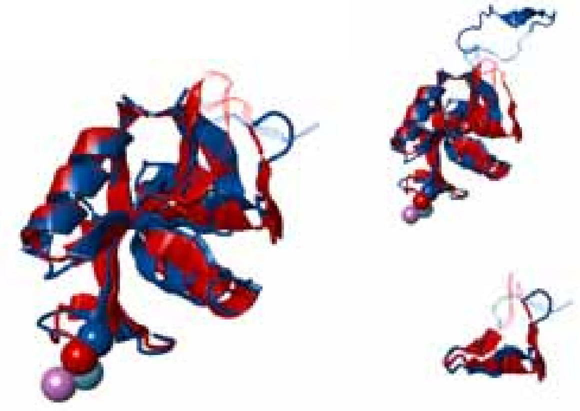 Early activation antigen CD69<br>EMS16 a chain
	1ukmA (131)	74-83 (10), 92-102 (11)	74-83 (10), 92-102 (11)	149-153 (5), 169-171 (3)						
1004	1e87A (117)	146-155 (10), 161-183 (23)	147-153 (7), 161-171 (11)	149-153 (5), 168-171 (4)	M	0.66	1.64	95.73% (112/117)	26.50% (31/117)	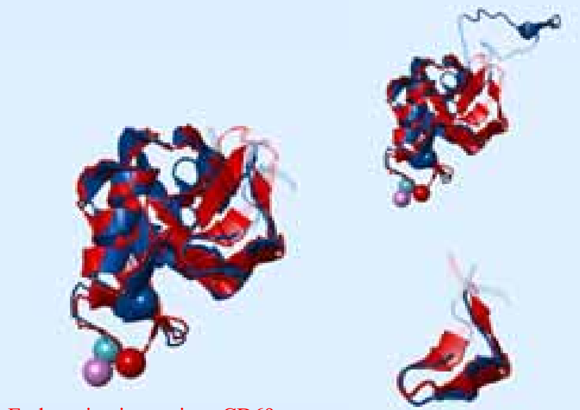 Early activation antigen CD69<br>Bitiscetin alpha chain
	1uexA (125)	71-83 (13), 89-114 (26)	72-81 (10), 89-98 (10)	149-153 (5), 168-171 (4)						

1005	1e87A (117)	147-155 (9), 161-172 (12)	147-152 (6), 161-172 (12)	147-152 (6), 168-172 (5)	M	0.69	1.62	96.58% (113/117)	23.08% (27/117)	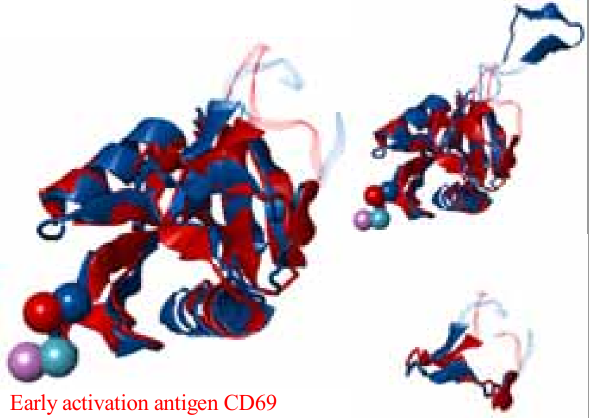 Early activation antigen CD69<br>Coagulation factor IX-binding protein B chain
	1j34B (123)	270-279 (10), 285-295 (11)	270-276 (7), 285-295 (11)	147-152 (6), 168-172 (5)						
1006	1e87A (117)	147-155 (9), 161-183 (23)	147-153 (7), 161-172 (12)	149-153 (5), 168-172 (5)	M	0.69	1.67	97.44% (114/117)	19.66% (23/117)	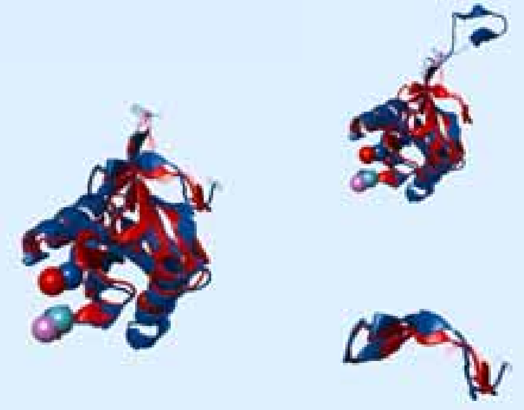 Early activation antigen CD69<br>Platelet aggregation inducer
	1jwiB (123)	72-81 (10), 87-110 (24)	72-79 (8), 87-97 (11)	149-153 (5), 168-172 (5)						
1007	1e87A (117)	133-155 (23), 158-189 (32)	146-153 (8), 161-172 (12)	147-153 (7), 167-172 (6)	M	0.66	1.81	98.29% (115/117)	22.22% (26/117)	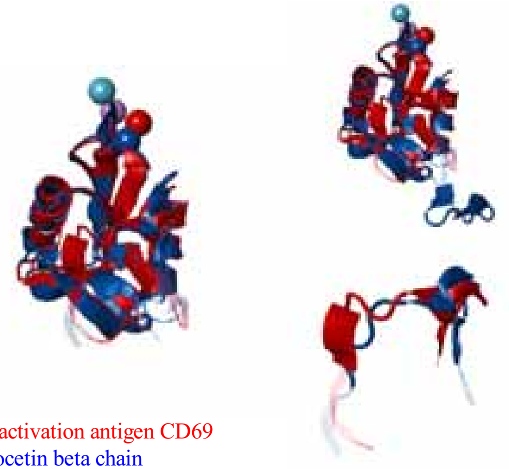 Early activation antigen CD69<br>Mucrocetin beta chain
	1v4lB (125)	254-281 (28), 284-316 (33)	271-279 (9), 287-297 (11)	147-153 (7), 167-172 (6)						

1008	1e87A (117)	147-154 (8), 161-171 (11)	147-154 (8), 162-171 (10)	149-152 (4), 169-171 (3)	M	0.65	1.50	94.87% (111/117)	23.08% (27/117)	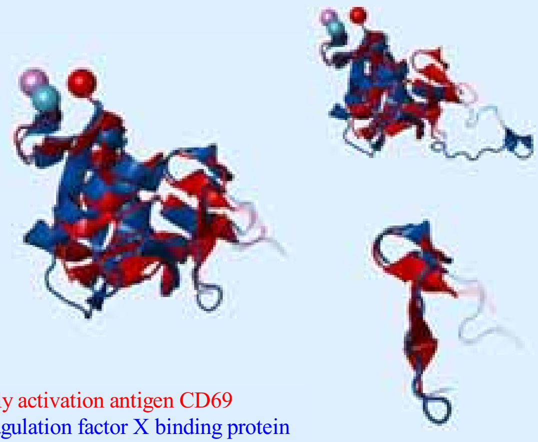 Early activation antigen CD69<br>Coagulation factor X binding protein
	1iodA (129)	72-82 (11), 89-100 (12)	72-82 (11), 90-100 (11)	149-152 (4), 169-171 (3)						
1009	1e87A (117)	144-153 (10), 161-183 (23)	144-153 (10), 161-183 (23)	147-153 (7), 169-171 (3)	M	0.65	1.58	97.44% (114/117)	26.50% (31/117)	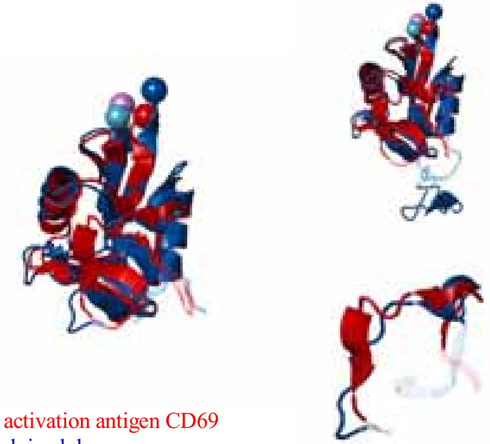 Early activation antigen CD69<br>Convulxin alpha
	1uosA (133)	69-81 (13), 89-116 (28)	69-81 (13), 89-116 (28)	147-153 (7), 169-171 (3)						
1010	1e87A (117)	144-153 (10), 162-183 (22)	144-153 (10), 162-171 (10)	147-153 (7), 166-171 (6)	M	0.63	1.50	93.16% (109/117)	21.37% (25/117)	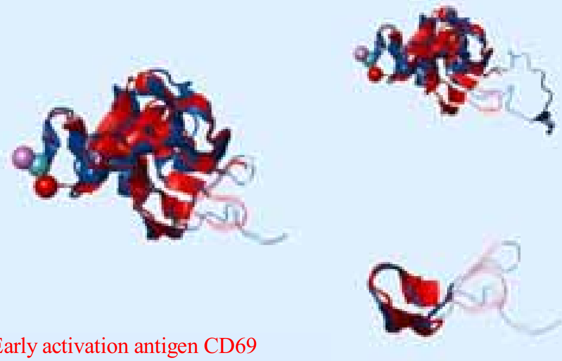 Early activation antigen CD69<br>Coagulation factor IX-binding protein a chain
	1j34A (129)	69-81 (13), 90-116 (27)	69-81 (13), 90-100 (11)	147-153 (7), 166-171 (6)						

1011	1p4lD (122)	197-208 (12), 213-232 (20)	198-208 (11), 213-232 (20)	202-208 (7), 220-232 (13)	M	0.51	2.10	90.16% (110/122)	19.67% (24/122)	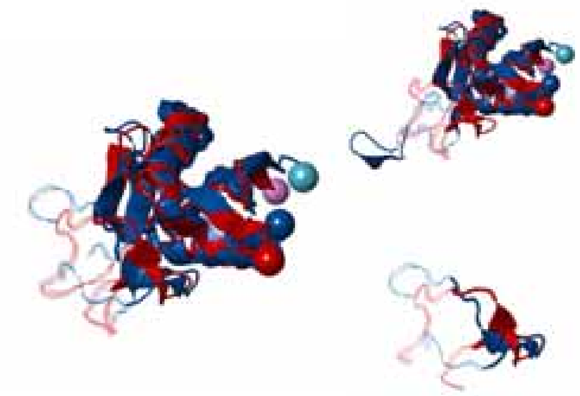 LY49-C EMS16 a chain
	1ukmA (131)	70-82 (13), 88-104 (17)	71-82 (12), 88-104 (17)	202-208 (7), 220-232 (13)						
1012	1p4lD (122)	197-207 (11), 210-256 (47)	198-207 (10), 216-232 (17)	201-207 (7), 223-232 (10)	M	0.54	2.06	89.34% (109/122)	15.57% (19/122)	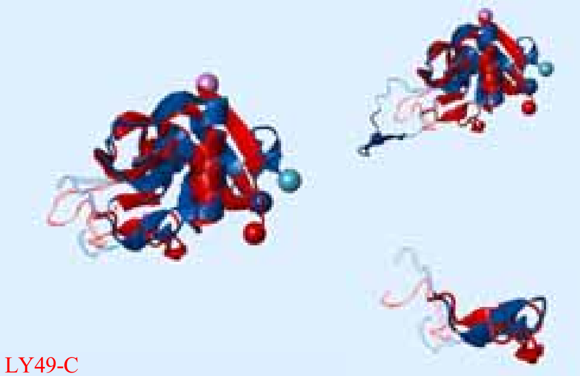 LY49-C Coagulation factor IX-binding protein B chain
	1j34B (123)	266-276 (11), 279-322 (44)	267-276 (10), 282-296 (15)	201-207 (7), 223-232 (10)						
1013	1p4lD (122)	198-210 (13), 213-232 (20)	199-207 (9), 213-232 (20)	206-207 (2), 219-232 (14)	M	0.61	1.70	90.16% (110/122)	16.39% (20/122)	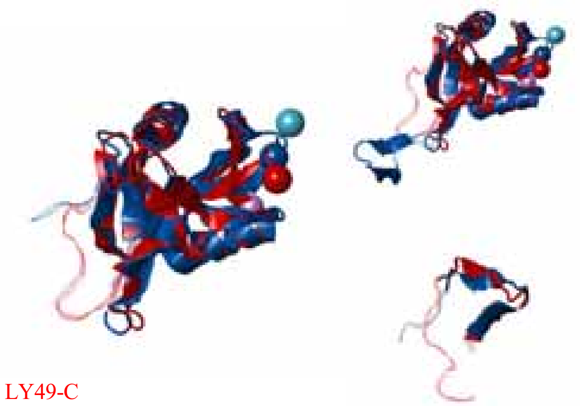 LY49-C Platelet aggregation inducer
	1jwiB (123)	69-81 (13), 84-98 (15)	70-78 (9), 84-98 (15)	206-207 (2), 219-232 (14)						

1014	1p4lD (122)	198-207 (10), 210-243 (34)	198-203 (6), 216-233 (18)	201-203 (3), 223-232 (10)	M	0.53	2.05	89.34% (109/122)	17.21% (21/122)	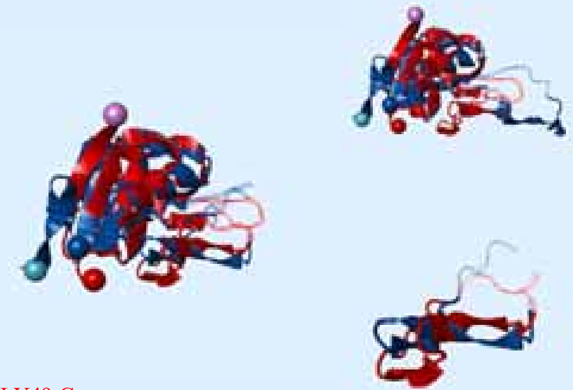 LY49-C<br>Mucrocetin beta chain
	1v4lB (125)	269-278 (10), 281-311 (31)	269-274 (6), 284-299 (16)	201-203 (3), 223-232 (10)						
1015	1p4lD (122)	187-224 (38), 206-254 (49)	198-209 (12), 213-232 (20)	202-207 (6), 220-232 (13)	M	0.51	2.09	89.34% (109/122)	19.67% (24/122)	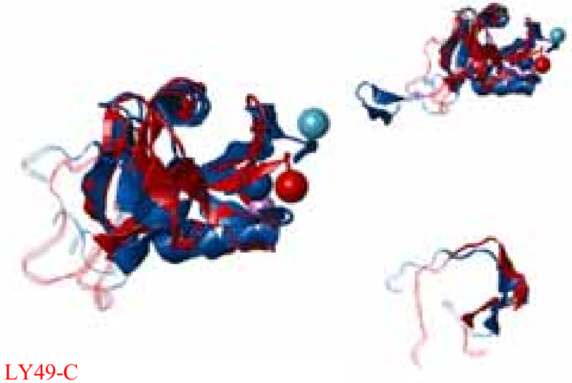 LY49-C<br>Coagulation factor X binding protein
	1iodA (129)	52-98 (47), 79-128 (50)	69-82 (14), 86-102 (17)	202-207 (6), 220-232 (13)						
1016	1r13A (145)	167-194 (28), 176-227 (52)	169-177 (9), 191-218 (28)	170-177 (8), 193-202 (10)	M	0.48	2.03	92.68% (114/123)	17.07% (21/123)	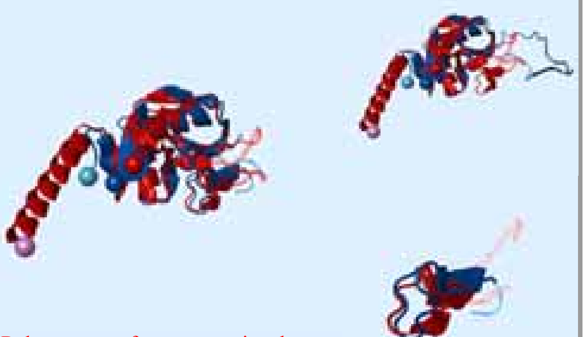 Pulmonary surfactant-associated protein a<br>Echicetin B-chain
	1oz7B (123)	66-93 (28), 74-120 (47)	68-75 (8), 90-111 (22)	170-177 (8), 193-202 (10)						

1017	1r13A (145)	160-180 (21), 190-205 (16)	168-180 (13), 190-205 (16)	169-178 (10), 192-203 (12)	M	0.48	1.93	88.71% (110/124)	19.35% (24/124)	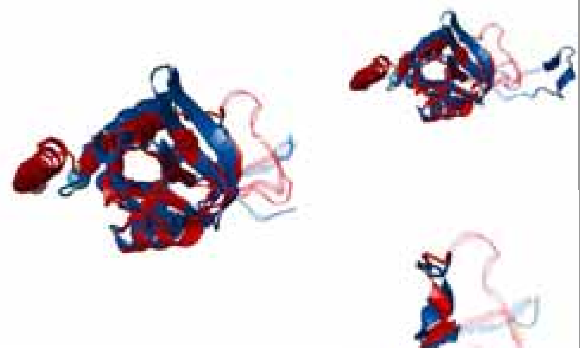 Pulmonary surfactant-associated protein a EMS16 B chain
	1ukmB (124)	56-78 (23), 88-96 (9)	66-78 (13), 88-96 (9)	169-178 (10), 192-203 (12)						
1018	1r13A (145)	167-178 (12), 194-211 (18)	168-178 (11), 194-211 (18)	172-178 (7), 194-202 (9)	M	0.50	1.86	87.79% (115/131)	18.32% (24/131)	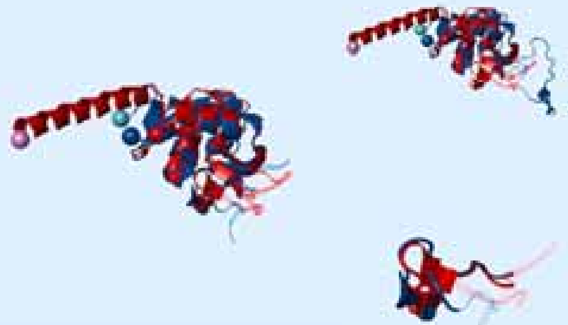 Pulmonary surfactant-associated protein a EMS16 a chain
	1ukmA (131)	70-82 (13), 99-114 (16)	71-82 (12), 99-114 (16)	172-178 (7), 194-202 (9)						
1019	1r13A (145)	167-180 (14), 186-212 (27)	167-178 (12), 192-211 (20)	172-176 (5), 192-203 (12)	M	0.52	1.79	90.40% (113/125)	16.80% (21/125)	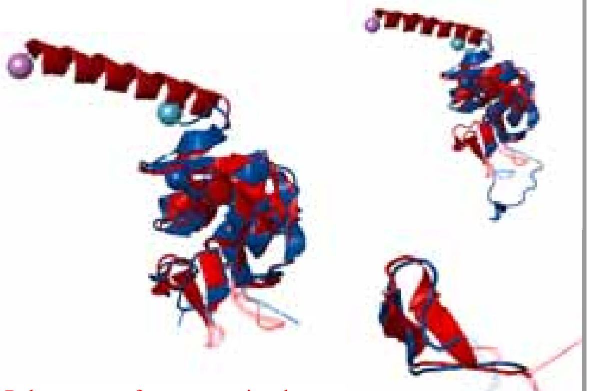 Pulmonary surfactant-associated protein a Bitiscetin alpha chain
	1uexA (125)	68-83 (16), 89-111 (23)	68-81 (14), 95-110 (16)	172-176 (5), 192-203 (12)						

1020	1r13A (145)	168-180 (13), 192-224 (33)	168-178 (11), 192-211 (20)	171-178 (8), 192-203 (12)	M	0.52	1.88	92.68% (114/123)	17.07% (21/123)	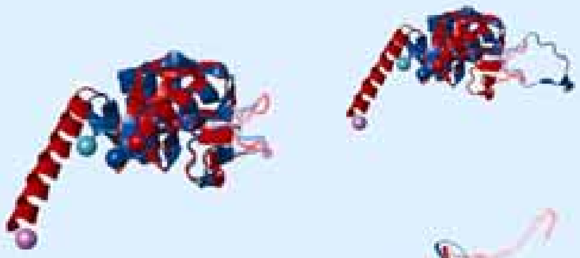 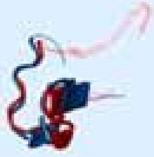 Pulmonary surfactant-associated protein a<br>Coagulation factor IX-binding protein B chain
	1j34B (123)	267-279 (13), 291-317 (27)	267-277 (11), 291-304 (14)	171-178 (8), 192-203 (12)						
1021	1r13A (145)	159-180 (22), 190-205 (16)	169-178 (10), 191-204 (14)	171-178 (8), 193-203 (11)	M	0.53	1.85	92.68% (114/123)	15.45% (19/123)	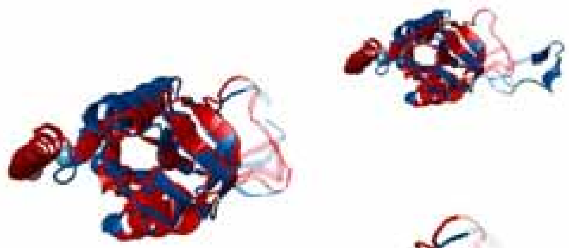 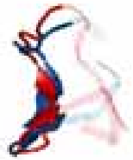 Pulmonary surfactant-associated protein a<br>Platelet aggregation inducer
	1jwiB (123)	58-81 (24), 91-99 (9)	70-79 (10), 92-98 (7)	171-178 (8), 193-203 (11)						
1022	1r13A (145)	155-180 (26), 183-218 (36)	167-178 (12), 192-207 (16)	170-176 (7), 192-203 (12)	M	0.49	2.01	90.40% (113/125)	19.20% (24/125)	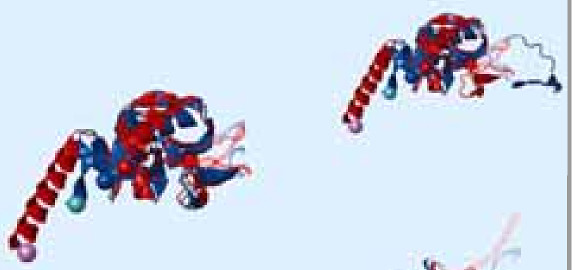 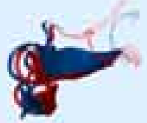 Pulmonary surfactant-associated protein a<br>Mucrocetin beta chain
	1v4lB (125)	254-281 (28), 284-313 (30)	268-279 (12), 293-301 (9)	170-176 (7), 192-203 (12)						

1023	1r13A (145)	167-179 (13), 192-218 (27)	172-177 (6), 193-218 (26)	172-177 (6), 193-202 (10)	M	0.51	1.94	89.92% (116/129)	18.60% (24/129)	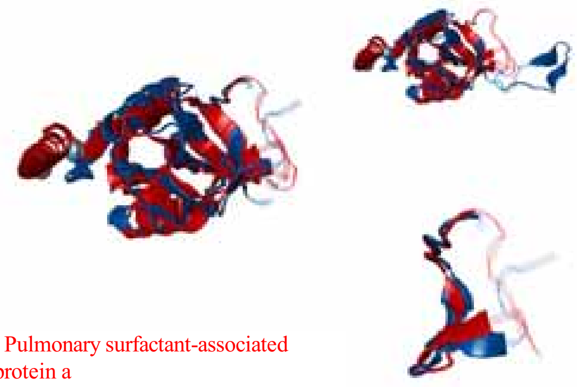 Pulmonary surfactant-associated protein a<br>Coagulation factor X binding protein
	1iodA (129)	68-82 (15), 95-119 (25)	73-80 (8), 96-119 (24)	172-177 (6), 193-202 (10)						
1024	1r13A (145)	167-179 (13), 192-220 (29)	172-178 (7), 192-204 (13)	172-178 (7), 193-201 (9)	M	0.51	1.84	89.15% (115/129)	19.38% (25/129)	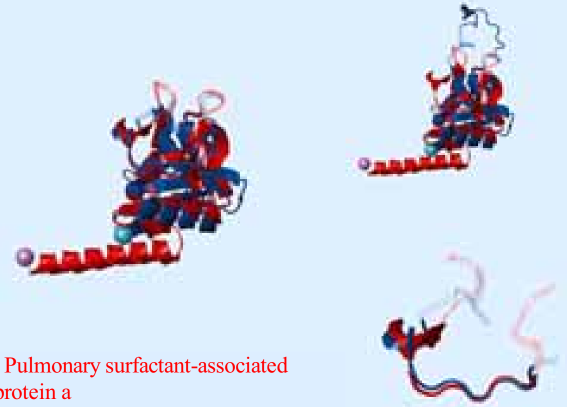 Pulmonary surfactant-associated protein a<br>Coagulation factor IX-binding protein a chain
	1j34A (129)	68-82 (15), 95-121 (27)	73-81 (9), 95-102 (8)	172-178 (7), 193-201 (9)						
1025	1tdqB (126)	51-72 (22), 79-112 (34)	53-71 (19), 80-101 (22)	65-71 (7), 84-98 (15)	M	0.67	1.36	91.06% (112/123)	29.27% (36/123)	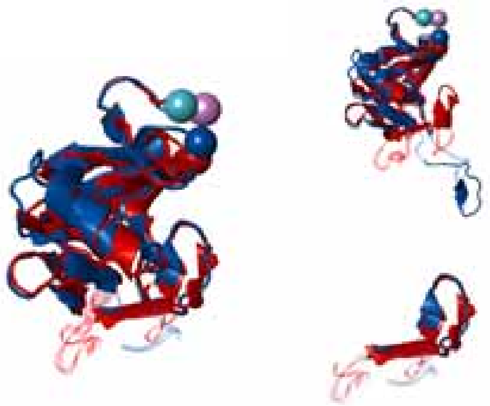 Aggrecan core protein<br>Echicetin B-chain
	1oz7B (123)	52-78 (27), 85-108 (24)	54-77 (24), 86-98 (13)	65-71 (7), 84-98 (15)						

1026	1tdqB (126)	61-69 (9), 79-100 (22)	62-69 (8), 81-98 (18)	62-69 (8), 91-98 (8)	M	0.62	1.54	89.52% (111/124)	32.26% (40/124)	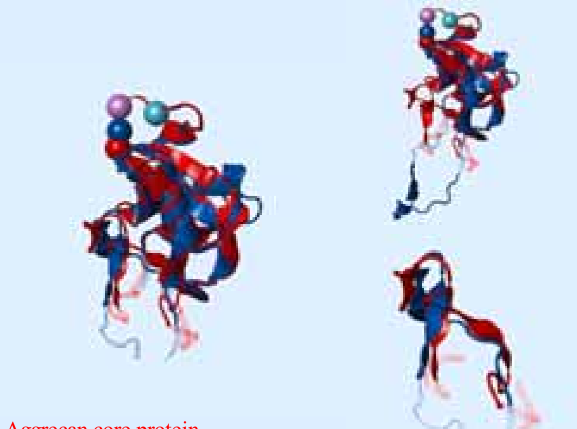 Aggrecan core protein<br>EMS16 B chain
	1ukmB (124)	66-74 (9), 84-96 (13)	67-74 (8), 85-94 (10)	62-69 (8), 91-98 (8)						
1027	1tdqB (126)	61-73 (13), 87-108 (22)	64-73 (10), 87-97 (11)	66-71 (6), 89-97 (9)	M	0.68	1.38	92.86% (117/126)	30.95% (39/126)	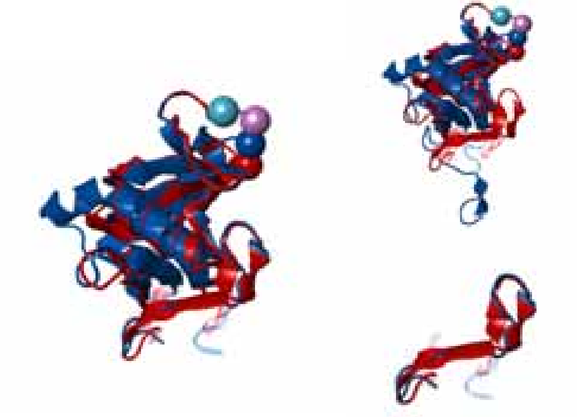 Aggrecan core protein<br>EMS16 a chain
	1ukmA (131)	71-85 (15), 99-114 (16)	74-85 (12), 99-102 (4)	66-71 (6), 89-97 (9)						
1028	1tdqB (126)	61-73 (13), 80-109 (30)	61-73 (13), 80-97 (18)	64-71 (8), 84-93 (10)	M	0.62	1.77	92.00% (115/125)	35.20% (44/125)	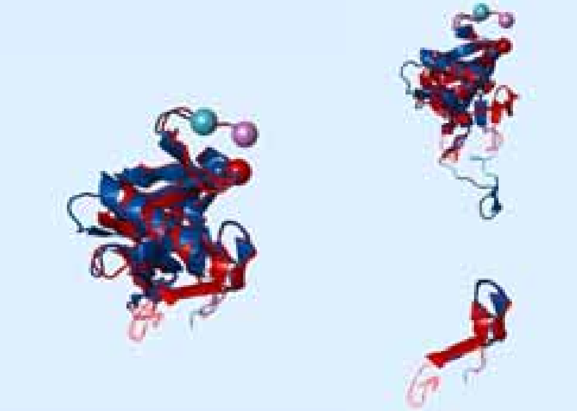 Aggrecan core protein<br>Bitiscetin alpha chain
	1uexA (125)	69-83 (15), 90-111 (22)	69-83 (15), 90-98 (9)	64-71 (8), 84-93 (10)						

1029	1tdqB (126)	61-70 (10), 80-98 (19)	61-70 (10), 80-98 (19)	64-69 (6), 84-98 (15)	M	0.68	1.36	91.87% (113/123)	32.52% (40/123)	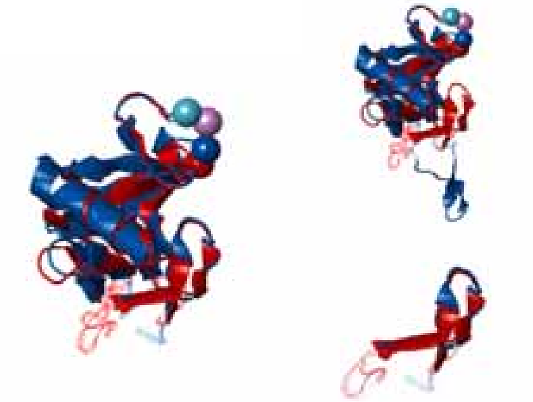 Aggrecan core protein<br>Platelet aggregation inducer
	1jwiB (123)	69-78 (10), 88-97 (10)	69-78 (10), 88-97 (10)	64-69 (6), 84-98 (15)						
1030	1tdqB (126)	53-70 (18), 76-109 (34)	55-69 (15), 81-109 (29)	62-69 (8), 81-97 (17)	M	0.64	1.59	91.20% (114/125)	28.80% (36/125)	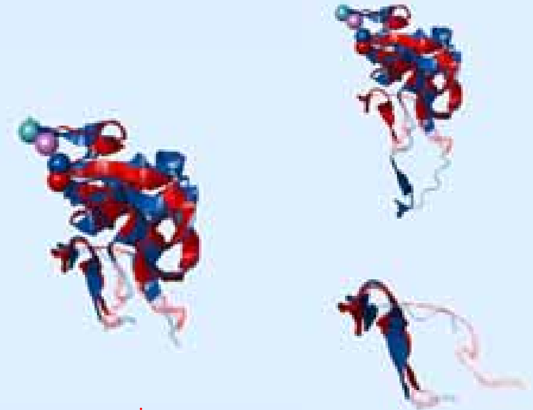 Aggrecan core protein<br>Mucrocetin beta chain
	1v4lB (125)	256-278 (23), 284-307 (24)	258-277 (20), 288-307 (20)	62-69 (8), 81-97 (17)						
1031	1tdqB (126)	62-76 (15), 80-103 (24)	64-72 (9), 85-98 (14)	66-70 (5), 86-96 (11)	M	0.68	1.43	92.06% (116/126)	31.75% (40/126)	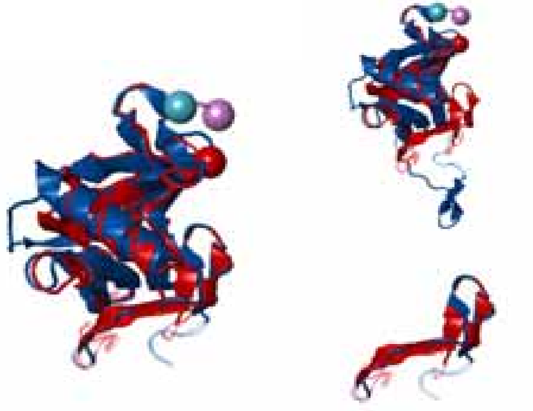 Aggrecan core protein<br>Coagulation factor X binding protein
	1iodA (129)	70-86 (17), 90-106 (17)	72-82 (11), 95-101 (7)	66-70 (5), 86-96 (11)						

1032	1tdqB (126)	63-73 (11), 82-108 (27)	64-71 (8), 82-108 (27)	64-70 (7), 90-96 (7)	M	0.61	1.55	90.48% (114/126)	28.57% (36/126)	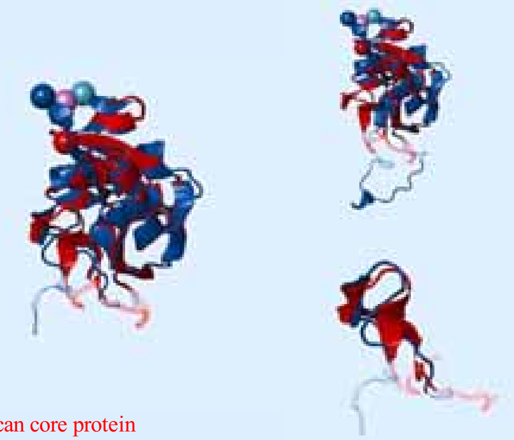 Aggrecan core protein<br>Convulxin alpha
	1uosA (133)	71-83 (13), 92-112 (21)	72-80 (9), 92-112 (21)	64-70 (7), 90-96 (7)						
1033	1tdqB (126)	64-73 (10), 80-98 (19)	64-71 (8), 85-98 (14)	64-71 (8), 86-96 (11)	M	0.67	1.51	92.86% (117/126)	34.92% (44/126)	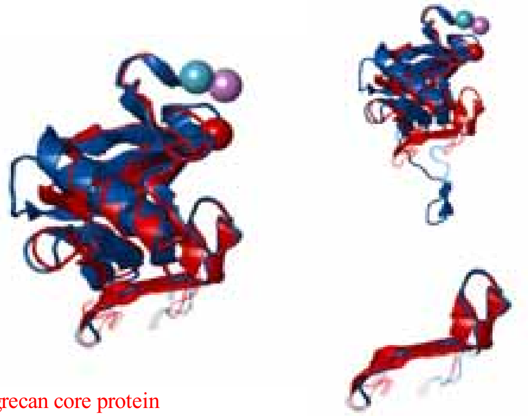 Aggrecan core protein<br>Coagulation factor IX-binding protein a chain
	1j34A (129)	72-83 (12), 90-101 (12)	72-80 (9), 95-101 (7)	64-71 (8), 86-96 (11)						
1034	3bc7C (128)	244-280 (37), 284-307 (24)	253-280 (28), 284-296 (13)	255-264 (10), 284-295 (12)	M	0.56	1.75	88.62% (109/123)	26.02% (32/123)	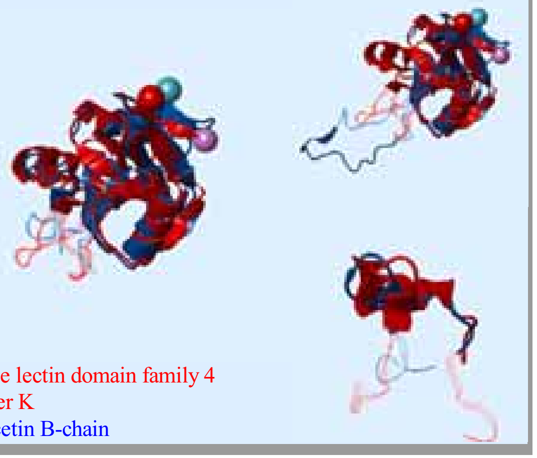 C-type lectin domain family 4 member K<br>Echicetin B-chain
	1oz7B (123)	53-89 (37), 92-107 (16)	66-89 (24), 92-97 (6)	255-264 (10), 284-295 (12)						

1035	3bc7C (128)	255-266 (12), 274-308 (35)	255-263 (9), 274-306 (33)	256-263 (8), 287-294 (8)	M	0.59	1.60	88.71% (110/124)	25.81% (32/124)	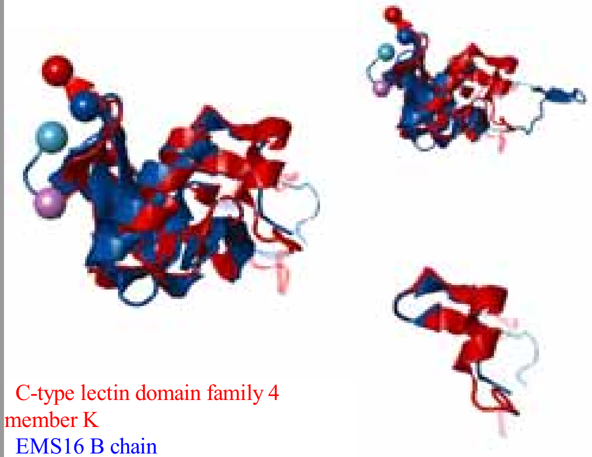 C-type lectin domain family 4 member K EMS16 B chain
	1ukmB (124)	67-78 (12), 85-109 (25)	67-75 (9), 85-107 (23)	256-263 (8), 287-294 (8)						
1036	3bc7C (128)	255-266 (12), 274-292 (19)	257-266 (10), 274-292 (19)	258-265 (8), 287-292 (6)	M	0.55	1.89	89.06% (114/128)	22.66% (29/128)	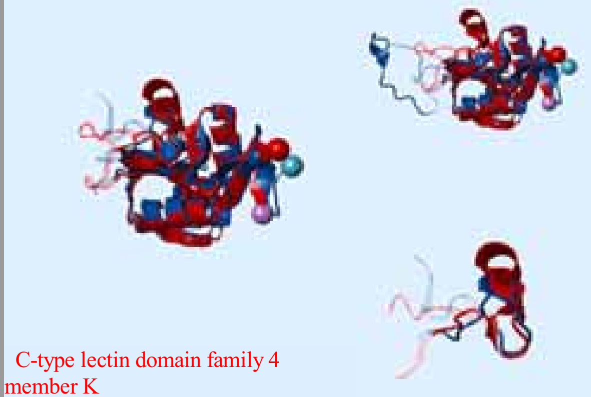 C-type lectin domain family 4 member K EMS16 a chain
	1ukmA (131)	72-85 (14), 92-101 (10)	74-85 (12), 92-101 (10)	258-265 (8), 287-292 (6)						
1037	3bc7C (128)	254-266 (13), 272-309 (38)	255-263 (9), 272-294 (23)	257-263 (7), 273-294 (22)	M	0.59	1.72	90.24% (111/123)	24.39% (30/123)	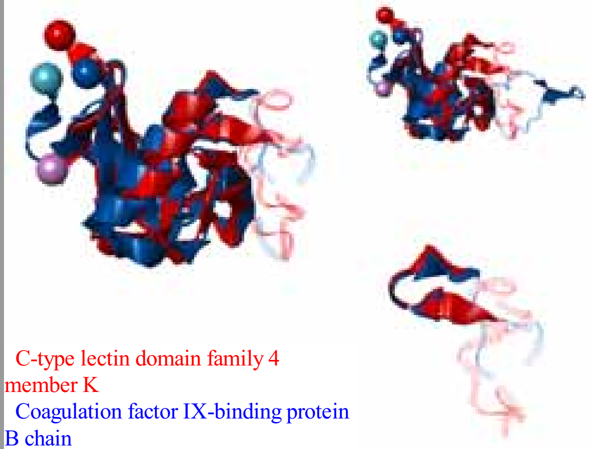 C-type lectin domain family 4 member K Coagulation factor IX-binding protein B chain
	1j34B (123)	267-279 (13), 285-309 (25)	268-276 (9), 285-295 (11)	257-263 (7), 273-294 (22)						

1038	3bc7C<br>(128)	253-266 (14),<br>270-294 (25)	254-265 (12),<br>270-294 (25)	257-263 (7),<br>287-294 (8)	M	0.57	1.80	90.24%<br>(111/123)	27.64%<br>(34/123)	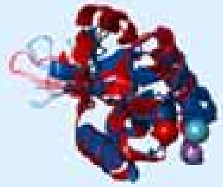 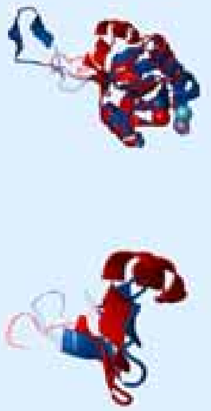 C-type lectin domain family 4 member K<br>Platelet aggregation inducer
	1jwiB<br>(123)	68-81 (14),<br>84-97 (14)	69-80 (12),<br>84-97 (14)	257-263 (7),<br>287-294 (8)						
1039	3bc7C<br>(128)	242-266 (25),<br>272-313 (42)	255-266 (12),<br>272-306 (35)	255-261 (7),<br>285-294 (10)	M	0.58	1.62	87.20%<br>(109/125)	22.40%<br>(28/125)	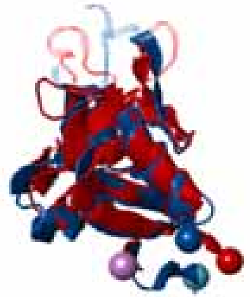 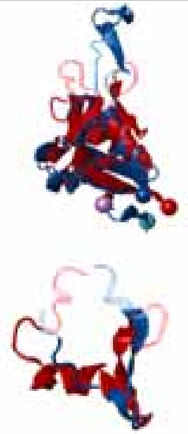 C-type lectin domain family 4 member K<br>Mucrocetin beta chain
	1v4lB<br>(125)	253-281 (29),<br>287-315 (29)	270-281 (12),<br>287-308 (22)	255-261 (7),<br>285-294 (10)						
1040	3bc7C<br>(128)	255-287 (33),<br>261-292 (32)	258-266 (9),<br>269-292 (24)	258-264 (7),<br>287-292 (6)	M	0.55	1.85	87.50%<br>(112/128)	22.66%<br>(29/128)	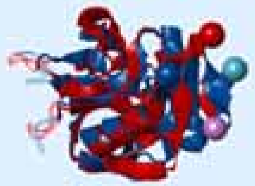 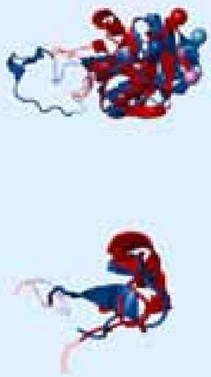 C-type lectin domain family 4 member K<br>Coagulation factor X binding protein
	1iodA<br>(129)	70-96 (27),<br>75-99 (25)	73-83 (11),<br>86-99 (14)	258-264 (7),<br>287-292 (6)						

1041	3bc7C (128)	258-288 (31), 262-308 (47)	258-288 (31), 262-292 (31)	258-265 (8), 287-292 (6)	M	0.46	1.77	88.28% (113/128)	20.31% (26/128)	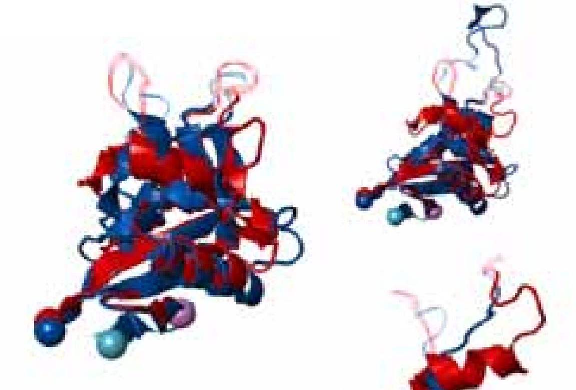 C-type lectin domain family 4 member K<br>Convulxin alpha
	1uosA (133)	73-96 (24), 76-116 (41)	73-96 (24), 76-99 (24)	258-265 (8), 287-292 (6)						
1042	3bc7C (128)	257-289 (33), 261-293 (33)	258-289 (32), 261-292 (32)	258-266 (9), 287-292 (6)	M	0.42	1.79	85.94% (110/128)	21.88% (28/128)	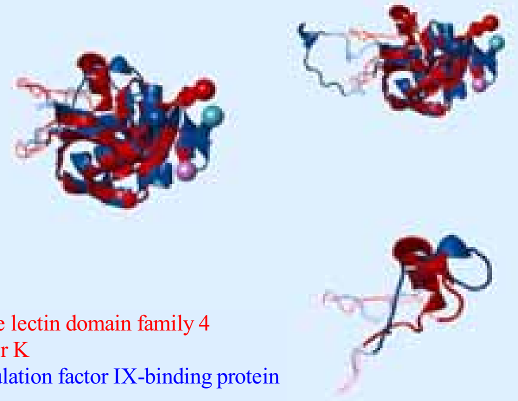 C-type lectin domain family 4 member K<br>Coagulation factor IX-binding protein a chain
	1j34A (129)	72-97 (26), 75-100 (26)	73-97 (25), 75-99 (25)	258-266 (9), 287-292 (6)						
1043	1byfA (123)	44-68 (25), 75-100 (26)	57-68 (12), 75-98 (24)	58-66 (9), 88-95 (8)	M	0.53	1.81	85.37% (105/123)	21.14% (26/123)	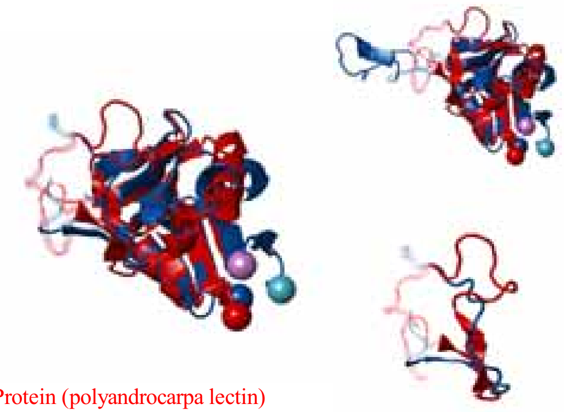 Protein (polyandrocarpa lectin)<br>Echicetin B-chain
	1oz7B (123)	53-78 (26), 85-100 (16)	68-78 (11), 85-98 (14)	58-66 (9), 88-95 (8)						

1044	1byfA (123)	56-66 (11), 72-94 (23)	56-66 (11), 76-92 (17)	58-64 (7), 88-92 (5)	M	0.51	1.96	85.37% (105/123)	12.20% (15/123)	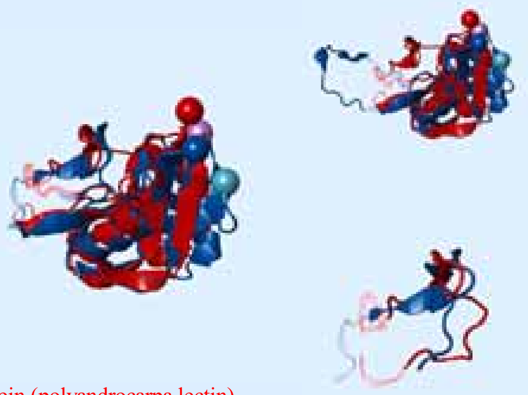<br>Protein (polyandrocarpa lectin)<br>EMS16 B chain
	1ukmB (124)	66-75 (10), 81-93 (13)	66-75 (10), 85-91 (7)	58-64 (7), 88-92 (5)						
1045	1byfA (123)	56-68 (13), 85-95 (11)	56-67 (12), 85-95 (11)	61-67 (7), 86-95 (10)	M	0.54	1.88	89.43% (110/123)	16.26% (20/123)	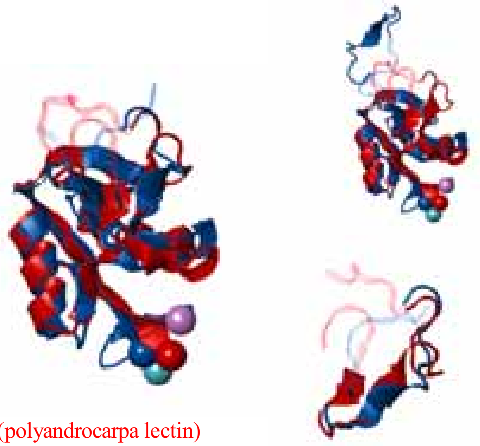<br>Protein (polyandrocarpa lectin)<br>EMS16 a chain
	1ukmA (131)	71-84 (14), 99-103 (5)	71-83 (13), 99-103 (5)	61-67 (7), 86-95 (10)						
1046	1byfA (123)	56-69 (14), 73-117 (45)	59-69 (11), 73-99 (27)	60-64 (5), 88-93 (6)	M	0.53	1.95	87.80% (108/123)	14.63% (18/123)	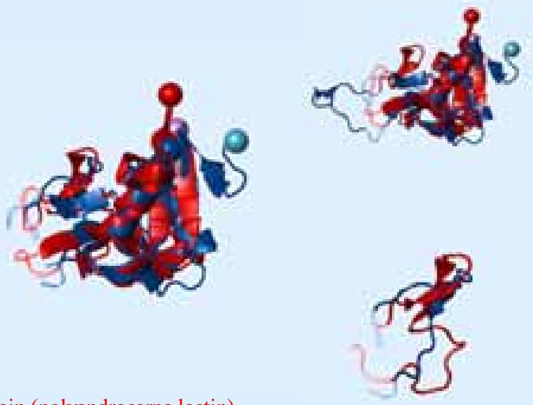<br>Protein (polyandrocarpa lectin)<br>Bitiscetin alpha chain
	1uexA (125)	69-83 (15), 86-123 (38)	72-83 (12), 86-103 (18)	60-64 (5), 88-93 (6)						

1047	1byfA (123)	56-65 (10), 75-94 (20)	57-65 (9), 76-94 (19)	59-65 (7), 76-94 (19)	M	0.51	2.10	86.99% (107/123)	18.70% (23/123)	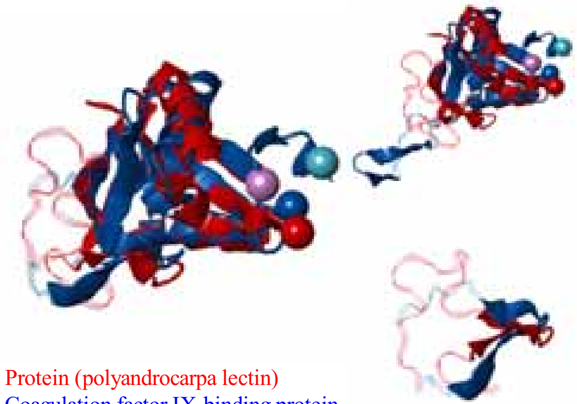<br>Protein (polyandrocarpa lectin)<br>Coagulation factor IX-binding protein B chain
	1j34B (123)	267-274 (8), 285-294 (10)	268-274 (7), 286-294 (9)	59-65 (7), 76-94 (19)						
1048	1byfA (123)	56-66 (11), 73-94 (22)	59-65 (7), 76-94 (19)	59-65 (7), 88-93 (6)	M	0.52	1.95	86.18% (106/123)	15.45% (19/123)	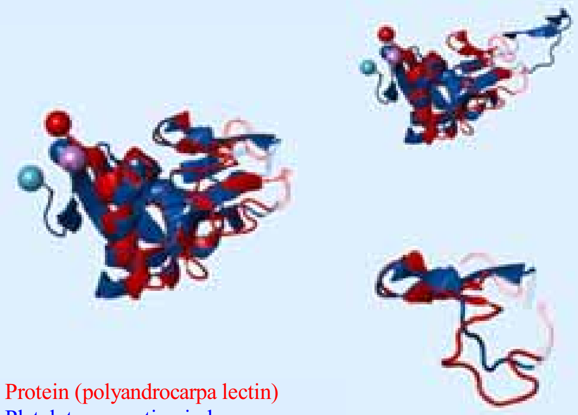<br>Protein (polyandrocarpa lectin)<br>Platelet aggregation inducer
	1jwiB (123)	69-78 (10), 85-96 (12)	72-75 (4), 88-96 (9)	59-65 (7), 88-93 (6)						
1049	1byfA (123)	56-78 (23), 81-114 (34)	56-78 (23), 92-94 (3)	58-64 (7), 92-93 (2)	M	0.45	2.46	87.80% (108/123)	17.07% (21/123)	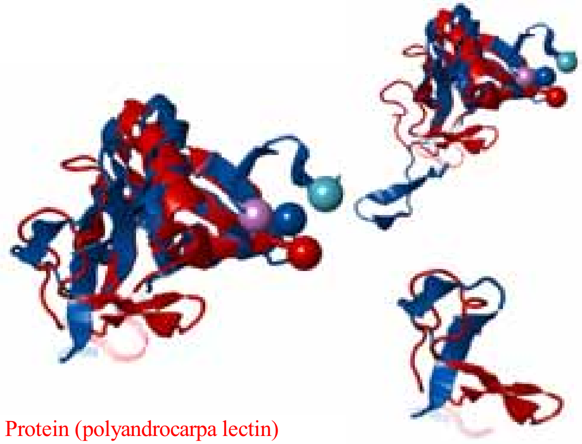<br>Protein (polyandrocarpa lectin)<br>Mucrocetin beta chain
	1v4lB (125)	269-280 (12), 283-316 (34)	269-280 (12), 294-296 (3)	58-64 (7), 92-93 (2)						

1050	1byfA (123)	56-68 (13), 71-95 (25)	56-68 (13), 76-95 (20)	61-67 (7), 84-95 (12)	M	0.53	1.89	87.80% (108/123)	16.26% (20/123)	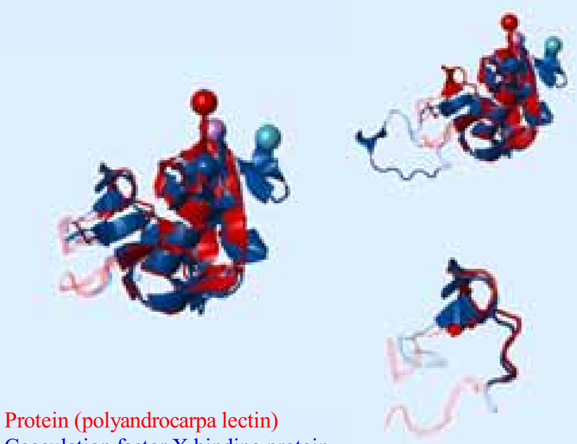<br>Protein (polyandrocarpa lectin)<br>Coagulation factor X binding protein
	1iodA (129)	69-82 (14), 85-101 (17)	69-82 (14), 90-101 (12)	61-67 (7), 84-95 (12)						
1051	1byfA (123)	56-69 (14), 76-100 (25)	56-67 (12), 84-100 (17)	59-64 (6), 84-96 (13)	M	0.50	2.04	88.62% (109/123)	14.63% (18/123)	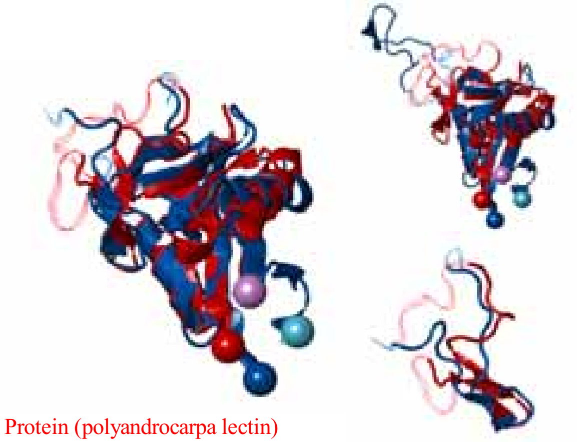<br>Protein (polyandrocarpa lectin)<br>Convulxin alpha
	1uosA (133)	69-83 (15), 90-106 (17)	69-81 (13), 96-106 (11)	59-64 (6), 84-96 (13)						
1052	1byfA (123)	56-68 (13), 76-95 (20)	56-67 (12), 76-95 (20)	59-67 (9), 76-95 (20)	M	0.51	2.01	87.80% (108/123)	13.82% (17/123)	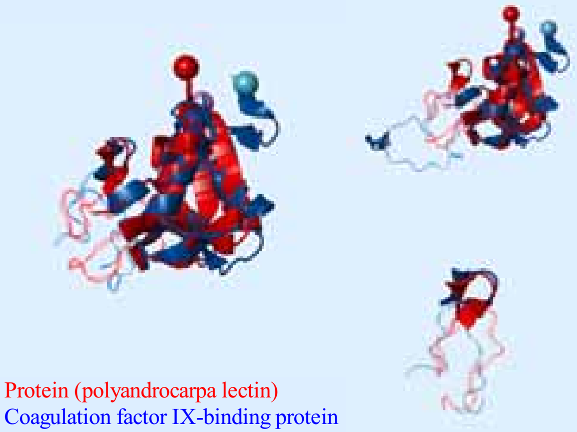<br>Protein (polyandrocarpa lectin)<br>Coagulation factor IX-binding protein a chain
	1j34A (129)	69-82 (14), 90-101 (12)	69-81 (13), 90-101 (12)	59-67 (9), 76-95 (20)						

1053	1kza1 (115)	142-182 (41), 185-210 (26)	165-175 (11), 185-210 (26)	167-173 (7), 188-200 (13)	M	0.60	1.68	92.17% (106/115)	19.13% (22/115)	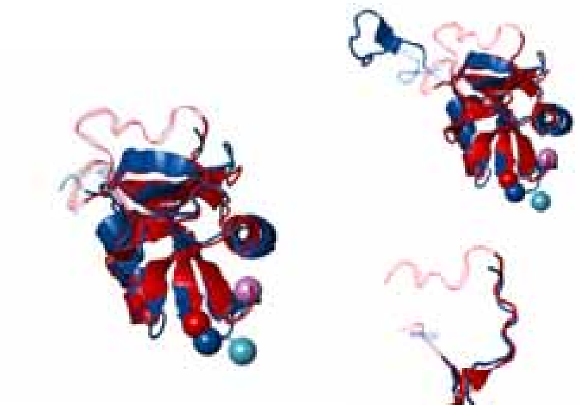 Mannose-binding protein C
	1oz7B (123)	41-86 (46), 89-107 (19)	69-79 (11), 89-107 (19)	167-173 (7), 188-200 (13)						Echicetin B-chain
1054	1kza1 (115)	163-172 (10), 175-211 (37)	163-172 (10), 185-201 (17)	165-172 (8), 187-199 (13)	M	0.61	1.64	92.17% (106/115)	16.52% (19/115)	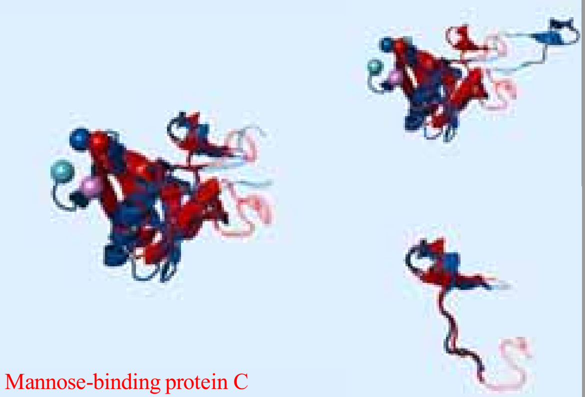 Mannose-binding protein C
	1ukmB (124)	66-75 (10), 78-109 (32)	66-75 (10), 88-96 (9)	165-172 (8), 187-199 (13)						EMS16 B chain
1055	1kza1 (115)	163-182 (20), 189-207 (19)	164-176 (13), 189-197 (9)	168-174 (7), 189-197 (9)	M	0.63	1.34	93.04% (107/115)	22.61% (26/115)	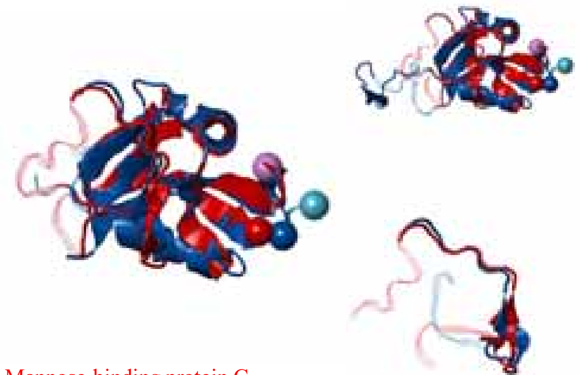 Mannose-binding protein C
	1ukmA (131)	71-92 (22), 99-114 (16)	72-86 (15), 99-101 (3)	168-174 (7), 189-197 (9)						EMS16 a chain

1056	1kza1 (115)	166-186 (21), 171-208 (38)	166-175 (10), 186-197 (12)	168-173 (6), 186-197 (12)	M	0.61	1.53	91.30% (105/115)	19.13% (22/115)	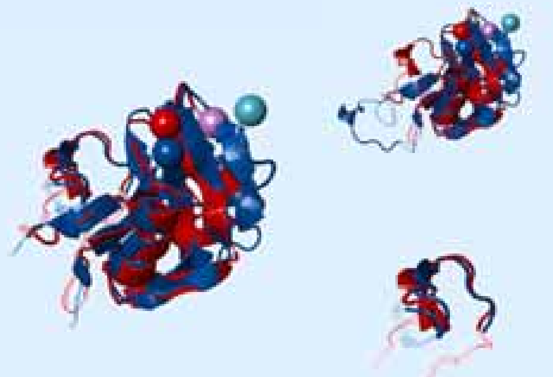 Mannose-binding protein C Bitiscetin alpha chain
	1uexA (125)	72-94 (23), 79-111 (33)	72-83 (12), 94-97 (4)	168-173 (6), 186-197 (12)						
1057	1kza1 (115)	166-172 (7), 186-212 (27)	166-172 (7), 186-207 (22)	166-171 (6), 186-199 (14)	M	0.59	1.68	91.30% (105/115)	17.39% (20/115)	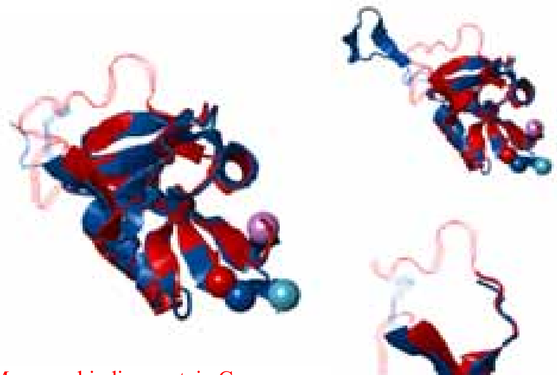 Mannose-binding protein C Coagulation factor IX-binding protein B chain
	1j34B (123)	270-276 (7), 290-309 (20)	270-276 (7), 290-304 (15)	166-171 (6), 186-199 (14)						
1058	1kza1 (115)	166-172 (7), 185-200 (16)	166-172 (7), 186-199 (14)	166-172 (7), 190-199 (10)	M	0.59	1.69	91.30% (105/115)	16.52% (19/115)	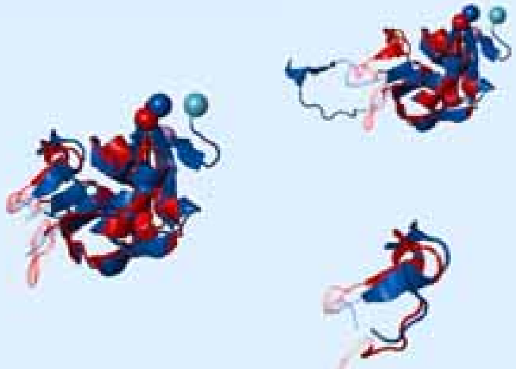 Mannose-binding protein C Platelet aggregation inducer
	1jwiB (123)	72-78 (7), 91-98 (8)	72-78 (7), 92-97 (6)	166-172 (7), 190-199 (10)						

1059	1kza1 (115)	154-176 (23), 179-208 (30)	163-172 (10), 187-207 (21)	166-172 (7), 187-199 (13)	M	0.62	1.56	92.17% (106/115)	17.39% (20/115)	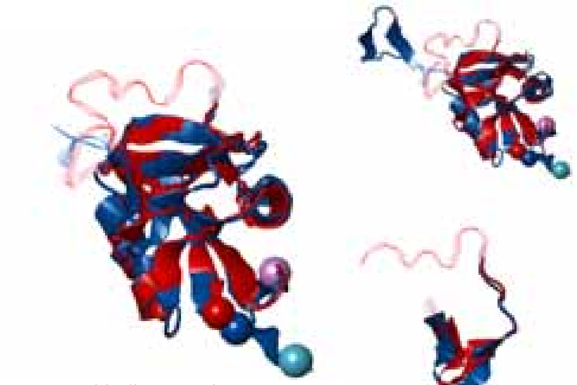 Mannose-binding protein C<br>Mucrocetin beta chain
	1v4lB (125)	255-282 (28), 285-307 (23)	269-278 (10), 293-306 (14)	166-172 (7), 187-199 (13)						
1060	1kza1 (115)	163-183 (21), 187-210 (24)	163-175 (13), 188-210 (23)	168-172 (5), 188-196 (9)	M	0.61	1.53	93.04% (107/115)	17.39% (20/115)	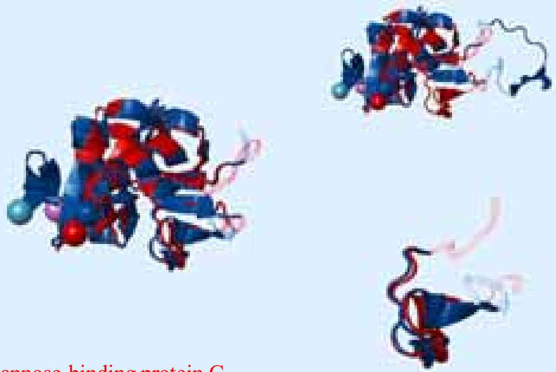 Mannose-binding protein C<br>Coagulation factor X binding protein
	1iodA (129)	69-91 (23), 95-115 (21)	69-83 (15), 96-115 (20)	168-172 (5), 188-196 (9)						
1061	1kza1 (115)	162-185 (24), 188-208 (21)	163-175 (13), 188-207 (20)	166-171 (6), 188-196 (9)	M	0.60	1.36	91.30% (105/115)	15.65% (18/115)	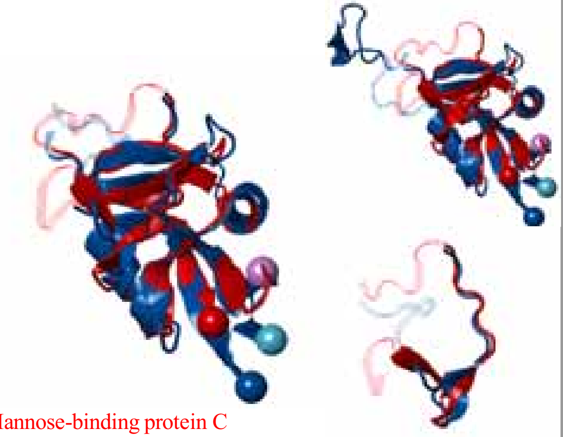 Mannose-binding protein C<br>Convulxin alpha
	1uosA (133)	68-93 (26), 96-113 (18)	69-83 (15), 96-112 (17)	166-171 (6), 188-196 (9)						

1062	1kza1 (115)	163-181 (19), 187-199 (13)	163-175 (13), 187-198 (12)	166-173 (8), 188-198 (11)	M	0.61	1.53	93.04% (107/115)	20.87% (24/115)	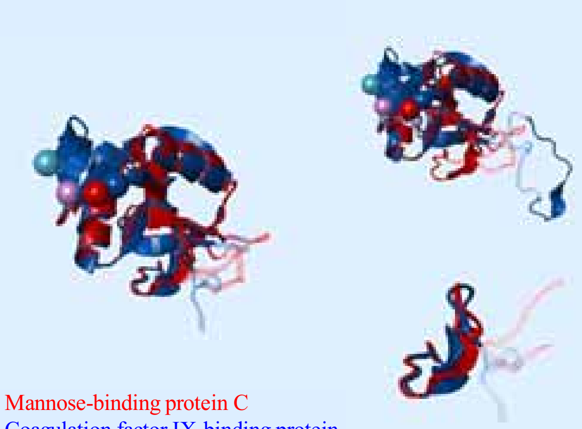 Mannose-binding protein C<br>Coagulation factor IX-binding protein a chain
	1j34A (129)	69-89 (21), 95-101 (7)	69-83 (15), 95-100 (6)	166-173 (8), 188-198 (11)						
1063	1yttA (115)	137-169 (33), 180-205 (26)	160-169 (10), 180-196 (17)	160-168 (9), 183-194 (12)	M	0.55	1.75	92.17% (106/115)	19.13% (22/115)	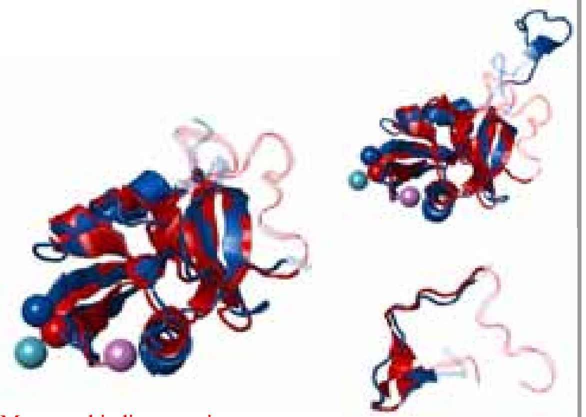 Mannose-binding protein a<br>Echicetin B-chain
	1oz7B (123)	41-78 (38), 89-107 (19)	69-78 (10), 89-97 (9)	160-168 (9), 183-194 (12)						
1064	1yttA (115)	157-166 (10), 180-206 (27)	158-166 (9), 180-196 (17)	159-166 (8), 182-194 (13)	M	0.56	1.80	90.43% (104/115)	17.39% (20/115)	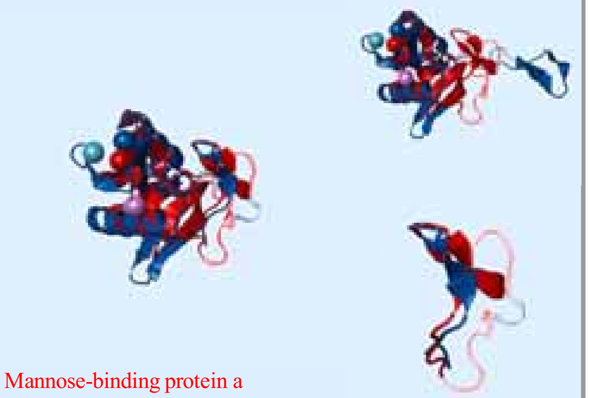 Mannose-binding protein a<br>EMS16 B chain
	1ukmB (124)	65-74 (10), 88-109 (22)	66-74 (9), 88-96 (9)	159-166 (8), 182-194 (13)						

1065	1yttA (115)	158-170 (13), 184-202 (19)	158-168 (11), 184-193 (10)	163-168 (6), 184-192 (9)	M	0.59	1.61	93.04% (107/115)	19.13% (22/115)	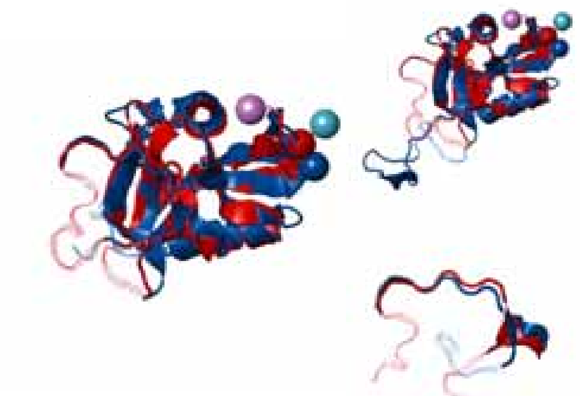 Mannose-binding protein a<br>EMS16 a chain
	1ukmA (131)	71-85 (15), 99-114 (16)	71-82 (12), 99-102 (4)	163-168 (6), 184-192 (9)						
1066	1yttA (115)	158-167 (10), 182-207 (26)	161-167 (7), 182-207 (26)	161-167 (7), 182-194 (13)	M	0.59	1.74	92.17% (106/115)	17.39% (20/115)	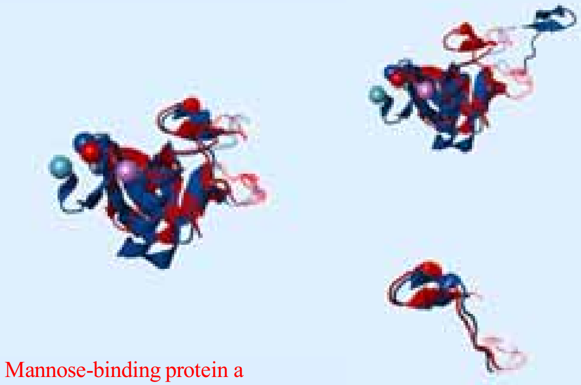 Mannose-binding protein a<br>Coagulation factor IX-binding protein B chain
	1j34B (123)	267-276 (10), 291-309 (19)	270-276 (7), 291-309 (19)	161-167 (7), 182-194 (13)						
1067	1yttA (115)	158-167 (10), 173-195 (23)	161-167 (7), 173-194 (22)	161-166 (6), 177-194 (18)	M	0.53	2.05	91.30% (105/115)	15.65% (18/115)	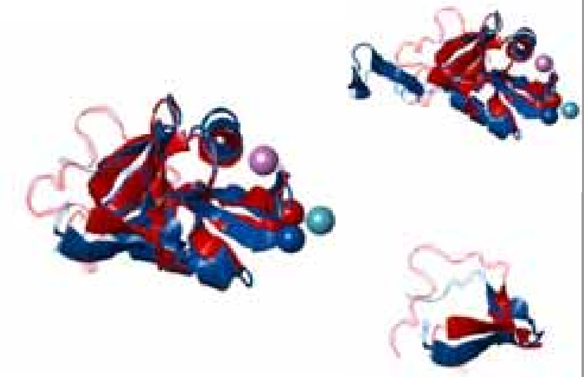 Mannose-binding protein a<br>Platelet aggregation inducer
	1jwiB (123)	69-78 (10), 84-98 (15)	72-78 (7), 84-97 (14)	161-166 (6), 177-194 (18)						

1068	1yttA (115)	148-167 (20), 179-203 (25)	158-167 (10), 182-203 (22)	161-167 (7), 182-194 (13)	M	0.59	1.72	92.17% (106/115)	16.52% (19/115)	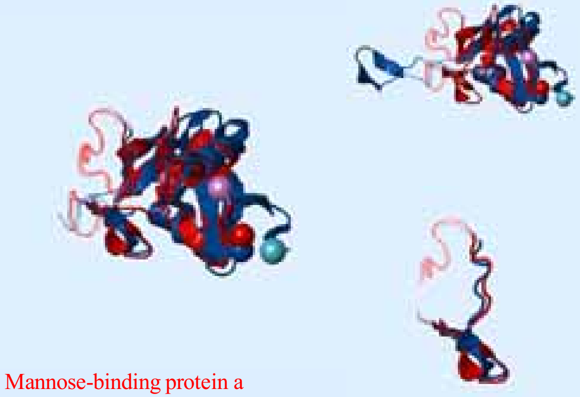 Mannose-binding protein a<br>Mucrocetin beta chain
	1v4lB (125)	254-278 (25), 290-307 (18)	269-278 (10), 293-307 (15)	161-167 (7), 182-194 (13)						
1069	1yttA (115)	158-169 (12), 182-207 (26)	158-169 (12), 182-205 (24)	163-167 (5), 183-193 (11)	M	0.57	1.69	92.17% (106/115)	12.17% (14/115)	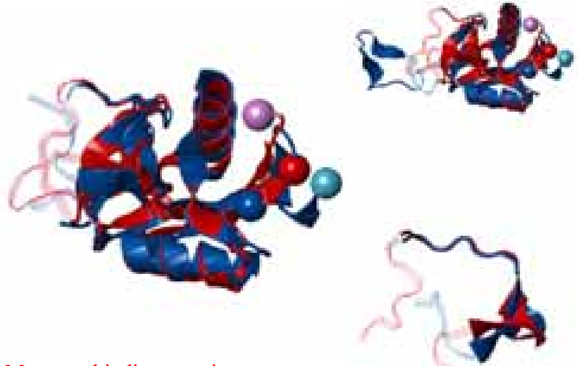 Mannose-binding protein a<br>Coagulation factor X binding protein
	1iodA (129)	69-82 (14), 95-117 (23)	69-82 (14), 95-115 (21)	163-167 (5), 183-193 (11)						
1070	1yttA (115)	157-170 (14), 183-202 (20)	158-168 (11), 183-202 (20)	161-166 (6), 183-191 (9)	M	0.56	1.80	93.91% (108/115)	14.78% (17/115)	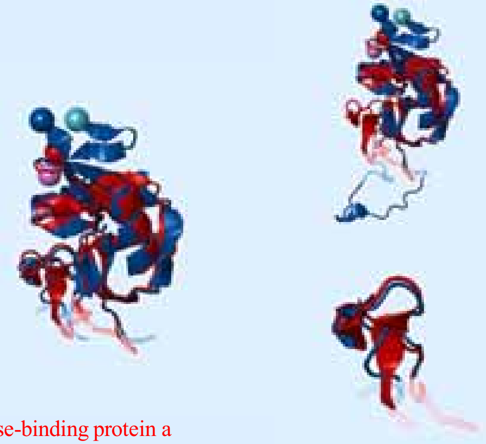 Mannose-binding protein a<br>Convulxin alpha
	1uosA (133)	68-83 (16), 96-112 (17)	69-81 (13), 96-112 (17)	161-166 (6), 183-191 (9)						

1071	1yttA (115)	158-169 (12), 182-195 (14)	158-168 (11), 182-194 (13)	161-168 (8), 183-193 (11)	M	0.58	1.59	91.30% (105/115)	14.78% (17/115)	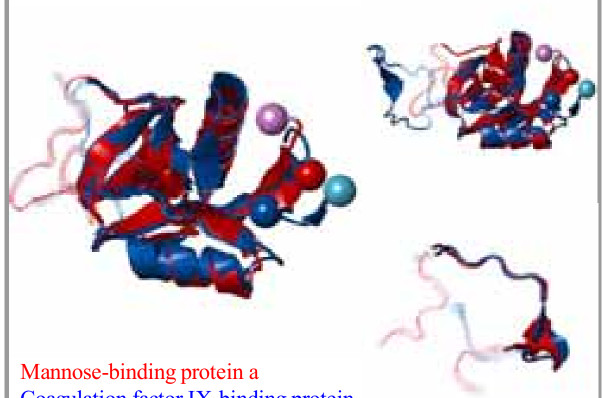 Mannose-binding protein a<br>Coagulation factor IX-binding protein a chain
	1j34A (129)	69-82 (14), 95-102 (8)	69-81 (13), 95-101 (7)	161-168 (8), 183-193 (11)						
1072	2cw1A (65)	42-64 (23)	51-64 (14)	54-58 (5)	C	0.57	2.11	95.08% (58/61)	19.67% (12/61)	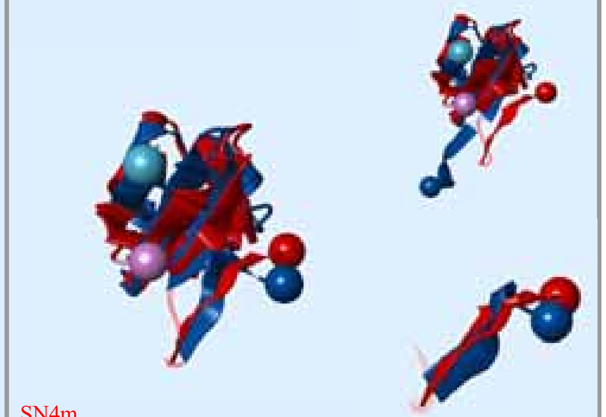 SN4m<br>Lambda CRO repressor
	1d1lA (61)	42-60 (19)	51-60 (10)	54-58 (5)						
1073	2cw1A (65)	42-62 (21)	51-62 (12)	55-59 (5)	C	0.55	2.14	95.00% (57/60)	20.00% (12/60)	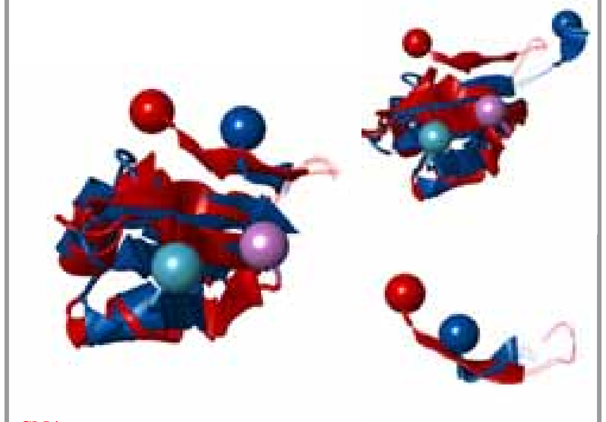 SN4m<br>Phage lambda Cro
	2ecsA (60)	42-59 (18)	51-59 (9)	55-59 (5)						

1074	2orcA (71)	43-63 (21)	49-63 (15)	56A-56B (1)	C	0.62	1.71	100.00% (66/66)	81.82% (54/66)	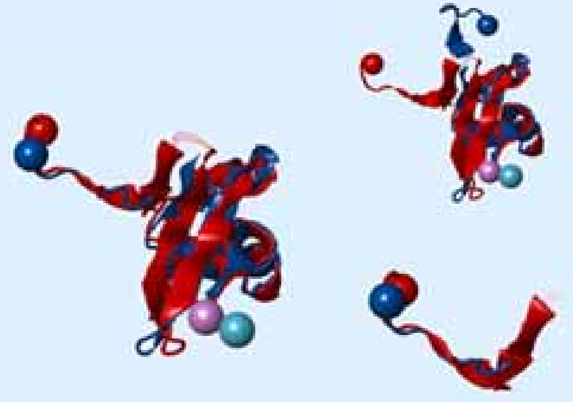 CRO repressor<br>Regulatory protein cro
	2a63A (66)	43-65 (23)	49-65 (17)	56A-56B (1)						
1075	1d1mB (65)	54-64 (11)	54-64 (11)	55-60 (6)	C	0.86	0.91	100.00% (61/61)	91.80% (56/61)	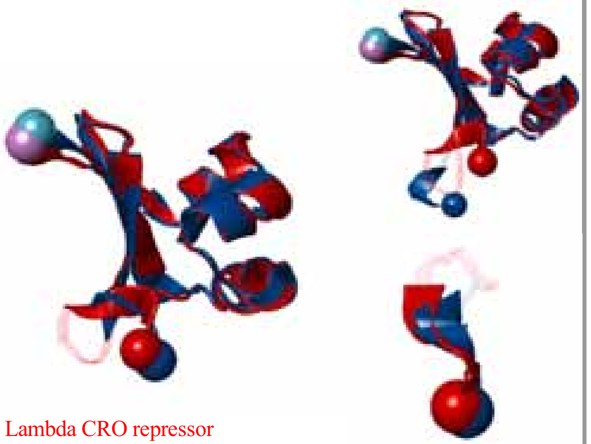 Lambda CRO repressor<br>Lambda CRO repressor
	1d1lA (61)	54-60 (7)	54-60 (7)	55-60 (6)						
1076	1d1mB (65)	45-64 (20)	46-64 (19)	55-56 (2)	C	0.70	1.37	98.46% (64/65)	81.54% (53/65)	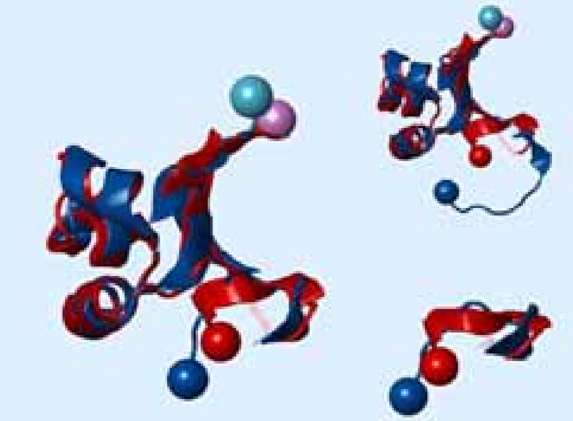 Lambda CRO repressor<br>Regulatory protein cro
	2a63A (66)	45-64 (20)	46-64 (19)	55-56 (2)						

1077	1gcpA (65)	627-635 (9)	628-635 (8)	628-635 (8)	N	0.62	1.46	91.53% (54/59)	18.64% (11/59)	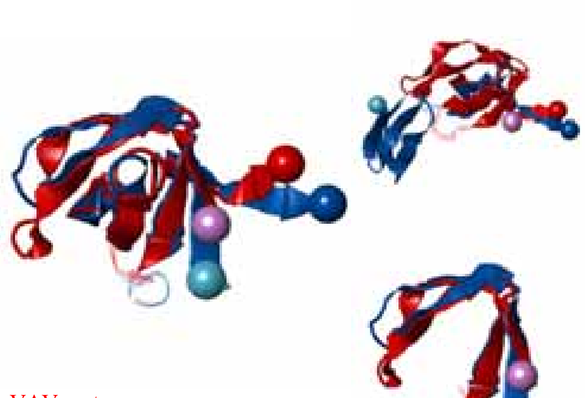 VAV proto-oncogene<br>Epidermal growth factor receptor kinase subst
	1i07A (59)	33-39 (7)	34-39 (6)	628-635 (8)						
1078	1ckaA (57)	162-169 (8)	162-169 (8)	162-168 (7)	C	0.78	0.82	92.98% (53/57)	26.32% (15/57)	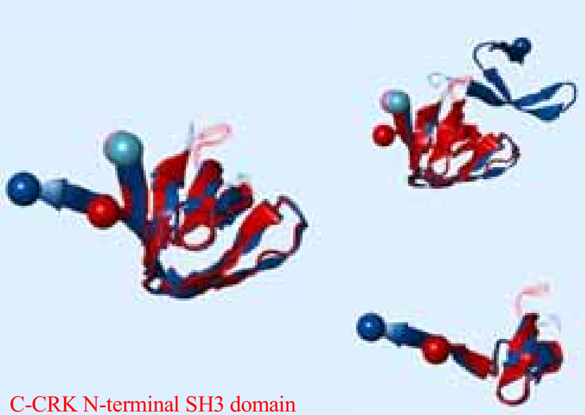 C-CRK N-terminal SH3 domain<br>Epidermal growth factor receptor kinase subst
	1i07A (59)	34-40 (7)	34-40 (7)	162-168 (7)						
1079	1m3aA (57)	136-190 (55)	162-169 (8)	162-169 (8)	C	0.65	1.59	92.98% (53/57)	26.32% (15/57)	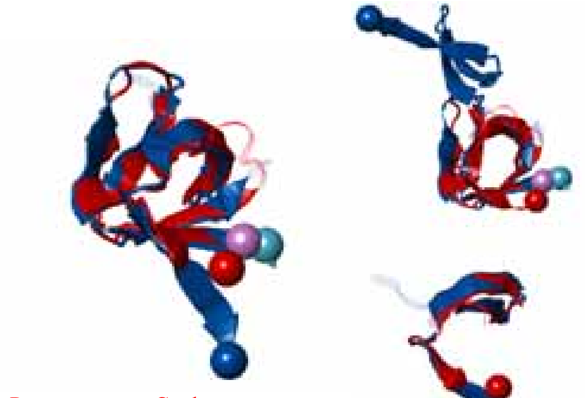 Proto-oncogene C-crk<br>Epidermal growth factor receptor kinase subst
	1i07A (59)	8-61 (54)	34-40 (7)	162-169 (8)						

1080	1zuyA (58)	30-36 (7)	30-36 (7)	30-36 (7)	C	0.68	1.53	93.10% (54/58)	24.14% (14/58)	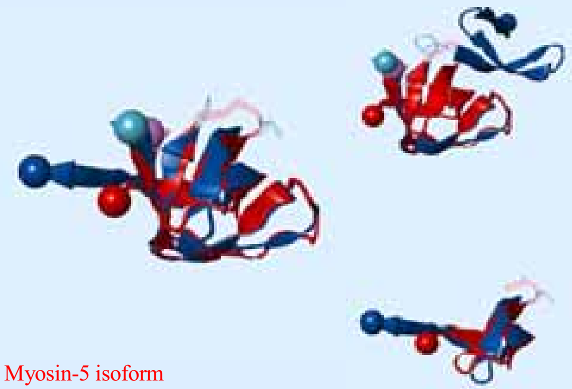 Myosin-5 isoform Epidermal growth factor receptor kinase subst
	1i07A (59)	34-39 (6)	34-39 (6)	30-36 (7)						
1081	1bu1C (56)	108-115 (8)	108-114 (7)	108-113 (6)	C	0.77	0.72	92.86% (52/56)	26.79% (15/56)	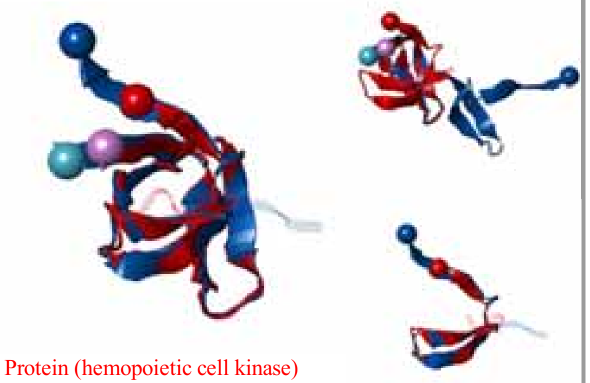 Protein (hemopoietic cell kinase) Epidermal growth factor receptor kinase subst
	1i07A (59)	34-41 (8)	34-40 (7)	108-113 (6)						
1082	1avzC (57)	102-120 (19)	105-120 (16)	112-119 (8)	C	0.73	1.13	92.98% (53/57)	28.07% (16/57)	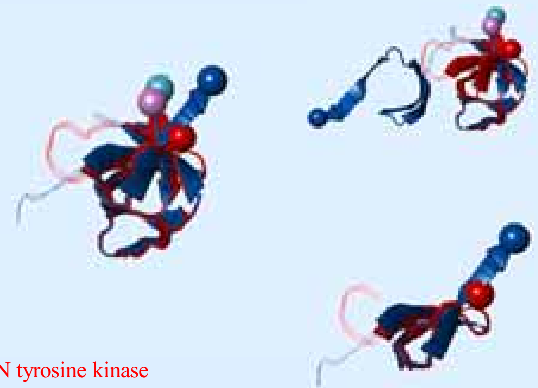 FYN tyrosine kinase Epidermal growth factor receptor kinase subst
	1i07A (59)	24-41 (18)	27-41 (15)	112-119 (8)						

1083	2ebpA (73)	13-56 (44)	39-56 (18)	39-46 (8)	C	0.55	1.55	93.22% (55/59)	16.95% (10/59)	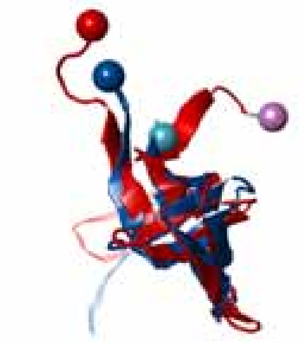 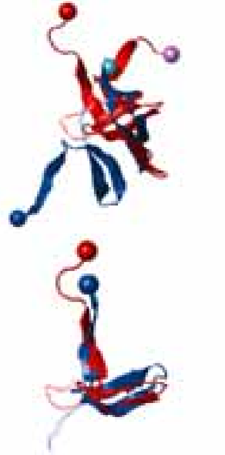<br>SAM and SH3 domain-containing protein 1<br>Epidermal growth factor receptor kinase subst
	1i07A (59)	10-51 (42)	34-51 (18)	39-46 (8)						
1084	2eyxA (67)	265-284 (20)	265-284 (20)	267-274 (8)	N	0.65	1.54	96.61% (57/59)	13.56% (8/59)	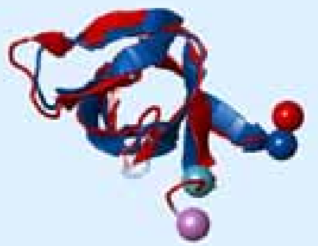 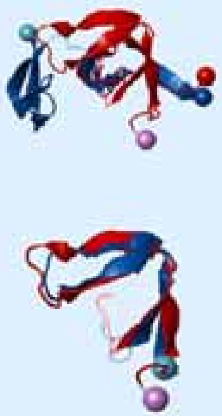<br>V-crk sarcoma virus CT10 oncogene homolog iso<br>Epidermal growth factor receptor kinase subst
	1i07A (59)	32-51 (20)	32-51 (20)	267-274 (8)						
1085	2vq9A (123)	21-25 (5)	21-25 (5)	23-25 (3)	N	0.62	1.66	91.06% (112/123)	30.89% (38/123)	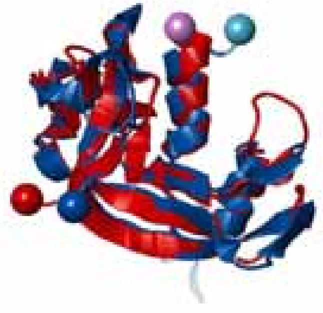 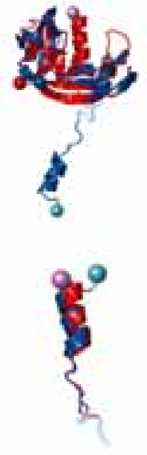<br>Rnase 1<br>Ribonuclease 1
	1h8xA (125)	117-125 (9)	117-125 (9)	23-25 (3)						

1086	1yv7A (102)	10-20 (11)	12-18 (7)	15-18 (4)	N	0.56	1.67	95.10% (97/102)	24.51% (25/102)	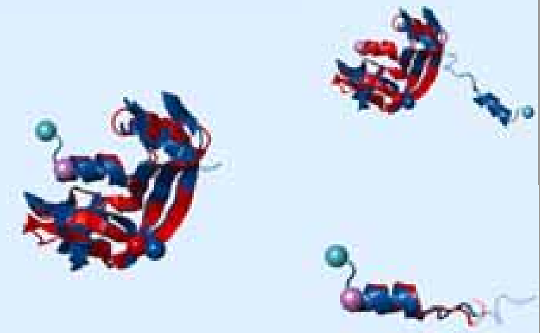 P-30 protein<br>Ribonuclease 1
	1h8xA (125)	112-127 (16)	114-125 (12)	15-18 (4)						
1087	1k5aA (118)	13-24 (12)	14-24 (11)	17-24 (8)	N	0.67	1.50	94.07% (111/118)	34.75% (41/118)	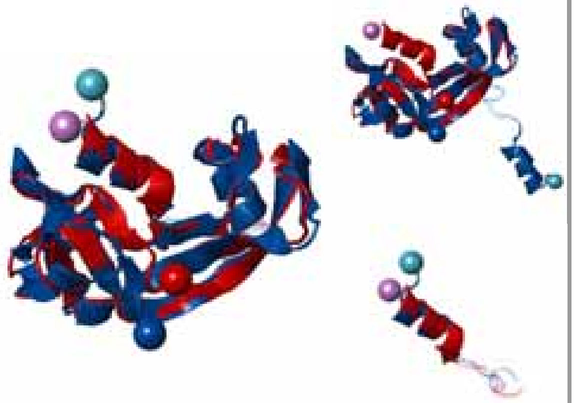 Angiogenin<br>Ribonuclease 1
	1h8xA (125)	112-124 (13)	113-124 (12)	17-24 (8)						
1088	1un5A (121)	13-24 (12)	15-24 (10)	17-24 (8)	N	0.66	1.52	92.56% (112/121)	35.54% (43/121)	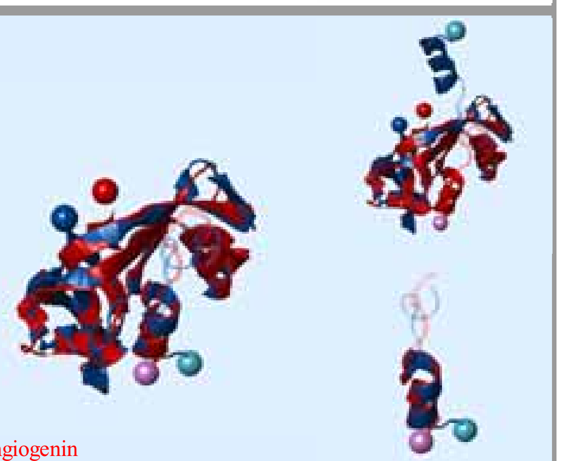 Angiogenin<br>Ribonuclease 1
	1h8xA (125)	112-124 (13)	114-124 (11)	17-24 (8)						

1089	1a5qA (124)	15-23 (9)	16-23 (8)	17-23 (7)	N	0.86	1.03	98.39% (122/124)	69.35% (86/124)	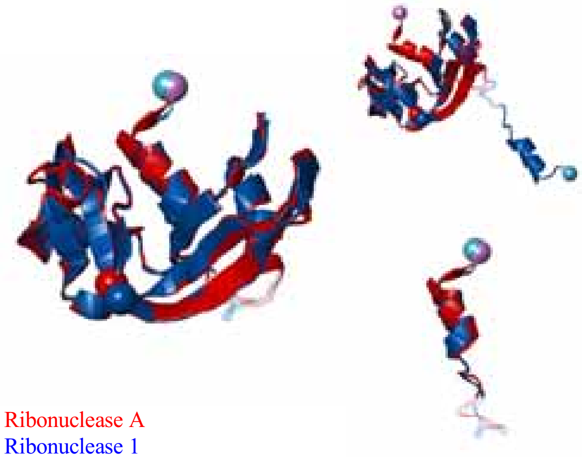 Ribonuclease A<br>Ribonuclease 1
	1h8xA (125)	115-123 (9)	116-123 (8)	17-23 (7)						
1090	1lbgA (124)	16-22 (7)	16-22 (7)	16-22 (7)	N	0.61	0.70	100.00% (122/122)	99.18% (121/122)	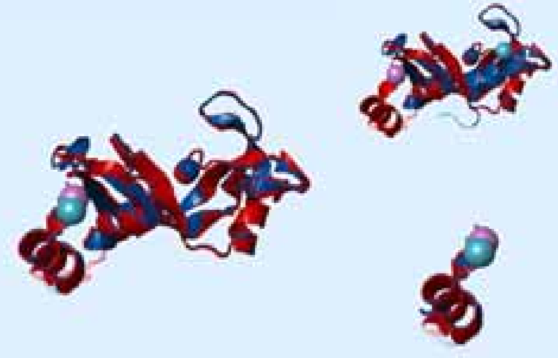 Protein (bovine seminal ribonuclease)<br>Ribonuclease, seminal
	1r3mB (122)	16-22 (7)	16-22 (7)	16-22 (7)						
1091	1lbgA (124)	15-22 (8)	15-22 (8)	15-22 (8)	N	0.90	0.90	99.19% (123/124)	80.65% (100/124)	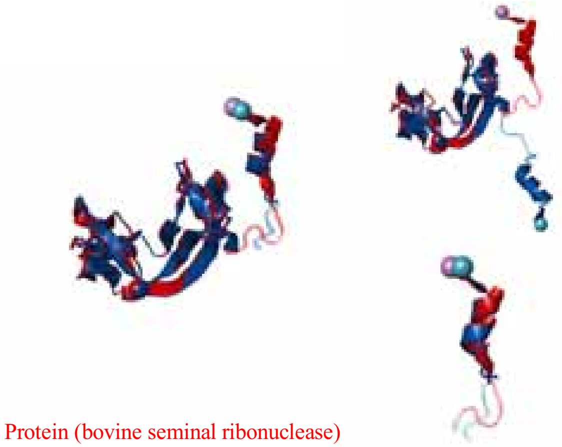 Protein (bovine seminal ribonuclease)<br>Ribonuclease A
	1a2wA (124)	15-22 (8)	15-22 (8)	15-22 (8)						

1092	11bgA (124)	15-24 (10)	15-23 (9)	15-23 (9)	N	0.86	1.05	98.39% (122/124)	71.77% (89/124)	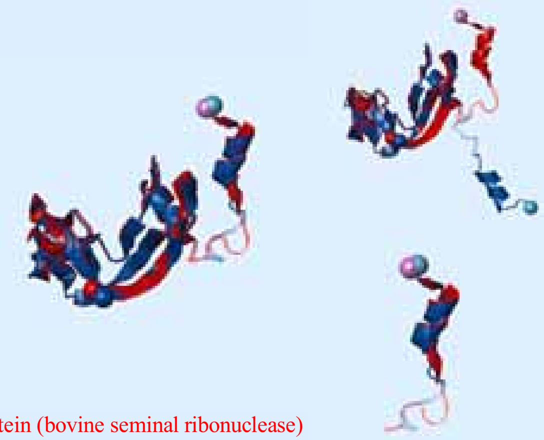 Protein (bovine seminal ribonuclease) Ribonuclease 1
	1h8xA (125)	115-124 (10)	115-123 (9)	15-23 (9)						
1093	11baA (124)	15-24 (10)	15-23 (9)	15-23 (9)	N	0.85	1.16	99.19% (123/124)	74.19% (92/124)	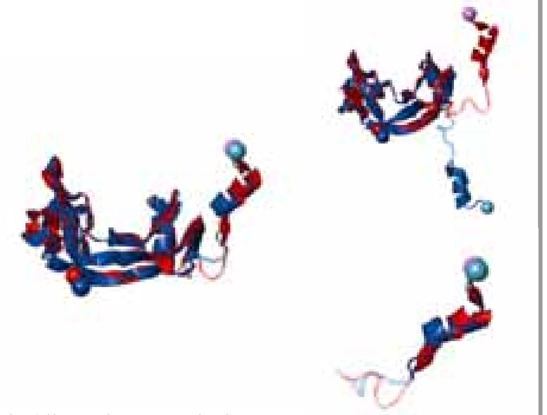 Protein (ribonuclease, seminal) Ribonuclease 1
	1h8xA (125)	115-124 (10)	115-123 (9)	15-23 (9)						
