# Supplementary material for: Detection and Alignment of 3D Domain Swapping Proteins Using Angle-Distance Image-Based Secondary Structural Matching Techniques
Source: PLoS One. 2010 Oct 14;5(10):e13361. doi: 10.1371/journal.pone.0013361 (PMC2955075; doi:10.1371/journal.pone.0013361)
Supplement: Table S5 — Structure-based sequence alignments for DSCO pairs in Datasets L and M performed by several protein structural comparison methods. The structure-based sequence alignments performed by TM-align [31], SARST [34] and the proposed DS-detecting method as well as the sequence alignments performed by BLAST [42] for the 1,093 DSCO pairs shown in Table S4 are listed here. (9.84 MB PDF) [file pone.0013361.s009.pdf]

Table S5. Structure-based sequence alignments for DS_{CO} pairs in Datasets L and M performed by several protein structural comparison methods

The structure-based sequence alignments performed by TM-align [31], SARST [34] and the proposed DS-detecting method as well as the sequence alignments performed by BLAST [42] for the 1,093 DS_{CO} pairs shown in Table S4 are listed here. In this table, hinge loops of the query and subject proteins are highlighted in red and blue, respectively. Aligned residues with identical amino acid types are highlighted in green while those with equal or positive substitution rates defined in the BLOSUM62 substitution matrix [58] are highlighted in light green. TM-align is an accurate protein structural comparison (PSC) method which also showed the best DS-detecting ability among the PSC methods treating protein structures as rigid bodies (Fig. 2c). SARST is a flexible PSC method which does not treat protein structures as rigid bodies and showed much better DS-detecting performance than conventional rigid PSC methods (Fig. 2c). BLAST is the most widely used amino acid sequence alignment method. As shown in this table, a rigid PSC method almost always gives very local alignments, which means it only aligns one pair of domains of a DS_{CO} pair. A flexible PSC method often produces alignments more global than a rigid PSC method by simultaneously aligning the main and swapped domains. A sequence alignment method can make quite global alignments for DS-related proteins; however, if the sequence identity of a DS_{CO} pair is <25%, in most cases the alignment will either be still very local or be not available at all (note that there are 55% DS_{CO} pairs in this table sharing <25% identities). Only the proposed method always simultaneously aligns the hinge loops, main domains and swapped domains.

No.	PDB entry (size)	DS type	Identity (%)	Alignment
1	1brnL (108)<br>1yvsA (108)	N	99.07	TM-align: aliSize=73 (resi) RMSD=0.97 (Å)<br>vintfdgvadylqtyhklpdnyitkseaqalgwva----------------------------------SKGNLADVAPGKSIGGDIFSNREGKLPGKSGRTWREADINYTSGFRNSDRILYSSDWLIYKTTDHYQTFTKIR<br>----------------------------------vintfdgvadylqtyhklpdnyitkseaqalgwvaSKGNLADVAPGKSIGGDIFSNREGKLPGKSGRTWREADINYTSGFRNSDRILYSSDWLIYKTTDHYQTFTKIR<br><br>SARST: aliSize=106 (resi) RMSD=17.16 (Å)<br>v-INTFDGVADYLQTYHKLPDNYITKSEAQALGWVASKGNLADVAPGKSIGGDIFSNREGKLPGKSGRTWREADINYTSGFRNSDRILYSSDWLIYKTTDHYQTFTKI-r<br>-vINTFDGVADYLQTYHKLPDNYITKSEAQALGWVASKGNLADVAPGKSIGGDIFSNREGKLPGKSGRTWREADINYTSGFRNSDRILYSSDWLIYKTTDHYQTFTKIr-<br><br>BLAST: aliSize=108 (resi) iden=100.00% (108/108) simi=100.00% (108/108)<br>VINTFDGVADYLQTYHKLPDNYITKSEAQALGWVASKGNLADVAPGKSIGGDIFSNREGKLPGKSGRTWREADINYTSGFRNSDRILYSSDWLIYKTTDHYQTFTKIR<br>VINTFDGVADYLQTYHKLPDNYITKSEAQALGWVASKGNLADVAPGKSIGGDIFSNREGKLPGKSGRTWREADINYTSGFRNSDRILYSSDWLIYKTTDHYQTFTKIR<br><br>Proposed: aliSize=107 (resi) RMSD=0.47 (Å)<br>VINTFDGVADYLQTYHKLPDNYITKSEAQALGWVAS-kGNLADVAPGKSIGGDIFSNREGKLPGKSGRTWREADINYTSGFRNSDRILYSSDWLIYKTTDHYQTFTKIR<br>VINTFDGVADYLQTYHKLPDNYITKSEAQALGWVASk-GNLADVAPGKSIGGDIFSNREGKLPGKSGRTWREADINYTSGFRNSDRILYSSDWLIYKTTDHYQTFTKIR
2	4icbA (76)<br>1ht9A (76)	C	96.05	TM-align: aliSize=48 (resi) RMSD=1.79 (Å)<br>MKSPEELKGIFEKYAAKEGDPNQLSKEELKLLLQTEFPSLLK-GPStld--ELfe--Eldkngdgevsfeefqvlvkkisq-----------------------<br>MKSPEELKGIFEKYAAKEGDPNNLSKEELKLLLQTEFPSLLKgMST---ldEL--feE-----------------------ldkngdgevsfeefqvlvkkisq<br><br>SARST: aliSize=69 (resi) RMSD=10.69 (Å)<br>mk--SPEELKGIFEKYAAKEGDPNQLSKEELKLLLQTEFPSLLKgPSTLDELFEEL-DKNGDGEVSFEEFQVLVK----kisq<br>--mkSPEELKGIFEKYAAKEGDPNNLSKEELKLLLQTEFPSLLK-GMSTLDELFEElDKNGDGEVSFEEFQVLVKkisq----<br><br>BLAST: aliSize=74 (resi) iden=97.37% (74/76) simi=97.37% (74/76)<br>MKSPEELKGIFEKYAAKEGDPNQLSKEELKLLLQTEFPSLLKGPSTLDELFEELDKNGDGEVSFEEFQVLVKKISQ<br>MKSPEELKGIFEKYAAKEGDPNNLSKEELKLLLQTEFPSLLKGMSTLDELFEELDKNGDGEVSFEEFQVLVKKISQ<br><br>Proposed: aliSize=74 (resi) RMSD=0.97 (Å)<br>MKSPEELKGIFEKYAAKEGDPNQLSKEELKLLLQTEFPSLLKG--psTLDELFEELDKNGDGEVSFEEFQVLVKKISQ<br>MKSPEELKGIFEKYAAKEGDPNNLSKEELKLLLQTEFPSLLKGms--TLDELFEELDKNGDGEVSFEEFQVLVKKISQ

3	1hngA (175)<br>1cdcA (96)	C	97.92	TM-align: aliSize=54 (resi) RMSD=1.17 (Å)<br>dsgtvwgalghginlnipnfqmtddidevrwergstlvaefkrk--MKPFLKSGAFEILANGDLKIKNLTRDDSGTYNVTVYSTNGTRILNKALDLRILemvskpmiywecsnatltcevlegTdvelklyqgkehlrslrqktmsyqwt<br>--gtvwgalghginlnipnfqmtddidevrwergstlvaefkrkMKPFLKSGAFEILANGDLKIKNLTRDDSGTYNVTVYSTNGTRILDKALDLRIL------------------------E--------------------------<br>nlrapfkckavnrvsqesemevvncpe<br>---------------------------<br><br>SARST: aliSize=92 (resi) RMSD=18.76 (Å)<br>dsg-TVWGALGHGINLNIPNFQMTDDIDEVRWERGSTLVAEFKRKMKPFLKSGAFEILANGDLKIKNLTRDDSGTYNVTVYSTNGTRILNKALDLR---ilemvskpmiywecsnatltcevlegtdvelklyqgkehlrslrqktmsyqwtnlrapfkckavnrvsqesemevvncpe<br>---gTVWGALGHGINLNIPNFQMTDDIDEVRWERGSTLVAEFKRKMKPFLKSGAFEILANGDLKIKNLTRDDSGTYNVTVYSTNGTRILDKALDLRile--<br><br>BLAST: aliSize=96 (resi) iden=98.96% (95/96) simi=100.00% (96/96)<br>dsGTVWGALGHGINLNIPNFQMTDDIDEVRWERGSTLVAEFKRKMKPFLKSGAFEILANGDLKIKNLTRDDSGTYNVTVYSTNGTRILNKALDLRILEmvskpmiywecsnatltcevlegtdvelklyqgkehlrslrqktmsyqwtnlrapfkckavnrvsqesemevvncpe<br>--GTVWGALGHGINLNIPNFQMTDDIDEVRWERGSTLVAEFKRKMKPFLKSGAFEILANGDLKIKNLTRDDSGTYNVTVYSTNGTRILDKALDLRILE---<br><br>Proposed: aliSize=96 (resi) RMSD=1.01 (Å)<br>dsGTVWGALGHGINLNIPNFQMTDDIDEVRWERGSTLVAEFKRKMKPFLKSGAFEILANGDLKIKNLTRDDSGTYNVTVYSTNGTRILNKALDLRILemvskpmiywecsnatltcevlegTdvelklyqgkehlrslrqktmsyqwtnlrapfkckavnrvsqesemevvncpe<br>--GTVWGALGHGINLNIPNFQMTDDIDEVRWERGSTLVAEFKRKMKPFLKSGAFEILANGDLKIKNLTRDDSGTYNVTVYSTNGTRILDKALDLRIL------------------------E---
4	1orcA (64)<br>5croA (61)	C	93.44	TM-align: aliSize=57 (resi) RMSD=1.69 (Å)<br>--QRITLKDYAMRFGQTKTAKDLGVYQSAINKAIHAGRKIFLTINADGSVYAEEVKDGEvkpfpsn--<br>meQRITLKDYAMRFGQTKTAKDLGVYQSAINKAIHAGRKIFLTINADGSVYAEEVKPFP-------sn<br><br>SARST: aliSize=55 (resi) RMSD=3.06 (Å)<br>qr----ITLKDYAMRFGQTKTAKDLGVYQSAINKAIHAGRKIFLTINADGSVYAEEvkdgeVKPFP-sn<br>--meqrITLKDYAMRFGQTKTAKDLGVYQSAINKAIHAGRKIFLTINADGSVYAEE-----VKPFPs--<br><br>BLAST: aliSize=58 (resi) iden=96.67% (58/61) simi=96.67% (58/61)<br>--QRITLKDYAMRFGQTKTAKDLGVYQSAINKAIHAGRKIFLTINADGSVYAEEVKdgevkPFPSn<br>meQRITLKDYAMRFGQTKTAKDLGVYQSAINKAIHAGRKIFLTINADGSVYAEEVK-----PFPS-<br><br>Proposed: aliSize=59 (resi) RMSD=1.24 (Å)<br>--QRITLKDYAMRFGQTKTAKDLGVYQSAINKAIHAGRKIFLTINADGSVYAEEVKdgeVKPfpSN<br>meQRITLKDYAMRFGQTKTAKDLGVYQSAINKAIHAGRKIFLTINADGSVYAEEVK---PFP--SN
5	2ezmA (101)<br>3ezmA (101)	C	100.00	TM-align: aliSize=51 (resi) RMSD=1.06 (Å)<br>LGKFSQTCYNSAIQGSVLTSTCERTNGGYNTSSIDLNSVIENVDGSLKWQPsnfietcrntqlagsselaaecktraqqfvstkinlddhianidgtlkye--<br>LGKFSQTCYNSAIQGSVLTSTCERTNGGYNTSSIDLNSVIENVDGSLKWQP--snfietcrntqlagsselaaecktraqqfvstkinlddhianidgtlkye<br><br>SARST: aliSize=99 (resi) RMSD=16.28 (Å)<br>l-GKFSQTCYNSAIQGSVLTSTCERTNGGYNTSSIDLNSVIENVDGSLKWQPSNFIETCRNTQLAGSSELAAECKTRAQQFVSTKINLDDHIANIDGTLKY-e<br>-lGKFSQTCYNSAIQGSVLTSTCERTNGGYNTSSIDLNSVIENVDGSLKWQPSNFIETCRNTQLAGSSELAAECKTRAQQFVSTKINLDDHIANIDGTLKYe-<br><br>BLAST: aliSize=101 (resi) iden=100.00% (101/101) simi=100.00% (101/101)<br>LGKFSQTCYNSAIQGSVLTSTCERTNGGYNTSSIDLNSVIENVDGSLKWQPSNFIETCRNTQLAGSSELAAECKTRAQQFVSTKINLDDHIANIDGTLKYE<br>LGKFSQTCYNSAIQGSVLTSTCERTNGGYNTSSIDLNSVIENVDGSLKWQPSNFIETCRNTQLAGSSELAAECKTRAQQFVSTKINLDDHIANIDGTLKYE<br><br>Proposed: aliSize=101 (resi) RMSD=0.82 (Å)<br>LGKFSQTCYNSAIQGSVLTSTCERTNGGYNTSSIDLNSVIENVDGSLKWQPSNFIETCRNTQLAGSSELAAECKTRAQQFVSTKINLDDHIANIDGTLKYE<br>LGKFSQTCYNSAIQGSVLTSTCERTNGGYNTSSIDLNSVIENVDGSLKWQPSNFIETCRNTQLAGSSELAAECKTRAQQFVSTKINLDDHIANIDGTLKYE

6	1mdtA (523)<br>1ddtA (523)	C	98.85	TM-align: aliSize=390 (resi) RMSD=2.34 (Å)<br>GADDVVDSSKSFVMENFSSYHGTKPGYVDSIQKGIQKPKSGTQGNYDDDWKGFYSTDNKYDAAGYSVDNENPLSGKAGGVVKVTYPGLTKVLALKVDNAETIKKELGLSLTEPLMEQVGTEEFIKRFGDGASRVVLSLPFAEGSSSVEYINNWEQAKALSVELEINFETRGKRGQDAMYEYMAQACASCI<br>GADDVVDSSKSFVMENFSSYHGTKPGYVDSIQKGIQKPKSGTQGNYDDDWKGFYSTDNKYDAAGYSVDNENPLSGKAGGVVKVTYPGLTKVLALKVDNAETIKKELGLSLTEPLMEQVGTEEFIKRFGDGASRVVLSLPFAEGSSSVEYINNWEQAKALSVELEINFETRGKRGQDAMYEYMAQACASCI<br>NLDWDVIRDKTKTKIESLKEHGPIKNKMSESPNKTVSEEKAKQYLEEFHQTALEHPELSELKTVTGTNPVFAGANYAAWAVNVAQVIDSETADNLEKTTAALSILPGIGSVMGIADGAVHHNTEEIVAQSIALSSLMVAQAIPLVGELVDIGFAAYNFVESIINLFQVVHNSYNRPAYSPGHKTQPFLHD<br>NLDWDVIRDKTKTKIESLKEHGPIKNKMSESPNKTVSEEKAKQYLEEFHQTALEHPELSELKTVTGTNPVFAGANYAAWAVNVAQVIDSETADNLEKTTAALSILPGIGSVMGIADGAVHHNTEEIVAQSIALSSLMVAQAIPLVGELVDIGFAAYNFVESIINLFQVVHNSYNRPAYSPGHKTQPFLHD<br>GYAVSWNTVedsiirtgfqgesghdikita--------------------Fntplpiagvllptipgkldvnkskthisvngrkirmrcraidgdvtfcrpkspvyvgngvhanlhvafhrsssekihsneissdsigvlgyqktvdhtkvnsklslffeiks--------------------------<br>GYAVSWNTV--------------------edsiirtgfqgesghdikitaE---ntplpiagvllptipgkldvnkskth<br>---<br>isvngrkirmrcraidgdvtfcrpkspvyvgngvhanlhvafhrsssekihsneissdsigvlgyqktvdhtkvnsklslffeiks<br>SARST: aliSize=521 (resi) RMSD=15.63 (Å)<br>g-ADDVVDSSKSFVMENFSSYHGTKPGYVDSIQKGIQKPKSGTQGNYDDDWKGFYSTDNKYDAAGYSVDNENPLSGKAGGVVKVTYPGLTKVLALKVDNAETIKKELGLSLTEPLMEQVGTEEFIKRFGDGASRVVLSLPFAEGSSSVEYINNWEQAKALSVELEINFETRGKRGQDAMYEYMAQACASC<br>-gADDVVDSSKSFVMENFSSYHGTKPGYVDSIQKGIQKPKSGTQGNYDDDWKGFYSTDNKYDAAGYSVDNENPLSGKAGGVVKVTYPGLTKVLALKVDNAETIKKELGLSLTEPLMEQVGTEEFIKRFGDGASRVVLSLPFAEGSSSVEYINNWEQAKALSVELEINFETRGKRGQDAMYEYMAQACASC<br>INLDWDVIRDKTKTKIESLKEHGPIKNKMSESPNKTVSEEKAKQYLEEFHQTALEHPELSELKTVTGTNPVFAGANYAAWAVNVAQVIDSETADNLEKTTAALSILPGIGSVMGIADGAVHHNTEEIVAQSIALSSLMVAQAIPLVGELVDIGFAAYNFVESIINLFQVVHNSYNRPAYSPGHKTQPFLH<br>INLDWDVIRDKTKTKIESLKEHGPIKNKMSESPNKTVSEEKAKQYLEEFHQTALEHPELSELKTVTGTNPVFAGANYAAWAVNVAQVIDSETADNLEKTTAALSILPGIGSVMGIADGAVHHNTEEIVAQSIALSSLMVAQAIPLVGELVDIGFAAYNFVESIINLFQVVHNSYNRPAYSPGHKTQPFLH<br>DGYAVSWNTVEDSIIRTGFQGESGHDIKITAENTPLPIAGVLLPTIPGKLDVNKSKTHISVNGRKIRMRCRAIDGDVTFCRPKSPVYVGNGVHANLHVAFHRSSSEKIHSNEISSDSIGVLGYQKTVDHTKVNSKLSLFFEIK-s<br>DGYAVSWNTVEDSIIRTGFQGESGHDIKITAENTPLPIAGVLLPTIPGKLDVNKSKTHISVNGRKIRMRCRAIDGDVTFCRPKSPVYVGNGVHANLHVAFHRSSSEKIHSNEISSDSIGVLGYQKTVDHTKVNSKLSLFFEIKs-<br>BLAST: aliSize=523 (resi) iden=100.00% (523/523) simi=100.00% (523/523)<br>GADDVVDSSKSFVMENFSSYHGTKPGYVDSIQKGIQKPKSGTQGNYDDDWKGFYSTDNKYDAAGYSVDNENPLSGKAGGVVKVTYPGLTKVLALKVDNAETIKKELGLSLTEPLMEQVGTEEFIKRFGDGASRVVLSLPFAEGSSSVEYINNWEQAKALSVELEINFETRGKRGQDAMYEYMAQACASCI<br>GADDVVDSSKSFVMENFSSYHGTKPGYVDSIQKGIQKPKSGTQGNYDDDWKGFYSTDNKYDAAGYSVDNENPLSGKAGGVVKVTYPGLTKVLALKVDNAETIKKELGLSLTEPLMEQVGTEEFIKRFGDGASRVVLSLPFAEGSSSVEYINNWEQAKALSVELEINFETRGKRGQDAMYEYMAQACASCI<br>NLDWDVIRDKTKTKIESLKEHGPIKNKMSESPNKTVSEEKAKQYLEEFHQTALEHPELSELKTVTGTNPVFAGANYAAWAVNVAQVIDSETADNLEKTTAALSILPGIGSVMGIADGAVHHNTEEIVAQSIALSSLMVAQAIPLVGELVDIGFAAYNFVESIINLFQVVHNSYNRPAYSPGHKTQPFLHD<br>NLDWDVIRDKTKTKIESLKEHGPIKNKMSESPNKTVSEEKAKQYLEEFHQTALEHPELSELKTVTGTNPVFAGANYAAWAVNVAQVIDSETADNLEKTTAALSILPGIGSVMGIADGAVHHNTEEIVAQSIALSSLMVAQAIPLVGELVDIGFAAYNFVESIINLFQVVHNSYNRPAYSPGHKTQPFLHD<br>GYAVSWNTVEDSIIRTGFQGESGHDIKITAENTPLPIAGVLLPTIPGKLDVNKSKTHISVNGRKIRMRCRAIDGDVTFCRPKSPVYVGNGVHANLHVAFHRSSSEKIHSNEISSDSIGVLGYQKTVDHTKVNSKLSLFFEIKS<br>GYAVSWNTVEDSIIRTGFQGESGHDIKITAENTPLPIAGVLLPTIPGKLDVNKSKTHISVNGRKIRMRCRAIDGDVTFCRPKSPVYVGNGVHANLHVAFHRSSSEKIHSNEISSDSIGVLGYQKTVDHTKVNSKLSLFFEIKS<br>Proposed: aliSize=517 (resi) RMSD=0.84 (Å)<br>GADDVVDSSKSFVMENFSSYHGTKPGYVDSIQKGIQKPKSGTQGNYDDDWKGFYSTDNKYDAAGYSVDNENPLSGKAGGVVKVTYPGLTKVLALKVDNAETIKKELGLSLTEPLMEQVGTEEFIKRFGDGASRVVLSLPFAEGSSSVEYINNWEQAKALSVELEINFETRGKRGQDAMYEYMAQACASCI<br>GADDVVDSSKSFVMENFSSYHGTKPGYVDSIQKGIQKPKSGTQGNYDDDWKGFYSTDNKYDAAGYSVDNENPLSGKAGGVVKVTYPGLTKVLALKVDNAETIKKELGLSLTEPLMEQVGTEEFIKRFGDGASRVVLSLPFAEGSSSVEYINNWEQAKALSVELEINFETRGKRGQDAMYEYMAQACASCI<br>NLDWDVIRDKTKTKIESLKEHGPIKNKMSESPNKTVSEEKAKQYLEEFHQTALEHPELSELKTVTGTNPVFAGANYAAWAVNVAQVIDSETADNLEKTTAALSILPGIGSVMGIADGAVHHNTEEIVAQSIALSSLMVAQAIPLVGELVDIGFAAYNFVESIINLFQVVHNSYNRPA------yspghkT<br>NLDWDVIRDKTKTKIESLKEHGPIKNKMSESPNKTVSEEKAKQYLEEFHQTALEHPELSELKTVTGTNPVFAGANYAAWAVNVAQVIDSETADNLEKTTAALSILPGIGSVMGIADGAVHHNTEEIVAQSIALSSLMVAQAIPLVGELVDIGFAAYNFVESIINLFQVVHNSYNRPAyspghk------T<br>QPFLHDGYAVSWNTVEDSIIRTGFQGESGHDIKITAENTPLPIAGVLLPTIPGKLDVNKSKTHISVNGRKIRMRCRAIDGDVTFCRPKSPVYVGNGVHANLHVAFHRSSSEKIHSNEISSDSIGVLGYQKTVDHTKVNSKLSLFFEIKS<br>QPFLHDGYAVSWNTVEDSIIRTGFQGESGHDIKITAENTPLPIAGVLLPTIPGKLDVNKSKTHISVNGRKIRMRCRAIDGDVTFCRPKSPVYVGNGVHANLHVAFHRSSSEKIHSNEISSDSIGVLGYQKTVDHTKVNSKLSLFFEIKS
7	1qlxA (104)<br>1i4mA (108)	C	92.31	TM-align: aliSize=70 (resi) RMSD=1.85 (Å)<br>------LGGYMLGSAMSRPIIHFGSDYEDRYYRENMHRYPNQVYYRPMDEYSNQNNFVHDCVNITIKQHTVTTttKGEnftetdvkmmervveqmcitqyeresqayyqr--------------------------------<br>gavvggLGGYMLGSAMSRPIIHFGSDYEDRYYRENMHRYPNQVYYRPMDEYSNQNNFVHDCVNITIKQHTVTT--TTK--------------------------------genftetdvkmmervveqmcitqyeresqayy<br>SARST: aliSize=95 (resi) RMSD=20.86 (Å)<br>l-------GGYMLGSAMSRPIIHFGSDYEDRYYRENMHRYPNQVYYRPMDEYSNQNNFVHDCVNITIKQHTV----TTTTkgenFTETDVKMMERVVEQMCITQYERESQA--yyqr<br>-gavvgglGGYMLGSAMSRPIIHFGSDYEDRYYRENMHRYPNQVYYRPMDEYSNQNNFVHDCVNITIKQHTVttttKGEN----FTETDVKMMERVVEQMCITQYERESQAyy----<br>BLAST: aliSize=102 (resi) iden=98.08% (102/104) simi=98.08% (102/104)<br>------LGGYMLGSAMSRPIIHFGSDYEDRYYRENMHRYPNQVYYRPMDEYSNQNNFVHDCVNITIKQHTVTTTTKGENFTETDVKMMERVVEQMCITQYERESQAYYqr<br>gavvggLGGYMLGSAMSRPIIHFGSDYEDRYYRENMHRYPNQVYYRPMDEYSNQNNFVHDCVNITIKQHTVTTTTKGENFTETDVKMMERVVEQMCITQYERESQAYY--<br>Proposed: aliSize=99 (resi) RMSD=1.56 (Å)<br>------LGGYMLGSAMSRPIIHFGSDYEDRYYRENMHRYPNQVYYRPMDEYSNQNNFVHDCVNITIKQHTVT---TTtkgENFTETDVKMMERVVEQMCITQYERESQAYyqR<br>gavvggLGGYMLGSAMSRPIIHFGSDYEDRYYRENMHRYPNQVYYRPMDEYSNQNNFVHDCVNITIKQHTVTtttKG---ENFTETDVKMMERVVEQMCITQYERESQAY--Y
8	1hz5A (72)<br>1jmlA (72)	C	88.89	TM-align: aliSize=60 (resi) RMSD=1.68 (Å)<br>--MHHHHHHamEEVTIKANLIFANGSTQTAEFKGTFEKATSEAYAYADTLKKDNGEWTVDVADKgytlnikfag----------<br>mhHHHHHGM--EEVTIKANLIFANGSTQTAEFKGTFEKATSEAYAYADTLKKDNGEWTVDVVPK----------aytlnikfag<br>SARST: aliSize=69 (resi) RMSD=11.31 (Å)<br>mh--HHHHHHAMEEVTIKANLIFANGSTQTAEFKGTFEKATSEAYAYADTLKKDNGEWTVDVADKGYTLNIKFA-g<br>--mhHHHHHHGMEEVTIKANLIFANGSTQTAEFKGTFEKATSEAYAYADTLKKDNGEWTVDVVPKAYTLNIKFAg-<br>BLAST: aliSize=68 (resi) iden=94.44% (68/72) simi=94.44% (68/72)<br>MHHHHHHAMEEVTIKANLIFANGSTQTAEFKGTFEKATSEAYAYADTLKKDNGEWTVDVADKGYTLNIKFAG<br>MHHHHHHGMEEVTIKANLIFANGSTQTAEFKGTFEKATSEAYAYADTLKKDNGEWTVDVVPKAYTLNIKFAG<br>Proposed: aliSize=70 (resi) RMSD=1.58 (Å)<br>--MHHHHHHamEEVTIKANLIFANGSTQTAEFKGTFEKATSEAYAYADTLKKDNGEWTVDVADKGYTLNIKFAG<br>mhHHHHHGM--EEVTIKANLIFANGSTQTAEFKGTFEKATSEAYAYADTLKKDNGEWTVDVVPKAYTLNIKFAG

<table>
<tr><td rowspan="2">9</td><td>5rsaA
(124)</td><td rowspan="2">N</td><td rowspan="2">100.00</td><td rowspan="2">TM-align: aliSize=105 (resi) RMSD=0.85 (Å)
ketaaakferqhmdssts a------------------ASSSNYCNQMMKSRNLTKDRCKPVNTFVHESLADVQAVCSQKNVACKNGQTNCYQSYSTMSITDCRETGSSKYPNCAYKTTQANKHIIVACEGNPYVPVHFDASV
------------------ketaaakferqhmdsstsaASSSNYCNQMMKSRNLTKDRCKPVNTFVHESLADVQAVCSQKNVACKNGQTNCYQSYSTMSITDCRETGSSKYPNCAYKTTQANKHIIVACEGNPYVPVHFDASV

SARST: aliSize=122 (resi) RMSD=14.18 (Å)
k-ETAAAKFERQHMDSSTSAASSSNYCNQMMKSRNLTKDRCKPVNTFVHESLADVQAVCSQKNVACKNGQTNCYQSYSTMSITDCRETGSSKYPNCAYKTTQANKHIIVACEGNPYVPVHFDAS-v
-kETAAAKFERQHMDSSTSAASSSNYCNQMMKSRNLTKDRCKPVNTFVHESLADVQAVCSQKNVACKNGQTNCYQSYSTMSITDCRETGSSKYPNCAYKTTQANKHIIVACEGNPYVPVHFDASv-

BLAST: aliSize=124 (resi) iden=100.00% (124/124) simi=100.00% (124/124)
KETAAAKFERQHMDSSTSAASSSNYCNQMMKSRNLTKDRCKPVNTFVHESLADVQAVCSQKNVACKNGQTNCYQSYSTMSITDCRETGSSKYPNCAYKTTQANKHIIVACEGNPYVPVHFDASV
KETAAAKFERQHMDSSTSAASSSNYCNQMMKSRNLTKDRCKPVNTFVHESLADVQAVCSQKNVACKNGQTNCYQSYSTMSITDCRETGSSKYPNCAYKTTQANKHIIVACEGNPYVPVHFDASV

Proposed: aliSize=124 (resi) RMSD=0.54 (Å)
KETAAAKFERQHMDSSTSAASSSNYCNQMMKSRNLTKDRCKPVNTFVHESLADVQAVCSQKNVACKNGQTNCYQSYSTMSITDCRETGSSKYPNCAYKTTQANKHIIVACEGNPYVPVHFDASV
KETAAAKFERQHMDSSTSAASSSNYCNQMMKSRNLTKDRCKPVNTFVHESLADVQAVCSQKNVACKNGQTNCYQSYSTMSITDCRETGSSKYPNCAYKTTQANKHIIVACEGNPYVPVHFDASV</td></tr>
<tr><td>1a2wA
(124)</td></tr>
<tr><td rowspan="2">10</td><td>5rsaA
(124)</td><td rowspan="2">C</td><td rowspan="2">100.00</td><td rowspan="2">TM-align: aliSize=115 (resi) RMSD=1.27 (Å)
KETAAAKFERQHMDSSTSAASSSNYCNQMMKSRNLTKDRCKPVNTFVHESLADVQAVCSQKNVACKNGQTNCYQSYSTMSITDCRETGSSKYPNCAYKTTQANKHIIVACEGNPYvpvhfdasv---------
KETAAAKFERQHMDSSTSAASSSNYCNQMMKSRNLTKDRCKPVNTFVHESLADVQAVCSQKNVACKNGQTNCYQSYSTMSITDCRETGSSKYPNCAYKTTQANKHIIVACEGNPY---------vpvhfdasv

SARST: aliSize=122 (resi) RMSD=8.84 (Å)
k-ETAAAKFERQHMDSSTSAASSSNYCNQMMKSRNLTKDRCKPVNTFVHESLADVQAVCSQKNVACKNGQTNCYQSYSTMSITDCRETGSSKYPNCAYKTTQANKHIIVACEGNPYVPVHFDAS-v
-kETAAAKFERQHMDSSTSAASSSNYCNQMMKSRNLTKDRCKPVNTFVHESLADVQAVCSQKNVACKNGQTNCYQSYSTMSITDCRETGSSKYPNCAYKTTQANKHIIVACEGNPYVPVHFDASv-

BLAST: aliSize=124 (resi) iden=100.00% (124/124) simi=100.00% (124/124)
KETAAAKFERQHMDSSTSAASSSNYCNQMMKSRNLTKDRCKPVNTFVHESLADVQAVCSQKNVACKNGQTNCYQSYSTMSITDCRETGSSKYPNCAYKTTQANKHIIVACEGNPYVPVHFDASV
KETAAAKFERQHMDSSTSAASSSNYCNQMMKSRNLTKDRCKPVNTFVHESLADVQAVCSQKNVACKNGQTNCYQSYSTMSITDCRETGSSKYPNCAYKTTQANKHIIVACEGNPYVPVHFDASV

Proposed: aliSize=124 (resi) RMSD=0.43 (Å)
KETAAAKFERQHMDSSTSAASSSNYCNQMMKSRNLTKDRCKPVNTFVHESLADVQAVCSQKNVACKNGQTNCYQSYSTMSITDCRETGSSKYPNCAYKTTQANKHIIVACEGNPYVPVHFDASV
KETAAAKFERQHMDSSTSAASSSNYCNQMMKSRNLTKDRCKPVNTFVHESLADVQAVCSQKNVACKNGQTNCYQSYSTMSITDCRETGSSKYPNCAYKTTQANKHIIVACEGNPYVPVHFDASV</td></tr>
<tr><td>1f0vA
(124)</td></tr>
<tr><td rowspan="2">11</td><td>5rsaA
(124)</td><td rowspan="2">C</td><td rowspan="2">100.00</td><td rowspan="2">TM-align: aliSize=115 (resi) RMSD=1.38 (Å)
KETAAAKFERQHMDSSTSAASSSNYCNQMMKSRNLTKDRCKPVNTFVHESLADVQAVCSQKNVACKNGQTNCYQSYSTMSITDCRETGSSKYPNCAYKTTQANKHIIVACEGNPYvpvhfdasv---------
KETAAAKFERQHMDSSTSAASSSNYCNQMMKSRNLTKDRCKPVNTFVHESLADVQAVCSQKNVACKNGQTNCYQSYSTMSITDCRETGSSKYPNCAYKTTQANKHIIVACEGNPY---------vpvhfdasv

SARST: aliSize=122 (resi) RMSD=6.27 (Å)
k-ETAAAKFERQHMDSSTSAASSSNYCNQMMKSRNLTKDRCKPVNTFVHESLADVQAVCSQKNVACKNGQTNCYQSYSTMSITDCRETGSSKYPNCAYKTTQANKHIIVACEGNPYVPVHFDAS-v
-kETAAAKFERQHMDSSTSAASSSNYCNQMMKSRNLTKDRCKPVNTFVHESLADVQAVCSQKNVACKNGQTNCYQSYSTMSITDCRETGSSKYPNCAYKTTQANKHIIVACEGNPYVPVHFDASv-

BLAST: aliSize=124 (resi) iden=100.00% (124/124) simi=100.00% (124/124)
KETAAAKFERQHMDSSTSAASSSNYCNQMMKSRNLTKDRCKPVNTFVHESLADVQAVCSQKNVACKNGQTNCYQSYSTMSITDCRETGSSKYPNCAYKTTQANKHIIVACEGNPYVPVHFDASV
KETAAAKFERQHMDSSTSAASSSNYCNQMMKSRNLTKDRCKPVNTFVHESLADVQAVCSQKNVACKNGQTNCYQSYSTMSITDCRETGSSKYPNCAYKTTQANKHIIVACEGNPYVPVHFDASV

Proposed: aliSize=124 (resi) RMSD=0.57 (Å)
KETAAAKFERQHMDSSTSAASSSNYCNQMMKSRNLTKDRCKPVNTFVHESLADVQAVCSQKNVACKNGQTNCYQSYSTMSITDCRETGSSKYPNCAYKTTQANKHIIVACEGNPYVPVHFDASV
KETAAAKFERQHMDSSTSAASSSNYCNQMMKSRNLTKDRCKPVNTFVHESLADVQAVCSQKNVACKNGQTNCYQSYSTMSITDCRETGSSKYPNCAYKTTQANKHIIVACEGNPYVPVHFDASV</td></tr>
<tr><td>1js0A
(124)</td></tr>
</table>

12	5rsaA (124)<br>1bsrA (124)	N	81.45	TM-align: aliSize=104 (resi) RMSD=0.79 (Å)<br>ketaaakferqhmdsstsaa--------------------SSSNYCNQMMKSRNLTKDRCKPVNTFVHESLADVQAVCSQKNVACKNGQTNCYQSYSTMSITDCRETGSSKYPNCAYKTTQANKHIIVACEGNPYVPVHFDASV<br>--------------------kesaaakferqhmdsgnspsSSSNYCNLMMCCRKMTQGKCKPVNTFVHESLADVKAVCSQKKVTCKNGQTNCYQSKSTMRITDCRETGSSKYPNCAYKTTQVEKHIIVACGGKPSVPVHFDASV<br><br>SARST: aliSize=121 (resi) RMSD=9.79 (Å)<br>k-ETAAAKFERQHMDsSTSAASSS-NYCNQMMKSRNLTKDRCKPVNTFVHESLADVQAVCSQKNVACKNGQTNCYQSYSTMSITDCRETGSSKYPNCAYKTTQANKHIIVACEGNPYVPVHFDAS-v<br>-kESAAAKFERQHMD-SGNSPSSSsNYCNLMMCCRKMTQGKCKPVNTFVHESLADVKAVCSQKKVTCKNGQTNCYQSKSTMRITDCRETGSSKYPNCAYKTTQVEKHIIVACGGKPSVPVHFDASv-<br><br>BLAST: aliSize=107 (resi) iden=81.45% (101/124) simi=86.29% (107/124)<br>KETAAAKFERQHMDSSTSAASSSNYCNQMMKSRNLTKDRCKPVNTFVHESLADVQAVCSQKNVACKNGQTNCYQSYSTMSITDCRETGSSKYPNCAYKTTQANKHIIVACEGNPYVPVHFDASV<br>KESAAAKFERQHMDSGNSPSSSSNYCNLMMCCRKMTQGKCKPVNTFVHESLADVKAVCSQKKVTCKNGQTNCYQSKSTMRITDCRETGSSKYPNCAYKTTQVEKHIIVACGGKPSVPVHFDASV<br><br>Proposed: aliSize=124 (resi) RMSD=0.91 (Å)<br>KETAAAKFERQHMDSSTSAASSSNYCNQMMKSRNLTKDRCKPVNTFVHESLADVQAVCSQKNVACKNGQTNCYQSYSTMSITDCRETGSSKYPNCAYKTTQANKHIIVACEGNPYVPVHFDASV<br>KESAAAKFERQHMDSGNSPSSSSNYCNLMMCCRKMTQGKCKPVNTFVHESLADVKAVCSQKKVTCKNGQTNCYQSKSTMRITDCRETGSSKYPNCAYKTTQVEKHIIVACGGKPSVPVHFDASV
13	1qmpA (126)<br>1dz3A (123)	C	89.43	TM-align: aliSize=106 (resi) RMSD=1.72 (Å)<br>SIKVCIADDNRELVSLLDEYISSQPDMEVIGTAYNGQDCLQMLEEKRPDILLL-IIMPHLDGLAVLERIRAGFEHQPNVIMLTA--FGQedVTKKAVELGAsYFILKPF-DMEnlahhirqvygktpvv-------------<br>SIKVCIADDNRELVSLLDEYISSQPDMEVIGTAYNGQDCLQMLEEKRPDILLLdIIMPHLDGLAVLERIRAGFEHQPNVIMLTAfgQED--VTKKAVELGA-SYFILKPfDME----------------nlahhirqvygkt<br><br>SARST: aliSize=116 (resi) RMSD=6.93 (Å)<br>s-IKVCIADDNRELVSLLDEYISSQPDMEVIGTAYNGQDCLQMLEEKRPDILLLI-IMPHLDGLAVLERIRAGFEHQPNVIMLTAFGQEDVTKKAVELGASYFILKPFD---MENLAHHIRQ--vygktpvv<br>-sIKVCIADDNRELVSLLDEYISSQPDMEVIGTAYNGQDCLQMLEEKRPDILLLDiIMPHLDGLAVLERIRAGFEHQPNVIMLTAFGQEDVTKKAVELGASYFILKPFDmenLAHHIRQVYGkt--------<br><br>BLAST: aliSize=122 (resi) iden=99.19% (122/123) simi=99.19% (122/123)<br>SIKVCIADDNRELVSLLDEYISSQPDMEVIGTAYNGQDCLQMLEEKRPDILLL-IIMPHLDGLAVLERIRAGFEHQPNVIMLTAFGQEDVTKKAVELGASYFILKPFDMENLAHHIRQVYGKTpvv<br>SIKVCIADDNRELVSLLDEYISSQPDMEVIGTAYNGQDCLQMLEEKRPDILLLdIIMPHLDGLAVLERIRAGFEHQPNVIMLTAFGQEDVTKKAVELGASYFILKPFDMENLAHHIRQVYGKT---<br><br>Proposed: aliSize=121 (resi) RMSD=1.33 (Å)<br>SIKVCIADDNRELVSLLDEYISSQPDMEVIGTAYNGQDCLQMLEEKRPDILLLPIIMPHLDGLAVLERIRAGFEHQPNVIMLTA-FGQEdVTKKAVELGAsYFILKP-FDMENLAHHIRQVYGKTpvv<br>SIKVCIADDNRELVSLLDEYISSQPDMEVIGTAYNGQDCLQMLEEKRPDILLLDIIMPHLDGLAVLERIRAGFEHQPNVIMLTAfGQED-VTKKAVELGA-SYFILKpFDMENLAHHIRQVYGKT---
14	1sncA (135)<br>1sndA (129)	C	99.22	TM-align: aliSize=111 (resi) RMSD=1.64 (Å)<br>LHKEPATLIKAIDGDTVKLMYKGQPMTFRLLLVDTPETKHPKKGVEKYGPEASAFTKKMVENAKKIEVEFDKGQRTDKYGRGLAYIYADGKMVNEALVRQGLAKVAYVYkpnnthEQhlrkseaqakkeklniws------------------<br>LHKEPATLIKAIDGDTVKLMYKGQPMTFRLLLVDTPETKHPKKGVEKYGPEASAFTKKMVENAKKIEVEFDKGQRTDKYGRGLAYIYADGKMVNEALVRQGLAKVAYTH------EQ------------------hlrkseaqakkeklniws<br><br>SARST: aliSize=127 (resi) RMSD=9.66 (Å)<br>l-HKEPATLIKAIDGDTVKLMYKGQPMTFRLLLVDTPETKHPKKGVEKYGPEASAFTKKMVENAKKIEVEFDKGQRTDKYGRGLAYIYADGKMVNEALVRQGLAKVAYVykpnntHEQHLRKSEAQAKKEKLNIW-s<br>-lHKEPATLIKAIDGDTVKLMYKGQPMTFRLLLVDTPETKHPKKGVEKYGPEASAFTKKMVENAKKIEVEFDKGQRTDKYGRGLAYIYADGKMVNEALVRQGLAKVAYT------HEQHLRKSEAQAKKEKLNIWs-<br><br>BLAST: aliSize=129 (resi) iden=100.00% (129/129) simi=100.00% (129/129)<br>LHKEPATLIKAIDGDTVKLMYKGQPMTFRLLLVDTPETKHPKKGVEKYGPEASAFTKKMVENAKKIEVEFDKGQRTDKYGRGLAYIYADGKMVNEALVRQGLAKVAYvykpnnTHEQHLRKSEAQAKKEKLNIWS<br>LHKEPATLIKAIDGDTVKLMYKGQPMTFRLLLVDTPETKHPKKGVEKYGPEASAFTKKMVENAKKIEVEFDKGQRTDKYGRGLAYIYADGKMVNEALVRQGLAKVAY------THEQHLRKSEAQAKKEKLNIWS<br><br>Proposed: aliSize=129 (resi) RMSD=0.93 (Å)<br>LHKEPATLIKAIDGDTVKLMYKGQPMTFRLLLVDTPETKHPKKGVEKYGPEASAFTKKMVENAKKIEVEFDKGQRTDKYGRGLAYIYADGKMVNEALVRQGLAKVAyvykpnNTHEQHLRKSEAQAKKEKLNIWS<br>LHKEPATLIKAIDGDTVKLMYKGQPMTFRLLLVDTPETKHPKKGVEKYGPEASAFTKKMVENAKKIEVEFDKGQRTDKYGRGLAYIYADGKMVNEALVRQGLAKVA------YTHEQHLRKSEAQAKKEKLNIWS

15	1wwwX (101)<br>1wwaX (105)	N	100.00	TM-align: aliSize=87 (resi) RMSD=1.23 (Å)<br>vsfpasvqlhtave--------------MHHWCIPFSVDGQPAPSLRWLFNGSVLNETSFIFTEFLEPAANETVRHGCLRLNQPTHVNNGNYTLLAANPFGQASASIMAAFMDNP----<br>--------------vsfpasvqlhtaveMHHWCIPFSVDGQPAPSLRWLFNGSVLNETSFIFTEFLEPAANETVRHGCLRLNQPTHVNNGNYTLLAANPFGQASASIMAAFMDNPfefn<br><br>SARST: aliSize=99 (resi) RMSD=15.39 (Å)<br>v-SFPASVQLHTAVEMHHWCIPFSVDGQPAPSLRWLFNGSVLNETSFIFTEFLEPAANETVRHGCLRLNQPTHVNNGNYTLLAANPFGQASASIMAAFMDN-----p<br>-vSFPASVQLHTAVEMHHWCIPFSVDGQPAPSLRWLFNGSVLNETSFIFTEFLEPAANETVRHGCLRLNQPTHVNNGNYTLLAANPFGQASASIMAAFMDNpfefn-<br><br>BLAST: aliSize=101 (resi) iden=100.00% (101/101) simi=100.00% (101/101)<br>VSFPASVQLHTAVEMHHWCIPFSVDGQPAPSLRWLFNGSVLNETSFIFTEFLEPAANETVRHGCLRLNQPTHVNNGNYTLLAANPFGQASASIMAAFMDNP----<br>VSFPASVQLHTAVEMHHWCIPFSVDGQPAPSLRWLFNGSVLNETSFIFTEFLEPAANETVRHGCLRLNQPTHVNNGNYTLLAANPFGQASASIMAAFMDNPfefn<br><br>Proposed: aliSize=101 (resi) RMSD=0.68 (Å)<br>VSFPASVQLHTAVEMHHWCIPFSVDGQPAPSLRWLFNGSVLNETSFIFTEFLEPAANETVRHGCLRLNQPTHVNNGNYTLLAANPFGQASASIMAAFMDNP----<br>VSFPASVQLHTAVEMHHWCIPFSVDGQPAPSLRWLFNGSVLNETSFIFTEFLEPAANETVRHGCLRLNQPTHVNNGNYTLLAANPFGQASASIMAAFMDNPfefn
16	1wwwX (101)<br>1wwbX (103)	N	45.54	TM-align: aliSize=84 (resi) RMSD=1.18 (Å)<br>vsfpasvqlhtavem---------------HHWCIPFSVDGQPAPSLRWLFNGSVLNETSFIFTEFLEPAanETVRHGCLRLNQPTHVNNGNYTLLAANPFGQASASIMAAFMDNP---<br>---------------vhfaptitflesptsdHHWCIPFTVKGNPKPALQWFYNGAILNESKYICTKIHVTN--HTEYHGCLQLDNPTHMNNGDYTLIAKNEYGKDEKQISAHFMGWPgid<br><br>SARST: aliSize=97 (resi) RMSD=15.51 (Å)<br>v-SFPASV-QLHTAVEMHHWCIPFSVDGQPAPSLRWLFNGSVLNETSFIFTEFLepAANETVRHGCLRLNQPTHVNNGNYTLLAANPFGQASASIMAAFMDN----p<br>-vHFAPTItFLESPTSDHHWCIPFTVKGNPKPALQWFYNGAILNESKYICTKIH--VTNHTEYHGCLQLDNPTHMNNGDYTLIAKNEYGKDEKQISAHFMGWpgid-<br><br>BLAST: aliSize=65 (resi) iden=47.52% (48/101) simi=64.36% (65/101)<br>VSF-PASVQLHTAVEMHHWCIPFSVDGQPAPSLRWLFNGSVLNETSFIFTEFlePAANETVRHGCLRLNQPTHVNNGNYTLLAANPFGQASASIMAAFMDNP---<br>VHFaPTITFLESPTSDHHWCIPFTVKGNPKPALQWFYNGAILNESKYICTKI--HVTNHTEYHGCLQLDNPTHMNNGDYTLIAKNEYGKDEKQISAHFMGWPgid<br><br>Proposed: aliSize=99 (resi) RMSD=1.10 (Å)<br>VSFPASV-QLHTAVEMHHWCIPFSVDGQPAPSLRWLFNGSVLNETSFIFTEFLEPAanETVRHGCLRLNQPTHVNNGNYTLLAANPFGQASASIMAAFMDNP---<br>VHFAPTItFLESPTSDHHWCIPFTVKGNPKPALQWFYNGAILNESKYICTKIHVTN--HTEYHGCLQLDNPTHMNNGDYTLIAKNEYGKDEKQISAHFMGWPgid
17	1wwwX (101)<br>1wwcA (105)	N	41.58	TM-align: aliSize=84 (resi) RMSD=1.68 (Å)<br>vsfpasvqlhtave----------------MHHWCIPFSVDGQPAPSLRWLFNGSVLNETSFIFTEFLEPaanETVRHGCLRLNQPTHVNNGNYTLLAANPFGQASASIMAAFMDNP-----<br>--------------tvyypprvvsleepelRLEHCIEFVVRGNPPPTLHWLHNGQPLRESKIIHVEYYQE---GEISEGCLLFNKPTHYNNGNYTLIAKNPLGTANQTINGHFLKEPfpvde<br><br>SARST: aliSize=96 (resi) RMSD=12.04 (Å)<br>v--SFPASV-QLHTAVEMHHWCIPFSVDGQPAPSLRWLFNGSVLNETSFIFTEFLEPAaneTVRHGCLRLNQPTHVNNGNYTLLAANPFGQASASIMAAFMDN------p<br>-tvYYPPRVvSLEEPELRLEHCIEFVVRGNPPPTLHWLHNGQPLRESKIIHVEYYQEG---EISEGCLLFNKPTHYNNGNYTLIAKNPLGTANQTINGHFLKEpfpvde-<br><br>BLAST: aliSize=54 (resi) iden=41.58% (42/101) simi=53.47% (54/101)<br>vsf-----PASVQLHTA-VEMHHwCIPFSVDGQPAPSLRWLFNGSVLNETSFIFTEFLEPAAnetVRHGCLRLNQPTHVNNGNYTLLAANPFGQASASIMAAFMDNP-----<br>---tvyypPRVVSLEEPeLRLEH-CIEFVVRGNPPPTLHWLHNGQPLRESKIIHVEYYQEGE---ISEGCLLFNKPTHYNNGNYTLIAKNPLGTANQTINGHFLKEPfpvde<br><br>Proposed: aliSize=97 (resi) RMSD=1.13 (Å)<br>-VSFPASV-QLHTAVEMHHWCIPFSVDGQPAPSLRWLFNGSVLNETSFIFTEFLEP-aaneTVRHGCLRLNQPTHVNNGNYTLLAANPFGQASASIMAAFMDNP-----<br>tVYYPPRVvSLEEPELRLEHCIEFVVRGNPPPTLHWLHNGQPLRESKIIHVEYYQEg----EISEGCLLFNKPTHYNNGNYTLIAKNPLGTANQTINGHFLKEPfpvde

18	1dksA<br>(76)<br><br>1cksA<br>(74)	C	83.78	TM-align: aliSize=61 (resi) RMSD=1.57 (Å)<br>s-KHQIYYSDKYDDEEFEYRHVMLPKDIAKLVPKTHLMSESEWRNLGVQQSQGWVHYMIHEPEphillfrrplpkkp------------<br>-aHKQIYYSDKYFDEHYEYRHVMLPRELSKQVPKTHLMSEEEWRRLGVQQSLGWVHYMIHEPE--------------phillfrrplpk<br><br>SARST: aliSize=69 (resi) RMSD=14.87 (Å)<br>skhq----IYYSDKYDDEEFEYRHVMLPKDIAKLVPKTHLMSESEWRNLGVQQSQGWVHYMIHEPEPHILLFRRPLP-kkp<br>----ahkqIYYSDKYFDEHYEYRHVMLPRELSKQVPKTHLMSEEEWRRLGVQQSLGWVHYMIHEPEPHILLFRRPLPk---<br><br>BLAST: aliSize=65 (resi) iden=81.08% (60/74) simi=87.84% (65/74)<br>skh---QIYYSDKYDDEEFEYRHVMLPKDIAKLVPKTHLMSESEWRNLGVQQSQGWVHYMIHEPEPHILLFRRPLPKkp<br>---ahkQIYYSDKYFDEHYEYRHVMLPRELSKQVPKTHLMSEEEWRRLGVQQSLGWVHYMIHEPEPHILLFRRPLPK--<br><br>Proposed: aliSize=73 (resi) RMSD=1.37 (Å)<br>s-HKQIYYSDKYDDEEFEYRHVMLPKDIAKLVPKTHLMSESEWRNLGVQQSQGWVHYMIHEPEPHILLFRRPLPKkp<br>-aHKQIYYSDKYFDEHYEYRHVMLPRELSKQVPKTHLMSEEEWRRLGVQQSLGWVHYMIHEPEPHILLFRRPLPK--
19	4gcrA<br>(174)<br><br>1blbA<br>(185)	N	36.78	TM-align: aliSize=87 (resi) RMSD=1.63 (Å)<br>------GKITFYEDRGFQGHCYECSSDCPNLQPY-FSRCNSIRVDSGCWMLYERPNYQGHQYFLRRGDYPDYQQWM--GFNDSIRSCRLIPQHTGTfrmriyerddfrgqmseitddcpslqdrfhltevhslnvlegswvlyempsyrgrqyllrpgeyrryldwgamnakvgslrrvmdfy-------<br>apqplnPKIIFEQENFQGHSHELNGPCPNLKETgVEKAGSVLVQAGPWVGYEQANCKGEQFVFEKGEYPRWDSWTssRRTDSLSSLRPIKVDSQE---hkitlye<br><br>--<br>npnftgkkmevidddvpsfhahgyqekvssvrvqsgtwvgyqypgyrglqyllekgdykdsgdfgapqpqvqsvrrirdmqw<br><br>SARST: aliSize=167 (resi) RMSD=16.15 (Å)<br>g-------KITFYEDRGFQGHCYECSSDCPNLQP-YFSRCNSIRVDSGCWMLYERPNYQGHQYFLRRGDYPDYQQW---MGFNDSiRSCRLIPQHTGTFRMRIYERDDFRGQMSEI-TDDCPSlQDRFHLTE-VHSLNVLEGSWVLYEMPSYRGRQYLLRPGEYRRYLDWGAMNAKVGSLRRV-----mdfy<br>-apqplnpKIIFEQENFQGHSHELNGPCPNLKEtGVEKAGSVLVQAGPWVGYEQANCKGEQFVFEKGEYPRWDSWtssRRTDSL-SSLRPIKVDSQEHKITLYENPNFTGKKMEViDDDVPS-FHAHGYQEkVSSVRVQSGTWVGYQYPGYRGLQYLLEKGDYKDSGDFGAPQPQVQSVRRIrdmqw----<br><br>BLAST: aliSize=97 (resi) iden=37.36% (65/174) simi=55.75% (97/174)<br>g-------KITFYEDRGFQGHCYECSSDCPNL-QPYFSRCNSIRVDSGCWMLYERPNYQGHQYFLRRGDYPDYQQWMGF--NDSIRSCRLIPQHTGTFRMRIYERDDFRG-QMSEITDDCPSLQDRFHLTEVHSLNVLEGSWVLYEMPSYRGRQYLLRPGEYRRYLDWGAMNAKVGSLRRVMD---fy<br>-apqplnpKIIFEQENFQGHSHELNGPCPNLkETGVEKAGSVLVQAGPWVGYEQANCKGEQFVFEKGEYPRWDSWTSSrrTDSLSSLRPIKVDSQEHKITLYENPNFTGkKMEVIDDDVPSFHAHGYQEKVSSVRVQSGTWVGYQYPGYRGLQYLLEKGDYKDSGDFGAPQPQVQSVRRIRDmqw--<br><br>Proposed: aliSize=171 (resi) RMSD=1.30 (Å)<br>------GKITFYEDRGFQGHCYECSSDCPNLQPY-FSRCNSIRVDSGCWMLYERPNYQGHQYFLRRGDYPDYQQWM--gF-NDSIRSCRLIPQHTGTFRMRIYERDDFRGQMSEIT-DDCPSLQDRFHLTEVHSLNVLEGSWVLYEMPSYRGRQYLLRPGEYRRYLDWGAMNAKVGSLRRVMD---fy<br>kpqplnPKIIFEQENFQGHSHELNGPCPNLKETgVEKAGSVLVQAGPWVGYEQANCKGEQFVFEKGEYPRWDSWTss-RrTDSLSSLRPIKVDSQEHKITLYENPNFTGKKMEVIdDDVPSFHAHGYQEKVSSVRVQSGTWVGYQYPGYRGLQYLLEKGDYKDSGDFGAPQPQVQSVRRIRDmqw--
20	1cewI<br>(108)<br><br>1g96A<br>(111)	N	39.81	TM-align: aliSize=58 (resi) RMSD=2.19 (Å)<br>gapvpvdendeglqralqfamaeynrasndkyssrvvrvisakrql---VSGIKYILQVEIGRTTCPKSSgdLQsCEFHDEpEM----AKYTTCTFVVYSIPWLNQIKLLESKCQ--<br>--vggpmdasveeegvrraldfavgeynkasndmyhsralqvvrarkqiVAGVNYFLDVELGRTTCTKTQ--PN-LDNCPF-HDqphlKRKAFCSFQIYAVPWQGTMTLSKSTCQda<br><br>SARST: aliSize=100 (resi) RMSD=19.74 (Å)<br>g--APVPVDENDEGLQRALQFAMAEYNRASNDKYSSRVVRVISAKRQLVSGIKYILQVEIGRTTCPKSSGDlqscefHDEPEMA------KYTTCTFVVYSIPWLNQIKLLESKC---q<br>-vgGPMDASVEEEGVRRALDFAVGEYNKASNDMYHSRALQVVRARKQIVAGVNYFLDVELGRTTCTKTQPN------LDNCPFHdqphlkRKAFCSFQIYAVPWQGTMTLSKSTCqda-<br><br>BLAST: aliSize=76 (resi) iden=45.37% (49/108) simi=70.37% (76/108)<br>-GAPVPVDENDEGLQRALQFAMAEYNRASNDKYSSRVVRVISAKRQLVSGIKYILQVEIGRTTCPKSSGDLQSCEFHDEPEMAKYTTCTFVVYSIPWLNQIKLLESKCQ--<br>vGGPMDASVEEEGVRRALDFAVGEYNKASNDMYHSRALQVVRARKQIVAGVNYFLDVELGRTTCTKTQPNLDNCPFHDQPHLKRKAFCSFQIYAVPWQGTMTLSKSTCQda<br><br>Proposed: aliSize=104 (resi) RMSD=1.45 (Å)<br>-GAPVPVDENDEGLQRALQFAMAEYNRASNDKYSSRVVRVISAKRQLVSGIKYILQVEIGRTTCPKSSGdLQscEFHDEpEM----AKYTTCTFVVYSIPWLNQIKLLESKCQ--<br>vGGPMDASVEEEGVRRALDFAVGEYNKASNDMYHSRALQVVRARKQIVAGVNYFLDVELGRTTCTKTQP-NL--DNCPF-HDqphlKRKAFCSFQIYAVPWQGTMTLSKSTCQda

21	2gmfA (121)<br><br>1hulA (108)	C	10.19	TM-align: aliSize=77 (resi) RMSD=3.07 (Å)<br>rspspstqpWEHVNAIQEARRLLNLSRDTa-AEMNETVEVISEMFDLqePTCLQTRLELYKQGL--RG---SLTKLKGPLTMMASHYKQHC--PPtpetscatqiitfesfkenlkdfllvipfdcw--------------E--P------<br>---------IPTSALVKETLALLSTHRTL-lIANETLRIPVPVHKNH--QLCTEEIFQGIGTLEsqTVqggTVERLFKNLSLIKKYIDGQKkkCG--------------------------------eerrrvnqfldylqeFlgVmntewi<br><br>SARST: aliSize=87 (resi) RMSD=14.73 (Å)<br>rspspstqp--------WEHVNAIQEARRLLNLsrdtaaeMNETVEVISEMFDLQEPTCLQTRLELYKQGLRG----SLTKLKGPLTMMASHYKQHcpptPETSCATQiitfESFKENLKDFL----------lvipfdcwep<br>---------iptsalvkETLALLSTHRTLLIAN-------ETLRIPVPVHKNHQLCTEEIFQGIGTLESQTVQggtvERLFKNLSLIKKYIDGQKK----KCGEERRR----VNQFLDYLQEFlgvmntewi----------<br><br>BLAST: aliSize=3 (resi) iden=2.78% (3/108) simi=2.78% (3/108)<br>rspspstqpwehvnaiqearrllnlsrdtaaemnetvevisemfdlqeptclqtrlelykqglrgsltklkgpltmmashykqhcpptpetscatqiitfesfkenlkdfl-------------------------LVIP---<br>---iptsalvketlallsthrtllianetLRIPvpvhknhqlcteeifqgigtlesqtvqggtverlfknlslikkyidgqk<br><br>-----------------------------fdcwep<br>kkcgeerrrvnqfldylqeflgvmntewi------<br><br>Proposed: aliSize=94 (resi) RMSD=2.26 (Å)<br>rspspstqp-WEhVNAIQEARRLLNLS-RD--taaEMNETVEVISEMFDLqePTCLQTRLELYKQGL--RG---SLTKLKGPLTMMASHYKQHcpptpetSCA-T-QIITFESFKENLKDFLLVI---PfdcWep<br>---------iPT-SALVKETLALLSTHrTLli---ANETLRIPVPVHKNH--QLCTEEIFQGIGTLEsqTVqggTVERLFKNLSLIKKYIDGQ-------KKKcGeERRRVNQFLDYLQEFLGVMnteW---I--
22	1msbA (115)<br><br>1ixxA (129)	M	13.04	TM-align: aliSize=90 (resi) RMSD=1.94 (Å)<br>--------SGKKFFVTNHERMPFSKVKALCSEL-R-GTVAIPRNAEENKAIQEVAK-----T--SAFLGITDEV-T-EGqfmyvtggrltysnwkkdepndh-----------------GSGEDCVTIV--DN-GLWNDISCQASHTAVCEFpa<br>dclsgwssYEGHCYKAFEKYKTWEDAERVCTEQaKgAHLVSIESSGEADFVAQLVTqnmkrLdfYIWIGLRVQGkVkQC----------------------nsewsdgssvsyenwieAESKTCLGLEkeTDfRKWVNIYCGQQNPFVCEA--<br><br>SARST: aliSize=91 (resi) RMSD=12.21 (Å)<br>s---------GKKFFVTNHERMPFSKVKALCSEL--RGTVAIPRNAEENKAIQEVAKTSAflgitdeVTEGQFMYVTGGRLTYSNWKKDEpndhgsgedcvtiVDNGL-------------------------WNDISCQASHTAVCE-fpa<br>-dclsgwssyEGHCYKAFEKYKTWEDAERVCTEQakGAHLVSIESSGEADFVAQLVTQNM-------KRLDFYIWIGLRVQGKVKQCNSE-------------WSDGSsvsyenwieaesktclgleketdfrkWVNIYCGQQNPFVCEa---<br><br>BLAST: aliSize=27 (resi) iden=13.04% (15/115) simi=23.48% (27/115)<br>sgkkffvtnhermpfskvkalcselrgtvaiprnaeenkaiqevaktsaf--LGITDEVTEGQFMYVTGGRLTYSNWKKDEpndhgsGEDCVTI---VDNGLWNDISCQASHTAVCE-fpa<br>---dclsgwssyeghcykafekyktwedaervcteqakgahlvsiessgeadfvaqlvtqnmkrldfyiwigLRVQGKVKQCNSEWSDGSSVSYENWIEAE------SKTCLGLeketDFRKWVNIYCGQQNPFVCEa---<br><br>Proposed: aliSize=105 (resi) RMSD=1.64 (Å)<br>--------SGKKFFVTNHERMPFSKVKALCSEL-R-GTVAIPRNAEENKAIQEVAK-----T--SAFLGITDEV---teGQF-MYVTGGRLTYSNWKKDepndhgSGEDCVTIV--DN-GLWNDISCQASHTAVCEFpa<br>dclsgwssYEGHCYKAFEKYKTWEDAERVCTEQaKgAHLVSIESSGEADFVAQLVTqnmkrLdfYIWIGLRVQGkvk--QCNsEWSDGSSVSYENWIEA------ESKTCLGLEkeTDfRKWVNIYCGQQNPFVCEA--
23	1mupA (157)<br><br>1obpA (158)	C	24.84	TM-align: aliSize=119 (resi) RMSD=2.55 (Å)<br>eeasst---GRNFNVEKINGEWHTIILASDKREKIEDNGNFRLFLEQIHVL--ENSLVLKFHTVRDEECSELSMVADKTEKAgEYSVTYDGFNTFTIPKTDyDNFLMAHLINEK-DGETFQLMGLYGREpdlssdikerfaqlceehgilreniidlsn------------------------------<br>------qeeEAEQNLSELSGPWRTVYIGSTNPEKIQENGPFRTYFRELVFDdeKGTVDFYFSVKRDGKWKNVHVKATKQDDG-TYVADYEGQNVFKIVSLS-RTHLVAHNINVDkHGQTTELTGLFVKL-----------------------------nvededlekfwkltedkgidkknvvnflene<br><br>--ANRC<br>dhPHPE<br><br>SARST: aliSize=143 (resi) RMSD=13.39 (Å)<br>eeasstgrn------FNVEKINGEWHTIILASDKREKIEDNGNFRLFLEQIHVLE--NSLVLKFHTVRDEECSELSMVADKTEKaGEYSVTYDGFNTFTIPKTdYDNFLMAHLINEK-DGETFQLMGLYGrEPDLSSDIKERFAQLCEEHGILRENIIDLSNAN------rc<br>---------qeeeaeQNLSELSGPWRTVYIGSTNPEKIQENGPFRTYFRELVFDDekGTVDFYFSVKRDGKWKNVHVKATKQDD-GTYVADYEGQNVFKIVSL-SRTHLVAHNINVDkHGQTTELTGLFV-KLNVEDEDLEKFWKLTEDKGIDKKNVVNFLENEdhphpe--<br><br>BLAST: aliSize=79 (resi) iden=31.21% (49/157) simi=50.32% (79/157)<br>eeasstgrnf-------NVEKINGEWHTIILASDKREKIEDNGNFRL-FLEQIHVLENSLV-LKFHTVRDEECSELSMVADKTEKaGEYSVTYDGFNTFTIPKTDYDNfLMAHLIN-EKDGETFQLMGLYGREPDLSSDIkERFAQLCEEHGILRENIID-LSN-------anrc<br>----------qeeeaeqNLSELSGPWRTVYIGSTNPEKIQENGPFRTyFRELVFDDEKGTVdFYFSVKRDGKWKNVHVKATKQDD-GTYVADYEGQNVFKIVSLSRTH-LVAHNINvDKHGQTTELTGLFVKLNVEDEDL-EKFWKLTEDKGIDKKNVVNfLENedhphpe----<br><br>Proposed: aliSize=145 (resi) RMSD=1.92 (Å)<br>eeasstg----RNFNVEKINGEWHTIILASDKREKIEDNGNFRLFLEQIHVL--ENSLVLKFHTVR-DeECSELSMVADKTEKAgEYSVTYDGFNTFTIPKTDYdNFLMAHLINEK-DGETFQLMGLYGREpDLSSDIKERFAQLCEEHGILRENIIDLSNA--N-rC--<br>-------qeeeAEQNLSELSGPWRTVYIGSTNPEKIQENGPFRTYFRELVFDdeKGTVDFYFSVKRdG-KWKNVHVKATKQDDG-TYVADYEGQNVFKIVSLSR-THLVAHNINVDkHGQTTELTGLFVKL-NVEDEDLEKFWKLTEDKGIDKKNVVNFLENedHp-Hpe

24	1f0vA (124)<br>1h8xA (125)	N	60.48	TM-align: aliSize=96 (resi) RMSD=2.14 (Å)<br>ketaaakferqhmdssts-----------------AAs-SSNYCNQMMKSRNLTKDRCKPVNTFVHESLADVQAVCSQKNVACKNGQTNCYQSYSTMSITDCRETGSSKYPNCAYKTTQANKHIIVACEGNPYvpvhfdasv----------<br>-----------------kesaaakferqhmdsgnsPS-sSSTYCNQMMRRRNMTQGRCKPVNTFVHEPLVDVQNVCFQEKVTCKNGQGNCYKSNSSMHITDCRLTNGSRYPNCAYRTSQKERHIIVACEGSPY---------vpvhfdasve<br><br>SARST: aliSize=98 (resi) RMSD=18.19 (Å)<br>k-ETAAAKFERQHMDSST---SAAsssNYCNQMMKSRNLTKDRCKPVNTFVHESLADVQAVCSQKNVACKN---------------------GQtncyqsystmsitdcretgssKYPNCAYKTTQANKHIIVACEGNPYVPVHFDAS--v<br>-kESAAAKFERQHMDSGNspsSSS---TYCNQMMRRRNMTQGRCKPVNTFVHEPLVDVQNVCFQEKVTCKNgqgncyksnssmhitdcrltnGS---------------------RYPNCAYRTSQKERHIIVACEGSPYVPVHFDASve-<br><br>BLAST: aliSize=104 (resi) iden=73.39% (91/124) simi=83.87% (104/124)<br>KETAAAKFERQHMDSSTSAASSSNYCNQMMKSRNLTKDRCKPVNTFVHESLADVQAVCSQKNVACKNGQTNCYQSYSTMSITDCRETGSSKYPNCAYKTTQANKHIIVACEGNPYVPVHFDASV-<br>KESAAAKFERQHMDSGNSPSSSSTYCNQMMRRRNMTQGRCKPVNTFVHEPLVDVQNVCFQEKVTCKNGQGNCYKSNSSMHITDCRLTNGSRYPNCAYRTSQKERHIIVACEGSPYVPVHFDASVe<br><br>Proposed: aliSize=111 (resi) RMSD=1.14 (Å)<br>KETAAAKFERQHMDSSTsAA-SSSNYCNQMMKSRNLTKDRCKPVNTFVHESLADVQAVCSQKNVAC-KNGQtNCYQSYSTMSITDCRETGSSKYPNCAYKTTQANKHIIVACEGN-----------pyvpvhfdasv<br>KESAAAKFERQHMDSGN-SPsSSSTYCNQMMRRRNMTQGRCKPVNTFVHEPLVDVQNVCFQEKVTCkNGQG-NCYKSNSSMHITDCRLTNGSRYPNCAYRTSQKERHIIVACEGSpyvpvhfdasve----------
25	1f0vA (124)<br>1h8xA (125)	C	57.26	TM-align: aliSize=96 (resi) RMSD=2.14 (Å)<br>ketaaakferqhmdssts-----------------AAs-SSNYCNQMMKSRNLTKDRCKPVNTFVHESLADVQAVCSQKNVACKNGQTNCYQSYSTMSITDCRETGSSKYPNCAYKTTQANKHIIVACEGNPYvpvhfdasv----------<br>-----------------kesaaakferqhmdsgnsPS-sSSTYCNQMMRRRNMTQGRCKPVNTFVHEPLVDVQNVCFQEKVTCKNGQGNCYKSNSSMHITDCRLTNGSRYPNCAYRTSQKERHIIVACEGSPY---------vpvhfdasve<br><br>SARST: aliSize=98 (resi) RMSD=18.19 (Å)<br>k-ETAAAKFERQHMDSST---SAAsssNYCNQMMKSRNLTKDRCKPVNTFVHESLADVQAVCSQKNVACKN---------------------GQtncyqsystmsitdcretgssKYPNCAYKTTQANKHIIVACEGNPYVPVHFDAS--v<br>-kESAAAKFERQHMDSGNspsSSS---TYCNQMMRRRNMTQGRCKPVNTFVHEPLVDVQNVCFQEKVTCKNgqgncyksnssmhitdcrltnGS---------------------RYPNCAYRTSQKERHIIVACEGSPYVPVHFDASve-<br><br>BLAST: aliSize=104 (resi) iden=73.39% (91/124) simi=83.87% (104/124)<br>KETAAAKFERQHMDSSTSAASSSNYCNQMMKSRNLTKDRCKPVNTFVHESLADVQAVCSQKNVACKNGQTNCYQSYSTMSITDCRETGSSKYPNCAYKTTQANKHIIVACEGNPYVPVHFDASV-<br>KESAAAKFERQHMDSGNSPSSSSTYCNQMMRRRNMTQGRCKPVNTFVHEPLVDVQNVCFQEKVTCKNGQGNCYKSNSSMHITDCRLTNGSRYPNCAYRTSQKERHIIVACEGSPYVPVHFDASVe<br><br>Proposed: aliSize=103 (resi) RMSD=1.20 (Å)<br>ketaaakferqhmdsstsaa------------------SS--SNYCNQMMKSRNLTKDRCKPVNTFVHESLADVQAVCSQKNVAC-KNGQtNCYQSYSTMSITDCRETGSSKYPNCAYKTTQANKHIIVACEGNPYVPVHFDASV-<br>--------------------kesaaakferqhmdsgnsPSssSTYCNQMMRRRNMTQGRCKPVNTFVHEPLVDVQNVCFQEKVTCkNGQG-NCYKSNSSMHITDCRLTNGSRYPNCAYRTSQKERHIIVACEGSPYVPVHFDASVe
26	1griA (211)<br>1fyrA (95)	C	100.00	TM-align: aliSize=66 (resi) RMSD=1.50 (Å)<br>meaiakydfkataddelsfkrgdilkvqnwykaelngkdgfipknyiemkpHPWFFGKIPRAKAEEMLSKQRHDGAFLIRESESAPGDFSLSVKFGNDVQHFKVLRDGAGKYFLWVVkfnslnelvdyhrstsvsrnqqiflrdieqvpqqptyvqalfdfdpqedgelgfrrgdfihvmdnsdpnwwkg<br>---HPWFFGKIPRAKAEEMLSKQRHDGAFLIRESESAPGDFSLSVKFGNDVQHFKVLRDGAGKYFLWVV---<br><br>achgqtgmfprnyvtpvnrnv-----------------------------<br>---------------------kfnslnelvdyhrstsvsrnqqiflrdie<br><br>SARST: aliSize=93 (resi) RMSD=11.34 (Å)<br>meaiakydfkataddelsfkrgdilkvqnwykaelngkdgfipknyiemkph-PWFFGKIPRAKAEEMLSKQRHDGAFLIRESESAPGDFSLSVKFGNDVQHFKVLRDGAGKYFLWVVKFNSLNELVDYHRSTSVSRNQQIFLRDI-eqvpqqptyvqalfdfdpqedgelgfrrgdfihvmdnsdpnww<br>--hPWFFGKIPRAKAEEMLSKQRHDGAFLIRESESAPGDFSLSVKFGNDVQHFKVLRDGAGKYFLWVVKFNSLNELVDYHRSTSVSRNQQIFLRDIe---<br><br>kgachgqtgmfprnyvtpvnrnv<br>-----------------------<br><br>BLAST: aliSize=95 (resi) iden=100.00% (95/95) simi=100.00% (95/95)<br>meaiakydfkataddelsfkrgdilkvqnwykaelngkdgfipknyiemkpHPWFFGKIPRAKAEEMLSKQRHDGAFLIRESESAPGDFSLSVKFGNDVQHFKVLRDGAGKYFLWVVKFNSLNELVDYHRSTSVSRNQQIFLRDIEqvpqqptyvqalfdfdpqedgelgfrrgdfihvmdnsdpnwwkg<br>---HPWFFGKIPRAKAEEMLSKQRHDGAFLIRESESAPGDFSLSVKFGNDVQHFKVLRDGAGKYFLWVVKFNSLNELVDYHRSTSVSRNQQIFLRDIE--<br><br>achgqtgmfprnyvtpvnrnv<br>---------------------<br><br>Proposed: aliSize=95 (resi) RMSD=1.35 (Å)<br>meaiakydfkataddelsfkrgdilkvqnwykaelngkdgfipknyiemkpHPWFFGKIPRAKAEEMLSKQRHDGAFLIRESESAPGDFSLSVKFGNDVQHFKVLRDGAGKYFLWVVKFNSLNELVDYHRSTSVSRNQQIFLRDIEqvpqqptyvqalfdfdpqedgelgfrrgdfihvmdnsdpnwwkg<br>---HPWFFGKIPRAKAEEMLSKQRHDGAFLIRESESAPGDFSLSVKFGNDVQHFKVLRDGAGKYFLWVVKFNSLNELVDYHRSTSVSRNQQIFLRDIE--<br><br>achgqtgmfprnyvtpvnrnv<br>---------------------

27	1fynA (62)<br>1aojA (60)	C	26.67	TM-align: aliSize=37 (resi) RMSD=2.49 (Å)<br>gtgVTLFVALYDYEARTEDDLSFHKGEKFQILN-SSEGDWwearslttgetg---------Yipsnyvapvd-------------<br>---KKYAKSKYDFVARNSSELSVMKDDVLEILDdRRQWWK------------vrnasgdsgF----------vpnnildimrtpe<br><br>SARST: aliSize=54 (resi) RMSD=12.51 (Å)<br>gtgvt--LFVALYDYEARTEDDLSFHKGEKFQIlnSSEGDWWEARSLTTGETGYIPSNYVAPV----d<br>-----kkYAKSKYDFVARNSSELSVMKDDVLEI--LDDRRQWWKVRNASGDSGFVPNNILDIMrtpe-<br><br>BLAST: aliSize=29 (resi) iden=26.67% (16/60) simi=48.33% (29/60)<br>gtgvtlfval-------YDYEARTEDDLSFHKGEKFQILNSSEgDWWEARSlTTGETGYIPSN---------yvapvd<br>----------kkyakskYDFVARNSSELSVMKDDVLEILDDRR-QWWKVRN-ASGDSGFVPNNildimrtpe------<br><br>Proposed: aliSize=54 (resi) RMSD=1.10 (Å)<br>gtgv-TLFVALYDYEARTEDDLSFHKGEKFQILN--sseGDWWEARSlTTGETGYIPSNYVAPVD---<br>----kKYAKSKYDFVARNSSELSVMKDDVLEILDdr---RQWWKVRN-ASGDSGFVPNNILDIMRtpe
28	1k3sA (109)<br>1k3eA (141)	N	12.84	TM-align: aliSize=86 (resi) RMSD=2.12 (Å)<br>esllnrlydalgldepllii--------------------------------DDGIQVYFNESD-HTLE-CCPF-PLPD-DILTLQHFLRLNYTS----AVTIGADADNTALVALYRLPQTSTE-EEALTGFELFISNVKOLKEHY-----A--------<br>-------------------mssrselllekfaekigigsisfnenrlcsfaiDEIYYISLSDANdEYMMiYGVCgKFPTdNSNFALEILNANLWFaengGPYLCYEAGAQSLLLALRFPLDDATpEKLENEIEVVVKSMENLYLVLhnqgiTlkieeiss<br><br>SARST: aliSize=91 (resi) RMSD=9.77 (Å)<br>esllnrlyd------------------------ALGLDEPLLLIIDdgIQVYFNE-SDHTLECCPFPLPDD------ILTLQHFLRLNYTSA-VTIGAD-ADNtalVALYRLP---OTSTEEEALTG---FELFISNVKQLKEHY-----------a<br>---------mssrselllekfaekigigsisfnENRLCSFAIDEI--YYISLSDaNDEYMMIYGVCGKFPtdnsnfALEILNANLWFAENGgPYLCYEaGAQ---SLLLALRfplDDATPEKLENEievVVKSMENLYLVLHNQgitlkieeiss-<br><br>BLAST: aliSize=18 (resi) iden=9.43% (10/109) simi=16.98% (18/109)<br>esllnrlydalgldeplliiddgiqvyfnesdhtleccpfplpddiltlqhflrlnytsavti--GADADNTALvalyRLP-OTSTEEEALTGFELFISNVKOL<br>---mssrselllekfaekigigsisfnenrlcsfaideiyyislsdandeymmiygvcgkfptdnsnfaleilnanlwfaenggpylcyeaGAQSLLLAL----RFPlDDATPEKLENEIEVVVKSMENL<br><br>------------------kehya<br>ylvlhnqgitlkieeiss-----<br><br>Proposed: aliSize=107 (resi) RMSD=1.75 (Å)<br>me------SLLNRLYDALGLD--E-P----LLIIDD-GIQVYFNESD-HTLEMCCPFMPLPD-DILTLQHFLRLNYTS----AVTIGADADNTALVALYRLPQTSTE-EEALTGFELFISNVKOLKEHY-----A--------<br>--mssrseLLLEKFAEKIGIGsiSfNenrlCSFAIDeIYYISLSDANdEYMMIYGVCGKFPTdNSNFALEILNANLWFaengGPYLCYEAGAQSLLLALRFPLDDATpEKLENEIEVVVKSMENLYLVLhnqgiTlkieeiss
29	1cunA (213)<br>2spcA (107)	C	15.89	TM-align: aliSize=103 (resi) RMSD=3.03 (Å)<br>mvhqffrdmddeeswikekkllvssedygrdltgvQN-LRKKHKRLEAELAAHEPAIQSVLD-TG-KKLsdDNTIGK-EEIQQRLAQFVDHWKELKQLAAARGQRLEESLEYQQFVANVEeEeawinekmtlvasedygdtlaaiqgllkkheafetdftvhkdrVndvcangedlikknnhhvenitak<br>-----------------------------------QNlDLQLYMRDCELAESWMSAREAFLNaDDdANA--GGNVEAlIKKHEDFDKAINGHEQKIAALQTVADQLIAQNHYASNLVDEK-R---K------------------------<br><br>mkgLKgKVsDLEKAAAQRKAKLDENSA<br>---QV-LE-RWRHLKEGLIEKRSRLGD<br><br>SARST: aliSize=103 (resi) RMSD=27.07 (Å)<br>mvhqffrdmddeeswikekkllvssedygrdltgvqnlrkkhkrleaelaahepaiqsvldtgkklsddntigkeeiqqrlaqfvdhwkelkqlaaargqr--LEESLEYQQFVANVEEEEAWINEKMTLVAsEDYGDTLAAIQGLLKKHEAFETDFTVHKDRVNDVCANGEDLIKKnnhhvENITAKMK<br>---qnLDLQLYMRDCELAESWMSAREAFLNADDD-ANAGGNVEALIKKHEDFDKAINGHEQKIAALQTVADQLIAQNH------YASNLVDE<br><br>GLKGKVSDLEKAAAQRKAKLDEN--sa<br>KRKQVLERWRHLKEGLIEKRSRLgd--<br><br>BLAST: aliSize=58 (resi) iden=20.56% (22/107) simi=54.21% (58/107)<br>mvhqffrdmddeeswikekkllvssedygrdltgvqnlrkkhkrleaelaahepaiqsvldtgkklsddntigkeeiqqrlaqfvdhwkelkqlaaargqrleESLEYQQFVANVEEEEAWINEKMTLVASEDYGDTLAAIQGLLKKHEAFETDFTVHKDRVNDVCANGEDLIKKnNHHVEN-ITAKMKG<br>---QNLDLQLYMRDCELAESWMSAREAFLNADDDANAGGNVEALIKKHEDFDKAINGHEQKIAALQTVADQLIAQ-NHYASNlVDEKRKQ<br><br>LKGKVSDLEKAAAQRKAKLDEnsa<br>VLERWRHLKEGLIEKRSRLGD---<br><br>Proposed: aliSize=105 (resi) RMSD=2.01 (Å)<br>mvhqffrdmddeeswikekkllvssedYgrdltgvqnlrkkhkrleaelaahepaiqsvldtgkklsddntigkeeiqqrlaqfvdhwkelkqlaaargqrleeSLEYQQFVANVEEEEAWINEKMTLVA-SE-DYGDtLAaIQGLLKKHEAFETDFTVHKDRVNDVCANGEDLIKKNnhhVENITAKMK<br>---------------------------Q--NLDLQLYMRDCELAESWMSAREAFLNaDDdANAG-GN-VEALIKKHEDFDKAINGHEQKIAALQTVADQLIAQN---HYASNLVDE<br><br>GLKGKVSDLEKAAAQRKAKLDENSA<br>KRKQVLERWRHLKEGLIEKRSRLGD

30	1wu3I (161)<br>1ilkA (151)	C	9.93	TM-align: aliSize=99 (resi) RMSD=3.88 (Å)<br>iny-----KQLqLQeRTNIRKCQELLEQLNGKINL-TYRADFKIPMEMTE-KMQkSYTAFAIQEMLQNVFL-VFRNNFSSTGWNETIVVRLLDELHQQTVFLKTV-LEEKQEERltwemsstalhlksyywrvqrylklmkynsyawmvvraeifrnfliirrltrnfqn--------------------<br>---nscthFPG-NL-PNMLRDLRDAFSRVKTFFQMkDQLDNLLLKESLLEdFKG-YLGCQALSEMIQFYLEeVMPQAENQDPDIKAHVNSLGENLKTLRLRLRRChRFLPCENK--skaveqvknafnklqekgiy<br>-----------------------<br>kamsefdifinyieaymtmkirn<br><br>SARST: aliSize=117 (resi) RMSD=20.12 (Å)<br>inykqlqlqertnirkcqelleqlngkinltyradfkipme-MTEKMQKSYTAFAIQEMLQN----VFLVFRN-NFSSTGWNETIVVRLLDELHQQTVFLKTVLEEKQEERLTWE--MSSTALHLKSYYWRVQRYLKLM-KYN---------SYAWMVVRAEIFRNFLIIRRLTRN--------------<br>---nSCTHFPGNLPNMLRDLRDAFsrvkTFFQMKDqLDNLLLKESLLEDFKGYLGCQALSEMIQFYLEEVMPQAENQdpDIKAHVNSLGENLKTLRLRLRRcHRFlpcenkskaVEQVKNAFNKLQEKGIYKAMSEFDifinyieaymtmki<br>--fqn<br>rn---<br><br>BLAST: aliSize=60 (resi) iden=19.21% (29/151) simi=39.74% (60/151)<br>inykql----------------------------QLQERT-NIRKCQELLEQLNGKINLTYRAdfkipmEMTEkmqksytaFAIQEML---QNVFLVFRNNFSSTGWN-ETIVVRlLDELHQqtvFLKTVLEEKQEERLtwEMSSTALHLKSYYWRVQRYLKLMKY-NSYAWMVVR-aeifrnfliirrl<br>------nscthfpgnlpnmlrdlrdafsrvktffQMKDQLdNLLLKESLLEDFKGYLGCQALS------EMIQ--------FYLEEVMpqaENQDPDIKAHVNSLGENlKTLRLR-LRRCHR---FLPCENKSKAVEQV--KNAFNKLQEKGIYKAMSEFDIFINYiEAYMTMKIRn-------------<br>trnfqn<br>------<br><br>Proposed: aliSize=131 (resi) RMSD=2.80 (Å)<br>iny------KQ-LqLQERTNIRKCQELLEqlNG-kIN--ltYRADFKIPMEMTE--kMQ-ksYTAFAIQEMLQNVFL-VFRNNFSSTGWNETIVVRLLDELHQQTVFLKT----vleekqEERLTwEmsstALHLKSYYWRVQRYlKlmkYnsyA-WMVVRAEIFRNFLIIRRLTRNFQ-N<br>---nscthfPGnL-PNMLRDLRDAFSRVK--TFf-QMkd--QLDNLLLKESLLEdf-KGy--LGCQALSEMIQFYLEeVMPQAENQDPDIKAHVNSLGENLKTLRLRLRRchrf------LPCEN-K----SKAVEQVKNAFNKL-Q---E---KgIYKAMSEFDIFINYIEAYMTMKIrN
31	1vjqA (73)<br>2i9sA (97)	C	9.59	TM-align: aliSize=63 (resi) RMSD=2.39 (Å)<br>----------------KTIFVIVPT-N---EeQV-AFLEALAKQ-D--ELNFDWqNPPTePGqPVVILI-PSD-VEWFLE-LKAK--GIPFTVYVEeggs-----<br>gpgkpesilkmtkkgkTLMMFVTVSgNpteK-ETeEITSLWQGSlFnaNYDVQR-FIVG-SD-RAIFMLrDGSyAWEIKDfLVSQdrCAEVTLEGQ----mypgk<br><br>SARST: aliSize=35 (resi) RMSD=4.22 (Å)<br>ktifvivptneeqvaflealakqdelnfdwqnpp-----------TEPGQPVVILIP-----------SDVEWFLELKAKGIPFTVYVEEG--------------------------------------gs<br>----------------------------------gpgkpesilkmTKKGKTLMMFVTvsgnpteketeEITSLWQGSLFNANYDVQRFIVGsdraifmlrdgsyaweikdflvsqdrcaevtlegqmypgk--<br><br>BLAST: aliSize=6 (resi) iden=7.04% (5/73) simi=8.45% (6/73)<br>ktifvivptneeqvaflealakqdelnfdwqnppte-PGQPVVIL--ipsdvewflelkakgipftvyveeggs<br>------------------------------------gPGKPESILkmtkkgktlmmfvtvsgnpteketeeitslwqgslfnanydvqrfivgsdraifmlrdgsyaweikdflvsqdrcaevtlegqmypgk---------------------------<br><br>Proposed: aliSize=72 (resi) RMSD=2.55 (Å)<br>---------------K-TIFVIVPT-N---EEQVAFLEALAKQ-D-ELNFDWQnPPTEPGQPVVILIPSDMVEWFLEMLKAK--GIPFTVYVEEG-GS<br>gpgkpesilkmtkkgKtLMMFVTVSgNpteKETEEITSLWQGSlFnANYDVQR-FIVGSDRAIFMLRDGSYAWEIKDFLVSQdrCAEVTLEGQMYpGK
32	2d8dA (80)<br>2gtvX (104)	N	11.25	TM-align: aliSize=65 (resi) RMSD=1.86 (Å)<br>eriqalrk------------------------EVdrVNreILrlLSERGRLVQEIGRLQTELGLPHYDPKREEEMLAYLTAEN--PGPFpDETIRKLFKEIFKASL-------------<br>--------mieklaeirkkideidnkilkarwPW--AE--KL--IAERNSLAKDVAEIKNQLGIPINDPEREKYIYDRIRKLCkeHNVD-ENIGIKIFQRLIEHNKalqkqyleetleh<br><br>SARST: aliSize=73 (resi) RMSD=9.32 (Å)<br>eriqal---RKEVDRVNREILRLL-------------SERGRLVQEIGRLQTELGLPHYDP--KREEEMLAYLTAENPGPFPDETIRKLFKEIFKAS-------------l<br>------mieKLAEIRKKIDEIDNKilkarwpwaekliAERNSLAKDVAEIKNQLGIPINDPerEKYIYDRIRKLCKEHNVDENIGIKIFQRLIEHNKalqkqyleetleh-<br><br>BLAST: aliSize=51 (resi) iden=30.00% (24/80) simi=63.75% (51/80)<br>--ERIQALRKEVDRVNREILR--------LLSERGRLVQEIGRLQTELGLPHYDPKREE---EMLAYLTAENPgpfPDETIR-KLFKEIFK----------------asl<br>miEKLAEIRKKIDEIDNKILKarwpwaekLIAERNSLAKDVAEIKNQLGIPINDPEREKyiyDRIRKLCKEHN---VDENIGiKIFQRLIEhnkalqkqyleetleh---<br><br>Proposed: aliSize=72 (resi) RMSD=2.28 (Å)<br>--ERIQALRKEVDRVNREI------------LRLLSERGRLVQEIGrLQtelgLPHYDpkrEEEMLAYLTAEN--PG-----P--FPDETIRKLFKEIFKASL---------<br>miEKLAEIRKKIDEIDNKIlkarwpwaekliAERNSLAKDVAEIKN-QL----GIPIN---DPEREKYIYDRIrkLCkehnvDenIGIKIFQRLIEHNKALQKqyleetleh

33	2d8dA (80)<br>1q6aA (107)	N	11.25	TM-align: aliSize=61 (resi) RMSD=2.40 (Å)<br>eriqalrkevdr---------------------------VNREilRLLSERGRLVQEIGRLQTelgLPHYD-PKREEEMLAYLTAEN--PGPfPDeTIRKLFKEIFKASL---------------<br>------------amarmspadkrklldelrsiyrtivleyFNTD--AKVNERIDEFVSKAFFAD---ISVSQvLEIHVELMDTFSKQLklEGR-SE-DILLDYRLTLIDVIahlcemyrrsiprev<br><br>SARST: aliSize=59 (resi) RMSD=11.11 (Å)<br>er----------------------------------IQALRKEVDRVNRE----ILRLLSERGRLVQEIGRLQTELGLP----HYDPKREEEMLAY----LTAENPG--pfpdetirklfkeifkasl<br>--amarmspadkrklldelrsiyrtivleyfntdakVNERIDEFVSKAFFadisVSQVLEIHVELMDTFSKQLKLEGRSedilLDYRLTLIDVIAHlcemYRRSIPRev-------------------<br><br>BLAST: aliSize=0 (resi) iden=0.00% (0/80) simi=0.00% (0/80)<br>eriqalrkevdrvnreilrllsergrlvqeigrlqtelglphydpkreeemlayltaenpgpfpdetirklfkeifkasl---<br>--amarmspadkrklldelrsiyrtivleyfntdakvneridefvskaffadisvsqvleihvelmdtfskqlklegrsedilldyrltlidviahlcemyrrsiprev<br><br>Proposed: aliSize=75 (resi) RMSD=1.73 (Å)<br>-------ERIQALRKEVDRVNREILRLL-------SERGRLVQEIGRLQTelgLPHYD-PKREEEMLAYLTAEN--PGPfPDeTIRKLFKEIFKASL---------------<br>amarmspADKRKLLDELRSIYRTIVLEYfntdakvNERIDEFVSKAFFAD---ISVSQvLEIHVELMDTFSKQLklEGR-SE-DILLDYRLTLIDVIahlcemyrrsiprev
34	2c5jA (82)<br>2c5iT (94)	C	62.20	TM-align: aliSize=56 (resi) RMSD=2.82 (Å)<br>DPFQQVVKDTKEQLNRINNYITRHNTa---EEEIQDILKDVEETIVDLDRSIIVMKRDENedvsgreaqvknikqqldalklrfd---------------------------------<br>DPFQQVVKDTKEQLNRINNYITRHNT-agdDDQEEEIQDILKDVEETIVDLDRSIIVKRD------------------------enedvsgreaqvknikqqldalklrfdrriqest<br><br>SARST: aliSize=80 (resi) RMSD=22.65 (Å)<br>d-PFQQVVKDTKEQLNRINNYITRHN--TAEEEIQDILKDVEETIVDLDRSIIVMKRDE------NEDVSGREAQVKNIKQQLDALKLRF----d<br>-dPFQQVVKDTKEQLNRINNYITRHNtaGDDDQEEEIQDILKDVEETIVDLDRSIIVKRdenedvSGREAQVKNIKQQLDALKLRFDRRIqest-<br><br>BLAST: aliSize=81 (resi) iden=98.78% (81/82) simi=98.78% (81/82)<br>DPFQQVVKDTKEQLNRINNYITRHNTA-----EEEIQDILKDVEETIVDLDRSIIVmKRDENEDVSGREAQVKNIKQQLDALKLRFD-------<br>DPFQQVVKDTKEQLNRINNYITRHNTAgdddqEEEIQDILKDVEETIVDLDRSIIV-KRDENEDVSGREAQVKNIKQQLDALKLRFDrriqest<br><br>Proposed: aliSize=81 (resi) RMSD=0.89 (Å)<br>DPFQQVVKDTKEQLNRINNYITRHNtA------EEEIQDILKDVEETIVDLDRSIIVMK-----RDENEDVSGREAQVKNIKQQLDALKLRFD--<br>DPFQQVVKDTKEQLNRINNYITRHN-TagdddqEEEIQDILKDVEETIVDLDRSIIVMKrdeneDVSGREAQVKNIKQQLDALKLRFDRRIQEst
35	2c5jA (82)<br>1lvfA (106)	C	15.85	TM-align: aliSize=63 (resi) RMSD=2.50 (Å)<br>--------DPFQQVVKDTKEQLNRINNYIT--RHNta-EEEIQDILKDVEETIVDLDRSIIVMKRDENEDVSGREaQvknikqqldalklrfd--------------------------------<br>edpffvvkGEVQKAVNTAQGLFQRWTELLQgpSAA--tREEIDWTTNELRNNLRSIEWDLEDLDETISIVEANPR-K----------------fnldatelsirkafitstrqivrdmkdqmsas<br><br>SARST: aliSize=80 (resi) RMSD=20.77 (Å)<br>d------PFQQVVKDTKEQLNRINNYITRH-------NTAEEEIQDILKDVEETIVDLDRSIIVMKRDENE----------DVSGREAQVKNIKQQLDALKLRF--d<br>-edpffvVKGEVQKAVNTAQGLFQRWTELLqgpsaatREEIDWTTNELRNNLRSIEWDLEDLDETISIVEAnprkfnldateLSIRKAFITSTRQIVRDMKDQMSas-<br><br>BLAST: aliSize=35 (resi) iden=25.61% (21/82) simi=42.68% (35/82)<br>-DPFQQVVKDTKEQLNRINNYITRHNTAEEEIQDILKD-VEETIVDLD---RSIIVMKRDENEDVSGREAQVKniKQQLDALKL------------------------rfd<br>eDPFFVVKGEVQKAVNTAQGLFQRWTELLQGPSAATREeIDWTTNELRnnlRSIEWDLEDLDETISIVEANPR--KFNLDATELsirkafitstrqivrdmkdqmsas---<br><br>Proposed: aliSize=82 (resi) RMSD=1.74 (Å)<br>-DPFQQVVKDTKEQLNRINNYITRHNTA---------------EEEIQDILKDVEETIVDLDRSIIVMK--------RDENEDVSGREAQVKNIKQQLDALKLRFD<br>eDPFFVVKGEVQKAVNTAQGLFQRWTELlqgpsaatreeidwtTNELRNNLRSIEWDLEDLDETISIVEanprkfnlDATELSIRKAFITSTRQIVRDMKDQMSAS

36	2c5jA<br>(82)<br>2jmhA<br>(117)	C	7.32	TM-align: aliSize=55 (resi) RMSD=2.29 (Å)<br>--DP-FQQVVKDTKEQLNRINNYITRHNta----EEEIQDILKDVEETIVDLDRSIIVM-KrDEneDVsgreaqvknikqqldalklrfd<br>qehkpkkddfrnefdhllieqanhaiekgehqllylqhqldelnenkskelqekiiRElDVVCAMIEGAQGALERELKRTDL--nileRFNYEEAQTLSKILLKDLKETEQKVkD-IQ--TQ----------------------<br><br>SARST: aliSize=80 (resi) RMSD=18.52 (Å)<br>d------------------PFQQV---VKDTKEQLNRINNYITRHNTAEEEIQDILKDVEETIVDLDRSIIVMKRDENED----VSGREAQVKNIKQQLDALKLRF------------d<br>-qehkpkkddfrnefdhllIEQANhaiEKGEHQLLYLQHQLDELNENKSKELQEKIIRELDVVCAMIEGAQGALERELKRtdlnILERFNYEEAQTLSKILLKDLKeteqkvkdiqtq-<br><br>BLAST: aliSize=19 (resi) iden=13.41% (11/82) simi=23.17% (19/82)<br>dpfqqvvkdt---KEQLNRIN-NYITRHNTAEEEIQD--ILKDVEET----------ivdldrsiivmkrdenedvsgreaqvknikqqldalklrfd<br>----------qehkpkkddfrnefdhllieqanhaiekgehqllylqhqldelnenkskelqekiireldvvcamiegaqgalERELKRTDINILERFNYEEAQTLSkiLLKDLKETeqkvkdiqtq---<br><br>Proposed: aliSize=73 (resi) RMSD=1.75 (Å)<br>--------------------------DPFQQVVKDTKEQLNRINNYItrhntaeeeIQDILKDVEETIV-DLDRSIIVMKRDENEDVS-----------GREAQVKNIKQQLDALKLRFD------<br>qehkpkkddfrnefdhllieqanhaiEKGEHQLLYLQHQLDELNENK---------SKELQEKIIRELDvVCAMIEGAQGALERELKRtdlnilerfnyEEAQTLSKILLKDLKETEQKVkdiqtq
37	2c5jA<br>(82)<br>1hx1B<br>(112)	C	4.88	TM-align: aliSize=43 (resi) RMSD=1.98 (Å)<br>dpfqqvvkdtkeqlnrinnyitrhntaeeEiqDILKDVEETIVDLDRSIIVMKRDENEDVSGREAQVKNIKqqLDAlklrfd---<br>-----------------------------G--NSPQEEVELKKLKHLEKSVEKIADQLEELNKELTGIQQG--FLP------kdlqaealckldrrvkatieqfmkileeidtlilpenfkdsrlkrkglvkkvqaflaecdtveqnicqe<br><br>SARST: aliSize=77 (resi) RMSD=18.87 (Å)<br>d--------------PFQQVVKDTKEQLNRINNYITRHN------TAEEEIQDILKDVEETIVDLDRSIIVMK---------RDENEDVSGREAQVKNIKQQLDALK------lrfd<br>-gnspqeevelkklkHLEKSVEKIADQLEELNKELTGIQqgflpkDLQAEALCKLDRRVKATIEQFMKILEEIdtlilpenfKDSRLKRKGLVKKVQAFLAECDTVEqnicqe----<br><br>BLAST: aliSize=21 (resi) iden=14.63% (12/82) simi=25.61% (21/82)<br>dpfqqv---VKDTKEQ----LNRINNYITRHNTAEEEI--QDILKDVEETIVDLD---------rsiivmkrdenedvsgreaqvknikqqldalklrfd<br>------gnspqeevelkklkhleksvekiadqleelnkeltgiqqgflpkdlqaealckldrrVKATIEQfmkiLEEIDTLILPENFKDSRLkrKGLVKKVQAFLAECDtveqnicqe------------------------------------<br><br>Proposed: aliSize=76 (resi) RMSD=1.70 (Å)<br>--------------------DPFQQVVKDTKEQLNRINNYitrH-ntaE-EEIQDILKDVEETIVDLDRSIIVMKRDENE----------DVSGREAQVKNIKQQLDALKLRFD----<br>gnspqeevelkklkhleksvEKIADQLEELNKELTGIQQG---Fl---PkDLQAEALCKLDRRVKATIEQFMKILEEIDTlilpenfkdsRLKRKGLVKKVQAFLAECDTVEQNicqe
38	1wz3A<br>(84)<br>1gnuA<br>(117)	C	17.86	TM-align: aliSize=54 (resi) RMSD=2.19 (Å)<br>--------------------------QKIVVHLRAT-GG-APILKQSKFKVSGSDKFANVIDFLRRQLHS-D--SLFVYVNSAfspnpdesvidlynnfgfdgklvvnyacsm----------------AW----------------<br>mkfvykeehpfekrrsegekirkkypDRVPVIVEKApKArIGDLDKKKYLVPSDLTVGQFYFLIRKRIHLrAedALFFFVNNV------------------------------ipptsatmgqlyqehhEEdfflyiaysdesvygl<br><br>SARST: aliSize=81 (resi) RMSD=13.64 (Å)<br>q---------------------------KIVVHLRATGGA--PILKQSKFKVSGSDKFANVIDFLRRQLHSDSLFVYVNSA--FSPNPDESVIDLYNNFGFD-GKLVVNYACSM----aw<br>-mkfvykeehpfekrrsegekirkkypdRVPVIVEKAPKAriGDLDKKKYLVPSDLTVGQFYFLIRKRIHLRAEDALFFFVnnVIPPTSATMGQLYQEHHEEdFFLYIAYSDESvygl--<br><br>BLAST: aliSize=28 (resi) iden=19.05% (16/84) simi=33.33% (28/84)<br>qkivvhlratgga-----------------------------PIL--KQSKFKVSGSDKFANVI--D-------FL-RRQLH---SDSLFVYVNSAFSP-------------------------------npdesvidlynnfgfdgklvvnyacsmaw<br>-------------mkfvykeehpfekrrsegekirkkypdrvPVIveKAPKARIGDLDKKKYLVpsDltvgqfyFLiRKRIHlraEDALFFFVNNVIPPtsatmgqlyqehheedfflyiaysdesvygl-----------------------------<br><br>Proposed: aliSize=82 (resi) RMSD=1.60 (Å)<br>--------------------------QKIVVHLRAT--GGAPILKQSKFKVSGSDKFANVIDFLRRQ-LHSD--SLFVYVNsAFSPNPDESVIDLYNNFGF-DGKLVVNYACS-MA--w<br>mkfvykeehpfekrrsegekirkkypDRVPVIVEKApkARIGDLDKKKYLVPSDLTVGQFYFLIRKRiHLRAedALFFFVN-NVIPPTSATMGQLYQEHHEeDFFLYIAYSDEsVYgl-

39	1wz3A (84)<br>1eo6A (116)	C	17.86	TM-align: aliSize=54 (resi) RMSD=2.15 (Å)<br>--------------------------QKIVVHLRAT--GGAPILKQSKFKVSGSDKFANVIDFLRRQL-HSD--SLFVYVNSAfspnpdesvidlynnfgfdgklvvnyacsm----------------AW--------------<br>mkwmfkedhslehrcvesakirakypDRVPVIVEKVsgSQIVDIDKRKYLVPSDITVAQFMWIIRKRIqLPSekAIFLFVDKT-----------------------------vpqssltmgqlyekekDEdgflyvaysgentfg<br><br>SARST: aliSize=78 (resi) RMSD=12.35 (Å)<br>q---------------------------KIVVHLRATGGA--PILKQSKFKVSGSDKFANVIDFLRRQLHSDSLFVYVNSA--FSPNPDESVIDLYNNFGF-DGKLVVNYA------csmaw<br>-mkwmfkedhslehrcvesakirakypdRVPVIVEKVSGSqiVDIDKRKYLVPSDITVAQFMWIIRKRIQLPSEKAIFLFVdkTVPQSSLTMGQLYEKEKDeDGFLYVAYSgentfg-----<br><br>BLAST: aliSize=39 (resi) iden=23.81% (20/84) simi=46.43% (39/84)<br>q---------------------------KIVVHLRATGGAPI--LKQSKFKVSGSDKFANVIDFLRR--QLHSD-SLFVYVNSAFsPNPDESVIDLY-NNFGFDGKLVVNYA------csmaw<br>-mkwmfkedhslehrcvesakirakypdRVPVIVEKVSGSQIvdIDKRKYLVPSDITVAQFMWIIRKriQLPSEkAIFLFVDKTV-PQSSLTMGQLYeKEKDEDGFLYVAYSgentfg-----<br><br>Proposed: aliSize=80 (resi) RMSD=1.46 (Å)<br>--------------------------QKIVVHLRAT--GGAPILKQSKFKVSGSDKFANVIDFLRRQL-HSD--SLFVYVNsAFSPNPDESVIDLYNNFGF-DGKLVVNYAC-S---maw<br>mkwmfkedhslehrcvesakirakypDRVPVIVEKVsgSQIVDIDKRKYLVPSDITVAQFMWIIRKRIqLPSekAIFLFVD-KTVPQSSLTMGQLYEKEKDeDGFLYVAYSGeNtfg---
40	1wz3A (84)<br>2r2qA (110)	C	21.43	TM-align: aliSize=54 (resi) RMSD=2.02 (Å)<br>-------------------------QKIVVHLRAT--GGAPILKQSKFKVSGSDKFANVIDFLRRQLHS-D-SLFVY-VNSAfspnpdesvidlynnfgfdgklvvnyacsm----------------AW----------<br>gfqykedhpfeyrkkegekirkkypDRVPVIVEKApkARVPDLDKRKYLVPSDLTVGQFYFLIRKRIHLrPeDALFFfVNNT-----------------------------ipptsatmgqlyednhEEdyflyvaysd<br><br>SARST: aliSize=78 (resi) RMSD=12.25 (Å)<br>q--------------------------KIVVHLRATGGA--PILKQSKFKVSGSDKFANVIDFLRRQLHS-DSLFVYVNSA-FSPNPDESVIDLYNNFGF-DGKLVVNYA-csmaw<br>-gfqykedhpfeyrkkegekirkkypdRVPVIVEKAPKArvPDLDKRKYLVPSDLTVGQFYFLIRKRIHLrPEDALFFFVNnTIPPTSATMGQLYEDNHEeDYFLYVAYSd-----<br><br>BLAST: aliSize=39 (resi) iden=28.57% (24/84) simi=46.43% (39/84)<br>qkivvhlratgga----------------------------PIL--KQSKFKVSGSDKFANVI--D-------FL-RRQLH---SDSLFVYVNSAFSPNpDESVIDLY-NNFGFDGKLVVNYA-csmaw<br>-------------gfqykedhpfeyrkkegekirkkypdrvPVIveKAPKARVPDLDKRKYLVpsDltvgqfyFLiRKRIHlrpEDALFFFVNNTIPPT-SATMGQLYeDNHEEDYFLYVAYSd-----<br><br>Proposed: aliSize=79 (resi) RMSD=1.35 (Å)<br>-------------------------QKIVVHLRAT--GGAPILKQSKFKVSGSDKFANVIDFLRRQLHS-D--SLFVYVNsAFSPNPDESVIDLYNNFGF-DGKLVVNYACsmaw<br>gfqykedhpfeyrkkegekirkkypDRVPVIVEKApkARVPDLDKRKYLVPSDLTVGQFYFLIRKRIHLrPedALFFFVN-NTIPPTSATMGQLYEDNHEeDYFLYVAYSD----
41	1wz3A (84)<br>1l7yA (94)	C	10.71	TM-align: aliSize=50 (resi) RMSD=2.42 (Å)<br>-----------QKIVVHLRATGGAPILkQSKFKVSGSDKFANVIDFLRRQLHSD--SLFVYV---NSafspnpdesvidlynnfgfdgklvvnyacsmaw---------------------------<br>msggtaattagSKVTFKITLTSDPKLP-FKVLSVPESTPFTAVLKFAAEEFKVPaaTSAIITndgVG---------------------------------vnpaqpagniflkhgselrliprdrvgh<br><br>SARST: aliSize=72 (resi) RMSD=12.54 (Å)<br>qk-----------IVVHLRATGG---APILkqSKFKVSGSDKFANVIDFLRRQLHS--DSLFVYVNSAFSPNPDESVIDLYNNFGfDGKLVVN------yacsmaw<br>--msggtaattagSKVTFKITLTsdpKLPF--KVLSVPESTPFTAVLKFAAEEFKVpaATSAIITNDGVGVNPAQPAGNIFLKHG-SELRLIPrdrvgh-------<br><br>BLAST: aliSize=0 (resi) iden=0.00% (0/84) simi=0.00% (0/84)<br>qkivvhlratggapilkqskfkvsgsdkfanvidflrrqlhsdslfvyvnsafspnpdesvidlynnfgfdgklvvnyacsmaw--<br>--msggtaattagskvtfkitltsdpklpfkvlsvpestpftavlkfaaeefkvpaatsaiitndgvgvnpaqpagniflkhgselrliprdrvgh<br><br>Proposed: aliSize=75 (resi) RMSD=2.31 (Å)<br>-----------QKIVVHLRATGGAPILkQSKFKVSGSDKFANVIDFLRRQLHSD--SLFVY---vNSAFSPNpdESVIDLYNNFGfdgKLVVNYACS---Maw<br>msggtaattagSKVTFKITLTSDPKLP-FKVLSVPESTPFTAVLKFAAEEFKVPaaTSAIItnd-GVGVNPA--QPAGNIFLKHG---SELRLIPRDrvgH--

42	1wz3A (84)<br>1lm8B (106)	C	5.95	TM-align: aliSize=48 (resi) RMSD=2.59 (Å)<br>QKIVVHLRATgGapILkqSKFKVSGSDKFANVIDFLRRQLHSD--SLFVYVNSAfspnpdesvidlynnfgf---Dgklvvnyacsmaw---------------<br>MDVFLMIRRH-K--TT--IFTDAKESSTVFELKRIVEGILKRPpdEQRLYKDDQ------------------llddgktlgecgftsqtarpqapatvglafraddtfealciE-------------pfssppelpdvmkpq<br><br>SARST: aliSize=72 (resi) RMSD=13.27 (Å)<br>qki-VVHLRATGGAPILkqsKFKVSGSDKFANVIDFLRRQLHS--DSLFVYVNsAFSPNPDESVIDL---YNNFGFDGKLVVNYA----------------------------csmaw<br>---mDVFLMIRRHKTTI---FTDAKESSTVFELKRIVEGILKRppDEQRLYKD-DQLLDDGKTLGECgftSQTARPQAPATVGLAfraddtfealciepfssppelpdvmkpq-----<br><br>BLAST: aliSize=8 (resi) iden=7.14% (6/84) simi=9.52% (8/84)<br>qkivvhlratggapilkqskfkvsgsdkfanvidflrrqlhsdslfvyvnsafspn--------------------------------------PDESVIDLYNNFGFDGK---lvvnyacsmaw<br>--mdvflmirrhkttiftdakesstvfelkrivegilkrpPDEQRLYKDDQLLDDGKtlgecgftsqtarpqapatvglafraddtfealciepfssppelpdvmkpq-----------<br><br>Proposed: aliSize=75 (resi) RMSD=2.33 (Å)<br>QKIVVHLRAT-ggapILkqSKFKVSGSDKFANVIDFLRRQLHSD-SLFVYV-nSAFSPNPDESVIDL-YN--nfGFDG--KLVVNYAC------SMAW-----------------<br>MDVFLMIRRHk----TT--IFTDAKESSTVFELKRIVEGILKRPpDEQRLYk-DDQLLDDGKTLGECgFTsq--TARPqaPATVGLAFraddtfEALCiepfssppelpdvmkpq
43	1wz3A (84)<br>2io1B (81)	C	6.17	TM-align: aliSize=48 (resi) RMSD=2.29 (Å)<br>QKIVVHLRATgGaPILkqSKFKVSGSDKFANVIDFLRRQLHSD-S-LFVYVNSAfspnpdesvidlynnfgfdgklvvnyacsmaw-------------------------------<br>DHINLKVAGQ-D-GSV--VQFKIKRHTPLSKLMKAYCERQGLSmRqIRFRFDGQ--------------------------------pinetdtpaqlemededtidvfqqqtggvpe<br><br>SARST: aliSize=69 (resi) RMSD=12.83 (Å)<br>q-KIVVHLRATGGApilkQSKFKVSGSDKFANVIDFLRRQLHS---DSLFVYVNSAFSpnPDESvidLYNNFGFDGKLVVNYA--------csmaw<br>-dHINLKVAGQDGS----VVQFKIKRHTPLSKLMKAYCERQGLsmrQIRFRFDGQPIN--ETDT---PAQLEMEDEDTIDVFQqqtggvpe-----<br><br>BLAST: aliSize=6 (resi) iden=4.94% (4/81) simi=7.41% (6/81)<br>qkivvhlratggapilkqskfkvsgsdkfanvidflrrqlhsdslfvyvnsafspnp--DESVIDLY---------nnfgfdgklvvnyacsmaw<br>---dhinlkvagqdgsvvqfkikrhtplsklmkaycerqglsmrqirfrfdgqpinetdtpaqlemeDEDTIDVFqqqtggvpe-------------------<br><br>Proposed: aliSize=73 (resi) RMSD=2.03 (Å)<br>QKIVVHLRATggAPILkqSKFKVSGSDKFANVIDFLRRQLHSD--SLFVYVNsAFSPNPDESVIDLynNFGfdGKLVVNYACSM------aw<br>DHINLKVAGQ--DGSV--VQFKIKRHTPLSKLMKAYCERQGLSmrQIRFRFD-GQPINETDTPAQL--EME--DEDTIDVFQQQtggvpe--
44	1wz3A (84)<br>2dziA (81)	C	6.17	TM-align: aliSize=48 (resi) RMSD=2.55 (Å)<br>q-----KIVVHLRATGGAPILkqSKFkVSGSDKFANVIDFLRRQLHSD--SLFVYVNSAfspnpdesvidlynnfgfdgklvvnyacsmaw--------------------------<br>-gssgsSGMQLTVKALQGREC--SLQ-VPEDELVSTLKQLVSEKLNVPvrQQRLLFKGK--------------------------------aladgkrlsdysigpnsklnlvvkpl<br><br>SARST: aliSize=69 (resi) RMSD=12.76 (Å)<br>qk-------IVVHLRATGGAPILkqskFKVSGSDKFANVIDFLRRQLHS--DSLFVYVNsAFSPNPDESvidLYNNFGFDGKLVVNYA---csmaw<br>--gssgssgMQLTVKALQGRECS----LQVPEDELVSTLKQLVSEKLNVpvRQQRLLFK-GKALADGKR---LSDYSIGPNSKLNLVVkpl-----<br><br>BLAST: aliSize=0 (resi) iden=0.00% (0/81) simi=0.00% (0/81)<br>qkivvhlratggapilkqskfkvsgsdkfanvidflrrqlhsdslfvyvnsafspnpdesvidlynnfgfdgklvvnyacsmaw---<br>--gssgssgmqltvkalqgrecslqvpedelvstlkqlvseklnvpvrqqrllfkgkaladgkrlsdysigpnsklnlvvkpl<br><br>Proposed: aliSize=72 (resi) RMSD=2.24 (Å)<br>-----QKIVVHLRATgGApILkqSKFKVSGSDKFANVIDFLRR-QLHS-DSLFVYVNsAFSPNPDESVIDLynNFGfdGKLVVNYACS--maw<br>gssgsSGMQLTVKAL-QG-RE--CSLQVPEDELVSTLKQLVSEkLNVPvRQQRLLFK-GKALADGKRLSDY--SIG--PNSKLNLVVKpl---

45	1wz3A (84)<br>1wjuA (100)	C	1.19	TM-align: aliSize=51 (resi) RMSD=2.73 (Å)<br>-------------QKIVVHLRATgGAPILK-QSKFKVSGSDKFANVIDFLRR--QLHSDSLFVYVNSAfspnpdesvidlynnfgfdgklvvnyacsmaw---------------------------------<br>gssgssgdnyrttGIATIEVFLP-PRLKKDrKNLLETRLHITGRELRSKIAEtfGLQENYIKIVINKK--------------------------------qlqlgktleeqgvahnvkamvlelkqssgpssg<br><br>SARST: aliSize=71 (resi) RMSD=12.92 (Å)<br>qk---------------IVVHLRAT---GGAPILkqsKFKVSGSDKFANVIDFLRRQLHS-DSLFVYVNSAFSPNPDESVIDLynnFGFDGKLVVNYA----------csmaw<br>--gssgssgdnyrttgiATIEVFLPprlKKDRKN---LLETRLHITGRELRSKIAETFGLqENYIKIVINKKQLQLGKTLEEQ---GVAHNVKAMVLElkqssgpssg-----<br><br>BLAST: aliSize=6 (resi) iden=7.14% (6/84) simi=7.14% (6/84)<br>qkivvhlratggapi---LKQSKFKVSGsdkfanvidflrrqlhsdslfvyvnsafspnpdesvidlynnfgfdgklvvnyacsmaw<br>---------------gssgssgdnyrttgiatievflpprlkkdrknlletrlhitgrelrskiaetfglqenyikivinkkqlqlgktleeqgvahnvkamvleLKQSSGPSSG---<br><br>Proposed: aliSize=73 (resi) RMSD=2.20 (Å)<br>-------------QKIVVHLRATgGAPILK--qSKFKVSGSDKFANVIDFLRR-QLHS-DSLFVYVNsAFSPNPDESVIDLynNFGfdGKLVVNYAC----------smaw<br>gssgssgdnyrttGIATIEVFLP-PRLKKDrk-NLLETRLHITGRELRSKIAEtFGLQeNYIKIVIN-KKQLQLGKTLEEQ--GVA--HNVKAMVLElkqssgpssg----
46	1wz3A (84)<br>1wx7A (106)	C	9.52	TM-align: aliSize=48 (resi) RMSD=2.47 (Å)<br>---------------QKIVVHLRATggApILkqSKFKVSGSDKFANVIDFLRRQLHSD-SLFVYVNSAFspnpdesvidlynnfgfdgklvvnyacsmaw--<br>gssgssgspapvqdpHLIKVTVKTP--K-DK--EDFSVTDTCTIQQLKEEISQRFKAHpDQLVLIFAGK-------------------------------ilkdpdslaqcgvrdgltvhlvikrqhramgnecpasgpssg<br><br>SARST: aliSize=74 (resi) RMSD=14.64 (Å)<br>qk----------------IVVHLRATGGAPILkqskFKVSGSDKFANVIDFLRRQLHS-DSLFVYVNSAFSPNPDESVIDlynNFGFDGKLVVNYACSMA----------------w<br>--gssgssgspapvqdphLIKVTVKTPKDKED----FSVTDTCTIQQLKEEISQRFKAhPDQLVLIFAGKILKDPDSLAQ---CGVRDGLTVHLVIKRQHramgnecpasgpssg-<br><br>BLAST: aliSize=9 (resi) iden=4.76% (4/84) simi=10.71% (9/84)<br>qkivvhlratggapilkqskfkvsgsdkfanvidflrrqlhsdslfvy---VNSAFSPNPDESVI---dlynnfgfdgklvvnyacsmaw<br>--gssgssgspapvqdphlikvtvktpkdkedfsvtdtctiqqlkeeISQRFKAHPDQLVLifagkilkdpdslaqcgvrdgltvhlvikrqhramgnecpasgpssg----------------------<br><br>Proposed: aliSize=71 (resi) RMSD=2.15 (Å)<br>---------------QKIVVHLRATggApILkqSKFKVSGSDKFANVIDFLRRQLHSD-SLFVYVNSAFSPNPDESVIDLynNFGfdGKLVVNYA-C------------------smaw<br>gssgssgspapvqdpHLIKVTVKTP--K-DK--EDFSVTDTCTIQQLKEEISQRFKAHpDQLVLIFAGKILKDPDSLAQC--GVR--DGLTVHLViKrqhramgnecpasgpssg----
47	1wz3A (84)<br>1se9A (101)	C	4.76	TM-align: aliSize=49 (resi) RMSD=2.65 (Å)<br>-----QKIVVHLRATgGAPILkqSKFKVSGSDKFANVIDFLRRQL-H------SD--SLFVYVNSAfspnpdesvidlynnfgfdgklvvnyacsmaw--------------------------------------<br>eaevhNQLEIKFRLT-DGSDI--GPKAFPDATTVSALKETVISEWpRekengpKTvkEVKLISAGK-------------------------------vlensktvkdyrspvsnlagavttmhviiqapvtekek<br><br>SARST: aliSize=71 (resi) RMSD=12.30 (Å)<br>qk-------IVVHLRATGGAPILkqsKFKVSGSDKFANVIDFLRRQL-----HSDSL---FVYVNSAFSPNPDESvidLYNNFGFDGKLVVNYA---------------csmaw<br>--eaevhnqLEIKFRLTDGSDIG---PKAFPDATTVSALKETVISEWprekeNGPKTvkeVKLISAGKVLENSKT---VKDYRSPVSNLAGAVTtmhviiqapvtekek-----<br><br>BLAST: aliSize=0 (resi) iden=0.00% (0/84) simi=0.00% (0/84)<br>qkivvhlratggapilkqskfkvsgsdkfanvidflrrqlhsdslfvyvnsafspnpdesvidlynnfgfdgklvvnyacsmaw---<br>--eaevhnqleikfrltdgsdigpkafpdattvsalketvisewprekengpktvkevklisagkvlensktvkdyrspvsnlagavttmhviiqapvtekek<br><br>Proposed: aliSize=70 (resi) RMSD=1.93 (Å)<br>q------KIVVHLRATgG-apILkQSKFKVSGSDKFANVIDFLRRQL-H------SD--SLFVYVNsAFSPNPDESVIDLYN-------nfgfdGKLVVNYACS--------maw<br>-eaevhnQLEIKFRLT-Dg--SD-IGPKAFPDATTVSALKETVISEWpRekengpKTvkEVKLISA-GKVLENSKTVKDYRSpvsnlag-----AVTTMHVIIQapvtekek---

48	1wz3A (84)<br>1wj4A (124)	C	9.52	TM-align: aliSize=47 (resi) RMSD=1.96 (Å)<br>---QKIVVHLRAtggAP-ILkqSKFKVSGSDKFANVIDFLRRQLHS-DSLFVYV-NSAfspnpdesvidlynnfgfdgklvvnyacsmaw---------------------------------<br>gssgssgtatnhqglpavdseilemppekadgvvegidvngPKAQLMLRY---PDgKR--EQITLPEQAKLLALVKHVQSKGYPnERFELLTnFPR--------------------------------rklshldyditlqeaglcpqetvfvqesgpssg<br><br>SARST: aliSize=48 (resi) RMSD=3.67 (Å)<br>q---KIVVHLRATGGAPILkqskFKVSGSDKFANVIDFLRRQLHS-DSLFVYVNSAfspN---------------------------------pdesvidlynnfgfdgklvvnyacsmaw<br>-gssgssgtatnhqglpavdseilemppekadgvvegidvngpKAQLMLRYPDGKREQ----ITLPEQAKLLALVKHVQSKGYPnERFELLTNFP---Rrklshldyditlqeaglcpqetvfvqesgpssg----------------------------<br><br>BLAST: aliSize=15 (resi) iden=8.33% (7/84) simi=17.86% (15/84)<br>qkivvhlratggapilkqskfkvsgsdkfanvidflrrqlhs------------------DSLFVyvnsAFSPNPDESVIDLYNNFGFDGKLVVNY--acsmaw<br>--gssgssgtatnhqglpavDSEIL----EMPPEKADGVVEGIDVNGPKAQLMLRYpdgkreqitlpeqakllalvkhvqskgypnerfelltnfprrklshldyditlqeaglcpqetvfvqesgpssg------<br><br>Proposed: aliSize=74 (resi) RMSD=2.41 (Å)<br>---QKIVVHLRAtggAPILKqSKFKVSGSDKFANVIDFLRRQLHS-DSLFVYV--NSAF-S-PNPDESVIDLynNFGfdGKLVVNYACSM----aw<br>gssgssgtatnhqglpavdseilemppekadgvvegidvngPKAQLMLRY---PDGKR-EQITLPEQAKLLALVKHVQSKGYPnERFELLTnfPRRKlShLDYDITLQEA--GLC--PQETVFVQESGpssg--
49	1wz3A (84)<br>1yqbA (88)	C	5.95	TM-align: aliSize=48 (resi) RMSD=2.65 (Å)<br>--------Q-KIVVHLRATgGapILkqSKFKVSGSDKFANVIDFLRRQLHSD-SLFVYVNSAFspnpdesvidlynnfgfdgklvvnyacsmaw------------------------------<br>sglvprgsPhLIKVTVKTP-K--DK--EDFSVTDTCTIQQLKEEISQRFKAHpDQLVLIFAGK-------------------------------ilkdpdslaqcgvrdgltvhlvikrqhram<br><br>SARST: aliSize=70 (resi) RMSD=12.46 (Å)<br>qk----------IVVHLRATGGAPILkqskFKVSGSDKFANVIDFLRRQLHS-DSLFVYVNSAFSPNPDESvidLYNNFGFDGKLVVNYA-------csmaw<br>--sglvprgsphLIKVTVKTPKDKED----FSVTDTCTIQQLKEEISQRFKAhPDQLVLIFAGKILKDPDS---LAQCGVRDGLTVHLVIkrqhram-----<br><br>BLAST: aliSize=9 (resi) iden=4.76% (4/84) simi=10.71% (9/84)<br>qkivvhlratggapilkqskfkvsgsdkfanvidflrrqlhsdslfvy---------------------------------------VNSAFSPNPDESVI-----------------------------------dlynnfgfdgklvvnyacsmaw<br>--sglvprgsphlikvtvktpkdkedfsvtdtctiqqlkeeISQRFKAHPDQLVLifagkilkdpdslaqcgvrdgltvhlvikrqhram----------------------<br><br>Proposed: aliSize=72 (resi) RMSD=2.34 (Å)<br>--------QKIVVHLRATGGaPILkqSKFKVsGSDKFANVIDFLRRQLHSD--SLFVYVNSaFSPNPDESVIDLynNFGfdGKLVVNYACS------maw<br>sglvprgsPHLIKVTVKTPK-DKE--DFSVT-DTCTIQQLKEEISQRFKAHpdQLVLIFAG-KILKDPDSLAQC--GVR--DGLTVHLVIKrqhram---
50	1wz3A (84)<br>1euvB (79)	C	10.13	TM-align: aliSize=48 (resi) RMSD=2.40 (Å)<br>--QKIVVHLRATggaPILkqSKFKVSGSDKFANVIDFLRRQLHSD--SLFVYVNSAfspnpdesvidlynnfgfdgklvvnyacsma-------------W--------------<br>peTHINLKVSDG---SSE--IFFKIKKTTPLRRLMEAFAKRQGKEmdSLRFLYDGI------------------------------riqadqtpedldmEdndiieahreqigg<br><br>SARST: aliSize=68 (resi) RMSD=12.31 (Å)<br>qkiv----VHLRATGGAPILkqsKFKVSGSDKFANVIDFLRRQLHS-DSLFVYVNSAFSPNPDESVIDlynNFGFDGKLVVNY------acsmaw<br>----pethINLKVSDGSSEI---FFKIKKTTPLRRLMEAFAKRQGKeMDSLRFLYDGIRIQADQTPED---LDMEDNDIIEAHreqigg------<br><br>BLAST: aliSize=30 (resi) iden=18.99% (15/79) simi=37.97% (30/79)<br>qkiv----VHLRATGGApilKQSKFKVSGSDKFANVID-FLRRQ-LHSDSL-FVYvnSAFSPNPDESVIDL-----------------ynnfgfdgklvvnyacsmaw<br>----pethINLKVSDGS---SEIFFKIKKTTPLRRLMEaFAKRQgKEMDSLrFLY--DGIRIQADQTPEDLdmedndiieahreqigg--------------------<br><br>Proposed: aliSize=72 (resi) RMSD=1.94 (Å)<br>--QKIVVHLRATggaPILkqSKFKVSGSDKFANVIDFLRRQLHSD--SLFVYVNsAFSPNPDESVIDLynNFGfdGKLVVNYACSM---aw<br>peTHINLKVSDG---SSE--IFFKIKKTTPLRRLMEAFAKRQGKEmdSLRFLYD-GIRIQADQTPEDL--DME--DNDIIEAHREQigg--

51	1wz3A (84)<br>1wh3A (87)	C	3.57	TM-align: aliSize=48 (resi) RMSD=2.48 (Å)<br>-----QKIVVHLRATgGApILkqSKFKVSGSDKFANVIDFLRRQLHSD-SLFVYVNSAfspnpdesvidlynnfgfdgklvvnyacsmaw---------------------------------<br>gssgsSGIQVFVKNP-DG-GS--YAYAINPNSFILGLKQQIEDQQGLPkKQQQLEFQG--------------------------------qvlqdwlglgiygiqdsdtlilskkkgsgpssg<br><br>SARST: aliSize=69 (resi) RMSD=12.16 (Å)<br>q-----KIVVHLRATGGAPILkqsKFKVSGSDKFANVIDFLRRQLHS--DSLFVYVNsAFSPNPDESVIdlyNNFGFDG--KLVVN---------yacsmaw<br>-gssgsSGIQVFVKNPDGGSY---AYAINPNSFILGLKQQIEDQQGLpkKQQQLEFQ-GQVLQDWLGLG---IYGIQDSdtLILSKkkgsgpssg-------<br><br>BLAST: aliSize=7 (resi) iden=4.76% (4/84) simi=8.33% (7/84)<br>qkivvhlratggapilkqskfkvsgsdkfanvidflrrqlh--SDSLFVYVNSAFSPN--pdesvidlynnfgfdgklvvnyacsmaw<br>---gssgssgiqvfvknpdggsyayainpnsfilglkqqiedqqglpkkqqqlefqgqvlqdwlglgiygiqdSDTLILSKKKGSGPSsg----------------------------<br><br>Proposed: aliSize=72 (resi) RMSD=2.31 (Å)<br>-----QKIVVHLRATggAPILkqSKFKVSGSDKFANVIDFLRRQLHSD--SLFVYVNsAFSPNPDESVIDLynNFGfdGKLVVNYAC-S-------maw<br>gssgsSGIQVFVKNP--DGGS--YAYAINPNSFILGLKQQIEDQQGLPkkQQQLEFQ-GQVLQDWLGLGIY--GIQ--DSDTLILSKkKgsgpssg---
52	1wz3A (84)<br>1m94A (73)	C	10.96	TM-align: aliSize=47 (resi) RMSD=2.64 (Å)<br>qKiVVHLRATGGAPILkqSKFkVSGSDKFANVIDFLRRQLHSD--SLFVYVNSAfspnpdesvidlynnfgfdgklvvnyacsmaw------------------------<br>-M-IEVVVNDRLGKKV--RVK-CLAEDSVGDFKKVLSLQIGTQpnKIVLQKGGS--------------------------------vlkdhisledyevhdqtnlelyyl<br><br>SARST: aliSize=57 (resi) RMSD=13.89 (Å)<br>qkivvhlratggapilkqs-------------KFKVSGSDKFANVIDFLRRQLH-SDSLFVYVNSAFSPNPDESVIDlynNFGFDGKLVVNYA-csmaw<br>------------------mievvvndrlgkkvRVKCLAEDSVGDFKKVLSLQIGtQPNKIVLQKGGSVLKDHISLED---YEVHDQTNLELYYl-----<br><br>BLAST: aliSize=0 (resi) iden=0.00% (0/73) simi=0.00% (0/73)<br>qkivvhlratggapilkqskfkvsgsdkfanvidflrrqlhsdslfvyvnsafspnpdesvidlynnfgfdgklvvnyacsmaw---<br>--mievvvndrlgkkvrvkclaedsvgdfkkvlslqigtqpnkivlqkggsvlkdhisledyevhdqtnlelyyl<br><br>Proposed: aliSize=70 (resi) RMSD=2.41 (Å)<br>qKIVVHLRATGGaPILkqSKFkVSGSDKFANVIDFLRRQLHSD--SLFVYVNsAFSPNPDESVIDLynNFGfdGKLVVNYAC-smaw<br>-MIEVVVNDRLG-KKV--RVK-CLAEDSVGDFKKVLSLQIGTQpnKIVLQKG-GSVLKDHISLEDY--EVH--DQTNLELYYl----
53	1wz3A (84)<br>1wxsA (88)	C	11.90	TM-align: aliSize=47 (resi) RMSD=2.74 (Å)<br>------QKIVVHLR-ATgGap--IlKQSKFKVSGSDKF-ANVIDFLRRQLHS-DSLFVYV--NSafspnpdesvidlynnfgfdgklvvnyacsmaw----------------------------<br>gplgsmSKVSFKITlTS-D--prL-PYKVLSVPESTPFtAVLKFAAEEFKVPaATSAIITndGI--------------------------------ginpaqtagnvflkhgselriiprdrvg<br><br>SARST: aliSize=70 (resi) RMSD=11.95 (Å)<br>q------KIVVHLRATGGA---PILkqsKFKVSGSDKFANVIDFLRRQLHSDSLFVYVNS--AFSPNPDESVIDLYNNFgfDGKLVV-------nyacsmaw<br>-gplgsmSKVSFKITLTSDprlPYK---VLSVPESTPFTAVLKFAAEEFKVPAATSAIITndGIGINPAQTAGNVFLKH--GSELRIiprdrvg--------<br><br>BLAST: aliSize=0 (resi) iden=0.00% (0/84) simi=0.00% (0/84)<br>qkivvhlratggapilkqskfkvsgsdkfanvidflrrqlhsdslfvyvnsafspnpdesvidlynnfgfdgklvvnyacsmaw--<br>--gplgsmskvsfkitltsdprlpykvlsvpestpftavlkfaaeefkvpaatsaiitndgiginpaqtagnvflkhgselriiprdrvg<br><br>Proposed: aliSize=72 (resi) RMSD=2.30 (Å)<br>------QKIVVHLRAtggaPI---LKQSKFKVSGSDKF-ANVIDFLRRQLHS-DSLFVY-vNSAFSPNPDESVIDLYNNFGfdgkLVVNYACS----maw<br>gplgsmSKVSFKITL----TSdprLPYKVLSVPESTPFtAVLKFAAEEFKVPaATSAIIt-NDGIGINPAQTAGNVFLKHG----SELRIIPRdrvg---

54	1wz3A<br>(84)<br><br>2gbjA<br>(81)	C	9.88	TM-align: aliSize=47 (resi) RMSD=2.98 (Å)<br>qKiVVHLRAT-G----G-Ap-ILkqSKFKVSGSDKFANVIDFLRRQLHSD--SLFVYVNSAfspnpdesvidlynnfgfdgklvvnyacsmaw-------------------------<br>-M-QIFVKTLtGggggGgG-gKT--ITLEVEPSDTIENVKAKIQDKEGIPpdQQRLIFAGK--------------------------------qledgrtlsdyniqkestlhlvlrl<br><br>SARST: aliSize=42 (resi) RMSD=7.20 (Å)<br>qkivvhlrat---------------GGAPILkqsKFKVSGSDKFANVIDFLRRQLHS--DSLFVYVnsafspnpdesvidlynnfgfDGKLVV----------------------nyacsmaw<br>----------mqifvktltggggggGGGKTI---TLEVEPSDTIENVKAKIQDKEGIppDQQRLIF--------------------AGKQLEdgrtlsdyniqkestlhlvlrl--------<br><br>BLAST: aliSize=9 (resi) iden=9.88% (8/81) simi=11.11% (9/81)<br>qkivvhlrat------------GGAPILKQSKFKVSGSDKFANV---idflrrqlhsdslfvyvnsafspnpdesvidlynnfgfdgklvvnyacsmaw<br>----------mqifvktltgggGGGGGGKTITLEVEPSDTIENVkakiqdkegippdqqrlifagkqledgrtlsdyniqkestlhlvlrl---<br><br>Proposed: aliSize=69 (resi) RMSD=2.34 (Å)<br>qKiVVHLRATGG--------apILkqSKFKVSGSDKFANVIDFLRRQLHSD--SLFVYVNsAFSPNPDESVIDLynNFGfdGKLVVNYAC--smaw<br>-M-QIFVKTLTGgggggggg--KT--ITLEVEPSDTIENVKAKIQDKEGIPpdQQRLIFA-GKQLEDGRTLSDY--NIQ--KESTLHLVLrl----
55	1wz3A<br>(84)<br><br>1we6A<br>(111)	C	5.95	TM-align: aliSize=50 (resi) RMSD=2.85 (Å)<br>------------------------Q-KIVVHLRATGGA-PILkqSKFKV-SGSDKFANVIDFLRRQLHSD--SLFVYVNSAfspnpdesvidlynnfgfdgklvvnyacsmaw--------------------------------<br>gssgssgkfdesalvpedqflaqhPgPATIRVSKPNENdGQF--MEITVqSLSENVGSLKEKIAGEIQIPanKQKLSGKAG--------------------------------flkdnmslahynvgageiltlslrersgpssg<br><br>SARST: aliSize=68 (resi) RMSD=13.34 (Å)<br>q-------------------------KIVVHLRATGG------APILKQSkfkvsgSDKFANVIDFLRRQLHSD--SLFVYVNsAFSPNPDESVIDlynNFGFDGKLVVNYA---------csmaw<br>-gssgssgkfdesalvpedqflaqhpgPATIRVSKPNEndgqfmEITVQSL------SENVGSLKEKIAGEIQIPanKQKLSGK-AGFLKDNMSLAH---YNVGAGEILTLSLrersgpssg-----<br><br>BLAST: aliSize=7 (resi) iden=7.14% (6/84) simi=8.33% (7/84)<br>qkivvhlrat---GGAPILKQSKFKVSG----sdkfanvidflrrqlhsdslfvyvnsafspnpdesvidlynnfgfdgklvvnyacsmaw<br>----------gssgssgkfdesalvpedqflaqhpgpatirvskpnendgqfmeitvqslsenvgslkekiageiqipankqklsgkagflkdnmslahynvGAGEILTLSLRERSGpssg--<br><br>Proposed: aliSize=71 (resi) RMSD=2.09 (Å)<br>------------------------Q-KIVVHLRATGG---apILkqSKFKV-SGSDKFANVIDFLRRQLHSD--SLFVYVNSaFSPNPDESVIDLyNnfG-fdGKLVVNYAC--S------maw<br>gssgssgkfdesalvpedqflaqhPgPATIRVSKPNEndg--QF--MEITVqSLSENVGSLKEKIAGEIQIPanKQKLSGKA-GFLKDNMSLAHY-N--Vg--AGEILTLSLreRsgpssg---
56	1wz3A<br>(84)<br><br>2ojrA<br>(110)	C	9.52	TM-align: aliSize=48 (resi) RMSD=2.46 (Å)<br>---------------------------------QKIVVHLRATggAPILkqSKFKVSGSDKFANVIDFLRRQLHSD--SLFVYVNSAfspnpdesvidlynnfgfdgklvvnyacsmaw---------------------------<br>gpgyidtnndgwiegdelyidtnndgwiegdelLAMQIFVKTL--TGKT--ITLEVEPSDTIENVKAKIQDKEGIPpdQQRLIFAGK--------------------------------qledgrtlsdyniqkestlhlvlrlrg<br><br>SARST: aliSize=50 (resi) RMSD=8.66 (Å)<br>qk----------------------------------IVVHLRATGGAPILkqsKFKVSGSDKFANVIDFLRRQLHS--------------------DSLFVYVNSAFSP------npdesvidlynnfgfdgklvvnyacsmaw<br>--gpgyidtnndgwiegdelyidtnndgwiegdellAMQIFVKTLTGKTI---TLEVEPSDTIENVKAKIQDKEGIppdqqrlifagkqledgrtlSDYNIQKESTLHLvlrlrg----------------------------<br><br>BLAST: aliSize=7 (resi) iden=7.14% (6/84) simi=8.33% (7/84)<br>qkivvhlratggapil---KQSKFKVSGSDKFANV---idflrrqlhsdslfvyvnsafspnpdesvidlynnfgfdgklvvnyacsmaw<br>----------------gpgyidtnndgwiegdelyidtnndgwiegdellamqifvktltgKTITLEVEPSDTIENVkakiqdkegippdqqrlifagkqledgrtlsdyniqkestlhlvlrlrg---<br><br>Proposed: aliSize=72 (resi) RMSD=2.31 (Å)<br>---------------------------------QKIVVHLRATggAPILkqSKFKVSGSDKFANVIDFLRR-QLHS-DSLFVYVNsAFSPNPDESVIDLynNFGfdGKLVVNYACS---maw<br>gpgyidtnndgwiegdelyidtnndgwiegdelLAMQIFVKTL--TGKT--ITLEVEPSDTIENVKAKIQDkEGIPpDQQRLIFA-GKQLEDGRTLSDY--NIQ--KESTLHLVLRlrg---

57	1wz3A<br>(84)<br><br>1t0yA<br>(90)	C	8.33	TM-align: aliSize=49 (resi) RMSD=2.03 (Å)<br>--QKIVVHLRATggAPILKqSKFKVSGSDKFANVIDFLRRQLHSD--SLFVYV------NSAfspnpdesvidlynnfgfdgklvvnyacsmaw-------------------------------<br>mtEVYDLEITTN--ATDFP-MEKKYPAGMSLNDLKKKLELVVGTTvdSMRIQLfdgddqLKG--------------------------------eltdgakslkdlgvrdgyrihavdvtggned<br><br>SARST: aliSize=72 (resi) RMSD=13.76 (Å)<br>q-KIVVHLRAT---GGAPILkqskFKVSGSDKFANVIDFLRRQLHS--DSLFVYV-----NSAFS-PNPDESVIDlynNFGFDGKLVVNYAC------smaw<br>-mTEVYDLEITtnaTDFPME----KKYPAGMSLNDLKKKLELVVGTtvDSMRIQLfdgddQLKGEITDGAKSLKD---LGVRDGYRIHAVDVtggned----<br><br>BLAST: aliSize=5 (resi) iden=5.95% (5/84) simi=5.95% (5/84)<br>qkivvhlra--TGGAPILK---------------------qskfkvsgsdkfanvidflrrqlhsdslfvyvnsafspnpdesvidlynnfgfdgklvvnyacsmaw<br>---------mtevydleittnatdfpmekkypagmslndlkkklelvvgttvdsmriqlfdgddqlkgelTDGAKSLKdlgvrdgyrihavdvtggned--<br><br>Proposed: aliSize=76 (resi) RMSD=2.08 (Å)<br>--QKIVVHLRATggAPILKqSKFKVSGSDKFANVIDFLRRQLHSD--SLFVYV------NSAFSPNPDESVIDLynNFGFDgkLVVNYAC----SMaW<br>mtEVYDLEITTN--ATDFP-MEKKYPAGMSLNDLKKKLELVVGTTvdSMRIQLfdgddqLKGELTDGAKSLKDL--GVRDG--YRIHAVDvtggNE-D
58	1wz3A<br>(84)<br><br>2gbkA<br>(82)	C	8.54	TM-align: aliSize=48 (resi) RMSD=2.90 (Å)<br>qKiVVHLRAT--GG--APILKqSKFKVSGSDKFANVIDFLRRQLHSD-SLFVYV--NSafspnpdesvidlynnfgfdgklvvnyacsmaw---------------------------<br>-M-QIFVKTLtqVRelVGGKT-ITLEVEPSDTIENVKAKIQDKEGIPpDQQRLIfaGK---------------------------------qledgrtlsdyniqkestlhlvlrlrg<br><br>SARST: aliSize=55 (resi) RMSD=13.41 (Å)<br>qkivvhlratggapilkqs--------------------KFKVSGSDKFANVIDFLRRQLHS--DSLFVYVNsAFSPNPDESVIDlynNFGFDGKLVVNY-----acsmaw<br>-------------------mqifvktltqvrelvggktiTLEVEPSDTIENVKAKIQDKEGIppDQQRLIFA-GKQLEDGRTLSD---YNIQKESTLHLVlrlrg------<br><br>BLAST: aliSize=7 (resi) iden=7.32% (6/82) simi=8.54% (7/82)<br>qkivvhlratggapil-----------------KQSKFKVSGSDKFANV--idflrrqlhsdslfvyvnsafspnpdesvidlynnfgfdgklvvnyacsmaw<br>----------------mqifvktltqvrelvggKTITLEVEPSDTIENVkakiqdkegippdqqrlifagkqledgrtlsdyniqkestlhlvlrlrg--<br><br>Proposed: aliSize=70 (resi) RMSD=2.25 (Å)<br>qkIVVHLRAT--GG----aPILKqSKFKVSGSDKFANVIDFLRRQLHSD--SLFVYVNsAFSPNPDESVIDLynNFGfdGKLVVNYAC----smaw<br>--MQIFVKTLtqVRelvg-GKT--ITLEVEPSDTIENVKAKIQDKEGIPpdQQRLIFA-GKQLEDGRTLSDY--NIQ--KESTLHLVLrlrg----
59	1wz3A<br>(84)<br><br>2jxxA<br>(78)	C	6.41	TM-align: aliSize=49 (resi) RMSD=2.57 (Å)<br>----QKIVVHLRATgGAPILKqSKFKVSGSDKFANVIDFLRR-QLH-SDSLFVYVNSAfspnpdesvidlynnfgfdgklvvnyacsmaw-----------------------<br>tetsQQLQLRVQGK-EKHQT--LEVSLSRDSPLKTLMSHYEEaMGLsGRKLSFFFDGT--------------------------------klsgrelpadlgmesgdlievwg<br><br>SARST: aliSize=56 (resi) RMSD=13.07 (Å)<br>qkivvhlratggapilk------------------QSKFKVSGSDKFANVIDFLRRQLHsdSLFVYVNSAF--SPNP-DESvidLYNNFGFDGKLVVNY-acsmaw<br>-----------------tetsqqlqlrvqgkekhqTLEVSLSRDSPLKTLMSHYEEAMG--LSGRKLSFFFdgTKLSgREL---PADLGMESGDLIEVWg------<br><br>BLAST: aliSize=10 (resi) iden=5.13% (4/78) simi=12.82% (10/78)<br>qkivvhlratggapilkqskfkvsgsdkfanvidflrrqlhsdslfvyvnsafsp---------------------------NPDESVIDLYNN-FGFDGK--------------------------------lvvnyacsmaw<br>--tetsqqlqlrvqgkekhqtlevslsrdSPLKTLMSHYEEaMGLSGRklsfffdgtklsgrelpadlgmesgdlievwg-----------<br><br>Proposed: aliSize=71 (resi) RMSD=2.19 (Å)<br>----QKIVVHLRATgG-aPILKqSKFKVSGSDKFANVIDFLRRQLHS-DSLFVYV-nSAFSPNPDESVIDLynNFGfdGKLVVNYACsmaw<br>tetsQQLQLRVQGK-Ek-HQT--LEVSLSRDSPLKTLMSHYEEAMGLsGRKLSFFf-DGTKLSGRELPADL--GME--SGDLIEVWG----

60	1wz3A<br>(84)<br><br>1sifA<br>(71)	C	8.45	TM-align: aliSize=46 (resi) RMSD=2.59 (Å)<br>qkiVVHLRATGGAPILkqSKFKVsGSDKFANVIDFLRRQLHSD--SLFVYVNSAfspnpdesvidlynnfgfdgklvvnyacsmaw-----------------------<br>---LQLFIKTLTGKTF--TVEME-PSDTIENLKAKIQDKEGIPpdQQRLIFAGK--------------------------------qledgrtlsdyniqkestlhlvl<br><br>SARST: aliSize=67 (resi) RMSD=12.30 (Å)<br>qki-VVHLRATGGAPILkqskFKVSGSDKFANVIDFLRRQLHS--DSLFVYVNsAFSPNPDESvidLYNNFGFDGKLVVNY-acsmaw<br>---lQLFIKTLTGKTFT----VEMEPSDTIENLKAKIQDKEGIppDQQRLIFA-GKQLEDGRT---LSDYNIQKESTLHLVl------<br><br>BLAST: aliSize=6 (resi) iden=4.23% (3/71) simi=8.45% (6/71)<br>qkivvhlratggapilkqskfkvsgsdkfanvidflrrqlhsdslfvyvnsafspnpdesvidlynnfgf---DGKLVVNY------------acsmaw<br>--lqlfiktltgktftvemepsdtienlkakiqdkegippdqqrlifagkqleDGRTLSDYniqkestlhlvl------<br><br>Proposed: aliSize=69 (resi) RMSD=2.22 (Å)<br>qkiVVHLRATGGAPILkqSKFKVsGSDKFANVIDFLRRQLHSD--SLFVYVNsAFSPNPDESVIDLynNFGfdGKLVVNYACsmaw<br>---LQLFIKTLTGKTF--TVEME-PSDTIENLKAKIQDKEGIPpdQQRLIFA-GKQLEDGRTLSDY--NIQ--KESTLHLVL----
61	1wz3A<br>(84)<br><br>1ud7A<br>(76)	C	7.89	TM-align: aliSize=47 (resi) RMSD=2.84 (Å)<br>qKiVVHLRATgGApILkqSKFKVSGSDKFANVIDFLRRQLHSD-SLFVYV-NSAfspnpdesvidlynn---------------------------Fgfdgklvvnyacsmaw<br>-M-QVFLKTL-TG-KT--VTIEVEPSDTVENFKAKIQDKEGIPpDQQRLIfAGK---------------qledgrtlsdyniqkestihlvlrlrgG----------------<br><br>SARST: aliSize=67 (resi) RMSD=11.74 (Å)<br>qkiv-VHLRATGGAPILkqsKFKVSGSDKFANVIDFLRRQLHS-DSLFVYVNSAFSPNPDESviDLYNNFGFDGKLVV-------nyacsmaw<br>----mQVFLKTLTGKTV---TIEVEPSDTVENFKAKIQDKEGIpPDQQRLIFAGKQLEDGRT--LSDYNIQKESTIHLvlrlrgg--------<br><br>BLAST: aliSize=6 (resi) iden=6.58% (5/76) simi=7.89% (6/76)<br>qkiv--VHL--RATGGapilkqskfkvsgsdkfanvidflrrqlhsdslfvyvnsafspnpdesvidlynnfgfdgklvvnyacsmaw<br>----mqvflktltgktvtievepsdtvenfkakiqdkegippdqqrlifagkqledgrtlsdyniqkestIHLvlRLRGG--<br><br>Proposed: aliSize=73 (resi) RMSD=2.55 (Å)<br>qkiVVHLRATGGAPILkqSKFKVsGSDKFANVIDFLRRQLHSD--SLFVYVNSaFSPNPDESVIDLyNNfG-fdGKLVVNYACSMAW<br>---MQVFLKTLTGKTV--TIEVE-PSDTVENFKAKIQDKEGIPpdQQRLIFAG-KQLEDGRTLSDY-NI-Qk--ESTIHLVLRLRGG
62	1wz3A<br>(84)<br><br>2dzmA<br>(100)	C	2.38	TM-align: aliSize=50 (resi) RMSD=2.91 (Å)<br>------QKIVVHLRATggAPILkQSKFkVSGSDKFANVIDFLRRQLHSD--SLFVYVNSAFspnpdesvidlynnfgfdgklvvnyacsm-------------Aw-----------------------------<br>gssgssGRMLDFRVEY--RDRN-VDVV-LEDTCTVGEIKQILENELQIPvsKMLLKGWKTG-----------------------------dvedstvlkslhlP-knnslyvltpdlpppssssshagalqesln<br><br>SARST: aliSize=63 (resi) RMSD=12.04 (Å)<br>q-KIVVHLRATGGAPILK--QSKFKVSGSDKFANVIDFLRRQLHSD--SLFVYVNSAFSPNPDESVIDL--------------------------------ynnfgfdgklvvnyacsmaw<br>-gSSGSSGRMLDFRVEYRdrNVDVVLEDTCTVGEIKQILENELQIPvsKMLLKGWKTGDVEDSTVLKSLhlpknnslyvltpdlpppssssshagalqesln--------------------<br><br>BLAST: aliSize=22 (resi) iden=17.86% (15/84) simi=26.19% (22/84)<br>qkivvhlratggapilkq--SKFKVSGSdKFANVIDF-LRRQLH---SDSLfvYVNSAFSPNPDES-----------vidlynnfgfdgklvvnyacsmaw<br>------------------gssgssgrmldfrveyrdrnvdvvledtctvgeikqilenelqipvSKMLLKGW-KTGDVEDStVLKSLHlpkNNSL--YVLTPDLPPPSSSshagalqesln------------------------<br><br>Proposed: aliSize=70 (resi) RMSD=2.19 (Å)<br>-------QKIVVHLRAtggAPILkqSKFKVSGSDKFANVIDFLRR-QLHS-DSLFVYVnsA--FSPNPDESVIDLyNnfGFdGKLVVNYACS-------------------maw<br>gssgssgRMLDFRVEY---RDRN--VDVVLEDTCTVGEIKQILENeLQIPvSKMLLKG--WktGDVEDSTVLKSL-H--LP-KNNSLYVLTPDlpppssssshagalqesln---

63	1wz3A (84)<br>1wx9A (86)	C	8.33	TM-align: aliSize=48 (resi) RMSD=3.03 (Å)<br>q-----KIVVHLRATGGAPILkqSKFKVSGsDKFANVIDFLRRQLHSD--SLFVYVNSAfspnpdesvidlynnfgfdgklvvnyacsmaw-------------------------------<br>-gssgsSGLEVLVKTLDSQTR--TFIVGAQ-MNVKEFKEHIAASVSIPseKQRLIYQGR--------------------------------vlqddkklqeynvggkvihlverapsgpssg<br><br>SARST: aliSize=70 (resi) RMSD=12.57 (Å)<br>q-----KIVVHLRATGGAPILkqsKFKVSGSDKFANVIDFLRRQLHS--DSLFVYVNsAFSPNPDESVIDLynNFGfdGKLVVNYA---------csmaw<br>-gssgsSGLEVLVKTLDSQTR---TFIVGAQMNVKEFKEHIAASVSIpsEKQRLIYQ-GRVLQDDKKLQEY--NVG--GKVIHLVErapsgpssg-----<br><br>BLAST: aliSize=10 (resi) iden=7.14% (6/84) simi=11.90% (10/84)<br>qkivvhlratggapilkqskfkvsgsdkfanvidflrrqlhsdslfvyvnsafspnp--DESVIDLYnNFGfdGKLV-------------vnyacsmaw<br>---gssgssglevlvktldsqtrtfivgaqmnvkefkehiaasvsipsekqrliyqgrvlqDDKKLQEY-NVG--GKVIhlverapsgpssg---------<br><br>Proposed: aliSize=71 (resi) RMSD=2.51 (Å)<br>q-----KIVVHLRATGGApILkqSKFKVSGSDKFANVIDFLRRQLHSD--SLFVYVNsAFSPNPDESVIDLYnnfGfdGKLVVNYAC--S------maw<br>-gssgsSGLEVLVKTLDS-QT--RTFIVGAQMNVKEFKEHIAASVSIPseKQRLIYQ-GRVLQDDKKLQEYN---V--GGKVIHLVEraPsgpssg---
64	1wz3A (84)<br>1wj6A (101)	C	7.14	TM-align: aliSize=54 (resi) RMSD=2.88 (Å)<br>-----Q------KIVVHLRATGGaPILkqSKFKVS-GSDKFANVIDFLRRQLHSDSLFVYVNSAFspnpdesvidlynn-Fgfdgklvvnyacs--------------M-AW------------------<br>gssgsSgphsmePQVTLNVTFKN-EIQ--SFLVSDpENTTWADIEAMVKVSFDLNTIQIKYLDEE-------------nE-------------evsinsqgeyeealkMaVKqgnqlqmqvhegsgpssg<br><br>SARST: aliSize=64 (resi) RMSD=12.49 (Å)<br>qk-------------IVVHLRATGGApilkqsKFKVS----GSDKFANVIDFLRRQLHSDslfvyVNSAFSPNPDES-----------VIDLYNNFgfDGKLVVNYA--------csmaw<br>--gssgssgphsmepQVTLNVTFKNE------IQSFLvsdpENTTWADIEAMVKVSFDLN-----TIQIKYLDEENEevsinsqgeyeeALKMAVKQ--GNQLQMQVHegsgpssg-----<br><br>BLAST: aliSize=2 (resi) iden=2.38% (2/84) simi=2.38% (2/84)<br>qkivvhlratggapilkqskfkvsgsdkfanvidflrrqlhsdslfvyvnsafspnpdesvi---DL--ynnfgfdgklvvnyacsmaw<br>--gssgssgphsmepqvtlnvtfkneiqsflvsdpenttwadieamvkvsfDLntiqikyldeeneevsinsqgeyeealkmavkqgnqlqmqvhegsgpssg--------------------<br><br>Proposed: aliSize=74 (resi) RMSD=2.19 (Å)<br>------------QKIVVHLRATgGapILkqSKFKV-SG-SDKFANVIDFLRRQLHSDSLFVYVNS----AFSPNPDESVIDLYNNfG--FDGKLVVNYAC-------smaw<br>gssgssgphsmePQVTLNVTFK-N--EI--QSFLVsDPeNTTWADIEAMVKVSFDLNTIQIKYLDeeneEVSINSQGEYEEALKM-AvkQGNQLQMQVHEgsgpssg----
65	1wz3A (84)<br>1p0rA (73)	C	8.22	TM-align: aliSize=46 (resi) RMSD=1.95 (Å)<br>qKIVVHLRATGGaPILkqSKFkVSGSDKFANVIDFLRRQLHSD--SLFVYVNSafspnpdesvidlynnfgfdgklvvnyacsmaw-------------------------<br>-MIEVVCNDRLG-KKV--RVK-CNTDDTIGDLKKLIAAQTGTRwnKIVLKKWY---------------------------------tifkdhvslgdyeihdgmnlelyyq<br><br>SARST: aliSize=59 (resi) RMSD=13.42 (Å)<br>qkivvhlratggapilk------------QSKFKVSGSDKFANVIDFLRRQLH-SDSLFVYVNSAFSPNPDESvidLYNNFGFDGKLVVNYA-csmaw<br>-----------------mievvcndrlgkKVRVKCNTDDTIGDLKKLIAAQTGtRWNKIVLKKWYTIFKDHVS---LGDYEIHDGMNLELYYq-----<br><br>BLAST: aliSize=0 (resi) iden=0.00% (0/73) simi=0.00% (0/73)<br>qkivvhlratggapilkqskfkvsgsdkfanvidflrrqlhsdslfvyvnsafspnpdesvidlynnfgfdgklvvnyacsmaw---<br>--mievvcndrlgkkvrvkcntddtigdlkkliaaqtgtrwnkivlkkwytifkdhvslgdyeihdgmnlelyyq<br><br>Proposed: aliSize=71 (resi) RMSD=2.21 (Å)<br>qKIVVHLRATGGaPILkqSKFkVSGSDKFANVIDFLRRQLHSD--SLFVYVNsAFSPNPDESViDLynNFGFdGKLVVNYACSmaw<br>-MIEVVCNDRLG-KKV--RVK-CNTDDTIGDLKKLIAAQTGTRwnKIVLKKW-YTIFKDHVSL-GD--YEIH-DGMNLELYYQ---

66	1wz3A (84)<br>2fazA (77)	C	7.79	TM-align: aliSize=49 (resi) RMSD=2.65 (Å)<br>qKIVVHLRATGGAPILkqSKFKVSGSDKFANVIDFLRRQLHSD--SLFVYVNSAfspnpdesvidlynnfgfdgklvvnyacsmaw-------------------------<br>-SMWIQVRTMDGRQTH--TVDSLSRLTKVEELRRKIQELFHVEpgLQRLFYRGK--------------------------------qmedghtlfdyevrlndtiqllvrqs<br><br>SARST: aliSize=57 (resi) RMSD=14.77 (Å)<br>qkivvhlratggapilkqs----------------KFKVSGSDKFANVIDFLRRQLHS--DSLFVYV-NSAFSPnpDESviDLYNNFGFDGKLVVNYAC-smaw<br>------------------smwiqvrtmdgrqthtVDSLSRLTKVEELRRKIQELFHVepGLQRLFYrGKQMED--GHT--LFDYEVRLNDTIQLLVRQs----<br><br>BLAST: aliSize=0 (resi) iden=0.00% (0/77) simi=0.00% (0/77)<br>qkivvhlratggapilkqskfkvsgsdkfanvidflrrqlhsdslfvyvnsafspnpdesvidlynnfgfdgklvvnyacsmaw---<br>--smwiqvrtmdgrqthtvdslsrltkveelrrkiqelfhvepglqrlfyrgkqmedghtlfdyevrlndtiqllvrqs<br><br>Proposed: aliSize=73 (resi) RMSD=2.33 (Å)<br>qKIVVHLRATGGAPILkqSKFKVSGSDKFANVIDFLRRQLHSD--SLFVYVNsAFSPNPDESVIDLynNFGfdGKLVVNYACS--maw<br>-SMWIQVRTMDGRQTH--TVDSLSRLTKVEELRRKIQELFHVEpgLQRLFYR-GKQMEDGHTLFDY--EVR--LNDTIQLLVRqs---
67	1wz3A (84)<br>2nptA (102)	C	11.90	TM-align: aliSize=55 (resi) RMSD=3.31 (Å)<br>----------QKIVVHLRAtggAPILkQSKFKVSGSDKFANVIDFLRRQLH-SDSLFVYVNSAFSpnpdesvidlynNFgfdgklvvnyacs------------------M--AW---------------<br>smalgpfpamQVLVIRIKI---PNSG-AVDWTVHSQLLFRDVLDVIGQVLPeATTTAFEYEDEDG------------DR-------------itvrsdeemkamlsyyysTvmEQqvngqlieplqifpra<br><br>SARST: aliSize=52 (resi) RMSD=6.81 (Å)<br>qk-----------IVVHLRATGGAPILkqsKFKVSGSDKFANVIDFLRRQlhsDSLFVYVNSAFSPNPDES--------------------------------------vidlynnfgfdgklvvnyacsmaw<br>--smalgpfpamqVLVIRIKIPNSGAV---DWTVHSQLLFRDVLDVIGQV---LPEATTTAFEYEDEDGDRitvrsdeemkamlsyyystvmeqqvngqlieplqifpra-----------------------<br><br>BLAST: aliSize=11 (resi) iden=9.52% (8/84) simi=13.10% (11/84)<br>qkivvhlratggapilkqskfkvsgsdkfanvidflrrq----------------------------LHSDSLFVYVNSAFSPNPDESVIDLYNNFGFDG--klvvnyacsmaw<br>---------------------------------------smalgpfpamqvlvirikipnsgavdwtVHSQLLFRDVLDVIGQVLPEATTTAFEYEDEDGdritvrsdeemkamlsyyystvmeqqvngqlieplqifpra------------<br><br>Proposed: aliSize=76 (resi) RMSD=2.89 (Å)<br>----------QKIVVHLRAtggAPILkQSKFKVSGSDKFANVIDFLRRQL-HSDSLFVYVNSA----FSPNPDESVIDLYNNFG--F---------DGKLVVNYACsmaw<br>smalgpfpamQVLVIRIKI---PNSG-AVDWTVHSQLLFRDVLDVIGQVLpEATTTAFEYEDEdgdrITVRSDEEMKAMLSYYYstVmeqqvngqlIEPLQIFPRA----
68	1wz3A (84)<br>1raxA (93)	C	10.71	TM-align: aliSize=54 (resi) RMSD=3.43 (Å)<br>---QKIVVHLRAT--GG-APILkqSKFkVSGSDKFANVIDFLRRQLHS--D---SLFVYVNSAfspnpdesvidlynn-FGfdgklvvnyacs-------------MAW------------<br>qqvGDCCIIRVSLdvDNgNMYK--SIL-VTSQDKAPAVIRKAMDKHNLeeEepeDYELLQILS---------------dDR------------klkipenanvfyamNSTanydfvlkkrtft<br><br>SARST: aliSize=72 (resi) RMSD=11.96 (Å)<br>q----KIVVHLRATGGAPILKQSKFKVSGSDKFANVIDFLRRQLHS------DSLFVYV---NSAFSPNPDESvidlYNNF----GFDGKLVVN----yacsmaw<br>-qqvgDCCIIRVSLDVDNGNMYKSILVTSQDKAPAVIRKAMDKHNLeeeepeDYELLQIlsdDRKLKIPENAN----VFYAmnstANYDFVLKKrtft-------<br><br>BLAST: aliSize=6 (resi) iden=5.95% (5/84) simi=7.14% (6/84)<br>qkivvhlratggapilkqskfk------------------------VSGSDKFANVI--dflrrqlhsdslfvyvnsafspnpdesvidlynnfgfdgklvvnyacsmaw<br>----------------------qqvgdcciirvsldvdngnmyksilVTSQDKAPAVIrkamdkhnleeeepedyellqilsddrklkipenanvfyamnstanydfvlkkrtft--<br><br>Proposed: aliSize=70 (resi) RMSD=2.67 (Å)<br>----QKIVVHLRATGG--apILKqSKFKVSGSDKFANVIDFLRR--QLHSD-------------slfvyVNSAFsPNPDESVIDLYnNF-GFdgKLVVNYA-CSMaw<br>qqvgDCCIIRVSLDVDng--NMY-KSILVTSQDKAPAVIRKAMDkhNLEEEepedyellqilsd-----DRKLK-IPENANVFYAM-NStAN--YDFVLKKrTFT--

69	1wz3A<br>(84)<br><br>2cu1A<br>(103)	C	4.76	TM-align: aliSize=53 (resi) RMSD=3.09 (Å)<br>---Q--KIVVHLRATgGAPILkqSKFkVSGSDKFANVIDFLRRQLHSDsLFVYVNSAFspnpdesvidlynNFgfdgklvvnyacs------------------MAW----------------------------<br>gssGssGDVRVKFEH-RGEKR--ILQ-FPRPVKLEDLRSKAKIAFGQS-MDLHYTNNE-------------LV--------------iplttqddldkavelldRSIhmkslkillvingstqatnlepsgpssg<br><br>SARST: aliSize=44 (resi) RMSD=2.72 (Å)<br>q------KIVVHLRATGGAPILkqskFKVSGSDKFANVIDFLRRQLHSDSLFVYV---nsafspnpdesvidlynnfgfdgklvvnyacsmaw<br>-gssgssGDVRVKFEHRGEKRI----LQFPRPVKLEDLRSKAKIAFGQSMDLHYTnnelviplttqddldkavelldrsihmkslkillvingstqatnlepsgpssg-----------------------------------<br><br>BLAST: aliSize=0 (resi) iden=0.00% (0/84) simi=0.00% (0/84)<br>qkivvhlratggapilkqskfkvsgsdkfanvidflrrqlhsdslfvyvnsafspnpdesvidlynnfgfdgklvvnyacsmaw---<br>--gssgssgdvrvkfehrgekrilqfprpvkledlrskakiafgqsmdlhytnnelviplttqddldkavelldrsihmkslkillvingstqatnlepsgpssg<br><br>Proposed: aliSize=73 (resi) RMSD=2.44 (Å)<br>------QKIVVHLRATgGapILkqSKFKVSGSDKFANVIDFLRRQLHSDsLFVYVNSA---FSPNPDESVIDLYNNfG----FDGKLVVNYAC-----------------smaw<br>gssgssGDVRVKFEHR-G--EK--RILQFPRPVKLEDLRSKAKIAFGQS-MDLHYTNNelvIPLTTQDDLDKAVEL-LdrsiHMKSLKILLVIngstqatnlepsgpssg----
70	1wz3A<br>(84)<br><br>1u4aA<br>(79)	C	5.06	TM-align: aliSize=48 (resi) RMSD=2.29 (Å)<br>-QKIVVHLRATggAPILkqSKFKVSGSDKFANVIDFLRRQLHSD--SLFVYVNSAfspnpdesvidlynnfgfdgklvvnyacsmaw----------------------------<br>nDHINLKVAGQ--DGSV--VQFKIKRHTPLSKLMKAYSERQGLSmrQIRFRFDGQ--------------------------------pinetdtpaqlemededtidvfqqqtgg<br><br>SARST: aliSize=71 (resi) RMSD=13.06 (Å)<br>q--KIVVHLRATGGAPILkqskFKVSGSDKFANVIDFLRRQLH-SDSLFVYVNSAFSPNPDESVIDLYNNFGFdgkLVVNYA-----csmaw<br>-ndHINLKVAGQDGSVVQ----FKIKRHTPLSKLMKAYSERQGlSMRQIRFRFDGQPINETDTPAQLEMEDED---TIDVFQqqtgg-----<br><br>BLAST: aliSize=6 (resi) iden=5.06% (4/79) simi=7.59% (6/79)<br>qkivvhlratggapilkqskfkvsgsdkfanvidflrrqlhsdslfvyvnsafspnp---DESVIDLY------nnfgfdgklvvnyacsmaw<br>---ndhinlkvagqdgsvvqfkikrhtplsklmkayserqglsmrqirfrfdgqpinetdtpaqlemeDEDTIDVFqqqtgg-------------------<br><br>Proposed: aliSize=71 (resi) RMSD=2.22 (Å)<br>-QKIVVHLRATggAPILkqSKFKVSGSDKFANVIDFLRRQLHSD--SLFVYVNsAFSPNPDESviDLYNNfGFdGKLVVNYAC-----smaw<br>nDHINLKVAGQ--DGSV--VQFKIKRHTPLSKLMKAYSERQGLSmrQIRFRFD-GQPINETDT--PAQLE-ME-DEDTIDVFQqqtgg----
71	1wz3A<br>(84)<br><br>1c3tA<br>(76)	C	9.21	TM-align: aliSize=46 (resi) RMSD=2.67 (Å)<br>qkiVVHLRATGGAPILkqSKFKVsGSDKFANVIDFLRRQLHSD--SLFVYVNSAfspnpdesvidlynnfgfdgklvvnyacsmaw----------------------------<br>---MQLFVKTLTGKTL--TVELE-PSDTVENLKAKIQDKEGIPpdQQRLIFAGK--------------------------------qledgrtlsdynlqkestihlvlrlrgg<br><br>SARST: aliSize=68 (resi) RMSD=12.14 (Å)<br>qkiv-VHLRATGGAPILkqsKFKVSGSDKFANVIDFLRRQLHSD-SLFVYVNSAFSPNPDESvidLYNNFGFDGKLVVNY------acsmaw<br>----mQLFVKTLTGKTL---TVELEPSDTVENLKAKIQDKEGIPpDQQRLIFAGKQLEDGRT---LSDYNLQKESTIHLVlrlrgg------<br><br>BLAST: aliSize=6 (resi) iden=6.58% (5/76) simi=7.89% (6/76)<br>qkiv--VHL--RATGGapilkqskfkvsgsdkfanvidflrrqlhsdslfvyvnsafspnpdesvidlynnfgfdgklvvnyacsmaw<br>----mqlfvktltgktltvelepsdtvenlkakiqdkegippdqqrlifagkqledgrtlsdynlqkestIHLvlRLRGG--<br><br>Proposed: aliSize=70 (resi) RMSD=2.38 (Å)<br>qkiVVHLRATGGAPILkqSKFkVSGSDKFANVIDFLRRQLHSD--SLFVYVNsAFSPNPDESVIDLynNFGfdGKLVVNYAC-S---maw<br>---MQLFVKTLTGKTL--TVE-LEPSDTVENLKAKIQDKEGIPpdQQRLIFA-GKQLEDGRTLSDY--NLQ--KESTIHLVLrLrgg---

72	1wz3A<br>(84)<br><br>2jwzA<br>(76)	C	9.21	TM-align: aliSize=47 (resi) RMSD=2.70 (Å)<br>qkiVVHLRATGGAPILkqSKFKVsGSDKFANVIDFLRRQLHSDS-LFVYVNSAFspnpdesvidlynnfgfdgklvvnyacsmaw----------------------------<br>---MQIFVKTLTGKTI--TLEVE-SSDTIDNVKSKIQDKEGIPPdQQRLIFAGK-------------------------------qledgrtlsdyniqkestlhsvlrlrgg<br><br>SARST: aliSize=69 (resi) RMSD=12.46 (Å)<br>qkiv-VHLRATGGAPILkqsKFKVSGSDKFANVIDFLRRQLHSD-SLFVYVNSAFSPNPDESvidLYNNFGFDGKLVVNYA-----csmaw<br>----mQIFVKTLTGKTI---TLEVESSDTIDNVKSKIQDKEGIPpDQQRLIFAGKQLEDGRT---LSDYNIQKESTLHSVLrlrgg-----<br><br>BLAST: aliSize=7 (resi) iden=7.89% (6/76) simi=9.21% (7/76)<br>qkivvhlratggapil----------KQSKFKVSGSDKFANV--idflrrqlhsdslfvyvnsafspnpdesvidlynnfgfdgklvvnyacsmaw<br>----------------mqifvktltgKTITLEVESSDTIDNVkskiqdkegippdqqrlifagkqledgrtlsdyniqkestlhsvlrlrgg--<br><br>Proposed: aliSize=71 (resi) RMSD=2.40 (Å)<br>qkIVVHLRAtgGAPILkqSKFKVSGSDKFANVIDFLRRQLHSD-SLFVYVNSAFSPNPDESVIDLynNFGfdGKLVVNYACS----maw<br>--MQIFVKT--LTGKT--ITLEVESSDTIDNVKSKIQDKEGIPpDQQRLIFAGKQLEDGRTLSDY--NIQ--KESTLHSVLRlrgg---
73	1wz3A<br>(84)<br><br>2byfA<br>(116)	C	8.33	TM-align: aliSize=60 (resi) RMSD=4.12 (Å)<br>---QKIVVHLRATgGAPIlkq----SKFKVsGsDKF-ANVIdFLRRQ--------L--HS-DSLFVYVNSAFSPNPDESVidLYnNFgfdgklvvnyacs------------------MAw-----------------<br>sseEESFFVQVHD-VSPE---qpltVIKAP-R-VSTaQDVI-QQTLCkakysysiLsnPNpSDYVLLEEVVKDTTNKKTT--TP-KS-------------sqrvlldqecvfqaqskwkGA-gkfilklkeqvqasredk<br><br>SARST: aliSize=68 (resi) RMSD=9.12 (Å)<br>q-----KIVVHLR--ATGGAPILkqskFKVSGSDKFANVIDFLRRQL---------HSDSLFVYVNSAFSPNPDEsvidlynNFGFDGKLVVNYAC--------------------------------smaw<br>-sseeeSFFVQVHdvSPEQPLTV----IKAPRVSTAQDVIQQTLCKAkysysilsnPNPSDYVLLEEVVKDTTNK-------KTTTPKSSQRVLLDqecvfqaqskwkgagkfilklkeqvqasredk----<br><br>BLAST: aliSize=11 (resi) iden=9.52% (8/84) simi=13.10% (11/84)<br>qkivvhlratggapilkqskfkvsgsdkfanvidflrrqlhs----DSLFVYVNSAfSPNPDESVI---dlynnfgfdgklvvnyacsmaw<br>--ssseeESFFVQVHDV-SPEQPLTVIkaprvstaqdviqqtlckakysysilsnpnpsdyvlleevvkdttnkkttpkssqrvlldqecvfqaqskwkgagkfilklkeqvqasredk---------------------<br><br>Proposed: aliSize=76 (resi) RMSD=2.93 (Å)<br>----QKIVVHLRAtggAPILK--qSKFKVSGSDKFANVIDFLRR-Q-----LHSD----SLFVYVN-----------------sAFSPNPdESVIDLYNNFGFDgKLVVNYA-CSMA------w<br>sseeESFFVQVHD---VSPEQpl-TVIKAPRVSTAQDVIQQTLCkAkysysILSNpnpsDYVLLEEvvkdttnkkttpkssq-RVLLDQ-ECVFQAQSKWKGA-GKFILKLkEQVQasredk-
74	1wz3A<br>(84)<br><br>2jrhA<br>(93)	C	8.33	TM-align: aliSize=51 (resi) RMSD=2.70 (Å)<br>-QKIVVHLRAtgGApILkqSKFKVSGSDKFANVIDFLRRQLHSdSLFVYVNSAFSpnpdesvidlynnfgfdgklvvnyacs------------------MAW----------------------<br>qSDVRIKFEH--NG-ER--RIIAFSRPVKYEDVEHKVTTVFGQ-PLDLHYMNNEL---------------------------silllknqddldkaidildRSSsmkslrilllsqdrnlehhhhhh<br><br>SARST: aliSize=70 (resi) RMSD=12.62 (Å)<br>qki---VVHLRATGGAPIlkqsKFKVSGSDKFANVIDFLRRQLHSDSLFVYVNSAFSPNPDESVIDLYN-------NFGfdGKLVVNYA-------------csmaw<br>---qsdVRIKFEHNGERR----IIAFSRPVKYEDVEHKVTTVFGQPLDLHYMNNELSILLKNQDDLDKAidildrsSSM--KSLRILLLsqdrnlehhhhhh-----<br><br>BLAST: aliSize=11 (resi) iden=9.52% (8/84) simi=13.10% (11/84)<br>qkivvhlratggapilkqskfk--VSGSDKFANVIDFLRRQLHSDSLFVYVNS------------afspnpdesvidlynnfgfdgklvvnyacsmaw<br>----------------------qsdvrikfehngerriiafsrpvkyedvehkvttvfgqpldlhymnnelsilLKNQDDLDKAIDILDRSSSMKSLRILLLSqdrnlehhhhhh----------------------------------<br><br>Proposed: aliSize=75 (resi) RMSD=2.63 (Å)<br>QKIVVHLRATGGaPILkqSKFKVSgSDKFANVIDFLRRQLhSDSLFVYVNS---AFSPNPDESVIDLYNNfG----FDGKLVVNYACS-----------maw<br>QSDVRIKFEHNG-ERR--IIAFSR-PVKYEDVEHKVTTVF-GQPLDLHYMNnelSILLKNQDDLDKAIDI-LdrssSMKSLRILLLSQdrnlehhhhhh---

75	1wz3A (84)<br>2bpsA (81)	C	11.11	TM-align: aliSize=50 (resi) RMSD=2.72 (Å)<br>--QKIVVHLRATGGAPILkqSKFKVSGSDKFANVIDFLRR-Q-LHSDSL--FVYV----NSAfspnpdesvidlynnfgfdgklvvnyacsmaw--------------------<br>hgSYIDITIDLKHYNGSV--FDLRLSDYHPVKKVIDIAWQaQsVSMPPRegHWIRvvnkDKV--------------------------------fsgecklsdcgitngdrleil<br><br>SARST: aliSize=68 (resi) RMSD=11.52 (Å)<br>qk--IVVHLRATG----GApilkQSKFKVSGSDKFANVIDFLRRQLHSDSLFVY--VNSA----FSPNPDESvidLYNNFGFDGKLVVN-yacsmaw<br>--hgSYIDITIDLkhynGS----VFDLRLSDYHPVKKVIDIAWQAQSVSMPPREghWIRVvnkdKVFSGECK---LSDCGITNGDRLEIl-------<br><br>BLAST: aliSize=13 (resi) iden=13.58% (11/81) simi=16.05% (13/81)<br>qkivvhlratggapilkqskfkvsgsdkfanvidflrrqlhsdslfvyvnsafspnpdesv--------IDL--YNNFGFDGKL----VVNYACSMAW---<br>---hgsyiditIDLkhYNGSVFDLRLsdyhPVKKVIDIAWqaqsvsmppreghwirvvnkdkvfsgecklsdcgitngdrleil<br><br>Proposed: aliSize=62 (resi) RMSD=2.54 (Å)<br>--QKIVVHLRATGgAPILKqSKFKVSGSDKFANVIDFLRR-Q-L---------------hsdslfvyvNSAFSPNpdEsviDLYNNFGFdGKLVVNYAcsmaw<br>hgSYIDITIDLKH-YNGSV-FDLRLSDYHPVKKVIDIAWQaQsVsmppreghwirvvnk---------DKVFSGE--C---KLSDCGIT-NGDRLEIL-----
76	1wz3A (84)<br>1lfdA (87)	C	8.33	TM-align: aliSize=55 (resi) RMSD=3.47 (Å)<br>-QKIVVHLRATgGAPILKQSKFKVSGSDKFANVIDF-LrR--QL-HSD-SLFVYVNSAfspnpdesvidlynnfgfdgkl-VVNyacsm----------------AW---------<br>gDCCIIRVSLD-VDNGNMYKSILVTSQDKAPTVIRKaM-DkhNLdEDEpEDYELLQII----------------------sEDH-----klkipenanvfyamnsAAnydfilkkr<br><br>SARST: aliSize=73 (resi) RMSD=12.80 (Å)<br>q----KIVVHLRATGGapiLKQSKFKVSGSDKFANVIDFLRRQLHSD-----SLFVYVN----SAFSPNPDESVIDLYNNFGFDGKLVVN-yacsmaw<br>-gdccIIRVSLDVDNG---NMYKSILVTSQDKAPTVIRKAMDKHNLDedepeDYELLQIisedHKLKIPENANVFYAMNSAANYDFILKKr-------<br><br>BLAST: aliSize=6 (resi) iden=5.95% (5/84) simi=7.14% (6/84)<br>qkivvhlratggapilkqskfk---------------------VSGSDKFANVI--dflrrqlhsdslfvyvnsafspnpdesvidlynnfgfdgklvvnyacsmaw<br>----------------------gdcciirvsldvdngnmyksilVTSQDKAPTVIrkamdkhnldedepedyellqiisedhklkipenanvfyamnsaanydfilkkr--<br><br>Proposed: aliSize=74 (resi) RMSD=2.62 (Å)<br>-QKIVVHLRATgGAP-I-LkqSKFKVSGSDKFANVIDFLRR--QL-HSD--SLFVYV----NSAFSPNPDESVIDLYnnFGFDgKLVVNYAC-smaw<br>gDCCIIRVSLD-VDNgNmY--KSILVTSQDKAPTVIRKAMDkhNLdEDEpeDYELLQiiseDHKLKIPENANVFYAM--NSAA-NYDFILKKr----
77	1wz3A (84)<br>1wmhB (82)	C	10.98	TM-align: aliSize=52 (resi) RMSD=3.15 (Å)<br>qkIVVHLRAT-GGAPILkqSKFKVSGSDKFANVIDFLRRQLHS--DSLFVYVNSAFspnpdesvidlynn-Fgfdgklvvnyacsm----------------AW----------<br>--SIVEVKSKfDAEFRR--FALPRASVSGFQEFSRLLRAVHQIpgLDVLLGYTDAH--------------gD--------------llpltnddslhralasGPpplrllvqkr<br><br>SARST: aliSize=71 (resi) RMSD=12.16 (Å)<br>qk-IVVHLRATGGAPILkqskFKVSGSDKF--ANVIDFLRRQLHsdSLFVYVNSAFSPNPDES-------VIDLYNNFGFDGKLVVNYA-csmaw<br>--sIVEVKSKFDAEFRR----FALPRASVSgfQEFSRLLRAVHQ--IPGLDVLLGYTDAHGDLlpltnddSLHRALASGPPPLRLLVQKr-----<br><br>BLAST: aliSize=0 (resi) iden=0.00% (0/82) simi=0.00% (0/82)<br>qkivvhlratggapilkqskfkvsgsdkfanvidflrrqlhsdslfvyvnsafspnpdesvidlynnfgfdgklvvnyacsmaw--<br>--sivevkskfdaefrrfalprasvsgfqefsrllravhqipgldvllgytdahgdllpltnddslhralasgppplrllvqkr<br><br>Proposed: aliSize=74 (resi) RMSD=2.51 (Å)<br>QKIVVHLRAtggAPILkqSKFKVSGS-DK-FANVIDFLRR--QLHSDSLFVYVN----SAFSPNPDESVIDLYNnfGFDGKLVVNYACSmaw<br>SIVEVKSKF---DAEF--RRFALPRAsVSgFQEFSRLLRAvhQIPGLDVLLGYTdahgDLLPLTNDDSLHRALA--SGPPPLRLLVQKR---

78	1wz3A<br>(84)<br>2al3A<br>(76)	C	11.84	TM-align: aliSize=45 (resi) RMSD=2.43 (Å)<br>qkIVVHLRATgGaPILkqSKFKVSGSDKFANVIDFLRR-QlHSD-SLFVYV-NSAfspnpdesvidlynnfgfdgklvvnyacsmaw---------------------------<br>--SAVSVLAP-N-GRR--HTVKVTPSTVLLQVLEDTCRrQ-DFNpSEYDLKfQRT-------------------------------vldlslqwrfanlpnnaklemvpvsrsr<br><br>SARST: aliSize=68 (resi) RMSD=12.98 (Å)<br>qkiv--VHLRATGGApilkQSKFKVSGSDKFANVIDFLRRQLHS-DSLFVYVNSAFSPNPDESvidLYNNFGF--DGKLVVNYA---csmaw<br>----saVSVLAPNGR----RHTVKVTPSTVLLQVLEDTCRRQDFnPSEYDLKFQRTVLDLSLQ---WRFANLPnnAKLEMVPVSrsr-----<br><br>BLAST: aliSize=14 (resi) iden=13.16% (10/76) simi=18.42% (14/76)<br>qkivvhlratgg------APILKQSKFKVSGSDKFANVI-DFLRRQ--lhsdslfvyvnsafspnpdesvidlynnfgfdgklvvnyacsmaw<br>------------savsvlAPNGRRHTVKVTPSTVLLQVLeDTCRRQdfnpseydlkfqrtvldlslqwrfanlpnnaklemvpvsrsr---<br><br>Proposed: aliSize=69 (resi) RMSD=2.55 (Å)<br>qkIVVHLRATgGaPILkqSKFKVSGSDKFANVIDFLRRQLHSD-SLFVYV-NSaFSPNPDESVIDLynNFGfdGKLVVNYAC-----smaw<br>--SAVSVLAP-N-GRR--HTVKVTPSTVLLQVLEDTCRRQDFNpSEYDLKfQR-TVLDLSLQWRFA--NLP--NNAKLEMVPvsrsr----
79	1wz3A<br>(84)<br>1v6eA<br>(95)	C	9.52	TM-align: aliSize=49 (resi) RMSD=2.88 (Å)<br>-----QKIVVHLRAtggAPILKQSKFKVSGSDKFANVIDFLRRQLHSD--SLFVYV------NSAfspnpdesvidlynnfgfdgklvvnyacsmaw--------------------------------<br>gssgsSGVMVFISS---SLNSFRSEKRYSRSLTIAEFKCKLELVVGSPasCMELELygaddkFYS-------------------------------kldqedallgsypvddgcrihvidhsgsgpssg<br><br>SARST: aliSize=58 (resi) RMSD=14.18 (Å)<br>qkivvhlratggapilkqs--------------------KFKVSGSDKFANVIDFLRRQLHS-DSLFVYVNSA------FSP-NPDESvidLYNNFGFDGKLVVNYAC--------smaw<br>------------------gssgssgvmvfissslnsfrsEKRYSRSLTIAEFKCKLELVVGSpASCMELELYGaddkfySKLdQEDAL---LGSYPVDDGCRIHVIDHsgsgpssg----<br><br>BLAST: aliSize=8 (resi) iden=5.95% (5/84) simi=9.52% (8/84)<br>qkivvhlratggapilkqskfkvsgsdkfanvidflrrqlhsdslfvyvnsafspnpdesv---IDLYN-NFGFDGKL-------------------------------vvnyacsmaw<br>--gssgssgvmvfissslnsfrsekrysrsltiaefkcklelvvgspascmeLELYGaDDKFYSKLdqedallgsypvddgcrihvidhsgsgpssg----------<br><br>Proposed: aliSize=72 (resi) RMSD=2.43 (Å)<br>-----QKIVVHLRAtgGAPI-LkqSKFKVSGSDKFANVIDFLRRQLHSD--SLFVYV-----NSAFSP-NPDESVIDLynNFGfdGKLVVNYAC---------smaw<br>gssgsSGVMVFISS--SLNSfR--SEKRYSRSLTIAEFKCKLELVVGSPasCMELELygaddKFYSKLdQEDALLGSY--PVD--DGCRIHVIDhsgsgpssg----
80	1wz3A<br>(84)<br>2cs4A<br>(95)	C	9.52	TM-align: aliSize=51 (resi) RMSD=3.15 (Å)<br>-----QKIVVHLRAtggAPILkqSKFK-VSGSDKFANVIDFLRR-QLHSDSLFVYVNSAfspnpdesvidlynnFgfdgklvvnyacs------------------MAW-------------------<br>gssgsSGMELKVWV---DGVQ--RIVCgVTEVTTCQEVVIALAQaIGRTGRYTLIEKWR---------------D-------------terhlaphenpiislnkwGQYasdvqlilrrtgpsgpssg<br><br>SARST: aliSize=62 (resi) RMSD=11.21 (Å)<br>q-----KIVVHLRATGGAPILKQSKFkvsGSDKFANVIDFLRRQLHSDSLFVYVNS----AFSPNPDESVIDLYN------------------------nfgfdgklvvnyacsmaw<br>-gssgsSGMELKVWVDGVQRIVCGVT---EVTTCQEVVIALAQAIGRTGRYTLIEKwrdtERHLAPHENPIISLNkwgqyasdvqlilrrtgpsgpssg------------------<br><br>BLAST: aliSize=11 (resi) iden=9.52% (8/84) simi=13.10% (11/84)<br>qkivv---HLrATGGAPILKQSKFKVSGSD----------------kfanvidflrrqlhsdslfvyvnsafspnpdesvidlynnfgfdgklvvnyacsmaw<br>-----gssgssgmelkvwvdgvqrivcgvtevttcqevvialaqaigrtgrytliekwrdterHL-APHENPIISLNKWGQYASDvqlilrrtgpsgpssg---<br><br>Proposed: aliSize=73 (resi) RMSD=2.71 (Å)<br>q------KIVVHLRAtggAPILkQSKFKVSGSDKFANVIDFLRR-QLHSDSLFVYV---NSAFSPNPDESVIDLYNNF---GFDgkLVVNYAC---------smaw<br>-gssgssGMELKVWV---DGVQ-RIVCGVTEVTTCQEVVIALAQaIGRTGRYTLIEkwrDTERHLAPHENPIISLNKWgqyASD--VQLILRRtgpsgpssg----

81	1wz3A (84)<br>1vd2A (89)	C	8.33	TM-align: aliSize=51 (resi) RMSD=2.95 (Å)<br>----QKIVVHLRATgGapILkqSKFKVSGSDKFANVIDFLRR--QLH-SDSLFVYVNSAFspnpdesvidlynnfgfdgklvvnyacs--------------------MAW-----------<br>gplgSQVRVKAYYR-G--DI--MITHFEPSISFEGLCNEVRDmcSFDnEQLFTMKWIDEE----------------------------gdpctvssqleleeafrlyeLNKdsellihvfpc<br><br>SARST: aliSize=70 (resi) RMSD=11.76 (Å)<br>qk-----IVVHLRATGGAPILkqskFKVSGSDKFANVIDFLRRQLHSDSLFVYVNSAfsPNPDES------------VIDLYNNFGFDGKLVVNY--acsmaw<br>--gplgsQVRVKAYYRGDIMI----THFEPSISFEGLCNEVRDMCSFDNEQLFTMKW--IDEEGDpctvssqleleeAFRLYELNKDSELLIHVFpc------<br><br>BLAST: aliSize=0 (resi) iden=0.00% (0/84) simi=0.00% (0/84)<br>qkivvhlratggapilkqskfkvsgsdkfanvidflrrqlhsdslfvyvnsafspnpdesvidlynnfgfdgklvvnyacsmaw---<br>--gplgsqvrvkayyrgdimithfepsisfeglcnevrdmcsfdneqlftmkwideegdpctvssqleleeafrlyelnkdsellihvfpc<br><br>Proposed: aliSize=73 (resi) RMSD=2.54 (Å)<br>----QKIVVHLRATgGapI-lkqSKFKVSGSDKFANVIDFLRR--QLH-SDSLFVYVN----SAFSPNPDESVIDLYNNfG--F-DGKLVVNYAC-smaw<br>gplgSQVRVKAYYR-G--Di---MITHFEPSISFEGLCNEVRDmcSFDnEQLFTMKWIdeegDPCTVSSQLELEEAFRL-YelNkDSELLIHVFPc----
82	1zvnA (99)<br>2omwB (105)	N	28.28	TM-align: aliSize=93 (resi) RMSD=2.22 (Å)<br>sgwv-------WNQFFVLEEYTGTDPLYVGKLHSDMDRGDgSIKYILSGEGA----GIVFTIDDTTGDIHAIQRLDREERSQYTLRAQALDrRTGRPMEPESEFIIKIQD<br>----gplgswvIPPISCPENEKGEFPKNLVQIKSNRDKET-KVFYSITGQGAdkppVGVFIIERETGWLKVTQPLDREAIAKYILYSHAVS-SNGNAVEDPMEIVITVTD<br><br>SARST: aliSize=94 (resi) RMSD=3.51 (Å)<br>s----GWVWNQFFVLEEYTGTDPLYVGKLHSDMdRGDGSIKYILSGEG-----AGiVFTIDDTTGDIHAIQRLDREERSQYTLRAQALDRrTGRPMEPESEFIIKIQ-d<br>-gplgSWVIPPISCPENEKGEFPKNLVQIKSNR-DKETKVFYSITGQGadkppVG-VFIIERETGWLKVTQPLDREAIAKYILYSHAVSS-NGNAVEDPMEIVITVTd-<br><br>BLAST: aliSize=48 (resi) iden=29.29% (29/99) simi=48.48% (48/99)<br>sg-----WVWNQFFVLEEYTGTDPLYVGKLHSDMDRgDGSIKYILSGEGAGI----VFTIDDTTGDIHAIQRLDREERSQYTLRAQALDrRTGRPMEPESEFIIKIQD<br>--gplgsWVIPPISCPENEKGEFPKNLVQIKSNRDK-ETKVFYSITGQGADKppvgVFIIERETGWLKVTQPLDREAIAKYILYSHAVS-SNGNAVEDPMEIVITVTD<br><br>Proposed: aliSize=95 (resi) RMSD=1.64 (Å)<br>-S-GWVWNQ-FFVLEEYTGTDPLYVGKLHSDMDR--gdgSIKYILSGEGA----GIVFTIDDTTGDIHAIQRLDREERSQYTLRAQALDrRTGRPMEPESEFIIKIQD-<br>gPlGSWVIPpISCPENEKGEFPKNLVQIKSNRDKet---KVFYSITGQGAdkppVGVFIIERETGWLKVTQPLDREAIAKYILYSHAVS-SNGNAVEDPMEIVITVTDa
83	2oyaA (102)<br>2oy3A (98)	N	97.96	TM-align: aliSize=90 (resi) RMSD=0.71 (Å)<br>aplaqrvrimgg--------TNRGRAEVYYNNEWGTICDDDWDNNDATVFCRMLGYSRGRALSSYGGGSGNIWLDNVNCRGTENSLWDCSKNSWGNHNCVHNEDAGVECS<br>------------qrvrimggTNRGRAEVYYNNEWGTICDDDWDNNDATVFCRMLGYSRGRALSSYGGGSGNIWLDNVNCRGTENSLWDCSKNSWGNHNCVHNEDAGVECS<br><br>SARST: aliSize=95 (resi) RMSD=4.01 (Å)<br>aplaqr--VRIMGGTNRGRAEVYYNNEWGTICDDDWDNNDATVFCRMLGYSRGRALSSYGGGSGNIWLDNVNCRGTENSLWDCSKNSWGNHNCVHNEDAGVEC-s<br>------qrVRIMGGTNRGRAEVYYNNEWGTICDDDWDNNDATVFCRMLGYSRGRALSSYGGGSGNIWLDNVNCRGTENSLWDCSKNSWGNHNCVHNEDAGVECs-<br><br>BLAST: aliSize=98 (resi) iden=100.00% (98/98) simi=100.00% (98/98)<br>aplaQRVRIMGGTNRGRAEVYYNNEWGTICDDDWDNNDATVFCRMLGYSRGRALSSYGGGSGNIWLDNVNCRGTENSLWDCSKNSWGNHNCVHNEDAGVECS<br>----QRVRIMGGTNRGRAEVYYNNEWGTICDDDWDNNDATVFCRMLGYSRGRALSSYGGGSGNIWLDNVNCRGTENSLWDCSKNSWGNHNCVHNEDAGVECS<br><br>Proposed: aliSize=98 (resi) RMSD=0.45 (Å)<br>apLaQrVRIMGGTNRGRAEVYYNNEWGTICDDDWDNNDATVFCRMLGYSRGRALSSYGGGSGNIWLDNVNCRGTENSLWDCSKNSWGNHNCVHNEDAGVECS<br>--Q-R-VRIMGGTNRGRAEVYYNNEWGTICDDDWDNNDATVFCRMLGYSRGRALSSYGGGSGNIWLDNVNCRGTENSLWDCSKNSWGNHNCVHNEDAGVECS

84	2oyaA (102)<br>1by2A (112)	N	47.06	TM-align: aliSize=91 (resi) RMSD=1.47 (Å)<br>aplaqrvrimg-------------GTNRGRAEVYYNNEWGTICDDDWDNNDATVFCRMLGYSRG-RALSS-Y-GGGSGNIWLDNVNCRGTENSLWDCSKNSWGNHNCVHNEDAGVECS-----<br>-----------avndgdmrladggATNQGRVEIFYRGQWGTVCDNLWDLTDASVVCRALGFENAtQALGRaAfGQGSGPIMLDEVQCTGTEASLADCKSLGWLKSNCRHERDAGVVCTnettl<br><br>SARST: aliSize=94 (resi) RMSD=4.15 (Å)<br>aplaqr------VRIMG--GTNRGRAEVYYNNEWGTICDDDWDNNDATVFCRMLGYSRG-RALSS--YGGGSGNIWLDNVNCRGTENSLWDCSKNSWGNHNCVHNEDAGVE-------cs<br>------avndgdMRLADggATNQGRVEIFYRGQWGTVCDNLWDLTDASVVCRALGFENAtQALGRaaFGQGSGPIMLDEVQCTGTEASLADCKSLGWLKSNCRHERDAGVVctnettl--<br><br>BLAST: aliSize=61 (resi) iden=47.06% (48/102) simi=59.80% (61/102)<br>aplaqrvrim-----------GGTNRGRAEVYYNNEWGTICDDDWDNNDATVFCRMLGYSRG-RAL--SSYGGGSGNIWLDNVNCRGTENSLWDCSKNSWGNHNCVHNEDAGVECS-----<br>----------avndgdmrladgGATNQGRVEIFYRGQWGTVCDNLWDLTDASVVCRALGFENAtQALgrAAFGQGSGPIMLDEVQCTGTEASLADCKSLGWLKSNCRHERDAGVVCTnettl<br><br>Proposed: aliSize=101 (resi) RMSD=1.18 (Å)<br>-APLAQrVRIMGG-T-NRGRAEVYYNNEWGTICDDDWDNNDATVFCRMLGYSRG-RALSSY--GGGSGNIWLDNVNCRGTENSLWDCSKNSWGNHNCVHNEDAGVECS-----<br>aVNDGD-MRLADGgAtNQGRVEIFYRGQWGTVCDNLWDLTDASVVCRALGFENAtQALGRAafGQGSGPIMLDEVQCTGTEASLADCKSLGWLKSNCRHERDAGVVCTnettl
85	1v9yA (103)<br>2pd7A (149)	N	15.53	TM-align: aliSize=97 (resi) RMSD=2.97 (Å)<br>----------GIFFp------------------ALeqNmMGAVLINE-N--DEVMFFNPAAEKLWGYKREEVIGNNIDMLiPR---DLR--P------A--HPEYIrHN-RE-----RELQLEKKDGSKIWTRFALSKVSA-E-GKVYYLALVRD<br>mhtlyapggyDIMG-yliqimnrpnpqvelgpvDT--S-CALILCDLkQkdTPIVYASEAFLYMTGYSNAEVLGRNCRFL-QSpdgMVKpkStrkyvdSntINTMR-KAiDRnaevqVEVVNFKKNGQRFVNFLTMIPVRDeTgEYRYSMGFQCE<br><br>SARST: aliSize=92 (resi) RMSD=4.38 (Å)<br>giffpaleqn----------------------------------MMGAVLINEN---DEVMFFNPAAEKLWGYKREEVIGNNIDMLIPRDL------------RPAHPEYIRHNRERE-----LQLEKKDGSKIWTRFALSKVSAE-GKVYYLALVR--d<br>----------mhtlyapggydimgyliqimnrpnpqvelgpvdtSCALILCDLKqkdTPIVYASEAFLYMTGYSNAEVLGRNCRFLQSPDGmvkpkstrkyvdSNTINTMRKAIDRNAevqveVVNFKKNGQRFVNFLTMIPVRDEtGEYRYSMGFQce-<br><br>BLAST: aliSize=41 (resi) iden=18.45% (19/103) simi=39.81% (41/103)<br>giffpaleqnmmgavl---INENDE-VMFFNPAAEKLWGYKREEVIGNNIDMLIPRD--LRP-AHPEYIRHNRERELQLEKKDGSKIWTRFALSKVSAEGKVYYLAL--VRD--------------<br>---------------mhtlyapggydimgyliqimnrpnpqvelgpvdtscalilcdLKQKDTpIVYASEAFLYMTGYSNAEVLGRNCRFLQSPDgmVKPkSTRKYVDSNTINTMRKAIDRNAEVQVEVVNFKKNGQRFVNFLTMipVRDetgeyrysmgfqce<br><br>Proposed: aliSize=99 (resi) RMSD=2.15 (Å)<br>------------GIFFPALEQ------------NMMGAVLINE---NDEVMFFNPAAEKLWGYKREEVIGNNIDMLiPR------------DL-rpA--HpEYIRHN-RE-----RELQLEKKDGSKIWTRFALSKVSA-E-GKVYYLALVRDh<br>mhtlyapggydiMGYLIQIMNrpnpqvelgpvdTSCALILCDLkqkDTPIVYASEAFLYMTGYSNAEVLGRNCRFL-QSpdgmvkpkstrkYVd--SntI-NTMRKAiDRnaevqVEVVNFKKNGQRFVNFLTMIPVRDeTgEYRYSMGFQCE-
86	1v9yA (103)<br>1f98A (125)	N	14.56	TM-align: aliSize=97 (resi) RMSD=2.16 (Å)<br>---------------GIFF--PaLEQNMMGAVLINENDEVMFFNPAAEKLWGYKREEVIGNNID--MLIPrdLRPA-HPEYIRHNRE-------RELQLEKkDGSKIWTRFALSKVSAegKVYYLALVRD-<br>mehvafgsedientlAKMDdgQ-LDGLAFGAIQLDGDGNILQYNAAEGDIVGRDPKQVIGKNFFkdVAPC--TDSPeFYGKFKEGVAsgnlntmFEYTFDY-QMTPTKVKVHMKKALS--GDSYWVFVKRv<br><br>SARST: aliSize=86 (resi) RMSD=13.35 (Å)<br>gif-------------------FPALEQNMMGAVLINENDEVMFFNPAAEKLWGYKREEVIGNNI-----DMLIPRDLRPAHPEYIrhnrerelqlekKDGSKIWTRFALSKVSAEGKVYYLALV---------------rd<br>---mehvafgsedientlakmdDGQLDGLAFGAIQLDGDGNILQYNAAEGDIVGRDPKQVIGKNFfkdvaPCTDSPEFYGKFKEGV------------ASGNLNTMFEYTFDYQMTPTKVKVHMKkalsgdsywvfvkrv--<br><br>BLAST: aliSize=26 (resi) iden=12.62% (13/103) simi=25.24% (26/103)<br>giffpaleqnmm----------------------------GAVLINENDEVMFFNPAAEKLWGYKREEVIGNNIDmlipRDLRPA--HPEY--irhnrerelqlekkdgskiwtrfalskvsaegkvyylalvrd<br>------------mehvafgsedientlakmddgqldglafGAIQLDGDGNILQYNAAEGDIVGRDPKQVIGKNFF----KDVAPCtdSPEFygkfkegvasgnlntmfeytfdyqmtptkvkvhmkkalsgdsywvfvkrv--<br><br>Proposed: aliSize=99 (resi) RMSD=1.93 (Å)<br>--------GIFFPA--------LEQNMMGAVLINENDEVMFFNPAAEKLWGYKREEVIGNNID-MLIPRdLRPA-HPEYIRHNRE-------RELQLEKkDGSKIWTRFALSKVSAegKVYYLALVRD-h<br>mehvafgsEDIENTlakmddgqLDGLAFGAIQLDGDGNILQYNAAEGDIVGRDPKQVIGKNFFkDVAPC-TDSPeFYGKFKEGVAsgnlntmFEYTFDY-QMTPTKVKVHMKKALS--GDSYWVFVKRv-

87	1v9yA (103)<br>1d7eA (119)	N	16.50	TM-align: aliSize=94 (resi) RMSD=2.05 (Å)<br>g-------------IFF-PALEQNMMGAVLINENDEVMFFNPAAEKLWGYKREEVIGNNID--MLIPrdLRPA-HPEYIRHNRE-------RELQLEKKDgSKIWTRFALSKVSaegkvYYLALVRD-<br>-vafgsedientlaKMDdGQLDGLAFGAIQLDGDGNILQYNAAEGDITGRDPKQVIGKNFFkdVAPC--TDSPeFYGKFKEGVAsgnlntmFEYTFDYQM-TPTKVKVHMKKAL-----SYWVFVKRv<br><br>SARST: aliSize=97 (resi) RMSD=5.75 (Å)<br>gif----------------FPALEQNMMGAVLINENDEVMFFNPAAEKLWGYKREEVIGNNI-----DMLIPRDLRPAHPEYIRHNRERELQLEKKDGSKIWTRFALSKVSAEGKVYYLAL-vrd<br>---vafgsedientlakmdDGQLDGLAFGAIQLDGDGNILQYNAAEGDITGRDPKQVIGKNFfkdvaPCTDSPEFYGKFKEGVASGNLNTMFEYTFDYQMTPTKVKVHMKKALSYWVFVKRv---<br><br>BLAST: aliSize=26 (resi) iden=12.62% (13/103) simi=25.24% (26/103)<br>giffpaleqnmm-------------------------GAVLINENDEVMFFNPAAEKLWGYKREEVIGNNIDmlipRDLRPA--HPEY---irhnrerelqlekkdgskiwtrfalskvsaegkvyylalvrd<br>------------vafgsedientlakmddgqldglafGAIQLDGDGNILQYNAAEGDITGRDPKQVIGKNFF----KDVAPCtdSPEFygkfkegvasgnlntmfeytfdyqmtptkvkvhmkkalsywvfvkrv--<br><br>Proposed: aliSize=96 (resi) RMSD=2.12 (Å)<br>G------IFFP-A------LEQNMMGAVLINENDEVMFFNPAAEKLWGYKREEVIGNNID-MLIPRdLRPA-HPEYIRHNRE-------RELQLEKKDgSKIWTRFALSKVSaegkvYYLALVRD-h<br>VafgsedIENTlAkmddgqLDGLAFGAIQLDGDGNILQYNAAEGDITGRDPKQVIGKNFFkDVAPC-TDSPeFYGKFKEGVAsgnlntmFEYTFDYQM-TPTKVKVHMKKAL-----SYWVFVKRv-
88	1lgqA (112)<br>1gxcA (116)	C	24.11	TM-align: aliSize=70 (resi) RMSD=2.32 (Å)<br>mqPWGRLLRLGAEegEPHVLLRKREWTIGRRRGCDLSFP----S----NKLVSGDHCRIVVDE---KSGQVTLEDTSTSGTVINKlkvvkkqtcplqtgdviylvyrknepehnvaylyesls-----------------------------------<br>--PWARLWALQDG--FANLECVNDNYWFGRDKSCEYCFDepllKrtdkYRTYSKKHFRIFREVgpkNSYIAYIEDHSGNGTFVNT--------------------------------------elvgkgkrrplnnnseialslsrnkvfvffdltvd<br><br>SARST: aliSize=100 (resi) RMSD=12.95 (Å)<br>mqpwg--RLLRLGAEeGEPHVLLRKREWTIGRRRGCDLSFpSN---------KLVSGDHCRIVV-DEKSG--QVTLEDTSTSGTVINKLKVVKKQTCPLQTGDVIYlvyrKNEPEHNVAYLYESL--s<br>-----pwARLWALQD-GFANLECVNDNYWFGRDKSCEYCF-DEpllkrtdkyRTYSKKHFRIFReVGPKNsyIAYIEDHSGNGTFVNTELVGKGKRRPLNNNSEIA----LSLSRNKVFVFFDLTvd-<br><br>BLAST: aliSize=49 (resi) iden=28.57% (32/112) simi=43.75% (49/112)<br>mqPWGRLLRLgaEEGEPHV-LLRKREWtIGRRRGCDLSF--P----SNK--LVSGDHCRIV--VDEKSGQVT-LEDTSTSGTVINKLKVVKKQTCPLQTGDVIYLVYRKNE----------pehnvaylyesls<br>--PWARLWAL--QDGFANLeCVNDNYW-FGRDKSCEYCFdePllkrTDKyrTYSKKHFRIFreVGPKNSYIAyIEDHSGNGTFVNTELVGKGKRRPLNNNSEIALSLSRNKvfvffdltvd-------------<br><br>Proposed: aliSize=100 (resi) RMSD=1.75 (Å)<br>mqPWGRLLRLGA--eegePHVLLRKREWTIGRRRGCDLSFP----S----NKLVSGDHCRIVVDE---KSGQVTLEDTSTSGTVINkLKVVK-KQTCPLQTGDVIYLVYRKnepehNVAYLYESLS--<br>--PWARLWALQDgf----ANLECVNDNYWFGRDKSCEYCFDepllKrtdkYRTYSKKHFRIFREVgpkNSYIAYIEDHSGNGTFVN-TELVGkGKRRPLNNNSEIALSLSR-----NKVFVFFDLTvd
89	1lgqA (112)<br>1dmzA (158)	C	16.96	TM-align: aliSize=72 (resi) RMSD=2.90 (Å)<br>-MQPWGRLLRLGAeeg---EPHVLLR--KREWTIGRRRGCDLSFpSNKLVSGDHCRIVVDEK-------------SGQVTLEDTSTSGTVINK-----------Lkvvkkqt---------------------------------------Cplqtgdviylvyrknepehnvaylyesls---------<br>gNGRFLTLKPLPD---siiQESLEIQqgVNPFFIGRSEDCNCKI-EDNRLSRVHCFIFKKRHavgksmyespaqgLDDIWYCHTGTNVSYLNNnrmiqgtkfllQ-------dgdeikiiwdknnkfvigfkveindttglfneglgmlqeQ-----------------------------rvvlkqtae<br>--------<br>ekdlvkkl<br><br>SARST: aliSize=89 (resi) RMSD=16.86 (Å)<br>mqpw-----------------GRLLRLGaeeGEPHVllrkrewTIGRRRGCDLSFpSNKLVSGDHCRIVVDeksGQVTLEDT---STSGTVINKLK-------------VVKKQTCPLQTGDVIYLVYRknePEHNVAYLYES------------------------------------ls<br>----gngrfltlkplpdsiiqESLEIQQ---GVNPF-------FIGRSEDCNCKI-EDNRLSRVHCFIFKK---RHAVGKSMyesPAQGLDDIWYChtgtnvsylnnnrMIQGTKFLLQDGDEIKIIWD---KNNKFVIGFKVeindttglfneglgmlqeqrvvlkqtaeekdlvkkl--<br><br>BLAST: aliSize=39 (resi) iden=21.43% (24/112) simi=34.82% (39/112)<br>mqpwgrllrlgaeegephvllrkrewt------------------------------IGRRRGCDLSFPSNKLvSGDHC-----RIVVDE---KSGQVTLED-----TSTSGTVINKLKVVKKQTCPLQTGDVIYLVYRKN---epeh<br>---------------------------gngrfltlkplpdsiiqesleiqqgvnpffIGRSEDCNCKIEDNRL-SRVHCfifkkRHAVGKsmyESPAQGLDDiwychTGTNVSYLNNNRMIQGTKFLLQDGDEIKIIWDKNnkfvigfkveindttglfneglgmlqeqrvvlkqtaeekdlvkkl----<br>nvaylyesls<br>----------<br><br>Proposed: aliSize=104 (resi) RMSD=2.14 (Å)<br>m--QPWGRLLRLGA---eegEPHVLLR--KREWTIGRRRGCDLSFPsNKLVSGDHCRIVVDeKS--------------GQVTLEDTSTSGTVINKLKVVKKQTCPLQTGDVIYLVYRkNE-pEHNVAYLYESL-S-------------------------------<br>-gnGRFLTLKPLPDsii---QESLEIQqgVNPFFIGRSEDCNCKIE-DNRLSRVHCFIFKK-RHavgksmyespaqglDDIWYCHTGTNVSYLNNNRMIQGTKFLLQDGDEIKIIWD-KNn-KFVIGFKVEINdTtglfneglgmlqeqrvvlkqtaeekdlvkkl

90	1lgqA (112)<br>2cswA (145)	C	24.11	TM-align: aliSize=71 (resi) RMSD=2.46 (Å)<br>------------M-QPWGRLLRLGAeeGEPHVLLR-KREWTIGRRRGCDLSFPS---NKLVSGDHCRIVVDeKSGQVTLEDT-STSGTVINKlkvvkkqtcplqtgdviylvyrknepehnvaylyesls--<br>gssgssgvtgdrAgGRSWCLRRVGM--SAGWLLLEdGCEVTVGRGFGVTYQLVSkicPLMISRNHCVLKQN-PEGQWTIMDNkSLNGVWLNR--------------------------------------arleplrvysihqgdyiqlgvplenkenaeyeyevteedwetiypclspksgpssg<br><br>SARST: aliSize=103 (resi) RMSD=12.95 (Å)<br>mqpwg------------------RLLRLGAEEGEPHVLLRKrEWTIGRRRGCDLSF---PSNKLVSGDHCRIVVDEKSGQVTLEDTSTSGTVINKLKVVKKQTCPLQTGDVIYLVYRKNEPE--HNVAYLYE-------------------sls<br>-----gssgssgvtgdraggrswCLRRVGMSAGWLLLEDGC-EVTVGRGFGVTYQLvskICPLMISRNHCVLKQNPEGQWTIMDNKSLNGVWLNRARLEPLRVYSIHQGDYIQLGVPLENKEnaEYEYEVTEedwetiypclspksgpssg---<br><br>BLAST: aliSize=54 (resi) iden=28.57% (32/112) simi=48.21% (54/112)<br>mqpwgr-------------------LLRLGAEEGepHVLLRKR-EWTIGRrrGCDLSFP-SNK----LVSGDHCrIVVDEKSGQVTLEDT-STSGTVINKLKVVKKQTCPLQTGDVIYL-VYRKNepEHNVAYLYE----------------------sls<br>------gssgssgvtgdraggrswcLRRVGMSAG--WLLLEDGcEVTVGR--GFGVTYQlVSKicplMISRNHC-VLKQNPEGQWTIMDNkSLNGVWLNRARLEPLRVYSIHQGDYIQLgVPLEN--KENAEYEYEvteedwetiypclspksgpssg---<br><br>Proposed: aliSize=102 (resi) RMSD=1.89 (Å)<br>mq----------------PwGRLLRLGAeeGEPHVLLR-KREWTIGRRRGCDLSFPS---NKLVSGDHCRIVVDeKSGQVTLED-TSTSGTVINkLKVVKK-QTCPLQTGDVIYLVYR---kNEPeHNVAYLYESL------------------s<br>--gssgssgvtgdraggrS-WCLRRVGM--SAGWLLLEdGCEVTVGRGFGVTYQLVSkicPLMISRNHCVLKQN-PEGQWTIMDnKSLNGVWLN-RARLEPlRVYSIHQGDYIQLGVPlen-KEN-AEYEYEVTEEdwetiypclspksgpssg-
91	1lgqA (112)<br>1r21A (100)	C	28.00	TM-align: aliSize=71 (resi) RMSD=2.75 (Å)<br>m-QPWGRLLRL-GAEEGEPHVLLRKREWTIGRRRGCDLSFpSNKLVSGDHCRIVVDEksGQVTLEDTSTS-GTVINKlkvvkkqtcplQtgdviylvyrknepehnvaylyesls--------------------------<br>-mWPTRRLVTIkRSGVDGPHFPLSLSTCLFGRGIECDIRI-QLPVVSKQHCKIEIHE--QEAILHNFSSTnPTQVNG-----------S--------------------------videpvrlkhgdvitiidrsfryene<br><br>SARST: aliSize=87 (resi) RMSD=11.85 (Å)<br>mqpwgrllr------------LGAEEgePHVLLRKREWTIGRRRGCDLSFpSNKLVSGDHCRIVVDEKsgQVTLEDTSTSGTVInKLKVVKKQTCPLQTGDVIYlvyrknePEHNVAYLYE-sls<br>---------mwptrrlvtikrSGVDG--PHFPLSLSTCLFGRGIECDIRI-QLPVVSKQHCKIEIHEQ--EAILHNFSSTNPTQ-VNGSVIDEPVRLKHGDVIT-------IIDRSFRYENe---<br><br>BLAST: aliSize=46 (resi) iden=29.00% (29/100) simi=46.00% (46/100)<br>MQPWGRLLRL--GAEEGePHVLLRKREWTIGRRRGCDLSFPSnKLVSGDHCRIVVDEKSGqVTLEDTSTSGTVINKlKVVkKQTCPLQTGDVIYLVYR-------knepehnvaylyesls<br>MWPTRRLVTIkrSGVDG-PHFPLSLSTCLFGRGIECDIRIQL-PVVSKQHCKIEIHEQEA-ILHNFSSTNPTQVNG-SVI-DEPVRLKHGDVITIIDRsfryene----------------<br><br>Proposed: aliSize=96 (resi) RMSD=1.98 (Å)<br>m-QPWGRLLRLGA--EEgEPHVLLRKREWTIGRRRGCDLSFpSNKLVSGDHCRIVVDEksGQVTLEDTST-SGTVINkLKVVKkQTCPLQTGDVIYLVyrknepehNVAYLYESLs<br>-mWPTRRLVTIKRsgVD-GPHFPLSLSTCLFGRGIECDIRI-QLPVVSKQHCKIEIHE--QEAILHNFSStNPTQVN-GSVID-EPVRLKHGDVITII--------DRSFRYENE-
92	1lgqA (112)<br>1uhtA (118)	C	21.43	TM-align: aliSize=67 (resi) RMSD=2.36 (Å)<br>m---------QPWGRLLRLGAeeg---EPHVLLR-KREWTIGRRRG-CDLSFpSNKLVSGDHCRIVVDEksGQVTLEDT-STSGTVINKlkvvkkqtcplqtgdviylvyrknepehnvaylyesls------------------------------------<br>-gssgssgmvTPSLRLVFVKG---preGDALDYKpGSTIRVGRIVRgNEIAI-KDAGISTKHLRIESDS--GNWVIQDLgSSNGTLLNS--------------------------------------naldpetsvnlgdgdviklgeytsilvnfvsgpssg<br><br>SARST: aliSize=85 (resi) RMSD=14.13 (Å)<br>mqpw-------------------------GRLLRLGAEEGEPHVLLRKREWTIgrrrgcdlsfpsNKLVSGDHCRIVVDEKsgQVTLEDT-STSGTVINKLKVVKKQTCPLQTGDVIYlvyrKNEPehnVAYLYES-------ls<br>----gssgssgmvtpslrlvfvkgpregdALDYKPGSTIRVGRIVRGNEIAIK------------DAGISTKHLRIESDSG--NWVIQDLgSSNGTLLNSNALDPETSVNLGDGDVIK----LGEY---TSILVNFvsgpssg--<br><br>BLAST: aliSize=37 (resi) iden=19.64% (22/112) simi=33.04% (37/112)<br>mqpwgrllrlgaeegephvllrkrewt------------------------------------IGRR-RGCDLSFpSNKLVSGDHCRIVVDekSGQVTLEDT-STSGTVINKLKVVKKQTCPLQTGDVIYL-----------------vyrknepehnvaylyesls<br>---------------------------gssgssgmvtpslrlvfvkgpregdaldykpgstirVGRIvRGNEIAI-KDAGISTKHLRIESD--SGNWVIQDLgSSNGTLLNSNALDPETSVNLGDGDVIKLgeytsilvnfvsgpssg-------------------<br><br>Proposed: aliSize=96 (resi) RMSD=1.70 (Å)<br>mq----------PWGRLLRLG---AeegEPHVLLR-KREWTIGRRRG-CDLSFpSNKLVSGDHCRIVVDEksGQVTLEDT-STSGTVINkLKVVKK-QTCPLQTGDVIYLVyrknepeHNVAYLYESLS-----<br>--gssgssgmvtPSLRLVFVKgprE---GDALDYKpGSTIRVGRIVRgNEIAI-KDAGISTKHLRIESDS--GNWVIQDLgSSNGTLLN-SNALDPeTSVNLGDGDVIKLG-------EYTSILVNFVSgpssg

93	1lgqA (112)<br>2jpeA (132)	C	17.86	TM-align: aliSize=73 (resi) RMSD=2.85 (Å)<br>----------------------MQPWGRLLRLGAe-EGEPHVLL-RKREWTIGRRRG-CDLSFpSNKLVSGDHCRIVVDEKSGQVTLEDT-STSGTVINKLkvvkkqtcplqtgdviylvyrknepehnvaylyesls---------------------------------<br>maaavnsgsslplfdcptwagkPPPGLHLDVVKG-dKLIEKLIIdEKKYYLFGRNPDlCDFTI-DHQSCSRVHAALVYHKHLKRVFLIDLnSTHGTFLGHI-------------------------------------rlephkpqqipidstvsfgastraytlrekpqt<br><br>SARST: aliSize=104 (resi) RMSD=12.50 (Å)<br>m----------------------QPWGRLLRL-GAEEGEPHVLLRKREWTIGRRRGCDLSFPSNKLVSGDHCRIVVDEKSGQVTLE-DTSTSGTVINKLKVVKKQTCPLQTGDviylVYRKnEPEHNVAYLYES---ls<br>-maaavnsgsslplfdcptwagkpPPGLHLDVVkGDKLIEKLIIDEKKYYLFGRNPDLCDFTIDHQSCSRVHAALVYHKHLKRVFLIdLNSTHGTFLGHIRLEPHKPQQIPIDS----TVSF-GASTRAYTLREKpqt--<br><br>BLAST: aliSize=35 (resi) iden=17.86% (20/112) simi=31.25% (35/112)<br>mqpwgrllrlgaeeg-------------------------------------EPHVLLRKREWTIGRRRG-CDLSFpSNKLVSGDHCRIVVDEKSGQVTLED-TSTSGTVIN--KLKVVKKQTCPLQT-------------------gdviylvyrknepehnvaylyesls<br>--------------maaavnsgsslplfdcptwagkpppglhldvvkgdkliEKLIIDEKKYYLFGRNPDlCDFTI-DHQSCSRVHAALVYHKHLKRVFLIDlNSTHGTFLGhiRLEPHKPQQIPIDStvsfgastraytlrekpqt-------------------------<br><br>Proposed: aliSize=101 (resi) RMSD=2.27 (Å)<br>--------------------M-QPWGRLLRL---G-AeegEpHVLL-RKREWTIGRRR-GCDLSFpSNKLVSGDHCRIVVDEKSGQVTLEDT-STSGTVINKLKVVKKQTCPLQTGDVIYLVYrknepeHNVAYLYESLS--<br>maaavnsgsslplfdcptwagKpPPGLHLDVVkgdKlI---E-KLIIdEKKYYLFGRNPdLCDFTI-DHQSCSRVHAALVYHKHLKRVFLIDLnSTHGTFLGHIRLEPHKPQQIPIDSTVSFGA------STRAYTLREKPqt
94	1lgqA (112)<br>1wlnA (120)	C	14.29	TM-align: aliSize=70 (resi) RMSD=2.36 (Å)<br>------MQ-PWGRLLRLG-----AEEGEPHVLLRKREWTIGRRrGC---DLSFPsNKLVSGDHCRIVVDekSGQVTLEDTSTS-GTVINKlkvvkkqtcplqtgdviylvyrknepehnvaylyesls----------------------------------<br>gssgssGPeKLPYLVELSpdgsdSRDKPKLYRLQLSVTEVGTE-KFddnSIQLF-GPGIQPHHCDLTNM--DGVVTVTPRSMDaETYVDG--------------------------------------qrisettmlqsgmrlqfgtshvfkfvdpsgpssg<br><br>SARST: aliSize=97 (resi) RMSD=12.25 (Å)<br>mqpw----------GRLLRL----GAEEG--EPHVLLRKREWTIGRRRGCDL-SFPSNKLVSGDHCRIVVDEksGQVTLEDTSTSGTVINkLKVVKKQTCPLQTGDVIYlvyRKNEpehnVAYLYESL------s<br>----gssgssgpekLPYLVElspdGSDSRdkPKLYRLQLSVTEVGTEKFDDNsIQLFGPGIQPHHCDLTNMD--GVVTVTPRSMDAETYV-DGQRISETTMLQSGMRLQ---FGTS----HVFKFVDPsgpssg-<br><br>BLAST: aliSize=19 (resi) iden=9.82% (11/112) simi=16.96% (19/112)<br>mqpwgrllrlgaeegephvllrkrewtigrrrgcdlsfpsnklvsgd--HCRIVVdeKSGQVTLEDTSTSG-TVINKLKVvkKQTCPLQTG----------------------dviylvyrknepehnvayl<br>---gssgssgpeklpylvelspdgsdsrdkpklyrlqlsvtevgtekfddnsiqlfgpgiqphHCDLTN--MDGVVTVTPRSMDAeTYVDGQRI--SETTMLQSGmrlqfgtshvfkfvdpsgpssg-------------------<br><br>yesls<br>-----<br><br>Proposed: aliSize=100 (resi) RMSD=1.97 (Å)<br>-------MQPWGRLLRLG-----AEEGEPHVLLRKREWTIGRRRGC--DLSFpSNKLVSGDHCRIVVDEksGQVTLEDTSTS-GTVINkLKVVKkQTCPLQTGDVIYLVYrknepehNVAYLYESLS-----<br>gssgssgPEKLPYLVELSpdgsdSRDKPKLYRLQLSVTEVGTEKFDdnSIQL-FGPGIQPHHCDLTNMD--GVVTVTPRSMDaETYVD-GQRIS-ETTMLQSGMRLQFGT-------SHVFKFVDPSgpssg
95	1lgqA (112)<br>2g1lA (103)	C	17.48	TM-align: aliSize=70 (resi) RMSD=2.78 (Å)<br>mqPWGRLLRLGAEEg----EPHVLLRKREWTIGRRrGCDLSFpSNKLVSGDHCRIVVDEKS---GQVTLEDTSTSGTVINKlkvvkkqtcplQtgdviylvyrknepehnvaylyesls--------------------------<br>--STPHLVNLNEDP-lmseCLLYHIKDGVTRVGQV-DMDIKL-TGQFIREQHCLFRSIPQPdgeVVVTLEPCEGAETYVNG-----------K--------------------------lvteplvlksgnrivmgknhvfrfnh<br><br>SARST: aliSize=75 (resi) RMSD=15.48 (Å)<br>mqpwgrllrlgaeegeph------------------VLLRKREWTIGRRRgCDLSFpSNKLVSGDHCRIvvdeksgqvtLEDTSTSGTVINKLKVVKKQTCpLQTGDVIY--------LVYRKNEPehnVAYLYE-sls<br>-----------------stphlvnlnedplmsecllYHIKDGVTRVGQVD-MDIKL-TGQFIREQHCLF----------RSIPQPDGEVVVTLEPCEGAET-YVNGKLVTeplvlksgNRIVMGKN---HVFRFNh---<br><br>BLAST: aliSize=30 (resi) iden=16.50% (17/103) simi=29.13% (30/103)<br>mqpwgrllrlgaeegephvllrkrewtigrrrgcdlsfp--------------------------------------SNKLVSGDHCRI-VVDEKSGQV--TLEDTSTSGTVINKlKVVKKQTCpLQTGDVIylVYRKN-------epehnvaylyesls<br>---------------------------------------stphlvnlnedplmsecllyhikdgvtrvgqvdmdiklTGQFIREQHCLFrSIPQPDGEVvvTLEPCEGAETYVNG-KLVTEPLV-LKSGNRI--VMGKNhvfrfnh--------------<br><br>Proposed: aliSize=93 (resi) RMSD=1.75 (Å)<br>mqp-WGRLLRLGAE------egePHVLLRKREWTIGRRrGCDLSFpSNKLVSGDHCRIVVDEKS---GQVTLEDTSTSGTVINkLKVVKkQTCPLQTGDVIYLVYrknepehNVAYLYESls<br>---sTPHLVNLNEDplmsec---LLYHIKDGVTRVGQV-DMDIKL-TGQFIREQHCLFRSIPQPdgeVVVTLEPCEGAETYVN-GKLVT-EPLVLKSGNRIVMGK-------NHVFRFNH--

96	1lgqA (112)<br>2pieA (132)	C	24.11	TM-align: aliSize=72 (resi) RMSD=2.74 (Å)<br>m------QPWGRLLRLGAeeGEPHVLLR-KREWTIGRRRGCDLSFPS---NKLVSGDHCRIVVDeKSGQVTLEDT-STSGTVINK-------------LKvvkkqtcplqtgdviylvyrknepehnvaylyesls------------------------------------<br>-gahmagGRSWCLRRVGM--SAGWLLLEdGCEVTVGRGFGVTYQLVSkicPLMISRNHCVLKQN-PEGQWTIMDNkSLNGVWLNRarleplrvysihqGD-----------------------------------yiqlgvplenkenaeyeyevteedwetiypclspkn<br><br>SARST: aliSize=91 (resi) RMSD=11.32 (Å)<br>mqpwg----------RLLRLGAEEGEPHVLLRKrEWTIGRRRGCDLSF---PSNKLVSGDHCRIVVDEkSGQVTLEDT-STSGTVINKLKVVKKQTCPLQTGD-VIYLVYRKN--------------------------epehnvaylyesls<br>-----gahmaggrswCLRRVGMSAGWLLLEDGC-EVTVGRGFGVTYQLvskICPLMISRNHCVLKQNP-EGQWTIMDNkSLNGVWLNRARLEPLRVYSIHQGDyIQLGVPLENkenaeyeyevteedwetiypclspkn--------------<br><br>BLAST: aliSize=54 (resi) iden=28.57% (32/112) simi=48.21% (54/112)<br>mqpwgr-----------LLRLGAEEGepHVLLRKR-EWTIGRrrGCDLSFP-SNK----LVSGDHCrIVVDEKSGQVTLEDT-STSGTVINKLKVVKKQTCPLQTGDVIYL-VYRKNepEHNVAYLYE------------------sls<br>------gahmaggrswcLRRVGMSAG--WLLLEDGcEVTVGR--GFGVTYQlVSKicplMISRNHC-VLKQNPEGQWTIMDNkSLNGVWLNRARLEPLRVYSIHQGDYIQLgVPLEN--KENAEYEYEvteedwetiypclspkn---<br><br>Proposed: aliSize=103 (resi) RMSD=2.02 (Å)<br>mq--------PwGRLLRLGAeeGEPHVLLR-KREWTIGRRRGCDLSFPS---NKLVSGDHCRIVVDeKSGQVTLEDT-STSGTVINkLKVVKK-QTCPLQTGDVIYLVYR--kNEPEHNVAYLYESL-------------s<br>--gahmaggrS-WCLRRVGM--SAGWLLLEdGCEVTVGRGFGVTYQLVSkicPLMISRNHCVLKQN-PEGQWTIMDNkSLNGVWLN-RARLEPlRVYSIHQGDYIQLGVPle-NKENAEYEYEVTEEdwetiypclspkn-
97	1lgqA (112)<br>2eh0A (130)	C	18.75	TM-align: aliSize=76 (resi) RMSD=3.06 (Å)<br>m-----QPWGRLLRL-GAEEG--EPHVLLRKREWTIGRRR---GCDLSFpSNKLVSGDHCRIVVDEKS---GQVTLEDTSTSGTVINK-----------LKv----------------------------VKkqtcplqtgdviylvyrknepehnvaylyesls<br>-gssgsSGTPHLVNLnEDPLMseCLLYYIKDGITRVGQADaerRQDIVL-SGAHIKEEHCIFRSERSNsgeVIVTLEPCERSETYVNGkrvsqpvqlrsGN-riimgknhvfrfnhpeqaraerektsgpsSG--------------------------------<br><br>SARST: aliSize=93 (resi) RMSD=11.88 (Å)<br>mq-----PWGRLLRLGAEEGEP----HVLLRKREWTIGrRRGCDLSFP---SNKLVSGDHCRIVV-DEKSGQVTLEDTSTSGTVInklkvvkkqtcpLQTGDvIYLVYRKN-------EPEHNVAYLYE-----------------sls<br>--gssgsSGTPHLVNLNEDPLMseclLYYIKDGITRVG-QADAERRQDivlSGAHIKEEHCIFRSeRSNSGEVIVTLEPCERSET------------YVNGK-RVSQPVQLrsgnriiMGKNHVFRFNHpeqaraerektsgpssg---<br><br>BLAST: aliSize=34 (resi) iden=20.54% (23/112) simi=30.36% (34/112)<br>mqpwgrllrlgaeegephvllrkrewtigrrrgcdlsfpsnkl--VSGDHCrIVVDEKS--GQV--TLEDTSTSGTVINKLKVvkKQTCPLQTGDVIYL----VYRKNEPE---------------hnvaylyesls<br>---gssgssgtphlvnlnedplmsecllyyikdgitrvgqadaerrqdivlsgahIKEEHC-IFRSERSnsGEVivTLEPCERSETYVNGKRV--SQPVQLRSGNRIIMgknhVFRFNHPEqaraerektsgpssg----------<br><br>Proposed: aliSize=98 (resi) RMSD=1.94 (Å)<br>mq------PWGRLLRL-G-AE---egEPHVLLRKREWTIGRRR---GCDLSFpSNKLVSGDHCRIVVDEKS---GQVTLEDTSTSGTVINkLKVVKkQTCPLQTGDVIYLVYrknepehNVAYLYESLS--------------<br>--gssgssGTPHLVNLnEdPLmse--CLLYYIKDGITRVGQADaerRQDIVL-SGAHIKEEHCIFRSERSNsgeVIVTLEPCERSETYVN-GKRVS-QPVQLRSGNRIIMGK-------NHVFRFNHPEqaraerektsgpssg
98	1lgqA (112)<br>1yj5C (105)	C	16.19	TM-align: aliSize=68 (resi) RMSD=3.54 (Å)<br>mq--PWGRLLRLgAEeGEPHVLLRK--REWTIGRRRgcdLSFPSNKLVSGDHCRIVVDEKSGQVTLEDTSTSGTVINKl-----------Kvvkkqtcplqtgdviylvyrknepehnvaylyesls---------------------<br>--srGRLWLQSP-TG-GPPPIFLPSdgQALVLGRGP---LTQVTDRKCSRNQVELIADPESRTVAVKQLGVNPSTVGV-helkpglsgslS------------------------------------lgdvlylvnglypltlrweels<br><br>SARST: aliSize=99 (resi) RMSD=14.23 (Å)<br>mqpw-----GRLLRLGAEEGEPHVLLRKrEWTIGRRRGCDLsfpSNKLVSGDHCRIVVDEKSGQVTLEDTSTSGTVINKLKVVKKQTCPLQTGDVIYLVYRKNepehNVAYLYESL-s<br>----srgrlWLQSPTGGPPPIFLPSDGQ-ALVLGRGPLTQV---TDRKCSRNQVELIADPESRTVAVKQLGVNPSTVGVHELKPGLSGSLSLGDVLYLVNGLY----PLTLRWEELs-<br><br>BLAST: aliSize=39 (resi) iden=19.05% (20/105) simi=37.14% (39/105)<br>mqpw--GRLLRLGAEEGEPHVLLRKREWTIGRRRGcDLSFPSNKLVSGDHCRIVVDEKSGQVTLEDTSTSGTVINKLKVVKKQTCPLQTGDVIYLV--------------yrknepehnvaylyesls<br>----srGRLWLQSPTGGPPPIFLPSDGQALVLGRG-PLTQVTDRKCSRNQVELIADPESRTVAVKQLGVNPSTVGVHELKPGLSGSLSLGDVLYLVnglypltlrweels------------------<br><br>Proposed: aliSize=94 (resi) RMSD=2.48 (Å)<br>mq--PWGRLLRLgAEegE-PHVLLRK--REWTIGRRRgcdLSFPSNKLVSGDHCRIVVD-eksG--QVTLEDTSTSGTVINkLKVVK-KQTCPLQTGDVIYLVYRknepehNVAYLYESLS--<br>--srGRLWLQSP-TG--GpPPIFLPSdgQALVLGRGP---LTQVTDRKCSRNQVELIADp---EsrTVAVKQLGVNPSTVG-VHELKpGLSGSLSLGDVLYLVNG------LYPLTLRWEEls

99	1lgqA (112)<br>1ujxA (119)	C	15.18	TM-align: aliSize=65 (resi) RMSD=3.11 (Å)<br>mq--------------PWGRLLRLgAEeGEPHVLLRK-R-EWTIGRRgcdLSFPSNKLVSGDHCRIVVDEksGQ--VTLEDTSTSGTVINKlkvvkkqtcplqtgdviylvyrknepehnvaylyesls------------------------------------<br>--gssgssgmsqlgsrGRLWLQSP-TG-GPPPIFLPSdGqALVLGRGP---LTQVTDRKCSRNQVELIADP--ESrtVAVKQLGVNPSTVGV-------------------------------------qelkpglsgslslgdvlylvnglypltlrwsgpssg<br><br>SARST: aliSize=97 (resi) RMSD=13.20 (Å)<br>mqpw-----------------GRLLRLGAEEGEPHVLLRKrEWTIGRRRGCDLsfpSNKLVSGDHCRIVVDEKSGQVTLEDTSTSGTVINKLKVVKKQTCPLQTGDVIYLVYRKNepehnVAYLYES-----ls<br>----gssgssgmsqlgsrgrlWLQSPTGGPPPIFLPSDGQ-ALVLGRGPLTQV---TDRKCSRNQVELIADPESRTVAVKQLGVNPSTVGVQELKPGLSGSLSLGDVLYLVNGLY-----PLTLRWSgpssg--<br><br>BLAST: aliSize=39 (resi) iden=17.86% (20/112) simi=34.82% (39/112)<br>mqpw--------------GRLLRLGAEEGEPHVLLRKREWTIGRRRGcDLSFPSNKLVSGDHCRIVVDEKSGQVTLEDTSTSGTVINKLKVVKKQTCPLQTGDVIYLV----------------yrknepehnvaylyesls<br>----gssgssgmsqlgsrGRLWLQSPTGGPPPIFLPSDGQALVLGRG-PLTQVTDRKCSRNQVELIADPESRTVAVKQLGVNPSTVGVQELKPGLSGSLSLGDVLYLVnglypltlrwsgpssg------------------<br><br>Proposed: aliSize=92 (resi) RMSD=2.29 (Å)<br>mq--------------PWGRLLRLgAEegE-PHVLLRK-R-EWTIGRrR-gcdLS-FpSNKLVSGDHCRIVVD-ekSG-QVTLEDTSTSGTVINkLKVVK-KQTCPLQTGDVIYLVYRknepehNVAYLYESL-----s<br>--gssgssgmsqlgsrGRLWLQSP-TG--GpPPIFLPSdGqALVLGR-Gp---LTqV-TDRKCSRNQVELIADp--ESrTVAVKQLGVNPSTVG-VQELKpGLSGSLSLGDVLYLVNG------LYPLTLRWSgpssg-
100	1lgqA (112)<br>1g3gA (164)	C	24.11	TM-align: aliSize=70 (resi) RMSD=3.03 (Å)<br>------------------------------MQPWGRLLRLGAeege--PHVLLRK-------------REWTIGRRRGCDLSFPSNKLVSGDHCRIVVDeKSGQVTLEDTSTSGTVINK-------------Lkvvkkqtcplqtgdviylvyrknepehnvaylyesls----------------------<br>genitqptqqstqatqrfliekfsqeqigENIVCRVICTTG----qiPIRDLSAdisqvlkekrsikKVWTFGRNPACDYHLGNISRLSNKHFQILLG-EDGNLLLNDISTNGTWLNGqkveknsnqllsqG-------------------------------------deitvgvgvesdilslvifin<br>-----------------<br>dkfkqcleqnkvdrir<br><br>SARST: aliSize=83 (resi) RMSD=13.46 (Å)<br>mqpwgrllrlgaeegephvllrkr--EWTIGRRRGCDLSFPSNKLVSGDHCRIVVDEKsGQVTLEDTSTSGTVINKLKVVKKQTCPLQTGDVIYlvYRKNEPEHNVAYLYES-----------------ls<br>------------------------genitqptqqstqatqrfliekfsqeqigenivcrvicttgqipirdlsadisqvlkekrsikkVWTFGRNPACDYHLGNISRLSNKHFQILLGED-GNLLLNDISTNGTWLNGQKVEKNSNQLLSQGDEIT--VGVGVESDILSLVIFIndkfkqcleqnkvdrir--<br><br>BLAST: aliSize=35 (resi) iden=23.21% (26/112) simi=31.25% (35/112)<br>mqpwgrllrlgaeegephvllr--KREWTIGRRRGCDLSFPSNKLVSGDHCRIVVDEkSGQVTLEDTSTSGTVINKLKVVKKQTCPLQTGDVI----------------------------------ylv<br>----------------------genitqptqqstqatqrfliekfsqeqigenivcrvicttgqipirdlsadisqvlkekrsiKKVWTFGRNPACDYHLGNISRLSNKHFQILLGE-DGNLLLNDISTNGTWLNGQKVEKNSNQLLSQGDEItvgvgvesdilslvifindkfkqcleqnkvdrir---<br>yrknepehnvaylyesls<br>------------------<br><br>Proposed: aliSize=104 (resi) RMSD=2.41 (Å)<br>m------------------------------QPWGRLLRLGA--eegePHVLLRK-------------REWTIGRRRGCDLSFPSNKLVSGDHCRIVVDeKSGQVTLEDTSTSGTVINKLKVVKKQTCPLQTGDVIYLVYRknEPEHNVAYLYESLS---------------<br>-genitqptqqstqatqrfliekfsqeqigeNIVCRVICTTGqi----PIRDLSAdisqvlkekrsikKVWTFGRNPACDYHLGNISRLSNKHFQILLG-EDGNLLLNDISTNGTWLNGQKVEKNSNQLLSQGDEITVGVG--VESDILSLVIFINDkfkqcleqnkvdrir
101	1lgqA (112)<br>2brfA (100)	C	18.00	TM-align: aliSize=66 (resi) RMSD=3.44 (Å)<br>mqPWGRLLRLgAEeGEPHVLLRK--REWTIGRRgcdLSFPSNKLVSGDHCRIVVDEk-SGQVTLEDTSTSGTVINKlkvvkkqtcplqtgdviylvyrknepehnvaylyesls-------------------------------<br>--GRLWLESP-PG-EAPPIFLPSdgQALVLGRGP---LTQVTDRKCSRTQVELVADP-eTRTVAVKQLGVNPSTTGQ--------------------------------------elkpglegslgvgdtlylvnglhpltlrwee<br><br>SARST: aliSize=95 (resi) RMSD=13.22 (Å)<br>mqpw---GRLLRLGAEEGEPHVLLRKREWTI-GRRRGCdlsfpSNKLVSGDHCRIVVDEKSGQVTLEDTSTsGTVINKLKVVKKQTCPLQTGDVIYLVYRKNepehNVAYLYE-sls<br>----grlWLESPPGEAPPIFLPSDGQALVLGrGPLTQV-----TDRKCSRTQVELVADPETRTVAVKQLGV-NPSTTGQELKPGLEGSLGVGDTLYLVNGLH----PLTLRWEe---<br><br>BLAST: aliSize=41 (resi) iden=24.00% (24/100) simi=41.00% (41/100)<br>mqpwGRLLrLGAEEGE-PHVLLRKREWTIGRRRGcDLSFPSNKLVSGDHCRIVVDEKSGQVTLEDTSTS-GTVINKLKVVKKQTcpLQTGDVIYLV------------yrknepehnvaylyesls<br>----GRLW-LESPPGEaPPIFLPSDGQALVLGRG-PLTQVTDRKCSRTQVELVADPETRTVAVKQLGVNpSTTGQELKPGLEGS--LGVGDTLYLVnglhpltlrwee------------------<br><br>Proposed: aliSize=91 (resi) RMSD=2.37 (Å)<br>mqPWGRLLRLgAEegE-PHVLLRK--REWTIGRrR-gcdLS-FpSNKLVSGDHCRIVVD-eksGQ--VTLEDTSTSGTVINklKVVK-KQTCPLQTGDVIYLVYRknepehNVAYLYESLS<br>--GRLWLESP-PG--EaPPIFLPSdgQALVLGR-Gp---LTqV-TDRKCSRTQVELVADp---ETrtVAVKQLGVNPSTTG--QELKpGLEGSLGVGDTLYLVNG------LHPLTLRWEE

102	2pfsA (125)<br>1jmvA (140)	M	27.20	TM-align: aliSize=109 (resi) RMSD=1.97 (Å)<br>sVYHHILLAVDFSSEDSQVVQKVRNLASQIGARLSLIHVLDtaipldtetty-------------------DALDVEKQKLSQIGNTL--GIDpaHRWLVWGEPREEIIRIAEQENVDLIVVGSH------S--TANSVLHYAKCDVLAVRL--<br>-MYKHILVAVDLSEESPILLKKAVGIAKRHDAKLSIIHVDV-----------nfsdlytglidvnmssmqdRISTETQKALLDLAESVdyPIS--EKLSGSGDLGQVLSDAIEQYDVDLLVTGHHqdfwskLmsSTRQVMNTIKIDMLVVPLrd<br><br>SARST: aliSize=119 (resi) RMSD=6.62 (Å)<br>svy--HHILLAVDFSSEDSQVVQKVRNLASQIGARLSLIHVLDTA--IPLDTETTYDALD------VEKQKLSQIGNTLGIDPAHRWLVWGEPREEIIRIAEQENVDLIVVGS--------HSTANSVLHYAKCDVLAVR---l<br>---myKHILVAVDLSEESPILLKKAVGIAKRHDAKLSIIHVDVNFsdLYTGLIDVNMSSMqdristETQKALLDLAESVDYPISEKLSGSGDLGQVLSDAIEQYDVDLLVTGHhqdfwsklMSSTRQVMNTIKIDMLVVPlrd-<br><br>BLAST: aliSize=68 (resi) iden=33.33% (41/125) simi=55.28% (68/125)<br>sVYHHILLAVDFSSEDSQVVQKVRNLASQIGARLSLIHV------LDTA-IPLDTETTYDALDVEKQK-LSQIGNTLGIDPAHRWLVWGEPREEIIRIAEQENVDLIVVGSH--------STANSVLHYAKCDVLAVRL--<br>-MYKHILVAVDLSEESPILLKKAVGIAKRHDAKLSIIHVdvnfsdLYTGlIDVNMSSMQDRISTETQKaLLDLAESVDYPISEKLSGSGDLGQVLSDAIEQYDVDLLVTGHHqdfwsklmSSTRQVMNTIKIDMLVVPLrd<br><br>Proposed: aliSize=119 (resi) RMSD=1.71 (Å)<br>msVYHHILLAVDFSSEDSQVVQKVRNLASQIGARLSLIHVLDtA---iPLDTETTYDAMLD------VEKQKLSQIGNTL--GIDpaHRWLVWGEPREEIIRIAEQENVDLIVVGSH------S--TANSVLHYAKCDVLAVRL--<br>--MYKHILVAVDLSEESPILLKKAVGIAKRHDAKLSIIHVDV-Nfsd-LYTGLIDVNMSSMqdristETQKALLDLAESVdyPIS--EKLSGSGDLGQVLSDAIEQYDVDLLVTGHHqdfwskLmsSTRQVMNTIKIDMLVVPLrd
103	2qntA (125)<br>2r6uA (130)	C	12.00	TM-align: aliSize=58 (resi) RMSD=3.03 (Å)<br>nlyfqgrfvnpipfvrdinrsksfyrdrlg--LKiledfgsfvlf---ETGfaihegrsleetiwrtssqeayg--RRNLLYFEHADVDAAFOIAPH--VELIHPLERQaWG-QRVFRFYDPDGHAIEV<br>-------------------------------lyFQ-----------gmtGRI-----------------------vhfeipfddgdrarafyrdafgwaiaeipdmdysmvttgpvgesgmpdepgyinggmmqrgevTTPVVTVDVESIESALERIESlgGKTVTGRTPV-GNmGFAAYFTDSEGNVVGL<br><br>GESL-<br>WETAr<br><br>SARST: aliSize=110 (resi) RMSD=14.09 (Å)<br>nlyf------QGRFVNPIPFVRDINRSKSFYRDRLGLKILEDFGSFVLFETGFAIHegrsleetIWR----------TSSQEAYGRRNLLYFEHADVDAAFOIA--PHVELIHPLERQAWGQRVFRFYDPDGHAIEVGES--l<br>----lyfqgmTGRIVHFEIPFDDGDRARAFYRDAFGWAIAEIPDMDYSMVTTGPVG--------ESGmpdepgyingGMMQRGEVTTPVVTVDVESIESALERIesLGGKTVTGRTPVGNMGFAAYFTDSEGNVVGLWETar-<br><br>BLAST: aliSize=29 (resi) iden=17.89% (22/125) simi=23.58% (29/125)<br>nLYFOG---RFVN-PIPFvRDINRSKSFYRDRLGLKILE--DFgSFVLFETG--faihegrsleetiwrtssqeaygrrnllyfehadvdaafqiaphvelihplerqawg<br>-LYFQGmtgRIVHfEIPF-DDGDRARAFYRDAFGWAIAEipDM-DYSMVTTGpvgesgmpdepgyinggmmqrgevttpvvtvdvesiesalerieslggktvtgrtpvgnmgfaayftdsegnvvglwetar--<br><br>qrvfrfydpdghaievgesl<br>--------------------<br><br>Proposed: aliSize=102 (resi) RMSD=2.24 (Å)<br>nlyf-----QGMRFVNPIPFVRDINRSKSFYRDRLGLKIlEDF--GSFVLFE-T--------------GFAIHEGR--sleetiwrtssqeaygrRNMLLYFEHADVDAAFOdIAP--HVELIHPLERQAWGQRVFRFYDPDGHAIEVGES--l<br>----lyfqgMTGRIVHFEIPFDDGDRARAFYRDAFGWAI-AEIpdMDYSMVTtGpvgesgmpdepgyiNGGMMQRGev-----------------TTPVVTVDVESIESALE-RIEslGGKTVTGRTPVGNMGFAAYFTDSEGNVVGLWETar-
104	2qntA (125)<br>2qh0A (129)	N	11.20	TM-align: aliSize=61 (resi) RMSD=3.24 (Å)<br>nlyfOGRFVNPIPFVRDINRSKSFYRdRLGLKILEDFG-------SFVLFE--T-GFAIHEgRSl--EEtiwRTSS--OEAYGRrNllvfehadvdaafqiaphvelihplerqawgqrvfrfydpdghaievgesl---<br>----SLKVHHIGYAVKNIDSALKKFK-RLGYVEESEVVrdevrkvYIQFVIngGyRVELVA-PD-geDS---PINKtiKKGSTP-Y---hicyevediqksieemsqigytlfkkaeiapaidnrkvaflfstdigliellek<br><br>SARST: aliSize=106 (resi) RMSD=12.71 (Å)<br>nlyfqgr---FVNPIPFVRDINRSKSFYrDRLGLKILED------FGSFVL--FETGFAIHEG-------RSLEETIWRTSSOEaygrrnlLYFEHADVDAAFOIAPH---VELIHPLERQAWG-ORVFRFYDPDGHAIEVGE-sl<br>-------slkVHHIGYAVKNIDSALKKF-KRLGYVEESEvvrdevRKVYIQfvINGGYRVELVapdgedsPINKTIKKGSTPYH-------ICYEVEDIQKSIEEMSQigyTLFKKAEIAPAIDnRKVAFLFSTDIGLIELLEk--<br><br>BLAST: aliSize=9 (resi) iden=4.88% (6/125) simi=7.32% (9/125)<br>nlyfqgrfvnpipfvrdinrsksfyrdrlglkiledfgsfvlfetgfaihegrsleetiwrtssqeaygrrnllyfehadvda--<br>--slkvhhigyavknidsalkkfkrlgyveesevvrdevrkvyiqfvinggyrvelvapdgedspinktikkgstpyhicyevediqksieemsqigytlfkkaeiapa<br><br>------AFQIAPHVELIHPLERqawgqrvfrfydpdghaievgesl<br>idnrkvAFLFSTDIGLIELLEK------------------------<br><br>Proposed: aliSize=109 (resi) RMSD=2.23 (Å)<br>nlyfqGMRFVNPIPFVRDINRSKSFYRdRLGLKILEDFG-------SFVLFE--TGFAIHEGRS---leEtiwrT-SS--OEAYG-rrnMLLYFEHADVDAAFO-dIAP-HVELIHPLERQAW--GORVFRFYDPDGHAIEVGESl<br>-----SLKVHHIGYAVKNIDSALKKFK-RLGYVEESEVVrdevrkvYIQFVIngGYRVELVAPDged--S----PiNKtiKKGSTp---YHICYEVEDIQKSIEe-MSQiGYTLFKKAEIAPAidNRKVAFLFSTDIGLIELLEK-

105	2rbbA (129)<br>2r6uA (130)	N	14.73	TM-align: aliSize=63 (resi) RMSD=2.97 (Å)<br>nadlsyvniftrdivasafyqqv--FGfqeiesirspifrgld---TGKScigfnaheayelqlaqfsets--GIKFLLNFDVdTkeAVDKLVPVAIAAGATLIKAPYETYYHWYQAVLLDPERNVFRINNVL-<br>-----------------------lyFQ----------------gmtGRIV--------------------hfeipfddgdrarafyrdafgwaiaeipdmdysmvttgpvgesgmpdepgyinggmmqrgeVTTPVVTVDV-E--SIESALERIESLGGKTVTGRTPVGNMGFAAYFTDSEGNVVGLWETAr<br><br>SARST: aliSize=105 (resi) RMSD=14.61 (Å)<br>nad---------LSYVNIFTRDIVASAFYQQV-FGFQEiesirspiFRGLDTGKSCIGFNAheayelQLAQF-----SETSGIKF--------LLNFDVDTKEAVdklVPVAIAAGATLIKAPYETYYHWYQAVLLDPERNVFRINNV--l<br>---lyfqgmtgrIVHFEIPFDDGDRARAFYRDaFGWAI--------AEIPDMDYSMVTTGP------VGESGmpdepGYINGGMMqrgevttpVVTVDVESIESA---LERIESLGGKTVTGRTPVGNMGFAAYFTDSEGNVVGLWETar-<br><br>BLAST: aliSize=14 (resi) iden=7.94% (10/129) simi=11.11% (14/129)<br>nadlsyvniftrdiv----------------------ASAFYQQVFGFqEIESIRSPIFRGLDTG--kscigfnaheayelqlaqfsetsgikfllnfdvdtkeavdklvp<br>---------------lyfqgmtgrivhfeipfddgdrARAFYRDAFGW-AIAEIPDMDYSMVTTGpvgesgmpdepgyinggmmqrgevttpvvtvdvesiesalerieslggktvtgrtpvgnmgfaayftdsegnvvglwetar--<br><br>vaiaagatlikapyetyyhwyqavlldpernvfrinnvl<br>---------------------------------------<br><br>Proposed: aliSize=110 (resi) RMSD=2.08 (Å)<br>-----NMADLSYVNIFTRDIVAMSAFYQQVFGFQEIEsiR--SPiFRGLDTG------------KSCIGFNAHEAYelmqlaqfSetsgiKFLLNFDVDtkeAVDKLVPVAIAAGATLIKAPYETYYHWYQAVLLDPERNVFRINNVL-<br>lyfqgMTGRIVHFEIPFDDGDRARAFYRDAFGWAIAE--IpdMD-YSMVTTGpvgesgmpdepgYINGGMMQRGEV--------T-----TPVVTVDVE---SIESALERIESLGGKTVTGRTPVGNMGFAAYFTDSEGNVVGLWETAr
106	2rbbA (129)<br>2qh0A (129)	C	10.08	TM-align: aliSize=67 (resi) RMSD=3.58 (Å)<br>NADLSYVNIFTRDIVA-SAFYQQvFGFQEIESiRSP------IFRGLDTGKSCIGFNAHEAYELqlAQFSE-TSGIKFLlnfdvdtkeavdklvpvaiaagatlikapyetyyhwyqavlldpernvfrinnvl---<br>SLKVHHIGYAVKNIDSaLKKFKR-LGYVEESE-VVRdevrkvYIQFVINGGYRVELVAPDGEDS--PINKTiKKGSTPY--hicyevediqksieemsqigytlfkkaeiapaidnrkvaflfstdigliellek<br><br>SARST: aliSize=119 (resi) RMSD=13.35 (Å)<br>na--DLSYVNIFTRDIVASAFYQQVFG------FQEIESIRSPIFRGLDTGKSCIGFNAHEAYELQLAQFSETSGIKFLLNFdVDTKEAVDKLVPvaiAAGATL--IKAPYETYYHWYQAVLLDPERNVFRINnVL<br>--slKVHHIGYAVKNIDSALKKFKRLGyveeseVVRDEVRKVYIQFVINGGYRVELVAPDGEDSPINKTIKKGSTPYHICYE-VEDIQKSIEEMS---QIGYTLfkKAEIAPAIDNRKVAFLFSTDIGLIELL-EK<br><br>BLAST: aliSize=46 (resi) iden=23.81% (30/129) simi=36.51% (46/129)<br>nad-----------------------LSYV--NIFTRDIVASAFYQQVF-GFQEIESIR-----SPIFRGLDTGKSCIgfnaHEAYELQLAQFS--ETSGIKFLLNFDVDTKEAVD-KLVPVAIAAGATLIKapyetyyhwyqavLLD-pernvfrinnvl<br>---slkvhhigyavknidsalkkfkrLGYVeeSEVVRDEVRKVYIQFVInGGYRVELVApdgedSPINKTIKKGSTPY----HICYEVEDIQKSieEMSQIGYTLFKKAEIAPAIDnRKVAFLFSTDIGLIE------------LLEk------------<br><br>Proposed: aliSize=112 (resi) RMSD=2.05 (Å)<br>nMADLSYVNIFTRDIVAMSAFYQQvFGFQEIEsIRSP------IFRGLDTGKSCIGFNAHE---aYelmqlAqFSEtsgI------KFLLNFDVDtkeAVDKLVPVAIAAGATLIKAPYETYY--HWYQAVLLDPERNVFRINNVl<br>-SLKVHHIGYAVKNIDSALKKFKR-LGYVEES-EVVRdevrkvYIQFVINGGYRVELVAPDged-S-----P-INK---TikkgstPYHICYEVE---DIQKSIEEMSQIGYTLFKKAEIAPAidNRKVAFLFSTDIGLIELLEK-
107	2p7oA (127)<br>2r6uA (130)	C	13.39	TM-align: aliSize=63 (resi) RMSD=3.99 (Å)<br>misglshitlivkdlnkttaflqni--FNaeeiytfslskekffl---IAGLwicimegdslqe--RTYNHIAFQIQseEVDEYTERIKALGVEMKPERPRVQGEGRSIYFYDFDNHLFELHAGTleerlk<br>-------------------------lyFQ----------------gmtGRIV------------hfeipfddgdrarafyrdafgwaiaeipdmdysmvttgpvgesgmpdepgyinggmmqrgeVTTPVVTVDVE--SIESALERIESLGGKTVTGRTPVGNMGFAAYFTDSEGNVVGLWETA------<br><br>ryh-<br>---r<br><br>SARST: aliSize=114 (resi) RMSD=13.53 (Å)<br>misg---------LSHITLIVKDLNKTTAFLQNIFNAEEIYTFSLSKEKFFLI-----AGLWICIMEGDSLQERTYN-HIAFQIQSEEVDEYTERIKALGVEMKPERPRVQGEGRSIYFYDFDNHLFELHAGT-leerlkryh<br>----lyfqgmtgrIVHFEIPFDDGDRARAFYRDAFGWAIAEIPDMDYSMVTTGpvgesGMPDEPGYINGGMMQRGEVtTPVVTVDVESIESALERIESLGGKTVTGRTPVGNMGFAAYFTDSEGNVVGLWETAr---------<br><br>BLAST: aliSize=27 (resi) iden=11.81% (15/127) simi=21.26% (27/127)<br>misglshitlivkdlnkttaflqnifnaeeiytfslskekffliaglwici---MEGDSLQERTYNHIAFQIQSEEVDEYTERIKALGVEMKPERPRVQGEGRSIYFYDFDNHLFEL-----hagtleerl<br>---lyfqgmtgrivhfeipfddgdrarafyrdafgwaiaeipdmdysmvttgpvgesgmpdepgyINGGMMQRGEVTTPVVTVDVESIESALERIESLGGKTVTGRTPVGNMGFAAYFTDSEGNVVGLwetar---------<br><br>kryh<br>----<br><br>Proposed: aliSize=101 (resi) RMSD=2.07 (Å)<br>------MIsGLSHITLIVKDLNKTTAFLQNIFNAEEI-Y-T-fslsKEKFFLIA------------GLWICIMEG---dslqerTYNHIAFQIQseEVDEYTERIKALGVEMKPE-RpR--vqGEGRSIYFYDFDNHLFELHAG--tleerlkryh<br>lyfqgmTG-RIVHFEIPFDDGDRARAFYRDAFGWAIAeIpDm----DYSMVTTGpvgesgmpdepgYINGGMMQRgev------TTPVVTVDVE--SIESALERIESLGGKTVTGrT-Pvg--NMGFAAYFTDSEGNVVGLWETAr----------

108	2p7oA<br>(127)<br>2qh0A<br>(129)	N	11.81	TM-align: aliSize=61 (resi) RMSD=2.29 (Å)<br>MIsGLSHITLIVKDLNKTTAFLONiFNAEEIY--TFS---LSKEKFFLIAGLWICIMEG-------DS-LOErtvnhiafqiqseevdeyterikalgvemkperprvqgegrsiyfydfDNHLfelhagtleerlkryh---<br>SL-KVHHIGYAVKNIDSALKKFKR-LGYVEESevVRDevrKVYIQFVINGGYRVELVAPdgedspiNKtIKK--GSTP-----------------yhicyevediqksieemsqigytlfkkaeiapaidnrkvaflfstdigli<br>-----<br>ellek<br>SARST: aliSize=107 (resi) RMSD=13.76 (Å)<br>misg---LSHITLIVKDLNKTTAFLONIF---NAEEIYTFSLSKEKFFLIAGLWICIMEGDSL-----------OERTYNHIAFQIOSEEvdeyTERIKALGVEMKPERPRVQ---GEGRSIYFYDFDNHLFELH--agtleerlkryh<br>----slkVHHIGYAVKNIDSALKKFKRLGyveESEVVRDEVRKVYIQFVINGGYRVELVAPDGedspinktikkGSTPYHICYEVEDIQK----SIEEMSQIGYTLFKKAEIApaiDNRKVAFLFSTDIGLIELLek------------<br>BLAST: aliSize=53 (resi) iden=22.05% (28/127) simi=41.73% (53/127)<br>misgls-----HITLIVKDLNKTTAFLONIFNAEE--IYTFSLSKEKF-FLIAG---LWICIMEG-DSLOERTYN------HIAFOIqsEEVDEYTERIKALGVEM--KPE-RPRVqgEGRSIYF-YDFDNHLFEL---hagtleerlkryh<br>------slkvhHIGYAVKNIDSALKKFKRLGYVEEseVVRDEVRKVYIqFVINGgyrVELVAPDGeDSPINKTIKkgstpyHICYEV--EDIQKSIEEMSQIGYTLfkKAEiAPAI--DNRKVAFlFSTDIGLIELlek-------------<br>Proposed: aliSize=106 (resi) RMSD=2.06 (Å)<br>MIsGLSHITLIVKDLNKTTAFLONIfNAEEIY---TFS--LSKEKFFLIAGLWICIME-------GD--SLO----ertvNHIAFOIOseEVDEYTERIKALGVEMKPERPR-----vqgEGRSIYFYDFDNHLFELHAGtleerlkryh<br>SL-KVHHIGYAVKNIDSALKKFKRL-GYVEESevvRDEvrKVYIQFVINGGYRVELVApdgedspINktIKKgstp----YHICYEVE--DIQKSIEEMSQIGYTLFKKAEIapaid---NRKVAFLFSTDIGLIELLEK----------
109	2f5gA<br>(130)<br>2fyxA<br>(130)	C	34.62	TM-align: aliSize=109 (resi) RMSD=1.55 (Å)<br>eLKSTRHTKYLCNYHFVWIPKHRRNTLVNEIAEYTKEVLKSIAEELGCEIIALEVMPDHIHLFVNCPPRYAPSYLANYFKGKSARLILKKFPQLNK----GKLWTRSYFVATAGnvssevikkyieeqwrkege-----------------<br>-MKKGRGYVYKLEYHLIWATKYRHQVLVDEVADGLKDILRDIATQNGLELVALEVMPDYVHLLLGATPQHVIPDFVKALKGASARRMFSAFPHLKQphwgGNLWNPSYCVLTVS--------------------ehtraqiqqyienqhaa<br>SARST: aliSize=120 (resi) RMSD=10.12 (Å)<br>el-KSTRHTKYLCNYHFVWIPKHRRNTLVNEIAEYTKEVLKSIAEELGCEIIALEVMPDHIHLFVNCPPRYAPSYLANYFKGKSARLILKKFPQL----NKGKLWTRSYFVATagnVSSEVIKKYIEEQW-----rkege<br>--mKKGRGYVYKLEYHLIWATKYRHQVLVDEVADGLKDILRDIATQNGLELVALEVMPDYVHLLLGATPQHVIPDFVKALKGASARRMFSAFPHLkqphWGGNLWNPSYCVLT---VSEHTRAQIQQYIEnqhaa-----<br>BLAST: aliSize=73 (resi) iden=37.69% (49/130) simi=56.15% (73/130)<br>eLKSTRHTKYLCNYHFVWIPKHRRNTLVNEIAEYTKEVLKSIAEELGCEIIALEVMPDHIHLFVNCPPRYA-PSYLaNYFKGKSARLILKKFPQLNK----GKLWTRSYFVATAGNVSSEVIKKYIEEQ---wrkege<br>-MKKGRGYVYKLEYHLIWATKYRHQVLVDEVADGLKDILRDIATQNGLELVALEVMPDYVHLLLGATPQHViPDFV-KALKGASARRMFSAFPHLKQphwgGNLWNPSYCVLTVSEHTRAQIQQYIENQhaa------<br>Proposed: aliSize=125 (resi) RMSD=1.46 (Å)<br>eLKSTRHTKYLCNYHFVWIPKHRRNTLVNEIAEYTKEVLKSIAEELGCEIIALEVMPDHIHLFVNCPPRYAPSYLANYFKGKSARLILKKFPQLNK---GK-LWTRSYFVATagnVSSEVIKKYIEEQWRKEG-e<br>-MKKGRGYVYKLEYHLIWATKYRHQVLVDEVADGLKDILRDIATQNGLELVALEVMPDYVHLLLGATPQHVIPDFVKALKGASARRMFSAFPHLKQphwGGnLWNPSYCVLT---VSEHTRAQIQQYIENQHAa-
110	2axwA<br>(134)<br>2fvnA<br>(142)	C	79.85	TM-align: aliSize=121 (resi) RMSD=2.96 (Å)<br>-AELHLESRGGSGTQLRDGAKVATGRIICrEAHT-GFHVWMNERQVDGRAERYVVQSKDGRHELRVRTGGDgWSPVKGEG-GKGVSRPGQEEQVFFDVMADGNQDIAPGEYRFSVGGACVVPQ-EKLaaalehhhhhh-----------------<br>sAELHLESRGGSGTQLRDGAKVATGRIIC-REAHtGFHVWMNERQVDGRAERYVVQSKDGRHELRVRTGGD-GWSPVKGEgGKGVSRPGQEEQVFFDVMADGNQDIAPGEYRFSVGGACVVPQeDNK-----------qgftpsgttgttkltvt<br>SARST: aliSize=125 (resi) RMSD=15.50 (Å)<br>aelhl---------ESRGGSgtqLRDGAKVATGRIICREAHTGFHVWMNERQVDGRAERYVVQSKDGRHELRVRTGGDGWSPVKGEGGKGVSRPGQEEQVFFDVMAD-GNQDIAPGEYRF-----SVGGACVVPQEKLAAALEHHHHH--h<br>-----saelhlesrGGSGTQ---LRDGAKVATGRIICREAHTGFHVWMNERQVDGRAERYVVQSKDGRHELRVRTGGDGWSPVKGEGGKGVSRPGQEEQVFFDVMADgNQDIAPGEYRFSvggacVVPQEDNKQGFTPSGTTGTTKLTvt-<br>BLAST: aliSize=121 (resi) iden=90.30% (121/134) simi=90.30% (121/134)<br>-AELHLESRGGSGTQLRDGAKVATGRIICREAHTGFHVWMNERQVDGRAERYVVQSKDGRHELRVRTGGDGWSPVKGEGGKGVSRPGQEEQVFFDVMADGNQDIAPGEYRFSVGGACVVPQE--------------------klaaalehhhhhh<br>sAELHLESRGGSGTQLRDGAKVATGRIICREAHTGFHVWMNERQVDGRAERYVVQSKDGRHELRVRTGGDGWSPVKGEGGKGVSRPGQEEQVFFDVMADGNQDIAPGEYRFSVGGACVVPQEdnkqgftpsgttgttkltvt-------------<br>Proposed: aliSize=129 (resi) RMSD=2.54 (Å)<br>-AELHLESRGGSGTQLRDGAKVATGRIICrEAHT-GFHVWMNERQVDGRAERYVVQSKDGRHELRVRTG-GdGWSPVkgeGG---KGVSRPGQEEQVFFDVMADGNQDIAPGEYRFSVGGACV-----VPQEKLAAALEHHHHHH--<br>sAELHLESRGGSGTQLRDGAKVATGRIIC-REAHtGFHVWMNERQVDGRAERYVVQSKDGRHELRVRTGgD-GWSPV---KGeggKGVSRPGQEEQVFFDVMADGNQDIAPGEYRFSVGGACVvpqedNKQGFTPSGTTGTTKLTvt

111	2pa7A (135)<br>1cauA (181)	N	11.11	TM-align: aliSize=90 (resi) RMSD=2.59 (Å)<br>enkvinfkkiidsrgslvaieenknipf--SIKRVYYIFdTKGEEPRGFHAHkKLEQVLVCLNGSCRVILDDGNIIQEITLDSPaVGLYVGPAVWHEMHDF-S-SDCVMMVLASD-----YYDetDYIRQ------Yd-Nfkkyiakinle-<br>----------------------------aqnnpylfrsnkfltlfknqhgslrllqrfnedteklenlRDYRVLEYC-SKPNTLLLPHHS-DSDLLVLVLEGQAILVLVNPDGRDTYKLDQG-DAIKIQAGTPFYLINPdNnQNLRILKFAITfrrpgTVE--DFFLSstkrlpS-yL-----------s<br><br>------------------------------------<br>afsknfleasydspydeieqtllqeeqegvivkmpk<br><br>SARST: aliSize=105 (resi) RMSD=10.52 (Å)<br>en-------KVINFKKI--IDSRGSLVAIEE-NKNIPFS---IKRVYYIFDTKGEEPRGFHAHKKLEqVLVCLNGSCRVILDDGNIIQEITLDSPaVGLYVGPAVWHEMHDFSSDCVMMVLA---sdyyd<br>--aqnnpylFRSNKFLTlfKNQHGSLRLLQRfNEDTEKLenlRDYRVLEYCSKPNTLLLPHHSDSDL-LVLVLEGQAILVLVNPDGRDTYKLDQG-DAIKIQAGTPFYLINPDNNQNLRILKfaitfrrpgtvedfflsstkrlpsylsafsknfleasydspydeieqtllqeeqegvivkmpk-----<br><br>etdyirqydnfkkyiakinle<br>---------------------<br><br>BLAST: aliSize=16 (resi) iden=7.41% (10/135) simi=11.85% (16/135)<br>enkvinfkkiidsrgslvaieenknipfsikrvyyifdtkgeeprgfhahkkl--EQVLVCLN--GSCRVILDDGNIIqEITLDSP----------------------------------<br>---aqnnpylfrsnkfltlfknqhgslrllqrfnedteklenlrdyrvleycskpntlllphhsdsdllvlvlegQAILVLVNpdGRDTYKLDQGDAI-KIQAGTPfylinpdnnqnlrilkfaitfrrpgtvedfflss<br><br>---avglyvgpavwhemhdfssdcvmmvlasdyydetdyirqydnfkkyiakinle<br>tkrlpsylsafsknfleasydspydeieqtllqeeqegvivkmpk---<br><br>Proposed: aliSize=113 (resi) RMSD=2.26 (Å)<br>e------NKVINFKKI--IDSRGSLVAIeenKNIP--------FSIKRVYYIFdTKGEEPRGFHAHkKLEQVLVCLNGSCRVILDDGNIIQEITLDSPaVGLYVGPAVWHEMHDF-S-SDCVMMVLASD-----YYDetDYIRQ--------ydN-----------------------------------<br>-aqnnpyLFRSNKFLTlfKNQHGSLRLL---QRFNedteklenLRDYRVLEYC-SKPNTLLLPHHS-DSDLLVLVLEGQAILVLVNPDGRDTYKLDQG-DAIKIQAGTPFYLINPdNnQNLRILKFAITfrrpgTVE--DFFLSstkrlpsy--Lsafsknfleasydspydeieqtllqeeqegvivkm<br><br>--fkkyiakinle<br>pk-----------
112	2pa7A (135)<br>1znpA (140)	N	8.89	TM-align: aliSize=96 (resi) RMSD=2.97 (Å)<br>enkvinfkkiidsrgslvaieenknipf---------------------------------SIKRVYYIFdTKGEEPRGFHAHKKLEQVLVCLNG-SCRVILDDG-NIIQEITLDSP-----AVGLYVGPAVWHEMHDFSSDCVMMVLASDYYDETDYIRQYd-NFKkyiakinle-<br>----------------------------dsaqaiirelglephpeggfyhqtfrdkaggerGHSTAIYYL-LEKGVRSHWHRVTDAVEVWHYYAGaPIALHLSQDgREVQTFTLGPAilegeRPQVIVPANCWQSAESLGDFTLVGCTVSPGFAFSSFVAEP-gWSP---------g<br><br>SARST: aliSize=105 (resi) RMSD=8.68 (Å)<br>enkvinfkkiid---------------SRGSLVAIE----ENKNipfSIKRVYYIFDTKGEEPRGFHAHKKLEQVLVCLNGSCRVILD--DGNIIQEITL------DSPAVGLYvGPAVWHEMHDFSSDCVMMVLASDYYDETDYIRQ------ydnfkkyiakinle<br>------------dsaqaiirelglephPEGGFYHQTfrdkAGGE---RGHSTAIYYLLEKGVRSHWHRVTDAVEVWHYYAGAPIALHLsqDGREVQTFTLgpaileGERPQVIV-PANCWQSAESLGDFTLVGCTVSPGFAFSSFVAEpgwspg--------------<br><br>BLAST: aliSize=24 (resi) iden=11.85% (16/135) simi=17.78% (24/135)<br>enkvinfkkiidsrgslvaieenknipfsikr--------------------------------------VYYIFDtKGEEPrgfHAHKKLEQVLVC-LNGSCRVIL---DDGNIIQEITLdSPAV--glyvgpavwhemhdf<br>--------------------------------dsaqaiirelglephpeggfyhqtfrdkaggerghstaIYYLLE-KGVRS---HWHRVTDAVEVWhYYAGAPIALhlsQDGREVQTFTL-GPAIlegerpqvivpancwqsaeslgdftlvgctvspgfafssfvaepgwspg--------------<br><br>ssdcvmmvlasdyydetdyirqydnfkkyiakinle<br>------------------------------------<br><br>Proposed: aliSize=107 (resi) RMSD=2.17 (Å)<br>enk---------VINFKKIIdsRGSLVAIEE----------nknipfsiKRVYYIFdTKGEEPRGFHAHKKLEQVLVCLNG-SCRVILDDG-NIIQEITLDS------pAVGLYVGPAVWHEMHDFSSDCVMMVLASDYYDETDYIRQ---Y---dnfkkyiakinle<br>---dmsaqaiirELGLEPHP--EGGFYHQTFrdkaggergh--------STAIYYL-LEKGVRSHWHRVTDAVEVWHYYAGaPIALHLSQDgREVQTFTLGPailege-RPQVIVPANCWQSAESLGDFTLVGCTVSPGFAFSSFVMAepgWspg-------------
113	2pa7A (135)<br>1lr5A (160)	N	11.85	TM-align: aliSize=94 (resi) RMSD=2.62 (Å)<br>enkvinfkkiidsrgslvaieenknipf--------------------------------------SIKRVYYIFdTKGEEPRGFHAHkKLEQVLVCLNGSCRVILDDGN----I-IQEITLDSPaVGLYVGPAVWHEMHDFS--SDCVMMVLASDYYDetDYIRQ---YDNFkKYiakinle-------<br>----------------------------scvrdnslvrdisqmpqssygieglshitvagalnhgmKEVEVWLQT-ISPGQRTPIHRH-SCEEVFTVLKGKGTLLMGSSSlkypGqPQEIPFFQN-TTFSIPVNDPHQVWNSDehEDLQVLVIISRPPA--KIFLYddwSMPH-TA-------avlkfpf<br><br>-----------<br>vwdedcfeaak<br><br>SARST: aliSize=85 (resi) RMSD=3.97 (Å)<br>enkvinfkkiidsrgslvaieenknipf-------------------------------------SIKRVYYIFDTKGEEPRGFHAHKKLEQVLvCLNGSCRVILDDGNI----IQEITLDSPAVGLYVGPAVWHEMHDFSSDCVMMVLA---SDYYD-------------------------------e<br>----------------------------scvrdnslvrdisqmpqssygieglshitvagalnhgMKEVEVWLQTISPGQRTPIHRHSCEEVFT-VLKGKGTLLMGSSSLkypgQPQEIPFFQNTTFSIPVNDPHQVWNSDEHEDLQVLViisRPPAKiflyddwsmphtaavlkfpfvwdedcfeaak-<br><br>tdyirqydnfkkyiakinle<br>--------------------<br><br>BLAST: aliSize=21 (resi) iden=9.63% (13/135) simi=15.56% (21/135)<br>enkvinfkkiidsrgslvaieen---KNIPFSIKRVYYIFDTKGEEPRGFHAHKKLeQVLVCLN-GSCRVIL-DD-------------------------gniiqe<br>-----------------------scvrdnslvrdisqmpqssygieglshitvagalnhgmkevevwlqtispgqrtpihrhsceevftvlkgkgtllmgssslkypgqpQEIPFFQNTTFSIPVNDPHQVWNSDEHEDL-QVLVIISrPPAKIFLyDDwsmphtaavlkfpfvwdedcfeaak------<br><br>itldspavglyvgpavwhemhdfssdcvmmvlasdyydetdyirqydnfkkyiakinle<br>---<br><br>Proposed: aliSize=119 (resi) RMSD=2.39 (Å)<br>------ENKVIN--FKKII--DSRGSLVA-IEEnKNIPFSIKRVYYIFdTKGEEPRGFHAHkKLEQVLVCLNGSCRVILDDGN------iIQEITLDSPaVGLYVGPAVWHEMHDFS--SDCVMMVLASDYYDetDYIRQ--Y-DNFkK-------------------yiakinle<br>scvrdnSLVRDIsqMPQSSygIEGLSHITvAGA-LNHGMKEVEVWLQT-ISPGQRTPIHRH-SCEEVFTVLKGKGTLLMGSSSlkypgq-PQEIPFFQN-TTFSIPVNDPHQVWNSDehEDLQVLVIISRPPA--KIFLYddWsMPH-Taavlkfpfvwdedcfeaak--------

114	1s60A (152)<br>2i79A (171)	C	10.53	TM-align: aliSize=138 (resi) RMSD=2.72 (Å)<br>lvpRGSHMDIRQMNKTHLEHWRGLRK-QL-------WP-G-HPDDaHLADGEEILQA--D-HLASFIAMADGVAIGFADASIRHD-YVngcdsSPVVFLeGIFVLPSFRQRGVAKQLIAAVQRWGTNKG-CREMASDTSPENTISQKVHQALGFEETERVIFYrkrc------------------<br>---MEYELLIREAEPKDAAELVAFLNrVSletdftsLDgDgILLT-SEEMEIFLNKQasSdNQITLLAFLNGKIAGIVNITADQRkRV-----RHIGDL-FIVIGKRYWNNGLGSLLLEEAIEWAQASGiLRRLQLTVQTRNQAAVHLYQKHGFVIEGSQERG----ayieegkfidvylmgkli<br><br>SARST: aliSize=129 (resi) RMSD=4.05 (Å)<br>lvprgsh----MDIRQMNKTH---LEHWRGLRKQLWP---------GHPDDAHLADGEEILQADHLASFIAMADGVAIGFADASIRHD-----YVNGCdsspvvflegIFVLPSFRQRGVAKQLIAA-VQRWGTNKGCREMASDTSPENTISQKVHQALGFEETERVI--------------------fy<br>-------meyeLLIREAEPKDaaeLVAFLNRVSLETDftsldgdgiLLTSEEMEIFLNKQASSDNQITLLAFLNGKIAGIVNITADQRkrvrhIGDLF----------IVIGKRYWNNGLGSLLLEEaIEWAQASGILRRLQLTVQTRNQAAVHLYQKHGFVIEGSQErgayieegkfidvylmgkli--<br><br>rkrc<br>----<br><br>BLAST: aliSize=41 (resi) iden=15.13% (23/152) simi=26.97% (41/152)<br>lvprgshmdirqmnkthlehwrglrkqlwpghpddahla------------------------------------DGeEILQADHLASFI---AMADG--VAIGFADASIRhDYVN-GCDS-SPVVFLEGIFVLPSFR--QRGVAKQLIAAVQRWGTNKG-CREMASDTSPENTISQKVHQALGF-----<br>---------------------------------------meyellireaepkdaaelvaflnrvsletdftsldgDG-ILLTSEEMEIFLnkqASSDNqiTLLAFLNGKIA-GIVNiTADQrKRVRHIGDLFIVIGKRywNNGLGSLLLEEAIEWAQASGiLRRLQLTVQTRNQAAVHLYQKHGFviegs<br><br>----------------------eetervifyrkrc<br>qergayieegkfidvylmgkli-------------<br><br>Proposed: aliSize=139 (resi) RMSD=2.15 (Å)<br>lvpr-GSHMDIRQMNKTHLEHWRGLRK-QL-------WP-G-HPDDahL-ADGEEILQA--D-HLASFIAMADGVAIGFADASIRH--DYvngcdsSPVVFLeGIFVLPSFRQRGVAKQLIAAVQRWGTNKG-CREMASDTSPENTISQKVHQALGFEETER---------VIFYRKRC-----c<br>----mEYELLIREAEPKDAAELVAFLNrVSletdftsLDgDgILLT--SeEMEIFLNKQasSdNQITLLAFLNGKIAGIVNITADQrkRV------RHIGDL-FIVIGKRYWNNGLGSLLLEEAIEWAQASGiLRRLQLTVQTRNQAAVHLYQKHGFVIEGSqergayieegKFIDVYLMgklia-
115	1s60A (152)<br>1wwzA (157)	C	8.55	TM-align: aliSize=136 (resi) RMSD=2.69 (Å)<br>lvprGSHMDIRQMNK-T--HLEHWRGLRKQ-L------WPGHPDDAHLADGEEIlQAdHL-ASFIAMADGVAIGFADAS-IRHDYVngCdSSPVVFLEGIFVLPSFRQRGVAKQLIAAVQRWGTNkGCREMASDTSPENTISQKVHQALGFEEtERVIFyrkrc---------<br>----MDEIKIEKLKKlDkkALNELIDVYMSgYegleeyGGEGRDYARNYIKWCW-KK-ASdGFFVAKVGDKIVGFIVCDkDWFSKY--E-GRIVGAIHEFVVDKKFQGKGIGRKLLITCLDFLGK-YNDTIELWVGEKNYGAMNLYEKFGFKK-VGKSG-----iwvrmikrq<br><br>SARST: aliSize=139 (resi) RMSD=4.60 (Å)<br>lvprg-SHMDIRQM---NKTHLEHWRGLRKQLWPG------HPDDAHLADGEEILQADHLASFIAMADGVAIGFADasIRHDYVN---GcdsSPVVFLEGIFVLPSFRQRGVAKQLIAAVQRWGTNKgCREMASDTSPENTISQKVHQALGFEETERV--IFYRK---rc<br>-----mDEIKIEKLkklDKKALNELIDVYMSGYEGleeyggEGRDYARNYIKWCWKKASDGFFVAKVGDKIVGFIV--CDKDWFSkyeG---RIVGAIHEFVVDKKFQGKGIGRKLLITCLDFLGKY-NDTIELWVGEKNYGAMNLYEKFGFKKVGKSgiWVRMIkrq--<br><br>BLAST: aliSize=37 (resi) iden=14.47% (22/152) simi=24.34% (37/152)<br>lvprgshmdirqmnkthlehwrglrkqlwpghpddahladgeeilqadhlasfiamadgvai---GFADASIrHDYVNG---CDSSPVVFLEG-I------FVL-PSFRQRGVA-KQLIAAVQRWGTNKGCREMAsdT<br>--mdeikieklkkldkkalnelidvymsgyegleeyggegrdyarnyikwcwkkasdGFFVAKV-GDKIVGfivCDKDWFSKYEGrIvgaiheFVVdKKFQGKGIGrKLLITCLDFLGKYNDTIELW--V<br><br>SPENTISQKVHQALGFEE--------------tervifyrkrc<br>GEKNYGAMNLYEKFGFKKvgksgiwvrmikrq-----------<br><br>Proposed: aliSize=139 (resi) RMSD=2.37 (Å)<br>lvprGSHMDIRQM-NKT--HLEHWRGLRKQ-L------WPGhPDDAHLADGEEILQAdHL-ASFIAMADGVAIGFADAS-IRHDYVngC-dsSPVVFLEGIFVLPSFRQRGVAKQLIAAVQRWGTNkGCREMASDTSPENTISQKVHQAlGFEETERV-----IFYRKR-cc<br>----MDEIKIEKLkKLDkkALNELIDVYMSgYegleeyGGE-GRDYARNYIKWCWKK-ASdGFFVAKVGDKIVGFIVCDkDWFSKY--Eg--RIVGAIHEFVVDKKFQGKGIGRKLLITCLDFLGK-YNDTIELWVGEKNYGAMNLYEK-FGFKKVGKsgiwvRMIKRQa--
116	1s60A (152)<br>2cnmA (151)	C	14.57	TM-align: aliSize=127 (resi) RMSD=2.27 (Å)<br>lvprgsHMDIRQMNKTHLEHWRGLRKQ-L-WPGHPdDAHLaDgeEIlQAdHLASFIAMADGVAIGFADASIRHdyvngcdssPVVFLEGIFVLPSFRQRGVAKQLIAAVQRWGTNKGCREMASDTSPENTISQKVHQALGFEETERVIFYrkrc---------------------<br>------MNTISILSTTDLPAAWQIEQRaHaFPWSE-KTFF-G--NQ-GE-RYLNLKLTADDRMAAFAITQVVL---------DEATLFNIAVDPDFQRRGLGRMLLEHLIDELETRGVVTLWLEVRASNAAAIALYESLGFNEATIRRNY----yptaqghedaiimalpismklh<br><br>SARST: aliSize=122 (resi) RMSD=2.93 (Å)<br>lvprgsh-MDIRQMNKTHLEHWRGLRKQLWPGHPDDAHLAdgeeilqaDH----LASFIAMADGVAIGFADASIRHDyvngcdsspVVFLEGIFVLPSFRQRGVAKQLIAAVQRWGTNKGCREMASDTSPENTISQKVHQALGFEETERVI-------------------------fyrkrc<br>-------mNTISILSTTDLPAAWQIEQRAHAFPWSEKTFF--------GNqgerYLNLKLTADDRMAAFAITQVVLD---------EATLFNIAVDPDFQRRGLGRMLLEHLIDELETRGVVTLWLEVRASNAAAIALYESLGFNEATIRRnyyptaqghedaiimalpismklh------<br><br>BLAST: aliSize=43 (resi) iden=15.89% (24/151) simi=28.48% (43/151)<br>lvprgshmdirqmnkthlehwrglrkqlwpghpddahladgeeil-----------------------------------QADHLASFIAMADGVAIGFADASIRHDyvngcdsSPVVFleGIFVLPSFRQRGVAKQLIA-AVQRWGTnKGCREMASDTSPENTISQKVHQALGFEE-TERVIFY-----<br>---mntisilsttdlpaawqieqrahafpwsektffgnQGERYLNLKLTADDRMAAFAITQVVLD-------EATLF--NIAVDPDFQRRGLGRMLLEhLIDELET-RGVVTLWLEVRASNAAAIALYESLGFNEaTIRRNYYptaqg<br><br>----------------rkrc<br>hedaiimalpismklh----<br><br>Proposed: aliSize=131 (resi) RMSD=1.97 (Å)<br>lvprgsHMDIRQMNKTHLEHWRGLRKQ-L-WPgHPDDAHLADgeeIlQAdHLASFIAMADGVAIGFADASIRHdyvngcdssPVVFLEGIFVLPSFRQRGVAKQLIAAVQRWGTNKGCREMASDTSPENTISQKVHQALGFEETER---V---------IFYRKRC------c<br>------MNTISILSTTDLPAAWQIEQRaHaFP-WSEKTFFGN---Q-GE-RYLNLKLTADDRMAAFAITQVVL---------DEATLFNIAVDPDFQRRGLGRMLLEHLIDELETRGVVTLWLEVRASNAAAIALYESLGFNEATIrrnYyptaqghedAIIMALPismklh-

117	1s60A<br>(152)<br><br>1wk4A<br>(174)	C	17.11	TM-align: aliSize=137 (resi) RMSD=2.04 (Å)<br>lvprgshMDIRQMNKTHLEHWRGLRKQ-LW------------P-GHPDDAHLADGEEILQA--DHLASFIAMAD-GVAIGFADASIRhDYVNgCDSSpVVFLEGIFVLPSFRQRGVAKQLIAAVQRWGTNKGCREmASDTSPENTISQKVHQALGFEETERVIFYrkrc--------------------<br>-------VRIRRAGLEDLPGVARVLVDtWRatyrgvvpeafLeGLSYEGQAERWAQRLKTptWPGRLFVAESEsGEVVGFAAFGPD-RASG-FPGY-TAELWAIYVLPTWQRKGLGRALFHEGARLLQAEGYGR-LVWVLKENPKGRGFYEHLGGVLLGEREIE----lggaklwevaygfdlgghkw<br><br>SARST: aliSize=136 (resi) RMSD=4.51 (Å)<br>lvprgshm-DIRQMNKTHLEHWRGLR-----KQLWPGHPDDAH-------LADGEEILQA-DHL-ASFIAMAD--GVAIGFADASIRHD-YVNGCDSSpvvfLEGIFVLPSFRQRGVAKQLIAAVQRWGTNKGCREMaSDTSPENTISQKVHQALGFEETERVIFYR-------------------krc<br>--------vRIRRAGLEDLPGVARVLvdtwrATYRGVVPEAFLeglsyegQAERWAQRLKtPTWpGRLFVAESesGEVVGFAAFGPDRAsGFPGYTAE----LWAIYVLPTWQRKGLGRALFHEGARLLQAEGYGRL-VWVLKENPKGRGFYEHLGGVLLGEREIELggaklwevaygfdlgghkw---<br><br>BLAST: aliSize=40 (resi) iden=13.82% (21/152) simi=26.32% (40/152)<br>lvprgshmdirqmnkthlehwrglrkqlwpghpddahladgeeilqadhlas--FIAMAD-GVAIGFAdaSIRHDYVNGCDSSPVVfLEGIFVLPSFRQRGVAKQLIAAVQRWGTNKGCREMASdTSPENTI<br>--vrirragledlpgvarvlvdtwratyrgvvpeafleglsyegqaerwaqrlktptwpgrlFVAESEsGEVVGFA--AFGPDRASGFPGYTAE-LWAIYVLPTWQRKGLGRALFHEGARLLQAEGYGRLVW-VLKENPK<br><br>SQKVHQALG------------------------------feetervifyrkrc<br>GRGFYEHLGgvllgereielggaklwevaygfdlgghkw--------------<br><br>Proposed: aliSize=142 (resi) RMSD=1.87 (Å)<br>lvprgshMDIRQMNKTHLEHWRGLRKQLW------------P-GHPDDAHLADGEEILQA--DHLASFIAMAD-GVAIGFADASIRhDYVNgCDSSPVvFLEGIFVLPSFRQRGVAKQLIAAVQRWGTNKGCREMASDTSPENTISQKVHQALGFEETERVI-----------FYRKR---C--c<br>-------VRIRRAGLEDLPGVARVLVDTWratyrgvvpeafLeGLSYEGQAERWAQRLKTptWPGRLFVAESEsGEVVGFAAFGPD-RASG-FPGYTA-ELWAIYVLPTWQRKGLGRALFHEGARLLQAEGYGRMLVWVLKENPKGRGFYEHLGGVLLGEREielggaklwevAYGFDlggHkw-
118	1s60A<br>(152)<br><br>1gheA<br>(170)	C	15.13	TM-align: aliSize=131 (resi) RMSD=2.38 (Å)<br>lvprgshMDIRQMN---K-THLEHWRGLRKQL--------WP-GH-PDDAHLADGEEILQADHLASFIAMAdG-VAIGFADASIRHDyvngcd--SSPVVFLEGIfVLPSFRQRGVAKQLiAAVQRWGTNKGCREMASDTSPeNTISQKVHQALGFEETERVI-FYrkrc-----------------<br>-------AQLRRVTaesFaHYRHGLAQLLFETvhggasvgFAdLDqQAYAWCDGLKADIAAGSLLLWVVAE-DdNVLASAQLSLCQK------pnGLNRAEVQKL-VLPSARGRGLGRQL-DEVEQVAVKHKRGLLHLDTEA-GSVAEAFYSALAYTRVGELPgYC----atpdgrlhptaiyfktl<br><br>SARST: aliSize=136 (resi) RMSD=6.28 (Å)<br>lvprgshm-DIRQMNKTHLEH--------WRGLRKQLWP-GHPD-----DAHLADGEEILQADHLASFIAMADGVAIGFADASIRHDYVNGcDSSPVvfleGIFVLPSFRQRGVaKQLIAAVQRWGTNKGCREMASDTSPENTISQKVHqALGFEETER-------------VIFYRKR-c<br>--------aQLRRVTAESFAHyrhglaqlLFETVHGGASvGFADldqqaYAWCDGLKADIAAGSLLLWVVAEDDNVLASAQLSLCQKPNGL-NRAEV----QKLVLPSARGRGL-GRQLDEVEQVAVKHKRGLLHLDTEAGSVAEAFYS-ALAYTRVGElpgycatpdgrlhpTAIYFKTl-<br><br>BLAST: aliSize=45 (resi) iden=17.76% (27/152) simi=29.61% (45/152)<br>lvprgshmdirqmnkthlehwrglrkqlwpghpddahla---DGEEI-LQADHLASFIAMADGVAIGFADASIRHDyVNGCDSSPVVFLegifVLPSFRQRGVAKQLIAAVQRWGTNK-GCREMasDTSpENTISQKVHQALGF----<br>---------------------------------------aqlrrvtaesfahyrhglaqllfetvhggasvgfadldqqayawcDGLKAdIAAGSLLLWVVAEDDNVLASAQLSLCQK-PNGLNRAEVQKL----VLPSARGRGLGRQLDEVEQVAVKHKrGLLHL--DTE-AGSVAEAFYSALAYtrvg<br><br>-----------------------eetervifyrkrc<br>elpgycatpdgrlhptaiyfktl-------------<br><br>Proposed: aliSize=136 (resi) RMSD=1.96 (Å)<br>lvprgshMDIRQMN---K-THLEHWRGLRKQLW-------PG--H---PDDAHLADGEEILQADHLASFIAMAdG-VAIGFADASIR-H--DyvngcdsSPVVFLEGIFVLPSFRQRGVAKQLIAAVQRWGTNKGCREMASDTSPENtISQKVHQALGFEETERV-------------IFYRKRC-c<br>-------AQLRRVTaesFaHYRHGLAQLLFETVhggasvgFMadLdmqQAYAWCDGLKADIAAGSLLLWVVAE-DdNVLASAQLSLCqKpnG-------LNRAEVQKLMVLPSARGRGLGRQLMDEVEQVAVKHKRGLLHLDTEAGS-VAEAFYSALAYTRVGELpgycatpdgrlhptAIYFKTLa-
119	1s60A<br>(152)<br><br>1u6mA<br>(189)	C	13.16	TM-align: aliSize=135 (resi) RMSD=2.89 (Å)<br>lvprgshMDIRQMNKTHLEHWRGLRKQLWP---G--H-------PDDAHLADGEEilQADH---LASFIAMADGVAIGFADASIRHD---------------------------YVNgCdsSpVVFLEGIFVLPSFRQRGVAKQLIAAVQRWGTNKGCREMASDTSPENTISQKVHQALGFEETERVIFY<br>-------SLIRSATKEDGQAIARLVLVILKdmeLpiLeevseeqMIDLLAEATAY--PTYRygyQRILVYEHAGEVAGIAVGYPAEDekiideplrevfkkhglaedvrlfieeETL-P--N-EWYLDTISVDERFRGMGIGSKLLDALPEVAKASGKQALGLNVDFDNPGARKLYASKGFKDVTTMTIS<br><br>rkrc------------<br>----ghlynhmqkeve<br><br>SARST: aliSize=138 (resi) RMSD=5.87 (Å)<br>lvprgshm-DIRQMNKTHLEHWRGLRKQLwPG---------HPDDAHLADGEEILQADHL-----ASFIAMADGVAIGFADAS--------------------IRHDY--VNGCDSSP-VVFLEGIFVLPSFRQRGVAKQLIAAVQRWGTNKGCREMASDTSPENTISQKVHQALGFEETERVIF-----<br>--------sLIRSATKEDGQAIARLVLVI-LKdmelpileeVSEEQMIDLLAEATAYPTYrygyqRILVYEHAGEVAGIAVGYpaedekiideplrevfkkhgLAEDVrlFIEEETLPnEWYLDTISVDERFRGMGIGSKLLDALPEVAKASGKQALGLNVDFDNPGARKLYASKGFKDVTTMTIsghly<br><br>-------yrkrc<br>nhmqkev-----<br><br>BLAST: aliSize=16 (resi) iden=6.58% (10/152) simi=10.53% (16/152)<br>lvprgshmdirqmnkthlehwrglrkqlwp---GHPDDAHLADGEEILQADHLASFIAMAD---GVAIG-------------------------------<br>------------------------------slirsatkedgqaiarlvlvilkdmelpileevseeqmidllaeataypty rygyqrilvyehagevagiavgypaedekiideplrevfkkhGLAEDVRLFIEEETLPNEWYLDTISVDErfrGMGIGsklldalpevakasgkqalglnvdfdnpgar<br><br>----------------------------fadasirhdyvngcdsspvvflegifvlpsfrqrgvakqliaavqrwgtnkgcremasdtspentisqkvhqalgfeetervifyrkrc<br>klyaskgfkdvttmtisghlynhmqkev---<br><br>Proposed: aliSize=134 (resi) RMSD=2.27 (Å)<br>lvprgshMDIRQMNKTHLEHWRGLRKQLWP--GH----------PDDAHLADGEEilQA-----dhLASFIAMADGVAIGFADASIRH-----------------------------dyVNgcdsSPVVFLEGIFVLP-SFRqRGVAKQLIAAVQRWGTNKGCREMASDTSPENTISQKVHQALGFEETE<br>-------SLIRSATKEDGQAIARLVLVILKdmELpileevseeqMIDLLAEATAY--PTyrygy--QRILVYEHAGEVAGIAVGYPAEdekiideplrevfkkhglaedvrlfieee--TL----PNEWYLDTISVDErFRG-MGIGSKLLDALPEVAKASGKQALGLNVDFDNPGARKLYASKGFKDVT<br><br>RVI----FYRKRC----c<br>TMTisghLYNHMQkeve-

120	1s60A (152)<br>2r7hA (157)	C	15.13	TM-align: aliSize=136 (resi) RMSD=2.57 (Å)<br>lvprgsHMDIRQMNK-THLEHWRGLRK-Q-LWPGHPDDAHLADGEEILQADHLASFIAMADGVaIGFADASIRHDyvngcdSSPVVFLEGIFVLPSFRQRGVAKQLIAAVQRWGTNKGCREMASDTSPE--NTISQKVHQALGFEETERVI-FYRkrc-------------<br>------AVAFRRQVLpQDALLVRRVVEsTgFFTPEEADVAQELVDEHLHACGYHFVFATEDDD-AGYACYGPTPA------TEGTYDLYWIAVAPHRQHSGLGRALLAEVVHDVRLTGGRLLFAETSGIrkYAPTRRFYERAGFSAEAVLKaFYR---agddkiiyrleva<br><br>SARST: aliSize=131 (resi) RMSD=3.96 (Å)<br>lvprgs-HMDIRQMNKTHLEHWRGL--RKQLWpghPDDAHLADGEEILQ--ADHLASFIAMADGVAIGFADASirhdYVNGcdSSPVVFLEGIFVLPSFRQRGVAKQLIAAVQRWGTNKGCREMASDT--SPENTISQKVHQALGFEETERVI-----------------fyrkrc<br>------aVAFRRQVLPQDALLVRRVveSTGFF---TPEEADVAQELVDEhlHACGYHFVFATEDDDAGYACYG----PTPA--TEGTYDLYWIAVAPHRQHSGLGRALLAEVVHDVRLTGGRLLFAETsgIRKYAPTRRFYERAGFSAEAVLKafyragddkiiyrleva------<br><br>BLAST: aliSize=54 (resi) iden=21.71% (33/152) simi=35.53% (54/152)<br>lvprgshmdirqmnkthlehwrglrkqlwpgh-------------------------------PDDAHLAdgEEILQaDHLasfiaMADGVAIGFADASIRHDYvnGCdSSPVVFLEG------IFVLPSFRQRGVAKQLIAAVQRWGTNKGCREMASDTS--PENTISQKVHQALGFeETERVI--FYR----<br>-------------------------------avafrrqvlpqdallvrrvvestgfftPEEADVA--QELVD-EHL-----HACGYHFVFATEDDDAGY--AC-YGPTPATEGtydlywIAVAPHRQHSGLGRALLAEVVHDVRLTGGRLLFAETSgiRKYAPTRRFYERAGF-SAEAVLkaFYRagdd<br><br>---------krc<br>kiiyrleva---<br><br>Proposed: aliSize=138 (resi) RMSD=2.18 (Å)<br>lvprgsHMDIRQMNK-THLEHWRGLRK-Q-LWPGH--PddAHLADGEEIL-QADHLASFIAMADGVAIGFADASIRHDyvngcdSSPVVFLEGIFVLPSFRQRGVAKQLIAAVQRWGTNKGCREMASDTSP-E-NTISQKVHQALGFEETERVI-------FYRKRC----c<br>------AVAFRRQVLpQDALLVRRVVEsTgFFTPEeaD--VAQELVDEHLmHACGYHFVFATEDDDMAGYACYGPTPA------TEGTYDLYWIAVAPHRQHSGLGRALLAEVVHDVRLTGGRLLFAETSGiRkYAPTRRFYERAGFSAEAVLKafyragdDKIIYRleva-
121	1s60A (152)<br>1yr0A (163)	C	17.11	TM-align: aliSize=133 (resi) RMSD=2.47 (Å)<br>lvprgsHMDIRQMNKTHLEHWrGLRKQLWP-------GHPDDaHLADGEEILQ--ADHLASFIAMADGVAIGFADASIRHDYvngcd--SSPVVFLeGIFVLPSFRQRGVAKQLiAAVQRWGTNKGCREMASDTSPENTISQKVHQALGFEETERVI-FYrkrc---------------<br>------SVELRDATVDDLSGI-EIYNDAVVnttaiwnEVVVD-LENRKDWFAArtSRGFPVIVAILDGKVAGYASYGDWRAF-----dgYRHTREH-SVYVHKDARGHGIGKRL-QALIDHAGGNDVHVLIAAIEAENTASIRLHESLGFRVVGRFSeVG----tkfgrwldltcelkl<br><br>SARST: aliSize=133 (resi) RMSD=4.36 (Å)<br>lvprgsh-MDIRQMNKTHLEH-----WRGLRKQLW---PGHPDDAHLADGEEILQaDHLASFIAMADGVAIGFADASirhdYVNGCDSSPVVFLEGIFVLPSFRQRGVaKQLIAAVQRWGTNKGCREMASDTSPENTISQKVHQALGFEETERVI------------------fyrkrc<br>-------sVELRDATVDDLSGieiynDAVVNTTAIwneVVVDLENRKDWFAARTS-RGFPVIVAILDGKVAGYASYG----DWRAFDGYRHTREHSVYVHKDARGHGI-GKRLQALIDHAGGNDVHVLIAAIEAENTASIRLHESLGFRVVGRFSevgtkfgrwldltcelkl------<br><br>BLAST: aliSize=39 (resi) iden=15.79% (24/152) simi=25.66% (39/152)<br>lvprgshmdirqmnkthlehwrglrkqlwpghpddahladgeeilqadhlasf---IAMADGVAIGFADASirhDYvNGCDSSPVVFLEGIFVLPSFRQRGVAKQLIAAVQRWGTNkGCREMASDTSPENTISQKVHQA<br>---svelrdatvddlsgieiyndavvnttaiwnevvvdlenrkdwfaartsrgfpviVAILDGKVAGYASYG---DW-RAFDGYRHTREHSVYVHKDARGHGIGKRLQALIDHAGGN-DVHVLIAAIEAENTASIRLHES<br><br>LGFEETER--------------------vifyrkrc<br>LGFRVVGRfsevgtkfgrwldltcelkl--------<br><br>Proposed: aliSize=137 (resi) RMSD=2.04 (Å)<br>lvprgsHMDIRQMNKTHLEHWRGLRKQLWP-------GHPDDahLADGEEILQA--DH-LASFIAMADGVAIGFADASIR-HD--YvngcdsSPVVFLeGIFVLPSFRQRGVAKQLIAAVQRWGTNKGCREMASDTSPENTISQKVHQALGFEETERV-----------IFYRKRC-c<br>------SVELRDATVDDLSGIMEIYNDAVVnttaiwnEVVVD--LENRKDWFAArtSRgFPVIVAILDGKVAGYASYGDWrAFdgY------RHTREH-SVYVHKDARGHGIGKRLMQALIDHAGGNDVHVLIAAIEAENTASIRLHESLGFRVVGRFsevgtkfgrwldLTCMELKl-
122	1s60A (152)<br>2fiaA (157)	C	13.16	TM-align: aliSize=129 (resi) RMSD=2.75 (Å)<br>lvprgshMDIRQMNKTHLEHWRGLRK-QL----------WPGHPDdAHlADGEEILQAdhLASFIAMADGVAIGFADASIRHdyvngcds-SPVVFLEGIFVLPSFRQRGVAKQLIAAVQRWGTNKGCREMASDTSPENTISQKVHQAlGFEETERVIFYRkrc---------------<br>-------MKIRVADEKELPMILQFLTeVKaymdvvgitqWTKDYP-SQ-GDIQEDITK--KRLYLLVHEEMIFSMATFCMEQ--------eQDFVWLKRFATSPNYIAKGYGSLLFHELEKRAVWEGRRKMYAQTNHTNHRMIRFFES-KGFTKIHESLQM---nrldfgsfylyvkele<br><br>SARST: aliSize=124 (resi) RMSD=6.15 (Å)<br>lvprgshmd--IRQMNKTHLEHWRGLRKqlwpghpDDAHLA--------------DGEEILQADHLASFIAMADGVAIGFadasIRHDYVNGCDSSPVVFLegiFVLPSFRQRGVAKQLIAAVQRWGTNKGCREMASDTSPENTISQKVHQALGFEETERVIF-----------------yrkrc<br>---------mkIRVADEKELPMILQFLT-------EVKAYMdvvgitqwtkdypsQGDIQEDITKKRLYLLVHEEMIFSM----ATFCMEQEQDFVWLKRF---ATSPNYIAKGYGSLLFHELEKRAVWEGRRKMYAQTNHTNHRMIRFFESKGFTKIHESLQmnrldfgsfylyvkele-----<br><br>BLAST: aliSize=34 (resi) iden=11.18% (17/152) simi=22.37% (34/152)<br>lvprgshmdirqmnkthlehwrglrkqlwpghpddahladgeeilqadhlasfiamadgvaigfadasirhdyvngcdsspv---VFLEGIFVLPSFRQRGVAKQLIAAVQRWGTNKG<br>--mkirvadekelpmilqfltevkaymdvvgitqwtkdypsqgdiqeditkkrlyllvheemifsmatfcmeqeqdfVWLKRFATSPNYIAKGYGSLLFHELEKRAVWEG<br><br>CREMASDTSPEN--TI----SQ---KVHQALGFEETERVIFY-------rkrc<br>RRKMYAQTNHTNhrMIrffeSKgftKIHESLQMNRLDFGSFYlyvkele----<br><br>Proposed: aliSize=129 (resi) RMSD=2.25 (Å)<br>lvprgshMDIRQMNKTHLEHWRGLRK-QL----------WPGH--pddAHlADGEEILQAdhLASFIAMADGVAIGFADASIR--hdyvngcdsSPVVFLEGIFVLPSFRQRGVAKQLIAAVQRWGTNKGCREMASDTSPENTISQKVHQAlGFEETERV-----------IFYRKRC-c<br>-------MKIRVADEKELPMILQFLTeVKaymdvvgitqWTKDyp---SQ-GDIQEDITK--KRLYLLVHEEMIFSMATFCMEqe---------QDFVWLKRFATSPNYIAKGYGSLLFHELEKRAVWEGRRKMYAQTNHTNHRMIRFFES-KGFTKIHEslqmnrldfgsfYLYVKELe-

123	1s60A (152)<br>1cm0B (161)	C	7.89	TM-align: aliSize=125 (resi) RMSD=2.76 (Å)<br>lvprgSHMDIRQMN-------K--T--HLEHWRGLRKQ-LW-PGHPDdAHlADGEEIlqAdhLASFIAMADGVAIGFADASIRHDyvngcdsSPVVFLEGIFVLPSFRQRGVAKQLIAAVQRWGTNKGCREMASDTSpeNtISQKVHQALGFEETErvi------------FYRkrc-----------<br>-----KVIEFHVVGnslnqkpNkkIlmWLVGLQNVFSHqLPrMPKEY-IT-RLVFDP--K--HKTLALIKDGRVIGGICFRMFPS-------QGFTEIVFCAVTSNEQVKGYGTHLMNHLKEYHIKHDILNFLTYAD--E-YAIGYFKKQGFSKEI---kipktkyvgyikDYE---gatlmgcelnp<br><br>SARST: aliSize=112 (resi) RMSD=3.24 (Å)<br>lvprgshmdirqmn--------------------KTHLEHWRGLRKQLWPGHPDDAHLADgeeiLQADHLASFIAMADGVAIGFADASIRHDyvNGcdsspVVFLEGIFVLPSFRQRGVAKQLIAAVQRWGTNKGCREMASDTSPEntiSQKVHQALGFE----------------------------e<br>-------------kviefhvvgnslnqkpnkkiLMWLVGLQNVFSHQLPRMPKEYITRL----VFDPKHKTLALIKDGRVIGGICFRMFPS--QG-----FTEIVFCAVTSNEQVKGYGTHLMNHLKEYHIKHDILNFLTYADEY---AIGYFKKQGFSkeikipktkyvgyikdyegatlmgcelnp-<br>tervifyrkrc<br>-----------<br><br>BLAST: aliSize=16 (resi) iden=6.58% (10/152) simi=10.53% (16/152)<br>lvprgshmdirqmnkthlehwrglrkqlwpghpdda--HLADGEEILQADH-LASFIAMADGVAIG------FA----------------------<br>------------------------------------kviefhvvgnslnqkpnkkilmwlvglqnvfshqlprmpkeyitrlvfdpkhktlalikdgrviggicfrmfpsqgfteivfcavtsneqvkgygtHLMNHLKEYHIKHdILNFLTYADEYAIGyfkkqgFSkeikipktkyvgyikdyegatl<br>-------dasirhdyvngcdsspvvflegifvlpsfrqrgvakqliaavqrwgtnkgcremasdtspentisqkvhqalgfeetervifyrkrc<br>mgcelnp---<br><br>Proposed: aliSize=130 (resi) RMSD=2.12 (Å)<br>lvprgSHMDIRQMN----------KT-HLEHWRGLRKQLWP--GHPddAHLADGEEilQAdhLASFIAMADGVAIGFADASIR-HdyvngcdsSPVVFLEGIFVLPSFRQRGVAKQLIAAVQRWGTNKGCREMASDTSpENtiSQKVHQALGFEET----------------ERVIFYRKRC-c<br>-----KVIEFHVVGnslnqkpnkkILmWLVGLQNVFSHQLPrmPKE--YITRLVFD--PK--HKTLALIKDGRVIGGICFRMFpS--------QGFTEIVFCAVTSNEQVKGYGTHLMNHLKEYHIKHDILNFLTYAD-EY--AIGYFKKQGFSKEikipktkyvgyikdyegATLMGCELNPc-
124	1s60A (152)<br>1mk4A (157)	C	19.74	TM-align: aliSize=132 (resi) RMSD=2.67 (Å)<br>lvprgsHMDIRQMNKTHLEHWRGLRK-QL---WPGHPddaHlaDGeEILQADHLASFIAMADGVAIGFADASIRHDYvngcdsSPVVFLEGIFVLPSFRQRGVAKQLIAAVQRWGTNKGCREMASDTSPENTISQKVHQALGFEETE---------RVI-FYRKrc-----------<br>------HMDIRTITSSDYEMVTSVLNeWWggrQLKEK---L--PR-LFFEHFQDTSFITSEHNSMTGFLIGFQSQSD------PETAYIHFSGVHPDFRKMQIGKQLYDVFIETVKQRGCTRVKCVTSPVNKVSIAYHTKLGFDIEKgtktvngisVFAnYDGP--gqdrvlfvkni<br><br>SARST: aliSize=126 (resi) RMSD=5.15 (Å)<br>lvprgsh-MDIRQMNKTHLEHWRGLRKQLWPGH------PDDAHLADGeeilqaDHLASFIAmaDGVAIGFADASIRHdyvngcDSSPVVFLEGIFVLPSFRQRGVAKQLIAAVQRWGTNKGCREMASDTSPENTISQKVHQALGFeetERVIFYRK------------------------rc<br>-------hMDIRTITSSDYEMVTSVLNEWWGGRqlkeklPRLFFEHFQ------DTSFITSE--HNSMTGFLIGFQSQ------SDPETAYIHFSGVHPDFRKMQIGKQLYDVFIETVKQRGCTRVKCVTSPVNKVSIAYHTKLGF---DIEKGTKTvngisvfanydgpgqdrvlfvkni--<br><br>BLAST: aliSize=54 (resi) iden=23.68% (36/152) simi=35.53% (54/152)<br>lvprgsHMDIRQMNKTHLEHWRGLRKQLWPGHpddaHLADGEEILQADHL--ASFIAMADGVAIGFAdasIRHDYVNGCDSSPVVFlEGIFvlPSFRQRGVAKQLIAAVQRWGTNKGCREMASDTSPENTISQKVHQALGFE--------------------------------etervifyrkrc<br>------HMDIRTITSSDYEMVTSVLNEWWGGR----QLKEKLPRLFFEHFqdTSFITSEHNSMTGFL---IGFQSQSDPETAYIHF-SGVH--PDFRKMQIGKQLYDVFIETVKQRGCTRVKCVTSPVNKVSIAYHTKLGFDiekgtktvngisvfanydgpgqdrvlfvkni-----------<br><br>Proposed: aliSize=128 (resi) RMSD=2.07 (Å)<br>lvprgsHMDIRQMNKTHLEHWRGLRK-QL---WPG-HpddaHlaDGeEILQAdhL--ASFIAMADGVAIGFADASIRHDyvngcdsSP-VVFLEGIFVLPSFRQRGVAKQLIAAVQRWGTNKGCREMASDTSPENTISQKVHQALGFEETE--------------------RVIFYRKrcc<br>------HMDIRTITSSDYEMVTSVLNeWWggrQLKeK----L--PR-LFFEH--FqdTSFITSEHNSMTGFLIGFQSQS-------DPeTAYIHFSGVHPDFRKMQIGKQLYDVFIETVKQRGCTRVKCVTSPVNKVSIAYHTKLGFDIEKgtktvngisvfanydgpgqdrVLFVKNI---
125	1s60A (152)<br>1z4rA (163)	C	7.24	TM-align: aliSize=126 (resi) RMSD=2.83 (Å)<br>lvprg-SHMDIRQMN----K-----T--HLEHWRGLRKQ-LWPGHPDdAHLADGEEilQAdhLASFIAMADGVAIGFADASIRHDyvngcdsSPVVFLEGIFVLPSFRQRGVAKQLIAAVQRWGTNKGCREMASDTSPeNtiSQKVHQALGFEETErvi------------FYRkrc-----------<br>-----sGIIEFHVIGnsltPkanrrVllWLVGLQNVFSHqLPRMPKE-YIARLVFD--PK--HKTLALIKDGRVIGGICFRMFPT-------QGFTEIVFCAVTSNEQVKGYGTHLMNHLKEYHIKHNILYFLTYADE-Y--AIGYFKKQGFSKDI---kvpksrylgyikDYE---gatlmecelnpr<br><br>SARST: aliSize=104 (resi) RMSD=3.31 (Å)<br>lvprgshmdirqmnkth------------------------LEHWRGLRKQLWPGHPddahLADGEEILQADHLASFIAMADGVAIGFADASIR--HdyvnGcdsspVVFLEGIFVLPSFRQRGVAKQLIAAVQRWGTNKGCREMASDTSPENTISQKVH-----------------------------<br>-----------------sgiiefhvignsltpkanrrvllwLVGLQNVFSHQLPRMP----KEYIARLVFDPKHKTLALIKDGRVIGGICFRMFptQ----G-----FTEIVFCAVTSNEQVKGYGTHLMNHLKEYHIKHNILYFLTYADEYAIGYFKKQgfskdikvpksrylgyikdyegatlmecel<br>---qalgfeetervifyrkrc<br>npr------------------<br><br>BLAST: aliSize=22 (resi) iden=6.58% (10/152) simi=14.47% (22/152)<br>lvprgshmdirqmnkthlehwrglrkqlwpghpddahladgeeilqadhlasfiamadgvaigfadasirhdyvngcdsspvvflegifvlps--FRQRGVAKQLIAAVQRWGTNKGCRE<br>---sgiiefhvignsltpkanrrvllwlvglqnvfshqlprmpkeyiarlvfdpkhktlalikdgrviggicfrmFPTQGFTEIVFCAVTSNEQVKGYGT<br>MASDTSPENTISQKVHQALGFEETERVIFYRKR---------------------------------c<br>HLMNHLKEYHIKHNILYFLTYADEYAIGYFKKQgfskdikvpksrylgyikdyegatlmecelnpr-<br><br>Proposed: aliSize=131 (resi) RMSD=2.26 (Å)<br>lvprg-SHMDIRQMN----------KT-HLEHWRGLRKQLWP-GHPDdAHLADGEEilQAdhLASFIAMADGVAIGFADASIR-HdyvngcdsSPVVFLEGIFVLPSFRQRGVAKQLIAAVQRWGTNKGCREMASDTSPeNtiSQKVHQALGFEET----------------ERVIFYRKRC--c<br>-----sGIIEFHVIGnsltpkanrrVLlWLVGLQNVFSHQLPrMPKE-YIARLVFD--PK--HKTLALIKDGRVIGGICFRMFpT--------QGFTEIVFCAVTSNEQVKGYGTHLMNHLKEYHIKHNILYFLTYADE-Y--AIGYFKKQGFSKDikvpksrylgyikdyegATLMECELNPra-

<table>
<tr><td rowspan="2">126</td><td>1s60A
(152)</td><td rowspan="2">C</td><td rowspan="2">13.16</td><td rowspan="2">TM-align: aliSize=135 (resi)   RMSD=2.70 (Å)
lvprgSHMDIRQMNKTHLEHWRGLRKQLWPGHp-Dd-AHlADGEEILQADHLASFIAMADGVAIGFADASIRHdYVNgcds------------------SPVVFLEGIFVLPSFRQRGVAKQLIAAVQ-RWGTNKGCREMASDTSPEN-----------------------TISQKVHQALGFEETERVIF
-----NYYNLRHPKIEDLRDLIALETLCWSEN-lQ-vDN-EEIYRRIFKIPQGQFILELEDKIVGAIYSQRID-NPQ----lldnktctqvpllhtesGVVVQLLAVNILPELQNQGLGDRLLEFMLqYCAQISGVEKVVAVTLCRNypdyspmpmaeyihqknesgllvDPLLRFHQIHGAKIEKLLPG
Yrkrc---------------------
Y----rpkdwenqtcgvlvsydiqhr

SARST: aliSize=136 (resi)   RMSD=4.90 (Å)
lvprgs-HMDIRQMNKTHLEHWRGLRKQLWP---GHPDDAHLADGEEILQAdhlaSFIAMADGVAIGFADASIR-----HDYVN-------GCDSSPVVFLEGIFVLPSFRQRGVAKQLIAAVQRWGTN-KGCREMASDT-----------SPENT------------ISQKVHQALGFEETERVI----
------nYYNLRHPKIEDLRDLIALETLCWSenlQVDNEEIYRRIFKIPQG----QFILELEDKIVGAIYSQRIdnpqlLDNKTctqvpllHTESGVVVQLLAVNILPELQNQGLGDRLLEFMLQYCAQiSGVEKVVAVTlcrnypdyspmPMAEYihqknesgllvdPLLRFHQIHGAKIEKLLPgyrp
-------------------fyrkrc
kdwenqtcgvlvsydiqhr------

BLAST: aliSize=32 (resi)   iden=11.18% (17/152)   simi=21.05% (32/152)
lvprgshmdirqmnkthlehwrglrkqlwpghpddahladgeeilqadhlasfiamadgvaigfadasirhdyvngc---DSSPVVFLEGIFVLPSFRQRGVAKQLIAAV
---nyynlrhpkiedlrdlialetlcwsenlqvdneeiyrrifkipqgqfileledkivgaiysqridnpqlldnktctqvpllhtESGVVVQLLAVNILPELQNQGLGDRLLEFM
QRW-----GTNKG-----CREMaSDTSPeNTISQKVHQ--algfeetervifyrkrc
LQYcaqisGVEKVvavtlCRNY-PDYSP-MPMAEYIHQknesgllvdpllrfhqihgakiekllpgyrpkdwenqtcgvlvsydiqhr-----------------

Proposed: aliSize=136 (resi)   RMSD=2.23 (Å)
lvprgSHMDIRQMNKTHLEHWRGLRKQLWPG---HpddAHlADGEEILQAdhL--ASFIAMADGVAIGFADASIRHdYVN-----------------gcdsSPVVFLEGIFVLPSFRQRGVAKQLIAAVQRWGT-NKGCREMASDTSPEN-----------------------TISQKVHQALGFEETER
-----NYYNLRHPKIEDLRDLIALETLCWSEnlqV---DN-EEIYRRIFK--IpqGQFILELEDKIVGAIYSQRID-NPQlldnktctqvpllhtes----GVVVQLLAVNILPELQNQGLGDRLLEFMLQYCAqISGVEKVVAVTLCRNypdyspmpmaeyihqknesgllvDPLLRFHQIHGAKIEKL
VI------FYR---KRC--------c
LPgyrpkdWENqtcGVLvsydiqhr-</td></tr>
<tr><td>2reeA
(199)</td></tr>
<tr><td rowspan="2">127</td><td>1s60A
(152)</td><td rowspan="2">N</td><td rowspan="2">11.18</td><td rowspan="2">TM-align: aliSize=137 (resi)   RMSD=2.90 (Å)
lvprg----------------SHMDIRQMNKTHLEHWRGLRK--Q--LWP---GHPD-DAHLADGEEILQA--D-HLASFIAMADGVAIGFADASIRHDyvngcdSSPVVFLEgIFVLPSFRQRGVAKQLIAAVQRWGT-NKGCREMASDTSPENTISQKVHQALGFEETERVI-FYRkrc---------
-----glvprgshmveiipvsTTLELRAADESHVPALHQLVLknKawLQQsldWPTSqEETRKHVQGNILLhqRgYAKMYLIFCQNEMAGVLSFNAIEP------INKAAYIG-YWLDESFQGQGIMSQSLQALMTHYArRGDIRRFVIKCRVDNQASNAVARRNHFTLEGCMKqAEY---lngdyhdvn
------
myarii

SARST: aliSize=131 (resi)   RMSD=3.93 (Å)
lvprgs----------------HMDIRQMNKTHLEHWRGL--------RKQLWPGHPDDAHLADGEEILQAD---HLASFIAMADGVAIGFAdasIRHDYV--NGCdsspVVFleGIFVLPSFRQRGVAKQLIAAVQRWGTNKG-CREMASDTSPENTISQKVHQALGFEETERVI------------
------glvprgshmveiipvstTLELRAADESHVPALHQLvlknkawlQQSLDWPTSQEETRKHVQGNILLHqrgYAKMYLIFCQNEMAGVL---SFNAIEpiNKA----AYI--GYWLDESFQGQGIMSQSLQALMTHYARRGdIRRFVIKCRVDNQASNAVARRNHFTLEGCMKqaeylngdyhdvn
------fyrkrc
myarii------

BLAST: aliSize=15 (resi)   iden=7.89% (12/152)   simi=9.87% (15/152)
-LVPRGSHM-DIRQMNKThLE--hwrglrkq
gLVPRGSHMvEIIPVSTT-LElraadeshvpalhqlvlknkawlqqsldwptsqeetrkhvqgnillhqrgyakmylifcqnemagvlsfnaiepinkaayigywldesfqgqgimsqslqalmthyarrgdirrfvikcrvdnqasnavarrnhftlegcmkqaeylngdyhdvnmyarii--------
lwpghpddahladgeeilqadhlasfiamadgvaigfadasirhdyvngcdsspvvflegifvlpsfrqrgvakqliaavqrwgtnkgcremasdtspentisqkvhqalgfeetervifyrkrc
----

Proposed: aliSize=135 (resi)   RMSD=2.24 (Å)
--L--------VPRGS-HMDIRQMNKTHLEHWRGLRK--Q--L-----WpgHPD-DAHLADGEEILQA--D-HLASFIAMADGVAIGFADASIRH--dyvngcdsSPVVFLEgIFVLPSFRQRGVAKQLIAAVQRWGT-N-kGCREMASDTSPENTISQKVHQALGFEETERVI-F-------------
glVprgshmveIIPVStTLELRAADESHVPALHQLVLknKawLqqsldW--PTSqEETRKHVQGNILLhqRgYAKMYLIFCQNEMAGVLSFNAIEpi--------NKAAYIG-YWLDESFQGQGIMSQSLQALMTHYArRg-DIRRFVIKCRVDNQASNAVARRNHFTLEGCMKqAeylngdyhdvnmya
---yrkrcc
rii------</td></tr>
<tr><td>1s7fA
(181)</td></tr>
<tr><td rowspan="2">128</td><td>1s60A
(152)</td><td rowspan="2">C</td><td rowspan="2">10.53</td><td rowspan="2">TM-align: aliSize=137 (resi)   RMSD=2.90 (Å)
lvprg----------------SHMDIRQMNKTHLEHWRGLRK--Q--LWP---GHPD-DAHLADGEEILQA--D-HLASFIAMADGVAIGFADASIRHDyvngcdSSPVVFLEgIFVLPSFRQRGVAKQLIAAVQRWGT-NKGCREMASDTSPENTISQKVHQALGFEETERVI-FYRkrc---------
-----glvprgshmveiipvsTTLELRAADESHVPALHQLVLknKawLQQsldWPTSqEETRKHVQGNILLhqRgYAKMYLIFCQNEMAGVLSFNAIEP------INKAAYIG-YWLDESFQGQGIMSQSLQALMTHYArRGDIRRFVIKCRVDNQASNAVARRNHFTLEGCMKqAEY---lngdyhdvn
------
myarii

SARST: aliSize=131 (resi)   RMSD=3.93 (Å)
lvprgs----------------HMDIRQMNKTHLEHWRGL--------RKQLWPGHPDDAHLADGEEILQAD---HLASFIAMADGVAIGFAdasIRHDYV--NGCdsspVVFleGIFVLPSFRQRGVAKQLIAAVQRWGTNKG-CREMASDTSPENTISQKVHQALGFEETERVI------------
------glvprgshmveiipvstTLELRAADESHVPALHQLvlknkawlQQSLDWPTSQEETRKHVQGNILLHqrgYAKMYLIFCQNEMAGVL---SFNAIEpiNKA----AYI--GYWLDESFQGQGIMSQSLQALMTHYARRGdIRRFVIKCRVDNQASNAVARRNHFTLEGCMKqaeylngdyhdvn
------fyrkrc
myarii------

BLAST: aliSize=15 (resi)   iden=7.89% (12/152)   simi=9.87% (15/152)
-LVPRGSHM-DIRQMNKThLE--hwrglrkq
gLVPRGSHMvEIIPVSTT-LElraadeshvpalhqlvlknkawlqqsldwptsqeetrkhvqgnillhqrgyakmylifcqnemagvlsfnaiepinkaayigywldesfqgqgimsqslqalmthyarrgdirrfvikcrvdnqasnavarrnhftlegcmkqaeylngdyhdvnmyarii--------
lwpghpddahladgeeilqadhlasfiamadgvaigfadasirhdyvngcdsspvvflegifvlpsfrqrgvakqliaavqrwgtnkgcremasdtspentisqkvhqalgfeetervifyrkrc
----

Proposed: aliSize=134 (resi)   RMSD=2.21 (Å)
lvprgs----------------HMDIRQMNKTHLEHWRGLRK--Q--L-----WpgHPD-DAHLADGEEILQA--D-HLASFIAMADGVAIGFADASIRH--dyvngcdsSPVVFLEgIFVLPSFRQRGVAKQLIAAVQRWGT-N-kGCREMASDTSPENTISQKVHQALGFEETERVI---FY----R
------glvprgshmveiipvstTLELRAADESHVPALHQLVLknKawLqqsldW--PTSqEETRKHVQGNILLhqRgYAKMYLIFCQNEMAGVLSFNAIEpi--------NKAAYIG-YWLDESFQGQGIMSQSLQALMTHYArRg-DIRRFVIKCRVDNQASNAVARRNHFTLEGCMKqaeYLngdyH
KRC------c
DVNmyarii-</td></tr>
<tr><td>1s7fA
(181)</td></tr>
</table>

129	1s60A (152)<br>1m1dA (163)	C	8.55	TM-align: aliSize=122 (resi) RMSD=2.58 (Å)<br>lvprgsHMDIRQM-------NKTHLEHWRGLRKQ-LW-PGHPDdAHlADGEEIlqAdhLASFIAMAD-GVAIGFADASIRHDyvngcdsSPVVFLEGIFVLPSFRQRGVAKQLIAAVQRWGTNKGCREMASDTSpENtiSQKVHQALGFEETErvi------------Fyrkrc-------------------<br>------LLDFDILtndgthrNMKLLIDLKNIFSRqLPkMPKEY-IV-KLVFDR--H--HESMVILKNkQKVIGGICFRQYKP-------QRFAEVAFLAVTANEQVRGYGTRLMNKFKDHMQKQNIEYLLTYAD-NF--AIGYFKKQGFTKEH---rmpqekwkgyikD-----ydggtlmecyihpyvdygr<br><br>SARST: aliSize=105 (resi) RMSD=9.67 (Å)<br>lvprgshmdirqmnkthlehwrglrkqlwpghpddah-----------------------------------LADGEEILQADHLASFIAMADG-VAIGFADASIR--HdyvnGCdsspvVFLEGIFVLPSFRQRGVAKQLIAAVQRWGTNKGCREMASDTSPENTISQK-----------VHQALG--FE<br>-------------------------------------lldfdiltndgthrnmkllidlknifsrqlpkmpKEYIVKLVFDRHHESMVILKNKqKVIGGICFRQYkpQ----RF-----AEVAFLAVTANEQVRGYGTRLMNKFKDHMQKQNIEYLLTYADNFAIGYFKkqgftkehrmpQEKWKGyiKD<br><br>ETERVIFYRKR--------c<br>YDGGTLMECYIhpyvdygr-<br><br>BLAST: aliSize=11 (resi) iden=3.95% (6/152) simi=7.24% (11/152)<br>lvprgshmdirqmnkthlehwrglrkqlwpghpddahladgeei---LQADHLASFIAMADGVAIGF------------------------<br>--lldfdiltndgthrnmkllidlknifsrqlpkmpkeyivklvfdrhhesmvilknkqkviggicfrqykpqrfaevaflavtaneqvrgygtrlmnkfkdhMQKQNIEYLLTYADNFAIGYfkkqgftkehrmpqekwkgyikdyd<br><br>-----------------adasirhdyvngcdsspvvflegifvlpsfrqrgvakqliaavqrwgtnkgcremasdtspentisqkvhqalgfeetervifyrkrc<br>ggtlmecyihpyvdygr---<br><br>Proposed: aliSize=130 (resi) RMSD=2.27 (Å)<br>lvprgsHMDIRQM-------NKTHLEHWRGLRKQLWP-GHPdDAHLADGEEilQAdhLASFIAMAD-GVAIGFADASIR-HdyvngcdsSPVVFLEGIFVLPSFRQRGVAKQLIAAVQRWGTNKGCREMASDTSpENtiSQKVHQALGFEET------------E----RVIFYRKRC------c<br>------LLDFDILtndgthrNMKLLIDLKNIFSRQLPkMPK-EYIVKLVFD--RH--HESMVILKNkQKVIGGICFRQYkP--------QRFAEVAFLAVTANEQVRGYGTRLMNKFKDHMQKQNIEYLLTYAD-NF--AIGYFKKQGFTKEhrmpqekwkgyikDydggTLMECYIHPyvdygr-
130	1s60A (152)<br>1yvoA (169)	C	15.13	TM-align: aliSize=132 (resi) RMSD=2.41 (Å)<br>lvprgshmDIRQMNKTHLEHWRGLRKQLWP--------GHPD-DAHLADGEEILQADhLASFIAMADG-VAIGFADASIRHDYvngcd--SSPVVFLeGIFVLPSFRQRGVAKQLIAAVQRWGTNKGCREmASDTSPENTISQKVHQALGFEETERVIFYrkrc---------------------<br>--------SIRDAGVADLPGILAIYNDAVGnttaiwneTPVDlANRQAWFDTRARQG-YPILVASDAAgEVLGYASYGDWRPF-----egFRGTVEH-SVYVRDDQRGKGLGVQLLQALIERARAQGLHV-VAAIESGNAASIGLHRRLGFEISGQPQVG----qkfgrwldltfqlnldptrsap<br><br>SARST: aliSize=135 (resi) RMSD=4.11 (Å)<br>lvprgshmd-IRQMNKTHLEHWRGLRK------QLW---PGHPDDAHLADGEEILQaDHLASFIAMAD-GVAIGFADASirhdYVNGCDSSPVVFLEGIFVLPSFRQRGVAKQLIAAVQRWGTNKGCREMAsDTSPENTISQKVHQALGFEETERVIFYRK-------------------rc<br>---------sIRDAGVADLPGILAIYNdavgntTAIwneTPVDLANRQAWFDTRAR-QGYPILVASDAaGEVLGYASYG----DWRPFEGFRGTVEHSVYVRDDQRGKGLGVQLLQALIERARAQGLHVVA-AIESGNAASIGLHRRLGFEISGQPQVGQKfgrwldltfqlnldptrsap--<br><br>BLAST: aliSize=27 (resi) iden=10.53% (16/152) simi=17.76% (27/152)<br>lvprgshmdirqmnkthlehwrglrkqlwpghpddahladgeeilqadhlasfiamadgvaigfadasirhdyvngcdsspvvfleg--IFVLPSFRQRGVAKQLIAA<br>---sirdagvadlpgilaiyndavgnttaiwnetpvdlanrqawfdtrarqgypilvasdaagevlgyasygdwrpfegfrgtvehsVYVRDDQRGKGLGVQLLQA<br><br>VQRWGTNKGCREMASDTSPeNTISQKVHQALGFE-----------------------------etervifyrkrc<br>LIERARAQGLHVVAAIESG-NAASIGLHRRLGFEisgqpqvgqkfgrwldltfqlnldptrsap-----------<br><br>Proposed: aliSize=136 (resi) RMSD=2.02 (Å)<br>lvprgshmDIRQMNKTHLEHWRGLRKQLWP--------GHPD-DAHLADGEEILQADhLASFIAMADG-VAIGFADASIR---HDYvngcdsSPVVFLeGIFVLPSFRQRGVAKQLIAAVQRWGTNKGCREMASDTSPENTISQKVHQALGFEETERV----------IFYRKRC----------c<br>--------SIRDAGVADLPGILAIYNDAVGnttaiwneTPVDlANRQAWFDTRARQG-YPILVASDAAgEVLGYASYGDWrpfEGF------RGTVEH-SVYVRDDQRGKGLGVQLLQALIERARAQGLHVMVAAIESGNAASIGLHRRLGFEISGQMpqvgqkfgrwLDLTFMQlnldptrsap-
131	1s60A (152)<br>2ob0A (154)	C	10.53	TM-align: aliSize=134 (resi) RMSD=2.77 (Å)<br>lvp-RGSHMDIRQMNKTHLEHWRGLRKQLWPGHPddAhLADGEEILQADhLASFIAMADGVAIGFADASIRHDYvngcdSSPVVFLeGIFVLPSFRQRGVAKQlIAAVQRWGT-NKGCREMASDTSPENTISQKVHQALGFEETERVIFYrkrc---------------<br>---sKGSRIELGDVTPHNIKQLKRLNQVIFPVSY--N-DKFYKDVLEVG-ELAKLAYFNDIAVGAVCCRVDHSQ-----NQKRLYI-TLGCLAPYRRLGIGTK-LNHVLNICEkDGTFDNIYLHVQISNESAIDFYRKFGFEIIETKKNY----ykriepadahvlqknl<br><br>SARST: aliSize=130 (resi) RMSD=3.36 (Å)<br>lvprgs----HMDIRQMNKTHLEHWRGLRKQLWPGHPDDAHLADGEEILQAdhlaSFIAMADGVAIGFADASIRHDyvNGCdsspVVFLEGIFVLPSFRQRGVAKQLIAAVQRWGTNKGCREMASDTSPENTISQKVHQALGFEETERVI------------------fyrkrc<br>------skgsRIELGDVTPHNIKQLKRLNQVIFPVSYNDKFYKDVLEVGEL----AKLAYFNDIAVGAVCCRVDHS--QNQ----KRLYITLGCLAPYRRLGIGTKLNHVLNICEKDGTFDNIYLHVQISNESAIDFYRKFGFEIIETKKnyykriepadahvlqknl------<br><br>BLAST: aliSize=65 (resi) iden=19.08% (29/152) simi=42.76% (65/152)<br>lvp-RGSHMDIRQMNKTHLEHWRGLRKQLWPGHPDDAHLADgeeILQADHLASfIAMADGVAIGFADASIRHDyvngcDSSPVVFLEgIFVLPSFRQRGVAKQL---IAAVQRWGTNKGCReMASDTSPENTISqkVHQALGFEETERVIFYRKR-------------c<br>---sKGSRIELGDVTPHNIKQLKRLNQVIFPVSYNDKFYKD---VLEVGELAK-LAYFNDIAVGAVCCRVDHS-----QNQKRLYIT-LGCLAPYRRLGIGTKLnhvLNICEKDGTFDNIY-LHVQISNESAID--FYRKFGFEIIETKKNYYKRiepadahvlqknl-<br><br>Proposed: aliSize=134 (resi) RMSD=2.13 (Å)<br>lvp--RgSHMDIRQMNKTHLEHWRGLRKQ-LWPGHpddAhLADGEEILQadhL--ASFIAMADGVAIGFADASIRHDY-vngcdsSPVVFLEGIFVLPSFRQRGVAKQLIAAVQRWGTNK-GCREMASDTSPENTISQKVHQALGFEETERVI------------FYRKR-cc<br>---skG-SRIELGDVTPHNIKQLKRLNQViFPVSY---N-DKFYKDVLE---VgeLAKLAYFNDIAVGAVCCRVDHSQn------QKRLYIMTLGCLAPYRRLGIGTKMLNHVLNICEKDgTFDNIYLHVQISNESAIDFYRKFGFEIIETKKnyykriepadahvLQKNLa--

132	1s60A (152)<br>2vi7A (163)	C	14.47	TM-align: aliSize=135 (resi) RMSD=2.91 (Å)<br>lvprgsHMDIRQMNKTHLEHWRGLRKQ---L----WPGHPD-DAHLADGEEIlQADHLASFIAMADGVAIGFADASIRHDYVngcdSSPVVFLE-GIFVLpsFRQRGVAKQLIAAVQRWGT-NKGCREMASDTSPENTISQKVHQALGFEETERVIFYrkrc------------------<br>------TIRLERYSERHVEGLTALYNDpavArqvlQMPYQSvEQRRKRLHDS-DDDRLLILVALHQGDVIGSASLEQHPRIR----RSHSGSIGmGVAVA--WQGKGVGSRLLGELLDIADnWMNLRRVELTVYTDNAPALALYRKFGFETEGEMRDY----avrdgrfvdvysmarlrr<br><br>SARST: aliSize=133 (resi) RMSD=3.73 (Å)<br>lvprgsh-MDIRQMNKTHLEHWRGLRK--------QLWPGHPDDAhLADGEEILQADHLASFIAMADGVAIGFADASIRHDYVNGcDSSpvVFLEGIFVlpSFRQRG-VAKQLIAAVQRWGTNKGCREMASDTSPENTISQKVHQALGFEETERVI--------------------fyrkrc<br>-------tIRLERYSERHVEGLTALYNdpavarqvLQMPYQSVEQ-RRKRLHDSDDDRLLILVALHQGDVIGSASLEQHPRIRRS-HSG--SIGMGVAV--AWQGKGvGSRLLGELLDIADNWMNLRRVELTVYTDNAPALALYRKFGFETEGEMRdyavrdgrfvdvysmarlrr------<br><br>BLAST: aliSize=48 (resi) iden=18.42% (28/152) simi=31.58% (48/152)<br>lvprgshmdirqmnkthlehwrgl---------------------------------------RKQLwpgHPDDahlaDgeeilqaDHLASFIAMADGVAIGFADASiRHDYVNGCDSSPVvfleGIFVLPSFRQRGVAKQLIAAVQRWGTN-KGCREMASDTSPENTISQKVHQALGFeETE-------<br>------------------------tirleryserhvegltalyndpavarqvlqmpyqsveqrRKRL---HDSD----D--------DRLLILVALHQGDVIGSASLE-QHPRIRRSHSGSI----GMGVAVAWQGKGVGSRLLGELLDIADNwMNLRRVELTVYTDNAPALALYRKFGF-ETEgemrdya<br><br>-----------------rvifyrkrc<br>vrdgrfvdvysmarlrr---------<br><br>Proposed: aliSize=137 (resi) RMSD=2.35 (Å)<br>lvprgsHMDIRQMNKTHLEHWRGLRKQ---L----WPGHPD-DAHLADGEEIlQADHLASFIAMADGVAIGFADASIRHD--Yvngcds SPVVFLE-GIFVLpsFRQRGVAKQLIAAVQRWGT-NKGCREMASDTSPENTISQKVHQALGFEETER------------VIFYRKRC--c<br>------TIRLERYSERHVEGLTALYNDpavArqvlQMPYQSvEQRRKRLHDS-DDDRLLILVALHQGDVIGSASLEQHPRirR------SHSGSIGmGVAVA--WQGKGVGSRLLGELLDIADnWMNLRRVELTVYTDNAPALALYRKFGFETEGEmrdyavrdgrfvDVYSMARLrr-
133	1s60A (152)<br>1q2yA (140)	C	12.86	TM-align: aliSize=121 (resi) RMSD=2.41 (Å)<br>lvprgshMDIRQMNK-THLEHWRGLRKQLWP-----GHPDdAHlADGeEIlqAdhLASFIAMADGVAIGFADASIRHdyvngcdssPVVFLEGIFVLPSFRQRGVAKQLIAAVQRWGTNKGCREMASDTSpeNTiSQKVHQALGFEETERVIFyrkrc-------------<br>-------MKAVIAKNeEQLKDAFYVREEVFVkeqnvPAEE-EI-DEL-EN--E--SEHIVVYDGEKPVGAGRWRMKD---------GYGKLERICVLKSHRSAGVGGIIMKALEKAAADGGASGFILNAQ--TQ-AVPFYKKHGYRVLSEKEF-----ldagiphlqmmkd<br><br>SARST: aliSize=90 (resi) RMSD=5.54 (Å)<br>lvprgshmdirqmnkthlehwrglrkqlwpghpddahladgeeilqad--HLASFIAMADGVAIGFADASirhdYVNGcdssPVVFLEGIFvLPSFRQRGVAKQLIAAVQRWGTNKGCREMASDTSPENTISQKVhqaLGFEETE-------<br>--mkaviakneeqlkdafyvreevfvkeqnvpaeeeidelenESEHIVVYDGEKPVGAGRWR----MKDG----YGKLERICV-LKSHRSAGVGGIIMKALEKAAADGGASGFILNAQTQAVPFYKK---HGYRVLSekeflda<br><br>-RVIFYRK--rc<br>gIPHLQMMkd--<br><br>BLAST: aliSize=34 (resi) iden=14.29% (20/140) simi=24.29% (34/140)<br>lvprgshmdirqmnkthlehwrglrkqlwpghpddahladg-------------------------------EEILQADHLASFIAMADG-VAIGFADASIRHDYvnGcdsspvvFLEGIFVLPSFRQRGVAKQLIAAVQRWGTNKG---<br>---mkaviakneeqlkdafyvreevfvkeqnvpaeEEIDELENESEHIVVYDGeKPVGAGRWRMKDGY--G-------KLERICVLKSHRSAGVGGIIMKALEKAAADGGasgfilnaqtqavpfykkhgyrvlsekefldagiphlqmmkd<br><br>cremasdtspentisqkvhqalgfeetervifyrkrc<br>-------------------------------------<br><br>Proposed: aliSize=125 (resi) RMSD=2.02 (Å)<br>lvprgshMDIRQMN-KTHLEHWRGLRKQLWP----GHPDdA-HlaD-geEIlQAdhLASFIAMADGVAIGFADASIRHdyvngcdssPVVFLEGIFVLPSFRQRGVAKQLIAAVQRWGTNKGCREMASDTSpeNTiSQKVHQALGFEETERV------IFYRKRC--c<br>-------MKAVIAKnEEQLKDAFYVREEVFVkeqnVPAE-EeI--De--LE-NE--SEHIVVYDGEKPVGAGRWRMKD---------GYGKLERICVLKSHRSAGVGGIIMKALEKAAADGGASGFILNAQ--TQ-AVPFYKKHGYRVLSEKefldagIPHLQMMkd-
134	1s60A (152)<br>1yk3A (198)	C	12.50	TM-align: aliSize=140 (resi) RMSD=2.90 (Å)<br>lvprg------------------------------SHMDiRQMNKTHLE-HWRGLRKQ--L----WPGHPDDAHLADGEEILQADHLASFIAMADGVAIGFADASIRHD---YVNGCDSSPVVFLeGIFV-LPSFR--QR--GVAKQLIAAVQRWgTNkGCREMASDTSPENTISQKVHQALGFEETER<br>-----addalvrlarerfdlpdqvrrlarppvpslePPYG-LRVAQLTDAeMLAEWMNRphLaaawEYDWPASRWRQHLNAQLEGTYSLPLIGSWHGTDGGYLELYWAAKdliSHYYDADPYDLGL-HAAIaDLSKVnrGFgpLLLPRIVASVFAN-EP-RCRRIMFDPDHRNTATRRLCEWAGCKFLGE<br><br>VIFYRkrc------------<br>HDTTN---rrmalyaleapt<br><br>SARST: aliSize=139 (resi) RMSD=5.55 (Å)<br>lvpr------------------------------GSHMDIRQMNKTHLEHW------RGLRKQLWPGHPDDAHLADGEEILQADHLASFIAMADGVAIGFADASI-------RHDYVNGCDSSPVVFLEgifvLPSFRQRGV--AKQLIAAVQRWGTNKGCREMASDTSPENTISQKVHQALGFEETERV<br>----addalvrlarerfdlpdqvrrlarppvpslEPPYGLRVAQLTDAEMLaewmnrPHLAAAWEYDWPASRWRQHLNAQLEGTYSLPLIGSWHGTDGGYLELYWaakdlisHYYDADPYDLGLHAAIA----DLSKVNRGFgpLLLPRIVASVFANEPRCRRIMFDPDHRNTATRRLCEWAGCKFLGEH<br><br>IF--------------yrkrc<br>DTtnrrmalyaleapt-----<br><br>BLAST: aliSize=48 (resi) iden=17.76% (27/152) simi=31.58% (48/152)<br>lvprgshmdirq--MNKTHL-EHWrglrKQLWPGHPDDAHLaDGEeiLQADHLASFIAMADGVAIGFADAS-IRHDYVNG-CDSSPV-VFLEGIFVLPSFRQRGVAKQLIAAV--QRWGTNKGCREMASDTSPENTISQKVH<br>------------addalvrlarerfdlpdqvrrlarppvpsleppyglrvaqltdaemlaewMNRPHLaAAW----EYDWPASRWRQHL-NAQ--LEGTYSLPLIGSWHGTDGGYLELYwAAKDLISHyYDADPYdLGLHAAIADLSKVNRGFGPLLLPRIvaSVFANEPRCRRIMFDPDHRNTATRRLC<br><br>QALG-----------------------feetervifyrkrc<br>EWAGckflgehdttnrrmalyaleapt--------------<br><br>Proposed: aliSize=143 (resi) RMSD=2.74 (Å)<br>lvprgs------------------------------HMDIRQMNKTHLEHWRGLRKQ--L----WPGHPDDAHLADGEEILQADHLASFIAMADGVAIGFADASIRHD---YVNGCDSSPVVFLeGIFV-LPSFR--QR--GVAKQLIAAVQRWgTNkGCREMASDTSPENTISQKVHQALGFEETER<br>------addalvrlarerfdlpdqvrrlarppvpslepPYGLRVAQLTDAEMLAEWMNRphLaaawEYDWPASRWRQHLNAQLEGTYSLPLIGSWHGTDGGYLELYWAAKdliSHYYDADPYDLGL-HAAIaDLSKVnrGFgpLLLPRIVASVFAN-EP-RCRRIMFDPDHRNTATRRLCEWAGCKFLGE<br><br>VIF----YRKRC-----c<br>HDTtnrrMALYAleapt-

135	1s60A (152)<br>1yghA (164)	C	7.24	TM-align: aliSize=124 (resi) RMSD=3.04 (Å)<br>lvprgsHMDIRQMN---K-THLEHWRGLRKQLW---P--GHPDdAHLADGeEIlqAdhLASFIAMADG-VAIGFADASIRHDyvngcdsSPVVFLEGIFVLPSFRQRGVAKQLIAAVQRWGT-NKGCREMASDTSpeNTiSQKVHQALGFEETErv-----------IFYrkrc------------------<br>------KIEFRVVNndnTkENMMVLTGLKNIFQkqlPkmPKEY-IARLVY-DR--S--HLSMAVIRKPlTVVGGITYRPFDK-------REFAEIVFCAISSTEQVRGYGAHLMNHLKDYVRnTSNIKYFLTYAD--NY-AIGYFKKQGFTKEI--tldksiwmgyiKDY----eggtlmqcsmlpriryld<br><br>SARST: aliSize=108 (resi) RMSD=4.66 (Å)<br>lvprgshmdirqmnkthle-----------------------HWRGLRKQLWPghpddahLADGEEILQADHLASFIAMAD-GVAIGFADASIR--HdyvnGCdsspvVFLEGIFVLPSFRQRGVAKQLIAAVQRWGTNK-GCREMASDTSPENTISQKVhqaLGF--EETE--------RVI-------<br>-------------------kiefrvvnndntkenmmvltglkNIFQKQLPKMP-------KEYIARLVYDRSHLSMAVIRKpLTVVGGITYRPFdkR----EF-----AEIVFCAISSTEQVRGYGAHLMNHLKDYVRNTsNIKYFLTYADNYAIGYFKK---QGFtkEITLdksiwmgyIKDyeggtlm<br><br>------------fyrkrc<br>qcsmlpriryld------<br><br>BLAST: aliSize=14 (resi) iden=5.92% (9/152) simi=9.21% (14/152)<br>lvprgshmdirqmnkthlehwrglrkqlwpghpdda--HLADgeEILQADHLASFIAMADGVAIGF------------------------------<br>------------------------------------kiefrvvnndntkenmmvltglknifqkqlpkmpkeyiarlvydrshlsmavirkpltvvggityrpfdkrefaeivfcaissteqvrgygahlmnHLKD--YVRNTSNIKYFLTYADNYAIGYfkkqgftkeitldksiwmgyikdyeggtlm<br><br>------------adasirhdyvngcdsspvvflegifvlpsfrqrgvakqliaavqrwgtnkgcremasdtspentisqkvhqalgfeetervifyrkrc<br>qcsmlpriryld--<br><br>Proposed: aliSize=130 (resi) RMSD=2.40 (Å)<br>lvprgsHMDIRQM-------NKTHLEHWRGLRKQLWP-GHPdDAHLADGEEilQAdhLASFIAMADG-VAIGFADASIR-HdyvngcdsSPVVFLEGIFVLPSFRQRGVAKQLIAAVQRWGT-NKGCREMASDTSpENtiSQKVHQALGFEET------------------ERVIFYRKR-C----c<br>------KIEFRVVnndntkeNMMVLTGLKNIFQKQLPkMPK-EYIARLVYD--RS--HLSMAVIRKPlTVVGGITYRPFdK--------REFAEIVFCAISSTEQVRGYGAHLMNHLKDYVRnTSNIKYFLTYAD-NY--AIGYFKKQGFTKEitldksiwmgyikdyeggTLMQCSMLPrIryld-
136	1s60A (152)<br>2bswA (145)	C	15.17	TM-align: aliSize=128 (resi) RMSD=2.60 (Å)<br>lvprgshMDIRQMNKthLEhWRGLRKQ-LWPGHPDDAHlaDGeEIlQADHLASFIAMADGVAIGFADASIRHDYvNgCDSSPVVFLEGiFVLPSFRQRGVAKQLIAAVQRWGTNKGCREMASDTSPeNtiSQKVHQALGFEETERVIFYrkrc-------------<br>-------IEVKPINA--ED-TYELRHRiLRPNQPIEAC--FE-SD-LLRGAFHLGGYYGGKLISIASFHQAEHS-E-LQGQKQYQLRG-ATLEGYREQKAGSSLIKHAEEILRKRGADLLWCNART-S--ASGYYKKLGFSEQGEVFDT----ppvgphilykrit<br><br>SARST: aliSize=129 (resi) RMSD=4.06 (Å)<br>lvprgshm-DIRQMNKTHLEHWRGLRKQLwpGHPDDAHLADgeeiLQADHLASFIAMADGVAIGFADASIRHDYVNGCDSSPVVfleGIFVLPSFRQRGVAKQLIAAVQRWGTNKGCREMASDTSPENTISQKvhqALGFEEteRVIFYRKR-----------c<br>--------iEVKPINAEDTYELRHRILRP--NQPIEACFES----DLLRGAFHLGGYYGGKLISIASFHQAEHSELQGQKQYQL---RGATLEGYREQKAGSSLIKHAEEILRKRGADLLWCNARTSASGYYK---KLGFSE--QGEVFDTPpvgphilykrit-<br><br>BLAST: aliSize=44 (resi) iden=19.01% (27/145) simi=30.99% (44/145)<br>lvprgshmdirqmnkthlehwrg-------------LRKQ-LWPGHPDDAHLaDGEEILQADHLASFIAmadGVAIGFAD-ASIRHDYVNGcdsSPVVFLEGIfVLPSFRQRGVAKQLIAAVQRWGTNKGCREMASDTspeNTISQKVHQALGFEETERV----------------ifyrkrc<br>-----------------------ievkpinaedtyeLRHRiLRPNQPIEACF-ESDLLRGAFHLGGYYG---GKLISIASfHQAEHSELQG---QKQYQLRGA-TLEGYREQKAGSSLIKHAEEILRKRGADLLWCNA---RTSASGYYKKLGFSEQGEVfdtppvgphilykrit-------<br><br>Proposed: aliSize=133 (resi) RMSD=2.21 (Å)<br>lvprgshMDIRQMNKthLEhWRGLRK-QLWPGHPDDAHlADgeE-IlQADHLASFIAMADGVAIGFADASIRHDYvNgCDSSPVVFLEGIFVLPSFRQRGVAKQLIAAVQRWGTNKGCREMASDTSPeNtiSQKVHQALGFEETER-----VIFYRKRC------c<br>-------IEVKPINA--ED-TYELRHrILRPNQPIEAC-MF--EsD-LLRGAFHLGGYYGGKLISIASFHQAEHS-E-LQGQKQYQLRGMATLEGYREQKAGSSLIKHAEEILRKRGADLLWCNART-S--ASGYYKKLGFSEQGEvfdtpPVGPHILMykritc-
137	1s60A (152)<br>2pc1A (173)	C	15.79	TM-align: aliSize=133 (resi) RMSD=2.82 (Å)<br>lvprgs----HMDIRQMNKTHLEHWrGLRKQLWP----------G---HPddAHLADGEEILQAdhLASFIAMADGvAIGFADASIRHDY-V--N----G-CDSSPVVFLEGIFVLPSFRQRGVAKQLIAAVQRWGTnkgCREMASDTSPENTIsQKVHQALGFEETERVIFYRkrc-----------<br>------nlyfQGQIRLAFPNEIDQI-LLIEEARAeiaktgsdqwQkedGY--PNRNDIIDDILN--GYAWVGIEDG-LATYAAVIDGHEEvYdaIyegkWlHDNHRYLTFHRIAISNQFRGRGLAQTFLQGLIEGHK---GPDFRCDTHEKNVT-QHILNKLGYQYCGKVPLDG---vrlayqkikek<br><br>SARST: aliSize=138 (resi) RMSD=7.74 (Å)<br>lvprg---SHMDIRQMNKTHLEH--------WRGLRKQLWPGHPD-----DAHLADGEEILQAdhlasFIAMADGVAIGFADASIR-----HDYVNGCDSSP-VVFLEGIFVLPSFRQRGVAKQLIAavQRWGTNKGCREMASDTSPEnTISQKVHQALGFEETERV-------IFYRKR--c<br>-----nlyFQGQIRLAFPNEIDQillieearAEIAKTGSDQWQKEdgypnRNDIIDDILNGYA-----WVGIEDGLATYAAVIDGHeevydAIYEGKWLHDNhRYLTFHRIAISNQFRGRGLAQTFL--QGLIEGHKGPDFRCDTHEK-NVTQHILNKLGYQYCGKVpldgvrlAYQKIKek-<br><br>BLAST: aliSize=66 (resi) iden=22.37% (34/152) simi=43.42% (66/152)<br>lvprgshmdir---------------------------QMNKTHLEHWRglrKQlwPGHPDDAHLADgeEILQA-------DHLASFIAMADGVAIGFaDASIRHDYVNgcDSSPVVFLEGIFVLPSFRQRGVAKQLIAAVQRwGtNKGcREMASDTSPENtISQKVHQALGFEETERV-----------<br>-----------nlyfqgqirlafpneidqillieearaEIAKTGSDQWQ---KE--DGYPNRNDIID--DILNGyawvgieDGLATYAAVIDGHEEVY-DAIYEGKWLH--DNHRYLTFHRIAISNQFRGRGLAQTFLQGLIE-G-HKG-PDFRCDTHEKN-VTQHILNKLGYQYCGKVpldgvrlayqk<br><br>----ifyrkrc<br>ikek-------<br><br>Proposed: aliSize=137 (resi) RMSD=2.30 (Å)<br>lvprgs-----HMDIRQMNKTHLEHWRGLRK-QL----------WPG-HP--ddaHLADGEEILQAdhLASFIAMADGVAIGFADASIRHDY-V--N----G-CDSSPVVFLEGIFVLPSFRQRGVAKQLIAAVQ-RWgTnkgCREMASDTSPENTISQKVHQALGFEETER------VIFYRKRC--c<br>------nlyfqGMQIRLAFPNEIDQIMLLIEeARaeiaktgsdqWQKeDGyp---NRNDIIDDILN--GYAWVGIEDGMLATYAAVIDGHEEvYdaIyegkWlHDNHRYLTFHRIAISNQFRGRGLAQTFLQGLIeGH-K---GPDFRCDTHEKNVTMQHILNKLGYQYCGKvpldgvRLAYQKIKek-

138	1s60A (152)<br>2q7bA (164)	C	14.47	TM-align: aliSize=131 (resi) RMSD=2.60 (Å)<br>lvprGSHmDIRQMNKT--HLEHWRGLRKQLWP------GHPddAHlADGEEILQ---ADHLASFIAMADGVAIGFADASIRHdyvngcdsSPVVFLEGIFVLPSFRQ--RGVAKQLIAAVqRWGTNKGCREMASDTSPENTISQKVHQALGFEEtERVI-------FYRkrc----------<br>----FQG-EIKEYENNpyHLAQLVDLINYCQNieakldIKA--EQ-DDIFQIENyyqNRKGQFWIALENEKVVGSIALLRID--------DKTAVLKKFFTYPKYRGnpVRLGRKLFERF-LFARASKFTRIVLDTPEKEKRSHFFYENQGFKQ-ITRDeldvdyiFPD---rdsriyvkll<br><br>SARST: aliSize=103 (resi) RMSD=6.76 (Å)<br>lvprgshmdirqmnkthlehwrglrkqlwpghpddahlad---GEEILQADHLASFIAMADGVAIGFAdasirhDYVNGCDSSPVVFLegIFVLPSFRQRGVAKQLIAAVQ-RWGTNKGCREMASDTSPENTISQKVHQALG--------<br>--fqgeikeyennpyhlaqlvdlinycqnieakldikaeqddifqIENYYQNRKGQFWIALENEKVVGSI------ALLRIDDKTAVLKK--FFTYPKYRGNPVRLGRKLFERfLFARASKFTRIVLDTPEKEKRSHFFYENQGfkqitrde<br><br>FEETERVIFYRKR------c<br>LDVDYIFPDRDSRiyvkll-<br><br>BLAST: aliSize=40 (resi) iden=14.47% (22/152) simi=26.32% (40/152)<br>lvprgshmdirqmnkthlehwrglrkqlwpghpddahladgeeilqadhlas---FIAMADGVAIGfADASIRHDyvngcDSSPVvfLEGIFVLPSFRQRGV--AKQLIAAVQRWGTNKGCReMASDTSPENTISQKV<br>--fqgeikeyennpyhlaqlvdlinycqnieakldikaeqddifqienyyqnrkgqfWIALENEKVVG-SIALLRID-----DKTAV--LKKFFTYPKYRGNPVrlGRKLFERFLFARASKFTR-IVLDTPEKEKRSHFF<br><br>HQALGFEETER---------------------vifyrkrc<br>YENQGFKQITRdeldvdyifpdrdsriyvkll--------<br><br>Proposed: aliSize=137 (resi) RMSD=2.08 (Å)<br>lvprGSHMDIRQMNKT--HLEHWRGLRKQLWP------GHPddA-HlADGEEILQ---ADHLASFIAMADGVAIGFADASIRHdyvngcdsSPVVFLEGIFVLPSFRQ--RGVAKQLIAAVQRWGTNKGCREMASDTSPENTISQKVHQALGFEE-------------TERVIFYRKRC-c<br>----FQGMEIKEYENNpyHLAQLVDLINYCQNieakldIKM--AeQ-DDIFQIENyyqNRKGQFWIALENEKVVGSIALLRID--------DKTAVLKKFFTYPKYRGnpVRLGRKLFERFMLFARASKFTRIVLDTPEKEKRSHFFYENQGFKQitrdeldvdyifpDRDSRIYVKLLf-
139	1s60A (152)<br>2fiwA (160)	C	14.47	TM-align: aliSize=123 (resi) RMSD=2.40 (Å)<br>lvprg---SHMDIRQMNKTHLEHWRGLRKQ-L---------------WPGHPDdAHLADGEEILqAdhLASFIAMADGVAIGFADASIrhdyvngcdsspVVFLEgIFVLPSFRQRGVAKQLIAAVQRWGTNKGCREMASDTSpeNtISQKVHQALGFEETERVIFYrkrc--------------<br>-----ghvSTPALRPYLPEDAAVTAAIFVAsIeqltaddyseeqqeaWASAAD-DEAKFAARLS-G--QLTLIATLQGVPVGFASLKG------------PDHID-LYVHPDYVGRDVGTTLIDALEKLAGARGALILTVDAS--D-NAAEFFAKRGYVAKQRNTVS----ingewlantttksl<br><br>SARST: aliSize=125 (resi) RMSD=6.02 (Å)<br>lvprgshm------DIRQMNKTHLEH-----WRGLRKQLWPGHPDDAHLAD-------GEEILQADHLASFIAMADGVAIGFADASIRHDYVNgcdsspvvflegiFVLPSFRQRGVAKQLIAAVQRWGTNKGCREMASDTSPentISQKVHQALGFEETERVIFYR-------------krc<br>--------ghvstpALRPYLPEDAAVtaaifVASIEQLTADDYSEEQQEAWasaaddeAKFAARLSGQLTLIATLQGVPVGFASLKGPDHIDL-------------YVHPDYVGRDVGTTLIDALEKLAGARGALILTVDASD---NAAEFFAKRGYVAKQRNTVSIngewlantttksl---<br><br>BLAST: aliSize=43 (resi) iden=14.47% (22/152) simi=28.29% (43/152)<br>lvprgshmdirqmnkthlehwrgl--------------------------------------RKQLWPGHPDDahlaDGEEILQADHLASFIAMADGVAIGFadASIR-HDYVNgcdsspvvflegIFVLPSFRQRGVAKQLIAAVQRWGTNKGCREMASDTS---------------------------<br>------------------------ghvstpalrpylpedaavtaaifvasieqltaddyseeQQEAWASAADD----EAKFAARLSGQLTLIATLQGVPVGF--ASLKgPDHID------------LYVHPDYVGRDVGTTLIDALEKLAGARGALILTVDASdnaaeffakrgyvakqrntvsingewl<br><br>--------pentisqkvhqalgfeetervifyrkrc<br>antttksl----------------------------<br><br>Proposed: aliSize=126 (resi) RMSD=1.88 (Å)<br>lvprgsh-------MDIRQMNKTHLEHWRGLRKQ-L---------------WPGHPDdAHLADGEEILqAdhLASFIAMADGVAIGFADASIrhdyvngcdsspVVFLEGIFVLPSFRQRGVAKQLIAAVQRWGTNKGCREMASDTSpeNtISQKVHQALGFEE---------TERVIFYRKRC---c<br>-------ghmvmstPALRPYLPEDAAVTAAIFVAsIeqltaddyseeqqeaWASAAD-DEAKFAARLS-G--QLTLIATLQGVPVGFASLKG------------PDHIDMLYVHPDYVGRDVGTTLIDALEKLAGARGALILTVDAS--D-NAAEFFAKRGYVAkqrntvsinGEWLANTTMTKsla-
140	1s60A (152)<br>2k5tA (128)	C	15.63	TM-align: aliSize=121 (resi) RMSD=2.62 (Å)<br>lvprgSHMDIRQMNKTHLEHWRGLRKQLWPgHPDdAhlAdGeEIlqADHLASFIAMADGVAIGFADASIRHdyvngcdssPVVFLEGIFVLPSFRQRGVAKQLIAAVQRWGTnkGCREMASDT-SPE-NTISQKVHQALGFEETERVifyrkrc-----<br>-----MKLTIIRLEKFSDQDRIDLQKIWPE-YSP-S--S-L-QV--DDNHRIYAARFNERLLAAVRVTLSG---------TEGALDSLRVREVTRRRGVGQYLLEEVLRNNP--GVSCWWMADaGVEdRGVMTAFMQALGFTTQQGG-------wekcg<br><br>SARST: aliSize=94 (resi) RMSD=6.34 (Å)<br>lvprgshmdirqmnkthlehwrglrkqlwpghpdda------------HLADGEEILQADHL-----------ASFIAMADGVAIGFADASIRhdyvngcdsSPVVFLEGIFVLPSFRQRGVAKQLIAavQRWGTNKGCREMASD--TSPENTISQKVHQALGFEE---------tervifyrkrc<br>------------------------------------mkltiirlekfsDQDRIDLQKIWPEYspsslqvddnhRIYAARFNERLLAAVRVTLS---------GTEGALDSLRVREVTRRRGVGQYLLE--EVLRNNPGVSCWWMAdaGVEDRGVMTAFMQALGFTTqqggwekcg-----------<br><br>BLAST: aliSize=24 (resi) iden=13.28% (17/128) simi=18.75% (24/128)<br>lvprgshmdirqmnkthlehwrglrkqlwpghpddahladgeeilqadhlasfiamadgvaigfadasirhdyvngcdsspvvf--LEGIFVLPSFRQRGVAKQLIAAVQRWGTNKGCREMASDTSPENT<br>--mkltiirlekfsdqdridlqkiwpeyspsslqvddnhriyaarfnerllaavrvtlsgtegaLDSLRVREVTRRRGVGQYLLEEVLRNNPGVSCWWMADAGVEDRG<br><br>ISQKVHQALGF-----------eetervifyrkrc<br>VMTAFMQALGFttqqggwekcg-------------<br><br>Proposed: aliSize=121 (resi) RMSD=2.06 (Å)<br>lvprgsH-MDIRQMNK-T--HLEHWRGLRKqlwPgHPDdAhlAdGeEIlqADHLASFIAMADGVAIGFADASIRHdyvngcdssPVVFLEGIFVLPSFRQRGVAKQLIAAVQRWGTnkGCREMASDT-S-pE-NTISQKVHQALGFEETErVIFYRKR-cc<br>------MkLTIIRLEKfSdqDRIDLQKIWP---E-YSP-S--S-L-QV--DDNHRIYAARFNERLLAAVRVTLSG---------TEGALDSLRVREVTRRRGVGQYLLEEVLRNNP--GVSCWWMADaGv-EdRGVMTAFMQALGFTTQQ-GGWEKCGc--

141	1s60A (152)<br>3d3sA (159)	C	13.82	TM-align: aliSize=132 (resi) RMSD=2.68 (Å)<br>lvprgsHMDIRQMNKTHLEHWRGLRK-Q--LWPgHpddAhLADGEEILQADHLASFIAMAD-GVAIGFADASIRHDYvngcdsSPVVFLEGIFVLPSFRQRGVAKQlIAAVQRW-GTNkGCREMASDTSPENTISQKVHQALGF----EETERVIF--YRKRc-------------<br>------RYHLRPPRRNDGAAIHQLVSeCppLDL-N---S-LYAYLLLCEHHAHTCVVAESPgGRIDGFVSAYLLPTR------PDVLFVWQVAVHSRARGHRLGRA-LGHILERqECR-HVRHLETTVGPDNQASRRTFAGLAGergaHVSEQPFFdrQAFG-gadhddellrigpf<br><br>SARST: aliSize=128 (resi) RMSD=5.30 (Å)<br>lvprgsh-MDIRQMNKTHLEHWRGLRKQLW--PGHPDDAHLADGEEILqadHLASFIAMADGVAIGFADASIRHdyvngcDSSPVVFLEGIFVLPSFRQRGVAKQLIAAVQRWGTNKgCREMASDTSPENTISQKVHQA----LGfeeterVIFYRKR----------------------c<br>-------rYHLRPPRRNDGAAIHQLVSECPplDLNSLYAYLLLCEHHA---HTCVVAESPGGRIDGFVSAYLLP------TRPDVLFVWQVAVHSRARGHRLGRALGHILERQECRH-VRHLETTVGPDNQASRRTFAGlageRG------AHVSEQPffdrqafggadhddellrigpf-<br><br>BLAST: aliSize=12 (resi) iden=4.61% (7/152) simi=7.89% (12/152)<br>lvprgshmdirqmnkthlehwrglrkqlwpghpddahladgeeilqadhlasfiamadgvaigfadasirhdyvngcdsspvvflegifvlpsfrqrgvakqliaavqrwgtnkgc--<br>--ryhlrpprrndgaaihqlvsecppldlnslyaylllcehhahtcvvaespggridgfvsayllptrpdvlfvwq<br><br>-----------------------------REMASDTSPENTISQKVHQALGfeeTER----------------------------vifyrkrc<br>vavhsrarghrlgralghilerqecrhvRHLETTVGPDNQASRRTFAGLA---GERgahvseqpffdrqafggadhddellrigpf--------<br><br>Proposed: aliSize=132 (resi) RMSD=2.08 (Å)<br>lvprgsHMDIRQMNKTHLEHWRGLRK-Q--LWPgHpddAhLADGEEILQAdhLA--SFIAMAD-GVAIGFADASIRHDYvngcdsSPVVFLEGIFVLPSFRQRGVAKQLIAAVQRW-GTNkGCREMASDTSPENTISQKVHQALGF----EETERV--------------IFYRKR-Cc<br>------RYHLRPPRRNDGAAIHQLVSeCppLDL-N---S-LYAYLLLCEH--HAhtCVVAESPgGRIDGFVSAYLLPTR------PDVLFVWQVAVHSRARGHRLGRAMLGHILERqECR-HVRHLETTVGPDNQASRRTFAGLAGergaHVSEQPffdrqafggadhddeMLLRIGpF-
142	1s60A (152)<br>2pr8A (173)	C	14.47	TM-align: aliSize=139 (resi) RMSD=3.35 (Å)<br>lvprGsHMDIRQMNKTHLEHWRGLRK-QLWP-----G-HPDD--AHLADGEeiLQ--AD-HLASFIAMADGVAIGFADASIRHD----YVNGCDSSPVVFLEGI-FV--LPSFRQR-GVAKQLIAAvQRwgTNKGCREMASDTSPENTISQKVHQALGFEETERVIFYRkrc-------------<br>----D-SVTLRLMTEHDLAMLYEWLNrSHIVewwggEeARPTlaDVQEQYL--PSvlAQeSVTPYIAMLNGEPIGYAQSYVALGsgdgWWEEETDPGVRGIDLSlANasQLGKGLGtKLVRALVEL-LF--NDPEVTKIQTDPSPSNLRAIRCYEKAGFERQGTVTTPD---gpavymvqtrqafe<br><br>SARST: aliSize=137 (resi) RMSD=5.21 (Å)<br>lvprgs-HMDIRQMNKTHLEHWRGLRKQLWP---------GHPDDAHLADGEEILQADH-LASFIAMADGVAIGFADAS--IRHDYVNGcDSSPV--VFLegiFVLPSFRQRG-----VAKQLIAAVQRWGTNKGCREMASDTSPENTISQKVHQALGFEETERVIF----------------yrkrc<br>------dSVTLRLMTEHDLAMLYEWLNRSHIvewwggeeaRPTLADVQEQYLPSVLAQEsVTPYIAMLNGEPIGYAQSYvaLGSGDGWW-EEETDpgVRG---IDLSLANASQlgkglGTKLVRALVELLFNDPEVTKIQTDPSPSNLRAIRCYEKAGFERQGTVTTpdgpavymvqtrqafe-----<br><br>BLAST: aliSize=66 (resi) iden=23.03% (35/152) simi=43.42% (66/152)<br>lvprgshmdirq-------------------MNKTHLEHWrglrkqlWPGHPDDAHLADGEE-----ILQADHLASFIAMADGVAIGFADASIRHDYVNGC---DSSPVVflEGIFV-LPSFRQ--RGVAKQLIAA-VQRWGTNKGCREMASDTSPENTISQKVHQALGFE-------------------<br>------------dsvtlrlmtehdlamlyewLNRSHIVEW-------WGGEEARPTLADVQEqylpsVLAQESVTPYIAMLNGEPIGYAQSYVALGSGDGWweeETDPGV--RGIDLsLANASQlgKGLGTKLVRAlVELLFNDPEVTKIQTDPSPSNLRAIRCYEKAGFErqgtvttpdgpavymvqtr<br><br>----etervifyrkrc<br>qafe------------<br><br>Proposed: aliSize=137 (resi) RMSD=2.74 (Å)<br>lvprgs-HMDIRQMNKTHLEHWRGLrK--QlwP-------G-HPDD--AHLADGEeiLQ-AD--HLASFIAMADGVAIGFADASIRHD-----YVNgCDSSPVVFLEGI-FV---LPSFRQRGVAKQLIAAVQRwgTNkGCREMASDTSPENTISQKVHQALGFEETERV------IFYRKRC-----c<br>------dSVTLRLMTEHDLAMLYEW-LnrS--HivewwggEeARPTlaDVQEQYL--PSvLAqeSVTPYIAMLNGEPIGYAQSYVALGsgdgwWEE-ETDPGVRGIDLSlANasqLGKGLGTKLVRALVELLFN--DP-EVTKIQTDPSPSNLRAIRCYEKAGFERQGTVttpdgpAVYMVQTrqafe-
143	1s60A (152)<br>2fl4A (147)	N	12.24	TM-align: aliSize=114 (resi) RMSD=3.29 (Å)<br>lvprgshmdirqmnkthlehwrglr---------------------KQl-WP-GHPDdAHLADGEEILQADHLASFIAMADGVAIGFADASIRHDyvngcdssPVVFLEGIFVLPSFRQRGVAKQLI-AAVQRWGTNKGCREMASDTSPENTISQKVHQALGFEETERVIFYRkrc---------<br>-------------------------gmeihfekvtsdnrkavenlqVF-aEQqAFIE-SMAENLKESDQFPEWESAGIYDGNQLIGYAMYGRWQD--------GRVWLDRFLIDQRFQGQGYGKAACrLLMLKLIEKYQTNKLYLSVYDTNSSAIRLYQQLGFVFNGELDTNG---ervmewthq<br><br>SARST: aliSize=128 (resi) RMSD=11.98 (Å)<br>lvprgs--HMDIRQMNKTHlehWRGLRKQLWPGHPDDAHLADGEEILQ---ADHLASFIAMADGVAIGFADASIrhdYVNGCdsspvVFLEGIFVLPSFRQRGVAKQLIAAVQRWGTN-KGCREMASDTSPENTISQKVHQALGFEETERV-------------ifyrkrc<br>------gmEIHFEKVTSDN---RKAVENLQVFAEQQAFIESMAENLKEsdqFPEWESAGIYDGNQLIGYAMYGR---WQDGR-----VWLDRFLIDQRFQGQGYGKAACRLLMLKLIEkYQTNKLYLSVYDTNSSAIRLYQQLGFVFNGELdtngervmewthq-------<br><br>BLAST: aliSize=32 (resi) iden=10.20% (15/147) simi=21.77% (32/147)<br>lvprgshmdirqmnkthlehwrglrkqlwpghpddahladgeeilqadhlasfiamadgvaigfadasirhdyvngcdsspv--VFLEGIFVLPSFRQRGVAKQ-----LIAAVQRWGTNKG<br>--gmeihfekvtsdnrkavenlqvfaeqqafiesmaenlkesdqfpewesagiydgnqligyamygrwqdgrVWLDRFLIDQRFQGQGYGKAacrllMLKLIEKYQTNKL<br><br>CREMAsDTspeNTISQKVHQALGF-------------------eetervifyrkrc<br>YLSVY-DT---NSSAIRLYQQLGFvfngeldtngervmewthq-------------<br><br>Proposed: aliSize=127 (resi) RMSD=2.48 (Å)<br>lvprGSHMDIRQMNKTH-LEHWRGLRK-Q--LW---pghpddaHLADGEEILQADHLASFIAMADGVAIGFADASIRHDyvngcdssPVVFLEGIFVLPSFRQRGVAKQLIAAVQRWGT-NKGCREMASDTSPENTISQKVHQALGFEETERV---iF---------yrkrcc<br>----GMEIHFEKVTSDNrKAVENLQVFaEqqAFies-------MAENLKESDQFPEWESAGIYDGNQLIGYAMYGRWQD--------GRVWLDRFLIDQRFQGQGYGKAACRLLMLKLIeKYQTNKLYLSVYDTNSSAIRLYQQLGFVFNGELdtn-Gervmewthq------

144	1s60A<br>(152)<br><br>2fl4A<br>(147)	C	11.56	TM-align: aliSize=114 (resi) RMSD=3.29 (Å)<br>lvprgshmdirqmnkthlehwrglr---------------------KQl-WP-GHPDdAHLADGEEILQADHLASFIAMADGVAIGFADASIRHDyvngcdssPVVFLEGIFVLPSFRQRGVAKQLI-AAVQRWGTNKGCREMASDTSPENTISQKVHQALGFEETERVIFYRkrc---------<br>-------------------------gmeihfekvtsdnrkavenlqVF-aEQqAFIE-SMAENLKESDQFPEWESAGIYDGNQLIGYAMYGRWQD--------GRVWLDRFLIDQRFQGQGYGKAACrLLMLKLIEKYQTNKLYLSVYDTNSSAIRLYQQLGFVFNGELDTNG---ervmewthq<br><br>SARST: aliSize=128 (resi) RMSD=11.98 (Å)<br>lvprgs--HMDIRQMNKTHlehWRGLRKQLWPGHPDDAHLADGEEILQ---ADHLASFIAMADGVAIGFADASIrhdYVNGCdsspvVFLEGIFVLPSFRQRGVAKQLIAAVQRWGTN-KGCREMASDTSPENTISQKVHQALGFEETERV-------------ifyrkrc<br>------gmEIHFEKVTSDN---RKAVENLQVFAEQQAFIESMAENLKEsdqFPEWESAGIYDGNQLIGYAMYGR---WQDGR-----VWLDRFLIDQRFQGQGYGKAACRLLMLKLIEkYQTNKLYLSVYDTNSSAIRLYQQLGFVFNGELdtngervmewthq-------<br><br>BLAST: aliSize=32 (resi) iden=10.20% (15/147) simi=21.77% (32/147)<br>lvprgshmdirqmnkthlehwrglrkqlwpghpddahladgeeilqadhlasfiamadgvaigfadasirhdyvngcdsspv--VFLEGIFVLPSFRQRGVAKQ-----LIAAVQRWGTNKG<br>--gmeihfekvtsdnrkavenlqvfaeqqafiesmaenlkesdqfpewesagiydgnqligyamygrwqdgrVWLDRFLIDQRFQGQGYGKAacrllMLKLIEKYQTNKL<br><br>CREMAsDTspeNTISQKVHQALGF-------------------eetervifyrkrc<br>YLSVY-DT---NSSAIRLYQQLGFvfngeldtngervmewthq-------------<br><br>Proposed: aliSize=111 (resi) RMSD=2.36 (Å)<br>lvprgshmdirqmnkthlehwrglrkqlw---------------------P-----ghP-DDAHLADGEEILQADHLASFIAMADGVAIGFADASIRHDyvngcdssPVVFLEGIFVLPSFRQRGVAKQLIAAVQRWGT-NKGCREMASDTSPENTISQKVHQALGFEETE--------RVIFYRKrcc<br>-----------------------------gmeihfekvtsdnrkavenlqVfaeqq--AfIESMAENLKESDQFPEWESAGIYDGNQLIGYAMYGRWQD--------GRVWLDRFLIDQRFQGQGYGKAACRLLMLKLIeKYQTNKLYLSVYDTNSSAIRLYQQLGFVFNGeldtngerVMEWTHQ---
145	1s60A<br>(152)<br><br>1b6bA<br>(168)	C	12.50	TM-align: aliSize=128 (resi) RMSD=3.13 (Å)<br>lvprgsHMDIRQMNKTHLEHWR-G--LRK------QLWPgHpddAHlaDGeEILQADHLASFIAMADGVAIGFADASIRHDYVNgcds-----------SPVVFLEGIFVLPSFRQRGVAKQLIAAVQ-RWGTNKGCREMASDTSPenTiSQKVHQALGFEETERVIFYrkrc-------------------<br>------ANEFRCLTPEDAAGVFeIerEAFisvsgnCPLN-L---DE--VQ-HFLTLCPELSLGWFVEGRLVAFIIGSLWDEERL----tqeslalhrprGHSAHLHALAVHRSFRQQGKGSVLLWRYLhHVGAQPAVRRAVLMCED--A-LVPFYQRFGFHPAGPCAIV----vgsltftemhcslrghaal<br><br>SARST: aliSize=97 (resi) RMSD=7.32 (Å)<br>lvprgshmdirqmnkthlehwrglrkqlwpghpddahladgeeilqadhla---SFIAMADGVAIGFADASIRHDYVN-------GCDSSPVVFLEGIFVLPSFRQRGVAKQLIAAVQRWGTN-KGCREMASDTSPEntiSQKVHQ<br>---anefrcltpedaagvfeiereafisvsgncplnldevqhfltlcpelSLGWFVEGRLVAFIIGSLWDEERLtqeslalHRPRGHSAHLHALAVHRSFRQQGKGSVLLWRYLHHVGAqPAVRRAVLMCEDA---LVPFYQ<br><br>ALGFEE---------TERVIFYRKR-------c<br>RFGFHPagpcaivvgSLTFTEMHCSlrghaal-<br><br>BLAST: aliSize=11 (resi) iden=5.26% (8/152) simi=7.24% (11/152)<br>lvprgshmdirqmnkthlehwrglrkqlwpghpddahladgeeilqadhlasfiamadgvaigfadasirhdyvngcdsspvvf---LEGIFVLPSFRQRGVAKQL<br>--anefrcltpedaagvfeiereafisvsgncplnldevqhfltlcpelslgwfvegrlvafiigslwdeerltqeslalhrprghsahLHALAVHRSFRQQGKGSVL<br><br>I---aavqrwgtnkgcremasdtspentisqkvhqalgfeetervifyrkrc<br>Lwrylhhvgaqpavrravlmcedalvpfyqrfgfhpagpcaivvgsltftemhcslrghaal--<br><br>Proposed: aliSize=126 (resi) RMSD=2.57 (Å)<br>lvprgsHMDIRQMNKTHLEHWR-G--LrkQ--------LW-pgHpddaH-laDGeEILQAdHL-ASFIAMADGVAIGFADASIRHDYVN-----------gcdsSPVVFLEGIFVL-pSFRQRGVAKQLIAAVQRWGTNK-GCREMASDTSPenTiSQKVHQALGFEETERVIF------Y-RKRC----<br>------ANEFRCLTPEDAAGVFeIerE--AfisvsgncPLn--L----De--VQ-HFLTL-CPeLSLGWFVEGRLVAFIIGSLWDEERLtqeslalhrpr----GHSAHLHALAVHr-SFRQQGKGSVLLWRYLHHVGAQpAVRRAVLMCED--A-LVPFYQRFGFHPAGPCAIvvgsltFtEMHCslrg<br><br>----c<br>haal-
146	1s60A<br>(152)<br><br>2pr1A<br>(152)	C	8.55	TM-align: aliSize=128 (resi) RMSD=3.10 (Å)<br>lvprgsHMDIRQMNKThLEHWRGLRKQLWPGHPDDAHLADG-----Ee--ILqadhLASFIAMADGVAIGFADASIRHD-YVNGCD-SSPVVFLEGIFVLPSFRQRGVAKQLIAAVQRwGTnkgCrEMASDTSpeNTIsQKVHQALGFEETER----VIFYrkrc-----------<br>------MTKVERLLIN-YKTLEEFKKFKEYGIQELSMLEELqdniiE-ndST----SPFYGIYFGDKLVARMSLYQVNGkSNPYFDnRQDYLELWKLEVLPGYQNRGYGRALVEFAKS-FK---M-PIRTNPR--MKS-AEFWNKMNFKTVKYdmarDKGE----dpliwhpdmdr<br><br>SARST: aliSize=130 (resi) RMSD=9.13 (Å)<br>lvprg-SHMDIRQMNkthleHWRGLRKQLWPGHPDDAHLADGEE---ILQADHLA-SFIAMADGVAIGFADASIR----HDYVNGcdSSPVVFLEGIFVLPSFRQRGVAKQLIaavqRWGTNKGCrEMASDTSPENTISQKV---------HQALGFeeteRVIFYRKR----c<br>-----mTKVERLLIN-----YKTLEEFKKFKEYGIQELSMLEELqdnIIENDSTSpFYGIYFGDKLVARMSLYQVngksNPYFDN--RQDYLELWKLEVLPGYQNRGYGRALV----EFAKSFKM-PIRTNPRMKSAEFWNKmnfktvkydMARDKG----EDPLIWHPdmdr-<br><br>BLAST: aliSize=14 (resi) iden=5.26% (8/152) simi=9.21% (14/152)<br>lvprgs---HMDIRQmnkTHLEHWRglrKQLWPGH---pddahladgeeilqadhlasfiamad<br>------mtkverllinyktleefkkfkeygiqelsmleelqdniiendstspfygiyfgdklvarmslyqvngksnpYFDNRQ---DYLELWK---LEVLPGYqnrgygralvefaksfkmpirtnprmksaefwnkmnfktvkydmardkgedpliwhpdmdr--------------------------<br><br>gvaigfadasirhdyvngcdsspvvflegifvlpsfrqrgvakqliaavqrwgtnkgcremasdtspentisqkvhqalgfeetervifyrkrc<br>--<br><br>Proposed: aliSize=126 (resi) RMSD=2.38 (Å)<br>lvprgsHMDIRQMNKthL-EHwRGLRK-QLWPGH-pDDAHL-A---------dgEeILqadhlASFIAMADGVAIGFADASIRHD-YVNGCD-SSPVVFLEGIFVLPSFRQRGVAKQLIAAVQRwGTnkgCrEMASDTspeNTISQKVHQALGFEET---E----R-VIFYRKRC----c<br>------MTKVERLLI--NyKT-LEEFKkFKEYGIq-ELSMLeElqdniiend--S-TS-----PFYGIYFGDKLVARMSLYQVNGkSNPYFDnRQDYLELWKLEVLPGYQNRGYGRALVEFAKS-FK---M-PIRTNP---RMKSAEFWNKMNFKTVkydMardkGeDPLIWHPDmdrc-

147	1s60A (152)<br>1xebA (149)	C	10.74	TM-align: aliSize=122 (resi) RMSD=2.63 (Å)<br>lvprgsHMDIRQM----NK-THLEHWRGLRKQLWP-----GHPddAHlADgeEILqADHLASFiAMADGVAIGFADASIRH-DYvngcdssPVVFLEGIFVLPSFRQRGVAKQLiAAVQRWGTNKGCR-EMASDTSPenTiSQKVHQAlGFEETERVIFyrkrc------------<br>------SLDWTCKhhadLTlKELYALLQLRTEVFVveqkcPYQ--EV-DG--LDL-VGDTHHL-AWRDGQLLAYLRLLDPVrHE-------GQVVIGRVVSSSAARGQGLGHQL-ERALQAAERLWLDtPVYLSAQA--H-LQAYYGR-YGFVAVTEVY-----leddiphigrra<br><br>SARST: aliSize=123 (resi) RMSD=8.19 (Å)<br>lvprgsh-MDIRQMNKTHLEHWRGLRkqlwpghpDDAHLADGEEILQADHL-----------ASFIAMADGVAIGFAdASIRHDYVNGcdsspVVFLEGIFVLPSFRQRGVAKQLIAAVQRWGTNK-GCrEMASDTSPENtisQKVHQALGFEETERVIFY----------rkrc<br>-------sLDWTCKHHADLTLKELYA--------LLQLRTEVFVVEQKCPYqevdgldlvgdTHHLAWRDGQLLAYL-RLLDPVRHEG-----QVVIGRVVSSSAARGQGLGHQLERALQAAERLWlDT-PVYLSAQAHL---QAYYGRYGFVAVTEVYLEddiphigrra----<br><br>BLAST: aliSize=16 (resi) iden=5.48% (8/149) simi=10.96% (16/149)<br>lvprgshmdirqmnkthlehwrglrkqlwpghpddahla---------------------------------------DGEEILQADHlasFIAMADGVAIGFADA--SIRHD---yv<br>---------------------------------------sldwtckhhadltlkelyallqlrtevfvveqkcpyqevDGLDLVGDTH---HLAWRDGQLLAYLRLldPVRHEgqvvigrvvsssaargqglghqleralqaaerlwldtpvylsaqahlqayygrygfvavtevyleddiphigrra--<br>ngcdsspvvflegifvlpsfrqrgvakqliaavqrwgtnkgcremasdtspentisqkvhqalgfeetervifyrkrc<br>--<br><br>Proposed: aliSize=126 (resi) RMSD=2.44 (Å)<br>lvprgsHMDIRQM----NK-THLEHWRGLRK-QLW----PGHPddAHlADgeEILqADHLASFIAMADGVAIGFADASIR--HDyvngcdssPVVFLEGIFVLPSFRQRGVAKQLIAAVQRWGT-NKGCREMASDTSPenTiSQKVHQAlGFEETERV----------IFYRKrcc<br>------SLDWTCKhhadLTlKELYALLQLRTeVFVveqkCPYQ--EV-DG--LDL-VGDTHHLMAWRDGQLLAYLRLLDPvrHE--------GQVVIGRVVSSSAARGQGLGHQLMERALQAAErLWLDTPVYLSAQA--H-LQAYYGR-YGFVAVTEvyleddiphiGMRRA---
148	1s60A (152)<br>2fckA (174)	N	8.55	TM-align: aliSize=136 (resi) RMSD=3.06 (Å)<br>lvprgs--------HMDIRQMNKTHLE-HWRGLRK-Q--LWP--GHPD-DAHLADGEEILQA---DHLA-SFIAMADGVAIGFADASIRHDyvngcdSSPVvFLEGIFVlPSFRQRGVAKQLIAAVQRWGT-NKGCREMASDTSPENTISQKVHQALGFEETERVIFY-----RKrc----------<br>------tpdfqivtQRLQLRLITADEAeELVQCIRqSqtLHQwvDWFSqQEAEQFIQATRLNwvkAEAYgFGVFERQTQTLVGVAINEFYH------TFNA-SLGYWIG-DRYQRQGYGKEALTALILFCFeRLELTRLEIVCDPENVPSQALALRCGANREQLAPNRflyagEP--kagivfslip<br><br>SARST: aliSize=129 (resi) RMSD=4.00 (Å)<br>lvprgs---------HMDIRQMNKTHLEHWRGL------RKQLWPGHPDDAH--LADGEEILQADHLASFIAMA---DGVAIGFADASIR--HDYVNgcdsspvvfleGIFVLPSFRQRGVAKQLIAAVQRW-GTNKGCREMASDTSPENTISQKVHQALGFEETERVI-------------------fy<br>------tpdfqivtqRLQLRLITADEAEELVQCirqsqtLHQWVDWFSQQEAeqFIQATRLNWVKAEAYGFGVFerqTQTLVGVAINEFYhtFNASL-----------GYWIGDRYQRQGYGKEALTALILFcFERLELTRLEIVCDPENVPSQALALRCGANREQLAPnrflyagepkagivfslip--<br>rkrc<br>----<br><br>BLAST: aliSize=24 (resi) iden=7.89% (12/152) simi=15.79% (24/152)<br>lvprgshmdirqmnkthlehwrglrkqlwpghpddahladgeeilqadhlasfiamadgvaigfadasirhdyvngcdsspvvfle---GIFVLPSFRQRGV<br>--tpdfqivtqrlqlrlitadeaeelvqcirqsqtlhqwvdwfsqqeaeqfiqatrlnwvkaeaygfgvferqtqtlvgvainefyhtfnaslGYWIGDRYQRQGY<br>AKQLIAAV-----QRWGTNKgcREMASDtsPENTISQ----------------------------------kvhqalgfeetervifyrkrc<br>GKEALTALilfcfERLELTR--LEIVCD--PENVPSQalalrcganreqlapnrflyagepkagivfslip---------------------<br><br>Proposed: aliSize=139 (resi) RMSD=2.60 (Å)<br>LV-PRGSH--MDIRQMNK-THLEHWRGLRK-Q--LWP---gHPD-DAHLADGEEILQA---D-HLASFIAMADGVAIGFADASIRHD--yvngcdSSPVvFLEGIFVlPSFRQRGVAKQLIAAVQRWGT-NKGCREMASDTSPENTISQKVHQALGFEETERVIF-------Y----------rkrcc<br>MTpDFQIVtqRLQLRLITaDEAEELVQCIRqSqtLHQwvd-WFSqQEAEQFIQATRLNwvkAeAYGFGVFERQTQTLVGMVAINEFYht------FNMA-SLGYWIG-DRYQRQGYGKEALTALILFCFeRLELTRLEIVCDPENVPSQALALRCGANREQLAPNrflyagePkagivfslip-----
149	1s60A (152)<br>2fckA (174)	C	9.21	TM-align: aliSize=136 (resi) RMSD=3.06 (Å)<br>lvprgs--------HMDIRQMNKTHLE-HWRGLRK-Q--LWP--GHPD-DAHLADGEEILQA---DHLA-SFIAMADGVAIGFADASIRHDyvngcdSSPVvFLEGIFVlPSFRQRGVAKQLIAAVQRWGT-NKGCREMASDTSPENTISQKVHQALGFEETERVIFY-----RKrc----------<br>------tpdfqivtQRLQLRLITADEAeELVQCIRqSqtLHQwvDWFSqQEAEQFIQATRLNwvkAEAYgFGVFERQTQTLVGVAINEFYH------TFNA-SLGYWIG-DRYQRQGYGKEALTALILFCFeRLELTRLEIVCDPENVPSQALALRCGANREQLAPNRflyagEP--kagivfslip<br><br>SARST: aliSize=129 (resi) RMSD=4.00 (Å)<br>lvprgs---------HMDIRQMNKTHLEHWRGL------RKQLWPGHPDDAH--LADGEEILQADHLASFIAMA---DGVAIGFADASIR--HDYVNgcdsspvvfleGIFVLPSFRQRGVAKQLIAAVQRW-GTNKGCREMASDTSPENTISQKVHQALGFEETERVI-------------------fy<br>------tpdfqivtqRLQLRLITADEAEELVQCirqsqtLHQWVDWFSQQEAeqFIQATRLNWVKAEAYGFGVFerqTQTLVGVAINEFYhtFNASL-----------GYWIGDRYQRQGYGKEALTALILFcFERLELTRLEIVCDPENVPSQALALRCGANREQLAPnrflyagepkagivfslip--<br>rkrc<br>----<br><br>BLAST: aliSize=24 (resi) iden=7.89% (12/152) simi=15.79% (24/152)<br>lvprgshmdirqmnkthlehwrglrkqlwpghpddahladgeeilqadhlasfiamadgvaigfadasirhdyvngcdsspvvfle---GIFVLPSFRQRGV<br>--tpdfqivtqrlqlrlitadeaeelvqcirqsqtlhqwvdwfsqqeaeqfiqatrlnwvkaeaygfgvferqtqtlvgvainefyhtfnaslGYWIGDRYQRQGY<br>AKQLIAAV-----QRWGTNKgcREMASDtsPENTISQ----------------------------------kvhqalgfeetervifyrkrc<br>GKEALTALilfcfERLELTR--LEIVCD--PENVPSQalalrcganreqlapnrflyagepkagivfslip---------------------<br><br>Proposed: aliSize=136 (resi) RMSD=2.58 (Å)<br>lvprgsh----------MDIRQMNK-THLEHWRGLRK-Q--LWP---gHPD-DAHLADGEEILQA---D-HLASFIAMADGVAIGFADASIRHD--yvngcdSSPVvFLEGIFVlPSFRQRGVAKQLIAAVQRWGT-NKGCREMASDTSPENTISQKVHQALGFEETERVIF------YRKRC-------c<br>-------mtpdfqivtqRLQLRLITaDEAEELVQCIRqSqtLHQwvd-WFSqQEAEQFIQATRLNwvkAeAYGFGVFERQTQTLVGMVAINEFYht------FNMA-SLGYWIG-DRYQRQGYGKEALTALILFCFeRLELTRLEIVCDPENVPSQALALRCGANREQLAPNrflyagEPKAGivfslip-

No.	Templates	Class	Score	Alignments
150	1tiyA (157)<br>2g84A (189)	C	26.11	TM-align: aliSize=116 (resi) RMSD=1.45 (Å)<br>------------------------NHETFLKRAVTLACEGVNAGiGGPFGAVIVK--DGAIIAEGQNNVTTSNDPTAHAEVTAIRKACKVLGAYQLD-----DCILYTSCEPCP-CLGAIYWARPKAVFYAAEHTDAAEAGFDDsfIYKeIDkpaeertipfyqvtltehlspfqawrnfankkeyl---<br>mndalhiglppflvqanneprvlaAPEARMGYVLELVRANIAAD-GGPFAAAVFErdSGLLIAAGTNRVVPGRCSAAHAEILALSLAQAKLDTHDLSadglpACELVTSAEPCVmCFGAVIWSGVRSLVCAARSDDVEAIGFDE--GPR-PE-------------------------------------nwm<br>--------------------------------------<br>ggleargitvttgllrdaacallreynacngviynarc<br><br>SARST: aliSize=143 (resi) RMSD=18.07 (Å)<br>n------------------------HETFLKRAVTLACEGVNAGiGGPFGAVIVK--DGAIIAEGQNNVTTSNDPTAHAEVTAIRKACKVLGAYQL-----DDCILYTSCEPCPCLGAIY-WARPKAVFYAAEHTDAAEAG------FDDSFIYKEIDKpaeertIPFYQVTLTEHLSPFQAWRNFANK<br>-mndalhiglppflvqanneprvlaaPEARMGYVLELVRANIAAD-GGPFAAAVFErdSGLLIAAGTNRVVPGRCSAAHAEILALSLAQAKLDTHDLsadglPACELVTSAEPCVMCFGAViWSGVRSLVCAARSDDVEAIGfdegprPENWMGGLEARG------ITVTTGLLRDAACALLREYNACNG<br>-------keyl<br>viynarc----<br><br>BLAST: aliSize=48 (resi) iden=23.23% (36/157) simi=30.97% (48/157)<br>nhetflkravtlacegvn---AGIGGPFGAVIV-KD-GAIIAEGQNNVTTSNDPTAHAEVTAIR----------------KACKvlgayqlddciLYTSCEPC-PCLGAIYWARPKAVFYAAEHTDAAEAGFDD-------------------<br>-----------------mndalhiglppflvqanneprvlaapearmgyvlelvraniAADGGPFAAAVFeRDsGLLIAAGTNRVVPGRCSAAHAEILALSlaqakldthdlsadglPACE-----------LVTSAEPCvMCFGAVIWSGVRSLVCAARSDDVEAIGFDEgprpenwmggleargitv<br>---------------------------sfiykeidkpaeertipfyqvtltehlspfqawrnfankkeyl<br>ttgllrdaacallreynacngviynarc--<br><br>Proposed: aliSize=146 (resi) RMSD=1.49 (Å)<br>-----------------------MNHETFLKRAVTLACEGVNAGiGGPFGAVIVK--DGAIIAEGQNNVTTSNDPTAHAEVTAIRKACKVLGAYQLD-----DCILYTSCEPCPMCLGAIYWARPKAVFYAAEHTDAAEAGFDD---sfivkeIDKPAEER--T-IPFYQVTL-TEHLSPFQAWRNFANK<br>mndalhiglppflvqanneprvlAAPEARMGYVLELVRANIAAD-GGPFAAAVFErdSGLLIAAGTNRVVPGRCSAAHAEILALSLAQAKLDTHDLSadglpACELVTSAEPCVMCFGAVIWSGVRSLVCAARSDDVEAIGFDEgpr------PENWMGGLeaRgITVTTGLLrDAACALLREYNACNGV<br>------keyl<br>iynarc----
151	1tiyA (157)<br>1wwrA (154)	C	29.22	TM-align: aliSize=108 (resi) RMSD=1.71 (Å)<br>----NHETFLKRAVTLACEGVNAGiGGPFGAVIVKDGAIIAEGQNNVTTSNDPTAHAEVTAIRKACKVLGAYQLDDCILYTSCEPCP-CLGAIYWARPKAVFYAAEHTdaaeagfDDSfi----YKe-Idkpaeertipfyqvtltehlspfqawrnfankkeyl--------------------------<br>gshmGKEYFLKVALREAKRAFEKG-EVPVGAIIVKEGEIISKAHNSVEELKDPTAHAEMLAIKEACRRLNTKYLEGCELYVTLEPCImCSYALVLSRIEKVIFSALDK-------KHG--gvvsVF-nI---------------------------------------ldeptlnhrvkweyypleeasells<br>-----------<br>effkklrnnii<br><br>SARST: aliSize=97 (resi) RMSD=0.97 (Å)<br>n-----HETFLKRAVTLACEGVNAGiGGPFGAVIVKDGAIIAEGQNNVTTSNDPTAHAEVTAIRKACKVLGAYQLDDCILYTSCEPC-PCLGAIYWARPKAVFYA---aehtdaaeagfddsfiykeidkpaeertipfyqv<br>-gshmgKEYFLKVALREAKRAFEKG-EVPVGAIIVKEGEIISKAHNSVEELKDPTAHAEMLAIKEACRRLNTKYLEGCELYVTLEPCiMCSYALVLSRIEKVIFSaldkkhggvvsvfnildeptlnhrvkweyypleeasellseffkklrnnii-----------------------------------<br>tltehlspfqawrnfankkeyl<br>----------------------<br><br>BLAST: aliSize=76 (resi) iden=33.12% (51/154) simi=49.35% (76/154)<br>nh------ETFLKRAVTLACEGVNAGiGGPFGAVIVKDGAIIAEGQNNVTTSNDPTAHAEVTAIRKACKVLGAYQLDdcilytSCE------PC-PCLGAIYWARPKAVFYAAehTDAAEAGFDDsfIYKEIDKPAEERTI--PFYQV-TLTEHLSP-FQAWRN---fankkeyl<br>--gshmgkEYFLKVALREAKRAFEKG-EVPVGAIIVKEGEIISKAHNSVEELKDPTAHAEMLAIKEACRRLNTKYLE------GCElyvtlePCiMCSYALVLSRIEKVIFSA--LDKKHGGVVS--VFNILDEPTLNHRVkwEYYPLeEASELLSEfFKKLRNnii--------<br><br>Proposed: aliSize=134 (resi) RMSD=1.72 (Å)<br>---MNHETFLKRAVTLACEGVNAGiGGPFGAVIVKDGAIIAEGQNNVTTSNDPTAHAEVTAIRKACKVLGAYQLDDCILYTSCEPCPMCLGAIYWARPKAVFYAAEH---------tdaaeagfddsfivkeIdkP-AEER-T-IPFYQVTLTEhLSPFQAWRNF-AnKKE----yl<br>gshMGKEYFLKVALREAKRAFEKG-EVPVGAIIVKEGEIISKAHNSVEELKDPTAHAEMLAIKEACRRLNTKYLEGCELYVTLEPCIMCSYALVLSRIEKVIFSALDkkhggvvsv----------------F--NiLDEPtLnHRVKWEYYPL-EEASELLSEFfK-KLRnnii--
152	1tiyA (157)<br>2b3jA (151)	C	29.14	TM-align: aliSize=108 (resi) RMSD=1.81 (Å)<br>--NHETFLKRAVTLACEGVNaGIGGPFGAVIVKDGAIIAEGQNNVTTSNDPTAHAEVTAIRKACKVLGAYQLDDCILYTSCEPCP-CLGAIYWARPKAVFYAAEHTdaaeagfDDSfi----YKeidkpaeertipfyqvtltehlspfqawrnfankkey---------L--------------------<br>mtNDIYFMTLAIEEAKKAAQ-LGEVPIGAIITKDDEVIARAHNLRETLQQPTAHAEHIAIERAAKVLGSWRLEGCTLYVTLEPCVmCAGTIVMSRIPRVVYGADDP-------KGG--csgsLM-------------------------------------nllqqsnfnHraivdkgvlkeacstlltt<br>--------<br>ffknlran<br><br>SARST: aliSize=107 (resi) RMSD=3.42 (Å)<br>n---HETFLKRAVTLACEGVNAGiGGPFGAVIVKDGAIIAEGQNNVTTSNDPTAHAEVTAIRKACKVLGAYQLDDCILYTSCEPCPCLGAIYWA-RPKAVFYAAEHTDAAEAG---------------------------------------fddsfiykeidkpaeertipfyqvtltehlspfqawr<br>-mtnDIYFMTLAIEEAKKAAQLG-EVPIGAIITKDDEVIARAHNLRETLQQPTAHAEHIAIERAAKVLGSWRLEGCTLYVTLEPCVMCAGTIVMsRIPRVVYGADDPKGGCSGslmnllqqsnfnhraivdkgvlkeacstllttffknlran-------------------------------------<br>nfankkeyl<br>---------<br><br>BLAST: aliSize=55 (resi) iden=28.48% (43/151) simi=36.42% (55/151)<br>nhetflkra-------VTLACEGVN--AGIGG-PFGAVIVKDGAIIAEGQNNVTTSNDPTAHAEVTAIRKACKVLGAYQLDDCILYTSCEPC-PCLGAIYWARPKAVFYAAE---htdaaeagfddsfiykeidkpaeertipfy<br>---------mtndiyfMTLAIEEAKkaAQLGEvPIGAIITKDDEVIARAHNLRETLQQPTAHAEHIAIERAAKVLGSWRLEGCTLYVTLEPCvMCAGTIVMSRIPRVVYGADdpkggcsgslmnllqqsnfnhraivdkgvlkeacstllttffknlran------------------------------<br>qvtltehlspfqawrnfankkeyl<br>------------------------<br><br>Proposed: aliSize=133 (resi) RMSD=1.59 (Å)<br>m--NHETFLKRAVTLACEGVNAGiGGPFGAVIVKDGAIIAEGQNNVTTSNDPTAHAEVTAIRKACKVLGAYQLDDCILYTSCEPCPMCLGAIYWARPKAVFYAAEH----------tdaaeagfddsfivkeidkP-AE-E-RTIPFYQVTLTEHLSPFQAWRNF-ANKK--eyl<br>-mtNDIYFMTLAIEEAKKAAQLG-EVPIGAIITKDDEVIARAHNLRETLQQPTAHAEHIAIERAAKVLGSWRLEGCTLYVTLEPCVMCAGTIVMSRIPRVVYGADDpkggcsgslm------------------NlLQqSnFNHRAIVDKGVLKEACSTLLTTFfKNLRan---

153	1tiyA (157)<br>2nx8A (168)	C	24.20	TM-align: aliSize=109 (resi) RMSD=2.05 (Å)<br>---------NHETFLKRAVTLACEGVNaGIGGPFGAVIVKDGAIIAEGQNNVTTSNDPTAHAEVTAIRKACKVLGAYQLDDCILYTSCEPCP-CLGAIYWARPKAVFYAAEHTdaaeagfDDSFi---YKe-Idkpaeertipfvqvtltehlspfqawrnfankkeyl----------------------<br>sflmpysleEQTYFMQEALKESEKSLQ-KAEIPIGCVIVKDGEIIGRGHNAREESNQAIMHAEMMAINEANAHEGNWRLLDTTLFVTIEPCVmCSGAIGLARIPHVIYGASNQ-------KFGG-vdsLY-qI------------------------------------ltderlnhrvqvergllaadc<br>-------------------------<br>animqtffrqgrerkkiakhlike<br>SARST: aliSize=140 (resi) RMSD=16.54 (Å)<br>n----------HETFLKRAVTLACEGVNAGiGGPFGAVIVKDGAIIAEGQNNVTTSNDPTAHAEVTAIRKACKVLGAYQLDDCILYTSCEPCPCLGAIY-WARPKAVFYAAEHTDaaeagFDDSFIY-KEIDKPAEERTIPFYQVTLTEHLSPFQAWRN----------------fankkeyl<br>-sflmpysleeQTYFMQEALKESEKSLQKA-EIPIGCVIVKDGEIIGRGHNAREESNQAIMHAEMMAINEANAHEGNWRLLDTTLFVTIEPCVMCSGAIgLARIPHVIYGASNQK-----FGGVDSLyQILTDERLNHRVQVERGLLAADCANIMQTFFrqgrerkkiakhlike--------<br>BLAST: aliSize=43 (resi) iden=23.23% (36/157) simi=27.74% (43/157)<br>nhetflkravtlacegvnagigg--------------------------------PFGAVIVKDGAIIAEGQNNVTTSNDPTAHAEVTAIRKACKVLGAYQLDDCILYTSCEPC-PCLGAIYWARPKAVFYAAEH---<br>------------------------sflmpysleeqtyfmqealkesekslqkaeiPIGCVIVKDGEIIGRGHNAREESNQAIMHAEMMAINEANAHEGNWRLLDTTLFVTIEPCvMCSGAIGLARIPHVIYGASNqkfggvdslyqiltderlnhrvqvergllaadcanimqtffrqgrerkkiakhlik<br>-tdaaeagfddsfiykeidkpaeertipfyqvtltehlspfqawrnfankkeyl<br>e--<br>Proposed: aliSize=132 (resi) RMSD=1.92 (Å)<br>--------MNHETFLKRAVTLACEGVNAGiGGPFGAVIVKDGAIIAEGQNNVTTSNDPTAHAEVTAIRKACKVLGAYQLDDCILYTSCEPCPMCLGAIYWARPKAVFYAAEH-------------T--DAAEagfD-dSFivkEidkpaeeRTIPFY--Q---V---tlteH-LSPFQAWRNFA--nkkeyl<br>sflmpyslEEQTYFMQEALKESEKSLQKA-EIPIGCVIVKDGEIIGRGHNAREESNQAIMHAEMMAINEANAHEGNWRLLDTTLFVTIEPCVMCSGAIGLARIPHVIYGASNqkfggvdslyqiltDerLNHR---Vq-VE---R-------GLLAADcaNimqTffr----QgRERKKIAKHLIke------
154	1tiyA (157)<br>1z3aA (156)	C	25.64	TM-align: aliSize=108 (resi) RMSD=1.79 (Å)<br>-----NHETFLKRAVTLACEGVNaGIGGPFGAVIVKDGAIIAEGQNNVTTSNDPTAHAEVTAIRKACKVLGAYQLDDCILYTSCEPCP-CLGAIYWARPKAVFYAAEHTdaaeagfDDSfi----YKe-Idkpaeertipfvqvtltehlspfqawrnfankkeyl-----------------------<br>sevefSHEYWMRHALTLAKRAWD-EREVPVGAVLVHNNRVIGEGWNRPIGRHDPTAHAEIMALRQGGLVMQNYRLIDATLYVTLEPCVmCAGAMIHSRIGRVVFGARDA-------KTG--aagsLM-dV-----------------------------------lhhpgmnhrveitegiladecaal<br>-------------<br>lsdffrmrrqeik<br>SARST: aliSize=99 (resi) RMSD=1.10 (Å)<br>n------HETFLKRAVTLACEGVNAGiGGPFGAVIVKDGAIIAEGQNNVTTSNDPTAHAEVTAIRKACKVLGAYQLDDCILYTSCEPC-PCLGAIYWARPKAVFYAAE---htdaaeagfddsfiykeidkpaeertipfyqv<br>-sevefsHEYWMRHALTLAKRAWDER-EVPVGAVLVHNNRVIGEGWNRPIGRHDPTAHAEIMALRQGGLVMQNYRLIDATLYVTLEPCvMCAGAMIHSRIGRVVFGARdaktgaagslmdvlhhpgmnhrveitegiladecaallsdffrmrrqeik------------------------------<br>tltehlspfqawrnfankkeyl<br>----------------------<br>BLAST: aliSize=60 (resi) iden=26.45% (41/156) simi=38.71% (60/156)<br>-----NHETFLKRAVTLACEGVNAGiGGPFGAVIVKDGAIIAEGQNNVTTSNDPTAHAEVTAIRKACKVLGAYQLDDCILYTSCEPC-PCLGAIYWARPKAVFYAAEHTDAAEAG---fddsfiykeidkpaeertipfyqvtltehlspf<br>sevefSHEYWMRHALTLAKRAWDER-EVPVGAVLVHNNRVIGEGWNRPIGRHDPTAHAEIMALRQGGLVMQNYRLIDATLYVTLEPCvMCAGAMIHSRIGRVVFGARDAKTGAAGslmdvlhhpgmnhrveitegiladecaallsdffrmrrqeik----------------------------------<br>qawrnfankkeyl<br>-------------<br>Proposed: aliSize=135 (resi) RMSD=1.62 (Å)<br>----MNHETFLKRAVTLACEGVNAGiGGPFGAVIVKDGAIIAEGQNNVTTSNDPTAHAEVTAIRKACKVLGAYQLDDCILYTSCEPCPMCLGAIYWARPKAVFYAAEHT---------daaeagfddsfivkeidkP-AE-E-RTIPFYQVTLTEHLSPFQAWRNF-ANKK----eyl<br>seveFSHEYWMRHALTLAKRAWDER-EVPVGAVLVHNNRVIGEGWNRPIGRHDPTAHAEIMALRQGGLVMQNYRLIDATLYVTLEPCVMCAGAMIHSRIGRVVFGARDAktgaagslm-----------------DvLHhPgMNHRVEITEGILADECAALLSDFfRMRRqeik---
155	2p1jA (164)<br>1j53A (174)	C	29.27	TM-align: aliSize=143 (resi) RMSD=1.75 (Å)<br>TFVVLDFETTGLD-----PQVDEIIEIGAVKIQGGQIV-DEYHTLIKPSREISRKSSEITGITQEMLENKRSIEEVLPEFLGFLEDSIIVAHNANFDYRFLRLWIKKV-MGLDWER---PYIDTLALAKSLLKLRSYSLDSVVEKLGLgPFRHHralddarvtaqvflrfvemm----------------<br>RQIVLDTETTGMNqigahYEGHKIIEIGAVEVVNRRLTgNNFHVYLKPDRLVDPEAFGVHGIADEFLLDKPTFAEVADEFMDYIRGAELVIHNAAFDIGFMDYEFSLLkRDIPKTNtfcKVTDSLAVARKMFPGKRNSLDALCARYEI-DNSKR--------------------tlhgalldaqilaevy<br>-----<br>lamtg<br>SARST: aliSize=155 (resi) RMSD=7.89 (Å)<br>t-FVVLDFETTG----LDPQ-VDEIIEIGAVKIQGGQIV-DEYHTLIKPSREISRKSSEITGITQEMLENKRSIEEVLPEFLGFLEDSIIVAHNANFDYRFLRLWIKKV-MGLDW---ERPYIDTLALAKSLL-KLrSYSLDSVVEKLGlgPFR-----HHRALDDARVTAQVFLR--fvemm<br>-rQIVLDTETTGmnqiGAHYeGHKIIEIGAVEVVNRRLTgNNFHVYLKPDRLVDPEAFGVHGIADEFLLDKPTFAEVADEFMDYIRGAELVIHNAAFDIGFMDYEFSLLkRDIPKtntFCKVTDSLAVARKMFpGK-RNSLDALCARYE--IDNskrtlHGALLDAQILAEVYLAMtg-----<br>BLAST: aliSize=91 (resi) iden=30.49% (50/164) simi=55.49% (91/164)<br>tf--VVLDFETTGLDP-----QVDEIIEIGAVKIQGGQIV-DEYHTLIKPSREISRKSSEITGITQEMLENKRSIEEVLPEFLGFLEDSIIVAHNANFDYRFL----RLWIKKVMGLDWERPYIDTLALAKSLLKLRSYSLDSVVEKLGLGPFRH--HRALDDARVTAQVFL----rfvemm<br>--rqIVLDTETTGMNQigahyEGHKIIEIGAVEVVNRRLTgNNFHVYLKPDRLVDPEAFGVHGIADEFLLDKPTFAEVADEFMDYIRGAELVIHNAAFDIGFMdyefSLLKRDIPKTNTFCKVTDSLAVARKMFPGKRNSLDALCARYEIDNSKRtlHGALLDAQILAEVYLamtg------<br>Proposed: aliSize=156 (resi) RMSD=1.49 (Å)<br>TFVVLDFETTGL-----DPQVDEIIEIGAVKIQGGQIV-DEYHTLIKPSREISRKSSEITGITQEMLENKRSIEEVLPEFLGFLEDSIIVAHNANFDYRFLRLWIKKV-MGLDW-E--RPYIDTLALAKSLLKLRSYSLDSVVEKLGL--------gpfrhhRALDDARVTAQVFLRFVEmm<br>RQIVLDTETTGMnqigaHYEGHKIIEIGAVEVVNRRLTgNNFHVYLKPDRLVDPEAFGVHGIADEFLLDKPTFAEVADEFMDYIRGAELVIHNAAFDIGFMDYEFSLLkRDIPKtNtfCKVTDSLAVARKMFPGKRNSLDALCARYEIdnskrtlh------GALLDAQILAEVYLAMTG--

156	2p1jA (164)<br>2iocB (220)	C	25.00	TM-align: aliSize=144 (resi) RMSD=2.48 (Å)<br>----TFVVLDFETTGLDPQVDEIIEIGAVKIQ-GG-----------------Q-IVDEYHTLIKPSREISRKSSEITGITQEMLE--NKRSIE-EVLPEFLGFLE---D-SIIVAHNAN-FDYRFLRLWIKKVMGLD-WE-RPYIDTLALAKSLLK--LRSYSLDSVVEKLGLGPFRHHralddarvtaq<br>hghqTLIFLDLEATGLPSSRPEVTELCLLAVHrRAlentsisqghpppvprpPrVVDKLSLCIAPGKACSPGASEITGLSKAELEvqGRQRFDdNLAILLRAFLQrqpQpCCLVAHNGDrYDFPLLQTELARLSTPSpLDgTFCVDSIAALKALEQasRKSYSLGSIYTRLYWQAPTDS-----------<br><br>vflrfvemm--<br>---------htaegdvltllsicqwkpqallqwvdeharpfstvkpyg<br><br>SARST: aliSize=156 (resi) RMSD=8.75 (Å)<br>t-----FVVLDFETTGLDPQVDEIIEIGAVKI-----------QGGQIV--------DEYHTLIKPSREISRKSSEITGITQEMLEN----KRSIEEVLPEFLGFLEDSIIVAH---NANFDYRFLRLWIKKVMGLDW---ERPYIDTLALAKSLL--KLRSYSLDSVVEKLGLGPF---RHHRALDDAR<br>-hghqtLIFLDLEATGLPSSRPEVTELCLLAVhrralentsisQGHPPPvprpprvvDKLSLCIAPGKACSPGASEITGLSKAELEVqgrqRFDDNLAILLRAFLQRQPQPCCLvahNGDRYDFPLLQTELARLSTPSpldGTFCVDSIAALKALEqaSRKSYSLGSIYTRLYWQAPtdsHTAEGDVLTL<br><br>VTAQVF-----------------------lrfvemm<br>LSICQWkpqallqwvdeharpfstvkpyg-------<br><br>BLAST: aliSize=82 (resi) iden=30.49% (50/164) simi=50.00% (82/164)<br>----TFVVLDFETTGLD---PQVDEIIEIGA-------VKIQGG---------QIVDEYHTLIKPSREISRKSSEITGITQEMLE--NKRSIEEVLPEFL-GFLEDS----IIVAHNAN-FDYRFLRLWIKKV---MGLDWERpYIDTLALAKSL--LKLRSYSLDSVVEKL-GLGPFRHHRALDD----<br>hghqTLIFLDLEATGLPssrPEVTELCLLAVhrralenTSISQGhpppvprppRVVDKLSLCIAPGKACSPGASEITGLSKAELEvqGRQRFDDNLAILLrAFLQRQpqpcCLVAHNGDrYDFPLLQTELARLstpSPLDGTF-CVDSIAALKALeqASRKSYSLGSIYTRLyWQAPTDSHTAEGDvltl<br><br>-----------------------------arvtaqvflrfvemm<br>lsicqwkpqallqwvdeharpfstvkpyg---------------<br><br>Proposed: aliSize=155 (resi) RMSD=2.06 (Å)<br>-----TFVVLDFETTGLD--pqVDEIIEIGAVKIQ-GG-----------------Q-IVDEYHTLIKPSREISRKSSEITGITQEMLE--NKRSIE-EVLPEFLGFLE---D-SIIVAHNAN-FDYRFLRLWIKKVMGLD-WE-RPYIDTLALAKSLLK--LRSYSLDSVVEKLG-----lgpfrhH--R<br>hghmqTLIFLDLEATGLPss--RPEVTELCLLAVHrRAlentsisqghpppvprpPrVVDKLSLCIAPGKACSPGASEITGLSKAELEvqGRQRFDdNLAILLRAFLQrqpQpCCLVAHNGDrYDFPLLQTELARLSTPSpLDgTFCVDSIAALKALEQasRKSYSLGSIYTRLYwqapt------DshT<br><br>ALDDARVTAQVFLRFVEM--------------------m<br>AEGDVLTLLSICQWKPQAllqwvdeharpfstvkpmyg-
157	2p1jA (164)<br>2d5rA (252)	C	18.90	TM-align: aliSize=140 (resi) RMSD=2.69 (Å)<br>------------------------TFVVLDFETTGL---------------------DPQVDEIIEIGAVKIQ-GGQIV---DEYHTLIKPS--REIS-RKSSEITG---ITQEML-ENKRSIEEVLPEFL-GFLE--D-SIIVAHNANFDYRFLRLWIKKVm-Gl---------DWE--R-PYIDTLAL<br>ricevwacnldeemkkirqvirkyNYVAMDTEFPGVvarpigefrsnadyqyqllrcNVDLLKIIQLGLTFMNeQGEYPpgtSTWQFNFKFNltEDMYaQDSIELLTtsgIQFKKHeEEGIETQYFAELLMtSGVVlcEgVKWLSFHSGYDFGYLIKILTNS-nL-peeeldffeILRlfFpVIYDVKYL<br><br>AKSLLKLrSYSLDSVVEKLGLGpFRHHralddarvtaqvflrfvemm---------------------------------------<br>MKSCKNL-KGGLQEVAEQLELE-RIGP--------------------qhqagsdslltgmaffkmremffedhiddakycghlygl<br><br>SARST: aliSize=136 (resi) RMSD=7.86 (Å)<br>tfvvldfettgld--POVDEIIEIGAVKIQ--GGQIVDEYHTLIKPSR----EIS----RKSSEITGITQEMLENKRSIEEVLPEFLGFLED-----SIIVAHNANFDYRFLRlwiKKVMGLDWE---------<br>-------------ricevwacnldeemkkirqvirkynyvamdtefpgvvarpigefrsnadyqyqllrcnVDLLKIIQLGLTFMNeqGEYPPGTSTWQFNFKFnlteDMYaqdsIELLTTSGIQFKKHEEEGIETQYFAELLMTSGVvlcegVKWLSFHSGYDFGYLI---KILTNSNLPeeeldffei<br><br>----RPYIDTLALAKSLLKLRSYSLDSVVEKLGLGPFRHH------------RALDDARV------------------taqvflrfvemm<br>lrlfFPVIYDVKYLMKSCKNLKGGLQEVAEQLELERIGPQhqagsdslltgmAFFKMREMffedhiddakycghlygl------------<br><br>BLAST: aliSize=27 (resi) iden=12.80% (21/164) simi=16.46% (27/164)<br>tfvvldfettgldpqvdeiieigavkiqggqivdeyhtlikpsreisrksseitgitqemlenkrsieevlpeflgfledsiivahnanfdyrflrlwikkvmgldwer---<br>---ricevwacnldeemkkirqvirkynyvamdtefpgvvarpigefrsnadyqyqllrcnvdllkiiqlgltfmneqgeyppg<br><br>--PYI-DTLALAKSLLKLRSySLDSVVEKLGL---GPfrHHRALDDARVTAQVFLRFVEM------------------m<br>tstwqfnfkfnltedmyaqdsiellttsgiqfkkheeegietqyfaellmtsgvvlcegvkwlsfhsgydfgylikiltnsnlpeeeldffeilrlffPVIyDVKYLMKSCKNLKG-GLQEVAEQLELeriGP--QHQAGSDSLLTGMAFFKMREMffedhiddakycghlygl-<br><br>Proposed: aliSize=153 (resi) RMSD=2.14 (Å)<br>------------------------TFVVLDFETTGL---------------------DPQVDEIIEIGAVKIQG-GQIV---DEYHTLIKPsrEI----S-RKSSEITG---ITQEML-ENKRSIEEVLPEFL-GFLE--D-SIIVAHNANFDYRFLRLWIKKV-----------mglD--WER-PYIDT<br>ricevwacnldeemkkirqvirkyNYVAMDTEFPGVvarpigefrsnadyqyqllrcNVDLLKIIQLGLTFMNEqGEYPpgtSTWQFNFKF--NLtedmYaQDSIELLTtsgIQFKKHeEEGIETQYFAELLMtSGVVlcEgVKWLSFHSGYDFGYLIKILTNSnlpeeeldffe---IlrLFFpVIYDV<br><br>LALAKSLLKLrSYSLDSVVEKLGL----gpfrhH--RALDDARVTAQVFLRFVEMM-----------------<br>KYLMKSCKNL-KGGLQEVAEQLELerig-----PqhQAGSDSLLTGMAFFKMREMFfedhiddakycghlygl
158	2p1jA (164)<br>2p51A (255)	C	17.68	TM-align: aliSize=138 (resi) RMSD=2.65 (Å)<br>----------------------------TFVVLDFETTGL---------------------DPQVDEIIEIGAVKIQGG-QIV--D-EYHTLIKPS--REIS-RKSSEITG---ITQEML-ENKRSIEEVLPEFL-GFLE-D--SIIVAHNANFDYRFLRLWIKKVmgl----------DW-E-R-PYI<br>sqispirdvwstnlqqemnlimslieryPVVSMDTEFPGVvarplgvfkssddyhyqtlraNVDSLKIIQIGLALSDEEgNAPveAcTWQFNFTFNlqDDMYaPESIELLTksgIDFKKHqEVGIEPADFAELLIgSGLVlQeeVTWITFHSGYDFAYLLKAMTQI---plpaeyeefykILcIyFpKNY<br><br>DTLALAK-SLlKLrSYSLDSVVEKLGLGpFRHHralddarvtaqvflrfvemm--------------------------------------<br>DIKYIMKsVL-NN-SKGLQDIADDLQIH-RIGP--------------------qhqagsdalltariffeirsryfdgsidsrmlnqlygl<br><br>SARST: aliSize=154 (resi) RMSD=11.61 (Å)<br>tf---VVLDFETTGLDPQ----------VDEIIEIGAVKIQ--GGQIV----DEYHTLIKPSREIS----RKSSEITGITQEMLENKRSIEEVLPEFLGFLE-----DSIIVAHNANfdyRFLRLWIKKVMGLDW--------------ERP<br>--sqispirdvwstnlqqemnlimslierypvvsmdtefpgvvARPLGVFKSSDDYhyqtlranvdSLKIIQIGLALSDeeGNAPVeactWQFNFTFNLQDDMYapesIELLTKSGIDFKKHQEVGIEPADFAELLIGSGlvlqeEVTWITFHSG---YDFAYLLKAMTQIPLpaeyeefykilciyFPK<br><br>YIDTLALAKSLLKLRSySLDSVVEKLGLGPFRH--HRALDDARVTAQVFLRF-------------------vemm<br>NYDIKYIMKSVLNNSK-GLQDIADDLQIHRIGPqhQAGSDALLTARIFFEIRsryfdgsidsrmlnqlygl----<br><br>BLAST: aliSize=32 (resi) iden=10.98% (18/164) simi=19.51% (32/164)<br>tfvvldfettgldpqvdeiieigavkiqggqivdeyhtlikpsreisrksseitgitqemlenkrsieevlpeflgfledsiivahnanfdyrflrlwik--<br>--sqispirdvwstnlqqemnlimslierypvvsmdtefpgvvarplgvfkssddyhyqtlranvdslkiiqiglalsdeegnapveactwq<br><br>--KVMGLDWERPYiDTLALAKSLLKlRSYSLDSVVEKLGL---GPfrHHRALDDARVTAQVF-----------------------lrfvemm<br>fnftfnlqddmyapesielltksgidfkkhqevgiepadfaelligsglvlqeevtwitfhsgydfayllkamtqiplpaeyeefyKILCIYFPKNY-DIKYIMKSVLN-NSKGLQDIADDLQIhriGP--QHQAGSDALLTARIFfeirsryfdgsidsrmlnqlygl-------<br><br>Proposed: aliSize=152 (resi) RMSD=2.23 (Å)<br>----------------------------TFVVLDFETTGL---------------------DPQVDEIIEIGAVKIQGG-QIV--D-EYHTLIKPsrEI----S-RKSSEITG---ITQEML-ENKRSIEEVLPEFLGF-LE-D--SIIVAHNANFDYRFLRLWIKKV-----------mglDW-E-RP-<br>sqispirdvwstnlqqemnlimslieryPVVSMDTEFPGVvarplgvfkssddyhyqtlraNVDSLKIIQIGLALSDEEgNAPveAcTWQFNFTF--NLqddmYaPESIELLTksgIDFKKHqEVGIEPADFAELLIGSgLVlQeeVTWITFHSGYDFAYLLKAMTQIplpaeyeefyk---ILcIyFPk<br><br>YIDTLALAK-SLlKLrSYSLDSVVEKLGL----gpfrhH--RALDDARVTAQVFLRFVEMM----------------<br>NYDIKYIMKsVL-NN-SKGLQDIADDLQIhrig-----PqhQAGSDALLTARIFFEIRSRYfdgsidsrmlnqlygl

159	2p1jA (164)<br>1wljA (168)	C	19.51	TM-align: aliSize=131 (resi) RMSD=2.97 (Å)<br>TFVVLDFETTGLDp--QVDEIIEIGAVKIqggQIVDEYHTLIKPSREISRKSSEITGITQEMLENKRSIEEVLPEFLGFLEDSIIVAHNANFDYRFLrlWIKkvmglDWERPYIDTLAL--AKSLLKLrsYSLDSVVEKLGLGPFRHHralddarvtaqvflrfvemm----------------------<br>EVVAMDCEMVGLG-phRESGLARCSLVNV---HGAVLYDKFIRPEGEITDYRTRVSGVTPQHMVGATPFAVARLEILQLLKGKLVVGHDLKHDFQAL--KED-----MSGYTIYDTSTDrlLWREAKL--VSLRVLSERLLHKSIQNS--------------------llghssvedaratmelyqisqr<br>-----------<br>irarrglprla<br><br>SARST: aliSize=149 (resi) RMSD=10.94 (Å)<br>t-FVVLDFETTGLDPQVDEIIEIGAVKIQGGQIVDeyHTLIKPSREISRKSSEITGITQEMLENKRSIEEVLPEFLGFLEDSIIVAHNANFDYRFlrlwikkvMGLD---WERPYIDTLALAKSLLKlrSYSLDSVVEKLGLGPFR-----HHRALDDARVTAQVFLRFVE----------mm<br>-eVVAMDCEMVGLGPHRESGLARCSLVNVHGAVLY--DKFIRPEGEITDYRTRVSGVTPQHMVGATPFAVARLEILQLLKGKLVVGHDLKHDFQA--------LKEDmsgYTIYDTSTDRLLWREAK--LVSLRVLSERLLHKSIQnsllgHSSVEDARATMELYQISQRIrarrglprla--<br><br>BLAST: aliSize=71 (resi) iden=21.34% (35/164) simi=43.29% (71/164)<br>tf--VVLDFETTGLDPQVDE-IIEIGAVKIQGGQIVDEYhtlIKPSREISRKSSEITGITQEMLENKRSIEEVLPEFLGFLEDSIIVAHNANFDYRFLRlwiKKVMGLDWERPYIDTLALAKS-LLKLRSYSlDSVVEKLGLGPFRHHRALDDARVTAQVF----------------lrfvemm<br>--evVAMDCEMVGLGPHRESgLARCSLVNVHGAVLYDKF---IRPEGEITDYRTRVSGVTPQHMVGATPFAVARLEILQLLKGKLVVGHDLKHDFQALK---EDMSGYTIYDTSTDRLLWREAkLVSLRVLS-ERLLHKSIQNSLLGHSSVEDARATMELYqisqrirarrglprla-------<br><br>Proposed: aliSize=146 (resi) RMSD=2.14 (Å)<br>TFVVLDFETTG-----lDpqvDEIIEIGAVKIqggQIVDEYHTLIKPSREISRKSSEITGITQEMLENKRSIEEVLPEFLGFLEDSIIVAHNANFDYRFLrlWIkkvmgldW--ERPYIDTLAL--AKSLLKLrsYSLDSVVEKLGLGPF----RHHRALDDARVTAQVFLRFVEMM---------<br>EVVAMDCEMVGlgphr-E---SGLARCSLVNV---HGAVLYDKFIRPEGEITDYRTRVSGVTPQHMVGATPFAVARLEILQLLKGKLVVGHDLKHDFQAL--KE-------DmsGYTIYDTSTDrlLWREAKL--VSLRVLSERLLHKSIqnslLGHSSVEDARATMELYQISQRIRarrglprla
160	2p1jA (164)<br>2gbzA (179)	C	15.85	TM-align: aliSize=132 (resi) RMSD=3.01 (Å)<br>--TFVVLDFETTGLDPQVDEIIEIGAVKIQGG-QIVDEY-HTLIKPS-REIS-R-K-SSEITG---ITqEML-ENKRSIEEVLPEFLGFL-----E-DSIIVAHNANFDYRFLRLWIKkvMgLDW-ER-PYIDTLALAKSlLKLRsysld-SVvEKLGLGpFRHHralddarvtaqvflrfvemm-----<br>ndRLIWIDLEMTGLDTDRDSIIEIATIVTDAQlNVLAEGpELAIAHSlETLEaMdEwNRNQHRrsgLW-QRVlDSQVTHAQAEAQTVAFLgewirAgASPMCGNSICQDRRFLHRQMS--R-LERyFHyRNLDVSTIKEL-ARRW-----aPA-VASGFA-KSSA--------------------htals<br>----------------------<br>dvrdsidelrhyrqfmgtlgg<br><br>SARST: aliSize=150 (resi) RMSD=10.12 (Å)<br>t---FVVLDFETTGLDPQVDEIIEIGAVKIQ-GGQIVDEYHTLIKPsRE------ISRKSSE---ITGitQEMLENKR--SIEEVLPEFLGF-----LED-SIIVAHNANFDYRFLRLW-IKKVMGLDweRPYIDTLALAKSLLklrsysLDSVVEKLGLGPFRHHRALDDARVTAQVFLRFVE-------mm<br>-ndrLIWIDLEMTGLDTDRDSIIEIATIVTDaQLNVLAEGPELAIA-HSletleaMDEWNRNqhrRSG--LWQRVLDSqvTHAQAEAQTVAFlgewiRAGaSPMCGNSICQDRRFLHRQmSRLERYFH--YRNLDVSTIKELAR------RWAPAVASGFAKSSAHTALSDVRDSIDELRHYRQfmgtlgg--<br><br>BLAST: aliSize=38 (resi) iden=16.46% (27/164) simi=23.17% (38/164)<br>tfvv------LDFETTGLDPQVDEIIEIGAVkIQGGQIvdeyHTLIK-PSREISRkSSEITGITQEMLENKRSIEEVLPEFLgfleDSIIVAHNANFD-YRFLRLWIK--<br>----ndrliwIDLEMTGLDTDRDSIIEIATI-VTDAQL----NVLAEgPELAIAH-SLETLEAMDEWNRNQHRRSGLWQRVL----DSQVTHAQAEAQtVAFLGEWIRagaspmcgnsicqdrrflhrqmsrleryfhyrnldvstikelarrwapavasgfakssahtalsdvrdsidelrhyrqfmgt<br>---kvmgldwerpyidtlalaksllklrsysldsvveklglgpfrhhralddarvtaqvflrfvemm<br>lgg--<br><br>Proposed: aliSize=136 (resi) RMSD=2.09 (Å)<br>--TFVVLDFETTGLDPQVDEIIEIGAVKIQ-GGQIVDEY-HTLIKPsRE-ISR---K-SSEITG---ITqEMLENK-RSIEEVLPEFLGFL-----E-DSIIVAHNANFDYRFLRLWIKkvmgL--dW-ER-PYIDTLALAKSLLK---------------lrsysldsvveklglgpfrhhRALDDARV<br>ndRLIWIDLEMTGLDTDRDSIIEIATIVTDaQLNVLAEGpELAIAH-SLeTLEamdEwNRNQHRrsgLW-QRVLDSqVTHAQAEAQTVAFLgewirAgASPMCGNSICQDRRFLHRQMS----Rle-RyFHyRNLDVSTIKELARRwapavasgfakssah--------------------TALSDVRD<br>TAQVFLRFVEMM-----<br>SIDELRHYRQFMgtlgg
161	2jevA (169)<br>1yreA (183)	C	10.65	TM-align: aliSize=141 (resi) RMSD=3.57 (Å)<br>a-------KFVIRPATAADCSDILRLIKELAK--YEYMEE-QVILTEKDLLEDGfGeHPFYHCLVAEVPKehwtpeghSIVGFAMYYFTYDPwiGKLLYLEDFFVMSDYRGFGIGSEILKNLSQVAM-RCRCSSMHFLVAEWNEPSINFYKRRGASDLSSeegWRLFKidkeyllkmate----------<br>-lpitlqrGALRLEPLVEADIPELVSLAEANReaLQYMDGpTRPDWYRQSLAEQ-R-EGRALPLAVRLGV--------QLVGTTRFAEFLPA--LPACEIGWTWLDQAQHGSGLNRMIKYLMLKHAFdNLRMVRVQLSTAASNLRAQGAIDKLGAQREGV---LRNHR------------rlaggrlddt<br>----------------------<br>fvysitdhewpqvkaaleasf<br><br>SARST: aliSize=146 (resi) RMSD=11.17 (Å)<br>ak---------FVIRPATAADCSDILRLIKELAKYEYmEEQVILTEKD------LLEDGFgehpfyHCLVAEVPKEHWtpeghsiVGFAMYYFTYdpWIGKLLYLEDFFVMSDYRGFGIGSEI-LKNLSQVAMRCRCSSMHFLVAEWNEPSINFYKRRGASDL---------ssEEGW----RLFKIDKE<br>--lpitlqrgaLRLEPLVEADIPELVSLAEANREALQ-YMDGPTRPDWyrqslaEQREGR------ALPLAVRLGVQL-------VGTTRFAEFL--PALPACEIGWTWLDQAQHGSGLNRMIkYLMLKHAFDNLRMVRVQLSTAASNLRAQGAIDKLGAQREgvlrnhrrl--AGGRlddtFVYSITDH<br>YLLKM--------ate<br>EWPQVkaaleasf---<br><br>BLAST: aliSize=23 (resi) iden=8.28% (14/169) simi=13.61% (23/169)<br>akfvirpataadcsdi---------LRLiKELAKYEyMEEQVILTE--KDLLE--DGFGEHPFYHCLVAE--<br>---------------lpitlqrgaLRL-EPLVEAD-IPELVSLAEanREALQymDGPTRPDWYRQSLAEqregralplavrlgvqlvgttrfaeflpalpaceigwtwldqaqhgsglnrmikylmlkhafdnlrmvrvqlstaasnlraqgaidklgaqregvlrnhrrlaggrlddtfvysitdhew<br>-----------vpkehwtpeghsivgfamyyftydpwigkllyledffvmsdyrgfgigseilknlsqvamrcrcssmhflvaewnepsinfykrrgasdlsseegwrlfkidkeyllkmate<br>pqvkaaleasf--<br><br>Proposed: aliSize=151 (resi) RMSD=2.82 (Å)<br>ak--------FVIRPATA-ADCSDILRLiKE-LAKYEYM-eEQ-VILT-EKDLLEDGfgEHPFYHCLVAEVPKehwtpeghSIVGFAMYYFTY--dpwiGKLLYLEDFFVMSDYRGFGIGSEILKNLSQVAM-RCRCSSMHFLVAEWNEPSINFYKRRGASDLS---S--------EEGWRLFKIDKEYL<br>--lpitlqrgALRLEPLVeADIPELVSL-AEaNREALQYm-DGpTRPDwYRQSLAEQ--REGRALPLAVRLGV--------QLVGTTRFAEFLpa----LPACEIGWTWLDQAQHGSGLNRMIKYLMLKHAFdNLRMVRVQLSTAASNLRAQGAIDKLGAQREGvlrNhrrlaggrLDDTFVYSITDHEW<br>LKMATE------<br>PQVKAAleasfc

162	1vguA (214)<br>1gq9A (241)	M	14.49	TM-align: aliSize=198 (resi) RMSD=2.80 (Å)<br>LDVCAVVP-A-----CP-KQYLSIGNQTILEHSVHALLAHPRVKRVVIAISpgDSRFAQLpLANHPQITVVDG-GDER-ADSVLAGLKAAGdAQWVLVHDAARPCLHQDDLARLLALSETS--RTGGILAAPVRDT-MKRAepgknaia--------------------HTVD--R--NGLWHALTPQFF<br>SKAVIVIPaRygssrLPgKPLLDIVGKPMIQHVYERALQVAGVAEVWVATD--DPRVEQA-VQAFGGKAIMTRnDHESgTDRLVEVMHKVE-ADIYINLQGDEPMIRPRDVETLLQGMRDDpaLPVATLCHAISAAeAAEP--------stvkvvvntrqdalyfsrspIPYPrnAekARYLKHVGIYAY<br><br>PRELLHDcLTRAlnEGA--T---ITDeASALEYCGFHPQLVEGRADNIKVTRPEDLALAEFYL-TR-<br>RRDVLQN-YSQL--PESmpEqaeSLE-QLRLMNAGINIRTFEVAATGPGVDTPACLEKVRALMaQEl<br><br>SARST: aliSize=203 (resi) RMSD=7.27 (Å)<br>l-DVCAVVPAC------PKQ-YLSIGNQTILEHSVHALLAHPRVKRVVIAI-SPGDSRFAqlPLANHPQITVVDGGDERADSVLAGLKAAGdAQWVLVHDAARPCLHQDDLARLLALSETSRTG---GILAAPVR--------DTMKRAEPGKN--AIAHTVD------RNGLWHALTPQFFPRELLHDC<br>-sKAVIVIPARygssrlPGKpLLDIVGKPMIQHVYERALQVAGVAEVWVATdDPRVEQAV--QAFGGKAIMTRNDHESGTDRLVEVMHKVE-ADIYINLQGDEPMIRPRDVETLLQGMRDDPALpvaTLCHAISAaeaaepstVKVVVNTRQDAlyFSRSPIPyprnaeKARYLKHVGIYAYRRDVLQNY<br><br>L-----TRALNEGatitDEASALEYCGFHPQLVEGRADNIKVTRPEDLALAEFY-----ltr<br>SqlpesMPEQAES----LEQLRLMNAGINIRTFEVAATGPGVDTPACLEKVRALmaqel---<br><br>BLAST: aliSize=23 (resi) iden=7.94% (17/214) simi=10.75% (23/214)<br>ldvcavvpacpkqylsignqtilehsvhallahprvkr----VVIAISPGDSRFAQLPLANHPQITVVDGGDERAdsvlagLKAAGDAQ-WVLVHD--<br>--------------------------------------skavIVIPARYGSSRLPGKPLLDIVGKPMIQHVYERA------LQVAGVAEvWVATDDprveqavqafggkaimtrndhesgtdrlvevmhkveadiyinlqgdepmirprdvetllqgmrddpalpvatlchaisaaeaaepstvkvvvnt<br><br>---aarpclhqddlarllalsetsrtggilaapvrdtmkraepgknaiahtvdrnglwhaltpqffprellhdcltralnegatitdeasaleycgfh<br>rqdalyfsrspipyprnaekarylkhvgiyayrrdvlqnysqlpesmpeqaesleqlrlmnaginirtfevaatgpgvdtpaclekvralmaqel---<br><br>pqlvegradnikvtrpedlalaefyltr<br>----------------------------<br><br>Proposed: aliSize=201 (resi) RMSD=2.12 (Å)<br>LDVCAVVP-A-----CP-KQYLSIGNQTILEHSVHALLAHPRVKRVVIAISpgDSRFAQLpLA--NHpqITVVDG-GDER-ADSVLAGLKAAGdAQWVLVHDAARPCLHQDDLARLLALSETS--RTGGILAAPVRDT---------MKRAEpGKNAIAHTVDRnG-----------LWHALTPQFFPREL<br>SKAVIVIPaRygssrLPgKPLLDIVGKPMIQHVYERALQVAGVAEVWVATD--DPRVEQA-VQafGG--KAIMTRnDHESgTDRLVEVMHKVE-ADIYINLQGDEPMIRPRDVETLLQGMRDDpaLPVATLCHAISAAeaaepstvKVVVN-TRQDALYFSRS-PipyprnaekarYLKHVGIYAYRRDV<br><br>LHDcLTRAlnEGA--T----ITdeASALEYCGFHPQLVEGRADNIKVTRPEDLALAEFYL-TR-<br>LQN-YSQL--PESmpEqaesLE--QLRLMNAGINIRTFEVAATGPGVDTPACLEKVRALMaQEl
163	1vguA (214)<br>2pa4A (294)	M	13.55	TM-align: aliSize=198 (resi) RMSD=3.12 (Å)<br>lDVCAVVP-A-CP-----------KQYLSIGNQTILEHSVHALLAHPrVKRVVIAISPGDSRFAQLpLAN-----------------------H--PQITVVDGG-DE-RADSVLAGLKAAG-DAQWVLVHDAARPCLHQDDLARLLALSETSRTGGILAAPVRDTMKRaepgknai-------------<br>-VKTVVVPaAgLGtrflpatktvpKELLPVVDTPGIELIAAEAAELG-ATRLAIITAPNKAGVLAH-FERsseleetlmergktdqveiirraAdlIKAVPVTQDkPLgLGHAVGLAESVLDdDEDVVAVMLPDDLVLPTGVMERMAQVRAEFGGSVLCAVEVSEADVS--------kygifeieadtkd<br><br>-------------AHTVDRnGLWHALTPQFFPReLLHDCLTRAlneGAT---ITDEASALEYC-G--FHPQLVEGRADNIKVTRPEDLALAEFYL--TR---------------------<br>sdvkkvkgmvekpAIEDAP-SRLAATGRYLLDR-KIFDALRRI---TPGaggELQLTDAIDLLiDegHPVHIVIHQGKRHDLGNPGGYIPACVDFglSHpvygaqlkdaikqilaeheaa<br><br>SARST: aliSize=191 (resi) RMSD=9.64 (Å)<br>ldvcavvpac----------------------PKQYLSIGNQTILEHSVHALLAhPRVKRVVIAISPGD----------------SRFAQLPLANHPQ--------ITVVDG--GDERADSVLAGLKAAGD-AQWVLVHDAARPC-LHQDDLARLLALSETSrtGGILAAPV-------RDTMKRAEPGK<br>----------vktvvvpaaglgtrflpatktvPKELLPVVDTPGIELIAAEAAE-LGATRLAIITAPNKagvlahfersseleetLMERGKTDQVEIIrraadlikAVPVTQdkPLGLGHAVGLAESVLDDdEDVVAVMLPDDLVlPTGVMERMAQVRAEFG--GSVLCAVEvseadvsKYGIFEIEADT<br><br>NAIAHTVDR---NGL--------WHALTPQFFPRELLHDCLTralnegATITD--------EASALEYCGFhPQLVEGRADNIKVT-RPEDLALAEFY--------------------------ltr<br>KDSDVKKVKgmvEKPaiedapsrLAATGRYLLDRKIFDALRR------ITPGAggelqltdAIDLLIDEGH-PVHIVIHQGKRHDLgNPGGYIPACVDfglshpvygaqlkdaikqilaeheaa---<br><br>BLAST: aliSize=51 (resi) iden=15.42% (33/214) simi=23.83% (51/214)<br>ldvcavvpacpkqylsignqtilehsvhallahprVKRVVIAISPGDSRFaqLPLANH-PQ--ITVVDG-GDERADSVLAGLKAAGDAQwVLVHDAARPCLHQDDLARLL-ALSETSRTGGIlaapvrDTMKRAEPGKNAIAHTVDRN-GLWHAL-----------------------------------<br>-----------------------------------VKTVVVPAAGLGTRF--LPATKTvPKelLPVVDTpGIELIAAEAAELGATRLAI-ITAPNKAGVLAHFERSSELEeTLMERGKTDQV------EIIRRAADLIKAVPVTQDKPlGLGHAVglaesvldddedvvavmlpddlvlptgvmermaqv<br><br>--tpqffprellhdcltralnegatitdeasaleycgfhpqlve<br>raefggsvlcavevseadvskygifeieadtkdsdvkkvkgmvekpaiedapsrlaatgrylldrkifdalrritpgaggelqltdaidllideghpvhivihqgkrhdlgnpggyipacvdfglshpvygaqlkdaikqilaeheaa--<br><br>gradnikvtrpedlalaefyltr<br>-----------------------<br><br>Proposed: aliSize=205 (resi) RMSD=2.29 (Å)<br>lDVCAVVP-A-CP-----------KQYLSIGNQTILEHSVHALLAHPrVKRVVIAISPGDSRFAQLpLAN-----------------------H--PQITVVDGG-DE-RADSVLAGLKAAG-DAQWVLVHDAARPCLHQDDLARLLALSETSRTGGILAAPV------RDTMKRAEPGK-----NAIAH<br>-VKTVVVPaAgLGtrflpatktvpKELLPVVDTPGIELIAAEAAELG-ATRLAIITAPNKAGVLAH-FERsseleetlmergktdqveiirraAdlIKAVPVTQDkPLgLGHAVGLAESVLDdDEDVVAVMLPDDLVLPTGVMERMAQVRAEFGGSVLCAVEVseadvsKYGIFEIEADTkdsdvKKVKG<br><br>TVDRNG------LWHALTPQFFPrELLHDCLTRAlneGAT----ITdEASALEYC-G--FHPQLVEGRADNIKVTRPEDLALAEFYL--T----------------------r<br>MVEKPAiedapsRLAATGRYLLD-RKIFDALRRI---TPGaggeLQ-LTDAIDLLiDegHPVHIVIHQGKRHDLGNPGGYIPACVDFglShpvygaqlkdaikqilaeheaa-
164	1vguA (214)<br>2cu2A (335)	M	19.16	TM-align: aliSize=199 (resi) RMSD=3.45 (Å)<br>LDVCAVVP-A-CP------------KQYLSI-GNQTILEHSVHALLAHPRVKRVVIAISPGD-SRFAqLpLAnHPqITVVDG-GDE-RADSVLAGLKAA--GDAQWVLVHDAARPC-LHQDDLARLLALSETSR--TGGILAAPVRDTMKraepgk------------------------NAIAHTVD-R<br>MKTYALVMaGgRGerlwplsredrpKPFLPLfEGKTLLEATLERLAPLVPPERTLLAVRRDQeAVAR-P-YA-DG-IRLLLEpLGRdTAGAVLLGVAEAlkEGAERLLVLPADHYVgDDEAYREALATMLEAAEegFVVALGLRPTRPET------eygyirlgpregawyrgegfvekpSYAEALEYiR<br><br>NGLWHALTPQFFPRELLHDCLTRALNEG--------------------ATITDEASALEYCGFhPQLVEGRADNIKVTRPEDLALAeFYltr--<br>KGYVWNGGVFAFAPATMAELFRRHLPSHhealerllagasleevyaglPKISIDYGVMEKAER-VRVVLGRFPWDDVGNWRALERV-FS---qdphenvvlgegrhvaldtfgcvvyadrgvvatlgvsglvvakvgdevlvvpkdwarevrevvkrlea<br><br>SARST: aliSize=185 (resi) RMSD=9.24 (Å)<br>ldvcavvpacpkqyls-------------------------------IGNQTILEHSVHALLA--HPRVkrVVIAISPGDSRFAqlPLANHPQITV---VDGGDERADSVLAGLKAAGDAQWVLVHDAARpcLH-----QDDLARLLALSETSRTGGILAAPVR-----DTMK-RAEPGKNA--IAHTVD<br>----------------mktyalvmaggrgerlwplsredrpkpflplFEGKTLLEATLERLAPlvPPER--TLLAVRRDQEAVA--RPYADGIRLLlepLGRDTAGAVLLGVAEALKEGAERLLVLPADH--YVgddeaYREALATMLEAAEEGFVVALGLRPTrpeteYGYIrLGPREGAWyrGEGFVE<br><br>-----------RNGLWHALTPQFFPRELLH----------DCLTRALNEGAT---------ITDEASALEYCGFHPQLVEGraDNIKVT--RPEDLALAE---fyltr<br>kpsyaealeyiRKGYVWNGGVFAFAPATMAelfrrhlpshHEALERLLAGASleevyaglpKISIDYGVMEKAERVRVVLG--RFPWDDvgNWRALERVFsqdphenvvlgegrhvaldtfgcvvyadrgvvatlgvsglvvakvgdevlvvpkdwarevrevvkrlea-----<br><br>BLAST: aliSize=64 (resi) iden=16.36% (35/214) simi=29.91% (64/214)<br>ldvcavvpac------------------------PKQYLSI-GNQTILEHSVHALLAHPRVKRVVIAISPGDSRFAQlPLANHPQITVVDGGDERADSVLAGLKAA--GDAQWVLVHDAARpCLHQDDLAR--LLALSETSRTGGILAAPVRDTMKRAEPGKNAIAhtvDRNGLWH--------------<br>----------mktyalvmaggrgerlwplsredrPKPFLPLfEGKTLLEATLERLAPLVPPERTLLAVRRDQEAVAR-PYADGIRLLLEPLGRDTAGAVLLGVAEAlkEGAERLLVLPADH-YVGDDEAYReaLATMLEAAEEGFVVALGLRPTRPETEYGYIRLG---PREGAWYrgegfvekpsyaea<br><br>--altpqffprellhdcltralnegatitdea<br>leyirkgyvwnggvfafapatmaelfrrhlpshhealerllagasleevyaglpkisidygvmekaervrvvlgrfpwddvgnwralervfsqdphenvvlgegrhvaldtfgcvvyadrgvvatlgvsglvvakvgdevlvvpkdwarevrevvkrlea------------------------------<br><br>saleycgfhpqlvegradnikvtrpedlalaefyltr<br>-------------------------------------<br><br>Proposed: aliSize=197 (resi) RMSD=2.45 (Å)<br>LDVCAVVP-A-CP------------KQYLSI-GNQTILEHSVHALLAHprVK--RVVIAISPGD-SRFAqLpLAnHPqITVVDG-GDE-RADSVLAGLKAA--GDAQWVLVHDAARPC-LHQDDLARLLALSETSR--TGGILAAPVRD-----TMKRAEPGK---NAIAHTV--D---------RNGLW<br>MKTYALVMaGgRGerlwplsredrpKPFLPLfEGKTLLEATLERLAPL--VPpeRTLLAVRRDQeAVAR-P-YA-DG-IRLLLEpLGRdTAGAVLLGVAEAlkEGAERLLVLPADHYVgDDEAYREALATMLEAAEegFVVALGLRPTRpeteyGYIRLGPREgawYRGEGFVekPsyaealeyiRKGYV<br><br>HALTPQFFPRELLHDClTR-ALNE-----------------------gatITDEaS-ALEYCGFhPQLVEGRADNIKVTRPEDLALAeF--yltr<br>WNGGVFAFAPATMAEL-FRrHLPShhealerllagasleevyaglpk---ISID-YgVMEKAER-VRVVLGRFPWDDVGNWRALERV-Fsqdphenvvlgegrhvaldtfgcvvyadrgvvatlgvsglvvakvgdevlvvpkdwarevrevvkrlea----

165	1vguA (214)<br>1h5rA (290)	M	11.21	TM-align: aliSize=194 (resi) RMSD=3.11 (Å)<br>LDVCAVVP-A--C------P----KQYLSIGNQTILEHSVHALLAHpRVKRVVIAISPGD-SRFAQLpLANHP----QITVVDGGD-E-RADSVLAGLKAAGDaQWVLVHDAARPCLHQDDLARLLALSETSRTGGILAAPVRDTMkraepgknaiah--------------------TVDRngLWHAL<br>KMRKGIILaGgsGtrlypvTmavsKQLLPIYDKPMIYYPLSTLMLA-GIRDILIISTPQDtPRFQQL-LGDGSqwglNLQYKVQPSpDgLAQAFIIGEEFIGG-DDCALVLGDNIFYGHDLPKLMEAAVNKESGATVFAYHVNDPE------------rygvvefdkngtaisleekplEPKS--NYAVT<br><br>TPQFFPReLLHDCLTRAlnEGAT--ITDEASALEYC-GFH-PQLVEGR--ADNIKVTRPEDLALAEFYLT-R--<br>GLYFYDN-DVVQMAKNL--KPSArgELEITDINRIYlEQGrLSVAMMGrgYAWLDTGTHQSLIEASNFIAtleerqglkvscpeeiafrkgfidveqvrklavpliknnygqylykmtkd<br><br>SARST: aliSize=188 (resi) RMSD=7.27 (Å)<br>ldvcavvpac-----------------------PKQYLSIGNQTILEHSVHALLAhPRVKRVVIAISPGDSRFAqlPLANH---PQITV-------VDGGDERADSVLAGLKaaGDAqWVLVHDAARP--CLHQDDLARLLALsetSRTGGILAAPV-----RDTMKRA----EPGKNAIAHTVDRNGLW<br>----------kmrkgiilaggsgtrlypvtmavSKQLLPIYDKPMIYYPLSTLML-AGIRDILIISTPQDTPRF--QQLLGdgsQWGLNlqykvqpSPDGLAQAFIIGEEFI--GGD-DCALVLGDNIfyGHDLPKLMEAAVN---KESGATVFAYHvndpeRYGVVEFdkngTAISLEEKPLEPKSNYA<br><br>HALTpQFFPREllhDCLTRAL----NEGATITDEASALEYCGFHPQLVEGRADNIKV--TRPEDLALAEFY-----------------------------------ltr<br>VTGL-YFYDND---VVQMAKNlkpsARGELEITDINRIYLEQGRLSVAMMGRGYAWLdtGTHQSLIEASNFiatieerqglkvscpeeiafrkgfidveqvrklavpliknnygqylykmtkd---<br><br>BLAST: aliSize=99 (resi) iden=25.70% (55/214) simi=46.26% (99/214)<br>ldvca------------------VVPACPKQYLSIGNQTILEHSVHALLAhPRVKRVVIAISPGDS-RFAQLpLAN------HPQITVVDGGDERADSVLAGLKAAGDAQWVLVhdAARPCLHQDDLARLLALSETSRTGG-ILAAPVRDTMKRA--EPGKNAIAHTVDRNGLW----HALTP-QFFPRE<br>-----kmrkgiilaggsgtrlypVTMAVSKQLLPIYDKPMIYYPLSTLML-AGIRDILIISTPQDTpRFQQL-LGDgsqwglNLQYKVQPSPDGLAQAFIIGEEFIGGDDCALV--LGDNIFYGHDLPKLMEAAVNKESGAtVFAYHVNDPERYGvvEFDKNGTAISLEEKPLEpksnYAVTGlYFYDND<br><br>LLHdcLTRALNEGAT----ITD------EASALE-------YC----GFHPQLVEG---------RADNIKVTRPEDLALAEFYL------------------------tr<br>VVQ--MAKNLKPSARgeleITDinriylEQGRLSvammgrgYAwldtGTHQSLIEAsnfiatieERQGLKVSCPEEIAFRKGFIdveqvrklavpliknnygqylykmtkd--<br><br>Proposed: aliSize=201 (resi) RMSD=2.37 (Å)<br>l-DVCAVVP-A-CP-----------KQYLSIGNQTILEHSVHALLAHpRVKRVVIAISPGD-SRFAQLpLANHP----QITVVDGGD-E-RADSVLAGLKAAGDaQWVLVHDAARPCLH-qDDLARLLALSETSRTGGILAAPV----RDTMKRAEpGKNAIAHTVDRNG----LWHALTPQFFPReLLH<br>-kMRKGIILaGgSGtrlypvtmavsKQLLPIYDKPMIYYPLSTLMLA-GIRDILIISTPQDtPRFQQL-LGDGSqwglNLQYKVQPSpDgLAQAFIIGEEFIGG-DDCALVLGDNIFYGh-DLPKLMEAAVNKESGATVFAYHVndpeRYGVVEFD-KNGTAISLEEKPLepksNYAVTGLYFYDN-DVV<br><br>DCLTRAlnEGA---TITdEASALEycGFH----PQLVEG---RaDNIKVTRPEDLALAEFYLT-R-------------------------------------<br>QMAKNL--KPSargELE-ITDINR--IYLeqgrLSVAMMgrgY-AWLDTGTHQSLIEASNFIAtleerqglkvscpeeiafrkgfidveqvrklavpliknnygqylykmtkd
166	1vguA (214)<br>1iimA (289)	M	11.21	TM-align: aliSize=194 (resi) RMSD=3.05 (Å)<br>-LDVCAVVP-A--C----------PKQYLSIGNQTILEHSVHALLAHPrVKRVVIAISPGD-SRFAQLpLANHP----QITVVDGGD-E-RADSVLAGLKAAGDaQWVLVHDAARPCLHQDDLARLLALSETSRTGGILAAPVRDTMkraepgknaia--------------------HTVDRngLWHAL<br>mKTRKGIILaGgsGtrlypvtmavSQQLLPIYDKPMIYYPLSTLMLAG-IRDILIISTPQDtPRFQQL-LGDGSqwglNLQYKVQPSpDgLAQAFIIGEEFIGH-DDCALVLGDNIFYGHDLPKLMEAAVNKESGATVFAYHVNDPE-----------rygvvefdqkgtavsleekpLQPKS--NYAVT<br><br>TPQFFPrELLHDCLTRAlnEGA---TITdEASALEYC-GFH-PQLVEGRA--DNIKVTRPEDLALAEFYL-TR----------------------------------<br>GLYFYD-NSVVEMAKNL--KPSargELE-ITDINRIYmEQGrLSVAMMGRgyAWLDTGTHQSLIEASNFIaTIeerqglkvscpeeiafrknfinaqqvielagplskndygkyllkmv<br><br>SARST: aliSize=164 (resi) RMSD=16.38 (Å)<br>ldvcavvpac-----------------------PKQYLSIGNQTILEHSVHALLAhPRVKRVVIAISPGDSRFAqlPLANHPQ-----ITVVDGG---DERADSVLAGLKAAGDAQWVLVhdaaRPCLH-----QDDLARLLALsetSRTGGILAAPV-----RDTMKRAE---------PGKNAIAHT<br>----------mktrkgiilaggsgtrlypvtmavSQQLLPIYDKPMIYYPLSTLML-AGIRDILIISTPQDTPRF--QQLLGDGsqwglNLQYKVQpspDGLAQAFIIGEEFIGHDDCALV----LGDNIfyghdLPKLMEAAVN---KESGATVFAYHvndpeRYGVVEFDqkgtavsleEKPLQPKSN<br><br>VDRNGLWHAL-----------------------------------TPQFFP--------------RELLHDCLTRALNEGATITDEASALEYCG---------------------------fhpqlvegradnikvtrpedlalaefyltr<br>YAVTGLYFYDnsvvemaknlkpsargeleitdinriymeqgrlSVAMMGrgyawldtgthqslIEASNFIATIEERQGLKVSCPEEIAFRKNfinaqqvielagplskndygkyllkmv-------------------------------<br><br>BLAST: aliSize=15 (resi) iden=3.27% (7/214) simi=7.01% (15/214)<br>ldvcavvpacpkqylsignqtilehsvhallahprvkrvviaispgdsrfaqlplanhpqitvvdggderadsvlaglkaagdaqwvlvhdaarpclhqddlarllalsetsrtggilaapvrdtmkraepgknaiahtvdrnglwhaltpqffprellhdcltralnegatitdeasaleycgfhpql-<br>--m<br><br>---<br>ktrkgiilaggsgtrlypvtmavsqqllpiydkpmiyyplstlmlagirdiliistpqdtprfqqllgdgsqwglnlqykvqpspdglaqafiigeefighddcalvlgdnifyghdlpklmeaavnkesgatvfayhvndperygvvefdqkgtavsleekplqpksnyavtglyfydnsvvemaknlk<br><br>---VEGRaDNIKVTRPEDLALAEFYL-------------------------tr<br>psargeleitdinriymeqgrlsvammgrgyawldtgthqslieasnfiatIEER-QGLKVSCPEEIAFRKNFInaqqvielagplskndygkyllkmv--<br><br>Proposed: aliSize=202 (resi) RMSD=2.40 (Å)<br>-LDVCAVVP-A--C----------PKQYLSIGNQTILEHSVHALLAHPrVKRVVIAISPGD-SRFAQLpLANHP----QITVVDGGD-E-RADSVLAGLKAAGDaQWVLVHDAARPCLHQDDLARLLAL-SETsRTGGILAAPV----RDTMKRAEpGKNAIAHTVDRNG----LWHALTPQFFPrELLH<br>mKTRKGIILaGgsGtrlypvtmavSQQLLPIYDKPMIYYPLSTLMLAG-IRDILIISTPQDtPRFQQL-LGDGSqwglNLQYKVQPSpDgLAQAFIIGEEFIGH-DDCALVLGDNIFYGHDLPKLMEAAvNKE-SGATVFAYHVndpeRYGVVEFD-QKGTAVSLEEKPLqpksNYAVTGLYFYD-NSVV<br><br>DCLTRAlnFGA---TITdEASALFycGFH----PQLVEG---RaDNIKVTRPEDLALAEFYLT-R---------------------------------<br>EMAKNL--KPSargELE-ITDINR--IYMeqgrLSVAMMgrgY-AWLDTGTHQSLIEASNFIAtleerqglkvscpeeiafrknfinaqqvielagplskndygkyllkmv
167	1vguA (214)<br>1lvwA (295)	M	14.49	TM-align: aliSize=197 (resi) RMSD=2.99 (Å)<br>-LDVCAVVP-A---------C---PKQYLSIGNQTILEHSVHALLAHpRVKRVVIAISPGD-SRFAQLpLANHP----QITVVDGG-DE-RADSVLAGLKAAGDaQWVLVHDAARPCLHQDDLARLLALSETSRTGGILAAPVRDTMKraepgknaiah------------------TVDRNGLWHALTP<br>gAHMKGIVLaGgsgtrlypiTravSKQLLPIYDKPMIYYPLSVLMLA-GIRDILIISTPRDlPLYRDL-LGDGSqfgvRFSYRVQEePRgIADAFIVGKDFIGD-SKVALVLGDNVFYGHRFSEILRRAASLEDGAVIFGYYVRDPRP-----------fgvvefdsegrvisieekPSRPKSNYVVPGL<br><br>QFFPReLLHDCLTRAlnEGAT--ITDEASALEYC-GFH-PQLVEGR--ADNIKVTRPEDLALAEFYL-TR------------------------------------<br>YFYDN-QVVEIARRI--EPSDrgELEITSVNEEYlRMGkLRVELMGrgMAWLDTGTHDGLLEASSFIeTIqkrqgfyiacleeiaynngwitredvlemaeklektdygkylrdlaegnfhg<br><br>SARST: aliSize=176 (resi) RMSD=10.15 (Å)<br>l----DVCAVVPACPKQYL-----------SIGNQTILEHSVHALLAhPRVKRVVIAISpgdsrfaqlplanhpqitvvdggderADSVLAGLKAAGDAQ----WVLVHD-------AARPCLHQDDL-------------------ARLLALSETSRTGGILAAPV-----RDTMKRA----EPGKNAI<br>-gahmKGIVLAGGSGTRLYpitravskqllPIYDKPMIYYPLSVLML-AGIRDILIIST---------------------------PRDLPLYRDLLGDGSqfgvRFSYRVqeeprgiADAFIVGKDFIgdskvalvlgdnvfyghrfSEILRRAASLEDGAVIFGYYvrdprPFGVVEFdsegRVISIEE<br><br>AHTVDRNGLWHALTpQFFPRELLHDCltralnEGATITD--------EASALEYCGFHPQLVEGRAD-NIKVTRPEDLALAEFY--ltr<br>KPSRPKSNYVVPGL-YFYDNQVVEIA------RRIEPSDrgeleitsVNEEYLRMGKLRVELMGRGMaWLDTGTHDGLLEASSFietiqkrqgfyiacleeiaynngwitredvlemaeklektdygkylrdlaegnfhg---<br><br>BLAST: aliSize=40 (resi) iden=9.35% (20/214) simi=18.69% (40/214)<br>ldvca------------------VVPACPKQYLSIGNQTILEHSVHALLAhPRVKRVVIAISPGDSRFAQLPLANHPQITVVDG----GDER--ADSVLAGLKAAGDAQWVLV---<br>-----gahmkgivlaggsgtrlypITRAVSKQLLPIYDKPMIYYPLSVLML-AGIRDILIISTPRDLPLYRDLLGDGSQFGVRFSyrvqEEPRgiADAFIVGKDFIGDSKVALVlgdnvfyghrfseilrraasledgavifgyyvrdprpfgvvefdsegrvisieekpsrpksnyvvpglyfydnqvv<br><br>---hdaarpclhqddlarllalsetsrtggilaapvrdtmkraepgknaiahtvdrnglwhaltpqffprellhdcltraln<br>eiarriepsdrgeleitsvneeylrmgklrvelmgrgmawldtgthdglleassfietiqkrqgfyiacleeiaynngwitredvlemaeklektdygkylrdlaegnfhg---<br><br>egatitdeasaleycgfhpqlvegradnikvtrpedlalaefyltr<br>--<br><br>Proposed: aliSize=201 (resi) RMSD=2.38 (Å)<br>-LDVCAVVP-A---------C---PKQYLSIGNQTILEHSVHALLAHPrVKRVVIAISPGD-SRFAQLpLANHP----QITVVDGG-DE-RADSVLAGLKAAGDAqWVLVHDAARPCL---HQDDLARLLAlSEtsRTGGILAAPVRD----TMKRAEpGKNAIAHTVD----RNGLWHALTPQFFPReL<br>gAHMKGIVLaGgsgtrlypiTravSKQLLPIYDKPMIYYPLSVLMLAG-IRDILIISTPRDlPLYRDL-LGDGSqfgvRFSYRVQEePRgIADAFIVGKDFIGDS-KVALVLGDNVFYghrFSEILRRAAS-LE--DGAVIFGYYVRDprpfGVVEFD-SEGRVISIEEkpsrPKSNYVVPGLYFYDN-Q<br><br>LHDCLTRAlnEGAT---ITdEASALEyCgFH----PQLVEGR--ADNIKVTRPEDLALAEFYL--TR------------------------------<br>VVEIARRI--EPSDrgeLE-ITSVNE-E-YLrmgkLRVELMGrgMAWLDTGTHDGLLEASSFIetIQkrqgfyiacleeiaynngwitredvlemaeklektdygkylrdlaegnfhg

168	1vguA (214)<br>1fxoA (292)	M	11.21	TM-align: aliSize=194 (resi) RMSD=3.13 (Å)<br>lDVCAVVP-A--C----------PKQYLSIGNQTILEHSVHALLAHpRVKRVVIAISPGD-SRFAQLpLANHP----QITVVDGGD-E-RADSVLAGLKAAGDaQWVLVHDAARPCLHQDDLARLLALSETSRTGGILAAPVRDTMkraepgknaia-------------------HTVDRngLWHALT<br>-KRKGIILaGgsGtrlhpatlaiSKQLLPVYDKPMIYYPLSTLMLA-GIREILIISTPQDtPRFQQL-LGDGSnwglDLQYAVQPSpDgLAQAFLIGESFIGN-DLSALVLGDNLYYGHDFHELLGSASQRQTGASVFAYHVLDPE-----------rygvvefdqggkaisleekpLEPKS--NYAVTG<br>PQFFPrELLHDCLTRAlnEGAT--ITDEASALEYC-GFH-PQLVEGR--ADNIKVTRPEDLALAEFYLT-R--<br>LYFYD-QQVVDIARDL--KPSPrgELEITDVNRAYlERGqLSVEIMGrgYAWLDTGTHDSLLEAGQFIAtLenrqglkvacpeeiayrqkwidaaqleklaaplakngygqylkrlltetvy<br>SARST: aliSize=165 (resi) RMSD=16.64 (Å)<br>ldvcavvpac---------------------PKQYLSIGNQTILEHSVHALLAhPRVKRVVIAISPGdSRFAQlPLANHPQ------ITVVD----GGDERADSVLAGLKaaGDAQWVLvHDAARP--CLHQDDLARLLALsetSRTGGILAAPV-----RDTMKRAE---------PGKNAIAHTVDR<br>----------krkgiilaggsgtrlhpatlaiSKQLLPVYDKPMIYYPLSTLML-AGIREILIISTPQ-DTPRF-QQLLGDGsnwgldLQYAVqpspDGLAQAFLIGESFI--GNDLSAL-VLGDNLyyGHDFHELLGSASQ---RQTGASVFAYHvldpeRYGVVEFDqggkaisleEKPLEPKSNYAV<br>NGLWH-----------ALTP--------------------QFFP-----------------RELLHDCLTRALNEGATITDEASALEYCG---------------------------------fhpqlvegradnikvtrpedlalaefyltr<br>TGLYFydqqvvdiardLKPSprgeleitdvnraylergqlSVEImgrgyawldtgthdslLEAGQFIATLENRQGLKVACPEEIAYRQKwidaaqleklaaplakngygqylkrlltetvy------------------------------<br>BLAST: aliSize=98 (resi) iden=26.64% (57/214) simi=45.79% (98/214)<br>ldvcavvp--------------------ACPKQYLSIGNQTILEHSVHALLAhPRVKRVVIAISPGDS-RFAQLpLANHP------QITVVDGGDERADSVLAGLKAAGDAQWVLVhdAARPCLHQDDLARLLALSETSRTGGILAA-----PVRDTMKRAEPGKNAIAhtVDRNGLW----HALTPQFF<br>--------krkgiilaggsgtrlhpatlAISKQLLPVYDKPMIYYPLSTLML-AGIREILIISTPQDTpRFQQL-LGDGSnwgldlQYAVQPSPDGLAQAFLIGESFIGNDLSALV--LGDNLYYGHDFHELLGSASQRQTGASVFAyhvldPERYGVVEFDQGGKAIS--LEEKPLEpksnYAVTGLYF<br>---------------PR-ELLHDCLTRALNEGATITDEASALEYC----GFHPQLVE-GR----ADN---IKVTRPEDLALAEFYL--------------------------------tr<br>ydqqvvdiardlkpsPRgELEITDVNRAYLERGQLSVEIMGRGYAwldtGTHDSLLEaGQfiatLENrqgLKVACPEEIAYRQKWIdaaqleklaaplakngygqylkrlltetvy--<br>Proposed: aliSize=200 (resi) RMSD=2.32 (Å)<br>lDVCAVVP-A--C----------PKQYLSIGNQTILEHSVHALLAHPrVKRVVIAISPGD-SRFAQLpLANHP----QITVVDGGD-E-RADSVLAGLKAAGDaQWVLVHDAARPCL---HQDDLARLLALSetsRTGGILAAPV----RDTMKRAEpGKNAIAHTVDRNG----LWHALTPQFFPrELL<br>-KRKGIILaGgsGtrlhpatlaiSKQLLPVYDKPMIYYPLSTLMLAG-IREILIISTPQDtPRFQQL-LGDGSnwglDLQYAVQPSpDgLAQAFLIGESFIGN-DLSALVLGDNLYYghdFHELLGSASQRQ---TGASVFAYHVldpeRYGVVEFD-QGGKAISLEEKPLepksNYAVTGLYFYD-QQV<br>HDCLTRAlnEGA---TITdEASALEyCgFH----PQLVEGR--ADNIKVTRPEDLALAEFYL--TR---<br>VDIARDL--KPSprgELE-ITDVNR-A-YLergqLSVEIMGrgYAWLDTGTHDSLLEAGQFIatLEnrqglkvacpeeiayrqkwidaaqleklaaplakngygqylkrlltetvy
169	2c49A (299)<br>1bx4A (342)	M	20.07	TM-align: aliSize=287 (resi) RMSD=2.82 (Å)<br>g-KMEKITCVGHTALDYIFNVEK-FPEPNT------------------SI-QIPSARKYYGGAAANTAVGIKKLGV----NSELLSCVGYDFKNSGYERYLKNLDINISkLYYSEEEETPKAWIFTDKdNNQITFFLWGAAKHYKEL--NP----PN-F-NTEIVHIA-TGDP--E-FNLKCAKKAYGN-<br>-vRENILFGMGNPLLDISAVVDKdFLDKYSlkpndqilaedkhkelfdELvKKFKVEYHAGGSTQNSIKVAQWMIQqphkAATFFGCIGIDKFGEILKRKAAEAHVDAH-YYEQNEQPTGTCAACITG-DNRSLIANLAAANCYKKEkhLDleknWMlVeKARVCYIAgFFLTvsPeSVLKVAHHASENn<br>NLVSFDPGQ-DLPQYSKEMLLEIIEHTNFLFMNKHEFERASNLLNF-------EIDDYLE--R-----VDALIVTKGSKGSVIYTKDKKIEIPCIK-A-GKVIDPTGAGDSYRAGFLSAYVKGYDLEKCGLIGAATASFVVEAKGCqTNLPTWdKVVerlekhr<br>RIFTLNLSApFISQFYKESLMKVMPYVDILFGNETEAATFAREQGFetkdikeIAKKTQAlpKmnskrQRIVIFTQGRDDTIMATESEVTAFAVLDqDqKEIIDTNGAGDAFVGGFLSQLVSDKPLTECIRAGHYAASIIIRRTGC-TFPEKP-DFH-------<br>SARST: aliSize=263 (resi) RMSD=5.12 (Å)<br>gkmek-----ITCVGHTALDYIFNV-------EKFPEPNTSIQIP-------------SARKYYGGAAAN-TAVGIKKLGVN---SELLSCVG---YDFknsGYERYLKNLDINIsKLYYSEEEETPKAWIFTDKDnNQITFFLWGAAKHykelnppNF--------------NTEIVHIATGD-----<br>-----vreniLFGMGNPLLDISAVVdkdfldkYSLKPNDQILAEDkhkelfdelvkkfKVEYHAGGSTQNsIKVAQWMIQQPhkaATFFGCIGidkFGE---ILKRKAAEAHVDA-HYYEQNEQPTGTCAACITGD-NRSLIANLAAANC-------YKkekhldleknwmlveKARVCYIAGFFltvsp<br>-PEFNLKCAKKAYGNNLVSFDPGQDLPQYSKEMLLEIIEHTNFLFMNKHEFERASNLLN---FEIDDYLER-----VD------ALIVTKGSKGSVIYTKDKKIEIPCIKAG--KVIDPTGAGDSYRAGFLSAYVKGYDLEKCGLIGAATASFVVEAKG----------cqtnlptwdkvverlekhr<br>eSVLKVAHHASENNRIFTLNLSAPFISQFYKESLMKVMPYVDILFGNETEAATFAREQGfetKDIKEIAKKtqalpKMnskrqrIVIFTQGRDDTIMATESEVTAFAVLDQDqkEIIDTNGAGDAFVGGFLSQLVSDKPLTECIRAGHYAASIIIRRTGctfpekpdfh------------------<br>BLAST: aliSize=117 (resi) iden=22.74% (68/299) simi=39.13% (117/299)<br>gkmekitcvghtaldyifnvekfpepntsiqipsar--KYYGGAAANTAVGIKKLGVN-----SELLSCVGYDFKNSGYERylKNLDINISKLYYSEEEE-TPKAWIFTDKDNNQITFFLwGAAKHYKE---LN-PP<br>--------------------------------vrenilfgmgnplldisavvdkdfldkyslkpndqilaedkhkelfdelvkkfkvEYHAGGSTQNSIKVAQWMIQqphkaATFFGCIGIDKFGEILKR--KAAEAHVDAHYYEQNEQpTGTCAACITGDNRSLIANL-AAANCYKKekhLDlEK<br>NF----NTEIVHIA----TGDPEFNLKCAKKAYGNNLVsFDPGQDLP---QYSKEMLLEIIEHTNFLFMNKHE---FERASNLLNFEIDDYLERVDAL-----------IVTKGSKGSVIYTKDKKIEIPCIKAGK--VIDPTGAGDSYRAGFLSAYVKGYDLEKCGLIGAATASFVVEAKGC-------<br>NWmlveKARVCYIAgfflTVSPESVLKVAHHASENNRI-FTLNLSAPfisQFYKESLMKVMPYVDILFGNETEaatFAREQGFETKDIKEIAKKTQALpkmnskrqrivIFTQGRDDTIMATESEVTAFAVLDQDQkeIIDTNGAGDAFVGGFLSQLVSDKPLTECIRAGHYAASIIIRRTGCtfpekpd<br>--qtnlptwdkvverlekhr<br>fh------------------<br>Proposed: aliSize=278 (resi) RMSD=2.11 (Å)<br>gkmE---KITCVGHTALDYIFNV-------eKFPEPNTSIQIP--------------SARKYYGGAAANTAVGIKKLGV----NSELLSCVGYD--FknSGYERYLKNLDINISkLYYSEEEETPKAWIFTDKdNNQITFFLWGAAKHYkELN--P-----P--NFNTEIVHIA-TGDP--E-FNLKCAK<br>---VrenILFGMGNPLLDISAVVdkdfldk-YSLKPNDQILAEdkhkelfdelvkkfKVEYHAGGSTQNSIKVAQWMIQqphkAATFFGCIGIDkfG--EILKRKAAEAHVDAH-YYEQNEQPTGTCAACITG-DNRSLIANLAAANCY-KKEkhLdleknWmlVEKARVCYIAgFFLTvsPeSVLKVAH<br>KAYGN-NLVSFDPGQ-DL-PQYSkEMLLEIIEHTNFLFMNKHEFERASNLLNF-------EIDDYLE--R-----VDALIVTKGSKGSVIYTKDKKIEIPCIK-A--gKVIDPTGAGDSYRAGFLSAYVKGYDLEKCGLIGAATASFVVEAKGCqTNlP-TWdKVVerlekhr<br>HASENnRIFTLNLSApFIsQFYK-ESLMKVMPYVDILFGNETEAATFAREQGFetkdikeIAKKTQAlpKmnskrQRIVIFTQGRDDTIMATESEVTAFAVLDqDqk-EIIDTNGAGDAFVGGFLSQLVSDKPLTECIRAGHYAASIIIRRTGC-TF-PeKP-DFH-------
170	1h1cA (329)<br>2qlrA (425)	N	12.16	TM-align: aliSize=288 (resi) RMSD=3.23 (Å)<br>liakraypyetekrdktylalnenpfpf---PEDL-VDFVFRRL-NSDALRIYYDS-PDEELIFKILSYL-DT-DF-LS-------KNNVSVGNGADFIIYV--L-FD---R-SVFFPPTYSCYRIFAKavGAKFLEVPLT<br>---------------------------mnyarfitaasaarnpspirtmtdilsrgpksmislagglpnpnmfpfktavITVEnGKTIQFGEeMMKRALQYSPSaGIPELLSWLKQLQiKLhNPpTIhyppsqgQMDLCVTSGSQQGLCKvfEmIInpgDnVLLDEPAYSGTLQSLH--PLGCNIINVA<br>KD--LRI--PE--VN--V----G------EGDVVFIPNP-NNPTGHVF---FRFEIERILK-TGAFVALDFAYYEFH----GESY-VDFLkKYENLAVIRTFSkAFsLAAQRVGYVVASFKFIDAYNRVRLP--FNVSYVSQ-FAKVAL--D---HR-EIFFERTKFIVEERFRKSA-LRF--G--YRIT<br>SDesGIVpdSLrdILsrWkpedAknpqknTPKFLYTVPNgNNPTGNSLtseRKKEIYELARkYDFLIIEDDPYYFLQfnkfRVPTfLSMD-VDGRVIRADSFS-II-SSGLRIGFLTGPKPLIERVILHIQVstLHPSTFNQlMISQLLheWgeeGFmAHVDRVIDFYSNQKDAILAaADKwlTglAEWH<br>DSR-GNFVFVFE--KFFK-FRLLFHLRTKNVAVRSF----------REGVRITIGKRFENDILRELFV-F-------<br>VPAaGMFLWIKVkgINDVkELIEEKAVKMGVLMLPGnafyvdssapSPYLRASFSSASPEQMDVAFQVlAqlikesl<br>SARST: aliSize=269 (resi) RMSD=8.87 (Å)<br>liakraypyetekrdktylal---NENPEPEPEDLVDEVF----RRLNSDALriyydspdFELIFKILSYLDTDF--LSKNNVSVGngadeiiyvlfdrsVFFPPTYSCYRIFAKAV----GAKFLEVPLT------<br>---------------------mnyarfitaasaarnpspirtmtdilsrgpksmislagglpnpnmfpfktavitveNGKTIQFGEEMMKRALqyspSAGIPELL--------SWLKQLQIKLHNPPTihYPPSQGQMD--------------LCVTSGSQQGLCKVFEMiinpGDNVLLDEPAysgtlq<br>---KDLRIPEVNVG-----------------------------EGDVVFIPNPNNPTGHVFERFEIFRILKT----GAFVALDFAYYEFHG-----ESYVDFLKKyENLAVIRTFSKAfsLAAQRVGYVVASFKFIDAYNRV-------RLPfnvsYVSQFAKVALDH-------REIFFERTKFIVEER<br>slhPLGCNIINVASdesgivpdslrdilsrwkpedaknpqkntPKFLYTVPNGNNPTGNSLTSERKKEIYELarkyDFLIIEDDPYYFLQFnkfrvPTFLSMDVD-GRVIRADSFSII--SSGLRIGFLTGPKPLIERVILHiqvstlhPST----FNQLMISQLLHEwgeegfmAHVDRVIDFYSNQKD<br>ERKSALRFGY----RITDSRGNFVFVFFKFE----KFRLLFHLRTKNVAVRSFREGV---------RITI---GKRFENDILRELE--------vf<br>AILAAADKWLtglaEWHVPAAGMFLWIKVKGindvKELIEEKAVKMGVLMLPGNAFYvdssapspyLRASfssASPEQMDVAFQVLaqlikesl--<br>BLAST: aliSize=60 (resi) iden=10.59% (34/329) simi=18.69% (60/329)<br>liakraypyetekrdktylalnenpfpfpedlvdevfrrlnsdalriyydspdeeliekilsyldtdflsknnvsvgngadeiiyvlfdrsvffpptyscyrifakavgakflevplt---------------------------------------<br>--mnyarfitaasaarnpspirtmtdilsrgpksmislagglpnpnmfpfktavitvengktiqfgeemmkral<br>--KDLRIPFVNVGFGdVVFIPNPNNPTGHVF--FRF-EIERILKTGAFVAL-DFAYY--FFHGESYVDFLKKYENLAVIRTFSK<br>qyspsagipellswlkqlqiklhnpptihyppsqgqmdlcvtsgsqqglckvfemiinpgdnvlldepaysgtlqslhplgcniinvasdesgivpdslrdilsrwkpEDAKNPQKNTPKF-LYTVPNGNNPTGNSLtsERKkEIYELARKYDFLIIeDDPYYflQFNKFRVPTFLSMDVDGRVIRADSF<br>AFSLAAQRVGYVVASFKFIDA---YNRVRL--P--FNVSYVSQ---fakvaldhreifeertkfiveererks<br>SIISSGLRIGFLTGPKPLIERvilHIQVSTlhPstFNQLMISQllhewgeegfmahvdrvidfysnqkdailaaadkwltglaewhvpaagmflwikvkgindvkelieekavkmgvlmlpgnafyvdssapspylrasfssaspeqmdvafqvlaqlikesl-------------------------<br>alregyritdsrgnfvfvfekeekerllehlrtknvavrsfregvritigkreendilrelevf<br>--<br>Proposed: aliSize=306 (resi) RMSD=2.67 (Å)<br>----LIA---KRAYPYF-TFKR---D-------KTYLALnENPFP-FPedLVDeVFrrLNSD---------------aLRiYYDS-PDEELIFKILSYL-DT-DF--L---------skNNVSVGNGADEIIYVMMLMFD---R-SVFFPPTYSCYRIFAKAvGAKFLEVPLTkD--LRI--PE--VN---<br>mnyaRFItaaSAARNPSpIRTMtdiLsrgpksmISLAGG-LPNPNmFP--FKT-AV--ITVEngktiqfgeemmkra-LQ-YSPSaGIPELLSWLKQLQiKLhNPptIhyppsqgq--MDLCVTSGSQQGLCKVFEMIInpgDnVLLDEPAYSGTLQSLHP-LGCNIINVAS-DesGIVpdSLrdILsrw<br>----------VGegDVVFIPNP-NNPTGHVFE---RFEIERILK-TGAFVALDFAYYEFH----GESY-VDFLkKYENLAVIRTFSKAFsLAAQRVGYVVASFKFIDAYNRvRLP---FNVSYVSQMFAKVALD-H----R-EIFFERTKFIVEERFRMKSALRF-MG--YRITDS-RGNFVFVFM--E<br>kpedaknpqknTP--KFLYTVPNgNNPTGNSLTserKKEIYELARkYDFLIIEDDPYYFLQfnkfRVPTfLSMD-VDGRVIRADSFSLII-SSGLRIGFLTGPKPLIERVIL-HIQvstLHPSTFNQLMISQLLHeWgeegFmAHVDRVIDFYSNQKDAILAAADKwLTglAEWHVPaAGMFLWIKVkgI<br>-KeeK-FRlL--FHlR-TKnVAVRSF----------REGVRITIGKrEFNDMILRELFV-F-------<br>nD--VkEL-IeeKA-VkMG-VLMLPGnafyvdssapSPYLRASFSS-ASPEQMDVAFQVlAqlikesl

171	1a5pA (124)<br>1a2wA (124)	N	98.39	TM-align: aliSize=105 (resi) RMSD=0.95 (Å)<br>ketaaakferqhmdsstsa-------------------ASSSNYCNQMMKSRNLTKDRAKPVNTFVHESLADVQAVCSQKNVACKNGQTNCYQSYSTMSITDCRETGSSKYPNAAYKTTQANKHIIVACEGNPYVPVHFDASV<br>-------------------ketaaakferqhmdsstsaASSSNYCNQMMKSRNLTKDRCKPVNTFVHESLADVQAVCSQKNVACKNGQTNCYQSYSTMSITDCRETGSSKYPNCAYKTTQANKHIIVACEGNPYVPVHFDASV<br><br>SARST: aliSize=121 (resi) RMSD=14.30 (Å)<br>k-ETAAAKFERQHMDSSTsAASS-SNYCNQMMKSRNLTKDRAKPVNTFVHESLADVQAVCSQKNVACKNGQTNCYQSYSTMSITDCRETGSSKYPNAAYKTTQANKHIIVACEGNPYVPVHFDAS-v<br>-kETAAAKFERQHMDSST-SAASsSNYCNQMMKSRNLTKDRCKPVNTFVHESLADVQAVCSQKNVACKNGQTNCYQSYSTMSITDCRETGSSKYPNCAYKTTQANKHIIVACEGNPYVPVHFDASv-<br><br>BLAST: aliSize=122 (resi) iden=98.39% (122/124) simi=98.39% (122/124)<br>KETAAAKFERQHMDSSTSAASSSNYCNQMMKSRNLTKDRAKPVNTFVHESLADVQAVCSQKNVACKNGQTNCYQSYSTMSITDCRETGSSKYPNAAYKTTQANKHIIVACEGNPYVPVHFDASV<br>KETAAAKFERQHMDSSTSAASSSNYCNQMMKSRNLTKDRCKPVNTFVHESLADVQAVCSQKNVACKNGQTNCYQSYSTMSITDCRETGSSKYPNCAYKTTQANKHIIVACEGNPYVPVHFDASV<br><br>Proposed: aliSize=124 (resi) RMSD=0.68 (Å)<br>KETAAAKFERQHMDSSTSAASSSNYCNQMMKSRNLTKDRAKPVNTFVHESLADVQAVCSQKNVACKNGQTNCYQSYSTMSITDCRETGSSKYPNAAYKTTQANKHIIVACEGNPYVPVHFDASV<br>KETAAAKFERQHMDSSTSAASSSNYCNQMMKSRNLTKDRCKPVNTFVHESLADVQAVCSQKNVACKNGQTNCYQSYSTMSITDCRETGSSKYPNCAYKTTQANKHIIVACEGNPYVPVHFDASV
172	1nloC (56)<br>1aojA (60)	C	25.00	TM-align: aliSize=34 (resi) RMSD=2.23 (Å)<br>--TFVALYDYESRTETDLSFKKGERLQIVN--NTEGDwwlahslttgqtg---------Yipsnyvaps-------------<br>kkYAKSKYDFVARNSSELSVMKDDVLEILDdrRQWWK-------------vrnasgdsgF---------vpnnildimrtpe<br><br>SARST: aliSize=52 (resi) RMSD=11.63 (Å)<br>t---FVALYDYESRTETDLSFKKGERLQIVNNteGDWWLAHSLTTGQTGYIPSNYVAP-----s<br>-kkyAKSKYDFVARNSSELSVMKDDVLEILDD--RRQWWKVRNASGDSGFVPNNILDImrtpe-<br><br>BLAST: aliSize=29 (resi) iden=25.00% (14/56) simi=51.79% (29/56)<br>tfval-------YDYESRTETDLSFKKGERLQIVNNTEgDWWLAHSlTTGQTGYIPSN---------yvaps<br>-----kkyakskYDFVARNSSELSVMKDDVLEILDDRR-QWWKVRN-ASGDSGFVPNNildimrtpe-----<br><br>Proposed: aliSize=52 (resi) RMSD=1.23 (Å)<br>--TFVALYDYESRTETDLSFKKGERLQIVN--nteGDWWLAHSlTTGQTGYIPSNYVAPS----<br>kkYAKSKYDFVARNSSELSVMKDDVLEILDdr---RQWWKVRN-ASGDSGFVPNNILDIMrtpe
173	1eydA (136)<br>1sndA (129)	C	100.00	TM-align: aliSize=111 (resi) RMSD=1.36 (Å)<br>kLHKEPATLIKAIDGDTVKLMYKGQPMTFRLLLVDTPETKHPKKGVEKYGPEASAFTKKMVENAKKIEVEFDKGQRTDKYGRGLAYIYADGKMVNEALVRQGLAKVAYVYkpnnthEQhlrkseaqakkeklniws-----------------<br>-LHKEPATLIKAIDGDTVKLMYKGQPMTFRLLLVDTPETKHPKKGVEKYGPEASAFTKKMVENAKKIEVEFDKGQRTDKYGRGLAYIYADGKMVNEALVRQGLAKVAYTH------EQ-----------------hlrkseaqakkeklniws<br><br>SARST: aliSize=127 (resi) RMSD=9.61 (Å)<br>kl-HKEPATLIKAIDGDTVKLMYKGQPMTFRLLLVDTPETKHPKKGVEKYGPEASAFTKKMVENAKKIEVEFDKGQRTDKYGRGLAYIYADGKMVNEALVRQGLAKVAYVykpnntHEQHLRKSEAQAKKEKLNIW-s<br>--lHKEPATLIKAIDGDTVKLMYKGQPMTFRLLLVDTPETKHPKKGVEKYGPEASAFTKKMVENAKKIEVEFDKGQRTDKYGRGLAYIYADGKMVNEALVRQGLAKVAYT------HEQHLRKSEAQAKKEKLNIWs-<br><br>BLAST: aliSize=129 (resi) iden=100.00% (129/129) simi=100.00% (129/129)<br>kLHKEPATLIKAIDGDTVKLMYKGQPMTFRLLLVDTPETKHPKKGVEKYGPEASAFTKKMVENAKKIEVEFDKGQRTDKYGRGLAYIYADGKMVNEALVRQGLAKVAYvykpnnTHEQHLRKSEAQAKKEKLNIWS<br>-LHKEPATLIKAIDGDTVKLMYKGQPMTFRLLLVDTPETKHPKKGVEKYGPEASAFTKKMVENAKKIEVEFDKGQRTDKYGRGLAYIYADGKMVNEALVRQGLAKVAY------THEQHLRKSEAQAKKEKLNIWS<br><br>Proposed: aliSize=129 (resi) RMSD=0.51 (Å)<br>kLHKEPATLIKAIDGDTVKLMYKGQPMTFRLLLVDTPETKHPKKGVEKYGPEASAFTKKMVENAKKIEVEFDKGQRTDKYGRGLAYIYADGKMVNEALVRQGLAKVAYvykpnnTHEQHLRKSEAQAKKEKLNIWS<br>-LHKEPATLIKAIDGDTVKLMYKGQPMTFRLLLVDTPETKHPKKGVEKYGPEASAFTKKMVENAKKIEVEFDKGQRTDKYGRGLAYIYADGKMVNEALVRQGLAKVAY------THEQHLRKSEAQAKKEKLNIWS

174	1qd0A (128)<br>1sjvA (102)	C	70.59	TM-align: aliSize=89 (resi) RMSD=1.65 (Å)<br>qvqlqeSgGGLVQAGGSLRLSCAAsgraasghGHYGMGWFRQVPGKEREFVAAIRWSGkETWYKDSVKGRFTISRDNAKTTVYLQMNSLKGEDTAVYYCAARPVRvadislpvgfdywgqgtqvtss-------------<br>------G-GGLVQAGESLKLSCAA--------SGGFMGWYRQAPGKQRELVATINSRG-ITNYADFVKGRFTISRDNAKKTVYLEMNSLEPEDTAVYYCYTHYFR----------------------sywgqgtqvtss<br><br>SARST: aliSize=98 (resi) RMSD=14.81 (Å)<br>qvqlqesgg--GLVQAGGSLRLSCAASGRAasghghygMGWFRQVPGKEREFVAAIRWSGkETWYKDSVKGRFTISRDNAKTTVYLQMNSLKGEDTAVYYCAARPVRVadislpvgfdYWGQGTQVTV--ss<br>---------ggGLVQAGESLKLSCAASGGF--------MGWYRQAPGKQRELVATINSRG-ITNYADFVKGRFTISRDNAKKTVYLEMNSLEPEDTAVYYCYTHYFRS----------YWGQGTQVTVss--<br><br>BLAST: aliSize=82 (resi) iden=75.49% (77/102) simi=80.39% (82/102)<br>qvqlqesGGGLVQAGGSLRLSCAASGraasghGHygMGWFRQVPGKEREFVAAIRWSGKeTWYKDSVKGRFTISRDNAKTTVYLQMNSLKGEDTAVYYCAARPVRvadislpvgfDYWGQGTQVTVSS<br>-------GGGLVQAGESLKLSCAASG------GF--MGWYRQAPGKQRELVATINSRGI-TNYADFVKGRFTISRDNAKKTVYLEMNSLEPEDTAVYYCYTHYFR----------SYWGQGTQVTVSS<br><br>Proposed: aliSize=102 (resi) RMSD=1.49 (Å)<br>qvqlqeSgGGLVQAGGSLRLSCAAsgraasghGHYGMGWFRQVPGKEREFVAAIRWSGkETWYKDSVKGRFTISRDNAKTTVYLQMNSLKGEDTAVYYCAARPVrvadislpvGFdYWGQGTQVTVSS<br>------G-GGLVQAGESLKLSCAA--------SGGFMGWYRQAPGKQRELVATINSRG-ITNYADFVKGRFTISRDNAKKTVYLEMNSLEPEDTAVYYCYTHYF---------RS-YWGQGTQVTVSS
175	1pv3A (146)<br>1k04A (142)	N	83.80	TM-align: aliSize=109 (resi) RMSD=2.62 (Å)<br>gspgisgggggirsndkvyenvtglvkaviem--------------------------------SSKIQpAPPEEYVPMVKEVGLALRTLLATVDESLPVLPASTHREIEMAQKLLNSDLAELINKMKLAQQYVMTSLQQEYKKQMLTAAHALAVDAKNLLDVIDQARLKMISQsrph-<br>--------------------------------eisppptanldrsndkvyenvtglvkaviemsSKIQP-APPEEYVPMVKEVGLALRTLLATVDETIPLLPASTHREIEMAQKLLNSDLGELINKMKLAQQYVMTSLQQEYKKQMLTAAHALAVDAKNLLDVIDQARLKMLGQ----t<br><br>SARST: aliSize=123 (resi) RMSD=8.28 (Å)<br>gspgisgggggirsndkv---------------YENVTGLVKAVIEMSSKIQPAPP--EEYVPMVKEVGLALRTLLATVDESLPVLPASTHREIEMAQKLLNSDLAELINKMKLAQQYVMTSLQQEYKKQMLTAAHALAVDAKNLLDVIDQARLKMIS--qsrp<br>-----------------eispppptanldrsndKVYENVTGLVKAVIEMSSKIQPAppEEYVPMVKEVGLALRTLLATVDETIPLLPASTHREIEMAQKLLNSDLGELINKMKLAQQYVMTSLQQEYKKQMLTAAHALAVDAKNLLDVIDQARLKMLGqt----<br><br>BLAST: aliSize=129 (resi) iden=87.32% (124/142) simi=90.85% (129/142)<br>gspgisgggggi-----------RSNDKVYENVTGLVKAVIEMSSKIQPAPPEEYVPMVKEVGLALRTLLATVDESLPVLPASTHREIEMAQKLLNSDLAELINKMKLAQQYVMTSLQQEYKKQMLTAAHALAVDAKNLLDVIDQARLKMISQSrp<br>------------eispppptanldRSNDKVYENVTGLVKAVIEMSSKIQPAPPEEYVPMVKEVGLALRTLLATVDETIPLLPASTHREIEMAQKLLNSDLGELINKMKLAQQYVMTSLQQEYKKQMLTAAHALAVDAKNLLDVIDQARLKMLGQT--<br><br>Proposed: aliSize=130 (resi) RMSD=2.02 (Å)<br>gspgis-------GggggIRSN--DKVYENVTGLVKAVIEMSSKIQP-aPPEEYVPMVKEVGLALRTLLATVDESLPVLPASTHREIEMAQKLLNSDLAELINKMKLAQQYVMTSLQQEYKKQMLTAAHALAVDAKNLLDVIDQARLKMIS--qsrph<br>------eispppptA----NLDRsnDKVYENVTGLVKAVIEMSSKIQPa-PPEEYVPMVKEVGLALRTLLATVDETIPLLPASTHREIEMAQKLLNSDLGELINKMKLAQQYVMTSLQQEYKKQMLTAAHALAVDAKNLLDVIDQARLKMLGqt-----
176	1b6bA (168)<br>1y9wA (140)	N	15.71	TM-align: aliSize=101 (resi) RMSD=2.62 (Å)<br>anefrcltpedaagvfeiereafisvsgncplnldevqhflt-----------------------------------LCPeLSLGWFVEGR-LVAFIIGSLwdeerltqeslalhrPrGHSAHLHALAVHRSFRQQGKGSVLLWRYLHHVGAQPaVRRAVLMCEDA-LVPFYQRFGFHPAGPcAIVVGS<br>--mymkhiengtriegeyiknkviqynmsiltdevkqpMEE-VSLVVKNEEGkIFGGVTGTM---------------Y-FYHLHIDFLWVDESVRHDGYGSQLLHEIEGIAKEKG-CRLILLDSFSFqAPEFYKKHGYREYGV-VEDHPK<br><br>-LTFTEMHCSLrghaal<br>gHSQHFFEKRL------<br><br>SARST: aliSize=100 (resi) RMSD=5.57 (Å)<br>anefrcltpedaagvfeiereafisvsgncplnldevqhfltlc---------------------------PELS----------LGWFV---EGRLVAFIIGSLWDeerltqeslalhrprgHSAHLHALAVHRSFRQQGKGSVLLWRYLHHVGAqPAVRRAVLMCEDAL-VPFYQRFGFHPAGPCAIVVG<br>--mymkhiengtriegeyiknkviqynMSILtdevkqpmeeVSLVVkneEGKIFGGVTGTMYF----------------YHLHIDFLWVDESVRHDGYGSQLLHEIEGIAKE-KGCRLILLDSFSFQaPEFYKKHGYREYGVVEDHPK<br><br>SLTFTEMHCS-lrghaal<br>GHSQHFFEKRl-------<br><br>BLAST: aliSize=45 (resi) iden=19.29% (27/140) simi=32.14% (45/140)<br>anefrcltpedaagvfeiereafisvsgncplnldevqhfltlcpelsl-------------GWFVEGRLVAFIIGSLWDEERLTQE--SLALHRPRG----------HSAHLHA--LAVHRSFRQQGKGSVLLWRyLHHVGAQPAVRRAVLMCEDALVP-FYQRFGFHPAG-----------------<br>---mymkhiengtrieGEYIKNKVIQYNMSILTDEVKQPMEevSLVVKNEEGkifggvtgtmYFYHLHIdfLWVDESVRHDGYGSQLLHE-IEGIAKEKGCRLILLDSFSFQAPeFYKKHGYREYGvvedhpkghsqhffekrl<br><br>pcaivvgsltftemhcslrghaal<br>------------------------<br><br>Proposed: aliSize=114 (resi) RMSD=2.03 (Å)<br>--ANEFRCL---T-PEDAAGVFEIE--------------reafisvsgncplnldevqhfltlcpeLSLGWFVEGR-LVAFIIGSLwdeerltqeslalhrpRGHSAHLHALAVH-rSFRQQGKGSVLLWRYLHHVGAQPaVRRAVLMCEDA-LVPFYQRFGFHPAGPcA--iVvGS-LTFTEMHCSLrg<br>myMKHIENGtriEgEYIKNKVIQYNmsiltdevkqpmee---------------------------VSLVVKNEEGkIFGGVTGTM----------------YFYHLHIDFLWVDe-SVRHDGYGSQLLHEIEGIAKEKG-CRLILLDSFSFqAPEFYKKHGYREYGV-Ved-H-PKgHSQHFFEKRL--<br><br>haal<br>----

177	1bsrA (124)<br>1awzA (123)	N	31.71	TM-align: aliSize=97 (resi) RMSD=2.01 (Å)<br>kesaaakferqhmdsgnsp-------------------SSSSNYCNLMMCCRKMTQgKCKPVNTFVHESLADVKAVCS---QKKVTckngqTNCYQSKSTMRITDCRETGSSKYPNCAYKTTQVEKHIIVACGGkpSVPVHFDA-SV---<br>-------------------qdnsrythfltqhydakpqGRDDRYCESIMRRRGLTS-PCKDINTFIHGNKRSIKAICEnknGNPHR-----ENLRISKSSFQVTTCKLHGGSPWPPCQYRATAGFRNVVVACEN--GLPVHLDQsIFrrp<br><br>SARST: aliSize=88 (resi) RMSD=5.96 (Å)<br>kes------------------------AAAKFERQhmdsgnspssssnycnlmmccRKMTQgKCKPVNTFVHESLADVKAVC---SQKKVtckngQTNCYQSKSTMRITDCRETGSSKYPNCAYKTTQVEKHIIVACGGkpSVPVHF--------dasv<br>---qdnsrythfltqhydakpqgrddrYCESIMRR---------------------RGLTS-PCKDINTFIHGNKRSIKAICenkNGNPH-----RENLRISKSSFQVTTCKLHGGSPWPPCQYRATAGFRNVVVACEN--GLPVHLdqsifrrp----<br><br>BLAST: aliSize=65 (resi) iden=34.15% (42/123) simi=52.85% (65/123)<br>kesaaak--------FERQHMDSgnSPSSSSN-YCNLMMCCRKMTQgKCKPVNTFVHESLADVKAVCSQkkvtcKNG---QTNCYQSKSTMRITDCRETGSSKYPNCAYKTTQVEKHIIVACggKPSVPVHFDASV----<br>-------qdnsrythFLTQHYDA--KPQGRDDrYCESIMRRRGLTS-PCKDINTFIHGNKRSIKAICEN-----KNGnphRENLRISKSSFQVTTCKLHGGSPWPPCQYRATAGFRNVVVAC--ENGLPVHLDQSIfrrp<br><br>Proposed: aliSize=112 (resi) RMSD=1.55 (Å)<br>kE--SAAAKFERQHMDSGnsPSS-SSNYCNLMMCCRKMTQgKCKPVNTFVHESLADVKAVCS---QKKVTckngqTNCYQSKSTMRITDCRETGSSKYPNCAYKTTQVEKHIIVACGGkpSVPVHFDA-S----v<br>-QdnSRYTHFLTQHYDAK--PQGrDDRYCESIMRRRGLTS-PCKDINTFIHGNKRSIKAICEnknGNPHR-----ENLRISKSSFQVTTCKLHGGSPWPPCQYRATAGFRNVVVACEN--GLPVHLDQsIfrrp-
178	1bsrA (124)<br>1e21A (119)	N	71.43	TM-align: aliSize=104 (resi) RMSD=1.33 (Å)<br>kesaaakferqhmdsgnsps------------SS--SNYCNLMMCCRKMTQGKCKPVNTFVHESLADVKAVCSQKKVTCKNGQTNCYQSKSTMRITDCRETGSSKYPNCAYKTTQVEKHIIVACGGKPSVPVHFDASV-<br>--------------------afqrqhmdsdssPSssSTYCNQMMRRRNMTQGRCKPVNTFVHEPLVDVQNVCFQEKVTCKNGQGNCYKSNSSMHITDCRLTNGSRYPNCAYRTSPKERHIIVACEGSPYVPVHFDASVe<br><br>SARST: aliSize=109 (resi) RMSD=5.16 (Å)<br>kesaaakfer--------QHMDSGnspsSSSNYCNLMMCCRKMTQGKCKPVNTFVHESLADVKAVCSQKKVTCKNGQTNCYQSKSTMRITDCRETGSSKYPNCAYKTTQVEKHIIVACGGKPSVPVHFDAS--v<br>----------afqrqhmdSDSSPS----SSSTYCNQMMRRRNMTQGRCKPVNTFVHEPLVDVQNVCFQEKVTCKNGQGNCYKSNSSMHITDCRLTNGSRYPNCAYRTSPKERHIIVACEGSPYVPVHFDASve-<br><br>BLAST: aliSize=96 (resi) iden=71.43% (85/119) simi=80.67% (96/119)<br>kesaaak-FERQHMDSGNSPSSSSNYCNLMMCCRKMTQGKCKPVNTFVHESLADVKAVCSQKKVTCKNGQTNCYQSKSTMRITDCRETGSSKYPNCAYKTTQVEKHIIVACGGKPSVPVHFDASV-<br>-------aFQRQHMDSDSSPSSSSTYCNQMMRRRNMTQGRCKPVNTFVHEPLVDVQNVCFQEKVTCKNGQGNCYKSNSSMHITDCRLTNGSRYPNCAYRTSPKERHIIVACEGSPYVPVHFDASVe<br><br>Proposed: aliSize=118 (resi) RMSD=1.35 (Å)<br>kesaaaKFERQHMDSGNSPSSSSNYCNLMMCCRKMTQGKCKPVNTFVHESLADVKAVCSQKKVTCKNGQTNCYQSKSTMRITDCRETGSSKYPNCAYKTTQVEKHIIVACGGKPSVPVHFDASV-<br>------AFQRQHMDSDSSPSSSSTYCNQMMRRRNMTQGRCKPVNTFVHEPLVDVQNVCFQEKVTCKNGQGNCYKSNSSMHITDCRLTNGSRYPNCAYRTSPKERHIIVACEGSPYVPVHFDASVe
179	1bsrA (124)<br>1gioA (125)	N	33.06	TM-align: aliSize=96 (resi) RMSD=1.93 (Å)<br>kesaaakferqhmdsgnsp-------------------SSSS-NYCNLMMCCRKMTQgKCKPVNTFVHESLADVKAVCS---QKKVTckngqTNCYQSKSTMRITDCRETGSSKyPN-CAYKTTQVEKHIIVACGGkpSVPVHFDA-S-V---<br>------------------aqddyryihfltqhydakpKGRNdEYCFNMMKNRRLTR-PCKDRNTFIHGNKNDIKAICEdrnGQPYR-----GDLRISKSEFQITICKHKGGSS-RPpCRYGATEDSRVIVVGCEN--GLPVHFDEsFiTprh<br><br>SARST: aliSize=85 (resi) RMSD=2.60 (Å)<br>kesaaakferqhmdsgnspssss------------------------NYCNLMMCCRKMTQ-GkcKPVNTFVHESLADVKAV---CSQKKVTckngqTNCYQSKSTMRITDCRETgsSKYPNCA--YKTTQVEKHIIVACGGKPSVP----------vhfdasv<br>-----------------------aqddyryihfltqhydakpkgrndEYCFNMMKNRRLTRpC--KDRNTFIHGNKNDIKAIcedRNGQPYR-----GDLRISKSEFQITICKHK--GGSSRPPcrYGATEDSRVIVVGCENGLPVHfdesfitprh-------<br><br>BLAST: aliSize=61 (resi) iden=34.68% (43/124) simi=49.19% (61/124)<br>kesaaak---------FERQHMDSgnSPSSSSN-YCNLMMCCRKMTQgKCKPVNTFVHESLADVKAVCSQKKVTCKNGQTNCyqSKSTMRITDCRETGSSKYPNCAYKTTQVEKHIIVACggKPSVPVHFDAS------v<br>-------aqddyryihFLTQHYDA--KPKGRNDeYCFNMMKNRRLTR-PCKDRNTFIHGNKNDIKAICEDRNGQPYRGDLRI--SKSEFQITICKHKGGSSRPPCRYGATEDSRVIVVGC--ENGLPVHFDESfitprh-<br><br>Proposed: aliSize=111 (resi) RMSD=1.89 (Å)<br>k---ESAAAKFERQHMDSGnsPSS---ssNYCNLMMCCRKMTQgKCKPVNTFVHESLADVKAVCS---QKKVTckngqTNCYQSKSTMRITDCRETGSSKYPNCAYKTTQVEKHIIVACGGkpSVPVHFDA-S-V---<br>-aqdDYRYIHFLTQHYDAK--PKGrnd--EYCFNMMKNRRLTR-PCKDRNTFIHGNKNDIKAICEdrnGQPYR-----GDLRISKSEFQITICKHKGGSSRPPCRYGATEDSRVIVVGCEN--GLPVHFDEsFiTprh

180	1bsrA (124)<br>1kvzA (107)	N	24.30	TM-align: aliSize=86 (resi) RMSD=2.69 (Å)<br>`kesaaakferqhmdsgnspss---------------SSNYCNLMMCCRKMTqgkCKPVNTFVHESLADVKAVCSQkkvtckngq---TNCYQSKSTMRITDCRETGSSKypnCAYKTTQVEKHIIVACGGkpSVPVHFDASV---`<br>`---------------------mqdwatfkkkhltdtWDVDCDNLMPTSLFD---CKDKNTFIYSLPGPVKALCRG---------vifSADVLSNSEFYLAECNVKPRKP---CKYKLKKSSNRICIRCEH--ELPVHFAGVGicp`<br><br>SARST: aliSize=99 (resi) RMSD=10.50 (Å)<br>`kesa---AAKFERQHMDSGNSpsSSSNYCNLMMCCRKmtqgkcKPVNTFVHESLADVKAVCSQKKVTCkngqtnCYQSKSTMRITDCRETgsskYPNC-AYKTTQVEKHIIVACGGkpSVPVHFDAS----v`<br>`----mqdWATFKKKHLTDTWD--VDCDNLMPTSLFDC------KDKNTFIYSLPGPVKALCRGVIFSA------DVLSNSEFYLAECNVK----PRKPcKYKLKKSSNRICIRCEH--ELPVHFAGVgicp-`<br><br>BLAST: aliSize=34 (resi) iden=21.50% (23/107) simi=31.78% (34/107)<br>`kesaaakferqhmdsgnspssssnycnlmmccrkmtqgk------------------------------CKPVNTFVHESLADVKAVCsqKKVTCKNGQTncyqSKSTMRITDCREtgSSKYPnCAYKTTQVEKHIIVACggKPSVPVHF-------dasv`<br>`---------------------------------------mqdwatfkkkhltdtwdvdcdnlmptslfdCKDKNTFIYSLPGPVKALC--RGVIFSADVL----SNSEFYLAECNV--KPRKP-CKYKLKKSSNRICIRC--EHELPVHFagvgicp----`<br><br>Proposed: aliSize=99 (resi) RMSD=2.06 (Å)<br>`KESaAAKFERQHMDSGNSPssssNyCNLMMCCrKMTQgkCKPVNTFVHESLADVKAVCS-----qkkvtckngqtNCYQSKSTMRITDCRETGSSkypNCAYKTTQVEKHIIVACGGkpSVPVHFDASV---`<br>`MQD-WATFKKKHLTDTWDV----D-CDNLMPT-SLFD--CKDKNTFIYSLPGPVKALCRgvifs-----------ADVLSNSEFYLAECNVKPRK---PCKYKLKKSSNRICIRCEH--ELPVHFAGVGicp`
181	1bsrA (124)<br>1pu3A (105)	N	23.81	TM-align: aliSize=84 (resi) RMSD=2.21 (Å)<br>`kesaaakferqhmdsgnspss----------------SsNYCNLMMCCRKMTqgkCKPVNTFVHESLADVKAVCSQkkvtckngq---TNCYQSKSTMRITDCRETGSSkypnCAYKTTQVEKHIIVACGgKpSVPVHFDASV--`<br>`---------------------mqdwltfqkkhitntrD-VDCDNILSTNLFH---CKDKNTFIYSRPEPVKAICKG---------iiaSKNVLTTSEFYLSDCNVTSRP----CKYKLKKSTNKFCVTCE-N-QAPVHFVGVGsc`<br><br>SARST: aliSize=96 (resi) RMSD=10.50 (Å)<br>`kesa---AAKFERQHMDSGNSpsSSSNYCNLMMCCRKmtqgkcKPVNTFVHESLADVKAvCSQkkvtcknGQT--NCYQSKSTMRITDCRETgsskyPNC-AYKTTQVEKHIIVACGGkpSVPVHFDAS---v`<br>`----mqdWLTFQKKHITNTRD--VDCDNILSTNLFHC------KDKNTFIYSRPEPVKA-ICK-------GIIasKNVLTTSEFYLSDCNVT-----SRPcKYKLKKSTNKFCVTCEN--QAPVHFVGVgsc-`<br><br>BLAST: aliSize=37 (resi) iden=25.71% (27/105) simi=35.24% (37/105)<br>`kesaaakferqhmdsgnspssssnycnlmmccrkmtqgk------------------------------CKPVNTFVHESLADVKAVCsQKKVTCKNGQTNcyqskSTMRITDCRETgsSKyPnCAYKTTQVEKHIIVACGGKpsVPVHF------dasv`<br>`---------------------------------------mqdwltfqkkhitntrdvdcdnilstnlfhCKDKNTFIYSRPEPVKAIC-KGIIASKNVLTT-----SEFYLSDCNVT--SR-P-CKYKLKKSTNKFCVTCENQ--APVHFvgvgsc----`<br><br>Proposed: aliSize=98 (resi) RMSD=1.79 (Å)<br>`kESAAAKFERQHMDSGnSPSsssnYCNLMMCCRKMTqgkCKPVNTFVHESLADVKAVCS-----qkkvtckngqtNCYQSKSTMRITDCRETGSSkypnCAYKTTQVEKHIIVACGgKpSVPVHFDASV--`<br>`-MQDWLTFQKKHITNT-RDV----DCDNILSTNLFH---CKDKNTFIYSRPEPVKAICKgiias-----------KNVLTTSEFYLSDCNVTSRP----CKYKLKKSTNKFCVTCE-N-QAPVHFVGVGsc`
182	1bsrA (124)<br>1rnfA (120)	N	42.50	TM-align: aliSize=100 (resi) RMSD=1.45 (Å)<br>`kesaaakferqhmdsgnsps--------------------SSSNYCNLMMCCRKMTQGKCKPVNTFVHESLADVKAVCSQKKVTCKNGQTNCYQSksTMRITDCRETGSSKYPNCAYKTTQVEKHIIVACGGKPSVPVHFDAsv`<br>`--------------------mqdgmyqrflrqhvhpeetgGSDRYCNLMMQRRKMTLYHCKRFNTFIHEDIWNIRSICSTTNIQCKNGKMNCHEG--VVKVTDCRDTGSSRAPNCRYRAIASTRRVVIACEGNPQVPVHFDG--`<br><br>SARST: aliSize=112 (resi) RMSD=9.33 (Å)<br>`kes----AAAKFERQHMD---SGNSPssssNYCNLMMCCRKMTQGKCKPVNTFVHESLADVKAVCSQKKVTCKNGQTNCYQskSTMRITDCRETGSSKYPNCAYKTTQVEKHIIVACGGKPSVPVHFD-asv`<br>`---mqdgMYQRFLRQHVHpeeTGGSD----RYCNLMMQRRKMTLYHCKRFNTFIHEDIWNIRSICSTTNIQCKNGKMNCHE--GVVKVTDCRDTGSSRAPNCRYRAIASTRRVVIACEGNPQVPVHFDg---`<br><br>BLAST: aliSize=76 (resi) iden=43.33% (52/120) simi=63.33% (76/120)<br>`kesaaa-------KFERQHMDSGNSpSSSSNYCNLMMCCRKMTQGKCKPVNTFVHESLADVKAVCSQKKVTCKNGQTNCYQskSTMRITDCRETGSSKYPNCAYKTTQVEKHIIVACGGKPSVPVHFD-asv`<br>`------mqdgmyqRFLRQHVHPEET-GGSDRYCNLMMQRRKMTLYHCKRFNTFIHEDIWNIRSICSTTNIQCKNGKMNCHE--GVVKVTDCRDTGSSRAPNCRYRAIASTRRVVIACEGNPQVPVHFDg---`<br><br>Proposed: aliSize=115 (resi) RMSD=1.19 (Å)<br>`ke---SAAAKFERQHMDSGnSPS--ssSNYCNLMMCCRKMTQGKCKPVNTFVHESLADVKAVCSQKKVTCKNGQTNCYQSksTMRITDCRETGSSKYPNCAYKTTQVEKHIIVACGGKPSVPVHFDAsv`<br>`--mqdGMYQRFLRQHVHPE-ETGgs--DRYCNLMMQRRKMTLYHCKRFNTFIHEDIWNIRSICSTTNIQCKNGKMNCHEG--VVKVTDCRDTGSSRAPNCRYRAIASTRRVVIACEGNPQVPVHFDG--`

183	1bsrA (124)<br>2aasA (124)	N	81.45	TM-align: aliSize=104 (resi) RMSD=1.40 (Å)<br>kesaaakferqhmdsgnsps--------------------SSSNYCNLMMCCRKMTQGKCKPVNTFVHESLADVKAVCSQKKVTCKNGQTNCYQSKSTMRITDCRETGSSKYPNCAYKTTQVEKHIIVACGGKPSVPVHFDASV<br>--------------------ketaaakferqhmdsstsaaSSSNYCNQMMKSRNLTKDRCKPVNTFVHESLADVQAVCSQKNVACKNGQTNCYQSYSTMSITDCRETGSSKYPNCAYKTTQANKHIIVACEGNPYVPVHFDASV<br><br>SARST: aliSize=118 (resi) RMSD=8.87 (Å)<br>kesa----AAKFERQHMDSgNSPSSS-SNYCNLMMCCRKMTQGKCKPVNTFVHESLADVKAVCSQKKVTCKNGQTNCYQSKSTMRITDCRETGSSKYPNCAYKTTQVEKHIIVACGGKPSVPVHFDAS-v<br>----ketaAAKFERQHMDS-STSAASsSNYCNQMMKSRNLTKDRCKPVNTFVHESLADVQAVCSQKNVACKNGQTNCYQSYSTMSITDCRETGSSKYPNCAYKTTQANKHIIVACEGNPYVPVHFDASv-<br><br>BLAST: aliSize=107 (resi) iden=81.45% (101/124) simi=86.29% (107/124)<br>KESAAAKFERQHMDSGNSPSSSSNYCNLMMCCRKMTQGKCKPVNTFVHESLADVKAVCSQKKVTCKNGQTNCYQSKSTMRITDCRETGSSKYPNCAYKTTQVEKHIIVACGGKPSVPVHFDASV<br>KETAAAKFERQHMDSSTSAASSSNYCNQMMKSRNLTKDRCKPVNTFVHESLADVQAVCSQKNVACKNGQTNCYQSYSTMSITDCRETGSSKYPNCAYKTTQANKHIIVACEGNPYVPVHFDASV<br><br>Proposed: aliSize=124 (resi) RMSD=1.40 (Å)<br>KESAAAKFERQHMDSGNSPSSSSNYCNLMMCCRKMTQGKCKPVNTFVHESLADVKAVCSQKKVTCKNGQTNCYQSKSTMRITDCRETGSSKYPNCAYKTTQVEKHIIVACGGKPSVPVHFDASV<br>KETAAAKFERQHMDSSTSAASSSNYCNQMMKSRNLTKDRCKPVNTFVHESLADVQAVCSQKNVACKNGQTNCYQSYSTMSITDCRETGSSKYPNCAYKTTQANKHIIVACEGNPYVPVHFDASV
184	1bsrA (124)<br>2hkyA (129)	N	35.48	TM-align: aliSize=97 (resi) RMSD=2.01 (Å)<br>kesaaakferqhmdsgnspssss---------------------NYCNLMMCcrKMTQ--GKCKPVNTFVHESLADVKAVCSQKKVTCkNG-QTNCYQSKSTMRITDCRETgSSKYPNCAYKTTQVEKHIIVACGGK--------PSVPVHFDASV<br>-----------------------mkpkgmtssqwfkiqhmqpspQACNSAMK--NINKhtKRCKDLNTFLHEPFSSVAATCQTPKIAC-KNgDKNCHQSHGPVSLTMCKLT-SGKYPNCRYKEKRQNKSYVVACKPPqkkdsqqfHLVPVHLDRVL<br><br>SARST: aliSize=115 (resi) RMSD=10.20 (Å)<br>k-----ESAAAKFERQHMDSGNSpsSSSNYCNLMMCCRKmtqgKCKPVNTFVHESLADVKAVCSQKKVTCKNGQTNCYQSKSTMRITDCReTGSSKYPNCAYKTTQVEKHIIVACG--------GKPSVPVHFDAS-v<br>-mkpkgMTSSQWFKIQHMQPSPQ--ACNSAMKNINKHTK----RCKDLNTFLHEPFSSVAATCQTPKIACKNGDKNCHQSHGPVSLTMCK-LTSGKYPNCRYKEKRQNKSYVVACKppqkkdsqQFHLVPVHLDRVl-<br><br>BLAST: aliSize=66 (resi) iden=41.94% (52/124) simi=53.23% (66/124)<br>kesaaak-----------FERQHMDSgnSPSSssnyCN-LMMCCRKMTQgKCKPVNTFVHESLADVKAVCSQKKVTCKNGQTNCYQSKSTMRITDCRETgSSKYPNCAYKTTQVEKHIIVAC---GGKPS-----VPVHFD---asv<br>-------mkpkgmtssqwFKIQHMQP--SPQA----CNsAMKNINKHTK-RCKDLNTFLHEPFSSVAATCQTPKIACKNGDKNCHQSHGPVSLTMCKLT-SGKYPNCRYKEKRQNKSYVVACkppQKKDSqqfhlVPVHLDrvl---<br><br>Proposed: aliSize=113 (resi) RMSD=1.85 (Å)<br>---K-ESAAAKFERQHMDSG-nsPssssnYCNLMMCcrKMTQ--GKCKPVNTFVHESLADVKAVCSQKKVTCkNG-QTNCYQSKSTMRITDCRETgSSKYPNCAYKTTQVEKHIIVACGGK--------PSVPVHFDASV<br>mkpKgMTSSQWFKIQHMQPSp--Q-----ACNSAMK--NINKhtKRCKDLNTFLHEPFSSVAATCQTPKIAC-KNgDKNCHQSHGPVSLTMCKLT-SGKYPNCRYKEKRQNKSYVVACKPPqkkdsqqfHLVPVHLDRVL
185	1bsrA (124)<br>2k11A (127)	N	70.16	TM-align: aliSize=104 (resi) RMSD=1.53 (Å)<br>kesaaakferqhmdsgnsps--------------------SSSNYCNLMMCCRKMTQGKCKPVNTFVHESLADVKAVCSQKKVTCKNGQTNCYQSKSTMRITDCRETGSSKYPNCAYKTTQVEKHIIVACGGKPSVPVHFDASV---<br>--------------------kesrakkfqrqhmdsdsspsSSSTYCNQMMRRRNMTQGRCKPVNTFVHEPLVDVQNVCFQEKVTCKNGQGNCYKSNSSMHITDCRLTNGSRYPNCAYRTSPKERHIIVACEGSPYVPVHFDASVeds<br><br>SARST: aliSize=118 (resi) RMSD=9.80 (Å)<br>k-ESAAAKFERQHMD---SGNSpSSssNYCNLMMCCRKMTQGKCKPVNTFVHESLADVKAVCSQKKVTCKNGQTNCYQSKSTMRITDCRETGSSKYPNCAYKTTQVEKHIIVACGGKPSVPVHFDA-----sv<br>-kESRAKKFQRQHMDsdsSPSS-SS--TYCNQMMRRRNMTQGRCKPVNTFVHEPLVDVQNVCFQEKVTCKNGQGNCYKSNSSMHITDCRLTNGSRYPNCAYRTSPKERHIIVACEGSPYVPVHFDAsveds--<br><br>BLAST: aliSize=101 (resi) iden=72.58% (90/124) simi=81.45% (101/124)<br>KESAAAKFERQHMDSGNSPSSSSNYCNLMMCCRKMTQGKCKPVNTFVHESLADVKAVCSQKKVTCKNGQTNCYQSKSTMRITDCRETGSSKYPNCAYKTTQVEKHIIVACGGKPSVPVHFDASV---<br>KESRAKKFQRQHMDSDSSPSSSSTYCNQMMRRRNMTQGRCKPVNTFVHEPLVDVQNVCFQEKVTCKNGQGNCYKSNSSMHITDCRLTNGSRYPNCAYRTSPKERHIIVACEGSPYVPVHFDASVeds<br><br>Proposed: aliSize=122 (resi) RMSD=1.40 (Å)<br>KESAAAKFERQHMDS--GNSPssSSNYCNLMMCCRKMTQGKCKPVNTFVHESLADVKAVCSQKKVTCKNGQTNCYQSKSTMRITDCRETGSSKYPNCAYKTTQVEKHIIVACGGKPSVPVHFDASV---<br>KESRAKKFQRQHMDSdsSPSS--SSTYCNQMMRRRNMTQGRCKPVNTFVHEPLVDVQNVCFQEKVTCKNGQGNCYKSNSSMHITDCRLTNGSRYPNCAYRTSPKERHIIVACEGSPYVPVHFDASVeds

186	1bsrA (124)<br>2p7sA (110)	N	26.36	TM-align: aliSize=85 (resi) RMSD=2.18 (Å)<br>kesaaakferqhmdsgnspss-----------------SSNYCNLMMCC--RK--MTqgkCKPVNTFVHESLADVKAVCSQkKvtckngq--TNCYQSKSTMRITDCRETgsSkypNCAYKTTQVEKHIIVACGGkpSVPVHFDASV--<br>--------------------drewekfktkhitsqsvADFNCNRTMNDpaYTpdGQ---CKPINTFIHSTTGPVKEICRR-A-------tgRVNKSSTQQFTLTTCKNP--I---RCKYSQSNTTNFICITCRD--NYPVHFVKTGkc<br><br>SARST: aliSize=98 (resi) RMSD=10.56 (Å)<br>kes--AAAKFERQHMDSGNSPSSSSNYCnlmmccrkMTQ------GKCKPVNTFVHESLADVKAVCSQKKVTCkngqtnCYQSKSTMRITDCRetgSSKYPncAYKTTQVEKHIIVACGGkpSVPVHFDA----sv<br>---drEWEKFKTKHITSQSVADFNCNRT--------MNDpaytpdGQCKPINTFIHSTTGPVKEICRRATGRV------NKSSTQQFTLTTCK---NPIRC--KYSQSNTTNFICITCRD--NYPVHFVKtgkc--<br><br>BLAST: aliSize=53 (resi) iden=32.73% (36/110) simi=48.18% (53/110)<br>kesaaa-----KFERQHMdsgNSPSSSSNYCNLMMCCRKMT-QGKCKPVNTFVHESLADVKAVCsqKKVTcknGQTNcYQSKSTMRITDCretgssKYP-NCAYKTTQVEKHIIVACggKPSVPVHF------dasv<br>------dreweKFKTKHI---TSQSVADFNCNRTMNDPAYTpDGQCKPINTFIHSTTGPVKEIC--RRAT---GRVN-KSSTQQFTLTTC------KNPiRCKYSQSNTTNFICITC--RDNYPVHFvktgkc----<br><br>Proposed: aliSize=99 (resi) RMSD=1.61 (Å)<br>ke-SAAAKFERQHMDSGnSPSSssNYCNLMMCC--RK--MTqgkCKPVNTFVHESLADVKAVCSQ----kkvtckngqtNCYQSKSTMRITDCRETgsSkypNCAYKTTQVEKHIIVACGGkpSVPVHFDASV--<br>--dREWEKFKTKHITSQ-SVAD--FNCNRTMNDpaYTpdGQ---CKPINTFIHSTTGPVKEICRRatgr---------VNKSSTQQFTLTTCKNP--I---RCKYSQSNTTNFICITCRD--NYPVHFVKTGkc
187	1cauA (181)<br>1dzrA (183)	N	10.50	TM-align: aliSize=96 (resi) RMSD=2.85 (Å)<br>aqnnpylfrsnkfltlfknqhgslrllqrfnedteklenl---RDYRVLEYCSKPNTLLLPHHS----DSDLLVLVLEGQAILVLVN-------PDGRDTYKLDQ--GDAIKIQAGTPFYLINPdNnQNLRILKFAITfrrpgTVED--<br>--------------------------------------mmiviktaipdvlilepkvfgdergfffesynqqtfeeligrkvTFVQDNHSKSKKNVLRGLHFQrgenAQGKLVRCAVGEVFDVAVDirkesptFGQWVGVNLSAenKRQLWIPEGFAHGFVTL-S-EYAEFLYKATN-----YYSPss<br><br>FFLSStkrlpsylsafsknfleasydspydeieqtllqe----------------EQEGVIVkmpk------------<br>EGSIL---------------------------------wndeaigiewpfsqlpELSAKDA----aaplldqallte<br><br>SARST: aliSize=107 (resi) RMSD=10.11 (Å)<br>aqnnpyl----------FRSNKFLTLFKNQHGSLRLLQRFNEdtekLENLRDY----RVLEYCSKPNTLLLPHHSD----SDLLVLVLEGQAILVLVNP-------DGRDTYKLD--QGDAIKIQAGTPFYLINPDNNQNLRILK--<br>-------mmiviktaipDVLILEPKVFGDERGFFFESYNQQT----FEELIGRkvtfvQDNHSKSKKNVLRGLHFQRgenaQGKLVRCAVGEVFDVAVDIrkesptfGQWVGVNLSaeNKRQLWIPEGFAHGFVTLSEYAEFLYKAtnyyspssegsilwndeaigiewpfsqlpelsakdaaaplldqa<br><br>----faitfrrpgtvedfflsstkrlpsylsafsknfleasydspydeieqtllqeeqegvivkmpk<br>llte---<br><br>BLAST: aliSize=25 (resi) iden=6.63% (12/181) simi=13.81% (25/181)<br>aqnnpylfrsnkfltlfknqhgslrllqrfnedteklenlrdyrvleycskpntlllphhsdsdllvlvlegqailvlvnpdgrdtykldqgdaikiqagtpfyl--<br>---mmiviktaipdvlilepkvfgdergfffesynqqtfeeligrkvtfvqdnhskskknvlrglhfqrgenaqgklvrcavgevfdv<br><br>------------------INPDNNQNLRILK-FAITFRRPGTVEDFFLSSTKRL-PSYLSAFSKNFLEASYDSPYDEIEQ------------------tllqeeqegvivkmpk<br>avdirkesptfgqwvgvnLSAENKRQLWIPEgFAHGFVTLSEYAEFLYKATNYYsPSSEGSILWNDEAIGIEWPFSQLPElsakdaaaplldqallte----------------<br><br>Proposed: aliSize=126 (resi) RMSD=2.39 (Å)<br>--A------qnnpYLFRSNKFLTlFKNQHGSLRLLQ---RFNEDTEKLENLrdyRVLEYCSKPNTLLLPHHS----DSDLLVLVLEGQAILVLVN-------PDGRDTYKLDQ--GDAIKIQAGTPFYLINPdNnQNLRILKFAITfrrpgTVE---DFFLS------------------stkrlpsyls<br>mmIviktai----PDVLILEPKV-FGDERGFFFESYnqqTFEELIGRKVTF--VQDNHSKSKKNVLRGLHFQrgenAQGKLVRCAVGEVFDVAVDirkesptFGQWVGVNLSAenKRQLWIPEGFAHGFVTL-S-EYAEFLYKATN-----YYSpssEGSILwndeaigiewpfsqlpel----------<br><br>afsknfleasydspydeieqtllqeeqEGVIV------------kmpk<br>---------------------------SAKDAaaplldqallte----
188	1cauA (181)<br>1j3rB (185)	C	6.63	TM-align: aliSize=123 (resi) RMSD=3.06 (Å)<br>aqn-NpYLFRS---------NKFLTLFKN----------------------QHGSLRLLQRfNedteklenl---RDYRVLEYCSKPN------TLLLPHHS-D--SDLLVLVLEGQAILVLVN-PDGRDTYKLDQGDAIKIQAGTPFYLINPdNNQNLRILKFAiTfrrpGTVEDFFLSStkrlpSyL<br>---yK-EPFGVkldfetgiiENAKKSVRRlsdmkgyfideeawkkmveegdpVVYEVYAIEQ-E---------ekeGDLNFATTVLYPGkvgnefFMTKGHYHsKidRAEVYFALKGKGGMLLQTpEGEARFIEMEPGTIVYVPPYWAHRTINT-GDKPFIFLALY-P----ADAGHDYGTI-----A-E<br><br>safsknfleasydspydeieqTLlqeeqegvivk-----MPK-----------<br>--------------------KG-----------fskivVEEngkvvvkdnpk<br><br>SARST: aliSize=100 (resi) RMSD=9.18 (Å)<br>aqnnpylfrsnkfltlfknqhgslrllqrfnedteklenlrd---YRVLEYCSKPNTL--LLP----HHS---DSDLLVLVLEGQAILVLVN-PDGRDTYKLDQGDAIKIQAGTPFYLINpDNNQNLRI<br>--ykepfgvkldfetgiienakksvrrlsdmkgyfideeawkkmveegdpvvyevyaieqeekegdLNFATTVLYPGKVgnEFFmtkgHYHskiDRAEVYFALKGKGGMLLQTpEGEARFIEMEPGTIVYVPPYWAHRTIN-TGDKPFIF<br><br>LKFAITF----------RRPGTVEDFFLSSTKrLPSYL-safsknfleasydspydeieqtllqeeqegvivkmpk<br>LALYPADaghdygtiaeKGFSKIVVEENGKVV-VKDNPk-----------------------------------<br><br>BLAST: aliSize=27 (resi) iden=9.39% (17/181) simi=14.92% (27/181)<br>aqnnpylfrsnkfltlfknqhgslrllqrfnedteklenlrdyrvleycskpntlllphhsdsdllvlvlegqailvlvnpdgrdt--------------------------------YKLDQgDAIK--IQAGTPF----YLINPDNNQNlrILKFAITFRRPGTV-EDFFLS----------------<br>---ykepfgvkldfetgiienakksvrrlsdmkgYFIDE-EAWKkmVEEGDPVvyevYAIEQEEKEG--DLNFATTVLYPGKVgNEFFMTkghyhskidraevyfal<br><br>---stkrlpsylsafsknfleasydspydeieqtllqeeqegvivkmpk<br>kgkggmllqtpegearfiemepgtivyvppywahrtintgdkpfiflalypadaghdygtiaekgfskivveengkvvvkdnpk---<br><br>Proposed: aliSize=126 (resi) RMSD=2.10 (Å)<br>aqnnp--YLFRS---------NKFLTLFKN----------------------QHGSLRLLQR----fnedteklenlRDYRVLEYCSKPN------TLLLPHHS-D--SDLLVLVLEGQAILVLVN-PDGRDTYKLDQGDAIKIQAGTPFYLINPdNNQNLRILKFAiTfrrpGTVEDFF-------ls<br>-----ykEPFGVkldfetgiiENAKKSVRRlsdmkgyfideeawkkmveegdpVVYEVYAIEQeeke-----------GDLNFATTVLYPGkvgnefFMTKGHYHsKidRAEVYFALKGKGGMLLQTpEGEARFIEMEPGTIVYVPPYWAHRTINT-GDKPFIFLALY-P----ADAGHDYgtiaekg--<br><br>stkrLPSvLSafsknfLEasYdSPYdeIEqtLL----qeeqegvivkmpk<br>----FSK-IV------VE--E-NGK--VV--VKdnpk------------

No.	Protein 1	Protein 2	Class	Score
189	1cauA (181)	1lr5B (159)	C	11.95

```
TM-align:  aliSize=129 (resi)      RMSD=2.99 (Å)
--AQNNPyLFRSN--KFLTLFKNQHGSLRLLQRFNEDTeklenLRDYRVLEYCSKPNTLLLPHHSDSDLLVLVLEGQAILVLVN-PD---G-RDTYKLDQGDAIKIQAGTPFYLINPDNNQNLRILKFAITfrrpGTVEDFFLSStkrlp-------SYLsafsknfleasydspydeieqtllqeeqeg
scVRDNS-LVRDIsqMPQSSYGIEGLSHITVAGALNHG-----MKEVEVWLQTISPGQRTPIHRHSCEEVFTVLKGKGTLLMGSsSLkypGqPQEIPFFQNTTFSIPVNDPHQVWNSDEHEDLQVLVIISR----PPAKIFLYDD-----wsmphtaAVL--------------------------------

vivkmpk--------------
-------kfpfvwdedcfeaa

SARST:     aliSize=89 (resi)       RMSD=2.34 (Å)
aqnnpylfrsnkfltlfknqhgslrllqrfnedteklenlr---------------------------------------DYRVLEYCSKPNTLLLPHHSDSDLLVLVLEGQAILVLVNPDG-----RDTYKLDQGDAIKIQAGTPFYLINPDNNQNLRILKFAitfrrPG--TVEDFFLS---------
-----------------------------------------scvrdnslvrdisqmpqssygieglshitvagalnhgmkEVEVWLQTISPGQRTPIHRHSCEEVFTVLKGKGTLLMGSSSLkypgqPQEIPFFQNTTFSIPVNDPHQVWNSDEHEDLQVLVII-----SRppAKIFLYDDwsmphtaav

---------------stkrlpsylsafsknfleasydspydeieqtllqeeqegvivkmpk
lkfpfvwdedcfeaa----------------------------------------------

BLAST:     aliSize=11 (resi)       iden=5.66% (9/159)         simi=6.92% (11/159)
aqnnpylfrsnkfltlfknqhgslrllqrfnedteklenlrdyrvleycskpntlll------------------------------------------------------------------------------------------------PH---HSD--SDLLVLVL-----------
---------------------------------------------------------scvrdnslvrdisqmpqssygieglshitvagalnhgmkevevwlqtispgqrtpihrhsceevftvlkgkgtllmgssslkypgqpqeipffqnttfsipvndPHqvwNSDehEDLQVLVIisrppakifly

---------------------------egqailvlvnpdgrdtykldqgdaikiqagtpfylinpdnnqnlrilkfaitfrrpgtvedfflsstkrlpsylsafsknfleasydspydeieqtllqeeqegvivkmpk
ddwsmphtaavlkfpfvwdedcfeaa----------------------------------------------------------------------------------------------------------

Proposed:  aliSize=137 (resi)      RMSD=2.44 (Å)
--AQNNpyLFRS-N--KFLT-lFKNQHGSLRLL-QR--FnEdteklenLRDYRVLEYCSKPNTLLLPHHSDSDLLVLVLEGQAILVLVN-PD---G-RDTYKLDQGDAIKIQAGTPFYLINPDNNQNLRILKFAITfrrpGTVEDFFLSSTKRL--PSYlsAFSkNFLEAS------ydspydeieqtll
scVRDN--SLVRdIsqMPQSs-YGIEGLSHITVaGAlnH-G-------MKEVEVWLQTISPGQRTPIHRHSCEEVFTVLKGKGTLLMGSsSLkypGqPQEIPFFQNTTFSIPVNDPHQVWNSDEHEDLQVLVIISR----PPAKIFLYDDWSMPhtAAV--LKF-PFVWDEdcfeaa-------------

qeeqegvivkmpk
-------------
```

No.	Protein 1	Protein 2	Class	Score
190	1cauA (181)	1x7nA (189)	C	5.52

```
TM-align:  aliSize=124 (resi)      RMSD=3.30 (Å)
aqnn---PYLFRS---------NKFLTLFKN-----------------------QHGSLRLLQRfNEdteklen-LRDYRVLEYCSKPN------TLLLPHHS-D--SDLLVLVLEGQAILVLVN-PDGRDTYKLDQGDAIKIQAGTPFYLINPdNNQNLRILKFAiTfrrpGTVEDFFLSStkrlpSyl
----mmyKEPFGVkvdfetgiiEGAKKSVRRlsdmegyfvderawkelvekedpVVYEVYAVEQ-EE-------kEGDLNFATTVLYPGkvgkefFFTKGHFHaKldRAEVYVALKGKGGMLLQTpEGDAKWISMEPGTVVYVPPYWAHRTVNI-GDEPFIFLAIY-P----ADAGHDYGTI-----A--

safsknfleasydspydeieqtLLqeeqegvivk------MPK-------------
----------------------EK----------gfskivIEEngevkvvdnprwk

SARST:     aliSize=89 (resi)       RMSD=16.86 (Å)
aqnnpylfrsnkfltlfknqhgslrllqrfnedteklenlr---------------------------------------DYRVLEYCSK--PNTL------LLPHHS---------------DSDLLVLVLEGQAILVLVNPDgrdtykldqgdaikiqaGTPFYLINPDNNQNLR-
-----------------------------------------mmykepfgvkvdfetgiiegakksvrrlsdmegyfvderawkelvekedpvVYEVYAVEQEekEGDLnfattvLYPGKVgkeffftkghfhaklDRAEVYVALKGKGGMLLQTPE-----------------GDAKWISMEPGTVVYVp

--ILKFAITFrrPGTVEdfflsSTKRLPSYLSAFSKNFLEA-------------------------sydspydeieqtllqeeqegvivkmpk
pyWAHRTVNI--GDEPF-----IFLAIYPADAGHDYGTIAEkgfskivieengevkvvdnprwk--------------------------

BLAST:     aliSize=12 (resi)       iden=4.97% (9/181)         simi=6.63% (12/181)
aqnnpylfrsnkfltlfknqhgslrllqrfnedteklenlrdyrvleycskpntlllphhsdsdllvlvlegqailvlvnpdgrdtykldqgdaikiqagtpfylinpdnnqnlri-------------------------------------------------------LKFAITFR
-----------------------------------------------------------------------------------------------------------------------mmykepfgvkvdfetgiiegakksvrrlsdmegyfvderawkelvekedpvvyevyaveqeekegdLNFATTVL

RPGTV-EDFFLS---------------------------------------------------------------------------------------stkrlpsylsafsknfleasydspydeieqtllqeeqegvivkmpk
YPGKVgKEFFFTkghfhakldraevyvalkgkggmllqtpegdakwismepgtvvyvppywahrtvnigdepfiflaiypadaghdygtiaekgfskivieengevkvvdnprwk--------------------------------------------

Proposed:  aliSize=132 (resi)      RMSD=2.40 (Å)
aqnnp----YLFRS---------NKFLTLFKN-----------------------QHGSLRLLQR----fnedteklenlRDYRVLEYCSKPN------TLLLPHHS-D--SDLLVLVLEGQAILVLVN-PDGRDTYKLDQGDAIKIQAGTPFYLINPdNNQNLRILKFAiTfrrpGTVEDFF-lsstkR
-----mmykEPFGVkvdfetgiiEGAKKSVRRlsdmegyfvderawkelvekedpVVYEVYAVEQeeke----------GDLNFATTVLYPGkvgkefFFTKGHFHaKldRAEVYVALKGKGGMLLQTpEGDAKWISMEPGTVVYVPPYWAHRTVNI-GDEPFIFLAIY-P----ADAGHDYg-----T

L--PSylS--afsKnfLEAsYD--SPYDeiEQTLlQEeqegvivkmpk
IaeKG--Fsk---I--VIE-ENgeVKVV--DNPR-WK-----------
```

No.	Protein 1	Protein 2	Class	Score
191	1cauA (181)	2ixkA (184)	N	9.94

```
TM-align:  aliSize=76 (resi)       RMSD=3.89 (Å)
aqnnpylfrsnkfltlfknqhgslrllqrfnedteklenlrdyrvleycs-------------------------------------------KPNTLLLPHHSD-SDLLVLVL---EGQAILVLV--------------------------NPDGRDTYKLDQGDAIKIQA---GTPFYLINPdnn
--------------------------------------------------smsmkatrlaipdvilfeprvfgddrgfffesynqrafeeacghpvSFVQDNHSRSARgVLRGLHYQirqAQGKLVRATlgevfdvavdlrrgsptfgqwvgerlSAENKRQMWIPAGFAHGFVVlseYAEFLYKTT---

QNLR---ILKFAI------------TFRRPGTVedfflsstkrlpsylsafsknfleasydspydeieqtllqeeqegvivkmpk-------------
DFWApehERCIVWndpelkidwplqDAPLLSEK-------------------------------------------------drqgkafadadcfp

SARST:     aliSize=139 (resi)      RMSD=12.65 (Å)
aqnnpylf---------------RSNKFLTlfKNQHGSLRLLQRFNEDteklENLRDY-----RVLEYCSKPNTLLLPHHSDS---DLLVLVLEGQAILVLVNP-------DGRDTYKLDQ--GDAIKIQAGTPFYLINPDNNQNLRILK-FAITFRRPgtvEDFFL-------SSTKRLPSylsaFSKN
--------smsmkatrlaipdviLFEPRVF--GDDRGFFFESYNQRAF----EEACGHpvsfvQDNHSRSARGVLRGLHYQIRqaqGKLVRATLGEVFDVAVDLrrgsptfGQWVGERLSAenKRQMWIPAGFAHGFVVLSEYAEFLYKTtDFWAPEHE---RCIVWndpelkiDWPLQDAP----LLSE

FLeaSYDSPYDE-----ieqtllqeeqegvivkmpk
KD--RQGKAFADadcfp-------------------

BLAST:     aliSize=36 (resi)       iden=9.39% (17/181)        simi=19.89% (36/181)
aqnnpylfrsnkfltlfknqhgslrllqrfnedteklenlrdyrvleycskpntlllphhsdsdllvlvlegqailvlvnpdgrdtykldqgd---------------------------------------------------------------------------------------------AIKIQAGTPFY
-----------------------------------------------------------------------------------------------smsmkatrlaipdvilfeprvfgddrgfffesynqrafeeacghpvsfvqdnhsrsargvlrglhyqirqaqgklvratlgevfdvAVDLRRGSPTF

------LINPDNNQNLRI-LKFAITFRRPGTVEDFFLSSTK-RLPSYLSAFSKNFLEASYDSPYDEiEQTLLQEEQEG----------vivkmpk
gqwvgeRLSAENKRQMWIpAGFAHGFVVLSEYAEFLYKTTDfWAPEHERCIVWNDPELKIDWPLQD-APLLSEKDRQGkafadadcfp-------

Proposed:  aliSize=127 (resi)      RMSD=2.24 (Å)
----AQNN---PYLFRSnKFLTlFKNQHGSLRLL-Q--RFNEDTEKlENL-rdYRVLEYCSKPNTLLLPHHS---DSDLLVLVLEGQAILVLVN-------PDGRDTYKLDQ--GDAIKIQAGTPFYLINPdNnQNLRILKFAITfrrpgTVE---DFFLS------------------stkrlpsylsa
smsmKATRlaiPDVILF-EPRV-FGDDRGFFFESyNqrAFEEACGH-PVSf--VQDNHSRSARGVLRGLHYQirqAQGKLVRATLGEVFDVAVDlrrgsptFGQWVGERLSAenKRQMWIPAGFAHGFVVL-S-EYAEFLYKTTD-----FWApehERCIVwndpelkidwplqdapll-----------

fsknfleasydspydeieqtllqeeqEGVI-------------vkmpk
---------------------------SEKDrqgkafadadcfp-----
```

<table>
<tr><td rowspan="2">192</td><td>1cauA
(181)</td><td rowspan="2">N</td><td rowspan="2">11.19</td><td rowspan="2">TM-align: aliSize=90 (resi) RMSD=2.66 (Å)
aqnnpylfrsnkfltlfknqhgslrllqrfnedteklenl----------------------------RDYRVLEYC-SKPNTLLLPHHS-DSDLLVLVLEGQAILVLVNPDGRDTYKLDQG-DAIKIQAGTPFYLINPdNnQNLRILKFAITfrrpgTVED--FFLSstkrlpSy-Lsafsknfleasy
--enkvinfkkiidsrgslvaieenknipfSIKRVYYIFdTKGEEPRGFHANkKLEQVLVCLNGSCRVILDDGNIIQEITLDSPaVGLYVGPAVWHEMHDF-S-SDCVMMVLASD-----YYDEtdYIRQ------Y-dN------------

dspydeieqtllqeeqegvivkmpk----------
-------------------------fkkyiakinl

SARST: aliSize=102 (resi) RMSD=10.36 (Å)
aqnnpyl--FRSNKFLTlfKNQHGSLRLLQRfNEDTeklenlR---DYRVLEYCSKPNTLLLPHHSDSDL-LVLVLEGQAILVLVNPDGRDTYKLDQG-DAIKIQAGTPFYLINPDNNQNLRILK------------------------faitfrrpgtvedfflsstkrlpsylsafsknfleasyds
-------enKVINFKKI--IDSRGSLVAIEE-NKNI------PfsiKRVYYIFDTKGEEPRGFHANKKLEqVLVCLNGSCRVILDDGNIIQEITLDSPaVGLYVGPAVWHEMHDFSSDCVMMVLAsdyydetdyirqydnfkkyiakinl--

pydeieqtllqeeqegvivkmpk

BLAST: aliSize=16 (resi) iden=7.46% (10/134) simi=11.94% (16/134)
aqnnpylfrsnkfltlfknqhgslrllqrfnedteklenlrdyrvleycskpntlllphhsdsdllvlvleg----------------------------------QAILVLVNpdGRDTYKLDQGDAI-KIQAGTP-------------------------------------
--enkvinfkkiidsrgslvaieenknipfsikrvyyifdtkgeeprgfhankklEQVLVCLN--GSCRVILDDGNIIqEITLDSPavglyvgpavwhemhdfssdcvmmvlasdyydet

------------------fylinpdnnqnlrilkfaitfrrpgtvedfflsstkrlpsylsafsknfleasydspydeieqtllqeeqegvivkmpk
dyirqydnfkkyiakinl---

Proposed: aliSize=113 (resi) RMSD=2.32 (Å)
aqnnpYLFRSnKFLTlFKNQHGSLRLL----qRFNedteklenLRDYRVLEYC-SKPNTLLLPHHS-DSDLLVLVLEGQAILVLVNPDGRDTYKLDQG-DAIKIQAGTPFYLINPdNnQNLRILKFAITfrrpgTVE--DFFLS--stkrlpsyL----------safsknfleasydspydeieqtllq
-----ENKVI-NFKK-IIDSRGSLVAIeenk-NIP--------FSIKRVYYIFdTKGEEPRGFHANkKLEQVLVCLNGSCRVILDDGNIIQEITLDSPaVGLYVGPAVWHEMHDF-S-SDCVMMVLASD-----YYDetDYIRQyd--------Nfkkyiakinl------------------------

eeqegvivkmpk
------------</td></tr>
<tr><td>2pakB
(134)</td></tr>
<tr><td rowspan="2">193</td><td>1cauA
(181)</td><td rowspan="2">N</td><td rowspan="2">9.63</td><td rowspan="2">TM-align: aliSize=90 (resi) RMSD=2.63 (Å)
aqnnpylfrsnkfltlfknqhgslrllqrfnedteklenl----------------------------RDYRVLEYC-SKPNTLLLPHHS-DSDLLVLVLEGQAILVLVNPDGRDTYKLDQG-DAIKIQAGTPFYLINPdNnQNLRILKFAITfrrpgTVED--F-FLSstkrlpsYLsafsknfleasy
--enkvinfkkiidsrgslvaieenknipfSIKRVYYIFdTKGEEPRGFNANkKLEQVLVCLNGSCRVILDDGNIIQEITLDSPaVGLYVGPAVWHEMHDF-S-SDCVMMVLASD-----YYDEtdYiRQY-------DN------------

dspydeieqtllqeeqegvivkmpk-----------
-------------------------fkkyiakinle

SARST: aliSize=94 (resi) RMSD=9.02 (Å)
aqnnpylfrsnkfltl---------FKNQHGSLRLLQRfNEDTeklenlrDYRVLEYCSKPNTL----LLPHHS-DSDLLVLVLEGQAILVLVNPDGRDTYKLDQ-GDAIKIQAGTPFYLINPDNNQNLRILK---------------------------faitfrrpgtvedfflsstkrlpsylsafsk
----------------enkvinfkkIIDSRGSLVAIEE-NKNI-------PFSIKRVYYIFDTKgeepRGFNANkKLEQVLVCLNGSCRVILDDGNIIQEITLDSpAVGLYVGPAVWHEMHDFSSDCVMMVLAsdyydetdyirqydnfkkyiakinle--------------------------------

nfleasydspydeieqtllqeeqegvivkmpk

BLAST: aliSize=16 (resi) iden=7.41% (10/135) simi=11.85% (16/135)
aqnnpylfrsnkfltlfknqhgslrllqrfnedteklenlrdyrvleycskpntlllphhsdsdllvlvleg----------------------------------QAILVLVNpdGRDTYKLDQGDAI-KIQAGTP-------------------------------------
--enkvinfkkiidsrgslvaieenknipfsikrvyyifdtkgeeprgfnankklEQVLVCLN--GSCRVILDDGNIIqEITLDSPavglyvgpavwhemhdfssdcvmmvlasdyydet

------------------fylinpdnnqnlrilkfaitfrrpgtvedfflsstkrlpsylsafsknfleasydspydeieqtllqeeqegvivkmpk
dyirqydnfkkyiakinle--

Proposed: aliSize=112 (resi) RMSD=2.14 (Å)
aqnnpy-LFRSnKFLTlFKNQHGSLRLL---QRFNedteklenLRDYRVLEYC-SKPNTLLLPHHS-DSDLLVLVLEGQAILVLVNPDGRDTYKLDQG-DAIKIQAGTPFYLINPdNnQNLRILKFAITfrrpgTVE---DFFL--sstkrlpsyL-----------safsknfleasydspydeieqtl
------eNKVI-NFKK-IIDSRGSLVAIeenKNIP--------FSIKRVYYIFdTKGEEPRGFNANkKLEQVLVCLNGSCRVILDDGNIIQEITLDSPaVGLYVGPAVWHEMHDF-S-SDCVMMVLASD-----YYDetdYIRQyd---------Nfkkyiakinle----------------------

lqeeqegvivkmpk
--------------</td></tr>
<tr><td>2pamA
(135)</td></tr>
<tr><td rowspan="2">194</td><td>1cm0B
(161)</td><td rowspan="2">M</td><td rowspan="2">6.83</td><td rowspan="2">TM-align: aliSize=145 (resi) RMSD=3.18 (Å)
KVIEFHVVGnSlnqKpNkkILMWLVGLQNVFSHQLPRMPKEYITRLVfDpKHKTLALIKDGRVIGGICFRMFP------SQGFTEIVFCAVTSNEQVKGYGTHLMNHLKEYHIKHdILNFLTYADEYAIGYFKKQGFSKEIKiPKtkYVGYik------DYEG-ATLMGCELNp-------------------
VHTARLVHT-A---D-L--DSETRQDIRQMVTGAFAGDFTETDWEHT-L-GGMHALIWHHGAIIAHAAVIQRRliyrgnALRCGYVEGVAVRADWRGQRLVSALLDAVEQVMRGA-YQLGALSSSARARRLYASRGWLPWHG-PT--SVLA--ptgpvrTPDDdGTVFVLPID-isldtsaelmcdwragdvw

SARST: aliSize=127 (resi) RMSD=13.22 (Å)
kviefhvvgnslnqkpnkki--------------LMWLVGLQNVFSHQLPRMPKEYITRLV-----------FDPKHKTL----ALIKDGRVIGGICFRMfpsqgfteivfCAVTSNEQVKGyGTHLMNHLKEYHIKHDILNFLTYADEYAIGYFKKQGFSkEIK-----------IPKTKYVGY-IKDYE
--------------------vhtarlvhtadldSETRQDIRQMVTGAFAGDFTETDWEHTlggmhaliwhhGAIIAHAAviqrRLIYRGNALRCGYVEG-----------VAVRADWRGQR-LVSALLDAVEQVMRGAYQLGALSSSARARRLYASRGWL-PWHgptsvlaptgpVRTPDDDGTvFVLPI

GATL---MGCELN-------p
DISLdtsAELMCDwragdvw-

BLAST: aliSize=11 (resi) iden=3.73% (6/161) simi=6.83% (11/161)
kviefhvvgnslnqkpnkkilmwlvglqnvfshqlprmpkeyitrlvfdpkhktlalikdgrviggicfrmfpsqgfteivfcavtsneqvkgygthlmnhlkeyhikhdilnfltyadeyaigyfkkqgfskeikipktkyv---
---vhtarlvhtadldsetrqdirqmvtgafagdftetdwehtlggmhal

----------------------------GYIK------DYEGATLMGCELN--p
iwhhgaiiahaaviqrrliyrgnalrcGYVEgvavraDWRGQRLVSALLDaveqvmrgayqlgalsssararrlyasrgwlpwhgptsvlaptgpvrtpdddgtvfvlpidisldtsaelmcdwragdvw-

Proposed: aliSize=142 (resi) RMSD=2.43 (Å)
kv-IEFHVVGNSlnqKpNkkILMWLVGLQNVFSHQLPRMPKEYITRLVfDpKHKTLALIKDGRVIGGICFRMFpS-------QGFTEIVFCAVTSNEQVKGYGTHLMNHLKEYHIKHdILNFLTYADEYAIGYFKKQGFSKEI------kipKTKyVGyIKDY--EGaTLMGCELN-------------
--vHTARLVHTA---D-L--DSETRQDIRQMVTGAFAGDFTETDWEHT-L-GGMHALIWHHGAIIAHAAVIQR-RliyrgnaLRCGYVEGVAVRADWRGQRLVSALLDAVEQVMRGA-YQLGALSSSARARRLYASRGWLPWHgptsvl---APT-GP-VRTPddDG-TVFVLPIDisldtsaelmcdwr

-----pc
agdvw--</td></tr>
<tr><td>1m44A
(177)</td></tr>
</table>

195	1cm0B (161)<br>1s3zB (145)	C	8.97	TM-align: aliSize=125 (resi) RMSD=2.77 (Å)<br>kvIEFHVVgnslnqkpnkkILMWLVGLQNVFSHQLPrMPKEYIT-RLVFD--PK--HKTLALIKDGRVIGGICFRMFPS-------QGFTEIVFCAVTSNEQVKGYGTHLMNHLKEYHIKHDILNFLTYAD-EY--AIGYFKKQGFSKEIkipktkyvgyik-DYEGatlmgcelnp----<br>--MDIRQM----------NKTHLEHWRGLRKQLWP-GHPDDAHlADGEEilQAdhLASFIAMADGVAIGFADASIRHDyvngcdsSPVVFLEGIFVLPSFRQRGVAKQLIAAVQRWGTNKGCREMASDTSpENtiSQKVHQALGFEETE------------rVIFY----------rkrc<br><br>SARST: aliSize=112 (resi) RMSD=3.18 (Å)<br>kviefhvvgnslnqkpnkki-------LMWLVGLQNVFSHQLPRMPK----EYITRLVFDPKHKTLALIKDGRVIGGICFRMFPSQG-------FTEIVFCAVTSNEQVKGYGTHLMNHLKEYHIKHDILNFLTYADEY---AIGYFKKQGFS-----------keikipktkyvgyikdyegatlmgc<br>--------------------mdirqmnKTHLEHWRGLRKQLWPGHPDdahlADGEEILQADHLASFIAMADGVAIGFADASIRHDYVngcdsspVVFLEGIFVLPSFRQRGVAKQLIAAVQRWGTNKGCREMASDTSPEntiSQKVHQALGFEetervifyrkrc-----------------------<br>elnp<br>----<br><br>BLAST: aliSize=16 (resi) iden=6.90% (10/145) simi=11.03% (16/145)<br>kviefhvvgnslnqkpnkkilmwlvglqnvfshqlprmpkeyitrlvfdpkhktlalikdgrviggicfrmfpsqgfteivfcavtsneqvkgygt-----------------------------HLMNHLKEYHIKHdILNFLTYADEYAIGyfkkqgFS-------------------------------<br>--mdirqmnkthlehwrglrkqlwpghpddaHLADGEEILQADH-LASFIAMADGVAIG------FAdasirhdyvngcdsspvvflegifvlpsf<br>---keikipktkyvgyikdyegatlmgcelnp<br>rqrgvakqliaavqrwgtnkgcremasdtspentisqkvhqalgfeetervifyrkrc----------------------------<br><br>Proposed: aliSize=130 (resi) RMSD=2.14 (Å)<br>kvIEFHVVgnslnqkpnkkILMWLVGLQNVFSHQLPRMPKEYITRLVFD--PK--HKTLALIKDGRVIGGICFRMFpS--------QGFTEIVFCAVTSNEQVKGYGTHLMNHLKEYHIKHDILNFLTYAD-EY--AIGYFKKQGFSKEikipktkyvgyikdyegATLMGCELNP-c<br>--MDIRQM-----------NKTHLEHWRGLRKQLWPGHPDDAHLADGEEilQAdhLASFIAMADGVAIGFADASIR-HdyvngcdsSPVVFLEGIFVLPSFRQRGVAKQLIAAVQRWGTNKGCREMASDTSpENtiSQKVHQALGFEET-----------------ERVIFYRKRCc-
196	1cm0B (161)<br>1s5kA (153)	C	7.84	TM-align: aliSize=126 (resi) RMSD=2.90 (Å)<br>------KVIEFHVVgnslnqkpnkkILMWLVGLQNVFSHqLPrMPKE-YIT-RLVFD--PK--HKTLALIKDGRVIGGICFRMFPS-------QGFTEIVFCAVTSNEQVKGYGTHLMNHLKEYHIKHDILNFLTYAD-EY--AIGYFKKQGFSKEIkipktkyvgyik-DYEGatlmgcelnp----<br>glvprgSHMDIRQM----------NKTHLEHWRGLRKQ-LW-PGHPdDAHlADGEEilQAdhLASFIAMADGVAIGFADASIRHDyvngcdsSPVVFLEGIFVLPSFRQRGVAKQLIAAVQRWGTNKGCREMASDTSpENtiSQKVHQALGFEETE------------rVIFY----------rkrc<br><br>SARST: aliSize=112 (resi) RMSD=3.20 (Å)<br>kviefhvvgnslnqkpnkki---------------LMWLVGLQNVFSHQLPRMPKEYITRLV----FDPKHKTLALIKDGRVIGGICFRMFPS--QG-----FTEIVFCAVTSNEQVKGYGTHLMNHLKEYHIKHDILNFLTYADEY---AIGYFKKQGFS-----------keikipktkyvgyikdy<br>-------------------glvprgshmdirqmnKTHLEHWRGLRKQLWPGHPDDAHLADGeeilQADHLASFIAMADGVAIGFADASIRHDyvNGcdsspVVFLEGIFVLPSFRQRGVAKQLIAAVQRWGTNKGCREMASDTSPEntiSQKVHQALGFEetervifyrkrc-----------------<br>egatlmgcelnp<br>------------<br><br>BLAST: aliSize=16 (resi) iden=6.54% (10/153) simi=10.46% (16/153)<br>kviefhvvgnslnqkpnkkilmwlvglqnvfshqlprmpkeyitrlvfdpkhktlalikdgrviggicfrmfpsqgfteivfcavtsneqvkgygt-------------------------------------HLMNHLKEYHIKHdILNFLTYADEYAIGyfkkqgFS--------------------<br>--glvprgshmdirqmnkthlehwrglrkqlwpghpddaHLADGEEILQADH-LASFIAMADGVAIG------FAdasirhdyvngcdsspvvfle<br>---keikipktkyvgyikdyegatlmgcelnp<br>gifvlpsfrqrgvakqliaavqrwgtnkgcremasdtspentisqkvhqalgfeetervifyrkrc----------------------------<br><br>Proposed: aliSize=130 (resi) RMSD=2.15 (Å)<br>------KVIEFHVVgnslnqkpnkkILMWLVGLQNVFSHQLPrmPKE--YITRLVFD--PK--HKTLALIKDGRVIGGICFRMFpS--------QGFTEIVFCAVTSNEQVKGYGTHLMNHLKEYHIKHDILNFLTYAD-EY--AIGYFKKQGFSKEikipktkyvgyikdyegATLMGCELNP-c<br>glvprgSHMDIRQM----------NKTHLEHWRGLRKQLWP--GHPddAHLADGEEilQAdhLASFIAMADGVAIGFADASIR-HdyvngcdsSPVVFLEGIFVLPSFRQRGVAKQLIAAVQRWGTNKGCREMASDTSpENtiSQKVHQALGFEET-----------------ERVIFYRKRCc-
197	1cm0B (161)<br>2vbqB (144)	C	8.33	TM-align: aliSize=124 (resi) RMSD=2.81 (Å)<br>kviEFHVVgnslnqkpnkkILMWLVGLQNVFSHQLPrMPKEYIT-RLVFD--PK--HKTLALIKDGRVIGGICFRMFPS-------QGFTEIVFCAVTSNEQVKGYGTHLMNHLKEYHIKHDILNFLTYAD-EY--AIGYFKKQGFSKEIkipktkyvgyik-DYEGatlmgcelnp----<br>---DIRQM----------NKTHLEHWRGLRKQLWP-GHPDDAHlADGEEilQAdhLASFIAMADGVAIGFADASIRHDyvngcdsSPVVFLEGIFVLPSFRQRGVAKQLIAAVQRWGTNKGCREMASDTSpENtiSQKVHQALGFEETE------------rVIFY----------rkrc<br><br>SARST: aliSize=112 (resi) RMSD=3.66 (Å)<br>kviefhvvgnslnqkpnkki------LMWLVGLQNVFSHQLPRMP----KEYITRLVFDPKHKTLALIKDGRVIGGICFRMFP----SQGFT---EIVFCAVTSNEQVKGYGTHLMNHLKEYHIKHDILNFLTYADEY---AIGYFKKQGFS-----------keikipktkyvgyikdyegatlmgcelnp<br>--------------------dirqmnKTHLEHWRGLRKQLWPGHPddahLADGEEILQADHLASFIAMADGVAIGFADASIRHdyvnGCDSSpvvFLEGIFVLPSFRQRGVAKQLIAAVQRWGTNKGCREMASDTSPEntiSQKVHQALGFEetervifyrkrc------------------------------<br><br>BLAST: aliSize=16 (resi) iden=6.94% (10/144) simi=11.11% (16/144)<br>kviefhvvgnslnqkpnkkilmwlvglqnvfshqlprmpkeyitrlvfdpkhktlalikdgrviggicfrmfpsqgfteivfcavtsneqvkgygt-----------------------------HLMNHLKEYHIKHdILNFLTYADEYAIGyfkkqgFS------------------------------<br>--dirqmnkthlehwrglrkqlwpghpddaHLADGEEILQADH-LASFIAMADGVAIG------FAdasirhdyvngcdsspvvflegifvlpsfr<br>--keikipktkyvgyikdyegatlmgcelnp<br>qrgvakqliaavqrwgtnkgcremasdtspentisqkvhqalgfeetervifyrkrc-----------------------------<br><br>Proposed: aliSize=128 (resi) RMSD=2.10 (Å)<br>kviEFHVVgnslnqkpnkkILMWLVGLQNVFSHQLPrMPKE-YITRLVFD--PK--HKTLALIKDGRVIGGICFRMFpS--------QGFTEIVFCAVTSNEQVKGYGTHLMNHLKEYHIKHDILNFLTYAD-EY--AIGYFKKQGFSKEikipktkyvgyikdyegATLMGCELNPc<br>---DIRQM-----------NKTHLEHWRGLRKQLWP-GHPDdAHLADGEEilQAdhLASFIAMADGVAIGFADASIR-HdyvngcdsSPVVFLEGIFVLPSFRQRGVAKQLIAAVQRWGTNKGCREMASDTSpENtiSQKVHQALGFEET-----------------ERVIFYRKRC-

198	1d7eA (119)<br>1p97A (114)	N	4.39	TM-align: aliSize=93 (resi) RMSD=2.17 (Å)<br>vafgsedientlakmddgqldgl-----AFGAIQLDGDGNILQYNAAEGDITGRDPKQVIGKNFFkDVAPC-TDSpEFYGKFKEGVaSGNLNTMFEYTFDY-QMTPTKVKVHMKKAL--------SYWVFVKRV------<br>-----------------------gamdsKTFLSRHSMDMKFTYCDDRITELIGYHPEELLGRSAY-EFYHAlDSE-NMTKSHQNLC-TKGQVVSGQYRMLAkHGGYVWLETQGTVIYnprnlqpqCIMCVNYVLseiekn<br><br>SARST: aliSize=87 (resi) RMSD=3.69 (Å)<br>vafgsedientlakmddgqldgla-------FGAIQLDGdGNILQYNAAEGDITGRDPKQVIGKNFfkdvaPCTDSPEFYGKFKEGVAS--GNLNTMFEYTFdYQM---TPTKVKVHMK--------KALSYWVFVKR-------v<br>------------------------gamdsktFLSRHSMD-MKFTYCDDRITELIGYHPEELLGRSA-----YEFYHALDSENMTKSHQNlcTKGQVVSGQYR-MLAkhgGYVWLETQGTviynprnlQPQCIMCVNYVlseiekn-<br><br>BLAST: aliSize=16 (resi) iden=8.77% (10/114) simi=14.04% (16/114)<br>vafgsedien---TLAKMDDGQLDGLA--FGAIQLDGDGNILqYN----------------------aaegditgrdpkqvigknffkdvapctdspefygkfkegvasgnlntmfeytfdyqmtptkvkvh<br>----------gamdsktflsrhsmdmkftycddriteligyhpeellgrsayefyhaldsenmtkshqnlcTKGQVVSGQYRMLAkhGGYVWLETQGTVI-YNprnlqpqcimcvnyvlseiekn---<br><br>mkkalsywvfvkrv<br>--------------<br><br>Proposed: aliSize=97 (resi) RMSD=2.12 (Å)<br>VafgsedienTLAkM-ddgqldglaFGAIQLDGDGNILQYNAAEGDITGRDPKQVIGKNFFkDVAPC-TDSpEFYGKFKEGVaSGNLNTMFEYTFDY-QMTPTKVKVHMKKAL--------SYWVFVKRV------<br>G---------AMD-Sk---------TFLSRHSMDMKFTYCDDRITELIGYHPEELLGRSAY-EFYHAlDSE-NMTKSHQNLC-TKGQVVSGQYRMLAkHGGYVWLETQGTVIYnprnlqpqCIMCVNYVLseiekn
199	1d7eA (119)<br>1s67L (119)	N	13.45	TM-align: aliSize=102 (resi) RMSD=2.15 (Å)<br>vafgsedientla-----KMDdGQLDGLAFGAIQLDGDGNILQYNAAEGDITGRDPKQVIGKNFFkdVAPC--TDSPeFYGKFKEGVASG---NLNTMFEYTFDYQM-TPTKVKVHMKKAL-----SYWVFVKRV-<br>-------------naadgIFF-PALEQNMMGAVLINENDEVMFFNPAAEKLWGYKREEVIGNNID--MLIPrdLRPA-HPEYIRHNREGGkarVEGMSRELQLEKKDgSKIWTRFALSKVSaegkvYYLALVRDAs<br><br>SARST: aliSize=105 (resi) RMSD=6.64 (Å)<br>vafgsedient-LAKMDDGQLD-GLAFGAIQLDGDGNILQYNAAEGDITGRDPKQVIGKNFFKDVAPCTdsPEFYGKFKEGVAS------GNLNTMFEYTFDYQ----MTPTKVKVHMKKALSYWVFVKR--v<br>------------nAADGIFFPALeQNMMGAVLINENDEVMFFNPAAEKLWGYKREEVIGNNIDMLIPRDL--RPAHPEYIRHNREggkarvEGMSRELQLEKKDGskiwTRFALSKVSAEGKVYYLALVRDas-<br><br>BLAST: aliSize=29 (resi) iden=12.61% (15/119) simi=24.37% (29/119)<br>vafgsedientlakmddgqldglaf----------------GAIQLDGDGNILQYNAAEGDITGRDPKQVIGKNFF----KDVAPCtdSPEFYGKFKEG---vasgnlntmfeytfdyqmtptkvkvhmkkalsywvfvkrv<br>-------------------------naadgiffpaleqnmmGAVLINENDEVMFFNPAAEKLWGYKREEVIGNNIDmlipRDLRPA--HPEYIRHNREGgkarvegmsrelqlekkdgskiwtrfalskvsaegkvyylalvrdas--<br><br>Proposed: aliSize=108 (resi) RMSD=2.24 (Å)<br>vAFgSEDieNTLAKmddgqLDGLAFGAIQLDGDGNILQYNAAEGDITGRDPKQVIGKNFFkDVAPC-TDSPeFYGKFKEGVASG--N-LNTMFEYTFDYQM-TPTKVKVHMKKAL-----SYWVFVKRV--<br>-NA-ADG--IFFPA-----LEQNMMGAVLINENDEVMFFNPAAEKLWGYKREEVIGNNID-MLIPRdLRPA-HPEYIRHNREGGkaRvEGMSRELQLEKKDgSKIWTRFALSKVSaegkvYYLALVRDAsh
200	1d7eA (119)<br>1s67U (115)	N	14.78	TM-align: aliSize=102 (resi) RMSD=2.17 (Å)<br>vafgsedientla-KMDdGQLDGLAFGAIQLDGDGNILQYNAAEGDITGRDPKQVIGKNFFkdVAPC--TDSPeFYGKFKEGVASGN-LNT--MFEYTFDYQM-TPTKVKVHMKKAL-----SYWVFVKRV-<br>-------------gIFF-PALEQNMMGAVLINENDEVMFFNPAAEKLWGYKREEVIGNNID--MLIPrdLRPA-HPEYIRHNREGGKaRVEgmSRELQLEKKDgSKIWTRFALSKVSaegkvYYLALVRDAs<br><br>SARST: aliSize=94 (resi) RMSD=7.58 (Å)<br>vafgsedientlakmd---DGQLDGLAFGAIQLDGDGNILQYNAAEGDITGRDPKQVIGKNFFKdvapcTDSPEFYGKFKEGVAS-----------GNLNTMF-----EYTFdyqMTPTKVKVHMKKALSYWVFVKR--v<br>----------------gifFPALEQNMMGAVLINENDEVMFFNPAAEKLWGYKREEVIGNNIDM-----LIPRDLRPAHPEYIRHnreggkarvegMSRELQLekkdgSKIW---TRFALSKVSAEGKVYYLALVRDas-<br><br>BLAST: aliSize=29 (resi) iden=13.04% (15/115) simi=25.22% (29/115)<br>vafgsedientlakmddgqldglaf------------GAIQLDGDGNILQYNAAEGDITGRDPKQVIGKNFF----KDVAPCtdSPEFYGKFKEG---vasgnlntmfeytfdyqmtptkvkvhmkkalsywvfvkrv<br>-------------------------giffpaleqnmmGAVLINENDEVMFFNPAAEKLWGYKREEVIGNNIDmlipRDLRPA--HPEYIRHNREGgkarvegmsrelqlekkdgskiwtrfalskvsaegkvyylalvrdas--<br><br>Proposed: aliSize=104 (resi) RMSD=2.16 (Å)<br>VAfgsedIenTLakmddgQLDGLAFGAIQLDGDGNILQYNAAEGDITGRDPKQVIGKNFFkDVAPC-TDSPeFYGKFKEGVASGN-LNT-MF-EYTFDYQM-TPTKVKVHMKKAL-----SYWVFVKRV--<br>GI-----F--FP------ALEQNMMGAVLINENDEVMFFNPAAEKLWGYKREEVIGNNID-MLIPRdLRPA-HPEYIRHNREGGKaRVEgMSrELQLEKKDgSKIWTRFALSKVSaegkvYYLALVRDAsh

201	1d7eA (119)<br>1v9zA (113)	N	14.16	TM-align: aliSize=101 (resi) RMSD=2.13 (Å)<br>vafgsedientla-KMDdGQLDGLAFGAIQLDGDGNILQYNAAEGDITGRDPKQVIGKNFFkdVAPC--TDSPeFYGKFKEGVASGN-LNT--MFEYTFDYQM-TPTKVKVHMKKAL-----SYWVFVKRv<br>-------------gIFF-PALEQNMMGAVLINENDEVMFFNPAAEKLWGYKREEVIGNNID--MLIPrdLRPA-HPEYIRHNREGGKaRVEgmSRELQLEKKDgSKIWTRFALSKVSaegkvYYLALVRD-<br><br>SARST: aliSize=98 (resi) RMSD=3.37 (Å)<br>vafgsedientlakmd---DGQLDGLAFGAIQLDGDGNILQYNAAEGDITGRDPKQVIGKNFFKDVAPCTdsPEFYGKFKEGVASGNLNTM----FEYTFDY--QmTPTKVKVHMKK-----ALSYWVFVK-rv<br>----------------gifFPALEQNMMGAVLINENDEVMFFNPAAEKLWGYKREEVIGNNIDMLIPRDL--RPAHPEYIRHNREGGKARVegmsRELQLEKkdG-SKIWTRFALSKvsaegKVYYLALVRd--<br><br>BLAST: aliSize=29 (resi) iden=13.27% (15/113) simi=25.66% (29/113)<br>vafgsedientlakmddgqldglaf------------GAIQLDGDGNILQYNAAEGDITGRDPKQVIGKNFF----KDVAPCtdSPEFYGKFKEG---vasgnlntmfeytfdyqmtptkvkvhmkkalsywvfvkrv<br>-------------------------giffpaleqnmmGAVLINENDEVMFFNPAAEKLWGYKREEVIGNNIDmlipRDLRPA--HPEYIRHNREGgkarvegmsrelqlekkdgskiwtrfalskvsaegkvyylalvrd--<br><br>Proposed: aliSize=103 (resi) RMSD=2.10 (Å)<br>vafgsedienTLaKMDdgQLDGLAFGAIQLDGDGNILQYNAAEGDITGRDPKQVIGKNFFkDVAPC-TDSPeFYGKFKEGVASGN--LNT-MFEYTFDYQM-TPTKVKVHMKKAL-----SYWVFVKR-v<br>----------GI-FFP--ALEQNMMGAVLINENDEVMFFNPAAEKLWGYKREEVIGNNID-MLIPRdLRPA-HPEYIRHNREGGKarVEGmSRELQLEKKDgSKIWTRFALSKVSaegkvYYLALVRDh-
202	1d7eA (119)<br>1vb6A (114)	N	14.04	TM-align: aliSize=102 (resi) RMSD=2.16 (Å)<br>vafgsedientla-KMDdGQLDGLAFGAIQLDGDGNILQYNAAEGDITGRDPKQVIGKNFFkdVAPC--TDSPeFYGKFKEGVASGN-LNT--MFEYTFDYQM-TPTKVKVHMKKAL-----SYWVFVKRV<br>-------------gIFF-PALEQNMMGAVLINENDEVMFFNPAAEKLWGYKREEVIGNNID--MLIPrdLRPA-HPEYIRHNREGGKaRVEgmSRELQLEKKDgSKIWTRFALSKVSaegkvYYLALVRDA<br><br>SARST: aliSize=94 (resi) RMSD=4.06 (Å)<br>vafgsedientlakmd---DGQLDGLAFGAIQLDGDGNILQYNAAEGDITGRDPKQVIGKNFfkdvaPCTDSPEFYGKFKEGVAS-----------GNlnTMFEYTFDYQmTPTKVKVHMKK-----ALSYWVFVKR-v<br>----------------gifFPALEQNMMGAVLINENDEVMFFNPAAEKLWGYKREEVIGNNI-----DMLIPRDLRPAHPEYIRHnreggkarvegMS--RELQLEKKDG-SKIWTRFALSKvsaegKVYYLALVRDa-<br><br>BLAST: aliSize=29 (resi) iden=13.16% (15/114) simi=25.44% (29/114)<br>vafgsedientlakmddgqldglaf------------GAIQLDGDGNILQYNAAEGDITGRDPKQVIGKNFF----KDVAPCtdSPEFYGKFKEG--vasgnlntmfeytfdyqmtptkvkvhmkkalsywvfvkrv<br>-------------------------giffpaleqnmmGAVLINENDEVMFFNPAAEKLWGYKREEVIGNNIDmlipRDLRPA--HPEYIRHNREGgkarvegmsrelqlekkdgskiwtrfalskvsaegkvyylalvrda--<br><br>Proposed: aliSize=104 (resi) RMSD=2.10 (Å)<br>vafgsedienTLAKMddgQLDGLAFGAIQLDGDGNILQYNAAEGDITGRDPKQVIGKNFFkDVAPC-TDSPeFYGKFKEGVASGN-LNT--MFEYTFDYQM-TPTKVKVHMKKAL-----SYWVFVKRV-<br>----------GIFFP---ALEQNMMGAVLINENDEVMFFNPAAEKLWGYKREEVIGNNID-MLIPRdLRPA-HPEYIRHNREGGKaRVEgmSRELQLEKKDgSKIWTRFALSKVSaegkvYYLALVRDAh
203	1d7eA (119)<br>2gj3A (119)	N	15.13	TM-align: aliSize=102 (resi) RMSD=2.39 (Å)<br>vafgsedient--La-KMDdGQLDGLAFGAIQLDGDGNILQYNAAEGDITGRDPKQVIGKNFFkdVAPC--TDSPEFyGKFKEGVASGNlNTMFEYTFDYQ-MTPTKVKVHMKKAL-------SYWVFVKRV----<br>-----------llP-eIFR-QTVEHAPIAISITDLKANILYANRAFRTITGYGSEEVLGKNES--ILSNgtTPRLVY-QALWGRLAQKK-PWSGVLVNRRKdKTLYLAELTVAPVLneagetiYYLGMHRDTselh<br><br>SARST: aliSize=101 (resi) RMSD=3.00 (Å)<br>vafgsedientlakmd------DGQLDGLAFGAIQLDGDGNILQYNAAEGDITGRDPKQVIGKNFFKDVApCTDSPEFYGKFKEGVASGNLNTMFEYTFDYQMTPTKVKVHMKK-------ALSYWVFVKR-----v<br>-----------------llpeifRQTVEHAPIAISITDLKANILYANRAFRTITGYGSEEVLGKNESILSN-GTTPRLVYQALWGRLAQKKPWSGVLVNRRKDKTLYLAELTVAPvlneageTIYYLGMHRDtselh-<br><br>BLAST: aliSize=19 (resi) iden=12.61% (15/119) simi=15.97% (19/119)<br>vafgsedientlakmddgqldglafg---------------AIQL-DGDGNILQYNAAEGDITGRDPKQVIGKN---ffkdvapctdspefygkfkegvasgnlntmfeytfdyqmtptkvk<br>--------------------------llpeifrqtvehapiAISItDLKANILYANRAFRTITGYGSEEVLGKNesilsngttprlvyqalwgrlaqkkpwsgvlvnrrkdktlylaeltvapvlneagetiyylgmhrdtselh---<br>vhmkkalsywvfvkrv<br>----------------<br><br>Proposed: aliSize=103 (resi) RMSD=1.85 (Å)<br>VafgSedIENTLAkmddgQLDGLAFGAIQLDGDGNILQYNAAEGDITGRDPKQVIGKNFFkdVAPC----tdSPEFyGKFKEGVASGNlNTMFEYTFDYQM-TPTKVKVHMKKAL-------SYWVFVKRV----<br>L---L--PEIFRQ-----TVEHAPIAISITDLKANILYANRAFRTITGYGSEEVLGKNES--ILSNgttp--RLVY-QALWGRLAQKK-PWSGVLVNRRKDkTLYLAELTVAPVLneagetiYYLGMHRDTselh

204	1d7eA (119)<br>2gj3B (119)	N	16.81	TM-align: aliSize=102 (resi) RMSD=2.34 (Å)<br>vafgsedientl----AKMDdGQLDGLAFGAIQLDGDGNILQYNAAEGDITGRDPKQVIGKNFFkdVAPCT-D-SPEFyGKFKEGVASGNlNTMFEYTFDYQ-MTPTKVKVHMKKAL-------SYWVFVKRV---<br>------------ellpEIFR-QTVEHAPIAISITDLKANILYANRAFRTITGYGSEEVLGKNES--ILSNGtTpRLVY-QALWGRLAQKK-PWSGVLVNRRKdKTLYLAELTVAPVLneagetiYYLGMHRDTsel<br><br>SARST: aliSize=101 (resi) RMSD=4.10 (Å)<br>vafgsedientlakmd-------DGQLDGLAFGAIQLDGDGNILQYNAAEGDITGRDPKQVIGKNFFKdVAPCTDSPEFYGKFKEGVASG-NLNTMFEYTFDYQ-MTPTKVKVHMKKA-----LSYWVFVKR----v<br>----------------ellpeifRQTVEHAPIAISITDLKANILYANRAFRTITGYGSEEVLGKNESI-LSNGTTPRLVYQALWGRLAQKkPWSGVLVNRRKDKtLYLAELTVAPVLNeagetIYYLGMHRDtsel-<br><br>BLAST: aliSize=19 (resi) iden=12.61% (15/119) simi=15.97% (19/119)<br>vafgsedientlakmddgqldglafg------------------AIQL-DGDGNILQYNAAEGDITGRDPKQVIGKN--ffkdvapctdspefygkfkegvasgnlntmfeytfdyqmtptkvk<br>--------------------------ellpeifrqtvehapiAISItDLKANILYANRAFRTITGYGSEEVLGKNesilsngttprlvyqalwgrlaqkkpwsgvlvnrrkdktlylaeltvapvlneagetiyylgmhrdtsel---<br><br>vhmkkalsywvfvkrv<br>----------------<br><br>Proposed: aliSize=105 (resi) RMSD=1.88 (Å)<br>vafgSEDIENtLAKmddgQLDGLAFGAIQLDGDGNILQYNAAEGDITGRDPKQVIGKNFFkdVAPC--T-dSPEfYGKFKEGVASGNlNTMFEYTFDYQ-MTPTKVKVHMKKAL-------SYWVFVKRV---<br>----ELLPEI-FRQ----TVEHAPIAISITDLKANILYANRAFRTITGYGSEEVLGKNES--ILSNgtTp-RLV-YQALWGRLAQKK-PWSGVLVNRRKdKTLYLAELTVAPVLneagetiYYLGMHRDTsel
205	1dgmA (346)<br>2c4eA (299)	M	16.39	TM-align: aliSize=284 (resi) RMSD=3.14 (Å)<br>tg---PMRVFAIGNPILDLVAeVPSSfLDEFFLk-RgdatlatpeqmrIysTLdqFnPTSLPGGSALNSVRVVQKLLRkpgSAGYMGAIGDdPRGQV-LKELCDKEGLAT-RFMVAPGQSTGVCAVLINEKERT-LCTHLGACGSFRLPEDWTtfAsGALIFYATaYTltATPkNAFEVAGYAHGIpnAI<br>--ggkMEKITCVGHTALDYIF-NVEK-FPEPNT-sI-----------Q--IP--S-ARKYYGGAAANTAVGIKKLGV---NSELLSCVGY-DFKNSgYERYLKNLDINIsKLYYSEEEEIPKAWIFTDKDNNQiTFFLWGAAKHYKELNPPN--F-NTEIVHIA-TG--DPE-FNLKCAKKAYGN--NL<br><br>FTLNLSApFCVELYKDAMQSLLLHTNILFGNEFEFAHLAKVHNLvtankehaveVCTGALRlLTagqntgatKLVVMTRGHNPVIAAEQtadgtVVVHEVGVPVVAaeKIVDTNGAGDAFVGGFLYALSQGKTVKQCIMCGNACAQDVIQHVGF-SLSFT-SLPC------<br>VSFDPGQ-DLPQYSKEMLLEIIEHTNFLFMNKHEFERASNLLNF----------EIDDYLE-RV--------DALIVTKGSKGSVIYTK-----DKKIEIPCIKAG--KVIDPTGAGDSYRAGFLSAYVKGYDLEKCGLIGAATASFVVEAKGCqTNLPTwDKVVerlekh<br><br>SARST: aliSize=261 (resi) RMSD=4.83 (Å)<br>tgpmr------VFAIGNPILDLVAEVpssfldEFFLKRGDATLAtpeqmriystldqFNPTSLPGGSALNSvRVVQKLLRKPgsAGYMGAIG----DDPrgqvLKELCDKEGLATRFMVAPG-QSTGVCAVLINE-KERTLCTHLGACGS-----FRLPedwttfasGALIFY---ATAytltatpknAF<br>-----ggkmekITCVGHTALDYIFNV------EKFPEPNTSIQI-------------PSARKYYGGAAANT-AVGIKKLGVN--SELLSCVGydfkNSG----YERYLKNLDINISKLYYSEeEEIPKAWIFTDKdNNQITFFLWGAAKHykelnPPNF--------NTEIVHiatGDP---------EF<br><br>FVAGYAHGIPNAIFTLNLsAPFCVELYKDAMQSLLLHTNILFGNEFEFAHLAKVHNLVtankehavevctGALRlLTagqntgaTKLVVMTRGHNPVIAAEqtADgtVVVHEVGVPVvaaEKIVDTNGAGDAFVGGFLYALSQGKTVKQCIMCGNACAQDVIQHVG------------------fslsft<br>NLKCAKKAYGNNLVSFDP-GQDLPQYSKEMLLEIIEHTNFLFMNKHEFERASNLLNFE------------IDDYLER-------VDALIVTKGSKGSVIYT--KD--KKIEIPCIKA---GKVIDPTGAGDSYRAGFLSAYVKGYDLEKCGLIGAATASFVVEAKGcqtnlptwdkvverlekh------<br><br>slpc<br>----<br><br>BLAST: aliSize=82 (resi) iden=16.39% (49/299) simi=27.42% (82/299)<br>tgpmrvfaignpildlvaevpssfldefflkrgdatlatpeqmriystldqfnptslpggsalnsvrvvqkllrkpgsagymgaigddprgqvlkelcdkeglatrfmvapgqstgvcavlinekertlcthlgacgsfrlpedwttfasgalifyataytl---------------------------------<br>---ggkmekitcvghtaldyifnvekfpepn<br><br>--TATPKNAFEVAGYAHGIPNAIFTLNLSAPfcvELYKDAMQSLLLHTNILFGNEFEFAhlaKVHNLVTANKEHAVEVcTGALr<br>tsiqipsarkyyggaaantavgikklgvnsellscvgydfknsgyerylknldinisklyyseeeetpkawiftdkdnnqitfflwgaakhykelnppnfnteivhiaTGDPEFNLKCAKKAYGNNLVSFDPGQDLP---QYSKEMLLEIIEHTNFLFMNKHEFE---RASNLLNFEIDDYLER-VDAL-<br><br>LLTAGQNtGAtklVVMTRghnpviaaeqtaDgtvvvHEVGVPVVAAEKIVDTNGAGDAFVGGFLYALSQGKTVKQCimcG---NACAQDVIQHVGFSlsfTSLP------------c<br>IVTKGSK-GS---VIYTK-----------D-----KKIEIPCIKAGKVIDPTGAGDSYRAGFLSAYVKGYDLEKC---GligAATASFVVEAKGCQ---TNLPtwdkvverlekh-<br><br>Proposed: aliSize=277 (resi) RMSD=2.34 (Å)<br>tgp----MRVFAIGNPILDLVA-evpssfldEFFLKRGDATLAtpeQMRiystldqfnPTSLPGGSALNSVRVVQKLLRkpgSAGYMGAIGDdPrGQ-V-LKELCDKEGLAT-RFMVAPGQSTGVCAVLINE-kE-RTLCTHLGACGSF--RLPedWTtfAsGALIFYATaYTLTatPkNAFEVAGYAHG<br>---ggkmEKITCVGHTALDYIFn--------VEKFPEPNTSIQ---IPS---------ARKYYGGAAANTAVGIKKLGV---NSELLSCVGY-D-FKnSgYERYLKNLDINIsKLYYSEEEETPKAWIFTDKd-NnQITFFLWGAAKHYkeLNP--PN--F-NTEIVHIA-TGDP--E-FNLKCAKKAYG<br><br>IpnAIFTLNLSA-PFCveLYKDAMQSLLLHTNILFGNEFEFAHLAKVHNLvtankehaveVCTGALRLLtagqntgatKLVVMTRGHNPVIAAEQtadgtVVVHEVGVPVVAaeKIVDTNGAGDAFVGGFLYALSQGKTVKQCIMCGNACAQDVIQHVGFS-LSFtS--LPC------<br>N--NLVSFDPGQdLPQ--YSKEMLLEIIEHTNFLFMNKHEFERASNLLNF----------EIDDYLERV---------DALIVTKGSKGSVIYTK-----DKKIEIPCIKAG--KVIDPTGAGDSYRAGFLSAYVKGYDLEKCGLIGAATASFVVEAKGCQtNLP-TwdKVVerlekh
206	1dmzA (158)<br>1g3gA (164)	C	13.29	TM-align: aliSize=114 (resi) RMSD=2.91 (Å)<br>---------------------------GN-GRFLTLKPLpdSIIQESLEIQQG----------VNPFFIGRSEDCNCKIEDN-RLSRVHCFIFKKRHavgksmyespaqglDDIWYCHTGTNVSYLNNNRMIQGTKFLLQDGDEIKIIWDKNnKFVIGFKVEIN-DTT-GLFneglgmlqeqrvvlkqt<br>genitqptqqstqatqrfliekfsqeqIGeNIVCRVICT--TGQIPIRDLSADisqvlkekrsiKKVWTFGRNPACDYHLGNIsRLSNKHFQILLGED--------------GNLLLNDISTNGTWLNGQKVEKNSNQLLSQGDEITVGVGVE-SDILSLVIFINdKFKqCLE-----------------<br><br>aeekdlvkkl---------<br>----------qnkvdrir<br><br>SARST: aliSize=72 (resi) RMSD=14.30 (Å)<br>gngrfltlkplpdsiiqesleiqqgvnpffigrsedcnckiednrlsrvh---CFIFKKR-----HAVGKSMYESPAQGLD------DIWYCHTGTNVSYLNNNRMIQGTKFLLQDGDEIK---IIwdkN<br>--genitqptqqstqatqrfliekfsqeqigenivcrvicttgqipirdlsadisqvlkekrsikKVWTFGRnpacdYHLGNISRLSNKHFQIllgedgNLLLNDISTNGTWLNGQKVEKNSNQLLSQGDEITvgvGV---E<br><br>NKFVIGFKVEIN--------------dttglfneglgmlqeqrvvlkqtaeekdlvkkl<br>SDILSLVIFINDkfkqcleqnkvdrir---------------------------------<br><br>BLAST: aliSize=59 (resi) iden=18.99% (30/158) simi=37.34% (59/158)<br>gngrfltlk---PLPD--SIIQESLEIQQGVNPFF-IGRSEDCNCKIED-NRLSRVHCFIFkkrhaVGksmyESPAQGLDDIwychtGTNVSYLNNNRMIQGTKFLLQDGDEIKI-IWDKNNkfVIGFKVEIND--------------t<br>---------genitqptqqstqatqrfliekfsqeqigenivcrvicttgqiPIRDlsADISQVLKEKRSIKKVWtFGRNPACDYHLGNiSRLSNKHFQIL-----LG----EDGNLLLNDI-----STNGTWLNGQKVEKNSNQLLSQGDEITVgVGVESD--ILSLVIFINDkfkqcleqnkvdrir-<br><br>tglfneglgmlqeqrvvlkqtaeekdlvkkl<br>-------------------------------<br><br>Proposed: aliSize=114 (resi) RMSD=2.28 (Å)<br>---------------------------GN-GRFLTLKPLpdSIIQESLEIQqgV-------------NPFFIGRSEDCNCKIE-dN-RLSRVHCFIFKKRHavgksmyespaqglDDIWYCHTGTNVSYLNNNRMIQGTKFLLQDGDEIKIIWDK-nnKFVIGFKVEINdttglfneglgmlqeqrvvlk<br>genitqptqqstqatqrfliekfsqeqIGeNIVCRVICT--TGQIPIRDLS--AdisqvlkekrsikKVWTFGRNPACDYHLGn-IsRLSNKHFQILLGED-------------GNLLLNDISTNGTWLNGQKVEKNSNQLLSQGDEITVGVGVe--SDILSLVIFIN--------------------<br><br>qtAEEKDLVKKL------<br>--DKFKQCLEQNkvdrir

207	1dmzA (158)<br>2cswA (145)	C	13.79	TM-align: aliSize=109 (resi) RMSD=2.73 (Å)<br>-----------GNG-RFLTLKPLPDsii-QESLEIQqGVNPFFIGRSEDCNCKIE----DNRLSRVHCFIFKKRHavgksmyespaqglDDIWYCHT-GTNVSYLNNNRMIQGTKFLLQDGDEIKIIWDKNNK-FV-IGFKVEINDttglfneglgmlqeqrvvlkqtaeekdlvkk-------------<br>gssgssgvtgdRAGgRSWCLRRVGM---sAGWLLLE-DGCEVTVGRGFGVTYQLVskicPLMISRNHCVLKQNPE--------------GQWTIMDNkSLNGVWLNRARLEPLRVYSIHQGDYIQLGVPLENKeNAeYEYEVTEED------------------------------wetiypclspksg<br>L---<br>Pssg<br>SARST: aliSize=78 (resi) RMSD=13.93 (Å)<br>gngrfltlkplpd-------------SIIQESLEIQQ---GVNPFF------IGRSEDCNCKIEDNRLSRvhcfifkkrhavgksmYESPAQGLDDI-------WYCHTGTNVSYLNNNRMIQGTKFLLQDGDEIK--------------------------------------iiwdknnkfvigfkve<br>-------------gssgssgvtgdraGGRSWCLRRVGmsaGWLLLEdgcevtVGRGFGVTYQLVSKICPL----------------MISRNHCVLKQnpegqwtIMDNKSLNGVWLNRARLEPLRVYSIHQGDYIQlgvplenkenaeyeyevteedwetiypclspksgpssg----------------<br>indttglfneglgmlqeqrvvlkqtaeekdlvkkl<br>----------------------------------<br>BLAST: aliSize=50 (resi) iden=15.86% (23/145) simi=34.48% (50/145)<br>gngrfltlkplpds------------------------------IIQESLEIQQGvNPFfiGRSEDCNCKIEDNRLSRVHCFIfkKRHAVGK-SMYESpaQGLDDIWychtgtnvsyLNNNRMIQGTKFLLQDGDEIKIIWDKNNKFVIGFKVEIND-------------------ttglfneglgmlqe<br>--------------gssgssgvtgdraggrswclrrvgmsagwlLLEDGCEVTVG-RGF--GVTYQLVSKICPLMISRNHCVL--KQNPEGQwTIMDN--KSLNGVW----------LNRARLEPLRVYSIHQGDYIQLGVPLENKENAEYEYEVTEedwetiypclspksgpssg--------------<br>qrvvlkqtaeekdlvkkl<br>------------------<br>Proposed: aliSize=122 (resi) RMSD=2.52 (Å)<br>------------GNGRFLTLKPLPD-siiQESLEIQqGVNPFFIGRSEDCNCKIE----DNRLSRVHCFIFKKRHavgksmyespaqglDDIWYCHT-GTNVSYLNNNRMIQGTKFLLQDGDEIKIIWDKNNK--FVIGFKVEI-ndttgLFNE-GLGMlqeQRVVLKQtaeE-kdlvkkl<br>gssgssgvtgdrAGGRSWCLRRVGMs---AGWLLLE-DGCEVTVGRGFGVTYQLVskicPLMISRNHCVLKQNPE--------------GQWTIMDNkSLNGVWLNRARLEPLRVYSIHQGDYIQLGVPLENKenAEYEYEVTEe-----DWETiYPCL---SPKSGPS---Sg-------
208	1dmzA (158)<br>2jqiA (151)	C	13.91	TM-align: aliSize=112 (resi) RMSD=2.21 (Å)<br>--------------G-NGRFLTLKPLpDSiIQESLEIQQG-----------VNPFFIGRSEDCNCKIE-DNRLSRVHCFIFKKRhavgksmyespaqgLDDIWYCHTGTNVSYLNNNRMIQGTKFLLQDGDEIKIIW-DKnnKFVIGFKVEIN-D-T-TGLfneglgmlqeqrvvlkqtaeekdlvkkl-<br>atqrfliekfsqeqIgENIVCRVICT-TG-QIPIRDLSADisqvlkekrsiKKVWTFGRNPACDYHLGnISRLSNKHFQILLGE-------------DGNLLLNDISTNGTWLNGQKVEKNSNQLLSQGDEITVGVgVE--SDILSLVIFINdKfKqCLE----------------------------q<br>-------<br>nkvdrir<br>SARST: aliSize=115 (resi) RMSD=14.36 (Å)<br>gn-----------------GRFLTLKPlPDSIIQESLEIQ----------QGVNPFFIGRSEDCNCK-IEDNRLSRVHCFIFKKrhavgksmyespAQGLDDIWYchTGTNVSYLNNNRMIqgtkfllqDGDEIKIIWdknnKFVigfkveinDTTGLFNEGLgmlqEQRVVLKQTAEEKDLVK------<br>--atqrfliekfsqeqigeNIVCRVIC-TTGQIPIRDLSAdisqvlkekrSIKKVWTFGRNPACDYHlGNISRLSNKHFQILLG------------EDGNLLLND--ISTNGTWLNGQKVE--------KNSNQLLSQ----GDE--------ITVGVGVESD----ILSLVIFINDKFKQCLEqnkvdr<br>--kl<br>ir--<br>BLAST: aliSize=59 (resi) iden=19.87% (30/151) simi=39.07% (59/151)<br>gngrfltlk------------------------------PLPD--SIIQESLEIQQGVNPFF-IGRSEDCNCKIED-NRLSRVHCFIFkkrhaVGksmyESPAQGLDDIwychtGTNVSYLNNNRMIQGTKFLLQDGDEIKI-IWDKNNkfVIGFKVEIND--------------ttglfneglgmlqe<br>---------atqrfliekfsqeqigenivcrvicttgqiPIRDlsADISQVLKEKRSIKKVWtFGRNPACDYHLGNiSRLSNKHFQIL-----LG----EDGNLLLNDI-----STNGTWLNGQKVEKNSNQLLSQGDEITVgVGVESD--ILSLVIFINDkfkqcleqnkvdrir-------------<br>qrvvlkqtaeekdlvkkl<br>------------------<br>Proposed: aliSize=115 (resi) RMSD=1.81 (Å)<br>--------------G-NGRFLTLKPLpDSiIQESLEIQqgV-------------NPFFIGRSEDCNCKIE-DNRLSRVHCFIFKKRhavgksmyespaqgLDDIWYCHTGTNVSYLNNNRMIQGTKFLLQDGDEIKIIW-DKnnKFVIGFKVEINdttglfneglgmlqeqrvvlkqtAEEKDLVKKL--<br>atqrfliekfsqeqIgENIVCRVICT-TG-QIPIRDLS--AdisqvlkekrsikKVWTFGRNPACDYHLGnISRLSNKHFQILLGE-------------DGNLLLNDISTNGTWLNGQKVEKNSNQLLSQGDEITVGVgVE--SDILSLVIFIN-----------------------DKFKQCLEQNkv<br>----<br>drir
209	1dmzA (158)<br>2jqjA (141)	C	17.02	TM-align: aliSize=105 (resi) RMSD=3.08 (Å)<br>g-------N-----GRFLTLKPLPDSii-QESLEIQqGVNPFFIGRSEDCNCKIEDNRLSRVHCFIFKKRHavgksmye-SPaqg---LDDIWYCHTGTNVSYLNNNRMIqGTKFLLQDGDEIKIIWdknnkfVIGFKVEINDTtglfneglgmlqeqrvvlkqtaeekdlvkkl---------------<br>-krqqrsnKpsseyTCLGHLVNLIPG--kEQKVEIT-NRNVTTIGRSRSCDVILSEPDISTFHAEFHLLQM--------dVD---nfqRNLINVIDKSRNGTFINGNRLV-KKDYILKNGDRIVFGK------SCSFLFKYASS-------------------------------sstdienddekvsse<br>----<br>srsy<br>SARST: aliSize=94 (resi) RMSD=5.06 (Å)<br>gngrfltlkplpdsi-------------------------IQESLEIQQGVnpFFIGRSEDCNCKIEDNRLSRVHCFIFKKRHAVGKSMyespaqgLDDIWYCHTGTNVSYLNNNRMIQGTKfLLQDGDEIKiIWdknNKFVIGFKVE---------------------indttglfneglgmlqeqrv<br>---------------krqqrsnkpsseytclghlvnlipgkEQKVEITNRNV--TTIGRSRSCDVILSEPDISTFHAEFHLLQMDVDNFQ-------RNLINVIDKSRNGTFINGNRLVKKDY-ILKNGDRIV-FG---KSCSFLFKYAsssstdienddekvssesrsy--------------------<br>vlkqtaeekdlvkkl<br>---------------<br>BLAST: aliSize=63 (resi) iden=19.86% (28/141) simi=44.68% (63/141)<br>gngrfltlkplpdsii--------------------------QESLEIQQGvNPFFIGRSEDCNCKIEDNRLSRVHCfifkKRHAVGKSMyESPAQGLDDIwyCHTGTNVSYLNNNRMIQgTKFLLQDGDeiKIIWDKNNKFVigFKVEINDTTGLFNEGLGMLQEQR--vvlkqtaeekdlvkkl<br>----------------krqqrsnkpsseytclghlvnlipgkEQKVEITNR-NVTTIGRSRSCDVILSEPDISTFHA----EFHLLQMDV-DNFQRNLINV--IDKSRNGTFINGNRLVK-KDYILKNGD--RIVFGKSCSFL--FKYASSSSTDIENDDEKVSSESRsy----------------<br>Proposed: aliSize=113 (resi) RMSD=2.34 (Å)<br>gn-------------GRFLTLKPLPD--siiQESLEIQqGVNPFFIGRSEDCNCKIEDNRLSRVHCFIFKK-rhavgksmyespaqgL-------DDIWYCHTGTNVSYLNNNRMI-qgTKFLLQDGDEIKIIWdknnkfVIGFKVEINDtTGLfNeGLGMLQEqrVVLKQ----taeekdlvkkl<br>--krqqrsnkpsseyTCLGHLVNLIPgk---EQKVEIT-NRNVTTIGRSRSCDVILSEPDISTFHAEFHLLq---------------MdvdnfqrNLINVIDKSRNGTFINGNRLVk--KDYILKNGDRIVFGK------SCSFLFKYAS-SSS-T-DIENDDE--KVSSEsrsy-----------

210	1dz3A<br>(123)<br><br>1natA<br>(119)	C	29.41	TM-align: aliSize=107 (resi) RMSD=2.17 (Å)<br>SIKVCIADDNRELVSLLDEYISSQPdMEViGTAYNGQDCLQMLEEKRPDILLLDIIMPHLDGLAVLERIRAgFEHQPNVIMLTAFGQEDVTKKAVELGASYFILKPFDMEnlahhirqvygkt------------<br>NEKILIVDDQYGIRILLNEVFNKEG-YQT-FQAANGLQALDIVTKERPDLVLLDMKIPGMDGIEILKRMKV-IDENIRVIIMTAYGELDMIQESKELGALTHFAKPFDID-------------eirdavkkylpl<br><br>SARST: aliSize=114 (resi) RMSD=7.16 (Å)<br>s-IKVCIADDNRELVSLLDEYISSqPDMEViGTAYNGQDCLQMLEEKRPDILLLDIIMPHLDGLAVLERIRAGFEhQPNVIMLTAFGQEDVTKKAVELGA-SYFILKpFDMenLAHHIRQVYG---kt<br>-nEKILIVDDQYGIRILLNEVFNK-EGYQT-FQAANGLQALDIVTKERPDLVLLDMKIPGMDGIEILKRMKVIDE-NIRVIIMTAYGELDMIQESKELGAlTHFAKP-FDI--DEIRDAVKKYlpl--<br><br>BLAST: aliSize=72 (resi) iden=30.25% (36/119) simi=60.50% (72/119)<br>si--KVCIADDNRELVSLLDEyISSQPDMEVIGTAyNGQDCLQMLEEKRPDILLLDIIMPHLDGLAVLERIRAgFEHQPNVIMLTAFGQEDVTKKAVELGASYFILKPFDMENLAHHIRQ----vygkt<br>--neKILIVDDQYGIRILLNE-VFNKEGYQTFQAA-NGLQALDIVTKERPDLVLLDMKIPGMDGIEILKRMKV-IDENIRVIIMTAYGELDMIQESKELGALTHFAKPFDIDEIRDAVKKylpl-----<br><br>Proposed: aliSize=116 (resi) RMSD=1.71 (Å)<br>SIKVCIADDNRELVSLLDEYISSQPdMEViGTAYNGQDCLQMLEEKRPDILLLDIIMPHLDGLAVLERIRAgFeH-QPNVIMLTAFGQEDVTKKAVELGASYFILKP-fdMENLAHHIRQVYGK-t<br>NEKILIVDDQYGIRILLNEVFNKEG-YQT-FQAANGLQALDIVTKERPDLVLLDMKIPGMDGIEILKRMKV-I-DeNIRVIIMTAYGELDMIQESKELGALTHFAKPf--DIDEIRDAVKKYLPl-
211	1dz3A<br>(123)<br><br>1peyC<br>(120)	C	29.17	TM-align: aliSize=106 (resi) RMSD=2.08 (Å)<br>-SIKVCIADDNRELVSLLDEYISSQPdMEViGTAYNGQDCLQMLEEKRPDILLLDIIMPHLDGLAVLERIRAgFEHQPNVIMLTAFGqED-VTKKAVELGASYFILKPFDMEnlahhirqvygkt------------<br>mNEKILIVDDQSGIRILLNEVFNKEG-YQT-FQAANGLQALDIVTKERPDLVLLDMKIPGMDGIEILKRMKV-IDENIRVIIMTAYG-ELdMIQESKELGALTHFAKPFDID-------------eirdavkkylpl<br><br>SARST: aliSize=113 (resi) RMSD=7.40 (Å)<br>s--IKVCIADDNRELVSLLDEYISSqpDMEVIGTAYNGQDCLQMLEEKRPDILLLDIIMPHLDGLAVLERIRAGFEHqPNVIMLTAFGQEDVTKKAVELGA-SYFILKpFDmenlAHHIRQVYGK----t<br>-mnEKILIVDDQSGIRILLNEVFNK--EGYQTFQAANGLQALDIVTKERPDLVLLDMKIPGMDGIEILKRMKVIDEN-IRVIIMTAYGELDMIQESKELGAlTHFAKP-FD----IDEIRDAVKKylpl-<br><br>BLAST: aliSize=72 (resi) iden=30.00% (36/120) simi=60.00% (72/120)<br>si---KVCIADDNRELVSLLDEyISSQPDMEVIGTAyNGQDCLQMLEEKRPDILLLDIIMPHLDGLAVLERIRAgFEHQPNVIMLTAFGQEDVTKKAVELGASYFILKPFDMENLAHHIRQ----vygkt<br>--mneKILIVDDQSGIRILLNE-VFNKEGYQTFQAA-NGLQALDIVTKERPDLVLLDMKIPGMDGIEILKRMKV-IDENIRVIIMTAYGELDMIQESKELGALTHFAKPFDIDEIRDAVKKylpl-----<br><br>Proposed: aliSize=116 (resi) RMSD=1.67 (Å)<br>-SIKVCIADDNRELVSLLDEYISSQPdMEViGTAYNGQDCLQMLEEKRPDILLLDIIMPHLDGLAVLERIRAgFeHQ-PNVIMLTAFGQEDVTKKAVELGASYFILKP-fdMENLAHHIRQVYGK-t<br>mNEKILIVDDQSGIRILLNEVFNKEG-YQT-FQAANGLQALDIVTKERPDLVLLDMKIPGMDGIEILKRMKV-I-DEnIRVIIMTAYGELDMIQESKELGALTHFAKPf--DIDEIRDAVKKYLPl-
212	1dz3A<br>(123)<br><br>1puxA<br>(124)	C	26.83	TM-align: aliSize=104 (resi) RMSD=2.87 (Å)<br>--SIKVCIADdNReLvSLLDEYISSQ-PDMEVIGTAYNGQDCLQMLEEKRPDILLLDIIMPHLDGLAVLERIRAGFEHQpNVIMLTAFGQEDVTKKAVELGA-SYFILKPfDm-Enlahhirqvygkt---------------<br>mmNEKILIVD-DQ-Y-GIRILLNEVFnKEGYQTFQAANGLQALDIVTKERPDLVLLDMKIPGMDGIEILKRMKVIDENI-RVIIMTAYGELDMIQESKELGAlTHFAKPF-D-iD-------------eirdavkkylplksn<br><br>SARST: aliSize=99 (resi) RMSD=2.79 (Å)<br>si----KVCIADDNRELVSLLDEYISSqPDMEViGTAYNGQDCLQMLEEKRPDILLLDIIMPHLDGLAVLERIRAGFEhQPNVIMLTAFGQEDVTKKAVELGASYFIL---------------------kpfdmenlahhirqvygkt<br>--mmneKILIVDDQYGIRILLNEVFNK-EGYQT-FQAANGLQALDIVTKERPDLVLLDMKIPGMDGIEILKRMKVIDE-NIRVIIMTAYGELDMIQESKELGALTHFAkpfdideirdavkkylplksn-------------------<br><br>BLAST: aliSize=64 (resi) iden=28.46% (35/123) simi=52.03% (64/123)<br>si----KVCIADDNRELVSLLDEYISSQpDMEVIGTAyNGQDCLQMLEEKRPDILLLDIIMPHLDGLAVLERIRAgFEHQPNVIMLTAFGQEDVTKKAVELGA------------------SYFILK--pfdmenlahhirqvygkt<br>--mmneKILIVDDQYGIRILLNEVFNKE-GYQTFQAA-NGLQALDIVTKERPDLVLLDMKIPGMDGIEILKRMKV-IDENIRVIIMTAYGELDMIQESKELGAlthfakpfdideirdavkKYLPLKsn------------------<br><br>Proposed: aliSize=114 (resi) RMSD=2.43 (Å)<br>--SIKVCIADDNRELVSLLDEYISSqP-dmEVIGTAYNGQDCLQMLEEKRPDILLLDIIMPHLDGLAVLERIRAGF-ehQPNVIMLTAFGQEDVTKKAVELGASYFI-lkPFDMENLAHHIRQVY-G----kt<br>mmNEKILIVDDQYGIRILLNEVFNK-Eg--YQTFQAANGLQALDIVTKERPDLVLLDMKIPGMDGIEILKRMKVIDe--NIRVIIMTAYGELDMIQESKELGALTHFa--KPFDIDEIRDAVKKYlPlksn--

213	1dz3A (123)<br>2hf9A (211)	C	9.76	TM-align: aliSize=98 (resi) RMSD=3.18 (Å)<br>---------------------SIKVCIADDNRELVSLLDEYISS-Q---P--DMEVIGTAYNGQDCLQmlEEKRPDILLLDII--MPh-LDGL--A-VLErIragfehqpNVIMLTA-F--------GQEDVTKKAVE<br>kdilkankrladknrkllnkhgVVAFDFMGAIGSGKTLLIEKLIDnLkdkYkiaciagdviakfdaermekhgakvvplntgkechldahlvghaledlnldeiDLLFIENVGNLICPAD--FDLGTHKRIVVISttEG-dDTIEkhPgIMK-T--------ADLIVINkIdladavgaDIKKMENDAKR<br>--LGASYFILKPFDMEnlahhirqvygkt-----------------<br>inPDAEVVLLSLKTME-------------gfdkvlefieksvkevk<br>SARST: aliSize=99 (resi) RMSD=7.73 (Å)<br>s-----------------------IKVCIADDN--RELVSLLDEYISSQPDMEVIGTAYNG--QDCLQMLEEKRPDILLLDIIMPHLD-GLAVLERIRA----GFEHQPNVIMLTafgqeDVTKKAVELGASYFILK--<br>-kdilkankrladknrkllnkhgvVAFDFMGAIgsGKTLLIEKLIDNLKDKYKIACIAGDViaKFDAERMEKHGAKVVPLNTGKECHLdAHLVGHALEDlnldEIDLLFIENVGN-----LICPADFDLGTHKRIVVisttegddtiekhpgimktadlivinkidladavgadikkmendakrinpdae<br>---------------------------pfdmenlahhirqvygkt<br>vvllslktmegfdkvlefieksvkevk------------------<br>BLAST: aliSize=9 (resi) iden=4.07% (5/123) simi=7.32% (9/123)<br>sikvc---------IADDNRELVS---<br>-----kdilkankrLADKNRKLLNkhgvvafdfmgaigsgktllieklidnlkdkykiaciagdviakfdaermekhgakvvplntgkechldahlvghaledlnldeidllfienvgnlicpadfdlgthkrivvisttegddtiekhpgimktadlivinkidladavgadikkmendakrinpdaev<br>-------------------------lldeyissqpdmevigtayngqdclqmleekrpdillldiimphldglavleriragfehqpnvimltafgqedvtkkavelgasyfilkpfdmenlahhirqvygkt<br>vllslktmegfdkvlefieksvkevk--<br>Proposed: aliSize=108 (resi) RMSD=2.50 (Å)<br>---------------------SIKVCIADDNRELVSLLDEYISS-Q---P--DMEVIGTAYNGQDCLQmLEeKRPDILLLDI-------IM-phLDgLAVLErIragfehqpNVIMLT--A---------FG-QEDVT<br>kdilkankrladknrkllnkhgVVAFDFMGAIGSGKTLLIEKLIDnLkdkYkiaciagdviakfdaermekhgakvvplntgkechldahlvghaledlnldeiDLLFIENVGNLICPAD-FD-LGTHKRIVVIsttegddTIe--KH-PGIMK-T--------ADLIVInkIdladavgadIKkMENDA<br>KKAVElGASYFILKPF--D-MENLAHHIRQVYGKT-<br>KRINP-DAEVVLLSLKtmEgFDKVLEFIEKSVKEVk
214	1dz3A (123)<br>2hf9B (209)	C	10.57	TM-align: aliSize=100 (resi) RMSD=3.21 (Å)<br>---------------------SIKVCI-ADDNReLVSLLDEYISS-Q---P--DMEVIGTAYNGQDCLqmLEEKRP-DILLLDII-MPh-LDGLAVlERI-RagfehQPNVIMLT-A--F-----GQEDVTKKAVE-LG<br>dilkankrladknrkllnkhgVVAFDFmGAIGS-GKTLLIEKLIDnLkdkYkiaciagdviakfdaermekhgakvvplntgkechldahlvghaledlnldeiDLLFIENVGNLICPA--DFDLGThKRIVVISTtEG-dDTIEKH-PGImK-----TADLIVINkIdlAdavgaDIKKMENDAKRiNP<br>-ASYFILKPFDMEnlahhirqvygkt-----------------<br>dAEVVLLSLKTME-------------gfdkvlefieksvkev<br>SARST: aliSize=69 (resi) RMSD=6.53 (Å)<br>sikvciaddnrelvslldeyissqpdmevigtayn---------GQDCLQMLEEKRPdILLLDIIMPHLDGLAVLERIR-AGFEHQPNVIMLTAFGQEDVTKKAVELGASYFILK--<br>-----------------------------------dilkankrlADKNRKLLNKHGV-VAFDFMGAIGSGKTLLIEKLIdNLKDKYKIACIAGDVIAKFDAERMEKHGAKVVPLNtgkechldahlvghaledlnldeidllfienvgnlicpadfdlgthkrivvisttegddtiekhpgimktadliv<br>---pfdmenlahhirqvygkt<br>inkidladavgadikkmendakrinpdaevvllslktmegfdkvlefieksvkev-----------------<br>BLAST: aliSize=9 (resi) iden=4.07% (5/123) simi=7.32% (9/123)<br>sikvc--------IADDNRELVS--<br>-----dilkankrLADKNRKLLNkhgvvafdfmgaigsgktllieklidnlkdkykiaciagdviakfdaermekhgakvvplntgkechldahlvghaledlnldeidllfienvgnlicpadfdlgthkrivvisttegddtiekhpgimktadlivinkidladavgadikkmendakrinpdaevv<br>------------------------lldeyissqpdmevigtayngqdclqmleekrpdillldiimphldglavleriragfehqpnvimltafgqedvtkkavelgasyfilkpfdmenlahhirqvygkt<br>llslktmegfdkvlefieksvkev---<br>Proposed: aliSize=108 (resi) RMSD=2.59 (Å)<br>----------------------SIKVCIADDNReLVSLLDEYIsS--Q---P--DMEVIGTAYNGQdclQMLEEKR-P-DILLLDII-MP--hlDGLAVlERI-RagfehQPNVIMLT-AF-G------QEDVTKKAVE<br>dilkankrladknrkllnkhgvVAFDFMGAIGS-GKTLLIEKL-IdnLkdkYkiaciagdviakfdaermekhgakvvplntgkechldahlvghaledlnldeiDLLFIENVGNLI---CPADFDLgThKRIVVISTtEGdd--TIEKH-PGImK-----TADLIVINkIDlAdavgadIKKMENDAKR<br>L-G-ASYFIL--kpFDMENLAHHIRQVYGKT---<br>InPdAEVVLLsl--KTMEGFDKVLEFIEKSVkev
215	1dz3A (123)<br>2j48A (119)	C	12.61	TM-align: aliSize=99 (resi) RMSD=2.81 (Å)<br>SIKVCIADDNRELVSLLDEYISSQPdmEVIGTAYNGQDCLQMLEEKRPDILLLDII--MPhLdGLAVLERIRAGF-E-HQPNVIMLTAfgQEDVtKKAVELGASYFILKpfdm-Enlahhirqvygkt---------------<br>AGHILLLEEEDEAATVVCEMLTAAG--FKVIWLVDGSTALDQLDLLQPIVILMAWPppDQ-S-CLLLLQHLREHQaDpHPPLVLFLGE--PPVD-PLLTAQASAILSKP----lD---------------pqllttlqglcppn<br>SARST: aliSize=111 (resi) RMSD=8.34 (Å)<br>si--KVCIADDNRELVSLLDEYISSqPDMEViGTAYNGQDCLQMLEEKRPDILLLDII-MPHLDGLAVLERIRAGFEHQPNVIMLTAFGQEDvtKKAVELGASYFILKpfdmENLAHHIRQVYG-----kt<br>--agHILLLEEEDEAATVVCEMLTA-AGFKV-IWLVDGSTALDQLDLLQPIVILMAWPpPDQSCLLLLQHLREHQADPHPPLVLFLGEPPVD--PLLTAQASAILSKP----LDPQLLLTTLQGlcppn--<br>BLAST: aliSize=52 (resi) iden=21.01% (25/119) simi=43.70% (52/119)<br>sik---VCIADDNRELVSLLDEYISSQpDMEVIGTAyNGQDCLQMLEEKRPDILLLDIIMPHLDGLAVLERIRagfEHQ--PNVIMLTAFGQEDVTKkAVELGASYFILKPFDMENL-----------ahhirqvygkt<br>---aghILLLEEEDEAATVVCEMLTAA-GFKVIWLV-DGSTALDQLDLLQPIVILMAWPPPDQSCLLLLQHLR---EHQadPHPPLVLFLGEPPVDP-LLTAQASAILSKPLDPQLLlttlqglcppn-----------<br>Proposed: aliSize=111 (resi) RMSD=2.40 (Å)<br>SIKVCIADDNRELVSLLDEYISSQPdMEViGTAYNGQDCLQMLEEKRPDILLLDII--MPhLdGLAVLERIRAGF-E-HQPNVIMLTAfgQEDVtKKAVELGASYFI--lkpfdMENLAHHIRQVYGKT--<br>AGHILLLEEEDEAATVVCEMLTAAG-FKV-IWLVDGSTALDQLDLLQPIVILMAWPppDQ-S-CLLLLQHLREHQaDpHPPLVLFLGE--PPVD-PLLTAQASAILSkp-----LDPQLLLTTLQGLCPpn

216	1dz3A (123)<br>3cu5B (129)	C	22.76	TM-align: aliSize=108 (resi) RMSD=2.43 (Å)<br>SIKVCIADDNRELVSLLDEYISSQPDME-VIGTAYNGQDCLQMLEEKRPDILLLDIIMPHLDGLAVLERIRAgFEHQPNVIMLTAFGQEDVTKKAVELG-ASYFILKPfDMEnlahhirqvygkt------------------<br>SLRILIVDDEKLTRDGLIANINWKALSFdQIDQADDGINAIQIALKHPPNVLLTDVRMPRMDGIELVDNILK-LYPDCSVIFMSGYSDKEYLKAAIKFRaIRYVEKPI-DPS-------------eimdalkqsiqtvlqhaq<br><br>SARST: aliSize=112 (resi) RMSD=8.11 (Å)<br>s-IKVCIADDNRELVSlldEYISSQ--PDMEVI--GTAYNGQDCLQMLEEKRPDILLLDIIMPHLDGLAVLERIRAGFEhQPNVIMLTAFGQEDVTKKAVE--LGASYFILKpfdmENLAHHIRQVYG----------kt<br>-sLRILIVDDEKLTRD---GLIANInwKALSFDqiDQADDGINAIQIALKHPPNVLLTDVRMPRMDGIELVDNILKLYP-DCSVIFMSGYSDKEYLKAAIKfrAIRYVEKPI----DPSEIMDALKQSiqtvlqhaq--<br><br>BLAST: aliSize=62 (resi) iden=22.76% (28/123) simi=50.41% (62/123)<br>SIKVCIADDNRELVSLLDEYISSQP-DMEVIGTAYNGQDCLQMLEEKRPDILLLDIIMPHLDGLAVLERIRAGFEhQPNVIMLTAFGQEDVTKKAVELGASYFILKPFDMENLAHHIRQ-----------vygkt<br>SLRILIVDDEKLTRDGLIANINWKAlSFDQIDQADDGINAIQIALKHPPNVLLTDVRMPRMDGIELVDNILKLYP-DCSVIFMSGYSDKEYLKAAIKFRAIRYVEKPIDPSEIMDALKQsiqtvlqhaq-----<br><br>Proposed: aliSize=121 (resi) RMSD=1.98 (Å)<br>SIKVCIADDNRELVSLLDEYISSQPDME-VIGTAYNGQDCLQMLEEKRPDILLLDIIMPHLDGLAVLERIRAgFeHQ-PNVIMLTAFGQEDVTKKAVELGASYFILKPFD---MENLAHHIRQVYGKT---<br>SLRILIVDDEKLTRDGLIANINWKALSFdQIDQADDGINAIQIALKHPPNVLLTDVRMPRMDGIELVDNILK-L-YPdCSVIFMSGYSDKEYLKAAIKFRAIRYVEKPIDpseIMDALKQSIQTVLQHqaq
217	1dz3A (123)<br>6chyA (128)	C	21.14	TM-align: aliSize=108 (resi) RMSD=2.27 (Å)<br>--S-IKVCIADDNRELVSLLDEYISSQPdMEVIGTAYNGQDCLQMLEEKRPDILLLDIIMPHLDGLAVLERIRAGFEHQ-PNVIMLTAFGQEDVTKKAVELGA-SYFILKPfDMEnlahhirqvygkt---------------<br>adKeLKFLVVDDFSTMRRIVRNLLKELG-FNNVEEAEDGVDALNKLQAGGYGFVISDWNMPNMDGLELLKTIRADGAMSaLPVLMVIAEAKKENIIAAAQAGAsGWVVKPF-TAA-------------tleeklnkifeklgm<br><br>SARST: aliSize=103 (resi) RMSD=2.58 (Å)<br>s----IKVCIADDNRELVSLLDEYISSqPDMEVIGTAYNGQDCLQMLEEKRPDILLLDIIMPHLDGLAVLERIRA-----GfehqPNVIMLTAFGQEDVTKKAVELGASYFILKPFDM----------------enlahhirqvygkt<br>-adkeLKFLVVDDFSTMRRIVRNLLKE-LGFNNVEEAEDGVDALNKLQAGGYGFVISDWNMPNMDGLELLKTIRAdgamsA----LPVLMVIAEAKKENIIAAAQAGASGWVVKPFTAatleeklnkifeklgm-------------<br><br>BLAST: aliSize=60 (resi) iden=25.20% (31/123) simi=48.78% (60/123)<br>s----IKVCIADDNRELVSLLDEYISsQPDMEVIGTAYNGQDCLQMLEEKRPDILLLDIIMPHLDGLAVLERIRA-GFEHQPNVIMLTAFGQEDVTKKAVELGASYFILKPFDMENLAHHIRQVYGK---t<br>-adkeLKFLVVDDFSTMRRIVRNLLK-ELGFNNVEEAEDGVDALNKLQAGGYGFVISDWNMPNMDGLELLKTIRAdGAMSALPVLMVIAEAKKENIIAAAQAGASGWVVKPFTAATLEEKLNKIFEKlgm-<br><br>Proposed: aliSize=120 (resi) RMSD=1.74 (Å)<br>--S-IKVCIADDNRELVSLLDEYISSQPdMEVIGTAYNGQDCLQMLEEKRPDILLLDIIMPHLDGLAVLERIRAGFEHQ-PNVIMLTAFGQEDVTKKAVELGA-SYFILK-pfDMENLAHHIRQVYGKT--<br>adKeLKFLVVDDFSTMRRIVRNLLKELG-FNNVEEAEDGVDALNKLQAGGYGFVISDWNMPNMDGLELLKTIRADGAMSaLPVLMVIAEAKKENIIAAAQAGAsGWVVKPf--TAATLEEKLNKIFEKLgm
218	1f0vA (124)<br>1a5pA (124)	C	98.39	TM-align: aliSize=115 (resi) RMSD=1.31 (Å)<br>KETAAAKFERQHMDSSTSAASSSNYCNQMMKSRNLTKDRCKPVNTFVHESLADVQAVCSQKNVACKNGQTNCYQSYSTMSITDCRETGSSKYPNCAYKTTQANKHIIVACEGNPYvpvhfdasv---------<br>KETAAAKFERQHMDSSTSAASSSNYCNQMMKSRNLTKDRAKPVNTFVHESLADVQAVCSQKNVACKNGQTNCYQSYSTMSITDCRETGSSKYPNAAYKTTQANKHIIVACEGNPY---------vpvhfdasv<br><br>SARST: aliSize=122 (resi) RMSD=8.84 (Å)<br>k-ETAAAKFERQHMDSSTSAASSSNYCNQMMKSRNLTKDRCKPVNTFVHESLADVQAVCSQKNVACKNGQTNCYQSYSTMSITDCRETGSSKYPNCAYKTTQANKHIIVACEGNPYVPVHFDAS-v<br>-kETAAAKFERQHMDSSTSAASSSNYCNQMMKSRNLTKDRAKPVNTFVHESLADVQAVCSQKNVACKNGQTNCYQSYSTMSITDCRETGSSKYPNAAYKTTQANKHIIVACEGNPYVPVHFDASv-<br><br>BLAST: aliSize=122 (resi) iden=98.39% (122/124) simi=98.39% (122/124)<br>KETAAAKFERQHMDSSTSAASSSNYCNQMMKSRNLTKDRCKPVNTFVHESLADVQAVCSQKNVACKNGQTNCYQSYSTMSITDCRETGSSKYPNCAYKTTQANKHIIVACEGNPYVPVHFDASV<br>KETAAAKFERQHMDSSTSAASSSNYCNQMMKSRNLTKDRAKPVNTFVHESLADVQAVCSQKNVACKNGQTNCYQSYSTMSITDCRETGSSKYPNAAYKTTQANKHIIVACEGNPYVPVHFDASV<br><br>Proposed: aliSize=124 (resi) RMSD=0.52 (Å)<br>KETAAAKFERQHMDSSTSAASSSNYCNQMMKSRNLTKDRCKPVNTFVHESLADVQAVCSQKNVACKNGQTNCYQSYSTMSITDCRETGSSKYPNCAYKTTQANKHIIVACEGNPYVPVHFDASV<br>KETAAAKFERQHMDSSTSAASSSNYCNQMMKSRNLTKDRAKPVNTFVHESLADVQAVCSQKNVACKNGQTNCYQSYSTMSITDCRETGSSKYPNAAYKTTQANKHIIVACEGNPYVPVHFDASV

219	1f0vA (124)<br>1a5qA (124)	C	99.19	TM-align: aliSize=115 (resi) RMSD=1.26 (Å)<br>KETAAAKFERQHMDSSTSAASSSNYCNQMMKSRNLTKDRCKPVNTFVHESLADVQAVCSQKNVACKNGQTNCYQSYSTMSITDCRETGSSKYPNCAYKTTQANKHIIVACEGNPYvpvhfdasv---------<br>KETAAAKFERQHMDSSTSAASSSNYCNQMMKSRNLTKDRCKPVNTFVHESLADVQAVCSQKNVACKNGQTNCYQSYSTMSITDCRETGSSKYANCAYKTTQANKHIIVACEGNPY---------vpvhfdasv<br><br>SARST: aliSize=122 (resi) RMSD=8.77 (Å)<br>k-ETAAAKFERQHMDSSTSAASSSNYCNQMMKSRNLTKDRCKPVNTFVHESLADVQAVCSQKNVACKNGQTNCYQSYSTMSITDCRETGSSKYPNCAYKTTQANKHIIVACEGNPYVPVHFDAS-v<br>-kETAAAKFERQHMDSSTSAASSSNYCNQMMKSRNLTKDRCKPVNTFVHESLADVQAVCSQKNVACKNGQTNCYQSYSTMSITDCRETGSSKYANCAYKTTQANKHIIVACEGNPYVPVHFDASv-<br><br>BLAST: aliSize=123 (resi) iden=99.19% (123/124) simi=99.19% (123/124)<br>KETAAAKFERQHMDSSTSAASSSNYCNQMMKSRNLTKDRCKPVNTFVHESLADVQAVCSQKNVACKNGQTNCYQSYSTMSITDCRETGSSKYPNCAYKTTQANKHIIVACEGNPYVPVHFDASV<br>KETAAAKFERQHMDSSTSAASSSNYCNQMMKSRNLTKDRCKPVNTFVHESLADVQAVCSQKNVACKNGQTNCYQSYSTMSITDCRETGSSKYANCAYKTTQANKHIIVACEGNPYVPVHFDASV<br><br>Proposed: aliSize=124 (resi) RMSD=0.45 (Å)<br>KETAAAKFERQHMDSSTSAASSSNYCNQMMKSRNLTKDRCKPVNTFVHESLADVQAVCSQKNVACKNGQTNCYQSYSTMSITDCRETGSSKYPNCAYKTTQANKHIIVACEGNPYVPVHFDASV<br>KETAAAKFERQHMDSSTSAASSSNYCNQMMKSRNLTKDRCKPVNTFVHESLADVQAVCSQKNVACKNGQTNCYQSYSTMSITDCRETGSSKYANCAYKTTQANKHIIVACEGNPYVPVHFDASV
220	1f0vA (124)<br>1awzA (123)	C	27.64	TM-align: aliSize=105 (resi) RMSD=1.97 (Å)<br>KE-TAAAKFERQHMDSsTsAAS-SSNYCNQMMKSRNLTKdRCKPVNTFVHESLADVQAVCS---QKNVAckngqTNCYQSYSTMSITDCRETGSSKYPNCAYKTTQANKHIIVACEGNpyvpvhfdasv-------------<br>QDnSRYTHFLTQHYDA-K-PQGrDDRYCESIMRRRGLTS-PCKDINTFIHGNKRSIKAICEnknGNPHR-----ENLRISKSSFQVTTCKLHGGSPWPPCQYRATAGFRNVVVACENG-----------lpvhldqsifrrp<br><br>SARST: aliSize=92 (resi) RMSD=10.72 (Å)<br>ket----AAAKFERQHMDSSTSAAsSSNYCNQMMKSRNLtkdrckpvntfvhesladvqavCSQKN---VAC--------KNGQT--------NCYQSYSTMSITDCRETGSSKYPNCAYKTTQANKHIIVACegNPYVPVHF--------dasv<br>---qdnsRYTHFLTQHYDAKPQGR-DDRYCESIMRRRGL----------------------TSPCKdinTFIhgnkrsikAICENkngnphreNLRISKSSFQVTTCKLHGGSPWPPCQYRATAGFRNVVVAC--ENGLPVHLdqsifrrp----<br><br>BLAST: aliSize=61 (resi) iden=32.52% (40/123) simi=49.59% (61/123)<br>ketaaak--------FERQHMDSSTSAASSsNYCNQMMKSRNLTKDrCKPVNTFVHESLADVQAVCSQKNVacKNGQTNCYQSYSTMSITDCRETGSSKYPNCAYKTTQANKHIIVACEGNpyVPVHFDASV----<br>-------qdnsrythFLTQHYDAKPQGRDD-RYCESIMRRRGLTSP-CKDINTFIHGNKRSIKAICENKNG--NPHRENLRISKSSFQVTTCKLHGGSPWPPCQYRATAGFRNVVVACENG--LPVHLDQSIfrrp<br><br>Proposed: aliSize=115 (resi) RMSD=1.75 (Å)<br>kE--TAAAKFERQHMDSST-saASSSNYCNQMMKSRNLTKdRCKPVNTFVHESLADVQAVCS---QKNVAckngqTNCYQSYSTMSITDCRETGSSKYPNCAYKTTQANKHIIVACEGNPYVPVHFD--ASV<br>-QdnSRYTHFLTQHYDAKPq--GRDDRYCESIMRRRGLTS-PCKDINTFIHGNKRSIKAICEnknGNPHR-----ENLRISKSSFQVTTCKLHGGSPWPPCQYRATAGFRNVVVACENGLPVHLDQSifRRP
221	1f0vA (124)<br>1b6vA (124)	C	94.35	TM-align: aliSize=115 (resi) RMSD=1.29 (Å)<br>KETAAAKFERQHMDSSTSAASSSNYCNQMMKSRNLTKDRCKPVNTFVHESLADVQAVCSQKNVACKNGQTNCYQSYSTMSITDCRETGSSKYPNCAYKTTQANKHIIVACEGNPYvpvhfdasv---------<br>KETAAAKFERQHMDSSTSAASSSNYCNQMMKSRNLTKDRCKPVNTFVHESLADVKAVCSQKKVTCKNGQTNCYQSKSTMRITDCRETGSSKYPNCAYKTTQANKHIIVACGGKPY---------vpvhfdasv<br><br>SARST: aliSize=122 (resi) RMSD=8.77 (Å)<br>k-ETAAAKFERQHMDSSTSAASSSNYCNQMMKSRNLTKDRCKPVNTFVHESLADVQAVCSQKNVACKNGQTNCYQSYSTMSITDCRETGSSKYPNCAYKTTQANKHIIVACEGNPYVPVHFDAS-v<br>-kETAAAKFERQHMDSSTSAASSSNYCNQMMKSRNLTKDRCKPVNTFVHESLADVKAVCSQKKVTCKNGQTNCYQSKSTMRITDCRETGSSKYPNCAYKTTQANKHIIVACGGKPYVPVHFDASv-<br><br>BLAST: aliSize=118 (resi) iden=94.35% (117/124) simi=95.16% (118/124)<br>KETAAAKFERQHMDSSTSAASSSNYCNQMMKSRNLTKDRCKPVNTFVHESLADVQAVCSQKNVACKNGQTNCYQSYSTMSITDCRETGSSKYPNCAYKTTQANKHIIVACEGNPYVPVHFDASV<br>KETAAAKFERQHMDSSTSAASSSNYCNQMMKSRNLTKDRCKPVNTFVHESLADVKAVCSQKKVTCKNGQTNCYQSKSTMRITDCRETGSSKYPNCAYKTTQANKHIIVACGGKPYVPVHFDASV<br><br>Proposed: aliSize=124 (resi) RMSD=0.56 (Å)<br>KETAAAKFERQHMDSSTSAASSSNYCNQMMKSRNLTKDRCKPVNTFVHESLADVQAVCSQKNVACKNGQTNCYQSYSTMSITDCRETGSSKYPNCAYKTTQANKHIIVACEGNPYVPVHFDASV<br>KETAAAKFERQHMDSSTSAASSSNYCNQMMKSRNLTKDRCKPVNTFVHESLADVKAVCSQKKVTCKNGQTNCYQSKSTMRITDCRETGSSKYPNCAYKTTQANKHIIVACGGKPYVPVHFDASV

222	1f0vA<br>(124)<br><br>1c8wA<br>(124)	C	99.19	TM-align: aliSize=115 (resi) RMSD=1.38 (Å)<br>KETAAAKFERQHMDSSTSAASSSNYCNQMMKSRNLTKDRCKPVNTFVHESLADVQAVCSQKNVACKNGQTNCYQSYSTMSITDCRETGSSKYPNCAYKTTQANKHIIVACEGNPYvpvhfdasv---------<br>KETAAAKFERQHMDSSTSAASSSNYCNQMMKSRNLTKDRCKPVNGFVHESLADVQAVCSQKNVACKNGQTNCYQSYSTMSITDCRETGSSKYPNCAYKTTQANKHIIVACEGNPY---------vpvhfdasv<br><br>SARST: aliSize=122 (resi) RMSD=8.84 (Å)<br>k-ETAAAKFERQHMDSSTSAASSSNYCNQMMKSRNLTKDRCKPVNTFVHESLADVQAVCSQKNVACKNGQTNCYQSYSTMSITDCRETGSSKYPNCAYKTTQANKHIIVACEGNPYVPVHFDAS-v<br>-kETAAAKFERQHMDSSTSAASSSNYCNQMMKSRNLTKDRCKPVNGFVHESLADVQAVCSQKNVACKNGQTNCYQSYSTMSITDCRETGSSKYPNCAYKTTQANKHIIVACEGNPYVPVHFDASv-<br><br>BLAST: aliSize=123 (resi) iden=99.19% (123/124) simi=99.19% (123/124)<br>KETAAAKFERQHMDSSTSAASSSNYCNQMMKSRNLTKDRCKPVNTFVHESLADVQAVCSQKNVACKNGQTNCYQSYSTMSITDCRETGSSKYPNCAYKTTQANKHIIVACEGNPYVPVHFDASV<br>KETAAAKFERQHMDSSTSAASSSNYCNQMMKSRNLTKDRCKPVNGFVHESLADVQAVCSQKNVACKNGQTNCYQSYSTMSITDCRETGSSKYPNCAYKTTQANKHIIVACEGNPYVPVHFDASV<br><br>Proposed: aliSize=124 (resi) RMSD=0.66 (Å)<br>KETAAAKFERQHMDSSTSAASSSNYCNQMMKSRNLTKDRCKPVNTFVHESLADVQAVCSQKNVACKNGQTNCYQSYSTMSITDCRETGSSKYPNCAYKTTQANKHIIVACEGNPYVPVHFDASV<br>KETAAAKFERQHMDSSTSAASSSNYCNQMMKSRNLTKDRCKPVNGFVHESLADVQAVCSQKNVACKNGQTNCYQSYSTMSITDCRETGSSKYPNCAYKTTQANKHIIVACEGNPYVPVHFDASV
223	1f0vA<br>(124)<br><br>1c9vA<br>(124)	C	99.19	TM-align: aliSize=114 (resi) RMSD=0.97 (Å)<br>KETAAAKFERQHMDSSTSAASSSNYCNQMMKSRNLTKDRCKPVNTFVHESLADVQAVCSQKNVACKNGQTNCYQSYSTMSITDCRETGSSKYPNCAYKTTQANKHIIVACEGNPyvpvhfdasv----------<br>KETAAAKFERQAMDSSTSAASSSNYCNQMMKSRNLTKDRCKPVNTFVHESLADVQAVCSQKNVACKNGQTNCYQSYSTMSITDCRETGSSKYPNCAYKTTQANKHIIVACEGNP----------yvpvhfdasv<br><br>SARST: aliSize=122 (resi) RMSD=8.85 (Å)<br>k-ETAAAKFERQHMDSSTSAASSSNYCNQMMKSRNLTKDRCKPVNTFVHESLADVQAVCSQKNVACKNGQTNCYQSYSTMSITDCRETGSSKYPNCAYKTTQANKHIIVACEGNPYVPVHFDAS-v<br>-kETAAAKFERQAMDSSTSAASSSNYCNQMMKSRNLTKDRCKPVNTFVHESLADVQAVCSQKNVACKNGQTNCYQSYSTMSITDCRETGSSKYPNCAYKTTQANKHIIVACEGNPYVPVHFDASv-<br><br>BLAST: aliSize=123 (resi) iden=99.19% (123/124) simi=99.19% (123/124)<br>KETAAAKFERQHMDSSTSAASSSNYCNQMMKSRNLTKDRCKPVNTFVHESLADVQAVCSQKNVACKNGQTNCYQSYSTMSITDCRETGSSKYPNCAYKTTQANKHIIVACEGNPYVPVHFDASV<br>KETAAAKFERQAMDSSTSAASSSNYCNQMMKSRNLTKDRCKPVNTFVHESLADVQAVCSQKNVACKNGQTNCYQSYSTMSITDCRETGSSKYPNCAYKTTQANKHIIVACEGNPYVPVHFDASV<br><br>Proposed: aliSize=124 (resi) RMSD=0.56 (Å)<br>KETAAAKFERQHMDSSTSAASSSNYCNQMMKSRNLTKDRCKPVNTFVHESLADVQAVCSQKNVACKNGQTNCYQSYSTMSITDCRETGSSKYPNCAYKTTQANKHIIVACEGNPYVPVHFDASV<br>KETAAAKFERQAMDSSTSAASSSNYCNQMMKSRNLTKDRCKPVNTFVHESLADVQAVCSQKNVACKNGQTNCYQSYSTMSITDCRETGSSKYPNCAYKTTQANKHIIVACEGNPYVPVHFDASV
224	1f0vA<br>(124)<br><br>1c9xA<br>(124)	C	99.19	TM-align: aliSize=115 (resi) RMSD=1.30 (Å)<br>KETAAAKFERQHMDSSTSAASSSNYCNQMMKSRNLTKDRCKPVNTFVHESLADVQAVCSQKNVACKNGQTNCYQSYSTMSITDCRETGSSKYPNCAYKTTQANKHIIVACEGNPYvpvhfdasv---------<br>KETAAAKFERQHMDSSTSAASSSNYCNQMMKSRNLTKDRCKPVNTFVHESLADVQAVCSQKNVACKNGQTNCYQSYSTMSITDCRETGSSKYPNCAYKTTQANKHIIVACEGNPY---------vpvafdasv<br><br>SARST: aliSize=122 (resi) RMSD=8.84 (Å)<br>k-ETAAAKFERQHMDSSTSAASSSNYCNQMMKSRNLTKDRCKPVNTFVHESLADVQAVCSQKNVACKNGQTNCYQSYSTMSITDCRETGSSKYPNCAYKTTQANKHIIVACEGNPYVPVHFDAS-v<br>-kETAAAKFERQHMDSSTSAASSSNYCNQMMKSRNLTKDRCKPVNTFVHESLADVQAVCSQKNVACKNGQTNCYQSYSTMSITDCRETGSSKYPNCAYKTTQANKHIIVACEGNPYVPVAFDASv-<br><br>BLAST: aliSize=123 (resi) iden=99.19% (123/124) simi=99.19% (123/124)<br>KETAAAKFERQHMDSSTSAASSSNYCNQMMKSRNLTKDRCKPVNTFVHESLADVQAVCSQKNVACKNGQTNCYQSYSTMSITDCRETGSSKYPNCAYKTTQANKHIIVACEGNPYVPVHFDASV<br>KETAAAKFERQHMDSSTSAASSSNYCNQMMKSRNLTKDRCKPVNTFVHESLADVQAVCSQKNVACKNGQTNCYQSYSTMSITDCRETGSSKYPNCAYKTTQANKHIIVACEGNPYVPVAFDASV<br><br>Proposed: aliSize=124 (resi) RMSD=0.48 (Å)<br>KETAAAKFERQHMDSSTSAASSSNYCNQMMKSRNLTKDRCKPVNTFVHESLADVQAVCSQKNVACKNGQTNCYQSYSTMSITDCRETGSSKYPNCAYKTTQANKHIIVACEGNPYVPVHFDASV<br>KETAAAKFERQHMDSSTSAASSSNYCNQMMKSRNLTKDRCKPVNTFVHESLADVQAVCSQKNVACKNGQTNCYQSYSTMSITDCRETGSSKYPNCAYKTTQANKHIIVACEGNPYVPVAFDASV

225	1f0vA<br>(124)<br><br>1e21A<br>(119)	C	68.91	TM-align: aliSize=108 (resi) RMSD=1.77 (Å)<br>ketaaaKFERQHMDSSTsAAS-SSNYCNQMMKSRNLTKDRCKPVNTFVHESLADVQAVCSQKNVACKNGQTNCYQSYSTMSITDCRETGSSKYPNCAYKTTQANKHIIVACEGNPYvpvhfdasv---------<br>------AFQRQHMDSDS-SPSsSSTYCNQMMRRRNMTQGRCKPVNTFVHEPLVDVQNVCFQEKVTCKNGQGNCYKSNSSMHITDCRLTNGSRYPNCAYRTSPKERHIIVACEGSPY---------vpvhfdasve<br><br>SARST: aliSize=116 (resi) RMSD=9.09 (Å)<br>ketaaak-FERQHMDSSTSAASSSNYCNQMMKSRNLTKDRCKPVNTFVHESLADVQAVCSQKNVACKNGQTNCYQSYSTMSITDCRETGSSKYPNCAYKTTQANKHIIVACEGNPYVPVHFDAS--v<br>-------aFQRQHMDSDSSPSSSSTYCNQMMRRRNMTQGRCKPVNTFVHEPLVDVQNVCFQEKVTCKNGQGNCYKSNSSMHITDCRLTNGSRYPNCAYRTSPKERHIIVACEGSPYVPVHFDASve-<br><br>BLAST: aliSize=97 (resi) iden=69.75% (83/119) simi=81.51% (97/119)<br>ketaaak-FERQHMDSSTSAASSSNYCNQMMKSRNLTKDRCKPVNTFVHESLADVQAVCSQKNVACKNGQTNCYQSYSTMSITDCRETGSSKYPNCAYKTTQANKHIIVACEGNPYVPVHFDASV-<br>-------aFQRQHMDSDSSPSSSSTYCNQMMRRRNMTQGRCKPVNTFVHEPLVDVQNVCFQEKVTCKNGQGNCYKSNSSMHITDCRLTNGSRYPNCAYRTSPKERHIIVACEGSPYVPVHFDASVe<br><br>Proposed: aliSize=117 (resi) RMSD=1.21 (Å)<br>ketaaaKFERQHMDSSTsAASSS-NYCNQMMKSRNLTKDRCKPVNTFVHESLADVQAVCSQKNVACKNGQTNCYQSYSTMSITDCRETGSSKYPNCAYKTTQANKHIIVACEGNPYVPVHFDASV-<br>------AFQRQHMDSDS-SPSSSsTYCNQMMRRRNMTQGRCKPVNTFVHEPLVDVQNVCFQEKVTCKNGQGNCYKSNSSMHITDCRLTNGSRYPNCAYRTSPKERHIIVACEGSPYVPVHFDASVe
226	1f0vA<br>(124)<br><br>1eicA<br>(124)	C	99.19	TM-align: aliSize=115 (resi) RMSD=1.32 (Å)<br>KETAAAKFERQHMDSSTSAASSSNYCNQMMKSRNLTKDRCKPVNTFVHESLADVQAVCSQKNVACKNGQTNCYQSYSTMSITDCRETGSSKYPNCAYKTTQANKHIIVACEGNPYvpvhfdasv---------<br>KETAAAKFERQHMDSSTSAASSSNYCNQMMKSRNLTKDRCKPVNTFVHESLADVQAVCSQKNVACKNGQTNCYQSYSTMSITDCRETGSSKYPNCAYKTTQANKHIIVACEGNPY---------vpvhadasv<br><br>SARST: aliSize=122 (resi) RMSD=8.81 (Å)<br>k-ETAAAKFERQHMDSSTSAASSSNYCNQMMKSRNLTKDRCKPVNTFVHESLADVQAVCSQKNVACKNGQTNCYQSYSTMSITDCRETGSSKYPNCAYKTTQANKHIIVACEGNPYVPVHFDAS-v<br>-kETAAAKFERQHMDSSTSAASSSNYCNQMMKSRNLTKDRCKPVNTFVHESLADVQAVCSQKNVACKNGQTNCYQSYSTMSITDCRETGSSKYPNCAYKTTQANKHIIVACEGNPYVPVHADASv-<br><br>BLAST: aliSize=123 (resi) iden=99.19% (123/124) simi=99.19% (123/124)<br>KETAAAKFERQHMDSSTSAASSSNYCNQMMKSRNLTKDRCKPVNTFVHESLADVQAVCSQKNVACKNGQTNCYQSYSTMSITDCRETGSSKYPNCAYKTTQANKHIIVACEGNPYVPVHFDASV<br>KETAAAKFERQHMDSSTSAASSSNYCNQMMKSRNLTKDRCKPVNTFVHESLADVQAVCSQKNVACKNGQTNCYQSYSTMSITDCRETGSSKYPNCAYKTTQANKHIIVACEGNPYVPVHADASV<br><br>Proposed: aliSize=124 (resi) RMSD=0.56 (Å)<br>KETAAAKFERQHMDSSTSAASSSNYCNQMMKSRNLTKDRCKPVNTFVHESLADVQAVCSQKNVACKNGQTNCYQSYSTMSITDCRETGSSKYPNCAYKTTQANKHIIVACEGNPYVPVHFDASV<br>KETAAAKFERQHMDSSTSAASSSNYCNQMMKSRNLTKDRCKPVNTFVHESLADVQAVCSQKNVACKNGQTNCYQSYSTMSITDCRETGSSKYPNCAYKTTQANKHIIVACEGNPYVPVHADASV
227	1f0vA<br>(124)<br><br>1eidA<br>(124)	C	99.19	TM-align: aliSize=115 (resi) RMSD=1.33 (Å)<br>KETAAAKFERQHMDSSTSAASSSNYCNQMMKSRNLTKDRCKPVNTFVHESLADVQAVCSQKNVACKNGQTNCYQSYSTMSITDCRETGSSKYPNCAYKTTQANKHIIVACEGNPYvpvhfdasv---------<br>KETAAAKFERQHMDSSTSAASSSNYCNQMMKSRNLTKDRCKPVNTFVHESLADVQAVCSQKNVACKNGQTNCYQSYSTMSITDCRETGSSKYPNCAYKTTQANKHIIVACEGNPY---------vpvhgdasv<br><br>SARST: aliSize=119 (resi) RMSD=5.65 (Å)<br>k-ETAAAKFERQHMDSSTSAASSSNYCNQMMKSRNLTKDRCKPVNTFVHESLADVQAVCSQKNVACKNGQTNCYQSYSTMSITDCRETGSSKYPNCAYKTTQANKHIIVACEGNPYVPVHF----dasv<br>-kETAAAKFERQHMDSSTSAASSSNYCNQMMKSRNLTKDRCKPVNTFVHESLADVQAVCSQKNVACKNGQTNCYQSYSTMSITDCRETGSSKYPNCAYKTTQANKHIIVACEGNPYVPVHGdasv----<br><br>BLAST: aliSize=123 (resi) iden=99.19% (123/124) simi=99.19% (123/124)<br>KETAAAKFERQHMDSSTSAASSSNYCNQMMKSRNLTKDRCKPVNTFVHESLADVQAVCSQKNVACKNGQTNCYQSYSTMSITDCRETGSSKYPNCAYKTTQANKHIIVACEGNPYVPVHFDASV<br>KETAAAKFERQHMDSSTSAASSSNYCNQMMKSRNLTKDRCKPVNTFVHESLADVQAVCSQKNVACKNGQTNCYQSYSTMSITDCRETGSSKYPNCAYKTTQANKHIIVACEGNPYVPVHGDASV<br><br>Proposed: aliSize=124 (resi) RMSD=0.61 (Å)<br>KETAAAKFERQHMDSSTSAASSSNYCNQMMKSRNLTKDRCKPVNTFVHESLADVQAVCSQKNVACKNGQTNCYQSYSTMSITDCRETGSSKYPNCAYKTTQANKHIIVACEGNPYVPVHFDASV<br>KETAAAKFERQHMDSSTSAASSSNYCNQMMKSRNLTKDRCKPVNTFVHESLADVQAVCSQKNVACKNGQTNCYQSYSTMSITDCRETGSSKYPNCAYKTTQANKHIIVACEGNPYVPVHGDASV

228	1f0vA (124)<br>1eieA (124)	C	99.19	TM-align: aliSize=115 (resi) RMSD=1.33 (Å)<br>KETAAAKFERQHMDSSTSAASSSNYCNQMMKSRNLTKDRCKPVNTFVHESLADVQAVCSQKNVACKNGQTNCYQSYSTMSITDCRETGSSKYPNCAYKTTQANKHIIVACEGNPYvpvhfdasv---------<br>KETAAAKFERQHMDSSTSAASSSNYCNQMMKSRNLTKDRCKPVNTFVHESLADVQAVCSQKNVACKNGQTNCYQSYSTMSITDCRETGSSKYPNCAYKTTQANKHIIVACEGNPY---------vpvhwdasv<br><br>SARST: aliSize=122 (resi) RMSD=8.81 (Å)<br>k-ETAAAKFERQHMDSSTSAASSSNYCNQMMKSRNLTKDRCKPVNTFVHESLADVQAVCSQKNVACKNGQTNCYQSYSTMSITDCRETGSSKYPNCAYKTTQANKHIIVACEGNPYVPVHFDAS-v<br>-kETAAAKFERQHMDSSTSAASSSNYCNQMMKSRNLTKDRCKPVNTFVHESLADVQAVCSQKNVACKNGQTNCYQSYSTMSITDCRETGSSKYPNCAYKTTQANKHIIVACEGNPYVPVHWDASv-<br><br>BLAST: aliSize=124 (resi) iden=99.19% (123/124) simi=100.00% (124/124)<br>KETAAAKFERQHMDSSTSAASSSNYCNQMMKSRNLTKDRCKPVNTFVHESLADVQAVCSQKNVACKNGQTNCYQSYSTMSITDCRETGSSKYPNCAYKTTQANKHIIVACEGNPYVPVHFDASV<br>KETAAAKFERQHMDSSTSAASSSNYCNQMMKSRNLTKDRCKPVNTFVHESLADVQAVCSQKNVACKNGQTNCYQSYSTMSITDCRETGSSKYPNCAYKTTQANKHIIVACEGNPYVPVHWDASV<br><br>Proposed: aliSize=124 (resi) RMSD=0.56 (Å)<br>KETAAAKFERQHMDSSTSAASSSNYCNQMMKSRNLTKDRCKPVNTFVHESLADVQAVCSQKNVACKNGQTNCYQSYSTMSITDCRETGSSKYPNCAYKTTQANKHIIVACEGNPYVPVHFDASV<br>KETAAAKFERQHMDSSTSAASSSNYCNQMMKSRNLTKDRCKPVNTFVHESLADVQAVCSQKNVACKNGQTNCYQSYSTMSITDCRETGSSKYPNCAYKTTQANKHIIVACEGNPYVPVHWDASV
229	1f0vA (124)<br>1gioA (125)	C	33.06	TM-align: aliSize=103 (resi) RMSD=2.17 (Å)<br>ke----TAAAKFERQHMDSsTsAASSS-NYCNQMMKSRNLTkDRCKPVNTFVHESLADVQAVCS---QKNVAckngqTNCYQSYSTMSITDCRETGSSKYPNCAYKTTQANKHIIVACEGNpyvpvhfdasv-------------<br>--aqddYRYIHFLTQHYDA-K-PKGRNdEYCFNMMKNRRLT-RPCKDRNTFIHGNKNDIKAICEdrnGQPYR-----GDLRISKSEFQITICKHKGGSSRPPCRYGATEDSRVIVVGCENG-----------lpvhfdesfitprh<br><br>SARST: aliSize=97 (resi) RMSD=6.94 (Å)<br>ketaaakferqhmdssts------------------AASSSNYCNQMMKSRNLTkDRCKPVNTFVHESLADVQAVCSQKNVACKNGqtNCYQSYSTMSITDCRETGSSKYPNCAYKTTQANKHIIVACE--GNPYVPV-------hfdasv<br>-----------------aqddyryihfltqhydakpKGRNDEYCFNMMKNRRLT-RPCKDRNTFIHGNKNDIKAICEDRNGQPYRG--DLRISKSEFQITICKHKGGSSRPPCRYGATEDSRVIVVGCEngLPVHFDEsfitprh------<br><br>BLAST: aliSize=60 (resi) iden=35.48% (44/124) simi=48.39% (60/124)<br>ketaaak---------FERQHMDSSTSAASSSnYCNQMMKSRNLTKDrCKPVNTFVHESLADVQAVCSQKNVACKNGQTNCyqSYSTMSITDCRETGSSKYPNCAYKTTQANKHIIVACEGNpyVPVHFDAS------v<br>-------aqddyryihFLTQHYDAKPKGRNDE-YCFNMMKNRRLTRP-CKDRNTFIHGNKNDIKAICEDRNGQPYRGDLRI--SKSEFQITICKHKGGSSRPPCRYGATEDSRVIVVGCENG--LPVHFDESfitprh-<br><br>Proposed: aliSize=111 (resi) RMSD=1.95 (Å)<br>ke-----TaAAKFERQHMDSsTsAASSS-NYCNQMMKSRNLTkDRCKPVNTFVHESLADVQAVCS---QKNVAckngqTNCYQSYSTMSITDCRETGSSKYPNCAYKTTQANKHIIVACEGnpYVPVHFD-ASV----<br>--aqddyR-YIHFLTQHYDA-K-PKGRNdEYCFNMMKNRRLT-RPCKDRNTFIHGNKNDIKAICEdrnGQPYR-----GDLRISKSEFQITICKHKGGSSRPPCRYGATEDSRVIVVGCEN--GLPVHFDeSFItprh
230	1f0vA (124)<br>1gv7A (123)	C	41.46	TM-align: aliSize=111 (resi) RMSD=2.01 (Å)<br>KETAAAKFERQHMDSSTSAAsSSNYCNQMMKSRNLTkDRCKPVNTFVHESLADVQAVCSQKNVACKNGQTNCYQSYSTMSITDCRETGSSKYPNCAYKTTQANKHIIVACEGNpyvpvhfdasv-----------<br>DNSRYTHFLTQHYDAKPQGR-DDRYCESIMRRRGLT-SPCKDINTFIHGNKRSIKAICSQKNVACKNGQTNCYISKSSFQVTTCKLHGGSPWPPCQYRATAGFRNVVVACENG-----------lpvhldqsifr<br><br>SARST: aliSize=113 (resi) RMSD=5.97 (Å)<br>ket---AAAKFERQHMDSSTSAaSSSNYCNQMMKSRNLTkDRCKPVNTFVHESLADVQAVCSQKNVACKNGQTNCYQSYSTMSITDCRETGSSKYPNCAYKTTQANKHIIVAceGNPYVPVHF------dasv<br>---dnsRYTHFLTQHYDAKPQG-RDDRYCESIMRRRGLT-SPCKDINTFIHGNKRSIKAICSQKNVACKNGQTNCYISKSSFQVTTCKLHGGSPWPPCQYRATAGFRNVVVA--CENGLPVHLdqsifr----<br><br>BLAST: aliSize=72 (resi) iden=42.62% (52/123) simi=59.02% (72/123)<br>ketaaak-------FERQHMDSSTSAASSsNYCNQMMKSRNLTKDrCKPVNTFVHESLADVQAVCSQKNVACKNGQTNCYQSYSTMSITDCRETGSSKYPNCAYKTTQANKHIIVACEGNpyVPVHFDASV--<br>-------dnsrythFLTQHYDAKPQGRDD-RYCESIMRRRGLTSP-CKDINTFIHGNKRSIKAICSQKNVACKNGQTNCYISKSSFQVTTCKLHGGSPWPPCQYRATAGFRNVVVACENG--LPVHLDQSIfr<br><br>Proposed: aliSize=117 (resi) RMSD=1.40 (Å)<br>ke---TAAAKFERQHMDSST-saASSSNYCNQMMKSRNLTkDRCKPVNTFVHESLADVQAVCSQKNVACKNGQTNCYQSYSTMSITDCRETGSSKYPNCAYKTTQANKHIIVACEGnpYVPVHFDA-SV-<br>--pdnSRYTHFLTQHYDAKPq--GRDDRYCESIMRRRGLT-SPCKDINTFIHGNKRSIKAICSQKNVACKNGQTNCYISKSSFQVTTCKLHGGSPWPPCQYRATAGFRNVVVACEN--GLPVHLDQsIFr

231	1f0vA (124)<br>1izpA (124)	C	99.19	TM-align: aliSize=115 (resi) RMSD=1.30 (Å)<br>KETAAAKFERQHMDSSTSAASSSNYCNQMMKSRNLTKDRCKPVNTFVHESLADVQAVCSQKNVACKNGQTNCYQSYSTMSITDCRETGSSKYPNCAYKTTQANKHIIVACEGNPYvpvhfdasv---------<br>KETAAAKFERQHMDSSTSAASSSNYCNQMMKSRNLTKDRCKPVNTLVHESLADVQAVCSQKNVACKNGQTNCYQSYSTMSITDCRETGSSKYPNCAYKTTQANKHIIVACEGNPY---------vpvhfdasv<br><br>SARST: aliSize=122 (resi) RMSD=8.84 (Å)<br>k-ETAAAKFERQHMDSSTSAASSSNYCNQMMKSRNLTKDRCKPVNTFVHESLADVQAVCSQKNVACKNGQTNCYQSYSTMSITDCRETGSSKYPNCAYKTTQANKHIIVACEGNPYVPVHFDAS-v<br>-kETAAAKFERQHMDSSTSAASSSNYCNQMMKSRNLTKDRCKPVNTLVHESLADVQAVCSQKNVACKNGQTNCYQSYSTMSITDCRETGSSKYPNCAYKTTQANKHIIVACEGNPYVPVHFDASv-<br><br>BLAST: aliSize=123 (resi) iden=99.19% (123/124) simi=99.19% (123/124)<br>KETAAAKFERQHMDSSTSAASSSNYCNQMMKSRNLTKDRCKPVNTFVHESLADVQAVCSQKNVACKNGQTNCYQSYSTMSITDCRETGSSKYPNCAYKTTQANKHIIVACEGNPYVPVHFDASV<br>KETAAAKFERQHMDSSTSAASSSNYCNQMMKSRNLTKDRCKPVNTLVHESLADVQAVCSQKNVACKNGQTNCYQSYSTMSITDCRETGSSKYPNCAYKTTQANKHIIVACEGNPYVPVHFDASV<br><br>Proposed: aliSize=124 (resi) RMSD=0.48 (Å)<br>KETAAAKFERQHMDSSTSAASSSNYCNQMMKSRNLTKDRCKPVNTFVHESLADVQAVCSQKNVACKNGQTNCYQSYSTMSITDCRETGSSKYPNCAYKTTQANKHIIVACEGNPYVPVHFDASV<br>KETAAAKFERQHMDSSTSAASSSNYCNQMMKSRNLTKDRCKPVNTLVHESLADVQAVCSQKNVACKNGQTNCYQSYSTMSITDCRETGSSKYPNCAYKTTQANKHIIVACEGNPYVPVHFDASV
232	1f0vA (124)<br>1izqA (124)	C	99.19	TM-align: aliSize=115 (resi) RMSD=1.32 (Å)<br>KETAAAKFERQHMDSSTSAASSSNYCNQMMKSRNLTKDRCKPVNTFVHESLADVQAVCSQKNVACKNGQTNCYQSYSTMSITDCRETGSSKYPNCAYKTTQANKHIIVACEGNPYvpvhfdasv---------<br>KETAAAKFERQHMDSSTSAASSSNYCNQMMKSRNLTKDRCKPVNTVVHESLADVQAVCSQKNVACKNGQTNCYQSYSTMSITDCRETGSSKYPNCAYKTTQANKHIIVACEGNPY---------vpvhfdasv<br><br>SARST: aliSize=122 (resi) RMSD=8.83 (Å)<br>k-ETAAAKFERQHMDSSTSAASSSNYCNQMMKSRNLTKDRCKPVNTFVHESLADVQAVCSQKNVACKNGQTNCYQSYSTMSITDCRETGSSKYPNCAYKTTQANKHIIVACEGNPYVPVHFDAS-v<br>-kETAAAKFERQHMDSSTSAASSSNYCNQMMKSRNLTKDRCKPVNTVVHESLADVQAVCSQKNVACKNGQTNCYQSYSTMSITDCRETGSSKYPNCAYKTTQANKHIIVACEGNPYVPVHFDASv-<br><br>BLAST: aliSize=123 (resi) iden=99.19% (123/124) simi=99.19% (123/124)<br>KETAAAKFERQHMDSSTSAASSSNYCNQMMKSRNLTKDRCKPVNTFVHESLADVQAVCSQKNVACKNGQTNCYQSYSTMSITDCRETGSSKYPNCAYKTTQANKHIIVACEGNPYVPVHFDASV<br>KETAAAKFERQHMDSSTSAASSSNYCNQMMKSRNLTKDRCKPVNTVVHESLADVQAVCSQKNVACKNGQTNCYQSYSTMSITDCRETGSSKYPNCAYKTTQANKHIIVACEGNPYVPVHFDASV<br><br>Proposed: aliSize=124 (resi) RMSD=0.52 (Å)<br>KETAAAKFERQHMDSSTSAASSSNYCNQMMKSRNLTKDRCKPVNTFVHESLADVQAVCSQKNVACKNGQTNCYQSYSTMSITDCRETGSSKYPNCAYKTTQANKHIIVACEGNPYVPVHFDASV<br>KETAAAKFERQHMDSSTSAASSSNYCNQMMKSRNLTKDRCKPVNTVVHESLADVQAVCSQKNVACKNGQTNCYQSYSTMSITDCRETGSSKYPNCAYKTTQANKHIIVACEGNPYVPVHFDASV
233	1f0vA (124)<br>1izrA (124)	C	99.19	TM-align: aliSize=115 (resi) RMSD=1.55 (Å)<br>KETAAAKFERQHMDSSTSAASSSNYCNQMMKSRNLTKDRCKPVNTFVHESLADVQAVCSQKNVACKNGQTNCYQSYSTMSITDCRETGSSKYPNCAYKTTQANKHIIVACEGNPYvpvhfdasv---------<br>KETAAAKFERQHMDSSTSAASSSNYCNQMMKSRNLTKDRCKPVNTAVHESLADVQAVCSQKNVACKNGQTNCYQSYSTMSITDCRETGSSKYPNCAYKTTQANKHIIVACEGNPY---------vpvhfdasv<br><br>SARST: aliSize=122 (resi) RMSD=8.86 (Å)<br>k-ETAAAKFERQHMDSSTSAASSSNYCNQMMKSRNLTKDRCKPVNTFVHESLADVQAVCSQKNVACKNGQTNCYQSYSTMSITDCRETGSSKYPNCAYKTTQANKHIIVACEGNPYVPVHFDAS-v<br>-kETAAAKFERQHMDSSTSAASSSNYCNQMMKSRNLTKDRCKPVNTAVHESLADVQAVCSQKNVACKNGQTNCYQSYSTMSITDCRETGSSKYPNCAYKTTQANKHIIVACEGNPYVPVHFDASv-<br><br>BLAST: aliSize=123 (resi) iden=99.19% (123/124) simi=99.19% (123/124)<br>KETAAAKFERQHMDSSTSAASSSNYCNQMMKSRNLTKDRCKPVNTFVHESLADVQAVCSQKNVACKNGQTNCYQSYSTMSITDCRETGSSKYPNCAYKTTQANKHIIVACEGNPYVPVHFDASV<br>KETAAAKFERQHMDSSTSAASSSNYCNQMMKSRNLTKDRCKPVNTAVHESLADVQAVCSQKNVACKNGQTNCYQSYSTMSITDCRETGSSKYPNCAYKTTQANKHIIVACEGNPYVPVHFDASV<br><br>Proposed: aliSize=124 (resi) RMSD=0.96 (Å)<br>KETAAAKFERQHMDSSTSAASSSNYCNQMMKSRNLTKDRCKPVNTFVHESLADVQAVCSQKNVACKNGQTNCYQSYSTMSITDCRETGSSKYPNCAYKTTQANKHIIVACEGNPYVPVHFDASV<br>KETAAAKFERQHMDSSTSAASSSNYCNQMMKSRNLTKDRCKPVNTAVHESLADVQAVCSQKNVACKNGQTNCYQSYSTMSITDCRETGSSKYPNCAYKTTQANKHIIVACEGNPYVPVHFDASV

234	1f0vA (124)<br>1j80B (101)	C	100.00	TM-align: aliSize=91 (resi) RMSD=1.07 (Å)<br>ketaaakferqhmdsstsaasssNYCNQMMKSRNLTKDRCKPVNTFVHESLADVQAVCSQKNVACKNGQTNCYQSYSTMSITDCRETGSSKYPNCAYKTTQANKHIIVACEGNPyvpvhfdasv----------<br>-----------------------NYCNQMMKSRNLTKDRCKPVNTFVHESLADVQAVCSQKNVACKNGQTNCYQSYSTMSITDCRETGSSKYPNCAYKTTQANKHIIVACEGNP----------yvpvhfdasv<br><br>SARST: aliSize=99 (resi) RMSD=9.76 (Å)<br>ketaaakferqhmdsstsaasssn-YCNQMMKSRNLTKDRCKPVNTFVHESLADVQAVCSQKNVACKNGQTNCYQSYSTMSITDCRETGSSKYPNCAYKTTQANKHIIVACEGNPYVPVHFDAS-v<br>------------------------nYCNQMMKSRNLTKDRCKPVNTFVHESLADVQAVCSQKNVACKNGQTNCYQSYSTMSITDCRETGSSKYPNCAYKTTQANKHIIVACEGNPYVPVHFDASv-<br><br>BLAST: aliSize=101 (resi) iden=100.00% (101/101) simi=100.00% (101/101)<br>ketaaakferqhmdsstsaasssNYCNQMMKSRNLTKDRCKPVNTFVHESLADVQAVCSQKNVACKNGQTNCYQSYSTMSITDCRETGSSKYPNCAYKTTQANKHIIVACEGNPYVPVHFDASV<br>-----------------------NYCNQMMKSRNLTKDRCKPVNTFVHESLADVQAVCSQKNVACKNGQTNCYQSYSTMSITDCRETGSSKYPNCAYKTTQANKHIIVACEGNPYVPVHFDASV<br><br>Proposed: aliSize=101 (resi) RMSD=0.71 (Å)<br>ketaaakferqhmdsstsaasssNYCNQMMKSRNLTKDRCKPVNTFVHESLADVQAVCSQKNVACKNGQTNCYQSYSTMSITDCRETGSSKYPNCAYKTTQANKHIIVACEGNPYVPVHFDASV<br>-----------------------NYCNQMMKSRNLTKDRCKPVNTFVHESLADVQAVCSQKNVACKNGQTNCYQSYSTMSITDCRETGSSKYPNCAYKTTQANKHIIVACEGNPYVPVHFDASV
235	1f0vA (124)<br>1kh8A (125)	C	97.58	TM-align: aliSize=114 (resi) RMSD=1.19 (Å)<br>-KETAAAKFERQHMDSSTSAASSSNYCNQMMKSRNLTKDRCKPVNTFVHESLADVQAVCSQKNVACKNGQTNCYQSYSTMSITDCRETGSSKYPNCAYKTTQANKHIIVACEGNPyvpvhfdasv----------<br>mKETAAAKFERQHMDSSTSAASSSNYCNQMMKSRNLTKDRCKPVNTFVHESLADVQAVCSQKNVACKNGQTNCYQSYSTMSITDCRETGSSKYPNCAYKTTQANKHIIVACEGNG----------yvpvhfdasv<br><br>SARST: aliSize=122 (resi) RMSD=8.83 (Å)<br>k--ETAAAKFERQHMDSSTSAASSSNYCNQMMKSRNLTKDRCKPVNTFVHESLADVQAVCSQKNVACKNGQTNCYQSYSTMSITDCRETGSSKYPNCAYKTTQANKHIIVACEGNPYVPVHFDAS-v<br>-mkETAAAKFERQHMDSSTSAASSSNYCNQMMKSRNLTKDRCKPVNTFVHESLADVQAVCSQKNVACKNGQTNCYQSYSTMSITDCRETGSSKYPNCAYKTTQANKHIIVACEGNGYVPVHFDASv-<br><br>BLAST: aliSize=123 (resi) iden=99.19% (123/124) simi=99.19% (123/124)<br>-KETAAAKFERQHMDSSTSAASSSNYCNQMMKSRNLTKDRCKPVNTFVHESLADVQAVCSQKNVACKNGQTNCYQSYSTMSITDCRETGSSKYPNCAYKTTQANKHIIVACEGNPYVPVHFDASV<br>mKETAAAKFERQHMDSSTSAASSSNYCNQMMKSRNLTKDRCKPVNTFVHESLADVQAVCSQKNVACKNGQTNCYQSYSTMSITDCRETGSSKYPNCAYKTTQANKHIIVACEGNGYVPVHFDASV<br><br>Proposed: aliSize=122 (resi) RMSD=0.73 (Å)<br>-KETAAAKFERQHMDSSTSAASSSNYCNQMMKSRNLTKDRCKPVNTFVHESLADVQAVCSQKNVACKNGQTNCYQSYSTMSITDCRETGSSKYPNCAYKTTQANKHIIVACE--gnPYVPVHFDASV<br>mKETAAAKFERQHMDSSTSAASSSNYCNQMMKSRNLTKDRCKPVNTFVHESLADVQAVCSQKNVACKNGQTNCYQSYSTMSITDCRETGSSKYPNCAYKTTQANKHIIVACEgn--GYVPVHFDASV
236	1f0vA (124)<br>1kvzA (107)	C	23.36	TM-align: aliSize=92 (resi) RMSD=2.74 (Å)<br>kETAAAKFERQHMDSsTsAASssnYCNQMMKSRNLTkdrCKPVNTFVHESLADVQAVCSQknvackngq---TNCYQSYSTMSITDCRETGSSKypnCAYKTTQANKHIIVACEGNpyvpvhfdasv-----------<br>-MQDWATFKKKHLTD-T-WDV---DCDNLMPTSLFD---CKDKNTFIYSLPGPVKALCRG---------vifSADVLSNSEFYLAECNVKPRKP---CKYKLKKSSNRICIRCEHE-----------lpvhfagvgicp<br><br>SARST: aliSize=99 (resi) RMSD=10.79 (Å)<br>keta---AAKFERQHMdssTSA-ASSSNYCNQMMKSRNltkdrcKPVNTFVHESLADVQAVCSQKNVACkngqtnCYQSYSTMSITDCRETGSSKYpncAYKTTQANKHIIVACEgnPYVPVHFDAS----v<br>----mqdWATFKKKHL---TDTwDVDCDNLMPTSLFDC------KDKNTFIYSLPGPVKALCRGVIFSA------DVLSNSEFYLAECNVKPRKPC---KYKLKKSSNRICIRCE--HELPVHFAGVgicp-<br><br>BLAST: aliSize=53 (resi) iden=27.10% (29/107) simi=49.53% (53/107)<br>ketaa----AKFERQHMDSSTSAAsssnyCNQMMKSrNLTkDrCKPVNTFVHESLADVQAVCsqKNVACKNGQTNCYQSYstmsITDCREtgSSKYPnCAYKTTQANKHIIVACEGNpyVPVHF-------dasv<br>-----mqdwATFKKKHLTDTWDVD-----CDNLMPT-SLF-D-CKDKNTFIYSLPGPVKALC--RGVIFSADVLSNSEFY----LAECNV--KPRKP-CKYKLKKSSNRICIRCEHE--LPVHFagvgicp----<br><br>Proposed: aliSize=96 (resi) RMSD=1.91 (Å)<br>ket--AAAKFERQHMDSsTsAAsssnYcnQMMKSrnLTKD--RCKPVNTFVHESLADVQAVCS----qknvackngqTNCYQSYSTMSITDCRETGSSKypnCAYKTTQANKHIIVACEGnpYVPVHFDASV---<br>---mqDWATFKKKHLTD-T-WD----V--DCDNL--MPTSlfDCKDKNTFIYSLPGPVKALCRgvif----------SADVLSNSEFYLAECNVKPRKP---CKYKLKKSSNRICIRCEH--ELPVHFAGVGicp

237	1f0vA<br>(124)<br>1pu3A<br>(105)	C	24.76	TM-align: aliSize=91 (resi) RMSD=2.37 (Å)<br>kETAAAKFERQHMDSsTsAASssnYCNQMMKSRNLTkdrCKPVNTFVHESLADVQAVCSQknvackngq---TNCYQSYSTMSITDCRETGSSkypnCAYKTTQANKHIIVACEGNpyvpvhfdasv----------<br>-MQDWLTFQKKHITN-T-RDV---DCDNILSTNLFH---CKDKNTFIYSRPEPVKAICKG---------iiaSKNVLTTSEFYLSDCNVTSRP----CKYKLKKSTNKFCVTCENQ-----------apvhfvgvgsc<br><br>SARST: aliSize=99 (resi) RMSD=10.84 (Å)<br>keta---AAKFERQHMDSsTSAASssnycnqMMKSRNLTKDRCKPVNTFVHESLADVQAVCsqknvaCKNGQTNCYQSYSTMSITDCRETGssKYPNcaYKTTQANKHiiVACEGNPYVPVHFDAS---v<br>----mqdWLTFQKKHITN-TRDVD-------CDNILSTNLFHCKDKNTFIYSRPEPVKAIC------KGIIASKNVLTTSEFYLSDCNVTS--RPCK--YKLKKSTNK--FCVTCENQAPVHFVGVgsc-<br><br>BLAST: aliSize=54 (resi) iden=32.38% (34/105) simi=51.43% (54/105)<br>ketaaak------FERQHMDSSTSAAsssnyCNQMMkSRNLTkdRCKPVNTFVHESLADVQAVCsQKNVACKNGQTNcyqsySTMSITDCRETgsSKyPnCAYKTTQANKHIIVACEGNpyVPVHF------dasv<br>-------mqdwltFQKKHITNTRDVD-----CDNIL-STNLF--HCKDKNTFIYSRPEPVKAIC-KGIIASKNVLTT-----SEFYLSDCNVT--SR-P-CKYKLKKSTNKFCVTCENQ--APVHFvgvgsc----<br><br>Proposed: aliSize=97 (resi) RMSD=1.68 (Å)<br>keTA-AAKFERQHMDSsTsAASssnYCNQMMKSRNLTkdrCKPVNTFVHESLADVQAVCS-----qknvackngqtNCYQSYSTMSITDCRETGSSkypnCAYKTTQANKHIIVACEGnpYVPVHFDASV--<br>--MQdWLTFQKKHITN-T-RDV---DCDNILSTNLFH---CKDKNTFIYSRPEPVKAICKgiias-----------KNVLTTSEFYLSDCNVTSRP----CKYKLKKSTNKFCVTCEN--QAPVHFVGVGsc
238	1f0vA<br>(124)<br>1qwqA<br>(124)	C	77.42	TM-align: aliSize=113 (resi) RMSD=2.45 (Å)<br>k-ETAAAKFERQHMDSSTSaASSSN-YCNQMMKSRNLTKDRCKPVNTFVHESLADVQAVCSQKNVACKNGQTNCYQSYSTMSITDCRETGSSKYPNCAYKTTQANKHIIVACEGNPYvpvhfdasv---------<br>-kESAAAKFERQHMDSGNS-PSSSSnYCNLMMCCRKMTQGKCKPVNTFVHESLADVKAVCSQKKVTCKDGQTNCYQSKSTMRITDCRETGSSKYPNCAYKTTQVEKHIIVACGGKPS---------vpvhfdasv<br><br>SARST: aliSize=120 (resi) RMSD=9.19 (Å)<br>k-ETAAAKFERQHmdSSTSAASSSNYCNQMMKS--RNLTKDRCKPVNTFVHESLADVQAVCSQKNVACKNGQTNCYQSYSTMSITDCRETGSSKYPNCAYKTTQANKHIIVACEGNPYVPVHFDAS-v<br>-kESAAAKFERQH--MDSGNSPSSSSNYCNLMMccRKMTQGKCKPVNTFVHESLADVKAVCSQKKVTCKDGQTNCYQSKSTMRITDCRETGSSKYPNCAYKTTQVEKHIIVACGGKPSVPVHFDASv-<br><br>BLAST: aliSize=107 (resi) iden=80.65% (100/124) simi=86.29% (107/124)<br>KETAAAKFERQHMDSSTSAASSSNYCNQMMKSRNLTKDRCKPVNTFVHESLADVQAVCSQKNVACKNGQTNCYQSYSTMSITDCRETGSSKYPNCAYKTTQANKHIIVACEGNPYVPVHFDASV<br>KESAAAKFERQHMDSGNSPSSSSNYCNLMMCCRKMTQGKCKPVNTFVHESLADVKAVCSQKKVTCKDGQTNCYQSKSTMRITDCRETGSSKYPNCAYKTTQVEKHIIVACGGKPSVPVHFDASV<br><br>Proposed: aliSize=120 (resi) RMSD=1.90 (Å)<br>ke--TAAAKFERQHMDSSTSaASSSN-YCNQMMKSRNLTKDRCKPVNTFVHESLADVQAVCSQKNVACKNGQTNCYQSYSTMSITDCRET-gSSKYPNCAYKTTQANKHIIVACEGNPYVPVHFDASV<br>--keSAAAKFERQHMDSGNS-PSSSSnYCNLMMCCRKMTQGKCKPVNTFVHESLADVKAVCSQKKVTCKDGQTNCYQSKSTMRITDCRETg-SSKYPNCAYKTTQVEKHIIVACGGKPSVPVHFDASV
239	1f0vA<br>(124)<br>1rasA<br>(123)	C	100.00	TM-align: aliSize=114 (resi) RMSD=1.29 (Å)<br>kETAAAKFERQHMDSSTSAASSSNYCNQMMKSRNLTKDRCKPVNTFVHESLADVQAVCSQKNVACKNGQTNCYQSYSTMSITDCRETGSSKYPNCAYKTTQANKHIIVACEGNPYvpvhfdasv---------<br>-ETAAAKFERQHMDSSTSAASSSNYCNQMMKSRNLTKDRCKPVNTFVHESLADVQAVCSQKNVACKNGQTNCYQSYSTMSITDCRETGSSKYPNCAYKTTQANKHIIVACEGNPY---------vpvhfdasv<br><br>SARST: aliSize=121 (resi) RMSD=8.85 (Å)<br>ke-TAAAKFERQHMDSSTSAASSSNYCNQMMKSRNLTKDRCKPVNTFVHESLADVQAVCSQKNVACKNGQTNCYQSYSTMSITDCRETGSSKYPNCAYKTTQANKHIIVACEGNPYVPVHFDAS-v<br>--eTAAAKFERQHMDSSTSAASSSNYCNQMMKSRNLTKDRCKPVNTFVHESLADVQAVCSQKNVACKNGQTNCYQSYSTMSITDCRETGSSKYPNCAYKTTQANKHIIVACEGNPYVPVHFDASv-<br><br>BLAST: aliSize=123 (resi) iden=100.00% (123/123) simi=100.00% (123/123)<br>kETAAAKFERQHMDSSTSAASSSNYCNQMMKSRNLTKDRCKPVNTFVHESLADVQAVCSQKNVACKNGQTNCYQSYSTMSITDCRETGSSKYPNCAYKTTQANKHIIVACEGNPYVPVHFDASV<br>-ETAAAKFERQHMDSSTSAASSSNYCNQMMKSRNLTKDRCKPVNTFVHESLADVQAVCSQKNVACKNGQTNCYQSYSTMSITDCRETGSSKYPNCAYKTTQANKHIIVACEGNPYVPVHFDASV<br><br>Proposed: aliSize=123 (resi) RMSD=0.47 (Å)<br>kETAAAKFERQHMDSSTSAASSSNYCNQMMKSRNLTKDRCKPVNTFVHESLADVQAVCSQKNVACKNGQTNCYQSYSTMSITDCRETGSSKYPNCAYKTTQANKHIIVACEGNPYVPVHFDASV<br>-ETAAAKFERQHMDSSTSAASSSNYCNQMMKSRNLTKDRCKPVNTFVHESLADVQAVCSQKNVACKNGQTNCYQSYSTMSITDCRETGSSKYPNCAYKTTQANKHIIVACEGNPYVPVHFDASV

240	1f0vA (124)<br>1rnfA (120)	C	43.33	TM-align: aliSize=112 (resi) RMSD=1.99 (Å)<br>K-ETAAAKFERQHMDSSTsAASSSNYCNQMMKSRNLTKDRCKPVNTFVHESLADVQAVCSQKNVACKNGQTNCYQSysTMSITDCRETGSSKYPNCAYKTTQANKHIIVACEGNPYvpvhfdasv-------<br>MqDGMYQRFLRQHVHPEE-TGGSDRYCNLMMQRRKMTLYHCKRFNTFIHEDIWNIRSICSTTNIQCKNGKMNCHEG--VVKVTDCRDTGSSRAPNCRYRAIASTRRVVIACEGNPQ---------vpvhfdg<br><br>SARST: aliSize=115 (resi) RMSD=6.88 (Å)<br>ket----AAAKFERQHMDSSTSAaSSSNYCNQMMKSRNLTKDRCKPVNTFVHESLADVQAVCSQKNVACKNGQTNCYQsySTMSITDCRETGSSKYPNCAYKTTQANKHIIVACEGNPYVPVHFD-asv<br>---mqdgMYQRFLRQHVHPEETG-GSDRYCNLMMQRRKMTLYHCKRFNTFIHEDIWNIRSICSTTNIQCKNGKMNCHE--GVVKVTDCRDTGSSRAPNCRYRAIASTRRVVIACEGNPQVPVHFDg---<br><br>BLAST: aliSize=78 (resi) iden=43.33% (52/120) simi=65.00% (78/120)<br>ketaaa-------KFERQHMDSSTSAASSsNYCNQMMKSRNLTKDRCKPVNTFVHESLADVQAVCSQKNVACKNGQTNCYQsySTMSITDCRETGSSKYPNCAYKTTQANKHIIVACEGNPYVPVHFD-asv<br>------mqdgmyqRFLRQHVHPEETGGSD-RYCNLMMQRRKMTLYHCKRFNTFIHEDIWNIRSICSTTNIQCKNGKMNCHE--GVVKVTDCRDTGSSRAPNCRYRAIASTRRVVIACEGNPQVPVHFDg---<br><br>Proposed: aliSize=117 (resi) RMSD=1.20 (Å)<br>ke---TAAAKFERQHMDSSTsAASSSNYCNQMMKSRNLTKDRCKPVNTFVHESLADVQAVCSQKNVACKNGQTNCYQSysTMSITDCRETGSSKYPNCAYKTTQANKHIIVACEGNPYVPVHFDAsv<br>--mqdGMYQRFLRQHVHPEE-TGGSDRYCNLMMQRRKMTLYHCKRFNTFIHEDIWNIRSICSTTNIQCKNGKMNCHEG--VVKVTDCRDTGSSRAPNCRYRAIASTRRVVIACEGNPQVPVHFDG--
241	1f0vA (124)<br>1rraA (124)	C	66.94	TM-align: aliSize=115 (resi) RMSD=1.51 (Å)<br>KETAAAKFERQHMDSSTSAASSSNYCNQMMKSRNLTKDRCKPVNTFVHESLADVQAVCSQKNVACKNGQTNCYQSYSTMSITDCRETGSSKYPNCAYKTTQANKHIIVACEGNPYvpvhfdasv---------<br>AESSADKFKRQHMDTEGPSKSSPTYCNQMMKRQGMTKGSCKPVNTFVHEPLEDVQAICSQGQVTCKNGRNNCHKSSSTLRITDCRLKGSSKYPNCDYTTTDSQKHIIACDGNPY---------vpvhfdasv<br><br>SARST: aliSize=122 (resi) RMSD=8.84 (Å)<br>k-ETAAAKFERQHMDSSTSAASSSNYCNQMMKSRNLTKDRCKPVNTFVHESLADVQAVCSQKNVACKNGQTNCYQSYSTMSITDCRETGSSKYPNCAYKTTQANKHIIVACEGNPYVPVHFDAS-v<br>-aESSADKFKRQHMDTEGPSKSSPTYCNQMMKRQGMTKGSCKPVNTFVHEPLEDVQAICSQGQVTCKNGRNNCHKSSSTLRITDCRLKGSSKYPNCDYTTTDSQKHIIACDGNPYVPVHFDASv-<br><br>BLAST: aliSize=98 (resi) iden=66.94% (83/124) simi=79.03% (98/124)<br>k-ETAAAKFERQHMDSSTSAASSSNYCNQMMKSRNLTKDRCKPVNTFVHESLADVQAVCSQKNVACKNGQTNCYQSYSTMSITDCRETGSSKYPNCAYKTTQANKHIIVACEGNPYVPVHFDASV<br>-aESSADKFKRQHMDTEGPSKSSPTYCNQMMKRQGMTKGSCKPVNTFVHEPLEDVQAICSQGQVTCKNGRNNCHKSSSTLRITDCRLKGSSKYPNCDYTTTDSQKHIIACDGNPYVPVHFDASV<br><br>Proposed: aliSize=124 (resi) RMSD=1.04 (Å)<br>KETAAAKFERQHMDSSTSAASSSNYCNQMMKSRNLTKDRCKPVNTFVHESLADVQAVCSQKNVACKNGQTNCYQSYSTMSITDCRETGSSKYPNCAYKTTQANKHIIVACEGNPYVPVHFDASV<br>AESSADKFKRQHMDTEGPSKSSPTYCNQMMKRQGMTKGSCKPVNTFVHEPLEDVQAICSQGQVTCKNGRNNCHKSSSTLRITDCRLKGSSKYPNCDYTTTDSQKHIIACDGNPYVPVHFDASV
242	1f0vA (124)<br>1ymnA (124)	C	99.19	TM-align: aliSize=115 (resi) RMSD=1.44 (Å)<br>KETAAAKFERQHMDSSTSAASSSNYCNQMMKSRNLTKDRCKPVNTFVHESLADVQAVCSQKNVACKNGQTNCYQSYSTMSITDCRETGSSKYPNCAYKTTQANKHIIVACEGNPYvpvhfdasv---------<br>KETAAAKFERQHMDSSTSAASSSNYCNQMMKSRNLTKDRCKPVNTFVHESLADVQAVCSQKNVACKNGQTNCYQSYSTMSITDCRETGSSKLPNCAYKTTQANKHIIVACEGNPY---------vpvhfdasv<br><br>SARST: aliSize=122 (resi) RMSD=8.88 (Å)<br>k-ETAAAKFERQHMDSSTSAASSSNYCNQMMKSRNLTKDRCKPVNTFVHESLADVQAVCSQKNVACKNGQTNCYQSYSTMSITDCRETGSSKYPNCAYKTTQANKHIIVACEGNPYVPVHFDAS-v<br>-kETAAAKFERQHMDSSTSAASSSNYCNQMMKSRNLTKDRCKPVNTFVHESLADVQAVCSQKNVACKNGQTNCYQSYSTMSITDCRETGSSKLPNCAYKTTQANKHIIVACEGNPYVPVHFDASv-<br><br>BLAST: aliSize=123 (resi) iden=99.19% (123/124) simi=99.19% (123/124)<br>KETAAAKFERQHMDSSTSAASSSNYCNQMMKSRNLTKDRCKPVNTFVHESLADVQAVCSQKNVACKNGQTNCYQSYSTMSITDCRETGSSKYPNCAYKTTQANKHIIVACEGNPYVPVHFDASV<br>KETAAAKFERQHMDSSTSAASSSNYCNQMMKSRNLTKDRCKPVNTFVHESLADVQAVCSQKNVACKNGQTNCYQSYSTMSITDCRETGSSKLPNCAYKTTQANKHIIVACEGNPYVPVHFDASV<br><br>Proposed: aliSize=124 (resi) RMSD=0.74 (Å)<br>KETAAAKFERQHMDSSTSAASSSNYCNQMMKSRNLTKDRCKPVNTFVHESLADVQAVCSQKNVACKNGQTNCYQSYSTMSITDCRETGSSKYPNCAYKTTQANKHIIVACEGNPYVPVHFDASV<br>KETAAAKFERQHMDSSTSAASSSNYCNQMMKSRNLTKDRCKPVNTFVHESLADVQAVCSQKNVACKNGQTNCYQSYSTMSITDCRETGSSKLPNCAYKTTQANKHIIVACEGNPYVPVHFDASV

243	1f0vA<br>(124)<br><br>1ymrA<br>(124)	C	99.19	TM-align: aliSize=115 (resi) RMSD=1.31 (Å)<br>KETAAAKFERQHMDSSTSAASSSNYCNQMMKSRNLTKDRCKPVNTFVHESLADVQAVCSQKNVACKNGQTNCYQSYSTMSITDCRETGSSKYPNCAYKTTQANKHIIVACEGNPYvpvhfdasv---------<br>KETAAAKFERQHMDSSTSAASSSNYCNQMMKSRNLTKDRCKPVNTFVHESLADVQAVCSQKNVACKNGQTNCYQSYSTMSITDCRETGSSKAPNCAYKTTQANKHIIVACEGNPY---------vpvhfdasv<br><br>SARST: aliSize=122 (resi) RMSD=8.86 (Å)<br>k-ETAAAKFERQHMDSSTSAASSSNYCNQMMKSRNLTKDRCKPVNTFVHESLADVQAVCSQKNVACKNGQTNCYQSYSTMSITDCRETGSSKYPNCAYKTTQANKHIIVACEGNPYVPVHFDAS-v<br>-kETAAAKFERQHMDSSTSAASSSNYCNQMMKSRNLTKDRCKPVNTFVHESLADVQAVCSQKNVACKNGQTNCYQSYSTMSITDCRETGSSKAPNCAYKTTQANKHIIVACEGNPYVPVHFDASv-<br><br>BLAST: aliSize=123 (resi) iden=99.19% (123/124) simi=99.19% (123/124)<br>KETAAAKFERQHMDSSTSAASSSNYCNQMMKSRNLTKDRCKPVNTFVHESLADVQAVCSQKNVACKNGQTNCYQSYSTMSITDCRETGSSKYPNCAYKTTQANKHIIVACEGNPYVPVHFDASV<br>KETAAAKFERQHMDSSTSAASSSNYCNQMMKSRNLTKDRCKPVNTFVHESLADVQAVCSQKNVACKNGQTNCYQSYSTMSITDCRETGSSKAPNCAYKTTQANKHIIVACEGNPYVPVHFDASV<br><br>Proposed: aliSize=124 (resi) RMSD=0.52 (Å)<br>KETAAAKFERQHMDSSTSAASSSNYCNQMMKSRNLTKDRCKPVNTFVHESLADVQAVCSQKNVACKNGQTNCYQSYSTMSITDCRETGSSKYPNCAYKTTQANKHIIVACEGNPYVPVHFDASV<br>KETAAAKFERQHMDSSTSAASSSNYCNQMMKSRNLTKDRCKPVNTFVHESLADVQAVCSQKNVACKNGQTNCYQSYSTMSITDCRETGSSKAPNCAYKTTQANKHIIVACEGNPYVPVHFDASV
244	1f0vA<br>(124)<br><br>1ymwA<br>(124)	C	99.19	TM-align: aliSize=115 (resi) RMSD=1.33 (Å)<br>KETAAAKFERQHMDSSTSAASSSNYCNQMMKSRNLTKDRCKPVNTFVHESLADVQAVCSQKNVACKNGQTNCYQSYSTMSITDCRETGSSKYPNCAYKTTQANKHIIVACEGNPYvpvhfdasv---------<br>KETAAAKFERQHMDSSTSAASSSNYCNQMMKSRNLTKDRCKPVNTFVHESLADVQAVCSQKNVACKNGQTNCYQSYSTMSITDCRETGSSKGPNCAYKTTQANKHIIVACEGNPY---------vpvhfdasv<br><br>SARST: aliSize=122 (resi) RMSD=8.85 (Å)<br>k-ETAAAKFERQHMDSSTSAASSSNYCNQMMKSRNLTKDRCKPVNTFVHESLADVQAVCSQKNVACKNGQTNCYQSYSTMSITDCRETGSSKYPNCAYKTTQANKHIIVACEGNPYVPVHFDAS-v<br>-kETAAAKFERQHMDSSTSAASSSNYCNQMMKSRNLTKDRCKPVNTFVHESLADVQAVCSQKNVACKNGQTNCYQSYSTMSITDCRETGSSKGPNCAYKTTQANKHIIVACEGNPYVPVHFDASv-<br><br>BLAST: aliSize=123 (resi) iden=99.19% (123/124) simi=99.19% (123/124)<br>KETAAAKFERQHMDSSTSAASSSNYCNQMMKSRNLTKDRCKPVNTFVHESLADVQAVCSQKNVACKNGQTNCYQSYSTMSITDCRETGSSKYPNCAYKTTQANKHIIVACEGNPYVPVHFDASV<br>KETAAAKFERQHMDSSTSAASSSNYCNQMMKSRNLTKDRCKPVNTFVHESLADVQAVCSQKNVACKNGQTNCYQSYSTMSITDCRETGSSKGPNCAYKTTQANKHIIVACEGNPYVPVHFDASV<br><br>Proposed: aliSize=124 (resi) RMSD=0.55 (Å)<br>KETAAAKFERQHMDSSTSAASSSNYCNQMMKSRNLTKDRCKPVNTFVHESLADVQAVCSQKNVACKNGQTNCYQSYSTMSITDCRETGSSKYPNCAYKTTQANKHIIVACEGNPYVPVHFDASV<br>KETAAAKFERQHMDSSTSAASSSNYCNQMMKSRNLTKDRCKPVNTFVHESLADVQAVCSQKNVACKNGQTNCYQSYSTMSITDCRETGSSKGPNCAYKTTQANKHIIVACEGNPYVPVHFDASV
245	1f0vA<br>(124)<br><br>1yv4A<br>(104)	C	26.92	TM-align: aliSize=90 (resi) RMSD=2.44 (Å)<br>ketAAAKFERQHMDsSTsAASssnYCNQMMKSRNLTkdrCKPVNTFVHESLADVQAVCSQkNvackngq--TNCYQSYSTMSITDCRETGSSkypnCAYKTTQANKHIIVACEGNpyvpvhfdasv----------<br>---DWLTFQKKHIT-NT-RDV---DCDNILSTNLFH---CKDKNTFIYSRPEPVKAICKG-I-------iaSKNVLTTSEFYLSDCNVTSRP----CKYKLKKSTNKFCVTCENQ----------apvhfvgvgsc<br><br>SARST: aliSize=97 (resi) RMSD=9.43 (Å)<br>keta-AAKFERQHMDSsTSAASssnycnqMMKSRNLTKDRCKPVNTFVHESLADVQAVCSQKNVACkngqtnCYQSYSTMSITDCRETgsskyPNC-AYKTTQANKHIIVACegNPYVPVHFDA----sv<br>----dWLTFQKKHITN-TRDVD-------CDNILSTNLFHCKDKNTFIYSRPEPVKAICKGIIASK------NVLTTSEFYLSDCNVT-----SRPcKYKLKKSTNKFCVTC--ENQAPVHFVGvgsc--<br><br>BLAST: aliSize=54 (resi) iden=33.01% (34/104) simi=52.43% (54/104)<br>ketaaak----FERQHMDSSTSAAsssnyCNQMMkSRNLTkdRCKPVNTFVHESLADVQAVCsQKNVACKNGQTNcyqsySTMSITDCRETgsSKyPnCAYKTTQANKHIIVACEGNpyVPVHF------dasv<br>-------dwltFQKKHITNTRDVD-----CDNIL-STNLF--HCKDKNTFIYSRPEPVKAIC-KGIIASKNVLTT-----SEFYLSDCNVT--SR-P-CKYKLKKSTNKFCVTCENQ--APVHFvgvgsc----<br><br>Proposed: aliSize=97 (resi) RMSD=1.78 (Å)<br>keTAAAKFERQHMDsSTsAASssnYCNQMMKSrNLTKdrCKPVNTFVHESLADVQAVCS-----qknvackngqtNCYQSYSTMSITDCRETGSSkypnCAYKTTQANKHIIVACEGnpYVPVHFDASV--<br>--PDWLTFQKKHIT-NT-RDV---DCDNILST-NLFH--CKDKNTFIYSRPEPVKAICKgiias-----------KNVLTTSEFYLSDCNVTSRP----CKYKLKKSTNKFCVTCEN--QAPVHFVGVGsc

246	1f0vA (124)<br>2aasA (124)	C	100.00	TM-align: aliSize=114 (resi) RMSD=1.49 (Å)<br>KETAAAKFERQHMDSSTSAASSSNYCNQMMKSRNLTKDRCKPVNTFVHESLADVQAVCSQKNVACKNGQTNCYQSYSTMSITDCRETGSSKYPNCAYKTTQANKHIIVACEGNPyvpvhfdasv----------<br>KETAAAKFERQHMDSSTSAASSSNYCNQMMKSRNLTKDRCKPVNTFVHESLADVQAVCSQKNVACKNGQTNCYQSYSTMSITDCRETGSSKYPNCAYKTTQANKHIIVACEGNP----------yvpvhfdasv<br><br>SARST: aliSize=122 (resi) RMSD=8.86 (Å)<br>k-ETAAAKFERQHMDSSTSAASSSNYCNQMMKSRNLTKDRCKPVNTFVHESLADVQAVCSQKNVACKNGQTNCYQSYSTMSITDCRETGSSKYPNCAYKTTQANKHIIVACEGNPYVPVHFDAS-v<br>-kETAAAKFERQHMDSSTSAASSSNYCNQMMKSRNLTKDRCKPVNTFVHESLADVQAVCSQKNVACKNGQTNCYQSYSTMSITDCRETGSSKYPNCAYKTTQANKHIIVACEGNPYVPVHFDASv-<br><br>BLAST: aliSize=124 (resi) iden=100.00% (124/124) simi=100.00% (124/124)<br>KETAAAKFERQHMDSSTSAASSSNYCNQMMKSRNLTKDRCKPVNTFVHESLADVQAVCSQKNVACKNGQTNCYQSYSTMSITDCRETGSSKYPNCAYKTTQANKHIIVACEGNPYVPVHFDASV<br>KETAAAKFERQHMDSSTSAASSSNYCNQMMKSRNLTKDRCKPVNTFVHESLADVQAVCSQKNVACKNGQTNCYQSYSTMSITDCRETGSSKYPNCAYKTTQANKHIIVACEGNPYVPVHFDASV<br><br>Proposed: aliSize=124 (resi) RMSD=1.13 (Å)<br>KETAAAKFERQHMDSSTSAASSSNYCNQMMKSRNLTKDRCKPVNTFVHESLADVQAVCSQKNVACKNGQTNCYQSYSTMSITDCRETGSSKYPNCAYKTTQANKHIIVACEGNPYVPVHFDASV<br>KETAAAKFERQHMDSSTSAASSSNYCNQMMKSRNLTKDRCKPVNTFVHESLADVQAVCSQKNVACKNGQTNCYQSYSTMSITDCRETGSSKYPNCAYKTTQANKHIIVACEGNPYVPVHFDASV
247	1f0vA (124)<br>2e0jA (128)	C	70.16	TM-align: aliSize=114 (resi) RMSD=1.65 (Å)<br>KETAAAKFERQHMDSS-TSAAsSSNYCNQMMKSRNLTKDRCKPVNTFVHESLADVQAVCSQKNVACKNGQTNCYQSYSTMSITDCRETGSSKYPNCAYKTTQANKHIIVACEGNPYvpvhfdasv-------------<br>KESRAKKFQRQHMDSDsSPSS-SSTYCNQMMLLRNMTQGRCKPVNTFVHEPLVDVQNVCFQEKVTCKNGQGNCYKSNSSMHITDCRLTNGSRYPNCAYRTSPKERHIIVACEGSPY---------vpvhfdasvedst<br><br>SARST: aliSize=122 (resi) RMSD=8.84 (Å)<br>k-ETAAAKFERQHMDSSTSAASSSNYCNQMMKSRNLTKDRCKPVNTFVHESLADVQAVCSQKNVACKNGQTNCYQSYSTMSITDCRETGSSKYPNCAYKTTQANKHIIVACEGNPYVPVHFDAS-----v<br>-kESRAKKFQRQHMDSDSSPSSSSTYCNQMMLLRNMTQGRCKPVNTFVHEPLVDVQNVCFQEKVTCKNGQGNCYKSNSSMHITDCRLTNGSRYPNCAYRTSPKERHIIVACEGSPYVPVHFDASvedst-<br><br>BLAST: aliSize=101 (resi) iden=70.16% (87/124) simi=81.45% (101/124)<br>KETAAAKFERQHMDSSTSAASSSNYCNQMMKSRNLTKDRCKPVNTFVHESLADVQAVCSQKNVACKNGQTNCYQSYSTMSITDCRETGSSKYPNCAYKTTQANKHIIVACEGNPYVPVHFDASV----<br>KESRAKKFQRQHMDSDSSPSSSSTYCNQMMLLRNMTQGRCKPVNTFVHEPLVDVQNVCFQEKVTCKNGQGNCYKSNSSMHITDCRLTNGSRYPNCAYRTSPKERHIIVACEGSPYVPVHFDASVedst<br><br>Proposed: aliSize=124 (resi) RMSD=1.13 (Å)<br>KETAAAKFERQHMDSSTSAASSSNYCNQMMKSRNLTKDRCKPVNTFVHESLADVQAVCSQKNVACKNGQTNCYQSYSTMSITDCRETGSSKYPNCAYKTTQANKHIIVACEGNPYVPVHFDASV----<br>KESRAKKFQRQHMDSDSSPSSSSTYCNQMMLLRNMTQGRCKPVNTFVHEPLVDVQNVCFQEKVTCKNGQGNCYKSNSSMHITDCRLTNGSRYPNCAYRTSPKERHIIVACEGSPYVPVHFDASVedst
248	1f0vA (124)<br>2e0lA (128)	C	69.35	TM-align: aliSize=115 (resi) RMSD=1.47 (Å)<br>KETAAAKFERQHMDSSTSAASSSNYCNQMMKSRNLTKDRCKPVNTFVHESLADVQAVCSQKNVACKNGQTNCYQSYSTMSITDCRETGSSKYPNCAYKTTQANKHIIVACEGNPYvpvhfdasv-------------<br>KESRAKKFQRQHMDSDSSPSSSSTYCNLMMLLRNMTQGRCKPVNTFVHEPLVDVQNVCFQEKVTCKNGQGNCYKSNSSMHITDCRLTNGSRYPNCAYRTSPKERHIIVACEGSPY---------vpvhfdasvedst<br><br>SARST: aliSize=121 (resi) RMSD=8.87 (Å)<br>ke--TAAAKFERQHMDSSTSAASSSNYCNQMMKSRNLTKDRCKPVNTFVHESLADVQAVCSQKNVACKNGQTNCYQSYSTMSITDCRETGSSKYPNCAYKTTQANKHIIVACEGNPYVPVHFDAS-----v<br>--keSRAKKFQRQHMDSDSSPSSSSTYCNLMMLLRNMTQGRCKPVNTFVHEPLVDVQNVCFQEKVTCKNGQGNCYKSNSSMHITDCRLTNGSRYPNCAYRTSPKERHIIVACEGSPYVPVHFDASvedst-<br><br>BLAST: aliSize=100 (resi) iden=69.35% (86/124) simi=80.65% (100/124)<br>KETAAAKFERQHMDSSTSAASSSNYCNQMMKSRNLTKDRCKPVNTFVHESLADVQAVCSQKNVACKNGQTNCYQSYSTMSITDCRETGSSKYPNCAYKTTQANKHIIVACEGNPYVPVHFDASV----<br>KESRAKKFQRQHMDSDSSPSSSSTYCNLMMLLRNMTQGRCKPVNTFVHEPLVDVQNVCFQEKVTCKNGQGNCYKSNSSMHITDCRLTNGSRYPNCAYRTSPKERHIIVACEGSPYVPVHFDASVedst<br><br>Proposed: aliSize=124 (resi) RMSD=0.86 (Å)<br>KETAAAKFERQHMDSSTSAASSSNYCNQMMKSRNLTKDRCKPVNTFVHESLADVQAVCSQKNVACKNGQTNCYQSYSTMSITDCRETGSSKYPNCAYKTTQANKHIIVACEGNPYVPVHFDASV----<br>KESRAKKFQRQHMDSDSSPSSSSTYCNLMMLLRNMTQGRCKPVNTFVHEPLVDVQNVCFQEKVTCKNGQGNCYKSNSSMHITDCRLTNGSRYPNCAYRTSPKERHIIVACEGSPYVPVHFDASVedst

249	1f0vA<br>(124)<br>2e0mA<br>(128)	C	69.35	TM-align: aliSize=115 (resi) RMSD=1.47 (Å)<br>KETAAAKFERQHMDSSTSAASSSNYCNQMMKSRNLTKDRCKPVNTFVHESLADVQAVCSQKNVACKNGQTNCYQSYSTMSITDCRETGSSKYPNCAYKTTQANKHIIVACEGNPYvpvhfdasv-------------<br>KESRAKKFQRQHMDSDSSPSSSSLYCNLMMLLRNMTQGRCKPVNTFVHEPLVDVQNVCFQEKVTCKNGQGNCYKSNSSMHITDCRLTNGSRYPNCAYRTSPKERHIIVACEGSPY---------vpvhfdasvedst<br><br>SARST: aliSize=121 (resi) RMSD=8.87 (Å)<br>ke--TAAAKFERQHMDSSTSAASSSNYCNQMMKSRNLTKDRCKPVNTFVHESLADVQAVCSQKNVACKNGQTNCYQSYSTMSITDCRETGSSKYPNCAYKTTQANKHIIVACEGNPYVPVHFDAS-----v<br>--keSRAKKFQRQHMDSDSSPSSSSLYCNLMMLLRNMTQGRCKPVNTFVHEPLVDVQNVCFQEKVTCKNGQGNCYKSNSSMHITDCRLTNGSRYPNCAYRTSPKERHIIVACEGSPYVPVHFDASvedst-<br><br>BLAST: aliSize=100 (resi) iden=69.35% (86/124) simi=80.65% (100/124)<br>KETAAAKFERQHMDSSTSAASSSNYCNQMMKSRNLTKDRCKPVNTFVHESLADVQAVCSQKNVACKNGQTNCYQSYSTMSITDCRETGSSKYPNCAYKTTQANKHIIVACEGNPYVPVHFDASV----<br>KESRAKKFQRQHMDSDSSPSSSSLYCNLMMLLRNMTQGRCKPVNTFVHEPLVDVQNVCFQEKVTCKNGQGNCYKSNSSMHITDCRLTNGSRYPNCAYRTSPKERHIIVACEGSPYVPVHFDASVedst<br><br>Proposed: aliSize=124 (resi) RMSD=0.85 (Å)<br>KETAAAKFERQHMDSSTSAASSSNYCNQMMKSRNLTKDRCKPVNTFVHESLADVQAVCSQKNVACKNGQTNCYQSYSTMSITDCRETGSSKYPNCAYKTTQANKHIIVACEGNPYVPVHFDASV----<br>KESRAKKFQRQHMDSDSSPSSSSLYCNLMMLLRNMTQGRCKPVNTFVHEPLVDVQNVCFQEKVTCKNGQGNCYKSNSSMHITDCRLTNGSRYPNCAYRTSPKERHIIVACEGSPYVPVHFDASVedst
250	1f0vA<br>(124)<br>2e0oA<br>(126)	C	68.55	TM-align: aliSize=114 (resi) RMSD=1.52 (Å)<br>KETAAAKFERQHMDSSTsAASS-SNYCNQMMKSRNLTKDRCKPVNTFVHESLADVQAVCSQKNVACKNGQTNCYQSYSTMSITDCRETGSSKYPNCAYKTTQANKHIIVACEGNPYvpvhfdasv-----------<br>KESRAKKFQRQHMDSDS-SPSSsSTYCNQMMRRRNMTQGRCKPVNTFVHEPLLLVQLVCLQEKVTCKNGQGNCYKSNSSMHITDCRLTNGSRYPNCAYRTSPKERHIIVACEGSPY---------vpvhfdasved<br><br>SARST: aliSize=122 (resi) RMSD=8.81 (Å)<br>k-ETAAAKFERQHMDSSTSAASSSNYCNQMMKSRNLTKDRCKPVNTFVHESLADVQAVCSQKNVACKNGQTNCYQSYSTMSITDCRETGSSKYPNCAYKTTQANKHIIVACEGNPYVPVHFDAS---v<br>-kESRAKKFQRQHMDSDSSPSSSSTYCNQMMRRRNMTQGRCKPVNTFVHEPLLLVQLVCLQEKVTCKNGQGNCYKSNSSMHITDCRLTNGSRYPNCAYRTSPKERHIIVACEGSPYVPVHFDASved-<br><br>BLAST: aliSize=101 (resi) iden=69.35% (86/124) simi=81.45% (101/124)<br>KETAAAKFERQHMDSSTSAASSSNYCNQMMKSRNLTKDRCKPVNTFVHESLADVQAVCSQKNVACKNGQTNCYQSYSTMSITDCRETGSSKYPNCAYKTTQANKHIIVACEGNPYVPVHFDASV--<br>KESRAKKFQRQHMDSDSSPSSSSTYCNQMMRRRNMTQGRCKPVNTFVHEPLLLVQLVCLQEKVTCKNGQGNCYKSNSSMHITDCRLTNGSRYPNCAYRTSPKERHIIVACEGSPYVPVHFDASVed<br><br>Proposed: aliSize=123 (resi) RMSD=0.92 (Å)<br>KETAAAKFERQHMDSSTsAASS-SNYCNQMMKSRNLTKDRCKPVNTFVHESLADVQAVCSQKNVACKNGQTNCYQSYSTMSITDCRETGSSKYPNCAYKTTQANKHIIVACEGNPYVPVHFDASV--<br>KESRAKKFQRQHMDSDS-SPSSsSTYCNQMMRRRNMTQGRCKPVNTFVHEPLLLVQLVCLQEKVTCKNGQGNCYKSNSSMHITDCRLTNGSRYPNCAYRTSPKERHIIVACEGSPYVPVHFDASVed
251	1f0vA<br>(124)<br>2e0oB<br>(125)	C	69.35	TM-align: aliSize=115 (resi) RMSD=1.61 (Å)<br>-KETAAAKFERQHMDSSTSAASSSNYCNQMMKSRNLTKDRCKPVNTFVHESLADVQAVCSQKNVACKNGQTNCYQSYSTMSITDCRETGSSKYPNCAYKTTQANKHIIVACEGNPYvpvhfdasv---------<br>mKESRAKKFQRQHMDSDSSPSSSSTYCNQMMRRRNMTQGRCKPVNTFVHEPLLLVQLVCLQEKVTCKNGQGNCYKSNSSMHITDCRLTNGSRYPNCAYRTSPKERHIIVACEGSPY---------vpvhfdasv<br><br>SARST: aliSize=122 (resi) RMSD=8.85 (Å)<br>k--ETAAAKFERQHMDSSTSAASSSNYCNQMMKSRNLTKDRCKPVNTFVHESLADVQAVCSQKNVACKNGQTNCYQSYSTMSITDCRETGSSKYPNCAYKTTQANKHIIVACEGNPYVPVHFDAS-v<br>-mkESRAKKFQRQHMDSDSSPSSSSTYCNQMMRRRNMTQGRCKPVNTFVHEPLLLVQLVCLQEKVTCKNGQGNCYKSNSSMHITDCRLTNGSRYPNCAYRTSPKERHIIVACEGSPYVPVHFDASv-<br><br>BLAST: aliSize=101 (resi) iden=69.35% (86/124) simi=81.45% (101/124)<br>-KETAAAKFERQHMDSSTSAASSSNYCNQMMKSRNLTKDRCKPVNTFVHESLADVQAVCSQKNVACKNGQTNCYQSYSTMSITDCRETGSSKYPNCAYKTTQANKHIIVACEGNPYVPVHFDASV<br>mKESRAKKFQRQHMDSDSSPSSSSTYCNQMMRRRNMTQGRCKPVNTFVHEPLLLVQLVCLQEKVTCKNGQGNCYKSNSSMHITDCRLTNGSRYPNCAYRTSPKERHIIVACEGSPYVPVHFDASV<br><br>Proposed: aliSize=124 (resi) RMSD=1.01 (Å)<br>-KETAAAKFERQHMDSSTSAASSSNYCNQMMKSRNLTKDRCKPVNTFVHESLADVQAVCSQKNVACKNGQTNCYQSYSTMSITDCRETGSSKYPNCAYKTTQANKHIIVACEGNPYVPVHFDASV<br>mKESRAKKFQRQHMDSDSSPSSSSTYCNQMMRRRNMTQGRCKPVNTFVHEPLLLVQLVCLQEKVTCKNGQGNCYKSNSSMHITDCRLTNGSRYPNCAYRTSPKERHIIVACEGSPYVPVHFDASV

252	1f0vA (124)<br>2hkyA (129)	C	41.13	TM-align: aliSize=104 (resi) RMSD=2.46 (Å)<br>--K--ETAAAKFERQHMDSSTsaAsssnYCNQMMKsrNLTK--DRCKPVNTFVHESLADVQAVCSQKNVACkNG-QTNCYQSYSTMSITDCRETgSSKYPNCAYKTTQANKHIIVACEGNp--------Yvpvhfdasv----------<br>mkPkgMTSSQWFKIQHMQPSP--Q----ACNSAMK--NINKhtKRCKDLNTFLHEPFSSVAATCQTPKIAC-KNgDKNCHQSHGPVSLTMCKLT-SGKYPNCRYKEKRQNKSYVVACKPP-qkkdsqqfH---------lvpvhldrvl<br><br>SARST: aliSize=100 (resi) RMSD=4.51 (Å)<br>keta-------AAKFERQHMDSSTSAASsSNYCNQMMKSRNltkdRCKPVNTFVHESLADVQAVCSQKNVACKNGQTNCYQSYSTMSITDCRE---TGsskyPNCAYKTTQANKHIIvaCEGNPY------------------vpvhfdasv<br>----mkpkgmtSSQWFKIQHMQPSPQAC-NSAMKNINKHTK----RCKDLNTFLHEPFSSVAATCQTPKIACKNGDKNCHQSHGPVSLTMCKLtsgKY----PNCRYKEKRQNKSYV--VACKPPqkkdsqqfhlvpvhldrvl---------<br><br>BLAST: aliSize=71 (resi) iden=43.55% (54/124) simi=57.26% (71/124)<br>ke------TAAAKFERQHMDSSTSAasssnyCNQMMKSRNLTKDRCKPVNTFVHESLADVQAVCSQKNVACKNGQTNCYQSYSTMSITDCRETgSSKYPNCAYKTTQANKHIIVAC------EGNPY--VPVHFD---asv<br>--mkpkgmTSSQWFKIQHMQPSPQA------CNSAMKNINKHTKRCKDLNTFLHEPFSSVAATCQTPKIACKNGDKNCHQSHGPVSLTMCKLT-SGKYPNCRYKEKRQNKSYVVACkppqkkDSQQFhlVPVHLDrvl---<br><br>Proposed: aliSize=112 (resi) RMSD=1.80 (Å)<br>--K--ETAAAKFERQHMDSS--tsaasssnYCNQMMKsrNLTK--DRCKPVNTFVHESLADVQAVCSQKNVACkNG-QTNCYQSYSTMSITDCRETgSSKYPNCAYKTTQANKHIIVACEGN-------P-YVPVHFDASV<br>mkPkgMTSSQWFKIQHMQPSpq--------ACNSAMK--NINKhtKRCKDLNTFLHEPFSSVAATCQTPKIAC-KNgDKNCHQSHGPVSLTMCKLT-SGKYPNCRYKEKRQNKSYVVACKPPqkkdsqqFhLVPVHLDRVL
253	1f0vA (124)<br>2i5sX (104)	C	27.88	TM-align: aliSize=90 (resi) RMSD=2.38 (Å)<br>ketAAAKFERQHMDsSTsAASssnYCNQMMKSRNLTkdrCKPVNTFVHESLADVQAVCSQkNvackngq--TNCYQSYSTMSITDCRETGSSkypnCAYKTTQANKHIIVACEGNpyvpvhfdasv----------<br>---DWLTFQKKHIT-NT-RDV---DCDNIMSTNLFH---CKDKNTFIYSRPEPVKAICKG-I-------iaSKNVLTTSEFYLSDCNVTSRP----CKYKLKKSTNKFCVTCENQ----------apvhfvgvgsc<br><br>SARST: aliSize=99 (resi) RMSD=10.62 (Å)<br>keta-AAKFERQHMdssTSAASSSNYCNQMMKSRNLtkdrcKPVNTFVHESLADVQAVCSQKNVACkngqtnCYQSYSTMSITDCRETGssKYPncAYKTTQANKHIIVACegNPYVPVHFDAS---v<br>----dWLTFQKKHI---TNTRDVDCDNIMSTNLFHC-----KDKNTFIYSRPEPVKAICKGIIASK------NVLTTSEFYLSDCNVTS--RPC--KYKLKKSTNKFCVTC--ENQAPVHFVGVgsc-<br><br>BLAST: aliSize=54 (resi) iden=33.98% (35/104) simi=52.43% (54/104)<br>ketaaak----FERQHMDSSTSAAsssnyCNQMMkSRNLTkdRCKPVNTFVHESLADVQAVCsQKNVACKNGQTNcyqsySTMSITDCRETgsSKyPnCAYKTTQANKHIIVACEGNpyVPVHF------dasv<br>-------dwltFQKKHITNTRDVD-----CDNIM-STNLF--HCKDKNTFIYSRPEPVKAIC-KGIIASKNVLTT-----SEFYLSDCNVT--SR-P-CKYKLKKSTNKFCVTCENQ--APVHFvgvgsc----<br><br>Proposed: aliSize=97 (resi) RMSD=1.75 (Å)<br>keTAAAKFERQHMDsSTsAASssnYCNQMMKSrNLTkdRCKPVNTFVHESLADVQAVCS-----qknvackngqtNCYQSYSTMSITDCRETGSSkypnCAYKTTQANKHIIVACEGnpYVPVHFDASV--<br>--PDWLTFQKKHIT-NT-RDV---DCDNIMST-NLF--HCKDKNTFIYSRPEPVKAICKgiias-----------KNVLTTSEFYLSDCNVTSRP----CKYKLKKSTNKFCVTCEN--QAPVHFVGVGsc
254	1f0vA (124)<br>2k11A (127)	C	69.35	TM-align: aliSize=114 (resi) RMSD=1.95 (Å)<br>KETAAAKFERQHMDSSTSAASSSNYCNQMMKSRNLTKDRCKPVNTFVHESLADVQAVCSQKNVACKNGQTNCYQSYSTMSITDCRETGSSKYPNCAYKTTQANKHIIVACEGNPyvpvhfdasv------------<br>KESRAKKFQRQHMDSDSSPSSSSTYCNQMMRRRNMTQGRCKPVNTFVHEPLVDVQNVCFQEKVTCKNGQGNCYKSNSSMHITDCRLTNGSRYPNCAYRTSPKERHIIVACEGSP----------yvpvhfdasveds<br><br>SARST: aliSize=121 (resi) RMSD=8.10 (Å)<br>k-ETAAAKFERQHMDSSTSAASSSNYCNQMMKSRNLTKDRCKPVNTFVHESLADVQAVCSQKNVACKNGQTNCYQSYSTMSITDCRETGSSKYPNCAYKTTQANKHIIVACEGNPYVPVHFDA-----sv<br>-kESRAKKFQRQHMDSDSSPSSSSTYCNQMMRRRNMTQGRCKPVNTFVHEPLVDVQNVCFQEKVTCKNGQGNCYKSNSSMHITDCRLTNGSRYPNCAYRTSPKERHIIVACEGSPYVPVHFDAsveds--<br><br>BLAST: aliSize=102 (resi) iden=70.16% (87/124) simi=82.26% (102/124)<br>KETAAAKFERQHMDSSTSAASSSNYCNQMMKSRNLTKDRCKPVNTFVHESLADVQAVCSQKNVACKNGQTNCYQSYSTMSITDCRETGSSKYPNCAYKTTQANKHIIVACEGNPYVPVHFDASV---<br>KESRAKKFQRQHMDSDSSPSSSSTYCNQMMRRRNMTQGRCKPVNTFVHEPLVDVQNVCFQEKVTCKNGQGNCYKSNSSMHITDCRLTNGSRYPNCAYRTSPKERHIIVACEGSPYVPVHFDASVeds<br><br>Proposed: aliSize=123 (resi) RMSD=1.56 (Å)<br>k-ETAAAKFERQHMDSSTSAASSSNYCNQMMKSRNLTKDRCKPVNTFVHESLADVQAVCSQKNVACKNGQTNCYQSYSTMSITDCRETGSSKYPNCAYKTTQANKHIIVACEGNPYVPVHFDASV---<br>-kESRAKKFQRQHMDSDSSPSSSSTYCNQMMRRRNMTQGRCKPVNTFVHEPLVDVQNVCFQEKVTCKNGQGNCYKSNSSMHITDCRLTNGSRYPNCAYRTSPKERHIIVACEGSPYVPVHFDASVeds

255	1f0vA (124)<br>2nuiA (124)	C	99.19	TM-align: aliSize=114 (resi) RMSD=0.95 (Å)<br>KETAAAKFERQHMDSSTSAASSSNYCNQMMKSRNLTKDRCKPVNTFVHESLADVQAVCSQKNVACKNGQTNCYQSYSTMSITDCRETGSSKYPNCAYKTTQANKHIIVACEGNPyvpvhfdasv----------<br>KETAAAKFERQHMDSSTSAASSSNYCNQMMKSRNLTKDRCKPVNTFVHESLADVQAVCSQKNVACKNGQTNCYQSYSTMSITACRETGSSKYPNCAYKTTQANKHIIVACEGNP----------yvpvhfdasv<br><br>SARST: aliSize=122 (resi) RMSD=8.86 (Å)<br>k-ETAAAKFERQHMDSSTSAASSSNYCNQMMKSRNLTKDRCKPVNTFVHESLADVQAVCSQKNVACKNGQTNCYQSYSTMSITDCRETGSSKYPNCAYKTTQANKHIIVACEGNPYVPVHFDAS-v<br>-kETAAAKFERQHMDSSTSAASSSNYCNQMMKSRNLTKDRCKPVNTFVHESLADVQAVCSQKNVACKNGQTNCYQSYSTMSITACRETGSSKYPNCAYKTTQANKHIIVACEGNPYVPVHFDASv-<br><br>BLAST: aliSize=123 (resi) iden=99.19% (123/124) simi=99.19% (123/124)<br>KETAAAKFERQHMDSSTSAASSSNYCNQMMKSRNLTKDRCKPVNTFVHESLADVQAVCSQKNVACKNGQTNCYQSYSTMSITDCRETGSSKYPNCAYKTTQANKHIIVACEGNPYVPVHFDASV<br>KETAAAKFERQHMDSSTSAASSSNYCNQMMKSRNLTKDRCKPVNTFVHESLADVQAVCSQKNVACKNGQTNCYQSYSTMSITACRETGSSKYPNCAYKTTQANKHIIVACEGNPYVPVHFDASV<br><br>Proposed: aliSize=124 (resi) RMSD=0.52 (Å)<br>KETAAAKFERQHMDSSTSAASSSNYCNQMMKSRNLTKDRCKPVNTFVHESLADVQAVCSQKNVACKNGQTNCYQSYSTMSITDCRETGSSKYPNCAYKTTQANKHIIVACEGNPYVPVHFDASV<br>KETAAAKFERQHMDSSTSAASSSNYCNQMMKSRNLTKDRCKPVNTFVHESLADVQAVCSQKNVACKNGQTNCYQSYSTMSITACRETGSSKYPNCAYKTTQANKHIIVACEGNPYVPVHFDASV
256	1f0vA (124)<br>2op2A (124)	C	98.39	TM-align: aliSize=115 (resi) RMSD=1.40 (Å)<br>KETAAAKFERQHMDSSTSAASSSNYCNQMMKSRNLTKDRCKPVNTFVHESLADVQAVCSQKNVACKNGQTNCYQSYSTMSITDCRETGSSKYPNCAYKTTQANKHIIVACEGNPYvpvhfdasv---------<br>KETAAAKFERQHMDSSTSAASSSNYCNQMMKSRNLTKDRCKPCNTFVHESLADVQAVCSQKNVACKNGQTNCYQSYSTMSITDCCETGSSKYPNCAYKTTQANKHIIVACEGNPY---------vpvhfdasv<br><br>SARST: aliSize=122 (resi) RMSD=8.79 (Å)<br>k-ETAAAKFERQHMDSSTSAASSSNYCNQMMKSRNLTKDRCKPVNTFVHESLADVQAVCSQKNVACKNGQTNCYQSYSTMSITDCRETGSSKYPNCAYKTTQANKHIIVACEGNPYVPVHFDAS-v<br>-kETAAAKFERQHMDSSTSAASSSNYCNQMMKSRNLTKDRCKPCNTFVHESLADVQAVCSQKNVACKNGQTNCYQSYSTMSITDCCETGSSKYPNCAYKTTQANKHIIVACEGNPYVPVHFDASv-<br><br>BLAST: aliSize=122 (resi) iden=98.39% (122/124) simi=98.39% (122/124)<br>KETAAAKFERQHMDSSTSAASSSNYCNQMMKSRNLTKDRCKPVNTFVHESLADVQAVCSQKNVACKNGQTNCYQSYSTMSITDCRETGSSKYPNCAYKTTQANKHIIVACEGNPYVPVHFDASV<br>KETAAAKFERQHMDSSTSAASSSNYCNQMMKSRNLTKDRCKPCNTFVHESLADVQAVCSQKNVACKNGQTNCYQSYSTMSITDCCETGSSKYPNCAYKTTQANKHIIVACEGNPYVPVHFDASV<br><br>Proposed: aliSize=124 (resi) RMSD=0.65 (Å)<br>KETAAAKFERQHMDSSTSAASSSNYCNQMMKSRNLTKDRCKPVNTFVHESLADVQAVCSQKNVACKNGQTNCYQSYSTMSITDCRETGSSKYPNCAYKTTQANKHIIVACEGNPYVPVHFDASV<br>KETAAAKFERQHMDSSTSAASSSNYCNQMMKSRNLTKDRCKPCNTFVHESLADVQAVCSQKNVACKNGQTNCYQSYSTMSITDCCETGSSKYPNCAYKTTQANKHIIVACEGNPYVPVHFDASV
257	1f0vA (124)<br>2p6zB (109)	C	26.61	TM-align: aliSize=92 (resi) RMSD=2.27 (Å)<br>keTAAAKFERQHMDsSTSAaSSsNYCNQMMKS--RN--LTkdrCKPVNTFVHESLADVQAVCSQkNvackngq--TNCYQSYSTMSITDCRETgSSkypnCAYKTTQANKHIIVACEGNpyvpvhfdasv-----------<br>--REWEKFKTKHIT-SQSV-AD-FNCNRTMNDpaYTpdGQ---CKPINTFIHSTTGPVKEICRR-A-------tgRVNKSSTQQFTLTTCKNP-IR----CKYSQSNTTNFICITCRDN-----------ypvhfvktgkc<br><br>SARST: aliSize=100 (resi) RMSD=9.42 (Å)<br>ket-AAAKFERQHMDSSTSaasssnYCNQMMKSRN---LTKDRC-KPVNTFVHESLADVQAVCSQKNVACkngqtnCYQSYSTMSITDCRETGSSKypncaYKTTQANKHIIVACEGnpYVPVHFDA----sv<br>---rEWEKFKTKHITSQSV------ADFNCNRTMNdpaYTPDGQcKPINTFIHSTTGPVKEICRRATGRV------NKSSTQQFTLTTCKNPIRCK-----YSQSNTTNFICITCRD--NYPVHFVKtgkc--<br><br>BLAST: aliSize=55 (resi) iden=33.94% (37/109) simi=50.46% (55/109)<br>ketaaa----KFERQHMdSSTSAASSSnyCNQMMKSRNLTKD-RCKPVNTFVHESLADVQAVCSQknvacKNGQTNcYQSYSTMSITDCretgssKYP-NCAYKTTQANKHIIVACEGNpYvPVHF------dasv<br>------reweKFKTKHI-TSQSVADFN--CNRTMNDPAYTPDgQCKPINTFIHSTTGPVKEICRR-----ATGRVN-KSSTQQFTLTTC------KNPiRCKYSQSNTTNFICITCRDN-Y-PVHFvktgkc----<br><br>Proposed: aliSize=98 (resi) RMSD=1.63 (Å)<br>keTAAAKFERQHMDsSTSAASSsnYCNQMMKS--RN--LTkdrCKPVNTFVHESLADVQAVCS-----qknvackngqtNCYQSYSTMSITDCRETgSSkypnCAYKTTQANKHIIVACEgnPYVPVHFDASV--<br>--REWEKFKTKHIT-SQSVADF--NCNRTMNDpaYTpdGQ---CKPINTFIHSTTGPVKEICRratgr-----------VNKSSTQQFTLTTCKNP-IR----CKYSQSNTTNFICITCR--DNYPVHFVKTGkc

258	1f0vA (124)<br>2q4gX (126)	C	69.35	TM-align: aliSize=114 (resi) RMSD=1.43 (Å)<br>kETAAAKFERQHMDSSTSAASSSNYCNQMMKSRNLTKDRCKPVNTFVHESLADVQAVCSQKNVACKNGQTNCYQSYSTMSITDCRETGSSKYPNCAYKTTQANKHIIVACEGNPYvpvhfdasv------------<br>-ESRAKKFQRQHMDSDSSPSSSSTYCNQMMRRRNMTQGRCKPVNTFVHEPLVDVQNVCFQEKVTCKNGQGNCYKSNSSMHITDCRLTNGSRYPNCAYRTSPKERHIIVACEGSPY---------vpvhfdasveds<br><br>SARST: aliSize=118 (resi) RMSD=9.00 (Å)<br>ketaa----AKFERQHMDSSTSAASSSNYCNQMMKSRNLTKDRCKPVNTFVHESLADVQAVCSQKNVACKNGQTNCYQSYSTMSITDCRETGSSKYPNCAYKTTQANKHIIVACEGNPYVPVHFDAS----v<br>-----esraKKFQRQHMDSDSSPSSSSTYCNQMMRRRNMTQGRCKPVNTFVHEPLVDVQNVCFQEKVTCKNGQGNCYKSNSSMHITDCRLTNGSRYPNCAYRTSPKERHIIVACEGSPYVPVHFDASveds-<br><br>BLAST: aliSize=101 (resi) iden=69.35% (86/124) simi=81.45% (101/124)<br>kETAAAKFERQHMDSSTSAASSSNYCNQMMKSRNLTKDRCKPVNTFVHESLADVQAVCSQKNVACKNGQTNCYQSYSTMSITDCRETGSSKYPNCAYKTTQANKHIIVACEGNPYVPVHFDASV---<br>-ESRAKKFQRQHMDSDSSPSSSSTYCNQMMRRRNMTQGRCKPVNTFVHEPLVDVQNVCFQEKVTCKNGQGNCYKSNSSMHITDCRLTNGSRYPNCAYRTSPKERHIIVACEGSPYVPVHFDASVeds<br><br>Proposed: aliSize=123 (resi) RMSD=0.81 (Å)<br>kETAAAKFERQHMDSSTSAASSSNYCNQMMKSRNLTKDRCKPVNTFVHESLADVQAVCSQKNVACKNGQTNCYQSYSTMSITDCRETGSSKYPNCAYKTTQANKHIIVACEGNPYVPVHFDASV---<br>-ESRAKKFQRQHMDSDSSPSSSSTYCNQMMRRRNMTQGRCKPVNTFVHEPLVDVQNVCFQEKVTCKNGQGNCYKSNSSMHITDCRLTNGSRYPNCAYRTSPKERHIIVACEGSPYVPVHFDASVeds
259	1f0vA (124)<br>3dh6A (124)	C	99.19	TM-align: aliSize=115 (resi) RMSD=1.30 (Å)<br>KETAAAKFERQHMDSSTSAASSSNYCNQMMKSRNLTKDRCKPVNTFVHESLADVQAVCSQKNVACKNGQTNCYQSYSTMSITDCRETGSSKYPNCAYKTTQANKHIIVACEGNPYvpvhfdasv---------<br>KETAAAKFERQHMDSSTSAASSSNYCNQMMKSRNLTKDRCKPVNTFAHESLADVQAVCSQKNVACKNGQTNCYQSYSTMSITDCRETGSSKYPNCAYKTTQANKHIIVACEGNPY---------vpvhfdasv<br><br>SARST: aliSize=122 (resi) RMSD=8.85 (Å)<br>k-ETAAAKFERQHMDSSTSAASSSNYCNQMMKSRNLTKDRCKPVNTFVHESLADVQAVCSQKNVACKNGQTNCYQSYSTMSITDCRETGSSKYPNCAYKTTQANKHIIVACEGNPYVPVHFDAS-v<br>-kETAAAKFERQHMDSSTSAASSSNYCNQMMKSRNLTKDRCKPVNTFAHESLADVQAVCSQKNVACKNGQTNCYQSYSTMSITDCRETGSSKYPNCAYKTTQANKHIIVACEGNPYVPVHFDASv-<br><br>BLAST: aliSize=123 (resi) iden=99.19% (123/124) simi=99.19% (123/124)<br>KETAAAKFERQHMDSSTSAASSSNYCNQMMKSRNLTKDRCKPVNTFVHESLADVQAVCSQKNVACKNGQTNCYQSYSTMSITDCRETGSSKYPNCAYKTTQANKHIIVACEGNPYVPVHFDASV<br>KETAAAKFERQHMDSSTSAASSSNYCNQMMKSRNLTKDRCKPVNTFAHESLADVQAVCSQKNVACKNGQTNCYQSYSTMSITDCRETGSSKYPNCAYKTTQANKHIIVACEGNPYVPVHFDASV<br><br>Proposed: aliSize=124 (resi) RMSD=0.49 (Å)<br>KETAAAKFERQHMDSSTSAASSSNYCNQMMKSRNLTKDRCKPVNTFVHESLADVQAVCSQKNVACKNGQTNCYQSYSTMSITDCRETGSSKYPNCAYKTTQANKHIIVACEGNPYVPVHFDASV<br>KETAAAKFERQHMDSSTSAASSSNYCNQMMKSRNLTKDRCKPVNTFAHESLADVQAVCSQKNVACKNGQTNCYQSYSTMSITDCRETGSSKYPNCAYKTTQANKHIIVACEGNPYVPVHFDASV
260	1f0vA (124)<br>3di7A (124)	C	99.19	TM-align: aliSize=115 (resi) RMSD=1.31 (Å)<br>KETAAAKFERQHMDSSTSAASSSNYCNQMMKSRNLTKDRCKPVNTFVHESLADVQAVCSQKNVACKNGQTNCYQSYSTMSITDCRETGSSKYPNCAYKTTQANKHIIVACEGNPYvpvhfdasv---------<br>KETAAAKFERQHMDSSTSAASSSNYCNQMMKSRNLTKDRCKPVNTFVHESLADAQAVCSQKNVACKNGQTNCYQSYSTMSITDCRETGSSKYPNCAYKTTQANKHIIVACEGNPY---------vpvhfdasv<br><br>SARST: aliSize=122 (resi) RMSD=8.84 (Å)<br>k-ETAAAKFERQHMDSSTSAASSSNYCNQMMKSRNLTKDRCKPVNTFVHESLADVQAVCSQKNVACKNGQTNCYQSYSTMSITDCRETGSSKYPNCAYKTTQANKHIIVACEGNPYVPVHFDAS-v<br>-kETAAAKFERQHMDSSTSAASSSNYCNQMMKSRNLTKDRCKPVNTFVHESLADAQAVCSQKNVACKNGQTNCYQSYSTMSITDCRETGSSKYPNCAYKTTQANKHIIVACEGNPYVPVHFDASv-<br><br>BLAST: aliSize=123 (resi) iden=99.19% (123/124) simi=99.19% (123/124)<br>KETAAAKFERQHMDSSTSAASSSNYCNQMMKSRNLTKDRCKPVNTFVHESLADVQAVCSQKNVACKNGQTNCYQSYSTMSITDCRETGSSKYPNCAYKTTQANKHIIVACEGNPYVPVHFDASV<br>KETAAAKFERQHMDSSTSAASSSNYCNQMMKSRNLTKDRCKPVNTFVHESLADAQAVCSQKNVACKNGQTNCYQSYSTMSITDCRETGSSKYPNCAYKTTQANKHIIVACEGNPYVPVHFDASV<br><br>Proposed: aliSize=124 (resi) RMSD=0.50 (Å)<br>KETAAAKFERQHMDSSTSAASSSNYCNQMMKSRNLTKDRCKPVNTFVHESLADVQAVCSQKNVACKNGQTNCYQSYSTMSITDCRETGSSKYPNCAYKTTQANKHIIVACEGNPYVPVHFDASV<br>KETAAAKFERQHMDSSTSAASSSNYCNQMMKSRNLTKDRCKPVNTFVHESLADAQAVCSQKNVACKNGQTNCYQSYSTMSITDCRETGSSKYPNCAYKTTQANKHIIVACEGNPYVPVHFDASV

261	1f0vA (124)<br>3di8A (124)	C	99.19	TM-align: aliSize=114 (resi) RMSD=0.93 (Å)<br>KETAAAKFERQHMDSSTSAASSSNYCNQMMKSRNLTKDRCKPVNTFVHESLADVQAVCSQKNVACKNGQTNCYQSYSTMSITDCRETGSSKYPNCAYKTTQANKHIIVACEGNPyvpvhfdasv----------<br>KETAAAKFERQHMDSSTSAASSSNYCNQMMKSRNLTKDRCKPVNTFVHESLADVQAACSQKNVACKNGQTNCYQSYSTMSITDCRETGSSKYPNCAYKTTQANKHIIVACEGNP----------yvpvhfdasv<br><br>SARST: aliSize=122 (resi) RMSD=8.86 (Å)<br>k-ETAAAKFERQHMDSSTSAASSSNYCNQMMKSRNLTKDRCKPVNTFVHESLADVQAVCSQKNVACKNGQTNCYQSYSTMSITDCRETGSSKYPNCAYKTTQANKHIIVACEGNPYVPVHFDAS-v<br>-kETAAAKFERQHMDSSTSAASSSNYCNQMMKSRNLTKDRCKPVNTFVHESLADVQAACSQKNVACKNGQTNCYQSYSTMSITDCRETGSSKYPNCAYKTTQANKHIIVACEGNPYVPVHFDASv-<br><br>BLAST: aliSize=123 (resi) iden=99.19% (123/124) simi=99.19% (123/124)<br>KETAAAKFERQHMDSSTSAASSSNYCNQMMKSRNLTKDRCKPVNTFVHESLADVQAVCSQKNVACKNGQTNCYQSYSTMSITDCRETGSSKYPNCAYKTTQANKHIIVACEGNPYVPVHFDASV<br>KETAAAKFERQHMDSSTSAASSSNYCNQMMKSRNLTKDRCKPVNTFVHESLADVQAACSQKNVACKNGQTNCYQSYSTMSITDCRETGSSKYPNCAYKTTQANKHIIVACEGNPYVPVHFDASV<br><br>Proposed: aliSize=124 (resi) RMSD=0.47 (Å)<br>KETAAAKFERQHMDSSTSAASSSNYCNQMMKSRNLTKDRCKPVNTFVHESLADVQAVCSQKNVACKNGQTNCYQSYSTMSITDCRETGSSKYPNCAYKTTQANKHIIVACEGNPYVPVHFDASV<br>KETAAAKFERQHMDSSTSAASSSNYCNQMMKSRNLTKDRCKPVNTFVHESLADVQAACSQKNVACKNGQTNCYQSYSTMSITDCRETGSSKYPNCAYKTTQANKHIIVACEGNPYVPVHFDASV
262	1f0vA (124)<br>3di9A (124)	C	99.19	TM-align: aliSize=115 (resi) RMSD=1.33 (Å)<br>KETAAAKFERQHMDSSTSAASSSNYCNQMMKSRNLTKDRCKPVNTFVHESLADVQAVCSQKNVACKNGQTNCYQSYSTMSITDCRETGSSKYPNCAYKTTQANKHIIVACEGNPYvpvhfdasv---------<br>KETAAAKFERQHMDSSTSAASSSNYCNQMMKSRNLTKDRCKPVNTFVHESLADVQAVCSQKNVACKNGQTNCYQSYSTMSATDCRETGSSKYPNCAYKTTQANKHIIVACEGNPY---------vpvhfdasv<br><br>SARST: aliSize=121 (resi) RMSD=7.81 (Å)<br>k-ETAAAKFERQHMDSSTSAASSSNYCNQMMKSRNLTKDRCKPVNTFVHESLADVQAVCSQKNVACKNGQTNCYQSYSTMSITDCRETGSSKYPNCAYKTTQANKHIIVACEGNPYVPVHFDA--sv<br>-kETAAAKFERQHMDSSTSAASSSNYCNQMMKSRNLTKDRCKPVNTFVHESLADVQAVCSQKNVACKNGQTNCYQSYSTMSATDCRETGSSKYPNCAYKTTQANKHIIVACEGNPYVPVHFDAsv--<br><br>BLAST: aliSize=123 (resi) iden=99.19% (123/124) simi=99.19% (123/124)<br>KETAAAKFERQHMDSSTSAASSSNYCNQMMKSRNLTKDRCKPVNTFVHESLADVQAVCSQKNVACKNGQTNCYQSYSTMSITDCRETGSSKYPNCAYKTTQANKHIIVACEGNPYVPVHFDASV<br>KETAAAKFERQHMDSSTSAASSSNYCNQMMKSRNLTKDRCKPVNTFVHESLADVQAVCSQKNVACKNGQTNCYQSYSTMSATDCRETGSSKYPNCAYKTTQANKHIIVACEGNPYVPVHFDASV<br><br>Proposed: aliSize=124 (resi) RMSD=0.55 (Å)<br>KETAAAKFERQHMDSSTSAASSSNYCNQMMKSRNLTKDRCKPVNTFVHESLADVQAVCSQKNVACKNGQTNCYQSYSTMSITDCRETGSSKYPNCAYKTTQANKHIIVACEGNPYVPVHFDASV<br>KETAAAKFERQHMDSSTSAASSSNYCNQMMKSRNLTKDRCKPVNTFVHESLADVQAVCSQKNVACKNGQTNCYQSYSTMSATDCRETGSSKYPNCAYKTTQANKHIIVACEGNPYVPVHFDASV
263	1f0vA (124)<br>3dibA (124)	C	99.19	TM-align: aliSize=115 (resi) RMSD=1.30 (Å)<br>KETAAAKFERQHMDSSTSAASSSNYCNQMMKSRNLTKDRCKPVNTFVHESLADVQAVCSQKNVACKNGQTNCYQSYSTMSITDCRETGSSKYPNCAYKTTQANKHIIVACEGNPYvpvhfdasv---------<br>KETAAAKFERQHMDSSTSAASSSNYCNQMMKSRNLTKDRCKPVNTFVHESLADVQAVCSQKNVACKNGQTNCYQSYSTMSITDCRETGSSKYPNCAYKTTQANKHAIVACEGNPY---------vpvhfdasv<br><br>SARST: aliSize=121 (resi) RMSD=8.89 (Å)<br>ke--TAAAKFERQHMDSSTSAASSSNYCNQMMKSRNLTKDRCKPVNTFVHESLADVQAVCSQKNVACKNGQTNCYQSYSTMSITDCRETGSSKYPNCAYKTTQANKHIIVACEGNPYVPVHFDAS-v<br>--keTAAAKFERQHMDSSTSAASSSNYCNQMMKSRNLTKDRCKPVNTFVHESLADVQAVCSQKNVACKNGQTNCYQSYSTMSITDCRETGSSKYPNCAYKTTQANKHAIVACEGNPYVPVHFDASv-<br><br>BLAST: aliSize=123 (resi) iden=99.19% (123/124) simi=99.19% (123/124)<br>KETAAAKFERQHMDSSTSAASSSNYCNQMMKSRNLTKDRCKPVNTFVHESLADVQAVCSQKNVACKNGQTNCYQSYSTMSITDCRETGSSKYPNCAYKTTQANKHIIVACEGNPYVPVHFDASV<br>KETAAAKFERQHMDSSTSAASSSNYCNQMMKSRNLTKDRCKPVNTFVHESLADVQAVCSQKNVACKNGQTNCYQSYSTMSITDCRETGSSKYPNCAYKTTQANKHAIVACEGNPYVPVHFDASV<br><br>Proposed: aliSize=124 (resi) RMSD=0.51 (Å)<br>KETAAAKFERQHMDSSTSAASSSNYCNQMMKSRNLTKDRCKPVNTFVHESLADVQAVCSQKNVACKNGQTNCYQSYSTMSITDCRETGSSKYPNCAYKTTQANKHIIVACEGNPYVPVHFDASV<br>KETAAAKFERQHMDSSTSAASSSNYCNQMMKSRNLTKDRCKPVNTFVHESLADVQAVCSQKNVACKNGQTNCYQSYSTMSITDCRETGSSKYPNCAYKTTQANKHAIVACEGNPYVPVHFDASV

264	1f0vA<br>(124)<br><br>3dicA<br>(124)	C	99.19	TM-align: aliSize=115 (resi) RMSD=1.30 (Å)<br>KETAAAKFERQHMDSSTSAASSSNYCNQMMKSRNLTKDRCKPVNTFVHESLADVQAVCSQKNVACKNGQTNCYQSYSTMSITDCRETGSSKYPNCAYKTTQANKHIIVACEGNPYvpvhfdasv---------<br>KETAAAKFERQHMDSSTSAASSSNYCNQMMKSRNLTKDRCKPVNTFVHESLADVQAVCSQKNVACKNGQTNCYQSYSTMSITDCRETGSSKYPNCAYKTTQANKHIIAACEGNPY---------vpvhfdasv<br><br>SARST: aliSize=122 (resi) RMSD=8.83 (Å)<br>k-ETAAAKFERQHMDSSTSAASSSNYCNQMMKSRNLTKDRCKPVNTFVHESLADVQAVCSQKNVACKNGQTNCYQSYSTMSITDCRETGSSKYPNCAYKTTQANKHIIVACEGNPYVPVHFDAS-v<br>-kETAAAKFERQHMDSSTSAASSSNYCNQMMKSRNLTKDRCKPVNTFVHESLADVQAVCSQKNVACKNGQTNCYQSYSTMSITDCRETGSSKYPNCAYKTTQANKHIIAACEGNPYVPVHFDASv-<br><br>BLAST: aliSize=123 (resi) iden=99.19% (123/124) simi=99.19% (123/124)<br>KETAAAKFERQHMDSSTSAASSSNYCNQMMKSRNLTKDRCKPVNTFVHESLADVQAVCSQKNVACKNGQTNCYQSYSTMSITDCRETGSSKYPNCAYKTTQANKHIIVACEGNPYVPVHFDASV<br>KETAAAKFERQHMDSSTSAASSSNYCNQMMKSRNLTKDRCKPVNTFVHESLADVQAVCSQKNVACKNGQTNCYQSYSTMSITDCRETGSSKYPNCAYKTTQANKHIIAACEGNPYVPVHFDASV<br><br>Proposed: aliSize=124 (resi) RMSD=0.48 (Å)<br>KETAAAKFERQHMDSSTSAASSSNYCNQMMKSRNLTKDRCKPVNTFVHESLADVQAVCSQKNVACKNGQTNCYQSYSTMSITDCRETGSSKYPNCAYKTTQANKHIIVACEGNPYVPVHFDASV<br>KETAAAKFERQHMDSSTSAASSSNYCNQMMKSRNLTKDRCKPVNTFVHESLADVQAVCSQKNVACKNGQTNCYQSYSTMSITDCRETGSSKYPNCAYKTTQANKHIIAACEGNPYVPVHFDASV
265	1f0vA<br>(124)<br><br>3rsdA<br>(124)	C	99.19	TM-align: aliSize=115 (resi) RMSD=1.27 (Å)<br>KETAAAKFERQHMDSSTSAASSSNYCNQMMKSRNLTKDRCKPVNTFVHESLADVQAVCSQKNVACKNGQTNCYQSYSTMSITDCRETGSSKYPNCAYKTTQANKHIIVACEGNPYvpvhfdasv---------<br>KETAAAKFERQHMDSSTSAASSSNYCNQMMKSRNLTKDRCKPVNTFVHESLADVQAVCSQKNVACKNGQTNCYQSYSTMSITDCRETGSSKYPNCAYKTTQANKHIIVACEGNPY---------vpvhfnasv<br><br>SARST: aliSize=122 (resi) RMSD=8.82 (Å)<br>k-ETAAAKFERQHMDSSTSAASSSNYCNQMMKSRNLTKDRCKPVNTFVHESLADVQAVCSQKNVACKNGQTNCYQSYSTMSITDCRETGSSKYPNCAYKTTQANKHIIVACEGNPYVPVHFDAS-v<br>-kETAAAKFERQHMDSSTSAASSSNYCNQMMKSRNLTKDRCKPVNTFVHESLADVQAVCSQKNVACKNGQTNCYQSYSTMSITDCRETGSSKYPNCAYKTTQANKHIIVACEGNPYVPVHFNASv-<br><br>BLAST: aliSize=124 (resi) iden=99.19% (123/124) simi=100.00% (124/124)<br>KETAAAKFERQHMDSSTSAASSSNYCNQMMKSRNLTKDRCKPVNTFVHESLADVQAVCSQKNVACKNGQTNCYQSYSTMSITDCRETGSSKYPNCAYKTTQANKHIIVACEGNPYVPVHFDASV<br>KETAAAKFERQHMDSSTSAASSSNYCNQMMKSRNLTKDRCKPVNTFVHESLADVQAVCSQKNVACKNGQTNCYQSYSTMSITDCRETGSSKYPNCAYKTTQANKHIIVACEGNPYVPVHFNASV<br><br>Proposed: aliSize=124 (resi) RMSD=0.46 (Å)<br>KETAAAKFERQHMDSSTSAASSSNYCNQMMKSRNLTKDRCKPVNTFVHESLADVQAVCSQKNVACKNGQTNCYQSYSTMSITDCRETGSSKYPNCAYKTTQANKHIIVACEGNPYVPVHFDASV<br>KETAAAKFERQHMDSSTSAASSSNYCNQMMKSRNLTKDRCKPVNTFVHESLADVQAVCSQKNVACKNGQTNCYQSYSTMSITDCRETGSSKYPNCAYKTTQANKHIIVACEGNPYVPVHFNASV
266	1f0vA<br>(124)<br><br>3rskA<br>(124)	C	97.58	TM-align: aliSize=115 (resi) RMSD=1.27 (Å)<br>KETAAAKFERQHMDSSTSAASSSNYCNQMMKSRNLTKDRCKPVNTFVHESLADVQAVCSQKNVACKNGQTNCYQSYSTMSITDCRETGSSKYPNCAYKTTQANKHIIVACEGNPYvpvhfdasv---------<br>KETAAAAFEAQHMDSSTSAASSSNYCNQMMKSRNLTKDRCKPVNTFVHESLADVQAVCSQKNVACANGQTNCYQSYSTMSITDCRETGSSKYPNCAYKTTQANKHIIVACEGNPY---------vpvhfdasv<br><br>SARST: aliSize=122 (resi) RMSD=8.84 (Å)<br>k-ETAAAKFERQHMDSSTSAASSSNYCNQMMKSRNLTKDRCKPVNTFVHESLADVQAVCSQKNVACKNGQTNCYQSYSTMSITDCRETGSSKYPNCAYKTTQANKHIIVACEGNPYVPVHFDAS-v<br>-kETAAAAFEAQHMDSSTSAASSSNYCNQMMKSRNLTKDRCKPVNTFVHESLADVQAVCSQKNVACANGQTNCYQSYSTMSITDCRETGSSKYPNCAYKTTQANKHIIVACEGNPYVPVHFDASv-<br><br>BLAST: aliSize=121 (resi) iden=97.58% (121/124) simi=97.58% (121/124)<br>KETAAAKFERQHMDSSTSAASSSNYCNQMMKSRNLTKDRCKPVNTFVHESLADVQAVCSQKNVACKNGQTNCYQSYSTMSITDCRETGSSKYPNCAYKTTQANKHIIVACEGNPYVPVHFDASV<br>KETAAAAFEAQHMDSSTSAASSSNYCNQMMKSRNLTKDRCKPVNTFVHESLADVQAVCSQKNVACANGQTNCYQSYSTMSITDCRETGSSKYPNCAYKTTQANKHIIVACEGNPYVPVHFDASV<br><br>Proposed: aliSize=124 (resi) RMSD=0.48 (Å)<br>KETAAAKFERQHMDSSTSAASSSNYCNQMMKSRNLTKDRCKPVNTFVHESLADVQAVCSQKNVACKNGQTNCYQSYSTMSITDCRETGSSKYPNCAYKTTQANKHIIVACEGNPYVPVHFDASV<br>KETAAAAFEAQHMDSSTSAASSSNYCNQMMKSRNLTKDRCKPVNTFVHESLADVQAVCSQKNVACANGQTNCYQSYSTMSITDCRETGSSKYPNCAYKTTQANKHIIVACEGNPYVPVHFDASV

267	1f0vA (124)<br>3rspA (124)	C	99.19	TM-align: aliSize=115 (resi) RMSD=1.31 (Å)<br>KETAAAKFERQHMDSSTSAASSSNYCNQMMKSRNLTKDRCKPVNTFVHESLADVQAVCSQKNVACKNGQTNCYQSYSTMSITDCRETGSSKYPNCAYKTTQANKHIIVACEGNPYvpvhfdasv---------<br>KETAAAKFERQHMDSSTSAASSSNYCNQMMKSRNLTKDRCKPVNTFVHESLADVQAVCSQKNVACKNGQTNCYQSYSTMSITDCRETGSSKYGNCAYKTTQANKHIIVACEGNPY---------vpvhfdasv<br><br>SARST: aliSize=122 (resi) RMSD=8.84 (Å)<br>k-ETAAAKFERQHMDSSTSAASSSNYCNQMMKSRNLTKDRCKPVNTFVHESLADVQAVCSQKNVACKNGQTNCYQSYSTMSITDCRETGSSKYPNCAYKTTQANKHIIVACEGNPYVPVHFDAS-v<br>-kETAAAKFERQHMDSSTSAASSSNYCNQMMKSRNLTKDRCKPVNTFVHESLADVQAVCSQKNVACKNGQTNCYQSYSTMSITDCRETGSSKYGNCAYKTTQANKHIIVACEGNPYVPVHFDASv-<br><br>BLAST: aliSize=123 (resi) iden=99.19% (123/124) simi=99.19% (123/124)<br>KETAAAKFERQHMDSSTSAASSSNYCNQMMKSRNLTKDRCKPVNTFVHESLADVQAVCSQKNVACKNGQTNCYQSYSTMSITDCRETGSSKYPNCAYKTTQANKHIIVACEGNPYVPVHFDASV<br>KETAAAKFERQHMDSSTSAASSSNYCNQMMKSRNLTKDRCKPVNTFVHESLADVQAVCSQKNVACKNGQTNCYQSYSTMSITDCRETGSSKYGNCAYKTTQANKHIIVACEGNPYVPVHFDASV<br><br>Proposed: aliSize=124 (resi) RMSD=0.58 (Å)<br>KETAAAKFERQHMDSSTSAASSSNYCNQMMKSRNLTKDRCKPVNTFVHESLADVQAVCSQKNVACKNGQTNCYQSYSTMSITDCRETGSSKYPNCAYKTTQANKHIIVACEGNPYVPVHFDASV<br>KETAAAKFERQHMDSSTSAASSSNYCNQMMKSRNLTKDRCKPVNTFVHESLADVQAVCSQKNVACKNGQTNCYQSYSTMSITDCRETGSSKYGNCAYKTTQANKHIIVACEGNPYVPVHFDASV
268	1f0vA (124)<br>4rsdA (124)	C	99.19	TM-align: aliSize=115 (resi) RMSD=1.34 (Å)<br>KETAAAKFERQHMDSSTSAASSSNYCNQMMKSRNLTKDRCKPVNTFVHESLADVQAVCSQKNVACKNGQTNCYQSYSTMSITDCRETGSSKYPNCAYKTTQANKHIIVACEGNPYvpvhfdasv---------<br>KETAAAKFERQHMDSSTSAASSSNYCNQMMKSRNLTKDRCKPVNTFVHESLADVQAVCSQKNVACKNGQTNCYQSYSTMSITDCRETGSSKYPNCAYKTTQANKHIIVACEGNPY---------vpvhfaasv<br><br>SARST: aliSize=122 (resi) RMSD=8.84 (Å)<br>k-ETAAAKFERQHMDSSTSAASSSNYCNQMMKSRNLTKDRCKPVNTFVHESLADVQAVCSQKNVACKNGQTNCYQSYSTMSITDCRETGSSKYPNCAYKTTQANKHIIVACEGNPYVPVHFDAS-v<br>-kETAAAKFERQHMDSSTSAASSSNYCNQMMKSRNLTKDRCKPVNTFVHESLADVQAVCSQKNVACKNGQTNCYQSYSTMSITDCRETGSSKYPNCAYKTTQANKHIIVACEGNPYVPVHFAASv-<br><br>BLAST: aliSize=123 (resi) iden=99.19% (123/124) simi=99.19% (123/124)<br>KETAAAKFERQHMDSSTSAASSSNYCNQMMKSRNLTKDRCKPVNTFVHESLADVQAVCSQKNVACKNGQTNCYQSYSTMSITDCRETGSSKYPNCAYKTTQANKHIIVACEGNPYVPVHFDASV<br>KETAAAKFERQHMDSSTSAASSSNYCNQMMKSRNLTKDRCKPVNTFVHESLADVQAVCSQKNVACKNGQTNCYQSYSTMSITDCRETGSSKYPNCAYKTTQANKHIIVACEGNPYVPVHFAASV<br><br>Proposed: aliSize=124 (resi) RMSD=0.59 (Å)<br>KETAAAKFERQHMDSSTSAASSSNYCNQMMKSRNLTKDRCKPVNTFVHESLADVQAVCSQKNVACKNGQTNCYQSYSTMSITDCRETGSSKYPNCAYKTTQANKHIIVACEGNPYVPVHFDASV<br>KETAAAKFERQHMDSSTSAASSSNYCNQMMKSRNLTKDRCKPVNTFVHESLADVQAVCSQKNVACKNGQTNCYQSYSTMSITDCRETGSSKYPNCAYKTTQANKHIIVACEGNPYVPVHFAASV
269	1f98A (125)<br>1s67L (119)	N	13.45	TM-align: aliSize=105 (resi) RMSD=2.25 (Å)<br>mehvafgsedientl----AKMDdgQ-LDGLAFGAIQLDGDGNILQYNAAEGDIVGRDPKQVIGKNFFkdVAPC--TDSPeFYGKFKEGVASG--N-LNTMFEYTFDY-QMTPTKVKVHMKKALS--GDSYWVFVKRV-<br>---------------naadGIFF--PaLEQNMMGAVLINENDEVMFFNPAAEKLWGYKREEVIGNNID--MLIPrdLRPA-HPEYIRHNREGGkaRvEGMSRELQLEKkDGSKIWTRFALSKVSAegKVYYLALVRDAs<br><br>SARST: aliSize=105 (resi) RMSD=5.97 (Å)<br>mehvafgsedient-LAKMDDG-QLDGLAFGAIQLDGDGNILQYNAAEGDIVGRDPKQVIGKNFFKDVAPCTdsPEFYGKFKEGVAS----GNLNTMFEYTF------DYQMTPTKVKVHMKKAlsgDSYWVFVKR--v<br>-------------nAADGIFFpALEQNMMGAVLINENDEVMFFNPAAEKLWGYKREEVIGNNIDMLIPRDL--RPAHPEYIRHNREggkaRVEGMSRELQLekkdgsKIWTRFALSKVSAEGK---VYYLALVRDas-<br><br>BLAST: aliSize=29 (resi) iden=12.61% (15/119) simi=24.37% (29/119)<br>mehvafgsedientlakmddgqldglaf----------------GAIQLDGDGNILQYNAAEGDIVGRDPKQVIGKNFF----KDVAPCtdSPEFYGKFKEG--vasgnlntmfeytfdyqmtptkvkvhmkkalsgdsywvfvkrv<br>---------------------------naadgiffpaleqnmmGAVLINENDEVMFFNPAAEKLWGYKREEVIGNNIDmlipRDLRPA--HPEYIRHNREGgkarvegmsrelqlekkdgskiwtrfalskvsaegkvyylalvrdas--<br><br>Proposed: aliSize=109 (resi) RMSD=2.10 (Å)<br>mehvafgsED-IEntLA-KMddgqLDGLAFGAIQLDGDGNILQYNAAEGDIVGRDPKQVIGKNFFkDVAPC-TDSPeFYGKFKEGVASG--N-LNTMFEYTFDYQM-TPTKVKVHMKKALS--GDSYWVFVKRV--<br>--------NAaDG--IFfPA----LEQNMMGAVLINENDEVMFFNPAAEKLWGYKREEVIGNNID-MLIPRdLRPA-HPEYIRHNREGGkaRvEGMSRELQLEKKDgSKIWTRFALSKVSAegKVYYLALVRDAsh

270	1f98A<br>(125)<br><br>1s67U<br>(115)	N	13.91	TM-align: aliSize=106 (resi) RMSD=2.30 (Å)<br>mehvafgsedientlAKMDdGQLDGLAFGAIQLDGDGNILQYNAAEGDIVGRDPKQVIGKNFFkdVAPC--TDSPeFYGKFKEGVASGN-LNT--MFEYTFDY-QMTPTKVKVHMKKALS--GDSYWVFVKRV-<br>---------------GIFF-PALEQNMMGAVLINENDEVMFFNPAAEKLWGYKREEVIGNNID--MLIPrdLRPA-HPEYIRHNREGGKaRVEgmSRELQLEKkDGSKIWTRFALSKVSAegKVYYLALVRDAs<br><br>SARST: aliSize=102 (resi) RMSD=3.50 (Å)<br>mehvafgsedientlakmd---DGQLDGLAFGAIQLDGDGNILQYNAAEGDIVGRDPKQVIGKNFFKDVAPCTdsPEFYGKFKEGVAS-GNLN-----TMFEYTFDYQmTPTKVKVHMKKALSGDS--YWVFVKR--v<br>-------------------gifFPALEQNMMGAVLINENDEVMFFNPAAEKLWGYKREEVIGNNIDMLIPRDL--RPAHPEYIRHNREgGKARvegmsRELQLEKKDG-SKIWTRFALSKVSAEGKvyYLALVRDas-<br><br>BLAST: aliSize=29 (resi) iden=13.04% (15/115) simi=25.22% (29/115)<br>mehvafgsedientlakmddgqldglaf------------GAIQLDGDGNILQYNAAEGDIVGRDPKQVIGKNFF----KDVAPCtdSPEFYGKFKEG---vasgnlntmfeytfdyqmtptkvkvhmkkalsgdsywvfvkrv<br>----------------------------giffpaleqnmmGAVLINENDEVMFFNPAAEKLWGYKREEVIGNNIDmlipRDLRPA--HPEYIRHNREGgkarvegmsrelqlekkdgskiwtrfalskvsaegkvyylalvrdas---<br><br>Proposed: aliSize=107 (resi) RMSD=2.23 (Å)<br>mEhVafgsedientLakMddGQLDGLAFGAIQLDGDGNILQYNAAEGDIVGRDPKQVIGKNFFkDVAPC-TDSPeFYGKFKEGVASGN-LNT--MFEYTFDYQM-TPTKVKVHMKKALS--GDSYWVFVKRV--<br>-G-I----------F--F--PALEQNMMGAVLINENDEVMFFNPAAEKLWGYKREEVIGNNID-MLIPRdLRPA-HPEYIRHNREGGKaRVEgmSRELQLEKKDgSKIWTRFALSKVSAegKVYYLALVRDAsh
271	1f98A<br>(125)<br><br>1v9zA<br>(113)	N	14.16	TM-align: aliSize=105 (resi) RMSD=2.26 (Å)<br>mehvafgsedientlAKMDdGQLDGLAFGAIQLDGDGNILQYNAAEGDIVGRDPKQVIGKNFFkdVAPC--TDSPeFYGKFKEGVASGN-LNT--MFEYTFDY-QMTPTKVKVHMKKALS--GDSYWVFVKRv<br>---------------GIFF-PALEQNMMGAVLINENDEVMFFNPAAEKLWGYKREEVIGNNID--MLIPrdLRPA-HPEYIRHNREGGKaRVEgmSRELQLEKkDGSKIWTRFALSKVSAegKVYYLALVRD-<br><br>SARST: aliSize=103 (resi) RMSD=3.01 (Å)<br>mehvafgsedientlakmd---DGQLDGLAFGAIQLDGDGNILQYNAAEGDIVGRDPKQVIGKNFFKDVAPCTDsPEFYGKFKEGVASGNLNTM---FEYTFDY-QMTPTKVKVHMKKALSGDSYWVFVK---rv<br>-------------------gifFPALEQNMMGAVLINENDEVMFFNPAAEKLWGYKREEVIGNNIDMLIPRDLR-PAHPEYIRHNREGGKARVEgmsRELQLEKkDGSKIWTRFALSKVSAEGKVYYLALvrd--<br><br>BLAST: aliSize=29 (resi) iden=13.27% (15/113) simi=25.66% (29/113)<br>mehvafgsedientlakmddgqldglaf------------GAIQLDGDGNILQYNAAEGDIVGRDPKQVIGKNFF----KDVAPCtdSPEFYGKFKEG---vasgnlntmfeytfdyqmtptkvkvhmkkalsgdsywvfvkrv<br>----------------------------giffpaleqnmmGAVLINENDEVMFFNPAAEKLWGYKREEVIGNNIDmlipRDLRPA--HPEYIRHNREGgkarvegmsrelqlekkdgskiwtrfalskvsaegkvyylalvrd---<br><br>Proposed: aliSize=106 (resi) RMSD=2.00 (Å)<br>mehvafgsEDIENTlakmddgqLDGLAFGAIQLDGDGNILQYNAAEGDIVGRDPKQVIGKNFFkDVAPC-TDSPeFYGKFKEGVASGN--LNT-MFEYTFDYQM-TPTKVKVHMKKALS--GDSYWVFVKR-v<br>--------GIFFPA--------LEQNMMGAVLINENDEVMFFNPAAEKLWGYKREEVIGNNID-MLIPRdLRPA-HPEYIRHNREGGKarVEGmSRELQLEKKDgSKIWTRFALSKVSAegKVYYLALVRDh-
272	1f98A<br>(125)<br><br>1vb6A<br>(114)	N	14.04	TM-align: aliSize=106 (resi) RMSD=2.29 (Å)<br>mehvafgsedientlAKMDdGQLDGLAFGAIQLDGDGNILQYNAAEGDIVGRDPKQVIGKNFFkdVAPC--TDSPeFYGKFKEGVASGN-LNT--MFEYTFDY-QMTPTKVKVHMKKALS--GDSYWVFVKRV<br>---------------GIFF-PALEQNMMGAVLINENDEVMFFNPAAEKLWGYKREEVIGNNID--MLIPrdLRPA-HPEYIRHNREGGKaRVEgmSRELQLEKkDGSKIWTRFALSKVSAegKVYYLALVRDA<br><br>SARST: aliSize=102 (resi) RMSD=3.53 (Å)<br>mehvafgsedientlakmd---DGQLDGLAFGAIQLDGDGNILQYNAAEGDIVGRDPKQVIGKNFFKDVAPCTDsPEFYGKFKEGVASGNLN-----TMFEYTFDYQmTPTKVKVHMKKALSGDSYWVFVK----rv<br>-------------------gifFPALEQNMMGAVLINENDEVMFFNPAAEKLWGYKREEVIGNNIDMLIPRDLR-PAHPEYIRHNREGGKARvegmsRELQLEKKDG-SKIWTRFALSKVSAEGKVYYLALvrda--<br><br>BLAST: aliSize=29 (resi) iden=13.16% (15/114) simi=25.44% (29/114)<br>mehvafgsedientlakmddgqldglaf------------GAIQLDGDGNILQYNAAEGDIVGRDPKQVIGKNFF----KDVAPCtdSPEFYGKFKEG--vasgnlntmfeytfdyqmtptkvkvhmkkalsgdsywvfvkrv<br>----------------------------giffpaleqnmmGAVLINENDEVMFFNPAAEKLWGYKREEVIGNNIDmlipRDLRPA--HPEYIRHNREGgkarvegmsrelqlekkdgskiwtrfalskvsaegkvyylalvrda---<br><br>Proposed: aliSize=107 (resi) RMSD=2.04 (Å)<br>mehvafgsEDIENTlakmddgqLDGLAFGAIQLDGDGNILQYNAAEGDIVGRDPKQVIGKNFFkDVAPC-TDSPeFYGKFKEGVASGN-LNT--MFEYTFDYQM-TPTKVKVHMKKALS--GDSYWVFVKRV-<br>--------GIFFPA--------LEQNMMGAVLINENDEVMFFNPAAEKLWGYKREEVIGNNID-MLIPRdLRPA-HPEYIRHNREGGKaRVEgmSRELQLEKKDgSKIWTRFALSKVSAegKVYYLALVRDAh

<table>
<tr><td rowspan="2">273</td><td>1fyrA
(95)</td><td rowspan="2">C</td><td rowspan="2">26.32</td><td rowspan="2">TM-align: aliSize=64 (resi) RMSD=1.92 (Å)
hPWFFGKIPRAKAEEMLSKQRhDGAFLIRESESAPGDFSLSVKFGNDVQHFKVLRD----------------GAGKYFLWVVkfnslnelvdyhrstsvsrnqqiflrdie---------------------------
-SWYWGRLSRQEAVALLQGQR-HGVFLVRDSSTSPGDYVLSVSENSRVSHYIINSSgprppvppspaqppppgvSPSRLRIGDQ---------------------------efdslpallefykihyldtttliepvsr

SARST: aliSize=73 (resi) RMSD=8.83 (Å)
hpwffg-----KIPRAKAEEMLSKQrHDGAFLIRESESAPGDFSLSVKFGNDVQHFKVLRDGA------------GKYFLWVV----KFNSLNELVDYHRS-------------tsvsrnqqiflrdie
------swywgRLSRQEAVALLQGQ-RHGVFLVRDSSTSPGDYVLSVSENSRVSHYIINSSGPrppvppspaqpppGVSPSRLRigdqEFDSLPALLEFYKIhyldtttliepvsr--------------

BLAST: aliSize=36 (resi) iden=23.16% (22/95) simi=37.89% (36/95)
hp-WFFGKIPRAKAEEMLSKQRHdGAFLIRESESAPGDFSLSVKFGNDVQHFKVLRDG--agkyflwvvkfnslnelvdyhrstsvsrnqqiflrdie
--sWYWGRLSRQEAVALLQGQRH-GVFLVRDSSTSPGDYVLSVSENSRVSHYIINSSGprppvppspaqppppgvspsrlrigdqefdslpallefykihyldtttliepvsr-------------------------------------

Proposed: aliSize=89 (resi) RMSD=1.64 (Å)
hPWFFGKIPRAKAEEMLSKQRhDGAFLIRESESAPGDFSLSVKFGNDVQHFKVLR-----------------dGAGKYFLWVVKFNSLNELVDYHRSTSVSrnQQiFLRDI--E
-SWYWGRLSRQEAVALLQGQR-HGVFLVRDSSTSPGDYVLSVSENSRVSHYIINSsgprppvppspaqppppgv-SPSRLRIGDQEFDSLPALLEFYKIHYLD--TT-TLIEPvsR</td></tr>
<tr><td>1ju5A
(109)</td></tr>
<tr><td rowspan="2">274</td><td>1gq9A
(241)</td><td rowspan="2">M</td><td rowspan="2">17.54</td><td rowspan="2">TM-align: aliSize=199 (resi) RMSD=2.70 (Å)
-SKAVIVIPARYGSSRLPGKPLLDIVGKPMIQHVYERALQVAGVAEVWVATDDPRVEQAVQAFGGKAIMTRNDH----ESGTDRLVEVMHKV-EADIYINLQGDEPMIRPRDVETLLQGMRDDPaLPVATLCHAISAAeAAe-Pstvkvvvntrqdalyfsrsp---------IPYPR--NAE----KA
pPHLAALVLARGGSKGIPLKNIKRLAGVPLIGWVLRAALDAGVFQSVWVSTDHDEIENVAKQFGAQVHRRSSETskdsSTSLDAIVEFLNYHnEVDIVGNIQATSPCLHPTDLQKVAEMIREEG-YDSVFSVVRRHQF-RW-sE-------------------iqkgvrevtePLNLNpaKRPrrqdWD
RYLKHVG-IYAYRRDVLQNYsqLpesmpeqaesleQlrLMnagINIRTFEVAA-TGPGVDTPACLEKVRALMAQE--L--
GELYENGsFYFAKRHLIEMG--Y-----------L--QG---GKMAYYEMRAeHSVDIDVDIDWPIAEQRVLRFgyFgk

SARST: aliSize=184 (resi) RMSD=8.10 (Å)
s--KAVIVIPARYGSSRLPGKPLLDIVGKPMIQHVYERALQVAGVAEVWVATDDPRVEQAVQAFGGKAIMTR--NDHE--SGTDRLVEVMHKV-EADIYINLQGDEPMIRPRDVETLLQGMRDdPALPVATLCHAIsaaeaaepsTVKVVVNTRQDAlyfsRSPIPYPRN-------AEKARYLKHV--G
-ppHLAALVLARGGSKGIPLKNIKRLAGVPLIGWVLRAALDAGVFQSVWVSTDHDEIENVAKQFGAQVHRRSseTSKDssTSLDAIVEFLNYHnEVDIVGNIQATSPCLHPTDLQKVAEMIRE-EGYDSVFSVVRR---------HQFRWSEIQKGV----REVTEPLNLnpakrprRQDWDGELYEngS
IYAYRRdvlqnysqlpesmpeqaesleqlRLMNAGINIRTFEVAATGP---------------------------gvdtpaclekvralmaqel
FYFAKR----------------------HLIEMGYLQGGKMAYYEMRaehsvdidvdidwpiaeqrvlrfgyfgk------------------

BLAST: aliSize=64 (resi) iden=17.11% (39/228) simi=28.07% (64/228)
sk----AVIVIpARYGSSRLPGKPLLDIVGKPMIQHVYERALQvAGVAE-VWVATDDPRVEQAVQAFGG----KAIMTRNDHESGTDRLVEVM-HKVEADIYINLQGDEPMIRPRDVETLLQGMRDD--
--pphlAALVL-ARGGSKGIPLKNIKRLAGVPLIGWVLRAALD-AGVFQsVWVSTDHDEIENVAKQFGAqvhrRSSETSKDSSTSLDAIVEFLnYHNEVDIVGNIQATSPCLHPTDLQKVAEMIREEgydsvfsvvrrhqfrwseiqkgvrevteplnlnpakrprrqdwdgelyengsfyfakrhliem
---palpvatlchaisaaeaaepstvkvvvntrqdalyfsrspipyprnaekarylkhvgiyayrrdvlqnysqlpesmpeqaesleqlrlmnaginirtfevaatgpgvdtpaclekvralmaqel
gylqggkmayyemraehsvdidvdidwpiaeqrvlrfgyfgk--

Proposed: aliSize=201 (resi) RMSD=2.30 (Å)
-SKAVIVIPARYGSSRLPGKPLLDIVGKPMIQHVYERALQVAGVAEVWVATDDPRVEQAVQAFGGKAIMTRNDH----ESGTDRLVEVMHKV-EADIYINLQGDEPMIRPRDVETLLQGMRDDPaLPVATLCHAISAAeaaepstVKV-----vvntRQDAlYFSRSPIPYP------rnaeKARYLKHV
pPHLAALVLARGGSKGIPLKNIKRLAGVPLIGWVLRAALDAGVFQSVWVSTDHDEIENVAKQFGAQVHRRSSETskdsSTSLDAIVEFLNYHnEVDIVGNIQATSPCLHPTDLQKVAEMIREEG-YDSVFSVVRRHQF-------RWSeiqkg----VREV-TEPLNLNPAKrprrqd----WDGELYEN
G-IYAYRRDVLQNYsqlpesmpeqaesleqlrL--mnagIN-IRTFEVAA-TGPGVD-TPAcLEKVRALMAQE--L--
GsFYFAKRHLIEMG------------------Ylq----GGkMAYYEMRAeHSVDIDvDID-WPIAEQRVLRFgyFgk</td></tr>
<tr><td>1qwjA
(228)</td></tr>
<tr><td rowspan="2">275</td><td>1h8xA
(125)</td><td rowspan="2">N</td><td rowspan="2">33.33</td><td rowspan="2">TM-align: aliSize=98 (resi) RMSD=2.12 (Å)
kesaaakferqhmdsgns------------------PSsSSTYCNQMMRRRNMTQgRCKPVNTFVHEPLVDVQNVCF---QEKVTckngqGNCYKSNSSMHITDCRLTNGSRYPNCAYRTSQKERHIIVACEGspYVPVHFDA-SV--E
-----------------qdnsrythfltqhydakpqGR-DDRYCESIMRRRGLTS-PCKDINTFIHGNKRSIKAICEnknGNPHR-----ENLRISKSSFQVTTCKLHGGSPWPPCQYRATAGFRNVVVACEN--GLPVHLDQsIFrrP

SARST: aliSize=90 (resi) RMSD=5.21 (Å)
kesaaakferqhmdsgnspssss----------------------TYCNQMMRRRNMTQgRCKPVNTFVHEPLVDVQNVCfqekvTCKNGQGN---CYKSNSSMHITDCRLTNGSRYPNCAYRTSQKERHIIVACEGspyvpVHFDASV-------e
----------------------qdnsrythfltqhydakpqgrddRYCESIMRRRGLTS-PCKDINTFIHGNKRSIKAIC-----ENKNGNPHrenLRISKSSFQVTTCKLHGGSPWPPCQYRATAGFRNVVVACEN-----GLPVHLDqsifrrp-

BLAST: aliSize=66 (resi) iden=36.59% (45/123) simi=53.66% (66/123)
kesaaak--------FERQHMDSgnSPSS-SSTYCNQMMRRRNMTQGrCKPVNTFVHEplvDVQNVcfqeKVTCKNGQGNCYKSN-----SSMHITDCRLTNGSRYPNCAYRTSQKERHIIVACEGSpyVPVHFDASV----e
-------qdnsrythFLTQHYDA--KPQGrDDRYCESIMRRRGLTSP-CKDINTFIHG---NKRSI----KAICENKNGNPHRENlriskSSFQVTTCKLHGGSPWPPCQYRATAGFRNVVVACENG--LPVHLDQSIfrrp-

Proposed: aliSize=112 (resi) RMSD=1.65 (Å)
kE--SAAAKFERQHMDSGN-SPSssSTYCNQMMRRRNMTQgRCKPVNTFVHEPLVDVQNVCF---QEKVTckngqGNCYKSNSSMHITDCRLTNGSRYPNCAYRTSQKERHIIVACEGspYVPVHFDA-S----ve
-QdnSRYTHFLTQHYDAKPqGRD--DRYCESIMRRRGLTS-PCKDINTFIHGNKRSIKAICEnknGNPHR-----ENLRISKSSFQVTTCKLHGGSPWPPCQYRATAGFRNVVVACEN--GLPVHLDQsIfrrp--</td></tr>
<tr><td>1awzA
(123)</td></tr>
</table>

276	1h8xA (125)<br>1e21A (119)	N	94.12	TM-align: aliSize=106 (resi) RMSD=0.84 (Å)<br>kesaaakferqhmdsgns------------PSSsSTYCNQMMRRRNMTQGRCKPVNTFVHEPLVDVQNVCFQEKVTCKNGQGNCYKSNSSMHITDCRLTNGSRYPNCAYRTSQKERHIIVACEGSPYVPVHFDASVE<br>-----------------afqrqhmdsdsspSSS-STYCNQMMRRRNMTQGRCKPVNTFVHEPLVDVQNVCFQEKVTCKNGQGNCYKSNSSMHITDCRLTNGSRYPNCAYRTSPKERHIIVACEGSPYVPVHFDASVE<br><br>SARST: aliSize=117 (resi) RMSD=9.42 (Å)<br>kesaaak-FERQHMDSGNSPSSSSTYCNQMMRRRNMTQGRCKPVNTFVHEPLVDVQNVCFQEKVTCKNGQGNCYKSNSSMHITDCRLTNGSRYPNCAYRTSQKERHIIVACEGSPYVPVHFDASV-e<br>-------aFQRQHMDSDSSPSSSSTYCNQMMRRRNMTQGRCKPVNTFVHEPLVDVQNVCFQEKVTCKNGQGNCYKSNSSMHITDCRLTNGSRYPNCAYRTSPKERHIIVACEGSPYVPVHFDASVe-<br><br>BLAST: aliSize=116 (resi) iden=95.80% (114/119) simi=97.48% (116/119)<br>kesaaak-FERQHMDSGNSPSSSSTYCNQMMRRRNMTQGRCKPVNTFVHEPLVDVQNVCFQEKVTCKNGQGNCYKSNSSMHITDCRLTNGSRYPNCAYRTSQKERHIIVACEGSPYVPVHFDASVE<br>-------aFQRQHMDSDSSPSSSSTYCNQMMRRRNMTQGRCKPVNTFVHEPLVDVQNVCFQEKVTCKNGQGNCYKSNSSMHITDCRLTNGSRYPNCAYRTSPKERHIIVACEGSPYVPVHFDASVE<br><br>Proposed: aliSize=117 (resi) RMSD=0.78 (Å)<br>kesaaaKFERQHMDSGNSPS--ssSTYCNQMMRRRNMTQGRCKPVNTFVHEPLVDVQNVCFQEKVTCKNGQGNCYKSNSSMHITDCRLTNGSRYPNCAYRTSQKERHIIVACEGSPYVPVHFDASVE<br>------AFQRQHMDSDSSPSss--STYCNQMMRRRNMTQGRCKPVNTFVHEPLVDVQNVCFQEKVTCKNGQGNCYKSNSSMHITDCRLTNGSRYPNCAYRTSPKERHIIVACEGSPYVPVHFDASVE
277	1h8xA (125)<br>1gioA (125)	N	30.40	TM-align: aliSize=98 (resi) RMSD=2.14 (Å)<br>kesaaakferqhmdsgns--------------------PSsSSTYCNQMMRRRNMTQgRCKPVNTFVHEPLVDVQNVCF---QEKVTCkngqgNCYKSNSSMHITDCRLTNGSRYPNCAYRTSQKERHIIVACEGspYVPVHFDA-SVE---<br>------------------aqddyryihfltqhydakpkGR-NDEYCFNMMKNRRLTR-PCKDRNTFIHGNKNDIKAICEdrnGQPYRG-----DLRISKSEFQITICKHKGGSSRPPCRYGATEDSRVIVVGCEN--GLPVHFDEsFITprh<br><br>SARST: aliSize=90 (resi) RMSD=2.61 (Å)<br>kesaaakferqhmdsgnspss----------------------SSTYCNQMMRRRNMTQgRCKPVNTFVHEPLVDVQNVC---FQEKVTCKngqgnCYKSNSSMHITDCRLTNGSRYPNCAYRTSQKERHIIVACEGSPYVP----------vhfdasve<br>---------------------aqddyryihfltqhydakpkgrNDEYCFNMMKNRRLTR-PCKDRNTFIHGNKNDIKAICedrNGQPYRGD-----LRISKSEFQITICKHKGGSSRPPCRYGATEDSRVIVVGCENGLPVHfdesfitprh--------<br><br>BLAST: aliSize=61 (resi) iden=36.00% (45/125) simi=48.80% (61/125)<br>kesaaak---------FERQHMDSgnSPSS-SSTYCNQMMRRRNMTQGrCKPVNTFVHEPLVDVQNVCfqEKvtcKNGQ---GNCYKSNSSMHITDCRLTNGSRYPNCAYRTSQKERHIIVACEGSpyVPVHFDAS------ve<br>-------aqddyryihFLTQHYDA--KPKGrNDEYCFNMMKNRRLTRP-CKDRNTFIHGNKNDIKAIC--ED---RNGQpyrGDLRISKSEFQITICKHKGGSSRPPCRYGATEDSRVIVVGCENG--LPVHFDESfitprh--<br><br>Proposed: aliSize=113 (resi) RMSD=1.90 (Å)<br>k---ESAAAKFERQHMDSGNSPSssS-TYCNQMMRRRNMTQgRCKPVNTFVHEPLVDVQNVCF---QEKVT-ckngqgNCYKSNSSMHITDCRLTNGSRYPNCAYRTSQKERHIIVACEGspYVPVHFDA-SVE---<br>-aqdDYRYIHFLTQHYDAKPKGR--NdEYCFNMMKNRRLTR-PCKDRNTFIHGNKNDIKAICEdrnGQPYRg------DLRISKSEFQITICKHKGGSSRPPCRYGATEDSRVIVVGCEN--GLPVHFDEsFITprh
278	1h8xA (125)<br>1k58A (123)	N	32.52	TM-align: aliSize=98 (resi) RMSD=2.16 (Å)<br>kesaaakferqhmdsgns------------------PSsSSTYCNQMMRRRNMTQgRCKPVNTFVHEPLVDVQNVCF---QEKVTCkngqgNCYKSNSSMHITDCRLTNGSRYPNCAYRTSQKERHIIVACEGspYVPVHFDA-SVE--<br>------------------dnsrythfltqhydakpqGR-DDRYCESIMRRRGLTS-PCKDINTFIHGNKRSIKAICEnknGNPHRE-----NLRISKSSFQVTTCKLHGGSPWPPCQYRATAGFRNVVVACEN--GLPVHLHQsIFRrp<br><br>SARST: aliSize=105 (resi) RMSD=13.38 (Å)<br>kes---AAAKFERQHMDSGNspssSST---YCNQMMRRRNMTQgRCKPVNTFVHEPLVDVQN---VCFQEKVTCKngqgnCYKSNSSMHITDCRLTNGSRYPNCAYRTSQKERHIIVACEGspYVPVHF--------dasve<br>---dnsRYTHFLTQHYDAKP----QGRddrYCESIMRRRGLTS-PCKDINTFIHGNKRSIKAiceNKNGNPHREN-----LRISKSSFQVTTCKLHGGSPWPPCQYRATAGFRNVVVACEN--GLPVHLhqsifrrp-----<br><br>BLAST: aliSize=65 (resi) iden=36.07% (44/123) simi=53.28% (65/123)<br>kesaaak-------FERQHMDSgnSPSS-SSTYCNQMMRRRNMTQGrCKPVNTFVHEplvDVQNVcfqeKVTCKNGQGNCYKSN-----SSMHITDCRLTNGSRYPNCAYRTSQKERHIIVACEGSpyVPVHFDASV----e<br>-------dnsrythFLTQHYDA--KPQGrDDRYCESIMRRRGLTSP-CKDINTFIHG---NKRSI----KAICENKNGNPHRENlriskSSFQVTTCKLHGGSPWPPCQYRATAGFRNVVVACENG--LPVHLHQSIfrrp-<br><br>Proposed: aliSize=111 (resi) RMSD=1.54 (Å)<br>ke---SAAAKFERQHMDSGN-SPSssSTYCNQMMRRRNMTQgRCKPVNTFVHEPLVDVQNVCF---QEKVTCkngqgNCYKSNSSMHITDCRLTNGSRYPNCAYRTSQKERHIIVACEGspYVPVHFD--AS---ve<br>--pdnSRYTHFLTQHYDAKPqGRD--DRYCESIMRRRGLTS-PCKDINTFIHGNKRSIKAICEnknGNPHRE-----NLRISKSSFQVTTCKLHGGSPWPPCQYRATAGFRNVVVACEN--GLPVHLHqsIFrrp--

279	1h8xA (125)<br>1kvzA (107)	N	23.36	TM-align: aliSize=87 (resi) RMSD=2.68 (Å)<br>kesaaakferqhmdsgnsp--------------SssSTYCNQMMRRRNMtQgrCKPVNTFVHEPLVDVQNVCFQekvtckngq---GNCYKSNSSMHITDCRLTNGSRypnCAYRTSQKERHIIVACEGspYVPVHFDASVE--<br>------------------mqdwatfkkkhltdtW--DVDCDNLMPTSLF-D--CKDKNTFIYSLPGPVKALCRG---------vifSADVLSNSEFYLAECNVKPRKP---CKYKLKKSSNRICIRCEH--ELPVHFAGVGIcp<br><br>SARST: aliSize=72 (resi) RMSD=3.06 (Å)<br>kesaaakferqhmdsgnspsssstycnqmmrrrnmtqgrc-------------------------------KPVNTFVHEPLVDVQNVCFQEKVTckngqgNCYKSNSSMHITDCRLTNGSrypNCAYRTSQKERHIIVACEGspYVPVHFDAS----ve<br>--mqdwatfkkkhltdtwdvdcdnlmptslfdcKDKNTFIYSLPGPVKALCRGVIFS------ADVLSNSEFYLAECNVKPRK---PCKYKLKKSSNRICIRCEH--ELPVHFAGVgicp--<br><br>BLAST: aliSize=37 (resi) iden=20.56% (22/107) simi=34.58% (37/107)<br>kesaaakferqhmdsgnspsssstycnqmmrrrnmtqgr------------------------------CKPVNTFVHEPLVDVQNVCfQEKVTCKNgqgncYKSNSSMHITDCRLTngSRYPnCAYRTSQKERHIIVACEGSpyVPVHF-------dasve<br>---------------------------------------mqdwatfkkkhltdtwdvdcdnlmptslfdCKDKNTFIYSLPGPVKALC-RGVIFSAD-----VLSNSEFYLAECNVK--PRKP-CKYKLKKSSNRICIRCEHE--LPVHFagvgicp-----<br><br>Proposed: aliSize=98 (resi) RMSD=1.94 (Å)<br>KESaAAKFERQHMDSgNSP--sssstyCNQMMRRrNMTQgrCKPVNTFVHEPLVDVQNVCF----qekvtckngqGNCYKSNSSMHITDCRLTNGSRypnCAYRTSQKERHIIVACEGspYVPVHFDASV---e<br>MQD-WATFKKKHLTD-TWDvd------CDNLMPT-SLFD--CKDKNTFIYSLPGPVKALCRgvif----------SADVLSNSEFYLAECNVKPRKP---CKYKLKKSSNRICIRCEH--ELPVHFAGVGicp-
280	1h8xA (125)<br>1oncA (104)	N	24.04	TM-align: aliSize=87 (resi) RMSD=2.59 (Å)<br>kesaaakferqhmdsgnsp-------------SssSTYCNQMMRRRNMTqgrCKPVNTFVHEPLVDVQNVCFQeKvtckngq--GNCYKSNSSMHITDCRLTNGSrypnCAYRTSQKERHIIVACEGspYVPVHFDASVE-<br>------------------�dwltfqkkhitntR--DVDCDNIMSTNLFH---CKDKNTFIYSRPEPVKAICKG-I-------iaSKNVLTTSEFYLSDCNVTSRP----CKYKLKKSTNKFCVTCEN--QAPVHFVGVGSc<br><br>SARST: aliSize=97 (resi) RMSD=12.76 (Å)<br>kesa--AAKFERQHMDsGNspsSSSTYCNQMM-RRRNMtqgrcKPVNTFVHEPLVDVQNVCFQEKVTckngqgNCYKSNSSMHITDCRLTngsryPNC-AYRTSQKERHIIVACEGspYVPVHFDAS---ve<br>----xdWLTFQKKHIT-NT---RDVDCDNIMStNLFHC-----KDKNTFIYSRPEPVKAICKGIIAS------KNVLTTSEFYLSDCNVT-----SRPcKYKLKKSTNKFCVTCEN--QAPVHFVGVgsc--<br><br>BLAST: aliSize=52 (resi) iden=28.85% (30/104) simi=50.00% (52/104)<br>kesaaak-----FERQHMDSgnspsSSSTYCNQMMRRrNMTQgrCKPVNTFVHEPLVDVQNVCfQEKVTCKNgqgncYKSNSSMHITDCRLTngSRyPnCAYRTSQKERHIIVACEGSpyVPVHF------dasve<br>-------xdwltFQKKHITN-----TRDVDCDNIMST-NLFH--CKDKNTFIYSRPEPVKAIC-KGIIASKN-----VLTTSEFYLSDCNVT--SR-P-CKYKLKKSTNKFCVTCENQ--APVHFvgvgsc-----<br><br>Proposed: aliSize=97 (resi) RMSD=1.65 (Å)<br>keSAAAKFERQHMDsGNSP-sssstYCNQMMRRrNMTQgrCKPVNTFVHEPLVDVQNVCF----qekvtckngqGNCYKSNSSMHITDCRLTNGSrypnCAYRTSQKERHIIVACEGspYVPVHFDASV--e<br>--PDWLTFQKKHIT-NTRDv-----DCDNIMST-NLFH--CKDKNTFIYSRPEPVKAICKgiia----------SKNVLTTSEFYLSDCNVTSRP----CKYKLKKSTNKFCVTCEN--QAPVHFVGVGsc-
281	1h8xA (125)<br>1qwqA (124)	N	70.97	TM-align: aliSize=104 (resi) RMSD=2.24 (Å)<br>kesaaakferqhmdsgns------------------PSSSSTYCNQMMRRRNMTQGRCKPVNTFVHEPLVDVQNVCFQEKVTCknGQ--GNCYKSNSSMHITDCRLTNGSRYPNCAYRTSQKERHIIVACEGSPYVPVHFDASVe<br>------------------kesaaakferqhmdsgnsPSSSSNYCNLMMCCRKMTQGKCKPVNTFVHESLADVKAVCSQKKVTC--KDgqTNCYQSKSTMRITDCRETGSSKYPNCAYKTTQVEKHIIVACGGKPSVPVHFDASV-<br><br>SARST: aliSize=99 (resi) RMSD=2.15 (Å)<br>kesaaakferqhmdsgnspsssst------------------------YCNQMMRRRNMTQGRCKPVNTFVHEPLVDVQNVCFQEKVTCKNGQGNCYKSNSSMHITDCRLTNGSRYPNCAYRTSQKERHIIVACEGSPYVPVHFDAS-ve<br>------------------------kesaaakferqhmdsgnspssssnYCNLMMCCRKMTQGKCKPVNTFVHESLADVKAVCSQKKVTCKDGQTNCYQSKSTMRITDCRETGSSKYPNCAYKTTQVEKHIIVACGGKPSVPVHFDASv--<br><br>BLAST: aliSize=105 (resi) iden=76.61% (95/124) simi=84.68% (105/124)<br>KESAAAKFERQHMDSGNSPSSSSTYCNQMMRRRNMTQGRCKPVNTFVHEPLVDVQNVCFQEKVTCKNGQGNCYKSNSSMHITDCRLTNGSRYPNCAYRTSQKERHIIVACEGSPYVPVHFDASVe<br>KESAAAKFERQHMDSGNSPSSSSNYCNLMMCCRKMTQGKCKPVNTFVHESLADVKAVCSQKKVTCKDGQTNCYQSKSTMRITDCRETGSSKYPNCAYKTTQVEKHIIVACGGKPSVPVHFDASV-<br><br>Proposed: aliSize=118 (resi) RMSD=1.68 (Å)<br>KESAAAKFERQHMDSGN-spsSSS--TYCNQMMRRRNMTQGRCKPVNTFVHEPLVDVQNVCFQEKVTCknGQ--GNCYKSNSSMHITDCRLT-nGSRYPNCAYRTSQKERHIIVACEGSPYVPVHFDASVe<br>KESAAAKFERQHMDSGNs---PSSssNYCNLMMCCRKMTQGKCKPVNTFVHESLADVKAVCSQKKVTC--KDgqTNCYQSKSTMRITDCRETg-SSKYPNCAYKTTQVEKHIIVACGGKPSVPVHFDASV-

282	1h8xA (125)<br>1yv7A (102)	N	24.51	TM-align: aliSize=86 (resi) RMSD=2.50 (Å)<br>kesaaakferqhmdsgnsp------------SssSTYCNQMMRRRNMTqgrCKPVNTFVHEPLVDVQNVCFQeKvtckngq--GNCYKSNSSMHITDCRLTNGSrypnCAYRTSQKERHIIVACEGspYVPVHFDASVe<br>------------------dwltfqkkhitntR--DVDCDNIMSTNLFH---CKDKNTFIYSRPEPVKAICKG-I-------iaSKNVLTTSEFYLSDCNVTSRP----CKYKLKKSTNKFSVTCEN--QAPVHFVGVG-<br><br>SARST: aliSize=96 (resi) RMSD=12.82 (Å)<br>kesa-AAKFERQHMDsGNspsSSSTYCNQMM-RRRNMtqgrcKPVNTFVHEPLVDVQNVCFQEKVTckngqgNCYKSNSSMHITDCRLTngsryPNC-AYRTSQKERHIIVACEGspYVPVHFDA--sve<br>----dWLTFQKKHIT-NT---RDVDCDNIMStNLFHC-----KDKNTFIYSRPEPVKAICKGIIAS------KNVLTTSEFYLSDCNVT-----SRPcKYKLKKSTNKFSVTCEN--QAPVHFVGvg---<br><br>BLAST: aliSize=52 (resi) iden=29.70% (30/102) simi=51.49% (52/102)<br>kesaaak----FERQHMDSgnspsSSSTYCNQMMRRrNMTQgrCKPVNTFVHEPLVDVQNVCfQEKVTCKNgqgncYKSNSSMHITDCRLTngSRyPnCAYRTSQKERHIIVACEGSpyVPVHF----dasve<br>-------dwltFQKKHITN-----TRDVDCDNIMST-NLFH--CKDKNTFIYSRPEPVKAIC-KGIIASKN-----VLTTSEFYLSDCNVT--SR-P-CKYKLKKSTNKFSVTCENQ--APVHFvgvg-----<br><br>Proposed: aliSize=97 (resi) RMSD=1.67 (Å)<br>keSAAAKFERQHMDSgNSP-sssstYCNQMMRRrNMTQgrCKPVNTFVHEPLVDVQNVCF----qekvtckngqGNCYKSNSSMHITDCRLTNGSrypnCAYRTSQKERHIIVACEGspYVPVHFDASVe<br>--PDWLTFQKKHITN-TRDv-----DCDNIMST-NLFH--CKDKNTFIYSRPEPVKAICKgiia----------SKNVLTTSEFYLSDCNVTSRP----CKYKLKKSTNKFSVTCEN--QAPVHFVGVG-
283	1h8xA (125)<br>2aasA (124)	N	70.16	TM-align: aliSize=105 (resi) RMSD=1.88 (Å)<br>kesaaakferqhmdsgnsp------------------SSSSTYCNQMMRRRNMTQGRCKPVNTFVHEPLVDVQNVCFQEKVTCKNGQGNCYKSNSSMHITDCRLTNGSRYPNCAYRTSQKERHIIVACEGSPYVPVHFDASVe<br>------------------ketaaakferqhmdsstsaASSSNYCNQMMKSRNLTKDRCKPVNTFVHESLADVQAVCSQKNVACKNGQTNCYQSYSTMSITDCRETGSSKYPNCAYKTTQANKHIIVACEGNPYVPVHFDASV-<br><br>SARST: aliSize=118 (resi) RMSD=14.39 (Å)<br>k-ESAAAKFERQHMDSGN----SPssssTYCNQMMRRRNMTQGRCKPVNTFVHEPLVDVQNVCFQEKVTCKNGQGNCYKSNSSMHITDCRLTNGSRYPNCAYRTSQKERHIIVACEGSPYVPVHFDAS-ve<br>-kETAAAKFERQHMDSSTsaasSS----NYCNQMMKSRNLTKDRCKPVNTFVHESLADVQAVCSQKNVACKNGQTNCYQSYSTMSITDCRETGSSKYPNCAYKTTQANKHIIVACEGNPYVPVHFDASv--<br><br>BLAST: aliSize=104 (resi) iden=73.39% (91/124) simi=83.87% (104/124)<br>KESAAAKFERQHMDSGNSPSSSSTYCNQMMRRRNMTQGRCKPVNTFVHEPLVDVQNVCFQEKVTCKNGQGNCYKSNSSMHITDCRLTNGSRYPNCAYRTSQKERHIIVACEGSPYVPVHFDASVe<br>KETAAAKFERQHMDSSTSAASSSNYCNQMMKSRNLTKDRCKPVNTFVHESLADVQAVCSQKNVACKNGQTNCYQSYSTMSITDCRETGSSKYPNCAYKTTQANKHIIVACEGNPYVPVHFDASV-<br><br>Proposed: aliSize=120 (resi) RMSD=1.32 (Å)<br>KESAAAKFERQHMDSGN-SPssSS--tYCNQMMRRRNMTQGRCKPVNTFVHEPLVDVQNVCFQEKVTCKnG-QGNCYKSNSSMHITDCRLTNGSRYPNCAYRTSQKERHIIVACEGSPYVPVHFDASVe<br>KETAAAKFERQHMDSSTsAA--SSsn-YCNQMMKSRNLTKDRCKPVNTFVHESLADVQAVCSQKNVACK-NgQTNCYQSYSTMSITDCRETGSSKYPNCAYKTTQANKHIIVACEGNPYVPVHFDASV-
284	1h8xA (125)<br>2hkyA (129)	N	33.60	TM-align: aliSize=95 (resi) RMSD=1.94 (Å)<br>kesaaakferqhmdsgnspssss--------------------TYCNQMMRrrNMTQ--GRCKPVNTFVHEPLVDVQNVCFQEKVTCkngQ--G-NCYKSNSSMHITDCRLTnGSRYPNCAYRTSQKERHIIVACEGS--------PYVPVHFDASVe<br>----------------------mkpkgmtssqwfkiqhmqpspQACNSAMK--NINKhtKRCKDLNTFLHEPFSSVAATCQTPKIAC---KngDkNCHQSHGPVSLTMCKLT-SGKYPNCRYKEKRQNKSYVVACKPPqkkdsqqfHLVPVHLDRVL-<br><br>SARST: aliSize=114 (resi) RMSD=14.12 (Å)<br>k-----ESAAAKFERQHMDS-GNSPSSsstYCNQMMRRRNMTQGRckpvNTFVHEPLVDVQNVCFQEKVTCKNGQGNCYKSNSSMHITDCRLTNGSRyPNCAYRTSQKERHIIVACE--------GSPYVPVHFDAS-ve<br>-mkpkgMTSSQWFKIQHMQPsPQACNS---AMKNINKHTKRCKDL----NTFLHEPFSSVAATCQTPKIACKNGDKNCHQSHGPVSLTMCKLTSGKY-PNCRYKEKRQNKSYVVACKppqkkdsqQFHLVPVHLDRVl--<br><br>BLAST: aliSize=68 (resi) iden=37.60% (47/125) simi=54.40% (68/125)<br>kesaaak----------FERQHMDSgnSPSSssstyCNQMMRRRNMTQGRCKPVNTFVHEPLVDVQNVCFQEKVTCKNGQGNCYKSNSSMHITDCRLTNGsRYPNCAYRTSQKERHIIVAC------EGSPY--VPVHFD---asve<br>-------mkpkgmtssqwFKIQHMQP--SPQA----CNSAMKNINKHTKRCKDLNTFLHEPFSSVAATCQTPKIACKNGDKNCHQSHGPVSLTMCKLTSG-KYPNCRYKEKRQNKSYVVACkppqkkDSQQFhlVPVHLDrvl----<br><br>Proposed: aliSize=111 (resi) RMSD=1.78 (Å)<br>---K-ESAAAKFERQHMDSG-NspssssstYCNQMMRrrNMTQ--GRCKPVNTFVHEPLVDVQNVCFQEKVTCkngQ--G-NCYKSNSSMHITDCRLTnGSRYPNCAYRTSQKERHIIVACEGS--------PYVPVHFDASVe<br>mkpKgMTSSQWFKIQHMQPSpQ-------ACNSAMK--NINKhtKRCKDLNTFLHEPFSSVAATCQTPKIAC---KngDkNCHQSHGPVSLTMCKLT-SGKYPNCRYKEKRQNKSYVVACKPPqkkdsqqfHLVPVHLDRVL-

285	1h8xA (125)<br>2k11A (127)	N	89.60	TM-align: aliSize=104 (resi) RMSD=1.73 (Å)<br>kesaaakferqhmdsgn-------------------SPSssSTYCNQMMRRRNMTQGRCKPVNTFVHEPLVDVQNVCFQEKVTCknGQ-G-NCYKSNSSMHITDCRLTNGSRYPNCAYRTSQKERHIIVACEGSPYVPVHFDASVE--<br>-----------------kesrakkfqrqhmdsdsspSSS--STYCNQMMRRRNMTQGRCKPVNTFVHEPLVDVQNVCFQEKVTC--KNgQgNCYKSNSSMHITDCRLTNGSRYPNCAYRTSPKERHIIVACEGSPYVPVHFDASVEds<br><br>SARST: aliSize=101 (resi) RMSD=2.03 (Å)<br>kesaaakferqhmdsgnspss---------------------SSTYCNQMMRRRNMTQGRCKPVNTFVHEPLVDVQNVCFQEKVTCKNGQGNCYKSNSSMHITDCRLTNGSRYPNCAYRTSQKERHIIVACEGSPYVPVHFDA-----sve<br>---------------------kesrakkfqrqhmdsdsspssSSTYCNQMMRRRNMTQGRCKPVNTFVHEPLVDVQNVCFQEKVTCKNGQGNCYKSNSSMHITDCRLTNGSRYPNCAYRTSPKERHIIVACEGSPYVPVHFDAsveds---<br><br>BLAST: aliSize=121 (resi) iden=95.20% (119/125) simi=96.80% (121/125)<br>KESAAAKFERQHMDSGNSPSSSSTYCNQMMRRRNMTQGRCKPVNTFVHEPLVDVQNVCFQEKVTCKNGQGNCYKSNSSMHITDCRLTNGSRYPNCAYRTSQKERHIIVACEGSPYVPVHFDASVE--<br>KESRAKKFQRQHMDSDSSPSSSSTYCNQMMRRRNMTQGRCKPVNTFVHEPLVDVQNVCFQEKVTCKNGQGNCYKSNSSMHITDCRLTNGSRYPNCAYRTSPKERHIIVACEGSPYVPVHFDASVEds<br><br>Proposed: aliSize=122 (resi) RMSD=1.58 (Å)<br>KESAAAKFERQHMDS-GNSpSSSSTYCNQMMRRRNMTQGRCKPVNTFVHEPLVDVQNVCFQEKVTCknGQ-G-NCYKSNSSMHITDCRLTNGSRYPNCAYRTSQKERHIIVACEGSPYVPVHFDASVE--<br>KESRAKKFQRQHMDSdSSP-SSSSTYCNQMMRRRNMTQGRCKPVNTFVHEPLVDVQNVCFQEKVTC--KNgQgNCYKSNSSMHITDCRLTNGSRYPNCAYRTSPKERHIIVACEGSPYVPVHFDASVEds
286	1jmlA (72)<br>1k52A (72)	C	87.50	TM-align: aliSize=60 (resi) RMSD=1.69 (Å)<br>mhHHHHHGM--EEVTIKANLIFANGSTQTAEFKGTFEKATSEAYAYADTLKKDNGEWTVDVVPKaytlnikfag----------<br>--MHHHHHHamEEVTIKANLIFANGSTQTAEFKGTFEKATSEAYAYADTLKKDNGEWTVDVADG----------gytlnikfag<br><br>SARST: aliSize=69 (resi) RMSD=11.31 (Å)<br>mh--HHHHHGMEEVTIKANLIFANGSTQTAEFKGTFEKATSEAYAYADTLKKDNGEWTVDVVPKAYTLNIKFA-g<br>--mhHHHHHAMEEVTIKANLIFANGSTQTAEFKGTFEKATSEAYAYADTLKKDNGEWTVDVADGGYTLNIKFAg-<br><br>BLAST: aliSize=67 (resi) iden=93.06% (67/72) simi=93.06% (67/72)<br>MHHHHHHGMEEVTIKANLIFANGSTQTAEFKGTFEKATSEAYAYADTLKKDNGEWTVDVVPKAYTLNIKFAG<br>MHHHHHHAMEEVTIKANLIFANGSTQTAEFKGTFEKATSEAYAYADTLKKDNGEWTVDVADGGYTLNIKFAG<br><br>Proposed: aliSize=70 (resi) RMSD=1.58 (Å)<br>mhHHHHHGM--EEVTIKANLIFANGSTQTAEFKGTFEKATSEAYAYADTLKKDNGEWTVDVVPKAYTLNIKFAG<br>--MHHHHHHamEEVTIKANLIFANGSTQTAEFKGTFEKATSEAYAYADTLKKDNGEWTVDVADGGYTLNIKFAG
287	1jmlA (72)<br>1k53A (72)	C	87.50	TM-align: aliSize=60 (resi) RMSD=1.67 (Å)<br>mhHHHHHGM--EEVTIKANLIFANGSTQTAEFKGTFEKATSEAYAYADTLKKDNGEWTVDVVPKaytlnikfag----------<br>--MHHHHHHamEEVTIKANLIFANASTQTAEFKGTFEKATSEAYAYADTLKKDNGEWTVDVADK----------gytlnikfag<br><br>SARST: aliSize=69 (resi) RMSD=11.32 (Å)<br>mh--HHHHHGMEEVTIKANLIFANGSTQTAEFKGTFEKATSEAYAYADTLKKDNGEWTVDVVPKAYTLNIKFA-g<br>--mhHHHHHAMEEVTIKANLIFANASTQTAEFKGTFEKATSEAYAYADTLKKDNGEWTVDVADKGYTLNIKFAg-<br><br>BLAST: aliSize=67 (resi) iden=93.06% (67/72) simi=93.06% (67/72)<br>MHHHHHHGMEEVTIKANLIFANGSTQTAEFKGTFEKATSEAYAYADTLKKDNGEWTVDVVPKAYTLNIKFAG<br>MHHHHHHAMEEVTIKANLIFANASTQTAEFKGTFEKATSEAYAYADTLKKDNGEWTVDVADKGYTLNIKFAG<br><br>Proposed: aliSize=70 (resi) RMSD=1.56 (Å)<br>mhHHHHHGM--EEVTIKANLIFANGSTQTAEFKGTFEKATSEAYAYADTLKKDNGEWTVDVVPKAYTLNIKFAG<br>--MHHHHHHamEEVTIKANLIFANASTQTAEFKGTFEKATSEAYAYADTLKKDNGEWTVDVADKGYTLNIKFAG

<table>
<tr><td rowspan="2">288</td><td>1jmlA
(72)</td><td rowspan="2">C</td><td rowspan="2">54.55</td><td rowspan="2">TM-align: aliSize=56 (resi)   RMSD=1.86 (Å)
mhhhhhhGMEEVTIKANLIFANGSTQTAEFKGTFEKATSEAYAYADTLKKDNGEWTVDVVPK-Aytlnikfag---------
-------PKEEVTIKVNLIFADGKIQTAEFKGTFEEATAEAYRYAALHAKVNGEWTADLEDGgN---------hmnikfagk

SARST: aliSize=49 (resi)   RMSD=1.23 (Å)
mhhhhhhg-MEEVTIKANLIFANGSTQTAEFKGTFEKATSEAYAYADTLKKDNGEWTV---------------dvvpkaytlnikfag
--------pKEEVTIKVNLIFADGKIQTAEFKGTFEEATAEAYRYAALHAKVNGEWTAdledggnhmnikfagk---------------

BLAST: aliSize=47 (resi)   iden=63.64% (42/66)   simi=71.21% (47/66)
mhhhhhhgm--EEVTIKANLIFANGSTQTAEFKGTFEKATSEAYAYADTLKKDNGEWTVDVVPKAYTLNIKFAG-
---------pkEEVTIKVNLIFADGKIQTAEFKGTFEEATAEAYRYAALHAKVNGEWTADLEDGGNHMNIKFAGk

Proposed: aliSize=65 (resi)   RMSD=1.40 (Å)
mhhhhhhGMEEVTIKANLIFANGSTQTAEFKGTFEKATSEAYAYADTLKKDNGEWTVDVVPKA-YTLNIKFAG
-------PKEEVTIKVNLIFADGKIQTAEFKGTFEEATAEAYRYAALHAKVNGEWTADLEDGGnHMNIKFAGK</td></tr>
<tr><td>1xcqL
(66)</td></tr>
<tr><td rowspan="2">289</td><td>1jmlA
(72)</td><td rowspan="2">C</td><td rowspan="2">66.13</td><td rowspan="2">TM-align: aliSize=52 (resi)   RMSD=1.42 (Å)
mhhhhhhgmeEVTIKANLIFANGSTQTAEFKGTFEKATSEAYAYADTLKKDNGEWTVDVVPKaytlnikfag----------
----------EVTIKVNLIFADGKIQTAEFKGTFEEATAEAYRYAALHAKVNGEWTADLEDG----------gnhmnikfag

SARST: aliSize=48 (resi)   RMSD=1.07 (Å)
mhhhhhhgmee-VTIKANLIFANGSTQTAEFKGTFEKATSEAYAYADTLKKDNGEWTVDV------------vpkaytlnikfag
-----------eVTIKVNLIFADGKIQTAEFKGTFEEATAEAYRYAALHAKVNGEWTADLedggnhmnikfag------------

BLAST: aliSize=46 (resi)   iden=66.13% (41/62)   simi=74.19% (46/62)
mhhhhhhgmeEVTIKANLIFANGSTQTAEFKGTFEKATSEAYAYADTLKKDNGEWTVDVVPKAYTLNIKFAG
----------EVTIKVNLIFADGKIQTAEFKGTFEEATAEAYRYAALHAKVNGEWTADLEDGGNHMNIKFAG

Proposed: aliSize=62 (resi)   RMSD=1.28 (Å)
mhhhhhhgmeEVTIKANLIFANGSTQTAEFKGTFEKATSEAYAYADTLKKDNGEWTVDVVPKAYTLNIKFAG
----------EVTIKVNLIFADGKIQTAEFKGTFEEATAEAYRYAALHAKVNGEWTADLEDGGNHMNIKFAG</td></tr>
<tr><td>1xcqM
(62)</td></tr>
<tr><td rowspan="2">290</td><td>1jmlA
(72)</td><td rowspan="2">C</td><td rowspan="2">65.63</td><td rowspan="2">TM-align: aliSize=54 (resi)   RMSD=1.58 (Å)
mhhhhhhgMEEVTIKANLIFANGSTQTAEFKGTFEKATSEAYAYADTLKKDNGEWTVDVVP--Kaytlnikfag--------
--------KEEVTIKVNLIFADGKIQTAEFKGTFEEATAEAYRYAALHAKVNGEWTADLEDggN----------hmnikfag

SARST: aliSize=46 (resi)   RMSD=1.05 (Å)
mhhhhhhgmee---VTIKANLIFANGSTQTAEFKGTFEKATSEAYAYADTLKKDNGEWTV--------------dvvpkaytlnikfag
-----------keeVTIKVNLIFADGKIQTAEFKGTFEEATAEAYRYAALHAKVNGEWTAdledggnhmnikfag--------------

BLAST: aliSize=47 (resi)   iden=65.62% (42/64)   simi=73.44% (47/64)
mhhhhhhgm-EEVTIKANLIFANGSTQTAEFKGTFEKATSEAYAYADTLKKDNGEWTVDVVPKAYTLNIKFAG
---------kEEVTIKVNLIFADGKIQTAEFKGTFEEATAEAYRYAALHAKVNGEWTADLEDGGNHMNIKFAG

Proposed: aliSize=62 (resi)   RMSD=1.28 (Å)
mhhhhhhgm-EEVTIKANLIFANGSTQTAEFKGTFEKATSEAYAYADTLKKDNGEWTVDVVP-kAYTLNIKFAG
---------kEEVTIKVNLIFADGKIQTAEFKGTFEEATAEAYRYAALHAKVNGEWTADLEDg-GNHMNIKFAG</td></tr>
<tr><td>1xcqN
(64)</td></tr>
</table>

291	1jmlA (72)<br>1xf5M (67)	C	55.22	TM-align: aliSize=53 (resi) RMSD=1.75 (Å)<br>mhhhhhhgme----EVTIKANLIFANGSTQTAEFKGTFEKATSEAYAYADTLKKDNGEWTVDVVPK-Aytlnikfag---------<br>----------epkeEVTIKVNLIFADGKIQTAEFKGTFEEATAEAYRYADLLAKVNGEWTADLEDGgN---------cmnikfagk<br><br>SARST: aliSize=59 (resi) RMSD=11.70 (Å)<br>mhhhhhhgmeev------TIKANLIFANGSTQTAEFKGTFEKATSEAYAYADTLKKDNGEWTVDVVPKAYTLNIKFA--g<br>------------epkeevTIKVNLIFADGKIQTAEFKGTFEEATAEAYRYADLLAKVNGEWTADLEDGGNCMNIKFAgk-<br><br>BLAST: aliSize=49 (resi) iden=65.67% (44/67) simi=73.13% (49/67)<br>mhhhhhhgm---EEVTIKANLIFANGSTQTAEFKGTFEKATSEAYAYADTLKKDNGEWTVDVVPKAYTLNIKFAG-<br>---------epkEEVTIKVNLIFADGKIQTAEFKGTFEEATAEAYRYADLLAKVNGEWTADLEDGGNCMNIKFAGk<br><br>Proposed: aliSize=62 (resi) RMSD=1.17 (Å)<br>mhhhhhhgme----EVTIKANLIFANGSTQTAEFKGTFEKATSEAYAYADTLKKDNGEWTVDVVPK-AYTLNIKFAG<br>----------epkeEVTIKVNLIFADGKIQTAEFKGTFEEATAEAYRYADLLAKVNGEWTADLEDGgNCMNIKFAGK
292	1js0A (124)<br>1a5pA (124)	C	98.39	TM-align: aliSize=114 (resi) RMSD=1.05 (Å)<br>KETAAAKFERQHMDSSTSAASSSNYCNQMMKSRNLTKDRCKPVNTFVHESLADVQAVCSQKNVACKNGQTNCYQSYSTMSITDCRETGSSKYPNCAYKTTQANKHIIVACEGNPyvpvhfdasv----------<br>KETAAAKFERQHMDSSTSAASSSNYCNQMMKSRNLTKDRAKPVNTFVHESLADVQAVCSQKNVACKNGQTNCYQSYSTMSITDCRETGSSKYPNAAYKTTQANKHIIVACEGNP----------yvpvhfdasv<br><br>SARST: aliSize=122 (resi) RMSD=6.23 (Å)<br>k-ETAAAKFERQHMDSSTSAASSSNYCNQMMKSRNLTKDRCKPVNTFVHESLADVQAVCSQKNVACKNGQTNCYQSYSTMSITDCRETGSSKYPNCAYKTTQANKHIIVACEGNPYVPVHFDAS-v<br>-kETAAAKFERQHMDSSTSAASSSNYCNQMMKSRNLTKDRAKPVNTFVHESLADVQAVCSQKNVACKNGQTNCYQSYSTMSITDCRETGSSKYPNAAYKTTQANKHIIVACEGNPYVPVHFDASv-<br><br>BLAST: aliSize=122 (resi) iden=98.39% (122/124) simi=98.39% (122/124)<br>KETAAAKFERQHMDSSTSAASSSNYCNQMMKSRNLTKDRCKPVNTFVHESLADVQAVCSQKNVACKNGQTNCYQSYSTMSITDCRETGSSKYPNCAYKTTQANKHIIVACEGNPYVPVHFDASV<br>KETAAAKFERQHMDSSTSAASSSNYCNQMMKSRNLTKDRAKPVNTFVHESLADVQAVCSQKNVACKNGQTNCYQSYSTMSITDCRETGSSKYPNAAYKTTQANKHIIVACEGNPYVPVHFDASV<br><br>Proposed: aliSize=124 (resi) RMSD=0.61 (Å)<br>KETAAAKFERQHMDSSTSAASSSNYCNQMMKSRNLTKDRCKPVNTFVHESLADVQAVCSQKNVACKNGQTNCYQSYSTMSITDCRETGSSKYPNCAYKTTQANKHIIVACEGNPYVPVHFDASV<br>KETAAAKFERQHMDSSTSAASSSNYCNQMMKSRNLTKDRAKPVNTFVHESLADVQAVCSQKNVACKNGQTNCYQSYSTMSITDCRETGSSKYPNAAYKTTQANKHIIVACEGNPYVPVHFDASV
293	1js0A (124)<br>1a5qA (124)	C	99.19	TM-align: aliSize=114 (resi) RMSD=1.02 (Å)<br>KETAAAKFERQHMDSSTSAASSSNYCNQMMKSRNLTKDRCKPVNTFVHESLADVQAVCSQKNVACKNGQTNCYQSYSTMSITDCRETGSSKYPNCAYKTTQANKHIIVACEGNPyvpvhfdasv----------<br>KETAAAKFERQHMDSSTSAASSSNYCNQMMKSRNLTKDRCKPVNTFVHESLADVQAVCSQKNVACKNGQTNCYQSYSTMSITDCRETGSSKYANCAYKTTQANKHIIVACEGNP----------yvpvhfdasv<br><br>SARST: aliSize=122 (resi) RMSD=6.21 (Å)<br>k-ETAAAKFERQHMDSSTSAASSSNYCNQMMKSRNLTKDRCKPVNTFVHESLADVQAVCSQKNVACKNGQTNCYQSYSTMSITDCRETGSSKYPNCAYKTTQANKHIIVACEGNPYVPVHFDAS-v<br>-kETAAAKFERQHMDSSTSAASSSNYCNQMMKSRNLTKDRCKPVNTFVHESLADVQAVCSQKNVACKNGQTNCYQSYSTMSITDCRETGSSKYANCAYKTTQANKHIIVACEGNPYVPVHFDASv-<br><br>BLAST: aliSize=123 (resi) iden=99.19% (123/124) simi=99.19% (123/124)<br>KETAAAKFERQHMDSSTSAASSSNYCNQMMKSRNLTKDRCKPVNTFVHESLADVQAVCSQKNVACKNGQTNCYQSYSTMSITDCRETGSSKYPNCAYKTTQANKHIIVACEGNPYVPVHFDASV<br>KETAAAKFERQHMDSSTSAASSSNYCNQMMKSRNLTKDRCKPVNTFVHESLADVQAVCSQKNVACKNGQTNCYQSYSTMSITDCRETGSSKYANCAYKTTQANKHIIVACEGNPYVPVHFDASV<br><br>Proposed: aliSize=124 (resi) RMSD=0.60 (Å)<br>KETAAAKFERQHMDSSTSAASSSNYCNQMMKSRNLTKDRCKPVNTFVHESLADVQAVCSQKNVACKNGQTNCYQSYSTMSITDCRETGSSKYPNCAYKTTQANKHIIVACEGNPYVPVHFDASV<br>KETAAAKFERQHMDSSTSAASSSNYCNQMMKSRNLTKDRCKPVNTFVHESLADVQAVCSQKNVACKNGQTNCYQSYSTMSITDCRETGSSKYANCAYKTTQANKHIIVACEGNPYVPVHFDASV

294	1js0A<br>(124)<br>1awzA<br>(123)	C	27.64	TM-align: aliSize=107 (resi) RMSD=2.31 (Å)<br>KE-TAAAKFERQHMDSSTSAAsSSNYCNQMMKSRNLTKdRCKPVNTFVHESLADVQAVCS---QKNVAckngqTNCYQSYSTMSITDCRETGSSKYPNCAYKTTQANKHIIVACEGNPyvpvhfdasv-----------<br>QDnSRYTHFLTQHYDAKPQGR-DDRYCESIMRRRGLTS-PCKDINTFIHGNKRSIKAICEnknGNPHR-----ENLRISKSSFQVTTCKLHGGSPWPPCQYRATAGFRNVVVACENGL----------pvhldqsifrrp<br><br>SARST: aliSize=104 (resi) RMSD=2.84 (Å)<br>ket----AAAKFERQHMDSSTSAAsSSNYCNQMMKSRNLTkDRCKPVNTFVHESLADVQAVCSQKNVACknGQTNCYQSYSTMSITDCRETGSSKYPNCAYKTTQANKHIIVACE---------------gnpyvpvhfdasv<br>---qdnsRYTHFLTQHYDAKPQGR-DDRYCESIMRRRGLT-SPCKDINTFIHGNKRSIKAICENKNGNP--HRENLRISKSSFQVTTCKLHGGSPWPPCQYRATAGFRNVVVACEnglpvhldqsifrrp------------<br><br>BLAST: aliSize=61 (resi) iden=32.52% (40/123) simi=49.59% (61/123)<br>ketaaak--------FERQHMDSSTSAASSsNYCNQMMKSRNLTKDrCKPVNTFVHESLADVQAVCSQKNVacKNGQTNCYQSYSTMSITDCRETGSSKYPNCAYKTTQANKHIIVACEGNpyVPVHFDASV----<br>-------qdnsrythFLTQHYDAKPQGRDD-RYCESIMRRRGLTSP-CKDINTFIHGNKRSIKAICENKNG--NPHRENLRISKSSFQVTTCKLHGGSPWPPCQYRATAGFRNVVVACENG--LPVHLDQSIfrrp<br><br>Proposed: aliSize=115 (resi) RMSD=1.74 (Å)<br>kE--TAAAKFERQHMDSST-saASSSNYCNQMMKSRNLTKdRCKPVNTFVHESLADVQAVCS---QKNVAckngqTNCYQSYSTMSITDCRETGSSKYPNCAYKTTQANKHIIVACEGNPYVPVHFD--ASV<br>-QdnSRYTHFLTQHYDAKPq--GRDDRYCESIMRRRGLTS-PCKDINTFIHGNKRSIKAICEnknGNPHR-----ENLRISKSSFQVTTCKLHGGSPWPPCQYRATAGFRNVVVACENGLPVHLDQSifRRP
295	1js0A<br>(124)<br>1b6vA<br>(124)	C	94.35	TM-align: aliSize=115 (resi) RMSD=1.39 (Å)<br>KETAAAKFERQHMDSSTSAASSSNYCNQMMKSRNLTKDRCKPVNTFVHESLADVQAVCSQKNVACKNGQTNCYQSYSTMSITDCRETGSSKYPNCAYKTTQANKHIIVACEGNPYvpvhfdasv---------<br>KETAAAKFERQHMDSSTSAASSSNYCNQMMKSRNLTKDRCKPVNTFVHESLADVKAVCSQKKVTCKNGQTNCYQSKSTMRITDCRETGSSKYPNCAYKTTQANKHIIVACGGKPY---------vpvhfdasv<br><br>SARST: aliSize=122 (resi) RMSD=6.20 (Å)<br>k-ETAAAKFERQHMDSSTSAASSSNYCNQMMKSRNLTKDRCKPVNTFVHESLADVQAVCSQKNVACKNGQTNCYQSYSTMSITDCRETGSSKYPNCAYKTTQANKHIIVACEGNPYVPVHFDAS-v<br>-kETAAAKFERQHMDSSTSAASSSNYCNQMMKSRNLTKDRCKPVNTFVHESLADVKAVCSQKKVTCKNGQTNCYQSKSTMRITDCRETGSSKYPNCAYKTTQANKHIIVACGGKPYVPVHFDASv-<br><br>BLAST: aliSize=118 (resi) iden=94.35% (117/124) simi=95.16% (118/124)<br>KETAAAKFERQHMDSSTSAASSSNYCNQMMKSRNLTKDRCKPVNTFVHESLADVQAVCSQKNVACKNGQTNCYQSYSTMSITDCRETGSSKYPNCAYKTTQANKHIIVACEGNPYVPVHFDASV<br>KETAAAKFERQHMDSSTSAASSSNYCNQMMKSRNLTKDRCKPVNTFVHESLADVKAVCSQKKVTCKNGQTNCYQSKSTMRITDCRETGSSKYPNCAYKTTQANKHIIVACGGKPYVPVHFDASV<br><br>Proposed: aliSize=124 (resi) RMSD=0.68 (Å)<br>KETAAAKFERQHMDSSTSAASSSNYCNQMMKSRNLTKDRCKPVNTFVHESLADVQAVCSQKNVACKNGQTNCYQSYSTMSITDCRETGSSKYPNCAYKTTQANKHIIVACEGNPYVPVHFDASV<br>KETAAAKFERQHMDSSTSAASSSNYCNQMMKSRNLTKDRCKPVNTFVHESLADVKAVCSQKKVTCKNGQTNCYQSKSTMRITDCRETGSSKYPNCAYKTTQANKHIIVACGGKPYVPVHFDASV
296	1js0A<br>(124)<br>1c8wA<br>(124)	C	99.19	TM-align: aliSize=115 (resi) RMSD=1.44 (Å)<br>KETAAAKFERQHMDSSTSAASSSNYCNQMMKSRNLTKDRCKPVNTFVHESLADVQAVCSQKNVACKNGQTNCYQSYSTMSITDCRETGSSKYPNCAYKTTQANKHIIVACEGNPYvpvhfdasv---------<br>KETAAAKFERQHMDSSTSAASSSNYCNQMMKSRNLTKDRCKPVNGFVHESLADVQAVCSQKNVACKNGQTNCYQSYSTMSITDCRETGSSKYPNCAYKTTQANKHIIVACEGNPY---------vpvhfdasv<br><br>SARST: aliSize=122 (resi) RMSD=6.20 (Å)<br>k-ETAAAKFERQHMDSSTSAASSSNYCNQMMKSRNLTKDRCKPVNTFVHESLADVQAVCSQKNVACKNGQTNCYQSYSTMSITDCRETGSSKYPNCAYKTTQANKHIIVACEGNPYVPVHFDAS-v<br>-kETAAAKFERQHMDSSTSAASSSNYCNQMMKSRNLTKDRCKPVNGFVHESLADVQAVCSQKNVACKNGQTNCYQSYSTMSITDCRETGSSKYPNCAYKTTQANKHIIVACEGNPYVPVHFDASv-<br><br>BLAST: aliSize=123 (resi) iden=99.19% (123/124) simi=99.19% (123/124)<br>KETAAAKFERQHMDSSTSAASSSNYCNQMMKSRNLTKDRCKPVNTFVHESLADVQAVCSQKNVACKNGQTNCYQSYSTMSITDCRETGSSKYPNCAYKTTQANKHIIVACEGNPYVPVHFDASV<br>KETAAAKFERQHMDSSTSAASSSNYCNQMMKSRNLTKDRCKPVNGFVHESLADVQAVCSQKNVACKNGQTNCYQSYSTMSITDCRETGSSKYPNCAYKTTQANKHIIVACEGNPYVPVHFDASV<br><br>Proposed: aliSize=124 (resi) RMSD=0.76 (Å)<br>KETAAAKFERQHMDSSTSAASSSNYCNQMMKSRNLTKDRCKPVNTFVHESLADVQAVCSQKNVACKNGQTNCYQSYSTMSITDCRETGSSKYPNCAYKTTQANKHIIVACEGNPYVPVHFDASV<br>KETAAAKFERQHMDSSTSAASSSNYCNQMMKSRNLTKDRCKPVNGFVHESLADVQAVCSQKNVACKNGQTNCYQSYSTMSITDCRETGSSKYPNCAYKTTQANKHIIVACEGNPYVPVHFDASV

297	1js0A (124)<br>1c9vA (124)	C	99.19	TM-align: aliSize=115 (resi) RMSD=1.38 (Å)<br>KETAAAKFERQHMDSSTSAASSSNYCNQMMKSRNLTKDRCKPVNTFVHESLADVQAVCSQKNVACKNGQTNCYQSYSTMSITDCRETGSSKYPNCAYKTTQANKHIIVACEGNPYvpvhfdasv---------<br>KETAAAKFERQAMDSSTSAASSSNYCNQMMKSRNLTKDRCKPVNTFVHESLADVQAVCSQKNVACKNGQTNCYQSYSTMSITDCRETGSSKYPNCAYKTTQANKHIIVACEGNPY---------vpvhfdasv<br><br>SARST: aliSize=122 (resi) RMSD=6.24 (Å)<br>k-ETAAAKFERQHMDSSTSAASSSNYCNQMMKSRNLTKDRCKPVNTFVHESLADVQAVCSQKNVACKNGQTNCYQSYSTMSITDCRETGSSKYPNCAYKTTQANKHIIVACEGNPYVPVHFDAS-v<br>-kETAAAKFERQAMDSSTSAASSSNYCNQMMKSRNLTKDRCKPVNTFVHESLADVQAVCSQKNVACKNGQTNCYQSYSTMSITDCRETGSSKYPNCAYKTTQANKHIIVACEGNPYVPVHFDASv-<br><br>BLAST: aliSize=123 (resi) iden=99.19% (123/124) simi=99.19% (123/124)<br>KETAAAKFERQHMDSSTSAASSSNYCNQMMKSRNLTKDRCKPVNTFVHESLADVQAVCSQKNVACKNGQTNCYQSYSTMSITDCRETGSSKYPNCAYKTTQANKHIIVACEGNPYVPVHFDASV<br>KETAAAKFERQAMDSSTSAASSSNYCNQMMKSRNLTKDRCKPVNTFVHESLADVQAVCSQKNVACKNGQTNCYQSYSTMSITDCRETGSSKYPNCAYKTTQANKHIIVACEGNPYVPVHFDASV<br><br>Proposed: aliSize=124 (resi) RMSD=0.66 (Å)<br>KETAAAKFERQHMDSSTSAASSSNYCNQMMKSRNLTKDRCKPVNTFVHESLADVQAVCSQKNVACKNGQTNCYQSYSTMSITDCRETGSSKYPNCAYKTTQANKHIIVACEGNPYVPVHFDASV<br>KETAAAKFERQAMDSSTSAASSSNYCNQMMKSRNLTKDRCKPVNTFVHESLADVQAVCSQKNVACKNGQTNCYQSYSTMSITDCRETGSSKYPNCAYKTTQANKHIIVACEGNPYVPVHFDASV
298	1js0A (124)<br>1c9xA (124)	C	99.19	TM-align: aliSize=115 (resi) RMSD=1.36 (Å)<br>KETAAAKFERQHMDSSTSAASSSNYCNQMMKSRNLTKDRCKPVNTFVHESLADVQAVCSQKNVACKNGQTNCYQSYSTMSITDCRETGSSKYPNCAYKTTQANKHIIVACEGNPYvpvhfdasv---------<br>KETAAAKFERQHMDSSTSAASSSNYCNQMMKSRNLTKDRCKPVNTFVHESLADVQAVCSQKNVACKNGQTNCYQSYSTMSITDCRETGSSKYPNCAYKTTQANKHIIVACEGNPY---------vpvafdasv<br><br>SARST: aliSize=122 (resi) RMSD=6.24 (Å)<br>k-ETAAAKFERQHMDSSTSAASSSNYCNQMMKSRNLTKDRCKPVNTFVHESLADVQAVCSQKNVACKNGQTNCYQSYSTMSITDCRETGSSKYPNCAYKTTQANKHIIVACEGNPYVPVHFDAS-v<br>-kETAAAKFERQHMDSSTSAASSSNYCNQMMKSRNLTKDRCKPVNTFVHESLADVQAVCSQKNVACKNGQTNCYQSYSTMSITDCRETGSSKYPNCAYKTTQANKHIIVACEGNPYVPVAFDASv-<br><br>BLAST: aliSize=123 (resi) iden=99.19% (123/124) simi=99.19% (123/124)<br>KETAAAKFERQHMDSSTSAASSSNYCNQMMKSRNLTKDRCKPVNTFVHESLADVQAVCSQKNVACKNGQTNCYQSYSTMSITDCRETGSSKYPNCAYKTTQANKHIIVACEGNPYVPVHFDASV<br>KETAAAKFERQHMDSSTSAASSSNYCNQMMKSRNLTKDRCKPVNTFVHESLADVQAVCSQKNVACKNGQTNCYQSYSTMSITDCRETGSSKYPNCAYKTTQANKHIIVACEGNPYVPVAFDASV<br><br>Proposed: aliSize=124 (resi) RMSD=0.58 (Å)<br>KETAAAKFERQHMDSSTSAASSSNYCNQMMKSRNLTKDRCKPVNTFVHESLADVQAVCSQKNVACKNGQTNCYQSYSTMSITDCRETGSSKYPNCAYKTTQANKHIIVACEGNPYVPVHFDASV<br>KETAAAKFERQHMDSSTSAASSSNYCNQMMKSRNLTKDRCKPVNTFVHESLADVQAVCSQKNVACKNGQTNCYQSYSTMSITDCRETGSSKYPNCAYKTTQANKHIIVACEGNPYVPVAFDASV
299	1js0A (124)<br>1e21A (119)	C	68.91	TM-align: aliSize=107 (resi) RMSD=1.60 (Å)<br>ketaaaKFERQHMDSSTsAAS-SSNYCNQMMKSRNLTKDRCKPVNTFVHESLADVQAVCSQKNVACKNGQTNCYQSYSTMSITDCRETGSSKYPNCAYKTTQANKHIIVACEGNPyvpvhfdasv-----------<br>------AFQRQHMDSDS-SPSsSSTYCNQMMRRRNMTQGRCKPVNTFVHEPLVDVQNVCFQEKVTCKNGQGNCYKSNSSMHITDCRLTNGSRYPNCAYRTSPKERHIIVACEGSP----------yvpvhfdasve<br><br>SARST: aliSize=116 (resi) RMSD=6.54 (Å)<br>ketaaak-FERQHMDSSTSAASSSNYCNQMMKSRNLTKDRCKPVNTFVHESLADVQAVCSQKNVACKNGQTNCYQSYSTMSITDCRETGSSKYPNCAYKTTQANKHIIVACEGNPYVPVHFDAS--v<br>-------aFQRQHMDSDSSPSSSSTYCNQMMRRRNMTQGRCKPVNTFVHEPLVDVQNVCFQEKVTCKNGQGNCYKSNSSMHITDCRLTNGSRYPNCAYRTSPKERHIIVACEGSPYVPVHFDASve-<br><br>BLAST: aliSize=97 (resi) iden=69.75% (83/119) simi=81.51% (97/119)<br>ketaaak-FERQHMDSSTSAASSSNYCNQMMKSRNLTKDRCKPVNTFVHESLADVQAVCSQKNVACKNGQTNCYQSYSTMSITDCRETGSSKYPNCAYKTTQANKHIIVACEGNPYVPVHFDASV-<br>-------aFQRQHMDSDSSPSSSSTYCNQMMRRRNMTQGRCKPVNTFVHEPLVDVQNVCFQEKVTCKNGQGNCYKSNSSMHITDCRLTNGSRYPNCAYRTSPKERHIIVACEGSPYVPVHFDASVe<br><br>Proposed: aliSize=117 (resi) RMSD=1.29 (Å)<br>ketaaaKFERQHMDSSTsAASSS-NYCNQMMKSRNLTKDRCKPVNTFVHESLADVQAVCSQKNVACKNGQTNCYQSYSTMSITDCRETGSSKYPNCAYKTTQANKHIIVACEGNPYVPVHFDASV-<br>------AFQRQHMDSDS-SPSSSsTYCNQMMRRRNMTQGRCKPVNTFVHEPLVDVQNVCFQEKVTCKNGQGNCYKSNSSMHITDCRLTNGSRYPNCAYRTSPKERHIIVACEGSPYVPVHFDASVe

300	1js0A (124)<br>1eidA (124)	C	99.19	TM-align: aliSize=115 (resi) RMSD=1.45 (Å)<br>KETAAAKFERQHMDSSTSAASSSNYCNQMMKSRNLTKDRCKPVNTFVHESLADVQAVCSQKNVACKNGQTNCYQSYSTMSITDCRETGSSKYPNCAYKTTQANKHIIVACEGNPYvpvhfdasv---------<br>KETAAAKFERQHMDSSTSAASSSNYCNQMMKSRNLTKDRCKPVNTFVHESLADVQAVCSQKNVACKNGQTNCYQSYSTMSITDCRETGSSKYPNCAYKTTQANKHIIVACEGNPY---------vpvhgdasv<br><br>SARST: aliSize=119 (resi) RMSD=4.31 (Å)<br>k-ETAAAKFERQHMDSSTSAASSSNYCNQMMKSRNLTKDRCKPVNTFVHESLADVQAVCSQKNVACKNGQTNCYQSYSTMSITDCRETGSSKYPNCAYKTTQANKHIIVACEGNPYVPVHF----dasv<br>-kETAAAKFERQHMDSSTSAASSSNYCNQMMKSRNLTKDRCKPVNTFVHESLADVQAVCSQKNVACKNGQTNCYQSYSTMSITDCRETGSSKYPNCAYKTTQANKHIIVACEGNPYVPVHGdasv----<br><br>BLAST: aliSize=123 (resi) iden=99.19% (123/124) simi=99.19% (123/124)<br>KETAAAKFERQHMDSSTSAASSSNYCNQMMKSRNLTKDRCKPVNTFVHESLADVQAVCSQKNVACKNGQTNCYQSYSTMSITDCRETGSSKYPNCAYKTTQANKHIIVACEGNPYVPVHFDASV<br>KETAAAKFERQHMDSSTSAASSSNYCNQMMKSRNLTKDRCKPVNTFVHESLADVQAVCSQKNVACKNGQTNCYQSYSTMSITDCRETGSSKYPNCAYKTTQANKHIIVACEGNPYVPVHGDASV<br><br>Proposed: aliSize=124 (resi) RMSD=0.76 (Å)<br>KETAAAKFERQHMDSSTSAASSSNYCNQMMKSRNLTKDRCKPVNTFVHESLADVQAVCSQKNVACKNGQTNCYQSYSTMSITDCRETGSSKYPNCAYKTTQANKHIIVACEGNPYVPVHFDASV<br>KETAAAKFERQHMDSSTSAASSSNYCNQMMKSRNLTKDRCKPVNTFVHESLADVQAVCSQKNVACKNGQTNCYQSYSTMSITDCRETGSSKYPNCAYKTTQANKHIIVACEGNPYVPVHGDASV
301	1js0A (124)<br>1eieA (124)	C	99.19	TM-align: aliSize=114 (resi) RMSD=1.16 (Å)<br>KETAAAKFERQHMDSSTSAASSSNYCNQMMKSRNLTKDRCKPVNTFVHESLADVQAVCSQKNVACKNGQTNCYQSYSTMSITDCRETGSSKYPNCAYKTTQANKHIIVACEGNPyvpvhfdasv----------<br>KETAAAKFERQHMDSSTSAASSSNYCNQMMKSRNLTKDRCKPVNTFVHESLADVQAVCSQKNVACKNGQTNCYQSYSTMSITDCRETGSSKYPNCAYKTTQANKHIIVACEGNP----------yvpvhwdasv<br><br>SARST: aliSize=122 (resi) RMSD=6.21 (Å)<br>k-ETAAAKFERQHMDSSTSAASSSNYCNQMMKSRNLTKDRCKPVNTFVHESLADVQAVCSQKNVACKNGQTNCYQSYSTMSITDCRETGSSKYPNCAYKTTQANKHIIVACEGNPYVPVHFDAS-v<br>-kETAAAKFERQHMDSSTSAASSSNYCNQMMKSRNLTKDRCKPVNTFVHESLADVQAVCSQKNVACKNGQTNCYQSYSTMSITDCRETGSSKYPNCAYKTTQANKHIIVACEGNPYVPVHWDASv-<br><br>BLAST: aliSize=124 (resi) iden=99.19% (123/124) simi=100.00% (124/124)<br>KETAAAKFERQHMDSSTSAASSSNYCNQMMKSRNLTKDRCKPVNTFVHESLADVQAVCSQKNVACKNGQTNCYQSYSTMSITDCRETGSSKYPNCAYKTTQANKHIIVACEGNPYVPVHFDASV<br>KETAAAKFERQHMDSSTSAASSSNYCNQMMKSRNLTKDRCKPVNTFVHESLADVQAVCSQKNVACKNGQTNCYQSYSTMSITDCRETGSSKYPNCAYKTTQANKHIIVACEGNPYVPVHWDASV<br><br>Proposed: aliSize=124 (resi) RMSD=0.73 (Å)<br>KETAAAKFERQHMDSSTSAASSSNYCNQMMKSRNLTKDRCKPVNTFVHESLADVQAVCSQKNVACKNGQTNCYQSYSTMSITDCRETGSSKYPNCAYKTTQANKHIIVACEGNPYVPVHFDASV<br>KETAAAKFERQHMDSSTSAASSSNYCNQMMKSRNLTKDRCKPVNTFVHESLADVQAVCSQKNVACKNGQTNCYQSYSTMSITDCRETGSSKYPNCAYKTTQANKHIIVACEGNPYVPVHWDASV
302	1js0A (124)<br>1gioA (125)	C	32.26	TM-align: aliSize=103 (resi) RMSD=2.39 (Å)<br>ke----TAAAKFERQHMDSsTsAASSS-NYCNQMMKSRNLTkDRCKPVNTFVHESLADVQAVCS---QKNVAckngqTNCYQSYSTMSITDCRETGSSKyPN-CAYKTTQANKHIIVACEGNPyvpvhfdasv-------------<br>--aqddYRYIHFLTQHYDA-K-PKGRNdEYCFNMMKNRRLT-RPCKDRNTFIHGNKNDIKAICEdrnGQPYR-----GDLRISKSEFQITICKHKGGSS-RPpCRYGATEDSRVIVVGCENGL----------pvhfdesfitprh<br><br>SARST: aliSize=93 (resi) RMSD=5.77 (Å)<br>ketaaakferqhmdsstsaass-----------------------SNYCNQMMKSRNLTkDRCKPVNTFVHESLADVQAVCSQKNVACKNGqtNCYQSYSTMSITDCRETGSSKYPNCAYKTTQANKHIivACEGNPYVPVHF---------dasv<br>----------------------aqddyryihfltqhydakpkgrnDEYCFNMMKNRRLT-RPCKDRNTFIHGNKNDIKAICEDRNGQPYRG--DLRISKSEFQITICKHKGGSSRPPCRYGATEDSRVI--VVGCENGLPVHFdesfitprh----<br><br>BLAST: aliSize=60 (resi) iden=35.48% (44/124) simi=48.39% (60/124)<br>ketaaak---------FERQHMDSSTSAASSSnYCNQMMKSRNLTKDrCKPVNTFVHESLADVQAVCSQKNVACKNGQTNCyqSYSTMSITDCRETGSSKYPNCAYKTTQANKHIIVACEGNpyVPVHFDAS------v<br>-------aqddyryihFLTQHYDAKPKGRNDE-YCFNMMKNRRLTRP-CKDRNTFIHGNKNDIKAICEDRNGQPYRGDLRI--SKSEFQITICKHKGGSSRPPCRYGATEDSRVIVVGCENG--LPVHFDESfitprh-<br><br>Proposed: aliSize=110 (resi) RMSD=1.92 (Å)<br>ke-----TaAAKFERQHMDSsTsAASSS-NYCNQMMKSRNLTkDRCKPVNTFVHESLADVQAVCS---QKNVAckngqTNCYQSYSTMSITDCRETGSSKyPN-CAYKTTQANKHIIVACEGnpYVPVHFDA-SV----<br>--aqddyR-YIHFLTQHYDA-K-PKGRNdEYCFNMMKNRRLT-RPCKDRNTFIHGNKNDIKAICEdrnGQPYR-----GDLRISKSEFQITICKHKGGSS-RPpCRYGATEDSRVIVVGCEN--GLPVHFDEsFItprh

303	1js0A (124)<br>1gv7A (123)	C	41.46	TM-align: aliSize=112 (resi) RMSD=2.19 (Å)<br>KETAAAKFERQHMDSSTSAAsSSNYCNQMMKSRNLTkDRCKPVNTFVHESLADVQAVCSQKNVACKNGQTNCYQSYSTMSITDCRETGSSKYPNCAYKTTQANKHIIVACEGNPyvpvhfdasv----------<br>DNSRYTHFLTQHYDAKPQGR-DDRYCESIMRRRGLT-SPCKDINTFIHGNKRSIKAICSQKNVACKNGQTNCYISKSSFQVTTCKLHGGSPWPPCQYRATAGFRNVVVACENGL----------pvhldqsifr<br><br>SARST: aliSize=113 (resi) RMSD=4.69 (Å)<br>ket---AAAKFERQHMDSSTSAaSSSNYCNQMMKSRNLTkDRCKPVNTFVHESLADVQAVCSQKNVACKNGQTNCYQSYSTMSITDCRETGSSKYPNCAYKTTQANKHIIVACegNPYVPVHF------dasv<br>---dnsRYTHFLTQHYDAKPQG-RDDRYCESIMRRRGLT-SPCKDINTFIHGNKRSIKAICSQKNVACKNGQTNCYISKSSFQVTTCKLHGGSPWPPCQYRATAGFRNVVVAC--ENGLPVHLdqsifr----<br><br>BLAST: aliSize=72 (resi) iden=42.62% (52/123) simi=59.02% (72/123)<br>ketaaak-------FERQHMDSSTSAASSsNYCNQMMKSRNLTKDrCKPVNTFVHESLADVQAVCSQKNVACKNGQTNCYQSYSTMSITDCRETGSSKYPNCAYKTTQANKHIIVACEGNpyVPVHFDASV--<br>-------dnsrythFLTQHYDAKPQGRDD-RYCESIMRRRGLTSP-CKDINTFIHGNKRSIKAICSQKNVACKNGQTNCYISKSSFQVTTCKLHGGSPWPPCQYRATAGFRNVVVACENG--LPVHLDQSIfr<br><br>Proposed: aliSize=117 (resi) RMSD=1.41 (Å)<br>ke---TAAAKFERQHMDSST-saASSSNYCNQMMKSRNLTkDRCKPVNTFVHESLADVQAVCSQKNVACKNGQTNCYQSYSTMSITDCRETGSSKYPNCAYKTTQANKHIIVACEGnpYVPVHFDA-SV-<br>--pdnSRYTHFLTQHYDAKPq--GRDDRYCESIMRRRGLT-SPCKDINTFIHGNKRSIKAICSQKNVACKNGQTNCYISKSSFQVTTCKLHGGSPWPPCQYRATAGFRNVVVACEN--GLPVHLDQsIFr
304	1js0A (124)<br>1izpA (124)	C	99.19	TM-align: aliSize=114 (resi) RMSD=1.06 (Å)<br>KETAAAKFERQHMDSSTSAASSSNYCNQMMKSRNLTKDRCKPVNTFVHESLADVQAVCSQKNVACKNGQTNCYQSYSTMSITDCRETGSSKYPNCAYKTTQANKHIIVACEGNPyvpvhfdasv----------<br>KETAAAKFERQHMDSSTSAASSSNYCNQMMKSRNLTKDRCKPVNTLVHESLADVQAVCSQKNVACKNGQTNCYQSYSTMSITDCRETGSSKYPNCAYKTTQANKHIIVACEGNP----------yvpvhfdasv<br><br>SARST: aliSize=122 (resi) RMSD=6.24 (Å)<br>k-ETAAAKFERQHMDSSTSAASSSNYCNQMMKSRNLTKDRCKPVNTFVHESLADVQAVCSQKNVACKNGQTNCYQSYSTMSITDCRETGSSKYPNCAYKTTQANKHIIVACEGNPYVPVHFDAS-v<br>-kETAAAKFERQHMDSSTSAASSSNYCNQMMKSRNLTKDRCKPVNTLVHESLADVQAVCSQKNVACKNGQTNCYQSYSTMSITDCRETGSSKYPNCAYKTTQANKHIIVACEGNPYVPVHFDASv-<br><br>BLAST: aliSize=123 (resi) iden=99.19% (123/124) simi=99.19% (123/124)<br>KETAAAKFERQHMDSSTSAASSSNYCNQMMKSRNLTKDRCKPVNTFVHESLADVQAVCSQKNVACKNGQTNCYQSYSTMSITDCRETGSSKYPNCAYKTTQANKHIIVACEGNPYVPVHFDASV<br>KETAAAKFERQHMDSSTSAASSSNYCNQMMKSRNLTKDRCKPVNTLVHESLADVQAVCSQKNVACKNGQTNCYQSYSTMSITDCRETGSSKYPNCAYKTTQANKHIIVACEGNPYVPVHFDASV<br><br>Proposed: aliSize=124 (resi) RMSD=0.61 (Å)<br>KETAAAKFERQHMDSSTSAASSSNYCNQMMKSRNLTKDRCKPVNTFVHESLADVQAVCSQKNVACKNGQTNCYQSYSTMSITDCRETGSSKYPNCAYKTTQANKHIIVACEGNPYVPVHFDASV<br>KETAAAKFERQHMDSSTSAASSSNYCNQMMKSRNLTKDRCKPVNTLVHESLADVQAVCSQKNVACKNGQTNCYQSYSTMSITDCRETGSSKYPNCAYKTTQANKHIIVACEGNPYVPVHFDASV
305	1js0A (124)<br>1izqA (124)	C	99.19	TM-align: aliSize=114 (resi) RMSD=1.10 (Å)<br>KETAAAKFERQHMDSSTSAASSSNYCNQMMKSRNLTKDRCKPVNTFVHESLADVQAVCSQKNVACKNGQTNCYQSYSTMSITDCRETGSSKYPNCAYKTTQANKHIIVACEGNPyvpvhfdasv----------<br>KETAAAKFERQHMDSSTSAASSSNYCNQMMKSRNLTKDRCKPVNTVVHESLADVQAVCSQKNVACKNGQTNCYQSYSTMSITDCRETGSSKYPNCAYKTTQANKHIIVACEGNP----------yvpvhfdasv<br><br>SARST: aliSize=122 (resi) RMSD=6.24 (Å)<br>k-ETAAAKFERQHMDSSTSAASSSNYCNQMMKSRNLTKDRCKPVNTFVHESLADVQAVCSQKNVACKNGQTNCYQSYSTMSITDCRETGSSKYPNCAYKTTQANKHIIVACEGNPYVPVHFDAS-v<br>-kETAAAKFERQHMDSSTSAASSSNYCNQMMKSRNLTKDRCKPVNTVVHESLADVQAVCSQKNVACKNGQTNCYQSYSTMSITDCRETGSSKYPNCAYKTTQANKHIIVACEGNPYVPVHFDASv-<br><br>BLAST: aliSize=123 (resi) iden=99.19% (123/124) simi=99.19% (123/124)<br>KETAAAKFERQHMDSSTSAASSSNYCNQMMKSRNLTKDRCKPVNTFVHESLADVQAVCSQKNVACKNGQTNCYQSYSTMSITDCRETGSSKYPNCAYKTTQANKHIIVACEGNPYVPVHFDASV<br>KETAAAKFERQHMDSSTSAASSSNYCNQMMKSRNLTKDRCKPVNTVVHESLADVQAVCSQKNVACKNGQTNCYQSYSTMSITDCRETGSSKYPNCAYKTTQANKHIIVACEGNPYVPVHFDASV<br><br>Proposed: aliSize=124 (resi) RMSD=0.66 (Å)<br>KETAAAKFERQHMDSSTSAASSSNYCNQMMKSRNLTKDRCKPVNTFVHESLADVQAVCSQKNVACKNGQTNCYQSYSTMSITDCRETGSSKYPNCAYKTTQANKHIIVACEGNPYVPVHFDASV<br>KETAAAKFERQHMDSSTSAASSSNYCNQMMKSRNLTKDRCKPVNTVVHESLADVQAVCSQKNVACKNGQTNCYQSYSTMSITDCRETGSSKYPNCAYKTTQANKHIIVACEGNPYVPVHFDASV

306	1js0A (124)<br>1izrA (124)	C	99.19	TM-align: aliSize=115 (resi) RMSD=1.64 (Å)<br>KETAAAKFERQHMDSSTSAASSSNYCNQMMKSRNLTKDRCKPVNTFVHESLADVQAVCSQKNVACKNGQTNCYQSYSTMSITDCRETGSSKYPNCAYKTTQANKHIIVACEGNPYvpvhfdasv---------<br>KETAAAKFERQHMDSSTSAASSSNYCNQMMKSRNLTKDRCKPVNTAVHESLADVQAVCSQKNVACKNGQTNCYQSYSTMSITDCRETGSSKYPNCAYKTTQANKHIIVACEGNPY---------vpvhfdasv<br><br>SARST: aliSize=122 (resi) RMSD=6.29 (Å)<br>k-ETAAAKFERQHMDSSTSAASSSNYCNQMMKSRNLTKDRCKPVNTFVHESLADVQAVCSQKNVACKNGQTNCYQSYSTMSITDCRETGSSKYPNCAYKTTQANKHIIVACEGNPYVPVHFDAS-v<br>-kETAAAKFERQHMDSSTSAASSSNYCNQMMKSRNLTKDRCKPVNTAVHESLADVQAVCSQKNVACKNGQTNCYQSYSTMSITDCRETGSSKYPNCAYKTTQANKHIIVACEGNPYVPVHFDASv-<br><br>BLAST: aliSize=123 (resi) iden=99.19% (123/124) simi=99.19% (123/124)<br>KETAAAKFERQHMDSSTSAASSSNYCNQMMKSRNLTKDRCKPVNTFVHESLADVQAVCSQKNVACKNGQTNCYQSYSTMSITDCRETGSSKYPNCAYKTTQANKHIIVACEGNPYVPVHFDASV<br>KETAAAKFERQHMDSSTSAASSSNYCNQMMKSRNLTKDRCKPVNTAVHESLADVQAVCSQKNVACKNGQTNCYQSYSTMSITDCRETGSSKYPNCAYKTTQANKHIIVACEGNPYVPVHFDASV<br><br>Proposed: aliSize=124 (resi) RMSD=1.05 (Å)<br>KETAAAKFERQHMDSSTSAASSSNYCNQMMKSRNLTKDRCKPVNTFVHESLADVQAVCSQKNVACKNGQTNCYQSYSTMSITDCRETGSSKYPNCAYKTTQANKHIIVACEGNPYVPVHFDASV<br>KETAAAKFERQHMDSSTSAASSSNYCNQMMKSRNLTKDRCKPVNTAVHESLADVQAVCSQKNVACKNGQTNCYQSYSTMSITDCRETGSSKYPNCAYKTTQANKHIIVACEGNPYVPVHFDASV
307	1js0A (124)<br>1j80B (101)	C	100.00	TM-align: aliSize=91 (resi) RMSD=1.23 (Å)<br>ketaaakferqhmdsstsaasssNYCNQMMKSRNLTKDRCKPVNTFVHESLADVQAVCSQKNVACKNGQTNCYQSYSTMSITDCRETGSSKYPNCAYKTTQANKHIIVACEGNPyvpvhfdasv----------<br>-----------------------NYCNQMMKSRNLTKDRCKPVNTFVHESLADVQAVCSQKNVACKNGQTNCYQSYSTMSITDCRETGSSKYPNCAYKTTQANKHIIVACEGNP----------yvpvhfdasv<br><br>SARST: aliSize=99 (resi) RMSD=6.89 (Å)<br>ketaaakferqhmdsstsaasssn-YCNQMMKSRNLTKDRCKPVNTFVHESLADVQAVCSQKNVACKNGQTNCYQSYSTMSITDCRETGSSKYPNCAYKTTQANKHIIVACEGNPYVPVHFDAS-v<br>-----------------------nYCNQMMKSRNLTKDRCKPVNTFVHESLADVQAVCSQKNVACKNGQTNCYQSYSTMSITDCRETGSSKYPNCAYKTTQANKHIIVACEGNPYVPVHFDASv-<br><br>BLAST: aliSize=101 (resi) iden=100.00% (101/101) simi=100.00% (101/101)<br>ketaaakferqhmdsstsaasssNYCNQMMKSRNLTKDRCKPVNTFVHESLADVQAVCSQKNVACKNGQTNCYQSYSTMSITDCRETGSSKYPNCAYKTTQANKHIIVACEGNPYVPVHFDASV<br>-----------------------NYCNQMMKSRNLTKDRCKPVNTFVHESLADVQAVCSQKNVACKNGQTNCYQSYSTMSITDCRETGSSKYPNCAYKTTQANKHIIVACEGNPYVPVHFDASV<br><br>Proposed: aliSize=101 (resi) RMSD=0.70 (Å)<br>ketaaakferqhmdsstsaasssNYCNQMMKSRNLTKDRCKPVNTFVHESLADVQAVCSQKNVACKNGQTNCYQSYSTMSITDCRETGSSKYPNCAYKTTQANKHIIVACEGNPYVPVHFDASV<br>-----------------------NYCNQMMKSRNLTKDRCKPVNTFVHESLADVQAVCSQKNVACKNGQTNCYQSYSTMSITDCRETGSSKYPNCAYKTTQANKHIIVACEGNPYVPVHFDASV
308	1js0A (124)<br>1k5aA (118)	C	31.36	TM-align: aliSize=105 (resi) RMSD=1.86 (Å)<br>kETAAAKFERQHMDSSTSAAsSSNYCNQMMKSRNLTkDRCKPVNTFVHESLADVQAVCS---QKNVAckngqTNCYQSYSTMSITDCRETGSSKYPNCAYKTTQANKHIIVACEGNpyvpvhfdasv----------<br>-NSRYTHFLTQHYDAKPQGR-DDRYCESIMRRRGLT-SPCKDINTFIHGNKRSIKAICEnknGNPHR-----ENLRISKSSFQVTTCKLHGGSPWPPCQYRATAGFRNVVVACENG-----------lpvhldqsaa<br><br>SARST: aliSize=108 (resi) RMSD=4.68 (Å)<br>ket--AAAKFERQHMDSsTSAASSSNYCNQMMKSRNLTkDRCKPVNTFVHESLADVQAVC---SQKNVAckngqTNCYQSYSTMSITDCRETGSSKYPNCAYKTTQANKHIIVACEGnpYVPVHF-----dasv<br>---nsRYTHFLTQHYDA-KPQGRDDRYCESIMRRRGLT-SPCKDINTFIHGNKRSIKAICenkNGNPHR-----ENLRISKSSFQVTTCKLHGGSPWPPCQYRATAGFRNVVVACEN--GLPVHLdqsaa----<br><br>BLAST: aliSize=60 (resi) iden=33.90% (40/118) simi=50.85% (60/118)<br>ketaaak------FERQHMDSSTSAASSsNYCNQMMKSRNLTKDrCKPVNTFVHESLADVQAVCSQKNVacKNGQTNCYQSYSTMSITDCRETGSSKYPNCAYKTTQANKHIIVACEGNpyVPVHFDAS--v<br>-------nsrythFLTQHYDAKPQGRDD-RYCESIMRRRGLTSP-CKDINTFIHGNKRSIKAICENKNG--NPHRENLRISKSSFQVTTCKLHGGSPWPPCQYRATAGFRNVVVACENG--LPVHLDQSaa-<br><br>Proposed: aliSize=113 (resi) RMSD=1.56 (Å)<br>ke-TAAAKFERQHMDSSTsAASSSNYCNQMMKSRNLTkDRCKPVNTFVHESLADVQAVCS---QKNVAckngqTNCYQSYSTMSITDCRETGSSKYPNCAYKTTQANKHIIVACEGnpYVPVHFDA-SV<br>--nSRYTHFLTQHYDAKP-QGRDDRYCESIMRRRGLT-SPCKDINTFIHGNKRSIKAICEnknGNPHR-----ENLRISKSSFQVTTCKLHGGSPWPPCQYRATAGFRNVVVACEN--GLPVHLDQsAA

309	1js0A (124)<br>1kh8A (125)	C	98.39	TM-align: aliSize=113 (resi) RMSD=0.92 (Å)<br>-KETAAAKFERQHMDSSTSAASSSNYCNQMMKSRNLTKDRCKPVNTFVHESLADVQAVCSQKNVACKNGQTNCYQSYSTMSITDCRETGSSKYPNCAYKTTQANKHIIVACE--G-Npyvpvhfdasv--------<br>mKETAAAKFERQHMDSSTSAASSSNYCNQMMKSRNLTKDRCKPVNTFVHESLADVQAVCSQKNVACKNGQTNCYQSYSTMSITDCRETGSSKYPNCAYKTTQANKHIIVACEgnGyV-----------pvhfdasv<br><br>SARST: aliSize=122 (resi) RMSD=6.37 (Å)<br>k--ETAAAKFERQHMDSSTSAASSSNYCNQMMKSRNLTKDRCKPVNTFVHESLADVQAVCSQKNVACKNGQTNCYQSYSTMSITDCRETGSSKYPNCAYKTTQANKHIIVACEGNPYVPVHFDAS-v<br>-mkETAAAKFERQHMDSSTSAASSSNYCNQMMKSRNLTKDRCKPVNTFVHESLADVQAVCSQKNVACKNGQTNCYQSYSTMSITDCRETGSSKYPNCAYKTTQANKHIIVACEGNGYVPVHFDASv-<br><br>BLAST: aliSize=123 (resi) iden=99.19% (123/124) simi=99.19% (123/124)<br>-KETAAAKFERQHMDSSTSAASSSNYCNQMMKSRNLTKDRCKPVNTFVHESLADVQAVCSQKNVACKNGQTNCYQSYSTMSITDCRETGSSKYPNCAYKTTQANKHIIVACEGNPYVPVHFDASV<br>mKETAAAKFERQHMDSSTSAASSSNYCNQMMKSRNLTKDRCKPVNTFVHESLADVQAVCSQKNVACKNGQTNCYQSYSTMSITDCRETGSSKYPNCAYKTTQANKHIIVACEGNGYVPVHFDASV<br><br>Proposed: aliSize=122 (resi) RMSD=0.66 (Å)<br>-KETAAAKFERQHMDSSTSAASSSNYCNQMMKSRNLTKDRCKPVNTFVHESLADVQAVCSQKNVACKNGQTNCYQSYSTMSITDCRETGSSKYPNCAYKTTQANKHIIVACE--GnpYVPVHFDASV<br>mKETAAAKFERQHMDSSTSAASSSNYCNQMMKSRNLTKDRCKPVNTFVHESLADVQAVCSQKNVACKNGQTNCYQSYSTMSITDCRETGSSKYPNCAYKTTQANKHIIVACEgnG--YVPVHFDASV
310	1js0A (124)<br>1kvzA (107)	C	26.17	TM-align: aliSize=90 (resi) RMSD=2.66 (Å)<br>kETAAAKFERQHMDSsTsAAsssnYcnQMMKSrnLTKD--RCKPVNTFVHESLADVQAVCSqknvackngq----TNCYQSYSTMSITDCRETGSSKypnCAYKTTQANKHIIVACEGNPyvpvhfdasv-----------<br>-MQDWATFKKKHLTD-T-WD----V--DCDNL--MPTSlfDCKDKNTFIYSLPGPVKALCR----------gvifSADVLSNSEFYLAECNVKPRKP---CKYKLKKSSNRICIRCEHEL----------pvhfagvgicp<br><br>SARST: aliSize=99 (resi) RMSD=8.20 (Å)<br>keta---AAKFERQHMdssTSA-ASSSNYCNQMMKSRNltkdrcKPVNTFVHESLADVQAVCSQKNVACkngqtnCYQSYSTMSITDCRETGSsKYPNCAYKTtqANKHIIVACEGNpyVPVHFDAS----v<br>----mqdWATFKKKHL---TDTwDVDCDNLMPTSLFDC------KDKNTFIYSLPGPVKALCRGVIFSA------DVLSNSEFYLAECNVKPR-KPCKYKLKK--SSNRICIRCEHE--LPVHFAGVgicp-<br><br>BLAST: aliSize=53 (resi) iden=27.10% (29/107) simi=49.53% (53/107)<br>ketaa----AKFERQHMDSSTSAAsssnyCNQMMKSrNLTkDrCKPVNTFVHESLADVQAVCsqKNVACKNGQTNCYQSYstmsITDCREtgSSKYPnCAYKTTQANKHIIVACEGNpyVPVHF-------dasv<br>-----mqdwATFKKKHLTDTWDVD-----CDNLMPT-SLF-D-CKDKNTFIYSLPGPVKALC--RGVIFSADVLSNSEFY----LAECNV--KPRKP-CKYKLKKSSNRICIRCEHE--LPVHFagvgicp----<br><br>Proposed: aliSize=98 (resi) RMSD=2.08 (Å)<br>ke-TAAAKFERQHMDSsTsAASssnYCNQMMKSrNLTKdrCKPVNTFVHESLADVQAVCS-----qknvackngqtNCYQSYSTMSITDCRETGSSkypNCAYKTTQANKHIIVACEGnpYVPVHFDASV---<br>--mQDWATFKKKHLTD-T-WDV---DCDNLMPT-SLFD--CKDKNTFIYSLPGPVKALCRgvifs-----------ADVLSNSEFYLAECNVKPRK---PCKYKLKKSSNRICIRCEH--ELPVHFAGVGicp
311	1js0A (124)<br>1m58A (106)	C	28.30	TM-align: aliSize=91 (resi) RMSD=2.73 (Å)<br>kETAAAKFERQHMDSsTsAASssnYCNQMMKSRNLTkdrCKPVNTFVHESLADVQAVC-SQknvackngq--TNCYQSYSTMSITDCRETGSSkypnCAYKTTQANKHIIVACEGNpyvpvhfdasv------------<br>-MQNWETFQKKHLTD-T-RDV---KCDAEMKKALFD---CKQKNTFIYARPGRVQALCkNI---------ivSKNVLSTDEFYLSDCNRIKLP----CHYKLKKSSNTICITCENK-----------lpvhfvaveecp<br><br>SARST: aliSize=78 (resi) RMSD=7.87 (Å)<br>ketaaakferqhmdsstsaasssn--------------------YCNQMMKSRNLtkdrcKPVNTFVHESLADVQAVCS---QKNvackngqtnCYQSYSTMSITDCREtgsSKYPNCAYKTtqaNKHIIVACEGNPYVPVHFDA-----sv<br>------------------------mqnwetfqkkhltdtrdvkcDAEMKKALFDC-----KQKNTFIYARPGRVQALCKniiVSK---------NVLSTDEFYLSDCNR---IKLPCHYKLK---KSSNTICITCENKLPVHFVAveecp--<br><br>BLAST: aliSize=52 (resi) iden=31.13% (33/106) simi=49.06% (52/106)<br>ketaaak------FERQHMDSSTSAAsssnyCNQMMKSRNLtkDrCKPVNTFVHESLADVQAVCsqKNVACKNGQTNCYQSYstmsITDCRETgssKYPnCAYKTTQANKHIIVACEGNpyVPVHFDA-----sv<br>-------mqnwetFQKKHLTDTRDVK-----CDAEMKKALF--D-CKQKNTFIYARPGRVQALC--KNIIVSKNVLSTDEFY----LSDCNRI---KLP-CHYKLKKSSNTICITCENK--LPVHFVAveecp--<br><br>Proposed: aliSize=97 (resi) RMSD=2.06 (Å)<br>ke-TAAAKFERQHMDsSTsAASssnYCNQMMKSrNLTKdrCKPVNTFVHESLADVQAVC-S----qknvackngqtNCYQSYSTMSITDCRETgSSkypNCAYKTTQANKHIIVACEGnpYVPVHFDASV---<br>--mQNWETFQKKHLT-DT-RDV---KCDAEMKK-ALFD--CKQKNTFIYARPGRVQALCkNiivs-----------KNVLSTDEFYLSDCNRI-KL---PCHYKLKKSSNTICITCEN--KLPVHFVAVEecp

312	1js0A (124)<br>1oj8A (105)	C	23.81	TM-align: aliSize=91 (resi) RMSD=2.56 (Å)<br>ketAAAKFERQHMDsSTsAASssnYCNQMMKSRNLTkdrCKPVNTFVHESLADVQAVCSQKNvackngq-TNCYQSYSTMSITDCRETgSSKypnCAYKTTQANKHIIVACEG-Npyvpvhfdasv-----------<br>---DWDTFQKKHLT-DT-KKV---KCDVEMKKALFD---CKKTNTFIFARPPRVQALCKNIK-------nNTNVLSRDVFYLPQCNRK-KLP---CHYRLDGSTNTICLTCMKeL-----------pihfagvgkcp<br><br>SARST: aliSize=99 (resi) RMSD=7.48 (Å)<br>keta-AAKFERQHMDSsTSAASssnycnqMMKSRNLTKDRCKPVNTFVHESLADVQAVCSQKNvackngQTNCYQSYSTMSITDCRETgsskYPNcAYKTTQANKHIIVACegNPYVPVHFDAS----v<br>----dWDTFQKKHLTD-TKKVK-------CDVEMKKALFDCKKTNTFIFARPPRVQALCKNIK------NNTNVLSRDVFYLPQCNRK---KLPC-HYRLDGSTNTICLTC--MKELPIHFAGVgkcp-<br><br>BLAST: aliSize=48 (resi) iden=31.73% (33/105) simi=46.15% (48/105)<br>ketaaak----FERQHMdSSTSAASssnyCNQMMKSRNLtkDrCKPVNTFVHESLADVQAVCsqKNVacKNgQTNCYqSYSTMSITDCREtgsSKYPnCAYKTTQANKHIIVACEGNpyVPVHF-------dasv<br>-------dwdtFQKKHL-TDTKKVK----CDVEMKKALF--D-CKKTNTFIFARPPRVQALC--KNI--KN-NTNVL-SRDVFYLPQCNR---KKLP-CHYRLDGSTNTICLTCMKE--LPIHFagvgkcp----<br><br>Proposed: aliSize=97 (resi) RMSD=1.84 (Å)<br>keTAAAKFERQHMDsSTsAASssnYCNQMMKSrNLTKdrCKPVNTFVHESLADVQAVCS-----qknvackngqtNCYQSYSTMSITDCRETgSSkypNCAYKTTQANKHIIVACEGnpYVPVHFDASV---<br>--PDWDTFQKKHLT-DT-KKV---KCDVEMKK-ALFD--CKKTNTFIFARPPRVQALCKniknn-----------TNVLSRDVFYLPQCNRK-KL---PCHYRLDGSTNTICLTCMK--ELPIHFAGVGkcp
313	1js0A (124)<br>1pu3A (105)	C	26.67	TM-align: aliSize=91 (resi) RMSD=2.44 (Å)<br>kETAAAKFERQHMDSsTsAASssnYCNQMMKSRNLTkdrCKPVNTFVHESLADVQAVCSQknvackngq---TNCYQSYSTMSITDCRETGSSkypnCAYKTTQANKHIIVACEGNpyvpvhfdasv-----------<br>-MQDWLTFQKKHITN-T-RDV---DCDNILSTNLFH---CKDKNTFIYSRPEPVKAICKG---------iiaSKNVLTTSEFYLSDCNVTSRP----CKYKLKKSTNKFCVTCENQ-----------apvhfvgvgsc<br><br>SARST: aliSize=97 (resi) RMSD=7.56 (Å)<br>keta---AAKFERQHMDSsTSAASssnycnqMMKSRNLTKDRCKPVNTFVHESLADVQAVCSqknvacknGQT--NCYQSYSTMSITDCRETGSSkypnCAYKTTQANKHIIVAceGNPYVPVHFDAS---v<br>----mqdWLTFQKKHITN-TRDVD-------CDNILSTNLFHCKDKNTFIYSRPEPVKAICK--------GIIasKNVLTTSEFYLSDCNVTSRP----CKYKLKKSTNKFCVT--CENQAPVHFVGVgsc-<br><br>BLAST: aliSize=54 (resi) iden=32.38% (34/105) simi=51.43% (54/105)<br>ketaaak------FERQHMDSSTSAAsssnyCNQMMkSRNLTkdRCKPVNTFVHESLADVQAVCsQKNVACKNGQTNcyqsySTMSITDCRETgsSKyPnCAYKTTQANKHIIVACEGNpyVPVHF------dasv<br>-------mqdwltFQKKHITNTRDVD-----CDNIL-STNLF--HCKDKNTFIYSRPEPVKAIC-KGIIASKNVLTT-----SEFYLSDCNVT--SR-P-CKYKLKKSTNKFCVTCENQ--APVHFvgvgsc----<br><br>Proposed: aliSize=97 (resi) RMSD=1.76 (Å)<br>keTA-AAKFERQHMDSsTsAASssnYCNQMMKSrNLTKdrCKPVNTFVHESLADVQAVCS-----qknvackngqtNCYQSYSTMSITDCRETGSSkypnCAYKTTQANKHIIVACEGnpYVPVHFDASV--<br>--MQdWLTFQKKHITN-T-RDV---DCDNILST-NLFH--CKDKNTFIYSRPEPVKAICKgiias-----------KNVLTTSEFYLSDCNVTSRP----CKYKLKKSTNKFCVTCEN--QAPVHFVGVGsc
314	1js0A (124)<br>1qwqA (124)	C	75.81	TM-align: aliSize=112 (resi) RMSD=2.52 (Å)<br>KETAAAKFERQHMDSSTSaASSSN-YCNQMMKSRNLTKDRCKPVNTFVHESLADVQAVCSQKNVACKNGQTNCYQSYSTMSITDCRET-GSSKyPNCAYKTTQANKHIIVACEGNPyvpvhfdasv----------<br>KESAAAKFERQHMDSGNS-PSSSSnYCNLMMCCRKMTQGKCKPVNTFVHESLADVKAVCSQKKVTCKDGQTNCYQSKSTMRITDCRETgSSKY-PNCAYKTTQVEKHIIVACGGKP----------svpvhfdasv<br><br>SARST: aliSize=119 (resi) RMSD=6.76 (Å)<br>k--ETAAAKFERQHmdsSTSAASSSNYCN--QMMKSRNLTKDRCKPVNTFVHESLADVQAVCSQKNVACKNGQTNCYQSYSTMSITDCRETGSSKYPNCAYKTTQANKHIIVACEGNPYVPVHFDAS-v<br>-keSAAAKFERQHM---DSGNSPSSSSNYcnLMMCCRKMTQGKCKPVNTFVHESLADVKAVCSQKKVTCKDGQTNCYQSKSTMRITDCRETGSSKYPNCAYKTTQVEKHIIVACGGKPSVPVHFDASv-<br><br>BLAST: aliSize=107 (resi) iden=80.65% (100/124) simi=86.29% (107/124)<br>KETAAAKFERQHMDSSTSAASSSNYCNQMMKSRNLTKDRCKPVNTFVHESLADVQAVCSQKNVACKNGQTNCYQSYSTMSITDCRETGSSKYPNCAYKTTQANKHIIVACEGNPYVPVHFDASV<br>KESAAAKFERQHMDSGNSPSSSSNYCNLMMCCRKMTQGKCKPVNTFVHESLADVKAVCSQKKVTCKDGQTNCYQSKSTMRITDCRETGSSKYPNCAYKTTQVEKHIIVACGGKPSVPVHFDASV<br><br>Proposed: aliSize=119 (resi) RMSD=1.92 (Å)<br>ke--TAAAKFERQHMDSSTSaASS-SNYCNQMMKSRNLTKDRCKPVNTFVHESLADVQAVCSQKNVACKNGQTNCYQSYSTMSITDCRET--gSSKyPNCAYKTTQANKHIIVACEGNPYVPVHFDASV<br>--keSAAAKFERQHMDSGNS-PSSsSNYCNLMMCCRKMTQGKCKPVNTFVHESLADVKAVCSQKKVTCKDGQTNCYQSKSTMRITDCRETgs-SKY-PNCAYKTTQVEKHIIVACGGKPSVPVHFDASV

315	1js0A (124)<br>1rasA (123)	C	100.00	TM-align: aliSize=113 (resi) RMSD=1.04 (Å)<br>kETAAAKFERQHMDSSTSAASSSNYCNQMMKSRNLTKDRCKPVNTFVHESLADVQAVCSQKNVACKNGQTNCYQSYSTMSITDCRETGSSKYPNCAYKTTQANKHIIVACEGNPyvpvhfdasv----------<br>-ETAAAKFERQHMDSSTSAASSSNYCNQMMKSRNLTKDRCKPVNTFVHESLADVQAVCSQKNVACKNGQTNCYQSYSTMSITDCRETGSSKYPNCAYKTTQANKHIIVACEGNP----------yvpvhfdasv<br><br>SARST: aliSize=121 (resi) RMSD=6.26 (Å)<br>ke-TAAAKFERQHMDSSTSAASSSNYCNQMMKSRNLTKDRCKPVNTFVHESLADVQAVCSQKNVACKNGQTNCYQSYSTMSITDCRETGSSKYPNCAYKTTQANKHIIVACEGNPYVPVHFDAS-v<br>--eTAAAKFERQHMDSSTSAASSSNYCNQMMKSRNLTKDRCKPVNTFVHESLADVQAVCSQKNVACKNGQTNCYQSYSTMSITDCRETGSSKYPNCAYKTTQANKHIIVACEGNPYVPVHFDASv-<br><br>BLAST: aliSize=123 (resi) iden=100.00% (123/123) simi=100.00% (123/123)<br>kETAAAKFERQHMDSSTSAASSSNYCNQMMKSRNLTKDRCKPVNTFVHESLADVQAVCSQKNVACKNGQTNCYQSYSTMSITDCRETGSSKYPNCAYKTTQANKHIIVACEGNPYVPVHFDASV<br>-ETAAAKFERQHMDSSTSAASSSNYCNQMMKSRNLTKDRCKPVNTFVHESLADVQAVCSQKNVACKNGQTNCYQSYSTMSITDCRETGSSKYPNCAYKTTQANKHIIVACEGNPYVPVHFDASV<br><br>Proposed: aliSize=123 (resi) RMSD=0.54 (Å)<br>kETAAAKFERQHMDSSTSAASSSNYCNQMMKSRNLTKDRCKPVNTFVHESLADVQAVCSQKNVACKNGQTNCYQSYSTMSITDCRETGSSKYPNCAYKTTQANKHIIVACEGNPYVPVHFDASV<br>-ETAAAKFERQHMDSSTSAASSSNYCNQMMKSRNLTKDRCKPVNTFVHESLADVQAVCSQKNVACKNGQTNCYQSYSTMSITDCRETGSSKYPNCAYKTTQANKHIIVACEGNPYVPVHFDASV
316	1js0A (124)<br>1rnfA (120)	C	43.33	TM-align: aliSize=111 (resi) RMSD=2.02 (Å)<br>K-ETAAAKFERQHMDSSTsAASSSNYCNQMMKSRNLTKDRCKPVNTFVHESLADVQAVCSQKNVACKNGQTNCYQSysTMSITDCRETGSSKYPNCAYKTTQANKHIIVACEGNPyvpvhfdasv--------<br>MqDGMYQRFLRQHVHPEE-TGGSDRYCNLMMQRRKMTLYHCKRFNTFIHEDIWNIRSICSTTNIQCKNGKMNCHEG--VVKVTDCRDTGSSRAPNCRYRAIASTRRVVIACEGNP----------qvpvhfdg<br><br>SARST: aliSize=115 (resi) RMSD=5.06 (Å)<br>ket----AAAKFERQHMDSSTSAAsSSNYCNQMMKSRNLTKDRCKPVNTFVHESLADVQAVCSQKNVACKNGQTNCYQsySTMSITDCRETGSSKYPNCAYKTTQANKHIIVACEGNPYVPVHFD-asv<br>---mqdgMYQRFLRQHVHPEETGG-SDRYCNLMMQRRKMTLYHCKRFNTFIHEDIWNIRSICSTTNIQCKNGKMNCHE--GVVKVTDCRDTGSSRAPNCRYRAIASTRRVVIACEGNPQVPVHFDg---<br><br>BLAST: aliSize=78 (resi) iden=43.33% (52/120) simi=65.00% (78/120)<br>ketaaa-------KFERQHMDSSTSAASSsNYCNQMMKSRNLTKDRCKPVNTFVHESLADVQAVCSQKNVACKNGQTNCYQsySTMSITDCRETGSSKYPNCAYKTTQANKHIIVACEGNPYVPVHFD-asv<br>------mqdgmyqRFLRQHVHPEETGGSD-RYCNLMMQRRKMTLYHCKRFNTFIHEDIWNIRSICSTTNIQCKNGKMNCHE--GVVKVTDCRDTGSSRAPNCRYRAIASTRRVVIACEGNPQVPVHFDg---<br><br>Proposed: aliSize=117 (resi) RMSD=1.31 (Å)<br>ke---TAAAKFERQHMDSSTSAAsSSNYCNQMMKSRNLTKDRCKPVNTFVHESLADVQAVCSQKNVACKNGQTNCYQSysTMSITDCRETGSSKYPNCAYKTTQANKHIIVACEGNPYVPVHFDAsv<br>--mqdGMYQRFLRQHVHPEETGG-SDRYCNLMMQRRKMTLYHCKRFNTFIHEDIWNIRSICSTTNIQCKNGKMNCHEG--VVKVTDCRDTGSSRAPNCRYRAIASTRRVVIACEGNPQVPVHFDG--
317	1js0A (124)<br>1rraA (124)	C	66.94	TM-align: aliSize=115 (resi) RMSD=1.66 (Å)<br>KETAAAKFERQHMDSSTSAASSSNYCNQMMKSRNLTKDRCKPVNTFVHESLADVQAVCSQKNVACKNGQTNCYQSYSTMSITDCRETGSSKYPNCAYKTTQANKHIIVACEGNPYvpvhfdasv---------<br>AESSADKFKRQHMDTEGPSKSSPTYCNQMMKRQGMTKGSCKPVNTFVHEPLEDVQAICSQGQVTCKNGRNNCHKSSSTLRITDCRLKGSSKYPNCDYTTTDSQKHIIIACDGNPY---------vpvhfdasv<br><br>SARST: aliSize=122 (resi) RMSD=6.28 (Å)<br>k-ETAAAKFERQHMDSSTSAASSSNYCNQMMKSRNLTKDRCKPVNTFVHESLADVQAVCSQKNVACKNGQTNCYQSYSTMSITDCRETGSSKYPNCAYKTTQANKHIIVACEGNPYVPVHFDAS-v<br>-aESSADKFKRQHMDTEGPSKSSPTYCNQMMKRQGMTKGSCKPVNTFVHEPLEDVQAICSQGQVTCKNGRNNCHKSSSTLRITDCRLKGSSKYPNCDYTTTDSQKHIIIACDGNPYVPVHFDASv-<br><br>BLAST: aliSize=98 (resi) iden=66.94% (83/124) simi=79.03% (98/124)<br>k-ETAAAKFERQHMDSSTSAASSSNYCNQMMKSRNLTKDRCKPVNTFVHESLADVQAVCSQKNVACKNGQTNCYQSYSTMSITDCRETGSSKYPNCAYKTTQANKHIIVACEGNPYVPVHFDASV<br>-aESSADKFKRQHMDTEGPSKSSPTYCNQMMKRQGMTKGSCKPVNTFVHEPLEDVQAICSQGQVTCKNGRNNCHKSSSTLRITDCRLKGSSKYPNCDYTTTDSQKHIIIACDGNPYVPVHFDASV<br><br>Proposed: aliSize=124 (resi) RMSD=1.07 (Å)<br>KETAAAKFERQHMDSSTSAASSSNYCNQMMKSRNLTKDRCKPVNTFVHESLADVQAVCSQKNVACKNGQTNCYQSYSTMSITDCRETGSSKYPNCAYKTTQANKHIIVACEGNPYVPVHFDASV<br>AESSADKFKRQHMDTEGPSKSSPTYCNQMMKRQGMTKGSCKPVNTFVHEPLEDVQAICSQGQVTCKNGRNNCHKSSSTLRITDCRLKGSSKYPNCDYTTTDSQKHIIIACDGNPYVPVHFDASV

318	1js0A<br>(124)<br><br>1ymnA<br>(124)	C	99.19	TM-align: aliSize=115 (resi) RMSD=1.53 (Å)<br>KETAAAKFERQHMDSSTSAASSSNYCNQMMKSRNLTKDRCKPVNTFVHESLADVQAVCSQKNVACKNGQTNCYQSYSTMSITDCRETGSSKYPNCAYKTTQANKHIIVACEGNPYvpvhfdasv---------<br>KETAAAKFERQHMDSSTSAASSSNYCNQMMKSRNLTKDRCKPVNTFVHESLADVQAVCSQKNVACKNGQTNCYQSYSTMSITDCRETGSSKLPNCAYKTTQANKHIIVACEGNPY---------vpvhfdasv<br><br>SARST: aliSize=122 (resi) RMSD=6.29 (Å)<br>k-ETAAAKFERQHMDSSTSAASSSNYCNQMMKSRNLTKDRCKPVNTFVHESLADVQAVCSQKNVACKNGQTNCYQSYSTMSITDCRETGSSKYPNCAYKTTQANKHIIVACEGNPYVPVHFDAS-v<br>-kETAAAKFERQHMDSSTSAASSSNYCNQMMKSRNLTKDRCKPVNTFVHESLADVQAVCSQKNVACKNGQTNCYQSYSTMSITDCRETGSSKLPNCAYKTTQANKHIIVACEGNPYVPVHFDASv-<br><br>BLAST: aliSize=123 (resi) iden=99.19% (123/124) simi=99.19% (123/124)<br>KETAAAKFERQHMDSSTSAASSSNYCNQMMKSRNLTKDRCKPVNTFVHESLADVQAVCSQKNVACKNGQTNCYQSYSTMSITDCRETGSSKYPNCAYKTTQANKHIIVACEGNPYVPVHFDASV<br>KETAAAKFERQHMDSSTSAASSSNYCNQMMKSRNLTKDRCKPVNTFVHESLADVQAVCSQKNVACKNGQTNCYQSYSTMSITDCRETGSSKLPNCAYKTTQANKHIIVACEGNPYVPVHFDASV<br><br>Proposed: aliSize=124 (resi) RMSD=0.88 (Å)<br>KETAAAKFERQHMDSSTSAASSSNYCNQMMKSRNLTKDRCKPVNTFVHESLADVQAVCSQKNVACKNGQTNCYQSYSTMSITDCRETGSSKYPNCAYKTTQANKHIIVACEGNPYVPVHFDASV<br>KETAAAKFERQHMDSSTSAASSSNYCNQMMKSRNLTKDRCKPVNTFVHESLADVQAVCSQKNVACKNGQTNCYQSYSTMSITDCRETGSSKLPNCAYKTTQANKHIIVACEGNPYVPVHFDASV
319	1js0A<br>(124)<br><br>1ymrA<br>(124)	C	99.19	TM-align: aliSize=115 (resi) RMSD=1.37 (Å)<br>KETAAAKFERQHMDSSTSAASSSNYCNQMMKSRNLTKDRCKPVNTFVHESLADVQAVCSQKNVACKNGQTNCYQSYSTMSITDCRETGSSKYPNCAYKTTQANKHIIVACEGNPYvpvhfdasv---------<br>KETAAAKFERQHMDSSTSAASSSNYCNQMMKSRNLTKDRCKPVNTFVHESLADVQAVCSQKNVACKNGQTNCYQSYSTMSITDCRETGSSKAPNCAYKTTQANKHIIVACEGNPY---------vpvhfdasv<br><br>SARST: aliSize=122 (resi) RMSD=6.27 (Å)<br>k-ETAAAKFERQHMDSSTSAASSSNYCNQMMKSRNLTKDRCKPVNTFVHESLADVQAVCSQKNVACKNGQTNCYQSYSTMSITDCRETGSSKYPNCAYKTTQANKHIIVACEGNPYVPVHFDAS-v<br>-kETAAAKFERQHMDSSTSAASSSNYCNQMMKSRNLTKDRCKPVNTFVHESLADVQAVCSQKNVACKNGQTNCYQSYSTMSITDCRETGSSKAPNCAYKTTQANKHIIVACEGNPYVPVHFDASv-<br><br>BLAST: aliSize=123 (resi) iden=99.19% (123/124) simi=99.19% (123/124)<br>KETAAAKFERQHMDSSTSAASSSNYCNQMMKSRNLTKDRCKPVNTFVHESLADVQAVCSQKNVACKNGQTNCYQSYSTMSITDCRETGSSKYPNCAYKTTQANKHIIVACEGNPYVPVHFDASV<br>KETAAAKFERQHMDSSTSAASSSNYCNQMMKSRNLTKDRCKPVNTFVHESLADVQAVCSQKNVACKNGQTNCYQSYSTMSITDCRETGSSKAPNCAYKTTQANKHIIVACEGNPYVPVHFDASV<br><br>Proposed: aliSize=124 (resi) RMSD=0.61 (Å)<br>KETAAAKFERQHMDSSTSAASSSNYCNQMMKSRNLTKDRCKPVNTFVHESLADVQAVCSQKNVACKNGQTNCYQSYSTMSITDCRETGSSKYPNCAYKTTQANKHIIVACEGNPYVPVHFDASV<br>KETAAAKFERQHMDSSTSAASSSNYCNQMMKSRNLTKDRCKPVNTFVHESLADVQAVCSQKNVACKNGQTNCYQSYSTMSITDCRETGSSKAPNCAYKTTQANKHIIVACEGNPYVPVHFDASV
320	1js0A<br>(124)<br><br>1ymwA<br>(124)	C	99.19	TM-align: aliSize=115 (resi) RMSD=1.40 (Å)<br>KETAAAKFERQHMDSSTSAASSSNYCNQMMKSRNLTKDRCKPVNTFVHESLADVQAVCSQKNVACKNGQTNCYQSYSTMSITDCRETGSSKYPNCAYKTTQANKHIIVACEGNPYvpvhfdasv---------<br>KETAAAKFERQHMDSSTSAASSSNYCNQMMKSRNLTKDRCKPVNTFVHESLADVQAVCSQKNVACKNGQTNCYQSYSTMSITDCRETGSSKGPNCAYKTTQANKHIIVACEGNPY---------vpvhfdasv<br><br>SARST: aliSize=122 (resi) RMSD=6.26 (Å)<br>k-ETAAAKFERQHMDSSTSAASSSNYCNQMMKSRNLTKDRCKPVNTFVHESLADVQAVCSQKNVACKNGQTNCYQSYSTMSITDCRETGSSKYPNCAYKTTQANKHIIVACEGNPYVPVHFDAS-v<br>-kETAAAKFERQHMDSSTSAASSSNYCNQMMKSRNLTKDRCKPVNTFVHESLADVQAVCSQKNVACKNGQTNCYQSYSTMSITDCRETGSSKGPNCAYKTTQANKHIIVACEGNPYVPVHFDASv-<br><br>BLAST: aliSize=123 (resi) iden=99.19% (123/124) simi=99.19% (123/124)<br>KETAAAKFERQHMDSSTSAASSSNYCNQMMKSRNLTKDRCKPVNTFVHESLADVQAVCSQKNVACKNGQTNCYQSYSTMSITDCRETGSSKYPNCAYKTTQANKHIIVACEGNPYVPVHFDASV<br>KETAAAKFERQHMDSSTSAASSSNYCNQMMKSRNLTKDRCKPVNTFVHESLADVQAVCSQKNVACKNGQTNCYQSYSTMSITDCRETGSSKGPNCAYKTTQANKHIIVACEGNPYVPVHFDASV<br><br>Proposed: aliSize=124 (resi) RMSD=0.67 (Å)<br>KETAAAKFERQHMDSSTSAASSSNYCNQMMKSRNLTKDRCKPVNTFVHESLADVQAVCSQKNVACKNGQTNCYQSYSTMSITDCRETGSSKYPNCAYKTTQANKHIIVACEGNPYVPVHFDASV<br>KETAAAKFERQHMDSSTSAASSSNYCNQMMKSRNLTKDRCKPVNTFVHESLADVQAVCSQKNVACKNGQTNCYQSYSTMSITDCRETGSSKGPNCAYKTTQANKHIIVACEGNPYVPVHFDASV

321	1js0A<br>(124)<br>1yv7A<br>(102)	C	28.43	TM-align: aliSize=90 (resi) RMSD=2.54 (Å)<br>ketAAAKFERQHMDsSTsAASssnYCNQMMKSRNLTkdrCKPVNTFVHESLADVQAVCSQkNvackngq--TNCYQSYSTMSITDCRETGSSkypnCAYKTTQANKHIIVACEGNpyvpvhfdasv--------<br>---DWLTFQKKHIT-NT-RDV---DCDNIMSTNLFH---CKDKNTFIYSRPEPVKAICKG-I-------iaSKNVLTTSEFYLSDCNVTSRP----CKYKLKKSTNKFSVTCENQ----------apvhfvgvg<br><br>SARST: aliSize=98 (resi) RMSD=7.20 (Å)<br>keta-AAKFERQHMDSsTSAAssSNYCNQMMKSRNLtkdrcKPVNTFVHESLADVQAVCSQKNVACkngqtnCYQSYSTMSITDCRETGSSkypnCAYKTTQANKHIIVACEGnpYVPVHFDA--sv<br>----dWLTFQKKHITN-TRDV--DCDNIMSTNLFHC-----KDKNTFIYSRPEPVKAICKGIIASK------NVLTTSEFYLSDCNVTSRP----CKYKLKKSTNKFSVTCEN--QAPVHFVGvg--<br><br>BLAST: aliSize=54 (resi) iden=34.65% (35/102) simi=53.47% (54/102)<br>ketaaak----FERQHMDSSTSAAsssnyCNQMMkSRNLTkdRCKPVNTFVHESLADVQAVCsQKNVACKNGQTNcyqsySTMSITDCRETgsSKyPnCAYKTTQANKHIIVACEGNpyVPVHF----dasv<br>-------dwltFQKKHITNTRDVD-----CDNIM-STNLF--HCKDKNTFIYSRPEPVKAIC-KGIIASKNVLTT-----SEFYLSDCNVT--SR-P-CKYKLKKSTNKFSVTCENQ--APVHFvgvg----<br><br>Proposed: aliSize=97 (resi) RMSD=1.83 (Å)<br>keTAAAKFERQHMDsSTsAASssnYCNQMMKSrNLTKdrCKPVNTFVHESLADVQAVCS-----qknvackngqtNCYQSYSTMSITDCRETGSSkypnCAYKTTQANKHIIVACEGnpYVPVHFDASV<br>--PDWLTFQKKHIT-NT-RDV---DCDNIMST-NLFH--CKDKNTFIYSRPEPVKAICKgiias-----------KNVLTTSEFYLSDCNVTSRP----CKYKLKKSTNKFSVTCEN--QAPVHFVGVG
322	1js0A<br>(124)<br>2aasA<br>(124)	C	100.00	TM-align: aliSize=114 (resi) RMSD=1.62 (Å)<br>KETAAAKFERQHMDSSTSAASSSNYCNQMMKSRNLTKDRCKPVNTFVHESLADVQAVCSQKNVACKNGQTNCYQSYSTMSITDCRETGSSKYPNCAYKTTQANKHIIVACEGNPyvpvhfdasv----------<br>KETAAAKFERQHMDSSTSAASSSNYCNQMMKSRNLTKDRCKPVNTFVHESLADVQAVCSQKNVACKNGQTNCYQSYSTMSITDCRETGSSKYPNCAYKTTQANKHIIVACEGNP----------yvpvhfdasv<br><br>SARST: aliSize=119 (resi) RMSD=6.44 (Å)<br>keta----AAKFERQHMDSSTSAASSSNYCNQMMKSRNLTKDRCKPVNTFVHESLADVQAVCSQKNVACKNGQTNCYQSYSTMSITDCRETGSSKYPNCAYKTTQANKHIIVACEGNPYVPVHFDAS-v<br>----ketaAAKFERQHMDSSTSAASSSNYCNQMMKSRNLTKDRCKPVNTFVHESLADVQAVCSQKNVACKNGQTNCYQSYSTMSITDCRETGSSKYPNCAYKTTQANKHIIVACEGNPYVPVHFDASv-<br><br>BLAST: aliSize=124 (resi) iden=100.00% (124/124) simi=100.00% (124/124)<br>KETAAAKFERQHMDSSTSAASSSNYCNQMMKSRNLTKDRCKPVNTFVHESLADVQAVCSQKNVACKNGQTNCYQSYSTMSITDCRETGSSKYPNCAYKTTQANKHIIVACEGNPYVPVHFDASV<br>KETAAAKFERQHMDSSTSAASSSNYCNQMMKSRNLTKDRCKPVNTFVHESLADVQAVCSQKNVACKNGQTNCYQSYSTMSITDCRETGSSKYPNCAYKTTQANKHIIVACEGNPYVPVHFDASV<br><br>Proposed: aliSize=124 (resi) RMSD=1.27 (Å)<br>KETAAAKFERQHMDSSTSAASSSNYCNQMMKSRNLTKDRCKPVNTFVHESLADVQAVCSQKNVACKNGQTNCYQSYSTMSITDCRETGSSKYPNCAYKTTQANKHIIVACEGNPYVPVHFDASV<br>KETAAAKFERQHMDSSTSAASSSNYCNQMMKSRNLTKDRCKPVNTFVHESLADVQAVCSQKNVACKNGQTNCYQSYSTMSITDCRETGSSKYPNCAYKTTQANKHIIVACEGNPYVPVHFDASV
323	1js0A<br>(124)<br>2e0jA<br>(128)	C	68.55	TM-align: aliSize=113 (resi) RMSD=1.50 (Å)<br>KETAAAKFERQHMDSS-TSAAsSSNYCNQMMKSRNLTKDRCKPVNTFVHESLADVQAVCSQKNVACKNGQTNCYQSYSTMSITDCRETGSSKYPNCAYKTTQANKHIIVACEGNPyvpvhfdasv-------------<br>KESRAKKFQRQHMDSDsSPSS-SSTYCNQMMLLRNMTQGRCKPVNTFVHEPLVDVQNVCFQEKVTCKNGQGNCYKSNSSMHITDCRLTNGSRYPNCAYRTSPKERHIIVACEGSP----------yvpvhfdasvedst<br><br>SARST: aliSize=122 (resi) RMSD=6.26 (Å)<br>k-ETAAAKFERQHMDSSTSAASSSNYCNQMMKSRNLTKDRCKPVNTFVHESLADVQAVCSQKNVACKNGQTNCYQSYSTMSITDCRETGSSKYPNCAYKTTQANKHIIVACEGNPYVPVHFDAS-----v<br>-kESRAKKFQRQHMDSDSSPSSSSTYCNQMMLLRNMTQGRCKPVNTFVHEPLVDVQNVCFQEKVTCKNGQGNCYKSNSSMHITDCRLTNGSRYPNCAYRTSPKERHIIVACEGSPYVPVHFDASvedst-<br><br>BLAST: aliSize=101 (resi) iden=70.16% (87/124) simi=81.45% (101/124)<br>KETAAAKFERQHMDSSTSAASSSNYCNQMMKSRNLTKDRCKPVNTFVHESLADVQAVCSQKNVACKNGQTNCYQSYSTMSITDCRETGSSKYPNCAYKTTQANKHIIVACEGNPYVPVHFDASV----<br>KESRAKKFQRQHMDSDSSPSSSSTYCNQMMLLRNMTQGRCKPVNTFVHEPLVDVQNVCFQEKVTCKNGQGNCYKSNSSMHITDCRLTNGSRYPNCAYRTSPKERHIIVACEGSPYVPVHFDASVedst<br><br>Proposed: aliSize=123 (resi) RMSD=1.08 (Å)<br>KETAAAKFERQHMDSS-TSAAsSSNYCNQMMKSRNLTKDRCKPVNTFVHESLADVQAVCSQKNVACKNGQTNCYQSYSTMSITDCRETGSSKYPNCAYKTTQANKHIIVACEGNPYVPVHFDASV----<br>KESRAKKFQRQHMDSDsSPSS-SSTYCNQMMLLRNMTQGRCKPVNTFVHEPLVDVQNVCFQEKVTCKNGQGNCYKSNSSMHITDCRLTNGSRYPNCAYRTSPKERHIIVACEGSPYVPVHFDASVedst

324	1js0A (124)<br>2e0lA (128)	C	69.35	TM-align: aliSize=115 (resi) RMSD=1.52 (Å)<br>KETAAAKFERQHMDSSTSAASSSNYCNQMMKSRNLTKDRCKPVNTFVHESLADVQAVCSQKNVACKNGQTNCYQSYSTMSITDCRETGSSKYPNCAYKTTQANKHIIVACEGNPYvpvhfdasv-------------<br>KESRAKKFQRQHMDSDSSPSSSSTYCNLMMLLRNMTQGRCKPVNTFVHEPLVDVQNVCFQEKVTCKNGQGNCYKSNSSMHITDCRLTNGSRYPNCAYRTSPKERHIIVACEGSPY---------vpvhfdasvedst<br><br>SARST: aliSize=120 (resi) RMSD=6.28 (Å)<br>ke--TAAAKFERQHMDSST-SAAsSSNYCNQMMKSRNLTKDRCKPVNTFVHESLADVQAVCSQKNVACKNGQTNCYQSYSTMSITDCRETGSSKYPNCAYKTTQANKHIIVACEGNPYVPVHFDAS-----v<br>--keSRAKKFQRQHMDSDSsPSS-SSTYCNLMMLLRNMTQGRCKPVNTFVHEPLVDVQNVCFQEKVTCKNGQGNCYKSNSSMHITDCRLTNGSRYPNCAYRTSPKERHIIVACEGSPYVPVHFDASvedst-<br><br>BLAST: aliSize=100 (resi) iden=69.35% (86/124) simi=80.65% (100/124)<br>KETAAAKFERQHMDSSTSAASSSNYCNQMMKSRNLTKDRCKPVNTFVHESLADVQAVCSQKNVACKNGQTNCYQSYSTMSITDCRETGSSKYPNCAYKTTQANKHIIVACEGNPYVPVHFDASV----<br>KESRAKKFQRQHMDSDSSPSSSSTYCNLMMLLRNMTQGRCKPVNTFVHEPLVDVQNVCFQEKVTCKNGQGNCYKSNSSMHITDCRLTNGSRYPNCAYRTSPKERHIIVACEGSPYVPVHFDASVedst<br><br>Proposed: aliSize=124 (resi) RMSD=0.88 (Å)<br>KETAAAKFERQHMDSSTSAASSSNYCNQMMKSRNLTKDRCKPVNTFVHESLADVQAVCSQKNVACKNGQTNCYQSYSTMSITDCRETGSSKYPNCAYKTTQANKHIIVACEGNPYVPVHFDASV----<br>KESRAKKFQRQHMDSDSSPSSSSTYCNLMMLLRNMTQGRCKPVNTFVHEPLVDVQNVCFQEKVTCKNGQGNCYKSNSSMHITDCRLTNGSRYPNCAYRTSPKERHIIVACEGSPYVPVHFDASVedst
325	1js0A (124)<br>2e0mA (128)	C	69.35	TM-align: aliSize=115 (resi) RMSD=1.53 (Å)<br>KETAAAKFERQHMDSSTSAASSSNYCNQMMKSRNLTKDRCKPVNTFVHESLADVQAVCSQKNVACKNGQTNCYQSYSTMSITDCRETGSSKYPNCAYKTTQANKHIIVACEGNPYvpvhfdasv-------------<br>KESRAKKFQRQHMDSDSSPSSSSLYCNLMMLLRNMTQGRCKPVNTFVHEPLVDVQNVCFQEKVTCKNGQGNCYKSNSSMHITDCRLTNGSRYPNCAYRTSPKERHIIVACEGSPY---------vpvhfdasvedst<br><br>SARST: aliSize=121 (resi) RMSD=6.27 (Å)<br>ke--TAAAKFERQHMDSSTSAASSSNYCNQMMKSRNLTKDRCKPVNTFVHESLADVQAVCSQKNVACKNGQTNCYQSYSTMSITDCRETGSSKYPNCAYKTTQANKHIIVACEGNPYVPVHFDAS-----v<br>--keSRAKKFQRQHMDSDSSPSSSSLYCNLMMLLRNMTQGRCKPVNTFVHEPLVDVQNVCFQEKVTCKNGQGNCYKSNSSMHITDCRLTNGSRYPNCAYRTSPKERHIIVACEGSPYVPVHFDASvedst-<br><br>BLAST: aliSize=100 (resi) iden=69.35% (86/124) simi=80.65% (100/124)<br>KETAAAKFERQHMDSSTSAASSSNYCNQMMKSRNLTKDRCKPVNTFVHESLADVQAVCSQKNVACKNGQTNCYQSYSTMSITDCRETGSSKYPNCAYKTTQANKHIIVACEGNPYVPVHFDASV----<br>KESRAKKFQRQHMDSDSSPSSSSLYCNLMMLLRNMTQGRCKPVNTFVHEPLVDVQNVCFQEKVTCKNGQGNCYKSNSSMHITDCRLTNGSRYPNCAYRTSPKERHIIVACEGSPYVPVHFDASVedst<br><br>Proposed: aliSize=124 (resi) RMSD=0.88 (Å)<br>KETAAAKFERQHMDSSTSAASSSNYCNQMMKSRNLTKDRCKPVNTFVHESLADVQAVCSQKNVACKNGQTNCYQSYSTMSITDCRETGSSKYPNCAYKTTQANKHIIVACEGNPYVPVHFDASV----<br>KESRAKKFQRQHMDSDSSPSSSSLYCNLMMLLRNMTQGRCKPVNTFVHEPLVDVQNVCFQEKVTCKNGQGNCYKSNSSMHITDCRLTNGSRYPNCAYRTSPKERHIIVACEGSPYVPVHFDASVedst
326	1js0A (124)<br>2e0oA (126)	C	68.55	TM-align: aliSize=113 (resi) RMSD=1.38 (Å)<br>KETAAAKFERQHMDSSTsAASS-SNYCNQMMKSRNLTKDRCKPVNTFVHESLADVQAVCSQKNVACKNGQTNCYQSYSTMSITDCRETGSSKYPNCAYKTTQANKHIIVACEGNPyvpvhfdasv-----------<br>KESRAKKFQRQHMDSDS-SPSSsSTYCNQMMRRRNMTQGRCKPVNTFVHEPLLLVQLVCLQEKVTCKNGQGNCYKSNSSMHITDCRLTNGSRYPNCAYRTSPKERHIIVACEGSP----------yvpvhfdasved<br><br>SARST: aliSize=122 (resi) RMSD=6.29 (Å)<br>k-ETAAAKFERQHMDSSTSAASSSNYCNQMMKSRNLTKDRCKPVNTFVHESLADVQAVCSQKNVACKNGQTNCYQSYSTMSITDCRETGSSKYPNCAYKTTQANKHIIVACEGNPYVPVHFDAS---v<br>-kESRAKKFQRQHMDSDSSPSSSSTYCNQMMRRRNMTQGRCKPVNTFVHEPLLLVQLVCLQEKVTCKNGQGNCYKSNSSMHITDCRLTNGSRYPNCAYRTSPKERHIIVACEGSPYVPVHFDASved-<br><br>BLAST: aliSize=101 (resi) iden=69.35% (86/124) simi=81.45% (101/124)<br>KETAAAKFERQHMDSSTSAASSSNYCNQMMKSRNLTKDRCKPVNTFVHESLADVQAVCSQKNVACKNGQTNCYQSYSTMSITDCRETGSSKYPNCAYKTTQANKHIIVACEGNPYVPVHFDASV--<br>KESRAKKFQRQHMDSDSSPSSSSTYCNQMMRRRNMTQGRCKPVNTFVHEPLLLVQLVCLQEKVTCKNGQGNCYKSNSSMHITDCRLTNGSRYPNCAYRTSPKERHIIVACEGSPYVPVHFDASVed<br><br>Proposed: aliSize=123 (resi) RMSD=1.02 (Å)<br>KETAAAKFERQHMDSSTsAASS-SNYCNQMMKSRNLTKDRCKPVNTFVHESLADVQAVCSQKNVACKNGQTNCYQSYSTMSITDCRETGSSKYPNCAYKTTQANKHIIVACEGNPYVPVHFDASV--<br>KESRAKKFQRQHMDSDS-SPSSsSTYCNQMMRRRNMTQGRCKPVNTFVHEPLLLVQLVCLQEKVTCKNGQGNCYKSNSSMHITDCRLTNGSRYPNCAYRTSPKERHIIVACEGSPYVPVHFDASVed

327	1js0A (124)<br>2e0oB (125)	C	69.35	TM-align: aliSize=115 (resi) RMSD=1.66 (Å)<br>-KETAAAKFERQHMDSSTSAASSSNYCNQMMKSRNLTKDRCKPVNTFVHESLADVQAVCSQKNVACKNGQTNCYQSYSTMSITDCRETGSSKYPNCAYKTTQANKHIIVACEGNPYvpvhfdasv---------<br>mKESRAKKFQRQHMDSDSSPSSSSTYCNQMMRRRNMTQGRCKPVNTFVHEPLLLVQLVCLQEKVTCKNGQGNCYKSNSSMHITDCRLTNGSRYPNCAYRTSPKERHIIVACEGSPY---------vpvhfdasv<br><br>SARST: aliSize=122 (resi) RMSD=6.30 (Å)<br>k--ETAAAKFERQHMDSSTSAASSSNYCNQMMKSRNLTKDRCKPVNTFVHESLADVQAVCSQKNVACKNGQTNCYQSYSTMSITDCRETGSSKYPNCAYKTTQANKHIIVACEGNPYVPVHFDAS-v<br>-mkESRAKKFQRQHMDSDSSPSSSSTYCNQMMRRRNMTQGRCKPVNTFVHEPLLLVQLVCLQEKVTCKNGQGNCYKSNSSMHITDCRLTNGSRYPNCAYRTSPKERHIIVACEGSPYVPVHFDASv-<br><br>BLAST: aliSize=101 (resi) iden=69.35% (86/124) simi=81.45% (101/124)<br>-KETAAAKFERQHMDSSTSAASSSNYCNQMMKSRNLTKDRCKPVNTFVHESLADVQAVCSQKNVACKNGQTNCYQSYSTMSITDCRETGSSKYPNCAYKTTQANKHIIVACEGNPYVPVHFDASV<br>mKESRAKKFQRQHMDSDSSPSSSSTYCNQMMRRRNMTQGRCKPVNTFVHEPLLLVQLVCLQEKVTCKNGQGNCYKSNSSMHITDCRLTNGSRYPNCAYRTSPKERHIIVACEGSPYVPVHFDASV<br><br>Proposed: aliSize=124 (resi) RMSD=1.12 (Å)<br>-KETAAAKFERQHMDSSTSAASSSNYCNQMMKSRNLTKDRCKPVNTFVHESLADVQAVCSQKNVACKNGQTNCYQSYSTMSITDCRETGSSKYPNCAYKTTQANKHIIVACEGNPYVPVHFDASV<br>mKESRAKKFQRQHMDSDSSPSSSSTYCNQMMRRRNMTQGRCKPVNTFVHEPLLLVQLVCLQEKVTCKNGQGNCYKSNSSMHITDCRLTNGSRYPNCAYRTSPKERHIIVACEGSPYVPVHFDASV
328	1js0A (124)<br>2hkyA (129)	C	41.94	TM-align: aliSize=105 (resi) RMSD=2.47 (Å)<br>---K-ETAAAKFERQHMDSSTsaaSssnYCNQMMKsrNLTK--DRCKPVNTFVHESLADVQAVCSQKNVACkNG-QTNCYQSYSTMSITDCRETgSSKYPNCAYKTTQANKHIIVACEGNPYvpvhfdasv-----------------<br>mkpKgMTSSQWFKIQHMQPSP---Q---ACNSAMK--NINKhtKRCKDLNTFLHEPFSSVAATCQTPKIAC-KNgDKNCHQSHGPVSLTMCKLT-SGKYPNCRYKEKRQNKSYVVACKPPQK---------kdsqqfhlvpvhldrvl<br><br>SARST: aliSize=113 (resi) RMSD=7.66 (Å)<br>keta-------AAKFERQHMDSSTSAASsSNYCNQMMKSRNltkdRCKPVNTFVHESLADVQAVCSQKNVACKNGQTNCYQSYSTMSITDCREtGSSKYPNCAYKTTQANKHIIVACEGNP--------YVPVHFDAS-v<br>----mkpkgmtSSQWFKIQHMQPSPQAC-NSAMKNINKHTK----RCKDLNTFLHEPFSSVAATCQTPKIACKNGDKNCHQSHGPVSLTMCKL-TSGKYPNCRYKEKRQNKSYVVACKPPQkkdsqqfhLVPVHLDRVl-<br><br>BLAST: aliSize=71 (resi) iden=43.55% (54/124) simi=57.26% (71/124)<br>ke------TAAAKFERQHMDSSTSAasssnyCNQMMKSRNLTKDRCKPVNTFVHESLADVQAVCSQKNVACKNGQTNCYQSYSTMSITDCRETgSSKYPNCAYKTTQANKHIIVAC------EGNPY--VPVHFD---asv<br>--mkpkgmTSSQWFKIQHMQPSPQA------CNSAMKNINKHTKRCKDLNTFLHEPFSSVAATCQTPKIACKNGDKNCHQSHGPVSLTMCKLT-SGKYPNCRYKEKRQNKSYVVACkppqkkDSQQFhlVPVHLDrvl---<br><br>Proposed: aliSize=112 (resi) RMSD=1.86 (Å)<br>---K-ETAAAKFERQHMDSS--tsaasssnYCNQMMKsrNLTK--DRCKPVNTFVHESLADVQAVCSQKNVACkNG-QTNCYQSYSTMSITDCRETgSSKYPNCAYKTTQANKHIIVACEGN-------P-YVPVHFDASV<br>mkpKgMTSSQWFKIQHMQPSpq--------ACNSAMK--NINKhtKRCKDLNTFLHEPFSSVAATCQTPKIAC-KNgDKNCHQSHGPVSLTMCKLT-SGKYPNCRYKEKRQNKSYVVACKPPqkkdsqqFhLVPVHLDRVL
329	1js0A (124)<br>2k11A (127)	C	69.35	TM-align: aliSize=113 (resi) RMSD=1.92 (Å)<br>KETAAAKFERQHMDSSTSAASSSNYCNQMMKSRNLTKDRCKPVNTFVHESLADVQAVCSQKNVACkN-GQTNCYQSYSTMSITDCRETGSSKYPNCAYKTTQANKHIIVACEGNPyvpvhfdasv-------------<br>KESRAKKFQRQHMDSDSSPSSSSTYCNQMMRRRNMTQGRCKPVNTFVHEPLVDVQNVCFQEKVTC-KnGQGNCYKSNSSMHITDCRLTNGSRYPNCAYRTSPKERHIIVACEGSP----------yvpvhfdasveds<br><br>SARST: aliSize=121 (resi) RMSD=5.81 (Å)<br>k-ETAAAKFERQHMDSSTSAASSSNYCNQMMKSRNLTKDRCKPVNTFVHESLADVQAVCSQKNVACKNGQTNCYQSYSTMSITDCRETGSSKYPNCAYKTTQANKHIIVACEGNPYVPVHFDA-----sv<br>-kESRAKKFQRQHMDSDSSPSSSSTYCNQMMRRRNMTQGRCKPVNTFVHEPLVDVQNVCFQEKVTCKNGQGNCYKSNSSMHITDCRLTNGSRYPNCAYRTSPKERHIIVACEGSPYVPVHFDAsveds--<br><br>BLAST: aliSize=102 (resi) iden=70.16% (87/124) simi=82.26% (102/124)<br>KETAAAKFERQHMDSSTSAASSSNYCNQMMKSRNLTKDRCKPVNTFVHESLADVQAVCSQKNVACKNGQTNCYQSYSTMSITDCRETGSSKYPNCAYKTTQANKHIIVACEGNPYVPVHFDASV---<br>KESRAKKFQRQHMDSDSSPSSSSTYCNQMMRRRNMTQGRCKPVNTFVHEPLVDVQNVCFQEKVTCKNGQGNCYKSNSSMHITDCRLTNGSRYPNCAYRTSPKERHIIVACEGSPYVPVHFDASVeds<br><br>Proposed: aliSize=123 (resi) RMSD=1.63 (Å)<br>k-ETAAAKFERQHMDSSTSAASSSNYCNQMMKSRNLTKDRCKPVNTFVHESLADVQAVCSQKNVACKNGQTNCYQSYSTMSITDCRETGSSKYPNCAYKTTQANKHIIVACEGNPYVPVHFDASV---<br>-kESRAKKFQRQHMDSDSSPSSSSTYCNQMMRRRNMTQGRCKPVNTFVHEPLVDVQNVCFQEKVTCKNGQGNCYKSNSSMHITDCRLTNGSRYPNCAYRTSPKERHIIVACEGSPYVPVHFDASVeds

330	1js0A (124)<br>2nuiA (124)	C	99.19	TM-align: aliSize=114 (resi) RMSD=1.06 (Å)<br>KETAAAKFERQHMDSSTSAASSSNYCNQMMKSRNLTKDRCKPVNTFVHESLADVQAVCSQKNVACKNGQTNCYQSYSTMSITDCRETGSSKYPNCAYKTTQANKHIIVACEGNPyvpvhfdasv----------<br>KETAAAKFERQHMDSSTSAASSSNYCNQMMKSRNLTKDRCKPVNTFVHESLADVQAVCSQKNVACKNGQTNCYQSYSTMSITACRETGSSKYPNCAYKTTQANKHIIVACEGNP----------yvpvhfdasv<br><br>SARST: aliSize=122 (resi) RMSD=6.25 (Å)<br>k-ETAAAKFERQHMDSSTSAASSSNYCNQMMKSRNLTKDRCKPVNTFVHESLADVQAVCSQKNVACKNGQTNCYQSYSTMSITDCRETGSSKYPNCAYKTTQANKHIIVACEGNPYVPVHFDAS-v<br>-kETAAAKFERQHMDSSTSAASSSNYCNQMMKSRNLTKDRCKPVNTFVHESLADVQAVCSQKNVACKNGQTNCYQSYSTMSITACRETGSSKYPNCAYKTTQANKHIIVACEGNPYVPVHFDASv-<br><br>BLAST: aliSize=123 (resi) iden=99.19% (123/124) simi=99.19% (123/124)<br>KETAAAKFERQHMDSSTSAASSSNYCNQMMKSRNLTKDRCKPVNTFVHESLADVQAVCSQKNVACKNGQTNCYQSYSTMSITDCRETGSSKYPNCAYKTTQANKHIIVACEGNPYVPVHFDASV<br>KETAAAKFERQHMDSSTSAASSSNYCNQMMKSRNLTKDRCKPVNTFVHESLADVQAVCSQKNVACKNGQTNCYQSYSTMSITACRETGSSKYPNCAYKTTQANKHIIVACEGNPYVPVHFDASV<br><br>Proposed: aliSize=124 (resi) RMSD=0.59 (Å)<br>KETAAAKFERQHMDSSTSAASSSNYCNQMMKSRNLTKDRCKPVNTFVHESLADVQAVCSQKNVACKNGQTNCYQSYSTMSITDCRETGSSKYPNCAYKTTQANKHIIVACEGNPYVPVHFDASV<br>KETAAAKFERQHMDSSTSAASSSNYCNQMMKSRNLTKDRCKPVNTFVHESLADVQAVCSQKNVACKNGQTNCYQSYSTMSITACRETGSSKYPNCAYKTTQANKHIIVACEGNPYVPVHFDASV
331	1js0A (124)<br>2op2A (124)	C	98.39	TM-align: aliSize=114 (resi) RMSD=1.21 (Å)<br>KETAAAKFERQHMDSSTSAASSSNYCNQMMKSRNLTKDRCKPVNTFVHESLADVQAVCSQKNVACKNGQTNCYQSYSTMSITDCRETGSSKYPNCAYKTTQANKHIIVACEGNPyvpvhfdasv----------<br>KETAAAKFERQHMDSSTSAASSSNYCNQMMKSRNLTKDRCKPCNTFVHESLADVQAVCSQKNVACKNGQTNCYQSYSTMSITDCCETGSSKYPNCAYKTTQANKHIIVACEGNP----------yvpvhfdasv<br><br>SARST: aliSize=122 (resi) RMSD=6.21 (Å)<br>k-ETAAAKFERQHMDSSTSAASSSNYCNQMMKSRNLTKDRCKPVNTFVHESLADVQAVCSQKNVACKNGQTNCYQSYSTMSITDCRETGSSKYPNCAYKTTQANKHIIVACEGNPYVPVHFDAS-v<br>-kETAAAKFERQHMDSSTSAASSSNYCNQMMKSRNLTKDRCKPCNTFVHESLADVQAVCSQKNVACKNGQTNCYQSYSTMSITDCCETGSSKYPNCAYKTTQANKHIIVACEGNPYVPVHFDASv-<br><br>BLAST: aliSize=122 (resi) iden=98.39% (122/124) simi=98.39% (122/124)<br>KETAAAKFERQHMDSSTSAASSSNYCNQMMKSRNLTKDRCKPVNTFVHESLADVQAVCSQKNVACKNGQTNCYQSYSTMSITDCRETGSSKYPNCAYKTTQANKHIIVACEGNPYVPVHFDASV<br>KETAAAKFERQHMDSSTSAASSSNYCNQMMKSRNLTKDRCKPCNTFVHESLADVQAVCSQKNVACKNGQTNCYQSYSTMSITDCCETGSSKYPNCAYKTTQANKHIIVACEGNPYVPVHFDASV<br><br>Proposed: aliSize=124 (resi) RMSD=0.79 (Å)<br>KETAAAKFERQHMDSSTSAASSSNYCNQMMKSRNLTKDRCKPVNTFVHESLADVQAVCSQKNVACKNGQTNCYQSYSTMSITDCRETGSSKYPNCAYKTTQANKHIIVACEGNPYVPVHFDASV<br>KETAAAKFERQHMDSSTSAASSSNYCNQMMKSRNLTKDRCKPCNTFVHESLADVQAVCSQKNVACKNGQTNCYQSYSTMSITDCCETGSSKYPNCAYKTTQANKHIIVACEGNPYVPVHFDASV
332	1js0A (124)<br>2q4gX (126)	C	69.35	TM-align: aliSize=114 (resi) RMSD=1.50 (Å)<br>kETAAAKFERQHMDSSTSAASSSNYCNQMMKSRNLTKDRCKPVNTFVHESLADVQAVCSQKNVACKNGQTNCYQSYSTMSITDCRETGSSKYPNCAYKTTQANKHIIVACEGNPYvpvhfdasv-----------<br>-ESRAKKFQRQHMDSDSSPSSSSTYCNQMMRRRNMTQGRCKPVNTFVHEPLVDVQNVCFQEKVTCKNGQGNCYKSNSSMHITDCRLTNGSRYPNCAYRTSPKERHIIVACEGSPY---------vpvhfdasveds<br><br>SARST: aliSize=121 (resi) RMSD=6.30 (Å)<br>ke-TAAAKFERQHMDSSTSAASSSNYCNQMMKSRNLTKDRCKPVNTFVHESLADVQAVCSQKNVACKNGQTNCYQSYSTMSITDCRETGSSKYPNCAYKTTQANKHIIVACEGNPYVPVHFDAS----v<br>--eSRAKKFQRQHMDSDSSPSSSSTYCNQMMRRRNMTQGRCKPVNTFVHEPLVDVQNVCFQEKVTCKNGQGNCYKSNSSMHITDCRLTNGSRYPNCAYRTSPKERHIIVACEGSPYVPVHFDASveds-<br><br>BLAST: aliSize=101 (resi) iden=69.35% (86/124) simi=81.45% (101/124)<br>kETAAAKFERQHMDSSTSAASSSNYCNQMMKSRNLTKDRCKPVNTFVHESLADVQAVCSQKNVACKNGQTNCYQSYSTMSITDCRETGSSKYPNCAYKTTQANKHIIVACEGNPYVPVHFDASV---<br>-ESRAKKFQRQHMDSDSSPSSSSTYCNQMMRRRNMTQGRCKPVNTFVHEPLVDVQNVCFQEKVTCKNGQGNCYKSNSSMHITDCRLTNGSRYPNCAYRTSPKERHIIVACEGSPYVPVHFDASVeds<br><br>Proposed: aliSize=123 (resi) RMSD=0.84 (Å)<br>kETAAAKFERQHMDSSTSAASSSNYCNQMMKSRNLTKDRCKPVNTFVHESLADVQAVCSQKNVACKNGQTNCYQSYSTMSITDCRETGSSKYPNCAYKTTQANKHIIVACEGNPYVPVHFDASV---<br>-ESRAKKFQRQHMDSDSSPSSSSTYCNQMMRRRNMTQGRCKPVNTFVHEPLVDVQNVCFQEKVTCKNGQGNCYKSNSSMHITDCRLTNGSRYPNCAYRTSPKERHIIVACEGSPYVPVHFDASVeds

333	1js0A (124)<br>3dh6A (124)	C	99.19	TM-align: aliSize=114 (resi) RMSD=1.07 (Å)<br>KETAAAKFERQHMDSSTSAASSSNYCNQMMKSRNLTKDRCKPVNTFVHESLADVQAVCSQKNVACKNGQTNCYQSYSTMSITDCRETGSSKYPNCAYKTTQANKHIIVACEGNPyvpvhfdasv----------<br>KETAAAKFERQHMDSSTSAASSSNYCNQMMKSRNLTKDRCKPVNTFAHESLADVQAVCSQKNVACKNGQTNCYQSYSTMSITDCRETGSSKYPNCAYKTTQANKHIIVACEGNP----------yvpvhfdasv<br><br>SARST: aliSize=122 (resi) RMSD=6.23 (Å)<br>k-ETAAAKFERQHMDSSTSAASSSNYCNQMMKSRNLTKDRCKPVNTFVHESLADVQAVCSQKNVACKNGQTNCYQSYSTMSITDCRETGSSKYPNCAYKTTQANKHIIVACEGNPYVPVHFDAS-v<br>-kETAAAKFERQHMDSSTSAASSSNYCNQMMKSRNLTKDRCKPVNTFAHESLADVQAVCSQKNVACKNGQTNCYQSYSTMSITDCRETGSSKYPNCAYKTTQANKHIIVACEGNPYVPVHFDASv-<br><br>BLAST: aliSize=123 (resi) iden=99.19% (123/124) simi=99.19% (123/124)<br>KETAAAKFERQHMDSSTSAASSSNYCNQMMKSRNLTKDRCKPVNTFVHESLADVQAVCSQKNVACKNGQTNCYQSYSTMSITDCRETGSSKYPNCAYKTTQANKHIIVACEGNPYVPVHFDASV<br>KETAAAKFERQHMDSSTSAASSSNYCNQMMKSRNLTKDRCKPVNTFAHESLADVQAVCSQKNVACKNGQTNCYQSYSTMSITDCRETGSSKYPNCAYKTTQANKHIIVACEGNPYVPVHFDASV<br><br>Proposed: aliSize=124 (resi) RMSD=0.62 (Å)<br>KETAAAKFERQHMDSSTSAASSSNYCNQMMKSRNLTKDRCKPVNTFVHESLADVQAVCSQKNVACKNGQTNCYQSYSTMSITDCRETGSSKYPNCAYKTTQANKHIIVACEGNPYVPVHFDASV<br>KETAAAKFERQHMDSSTSAASSSNYCNQMMKSRNLTKDRCKPVNTFAHESLADVQAVCSQKNVACKNGQTNCYQSYSTMSITDCRETGSSKYPNCAYKTTQANKHIIVACEGNPYVPVHFDASV
334	1js0A (124)<br>3di7A (124)	C	99.19	TM-align: aliSize=114 (resi) RMSD=1.11 (Å)<br>KETAAAKFERQHMDSSTSAASSSNYCNQMMKSRNLTKDRCKPVNTFVHESLADVQAVCSQKNVACKNGQTNCYQSYSTMSITDCRETGSSKYPNCAYKTTQANKHIIVACEGNPyvpvhfdasv----------<br>KETAAAKFERQHMDSSTSAASSSNYCNQMMKSRNLTKDRCKPVNTFVHESLADAQAVCSQKNVACKNGQTNCYQSYSTMSITDCRETGSSKYPNCAYKTTQANKHIIVACEGNP----------yvpvhfdasv<br><br>SARST: aliSize=122 (resi) RMSD=6.25 (Å)<br>k-ETAAAKFERQHMDSSTSAASSSNYCNQMMKSRNLTKDRCKPVNTFVHESLADVQAVCSQKNVACKNGQTNCYQSYSTMSITDCRETGSSKYPNCAYKTTQANKHIIVACEGNPYVPVHFDAS-v<br>-kETAAAKFERQHMDSSTSAASSSNYCNQMMKSRNLTKDRCKPVNTFVHESLADAQAVCSQKNVACKNGQTNCYQSYSTMSITDCRETGSSKYPNCAYKTTQANKHIIVACEGNPYVPVHFDASv-<br><br>BLAST: aliSize=123 (resi) iden=99.19% (123/124) simi=99.19% (123/124)<br>KETAAAKFERQHMDSSTSAASSSNYCNQMMKSRNLTKDRCKPVNTFVHESLADVQAVCSQKNVACKNGQTNCYQSYSTMSITDCRETGSSKYPNCAYKTTQANKHIIVACEGNPYVPVHFDASV<br>KETAAAKFERQHMDSSTSAASSSNYCNQMMKSRNLTKDRCKPVNTFVHESLADAQAVCSQKNVACKNGQTNCYQSYSTMSITDCRETGSSKYPNCAYKTTQANKHIIVACEGNPYVPVHFDASV<br><br>Proposed: aliSize=124 (resi) RMSD=0.64 (Å)<br>KETAAAKFERQHMDSSTSAASSSNYCNQMMKSRNLTKDRCKPVNTFVHESLADVQAVCSQKNVACKNGQTNCYQSYSTMSITDCRETGSSKYPNCAYKTTQANKHIIVACEGNPYVPVHFDASV<br>KETAAAKFERQHMDSSTSAASSSNYCNQMMKSRNLTKDRCKPVNTFVHESLADAQAVCSQKNVACKNGQTNCYQSYSTMSITDCRETGSSKYPNCAYKTTQANKHIIVACEGNPYVPVHFDASV
335	1js0A (124)<br>3di8A (124)	C	99.19	TM-align: aliSize=115 (resi) RMSD=1.38 (Å)<br>KETAAAKFERQHMDSSTSAASSSNYCNQMMKSRNLTKDRCKPVNTFVHESLADVQAVCSQKNVACKNGQTNCYQSYSTMSITDCRETGSSKYPNCAYKTTQANKHIIVACEGNPYvpvhfdasv---------<br>KETAAAKFERQHMDSSTSAASSSNYCNQMMKSRNLTKDRCKPVNTFVHESLADVQAACSQKNVACKNGQTNCYQSYSTMSITDCRETGSSKYPNCAYKTTQANKHIIVACEGNPY---------vpvhfdasv<br><br>SARST: aliSize=122 (resi) RMSD=6.25 (Å)<br>k-ETAAAKFERQHMDSSTSAASSSNYCNQMMKSRNLTKDRCKPVNTFVHESLADVQAVCSQKNVACKNGQTNCYQSYSTMSITDCRETGSSKYPNCAYKTTQANKHIIVACEGNPYVPVHFDAS-v<br>-kETAAAKFERQHMDSSTSAASSSNYCNQMMKSRNLTKDRCKPVNTFVHESLADVQAACSQKNVACKNGQTNCYQSYSTMSITDCRETGSSKYPNCAYKTTQANKHIIVACEGNPYVPVHFDASv-<br><br>BLAST: aliSize=123 (resi) iden=99.19% (123/124) simi=99.19% (123/124)<br>KETAAAKFERQHMDSSTSAASSSNYCNQMMKSRNLTKDRCKPVNTFVHESLADVQAVCSQKNVACKNGQTNCYQSYSTMSITDCRETGSSKYPNCAYKTTQANKHIIVACEGNPYVPVHFDASV<br>KETAAAKFERQHMDSSTSAASSSNYCNQMMKSRNLTKDRCKPVNTFVHESLADVQAACSQKNVACKNGQTNCYQSYSTMSITDCRETGSSKYPNCAYKTTQANKHIIVACEGNPYVPVHFDASV<br><br>Proposed: aliSize=124 (resi) RMSD=0.59 (Å)<br>KETAAAKFERQHMDSSTSAASSSNYCNQMMKSRNLTKDRCKPVNTFVHESLADVQAVCSQKNVACKNGQTNCYQSYSTMSITDCRETGSSKYPNCAYKTTQANKHIIVACEGNPYVPVHFDASV<br>KETAAAKFERQHMDSSTSAASSSNYCNQMMKSRNLTKDRCKPVNTFVHESLADVQAACSQKNVACKNGQTNCYQSYSTMSITDCRETGSSKYPNCAYKTTQANKHIIVACEGNPYVPVHFDASV

336	1js0A (124)<br>3di9A (124)	C	99.19	TM-align: aliSize=114 (resi) RMSD=1.13 (Å)<br>KETAAAKFERQHMDSSTSAASSSNYCNQMMKSRNLTKDRCKPVNTFVHESLADVQAVCSQKNVACKNGQTNCYQSYSTMSITDCRETGSSKYPNCAYKTTQANKHIIVACEGNPyvpvhfdasv----------<br>KETAAAKFERQHMDSSTSAASSSNYCNQMMKSRNLTKDRCKPVNTFVHESLADVQAVCSQKNVACKNGQTNCYQSYSTMSATDCRETGSSKYPNCAYKTTQANKHIIVACEGNP----------yvpvhfdasv<br><br>SARST: aliSize=121 (resi) RMSD=5.50 (Å)<br>k-ETAAAKFERQHMDSSTSAASSSNYCNQMMKSRNLTKDRCKPVNTFVHESLADVQAVCSQKNVACKNGQTNCYQSYSTMSITDCRETGSSKYPNCAYKTTQANKHIIVACEGNPYVPVHFDA--sv<br>-kETAAAKFERQHMDSSTSAASSSNYCNQMMKSRNLTKDRCKPVNTFVHESLADVQAVCSQKNVACKNGQTNCYQSYSTMSATDCRETGSSKYPNCAYKTTQANKHIIVACEGNPYVPVHFDAsv--<br><br>BLAST: aliSize=123 (resi) iden=99.19% (123/124) simi=99.19% (123/124)<br>KETAAAKFERQHMDSSTSAASSSNYCNQMMKSRNLTKDRCKPVNTFVHESLADVQAVCSQKNVACKNGQTNCYQSYSTMSITDCRETGSSKYPNCAYKTTQANKHIIVACEGNPYVPVHFDASV<br>KETAAAKFERQHMDSSTSAASSSNYCNQMMKSRNLTKDRCKPVNTFVHESLADVQAVCSQKNVACKNGQTNCYQSYSTMSATDCRETGSSKYPNCAYKTTQANKHIIVACEGNPYVPVHFDASV<br><br>Proposed: aliSize=124 (resi) RMSD=0.71 (Å)<br>KETAAAKFERQHMDSSTSAASSSNYCNQMMKSRNLTKDRCKPVNTFVHESLADVQAVCSQKNVACKNGQTNCYQSYSTMSITDCRETGSSKYPNCAYKTTQANKHIIVACEGNPYVPVHFDASV<br>KETAAAKFERQHMDSSTSAASSSNYCNQMMKSRNLTKDRCKPVNTFVHESLADVQAVCSQKNVACKNGQTNCYQSYSTMSATDCRETGSSKYPNCAYKTTQANKHIIVACEGNPYVPVHFDASV
337	1js0A (124)<br>3dibA (124)	C	99.19	TM-align: aliSize=115 (resi) RMSD=1.40 (Å)<br>KETAAAKFERQHMDSSTSAASSSNYCNQMMKSRNLTKDRCKPVNTFVHESLADVQAVCSQKNVACKNGQTNCYQSYSTMSITDCRETGSSKYPNCAYKTTQANKHIIVACEGNPYvpvhfdasv---------<br>KETAAAKFERQHMDSSTSAASSSNYCNQMMKSRNLTKDRCKPVNTFVHESLADVQAVCSQKNVACKNGQTNCYQSYSTMSITDCRETGSSKYPNCAYKTTQANKHAIVACEGNPY---------vpvhfdasv<br><br>SARST: aliSize=121 (resi) RMSD=6.28 (Å)<br>ke--TAAAKFERQHMDSSTSAASSSNYCNQMMKSRNLTKDRCKPVNTFVHESLADVQAVCSQKNVACKNGQTNCYQSYSTMSITDCRETGSSKYPNCAYKTTQANKHIIVACEGNPYVPVHFDAS-v<br>--keTAAAKFERQHMDSSTSAASSSNYCNQMMKSRNLTKDRCKPVNTFVHESLADVQAVCSQKNVACKNGQTNCYQSYSTMSITDCRETGSSKYPNCAYKTTQANKHAIVACEGNPYVPVHFDASv-<br><br>BLAST: aliSize=123 (resi) iden=99.19% (123/124) simi=99.19% (123/124)<br>KETAAAKFERQHMDSSTSAASSSNYCNQMMKSRNLTKDRCKPVNTFVHESLADVQAVCSQKNVACKNGQTNCYQSYSTMSITDCRETGSSKYPNCAYKTTQANKHIIVACEGNPYVPVHFDASV<br>KETAAAKFERQHMDSSTSAASSSNYCNQMMKSRNLTKDRCKPVNTFVHESLADVQAVCSQKNVACKNGQTNCYQSYSTMSITDCRETGSSKYPNCAYKTTQANKHAIVACEGNPYVPVHFDASV<br><br>Proposed: aliSize=124 (resi) RMSD=0.63 (Å)<br>KETAAAKFERQHMDSSTSAASSSNYCNQMMKSRNLTKDRCKPVNTFVHESLADVQAVCSQKNVACKNGQTNCYQSYSTMSITDCRETGSSKYPNCAYKTTQANKHIIVACEGNPYVPVHFDASV<br>KETAAAKFERQHMDSSTSAASSSNYCNQMMKSRNLTKDRCKPVNTFVHESLADVQAVCSQKNVACKNGQTNCYQSYSTMSITDCRETGSSKYPNCAYKTTQANKHAIVACEGNPYVPVHFDASV
338	1js0A (124)<br>3dicA (124)	C	99.19	TM-align: aliSize=114 (resi) RMSD=1.08 (Å)<br>KETAAAKFERQHMDSSTSAASSSNYCNQMMKSRNLTKDRCKPVNTFVHESLADVQAVCSQKNVACKNGQTNCYQSYSTMSITDCRETGSSKYPNCAYKTTQANKHIIVACEGNPyvpvhfdasv----------<br>KETAAAKFERQHMDSSTSAASSSNYCNQMMKSRNLTKDRCKPVNTFVHESLADVQAVCSQKNVACKNGQTNCYQSYSTMSITDCRETGSSKYPNCAYKTTQANKHIIAACEGNP----------yvpvhfdasv<br><br>SARST: aliSize=122 (resi) RMSD=6.23 (Å)<br>k-ETAAAKFERQHMDSSTSAASSSNYCNQMMKSRNLTKDRCKPVNTFVHESLADVQAVCSQKNVACKNGQTNCYQSYSTMSITDCRETGSSKYPNCAYKTTQANKHIIVACEGNPYVPVHFDAS-v<br>-kETAAAKFERQHMDSSTSAASSSNYCNQMMKSRNLTKDRCKPVNTFVHESLADVQAVCSQKNVACKNGQTNCYQSYSTMSITDCRETGSSKYPNCAYKTTQANKHIIAACEGNPYVPVHFDASv-<br><br>BLAST: aliSize=123 (resi) iden=99.19% (123/124) simi=99.19% (123/124)<br>KETAAAKFERQHMDSSTSAASSSNYCNQMMKSRNLTKDRCKPVNTFVHESLADVQAVCSQKNVACKNGQTNCYQSYSTMSITDCRETGSSKYPNCAYKTTQANKHIIVACEGNPYVPVHFDASV<br>KETAAAKFERQHMDSSTSAASSSNYCNQMMKSRNLTKDRCKPVNTFVHESLADVQAVCSQKNVACKNGQTNCYQSYSTMSITDCRETGSSKYPNCAYKTTQANKHIIAACEGNPYVPVHFDASV<br><br>Proposed: aliSize=124 (resi) RMSD=0.61 (Å)<br>KETAAAKFERQHMDSSTSAASSSNYCNQMMKSRNLTKDRCKPVNTFVHESLADVQAVCSQKNVACKNGQTNCYQSYSTMSITDCRETGSSKYPNCAYKTTQANKHIIVACEGNPYVPVHFDASV<br>KETAAAKFERQHMDSSTSAASSSNYCNQMMKSRNLTKDRCKPVNTFVHESLADVQAVCSQKNVACKNGQTNCYQSYSTMSITDCRETGSSKYPNCAYKTTQANKHIIAACEGNPYVPVHFDASV

339	1js0A<br>(124)<br><br>3rsdA<br>(124)	C	99.19	TM-align: aliSize=114 (resi) RMSD=1.08 (Å)<br>KETAAAKFERQHMDSSTSAASSSNYCNQMMKSRNLTKDRCKPVNTFVHESLADVQAVCSQKNVACKNGQTNCYQSYSTMSITDCRETGSSKYPNCAYKTTQANKHIIVACEGNPyvpvhfdasv----------<br>KETAAAKFERQHMDSSTSAASSSNYCNQMMKSRNLTKDRCKPVNTFVHESLADVQAVCSQKNVACKNGQTNCYQSYSTMSITDCRETGSSKYPNCAYKTTQANKHIIVACEGNP----------yvpvhfnasv<br><br>SARST: aliSize=122 (resi) RMSD=6.24 (Å)<br>k-ETAAAKFERQHMDSSTSAASSSNYCNQMMKSRNLTKDRCKPVNTFVHESLADVQAVCSQKNVACKNGQTNCYQSYSTMSITDCRETGSSKYPNCAYKTTQANKHIIVACEGNPYVPVHFDAS-v<br>-kETAAAKFERQHMDSSTSAASSSNYCNQMMKSRNLTKDRCKPVNTFVHESLADVQAVCSQKNVACKNGQTNCYQSYSTMSITDCRETGSSKYPNCAYKTTQANKHIIVACEGNPYVPVHFNASv-<br><br>BLAST: aliSize=124 (resi) iden=99.19% (123/124) simi=100.00% (124/124)<br>KETAAAKFERQHMDSSTSAASSSNYCNQMMKSRNLTKDRCKPVNTFVHESLADVQAVCSQKNVACKNGQTNCYQSYSTMSITDCRETGSSKYPNCAYKTTQANKHIIVACEGNPYVPVHFDASV<br>KETAAAKFERQHMDSSTSAASSSNYCNQMMKSRNLTKDRCKPVNTFVHESLADVQAVCSQKNVACKNGQTNCYQSYSTMSITDCRETGSSKYPNCAYKTTQANKHIIVACEGNPYVPVHFNASV<br><br>Proposed: aliSize=124 (resi) RMSD=0.62 (Å)<br>KETAAAKFERQHMDSSTSAASSSNYCNQMMKSRNLTKDRCKPVNTFVHESLADVQAVCSQKNVACKNGQTNCYQSYSTMSITDCRETGSSKYPNCAYKTTQANKHIIVACEGNPYVPVHFDASV<br>KETAAAKFERQHMDSSTSAASSSNYCNQMMKSRNLTKDRCKPVNTFVHESLADVQAVCSQKNVACKNGQTNCYQSYSTMSITDCRETGSSKYPNCAYKTTQANKHIIVACEGNPYVPVHFNASV
340	1js0A<br>(124)<br><br>3rskA<br>(124)	C	97.58	TM-align: aliSize=115 (resi) RMSD=1.34 (Å)<br>KETAAAKFERQHMDSSTSAASSSNYCNQMMKSRNLTKDRCKPVNTFVHESLADVQAVCSQKNVACKNGQTNCYQSYSTMSITDCRETGSSKYPNCAYKTTQANKHIIVACEGNPYvpvhfdasv---------<br>KETAAAAFEAQHMDSSTSAASSSNYCNQMMKSRNLTKDRCKPVNTFVHESLADVQAVCSQKNVACANGQTNCYQSYSTMSITDCRETGSSKYPNCAYKTTQANKHIIVACEGNPY---------vpvhfdasv<br><br>SARST: aliSize=122 (resi) RMSD=6.22 (Å)<br>k-ETAAAKFERQHMDSSTSAASSSNYCNQMMKSRNLTKDRCKPVNTFVHESLADVQAVCSQKNVACKNGQTNCYQSYSTMSITDCRETGSSKYPNCAYKTTQANKHIIVACEGNPYVPVHFDAS-v<br>-kETAAAAFEAQHMDSSTSAASSSNYCNQMMKSRNLTKDRCKPVNTFVHESLADVQAVCSQKNVACANGQTNCYQSYSTMSITDCRETGSSKYPNCAYKTTQANKHIIVACEGNPYVPVHFDASv-<br><br>BLAST: aliSize=121 (resi) iden=97.58% (121/124) simi=97.58% (121/124)<br>KETAAAKFERQHMDSSTSAASSSNYCNQMMKSRNLTKDRCKPVNTFVHESLADVQAVCSQKNVACKNGQTNCYQSYSTMSITDCRETGSSKYPNCAYKTTQANKHIIVACEGNPYVPVHFDASV<br>KETAAAAFEAQHMDSSTSAASSSNYCNQMMKSRNLTKDRCKPVNTFVHESLADVQAVCSQKNVACANGQTNCYQSYSTMSITDCRETGSSKYPNCAYKTTQANKHIIVACEGNPYVPVHFDASV<br><br>Proposed: aliSize=124 (resi) RMSD=0.53 (Å)<br>KETAAAKFERQHMDSSTSAASSSNYCNQMMKSRNLTKDRCKPVNTFVHESLADVQAVCSQKNVACKNGQTNCYQSYSTMSITDCRETGSSKYPNCAYKTTQANKHIIVACEGNPYVPVHFDASV<br>KETAAAAFEAQHMDSSTSAASSSNYCNQMMKSRNLTKDRCKPVNTFVHESLADVQAVCSQKNVACANGQTNCYQSYSTMSITDCRETGSSKYPNCAYKTTQANKHIIVACEGNPYVPVHFDASV
341	1js0A<br>(124)<br><br>3rspA<br>(124)	C	99.19	TM-align: aliSize=115 (resi) RMSD=1.41 (Å)<br>KETAAAKFERQHMDSSTSAASSSNYCNQMMKSRNLTKDRCKPVNTFVHESLADVQAVCSQKNVACKNGQTNCYQSYSTMSITDCRETGSSKYPNCAYKTTQANKHIIVACEGNPYvpvhfdasv---------<br>KETAAAKFERQHMDSSTSAASSSNYCNQMMKSRNLTKDRCKPVNTFVHESLADVQAVCSQKNVACKNGQTNCYQSYSTMSITDCRETGSSKYGNCAYKTTQANKHIIVACEGNPY---------vpvhfdasv<br><br>SARST: aliSize=122 (resi) RMSD=6.23 (Å)<br>k-ETAAAKFERQHMDSSTSAASSSNYCNQMMKSRNLTKDRCKPVNTFVHESLADVQAVCSQKNVACKNGQTNCYQSYSTMSITDCRETGSSKYPNCAYKTTQANKHIIVACEGNPYVPVHFDAS-v<br>-kETAAAKFERQHMDSSTSAASSSNYCNQMMKSRNLTKDRCKPVNTFVHESLADVQAVCSQKNVACKNGQTNCYQSYSTMSITDCRETGSSKYGNCAYKTTQANKHIIVACEGNPYVPVHFDASv-<br><br>BLAST: aliSize=123 (resi) iden=99.19% (123/124) simi=99.19% (123/124)<br>KETAAAKFERQHMDSSTSAASSSNYCNQMMKSRNLTKDRCKPVNTFVHESLADVQAVCSQKNVACKNGQTNCYQSYSTMSITDCRETGSSKYPNCAYKTTQANKHIIVACEGNPYVPVHFDASV<br>KETAAAKFERQHMDSSTSAASSSNYCNQMMKSRNLTKDRCKPVNTFVHESLADVQAVCSQKNVACKNGQTNCYQSYSTMSITDCRETGSSKYGNCAYKTTQANKHIIVACEGNPYVPVHFDASV<br><br>Proposed: aliSize=124 (resi) RMSD=0.69 (Å)<br>KETAAAKFERQHMDSSTSAASSSNYCNQMMKSRNLTKDRCKPVNTFVHESLADVQAVCSQKNVACKNGQTNCYQSYSTMSITDCRETGSSKYPNCAYKTTQANKHIIVACEGNPYVPVHFDASV<br>KETAAAKFERQHMDSSTSAASSSNYCNQMMKSRNLTKDRCKPVNTFVHESLADVQAVCSQKNVACKNGQTNCYQSYSTMSITDCRETGSSKYGNCAYKTTQANKHIIVACEGNPYVPVHFDASV

342	1js0A (124)<br>4rsdA (124)	C	99.19	TM-align: aliSize=115 (resi) RMSD=1.41 (Å)<br>KETAAAKFERQHMDSSTSAASSSNYCNQMMKSRNLTKDRCKPVNTFVHESLADVQAVCSQKNVACKNGQTNCYQSYSTMSITDCRETGSSKYPNCAYKTTQANKHIIVACEGNPYvpvhfdasv---------<br>KETAAAKFERQHMDSSTSAASSSNYCNQMMKSRNLTKDRCKPVNTFVHESLADVQAVCSQKNVACKNGQTNCYQSYSTMSITDCRETGSSKYPNCAYKTTQANKHIIVACEGNPY---------vpvhfaasv<br><br>SARST: aliSize=122 (resi) RMSD=6.24 (Å)<br>k-ETAAAKFERQHMDSSTSAASSSNYCNQMMKSRNLTKDRCKPVNTFVHESLADVQAVCSQKNVACKNGQTNCYQSYSTMSITDCRETGSSKYPNCAYKTTQANKHIIVACEGNPYVPVHFDAS-v<br>-kETAAAKFERQHMDSSTSAASSSNYCNQMMKSRNLTKDRCKPVNTFVHESLADVQAVCSQKNVACKNGQTNCYQSYSTMSITDCRETGSSKYPNCAYKTTQANKHIIVACEGNPYVPVHFAASv-<br><br>BLAST: aliSize=123 (resi) iden=99.19% (123/124) simi=99.19% (123/124)<br>KETAAAKFERQHMDSSTSAASSSNYCNQMMKSRNLTKDRCKPVNTFVHESLADVQAVCSQKNVACKNGQTNCYQSYSTMSITDCRETGSSKYPNCAYKTTQANKHIIVACEGNPYVPVHFDASV<br>KETAAAKFERQHMDSSTSAASSSNYCNQMMKSRNLTKDRCKPVNTFVHESLADVQAVCSQKNVACKNGQTNCYQSYSTMSITDCRETGSSKYPNCAYKTTQANKHIIVACEGNPYVPVHFAASV<br><br>Proposed: aliSize=124 (resi) RMSD=0.72 (Å)<br>KETAAAKFERQHMDSSTSAASSSNYCNQMMKSRNLTKDRCKPVNTFVHESLADVQAVCSQKNVACKNGQTNCYQSYSTMSITDCRETGSSKYPNCAYKTTQANKHIIVACEGNPYVPVHFDASV<br>KETAAAKFERQHMDSSTSAASSSNYCNQMMKSRNLTKDRCKPVNTFVHESLADVQAVCSQKNVACKNGQTNCYQSYSTMSITDCRETGSSKYPNCAYKTTQANKHIIVACEGNPYVPVHFAASV
343	1lgqA (112)<br>1qu5A (182)	C	19.64	TM-align: aliSize=74 (resi) RMSD=2.63 (Å)<br>-------------------------MQPWGRLLRLGAeeg---EPHVLLR--KREWTIGRRRGCDLSFPsNKLVSGDHCRIVVDeKS--------------GQVTLEDTSTSGTVINK--------------L--------------------------------KVVkkqTcplqtgdviylvyrkne<br>eaetreqkllhsnntenvksskkkgNGRFLTLKPLPD---siiQESLEIQqgVNPFFIGRSEDCNCKIE-DNRLSRVHCFIFKK-RHavgksmyespaqglDDIWYCHTGTNVSYLNNnrmiqgtkfllqdgDeikiiwdknnkfvigfkveindttglfneglgmLQE---Q-----------------<br><br>pehnvaylyesls-----------------<br>-------------rvvlkqtaeekdlvkkl<br><br>SARST: aliSize=100 (resi) RMSD=15.61 (Å)<br>mqpwgr-----------------------------LLRLGA----EEGEPHVLLRKREWTIGRRRGCDLS--FPSNK-----------LVSGDHCRIVVDEKsGQVTLEDTSTSGTVINKLKVVKKQTCPLQTGDVIYLVYRknePEHNVAYLYES------------------------------------<br>------eaetreqkllhsnntenvksskkkgngrfLTLKPLpdsiIQESLEIQQGVNPFFIGRSEDCNCKieDNRLSrvhcfifkkrhAVGKSMYESPAQGL-DDIWYCHTGTNVSYLNNNRMIQGTKFLLQDGDEIKIIWD---KNNKFVIGFKVeindttglfneglgmlqeqrvvlkqtaeekdlvk<br><br>--ls<br>kl--<br><br>BLAST: aliSize=39 (resi) iden=21.43% (24/112) simi=34.82% (39/112)<br>mqpwgrllrlgaeegephvllrkrewt---IGRRRGCDLSFPSNKLvSGDHC-----RIVVDE---KSGQVTLED-----TSTSGTVINKLKVVKKQTCPLQTGDVIYLVYRKN--------------------------<br>---------------------------eaetreqkllhsnntenvksskkkgngrfltlkplpdsiiqesleiqqgvnpffIGRSEDCNCKIEDNRL-SRVHCfifkkRHAVGKsmyESPAQGLDDiwychTGTNVSYLNNNRMIQGTKFLLQDGDEIKIIWDKNnkfvigfkveindttglfneglgml<br><br>--------------------epehnvaylyesls<br>qeqrvvlkqtaeekdlvkkl--------------<br><br>Proposed: aliSize=104 (resi) RMSD=1.87 (Å)<br>-------------------------MQPWGRLLRLG----AeegEpHVLLR--KREWTIGRRRGCDLSFPsNKLVSGDHCRIVVDeKS--------------GQVTLEDTSTSGTVINkLKVVK-KQTCPLQTGDVIYLVYRKNEpEHNVAYLYESL-S--------------------------------<br>eaetreqkllhsnntenvksskkkgNGRFLTLKPLPdsiiQ---E-SLEIQqgVNPFFIGRSEDCNCKIE-DNRLSRVHCFIFKK-RHavgksmyespaqglDDIWYCHTGTNVSYLN-NNRMIqGTKFLLQDGDEIKIIWDKNN-KFVIGFKVEINdTtglfneglgmlqeqrvvlkqtaeekdlvkkl
344	1lr5A (160)<br>1dgwA (178)	C	10.63	TM-align: aliSize=127 (resi) RMSD=3.04 (Å)<br>scvrd-NSLVRDIsQMPQSSYGIEGLSHITVAGAlNHG------MKEVEVWLQTISPGQRTPIHRHSCEEVFTVLKGKGTLLMGSsSLkypGqPQEIPFFQNTTFSIPVNDPHQVWNSDEHEDLQVLVIISR----PPAKIFLYDDwsmphta-----AVLkfpf-------------------------<br>-----nNPYLFRS-NKFLTLFKNQHGSLRLLQRF-NEDteklenLRDYRVLEYCSKPNTLLLPHHSDSDLLVLVLEGQAILVLVN-PD---G-RDTYKLDQGDAIKIQAGTPFYLINPDNNQNLRILKFAITfrrpGTVEDFFLSS-------tkrlpSYL----safsknfleasydspydeieqtllq<br><br>----Vwdedcfeaak------<br>eeqeG----------vivkmp<br><br>SARST: aliSize=127 (resi) RMSD=5.55 (Å)<br>scvrdnsl----VRDISQMP--QSSYGIEGLSHITVA--GALNHGMKEVEVWLQTISPGQRTPIHRHSCEEVFTVLKGKGTLLMGSSSLkypgqPQEIPFFQNTTFSIPVNDPHQVWNSDEHEDLQVLVII-----SRPPAKIFlyddwSMPHTAAVL-----------------------------------<br>--------nnpyLFRSNKFLtlFKNQHGSLRLLQRFNedTEKLENLRDYRVLEYCSKPNTLLLPHHSDSDLLVLVLEGQAILVLVNPDG-----RDTYKLDQGDAIKIQAGTPFYLINPDNNQNLRILKFAitfrrPGTVEDFF-----LSSTKRLPSylsafsknfleasydspydeieqtllqeeqeg<br><br>------kfpfvwdedcfeaak<br>vivkmp---------------<br><br>BLAST: aliSize=11 (resi) iden=5.62% (9/160) simi=6.88% (11/160)<br>scvrdnslvrdisqmpqssygieglshitvagalnhgmkevevwlqtispgqrtpihrhsceevftvlkgkgtllmgssslkypgqpqeipffqnttfsipvnd--PHqvwNSDehEDLQVLVI-------------<br>---nnpylfrsnkfltlfknqhgslrllqrfnedteklenlrdyrvleycskpntlllPH---HSD--SDLLVLVLegqailvlvnpdg<br><br>--isrppakiflyddwsmphtaavlkfpfvwdedcfeaak<br>rdtykldqgdaikiqagtpfylinpdnnqnlrilkfaitfrrpgtvedfflsstkrlpsylsafsknfleasydspydeieqtllqeeqegvivkmp-------------------------------------<br><br>Proposed: aliSize=140 (resi) RMSD=2.32 (Å)<br>scvrdn--SLVRDIsQMPQS-sYGIEGLSHITVaGAlnH-G-------MKEVEVWLQTISPGQRTPIHRHSCEEVFTVLKGKGTLLMGSsSLkypGqPQEIPFFQNTTFSIPVNDPHQVWNSDEHEDLQVLVIISR----PPAKIFLY---------D--DWS--M-------PHTAA--V-LKFPFVWd<br>------nnPYLFRS-NKFLTl-FKNQHGSLRLL-QR--FnEdteklenLRDYRVLEYCSKPNTLLLPHHSDSDLLVLVLEGQAILVLVN-PD---G-RDTYKLDQGDAIKIQAGTPFYLINPDNNQNLRILKFAITfrrpGTVEDFFLsstkrlpsyLsaFSKnfLeasydspYDEIEqtLlQEEQEGV-<br><br>EDcFEaaK<br>IV-KM--P

345	1lr5A<br>(160)<br><br>1dzrA<br>(183)	N	8.13	TM-align: aliSize=103 (resi) RMSD=2.76 (Å)<br>scvrdnslvrdisqmpqssygieglshitvagalnhgm---KEVEVWLQTISPGQRTPIHRH----SCEEVFTVLKGKGTLLMGSS---SLKYPGqPQEIPFFQ--NTTFSIPVNDPHQVWNSDehEDLQVLVIISRPPA--KIFLYD-<br>-------------------------------------mmiviktaipdvlilepkvfgdergfffesynqqtfeeligrkvTFVQDNHSKSKKNVLRGLHFQrgenAQGKLVRCAVGEVFDVAVDIrkeSPTFGQ-WVGVNLSAenKRQLWIPEGFAHGFVTLS--EYAEFLYKATNYYSpsSEGSILw<br>DW-S---------------------MPHtAAVlkfpfvwdedcfeaak--<br>NDeAigiewpfsqlpelsakdaaapLLD-QALl---------------te<br>SARST: aliSize=100 (resi) RMSD=5.31 (Å)<br>scvrdnslvrdisqmpqssygieglshitvagalnhgmkev--EVWLQTISPGQRTPIHRHSC----EEVFTVLKGKGTLLMGSS--SLKYPGQPQEIPFF--QNTTFSIPVNDPHQVWNSDEhedLQVLVIISRPPAK---IFL<br>--mmiviktaipdvlilepkvfgdergfffesynqqtfeeligrkvtfvQDNHSKSKKNVLRGLHFQRGenaqGKLVRCAVGEVFDVAVDIrkESPTFGQWVGVNLSaeNKRQLWIPEGFAHGFVTLSE---YAEFLYKATNYYSpssEGS<br>YdDWSMPHTAAVL------------------------kfpfvwdedcfeaak<br>I-LWNDEAIGIEWpfsqlpelsakdaaaplldqallte---------------<br>BLAST: aliSize=17 (resi) iden=5.00% (8/160) simi=10.62% (17/160)<br>scvrdnslvrdisqmpqssygieglshitvagalnhgmkevevwlqtispgqrtpihrhsceevftvlkgkgtllmgssslkypgqpqeipf--<br>--mmiviktaipdvlilepkvfgdergfffesynqqtfeeligrkvtfvqdnhskskknvlrglhfqrgenaqgklvrcavgevfdvavdirkesptfgq<br>-------------------------FQNTTFSIPVNDPHQVWNsDEHEDLQ-VLVIISRPPAK--------------iflyddwsmphtaavlkfpfvwdedcfeaak<br>wvgvnlsaenkrqlwipegfahgfvtlseyaeflYKATNYYSPSSEGSILWN-DEAIGIEwPFSQLPELSAKdaaaplldqallte-------------------------------<br>Proposed: aliSize=129 (resi) RMSD=2.39 (Å)<br>SCVR-D---nSLVRDisqMPQSSyGiE----GLSHIT----vaGALNHGMKE--VEVWLQTISPGQRTPIHRH----SCEEVFTVLKGKGTLLMGSS---S--LkyPGQPQEIPFFQ--NTTFSIPVNDPHQVWNSDehEDLQVLVIISRPPA--KIFLYD-DW-S---------------------MP<br>MMIViKtai-PDVLI---LEPKV-F-GdergFFFESYnqqt--FEELIGRKVtfVQDNHSKSKKNVLRGLHFQrgenAQGKLVRCAVGEVFDVAVDIrkeSptF--GQ-WVGVNLSAenKRQLWIPEGFAHGFVTLS--EYAEFLYKATNYYSpsSEGSILwNDeAigiewpfsqlpelsakdaaaplLD<br>htAAV---lkfpfvwdedcfeaak<br>--QALlte-----------------
346	1lr5A<br>(160)<br><br>1ep0A<br>(183)	N	8.13	TM-align: aliSize=102 (resi) RMSD=2.75 (Å)<br>scvrdnslvrdisqmpqssygieglshitvagalnhgmk---EVEVWLQTISPGQRTPIHRH---SCEEVFTVLKGKGTLLMGSS---SLKYPGqPQEIPFFQ--NTTFSIPVNDPHQVWNSDehEDLQVLVIISRPPA--KIFLYD-D<br>--------------------------------------efrfiktsldgaiiiepevytdergyfmetfneaifqenglevrFVQDNESMSVRGVLRGLHFQrekPQGKLVRVIRGEIFDVAVDLrknSDTYGE-WTGVRLSDenRREFFIPEGFAHGFLALS--DECIVNYKCTELYHpeYDSGIPwD<br>W-S-----------------------MPHTAAVlkfpfvwdedcfeaak--<br>DpDigidwplemvddliisekdrnwkPLRENPV-----------------yl<br>SARST: aliSize=107 (resi) RMSD=9.60 (Å)<br>scvrdnslvrdisqm-------------------PQSSYGieglSHITVAGALN---HGMKEV--EVWLQTISPGQRTPIHRHS---CEEVFTVLKGKGTLLMGSSSLKYPGQPQEIPFFQ----NTTFSIPVNDPHQVWNSDEhedLQVLVIISRPPAK-----------------------------------<br>---------------efrfiktsldgaiiiepevYTDERG----YFMETFNEAIfqeNGLEVRfvQDNESMSVRGVLRGLHFQRekpQGKLVRVIRGEIFDVAVDLRKNSDTYGEWTGVRLsdenRREFFIPEGFAHGFLALSD---ECIVNYKCTELYHpeydsgipwddpdigidwplemvddliise<br>----------------iflyddwsmphtaavlkfpfvwdedcfeaak<br>kdrnwkplrenpvyl----------------------------------<br>BLAST: aliSize=5 (resi) iden=2.50% (4/160) simi=3.12% (5/160)<br>scvrdnslvrdisqmpqssygieglshitvagalnhgmkevevwlqtispgqrtpihrhsceevftvlkgkgtllmgssslkypgqpqeipffqnttfs---<br>---efrfiktsldgaiiiepevytdergyfmetfneaifqenglevrfvqdnesmsvrgvlrglhfqrekpqgklvrvirgeifdvavdlrkns<br>---IPVNDP---------------------------------hqvwnsdehedlqvlviisrppakiflyddwsmphtaavlkfpfvwdedcfeaak<br>dtygewtgvrlsdenrreffipegfahgflalsdecivnykctelyhpeydsgIPWDDPdigidwplemvddliisekdrnwkplrenpvyl---<br>Proposed: aliSize=128 (resi) RMSD=2.30 (Å)<br>scVRDNS-------LVRdIsqMPQSS-ygiEGLSHITVAGALNHG-----MKEVEVWLQTISPGQRTPIHRH---SCEEVFTVLKGKGTLLMGSS---S--LkyPGQPQEIPFFQ--NTTFSIPVNDPHQVWNSDehEDLQVLVIISRPPA--KIFLYD-DW-S-----------------------MP-<br>--EFRFIktsldgaIII-E--PEVYTd---ERGYFMETFNEAIFQenglEVRFVQDNESMSVRGVLRGLHFQrekPQGKLVRVIRGEIFDVAVDLrknSdtY--GE-WTGVRLSDenRREFFIPEGFAHGFLALS--DECIVNYKCTELYHpeYDSGIPwDDpDigidwplemvddliisekdrnwkPLr<br>--HT--aavlkfpfvwdedcfeaak<br>enPVyl-------------------
347	1lr5A<br>(160)<br><br>1nxmA<br>(194)	N	4.38	TM-align: aliSize=102 (resi) RMSD=2.68 (Å)<br>scvrdnslvrdisqmpqssygieglshitvagalnhgm---KEVEVWLQTISPGQRTPIHRHSCEEVFTVLK-GKGTLLMGS-S-SLKYPGqPQEIPFFQNTTFSIPVNDPHQVWNsDeHEDLQVLVIISRPPA---K-<br>-------------------------------------nffgktlaarpveaipgmlefdipvhgdnrgwfkenfqkekmlplgfpesffaeGKLQNNVSFSRKNVLRGLHAEPWDKYISVADgGKVLGTWVDlReGETFGN-TYQTVIDASKSIFVPRGVANGFQV-L-SDFVAYSYLVNDYWAlelKp<br>-IFLYD--D--W---------------------SM-PHTAAvlkfpfvwdedcfeaak----<br>kYAFVNyaDpsLdikwenleeaevseadenhpFLkDVKPL-----------------rkedl<br>SARST: aliSize=105 (resi) RMSD=9.92 (Å)<br>scvrdnslvrdisqmpqssyg--------------IEGLSHITVAG-----------------ALNHGM-------KEVEVWLQTISPGQRTPIHRHSCEEVFTVLKGKGTLLMGSSS--LKYPGQPQEIPFFQNTTFSIPVNDPHQVWNSDEhedLQVLVIISRPPAK------------------------<br>---------------------nffgktlaarpveaIPGMLEFDIPVhgdnrgwfkenfqkekMLPLGFpesffaeGKLQNNVSFSRKNVLRGLHAEPWDKYISVADGGKVLGTWVDLreGETFGNTYQTVIDASKSIFVPRGVANGFQVLSD---FVAYSYLVNDYWAlelkpkyafvnyadpsldikwe<br>------------------------------iflyddwsmphtaavlkfpfvwdedcfeaak<br>nleeaevseadenhpflkdvkplrkedl----------------------------------<br>BLAST: aliSize=6 (resi) iden=3.12% (5/160) simi=3.75% (6/160)<br>scvrdnslvrdisqmpqssygieglshitvagalnhgmkevevwlqtispgqrtpihrhsceevftvlkgkgtllmgssslkypgqpqeip-FFQNTTFSIPV--<br>---nFFGKTLAARPVeaipgmlefdipvhgdnrgwfkenfqkekmlplgfpesffaegklqnnvsfsrknvlrglhaepwdkyisvadggkvlgtwvdlreg<br>--ndphqvwnsdehedlqvlviisrppakiflyddwsmphtaavlkfpfvwdedcfeaak<br>etfgntyqtvidaskslfvprgvangfqvlsdfvaysylvndywalelkpkyafvnyadpsldikwenleeaevseadenhpflkdvkplrkedl--<br>Proposed: aliSize=129 (resi) RMSD=2.45 (Å)<br>-----SCVRDNS----LVRDisqMPQSSYgiE----GLSHIT----vaGALNHGMK------EVEVWLQTISPGQRTPIHRHSCEEVFTVLK-GKGTLLMGS-S--SLKyPGqPQEIPFFQNTTFSIPVNDPHQVWNSDehEDLQVLVIISRPP----aK--IFLYD--D--W------------------<br>nffgkTLAARPVeaipGMLE---FDIPVH--GdnrgWFKENFqkek--MLPLGFPEsffaegkLQNNVSFSRKNVLRGLHAEPWDKYISVADgGKVLGTWVDlRegETF-GN-TYQTVIDASKSIFVPRGVANGFQVLS--DFVAYSYLVNDYWalel-KpkYAFVNyaDpsLdikwenleeaevseade<br>---SMP-HT-------aavlkfpfvwdedcfeaak<br>nhpFLKdVKplrkedl-------------------

348	1lr5A (160) / 1wa4A (205)	N	9.38	

```
TM-align:  aliSize=103 (resi)      RMSD=2.73 (Å)
scvrdnslvrdisqmpqssygieglshitvagalnhgm--------------------------------------------KEVEVWLQTISPGQRTPIHRH----SCEEVFTVLKGKGTLLMGSSSLKY--PGQPQEIPFFQ--NTTFSIPVNDPHQVWNsDeHEDLQVLVIISRPPA--KIFLY---
--------------------------------------mqarklavdgaieftprvfaddrgllilpyqeeafveahggplfRVAQTIHSMSKRGVVRGIHYTvtppGTAKYVVCARGKAMDIVIDIRVGSptFGQWDSVLMDQqdPRAVYLPVGVGHAFVA-L-EDDTVFSYMASRSYVtqDELALsal

----------------------D-DWSMPH--TAAvlkfpfvwdedcfeaak--------------------
dpalglpidigvepivsdrdrvAiTLAEAQrqGLL-----------------pdyttsqeierrltavpvst

SARST:     aliSize=122 (resi)      RMSD=10.37 (Å)
scvrdnslvrdisq-----------------MPQSSYGieglSHITVAGALN----HG----MKEVEVWLQTISPGQRTPIHRHS---CEEVFTVLKGKGTLLMGSSSLKYP--GQPQEIPFF--QNTTFSIPVNDPHQVWNSdehEDLQVLVIISRPPAK---IFLYdDWSMPHTAAV-----------
--------------mqarklavdgaieftprVFADDRG----LLILPYQEEAfveaHGgplfRVAQTIHSMSKRGVVRGIHYTVTppgTAKYVVCARGKAMDIVIDIRVGSPtfGQWDSVLMDqqDPRAVYLPVGVGHAFVAL---EDDTVFSYMASRSYVtqdELAL-SALDPALGLPidigvepivsd

-------------------------------------lkfpfvwdedcfeaak
rdrvaitlaeaqrqgllpdyttsqeierrltavpvst-----------------

BLAST:     aliSize=7 (resi)        iden=2.50% (4/160)        simi=4.38% (7/160)
scvrdnslvrdisqmpqs----------------------------------------------------------------------------------------------------------------------SYgIEGLSHIT------------------------------------
-----------------mqarklavdgaieftprvfaddrgllilpyqeeafveahggplfrvaqtihsmskrgvvrgihytvtppgtakyvvcargkamdividirvgsptfgqwdsvlmdqqdpravylpvgvghafvaleddtvfSY-MASRSYVTqdelalsaldpalglpidigvepivsdrdr

----------------------------------vagalnhgmkevevwlqtispgqrtpihrhsceevftvlkgkgtllmgssslkypgqpqeipffqnttfsipvndphqvwnsdehedlqvlviisrppakiflyddwsmphtaavlkfpfvwdedcfeaak
vaitlaeaqrqgllpdyttsqeierrltavpvst---------------------------------------------------------------------------------------------------------------------------------

Proposed:  aliSize=132 (resi)      RMSD=2.43 (Å)
scVRDNS-----LVRDisQMPQSSygIEGLSHITVAGALNHG-----MK-E-VEVWLQTISPGQRTPIHRH----SCEEVFTVLKGKGTLLMGS-S--S--LkyPGqPQEIPFFQ--NTTFSIPVNDPHQVWNsDeHEDLQVLVIISRPPA--KIFLYD--D--W--------------------SMP-H
--MQARKlavdgAIEF--TPRVFA--DDRGLLILPYQEEAFVeahggPLfRvAQTIHSMSKRGVVRGIHYTvtppGTAKYVVCARGKAMDIVIDiRvgSptF--GQ-WDSVLMDQqdPRAVYLPVGVGHAFVA-L-EDDTVFSYMASRSYVtqDELALSalDpaLglpidigvepivsdrdrvaiTLAeA

T-A-A-------------------vlkfpfvwdedcfeaak
QrQgLlpdyttsqeierrltavpvst----------------
```

349	1lr5A (160) / 2ixlA (196)	N	5.63	

```
TM-align:  aliSize=100 (resi)      RMSD=2.50 (Å)
scvrdnslvrdisqmpqssygieglshitvagalnhgm-------------------------------------------------------KEVEVWLQTISPGQRTPIHRHSCEEVFTVLK-GKGTLLMGSS----SLkyPGqPQEIPFFQNTTFSIPVNDPHQVWNsDeHEDLQVLVIISRPPA-
--------------------------------------tenffgktlaarpveaipgmlefdipvhgdnrgwfkenfqkekmlplgfpesffaeGKLQNNVSFSRKNVLRGLHAEPWDKYISVADgGKVLGTWVDLregeTF--GN-TYQTVIDASKSIFVPRGVANGFQV-L-SDFVAYSYLVNDYWAl

--K--IFLYD--D--W--------------------SMP-HTAAvlkfpfvwdedcfeaak-----
elKpkYAFVNyaDpsLdikwenleeaevseadenhpFLKdVKPL------------------rkedl

SARST:     aliSize=102 (resi)      RMSD=8.06 (Å)
scvrdnslvrdisqmpqssygieg--------------------------------LSHITVAG---ALNHGM-------KEVEVWLQTISPGQRTPIHRHSCEEVFTVLKGKGTLLMGSSS--LKYPGQPQEIPFFQNTTFSIPVNDPHQVWNSDEhedLQVLVIISRPPAK-----------------
------------------------tenffgktlaarpveaipgmlefdipvhgdnrGWFKENFQkekMLPLGFpesffaeGKLQNNVSFSRKNVLRGLHAEPWDKYISVADGGKVLGTWVDLreGETFGNTYQTVIDASKSIFVPRGVANGFQVLSD---FVAYSYLVNDYWAlelkpkyafvnyadpsl

---------------------------------iflyddwsmphtaavlkfpfvwdedcfeaak
dikwenleeaevseadenhpflkdvkplrkedl-------------------------------

BLAST:     aliSize=6 (resi)        iden=3.12% (5/160)        simi=3.75% (6/160)
scvrdnslvrdisqmpqssygieglshitvagalnhgmkevevwlqtispgqrtpihrhsceevftvlkgkgtllmgssslkypgqpqeip---FFQNTTFSIPV--------------------------------------------------------------------------------------------
-------------------------------------------------------------------------------------------tenFFGKTLAARPVeaipgmlefdipvhgdnrgwfkenfqkekmlplgfpesffaegklqnnvsfsrknvlrglhaepwdkyisvadggkvlgtwvdlr

----------------------------------------------------------------------------------------------------ndphqvwnsdehedlqvlviisrppakiflyddwsmphtaavlkfpfvwdedcfeaak
egetfgntyqtvidasksifvprgvangfqvlsdfvaysylvndywalelkpkyafvnyadpsldikwenleeaevseadenhpflkdvkplrkedl--------------------------------------------------------------

Proposed:  aliSize=131 (resi)      RMSD=2.53 (Å)
------SCVRD-nS-----LVRDisQMPQSSYG-iEGLSHITVAGALNHG----------mkEVEVWLQTISPGQRTPIHRHSCEEVFTVLK-GKGTLLMGS-S-S-LKyPGqPQEIPFFQNTTFSIPVNDPHQVWNsDeHEDLQVLVIISRPPA---K--IFLYD--D--W-----------------
tenffgKTLAAr-PveaipGMLE--FDIPVHGDn-RGWFKENFQKEKMLPlgfpesffaeg--KLQNNVSFSRKNVLRGLHAEPWDKYISVADgGKVLGTWVDlReGeTF-GN-TYQTVIDASKSIFVPRGVANGFQV-L-SDFVAYSYLVNDYWAlelKpkYAFVNyaDpsLdikwenleeaevseade

---SMP-HT-------aavlkfpfvwdedcfeaak
nhpFLKdVKplrkedl-------------------
```

350	1lr5A (160) / 2ixlB (195)	N	5.63	

```
TM-align:  aliSize=102 (resi)      RMSD=2.65 (Å)
scvrdnslvrdisqmpqssygieglshitvagalnhgm------------------------------------------------------KEVEVWLQTISPGQRTPIHRHSCEEVFTVLK-GKGTLLMGS-S-SLKYPGqPQEIPFFQNTTFSIPVNDPHQVWNsDeHEDLQVLVIISRPPA---K
--------------------------------------enffgktlaarpveaipgmlefdipvhgdnrgwfkenfqkekmlplgfpesffaeGKLQNNVSFSRKNVLRGLHAEPWDKYISVADgGKVLGTWVDlReGETFGN-TYQTVIDASKSIFVPRGVANGFQV-L-SDFVAYSYLVNDYWAlelK

--IFLYD--D--W--------------------SMP-HTAAvlkfpfvwdedcfeaak-----
pkYAFVNyaDpsLdikwenleeaevseadenhpFLKdVKPL------------------rkedl

SARST:     aliSize=101 (resi)      RMSD=10.05 (Å)
scvrdnslvrdisqm--------------------------PQSSYGieglSHITVAG---ALNHGM-------KEVEVWLQTISPGQrtpihrHSC------EEVFTVLKGKGTLLMGSSSLKYP--GQPQEIPFFQNTTFSIPVNDPHQVWNSDEhedLQVLVIISRPPAK-----------------
---------------enffgktlaarpveaipgmlefdipvHGDNRG----WFKENFQkekMLPLGFpesffaeGKLQNNVSFSRKNV------LRGlhaepwDKYISVADGGKVLGTWVDLREGEtfGNTYQTVIDASKSIFVPRGVANGFQVLSD---FVAYSYLVNDYWAlelkpkyafvnyadpsl

---------------------------------iflyddwsmphtaavlkfpfvwdedcfeaak
dikwenleeaevseadenhpflkdvkplrkedl-------------------------------

BLAST:     aliSize=6 (resi)        iden=3.12% (5/160)        simi=3.75% (6/160)
scvrdnslvrdisqmpqssygieglshitvagalnhgmkevevwlqtispgqrtpihrhsceevftvlkgkgtllmgssslkypgqpqeip--FFQNTTFSIPV---------------------------------------------------------------------------------------------
--------------------------------------------------------------------------------------------enFFGKTLAARPVeaipgmlefdipvhgdnrgwfkenfqkekmlplgfpesffaegklqnnvsfsrknvlrglhaepwdkyisvadggkvlgtwvdlre

---------------------------------------------------------------------------------------------------ndphqvwnsdehedlqvlviisrppakiflyddwsmphtaavlkfpfvwdedcfeaak
getfgntyqtvidasksifvprgvangfqvlsdfvaysylvndywalelkpkyafvnyadpsldikwenleeaevseadenhpflkdvkplrkedl--------------------------------------------------------------

Proposed:  aliSize=132 (resi)      RMSD=2.58 (Å)
-----SCVRDNS-----LVRDisQMPQSSYG-iEGLSHITVAGALNHG--M--------kEVEVWLQTISPGQRTPIHRHSCEEVFTVLK-GKGTLLMGS-S---SLkyPGqPQEIPFFQNTTFSIPVNDPHQVWNsDeHEDLQVLVIISRPPA---K--IFLYD--D--W-------------------
enffgKTLAARPveaipGMLE--FDIPVHGDn-RGWFKENFQKEKMLPlgFpesffaeg-KLQNNVSFSRKNVLRGLHAEPWDKYISVADgGKVLGTWVDlRegeTF--GN-TYQTVIDASKSIFVPRGVANGFQV-L-SDFVAYSYLVNDYWAlelKpkYAFVNyaDpsLdikwenleeaevseadenh

--SMPHT-------aavlkfpfvwdedcfeaak
pfLKDVKplrkedl-------------------
```

<table>
<tr><td rowspan="2">351</td><td>1lr5A
(160)</td><td rowspan="2">N</td><td rowspan="2">3.75</td><td rowspan="2">TM-align: aliSize=102 (resi) RMSD=2.66 (Å)
scvrdnslvrdisqmpqssygieglshitvagalnhgm--KEVEVWLQTISPGQRTPIHRHSCEEVFTVLK-GKGTLLMGS-S-SLKYPGqPQEIPFFQNTTFSIPVNDPHQVWNsDeHEDLQVLVIISRPPA--
--------------------------------------mtenffgktlaarpveaipgmlefdipvhgdnrgwfkenfqkekmlplgfpesffaeGKLQNNVSFSRKNVLRGLHAEPWDKYISVADgGKVLGTWVDlReGETFGN-TYQTVIDASKSIFVPRGVANGFQV-L-SDFVAYSYLVNDYWAle

K---IFLYD--D--W--------------------SM-PHTAAvlkfpfvwdedcfeaak-----
LkpkYAFVNyaDpsLdikwenleeaevseadenhpFLkDVKPL-----------------rkedl

SARST: aliSize=118 (resi) RMSD=9.88 (Å)
scvrdnslvrdisq---------------------------MPQSSYGieglSHITVAG---ALNHGMKE-------VEVWLQTISPGQRTPIHRHSCEEVFTVLKGKGTLLMGSSSLKYP--GQPQEIPFFQNTTFSIPVNDPHQVWNSDEhedLQVLVIISRPPAkiflydDWSMpHTAAVLK-----
--------------mtenffgktlaarpveaipgmlefdipVHGDNRG----WFKENFQkekMLPLGFPEsffaegkLQNNVSFSRKNVLRGLHAEPWDKYISVADGGKVLGTWVDLREGEtfGNTYQTVIDASKSIFVPRGVANGFQVLSD---FVAYSYLVNDYW------ALEL-KPKYAFVnyadp

-----------------------------------fpfvwdedcfeaak
sldikwenleeaevseadenhpflkdvkplrkedl--------------

BLAST: aliSize=6 (resi) iden=3.12% (5/160) simi=3.75% (6/160)
scvrdnslvrdisqmpqssygieglshitvagalnhgmkevevwlqtispgqrtpihrhsceevftvlkgkgtllmgssslkypgqpqeip----FFQNTTFSIPV--
---mtenFFGKTLAARPVeaipgmlefdipvhgdnrgwfkenfqkekmlplgfpesffaegklqnnvsfsrknvlrglhaepwdkyisvadggkvlgtwvdl

--ndphqvwnsdehedlqvlviisrppakiflyddwsmphtaavlkfpfvwdedcfeaak
regetfgntyqtvidasksifvprgvangfqvlsdfvaysylvndywalelkpkyafvnyadpsldikwenleeaevseadenhpflkdvkplrkedl--

Proposed: aliSize=132 (resi) RMSD=2.81 (Å)
-------SCVRDNS-----LVRDisQMPQSSYG-iEGLSHITVAGALNHG--M--------kEVEVWLQTISPGQRTPIHRHSCEEVFTVL--KGKGTLLMGSSSL--kyPGqPQEIPFFQN-TTFSIPVNDpHQVWNSDeHEDLQVLVIISRPPA--KI--FLY-------------------------
mtenffgKTLAARPveaipGMLE--FDIPVHGDn-RGWFKENFQKEKMLPlgFpesffaeg-KLQNNVSFSRKNVLRGLHAEPWDKYISVAdgGKVLGTWVDLREGet--FG-NTYQTVIDAsKSIFVPRGV-ANGFQVL-SDFVAYSYLVNDYWAleLKpkYAFvnyadpsldikwenleeaevseade

--DDWS-MPHT-----aavlkfpfvwdedcfeaak
nhPFLKdVKPLrkedl-------------------</td></tr>
<tr><td>2ixlC
(197)</td></tr>
<tr><td rowspan="2">352</td><td>1lr5A
(160)</td><td rowspan="2">N</td><td rowspan="2">11.94</td><td rowspan="2">TM-align: aliSize=94 (resi) RMSD=2.64 (Å)
scvrdnslvrdisqmpqssygieglshitvagalnhgm----------------------------KEVEVWLQT-ISPGQRTPIHRH-SCEEVFTVLKGKGTLLMGSSSlkypGqPQEIPFFQN-TTFSIPVNDPHQVWnSDeHEDLQVLVIISRPPA--KIFLYddWsMPH-TAavlkfpfvwdedcf
--------------------------------------enkvinfkkiidsrgslvaieenknipfSIKRVYYIFdTKGEEPRGFHAHkKLEQVLVCLNGSCRVILDDGN----I-IQEITLDSPaVGLYVGPAVWHEMH-DF-SSDCVMMVLASDYYDetDYIRQ--Y-DNFkKY--------------

eaak------
----iakinl

SARST: aliSize=120 (resi) RMSD=13.33 (Å)
scvrdnslvrdisqm---------PQSSYGIEGLSHITVAGalnHGMKEVEVWLQtispGQRTPIHRHSCEE-----VFTVLKGKGTLLMGSSSLkypgQPQEIPFFQNTTFSIPVNDPHQVWNSDEHEDLQVLViisRPPAKiflyddwsmphTAAVLKFPFVWDEDCFEAAK
---------------enkvinfkkIIDSRGSLVAIEENKNI---PFSIKRVYYIF----DTKGEEPRGFHAHkkleqVLVCLNGSCRVILDDGNI----IQEITLDSPAVGLYVGPAVWHEMHDFSSDCVMMVLA---SDYYD-----------ETDYIRQYDNFKKYIAKINL

BLAST: aliSize=21 (resi) iden=9.70% (13/134) simi=15.67% (21/134)
scvrdnslvrdisqmpqssygieglshitvagalnhgmkevevwlqtispgqrtpihrhsceevftvlkgkgtllmgssslkypgqp-----------------------QEIPFFQNTTFSIPVNDPHQVWNSDEHEDL-QVLVIISrPPAKIFLyDD-------------------------------
--enkvinfkkiidsrgslvaieenKNIPFSIKRVYYIFDTKGEEPRGFHAHKKLeQVLVCLN-GSCRVIL-DDgniiqeitldspavglyvgpavwhemhdfss

---------------------------------wsmphtaavlkfpfvwdedcfeaak
dcvmmvlasdyydetdyirqydnfkkyiakinl-------------------------

Proposed: aliSize=118 (resi) RMSD=2.38 (Å)
scvrdns-LVRDIsqMPQSSygIEGLSHITvAGA-LNHGMKEVEVWLQT-ISPGQRTPIHRH-SCEEVFTVLKGKGTLLMGSSS-lkypgqPQEIPFFQN-TTFSIPVNDPHQVWNSDehEDLQVLVIISRPPA--KIFLYddWsMPH-T-------aavlkfpfvwdedcfeaak
-------eNKVIN--FKKII--DSRGSLVA-IEEnKNIPFSIKRVYYIFdTKGEEPRGFHAHkKLEQVLVCLNGSCRVILDDGNi------IQEITLDSPaVGLYVGPAVWHEMHDFS--SDCVMMVLASDYYDetDYIRQ--Y-DNFkKyiakinl-------------------</td></tr>
<tr><td>2pa7B
(134)</td></tr>
<tr><td rowspan="2">353</td><td>1lr5A
(160)</td><td rowspan="2">C</td><td rowspan="2">11.94</td><td rowspan="2">TM-align: aliSize=94 (resi) RMSD=2.64 (Å)
scvrdnslvrdisqmpqssygieglshitvagalnhgm----------------------------KEVEVWLQT-ISPGQRTPIHRH-SCEEVFTVLKGKGTLLMGSSSlkypGqPQEIPFFQN-TTFSIPVNDPHQVWnSDeHEDLQVLVIISRPPA--KIFLYddWsMPH-TAavlkfpfvwdedcf
--------------------------------------enkvinfkkiidsrgslvaieenknipfSIKRVYYIFdTKGEEPRGFHAHkKLEQVLVCLNGSCRVILDDGN----I-IQEITLDSPaVGLYVGPAVWHEMH-DF-SSDCVMMVLASDYYDetDYIRQ--Y-DNFkKY--------------

eaak------
----iakinl

SARST: aliSize=120 (resi) RMSD=13.33 (Å)
scvrdnslvrdisqm---------PQSSYGIEGLSHITVAGalnHGMKEVEVWLQtispGQRTPIHRHSCEE-----VFTVLKGKGTLLMGSSSLkypgQPQEIPFFQNTTFSIPVNDPHQVWNSDEHEDLQVLViisRPPAKiflyddwsmphTAAVLKFPFVWDEDCFEAAK
---------------enkvinfkkIIDSRGSLVAIEENKNI---PFSIKRVYYIF----DTKGEEPRGFHAHkkleqVLVCLNGSCRVILDDGNI----IQEITLDSPAVGLYVGPAVWHEMHDFSSDCVMMVLA---SDYYD-----------ETDYIRQYDNFKKYIAKINL

BLAST: aliSize=21 (resi) iden=9.70% (13/134) simi=15.67% (21/134)
scvrdnslvrdisqmpqssygieglshitvagalnhgmkevevwlqtispgqrtpihrhsceevftvlkgkgtllmgssslkypgqp-----------------------QEIPFFQNTTFSIPVNDPHQVWNSDEHEDL-QVLVIISrPPAKIFLyDD-------------------------------
--enkvinfkkiidsrgslvaieenKNIPFSIKRVYYIFDTKGEEPRGFHAHKKLeQVLVCLN-GSCRVIL-DDgniiqeitldspavglyvgpavwhemhdfss

---------------------------------wsmphtaavlkfpfvwdedcfeaak
dcvmmvlasdyydetdyirqydnfkkyiakinl-------------------------

Proposed: aliSize=94 (resi) RMSD=2.04 (Å)
scvrdnslvrdisqmpqssygieglshitvagalnhgmk-----------------------------EVEVWLQT-ISPGQRTPIHRH-SCEEVFTVLKGKGTLLMGSSS-lkypgqPQEIPFFQN-TTFSIPVNDPHQVWNSDehEDLQVLVIISRPPA--KIFLYddwsmphtaavlkfpfvwdEDC
---------------------------------------enkvinfkkiidsrgslvaieenknipfsIKRVYYIFdTKGEEPRGFHAHkKLEQVLVCLNGSCRVILDDGNi------IQEITLDSPaVGLYVGPAVWHEMHDFS--SDCVMMVLASDYYDetDYIRQ-------------------YDN

FEAAK-----
FKKYIakinl</td></tr>
<tr><td>2pa7B
(134)</td></tr>
</table>

354	1lr5A (160)<br>2paeA (136)	N	11.03	TM-align: aliSize=95 (resi) RMSD=2.77 (Å)<br>scvrdnslvrdisqmpqssygieglshitvagalnhgm-----------------------------KEVEVWLQT-ISPGQRTPIHRH-SCEEVFTVLKGKGTLLMGSSSlkypGqPQEIPFFQN-TTFSIPVNDPHQVWnSDeHEDLQVLVIISRPPA--KIFLYdDwsMPH-TAAvlkfpfvwdedc<br>--------------------------------------menkvinfkkiidsrgslvaieenknipfSIKRVYYIFdTKGEEPRGFNAHkKLEQVLVCLNGSCRVILDDGN----I-IQEITLDSPaVGLYVGPAVWHEMH-DF-SSDCVMMVLASDYYDetDYIRQ-Y--DNFkKYI------------<br>feaak------<br>-----akinle<br><br>SARST: aliSize=93 (resi) RMSD=15.81 (Å)<br>scvrdnslvrdisqmpqssygieglshitvagalnhgmkeve---------------VWLQTISPG----QRTPIHRHSCEEVFTVLKGKGTllmgssslkYPGQPQEIPFFQNTTFSIPVND--PHQVWNSDEHedlQVLVIISRPPAKIFlyddwSMPHTAAVLKF----PFVWD-------------<br>--menkvinfkkiidsrGSLVAIEENknipFSIKRVYYIFDTKGEEPRGFNA---------HKKLEQVLVCLNGSCRVILDDGniIQEITLDSPA---VGLYVGPAVWHEMH-----DFSSDCVMMVLasdyYDETDyirqydnfkkyia<br>-----edcfeaak<br>kinle--------<br><br>BLAST: aliSize=32 (resi) iden=14.71% (20/136) simi=23.53% (32/136)<br>scvrdnslvrdisqmpqssygieglshitvagalnhgmkevevwlqtispgqrtpihrhsceevftvlkgkgtllmgssslkypgqp------------------------QEIPFFQNTTFSIPVNDPHQVWNSDEHEDL-QVLVIISRPpAKIFLYD-----DWSMPHTAAVLKF-PFVWDE------<br>---menkvinfkkiidsrgslvaieenKNIPFSIKRVYYIFDTKGEEPRGFNAHKKLeQVLVCLNGS-CRVILDDgniiqEITLDSPAVGLYVgPAVWHEmhdfss<br>DC--------------------------------feaak<br>DCvmmvlasdyydetdyirqydnfkkyiakinle-----<br><br>Proposed: aliSize=119 (resi) RMSD=2.39 (Å)<br>scvrdn-SLVRDIsqMPQSSyGiEGLSHITvAGA-LNHGMKEVEVWLQT-ISPGQRTPIHRH-SCEEVFTVLKGKGTLLMGSSS-lkypgqPQEIPFFQN-TTFSIPVNDPHQVWNSDehEDLQVLVIISRPPA--KIFLYdDwsMPH-T--------aavlkfpfvwdedcfeaak<br>------mENKVIN--FKKII-D-SRGSLVA-IEEnKNIPFSIKRVYYIFdTKGEEPRGFNAHkKLEQVLVCLNGSCRVILDDGNi------IQEITLDSPaVGLYVGPAVWHEMHDFS--SDCVMMVLASDYYDetDYIRQ-Y--DNFkKyiakinle------------------
355	1lr5A (160)<br>2paeA (136)	C	11.03	TM-align: aliSize=95 (resi) RMSD=2.77 (Å)<br>scvrdnslvrdisqmpqssygieglshitvagalnhgm-----------------------------KEVEVWLQT-ISPGQRTPIHRH-SCEEVFTVLKGKGTLLMGSSSlkypGqPQEIPFFQN-TTFSIPVNDPHQVWnSDeHEDLQVLVIISRPPA--KIFLYdDwsMPH-TAAvlkfpfvwdedc<br>--------------------------------------menkvinfkkiidsrgslvaieenknipfSIKRVYYIFdTKGEEPRGFNAHkKLEQVLVCLNGSCRVILDDGN----I-IQEITLDSPaVGLYVGPAVWHEMH-DF-SSDCVMMVLASDYYDetDYIRQ-Y--DNFkKYI------------<br>feaak------<br>-----akinle<br><br>SARST: aliSize=93 (resi) RMSD=15.81 (Å)<br>scvrdnslvrdisqmpqssygieglshitvagalnhgmkeve---------------VWLQTISPG----QRTPIHRHSCEEVFTVLKGKGTllmgssslkYPGQPQEIPFFQNTTFSIPVND--PHQVWNSDEHedlQVLVIISRPPAKIFlyddwSMPHTAAVLKF----PFVWD-------------<br>--menkvinfkkiidsrGSLVAIEENknipFSIKRVYYIFDTKGEEPRGFNA---------HKKLEQVLVCLNGSCRVILDDGniIQEITLDSPA---VGLYVGPAVWHEMH-----DFSSDCVMMVLasdyYDETDyirqydnfkkyia<br>-----edcfeaak<br>kinle--------<br><br>BLAST: aliSize=32 (resi) iden=14.71% (20/136) simi=23.53% (32/136)<br>scvrdnslvrdisqmpqssygieglshitvagalnhgmkevevwlqtispgqrtpihrhsceevftvlkgkgtllmgssslkypgqp------------------------QEIPFFQNTTFSIPVNDPHQVWNSDEHEDL-QVLVIISRPpAKIFLYD-----DWSMPHTAAVLKF-PFVWDE------<br>---menkvinfkkiidsrgslvaieenKNIPFSIKRVYYIFDTKGEEPRGFNAHKKLeQVLVCLNGS-CRVILDDgniiqEITLDSPAVGLYVgPAVWHEmhdfss<br>DC--------------------------------feaak<br>DCvmmvlasdyydetdyirqydnfkkyiakinle-----<br><br>Proposed: aliSize=94 (resi) RMSD=2.02 (Å)<br>scvrdnslvrdisqmpqssygieglshitvagalnhgmk-----------------------------E-VEVWLQT-ISPGQRTPIHRH-SCEEVFTVLKGKGTLLMGSSS-lkypgqPQEIPFFQN-TTFSIPVNDPHQVWNSDehEDLQVLVIISRPPA--KIFLYddwsmphtaavlkfpfvwdED<br>---------------------------------------menkvinfkkiidsrgslvaieenknipfSiKRVYYIFdTKGEEPRGFNAHkKLEQVLVCLNGSCRVILDDGNi------IQEITLDSPaVGLYVGPAVWHEMHDFS--SDCVMMVLASDYYDetDYIRQ-------------------YD<br>CFEAAK------<br>NFKKYIakinle
356	1lr5A (160)<br>2paeB (134)	N	11.19	TM-align: aliSize=94 (resi) RMSD=2.64 (Å)<br>scvrdnslvrdisqmpqssygieglshitvagalnhgm----------------------------KEVEVWLQT-ISPGQRTPIHRH-SCEEVFTVLKGKGTLLMGSSSlkypGqPQEIPFFQN-TTFSIPVNDPHQVWNSDehEDLQVLVIISRPPA--KIFLYddWsMPH-TAavlkfpfvwdedcf<br>--------------------------------------enkvinfkkiidsrgslvaieenknipfSIKRVYYIFdTKGEEPRGFNAHkKLEQVLVCLNGSCRVILDDGN----I-IQEITLDSPaVGLYVGPAVWHEMHDFS--SDCVMMVLASDYYDetDYIRQ--Y-DNFkKY-------------<br>eaak------<br>----iakinl<br><br>SARST: aliSize=83 (resi) RMSD=3.55 (Å)<br>scvrdnslvrdisqmpqssygieglshitvagalnhgmk------------------------------EVEVWLQTISPGQRTPIHRH-SCEEVFTVLKGKGTLLMGSSSLkypgQPQEIPFFQNTTFSIPVNDPHQVWNSDEHEDLQVLViisRPPAK-------------------iflyddwsmp<br>---------------------------------------enkvinfkkiidsrgslvaieenknipfsiKRVYYIFDTKGEEPRGFNAHkKLEQVLVCLNGSCRVILDDGNI----IQEITLDSPAVGLYVGPAVWHEMHDFSSDCVMMVLA---SDYYDetdyirqydnfkkyiakinl----------<br>htaavlkfpfvwdedcfeaak<br>---------------------<br><br>BLAST: aliSize=32 (resi) iden=14.93% (20/134) simi=23.88% (32/134)<br>scvrdnslvrdisqmpqssygieglshitvagalnhgmkevevwlqtispgqrtpihrhsceevftvlkgkgtllmgssslkypgqp-----------------------QEIPFFQNTTFSIPVNDPHQVWNSDEHEDL-QVLVIISRPpAKIFLYD-----DWSMPHTAAVLKF-PFVWDE------D<br>---enkvinfkkiidsrgslvaieenKNIPFSIKRVYYIFDTKGEEPRGFNAHKKLeQVLVCLNGS-CRVILDDgniiqEITLDSPAVGLYVgPAVWHEmhdfssD<br>C-------------------------------feaak<br>Cvmmvlasdyydetdyirqydnfkkyiakinl-----<br><br>Proposed: aliSize=118 (resi) RMSD=2.41 (Å)<br>scvrdn-SlVRDisQMPQSSygIEGLSHITvAGA-LNHGMKEVEVWLQT-ISPGQRTPIHRH-SCEEVFTVLKGKGTLLMGSSS-lkypgqPQEIPFFQN-TTFSIPVNDPHQVWNSDehEDLQVLVIISRPPA--KIFLYddWsMPH-T-------aavlkfpfvwdedcfeaak<br>------eN-KVI--NFKKII--DSRGSLVA-IEEnKNIPFSIKRVYYIFdTKGEEPRGFNAHkKLEQVLVCLNGSCRVILDDGNi------IQEITLDSPaVGLYVGPAVWHEMHDFS--SDCVMMVLASDYYDetDYIRQ--Y-DNFkKyiakinl-------------------

357	1lr5A (160) / 2paeB (134)	C	11.19	see alignments below

```
TM-align:  aliSize=94 (resi)       RMSD=2.64 (Å)
scvrdnslvrdisqmpqssygieglshitvagalnhgm----------------------------KEVEVWLQT-ISPGQRTPIHRH-SCEEVFTVLKGKGTLLMGSSSlkypGqPQEIPFFQN-TTFSIPVNDPHQVWNSDehEDLQVLVIISRPPA--KIFLYddWsMPH-TAavlkfpfvwdedcf
--------------------------------------enkvinfkkiidsrgslvaieenknipfSIKRVYYIFdTKGEEPRGFNAHkKLEQVLVCLNGSCRVILDDGN----I-IQEITLDSPaVGLYVGPAVWHEMHDFS--SDCVMMVLASDYYDetDYIRQ--Y-DNFkKY--------------

eaak------
----iakinl

SARST:     aliSize=83 (resi)       RMSD=3.55 (Å)
scvrdnslvrdisqmpqssygieglshitvagalnhgmk------------------------------EVEVWLQTISPGQRTPIHRH-SCEEVFTVLKGKGTLLMGSSSLkypgQPQEIPFFQNTTFSIPVNDPHQVWNSDEHEDLQVLVViisRPPAK-------------------iflyddwsmp
---------------------------------------enkvinfkkiidsrgslvaieenknipfsiKRVYYIFDTKGEEPRGFNAHkKLEQVLVCLNGSCRVILDDGNI----IQEITLDSPAVGLYVGPAVWHEMHDFSSDCVMMVLA---SDYYDetdyirqydnfkkyiakinl----------

htaavlkfpfvwdedcfeaak
---------------------

BLAST:     aliSize=32 (resi)       iden=14.93% (20/134)       simi=23.88% (32/134)
scvrdnslvrdisqmpqssygieglshitvagalnhgmkevevwlqtispgqrtpihrhsceevftvlkgkgtllmgssslkypgqp-----------------------QEIPFFQNTTFSIPVNDPHQVWNSDEHEDL-QVLVIISRPpAKIFLYD-----DWSMPHTAAVLKF-PFVWDE------D
---------------------------------------------------------------------------------------enkvinfkkiidsrgslvaieenKNIPFSIKRVYYIFDTKGEEPRGFNAHKKLeQVLVCLNGS-CRVILDDgniiqEITLDSPAVGLYVgPAVWHEmhdfssD

C-------------------------------feaak
Cvmmvlasdyydetdyirqydnfkkyiakinl-----

Proposed:  aliSize=94 (resi)       RMSD=2.04 (Å)
scvrdnslvrdisqmpqssygieglshitvagalnhgmk-----------------------------EVEVWLQT-ISPGQRTPIHRH-SCEEVFTVLKGKGTLLMGSSS-lkypgqPQEIPFFQN-TTFSIPVNDPHQVWNSDehEDLQVLVIISRPPA--KIFLYddwsmphtaavlkfpfvwdEDC
---------------------------------------enkvinfkkiidsrgslvaieenknipfsIKRVYYIFdTKGEEPRGFNAHkKLEQVLVCLNGSCRVILDDGNi------IQEITLDSPaVGLYVGPAVWHEMHDFS--SDCVMMVLASDYYDetDYIRQ-------------------YDN

FEAAK-----
FKKYIakinl
```

358	1lr5A (160) / 2pakA (135)	N	11.11	see alignments below

```
TM-align:  aliSize=94 (resi)       RMSD=2.59 (Å)
scvrdnslvrdisqmpqssygieglshitvagalnhgm----------------------------KEVEVWLQT-ISPGQRTPIHRH-SCEEVFTVLKGKGTLLMGSSSlkypGqPQEIPFFQN-TTFSIPVNDPHQVWNSDehEDLQVLVIISRPPA--KIFLYddWsMPH-TAavlkfpfvwdedcf
--------------------------------------enkvinfkkiidsrgslvaieenknipfSIKRVYYIFdTKGEEPRGFHANkKLEQVLVCLNGSCRVILDDGN----I-IQEITLDSPaVGLYVGPAVWHEMHDFS--SDCVMMVLASDYYDetDYIRQ--Y-DNFkKY--------------

eaak-------
----iakinle

SARST:     aliSize=78 (resi)       RMSD=3.49 (Å)
scvrdnslvrdisqmpqssygieglshitvagalnhgmk------------------------------EVEVWLQTISPGQRTPIHRH-SCEEVFTVLKGKGTLLMGSSSLkypgQPQEIPFFQNTTFSIPVNDPHQVWNSDEHEDLQVLV--------------------------iisrppakifly
---------------------------------------enkvinfkkiidsrgslvaieenknipfsiKRVYYIFDTKGEEPRGFHANkKLEQVLVCLNGSCRVILDDGNI----IQEITLDSPAVGLYVGPAVWHEMHDFSSDCVMMVLAsdyydetdyirqydnfkkyiakinle-----------

ddwsmphtaavlkfpfvwdedcfeaak
---------------------------

BLAST:     aliSize=6 (resi)        iden=4.44% (6/135)         simi=4.44% (6/135)
scvrdnslvrdisqmpqssygieglshitvagalnhgmkevevwlqtispgqrtpihrhsceevftvlkgkgtllmgssslkypgqpqeipffqnttfsipvndphqvwnsdehedlqvlviisrppakiflyddwsmphtaavlkf-------------------------------------------
---------------------------------------------------------------------------------------------------------------------------------------------------enkvinfkkiidsrgslvaieenknipfsikrvyyifdtkgee

----------------------------------------------PFVWDE------DC--------------------------------feaak
prgfhankkleqvlvclngscrvilddgniiqeitldspavglyvgPAVWHEmhdfssDCvmmvlasdyydetdyirqydnfkkyiakinle-----

Proposed:  aliSize=118 (resi)      RMSD=2.33 (Å)
scvrdn-SlVRDisQMPQSSygIEGLSHITvAGA-LNHGMKEVEVWLQT-ISPGQRTPIHRH-SCEEVFTVLKGKGTLLMGSSS-lkypgqPQEIPFFQN-TTFSIPVNDPHQVWNSDehEDLQVLVIISRPPA--KIFLYddWsMPH-T--------aavlkfpfvwdedcfeaak
------eN-KVI--NFKKII--DSRGSLVA-IEEnKNIPFSIKRVYYIFdTKGEEPRGFHANkKLEQVLVCLNGSCRVILDDGNi------IQEITLDSPaVGLYVGPAVWHEMHDFS--SDCVMMVLASDYYDetDYIRQ--Y-DNFkKyiakinle------------------
```

359	1lr5A (160) / 2pakA (135)	C	11.11	see alignments below

```
TM-align:  aliSize=94 (resi)       RMSD=2.59 (Å)
scvrdnslvrdisqmpqssygieglshitvagalnhgm----------------------------KEVEVWLQT-ISPGQRTPIHRH-SCEEVFTVLKGKGTLLMGSSSlkypGqPQEIPFFQN-TTFSIPVNDPHQVWNSDehEDLQVLVIISRPPA--KIFLYddWsMPH-TAavlkfpfvwdedcf
--------------------------------------enkvinfkkiidsrgslvaieenknipfSIKRVYYIFdTKGEEPRGFHANkKLEQVLVCLNGSCRVILDDGN----I-IQEITLDSPaVGLYVGPAVWHEMHDFS--SDCVMMVLASDYYDetDYIRQ--Y-DNFkKY--------------

eaak-------
----iakinle

SARST:     aliSize=78 (resi)       RMSD=3.49 (Å)
scvrdnslvrdisqmpqssygieglshitvagalnhgmk------------------------------EVEVWLQTISPGQRTPIHRH-SCEEVFTVLKGKGTLLMGSSSLkypgQPQEIPFFQNTTFSIPVNDPHQVWNSDEHEDLQVLV--------------------------iisrppakifly
---------------------------------------enkvinfkkiidsrgslvaieenknipfsiKRVYYIFDTKGEEPRGFHANkKLEQVLVCLNGSCRVILDDGNI----IQEITLDSPAVGLYVGPAVWHEMHDFSSDCVMMVLAsdyydetdyirqydnfkkyiakinle-----------

ddwsmphtaavlkfpfvwdedcfeaak
---------------------------

BLAST:     aliSize=6 (resi)        iden=4.44% (6/135)         simi=4.44% (6/135)
scvrdnslvrdisqmpqssygieglshitvagalnhgmkevevwlqtispgqrtpihrhsceevftvlkgkgtllmgssslkypgqpqeipffqnttfsipvndphqvwnsdehedlqvlviisrppakiflyddwsmphtaavlkf-------------------------------------------
---------------------------------------------------------------------------------------------------------------------------------------------------enkvinfkkiidsrgslvaieenknipfsikrvyyifdtkgee

----------------------------------------------PFVWDE------DC--------------------------------feaak
prgfhankkleqvlvclngscrvilddgniiqeitldspavglyvgPAVWHEmhdfssDCvmmvlasdyydetdyirqydnfkkyiakinle-----

Proposed:  aliSize=94 (resi)       RMSD=2.02 (Å)
scvrdnslvrdisqmpqssygieglshitvagalnhgmk-----------------------------E-VEVWLQT-ISPGQRTPIHRH-SCEEVFTVLKGKGTLLMGSSS-lkypgqPQEIPFFQN-TTFSIPVNDPHQVWNSDehEDLQVLVIISRPPA--KIFLYddwsmphtaavlkfpfvwdEDC
---------------------------------------enkvinfkkiidsrgslvaieenknipfSIKRVYYIFdTKGEEPRGFHANkKLEQVLVCLNGSCRVILDDGNi------IQEITLDSPaVGLYVGPAVWHEMHDFS--SDCVMMVLASDYYDetDYIRQ-------------------YDN

FEAAK------
FKKYIakinle
```

360	1lr5A (160)<br>2pakB (134)	N	11.19	TM-align: aliSize=94 (resi) RMSD=2.61 (Å)<br>scvrdnslvrdisqmpqssygieglshitvagalnhgm----------------------------KEVEVWLQT-ISPGQRTPIHRH-SCEEVFTVLKGKGTLLMGSSSlkypGqPQEIPFFQN-TTFSIPVNDPHQVWNSDehEDLQVLVIISRPPA--KIFLYddWsMPH-TAavlkfpfvwdedcf<br>--------------------------------------enkvinfkkiidsrgslvaieenknipfSIKRVYYIFdTKGEEPRGFHANkKLEQVLVCLNGSCRVILDDGN----I-IQEITLDSPaVGLYVGPAVWHEMHDFS--SDCVMMVLASDYYDetDYIRQ--Y-DNFkKY--------------<br><br>eaak------<br>----iakinl<br><br>SARST: aliSize=122 (resi) RMSD=13.14 (Å)<br>scvrdnslvrdisq--------MPQSSYGIEGLSHITVAGAlnhGMKEVEVWLQTISPGQRTPIHRHSCEE-VFTVLKGKGTLLMGSSSLkypgQPQEIPFFQNTTFSIPVNDPHQVWNSDEHEDLQVLVIISrppakiflyddwsMPHTAAVLKfpfVWDEDCFEAA---k<br>--------------enkvinfkKIIDSRGSLVAIEENKNIP---FSIKRVYYIFDTKGEEPRGFHANKKLEqVLVCLNGSCRVILDDGNI----IQEITLDSPAVGLYVGPAVWHEMHDFSSDCVMMVLASDY-------------YDETDYIRQ---YDNFKKYIAKinl-<br><br>BLAST: aliSize=6 (resi) iden=4.48% (6/134) simi=4.48% (6/134)<br>scvrdnslvrdisqmpqssygieglshitvagalnhgmkevevwlqtispgqrtpihrhsceevftvlkgkgtllmgssslkypgqpqeipffqnttfsipvndphqvwnsdehedlqvlviisrppakiflyddwsmphtaavlkf---<br>--enkvinfkkiidsrgslvaieenknipfsikrvyyifdtkgee<br><br>--PFVWDE------DC-------------------------------feaak<br>prgfhankkleqvlvclngscrvilddgniiqeitldspavglyvgPAVWHEmhdfssDCvmmvlasdyydetdyirqydnfkkyiakinl-----<br><br>Proposed: aliSize=117 (resi) RMSD=2.33 (Å)<br>scvrdnsl--VRDIsqMPQSSygIEGLSHITvAGA-LNHGMKEVEVWLQT-ISPGQRTPIHRH-SCEEVFTVLKGKGTLLMGSSS-lkypgqPQEIPFFQN-TTFSIPVNDPHQVWNSDehEDLQVLVIISRPPA--KIFLYddWsMPH--T------aavlkfpfvwdedcfeaak<br>--------enKVIN--FKKII--DSRGSLVA-IEEnKNIPFSIKRVYYIFdTKGEEPRGFHANkKLEQVLVCLNGSCRVILDDGNi------IQEITLDSPaVGLYVGPAVWHEMHDFS--SDCVMMVLASDYYDetDYIRQ--Y-DNFkkYiakinl-------------------
361	1lr5A (160)<br>2pakB (134)	C	11.19	TM-align: aliSize=94 (resi) RMSD=2.61 (Å)<br>scvrdnslvrdisqmpqssygieglshitvagalnhgm----------------------------KEVEVWLQT-ISPGQRTPIHRH-SCEEVFTVLKGKGTLLMGSSSlkypGqPQEIPFFQN-TTFSIPVNDPHQVWNSDehEDLQVLVIISRPPA--KIFLYddWsMPH-TAavlkfpfvwdedcf<br>--------------------------------------enkvinfkkiidsrgslvaieenknipfSIKRVYYIFdTKGEEPRGFHANkKLEQVLVCLNGSCRVILDDGN----I-IQEITLDSPaVGLYVGPAVWHEMHDFS--SDCVMMVLASDYYDetDYIRQ--Y-DNFkKY--------------<br><br>eaak------<br>----iakinl<br><br>SARST: aliSize=122 (resi) RMSD=13.14 (Å)<br>scvrdnslvrdisq--------MPQSSYGIEGLSHITVAGAlnhGMKEVEVWLQTISPGQRTPIHRHSCEE-VFTVLKGKGTLLMGSSSLkypgQPQEIPFFQNTTFSIPVNDPHQVWNSDEHEDLQVLVIISrppakiflyddwsMPHTAAVLKfpfVWDEDCFEAA---k<br>--------------enkvinfkKIIDSRGSLVAIEENKNIP---FSIKRVYYIFDTKGEEPRGFHANKKLEqVLVCLNGSCRVILDDGNI----IQEITLDSPAVGLYVGPAVWHEMHDFSSDCVMMVLASDY-------------YDETDYIRQ---YDNFKKYIAKinl-<br><br>BLAST: aliSize=6 (resi) iden=4.48% (6/134) simi=4.48% (6/134)<br>scvrdnslvrdisqmpqssygieglshitvagalnhgmkevevwlqtispgqrtpihrhsceevftvlkgkgtllmgssslkypgqpqeipffqnttfsipvndphqvwnsdehedlqvlviisrppakiflyddwsmphtaavlkf---<br>--enkvinfkkiidsrgslvaieenknipfsikrvyyifdtkgee<br><br>--PFVWDE------DC-------------------------------feaak<br>prgfhankkleqvlvclngscrvilddgniiqeitldspavglyvgPAVWHEmhdfssDCvmmvlasdyydetdyirqydnfkkyiakinl-----<br><br>Proposed: aliSize=94 (resi) RMSD=2.04 (Å)<br>scvrdnslvrdisqmpqssygieglshitvagalnhgmk-----------------------------EVEVWLQT-ISPGQRTPIHRH-SCEEVFTVLKGKGTLLMGSSS-lkypgqPQEIPFFQN-TTFSIPVNDPHQVWNSDehEDLQVLVIISRPPA--KIFLYddwsmphtaavlkfpfvwdEDC<br>---------------------------------------enkvinfkkiidsrgslvaieenknipfsIKRVYYIFdTKGEEPRGFHANkKLEQVLVCLNGSCRVILDDGNi------IQEITLDSPaVGLYVGPAVWHEMHDFS--SDCVMMVLASDYYDetDYIRQ-------------------YDN<br><br>FEAAK-----<br>FKKYIakinl
362	1lr5A (160)<br>2pamA (135)	N	10.37	TM-align: aliSize=94 (resi) RMSD=2.59 (Å)<br>scvrdnslvrdisqmpqssygieglshitvagalnhgm----------------------------KEVEVWLQT-ISPGQRTPIHRH-SCEEVFTVLKGKGTLLMGSSSlkypGqPQEIPFFQN-TTFSIPVNDPHQVWNSDehEDLQVLVIISRPPA--KIFLYdDwsMPH-TAavlkfpfvwdedcf<br>--------------------------------------enkvinfkkiidsrgslvaieenknipfSIKRVYYIFdTKGEEPRGFNANkKLEQVLVCLNGSCRVILDDGN----I-IQEITLDSPaVGLYVGPAVWHEMHDFS--SDCVMMVLASDYYDetDYIRQ-Y--DNFkKY--------------<br><br>eaak-------<br>----iakinle<br><br>SARST: aliSize=81 (resi) RMSD=5.68 (Å)<br>scvrdnslvrdisqmpqssygieglshitvagalnhgmk--------------------------EVEVWLQTISPGQRTPIHRHSCEE-----VFTVLKGKGTLLMGSSSLkypgQPQEIPFFQNTTFSIPVNDPHQVWNSDEHEDLQVLVIIS-----------------------rppakiflyddw<br>---------------------------------------enkvinfkkiidsrgslvaieenkniPFSIKRVYYIFDTKGEEPRGFNANkkleqVLVCLNGSCRVILDDGNI----IQEITLDSPAVGLYVGPAVWHEMHDFSSDCVMMVLASDYydetdyirqydnfkkyiakinle------------<br><br>smphtaavlkfpfvwdedcfeaak<br>------------------------<br><br>BLAST: aliSize=25 (resi) iden=8.89% (12/135) simi=18.52% (25/135)<br>scvrdnslvrdisqmpqssygieglshitvagalnhgmkevevwlqtispgqrtpihrhsceevftvlkgkgtllmgssslkypgqp-----------------------QEIPFFQNTTFSI--PVNDPHQVWNSDEHEDlQVLVIISrPPAKIFLyDD------------------------------<br>--enkvinfkkiidsrgslvaieenKNIPFSIKRVYYIfdTKGEEPRGFNANKKLE-QVLVCLN-GSCRVIL-DDgniiqeitldspavglyvgpavwhemhdfs<br><br>-----------------------------------wsmphtaavlkfpfvwdedcfeaak<br>sdcvmmvlasdyydetdyirqydnfkkyiakinle-------------------------<br><br>Proposed: aliSize=119 (resi) RMSD=2.41 (Å)<br>scvrdnSLVRDIsqMPQSSygIEGLSHITvAGA-LNHGMKEVEVWLQT-ISPGQRTPIHRH-SCEEVFTVLKGKGTLLMGSSS-lkypgqPQEIPFFQN-TTFSIPVNDPHQVWNSDehEDLQVLVIISRPPA--KIFLYdDwsMPH-T--------aavlkfpfvwdedcfeaak<br>------ENKVIN--FKKII--DSRGSLVA-IEEnKNIPFSIKRVYYIFdTKGEEPRGFNANkKLEQVLVCLNGSCRVILDDGNi------IQEITLDSPaVGLYVGPAVWHEMHDFS--SDCVMMVLASDYYDetDYIRQ-Y--DNFkKyiakinle-------------------

363	1lr5A (160)<br>2pamA (135)	C	10.37	TM-align: aliSize=94 (resi) RMSD=2.59 (Å)<br>scvrdnslvrdisqmpqssygieglshitvagalnhgm-----------------------------KEVEVWLQT-ISPGQRTPIHRH-SCEEVFTVLKGKGTLLMGSSSlkypGqPQEIPFFQN-TTFSIPVNDPHQVWNSDehEDLQVLVIISRPPA--KIFLYdDwsMPH-TAavlkfpfvwdedcf<br>--enkvinfkkiidsrgslvaieenknipfSIKRVYYIFdTKGEEPRGFNANkKLEQVLVCLNGSCRVILDDGN----I-IQEITLDSPaVGLYVGPAVWHEMHDFS--SDCVMMVLASDYYDetDYIRQ-Y--DNFkKY-------------<br>eaak-------<br>----iakinle<br><br>SARST: aliSize=81 (resi) RMSD=5.68 (Å)<br>scvrdnslvrdisqmpqssygieglshitvagalnhgmk--------------------------EVEVWLQTISPGQRTPIHRHSCEE-----VFTVLKGKGTLLMGSSSLkypgQPQEIPFFQNTTFSIPVNDPHQVWNSDEHEDLQVLVIIS-------------------------rppakiflyddw<br>--enkvinfkkiidsrgslvaieenkniPFSIKRVYYIFDTKGEEPRGFNANkkleqVLVCLNGSCRVILDDGNI----IQEITLDSPAVGLYVGPAVWHEMHDFSSDCVMMVLASDYydetdyirqydnfkkyiakinle-----------<br>smphtaavlkfpfvwdedcfeaak<br>------------------------<br><br>BLAST: aliSize=25 (resi) iden=8.89% (12/135) simi=18.52% (25/135)<br>scvrdnslvrdisqmpqssygieglshitvagalnhgmkevevwlqtispgqrtpihrhsceevftvlkgkgtllmgssslkypgqp-----------------------QEIPFFQNTTFSI--PVNDPHQVWNSDEHEDlQVLVIISrPPAKIFLyDD-------------------------------<br>---enkvinfkkiidsrgslvaieenKNIPFSIKRVYYIfdTKGEEPRGFNANKKLE-QVLVCLN-GSCRVIL-DDgniiqeitldspavglyvgpavwhemhdfs<br>------------------------------------wsmphtaavlkfpfvwdedcfeaak<br>sdcvmmvlasdyydetdyirqydnfkkyiakinle-------------------------<br><br>Proposed: aliSize=94 (resi) RMSD=2.01 (Å)<br>scvrdnslvrdisqmpqssygieglshitvagalnhgmk---------------------------EVEVWLQT-ISPGQRTPIHRH-SCEEVFTVLKGKGTLLMGSSS-lkypgqPQEIPFFQN-TTFSIPVNDPHQVWNSDehEDLQVLVIISRPPA--KIFLYddwsmphtaavlkfpfvwdEDC<br>--enkvinfkkiidsrgslvaieenknipfsIKRVYYIFdTKGEEPRGFNANkKLEQVLVCLNGSCRVILDDGNi------IQEITLDSPaVGLYVGPAVWHEMHDFS--SDCVMMVLASDYYDetDYIRQ------------------YDN<br>FEAAK------<br>FKKYIakinle
364	1lr5A (160)<br>2pamB (134)	N	10.45	TM-align: aliSize=94 (resi) RMSD=2.59 (Å)<br>scvrdnslvrdisqmpqssygieglshitvagalnhgm-----------------------------KEVEVWLQT-ISPGQRTPIHRH-SCEEVFTVLKGKGTLLMGSSSlkypGqPQEIPFFQN-TTFSIPVNDPHQVWNSDehEDLQVLVIISRPPA--KIFLYddWsMPH-TAavlkfpfvwdedcf<br>--enkvinfkkiidsrgslvaieenknipfSIKRVYYIFdTKGEEPRGFNANkKLEQVLVCLNGSCRVILDDGN----I-IQEITLDSPaVGLYVGPAVWHEMHDFS--SDCVMMVLASDYYDetDYIRQ--Y-DNFkKY-------------<br>eaak------<br>----iakinl<br><br>SARST: aliSize=90 (resi) RMSD=5.59 (Å)<br>scvrdnslvrdisqmpqssygieglshitvagalnhgmk-----------------------------EVEVWLQTISPGQRTPIHRH-SCEEVFTVLKGKGTLLMGSSSLkypgQPQEIPFFQNTTFSIPVNDPHQVWNSDEHEDLQVLVIISrppakiflyddwsMPHTAAVLK-------------<br>--enkvinfkkiidsrgslvaieenknipfsiKRVYYIFDTKGEEPRGFNANkKLEQVLVCLNGSCRVILDDGNI----IQEITLDSPAVGLYVGPAVWHEMHDFSSDCVMMVLASDY-------------YDETDYIRQydnfkkyiakinl<br>fpfvwdedcfeaak<br>--------------<br><br>BLAST: aliSize=25 (resi) iden=8.96% (12/134) simi=18.66% (25/134)<br>scvrdnslvrdisqmpqssygieglshitvagalnhgmkevevwlqtispgqrtpihrhsceevftvlkgkgtllmgssslkypgqp-----------------------QEIPFFQNTTFSI--PVNDPHQVWNSDEHEDlQVLVIISrPPAKIFLyDD------------------------------<br>---enkvinfkkiidsrgslvaieenKNIPFSIKRVYYIfdTKGEEPRGFNANKKLE-QVLVCLN-GSCRVIL-DDgniiqeitldspavglyvgpavwhemhdfs<br>-----------------------------------wsmphtaavlkfpfvwdedcfeaak<br>sdcvmmvlasdyydetdyirqydnfkkyiakinl------------------------<br><br>Proposed: aliSize=117 (resi) RMSD=2.30 (Å)<br>scvrdnsl--VRDIsqMPQSSygIEGLSHITvAGA-LNHGMKEVEVWLQT-ISPGQRTPIHRH-SCEEVFTVLKGKGTLLMGSSS-lkypgqPQEIPFFQN-TTFSIPVNDPHQVWNSDehEDLQVLVIISRPPA--KIFLYddWsMPH--T------aavlkfpfvwdedcfeaak<br>--------enKVIN--FKKII--DSRGSLVA-IEEnKNIPFSIKRVYYIFdTKGEEPRGFNANkKLEQVLVCLNGSCRVILDDGNi------IQEITLDSPaVGLYVGPAVWHEMHDFS--SDCVMMVLASDYYDetDYIRQ--Y-DNFkkYiakinl-------------------
365	1lr5A (160)<br>2pamB (134)	C	10.45	TM-align: aliSize=94 (resi) RMSD=2.59 (Å)<br>scvrdnslvrdisqmpqssygieglshitvagalnhgm-----------------------------KEVEVWLQT-ISPGQRTPIHRH-SCEEVFTVLKGKGTLLMGSSSlkypGqPQEIPFFQN-TTFSIPVNDPHQVWNSDehEDLQVLVIISRPPA--KIFLYddWsMPH-TAavlkfpfvwdedcf<br>--enkvinfkkiidsrgslvaieenknipfSIKRVYYIFdTKGEEPRGFNANkKLEQVLVCLNGSCRVILDDGN----I-IQEITLDSPaVGLYVGPAVWHEMHDFS--SDCVMMVLASDYYDetDYIRQ--Y-DNFkKY-------------<br>eaak------<br>----iakinl<br><br>SARST: aliSize=90 (resi) RMSD=5.59 (Å)<br>scvrdnslvrdisqmpqssygieglshitvagalnhgmk-----------------------------EVEVWLQTISPGQRTPIHRH-SCEEVFTVLKGKGTLLMGSSSLkypgQPQEIPFFQNTTFSIPVNDPHQVWNSDEHEDLQVLVIISrppakiflyddwsMPHTAAVLK-------------<br>--enkvinfkkiidsrgslvaieenknipfsiKRVYYIFDTKGEEPRGFNANkKLEQVLVCLNGSCRVILDDGNI----IQEITLDSPAVGLYVGPAVWHEMHDFSSDCVMMVLASDY-------------YDETDYIRQydnfkkyiakinl<br>fpfvwdedcfeaak<br>--------------<br><br>BLAST: aliSize=25 (resi) iden=8.96% (12/134) simi=18.66% (25/134)<br>scvrdnslvrdisqmpqssygieglshitvagalnhgmkevevwlqtispgqrtpihrhsceevftvlkgkgtllmgssslkypgqp-----------------------QEIPFFQNTTFSI--PVNDPHQVWNSDEHEDlQVLVIISrPPAKIFLyDD------------------------------<br>---enkvinfkkiidsrgslvaieenKNIPFSIKRVYYIfdTKGEEPRGFNANKKLE-QVLVCLN-GSCRVIL-DDgniiqeitldspavglyvgpavwhemhdfs<br>-----------------------------------wsmphtaavlkfpfvwdedcfeaak<br>sdcvmmvlasdyydetdyirqydnfkkyiakinl------------------------<br><br>Proposed: aliSize=94 (resi) RMSD=2.00 (Å)<br>scvrdnslvrdisqmpqssygieglshitvagalnhgmk---------------------------EVEVWLQT-ISPGQRTPIHRH-SCEEVFTVLKGKGTLLMGSSS-lkypgqPQEIPFFQN-TTFSIPVNDPHQVWNSDehEDLQVLVIISRPPA--KIFLYddwsmphtaavlkfpfvwdEDC<br>--enkvinfkkiidsrgslvaieenknipfsIKRVYYIFdTKGEEPRGFNANkKLEQVLVCLNGSCRVILDDGNi------IQEITLDSPaVGLYVGPAVWHEMHDFS--SDCVMMVLASDYYDetDYIRQ------------------YDN<br>FEAAK-----<br>FKKYIakinl

366	1m1dA (163)<br>1i1dD (161)	C	11.18	TM-align: aliSize=124 (resi) RMSD=3.21 (Å)<br>-------LLDFDILtndgthrNMKLLIDLKNIFSrQLpkMPKEYIV--Klv---F--DR---------------HHESMVILKN-KQKVIGGICFRQYKP-----QRFAEVAFLAVTANEQVRGYGTRLMNKFKDHMQKQNIEYLLTYADNFAIGYFKKQGFTKEHrmpqekwkgy-IKDYDGgtlmecy<br>gsmslpdGFYIRRM-------EEGDLEQVTETLK-VL--TTVGTITpeS--fckLikYWneatvwndnedkkimQYNPMVIVDKrTETVAATGNIIIERKiihelGLCGHIEDIAVNSKYQGQGLGKLLIDQLVTIGFDYGCYKIILDCDEKNVKFYEKCGFSNAG----------vEMQIR--------<br>ihpyvdygr-<br>---------k<br><br>SARST: aliSize=119 (resi) RMSD=7.51 (Å)<br>l--------LDFDILTNDGTHRNMKLLIDL-------KNIFSrqlpkmpkEYIVKLVFD------RHHES--MVILK-----NKQK-VIGGICFRQYKPQR--FAEVAF-LAVTANEQVRGYGTRLMNKFKDHMQKQNIEYLLTYADNFAIGYFKKQGFT----------kehrmpqekwkgyikdydgg<br>-gsmslpdgFYIRRMEEGDLEQVTETLKVLttvgtitPESFC--------KLIKYWNEAtvwndnEDKKImqYNPMVivdkrTETVaATGNIIIERKIIHElgLCGHIEdIAVNSKYQGQGLGKLLIDQLVTIGFDYGCYKIILDCDEKNVKFYEKCGFSnagvemqirk-------------------<br>tlmecyihpyvdygr<br>---------------<br><br>BLAST: aliSize=55 (resi) iden=16.15% (26/161) simi=34.16% (55/161)<br>lldfd---------------------------ILTNDGT--HRNMKLLIDLKN---IFSRQLPKMPKEYIVKLVFDRHHESMVILKN---KQKVIG--GICfrqykpqrfAEVAFLAVTANEQVRGYGTRLMNKFKDHMQKQNIEYLLTYADNFAIGYFKKQGFT----------kehrmpqekwkgyik<br>-----gsmslpdgfyirrmeegdleqvtetlkVLTTVGTitPESFCKLIKYWNeatVWNDNEDKKIMQYNPMVIVDKRTETVAATGNiiiERKIIHelGLC---------GHIEDIAVNSKYQGQGLGKLLIDQLVTIGFDYGCYKIILDCDEKNVKFYEKCGFSnagvemqirk---------------<br>dydggtlmecyihpyvdygr<br>--------------------<br><br>Proposed: aliSize=121 (resi) RMSD=2.03 (Å)<br>-------LLDFDILtndgthrNMKLLIDLKNIFSrQLpkMPKE---------yivklvF--DR---------------HHESMVILKN--kQKVIGGICFRQYKP-----QRFAEVAFLAVTANEQVRGYGTRLMNKFKDHMQKQNIEYLLTYADNFAIGYFKKQGFTKEhrmpqekwkgyikdydgGTL<br>gsmslpdGFYIRRM-------EEGDLEQVTETLK-VL--TTVGtitpesfck------LikYWneatvwndnedkkimQYNPMVIVDKrt-ETVAATGNIIIERKiihelGLCGHIEDIAVNSKYQGQGLGKLLIDQLVTIGFDYGCYKIILDCDEKNVKFYEKCGFSNA-----------------GVE<br>MECYIhpyvdygr<br>MQIRK--------
367	1m1dA (163)<br>1mk4A (157)	M	14.01	TM-align: aliSize=132 (resi) RMSD=2.51 (Å)<br>LLDFDILTNdgthrnMkLLIDLKNIFSRQL--PKMPkeYIVKlvFD--RHHE--SMVILKNkQKVIGGICFRQYKPQ-RFAEVAFLAVTANEQVRGYGTRLMNKFKDHMQKQNIEYLLTYAD--N-FAIGYFKKQGFTKEHrmpqekwkgyi-------------KDY--DGGTLMECYIhpyvdygr<br>HMDIRTITS------S-DYEMVTSVLNEWWggRQLK--EKLP--RLffEHFQdtSFITSEH-NSMTGFLIGFQSQSDpETAYIHFSGVHPDFRKMQIGKQLYDVFIETVKQRGCTRVKCVTSpvNkVSIAYHTKLGFDIEK-----------gtktvngisvfanYDGpgQDRVLFVKNI--------<br><br>SARST: aliSize=112 (resi) RMSD=6.62 (Å)<br>lldfdiltndgthrnmkllidlknifsrqlpkmpkey---------------------------------IVKLVFDRHHESMVILknKQKVIGGICFRQYKP-QRFAEVAFLAVTANEQVRGYGTRLMNKFKDHMQKQNIEYLLTYADNFAIG---YFKKQGFTKEHRMPqekWKGYIKDYDG------<br>-------------------------------------hmdirtitssdyemvtsvlnewwggrqlkeklpRLFFEHFQDTSFITSE--HNSMTGFLIGFQSQSdPETAYIHFSGVHPDFRKMQIGKQLYDVFIETVKQRGCTRVKCVTSPVNKVsiaYHTKLGFDIEKGTK---TVNGISVFANydgpgq<br>-GTLMECY-ihpyvdygr<br>dRVLFVKNi---------<br><br>BLAST: aliSize=47 (resi) iden=14.01% (22/157) simi=29.94% (47/157)<br>lldfdiltndgthrnmkllidlknifsrqlpkmp----------------------------KEYIVKLVFDRHHESMVILKNKQKVIGGIC-FRQYKPQRFAEVAFLAVTANEQVRGYGTRLMNKFKDHMQKQN---IEYLLTYADNFAIGYFKKQGFTKEHRMPQEKWKGYIKDYDG-----------<br>----------------------------------hmdirtitssdyemvtsvlnewwggrqlKEKLPRLFFEHFQDTSFITSEHNSMTGFLIgFQSQSDPETAYIHFSGVHPDFRKMQIGKQLYDVFIETVKQRGctrVKCVTSPVNKVSIAYHTKLGFDIEKGTKTVNGISVFANYDGpgqdrvlfvkn<br>-gtlmecyihpyvdygr<br>i----------------<br><br>Proposed: aliSize=141 (resi) RMSD=2.36 (Å)<br>LLDFDILTNdgthrnMkLLIDLKNIFSRQL--PKMPkeYIVKlvFD--RHHE--SMVILKNkQKVIGGICFRQYKPQR-FAEVAFLAVTANEQVRGYGTRLMNKFKDHMQKQNIEYLLTYAD--N-FAIGYFKKQGFTKEH----RMPQEKWKGYIKDY--dgGTLMECYIhpyvdygr<br>HMDIRTITS------S-DYEMVTSVLNEWWggRQLK--EKLP--RLffEHFQdtSFITSEH-NSMTGFLIGFQSQSDPeTAYIHFSGVHPDFRKMQIGKQLYDVFIETVKQRGCTRVKCVTSpvNkVSIAYHTKLGFDIEKgtktVNGISVFANYDGPGqd--RVLFVKNI--------
368	1m1dA (163)<br>1qsmA (150)	C	13.33	TM-align: aliSize=128 (resi) RMSD=2.96 (Å)<br>-LLDFDILTndgThRNMKLLIDLKNIFSRqLPKMPKEY-IVKLVFDR-----H-HESMVILKN-KQKVIGGICFRQYK----PQRFAEVAFLAVTANEQVRGYGTRLMNKFKDHMQKQNIEYLLTYADN-F--AIGYFKKQGFTKEHrmpqekw-Kgyik-Dydggtlmecyihpyvdygr----<br>dNITVRFVT---E-NDKEGWQRLWKSYQD-FYEVSFPDdLDDFNFGRfldpnIkMWAAVAVESsSEKIIGMINFFNHMttwdFKDKIYINDLYVDENSRVKGAGGKLIQFVYDEADKLGTPSVYWCTDEsNhrAQLLYVKVGYKAPK-------iL----yK-------------------rkgy<br><br>SARST: aliSize=136 (resi) RMSD=7.88 (Å)<br>l--LDFDILTNDGthrNMKLLIDLKNIFSRQLPKMPK---EYIVKLVFDRHHESMVILKNK---QKVIGGICFRQYKPQRF----AEVAFLAVTANEQVRGYGTRLMNKFKDHMQKQNIEYLLTYADNFAIGYFKKQgftkehrmpqekwkGYIKDYDGGTLMECY--ihpyvdygr<br>-dnITVRFVTEND---KEGWQRLWKSYQDFYEVSFPDdldDFNFGRFLDPNIKMWAAVAVEsssEKIIGMINFFNHMTTWDfkdkIYINDLYVDENSRVKGAGGKLIQFVYDEADKLGTPSVYWCTDESNHRAQLLY--------------VKVGYKAPKILYKRKgy---------<br><br>BLAST: aliSize=32 (resi) iden=12.00% (18/150) simi=21.33% (32/150)<br>lldfdiltndgthrnmkllidlknifsrqlpkmpkeyivklvfdrhhe--SMVILKNKQKVIGGICFRQYKPQ-RFAEVAF---LAVTANEQVRGYGTRLMNKFKDHMQKQNIEYLLTYADNfaigyfkkqgftKEHR--<br>--dnitvrfvtendkegwqrlwksyqdfyevsfpddlddfnfgrfldpnikmwaAVAVESSSEKIIGMINFFNHMTTwDFKDKIYindLYVDENSRVKGAGGKLIQFVYDEADKLGTPSVYWCTDE------------SNHRaq<br>-------------------mpqekwkgyikdydggtlmecyihpyvdygr<br>llyvkvgykapkilykrkgy------------------------------<br><br>Proposed: aliSize=129 (resi) RMSD=2.29 (Å)<br>-LLDFDILtndgthrNMKLLIDLKNIFSRQLP--KMPKE--YIVKLVFD--R--H-HESMVILKN-KQKVIGGICFRQYkP-----QRFAEVAFLAVTANEQVRGYGTRLMNKFKDHMQKQNIEYLLTYADN-F--AIGYFKKQGFTkehrmpqekwkgyikdyDGGTLMECY---ihpyvdygr<br>dNITVRFV-------TENDKEGWQRLWKSYQDfyEVSFPddLDDFNFGRflDpnIkMWAAVAVESsSEKIIGMINFFNH-MttwdfKDKIYINDLYVDENSRVKGAGGKLIQFVYDEADKLGTPSVYWCTDEsNhrAQLLYVKVGYK-----------------APKILYKRKgya---------

369	1m1dA (163)<br>1qsmB (149)	C	13.42	TM-align: aliSize=128 (resi) RMSD=2.99 (Å)<br>LLDFDILTndgThRNMKLLIDLKNIFSRqLPKMPKEY-IVKLVFDR-----H-HESMVILKN-KQKVIGGICFRQYK----PQRFAEVAFLAVTANEQVRGYGTRLMNKFKDHMQKQNIEYLLTYADN-F--AIGYFKKQGFTKEHrmpqekw-Kgyik-Dydggtlmecyihpyvdygr----<br>NITVRFVT---E-NDKEGWQRLWKSYQD-FYEVSFPDdLDDFNFGRfldpnIkMWAAVAVESsSEKIIGMINFFNHMttwdFKDKIYINDLYVDENSRVKGAGGKLIQFVYDEADKLGTPSVYWCTDEsNhrAQLLYVKVGYKAPK-------iL----yK-------------------rkgy<br><br>SARST: aliSize=120 (resi) RMSD=4.86 (Å)<br>lldf-DILTNDGTHRNMKLLIDLKNIFSRQLPKMPK---EYIVKLVFDRHH-ESMVILKN--KQKVIGGICFRQYKPQRF----AEVAFLAVTANEQVRGYGTRLMNKFKDHMQKQNIEYLLTYADNFAIGYFKK-----------------qgftkehrmpqekwkgyikdydggtlmecyihpyvdygr<br>----nITVRFVTENDKEGWQRLWKSYQDFYEVSFPDdldDFNFGRFLDPNIkMWAAVAVEssSEKIIGMINFFNHMTTWDfkdkIYINDLYVDENSRVKGAGGKLIQFVYDEADKLGTPSVYWCTDESNHRAQLLyvkvgykapkilykrkgy---------------------------------------<br><br>BLAST: aliSize=32 (resi) iden=12.08% (18/149) simi=21.48% (32/149)<br>lldfdiltndgthrnmkllidlknifsrqlpkmpkeyivklvfdrhhe--SMVILKNKQKVIGGICFRQYKPQ-RFAEVAF---LAVTANEQVRGYGTRLMNKFKDHMQKQNIEYLLTYADNfaigyfkkqgftKEHR---<br>---nitvrfvtendkegwqrlwksyqdfyevsfpddlddfnfgrfldpnikmwaAVAVESSSEKIIGMINFFNHMTTwDFKDKIYindLYVDENSRVKGAGGKLIQFVYDEADKLGTPSVYWCTDE-----------SNHRaql<br><br>------------------mpqekwkgyikdydggtlmecyihpyvdygr<br>lyvkvgykapkilykrkgy------------------------------<br><br>Proposed: aliSize=129 (resi) RMSD=2.22 (Å)<br>LLDFDILtndgthrNMKLLIDLKNIFSRQLP--KMPKE--YiV-KLVFD--R--H-HESMVILKN--kQKVIGGICFRQYkPQ-----RFAEVAFLAVTANEQVRGYGTRLMNKFKDHMQKQNIEYLLTYADN-F--AIGYFKKQGFTKehrmpqekwkgyikdydggtLMECYIHPYV-dygr<br>NITVRFV-------TENDKEGWQRLWKSYQDfyEVSFPddL-DdFNFGRflDpnIkMWAAVAVESss-EKIIGMINFFNH-MTtwdfkDKIYINDLYVDENSRVKGAGGKLIQFVYDEADKLGTPSVYWCTDEsNhrAQLLYVKVGYKA-------------------PKILYKRKGYa----
370	1m1dA (163)<br>1qsmD (152)	C	13.16	TM-align: aliSize=127 (resi) RMSD=2.88 (Å)<br>---LLDFDILTndgThRNMKLLIDLKNIFSRqLPKMPKEY-IVKLVFDR-----H-HESMVILK--NKqKVIGGICFRQYK----PQRFAEVAFLAVTANEQVRGYGTRLMNKFKDHMQKQNIEYLLTYADN-F--AIGYFKKQGFTKEHrmpqekw-Kgyik-Dydggtlmecyihpyvdygr----<br>sedNITVRFVT---E-NDKEGWQRLWKSYQD-FYEVSFPDdLDDFNFGRfldpnIkMWAAVAVEssSE-KIIGMINFFNHMttwdFKDKIYINDLYVDENSRVKGAGGKLIQFVYDEADKLGTPSVYWCTDEsNhrAQLLYVKVGYKAPK-------iL----yK-------------------rkgy<br><br>SARST: aliSize=136 (resi) RMSD=7.79 (Å)<br>lldf----DILTNDGTHRNMKLLIDLKNIFSRQLPKMPKEYIVKLV---FDRH--HESMVILK-NKQKVIGGICFRQYKPQRF----AEVAFLAVTANEQVRGYGTRLMNKFKDHMQKQNIEYLLTYADNFAIGYFKKqgftkehrmpQEKWkgyiKDYDGGTLMECY--ihpyvdygr<br>----sednITVRFVTENDKEGWQRLWKSYQDFYEVSFPDDLDDFNFgrfLDPNikMWAAVAVEsSSEKIIGMINFFNHMTTWDfkdkIYINDLYVDENSRVKGAGGKLIQFVYDEADKLGTPSVYWCTDESNHRAQLL----------YVKV----GYKAPKILYKRKgy---------<br><br>BLAST: aliSize=32 (resi) iden=11.84% (18/152) simi=21.05% (32/152)<br>lldfdiltndgthrnmkllidlknifsrqlpkmpkeyivklvfdrhhe---SMVILKNKQKVIGGICFRQYKPQ-RFAEVAF---LAVTANEQVRGYGTRLMNKFKDHMQKQNIEYLLTYADNfaigyfkkqgftKEHR<br>---sednitvrfvtendkegwqrlwksyqdfyevsfpddlddfnfgrfldpnikmwaAVAVESSSEKIIGMINFFNHMTTwDFKDKIYindLYVDENSRVKGAGGKLIQFVYDEADKLGTPSVYWCTDE-----------SNHR<br><br>---------------------mpqekwkgyikdydggtlmecyihpyvdygr<br>aqllyvkvgykapkilykrkgy------------------------------<br><br>Proposed: aliSize=129 (resi) RMSD=2.25 (Å)<br>---LLDFDILtndgthrNMKLLIDLKNIFSRQLP--KMPKE--YIVKLVFD--R--H-HESMVILK-NKQKVIGGICFRQYkP-----QRFAEVAFLAVTANEQVRGYGTRLMNKFKDHMQKQNIEYLLTYADN-F--AIGYFKKQGFTkehrmpqekwkgyikdyDGGTLMECY---ihpyvdygr<br>sedNITVRFV-------TENDKEGWQRLWKSYQDfyEVSFPddLDDFNFGRflDpnIkMWAAVAVEsSSEKIIGMINFFNH-MttwdfKDKIYINDLYVDENSRVKGAGGKLIQFVYDEADKLGTPSVYWCTDEsNhrAQLLYVKVGYK----------------APKILYKRKgya---------
371	1m1dA (163)<br>1s3zB (145)	C	9.66	TM-align: aliSize=122 (resi) RMSD=2.48 (Å)<br>lLDFDILtndgthrNMKLLIDLKNIFSRQLPkMPKEYIV-KLVFD--RH--HESMVILKNkQKVIGGICFRQYKP-------QRFAEVAFLAVTANEQVRGYGTRLMNKFKDHMQKQNIEYLLTYAD-NF--AIGYFKKQGFTKEHrmpqekwkgyik---Dydggtlmecyihpyvdygr-----<br>-MDIRQM-------NKTHLEHWRGLRKQLWP-GHPDDAHlADGEEilQAdhLASFIAMAD-GVAIGFADASIRHDyvngcdsSPVVFLEGIFVLPSFRQRGVAKQLIAAVQRWGTNKGCREMASDTSpENtiSQKVHQALGFEETE-------------rviF-------------------yrkrc<br><br>SARST: aliSize=118 (resi) RMSD=6.01 (Å)<br>lldfdiltndgthrnmkl----------LIDLKNIFSRQLPKMPK----EYIVKLVFDRHHESMVILKNKqKVIGGICFRQYkpQ----R-----FAEVAFLAVTANEQVRGYGTRLMNKFKDHMQKQNIEYLLTYADNFA---IGYFKKQGFTkeHRMPqekwkgyikdydgGTLMECY-ihpyvdygr<br>-----------------mdirqmnkthLEHWRGLRKQLWPGHPDdahlADGEEILQADHLASFIAMADG-VAIGFADASIR--HdyvnGcdsspVVFLEGIFVLPSFRQRGVAKQLIAAVQRWGTNKGCREMASDTSPENtisQKVHQALGFE--ETER-------------VIFYRKRc---------<br><br>BLAST: aliSize=11 (resi) iden=4.14% (6/145) simi=7.59% (11/145)<br>lldfdiltndgthrnmkllidlknifsrqlpkmpkeyivklvfdrhhesmvilknkqkviggicfrqykpqrfaevaflavtaneqvrgygtrlmnkfkdh------------------------------------MQKQNIEYLLTYADNFAIGY------------------------------<br>---mdirqmnkthlehwrglrkqlwpghpddahladgeeiLQADHLASFIAMADGVAIGFadasirhdyvngcdsspvvflegifvlpsfrq<br><br>---fkkqgftkehrmpqekwkgyikdydggtlmecyihpyvdygr<br>rgvakqliaavqrwgtnkgcremasdtspentisqkvhqalgfeetervifyrkrc---<br><br>Proposed: aliSize=130 (resi) RMSD=2.20 (Å)<br>lLDFDILtndgthrNMKLLIDLKNIFSRQLPKMPKEYIVKLVFD--RH--HESMVILKNkQKVIGGICFRQYkP-------QRFAEVAFLAVTANEQVRGYGTRLMNKFKDHMQKQNIEYLLTYAD-NF--AIGYFKKQGFTKEhrmpqekwkgyikDydggTLMECYIHP-yvdygr<br>-MDIRQM-------NKTHLEHWRGLRKQLWPGHPDDAHLADGEEilQAdhLASFIAMAD-GVAIGFADASIR-HdyvngcdsSPVVFLEGIFVLPSFRQRGVAKQLIAAVQRWGTNKGCREMASDTSpENtiSQKVHQALGFEET------------E----RVIFYRKRCc------

372	1m1dA (163)<br>1s5kA (153)	C	8.50	TM-align: aliSize=122 (resi) RMSD=2.53 (Å)<br>-------LLDFDILtndgthrNMKLLIDLKNIFSRQLPkmPKE-YIV-KLVFD--RH--HESMVILKNkQKVIGGICFRQYKP-------QRFAEVAFLAVTANEQVRGYGTRLMNKFKDHMQKQNIEYLLTYADN-F--AIGYFKKQGFTKEHrmpqekwkgyik---Dydggtlmecyihpyvdygr-<br>glvprgsHMDIRQM-------NKTHLEHWRGLRKQLWP--GHPdDAHlADGEEilQAdhLASFIAMAD-GVAIGFADASIRHDyvngcdsSPVVFLEGIFVLPSFRQRGVAKQLIAAVQRWGTNKGCREMASDTSPeNtiSQKVHQALGFEETE------------rviF-------------------y<br>----<br>rkrc<br><br>SARST: aliSize=117 (resi) RMSD=3.26 (Å)<br>l--------LDFDILTndgthrnMKLLIDLKNIFSRQLPKMPKEY----IVKLVFDRHHESMVILKNKqKVIGGICFRQYkpQ----RF-----AEVAFLAVTANEQVRGYGTRLMNKFKDHMQKQNIEYLLTYADNF---AIGYFKKQGFT-----------kehrmpqekwkgyikdydggtlmecy<br>-glvprgshMDIRQMN-------KTHLEHWRGLRKQLWPGHPDDAhladGEEILQADHLASFIAMADG-VAIGFADASIR--HdyvnGCdsspvVFLEGIFVLPSFRQRGVAKQLIAAVQRWGTNKGCREMASDTSPEntiSQKVHQALGFEetervifyrkrc------------------------<br>ihpyvdygr<br>---------<br><br>BLAST: aliSize=11 (resi) iden=3.92% (6/153) simi=7.19% (11/153)<br>lldfdiltndgthrnmkllidlknifsrqlpkmpkeyivklvfdrhhesmvilknkqkviggicfrqykpqrfaevaflavtaneqvrgygtrlmnkfkdh-------------------------------------MQKQNIEYLLTYADNFAIGY-----------------------<br>---glvprgshmdirqmnkthlehwrglrkqlwpghpddahladgeeiLQADHLASFIAMADGVAIGFadasirhdyvngcdsspvvflegi<br><br>---fkkqgftkehrmpqekwkgyikdydggtlmecyihpyvdygr<br>fvlpsfrqrgvakqliaavqrwgtnkgcremasdtspentisqkvhqalgfeetervifyrkrc---<br><br>Proposed: aliSize=129 (resi) RMSD=2.16 (Å)<br>-------LLDFDILtndgthrNMKLLIDLKNIFSRQLPkmPKE--YIVKLVFD--RH--HESMVILKNkQKVIGGICFRQYkP-------QRFAEVAFLAVTANEQVRGYGTRLMNKFKDHMQKQNIEYLLTYAD-NF--AIGYFKKQGFTKEhrmpqekwkgyikDydggTLMECYIHP-yvdygr<br>glvprgsHMDIRQM-------NKTHLEHWRGLRKQLWP--GHPddAHLADGEEilQAdhLASFIAMAD-GVAIGFADASIR-HdyvngcdsSPVVFLEGIFVLPSFRQRGVAKQLIAAVQRWGTNKGCREMASDTSpENtiSQKVHQALGFEET------------E----RVIFYRKRCc------
373	1m1dA (163)<br>1ufhA (155)	C	9.68	TM-align: aliSize=126 (resi) RMSD=2.96 (Å)<br>LLDFDILtndgthrNMKLLIDLKNIFSRQLPKM------------P--KEYIVK-LV--FD-RH-HESMVILKNKQKVIGGICFRQYKPQ--RFAEVAFLAVTANEQVRGYGTRLMNKFKDHMQKQNIEYLLTYADN-F--AIGYFKKQGFTKEHrmpqekwkgyikdydggTLmecyihpyvdygr-----<br>TIMLTPM-------QTEEFRSYLTYTTKHYAEEkvkagtwlpedaQllSKQVFTdLLprGLeTPhHHLWSLKLNEKDIVGWLWIHAEPEHpqQEAFIYDFGLYEPYRGKGYAKQALAALDQAARSMGIRKLSLHVFAhNqtARKLYEQTGFQETD-----------------VV-------------mskkl<br><br>SARST: aliSize=124 (resi) RMSD=7.33 (Å)<br>lldfdiltndgt-------------HRNMKLLIDLKNIFSRQLPkMPKEYIVKL------------VFDRHHESMVILKNKQKVIGGICFRQYK--PQRFAEVAFLAVTANEQVRGYGTRLMNKFKDHMQKQNIEYLLTYADNFAIGYFK---KQGFTkeHRmpqekwkgyikdydgGTLMECY-ihpyv<br>------------timltpmqteefrSYLTYTTKHYAEEKVKAGT-WLPEDAQLLskqvftdllprgLETPHHHLWSLKLNEKDIVGWLWIHAEPehPQQEAFIYDFGLYEPYRGKGYAKQALAALDQAARSMGIRKLSLHVFAHNQTARKlyeQTGFQ--ET--------------DVVMSKKl-----<br>dygr<br>----<br><br>BLAST: aliSize=35 (resi) iden=11.61% (18/155) simi=22.58% (35/155)<br>lldfdiltndgthrnmkllidlknifsrqlpkmpkeyivklvfdr--HHESMVILKNKQKVIGGICFRQyKPQRFAEVAFLA-VTANEQVRG--YGTRLMNKFKDHMQKQNIEYLL--TYADN-FAIGYFKKQGF<br>---timltpmqteefrsyltyttkhyaeekvkagtwlpedaqllskqvftdllprgletpHHHLWSLKLNEKDIVGWLWIHA-EPEHPQQEAFIYdFGLYEPYRGkgYAKQALAALDQAARSMGIRKLSlhVFAHNqTARKLYEQTGF<br><br>-----------tkehrmpqekwkgyikdydggtlmecyihpyvdygr<br>qetdvvmskkl------------------------------------<br><br>Proposed: aliSize=129 (resi) RMSD=2.46 (Å)<br>l-LDFDILtndgthrNMKLLIDLKNIFSRQL-PKM--------------PKEYIVKLV--F--DRHHESMVIL-KNKqKVIGGICFRQYKP-Q-RFAEVAFLAVTANEQVRGYGTRLMNKFKDHMQKQNIEYLLTYADN-F--AIGYFKKQGFTKehrmpqekwkgyikdydGGTLMECYIhpyvdygr<br>-tIMLTPM-------QTEEFRSYLTYTTKHYaEEKvkagtwlpedaqllSKQVFTDLLprGleTPHHHLWSLKlNEK-DIVGWLWIHAEPEhPqQEAFIYDFGLYEPYRGKGYAKQALAALDQAARSMGIRKLSLHVFAhNqtARKLYEQTGFQE-----------------TDVVMSKKL--------
374	1m1dA (163)<br>1ufhB (154)	C	9.09	TM-align: aliSize=126 (resi) RMSD=2.75 (Å)<br>lLDFDILtndgthrNMKLLIDLKNIFSRQL-PKM--------------PKEYIVKLV--F-DRH-HESMVILKNKQKVIGGICFRQYKPQ--RFAEVAFLAVTANEQVRGYGTRLMNKFKDHMQKQNIEYLLTYADN-F--AIGYFKKQGFTKEHrmpqekwkgyikdYDGgtlmecyihpyvdygr----<br>-IMLTPM-------QTEEFRSYLTYTTKHYaEEKvkagtwlpedaqllSKQVFTDLLprGlETPhHHLWSLKLNEKDIVGWLWIHAEPEHpqQEAFIYDFGLYEPYRGKGYAKQALAALDQAARSMGIRKLSLHVFAhNqtARKLYEQTGFQETD-------------VVM-----------------skkl<br><br>SARST: aliSize=115 (resi) RMSD=5.73 (Å)<br>lldfdiltndgt------------HRNMKLLIDLKNIFSRQLPkMPKEYIVKL---------VFDRHH--ESMVILKN-KQKVIGGICFRQYK--PQRFAEVAFLAVTANEQVRGYGTRLMNKFKDHMQKQNIEYLLTYADNFAIGYFK---KQGFT----------kehrmpqekwkgyikdydggtlm<br>------------imltpmqteefrSYLTYTTKHYAEEKVKAGT-WLPEDAQLLskqvftdllPRGLETphHHLWSLKLnEKDIVGWLWIHAEPehPQQEAFIYDFGLYEPYRGKGYAKQALAALDQAARSMGIRKLSLHVFAHNQTARKlyeQTGFQetdvvmskkl-------------------------<br>ecyihpyvdygr<br>------------<br><br>BLAST: aliSize=35 (resi) iden=11.69% (18/154) simi=22.73% (35/154)<br>lldfdiltndgthrnmkllidlknifsrqlpkmpkeyivklvfdr---HHESMVILKNKQKVIGGICFRQyKPQRFAEVAFLA-VTANEQVRG--YGTRLMNKFKDHMQKQNIEYLL--TYADN-FAIGYFKKQGF-<br>---imltpmqteefrsyltyttkhyaeekvkagtwlpedaqllskqvftdllprgletpHHHLWSLKLNEKDIVGWLWIHA-EPEHPQQEAFIYdFGLYEPYRGkgYAKQALAALDQAARSMGIRKLSlhVFAHNqTARKLYEQTGFq<br><br>----------tkehrmpqekwkgyikdydggtlmecyihpyvdygr<br>etdvvmskkl------------------------------------<br><br>Proposed: aliSize=128 (resi) RMSD=2.08 (Å)<br>lLDFDILtndgthrNMKLLIDLKNIFSRQL-PKM--------------PKEYIVKLV--F--DRHHESMVILK--NKqKvIGGICFRQYKP-Q-RFAEVAFLAVTANEQVRGYGTRLMNKFKDHMQKQNIEYLLTYADN-F--AIGYFKKQGFTKehrmpqekwkgyikdydGGTLMECYIhpyvdygr<br>-IMLTPM-------QTEEFRSYLTYTTKHYaEEKvkagtwlpedaqllSKQVFTDLLprGleTPHHHLWSLKLneKD-I-VGWLWIHAEPEhPqQEAFIYDFGLYEPYRGKGYAKQALAALDQAARSMGIRKLSLHVFAhNqtARKLYEQTGFQE-----------------TDVVMSKKL--------

375	1m1dA (163)<br>1vkcA (149)	C	12.08	TM-align: aliSize=126 (resi) RMSD=2.64 (Å)<br>LLDFDILTndgthrNmkLLIDLKNIFSRQLP-KM--------P---KEYIVKLVFDRH-HESMVILKNKQKVIGGICFRQYK----PQRFAEVAFLAVTANEQVRGYGTRLMNKFKDHMQKQNIEYLLTYADN-F-AIGYFKKQGFTKEHrmpqekwkgyik-DYDggtlmecyihpyvdygr---<br>EYTIVDGE------E--YIEEIKKLDREISYsFVrfpisyeeYeerHEELFESLLSQGeHKFFVALNERSELLGHVWICITLdtvdYVKIAYIYDIEVVKWARGLGIGSALLRKAEEWAKERGAKKIVLRVEIdNpAVKWYEERGYKARA------------lIME-----------------kpi<br><br>SARST: aliSize=120 (resi) RMSD=4.22 (Å)<br>lldf-DILTNDgthrNMKLLIDLKNIFSRQLPKMP-----------KEYIVKLVFDRHHESMVILKNKQKVIGGICFRQY--KPQRF--AEVAFLAVTANEQVRGYGTRLMNKFKDHMQKQNIEYLLTYADNFAI--GYFKKQGFT----------kehrmpqekwkgyikdydggtlmecyihpyvdygr<br>----eYTIVDG----EEYIEEIKKLDREISYSFVRfpisyeeyeerhEELFESLLSQGEHKFFVALNERSELLGHVWICITldTVDYVkiAYIYDIEVVKWARGLGIGSALLRKAEEWAKERGAKKIVLRVEIDNPavKWYEERGYKaralimekpi-----------------------------------<br><br>BLAST: aliSize=52 (resi) iden=14.77% (22/149) simi=34.90% (52/149)<br>lldfdiltndgthrnmkllidlknifsrqlpkmp-----------------------------------KEYIVKLVFDRHHESMVILKNKQKVIGG--ICFRqYKPQRFAEVAFLA-VTANEQVRG--YGTRLMNKFKDHMQKQNIE--YLLTYADNFAIGYFKKQGFtKEHRMPQEK--wkgyi<br>----------------------------------eytivdgeeyieeikkldreisysfvrfpisyeeyeerhEELFESLLSQGEHKFFVALNERSELLGHvwICIT-LDTVDYVKIAYIYdIEVVKWARGlgIGSALLRKAEEWAKERGAKkiVLRVEIDNPAVKWYEERGY-KARALIMEKpi-----<br><br>kdydggtlmecyihpyvdygr<br>---------------------<br><br>Proposed: aliSize=128 (resi) RMSD=2.10 (Å)<br>LLDFDILTndgthrNmkLLIDLKNIFSRQLP-KM--------P---KE-YIVKLvFDRH-HESMVILKNKQKVIGGICFRQYkP-----QRFAEVAFLAVTANEQVRGYGTRLMNKFKDHMQKQNIEYLLTYADN-F-AIGYFKKQGFTKehrmpqekwkgyikdydGGTLMECYIhpyvdygr<br>EYTIVDGE------E--YIEEIKKLDREISYsFVrfpisyeeYeerHEeLFESL-LSQGeHKFFVALNERSELLGHVWICIT-LdtvdyVKIAYIYDIEVVKWARGLGIGSALLRKAEEWAKERGAKKIVLRVEIdNpAVKWYEERGYKA-----------------RALIMEKPI--------
376	1m1dA (163)<br>2vxkA (165)	C	10.43	TM-align: aliSize=125 (resi) RMSD=3.21 (Å)<br>---------------------LLDFDILtndgthrNMKLLI-DLKNIFSrQLpkMPKEYI---VKl--VF-DR--H---HESMVILKNKQKVIGGICFRQYKP-----QRFAEVAFLAVTANEQVRGYGTRLMNKFKDHMQKQNIEYLLTYADNFAIGYFKKQGFTKEHrmpqekwkgy-IKDYDggtlm<br>entplfspslispdvlavlpaDYTIRPL-------CRSDYKrGYLDVLR-VL--TTVGDIneeQW-nsRYeWIraRsdeYYLLVVCDGEGRIVGTGSLVVERKfihslGMVGHIEDIAVEKGQQGKKLGLRIIQALDYVAEKVGCYKTILDCSEANEGFYIKCGFKRAG----------IEMAHY-----<br><br>ecyihpyvdygr-<br>------------y<br><br>SARST: aliSize=93 (resi) RMSD=13.94 (Å)<br>lldfdiltndgthrnm---KLLIDLKNIFSRQLPK-------MPKeyivklVFDRHHESMVILK----NKQkvigGICFRQYkpqRFAevaflaVTANEQVRGYGTRLMNKFKDHMQKQNIEYLLTYADNFAIGYFKKQGFT<br>----------------entplfspslispdvlavlpadytirplcrsdykrgyldvlrvlttvgdinEEQWNSRYEWIRARSDeyyllvvCDG------EGRIVGTGSLVVErkfiHSL----GMVGHIE---DIA------VEKGQQGKKLGLRIIQALDYVAEKVGCYKTILDCSEANEGFYIKCGFK<br><br>----------kehrmpqekwkgyikdydggtlmecyihpyvdygr<br>raglemahyy-----------------------------------<br><br>BLAST: aliSize=11 (resi) iden=3.07% (5/163) simi=6.75% (11/163)<br>lldfdiltndgthrnmkllidlknifsrqlpkmpkeyivklvfdrhhesmvilknkqkvigg---------------------------ICFRQYKpQRFAEVAFLAVTANE--<br>--entplfspslispdvlavlpadytirpLCRSDYK-RGYLDVLRVLTTVGDineeqwnsryewirarsdeyyllvvcdgegrivgtgslvverkfihslgmvghiediavekgqqgkklglriiqaldy<br><br>--------------------------------------qvrgygtrlmnkfkdhmqkqnieylltyadnfaigyfkkqgftkehrmpqekwkgyikdydggtlmecyihpyvdygr<br>vaekvgcyktildcseanegfyikcgfkraglemahyy--<br><br>Proposed: aliSize=120 (resi) RMSD=1.92 (Å)<br>---------------------LLDFDILtndgthrNMKLlI--DLKNIFSrQLpkMPKE---------yivklvF--DR--H---HESMVILKN-kQKVIGGICFRQYKP-----QRFAEVAFLAVTANEQVRGYGTRLMNKFKDHMQKQNIEYLLTYADNFAIGYFKKQGFTKEhrmpqekwkgyikdy<br>entplfspslispdvlavlpaDYTIRPL-------CRSD-YkrGYLDVLR-VL--TTVGdineeqwns------RyeWIraRsdeYYLLVVCDGe-GRIVGTGSLVVERKfihslGMVGHIEDIAVEKGQQGKKLGLRIIQALDYVAEKVGCYKTILDCSEANEGFYIKCGFKRA--------------<br><br>dgGTLMECYI-hpyvdygr<br>--GLEMAHYYc--------
377	1mk4A (157)<br>1s3zB (145)	C	20.69	TM-align: aliSize=130 (resi) RMSD=2.46 (Å)<br>hMDIRTITSSDYEMVTSVLNeWWggrQLKEK---L--PR-LFFEHFQDTSFITSEHNSMTGFLIGFQSQS-------DPeTAYIHFSGVHPDFRKMQIGKQLYDVFIETVKQRGCTRVKCVTSPVNKVSIAYHTKLGFDIEKgtktvngisVFAnYDGPgqdrvlfvkni--<br>-MDIRQMNKTHLEHWRGLRK-QL---WPGHPddaHlaDGeEILQADHLASFIAMADGVAIGFADASIRHDyvngcdsSP-VVFLEGIFVLPSFRQRGVAKQLIAAVQRWGTNKGCREMASDTSPENTISQKVHQALGFEETE---------RVI-FYRK-----------rc<br><br>SARST: aliSize=125 (resi) RMSD=4.50 (Å)<br>hm-DIRTITSSDYEMVTSVLNEWWGGRqlkeklPRLFFEHFQ------DTSFITS--EHNSMTGFLIGFQSQ------SDPETAYIHFSGVHPDFRKMQIGKQLYDVFIETVKQRGCTRVKCVTSPVNKVSIAYHTKLGFdIEKGTKTV-----ngisvfanydgpgqdrvlfvkni<br>--mDIRQMNKTHLEHWRGLRKQLWPGH------PDDAHLADGeeilqaDHLASFIamADGVAIGFADASIRHdyvngcDSSPVVFLEGIFVLPSFRQRGVAKQLIAAVQRWGTNKGCREMASDTSPENTISQKVHQALGF-EETERVIFyrkrc-----------------------<br><br>BLAST: aliSize=53 (resi) iden=24.14% (35/145) simi=36.55% (53/145)<br>hMDIRTITSSDYEMVTSVLNEWWGGR----QLKEKLPRLFFEHFqdTSFITSEHNSMTGFL---IGFQSQSDPETAYIHF-SGVH--PDFRKMQIGKQLYDVFIETVKQRGCTRVKCVTSPVNKVSIAYHTKLGFD-----------iekgtktvngisvfanydgpgqdrvlfvkni<br>-MDIRQMNKTHLEHWRGLRKQLWPGHpddaHLADGEEILQADHL--ASFIAMADGVAIGFAdasIRHDYVNGCDSSPVVFlEGIFvlPSFRQRGVAKQLIAAVQRWGTNKGCREMASDTSPENTISQKVHQALGFEetervifyrkrc-------------------------------<br><br>Proposed: aliSize=130 (resi) RMSD=2.01 (Å)<br>hMDIRTITSSDYEMVTSVLNeWWggrQLKEK---L--PR-LFFEH--FQdtSFITSEHNSMTGFLIGFQSQS-------DPeTAYIHFSGVHPDFRKMQIGKQLYDVFIETVKQRGCTRVKCVTSPVNKVSIAYHTKLGFDIEkgtktvngisvfanydgpgQDRVLFVKNI-<br>-MDIRQMNKTHLEHWRGLRK-QL---WPGHPddaHlaDGeEILQAdhLA--SFIAMADGVAIGFADASIRHDyvngcdsSP-VVFLEGIFVLPSFRQRGVAKQLIAAVQRWGTNKGCREMASDTSPENTISQKVHQALGFEET------------------ERVIFYRKRCc

378	1mk4A (157)<br>1s5kA (153)	C	19.61	TM-align: aliSize=132 (resi) RMSD=2.63 (Å)<br>-------HMDIRTITSSDYEMVTSVLNeWWggrQLKE---KL--PR-LFFEHFQDTSFITSEHNSMTGFLIGFQSQSD------PETAYIHFSGVHPDFRKMQIGKQLYDVFIETVKQRGCTRVKCVTSPVNKVSIAYHTKLGFDIEKgtktvngisVFAnYDGPgqdrvlfvkni--<br>glvprgsHMDIRQMNKTHLEHWRGLRK-QL---WPGHpddAHlaDGeEILQADHLASFIAMADGVAIGFADASIRHDYvngcdsSPVVFLEGIFVLPSFRQRGVAKQLIAAVQRWGTNKGCREMASDTSPENTISQKVHQALGFEETE---------RVI-FYRK-----------rc<br><br>SARST: aliSize=130 (resi) RMSD=5.69 (Å)<br>h--------MDIRTITSSDYEMVTSVLNEWWGGRqlkeklPRLFFEHFQD------TSFITSE--HNSMTGFLIGFQSQ------SDPETAYIHFSGVHPDFRKMQIGKQLYDVFIETVKQRGCTRVKCVTSPVNKVSIAYHTKLGFdieKGTKTVnGISVFA-nydgpgqdrvlfvkni<br>-glvprgshMDIRQMNKTHLEHWRGLRKQLWPGH------PDDAHLADGEeilqadHLASFIAmaDGVAIGFADASIRHdyvngcDSSPVVFLEGIFVLPSFRQRGVAKQLIAAVQRWGTNKGCREMASDTSPENTISQKVHQALGF---EETERV-IFYRKRc---------------<br><br>BLAST: aliSize=54 (resi) iden=23.53% (36/153) simi=35.29% (54/153)<br>-------HMDIRTITSSDYEMVTSVLNEWWGGR----QLKEKLPRLFFEHFqdTSFITSEHNSMTGFL---IGFQSQSDPETAYIHF-SGVH--PDFRKMQIGKQLYDVFIETVKQRGCTRVKCVTSPVNKVSIAYHTKLGFD------------iekgtktvngisvfanydgpgqdrvlfvkni<br>glvprgsHMDIRQMNKTHLEHWRGLRKQLWPGHpddaHLADGEEILQADHL--ASFIAMADGVAIGFAdasIRHDYVNGCDSSPVVFlEGIFvlPSFRQRGVAKQLIAAVQRWGTNKGCREMASDTSPENTISQKVHQALGFEetervifyrkrc------------------------------<br><br>Proposed: aliSize=128 (resi) RMSD=2.04 (Å)<br>-------HMDIRTITSSDYEMVTSVLNeWWggrQLKeK----L--PR-LFFEH--FQdtSFITSEHNSMTGFLIGFQSQS-------DPeTAYIHFSGVHPDFRKMQIGKQLYDVFIETVKQRGCTRVKCVTSPVNKVSIAYHTKLGFDIEKgtktvngisvfanydgpgqdrVLFVKNI---<br>glvprgsHMDIRQMNKTHLEHWRGLRK-QL---WPG-HpddaHlaDGeEILQAdhLA--SFIAMADGVAIGFADASIRHDyvngcdsSP-VVFLEGIFVLPSFRQRGVAKQLIAAVQRWGTNKGCREMASDTSPENTISQKVHQALGFEETE--------------------RVIFYRKrcc
379	1mk4A (157)<br>1ufhA (155)	C	9.68	TM-align: aliSize=127 (resi) RMSD=3.13 (Å)<br>HMDIRTITSSDYEMVTSVLNewwggrQL-K------------------EK--LP--R-LFFEHFQDTSFITS-EHNSMTGFLIGFQS-QSDPETAYIHFSGVHPDFRKMQIGKQLYDVFIETVKQRGCTRVKCVTSPVNKVSIAYHTKLGFDIEkGtktvngisVFAnydgpGqdrvlfvkni---<br>TIMLTPMQTEEFRSYLTYTT------KHyAeekvkagtwlpedaqllSKqvFTdlLpRGLETPHHHLWSLKlNEKDIVGWLWIHAEpEHPQQEAFIYDFGLYEPYRGKGYAKQALAALDQAARSMGIRKLSLHVFAHNQTARKLYEQTGFQET-D--------VVM-----S----------kkl<br><br>SARST: aliSize=109 (resi) RMSD=8.37 (Å)<br>hmdirtitssd---------------------------------------YEMVTSVLNEWWGGRQLKEKLprlffehfqdtSFITSEHNSMTGFLIGFQSQSDPE-TAYIHFSGVHPDFRKMQIGKQLYDVFIETVKQRGCTRVKCVTSPVNKVSIAYHTKLGfdiEKGTKTV-----ngisvfanyd<br>-----------timltpmqteefrsyltyttkhyaeekvkagtwlpedaqlLSKQVFTDLLPRGLETPHHHL-----------WSLKLNEKDIVGWLWIHAEPEHPQqEAFIYDFGLYEPYRGKGYAKQALAALDQAARSMGIRKLSLHVFAHNQTARKLYEQTG---FQETDVVmskkl----------<br><br>gpgqdrvlfvkni<br>-------------<br><br>BLAST: aliSize=40 (resi) iden=12.90% (20/155) simi=25.81% (40/155)<br>hmdirtitssdyemvtsvlnewwggrqlkek---LPR-LFFEHFQDTSFITSEHNsMTGFL-IGFQSQSDPETAYIHFSGVHPDFRKMQIGKQLYDVFIETVKQRGCTRVKCVTSPVNKVSIAYHTKLGF-----------die<br>-------------------------------timltpmqteefrsyltyttkhyaeekvkagtwlpedaqllskqvftdlLPRgLETPHHHLWSLKLNEKD-IVGWLwIHAEPEHPQQEAFIYDFGLYEPYRGKGYAKQALAALDQAARSMGIRKLSLHVFAHNQTARKLYEQTGFqetdvvmskkl---<br><br>kgtktvngisvfanydgpgqdrvlfvkni<br>-----------------------------<br><br>Proposed: aliSize=125 (resi) RMSD=2.56 (Å)<br>hm--DIRTITsS-DYEMVTSVL-newwggrQLKE-----------------K---LP--RL-FFE-HFQdTSFITS-EHNSMTGFLIGFQS-QSDPETAYIHFSGVHPDFRKMQIGKQLYDVFIETVKQRGCTRVKCVTSPVNKVSIAYHTKLGFDIEkgtktvngisvfanydgpgqdRVLFVKNI<br>--tiMLTPMQ-TeEFRSYLTYTt-------KHYAeekvkagtwlpedaqllSkqvFTdlLPrGLEtPHH-HLWSLKlNEKDIVGWLWIHAEpEHPQQEAFIYDFGLYEPYRGKGYAKQALAALDQAARSMGIRKLSLHVFAHNQTARKLYEQTGFQET--------------------DVVMSKKL
380	1mk4A (157)<br>1ufhB (154)	C	10.39	TM-align: aliSize=130 (resi) RMSD=3.20 (Å)<br>hMDIRTITSSDYEMVTSVLNEwWgg-RQLK---------------EK--LP--RL-FFEHFQDTSFITSEH-NSMTGFLIGFQS-QSDPETAYIHFSGVHPDFRKMQIGKQLYDVFIETVKQRGCTRVKCVTSPVNKVSIAYHTKLGFDIEkGtktvngisVfANydgpGQdrvlfvkni--<br>-IMLTPMQTEEFRSYLTYTTK-H--yAEEKvkagtwlpedaqllSKqvFTdlLPrGLETPHHHLWSLKLNeKDIVGWLWIHAEpEHPQQEAFIYDFGLYEPYRGKGYAKQALAALDQAARSMGIRKLSLHVFAHNQTARKLYEQTGFQET-D--------V-VM----SK---------kl<br><br>SARST: aliSize=109 (resi) RMSD=8.25 (Å)<br>hmdirtitssd---------------------------------------YEMVTSVLNEWWGGRQLKEKLprlffehfqdtSFITSEHNSMTGFLIGFQSQSDPE-TAYIHFSGVHPDFRKMQIGKQLYDVFIETVKQRGCTRVKCVTSPVNKVSIAYHTKLGfdiEKGTKTV-----ngisvfanydg<br>-----------imltpmqteefrsyltyttkhyaeekvkagtwlpedaqlLSKQVFTDLLPRGLETPHHHL-----------WSLKLNEKDIVGWLWIHAEPEHPQqEAFIYDFGLYEPYRGKGYAKQALAALDQAARSMGIRKLSLHVFAHNQTARKLYEQTG---FQETDVVmskkl-----------<br><br>pgqdrvlfvkni<br>------------<br><br>BLAST: aliSize=40 (resi) iden=12.99% (20/154) simi=25.97% (40/154)<br>hmdirtitssdyemvtsvlnewwggrqlkek--LPR-LFFEHFQDTSFITSEHNsMTGFL-IGFQSQSDPETAYIHFSGVHPDFRKMQIGKQLYDVFIETVKQRGCTRVKCVTSPVNKVSIAYHTKLGF-----------diek<br>-------------------------------imltpmqteefrsyltyttkhyaeekvkagtwlpedaqllskqvftdlLPRgLETPHHHLWSLKLNEKD-IVGWLwIHAEPEHPQQEAFIYDFGLYEPYRGKGYAKQALAALDQAARSMGIRKLSLHVFAHNQTARKLYEQTGFqetdvvmskkl----<br><br>gtktvngisvfanydgpgqdrvlfvkni<br>----------------------------<br><br>Proposed: aliSize=127 (resi) RMSD=2.51 (Å)<br>hMDIRTITSSDYEMVTSVL-NewwggrQLK-----------------EK--LP--R-LFFEH--FqdTSFITS-EHNSMTGFLIGFQS-QSDPETAYIHFSGVHPDFRKMQIGKQLYDVFIETVKQRGCTRVKCVTSPVNKVSIAYHTKLGFDIEkgtktvngisvfanydgpgqdRVLFVKNI<br>-IMLTPMQTEEFRSYLTYTtK------HYAeekvkagtwlpedaqllSKqvFTdlLpRGLETphH--HLWSLKlNEKDIVGWLWIHAEpEHPQQEAFIYDFGLYEPYRGKGYAKQALAALDQAARSMGIRKLSLHVFAHNQTARKLYEQTGFQET--------------------DVVMSKKL

381	1mk4A<br>(157)<br><br>1z4eA<br>(150)	C	18.67	TM-align: aliSize=134 (resi) RMSD=2.82 (Å)<br>HMDIRTITSSDYEMVTSVLNEwWGGRQLKEK--------L--PR-LFFEHFQDTSFITSEHNSMTGFLIGFQSQSD----PETAYIHFSGVHPDFRKMQIGKQLYDVFIETVKQRGCTRVKCVTSPVNKVSIAYHTKLGFDIEKgtktvngisVFanYDGPgqdrvlfvkni-<br>HVTIREATEGDLEQMVHMLAD-DVLGRKRERyekplpvsYvrAFkEIKKDKNNELIVACNGEEIVGMLQVTFTPYLtyqgSWRATIEGVRTHSAARGQGIGSQLVCWAIERAKERGCHLIQLTTDKQRPDALRFYEQLGFKASH---------EG--LKMH-----------f<br><br>SARST: aliSize=129 (resi) RMSD=6.83 (Å)<br>h-MDIRTITSSDYEMV-------TSVLNEWWGGRQLkeklPRLFFEHFQ-----DTSFITS--EHNSMTGF--LIGFQSQSDPETAYIHFS--GVHPDFRKMQIGKQLYDVFIETVKQRGCTRVKCVTSPVNKVSIAYHTKLGFDiekGTKtvngisvfanydgpgqdrvLFVKN--i<br>-hVTIREATEGDLEQMvhmladdVLGRKRERYEKPL----PVSYVRAFKeikkdKNNELIVacNGEEIVGMlqVTFTPYLTYQGSWRATIEgvRTHSAARGQGIGSQLVCWAIERAKERGCHLIQLTTDKQRPDALRFYEQLGFK---ASH------------------EGLKMhf-<br><br>BLAST: aliSize=63 (resi) iden=26.67% (40/150) simi=42.00% (63/150)<br>HMDIRTITSSDYEMVTSVLNEWWGGRQLK--EK-LP----RLFFEHFQDTS---FITSEHNSMTGFL-IGFQSQSDPETAY-IHFSGV--HPDFRKMQIGKQLYDVFIETVKQRGCTRVKCVTSPVNKVSIAYHTKLGFDI-EKGTK---tvngisvfanydgpgqdrvlfvkni<br>HVTIREATEGDLEQMVHMLADDVLGRKRERyEKpLPvsyvRAFKEIKKDKNnelIVACNGEEIVGMLqVTFTPYLTYQGSWrATIEGVrtHSAARGQGIGSQLVCWAIERAKERGCHLIQLTTDKQRPDALRFYEQLGFKAsHEGLKmhf------------------------<br><br>Proposed: aliSize=131 (resi) RMSD=2.26 (Å)<br>HMDIRTITSSDYEMVTSVLNEwWGgR-QLKeK---------L--PR-LFFEH--FQdtSFITSEHNSMTGFLIGFQSQSD----PETAYIHFSGVHPDFRKMQIGKQLYDVFIETVKQRGCTRVKCVTSPVNKVSIAYHTKLGFDIEkgtktvngisvfanydgpgqDRVLFVKNi<br>HVTIREATEGDLEQMVHMLAD-DV-LgRKR-EryekplpvsYvrAFkEIKKDknNE--LIVACNGEEIVGMLQVTFTPYLtyqgSWRATIEGVRTHSAARGQGIGSQLVCWAIERAKERGCHLIQLTTDKQRPDALRFYEQLGFKAS-------------------HEGLKMHF-
382	1mk4A<br>(157)<br><br>2d4pA<br>(130)	C	13.85	TM-align: aliSize=122 (resi) RMSD=2.61 (Å)<br>hMDIRTITSSDYEMVTSVLNewwggrOlkEKLPRLFFEHFQ--DTSFITSEHNSMTGFLIGFQSqSD--PETAYIHFSGVHpdfrKMOIGKQLYDVFIETVKORGCTRVKCVTSpVNKVSIAY-HTKLGFD-IEKGtktvngisVFanYDGPGqdrvlfvkni--<br>-MRFRPFTEEDLDRLNRLAG------K--RPVSLGALRFFArtGHSFLAEEGEEPMGFALAQAV-WQgeATTVLVTRIEGR----SVEALRGLLRAVVKSAYDAGVYEVALHLD-PERKELEEaLKAEGFAlGPLV--------LA--VRVLG----------sr<br><br>SARST: aliSize=125 (resi) RMSD=4.02 (Å)<br>hm-DIRTITSSDYEMVTSVLNEwwggrqlkEKLPRLFFE--HFQDTSFITSEHNSMTGFLIGFQSQSDPETAYI-HFSGVhpDFrkMOIGKQLYDVFIETVKORGCTRVKCVTSPVNKVSIAYHTKLGFdiEKGTKTvngISVFAnyDGP-gqdrvlfvkni<br>--mRFRPFTEEDLDRLNRLAGK--------RPVSLGALRffARTGHSFLAEEGEEPMGFALAQAVWQGEATTVLvTRIEG--RS--VEALRGLLRAVVKSAYDAGVYEVALHLDPERKELEEALKAEGF--ALGPLV---LAVRV--LGSr-----------<br><br>BLAST: aliSize=20 (resi) iden=12.31% (16/130) simi=15.38% (20/130)<br>hMDIRTITSSDYEMvtsvLNEWWGGRQLKEKLPRlFFEHfQDTSFITSEHNSMTGFLI--gfqsqsdpetayihfsgvhpdfrkmqigkqlydvfietvkqrgctrvkcvtsp<br>-MRFRPFTEEDLDR----LNRLAGKRPVSLGALR-FFAR-TGHSFLAEEGEEPMGFALaqavwqgeattvlvtriegrsvealrgllravvksaydagvyevalhldperkeleealkaegfalgplvlavrvlgsr--<br><br>vnkvsiayhtklgfdiekgtktvngisvfanydgpgqdrvlfvkni<br>--<br><br>Proposed: aliSize=122 (resi) RMSD=2.25 (Å)<br>hMDIRTITSSDYEMVTSVLNewwggrOlkEKLPRLFFEHFQ--DTSFITSEHNSMTGFLIGFQSqSD--PETAYIHFSGVHpdfrKMOIGKQLYDVFIETVKORGCTRVKCVTSpVNKV-SIAYHTKLGFD---iekgtkTVngiSVfanydgpgqdrVLFVKNI<br>-MRFRPFTEEDLDRLNRLAG------K--RPVSLGALRFFArtGHSFLAEEGEEPMGFALAQAV-WQgeATTVLVTRIEGR----SVEALRGLLRAVVKSAYDAGVYEVALHLD-PERKeLEEALKAEGFAlgp------LV---LA-----------VRVLGSR
383	1mk4A<br>(157)<br><br>2g3tA<br>(169)	C	13.38	TM-align: aliSize=134 (resi) RMSD=2.85 (Å)<br>HMDIRTITSSDYEMVTSVLNEW-WGGRQLKEK-L---PR-LFFE---H-FQdtSFITSEHN--------SMTGFLIGFQSqSD---PETAYIHFSGVHPDFRKMQIGKQLYDVFIETVKQRGCTRVKCVTSPVNKVSIAYHTKLGFDIE--KGTktvngisvFAnYDGPgqdrvlfvkni------------<br>KFVIRPATAADCSDILRLIKELaKYEYMEEQViLtekDLlEDGFgehPfYH--CLVAEVPKehwtpeghSIVGFAMYYFT-YDpwiGKLLYLEDFFVMSDYRGFGIGSEILKNLSQVAMRCRCSSMHFLVAEWNEPSINFYKRRGASDLssEEG--------WR-LFKI-----------dkeyllkmatee<br><br>SARST: aliSize=124 (resi) RMSD=4.62 (Å)<br>h-MDIRTITSSD----YEMVTSVLNEWWGGRQLKEKLPRLFFEHF-Q--DTSFITSE--------HNS-MTGFLIGFQS--QSDPETAYIHFSGVHPDFRKMQIGKQLYDVFIETVKQRGCTRVKCVTSPVNKVSIAYHTKLGF---------------------------diekgtktvngisvfanydg<br>-kFVIRPATAADcsdiLRLIKELAKYEYMEEQVILTEKDLLEDGFgEhpFYHCLVAEvpkehwtpEGHsIVGFAMYYFTydPWIGKLLYLEDFFVMSDYRGFGIGSEILKNLSQVAMRCRCSSMHFLVAEWNEPSINFYKRRGAsdlsseegwrlfkidkeyllkmatee--------------------<br><br>pgqdrvlfvkni<br>------------<br><br>BLAST: aliSize=36 (resi) iden=10.83% (17/157) simi=22.93% (36/157)<br>hmdirtitssdyemvtsvlnewwggrqlkeklprlffehfqdtsfi---TSEHNSMTGFLIGFQSQsDPETAYIHFSgvhPDF------RKMQIGKQLYDVFIETVKQRGCTRVKCVTSPVNKVSIAYHTK<br>--kfvirpataadcsdilrlikelakyeymeeqviltekdlledgfgehpfyhclvaevpkehwTPEGHSIVGFAMYYFTY-DPWIGKLLYL---EDFfvmsdyRGFGIGSEILKNLSQVAMRCRCSSMHFLVAEWNEPSINFYKR<br><br>LG---------------------------fdiekgtktvngisvfanydgpgqdrvlfvkni<br>RGasdlsseegwrlfkidkeyllkmatee---------------------------------<br><br>Proposed: aliSize=137 (resi) RMSD=2.50 (Å)<br>HMDIRTITSSDYEMVTSVLNEW-WGgR--QLKeK-L---PR--LfFE---H-FQdtSFITSEHN--------SMTGFLIGFQSqSD---PETAYIHFSGVHPDFRK-mQIGKQLYDVFIETVKQRGCTRVKCVTSPVNKVSIAYHTKLGFDIE--KGT-ktvNGISVFA-NYDGPG---qdrvlfvkni<br>KFVIRPATAADCSDILRLIKELaKY-EymEEQ-ViLtekDLleD-GFgehPfYH--CLVAEVPKehwtpeghSIVGFAMYYFT-YDpwiGKLLYLEDFFVMSDYRGf-GIGSEILKNLSQVAMRCRCSSMHFLVAEWNEPSINFYKRRGASDLssEEGw---RLFKIDKeYLLKMAtee----------

384	1mk4A<br>(157)<br><br>2jevA<br>(169)	C	13.38	TM-align: aliSize=135 (resi) RMSD=2.97 (Å)<br>-HMDIRTITSSDYEMVTSVLNEW-WGGRQLKEK-L---PR-LFFE---HFQdTSFITSEHN--------SMTGFLIGFQSqSD---PETAYIHFSGVHPDFRKMQIGKQLYDVFIETVKQRGCTRVKCVTSPVNKVSIAYHTKLGFDIE--KGTktvngisvFAnYDGPgqdrvlfvkni-----------<br>aKFVIRPATAADCSDILRLIKELaKYEYMEEQViLtekDLlEDGFgehPFY-HCLVAEVPKehwtpeghSIVGFAMYYFT-YDpwiGKLLYLEDFFVMSDYRGFGIGSEILKNLSQVAMRCRCSSMHFLVAEWNEPSINFYKRRGASDLssEEG--------WR-LFKI-----------dkeyllkmate<br><br>SARST: aliSize=124 (resi) RMSD=4.49 (Å)<br>h--MDIRTITSSDYEMVTSV----LNEWWGGRQLKEKlpRLFFEHFQD----TSFITSE----------HNSMTGFLIGFQS--QSDPETAYIHFSGVHPDFRKMQIGKQLYDVFIETVKQRGCTRVKCVTSPVNKVSIAYHTKLGFDI-----------------------ekgtktvngisvfanydg<br>-akFVIRPATAADCSDILRLikelAKYEYMEEQVILT--EKDLLEDGFgehpFYHCLVAevpkehwtpeGHSIVGFAMYYFTydPWIGKLLYLEDFFVMSDYRGFGIGSEILKNLSQVAMRCRCSSMHFLVAEWNEPSINFYKRRGASDlsseegwrlfkidkeyllkmate------------------<br><br>pgqdrvlfvkni<br>------------<br><br>BLAST: aliSize=36 (resi) iden=10.83% (17/157) simi=22.93% (36/157)<br>hmdirtitssdyemvtsvlnewwggrqlkeklprlffehfqdtsfi---TSEHNSMTGFLIGFQSQsDPETAYIHFSgvhPDF------RKMQIGKQLYDVFIETVKQRGCTRVKCVTSPVNKVSIAYHT<br>--akfvirpataadcsdilrlikelakyeymeeqviltekdlledgfgehpfyhclvaevpkehwTPEGHSIVGFAMYYFTY-DPWIGKLLYL---EDFfvmsdyRGFGIGSEILKNLSQVAMRCRCSSMHFLVAEWNEPSINFYK<br><br>KLG---------------------------fdiekgtktvngisvfanydgpgqdrvlfvkni<br>RRGasdlsseegwrlfkidkeyllkmate----------------------------------<br><br>Proposed: aliSize=129 (resi) RMSD=2.23 (Å)<br>-HMDIRTITSSDYEMVTSVLNEW-WGgR--QlK-eK-lP----R--LfFE---H-FQdtSFITSEHN--------SMTGFLIGFQSqSD---PETAYIHFSGVHPD-FR-kmQIGKQLYDVFIETVKQRGCTRVKCVTSPVNKVSIAYHTKLGFDIE--KGTktvngisvfanydgpgqdRVLFVKNI--<br>aKFVIRPATAADCSDILRLIKELaKY-EymE-Eq-Vi-LtekdLleD-GFgehPfYH--CLVAEVPKehwtpeghSIVGFAMYYFT-YDpwiGKLLYLEDFFVMSDyRGf--GIGSEILKNLSQVAMRCRCSSMHFLVAEWNEPSINFYKRRGASDLssEEG-----------------WRLFKIDKey<br><br>-------<br>llkmate
385	1mk4A<br>(157)<br><br>2vbqA<br>(147)	C	20.41	TM-align: aliSize=132 (resi) RMSD=2.75 (Å)<br>-HMDIRTITSSDYEMVTSVLNEwWggRQLKE--KL--PR-LFFEHFQDTSFITSEHNSMTGFLIGFQSQS-------DPeTAYIHFSGVHPDFRKMQIGKQLYDVFIETVKQRGCTRVKCVTSPVNKVSIAYHTKLGFDIEKgtktvngisVFANYdGPgqdrvlfvkni--<br>sHMDIRQMNKTHLEHWRGLRKQ-L--WPGHPddAHlaDGeEILQADHLASFIAMADGVAIGFADASIRHDyvngcdsSP-VVFLEGIFVLPSFRQRGVAKQLIAAVQRWGTNKGCREMASDTSPENTISQKVHQALGFEETE---------RVIFY-RK-----------rc<br><br>SARST: aliSize=126 (resi) RMSD=4.47 (Å)<br>h--MDIRTITSSDYEMVTSVLNEWWGGRqlkeklPRLFFEHFQ------DTSFIT--SEHNSMTGFLIGFQSQ------SDPETAYIHFSGVHPDFRKMQIGKQLYDVFIETVKQRGCTRVKCVTSPVNKVSIAYHTKLGFdIEKGTKTV-----ngisvfanydgpgqdrvlfvkni<br>-shMDIRQMNKTHLEHWRGLRKQLWPGH------PDDAHLADGeeilqaDHLASFiaMADGVAIGFADASIRHdyvngcDSSPVVFLEGIFVLPSFRQRGVAKQLIAAVQRWGTNKGCREMASDTSPENTISQKVHQALGF-EETERVIFyrkrc-----------------------<br><br>BLAST: aliSize=54 (resi) iden=24.49% (36/147) simi=36.73% (54/147)<br>-HMDIRTITSSDYEMVTSVLNEWWGGR----QLKEKLPRLFFEHFqdTSFITSEHNSMTGFL---IGFQSQSDPETAYIHF-SGVH--PDFRKMQIGKQLYDVFIETVKQRGCTRVKCVTSPVNKVSIAYHTKLGFD------------iekgtktvngisvfanydgpgqdrvlfvkni<br>sHMDIRQMNKTHLEHWRGLRKQLWPGHpddaHLADGEEILQADHL--ASFIAMADGVAIGFAdasIRHDYVNGCDSSPVVFlEGIFvlPSFRQRGVAKQLIAAVQRWGTNKGCREMASDTSPENTISQKVHQALGFEetervifyrkrc------------------------------<br><br>Proposed: aliSize=128 (resi) RMSD=2.16 (Å)<br>-HMDIRTITSSDYEMVTSVLNEwWggrQLKeK----L--PR-LFFEH--FQdtSFITSEHNSMTGFLIGFQSQS-------DPeTAYIHFSGVHPDFRKMQIGKQLYDVFIETVKQRGCTRVKCVTSPVNKVSIAYHTKLGFDIEKgtktvngisvfanydgpgqdrVLFVKNI--<br>sHMDIRQMNKTHLEHWRGLRKQ-L---WPG-HpddaHlaDGeEILQAdhLA--SFIAMADGVAIGFADASIRHDyvngcdsSP-VVFLEGIFVLPSFRQRGVAKQLIAAVQRWGTNKGCREMASDTSPENTISQKVHQALGFEETE--------------------RVIFYRKrc
386	1mk4A<br>(157)<br><br>2vbqB<br>(144)	C	19.44	TM-align: aliSize=130 (resi) RMSD=2.58 (Å)<br>hmDIRTITSSDYEMVTSVLNeWWggrQLKEK--LPRLFFEHFQ----DTSFITSEHNSMTGFLIGFQSQSD------PETAYIHFSGVHPDFRKMQIGKQLYDVFIETVKQRGCTRVKCVTSPVNKVSIAYHTKLGFDIEKgtktvngisVFAnYDGPgqdrvlfvkni--<br>--DIRQMNKTHLEHWRGLRK-QL---WPGHPddAHLADGEEILqadhLASFIAMADGVAIGFADASIRHDYvngcdsSPVVFLEGIFVLPSFRQRGVAKQLIAAVQRWGTNKGCREMASDTSPENTISQKVHQALGFEETE---------RVI-FYRK-----------rc<br><br>SARST: aliSize=127 (resi) RMSD=4.44 (Å)<br>hmd-IRTITSSDYEMVTSVLNEWWGGRQLKEKlpRLFFEHFQDTS-----FITSEHNSMTGFLIGFQSQ------SDPETAYIHFSGVHPDFRKMQIGKQLYDVFIETVKQRGCTRVKCVTSPVNKVSIAYHTKLGFdIEKGTKTV-----ngisvfanydgpgqdrvlfvkni<br>---dIRQMNKTHLEHWRGLRKQLWPGHPDDA---HLADGEEILQAdhlasFIAMADGVAIGFADASIRHdyvngcDSSPVVFLEGIFVLPSFRQRGVAKQLIAAVQRWGTNKGCREMASDTSPENTISQKVHQALGF-EETERVIFyrkrc-----------------------<br><br>BLAST: aliSize=52 (resi) iden=23.61% (34/144) simi=36.11% (52/144)<br>hmDIRTITSSDYEMVTSVLNEWWGGR----QLKEKLPRLFFEHFqdTSFITSEHNSMTGFL---IGFQSQSDPETAYIHF-SGVH--PDFRKMQIGKQLYDVFIETVKQRGCTRVKCVTSPVNKVSIAYHTKLGFD------------iekgtktvngisvfanydgpgqdrvlfvkni<br>--DIRQMNKTHLEHWRGLRKQLWPGHpddaHLADGEEILQADHL--ASFIAMADGVAIGFAdasIRHDYVNGCDSSPVVFlEGIFvlPSFRQRGVAKQLIAAVQRWGTNKGCREMASDTSPENTISQKVHQALGFEetervifyrkrc------------------------------<br><br>Proposed: aliSize=127 (resi) RMSD=2.09 (Å)<br>hmDIRTITSSDYEMVTSV-LN-ewwggrqLKEK---LPRLFFEHFQ----DTSFITSEHNSMTGFLIGFQSQSD------PETAYIHFSGVHPDFRKMQIGKQLYDVFIETVKQRGCTRVKCVTSPVNKVSIAYHTKLGFDIEKgtktvngisvfanydgpgqdrVLFVKNI--<br>--DIRQMNKTHLEHWRGLrKQl-------WPGHpddAHLADGEEILqadhLASFIAMADGVAIGFADASIRHDYvngcdsSPVVFLEGIFVLPSFRQRGVAKQLIAAVQRWGTNKGCREMASDTSPENTISQKVHQALGFEETE--------------------RVIFYRKrc

387	1obpA (158)<br>1cj5A (162)	C	14.56	TM-align: aliSize=121 (resi) RMSD=3.37 (Å)<br>qeee-------AEQNLSELSGPWRTVYIGSTNPEKI-QENGPFRTYFRELVFDDEKgTVDFYFSVKRDGKWKNVHVKATKQDDG-TYVADYEGQNVFKIVSLSR-THLVAHNINVdkhGQT--TELTGLFVKLNvededlekfwkltedkgidkknvvnf----------------------------L<br>----ayvtqtmKGLDIQKVAGTWYSLAMAASDISLLdAQSAPLRVYVEELKPTPEG-DLEILLQKWENDECAQKKIIAEKTKIPaVFKIDALNENKVLVLDTDYkKYLLFCMENS---AEPeqSLVCQCLVRTP-------------------------evddealekfdkalkalpmhirlsfnptqL<br>ENedHPHPe<br>EE--QCHI-<br><br>SARST: aliSize=118 (resi) RMSD=5.19 (Å)<br>qe-----EEAEQNLSELSGPWRTVYIGSTNPEKIQE-NGPFRTYFRELVFDDEKgTVDFYFSVKRDGKWKNVHVKATKQD-DGTYVADYEGQNVFKIVSLSRT-HLVAHNIN--VDKHGQTTELTG------------------------LFVKL---------nvededlekfwkltedkgidkknvvn<br>--ayvtqTMKGLDIQKVAGTWYSLAMAASDISLLDAqSAPLRVYVEELKPTPEG-DLEILLQKWENDECAQKKIIAEKTKiPAVFKIDALNENKVLVLDTDYKkYLLFCMENsaEPEQSLVCQCLVrtpevddealekfdkalkalpmhiRLSFNptqleeqch--------------------------<br>flenedhphpe<br>-----------<br><br>BLAST: aliSize=54 (resi) iden=15.82% (25/158) simi=34.18% (54/158)<br>qeeeaeq----------NLSELSGPWRTVYIGSTNPEKIQ-ENGPFRTYFRELVFDDEkGTVDFYFSVKRDGKWKNVHVKATKQD-DGTYVADYEGQNVFKIVSLS-RTHLVAHNINVDKHGQTTELTGLFVKLNVEDEDLEKFWK-----------------------ltedkgidkknvvnflenedh<br>-------ayvtqtmkglDIQKVAGTWYSLAMAASDISLLDaQSAPLRVYVEELKPTPE-GDLEILLQKWENDECAQKKIIAEKTKiPAVFKIDALNENKVLVLDTDyKKYLLFCMENSAEPEQSLVCQCLVRTPEVDDEALEKFDKalkalpmhirlsfnptqleeqch--------------------<br>phpe<br>----<br><br>Proposed: aliSize=140 (resi) RMSD=2.77 (Å)<br>qeee--------A-eQnLSELSGPWRTVYIGSTNPEKI-QENGPFRTYFRELVFDdEKGTVDFYFSVKRDGKWKNVHVKATKQD-dG-TYVADYEGQNVFKIVSLSR-THLVAHNINVdkhGQT--TELTGLFVK--lNVEDEDLEKFWKLTEDKGIDkKNVVNFL-E----nedHPHpe<br>----ayvtqtmkGl-D-IQKVAGTWYSLAMAASDISLLdAQSAPLRVYVEELKPT-PEGDLEILLQKWENDECAQKKIIAEKTKi-PaVFKIDALNENKVLVLDTDYkKYLLFCMENS---AEPeqSLVCQCLVRtp-EVDDEALEKFDKALKALPMH-IRLSFNPtQleeq---CHI--
388	1obpA (158)<br>1dv9A (162)	C	15.19	TM-align: aliSize=120 (resi) RMSD=2.92 (Å)<br>qeee-------AEQNLSELSGPWRTVYIGSTNPEKIQE-NGPFRTYFRELVFDdEKGTVDFYFSVKRDGKWKNVHVKATKQDDG-TYVADYEGQNVFKIVSLS-RTHLVAHNINVDkhGQ-TTELTGLFVKLNvededlekfwkltedkgidkknvvnflened-------------------------<br>----ayvtqtmKGLDIQKVAGTWYSLAMAASDISLLDAqSAPLRVYVEELKPT-PEGDLEILLQKWENDECAQKKIIAEKTKIPaVFKIDALNENKVLVLDTDyKKYLLFCMENSA--EPeQSLVCQCLVRTP-----------------------------evddealekfdkalkalpmhirlsfn<br>----HPHPE-<br>ptqlEEQCHi<br><br>SARST: aliSize=124 (resi) RMSD=11.51 (Å)<br>qeeeaeqnls-------------ELSGPWRTVYIGSTNPEK-IQENGPFRTYFRELVFDDeKGTVDFYFSVKRDGKWKNVHVKATKQD-DGTYVADYEGQNVFKIVSLSRTHLVAHNIN---VDKHGQtteLTGLFVKLNVEDEDLEKFWKLTEDK--------------------gidkknvvnflene<br>----------ayvtqtmkgldiqKVAGTWYSLAMAASDISLlDAQSAPLRVYVEELKPTP-EGDLEILLQKWENDECAQKKIIAEKTKiPAVFKIDALNENKVLVLDTDYKKYLLFCMEnsaEPEQSL---VCQCLVRTPEVDDEALEKFDKALKAlpmhirlsfnptqleeqchi--------------<br>dhphpe<br>------<br><br>BLAST: aliSize=54 (resi) iden=15.82% (25/158) simi=34.18% (54/158)<br>qeeeaeq----------NLSELSGPWRTVYIGSTNPEKIQ-ENGPFRTYFRELVFDDEkGTVDFYFSVKRDGKWKNVHVKATKQD-DGTYVADYEGQNVFKIVSLS-RTHLVAHNINVDKHGQTTELTGLFVKLNVEDEDLEKFWK-----------------------ltedkgidkknvvnflened<br>-------ayvtqtmkglDIQKVAGTWYSLAMAASDISLLDaQSAPLRVYVEELKPTPE-GDLEILLQKWENDECAQKKIIAEKTKiPAVFKIDALNENKVLVLDTDyKKYLLFCMENSAEPEQSLVCQCLVRTPEVDDEALEKFDKalkalpmhirlsfnptqleeqchi-------------------<br>hphpe<br>-----<br><br>Proposed: aliSize=142 (resi) RMSD=2.11 (Å)<br>qeee-------AEQNLSELSGPWRTVYIGSTNPEKIQE-NGPFRTYFRELVFDdEKGTVDFYFSVKR--dgKWKNVHVKATKQDD-GTYVADYEGQNVFKIVSLS-RTHLVAHNIN-VdkhgQ--TTELTGLFVK-LNVEDEDLEKFWKLTEDKGIDkkNVVNFL-EN---edhPHPE<br>----ayvtqtmKGLDIQKVAGTWYSLAMAASDISLLDAqSAPLRVYVEELKPT-PEGDLEILLQKWEnd--ECAQKKIIAEKTKIpAVFKIDALNENKVLVLDTDyKKYLLFCMENsA----EpeQSLVCQCLVRtPEVDDEALEKFDKALKALPMH--IRLSFNpTQlee---QCHI
389	1obpA (158)<br>1pboA (157)	N	91.72	TM-align: aliSize=147 (resi) RMSD=0.99 (Å)<br>qeeeaeq--------NLSELSGPWRTVYIGSTNPEKIQENGPFRTYFRELVFDDEKGTVDFYFSVKRDGKWKNVHVKATKQDDGTYVADYEGQNVFKIVSLSRTHLVAHNINVDKHGQTTELTGLFVKLNVEDEDLEKFWKLTEDKGIDKKNVVNFLEN--EDHphpe<br>-------aqeeeaeqNLSELSGPWRTVYIGSTNPEKIQENGPFRTYFRELVFDDEKGTVDFYFSVKRDGKWKNVHVKATKQDDGTYVADYEGQNVFKIVSLSRTHLVAHNINVDKHGQKTELTGLFVKLNVEDEDLEKFWKLTEDKGIDKKNVVNFLENedHPH----<br><br>SARST: aliSize=147 (resi) RMSD=4.00 (Å)<br>qe---EEAEQNLSELSGPWRTVYIGSTNPEKIQENGPFRTYFRELVFDDEKGTVDFYFSVKRDGKWKNVHVKATKQDDGTYVADYEGQNVFKIVSLSRTHLVAHNINVDKHGQTTELTGLFVKLNVEDEDLEKFWKLTEDKGIDKKNVVNFL-------enedhphpe<br>--aqeEEAEQNLSELSGPWRTVYIGSTNPEKIQENGPFRTYFRELVFDDEKGTVDFYFSVKRDGKWKNVHVKATKQDDGTYVADYEGQNVFKIVSLSRTHLVAHNINVDKHGQKTELTGLFVKLNVEDEDLEKFWKLTEDKGIDKKNVVNFLenedhph---------<br><br>BLAST: aliSize=155 (resi) iden=98.73% (155/157) simi=98.73% (155/157)<br>-QEEEAEQNLSELSGPWRTVYIGSTNPEKIQENGPFRTYFRELVFDDEKGTVDFYFSVKRDGKWKNVHVKATKQDDGTYVADYEGQNVFKIVSLSRTHLVAHNINVDKHGQTTELTGLFVKLNVEDEDLEKFWKLTEDKGIDKKNVVNFLENEDHPHpe<br>aQEEEAEQNLSELSGPWRTVYIGSTNPEKIQENGPFRTYFRELVFDDEKGTVDFYFSVKRDGKWKNVHVKATKQDDGTYVADYEGQNVFKIVSLSRTHLVAHNINVDKHGQKTELTGLFVKLNVEDEDLEKFWKLTEDKGIDKKNVVNFLENEDHPH--<br><br>Proposed: aliSize=153 (resi) RMSD=0.81 (Å)<br>QEEEAEQN-LSELSGPWRTVYIGSTNPEKIQENGPFRTYFRELVFDDEKGTVDFYFSVKRDGKWKNVHVKATKQDDGTYVADYEGQNVFKIVSLSRTHLVAHNINVDKHGQTTELTGLFVKLNVEDEDLEKFWKLTEDKGIDKKNVVNFLEN---EDhphpe<br>AQEEEAEQnLSELSGPWRTVYIGSTNPEKIQENGPFRTYFRELVFDDEKGTVDFYFSVKRDGKWKNVHVKATKQDDGTYVADYEGQNVFKIVSLSRTHLVAHNINVDKHGQKTELTGLFVKLNVEDEDLEKFWKLTEDKGIDKKNVVNFLENedhPH-----

390	1obpA (158)<br>1sy2A (184)	C	7.59

```
TM-align:  aliSize=119 (resi)      RMSD=3.64 (Å)
qeee-----------AEQNLSELSGPWRTVYIGSTNPEKIQENgpfRTYFrELVFDDEKGTVDFYFSVKRDGKWKNVHVKATKQDDG-TYVADY---------------EGQNVFKIVSLSRTHLVAHNINVDKHGQTTELTGLFVKLNvededlekfwkltedkgidkknvvn----------------
----actknaiaqtgFNKDKYFNGDVWYVTDYLDLEPDDVPKR---YCAA-LAAGTASGKLKEALYHYDPKTQDTFYDVSELQVESLgKYTANFkkvdkngnvkvavtaGNYYTFTVMYADDSSALIHTCLHKGNKAAGDLYAVLNRNK-------------------------daaagdkvksavsaat

----------------FLENEdhphpe-------
lefskfistkennca YDNDS------lkslltk

SARST:     aliSize=121 (resi)      RMSD=16.09 (Å)
qeeeaeqnlselsgpwrtvyigstnpeki--------QENG-----PF--RTYFRELVFdDEKG-----TVDFYFSVKRDGKWKNVHVKATKqDDGTYVADY---------------------------------EGQNVFKIVSLSRTHLVAHNINVDKHGQTTELTGLFV---KLNVEDEDLEKFWklteD
-----------------------------actknaiaQTGFnkdkyFNgdVWYVTDYLD-LEPDdvpkrYCAALAAGTASGKLKEALYHYDP-KTQDTFYDVselqveslgkytanfkkvdkngnvkvavtaGNYYTFTVMYADDSSALIHTCLHKGNKAAGDLYAVLNrnkDAAAGDKVKSAVS----A

KGIDKKNVVNFLENEDHPH----------pe
ATLEFSKFISTKENNCAYDndslkslltk--

BLAST:     aliSize=19 (resi)       iden=8.86% (14/158)        simi=12.03% (19/158)
qeeeaeqnlselsgpwrtvyigstnpekiqengpfrtyfrelvfddekgtvdfyfsvkrdgkwknvhvkat--------------------------------------------------KQDDGTYVADYEGQNVFKIVS-LSRTHLVAHNIN---VDKHG------------------------------
-----------------------------------------------------------------------actknaiaqtgfnkdkyfngdvwyvtdyldlepddvpkrycaalaagtasgKLKEALYHYDPKTQDTFYDVSeLQVESLGKYTANfkkVDKNGnvkvavtagnyytftvmyaddssali

------------------------------------------------------------qtteltglfvklnvededlekfwkltedkgidkknvvnflenedhphpe
htclhkgnkaagdlyavlnrnkdaaagdkvksavsaatlefskfistkenncaydndslkslltk----------------------------------------------

Proposed:  aliSize=132 (resi)      RMSD=2.38 (Å)
qeeeae-------------QNL-SElSGPWRTVYIGSTNPEKIQ-EngpfRTYFrELVFD--dEKgTVDFYFSVK--rdGKWKNVHVKATKQDDG-TYVADY----------------eGQNVFKIVSLSRTHLVAHNINVDKHGQTTELTGLFVK-LNVEdeDLEKFWKLTEDKGIDKKNVVNFLEN--
------actknaiaqtgfnKDKyFN-GDVWYVTDYLDLEPDDVPkR----YCAA-LAAGTas-GK-LKEALYHYDpk--TQDTFYDVSELQVESLgKYTANFkkvdkngnvkvavtag-NYYTFTVMYADDSSALIHTCLHKGNKAAGDLYAVLNRnKDAA--AGDKVKSAVSAATLEFSKFISTKENnc

--------------edhphpe
aydndslkslltkh-------
```

391	1obpA (158)<br>1u17A (185)	C	6.96

```
TM-align:  aliSize=119 (resi)      RMSD=3.91 (Å)
qeee-----------AEQNLSELSGPWRTVYIGSTNPEKIQENgpfRTYFrELVFDDEKGTVDFYFSVKRDGKWKNVHVKATKQDDG-TYVADY-E-------------GQNVFKIVSLSRTHLVAHNINVDKHGQTTELTGLFVKLNvededlekfwkltedkgidkknvvn--------------
----mkctknalaqtgFNKDKYFNGDVWYVTDYLDLEPDDVPKR---YCAA-LAAGTASGKLKEALYCYDPKTQDTFYDVSELQEESPgKYTANFkKveknqnvkvdvtsgNYYTFTVMYADDSSALIHTCLHKGNKDLGDLYAVLNRNK-------------------------dtnagdkvkgavtaa

----------------FLENEdhphpe-------
slkfsdfistkdnkceYDNVS------lkslltk

SARST:     aliSize=142 (resi)      RMSD=16.23 (Å)
qeeeaeqnl------------------SELSGPWRTVYIGSTNPEKIQENG---------PFRTYFRELVFDDEKGTVDFYFSVKRDGKW-KNVHVKATKQDDGTY-VADYEGQNVF-KIVSLSRTHLVAHNINVDkHGQTTELTGLFVK----LNVEDEDLEKFWklteDKGIDKKNVVNFLENEDHPH-
---------mkctknalaqtgfnkdkYFNGDVWYVTDYLDLEPDDVPKRYcaalaagtaSGKLKEALYCYDPKTQDTFYDVSELQEESPgKYTANFKKVEKNGNVkVDVTSGNYYTfTVMYADDSSALIHTCLHK-GNKDLGDLYAVLNrnkdTNAGDKVKGAVT----AASLKFSDFISTKDNKCEYDn

---------pe
vslkslltk--

BLAST:     aliSize=5 (resi)        iden=3.16% (5/158)         simi=3.16% (5/158)
qeeeaeqnlselsgpwrtvyigstnpekiqengpfrtyfrelvfddekgtvdfyfsvkrdgkwknvhvkatkqd----------------------DGTYVADY-------------------------------------------------------------------------------------------
--------------------------------------------------------------------------mkctknalaqtgfnkdkyfngDVWYVTDYldlepddvpkrycaalaagtasgklkealycydpktqdtfydvselqeespgkytanfkkveknqnvkvdvtsgnyytftvmyadds

-----------------------------------------------------------egqnvfkivslsrthlvahninvdkhgqtteltglfvklnvededlekfwkltedkgidkknvvnflenedhphpe
salihtclhkgnkdlgdlyavlnrnkdtnagdkvkgavtaaslkfsdfistkdnkceydnvslkslltk----------------------------------------------------------------

Proposed:  aliSize=129 (resi)      RMSD=2.55 (Å)
qeee-----------AeQNLSEL-S-GPWRTVYIGSTNPE---kiQEngpfRTYFRELVfD---deKgTvDFYFSVK---rdG-KWKNVHVKATKQdDG-TYVADY----------------eGQNVFKIVSLSRTHLVAHNINVDKHGQTTELTGLFVK-LNVEdeDLEKFWKLTEDKGIDKKNVVNFL
----mkctknalaqtG-FNKDKYfNgDVWYVTDYLDLEPDdvp--KR----YCAALAAG-Tasg--K-L-KEALYCYdpk--TqDTFYDVSELQEE-SPgKYTANFkkveknqnvkvdvtsg-NYYTFTVMYADDSSALIHTCLHKGNKDLGDLYAVLNRnKDTN--AGDKVKGAVTAASLKFSDFISTK

EN----------------edhphpe
DNkceydnvslkslltkh-------
```

392	1q2yA (140)<br>1s3zB (145)	C	12.14

```
TM-align:  aliSize=122 (resi)      RMSD=2.41 (Å)
MKAVIAKnEEQLKDAFYVREEVFVkeqnVPAEEEIDELE-N---E--SEHIVVYDGEKPVGAGRWRMKD---------GYGKLERICVLKSHRSAGVGGIIMKALEKAAADGGASGFILNAQ--TQ-AVPFYKKHGYRVLSEKEFldagiphlqmmkd-----
MDIRQMN-KTHLEHWRGLRKQLWP----GHPDDAHLADGeEilqAdhLASFIAMADGVAIGFADASIRHdyvngcdssPVVFLEGIFVLPSFRQRGVAKQLIAAVQRWGTNKGCREMASDTSpeNTiSQKVHQALGFEETERVIF-------------yrkrc

SARST:     aliSize=119 (resi)      RMSD=6.00 (Å)
m-KAVIAKNEEQLKDAFYVREEVFvkeqNVPAEEE---------IdeleNESEHIVVYDGEKPVGAGRWRmKDGYGKLeRI-----------CVLKSHRSAGVGGIIMKALEKAAADGGASGFILNAQTQA---VPFYKKHGYRVLSekefldagiPHLQMMK--d
-mDIRQMNKTHLEHWRGLRKQLWP----GHPDDAHladgeeilqA----DHLASFIAMADGVAIGFADAS-IRHDYVN-GCdsspvvflegiFVLPSFRQRGVAKQLIAAVQRWGTNKGCREMASDTSPENtisQKVHQALGFEETE---------RVIFYRKrc-

BLAST:     aliSize=34 (resi)       iden=14.29% (20/140)       simi=24.29% (34/140)
mkaviakneeqlkdafyvreevfvkeqnvpae------------------------------------EEIDELENESEHIVVYDGeKPVGAGRWRMKDGY--G-------KLERICVLKSHRSAGVGGIIMKALEKAAADGG---------------------------------------asgfilnaqtqa
------------------------------mdirqmnkthlehwrglrkqlwpghpddahladgEEILQADHLASFIAMADG-VAIGFADASIRHDYvnGcdsspvvFLEGIFVLPSFRQRGVAKQLIAAVQRWGTNKGcremasdtspentisqkvhqalgfeetervifyrkrc------------

vpfykkhgyrvlsekefldagiphlqmmkd
------------------------------

Proposed:  aliSize=125 (resi)      RMSD=2.02 (Å)
MKAVIAKnEEQLKDAFYVREEVFVkeqnVPAE-EeI--D--eLE-NE--SEHIVVYDGEKPVGAGRWRMKD---------GYGKLERICVLKSHRSAGVGGIIMKALEKAAADGGASGFILNAQT-Q--AVPFYKKHGYRVLSEKefldaGIPHLQM-mkd
MDIRQMN-KTHLEHWRGLRKQLWP----GHPDdA-HlaDge-EIlQAdhLASFIAMADGVAIGFADASIRHdyvngcdssPVVFLEGIFVLPSFRQRGVAKQLIAAVQRWGTNKGCREMASDTSPeNtiSQKVHQALGFEETERV-----IFYRKRCc---
```

393	1q2yA (140)<br>2o28A (182)	C	15.71	TM-align: aliSize=121 (resi) RMSD=3.18 (Å)<br>------------------------------------MKAVIAkNEEQlK-D-AFYVREEVFVkeqnvPAEee-----Id--EL-EN--E--SEHIVVYDG--EKPVGAGRWRMK-D------GYGKLERICVLKSHRSAGVGGIIMKALEKAAADGGASGFILNAQTQAVPFYKKHGYRVLSEKEFLDAG<br>pdetpmfdpsllkevdwsqntatfspaispthpgegLVLRPL-CTAD-LnRgFFKVLGQLTE-----TGV--vspeqF-mkSFeHMkkSgdYYVTVVEDVtlGQIVATATLIIEhKfihscaKRGRVEDVVVSDECRGKQLGKLLLSTLTLLSKKLNCYKITLECLPQNVGFYKKFGYTVSEENYMCRRF<br><br>iphlqmmkd--<br>---------lk<br><br>SARST: aliSize=109 (resi) RMSD=7.47 (Å)<br>m--KAVIAKNEEQLKDAFYVREEvfvkeqnvpaeeeidelENESEHIVVYD--GEKPVGAGRWRMK-----DGYG--KLERICVLKSHRSAGVGGIIMKALEKAAADGGASGFILNAQTQAVPFYKKHGYRVlS<br>-pdetpmfdpsllkevdwsqntatfspaispthpgeglvlrplctadlnrgffkvlgqlTETGVVSPEQFMKSFEHMKK-----------------SGDYYVTVVEDvtLGQIVATATLIIEhkfihSCAKrgRVEDVVVSDECRGKQLGKLLLSTLTLLSKKLNCYKITLECLPQNVGFYKKFGYTV-S<br><br>EKEFL------dagiphlqmmkd<br>EENYMcrrflk------------<br><br>BLAST: aliSize=29 (resi) iden=12.86% (18/140) simi=20.71% (29/140)<br>mkaviakneeqlkdafyvreevfvkeqnvpaeeeidelenesehivvydgekpvgagrwrmkdgy--GKLERICVLKS<br>---pdetpmfdpsllkevdwsqntatfspaispthpgeglvlrplctadlnrgffkvlgqltetgvvspeqfmksfehmkksgdyyvtvvedvtlgqivatatliiehkfihscakrGRVEDVVVSDE<br><br>HRSAGVGGIIMKALEKAAADGGASGFILNAQTQAVPFYKKHGYRVlSEKEFL------dagiphlqmmkd<br>CRGKQLGKLLLSTLTLLSKKLNCYKITLECLPQNVGFYKKFGYTV-SEENYMcrrflk------------<br><br>Proposed: aliSize=117 (resi) RMSD=2.20 (Å)<br>------------------------------------MKAVIAkNEEQlK-D-AFYVREEVFVkeqnvpAE---------eeidelE---N--E--SEHIVVYDG--EKPVGAGRWRMK-D------GYGKLERICVLKSHRSAGVGGIIMKALEKAAADGGASGFILNAQTQAVPFYKKHGYRVLsekef<br>pdetpmfdpsllkevdwsqntatfspaispthpgegLVLRPL-CTAD-LnRgFFKVLGQLTE------TGvvspeqfmk------SfehMkkSgdYYVTVVEDVtlGQIVATATLIIEhKfihscaKRGRVEDVVVSDECRGKQLGKLLLSTLTLLSKKLNCYKITLECLPQNVGFYKKFGYTVS-----<br><br>ldagIPHLQMMKD---<br>----EENYMCRRFlkc
394	1q2yA (140)<br>2vbqB (144)	C	12.14	TM-align: aliSize=122 (resi) RMSD=2.55 (Å)<br>mKAVIAKnEEQLKDAFYVREEVFVkeqnVPAEEEI-DEL-EN--E--SEHIVVYDGEKPVGAGRWRMKD---------GYGKLERICVLKSHRSAGVGGIIMKALEKAAADGGASGFILNAQ--TQ-AVPFYKKHGYRVLSEKEFLdagiphlqmmkd----<br>-DIRQMN-KTHLEHWRGLRKQLWP----GHPDDAHlADGeEIlqAdhLASFIAMADGVAIGFADASIRHdyvngcdssPVVFLEGIFVLPSFRQRGVAKQLIAAVQRWGTNKGCREMASDTSpeNTiSQKVHQALGFEETERVIFY------------rkrc<br><br>SARST: aliSize=90 (resi) RMSD=5.48 (Å)<br>mkaviakneeqlkdafyvreevfvkeqnvpaeeeidelen--ESEHIVVYDGEKPVGAGRWRMKD----G----YGKLE-RICVLKSHRSAGVGGIIMKALEKAAADGGASGFILNAQTQAVPFYK---KHGYRVLSekefldagiPHLQMM<br>--dirqmnkthlehwrglrkqlwpghpddahladgeeilqadHLASFIAMADGVAIGFADASIRHdyvnGcdssPVVFLeGIFVLPSFRQRGVAKQLIAAVQRWGTNKGCREMASDTSPENTISQKvhqALGFEETE---------RVIFYR<br><br>K--d<br>Krc-<br><br>BLAST: aliSize=34 (resi) iden=14.29% (20/140) simi=24.29% (34/140)<br>mkaviakneeqlkdafyvreevfvkeqnvpae---------------------------------EEIDELENESEHIVVYDGeKPVGAGRWRMKDGY--G-------KLERICVLKSHRSAGVGGIIMKALEKAAADGG---------------------------------------asgfilnaqtqav<br>--------------------------------dirqmnkthlehwrglrkqlwpghpddahladgEEILQADHLASFIAMADG-VAIGFADASIRHDYvnGcdsspvvFLEGIFVLPSFRQRGVAKQLIAAVQRWGTNKGcremasdtspentisqkvhqalgfeetervifyrkrc-------------<br><br>pfykkhgyrvlsekefldagiphlqmmkd<br>-----------------------------<br><br>Proposed: aliSize=124 (resi) RMSD=2.02 (Å)<br>mKAVIAKnEEQLKDAFYVREEVFVkeqnVPAE-EeI--D--eLE-NE--SEHIVVYDGEKPVGAGRWRMKD---------GYGKLERICVLKSHRSAGVGGIIMKALEKAAADGGASGFILNAQT-Q--AVPFYKKHGYRVLSEKefldagIPHLQMMkd<br>-DIRQMN-KTHLEHWRGLRKQLWP----GHPDdA-HlaDge-EIlQAdhLASFIAMADGVAIGFADASIRHdyvngcdssPVVFLEGIFVLPSFRQRGVAKQLIAAVQRWGTNKGCREMASDTSPeNtiSQKVHQALGFEETERV------IFYRKRC--
395	1q2yA (140)<br>2vxkA (165)	C	18.57	TM-align: aliSize=120 (resi) RMSD=2.89 (Å)<br>----------------------MKAVIAkNEEQlK-D-AFYVReEVFVkeqnVPAEEe----Id--EL-EN--E---SEHIVVYDG-EKPVGAGRWRMK-D------GYGKLERICVLKSHRSAGVGGIIMKALEKAAADGGASGFILNAQTQAVPFYKKHGYRVLSEKEFLDagiphlqmmkd-<br>entplfspslispdvlavlpadYTIRPL-CRSD-YkRgYLDVL-RVLT----TVGDI-neeqW-nsRYeWIraRsdeYYLLVVCDGeGRIVGTGSLVVErKfihslgMVGHIEDIAVEKGQQGKKLGLRIIQALDYVAEKVGCYKTILDCSEANEGFYIKCGFKRAGLEMAHY-----------y<br><br>SARST: aliSize=107 (resi) RMSD=6.12 (Å)<br>mkavi---AK---NEEQLKDAFYVREevfvkeqnvpaeeeidELENESEHIVVYDGE-KPVGAGRWRMK-----DGY--GKLERICVLKSHRSAGVGGIIMKALEKAAADGGASGFILNAQTQAVPFYKKHGYRVLSEKEFL--dagi<br>-----entplfspslispdvlavlpadytirplcrsdykrgyldvlrvltTVgdiNEEQWNSRYEWIR----------------ARSDEYYLLVVCDGEgRIVGTGSLVVErkfihSLGmvGHIEDIAVEKGQQGKKLGLRIIQALDYVAEKVGCYKTILDCSEANEGFYIKCGFKRAGLEMAHyy----<br><br>phlqmmkd<br>--------<br><br>BLAST: aliSize=39 (resi) iden=17.86% (25/140) simi=27.86% (39/140)<br>mkaviakneeqlkdafyvreevfvkeqnvpaeeeideleneseh---IVVYDGE-KPVGAGRWRMKDGY-------GKLERICVLKSHRSAGVGGIIMKALEKAAADGGASGFILNAQTQAV<br>--entplfspslispdvlavlpadytirplcrsdykrgyldvlrvlttvgdineeqwnsryewirarsdeyylLVVCDGEgRIVGTGSLVVERKFihslgmvGHIEDIAVEKGQQGKKLGLRIIQALDYVAEKVGCYKTILDCSEANE<br><br>PFYKKHGYR----------vlsekefldagiphlqmmkd<br>GFYIKCGFKraglemahyy-------------------<br><br>Proposed: aliSize=115 (resi) RMSD=2.00 (Å)<br>----------------------MKAVIAkNEEQlK-D-AFYVREEVFvkeqnVPAE--------eeidelE---N--E---SEHIVVYDG-EKPVGAGRWRMK-D------GYGKLERICVLKSHRSAGVGGIIMKALEKAAADGGASGFILNAQTQAVPFYKKHGYRVLSekefldagiphlQMMKD---<br>entplfspslispdvlavlpadYTIRPL-CRSD-YkRgYLDVLRVLT-----TVGDineeqwns------RyewIraRsdeYYLLVVCDGeGRIVGTGSLVVErKfihslgMVGHIEDIAVEKGQQGKKLGLRIIQALDYVAEKVGCYKTILDCSEANEGFYIKCGFKRAG------------LEMAHyyc

396	1s60A (152)<br>1b87A (181)	C	13.82

```
TM-align:  aliSize=132 (resi)      RMSD=2.09 (Å)
lvprgshMDIRQM-NKT--HLEHWRGLRKQLWPG-HPDdAHLADGEEILqADHLASFIAMADGVAIGFADASIRHDyvngcdsSPVVFLEGIFVLPSFRQRGVAKQLIAAVQRWGTNKGCREMASDTSPE--N-----------------------TISQKVHQALGFEETERVI-FYrkrc--------
-------MIISEFdRNNpvLKDQLSDLLRLTWPEeYGD-SSAEEVEEMM-NPERIAVAAVDQDELVGFIGAIPQYG-------ITGWELHPLVVESSRRKNQIGTRLVNYLEKEVASRGGITIYLGTDDLdhGttlsqtdlyehtfdkvasiqnlrEHPYEFYEKLGYKIVGVLPnAN----gwdkpdiw

-----------
maktiiprpds

SARST:     aliSize=108 (resi)      RMSD=3.30 (Å)
lvprgshm-DIRQMNKTHLEHWR---GLRKQLWPGHPDDAHLADGEEIlQADHLASFIAMADGVAIGFADASirhdYVNGCDsspVVFLEGIFVLPSFRQRGVAKQLIAAVQRWGTNKGCREMASDTS--------------------------------------------------------------
--------mIISEFDRNNPVLKDqlsDLLRLTWPEEYGDSSAEEVEEM-MNPERIAVAAVDQDELVGFIGAI----PQYGIT---GWELHPLVVESSRRKNQIGTRLVNYLEKEVASRGGITIYLGTDdldhgttlsqtdlyehtfdkvasiqnlrehpyefyeklgykivgvlpnangwdkpdiwmakt

-------pentisqkvhqalgfeetervifyrkrc
iiprpds----------------------------

BLAST:     aliSize=56 (resi)       iden=18.42% (28/152)       simi=36.84% (56/152)
lvprgshmdirqmnkthlehwrg-------------------LRKQLWPGHPDDAHLADGEEILQADHLAsfIAMAD-GVAIGFADASIRHDyVNGCDSSPVVflegifVLPSFRQRGVAKQLIAAVQRWGTNKGCREM---ASDTSPENTISQKVHQALGFEETERVI--------FYRK---------
-----------------------miisefdrnnpvlkdqlsdLLRLTWPEEYGDSSAEEVEEMMNPERIA--VAAVDqDELVGFIGAIPQYG-ITGWELHPLV------VESSRRKNQIGTRLVNYLEKEVASRGGITIylgTDDLDHGTTLSQTDLYEHTFDKVASIQnlrehpyeFYEKlgykivgvl

-----------------------rc
pnangwdkpdiwmaktiiprpds--

Proposed:  aliSize=135 (resi)      RMSD=1.73 (Å)
lvprgshMDIRQM-NKT--HLEHWRGLRKQLWPG-HPDdAHLADGEEILqADHLASFIAMADGVAIGFADASIRHdyvngcdsS-PVVFLEGIFVLPSFRQRGVAKQLIAAVQRWGTNKGCREMASDTSPE--N-----------------------TISQKVHQALGFEETERV-----------IFYR
-------MIISEFdRNNpvLKDQLSDLLRLTWPEeYGD-SSAEEVEEMM-NPERIAVAAVDQDELVGFIGAIPQY--------GiTGWELHPLVVESSRRKNQIGTRLVNYLEKEVASRGGITIYLGTDDLdhGttlsqtdlyehtfdkvasiqnlrEHPYEFYEKLGYKIVGVLpnangwdkpdiWMAK

K-RC-----c
TiIPrpdsa-
```

397	1s60A (152)<br>1bo4B (136)	C	8.82

```
TM-align:  aliSize=122 (resi)      RMSD=2.93 (Å)
lvprgSHMDIRQMN--K-THLEHWRGLRKQ-LWP-GHPDDAHLA--DGEEILQADHLASFIAMADGVAIGFADASIRHDYvngc-DSSPVVFLEGIFVLPSFRQRGVAKQLIAAVQRWGTNKGCREMASDTSpENTisqkv------HQalGFeetervifyrkrc
-----GIIRTCRLGpdQvKSMRAALDLFGReFGDvATYSQHQPDsdYLGNLLRSKTFIALAAFDQEAVVGALAAYVLPKF----eQPRSEIYIYDLAVSGEHRRQGIATALINLLKHEANALGAYVIYVQAD-YGD-----dpavalYT--KL-------------

SARST:     aliSize=107 (resi)      RMSD=3.37 (Å)
lvprgs-HMDIRQMNKTHLEHWRGLR------------KQLWPGHpddahLADGEEILQADHLASFIAMADGVAIGFADASIRHDYVNgcdSSPVVFLEGIFVLPSFRQRGVAKQLIAAVQRWGTNKGCREMAS---------------dtspentisqkvhqalgfeetervifyrkrc
------gIIRTCRLGPDQVKSMRAALdlfgrefgdvatYSQHQPD-----SDYLGNLLRSKTFIALAAFDQEAVVGALAAYVLPKFEQ---PRSEIYIYDLAVSGEHRRQGIATALINLLKHEANALGAYVIYVqadygddpavalytk-------------------------------

BLAST:     aliSize=9 (resi)        iden=5.93% (8/136)         simi=6.67% (9/136)
lvprgshmdirqmnkthlehwrglrkqlwpghpddahladgeeilqadhlasfiamadgvaigfadasirhdyvngc----------------------------------------------------------DSSPVVFLEGIFVLPSFRQ------------------------------------
-----------------------------------------------------------------------------giirtcrlgpdqvksmraaldlfgrefgdvatysqhqpdsdylgnllrsktfialaafDQEAVVGALAAYVLPKFEQprseiyiydlavsgehrrqgiatalinllkheanal

----------------------rgvakqliaavqrwgtnkgcremasdtspentisqkvhqalgfeetervifyrkrc
gayviyvqadygddpavalytk--------------------------------------------------------

Proposed:  aliSize=120 (resi)      RMSD=2.14 (Å)
lvprgSHMDIRQMN-KT--HLEHWRGLRKQ-L-WPGHPDDAHLA--DGEEILQADHLASFIAMADGVAIGFADASIRHD----yvngcdsSPVVFLEGIFVLPSFRQRGVAKQLIAAVQRWGTNKGCREMASDT--spentisqkvhqalgfeETE--RVIFYR--krcc
-----GIIRTCRLGpDQvkSMRAALDLFGReFgDVATYSQHQPDsdYLGNLLRSKTFIALAAFDQEAVVGALAAYVLPKfeqp-------RSEIYIYDLAVSGEHRRQGIATALINLLKHEANALGAYVIYVQAdy-----------------GDDpaVALYTKlc----
```

398	1s60A (152)<br>1kuxA (166)	C	13.16

```
TM-align:  aliSize=134 (resi)      RMSD=2.83 (Å)
lvprg---SHMDIRQMNKTHLEHWRGLRKQLWP---GHPdd-AHLADGEEILQADHLASFIAMADGVAIGFADASIRHDYVNgcds-----------SPVVFLEGIFVLPSFRQRGVAKQLIAAVQ-RWGTNKGCREMASDTSPenTiSQKVHQALGFEEtERVIFYRkrc-------------
-----htlPANEFRCLTPEDAAGVFEIEREAFIsvsGNC--pLNLDEVQHFLTLCPELSLGWFVEGRLVAFIIGSLWDEERL----tqeslalhrprGHSAHLHALAVHRSFRQQGKGSVLLWRYLhHVGAQPAVRRAVLMCED--A-LVPFYQRFGFHP-AGPCAIV---vgsltftemhcsl

SARST:     aliSize=137 (resi)      RMSD=6.70 (Å)
lvprgsh-----MDIRQMNKTHLEHWRGLR------KQLWPGHPddaHLADGEEILQADHLASFiAMADGVAIGFADASIRHDYVN-------GCDSSPVVFLEGIFVLPSFRQRGVAKQLIAAVQRWGTN-KGCREMASDTSPEntiSQKVHQALGFEE---------TERVIFYRKR-c
-------htlpaNEFRCLTPEDAAGVFEIEreafisVSGNCPLN---LDEVQHFLTLCPELSLG-WFVEGRLVAFIIGSLWDEERLtqeslalHRPRGHSAHLHALAVHRSFRQQGKGSVLLWRYLHHVGAqPAVRRAVLMCEDA---LVPFYQRFGFHPagpcaivvgSLTFTEMHCSl-

BLAST:     aliSize=11 (resi)       iden=5.26% (8/152)         simi=7.24% (11/152)
lvprgshmdirqmnkthlehwrglrkqlwpghpddahladgeeilqadhlasfiamadgvaigfadasirhdyvngcdsspvvf-------------------------------------------------------------------------------------------LEGIFVLPSFRQRGV
------------------------------------------------------------------------------------htlpanefrcltpedaagvfeiereafisvsgncplnldevqhfltlcpelslgwfvegrlvafiigslwdeerltqeslalhrprghsahLHALAVHRSFRQQGK

AKQLI-------------------------------------------------------aavqrwgtnkgcremasdtspentisqkvhqalgfeetervifyrkrc
GSVLLwrylhhvgaqpavrravlmcedalvpfyqrfgfhpagpcaivvgsltftemhcsl------------------------------------------------

Proposed:  aliSize=134 (resi)      RMSD=2.25 (Å)
lvprgs----HMDIRQMNKTHLEHWRGLRKQLWP---GH---pddaHLADGEEILQADhL-ASFIAMADGVAIGFADASIRHDYVN-----------gcdsSPVVFLEGIFVLPSFRQRGVAKQLIAAVQRWGT-NKGCREMASDTSPenTiSQKVHQALGFEE------TE---RVIFYRKRC-c
------htlpANEFRCLTPEDAAGVFEIEREAFIsvsGNcpl----NLDEVQHFLTLC-PeLSLGWFVEGRLVAFIIGSLWDEERLtqeslalhrpr----GHSAHLHALAVHRSFRQQGKGSVLLWRYLHHVGaQPAVRRAVLMCED--A-LVPFYQRFGFHPagpcaiVVgslTFTEMHCSLc-
```

399	1s60A (152)	C	11.84	TM-align: aliSize=134 (resi) RMSD=2.83 (Å)<br>lvprg---SHMDIRQMNKTHLEHWRGLRKQLWP---GHPdd-AHLADGEEILQADHLASFIAMADGVAIGFADASIRHDYVNgcds-----------SPVVFLEGIFVLPSFRQRGVAKQLIAAVQ-RWGTNKGCREMASDTSPenTiSQKVHQALGFEEtERVIFYRkrc-------------<br>-----htlPANEFRCLTPEDAAGVFEIEREAFIsvsGNC--pLNLDEVQHFLTLCPELSLGWFVEGRLVAFIIGSLWDEERL----tqeslalhrprGHSAHLHALAVHRSFRQQGKGSVLLWRYLhHVGAQPAVRRAVLMCED--A-LVPFFQRFGFHP-AGPCAIV---vgsltftemhcsl<br><br>SARST: aliSize=140 (resi) RMSD=6.66 (Å)<br>lvprgsh-----MDIRQMNKTHLEHWRGLRKQ---LWPGHPDDAHLADGEEILQADHLASFiAMADGVAIGFADASIRHDYVN-------GCDSSPVVFLEGIFVLPSFRQRGVAKQLIAAVQRWGTNKG-CREMASDTSPENtisQKVHQALGFEE---------TERVIFYRKR-c<br>-------htlpaNEFRCLTPEDAAGVFEIEREafiSVSGNCPLNLDEVQHFLTLCPELSLG-WFVEGRLVAFIIGSLWDEERLtqeslalHRPRGHSAHLHALAVHRSFRQQGKGSVLLWRYLHHVGAQPaVRRAVLMCEDAL---VPFFQRFGFHPagpcaivvgSLTFTEMHCSl-<br><br>BLAST: aliSize=11 (resi) iden=5.26% (8/152) simi=7.24% (11/152)<br>lvprgshmdirqmnkthlehwrglrkqlwpghpddahladgeeilqadhlasfiamadgvaigfadasirhdyvngcdsspvvf---LEGIFVLPSFRQRGV<br>--htlpanefrcltpedaagvfeiereafisvsgncplnldevqhfltlcpelslgwfvegrlvafiigslwdeerltqeslalhrprghsahLHALAVHRSFRQQGK<br><br>AKQLI---aavqrwgtnkgcremasdtspentisqkvhqalgfeetervifyrkrc<br>GSVLLwrylhhvgaqpavrravlmcedalvpffqrfgfhpagpcaivvgsltftemhcsl--<br><br>Proposed: aliSize=133 (resi) RMSD=2.29 (Å)<br>lvprgs----HMDIRQMNKTHLEHWRGLRKQLWP---GH---pddaHLADGEEILQADhL-ASFIAMADGVAIGFADASIRHDYVN-----------gcdsSPVVFLEGIFVLPSFRQRGVAKQLIAAVQRWGT-NKGCREMASDTSPenTiSQKVHQAlGFEETERVIF------YRKRC----c<br>------htlpANEFRCLTPEDAAGVFEIEREAFIsvsGNcpl----NLDEVQHFLTLC-PeLSLGWFVEGRLVAFIIGSLWDEERLtqeslalhrpr----GHSAHLHALAVHRSFRQQGKGSVLLWRYLHHVGaQPAVRRAVLMCED--A-LVPFFQR-FGFHPAGPCAivvgslTFTEMhcsl-
	1l0cA (166)			
400	1s60A (152)	C	13.82	TM-align: aliSize=132 (resi) RMSD=2.08 (Å)<br>lvprgshMDIRQM-NKT--HLEHWRGLRKQLWPG-HPDdAHLADGEEILqADHLASFIAMADGVAIGFADASIRHDyvngcdsSPVVFLEGIFVLPSFRQRGVAKQLIAAVQRWGTNKGCREMASDTSP--EN-----------------------TISQKVHQALGFEETERVI-FYrkrc--------<br>-------MIISEFdRNNpvLKDQLSDLLRLTWPEeYGD-SSAEEVEEMM-NPERIAVAAVDQDELVGFIGAIPQYG-------ITGWELHPLVVESSRRKNQIGTRLVNYLEKEVASRGGITIYLGTDDldHGttlsqtdlyehtfdkvasiqnlrEHPYEFYEKLGYKIVGVLPnAN----gwdkpdiw<br><br>----------<br>maktiiprpd<br><br>SARST: aliSize=104 (resi) RMSD=3.52 (Å)<br>lvprgshm-DIRQMNKTHLEHWRGLR-------KQLWpghpdDAHLADGEEILQADHLASFIAMADGVAIGFAdASIRHDYVNgcdsspVVFLEGIFVLPSFRQRGVAKQLIAAVQRWGTNKGCREMASDTS--<br>--------mIISEFDRNNPVLKDQLSdllrltwPEEY-----GDSSAEEVEEMMNPERIAVAAVDQDELVGFI-GAIPQYGIT------GWELHPLVVESSRRKNQIGTRLVNYLEKEVASRGGITIYLGTDdldhgttlsqtdlyehtfdkvasiqnlrehpyefyeklgykivgvlpnangwdkpdiw<br><br>----------pentisqkvhqalgfeetervifyrkrc<br>maktiiprpd----------------------------<br><br>BLAST: aliSize=56 (resi) iden=18.42% (28/152) simi=36.84% (56/152)<br>lvprgshmdirqmnkthlehwrg-------------------LRKQLWPGHPDDAHLADGEEILQADHLAsfIAMAD-GVAIGFADASIRHDyVNGCDSSPVVflegifVLPSFRQRGVAKQLIAAVQRWGTNKGCREM---ASDTSPENTISQKVHQALGFEETERVI--------FYRK---------<br>-----------------------miisefdrnnpvlkdqlsdLLRLTWPEEYGDSSAEEVEEMMNPERIA--VAAVDqDELVGFIGAIPQYG-ITGWELHPLV------VESSRRKNQIGTRLVNYLEKEVASRGGITIylgTDDLDHGTTLSQTDLYEHTFDKVASIQnlrehpyeFYEKlgykivgvl<br><br>----------------------rc<br>pnangwdkpdiwmaktiiprpd--<br><br>Proposed: aliSize=135 (resi) RMSD=1.76 (Å)<br>lvprgshMDIRQM-NKT--HLEHWRGLRKQLWPG-HPDdAHLADGEEILqADHLASFIAMADGVAIGFADASIRHdyvngcdsS-PVVFLEGIFVLPSFRQRGVAKQLIAAVQRWGTNKGCREMASDTSP--EN-----------------------TISQKVHQALGFEETERVI---------FYRKR<br>-------MIISEFdRNNpvLKDQLSDLLRLTWPEeYGD-SSAEEVEEMM-NPERIAVAAVDQDELVGFIGAIPQY--------GiTGWELHPLVVESSRRKNQIGTRLVNYLEKEVASRGGITIYLGTDDldHGttlsqtdlyehtfdkvasiqnlrEHPYEFYEKLGYKIVGVLPnangwdkpdIWMAK<br><br>C-------c<br>Tiiprpdc-
	1n71A (180)			
401	1s60A (152)	C	13.82	TM-align: aliSize=132 (resi) RMSD=2.13 (Å)<br>lvprgshMDIRQM-NKT--HLEHWRGLRKQLWPG-HPDdAHLADGEEILqADHLASFIAMADGVAIGFADASIRHDyvngcdsSPVVFLEGIFVLPSFRQRGVAKQLIAAVQRWGTNKGCREMASDTSP--EN-----------------------TISQKVHQALGFEETERVI-FYrkrc--------<br>-------MIISEFdRNNpvLKDQLSDLLRLTWPEeYGD-SSAEEVEEMM-NPERIAVAAVDQDELVGFIGAIPQYG-------ITGWELHPLVVESSRRKNQIGTRLVNYLEKEVASRGGITIYLGTDDldHGttlsqtdlyehtfdkvasiqnlrEHPYEFYEKLGYKIVGVLPnAN----gwdkpdiw<br><br>---------<br>maktiiprp<br><br>SARST: aliSize=105 (resi) RMSD=3.41 (Å)<br>lvprgshm-DIRQMNKTHL---EHWRGLRKQLWpghpDD---AHLADGEEILQADHLASFIAMADGVAIGFAdASIRHDYVNgcdsspVVFLEGIFVLPSFRQRGVAKQLIAAVQRWGTNKGCREMASDTS---<br>--------mIISEFDRNNPvlkDQLSDLLRLTW----PEeygDSSAEEVEEMMNPERIAVAAVDQDELVGFI-GAIPQYGIT------GWELHPLVVESSRRKNQIGTRLVNYLEKEVASRGGITIYLGTDdldhgttlsqtdlyehtfdkvasiqnlrehpyefyeklgykivgvlpnangwdkpdiwm<br><br>--------pentisqkvhqalgfeetervifyrkrc<br>aktiiprp----------------------------<br><br>BLAST: aliSize=56 (resi) iden=18.42% (28/152) simi=36.84% (56/152)<br>lvprgshmdirqmnkthlehwrg-------------------LRKQLWPGHPDDAHLADGEEILQADHLAsfIAMAD-GVAIGFADASIRHDyVNGCDSSPVVflegifVLPSFRQRGVAKQLIAAVQRWGTNKGCREM---ASDTSPENTISQKVHQALGFEETERVI--------FYRK---------<br>-----------------------miisefdrnnpvlkdqlsdLLRLTWPEEYGDSSAEEVEEMMNPERIA--VAAVDqDELVGFIGAIPQYG-ITGWELHPLV------VESSRRKNQIGTRLVNYLEKEVASRGGITIylgTDDLDHGTTLSQTDLYEHTFDKVASIQnlrehpyeFYEKlgykivgvl<br><br>---------------------rc<br>pnangwdkpdiwmaktiiprp--<br><br>Proposed: aliSize=135 (resi) RMSD=1.84 (Å)<br>lvprgshMDIRQM-NKT--HLEHWRGLRKQLWPG-HPDdAHLADGEEILqADHLASFIAMADGVAIGFADASIRHdyvngcdsS-PVVFLEGIFVLPSFRQRGVAKQLIAAVQRWGTNKGCREMASDTSP--EN-----------------------TISQKVHQALGFEETERVI----FYRKRC----<br>-------MIISEFdRNNpvLKDQLSDLLRLTWPEeYGD-SSAEEVEEMM-NPERIAVAAVDQDELVGFIGAIPQY--------GiTGWELHPLVVESSRRKNQIGTRLVNYLEKEVASRGGITIYLGTDDldHGttlsqtdlyehtfdkvasiqnlrEHPYEFYEKLGYKIVGVLPnangWDKPDIwmak<br><br>-------c<br>tiiprpc-
	1n71B (179)			

402	1s60A (152)<br>1q2dA (161)	C	7.89	TM-align: aliSize=124 (resi) RMSD=3.02 (Å)<br>lvprgshMDIRQMN---K-THLEHWRGLRKQLW---P--GHPDdAHLADGeEIlqAdhLASFIAMAD-GVAIGFADASIRHDyvngcdsSPVVFLEGIFVLPSFRQRGVAKQLIAAVQRWGTNKGCREMASDTSPenTiSQKVHQALGFEETE------------RVIFyrkrc---------------<br>-------LDFDILTndgThRNMKLLIDLKNIFSrqlPkmPKEY-IVKLVF-DR--H--HESMVILKNkQKVIGGICFRQYKP-------QRFAEVAFLAVTANEQVRGYGTRLMNKFKDHMQKQNIEYLLTYADN--F-AIGYFKKQGFTKEHrmpqekwkgyikDYDG-----gtlmecyihpyvdyg<br><br>SARST: aliSize=115 (resi) RMSD=6.35 (Å)<br>lvprgshmdirqmnkth--------------------LEHWRGLRKQLWPghpddahLADGEEILQADHLASFIAMADG-VAIGFADASIR--HdyvnGCdsspvVFLEGIFVLPSFRQRGVAKQLIAAVQRWGTNKGCREMASDTSPEntiSQKVHQALGF--EETER-------------VIFYRKR-<br>-----------------ldfdiltndgthrnmkllidLKNIFSRQLPKMP-------KEYIVKLVFDRHHESMVILKNKqKVIGGICFRQYkpQ----RF-----AEVAFLAVTANEQVRGYGTRLMNKFKDHMQKQNIEYLLTYADNF---AIGYFKKQGFtkEHRMPqekwkgyikdydgGTLMECYi<br><br>-------c<br>hpyvdyg-<br><br>BLAST: aliSize=11 (resi) iden=3.95% (6/152) simi=7.24% (11/152)<br>lvprgshmdirqmnkthlehwrglrkqlwpghpddahladgeei--LQADHLASFIAMADGVAIGF--------------------------<br>--ldfdiltndgthrnmkllidlknifsrqlpkmpkeyivklvfdrhhesmvilknkqkviggicfrqykpqrfaevaflavtaneqvrgygtrlmnkfkdhMQKQNIEYLLTYADNFAIGYfkkqgftkehrmpqekwkgyikdydg<br><br>---------------adasirhdyvngcdsspvvflegifvlpsfrqrgvakqliaavqrwgtnkgcremasdtspentisqkvhqalgfeetervifyrkrc<br>gtlmecyihpyvdyg--<br><br>Proposed: aliSize=129 (resi) RMSD=2.40 (Å)<br>lvprgshMDIRQMN------KT-HLEHWRGLRKQLWP-GHPDdAHLADGEEilQAdhLASFIAMAD-GVAIGFADASIR-HdyvngcdsSPVVFLEGIFVLPSFRQRGVAKQLIAAVQRWGTNKGCREMASDTSpENtiSQKVHQALGFEET---------------E--RVIFYRKRC------c<br>-------LDFDILTndgthrNMkLLIDLKNIFSRQLPkMPKE-YIVKLVFD--RH--HESMVILKNkQKVIGGICFRQYkP--------QRFAEVAFLAVTANEQVRGYGTRLMNKFKDHMQKQNIEYLLTYAD-NF--AIGYFKKQGFTKEhrmpqekwkgyikdyDggTLMECYIHPyvdygc-
403	1s60A (152)<br>1s7nA (177)	C	11.84	TM-align: aliSize=136 (resi) RMSD=2.64 (Å)<br>lvprg-------SHMDIRQMNKTHLEHWRGLRK--Q--L-----WPGHPDD--AHLADGEEILQA--D-HLASFIAMADGVAIGFADASIRHDyvngcdSSPVVFLeGIFVLPSFRQRGVAKQLIAAVQRWGT-NKGCREMASDTSPENTISQKVHQALGFEETERVI-FYrkrc------------------<br>-----veiipvsTTLELRAADESHVPALHQLVLknKawLqqsldWPQYVTSqeETRKHVQGNILLhqRgYAKMYLIFCQNEMAGVLSFNAIEP------INKAAYI-GYWLDESFQGQGIMSQSLQALMTHYArRGDIRRFVIKCRVDNQASNAVARRNHFTLEGCMKqAE----ylngdyhdvnmyariida<br><br>SARST: aliSize=129 (resi) RMSD=3.97 (Å)<br>lvprgs--------HMDIRQMNKTHLEHWRGLR------------KQLWPGHPDDAHLADGEEILQADH--LASFIAMADGVAIGFADasirhdyvngCDSSP----VVFLeGIFVLPSFRQRGVAKQLIAAVQRWGTNKG-CREMASDTSPENTISQKVHQALGFEETERVI-----------------<br>------veiipvstTLELRAADESHVPALHQLVlknkawlqqsldWPQYVTSQEETRKHVQGNILLHQRgyAKMYLIFCQNEMAGVLS----------FNAIEpinkAAYI-GYWLDESFQGQGIMSQSLQALMTHYARRGdIRRFVIKCRVDNQASNAVARRNHFTLEGCMKqaeylngdyhdvnmyar<br><br>----fyrkrc<br>iida------<br><br>BLAST: aliSize=7 (resi) iden=3.95% (6/152) simi=4.61% (7/152)<br>lvprgshmdirqmnkthlehwrglrkqlwpghpddahladgeeilqadhlasfiamadgvaigfadasirhdyvngcdsspvvflegifvlpsfr---QRGVAKQLIAAVQ---------------------<br>---veiipvsttlelraadeshvpalhqlvlknkawlqqsldwpqyvtsqeetrkhvqgnillhQRGYAKMYLIFCQnemagvlsfnaiepinkaayi<br><br>--rwgtnkgcremasdtspentisqkvhqalgfeetervifyrkrc<br>gywldesfqgqgimsqslqalmthyarrgdirrfvikcrvdnqasnavarrnhftlegcmkqaeylngdyhdvnmyariida--<br><br>Proposed: aliSize=137 (resi) RMSD=2.18 (Å)<br>lvprgs--------HMDIRQMNKTHLEHWRGLRK-Q---L-----WPGHPDD--AHLADGEEILQA--D-HLASFIAMADGVAIGFADASIRH--dyvngcdsSPVVFLeGIFVLPSFRQRGVAKQLIAAVQRWGT-NKGCREMASDTSPENTISQKVHQALGFEETERVI--------FYRKRC-----<br>------veiipvstTLELRAADESHVPALHQLVLkNkawLqqsldWPQYVTSqeETRKHVQGNILLhqRgYAKMYLIFCQNEMAGVLSFNAIEpi--------NKAAYI-GYWLDESFQGQGIMSQSLQALMTHYArRGDIRRFVIKCRVDNQASNAVARRNHFTLEGCMKqaeylngdYHDVNMyarii<br><br>---c<br>dac-
404	1s60A (152)<br>1s7nD (177)	C	11.84	TM-align: aliSize=136 (resi) RMSD=2.63 (Å)<br>lvprg------SHMDIRQMNKTHLEHWRGLRK--Q--L-----WPGHPDD--AHLADGEEILQA--D-HLASFIAMADGVAIGFADASIRHDyvngcdSSPVVFLeGIFVLPSFRQRGVAKQLIAAVQRWGT-NKGCREMASDTSPENTISQKVHQALGFEETERVI-FYrkrc-------------------<br>-----eiipvsTTLELRAADESHVPALHQLVLknKawLqqsldWPQYVTSqeETRKHVQGNILLhqRgYAKMYLIFCQNEMAGVLSFNAIEP------INKAAYI-GYWLDESFQGQGIMSQSLQALMTHYArRGDIRRFVIKCRVDNQASNAVARRNHFTLEGCMKqAE----ylngdyhdvnmyariidad<br><br>SARST: aliSize=127 (resi) RMSD=3.94 (Å)<br>lvprgs-------HMDIRQMNKTHLEHWRG-------LRKQLW-----PGhPDDAHLADGEEILQAD---HLASFIAMADGVAIGFADasirhdyvngCDSS-----PVVFleGIFVLPSFRQRGVAKQLIAAVQRWGTNKG-CREMASDTSPENTISQKVHQALGFEETERVI----------------<br>------eiipvstTLELRAADESHVPALHQlvlknkaWLQQSLdwpqyVT-SQEETRKHVQGNILLHqrgYAKMYLIFCQNEMAGVLS----------FNAIepinkAAYI--GYWLDESFQGQGIMSQSLQALMTHYARRGdIRRFVIKCRVDNQASNAVARRNHFTLEGCMKqaeylngdyhdvnmya<br><br>------fyrkrc<br>riidad------<br><br>BLAST: aliSize=7 (resi) iden=3.95% (6/152) simi=4.61% (7/152)<br>lvprgshmdirqmnkthlehwrglrkqlwpghpddahladgeeilqadhlasfiamadgvaigfadasirhdyvngcdsspvvflegifvlpsfr--QRGVAKQLIAAVQ----------------------<br>---eiipvsttlelraadeshvpalhqlvlknkawlqqsldwpqyvtsqeetrkhvqgnillhQRGYAKMYLIFCQnemagvlsfnaiepinkaayig<br><br>--rwgtnkgcremasdtspentisqkvhqalgfeetervifyrkrc<br>ywldesfqgqgimsqslqalmthyarrgdirrfvikcrvdnqasnavarrnhftlegcmkqaeylngdyhdvnmyariidad--<br><br>Proposed: aliSize=137 (resi) RMSD=2.19 (Å)<br>lvprgs-------HMDIRQMNKTHLEHWRGLRK--Q--L-----WPGHPDD--AHLADGEEILQA--D-HLASFIAMADGVAIGFADASIRH--dyvngcdsSPVVFLeGIFVLPSFRQRGVAKQLIAAVQRWGT-NKGCREMASDTSPENTISQKVHQALGFEETERVI-F---------YRKRC----<br>------eiipvstTLELRAADESHVPALHQLVLknKawLqqsldWPQYVTSqeETRKHVQGNILLhqRgYAKMYLIFCQNEMAGVLSFNAIEpi--------NKAAYI-GYWLDESFQGQGIMSQSLQALMTHYArRGDIRRFVIKCRVDNQASNAVARRNHFTLEGCMKqAeylngdyhdVNMYAriid<br><br>---c<br>adc-

405	1s60A<br>(152)<br><br>1wwzB<br>(157)	C	11.18	TM-align: aliSize=136 (resi) RMSD=2.63 (Å)<br>lvprgsHMDIRQM-NKT--HLEHWRGLRKQLW--P--G--HPDDAHLADGEEILQAdHL-ASFIAMADGVAIGFADAS-IRHDYVngCdSSPVVFLEGIFVLPSFRQRGVAKQLIAAVQRWGTNkGCREMASDTSPENTISQKVHQALGFeETERVIFYrkrc----------<br>------EIKIEKLkKLDkkALNELIDVYMSGYegLeeYggEGRDYARNYIKWCWKK-ASdGFFVAKVGDKIVGFIVCDkDWFSKY--E-GRIVGAIHEFVVDKKFQGKGIGRKLLITCLDFLGK-YNDTIELWVGEKNYGAMNLYEKFGF-KKVGKSGI----wvrmikrqnl<br><br>SARST: aliSize=139 (resi) RMSD=6.23 (Å)<br>lvprgshm--DIRQM---NKTHLEHWRGLRKQLWPGHPD-------DAHLADGEEILQADHlASFIAMADGVAIGFADasIRHDYVNGCDSSPVVFLEGIFVLPSFRQRGVAKQLIAAVQRWGTNKgCREMASDTSPENTISQKVHQALGFEETE----RVIFYRKR--c<br>--------eiKIEKLkklDKKALNELIDVYMSGYEGLEEyggegrdYARNYIKWCWKKASD-GFFVAKVGDKIVGFIV--CDKDWFSKYEGRIVGAIHEFVVDKKFQGKGIGRKLLITCLDFLGKY-NDTIELWVGEKNYGAMNLYEKFGFKKVGksgiWVRMIKRQnl-<br><br>BLAST: aliSize=37 (resi) iden=14.47% (22/152) simi=24.34% (37/152)<br>lvprgshmdirqmnkthlehwrglrkqlwpghpddahladgeeilqadhlasfiamadgvai---GFADASIrHDYVNG---CDSSPVVFLEG-I------FVL-PSFRQRGVA-KQLIAAVQRWGTNKGCREMAsdTSP<br>--eikieklkkldkkalnelidvymsgyegleeyggegrdyarnyikwcwkkasdGFFVAKV-GDKIVGfivCDKDWFSKYEGrIvgaiheFVVdKKFQGKGIGrKLLITCLDFLGKYNDTIELW--VGE<br><br>ENTISQKVHQALGFEE----------------tervifyrkrc<br>KNYGAMNLYEKFGFKKvgksgiwvrmikrqnl-----------<br><br>Proposed: aliSize=139 (resi) RMSD=2.36 (Å)<br>lvprgsHMDIRQM-NKT--HLEHWRGLRKQLW-PG--H---PDDAHLADGEEILQAdHL-ASFIAMADGVAIGFADAS-IRHDYVngC-dsSPVVFLEGIFVLPSFRQRGVAKQLIAAVQRWGTNkGCREMASDTSPENTISQKVHQALGFeETERV----IFYRKRC---c<br>------EIKIEKLkKLDkkALNELIDVYMSGYeGLeeYggeGRDYARNYIKWCWKK-ASdGFFVAKVGDKIVGFIVCDkDWFSKY--Eg--RIVGAIHEFVVDKKFQGKGIGRKLLITCLDFLGK-YNDTIELWVGEKNYGAMNLYEKFGF-KKVGKsgiwVRMIKRQnla-
406	1s60A<br>(152)<br><br>1y9wA<br>(140)	N	11.43	TM-align: aliSize=90 (resi) RMSD=2.32 (Å)<br>lvprgshmdirqmnkthlehwrglrkqlwpghpddahladgeeilq-----------------------------------ADHLASFIAMADG-VAIGFADASIRHdyvngcdssPVVFLEGIFVLPSFRQRGVAKQLIAAVQRWGTNKGCREMASDTSpENtISQKVHQALGFEETERVI-Fyrkrc-<br>--mymkhiengtriegeyiknkviqynmsiltdevkqPMEEVSLVVKNEEgKIFGGVTGTMYF---------YHLHIDFLWVDESVRHDGYGSQLLHEIEGIAKEKGCRLILLDSF-SF-QAPEFYKKHGYREYGVVEdH-----p<br><br>------------<br>kghsqhffekrl<br><br>SARST: aliSize=102 (resi) RMSD=7.21 (Å)<br>lvprgshmdirqmnkthlehwrglrkqlwpghp--------------DDAHLADGEEILQ--ADH------LASFIAMA-DGVAIGFADASIRHdyvnGCdsspvVFLEGIFVLPSFRQRGVAKQLIAAVQRWGTNKGCREMASDTSPENTISQKVHqaLGFEETERVI---------------fyrkrc<br>---------------------------------mymkhiengtriegEYIKNKVIQYNMSilTDEvkqpmeEVSLVVKNeEGKIFGGVTGTMYF----YH-----LHIDFLWVDESVRHDGYGSQLLHEIEGIAKEKGCRLILLDSFSFQAPEFYKK--HGYREYGVVEdhpkghsqhffekrl------<br><br>BLAST: aliSize=20 (resi) iden=7.86% (11/140) simi=14.29% (20/140)<br>lvprgshmdirqmnkthlehwrglrkqlwpghpddahladgeeilqadhlasfiamadgvaigfadasirhdyvngcdsspvvf---LEGIFVLPSFRQRGVAKQLIAAVQRWGTNKGCREMASDT--<br>--mymkhiengtriegeyiknkviqynmsiltdevkqpmeevslvvkneegkifggvtgtmyfyhlhIDFLWVDESVRHDGYGSQLLHEIEGIAKEKGCRLILLDSfs<br><br>----------------------------------spentisqkvhqalgfeetervifyrkrc<br>fqapefykkhgyreygvvedhpkghsqhffekrl-----------------------------<br><br>Proposed: aliSize=113 (resi) RMSD=1.95 (Å)<br>lVprGSHMD-------irqmNKTHLEHWRGLRKQLWPG-HP-dDAhladgeeilqadHLASFIAMaDG--VAIGFADASIRHdyvngcdssPVVFLEGIFVLPSFRQRGVAKQLIAAVQRWGTNKGCREMASDTSPENtiSQKVHQALGFEETERV----------------ifyrkrcc<br>-M--YMKHIengtrie----GEYIKNKVIQYNMSILTDeVKq-PM------------EEVSLVVK-NEegKIFGGVTGTMYF---------YHLHIDFLWVDESVRHDGYGSQLLHEIEGIAKEKGCRLILLDSFSFQ--APEFYKKHGYREYGVVedhpkghsqhffekrl--------
407	1s60A<br>(152)<br><br>1y9wA<br>(140)	C	12.86	TM-align: aliSize=90 (resi) RMSD=2.32 (Å)<br>lvprgshmdirqmnkthlehwrglrkqlwpghpddahladgeeilq-----------------------------------ADHLASFIAMADG-VAIGFADASIRHdyvngcdssPVVFLEGIFVLPSFRQRGVAKQLIAAVQRWGTNKGCREMASDTSpENtISQKVHQALGFEETERVI-Fyrkrc-<br>--mymkhiengtriegeyiknkviqynmsiltdevkqPMEEVSLVVKNEEgKIFGGVTGTMYF---------YHLHIDFLWVDESVRHDGYGSQLLHEIEGIAKEKGCRLILLDSF-SF-QAPEFYKKHGYREYGVVEdH-----p<br><br>------------<br>kghsqhffekrl<br><br>SARST: aliSize=102 (resi) RMSD=7.21 (Å)<br>lvprgshmdirqmnkthlehwrglrkqlwpghp--------------DDAHLADGEEILQ--ADH------LASFIAMA-DGVAIGFADASIRHdyvnGCdsspvVFLEGIFVLPSFRQRGVAKQLIAAVQRWGTNKGCREMASDTSPENTISQKVHqaLGFEETERVI---------------fyrkrc<br>---------------------------------mymkhiengtriegEYIKNKVIQYNMSilTDEvkqpmeEVSLVVKNeEGKIFGGVTGTMYF----YH-----LHIDFLWVDESVRHDGYGSQLLHEIEGIAKEKGCRLILLDSFSFQAPEFYKK--HGYREYGVVEdhpkghsqhffekrl------<br><br>BLAST: aliSize=20 (resi) iden=7.86% (11/140) simi=14.29% (20/140)<br>lvprgshmdirqmnkthlehwrglrkqlwpghpddahladgeeilqadhlasfiamadgvaigfadasirhdyvngcdsspvvf---LEGIFVLPSFRQRGVAKQLIAAVQRWGTNKGCREMASDT--<br>--mymkhiengtriegeyiknkviqynmsiltdevkqpmeevslvvkneegkifggvtgtmyfyhlhIDFLWVDESVRHDGYGSQLLHEIEGIAKEKGCRLILLDSfs<br><br>----------------------------------spentisqkvhqalgfeetervifyrkrc<br>fqapefykkhgyreygvvedhpkghsqhffekrl-----------------------------<br><br>Proposed: aliSize=93 (resi) RMSD=1.50 (Å)<br>lvprgshmdirqmnkthlehwrglrkqlwpghpddahladgeeilqa------------------------------------DHLASFIAMaDG--VAIGFADASIRHdyvngcdssPVVFLEGIFVLPSFRQRGVAKQLIAAVQRWGTNKGCREMASDTSPENtiSQKVHQALGFEETER--------<br>---mymkhiengtriegeyiknkviqynmsiltdevkqpMEEVSLVVK-NEegKIFGGVTGTMYF---------YHLHIDFLWVDESVRHDGYGSQLLHEIEGIAKEKGCRLILLDSFSFQ--APEFYKKHGYREYGVvedhpkgh<br><br>-VIFYRKRCc<br>sQHFFEKRL-

408	1s60A<br>(152)<br>1yx0A<br>(151)	C	11.92	TM-align: aliSize=122 (resi) RMSD=3.24 (Å)<br>lvprgshMDIRQMNKtHLEHWRGLRKQLWP-----------G-HPddAHl-ADgeEIlqAdhLASFIAMADGVAIGFADASIRHdyvngcdsSPVVFLEGIFVLP-SFRqRGVAKQLIAAVQRWGTNKGCREMASDTSPE--NTISQKVHQALGFEETERV--IFYrkrc-----------<br>-------MHIKIDDL-TGRQVVSLVNEHLHsmtlmsppesiHaLG--LE-kLR--GP--E--ITFWSAWEGDELAGCGALKELD--------TRHGEIKSMRTSAsHLR-KGVAKQVLQHIIEEAEKRGYERLSLETGSMasFEPARKLYESFGFQYCEPFadYGE----dpnsvfmtkkl<br><br>SARST: aliSize=97 (resi) RMSD=9.91 (Å)<br>lvp--RGSHMDIRqmnkthlehwrglrkqlwpghpddahladgeeilqadhlasfiAMADGVAIGFADASirhdyvNGCDSSPVVFleGIFVLPSFRQRGVAKQLIAAVQRWGTNKGCREMASDTS--PENTISQKVHQALGFEE-----<br>---mhikiddltgrqvvslvnehlhsmtlmsppesihalgleklrGPEITFWS--AWEGDELAGCGALK------ELDTRHGEIK--SMRTSASHLRKGVAKQVLQHIIEEAEKRGYERLSLETGsmASFEPARKLYESFGFQYcepfa<br><br>TERV---IFYRKR--c<br>DYGEdpnSVFMTKkl-<br><br>BLAST: aliSize=56 (resi) iden=19.87% (30/151) simi=37.09% (56/151)<br>lvprgshmdi----------RQMNKTHLEHwrgLRKQLWPGHPDDAHlADGEEILQADHLASFIAM-ADGVA-IG-FADASIRHDYVNGCDSSpvvflegifvlPSFRQRGVAKQLIAAVQRWGTNKGCREMASDTSPENTI--SQKVHQALGFEETE------------------rvifyrkrc<br>----------mhikiddltgRQVVSLVNEH---LHSMTLMSPPESIH-ALGLEKLRGPEITFWSAWeGDELAgCGaLKELDTRHGEIKSMRTS-----------ASHLRKGVAKQVLQHIIEEAEKRGYERLSLETGSMASFepARKLYESFGFQYCEpfadygedpnsvfmtkkl---------<br><br>Proposed: aliSize=121 (resi) RMSD=2.48 (Å)<br>lvprgshMDIRQMNKtHLEHWRGLRKQ----------------lW-pgHpddA-HlADgeEilqAdhLASFIAMADGVAIGFADASIRHdyvngcdsSPVVFLEGIFVLP-SFRqRGVAKQLIAAVQRWGTNKGCREMASDTSPE--NTISQKVHQALGFEET--------E-RVIFYRKRCc<br>-------MHIKIDDL-TGRQVVSLVNEhlhsmtlmsppesiha-Lg--L---EkL-RG--P---E--ITFWSAWEGDELAGCGALKELD--------TRHGEIKSMRTSAsHLR-KGVAKQVLQHIIEEAEKRGYERLSLETGSMasFEPARKLYESFGFQYCepfadygeDpNSVFMTKKL-
409	1s60A<br>(152)<br>2cntA<br>(151)	C	14.57	TM-align: aliSize=127 (resi) RMSD=2.30 (Å)<br>lvprgsHMDIRQMNKTHLEHWRGLRKQ-L-WPGHPddAHLADgeEIlQAdHLASFIAMADGVAIGFADASIRHdyvngcdssPVVFLEGIFVLPSFRQRGVAKQLIAAVQRWGTNKGCREMASDTSPENTISQKVHQALGFEETERVI-FYrkrc--------------------<br>------MNTISILSTTDLPAAWQIEQRaHaFPWSE--KTFFG--NQ-GE-RYLNLKLTADDRMAAFAITQVVL---------DEATLFNIAVDPDFQRRGLGRMLLEHLIDELETRGVVTLWLEVRASNAAAIALYESLGFNEATIRRnYY----ptaqghedaiimalpismklh<br><br>SARST: aliSize=126 (resi) RMSD=2.52 (Å)<br>lvprgsh-MDIRQMNKTHLEHWRGLRKQLWPGHPDdahLADGEEILQAdHLASFIAMADGVAIGFADASIRhdyvngcdsSPVVFLEGIFVLPSFRQRGVAKQLIAAVQRWGTNKGCREMASDTSPENTISQKVHQALGFEETERVI------------------------fyrkrc<br>-------mNTISILSTTDLPAAWQIEQRAHAFPWS---EKTFFGNQGE-RYLNLKLTADDRMAAFAITQVV---------LDEATLFNIAVDPDFQRRGLGRMLLEHLIDELETRGVVTLWLEVRASNAAAIALYESLGFNEATIRRnyyptaqghedaiimalpismklh------<br><br>BLAST: aliSize=43 (resi) iden=15.89% (24/151) simi=28.48% (43/151)<br>lvprgshmdirqmnkthlehwrglrkqlwpghpddahladgeeil-----------------------------------QADHLASFIAMADGVAIGFADASIRHDyvngcdsSPVVFleGIFVLPSFRQRGVAKQLIA-AVQRWGTnKGCREMASDTSPENTISQKVHQALGFEE-TERVIFY-----<br>---mntisilsttdlpaawqieqrahafpwsektffgnQGERYLNLKLTADDRMAAFAITQVVLD-------EATLF--NIAVDPDFQRRGLGRMLLEhLIDELET-RGVVTLWLEVRASNAAAIALYESLGFNEaTIRRNYYptaqg<br><br>----------------rkrc<br>hedaiimalpismklh----<br><br>Proposed: aliSize=130 (resi) RMSD=1.91 (Å)<br>lvprgsHMDIRQMNKTHLEHWRGLRKQ-L-WPGHPddA-HlADgeEIlQAdHLASFIAMADGVAIGFADASIRHdyvngcdssPVVFLEGIFVLPSFRQRGVAKQLIAAVQRWGTNKGCREMASDTSPENTISQKVHQALGFEETERV------------IFYRKRC-------c<br>------MNTISILSTTDLPAAWQIEQRaHaFPWSE--KtF-FG--NQ-GE-RYLNLKLTADDRMAAFAITQVVL---------DEATLFNIAVDPDFQRRGLGRMLLEHLIDELETRGVVTLWLEVRASNAAAIALYESLGFNEATIRrnyyptaqghedAIIMALPismklhc-
410	1s60A<br>(152)<br>2j8mA<br>(171)	C	17.76	TM-align: aliSize=136 (resi) RMSD=2.57 (Å)<br>lvprgsHMDIRQMNKTHLEHWRGLRKQLWP-------GHPDDaHLADGEEILQA-DH-LASFIAMADG-VAIGFADASIRHDYvngc-DSSPVVFLeGIFVLPSFRQRGVAKQLIAAVQRWGTNKGCREMASDTSPENTISQKVHQALGFEETERVI-FYrkrc----------------------<br>------SASIRDAGVADLPGILAIYNDAVGnttaiwnETPVD-LANRQAWFDARaRQgYPILVASDAAgEVLGYASYGDWRPF----eGFRGTVEH-SVYVRDDQRGKGLGVQLLQALIERARAQGLHVMVAAIESGNAASIGLHRRLGFEISGQMPqVG----qkfgrwldltfmqlnldptrsap<br><br>SARST: aliSize=133 (resi) RMSD=4.05 (Å)<br>lvprgsh-MDIRQMNKTHLEHWRGLRK------QLW---PGHPDDAHLADGEEILQaDHLASFIAMA-DGVAIGFADASirhdYVNGC----DSSPVvfleGIFVLPSFRQRGVAKQLIAAVQRWGTNKGCREMASDTSPENTISQKVHQALGFEETERVI--FYR---------------------krc<br>-------sASIRDAGVADLPGILAIYNdavgntTAIwneTPVDLANRQAWFDARAR-QGYPILVASDaAGEVLGYASYG----DWRPFegfrGTVEH----SVYVRDDQRGKGLGVQLLQALIERARAQGLHVMVAAIESGNAASIGLHRRLGFEISGQMPqvGQKfgrwldltfmqlnldptrsap---<br><br>BLAST: aliSize=25 (resi) iden=9.87% (15/152) simi=16.45% (25/152)<br>lvprgshmdirqmnkthlehwrglrkqlwpghpddahladgeeilqadhlasfiamadgvaigfadasirhdyvngcdsspvvfleg--IFVLPSFRQRGVAKQLI<br>---sasirdagvadlpgilaiyndavgnttaiwnetpvdlanrqawfdararqgypilvasdaagevlgyasygdwrpfegfrgtvehsVYVRDDQRGKGLGVQLL<br><br>AAVQRWGTNKGCREMASDTSPENTISQKVHQALGFE---------------------------------etervifyrkrc<br>QALIERARAQGLHVMVAAIESGNAASIGLHRRLGFEisgqmpqvgqkfgrwldltfmqlnldptrsap------------<br><br>Proposed: aliSize=137 (resi) RMSD=2.06 (Å)<br>lvprgsHMDIRQMNKTHLEHWRGLRKQLWP-------GHPDDahLADGEEILQA--DH-LASFIAMADG-VAIGFADASIR-HD--YvngcdsSPVVFLeGIFVLPSFRQRGVAKQLIAAVQRWGTNKGCREMASDTSPENTISQKVHQALGFEETERV----------IFYRKRC----------c<br>------SASIRDAGVADLPGILAIYNDAVGnttaiwnETPVD--LANRQAWFDAraRQgYPILVASDAAgEVLGYASYGDWrPFegF------RGTVEH-SVYVRDDQRGKGLGVQLLQALIERARAQGLHVMVAAIESGNAASIGLHRRLGFEISGQMpqvgqkfgrwLDLTFMQlnldptrsap-

411	1s60A (152)<br>2j8mB (170)	C	15.79	TM-align: aliSize=134 (resi) RMSD=2.47 (Å)<br>lvprgshMDIRQMNKTHLEHWRGLRKQLWP--------GHPD-DAHLADGEEIlQADHLASFIAMADG-VAIGFADASIRH-D-YVngcdsSPVVFLeGIFVLPSFRQRGVAKQLIAAVQRWGTNKGCREMASDTSPENTISQKVHQALGFEETERVI-FYrkrc----------------------<br>-------ASIRDAGVADLPGILAIYNDAVGnttaiwneTPVDlANRQAWFDAR-ARQGYPILVASDAAgEVLGYASYGDWRpFeGF-----RGTVEH-SVYVRDDQRGKGLGVQLLQALIERARAQGLHVMVAAIESGNAASIGLHRRLGFEISGQMPqVG----qkfgrwldltfmqlnldptrsap<br><br>SARST: aliSize=132 (resi) RMSD=4.06 (Å)<br>lvprgshm-DIRQMNKTHLEHWRGLRK------QLW---PGHPDDAHLADGEEILQaDHLASFIAMA-DGVAIGFADASirhdYVNGC----DSSPVvfleGIFVLPSFRQRGVAKQLIAAVQRWGTNKGCREMASDTSPENTISQKVHQALGFEETERVI--FYR----------------------krc<br>--------aSIRDAGVADLPGILAIYNdavgntTAIwneTPVDLANRQAWFDARAR-QGYPILVASDaAGEVLGYASYG----DWRPFegfrGTVEH----SVYVRDDQRGKGLGVQLLQALIERARAQGLHVMVAAIESGNAASIGLHRRLGFEISGQMPqvGQKfgrwldltfmqlnldptrsap---<br><br>BLAST: aliSize=25 (resi) iden=9.87% (15/152) simi=16.45% (25/152)<br>lvprgshmdirqmnkthlehwrglrkqlwpghpddahladgeeilqadhlasfiamadgvaigfadasirhdyvngcdsspvvfleg--IFVLPSFRQRGVAKQLIA<br>--asirdagvadlpgilaiyndavgnttaiwnetpvdlanrqawfdararqgypilvasdaagevlgyasygdwrpfegfrgtvehsVYVRDDQRGKGLGVQLLQ<br>AVQRWGTNKGCREMASDTSPENTISQKVHQALGFE-------------------------------etervifyrkrc<br>ALIERARAQGLHVMVAAIESGNAASIGLHRRLGFEisgqmpqvgqkfgrwldltfmqlnldptrsap------------<br><br>Proposed: aliSize=137 (resi) RMSD=2.11 (Å)<br>lvprgshMDIRQMNKTHLEHWRGLRKQLWP--------GHPD-DAHLADGEEIlQADHLASFIAMADG-VAIGFADASIR---HDYvngcdsSPVVFLeGIFVLPSFRQRGVAKQLIAAVQRWGTNKGCREMASDTSPENTISQKVHQALGFEETERV----------IFYRKRC----------c<br>-------ASIRDAGVADLPGILAIYNDAVGnttaiwneTPVDlANRQAWFDAR-ARQGYPILVASDAAgEVLGYASYGDWrpfEGF------RGTVEH-SVYVRDDQRGKGLGVQLLQALIERARAQGLHVMVAAIESGNAASIGLHRRLGFEISGQMpqvgqkfgrwLDLTFMQlnldptrsap-
412	1s60A (152)<br>2j8nA (169)	C	16.45	TM-align: aliSize=133 (resi) RMSD=2.40 (Å)<br>lvprgshmDIRQMNKTHLEHWRGLRKQLWP--------GHPD-DAHLADGEEIlQADHLASFIAMAD-GVAIGFADASIRH-D-YVngcdsSPVVFLeGIFVLPSFRQRGVAKQLIAAVQRWGTNKGCREMASDTSPENTISQKVHQALGFEETERVI-FYrkrc----------------------<br>--------SIRDAGVADLPGILAIYNDAVGnttaiwneTPVDlANRQAWFDAR-ARQGYPILVASDAaGEVLGYASYGDWRpFeGF-----RGTVEH-SVYVRDDQRGKGLGVQLLQALIERARAQGLHVMVAAIESGNAASIGLHRRLGFEISGQMPqVG----qkfgrwldltfmqlnldptrsap<br><br>SARST: aliSize=136 (resi) RMSD=4.01 (Å)<br>lvprgshmd-IRQMNKTHLEHWRGLRK------QLW---PGHPDDAHLADGEEILQaDHLASFIAMA-DGVAIGFADASirhdYVNGCDSSPVVFLEGIFVLPSFRQRGVAKQLIAAVQRWGTNKGCREMASDTSPENTISQKVHQALGFEETERVIFYRK----------------------rc<br>---------sIRDAGVADLPGILAIYNdavgntTAIwneTPVDLANRQAWFDARAR-QGYPILVASDaAGEVLGYASYG----DWRPFEGFRGTVEHSVYVRDDQRGKGLGVQLLQALIERARAQGLHVMVAAIESGNAASIGLHRRLGFEISGQMPQVGQkfgrwldltfmqlnldptrsap--<br><br>BLAST: aliSize=25 (resi) iden=9.87% (15/152) simi=16.45% (25/152)<br>lvprgshmdirqmnkthlehwrglrkqlwpghpddahladgeeilqadhlasfiamadgvaigfadasirhdyvngcdsspvvfleg---IFVLPSFRQRGVAKQLIAA<br>--sirdagvadlpgilaiyndavgnttaiwnetpvdlanrqawfdararqgypilvasdaagevlgyasygdwrpfegfrgtvehsVYVRDDQRGKGLGVQLLQA<br>VQRWGTNKGCREMASDTSPENTISQKVHQALGFE-------------------------------etervifyrkrc<br>LIERARAQGLHVMVAAIESGNAASIGLHRRLGFEisgqmpqvgqkfgrwldltfmqlnldptrsap------------<br><br>Proposed: aliSize=136 (resi) RMSD=2.05 (Å)<br>lvprgshmDIRQMNKTHLEHWRGLRKQLWP--------GHPD-DAHLADGEEIlQADHLASFIAMAD-GVAIGFADASIR---HDYvngcdsSPVVFLeGIFVLPSFRQRGVAKQLIAAVQRWGTNKGCREMASDTSPENTISQKVHQALGFEETER-----------VIFYRKRC--------c<br>--------SIRDAGVADLPGILAIYNDAVGnttaiwneTPVDlANRQAWFDAR-ARQGYPILVASDAaGEVLGYASYGDWrpfEGF------RGTVEH-SVYVRDDQRGKGLGVQLLQALIERARAQGLHVMVAAIESGNAASIGLHRRLGFEISGQmpqvgqkfgrwlDLTFMQLNldptrsap-
413	1s60A (152)<br>2jdcA (145)	C	15.17	TM-align: aliSize=130 (resi) RMSD=2.63 (Å)<br>lvprgshMDIRQMNKthLEhWRGLRKQ-LWPGHPDDAHlADGeEIlQADHLASFIAMADGVAIGFADASIRhDYVNgCDSSPVVFLEGIFVLPSFRQRGVAKQLIAAVQRWGTNKGCREMASDTSPeNtiSQKVHQALGFEETERVIFYrkrc--------------<br>-------IEVKPINA--ED-TYELRHRiLRPNQPIEAC-MFE-SD-LLRGAFHLGGYYGGKLISIASFHQA-EHSE-LQGQKQYQLRGMATLEGYREQKAGSSLIKHAEEILRKRGADLLWCNART-S--ASGYYKKLGFSEQGEVFDT----ppvgphilmykrit<br><br>SARST: aliSize=127 (resi) RMSD=4.72 (Å)<br>lvprgshm-DIRQMNKTHLEHWRGLRKQLWP----GHPDDahladgeeiLQADHLASFIAMADGVAIGFADASIRHDYVNGCDSSPVVFLegIFVLPSFRQRGVAKQLIAAVQRWGTNKGCREMASDTSPENTISQKvhqALGFEEteRVIFYRKR------------c<br>--------iEVKPINAEDTYELRHRILRPNQpieaCMFES---------DLLRGAFHLGGYYGGKLISIASFHQAEHSELQGQKQYQLRG--MATLEGYREQKAGSSLIKHAEEILRKRGADLLWCNARTSASGYYK---KLGFSE--QGEVFDTPpvgphilmykrit-<br><br>BLAST: aliSize=46 (resi) iden=18.62% (27/145) simi=31.72% (46/145)<br>lvprgshmdirqmnkthlehwrg-------------LRKQ-LWPGHPDDAHLADGEEILQADHLASFIAmadGVAIGFADA-SIRHDYVNGCDSSPvvfLEGIFVLPSFRQRGVAKQLIAAVQRWGTNKGCREMASDTspeNTISQKVHQALGFEETERV-----------------ifyrkrc<br>-----------------------ievkpinaedtyeLRHRiLRPNQPIEACMFESDLLRGAFHLGGYYG---GKLISIASFhQAEHSELQGQKQYQ---LRGMATLEGYREQKAGSSLIKHAEEILRKRGADLLWCNA---RTSASGYYKKLGFSEQGEVfdtppvgphilmykrit-------<br><br>Proposed: aliSize=133 (resi) RMSD=2.19 (Å)<br>lvprgshMDIRQMNKthLEhWRGLRK-QLWPGHPDDAHlADgeE-IlQADHLASFIAMADGVAIGFADASIRHDYvNgCDSSPVVFLEGIFVLPSFRQRGVAKQLIAAVQRWGTNKGCREMASDTSPeNtiSQKVHQALGFEETER--------VIFYRKRC---c<br>-------IEVKPINA--ED-TYELRHrILRPNQPIEAC-MF--EsD-LLRGAFHLGGYYGGKLISIASFHQAEHS-E-LQGQKQYQLRGMATLEGYREQKAGSSLIKHAEEILRKRGADLLWCNART-S--ASGYYKKLGFSEQGEvfdtppvgPHILMYKRitc-

<table>
<tr><td>414</td><td>1s60A
(152)

2z0zA
(194)</td><td>N</td><td>10.53</td><td>TM-align: aliSize=130 (resi)   RMSD=3.16 (Å)
lvprgs----------HMDIRQMNktHLEHWRGLR--K---QL-----WPG-----HPDDAHLADGeEIlqAdHLASFIAMADGVAIGFADASIRHDyvngcdSSPVVFLeGIFVLPSFRQRGVAKQLIAAVQRWGT-NKGCREMASDTSPENTISQKVHQALGFEETERVI-FYRkrc-----------
------mwafperfegRHVRLEPL--ALAHLPAFLrhYdpeVYrflsrAPVapteeALRAHLEGLL-GE--P-GRVNWAILFGKEVAGRISVIAPEP------EHAKLEL-GTMLFKPFWGSPANKEAKYLLLRHAFeVLRAERVQFKVDLRNERSQRALEALGAVREGVLRkNRR---lpdgafrddvv

----
ysvlkeewpgvkarlearlygasgnp

SARST: aliSize=138 (resi)   RMSD=4.84 (Å)
lvpr---------GSHMDIRQMNKTHLEHWR----GLRKQLWPGHPD----DAHLADGEEILQADHLASFIAMADGVAIGFADASIrhdYV--NGcdSSPVvfleGIFVLPSFRQRGVAKQLIAAV-QRWGTNKGCREMASDTSPENTISQKVHQALGFEETERVIFYRKR-------------------
----mwafperfeGRHVRLEPLALAHLPAFLrhydPEVYRFLSRAPVapteEALRAHLEGLLGEPGRVNWAILFGKEVAGRISVIA---PEpeHA--KLEL----GTMLFKPFWGSPANKEAKYLLlRHAFEVLRAERVQFKVDLRNERSQRALEALGAVREGVLRKNRRLpdgafrddvvysvlkeewp

-----------------c
gvkarlearlygasgnp-

BLAST: aliSize=23 (resi)   iden=8.55% (13/152)   simi=15.13% (23/152)
lvpr---------GSHMDIRQMNKTHLEHW-RGLRKQLWPgHPDDAHLADGEEILQAdHLASFI---
----mwafperfeGRHVRLEPLALAHLPAFlRHYDPEVYR-FLSRAPVAPTEEALRA-HLEGLLgepgrvnwailfgkevagrisviapepehaklelgtmlfkpfwgspankeakylllrhafevlraervqfkvdlrnersqralealgavregvlrknrrlpdgafrddvvysvlkeewpgvkarle

----------amadgvaigfadasirhdyvngcdsspvvflegifvlpsfrqrgvakqliaavqrwgtnkgcremasdtspentisqkvhqalgfeetervifyrkrc
arlygasgnp--

Proposed: aliSize=128 (resi)   RMSD=2.43 (Å)
l----VPRGSHM-DIRQMNKthLEHwR-GL---rK---QL-----WPG-----hP-DDAHLADGeEI-lqadHLASFIAMADGVAIGFADASIRH--dyvngcdsSPVVFLeGIFVLPSFRQRGVAKQLIAAVQRWGT-NKGCREMASDTSPENTISQKVHQALGFEETERVIFY---------------
-mwafPERFEGRhVRLEPLA--LAH-LpAFlrh-YdpeVYrflsrAPVaptee-AlRAHLEGLL-GEp----GRVNWAILFGKEVAGRISVIAPEpe--------HAKLEL-GTMLFKPFWGSPANKEAKYLLLRHAFeVLRAERVQFKVDLRNERSQRALEALGAVREGVLRKNrrlpdgafrddvvys

------------------------rkrcc
vlkeewpgvkarlearlygasgnp-----</td></tr>
<tr><td>415</td><td>1s60A
(152)

5gcnA
(166)</td><td>C</td><td>7.89</td><td>TM-align: aliSize=124 (resi)   RMSD=3.15 (Å)
lvprg--SHMDIRQMN---K-THLEHWRGLRKQLW------PGHpdd--AHl-ADGeEIlqadhLASFIAMADG-VAIGFADASIRHDYvngcdssPVVFLEGIFVLPSFRQRGVAKQLIAAVQRWGTNKGCrEMASDTSpeNTISQKVHQALGFE-ET-E-R--VIF-YRkrc----------------
-----mkGLLDFDILTndgThRNMKLLIDLKNIFSrqlpkmPKE---yiVK-lVFD-RH-----HESMVILKNKqKVIGGICFRQYKPQ-------RFAEVAFLAVTANEQVRGYGTRLMNKFKDHMQKQNI-EYLLTYA--DNFAIGYFKKQGFTkEHrMpQekWKGyIK---dydggtlmecyihpyv

----
dygn

SARST: aliSize=119 (resi)   RMSD=5.04 (Å)
lvprgshm-----DIRQMNKTHLEHWRGLR-------KQLWPGHPddaHLADGEEILQADHLASFIAMADGVAIGFADASIRHDyvNGcdsspVVFLEGIFVLPSFRQRGVAKQLIAAVQRWGTNKGCReMASDTSpeNTISQKVHQALGFE----------------------------------ete
--------mkgllDFDILTNDGTHRNMKLLidlknifSRQLPKMP---KEYIVKLVFDRHHESMVILKNKQKVIGGICFRQYKP--QR-----FAEVAFLAVTANEQVRGYGTRLMNKFKDHMQKQNIE-YLLTYA--DNFAIGYFKKQGFTkehrmpqekwkgyikdydggtlmecyihpyvdygn---

rvifyrkrc

BLAST: aliSize=11 (resi)   iden=3.95% (6/152)   simi=7.24% (11/152)
lvprgshmdirqmnkthlehwrglrkqlwpghpddahladgeei---LQADHLASFIAMADGVAIGF----------------------
---mkglldfdiltndgthrnmkllidlknifsrqlpkmpkeyivklvfdrhhesmvilknkqkviggicfrqykpqrfaevaflavtaneqvrgygtrlmnkfkdhMQKQNIEYLLTYADNFAIGYfkkqgftkehrmpqekwkgyik

--------------------adasirhdyvngcdsspvvflegifvlpsfrqrgvakqliaavqrwgtnkgcremasdtspentisqkvhqalgfeetervifyrkrc
dydggtlmecyihpyvdygn--

Proposed: aliSize=120 (resi)   RMSD=2.37 (Å)
lvprgs---HMDIRQMN---K-THLEHWRGLRKQ-L------WPgH--pddaH---laD-geEIlqadhLASFIAMAD-GVAIGFADASIR-HDyvngcdssPVVFLEGIFVLPSFRQRGVAKQLIAAVQRWGTNKGCrEMASDTSpeNTISQKVHQALGFE----------eTE--------RVIFYRK
------mkgLLDFDILTndgThRNMKLLIDLKNIfSrqlpkmPK-Eyi----Vklv--Fd--RH-----HESMVILKNkQKVIGGICFRQYkPQ--------RFAEVAFLAVTANEQVRGYGTRLMNKFKDHMQKQNI-EYLLTYA--DNFAIGYFKKQGFTkehrmpqekw-KGyikdydggTLMECYI

RC-------c
HPyvdygnc-</td></tr>
<tr><td>416</td><td>1s7fA
(181)

2f5iA
(167)</td><td>C</td><td>10.18</td><td>TM-align: aliSize=142 (resi)   RMSD=3.37 (Å)
glvprgshmveiipvsTTLELRAADeSHVPALHQLVLKNKAWLQQSldWPTSqeETRKHVQGNILLhQRGYAKMYLIFCQN--------EMAGVLSFNAIEP--INKAAYIG-YWLDESFQGQGIMSQSLQALMTHYArRGDIRRFVIKCRVDNQASNAVARRNHFTLEGC---MKqAEYlngdyhdvnm
----------------KFVIRPATA-ADCSDILRLIKELAKYEYME--EQVI--LTEKDLLEDGFG-EHPFYHCLVAEVPKehwtpeghSIVGFAMYYFTYDpwIGKLLYLEdFFVMSDYRGFGIGSEILKNLSQVAM-RCRCSSMHFLVAEWNEPSINFYKRRGASDLSSeegWR-LFK----------

yarii-----------
-----idkeyllkmat

SARST: aliSize=128 (resi)   RMSD=4.67 (Å)
glvprgshmveiipvstt-LELRAADESHVPALHQLVLKNKAWLQQSLDWPTSqeetrkhvqGNILLHQRG----YAKMYLIFC-------QNE-MAGVL--SFnAIEPINKAAYI--GYWLDESFQGQGIMSQSLQALMTHYARRGdIRRFVIKCRVDNQASNAVARRNHFTL-----------------
------------------kFVIRPATAADCSDILRLIKELAKYEYMEEQVILT---------EKDLLEDGFgehpFYHCLVAEVpkehwtpEGHsIVGFAmyYF-TYDPWIGKLLYleDFFVMSDYRGFGIGSEILKNLSQVAMRCR-CSSMHFLVAEWNEPSINFYKRRGASDlsseegwrlfkidkey

------egcmkqaeylngdyhdvnmyarii
llkmat------------------------

BLAST: aliSize=29 (resi)   iden=9.58% (16/167)   simi=17.37% (29/167)
glvprgshmveiipvsttlelraadeshvpalhqlvlknkawlqqsldwptsqeetrkhvqgnillhqrgyakmylifcqnemagvlsfnai--EP-INKAAYI-GYWLDESF
--kfvirpataadcsdilrlikelakyeymeeqviltekdlledgfgehpfyhclvaevpkehwtpeghsivgfamyyftyDPwIGKLLYLeDFFVMSDY

QGQGIMSQSLQALMTHYAR-RGDIRRFVIkCRVDNQASNAVARR--------------------------nhftlegcmkqaeylngdyhdvnmyarii
RGFGIGSEILKNLSQVAMRcRCSSMHFLV-AEWNEPSINFYKRRgasdlsseegwrlfkidkeyllkmat-----------------------------

Proposed: aliSize=140 (resi)   RMSD=2.84 (Å)
glvprgshmveiipvsTTLELRAADeSHVPALHQLVLKNKAWLQQ---sldwPTsqeETRKHVQ-GNIL-LHqrgYAKMYLIFCQN--------EMAGVLSFNAIE----piNKAAYIG-YWLDESFQGQGIMSQSLQALMTHYArRGDIRRFVIKCRVDNQASNAVARRNHFTLEG----cmkQaeyln
----------------KFVIRPATA-ADCSDILRLIKELAKYEYMeeq----VI---LTEKDLLeDGFGeHP---FYHCLVAEVPKehwtpeghSIVGFAMYYFTYdpwi--GKLLYLEdFFVMSDYRGFGIGSEILKNLSQVAM-RCRCSSMHFLVAEWNEPSINFYKRRGASDLSseeg---W-----

gdYHDVNM---YAR--Ii
--RLFKIDkeyLLKmaT-</td></tr>
</table>

417	1s7fA (181) / 2fe7B (166)	N	10.24

```
TM-align:  aliSize=143 (resi)      RMSD=3.28 (Å)
glvprgshmveiipvs--------TTLELRAADESHVPALHQLVLkNKAWLQQSLd-WPTsqEETRKHVQGNILlhQRGYAKMYLIFCQNEMAGVLSFNAIEP---INKAAYIG-YWLDESFQGQGIMSQSLQALMTHYARRGdIRRFVIKCRVDNQASNAVARRNHFTLEGCMKQAEYlngdyhdvnmy
-----------------enlyfqghMTLEIRPAVPADAEQILAFII-ELADYERAR-hEVV--TDVEGIRRSLFA--EGSPTRALMCLSEGRPIGYAVFFYSYStwlGRNGIYLEdLYVTPEYRGVGAGRRLLRELAREAVAND-CGRLEWSVLDWNQPAIDFYRSIGALPQDEWVRYRL-----------

arii----------
----dgealrkmae

SARST:     aliSize=146 (resi)      RMSD=7.40 (Å)
glvprgs---HMVEIIPvsttLELRAADESHVPALHQLVLKNKAWLQQSLDWPTSqeetrkhvQGNILLHQR--G-YAKMYLIFCQNEMAGV-LSFNAIEPINKAAYI---GYWLDESFQGQGIMSQSLQALMTHYARRGdIRRFVIKCRVDNQASNAVARRNHFTL--EGCMKqaEYLN--------gd
-------enlYFQGHMT----LEIRPAVPADAEQILAFIIELADYERARHEVVTD--------VEGIRRSLFaeGsPTRALMCLSEGRPIGYaVFFYSYSTWLGRNGIyleDLYVTPEYRGVGAGRRLLRELAREAVAND-CGRLEWSVLDWNQPAIDFYRSIGALPqdEWVRY--RLDGealrkmae--

yhdvnmyarii
-----------

BLAST:     aliSize=47 (resi)       iden=18.67% (31/166)        simi=28.31% (47/166)
glvprgshmveiipvst---------TLELRAAdeshVPALHQLVLknkAWLQQSLDWPTSQEETRKHVQG-NILLHQRGYAKMYLIfCQNEmaGvlsfnaiEPINKAAYI-GY--WLDESfqgqGIMSQSLQALMTHYA----RR-------------------------------------------
-----------------enlyfqghmTLEIRPA----VPADAEQIL---AFIIELADYERARHEVVTDVEGiRRSLFAEGSPTRALM-CLSE--G-------RPIGYAVFFySYstWLGRN----GIYLEDLYVTPEYRGvgagRRllrelareavandcgrlewsvldwnqpaidfyrsigalpqdewv

-------------gdirrfvikcrvdnqasnavarrnhftlegcmkqaeylngdyhdvnmyarii
ryrldgealrkmae----------------------------------------------------

Proposed:  aliSize=143 (resi)      RMSD=2.61 (Å)
glvprg-SHMVEIIPvstTLELRAADESHVPALHQLVLkN-KAWLQQ---sldwPTSqeETRKHVQGNILlhQR-gYAKMYLIFCQNEMAGVLSFNAIE----pINKAAYIG-YWLDESFQGQGIMSQSLQALMTHYArRGDIRRFVIKCRVDNQASNAVARRNHFTLEGCMKqAE------------yl
------eNLYFQGHM---TLEIRPAVPADAEQILAFII-ElADYERArhe----VVT--DVEGIRRSLFA--EGs-PTRALMCLSEGRPIGYAVFFYSYstwl-GRNGIYLEdLYVTPEYRGVGAGRRLLRELAREAV-ANDCGRLEWSVLDWNQPAIDFYRSIGALPQDEWV-RYrldgealrkmae--

ngdyhdvnmyarii
--------------
```

418	1s7fA (181) / 2fe7B (166)	C	10.84

```
TM-align:  aliSize=143 (resi)      RMSD=3.28 (Å)
glvprgshmveiipvs--------TTLELRAADESHVPALHQLVLkNKAWLQQSLd-WPTsqEETRKHVQGNILlhQRGYAKMYLIFCQNEMAGVLSFNAIEP---INKAAYIG-YWLDESFQGQGIMSQSLQALMTHYARRGdIRRFVIKCRVDNQASNAVARRNHFTLEGCMKQAEYlngdyhdvnmy
-----------------enlyfqghMTLEIRPAVPADAEQILAFII-ELADYERAR-hEVV--TDVEGIRRSLFA--EGSPTRALMCLSEGRPIGYAVFFYSYStwlGRNGIYLEdLYVTPEYRGVGAGRRLLRELAREAVAND-CGRLEWSVLDWNQPAIDFYRSIGALPQDEWVRYRL-----------

arii----------
----dgealrkmae

SARST:     aliSize=146 (resi)      RMSD=7.40 (Å)
glvprgs---HMVEIIPvsttLELRAADESHVPALHQLVLKNKAWLQQSLDWPTSqeetrkhvQGNILLHQR--G-YAKMYLIFCQNEMAGV-LSFNAIEPINKAAYI---GYWLDESFQGQGIMSQSLQALMTHYARRGdIRRFVIKCRVDNQASNAVARRNHFTL--EGCMKqaEYLN--------gd
-------enlYFQGHMT----LEIRPAVPADAEQILAFIIELADYERARHEVVTD--------VEGIRRSLFaeGsPTRALMCLSEGRPIGYaVFFYSYSTWLGRNGIyleDLYVTPEYRGVGAGRRLLRELAREAVAND-CGRLEWSVLDWNQPAIDFYRSIGALPqdEWVRY--RLDGealrkmae--

yhdvnmyarii
-----------

BLAST:     aliSize=47 (resi)       iden=18.67% (31/166)        simi=28.31% (47/166)
glvprgshmveiipvst---------TLELRAAdeshVPALHQLVLknkAWLQQSLDWPTSQEETRKHVQG-NILLHQRGYAKMYLIfCQNEmaGvlsfnaiEPINKAAYI-GY--WLDESfqgqGIMSQSLQALMTHYA----RR-------------------------------------------
-----------------enlyfqghmTLEIRPA----VPADAEQIL---AFIIELADYERARHEVVTDVEGiRRSLFAEGSPTRALM-CLSE--G-------RPIGYAVFFySYstWLGRN----GIYLEDLYVTPEYRGvgagRRllrelareavandcgrlewsvldwnqpaidfyrsigalpqdewv

-------------gdirrfvikcrvdnqasnavarrnhftlegcmkqaeylngdyhdvnmyarii
ryrldgealrkmae----------------------------------------------------

Proposed:  aliSize=139 (resi)      RMSD=2.52 (Å)
glvprgshmveiipvst---------TLELRAADESHVPALHQLVLkN-KAWLQQ---sldwPTSqeETRKHVQGNILlhQR-gYAKMYLIFCQNEMAGVLSFNAIE----pINKAAYIG-YWLDESFQGQGIMSQSLQALMTHYArRGDIRRFVIKCRVDNQASNAVARRNHFTLEGcmkqaeylngdy
-----------------enlyfqghmTLEIRPAVPADAEQILAFII-ElADYERArhe----VVT--DVEGIRRSLFA--EGs-PTRALMCLSEGRPIGYAVFFYSYstwl-GRNGIYLEdLYVTPEYRGVGAGRRLLRELAREAV-ANDCGRLEWSVLDWNQPAIDFYRSIGALPQD-----------

hDVNMYARI---I-----
-EWVRYRLDgeaLrkmae
```

419	1s7fA (181) / 2g3tA (169)	C	11.24

```
TM-align:  aliSize=142 (resi)      RMSD=3.39 (Å)
glvprgshmveiipvsTTLELRAADeSHVPALHQLVLKNKAWLQQSldWPTSqeETRKHVQGNILLhQRGYAKMYLIFCQN--------EMAGVLSFNAIEP--INKAAYIG-YWLDESFQGQGIMSQSLQALMTHYArRGDIRRFVIKCRVDNQASNAVARRNHFTLEGC---MKqAEYlngdyhdvnm
-----------------KFVIRPATA-ADCSDILRLIKELAKYEYME--EQVI--LTEKDLLEDGFG-EHPFYHCLVAEVPKehwtpeghSIVGFAMYYFTYDpwIGKLLYLEdFFVMSDYRGFGIGSEILKNLSQVAM-RCRCSSMHFLVAEWNEPSINFYKRRGASDLSSeegWR-LFK----------

yarii-------------
-----idkeyllkmatee

SARST:     aliSize=136 (resi)      RMSD=6.14 (Å)
glvprgshmveiipvstt-LELRAADESHVPALHQLVLKNKAWLQQSLDWPTSqeetrkhvqGNILLHQRG----YAKMYLIFC-------QNE-MAGVL--SFnAIEPINKAAYI--GYWLDESFQGQGIMSQSLQALMTHYARRGdIRRFVIKCRVDNQASNAVARRNHFTLE-----GCMkqaeyln
------------------kFVIRPATAADCSDILRLIKELAKYEYMEEQVILT---------EKDLLEDGFgehpFYHCLVAEVpkehwtpEGHsIVGFAmyYF-TYDPWIGKLLYleDFFVMSDYRGFGIGSEILKNLSQVAMRCR-CSSMHFLVAEWNEPSINFYKRRGASDLsseegWRL-------

gdyhdvnmYARI-----------i
--------FKIDkeyllkmatee-

BLAST:     aliSize=29 (resi)       iden=9.47% (16/169)         simi=17.16% (29/169)
glvprgshmveiipvsttlelraadeshvpalhqlvlknkawlqqsldwptsqeetrkhvqgnillhqrgyakmylifcqnemagvlsfnai------------------------------------------------------------------------------------EP-INKAAYI-GYWLDESF
--------------------------------------------------------------------------------------------kfvirpataadcsdilrlikelakyeymeeqviltekdlledgfgehpfyhclvaevpkehwtpeghsivgfamyyftyDPwIGKLLYLeDFFVMSDY

QGQGIMSQSLQALMTHYAR-RGDIRRFVIkCRVDNQASNAVARR---------------------------nhftlegcmkqaeylngdyhdvnmyarii
RGFGIGSEILKNLSQVAMRcRCSSMHFLV-AEWNEPSINFYKRRgasdlsseegwrlfkidkeyllkmatee-----------------------------

Proposed:  aliSize=140 (resi)      RMSD=2.70 (Å)
glvprgshmveiipvst-TLELRAaDESHVPALHQLVLKNKAWLQQ---sldwPTsqeETRKHVQ-GNIL-LHQrgyAKMYLIFCQN---------EMAGVLSFNAIE----piNKAAYIGYWL-DESFQGQGIMSQSLQALMTHYArRGDIRRFVIKCRVDNQASNAVARRNHFTLEG--cmkqaeyln
-----------------kFVIRPA-TAADCSDILRLIKELAKYEYMeeq----VI---LTEKDLLeDGFGeHP----FYHCLVAEVPKehwtpeghSIVGFAMYYFTYdpwi--GKLLYLEDFFvMSDYRGFGIGSEILKNLSQVAM-RCRCSSMHFLVAEWNEPSINFYKRRGASDLSse---------

GDYHDVNM---YA-R--II-
EGWRLFKIdkeYLlKmaTEe
```

420	1s7fA (181)<br>2jevA (169)	C	11.24	TM-align: aliSize=143 (resi) RMSD=3.55 (Å)<br>glvprgshmveiipvSTTLELRAADeSHVPALHQLVLKNKAWLQQSldWPTSqeETRKHVQGNILLhQRGYAKMYLIFCQN--------EMAGVLSFNAIEP--INKAAYIG-YWLDESFQGQGIMSQSLQALMTHYArRGDIRRFVIKCRVDNQASNAVARRNHFTLEGC---MKqAEYlngdyhdvnm<br>---------------AKFVIRPATA-ADCSDILRLIKELAKYEYME--EQVI--LTEKDLLEDGFG-EHPFYHCLVAEVPKehwtpeghSIVGFAMYYFTYDpwIGKLLYLEdFFVMSDYRGFGIGSEILKNLSQVAM-RCRCSSMHFLVAEWNEPSINFYKRRGASDLSSeegWR-LFK----------<br>yarii------------<br>-----idkeyllkmate<br><br>SARST: aliSize=129 (resi) RMSD=4.68 (Å)<br>glvprgshmveiipvstt--LELRAADESHVPALHQLVLKnKAWLQQSLDWPTSQEetrkhvqgNILLHQRG----YAKMYLIFC-------QNE-MAGVL--SFnAIEPINKAAYI--GYWLDESFQGQGIMSQSLQALMTHYARRGdIRRFVIKCRVDNQASNAVARRNHFTLE-------------<br>------------------akFVIRPATAADCSDILRLIKE-LAKYEYMEEQVILTE--------KDLLEDGFgehpFYHCLVAEVpkehwtpEGHsIVGFAmyYF-TYDPWIGKLLYleDFFVMSDYRGFGIGSEILKNLSQVAMRCR-CSSMHFLVAEWNEPSINFYKRRGASDLsseegwrlfkidke<br>--------gcmkqaeylngdyhdvnmyarii<br>yllkmate-----------------------<br><br>BLAST: aliSize=29 (resi) iden=9.47% (16/169) simi=17.16% (29/169)<br>glvprgshmveiipvsttlelraadeshvpalhqlvlknkawlqqsldwptsqeetrkhvqgnillhqrgyakmylifcqnemagvlsfnai--EP-INKAAYI-GYWLDES<br>---akfvirpataadcsdilrlikelakyeymeeqviltekdlledgfgehpfyhclvaevpkehwtpeghsivgfamyyftyDPwIGKLLYLeDFFVMSD<br>FQGQGIMSQSLQALMTHYAR-RGDIRRFVIkCRVDNQASNAVARR---------------------------nhftlegcmkqaeylngdyhdvnmyarii<br>YRGFGIGSEILKNLSQVAMRcRCSSMHFLV-AEWNEPSINFYKRRgasdlsseegwrlfkidkeyllkmate----------------------------<br><br>Proposed: aliSize=142 (resi) RMSD=2.88 (Å)<br>glvprgshmveiipvs-TTLELRAaDESHVPALHQLVLKNKAWLQQ---sldwPTsqeETRKHVQ-GNIL-LHqrgYAKMYLIFcQN--------EMAGVLSFNAIE----piNKAAYIGYWL-DESFQGQGIMSQSLQALMTHYArRGDIRRFVIKCRVDNQASNAVARRNHFTLEG--cmkqaeylN<br>----------------aKFVIRPA-TAADCSDILRLIKELAKYEYMeeq----VI---LTEKDLLeDGFGeHP---FYHCLVAE-VPkehwtpeghSIVGFAMYYFTYdpwi--GKLLYLEDFFvMSDYRGFGIGSEILKNLSQVAM-RCRCSSMHFLVAEWNEPSINFYKRRGASDLSse--------E<br>GDYHDVNM--YA-R--II<br>GWRLFKIDkeYLlKmaTE
421	1sjvA (102)<br>1dqlH (123)	C	62.75	TM-align: aliSize=90 (resi) RMSD=1.88 (Å)<br>-----G-GGLVQAGESLKLSCAA---S--GGFMGWYRQAPGKQRELVATINSRG-ITNYADFVKGRFTISRDNAKKTVYLEMNSLEPEDTAVYYCYTHYFRSywgqgtqvtvss-------------------<br>vqlveSgGGLVQPGGSLRLSCAAsgfTfsSYAMHWVRQAPGKGLEWVAVISSDGgNKYYTDSVKGRFTISRNDSKNTLYLQMNSLRTEDTAVFYCARGNPPY------------ssgwgggdywgqgtmvtvss<br><br>SARST: aliSize=98 (resi) RMSD=14.71 (Å)<br>gg--------GLVQAGESLKLSCAASG-----GFMGWYRQAPGKQRELVATINSRG-ITNYADFVKGRFTISRDNAKKTVYLEMNSLEPEDTAVYYCYTHYFRSY--------WGQGTQVTV--ss<br>--vqlvesggGLVQPGGSLRLSCAASGftfssYAMHWVRQAPGKGLEWVAVISSDGgNKYYTDSVKGRFTISRNDSKNTLYLQMNSLRTEDTAVFYCARGNPPYSsgwgggdyWGQGTMVTVss--<br><br>BLAST: aliSize=79 (resi) iden=67.65% (69/102) simi=77.45% (79/102)<br>------GGGLVQAGESLKLSCAASG-GF----MGWYRQAPGKQRELVATINSRGITNY-ADFVKGRFTISRDNAKKTVYLEMNSLEPEDTAVYYC------YTHYF--RSYWGQGTQVTVSS<br>vqlvesGGGLVQPGGSLRLSCAASGfTFssyaMHWVRQAPGKGLEWVAVISSDGGNKYyTDSVKGRFTISRNDSKNTLYLQMNSLRTEDTAVFYCargnppYSSGWggGDYWGQGTMVTVSS<br><br>Proposed: aliSize=102 (resi) RMSD=1.65 (Å)<br>------G-GGLVQAGESLKLSCAA---S--GGFMGWYRQAPGKQRELVATINSRG-ITNYADFVKGRFTISRDNAKKTVYLEMNSLEPEDTAVYYCYT----HY---FRS-YWGQGTQVTVSS<br>pvqlveSgGGLVQPGGSLRLSCAAsgfTfsSYAMHWVRQAPGKGLEWVAVISSDGgNKYYTDSVKGRFTISRNDSKNTLYLQMNSLRTEDTAVFYCARgnppYSsgwGGGdYWGQGTMVTVSS
422	1sjvA (102)<br>1f2xK (126)	C	69.61	TM-align: aliSize=88 (resi) RMSD=1.65 (Å)<br>------G-GGLVQAGESLKLSCAA---S--GGFMGWYRQAPGKQRELVATINSrGITNYADFVKGRFTISRDNAKKTVYLEMNSLEPEDTAVYYCYTHYFRsywgqgtqvtvss--------------------------<br>qvqlveSgGGSVQAGGSLRLSCAAsgyTvsTYCMGWFRQAPGKEREGVATILG-GSTYYGDSVKGRFTISQDNAKNTVYLQMNSLKPEDTAIYYCAGSTVA-------------stgwcsrlrpydyhyrgqgtqvtvss<br><br>SARST: aliSize=99 (resi) RMSD=16.12 (Å)<br>g--------GGLVQAGESLKLSCAASG-----GFMGWYRQAPGKQRELVATINSRgITNYADFVKGRFTISRDNAKKTVYLEMNSLEPEDTAVYYCYTHYFRS-------------YWGQGTQVTVS-s<br>-qvqlvesgGGSVQAGGSLRLSCAASGytvstYCMGWFRQAPGKEREGVATILGG-STYYGDSVKGRFTISQDNAKNTVYLQMNSLKPEDTAIYYCAGSTVAStgwcsrlrpydyhYRGQGTQVTVSs-<br><br>BLAST: aliSize=82 (resi) iden=73.53% (75/102) simi=80.39% (82/102)<br>-------GGGLVQAGESLKLSCAASGGF-----MGWYRQAPGKQRELVATINSrGITNYADFVKGRFTISRDNAKKTVYLEMNSLEPEDTAVYYC-------------YTHYFRSYWGQGTQVTVSS<br>qvqlvesGGGSVQAGGSLRLSCAASGYTvstycMGWFRQAPGKEREGVATILG-GSTYYGDSVKGRFTISQDNAKNTVYLQMNSLKPEDTAIYYCagstvastgwcsrLRPYDYHYRGQGTQVTVSS<br><br>Proposed: aliSize=100 (resi) RMSD=1.34 (Å)<br>g--------GGLVQAGESLKLSCAA---S--GGFMGWYRQAPGKQRELVATINSrGITNYADFVKGRFTISRDNAKKTVYLEMNSLEPEDTAVYYCYTHYF------------RSYWGQGTQVTVSS<br>-qvqlvesgGGSVQAGGSLRLSCAAsgyTvsTYCMGWFRQAPGKEREGVATILG-GSTYYGDSVKGRFTISQDNAKNTVYLQMNSLKPEDTAIYYCAGSTVastgwcsrlrpydYHYRGQGTQVTVSS

423	1sjvA (102)<br>1f2xL (126)	C	69.61	TM-align: aliSize=88 (resi) RMSD=1.55 (Å)<br>-----G-GGLVQAGESLKLSCAA---S--GGFMGWYRQAPGKQRELVATINSrGITNYADFVKGRFTISRDNAKKTVYLEMNSLEPEDTAVYYCYTHYFRsywgqgtqvtvss---------------------------<br>vqlveSgGGSVQAGGSLRLSCAAsgyTvsTYCMGWFRQAPGKEREGVATILG-GSTYYGDSVKGRFTISQDNAKNTVYLQMNSLKPEDTAIYYCAGSTVA-------------stgwcsrlrpydyhyrgqgtqvtvssr<br><br>SARST: aliSize=99 (resi) RMSD=16.15 (Å)<br>g-------GGLVQAGESLKLSCAASG-----GFMGWYRQAPGKQRELVATINsRGITNYADFVKGRFTISRDNAKKTVYLEMNSLEPEDTAVYYCYTHYFRS-------------YWGQGTQVTVS--s<br>-vqlvesgGGSVQAGGSLRLSCAASGytvstYCMGWFRQAPGKEREGVATIL-GGSTYYGDSVKGRFTISQDNAKNTVYLQMNSLKPEDTAIYYCAGSTVAStgwcsrlrpydyhYRGQGTQVTVSsr-<br><br>BLAST: aliSize=82 (resi) iden=73.53% (75/102) simi=80.39% (82/102)<br>------GGGLVQAGESLKLSCAASGGF-----MGWYRQAPGKQRELVATINSrGITNYADFVKGRFTISRDNAKKTVYLEMNSLEPEDTAVYYC-------------YTHYFRSYWGQGTQVTVSS-<br>vqlvesGGGSVQAGGSLRLSCAASGYTvstycMGWFRQAPGKEREGVATILG-GSTYYGDSVKGRFTISQDNAKNTVYLQMNSLKPEDTAIYYCagstvastgwcsrLRPYDYHYRGQGTQVTVSSr<br><br>Proposed: aliSize=101 (resi) RMSD=1.43 (Å)<br>-----G-GGLVQAGESLKLSCAA---S--GGFMGWYRQAPGKQRELVATINSrGITNYADFVKGRFTISRDNAKKTVYLEMNSLEPEDTAVYYCYTHYF-------------RSYWGQGTQVTVSS-<br>vqlveSgGGSVQAGGSLRLSCAAsgyTvsTYCMGWFRQAPGKEREGVATILG-GSTYYGDSVKGRFTISQDNAKNTVYLQMNSLKPEDTAIYYCAGSTVastgwcsrlrpydYHYRGQGTQVTVSSr
424	1sjvA (102)<br>1i3vA (129)	C	67.65	TM-align: aliSize=88 (resi) RMSD=1.48 (Å)<br>------G-GGLVQAGESLKLSCAA-----SGGFMGWYRQAPGKQRELVATINSR------GITNYADFVKGRFTISRDNAKKTVYLEMNSLEPEDTAVYYCYTHYFrsywgqgtqvtvss-----------------------<br>qvqlqeSgGGLVQAGDSLKLSCEAsgdsiGTYVIGWFRQAPGKERIYLATIGRNlvgpsdFYTRYADSVKGRFAVSRDNAKNTVNLQMNSLKPEDTAVYYCAAKTT--------------twggndpnnwnywgqgtqvtvss<br><br>SARST: aliSize=98 (resi) RMSD=14.83 (Å)<br>gg---------GLVQAGESLKLSCA-----ASGGFMGWYRQAPGKQRELVATINS----RGIT--NYADFVKGRFTISRDNAKKTVYLEMNSLEPEDTAVYYCYTHYFRS---------YWGQGTQVTV--ss<br>--qvqlqesggGLVQAGDSLKLSCEasgdsIGTYVIGWFRQAPGKERIYLATIGRnlvgPSDFytRYADSVKGRFAVSRDNAKNTVNLQMNSLKPEDTAVYYCAAKTTTWggndpnnwnYWGQGTQVTVss--<br><br>BLAST: aliSize=84 (resi) iden=73.53% (75/102) simi=82.35% (84/102)<br>-------GGGLVQAGESLKLSCAASG---G--FMGWYRQAPGKQRELVATI--NSRG----ITNYADFVKGRFTISRDNAKKTVYLEMNSLEPEDTAVYYCYTHYFR---------SYWGQGTQVTVSS<br>qvqlqesGGGLVQAGDSLKLSCEASGdsiGtyVIGWFRQAPGKERIYLATIgrNLVGpsdfYTRYADSVKGRFAVSRDNAKNTVNLQMNSLKPEDTAVYYCAAKTTTwggndpnnwNYWGQGTQVTVSS<br><br>Proposed: aliSize=101 (resi) RMSD=1.50 (Å)<br>------G-GGLVQAGESLKLSCAA-----SGGFMGWYRQAPGKQRELVATINSR------GITNYADFVKGRFTISRDNAKKTVYLEMNSLEPEDTAVYYCYTHyF----------RSYWGQGTQVTVSS<br>qvqlqeSgGGLVQAGDSLKLSCEAsgdsiGTYVIGWFRQAPGKERIYLATIGRNlvgpsdFYTRYADSVKGRFAVSRDNAKNTVNLQMNSLKPEDTAVYYCAAK-TttwggndpnnWNYWGQGTQVTVSS
425	1sjvA (102)<br>1i8kB (119)	C	57.84	TM-align: aliSize=89 (resi) RMSD=1.59 (Å)<br>------G-GGLVQAGESLKLSCAA---S--GGFMGWYRQAPGKQRELVATINSRG-ITNYADFVKGRFTISRDNAKKTVYLEMNSLEPEDTAVYYCYTHYFRsywgqgtqvtvss-----------------<br>qvklqqSgGGLVKPGASLKLSCVTsgfTfrKFGMSWVRQTSDKCLEWVASISTGGyNTYYSDNVKGRFTISRENAKNTLYLQMSSLKSEDTALYYCTRGYSS-------------tsyamdywgqgttvtvs<br><br>SARST: aliSize=99 (resi) RMSD=14.75 (Å)<br>g--------GGLVQAGESLKLSC--AASG---GFMGWYRQAPGKQRELVATINSRG-ITNYADFVKGRFTISRDNAKKTVYLEMNSLEPEDTAVYYCYTHYFRSY-----WGQGTQVTV-ss<br>-qvklqqsgGGLVKPGASLKLSCvtSGFTfrkFGMSWVRQTSDKCLEWVASISTGGyNTYYSDNVKGRFTISRENAKNTLYLQMSSLKSEDTALYYCTRGYSSTSyamdyWGQGTTVTVs--<br><br>BLAST: aliSize=77 (resi) iden=64.71% (66/102) simi=75.49% (77/102)<br>-------GGGLVQAGESLKLSCAASG----GF-MGWYRQAPGKQRELVATINSRGI-TNYADFVKGRFTISRDNAKKTVYLEMNSLEPEDTAVYYC---Y--THYFRSYWGQGTQVTVSs<br>qvklqqsGGGLVKPGASLKLSCVTSGftfrKFgMSWVRQTSDKCLEWVASISTGGYnTYYSDNVKGRFTISRENAKNTLYLQMSSLKSEDTALYYCtrgYssTSYAMDYWGQGTTVTVS-<br><br>Proposed: aliSize=100 (resi) RMSD=1.63 (Å)<br>------G-GGLVQAGESLKLSCAA---S--GGFMGWYRQAPGKQRELVATINSRG-ITNYADFVKGRFTISRDNAKKTVYLEMNSLEPEDTAVYYCYT--hY--F-RS-YWGQGTQVTVSs<br>qvklqqSgGGLVKPGASLKLSCVTsgfTfrKFGMSWVRQTSDKCLEWVASISTGGyNTYYSDNVKGRFTISRENAKNTLYLQMSSLKSEDTALYYCTRgy-SstSyAMdYWGQGTTVTVS-

426	1sjvA (102)<br>1ic4H (114)	C	40.20	TM-align: aliSize=87 (resi) RMSD=1.75 (Å)<br>------G-GGLVQAGESLKLSCAA---SG--GFMGWYRQAPGKQRELVATINSRGITNYADFVKGRFTISRDNAKKTVYLEMNSLEPEDTAVYYCYTHYfrsywgqgtqvtvss---------------<br>dvqlqeSgPSLVKPSQTLSLTCSVtgdSItsAYWSWIRKFPGNRLEYMGYVSYSGSTYYNPSLKSRISITRDTSKNQYLDLNSVTTEDTATYYCANWD---------------gdywgqgtlvtvsaa<br><br>SARST: aliSize=81 (resi) RMSD=1.95 (Å)<br>ggg----------LVQAGESLKLSCAASG-----GFMGWYRQAPGKQRELVATINSRGITNYADFVKGRFTISRDNAKKTVYLEMNSLEPEDTAVYYCY------------------thyfrsywgqgtqvtvss<br>---dvqlqesgpsLVKPSQTLSLTCSVTGdsitsAYWSWIRKFPGNRLEYMGYVSYSGSTYYNPSLKSRISITRDTSKNQYLDLNSVTTEDTATYYCAnwdgdywgqgtlvtvsaa------------------<br><br>BLAST: aliSize=64 (resi) iden=41.18% (42/102) simi=62.75% (64/102)<br>-------GGGLVQAGESLKLSCAASG-----GFMGWYRQAPGKQRELVATINSRGITNYADFVKGRFTISRDNAKKTVYLEMNSLEPEDTAVYYCyTHYFRSYWGQGTQVTVSS-<br>dvqlqesGPSLVKPSQTLSLTCSVTGdsitsAYWSWIRKFPGNRLEYMGYVSYSGSTYYNPSLKSRISITRDTSKNQYLDLNSVTTEDTATYYC-ANWDGDYWGQGTLVTVSAa<br><br>Proposed: aliSize=100 (resi) RMSD=1.71 (Å)<br>------G-GGLVQAGESLKLSCAA---SG--GFMGWYRQAPGKQRELVATINSRGITNYADFVKGRFTISRDNAKKTVYLEMNSLEPEDTAVYYCYT-hyFRSYWGQGTQVTVSS-<br>dvqlqeSgPSLVKPSQTLSLTCSVtgdSItsAYWSWIRKFPGNRLEYMGYVSYSGSTYYNPSLKSRISITRDTSKNQYLDLNSVTTEDTATYYCANw--DGDYWGQGTLVTVSAa
427	1sjvA (102)<br>1ic5H (114)	C	40.20	TM-align: aliSize=87 (resi) RMSD=1.71 (Å)<br>------G-GGLVQAGESLKLSCAA-----SGGFMGWYRQAPGKQRELVATINSRGITNYADFVKGRFTISRDNAKKTVYLEMNSLEPEDTAVYYCYTHYfrsywgqgtqvtvss---------------<br>dvqlqeSgPSLVKPSQTLSLTCSVtgdsiTSDYWSWIRKFPGNRLEYMGYVSYSGSTYYNPSLKSRISITRDTSKNQYLDLNSVTTEDTATYYCANWA---------------gdywgqgtlvtvsaa<br><br>SARST: aliSize=93 (resi) RMSD=16.21 (Å)<br>ggg----------LVQAGESLKLSCAASGGF-----MGWYRQAPGKQRELVATINSRGITNYADFVKGRFTISRDNAKKTVYLEMNSLEPEDTAVYycythYFRS----YWGQGTQVTVS--s<br>---dvqlqesgpsLVKPSQTLSLTCSVTGDSitsdyWSWIRKFPGNRLEYMGYVSYSGSTYYNPSLKSRISITRDTSKNQYLDLNSVTTEDTATY-----YCANwagdYWGQGTLVTVSaa-<br><br>BLAST: aliSize=64 (resi) iden=41.18% (42/102) simi=62.75% (64/102)<br>-------GGGLVQAGESLKLSCAASGG-----FMGWYRQAPGKQRELVATINSRGITNYADFVKGRFTISRDNAKKTVYLEMNSLEPEDTAVYYCyTHYFRSYWGQGTQVTVSS-<br>dvqlqesGPSLVKPSQTLSLTCSVTGDsitsdYWSWIRKFPGNRLEYMGYVSYSGSTYYNPSLKSRISITRDTSKNQYLDLNSVTTEDTATYYC-ANWAGDYWGQGTLVTVSAa<br><br>Proposed: aliSize=100 (resi) RMSD=1.66 (Å)<br>------G-GGLVQAGESLKLSCAA---S--GGFMGWYRQAPGKQRELVATINSRGITNYADFVKGRFTISRDNAKKTVYLEMNSLEPEDTAVYYCYT-hyFRSYWGQGTQVTVSS-<br>dvqlqeSgPSLVKPSQTLSLTCSVtgdSitSDYWSWIRKFPGNRLEYMGYVSYSGSTYYNPSLKSRISITRDTSKNQYLDLNSVTTEDTATYYCANw--AGDYWGQGTLVTVSAa
428	1sjvA (102)<br>1ic7H (114)	C	39.22	TM-align: aliSize=88 (resi) RMSD=1.93 (Å)<br>------G-GGLVQAGESLKLSCAA-----SGGFMGWYRQAPGKQRELVATINSRGITNYADFVKGRFTISRDNAKKTVYLEMNSLEPEDTAVYYCYTHYFrsywgqgtqvtvss--------------<br>dvqlqeSgPSLVKPSQTLSLTCSVtgdsiTSAYWSWIRKFPGNRLEYMGYVSYSGSTYYNPSLKSRISITRDTSKNQYLDLNSVTTEDTATYYCANWAG--------------dywgqgtlvtvsaa<br><br>SARST: aliSize=93 (resi) RMSD=16.19 (Å)<br>ggg----------LVQAGESLKLSCAASGGF-----MGWYRQAPGKQRELVATINSRGITNYADFVKGRFTISRDNAKKTVYLEMNSLEPEDTAVYycythYFRS----YWGQGTQVTVS--s<br>---dvqlqesgpsLVKPSQTLSLTCSVTGDSitsayWSWIRKFPGNRLEYMGYVSYSGSTYYNPSLKSRISITRDTSKNQYLDLNSVTTEDTATY-----YCANwagdYWGQGTLVTVSaa-<br><br>BLAST: aliSize=64 (resi) iden=41.18% (42/102) simi=62.75% (64/102)<br>-------GGGLVQAGESLKLSCAASG-----GFMGWYRQAPGKQRELVATINSRGITNYADFVKGRFTISRDNAKKTVYLEMNSLEPEDTAVYYCyTHYFRSYWGQGTQVTVSS-<br>dvqlqesGPSLVKPSQTLSLTCSVTGdsitsAYWSWIRKFPGNRLEYMGYVSYSGSTYYNPSLKSRISITRDTSKNQYLDLNSVTTEDTATYYC-ANWAGDYWGQGTLVTVSAa<br><br>Proposed: aliSize=100 (resi) RMSD=1.63 (Å)<br>------G-GGLVQAGESLKLSCAA-----SGGFMGWYRQAPGKQRELVATINSRGITNYADFVKGRFTISRDNAKKTVYLEMNSLEPEDTAVYYCYT-hyFRSYWGQGTQVTVSS-<br>dvqlqeSgPSLVKPSQTLSLTCSVtgdsiTSAYWSWIRKFPGNRLEYMGYVSYSGSTYYNPSLKSRISITRDTSKNQYLDLNSVTTEDTATYYCANw--AGDYWGQGTLVTVSAa

429	1sjvA (102)<br>1jhlH (116)	C	41.18	TM-align: aliSize=89 (resi) RMSD=1.77 (Å)<br>-------GGGLVQAGESLKLSCAA---S--GGFMGWYRQAPGKQRELVATINS-RGITNYADFVKGRFTISRDNAKKTVYLEMNSLEPEDTAVYYCYTHYFRsywgqgtqvtvss-------------<br>qvqlqqsGAELVRPGASVKLSCKAsgyTfiSYWINWVKQRPGQGLEWIGNIYPsDSYTNYNQKFKDKATLTVDKSSSTAYMQLSSPTSEDSAVYYCTRDDNY-------------gamdywgqgttvtv<br><br>SARST: aliSize=83 (resi) RMSD=2.03 (Å)<br>gg---------GLVQAGESLKLSCAASG-----GFMGWYRQAPGKQRELVATINSR-GITNYADFVKGRFTISRDNAKKTVYLEMNSLEPEDTAVYYCYT-----------------hyfrsywgqgtqvtvss<br>--qvqlqqsgaELVRPGASVKLSCKASGytfisYWINWVKQRPGQGLEWIGNIYPSdSYTNYNQKFKDKATLTVDKSSSTAYMQLSSPTSEDSAVYYCTRddnygamdywgqgttvtv----------------<br><br>BLAST: aliSize=62 (resi) iden=44.12% (45/102) simi=60.78% (62/102)<br>-------GGGLVQAGESLKLSCAASGG-----FMGWYRQAPGKQRELVATIN-SRGITNYADFVKGRFTISRDNAKKTVYLEMNSLEPEDTAVYYCY--THY-FRSYWGQGTQVTVss<br>qvqlqqsGAELVRPGASVKLSCKASGYtfisyWINWVKQRPGQGLEWIGNIYpSDSYTNYNQKFKDKATLTVDKSSSTAYMQLSSPTSEDSAVYYCTrdDNYgAMDYWGQGTTVTV--<br><br>Proposed: aliSize=100 (resi) RMSD=1.65 (Å)<br>-------GGGLVQAGESLKLSCAA---S--GGFMGWYRQAPGKQRELVATINS-RGITNYADFVKGRFTISRDNAKKTVYLEMNSLEPEDTAVYYCYTH--YFRS-YWGQGTQVTVss<br>qvqlqqsGAELVRPGASVKLSCKAsgyTfiSYWINWVKQRPGQGLEWIGNIYPsDSYTNYNQKFKDKATLTVDKSSSTAYMQLSSPTSEDSAVYYCTRDdnYGAMdYWGQGTTVTV--
430	1sjvA (102)<br>1jtoA (132)	C	72.55	TM-align: aliSize=89 (resi) RMSD=1.71 (Å)<br>------GGGLVQAGESLKLSCAA-----SGGFMGWYRQAPGKQRELVATINS-RGITNYADFVKGRFTISRD-NAkKTVYLEMNSLEPEDTAVYYCYTHYFRSywgqgtqvtvss-----------------------------<br>vqlqasGGGSVQAGGSLRLSCAAsgytiGPYCMGWFRQAPGKEREGVAAINMgGGITYYADSVKGRFTISQDnAK-NTVYLLMNSLEPEDTAIYYCAADSTIY------------asyyecghglstggygydswgqgtqvtvss<br><br>SARST: aliSize=100 (resi) RMSD=16.11 (Å)<br>g-------GGLVQAGESLKLSCA---ASGGF--MGWYRQAPGKQRELVATI-NSRGITNYADFVKGRFTISRDNAKKTVYLEMNSLEPEDTAVYYCYTHYFRS-----------------YWGQGTQVTVS-s<br>-vqlqasgGGSVQAGGSLRLSCAasgYTIGPycMGWFRQAPGKEREGVAAInMGGGITYYADSVKGRFTISQDNAKNTVYLLMNSLEPEDTAIYYCAADSTIYasyyecghglstggygydSWGQGTQVTVSs-<br><br>BLAST: aliSize=87 (resi) iden=79.41% (81/102) simi=85.29% (87/102)<br>------GGGLVQAGESLKLSCAASG---G--FMGWYRQAPGKQRELVATIN-SRGITNYADFVKGRFTISRDNAKKTVYLEMNSLEPEDTAVYYC------YTHYFR--------SY----WGQGTQVTVSS<br>vqlqasGGGSVQAGGSLRLSCAASGytiGpyCMGWFRQAPGKEREGVAAINmGGGITYYADSVKGRFTISQDNAKNTVYLLMNSLEPEDTAIYYCaadstiYASYYEcghglstgGYgydsWGQGTQVTVSS<br><br>Proposed: aliSize=101 (resi) RMSD=1.34 (Å)<br>g-------GGLVQAGESLKLSCAA-----SGGFMGWYRQAPGKQRELVATINS-RGITNYADFVKGRFTISRDNAKKTVYLEMNSLEPEDTAVYYCYTHYF----------------RS-YWGQGTQVTVSS<br>-vqlqasgGGSVQAGGSLRLSCAAsgytiGPYCMGWFRQAPGKEREGVAAINMgGGITYYADSVKGRFTISQDNAKNTVYLLMNSLEPEDTAIYYCAADSTiyasyyecghglstggyGYdSWGQGTQVTVSS
431	1sjvA (102)<br>1jtpA (135)	C	72.55	TM-align: aliSize=90 (resi) RMSD=1.63 (Å)<br>-------GGGLVQAGESLKLSCAA-----SGGFMGWYRQAPGKQRELVATIN-SRGITNYADFVKGRFTISRDNAKKTVYLEMNSLEPEDTAVYYCYTHYFRSywgqgtqvtvss-------------------------------<br>dvqlqasGGGSVQAGGSLRLSCAAsgytiGPYCMGWFRQAPGKEREGVAAINmGGGITYYADSVKGRFTISQDNAKNTVYLLMNSLEPEDTAIYYCAADSTIY------------asyyecghglstggygydswgqgtqvtvssrr<br><br>SARST: aliSize=85 (resi) RMSD=1.74 (Å)<br>g--------GGLVQAGESLKLSCA---ASGGF--MGWYRQAPGKQRELVATINS-RGITNYADFVKGRFTISRDNAKKTVYLEMNSLEPEDTAVYYCYTH----------------------------------yfrsywgqgtqvtvss<br>-dvqlqasgGGSVQAGGSLRLSCAasgYTIGPycMGWFRQAPGKEREGVAAINMgGGITYYADSVKGRFTISQDNAKNTVYLLMNSLEPEDTAIYYCAADstiyasyyecghglstggygydswgqgtqvtvssrr---------------<br><br>BLAST: aliSize=87 (resi) iden=79.41% (81/102) simi=85.29% (87/102)<br>-------GGGLVQAGESLKLSCAASG---G--FMGWYRQAPGKQRELVATIN-SRGITNYADFVKGRFTISRDNAKKTVYLEMNSLEPEDTAVYYC------YTHYFR--------SY----WGQGTQVTVSS--<br>dvqlqasGGGSVQAGGSLRLSCAASGytiGpyCMGWFRQAPGKEREGVAAINmGGGITYYADSVKGRFTISQDNAKNTVYLLMNSLEPEDTAIYYCaadstiYASYYEcghglstgGYgydsWGQGTQVTVSSrr<br><br>Proposed: aliSize=101 (resi) RMSD=1.13 (Å)<br>g--------GGLVQAGESLKLSCAA-----SGGFMGWYRQAPGKQRELVATIN-SRGITNYADFVKGRFTISRDNAKKTVYLEMNSLEPEDTAVYYCYTHYF----------------R-SYWGQGTQVTVSS--<br>-dvqlqasgGGSVQAGGSLRLSCAAsgytiGPYCMGWFRQAPGKEREGVAAINmGGGITYYADSVKGRFTISQDNAKNTVYLLMNSLEPEDTAIYYCAADSTiyasyyecghglstggyGyDSWGQGTQVTVSSrr

432	1sjvA (102)<br>1jtpB (133)	C	73.53	TM-align: aliSize=91 (resi) RMSD=1.84 (Å)<br>------G-GGLVQAGESLKLSCAA----SG-GFMGWYRQAPGKQRELVATINS-RGITNYADFVKGRFTISRDNAKKTVYLEMNSLEPEDTAVYYCYTHYFRSYwgqgtqvtss----------------------------<br>dvqlqaSgGGSVQAGGSLRLSCAAsgytIGpYCMGWFRQAPGKEREGVAAINMgGGITYYADSVKGRFTISQDNAKNTVYLLMNSLEPEDTAIYYCAADSTIYA----------syyecghglstggygydswgqgtqvtss<br><br>SARST: aliSize=85 (resi) RMSD=1.76 (Å)<br>g--------GGLVQAGESLKLSCAAS---GGF--MGWYRQAPGKQRELVATINS-RGITNYADFVKGRFTISRDNAKKTVYLEMNSLEPEDTAVYYCYTH--------------------------------yfrsywgqgtqvtss<br>-dvqlqasgGGSVQAGGSLRLSCAASgytIGPycMGWFRQAPGKEREGVAAINMgGGITYYADSVKGRFTISQDNAKNTVYLLMNSLEPEDTAIYYCAADstiyasyyecghglstggygydswgqgtqvtss--------------<br><br>BLAST: aliSize=87 (resi) iden=79.41% (81/102) simi=85.29% (87/102)<br>-------GGGLVQAGESLKLSCAASG---G--FMGWYRQAPGKQRELVATIN-SRGITNYADFVKGRFTISRDNAKKTVYLEMNSLEPEDTAVYYC------YTHYFR--------SY----WGQGTQVTSS<br>dvqlqasGGGSVQAGGSLRLSCAASGytiGpyCMGWFRQAPGKEREGVAAINmGGGITYYADSVKGRFTISQDNAKNTVYLLMNSLEPEDTAIYYCaadstiYASYYEcghglstgGYgydsWGQGTQVTSS<br><br>Proposed: aliSize=101 (resi) RMSD=1.13 (Å)<br>g--------GGLVQAGESLKLSCAA----SG-GFMGWYRQAPGKQRELVATINS-RGITNYADFVKGRFTISRDNAKKTVYLEMNSLEPEDTAVYYCYTHYF-----------------R-SYWGQGTQVTSS<br>-dvqlqasgGGSVQAGGSLRLSCAAsgytIGpYCMGWFRQAPGKEREGVAAINMgGGITYYADSVKGRFTISQDNAKNTVYLLMNSLEPEDTAIYYCAADSTiyasyyecghglstggyGyDSWGQGTQVTSS
433	1sjvA (102)<br>1kxqE (120)	C	66.67	TM-align: aliSize=88 (resi) RMSD=1.70 (Å)<br>------G-GGLVQAGESLKLSCAA--SGGFMGWYRQAPGKQRELVATIN-SRGITNYADFVKGRFTISRDNAKKTVYLEMNSLEPEDTAVYYCYTHYFrsywgqgtqvtss---------------------<br>qvqlveSgGGSVQAGGSLSLSCAAstYTDTVGWFRQAPGKEREGVAAIYrRTGYTYSADSVKGRFTLSQDNNKNTVYLQMNSLKPEDTGIYYCATGNS-------------vrlaswegyfywgqgtqvtss<br><br>SARST: aliSize=99 (resi) RMSD=14.70 (Å)<br>g--------GGLVQAGESLKLSCAASGGF--MGWYRQAPGKQRELVATINS-RGITNYADFVKGRFTISRDNAKKTVYLEMNSLEPEDTAVYYCYTHYFRS--------YWGQGTQVTV--ss<br>-qvqlvesgGGSVQAGGSLSLSCAASTYTdtVGWFRQAPGKEREGVAAIYRrTGYTYSADSVKGRFTLSQDNNKNTVYLQMNSLKPEDTGIYYCATGNSVRlaswegyfYWGQGTQVTVss--<br><br>BLAST: aliSize=81 (resi) iden=71.57% (73/102) simi=79.41% (81/102)<br>-------GGGLVQAGESLKLSCAASG--GFMGWYRQAPGKQRELVATINSR-GITNYADFVKGRFTISRDNAKKTVYLEMNSLEPEDTAVYYCYT----------HYFrsYWGQGTQVTSS<br>qvqlvesGGGSVQAGGSLSLSCAASTytDTVGWFRQAPGKEREGVAAIYRRtGYTYSADSVKGRFTLSQDNNKNTVYLQMNSLKPEDTGIYYCATgnsvrlasweGYF--YWGQGTQVTSS<br><br>Proposed: aliSize=100 (resi) RMSD=1.43 (Å)<br>g--------GGLVQAGESLKLSCAA--SGGFMGWYRQAPGKQRELVATIN-SRGITNYADFVKGRFTISRDNAKKTVYLEMNSLEPEDTAVYYCYTHyF--------RS-YWGQGTQVTSS<br>-qvqlvesgGGSVQAGGSLSLSCAAstYTDTVGWFRQAPGKEREGVAAIYrRTGYTYSADSVKGRFTLSQDNNKNTVYLQMNSLKPEDTGIYYCATG-NsvrlasweGYfYWGQGTQVTSS
434	1sjvA (102)<br>1mvfA (126)	C	68.63	TM-align: aliSize=89 (resi) RMSD=1.41 (Å)<br>------G-GGLVQAGESLKLSCAA----S-GGFMGWYRQAPGKQRELVATINS-RGITNYADFVKGRFTISRDNAKKTVYLEMNSLEPEDTAVYYCYTHYFRsywgqgtqvtss-----------------------<br>qvqlveSgGGSVQAGGSLRLSCAAsgftYsRKYMGWFRQAPGKEREGVAAIFIdNGNTIYADSVQGRFTISQDNAKNTVYLQMNSLKPEDTAMYYCAASSRW------------mdysaltakaynswgqgtqvtss<br><br>SARST: aliSize=99 (resi) RMSD=16.00 (Å)<br>gg---------GLVQAGESLKLSCA-----ASGGFMGWYRQAPGKQRELVATIN-SRGITNYADFVKGRFTISRDNAKKTVYLEMNSLEPEDTAVYYCYTHYF-----------RSYWGQGTQVTVS-s<br>--qvqlvesggGSVQAGGSLRLSCAasgftYSRKYMGWFRQAPGKEREGVAAIFiDNGNTIYADSVQGRFTISQDNAKNTVYLQMNSLKPEDTAMYYCAASSRwmdysaltakaYNSWGQGTQVTVSs-<br><br>BLAST: aliSize=87 (resi) iden=73.53% (75/102) simi=85.29% (87/102)<br>-------GGGLVQAGESLKLSCAASG-----GFMGWYRQAPGKQRELVATIN-SRGITNYADFVKGRFTISRDNAKKTVYLEMNSLEPEDTAVYYC-----YTHY----FRSY--WGQGTQVTSS<br>qvqlvesGGGSVQAGGSLRLSCAASGftysrKYMGWFRQAPGKEREGVAAIFiDNGNTIYADSVQGRFTISQDNAKNTVYLQMNSLKPEDTAMYYCaassrWMDYsaltAKAYnsWGQGTQVTSS<br><br>Proposed: aliSize=101 (resi) RMSD=1.12 (Å)<br>g--------GGLVQAGESLKLSCAA----S-GGFMGWYRQAPGKQRELVATINS-RGITNYADFVKGRFTISRDNAKKTVYLEMNSLEPEDTAVYYCYTHYF-----------RSYWGQGTQVTSS<br>-qvqlvesgGGSVQAGGSLRLSCAAsgftYsRKYMGWFRQAPGKEREGVAAIFIdNGNTIYADSVQGRFTISQDNAKNTVYLQMNSLKPEDTAMYYCAASSRwmdysaltakaYNSWGQGTQVTSS

435	1sjvA (102)<br>1n4xH (120)	C	44.12	TM-align: aliSize=89 (resi) RMSD=1.78 (Å)<br>-------GGGLVQAGESLKLSCAA---S--GGFMGWYRQAPGKQRELVATIN-SRGITNYADFVKGRFTISRDNAKKTVYLEMNSLEPEDTAVYYCYTHYFRsywgqgtqvtvss-----------------<br>evqlqqsGPELKKPGETVKISCKAtnyAftDYSMHWVKQAPGGDLKYVGWINtETDEPTFADDFKGRFAFSLDTSTSTAFLQINNLKNEDTATYFCVRDRHD------------ygeiftywgqgttvtvsa<br><br>SARST: aliSize=84 (resi) RMSD=2.10 (Å)<br>gg---------GLVQAGESLKLSCAASG-----GFMGWYRQAPGKQRELVATINS-RGITNYADFVKGRFTISRDNAKKTVYLEMNSLEPEDTAVYYCYTH-------------------yfrsywgqgtqvtvss<br>--evqlqqsgpELKKPGETVKISCKATNyaftdYSMHWVKQAPGGDLKYVGWINTeTDEPTFADDFKGRFAFSLDTSTSTAFLQINNLKNEDTATYFCVRDrhdygeiftywgqgttvtvsa---------------<br><br>BLAST: aliSize=66 (resi) iden=47.06% (48/102) simi=64.71% (66/102)<br>-------GGGLVQAGESLKLSCAASG-GF----MGWYRQAPGKQRELVATINSR-GITNYADFVKGRFTISRDNAKKTVYLEMNSLEPEDTAVYYC------YTHYFrSYWGQGTQVTVSS<br>evqlqqsGPELKKPGETVKISCKATNyAFtdysMHWVKQAPGGDLKYVGWINTEtDEPTFADDFKGRFAFSLDTSTSTAFLQINNLKNEDTATYFCvrdrhdYGEIF-TYWGQGTTVTVSA<br><br>Proposed: aliSize=102 (resi) RMSD=1.55 (Å)<br>-------GGGLVQAGESLKLSCAA---S--GGFMGWYRQAPGKQRELVATIN-SRGITNYADFVKGRFTISRDNAKKTVYLEMNSLEPEDTAVYYCYTHYF----RS-YWGQGTQVTVSS<br>evqlqqsGPELKKPGETVKISCKAtnyAftDYSMHWVKQAPGGDLKYVGWINtETDEPTFADDFKGRFAFSLDTSTSTAFLQINNLKNEDTATYFCVRDRHdygeIFtYWGQGTTVTVSA
436	1sjvA (102)<br>1n4xI (119)	C	35.29	TM-align: aliSize=89 (resi) RMSD=1.77 (Å)<br>-------GGGLVQAGESLKLSCAA---S--GGFMGWYRQAPGKQRELVATIN-SRGITNYADFVKGRFTISRDNAKKTVYLEMNSLEPEDTAVYYCYTHYFRsywgqgtqvtvss----------------<br>evqlqqsGPELKKPGETVKISCKAtnyAftDYSMHWVKQAPGGDLKYVGWINtETDEPTFADDFKGRFAFSLDTSTSTAFLQINNLKNEDTATYFCVRDRHD------------ygeiftywgqgttvtvs<br><br>SARST: aliSize=76 (resi) RMSD=16.51 (Å)<br>gg---------GLVQAGESLKLSCAASGG-----FMGWYRQAPGkqrelvatinsrgitnyadfvkG----------------------RFTISRDNAKKTVYLEMNSLEPEDTAVYYCYTH-----YFRSYWGQGTQVTV-ss<br>--evqlqqsgpELKKPGETVKISCKATNYaftdySMHWVKQAPG---------------------GdlkyvgwintetdeptfaddfkgRFAFSLDTSTSTAFLQINNLKNEDTATYFCVRDrhdygEIFTYWGQGTTVTVs--<br><br>BLAST: aliSize=65 (resi) iden=47.06% (48/102) simi=63.73% (65/102)<br>-------GGGLVQAGESLKLSCAASG-GF----MGWYRQAPGKQRELVATINSR-GITNYADFVKGRFTISRDNAKKTVYLEMNSLEPEDTAVYYC------YTHYFrSYWGQGTQVTVSs<br>evqlqqsGPELKKPGETVKISCKATNyAFtdysMHWVKQAPGGDLKYVGWINTEtDEPTFADDFKGRFAFSLDTSTSTAFLQINNLKNEDTATYFCvrdrhdYGEIF-TYWGQGTTVTVS-<br><br>Proposed: aliSize=102 (resi) RMSD=1.72 (Å)<br>-------GGGLVQAGESLKLSCAA---S--GGFMGWYRQAPGKQRELVATIN-SRGITNYADFVKGRFTISRDNAKKTVYLEMNSLEPEDTAVYYCYTHYF----RSYWGQGTQVTVSS<br>evqlqqsGPELKKPGETVKISCKAtnyAftDYSMHWVKQAPGGDLKYVGWINtETDEPTFADDFKGRFAFSLDTSTSTAFLQINNLKNEDTATYFCVRDRHdygeIFTYWGQGTTVTVS
437	1sjvA (102)<br>1nmcH (122)	C	41.18	TM-align: aliSize=89 (resi) RMSD=1.80 (Å)<br>-------GGGLVQAGESLKLSCAA---S--GGFMGWYRQAPGKQRELVATIN-SRGITNYADFVKGRFTISRDNAKKTVYLEMNSLEPEDTAVYYCYTHYFRsywgqgtqvtvss-------------------<br>qvqlqqsGAELVKPGASVRMSCKAsgyTftNYNMYWVKQSPGQGLEWIGIFYpGNGDTSYNQKFKDKATLTADKSSNTAYMQLSSLTSEDSAVYYCARSGGS------------yrydggfdywgqgttvtvss<br><br>SARST: aliSize=83 (resi) RMSD=5.64 (Å)<br>gg---------GLVQAGESLKLSCAASGG-----FMGWYRQAPGKQRELVATINS-RGITNYADFVKGRFTISRDNAKKTVYLEMNSLEPEDTAVYYCYT----------------------hyfrsywgqgtqvtvss<br>--qvqlqqsgaELVKPGASVRMSCKASGYtftnyNMYWVKQSPGQGLEWIGIFYPgNGDTSYNQKFKDKATLTADKSSNTAYMQLSSLTSEDSAVYYCARsggsyrydggfdywgqgttvtvss----------------<br><br>BLAST: aliSize=65 (resi) iden=46.08% (47/102) simi=63.73% (65/102)<br>-------GGGLVQAGESLKLSCAASG-GF----MGWYRQAPGKQRELVATIN-SRGITNYADFVKGRFTISRDNAKKTVYLEMNSLEPEDTAVYYC--------YTHYFrSYWGQGTQVTVSS<br>qvqlqqsGAELVKPGASVRMSCKASGyTFtnynMYWVKQSPGQGLEWIGIFYpGNGDTSYNQKFKDKATLTADKSSNTAYMQLSSLTSEDSAVYYCarsggsyrYDGGF-DYWGQGTTVTVSS<br><br>Proposed: aliSize=102 (resi) RMSD=1.69 (Å)<br>-------GGGLVQAGESLKLSCAA---S--GGFMGWYRQAPGKQRELVATIN-SRGITNYADFVKGRFTISRDNAKKTVYLEMNSLEPEDTAVYYCYTH-Y-----FRS-YWGQGTQVTVSS<br>qvqlqqsGAELVKPGASVRMSCKAsgyTftNYNMYWVKQSPGQGLEWIGIFYpGNGDTSYNQKFKDKATLTADKSSNTAYMQLSSLTSEDSAVYYCARSgGsyrydGGFdYWGQGTTVTVSS

438	1sjvA (102)<br>1nqbA (232)	C	26.47	TM-align: aliSize=89 (resi) RMSD=1.60 (Å)<br>------GGGLVQAGESLKLSCAA---S--GGFMGWYRQAPGKQRELVATIN-SRGITNYADFVKGRFTISRDNAKKTVYLEMNSLEPEDTAVYYCYTHYFRsywgqgtqvtvss--<br>vqlqqsGAELVKPGASVKLSCKAsgyTftSYWMHWVKQRPGRGLEWIGRIDpNSGGTKYNEKFKSKATLTVDKPSSTAYMQLSSLTSEDSAVYYCARYDYY-------------gssyfdywgqgttvtvssdieltqtplslpvslgdqasiscrssqsivhsngntylewylqkpgqspklliykvsn<br><br>--<br>rfsgvpdrfsgsgsgtdftlkisrveaedlgvyycfqgshvpytfgggtkleikr<br><br>SARST: aliSize=97 (resi) RMSD=18.32 (Å)<br>gg-GLVQA------GESLKLSC--AASG---GFMGWYRQAPGKQRELVATINS-RGITNYADFVKGRFTISRDNAKKTVYLEMNSLEPEDTAVYYCY---------------THYFRSYWGQGTQVT---<br>--qLQQSGaelvkpGASVKLSCkaSGYTftsYWMHWVKQRPGRGLEWIGRIDPnSGGTKYNEKFKSKATLTVDKPSSTAYMQLSSLTSEDSAVYYCArydyygssyfdywgqGTTVTVSSDIELTQTplslpvslgdqasiscrssqsivhsngntylewylqkpgqspklliykvsnrfsgvpdrfsgs<br><br>--vss<br>gsgtdftlkisrveaedlgvyycfqgshvpytfgggtkleikr---<br><br>BLAST: aliSize=68 (resi) iden=50.00% (51/102) simi=66.67% (68/102)<br>-----GGGLVQAGESLKLSCAASGG-----FMGWYRQAPGKQRELVATI--NSRGiTNYADFVKGRFTISRDNAKKTVYLEMNSLEPEDTAVYYC--YTHY---FRSYWGQGTQVTVSS---<br>qlqqsGAELVKPGASVKLSCKASGYtftsyWMHWVKQRPGRGLEWIGRIdpNSGG-TKYNEKFKSKATLTVDKPSSTAYMQLSSLTSEDSAVYYCarYDYYgssYFDYWGQGTTVTVSSdieltqtplslpvslgdqasiscrssqsivhsngntylewylqkpgqspklliykvsnrfsgvpdrfsgsg<br><br>--<br>sgtdftlkisrveaedlgvyycfqgshvpytfgggtkleikr<br><br>Proposed: aliSize=96 (resi) RMSD=1.89 (Å)<br>--GGGLVQAGESLKLSCAA--SG------GFMGWYRQAPGKQRELVATINsrgiTNYADFVKGRF<br>vqlqqsgaelvkpgasvklsckasgytftsywmhwvkqrpgrglewigridpnsggtkynekfkskatltvdkpsstaymqlssltsedsavyycarydyygssyfdywgqgttvtvssdieltqtpLSLPVSLGDQASISCRSsqSIvhsngnTYLEWYLQKPGQSPKLLIYKV----SNRFSGVPDRF<br><br>TISRDNakKTVYLEMNSLEPEDTAVYYCYTH-YFRSYWGQGTQVTVSS<br>SGSGSG--TDFTLKISRVEAEDLGVYYCFQGsHVPYTFGGGTKLEIKR
439	1sjvA (102)<br>1ri8A (124)	C	67.65	TM-align: aliSize=89 (resi) RMSD=1.76 (Å)<br>-----G-GGLVQAGESLKLSCAA----SG-GFMGWYRQAPGKQRELVATINSRGITNYADFVKGRFTISRDNAKKTVYLEMNSLEPEDTAVYYCYTHYFRsywgqgtqvtvss------------------------<br>vqlveSgGGSVQAGGSLRLSCAVsgykDRnYCMGWFRRAPGKEREGVAVIDSSGRTAYADSVKGRFTISRDVALDTAYLQMNSLKPEDTAMYYCAAGWSS------------lgscgtnrnrynywgqgtqvtvss<br><br>SARST: aliSize=100 (resi) RMSD=16.27 (Å)<br>g-------GGLVQAGESLKLSCA-----ASGGFMGWYRQAPGKQRELVATINSRGITNYADFVKGRFTISRDNAKKTVYLEMNSLEPEDTAVYYCYTHY----FRS-------YWGQGTQVTVS-s<br>-vqlvesgGGSVQAGGSLRLSCAvsgykDRNYCMGWFRRAPGKEREGVAVIDSSGRTAYADSVKGRFTISRDVALDTAYLQMNSLKPEDTAMYYCAAGWsslgSCGtnrnrynYWGQGTQVTVSs-<br><br>BLAST: aliSize=85 (resi) iden=72.55% (74/102) simi=83.33% (85/102)<br>------GGGLVQAGESLKLSCAASG----GF-MGWYRQAPGKQRELVATINSRGITNYADFVKGRFTISRDNAKKTVYLEMNSLEPEDTAVYYCY---------THYFR-SYWGQGTQVTVSS<br>vqlvesGGGSVQAGGSLRLSCAVSGykdrNYcMGWFRRAPGKEREGVAVIDSSGRTAYADSVKGRFTISRDVALDTAYLQMNSLKPEDTAMYYCAagwsslgscgTNRNRyNYWGQGTQVTVSS<br><br>Proposed: aliSize=100 (resi) RMSD=1.35 (Å)<br>g-------GGLVQAGESLKLSCAA----SG-GFMGWYRQAPGKQRELVATINSRGITNYADFVKGRFTISRDNAKKTVYLEMNSLEPEDTAVYYCYTHyF-----------RSYWGQGTQVTVSS<br>-vqlvesgGGSVQAGGSLRLSCAVsgykDRnYCMGWFRRAPGKEREGVAVIDSSGRTAYADSVKGRFTISRDVALDTAYLQMNSLKPEDTAMYYCAAG-WsslgscgtnrnrYNYWGQGTQVTVSS
440	1sjvA (102)<br>1t2jA (116)	C	62.75	TM-align: aliSize=86 (resi) RMSD=1.32 (Å)<br>------G-GGLVQAGESLKLSCAA---S--GGFMGWYRQAPGKQRELVATINSRG-ITNYADFVKGRFTISRDNAKKTVYLEMNSLEPEDTAVYYCYTHyfrsywgqgtqvtvss-----------------<br>qvqlqeSgGGLVQPGGSLRLSCAAsgfTfsNSAMSWVRQAPGKGLEWVSSISGSGgNTYSADSVKGRFTISRDNAKNSLYLQMNSLRAEDTAVYYCARD---------------wygmdvwgqgttvtvss<br><br>SARST: aliSize=100 (resi) RMSD=16.79 (Å)<br>g--------GGLVQAGESLKLSCAASGG-----FMGWYRQAPGKQRELVATINSRG-ITNYADFVKGRFTISRDNAKKTVYLEMNSLEPEDTAVYYCYTH-YFRSYWGQGTQVTVS-s<br>-qvqlqesgGGLVQPGGSLRLSCAASGFtfsnsAMSWVRQAPGKGLEWVSSISGSGgNTYSADSVKGRFTISRDNAKNSLYLQMNSLRAEDTAVYYCARDwYGMDVWGQGTTVTVSs-<br><br>BLAST: aliSize=77 (resi) iden=66.67% (68/102) simi=75.49% (77/102)<br>-------GGGLVQAGESLKLSCAASG-----GFMGWYRQAPGKQRELVATINSRGITNY-ADFVKGRFTISRDNAKKTVYLEMNSLEPEDTAVYYCYTHYF-RSYWGQGTQVTVSS<br>qvqlqesGGGLVQPGGSLRLSCAASGftfsnSAMSWVRQAPGKGLEWVSSISGSGGNTYsADSVKGRFTISRDNAKNSLYLQMNSLRAEDTAVYYCARDWYgMDVWGQGTTVTVSS<br><br>Proposed: aliSize=99 (resi) RMSD=1.54 (Å)<br>------G-GGLVQAGESLKLSCAA---S--GGFMGWYRQAPGKQRELVATINSRG-ITNYADFVKGRFTISRDNAKKTVYLEMNSLEPEDTAVYYCYThyfR---SYW-GQGTQVTVSS<br>qvqlqeSgGGLVQPGGSLRLSCAAsgfTfsNSAMSWVRQAPGKGLEWVSSISGSGgNTYSADSVKGRFTISRDNAKNSLYLQMNSLRAEDTAVYYCAR---DwygMDVwGQGTTVTVSS

441	1sjvA (102)<br>1uacH (114)	C	41.18	TM-align: aliSize=87 (resi) RMSD=1.73 (Å)<br>------G-GGLVQAGESLKLSCAA---S--GGFMGWYRQAPGKQRELVATINSRGITNYADFVKGRFTISRDNAKKTVYLEMNSLEPEDTAVYYCYTHYfrsywgqgtqvtss--------------<br>dvqlqeSgPSLVKPSQTLSLTCSVtgdSitSDYWSWIRKFPGNRLEYMGYVSSFGSTFYNPSLKSRISITRDTSKNQYYLDLNSVTTEDTATYYCANWD--------------gdywgqgtlvtvsaa<br><br>SARST: aliSize=81 (resi) RMSD=2.43 (Å)<br>ggg----------LVQAGESLKLSCAASGGF-----MGWYRQAPGKQRELVATINSRGITNYADFVKGRFTISRDNAKKTVYLEMNSLEPEDTAVYYCY-----------------thyfrsywgqgtqvtss<br>---dvqlqesgpsLVKPSQTLSLTCSVTGDSitsdyWSWIRKFPGNRLEYMGYVSSFGSTFYNPSLKSRISITRDTSKNQYYLDLNSVTTEDTATYYCAnwdgdywgqgtlvtvsaa-----------------<br><br>BLAST: aliSize=65 (resi) iden=42.16% (43/102) simi=63.73% (65/102)<br>-------GGGLVQAGESLKLSCAASGG-----FMGWYRQAPGKQRELVATINSRGITNYADFVKGRFTISRDNAKKTVYLEMNSLEPEDTAVYYCyTHYFRSYWGQGTQVTVSS-<br>dvqlqesGPSLVKPSQTLSLTCSVTGDsitsdYWSWIRKFPGNRLEYMGYVSSFGSTFYNPSLKSRISITRDTSKNQYYLDLNSVTTEDTATYYC-ANWDGDYWGQGTLVTVSAa<br><br>Proposed: aliSize=99 (resi) RMSD=1.56 (Å)<br>g--------GGLVQAGESLKLSCAA---S--GGFMGWYRQAPGKQRELVATINSRGITNYADFVKGRFTISRDNAKKTVYLEMNSLEPEDTAVYYCYT-hyFRSYWGQGTQVTVSS-<br>-dvqlqesgPSLVKPSQTLSLTCSVtgdSitSDYWSWIRKFPGNRLEYMGYVSSFGSTFYNPSLKSRISITRDTSKNQYYLDLNSVTTEDTATYYCANw--DGDYWGQGTLVTVSAa
442	1sjvA (102)<br>1wt5A (115)	C	38.24	TM-align: aliSize=89 (resi) RMSD=1.73 (Å)<br>-------GGGLVQAGESLKLSCAA---S--GGFMGWYRQAPGKQRELVATIN-SRGITNYADFVKGRFTISRDNAKKTVYLEMNSLEPEDTAVYYCYTHYFRsywgqgtqvtss------------<br>qvqlvqsGAEVKKPGASVKVSCKAsgyTftSYWMHWVRQAPGQGLEWMGNIYpGSGGTNYAEKFKNRVTMTRDTSISTAYMELSRLRSDDTAVYYCARSGGP------------yffdywgqgtlvt<br><br>SARST: aliSize=96 (resi) RMSD=11.88 (Å)<br>gg---------GLVQAGESLKLSCAAS-----GGFMGWYRQAPGKQRELVATINS-RGITNYADFVKGRFTISRDNAKKTVYLEMNSLEPEDTAVYYCYTH---YFRSYWGQGTQV-tvss<br>--qvqlvqsgaEVKKPGASVKVSCKASgytftSYWMHWVRQAPGQGLEWMGNIYPgSGGTNYAEKFKNRVTMTRDTSISTAYMELSRLRSDDTAVYYCARSggpYFFDYWGQGTLVt----<br><br>BLAST: aliSize=64 (resi) iden=48.04% (49/102) simi=62.75% (64/102)<br>-------GGGLVQAGESLKLSCAASGG-----FMGWYRQAPGKQRELVATIN-SRGITNYADFVKGRFTISRDNAKKTVYLEMNSLEPEDTAVYYCYTH---YFRSYWGQGTQVTvss<br>qvqlvqsGAEVKKPGASVKVSCKASGYtftsyWMHWVRQAPGQGLEWMGNIYpGSGGTNYAEKFKNRVTMTRDTSISTAYMELSRLRSDDTAVYYCARSggpYFFDYWGQGTLVT---<br><br>Proposed: aliSize=101 (resi) RMSD=1.71 (Å)<br>-------GGGLVQAGESLKLSCAA---S--GGFMGWYRQAPGKQRELVATIN-SRGITNYADFVKGRFTISRDNAKKTVYLEMNSLEPEDTAVYYCYTHY-fRSYWGQGTQVTVSS<br>qvqlvqsGAEVKKPGASVKVSCKAsgyTftSYWMHWVRQAPGQGLEWMGNIYpGSGGTNYAEKFKNRVTMTRDTSISTAYMELSRLRSDDTAVYYCARSGg-PYFFDYWGQGTLVT
443	1sjvA (102)<br>1xfpA (131)	C	61.76	TM-align: aliSize=90 (resi) RMSD=1.69 (Å)<br>-----G-GGLVQAGESLKLSCAA-----SGGFMGWYRQAPGKQRELVATIN-SRGITNYADFVKGRFTISRDNAKKTVYLEMNSLEPEDTAVYYCYTHYFRSywgqgtqvtss-----------------------------<br>vqlqaSgGGSVQAGGSLRLSCAAsgytiGPYCMGWFRQAPGKEREGVAAINsGGGSTYYADSVKGRFTISQDNAKNTVYLLMNSLEPEDTAIYYCAADSTIY------------asyyecghglstggygydswgqgtqvtvs<br><br>SARST: aliSize=99 (resi) RMSD=14.63 (Å)<br>g-------GGLVQAGESLKLSCAASGG-----FMGWYRQAPGKQRELVATINS-RGITNYADFVKGRFTISRDNAKKTVYLEMNSLEPEDTAVYYCYTHYFRS-----------------YWGQGTQVTV-ss<br>-vqlqasgGGSVQAGGSLRLSCAASGYtigpyCMGWFRQAPGKEREGVAAINSgGGSTYYADSVKGRFTISQDNAKNTVYLLMNSLEPEDTAIYYCAADSTIYasyyecghglstggygydSWGQGTQVTVs--<br><br>BLAST: aliSize=86 (resi) iden=78.43% (80/102) simi=84.31% (86/102)<br>------GGGLVQAGESLKLSCAASG---G--FMGWYRQAPGKQRELVATINSRG-ITNYADFVKGRFTISRDNAKKTVYLEMNSLEPEDTAVYYC------YTHYFR--------SY----WGQGTQVTVSs<br>vqlqasGGGSVQAGGSLRLSCAASGytiGpyCMGWFRQAPGKEREGVAAINSGGgSTYYADSVKGRFTISQDNAKNTVYLLMNSLEPEDTAIYYCaadstiYASYYEcghglstgGYgydsWGQGTQVTVS-<br><br>Proposed: aliSize=101 (resi) RMSD=1.45 (Å)<br>g-------GGLVQAGESLKLSCAA-----SGGFMGWYRQAPGKQRELVATIN-SRGITNYADFVKGRFTISRDNAKKTVYLEMNSLEPEDTAVYYCYTHYF----------------RSYWGQGTQVTVSS<br>-vqlqasgGGSVQAGGSLRLSCAAsgytiGPYCMGWFRQAPGKEREGVAAINsGGGSTYYADSVKGRFTISQDNAKNTVYLLMNSLEPEDTAIYYCAADSTiyasyyecghglstggyGYDSWGQGTQVTVS

444	1sjvA (102)<br>1zmyA (132)	C	69.61	TM-align: aliSize=90 (resi) RMSD=1.90 (Å)<br>------GGGLVQAGESLKLSCAA-----SGGFMGWYRQAPGKQRELVATIN-SRGITNYADFVKGRFTISRDNAKKTVYLEMNSLEPEDTAVYYCYTHYFRSywgqgtqvtss-----------------------------<br>vqlvesGGGSVQAGGSLRLSCTAsgytiGPYCMGWFRQAPGGEREAVAAINmGGGITYYADSVKGRFTISRDNAKNTVTLQMNSLKPEDTAMYYCAADSTIY------------asyyecghglstggygydswgqgtqvtss<br><br>SARST: aliSize=85 (resi) RMSD=4.26 (Å)<br>g-------GGLVQAGESLKLSCAAS---GGF--MGWYRQAPGKQRELVATINS-RGITNYADFVKGRFTISRDNAKKTVYLEMNSLEPEDTAVYYCYTH---------------------------------yfrsywgqgtqvtss<br>-vqlvesgGGSVQAGGSLRLSCTASgytIGPycMGWFRQAPGGEREAVAAINMgGGITYYADSVKGRFTISRDNAKNTVTLQMNSLKPEDTAMYYCAADstiyasyyecghglstggygydswgqgtqvtss---------------<br><br>BLAST: aliSize=85 (resi) iden=76.47% (78/102) simi=83.33% (85/102)<br>------GGGLVQAGESLKLSCAASG---G--FMGWYRQAPGKQRELVATIN-SRGITNYADFVKGRFTISRDNAKKTVYLEMNSLEPEDTAVYYC------YTHYFR--------SY----WGQGTQVTSS<br>vqlvesGGGSVQAGGSLRLSCTASGytiGpyCMGWFRQAPGGEREAVAAINmGGGITYYADSVKGRFTISRDNAKNTVTLQMNSLKPEDTAMYYCaadstiYASYYEcghglstgGYgydsWGQGTQVTSS<br><br>Proposed: aliSize=101 (resi) RMSD=1.43 (Å)<br>g-------GGLVQAGESLKLSCAA-----SGGFMGWYRQAPGKQRELVATIN-SRGITNYADFVKGRFTISRDNAKKTVYLEMNSLEPEDTAVYYCYTHYF-----------------RS-YWGQGTQVTSS<br>-vqlvesgGGSVQAGGSLRLSCTAsgytiGPYCMGWFRQAPGGEREAVAAINmGGGITYYADSVKGRFTISRDNAKNTVTLQMNSLKPEDTAMYYCAADSTiyasyyecghglstggyGYdSWGQGTQVTSS
445	1sjvA (102)<br>1zv5A (119)	C	69.61	TM-align: aliSize=88 (resi) RMSD=1.67 (Å)<br>-----G-GGLVQAGESLKLSCAA---SG--GFMGWYRQAPGKQRELVATINSRGITNY-ADFVKGRFTISRDNAKKTVYLEMNSLEPEDTAVYYCYTHYFrsywgqgtqvtss-------------------<br>vqlveSgGGSVQAGESLRLSCAAsgvTYknYCIGWFRQAPGKDREGVVFINSDGGITYyADSVKGRFTISQDNAKNTVYLQMNSLKPEDTASYYCAAGYR-------------nygqcatrywgqgtqvtvs<br><br>SARST: aliSize=98 (resi) RMSD=14.69 (Å)<br>gg--------GLVQAGESLKLSC--AASGGF---MGWYRQAPGKQRELVATINSRGITNY-ADFVKGRFTISRDNAKKTVYLEMNSLEPEDTAVYYCYTHY------FRSYWGQGTQVTV-ss<br>--vqlvesggGSVQAGESLRLSCaaSGVTYKnycIGWFRQAPGKDREGVVFINSDGGITYyADSVKGRFTISQDNAKNTVYLQMNSLKPEDTASYYCAAGYrnygqcATRYWGQGTQVTVs--<br><br>BLAST: aliSize=87 (resi) iden=76.47% (78/102) simi=85.29% (87/102)<br>------GGGLVQAGESLKLSCAASG----GF-MGWYRQAPGKQRELVATINSRG-ITNYADFVKGRFTISRDNAKKTVYLEMNSLEPEDTAVYYC---YTHYFR---SYWGQGTQVTVSs<br>vqlvesGGGSVQAGESLRLSCAASGvtykNYcIGWFRQAPGKDREGVVFINSDGgITYYADSVKGRFTISQDNAKNTVYLQMNSLKPEDTASYYCaagYRNYGQcatRYWGQGTQVTVS-<br><br>Proposed: aliSize=100 (resi) RMSD=1.54 (Å)<br>-----G-GGLVQAGESLKLSCAA---SG--GFMGWYRQAPGKQRELVATINSRGITNY-ADFVKGRFTISRDNAKKTVYLEMNSLEPEDTAVYYCYTHyF------RS-YWGQGTQVTVSs<br>vqlveSgGGSVQAGESLRLSCAAsgvTYknYCIGWFRQAPGKDREGVVFINSDGGITYyADSVKGRFTISQDNAKNTVYLQMNSLKPEDTASYYCAAG-YrnygqcATrYWGQGTQVTVS-
446	1sjvA (102)<br>1zvhA (124)	C	64.71	TM-align: aliSize=89 (resi) RMSD=1.45 (Å)<br>------G-GGLVQAGESLKLSCAA----S-GGFMGWYRQAPGKQRELVATIN-SRGITNYADFVKGRFTISRDNAKKTVYLEMNSLEPEDTAVYYCYTHYFRsywgqgtqvtss----------------------<br>dvqlveSgGGSVQAGGSLRLSCAAsgyiAsINYLGWFRQAPGKEREGVAAVSpAGGTPYYADSVKGRFTVSLDNAENTVYLQMNSLKPEDTALYYCAAARQG------------wyiplnsygynywgqgtqvtvs<br><br>SARST: aliSize=99 (resi) RMSD=14.47 (Å)<br>g--------GGLVQAGESLKLSCAAS-----GGFMGWYRQAPGKQRELVATINS-RGITNYADFVKGRFTISRDNAKKTVYLEMNSLEPEDTAVYYCYTHYF----------RSYWGQGTQVTV-ss<br>-dvqlvesgGGSVQAGGSLRLSCAASgyiasINYLGWFRQAPGKEREGVAAVSPaGGTPYYADSVKGRFTVSLDNAENTVYLQMNSLKPEDTALYYCAAARQgwyiplnsygYNYWGQGTQVTVs--<br><br>BLAST: aliSize=85 (resi) iden=70.59% (72/102) simi=83.33% (85/102)<br>-------GGGLVQAGESLKLSCAASG-----GFMGWYRQAPGKQRELVATINSRGIT-NYADFVKGRFTISRDNAKKTVYLEMNSLEPEDTAVYYC-------Y---THYFRSYWGQGTQVTVSs<br>dvqlvesGGGSVQAGGSLRLSCAASGyiasiNYLGWFRQAPGKEREGVAAVSPAGGTpYYADSVKGRFTVSLDNAENTVYLQMNSLKPEDTALYYCaaarqgwYiplNSYGYNYWGQGTQVTVS-<br><br>Proposed: aliSize=100 (resi) RMSD=1.22 (Å)<br>g--------GGLVQAGESLKLSCAA----S-GGFMGWYRQAPGKQRELVATIN-SRGITNYADFVKGRFTISRDNAKKTVYLEMNSLEPEDTAVYYCYTHY-F--------RS-YWGQGTQVTVSs<br>-dvqlvesgGGSVQAGGSLRLSCAAsgyiAsINYLGWFRQAPGKEREGVAAVSpAGGTPYYADSVKGRFTVSLDNAENTVYLQMNSLKPEDTALYYCAAARqGwyiplnsyGYnYWGQGTQVTVS-

447	1sjvA (102)<br>1zvyA (125)	C	52.94	TM-align: aliSize=89 (resi) RMSD=1.45 (Å)<br>------G-GGLVQAGESLKLSCAA-----SGGFMGWYRQAPGKQRELVATIN-SRGITNYADFVKGRFTISRDNAKKTVYLEMNSLEPEDTAVYYCYTHYFRsywgqgtqvtvss----------------------<br>dvqlveSgGGSVQAGGSLRLSCAAsgstdSIEYMTWFRQAPGKAREGVAALYtHTGNTYYTDSVKGRFTISQDKAKNMAYLRMDSVKSEDTAIYTCGATRKY-------------vpvrfaldqssydywgqgtqvtv<br><br>SARST: aliSize=98 (resi) RMSD=12.78 (Å)<br>g-------GGLVQAGESLKLSCAAS-----GGFMGWYRQAPGKQRELVATINSR-GITNYADFVKGRFTISRDNAKKTVYLEMNSLEPEDTAVYYCYTHYFRS------------YWGQGTQVT-vss<br>-vqlvesgGGSVQAGGSLRLSCAASgstdsIEYMTWFRQAPGKAREGVAALYTHtGNTYYTDSVKGRFTISQDKAKNMAYLRMDSVKSEDTAIYTCGATRKYVpvrfaldqssydYWGQGTQVTv---<br><br>BLAST: aliSize=76 (resi) iden=63.73% (65/102) simi=74.51% (76/102)<br>------GGGLVQAGESLKLSCAASGG-----FMGWYRQAPGKQRELVATINSR-GITNYADFVKGRFTISRDNAKKTVYLEMNSLEPEDTAVYYC-YTHYF---R--------SYWGQGTQVTVss<br>vqlvesGGGSVQAGGSLRLSCAASGStdsieYMTWFRQAPGKAREGVAALYTHtGNTYYTDSVKGRFTISQDKAKNMAYLRMDSVKSEDTAIYTCgATRKYvpvRfaldqssyDYWGQGTQVTV--<br><br>Proposed: aliSize=100 (resi) RMSD=1.41 (Å)<br>g--------GGLVQAGESLKLSCAA-----SGGFMGWYRQAPGKQRELVATIN-SRGITNYADFVKGRFTISRDNAKKTVYLEMNSLEPEDTAVYYCYTHYfRS----------YWGQGTQVTVSS<br>-dvqlvesgGGSVQAGGSLRLSCAAsgstdSIEYMTWFRQAPGKAREGVAALYtHTGNTYYTDSVKGRFTISQDKAKNMAYLRMDSVKSEDTAIYTCGATR-KYvpvrfaldqssYDYWGQGTQVTV
448	1sjvA (102)<br>2bseD (122)	C	64.71	TM-align: aliSize=88 (resi) RMSD=1.66 (Å)<br>------GGGLVQAGESLKLSCAA--S-G--GFMGWYRQAPGKQRELVATINS-RGITNYADFVKGRFTISRDNAKKTVYLEMNSLEPEDTAVYYCYTHYFrsywgqgtqvtvss---------------------<br>vqlqesGGGLVQAGGSLRLSCTAsrRtGsnWCMGWFRQLAGKEPELVVALNFdYDMTYYADSVKGRFTVSRDSGKNTVYLQMNSLKPEDTAIYYCAARSG-------------gfssnrelydgwgqgtqvtvss<br><br>SARST: aliSize=99 (resi) RMSD=14.67 (Å)<br>g-------GGLVQAGESLKLSCAASG-----GFMGWYRQAPGKQRELVATINS-RGITNYADFVKGRFTISRDNAKKTVYLEMNSLEPEDTAVYYCYTHYFRS--------YWGQGTQVTV--ss<br>-vqlqesgGGLVQAGGSLRLSCTASRrtgsnWCMGWFRQLAGKEPELVVALNFdYDMTYYADSVKGRFTVSRDSGKNTVYLQMNSLKPEDTAIYYCAARSGGFssnrelydGWGQGTQVTVss--<br><br>BLAST: aliSize=81 (resi) iden=69.61% (71/102) simi=79.41% (81/102)<br>------GGGLVQAGESLKLSCAAS---GG--FMGWYRQAPGKQRELVATIN-SRGITNYADFVKGRFTISRDNAKKTVYLEMNSLEPEDTAVYYCYTHY--FRS----Y--WGQGTQVTVSS<br>vqlqesGGGLVQAGGSLRLSCTASrrtGSnwCMGWFRQLAGKEPELVVALNfDYDMTYYADSVKGRFTVSRDSGKNTVYLQMNSLKPEDTAIYYCAARSggFSSnrelYdgWGQGTQVTVSS<br><br>Proposed: aliSize=101 (resi) RMSD=1.47 (Å)<br>g-------GGLVQAGESLKLSCAA--S-G--GFMGWYRQAPGKQRELVATINS-RGITNYADFVKGRFTISRDNAKKTVYLEMNSLEPEDTAVYYCYTHYF-------RS-YWGQGTQVTVSS<br>-vqlqesgGGLVQAGGSLRLSCTAsrRtGsnWCMGWFRQLAGKEPELVVALNFdYDMTYYADSVKGRFTVSRDSGKNTVYLQMNSLKPEDTAIYYCAARSGgfssnreLYdGWGQGTQVTVSS
449	1sjvA (102)<br>2dqcH (114)	C	39.22	TM-align: aliSize=88 (resi) RMSD=1.99 (Å)<br>------G-GGLVQAGESLKLSCAA---S--GGFMGWYRQAPGKQRELVATINSRGITNYADFVKGRFTISRDNAKKTVYLEMNSLEPEDTAVYYCYTHYFrsywgqgtqvtvss-------------<br>dvqlqeSgPSLVKPSQTLSLTCSVtgdSitSDFWSWIRKFPGNRLEYMGYVSYSGSTYYNPSLKSRISITRDTSKNQYYLDLNSVTTEDTATYYCANWDG-------------dywgqgtlvtvsaa<br><br>SARST: aliSize=93 (resi) RMSD=16.19 (Å)<br>ggg----------LVQAGESLKLSCAASGGF-----MGWYRQAPGKQRELVATINSRGITNYADFVKGRFTISRDNAKKTVYLEMNSLEPEDTAVYycythYFRS----YWGQGTQVTVS--s<br>---dvqlqesgpsLVKPSQTLSLTCSVTGDSitsdfWSWIRKFPGNRLEYMGYVSYSGSTYYNPSLKSRISITRDTSKNQYYLDLNSVTTEDTATY-----YCANwdgdYWGQGTLVTVSaa-<br><br>BLAST: aliSize=64 (resi) iden=42.16% (43/102) simi=62.75% (64/102)<br>-------GGGLVQAGESLKLSCAASGG-----FMGWYRQAPGKQRELVATINSRGITNYADFVKGRFTISRDNAKKTVYLEMNSLEPEDTAVYYCyTHYFRSYWGQGTQVTVSS-<br>dvqlqesGPSLVKPSQTLSLTCSVTGDsitsdFWSWIRKFPGNRLEYMGYVSYSGSTYYNPSLKSRISITRDTSKNQYYLDLNSVTTEDTATYYC-ANWDGDYWGQGTLVTVSAa<br><br>Proposed: aliSize=99 (resi) RMSD=1.60 (Å)<br>--------GGGLVQaGESLKLSCAA---S--GGFMGWYRQAPGKQRELVATINSRGITNYADFVKGRFTISRDNAKKTVYLEMNSLEPEDTAVYYCYT-hyFRSYWGQGTQVTVSS-<br>dvqlqesgPSLVKP-SQTLSLTCSVtgdSitSDFWSWIRKFPGNRLEYMGYVSYSGSTYYNPSLKSRISITRDTSKNQYYLDLNSVTTEDTATYYCANw--DGDYWGQGTLVTVSAa

450	1sjvA (102)<br>2dqdH (114)	C	40.20	TM-align: aliSize=88 (resi) RMSD=1.99 (Å)<br>------G-GGLVQAGESLKLSCAA---S--GGFMGWYRQAPGKQRELVATINSRGITNYADFVKGRFTISRDNAKKTVYLEMNSLEPEDTAVYYCYTHYFrsywgqgtqvtvss--------------<br>dvqlqeSgPSLVKPSQTLSLTCSVtgdSitSDYWSWIRKFPGNRLEYMGFVSYSGSTYYNPSLKSRISITRDTSKNQYYLDLNSVTTEDTATYYCANWDG--------------dywgqgtlvtvsaa<br><br>SARST: aliSize=93 (resi) RMSD=16.17 (Å)<br>ggg----------LVQAGESLKLSCAASGGF-----MGWYRQAPGKQRELVATINSRGITNYADFVKGRFTISRDNAKKTVYLEMNSLEPEDTAVYycythYFRS----YWGQGTQVTVS--s<br>---dvqlqesgpsLVKPSQTLSLTCSVTGDSitsdyWSWIRKFPGNRLEYMGFVSYSGSTYYNPSLKSRISITRDTSKNQYYLDLNSVTTEDTATY-----YCANwdgdYWGQGTLVTVSaa-<br><br>BLAST: aliSize=64 (resi) iden=41.18% (42/102) simi=62.75% (64/102)<br>-------GGGLVQAGESLKLSCAASGG-----FMGWYRQAPGKQRELVATINSRGITNYADFVKGRFTISRDNAKKTVYLEMNSLEPEDTAVYYCyTHYFRSYWGQGTQVTVSS-<br>dvqlqesGPSLVKPSQTLSLTCSVTGDsitsdYWSWIRKFPGNRLEYMGFVSYSGSTYYNPSLKSRISITRDTSKNQYYLDLNSVTTEDTATYYC-ANWDGDYWGQGTLVTVSAa<br><br>Proposed: aliSize=99 (resi) RMSD=1.56 (Å)<br>g--------GGLVQAGESLKLSCAA---S--GGFMGWYRQAPGKQRELVATINSRGITNYADFVKGRFTISRDNAKKTVYLEMNSLEPEDTAVYYCYT-hyFRSYWGQGTQVTVSS-<br>-dvqlqesgPSLVKPSQTLSLTCSVtgdSitSDYWSWIRKFPGNRLEYMGFVSYSGSTYYNPSLKSRISITRDTSKNQYYLDLNSVTTEDTATYYCANw--DGDYWGQGTLVTVSAa
451	1sjvA (102)<br>2dqeH (114)	C	38.24	TM-align: aliSize=88 (resi) RMSD=1.99 (Å)<br>------G-GGLVQAGESLKLSCAA---S--GGFMGWYRQAPGKQRELVATINSRGITNYADFVKGRFTISRDNAKKTVYLEMNSLEPEDTAVYYCYTHYFrsywgqgtqvtvss--------------<br>dvqlqeSgPSLVKPSQTLSLTCSVtgdSitSDYWSWIRKFPGNRLEYMGYVSASGSTYYNPSLKSRISITRDTSKNQYYLDLNSVTTEDTATYYCANWDG--------------dywgqgtlvtvsaa<br><br>SARST: aliSize=93 (resi) RMSD=16.17 (Å)<br>ggg----------LVQAGESLKLSCAASGGF-----MGWYRQAPGKQRELVATINSRGITNYADFVKGRFTISRDNAKKTVYLEMNSLEPEDTAVYycythYFRS----YWGQGTQVTVS--s<br>---dvqlqesgpsLVKPSQTLSLTCSVTGDSitsdyWSWIRKFPGNRLEYMGYVSASGSTYYNPSLKSRISITRDTSKNQYYLDLNSVTTEDTATY-----YCANwdgdYWGQGTLVTVSaa-<br><br>BLAST: aliSize=65 (resi) iden=41.18% (42/102) simi=63.73% (65/102)<br>-------GGGLVQAGESLKLSCAASGG-----FMGWYRQAPGKQRELVATINSRGITNYADFVKGRFTISRDNAKKTVYLEMNSLEPEDTAVYYCyTHYFRSYWGQGTQVTVSS-<br>dvqlqesGPSLVKPSQTLSLTCSVTGDsitsdYWSWIRKFPGNRLEYMGYVSASGSTYYNPSLKSRISITRDTSKNQYYLDLNSVTTEDTATYYC-ANWDGDYWGQGTLVTVSAa<br><br>Proposed: aliSize=99 (resi) RMSD=1.61 (Å)<br>--------GGGLVQaGESLKLSCAA---S--GGFMGWYRQAPGKQRELVATINSRGITNYADFVKGRFTISRDNAKKTVYLEMNSLEPEDTAVYYCYT-hyFRSYWGQGTQVTVSS-<br>dvqlqesgPSLVKP-SQTLSLTCSVtgdSitSDYWSWIRKFPGNRLEYMGYVSASGSTYYNPSLKSRISITRDTSKNQYYLDLNSVTTEDTATYYCANw--DGDYWGQGTLVTVSAa
452	1sjvA (102)<br>2dqfB (114)	C	40.20	TM-align: aliSize=88 (resi) RMSD=1.99 (Å)<br>------G-GGLVQAGESLKLSCAA---S--GGFMGWYRQAPGKQRELVATINSRGITNYADFVKGRFTISRDNAKKTVYLEMNSLEPEDTAVYYCYT-HYFrsywgqgtqvtvss-------------<br>dvqlqeSgPSLVKPSQTLSLTCSVtgdSitSDAWSWIRKFPGNRLEYMGYVSASGSTYYNPSLKSRISITRDTSKNQYYLDLNSVTTEDTATYYCANwDGD--------------ywgqgtlvtvsaa<br><br>SARST: aliSize=71 (resi) RMSD=16.72 (Å)<br>ggg----------LVQAGESLKLSCAASG-----GFMGWYRQAPGKQRELVATINS---------------------RGitnyadfvkgrftisrdnakkTVYLEMNSLEPEDTAVYYcythyFRS----YWGQGTQVTV---ss<br>---dvqlqesgpsLVKPSQTLSLTCSVTGdsitsDAWSWIRKFPGNRLEYMGYVSAsgstyynpslksrisitrdtsKN---------------------QYYLDLNSVTTEDTATYY-----CANwdgdYWGQGTLVTVsaa--<br><br>BLAST: aliSize=65 (resi) iden=41.18% (42/102) simi=63.73% (65/102)<br>-------GGGLVQAGESLKLSCAASGGFM-----GWYRQAPGKQRELVATINSRGITNYADFVKGRFTISRDNAKKTVYLEMNSLEPEDTAVYYCyTHYFRSYWGQGTQVTVSS-<br>dvqlqesGPSLVKPSQTLSLTCSVTGDSItsdawSWIRKFPGNRLEYMGYVSASGSTYYNPSLKSRISITRDTSKNQYYLDLNSVTTEDTATYYC-ANWDGDYWGQGTLVTVSAa<br><br>Proposed: aliSize=99 (resi) RMSD=1.56 (Å)<br>g--------GGLVQAGESLKLSCAA---S--GGFMGWYRQAPGKQRELVATINSRGITNYADFVKGRFTISRDNAKKTVYLEMNSLEPEDTAVYYCYT-hyFRSYWGQGTQVTVSS-<br>-dvqlqesgPSLVKPSQTLSLTCSVtgdSitSDAWSWIRKFPGNRLEYMGYVSASGSTYYNPSLKSRISITRDTSKNQYYLDLNSVTTEDTATYYCANw--DGDYWGQGTLVTVSAa

453	1sjvA (102)<br>2dqgH (114)	C	41.18	TM-align: aliSize=88 (resi) RMSD=1.99 (Å)<br>------G-GGLVQAGESLKLSCAA---S--GGFMGWYRQAPGKQRELVATINSRGITNYADFVKGRFTISRDNAKKTVYLEMNSLEPEDTAVYYCYTHYFrsywgqgtqvtvss-------------<br>dvqlqeSgPSLVKPSQTLSLTCSVtgdSitSDYWSWIRKFPGNRLEYMGYVSFSGSTYYNPSLKSRISITRDTSKNQYYLDLNSVTTEDTATYYCANWDG-------------dywgqgtlvtvsaa<br><br>SARST: aliSize=93 (resi) RMSD=16.20 (Å)<br>ggg---------LVQAGESLKLSCAASGGF-----MGWYRQAPGKQRELVATINSRGITNYADFVKGRFTISRDNAKKTVYLEMNSLEPEDTAVYYcythyFRS----YWGQGTQVTVS--s<br>---dvqlqesgpsLVKPSQTLSLTCSVTGDSitsdyWSWIRKFPGNRLEYMGYVSFSGSTYYNPSLKSRISITRDTSKNQYYLDLNSVTTEDTATYY-----CANwdgdYWGQGTLVTVSaa-<br><br>BLAST: aliSize=64 (resi) iden=41.18% (42/102) simi=62.75% (64/102)<br>-------GGGLVQAGESLKLSCAASGG-----FMGWYRQAPGKQRELVATINSRGITNYADFVKGRFTISRDNAKKTVYLEMNSLEPEDTAVYYCyTHYFRSYWGQGTQVTVSS-<br>dvqlqesGPSLVKPSQTLSLTCSVTGDsitsdYWSWIRKFPGNRLEYMGYVSFSGSTYYNPSLKSRISITRDTSKNQYYLDLNSVTTEDTATYYC-ANWDGDYWGQGTLVTVSAa<br><br>Proposed: aliSize=100 (resi) RMSD=1.69 (Å)<br>-------GGGLVQAGESLKLSCAA---S--GGFMGWYRQAPGKQRELVATINSRGITNYADFVKGRFTISRDNAKKTVYLEMNSLEPEDTAVYYCYT-hyFRSYWGQGTQVTVSS-<br>dvqlqesGPSLVKPSQTLSLTCSVtgdSitSDYWSWIRKFPGNRLEYMGYVSFSGSTYYNPSLKSRISITRDTSKNQYYLDLNSVTTEDTATYYCANw--DGDYWGQGTLVTVSAa
454	1sjvA (102)<br>2dqhH (114)	C	38.24	TM-align: aliSize=88 (resi) RMSD=2.02 (Å)<br>------G-GGLVQAGESLKLSCAA---S--GGFMGWYRQAPGKQRELVATINSRGITNYADFVKGRFTISRDNAKKTVYLEMNSLEPEDTAVYYCYT-HYFrsywgqgtqvtvss-------------<br>dvqlqeSgPSLVKPSQTLSLTCSVtgdSitSDYWSWIRKFPGNRLEYMGYVSYSGSTAYNPSLKSRISITRDTSKNQYYLDLNSVTTEDTATYYCANwDGD-------------ywgqgtlvtvsaa<br><br>SARST: aliSize=93 (resi) RMSD=16.17 (Å)<br>ggg---------LVQAGESLKLSCAASGGF-----MGWYRQAPGKQRELVATINSRGITNYADFVKGRFTISRDNAKKTVYLEMNSLEPEDTAVYYcythyFRS----YWGQGTQVTVS--s<br>---dvqlqesgpsLVKPSQTLSLTCSVTGDSitsdyWSWIRKFPGNRLEYMGYVSYSGSTAYNPSLKSRISITRDTSKNQYYLDLNSVTTEDTATYY-----CANwdgdYWGQGTLVTVSaa-<br><br>BLAST: aliSize=64 (resi) iden=41.18% (42/102) simi=62.75% (64/102)<br>-------GGGLVQAGESLKLSCAASGG-----FMGWYRQAPGKQRELVATINSRGITNYADFVKGRFTISRDNAKKTVYLEMNSLEPEDTAVYYCyTHYFRSYWGQGTQVTVSS-<br>dvqlqesGPSLVKPSQTLSLTCSVTGDsitsdYWSWIRKFPGNRLEYMGYVSYSGSTAYNPSLKSRISITRDTSKNQYYLDLNSVTTEDTATYYC-ANWDGDYWGQGTLVTVSAa<br><br>Proposed: aliSize=99 (resi) RMSD=1.62 (Å)<br>--------GGGLVQaGESLKLSCAA---S--GGFMGWYRQAPGKQRELVATINSRGITNYADFVKGRFTISRDNAKKTVYLEMNSLEPEDTAVYYCYT-hyFRSYWGQGTQVTVSS-<br>dvqlqesgPSLVKP-SQTLSLTCSVtgdSitSDYWSWIRKFPGNRLEYMGYVSYSGSTAYNPSLKSRISITRDTSKNQYYLDLNSVTTEDTATYYCANw--DGDYWGQGTLVTVSAa
455	1sjvA (102)<br>2dqjH (114)	C	38.24	TM-align: aliSize=88 (resi) RMSD=1.98 (Å)<br>------G-GGLVQAGESLKLSCAA---S--GGFMGWYRQAPGKQRELVATINSRGITNYADFVKGRFTISRDNAKKTVYLEMNSLEPEDTAVYYCYTHYFrsywgqgtqvtvss-------------<br>dvqlqeSgPSLVKPSQTLSLTCSVtgdSitSDYWSWIRKFPGNRLEYMGYVSYSGSTYYNPSLKSRISITRDTSKNQYYLDLNSVTTEDTATYYCANWDG-------------dywgqgtlvtvsaa<br><br>SARST: aliSize=93 (resi) RMSD=16.18 (Å)<br>ggg---------LVQAGESLKLSCAASGGF-----MGWYRQAPGKQRELVATINSRGITNYADFVKGRFTISRDNAKKTVYLEMNSLEPEDTAVYycythYFRS----YWGQGTQVTVS--s<br>---dvqlqesgpsLVKPSQTLSLTCSVTGDSitsdyWSWIRKFPGNRLEYMGYVSYSGSTYYNPSLKSRISITRDTSKNQYYLDLNSVTTEDTATY-----YCANwdgdYWGQGTLVTVSaa-<br><br>BLAST: aliSize=64 (resi) iden=41.18% (42/102) simi=62.75% (64/102)<br>-------GGGLVQAGESLKLSCAASGG-----FMGWYRQAPGKQRELVATINSRGITNYADFVKGRFTISRDNAKKTVYLEMNSLEPEDTAVYYCyTHYFRSYWGQGTQVTVSS-<br>dvqlqesGPSLVKPSQTLSLTCSVTGDsitsdYWSWIRKFPGNRLEYMGYVSYSGSTYYNPSLKSRISITRDTSKNQYYLDLNSVTTEDTATYYC-ANWDGDYWGQGTLVTVSAa<br><br>Proposed: aliSize=99 (resi) RMSD=1.60 (Å)<br>--------GGGLVQaGESLKLSCAA---S--GGFMGWYRQAPGKQRELVATINSRGITNYADFVKGRFTISRDNAKKTVYLEMNSLEPEDTAVYYCYT-hyFRSYWGQGTQVTVSS-<br>dvqlqesgPSLVKP-SQTLSLTCSVtgdSitSDYWSWIRKFPGNRLEYMGYVSYSGSTYYNPSLKSRISITRDTSKNQYYLDLNSVTTEDTATYYCANw--DGDYWGQGTLVTVSAa

456	1sjvA<br>(102)<br><br>2yssB<br>(113)	C	41.18	TM-align: aliSize=88 (resi) RMSD=1.98 (Å)<br>------G-GGLVQAGESLKLSCAA-----SGGFMGWYRQAPGKQRELVATINSRGITNYADFVKGRFTISRDNAKKTVYLEMNSLEPEDTAVYYCYTHYFrsywgqgtqvtss------------<br>qvqlqeSgPGLMKPSETLSLTCSVsgdsiRSDYWSWIRKPPGKGLEYIGYVSYSGSTYYNPSLKSRVTISVDTSKNRFSLKLNSVTAADTAVYYCARWDG------------dywgqgilvtvss<br><br>SARST: aliSize=93 (resi) RMSD=16.18 (Å)<br>ggg----------LVQAGESLKLSC--AASG---GFMGWYRQAPGKQRELVATINSRGITNYADFVKGRFTISRDNAKKTVYLEMNSLEPEDTAVYycythYFRS----YWGQGTQVTVS-s<br>---qvqlqesgpgLMKPSETLSLTCsvSGDSirsDYWSWIRKPPGKGLEYIGYVSYSGSTYYNPSLKSRVTISVDTSKNRFSLKLNSVTAADTAVY-----YCARwdgdYWGQGILVTVSs-<br><br>BLAST: aliSize=61 (resi) iden=44.12% (45/102) simi=59.80% (61/102)<br>-------GGGLVQAGESLKLSCAASGG-----FMGWYRQAPGKQRELVATINSRGITNYADFVKGRFTISRDNAKKTVYLEMNSLEPEDTAVYYCyTHYFRSYWGQGTQVTVSS<br>qvqlqesGPGLMKPSETLSLTCSVSGDsirsdYWSWIRKPPGKGLEYIGYVSYSGSTYYNPSLKSRVTISVDTSKNRFSLKLNSVTAADTAVYYC-ARWDGDYWGQGILVTVSS<br><br>Proposed: aliSize=100 (resi) RMSD=1.71 (Å)<br>------G-GGLVQAGESLKLSCAA-----SGGFMGWYRQAPGKQRELVATINSRGITNYADFVKGRFTISRDNAKKTVYLEMNSLEPEDTAVYYCYT-hyFRSYWGQGTQVTVSS<br>qvqlqeSgPGLMKPSETLSLTCSVsgdsiRSDYWSWIRKPPGKGLEYIGYVSYSGSTYYNPSLKSRVTISVDTSKNRFSLKLNSVTAADTAVYYCARw--DGDYWGQGILVTVSS
457	1sjvA<br>(102)<br><br>3cx5J<br>(127)	C	45.10	TM-align: aliSize=90 (resi) RMSD=2.02 (Å)<br>------G-GGLVQAGESLKLSCAA------SGGFMGWYRQAPGKQRELVATINSRGITNYADFVKGRFTISRDNAKKTVYLEMNSLEPEDTAVYYCYTHYFRSywgqgtqvtss------------------------<br>evklqeSgAGLVQPSQSLSLTCSVtgysitSGYYWNWIRLFPGNKLEWVGYISNVGDNNYNPSLKDRLSITRDTSKNQFFLKLNSVTTEDTATYYCARSEYYS-----------vtgyamdywgqgttvtvssawrhp<br><br>SARST: aliSize=86 (resi) RMSD=2.00 (Å)<br>ggg----------LVQAGESLKLSCA------ASGGFMGWYRQAPGKQRELVATINSRGITNYADFVKGRFTISRDNAKKTVYLEMNSLEPEDTAVYYCYTHYFR------------------------sywgqgtqvtss<br>---evklqesgagLVQPSQSLSLTCSvtgysiTSGYYWNWIRLFPGNKLEWVGYISNVGDNNYNPSLKDRLSITRDTSKNQFFLKLNSVTTEDTATYYCARSEYYsvtgyamdywgqgttvtvssawrhp------------<br><br>BLAST: aliSize=67 (resi) iden=50.00% (51/102) simi=65.69% (67/102)<br>-------GGGLVQAGESLKLSCAASG-----GFM-GWYRQAPGKQRELVATINSRGITNYADFVKGRFTISRDNAKKTVYLEMNSLEPEDTAVYYC-----Y--THYFRSYWGQGTQVTVSS-----<br>evklqesGAGLVQPSQSLSLTCSVTGysitsGYYwNWIRLFPGNKLEWVGYISNVGDNNYNPSLKDRLSITRDTSKNQFFLKLNSVTTEDTATYYCarseyYsvTGYAMDYWGQGTTVTVSSawrhp<br><br>Proposed: aliSize=101 (resi) RMSD=1.61 (Å)<br>g--------GGLVQAGESLKLSCAA------SGGFMGWYRQAPGKQRELVATINSRGITNYADFVKGRFTISRDNAKKTVYLEMNSLEPEDTAVYYCYT-----HYF-RSY-WGQGTQVTVSS-----<br>-evklqesgAGLVQPSQSLSLTCSVtgysitSGYYWNWIRLFPGNKLEWVGYISNVGDNNYNPSLKDRLSITRDTSKNQFFLKLNSVTTEDTATYYCARseyysVTGyAMDyWGQGTTVTVSSawrhp
458	1sndA<br>(129)<br><br>1nsnS<br>(138)	C	99.22	TM-align: aliSize=111 (resi) RMSD=1.66 (Å)<br>---LHKEPATLIKAIDGDTVKLMYKGQPMTFRLLLVDTPETKHPKKGVEKYGPEASAFTKKMVENAKKIEVEFDKGQRTDKYGRGLAYIYADGKMVNEALVRQGLAKVAYTH------EQhlrkseaqakkeklniws-----------------<br>tkkLHKEPATLIKAIDGDTVKLMYKGQPMTFRLLLVDTPETKHPKKGVEKYGPEASAFTKKMVENAKKIEVEFDKGQRTDKYGRGLAYIYADGKMVNEALVRQGLAKVAYVYkpnnthEQ-----------------hlrkseaqakkeklniws<br><br>SARST: aliSize=126 (resi) RMSD=9.35 (Å)<br>l----HKEPATLIKAIDGDTVKLMYKGQPMTFRLLLVDTPETKHPKKGVEKYGPEASAFTKKMVENAKKIEVEFDKGQRTDKYGRGLAYIYADGKMVNEALVRQGLAKVAYT------HEQHLRKSEAQAKKEKLNI--ws<br>-tkklHKEPATLIKAIDGDTVKLMYKGQPMTFRLLLVDTPETKHPKKGVEKYGPEASAFTKKMVENAKKIEVEFDKGQRTDKYGRGLAYIYADGKMVNEALVRQGLAKVAYVykpnntHEQHLRKSEAQAKKEKLNIws--<br><br>BLAST: aliSize=129 (resi) iden=100.00% (129/129) simi=100.00% (129/129)<br>---LHKEPATLIKAIDGDTVKLMYKGQPMTFRLLLVDTPETKHPKKGVEKYGPEASAFTKKMVENAKKIEVEFDKGQRTDKYGRGLAYIYADGKMVNEALVRQGLAKVAY------THEQHLRKSEAQAKKEKLNIWS<br>tkkLHKEPATLIKAIDGDTVKLMYKGQPMTFRLLLVDTPETKHPKKGVEKYGPEASAFTKKMVENAKKIEVEFDKGQRTDKYGRGLAYIYADGKMVNEALVRQGLAKVAYvykpnnTHEQHLRKSEAQAKKEKLNIWS<br><br>Proposed: aliSize=129 (resi) RMSD=1.07 (Å)<br>---LHKEPATLIKAIDGDTVKLMYKGQPMTFRLLLVDTPETKHPKKGVEKYGPEASAFTKKMVENAKKIEVEFDKGQRTDKYGRGLAYIYADGKMVNEALVRQGLAKVAYT------HEQHLRKSEAQAKKEKLNIWS<br>tkkLHKEPATLIKAIDGDTVKLMYKGQPMTFRLLLVDTPETKHPKKGVEKYGPEASAFTKKMVENAKKIEVEFDKGQRTDKYGRGLAYIYADGKMVNEALVRQGLAKVAYVykpnntHEQHLRKSEAQAKKEKLNIWS

459	1tiyA (157)<br>2a8nA (130)	C	30.00	TM-align: aliSize=109 (resi) RMSD=1.92 (Å)<br>-NHETFLKRAVTLACEGVNaGIGGPFGAVIVKDGAIIAEGQNNVTTSNDPTAHAEVTAIRKACKVLGAYQLDDCILYTSCEPCP-CLGAIYWARPKAVFYAAEHTdaaeagfDDSfi----YKe-Idkpaeertipfyqvtltehlspfqawrnfankkey-------L-------<br>aERTHFMELALVEARSAGE-RDEVPIGAVLVLDGRVIARSGNRTRELNDVTAHAEIAVIRMACEALGQERLPGADLYVTLEPCTmCAAAISFARIRRLYYGAQDP-------KGG--avesGV-rF--fsqptchHapdvysg<br><br>SARST: aliSize=99 (resi) RMSD=1.11 (Å)<br>n--HETFLKRAVTLACEGVNAGiGGPFGAVIVKDGAIIAEGQNNVTTSNDPTAHAEVTAIRKACKVLGAYQLDDCILYTSCEPC-PCLGAIYWARPKAVFYAAE--------------------------htdaaeagfddsfiykeidkpaeertipfyqvtltehlspfqawrnfankkeyl<br>-aeRTHFMELALVEARSAGERD-EVPIGAVLVLDGRVIARSGNRTRELNDVTAHAEIAVIRMACEALGQERLPGADLYVTLEPCtMCAAAISFARIRRLYYGAQdpkggavesgvrffsqptchhapdvysg--<br><br>BLAST: aliSize=51 (resi) iden=30.00% (39/130) simi=39.23% (51/130)<br>nhetflkravtlacegvnagigg-----------------------PFGAVIVKDGAIIAEGQNNVTTSNDPTAHAEVTAIRKACKVLGAYQLDDCILYTSCEPCP-CLGAIYWARPKAVFYAAEHT--DAAEAG------------------fddsfiykeidkpaeertipfyqvtltehlspfqawr<br>-----------------------aerthfmelalvearsagerdevPIGAVLVLDGRVIARSGNRTRELNDVTAHAEIAVIRMACEALGQERLPGADLYVTLEPCTmCAAAISFARIRRLYYGAQDPkgGAVESGvrffsqptchhapdvysg-------------------------------------<br><br>nfankkeyl<br>---------<br><br>Proposed: aliSize=119 (resi) RMSD=1.53 (Å)<br>MNHETFLKRAVTLACEGVNAGiGGPFGAVIVKDGAIIAEGQNNVTTSNDPTAHAEVTAIRKACKVLGAYQLDDCILYTSCEPCPMCLGAIYWARPKAVFYAAEHtdaaeagfdDSFI---YKEIdK-PAEertiPFYQ-------vtltehlspfqawrnfankkeyl<br>AERTHFMELALVEARSAGERD-EVPIGAVLVLDGRVIARSGNRTRELNDVTAHAEIAVIRMACEALGQERLPGADLYVTLEPCTMCAAAISFARIRRLYYGAQD---------PKGGaveSGVR-FfSQP----TCHHapdvysg-----------------------
460	1tiyA (157)<br>2b3jD (150)	C	29.33	TM-align: aliSize=109 (resi) RMSD=2.05 (Å)<br>NHETFLKRAVTLACEGVNaGIGGPFGAVIVKDGAIIAEGQNNVTTSNDPTAHAEVTAIRKACKVLGAYQLDDCILYTSCEPCP-CLGAIYWARPKAVFYAAEHTdaaeagfDDSFi---YKe-Idkpaeertipfyqvtltehlspfqawrnfankkeyl------------------------------<br>NDIYFMTLAIEEAKKAAQ-LGEVPIGAIITKDDEVIARAHNLRETLQQPTAHAEHIAIERAAKVLGSWRLEGCTLYVTLEPCVmCAGTIVMSRIPRVVYGADDP-------KGGC-sgsLM-nL------------------------------------lqqsnfnhraivdkgvlkeacstllttffk<br><br>------<br>nlrank<br><br>SARST: aliSize=107 (resi) RMSD=3.36 (Å)<br>n-HETFLKRAVTLACEGVNAGiGGPFGAVIVKDGAIIAEGQNNVTTSNDPTAHAEVTAIRKACKVLGAYQLDDCILYTSCEPCPCLGAIY-WARPKAVFYAAEHTDAAEAG---fddsfiykeidkpaeertipfyqvtltehlspfqawrn<br>-nDIYFMTLAIEEAKKAAQLG-EVPIGAIITKDDEVIARAHNLRETLQQPTAHAEHIAIERAAKVLGSWRLEGCTLYVTLEPCVMCAGTIvMSRIPRVVYGADDPKGGCSGslmnllqqsnfnhraivdkgvlkeacstllttffknlrank--------------------------------------<br><br>fankkeyl<br>--------<br><br>BLAST: aliSize=55 (resi) iden=28.67% (43/150) simi=36.67% (55/150)<br>nhetflkra-----VTLACEGVN--AGIGG-PFGAVIVKDGAIIAEGQNNVTTSNDPTAHAEVTAIRKACKVLGAYQLDDCILYTSCEPC-PCLGAIYWARPKAVFYAAE---htdaaeagfddsfiykeidkpaeertipfyq<br>---------ndiyfMTLAIEEAKkaAQLGEvPIGAIITKDDEVIARAHNLRETLQQPTAHAEHIAIERAAKVLGSWRLEGCTLYVTLEPCvMCAGTIVMSRIPRVVYGADdpkggcsgslmnllqqsnfnhraivdkgvlkeacstllttffknlrank-----------------------------<br><br>vtltehlspfqawrnfankkeyl<br>-----------------------<br><br>Proposed: aliSize=134 (resi) RMSD=1.71 (Å)<br>mNHETFLKRAVTLACEGVNAGiGGPFGAVIVKDGAIIAEGQNNVTTSNDPTAHAEVTAIRKACKVLGAYQLDDCILYTSCEPCPMCLGAIYWARPKAVFYAAEH---------tdaaeagfddsfiykeIdkP-AEER-T-IPFYQVTLTEHLSPFQAWRNF-ANKK---eyl<br>-NDIYFMTLAIEEAKKAAQLG-EVPIGAIITKDDEVIARAHNLRETLQQPTAHAEHIAIERAAKVLGSWRLEGCTLYVTLEPCVMCAGTIVMSRIPRVVYGADDpkggcsgsl----------------M--NlLQQSnFnHRAIVDKGVLKEACSTLLTTFfKNLRank---
461	1tiyA (157)<br>2g84B (182)	C	27.39	TM-align: aliSize=116 (resi) RMSD=1.36 (Å)<br>--------------------------NHETFLKRAVTLACEGVNAGiGGPFGAVIVK--DGAIIAEGQNNVTTSNDPTAHAEVTAIRKACKVLGAYQLD-----DCILYTSCEPCP-CLGAIYWARPKAVFYAAEHTDAAEAGFDDSfIYkeIDkpaeertipfyqvtltehlspfqawrnfankkeyl-<br>ghmndalhiglppflvqanneprvlaAPEARMGYVLELVRANIAAD-GGPFAAAVFErdSGLLIAAGTNRVVPGRCSAAHAEILALSLAQAKLDTHDLSadglpACELVTSAEPCVmCFGAVIWSGVRSLVCAARSDDVEAIGFDEG-PR--PE-----------------------------------n<br><br>-------------------------------<br>wmggleargitvttgllrdaacallreynac<br><br>SARST: aliSize=118 (resi) RMSD=4.16 (Å)<br>n---------------------------HETFLKRAVTLACEGVNAGiGGPFGAVIVK--DGAIIAEGQNNVTTSNDPTAHAEVTAIRKACKVLGAYQL-----DDCILYTSCEPC-PCLGAIYWARPKAVFYAAEHTDAAEAGFDD------SFIYKEID-----------------------kpaeer<br>-ghmndalhiglppflvqanneprvlaaPEARMGYVLELVRANIAAD-GGPFAAAVFErdSGLLIAAGTNRVVPGRCSAAHAEILALSLAQAKLDTHDLsadglPACELVTSAEPCvMCFGAVIWSGVRSLVCAARSDDVEAIGFDEgprpenWMGGLEARgitvttgllrdaacallreynac------<br><br>tipfyqvtltehlspfqawrnfankkeyl<br>-----------------------------<br><br>BLAST: aliSize=48 (resi) iden=23.23% (36/157) simi=30.97% (48/157)<br>nhetflkravtlacegvn---AGIGGPFGAVIV-KD-GAIIAEGQNNVTTSNDPTAHAEVTAIR----------------KACKvlgayqlddciLYTSCEPC-PCLGAIYWARPKAVFYAAEHTDAAEAGFDD----------------<br>------------------ghmndalhiglppflvqanneprvlaapearmgyvlelvraniAADGGPFAAAVFeRDsGLLIAAGTNRVVPGRCSAAHAEILALSlaqakldthdlsadglPACE-----------LVTSAEPCvMCFGAVIWSGVRSLVCAARSDDVEAIGFDEgprpenwmggleargi<br><br>--------------------sfiykeidkpaeertipfyqvtltehlspfqawrnfankkeyl<br>tvttgllrdaacallreynac--<br><br>Proposed: aliSize=144 (resi) RMSD=1.59 (Å)<br>-------------------------MNHETFLKRAVTLACEGVNAGiGGPFGAVIVK--DGAIIAEGQNNVTTSNDPTAHAEVTAIRKACKVLGAYQLD-----DCILYTSCEPCPMCLGAIYWARPKAVFYAAEHTDAAEAGFDD---sfiykeIDKPAE--ERTIPFYQVTLtEHLSPFQAWRNF-AN<br>ghmndalhiglppflvqanneprvlAAPEARMGYVLELVRANIAAD-GGPFAAAVFErdSGLLIAAGTNRVVPGRCSAAHAEILALSLAQAKLDTHDLSadglpACELVTSAEPCVMCFGAVIWSGVRSLVCAARSDDVEAIGFDEgpr------PENWMGglEARGITVTTGL-LRDAACALLREYnAC<br><br>kkeyl<br>-----

462	1tiyA (157)<br>2hvvA (143)	C	18.88	TM-align: aliSize=101 (resi) RMSD=2.74 (Å)<br>--NHETFLKRAVTLAcEGVnagIG--GPFGAVIVKDGAIIAEGQNNV----TTS-----N------DPTAHAEVTAIRKACKVlGAyQLDDCILYTSCEPCP-CLGAIYWARPKAVFYAAEHTdaaeagfddSfiYKeIdkpaeertipfyqvtltehlspfqawrnfankkeyl---------------<br>lsWQDYFMANAELIS-KRS---TCnrAYVGAVLVKNNRIIATGYNGGvadtDNCddvghEmedghcIRTVHAEMNALIQCAKE-GI-SANNTEIYVTHFPCInCTKALLQAGVKKITYNTAYR---------I--HP-F------------------------------------aielmtqkeveyvqh<br>-------<br>dvprvkl<br><br>SARST: aliSize=106 (resi) RMSD=7.49 (Å)<br>nhet--FLKRAVTLACEGVNAGIG--GPFGAVIVKDGAIIAEGQNN-------VTTS------NDPTAH-AEVTAIRKACKVLGAyQLDDCILYTSCEPC-PCLGAIYWARPKAVFYAAE-------HTDAAEAGFD-----------dsfiykeidkpaeertipfyqvtltehlspfqawrnfankkeyl<br>----lsWQDYFMANAELISKRSTCnrAYVGAVLVKNNRIIATGYNGgvadtdnCDDVghemedGHCIRTvHAEMNALIQCAKEGI-SANNTEIYVTHFPCiNCTKALLQAGVKKITYNTAyrihpfaIELMTQKEVEyvqhdvprvkl---<br><br>BLAST: aliSize=50 (resi) iden=20.98% (30/143) simi=34.97% (50/143)<br>nhetflkravtlacegvnagiggpf-------------------------GAVIVKDGAIIAEGQNNVTTSNDPtahaevtairkaCKVLGaYQLDD--CILYTSCEpcpcLGAIYWARPKAVfyAAEHTDAAEAGFDDSFIYKEIDKPAEERTIpfYQVTLTEHlsPFqAWRNFANKK-EYL---------<br>-------------------------lswqdyfmanaeliskrstcnrayvGAVLVKNNRIIATGYNGGVADTDN------------CDDVG-HEMEDghCIRTVHAE----MNALIQCAKEGI--SANNTEIYVTHFPCINCTKALLQAGVKKIT--YNTAYRIH--PF-AIELMTQKEvEYVqhdvprvkl<br><br>Proposed: aliSize=112 (resi) RMSD=1.97 (Å)<br>-MNHETFLKRAVTLACEGvnagIG--GPFGAVIVKDGAIIAEGQNNV-----TT-------------sNDptAHAEVTAIRKACKVLGAyqLDDCILYTSCEPCPMCLGAIYWARPKAVFYAAEHtdaaeagF-DDSFIYKEIDKPaeERTI---------pfyqvtltehlspfqawrnfankkeyl<br>lSWQDYFMANAELISKRS----TCnrAYVGAVLVKNNRIIATGYNGGvadtdNCddvghemedghci-RT--VHAEMNALIQCAKEGIS--ANNTEIYVTHFPCINCTKALLQAGVKKITYNTAY-------RiHPFAIELMTQKE--VEYVqhdvprvkl---------------------------
463	1u6mA (189)<br>1vkcA (149)	C	20.81	TM-align: aliSize=135 (resi) RMSD=3.06 (Å)<br>-SLIRSATKeDGQAIARLVLVILKDME-L-PILEEVsEeqMid--LLae--AtaYpTYRy-Gy-QRILVYEHA-GEVAGIAVGYPAEdekiideplrevfkkhglaedvrlfieeeTL--PN-EWYLDTISVDERFRGMGIGSKLLDALPEVAKASGKQALGLNVDFDNpGARKLYASKGFKDVTTMTIS<br>eYTIVDGEE-YIEEIKKLDREISYSFVrFpISYEEY-E--E--rhEE--lfE--S-LLS-qG-eHKFFVALNErSELLGHVWICITL-----------------------------DTvdYVkIAYIYDIEVVKWARGLGIGSALLRKAEEWAKERGAKKIVLRVEIDN-PAVKWYEERGYKARALIMEK<br>Ghlynhmqkeve-<br>P-----------i<br><br>SARST: aliSize=135 (resi) RMSD=6.08 (Å)<br>s-LIRSATKEDGQAIARLVLVILkdmelPILEEVS------EEQMIDLLAEATAYptyrygyqRILVYEH----AGEVAGIAVGYpaedekiideplrevfkkhglaedvRLFIEEETLPnEWYLDTISVDERFRGMGIGSKLLDALPEVAKASGKQALGLNVDFDNpGARKLYASKGFKDVTTMTI---<br>-eYTIVDGEEYIEEIKKLDREIS-----YSFVRFPisyeeyEERHEELFESLLSQ--------GEHKFFValneRSELLGHVWIC-----------------------ITLDTVDYVK-IAYIYDIEVVKWARGLGIGSALLRKAEEWAKERGAKKIVLRVEIDN-PAVKWYEERGYKARALIMEkpi<br>sghlynhmqkev<br>------------<br><br>BLAST: aliSize=33 (resi) iden=16.78% (25/149) simi=22.15% (33/149)<br>slirsatkedgqaiarlvlvilkdmelpileevseeqmidllaeataypt yrygyqrilvyehagevagiavgypaedekiideplrevfkkhglaedvrlfieeetlpnew---<br>---eytivdgeeyieeikkldreisysfvrfpisyeeyeerheelfesllsqgehkffvalnersellghvwicitldtvd<br>-----YLDTISVDERFRGMGIGSKLLDALPEVAKASGKQALGLNVDFDNPgARKLYASKGFK----------dvttmtisghlynhmqkev<br>yvkiaYIYDIEVVKWARGLGIGSALLRKAEEWAKERGAKKIVLRVEIDNP-AVKWYEERGYKaralimekpi-------------------<br><br>Proposed: aliSize=131 (resi) RMSD=2.42 (Å)<br>-SLIRSATKeDGQAIARLVL-VILKDMEL-PiLEEV-seeqM---idL--LaeAtayptYRyG----yQRILVYEHA-GEVAGIAVGYPAEdekiideplrevfkkhglaedvrlfieeetLP---N-EWYLDTISVDERFRGMGIGSKLLDALPEVAKASGKQALGLNVDFDNpGARKLYASKGFKDvT<br>eYTIVDGEE-YIEEIKKLDReISYSFVRFpI-SYEEy----Eerh--EelF--E-----SL-Lsqge-HKFFVALNErSELLGHVWICITL-----------------------------DTvdyVkIAYIYDIEVVKWARGLGIGSALLRKAEEWAKERGAKKIVLRVEIDN-PAVKWYEERGYKA-R<br>TMTisghlynhmQKEVE<br>ALI---------MEKPI
464	1u6mA (189)<br>2d4pA (130)	C	16.15	TM-align: aliSize=119 (resi) RMSD=2.84 (Å)<br>SLIRSATKEDGQAIARLVlVILkdmelpileevseeqMIdlLAeATAYptYryGYQR--ILVYEHAGEVAGIAVGYPAEdekiideplrevfkkhglaedvrlfieeetlP---NEWYLDTISVderfRGMGIGSKLLDALPEVAKASGKQALGLNVDfDNPG-ARKLYASKGFK-D-VTTMTISGhlyn<br>MRFRPFTEEDLDRLNRLA-GKR---------------PV--SL-GALR--F--FARTghSFLAEEGEEPMGFALAQAVW-------------------------------QgeaTTVLVTRIEG----RSVEALRGLLRAVVKSAYDAGVYEVALHLD-PERKeLEEALKAEGFAlGpLVLAVRVL----<br>hmqkeve---<br>-------gsr<br><br>SARST: aliSize=121 (resi) RMSD=6.46 (Å)<br>s-LIRSATKEDGQAIARLvlvilkdmelPILEEVSEEQMIDLLAEataypt yrygyQRILVYEHAGEVAGIAVGYPaedekiideplrevfkkhglaedvrlFIEEETLpnEWYLDTISV-DERfrgmgIGSKLLDALPEVAKASGKQALGLNVDFDNPGARKLYASKGFK-DVTTMTI------sghly<br>-mRFRPFTEEDLDRLNRL----------AGKRPVSLGALRFFART-----------GHSFLAEEGEEPMGFALAQA--------------------------VWQGEAT--TVLVTRIEGrSVE-----ALRGLLRAVVKSAYDAGVYEVALHLDPERKELEEALKAEGFAlGPLVLAVrvlgsr-----<br>nhmqkev<br>-------<br><br>BLAST: aliSize=26 (resi) iden=10.00% (13/130) simi=20.00% (26/130)<br>slirsatkedgqaiarlvlvilkdmelpileevseeqmidllaeataypt yrygyqrilvyehagevagiavgypaedekiideplrevfkkhglaedvrlfieeetlpnewyld--TISVDeRFRGMGIG<br>--mrfrpfteedldrlnrlagkrpvslgalrffartghsflaeegeepmgfalaqavwqgeatTVLVT-RIEGRSVE<br>SK--LLDALPEVAKASGKQALGLNVDFDNPGARKLYASKGF---------------kdvttmtisghlynhmqkev<br>ALrgLLRAVVKSAYDAGVYEVALHLDPERKELEEALKAEGFalgplvlavrvlgsr--------------------<br><br>Proposed: aliSize=121 (resi) RMSD=2.53 (Å)<br>SLIRSATKEDGQAIARLVlVILkdmelpileevseeqmIdlLAEATAYptYryGYQR--ILVYEHAGEVAGIAVGYPAEdekiideplrevfkkhglaedvrlfieeetlP---NEWYLDTISVderfRGMGIGSKLLDALPEVAKASGKQALGLNVDfDN-pG-ARKLYASKGFK-D-VTTMTisghLY<br>MRFRPFTEEDLDRLNRLA-GKR---------------P--VSLGALR--F--FARTghSFLAEEGEEPMGFALAQAVW-------------------------------QgeaTTVLVTRIEG----RSVEALRGLLRAVVKSAYDAGVYEVALHLD-PEr-KeLEEALKAEGFAlGpLVLAV----RV<br>NHMQkeve<br>LGSR----

465	1u6mA (189)<br>2f5iA (167)	C	21.56	TM-align: aliSize=133 (resi) RMSD=2.53 (Å)<br>-SLIRSATKEDGOAIARLVLVILK-D-MELpiLeevsEeqMidLLAEATAY-P----TYRygyqRILVYEHAG--------EVAGIAVGYPAEdekiideplrevfkkhglaedvrlfieeet---LPNEWYLDTISVDERFRGMGIGSKLLDALPEVAKASGKOALGLNVDFDNPGARKLYASKGFKDV<br>kFVIRPATAADCSDILRLIKELAKyEyMEE--Q----V--I--LTEKDLLEdGfgehPFY----HCLVAEVPKehwtpeghSIVGFAMYYFTY-------------------------------dpwIGKLLYLEDFFVMSDYRGFGIGSEILKNLSQVAMRCRCSSMHFLVAEWNEPSINFYKRRGASDL<br>TT---MTISghlynhmqkeve------------<br>SSeegWRLF------------kidkeyllkmat<br>SARST: aliSize=135 (resi) RMSD=6.09 (Å)<br>s--LIRSATKEDGOAIARLVLVILKDMELPILEEVSEEOMIDLlaeatayPTYRYGYQRILVYEH-------AGE-VAGIAVGYPaedekiideplrevfkkhglaedvRLFIEEETLpneWYLDTISVDERFRGMGIGSKLLDALPEVAKASGKOALGLNVDFDNPGARKLYASKGFKD----------<br>-kfVIRPATAADCSDILRLIKELAKYEYMEEQVILTEKDLLED-------GFGEHPFYHCLVAEVpkehwtpEGHsIVGFAMYYF------------------------TYDPWIGKL---LYLEDFFVMSDYRGFGIGSEILKNLSQVAMRCRCSSMHFLVAEWNEPSINFYKRRGASDlsseegwrlf<br>------------vttmtisghlynhmqkev<br>kidkeyllkmat------------------<br>BLAST: aliSize=71 (resi) iden=27.54% (46/167) simi=42.51% (71/167)<br>s--LIRSATKEDGOAIARLVLVILKdmelpilEEVSEEOMI----DLLAEATA-YPTYrygyqRILVYEHAGEvagiavGYPAEDEKIIdeplrevfkkhGLAedVRLFIEEETLPNEWYLDTISVDERFRGMGIGSKLLDALPEVAKASGKOALG-LNVDFDNPGArKLYASKGFKDVTT---------<br>-kfVIRPATAADCSDILRLIKELAK-------YEYMEEQVIltekDLLEDGFGeHPFY-----HCLVAEVPKE------HWTPEGHSIV-----------GFA--MYYFTYDPWIGKLLYLEDFFVMSDYRGFGIGSEILKNLSQVAMRCRCSSMHfLVAEWNEPSI-NFYKRRGASDLSSeegwrlfki<br>----------mtisghlynhmqkev<br>dkeyllkmat--------------<br>Proposed: aliSize=137 (resi) RMSD=2.04 (Å)<br>-SLIRSATKEDGOAIARLVLVILK-D-MELpiL-eevseeqMidLLAEATA-YP----TYRygyqRILVYEHAG--------EVAGIAVGYPAEdekiideplrevfkkhglaedvrlfieeetlP----NEWYLDTISVDERFRGMGIGSKLLDALPEVAKASGKOALGLNVDFDNPGARKLYASKGFK<br>kFVIRPATAADCSDILRLIKELAKyEyMEE--Qv-------I--LTEKDLLeDGfgehPFY----HCLVAEVPKehwtpeghSIVGFAMYYFTY--------------------------------DpwigKLLYLEDFFVMSDYRGFGIGSEILKNLSQVAMRCRCSSMHFLVAEWNEPSINFYKRRGAS<br>dvttmtIS--GHLYNHMOKEVE-------<br>------DLssEEGWRLFKIDKEyllkmat
466	1u6mA (189)<br>2g3tB (168)	C	22.02	TM-align: aliSize=133 (resi) RMSD=2.50 (Å)<br>-SLIRSATKEDGOAIARLVLVILK-D-MELPiLeevseeqMidLLAEATAY-P----TYRygyqRILVYEHAG--------EVAGIAVGYPAEdekiideplrevfkkhglaedvrlfieeet---LPNEWYLDTISVDERFRGMGIGSKLLDALPEVAKASGKOALGLNVDFDNPGARKLYASKGFKDV<br>kFVIRPATAADCSDILRLIKELAKyEyMEEQ-V-------I--LTEKDLLEdGfgehPFY----HCLVAEVPKehwtpeghSIVGFAMYYFTY-------------------------------dpwIGKLLYLEDFFVMSDYRGFGIGSEILKNLSQVAMRCRCSSMHFLVAEWNEPSINFYKRRGASDL<br>T----TMTISghlynhmqkeve------------<br>SseegWRLFK------------idkeyllkmate<br>SARST: aliSize=132 (resi) RMSD=6.20 (Å)<br>s--LIRSATKEDGOAIARLVLVIlkDMELPI---LEEVseeqmiDLLAEATAypTYRYGYQRILVYEH--------AGEVAGIAVGYpaedekiideplrevfkkhglaedVRLFIEEETLpneWYLDTISVDERFRGMGIGSKLLDALPEVAKASGKOALGLNVDFDNPGARKLYASKGFKD-------<br>-kfVIRPATAADCSDILRLIKEL--AKYEYMeeqVILT------EKDLLEDG--FGEHPFYHCLVAEVpkehwtpeGHSIVGFAMYY-------------------------FTYDPWIGKL---LYLEDFFVMSDYRGFGIGSEILKNLSQVAMRCRCSSMHFLVAEWNEPSINFYKRRGASDlsseegw<br>---------------vttmtisghlynhmqkev<br>rlfkidkeyllkmate-----------------<br>BLAST: aliSize=71 (resi) iden=27.38% (46/168) simi=42.26% (71/168)<br>s--LIRSATKEDGOAIARLVLVILKdmelpilEEVSEEOMI----DLLAEATA-YPTYrygyqRILVYEHAGEvagiavGYPAEDEKIIdeplrevfkkhGLAedVRLFIEEETLPNEWYLDTISVDERFRGMGIGSKLLDALPEVAKASGKOALG-LNVDFDNPGArKLYASKGFKDVTT---------<br>-kfVIRPATAADCSDILRLIKELAK-------YEYMEEQVIltekDLLEDGFGeHPFY-----HCLVAEVPKE------HWTPEGHSIV-----------GFA--MYYFTYDPWIGKLLYLEDFFVMSDYRGFGIGSEILKNLSQVAMRCRCSSMHfLVAEWNEPSI-NFYKRRGASDLSSeegwrlfki<br>-----------mtisghlynhmqkev<br>dkeyllkmate---------------<br>Proposed: aliSize=138 (resi) RMSD=2.00 (Å)<br>-SLIRSATKEDGOAIARLVLVILK-D-MELpiL-eevseeqMidLLAEATA-YP----TYRygyqRILVYEHAG--------EVAGIAVGYPAEdekiideplrevfkkhglaedvrlfieeetlP----NEWYLDTISVDERFRGMGIGSKLLDALPEVAKASGKOALGLNVDFDNPGARKLYASKGFK<br>kFVIRPATAADCSDILRLIKELAKyEyMEE--Qv-------I--LTEKDLLeDGfgehPFY----HCLVAEVPKehwtpeghSIVGFAMYYFTY--------------------------------DpwigKLLYLEDFFVMSDYRGFGIGSEILKNLSQVAMRCRCSSMHFLVAEWNEPSINFYKRRGAS<br>D-vttMTisGHLYNHMOKEVE--------<br>Dl---SS--EEGWRLFKIDKEyllkmate
467	1u6mA (189)<br>2jevA (169)	C	20.71	TM-align: aliSize=133 (resi) RMSD=2.56 (Å)<br>--SLIRSATKEDGOAIARLVLVILK-D-MELpILeevseeqMidLLAEATAY-P----TYRygyqRILVYEHAG--------EVAGIAVGYPAEdekiideplrevfkkhglaedvrlfieeet---LPNEWYLDTISVDERFRGMGIGSKLLDALPEVAKASGKOALGLNVDFDNPGARKLYASKGFKD<br>akFVIRPATAADCSDILRLIKELAKyEyMEE-QV-------I--LTEKDLLEdGfgehPFY----HCLVAEVPKehwtpeghSIVGFAMYYFTY-------------------------------dpwIGKLLYLEDFFVMSDYRGFGIGSEILKNLSQVAMRCRCSSMHFLVAEWNEPSINFYKRRGASD<br>VTT---MTISghlynhmqkeve-------------<br>LSSeegWRLF------------kidkeyllkmate<br>SARST: aliSize=131 (resi) RMSD=6.15 (Å)<br>s---LIRSATKEDGOAIARLVLVIlkDMELPIL---EEVseeqmiDLLAEATAypTYRYGyqRILVYEH---------AGE-VAGIAVGYpaedekiideplrevfkkhglaedvRLFIEEETLpnEWYLDTISVDERFRGMGIGSKLLDALPEVAKASGKOALGLNVDFDNPGARKLYASKGFKDV---<br>-akfVIRPATAADCSDILRLIKEL--AKYEYMEeqvILT------EKDLLEDG--FGEHP--FYHCLVAevpkehwtpEGHsIVGFAMYY-------------------------FTYDPWIGK--LLYLEDFFVMSDYRGFGIGSEILKNLSQVAMRCRCSSMHFLVAEWNEPSINFYKRRGASDLsse<br>------------------ttmtisghlynhmqkev<br>egwrlfkidkeyllkmate----------------<br>BLAST: aliSize=71 (resi) iden=27.22% (46/169) simi=42.01% (71/169)<br>s---LIRSATKEDGOAIARLVLVILKdmelpilEEVSEEOMI----DLLAEATA-YPTYrygyqRILVYEHAGEvagiavGYPAEDEKIIdeplrevfkkhGLAedVRLFIEEETLPNEWYLDTISVDERFRGMGIGSKLLDALPEVAKASGKOALG-LNVDFDNPGArKLYASKGFKDVTT--------<br>-akfVIRPATAADCSDILRLIKELAK-------YEYMEEQVIltekDLLEDGFGeHPFY-----HCLVAEVPKE------HWTPEGHSIV-----------GFA--MYYFTYDPWIGKLLYLEDFFVMSDYRGFGIGSEILKNLSQVAMRCRCSSMHfLVAEWNEPSI-NFYKRRGASDLSSeegwrlfk<br>-----------mtisghlynhmqkev<br>idkeyllkmate--------------<br>Proposed: aliSize=136 (resi) RMSD=2.07 (Å)<br>--SLIRSATKEDGOAIARLVLVILK-D-MELpiL-eevseeqMidLLAEATA-YP----TYRygyqRILVYEHAG--------EVAGIAVGYPAEdekiideplrevfkkhglaedvrlfieeetlP----NEWYLDTISVDERFRGMGIGSKLLDALPEVAKASGKOALGLNVDFDNPGARKLYASKGF<br>akFVIRPATAADCSDILRLIKELAKyEyMEE--Qv-------I--LTEKDLLeDGfgehPFY----HCLVAEVPKehwtpeghSIVGFAMYYFTY--------------------------------DpwigKLLYLEDFFVMSDYRGFGIGSEILKNLSQVAMRCRCSSMHFLVAEWNEPSINFYKRRGA<br>--kdvttMT-isGHLYNHMOKE---VE-----<br>sd-----LSs--EEGWRLFKIDkeyLLkmate

468	1v9yA (103)<br>1f9iA (125)	N	15.53	TM-align: aliSize=96 (resi) RMSD=2.10 (Å)<br>g--------------I-FF---PaLEQNMMGAVLINENDEVMFFNPAAEKLWGYKREEVIGNNID--MLIPrdLRPA-HPEYIRHNRE-------RELQLEKkDGSKIWTRFALSKVSAegKVYYLALVRD-<br>-mehvafgsedientLaKMddgQ-LDGLAFGAIQLDGDGNILQFNAAEGDITGRDPKQVIGKNFFkdVAPC--TDSPeFYGKFKEGVAsgnlntmFEYTFDY-QMTPTKVKVHMKKALS--GDSYWVFVKRv<br><br>SARST: aliSize=86 (resi) RMSD=13.30 (Å)<br>gif-------------------FPALEQNMMGAVLINENDEVMFFNPAAEKLWGYKREEVIGNNI-----DMLIPRDLRPAHPEYIrhnrerelqlekKDGSKIWTRFALSKVSAEGKVYYLALV---------------rd<br>---mehvafgsedientlakmdDGQLDGLAFGAIQLDGDGNILQFNAAEGDITGRDPKQVIGKNFfkdvaPCTDSPEFYGKFKEGV------------ASGNLNTMFEYTFDYQMTPTKVKVHMKkalsgdsywvfvkrv--<br><br>BLAST: aliSize=26 (resi) iden=13.59% (14/103) simi=25.24% (26/103)<br>giffpaleqnmm----------------------------GAVLINENDEVMFFNPAAEKLWGYKREEVIGNNIDmlipRDLRPA--HPEY--irhnrerelqlekkdgskiwtrfalskvsaegkvyylalvrd<br>------------mehvafgsedientlakmddgqldglafGAIQLDGDGNILQFNAAEGDITGRDPKQVIGKNFF----KDVAPCtdSPEFygkfkegvasgnlntmfeytfdyqmtptkvkvhmkkalsgdsywvfvkrv--<br><br>Proposed: aliSize=99 (resi) RMSD=1.95 (Å)<br>-------------GIFFPA---LEQNMMGAVLINENDEVMFFNPAAEKLWGYKREEVIGNNID-MLIPRdLRPA-HPEYIRHNRE-------RELQLEKkDGSKIWTRFALSKVSAegKVYYLALVRD-h<br>mehvafgsedienTLAKMDdgqLDGLAFGAIQLDGDGNILQFNAAEGDITGRDPKQVIGKNFFkDVAPC-TDSPeFYGKFKEGVAsgnlntmFEYTFDY-QMTPTKVKVHMKKALS--GDSYWVFVKRv-
469	1v9yA (103)<br>1gsvA (122)	N	15.53	TM-align: aliSize=97 (resi) RMSD=2.04 (Å)<br>g-------------IFF-PALEQNMMGAVLINENDEVMFFNPAAEKLWGYKREEVIGNNID--MLIPrdLRPA-HPEYIRHNRE-------RELQLEKkDGSKIWTRFALSKVSAegKVYYLALVRD-<br>-vafgsedientlaKMDdGQLDGLAFGAIQLDGDGNILQYNAAESDITGRDPKQVIGKNFFkdVAPC--TDSPeFYGKFKEGVAsgnlntmFEYTFDY-QMTPTKVKVHMKKALS--GDSYWVFVKRv<br><br>SARST: aliSize=86 (resi) RMSD=13.32 (Å)<br>gif----------------FPALEQNMMGAVLINENDEVMFFNPAAEKLWGYKREEVIGNNI-----DMLIPRDLRPAHPEYIrhnrerelqlekKDGSKIWTRFALSKVSAEGKVYYLALV---------------rd<br>---vafgsedientlakmdDGQLDGLAFGAIQLDGDGNILQYNAAESDITGRDPKQVIGKNFfkdvaPCTDSPEFYGKFKEGV------------ASGNLNTMFEYTFDYQMTPTKVKVHMKkalsgdsywvfvkrv--<br><br>BLAST: aliSize=26 (resi) iden=12.62% (13/103) simi=25.24% (26/103)<br>giffpaleqnmm-------------------------GAVLINENDEVMFFNPAAEKLWGYKREEVIGNNIDmlipRDLRPA--HPEY--irhnrerelqlekkdgskiwtrfalskvsaegkvyylalvrd<br>------------vafgsedientlakmddgqldglafGAIQLDGDGNILQYNAAESDITGRDPKQVIGKNFF----KDVAPCtdSPEFygkfkegvasgnlntmfeytfdyqmtptkvkvhmkkalsgdsywvfvkrv--<br><br>Proposed: aliSize=99 (resi) RMSD=2.05 (Å)<br>GI-----F--FPA------LEQNMMGAVLINENDEVMFFNPAAEKLWGYKREEVIGNNID-MLIPRdLRPA-HPEYIRHNRE-------RELQLEKKDgSKIWTRFALSKVSAegKVYYLALVRD-h<br>VAfgsedIenTLAkmddgqLDGLAFGAIQLDGDGNILQYNAAESDITGRDPKQVIGKNFFkDVAPC-TDSPeFYGKFKEGVAsgnlntmFEYTFDYQM-TPTKVKVHMKKALS--GDSYWVFVKRv-
470	1v9yA (103)<br>1gswA (122)	N	15.53	TM-align: aliSize=97 (resi) RMSD=2.07 (Å)<br>g-------------IFF-PALEQNMMGAVLINENDEVMFFNPAAEKLWGYKREEVIGNNID--MLIPrdLRPA-HPEYIRHNRE-------RELQLEKKDgSKIWTRFALSKVSAegKVYYLALVRD-<br>-vafgsedientlaKMDdGQLDGLAFGAIQLDGDGNILQYNAAEGDITSRDPKQVIGKNFFkdVAPC--TDSPeFYGKFKEGVAsgnlntmFEYTFDYQM-TPTKVKVHMKKALS--GDSYWVFVKRv<br><br>SARST: aliSize=86 (resi) RMSD=13.30 (Å)<br>gif----------------FPALEQNMMGAVLINENDEVMFFNPAAEKLWGYKREEVIGNNI-----DMLIPRDLRPAHPEYIrhnrerelqlekKDGSKIWTRFALSKVSAEGKVYYLALV---------------rd<br>---vafgsedientlakmdDGQLDGLAFGAIQLDGDGNILQYNAAEGDITSRDPKQVIGKNFfkdvaPCTDSPEFYGKFKEGV------------ASGNLNTMFEYTFDYQMTPTKVKVHMKkalsgdsywvfvkrv--<br><br>BLAST: aliSize=25 (resi) iden=11.65% (12/103) simi=24.27% (25/103)<br>giffpaleqnmm-------------------------GAVLINENDEVMFFNPAAEKLWGYKREEVIGNNIDmlipRDLRPA--HPEY--irhnrerelqlekkdgskiwtrfalskvsaegkvyylalvrd<br>------------vafgsedientlakmddgqldglafGAIQLDGDGNILQYNAAEGDITSRDPKQVIGKNFF----KDVAPCtdSPEFygkfkegvasgnlntmfeytfdyqmtptkvkvhmkkalsgdsywvfvkrv--<br><br>Proposed: aliSize=99 (resi) RMSD=2.04 (Å)<br>G------IFFP-A------LEQNMMGAVLINENDEVMFFNPAAEKLWGYKREEVIGNNID-MLIPRdLRPA-HPEYIRHNRE-------RELQLEKKDgSKIWTRFALSKVSAegKVYYLALVRD-h<br>VafgsedIENTlAkmddgqLDGLAFGAIQLDGDGNILQYNAAEGDITSRDPKQVIGKNFFkDVAPC-TDSPeFYGKFKEGVAsgnlntmFEYTFDYQM-TPTKVKVHMKKALS--GDSYWVFVKRv-

471	1v9yA (103)<br>1gsxA (122)	N	13.59	TM-align: aliSize=97 (resi) RMSD=2.12 (Å)<br>g-------------IFF-PALEQNMMGAVLINENDEVMFFNPAAEKLWGYKREEVIGNNID--MLIPrdLRPA-HPEYIRHNRE-------RELQLEKkDGSKIWTRFALSKVSAeGKvYYLALVRD-<br>-vafgsedientlaKMDdGQLDGLAFGAIQLDGDGNILQYNAAESDITSRDPKQVIGKNFFkdVAPC--TDSPeFYGKFKEGVAsgnlntmFEYTFDY-QMTPTKVKVHMKKALS-GD-SYWVFVKRv<br><br>SARST: aliSize=99 (resi) RMSD=4.85 (Å)<br>gif----------------FPALEQNMMGAVLINENDEVMFFNPAAEKLWGYKREEVIGNNI-----DMLIPRDLRPAHPEYIRHNRERELQLEKKDGSKIWTRFALSKVSAEGKVYYLALVR--d<br>---vafgsedientlakmdDGQLDGLAFGAIQLDGDGNILQYNAAESDITSRDPKQVIGKNFfkdvaPCTDSPEFYGKFKEGVASGNLNTMFEYTFDYQMTPTKVKVHMKKALSGDSYWVFVKrv-<br><br>BLAST: aliSize=25 (resi) iden=11.65% (12/103) simi=24.27% (25/103)<br>giffpaleqnmm-------------------------GAVLINENDEVMFFNPAAEKLWGYKREEVIGNNIDmlipRDLRPA--HPEY--irhnrerelqlekkdgskiwtrfalskvsaegkvyylalvrd<br>------------vafgsedientlakmddgqldglafGAIQLDGDGNILQYNAAESDITSRDPKQVIGKNFF----KDVAPCtdSPEFygkfkegvasgnlntmfeytfdyqmtptkvkvhmkkalsgdsywvfvkrv--<br><br>Proposed: aliSize=99 (resi) RMSD=2.06 (Å)<br>GI-----F-FPA-------LEQNMMGAVLINENDEVMFFNPAAEKLWGYKREEVIGNNID-MLIPRdLRPA-HPEYIRHNRE-------RELQLEKKDgSKIWTRFALSKVSAegKVYYLALVRD-h<br>VAfgsedIeNTLakmddgqLDGLAFGAIQLDGDGNILQYNAAESDITSRDPKQVIGKNFFkDVAPC-TDSPeFYGKFKEGVAsgnlntmFEYTFDYQM-TPTKVKVHMKKALS--GDSYWVFVKRv-
472	1v9yA (103)<br>1mzuB (114)	N	13.59	TM-align: aliSize=98 (resi) RMSD=2.32 (Å)<br>---G--IFF-PALEQNMMGAVLINENDEVMFFNPAAEKLWGYKREEVIGNNID--MLIPrdLRPA-HPEYIRHNRE-------RELQLEKkDGSKIWTRFALSKVSAegKVYYLALVRD<br>irgTidGMGtAEFDALPVGAIQVDGSGVIHRYNRTESRLSGRIPERVIGRNFFteVAPC--TNIPaFSGRFMDGVTsgtldarFDFVFDF-QMAPVRVQIRMQNAGV--PDRYWIFVRK<br><br>SARST: aliSize=97 (resi) RMSD=3.81 (Å)<br>gif---------FPALEQNMMGAVLINENDEVMFFNPAAEKLWGYKREEVIGNNIDMLIPRDL--RPAHPEYIRHNRER------ELQLEKKDGSKIWTRFALSKVSAEgkvYYLALVRD<br>---irgtidgmgTAEFDALPVGAIQVDGSGVIHRYNRTESRLSGRIPERVIGRNFFTEVAPCTniPAFSGRFMDGVTSGtldarfDFVFDFQMAPVRVQIRMQNAGVPD---RYWIFVRK<br><br>BLAST: aliSize=18 (resi) iden=9.71% (10/103) simi=17.48% (18/103)<br>giffpaleqnm-----------------MGAVLINENDEVMFFNPAAEKLWGYKREEVIGNN---idmliprdlrpahpeyirhnrerelqlekkdgskiwtrfalskvsaegkvyylalvrd<br>-----------irgtidgmgtaefdalpVGAIQVDGSGVIHRYNRTESRLSGRIPERVIGRNfftevapctnipafsgrfmdgvtsgtldarfdfvfdfqmapvrvqirmqnagvpdrywifvrk--<br><br>Proposed: aliSize=98 (resi) RMSD=1.92 (Å)<br>--GIFFP----ALEQNMMGAVLINENDEVMFFNPAAEKLWGYKREEVIGNNID--MLIPrdLRPA-HPEYIRHNRE-------RELQLEKkDGSKIWTRFALSKVSAegKVYYLALVRDh<br>irGTIDGmgtaEFDALPVGAIQVDGSGVIHRYNRTESRLSGRIPERVIGRNFFteVAPC--TNIPaFSGRFMDGVTsgtldarFDFVFDF-QMAPVRVQIRMQNAGV--PDRYWIFVRK-
473	1v9yA (103)<br>1ot6A (125)	N	14.56	TM-align: aliSize=96 (resi) RMSD=2.05 (Å)<br>g-I--------------FF---PaLEQNMMGAVLINENDEVMFFNPAAEKLWGYKREEVIGNNID--MLIPrdLRPA-HPEYIRHNRE-------RELQLEKkDGSKIWTRFALSKVSAEgkVYYLALVRD-<br>-mEhvafgsedientlaKMddgQ-LDGLAFGAIQLDGDGNILQYNAAQGDITGRDPKQVIGKNFFkdVAPC--TDSPeFYGKFKEGVAsgnlntmFEYTFDY-QMTPTKVKVHMKKALSG--DSYWVFVKRv<br><br>SARST: aliSize=86 (resi) RMSD=13.34 (Å)<br>gif-------------------FPALEQNMMGAVLINENDEVMFFNPAAEKLWGYKREEVIGNNI-----DMLIPRDLRPAHPEYIrhnrerelqlekKDGSKIWTRFALSKVSAEGKVYYLALV---------------rd<br>---mehvafgsedientlakmdDGQLDGLAFGAIQLDGDGNILQYNAAQGDITGRDPKQVIGKNFfkdvaPCTDSPEFYGKFKEGV------------ASGNLNTMFEYTFDYQMTPTKVKVHMKkalsgdsywvfvkrv--<br><br>BLAST: aliSize=26 (resi) iden=12.62% (13/103) simi=25.24% (26/103)<br>giffpaleqnmm----------------------------GAVLINENDEVMFFNPAAEKLWGYKREEVIGNNIDmlipRDLRPA--HPEY--irhnrerelqlekkdgskiwtrfalskvsaegkvyylalvrd<br>------------mehvafgsedientlakmddgqldglafGAIQLDGDGNILQYNAAQGDITGRDPKQVIGKNFF----KDVAPCtdSPEFygkfkegvasgnlntmfeytfdyqmtptkvkvhmkkalsgdsywvfvkrv--<br><br>Proposed: aliSize=99 (resi) RMSD=2.03 (Å)<br>---G---------IFFPA----LEQNMMGAVLINENDEVMFFNPAAEKLWGYKREEVIGNNID-MLIPRdLRPA-HPEYIRHNR-E------RELQLEKkDGSKIWTRFALSKVSAegKVYYLALVRD-h<br>mehVafgsedienTLAKMddgqLDGLAFGAIQLDGDGNILQYNAAQGDITGRDPKQVIGKNFFkDVAPC-TDSPeFYGKFKEGVaSgnlntmFEYTFDY-QMTPTKVKVHMKKALS--GDSYWVFVKRv-

474	1v9yA<br>(103)<br><br>1uguA<br>(122)	N	14.56	TM-align: aliSize=97 (resi) RMSD=2.07 (Å)<br>g-------------IFF-PALEQNMMGAVLINENDEVMFFNPAAEKLWGYKREEVIGNNID--MLIPrdLRPA-HPEYIRHNRE-------RELQLEKKDgSKIWTRFALSKVSAEgKvYYLALVRD-<br>-vafgsedientlaKMDdGQLDGLAFGAIQLDGDGNILQYNAAQGDITGRDPKQVIGKNFFkdVAPC--TDSPeFYGKFKEGVAsgnlntmFEYTFDYQM-TPTKVKVHMKKALSG-D-SYWVFVKRv<br><br>SARST: aliSize=97 (resi) RMSD=5.09 (Å)<br>gif----------------FPALEQNMMGAVLINENDEVMFFNPAAEKLWGYKREEVIGNNI-----DMLIPRDLRPAHPEYIRHNRERELQLEKKDGSKIWTRFALSKVSA--EGKVYYLAL--vrd<br>---vafgsedientlakmdDGQLDGLAFGAIQLDGDGNILQYNAAQGDITGRDPKQVIGKNFfkdvaPCTDSPEFYGKFKEGVASGNLNTMFEYTFDYQMTPTKVKVHMKKAlsGDSYWVFVKrv---<br><br>BLAST: aliSize=26 (resi) iden=12.62% (13/103) simi=25.24% (26/103)<br>giffpaleqnmm-------------------------GAVLINENDEVMFFNPAAEKLWGYKREEVIGNNIDmlipRDLRPA--HPEY--irhnrerelqlekkdgskiwtrfalskvsaegkvyylalvrd<br>------------vafgsedientlakmddgqldglafGAIQLDGDGNILQYNAAQGDITGRDPKQVIGKNFF----KDVAPCtdSPEFygkfkegvasgnlntmfeytfdyqmtptkvkvhmkkalsgdsywvfvkrv--<br><br>Proposed: aliSize=99 (resi) RMSD=2.05 (Å)<br>----------GI-FFP--ALEQNMMGAVLINENDEVMFFNPAAEKLWGYKREEVIGNNID-MLIPRdLRPA-HPEYIRHNRE-------RELQLEKKDgSKIWTRFALSKVSAegKVYYLALVRD-h<br>vafgsedienTLaKMDdgQLDGLAFGAIQLDGDGNILQYNAAQGDITGRDPKQVIGKNFFkDVAPC-TDSPeFYGKFKEGVAsgnlntmFEYTFDYQM-TPTKVKVHMKKALS--GDSYWVFVKRv-
475	1v9yA<br>(103)<br><br>2d01A<br>(124)	N	15.53	TM-align: aliSize=97 (resi) RMSD=2.07 (Å)<br>g---------------IFF-PALEQNMMGAVLINENDEVMFFNPAAEKLWGYKREEVIGNNID--MLIPrdLRPA-HPEYIRHNRE-------RELQLEKKDgSKIWTRFALSKVSAEgKvYYLALVRD-<br>-ehvafgsedientlaKMDdGQLDGLAFGAIQLDGDGNILQYNAAEGDITGRDPKQVIGKNFFkdVAPC--TDSPeFYGKFKEGVAsgnlntmFEYTFDYQM-TPTKVKVHMKKALSG-D-SYWVFVKRv<br><br>SARST: aliSize=95 (resi) RMSD=4.91 (Å)<br>gif------------------FPALEQNMMGAVLINENDEVMFFNPAAEKLWGYKREEVIGNNI-----DMLIPRDLrpAHPEYIRHNRERELQL---EKKDG-SKIWTRFALSKVSAEGKVYYLAL--vrd<br>---ehvafgsedientlakmdDGQLDGLAFGAIQLDGDGNILQYNAAEGDITGRDPKQVIGKNFfkdvaPCTDSPEF--YGKFKEGVASGNLNTMfeyTFDYQmTPTKVKVHMKKALSGDSYWVFVKrv---<br><br>BLAST: aliSize=26 (resi) iden=12.62% (13/103) simi=25.24% (26/103)<br>giffpaleqnmm---------------------------GAVLINENDEVMFFNPAAEKLWGYKREEVIGNNIDmlipRDLRPA--HPEY--irhnrerelqlekkdgskiwtrfalskvsaegkvyylalvrd<br>------------ehvafgsedientlakmddgqldglafGAIQLDGDGNILQYNAAEGDITGRDPKQVIGKNFF----KDVAPCtdSPEFygkfkegvasgnlntmfeytfdyqmtptkvkvhmkkalsgdsywvfvkrv--<br><br>Proposed: aliSize=99 (resi) RMSD=1.99 (Å)<br>--------GIFFPA-------LEQNMMGAVLINENDEVMFFNPAAEKLWGYKREEVIGNNID-MLIPRdLRPA-HPEYIRHNRE-------RELQLEKKDgSKIWTRFALSKVSAegKVYYLALVRD-h<br>ehvafgseDIENTLakmddgqLDGLAFGAIQLDGDGNILQYNAAEGDITGRDPKQVIGKNFFkDVAPC-TDSPeFYGKFKEGVAsgnlntmFEYTFDYQM-TPTKVKVHMKKALS--GDSYWVFVKRv-
476	1v9yA<br>(103)<br><br>2qj7A<br>(123)	N	15.53	TM-align: aliSize=97 (resi) RMSD=2.12 (Å)<br>g--------------IFF-PALEQNMMGAVLINENDEVMFFNPAAEKLWGYKREEVIGNNID--MLIPrdLRPA-HPEYIRHNRE-------RELQLEKKDgSKIWTRFALSKVSAEgKvYYLALVRD-<br>-hvafgsedientlaKMDdGQLDGLAFGAIQLDGDGNILQYNAAEGDITGRDPKQVIGKNFFkdVAPC--TDSPeFYGKFKEGVAsgnlntmFEYTFDYQM-TPTKVKVHMKKALSG-D-SYWVFVKRv<br><br>SARST: aliSize=86 (resi) RMSD=13.33 (Å)<br>gif-----------------FPALEQNMMGAVLINENDEVMFFNPAAEKLWGYKREEVIGNNI-----DMLIPRDLRPAHPEYIrhnrerelqlekKDGSKIWTRFALSKVSAEGKVYYLALV---------------rd<br>---hvafgsedientlakmdDGQLDGLAFGAIQLDGDGNILQYNAAEGDITGRDPKQVIGKNFfkdvaPCTDSPEFYGKFKEGV------------ASGNLNTMFEYTFDYQMTPTKVKVHMKkalsgdsywvfvkrv--<br><br>BLAST: aliSize=26 (resi) iden=12.62% (13/103) simi=25.24% (26/103)<br>giffpaleqnmm--------------------------GAVLINENDEVMFFNPAAEKLWGYKREEVIGNNIDmlipRDLRPA--HPEY--irhnrerelqlekkdgskiwtrfalskvsaegkvyylalvrd<br>------------hvafgsedientlakmddgqldglafGAIQLDGDGNILQYNAAEGDITGRDPKQVIGKNFF----KDVAPCtdSPEFygkfkegvasgnlntmfeytfdyqmtptkvkvhmkkalsgdsywvfvkrv--<br><br>Proposed: aliSize=99 (resi) RMSD=2.14 (Å)<br>-G------IFF--P-----ALEQNMMGAVLINENDEVMFFNPAAEKLWGYKREEVIGNNID-MLIPRdLRPA-HPEYIRHNRE-------RELQLEKKDgSKIWTRFALSKVSAegKVYYLALVRD-h<br>hVafgsedIENtlAkmddgQLDGLAFGAIQLDGDGNILQYNAAEGDITGRDPKQVIGKNFFkDVAPC-TDSPeFYGKFKEGVAsgnlntmFEYTFDYQM-TPTKVKVHMKKALS--GDSYWVFVKRv-

477	1v9yA (103)<br>3phyA (125)	N	15.53	TM-align: aliSize=96 (resi) RMSD=2.48 (Å)<br>g------------IF---FP--ALEQNMMGAVLINENDEVMFFNPAAEKLWGYKREEVIGNNI-D-MLIPrdLRPA-HPeY-IRHNRE-------RELQLEKkDGSKIWTRFALSKVSAEgKvYYLALVRD-<br>-mehvafgsedieNTlakMDdgQLDGLAFGAIQLDGDGNILQYNAAEGDITGRDPKQVIGKNFfKdVAPC--TDSPeFY-GkFKEGVAsgnlntmFEYTFDY-QMTPTKVKVHMKKALSG-D-SYWVFVKRv<br><br>SARST: aliSize=60 (resi) RMSD=10.93 (Å)<br>giffpale------------------------QNMMGAVLINENDEVMFFNPAA----------------------------------EKLWGYKreevignnidmliprdlrpahpeyirhnrERELQLEKKDGSkiwTRFALSKVSA----EGKVYYLAL--vrd<br>--------mehvafgsedientlakmddgqldGLAFGAIQLDGDGNILQYNAAEgditgrdpkqvigknffkdvapctdspefygkfkeGVASGNL----------------------------NTMFEYTFDYQM---TPTKVKVHMKkalsGDSYWVFVKrv---<br><br>BLAST: aliSize=26 (resi) iden=12.62% (13/103) simi=25.24% (26/103)<br>giffpaleqnmm----------------------------GAVLINENDEVMFFNPAAEKLWGYKREEVIGNNIDmlipRDLRPA--HPEY---irhnrerelqlekkdgskiwtrfalskvsaegkvyylalvrd<br>------------mehvafgsedientlakmddgqldglafGAIQLDGDGNILQYNAAEGDITGRDPKQVIGKNFF----KDVAPCtdSPEFygkfkegvasgnlntmfeytfdyqmtptkvkvhmkkalsgdsywvfvkrv--<br><br>Proposed: aliSize=99 (resi) RMSD=2.29 (Å)<br>--------G-IFFPA-------LEQNMMGAVLINENDEVMFFNPAAEKLWGYKREEVIGNNID-MLIPRdLRPA-HPEYIRHNRE-------RELQLEKkDGSKIWTRFALSKVSAEgKvYYLALVRD-h<br>mehvafgsEdIENTLakmddgqLDGLAFGAIQLDGDGNILQYNAAEGDITGRDPKQVIGKNFFkDVAPC-TDSPeFYGKFKEGVAsgnlntmFEYTFDY-QMTPTKVKVHMKKALSG-D-SYWVFVKRv-
478	1wk4A (174)<br>1s3zB (145)	C	16.55	TM-align: aliSize=137 (resi) RMSD=1.99 (Å)<br>VRIRRAGLEDLPGVARVLVDTWratyrgvvpeafLeGLSYEGQAERWAQRLKTptWPGRLFVAESEsGEVVGFAAFGPD-RASG-FPGY-TAELWAIYVLPTWQRKGLGRALFHEGARLLQAEGYGR-LVWVLKENPKGRGFYEHLGGVLLGEREIElggaklwevaygfdlgghkw----<br>MDIRQMNKTHLEHWRGLRKQLW------------P-GHPDDAHLADGEEILQA--DHLASFIAMAD-GVAIGFADASIRhDYVNgCDSSpVVFLEGIFVLPSFRQRGVAKQLIAAVQRWGTNKGCREmASDTSPENTISQKVHQALGFEETERVIFY--------------------rkrc<br><br>SARST: aliSize=136 (resi) RMSD=3.06 (Å)<br>v-RIRRAGLEDLPGVARVLVDTwratyrgvvPEafleGLSYEGQAERWAQRLKTPTWpgRLFVAESEsGEVVGFAAFGPDRAsGFPGYTAE----LWAIYVLPTWQRKGLGRALFHEGARLLQAEGYGRLV-WVLKENPKGRGFYEHLGGVLLGEREIEL---ggaklwevaygfdlgghkw<br>-mDIRQMNKTHLEHWRGLRKQL---------WP----GHPDDAHLADGEEILQADHL--ASFIAMAD-GVAIGFADASIRHD-YVNGCDSSpvvfLEGIFVLPSFRQRGVAKQLIAAVQRWGTNKGCREMAsDTSPENTISQKVHQALGFEETERVIFYRkrc------------------<br><br>BLAST: aliSize=40 (resi) iden=14.48% (21/145) simi=27.59% (40/145)<br>vrirragledlpgvarvlvdtwratyrgvvpeafleglsyegqaerwaqrlktptwpgrl--FVAESEsGEVVGFA--AFGPDRASGFPGYTAE-LWAIYVLPTWQRKGLGRALFHEGARLLQAEGYGRLVW-VLKENPKGRGFYEH<br>--mdirqmnkthlehwrglrkqlwpghpddahladgeeilqadhlasFIAMAD-GVAIGFAdaSIRHDYVNGCDSSPVVfLEGIFVLPSFRQRGVAKQLIAAVQRWGTNKGCREMASdTSPENTISQKVHQA<br>LG--------------gvllgereielggaklwevaygfdlgghkw<br>LGfeetervifyrkrc---------------------------------<br><br>Proposed: aliSize=142 (resi) RMSD=1.85 (Å)<br>VRIRRAGLEDLPGVARVLVDTWRatyrgvvpeafLegLSYEGQAERWAQRLKTptWPGRLFVAESEsGEVVGFAAFGPD-RASG-FPGYTA-ELWAIYVLPTWQRKGLGRALFHEGARLLQAEGYGRMLVWVLKENPKGRGFYEHLGGVLLGEREielggaklwEVAYGF-dlgghkw<br>MDIRQMNKTHLEHWRGLRKQLWP-----------G--HPDDAHLADGEEILQA--DHLASFIAMAD-GVAIGFADASIRhDYVNgCDSSPVvFLEGIFVLPSFRQRGVAKQLIAAVQRWGTNKGCREMASDTSPENTISQKVHQALGFEETERVI---------FYRKRCc-------
479	1wk4A (174)<br>1ufhA (155)	C	10.32	TM-align: aliSize=142 (resi) RMSD=3.86 (Å)<br>VRI-RRAGLEDLPGVARVLVDtWRa-TYRGVV---PEA-FLEGLSye-GQAERWAQRLKTptWPGRLFVAESESGEVVGFAAFGPDrasgf---PGYTAELWAIYVLPTWQRKGLGRALFHEGARLLQAEGYGR-LVWVLKENPKGRGFYEHLGGVLLGEREielggaKLwevaygfdlgghkw--<br>TIMlTPMQTEEFRSYLTYTTK-HY-aEEKVKAgtwLPEdAQLLSK--qVFTDLLPRGLET--PHHHLWSLKLNEKDIVGWLWIHAE-----pehPQQEAFIYDFGLYEPYRGKGYAKQALAALDQAARSMGIRKlSLHVFAHNQTARKLYEQTGFQETDVVM------SK--------------kl<br><br>SARST: aliSize=135 (resi) RMSD=5.30 (Å)<br>vrirrag-----------LEDLPGVARVLVDTWRATYrGVVPEAFLeglsYEGQAERWAQRlkTPT----WPGRLFVAESESGEVVGFAAFGPDRA--SGFpgytAELWAIYVLPTWQRKGLGRALFHEGARLLQAEGYGRLV-WVLKENPKGRGFYEHLGGVLLGEREIE--lggaklwevaygfdlgghkw<br>-------timltpmqteeFRSYLTYTTKHYAEEKVKA-GTWLPEDA----QLLSKQVFTDL--LPRgletPHHHLWSLKLNEKDIVGWLWIHAEPEhpQQE----AFIYDFGLYEPYRGKGYAKQALAALDQAARSMGIRKLSlHVFAHNQTARKLYEQTGFQETDVVMSKkl--------------------<br><br>BLAST: aliSize=24 (resi) iden=9.68% (15/155) simi=15.48% (24/155)<br>vrirragledlpgvarvlvdtwratyrgvvpeafleglsyegqaerwaqrlktptwpgrlfvaesesgevvgfaafgpdrasgfpgytaelwaiyvlptwqrkglgralfh-----------------------------------EGARLLQAEGYGRLVWVLKENPkgrgfYEHLGGVLLGEREIeLG<br>--timltpmqteefrsyltyttkhyaeekvkagtwlpEDAQLLSKQVFTDLLPRGLETP-----HHHLWSLKLNEKDI-VG<br>GakLW---evaygfdlgghkw<br>W--LWihaepehpqqeafiydfglyepyrgkgyakqalaaldqaarsmgirklslhvfahnqtarklyeqtgfqetdvvmskkl-------------<br><br>Proposed: aliSize=137 (resi) RMSD=2.96 (Å)<br>v-RI-RRAGLEDLPGVARVLVDTW-RatY-RGVV--PEAFLEG-LS--yegQaeR--WAQRLKTptWPGRLFVAE---SEsgeVVGFAAFGP---D-rasgfpGYTAELWAIYVLPTWQRKGLGRALFHEGARLLQAEGYGRMLVWVLKENPKGRGFYEHLGGVLLGEREIELGgaklwevaygfdlgghkw<br>-tIMlTPMQTEEFRSYLTYTTKHYaE--EkVKAGtwLPEDAQLlSKqv---F--TdlLPRGLET--PHHHLWSLKlneKD---IVGWLWIHAepeHp------QQEAFIYDFGLYEPYRGKGYAKQALAALDQAARSMGIRKLSLHVFAHNQTARKLYEQTGFQETDVVMSKKL-----------------

480	1wk4A (174)<br>2d4pA (130)	C	16.92	TM-align: aliSize=124 (resi) RMSD=2.89 (Å)<br>VRIRRAGLEDLPGVARVLVdtwratyrgvvpeafLegLsYegQAERWAQRLKtptWPGRLFVAESEsGEVVGFAAFGPDRasGf-PGYTAELWAIYVLptwQRkGLGRALFHEGARLLQAEGYGR-LVWVLKeNPK-GRGFYEHLGGV-L-LGEREIELGGaklwevaygfdlgghkw-<br>MRFRPFTEEDLDRLNRLAG---------------K--R-P--VSLGALRFFA---RTGHSFLAEEG-EEPMGFALAQAVW--Q-gEATTVLVTRIEGR---SV-EALRGLLRAVVKSAYDAGVYEvALHLDP-ERKeLEEALKAEGFAlGpLVLAVRVLGS----------------r<br><br>SARST: aliSize=121 (resi) RMSD=5.34 (Å)<br>v-RIRRAGLEDLPGVARvlvdtwratYRGVVPeaflegLSYEGQAERWAQrlktptwpGRLFVAESEsGEVVGFAAFGPDRAsGFPGYtAELWAIYV-LPTwqrkgLGRALFHEGARLLQAEGYG-RLVWVLKENPKGRGFYEHLGgVLLGEREIEL------ggaklwevaygfdlgghkw<br>-mRFRPFTEEDLDRLNR---------LAGKRP------VSLGALRFFART--------GHSFLAEEG-EEPMGFALAQAVWQ-GEATT-VLVTRIEGrSVE-----ALRGLLRAVVKSAYDAGVYeVALHLDPERKELEEALKAEG-FALGPLVLAVrvlgsr-------------------<br><br>BLAST: aliSize=35 (resi) iden=20.00% (26/130) simi=26.92% (35/130)<br>vrirragledlpgvarvlvdtwratyrgvvpeafleg--------------------------------------LSYEGQaERWAQRLKTPTWPGR---LFVAESESGEVVgfAAFGPDRASGFPGYTAELW--AIYVLPtwQRKGLGRALFHEGARL-------------lqaegygrlvwvlkenpk<br>-------------------------------------mrfrpfteedldrlnrlagkrpvslgalrffartghsfLAEEGE-EPMGFALAQAVWQGEattVLVTRIEGRSVE--ALRGLLRAVVKSAYDAGVYevALHLDP--ERKELEEALKAEGFALgplvlavrvlgsr------------------<br><br>grgfyehlggvllgereielggaklwevaygfdlgghkw<br>---------------------------------------<br><br>Proposed: aliSize=123 (resi) RMSD=2.25 (Å)<br>VRIRRAGLEDLPGVARVLVdtwratyrgvvpeafLegLSyegQAERWAQR-LktptWPGRLFVAESEsGEVVGFAAFGPD-rasgfP--GYTAELWAIYVLptwQRkGLGRALFHEGARLLQAEGYGRMLVWVLKeNPK-GRGFYEHLGGV-L-LGEREielggaklWEVAYGfdlgghkw<br>MRFRPFTEEDLDRLNRLAG---------------K--RP---VSLGALRFfA----RTGHSFLAEEG-EEPMGFALAQAVw-----QgeATTVLVTRIEGR---SV-EALRGLLRAVVKSAYDAGVYEVALHLDP-ERKeLEEALKAEGFAlGpLVLAV--------RVLGSR--------
481	1wk4A (174)<br>2f5iA (167)	C	14.97	TM-align: aliSize=142 (resi) RMSD=2.47 (Å)<br>-VRIRRAGLEDLPGVARVLVDTWRaTYrgVVPeaFleGLSyeGQAERWAQRLKTPTWPGRLFVAESES--G-----EVVGFAAFGPDRasGFPG-YTAELWAIYVLPTWQRKGLGRALFHEGARLLQAEGYGR-LVWVLKENPKGRGFYEHLGGVLLG----EREIElggaklwevaygfdlgghkw---<br>kFVIRPATAADCSDILRLIKELAK-YE--YME--E--QVI--LTEKDLLEDGFGEHPFYHCLVAEVPKehWtpeghSIVGFAMYYFTY--DPWIgKLLYLEDFFVMSDYRGFGIGSEILKNLSQVAMRCRCSSmHFLVAEWNEPSINFYKRRGASDLSseegWRLFK--------------------idk<br><br>--------<br>eyllkmat<br><br>SARST: aliSize=130 (resi) RMSD=3.93 (Å)<br>v--RIRRAGLEDLPGVARVLVDtwRATYRGV--VPeafleGLSYEGQAERWAqrlkTPTWPGRLFVAE------SESGE-VVGFAAFGPD---RAsgfpGYTAELWAIYVLPTWQRKGLGRALFHEGARLLQAEGYGRLV-WVLKENPKGRGFYEHLGGVL---------------------lgereie<br>-kfVIRPATAADCSDILRLIKE--LAKYEYMeeQV-----ILTEKDLLEDGF----GEHPFYHCLVAEvpkehwTPEGHsIVGFAMYYFTydpWI----GKLLYLEDFFVMSDYRGFGIGSEILKNLSQVAMRCRCSSMHfLVAEWNEPSINFYKRRGASDlsseegwrlfkidkeyllkmat-------<br><br>lggaklwevaygfdlgghkw<br>--------------------<br><br>BLAST: aliSize=56 (resi) iden=17.96% (30/167) simi=33.53% (56/167)<br>vr---IRRAGLEDLPGVARVLVDTwrATYRGVVPEAFL--EGLSYEGQAERWAQRLKTPTWPGRLFVAESESgeVVGFAAFgPDRASGFPGYTAELWAIYVLPTWQRKGLGRALFHEGARLLQAEGYGRLVWVLKE-NPKGRGFYEHLGGVLLGERE-----------------ielggaklwevaygfd<br>--kfvIRPATAADCSDILRLIKEL--AKYEYMEEQVILteKDLLEDGFGEHPFYHCLVAEVPKEHWTPEGHS--IVGFAMY-YFTYDPWIGKLLYLEDFFVMSDYRGFGIGSEILKNLSQVAMRCRCSSMHFLVAEwNEPSINFYKRRGASDLSSEEgwrlfkidkeyllkmat---------------<br><br>lgghkw<br>------<br><br>Proposed: aliSize=152 (resi) RMSD=2.17 (Å)<br>-VRIRRAGLEDLPGVARVLVDTWRAtYRgvVpEaFleGLSyeGQAERWAQRLKTPTW-pGRLFVAESES-------GEVVGFAAFGPDRasgF-PG-YTAELWAIYVLPTWQRKGLGRALFHEGARLLQAEGYGRMLVWVLKENPKGRGFYEHLGGVLLGerEielggaKLWEVAYGFDlGGH--KW--<br>kFVIRPATAADCSDILRLIKELAKY-EY--M-E-E--QVI--LTEKDLLEDGFGEHPf-YHCLVAEVPKehwtpegHSIVGFAMYYFTY---DpWIgKLLYLEDFFVMSDYRGFGIGSEILKNLSQVAMRCRCSSMHFLVAEWNEPSINFYKRRGASDLS--S------EEGWRLFKID-KEYllKMat
482	1wk4A (174)<br>2fe7B (166)	C	24.10	TM-align: aliSize=141 (resi) RMSD=2.54 (Å)<br>----------VRIRRAGLEDLPGVARVLVDTWRaTYRgvVpeafLEGLSyEGQAERWAQRLKTPtWPGRLFVAESEsGEVVGFAAFGPDRasGFPG--YTAELWAIYVLPTWQRKGLGRALFHEGARLLQAEGYGR-LVWVLKENPKGRGFYEHLGGVLLG-EREIElggaklwevaygfdlgghkw---<br>enlyfqghmtLEIRPAVPADAEQILAFIIELAD-YER--A----RHEVV-TDVEGIRRSLFAEG-SPTRALMCLSE-GRPIGYAVFFYSY--STWLgrNGIYLEDLYVTPEYRGVGAGRRLLRELAREAVANDCGRlEWSVLDWNQPAIDFYRSIGALPQDeWVRYR--------------------ldg<br><br>--------<br>ealrkmae<br><br>SARST: aliSize=133 (resi) RMSD=3.59 (Å)<br>v-----------RIRRAGLEDLPGVARVLVDTWRATyrGVvpeAFLEGLSYEGQAERwaqrLKTPTWPGRLFVAESESGEvVGFAAFGP--DRASGFPGytAELWAIYVLPTWQRKGLGRALFHEGARLLQAEGYGRLV-WVLKENPKGRGFYEHLGGVL-------------------lgereielgga<br>-enlyfqghmtlEIRPAVPADAEQILAFIIELADYE--RA---RHEVVTDVEGIRRS----LFAEGSPTRALMCLSEGRP-IGYAVFFYsySTWLGRNG--IYLEDLYVTPEYRGVGAGRRLLRELAREAVANDCGRLEwSVLDWNQPAIDFYRSIGALPqdewvryrldgealrkmae----------<br><br>klwevaygfdlgghkw<br>----------------<br><br>BLAST: aliSize=47 (resi) iden=21.08% (35/166) simi=28.31% (47/166)<br>vrirragledlpgvarvlvdtwratyrgvvpeaflegl syegqaerwaqrlktptw--PGRLFVAESEsGEVVGFAAFGPDRASGFPGYTAELWAIYVLPTWQRKGLGRALFHEGARLLQAEGYGRLVW-VLKEN<br>--enlyfqghmtleirpavpadaeqilafiieladyerarhevvtdvegirrslfaegsPTRALMCLSE-GRPIGYAVFFYSYSTWLGRNGIYLEDLYVTPEYRGVGAGRRLLRELAREAVANDCGRLEWsVLDWN<br><br>PKGRGFYEHLGGVLLGE-REIELGGAKLWEVA-ygfdlgghkw<br>QPAIDFYRSIGALPQDEwVRYRLDGEALRKMAe----------<br><br>Proposed: aliSize=148 (resi) RMSD=2.13 (Å)<br>----------VRIRRAGLEDLPGVARVLVDTWRaTYRgvVpeafLEGLSyeGQAERWAQRLKTPTWPGRLFVAESEsGEVVGFAAFGPD-rasgF-PG--YTAELWAIYVLPTWQRKGLGRALFHEGARLLQAEGYGRMLVWVLKENPKGRGFYEHLGGVLLGereielggaklWEVAYGFDlGGH--KW--<br>enlyfqghmtLEIRPAVPADAEQILAFIIELAD-YER--A----RHEVV--TDVEGIRRSLFAEGSPTRALMCLSE-GRPIGYAVFFYSy----StWLgrNGIYLEDLYVTPEYRGVGAGRRLLRELAREAVANDCGRLEWSVLDWNQPAIDFYRSIGALPQD----------EWVRYRLD-GEAlrKMae

483	1wk4A (174)<br>2g3tA (169)	C	13.61	TM-align: aliSize=142 (resi) RMSD=2.46 (Å)<br>-VRIRRAGLEDLPGVARVLVDTWRAtYRgvVPeaFleGLSyeGQAERWAQRLKTPTWPGRLFVAESES--G-----EVVGFAAFGPDRasGFPG-YTAELWAIYVLPTWQRKGLGRALFHEGARLLQAEGYGR-LVWVLKENPKGRGFYEHLGGVLLG----EREIElggaklwevaygfdlgghkw---<br>kFVIRPATAADCSDILRLIKELAKY-EY--ME--E--QVI--LTEKDLLEDGFGEHPFYHCLVAEVPKehWtpeghSIVGFAMYYFTY--DPWIgKLLYLEDFFVMSDYRGFGIGSEILKNLSQVAMRCRCSSmHFLVAEWNEPSINFYKRRGASDLSseegWRLFK--------------------idk<br>----------<br>eyllkmatee<br><br>SARST: aliSize=133 (resi) RMSD=4.08 (Å)<br>v--RIRRAGLEDLPGVARVLVDTWRATYRGVVPeafleGLSYEGQAERWAqrlkTPTWPGRLFVAE------SESGE-VVGFAAFGPD---RASGFpgytAELWAIYVLPTWQRKGLGRALFHEGARLLQAEGYGRLV-WVLKENPKGRGFYEHLGGVLL----------------------gereiel<br>-kfVIRPATAADCSDILRLIKELAKYEYMEEQV-----ILTEKDLLEDGF----GEHPFYHCLVAEvpkehwTPEGHsIVGFAMYYFTydpWIGKL----LYLEDFFVMSDYRGFGIGSEILKNLSQVAMRCRCSSMHfLVAEWNEPSINFYKRRGASDLsseegwrlfkidkeyllkmatee-------<br>ggaklwevaygfdlgghkw<br>-------------------<br><br>BLAST: aliSize=56 (resi) iden=17.75% (30/169) simi=33.14% (56/169)<br>vr---IRRAGLEDLPGVARVLVDTwrATYRGVVPEAFL--EGLSYEGQAERWAQRLKTPTWPGRLFVAESESgeVVGFAAFgPDRASGFPGYTAELWAIYVLPTWQRKGLGRALFHEGARLLQAEGYGRLVWVLKE-NPKGRGFYEHLGGVLLGERE-----------------ielggaklwevayg<br>--kfvIRPATAADCSDILRLIKEL--AKYEYMEEQVILteKDLLEDGFGEHPFYHCLVAEVPKEHWTPEGHS--IVGFAMY-YFTYDPWIGKLLYLEDFFVMSDYRGFGIGSEILKNLSQVAMRCRCSSMHFLVAEwNEPSINFYKRRGASDLSSEEgwrlfkidkeyllkmatee-------------<br>fdlgghkw<br>--------<br><br>Proposed: aliSize=154 (resi) RMSD=2.30 (Å)<br>-VRIRRAGLEDLPGVARVLVDTWRAtYRgvVpEaFleGLSyeGQAERWAQRLKTPTWPGRLFVAESES--G-----EVVGFAAFGPD-rasgF-PG-YTAELWAIYVLPTWQRKGLGRALFHEGARLLQAEGYGRMLVWVLKENPKGRGFYEHLGGVLLGereiElgGAKLWEVAY---GFD-LGgHKW<br>kFVIRPATAADCSDILRLIKELAKY-EY--M-E-E--QVI--LTEKDLLEDGFGEHPFYHCLVAEVPKehWtpeghSIVGFAMYYFTy----DpWIgKLLYLEDFFVMSDYRGFGIGSEILKNLSQVAMRCRCSSMHFLVAEWNEPSINFYKRRGASDLS----S--EEGWRLFKIdkeYLLkMA-TEE
484	1wk4A (174)<br>2g3tB (168)	C	13.69	TM-align: aliSize=142 (resi) RMSD=2.41 (Å)<br>-VRIRRAGLEDLPGVARVLVDTWRAtYRgvVPeaFleGLSyeGQAERWAQRLKTPTWPGRLFVAESES-------GEVVGFAAFGPDRasGFPG-YTAELWAIYVLPTWQRKGLGRALFHEGARLLQAEGYGR-LVWVLKENPKGRGFYEHLGGVLLG----EREIElggaklwevaygfdlgghkw---<br>kFVIRPATAADCSDILRLIKELAKY-EY--ME--E--QVI--LTEKDLLEDGFGEHPFYHCLVAEVPKehwtpegHSIVGFAMYYFTY--DPWIgKLLYLEDFFVMSDYRGFGIGSEILKNLSQVAMRCRCSSmHFLVAEWNEPSINFYKRRGASDLSseegWRLFK--------------------idk<br>---------<br>eyllkmate<br><br>SARST: aliSize=132 (resi) RMSD=4.06 (Å)<br>v--RIRRAGLEDLPGVARVLVDTWRATYRGVVPeafleGLSYEGQAERWAqrlkTPTWPGRLFVAE------SESGE-VVGFAAFGPD---RASGFpgytAELWAIYVLPTWQRKGLGRALFHEGARLLQAEGYGRLV-WVLKENPKGRGFYEHLGGVL----------------------lgereiel<br>-kfVIRPATAADCSDILRLIKELAKYEYMEEQV-----ILTEKDLLEDGF----GEHPFYHCLVAEvpkehwTPEGHsIVGFAMYYFTydpWIGKL----LYLEDFFVMSDYRGFGIGSEILKNLSQVAMRCRCSSMHfLVAEWNEPSINFYKRRGASDlsseegwrlfkidkeyllkmate--------<br>ggaklwevaygfdlgghkw<br>-------------------<br><br>BLAST: aliSize=56 (resi) iden=17.86% (30/168) simi=33.33% (56/168)<br>vr---IRRAGLEDLPGVARVLVDTwrATYRGVVPEAFL--EGLSYEGQAERWAQRLKTPTWPGRLFVAESESgeVVGFAAFgPDRASGFPGYTAELWAIYVLPTWQRKGLGRALFHEGARLLQAEGYGRLVWVLKE-NPKGRGFYEHLGGVLLGERE-----------------ielggaklwevaygf<br>--kfvIRPATAADCSDILRLIKEL--AKYEYMEEQVILteKDLLEDGFGEHPFYHCLVAEVPKEHWTPEGHS--IVGFAMY-YFTYDPWIGKLLYLEDFFVMSDYRGFGIGSEILKNLSQVAMRCRCSSMHFLVAEwNEPSINFYKRRGASDLSSEEgwrlfkidkeyllkmate--------------<br>dlgghkw<br>-------<br><br>Proposed: aliSize=152 (resi) RMSD=2.22 (Å)<br>-VRIRRAGLEDLPGVARVLVDTWRAtYRgvVpEaFleGLSyeGQAERWAQRLKTPTW-pGRLFVAESES-------GEVVGFAAFGPDRasgF-PG-YTAELWAIYVLPTWQRKGLGRALFHEGARLLQAEGYGRMLVWVLKENPKGRGFYEHLGGVLLGereiElgGAKLWEVAYG---FD--LggHkW<br>kFVIRPATAADCSDILRLIKELAKY-EY--M-E-E--QVI--LTEKDLLEDGFGEHPf-YHCLVAEVPKehwtpegHSIVGFAMYYFTY---DpWIgKLLYLEDFFVMSDYRGFGIGSEILKNLSQVAMRCRCSSMHFLVAEWNEPSINFYKRRGASDLS----S--EEGWRLFKIDkeyLLkmA--T-E
485	1wk4A (174)<br>2jevA (169)	C	14.79	TM-align: aliSize=142 (resi) RMSD=2.52 (Å)<br>--VRIRRAGLEDLPGVARVLVDTWRAtYRgvVPeaFleGLSyeGQAERWAQRLKTPTWPGRLFVAESES--G-----EVVGFAAFGPDRasGFPG-YTAELWAIYVLPTWQRKGLGRALFHEGARLLQAEGYGR-LVWVLKENPKGRGFYEHLGGVLLG----EREIElggaklwevaygfdlgghkw--<br>akFVIRPATAADCSDILRLIKELAKY-EY--ME--E--QVI--LTEKDLLEDGFGEHPFYHCLVAEVPKehWtpeghSIVGFAMYYFTY--DPWIgKLLYLEDFFVMSDYRGFGIGSEILKNLSQVAMRCRCSSmHFLVAEWNEPSINFYKRRGASDLSseegWRLFK--------------------id<br>----------<br>keyllkmate<br><br>SARST: aliSize=133 (resi) RMSD=4.93 (Å)<br>v---RIRRAGLEDLPGVARVLVDTWRATYRGVVPeafleGLSYEGQAERWAqrlkTPTWPGRLFVAE------SESGE-VVGFAAFGPD---RASGFPGYTAElwaiYVLPTWQRKGLGRALFHEGARLLQAEGYGRL-VWVLKENPKGRGFYEHLGGVLL----------------------gereiel<br>-akfVIRPATAADCSDILRLIKELAKYEYMEEQV-----ILTEKDLLEDGF----GEHPFYHCLVAEvpkehwTPEGHsIVGFAMYYFTydpWIGKLLYLEDF----FVMSDYRGFGIGSEILKNLSQVAMRCRCSSMhFLVAEWNEPSINFYKRRGASDLsseegwrlfkidkeyllkmate-------<br>ggaklwevaygfdlgghkw<br>-------------------<br><br>BLAST: aliSize=56 (resi) iden=17.75% (30/169) simi=33.14% (56/169)<br>vr----IRRAGLEDLPGVARVLVDTwrATYRGVVPEAFL--EGLSYEGQAERWAQRLKTPTWPGRLFVAESESgeVVGFAAFgPDRASGFPGYTAELWAIYVLPTWQRKGLGRALFHEGARLLQAEGYGRLVWVLKE-NPKGRGFYEHLGGVLLGERE-----------------ielggaklwevayg<br>--akfvIRPATAADCSDILRLIKEL--AKYEYMEEQVILteKDLLEDGFGEHPFYHCLVAEVPKEHWTPEGHS--IVGFAMY-YFTYDPWIGKLLYLEDFFVMSDYRGFGIGSEILKNLSQVAMRCRCSSMHFLVAEwNEPSINFYKRRGASDLSSEEgwrlfkidkeyllkmate-------------<br>fdlgghkw<br>--------<br><br>Proposed: aliSize=152 (resi) RMSD=2.21 (Å)<br>--VRIRRAGLEDLPGVARVLVDTWRAtYRgvVpEaFleGLSyeGQAERWAQRLKTPTW-pGRLFVAESES--G-----EVVGFAAFGPDRasgF-PG-YTAELWAIYVLPTWQRKGLGRALFHEGARLLQAEGYGRMLVWVLKENPKGRGFYEHLGGVLLGerEielggaKLWEVAYGFDlGGH--KW---<br>akFVIRPATAADCSDILRLIKELAKY-EY--M-E-E--QVI--LTEKDLLEDGFGEHPf-YHCLVAEVPKehWtpeghSIVGFAMYYFTY---DpWIgKLLYLEDFFVMSDYRGFGIGSEILKNLSQVAMRCRCSSMHFLVAEWNEPSINFYKRRGASDLS--S------EEGWRLFKID-KEYllKMate

486	1wk4A (174)<br>2vbqA (147)	C	17.01	TM-align: aliSize=137 (resi) RMSD=2.14 (Å)<br>--VRIRRAGLEDLPGVARVLVDTWratyrgvvpeafLeGLSyEGQAERWAQRLKTPtWPGRLFVAESEsGEVVGFAAFGPD-RASG-FPGY-TAELWAIYVLPTWQRKGLGRALFHEGARLLQAEGYGR-LVWVLKENPKGRGFYEHLGGVLLGEREIElggaklwevaygfdlgghkw----<br>shMDIRQMNKTHLEHWRGLRKQLW------------P-GHP-DDAHLADGEEILQA-DHLASFIAMAD-GVAIGFADASIRhDYVNgCDSSpVVFLEGIFVLPSFRQRGVAKQLIAAVQRWGTNKGCREmASDTSPENTISQKVHQALGFEETERVIFY-------------------rkrc<br><br>SARST: aliSize=136 (resi) RMSD=4.58 (Å)<br>v---RIRRAGLEDLPGVARVLvdtwrATYRGVVPEAFLeglsYEGQAERwaqrlktPTWPGRLFVAESESGEVVGFAAFGPDRAsGFPGYTAE----LWAIYVLPTWQRKGLGRALFHEGARLLQAEGYGRLV-WVLKENPKGRGFYEHLGGVLLGEREIEL---ggaklwevaygfdlgghkw<br>-shmDIRQMNKTHLEHWRGLR-----KQLWPGHPDDAH----LADGEEI-------LQADHLASFIAMADGVAIGFADASIRHD-YVNGCDSSpvvfLEGIFVLPSFRQRGVAKQLIAAVQRWGTNKGCREMAsDTSPENTISQKVHQALGFEETERVIFYRkrc------------------<br><br>BLAST: aliSize=40 (resi) iden=14.29% (21/147) simi=27.21% (40/147)<br>vrirragledlpgvarvlvdtwratyrgvvpeafleglsyegqaerwaqrlktptwpgrl---FVAESEsGEVVGFA--AFGPDRASGFPGYTAE-LWAIYVLPTWQRKGLGRALFHEGARLLQAEGYGRLVW-VLKENPKGRGFY<br>--shmdirqmnkthlehwrglrkqlwpghpddahladgeeilqadhlasFIAMAD-GVAIGFAdaSIRHDYVNGCDSSPVVfLEGIFVLPSFRQRGVAKQLIAAVQRWGTNKGCREMASdTSPENTISQKVH<br><br>EHLG--------------gvllgereielggaklwevaygfdlgghkw<br>QALGfeetervifyrkrc------------------------------<br><br>Proposed: aliSize=142 (resi) RMSD=2.01 (Å)<br>--VRIRRAGLEDLPGVARVLVDTWratyrgvvpeafLeGLSYEGQAERWAQRLKTptWPGRLFVAESEsGEVVGFAAFGPD-RASG-FPGY-TAELWAIYVLPTWQRKGLGRALFHEGARLLQAEGYGRMLVWVLKENPKGRGFYEHLGGVLLGEREielggaklwevaYGFdlggHKW<br>shMDIRQMNKTHLEHWRGLRKQLW------------P-GHPDDAHLADGEEILQA--DHLASFIAMAD-GVAIGFADASIRhDYVNgCDSSpVVFLEGIFVLPSFRQRGVAKQLIAAVQRWGTNKGCREMASDTSPENTISQKVHQALGFEETERVI------------FYR----KRC
487	1wk4A (174)<br>2vbqB (144)	C	16.67	TM-align: aliSize=136 (resi) RMSD=2.03 (Å)<br>vRIRRAGLEDLPGVARVLVDTWratyrgvvpeafLeGLSyEGQAERWAQRLKTPtWPGRLFVAESEsGEVVGFAAFGPD-RASG-FPGY-TAELWAIYVLPTWQRKGLGRALFHEGARLLQAEGYGR-LVWVLKENPKGRGFYEHLGGVLLGEREIElggaklwevaygfdlgghkw----<br>-DIRQMNKTHLEHWRGLRKQLW------------P-GHP-DDAHLADGEEILQA-DHLASFIAMAD-GVAIGFADASIRhDYVNgCDSSpVVFLEGIFVLPSFRQRGVAKQLIAAVQRWGTNKGCREmASDTSPENTISQKVHQALGFEETERVIFY-------------------rkrc<br><br>SARST: aliSize=135 (resi) RMSD=3.12 (Å)<br>vr-IRRAGLEDLPGVARVLVDTwratyrgvvPEAflegLSYEGQAERWAQRLKTPTWpgRLFVAESEsGEVVGFAAFGPDRAsGFPGYTAE----LWAIYVLPTWQRKGLGRALFHEGARLLQAEGYGRLV-WVLKENPKGRGFYEHLGGVLLGEREIEL---ggaklwevaygfdlgghkw<br>--dIRQMNKTHLEHWRGLRKQL---------WPG----HPDDAHLADGEEILQADHL--ASFIAMAD-GVAIGFADASIRHD-YVNGCDSSpvvfLEGIFVLPSFRQRGVAKQLIAAVQRWGTNKGCREMAsDTSPENTISQKVHQALGFEETERVIFYRkrc------------------<br><br>BLAST: aliSize=40 (resi) iden=14.58% (21/144) simi=27.78% (40/144)<br>vrirragledlpgvarvlvdtwratyrgvvpeafleglsyegqaerwaqrlktptwpgrl--FVAESEsGEVVGFA--AFGPDRASGFPGYTAE-LWAIYVLPTWQRKGLGRALFHEGARLLQAEGYGRLVW-VLKENPKGRGFYEHL<br>--dirqmnkthlehwrglrkqlwpghpddahladgeeilqadhlasFIAMAD-GVAIGFAdaSIRHDYVNGCDSSPVVfLEGIFVLPSFRQRGVAKQLIAAVQRWGTNKGCREMASdTSPENTISQKVHQAL<br><br>G--------------gvllgereielggaklwevaygfdlgghkw<br>Gfeetervifyrkrc------------------------------<br><br>Proposed: aliSize=141 (resi) RMSD=1.86 (Å)<br>vRIRRAGLEDLPGVARVLVDTWRatyrgvvpeafLegLSYEGQAERWAQRLKTptWPGRLFVAESEsGEVVGFAAFGPD-RASG-FPGYTA-ELWAIYVLPTWQRKGLGRALFHEGARLLQAEGYGRMLVWVLKENPKGRGFYEHLGGVLLGEREielggaklWEVAYGfdlgghkw<br>-DIRQMNKTHLEHWRGLRKQLWP-----------G--HPDDAHLADGEEILQA--DHLASFIAMAD-GVAIGFADASIRhDYVNgCDSSPVvFLEGIFVLPSFRQRGVAKQLIAAVQRWGTNKGCREMASDTSPENTISQKVHQALGFEETERVI--------FYRKRC--------
488	1wwaX (105)<br>2dm2A (110)	N	18.10	TM-align: aliSize=82 (resi) RMSD=2.37 (Å)<br>vsfpasvqlhtave----------------------MHHWCIPFSVDGQPAPSLRWLFNGSVLNE-TSFIFTEFLEPaanetvRHGCLRLNQPTHVNNGNYTLLAANPFGQASASIMAAfMDNPFEfn-----<br>--------------gssgssgapffemklkhykifeGMPVTFTCRVAGNPKPKIYWFKDGKQISPkSDHYTIQRDLD------GTCSLHTTASTLDDDGNYTIMAANPQGRISCTGRLM-VQAVNS--gpssg<br><br>SARST: aliSize=91 (resi) RMSD=13.32 (Å)<br>vsf-------PASV----QLHTAVEMHHWCIPFSVDGQPAPSLRWLFNGSVLN-ETSFIFTEFLePAAnetvrHGCLRLNQPTHVNNGNYTLLAANPFGQASASIMAA---FMDN----pfefn<br>---gssgssgAPFFemklKHYKIFEGMPVTFTCRVAGNPKPKIYWFKDGKQISpKSDHYTIQRD-LDG-----TCSLHTTASTLDDDGNYTIMAANPQGRISCTGRLMvqaVNSGpssg-----<br><br>BLAST: aliSize=36 (resi) iden=20.95% (22/105) simi=34.29% (36/105)<br>vsfpasvqlhtavemhhwcipfs-------------------------------VDGQPAPSLRWLFNGSVLNETSFIFTefLEPAANETvrhgC-LRLNQPTHVNNGNYTLLAANPFGQASAS---------------imaafmdnpfefn<br>-----------------------gssgssgapffemklkhykifegmpvtftcrVAGNPKPKIYWFKDGKQISPKSDHYT--IQRDLDGT----CsLHTTASTLDDDGNYTIMAANPQGRISCTgrlmvqavnsgpssg-------------<br><br>Proposed: aliSize=90 (resi) RMSD=1.94 (Å)<br>v---SFP-ASVQ--LHTAVE------mhhWCIPFSVDGQPAPSLRWLFNGSVLNE-TSFIFTEFLEPaanetvRHGCLRLNQPTHVNNGNYTLLAANPFGQASASIMAAfMDN--P-----fefn<br>-gssGSSgAPFFemKLKHYKifegmp---VTFTCRVAGNPKPKIYWFKDGKQISPkSDHYTIQRDLD------GTCSLHTTASTLDDDGNYTIMAANPQGRISCTGRLM-VQAvnSgpssg----

489	1wwaX (105)<br>2i26O (117)	N	9.52	TM-align: aliSize=82 (resi) RMSD=2.49 (Å)<br>vsfpasvqlhtave-------------MHHWCIPFSVD--GQPAPSLRWLFN------GSVLNETSFIFTEFLEPaanetVRHGCLRLNQPTHVNNGNYTLLAANP-------FG--Q-ASA-SIMAAfMDNPFefn---<br>--------------rvdqtpqtitketGESLTINCVLRdsNCALSSTYWYRKksgstnEESISKGGRYVETVNSG-----SKSFSLRINDLTVEDSGTYRCKPESRygsydaeCAalNdQYGgGTVVT-VNAAA---hhh<br><br>SARST: aliSize=78 (resi) RMSD=13.44 (Å)<br>v---SFPASVQLHTAVEMHHWCIPF-SVDGQPAPSLRWLFNG----SVLNETSFIFTEFLepaaNETVRHGCLRLnQPTHVNNGNYTLLAAN-------------------------------pfgqasasimaafmdnpfefn<br>-rvdQTPQTITKETGESLTINCVLRdSNCALSSTYWYRKKSGstneESISKGGRYVETVN----SGSKSFSLRIN-DLTVEDSGTYRCKPESrygsydaecaalndqyggg tvvtvnaaahhh--------------------<br><br>BLAST: aliSize=27 (resi) iden=17.14% (18/105) simi=25.71% (27/105)<br>vsfpasvqlhtavemhhwcipfsvdgqpapslrwlf---------------------------------------NGSVlNETSFI-FTEFLEpAANETVRHGCLRLNQPTHVNNGNYTLLAANPFGQASASiMAAFMD-----------------npfefn<br>-----------------------------------rvdqtpqtitketgesltincvlrdsncalsstywyrkkSGST-NEESISkGGRYVE-TVNSGSKSFSLRINDLTVEDSGTYRCKPESRYGSYDAE-CAALNDqyggg tvvtvnaaahhh------<br><br>Proposed: aliSize=92 (resi) RMSD=2.18 (Å)<br>vsfPASVQ-LHTAVE-MHHWCIPFSV--DGQPAPSLRWLFN------GSVLNETSFIFTEFL-ePaanetVRHGCLRLNQPTHVNNGNYTLLAANP-------FG--Q-ASASIMAAFMDnPF----efn<br>---RVDQTpQTITKEtGESLTINCVLrdSNCALSSTYWYRKksgstnEESISKGGRYVETVNs-G-----SKSFSLRINDLTVEDSGTYRCKPESRygsydaeCAalNdQYGGGTVVTVN-AAahhh---
490	1wwaX (105)<br>2i26P (114)	N	8.57	TM-align: aliSize=74 (resi) RMSD=3.86 (Å)<br>vsfpasvqlhtavem------------HHWCIPFSVDG------Q-PAPSLRWLfnG-S--VLNETsf--IFTEFLePAANETVRHGCLRLnqPthvNNGNYTLLAAN----------------PFGQASASIMAAFmDNpfefn<br>---------------rvdqtpqtitkeTGESLTINCVLrdsncaLsSTYWYRKK--SgStnEESIS--kgGRYVET-VNSGSKSFSLRIND--L---TVEDSGTYRCKpesrygsydaecaalnDQYGGGTVVTVNA-AA-----<br><br>SARST: aliSize=78 (resi) RMSD=13.35 (Å)<br>v---SFPASVQLHT-AVEMHHWCIPFSVDGQPAPSLRWLFNG----SVLNETSFIFTEFLEPAAnetvrHGCLRLNQPTHVNNGNYTLLAAN----------------------------pfgqasasimaafmdnpfefn<br>-rvdQTPQTITKETgESLTINCVLRDSNCALSSTYWYRKKSGstneESISKGGRYVETVNSGSK-----SFSLRINDLTVEDSGTYRCKPESrygsydaecaalndqyggg tvvtvnaaa--------------------<br><br>BLAST: aliSize=27 (resi) iden=17.14% (18/105) simi=25.71% (27/105)<br>vsfpasvqlhtavemhhwcipfsvdgqpapslrwlf---------------------------------------NGSVlNETSFI-FTEFLEpAANETVRHGCLRLNQPTHVNNGNYTLLAANPFGQASASiMAAFMD--------------npfefn<br>-----------------------------------rvdqtpqtitketgesltincvlrdsncalsstywyrkkSGST-NEESISkGGRYVE-TVNSGSKSFSLRINDLTVEDSGTYRCKPESRYGSYDAE-CAALNDqyggg tvvtvnaaa------<br><br>Proposed: aliSize=90 (resi) RMSD=2.23 (Å)<br>vSFPAS-VQLHTAVeM--hhWCIPFSV--DGQPAPSLRWLFN------GSVLNETSFIFTEFL-ePaanetVRHGCLRLNQPTHVNNGNYTLLAANP--------FG-Q-ASA-SIMAAfMDnPF-efn<br>-RVDQTpQTITKET-Ges--LTINCVLrdSNCALSSTYWYRKksgstnEESISKGGRYVETVNs-G-----SKSFSLRINDLTVEDSGTYRCKPESRygsydaecAAlNdQYGgGTVVT-VN-AAa---
491	1wwbX (103)<br>1hngA (175)	N	10.68	TM-align: aliSize=76 (resi) RMSD=2.45 (Å)<br>vhfaptitflespts---------DHHWCIPF--TVKGNPKPALOWFYNGAILN---------E-SKYICTKIhvtnhteyHGCLOLDNPTHMNNGDYTLIAKNEY-GK-DEKQISAHFMGwp-----------------------Gid---<br>---------------dsgtvwgalGHGINLNIpnFQMTDDIDEVRWERGSTLVAefkrkmkpfLkSGAFEILA--------NGDLKIKNLTRDDSGTYNVTVYSTNgTRiLNKALDLRILE--mvskpmiywecsnatltcevlegT--dvelklyqgkehlrslrqktmsyqwtnlrapfkckavnrvs<br><br>------------<br>qesemevvncpe<br><br>SARST: aliSize=81 (resi) RMSD=17.35 (Å)<br>vhfaptitflespts---DHHWCIPFTVKGnPKPALQWFY--NGAILNESKYIC-TKIHVTNHTEYHGCL---QLDNPTHMNNGDYTLIAKNEYGKDEKOISahfm<br>---------------dsgtvwgalghginlnipnfqmtddidevrwergstlvaefkrkmkpflksgafeilangdlkiknltrddsgtynvtvystngtriLNKALDLRILEM-VSKPMIYWEcsNATLTCEVLEGTdVELKLYQGKEHLRSLrqkTMSYQWTNLRAPFKCKAVNRVSQESEMEV----<br><br>GWPG-id<br>VNCPe--<br><br>BLAST: aliSize=23 (resi) iden=10.68% (11/103) simi=22.33% (23/103)<br>vhfaptitflesptsdhhwcipftvkgnpkpa-----------------------------LOWFYNGAILNESKYICTKIHVTNHTEY--HGCLOLDNPTHMNNGDYTLIAKNEYG--<br>--------------------------------dsgtvwgalghginlnipnfqmtddideVRWERGSTLVAEFKRKMKPFLKSGAFEIlaNGDLKIKNLTRDDSGTYNVTVYSTNGtrilnkaldlrilemvskpmiywecsnatltcevlegtdvelklyqgkehlrslrqktmsyqwtnlrapfkcka<br><br>-----------------kdekqisahfmgwpgid<br>vnrvsqesemevvncpe-----------------<br><br>Proposed: aliSize=84 (resi) RMSD=2.15 (Å)<br>vhFAPTItFLEsPTsdHHWCIPF--TVKGNPKPALOWFYNGAILN---------E-SKYICTKIhvtnhteYhGCLOLDNPTHMNNGDYTLIAKNEY-GK-DEKQISAHFMG---w<br>--DSGTV-WGA-LG--HGINLNIpnFQMTDDIDEVRWERGSTLVAefkrkmkpfLkSGAFEILA-------N-GDLKIKNLTRDDSGTYNVTVYSTNgTRiLNKALDLRILEmvskpmiywecsnatltcevlegtdvelklyqgkehlrslrqktmsyqwtnlrapfkckavnrvsqesemevvncpe-<br><br>pgid<br>----

492	1wwbX (103)<br>1ie5A (107)	N	13.59	TM-align: aliSize=78 (resi) RMSD=2.40 (Å)<br>vhfaptitflespt--------------------------SDHHWCIPFTVKGNPKPALQWFYNGAILN---ESKYICTKIhvtnhteYHGCLQLDNPTHMNNGDYTLIAKNEYGKDEKQISAHfMGWpGid<br>--------------gkdiqvivnvppsvrarqstmnatanLSQSVTLACDADGFPEPTMTWTKDGEPIEqedNEEKYSFNY-------DGSELIIKKVDKSDEAEYICIAENKAGEQDATIHLK-VFA-K--<br><br>SARST: aliSize=78 (resi) RMSD=16.18 (Å)<br>vhfaptit-------FLESPTSDH-------------HWCIPFTVKGNPKPALQWFYNGAILNesKYIctkihvTNHTEYHGC----LQLDNPTHMNNGDYTLIAKNEYGKDEKQISA-----hfmgwpgid<br>--------gkdiqviVNVPPSVRArqstmnatanlsqSVTLACDADGFPEPTMTWTKDGEPIE--QED------NEEKYSFNYdgseLIIKKVDKSDEAEYICIAENKAGEQDATIHLkvfak---------<br><br>BLAST: aliSize=29 (resi) iden=17.48% (18/103) simi=28.16% (29/103)<br>vhfaptitflesptsdhhwcipftvk--------------------------------------GNPKPALQWFYNGAIL----NESKYictkihvtnHTEYHGC-LQLDNPTHMNNGDYTLIAKNEYGKDEKQI-------sahfmgwpgid<br>--------------------------gkdiqvivnvppsvrarqstmnatanlsqsvtlacdadGFPEPTMTWTKDGEPIeqedNEEKY---------SFNYDGSeLIIKKVDKSDEAEYICIAENKAGEQDATIhlkvfak-----------<br><br>Proposed: aliSize=90 (resi) RMSD=2.04 (Å)<br>--V-----HFAPTItFL-ESPTSD-----HHWCIPFTVKGNPKPALQWFYNGAILN---ESKYICTKIhvtnhteYHGCLQLDNPTHMNNGDYTLIAKNEYGKDEKQISAHfMGW-pgid<br>gkDiqvivNVPPSV-RArQSTMNAtanlsQSVTLACDADGFPEPTMTWTKDGEPIEqedNEEKYSFNY-------DGSELIIKKVDKSDEAEYICIAENKAGEQDATIHLK-VFAk----
493	1wwbX (103)<br>1kxqE (120)	N	9.71	TM-align: aliSize=77 (resi) RMSD=2.40 (Å)<br>vhfaptitflespts-------------DHHWCIPFTV-KGnpKPALQWFYN-----GAILN----------E----SKYICTKIHVTnhtEYHGCLQLDNPTHMNNGDYTLIAKNEy--------GKDEK-QISAHfMGwPgid<br>---------------qvqlvesgggsvqaGGSLSLSCAAsTY--TDTVGWFRQapgkeREGVAaiyrrtgytySadsvKGRFTLSQDNN---KNTVYLQMNSLKPEDTGIYYCATGNS-vrlaswegYFYWGqGTQVT-VS-S---<br><br>SARST: aliSize=81 (resi) RMSD=17.34 (Å)<br>v--HFAPTITFLESP-TSDHHWCIP------FTVKGNPKPAL----QWFY--NGAILNESKYICTKIHVTNHTEYHGCLQLDNPTHMNNGDYTLIAK-----------------------neygkdekqisahfmgwpgid<br>-qvQLVESGGGSVQAgGSLSLSCAAstytdtVGWFRQAPGKEregvAAIYrrTGYTYSADSVKGRFTLSQDNNKNTVYLQMNSLKPEDTGIYYCATGnsvrlaswegyfywgqgtqvtvss---------------------<br><br>BLAST: aliSize=8 (resi) iden=6.80% (7/103) simi=7.77% (8/103)<br>vhfaptitflesptsdhhwc--IPFTVKGNPKPALQW---FYNG---------ailneskyictkihvtnhteyhgclqldnpthmnngdytliakneygkde<br>-------------------qvqlvesgggsvqaggslslscaastytdtvgwfrqapgkeregvaaiyrrtgytysadsvkgrftlsqdnnkntvylqmnslkpedtgIYYCATGNSVRLASWegyFYWGqgtqvtvss--<br><br>kqisahfmgwpgid<br>--------------<br><br>Proposed: aliSize=88 (resi) RMSD=2.06 (Å)<br>vHFAPTITFLESPTSD--hhWCIPFTV-KGnpKPALQWFYN-----GAILN----------E----SKYICTKIhVTNhtEYHGCLQLDNPTHMNNGDYTLIAKN----------eygKDEKQISAHFMGwPgid<br>-QVQLVESGGGSVQAGgs--LSLSCAAsTY--TDTVGWFRQapgkeREGVAaiyrrtgytySadsvKGRFTLSQ-DNN--KNTVYLQMNSLKPEDTGIYYCATGNsvrlaswegy---FYWGQGTQVTVS-S---
494	1wwbX (103)<br>1sq2N (112)	N	7.77	TM-align: aliSize=79 (resi) RMSD=2.55 (Å)<br>vhfaptitflespts-------------DHHWCIPFTVK-G-NPKPALQWFYN------GAILNESKYICTKIhvTNHtEYHGCLQLDNPTHMNNGDYTLIAKNE-----------YGKDEK-QISAHfMGwpgid<br>---------------rvdqtprsvtketGESLTINCVLRdAsYALGSTCWYRKksgegnEESISKGGRYVETV--NSG-SKSFSLRINDLTVEDGGTYRCGLGVAggycdyalcssRYAECGdGTAVT-VN-----<br><br>SARST: aliSize=84 (resi) RMSD=8.09 (Å)<br>vhfaptit--------FLESP-TSDHHWCIPFTVKGNPKPALQWFYNG-----AILNESKYICTKIhvTNHTeYHGCLQLDNPTHMNNGDYTLIAKN------------EYGKDE-KQISAH-fmgwpgid<br>--------rvdqtprsVTKETgESLTINCVLRDASYALGSTCWYRKKSgegneESISKGGRYVETV--NSGS-KSFSLRINDLTVEDGGTYRCGLGVaggycdyalcssRYAECGdGTAVTVn--------<br><br>BLAST: aliSize=2 (resi) iden=1.94% (2/103) simi=1.94% (2/103)<br>vhfaptitflesptsdhhwcipftvkgnpkpalqwfyngail---------------------------------------NE---skyictkihvtnhteyhgclqldnpthmnngdytli<br>--rvdqtprsvtketgesltincvlrdasyalgstcwyrkksgegNEesiskggryvetvnsgsksfslrindltvedggtyrcglgvaggycdyalcssryaecgdgtavtvn-----------------------------------<br><br>akneygkdekqisahfmgwpgid<br>-----------------------<br><br>Proposed: aliSize=87 (resi) RMSD=2.29 (Å)<br>vHFAPTITFLES----ptsdhHWCIPFTVK-G-NPKPALQWFYN------GAILNESKYICTKIhVTNhtEYHGCLQLDNPTHMNNGDYTLIAKN-------------eyGKDEKQISAHFMGwpgid<br>-RVDQTPRSVTKetge-----SLTINCVLRdAsYALGSTCWYRKksgegnEESISKGGRYVETV-NSG--SKSFSLRINDLTVEDGGTYRCGLGVaggycdyalcssr--YAECGDGTAVTVN-----

495	1wwbX (103)<br>2aw2A (110)	N	13.59	TM-align: aliSize=84 (resi) RMSD=2.54 (Å)<br>vhfaptitflespts-----------------DHHWCIPFTVKG-NPKPALQWFYN---GAILNESkYiCTKIHVTNHtEYHGCLQLDNPTHMNNGDYTLIAKNEYGKDEK-QISAHFMGW-PGId---<br>---------------cdvqlyikrqsehsilaGDPFELECPVKYcANRPHVTWCKLngtTCVKLED-R-QTSWKEEKN-ISFFILHFEPVLPNDNGSYRCSANFQSNLIEShSTTLYVTDVkHHH-hhh<br><br>SARST: aliSize=90 (resi) RMSD=12.71 (Å)<br>vhf-------APTITFLESPTSDHHWCIPFTVKGNPKPALQWFYNGAILNESKYICTKIhVTNHTEYHGCLQLDNPTHMNNGDYTLIAKNEYGKDEKQISA-------------hfmgwpgid<br>---cdvqlyiKRQSEHSILAGDPFELECPVKYCANRPHVTWCKLNGTTCVKLEDRQTSW-KEEKNISFFILHFEPVLPNDNGSYRCSANFQSNLIESHSTTlyvtdvkhhhhhh---------<br><br>BLAST: aliSize=15 (resi) iden=7.77% (8/103) simi=14.56% (15/103)<br>vhfaptitflesptsdhhwcipftvkgnpkpalqwfyngailnes----------------------------KYICTKIHVTnHTEYHG--CLQLDN--pthmnngdytliakneygkdekqisahfmgwpgid<br>--cdvqlyikrqsehsilagdpfelecpvKYCANRPHVT-WCKLNGttCVKLEDrqtswkeeknisffilhfepvlpndngsyrcsanfqsnlieshsttlyvtdvkhhhhhh-----------------------------------<br><br>Proposed: aliSize=97 (resi) RMSD=2.25 (Å)<br>VHFAPTIT--FLESPTSDHHWCIPFTVKG-NPKPALQWFYN---GAILNESkYiCTKIHVTNHtEYHGCLQLDNPTHMNNGDYTLIAKNEYGKDEK-QISAHfMGW--PG----id<br>CDVQLYIKrqSEHSILAGDPFELECPVKYcANRPHVTWCKLngtTCVKLED-R-QTSWKEEKN-ISFFILHFEPVLPNDNGSYRCSANFQSNLIEShSTTLY-VTDvkHHhhhh--
496	1wwbX (103)<br>2aw2X (107)	N	13.59	TM-align: aliSize=81 (resi) RMSD=2.27 (Å)<br>vhfaptitflesptsDHHWCIPFTVKG-NPKPALQWFYN---GAILNESkyICTKIHVTNHtEYHGCLQLDNPTHMNNGDYTLIAKNEYGKDEK-QISAHfMGWPgid----<br>---------------cdvqlyikrqsehsilaGDPFELECPVKYcANRPHVTWCKLngtTCVKLED--RQTSWKEEKN-ISFFILHFEPVLPNDNGSYRCSANFQSNLIEShSTTLY-VTDV---khhh<br><br>SARST: aliSize=64 (resi) RMSD=9.47 (Å)<br>vhfaptitflesptsdhhwcip-FTVKGNPKPALQWFYNGailNESKYICTKIhvTNHTEYHGCLQLDN-----PTHMNNGDYTLIAKneyGKDEKQISA------------------------------------hfmgwpgid<br>---------------------cDVQLYIKRQSEHSILAG---DPFELECPVK--YCANRPHVTWCKLNgttcvKLEDRQTSWKEEKN---ISFFILHFEpvlpndngsyrcsanfqsnlieshsttlyvtdvkhhh---------<br><br>BLAST: aliSize=15 (resi) iden=7.77% (8/103) simi=14.56% (15/103)<br>vhfaptitflesptsdhhwcipftvkgnpkpalqwfyngailnes----------------------------KYICTKIHVTnHTEYHG--CLQLDN--pthmnngdytliakneygkdekqisahfmgwpgid<br>--cdvqlyikrqsehsilagdpfelecpvKYCANRPHVT-WCKLNGttCVKLEDrqtswkeeknisffilhfepvlpndngsyrcsanfqsnlieshsttlyvtdvkhhh-----------------------------------<br><br>Proposed: aliSize=95 (resi) RMSD=2.14 (Å)<br>VHFAPTIT--FLESPTSDHHWCIPFTVKG-NPKPALQWFYN---GAILNESkyICTKIHVTNHtEYHGCLQLDNPTHMNNGDYTLIAKNEYGKDEK-QISAHfMGwP-----gid<br>CDVQLYIKrqSEHSILAGDPFELECPVKYcANRPHVTWCKLngtTCVKLED--RQTSWKEEKN-ISFFILHFEPVLPNDNGSYRCSANFQSNLIEShSTTLY-VT-Dvkhhh---
497	1wwbX (103)<br>2cdeA (192)	N	6.80	TM-align: aliSize=84 (resi) RMSD=2.49 (Å)<br>vhfaptitflespts--------------DHHWCIPFTVKGNPKPALQWFYN----GAILNE----------SKYICTKIhVTnHtEYHGCLQLDNPTHMNNGDYTLIAKNE----YGKDEK-QISAHfMGW---P<br>--------------nqveqspqsliileGKNCTLQCNYTVSPFSNLRWYKQdtgrGPVSLTimtfsentksNGRYTATL-DA-D-TKQSSLHITASQLSDSASYICVVSDRgstlGRLYFGrGTQLT-VWPdiqkpdpavyqlrdskssdksvclftdfdsqtnvsqskdsdvyitdktvldmrsmdfK<br>GID-------------------<br>SNSavawsnksdfacanafnn<br><br>SARST: aliSize=85 (resi) RMSD=12.79 (Å)<br>vhfaptitflesptsd-HHWCIPFTVKGNPKPALQWFY-----------------NGAILN---ESK--YICTKIHVTN---HTEYHGCLQLDNPTHMNNGDYTLIAKN---EYGKDEKQ--ISAHFMGWPG--<br>---------------nQVEQSPQSLIILEGKNCTLQCnytvspfsnlrwykqdtGRGPVSltiMTFseNTKSNGRYTAtldADTKQSSLHITASQLSDSASYICVVSDrgsTLGRLYFGrgTQLTVWPDIQkpdpavyqlrdskssdksvclftdfdsqtnvsqskdsdvyitdktvldmrsmdfksns<br>------------------id<br>avawsnksdfacanafnn--<br><br>BLAST: aliSize=17 (resi) iden=11.65% (12/103) simi=16.50% (17/103)<br>vhfaptitfle-------------------------SPTSDHHWCIPFTVKGNPKPALQWFYNGAILNeSKYICTKIHVTNHTEYH---<br>-----------nqveqspqsliilegknctlqcnytvSPFSNLRWYKQDTGRGPVSLTIMTFSENTKSN-GRYTATLDADTKQSSLHitasqlsdsasyicvvsdrgstlgrlyfgrgtqltvwpdiqkpdpavyqlrdskssdksvclftdfdsqtnvsqskdsdvyitdktvldmrsmdfksnsavaw<br>--------------gclqldnpthmnngdytliakneygkdekqisahfmgwpgid<br>snksdfacanafnn--<br><br>Proposed: aliSize=97 (resi) RMSD=2.42 (Å)<br>vHFAPTITFLESPTSD-hHWCIPFTVKGNPKPALQWFY-N---GAILNE----------SKYICTKIhVTNhtEYHGCLQLDNPTHMNNGDYTLIAKN-E---YGKDEK-QISAHfMGWPG-----------------------ID--<br>-NQVEQSPQSLIILEGk-NCTLQCNYTVSPFSNLRWYKqDtgrGPVSLTimtfsentksNGRYTATL-DAD--TKQSSLHITASQLSDSASYICVVSDrGstlGRLYFGrGTQLT-VWPDIqkpdpavyqlrdskssdksvclfTDfdsqtnvsqskdsdvyitdktvldmrsmdfksnsavawsnksdf<br>--------<br>acanafnn

498	1wwbX (103)<br>2i26P (114)	N	6.80	TM-align: aliSize=83 (resi) RMSD=2.56 (Å)<br>vhfaptitflespts-------------DHHWCIPFTV--KGNPKPALQWFYN------GAILNESKYICTKIhVTnHtEYHGCLQLDNPTHMNNGDYTLIAKNE-------YG--K-DEKQISAHFMGwPGId<br>---------------rvdqtpqtitketGESLTINCVLrdSNCALSSTYWYRKksgstnEESISKGGRYVETV-NS-G-SKSFSLRINDLTVEDSGTYRCKPESRygsydaeCAalNdQYGGGTVVTVN-AAA-<br><br>SARST: aliSize=75 (resi) RMSD=16.28 (Å)<br>v---HFAPTITFLESPTSDHHWCIP--------FTVKGNPKPALQWFyngaILNESKYICTKihVTNHTeYHGCLQLDNPTHMNNGDYTLIAKN---------------------------eygkdekqisahfmgwpgid<br>-rvdQTPQTITKETGESLTINCVLRdsncalssTYWYRKKSGSTNEE----SISKGGRYVET--VNSGS-KSFSLRINDLTVEDSGTYRCKPESrygsydaecaalndqygggtvvtvnaaa-------------------<br><br>BLAST: aliSize=24 (resi) iden=10.68% (11/103) simi=23.30% (24/103)<br>vhfaptitflesptsdhhwcipftvkgnpkpalq----------------------------------WFYN-GAILNESKYI--CTKIHVTNHTEYHGCLQLDNPTHMNNGDYTLIAKNEYGKDEKQISA-----------------hfmgwpgid<br>----------------------------------rvdqtpqtitketgesltincvlrdsncalsstyWYRKkSGSTNEESISkgGRYVETVNSGSKSFSLRINDLTVEDSGTYRCKPESRYGSYDAECAAlndqygggtvvtvnaaa---------<br><br>Proposed: aliSize=93 (resi) RMSD=2.21 (Å)<br>vhfAPTITFLESPTSDH--hWCIPFTV--KGNPKPALQWFYN------GAILNESKYICTKIhVTNhtEYHGCLQLDNPTHMNNGDYTLIAKNE-------YG--K-DEKQISAHFMGwPG-id<br>---RVDQTPQTITKETGes-LTINCVLrdSNCALSSTYWYRKksgstnEESISKGGRYVETV-NSG--SKSFSLRINDLTVEDSGTYRCKPESRygsydaeCAalNdQYGGGTVVTVN-AAa--
499	1wwbX (103)<br>2rheA (114)	N	10.68	TM-align: aliSize=81 (resi) RMSD=2.44 (Å)<br>vhfaptitflespts--------------DHHWCIPFTVK-GN-PKPALQWFYN-----GAILN-----E----SKYICTKIHvTnhteYHGCLQLDNPTHMNNGDYTLIAKNE--YGKDEK-QISAHfMGWPGId<br>---------------esvltqppsasgtpGQRVTISCTGSaTDiGSNSVIWYQQvpgkaPKLLIyyndlLpsgvSDRFSASKS-G----TSASLAISGLESEDEADYYCAAWNDslDEPGFGgGTKLT-VLGQPK-<br><br>SARST: aliSize=89 (resi) RMSD=15.69 (Å)<br>v----HFAPTITFLESPTSDHHWC---------IPFTVKGNPKPALQW-FYNG--AILNESKYICTKihVTNHteyHGCLQLDNPTHMNNGDYTLIAKNEY--GKDE--KQISAH-----fmgwpgid<br>-esvlTQPPSASGTPGQRVTISCTgsatdigsnSVIWYQQVPGKAPKLlIYYNdlLPSGVSDRFSAS--KSGT---SASLAISGLESEDEADYYCAAWNDSldEPGFggGTKLTVlgqpk--------<br><br>BLAST: aliSize=5 (resi) iden=4.85% (5/103) simi=4.85% (5/103)<br>vhfaptitflesptsdhhwcipf---TVKGNPKpalqwfyngailneskyictkihvtnhteyhgclqldnpthmnngdytliakn<br>-----------------------esvltqppsasgtpgqrvtisctgsatdigsnsviwyqqvpgkapklliyyndllpsgvsdrfsasksgtsaslaisglesedeadyycaawndsldepgfgggtklTVLGQPK---<br>eygkdekqisahfmgwpgid<br>--------------------<br><br>Proposed: aliSize=91 (resi) RMSD=2.24 (Å)<br>vh--FAPTITFLESP--tsdHHWCIPFTVKGN--PKPALQWFYNG---AILNE-----S-----KYICTKIhVTnhteYHGCLQLDNPTHMNNGDYTLIAKNE--YGKDEK-QISAHFMGWPGid<br>--esVLTQPPSASGTpg---QRVTISCTGSATdiGSNSVIWYQQVpgkaPKLLIyyndlLpsgvsDRFSASK-SG----TSASLAISGLESEDEADYYCAAWNDslDEPGFGgGTKLTVLGQPK--
500	1wwbX (103)<br>2ywzA (111)	N	8.74	TM-align: aliSize=79 (resi) RMSD=2.14 (Å)<br>vhfaptitflespts--------------DHHWCIPFTVK-G-NPKPALQWFYN------GAILNESKYICTKIhVTNhtEYHGCLQLDNPTHMNNGDYTLIAKNEY---------GKDEK-QISAHfMGwpgid<br>---------------awvdqtprtitketGESLTIKCVLKdHsCGLSSTTWYRTqlgstnEKTISIGGRYDETV-DKG--SKSFSLRISDLRVEDSGTYKCQADYSPscysypsleSAVEGaGTVLT-VK-----<br><br>SARST: aliSize=77 (resi) RMSD=13.18 (Å)<br>v----HFAPTitFLESP-TSDHHWCIPFTVKGNPKPALQWFYN-G----AILNESKYICTKIhVTNHTEyhGCLQLDNPTHMNNGDYTLIAKN------------------------eygkdekqisahfmgwpgid<br>-awvdQTPRT--ITKETgESLTIKCVLKDHSCGLSSTTWYRTQlGstneKTISIGGRYDETV-DKGSKS--FSLRISDLRVEDSGTYKCQADYspscysypslesavegagtvltvk--------------------<br><br>BLAST: aliSize=8 (resi) iden=4.85% (5/103) simi=7.77% (8/103)<br>vhfaptitflesptsdhhwcipftvkgnpkpalqwfyngailneskyictkihvtnhteyhgclqldnpthmnngdytlia-------------------------------------KNEYGK-DEKQIS---<br>---awvdqtprtitketgesltikcvlkdhscglssttwyRTQLGStNEKTISiggrydetvdkgsksfslrisdlrvedsgtykcqadyspscysypslesavegagtvlt<br>--ahfmgwpgid<br>vk----------<br><br>Proposed: aliSize=90 (resi) RMSD=2.15 (Å)<br>vHFAPTIT-FLESPTS--dhhWCIPFTVK-G-NPKPALQWFYN------GAILNESKYICTKIhVTNhtEYHGCLQLDNPTHMNNGDYTLIAKNEY---------GKDEK-QISAHfMGwpgid<br>-AWVDQTPrTITKETGes---LTIKCVLKdHsCGLSSTTWYRTqlgstnEKTISIGGRYDETV-DKG--SKSFSLRISDLRVEDSGTYKCQADYSPscysypsleSAVEGaGTVLT-VK-----

501	1wwbX (103)<br>3darA (99)	N	16.16	TM-align: aliSize=75 (resi) RMSD=2.08 (Å)<br>vhfaptitflespts---------------------DHHWCIPFTVKGNPKPALQWFYNGAILNES---KYICTKIHvtnhteyHGCLQLDNPTHMNNGDYTLIAKNEYGKDEKQISAHfMGwpgid<br>---------------krapywtntekmekrlhavpaANTVKFRCPAGGNPMPTMRWLKNGKEFKQEhriGGYKVRNQ-------HWSLIMESVVPSDKGNYTCVVENEYGSINHTYHLD-VV-----<br><br>SARST: aliSize=87 (resi) RMSD=15.88 (Å)<br>vh-FAPTITF-------LESPTSDHHWCIPFTVKGNPKPALQWFYNGAILN--ESKYICTKIHvtNHTeyhgCLQLDNPTHMNNGDYTLIAKNEYGKDEKQISAH--fmgwpgid<br>--kRAPYWTNtekmekrLHAVPAANTVKFRCPAGGNPMPTMRWLKNGKEFKqeHRIGGYKVRN--QHW----SLIMESVVPSDKGNYTCVVENEYGSINHTYHLDvv--------<br><br>BLAST: aliSize=29 (resi) iden=19.19% (19/99) simi=29.29% (29/99)<br>vhfaptitflesptsdhhwcipftvk--------------------------------GNPKPALQWFYNGAILNESKYIcTKIHVTNHteyHGCLQLDNPTHMNNGDYTLIAKNEYG-----------kdekqisahfmgwpgid<br>-------------------------krapywtntekmekrlhavpaantvkfrcpagGNPMPTMRWLKNGKEFKQEHRI-GGYKVRNQ---HWSLIMESVVPSDKGNYTCVVENEYGsinhtyhldvv-----------------<br><br>Proposed: aliSize=88 (resi) RMSD=2.00 (Å)<br>v-HFAPT--IT-FLE-SPTSDH--HWCIPFTVKGNPKPALQWFYNGAILNeS----KYICTKIHvtnhteyHGCLQLDNPTHMNNGDYTLIAKNEYGKDEKQISAHfMGwpgid<br>-kRAPYWtnTEkMEKrLHAVPAanTVKFRCPAGGNPMPTMRWLKNGKEFK-QehriGGYKVRNQ-------HWSLIMESVVPSDKGNYTCVVENEYGSINHTYHLD-VV-----
502	1wwcA (105)<br>1a6wL (109)	N	5.71	TM-align: aliSize=76 (resi) RMSD=2.50 (Å)<br>tvyyypprvvsleepelr---------------LEHCIEFVV-R--GNPPPTLHWLHN-----GQPL----------RESkIIHVEYYQegeiSEGCLLFNKPTHYNNGNYTLIAKNPLGTANQ-TINGHFLKepfpvde<br>-----------------avvtqesalttspgETVTLTCRSsTgaVTTSNYANWVQEkpdhlFTGLiggtnnrapgVPA-RFSGSLIG----NKAALTITGAQTEDEAIYFCALWYSNHWVFGgGTKLTVLE-------<br><br>SARST: aliSize=82 (resi) RMSD=12.06 (Å)<br>tvyyypprvv----------SLEEPELRLEHCI----------EFVVRGNPPPTLHW-LHNGQPLRE--SKIIHVEYyqEGEisEGCLLFNKPTHYNNGNYTLIAKNPLGTANQ---TINGH-flkepfpvde<br>---------avvtqesaltTSPGETVTLTCRSstgavttsnyANWVQEKPDHLFTGlIGGTNNRAPgvPARFSGSL--IGN--KAALTITGAQTEDEAIYFCALWYSNHWVFGggtKLTVLe----------<br><br>BLAST: aliSize=2 (resi) iden=1.90% (2/105) simi=1.90% (2/105)<br>tvyyypprvvsleepelrlehciefvvrgnppptlhwlhngqplreskiihveyyqegeisegcllfnkpthynngnytliaknplgtanqtinghflkepfpv---DE---<br>--avvtqesalttspgetvtltcrsstgavttsnyanwvqekpdhlftgliggtnnrapgvparfsgslignkaaltitgaqteDEaiy<br><br>----------------------<br>fcalwysnhwvfgggtkltvle<br><br>Proposed: aliSize=89 (resi) RMSD=2.31 (Å)<br>tvYYPPRVVSLEEPELRLEHCIEFVVRG-----npPPTLHWLHN-----GQPL-------R--ESKIIHVEYYQegeiSEGCLLFNKPTHYNNGNYTLIAKNPLGTANQ-TINGHFLKepfpvde<br>--AVVTQESALTTSPGE-TVTLTCRSSTgavtt--SNYANWVQEkpdhlFTGLiggtnnrApgVPARFSGSLIG----NKAALTITGAQTEDEAIYFCALWYSNHWVFGgGTKLTVLE-------
503	1wwcA (105)<br>1hxmA (206)	N	4.76	TM-align: aliSize=62 (resi) RMSD=3.39 (Å)<br>tvyyypprvvsleepelr--L--EHCIEFVVRGnpPPTLHWL-HNgqpl--ResKIIH-V<br>----------------aielvpehqtvpvsigvpatlrcsmkgeaignyyinwyrktqgntmtfiyrekdiygpgfkdnfqgdidiaknlavlkilapserdegsyycacdtlgmggeytdklifgkgtrvtveprsqphtkpsvfvmkNgtNVACLVKEFY--PKDIRINlVS----skK--ITEFdP<br><br>EYYQEg--EISEGCLLFnKPTHYNNGNYTLIAKnplgTANQTINghflkepfpvde---<br>AIVIS-psGKYNAVKLG-KYEDSNSVTCSVQHD----NKTVHST------------dfe<br><br>SARST: aliSize=91 (resi) RMSD=14.68 (Å)<br>t---VYYPPR------VVSLEEPELRLEHCIEFVVRGNPPPTLHWLHNGQ----PLRESKIIHVEYyQEGEIS-EGCLLFNKPTHYNNGNYTLIAKN--PLG<br>-aielvpehqtvpvsigvpatlrcsmkgeaignyyinwyrktqgntmtfiyrekdiygpgfkdnfqgdidiaknlavlkilapserdegsyyCACDTLgmggeyTDKLIFGKGTRVTVEPRSQPHTKPSVFVMKNGTNvaclVKEFYPKDIRIN-LVSSKKiTEFDPAIVISPSGKYNAVKLGKYedSNS<br><br>TANQTIN-----------ghflkepfpvde<br>VTCSVQHdnktvhstdfe------------<br><br>BLAST: aliSize=25 (resi) iden=12.38% (13/105) simi=23.81% (25/105)<br>tvyyypprvvsleep--ELRLEHCIEFVVRGnPPPTLHWLHNGQPLREskiIHVEYYQEGEISEGCLLFNKPTHYNNG---<br>-------------aiELVPEHQTVPVSIG-VPATLRCSMKGEAIGN---YYINWYRKTQGNTMTFIYREKDIYGPGfkdnfqgdidiaknlavlkilapserdegsyycacdtlgmggeytdklifgkgtrvtveprsqphtkpsvfvmkngtnvaclvkefypkdirinlvsskkitefdpaivisps<br><br>----------------------------------nytliaknplgtanqtinghflkepfpvde<br>gkynavklgkyedsnsvtcsvqhdnktvhstdfe------------------------------<br><br>Proposed: aliSize=96 (resi) RMSD=2.45 (Å)<br>tvYYPPRVVS-LEEPELRLEHCIEFVV-RGN--pPPTLHWLHN-----GQPL-------R--ESKIIHVEYYQ-egeISEGCLLFNKPTHYNNGNYTLIAKN------PL-GTANQ-TINGHFLK--EP------FP-------<br>--AIELVPEHqTVPVSIGVPATLRCSMkGEAig-NYYINWYRKtqgntMTFIyrekdiyGpgFKDNFQGDIDIa---KNLAVLKILAPSERDEGSYYCACDTlgmggeYTdKLIFGkGTRVTVEPrsqphtkpsvfvmkngtnvaclvkefypkdirinlvsskkitefdpaiVIspsgkyNAvklgkye<br><br>----------------------vde<br>dsnsvtcsvqhdnktvhstdfe---

504	1wwcA (105)<br>1sq2N (112)	N	10.48	TM-align: aliSize=78 (resi) RMSD=2.45 (Å)<br>tvyypprvvsleepelr---------------LEHCIEFVVR-G-NPPPTLHWLHN------GQPLRESKIIHVEYyQEGeISEGCLLFNKPTHYNNGNYTLIAKNP-----------LGTANQ-TINGHfLKepfpvde<br>-----------------rvdqtprsvtketgESLTINCVLRdAsYALGSTCWYRKksgegnEESISKGGRYVETV-NSG-SKSFSLRINDLTVEDGGTYRCGLGVAggycdyalcssRYAECGdGTAVT-VN-------<br><br>SARST: aliSize=84 (resi) RMSD=8.30 (Å)<br>tvyypprvv--------SLEEP-ELRLEHCIEFVVRGNPPPTLHWLHNG-----QPLRESKIIHVEYyqEGEISEGCLLFNKPTHYNNGNYTLIAKN------------PLGTAN-QTINGH-flkepfpvde<br>---------rvdqtprsVTKETgESLTINCVLRDASYALGSTCWYRKKSgegneESISKGGRYVETV--NSGSKSFSLRINDLTVEDGGTYRCGLGVaggycdyalcssRYAECGdGTAVTVn----------<br><br>BLAST: aliSize=9 (resi) iden=5.71% (6/105) simi=8.57% (9/105)<br>tvyyp-----PRVVSLEEPELRLEHCI---efvvrgnppptlhwlhngqplreskiihveyyqegeisegcllfnkpthynngnytliaknplgtanqtingh<br>-----rvdqtPRSVTKETGESLTINCVlrdasyalgstcwyrkksgegneesiskggryvetvnsgsksfslrindltvedggtyrcglgvaggycdyalcssryaecgdgtavtvn---<br>flkepfpvde<br>----------<br><br>Proposed: aliSize=89 (resi) RMSD=2.11 (Å)<br>tvyyPPRVVS-LEEPELR-lEHCIEFVVR-G-NPPPTLHWLHN------GQPLRESKIIHVEYyQEGeISEGCLLFNKPTHYNNGNYTLIAKN-------------plGTANQTINGHFLKepfpvde<br>----RVDQTPrSVTKETGe-SLTINCVLRdAsYALGSTCWYRKksgegnEESISKGGRYVETV-NSG-SKSFSLRINDLTVEDGGTYRCGLGVaggycdyalcssr--YAECGDGTAVTVN-------
505	1wwcA (105)<br>2aw2A (110)	N	13.33	TM-align: aliSize=78 (resi) RMSD=2.48 (Å)<br>tvyypprvvsleepelr------------------LEHCIEFVVRG-NPPPTLHWLHN----GQPLRESkiiHVEYYQEg-EISEGCLLFNKPTHYNNGNYTLIAKNPLGTANQ-TINGHFLKe--Pfpvde-----<br>-----------------cdvqlyikrqsehsilagDPFELECPVKYcANRPHVTWCKLngttCVKLEDR---QTSWKEE-kNISFFILHFEPVLPNDNGSYRCSANFQSNLIEShSTTLYVTD-vkH-----hhhhh<br><br>SARST: aliSize=90 (resi) RMSD=7.64 (Å)<br>tvyypprvv------------SLEEPELRLEHCIEFVVRGNPPPTLHWLHNGQPLRESKIIHVEYYQEGEISEGCLLFNKPTHYNNGNYTLIAKNPLGTANQTINGHFLKE--------pfpvde<br>---------cdvqlyikrqseHSILAGDPFELECPVKYCANRPHVTWCKLNGTTCVKLEDRQTSWKEEKNISFFILHFEPVLPNDNGSYRCSANFQSNLIESHSTTLYVTDvkhhhhhh------<br><br>BLAST: aliSize=19 (resi) iden=11.43% (12/105) simi=18.10% (19/105)<br>tvyypprvvsleepelrlehciefvvrgnpp---------------------------------PTLHWLH-NGQPLRESKIIHVEYYQEGEISEGCLLFNKPTHYNNGNY------------------------------tliaknplgtanqtinghflkepfpvde<br>-------------------------------cdvqlyikrqsehsilagdpfelecpvkycanrPHVTWCKlNGTTCVKLEDRQTSWKEEKNISFFILHFEPVLPNDNGSYrcsanfqsnlieshsttlyvtdvkhhhhhh----------------------------<br><br>Proposed: aliSize=91 (resi) RMSD=2.18 (Å)<br>TVYYpPRVV--SLEEPELRLEHCIEFVVRG-NPPPTLHWLHN----GQPLRESkiiHVEYYQ---egeISEGCLLFNKPTHYNNGNYTLIAKNPLGTANQ-TINGHFLK--------epfpvde<br>CDVQ-LYIKrqSEHSILAGDPFELECPVKYcANRPHVTWCKLngttCVKLEDR---QTSWKEekn---ISFFILHFEPVLPNDNGSYRCSANFQSNLIEShSTTLYVTDvkhhhhhh-------
506	1wwcA (105)<br>2dkuA (103)	N	9.71	TM-align: aliSize=71 (resi) RMSD=1.90 (Å)<br>tvyypprvvsleepelr----------------------LEHCIEFVVRGnpPP-TLHWLHNGQPLRESKIIHVEYyQEgeIsEGCLLFNKPTHYNNGNYTLIAKnplgTANQTINGHFLKepfpvde--------<br>-----------------gssgssgancfteeltnlqveekGTAVFTCKTEH--PAaTVTWRKGLLELRASGKHQPSQ-EG--L-TLRLTISALEKADSDTYTCDIG----QAQSRAQLLVQG-------rrsgpssg<br><br>SARST: aliSize=80 (resi) RMSD=11.84 (Å)<br>tvy------------YPPRVvSLEEPELRLEHCIEFVVrgnPPPTLHWLHNGQPLRESKIIHVeyyQEGEISEGcLLFNKPTHYNNGNYTLIaknpLGTANQTINGH-----------flkepfpvde<br>---gssgssgancftEELTN-LQVEEKGTAVFTCKTEH---PAATVTWRKGLLELRASGKHQP---SQEGLTLR-LTISALEKADSDTYTCD----IGQAQSRAQLLvqgrrsgpssg----------<br><br>BLAST: aliSize=4 (resi) iden=2.91% (3/103) simi=3.88% (4/103)<br>tvyypprvvsleepelrlehciefvvrgnppptlhwlhng--QPLRE--skiihveyyqegeisegcllfnkpthynngnytliaknplgtanqti<br>--gssgssgancfteeltnlqveekgtavftcktehpaatvtwrkgllelrasgkhQPSQEgltlrltisalekadsdtytcdigqaqsraqllvqgrrsgpssg--<br>nghflkepfpvde<br>-------------<br><br>Proposed: aliSize=88 (resi) RMSD=1.98 (Å)<br>---TVYYPPRVVSLEEPELR---LEHCIEFVVRGnpPP-TLHWLHNGQPLRESKIIHVEYyQEgeIsEGCLLFNKPTHYNNGNYTLIAKnplgTANQTINGHFLK--------epfpvde<br>gssGSSGANCFTEELTNLQVeekGTAVFTCKTEH--PAaTVTWRKGLLELRASGKHQPSQ-EG--L-TLRLTISALEKADSDTYTCDIG----QAQSRAQLLVQGrrsgpssg-------

507	1wwcA (105)<br>2rheA (114)	N	10.48	TM-align: aliSize=81 (resi) RMSD=2.62 (Å)<br>tvyypprvvsleepel---------------RLEHCIEFVVRGNP--PPTLHWLHN-----GQPLR-----E----SKIIHVEYyQEgeIsEGCLLFNKPTHYNNGNYTLIAKNP--LGTANQ-TINGHFLKEPFpvde<br>----------------esvltqppsasgtpGQRVTISCTGSATDigSNSVIWYQQvpgkaPKLLIyyndlLpsgvSDRFSASK-SG--T-SASLAISGLESEDEADYYCAAWNDslDEPGFGgGTKLTVLGQPK----<br><br>SARST: aliSize=76 (resi) RMSD=15.97 (Å)<br>tv---------YYPPRVVSLEEpELRLEH------CIEFVVRGNP--PPTLHWLHNGQPLR-ESKIIHVEYyqEGEisEGCLLFNKPTHYNNGNYTLIaknplgtanqtinghfLKE---------------------pfpvde<br>--esvltqppSASGTPGQRVT-ISCTGSatdigsNSVIWYQQVPgkAPKLLIYYNDLLPSgVSDRFSASK--SGT--SASLAISGLESEDEADYYCA----------------AWNdsldepgfgggtkltvlgqpk------<br><br>BLAST: aliSize=5 (resi) iden=3.81% (4/105) simi=4.76% (5/105)<br>tvyypprvv---SLEEP---------------elrlehciefvvrgnppptlhwlhngqplreskiihveyyqegeisegcllfnkpthynngnytlia<br>---------esvltqppsasgtpgqrvtisctgsatdigsnsviwyqqvpgkapklliyyndllpsgvsdrfsasksgtsaslaisglesedeadyycaawndSLDEPgfgggtkltvlgqpk--<br>knplgtanqtinghflkepfpvde<br>------------------------<br><br>Proposed: aliSize=91 (resi) RMSD=2.31 (Å)<br>tv-YYPPRVVSLEEP---elrlEHCIEFVVRGNP--PPTLHWLHNG----QPLR----------ESKIIHVEYyQEgeIsEGCLLFNKPTHYNNGNYTLIAKNP--LGTANQ-TINGHFLKEPFpvde<br>--eSVLTQPPSASGTpgq----RVTISCTGSATDigSNSVIWYQQVpgkaPKLLiyyndllpsgVSDRFSASK-SG--T-SASLAISGLESEDEADYYCAAWNDslDEPGFGgGTKLTVLGQPK----
508	1wwcA (105)<br>2yuvA (100)	N	12.00	TM-align: aliSize=72 (resi) RMSD=2.01 (Å)<br>tvyypprvvsleepelr-----------------------LEHCIEFVVRGnPPPTLHWLHNGQPLRESKIIHVEYyQEgeiSEGCLLFNKPTHYNNGNYTLIAKnplgTANQTINGHFLKepfpvde-----<br>-----------------gssgssgaafakildpayqvdkgGRVRFVVELAD-PKLEVKWYKNGQEIRPSTKYIFEH-KG---CQRILFINNCQMTDDSEYYVTAG----DEKCSTELFVRS-------gpssg<br><br>SARST: aliSize=77 (resi) RMSD=10.34 (Å)<br>tvyypprvvs-----------LEEPELRLEHCIEFVVRGNPP----PTLHWLHNGQPLRESKIIHVEYyqEGEISegCLLFNKPTHYNNGNYTLIAknplGTANQTINGH--------flkepfpvde<br>----------gssgssgaafaKILDPAYQVDKGGRVRFVVELadpkLEVKWYKNGQEIRPSTKYIFEH--KGCQR--ILFINNCQMTDDSEYYVTA----GDEKCSTELFvrsgpssg----------<br><br>BLAST: aliSize=26 (resi) iden=16.00% (16/100) simi=26.00% (26/100)<br>tvyypprvvsleepelrlehc-------------------------IEFVVR-GNPPPTLHWLHNGQPLRESkiihVEYYQEGEISEGCLLFNKPTHYNNGNYTLIA------------------knplgtanqtinghflkepfpvde<br>---------------------gssgssgaafakildpayqvdkggrVRFVVElADPKLEVKWYKNGQEIRPS----TKYIFEHKGCQRILFINNCQMTDDSEYYVTAgdekcstelfvrsgpssg------------------------<br><br>Proposed: aliSize=86 (resi) RMSD=2.02 (Å)<br>tvy----YP-PRVVSL-EEPELR---LEHCIEFVVRGnPPPTLHWLHNGQPLRESKIIHVEYyQEgeiSEGCLLFNKPTHYNNGNYTLIAKnplgTANQTINGHFLK-----epfpvde<br>---gssgSSgAAFAKIlDPAYQVdkgGRVRFVVELAD-PKLEVKWYKNGQEIRPSTKYIFEH-KG---CQRILFINNCQMTDDSEYYVTAG----DEKCSTELFVRSgpssg-------
509	1wwcA (105)<br>2ywyA (113)	N	8.57	TM-align: aliSize=80 (resi) RMSD=2.31 (Å)<br>tvyypprvvsleepelr---------------LEHCIEFVVR-G-NPPPTLHWLHN------GQPLRESKIIHVEYyQEGeISEGCLLFNKPTHYNNGNYTLIAKNP---------LGTANQTINGHFLKEpfpvde-<br>-----------------awvdqtprsvtketgESLTINCALKnAaDDLERTDWYRTtlgstnEQKISIGGRYVETV-NKG-SKSFSLRIRDLRVEDSGTYKCGAYFSdamsnysypIPGEKGAGTVLTVKA------a<br><br>SARST: aliSize=90 (resi) RMSD=12.69 (Å)<br>tv-YYPPRVVSLEEpELRLEHCIEFVVRGN--PPPTLHWLHN--GQ----PLRESKIIHVEYyqEGEISEGCLLFNKPTHYNNGNYTLIAKN------PLGTANQTI------NGH--flkepfpvde<br>--aWVDQTPRSVTK-ETGESLTINCALKNAadDLERTDWYRTtlGStneqKISIGGRYVETV--NKGSKSFSLRIRDLRVEDSGTYKCGAYFsdamsnYSYPIPGEKgagtvlTVKaa----------<br><br>BLAST: aliSize=10 (resi) iden=6.67% (7/105) simi=9.52% (10/105)<br>---TVYYPPRVVSLEEPELRLE--hciefvvrgnppptlhwlhngqplreskiihveyyqegeisegcllfnkpthynngnytliaknplgtanqtinghf<br>awvdqtprsvtketgesltincalknaaddlertdwyrttlgstneqkisiggryveTVNKGSKSFSLRIRDLRVEdsgtykcgayfsdamsnysypipgekgagtvltvkaa---<br>lkepfpvde<br>---------<br><br>Proposed: aliSize=91 (resi) RMSD=1.94 (Å)<br>tvyYPPRVVS-LEEPELR-lEHCIEFVVR-GNPPP-TLHWLHN------GQPLRESKIIHVEYyQEGeISEGCLLFNKPTHYNNGNYTLIAKNP----------lGTANQ-TINGHFLK-epfpvde<br>---AWVDQTPrSVTKETGe-SLTINCALKnAADDLeRTDWYRTtlgstnEQKISIGGRYVETV-NKG-SKSFSLRIRDLRVEDSGTYKCGAYFSdamsnysypi-PGEKGaGTVLTVKAa-------

510	1wwcA (105)<br>2ywzA (111)	N	8.57	TM-align: aliSize=78 (resi) RMSD=2.02 (Å)<br>tvyypprvvsleepelr----------------LEHCIEFVVR-GNPPP-TLHWLHN------GQPLRESKIIHVEYyQEGeISEGCLLFNKPTHYNNGNYTLIAKNPL---------GTANQ-TINGHfLKepfpvde<br>-----------------awvdqtprtitketgESLTIKCVLKdHSCGLsSTTWYRTqlgstnEKTISIGGRYDETV-DKG-SKSFSLRISDLRVEDSGTYKCQADYSPscysypsleSAVEGaGTVLT-VK-------<br><br>SARST: aliSize=91 (resi) RMSD=12.76 (Å)<br>tv-YYPPRVVSLEEP--ELRLEHCIEFVVRGNPPPTLHWLHN-G----QPLRESKIIHVEYyQEGEISEgCLLFNKPTHYNNGNYTLIA-KNPL------GTANQT---INGH--flkepfpvde<br>--aWVDQTPRTITKEtgESLTIKCVLKDHSCGLSSTTWYRTQlGstneKTISIGGRYDETV-DKGSKSF-SLRISDLRVEDSGTYKCQAdYSPScysypsLESAVEgagTVLTvk----------<br><br>BLAST: aliSize=8 (resi) iden=4.76% (5/105) simi=7.62% (8/105)<br>tvyyp------PRVVSLEEPELRLEHCI--efvvrgnppptlhwlhngqplreskiihveyyqegeisegcllfnkpthynngnytliaknplgtanqtinghf<br>-----awvdqtPRTITKETGESLTIKCVlkdhscglssttwyrtqlgstnektisiggrydetvdkgsksfslrisdlrvedsgtykcqadyspscysypslesavegagtvltvk--<br>lkepfpvde<br>---------<br><br>Proposed: aliSize=91 (resi) RMSD=1.93 (Å)<br>tvyYPPRVVS-LEEPELR-lEHCIEFVVR-GNPPP-TLHWLHN------GQPLRESKIIHVEYyQEGeISEGCLLFNKPTHYNNGNYTLIAKNPL---------GTANQ-TINGHFLkepfpvde<br>---AWVDQTPrTITKETGe-SLTIKCVLKdHSCGLsSTTWYRTqlgstnEKTISIGGRYDETV-DKG-SKSFSLRISDLRVEDSGTYKCQADYSPscysypsleSAVEGaGTVLTVK--------
511	1wwrA (154)<br>1wkqB (155)	C	29.87	TM-align: aliSize=111 (resi) RMSD=2.07 (Å)<br>gshmGKEYFLKVALREAKRAFEKG-EVPVGAIIVKEGEIISKAHNSVEELKDPTAHAEMLAIKEACRRLNTKYLEGCELYVTLEPCIMCSYALVLSRIEKVIFSALDK--K-----HGGvvSVFn-Ildeptlnhrvkweyypleeasellseffkklrnnii--------------------------<br>----NHETFLKRAVTLACEGVNAGiGGPFGAVIVKDGAIIAEGQNNVTTSNDPTAHAEVTAIRKACKVLGAYQLDDCILYTSCEPCPMCLGAIYWARPKAVFYAAEHTdaAeagfdDSF--IYK-eI-------------------------------------dkpaeertipfyqvtltehlspfqawr<br>--------<br>nfankkey<br><br>SARST: aliSize=136 (resi) RMSD=15.57 (Å)<br>gshmg-KEYFLKVALREAKRAFEKG-EVPVGAIIVKEGEIISKAHNSVEELKDPTAHAEMLAIKEACRRLNTKYLEGCELYVTLEPCIMCSYALVLSRIEKVIFSALDK----KHGGVVSV-----FNILDEPtlnhrVKWEYYPLEEASELLSEFF--------kklrnnii<br>-----nHETFLKRAVTLACEGVNAGiGGPFGAVIVKDGAIIAEGQNNVTTSNDPTAHAEVTAIRKACKVLGAYQLDDCILYTSCEPCPMCLGAIYWARPKAVFYAAEHTdaaeAGFDDSFIykeidKPAEERT-----IPFYQVTLTEHLSPFQAWRnfankkey--------<br><br>BLAST: aliSize=80 (resi) iden=35.06% (54/154) simi=51.95% (80/154)<br>gshmgk--EYFLKVALREAKRAFEKG-EVPVGAIIVKEGEIISKAHNSVEELKDPTAHAEMLAIKEACRRLNTKYLEGCELYVTLEPCIMCSYALVLSRIEKVIFSA--LDKKHGGVVS--VFNILDEPTLNHRVkwEYYPLeEASELLSEfFKKLRN-------nii<br>------nhETFLKRAVTLACEGVNAGiGGPFGAVIVKDGAIIAEGQNNVTTSNDPTAHAEVTAIRKACKVLGAYQLDDCILYTSCEPCPMCLGAIYWARPKAVFYAAehTDAAEAGFDDsfIYKEIDKPAEERTI--PFYQV-TLTEHLSP-FQAWRNfankkey---<br><br>Proposed: aliSize=133 (resi) RMSD=1.56 (Å)<br>gshmGKEYFLKVALREAKRAFEKG-EVPVGAIIVKEGEIISKAHNSVEELKDPTAHAEMLAIKEACRRLNTKYLEGCELYVTLEPCIMCSYALVLSRIEKVIFSALDK---------------khggvvsvFNI-L--DeptlnhRVKWEYYPLEEASELLSEFFKKlrNNI---i<br>----NHETFLKRAVTLACEGVNAGiGGPFGAVIVKDGAIIAEGQNNVTTSNDPTAHAEVTAIRKACKVLGAYQLDDCILYTSCEPCPMCLGAIYWARPKAVFYAAEHTdaaeagfddsfiyke--------IDKpAeeR------TIPFYQVTLTEHLSPFQAWRNF--ANKkey-
512	1wwrA (154)<br>3b8fB (142)	C	14.08	TM-align: aliSize=107 (resi) RMSD=3.23 (Å)<br>gsHMGKEYFLKVALREAKRAFEKGeVPVGAIIVKEGEIISKAHNSvEELK--DPTaHAEMLAIKEACRRLnTkyLeGCELYVT-----------LEPCIMCSYALVLSR-IEKVIFsALdkkhggv-------Vsvf---NILDEPTL---N--HRvkweyypleeasellseffkklrnnii------<br>--LNIEQQLYDVVKQLIEQRYPND-WGGAAAIRVEDGTIYTSVAP-DVINasTEL-CMETGAILEAHKFQ-K--K-VTHSICLarenehselkvLSPCGVCQERLFYWGpEVQCAI-TN-------akqdiifK---plkELQPYHWTeayHdeMV---------------------------kewstr<br><br>SARST: aliSize=100 (resi) RMSD=6.31 (Å)<br>gshmgk--EYFLKVALREAKRAFEKGEVPVGAIIVK--EGEIISKAHNSveeLKDPTAH-AEMLAIKEACRRLNTKYLEGCELYVTL--------EPCIMCSYALVLSRIEKVIFsALDKKHG---------------------------gvvsvfnildeptlnhrvkweyypleeasellseffkk<br>------lnIEQQLYDVVKQLIEQRYPNDWGGAAAIRveDGTIYTSVAPD---VINASTElCMETGAILEAHKFQKKVTHSICLARENehselkvlSPCGVCQERLFYWGPEVQCA-ITNAKQDiifkplkelqpyhwteayhdemvkewstr------------------------------------<br>lrnnii<br>------<br><br>BLAST: aliSize=26 (resi) iden=14.79% (21/142) simi=18.31% (26/142)<br>gshmgkeyflkvalreakrafekgevpvg---------------------------AIIVKEGEIISKAHNSVEELKDPTAhAEMLAIKEAcRRLNTKYLEG-C--------ELYVtLEPCIMCSYAL---vlsriekvifsaldkk<br>---------------------------lnieqqlydvvkqlieqrypndwggaaAIRVEDGTIYTSVAPDVINASTELC-METGAILEA-HKFQKKVTHSiClarenehsELKV-LSPCGVCQERLfywgpevqcaitnakqdiifkplkelqpyhwteayhdemvkewstr---------------<br>hggvvsvfnildeptlnhrvkweyypleeasellseffkklrnnii<br>--<br><br>Proposed: aliSize=114 (resi) RMSD=2.80 (Å)<br>gsHMGKEYFLKVALREAKRAFEKGEVPVGAIIVKEGEIiSKAHNSveELK---DPTaHAEMLAIKEACRrLnTkyLEgCELYVT-----------LEPCIMCSYALVLsRI-EKVIFsaldkkhggvvsvfnILDePTL----------nhrvkweYYPLeEASELLSEFFKK-LR-nnii<br>--LNIEQQLYDVVKQLIEQRYPNDWGGAAAIRVEDGTI-YTSVAP--DVInasTEL-CMETGAILEAHK-F-Q--KK-VTHSIClarenehselkvLSPCGVCQERLFY-WGpEVQCA---------------ITN-AKQdiifkplkel-------QPYH-WTEAYHDEMVKEwSTr----

513	1wwrA (154)<br>3b8fC (140)	C	14.29	TM-align: aliSize=106 (resi) RMSD=3.28 (Å)<br>gshMGKEYFLKVALREAKRAFEKGeVPVGAIIVKEGEIISKAHNSvEELK--DPTaHAEMLAIKEACRRLntkyLEGCELYVT-----------LEPCIMCSYALVlSRI--EKVIFsALDkkhggv------Vsvf---NILDEPTLN-----HRvkweyypleeasellseffkklrnnii-----<br>---NIEQQLYDVVKQLIEQRYPND-WGGAAAIRVEDGTIYTSVAP-DVINasTEL-CMETGAILEAHKFQ----KKVTHSICLarenehselkvLSPCGVCQERLF-YWGpeVQCAI-TNA------kqdiifK---plkELQPYHWTEayhdeMV---------------------------kewst<br><br>SARST: aliSize=92 (resi) RMSD=7.44 (Å)<br>gshmgk-EYFLKVALREAKRAFEKGEVPVGAIIVK--EGEIISKAHNSveeLKDPTAH-AEMLAIKEACRRLNTKYLEGCELYVTL--------EPCIMCSYALV------------LSRIEKVI-----------------------fsaldkkhggvvsvfnildeptlnhrvkweyypleeasell<br>------nIEQQLYDVVKQLIEQRYPNDWGGAAAIRveDGTIYTSVAPD---VINASTElCMETGAILEAHKFQKKVTHSICLARENehselkvlSPCGVCQERLFywgpevqcaitnAKQDIIFKplkelqpyhwteayhdemvkewst---<br>seffkklrnnii<br>------------<br><br>BLAST: aliSize=26 (resi) iden=15.00% (21/140) simi=18.57% (26/140)<br>gshmgkeyflkvalreakrafekgevpvg--------------------------AIIVKEGEIISKAHNSVEELKDPTAhAEMLAIKEAcRRLNTKYLEG-C--------ELYVtLEPCIMCSYAL------------------------------------vlsriekvifsaldkkhg<br>----------------------------nieqqlydvvkqlieqrypndwggaaAIRVEDGTIYTSVAPDVINASTELC-METGAILEA-HKFQKKVTHSiClarenehsELKV-LSPCGVCQERLfywgpevqcaitnakqdiifkplkelqpyhwteayhdemvkewst-----------------<br>gvvsvfnildeptlnhrvkweyypleeasellseffkklrnnii<br>--<br><br>Proposed: aliSize=114 (resi) RMSD=2.88 (Å)<br>gshMGKEYFLKVALREAKRAFEKGEVPVGAIIVKEGEIiSKAHNSveELK---DPTaHAEMLAIKEACRrLnTkyLEgCELYVT------------LEPCIMCSYALVLsRI-EKVIFsaldkkhggvvsvfnILDEPT----------lnhrvkweYYPLeEASELLSEFFKKLRnNii<br>---NIEQQLYDVVKQLIEQRYPNDWGGAAAIRVEDGTI-YTSVAP--DVInasTEL-CMETGAILEAHK-F-Q--KK-VTHSIClarenehselkvLSPCGVCQERLFY-WGpEVQCA---------------ITNAKQdiifkplkel--------QPYH-WTEAYHDEMVKEWS-T--
514	1wwzA (157)<br>1qsmA (150)	C	18.00	TM-align: aliSize=135 (resi) RMSD=2.80 (Å)<br>mDEIKIEKLKKlDkkALNELIDVYMSGYEGLEeygGEgrdYAR-NYIKWCWK-----KASdGFFVAKV--GDKIVGFIVCDkDWFSKyEGR-IVGAIHEFVVDKKFQGKGIGRKLLITCLDFLGK-YNDTIELWVGEKNYGAMNLYEKFGFKK-VGKSGiwvrmikrq----<br>-DNITVRFVTE-N--DKEGWQRLWKSYQDFYE---VS---FPDdLDDFNFGRfldpnIKM-WAAVAVEssSEKIIGMINFF-NHMTT-WDFkDKIYINDLYVDENSRVKGAGGKLIQFVYDEADKlGTPSVYWCTDESNHRAQLLYVKVGYKApKILYK---------rkgy<br><br>SARST: aliSize=132 (resi) RMSD=6.39 (Å)<br>mdeikieklkkld------------KKALNELIDVYMSGYegleeYGGEGRDYARNYIKWCWKKASDGFFVAKVG---DKIVGFIVCDkDWFSKYEGRIVGAIHEFVVDKKFQGKGIGRKLLITCLDFLGKY-NDTIELWVGEKNYGAMNLYEKfgfkkVGKSGIWVRMIKR--q<br>-------------dnitvrfvtendKEGWQRLWKSYQDFY-----EVSFPDDLDDFNFGRFLDPNIKMWAAVAVEsssEKIIGMINFF-NHMTTWDFKDKIYINDLYVDENSRVKGAGGKLIQFVYDEADKLgTPSVYWCTDESNHRAQLLYVK-----VGYKAPKILYKRKgy-<br><br>BLAST: aliSize=61 (resi) iden=26.67% (40/150) simi=40.67% (61/150)<br>mDEIKIEKLKKLDKKALNELIDVYMSGYE-----GLEEYgGEGRDYARNyIKwCWKKASdgffVAKVGDKIVGFIVCDKDWFS-KYEGRIVgaIHEFVVDKKFQGKGIGRKLLITCLDFLGKYNDTIELW-VGEKNYGAMNLYEKFGFK-----------kvgksgiwvrmikrq<br>-DNITVRFVTENDKEGWQRLWKSYQDFYEvsfpdDLDDF-NFGRFLDPN-IK-MWAAVA----VESSSEKIIGMINFFNHMTTwDFKDKIY--INDLYVDENSRVKGAGGKLIQFVYDEADKLGTPSVYWcTDESNHRAQLLYVKVGYKapkilykrkgy--------------<br><br>Proposed: aliSize=137 (resi) RMSD=2.54 (Å)<br>md-EIKIEKLKKlDkkALNELIDVYMSGYEGLEeygGEgrDYaRNYIKWCWKKAS-D----GFFVAKV--GDKIVGFIVCDkDWFskyEG-R--IVGAIHEFVVDKKFQGKGIGRKLLITCLDFLGK-YNDTIELWVGEKNYGAMNLYEKFGFKK-VGKsGIWVRM-ikrqa<br>--dNITVRFVTE-N--DKEGWQRLWKSYQDFYE---VS--FP-DDLDDFNFGRFLdPnikmWAAVAVEssSEKIIGMINFF-NHM---TTwDfkDKIYINDLYVDENSRVKGAGGKLIQFVYDEADKlGTPSVYWCTDESNHRAQLLYVKVGYKApKIL-YKRKGYa-----
515	1wwzA (157)<br>1qsmB (149)	C	19.46	TM-align: aliSize=137 (resi) RMSD=2.85 (Å)<br>mdEIKIEKLkKLDkkALNELIDVYMSGYEGLEeygGEGRDYARNYIKWCW-K--KASdGFFVAKV--GDKIVGFIVCDkDWFSKyEGR-IVGAIHEFVVDKKFQGKGIGRKLLITCLDFLGK-YNDTIELWVGEKNYGAMNLYEKFGFKK-VGKSGiwvrmikrq----<br>--NITVRFV-TEN--DKEGWQRLWKSYQDFYE---VSFPDDLDDFNFGRFlDpnIKM-WAAVAVEssSEKIIGMINFF-NHMTT-WDFkDKIYINDLYVDENSRVKGAGGKLIQFVYDEADKlGTPSVYWCTDESNHRAQLLYVKVGYKApKILYK---------rkgy<br><br>SARST: aliSize=131 (resi) RMSD=6.49 (Å)<br>mdeikieklkkld-----------KKALNELIDVYMSGYegleeyGGEGRDYARNYIKWCWKKASDGFFVAKV----GDKIVGFIVCDkDWFSKYEGRIVGAIHEFVVDKKFQGKGIGRKLLITCLDFLGKYND-TIELWVGEKNYGAMNLYEKfgfkkVGKSGIWVRMIKR--q<br>-------------nitvrfvtendKEGWQRLWKSYQDFY------EVSFPDDLDDFNFGRFLDPNIKMWAAVAvessSEKIIGMINFF-NHMTTWDFKDKIYINDLYVDENSRVKGAGGKLIQFVYDEADKLGTpSVYWCTDESNHRAQLLYVK-----VGYKAPKILYKRKgy-<br><br>BLAST: aliSize=60 (resi) iden=26.17% (39/149) simi=40.27% (60/149)<br>mde-IKIEKLKKLDKKALNELIDVYMSGYE-----GLEEYgGEGRDYARNyIKwCWKKASdgffVAKVGDKIVGFIVCDKDWFS-KYEGRIVgaIHEFVVDKKFQGKGIGRKLLITCLDFLGKYNDTIELW-VGEKNYGAMNLYEKFGFK-----------kvgksgiwvrmikrq<br>---nITVRFVTENDKEGWQRLWKSYQDFYEvsfpdDLDDF-NFGRFLDPN-IK-MWAAVA----VESSSEKIIGMINFFNHMTTwDFKDKIY--INDLYVDENSRVKGAGGKLIQFVYDEADKLGTPSVYWcTDESNHRAQLLYVKVGYKapkilykrkgy--------------<br><br>Proposed: aliSize=140 (resi) RMSD=2.74 (Å)<br>mdEIKIEKLkKlDkKALNELIDVYMSGYEGLEEygGEGRDYARNYIKWCWK---KASdgFFVAKV--GDKIVGFIVCDkDWFSkyEGR--IVGAIHEFVVDKKFQGKGIGRKLLITCLDFLGK-YNDTIELWVGEKNYGAMNLYEKFGFKK-VGKSGiWVRM-ikrqa<br>--NITVRFV-T-E-NDKEGWQRLWKSYQDFYEV--SFPDDLDDFNFGRFLDpniKMW--AAVAVEssSEKIIGMINFF-NHMT--TWDfkDKIYINDLYVDENSRVKGAGGKLIQFVYDEADKlGTPSVYWCTDESNHRAQLLYVKVGYKApKILYK-RKGYa-----

516	1wwzA (157)<br>1qsmD (152)	C	19.74	TM-align: aliSize=139 (resi) RMSD=2.84 (Å)<br>M-DEIKIEKLkKlDkKALNELIDVYMSGYEGLEeygGEGRDYARNYIKWCW-K--KASdGFFVAKV--GDKIVGFIVCDkDWFSKyEGR-IVGAIHEFVVDKKFQGKGIGRKLLITCLDFLGK-YNDTIELWVGEKNYGAMNLYEKFGFKK-VGKSGiwvrmikrq----<br>SeDNITVRFV-T-E-NDKEGWQRLWKSYQDFYE---VSFPDDLDDFNFGRFlDpnIKM-WAAVAVEssSEKIIGMINFF-NHMTT-WDFkDKIYINDLYVDENSRVKGAGGKLIQFVYDEADKlGTPSVYWCTDESNHRAQLLYVKVGYKApKILYK---------rkgy<br><br>SARST: aliSize=139 (resi) RMSD=5.25 (Å)<br>mde----IKIEKLkklDKKALNELIDVYMSGYeglEEYGGEGRDYARNYIKWCWKKASDGFFVAKV----GDKIVGFIVCDkDWFSKYEGRIVGAIHEFVVDKKFQGKGIGRKLLITCLDFLGKY-NDTIELWVGEKNYGAMNLYEKfgfkkVGKSGIWVRMI----krq<br>---sednITVRFV---TENDKEGWQRLWKSYQ---DFYEVSFPDDLDDFNFGRFLDPNIKMWAAVAvessSEKIIGMINFF-NHMTTWDFKDKIYINDLYVDENSRVKGAGGKLIQFVYDEADKLgTPSVYWCTDESNHRAQLLYVK-----VGYKAPKILYKrkgy---<br><br>BLAST: aliSize=61 (resi) iden=26.32% (40/152) simi=40.13% (61/152)<br>m--DEIKIEKLKKLDKKALNELIDVYMSGYE-----GLEEYgGEGRDYARNyIKwCWKKASdgffVAKVGDKIVGFIVCDKDWFS-KYEGRIVgaIHEFVVDKKFQGKGIGRKLLITCLDFLGKYNDTIELW-VGEKNYGAMNLYEKFGFK-----------kvgksgiwvrmikrq<br>-seDNITVRFVTENDKEGWQRLWKSYQDFYEvsfpdDLDDF-NFGRFLDPN-IK-MWAAVA----VESSSEKIIGMINFFNHMTTwDFKDKIY--INDLYVDENSRVKGAGGKLIQFVYDEADKLGTPSVYWcTDESNHRAQLLYVKVGYKapkilykrkgy--------------<br><br>Proposed: aliSize=142 (resi) RMSD=2.68 (Å)<br>M-DEIKIEKLkKlDkKALNELIDVYMSGYEGLEEygGEGRDYARNYIKWCWK---KASdgFFVAKV--GDKIVGFIVCDkDWFSkyEGR--IVGAIHEFVVDKKFQGKGIGRKLLITCLDFLGK-YNDTIELWVGEKNYGAMNLYEKFGFKkvgksGIWVRMIKRQ--a<br>SeDNITVRFV-T-E-NDKEGWQRLWKSYQDFYEV--SFPDDLDDFNFGRFLDpniKMW--AAVAVEssSEKIIGMINFF-NHMT--TWDfkDKIYINDLYVDENSRVKGAGGKLIQFVYDEADKlGTPSVYWCTDESNHRAQLLYVKVGYK-----APKILYKRKGya-
517	1wwzA (157)<br>1s3zB (145)	C	12.41	TM-align: aliSize=135 (resi) RMSD=2.78 (Å)<br>mdeIKIEKLKKlDkkALNELIDVYMSgYegleeyGGEGRDYARNYIKWCW-KKASDGFFVAKVGDKIVGFIVCDkDWFSK-Y--EGRIVGAIHEFVVDKKFQGKGIGRKLLITCLDFLGK-YNDTIELWVGEKNYGAMNLYEKFGFKKVGKSGIwvrmikrq-----<br>---MDIRQMNK-T--HLEHWRGLRKQ-L------WPGHPDDAHLADGEEIlQADHLASFIAMADGVAIGFADAS-IRHDYvNgcDSSPVVFLEGIFVLPSFRQRGVAKQLIAAVQRWGTNkGCREMASDTSPENTISQKVHQALGFEETERVIF--------yrkrc<br><br>SARST: aliSize=140 (resi) RMSD=5.62 (Å)<br>mdei-KIEKLkklDKKALNELIDVYMSGyegleeYGGEGRDYARNYIKWCWKKASDGFFVAKVGDKIVGFIV--CDKDWFSKYEGRIVGAIHEFVVDKKFQGKGIGRKLLITCLDFLGKY-NDTIELWVGEKNYGAMNLYEKFGFKKVGKsgiWVRMIKR-q<br>----mDIRQM---NKTHLEHWRGLRKQL------WPGHPDDAHLADGEEILQADHLASFIAMADGVAIGFADasIRHDYVNGCDSSPVVFLEGIFVLPSFRQRGVAKQLIAAVQRWGTNKgCREMASDTSPENTISQKVHQALGFEETER---VIFYRKRc-<br><br>BLAST: aliSize=37 (resi) iden=15.17% (22/145) simi=25.52% (37/145)<br>mdeikieklkkldkkalnelidvymsgyegleeyggegrdyarnyikwcwkkasd---GFFVAKV-GDKIVGfivCDKDWFSKYEGrIvgaiheFVVdKKFQGKGIGrKLLITCLDFLGKYNDTIELW--VGEKNYGA<br>---mdirqmnkthlehwrglrkqlwpghpddahladgeeilqadhlasfiamadgvaiGFADASIrHDYVNG---CDSSPVVFLEG-I------FVL-PSFRQRGVA-KQLIAAVQRWGTNKGCREMAsdTSPENTIS<br><br>MNLYEKFGFKK-----------vgksgiwvrmikrq<br>QKVHQALGFEEtervifyrkrc--------------<br><br>Proposed: aliSize=140 (resi) RMSD=2.44 (Å)<br>mdeIKIEKLkKLDkkALNELIDVYMSGYeglEeygGEGRDYARNYIKWCWKK-ASdGFFVAKVGDKIVGFIVCDkDWFSKY---EGRIVGAIHEFVVDKKFQGKGIGRKLLITCLDFLGK-YNDTIELWVGEKNYGAMNLYEKFGFKKVGksgIWVRMIKRQ-a<br>---MDIRQM-NKT--HLEHWRGLRKQLW---P---GHPDDAHLADGEEILQAdHL-ASFIAMADGVAIGFADAS-IRHDYVngcDSSPVVFLEGIFVLPSFRQRGVAKQLIAAVQRWGTNkGCREMASDTSPENTISQKVHQALGFEETE---RVIFYRKRCc-
518	1wwzA (157)<br>1z4eA (150)	C	17.33	TM-align: aliSize=138 (resi) RMSD=3.10 (Å)<br>mdEIKIEKLkKlDkKALNELIDVYMSgYeGLEEyGGEGR----DYARNYIKWCWK-K--ASdgFFVAKVGDKIVGFIVCDKDWFSKy-EGRIVGAIHEFVVDKKFQGKGIGRKLLITCLDFLGK-YNDTIELWVGEKNYGAMNLYEKFGFKK-VGKSGIwvrmikrq--<br>--HVTIREA-T-E-GDLEQMVHMLAD-D-VLGR-KRERYekplPVSYVRAFKEIKkDknNE--LIVACNGEEIVGMLQVTFTPYLT-yQGSWRATIEGVRTHSAARGQGIGSQLVCWAIERAKErGCHLIQLTTDKQRPDALRFYEQLGFKAsHEGLKM--------hf<br><br>SARST: aliSize=132 (resi) RMSD=5.46 (Å)<br>mdeikieklkkldkkaln-----------ELIDVYMSGYEGLEEYG---GEGRDYARNYIKWCWKKASDG-FFVAKVGDKIVGFIVCDkDWFSKYEGRIVGAIHE-FVVDKKFQGKG-IGRKLLITCLDFLGKYNDTIELWVGEKNYGAMNLYEKFGFKKVGksgiwVRMIKR-q<br>------------------hvtireategdLEQMVHMLADDVLGRKReryEKPLPVSYVRAFKEIKKDKNNeLIVACNGEEIVGMLQVT-FTPYLTYQGSWRATIEgVRTHSAARGQGiGSQLVCWAIERAKERGCHLIQLTTDKQRPDALRFYEQLGFKASH-----EGLKMHf-<br><br>BLAST: aliSize=46 (resi) iden=17.33% (26/150) simi=30.67% (46/150)<br>mdeikieklkkldkkalnelidvymsgyegleeyggegrd--------------------------------------YARNYiKWCWKKASDGFFVAKVGDKIVGFIVCDKDWFSKYEGRIVGAIHEFVVDKKFQGKGIGRKLLITCLDFLGKYN-DTIELWVGEKNYGAMNLYEKFGFK---------<br>--hvtireategdleqmvhmladdvlgrkreryekplpvsYVRAF-KEIKKDKNNELIVACNGEEIVGMLQVTFTPYLTYQGSWRATIEGVRTHSAARGQGIGSQLVCWAIERAKERGcHLIQLTTDKQRPDALRFYEQLGFKasheglkmh<br><br>-kvgksgiwvrmikrq<br>f---------------<br><br>Proposed: aliSize=136 (resi) RMSD=2.32 (Å)<br>mdEIKIEKLkkLDkKALNELIDVYMSgYeGLE-EygGEGR----D-YaR-NYIKWCWKK--ASdgFFVAKVGDKIVGFIVCDkDWFS-kyeG--R-IVGAIHEFVVDKKFQGKGIGRKLLITCLDFLGK-YNDTIELWVGEKNYGAMNLYEKFGFKKvgksGiWVRMIKRQa<br>--HVTIREA--TE-GDLEQMVHMLAD-D-VLGrK--RERYekplPvS-YvRAFKEIKKDknNE--LIVACNGEEIVGMLQVT-FTPYl---TyqGsWRATIEGVRTHSAARGQGIGSQLVCWAIERAKErGCHLIQLTTDKQRPDALRFYEQLGFKA----S-HEGLKMHF-

519	1wwzA (157)<br>2g3tA (169)	C	15.29	TM-align: aliSize=136 (resi) RMSD=2.80 (Å)<br>mdEIKIEKLKKlDkkALNELIDVYMSGYEGLEEygGEGRDYaRNy-IK-WCW----KKASdgFFVAKVGD--------KIVGFIVCDkDWFSKyEGRIVGAIHEFVVDKKFOGKGIGRKLLITCLDFLGK-YNDTIELWVGEKNYGAMNLYEKFGFKKVG----KSGIwvrmikrq-------------<br>--KFVIRPATA-A--DCSDILRLIKELAKYEYM--EEQVIL-TE-kDLlEDGfgehPFYH--CLVAEVPKehwtpeghSIVGFAMYY-FTYDP-WIGKLLYLEDFFVMSDYRGFGIGSEILKNLSQVAMRcRCSSMHFLVAEWNEPSINFYKRRGASDLSseegWRLF--------kidkeyllkmatee<br><br>SARST: aliSize=121 (resi) RMSD=4.90 (Å)<br>mdeikieklkkld----------KKALNELIDVYMSGYEGLE-EYGGEgrdyarnyIKWCWKKASDGFF------VAKVGDK-------IVGFIVCDkDWFSKYEGrIVGAIHEFVVDKKFOGKGIGRKLLITCLDFLGKY-NDTIELWVGEKNYGAMNLYEKFGFKKV--------------------<br>------------kfvirpataaDCSDILRLIKELAKYEYMEeQVILT--------EKDLLEDGFGEHPfyhclvAEVPKEHwtpeghsIVGFAMYY-FTYDPWIG-KLLYLEDFFVMSDYRGFGIGSEILKNLSQVAMRCrCSSMHFLVAEWNEPSINFYKRRGASDLsseegwrlfkidkeyllkmat<br><br>--gksgiwvrmikrq<br>ee-------------<br><br>BLAST: aliSize=45 (resi) iden=14.65% (23/157) simi=28.66% (45/157)<br>mdeikieklkkldkkalnelidvymsgyegleeyggegrdyarnyikwcwkkasdgffvakv---GDKIVGFIVcdkdWFSKYE---GRIVgAIHEFVVDKKFOGKGIGRKLLITCLDFLGKYN-DTI<br>--kfvirpataadcsdilrlikelakyeymeeqviltekdlledgfgehpfyhclvaevpkehwtpeGHSIVGFAM----YYFTYDpwiGKLL-YLEDFFVMSDYRGFGIGSEILKNLSQVAMRCRcSSM<br><br>ELWVGEKNYGAMNLYEKFGFKKVGKSGIWvRMIK-------------rq<br>HFLVAEWNEPSINFYKRRGASDLSSEEGW-RLFKidkeyllkmatee--<br><br>Proposed: aliSize=140 (resi) RMSD=2.31 (Å)<br>mdEIKIEKLkKLDkkALNELIDVYMSGYEGLEEygGEGRDYaR--NyiK--WcW----KKASdgFFVAKVGD--------KIVGFIVCDkDWFSkyEG-RIVGAIHEFVVDKKFOGKGIGRKLLITCLDFLGK-YNDTIELWVGEKNYGAMNLYEKFGFKKVGkSG-IWVRMIKRQ----------a<br>--KFVIRPA-TAA--DCSDILRLIKELAKYEYM--EEQVIL-TekD--LleD-GfgehPFYH--CLVAEVPKehwtpeghSIVGFAMYY-FTYD--PWiGKLLYLEDFFVMSDYRGFGIGSEILKNLSQVAMRcRCSSMHFLVAEWNEPSINFYKRRGASDLS-SEeGWRLFKIDKeyllkmatee-
520	1wx7A (106)<br>1wx8A (96)	N	35.42	TM-align: aliSize=81 (resi) RMSD=2.01 (Å)<br>gssgssgspapv-----------QDPHLIKVTVKTPKDKEDFSVTDTCTIQQLKEEISQRFKAHPDQLVLIFAGKILKDPDSLAQCGVRDGLTVHLVIKRQ--H-RAmgnecpasgpssg<br>------------gssgssgvsgrePSSRIIRVSVKTPQDCHEFFLAENSNVRRFKKQISKYLHCNADRLVLIFTGKILRDQDILSQRGILDGSTVHVVVRSHsgPsSG-------------<br><br>SARST: aliSize=86 (resi) RMSD=6.15 (Å)<br>gs------SGSSGSPAPVQDphliKVTVKTPKDKEDFSVTDTCTIQQLKEEISQRFKAHPDQLVLIFAGKILKDPDSLAQCGVRDGLTVHLVIkrqHRAMG----necpasgpssg<br>--gssgssGVSGREPSSRII----RVSVKTPQDCHEFFLAENSNVRRFKKQISKYLHCNADRLVLIFTGKILRDQDILSQRGILDGSTVHVVV---RSHSGpssg-----------<br><br>BLAST: aliSize=67 (resi) iden=48.96% (47/96) simi=69.79% (67/96)<br>GSSGSSGSPAPVQDPHLIKVTVKTPKDKEDFSVTDTCTIQQLKEEISQRFKAHPDQLVLIFAGKILKDPDSLAQCGVRDGLTVHLVIkRQHramgnecpaSGPSSG<br>GSSGSSGVSGREPSSRIIRVSVKTPQDCHEFFLAENSNVRRFKKQISKYLHCNADRLVLIFTGKILRDQDILSQRGILDGSTVHVVV-RSH---------SGPSSG<br><br>Proposed: aliSize=93 (resi) RMSD=1.93 (Å)<br>-GSSGSSgSPAPVQDPHLIKVTVKTPKDKEDFSVTDTCTIQQLKEEISQRFKAHPDQLVLIFAGKILKDPDSLAQCGVRDGLTVHLVIKRQHR--AMgnecpasgpssg<br>gSSGSSG-VSGREPSSRIIRVSVKTPQDCHEFFLAENSNVRRFKKQISKYLHCNADRLVLIFTGKILRDQDILSQRGILDGSTVHVVVRSHSGpsSG------------
521	1yghA (164)<br>1i1dD (161)	C	11.18	TM-align: aliSize=125 (resi) RMSD=3.45 (Å)<br>-------KIEFRVVnndntkeNMMVLTGLKNIFOKQLPkmpKEYIA--RLv--Y--DR---------------SHLSMAVIRK-PLTVVGGITYRPFD-----KREFAEIVFCAISSTEOVRGYGAHLMNHLKDYVRNtSNIKYFLTYADNYAIGYFKKQGFTKEItldksiwmgy-IKDYEggtlmqcs<br>gsmslpdGFYIRRM-------EEGDLEQVTETLKVLTT---VGTITpeSF-ckLikYWneatvwndnedkkimQYNPMVIVDKrTETVAATGNIIIERkiiheLGLCGHIEDIAVNSKYQGQGLGKLLIDQLVTIGFD-YGCYKIILDCDEKNVKFYEKCGFSNAG----------vEMQIR--------<br><br>mlpriryld-<br>---------k<br><br>SARST: aliSize=110 (resi) RMSD=6.63 (Å)<br>kiefrvvnndntkenmmv------------------LTGLKNIFOKQ--LPKMPKEYIARLV-----------YDRSHLSM----AVIRKP--LTVV--GGITYRPFD-KREFAEIVFC--AISSTEOVRGYGAHLMNHLKDYVRNTsNIKYFLTYADNYAIGYFKKQGFT----------keitldksiw<br>------------------gsmslpdgfyirrmeegdLEQVTETLKVLttVGTITPESFCKLIkywneatvwnDNEDKKIMqynpMVIVDKrtETVAatGNIIIERKIiHELGLCGHIEdiAVNSKYQGQGLGKLLIDQLVTIGFDY-GCYKIILDCDEKNVKFYEKCGFSnagvemqirk----------<br><br>mgyikdyeggtlmqcsmlpriryld<br>-------------------------<br><br>BLAST: aliSize=42 (resi) iden=15.53% (25/161) simi=26.09% (42/161)<br>kiefrvvnn-------------------DNTKENMMVLTGLKNIFQKOLPKMPKEYIARLVYDRSHLSMAVIRKPLTVVggityrpfDKR-EFAEIVFCAISSTEQVRGYGahLMNHLKDYVRNTsniKYFLTYADNYAIGYFKKQGF-------------------------------------tkeitld<br>---------gsmslpdgfyirrmeegdlEQVTETLKVLTTVGTITPESFCKLIKYWNEATVWNDNEDKKIMQYNPMVIV--------DKRtETVAATGNIIIERKIIHELG--LCGHIEDIAVNS---KYQGQGLGKLLIDQLVTIGFdygcykiildcdeknvkfyekcgfsnagvemqirk-------<br><br>ksiwmgyikdyeggtlmqcsmlpriryld<br>-----------------------------<br><br>Proposed: aliSize=122 (resi) RMSD=2.43 (Å)<br>-------KIEFRVVnndntkeNMMVLTGLKNIFOKQLpkmPKE-yiA----rlvY--D--RS----------------HLSMAVIRK-PLTVVGGITYRPFdKR------EFAEIVFCAISSTEOVRGYGAHLMNHLKDYVRNtSNIKYFLTYADNYAIGYFKKQGFTKEitldksiwmgyikdyeggTL<br>gsmslpdGFYIRRM-------EEGDLEQVTETLKVLT---TVGt--Itpes---FckLikYWneatvwndnedkkimqYNPMVIVDKrTETVAATGNIIIE-RKiihelgLCGHIEDIAVNSKYQGQGLGKLLIDQLVTIGFD-YGCYKIILDCDEKNVKFYEKCGFSNA-----------------GV<br><br>MOCSMLpriryld<br>EMQIRK-------

522	1yghA (164)<br>1s3zB (145)	C	8.28	TM-align: aliSize=123 (resi) RMSD=2.69 (Å)<br>kIEFRVVnndntkeNMMVLTGLKNIFQKQLPkMPKEYIA-RLVYD--RS--HLSMAVIRKPlTVVGGITYRPFDK-------REFAEIVFCAISSTEQVRGYGAHLMNHLKDYVRnTSNIKYFLTYAD-NY--AIGYFKKQGFTKEItldksiwmgyik---DYeggtlmqcsmlpriryld----<br>-MDIRQM-------NKTHLEHWRGLRKQLWP-GHPDDAHlADGEEilQAdhLASFIAMADG-VAIGFADASIRHDyvngcdsSPVVFLEGIFVLPSFRQRGVAKQLIAAVQRWGT-NKGCREMASDTSpENtiSQKVHQALGFEETE------------rviFY------------------rkrc<br><br>SARST: aliSize=107 (resi) RMSD=3.37 (Å)<br>kiefrvvnndntkenmmv----------LTGLKNIFQKQLPKMPKE----YIARLVYDRSHLSMAVIRKpLTVVGGITYRPFdkR----E-----FAEIVFCAISSTEQVRGYGAHLMNHLKDYVRNTsNIKYFLTYADNYA---IGYFKKQGFT-----------keitldksiwmgyikdyeggtlm<br>-----------------mdirqmnkthLEHWRGLRKQLWPGHPDDahlaDGEEILQADHLASFIAMAD-GVAIGFADASIR--HdyvnGcdsspVVFLEGIFVLPSFRQRGVAKQLIAAVQRWGTNK-GCREMASDTSPENtisQKVHQALGFEetervifyrkrc----------------------<br>qcsmlpriryld<br>------------<br><br>BLAST: aliSize=14 (resi) iden=6.21% (9/145) simi=9.66% (14/145)<br>kiefrvvnndntkenmmvltglknifqkqlpkmpkeyiarlvydrshlsmavirkpltvvggityrpfdkrefaeivfcaissteqvrgygahlmn----------------------------HLKD--YVRNTSNIKYFLTYADNYAIGY--<br>---mdirqmnkthlehwrglrkqlwpghpddaHLADgeEILQADHLASFIAMADGVAIGFadasirhdyvngcdsspvvflegifvlpsfrqrgvak<br>--fkkqgftkeitldksiwmgyikdyeggtlmqcsmlpriryld<br>qliaavqrwgtnkgcremasdtspentisqkvhqalgfeetervifyrkrc--<br><br>Proposed: aliSize=130 (resi) RMSD=2.33 (Å)<br>kIEFRVVnndntkeNMMVLTGLKNIFQKQLPKMPKEYIARLVYD--RS--HLSMAVIRKpLTVVGGITYRPFdK-------REFAEIVFCAISSTEQVRGYGAHLMNHLKDYVRnTSNIKYFLTYAD-NY--AIGYFKKQGFTKEitldksiwmgyikdyeggTLMQCSMLPrI-ryld<br>-MDIRQM-------NKTHLEHWRGLRKQLWPGHPDDAHLADGEEilQAdhLASFIAMAD-GVAIGFADASIR-HdyvngcdsSPVVFLEGIFVLPSFRQRGVAKQLIAAVQRWGT-NKGCREMASDTSpENtiSQKVHQALGFEET-----------------ERVIFYRKR-Cc----
523	1yghA (164)<br>1s5kA (153)	C	7.19	TM-align: aliSize=123 (resi) RMSD=2.77 (Å)<br>-------KIEFRVVnndntkeNMMVLTGLKNIFQKQLPkmPKE-YIA-RLVYDR--S--HLSMAVIRKPlTVVGGITYRPFDK-------REFAEIVFCAISSTEQVRGYGAHLMNHLKDYVRnTSNIKYFLTYAD-NY--AIGYFKKQGFTKEItldksiwmgyik---DYeggtlmqcsmlpriryld<br>glvprgsHMDIRQM-------NKTHLEHWRGLRKQLWP--GHPdDAHlADGEEIlqAdhLASFIAMADG-VAIGFADASIRHDyvngcdsSPVVFLEGIFVLPSFRQRGVAKQLIAAVQRWGT-NKGCREMASDTSpENtiSQKVHQALGFEETE------------rviFY------------------<br>----<br>rkrc<br><br>SARST: aliSize=117 (resi) RMSD=3.93 (Å)<br>k--------IEFRVVNndntkenMMVLTGLKNIFQKQLPKMP----KEYIARLVYDRSHLSMAVIRKpLTVVGGITYR----PFdkRE-----FAEIVFCAISSTEQVRGYGAHLMNHLKDYVRNTsNIKYFLTYADNYAIG---YFKKQGFT-----------keitldksiwmgyikdyeggtlmqc<br>-glvprgshMDIRQMN-------KTHLEHWRGLRKQLWPGHPddahLADGEEILQADHLASFIAMAD-GVAIGFADASirhdYV--NGcdsspVVFLEGIFVLPSFRQRGVAKQLIAAVQRWGTNK-GCREMASDTSPENTIsqkVHQALGFEetervifyrkrc-------------------------<br>smlpriryld<br>----------<br><br>BLAST: aliSize=14 (resi) iden=5.88% (9/153) simi=9.15% (14/153)<br>kiefrvvnndntkenmmvltglknifqkqlpkmpkeyiarlvydrshlsmavirkpltvvggityrpfdkrefaeivfcaissteqvrgygahlmn-------------------------------------HLKD--YVRNTSNIKYFLTYADNYAIGY---------------------------------<br>---glvprgshmdirqmnkthlehwrglrkqlwpghpddaHLADgeEILQADHLASFIAMADGVAIGFadasirhdyvngcdsspvvflegifvlps<br>--fkkqgftkeitldksiwmgyikdyeggtlmqcsmlpriryld<br>frqrgvakqliaavqrwgtnkgcremasdtspentisqkvhqalgfeetervifyrkrc--<br><br>Proposed: aliSize=129 (resi) RMSD=2.32 (Å)<br>-------KIEFRVVnndntkeNMMVLTGLKNIFQKQLPkmPKE--YIARLVYD--RS--HLSMAVIRKPlTVVGGITYRPFdK-------REFAEIVFCAISSTEQVRGYGAHLMNHLKDYVRnTSNIKYFLTYAD-NY--AIGYFKKQGFTKEitldksiwmgyikdyeggTLMQCSMLPrI-ryld<br>glvprgsHMDIRQM-------NKTHLEHWRGLRKQLWP--GHPddAHLADGEEilQAdhLASFIAMADG-VAIGFADASIR-HdyvngcdsSPVVFLEGIFVLPSFRQRGVAKQLIAAVQRWGT-NKGCREMASDTSpENtiSQKVHQALGFEET------------------ERVIFYRKR-Cc----
524	1yghA (164)<br>1ufhA (155)	C	10.97	TM-align: aliSize=125 (resi) RMSD=2.85 (Å)<br>kIEFRVVNndntkeNMMVLTGLKNIFQKQLPK-M-----------P--KE-YIARLV--YD-RS-HLSMAVI-RKPlTVVGGITYRPFDKR--EFAEIVFCAISSTEQVRGYGAHLMNHLKDYVRNtSNIKYFLTYADN-Y--AIGYFKKQGFTKEItldksiwmgyikdye-GGtlmqcsmlpriryld<br>-TIMLTPM------QTEEFRSYLTYTTKHYAEeKvkagtwlpedaQllSKqVFTDLLprGLeTPhHHLWSLKlNEK-DIVGWLWIHAEPEHpqQEAFIYDFGLYEPYRGKGYAKQALAALDQAARS-MGIRKLSLHVFAhNqtARKLYEQTGFQETD---------------vVM--------------<br>----<br>skkl<br><br>SARST: aliSize=128 (resi) RMSD=9.95 (Å)<br>kiefrvvnndnt------------KENMMVLTGLKNIFQKQLPKMPKEYIARLV---------YDRSHLSMAVIRK---PLTVVGGITYRPFD--KREFAEIVFCAISSTEQVRGYGAHLMNHLKDYVRNTsNIKYFLTYADNYAIGYFKKqgftkeitldkSIWmgyikdyEGGTLMQCSMLPR-iryld<br>------------timltpmqteefRSYLTYTTKHYAEEKVKAGTWLPEDAQLLSkqvftdllpRGLETPHHHLWSLklnEKDIVGWLWIHAEPehPQQEAFIYDFGLYEPYRGKGYAKQALAALDQAARSM-GIRKLSLHVFAHNQTARKL-----------YEQ-------TGFQETDVVMSKKl-----<br><br>BLAST: aliSize=25 (resi) iden=9.03% (14/155) simi=16.13% (25/155)<br>kiefrvvnndntkenmmvltglknifqkqlpkmpkeyiarlvydrshlsmavirkpltvvggityrpfdkrefaeivfcaisst---EQVRG--YGA<br>---timltpmqteefrsyltyttkhyaeekvkagtwlpedaqllskqvftdllprgletphhhlwslklnekdivgwlwihaepehpqqeafiydfglyEPYRGkgYAK<br>HLMNHLKDYVRNTSNIKYFL-TYADNY-AIGYFKKQGFTK-EITLDKSIwmgyikdyeggtlmqcsmlpriryld<br>QALAALDQAARSMGIRKLSLhVFAHNQtARKLYEQTGFQEtDVVMSKKL---------------------------<br><br>Proposed: aliSize=128 (resi) RMSD=2.41 (Å)<br>k-IEFRVVnndntkeNMMVLTGLKNIFQKQLPK-M------------PKE-YIaR-LV--Y--DRSHLSMAVI-RKPlTVVGGITYRPFDKR--EFAEIVFCAISSTEQVRGYGAHLMNHLKDYVRNtSNIKYFLTYADN-Y--AIGYFKKQGFTKeitldksiwmgyikdyEGGtLMQCSMlpriryld<br>-tIMLTPM-------QTEEFRSYLTYTTKHYAEeKvkagtwlpedaqlLSKqVF-TdLLprGleTPHHHLWSLKlNEK-DIVGWLWIHAEPEHpqQEAFIYDFGLYEPYRGKGYAKQALAALDQAARS-MGIRKLSLHVFAhNqtARKLYEQTGFQE----------------TDV-VMSKKL--------

525	1yghA (164)<br>1ufhB (154)	C	11.04	TM-align: aliSize=125 (resi) RMSD=2.66 (Å)<br>kIEFRVVnndntkeNMMVLTGLKNIFQKQLPK-M------------PKEYIAR-LV--YD-RS-HLSMAVIR-KPlTVVGGITYRPFDKR--EFAEIVFCAISSTEQVRGYGAHLMNHLKDYVRNtSNIKYFLTYADN-Y--AIGYFKKQGFTKEItldksiwmgyikDyeGGtlmqcsmlpriryld----<br>-IMLTPM-------QTEEFRSYLTYTTKHYAEeKvkagtwlpedaqlLSKQVFTdLLprGLeTPhHHLWSLKLnEK-DIVGWLWIHAEPEHpqQEAFIYDFGLYEPYRGKGYAKQALAALDQAARS-MGIRKLSLHVFAhNqtARKLYEQTGFQETD-----------V--VM--------------skkl<br><br>SARST: aliSize=116 (resi) RMSD=5.59 (Å)<br>kiefrvvnndnt----------KENMMVLTGLKNIFQKQLPKMPKEYIARLV--------YDRSHLSMAVIRK---PLTVVGGITYRPFD--KREFAEIVFCAISSTEQVRGYGAHLMNHLKDYVRNTsNIKYFLTYADNYAIGY---FKKQGFT---------keitldksiwmgyikdyeggtlm<br>-----------imltpmqteefRSYLTYTTKHYAEEKVKAGTWLPEDAQLLSkqvftdllpRGLETPHHHLWSLklnEKDIVGWLWIHAEPehPQQEAFIYDFGLYEPYRGKGYAKQALAALDQAARSM-GIRKLSLHVFAHNQTArklYEQTGFQetdvvmskkl---------------------<br><br>qcsmlpriryld<br>------------<br><br>BLAST: aliSize=25 (resi) iden=9.09% (14/154) simi=16.23% (25/154)<br>kiefrvvnndntkenmmvltglknifqkqlpkmpkeyiarlvydrshlsmavirkpltvvggityrpfdkrefaeivfcaisst---EQVRG--YGAH<br>--imltpmqteefrsyltyttkhyaeekvkagtwlpedaqllskqvftdllprgletphhhlwslklnekdivgwlwihaepehpqqeafiydfglyEPYRGkgYAKQ<br><br>LMNHLKDYVRNTSNIKYFL-TYADNY-AIGYFKKQGFTK-EITLDKSIwmgyikdyeggtlmqcsmlpriryld<br>ALAALDQAARSMGIRKLSLhVFAHNQtARKLYEQTGFQEtDVVMSKKL------------------------<br><br>Proposed: aliSize=129 (resi) RMSD=2.25 (Å)<br>kIEFRVVnndntkeNMMVLTGLKNIFQKQLPK-M------------PKEYIAR-LV--Y--DRSHLSMAVIR-KPlTVVGGITYRPFDKR--EFAEIVFCAISSTEQVRGYGAHLMNHLKDYVRNTSnIKYFLTYADN-Y--AIGYFKKQGFTKeitldksiwmgyikdyEGGtLMQCSMlpriryld<br>-IMLTPM-------QTEEFRSYLTYTTKHYAEeKvkagtwlpedaqlLSKQVFTdLLprGleTPHHHLWSLKLnEK-DIVGWLWIHAEPEHpqQEAFIYDFGLYEPYRGKGYAKQALAALDQAARSMG-IRKLSLHVFAhNqtARKLYEQTGFQE---------------TDV-VMSKKL--------
526	1yghA (164)<br>2d4pA (130)	C	7.69	TM-align: aliSize=117 (resi) RMSD=3.34 (Å)<br>kIEFRVVnndntkeNMMVLTGLKNIFqKqlPkmpKEyIARLVYDRS--H-LSMAVIRKpLTVVGGITYRPFD--KREFAEIVFCAISsteQvRGYGAHLMNHLKDYVRNtSNIKYFLTYADNYA--IGY-FKKQ-GFTKEITldksiw-MgyiKDYEggtlmqcsmlpriryld---<br>-MRFRPF-------TEEDLDRLNRLA-G--K---RP-VSLGALRFFarTgHSFLAEEG-EEPMGFALAQAVWqgEATTVLVTRIEGR---S-VEALRGLLRAVVKSAYD-AGVYEVALHLDPERkeLEEaLKAEgFALGPLV------lA---VRVL----------------gsr<br><br>SARST: aliSize=100 (resi) RMSD=4.69 (Å)<br>kiefrvvnndntkenmmvltgl---------KNIFQKQLPKMP--KEYIARLVYDRSHLSMAVirkPLTVVGGITYRPFDKREFAEI--VFCAISsteqvRGYGAHLMNHLKDYVRNTSNIKYFLTYADNYAIGYFK---KQGFTK------------eitldksiwmgyikdyeggtlmqcsmlpri<br>--------------------mrfrpfteedLDRLNRLAGKRPvsLGALRFFARTGHSFLAEE---GEEPMGFALAQAVWQGEATTVlvTRIEGR-----SVEALRGLLRAVVKSAYDAGVYEVALHLDPERKELEEalkAEGFALgplvlavrvlgsr----------------------------<br><br>ryld<br>----<br><br>BLAST: aliSize=8 (resi) iden=3.08% (4/130) simi=6.15% (8/130)<br>kiefrvvnndntkenmmvltglknifqkqlpkmpkey--IARLVYDRSHLSMAVIRKP-----------------------------ltvvggityrpfdkrefaeivfc<br>-------------------------------------mrfrpfteedldrlnrlagkrpvslgalrffartghsflaeegeepmgfalaqavwqgeattvlvtriegrsvealrgllraVVKSAYDAGVYEVALHLDPerkeleealkaegfalgplvlavrvlgsr-----------------------<br><br>aissteqvrgygahlmnhlkdyvrntsnikyfltyadnyaigyfkkqgftkeitldksiwmgyikdyeggtlmqcsmlpriryld<br>---<br><br>Proposed: aliSize=119 (resi) RMSD=2.58 (Å)<br>kIEFRVVnndntkeNMMVLTGLKNIFqKqlPkMpKeyIA-RLVYDR-SH-LSMAVIRKpLTVVGGITYRPFdKR---EFAEIVFCAISsteQvRGYGAHLMNHLKDYVRNtSNIKYFLTYADNYAiG-YF--KK-Q-GFTKEItldksiwmgyikdyeggtLMQCSMLPRIryld<br>-MRFRPF-------TEEDLDRLNRLA-G--K-R-P--VSlGALRFFaRTgHSFLAEEG-EEPMGFALAQAV-WQgeaTTVLVTRIEGR---S-VEALRGLLRAVVKSAYD-AGVYEVALHLDPER-KeLEeaLKaEgFALGPL-----------------VLAVRVLGSR----
527	1yghA (164)<br>2f5iA (167)	C	9.15	TM-align: aliSize=132 (resi) RMSD=3.21 (Å)<br>KIEFRVVNndnTkENMMVLTGLKNIFQKQLPKMPKEYiARl--VY--DR-----SHLSMAVIRKP-------LTVVGGITYRPFDK---REFAEIVFCAISSTEQVRGYGAHLMNHLKDYVRNtSNIKYFLTYAD-NY--AIGYFKKQGFTKEItl--DKsiwMgyIKDYeggtlmqcsmlpriryld--<br>KFVIRPAT---A-ADCSDILRLIKELAKYEYMEEQVI-LT-ekDLleDGfgehpFYHCLVAEVPKehwtpegHSIVGFAMYYFTYDpwiGKLLYLEDFFVMSDYRGFGIGSEILKNLSQVAMR-CRCSSMHFLVAeWNepSINFYKRRGASDLS--seEG---W--RLFK------------------id<br><br>---------<br>keyllkmat<br><br>SARST: aliSize=138 (resi) RMSD=7.00 (Å)<br>kie-FRVVNNDNTKENMMVLTGLKNIFQKQLP---KMPKEYIARLVYDR--SHLSMAVIRKP-------LTVVGGITYRP--FDKRE-FAEIVFCAISSTEQVRGYGAHLMNHLKDYVRNTsNIKYFLTYADNYAIGYF---KKQGFTkeitlDKSIwmgyikdYEGGTLMQC---------smlpriryld<br>---kFVIRPATAADCSDILRLIKELAKYEYMEeqvILTEKDLLEDGFGEhpFYHCLVAEVPKehwtpegHSIVGFAMYYFtyDPWIGkLLYLEDFFVMSDYRGFGIGSEILKNLSQVAMRC-RCSSMHFLVAEWNEPSInfyKRRGAS-----DLSS-------EEGWRLFKIdkeyllkmat----------<br><br>BLAST: aliSize=42 (resi) iden=12.20% (20/164) simi=25.61% (42/164)<br>kiefrvvnndntkenmmvltglknifqkqlpkmpkeyiarlvydrshlsmavirkpltvvggi---TYRPF-DKREFAEIVFcaISSTEQVRGYGAHLMNHLKDY-VRNTSNIKYF<br>---kfvirpataadcsdilrlikelakyeymeeqviltekdlledgfgehpfyhclvaevpkehwtpeghsivgfamyyfTYDPWiGKLLYLEDFF--VMSDYRGFGIGSEILKNLSQVaMRCRCSSMHF<br><br>LTYADNY-AIGYFKKQGfTKEITLDKSiWMGYIKDYE-------ggtlmqcsmlpriryld<br>LVAEWNEpSINFYKRRG-ASDLSSEEG-WRLFKIDKEyllkmat-----------------<br><br>Proposed: aliSize=136 (resi) RMSD=2.36 (Å)<br>KIEFRVVNndnTkENMMVLTGLKNIFQKQLPKMPKEyiAR----lvY--DR-----SHLSMAVIRKP-------LTVVGGITYRPFdK----REFAEIVFCAISSTEQVRGYGAHLMNHLKDYVRNtSNIKYFLTYAD-NY--AIGYFKKQGFTK---eitldksiwmgyikdyeGGTLMQCSMLP--RI<br>KFVIRPAT---A-ADCSDILRLIKELAKYEYMEEQV--ILtekd--LleDGfgehpFYHCLVAEVPKehwtpegHSIVGFAMYYFT-YdpwiGKLLYLEDFFVMSDYRGFGIGSEILKNLSQVAMR-CRCSSMHFLVAeWNepSINFYKRRGASDlss----------------EEGWRLFKIDKeyLL<br><br>R-YLd<br>KmAT-

528	1yghA (164)<br>2g3tA (169)	C	10.37	TM-align: aliSize=132 (resi) RMSD=3.22 (Å)<br>KIEFRVVNndnTkENMMVLTGLKNIFQKQLPKMPKEYiARl--VY--DR-----SHLSMAVIRKP-------LTVVGGITYRPFDK---REFAEIVFCAISSTEQVRGYGAHLMNHLKDYVRNtSNIKYFLTYAD-NY--AIGYFKKQGFTKEItl--DKsiwMgvIKDYeggtlmqcsmlpriryld--<br>KFVIRPAT---A-ADCSDILRLIKELAKYEYMEEQVI-LT-ekDLleDGfgehpFYHCLVAEVPKehwtpegHSIVGFAMYYFTYDpwiGKLLYLEDFFVMSDYRGFGIGSEILKNLSQVAMR-CRCSSMHFLVAeWNepSINFYKRRGASDLS--seEG---W--RLFK------------------id<br><br>-----------<br>keyllkmatee<br><br>SARST: aliSize=128 (resi) RMSD=7.32 (Å)<br>kiefrvvnndntke-----------NMMVLTGLKNIFQKQLP----KMPKEYIARLVYDR--SHLSMAVIRKPLT-------VVGGITYRP--FDKRE-FAEIVFCAISSTEQVRGYGAHLMNHLKDYVRNTsNIKYFLTYADNYAIGYFK---KQGFTkeitlDKSIwmgvikdYEGGTLMQCS-----<br>-------------kfvirpataadCSDILRLIKELAKYEYMeeqvILTEKDLLEDGFGEhpFYHCLVAEVPKEHwtpeghsIVGFAMYYFtyDPWIGkLLYLEDFFVMSDYRGFGIGSEILKNLSQVAMRC-RCSSMHFLVAEWNEPSINfykRRGAS-----DLSS-------EEGWRLFKIDkeyll<br><br>------mlpriryld<br>kmatee---------<br><br>BLAST: aliSize=42 (resi) iden=12.20% (20/164) simi=25.61% (42/164)<br>kiefrvvnndntkenmmvltglknifqkqlpkmpkeyiarlvydrshlsmavirkpltvvggi--TYRPF-DKREFAEIVFcaISSTEQVRGYGAHLMNHLKDY-VRNTSNIKYF<br>--kfvirpataadcsdilrlikelakyeymeeqviltekdlledgfgehpfyhclvaevpkehwtpeghsivgfamyyfTYDPWiGKLLYLEDFF--VMSDYRGFGIGSEILKNLSQVaMRCRCSSMHF<br><br>LTYADNY-AIGYFKKQGfTKEITLDKSiWMGYIKDYE---------ggtlmqcsmlpriryld<br>LVAEWNEpSINFYKRRG-ASDLSSEEG-WRLFKIDKEyllkmatee-----------------<br><br>Proposed: aliSize=137 (resi) RMSD=2.48 (Å)<br>KIEFRVVNndnTkENMMVLTGLKNIFQKQLPKMPKEyiAR----lvY--DR-----SHLSMAVIRKP-------LTVVGGITYRPFdK----REFAEIVFCAISSTEQVRGYGAHLMNHLKDYVRNtSNIKYFLTYAD-NY--AIGYFKKQGFTKeitldksiwmgvikD--YEGGtLMQCSM--L-P--<br>KFVIRPAT---A-ADCSDILRLIKELAKYEYMEEQV--ILtekd--LleDGfgehpFYHCLVAEVPKehwtpegHSIVGFAMYYFT-YdpwiGKLLYLEDFFVMSDYRGFGIGSEILKNLSQVAMR-CRCSSMHFLVAeWNepSINFYKRRGASD-------------LssEEGW-RLFKIDkeYlLkm<br><br>RIRYld<br>ATEE--
529	1yghA (164)<br>2g3tB (168)	C	9.15	TM-align: aliSize=132 (resi) RMSD=3.22 (Å)<br>KIEFRVVNndnTkENMMVLTGLKNIFQKQLPKMPKEYiARl--VY--DR-----SHLSMAVIRKP-------LTVVGGITYRPFDK---REFAEIVFCAISSTEQVRGYGAHLMNHLKDYVRNtSNIKYFLTYAD-NY--AIGYFKKQGFTKEItl--DKsiwMgyIKDYeggtlmqcsmlpriryld--<br>KFVIRPAT---A-ADCSDILRLIKELAKYEYMEEQVI-LT-ekDLleDGfgehpFYHCLVAEVPKehwtpegHSIVGFAMYYFTYDpwiGKLLYLEDFFVMSDYRGFGIGSEILKNLSQVAMR-CRCSSMHFLVAeWNepSINFYKRRGASDLS--seEG---W--RLFK------------------id<br><br>----------<br>keyllkmate<br><br>SARST: aliSize=136 (resi) RMSD=6.64 (Å)<br>kie-FRVVNNdnTKENMMVLTGLKNIFQKQLP-----KMPKEYIARLVYDR--SHLSMAVIRKP-------LTVVGGITYRP--FDKREFAE-IVFCAISSTEQVRGYGAHLMNHLKDYVRNTsNIKYFLTYADNYAIGYFK---KQGFTkeitlDKSIwmgvikdYEGGTLMQC-----------smlp<br>---kFVIRPA--TAADCSDILRLIKELAKYEYmeeqvILTEKDLLEDGFGEhpFYHCLVAEVPKehwtpegHSIVGFAMYYFtyDPWIGKLLyLEDFFVMSDYRGFGIGSEILKNLSQVAMRC-RCSSMHFLVAEWNEPSINfykRRGAS-----DLSS-------EEGWRLFKIdkeyllkmate----<br><br>riryld<br>------<br><br>BLAST: aliSize=42 (resi) iden=12.20% (20/164) simi=25.61% (42/164)<br>kiefrvvnndntkenmmvltglknifqkqlpkmpkeyiarlvydrshlsmavirkpltvvggi--TYRPF-DKREFAEIVFcaISSTEQVRGYGAHLMNHLKDY-VRNTSNIKYF<br>--kfvirpataadcsdilrlikelakyeymeeqviltekdlledgfgehpfyhclvaevpkehwtpeghsivgfamyyfTYDPWiGKLLYLEDFF--VMSDYRGFGIGSEILKNLSQVaMRCRCSSMHF<br><br>LTYADNY-AIGYFKKQGfTKEITLDKSiWMGYIKDYE--------ggtlmqcsmlpriryld<br>LVAEWNEpSINFYKRRG-ASDLSSEEG-WRLFKIDKEyllkmate-----------------<br><br>Proposed: aliSize=136 (resi) RMSD=2.35 (Å)<br>KIEFRVVNndnTkENMMVLTGLKNIFQKQLPKMPKEyiAR----lvY--DR-----SHLSMAVIRKP-------LTVVGGITYRPFdK----REFAEIVFCAISSTEQVRGYGAHLMNHLKDYVRNtSNIKYFLTYAD-NY--AIGYFKKQGFTK---eitldksiwmgvikdveGGTLMQCSMLP--RI<br>KFVIRPAT---A-ADCSDILRLIKELAKYEYMEEQV--ILtekd--LleDGfgehpFYHCLVAEVPKehwtpegHSIVGFAMYYFT-YdpwiGKLLYLEDFFVMSDYRGFGIGSEILKNLSQVAMR-CRCSSMHFLVAeWNepSINFYKRRGASDlss----------------EEGWRLFKIDKeyLL<br><br>R-YL-d<br>KmATe-
530	1yghA (164)<br>2o28B (180)	C	10.37	TM-align: aliSize=126 (resi) RMSD=3.39 (Å)<br>-----------------------------------KIEFRVVnndntkeNMMVLT-GLKNIFQKQLPkmpKEYI---ARl--VY-DR--S--HLSMAVIRKP-LTVVGGITYRPFD-----KREFAEIVFCAISSTEQVRGYGAHLMNHLKDYVRNtSNIKYFLTYADNYAIGYFKKQGFTKEITldksi<br>pdetpmfdpsllkevdwsqntatfspaispthpgeGLVLRPL-------CTADLNrGFFKVLGQLTE---TGVVspeQF-mkSFeHMkkSgdYYVTVVEDVTlGQIVATATLIIEHkfihsCAKRGRVEDVVVSDECRGKQLGKLLLSTLTLLSKK-LNCYKITLECLPQNVGFYKKFGYTVSEE-----<br><br>wmgy-IKDYEggtlmqcsmlpriryld-<br>----nYMCRR-----------------f<br><br>SARST: aliSize=109 (resi) RMSD=9.32 (Å)<br>kiefrvv--NNDNTKENMMVLTGLKNIFQKQLpkmpkeyiarlvydrshLSMAVIRK--PLTVVGGITYRPF--DKRE---FAEIVFCAISSTEQVRGYGAHLMNHLKDYVRNTsNIKYFLTYADNYAIGYFKK<br>-------pdetpmfdpsllkevdwsqntatfspaispthpgeglvlrplctadlnrgffkvlgqlTETGVVSPEQFMKSFEHMKKSGD-----------------YYVTVVEDvtLGQIVATATLIIEhkFIHScakRGRVEDVVVSDECRGKQLGKLLLSTLTLLSKKL-NCYKITLECLPQNVGFYKK<br><br>QGFTkeitldksiwmgyikdyeggtlmqcSMLPR------iryld<br>FGYT-------------------------VSEENymcrrf-----<br><br>BLAST: aliSize=20 (resi) iden=7.32% (12/164) simi=12.20% (20/164)<br>kiefrvvnndntkenmmvltglknifqkqlpkmpkeyiarlvydr------------------------------SHLSMAVIRKPLTVVGgiTYRPFDKrEFAEIVFCAISSTEQVRGYGAHLMNHLKDYV--<br>---pdetpmfdpsllkevdwsqntatfspaispTHPGEGLVLRPLCTAD--LNRGFFK-VLGQLTETGVVSPEQFMKSFEHMKKSGDYYVtvvedvtlgqivatatliiehkfihscakrgrvedvvvsdecrgkqlgklllstltll<br><br>---------------------------------------rntsnikyfltyadnyaigyfkkqgftkeitldksiwmgyikdyeggtlmqcsmlpriryld<br>skklncykitleclpqnvgfykkfgytvseenymcrrf---<br><br>Proposed: aliSize=123 (resi) RMSD=2.43 (Å)<br>-----------------------------------KIEFRVVnndntkeNMMVlT--GLKNIFQKQLpkmPKE-yiA----rlvY--D--RS-----HLSMAVIRKP-LTVVGGITYRPFDK-----REFAEIVFCAISSTEQVRGYGAHLMNHLKDYVRNtSNIKYFLTYADNYAIGYFKKQGFTKEit<br>pdetpmfdpsllkevdwsqntatfspaispthpgeGLVLRPL-------CTAD-LnrGFFKVLGQLT---ETGv--Vspeq---FmkSfeHMkksgdYYVTVVEDVTlGQIVATATLIIEHKfihscAKRGRVEDVVVSDECRGKQLGKLLLSTLTLLSKK-LNCYKITLECLPQNVGFYKKFGYTVS--<br><br>ldksiwmgyikdyegGTLMQCSML-priryld<br>---------------EENYMCRRFc-------

<table>
<tr><td rowspan="2">531</td><td>1yghA
(164)</td><td rowspan="2">C</td><td rowspan="2">8.33</td><td rowspan="2">TM-align: aliSize=122 (resi) RMSD=2.72 (Å)
kiEFRVVnndntkeNMMVLTGLKNIFQKQLPkMPKEYIA-RLVYDR--S--HLSMAVIRKpLTVVGGITYRPFDK-------REFAEIVFCAISSTEQVRGYGAHLMNHLKDYVRnTSNIKYFLTYAD-NY--AIGYFKKQGFTKEItldksiwmgyik---DYeggtlmqcsmlpriryld----
--DIRQM-------NKTHLEHWRGLRKQLWP-GHPDDAHlADGEEIlqAdhLASFIAMAD-GVAIGFADASIRHDyvngcdsSPVVFLEGIFVLPSFRQRGVAKQLIAAVQRWGT-NKGCREMASDTSpENtiSQKVHQALGFEETE------------rviFY------------------rkrc

SARST: aliSize=109 (resi) RMSD=4.19 (Å)
kiefrvvnndntkenmmv---------LTGLKNIFQKQLPKMPKEYIARLV----YDRSHLSMAVIRKpLTVVGGITYR----PFDKREFA---EIVFCAISSTEQVRGYGAHLMNHLKDYVRNTsNIKYFLTYADNYAIGYFK---KQGFT------------keitldksiwmgyikdyeggtlmqcs
------------------dirqmnkthLEHWRGLRKQLWPGHPDDAHLADGeeilQADHLASFIAMAD-GVAIGFADASirhdYVNGCDSSpvvFLEGIFVLPSFRQRGVAKQLIAAVQRWGTNK-GCREMASDTSPENTISQKvhqALGFEetervifyrkrc-------------------------

mlpriryld

BLAST: aliSize=14 (resi) iden=6.25% (9/144) simi=9.72% (14/144)
kiefrvvnndntkenmmvltglknifqkqlpkmpkeyiarlvydrshlsmavirkpltvvggityrpfdkrefaeivfcaissteqvrgygahlmn----------------------------HLKD--YVRNTSNIKYFLTYADNYAIGY--------------------------------------
--dirqmnkthlehwrglrkqlwpghpddaHLADgeEILQADHLASFIAMADGVAIGFadasirhdyvngcdsspvvflegifvlpsfrqrgvakq

--fkkqgftkeitldksiwmgyikdyeggtlmqcsmlpriryld
liaavqrwgtnkgcremasdtspentisqkvhqalgfeetervifyrkrc--

Proposed: aliSize=129 (resi) RMSD=2.37 (Å)
kiEFRVVnndntkeNMMVLTGLKNIFQKQLPKMPKEYIARLVYD--RS--HLSMAVIRKpLTVVGGITYRPFdK-------REFAEIVFCAISSTEQVRGYGAHLMNHLKDYVRnTSNIKYFLTYAD-NY--AIGYFKKQGFTKEitldksiwmgyikdyeggTLMQCSMLPrIryld
--DIRQM-------NKTHLEHWRGLRKQLWPGHPDDAHLADGEEilQAdhLASFIAMAD-GVAIGFADASIR-HdyvngcdsSPVVFLEGIFVLPSFRQRGVAKQLIAAVQRWGT-NKGCREMASDTSpENtiSQKVHQALGFEET------------------ERVIFYRKR-C----</td></tr>
<tr><td>2vbqB
(144)</td></tr>
<tr><td rowspan="2">532</td><td>1yghA
(164)</td><td rowspan="2">C</td><td rowspan="2">10.37</td><td rowspan="2">TM-align: aliSize=124 (resi) RMSD=3.22 (Å)
---------------------KIEFRVVnndntkeNMMVLT-GLKNIFQKQLPkmpKEy-I---ARl--VY-DR--S---HLSMAVIRKPLTVVGGITYRPFD-----KREFAEIVFCAISSTEQVRGYGAHLMNHLKDYVRNtSNIKYFLTYADNYAIGYFKKQGFTKEItldksiwmgy-IKDYEggt
entplfspslispdvlavlpaDYTIRPL-------CRSDYKrGYLDVLRVLTT---VG-dIneeQW-nsRYeWIraRsdeYYLLVVCDGEGRIVGTGSLVVERkfihsLGMVGHIEDIAVEKGQQGKKLGLRIIQALDYVAEK-VGCYKTILDCSEANEGFYIKCGFKRAG----------lEMAHY---

lmqcsmlpriryld-
--------------y

SARST: aliSize=108 (resi) RMSD=9.62 (Å)
kiefrvvnndntkenm---MVLTGLKNIFQKQLPKmpkeyiarlvydrSHLSMAVIRKPLTVVGGITYRPF---DKREFAEIVFC--AISSTEQVRGYGAHLMNHLKDYVRNTsNIKYFLTYADNYAIGYFKKQGFTkeitl
----------------entplfspslispdvlavlpadytirplcrsdykrgyldvlrvlttvgdinEEQWNSRYEWIRARSD-------------EYYLLVVCDGEGRIVGTGSLVVErkfIHSLGMVGHIEdiAVEKGQQGKKLGLRIIQALDYVAEKV-GCYKTILDCSEANEGFYIKCGFK-----

dksiwmgyikdyeggtLMQCSMLPR-iryld
----------------RAGLEMAHYy-----

BLAST: aliSize=25 (resi) iden=8.54% (14/164) simi=15.24% (25/164)
kiefrvvnndntkenmmvltglknifqkqlpkmpkeyiarlvydrshlsmavirkpltvvggityrpfdkrefaeivfc--AISST
---entplfspslispdvlavlpadytirplcrsdykrgyldvlrvlttvgdineeqwnsryewirarsdeyyllvvcdgegrivgtgslvverkfihslgmvghiediAVEKG

EQVRGYGAHLMNHLkDYVrnTSNIKYFLTYAD-NYAI-GYFKKQGFTK---------eitldksiwmgyikdyeggtlmqcsmlpriryld
QQGKKLGLRIIQAL-DYV--AEKVGCYKTILDcSEANeGFYIKCGFKRaglemahyy-----------------------------------

Proposed: aliSize=121 (resi) RMSD=2.28 (Å)
---------------------KIEFRVVnndntkeNMMVlT--GLKNIFQKQLPkmpKE---------yiaRlvY-DR--S---HLSMAVIRKPLTVVGGITYRPFdK------REFAEIVFCAISSTEQVRGYGAHLMNHLKDYVRNtSNIKYFLTYADNYAIGYFKKQGFTKEitldksiwmgyikdy
entplfspslispdvlavlpaDYTIRPL-------CRSD-YkrGYLDVLRVLTT---VGdineeqwns---R--YeWIraRsdeYYLLVVCDGEGRIVGTGSLVVE-RkfihslGMVGHIEDIAVEKGQQGKKLGLRIIQALDYVAEK-VGCYKTILDCSEANEGFYIKCGFKRA--------------

eggTLMQCSML-priryld
---GLEMAHYYc-------</td></tr>
<tr><td>2vxkA
(165)</td></tr>
<tr><td rowspan="2">533</td><td>1yk3A
(198)</td><td rowspan="2">C</td><td rowspan="2">12.41</td><td rowspan="2">TM-align: aliSize=141 (resi) RMSD=2.91 (Å)
addalvrlarerfdlpdqvrrlarppvpslepPYGLRVAQLTDaEMLAEWMNRphLAAawEYdWPaSRWRQHLNAQLEGTYSLPLIGSWHGTDGGYLELYWAAKdliSHYYDADPYDLGL-HAAIaDLSKVnRGFGPLLLPRIVASVFANEpRCRRIMFDPDHRNTATRRLCEWAGCKFLGEHDTTNrrm
--------------------------------MDIRQMNKTHL-EHWRGLRKQ--LWP--GH-PD-DAHLADGEEILQADHLASFIAMADGVAIGFADASIRHD---YVNGCDSSPVVFLeGIFV-LPSFR-QRGVAKQLIAAVQRWGTNK-GCREMASDTSPENTISQKVHQALGFEETERVIFYR---

alyaleapt---
---------krc

SARST: aliSize=139 (resi) RMSD=7.35 (Å)
addalvrlarerfdlpdqvrrlarppvpsleppy-GLRVAQLTDAEMLAEWmnrPHLAAAWeydWPASRWRQHLNAQLEGTYSLPLIGSWHGTDGGYLELYWaakdlisHYYDADPYDLGLHAAIA----DLSKVNRGFGplLLPRIVASVFANEPRCRRIMFDPDHRNTATRRLCEWAGCKFlgehdtt
----------------------------------mDIRQMNKTHLEHWRGL---RKQLWPG---HPDDAHLADGEEILQADHLASFIAMADGVAIGFADASI-------RHDYVNGCDSSPVVFLEgifvLPSFRQRGVA--KQLIAAVQRWGTNKGCREMASDTSPENTISQKVHQALGFEE-------

NRRMALYALE-apt
TERVIFYRKRc---

BLAST: aliSize=48 (resi) iden=18.62% (27/145) simi=33.10% (48/145)
addalvrlarerfdlpdqvrrlarppvpsleppyglrvaqltdaemlaew-----MNRPHLaAAW----EYDWPASRWRQHL-NAQ--LEGTYSLPLIGSWHGTDGGYLELYwAAKDLISHyYDADPYdLGLHAAIADLSKVNRGFGPLLLPRIvaSVFANEPRCRRIMFDPDHRNTATRRLCEWAG---
---mdirqMNKTHL-EHWrglrKQLWPGHPDDAHLaDGEeiLQADHLASFIAMADGVAIGFADAS-IRHDYVNG-CDSSPV-VFLEGIFVLPSFRQRGVAKQLIAAV--QRWGTNKGCREMASDTSPENTISQKVHQALGfee

-----------ckflgehdttnrrmalyaleapt
tervifyrkrc-----------------------

Proposed: aliSize=142 (resi) RMSD=2.61 (Å)
addalvrlarerfdlpdqvrrlarppvpslepPYGLRVAQlTDAEMLAEWMNRphLAAawEYdWPaSRWRQHLNAQLEGTYSLPLIGSWHGTDGGYLELYWAAKdliSHYYDADPYDLGL-HAAIaDLSKVnrGFgpLLLPRIVASVFAN-EP-RCRRIMFDPDHRNTATRRLCEWAGCKFLGEHDtTNR
--------------------------------MDIRQMNK-THLEHWRGLRKQ--LWP--GH-PD-DAHLADGEEILQADHLASFIAMADGVAIGFADASIRHD---YVNGCDSSPVVFLeGIFV-LPSFR--QR--GVAKQLIAAVQRWgTNkGCREMASDTSPENTISQKVHQALGFEETERVI-FYR

RMA-lyaleapt
KRCc--------</td></tr>
<tr><td>1s3zB
(145)</td></tr>
</table>

534	1yk3A (198)<br>1s5kA (153)	C	13.07	TM-align: aliSize=141 (resi) RMSD=2.70 (Å)<br>addalvrlarerfdlpdqvrrlarppvpsl----EP-P-YGLRVAQLTDAeMLAEWMNRphLaaawEYDWPASRWRQHLNAQLEGTYSLPLIGSWHGTDGGYLELYWAAKdliSHYYDADPYDLGL-HAAIaDLSKVnrGFgpLLLPRIVASVFAN-EP-RCRRIMFDPDHRNTATRRLCEWAGCKFLGE<br>------------------------------glvpRGsHmDIRQMNKTHLE-HWRGLRKQ--L----WPGHPDDAHLADGEEILQADHLASFIAMADGVAIGFADASIRHD---YVNGCDSSPVVFLeGIFV-LPSFR--QR--GVAKQLIAAVQRWgTNkGCREMASDTSPENTISQKVHQALGFEETER<br>HDTTNrrmalyaleapt---<br>VIFYR------------krc<br>SARST: aliSize=141 (resi) RMSD=7.38 (Å)<br>addalvrlarerfdlpdqvrrlarppvpslep-------PYGLRVAQLTDAEMLaewmnrPHLAAAWEYDWPASRWRQHLNAQLEGTYSLPLIGSWHGTDGGYLELYWaakdlisHYYDADPYDLGLHAAIA----DLSKVNRGFgpLLLPRIVASVFANEPRCRRIMFDPDHRNTATRRLCEWAGCKFl<br>--------------------------------glvprgsHMDIRQMNKTHLEHW------RGLRKQLWPGHPDDAHLADGEEILQADHLASFIAMADGVAIGFADASI-------RHDYVNGCDSSPVVFLEgifvLPSFRQRGV--AKQLIAAVQRWGTNKGCREMASDTSPENTISQKVHQALGFEE-<br>gehdttNRRMALYALE-apt<br>------TERVIFYRKRc---<br>BLAST: aliSize=48 (resi) iden=17.65% (27/153) simi=31.37% (48/153)<br>addalvrlarerfdlpdqvrrlarppvpsleppyglrvaqltdaemlaew-------------MNRPHLAAaW----EYDWPASRWRQHL-NAQ--LEGTYSLPLIGSWHGTDGGYLELYwAAKDLISHyYDADPYdLGLHAAIADLSKVNRGFGPLLLPRIvaSVFANEPRCRRIMFDPDHRNTATRRL<br>---glvprgshmdirqMNKTHLEH-WrglrKQLWPGHPDDAHLaDGEeiLQADHLASFIAMADGVAIGFADAS-IRHDYVNG-CDSSPV-VFLEGIFVLPSFRQRGVAKQLIAAV--QRWGTNKGCREMASDTSPENTISQKV<br>CEWAG---------------ckflgehdttnrrmalyaleapt<br>HQALGfeetervifyrkrc-----------------------<br>Proposed: aliSize=145 (resi) RMSD=2.58 (Å)<br>addalvrlarerfdlpdqvrrlarppvpsl----EP-PYGLRVAQLTDAEMLAEWMNRphLaaaWEyDWPASRWRQHLNAQLEGTYSLPLIGSWHGTDGGYLELYWAAKdliSHYYDADPYDLGL-HAAIaDLSKVnrGFgpLLLPRIVASVFAN-EP-RCRRIMFDPDHRNTATRRLCEWAGCKFLGEH<br>------------------------------glvpRGsHMDIRQMNKTHLEHWRGLRKQ--L---WP-GHPDDAHLADGEEILQADHLASFIAMADGVAIGFADASIRHD---YVNGCDSSPVVFLeGIFV-LPSFR--QR--GVAKQLIAAVQRWgTNkGCREMASDTSPENTISQKVHQALGFEETERV<br>DTtnrrMALYA-leapt<br>IF----YRKRCc-----
535	1yk3A (198)<br>2vbqB (144)	C	13.19	TM-align: aliSize=138 (resi) RMSD=2.76 (Å)<br>addalvrlarerfdlpdqvrrlarppvpsleppYGLRVAQLTDAeMLAEWMNRphLAAawEYdWPaSRWRQHLNAQLEGTYSLPLIGSWHGTDGGYLELYWAAKdliSHYYDADPYDLGL-HAAIaDLSKVnrGFgpLLLPRIVASVFAN-EP-RCRRIMFDPDHRNTATRRLCEWAGCKFLGEHDTTNr<br>----------------------------------DIRQMNKTHLE-HWRGLRKQ--LWP--GH-PD-DAHLADGEEILQADHLASFIAMADGVAIGFADASIRHD---YVNGCDSSPVVFLeGIFV-LPSFR--QR--GVAKQLIAAVQRWgTNkGCREMASDTSPENTISQKVHQALGFEETERVIFYR-<br>rmalyaleapt---<br>-----------krc<br>SARST: aliSize=138 (resi) RMSD=7.42 (Å)<br>addalvrlarerfdlpdqvrrlarppvpsleppyg-LRVAQLTDAEMLaewmnrPHLAAAWEYDWPASRWRQHLNAQLEGTYSLPLIGSWHGTDGGYLELYWaakdlisHYYDADPYDLGLHAAIA----DLSKVNRGFGPLLLPRIVASVFAnePRCRRIMFDPDHRNTATRRLCEWAGCKFlgehdtt<br>-----------------------------------dIRQMNKTHLEHW------RGLRKQLWPGHPDDAHLADGEEILQADHLASFIAMADGVAIGFADASI-------RHDYVNGCDSSPVVFLEgifvLPSFRQRGVAKQLIAAVQRWGTN--KGCREMASDTSPENTISQKVHQALGFEE-------<br>NRRMALYALE-apt<br>TERVIFYRKRc---<br>BLAST: aliSize=48 (resi) iden=18.75% (27/144) simi=33.33% (48/144)<br>addalvrlarerfdlpdqvrrlarppvpsleppyglrvaqltdaemlaew----MNRPHLaAAW----EYDWPASRWRQHL-NAQ--LEGTYSLPLIGSWHGTDGGYLELYwAAKDLISHyYDADPYdLGLHAAIADLSKVNRGFGPLLLPRIvaSVFANEPRCRRIMFDPDHRNTATRRLCEWAG----<br>--dirqMNKTHL-EHWrglrKQLWPGHPDDAHLaDGEeiLQADHLASFIAMADGVAIGFADAS-IRHDYVNG-CDSSPV-VFLEGIFVLPSFRQRGVAKQLIAAV--QRWGTNKGCREMASDTSPENTISQKVHQALGfeet<br>----------ckflgehdttnrrmalyaleapt<br>ervifyrkrc-----------------------<br>Proposed: aliSize=141 (resi) RMSD=2.60 (Å)<br>addalvrlarerfdlpdqvrrlarppvpsleppYGLRVAQlTDAEMLAEWMNRphLaaaWEYDWPaSRWRQHLNAQLEGTYSLPLIGSWHGTDGGYLELYWAAKdliSHYYDADPYDLGL-HAAIaDLSKVnrGFgpLLLPRIVASVFAN-EP-RCRRIMFDPDHRNTATRRLCEWAGCKFLGEHDtTNR<br>----------------------------------DIRQMNK-THLEHWRGLRKQ--L---WPGHPD-DAHLADGEEILQADHLASFIAMADGVAIGFADASIRHD---YVNGCDSSPVVFLeGIFV-LPSFR--QR--GVAKQLIAAVQRWgTNkGCREMASDTSPENTISQKVHQALGFEETERVI-FYR<br>RMAlyaleapt<br>KRC--------
536	1yr0A (163)<br>1s3zB (145)	C	16.55	TM-align: aliSize=133 (resi) RMSD=2.51 (Å)<br>sVELRDATVDDLSGI-EIYNDAVVnttaiwnEVVVD-LENRKDWFAArtSRGFPVIVAILDGKVAGYASYGDWRAFd----GYRHTREH-SVYVHKDARGHGIGKRL-QALIDHAGGNDVHVLIAAIEAENTASIRLHESLGFRVVGRFSeVGtkfgrwldltcelkl----<br>-MDIRQMNKTHLEHWrGLRKQLWP-------GHPDDaHLADGEEILQ--ADHLASFIAMADGVAIGFADASIRHDY-vngcDSSPVVFLeGIFVLPSFRQRGVAKQLiAAVQRWGTNKGCREMASDTSPENTISQKVHQALGFEETERVI-FY---------------rkrc<br>SARST: aliSize=132 (resi) RMSD=4.12 (Å)<br>sv-ELRDATVDDLSGIEIYNDAVvnttaiWNEVVvdLENRKDWFAARTS-RGFPVIVAILDGKVAGYASYG----DWRAFdgyrHTREH----SVYVHKDARGHGIGKRLQALID-HAGGNDVHVLIAAIEAENTASIRLHESLGFRVVGRFSEVGT--kfgrwldltcelkl<br>--mDIRQMNKTHLEHWRGLRKQL------WPGHP--DDAHLADGEEILQaDHLASFIAMADGVAIGFADASirhdYVNGC----DSSPVvfleGIFVLPSFRQRGVAKQLIAAVQrWGTNKGCREMASDTSPENTISQKVHQALGFEETERVIFYRKrc-------------<br>BLAST: aliSize=39 (resi) iden=16.55% (24/145) simi=26.90% (39/145)<br>svelrdatvddlsgieiyndavvnttaiwnevvvdlenrkdwfaartsrgfpvi--VAILDGKVAGYASYG---DW-RAFDGYRHTREHSVYVHKDARGHGIGKRLQALIDHAGGN-DVHVLIAAIEAENTASIRLHESLGFRVVG<br>--mdirqmnkthlehwrglrkqlwpghpddahladgeeilqadhlasfIAMADGVAIGFADASirhDYvNGCDSSPVVFLEGIFVLPSFRQRGVAKQLIAAVQRWGTNkGCREMASDTSPENTISQKVHQALGFEETE<br>R--------fsevgtkfgrwldltcelkl<br>Rvifyrkrc--------------------<br>Proposed: aliSize=137 (resi) RMSD=2.04 (Å)<br>sVELRDATVDDLSGIMEIYNDAVVnttaiwnEVVVD-LENRKDWFAARtSRgFPVIVAILDGKVAGYASYGDWrAFdgY------RHTREH-SVYVHKDARGHGIGKRLMQALIDHAGGNDVHVLIAAIEAENTASIRLHESLGFRVVGRFsevgtkfgrwldlTCMELKL-<br>-MDIRQMNKTHLEHWRGLRKQLWP-------GHPDDaHLADGEEILQA-DH-LASFIAMADGVAIGFADASIR-HD--YvngcdsSPVVFLeGIFVLPSFRQRGVAKQLIAAVQRWGTNKGCREMASDTSPENTISQKVHQALGFEETERV-------------IFYRKRCc

<table>
<tr><td rowspan="2">537</td><td>1yr0A
(163)</td><td rowspan="2">C</td><td rowspan="2">16.99</td><td rowspan="2">TM-align: aliSize=133 (resi) RMSD=2.43 (Å)
-------SVELRDATVDDLSGI-EIYNDAVVnttaiwnEVVVD-LENRKDWFAArtSRGFPVIVAILDGKVAGYASYGDWRAFdg-----YRHTREH-SVYVHKDARGHGIGKRL-QALIDHAGGNDVHVLIAAIEAENTASIRLHESLGFRVVGRFSeVGtkfgrwldltcelkl----
glvprgsHMDIRQMNKTHLEHWrGLRKQLWP-------GHPDDaHLADGEEILQ--ADHLASFIAMADGVAIGFADASIRHDY--vngcdSSPVVFLeGIFVLPSFRQRGVAKQLiAAVQRWGTNKGCREMASDTSPENTISQKVHQALGFEETERVI-FY---------------rkrc

SARST: aliSize=137 (resi) RMSD=4.08 (Å)
s--------VELRDATVDDLSGIEIYNDavvntTAIwneVVVDLENRKDWFAARTS-RGFPVIVAILDGKVAGYASYG-DWRAFDGYRHTREH---SVYVHKDARGHGIGKRLQAL-IDHAGGNDVHVLIAAIEAENTASIRLHESLGFRVVGRFSEVGT--kfgrwldltcelkl
-glvprgshMDIRQMNKTHLEHWRGLRK-----QLW---PGHPDDAHLADGEEILQaDHLASFIAMADGVAIGFADASiRHDYVNGCDSSPVVfleGIFVLPSFRQRGVAKQLIAAvQRWGTNKGCREMASDTSPENTISQKVHQALGFEETERVIFYRKrc--------------

BLAST: aliSize=39 (resi) iden=15.69% (24/153) simi=25.49% (39/153)
svelrdatvddlsgieiyndavvnttaiwnevvvdlenrkdwfaartsrgfpvi--VAILDGKVAGYASYG---DW-RAFDGYRHTREHSVYVHKDARGHGIGKRLQALIDHAGGN-DVHVLIAAIEAENTASIRLHE
--glvprgshmdirqmnkthlehwrglrkqlwpghpddahladgeeilqadhlasfIAMADGVAIGFADASirhDYvNGCDSSPVVFLEGIFVLPSFRQRGVAKQLIAAVQRWGTNkGCREMASDTSPENTISQKVHQ

SLGFRVVGR--------fsevgtkfgrwldltcelkl
ALGFEETERvifyrkrc-------------------

Proposed: aliSize=137 (resi) RMSD=2.02 (Å)
-------SVELRDATVDDLSGIMEIYNDAVVnttaiwnEVVVD--LENRKDWFAArtSRgFPVIVAILDGKVAGYASYGDWrAFdgY------RHTREH-SVYVHKDARGHGIGKRLMQALIDHAGGNDVHVLIAAIEAENTASIRLHESLGFRVVGRFsevgtkfgrwldlTCMELKL-
glvprgsHMDIRQMNKTHLEHWRGLRKQLWP-------GHPDDahLADGEEILQA--DH-LASFIAMADGVAIGFADASIR-HD--YvngcdsSPVVFLeGIFVLPSFRQRGVAKQLIAAVQRWGTNKGCREMASDTSPENTISQKVHQALGFEETERV-------------IFYRKRCc</td></tr>
<tr><td>1s5kA
(153)</td></tr>
<tr><td rowspan="2">538</td><td>1yr0A
(163)</td><td rowspan="2">C</td><td rowspan="2">19.33</td><td rowspan="2">TM-align: aliSize=139 (resi) RMSD=3.00 (Å)
SVELRDATVDDLSGI-EIYNDAvvNTtaIWNEVVV---DLENRKDWFAARTSRG-FPVIVAILDGKVAGYASYGDWraFDGY---RHTREHS-VYVHKDARGHGIGKRL-QALIDHAGGNDVHVLIAAIEAENTASIRLHESLGFRVVGRFSeVGTkfgrwldltcelkl-
HVTIREATEGDLEQMvHMLADD--VL--GRKRERYekpLPVSYVRAFKEIKKDKnNELIVACNGEEIVGMLQVTFT--PYLTyqgSWRATIEgVRTHSAARGQGIGSQLvCWAIERAKERGCHLIQLTTDKQRPDALRFYEQLGFKASHEGL-KMH--------------f

SARST: aliSize=137 (resi) RMSD=4.59 (Å)
s-VELRDATVDDLSGIEIYND----AVVNTtaIWNeVVVDLENRKDWFAARTSRGFPVIVAILDGKVAGYASYGDwRAFDGYRHTREHSVY---VHKDARGHG-IGKRLQALIDHAGGNDVHVLIAAIEAENTASIRLHESLGFRVVGRFS----evgtkfgrwldltcelkl
-hVTIREATEGDLEQMVHMLAddvlGRKRE--RYE-KPLPVSYVRAFKEIKKDKNNELIVACNGEEIVGMLQVTF-TPYLTYQGSWRATIEgvrTHSAARGQGiGSQLVCWAIERAKERGCHLIQLTTDKQRPDALRFYEQLGFKASHEGLkmhf------------------

BLAST: aliSize=63 (resi) iden=22.67% (34/150) simi=42.00% (63/150)
s-VELRDATVDDLSG-IEIYNDAVVNTTAIWNEVVVDLENRKDWFAARTSRGFPVIVAILDGKVAGY--ASYGDWRAFDG-YRHTREhSVYVHKDARGHGIGKRLQA-LIDHAGGNDVHVLIAAIEAENTASIRLHESLGFR----------vvgrfsevgtkfgrwldltcelkl
-hVTIREATEGDLEQmVHMLADDVLGRKRERYEKPLPVSYVRAFKEIKKDKNNELIVACNGEEIVGMlqVTFTPYLTYQGsWRATIE-GVRTHSAARGQGIGSQLVCwAIERAKERGCHLIQLTTDKQRPDALRFYEQLGFKasheglkmhf------------------------

Proposed: aliSize=136 (resi) RMSD=2.05 (Å)
SVELRDATVDDLSGIMEIYNDAvvNTtaI-wNEVVV---DLENRKDWFAARTSRG-FPVIVAILDGKVAGYASYG-dwrafdgYR-------HTREHS-VYVHKDARGHGIGKRLMQALIDHAGGNDVHVLIAAIEAENTASIRLHESLGFRVVgrFsevgtkfgrwldLTCMELKl
HVTIREATEGDLEQMVHMLADD--VL--Gr-KRERYekpLPVSYVRAFKEIKKDKnNELIVACNGEEIVGMLQVTf-------TPyltyqgsWRATIEgVRTHSAARGQGIGSQLVCWAIERAKERGCHLIQLTTDKQRPDALRFYEQLGFKAS--H------------EGLKMHF-</td></tr>
<tr><td>1z4eA
(150)</td></tr>
<tr><td rowspan="2">539</td><td>1yr0A
(163)</td><td rowspan="2">C</td><td rowspan="2">16.56</td><td rowspan="2">TM-align: aliSize=140 (resi) RMSD=3.00 (Å)
SVELRDATVDDLSGI-EIYNDAVVNTTaiWNEVV-VDLENRKDWFAArTSRGFPVIVAIL---DG-----KVAGYASYGDWraFDG-YRHTREH-SVYVHKDARGHGIGKRL-QALIDHAGGNDVHVLIAAIEAENTASIRLHESLGFRVVGR--FSEVGtkfgrwldltcelkl------------
KFVIRPATAADCSDIlRLIKELAKYEY--MEEQViLTEKDLLEDGFG-EHPFYHCLVAEVpkeHWtpeghSIVGFAMYYFT--YDPwIGKLLYLeDFFVMSDYRGFGIGSEIlKNLSQVAMRCRCSSMHFLVAEWNEPSINFYKRRGASDLSSeeGWRLF---------------kidkeyllkmat

SARST: aliSize=127 (resi) RMSD=4.30 (Å)
sv--ELRDATVDDLSGIEIYNDAV-VNTTAIWNEVVVdlenrkdwFAARTSRGFP------VIVAIL-------DGK-VAGYASYGDwRAFDGYRHTREH-SVYVHKDARGHGIGKRLQALIDHAGG-NDVHVLIAAIEAENTASIRLHESLGFRVV--------------------grfsevgtkfgr
--kfVIRPATAADCSDILRLIKELaKYEYMEEQVILT--------EKDLLEDGFGehpfyhCLVAEVpkehwtpEGHsIVGFAMYYF-TYDPWIGKLLYLeDFFVMSDYRGFGIGSEILKNLSQVAMrCRCSSMHFLVAEWNEPSINFYKRRGASDLsseegwrlfkidkeyllkmat-----------

wldltcelkl

BLAST: aliSize=33 (resi) iden=12.50% (20/163) simi=20.62% (33/163)
svelrdatvddlsgieiyndavvnttaiwnevvvdlenrkdwfaartsrgfpvivaildgk--VAGYASYgdWRAFDGY--RHTREHSVYVHKDARGHGIG----KRLQALIDHAGGNDVHVLI
---kfvirpataadcsdilrlikelakyeymeeqviltekdlledgfgehpfyhclvaevpkehwtpeghsIVGFAMY--YFTYDPWigKLLYLEDFFVMSDYRGFGIGseilKNLSQVAMRCRCSSMHFLV

AaieAENTASIRLHESLG-------------------------frvvgrfsevgtkfgrwldltcelkl
A---EWNEPSINFYKRRGasdlsseegwrlfkidkeyllkmat--------------------------

Proposed: aliSize=145 (resi) RMSD=2.22 (Å)
SVELRDATVDDLSGIMEIYNDAVVNTTaiWNEVV-VDLENRKDWFA---ARtsrgFPVIVAIL---DG-----KVAGYASYGDWrafdGY---RHTREH-SVYVHKDARGHGIGKRLMQALIDHAGGNDVHVLIAAIEAENTASIRLHESLGFRVVG-rfsevgtkFGRWLDLTC---ME-L-KL
KFVIRPATAADCSDILRLIKELAKYEY--MEEQViLTEKDLLEDGFgehPF----YHCLVAEVpkeHWtpeghSIVGFAMYYFT----YDpwiGKLLYLeDFFVMSDYRGFGIGSEILKNLSQVAMRCRCSSMHFLVAEWNEPSINFYKRRGASDLSs--------EEGWRLFKIdkeYLlKmAT</td></tr>
<tr><td>2f5iA
(167)</td></tr>
</table>

540	1yr0A (163)<br>2g3tA (169)	C	16.56	TM-align: aliSize=141 (resi) RMSD=3.11 (Å)<br>SVELRDATVDDLSGI-EIYNDAVVNTTaIWNEVVVDLENRKDWFAArTSRGFPVIVAIL---DG-----KVAGYASYGDWraFDG-YRHTREH-SVYVHKDARGHGIGKRL-QALIDHAGGNDVHVLIAAIEAENTASIRLHESLGFRVVGR--FSEVGtkfgrwldltcelkl-------------<br>KFVIRPATAADCSDIlRLIKELAKYEY-MEEQVILTEKDLLEDGFG-EHPFYHCLVAEVpkeHWtpeghSIVGFAMYYFT--YDPwIGKLLYLeDFFVMSDYRGFGIGSEIlKNLSQVAMRCRCSSMHFLVAEWNEPSINFYKRRGASDLSSeeGWRLF---------------kidkeyllkmatee<br><br>SARST: aliSize=128 (resi) RMSD=4.15 (Å)<br>sv--ELRDATVDDLSGIEIYNDAVVN-TTAIWNEVVVdlenrkdWFAARTSRGF-----PVIVAIL-------DGK-VAGYASYGDwRAFDGYRHTREH-SVYVHKDARGHGIGKRLQALID-HAGGNDVHVLIAAIEAENTASIRLHESLGFRVV-----------------------grfsevgtkfg<br>--kfVIRPATAADCSDILRLIKELAKyEYMEEQVILT-------EKDLLEDGFGehpfyHCLVAEVpkehwtpEGHsIVGFAMYYF-TYDPWIGKLLYLeDFFVMSDYRGFGIGSEILKNLSqVAMRCRCSSMHFLVAEWNEPSINFYKRRGASDLsseegwrlfkidkeyllkmatee-----------<br>rwldltcelkl<br>-----------<br><br>BLAST: aliSize=33 (resi) iden=12.50% (20/163) simi=20.62% (33/163)<br>svelrdatvddlsgieiyndavvnttaiwnevvvdlenrkdwfaartsrgfpvivaildgk--VAGYASYgdWRAFDGY--RHTREHSVYVHKDARGHGIG----KRLQALIDHAGGNDVHVLI<br>---kfvirpataadcsdilrlikelakyeymeeqviltekdlledgfgehpfyhclvaevpkehwtpeghsIVGFAMY--YFTYDPWigKLLYLEDFFVMSDYRGFGIGseilKNLSQVAMRCRCSSMHFLV<br>AaieAENTASIRLHESLG---------------------------frvvgrfsevgtkfgrwldltcelkl<br>A---EWNEPSINFYKRRGasdlsseegwrlfkidkeyllkmatee--------------------------<br><br>Proposed: aliSize=144 (resi) RMSD=2.21 (Å)<br>SVELRDATVDDLSGIMEIYNDAVVNT-T-aiwNEVVVDLENRKDWFA---ARtsrgFPVIVAIL---DG-----KVAGYASYGDWrafdGY---RHTREH-SVYVHKDARGHGIGKRLMQALIDHAGGNDVHVLIAAIEAENTASIRLHESLGFRVVG--rfsevgtkFGRWLDLTC--M-EL--KL-<br>KFVIRPATAADCSDILRLIKELAKYEyMe---EQVILTEKDLLEDGFgehPF----YHCLVAEVpkeHWtpeghSIVGFAMYYFT----YDpwiGKLLYLeDFFVMSDYRGFGIGSEILKNLSQVAMRCRCSSMHFLVAEWNEPSINFYKRRGASDLSse--------EGWRLFKIDkeYlLKmaTEe
541	1yr0A (163)<br>2g3tB (168)	C	15.95	TM-align: aliSize=141 (resi) RMSD=3.04 (Å)<br>SVELRDATVDDLSGI-EIYNDAVVNTTaIWNEVVVDLENRKDWFAArTSRGFPVIVAILDG--------KVAGYASYGDWraFDG-YRHTREH-SVYVHKDARGHGIGKRL-QALIDHAGGNDVHVLIAAIEAENTASIRLHESLGFRVVGR--FSEVGtkfgrwldltcelkl-------------<br>KFVIRPATAADCSDIlRLIKELAKYEY-MEEQVILTEKDLLEDGFG-EHPFYHCLVAEVPKehwtpeghSIVGFAMYYFT--YDPwIGKLLYLeDFFVMSDYRGFGIGSEIlKNLSQVAMRCRCSSMHFLVAEWNEPSINFYKRRGASDLSSeeGWRLF---------------kidkeyllkmate<br><br>SARST: aliSize=131 (resi) RMSD=4.05 (Å)<br>sv--ELRDATVDDLSGIEIYNDAVVNTTAIWNEVVVDLENRKDWFAartsRGFPVIVAIL-----------DGKVAGYASYGDwRAFDGYRHTREH-SVYVHKDAR-GHGIGKRLQALIDHAGGNDVHVLIAAIEAENTASIRLHESLGFRVV---------------------grfsevgtkfgrwld<br>--kfVIRPATAADCSDILRLIKELAKYEYMEEQVILTEKDLLEDGF----GEHPFYHCLVaevpkehwtpeGHSIVGFAMYYF-TYDPWIGKLLYLeDFFVMSDYRgFGIGSEILKNLSQVAMRCRCSSMHFLVAEWNEPSINFYKRRGASDLsseegwrlfkidkeyllkmate-------------<br>ltcelkl<br>-------<br><br>BLAST: aliSize=33 (resi) iden=12.50% (20/163) simi=20.62% (33/163)<br>svelrdatvddlsgieiyndavvnttaiwnevvvdlenrkdwfaartsrgfpvivaildgk--VAGYASYgdWRAFDGY--RHTREHSVYVHKDARGHGIG----KRLQALIDHAGGNDVHVLI<br>---kfvirpataadcsdilrlikelakyeymeeqviltekdlledgfgehpfyhclvaevpkehwtpeghsIVGFAMY--YFTYDPWigKLLYLEDFFVMSDYRGFGIGseilKNLSQVAMRCRCSSMHFLV<br>AaieAENTASIRLHESLG--------------------------frvvgrfsevgtkfgrwldltcelkl<br>A---EWNEPSINFYKRRGasdlsseegwrlfkidkeyllkmate--------------------------<br><br>Proposed: aliSize=144 (resi) RMSD=2.11 (Å)<br>SVELRDATVDDLSGIMEIYNDAVVNTTaiW-NEVVVDLENRKDWFA---ARtsrgFPVIVAILDG--------KVAGYASYGDWrafdGY---RHTREH-SVYVHKDARGHGIGKRLMQALIDHAGGNDVHVLIAAIEAENTASIRLHESLGFRVVGrfsevgtkfG--RWLDLTC---ME-L--KL<br>KFVIRPATAADCSDILRLIKELAKYEY--MeEQVILTEKDLLEDGFgehPF----YHCLVAEVPKehwtpeghSIVGFAMYYFT----YDpwiGKLLYLeDFFVMSDYRGFGIGSEILKNLSQVAMRCRCSSMHFLVAEWNEPSINFYKRRGASDLS---------SeeGWRLFKIdkeYLlKmaTE
542	1yr0A (163)<br>2vbqA (147)	C	17.01	TM-align: aliSize=133 (resi) RMSD=2.46 (Å)<br>S-VELRDATVDDLSGI-EIYNDAVVnttaiwnEVVVD-LENRKDWFAArtSRGFPVIVAILDGKVAGYASYGDWRAFdg-----YRHTREH-SVYVHKDARGHGIGKRL-QALIDHAGGNDVHVLIAAIEAENTASIRLHESLGFRVVGRFSeVGtkfgrwldltcelkl----<br>ShMDIRQMNKTHLEHWrGLRKQLWP-------GHPDDaHLADGEEILQ--ADHLASFIAMADGVAIGFADASIRHDY--vngcdSSPVVFLeGIFVLPSFRQRGVAKQLiAAVQRWGTNKGCREMASDTSPENTISQKVHQALGFEETERVI-FY---------------rkrc<br><br>SARST: aliSize=137 (resi) RMSD=4.23 (Å)<br>s--VELRDATVDDLSGIEIYNDAVvnttaiwnEVVVDLENRKDWFAARTS-RGFPVIVAILDGKVAGYASYGD-WRAFDGYRHTREH---SVYVHKDARGHGIGKRLQALIDHAG-GNDVHVLIAAIEAENTASIRLHESLGFRVVGRFSEVGT--kfgrwldltcelkl<br>-shMDIRQMNKTHLEHWRGLRKQL---------WPGHPDDAHLADGEEILQaDHLASFIAMADGVAIGFADASIrHDYVNGCDSSPVVfleGIFVLPSFRQRGVAKQLIAAVQRWGtNKGCREMASDTSPENTISQKVHQALGFEETERVIFYRKrc-------------<br><br>BLAST: aliSize=39 (resi) iden=16.33% (24/147) simi=26.53% (39/147)<br>svelrdatvddlsgieiyndavvnttaiwnevvvdlenrkdwfaartsrgfpvi--VAILDGKVAGYASYG---DW-RAFDGYRHTREHSVYVHKDARGHGIGKRLQALIDHAGGN-DVHVLIAAIEAENTASIRLHESLGFRV<br>--shmdirqmnkthlehwrglrkqlwpghpddahladgeeilqadhlasfIAMADGVAIGFADASirhDYvNGCDSSPVVFLEGIFVLPSFRQRGVAKQLIAAVQRWGTNkGCREMASDTSPENTISQKVHQALGFEE<br>VGR--------fsevgtkfgrwldltcelkl<br>TERvifyrkrc--------------------<br><br>Proposed: aliSize=138 (resi) RMSD=2.13 (Å)<br>S-VELRDATVDDLSGIMEIYNDAVVnttaiwnEVVVD-LENRKDWFAARtSRgFPVIVAILDGKVAGYASYGDWrAFdgY------RHTREH-SVYVHKDARGHGIGKRLMQALIDHAGGNDVHVLIAAIEAENTASIRLHESLGFRVVGRFsevgtkfgrwldlTCMELKL<br>ShMDIRQMNKTHLEHWRGLRKQLWP-------GHPDDaHLADGEEILQA-DH-LASFIAMADGVAIGFADASIR-HD--YvngcdsSPVVFLeGIFVLPSFRQRGVAKQLIAAVQRWGTNKGCREMASDTSPENTISQKVHQALGFEETERV-------------IFYRKRC

543	1yr0A (163)<br>2vxkA (165)	C	14.72	TM-align: aliSize=132 (resi) RMSD=2.57 (Å)<br>--------------------S-VELRDATVDDLS-GI-EIYNDAVVnttaiwneVVVD-LENRKDWFAARTSR-G-FPVIVAIL-DGKVAGYASYGDWraFDGY---RHTREHS-VYVHKDARGHGIGKRL-QALIDHAGGNDVHVLIAAIeAENTasIRLHESLGFRVVGRFSeVGTkfgrwldltcelkl-<br>entplfspslispdvlavlpAdYTIRPLCRSDYKrGYlDVLRVLTT--------VGDInEEQWNSRYEWIRARsDeYYLLVVCDgEGRIVGTGSLVVE--RKFIhslGMVGHIEdIAVEKGQQGKKLGLRIiQALDYVAEKVGCYKTILDC-SEAN--EGFYIKCGFKRAGLEM-AHY--------------y<br><br>SARST: aliSize=132 (resi) RMSD=3.82 (Å)<br>s----------------------VELRDATVDDLSGIEIYNDavvntTAIWNEVVVDLENRKDWFAARTS-RGFPVIVAIL--DGKVAGYASYGDwRAFDGYRHTREHSV---YVHKDARGHGIGKRLQAL-IDHAGGNDVHVLIAAIEAENTasiRLHESLGFRVVGRFS----evgtkfgrwldltcelkl<br>-entplfspslispdvlavlpadYTIRPLCRSDYKRGYLDVL-----RVLTTVGDINEEQWNSRYEWIRArSDEYYLLVVCdgEGRIVGTGSLVV-ERKFIHSLGMVGHIediAVEKGQQGKKLGLRIIQAlDYVAEKVGCYKTILDCSEANE---GFYIKCGFKRAGLEMahyy------------------<br><br>BLAST: aliSize=42 (resi) iden=14.38% (23/163) simi=26.25% (42/163)<br>svelrdatvddlsgieiyndavvnttaiwnevvvdlenrkdwfaarts----------------------------------RGFPVIVAILDgkVAGYASYGDWRA-FDGYR-HTREHSVYVHKDARGH--GIGKRL-QALIDHAGGNDVHVLIAAIEAENTAsirlhESLGFRVVGRFSEVGTKFG--<br>--entplfspslispdvlavlpadytirplcrsdykRGYLDVLRVLT--TVGDINEEQWNSrYEWIRaRSDEYYLLVVCDGEGRivGTGSLVvERKFIHSLGMVGHIEDIAVEKGQQG-----KKLGLRIIQALDYVAEKVGcy<br><br>------------------------------rwldltcelkl<br>ktildcseanegfyikcgfkraglemahyy-----------<br><br>Proposed: aliSize=134 (resi) RMSD=2.32 (Å)<br>--------------------S-VELRDATVDDLS-GIMEIYNDAVVnttaiwneVVVD-LENRKDWFAARTSR-G-FPVIVAIL-DGKVAGYASYGDWrafDGY----RHTREHS-VYVHKDARGHGIGKRLMQALIDHAGGNDVHVLIAAIeAENTasIRLHESLGFRVVGRFSEvgTKfG-rwldltc<br>entplfspslispdvlavlpAdYTIRPLCRSDYKrGYLDVLRVLTT--------VGDInEEQWNSRYEWIRARsDeYYLLVVCDgEGRIVGTGSLVVE---RKFihslGMVGHIEdIAVEKGQQGKKLGLRIIQALDYVAEKVGCYKTILDC-SEAN--EGFYIKCGFKRAGLEMA--HY-Yc-------<br><br>melkl<br>-----
544	1yvoA (169)<br>1z4eA (150)	C	18.67	TM-align: aliSize=138 (resi) RMSD=2.74 (Å)<br>--SIRDAGVADLPGILAIYNDAvgNTtaIWNETPV---DLANRQAWFDTRARQG-YPILVASDAaGEVLGYASYGDWrpFEGF---RGTVEHS-VYVRDDQRGKGLGVQLLQALIERARAQGLHV-VAAIESGNAASIGLHRRLGFEISGQPQVGQkfgrwldltfqlnldptrsap-<br>hvTIREATEGDLEQMVHMLADD--VL--GRKRERYekpLPVSYVRAFKEIKKDKnNELIVACNG-EEIVGMLQVTFT--PYLTyqgSWRATIEgVRTHSAARGQGIGSQLVCWAIERAKERGCHLiQLTTDKQRPDALRFYEQLGFKASHEGLKMH--------------------f<br><br>SARST: aliSize=136 (resi) RMSD=4.37 (Å)<br>s---IRDAGVADLPGILAIYNDAVGNTTAIWNETPVDLANRQAWFDTRARQGYPILVASDAAgEVLGYASYGDwRPFEGFRGTVEHSVY---VRDDQRGKGLGVQLLQALIERARAQGLHVVA-AIESGNAASIGLHRRLGFEISG-------qpqvgqkfgrwldltfqlnldptrsap<br>-hvtIREATEGDLEQMVHMLADDVLGRKRERYEKPLPVSYVRAFKEIKKDKNNELIVACNGE-EIVGMLQVTF-TPYLTYQGSWRATIEgvrTHSAARGQGIGSQLVCWAIERAKERGCHLIQlTTDKQRPDALRFYEQLGFKASHeglkmhf---------------------------<br><br>BLAST: aliSize=63 (resi) iden=21.33% (32/150) simi=42.00% (63/150)<br>--SIRDAGVADLPGIL-AIYNDAVGNTTAIWnETPVDLANRQAWFDTRARQGYPILVASDAAgEVLGY--ASYGDWRPFEG-FRGTVEhsvyvrddqrgkglGVQLLQAlierARAQGL--HVVA-AIEsgNAASIGLH----------------------------------rrlgfeisgqpqvgqkfg<br>hvTIREATEGDLEQMVhMLADDVLGRKRERY-EKPLPVSYVRAFKEIKKDKNNELIVACNGE-EIVGMlqVTFTPYLTYQGsWRATIE-------------GVRTHSA----ARGQGIgsQLVCwAIE--RAKERGCHliqlttdkqrpdalrfyeqlgfkasheglkmhf-----------------<br><br>rwldltfqlnldptrsap<br>------------------<br><br>Proposed: aliSize=136 (resi) RMSD=1.92 (Å)<br>--SIRDAGVADLPGILAIYNDAvgNTtaI-wnETPV----DLANRQAWFDTRARQG-YPILVASDAaGEVLGYASYG-DwrpfEGF----RGTVEHS-VYVRDDQRGKGLGVQLLQALIERARAQGLHVMVAAIESGNAASIGLHRRLGFEISGqmpqvgqkfgrWLDLTFMqlnldptrsap<br>hvTIREATEGDLEQMVHMLADD--VL--Gr--KRERyekpLPVSYVRAFKEIKKDKnNELIVACNG-EEIVGMLQVTfT----PYLtyqgSWRATIEgVRTHSAARGQGIGSQLVCWAIERAKERGCHLIQLTTDKQRPDALRFYEQLGFKASH-----------EGLKMHF-----------
545	1yvoA (169)<br>2f5iA (167)	C	17.96	TM-align: aliSize=139 (resi) RMSD=2.93 (Å)<br>--SIRDAGVADLPGILAIYNDAVGNTTaiWNETP-VDLANRQAWFDTrARQGYPILVASDAA-------GEVLGYASYGDWrpFEG-FRGTVEH-SVYVRDDQRGKGLGVQLLQALIERARAQGLHV-VAAIESGNAASIGLHRRLGFEISGQ---PQVGqkfgrwldltfqlnldptrsap--------<br>kfVIRPATAADCSDILRLIKELAKYEY--MEEQViLTEKDLLEDGFG-EHPFYHCLVAEVPKehwtpegHSIVGFAMYYFT--YDPwIGKLLYLeDFFVMSDYRGFGIGSEILKNLSQVAMRCRCSSmHFLVAEWNEPSINFYKRRGASDLSSeegWRLF-----------------------kidkeyll<br><br>----<br>kmat<br><br>SARST: aliSize=128 (resi) RMSD=3.88 (Å)<br>s---IRDAGVADLPGILAIYNDAVGNTTAIWNETPVdlanrqaWFDTRARQG------YPILVASDAAGEV------LGYASYGDwRPFEGFRGTVEH-SVYVRDDQRGKGLGVQLLQALIERARAQGLHVVA-AIESGNAASIGLHRRLGFEI-----------------------sgqpqvgqkfgrwl<br>-kfvIRPATAADCSDILRLIKELAKYEYMEEQVILT-------EKDLLEDGFgehpfyHCLVAEVPKEHWTpeghsiVGFAMYYF-TYDPWIGKLLYLeDFFVMSDYRGFGIGSEILKNLSQVAMRCRCSSMHfLVAEWNEPSINFYKRRGASDlsseegwrlfkidkeyllkmat-------------<br><br>dltfqlnldptrsap<br>---------------<br><br>BLAST: aliSize=59 (resi) iden=22.89% (38/167) simi=35.54% (59/167)<br>s---IRDAGVADLPGILAIYNDAVGNTtaiWNETPVDLANRQAWFDTRARQG-YPILVA-------SDAAGEVLGYAS-YGDWRPFEGFRGTVEhSVYVRDDQRGKGLGVQLLQALIE---RARAQGLHVVAAieSGNAASIGLHRRLG------------------------feisgqpqvgqkfgrw<br>-kfvIRPATAADCSDILRLIKELAKYE---YMEEQVILTEKDLLEDGFGEHPfYHCLVAevpkehwTPEGHSIVGFAMyYFTYDPWIGKLLYLE-DFFVMSDYRGFGIGSEILKNLSQvamRCRCSSMHFLVA--EWNEPSINFYKRRGasdlsseegwrlfkidkeyllkmat----------------<br><br>ldltfqlnldptrsap<br>----------------<br><br>Proposed: aliSize=147 (resi) RMSD=2.35 (Å)<br>--SIRDAGVADLPGILAIYNDAVGNTTaiWNETPV-DLANRQAWFD--TraRQgYPILVASDAA--------gEVLGYASYGDWrpfEGF--RGTVEH-SVYVRDDQRGKGLGVQLLQALIERARAQGLHVMVAAIESGNAASIGLHRRLGFEISGqmpqvgqkfgRW-LDLTFMQLNLD-PtR--Sap<br>kfVIRPATAADCSDILRLIKELAKYEY--MEEQVIlTEKDLLEDGFgeH--PF-YHCLVAEVPKehwtpegh-SIVGFAMYYFT---YDPwiGKLLYLeDFFVMSDYRGFGIGSEILKNLSQVAMRCRCSSMHFLVAEWNEPSINFYKRRGASDLS----------SEeGWRLFKIDKEYlL-KmaT--

546	1yvoA (169)<br>2fe7B (166)	C	18.07	TM-align: aliSize=139 (resi) RMSD=3.03 (Å)<br>-----------SIRDAGVADLPGILAIYNDAVgNTTAIWNETPVDLANRQAWFDTrARQGYPILVASDAaGEVLGYASYGDWRpfEG--FRGTVEH-SVYVRDDQRGKGLGVQLLQALIERARAQGLHV-VAAIESGNAASIGLHRRLGFEISGQPQVGqkfgrwldltfqlnldptrsap-----------<br>enlyfqghmtlEIRPAVPADAEQILAFIIELA-DYERARHEVVTDVEGIRRSLFA-EGSPTRALMCLSE-GRPIGYAVFFYSY--STwlGRNGIYLeDLYVTPEYRGVGAGRRLLRELAREAVANDCGRlEWSVLDWNQPAIDFYRSIGALPQDEWVRY----------------------rldgealrkmae<br><br>SARST: aliSize=130 (resi) RMSD=4.26 (Å)<br>s------------IRDAGVADLPG-ILAIYNDAVGNTTAIWNETPvdlanRQAWFDTRARQGYPILVASDAAGE-VLGYASYGDWRpFEGFRGTVEH--SVYVRDDQRGKGLGVQLLQALIERARAQGLHVVA-AIESGNAASIGLHRRLGFEI-------------------sgqpqvgqkfgrwldlt<br>-enlyfqghmtleIRPAVPADAEQiLAFIIELADYERARHEVVTD-----VEGIRRSLFAEGSPTRALMCLSEGrPIGYAVFFYSY-STWLGRNGIYleDLYVTPEYRGVGAGRRLLRELAREAVANDCGRLEwSVLDWNQPAIDFYRSIGALPqdewvryrldgealrkmae-----------------<br>fqlnldptrsap<br>------------<br><br>BLAST: aliSize=62 (resi) iden=25.30% (42/166) simi=37.35% (62/166)<br>s------------IRDAGVADLPGILAIYNDAVGNTTAIwNETPVDLAN-RQAWFdtraRQGYP---ILVASDaaGEVLGYA----SYGDWRpfeGFRGTVEHSVYVRDDQRGKGLGVQLLQALIERARAQGL-HVVAAIESGNAASIGLHRRLGfeisGQPQ----------------vgqkfgrwl<br>-enlyfqghmtleIRPAVPADAEQILAFIIELADYERAR-HEVVTDVEGiRRSLF----AEGSPtraLMCLSE--GRPIGYAvffySYSTWL---GRNGIYLEDLYVTPEYRGVGAGRRLLRELAREAVANDCgRLEWSVLDWNQPAIDFYRSIG----ALPQdewvryrldgealrkmae---------<br>dltfqlnldptrsap<br>---------------<br><br>Proposed: aliSize=146 (resi) RMSD=2.38 (Å)<br>-----------SIRDAGVADLPGILAIYNDAVGNTTaiWNETP-VDLANRQAWFDTrARQGYPILVASDAaGEVLGYASYGDWrpfeGF----RGTVEH-SVYVRDDQRGKGLGVQLLQALIERARAQGLHVMVAAIESGNAASIGLHRRLGFEISGampQvgqkfgrwlDLTFMQLNLD-PtR--Sap<br>enlyfqghmtlEIRPAVPADAEQILAFIIELADYER--ARHEVvTDVEGIRRSLFA-EGSPTRALMCLSE-GRPIGYAVFFYS----YStwlgRNGIYLeDLYVTPEYRGVGAGRRLLRELAREAVANDCGRLEWSVLDWNQPAIDFYRSIGALPQD---E---------WVRYRLDGEAlR-KmaE--
547	1yvoA (169)<br>2g3tB (168)	C	18.45	TM-align: aliSize=139 (resi) RMSD=2.91 (Å)<br>--SIRDAGVADLPGILAIYNDAVGNTTaiWNETP-VDLANRQAWFDTrARQGYPILVASDAA-------GEVLGYASYGDWRpFeG-FRGTVEH-SVYVRDDQRGKGLGVQLLQALIERARAQGLHV-VAAIESGNAASIGLHRRLGFEISGQ---PQVGqkfgrwldltfqlnldptrsap--------<br>kfVIRPATAADCSDILRLIKELAKYEY--MEEQViLTEKDLLEDGFG-EHPFYHCLVAEVPKehwtpegHSIVGFAMYYFTY-D-PwIGKLLYLeDFFVMSDYRGFGIGSEILKNLSQVAMRCRCSSmHFLVAEWNEPSINFYKRRGASDLSSeegWRLF----------------------kidkeyll<br>-----<br>kmate<br><br>SARST: aliSize=128 (resi) RMSD=3.91 (Å)<br>s---IRDAGVADLPGILAIYNDAVGNTTAIWNETPVdlanrqaWFDTRARQG------YPILVASDAAGEV------LGYASYGDwRPFEGFRG-TVEHSVYVRDDQRGKGLGVQLLQALIERARAQGLHVVA-AIESGNAASIGLHRRLGFEI-----------------------sgqpqvgqkfgrw<br>-kfvIRPATAADCSDILRLIKELAKYEYMEEQVILT-------EKDLLEDGFgehpfyHCLVAEVPKEHWTpeghsiVGFAMYYF-TYDPWIGKlLYLEDFFVMSDYRGFGIGSEILKNLSQVAMRCRCSSMHfLVAEWNEPSINFYKRRGASDlsseegwrlfkidkeyllkmate------------<br>ldltfqlnldptrsap<br>----------------<br><br>BLAST: aliSize=59 (resi) iden=22.89% (38/168) simi=35.54% (59/168)<br>s---IRDAGVADLPGILAIYNDAVGNTtaiWNETPVDLANRQAWFDTRARQG-YPILVA-------SDAAGEVLGYAS-YGDWRPFEGFRGTVEhSVYVRDDQRGKGLGVQLLQALIE---RARAQGLHVVAAieSGNAASIGLHRRLG-------------------------feisgqpqvgqkfgr<br>-kfvIRPATAADCSDILRLIKELAKYE---YMEEQVILTEKDLLEDGFGEHPfYHCLVAevpkehwTPEGHSIVGFAMyYFTYDPWIGKLLYLE-DFFVMSDYRGFGIGSEILKNLSQvamRCRCSSMHFLVA--EWNEPSINFYKRRGasdlsseegwrlfkidkeyllkmate-------------<br>wldltfqlnldptrsap<br>-----------------<br><br>Proposed: aliSize=149 (resi) RMSD=2.34 (Å)<br>--SIRDAGVADLPGILAIYNDAVGNTTaiW-nETP-VDLANRQAWFDT-raRQ-gYPILVASDAA--------gEVLGYASYGDWrpfEGF--RGTVEH-SVYVRDDQRGKGLGVQLLQALIERARAQGLHVMVAAIESGNAASIGLHRRLGFEISG-ampQVGqkfgrwlDLTFMQLNLDPTR-SAP<br>kfVIRPATAADCSDILRLIKELAKYEY--Me-EQViLTEKDLLEDGFGe--HPf-YHCLVAEVPKehwtpegh-SIVGFAMYYFT---YDPwiGKLLYLeDFFVMSDYRGFGIGSEILKNLSQVAMRCRCSSMHFLVAEWNEPSINFYKRRGASDLSs---EEG-------WRLFKIDKEYLLKmATE
548	1yvoA (169)<br>2jevA (169)	C	17.75	TM-align: aliSize=139 (resi) RMSD=3.01 (Å)<br>---SIRDAGVADLPGILAIYNDAVGNTTaiWNETP-VDLANRQAWFDTrARQGYPILVASDAA-------GEVLGYASYGDWrpFEG-FRGTVEH-SVYVRDDQRGKGLGVQLLQALIERARAQGLHV-VAAIESGNAASIGLHRRLGFEISGQ---PQVGqkfgrwldltfqlnldptrsap-------<br>akfVIRPATAADCSDILRLIKELAKYEY--MEEQViLTEKDLLEDGFG-EHPFYHCLVAEVPKehwtpegHSIVGFAMYYFT--YDPwIGKLLYLeDFFVMSDYRGFGIGSEILKNLSQVAMRCRCSSmHFLVAEWNEPSINFYKRRGASDLSSeegWRLF----------------------kidkeyl<br>------<br>lkmate<br><br>SARST: aliSize=129 (resi) RMSD=3.71 (Å)<br>s----IRDAGVADLPGILAIYNDAVGNTtAIWNETPVDLanrqawFDTRARQG------YPILVASDAAGE------VLGYASYGDwRPFEGFRGTVEH-SVYVRDDQRGKGLGVQLLQALIERARAQGLHVVA-AIESGNAASIGLHRRLGFEIS----------------------gqpqvgqkfgrw<br>-akfvIRPATAADCSDILRLIKELAKYE-YMEEQVILTE------KDLLEDGFgehpfyHCLVAEVPKEHWtpeghsIVGFAMYYF-TYDPWIGKLLYLeDFFVMSDYRGFGIGSEILKNLSQVAMRCRCSSMHfLVAEWNEPSINFYKRRGASDLsseegwrlfkidkeyllkmate-----------<br>ldltfqlnldptrsap<br>----------------<br><br>BLAST: aliSize=59 (resi) iden=22.89% (38/169) simi=35.54% (59/169)<br>s----IRDAGVADLPGILAIYNDAVGNTtaiWNETPVDLANRQAWFDTRARQG-YPILVA-------SDAAGEVLGYAS-YGDWRPFEGFRGTVEhSVYVRDDQRGKGLGVQLLQALIE---RARAQGLHVVAAieSGNAASIGLHRRLG-------------------------feisgqpqvgqkfg<br>-akfvIRPATAADCSDILRLIKELAKYE---YMEEQVILTEKDLLEDGFGEHPfYHCLVAevpkehwTPEGHSIVGFAMyYFTYDPWIGKLLYLE-DFFVMSDYRGFGIGSEILKNLSQvamRCRCSSMHFLVA--EWNEPSINFYKRRGasdlsseegwrlfkidkeyllkmate-------------<br>rwldltfqlnldptrsap<br>------------------<br><br>Proposed: aliSize=147 (resi) RMSD=2.30 (Å)<br>---SIRDAGVADLPGILAIYNDAVGNTT--aiwnETP-VDLANRQAWFD---T-RarqgyPILVASDAA--------gEVLGYASYGDWRpfeGF--RGTVEH-SVYVRDDQRGKGLGVQLLQALIERARAQGLHVMVAAIESGNAASIGLHRRLGFEISGamPQVGqkfgrwlDLTFMQLNLDPTR-SAP<br>akfVIRPATAADCSDILRLIKELAKYEYme----EQViLTEKDLLEDGFgehPfY-----HCLVAEVPKehwtpegh-SIVGFAMYYFTY---DPwiGKLLYLeDFFVMSDYRGFGIGSEILKNLSQVAMRCRCSSMHFLVAEWNEPSINFYKRRGASDLS--SEEG-------WRLFKIDKEYLLKmATE

549	1yvsA<br>(108)<br><br>1boxA<br>(95)	N	22.11	TM-align: aliSize=63 (resi) RMSD=2.60 (Å)<br>vintfdgvadylqtyhklpdnyitkseaqalgwvaskgnladvap-------------------------GKSI--GGDIFSNREGKLPGKSGRTWREADINYT-SGFRNSDRILYSSDW-LIYKTTDHYQTFTKIR---<br>---vsgtvclsalppeatdtlnliasdgPFPYsqDGVVFQSRESVLPTQSYGYYHEYTVITPgARTRGTRRIITGEATqEDYYTGDHYATFSLIDqtc<br><br>SARST: aliSize=68 (resi) RMSD=13.18 (Å)<br>vintfdgvadylqtyhklpdnyit----------------KSEAQALGWVA-SKGNladvapgksigGDIFSNREGKLPGK-SG-RTWREADI-NYTS-GfrnSDRILYSSD-WLIYKTTDHYQTFTK-----ir<br>------------------------vsgtvclsalppeatdTLNLIASDGPFpYSQD-----------GVVFQSRESVLPTQsYGyYHEYTVITpGARTrG---TRRIITGEAtQEDYYTGDHYATFSLidqtc--<br><br>BLAST: aliSize=30 (resi) iden=22.11% (21/95) simi=31.58% (30/95)<br>vintfdgvadylqtyhklpdnyitkseaqalgwvaskgnladvapgksig--------------------------------GDIFSNREGKLPGKSGRTWREADInYTSGFRN--SDRILYS-SDWLIYKTTDHYQTFTKI----r<br>--vsgtvclsalppeatdtlnliasdgpfpysqdGVVFQSRESVLPTQSYGYYHEYTV-ITPGARTrgTRRIITGeATQEDYYTGDHYATFSLIdqtc-<br><br>Proposed: aliSize=82 (resi) RMSD=2.48 (Å)<br>viNtfdgvadylqtyhklpdNYITK-SeaQalgWVAS-KGN-LADVAPGKS----iGGDIFSNREGKLPGKS-gRTWREADINYT-SGFRNSDRILYSSDW-LIYKTTDHYQTFTKIR---<br>--V-----------------SGTVClS--A---LPPEaTDTlNLIASDGPFpysq-DGVVFQSRESVLPTQSy-GYYHEYTVITPgARTRGTRRIITGEATqEDYYTGDHYATFSLIDqtc
550	1yvsA<br>(108)<br><br>1c54A<br>(96)	N	20.83	TM-align: aliSize=63 (resi) RMSD=2.42 (Å)<br>vintfdgvadylqtyhklpdnyitkseaqalgwvaskgnladvap---------------------------GKSI-GGDIFSNREGKLPGKSGRTWREADINYT-SGFRNSDRILYSSDW-LIYKTTDHYQTFTKIR---<br>---dvsgtvclsalppeatdtlnliasdgpFPYSqDGVVFQNRESVLPTQSYGYYHEYTVITPgARTRGTRRIITGEATqEDYYTGDHYATFSLIDqtc<br><br>SARST: aliSize=75 (resi) RMSD=16.61 (Å)<br>vintf--------------DGVADYLQTYHKLPDnyitkseaqalgwvaSKgnladvapgksIGGDIFSNREGKLPG-KSGRTWREADINYTSGFRNSDRILYSS-DWLIYKTTDHYQTFTK-----ir<br>-----dvsgtvclsalppeATDTLNLIASDGPFP---------------YS-----------QDGVVFQNRESVLPTqSYGYYHEYTVITPGARTRGTRRIITGEaTQEDYYTGDHYATFSLidqtc--<br><br>BLAST: aliSize=30 (resi) iden=22.92% (22/96) simi=31.25% (30/96)<br>vintfdgvadylqtyhklpdnyitkseaqalgwvaskgnladvapgksig---------------------------------GDIFSNREGKLPGKSGRTWREADInYTSGFRN--SDRILYS-SDWLIYKTTDHYQTFTKI----r<br>--dvsgtvclsalppeatdtlnliasdgpfpysqdGVVFQNRESVLPTQSYGYYHEYTV-ITPGARTrgTRRIITGeATQEDYYTGDHYATFSLIdqtc-<br><br>Proposed: aliSize=80 (resi) RMSD=2.42 (Å)<br>vINtfdgvadylqtyhklpDNYITKSEAQalGWVAS--KgNlADVA---------pgksiggDIFSNREGKLPGKSGRTWREADINYT-SGFRNSDRILYSSDW-LIYKTTDHYQTFTKIR---<br>-DV----------------SGTVCLSALP--PEATDtlN-L-IASDgpfpysqdg--------VVFQNRESVLPTQSYGYYHEYTVITPgARTRGTRRIITGEATqEDYYTGDHYATFSLIDqtc
551	1yvsA<br>(108)<br><br>1gmpA<br>(96)	N	20.83	TM-align: aliSize=63 (resi) RMSD=2.40 (Å)<br>vintfdgvadylqtyhklpdnyitkseaqalgwvaskgnladvap---------------------------GKS-IGGDIFSNREGKLPGKSGRTWREADINYT-SGFRNSDRILYSSDW-LIYKTTDHYQTFTKIR---<br>---dvsgtvclsalppeatdtlnliasdgpFPYsQDGVVFQNRESVLPTQSYGYYHEYTVITPgARTRGTRRIICGEATqEDYYTGDHYATFSLIDqtc<br><br>SARST: aliSize=71 (resi) RMSD=12.90 (Å)<br>vintfdgvadylqtyhklpdnyit-----------------KSEAQALGWVAskGNLAdvapgksiGGDIFSNREGKLPGK-SG-RTWREADInYTSGFRNSDRILYSSD-WLIYKTTDHYQTFTK-----ir<br>------------------------dvsgtvclsalppeatdTLNLIASDGPF--PYSQ--------DGVVFQNRESVLPTQsYGyYHEYTVIT-PGARTRGTRRIICGEAtQEDYYTGDHYATFSLidqtc--<br><br>BLAST: aliSize=30 (resi) iden=22.92% (22/96) simi=31.25% (30/96)<br>vintfdgvadylqtyhklpdnyitkseaqalgwvaskgnladvapgksig---------------------------------GDIFSNREGKLPGKSGRTWREADInYTSGFRN--SDRILYS-SDWLIYKTTDHYQTFTKI----r<br>--dvsgtvclsalppeatdtlnliasdgpfpysqdGVVFQNRESVLPTQSYGYYHEYTV-ITPGARTrgTRRIICGeATQEDYYTGDHYATFSLIdqtc-<br><br>Proposed: aliSize=80 (resi) RMSD=2.16 (Å)<br>vINtfdgvadylqtyhklpDNYITKSEAqalGWV-AS--KgNlADVA-------pgksiGGDIFSNREGKLPGKsG-RTWREADINYT-SGFRNSDRILYSSDW-LIYKTTDHYQTFTKIR---<br>-DV----------------SGTVCLSAL---PPEaTDtlN-L-IASDgpfpysq-----DGVVFQNRESVLPTQ-SyGYYHEYTVITPgARTRGTRRIICGEATqEDYYTGDHYATFSLIDqtc

552	1yvsA (108)<br>1i70A (96)	N	22.92	TM-align: aliSize=63 (resi) RMSD=2.38 (Å)<br>vintfdgvadylqtyhklpdnyitkseaqalgwvaskgnladvap---------------------------GKS-IGGDIFSNREGKLPGKSGRTWREADINYT-SGFRNSDRILYSSDW-LIYKTTDHYQTFTKIR---<br>---dvsgtvclsalppeatdtlnliasdgpFPYsQDGVVFQNRESVLPTQSYGYYHEYTVITPgARTRGTRRIITGEATqEDYYTGDHFATFSLIDqtc<br><br>SARST: aliSize=71 (resi) RMSD=12.76 (Å)<br>vintfdgvadylqtyhklpdnyit-----------------KSEAQALGWvaSKGNLAdvapgksiGGDIFSNREGKLPGK-SG-RTWREADInYTSGFRNSDRILYSSD-WLIYKTTDHYQTFTK-----ir<br>------------------------dvsgtvclsalppeatdTLNLIASDG--PFPYSQ--------DGVVFQNRESVLPTQsYGyYHEYTVIT-PGARTRGTRRIITGEAtQEDYYTGDHFATFSLidqtc--<br><br>BLAST: aliSize=30 (resi) iden=21.88% (21/96) simi=31.25% (30/96)<br>vintfdgvadylqtyhklpdnyitkseaqalgwvaskgnladvapgksig---------------------------------GDIFSNREGKLPGKSGRTWREADInYTSGFRN--SDRILYS-SDWLIYKTTDHYQTFTKI----r<br>--dvsgtvclsalppeatdtlnliasdgpfpysqdGVVFQNRESVLPTQSYGYYHEYTV-ITPGARTrgTRRIITGeATQEDYYTGDHFATFSLIdqtc-<br><br>Proposed: aliSize=82 (resi) RMSD=2.33 (Å)<br>vINtfdgvadylqtyhklpDNYITKSeAQalgWV-ASKgN--LaDVAPgKS-----iGGDIFSNREGKLPGKsG-RTWREADINYT-SGFRNSDRILYSSDW-LIYKTTDHYQTFTKIR---<br>-DV----------------SGTVCLS-AL---PPeATD-TlnL-IASD-GPfpysq-DGVVFQNRESVLPTQ-SyGYYHEYTVITPgARTRGTRRIITGEATqEDYYTGDHFATFSLIDqtc
553	1yvsA (108)<br>1i8vA (96)	N	19.79	TM-align: aliSize=63 (resi) RMSD=2.39 (Å)<br>vintfdgvadylqtyhklpdnyitkseaqalgwvaskgnladvap---------------------------GKS-IGGDIFSNREGKLPGKSGRTWREADINYT-SGFRNSDRILYSSDW-LIYKTTDHYQTFTKIR---<br>---dvsgtvclsalppeatdtlnliasdgpFPYsQDGVVFQNRESVLPTQSYGYYHEYTVITPgARTRGTRRIITGEATqEDFYTGDHYATFSLIDqtc<br><br>SARST: aliSize=71 (resi) RMSD=12.77 (Å)<br>vintfdgvadylqtyhklpdnyit-----------------KSEAQALGWvaSKGNLAdvapgksiGGDIFSNREGKLPGK-SG-RTWREADInYTSGFRNSDRILYSSD-WLIYKTTDHYQTFTK-----ir<br>------------------------dvsgtvclsalppeatdTLNLIASDG--PFPYSQ--------DGVVFQNRESVLPTQsYGyYHEYTVIT-PGARTRGTRRIITGEAtQEDFYTGDHYATFSLidqtc--<br><br>BLAST: aliSize=30 (resi) iden=21.88% (21/96) simi=31.25% (30/96)<br>vintfdgvadylqtyhklpdnyitkseaqalgwvaskgnladvapgksig---------------------------------GDIFSNREGKLPGKSGRTWREADInYTSGFRN--SDRILYS-SDWLIYKTTDHYQTFTKI----r<br>--dvsgtvclsalppeatdtlnliasdgpfpysqdGVVFQNRESVLPTQSYGYYHEYTV-ITPGARTrgTRRIITGeATQEDFYTGDHYATFSLIdqtc-<br><br>Proposed: aliSize=81 (resi) RMSD=2.27 (Å)<br>vINtfdgvadylqtyhklpdNYITK--seaqalgwVASKGN-LADVAP---GKSI---GGDIFSNREGKLPGKsG-RTWREADINYT-SGFRNSDRILYSSDW-LIYKTTDHYQTFTKIR---<br>-DV-----------------SGTVCls--------ALPPEAtDTLNLIasdGPFPysqDGVVFQNRESVLPTQ-SyGYYHEYTVITPgARTRGTRRIITGEATqEDFYTGDHYATFSLIDqtc
554	1yvsA (108)<br>1lovA (104)	N	11.54	TM-align: aliSize=57 (resi) RMSD=2.88 (Å)<br>vintfdgvadylqtyhklpdnyitkseaqalgwvaskgnladvapg--------------------------------KS--IGGDIFSNREGKLPGKSgRTWREADIN-----YTSGFRNSDRILYSSDWLI-YKTtDH-------YQTFTkir<br>--acdytcgsncysssdvstaqaagyklhedgetVGsnSYPHKYNNYEGFDFSVS-SPYYAWPILssgdvYSGGSPGADRVVFNENNQLaGVI-THtgasgnnFVECT---<br><br>SARST: aliSize=80 (resi) RMSD=17.21 (Å)<br>vintfdgvadylqtyh----KLPDNYIT-KSEA---------QALGWVASKGnladvapGKSIggdIFSNREGKLPGKSGRTWREADIN----YTSGFRNSDRILYSSDWL--IYKTTDH--YQTFTK--ir<br>----------------acdyTCGSNCYSsSDVStaqaagyklHEDGETVGSN-------SYPH---KYNNYEGFDFSVSSPYYAWPILSsgdvYSGGSPGADRVVFNENNQlaGVITHTGasGNNFVEct--<br><br>BLAST: aliSize=24 (resi) iden=17.31% (18/104) simi=23.08% (24/104)<br>vintf----------------------------DGVADYLQTYHKLPDNY--ITKS-EAQALGW-VASKGnlaDVAPGKSIGGD-IFSNREGKLPG----------------ksgrtwreadinytsgfrnsdrilyssdwliykttdhyqtftkir<br>-----acdytcgsncysssdvstaqaagyklheDGETVGSNSYPHKYNNYegFDFSvSSPYYAWpILSSG---DVYSGGSPGADrVVFNENNQLAGvithtgasgnnfvect---<br><br>Proposed: aliSize=79 (resi) RMSD=2.84 (Å)<br>vint--------FDgVadYlqtyhKLP-DNYIT-KSEAQALGWVASkGN-ladvapgksiGGDIFSNREGKLPG-KsgRTWREADIN-----YTSGFRNSDRILYSSDWLI-YKTtDH-------YQTFTkir<br>----acdytcgsNC-Y--S-----SSDvSTAQAaGYKLHEDGETVG-SNs----------YPHKYNNYEGFDFSvS--SPYYAWPILssgdvYSGGSPGADRVVFNENNQLaGVI-THtgasgnnFVECT---

555	1yvsA (108)<br>1mgrA (97)	N	22.68	TM-align: aliSize=63 (resi) RMSD=2.41 (Å)<br>vintfdgvadylqtyhklpdnyitkseaqalgwvaskgnladvap---------------------------GKSI--GGDIFSNREGKLPGKSGRTWREADINYT-SGFRNSDRILYSSDW-LIYKTTDHYQTFTKIR---<br>---vkavgrvcysalpsqahdtldlideggPFPYsqDGVVFQNREGLLPAHSTGYYHEYTVITPgSPTRGARRIITGQQWqEDYYTADHYASFRRVDfac<br><br>SARST: aliSize=69 (resi) RMSD=13.09 (Å)<br>vintfdgvadylqtyhklpdnyit------------------KSEAQALGWvaSKGNLAdvapgksiGGDIFSNREGKLPGKS-G-RTWREADI-NYTS-GfrnSDRILYSSD-WLIYKTTDHYQTFTK-----ir<br>------------------------vkavgrvcysalpsqahdTLDLIDEGG--PFPYSQ--------DGVVFQNREGLLPAHStGyYHEYTVITpGSPTrG---ARRIITGQQwQEDYYTADHYASFRRvdfac--<br><br>BLAST: aliSize=30 (resi) iden=22.68% (22/97) simi=30.93% (30/97)<br>vintfdgvadylqtyhklpdnyitkseaqalgwvaskgnladvapgksig----------------------------------GDIFSNREGKLPGKSGRTWREADInYTSGF--RNSDRILYSSDWL-IYKTTDHYQTFTKI----r<br>--vkavgrvcysalpsqahdtldlideggpfpysqdGVVFQNREGLLPAHSTGYYHEYTV-ITPGSptRGARRIITGQQWQeDYYTADHYASFRRVdfac-<br><br>Proposed: aliSize=84 (resi) RMSD=2.43 (Å)<br>-VINtfdgvadylqtyhklpdNYITKSeaQalgWVAS-KGN-LADVAPgKS-----iGGDIFSNREGKLPGKSGRTWREADINYT-SGFRNSDRILYSSDW-LIYKTTDHYQTFTKIR---<br>vKAV-----------------GRVCYS--A---LPSQaHDTlDLIDEG-GPfpysq-DGVVFQNREGLLPAHSTGYYHEYTVITPgSPTRGARRIITGQQWqEDYYTADHYASFRRVDfac
556	1yvsA (108)<br>1mgwA (99)	N	22.22	TM-align: aliSize=63 (resi) RMSD=2.47 (Å)<br>vintfdgvadylqtyhklpdnyitkseaqalgwvaskgnladvap-----------------------------GKSI--GGDIFSNREGKLPGKSGRTWREADINYT-SGFRNSDRILYSSDW-LIYKTTDHYQTFTKIR---<br>---asvkavgrvcysalpsqahdtldlideggPFPYsqDGVVFQNREGLLPAHSTGYYHEYTVITPgSPTRGARRIITGQQWqEDYYTADHYASFRRVDfac<br><br>SARST: aliSize=69 (resi) RMSD=13.13 (Å)<br>vintfdgvadylqtyhklpdnyit--------------------KSEAQALGWVAskGNLAdvapgksiGGDIFSNREGKLPGK-SG-RTWREADI-NYTS-GfrnSDRILYSSD-WLIYKTTDHYQTFTK-----ir<br>------------------------asvkavgrvcysalpsqahdTLDLIDEGGPF--PYSQ--------DGVVFQNREGLLPAHsTGyYHEYTVITpGSPTrG---ARRIITGQQwQEDYYTADHYASFRRvdfac--<br><br>BLAST: aliSize=30 (resi) iden=22.22% (22/99) simi=30.30% (30/99)<br>vintfdgvadylqtyhklpdnyitkseaqalgwvaskgnladvapgksig------------------------------------GDIFSNREGKLPGKSGRTWREADInYTSGF--RNSDRILYSSDWL-IYKTTDHYQTFTKI----r<br>--asvkavgrvcysalpsqahdtldlideggpfpysqdGVVFQNREGLLPAHSTGYYHEYTV-ITPGSptRGARRIITGQQWQeDYYTADHYASFRRVdfac-<br><br>Proposed: aliSize=84 (resi) RMSD=2.43 (Å)<br>vintfdgva-DYLqtyhKLpdnYITK-SeaQalgWVAS-KGN-LADVAPgKS-----iGGDIFSNREGKLPGKS-gRTWREADINYT-SGFRNSDRILYSSDW-LIYKTTDHYQTFTKIR---<br>---------aSVK----AV---GRVCyS--A---LPSQaHDTlDLIDEG-GPfpysq-DGVVFQNREGLLPAHSt-GYYHEYTVITPgSPTRGARRIITGQQWqEDYYTADHYASFRRVDfac
557	1yvsA (108)<br>1pylA (96)	N	20.83	TM-align: aliSize=63 (resi) RMSD=2.34 (Å)<br>vintfdgvadylqtyhklpdnyitkseaqalgwvaskgnladvap---------------------------GKSI--GGDIFSNREGKLPGKSGRTWREADINYT-SGFRNSDRILYSSDWLIYKTTDHYQTFTKIR---<br>---dpaladvcrtklpsqaqdtlaliakngPYPYnrDGVVFENRESRLPKKGNGYYHEFTVVTPgSNDRGTRRVVTGGYGEQYWSPDHYATFQEIDprc<br><br>SARST: aliSize=71 (resi) RMSD=12.85 (Å)<br>vintfdgvadylqtyhklpdnyit-----------------KSEAQALGWvaSKGNLAdvapgksiGGDIFSNREGKLPGKSG--RTWREADINyTSGFRNSDRILYSSDWLIYKTTDHYQTFTK-----ir<br>------------------------paladvcrtklpsqaqdTLALIAKNG--PYPYNR--------DGVVFENRESRLPKKGNgyYHEFTVVTP-GSNDRGTRRVVTGGYGEQYWSPDHYATFQEidprc--<br><br>BLAST: aliSize=46 (resi) iden=34.74% (33/96) simi=48.42% (46/96)<br>vintfdg--VADYLQTyhKLPdnyitkSEAQ-ALGWVASKGNLadvaPGKSiGGDIFSNREGKLPGKSGRTWREADInYTSGF--RNSDRILYSSDWLIYKTTDHYQTFTKI----r<br>-------paLADVCRT--KLP------SQAQdTLALIAKNGPY----PYNR-DGVVFENRESRLPKKGNGYYHEFTV-VTPGSndRGTRRVVTGGYGEQYWSPDHYATFQEIdprc-<br><br>Proposed: aliSize=82 (resi) RMSD=2.19 (Å)<br>vintfDGVadylqtyhklPdNYITKSEAqaLG-WVAS-KGNlaDV--------apgksiGGDIFSNREGKLPGKSGRTWREADINYT-SGFRNSDRILYSSDWLIYKTTDHYQTFTKIR---<br>-----DPA----------L-ADVCRTKL--PSqAQDTlALI--AKngpypynr------DGVVFENRESRLPKKGNGYYHEFTVVTPgSNDRGTRRVVTGGYGEQYWSPDHYATFQEIDprc

558	1yvsA (108)<br>1t2iA (96)	N	22.92	TM-align: aliSize=63 (resi) RMSD=2.48 (Å)<br>vintfdgvadylqtyhklpdnyitkseaqalgwvaskgnladvap--------------------------GKSI--GGDIFSNREGKLPGKSGRTWREADINYT-SGFRNSDRILYSSDW-LIYKTTDHYQTFTKIR---<br>---dvsgtvclsalppeatdtlnliasdgPFPYSqDGVVFQNRESVLPTQSYGYYHEYTVITPgARTRGTRRIITGEAWqEDYYTGDHYATFSLIDqtc<br><br>SARST: aliSize=81 (resi) RMSD=18.10 (Å)<br>vintfdgvadylqt--YHKLPDNYIT-----KSEAQALGWVAskGNLAdvapgksiGGDIFSNREGKLPGK-SG-RTWREADInYTSGFRNSDRILYSSD-WLIYKTTDHYQTFTK-----ir<br>--------------dvSGTVCLSALPpeatdTLNLIASDGPF--PYSQ--------DGVVFQNRESVLPTQsYGyYHEYTVIT-PGARTRGTRRIITGEAwQEDYYTGDHYATFSLidqtc--<br><br>BLAST: aliSize=30 (resi) iden=23.96% (23/96) simi=31.25% (30/96)<br>vintfdgvadylqtyhklpdnyitkseaqalgwvaskgnladvapgksig---------------------------------GDIFSNREGKLPGKSGRTWREADInYTSGFRN--SDRILYSSDWLI-YKTTDHYQTFTKI----r<br>--dvsgtvclsalppeatdtlnliasdgpfpysqdGVVFQNRESVLPTQSYGYYHEYTV-ITPGARTrgTRRIITGEAWQEdYYTGDHYATFSLIdqtc-<br><br>Proposed: aliSize=84 (resi) RMSD=2.53 (Å)<br>vINtfdgvadylqtyhklpDNYITKSeAQalgWVASKGN-LADVAPgKS-----iGGDIFSNREGKLPGKsG-RTWREADINYT-SGFRNSDRILYSSDW-LIYKTTDHYQTFTKIR---<br>-DV----------------SGTVCLS-AL---PPEATDTlNLIASD-GPfpysq-DGVVFQNRESVLPTQ-SyGYYHEYTVITPgARTRGTRRIITGEAWqEDYYTGDHYATFSLIDqtc
559	1yvsA (108)<br>1uciA (96)	N	21.88	TM-align: aliSize=63 (resi) RMSD=2.40 (Å)<br>vintfdgvadylqtyhklpdnyitkseaqalgwvaskgnladvap--------------------------GKSI-GGDIFSNREGKLPGKSGRTWREADINYT-SGFRNSDRILYSSDW-LIYKTTDHYQTFTKIR---<br>---dtsgtvclsalppeatdtlnliasdgpFPYSqDGVVFQNRESVLPTQSYGYYHEYTVITPgARTRGTRRIITGEATqEDYYTGDHYATFSLIDqtc<br><br>SARST: aliSize=83 (resi) RMSD=16.99 (Å)<br>vintfdgvadylqt--YHKLPDNYITKSEAQALgwvaskgnLADVAPGKS---IGGDIFSNREGK-LPGKSG-RTWREADInYTSGFRNSDRILYSSD-WLIYKTTDHYQTFTK-----ir<br>--------------dtSGTVCLSALPPEATDTL--------NLIASDGPFpysQDGVVFQNRESVlPTQSYGyYHEYTVIT-PGARTRGTRRIITGEAtQEDYYTGDHYATFSLidqtc--<br><br>BLAST: aliSize=30 (resi) iden=22.92% (22/96) simi=31.25% (30/96)<br>vintfdgvadylqtyhklpdnyitkseaqalgwvaskgnladvapgksig---------------------------------GDIFSNREGKLPGKSGRTWREADInYTSGFRN--SDRILYS-SDWLIYKTTDHYQTFTKI----r<br>--dtsgtvclsalppeatdtlnliasdgpfpysqdGVVFQNRESVLPTQSYGYYHEYTV-ITPGARTrgTRRIITGeATQEDYYTGDHYATFSLIdqtc-<br><br>Proposed: aliSize=81 (resi) RMSD=2.27 (Å)<br>vINtfdgvadylqtyhklpDNYITKSEAqalGWV-AS--KgNlADVA-------pgksiGGDIFSNREGKLPGKSGRTWREADINYT-SGFRNSDRILYSSDW-LIYKTTDHYQTFTKIR---<br>-DT----------------SGTVCLSAL---PPEaTDtlN-L-IASDgpfpysq-----DGVVFQNRESVLPTQSYGYYHEYTVITPgARTRGTRRIITGEATqEDYYTGDHYATFSLIDqtc
560	1yvsA (108)<br>1ucjA (96)	N	20.83	TM-align: aliSize=63 (resi) RMSD=2.49 (Å)<br>vintfdgvadylqtyhklpdnyitkseaqalgwvaskgnladvap--------------------------GKSI--GGDIFSNREGKLPGKSGRTWREADINYT-SGFRNSDRILYSSDW-LIYKTTDHYQTFTKIR---<br>---dvsgtvclsalppeatdtlnliasdgPFPYSqDGVTFQNRESVLPTQSYGYYHEYTVITPgARTRGTRRIITGEATqEDYYTGDHYATFSLIDqtc<br><br>SARST: aliSize=79 (resi) RMSD=18.30 (Å)<br>vintfdgvadylqt--YHKLPDNYIT-----KSEAQALGWvaSKGNLAdvapgksiGGDIFSNREGKLPGK-SG-RTWREADI-NYTS-GfrnSDRILYSSD-WLIYKTTDHYQTFTK-----ir<br>--------------dvSGTVCLSALPpeatdTLNLIASDG--PFPYSQ--------DGVTFQNRESVLPTQsYGyYHEYTVITpGARTrG---TRRIITGEAtQEDYYTGDHYATFSLidqtc--<br><br>BLAST: aliSize=29 (resi) iden=22.92% (22/96) simi=30.21% (29/96)<br>vintfdgvadylqtyhklpdnyitkseaqalgwvaskgnladvapgksig---------------------------------GDIFSNREGKLPGKSGRTWREADInYTSGFRN--SDRILYS-SDWLIYKTTDHYQTFTKI----r<br>--dvsgtvclsalppeatdtlnliasdgpfpysqdGVTFQNRESVLPTQSYGYYHEYTV-ITPGARTrgTRRIITGeATQEDYYTGDHYATFSLIdqtc-<br><br>Proposed: aliSize=80 (resi) RMSD=2.13 (Å)<br>VIntfdgvadylqtyhklpDNYITKSEAqalGWV-AS--KgNlADVaP-------gksiGGDIFSNREGKLPGKsG-RTWREADINYT-SGFRNSDRILYSSDW-LIYKTTDHYQTFTKIR---<br>DV-----------------SGTVCLSAL---PPEaTDtlN-L-IAS-Dgpfpysq----DGVTFQNRESVLPTQ-SyGYYHEYTVITPgARTRGTRRIITGEATqEDYYTGDHYATFSLIDqtc

561	1yvsA (108)<br>1uckA (96)	N	21.88	TM-align: aliSize=63 (resi) RMSD=2.38 (Å)<br>vintfdgvadylqtyhklpdnyitkseaqalgwvaskgnladvap----------------------------GKSI-GGDIFSNREGKLPGKSGRTWREADINYT-SGFRNSDRILYSSDW-LIYKTTDHYQTFTKIR---<br>--dvsgtvclsalppeatdtlnliasdgpFPYSqDGVVFQNRESTLPTQSYGYYHEYTVITPgARTRGTRRIITGEATqEDYYTGDHYATFSLIDqtc<br><br>SARST: aliSize=83 (resi) RMSD=17.06 (Å)<br>vintfdgvadylqt--YHKLPDNYITKSEAQALgwvaskgnLADVAPGKS---IGGDIFSNREGKLPGK-SG-RTWREADInYTSGFRNSDRILYSSD-WLIYKTTDHYQTFTK-----ir<br>-------------dvSGTVCLSALPPEATDTL--------NLIASDGPFpysQDGVVFQNRESTLPTQsYGyYHEYTVIT-PGARTRGTRRIITGEAtQEDYYTGDHYATFSLidqtc--<br><br>BLAST: aliSize=30 (resi) iden=22.92% (22/96) simi=31.25% (30/96)<br>vintfdgvadylqtyhklpdnyitkseaqalgwvaskgnladvapgksig--------------------------------GDIFSNREGKLPGKSGRTWREADInYTSGFRN--SDRILYS-SDWLIYKTTDHYQTFTKI----r<br>--dvsgtvclsalppeatdtlnliasdgpfpysqdGVVFQNRESTLPTQSYGYYHEYTV-ITPGARTrgTRRIITGeATQEDYYTGDHYATFSLIdqtc-<br><br>Proposed: aliSize=83 (resi) RMSD=2.36 (Å)<br>vINtfdgvadylqtyhklpdNYITK-SeaQalgWVAS-KGN-LADVAPgKS-----iGGDIFSNREGKLPGKSGRTWREADINYT-SGFRNSDRILYSSDW-LIYKTTDHYQTFTKIR---<br>-DV-----------------SGTVClS--A---LPPEaTDTlNLIASD-GPfpysq-DGVVFQNRESTLPTQSYGYYHEYTVITPgARTRGTRRIITGEATqEDYYTGDHYATFSLIDqtc
562	1yvsA (108)<br>1ynvX (96)	N	21.88	TM-align: aliSize=62 (resi) RMSD=2.48 (Å)<br>vintfdgvadylqtyhklpdnyitkseaqalgwvaskgnladvap-------------------------GKSI--GGDIFSNREGKLPGKSGRTWREADINYT-SGFRNSDRILYSsDW--LIYKTTDHYQTFTKIR---<br>--dvsgtvclsalppeatdtlnliasdgPFPYsqDGVVFQNRESVLPTQSYGYYHEYTVITPgARTRGTRRIITG-EAtqEDYYTGDHYATFSLIDktc<br><br>SARST: aliSize=71 (resi) RMSD=12.94 (Å)<br>vintfdgvadylqtyhklpdnyit-----------------KSEAQALGWVAskGNLAdvapgksiGGDIFSNREGKLPGK-SG-RTWREADInYTSGFRNSDRILY-SSDWLIYKTTDHYQTFTK-----ir<br>-----------------------dvsgtvclsalppeatdTLNLIASDGPF--PYSQ--------DGVVFQNRESVLPTQsYGyYHEYTVIT-PGARTRGTRRIITgEATQEDYYTGDHYATFSLidktc--<br><br>BLAST: aliSize=30 (resi) iden=22.92% (22/96) simi=31.25% (30/96)<br>vintfdgvadylqtyhklpdnyitkseaqalgwvaskgnladvapgksig--------------------------------GDIFSNREGKLPGKSGRTWREADInYTSGFRN--SDRILYS-SDWLIYKTTDHYQTFTKI----r<br>--dvsgtvclsalppeatdtlnliasdgpfpysqdGVVFQNRESVLPTQSYGYYHEYTV-ITPGARTrgTRRIITGeATQEDYYTGDHYATFSLIdktc-<br><br>Proposed: aliSize=81 (resi) RMSD=2.37 (Å)<br>vINtfdgvadylqtyhklPDnYITKSEAQalGWVA-S-KGNlaDVA-------pgksiGGDIFSNREGKLPGKS-gRTWREADINYT-SGFRNSDRILYSSDW-LIYKTTDHYQTFTKIR---<br>-DV---------------SG-TVCLSALP--PEATdTlNLI--ASDgpfpysq-----DGVVFQNRESVLPTQSy-GYYHEYTVITPgARTRGTRRIITGEATqEDYYTGDHYATFSLIDktc
563	1z3aA (156)<br>1wkqB (155)	C	26.45	TM-align: aliSize=111 (resi) RMSD=2.14 (Å)<br>sevefSHEYWMRHALTLAKRAWD-EREVPVGAVLVHNNRVIGEGWNRPIGRHDPTAHAEIMALRQGGLVMQNYRLIDATLYVTLEPCVMCAGAMIHSRIGRVVFGARDA--K-----TGAagSLMd-VIhhpgmnhrveitegiladecaallsdffrmrrqeik-------------------------<br>-----NHETFLKRAVTLACEGVNaGIGGPFGAVIVKDGAIIAEGQNNVTTSNDPTAHAEVTAIRKACKVLGAYQLDDCILYTSCEPCPMCLGAIYWARPKAVFYAAEHTdaAeagfdDSF--IYK-eI------------------------------------dkpaeertipfyqvtltehlspfqa<br>----------<br>wrnfankkey<br><br>SARST: aliSize=136 (resi) RMSD=14.81 (Å)<br>sevefs-HEYWMRHALTLAKRAWDER-EVPVGAVLVHNNRVIGEGWNRPIGRHDPTAHAEIMALRQGGLVMQNYRLIDATLYVTLEPCVMCAGAMIHSRIGRVVFGARDAKTGAAG---------SLMDVLHHPGMnhRVEITEGILADECAALL--------sdffrmrrqeik<br>------nHETFLKRAVTLACEGVNAGiGGPFGAVIVKDGAIIAEGQNNVTTSNDPTAHAEVTAIRKACKVLGAYQLDDCILYTSCEPCPMCLGAIYWARPKAVFYAAEHTDAAEAGfddsfiykeIDKPAEERTIP--FYQVTLTEHLSPFQAWRnfankkey------------<br><br>BLAST: aliSize=61 (resi) iden=27.10% (42/155) simi=39.35% (61/155)<br>sevefSHEYWMRHALTLAKRAWDER-EVPVGAVLVHNNRVIGEGWNRPIGRHDPTAHAEIMALRQGGLVMQNYRLIDATLYVTLEPCVMCAGAMIHSRIGRVVFGARDAKTGAAG---slmdvlhhpgmnhrveitegiladecaall<br>-----NHETFLKRAVTLACEGVNAGiGGPFGAVIVKDGAIIAEGQNNVTTSNDPTAHAEVTAIRKACKVLGAYQLDDCILYTSCEPCPMCLGAIYWARPKAVFYAAEHTDAAEAGfddsfiykeidkpaeertipfyqvtltehlspfqawrnfankkey-----------------------------<br>sdffrmrrqeik<br>------------<br><br>Proposed: aliSize=134 (resi) RMSD=1.70 (Å)<br>seveFsHEYWMRHALTLAKRAWDER-EVPVGAVLVHNNRVIGEGWNRPIGRHDPTAHAEIMALRQGGLVMQNYRLIDATLYVTLEPCVMCAGAMIHSRIGRVVFGARDA---------------ktgaagslmdV---LHhPgMNHRVEITEGILADECAALLSDFfRMRR--qeik<br>----N-HETFLKRAVTLACEGVNAGiGGPFGAVIVKDGAIIAEGQNNVTTSNDPTAHAEVTAIRKACKVLGAYQLDDCILYTSCEPCPMCLGAIYWARPKAVFYAAEHTdaaeagfddsfiyke----------IdkpAE-E-RTIPFYQVTLTEHLSPFQAWRNF-ANKKey----

No.	Proteins	Class	Score	Alignments
564	1z4rA (163)<br>1qsmD (152)	C	14.47	TM-align: aliSize=130 (resi) RMSD=3.20 (Å)<br>s--GIIEFHVIGnsltpkaNrRVLLWLVGLQNVFSHqLPRMPKEY-IARLVFDP-----K-HKTLALIK--DGRVIGGICFRMFP----TQGFTEIVFCAVTSNEQVKGYGTHLMNHLKEYHIKHNILYFLTYADE-Y--AIGYFKKQGFSKDIkvpksry--LgyikDYEgatlmecelnpr--<br>-seDNITVRFVT-------E-NDKEGWQRLWKSYQD-FYEVSFPDdLDDFNFGRfldpnIkMWAAVAVEssSEKIIGMINFFNHMttwdFKDKIYINDLYVDENSRVKGAGGKLIQFVYDEADKLGTPSVYWCTDEsNhrAQLLYVKVGYKAPK-------ilY----KRK------------gy<br><br>SARST: aliSize=110 (resi) RMSD=4.14 (Å)<br>sgiiefhvignsltpkanrr--------------VLLWLVGLQNVFSHQLPRMP---KEYIARLVFDPK--HKTLALIK--DGRVIGGICFRMFPTQGF----TEIVFCAVTSNEQVKGYGTHLMNHLKEYHIKHNILYFLTYADEYAIGYFKKQ-----------------gfskdikvpksrylgyik<br>--------------------sednitvrfvtendKEGWQRLWKSYQDFYEVSFPddlDDFNFGRFLDPNikMWAAVAVEssSEKIIGMINFFNHMTTWDfkdkIYINDLYVDENSRVKGAGGKLIQFVYDEADKLGTPSVYWCTDESNHRAQLLYvkvgykapkilykrkgy-----------------<br><br>dyegatlmecelnpr<br>---------------<br><br>BLAST: aliSize=17 (resi) iden=6.58% (10/152) simi=11.18% (17/152)<br>sgiiefhvignsltpkanrrvllwlvglqnvfshqlprmpkeyiarlvfdpkhktlalikdgrviggicfrmfptqgfteivfcav---TSNEQVKGYgTHLMNHLKEYHIKHNILYFLTYADE----------<br>--sednitvrfvtendkegwqrlwksyqdfyevsfpddlddfnfgrfldpnikmwaavaveSSSEKIIGM-INFFNHMTTWDFKDKIYINDLYVDEnsrvkgaggk<br><br>---yaigyfkkqgfskdikvpksrylgyikdyegatlmecelnpr<br>liqfvydeadklgtpsvywctdesnhraqllyvkvgykapkilykrkgy--<br><br>Proposed: aliSize=129 (resi) RMSD=2.19 (Å)<br>sg---IIEFHVIgnsltpkanrrVLLWLVGLQNVFSHQLP--RMPKE--YIARLVFD--P--K-HKTLALIK--DGRVIGGICFRMFpTQ-----GFTEIVFCAVTSNEQVKGYGTHLMNHLKEYHIKHNILYFLTYADE-Y--AIGYFKKQGFSkdikvpksrylgyikdyEGATLMECEL--npra<br>--sedNITVRFV-----------TENDKEGWQRLWKSYQDfyEVSFPddLDDFNFGRflDpnIkMWAAVAVEssSEKIIGMINFFNH-MTtwdfkDKIYINDLYVDENSRVKGAGGKLIQFVYDEADKLGTPSVYWCTDEsNhrAQLLYVKVGYK----------------APKILYKRKGya----
565	1znpA (140)<br>1ep0A (183)	N	10.71	TM-align: aliSize=99 (resi) RMSD=2.19 (Å)<br>dsaqaiirelglephpeggfyhqtfrdkaggerg---HSTAIYYLLEKGVRSHWHRVT--DAVEVWHYYAgAPIALHLSQD------GREVQTFTLGPAIlegERpQVIVPANCWQSAESLGDFTLVGCTVSPGFAFSSFVAEPGWSPG<br>----------------------------------efrfiktsldgaiiiepevytdergyfmetfneaifqenglevrFVQDNESMSVRGVLRGLHFQRekPQGKLVRVIR-GEIFDVAVDLrknsdtYGEWTGVRLSDEN---RR-EFFIPEGFAHGFLALSDECIVNYKCTELYHPEYDSGIPWDDPD<br><br>----------------------------------<br>igidwplemvddliisekdrnwkplrenpvyl<br><br>SARST: aliSize=118 (resi) RMSD=10.34 (Å)<br>dsaqaiirelg-----------------LEPHPEGGFYHQTFRDKAGGERGHST----AIYYLLEKGVRSHWHRVTD--AVEVWHYYAGAPIALHLS-----QDGREVQTFTLGpaILEGErpQVIVPANCWQSAESLGDFTLVGCTVSPGFAFSSFVAEP------------------------------<br>-----------efrfiktsldgaiiiepEVYTDERGYFMETFNEAIFQENGLEVrfvqDNESMSVRGVLRGLHFQREkpQGKLVRVIRGEIFDVAVDlrknsDTYGEWTGVRLS--DENRR--EFFIPEGFAHGFLALSDECIVNYKCTELYHPEYDSGIPwddpdigidwplemvddliisekdrnwkp<br><br>--------gwspg<br>lrenpvyl-----<br><br>BLAST: aliSize=19 (resi) iden=7.97% (11/140) simi=13.77% (19/140)<br>dsa----------------------------QAIIRELGLEPHpeggFYHQtfrDKAGGERGHSTAIYYLLEK---<br>---efrfiktsldgaiiiepevytdergyfmetfnEAIFQENGLEVR----FVQD--NESMSVRGVLRGLHFQREKpqgklvrvirgeifdvavdlrknsdtygewtgvrlsdenrreffipegfahgflalsdecivnykctelyhpeydsgipwddpdigidwplemvddliisekdrnwkplrenp<br><br>---gvrshwhrvtdavevwhyyagapialhlsqdgrevqtftlgpailegerpqvivpancwqsaeslgdftlvgctvspgfafssfvaepgwspg<br>vyl---<br><br>Proposed: aliSize=116 (resi) RMSD=2.22 (Å)<br>dmsaqaii--R-----------ELGLEPHP-EGGFYHQTF-------------rdkaggerghSTAIYYLLEKGVRSHWHRVT--DAVEVWHYYAgAPIALHLSQD------GREVQTFTLGPAIlegeRPQVIVPANCWQSAESLGDFTLVGCTVSPGFAFSSFVMAeP-GWSPG--------------<br>--------efRfiktsldgaiiIEPEVYTDeRGYFMETFNeaifqenglevrf----------VQDNESMSVRGVLRGLHFQRekPQGKLVRVIR-GEIFDVAVDLrknsdtYGEWTGVRLSDEN----RREFFIPEGFAHGFLALSDECIVNYKCTELYHPEYDSGI-PwDDPDIgidwplemvddlii<br><br>------------------<br>sekdrnwkplrenpvyl
566	1znpA (140)<br>1pm7A (199)	N	8.57	TM-align: aliSize=100 (resi) RMSD=2.28 (Å)<br>dsaqaiirelglephpeggfyhqtfrdkagger---GHSTAIYYLLEKGVRSHWHRVT---DAVEVWHYYAgAPIALHLSQD------GREVQTFTLGPAIlegERpQVIVPANCWQSAESLGDFTLVGCTVSPGFAFSSFV-AEPGWS-<br>----------------------------------mkareldvpgaweitptihvdsrglffewltdhgfrafaghslDVRQVNCSVSSAGVLRGLHFAQlppSQAKYVTCVS-GSVFDVVVDIregsptFGRWDSVLLDDQD---RR-TIYVSEGLAHGFLALQDNSTVMYLCSAEYNPQREHtICATDPt<br><br>PG--<br>LAvdwplvdgaapslsdrdaaapsfedvrasgllprweqtqrfigem<br><br>SARST: aliSize=108 (resi) RMSD=14.84 (Å)<br>dsaqaiirelg-----------------LEPHPEGGFYHQTFRD-----KAGGERGHSTAIYYLLEKGVRSHWHRV---TDAVEVWHYYAGAPIALHLS-----QDGREVQTFTLGPAILE------GERPQVIV-PANCWQSAESLGDFT---LVGCT----------------------------------<br>-----------mkareldvpgaweitpTIHVDSRGLFFEWLTDhgfraFAGHSLDVRQVNCSVSSAGVLRGLHFAqlpPSQAKYVTCVSGSVFDVVVDiregsPTFGRWDSVLLDDQDRRtiyvseGLAHGFLAlQDNSTVMYLCSAEYNpqrEHTICatdptlavdwplvdgaapslsdrdaaapsfed<br><br>--------------------vspgfafssfvaepgwspg<br>vrasgllprweqtqrfigem-------------------<br><br>BLAST: aliSize=23 (resi) iden=10.14% (14/140) simi=16.67% (23/140)<br>dsaqaiirelglephpeggfyhqtfrdkaggerghs--------------TAIYYLLEKGVRSHWhrVTDavEVWHYYAGapialHlSQDGREVQTFTLGPAILEG--<br>-------------------------------------mkareldvpgaweiTPTIHVDSRGLFFEW--LTD--HGFRAFAG-----H-SLDVRQVNCSVSSAGVLRGlhfaqlppsqakyvtcvsgsvfdvvvdiregsptfgrwdsvllddqdrrtiyvseglahgflalqdnstvmylcsaeynpqreh<br><br>--erpqvivpancwqsaeslgdftlvgctvspgfafssfvaepgwspg<br>ticatdptlavdwplvdgaapslsdrdaaapsfedvrasgllprweqtqrfigem---<br><br>Proposed: aliSize=118 (resi) RMSD=2.23 (Å)<br>dmsaqa----IIRelgL-ephpegGFYHQTFRDKA----gGERG-----------------hSTAIYYLLEKGVRSHWHRVT---DAVEVWHYYAgAPIALHLSQD------GREVQTFTLGPAIlegeRPQVIVPANCWQSAESLGDFTLVGCTVSPGFAFSSFVMAEPGWSPG---------------<br>------mkarELD---Vp------GAWEITPTIHVdsrg-LFFEwltdhgfrafaghsldv-RQVNCSVSSAGVLRGLHFAQlppSQAKYVTCVS-GSVFDVVVDIregsptFGRWDSVLLDDQD----RRTIYVSEGLAHGFLALQDNSTVMYLCSAEYNPQREHTICATDPTLavdwplvdgaapsls<br><br>------------------------------<br>drdaaapsfedvrasgllprweqtqrfigem

567	1znpA (140)<br>2ixcA (198)	N	8.57	TM-align: aliSize=100 (resi) RMSD=2.25 (Å)<br>dsaqaiirelglephpeggfyhqtfrdkagger---GHSTAIYYLLEKGVRSHWHRVT---DAVEVWHYYAgAPIALHLSQD------GREVQTFTLGPAIlegERpQVIVPANCWQSAESLGDFTLVGCTVSPGFAFSSFV-AEPGWS-<br>---------------------------------mkareldvpgaweitptihvdsrglffewltdhgfrafaghslDVRQVNCSVSSAGVLRGLHFAQlppSQAKYVTCVS-GSVFDVVVDIregsptFGRWDSVLLDDQD---RR-TIYVSEGLAHGFLALQDNSTVMYLCSAEYNPQREHtICATDPt<br>PG--------------------------------------<br>LAvdwplvdgaapslsdrdaaapsfedvrasgllprweqtqrfige<br>SARST: aliSize=112 (resi) RMSD=10.31 (Å)<br>ds--------------------------------AQAIIRELGLEPHpeggfyhQTFrdkaggerghstAIYYLLEKGVRSHWHRV---TDAVEVWHYYAGAPIALHLS-----QDGREVQTFTLGPAilEGERpqVIVPANCWQSAESLGDFTLVGCTVSPGFAFSSFVAEP--------------GWSP<br>--mkareldvpgaweitptihvdsrglffewltDHGFRAFAGHSLD-------VRQ------------VNCSVSSAGVLRGLHFAqlpPSQAKYVTCVSGSVFDVVVDiregsPTFGRWDSVLLDDQ--DRRT--IYVSEGLAHGFLALQDNSTVMYLCSAEYNPQREHTICatdptlavdwplvdGAAP<br>---------------------------------g<br>slsdrdaaapsfedvrasgllprweqtqrfige-<br>BLAST: aliSize=23 (resi) iden=10.14% (14/140) simi=16.67% (23/140)<br>dsaqaiirelglephpeggfyhqtfrdkaggerghs-------------TAIYYLLEKGVRSHWhrVTDavEVWHYYAGapialHlSQDGREVQTFTLGPAILEG--<br>-----------------------------------mkareldvpgaweiTPTIHVDSRGLFFEW--LTD--HGFRAFAG-----H-SLDVRQVNCSVSSAGVLRGlhfaqlppsqakyvtcvsgsvfdvvvdiregsptfgrwdsvllddqdrrtiyvseglahgflalqdnstvmylcsaeynpqreh<br>---erpqvivpancwqsaeslgdftlvgctvspgfafssfvaepgwspg<br>ticatdptlavdwplvdgaapslsdrdaaapsfedvrasgllprweqtqrfige---<br>Proposed: aliSize=118 (resi) RMSD=2.21 (Å)<br>dmsaqa----IIRelgL-ephpegGFYHQTFRDKA----gGERG-----------------hSTAIYYLLEKGVRSHWHRVT---DAVEVWHYYAgAPIALHLSQD------GREVQTFTLGPAIlegeRPQVIVPANCWQSAESLGDFTLVGCTVSPGFAFSSFVMAEPGWSPG---------------<br>------mkarELD---Vp------GAWEITPTIHVdsrg-LFFEwltdhgfrafaghsldv-RQVNCSVSSAGVLRGLHFAQlppSQAKYVTCVS-GSVFDVVVDIregsptFGRWDSVLLDDQD----RRTIYVSEGLAHGFLALQDNSTVMYLCSAEYNPQREHTICATDPTLavdwplvdgaapsls<br>--------------------------------<br>drdaaapsfedvrasgllprweqtqrfige
568	1znpA (140)<br>2ixhA (184)	N	6.43	TM-align: aliSize=100 (resi) RMSD=2.43 (Å)<br>dsaqaiirelglephpeggfyhqtfrdkagger--GHSTAIYYLLEKGVRSHWHRVT--DAVEVWHYYAGaPIALHLSQD------GREVQTFTLGPAIlegERpQVIVPANCWQSAESLGDFTLVGCTVSPGFAFSSFVAEPGWS<br>---------------------------------smamkatrlaipdvilfeprvfgddrgfffesynqrafeeacghpvSFVQDNHSRSARGVLRGLHYQIrqAQGKLVRATLG-EVFDVAVDLrrgsptFGQWVGERLSAEN---KR-QMWIPAGFAHGFVVLSEYAEFLYKTTDFWAPEHERCIVWND<br>--P--------------------G-------<br>peLkidwplqdapllsekdrqgkaFadadcfp<br>SARST: aliSize=105 (resi) RMSD=16.14 (Å)<br>ds-----------------------------------AQAIIRELG-LEPHPeggfYHQTFRDKAGGerghsTAIYYLLEKGVRSHWHRVTDAVEVWHY--------YAGAPIALHLSQ-DGrEVqtftlgpailegerpqVIVPANCWQSAESLGDFTLVGCTVSPGFAFSSFVAEP-------------<br>--smamkatrlaipdvilfeprvfgddrgfffesynQRAFEEACGhPVSFV----QDNHSRSARGV-----LRGLHYQIRQAQGKLVRATLGEVFDVAvdlrrgspTFGQWVGERLSAeNK-RQ----------------MWIPAGFAHGFVVLSEYAEFLYKTTDFWAPEHERCIVwndpelkidwplq<br>------------------------gwspg<br>dapllsekdrqgkafadadcfp-----<br>BLAST: aliSize=14 (resi) iden=6.52% (9/140) simi=10.14% (14/140)<br>dsaqaiirelgl-----------------EPHPEG---GFYHQTFRDKAGGER-GH--<br>------------smamkatrlaipdvilfEPRVFGddrGFFFESYNQRAFEEAcGHpvsfvqdnhsrsargvlrglhyqirqaqgklvratlgevfdvavdlrrgsptfgqwvgerlsaenkrqmwipagfahgfvvlseyaeflykttdfwapehercivwndpelkidwplqdapllsekdrqgkafa<br>------staiyyllekgvrshwhrvtdavevwhyyagapialhlsqdgrevqtftlgpailegerpqvivpancwqsaeslgdftlvgctvspgfafssfvaepgwspg<br>dadcfp---<br>Proposed: aliSize=117 (resi) RMSD=2.24 (Å)<br>dmsaqa------IIR-----elglephpegGFYHQtFRDKAGGERG-------------------hSTAIYYLLEKGVRSHWHRVT--DAVEVWHYYAgAPIALHLSQD------GREVQTFTLGPAIlegeRPQVIVPANCWQSAESLGDFTLVGCTVSPGFAFSSFVMAEPGWSPG------------<br>------smamkaTRLaipdv----------ILFEP-RVFGDDRGFFfesynqrafeeacghpvsf-VQDNHSRSARGVLRGLHYQIrqAQGKLVRATL-GEVFDVAVDLrrgsptFGQWVGERLSAEN----KRQMWIPAGFAHGFVVLSEYAEFLYKTTDFWAPEHERCIVWNDPELkidwplqdapll<br>-----------------<br>sekdrqgkafadadcfp
569	1znpA (140)<br>2paeB (134)	N	7.46	TM-align: aliSize=96 (resi) RMSD=3.00 (Å)<br>dsaqaiirelglephpeggfyhqtfrdkagger------------------------------GHSTAIYYL-LEKGVRSHWHRVTDAVEVWHYYAGaPIALHLSQDgREVQTFTLGPAilegeRPQVIVPANCWQSAESLGDFTLVGCTVSPGFAFSSFVAEPg-WSPg--------<br>---------------------------------enkvinfkkiidsrgslvaieenknipfSIKRVYYIFdTKGEEPRGFNAHKKLEQVLVCLNG-SCRVILDDG-NIIQEITLDSP-----AVGLYVGPAVWHEMHDFSSDCVMMVLASDYYDETDYIRQY-dNFK-kyiakinl<br>SARST: aliSize=100 (resi) RMSD=8.88 (Å)<br>dsaqaiirelgleph------------PEGGFYHQ------TFRDKaggerghSTAIYYLLEKGVRSHWHRVTDAVEVWHYYAGAPIALHLsqDGREVQTFTLgpAILEGErpqVIVPANCWQSAESLGDFTLVGCTVSPGFAFSSF----------------vaepgwspg<br>---------------enkvinfkkiidSRGSLVAIeenkniPFSIK-------RVYYIFDTKGEEPRGFNAHKKLEQVLVCLNGSCRVILD--DGNIIQEITL--DSPAVG---LYVGPAVWHEMHDFSSDCVMMVLASDYYDETDYirqydnfkkyiakinl---------<br>BLAST: aliSize=9 (resi) iden=5.22% (7/134) simi=6.72% (9/134)<br>dsaqaiirelglephpeggfyhqtfrdkaggerghstaiyyllekgvrshwhrvtdavevwhyyagapialhlsq--DGREVQTFTL-GPAI-----------------------------------<br>---enkvinfkkiidsrgslvaieenknipfsikrvyyifdtkgeeprgfnahkkleqvlvclngscrvildDGNIIQEITLdSPAVglyvgpavwhemhdfssdcvmmvlasdyyde<br>------------------legerpqvivpancwqsaeslgdftlvgctvspgfafssfvaepgwspg<br>tdyirqydnfkkyiakinl---<br>Proposed: aliSize=107 (resi) RMSD=2.23 (Å)<br>dmsaqaiir----ELGLEPHP-EGGFYHQTF---------rdkaggerghSTAIYYL-LEKGVRSHWHRVTDAVEVWHYYAgAPIALHLSQDgREVQTFTLGP-ailegeRPQVIVPANCWQSAESLGDFTLVGCTVSPGFAFSSFVMAepgW------------spg<br>---------enkvINFKKIIDsRGSLVAIEEnknipfsi-----------KRVYYIFdTKGEEPRGFNAHKKLEQVLVCLN-GSCRVILDDG-NIIQEITLDSp------AVGLYVGPAVWHEMHDFSSDCVMMVLASDYYDETDYIRQ---Ydnfkkyiakinl---

570	1zvnA (99)<br>2h7wA (108)	N	7.07	TM-align: aliSize=77 (resi) RMSD=3.65 (Å)<br>sgw-----VWNQFFVLEEyTGtdPLYVGKLHSdmDrgdGSI-KYILSGEg--AGI------VFTIDDT-----------TGDIHAIQRLDReerSQYTLRAQALdrrt-GRpme----PESEFIIKIQD-<br>---hkvtkAHNGATLTVA-VG--ELVEIQLPS--N---PTTgFAWYFEG-gtKESpnesmfTVENKYFppdskllgaggTEHFHVTVKAAG---THAVNLTYMR----pWT---gpshDSERFIVYLKAn<br><br>SARST: aliSize=60 (resi) RMSD=9.00 (Å)<br>sgwvwnqffvleey-------------TGTDPLYVGKLHSDMdRGdgsikYILSGEGAgivftiddttgdihAIQRLDREerSQYTLRAQALDRRtGRPMEPESEFIIKI----------------------------------qd<br>-------------hkvtkahngatltvAVGELVEIQLPSNPT-TG-----FAWYFEGG-------------TKESPNES--MFTVENKYFPPDS-KLLGAGGTEHFHVTvkaagthavnltymrpwtgpshdserfivylkan--<br><br>BLAST: aliSize=11 (resi) iden=7.07% (7/99) simi=11.11% (11/99)<br>sgwvwnqffvleeytgtdplyvgklhsdmdrg-------DGSIKYILSGEGAGIVFTIDDTTG---dihaiqrldreersqytlraqaldrrtgrpmepesefiikiqd<br>--------------------------------hkvtkahNGATLTVAVGELVEIQLPSNPTTGfawyfeggtkespnesmftvenkyfppdskllgaggtehfhvtvkaagthavnltymrpwtgpshdserfivylkan---<br><br>Proposed: aliSize=79 (resi) RMSD=3.18 (Å)<br>SGWV---WNQFFVLEeyTG-tdpLYVGKLHsdmDRgdGS--IKYILS-----geGAGI--VFTIDDT-----------TGDIHAIQRLDreeRsQYTLRAQALD-rrtGR-P--mEPESEFIIKIQD<br>HKVTkahNGATLTVA--VGe---LVEIQLP---SN--PTtgFAWYFEggtke--SPNEsmFTVENKYfppdskllgaggTEHFHVTVKAA---G-THAVNLTYMRp---WTgPsh-DSERFIVYLKAN
571	2b3jA (151)<br>1wkqA (158)	C	29.80	TM-align: aliSize=112 (resi) RMSD=2.22 (Å)<br>m--TNDIYFMTLAIEEAKKAAQ-LGEVPIGAIITKDDEVIARAHNLRETLQQPTAHAEHIAIERAAKVLGSWRLEGCTLYVTLEPCVMCAGTIVMSRIPRVVYGADDP--K-----GGCsgSLMn-Llqqsnfnhraivdkgvlkeacstllttffknlran----------------------------<br>-haMNHETFLKRAVTLACEGVNaGIGGPFGAVIVKDGAIIAEGQNNVTTSNDPTAHAEVTAIRKACKVLGAYQLDDCILYTSCEPCPMCLGAIYWARPKAVFYAAEHTdaAeagfdDSF--IYK-eI-----------------------------------dkpaeertipfyqvtltehlspfqawrn<br>-------<br>fankkey<br><br>SARST: aliSize=99 (resi) RMSD=0.97 (Å)<br>mtn----DIYFMTLAIEEAKKAAQLG-EVPIGAIITKDDEVIARAHNLRETLQQPTAHAEHIAIERAAKVLGSWRLEGCTLYVTLEPCVMCAGTIVMSRIPRVVYGA--ddpkggcsgslmnllqqsnfnhraivdkg<br>---hamnHETFLKRAVTLACEGVNAGiGGPFGAVIVKDGAIIAEGQNNVTTSNDPTAHAEVTAIRKACKVLGAYQLDDCILYTSCEPCPMCLGAIYWARPKAVFYAAehtdaaeagfddsfiykeidkpaeertipfyqvtltehlspfqawrnfankkey-----------------------------<br>vlkeacstllttffknlran<br>--------------------<br><br>BLAST: aliSize=56 (resi) iden=29.14% (44/151) simi=37.09% (56/151)<br>mtndiyf------------MTLAIEEAKkaAQLGEvPIGAIITKDDEVIARAHNLRETLQQPTAHAEHIAIERAAKVLGSWRLEGCTLYVTLEPCVMCAGTIVMSRIPRVVYGAD---dpkggcsgslmnllqqsnfnhr<br>-------hamnhetflkraVTLACEGVN--AGIGG-PFGAVIVKDGAIIAEGQNNVTTSNDPTAHAEVTAIRKACKVLGAYQLDDCILYTSCEPCPMCLGAIYWARPKAVFYAAEhtdaaeagfddsfiykeidkpaeertipfyqvtltehlspfqawrnfankkey----------------------<br>aivdkgvlkeacstllttffknlran<br>--------------------------<br><br>Proposed: aliSize=136 (resi) RMSD=1.84 (Å)<br>m--TNDIYFMTLAIEEAKKAAQLG-EVPIGAIITKDDEVIARAHNLRETLQQPTAHAEHIAIERAAKVLGSWRLEGCTLYVTLEPCVMCAGTIVMSRIPRVVYGADDP---------------kggcsgslM--NlLQQSnFnHRAIVDKGVLKEACSTLLTTFfKNLR--an<br>-haMNHETFLKRAVTLACEGVNAGiGGPFGAVIVKDGAIIAEGQNNVTTSNDPTAHAEVTAIRKACKVLGAYQLDDCILYTSCEPCPMCLGAIYWARPKAVFYAAEHTdaaeagfddsfiyke--------IdkP-AEER-T-IPFYQVTLTEHLSPFQAWRNF-ANKKey--
572	2bswA (145)<br>1vkcA (149)	C	13.79	TM-align: aliSize=129 (resi) RMSD=2.37 (Å)<br>-IEVKPIN--AEDTYELRHRILRP-------N--QPIEAC--F-ESDLLRGAFHLGGYYGG-KLISIASFHQAEHSElQGQKQYQLRG-ATLEGYREQKAGSSLIKHAEEILRKRGADLLWCNART-S-ASGYYKKLGFSEQGEVFDTPpvgphilykrit-<br>eYTIVDGEeyIEEIKKLDREISYSfvrfpisYeeYEERHEelFeSLLSQGEHKFFVALNERsELLGHVWICITLDTV-DYVKIAYIYDiEVVKWARGLGIGSALLRKAEEWAKERGAKKIVLRVEIdNpAVKWYEERGYKARALIMEKP------------i<br><br>SARST: aliSize=126 (resi) RMSD=4.23 (Å)<br>ie-VKPINAEDTYELRHRIL--------RPNQPIEA--------CFESDLLRGAFHLGGYY-GGKLISIASFHQAEHSE--LQgqKQYQLRGATLEGYREQKAGSSLIKHAEEILRKRGADLLWCNART--SASGYYKKLGFSEQGEVFDTP-pvgphilykrit<br>--eYTIVDGEEYIEEIKKLDreisysfvRFPISYEEyeerheelFESLLSQGEHKFFVALNeRSELLGHVWICITLDTVdyVK--IAYIYDIEVVKWARGLGIGSALLRKAEEWAKERGAKKIVLRVEIdnPAVKWYEERGYKARALIMEKPi------------<br><br>BLAST: aliSize=58 (resi) iden=21.83% (31/145) simi=40.85% (58/145)<br>ievk----------------------------PINAEDtYELRHrilrpnqpiEACFESDLLRG--AFHLGGYYGGKLIS----IASFHQAEHSELqgQKQYQLRgaTLEGYREQKAGSSLIKHAEEILRKRGAD--LLWCNARTSASGYYKKLGFSEQGEVFDTP-pvgphilykrit<br>----eytivdgeeyieeikkldreisysfvrfPISYEE-YEERH---------EELFESLLSQGehKFFVALNERSELLGhvwiCITLDTVDYVKI--AYIYDIE--VVKWARGLGIGSALLRKAEEWAKERGAKkiVLRVEIDNPAVKWYEERGYKARALIMEKPi------------<br><br>Proposed: aliSize=132 (resi) RMSD=1.95 (Å)<br>-IEVKPIN--AEDTYELRHRILRP-------N--QPIEAC--MFESDLLRGAFHLGGYYGG-KLISIASFHQAEHSElQGQKQYQLRGMATLEGYREQKAGSSLIKHAEEILRKRGADLLWCNART-S-ASGYYKKLGFSEQGevfdtppvgphILMYKRItc<br>eYTIVDGEeyIEEIKKLDREISYSfvrfpisYeeYEERHEelFESLLSQGEHKFFVALNERsELLGHVWICITLDTV-DYVKIAYIYDIEVVKWARGLGIGSALLRKAEEWAKERGAKKIVLRVEIdNpAVKWYEERGYKARA-----------LIMEKPI--

573	2bswA<br>(145)<br><br>2vbqA<br>(147)	C	14.48	TM-align: aliSize=128 (resi) RMSD=2.60 (Å)<br>--IEVKPINA--ED-TYELRHRiLRPNQPIEAC--FE-SD-LLRGAFHLGGYYGGKLISIASFHQAEHS-E-LQGQKQYQLRG-ATLEGYREQKAGSSLIKHAEEILRKRGADLLWCNART-S--ASGYYKKLGFSEQGEVFDTppvgphilykrit----<br>shMDIRQMNKthLEhWRGLRKQ-LWPGHPDDAHlaDGeEIlQADHLASFIAMADGVAIGFADASIRHDYvNgCDSSPVVFLEGiFVLPSFRQRGVAKQLIAAVQRWGTNKGCREMASDTSPeNtiSQKVHQALGFEETERVIFY-------------rkrc<br><br>SARST: aliSize=125 (resi) RMSD=4.28 (Å)<br>i---EVKPINaEDTYELRHRILRPNQP-------IEACFESDLLRGAFHLGGYYGGKLISIASFH----QAEHSELQGqKQYQLRGATLEGYREQKAGSSLIKHAEEILRKRGADLLWCNAR---TSASGYYKKLGFSEqGEVFDT-----ppvgphilykrit<br>-shmDIRQMN-KTHLEHWRGLRKQLWPghpddahLADGEEILQADHLASFIAMADGVAIGFADASirhdYVNGCDSSP-VVFLEGIFVLPSFRQRGVAKQLIAAVQRWGTNKGCREMASDTSpenTISQKVHQALGFEE-TERVIFyrkrc------------<br><br>BLAST: aliSize=44 (resi) iden=19.01% (27/145) simi=30.99% (44/145)<br>ievkpinaedtye-----------------LRHRiLRPNQPIEACF-ESDLLRGAFHLGGYYG---GKLISIASfHQAEHSELQG---QKQYQLRGA-TLEGYREQKAGSSLIKHAEEILRKRGADLLWCNA---RTSASGYYKKLGFSEQGEV-------fdtppvgphilykrit<br>------------shmdirqmnkthlehwrgLRKQ-LWPGHPDDAHLaDGEEILQADHLASFIAmadGVAIGFAD-ASIRHDYVNGcdsSPVVFLEGIfVLPSFRQRGVAKQLIAAVQRWGTNKGCREMASDTspeNTISQKVHQALGFEETERVifyrkrc---------------<br><br>Proposed: aliSize=131 (resi) RMSD=2.05 (Å)<br>--IEVKPIN---aED-TYELRHRiLRPNQPIEAC-MF--esDL--LRGAFHLGGYYGGKLISIASFHQAEHS-E-LQGQKQYQLRGMATLEGYREQKAGSSLIKHAEEILRKRGADLLWCNART-S--ASGYYKKLGFSEQGEvfdtppvgPHILMYKRitc<br>shMDIRQMNkth-LEhWRGLRKQ-LWPGHPDDAHlADge--EIlqADHLASFIAMADGVAIGFADASIRHDYvNgCDSSPVVFLEGIFVLPSFRQRGVAKQLIAAVQRWGTNKGCREMASDTSPeNtiSQKVHQALGFEETER--------VIFYRKRC---
574	2bswA<br>(145)<br><br>2vbqB<br>(144)	C	15.28	TM-align: aliSize=127 (resi) RMSD=2.53 (Å)<br>iEVKPINA--ED-TYELRHRiLRPNQPIEAC--FE-SD-LLRGAFHLGGYYGGKLISIASFHQA-EHSE-LQGQKQYQLRG-ATLEGYREQKAGSSLIKHAEEILRKRGADLLWCNART-S--ASGYYKKLGFSEQGEVFDTppvgphilykrit----<br>-DIRQMNKthLEhWRGLRKQ-LWPGHPDDAHlaDGeEIlQADHLASFIAMADGVAIGFADASIRhDYVNgCDSSPVVFLEGiFVLPSFRQRGVAKQLIAAVQRWGTNKGCREMASDTSPeNtiSQKVHQALGFEETERVIFY-------------rkrc<br><br>SARST: aliSize=125 (resi) RMSD=3.94 (Å)<br>ie-VKPINAEDTYELRHRILR--PNQP----IEACFESDLLRGAFHLGGYYGGKLISIASFH----QAEHSELQGqKQYQLRGATLEGYREQKAGSSLIKHAEEILRKRGADLLWCNAR---TSASGYYKKLGFSEqGEVFDT-----ppvgphilykrit<br>--dIRQMNKTHLEHWRGLRKQlwPGHPddahLADGEEILQADHLASFIAMADGVAIGFADASirhdYVNGCDSSP-VVFLEGIFVLPSFRQRGVAKQLIAAVQRWGTNKGCREMASDTSpenTISQKVHQALGFEE-TERVIFyrkrc------------<br><br>BLAST: aliSize=44 (resi) iden=19.01% (27/144) simi=30.99% (44/144)<br>ievkpinaedtye---------------LRHRiLRPNQPIEACF-ESDLLRGAFHLGGYYG---GKLISIASfHQAEHSELQG---QKQYQLRGA-TLEGYREQKAGSSLIKHAEEILRKRGADLLWCNA---RTSASGYYKKLGFSEQGEV-------fdtppvgphilykrit<br>------------dirqmnkthlehwrgLRKQ-LWPGHPDDAHLaDGEEILQADHLASFIAmadGVAIGFAD-ASIRHDYVNGcdsSPVVFLEGIfVLPSFRQRGVAKQLIAAVQRWGTNKGCREMASDTspeNTISQKVHQALGFEETERVifyrkrc---------------<br><br>Proposed: aliSize=132 (resi) RMSD=2.15 (Å)<br>iEVKPINA--ED-TYELRHrILRPNQPIEAC-MF--EsD-LLRGAFHLGGYYGGKLISIASFHQAEHS-E-LQGQKQYQLRGMATLEGYREQKAGSSLIKHAEEILRKRGADLLWCNART-S--ASGYYKKLGFSEQGEvfDtppvgPHILMYKRitc<br>-DIRQMNKthLEhWRGLRK-QLWPGHPDDAHlADgeE-IlQADHLASFIAMADGVAIGFADASIRHDYvNgCDSSPVVFLEGIFVLPSFRQRGVAKQLIAAVQRWGTNKGCREMASDTSPeNtiSQKVHQALGFEETER--V-----IFYRKRC----
575	2c49A<br>(299)<br><br>2i6bB<br>(326)	M	17.39	TM-align: aliSize=265 (resi) RMSD=3.61 (Å)<br>g-KMF-KITCVGHTALDYIFNVekf----Pep--NTSIQI-------------------PSARKYY-GGAAANTAVGIKKLG-V---NSELLSCVGYDFKNSGYFRYLKNLDINISkLYYSFFFEIPKAWIFTDKDNnQITFFLWGAAKHYKFL--NP----PN-FN-TEIVHIA-TG--DPE-FNLKCAK<br>-sVREnILFGMGNPLLDISAVV---dkdfL--dkYSLKPNdqilaedkhkelfdelvkKFKVEYHaGGSTQNSIKVAQWMIqQphkAATFFGCIGIDKFGEILKRKAAEAHVDAH-YYEQNEQPTGTCAACITGDN-RSLIANLAAANCYKKEkhLDlekn WMlVEkARVCYIAgFFltVSPeSVLKVAH<br><br>KAYGN-NLVSFDPGQ-DLPQYSKFMILEIIEHTNFLFMNKHFFERASNLLNF-------EIDDYLE--R-----VDALIVTKGSKGSVIYTKDKKIEIPCIKAG-KVI-DPTGAGDSYRAGFLSAYVKGYDLEKCGLIGAATASfvveakgcqtnlptwdkvverlekhr<br>HASENnRIFTLNLSApFISQFYKESLMKVMPYVDILFGNETEAATFAREQGFetkdikeIAKKTQAlpKmnskrQRIVIFTQGRDDTIMATESEVTAFAVLDQDqKEIiDTNGAGDAFVGGFLSQLVSDKPLTECIRAGHYAAS--------------------------<br><br>SARST: aliSize=213 (resi) RMSD=5.30 (Å)<br>gkmekitcvghtaldyifnvekfpepntsiqipsa---RKYYGGAAANTAVG-IKKLGVNS---ELLSCVGydfkNSGYER----YLKNLDINIsKLYYSFFFEIPKAWIfTDKDNNQITFFLWG------------<br>-----------------------------------svrenilfgmgnplldisavvdkdfldkyslkpndqilaedkhkelfdelvkkfkvEYHAGGSTQNSIKVaQWMIQQPHkaaTFFGCIG----IDKFGEilkrKAAEAHVDA-HYYEQNEQPTGTCAA-CITGDNRSLIANLAaancykkekhld<br><br>--AAKHYKFlnppnfNTEIVHIAT----GDPF-FNLKCAKKAYGNNLVSFDPG-QDLPQYSKFMILEIIEHTNFLFMNKHFFERASNL--LNF-FIDDYLFR-----------VDALIVTKGSKGSVIYTKDKKIEIPCIKAGKVIDPT--GAGDSYRAGFLSAYVKGYD--------------lekcgli<br>leKNWMLVE------KARVCYIAGffltVSPEsVLKVAHHASENNRIFTLNLSaPFISQFYKESLMKVMPYVDILFGNETEAATFAREqgFETkDIKEIAKKtqalpkmnskrQRIVIFTQGRDDTIMATESEVTAFAVLDQDQKEIIDtnGAGDAFVGGFLSQLVSDKPlteciraghyaas-------<br><br>gaatasfvveakgcqtnlptwdkvverlekhr<br>--------------------------------<br><br>BLAST: aliSize=113 (resi) iden=22.07% (66/299) simi=37.79% (113/299)<br>gkmekitcvghtaldyifnvekfpepntsiqipsar--KYYGGAAANTAVGIKKLGVN-----SELLSCVGYDFKNSGYFRylKNLDINISKLYYSFFFF-IPKAWIFTDKDNNQITFFLwGAAKHYKF---LN-P<br>-----------------------------------svrenilfgmgnplldisavvdkdfldkyslkpndqilaedkhkelfdelvkkfkvEYHAGGSTQNSIKVAQWMIQqphkaATFFGCIGIDKFGEILKR--KAAEAHVDAHYYEQNEQpTGTCAACITGDNRSLIANL-AAANCYKKekhLDIE<br><br>PNF----NTEIVHIA----TGDPFFNLKCAKKAYGNNLVsFDPGQDLP---QYSKFMILEIIEHTNFLFMNKHF---FERASNLLNFFIDDYLFRVDAL-----------IVTKGSKGSVIYTKDKKIEIPCIKAGK--VIDPTGAGDSYRAGFLSAYVKGYDLEKCGLIGAATASfvveakgcqtnlpt<br>KNWmlveKARVCYIAgfflTVSPESVLKVAHHASENNRI-FTLNLSAPfisQFYKESLMKVMPYVDILFGNETEaatFAREQGFETKDIKEIAKKTQALpkmnskrqrivIFTQGRDDTIMATESEVTAFAVLDQDQkeIIDTNGAGDAFVGGFLSQLVSDKPLTECIRAGHYAAS--------------<br><br>wdkvverlekhr<br>------------<br><br>Proposed: aliSize=255 (resi) RMSD=2.31 (Å)<br>gkm--F--KITCVGHTALD--YIF---NVF---KFPFPNTSI---Q--IP-SAR--KY-----vGGAAANTAVGIKKLG-V---NSELLSCVGY--DFknSGYFRYLKNLDINISkLYYSFFFEIPKAW-------IFtdkdnnqiTFFLWGAAKHYkFLN--PP----N--FN-TEIVHIA-TGDP--E<br>---svRenILFGMGNPLLDisAVVdkdFLDkysLKPNDQILAedkHkeLFdELVkkFKveyha-GGSTQNSIKVAQWMIqQPhkAATFFGCIGIdkFG--EILKRKAAEAHVDAH-YYEQNEQPTGTCAacitgdnRS--------LIANLAAANCY-KKEkhLDleknWmlVEkARVCYIAgFFLTvsP<br><br>-FNLKCAKKAYGN-NLVSFDPGQdLPQ--YSKFMILEIIEHTNFLFMNKHFFERASNLLNF-------EIDDYLE--R-----VDALIVTKGSKGSVIYTKDKKIEIPCIKA--gKVI-DPTGAGDSYRAGFLSAYVKGYDLEKCGLIGAATASfvveakgcqtnlptwdkvverlekhr<br>eSVLKVAHHASENnRIFTLNLSA-PFIsqFYKESLMKVMPYVDILFGNETEAATFAREQGFetkdikeIAKKTQAlpKmnskrQRIVIFTQGRDDTIMATESEVTAFAVLDQdq-KEIiDTNGAGDAFVGGFLSQLVSDKPLTECIRAGHYAAS--------------------------

576	2cnmA (151)<br>1s3zB (145)	C	14.48	TM-align: aliSize=127 (resi) RMSD=2.16 (Å)<br>mNTISILSTTDLPAAWQIEQRaHAFPWSEKTFF-G--NQ-GE-RYLNLKLTADDRMAAFAITQVVL--------DEATLFNIAVDPDFQRRGLGRMLLEHLIDELETRGVVTLWLEVRASNAAAIALYESLGFNEATIRRNYyptaqghedaiimalpismklh----<br>-MDIRQMNKTHLEHWRGLRKQ-LWPGHPDDAHLaDgeEIlQAdHLASFIAMADGVAIGFADASIRHdyvngcdssPVVFLEGIFVLPSFRQRGVAKQLIAAVQRWGTNKGCREMASDTSPENTISQKVHQALGFEETERVIFY-----------------------rkrc<br><br>SARST: aliSize=121 (resi) RMSD=3.02 (Å)<br>mn-TISILSTTDLPAAWQIEQRAHAFPWSEKTFF--------GNQGerylNLKLTADDRMAAFAITQVVL------DEA---TLFNIAVDPDFQRRGLGRMLLEHLIDELETRGVVTLWLEVRASNAAAIALYESLGFNEATIRR------nyyptaqghedaiimalpismklh<br>--mDIRQMNKTHLEHWRGLRKQLWPGHPDDAHLAdgeeilqaDHLA----SFIAMADGVAIGFADASIRHdyvngcDSSpvvFLEGIFVLPSFRQRGVAKQLIAAVQRWGTNKGCREMASDTSPENTISQKVHQALGFEETERVIfyrkrc------------------------<br><br>BLAST: aliSize=43 (resi) iden=16.55% (24/145) simi=29.66% (43/145)<br>mntisilsttdlpaawqieqrahafpwsektffgn-------------------------------------QGERYLNLKLTADDRMAAFAITQVVLD-------EATLF--NIAVDPDFQRRGLGRMLLEhLIDELET-RGVVTLWLEVRASNAAAIALYESLGFNEaTIRRNYY----ptaqghed<br>----------------------------------mdirqmnkthlehwrglrkqlwpghpddahladgeeilQADHLASFIAMADGVAIGFADASIRHDyvngcdsSPVVFleGIFVLPSFRQRGVAKQLIA-AVQRWGTnKGCREMASDTSPENTISQKVHQALGFEE-TERVIFYrkrc--------<br><br>aiimalpismklh<br>-------------<br><br>Proposed: aliSize=131 (resi) RMSD=1.88 (Å)<br>mNTISILSTTDLPAAWQIEQRAHaFPWSEKTFFGN---Q-GE-RYLNLKLTADDRMAAFAITQVVL--------DEATLFNIAVDPDFQRRGLGRMLLEHLIDELETRGVVTLWLEVRASNAAAIALYESLGFNEATIRrnyyptaqgheDAIIMAL-pismklh<br>-MDIRQMNKTHLEHWRGLRKQLW-PGHPDDAHLADgeeIlQAdHLASFIAMADGVAIGFADASIRHdyvngcdssPVVFLEGIFVLPSFRQRGVAKQLIAAVQRWGTNKGCREMASDTSPENTISQKVHQALGFEETERV-----------IFYRKRCc-------
577	2cnmA (151)<br>2vbqA (147)	C	13.61	TM-align: aliSize=128 (resi) RMSD=2.31 (Å)<br>-MNTISILSTTDLPAAWQIEQRAHaFPWSEKTFF-G--NQ-GE-RYLNLKLTADDRMAAFAITQVVL--------DEATLFNIAVDPDFQRRGLGRMLLEHLIDELETRGVVTLWLEVRASNAAAIALYESLGFNEATIRRNYyptaqghedaiimalpismklh----<br>sHMDIRQMNKTHLEHWRGLRKQLW-PGHPDDAHLaDgeEIlQAdHLASFIAMADGVAIGFADASIRHdyvngcdssPVVFLEGIFVLPSFRQRGVAKQLIAAVQRWGTNKGCREMASDTSPENTISQKVHQALGFEETERVIFY-----------------------rkrc<br><br>SARST: aliSize=122 (resi) RMSD=3.26 (Å)<br>m--NTISILSTTDLPAAWQIEQRahAFPWS--------EKTFFGnqGERYLNLKLTADDRMAAFAITQVV------LDEA---TLFNIAVDPDFQRRGLGRMLLEHLIDELETRGVVTLWLEVRASNAAAIALYESLGFNEATIRR------nyyptaqghedaiimalpismklh<br>-shMDIRQMNKTHLEHWRGLRKQ--LWPGHpddahladGEEILQ--ADHLASFIAMADGVAIGFADASIRhdyvngCDSSpvvFLEGIFVLPSFRQRGVAKQLIAAVQRWGTNKGCREMASDTSPENTISQKVHQALGFEETERVIfyrkrc------------------------<br><br>BLAST: aliSize=43 (resi) iden=16.33% (24/147) simi=29.25% (43/147)<br>mntisilsttdlpaawqieqrahafpwsektffgn-------------------------------------QGERYLNLKLTADDRMAAFAITQVVLD-------EATLF--NIAVDPDFQRRGLGRMLLEhLIDELET-RGVVTLWLEVRASNAAAIALYESLGFNEaTIRRNYY----ptaqgh<br>-----------------------------------shmdirqmnkthlehwrglrkqlwpghpddahladgeeilQADHLASFIAMADGVAIGFADASIRHDyvngcdsSPVVFleGIFVLPSFRQRGVAKQLIA-AVQRWGTnKGCREMASDTSPENTISQKVHQALGFEE-TERVIFYrkrc------<br><br>edaiimalpismklh<br>---------------<br><br>Proposed: aliSize=131 (resi) RMSD=2.00 (Å)<br>-MNTISILSTTDLPAAWQIEQRAHaFPWSEKTFFGN---Q-GE-RYLNLKLTADDRMAAFAITQVVL--------DEATLFNIAVDPDFQRRGLGRMLLEHLIDELETRGVVTLWLEVRASNAAAIALYESLGF-NEATIRrnyyptaqGHEDAIimalpismklh<br>sHMDIRQMNKTHLEHWRGLRKQLW-PGHPDDAHLADgeeIlQAdHLASFIAMADGVAIGFADASIRHdyvngcdssPVVFLEGIFVLPSFRQRGVAKQLIAAVQRWGTNKGCREMASDTSPENTISQKVHQALGFeETERVI--------FYRKRC-----------
578	2cswA (145)<br>1j4kA (158)	C	13.79	TM-align: aliSize=109 (resi) RMSD=2.55 (Å)<br>gssgssgvTgdrAGgRSWCLRRVGM--SAGWLLLEDGC-EVTVGRGFGVTYQLVskicPLMISRNHCVLKQNPE--------------GQWTIMDNkSLNGVWLNRARLEPLRVYSIHQGDYIQLGVPLENKeNAeYEYEVTEEDwetiypclspksgpssg--------------------------------<br>--------G---NG-RFLTLKPLPDsiIQESLEIQQGVnPFFIGRSEDCNCKIE----DNRLSRVHCFIFKKRHavgksmyespaqglDDIWYCHT-GTNVSYLNNNRMIQGTKFLLQDGDEIKIIWDKNNK-FV-IGFKVEIND-----------------ttglfneglgmlqeqrvvlkqtaeekdl<br><br>----<br>vkkl<br><br>SARST: aliSize=95 (resi) RMSD=9.39 (Å)<br>gssgssgvtgdr--AGGRSWCLRR-VGMSAGWLLLEDGCEVTVGRGFGVTYQLVSKICPLM-----ISRN--HCVLKQNPEGQWT--IMDNKSLNGVWLNRARLEPLRVYSIHQGDYIQ---lgvplenkenaeyeyevtee<br>------------gnGRFLTLKPLPdSIIQESLEIQQGVNPFFIGRSEDCNCKIEDNRLSRVhcfifKKRHavGKSMYESPAQGLDdiWYCHTGTNVSYLNNNRMIQGTKFLLQDGDEIKiiwdknnkfvigfkveindttglfneglgmlqeqrvvlkqtaeekdlvkkl--------------------<br><br>dwetiypclspksgpssg<br>------------------<br><br>BLAST: aliSize=50 (resi) iden=15.86% (23/145) simi=34.48% (50/145)<br>gssgssgvtgdraggrswclrrvgmsagwl--------------LLEDGCEVTVG-RGF--GVTYQLVSKICPLMISRNHCVL--KQNPEGQwTIMDN--KSLNGVW----------LNRARLEPLRVYSIHQGDYIQLGVPLENKENAEYEYEVTE-------------------------------e<br>-----------------------------gngrfltlkplpdsIIQESLEIQQGvNPFfiGRSEDCNCKIEDNRLSRVHCFIfkKRHAVGK-SMYESpaQGLDDIWychtgtnvsyLNNNRMIQGTKFLLQDGDEIKIIWDKNNKFVIGFKVEINDttglfneglgmlqeqrvvlkqtaeekdlvkkl-<br><br>dwetiypclspksgpssg<br>------------------<br><br>Proposed: aliSize=120 (resi) RMSD=2.22 (Å)<br>gssgssgvtgdrAG-gRSWCLRRVG----MsaGWLLLEDGC-EVTVGRGFGVTYQLVskicPLMISRNHCVLKQNPE--------------GQWTIMDNkSLNGVWLNRARLEPLRVYSIHQGDYIQLGVPLENKenAEYEYEVTE----eDwETIYPCLS----PKSGPS---S-------g<br>------------GNg-RFLTLKPLPdsiiQ--ESLEIQQGVnPFFIGRSEDCNCKIE----DNRLSRVHCFIFKKRHavgksmyespaqglDDIWYCHT-GTNVSYLNNNRMIQGTKFLLQDGDEIKIIWDKNNK--FVIGFKVEIndtt-G-LFNEGLGMlqeqRVVLKQtaeEkdlvkkl-

579	2cu2A (335)<br>1vpaA (221)	M	18.55	TM-align: aliSize=207 (resi) RMSD=3.08 (Å)<br>-MKTYALVMAGGRGERLWplSRedRPKPFLPLfEGKTLLEATLERLAPLVPPERTLLAVRRDQEAVARPYADG--I-RLLLEpLGrDTAGAVLLGVAEALKEGAERLLVLPADHYVgdDeaYREALATMLEAAEEGFVVALGLRPTRPEteygyirlgpregawyrgegfvekPSY----AEaLEYiRKG<br>hHMNVAILLAAGKGERMS--EN--VPKQFLEI-EGRMLFEYPLSTFLKSEAIDGVVIVTRREWFEVVEKRVFHekVlGIVEG-GD-TRSQSVRSALEFLEKFSPSYVLVHDSARPF--L--RKKHVSEVLRRARETGAATLALKNSDAL---------------------------VRVendrIE-YIP-RKG<br>YVWNGGVFAFAPATMAELFRrhlPsHhealerllagasleevyaglPkISIDY-GVMEKAER-VRVVLGRFPWDDVGNWRALERVFSQdphenvv-Lgegrhvaldtfgcvvyadrgvvatlgvsglvvakvgdevlvvpkdwarevrevvkrlea---<br>VYRILTPQAFSYEILKKAHE---N-G--------------------G-EWADDtEPVQKLGVkIALVEGDPLCFKVTFKEDLELARII-------aR---ewe<br><br>SARST: aliSize=166 (resi) RMSD=11.05 (Å)<br>m--KTYALVMAGGRGERLWPLsredRPKPFLPlFEGKTLLEAtlERLAPLVPPE--RTLLAVRRDQEAVARPYAD--GIRLLLEpLGRDTAGAVLLGVAEALKEGAERLLVLPADHYVGDDEAYREALATMLEAAEegfvVALGLRPTRpeteYGYIRL-----GPREGAWYRGEGFVEkPSYAEALEYI<br>-hhMNVAILLAAGKGERMSEN----VPKQFLE-IEGRMLFEY--PLSTFLKSEAidGVVIVTRREWFEVVEKRVFheKVLGIVE-GGDTRSQSVRSALEFLEKFSPSYVLVHDSARPFLRKKHVSEVLRRARETGA----ATLALKNSD----ALVRVEndrieYIPRKGVYRILTPQA-FSYEILKKAH<br>RKGYV--wnggvfafapatmaelfrrhlpshhealerllagasleevyaglpkisidygvmekaervrvvlgrfpwddvgnwralervfsqdphenvvlgegrhvaldtfgcvvyadrgvvatlgvsglvvakvgdevlvvpkdware<br>ENGGEwaddtepvqklgvkialvegdplcfkvtfkedlelariiarewe---<br>vrevvkrlea<br>----------<br><br>BLAST: aliSize=104 (resi) iden=26.70% (59/221) simi=47.06% (104/221)<br>mkty-----ALVMAGGRGERLwplsREDRPKPFLPLfEGKTLLEATLERLAPLVPPERTLLAVRRDQ-EAVA-RPYADGIRLLLEplGRDTAGAVLLGVAEALKEGAERLLVLpadHYVGDDEAYREALATMLEAAEEGFVVALGLRPTRP--ETEYGYIRLGPREGAwYRgegfVEKPSyAEALEYIRK<br>----hhmnvAILLAAGKGERM----SENVPKQFLEI-EGRMLFEYPLSTFLKSEAIDGVVIVTRREWfEVVEkRVFHEKVLGIVE--GGDTRSQSVRSALEFLEKFSPSYVLV---HDSARPFLRKKHVSEVLRRARETGAATLALKNSDAlvRVENDRIEYIPRKGV-YR----ILTPQ-AFSYEILKK<br>GYVwNGGVFAfapatmaelfrrhlpSHHEALERLLAGASLEEvyaGLPkISIDYGVMEKAERVRVVlGRfpwddvgNW-ralervfsqdphenvvlgegrhvaldtfgcvvyadrgvvatlgvsglvvakvgdevlvvpkdwarevrevvkrlea<br>AHE-NGGEWA---------------DDTEPVQKLGVKIALVE---GDP-LCFKVTFKEDLELARII-AR-------EWe--<br><br>Proposed: aliSize=205 (resi) RMSD=2.39 (Å)<br>-MKTYALVMAGGRGERLWplSRedRPKPFLPLfEGKTLLEATLERLAPL--VPpeRTLLAVRRDQEAVARPYADG--I-RLLLEpLGrDTAGAVLLGVAEALKEGAERLLVLPADHYVgdDeaYREALATMLEAAEEGFVVALGLRPTRpeteyGYIRLGPRegawyRGEGFVEkpsyaealeyirKGYV<br>hHMNVAILLAAGKGERMS--EN--VPKQFLEI-EGRMLFEYPLSTFLKSeaID--GVVIVTRREWFEVVEKRVFHekVlGIVEG-GD-TRSQSVRSALEFLEKFSPSYVLVHDSARPF--L--RKKHVSEVLRRARETGAATLALKNSD-----ALVRVEND-----RIEYIPR-----------KGVY<br>WNGGVFAFAPATMAELFRrhlPShhealerllagasleevyaglPkIS-ID-YgVMEKAE-RVRVVLGRFPWDDVGNWRALERV-F------sqdphenvvlgegrhvaldtfgcvvyadrgvvatlgvsglvvakvgdevlvvpkdwarevrevvkrlea<br>RILTPQAFSYEILKKAHE---NG----------------------G-EWaDDtE-PVQKLGvKIALVEGDPLCFKVTFKEDLELArIiarewe--
580	2cu2A (335)<br>1vpaB (220)	M	16.82	TM-align: aliSize=207 (resi) RMSD=3.09 (Å)<br>-MKTYALVMAGGRGERLWplSRedRPKPFLPLfEGKTLLEATLERLAPLVPPERTLLAVRRDQEAVARPYAD-G-I-RLLLEpLGrDTAGAVLLGVAEALKEGAERLLVLPADHYVgdDeaYREALATMLEAAEEGFVVALGLRPTRPETeygyirlgpregawyrgegfvekPS----YAEAlEYirKG<br>hHMNVAILLAAGKGERMS--EN--VPKQFLEI-EGRMLFEYPLSTFLKSEAIDGVVIVTRREWFEVVEKRVFhEkVlGIVEG-GD-TRSQSVRSALEFLEKFSPSYVLVHDSARPF--L--RKKHVSEVLRRARETGAATLALKNSDALV-----------------------RVendrIEYI-PR--KG<br>YVWNGGVFAFAPATMAELFRrhlPShhealerllagasleevyaglPkISIDY-GVMEKAE-RVRVVLGRFPWDDVGNWRALERVFSQdphenvv-Lgegrhvaldtfgcvvyadrgvvatlgvsglvvakvgdevlvvpkdwarevrevvkrlea--<br>VYRILTPQAFSYEILKKAHE---NG---------------------G-EWADDtEPVQKLGvKIALVEGDPLCFKVTFKEDLELARII-------aR---ew<br><br>SARST: aliSize=176 (resi) RMSD=9.63 (Å)<br>mk-TYALVMAG--GRGERLWPlsreDRPKPFLPlFEGKTLLEATLERLAP--LVppERTLLAVRRDQEAVARPY----ADGIRLLLEPLgRDTAGAVLlgVAEALKEGAERLLVLPADHYVGDDEAYREALATMLEAAeegfVVALGLRPTrpeteygyIRLGPREGAWyrgegFVEKPsyaealeyiRK<br>--hHMNVAILLaaGKGERMSE----NVPKQFLE-IEGRMLFEYPLSTFLKseAI--DGVVIVTRREWFEVVEKRvfheKVLGIVEGGDT-RSQSVRSA--LEFLEKFSPSYVLVHDSARPFLRKKHVSEVLRRARETG----AATLALKNS--------DALVRVENDR-----IEYIP---------RK<br>GYVWNGGVFAFAPATMAELFRR------HlpshheALERLLAGAS-----------------------------leevyaglpkisidygvmekaervrvvlgrfpwddvgnwralervfsqdphenvvlgegrhvaldtfgcvvyadrgvvatlgvsglvvakvgdevlvvpkdwarevrevvkrlea<br>GVYRILTPQAFSYEILKKAHENggewadD------TEPVQKLGVKialvegdplcfkvtfkedlelariiarew---<br><br>BLAST: aliSize=104 (resi) iden=26.82% (59/220) simi=47.27% (104/220)<br>mkty-----ALVMAGGRGERLwplsREDRPKPFLPLfEGKTLLEATLERLAPLVPPERTLLAVRRDQ-EAVA-RPYADGIRLLLEplGRDTAGAVLLGVAEALKEGAERLLVLpadHYVGDDEAYREALATMLEAAEEGFVVALGLRPTRP--ETEYGYIRLGPREGAwYRgegfVEKPSyAEALEYIRK<br>----hhmnvAILLAAGKGERM----SENVPKQFLEI-EGRMLFEYPLSTFLKSEAIDGVVIVTRREWfEVVEkRVFHEKVLGIVE--GGDTRSQSVRSALEFLEKFSPSYVLV---HDSARPFLRKKHVSEVLRRARETGAATLALKNSDAlvRVENDRIEYIPRKGV-YR----ILTPQ-AFSYEILKK<br>GYVwNGGVFAfapatmaelfrrhlpSHHEALERLLAGASLEEvyaGLPkISIDYGVMEKAERVRVVlGRfpwddvgNWralervfsqdphenvvlgegrhvaldtfgcvvyadrgvvatlgvsglvvakvgdevlvvpkdwarevrevvkrlea<br>AHE-NGGEWA---------------DDTEPVQKLGVKIALVE---GDP-LCFKVTFKEDLELARII-AR-------EW--<br><br>Proposed: aliSize=204 (resi) RMSD=2.44 (Å)<br>-MKTYALVMAGGRGERLWplSRedRPKPFLPLfEGKTLLEATLERLA-P-LVPpeRTLLAVRRDQEAVARPYAD-G-I-RLLLEplGRDTAGAVLLGVAEALKEGAERLLVLPADHYVgDDEAYREALATMLEAaEegFVVALGLRPTRpeteyGYIRLGPregawYRGeGFVEkpsyaealeyiRKGYV<br>hHMNVAILLAAGKGERMS--EN--VPKQFLEI-EGRMLFEYPLSTFLKSeAID--GVVIVTRREWFEVVEKRVFhEkVlGIVEG--GDTRSQSVRSALEFLEKFSPSYVLVHDSARPF-LRKKHVSEVLRRARE-T--GAATLALKNSD-----ALVRVEN-----DRI-EYIP-----------RKGVY<br>WNGGVFAFAPATMAELFRrhlPsHhealerllagasleevyaglPkIS-ID-YgVMEKAERVRVVLGRF-PWDDVGNWRALErV-fsqdphenvvL------gegrhvaldtfgcvvyadrgvvatlgvsglvvakvgdevlvvpkdwarevrevvkrlea<br>RILTPQAFSYEILKKAHE---N-G---------------------G-EWaDDtE-PVQKLGVKIALVEGDpLCFKVTFKEDLE-La----------Riiarew---
581	2e3dA (286)<br>1iniA (225)	M	15.11	TM-align: aliSize=208 (resi) RMSD=2.74 (Å)<br>NTKVKKAVIPVAGLGTRMLPatKaIPKEMLPLVDKPLIQYVVNECIAAG-ITEIVLVTHSSKNSIENH-FDTsfeleamlerqlldevqsiCpphVTIMQVRQGlAkGLGHAVLCAHPVVGD-EPVAVILPDVILDEyesdLsqDNLAEMIRRFDETGHSQIMVEPVAD-VTAygvvdckgvelapgesv<br>THLDVCAVVPAAGFGRRMQT--E-CPKQYLSIGNQTILEHSVHALLAHPrVKRVVIAISPGDSRFAQLpLAN-------------------H---PQITVVDGG-D-ERADSVLAGLKAAGDaQWVLVHDAARPCLH----Q--DDLARLLALSETSRTGGILAAPVRDtMKR-----------------<br>pmvgvvek--------PKADVAPSNLAIVGRYVLSA-DIWPLLAK--TPPEIQ-L-TDAIDMLiEKeTVEAYHMKGKSHDCGNKLGYMQAFVEYgiRhntlgtefkawleeem<br>--------aepgknaiAHTVDRNGLWHALTPQFFPReLLHDCLTRalNEGATItDeASALEYC-GF-HPQLVEGRADNIKVTRPEDLALAEFYL--T----------------<br><br>SARST: aliSize=212 (resi) RMSD=9.00 (Å)<br>nt--KVKKAVIPVAGlgtRMLPATKAIPKEMLPLVDKPLIQYVVNECIA-AGITEIVLVTHSSKNSienhfdtsfeleAMLErqlldevQSICPPHVTIMqvrqGLAKGLGHAVLCAHPVVGDE-PVAVILPDVildeyesdlSQDNLAEMIRRFDETGHSQIMVEPVadvtaygVVDCKGVELAPGESV<br>--thLDVCAVVPAAG---FGRRMQTECPKQYLSIGNQTILEHSVHALLAhPRVKRVVIAISPGDSR-----------FAQL-------PLANHPQITVV----DGGDERADSVLAGLKAAGDAqWVLVHDAAR---------PCLHQDDLARLLALSETSRTGGILA-------APVRDTMKRAEPGKN<br>PMVGVVekpkadvAPSNLAIVGRYVLS-------ADIWPLLAKTPPEIqltDAIDMLIEKETVEAYHMKGKSHDCGNKLGYMQAFVE--ygirhntlgtefkawleeem<br>AIAHTV-------DRNGLWHALTPQFFprellhdCLTRALNEGATITD---EASALEYCGFHPQLVEGRADNIKVTRPEDLALAEFYlt--------------------<br><br>BLAST: aliSize=70 (resi) iden=18.67% (42/225) simi=31.11% (70/225)<br>ntkvkk------AVIPVAGLGTRMlpATKAiPKEMLPLVDKPLIQYVVNECIAAGITEIVLVTHSSKnsienhfDTSFeleAMLErqLLDEvqsicpPHVTImqVRQGLAKGlgHAVLCAHPVVGDepVAVILpdvILDEYESDLSQDNLAEMIrRFDETGHS-QIMVEPVAD-----------------<br>------thldvcAVVPAAGFGRRM--QTEC-PKQYLSIGNQTILEHSVHALLAHPRVKRVVIAISPG-------DSRF---AQLP--LANH------PQITV--VDGGDERA--DSVLAGLKAAGD--AQWVL---VHDAARPCLHQDDLARLL-ALSETSRTgGILAAPVRDtmkraepgknaiahtvd<br>--vtaygvvdckgvelapgesvpmvgvvekpkadvapsnlaivgryvlsadiwpllaktppeiqltdaidmlieketveayhmkgkshdcgnklgymqafveygirhntlgtefkawlee<br>rnglwhaltpqffprellhdcltralnegatitdeasaleycgfhpqlvegradnikvtrpedlalaefylt--<br>em<br>--<br><br>Proposed: aliSize=216 (resi) RMSD=2.47 (Å)<br>NTKVKKAVIPVAGLGTRMLPatKaIPKEMLPLVDKPLIQYVVNECIAAG-ITEIVLVTHSSKNSIENH-FDTsfeleamlerqlldevqsiCpphVTIMQVRQGlAkGLGHAVLCAHPVVGD-EPVAVILPDVILDEyesdLsqDNLAEMIRRFDETGHSQIMVEPVADvtaYGVVDCKGVElapgesVp<br>THLDVCAVVPAAGFGRRMQT--E-CPKQYLSIGNQTILEHSVHALLAHPrVKRVVIAISPGDSRFAQLpLAN-------------------H---PQITVVDGG-D-ERADSVLAGLKAAGDaQWVLVHDAARPCLH----Q--DDLARLLALSETSRTGGILAAPVRD---TMKRAEPGKN------A-<br>MVGvvekpkADVAPSNLAIVGRYVLS-ADIWPLIAK--TPPEIQ-L-TDAIDMLiEKeTVFAYHMkG-KSHDCGNKLGYMQAFVEYgiRhntlgtefkawleeem<br>IAH------TVDRNGLWHALTPQFFPrELLHDCLTRalNEGATItDeASALEYC-GF-HPQLVEG-RaDNIKVTRPEDLALAEFYL--T----------------

582	2e3dA (286)<br>1vpaA (221)	M	14.48	TM-align: aliSize=207 (resi) RMSD=2.67 (Å)<br>NTKVkKAVIPVAGLGTRMLPATkaiPKEMLPLVDKPLIQYVVNECIAAG-ITEIVLVTHSSKN-SIENHFDtsfeleamlerqlldevqsiCppHVTIMQVRQGlAkGLGHAVLCAHPVVG--DE-PVAVILPDVILDeyesdLSqDNLAEMIRRFDeTGHSQIMVEPV-ADVTAygvvdckgvelapge<br>HHMN-VAILLAAGKGERMSENV---PKQFLEIEGRMLFEYPLSTFLKSEaIDGVVIVTRREWFeVVEKRVF--------------------H--EKVLGIVEGG-D-TRSQSVRSALEFLEkfSPsYVLVHDSARPFL-----RK-KHVSEVLRRAR-ETGAATLALKNsDALVR--------------<br><br>svpmvgvvek------PKADvAPSNLAIVGRYVLS-ADIWPLLAKTPpEIQ-LTDAIDMLiEkETVEAYHMKGKSHDCGNKLGYMQAFVEYGiRHNtlgtefkawleeem<br>----------vendriEYIP-RKGVYRILTPQAFSyEILKKAHENGG-EWAdDTEPVQKL-G-VKIALVEGDPLCFKVTFKEDLELARIIAR-EWE--------------<br><br>SARST: aliSize=209 (resi) RMSD=7.50 (Å)<br>ntkvk----KAVIPVAGLGTRMLPAtkaIPKEMLPLVDKPLIQYVVNECIA-AGITEIVLVTHSSKNSIENHFDTSFEleamlerqlldevqsicPPhvtimqVRQGLAKGLGHAVLCAHPVV---GDEPVAVILPDVildeYESDLSQdnLAEMIRRFDETGHsQIMVEPVADvtaYGVVDCKGVELAP<br>-----hhmnVAILLAAGKGERMSEN---VPKQFLEIEGRMLFEYPLSTFLKsEAIDGVVIVTRREWFEVVEKRVFHEK------------------VL------GIVEGGDTRSQSVRSALEFLekfSPSYVLVHDSAR----PFLRKKH--VSEVLRRARETGA-ATLALKNSD---ALVRVENDRIEYI<br><br>GESVPMVGVVEkpkadvapsnlaivgrYVLSADIWPLLA---KTPPEiqltDAIDMLIEKETVEAYHMKGKSHDCGNKLGYMQAFVEYGIR-hntlgtefkawleeem<br>PRKGVYRILTP---------------QAFSYEILKKAHengGEWAD----DTEPVQKLGVKIALVEGDPLCFKVTFKEDLELARIIAREWe----------------<br><br>BLAST: aliSize=30 (resi) iden=6.79% (15/221) simi=13.57% (30/221)<br>ntkvkk-----AVIPVAGLGTRMlpaTKAIPKEMLPLVDKPLIQYVVNECI-AAGITEIVLVT--<br>------hhmnvAILLAAGKGERM---SENVPKQFLEIEGRMLFEYPLSTFLkSEAIDGVVIVTrrewfevvekrvfhekvlgiveggdtrsqsvrsaleflekfspsyvlvhdsarpflrkkhvsevlrraretgaatlalknsdalvrvendrieyiprkgvyriltpqafsyeilkkahenggewadd<br><br>--hssknsienhfdtsfeleamlerqlldevqsicpphvtimqvrqglakglghavlcahpvvgdepvavilpdvildeyesdlsqdnlaemirrfdetghsqimvepvadvtaygvvdckgvelapgesvpmvgvvekpkadvapsnlaiv<br>tepvqklgvkialvegdplcfkvtfkedlelariiarewe---<br><br>gryvlsadiwpllaktppeiqltdaidmlieketveayhmkgkshdcgnklgymqafveygirhntlgtefkawleeem<br>---<br><br>Proposed: aliSize=212 (resi) RMSD=2.35 (Å)<br>NTKVkKAVIPVAGLGTRMLPATkaiPKEMLPLVDKPLIQYVVNECIAAG-ITEIVLVTHSSKN-SIENHFDtsfeleamlerqlldevqsiCppHVTIMQVRQGlAkGLGHAVLCAHPVVG--DE-PVAVILPDVILDeyesdLSqDNLAEMIRRFDeTGHSQIMVEPVadvtAYGVVDCKgVelapges<br>HHMN-VAILLAAGKGERMSENV---PKQFLEIEGRMLFEYPLSTFLKSEaIDGVVIVTRREWFeVVEKRVF--------------------H--EKVLGIVEGG-D-TRSQSVRSALEFLEkfSPsYVLVHDSARPFL-----RK-KHVSEVLRRAR-ETGAATLALKN----SDALVRVE-N-------<br><br>VPMVGVVekpkadvAPSNLAIVGRYVLS-ADIWPLLAKTPpeIQ--LTDAIDMLiEkETVEAYHMkG-KSHDCGNKLGYMQAFVEYGiRHNtlgtefkawleeem<br>DRIEYIP-------RKGVYRILTPQAFSyEILKKAHENGG--EWadDTEPVQKL-G-VKIALVEG-DpLCFKVTFKEDLELARIIAR-EWE--------------
583	2f5gA (130)<br>1x0pE (141)	C	5.38	TM-align: aliSize=83 (resi) RMSD=3.00 (Å)<br>elkstrhtkYLCNYHFVWIPKhRrnTLVNEIAEYTKEVLKSIAEELGCEIiALEVMPDHIHLFVNCPPRYAPsYLANYFKGksArlilkkfpqlnkgKLWTrSYFV-ATAGNVSsevikkyieeqwrkege--<br>---------GLHRLIYLSCAT-D--GLSYPDLRDIMAKSEVNNLRDGITG-MLCYGNGMFLQTLEGDRQKVS-ETYARILK--D-------------PRHH-SAEIvEFKAIEE-----------------rtfinwsmrlvqlgemdsdtirrlrlkyspaatfqprsmtaeqcfrflkelydmsqg<br><br>SARST: aliSize=98 (resi) RMSD=12.20 (Å)<br>elkstrhtk--YLCNYHFVWIPkhrrnTLVNEIAEYTKEVLKSIAEELGCEIiALEVMPDHIHLFVNCPpryapsylanYFKGKSARLILKKFPQLNKG-KLWTRSY----------FVATAGNVSSEVIKKYIEE--------------------------------qwrkege<br>---------glHRLIYLSCATD-----GLSYPDLRDIMAKSEVNNLRDGITG-MLCYGNGMFLQTLEGD----------RQKVSETYARILKDPRHHSAeIVEFKAIeertfinwsmRLVQLGEMDSDTIRRLRLKyspaatfqprsmtaeqcfrflkelydmsqg-------<br><br>BLAST: aliSize=23 (resi) iden=10.77% (14/130) simi=17.69% (23/130)<br>elkstrhtkylcnyhfvwipkhrrntlvn--EIAEYTKEVLKSIAEElGCEIIALEVMPDHIhlFVNCPPRYAPsvLANYFKGKSARLILKKFP----------------------------qlnkgklwtrsyfva<br>-----------------------------glhrliylscatdglsypdlrdimaksevnnlrdgitgmlcygngmflqtlegdrqKVSETYARILKDPRHH-SAEIVEFKAIEERT--FINWSMRLVQ--LGEMDSDTIRRLRLKYSPaatfqprsmtaeqcfrflkelydmsqg---------------<br><br>tagnvssevikkyieeqwrkege<br>-----------------------<br><br>Proposed: aliSize=94 (resi) RMSD=2.62 (Å)<br>elkstrhtkYLCNYHFVWIPKhRrnTLVNEIAEYTKEVLKSIAEELGCEIiALEVMPDHIHLFVNCPPRYAPsYLANYFKGksArlilkkfpqlnkgKLWTRSYFVAT-------------------agnvssEvIKKYIEEQWRKEG--------------E-------------<br>---------GLHRLIYLSCAT-D--GLSYPDLRDIMAKSEVNNLRDGITG-MLCYGNGMFLQTLEGDRQKVS-ETYARILK--D-------------PRHHSAEIVEFkaieertfinwsmrlvqlg------E-MDSDTIRRLRLKYspaatfqprsmtaeQcfrflkelydmsqg
584	2fckA (174)<br>2g3tA (169)	C	11.24	TM-align: aliSize=141 (resi) RMSD=3.54 (Å)<br>tpdfqivtQRLQLRLITADEAeELVQCIRQS-QTLHQWVDWFsqqEAEQFIQ-ATRLnwVKAeAYGFGVFERQT--------QTLVGVAINEFYHT-FN-A-SLGYWIGDR-YQRQGYGKEALTALILFCFeRLELTRLEIVCDPENVPSQALALRcGANREQL----APNRfLvAGEpkagivfslip-<br>--------KFVIRPATAADCS-DILRLIKELaKYEYMEEQVI---LTEKDLLeDGFG--EHP-FYHCLVAEVPKehwtpeghSIVGFAMYYFTYDPwIGkLlYLEDFFVMSdYRGFGIGSEILKNLSQVAM-RCRCSSMHFLVAEWNEPSINFYKR-RGASDLSseegWRLF-K-IDK-----------e<br><br>---------<br>yllkmatee<br><br>SARST: aliSize=127 (resi) RMSD=6.13 (Å)<br>tpdfqivtqr-LQLRLITADEAEELVQCIRQSQTLHQWVdWFSQ--QEAEQFiqatrlnwvKAE------AYGFGVFERQTQTLVGVAIN-------EFYHTFNASL---GYWIGDRYQRQGYGKEALTAlILFCFERLELTRLEIVCDPENVPSQALALRCGANRE-----------------------<br>----------kFVIRPATAADCSDILRLIKELAKYEYME-EQVIltEKDLLE---------DGFgehpfyHCLVAEVPKEHWTPEGHSIVgfamyyfTYDPWIGKLLyleDFFVMSDYRGFGIGSEILKN-LSQVAMRCRCSSMHFLVAEWNEPSINFYKRRGASDLsseegwrlfkidkeyllkmatee<br><br>qlapnrflyagepkagivfslip<br>-----------------------<br><br>BLAST: aliSize=34 (resi) iden=11.24% (19/169) simi=20.12% (34/169)<br>tpdfqivtqrlqlrlitadeaeelvqcirqsqtlhqwvdwfsqqeaeqfiqatrlnwvkaeaygfgvferqtq--TLVGVAIneFYHTFNASLG-------YWIGDRYQRQGYGKEALTALILFC<br>--kfvirpataadcsdilrlikelakyeymeeqviltekdlledgfgehpfyhclvaevpkehwtpeghSIVGFAM--YYFTYDPWIGkllyledFFVMSDYRGFGIGSEILKNLSQVA<br><br>FeRLELTRLEIVCDPENVPSQALALRCGAN-------------------------reqlapnrflyagepkagivfslip<br>M-RCRCSSMHFLVAEWNEPSINFYKRRGASdlsseegwrlfkidkeyllkmatee-------------------------<br><br>Proposed: aliSize=143 (resi) RMSD=3.06 (Å)<br>mtpdfqivtq-RLQLRLITaDEAEELVQCIRQS-QTL--HQ-wvdWFsqqEAE-QFIQATRL-NWvkaeAYGFGVFERQT--------QTLVGMVAINEFYhT-FNMA-SLGYWIGDR-YQRQGYGKEALTALILFCFeRLELTRLEIVCDPENVPSQALALRCGANREQL---APN-RFlvagepKAG-<br>----------kFVIRPATA-ADCSDILRLIKELaKYEymEEq---VI---LTEkDLLEDGFGeHP----FYHCLVAEVPKehwtpeghSIVGFAMYYFTYD-PwIGKLlYLEDFFVMSdYRGFGIGSEILKNLSQVAM-RCRCSSMHFLVAEWNEPSINFYKRRGASDLSSeegWRLfKI------DKEy<br><br>-IV--FSLip<br>lLKmaTEE--

585	2fckA (174)<br>2jevA (169)	C	9.47	TM-align: aliSize=143 (resi) RMSD=3.73 (Å)<br>tpdfqivt-QRLQLRLITADEAeELVQCIRQS-QTLHQWVDWFsqQEAEQFIQATRLnwVKAeAYGFGVFERQT--------QTLVGVAINEFYHT-FN-A-SLGYWIGDR-YQRQGYGKEALTALILFCFeRLELTRLEIVCDPENVPSQALALRcGANREQL----APNRfLYAGEpkagivfslip-<br>--------aKFVIRPATAADCS-DILRLIKELaKYEYMEEQVI--LTEKDLLEDGFG--EHP-FYHCLVAEVPKehwtpeghSIVGFAMYYFTYDPwIGkLlYLEDFFVMSdYRGFGIGSEILKNLSQVAM-RCRCSSMHFLVAEWNEPSINFYKR-RGASDLSseegWRLF-KIDKE-----------y<br>-------<br>llkmate<br><br>SARST: aliSize=126 (resi) RMSD=6.80 (Å)<br>tpdfqivtqr--LQLRLITADEAEELVQCIRQsqtLHQWVDWFS-----QqeaeqfiqATRLNWVKAE----AYGFGVFERQTQTLVGVAINEFY-------HTFNASL---GYWIGDRYQRQGYGKEALTAlILFCFERLELTRLEIVCDPENVPSQALALRCGANRE-----------------------<br>----------akFVIRPATAADCSDILRLIKE---LAKYEYMEEqviltE--------KDLLEDGFGEhpfyHCLVAEVPKEHWTPEGHSIVGFAmyyftydPWIGKLLyleDFFVMSDYRGFGIGSEILKN-LSQVAMRCRCSSMHFLVAEWNEPSINFYKRRGASDLsseegwrlfkidkeyllkmat<br>-qlapnrflyagepkagivfslip<br>e------------------------<br><br>BLAST: aliSize=34 (resi) iden=11.24% (19/169) simi=20.12% (34/169)<br>tpdfqivtqrlqlrlitadeaeelvqcirqsqtlhqwvdwfsqqeaeqfiqatrlnwvkaeaygfgvferqtq--TLVGVAIneFYHTFNASLG-------YWIGDRYQRQGYGKEALTALILF<br>---akfvirpataadcsdilrlikelakyeymeeqviltekdlledgfgehpfyhclvaevpkehwtpeghSIVGFAM--YYFTYDPWIGkllyledFFVMSDYRGFGIGSEILKNLSQV<br>CFeRLELTRLEIVCDPENVPSQALALRCGAN------------------------reqlapnrflyagepkagivfslip<br>AM-RCRCSSMHFLVAEWNEPSINFYKRRGASdlsseegwrlfkidkeyllkmate-------------------------<br><br>Proposed: aliSize=141 (resi) RMSD=3.04 (Å)<br>mtpdfqivt--QRLQLRL--ITaDE-AeEL-VqCI-RqsqTLHQ-wvDWFsqqEaeQFIQATRLNW-V-KAEAYGFgVFERQ---T-----QTLVgMVAINEFYHT--FNMASLGYWIG-DRYQRQGYGKEALTALILFCFeRLELTRLEIVCDPENVPSQALALRcGANREQL---APNR-fLYagEPK<br>---------akFVIRPATaaDC-SDiL-RLiK-ELaK---YEYMe--EQV---I--LTEKDLLEDGfGeHPFYHCL-VAEVPkehWtpeghSIVG-FAMYYFTYDPwiGKLLYLEDFFVmSDYRGFGIGSEILKNLSQVAM-RCRCSSMHFLVAEWNEPSINFYKR-RGASDLSseeGWRLf-KI--DKE<br>--AGI-VFslip<br>ylLKMaTE----
586	2fiaA (157)<br>1ufhB (154)	C	14.94	TM-align: aliSize=136 (resi) RMSD=3.22 (Å)<br>MKIRVADEKELPMILQFLTeVKAy-MDvv-GIT-QW----TK-DYP-SQG-DIQEDITK--KRLYLLV--HEeMIFSMATFCMEQEQ--DFVWLKRFATSPNYIAKGYGSLLFHELEKRAVWEGRRKMYAQTNHTNHRMIRFFESKGFTKIHESLqmnrldfGSfylyvkele--<br>IMLTPMQTEEFRSYLTYTT-KHY-aEE--kVKAgTWlpedAQlLSKqVFTdLLPRGLETphHHLWSLKlnEK-DIVGWLWIHAEPEHpqQEAFIYDFGLYEPYRGKGYAKQALAALDQAARSMGIRKLSLHVFAHNQTARKLYEQTGFQETDVVM-------SK---------kl<br><br>SARST: aliSize=122 (resi) RMSD=5.29 (Å)<br>mkirvadekelpm--------------ILQFLTEVKAYMDVVGitQWTKDypsQGDIQEDITK-----------KRLYLLVHE--EMIFSMATFCMEQEQD--FVWLKRFATSPNYIAKGYGSLLFHELEKRAVWEGRRKMYAQTNHTNHRMIRFFESKGFTKIHESLQ---mnrldfgsfylyvkele<br>-------------imltpmqteefrsyLTYTTKHYAEEKVKAG--TWLPE---DAQLLSKQVFtdllprgletpHHHLWSLKLneKDIVGWLWIHAEPEHPqqEAFIYDFGLYEPYRGKGYAKQALAALDQAARSMGIRKLSLHVFAHNQTARKLYEQTGFQETDVVMSkkl-----------------<br><br>BLAST: aliSize=25 (resi) iden=10.39% (16/154) simi=16.23% (25/154)<br>mkirvadekelpmilqfltevkaymdvvgitqwtkdypsqgdiqeditkkrlyllvheemifsmatfcme--QEQDFVWlkRFATSPNYIAKGYGSLLFHELEKRAVWE<br>--imltpmqteefrsyltyttkhyaeekvkagtwlpedaqllskqvftdllprgletphhhlwsklnekdivgwlwihaepehpQQEAFIY--DFGLYEPYRGKGYAKQALAALDQAARSM<br>GRRKMYAQTNHTNHRMIRFFESKGF-----------tkiheslqmnrldfgsfylyvkele<br>GIRKLSLHVFAHNQTARKLYEQTGFqetdvvmskkl-------------------------<br><br>Proposed: aliSize=131 (resi) RMSD=2.43 (Å)<br>MKIRVADEKELPMILQFL-TevK--aymDvvgI--TQ-------wTK-DYpS--QG-DIQEDITK--KRLYLLV---HEemIFSMATFCMEQEQ--DFVWLKRFATSPNYIAKGYGSLLFHELEKRAVWEGRRKMYAQTNHTNHRMIRFFESKGFTKIHEslqmnrLdFgSFYLyvkele<br>IMLTPMQTEEFRSYLTYTtK--Hya---E---EkvKAgtwlped-AQlLS-KqvFTdLLPRGLETphHHLWSLKlneKD--IVGWLWIHAEPEHpqQEAFIYDFGLYEPYRGKGYAKQALAALDQAARSMGIRKLSLHVFAHNQTARKLYEQTGFQETDV------V-M-SKKL------
587	2fl4A (147)<br>1qsmD (152)	N	12.24	TM-align: aliSize=112 (resi) RMSD=3.08 (Å)<br>gmeihfekvtsdnrkavenl-------------------------QVFa-EqqAFiESMAENLKESDQF----PEWESAGIYDG--NQLIGYAMYGRWQD-----GRVWLDRFLIDQRFQGQGYGKAACRLLMLKLIEkYQTNKLYLSVYDTNSSAIRLYQQLgFVFNGELDTNGervmewthq---<br>--------------------sednitvrfvtendkegwqrlwksyQDF-yE--VS-FPDDLDDFNFGRFldpnIKMWAAVAVESssEKIIGMINFFNHMTtwdfkDKIYINDLYVDENSRVKGAGGKLIQFVYDEADK-LGTPSVYWCTDESNHRAQLLYVKV-GYKAPKILYKR---------kgy<br><br>SARST: aliSize=120 (resi) RMSD=11.19 (Å)<br>gmei-----HFEKVTSDNRKAVENlqvfaeQQAFIESMA---------ENLKESDQFPEW-ESAGIYDG---NQLIGYAMYGRWQDG-----RVWLDRFLIDQRFQGQGYGKAACRLLMLKLIEKYqTNKLYLSVYDTNSSAIRLYQQLGFVFN---------geldtngervmewthq<br>----sedniTVRFVTENDKEGWQR------LWKSYQDFYevsfpddldDFNFGRFLDPNIkMWAAVAVEsssEKIIGMINFFNHMTTwdfkdKIYINDLYVDENSRVKGAGGKLIQFVYDEADKLG-TPSVYWCTDESNHRAQLLYVKVGYKAPkilykrkgy----------------<br><br>BLAST: aliSize=61 (resi) iden=17.01% (25/147) simi=41.50% (61/147)<br>gmeihfek---------VTSDNRKAVENLqvFAEQQAFIE-SMAENLKESD--QF--PE---WESAGIYDGNQ-LIGYAMY----GRWQ-DGRVWLDRFLIDQRFQGQGYGKAACRLLMLKlIEKYQTNKLYLSVYDTNSSAIRLYQQLGF------------vfngeldtngervmewthq<br>--------sednitvrfVTENDKEGWQRL--WKSYQDFYEvSFPDDLDDFNfgRFldPNikmWAAVAVESSSEkIIGMINFfnhmTTWDfKDKIYINDLYVDENSRVKGAGGKLIQFVYDE-ADKLGTPSVYWCTDESNHRAQLLYVKVGYkapkilykrkgy-------------------<br><br>Proposed: aliSize=127 (resi) RMSD=2.33 (Å)<br>-GMEIHFEKVTSDNRKAVENL-QV-faEQQAFI-esM-----aenlKESDQF-----PEWESAGIYDG--NQLIGYAMYGRWQD-----GRVWLDRFLIDQRFQGQGYGKAACRLLMLKLIEkYQTNKLYLSVYDTNSSAIRLYQQLGFVFNGELDT-----ngervmewthq<br>sEDNITVRFVTENDKEGWQRLwKSy--QDFYEVs--Fpddld----DFNFGRfldpnIKMWAAVAVESssEKIIGMINFFNHMTtwdfkDKIYINDLYVDENSRVKGAGGKLIQFVYDEADK-LGTPSVYWCTDESNHRAQLLYVKVGYKAPKILYKrkgya-----------

<table>
<tr><td rowspan="2">588</td><td>2fl4A
(147)</td><td rowspan="2">C</td><td rowspan="2">9.52</td><td rowspan="2">TM-align: aliSize=112 (resi) RMSD=3.08 (Å)
gmeihfekvtsdnrkavenl-------------------------QVFa-EqqAFiESMAENLKESDQF----PEWESAGIYDG--NQLIGYAMYGRWQD-----GRVWLDRFLIDQRFQGQGYGKAACRLLMLKLIEkYQTNKLYLSVYDTNSSAIRLYQQLgFVFNGELDTNGervmewthq---
--------------------sednitvrfvtendkegwqrlwksyQDF-yE--VS-FPDDLDDFNFGRFldpnIKMWAAVAVESssEKIIGMINFFNHMTtwdfkDKIYINDLYVDENSRVKGAGGKLIQFVYDEADK-LGTPSVYWCTDESNHRAQLLYVKV-GYKAPKILYKR---------kgy

SARST: aliSize=120 (resi) RMSD=11.19 (Å)
gmei-----HFEKVTSDNRKAVENlqvfaeQQAFIESMA---------ENLKESDQFPEW-ESAGIYDG---NQLIGYAMYGRWQDG-----RVWLDRFLIDQRFQGQGYGKAACRLLMLKLIEKYqTNKLYLSVYDTNSSAIRLYQQLGFVFN---------geldtngervmewthq
----sedniTVRFVTENDKEGWQR------LWKSYQDFYevsfpddldDFNFGRFLDPNIkMWAAVAVEsssEKIIGMINFFNHMTTwdfkdKIYINDLYVDENSRVKGAGGKLIQFVYDEADKLG-TPSVYWCTDESNHRAQLLYVKVGYKAPkilykrkgy----------------

BLAST: aliSize=61 (resi) iden=17.01% (25/147) simi=41.50% (61/147)
gmeihfek---------VTSDNRKAVENLqvFAEQQAFIE-SMAENLKESD--QF--PE---WESAGIYDGNQ-LIGYAMY----GRWQ-DGRVWLDRFLIDQRFQGQGYGKAACRLLMLKlIEKYQTNKLYLSVYDTNSSAIRLYQQLGF------------vfngeldtngervmewthq
--------sednitvrfVTENDKEGWQRL--WKSYQDFYEvSFPDDLDDFNfgRFldPNikmWAAVAVESSSEkIIGMINFfnhmTTWDfKDKIYINDLYVDENSRVKGAGGKLIQFVYDE-ADKLGTPSVYWCTDESNHRAQLLYVKVGYkapkilykrkgy------------------

Proposed: aliSize=109 (resi) RMSD=2.29 (Å)
gmeihfekvtsdnrkavenlqvfaeqqaf-------------------I-eSM-----------AENLKESDQF-----PEWESAGIYDG--NQLIGYAMYGRWQD-----GRVWLDRFLIDQRFQGQGYGKAACRLLMLKLIEkYQTNKLYLSVYDTNSSAIRLYQQLGFVFNGELdTNgERVM-ewthq
----------------------------sednitvrfvtendkegwqRl-WKsyqdfyevsfpDDLDDFNFGRfldpnIKMWAAVAVESssEKIIGMINFFNHMTtwdfkDKIYINDLYVDENSRVKGAGGKLIQFVYDEADK-LGTPSVYWCTDESNHRAQLLYVKVGYKAPKIL-YK-RKGYa-----</td></tr>
<tr><td>1qsmD
(152)</td></tr>
<tr><td rowspan="2">589</td><td>2fl4A
(147)</td><td rowspan="2">N</td><td rowspan="2">14.97</td><td rowspan="2">TM-align: aliSize=110 (resi) RMSD=3.21 (Å)
gmeihfekvtsd---------------------------NRKAVENLqvfaeqqafi-------Esm--AE-NLKESDQFPEWESAGIYDGN-QLIGYAMYGRWQDG---RVWLDRFLIDQRFQGQGYGKAACRLLMLKLIEKYqTNKLYLSVYDTNSSAIRLYQQLGFVFNgELdtnGErvmewthq----
------------timltpmqteefrsyltyttkhyaeekVKAGTWLP----------edaqllsK--qvFTdLLPRGLETPHHHLWSLKLNEkDIVGWLWIHAEPEHpqqEAFIYDFGLYEPYRGKGYAKQALAALDQAARSMG-IRKLSLHVFAHNQTARKLYEQTGFQET-DV---VM--------skkl

SARST: aliSize=108 (resi) RMSD=4.21 (Å)
gmeihfekvtsdnrkavenlqvfaeq---------------------------------------QAFIESMAENLKES--DQFPEWESAGIY-DGNQLIGYAMYGR---WQDGRVWLDRFLIDQRFQGQGYGKAACRLLMLKLIEKYqTNKLYLSVYDTNSSAIRLYQQLGFVFNGELD----tngervme
--------------------------timltpmqteefrsyltyttkhyaeekvkagtwlpedAQLLSKQVFTDLLPrgLETPHHHLWSLKlNEKDIVGWLWIHAepeHPQQEAFIYDFGLYEPYRGKGYAKQALAALDQAARSMG-IRKLSLHVFAHNQTARKLYEQTGFQETDVVMskkl--------

wthq

BLAST: aliSize=54 (resi) iden=19.05% (28/147) simi=36.73% (54/147)
gmeihfekvtsdnrkav-----------------------------------ENLQVFAEQqAFIESMAENLkESDQFPEWeSAGIYDgNQLIGYaMYGRWQDGRVWLDRFLID----QRFQGQGYGKAACRLLMlKLIEKYQTNKLYLSVYDTNSSAIRLYQQLGF-----------vfngeldtnger
-----------------timltpmqteefrsyltyttkhyaeekvkagtwlpEDAQLLSKQ-VFTDLLPRGL-ETPHHHLW-SLKLNE-KDIVGW-LWIHAEPEHPQQEAFIYDfglyEPYRGKGYAKQALAALD-QAARSMGIRKLSLHVFAHNQTARKLYEQTGFqetdvvmskkl-----------

vmewthq

Proposed: aliSize=126 (resi) RMSD=2.47 (Å)
GmEIHFEKVTSdNRKAVENLQ-VF--AEQ----Q--------aFIeSMAENlK--E--sDQFPEWESAGIYDGN-QLIGYAMYGRW--qD--GRVWLDRFLIDQRFQGQGYGKAACRLLMLKLIEKYqTNKLYLSVYDTNSSAIRLYQQLGFVFNgELdtnG-----ervmewthq
T-IMLTPMQTE-EFRSYLTYTtKHyaEEKvkagTwlpedaql-LS-KQVFT-DllPrg-LETPHHHLWSLKLNEkDIVGWLWIHAEpe-HpqQEAFIYDFGLYEPYRGKGYAKQALAALDQAARSMG-IRKLSLHVFAHNQTARKLYEQTGFQET-DV---Vmskkl---------</td></tr>
<tr><td>1ufhA
(155)</td></tr>
<tr><td rowspan="2">590</td><td>2fl4A
(147)</td><td rowspan="2">C</td><td rowspan="2">14.97</td><td rowspan="2">TM-align: aliSize=110 (resi) RMSD=3.21 (Å)
gmeihfekvtsd---------------------------NRKAVENLqvfaeqqafi-------Esm--AE-NLKESDQFPEWESAGIYDGN-QLIGYAMYGRWQDG---RVWLDRFLIDQRFQGQGYGKAACRLLMLKLIEKYqTNKLYLSVYDTNSSAIRLYQQLGFVFNgELdtnGErvmewthq----
------------timltpmqteefrsyltyttkhyaeekVKAGTWLP----------edaqllsK--qvFTdLLPRGLETPHHHLWSLKLNEkDIVGWLWIHAEPEHpqqEAFIYDFGLYEPYRGKGYAKQALAALDQAARSMG-IRKLSLHVFAHNQTARKLYEQTGFQET-DV---VM--------skkl

SARST: aliSize=108 (resi) RMSD=4.21 (Å)
gmeihfekvtsdnrkavenlqvfaeq---------------------------------------QAFIESMAENLKES--DQFPEWESAGIY-DGNQLIGYAMYGR---WQDGRVWLDRFLIDQRFQGQGYGKAACRLLMLKLIEKYqTNKLYLSVYDTNSSAIRLYQQLGFVFNGELD----tngervme
--------------------------timltpmqteefrsyltyttkhyaeekvkagtwlpedAQLLSKQVFTDLLPrgLETPHHHLWSLKlNEKDIVGWLWIHAepeHPQQEAFIYDFGLYEPYRGKGYAKQALAALDQAARSMG-IRKLSLHVFAHNQTARKLYEQTGFQETDVVMskkl--------

wthq

BLAST: aliSize=54 (resi) iden=19.05% (28/147) simi=36.73% (54/147)
gmeihfekvtsdnrkav-----------------------------------ENLQVFAEQqAFIESMAENLkESDQFPEWeSAGIYDgNQLIGYaMYGRWQDGRVWLDRFLID----QRFQGQGYGKAACRLLMlKLIEKYQTNKLYLSVYDTNSSAIRLYQQLGF-----------vfngeldtnger
-----------------timltpmqteefrsyltyttkhyaeekvkagtwlpEDAQLLSKQ-VFTDLLPRGL-ETPHHHLW-SLKLNE-KDIVGW-LWIHAEPEHPQQEAFIYDfglyEPYRGKGYAKQALAALD-QAARSMGIRKLSLHVFAHNQTARKLYEQTGFqetdvvmskkl-----------

vmewthq

Proposed: aliSize=105 (resi) RMSD=1.80 (Å)
gmeihfekvtsdn--------------------------R--KA-V-enL----------qvfaeqqafiesmaeN---lkE--SDQFPEWESAGIYDGN-QLIGYAMYGRW--qD--GRVWLDRFLIDQRFQGQGYGKAACRLLMLKLIEKYqTNKLYLSVYDTNSSAIRLYQQLGFVFNgeldtngER
------------timltpmqteefrsyltyttkhyaeeKvkAGtWl--Pedaqllskqv---------------Ftdl--LprGLETPHHHLWSLKLNEkDIVGWLWIHAEpe-HpqQEAFIYDFGLYEPYRGKGYAKQALAALDQAARSMG-IRKLSLHVFAHNQTARKLYEQTGFQET-------DV

VMEWTHq
VMSKKL-</td></tr>
<tr><td>1ufhA
(155)</td></tr>
</table>

591	2fl4A<br>(147)<br><br>1ufhB<br>(154)	N	14.29	TM-align: aliSize=113 (resi) RMSD=3.39 (Å)<br>gmeihfekvtsd---------------------------N-RKAVeNLQvfa--EqqAfi--ESm-AE-NLKESDQFPEWESAGIYDG-NQLIGYAMYGRWQDG---RVWLDRFLIDQRFQGQGYGKAACRLLMLKLIEKYqTNKLYLSVYDTNSSAIRLYQQLGFVFNgELdtnGErvmewthq----<br>------------imltpmqteefrsyltyttkhyaeekVkAGTW-LPE---daQ--L--lsKQ-vFTdLLPRGLETPHHHLWSLKLNeKDIVGWLWIHAEPEHpqqEAFIYDFGLYEPYRGKGYAKQALAALDQAARSMG-IRKLSLHVFAHNQTARKLYEQTGFQET-DV---VM--------skkl<br><br>SARST: aliSize=125 (resi) RMSD=11.41 (Å)<br>gmei-HFEKVTSDNRKAVENlqvfaEQQAFIESMA--------------ENLKESDQFPE-------WESAGIYDGNQLIGYAMYGRWQD---GRVWLDRFLIDQRFQGQGYGKAACRLLMLKLiEKYQTNKLYLSVYDTNSSAIRLYQQLGFVFNGELD----tngervmewthq<br>----iMLTPMQTEEFRSYLT-----YTTKHYAEEKvkagtwlpedaqllSKQVFTDLLPRgletphhHLWSLKLNEKDIVGWLWIHAEPEhpqQEAFIYDFGLYEPYRGKGYAKQALAALDQAA-RSMGIRKLSLHVFAHNQTARKLYEQTGFQETDVVMskkl------------<br><br>BLAST: aliSize=54 (resi) iden=19.05% (28/147) simi=36.73% (54/147)<br>gmeihfekvtsdnrkav-----------------------------------ENLQVFAEQqAFIESMAENLkESDQFPEWeSAGIYDgNQLIGYaMYGRWQDGRVWLDRFLID----QRFQGQGYGKAACRLLMlKLIEKYQTNKLYLSVYDTNSSAIRLYQQLGF-----------vfngeldtngerv<br>-----------------imltpmqteefrsyltyttkhyaeekvkagtwlpEDAQLLSKQ-VFTDLLPRGL-ETPHHHLW-SLKLNE-KDIVGW-LWIHAEPEHPQQEAFIYDfglyEPYRGKGYAKQALAALD-QAARSMGIRKLSLHVFAHNQTARKLYEQTGFqetdvvmskkl------------<br><br>mewthq<br>------<br><br>Proposed: aliSize=127 (resi) RMSD=2.56 (Å)<br>gMEIHFEKVTsdNRKAVENLQ-VF--AEQ----Q-----aF--IESMAENlK--E--sDQFPEWESAGIYDG-NQLIGYAMYGRW--QD-GRVWLDRFLIDQRFQGQGYGKAACRLLMLKLIEKYqTNKLYLSVYDTNSSAIRLYQQLGFVFNgELdtnG-----ervmewthq<br>-IMLTPMQTE--EFRSYLTYTtKHyaEEKvkagTwlped-AqlLSKQVFT-DllPrg-LETPHHHLWSLKLNeKDIVGWLWIHAEpeHPqQEAFIYDFGLYEPYRGKGYAKQALAALDQAARSMG-IRKLSLHVFAHNQTARKLYEQTGFQET-DV---Vmskkl---------
592	2fl4A<br>(147)<br><br>1ufhB<br>(154)	C	13.61	TM-align: aliSize=113 (resi) RMSD=3.39 (Å)<br>gmeihfekvtsd---------------------------N-RKAVeNLQvfa--EqqAfi--ESm-AE-NLKESDQFPEWESAGIYDG-NQLIGYAMYGRWQDG---RVWLDRFLIDQRFQGQGYGKAACRLLMLKLIEKYqTNKLYLSVYDTNSSAIRLYQQLGFVFNgELdtnGErvmewthq----<br>------------imltpmqteefrsyltyttkhyaeekVkAGTW-LPE---daQ--L--lsKQ-vFTdLLPRGLETPHHHLWSLKLNeKDIVGWLWIHAEPEHpqqEAFIYDFGLYEPYRGKGYAKQALAALDQAARSMG-IRKLSLHVFAHNQTARKLYEQTGFQET-DV---VM--------skkl<br><br>SARST: aliSize=125 (resi) RMSD=11.41 (Å)<br>gmei-HFEKVTSDNRKAVENlqvfaEQQAFIESMA--------------ENLKESDQFPE-------WESAGIYDGNQLIGYAMYGRWQD---GRVWLDRFLIDQRFQGQGYGKAACRLLMLKLiEKYQTNKLYLSVYDTNSSAIRLYQQLGFVFNGELD----tngervmewthq<br>----iMLTPMQTEEFRSYLT-----YTTKHYAEEKvkagtwlpedaqllSKQVFTDLLPRgletphhHLWSLKLNEKDIVGWLWIHAEPEhpqQEAFIYDFGLYEPYRGKGYAKQALAALDQAA-RSMGIRKLSLHVFAHNQTARKLYEQTGFQETDVVMskkl------------<br><br>BLAST: aliSize=54 (resi) iden=19.05% (28/147) simi=36.73% (54/147)<br>gmeihfekvtsdnrkav-----------------------------------ENLQVFAEQqAFIESMAENLkESDQFPEWeSAGIYDgNQLIGYaMYGRWQDGRVWLDRFLID----QRFQGQGYGKAACRLLMlKLIEKYQTNKLYLSVYDTNSSAIRLYQQLGF-----------vfngeldtngerv<br>-----------------imltpmqteefrsyltyttkhyaeekvkagtwlpEDAQLLSKQ-VFTDLLPRGL-ETPHHHLW-SLKLNE-KDIVGW-LWIHAEPEHPQQEAFIYDfglyEPYRGKGYAKQALAALD-QAARSMGIRKLSLHVFAHNQTARKLYEQTGFqetdvvmskkl------------<br><br>mewthq<br>------<br><br>Proposed: aliSize=108 (resi) RMSD=2.14 (Å)<br>gmeihfekvtsdnr-----------------------------KAVE----nL----qvfaeqqafieS---maE--NlKESDQFPEWESAGIYDG-NQLIGYAMYGRW--QD-GRVWLDRFLIDQRFQGQGYGKAACRLLMLKLIEKYqTNKLYLSVYDTNSSAIRLYQQLGFVFngeldtngeRVMEWTHQ-<br>--------------imltpmqteefrsyltyttkhyaeekvkAGTWlped-Aqlls-----------Kqvf--TdlL-PRGLETPHHHLWSLKLNeKDIVGWLWIHAEpeHPqQEAFIYDFGLYEPYRGKGYAKQALAALDQAARSMG-IRKLSLHVFAHNQTARKLYEQTGFQE---------TDVVMSKKl
593	2fl4A<br>(147)<br><br>1vkcA<br>(149)	N	11.56	TM-align: aliSize=113 (resi) RMSD=3.19 (Å)<br>gmeihfekvtsdnrkav------------------------ENLQVfa--EQQAFIESmAENLKESDQFpEWESAGIYDG--NQLIGYAMYGRWQ----D-GRVWLDRFLIDQRFQGQCYGKAACRLLMLKLIEKyQTNKLYLSVYDTNSsAIRLYQQLGFVFNgELdtNGErvmewthq---<br>-----------------eytivdgeeyieeikkldreisysFVRFP--isYEEYEERH-EELFESLLSQ-GEHKFFVALNerSELLGHVWICITLdtvdYvKIAYIYDIEVVKWARGLGIGSALLRKAEEWAKER-GAKKIVLRVEIDNP-AVKWYEERGYKAR-AL--IME--------kpi<br><br>SARST: aliSize=128 (resi) RMSD=11.52 (Å)<br>gmei-HFEKVTSDNRKAV---------ENLQVFAEQQAFIESMAENLKESDQFPEWESAGIYDGN-QLIGYAMYGRWQDG-----RVWLDRFLIDQRFQGQCYGKAACRLLMLKLIEKyQTNKLYLSVYDTNsSAIRLYQQLGFVFNGEL-----dtngervmewthq<br>----eYTIVDGEEYIEEIkkldreisySFVRFPISYEEYEERHEELFESLLSQGEHKFFVALNERsELLGHVWICITLDTvdyvkIAYIYDIEVVKWARGLGIGSALLRKAEEWAKER-GAKKIVLRVEIDN-PAVKWYEERGYKARALImekpi------------<br><br>BLAST: aliSize=33 (resi) iden=11.56% (17/147) simi=22.45% (33/147)<br>gmeihfekvtsdnrkavenlqvfaeqqafiesmaenlkesdqfpewesagiydgnqligyamygrwqd--GRVW----LD-------RFLID----QRFQGQCYGKAACRLLMlKLIEKYQTNKLYL<br>--eytivdgeeyieeikkldreisysfvrfpisyeeyeerheelfesllsqgehkffvalnersellGHVWicitLDtvdyvkiAYIYDievvKWARGLGIGSALLRKAE-EWAKERGAKKIVL<br><br>SVyDTNSSAIRLYQQLGF-----------vfngeldtngervmewthq<br>RV-EIDNPAVKWYEERGYkaralimekpi-------------------<br><br>Proposed: aliSize=121 (resi) RMSD=2.56 (Å)<br>GMEIHFEKVtsdNRKAVEnLQ-VFAE-QQafI------------eS--maeN-lKESDQFpEWESAGIYDG--NQLIGYAMYGRWQ----D-GRVWLDRFLIDQRFQGQCYGKAACRLLMLKLIEKyQTNKLYLSVYDTNSsAIRLYQQLGFVFNgELdtNG----ervmewthq<br>EYTIVDGEE---YIEEIK-KLdREISySF--Vrfpisyeeyeer-Hee---Lf-ESLLSQ-GEHKFFVALNerSELLGHVWICITLdtvdYvKIAYIYDIEVVKWARGLGIGSALLRKAEEWAKER-GAKKIVLRVEIDNP-AVKWYEERGYKAR-AL--IMekpi---------

594	2fl4A<br>(147)<br><br>1vkcA<br>(149)	C	12.93	TM-align: aliSize=113 (resi) RMSD=3.19 (Å)<br>gmeihfekvtsdnrkav------------------------ENLQVfa--EQQAFIESmAENLKESDQFpEWESAGIYDG--NQLIGYAMYGRWQ----D-GRVWLDRFLIDQRFQGQGYGKAACRLLMLKLIEKyQTNKLYLSVYDTNSsAIRLYQQLGFVFNgELdtNGErvmewthq---<br>-----------------eytivdgeeyieeikkldreisysFVRFP--isYEEYEERH-EELFESLLSQ-GEHKFFVALNerSELLGHVWICITLdtvdYvKIAYIYDIEVVKWARGLGIGSALLRKAEEWAKER-GAKKIVLRVEIDNP-AVKWYEERGYKAR-AL--IME--------kpi<br><br>SARST: aliSize=128 (resi) RMSD=11.52 (Å)<br>gmei-HFEKVTSDNRKAV---------ENLQVFAEQQAFIESMAENLKESDQFPEWESAGIYDGN-QLIGYAMYGRWQDG-----RVWLDRFLIDQRFQGQGYGKAACRLLMLKLIEKyQTNKLYLSVYDTNsSAIRLYQQLGFVFNGEL-----dtngervmewthq<br>----eYTIVDGEEYIEEIkkldreisySFVRFPISYEEYEERHEELFESLLSQGEHKFFVALNERsELLGHVWICITLDTvdyvkIAYIYDIEVVKWARGLGIGSALLRKAEEWAKER-GAKKIVLRVEIDN-PAVKWYEERGYKARALImekpi-------------<br><br>BLAST: aliSize=33 (resi) iden=11.56% (17/147) simi=22.45% (33/147)<br>gmeihfekvtsdnrkavenlqvfaeqqafiesmaenlkesdqfpewesagiydgnqligyamygrwqd---GRVW----LD-------RFLID----QRFQGQGYGKAACRLLMlKLIEKYQTNKLYL<br>--eytivdgeeyieeikkldreisysfvrfpisyeeyeerheelfesllsqgehkffvalnersellGHVWicitLDtvdyvkiAYIYDievvKWARGLGIGSALLRKAE-EWAKERGAKKIVL<br><br>SVyDTNSSAIRLYQQLGF-----------vfngeldtngervmewthq<br>RV-EIDNPAVKWYEERGYkaralimekpi-------------------<br><br>Proposed: aliSize=110 (resi) RMSD=2.38 (Å)<br>gmeihfekvtsdnrkave------------------------NLQ----vfaE--qqAfiE--SmAENLKESDQFpEWESAGIYDG--NQLIGYAMYGRWQ----D-GRVWLDRFLIDQRFQGQGYGKAACRLLMLKLIEKyQTNKLYLSVYDTNSsAIRLYQQLGFVFngeldtnGERVMEWTHq<br>------------------eytivdgeeyieeikkldreisysFVRfpis---Yee--Y--EerH-EELFESLLSQ-GEHKFFVALNerSELLGHVWICITLdtvdYvKIAYIYDIEVVKWARGLGIGSALLRKAEEWAKER-GAKKIVLRVEIDNP-AVKWYEERGYKA-------RALIMEKPI-
595	2fl4A<br>(147)<br><br>1z4eA<br>(150)	N	14.29	TM-align: aliSize=117 (resi) RMSD=2.96 (Å)<br>gmeihfekvtsdnrkave-----------------------NLQVFAEQQAf-IE-SMAENLKESDQFPEWESAGIYDGNQLIGYAMYGRWQD------GRVWLDRFLIDQRFQGQGYGKAACRLLMLKLIeKYQTNKLYLSVYDTNSSAIRLYQQLGFVFNGELDtNGervmewthq--<br>------------------hvtireategdleqmvhmladdvLGRKRERYEK-pLPvSYVRAFKEIKKDKNNELIVACNGEEIVGMLQVTFTPYltyqgsWRATIEGVRTHSAARGQGIGSQLVCWAIERAK-ERGCHLIQLTTDKQRPDALRFYEQLGFKASHEGL-KM---------hf<br><br>SARST: aliSize=128 (resi) RMSD=11.20 (Å)<br>gmei--HFEKVTSDN----RKAVENLqvfaeQQAFI---------ESMAENLKESDQFPEWESAGIYDGNQLIGYAMYGRWQD----GRVWLDRF--LIDQRFQGQGYGKAACRLLMLKLIEKYqTNKLYLSVYDTNSSAIRLYQQLGFVfngeldtNGERVMEWT-hq<br>----hvTIREATEGDleqmVHMLADD-----VLGRKreryekplpVSYVRAFKEIKKDKNNELIVACNGEEIVGMLQVTFTPYltyqGSWRATIEgvRTHSAARGQGIGSQLVCWAIERAKERG-CHLIQLTTDKQRPDALRFYEQLGFK-------ASHEGLKMHf--<br><br>BLAST: aliSize=31 (resi) iden=14.29% (21/147) simi=21.09% (31/147)<br>gmeihfekvtsdnrkavenlqvfaeqqafiesmaenlkesdqfpewesagiydgnqlig---YAMY-GRWqdgRVWLDRFLIDQRFQGQGYGKAacrlLMLKLIEKYQTNKLYLSVYDTN<br>--hvtireategdleqmvhmladdvlgrkreryekplpvsyvrafkeikkdknnelivacngeeivgmlqvtftpYLTYqGSW---RATIEGVRTHSAARGQGIGSQ----LVCWAIERAKERGCHLIQLTTD<br><br>SS---AIRLYQQLGF-----------vfngeldtngervmewthq<br>KQrpdALRFYEQLGFkasheglkmhf-------------------<br><br>Proposed: aliSize=127 (resi) RMSD=2.49 (Å)<br>GMEIHFEKVtsdNrkAVENL-QV-FAEQQAFIES-------------maenLKESDQFPEWESAGIYDGNQLIGYAMYGRWQD------GRVWLDRFLIDQRFQGQGYGKAACRLLMLKLIeKYQTNKLYLSVYDTNSSAIRLYQQLGFVFNGELDtNG--ervmewthq<br>HVTIREATE---G--DLEQMvHMlADDVLGRKREryekplpvsyvra----FKEIKKDKNNELIVACNGEEIVGMLQVTFTPYltyqgsWRATIEGVRTHSAARGQGIGSQLVCWAIERAK-ERGCHLIQLTTDKQRPDALRFYEQLGFKASHEGL-KMhf---------
596	2fl4A<br>(147)<br><br>1z4eA<br>(150)	C	12.93	TM-align: aliSize=117 (resi) RMSD=2.96 (Å)<br>gmeihfekvtsdnrkave-----------------------NLQVFAEQQAf-IE-SMAENLKESDQFPEWESAGIYDGNQLIGYAMYGRWQD------GRVWLDRFLIDQRFQGQGYGKAACRLLMLKLIeKYQTNKLYLSVYDTNSSAIRLYQQLGFVFNGELDtNGervmewthq--<br>------------------hvtireategdleqmvhmladdvLGRKRERYEK-pLPvSYVRAFKEIKKDKNNELIVACNGEEIVGMLQVTFTPYltyqgsWRATIEGVRTHSAARGQGIGSQLVCWAIERAK-ERGCHLIQLTTDKQRPDALRFYEQLGFKASHEGL-KM---------hf<br><br>SARST: aliSize=128 (resi) RMSD=11.20 (Å)<br>gmei--HFEKVTSDN----RKAVENLqvfaeQQAFI---------ESMAENLKESDQFPEWESAGIYDGNQLIGYAMYGRWQD----GRVWLDRF--LIDQRFQGQGYGKAACRLLMLKLIEKYqTNKLYLSVYDTNSSAIRLYQQLGFVfngeldtNGERVMEWT-hq<br>----hvTIREATEGDleqmVHMLADD-----VLGRKreryekplpVSYVRAFKEIKKDKNNELIVACNGEEIVGMLQVTFTPYltyqGSWRATIEgvRTHSAARGQGIGSQLVCWAIERAKERG-CHLIQLTTDKQRPDALRFYEQLGFK-------ASHEGLKMHf--<br><br>BLAST: aliSize=31 (resi) iden=14.29% (21/147) simi=21.09% (31/147)<br>gmeihfekvtsdnrkavenlqvfaeqqafiesmaenlkesdqfpewesagiydgnqlig---YAMY-GRWqdgRVWLDRFLIDQRFQGQGYGKAacrlLMLKLIEKYQTNKLYLSVYDTN<br>--hvtireategdleqmvhmladdvlgrkreryekplpvsyvrafkeikkdknnelivacngeeivgmlqvtftpYLTYqGSW---RATIEGVRTHSAARGQGIGSQ----LVCWAIERAKERGCHLIQLTTD<br><br>SS---AIRLYQQLGF-----------vfngeldtngervmewthq<br>KQrpdALRFYEQLGFkasheglkmhf-------------------<br><br>Proposed: aliSize=115 (resi) RMSD=2.37 (Å)<br>gmeihfekvtsdnrkave-----------------------N--lQV-faEQQ--afIE-SMAENLKESDQFPEWESAGIYDGNQLIGYAMYGRWQD------GRVWLDRFLIDQRFQGQGYGKAACRLLMLKLIeKYQTNKLYLSVYDTNSSAIRLYQQLGFVFNGeldtngeRVMEWTHq<br>------------------hvtireategdleqmvhmladdvLgr-KRe--RYEkp--LPvSYVRAFKEIKKDKNNELIVACNGEEIVGMLQVTFTPYltyqgsWRATIEGVRTHSAARGQGIGSQLVCWAIERAK-ERGCHLIQLTTDKQRPDALRFYEQLGFKASH-------EGLKMHF-

597	2fyxA<br>(130)<br><br>2a6mA<br>(130)	C	33.08	TM-align: aliSize=110 (resi) RMSD=1.47 (Å)<br>---MKKGRGYVYKLEYHLIWATKYRHQVLVDEVADGLKDILRDIATQNGLELVALEVMPDYVHLLLGATPQHVIPDFVKALKGASARRMFSAFPHLK-QPhwGGnLWNPSYCVLTVSehtraqiqqyienqhaa----------------<br>avlYKSNHNVVYSCKYHIVWCPKYRRKVLVGAVEMRLKEIIQEVAKELRVEIIEMQTDKDHIHILADIDPSFGVMKFIKTAKGRSSRILRQEFNHLKtKL--PT-LWTNSCFISTVG-----------------gaplnvvkqyienqqn<br><br>SARST: aliSize=109 (resi) RMSD=2.13 (Å)<br>m----KKGRGYVYKLEYHLIWATKYRHQVLVDEVADGLKDILRDIATQNGLELVALEVMPDYVHLLLGATPQHVIPDFVKALKGASARRMFSAFPHLkqpHWGGNL-WNPSYCVLTVS----------------ehtraqiqqyienqhaa<br>-avlyKSNHNVVYSCKYHIVWCPKYRRKVLVGAVEMRLKEIIQEVAKELRVEIIEMQTDKDHIHILADIDPSFGVMKFIKTAKGRSSRILRQEFNHL---KTKLPTlWTNSCFISTVGgaplnvvkqyienqqn-----------------<br><br>BLAST: aliSize=68 (resi) iden=33.08% (43/130) simi=52.31% (68/130)<br>m----KKGRGYVYKLEYHLIWATKYRHQVLVDEVADGLKDILRDIATQNGLELVALEVMPDYVHLLLGATPQHVIPDFVKALKGASARRMFSAFPHLKQPHwgGNLWNPSYCVLTVSEHTRAQIQQYIENQ--haa<br>-avlyKSNHNVVYSCKYHIVWCPKYRRKVLVGAVEMRLKEIIQEVAKELRVEIIEMQTDKDHIHILADIDPSFGVMKFIKTAKGRSSRILRQEFNHLKTKL--PTLWTNSCFISTVGGAPLNVVKQYIENQqn---<br><br>Proposed: aliSize=127 (resi) RMSD=1.51 (Å)<br>---MKKGRGYVYKLEYHLIWATKYRHQVLVDEVADGLKDILRDIATQNGLELVALEVMPDYVHLLLGATPQHVIPDFVKALKGASARRMFSAFPHLKQPhWGGnLWNPSYCVLTVSEHTRAQIQQYIENQHAa<br>avlYKSNHNVVYSCKYHIVWCPKYRRKVLVGAVEMRLKEIIQEVAKELRVEIIEMQTDKDHIHILADIDPSFGVMKFIKTAKGRSSRILRQEFNHLKTK-LPT-LWTNSCFISTVGGAPLNVVKQYIENQQN-
598	2fyxA<br>(130)<br><br>2ec2A<br>(130)	C	40.00	TM-align: aliSize=111 (resi) RMSD=1.68 (Å)<br>--MKKGRGYVYKLEYHLIWATKYRHQVLVDEVADGLKDILRDIATQNGLELVALEVMPDYVHLLLGATPQHVIPDFVKALKGASARRMFSAFPHLK-QPhwGGNLWNPSYCVLTVSehtraqiqqyienqhaa----------------<br>meYKSTRHAKYLCNYHFVWIPKYRRKVLTGEVAEYTKEVLRTIAEELGCEVLALEVMPDHIHLFVNCPPRYAPSYLANYFKGKSARLILKKFQELKkST--NGKLWTRSYFVSTSG-----------------nvssetikkyieeqwa<br><br>SARST: aliSize=123 (resi) RMSD=9.86 (Å)<br>m---KKGRGYVYKLEYHLIWATKYRHQVLVDEVADGLKDILRDIATQNGLELVALEVMPDYVHLLLGATPQHVIPDFVKALKGASARRMFSAFPHLkqPHWGGNL-WNPSYCVLTVSEHTRAQIQQYIEN---qhaa<br>-meyKSTRHAKYLCNYHFVWIPKYRRKVLTGEVAEYTKEVLRTIAEELGCEVLALEVMPDHIHLFVNCPPRYAPSYLANYFKGKSARLILKKFQEL--KKSTNGKlWTRSYFVSTSGNVSSETIKKYIEEqwa----<br><br>BLAST: aliSize=73 (resi) iden=40.77% (53/130) simi=56.15% (73/130)<br>m---KKGRGYVYKLEYHLIWATKYRHQVLVDEVADGLKDILRDIATQNGLELVALEVMPDYVHLLLGATPQHViPDFV-KALKGASARRMFSAFPHLKQpHWGGNLWNPSYCVLTVSEHTRAQIQQYIENQHAa<br>-meyKSTRHAKYLCNYHFVWIPKYRRKVLTGEVAEYTKEVLRTIAEELGCEVLALEVMPDHIHLFVNCPPRYA-PSYLaNYFKGKSARLILKKFQELKK-STNGKLWTRSYFVSTSGNVSSETIKKYIEEQWA-<br><br>Proposed: aliSize=127 (resi) RMSD=1.68 (Å)<br>--MKKGRGYVYKLEYHLIWATKYRHQVLVDEVADGLKDILRDIATQNGLELVALEVMPDYVHLLLGATPQHVIPDFVKALKGASARRMFSAFPHLKQPhWGGNLWNPSYCVLT-VSeHTRAQIQQYIENQHAa<br>meYKSTRHAKYLCNYHFVWIPKYRRKVLTGEVAEYTKEVLRTIAEELGCEVLALEVMPDHIHLFVNCPPRYAPSYLANYFKGKSARLILKKFQELKKS-TNGKLWTRSYFVSTsGN-VSSETIKKYIEEQWA-
599	2fyxA<br>(130)<br><br>2vhgA<br>(126)	C	34.13	TM-align: aliSize=112 (resi) RMSD=1.71 (Å)<br>-MKKGRGYVYKLEYHLIWATKYRHQVLVDEVADGLKDILRDIATQNGLELVALEVMPDYVHLLLGATPQHVIPDFVKALKGASARRMFSAFPHLKQpHWGGnLWNPSYCVLTVSEhtraqiqqyienqhaa-------------<br>lYKSNHNVVYSCKYHIVWCPKYRRKVLVGAVEMRLKEIIQEVAKELRVEIIEMQTDKDHIHILADIDPSFGVMKFIKTAKGRSSRILRQEFNHLKT-KLPT-LWTNSCFISTVGG----------------aplnvvkqyienq<br><br>SARST: aliSize=115 (resi) RMSD=7.72 (Å)<br>m--KKGRGYVYKLEYHLIWATKYRHQVLVDEVADGLKDILRDIATQNGLELVALEVMPDYVHLLLGATPQHVIPDFVKALKGASARRMFSAFPHLkqPHWggnL---WNPSYCVLTVS----EHTRAQIQ--qyienqhaa<br>-lyKSNHNVVYSCKYHIVWCPKYRRKVLVGAVEMRLKEIIQEVAKELRVEIIEMQTDKDHIHILADIDPSFGVMKFIKTAKGRSSRILRQEFNHL--KTK---LptlWTNSCFISTVGgaplNVVKQYIEnq---------<br><br>BLAST: aliSize=68 (resi) iden=34.13% (43/126) simi=53.97% (68/126)<br>m--KKGRGYVYKLEYHLIWATKYRHQVLVDEVADGLKDILRDIATQNGLELVALEVMPDYVHLLLGATPQHVIPDFVKALKGASARRMFSAFPHLKQPHwgGNLWNPSYCVLTVSEHTRAQIQQYIENQhaa<br>-lyKSNHNVVYSCKYHIVWCPKYRRKVLVGAVEMRLKEIIQEVAKELRVEIIEMQTDKDHIHILADIDPSFGVMKFIKTAKGRSSRILRQEFNHLKTKL--PTLWTNSCFISTVGGAPLNVVKQYIENQ---<br><br>Proposed: aliSize=125 (resi) RMSD=1.42 (Å)<br>-MKKGRGYVYKLEYHLIWATKYRHQVLVDEVADGLKDILRDIATQNGLELVALEVMPDYVHLLLGATPQHVIPDFVKALKGASARRMFSAFPHLKQpHWGGnLWNPSYCVLTVSEHTRAQIQQYIENQhaa<br>lYKSNHNVVYSCKYHIVWCPKYRRKVLVGAVEMRLKEIIQEVAKELRVEIIEMQTDKDHIHILADIDPSFGVMKFIKTAKGRSSRILRQEFNHLKT-KLPT-LWTNSCFISTVGGAPLNVVKQYIENQ---

600	2fyxA (130)<br>2vihA (151)	C	30.00	TM-align: aliSize=112 (resi) RMSD=1.72 (Å)<br>--MKKGRGYVYKLEYHLIWATKYRHQVLVDEVADGLKDILRDIATQNGLELVALEVMPDYVHLLLGATPQHVIPDFVKALKGASARRMFSAFPHLKQPhWGGnLWNPSYCVLTVS--Ehtraqiqqyienqhaa---------------------------------<br>vlYKSNHNVVYSCKYHIVWCPKYRRKVLVGAVEMRLKEIIQEVAKELRVEIIEMQTDKDHIHILADIDPSFGVMKFIKTAKGRSSRILRQEFNHLKTK-LPT-LWTNSCFISTVGgaP---------------lnvvkqyienqqnsnrpkqkekwksyvdnlqtkal<br><br>SARST: aliSize=122 (resi) RMSD=7.62 (Å)<br>m---KKGRGYVYKLEYHLIWATKYRHQVLVDEVADGLKDILRDIATQNGLELVALEVMPDYVHLLLGATPQHVIPDFVKALKGASARRMFSAFPHLkqpHWGGNL-WNPSYCVLTVSEHTRAQIQQYIEN------------------------qhaa<br>-vlyKSNHNVVYSCKYHIVWCPKYRRKVLVGAVEMRLKEIIQEVAKELRVEIIEMQTDKDHIHILADIDPSFGVMKFIKTAKGRSSRILRQEFNHL---KTKLPTlWTNSCFISTVGGAPLNVVKQYIENqqnsnrpkqkekwksyvdnlqtkal----<br><br>BLAST: aliSize=68 (resi) iden=33.08% (43/130) simi=52.31% (68/130)<br>m---KKGRGYVYKLEYHLIWATKYRHQVLVDEVADGLKDILRDIATQNGLELVALEVMPDYVHLLLGATPQHVIPDFVKALKGASARRMFSAFPHLKQPHwgGNLWNPSYCVLTVSEHTRAQIQQYIENQ----------------------haa<br>-vlyKSNHNVVYSCKYHIVWCPKYRRKVLVGAVEMRLKEIIQEVAKELRVEIIEMQTDKDHIHILADIDPSFGVMKFIKTAKGRSSRILRQEFNHLKTKL--PTLWTNSCFISTVGGAPLNVVKQYIENQqnsnrpkqkekwksyvdnlqtkal---<br><br>Proposed: aliSize=126 (resi) RMSD=1.54 (Å)<br>--MKKGRGYVYKLEYHLIWATKYRHQVLVDEVADGLKDILRDIATQNGLELVALEVMPDYVHLLLGATPQHVIPDFVKALKGASARRMFSAFPHLKQPHWGGnLWNPSYCVLTVS-EHTRAQIQQYIENQH---------------------aa<br>vlYKSNHNVVYSCKYHIVWCPKYRRKVLVGAVEMRLKEIIQEVAKELRVEIIEMQTDKDHIHILADIDPSFGVMKFIKTAKGRSSRILRQEFNHLKTK-LPT-LWTNSCFISTVGgaPLNVVKQYIENQQNsnrpkqkekwksyvdnlqtkal--
601	2fyxA (130)<br>2vihB (129)	C	33.33	TM-align: aliSize=112 (resi) RMSD=1.70 (Å)<br>--MKKGRGYVYKLEYHLIWATKYRHQVLVDEVADGLKDILRDIATQNGLELVALEVMPDYVHLLLGATPQHVIPDFVKALKGASARRMFSAFPHLKQPhWGGnLWNPSYCVLTVS--Ehtraqiqqyienqhaa------------<br>vlYKSNHNVVYSCKYHIVWCPKYRRKVLVGAVEMRLKEIIQEVAKELRVEIIEMQTDKDHIHILADIDPSFGVMKFIKTAKGRSSRILRQEFNHLKTK-LPT-LWTNSCFISTVGgaP---------------lnvvkqyienqqn<br><br>SARST: aliSize=122 (resi) RMSD=7.64 (Å)<br>m---KKGRGYVYKLEYHLIWATKYRHQVLVDEVADGLKDILRDIATQNGLELVALEVMPDYVHLLLGATPQHVIPDFVKALKGASARRMFSAFPHLkqpHWGGNL-WNPSYCVLTVSEHTRAQIQQYIEN---qhaa<br>-vlyKSNHNVVYSCKYHIVWCPKYRRKVLVGAVEMRLKEIIQEVAKELRVEIIEMQTDKDHIHILADIDPSFGVMKFIKTAKGRSSRILRQEFNHL---KTKLPTlWTNSCFISTVGGAPLNVVKQYIENqqn----<br><br>BLAST: aliSize=68 (resi) iden=33.33% (43/129) simi=52.71% (68/129)<br>m---KKGRGYVYKLEYHLIWATKYRHQVLVDEVADGLKDILRDIATQNGLELVALEVMPDYVHLLLGATPQHVIPDFVKALKGASARRMFSAFPHLKQPHwgGNLWNPSYCVLTVSEHTRAQIQQYIENQ--haa<br>-vlyKSNHNVVYSCKYHIVWCPKYRRKVLVGAVEMRLKEIIQEVAKELRVEIIEMQTDKDHIHILADIDPSFGVMKFIKTAKGRSSRILRQEFNHLKTKL--PTLWTNSCFISTVGGAPLNVVKQYIENQqn---<br><br>Proposed: aliSize=125 (resi) RMSD=1.51 (Å)<br>--MKKGRGYVYKLEYHLIWATKYRHQVLVDEVADGLKDILRDIATQNGLELVALEVMPDYVHLLLGATPQHVIPDFVKALKGASARRMFSAFPHLKQPHWGGnLWNPSYCVLTV-SeHTRAQIQQYIENQH-aa<br>vlYKSNHNVVYSCKYHIVWCPKYRRKVLVGAVEMRLKEIIQEVAKELRVEIIEMQTDKDHIHILADIDPSFGVMKFIKTAKGRSSRILRQEFNHLKTK-LPT-LWTNSCFISTVgG-APLNVVKQYIENQQn--
602	2fyxA (130)<br>2vjvA (125)	C	33.60	TM-align: aliSize=111 (resi) RMSD=1.66 (Å)<br>MKKG-RGYVYKLEYHLIWATKYRHQVLVDEVADGLKDILRDIATQNGLELVALEVMPDYVHLLLGATPQHVIPDFVKALKGASARRMFSAFPHLKQPhWGGnLWNPSYCVLTVSehtraqiqqyienqhaa------------<br>LYKSnHNVVYSCKYHIVWCPKYRRKVLVGAVEMRLKEIIQEVAKELRVEIIEMQTDKDHIHILADIDPSFGVMKFIKTAKGRSSRILRQEFNHLKTK-LPT-LWTNSCFISTVG----------------gaplnvvkqyien<br><br>SARST: aliSize=110 (resi) RMSD=2.47 (Å)<br>m--KKGRGYVYKLEYHLIWATKYRHQVLVDEVADGLKDILRDIATQNGLELVALEVMPDYVHLLLGATPQHVIPDFVKALKGASARRMFSAFPHLkqpHWGGNL-WNPSYCVLTVSE-----------htraqiqqyienqhaa<br>-lyKSNHNVVYSCKYHIVWCPKYRRKVLVGAVEMRLKEIIQEVAKELRVEIIEMQTDKDHIHILADIDPSFGVMKFIKTAKGRSSRILRQEFNHL---KTKLPTlWTNSCFISTVGGaplnvvkqyien---------------<br><br>BLAST: aliSize=67 (resi) iden=33.60% (42/125) simi=53.60% (67/125)<br>m--KKGRGYVYKLEYHLIWATKYRHQVLVDEVADGLKDILRDIATQNGLELVALEVMPDYVHLLLGATPQHVIPDFVKALKGASARRMFSAFPHLKQPHwgGNLWNPSYCVLTVSEHTRAQIQQYIENqhaa<br>-lyKSNHNVVYSCKYHIVWCPKYRRKVLVGAVEMRLKEIIQEVAKELRVEIIEMQTDKDHIHILADIDPSFGVMKFIKTAKGRSSRILRQEFNHLKTKL--PTLWTNSCFISTVGGAPLNVVKQYIEN----<br><br>Proposed: aliSize=122 (resi) RMSD=1.58 (Å)<br>-MKKGRGYVYKLEYHLIWATKYRHQVLVDEVADGLKDILRDIATQNGLELVALEVMPDYVHLLLGATPQHVIPDFVKALKGASARRMFSAFPHLKQPHWGGnLWNPSYCVLTV--SeHTRAQIQQYIENqhaa<br>lYKSNHNVVYSCKYHIVWCPKYRRKVLVGAVEMRLKEIIQEVAKELRVEIIEMQTDKDHIHILADIDPSFGVMKFIKTAKGRSSRILRQEFNHLKTK-LPT-LWTNSCFISTVggA--PLNVVKQYIEN----

603	2g84A (189)<br>1wkqA (158)	C	27.22	TM-align: aliSize=120 (resi) RMSD=1.70 (Å)<br>mndalhiglppflvqanneprVLAAPEARMGYVLELVRANIAAD-GGPFAAAVFErdSGLLIAAGTNRVVPGRCSAAHAEILALSLAQAKLDTHDLSadglpACELVTSAEPCVMCFGAVIWSGVRSLVCAARSDDVEAIGFDE--GPR-PEnwmggleargitvttgllrdaacallreynacngviyn<br>---------------------HAMNHETFLKRAVTLACEGVNAGiGGPFGAVIVK--DGAIIAEGQNNVTTSNDPTAHAEVTAIRKACKVLGAYQLD-----DCILYTSCEPCPMCLGAIYWARPKAVFYAAEHTDAAEAGFDDsfIYKeID--------------------------------------<br><br>arc----------------------------------<br>---kpaeertipfyqvtltehlspfqawrnfankkey<br><br>SARST: aliSize=121 (resi) RMSD=5.79 (Å)<br>mndalhiglppflvqanneprvlaa----PEARMGYVLELVRANIAAD-GGPFAAAVFErdSGLLIAAGTNRVVPGRCSAAHAEILALSLAQAKLDTHDLsaDGLpacELVTSAEPCVMCFGAVIWSGVRSLVCAARSDDVEAIGfdegprpENWMGGLEARGIT-------------------------<br>-------------------------hamnHETFLKRAVTLACEGVNAGiGGPFGAVIVK--DGAIIAEGQNNVTTSNDPTAHAEVTAIRKACKVLGAYQL--DDC---ILYTSCEPCPMCLGAIYWARPKAVFYAAEHTDAAEAG-------FDDSFIYKEIDKPaeertipfyqvtltehlspfqawrn<br><br>-------vttgllrdaacallreynacngviynarc<br>fankkey-----------------------------<br><br>BLAST: aliSize=53 (resi) iden=25.95% (41/158) simi=33.54% (53/158)<br>mndalhiglppflvqanneprvlaapearmgyvlelvrani---------------------AADGGPFAAAVFeRDsGLLIAAGTNRVVPGRCSAAHAEILALSLAqAKLdthdLSADGLPACELVTSAEPCVMCFGAVIWSGVRSLVCAARSDDVEAIGFDE--------------------------<br>---hamnhetflkravtlacegvnAGIGGPFGAVIV-KD-GAIIAEGQNNVTTSNDPTAHAEVTAIRKA-CKV----LGAYQLDDCILYTSCEPCPMCLGAIYWARPKAVFYAAEHTDAAEAGFDDsfiykeidkpaeertipfyqvtlteh<br><br>----------------gprpenwmggleargitvttgllrdaacallreynacngviynarc<br>lspfqawrnfankkey--<br><br>Proposed: aliSize=145 (resi) RMSD=1.44 (Å)<br>mndalhiglppflvqanneprvl--AAPEARMGYVLELVRANIAAD-GGPFAAAVFErdSGLLIAAGTNRVVPGRCSAAHAEILALSLAQAKLDTHDLSadglpACELVTSAEPCVMCFGAVIWSGVRSLVCAARSDDVEAIGFDE------gprPENWMGglEARgITVTTGLLrDAACALLREYNACn<br>-----------------------haMNHETFLKRAVTLACEGVNAGiGGPFGAVIVK--DGAIIAEGQNNVTTSNDPTAHAEVTAIRKACKVLGAYQLD-----DCILYTSCEPCPMCLGAIYWARPKAVFYAAEHTDAAEAGFDDsfiyke---IDKPAE--ERT-IPFYQVTL-TEHLSPFQAWRNF-<br><br>GV----iynarc<br>ANkkey------
604	2gbzA (179)<br>2p1jB (171)	C	12.87	TM-align: aliSize=132 (resi) RMSD=3.21 (Å)<br>ndRLIWIDLEMTGLDTDRDSIIEIATIVTDAQlNVLAEGpELAIAHSlETLEAMdewNRNQHRrsgLW-QRVLDSqVTHAQAEAQTVAFLgewirAgASPMCGNSICQDRRFLHRQMS--R-LERyfHYRNLDVSTIKEL-ARRWa-----PA-VASGFAKSSAhtalsdvrdsidelrhyrqfmgtlgg<br>--TFVVLDFETTGLDPQVDEIIEIGAVKIQGG-QIVDEY-HTLIKPS-REISRK---SSEITG---ITqEMLENK-RSIEEVLPEFLGFL-----E-DSIIVAHNANFDYRFLRLWIKkvMgLDW--ERPYIDTLALAKSlLKLR-sysldSVvEKLGLGPFRH--------------------------<br><br>----------------------------<br>hralddarvtaqvflrfvemmkkeghhh<br><br>SARST: aliSize=148 (resi) RMSD=13.11 (Å)<br>ndr-LIWIDLEMTGLDTDRDSIIEIATIVTDaQLNVLAEGPELAIAhsletLEAMDewnRNQHRRSGlwq--RVLDSQVTHAQAEAQTVAFLGewiraGASPMCGNSICQDRRFLHRQ--------------MSRLERYFHYRNLDVSTIKELARRwapavaSGFAKSSAHTALSDVRDSIDELRHYRQF<br>---tFVVLDFETTGLDPQVDEIIEIGAVKIQ-GGQIVDEYHTLIKP-----SREIS---RKSSEITG---itQEMLENKRSIEEVLPEFLGFL-----EDSIIVAHNANFDYRFLRLWikkvmgldwerpyiDTLALAKSLLKLRSYSLDSVVEKL------GLGPFRHHRALDDARVTAQVFLRFVEMM<br><br>M------gtlgg<br>Kkeghhh-----<br><br>BLAST: aliSize=38 (resi) iden=15.79% (27/171) simi=22.22% (38/171)<br>ndrliw----IDLEMTGLDTDRDSIIEIATI-VTDAQL----NVLAEgPELAIAH-SLETLEAMDEWNRNQHRRSGLWQRVL----DSQVTHAQAEAQtVAFLGEWIR---agaspmcgnsi<br>------tfvvLDFETTGLDPQVDEIIEIGAVkIQGGQIvdeyHTLIK-PSREISRkSSEITGITQEMLENKRSIEEVLPEFLgfleDSIIVAHNANFD-YRFLRLWIKkvmgldwerpyidtlalaksllklrsysldsvveklglgpfrhhralddarvtaqvflrfvemmkkeghhh----------<br><br>cqdrrflhrqmsrleryfhyrnldvstikelarrwapavasgfakssahtalsdvrdsidelrhyrqfmgtlgg<br>--<br><br>Proposed: aliSize=134 (resi) RMSD=2.07 (Å)<br>ndRLIWIDLEMTGLDTDRDSIIEIATIVTDAQlNVLAEGPELAIAHSLeTLEamdEwNRNQHRrsgLW-QRVLDSqVTHAQAEAQTVAFLgewirAgASPMCGNSICQDRRFLHRQMS----R-lERyfHyRNLDVST-----------------------------ikelarrwapavasgfakssah<br>--TFVVLDFETTGLDPQVDEIIEIGAVKIQGG-QIVDEYHTLIKPSRE-ISR---K-SSEITG---ITqEMLENK-RSIEEVLPEFLGFL-----E-DSIIVAHNANFDYRFLRLWIKkvmgLd-WE--R-PYIDTLAlaksllklrsysldsvveklglgpfrhhra---------------------<br><br>TALSDVRDSIDELRHYRQFMGTlGG-<br>LDDARVTAQVFLRFVEMMKKEG-HHh
605	2gtvX (104)<br>1ecmA (91)	N	31.87	TM-align: aliSize=77 (resi) RMSD=1.79 (Å)<br>mieklaeirkkideidnkilkarw--------PW--AE--KL--IAERNSLAKDVAEIKNQLGIPINDPEREKYIYDRIRKLCKEHNVDENIGIKIFQRLIEHNKALQKQYLEEtLeh<br>------------------------npllalreKIsaLDekLLalLAERRELAVEVGKAKLLSHRPVRDIDRERDLLERLITLGKAHHLDAHYITRLFQLIIEDSVLTQQALLQQ-H--<br><br>SARST: aliSize=85 (resi) RMSD=9.85 (Å)<br>mie---KLAEIRKKIDEIDNKILkarwpwaeklIAERNSLAKDVAEIKNQLGIPINDPEREKYIYDRIRKLCKEHNVDENIGIKIFQRLIEHNKALQKQYL---eetleh<br>---nplLALREKISALDEKLLAL----------LAERRELAVEVGKAKLLSHRPVRDIDRERDLLERLITLGKAHHLDAHYITRLFQLIIEDSVLTQQALLqqh------<br><br>BLAST: aliSize=53 (resi) iden=32.97% (30/91) simi=58.24% (53/91)<br>miek--LAEIRKKIDEIDNKILKarwpwaekLIAERNSLAKDVAEIKNQLGIPINDPEREKYIYDRIRKLCKEHNVDENIGIKIFQRLIEHNKALQKQYLEE-tleh<br>----npLLALREKISALDEKLLA--------LLAERRELAVEVGKAKLLSHRPVRDIDRERDLLERLITLGKAHHLDAHYITRLFQLIIEDSVLTQQALLQQh----<br><br>Proposed: aliSize=91 (resi) RMSD=1.20 (Å)<br>miEKLAEIRKKIDEIDNKILKarwpwAeklIAERNSLAKDVAEIKNQLGIPINDPEREKYIYDRIRKLCKEHNVDENIGIKIFQRLIEHNKALQKQYLEEtLeh<br>--NPLLALREKISALDEKLLA-----L---LAERRELAVEVGKAKLLSHRPVRDIDRERDLLERLITLGKAHHLDAHYITRLFQLIIEDSVLTQQALLQQ-H--

606	2gtvX (104)<br>1ecmB (95)	N	27.37	TM-align: aliSize=80 (resi) RMSD=2.09 (Å)<br>mieklaeirkkideidnkilkarw-------PW--AE--KL--IAERNSLAKDVAEIKNQLGIPINDPEREKYIYDRIRKLCKEHNVDENIGIKIFQRLIEHNKALQKQYLEETL-EH-<br>-----------------------pllalreKIsaLDekLLalLAERRELAVEVGKAKLLSHRPVRDIDRERDLLERLITLGKAHHLDAHYITRLFQLIIEDSVLTQQALLQQHLnKIn<br><br>SARST: aliSize=90 (resi) RMSD=9.63 (Å)<br>mie--KLAEIRKKIDEIDNKILkarwpwaeklIAERNSLAKDVAEIKNQLGIPINDPEREKYIYDRIRKLCKEHNVDENIGIKIFQRLIEHNKALQKQYLEETLE---h<br>---plLALREKISALDEKLLAL----------LAERRELAVEVGKAKLLSHRPVRDIDRERDLLERLITLGKAHHLDAHYITRLFQLIIEDSVLTQQALLQQHLNkin-<br><br>BLAST: aliSize=54 (resi) iden=32.63% (31/95) simi=56.84% (54/95)<br>miek-LAEIRKKIDEIDNKILKarwpwaekLIAERNSLAKDVAEIKNQLGIPINDPEREKYIYDRIRKLCKEHNVDENIGIKIFQRLIEHNKALQKQYLEETL----eh<br>----pLLALREKISALDEKLLA--------LLAERRELAVEVGKAKLLSHRPVRDIDRERDLLERLITLGKAHHLDAHYITRLFQLIIEDSVLTQQALLQQHLnkin--<br><br>Proposed: aliSize=93 (resi) RMSD=1.47 (Å)<br>miEKLAEIRKKIDEIDNKILKarwpwAekliAERNSLAKDVAEIKNQLGIPINDPEREKYIYDRIRKLCKEHNVDENIGIKIFQRLIEHNKALQKQYLEETLEH--<br>--PLLALREKISALDEKLLAL-----L----AERRELAVEVGKAKLLSHRPVRDIDRERDLLERLITLGKAHHLDAHYITRLFQLIIEDSVLTQQALLQQHLNKin
607	2gtvX (104)<br>2h9dD (100)	N	16.00	TM-align: aliSize=77 (resi) RMSD=2.26 (Å)<br>mieklaeirkkideidnkilkarw---------------PW--AE--KL--IAERNSLAKDVAEIKNQlg-IPINDPEREKYIYDRIRKLCKEHNVDENIGIKIFQRLIEHNKALQKQYLEET-LEh<br>-----------------------mktpedctgladireAIdrIDldIVqaLGRRMDYVKAASRFKAS--eAAIPAPERVAAMLPERARWAEENGLDAPFVEGLFAQIIHWYIAEQIKYWRQTrGA-<br><br>SARST: aliSize=83 (resi) RMSD=9.13 (Å)<br>miek---------LAEIRKKIDEIDNKIlkarwpwaekliaERNSLAKDVAEIKNQLGI----PINDPEREKYIYDRIRKLCKEHNvDENIGIKIFQRLIEHNKALQKQYLEET----leh<br>----mktpedctgLADIREAIDRIDLDI-------------VQALGRRMDYVKAASRFKaseaAIPAPERVAAMLPERARWAEENG-LDAPFVEGLFAQIIHWYIAEQIKYWRQtrga---<br><br>BLAST: aliSize=44 (resi) iden=26.00% (26/100) simi=44.00% (44/100)<br>miek---------LAEIRKKIDEIDNKILKArwpwaeklIAERNSLAKDVAEIK-NQLGIPIndPEREKYIYDRIRKLCKEHNVDENIGIKIFQRLIEHNKALQKQYLEET---leh<br>----mktpedctgLADIREAIDRIDLDIVQA--------LGRRMDYVKAASRFKaSEAAIPA--PERVAAMLPERARWAEENGLDAPFVEGLFAQIIHWYIAEQIKYWRQTrga---<br><br>Proposed: aliSize=89 (resi) RMSD=1.69 (Å)<br>mie-------KLAEIRKKIDEIDNKILKarwpWA-ekliAERNSLAKDVAEIKNqlgI--PINDPEREKYIYDRIRKLCKEHNVDENIGIKIFQRLIEHNKALQKQYLEET-LEh<br>---mktpedcTGLADIREAIDRIDLDIV----QAl----GRRMDYVKAASRFKA---SeaAIPAPERVAAMLPERARWAEENGLDAPFVEGLFAQIIHWYIAEQIKYWRQTrGA-
608	2i79A (171)<br>2fe7B (166)	C	19.28	TM-align: aliSize=145 (resi) RMSD=3.16 (Å)<br>------MEYELLIREAEPKDAAELVAFLNRVSLeTDFtslDGDGILLTSEEMEIFLNKqASSdNQITLLAFLNGKIAGIVNITADqRKR--VRHIGDL-FIVIGKRYWNNGLGSLLLEEAIEWAQASGiLRRLQLTVQTRNQAAVHLYQKHGFVIEGSQERGayieegkfidvylmgkli------------<br>enlyfqGHMTLEIRPAVPADAEQILAFIIELAD-YER---ARHEVVTDVEGIRRSLFA-EGS-PTRALMCLSEGRPIGYAVFFYS-YSTwlGRNGIYLeDLYVTPEYRGVGAGRRLLRELAREAVAND-CGRLEWSVLDWNQPAIDFYRSIGALPQDEWVRY------------------rldgealrkmae<br><br>SARST: aliSize=133 (resi) RMSD=4.71 (Å)<br>m-------EYELLIREAEPKDAAELVAFLNRVSLET---DFTSLDGdgillltseemeIFLNKQASSD--NQITLLAFLNGKIAGIVNITADQRKRVRHIGDL--FIVIGKRYWNNGLGSLLLEEAIEWAQASGiLRRLQLTVQTRNQAAVHLYQKHGFVI------------------egsqergayie<br>-enlyfqgHMTLEIRPAVPADAEQILAFIIELADYErarHEVVTDV-----------EGIRRSLFAEgsPTRALMCLSEGRPIGYAVFFYSYSTWLGRNGIYleDLYVTPEYRGVGAGRRLLRELAREAVAND-CGRLEWSVLDWNQPAIDFYRSIGALPqdewvryrldgealrkmae-----------<br><br>egkfidvylmgkli<br>--------------<br><br>BLAST: aliSize=61 (resi) iden=22.29% (37/166) simi=36.75% (61/166)<br>meye----------LLIREAEPKDAAELVAFLnrVSLeTDFTSLDGDgILLTSEEMEIFLNKQASSDNQITLLAflNGKIAGIVNI-----TADQRKRVrHIGDLFIVigKRYWNNGLGSLLLEEAIEWAQASGIlRRLQLTVQTRNQAAVHLYQKHG-----------------------fviegsqerg<br>----enlyfqghmtLEIRPAVPADAEQILAFI--IEL-ADYERARHE-VVTDVEGIRRSLFAEGSPTRALMCLS--EGRPIGYAVFfysysTWLGRNGI-YLEDLYVT--PEYRGVGAGRRLLRELAREAVANDC-GRLEWSVLDWNQPAIDFYRSIGalpqdewvryrldgealrkmae----------<br><br>ayieegkfidvylmgkli<br>------------------<br><br>Proposed: aliSize=144 (resi) RMSD=2.32 (Å)<br>------MEYELLIREAEPKDAAELVAFLNRVSLETDftslDGDGILLTSEEMEIFLNK-qaS-SdnQITLLAFLNGKIAGIVNITADqrkrV------rHIGDLF-IVIGKRYWNNGLGSLLLEEAIEWAQASGiLRRLQLTVQTRNQAAVHLYQKHGFVIEG--sqergayieEGKFID---VY--LMg<br>enlyfqGHMTLEIRPAVPADAEQILAFIIELADYER----ARHEVVTDVEGIRRSLFAe--GsP--TRALMCLSEGRPIGYAVFFYS----Ystwlgr-NGIYLEdLYVTPEYRGVGAGRRLLRELAREAVAND-CGRLEWSVLDWNQPAIDFYRSIGALPQDew---------VRYRLDgeaLRkmAE-<br><br>klia<br>----

609	2i9sA (97)<br>2acyA (98)	C	6.19	TM-align: aliSize=77 (resi) RMSD=2.84 (Å)<br>gpgkpesiLKMTKkGKtLMMFVTVSGNptEKEteEITSLWQGSLFnANYDVQRFIVG--SDRAIFMLRDGSYAWEIKDFLVSQ-----DRCAEVTLEGQmypgk--------------<br>--------AEGDT-LI-SVDYEIFGKV--QGV--FFRKYTQAEGK-KLGLVGWVQNTdqGTVQGQLQGPASKVRHMQEWLETKgspksHIDRASFHNEK-----vivkldytdfqivk<br><br>SARST: aliSize=73 (resi) RMSD=5.63 (Å)<br>gpgkpesilkmt--KKGKTLMMFVTVSgNPTEKETEEITSLWQGSlfnANYDVQRFIVGSDRAIFMLRDGSYAWEIKDFLVSQDRCAEVTL-----------------------egqmypgk<br>------------aeGDTLISVDYEIFG-KVQGVFFRKYTQAEGKK---LGLVGWVQNTDQGTVQGQLQGPASKVRHMQEWLETKGSPKSHIdrasfhnekvivkldytdfqivk--------<br><br>BLAST: aliSize=8 (resi) iden=6.19% (6/97) simi=8.25% (8/97)<br>gpgkpesilkmtkkgktlmmfvtvsgnpteketeeitslwqgslfnanydvqrfivgs---DRAIFMLRDGSYAWEIKDFLV--sqdrcaevtlegqmypgk<br>--aegdtlisvdyeifgkvqgvffrkytqaegkklglvgwvqntdqgtvqgqlqgpaskvrhmqewletkgspkshiDRASFHNEKVIVKLDYTDFQIvk------------------<br><br>Proposed: aliSize=80 (resi) RMSD=2.43 (Å)<br>gpgkpesILKmtKkGKtLMMFVTVSGNptekETE-EITSLWQGSLFnAnYDVQRFIVGS---DRAIFMLRDGSYAWEIKDFLVSQ------DRCAEVTLEG-----QM-YPG-K-<br>-------AEG--D-TL-ISVDYEIFGK----VQGvFFRKYTQAEGK-K-LGLVGWVQNTdqgTVQGQLQGPASKVRHMQEWLETKgspkshIDRASFHNEKvivklDYtDFQiVk
610	2jevA (169)<br>1wwzB (157)	C	15.92	TM-align: aliSize=137 (resi) RMSD=3.03 (Å)<br>aKFVIRPA-TAA--DCSDILRLIKELAKYEyMEE---QVILTEKDLLEDGfgehPFYH--CLVAEVPKehwtpeghSIVGFAMYY-FTYDP-WIGKLLYLEDFFVMSDYRGFGIGSEILKNLSQVAMRcRCSSMHFLVAEWNEPSINFYKRRGASDLSseegWRLFkidkeyllkmate----------<br>-EIKIEKLkKLDkkALNELIDVYMSGYEGL-EEYggeGRDYARNYIKWCW----KKASdgFFVAKVGD--------KIVGFIVCDkDWFSKyEGRIVGAIHEFVVDKKFQGKGIGRKLLITCLDFLGK-YNDTIELWVGEKNYGAMNLYEKFGFKKVG----KSGI-------------wvrmikrqnl<br><br>SARST: aliSize=108 (resi) RMSD=6.56 (Å)<br>akfvirpat---------------------------------AADCSDILRLIKELAkyeymeeqvilteKDLLEDGfgehpfyhCLVAEVPkeHWtpeghsIVGFAMYY-FTYDPWIG-KLLYLEDFFVMSDYRGFGIGSEILKnLSQVAMRCRCSSMHFLVAEWNEPSINFYKRRGASDL---------<br>---------eikieklkkldkkalnelidvymsgyegleeyGGEGRDYARNYIKWC-------------WKKASDG--------FFVAKVG--DK------IVGFIVCDkDWFSKYEGrIVGAIHEFVVDKKFQGKGIGRKLLI-TCLDFLGKYNDTIELWVGEKNYGAMNLYEKFGFKKVgksgiwvrm<br><br>------sseegwrlfkidkeyllkmate<br>ikrqnl----------------------<br><br>BLAST: aliSize=45 (resi) iden=14.65% (23/157) simi=28.66% (45/157)<br>akfvirpataadcsdilrlikelakyeymeeqviltekdlledgfgehpfyhclvaevpkehwtpe--GHSIVGFAM----YYFTYDpwiGKLL-YLEDFFVMSDYRGFGIGSEILKNLSQVAMRCRcSSMH<br>--eikieklkkldkkalnelidvymsgyegleeyggegrdyarnyikwcwkkasdgffvakvGDKIVGFIVcdkdWFSKYE---GRIVgAIHEFVVDKKFQGKGIGRKLLITCLDFLGKYN-DTIE<br><br>FLVAEWNEPSINFYKRRGASDLSSEEGW-RLFK----idkeyllkmate<br>LWVGEKNYGAMNLYEKFGFKKVGKSGIWvRMIKrqnl------------<br><br>Proposed: aliSize=138 (resi) RMSD=2.31 (Å)<br>aKFVIRPA-TAA--DCSDILRLIKELAKY-eymEEQ---V-IL-TekD--LleD-GfgehPFYH--CLVAEVPKehwtpeghSIVGFAMYY-FTYDP--WiGKLLYLEDFFVMSDYRGFGIGSEILKNLSQVAMRcRCSSMHFLVAEWNEPSINFYKRRGASDLS--sEEGWRLFKIDKE-yllkmate<br>-EIKIEKLkKLDkkALNELIDVYMSGYEGl---EEYggeGrDYaR--NyiK--WcW----KKASdgFFVAKVGD--------KIVGFIVCDkDWFSKyeG-RIVGAIHEFVVDKKFQGKGIGRKLLITCLDFLGK-YNDTIELWVGEKNYGAMNLYEKFGFKKVGks-GIWVRMIKRQNLa--------
611	2jevA (169)<br>2j8mB (170)	C	18.34	TM-align: aliSize=142 (resi) RMSD=3.09 (Å)<br>akFVIRPATAADCSDILRLIKELAKYEY--MEEQViLTEKDLLEDGFG-EHPFYHCLVAEVPKehwtpegHSIVGFAMYYFT--YDPwIGKLLYLeDFFVMSDYRGFGIGSEILKNLSQVAMRCRCSSMHFLVAEWNEPSINFYKRRGASDLSSeeGWRLFkidkeyllkmate----------------<br>--ASIRDAGVADLPGILAIYNDAVGNTTaiWNETP-VDLANRQAWFDArARQGYPILVASDAA-------GEVLGYASYGDWrpFEG-FRGTVEH-SVYVRDDQRGKGLGVQLLQALIERARAQGLHVMVAAIESGNAASIGLHRRLGFEISGQ--MPQVG-------------qkfgrwldltfmqlnl<br><br>-------<br>dptrsap<br><br>SARST: aliSize=134 (resi) RMSD=3.86 (Å)<br>akf-VIRPATAADCSDILRLIKELAKYE-YMEEQVILTEKDLLEDGF---GEHPFYHCLVaevpkehwTPEGHsIVGFAMYYF-TYDPWIGKLLYLeDFFVMSDYRGFGIGSEILKNLSQVAMRCRCSSMHFLVAEWNEPSINFYKRRGASDL-------------------------------sseegwr<br>---aSIRDAGVADLPGILAIYNDAVGNTtAIWNETPVDLANRQAWFDararRQGYPILVAS--------DAAGE-VLGYASYGDwRPFEGFRGTVEH-SVYVRDDQRGKGLGVQLLQALIERARAQGLHVMVAAIESGNAASIGLHRRLGFEISgqmpqvgqkfgrwldltfmqlnldptrsap-------<br><br>lfkidkeyllkmate<br>---------------<br><br>BLAST: aliSize=60 (resi) iden=23.08% (39/169) simi=35.50% (60/169)<br>akfv--IRPATAADCSDILRLIKELAKYE---YMEEQVILTEKDLLEDGFGEHPfYHCLVAevpkehwTPEGHSIVGFAMyYFTYDPWIGKLLYLE-DFFVMSDYRGFGIGSEILKNLSQvamRCRCSSMHFLVA---EWNEPSINFYKRRG-------------------------------------asdl<br>----asIRDAGVADLPGILAIYNDAVGNTtaiWNETPVDLANRQAWFDARARQG-YPILVA-------SDAAGEVLGYAS-YGDWRPFEGFRGTVEhSVYVRDDQRGKGLGVQLLQALIE---RARAQGLHVMVAaieSGNAASIGLHRRLGfeisgqmpqvgqkfgrwldltfmqlnldptrsap----<br><br>sseegwrlfkidkeyllkmate<br>----------------------<br><br>Proposed: aliSize=148 (resi) RMSD=2.29 (Å)<br>akFVIRPATAADCSDILRLIKELAKYEY----meEQViLTEKDLLEDGFgehPfY-----HCLVAEVPK-ehwtpeghSIVGFAMYYFT---YDPwiGKLLYLeDFFVMSDYRGFGIGSEILKNLSQVAMRCRCSSMHFLVAEWNEPSINFYKRRGASDLS--SEEG-------WRLFKIDKEYLLKmATE<br>--ASIRDAGVADLPGILAIYNDAVGNTTaiwn--ETP-VDLANRQAWFD---A-RarqgyPILVASDAAg--------EVLGYASYGDWrpfEGF--RGTVEH-SVYVRDDQRGKGLGVQLLQALIERARAQGLHVMVAAIESGNAASIGLHRRLGFEISGqmPQVGqkfgrwlDLTFMQLNLDPTR-SAP

612	2jevA (169)<br>2j8nA (169)	C	18.34	TM-align: aliSize=141 (resi) RMSD=3.04 (Å)<br>akfVIRPATAADCSDILRLIKELAKYEY--MEEQViLTEKDLLEDGFG-EHPFYHCLVAEVPKehwtpegHSIVGFAMYYFTY--DPwIGKLLYLeDFFVMSDYRGFGIGSEILKNLSQVAMRCRCSSMHFLVAEWNEPSINFYKRRGASDLSSeeGWRLFkidkeyllkmate---------------<br>---SIRDAGVADLPGILAIYNDAVGNTTaiWNETP-VDLANRQAWFDArARQGYPILVASDAA-------GEVLGYASYGDWRpfEG-FRGTVEH-SVYVRDDQRGKGLGVQLLQALIERARAQGLHVMVAAIESGNAASIGLHRRLGFEISGQ--MPQVG------------qkfgrwldltfmqlnl<br>-------<br>dptrsap<br><br>SARST: aliSize=130 (resi) RMSD=3.40 (Å)<br>akfv-IRPATAADCSDILRLIKELAKYE-YMEEQVILTEKDLLED------GFGehpfyhCLVAEVPKEHWtpeghsIVGFAMYYF-TYDPWIGKLLYLeDFFVMSDYRGFGIGSEILKNLSQVAMRCRCSSMHFLVAEWNEPSINFYKRRGASDL------------------------------ssee<br>----sIRDAGVADLPGILAIYNDAVGNTtAIWNETPVDLANRQAWfdararQGY------PILVASDAAGE------VLGYASYGDwRPFEGFRGTVEH-SVYVRDDQRGKGLGVQLLQALIERARAQGLHVMVAAIESGNAASIGLHRRLGFEISgqmpqvgqkfgrwldltfmqlnldptrsap----<br>gwrlfkidkeyllkmate<br>------------------<br><br>BLAST: aliSize=60 (resi) iden=23.08% (39/169) simi=35.50% (60/169)<br>akfv-IRPATAADCSDILRLIKELAKYE---YMEEQVILTEKDLLEDGFGEHPfYHCLVAevpkehwTPEGHSIVGFAMyYFTYDPWIGKLLYLE-DFFVMSDYRGFGIGSEILKNLSQvamRCRCSSMHFLVA---EWNEPSINFYKRRG----------------------------------asdls<br>----sIRDAGVADLPGILAIYNDAVGNTtaiWNETPVDLANRQAWFDARARQG-YPILVA-------SDAAGEVLGYAS-YGDWRPFEGFRGTVEhSVYVRDDQRGKGLGVQLLQALIE---RARAQGLHVMVAaieSGNAASIGLHRRLGfeisgqmpqvgqkfgrwldltfmqlnldptrsap-----<br>seegwrlfkidkeyllkmate<br>---------------------<br><br>Proposed: aliSize=147 (resi) RMSD=2.25 (Å)<br>akfVIRPATAADCSDILRLIKELAKYEY----meEQViLTEKDLLEDGFgehPfY-----HCLVAEVPKehwtpegHSIVGFAMYYFT---YDPwiGKLLYLeDFFVMSDYRGFGIGSEILKNLSQVAMRCRCSSMHFLVAEWNEPSINFYKRRGASDLS---sEEG-------WRLFKIDKEYLLKmATE<br>---SIRDAGVADLPGILAIYNDAVGNTTaiwn--ETP-VDLANRQAWFD---A-RarqgyPILVASDAA-------GEVLGYASYGDWrpfEGF--RGTVEH-SVYVRDDQRGKGLGVQLLQALIERARAQGLHVMVAAIESGNAASIGLHRRLGFEISGqmp-QVGqkfgrwlDLTFMQLNLDPTR-SAP
613	2ob0A (154)<br>1s3zB (145)	C	11.03	TM-align: aliSize=130 (resi) RMSD=2.35 (Å)<br>skgsrIELGDVTPHNIKQLKRLNQVIFPVSY--N-DKFYKDVLEVG-ELAKLAYFNDIAVGAVCCRVDHSQ-----NQKRLYI-TLGCLAPYRRLGIGTK-LNHVLNICEkDGTFDNIYLHVQISNESAIDFYRKFGFEIIETKKNYykriepadahvlqknl----<br>-----MDIRQMNKTHLEHWRGLRKQLWPGHPddAhLADGEEILQADhLASFIAMADGVAIGFADASIRHDYvngcdSSPVVFLeGIFVLPSFRQRGVAKQlIAAVQRWGT-NKGCREMASDTSPENTISQKVHQALGFEETERVIFY----------------rkrc<br><br>SARST: aliSize=126 (resi) RMSD=3.05 (Å)<br>skgsri-ELGDVTPHNIKQLKRLNQVIFPVSYNDKFYKDV------LEVGelAKLAYFNDIAVGAVCCRVDHS--QN----QKRLYITLGCLAPYRRLGIGTKLNHVLNICEKDGTFDNIYLHVQISNESAIDFYRKFGFEIIETKK------nyykriepadahvlqknl<br>------mDIRQMNKTHLEHWRGLRKQLWPGHPDDAHLADGeeilqaDHLA--SFIAMADGVAIGFADASIRHDyvNGcdssPVVFLEGIFVLPSFRQRGVAKQLIAAVQRWGTNKGCREMASDTSPENTISQKVHQALGFEETERVIfyrkrc-----------------<br><br>BLAST: aliSize=62 (resi) iden=18.62% (27/145) simi=42.76% (62/145)<br>skgsrIELGDVTPHNIKQLKRLNQVIFPVSYNDKFYKD---VLEVGELAK-LAYFNDIAVGAVCCRVDHS-----QNQKRLYIT-LGCLAPYRRLGIGTKLnhvLNICEKDGTFDNIY-LHVQISNESAID--FYRKFGFEIIETKKNYYKR-iepadahvlqknl<br>-----MDIRQMNKTHLEHWRGLRKQLWPGHPDDAHLADgeeILQADHLASfIAMADGVAIGFADASIRHDyvngcDSSPVVFLEgIFVLPSFRQRGVAKQL---IAAVQRWGTNKGCReMASDTSPENTISqkVHQALGFEETERVIFYRKRc------------<br><br>Proposed: aliSize=132 (resi) RMSD=1.87 (Å)<br>skgsrIELGDVTPHNIKQLKRLNQVIFPVSY--N-DKFYKDVLE---VgeLAKLAYFNDIAVGAVCCRVDHSQ------nQKRLYIMTLGCLAPYRRLGIGTKMLNHVLNICEKDgTFDNIYLHVQISNESAIDFYRKFGFEIIETKnyykriepadahvLQKNL--a<br>-----MDIRQMNKTHLEHWRGLRKQLWPGHPddAhLADGEEILQadhL--ASFIAMADGVAIGFADASIRHDYvngcds-SPVVFLEGIFVLPSFRQRGVAKQLIAAVQRWGTNK-GCREMASDTSPENTISQKVHQALGFEETERVI------------FYRKRcc-
614	2ob0A (154)<br>1s5kA (153)	C	10.46	TM-align: aliSize=135 (resi) RMSD=2.66 (Å)<br>---SKGSRIELGDVTPHNIKQLKRLNQVIFPVSY--N-DKFYKDVLEVG-ELAKLAYFNDIAVGAVCCRVDHSQ-----NQKRLYI-TLGCLAPYRRLGIGTK-LNHVLNICEkDGTFDNIYLHVQISNESAIDFYRKFGFEIIETKKNYykriepadahvlqknl----<br>glvPRGSHMDIRQMNKTHLEHWRGLRKQLWPGHPddAhLADGEEILQADhLASFIAMADGVAIGFADASIRHDYvngcdSSPVVFLeGIFVLPSFRQRGVAKQlIAAVQRWGT-NKGCREMASDTSPENTISQKVHQALGFEETERVIFY----------------rkrc<br><br>SARST: aliSize=133 (resi) RMSD=3.45 (Å)<br>s----KGSRIELGDVTPHNIKQLKRLNQVIFPVSYNDKFYKDVL--EVGELAK--LAYFNDIAVGAVCCRVD--HSQNQ----KRLYITLGCLAPYRRLGIGTKLNHVLNICEKDGTFDNIYLHVQISNESAIDFYRKFGFEIIETKK------nyykriepadahvlqknl<br>-glvpRGSHMDIRQMNKTHLEHWRGLRKQLWPGHPDDAHLADGEeiLQADHLAsfIAMADGVAIGFADASIRhdYVNGCdsspVVFLEGIFVLPSFRQRGVAKQLIAAVQRWGTNKGCREMASDTSPENTISQKVHQALGFEETERVIfyrkrc-----------------<br><br>BLAST: aliSize=65 (resi) iden=19.08% (29/153) simi=42.76% (65/153)<br>s----KGSRIELGDVTPHNIKQLKRLNQVIFPVSYNDKFYKD---VLEVGELAK-LAYFNDIAVGAVCCRVDHS-----QNQKRLYIT-LGCLAPYRRLGIGTKLnhvLNICEKDGTFDNIY-LHVQISNESAID--FYRKFGFEIIETKKNYYKR-iepadahvlqknl<br>-glvpRGSHMDIRQMNKTHLEHWRGLRKQLWPGHPDDAHLADgeeILQADHLASfIAMADGVAIGFADASIRHDyvngcDSSPVVFLEgIFVLPSFRQRGVAKQL---IAAVQRWGTNKGCReMASDTSPENTISqkVHQALGFEETERVIFYRKRc------------<br><br>Proposed: aliSize=136 (resi) RMSD=2.00 (Å)<br>s--KG-S-RIELGDVTPHNIKQLKRLNQViFPVSY---N-DKFYKDVLE---VgeLAKLAYFNDIAVGAVCCRVDHSQ------nQKRLYIMTLGCLAPYRRLGIGTKMLNHVLNICEKDgTFDNIYLHVQISNESAIDFYRKFGFEIIETKknyykriepadaHVLQKNL-a<br>-glVPrGsHMDIRQMNKTHLEHWRGLRKQ-LWPGHpddAhLADGEEILQadhL--ASFIAMADGVAIGFADASIRHDYvngcds-SPVVFLEGIFVLPSFRQRGVAKQLIAAVQRWGTNK-GCREMASDTSPENTISQKVHQALGFEETERV-----------IFYRKRCc-

615	2ob0A<br>(154)<br><br>2vbqA<br>(147)	C	11.56	TM-align: aliSize=132 (resi) RMSD=2.52 (Å)<br>skgSRIELGDVTPHNIKQLKRLNQVIFPVSY--N-DKFYKDVLEVG-ELAKLAYFNDIAVGAVCCRVDHSQ-----NQKRLYI-TLGCLAPYRRLGIGTK-LNHVLNICEKDgTFDNIYLHVQISNESAIDFYRKFGFEIIETKKNYykriepadahvlqknl----<br>---SHMDIRQMNKTHLEHWRGLRKQLWPGHPddAhLADGEEILQADhLASFIAMADGVAIGFADASIRHDYvngcdSSPVVFLeGIFVLPSFRQRGVAKQlIAAVQRWGTNK-GCREMASDTSPENTISQKVHQALGFEETERVIFY----------------rkrc<br><br>SARST: aliSize=129 (resi) RMSD=3.53 (Å)<br>skgsr--IELGDVTPHNIKQLKRLNQVIFPVSYN--DKFYKDVLEVGELAKLA--YFNDIAVGAVCCRVD--HSQN----QKRLYITLGCLAPYRRLGIGTKLNHVLNICEKDGTFDNIYLHVQISNESAIDFYRKFGFEIIETKK------nyykriepadahvlqknl<br>-----shMDIRQMNKTHLEHWRGLRKQLWPGHPDdaHLADGEEILQADHLASFiaMADGVAIGFADASIRhdYVNGcdssPVVFLEGIFVLPSFRQRGVAKQLIAAVQRWGTNKGCREMASDTSPENTISQKVHQALGFEETERVIfyrkrc------------------<br><br>BLAST: aliSize=63 (resi) iden=19.05% (28/147) simi=42.86% (63/147)<br>skgSRIELGDVTPHNIKQLKRLNQVIFPVSYNDKFYKD---VLEVGELAK-LAYFNDIAVGAVCCRVDHS-----QNQKRLYIT-LGCLAPYRRLGIGTKLnhvLNICEKDGTFDNIY-LHVQISNESAID--FYRKFGFEIIETKKNYYKR-iepadahvlqknl<br>---SHMDIRQMNKTHLEHWRGLRKQLWPGHPDDAHLADgeeILQADHLASfIAMADGVAIGFADASIRHDyvngcDSSPVVFLEgIFVLPSFRQRGVAKQL---IAAVQRWGTNKGCReMASDTSPENTISqkVHQALGFEETERVIFYRKRc-------------<br><br>Proposed: aliSize=134 (resi) RMSD=2.06 (Å)<br>skgSRIELGDVTPHNIKQLKRLNQVIFPVS---YNDKFYKDVLE---VgeLAKLAYFNDIAVGAVCCRVDHSQ------nQKRLYIMTLGCLAPYRRLGIGTKMLNHVLNICEKDgTFDNIYLHVQISNESAIDFYRKFGFEIIETkknyykriepadaHVLQKNL-a<br>---SHMDIRQMNKTHLEHWRGLRKQLWPGHpddAHLADGEEILQadhL--ASFIAMADGVAIGFADASIRHDYvngcds-SPVVFLEGIFVLPSFRQRGVAKQLIAAVQRWGTNK-GCREMASDTSPENTISQKVHQALGFEETER------------VIFYRKRc-
616	2pa4A<br>(294)<br><br>1i52A<br>(225)	M	15.11	TM-align: aliSize=208 (resi) RMSD=3.05 (Å)<br>--VKTVVVPAAGLGTRFLpAtKtVPKELLPVVDTPGIELIAAEAAELG-ATRLAIITAPNKAGVLAHFERsseleetlmergktdqveiirraADlIKAVPVTQdKPlGLGHAVGLAESVLDDdEDVVAVMLPDDLVLPTGVMERMAQVRAEFGGSVLCAVEVSEADVSkygifeieadtkdsdvkkvkg<br>hlDVCAVVPAAGFGRRMQ-T-E-CPKQYLSIGNQTILEHSVHALLAHPrVKRVVIAISPGDSRFAQLPLA------------------------NH-PQITVVDG-GD-ERADSVLAGLKAAGD-AQWVLVHDAARPCLHQDDLARLLALSETSRTGGILAAPVRDTMKR--------------------<br><br>mvekpa--------IEDAPS--RLAATGRYLLD-RKIFDALRRI---TPGAggeLQLTDAIDLLidEgHPVHIVIHQGKRHDLGNPGGYIPACVDFGLshpvygaqlkdaikqilaeheaa<br>------aepgknaiAHTVDRngLWHALTPQFFPrELLHDCLTRAlneGATI---TDEASALEYC--G-FHPQLVEGRADNIKVTRPEDLALAEFYLTR----------------------<br><br>SARST: aliSize=207 (resi) RMSD=9.59 (Å)<br>vk----TVVVPAAGLGTRFLPAtktVPKELLPVVDTPGIELIAAEAAE-LGATRLAIITApnkagvlahfersseleetlmeRGKTDQVeiIRRAAD-LIKAVPVTQDKPLGLGHAVGLAESVldddEDVVAVMLPDDLVlpTGVMERMAQVRAE----FGGSVLCAVevseadvsKYGIFEIEADTKDS<br>--hldvCAVVPAAGFGRRMQTE---CPKQYLSIGNQTILEHSVHALLAhPRVKRVVIAIS----------------------PGDSRFA--QLPLANhPQITVVDGGDERADSVLAGLKAAGD----AQWVLVHDAARPC--LHQDDLARLLALSetsrTGGILAAPV--------RDTMKRAEPGKNAI<br><br>DVKKVKgmvEKPaiedapsrLAATGRYLLDRKIFDALRR------ITPGAggelqltdAIDLLIDEGHPVHIVIHQGKRHDLGNPGGYIPACVDF--glshpvygaqlkdaikqilaeheaa<br>AHTVDR---NGL--------WHALTPQFFPRELLHDCLTralnegATITD--------EASALEYCGFHPQLVEGRADNIKVTRPEDLALAEFYLtr--------------------------<br><br>BLAST: aliSize=64 (resi) iden=16.44% (37/225) simi=28.44% (64/225)<br>vktv------VVPAAGLGTRFlpATKTvPKELLPVVDTPGIE-LIAAEAAELGATRLAIITAPNKAGvLAHFERSSELEETLMERGktDQveiirRAADLIKAVPVTQDKPLGLGHAVglAESVLDDDeDVVAVMLPDDLVLPTGVME---RMAQVRAEFGGSVL-------------------------<br>----hldvcaVVPAAGFGRRM--QTEC-PKQYLSIGNQTILEhSVHALLAHPRVKRVVIAISPGDSR-FAQLPLANHPQITVVDGG--DE-----RADSVLAGLKAAGDAQWVLVHDA--ARPCLHQD-DLARLLALSETSRTGGILAapvRDTMKRAEPGKNAIahtvdrnglwhaltpqffprellhd<br><br>---cavevseadvskygifeieadtkdsdvkkvkgmvekpaiedapsrlaatgrylldrkifdalrritpgaggelqltdaidllideghpvhivihqgkrhdlgnpggyipacvdfglshpvygaqlkdaikqilaehe<br>cltralnegatitdeasaleycgfhpqlvegradnikvtrpedlalaefyltr---<br><br>aa<br>--<br><br>Proposed: aliSize=216 (resi) RMSD=2.27 (Å)<br>--VKTVVVPAAGLGTRFLpAtKtVPKELLPVVDTPGIELIAAEAAELG-ATRLAIITAPNKAGVLAH-FErsseleetlmergktdqveiirraADlIKAVPVTQdKPlGLGHAVGLAESVLDDdEDVVAVMLPDDLVLPTGVMERMAQVRAEFGGSVLCAVEVseadvsKYGIFEIEADTkdsdvKKVK<br>hlDVCAVVPAAGFGRRMQ-T-E-CPKQYLSIGNQTILEHSVHALLAHPrVKRVVIAISPGDSRFAQLpLA------------------------NH-PQITVVDG-GD-ERADSVLAGLKAAGD-AQWVLVHDAARPCLHQDDLARLLALSETSRTGGILAAPV------RDTMKRAEPGK-----NAIA<br><br>GMVEKPAiedapsRLAATGRYLLD-RKIFDALRRI---TPGaggeLQLT-DAIDLLidEgHPVHIVIHQGKRHDLGNPGGYIPACVDFGLshpvygaqlkdaikqilaeheaa<br>HTVDRNG------LWHALTPQFFPrELLHDCLTRAlneGAT----ITDEaSALEYC--G-FHPQLVEGRADNIKVTRPEDLALAEFYLTR----------------------
617	2pa7A<br>(135)<br><br>1j3qA<br>(187)	N	15.56	TM-align: aliSize=95 (resi) RMSD=3.04 (Å)<br>enkvinfkkiidsrgslvaieenknipf---SIKRVYYIFDTKGE-----EPRGFHAH--KKLEQVLVCLNGSCRVILDD-GNIIQEITLDSPaVGLYVGPAVWHEMHDFS-SDCVMMVLASDYYDetD<br>-----------------------------mkykepfgvkldfetgiienakksvrrlsdmkgyfideeawkkmveegdpvvyevyaieqeekeGDLNFATTVLYPGKvgnefFMTKGHYHskIDRAEVYFALKGKGGMLLQTpEGEARFIEMEPG-TIVYVPPYWAHRTINTGdKPFIFLALYPADAG--H<br><br>YIRQY--DNFKkyiakinle------------------<br>DYGTIaeKGFS---------kivveengkvvvkdnpk<br><br>SARST: aliSize=89 (resi) RMSD=7.13 (Å)<br>enkvinfkkiids---RGSLVAI----EENKNIpfsikRVYYIFDTK--GEEPRG--FHAHK--KLEQVLVCLNGSCRVILD--DGniIQEITLDSPAVGLYVGPAVWHEMHDFSSDCVMMVLA------------------<br>-------------mkykepfgvkldfetgiienakksvrrlsdmkgyfideeawkkmveegdpvVYEVYAIeqeeKEGDLN-----FATTVLYPGkvGNEFFMtkGHYHSkiDRAEVYFALKGKGGMLLQtpEG--EARFIEMEPGTIVYVPPYWAHRTINTGDKPFIFLALypadaghdygtiaekgfs<br><br>-----------------sdyydetdyirqydnfkkyiakinle<br>kivveengkvvvkdnpk--------------------------<br><br>BLAST: aliSize=21 (resi) iden=8.15% (11/135) simi=15.56% (21/135)<br>------------------ENKVINFKKIIDSRGSLVAIEE-NKNIPFSIKRVYYIFDTKGEEPRG--fha<br>mkykepfgvkldfetgiiENAKKSVRRLSDMKGYFIDEEAwKKMVEEGDPVVYEVYAIEQEEKEGdlnfattvlypgkvgneffmtkghyhskidraevyfalkgkggmllqtpegearfiemepgtivyvppywahrtintgdkpfiflalypadaghdygtiaekgfskivveengkvvvkdnpk---<br><br>hkkleqvlvclngscrvilddgniiqeitldspavglyvgpavwhemhdfssdcvmmvlasdyydetdyirqydnfkkyiakinle<br>--<br><br>Proposed: aliSize=113 (resi) RMSD=2.42 (Å)<br>e------NKV---------INFKKII-D-----------------------SRGSLVAIEEN---knipfSIKRVYYIFDTKGE-----EPRGFHAHKK--LEQVLVCLNGSCRVILDDGN-IIQEITLDSPaVGLYVGPAVWHEMHDFS-SDCVMMVLASDY-yDetDYIRQY--D------------<br>-mkykepFGVkldfetgiiENAKKSVrRlsdmkgyfideeawkkmveegdpVVYEVYAIEQEeke-----GDLNFATTVLYPGKvgnefFMTKGHYHSKidRAEVYFALKGKGGMLLQTPEgEARFIEMEPG-TIVYVPPYWAHRTINTGdKPFIFLALYPADa-G--HDYGTIaeKgfskivveengkv<br><br>-------nfkkyiakinle<br>vvkdnpk------------

618	2pa7A (135)<br><br>1j3rB (185)	N	15.56	TM-align: aliSize=96 (resi) RMSD=3.05 (Å)<br>enkvinfkkiidsrgslvaieenknip---FSIKRVYYIFDTKGE-----EPRGFHAHKK--LEQVLVCLNGSCRVILDDGN-IIQEITLDSPaVGLYVGPAVWHEMHDFS-SDCVMMVLASDYYDetDYIR<br>---------------------------ykepfgvkldfetgiienakksvrrlsdmkgyfideeawkkmveegdpvvyevyaieqeekEGDLNFATTVLYPGKvgnefFMTKGHYHSKidRAEVYFALKGKGGMLLQTPEgEARFIEMEPG-TIVYVPPYWAHRTINTGdKPFIFLALYPADAG--HDYG<br><br>QY-DNFKkyiakinle------------------<br>TIaEKGF---------skivveengkvvvkdnpk<br><br>SARST: aliSize=95 (resi) RMSD=7.21 (Å)<br>enkvinfkkiids--RGSLVAIEENKNIPFSIkRVYYIFDTK--GEEP--RGFHA--HKKLEQVLVCLNGSCRVILDDGNIIQEITLDSPAVGLYVGPAVWHEMHDFSSDCVMMVLA---------------------------<br>------------ykepfgvkldfetgiienakksvrrlsdmkgyfideeawkkmveegdpvVYEVYAIEQEEKEGDLN-FATTVLYPGkvGNEFfmTKGHYhsKIDRAEVYFALKGKGGMLLQTPEGEARFIEMEPGTIVYVPPYWAHRTINTGDKPFIFLALypadaghdygtiaekgfskivveeng<br><br>---------sdyydetdyirqydnfkkyiakinle<br>kvvvkdnpk--------------------------<br><br>BLAST: aliSize=21 (resi) iden=8.15% (11/135) simi=15.56% (21/135)<br>----------------ENKVINFKKIIDSRGSLVAIEE-NKNIPFSIKRVYYIFDTKGEEPRG--fhahk<br>ykepfgvkldfetgiiENAKKSVRRLSDMKGYFIDEEAwKKMVEEGDPVVYEVYAIEQEEKEGdlnfattvlypgkvgneffmtkghyhskidraevyfalkgkggmllqtpegearfiemepgtivyvppywahrtintgdkpfiflalypadaghdygtiaekgfskivveengkvvvkdnpk-----<br><br>kleqvlvclngscrvilddgniiqeitldspavglyvgpavwhemhdfssdcvmmvlasdyydetdyirqydnfkkyiakinle<br>---<br><br>Proposed: aliSize=113 (resi) RMSD=2.37 (Å)<br>e----NKV---------INFKKIID------------------------SRGSLVAIEEN---knipfSIKRVYYIFDTKGE-----EPRGFHAHKK--LEQVLVCLNGSCRVILDDGN-IIQEITLDSPaVGLYVGPAVWHEMHDFS-SDCVMMVLASDY-yDetDYIRQY--D--------------<br>-ykepFGVkldfetgiiENAKKSVRrlsdmkgyfideeawkkmveegdpVVYEVYAIEQEeke-----GDLNFATTVLYPGKvgnefFMTKGHYHSKidRAEVYFALKGKGGMLLQTPEgEARFIEMEPG-TIVYVPPYWAHRTINTGdKPFIFLALYPADa-G--HDYGTIaeKgfskivveengkvvv<br><br>-----nfkkyiakinle<br>kdnpk------------
619	2pa7A (135)<br><br>1qxrA (187)	N	14.07	TM-align: aliSize=95 (resi) RMSD=3.01 (Å)<br>enkvinfkkiidsrgslvaieenknip--FSIKRVYYIFDTKGE-----EPRGFHAHKK--LEQVLVCLNGSCRVILDD-GNIIQEITLDSPaVGLYVGPAVWHEMHDFS-SDCVMMVLASDYYDetDYI<br>---------------------------mykepfgvkvdfetgiiegakksvrrlsdmegyfvderawkelvekedpvvyevyaveqeekEGDLNFATTVLYPGKvgkefFFTKGHFHAKldRAEVYVALKGKGGMLLQTpEGDAKWISMEPG-TVVYVPPYWAHRTVNIGdEPFIFLAIYPADAG--HDY<br><br>RQY-DNFkkyiakinle--------------------<br>GTIaEKG----------fskivieengevkvvdnprw<br><br>SARST: aliSize=102 (resi) RMSD=8.78 (Å)<br>enkvinfkkiids---RGSLVAI----EENKNIPFSIKRVYYIFDTKGeEPRGFHAHK--KLEQVLVCLNGSCRVILD--DGNIIQEITldSPAVGLYVGPAVWHEMHDFSSDCVMMVLAsdYYDETDYIR-----------<br>------------mykepfgvkvdfetgiiegakksvrrlsdmegyfvderawkelvekedpvVYEVYAVeqeeKEGDLNFATTVLYPGKVGKEF-FFTKGHFHAklDRAEVYVALKGKGGMLLQtpEGDAKWISM--EPGTVVYVPPYWAHRTVNIGDEPFIFLAI--YPADAGHDYgtiaekgfskiv<br><br>---------------qydnfkkyiakinle<br>ieengevkvvdnprw---------------<br><br>BLAST: aliSize=29 (resi) iden=14.07% (19/135) simi=21.48% (29/135)<br>enkvinfkkiidsrgslvaieenknipfsikrvyyi--FDTKGEeprgFHAHKKLEQVLVCLNGSCRVILD--DGNiIQEITLDsPAVGLYVgPAVW------------<br>------------------------------------mykepfgvkvdfetgiiegakksvrrlsdmegyfvderawkelvekedpvvyevyaveqeekegdlnfattvlypgkvgkefFFTKGH----FHAKLDRAEVYVALKGKGGMLLQtpEGD-AKWISME-PGTVVYV-PPYWahrtvnigdepfi<br><br>--hemhdfssdcvmmvlasdyydetdyirqydnfkkyiakinle<br>flaiypadaghdygtiaekgfskivieengevkvvdnprw---<br><br>Proposed: aliSize=113 (resi) RMSD=2.43 (Å)<br>e-----NKV---------INFKKII-D------------------------SRGSLVAIEEN---knipfSIKRVYYIFDTKGE-----EPRGFHAHKK--LEQVLVCLNGSCRVILDD-GNIIQEITLDSPaVGLYVGPAVWHEMHDFS-SDCVMMVLASDY-yDetDYIRQY--D-------------<br>-mykepFGVkvdfetgiiEGAKKSVrRlsdmegyfvderawkelvekedpVVYEVYAVEQEeke-----GDLNFATTVLYPGKvgkefFFTKGHFHAKldRAEVYVALKGKGGMLLQTpEGDAKWISMEPG-TVVYVPPYWAHRTVNIGdEPFIFLAIYPADa-G--HDYGTIaeKgfskivieengevk<br><br>-------nfkkyiakinle<br>vvdnprw------------
620	2pa7A (135)<br><br>1zvfA (176)	C	10.37	TM-align: aliSize=89 (resi) RMSD=2.84 (Å)<br>enkvinfkkiidsrgslvaieenknipfs---------------------------------IKRVYYIFdTKGeEPRGFHAHKkLEQVLVCLNGSCRVILDDGN----IIQEITLDSPaVGLYVGPAVWHEMHdFSSDCVMMVLAsDYYDe--TDYIRQY-------D--NFkkyiakinle-------<br>-----------------------------amfnttpinidkwlkenegllkppvnnyclhkgGFTVMIVG-GPN-ERTDYHINP-TPEWFYQKKGSMLLKVVDETdaepKFIDIIINEG-DSYLLPGNVPHSPV-RFADTVGIVVE-QDRP-ggENDKIRWycshcrqVvhES----------elqmldl<br><br>--------------------------------<br>gtqvkeaildfendvekrtcfhcktlnyarpq<br><br>SARST: aliSize=88 (resi) RMSD=5.71 (Å)<br>enkvinfkkiidsrgslvaieenkn---------------------------IPFSIKRVYYIFdtKGEEPRGFHAHKKLEQVLVCLNGSCRVILDDGNI---IQEITLDSPAVGLYVGPAVWHEMHDFssdCVMMVLASDYYDETDY--<br>------------------------amfnttpinidkwlkenegllkppvnnyCLHKGGFTVMIV--GGPNERTDYHINPTPEWFYQKKGSMLLKVVDETDaepKFIDIIINEGDSYLLPGNVPHSPVRF---ADTVGIVVEQDRPGGEndkirwycshcrqvvheselqmldlgtqvkeaildfendve<br><br>----------------irqydnfkkyiakinle<br>krtcfhcktlnyarpq-----------------<br><br>BLAST: aliSize=9 (resi) iden=4.44% (6/135) simi=6.67% (9/135)<br>enkvinfkkiidsrgslvaieenknipfsikrvyyifdtkgeeprgfhahkkleqvlvclngscrv---<br>--amfnttpinidkwlkenegllkppvnnyclhkggftvmivggpnertdyhinptpewfyqkkgsmllkvvdetdaepkfidiiinegdsyllpgnvphspvrfadtvgivveqdrpggendkir<br><br>----------------ILDDGNIIQEITLD----------------------spavglyvgpavwhemhdfssdcvmmvlasdyydetdyirqydnfkkyiakinle<br>wycshcrqvvheselqMLDLGTQVKEAILDfendvekrtcfhcktlnyarpq---<br><br>Proposed: aliSize=95 (resi) RMSD=1.88 (Å)<br>enkvinfkkiidsrgslvaieenknipfsi----------------------------------KRVYYIFdTKgeEPR-GFHAHkKLEQVLVCLNGSCRVILDDG----NIIQEITLDSPaVGLYVGPAVWHEMHdFSSDCVMMVLAsDYyD----etDYIRQ-------------------YDNFKK<br>-----------------------------amfnttpinidkwlkenegllkppvnnyclhkggFTVMIVG-GP--NERtDYHIN-PTPEWFYQKKGSMLLKVVDEtdaePKFIDIIINEG-DSYLLPGNVPHSPV-RFADTVGIVVE-QD-Rpgge--NDKIRwycshcrqvvheselqmldlGTQVKE<br><br>YIAKINLE------------------<br>AILDFENDvekrtcfhcktlnyarpq

621	2pa7A (135)<br>2b5hA (186)	N	13.33	TM-align: aliSize=108 (resi) RMSD=3.26 (Å)<br>enkvinf--KKIidsrgSLV--A--IE--E------N-KNIPf-------------------------------S--IKRVYYIFdTKGEEPRGFHAHKKLEQVLVCLNGSCRVILDDGN--------IIQEITLDSPaVGLYVGPA-VWHEMHDFS--SDCVMMVLASDYYDEtdYIRQY---DNFkky<br>-------elLKP-----RTLadLirILheLfagdevNvEEVQ-avleayesnpaewalyakfdqyrytrnlvdqGngKFNLMILC-WGEGHGSSIHDHTDSHCFLKLLQGNLKETLFDWPdkksnemiKKSERTLREN-QCAYINDSiGLHRVENVShtEPAVSLHLYSPPFDT--CHAFDqrtGHK---<br><br>iakinle----------------<br>-------nkvtmtfhskfgirtp<br><br>SARST: aliSize=95 (resi) RMSD=13.94 (Å)<br>enkvinfk--KIIDSRGSLVAIEENKNIPFSIKRVYYIFDTKGEEPRGFHAhkkLEQVLvCLNGSCRVILDDGNIIQEITLdSPAVGLYVGPAVWHEMHDfsSDCVMMVLAS-----------------------------------<br>--------ellkprtladlirilhelfagdevnveevqavleayesnpaewalyAKFDQYRYTRNLVDQGNGKFNLMILCWGEGHGSSIHDHTDS---HCFLK-LLQGNLKETLFDWPDKKSNEM-IKKSERTLRENQCAYIND--SIGLHRVENVshtepavslhlysppfdtchafdqrtghknkvtm<br><br>-----------dyydetdyirqydnfkkyiakinle<br>tfhskfgirtp-------------------------<br><br>BLAST: aliSize=22 (resi) iden=9.63% (13/135) simi=16.30% (22/135)<br>enkvinfkkiidsrgslvaieenknipfsikrvyyifdtkgeeprgfhah---KKLEQVLvcLNGSCRVILDDGNI--I<br>--ellkprtladlirilhelfagdevnveevqavleayesnpaewalyakfdqyrytrnlvdqgngkfnlmilcwgeghgssihdhtdshcflkllqgnlketlfdwpdkksnemiKKSERTL--RENQCAYINDSIGLhrV<br><br>QEITLDSPAVGLYVGPAVWHEMHDF-----------------------ssdcvmmvlasdyydetdyirqydnfkkyiakinle<br>ENVSHTEPAVSLHLYSPPFDTCHAFdqrtghknkvtmtfhskfgirtp------------------------------------<br><br>Proposed: aliSize=116 (resi) RMSD=2.52 (Å)<br>-----EN----------------------------KVI-N--F--KKIIDSRGSLVAIEENknipFS-IKRVYYIFdTKGEEPRGFHAHKKLEQVLVCLNGSCRVILDDG----------niIQEITLDSPaVGLYVGPA-VWHEMHDFS--SDCVMMVLASDYYDEtDYIRQY--DNF-----------<br>ellkpRTladlirilhelfagdevnveevqavleaYESnPaeWalYAKFDQYRYTRNLVDQ----GNgKFNLMILC-WGEGHGSSIHDHTDSHCFLKLLQGNLKETLFDWpdkksnemik--KSERTLREN-QCAYINDSiGLHRVENVShtEPAVSLHLYSPPFDT-CHAFDQrtGHKnkvtmtfhskf<br><br>-----kkyiakinle<br>girtp----------
622	2pa7A (135)<br>2gc1A (188)	N	13.33	TM-align: aliSize=95 (resi) RMSD=3.06 (Å)<br>enkvinfkkiidsrgslvaieenknip---FSIKRVYYIFDTKGE-----EPRGFHAHKK--LEQVLVCLNGSCRVILDDGN-IIQEITLDSPaVGLYVGPAVWHEMHDFS-SDCVMMVLASDYYDetDY<br>---------------------------mmykepfgvkvdfetgiiegakksvrrlsdmegyfvderawkelvekedpvvyevyaveqeekEGDLNFATTVLYPGKvgkefFFTKGHFHAKldRAEVYVALKGKGGMLLQTPEgDAKWISMEPG-TVVYVPPYWAHRTVNIGdEPFIFLAIYPADAG--HD<br><br>IRQYDNFkkyiakinle--------------------<br>YGTIAEK---------gfskivieengevkvvdnprw<br><br>SARST: aliSize=98 (resi) RMSD=8.54 (Å)<br>enkvinfkkiids--RGSLVAIEENKNIPFSIKRVYYIFDTK-GEEP-RGFHAH---KKLEQVLVCLNGSCRVILD--DGniIQEITLDSPAVGLYVGPAVWHEMHDFSSDCVMMVLAS----------------------<br>-------------mmykepfgvkvdfetgiiegakksvrrlsdmegyfvderawkelvekedpvVYEVYAVEQEEKEGDLNFATTVLYPGKvGKEFfFTKGHFhakLDRAEVYVALKGKGGMLLQtpEG--DAKWISMEPGTVVYVPPYWAHRTVNIGDEPFIFLAIYpadaghdygtiaekgfskivie<br><br>-------DYY---detdyirqydnfkkyiakinle<br>engevkvVDNprw----------------------<br><br>BLAST: aliSize=29 (resi) iden=14.07% (19/135) simi=21.48% (29/135)<br>enkvinfkkiidsrgslvaieenknipfsikrvyyi---FDTKGEeprgFHAHKKLEQVLVCLNGSCRVILD--DGNiIQEITLDsPAVGLYVgPAVW------------<br>------------------------------------mmykepfgvkvdfetgiiegakksvrrlsdmegyfvderawkelvekedpvvyevyaveqeekegdlnfattvlypgkvgkefFFTKGH----FHAKLDRAEVYVALKGKGGMLLQtpEGD-AKWISME-PGTVVYV-PPYWahrtvnigdepf<br><br>---hemhdfssdcvmmvlasdyydetdyirqydnfkkyiakinle<br>iflaiypadaghdygtiaekgfskivieengevkvvdnprw--<br><br>Proposed: aliSize=113 (resi) RMSD=2.49 (Å)<br>e-----NKVI----------NFKKIID------------------------SRGSLVAIEEN-knipfS--IKRVYYIFDTKGE-----EPRGFHAHKK--LEQVLVCLNGSCRVILDDGN-IIQEITLDSPaVGLYVGPAVWHEMHDFS-SDCVMMVLASDY-yDetDYIRQY--D------------<br>-mmykePFGVkvdfetgiieGAKKSVRrlsdmegyfvderawkelvekedpVVYEVYAVEQEe-----KegDLNFATTVLYPGKvgkefFFTKGHFHAKldRAEVYVALKGKGGMLLQTPEgDAKWISMEPG-TVVYVPPYWAHRTVNIGdEPFIFLAIYPADa-G--HDYGTIaeKgfskivieengev<br><br>--------nfkkyiakinle<br>kvvdnprw------------
623	2pc1A (173)<br>1s3zB (145)	C	15.86	TM-align: aliSize=133 (resi) RMSD=2.67 (Å)<br>nlyfqGQIRLAFPNEIDQI-LLIEEARaeiaktgsdqWQkeDGY-PNRNDIIDDILN--GYAWVGIEDG-LATYAAVIDGHEEvYdaIyegkWlHDNHRYLTFHRIAISNQFRGRGLAQTFLQGLIEGHK---GPDFRCDTHEKNVT-QHILNKLGYQYCGKVPLDGvrlayqkikek---<br>-----MDIRQMNKTHLEHWrGLRKQLW----------PG--HPDdAHLADGEEILQAdhLASFIAMADGvAIGFADASIRHDY-V--N----G-CDSSPVVFLEGIFVLPSFRQRGVAKQLIAAVQRWGTnkgCREMASDTSPENTIsQKVHQALGFEETERVIFYR-----------krc<br><br>SARST: aliSize=129 (resi) RMSD=6.86 (Å)<br>nlyfqgq--IRLAFPNEIDQILLIEEARaeiaktgsdqwqKEDGYPNRNDIIDDILN--GYAWVGIE---DGLATYAAVIDGheevydAI----YEGKWlhdnhrYLTFHRIAISNQFR-GRGLAQTFLQG--LIEGHKGPDFRCDTHEKNVTQHILNK-LGYQYCGkvpldgVRLAYQKI-kek<br>-------mdIRQMNKTHLEHWRGLRKQL------------WPGHPDDAHLADGEEILqaDHLASFIAmadGVAIGFADASIR------HDyvngCDSSP------VVFLEGIFVLPSFRqRGVAKQLIAAVqrWGTNKGCREMASDTSPENTISQKVHQaLGFEETE------RVIFYRKRc---<br><br>BLAST: aliSize=66 (resi) iden=23.45% (34/145) simi=45.52% (66/145)<br>nlyfqgqirlafpneidqillieeara----EIAKTGSDQWQ---KE--DGYPNRNDIID--DILNGyawvgieDGLATYAAVIDGHEEVY-DAIYEGKWLH--DNHRYLTFHRIAISNQFRGRGLAQTFLQGLIE-G-HKG-PDFRCDTHEKN-VTQHILNKLGYQYCGKV-------pldgvrlayqk<br>---------------------------mdirQMNKTHLEHWRglrKQlwPGHPDDAHLADgeEILQA-------DHLASFIAMADGVAIGFaDASIRHDYVNgcDSSPVVFLEGIFVLPSFRQRGVAKQLIAAVQRwGtNKGcREMASDTSPENtISQKVHQALGFEETERVifyrkrc----------<br><br>ikek<br>----<br><br>Proposed: aliSize=137 (resi) RMSD=2.23 (Å)<br>nlyfqgMQIRLAFPNEIDQIMLLIEEARaeiaktgsdqWQkeDG--yPNRNDIIDDILN--GYAWVGIEDGMLATYAAVIDGHEEvYdaIyegkWlHDNHRYLTFHRIAISNQFRGRGLAQTFLQGLIeGH-K---GPDFRCDTHEKNVTMQHILNKLGYQYCGKvpldgvRLAYQKIK-ek<br>------MDIRQMNKTHLEHWRGLRKQLW----------PG--HPdd-AHLADGEEILQAdhLASFIAMADGVAIGFADASIRHDY-V--N----G-CDSSPVVFLEGIFVLPSFRQRGVAKQLIAAVQ-RWgTnkgCREMASDTSPENTISQKVHQALGFEETER------VIFYRKRCc--

624	2pc1A (173)<br>2d4pA (130)	C	8.46	TM-align: aliSize=115 (resi) RMSD=2.66 (Å)<br>nlyfqGQIRLAFPNEIDQI-LLIEeaRaeiaktgsdqwqkedgyPNRNdiIDDILN--GY--AWVGIEDG-LATYAAVIDGHEevydaiyegkwlhDNHRYLTFHRIAISnqfRgRGLAQTFLQGLIEGHK---GPDFRCDTHEKNVT-QHILNKLGYQY--CGKVPLDGvrlayqkikek---<br>-----MRFRPFTEEDLDRLnRLAG--K-----------------RPVS--LGALRFfaRTghSFLAEEGEePMGFALAQAVWQ-------------GEATTVLVTRIEGR---S-VEALRGLLRAVVKSAYdagVYEVALHLDPERKElEEALKAEGFALgpLVLAVRVL-----------gsr<br><br>SARST: aliSize=113 (resi) RMSD=6.00 (Å)<br>nlyfqgq--IRLAFPNEIDQillieearaeIAKTGSDQwqkedgYPNRNDIIDDILNGYAWVGIEDGLATYAAVIDGHEEvydaiyEGKwlhdnhryLTFHRIAISNQFRgrGLAQTFLQGLIEGHK---GPDFRCDTHEKNVTQHILNKLGYQYCG------------kvpldgvrlayqkikek<br>-------mrFRPFTEEDLDR-----------LNRLAGKR------PVSLGALRFFARTGHSFLAEEGEEPMGFALAQAVWQ------GEA---------TTVLVTRIEGRSV--EALRGLLRAVVKSAYdagVYEVALHLDPERKELEEALKAEGFALGplvlavrvlgsr-----------------<br><br>BLAST: aliSize=6 (resi) iden=4.62% (6/130) simi=4.62% (6/130)<br>nlyfqgqirlafpneidqillieearaeiaktgsdqwqkedgypnrndiiddilngyawvgiedglatyaavidgheevydaiyegkwlhdnhryltfhriaisnqfr--GRGLAQTFLQG------------------------<br>---mrfrpfteedldrlnrlagkrpvslgalrffartghsflaeegeepmGFALAQAVWQGeattvlvtriegrsvealrgllra<br>---lieghkgpdfrcdtheknvtqhilnklgyqycgkvpldgvrlayqkikek<br>vvksaydagvyevalhldperkeleealkaegfalgplvlavrvlgsr---<br><br>Proposed: aliSize=119 (resi) RMSD=2.11 (Å)<br>nlyfqgMQIRLAFPNEIDQIMLLIEeaRaeiaktgsdqwqkedgyPNRNdiIDDILN--GY--AWVGIEDGMLATYAAVIDGHeevydaiyegkwlhdNH--RYLTFHRIAISnqfRgRGLAQTFLQGLIEGHK---GPDFRCDTHEKNVTMQHILNKLGYQY--CGKVPldgvRLAYQKikek<br>------MRFRPFTEEDLDRLNRLAG--K-----------------RPVS--LGALRFfaRTghSFLAEEGEEPMGFALAQAVW---------------QGeaTTVLVTRIEGR---S-VEALRGLLRAVVKSAYdagVYEVALHLDPERKELEEALKAEGFALgpLVLAV----RVLGSR----
625	2pc1A (173)<br>2vbqA (147)	C	14.97	TM-align: aliSize=134 (resi) RMSD=2.89 (Å)<br>nlyf-QGQIRLAFPNEIDQI-LLIEEARAeiaktgsdqwQkeDGY-PNRNDIIDDILN--GYAWVGIEDG-LATYAAVIDGHEEvYdaIyegkWlHDNHRYLTFHRIAISNQFRGRGLAQTFLQGLIEGHK---GPDFRCDTHEKNVT-QHILNKLGYQYCGKVPLDGvrlayqkikek---<br>----sHMDIRQMNKTHLEHWrGLRKQLWP----------G--HPDdAHLADGEEILQAdhLASFIAMADGvAIGFADASIRHDY-V--N----G-CDSSPVVFLEGIFVLPSFRQRGVAKQLIAAVQRWGTnkgCREMASDTSPENTIsQKVHQALGFEETERVIFYR-----------krc<br><br>SARST: aliSize=129 (resi) RMSD=7.07 (Å)<br>nlyfqgq----IRLAFPNEIDQILLIEEARaeiaktgsDQWQKEDGypnRNDIIDDILNGYAWVGIE----DGLATYAAVIDGheevydAIY----EGKWlhdnhrYLTFHRIAIS-NQFRGRGLAQTFLQGLIEGhKGP---DFRCDTHEKNVTQHILNK-LGYQYCGkvpldgVRLAYQKI-kek<br>-------shmdIRQMNKTHLEHWRGLRKQL--------WPGHPDDA---HLADGEEILQADHLASFIamadGVAIGFADASIR------HDYvngcDSSP------VVFLEGIFVLpSFRQRGVAKQLIAAVQRWG-TNKgcrEMASDTSPENTISQKVHQaLGFEETE------RVIFYRKRc---<br><br>BLAST: aliSize=66 (resi) iden=23.13% (34/147) simi=44.90% (66/147)<br>nlyfqgqirlafpneidqillieeara------EIAKTGSDQWQ---KE--DGYPNRNDIID--DILNGyawvgieDGLATYAAVIDGHEEVY-DAIYEGKWLH--DNHRYLTFHRIAISNQFRGRGLAQTFLQGLIE-G-HKG-PDFRCDTHEKN-VTQHILNKLGYQYCGKV-------pldgvrlay<br>---------------------------shmdirQMNKTHLEHWRglrKQlwPGHPDDAHLADgeEILQA-------DHLASFIAMADGVAIGFaDASIRHDYVNgcDSSPVVFLEGIFVLPSFRQRGVAKQLIAAVQRwGtNKGcREMASDTSPENtISQKVHQALGFEETERVifyrkrc---------<br>qkikek<br>------<br><br>Proposed: aliSize=137 (resi) RMSD=2.39 (Å)<br>nlyfq-GMQIRLAFPNEIDQIMLLIEEARAeiaktgsdqwQkeDG--YpNRNDIIDDILNG--YAWVGIEDGMLATYAAVIDGHEEvYdaIyegkWlHDNHRYLTFHRIAISNQFRGRGLAQTFLQGLIeGH-K---GPDFRCDTHEKNVTMQHILNKLGYQYCGKV-PldgvrLAYQKikek<br>-----sHMDIRQMNKTHLEHWRGLRKQLWP----------G--HPddA-HLADGEEILQADhlASFIAMADGVAIGFADASIRHDY-V--N----G-CDSSPVVFLEGIFVLPSFRQRGVAKQLIAAVQ-RWgTnkgCREMASDTSPENTISQKVHQALGFEETERViF-----YRKRC----
626	2pd7A (149)<br>2gj3B (119)	N	23.53	TM-align: aliSize=105 (resi) RMSD=2.02 (Å)<br>mhtlyapggy----DIMGyliqimnrpnpqvelgpv-DT--S-CALILCDLkqkDTPIVYASEAFLYMTGYSNAEVLGRNCRFLQSpdgmvkpkstRkYVDSNTINTMRKAIDRNAEVQVEVVNFKKNGQRFVNFLTMIPVRDetG--EYRYSMGFQCE----<br>----------ellpEIFR-------------------qTVehApIAISITDL---KANILYANRAFRTITGYGSEEVLGKNESILSN----------G-TTPRLVYQALWGRLAQKKPWSGVLVNRRKDKTLYLAELTVAPVLN--EagETIYYLGMHRDtsel<br><br>SARST: aliSize=109 (resi) RMSD=4.90 (Å)<br>mhtlyapggydim-----GYLIQIMNRPnpqvelgpvdtsCALILCDLKqkdTPIVYASEAFLYMTGYSNAEVLGRNCRFLqspdgmvkpksTRKYVDSNTINTMRKAIDRNAEVQVEVVNFKKNGQRFVNFLTMIPVRDETGEYRYSMGFQC-----e<br>-------------ellpeIFRQTVEHAP-------------IAISITDLK---ANILYANRAFRTITGYGSEEVLGKNESIL------------SNGTTPRLVYQALWGRLAQKKPWSGVLVNRRKDKTLYLAELTVAPVLNEAGETIYYLGMHRdtsel-<br><br>BLAST: aliSize=58 (resi) iden=30.25% (36/119) simi=48.74% (58/119)<br>mhtlyapggydimgyliqimnrpnpq----------VELGPVdtscALILCDLKQKdtpIVYASEAFLYMTGYSNAEVLGRNCRFLQSpdGmvkpkSTRKYVdsntINTMRKAIDRNAEVQVEVVNFKKNGQRFVNFLTMIPVRDETGEYRYSMG--------fqce<br>-------------------------ellpeifrqtVEHAPI----AISITDLKAN---ILYANRAFRTITGYGSEEVLGKNESILSN--G-----TTPRLV----YQALWGRLAQKKPWSGVLVNRRKDKTLYLAELTVAPVLNEAGETIYYLGmhrdtsel----<br><br>Proposed: aliSize=114 (resi) RMSD=1.52 (Å)<br>mhtlyapgGYDIMGYLIQIMNRPnpqvelgpvdtsCALILCDLkqkDTPIVYASEAFLYMTGYSNAEVLGRNCRFLQSpdgmvkpkstRkYVDSNTINTMRKAIDRNAEVQVEVVNFKKNGQRFVNFLTMIPVRDeTG-EYRYSMGFQCE----<br>--------ELLPEIFRQTVEHAP------------IAISITDL---KANILYANRAFRTITGYGSEEVLGKNESILSN----------G-TTPRLVYQALWGRLAQKKPWSGVLVNRRKDKTLYLAELTVAPVLN-EAgETIYYLGMHRDtsel

627	2pd7A (149)<br>2z6cA (121)	N	30.58	TM-align: aliSize=104 (resi) RMSD=1.77 (Å)<br>mhtlyapggydimgyliqimnrpnpqvelgpvdt-----------SCALILCDLKQKDTPIVYASEAFLYMTGYSNAEVLGRNCRFLQSpdgmvkpkstrKYVDSNTINTMRKAIDRNAEVQVEVVNFKKNGQRFVNFLTMIPVRDETGEYRYSMGFQCE-----<br>----------------------------------vsedlkdalstfQQTFVVSDATKPDYPIMYASAGFFNMTGYTSKEVVGRNCRFLQG-----------SGTDADELAKIRETLAAGNNYCGRILNYKKDGTSFWNLLTIAPIKDESGKVLKFIGMQVEvskht<br><br>SARST: aliSize=113 (resi) RMSD=9.23 (Å)<br>mhtlyapggydimg--YLIQIMNRpnpqvELGPVDtscalILCDLKQKDTPIVYASEAFLYMTGYSNAEVLGRNCRFLQSPDgmvkpkstrkyVDSNTINTMRKAIDRNAEVQVEVVNFKKNGQRFVNFLTMIPVRDETGEYRYSMGFQC------e<br>--------------vsEDLKDALS-----TFQQTF-----VVSDATKPDYPIMYASAGFFNMTGYTSKEVVGRNCRFLQGSG-----------TDADELAKIRETLAAGNNYCGRILNYKKDGTSFWNLLTIAPIKDESGKVLKFIGMQVevskht-<br><br>BLAST: aliSize=63 (resi) iden=33.06% (40/121) simi=52.07% (63/121)<br>mhtlyapggydimgyliqimnrpnpqvelgpvdtscal---------------ILCDLKQKDTPIVYASEAFLYMTGYSNAEVLGRNCRFLQSpDGmvkpkstrkyVDSNTINTMRKAIDRNAEVQVEVVNFKKNGQRFVNFLTMIPVRDETGEYRYSMGFQCE-----<br>--------------------------------------vsedlkdalstfqqtfVVSDATKPDYPIMYASAGFFNMTGYTSKEVVGRNCRFLQG-SG----------TDADELAKIRETLAAGNNYCGRILNYKKDGTSFWNLLTIAPIKDESGKVLKFIGMQVEvskht<br><br>Proposed: aliSize=115 (resi) RMSD=1.57 (Å)<br>mhtlyapggYdIMGYLIQIMnRpnPqVElgpvdtscALILCDLKQKDTPIVYASEAFLYMTGYSNAEVLGRNCRFLQSpdgmvkpkstrKYVDSNTINTMRKAIDRNAEVQVEVVNFKKNGQRFVNFLTMIPVRDeTG-EYRYSMGFQCE-----<br>---------V-SEDLKDALS-T--F-QQ--------TFVVSDATKPDYPIMYASAGFFNMTGYTSKEVVGRNCRFLQG-----------SGTDADELAKIRETLAAGNNYCGRILNYKKDGTSFWNLLTIAPIKD-ESgKVLKFIGMQVEvskht
628	2pieA (132)<br>1j4kA (158)	C	15.15	TM-align: aliSize=109 (resi) RMSD=2.71 (Å)<br>gaHMagGRSWCLRRVGM--SAGWLLLEDGC-EVTVGRGFGVTYQLVSkicpLMISRNHCVLKQNPE---------------GQWTIMDNkSLNGVWLNRARLEPLRVYSIHQGDYIQLGVPLENKeNAeYEYEVTEEDwetiypclspkn-------------------------------<br>--GN--GRFLTLKPLPDsiIQESLEIQQGVnPFFIGRSEDCNCKIED----NRLSRVHCFIFKKRHavgksmyespaqglDDIWYCHT-GTNVSYLNNNRMIQGTKFLLQDGDEIKIIWDKNNK-FV-IGFKVEIND------------ttglfneglgmlqeqrvvlkqtaeekdlvkkl<br><br>SARST: aliSize=108 (resi) RMSD=13.81 (Å)<br>gahmaggr-----SWCLRRVGMSAGWLLLEDG--CEVTVGRGFGVTYQLVSKIcplmISRNHCVLKQNPEGQWTIMDNKSLNGVWLNRARLEP--LRVYSIHQGDYIQLGVPLENKENAEYEY----EVTEED------------------------------------wetiypclspkn<br>--------gngrfLTLKPLPDSIIQESLEIQQgvNPFFIGRSEDCNCKIEDNR----LSRVHCFIFKKRHAVGKSMYESPAQGLDDIWYCHTGtnVSYLNNNRMIQGTKFLLQDGDEIKIIWDknnkFVIGFKveindttglfneglgmlqeqrvvlkqtaeekdlvkkl------------<br><br>BLAST: aliSize=50 (resi) iden=17.42% (23/132) simi=37.88% (50/132)<br>gahmaggrswclrrvgmsagwl---------------LLEDGCEVTVG-RGF--GVTYQLVSKICPLMISRNHCVL--KQNPEGQwTIMDN--KSLNGVW----------LNRARLEPLRVYSIHQGDYIQLGVPLENKENAEYEYEVTE---------------------------------edwetiypc<br>---------------------gngrfltlkplpdsIIQESLEIQQGvNPFfiGRSEDCNCKIEDNRLSRVHCFIfkKRHAVGK-SMYESpaQGLDDIWychtgtnvsyLNNNRMIQGTKFLLQDGDEIKIIWDKNNKFVIGFKVEINDttglfneglgmlqeqrvvlkqtaeekdlvkkl---------<br><br>lspkn<br>-----<br><br>Proposed: aliSize=112 (resi) RMSD=2.01 (Å)<br>gahMagG--rSWCLRRVG----MsaGWLLLEDGC-EVTVGRGFGVTYQLVSkicPlMISRNHCVLKQNPE--------------GQWTIMDNkSLNGVWLNRARLEPLRVYSIHQGDYIQLGVPLENKenAEYEYEVTE-------------------------eDWETIYPCLspkn<br>---G--Ngr-FLTLKPLPdsiiQ--ESLEIQQGVnPFFIGRSEDCNCKIED---N-RLSRVHCFIFKKRHavgksmyespaqglDDIWYCHT-GTNVSYLNNNRMIQGTKFLLQDGDEIKIIWDKNNK--FVIGFKVEIndttglfneglgmlqeqrvvlkqta-EEKDLVKKL----
629	2prlA (152)<br>1qsmA (150)	C	9.33	TM-align: aliSize=123 (resi) RMSD=2.90 (Å)<br>M-TKVERLLIN-YKTLEEFKKFKEY----GIQELSMLEELqdniiEnd-ST-----SPFYGIYF--GDKLVARMSLYQVNGKsnpyfdNR-QDYLELWKLEVLPGYQNRGYGRALVEFAKS-FK----MPIRTNPR-MK--SAEFWNKMNFKTVKYdmarDKGedpliwhpdmdr----<br>DnITVRFVTENdKEGWQRLWKSYQDfyevSFPDDLDDFNF-----G--rFLdpnikMWAAVAVEssSEKIIGMINFFNHMTT------WDfKDKIYINDLYVDENSRVKGAGGKLIQFVYDeADklgtPSVYWCTDeSNhrAQLLYVKVGYKAPKI----LYK------------rkgy<br><br>SARST: aliSize=122 (resi) RMSD=8.84 (Å)<br>mtkverllin-------------------YKTLEEFKKFkEYGIQELSMLEELQDNIiENDSTSpfyGIYFG---DKLVARMSLYQVNGKSNpyfdnRQDYLELWKLEVLPGYQNRG----YGRALVEFAKSFKM-PIRTNPRMKSAEFWNKMNFKTVKYDmardkgeDPLIWHPD-mdr<br>----------dnitvrfvtendkegwqrlWKSYQDFYEV-SFPDDLDDFNFGRFLDP-NIKMWA---AVAVEsssEKIIGMINFFNHMTTWD-----FKDKIYINDLYVDENSRVKGaggkLIQFVYDEADKLGTpSVYWCTDESNHRAQLLYVKVGYKAP-------KILYKRKGy---<br><br>BLAST: aliSize=9 (resi) iden=4.00% (6/150) simi=6.00% (9/150)<br>mtkverllinyktleefkkfkeygiqelsmleelqdniiendstspfygiyfgdklvarmslyqvngksnpyfdnrqdylelwklevlpgyqnrgygralvefaksfkmpirtnprmksaefwnkmnfk---TVKYDMARDKGEDPLIWHPDMD--<br>---dniTVRFVTENDKEGWQRLWKSYQDfyevsfpddlddfnfgrfldpnikmwaavavessse<br><br>---r<br>kiigminffnhmttwdfkdkiyindlyvdensrvkgaggkliqfvydeadklgtpsvywctdesnhraqllyvkvgykapkilykrkgy-<br><br>Proposed: aliSize=119 (resi) RMSD=2.24 (Å)<br>M-TKVERLLINykTLEE-FKKF-KEY------GIqELSMLeE----lqdniiendsTS------PFYGIYF--GDKLVARMSLYQVNGKsnpyfdNR-QDYLELWKLEVLPGYQNRGYGRALVEFAKS-FK----MPIRTNPR-MK--SAEFWNKMNFKTVKYdmardkgedplIWHPDM--drc<br>DnITVRFVTEN--DKEGwQRLWkSYQdfyevsFP-DDLDD-Fnfgr----------FLdpnikmWAAVAVEssSEKIIGMINFFNHMTT------WDfKDKIYINDLYVDENSRVKGAGGKLIQFVYDeADklgtPSVYWCTDeSNhrAQLLYVKVGYKAPKI-----------LYKRKGya---

630	2pr1A (152)<br>1qsmB (149)	C	10.07	TM-align: aliSize=123 (resi) RMSD=2.91 (Å)<br>MTKVERLLIN-YKTLEEFKKFKEY----GIQELSMLEELqdniiEnd-STS-----PFYGIYF--GDKLVARMSLYQVNGKsnpyfdNR-QDYLELWKLEVLPGYQNRGYGRALVEFAKS-FK----MPIRTNPR-MK--SAEFWNKMNFKTVKYdmarDKGedpliwhpdmdr----<br>NITVRFVTENdKEGWQRLWKSYQDfyevSFPDDLDDFNF-----G--rFLDpnikmWAAVAVEssSEKIIGMINFFNHMTT------WDfKDKIYINDLYVDENSRVKGAGGKLIQFVYDeADklgtPSVYWCTDeSNhrAQLLYVKVGYKAPKI----LYK------------rkgy<br><br>SARST: aliSize=103 (resi) RMSD=6.44 (Å)<br>mtkverllin-----------------YKTLEEFKKFKEYGIQELSMLEELQDNIIendstSPFYGIYF----GDKLVARMSLYQVNGKSNpyfdnRQDYLELWKLEVLPGYQNRGYGR----ALVEFAKSFKM-PIRTNPRMKSaefWNKM---------------------nfktvkydmardkgedpl<br>----------nitvrfvtendkegwqrLWKSYQDFYEVSFPDDLDDFNFGRFLDPN-----IKMWAAVAvessSEKIIGMINFFNHMTTWD-----FKDKIYINDLYVDENSRVKGAGGkliqFVYDEADKLGTpSVYWCTDESN---HRAQllyvkvgykapkilykrkgy------------------<br>iwhpdmdr<br>--------<br><br>BLAST: aliSize=9 (resi) iden=4.03% (6/149) simi=6.04% (9/149)<br>mtkverllinyktleefkkfkeygiqelsmleelqdniiendstspfygiyfgdklvarmslyqvngksnpyfdnrqdylelwklevlpgyqnrgygralvefaksfkmpirtnprmksaefwnkmnfk--TVKYDMARDKGEDPLIWHPDMD--------------------------------------<br>---niTVRFVTENDKEGWQRLWKSYQDfyevsfpddlddfnfgrfldpnikmwaavavesssek<br>---r<br>iigminffnhmttwdfkdkiyindlyvdensrvkgaggkliqfvydeadklgtpsvywctdesnhraqllyvkvgykapkilykrkgy-<br><br>Proposed: aliSize=122 (resi) RMSD=2.41 (Å)<br>MTKVERLLIN-YKTLEEFKKFKeY------GIqELSMLE--E-lqdniiendsTS------PFYGIYF--GDKLVARMSLYQVNGKsnpyfdNR-QDYLELWKLEVLPGYQNRGYGRALVEFAKS-FK----MPIRTNPRM-K--SAEFWNKMNFKTVKydmardkgedPLIWHPDM-drc<br>NITVRFVTENdKEGWQRLWKSY-QdfyevsFP-DDLDDFnfGr----------FLdpnikmWAAVAVEssSEKIIGMINFFNHMTT------WDfKDKIYINDLYVDENSRVKGAGGKLIQFVYDeADklgtPSVYWCTDEsNhrAQLLYVKVGYKAPK----------ILYKRKGYa---
631	2pr1A (152)<br>1qsmD (152)	C	9.21	TM-align: aliSize=123 (resi) RMSD=2.92 (Å)<br>---MTKVERLLIN-YKTLEEFKKFKEY----GIQELSMLEELqdniiEnd-ST-----SPFYGIYF--GDKLVARMSLYQVNGKsnpyfdNR-QDYLELWKLEVLPGYQNRGYGRALVEFAKS-FK----MPIRTNPR--MK-SAEFWNKMnFKTVKYDmarDKGedpliwhpdmdr----<br>sedNITVRFVTENdKEGWQRLWKSYQDfyevSFPDDLDDFNF-----G--rFLdpnikMWAAVAVEssSEKIIGMINFFNHMTT------WDfKDKIYINDLYVDENSRVKGAGGKLIQFVYDeADklgtPSVYWCTDesNHrAQLLYVKV-GYKAPKI---LYK------------rkgy<br><br>SARST: aliSize=121 (resi) RMSD=8.76 (Å)<br>mtkverllin--------------------YKTLEEFKKFkEYGIQELSMLEELQDNIIendstSPFYGIYF----GDKLVARMSLYQVNGKSNPyfdnrQDYLELWKLEVLPGYQNRG----YGRALVEFAKSFKM-PIRTNPRMKSAEFWNKMNFKTVKYDmardkgeDPLIWHPD-mdr<br>----------sednitvrfvtendkegwqrlWKSYQDFYEV-SFPDDLDDFNFGRFLDPN-----IKMWAAVAvessSEKIIGMINFFNHMTTWDF-----KDKIYINDLYVDENSRVKGaggkLIQFVYDEADKLGTpSVYWCTDESNHRAQLLYVKVGYKAP-------KILYKRKGy---<br><br>BLAST: aliSize=9 (resi) iden=3.95% (6/152) simi=5.92% (9/152)<br>mtkverllinyktleefkkfkeygiqelsmleelqdniiendstspfygiyfgdklvarmslyqvngksnpyfdnrqdylelwklevlpgyqnrgygralvefaksfkmpirtnprmksaefwnkmnfk-----TVKYDMARDKGEDPLIWHPDMD----------------------------------<br>--sedniTVRFVTENDKEGWQRLWKSYQDfyevsfpddlddfnfgrfldpnikmwaavavess<br>---r<br>sekiigminffnhmttwdfkdkiyindlyvdensrvkgaggkliqfvydeadklgtpsvywctdesnhraqllyvkvgykapkilykrkgy-<br><br>Proposed: aliSize=119 (resi) RMSD=2.18 (Å)<br>---MTKVERLLINykTLEEFKKF--KEY------GI--QELS-MleE-lqdniiendsTS------PFYGIYF--GDKLVARMSLYQVNGKsnpyfdNR-QDYLELWKLEVLPGYQNRGYGRALVEFAKS-FK----MPIRTNPR-MK--SAEFWNKMNFKTVKYdmardkgedPLIWHP--dmdrc<br>sedNITVRFVTEN--DKEGWQRLwkSYQdfyevsFPddLDDFnF--Gr----------FLdpnikmWAAVAVEssSEKIIGMINFFNHMTT------WDfKDKIYINDLYVDENSRVKGAGGKLIQFVYDeADklgtPSVYWCTDeSNhrAQLLYVKVGYKAPKI---------LYKRKGya-----
632	2pr1A (152)<br>1s3zB (145)	C	8.97	TM-align: aliSize=126 (resi) RMSD=2.94 (Å)<br>mTKVERLLIN-YKTLEEFKKFKEY-GIqELSMLEELqdniiEnd-ST----SPFYGIYFGDKLVARMSLYQVNGkSNPYFDnRQDYLELWKLEVLPGYQNRGYGRALVEFAKS-FK---M-PIRTNPR--MKS-AEFWNKMNFKTVKYdmarDKGEdpliwhpdmdr----<br>-MDIRQMNKThLEHWRGLRKQLWPgHP-DDAHLADG-----E--eILqadhLASFIAMADGVAIGFADASIRHD-YVNGCD-SSPVVFLEGIFVLPSFRQRGVAKQLIAAVQRwGTnkgCrEMASDTSpeNTIsQKVHQALGFEETER----VIFY-----------rkrc<br><br>SARST: aliSize=123 (resi) RMSD=7.28 (Å)<br>mtkverllin-----------YKTLEEFKKFKEYGIQELSMLEELqdnIIENDSTSpFYGIYFGDKLVARMSLYQVngkSNPYFDNRQDYLELWKLEVLP-GYQNRGYGRALVEFAK----SFKM-PIRTNPRMKSAEFWNK---MNFKTVKydmardkgEDPLIWHP-dmdr<br>----------mdirqmnkthleHWRGLRKQLWPGHPDDAHLADGEE---ILQADHLA-SFIAMADGVAIGFADASIR---HDYVNGCDSSPVVFLEGIFVLpSFRQRGVAKQLIAAVQrwgtNKGCrEMASDTSPENTISQKVhqaLGFEETE--------RVIFYRKRc----<br><br>BLAST: aliSize=10 (resi) iden=3.45% (5/145) simi=6.90% (10/145)<br>mtkverllinyktleefkkfkeygiqelsmleelqdniiendstspfygiyfgdklvarmslyqvngksnpyfdnrqd---------YLELWK---LEVLPGY--<br>--mdirqmnktHLEHWRglrKQLWPGHpddahladgeeilqadhlasfiamadgvaigfadasirhdyvngcdsspvvflegifvlpsfrqrgvakqliaavqrwgtnkgcrem<br>---------------------------------qnrgygralvefaksfkmpirtnprmksaefwnkmnfktvkydmardkgedpliwhpdmdr<br>asdtspentisqkvhqalgfeetervifyrkrc---<br><br>Proposed: aliSize=125 (resi) RMSD=2.28 (Å)<br>mTKVERLLINykTLEE-FKKF-KEY--GIqELSMLE--E-lqdniiendsTS-----PFYGIYFGDKLVARMSLYQVNGkSNPYFDnRQDYLELWKLEVLPGYQNRGYGRALVEFAKS-FK---M-PIRTNPR--MKS-AEFWNKMNFKTVKydmardkgEDPLIWHPD-mdrc<br>-MDIRQMNKT--HLEHwRGLRkQLWpgHP-DDAHLAdgEe----------ILqadhlASFIAMADGVAIGFADASIRHD-YVNGCD-SSPVVFLEGIFVLPSFRQRGVAKQLIAAVQRwGTnkgCrEMASDTSpeNTIsQKVHQALGFEETE--------RVIFYRKRCc----

633	2pr1A (152)<br>1s5kA (153)	C	8.55	TM-align: aliSize=127 (resi) RMSD=3.05 (Å)<br>-----M--TKVERLLINyKTLEEFKKF-KEYG-IQELSMLEELqdniiEnd-ST----SPFYGIYFGDKLVARMSLYQVNGkSNPYFDnRQDYLELWKLEVLPGYQNRGYGRALVEFAKS-FK---M-PIRTNPR--MKS-AEFWNKMNFKTVKYdmarDKGEdpliwhpdmdr----<br>glvprGshMDIRQMNKT-HLEHWRGLRkQLWPgHPDDAHLADG-----E--eILqadhLASFIAMADGVAIGFADASIRHD-YVNGCD-SSPVVFLEGIFVLPSFRQRGVAKQLIAAVQRwGTnkgCrEMASDTSpeNTIsQKVHQALGFEETER----VIFY-----------rkrc<br><br>SARST: aliSize=129 (resi) RMSD=7.73 (Å)<br>mtk--------VERLLINYKTLEE-----FKKFKEYGIQELSMLEELqdnIIENDSTSpFYGIYFGDKLVARMSLYqVNGKSNPYFdNRQDYLELWKLEVLPGYQNRGYGRAL----VEFAKSFKM-PIRTNPRMKSAEFWNKmnfktvkydmARDKGEDPLIWHP------dmdr<br>---glvprgshMDIRQMNKTHLEHwrglrKQLWPGHPDDAHLADGEE---ILQADHLA-SFIAMADGVAIGFADAS-IRHDYVNGC-DSSPVVFLEGIFVLPSFRQRGVAKQLiaavQRWGTNKGCrEMASDTSPENTISQKV----------HQALGFEETERVIfyrkrc----<br><br>BLAST: aliSize=14 (resi) iden=5.26% (8/152) simi=9.21% (14/152)<br>mtkverllinyktleefkkfkeygiqelsmleelqdniiendstspfygiyfgdklvarmslyqvngksnp-------YFDNRQ---DYLELWK---LEVLPGY--<br>---glvprgsHMDIRQmnkTHLEHWRglrKQLWPGHpddahladgeeilqadhlasfiamadgvaigfadasirhdyvngcdsspvvflegifvlpsfrqrgvakqliaavqrwgtnkgcre<br><br>----------------------------------qnrgygralvefaksfkmpirtnprmksaefwnkmnfktvkydmardkgedpliwhpdmdr<br>masdtspentisqkvhqalgfeetervifyrkrc--<br><br>Proposed: aliSize=125 (resi) RMSD=2.28 (Å)<br>-----M--TKVERLLINykTLEE-FKKF-KEY--G---IqELSMLeE--lqdniiendsT----SPFYGIYFGDKLVARMSLYQVNGkSNPYFDnRQDYLELWKLEVLPGYQNRGYGRALVEFAKS-FK----MPIRTNPR--MKS-AEFWNKMNFKTVKydmardkgEDPLIWHPD-mdrc<br>glvprGshMDIRQMNKT--HLEHwRGLRkQLWpgHpddA-HLADG-Eei----------LqadhLASFIAMADGVAIGFADASIRHD-YVNGCD-SSPVVFLEGIFVLPSFRQRGVAKQLIAAVQRwGTnkgcREMASDTSpeNTIsQKVHQALGFEETE--------RVIFYRKRCc----
634	2pr1A (152)<br>1ufhA (155)	C	5.26	TM-align: aliSize=116 (resi) RMSD=3.31 (Å)<br>MTKVERLLiNyKTLEEF-KKF--KEY-----------------GI--QELSMlEELqdnii-Ends--TSPFYGIYF-GDKLVARMSLYQV-NGKsnpyfdnrQDYLELWKLEVLPGYQNRGYGRALVEFAKS-FK----MPIRTNPRM-K--SAEFWNKMNFKTVkYdmarDkGEdpliwhpdmdr----<br>TIMLTPMQ-T-EEFRSYlTYTtkHYAeekvkagtwlpedaqllSKqvFTDLL-PRG-----lE---tpHHHLWSLKLnEKDIVGWLWIHAEpEHP--------QQEAFIYDFGLYEPYRGKGYAKQALAALDQaARsmgiRKLSLHVFAhNqtARKLYEQTGFQET-D----V-VM-----------skkl<br><br>SARST: aliSize=115 (resi) RMSD=8.25 (Å)<br>mtkverllin---------------------YKTLEEFKK--FKEyGIQELSMLEELQDNIiENDSTSPFYGIYF---GDKLVARMSLYqvngksNPYFDNRQDYLELWkLEVLPGYQNRGYGRAL----VEFAKSFKM-PIRTNPRMKSAEFWNK---MNFKTVKYD----mardkgedpliwhpdmdr<br>----------timltpmqteefrsyltyttkhYAEEKVKAGtwLPE-DAQLLSKQVFTDLLP-RGLETPHHHLWSLklnEKDIVGWLWIH------AEPEHPQQEAFIYD-FGLYEPYRGKGYAKQAlaalDQAARSMGIrKLSLHVFAHNQTARKLyeqTGFQETDVVmskkl-----------------<br><br>BLAST: aliSize=8 (resi) iden=2.63% (4/152) simi=5.26% (8/152)<br>mtkverllinyktleefkkf--KEYGIQELSM-----------------------------leelqdniiendsts<br>--------------------timltpmqteefrsyltyttkhyaeekvkagtwlpedaqllskqvftdllprgletphhhlwslklnekdivgwlwihaepehpqqeafiydfglyepyrgkgyakqalaaldqaaRSMGIRKLSLhvfahnqtarklyeqtgfqetdvvmskkl---------------<br><br>pfygiyfgdklvarmslyqvngksnpyfdnrqdylelwklevlpgyqnrgygralvefaksfkmpirtnprmksaefwnkmnfktvkydmardkgedpliwhpdmdr<br>---<br><br>Proposed: aliSize=113 (resi) RMSD=2.42 (Å)<br>MTKVERLLiNyKTLEEF-KKF--KE-----------------yG--IQeL---S-MleelqdniiendsTS----PFYGIYF--GDkLVARMSLYQV-NGKsnpyfdnrQDYLELWKLEVLPGYQNRGYGRALVEFAKS-FK----MPIRTNPRM-K--SAEFWNKMNFKTVkydmardkgeDPLIWHP<br>TIMLTPMQ-T-EEFRSYlTYTtkHYaeekvkagtwlpedaqll-SkqVF-TdllPrG------------LEtphhHLWSLKLneKD-IVGWLWIHAEpEHP--------QQEAFIYDFGLYEPYRGKGYAKQALAALDQaARsmgiRKLSLHVFAhNqtARKLYEQTGFQET----------DVVMSKK<br><br>Dmdrc<br>L----
635	2pr1A (152)<br>1z4eA (150)	C	11.33	TM-align: aliSize=122 (resi) RMSD=2.78 (Å)<br>MTKVERLLINykTLEEFKKF---K--E--YGIq-----ELSMLEELqdniiEnd--ST---SPFYGIYFGDKLVARMSLYQVNGKsnPyfDNRQDYLELWKLEVLPGYQNRGYGRALVEFAKSF-K---M-PIRTNPRM-K--SAEFWNKMNFKTVKYdmarDkGEdpliwhpdmdr---<br>HVTIREATEG--DLEQMVHMladDvlGrkRER-yekplPVSYVRAF-----K--eiKKdknNELIVACNGEEIVGMLQVTFTPYL--T--YQGSWRATIEGVRTHSAARGQGIGSQLVCWAIERakergChLIQLTTDKqRpdALRFYEQLGFKASHE----G-LK-----------mhf<br><br>SARST: aliSize=110 (resi) RMSD=5.49 (Å)<br>mtkverllin------------YKTLEEFKKFKE------YG----IQELSMLEELQDNIIENdstspFYGIYFGDKLVARMSLYQVNgksnPYFDNRQDYLELWKLEVLPGYQNRGYGRALVEFAK----SFKMPIRTNP----RMKSAEFWNKMNFK----------tvkydmardkgedpliwhpdmdr<br>----------hvtireategdlEQMVHMLADDVLgrkrerYEkplpVSYVRAFKEIKKDKNNE-----LIVACNGEEIVGMLQVTFTP----YLTYQGSWRATIEGVRTHSAARGQGIGSQLVCWAIerakERGCHLIQLTtdkqRPDALRFYEQLGFKasheglkmhf----------------------<br><br>BLAST: aliSize=14 (resi) iden=5.33% (8/150) simi=9.33% (14/150)<br>mtkverllinyktleefkkfkeygiqelsmleelqdniiendstspfygiyfgdklvarmslyqvngksnpyfdnrqdylelwkl---------------------EVLpGYQNRGYGRAL-VEFAKSFK--<br>---hvtireategdleqmvhmladDVL-GRKRERYEKPLpVSYVRAFKeikkdknnelivacngeeivgmlqvtftpyltyqgswratiegvrthsaargqgigsqlv<br><br>--mpirtnprmksaefwnkmnfktvkydmardkgedpliwhpdmdr<br>cwaierakergchliqlttdkqrpdalrfyeqlgfkasheglkmhf--<br><br>Proposed: aliSize=118 (resi) RMSD=1.87 (Å)<br>MTKVERLLINykTLEEFKKF---K--E---Y-------giqeLSMLE--E--lqdniiendstS-----PFYGIYFGDKLVARMSLYQVNGKsnPyfDNRQDYLELWKLEVLPGYQNRGYGRALVEFAKSF-K---M-PIRTNPRM-K--SAEFWNKMNFKTVkydmardkgEDPLIWHPdmdrc<br>HVTIREATEG--DLEQMVHMladDvlGrkrEryekplp----VSYVRafKei-----------KkdknnELIVACNGEEIVGMLQVTFTPYL--T--YQGSWRATIEGVRTHSAARGQGIGSQLVCWAIERakergChLIQLTTDKqRpdALRFYEQLGFKAS---------HEGLKMHF-----

636	2pr1A (152)<br>2f5iA (167)	C	10.53	TM-align: aliSize=126 (resi) RMSD=3.14 (Å)<br>MTKVERLLIN-YKT-LEEFKKFK--E--YGIqELSMLEELqdNiiEn-DST----SPFYGIYFG--------DKLVARMSLYQVNGKsnpyfdNRQDYLELWKLEVLPGYQNRGYGRALVEFAKS-FK---M-PIRTNPR-MK--SAEFWNKMNFKTV--KYDmarDKGEdpliwhpdmdr-----------<br>KFVIRPATAAdCSDiLRLIKELAkyEymEEQ-VILTEKDL--L--E-dGFGehpfYHCLVAEVPkehwtpegHSIVGFAMYYFTYDP------WIGKLLYLEDFFVMSDYRGFGIGSEILKNLSQvAMrcrCsSMHFLVAeWNepSINFYKRRGASDLssEEG---WRLF-----------kidkeyllkmat<br><br>SARST: aliSize=121 (resi) RMSD=7.60 (Å)<br>mtkverllin----------------YKTLEEFKKFK-----EYGIQELSMleelQDNIIENDSTSpfYGIYFG--DKL------VARMSLYQVNGKSnpyfdnRQDYLELWKLEVLPGYQNRG----YGRALVEFAKSFKM-PIRTNPRMKSA---EFWNKMNFKTvkydMARDKGEDPLIWH------<br>----------kfvirpataadcsdilRLIKELAKYEYmeeqvILTEKDLLE----DGFGEHPFYHC--LVAEVPkeHWTpeghsiVGFAMYYFTYDPW------IGKLLYLEDFFVMSDYRGFGigseILKNLSQVAMRCRCsSMHFLVAEWNEpsiNFYKRRGASD----LSSEEGWRLFKIDkeyllk<br><br>---pdmdr<br>mat-----<br><br>BLAST: aliSize=60 (resi) iden=21.05% (32/152) simi=39.47% (60/152)<br>mtkverllinyktleefkkfkeyg-------------------IQELSMLEELQDNII--ENDSTSPFYGIY-FGDKLVARM--SLYQVNGKS----NPYFDNRQDYL-ELWKLE---VLPGYQNRGYGRALVEFAKSFKMPIR-TNPRMKSAEfWNK--MNF--KTVKYDMARDKG-------------<br>------------------------kfvirpataadcsdilrlIKELAKYEYMEEQVIltEKDLLEDGFGEHpFYHCLVAEVpkEHWTPEGHSivgfAMYYFTYDPWIgKLLYLEdffVMSDYRGFGIGSEILKNLSQVAMRCRcSSMHFLVAE-WNEpsINFykRRGASDLSSEEGwrlfkidkeyllkm<br><br>--edpliwhpdmdr<br>at------------<br><br>Proposed: aliSize=129 (resi) RMSD=2.47 (Å)<br>MTKVERLLI--NYKTLEEFKKFK--E-----yGiqeLSMLEE-LqdniiE-ndST-----SPFYGIYF----G----DKLVARMSLYQVNGKsnpyfdNRQDYLELWKLEVLPGYQNRGYGRALVEFAKS-FK---M-PIRTNPR-MK--SAEFWNKMNFKTV--KYdmardkGEDPLIWHPDMDR----c<br>KFVIRPATAadCSDILRLIKELAkyEymeeq-V---ILTEKDlL-----Ed--GFgehpfYHCLVAEVpkehWtpegHSIVGFAMYYFTYDP------WIGKLLYLEDFFVMSDYRGFGIGSEILKNLSQvAMrcrCsSMHFLVAeWNepSINFYKRRGASDLssEE------GWRLFKIDKEYLLkmat-
637	2pr1A (152)<br>2g3tA (169)	C	10.53	TM-align: aliSize=126 (resi) RMSD=3.14 (Å)<br>MTKVERLLIN-YKT-LEEFKKFK--E--YGIqELSMLEELqdNiiEn-DS---T-SPFYGIYFG--------DKLVARMSLYQVNGKsnpyfdNRQDYLELWKLEVLPGYQNRGYGRALVEFAKSF-K---M-PIRTNPR--MK-SAEFWNKMNFKTV--KYDmarDKGEdpliwhpdmdr---------<br>KFVIRPATAAdCSDiLRLIKELAkyEymEEQ-VILTEKDL--L--E-dGFgehPfYHCLVAEVPkehwtpegHSIVGFAMYYFTYDP------WIGKLLYLEDFFVMSDYRGFGIGSEILKNLSQVaMrcrCsSMHFLVAewNEpSINFYKRRGASDLssEEG---WRLF-----------kidkeyllk<br><br>-----<br>matee<br><br>SARST: aliSize=120 (resi) RMSD=6.77 (Å)<br>mtkverllin----------------YKTLEEFKKFKEYG-----IQELSmleeLQDNIIENDSTSpfYGIYFG--DKL------VARMSLYQVNGKSnpyfdnRQDYLELWKLEVLPGYQNRGYGRALVEFAKS----FKM-PIRTNPRMKSAEFWNK---MNFKTvkydMARDKGEDPLIW-------<br>----------kfvirpataadcsdilRLIKELAKYEYMEEqviltEKDLL----EDGFGEHPFYHC--LVAEVPkeHWTpeghsiVGFAMYYFTYDPW------IGKLLYLEDFFVMSDYRGFGIGSEILKNLSQvamrCRCsSMHFLVAEWNEPSINFykrRGASD----LSSEEGWRLFKIdkeyllk<br><br>-----hpdmdr<br>matee------<br><br>BLAST: aliSize=60 (resi) iden=21.05% (32/152) simi=39.47% (60/152)<br>mtkverllinyktleefkkfkeyg-------------------IQELSMLEELQDNII--ENDSTSPFYGIY-FGDKLVARM--SLYQVNGKS----NPYFDNRQDYL-ELWKLE---VLPGYQNRGYGRALVEFAKSFKMPIR-TNPRMKSAEfWNK--MNF--KTVKYDMARDKG-------------<br>------------------------kfvirpataadcsdilrlIKELAKYEYMEEQVIltEKDLLEDGFGEHpFYHCLVAEVpkEHWTPEGHSivgfAMYYFTYDPWIgKLLYLEdffVMSDYRGFGIGSEILKNLSQVAMRCRcSSMHFLVAE-WNEpsINFykRRGASDLSSEEGwrlfkidkeyllkm<br><br>----edpliwhpdmdr<br>atee------------<br><br>Proposed: aliSize=130 (resi) RMSD=2.63 (Å)<br>MTKVERLLI--NYKTLEEFKKF--KE----yGIqeLSMLEE-LqdniiE-ndST-----SPFYGIYFG--------DKLVARMSLYQVNGKsnpyfdNRQDYLELWKLEVLPGYQNRGYGRALVEFAKS-FK---M-PIRTNPR-MK--SAEFWNKMNFKTV--KYDmardkgEDPLIW---H-PDMDR--c<br>KFVIRPATAadCSDILRLIKELakYEymee-QV--ILTEKDlL-----Ed--GFgehpfYHCLVAEVPkehwtpegHSIVGFAMYYFTYDP------WIGKLLYLEDFFVMSDYRGFGIGSEILKNLSQvAMrcrCsSMHFLVAeWNepSINFYKRRGASDLssEEG------WRLFKIdkeYlLKMATee-
638	2pr1A (152)<br>2jevA (169)	C	9.87	TM-align: aliSize=127 (resi) RMSD=3.22 (Å)<br>-MTKVERLLIN-YKT-LEEFKKF--KE--YGIqELSMLEELqdniIEn-DST----SPFYGIYFG--------DKLVARMSLYQVNGKsnpyfdNRQDYLELWKLEVLPGYQNRGYGRALVEFAKS-FK---M-PIRTNPR--M-KSAEFWNKMNFKTV-KYDMarDKGEdpliwhpdmdr---------<br>aKFVIRPATAAdCSDiLRLIKELakYEymEEQ-VILTEKDL----LE-dGFGehpfYHCLVAEVPkehwtpegHSIVGFAMYYFTYDP------WIGKLLYLEDFFVMSDYRGFGIGSEILKNLSQvAMrcrCsSMHFLVAewNePSINFYKRRGASDLsSEEG--WRLF-----------kidkeyllk<br><br>----<br>mate<br><br>SARST: aliSize=125 (resi) RMSD=17.64 (Å)<br>mt-KVERLLINYKTLEEfkkfkeygiQELSMLEELQDNIieNDSTSPFYgiyfG---------DKLVARMSLYQVNGKSNPYFDNRQDYLELWKLE----------------VLPGYQNRGYGRA----LVEFAKSFKM-PIRTNPRMKSAEFWNK---MNFKtvkyDMARDKGEDPLIW-----------<br>--aKFVIRPATAADCSD---------ILRLIKELAKYEY--MEEQVILT----EkdlledgfgEHPFYHCLVAEVPKEHWTPEGHSIVGFAMYYFTydpwigkllyledffVMSDYRGFGIGSEilknLSQVAMRCRCsSMHFLVAEWNEPSINFykrRGAS----DLSSEEGWRLFKIdkeyllkmate<br><br>hpdmdr<br>------<br><br>BLAST: aliSize=60 (resi) iden=21.05% (32/152) simi=39.47% (60/152)<br>mtkverllinyktleefkkfkeyg-------------------IQELSMLEELQDNII--ENDSTSPFYGIY-FGDKLVARM--SLYQVNGKS----NPYFDNRQDYL-ELWKLE---VLPGYQNRGYGRALVEFAKSFKMPIR-TNPRMKSAEfWNK--MNF--KTVKYDMARDKG-------------<br>------------------------akfvirpataadcsdilrlIKELAKYEYMEEQVIltEKDLLEDGFGEHpFYHCLVAEVpkEHWTPEGHSivgfAMYYFTYDPWIgKLLYLEdffVMSDYRGFGIGSEILKNLSQVAMRCRcSSMHFLVAE-WNEpsINFykRRGASDLSSEEGwrlfkidkeyllk<br><br>----edpliwhpdmdr<br>mate------------<br><br>Proposed: aliSize=130 (resi) RMSD=2.56 (Å)<br>-MTKVERLLI--NYKTLEEFKKFK--E----yGIqeLSMLEE-LqdniiE-ndST-----SPFYGIYFG--------DKLVARMSLYQVNGKsnpyfdNRQDYLELWKLEVLPGYQNRGYGRALVEFAKS-FK---M-PIRTNPR-MK--SAEFWNKMNFKTV--KYDmardkgEDPLIWHPDMDR-----c<br>aKFVIRPATAadCSDILRLIKELAkyEymee-QV--ILTEKDlL-----Ed--GFgehpfYHCLVAEVPkehwtpegHSIVGFAMYYFTYDP------WIGKLLYLEDFFVMSDYRGFGIGSEILKNLSQvAMrcrCsSMHFLVAeWNepSINFYKRRGASDLssEEG------WRLFKIDKEYLLkmate-

639	2pr1A (152)<br>2o28A (182)	C	8.55	TM-align: aliSize=127 (resi) RMSD=3.35 (Å)<br>-------------------------------MT----KVERLLIN-YKTlEEFKKF-KEYGIQELSMLEELqDNii-End-ST----SPFYGIYFG--DKLVARMSLYQVNGKSnpyfdNR-QDYLELWKLEVLPGYONRGYGRALVEFAKSF-K---M-PIRTNPRMKSAEFWNKMNFKTVKYDmarDK<br>pdetpmfdpsllkevdwsqntatfspaisptHPgeglVLRPLCTAdLNR-GFFKVLgQLTETGVVSPEQFM-KS--fE--hMKksgdYYVTVVEDVtlGQIVATATLIIEHKFI-----HScAKRGRVEDVVVSDECRGKQLGKLLLSTLTLLsKklnCyKITLECLPQNVGFYKKFGYTVSEEN---YM<br>GEdpliwhpdmdr----<br>CR-----------rflk<br>SARST: aliSize=114 (resi) RMSD=5.70 (Å)<br>mtkverlliny--KTLEEFKKFKE--YGIOELSMLEELqdnIIENDSTSpFYGIYF--GDKLVARMSLYQVngksNPYFDNRQDYLELWKLEVLPGYONRGYGRALVEFAKS----FKM-PIRTNPRMKSAEFWNKMNFKTV<br>-----------pdetpmfdpsllkevdwsqntatfspaispthpgeglvlrplctadlnrgFFKVLGQLTETgvVSPEQFMKSFEH---MKKSGDYY-VTVVEDvtLGQIVATATLIIE----HKFIHSCAKRGRVEDVVVSDECRGKQLGKLLLSTLTLlskkLNCyKITLECLPQNVGFYKKFGYTVS<br>KY---------dmardkgedpliwhpdmdr<br>EEnymcrrflk-------------------<br>BLAST: aliSize=20 (resi) iden=7.89% (12/152) simi=13.16% (20/152)<br>mtkverllinyktleefkkfkeygiqelsmleelqdniiendstspfygiyfgdklvarmslyqvngksnpyfdnrqdylelwklevlpgyq---NRGYGRALVEFAKSFKmpIRTNPRMKSAEFWNKMN--FKTVKYDM------<br>--pdetpmfdpsllkevdwsqntatfspaispthpgeglvlrplctadlNRGFFKVLGQLTETGV--VSPEQFMKSFEHMKKSGdyYVTVVEDVtlgqiv<br>--ardkgedpliwhpdmdr<br>atatliiehkfihscakrgrvedvvvsdecrgkqlgklllstltllskklncykitleclpqnvgfykkfgytvseenymcrrflk-----------<br>Proposed: aliSize=124 (resi) RMSD=2.32 (Å)<br>-------------------------------MT----KVERLLIN-YKTlEEFKKF-KEYGiqE-LsmLEeLOD-niiE--ndsT--S-----P-FYGIYF-G-DKLVARMSLYQVNGKsnpyfdNR--QDYLELWKLEVLPGYONRGYGRALVEFA-KSFK---M-PIRTNPRMKSAEFWNKMNFKTVK<br>pdetpmfdpsllkevdwsqntatfspaisptHPgeglVLRPLCTAdLNR-GFFKVLgQLTE--TgV--VS-PEQf---Mks---FehMkksgdYyVTVVEDvTlGQIVATATLIIEHKF------IHscAKRGRVEDVVVSDECRGKQLGKLLLSTLtLLSKklnCyKITLECLPQNVGFYKKFGYTVSE<br>YdmardkgeDPLIWHPDM-drc<br>E--------NYMCRRFLKc---
640	2pr1A (152)<br>2o28B (180)	C	8.55	TM-align: aliSize=127 (resi) RMSD=3.36 (Å)<br>-------------------------------MT----KVERLLIN-YKTlEEFKKF-KEYGIQELSMLEELqDNii-End-ST----SPFYGIYF-G-DKLVARMSLYQVNGKSnpyfdNR-QDYLELWKLEVLPGYONRGYGRALVEFAKSF-K---M-PIRTNPRMKSAEFWNKMNFKTVKYDmarDK<br>pdetpmfdpsllkevdwsqntatfspaisptHPgeglVLRPLCTAdLNR-GFFKVLgQLTETGVVSPEQFM-KS--fE--hMKksgdYYVTVVEDvTlGQIVATATLIIEHKFI-----HScAKRGRVEDVVVSDECRGKQLGKLLLSTLTLLsKklnCyKITLECLPQNVGFYKKFGYTVSEEN---YM<br>GEdpliwhpdmdr--<br>CR-----------rf<br>SARST: aliSize=120 (resi) RMSD=7.62 (Å)<br>mtkverlliny--KTLEEFKKFKE--YGIOELSMLEELODNIIENDstspFYGIYFG--DKLVARMSLYQVngksNPYFDNRQDYLELWKLEVLPGYONRGYGRAL----VEFAKSFKM-PIRTNPRMKSAEFWNKMNFKTV<br>-----------pdetpmfdpsllkevdwsqntatfspaispthpgeglvlrplctadlnrgFFKVLGQLTETgvVSPEQFMKSFEHMKKSGDYY----VTVVEDVtlGQIVATATLIIE----HKFIHSCAKRGRVEDVVVSDECRGKQLGKLLlstlTLLSKKLNCyKITLECLPQNVGFYKKFGYTVS<br>KYdmardkgedPLIWHP-dmdr<br>EE---------NYMCRRf----<br>BLAST: aliSize=20 (resi) iden=7.89% (12/152) simi=13.16% (20/152)<br>mtkverllinyktleefkkfkeygiqelsmleelqdniiendstspfygiyfgdklvarmslyqvngksnpyfdnrqdylelwklevlpgyq---NRGYGRALVEFAKSFKmpIRTNPRMKSAEFWNKMN--FKTVKYDM------<br>--pdetpmfdpsllkevdwsqntatfspaispthpgeglvlrplctadlNRGFFKVLGQLTETGV--VSPEQFMKSFEHMKKSGdyYVTVVEDVtlgqiv<br>---ardkgedpliwhpdmdr<br>atatliiehkfihscakrgrvedvvvsdecrgkqlgklllstltllskklncykitleclpqnvgfykkfgytvseenymcrrf----------<br>Proposed: aliSize=122 (resi) RMSD=2.36 (Å)<br>-------------------------------MT----KVERLLIN-YKTlEEFKKF-KEYGiqEL-smLEeLOD-niiE--ndsT--S-----P-FYGIYF-G-DKLVARMSLYQVNGKsnpyfdNR--QDYLELWKLEVLPGYONRGYGRALVEFA-KSFK---M-PIRTNPRMKSAEFWNKMNFKTVK<br>pdetpmfdpsllkevdwsqntatfspaisptHPgeglVLRPLCTAdLNR-GFFKVLgQLTE--TGv--VS-PEQf---Mks---FehMkksgdYyVTVVEDvTlGQIVATATLIIEHKF------IHscAKRGRVEDVVVSDECRGKQLGKLLLSTLtLLSKklnCyKITLECLPQNVGFYKKFGYTVSE<br>YdmardkgedPLIWHPD-mdrc<br>E---------NYMCRRFc----
641	2pr1A (152)<br>2vbqB (144)	C	9.03	TM-align: aliSize=124 (resi) RMSD=2.88 (Å)<br>mtKVERLLINyKTLEEFKKF-KEY--GIqELSMLEELqdniiEnd-ST----SPFYGIYFGDKLVARMSLYQVNGkSNPYFDnRODYLELWKLEVLPGYONRGYGRALVEFAKS-FK---M-PIRTNPR--MKS-AEFWNKMNFKTVKYdmarDKGEdpliwhpdmdr----<br>--DIRQMNKT-HLEHWRGLRkQLWpgHP-DDAHLADG-----E--eILqadhLASFIAMADGVAIGFADASIRHD-YVNGCD-SSPVVFLEGIFVLPSFRQRGVAKQLIAAVQRwGTnkgCrEMASDTSpeNTIsQKVHQALGFEETER----VIFY-----------rkrc<br>SARST: aliSize=121 (resi) RMSD=8.71 (Å)<br>mtkverllin-----------YKTLEEFKKFKEYGIOELSMLEEL-QDNIIendSTSpfYGIYFGDKLVARMSLYQVngkSNPYFDNRODYLELWKLEVLP-GYONRG----YGRALVEFAKSFKMPIRTNPRMKSAEFWNKmnfktvkydMARDKGEDPLIWHP------dmdr<br>----------dirqmnkthleHWRGLRKQLWPGHPDDAHLADGEEiLQADH---LAS--FIAMADGVAIGFADASIR---HDYVNGCDSSPVVFLEGIFVLpSFRQRGvakqLIAAVQRWGTNKGCREMASDTSPENTISQK---------VHQALGFEETERVIfyrkrc----<br>BLAST: aliSize=10 (resi) iden=3.47% (5/144) simi=6.94% (10/144)<br>mtkverllinyktleefkkfkeygiqelsmleelqdniiendstspfygiyfgdklvarmslyqvngksnpyfdnrqd--------YLELWK---LEVLPGY--<br>--dirqmnktHLEHWRglrKQLWPGHpddahladgeeilqadhlasfiamadgvaigfadasirhdyvngcdsspvvflegifvlpsfrqrgvakqliaavqrwgtnkgcrema<br>--------------------------------qnrgygralvefaksfkmpirtnprmksaefwnkmnfktvkydmardkgedpliwhpdmdr<br>sdtspentisqkvhqalgfeetervifyrkrc---<br>Proposed: aliSize=124 (resi) RMSD=2.27 (Å)<br>mtKVERLLINykTLEE-FKKF-KEY--GI--QELSMLeE--lqdniiendsT----SPFYGIYFGDKLVARMSLYQVNGkSNPYFDnRODYLELWKLEVLPGYONRGYGRALVEFAKS-FK----MPIRTNPR--MKS-AEFWNKMNFKTVKydmardkgEDPLIWHPDmdrc<br>--DIRQMNKT--HLEHwRGLRkQLWpgHPddAHLADG-Eei----------LqadhLASFIAMADGVAIGFADASIRHD-YVNGCD-SSPVVFLEGIFVLPSFRQRGVAKQLIAAVQRwGTnkgcREMASDTSpeNTIsQKVHQALGFEETE--------RVIFYRKRC----

642	2pr1A (152)<br>2vxkA (165)	C	10.53	TM-align: aliSize=127 (resi) RMSD=3.35 (Å)<br>------------------M---TKVERLLIN-YKTlEEFKKFK--EYGIqELSMLEELQDNiiEnd--S--T--SPFYGIYF-GDKLVARMSLYQVNGKsnPyfDNRQDYLELWKLEVLPGYQNRGYGRALVEFAKSF-K---M-PIRTNPRMKSAEFWNKMNFKTVKYdmarDKGEdpliwhpdmdr--<br>entplfspslispdvlavLpadYTIRPLCRSdYKR-GYLDVLRvlTTVG-DINEEQWNSRY--E--wiRarSdeYYLLVVCDgEGRIVGTGSLVVERKF--I--HSLGMVGHIEDIAVEKGQQGKKLGLRIIQALDYVaEkvgCyKTILDCSEANEGFYIKCGFKRAGL----EMAH-----------yy<br><br>SARST: aliSize=94 (resi) RMSD=6.73 (Å)<br>mtkverllin---YK-TLEEFKKFKEYGiqelsmleelqdniiendstspFYGIYFGD-KLVARMSLYQVNgksnPYFDNRQDYLELWKLEVLPGYQNRG----YGRALVEFAKSFKM-PIRTNPRMKSAEFWNKMNF<br>----------entplfspslispdvlavlpadytirplcrsdykrgyldvlrvlttvgdineeqwNSrYEWIRARSDEYY----------------------LLVVCDGEgRIVGTGSLVVER----KFIHSLGMVGHIEDIAVEKGQQGKKlglrIIQALDYVAEKVGCyKTILDCSEANEGFYIKCGF<br><br>KT---------vkydmardkgedpliwhpdmdr<br>KRaglemahyy----------------------<br><br>BLAST: aliSize=8 (resi) iden=3.95% (6/152) simi=5.26% (8/152)<br>mtkverllinyktleefkkfkeygiqelsmleelqdniiendstspfygiyfgdklvarmslyqvngksnpyfdnrqdylelwklevlpgyqnrgygralvefaksfkmpirtnprmksae---<br>---entplfspslispdvlavlpadytirplcrsdykrgyldvlrvlttvgdineeqwnsryewirarsdey<br><br>--FWNKMNFKTVKYDMA---rdkgedpliwhpdmdr<br>yllvvcdgegrivgtgslvverkfihslgmvghiediavekgqqgkklglriiqaldyvaekvgcyktildcseanegFYIKCGFKRAGLEMAhyy----------------<br><br>Proposed: aliSize=121 (resi) RMSD=2.34 (Å)<br>-----------------MT----KVERLLIN-YKTlEEFKKFK--EYG--iqeL---SMLEELqdniiE--ndsT--S---PFYGIYF-GDKLVARMSLYQVNGKsnpyfdNR--QDYLELWKLEVLPGYQNRGYGRALVEFAKSF-K---M-PIRTNPRMKSAEFWNKMNFKTVkydmardkgEDPLIW<br>entplfspslispdvlaVLpadYTIRPLCRSdYKR-GYLDVLRvlTTVgd---IneeQWNSRY-----Ewi---RarSdeyYLLVVCDgEGRIVGTGSLVVERKF------IHslGMVGHIEDIAVEKGQQGKKLGLRIIQALDYVaEkvgCyKTILDCSEANEGFYIKCGFKRA---------GLEMAH<br><br>HP-dmdrc<br>YYc-----
643	2pr8A (173)<br>1ufhA (155)	C	12.26	TM-align: aliSize=131 (resi) RMSD=3.36 (Å)<br>dSVTLRLMtehDLAMLYEWLNRS-HI-VE--W------WG-GEEar--Ptla---Dvq--EQYLPSvLAqeSVTPYIAMLNG-EPIGYAQSYVALGsgdgwweeeTDPGVRGIDLSlANaSQLGKG--LGTKLVRALVELLFNDPeVTKIQTDPSPSNLRAIRCYEKAGFERQGTVTtpdGpavymvqtr<br>-TIMLTPM---QTEEFRSYLTYTtKHyAEekVkagtwlPEdAQL--lsK---qvfT--dlLPRGLE-TP--HHHLWSLKLNEkDIVGWLWIHAEPE---------HPQQEAFIYDF-GL-YEPYRGkgYAKQALAALDQAARSMG-IRKLSLHVFAHNQTARKLYEQTGFQETDVVM---S---------<br><br>qafe---<br>----kkl<br><br>SARST: aliSize=121 (resi) RMSD=11.08 (Å)<br>ds-VTLRLMTEHDL----------AMLYEWLNRShiVEWWGGEEArptLADVQEQYLPSVLAQESVTPYIAM-LNGEPIGYAQSYValgsgdgwWEEEtdPGVRGIDL-----SLANASQlgkglgtKLVRALVELLfndpevtkiqtdpspsnlraiRCYEKAGFerQGTVTT-------------PDG<br>--tIMLTPMQTEEFrsyltyttkhYAEEKVKAGT--WLPEDAQLL---SKQVFTDLLPRGLETPHHHLWSLKlNEKDIVGWLWIHA--------EPEH--PQQEAFIYdfglyEPYRGKG-------YAKQALAALD---------------------QAARSMGI--RKLSLHvfahnqtarklyeQTG<br><br>P---AVYMVQT-rqafe<br>FqetDVVMSKKl-----<br><br>BLAST: aliSize=29 (resi) iden=12.90% (20/155) simi=18.71% (29/155)<br>dsvtlrlmtehdlamlyewlnrshivewwggeearptladvqeqylpsvlaqesvtpyiamlngepigyaqsyvalgsgd--GW-W--EEETDPGVRGI--DLSLANASQlGKGLGTKLV<br>--timltpmqteefrsyltyttkhyaeekvkagtwlpedaqllskqvftdllprgletphhhlwslklnekdivGWlWihAEPEHPQQEAFiyDFGLYEPYR-GKGYAKQAL<br><br>RALVELLfNDPEVTKIQTDPSPSNLRAIRCYEKAGFERQGTV-----ttpdgpavymvqtrqafe<br>AALDQAA-RSMGIRKLSLHVFAHNQTARKLYEQTGFQETDVVmskkl------------------<br><br>Proposed: aliSize=128 (resi) RMSD=2.56 (Å)<br>dSVTLRLMtehDLAMLYEWLNrS--HI-VE---------WWGGEE--A-------rptladvqEQYLPsvLAqeSVTPYIAMLNG-EPIGYAQSYVA--LgsgdgwweeeTDpGVRGIDLSlANasQLGKG---LGTKLVRALVELLFNDpEVTKIQTDPSPSNLRAIRCYEKAGFERqgtvttpdGPAV<br>-TIMLTPM---QTEEFRSYLT-YttKHyAEekvkagtwlPEDAQLlsKqvftdll--------PRGLE--TP--HHHLWSLKLNEkDIVGWLWIHAEpeH----------PQ-QEAFIYDF-GL--YEPYRgkgYAKQALAALDQAARSM-GIRKLSLHVFAHNQTARKLYEQTGFQE--------TDVV<br><br>YMVQtrqAfe<br>MSKK---L--
644	2pr8A (173)<br>1vkcA (149)	C	12.75	TM-align: aliSize=138 (resi) RMSD=3.40 (Å)<br>DSVTLRLMTeHDLaMLYEWLNRS-HIVEWWGGEE-ARPTla-DVQEQYLpsVLaQESVTPYIAMLN--GEPIGYAQSYVALgsgdgwWEEETDpGVRGIDLSlANasQLGKG---LGTKLVRALVELLFNDpEVTKIQTDPSPSNLrAIRCYEKAgFERQGtVTTpdGPavymvqtrqafe---<br>EYTIVDGEE-YIE-EIKKLDREIsYSFVRFPISYeEYEE--rHEELFES--LL-SQGEHKFFVALNerSELLGHVWICITL------DTVDYV-KIAYIYDI-EV--VKWARglgIGSALLRKAEEWAKER-GAKKIVLRVEIDNP-AVKWYEER-GYKAR-ALI--ME------------kpi<br><br>SARST: aliSize=122 (resi) RMSD=11.29 (Å)<br>dsv-TLRLMTEHDLAMLYEW------LNRSHIVEWWggeearptLADVQEQYLPSVLAQESVTPYIAML--NGEPIGYAQSYVALGSGDGWweeETDpgVR--GIDLSLANASQlgkglgtklvRALVELLfndpevtkiqtdpspsnlRAIRCYEKAGfeRQGTVTT------------PDGP---AVY<br>---eYTIVDGEEYIEEIKKLdreisySFVRFPISYE--------EYEERHEELFESLLSQGEHKFFVALneRSELLGHVWICITLDTVDYV---KIA--YIydIEVVKWARGLG----------IGSALLR------------------KAEEWAKERG--AKKIVLRveidnpavkwyeERGYkarALI<br><br>MVQT-rqafe<br>MEKPi-----<br><br>BLAST: aliSize=24 (resi) iden=8.72% (13/149) simi=16.11% (24/149)<br>dsvtlrlmtehdlamlyewlnrshivewwggeearptladvqeqylpsvlaqesvtpyiamlngepigyaqsyvalgsgdgwweeetdpgvrgidlslanasql--<br>--eytivdgeeyieeikkldreisysfvrfpisyeeyeerheelfesllsqgehkffvalnersellghvwicitldtvdyvkiayiy<br><br>---------GKGLGTKLVRALVELLfNDPEVTKIQTDPSPSNlRAIRCYEKAGFERQGTV-----ttpdgpavymvqtrqafe<br>dievvkwarGLGIGSALLRKAEEWA-KERGAKKIVLRVEIDN-PAVKWYEERGYKARALImekpi------------------<br><br>Proposed: aliSize=138 (resi) RMSD=3.09 (Å)<br>DSVTLRLMTehDLAMLYEWLNRS-HIVEwWGGEE--ARPT-laDVqEQYLpsV-LAqESVTPYIAMLN--GEPIGYAQSYVALgsgdgwWEEETDpGVRGIDLSlANasQLGKG---LGTKLVRALVELLFNDpEVTKIQTDPSPSNLrAIRCYEKAGFERQGTVTTPD-gpavymvqtrqafe<br>EYTIVDGEE--YIEEIKKLDREIsYSFV-RFPISyeEYEEr--HE-ELFE--SlLS-QGEHKFFVALNerSELLGHVWICITL------DTVDYV-KIAYIYDI-EV--VKWARglgIGSALLRKAEEWAKER-GAKKIVLRVEIDNP-AVKWYEERGYKARALIMEKPi--------------

645	2pr8A (173)<br>2vbqA (147)	C	14.29	TM-align: aliSize=139 (resi) RMSD=3.24 (Å)<br>DSVTLRLMTEHDLAMLYEWLNRSHIvewwggEEArPTlaDVQEQYL--PSvlAQeSVTPYIAMLNGEPIGYAQSYVALGsgdgWWEEETDPGVRGIDLSlANasQLGKGLGtKLVRALVEL-LF--NDPEVTKIQTDPSPSNLRAIRCYEKAGFERQGTVTTPDgpavymvqtrqafe---<br>SHMDIRQMNKTHLEHWRGLRKQLWP------GHP-DD--AHLADGEeiLQ--AD-HLASFIAMADGVAIGFADASIRHD----YVNGCDSSPVVFLEGI-FV--LPSFRQR-GVAKQLIAAvQRwgTNKGCREMASDTSPENTISQKVHQALGFEETERVIFYR--------------krc<br><br>SARST: aliSize=140 (resi) RMSD=7.02 (Å)<br>d-SVTLRLMTEHDLAMLYEWLNRSHIvewwggeeaRPTLADVQEQYlPSVLAQESVTPYIAMLNGEPIGYAQSYvaLGSGDGWWEEETdpgVRGIDL-----SLANASQlgkglGTKLVRALVELLFNDPEVTKIQTDPSPSNLRAIRCYEKAGFERQGTvttpdgpAVYMVQT-rqafe<br>-sHMDIRQMNKTHLEHWRGLRKQLWP---------GHPDDAHLADG-EEILQADHLASFIAMADGVAIGFADAS--IRHDYVNGCDSS---PVVFLEgifvlPSFRQRG-----VAKQLIAAVQRWGTNKGCREMASDTSPENTISQKVHQALGFEETER-------VIFYRKRc-----<br><br>BLAST: aliSize=66 (resi) iden=23.81% (35/147) simi=44.90% (66/147)<br>dsvtlrlmtehdlamlyew-------LNRSHIVEW-------WGGEEARPTLADVQEqylpsVLAQESVTPYIAMLNGEPIGYAQSYVALGSGDGWweeETDPGV--RGIDLsLANASQlgKGLGTKLVRAlVELLFNDPEVTKIQTDPSPSNLRAIRCYEKAGFE------------rqgtvttpdgpa<br>-------------------shmdirqMNKTHLEHWrglrkqlWPGHPDDAHLADGEE-----ILQADHLASFIAMADGVAIGFADASIRHDYVNGC---DSSPVVflEGIFV-LPSFRQ--RGVAKQLIAA-VQRWGTNKGCREMASDTSPENTISQKVHQALGFEeterv ifyrkrc-----------<br>vymvqtrqafe<br>-----------<br><br>Proposed: aliSize=139 (resi) RMSD=2.78 (Å)<br>DSVTLRLMTEHDLAMLYEWLNrS--HIvewwggEeaRPTlaDVQEQYL-PSVLAqeSVTPYIAMLNGEPIGYAQSYVALGsgdgwWEE-ETDPGVRGIDLSlANasqLGKGLG-tKLVRALVELLFN--DP-EVTKIQTDPSPSNLRAIRCYEKAGFERQGTvttpdgpaVYMVQTrqaFE<br>SHMDIRQMNKTHLEHWRGLRK-QlwPG------H--PDD--AHLADGEeILQAD--HLASFIAMADGVAIGFADASIRHD-----YVNgCDSSPVVFLEGI-FV---LPSFRQr-GVAKQLIAAVQRwgTNkGCREMASDTSPENTISQKVHQALGFEETER--------VIFYRK---RC
646	2qlrA (425)<br>1ahxA (396)	N	13.38	TM-align: aliSize=331 (resi) RMSD=3.06 (Å)<br>mnyarfitaasaarnpspirtmtdilsrgpksmislagglpnpnmfpfktavi---TVENG-KT-IQFGFeMMKRaLQYSPSAGIPELLSWLKQLQIKLhNPPTIHYPpsqgQMDLCVTSGSQQGLCKVFEMIIN--PGDNVLLDEPAY<br>--mfenitaapadpilgladlfraderpgkinlgiglyydetgkipVLTSVkKAeQYLLE-NETT-KLYLGIDGIPEFGRCTQELLFGK-GSALINDK----RARTAQTPGGSGALRVAADFLAKntSVKRVWVSNPSW<br><br>SGTLQSLHP-LGCNIINVASD---ESGIVpDSLRDILSRWkpedaknpqkNTPKFLYTVPNGNNPTGNSLTSERKKEIYELARKYDFLIIEDDPYYFLQFNKFRV-PTFLSMD-VDGRVIRADSFS-II-SSGLRIGFLTGP------KP-LIERVILHIQVSTLHPSTFNQLMISQLLHEWG-EEGFMA<br>PNHKSVFNSaGLEVREYAYYDaenHTLDF-DALINSLNEA---------QAGDVVLFHGCCHNPTGIDPTLEQWQTLAQLSVEKGWLPLFDFAYQGFARGLEEDaEGLRAFAaMHKELIVASSYSKNFgLYNERVGACTLVaadsetVDrAFSQMKAAIRANYSSPPAHGASVVATILSNDAIRAIWEQ<br><br>HVDRVIDFYSNQKDAILAAADKWLTGLAE-WHVPAAGMFLWIKVkgindVKELIEEKAVKMGVLMLPGnafyvdssapspyLRASFSSASPEQMDVAFQVLAQLIkesl<br>ELTDMRQRIQRMRQLFVNTLQEKGANRDFsFIIKQNGMFSFSGL-----TKEQVLRLREEFGVYAVAS------------GRVNVAGMTPDNMAPLCEAIVAVL----<br><br>SARST: aliSize=340 (resi) RMSD=13.08 (Å)<br>mnyarfit-----------------AASAARNPSpirtmtdilsrgpkSMISLAGGLPNPNMfpfktavitveNGKTiqFGFEMMKRA--------LQYSPSAGIPELLSWLKQLQiklhnpPTIHYPP-SQGQMDLCVTSGS--QQGLCKVFEMIINPGDNVLLDEPAYSGTLQSLHPLGCNIINVAS<br>--------mfenitaapadpilglaDLFRADERP-------------GKINLGIGLYYDET----------GKIP--VLTSVKKAEqyllenettKLYLGIDGIPEFGRCTQELL------FGKGSALiNDKRARTAQTPGGsgALRVAADFLAKNTSVKRVWVSNPSWPNHKSVFNSAGLEVREYAY<br><br>D---ESGIVPDSLRDILSRWKpedaknpqKNTPkfLYTVPNGNNPTGNSLTSERKKEIYELARKYDFLIIEDDPYYFLQFNKfrvptFLSM------DVDGRVIRADS---FSIISSGLRIGFLTGP-------KPLIERVILHIQVSTLHPSTFNQLMISQLLHEWGEEGFMAHVDRVIDFYSNQKDAI<br>YdaeNHTLDFDALINSLNEAQ--------AGDV--VLFHGCCHNPTGIDPTLEQWQTLAQLSVEKGWLPLFDFAYQGFARGL-----EEDAeglrafAAMHKELIVASsysKNFGLYNERVGACTLVaadsetvDRAFSQMKAAIRANYSSPPAHGASVVATILSNDALRAIWEQELTDMRQRIQRMRQL<br><br>LAADKWLTGlaewhvPAA-----GMFLW-IKVKGIndVKELIEEKAVKMGVLMLpgNAFyvdssapspylRASFSSASPEQMDVAFQVLAQ--likesl<br>FVNTLQE-KG------ANRdfsfiIKQNGmFSFSGL--TKEQVLRLREEFGVYAV--ASG----------RVNVAGMTPDNMAPLCEAIVAvl------<br><br>BLAST: aliSize=83 (resi) iden=10.86% (43/396) simi=20.96% (83/396)<br>mnyarfitaasaarnpspirtmtdilsrgpksmislagglpnpnmfpfktavitvengktiqfgeemmkralqyspsagipellswlkqlqiklhnpptihyppsqgqmdlcvtsgsqqglckvfemiinpgdnvlldepaysgtlqslhplgcniinvasdesgivpdslrdilsrwkpedaknpqknt<br>--<br><br>pkflytvpng--<br>----------mfenitaapadpilgladlfraderpgkinlgiglyydetgkipvltsvkkaeqyllenettklylgidgipefgrctqellfgkgsalindkrartaqtpggsgalrvaadflakntsvkrvwvsnpswpnhksvfnsaglevreyayydaenhtldfdalinslneaqagdvvlfhgc<br><br>-NNPTGNSLTSERKKEIYELARKYDFLIIEDDPYY-F---IQFNKFRVPTFLSMDVDgrVIRADSFSIiSSGL---RIGFLTgpkplierviLHIQVSTLHPStFNQlMISQLLHEWGEEGfmAHVDRVI-DFYSNqkDAILAADKWLTGLAEWHVPAAGMFL-WIKVKGIN-DVKELIEEKAV-----<br>cHNPTGIDPTLEQWQTLAQLSVEKGWLPLFDFAYQgFargLEEDAEGLRAFAAMHKE--LIVASSYSK-NFGLyneRVGACT---------LVAADSETVDRA-FSQ-MKAAIRANYSSPP--AHGASVVaTILSN--DALRAIWEQELTDMRQRIQRMRQLFVnTLQEKGANrDFSFIIKQNGMfsfsg<br><br>---kmgvlmlpgnafyvdssapspylrasfssaspeqmdvafqvlaqlikesl<br>ltkeqvlrlreefgvyavasgrvnvagmtpdnmaplceaivavl---<br><br>Proposed: aliSize=354 (resi) RMSD=2.63 (Å)<br>mNYA-rfitaasAARNPSP--IRtMTDILSR-GPKSMISLaGgLPNPN---------mfnpfKT-A-VITvengkTIQFGeeMMKRaLQYSPSAGIPELLSWLKQLQ-IKlhNPPTIHYPpsqgQMDLCVTSGSQQGLCKVFEMII----NpgDNVLLDEPAYSGTLQSLHP-LGCNIINVASD----ESgi<br>-MFEn--------ITAAPADpiLG-LADLFRAdERPGKINL-G-IGLYYdetgkipv----LTsVkKAE-----QYLLE--NETT-KLYLGIDGIPEFGRCTQELLfGK--GSALINDK----RARTAQTPGGSGALRVAADFLAkntsV--KRVWVSNPSWPNHKSVFNSaGLEVREYAYYDaenhTL--<br><br>VPDSLRDILSR-wkpedaknpqkNTPKFLYTVPNGNNPTGNSLTSERKKEIYELARKYDFLIIEDDPYYFLQfNK--fRV-PTFLSMDV-DGRVIRADSFSLII-SSGLRIGFLTGP------KP-LIERVILHIQVSTLHPSTFNQLMISQLLHF--WGeEGFMAHVDRVIDFYSNQKDAILAAADK-W<br>DFDALINSLNEa-----------QAGDVVLFHGCCHNPTGIDPTLEQWQTLAQLSVEKGWLPLFDFAYQGFA-RGle-EDaEGLRAFAAmHKELIVASSYSKNFgLYNERVGACTLVaadsetVDrAFSQMKAAIRANYSSPPAHGASVVATILSNdaLR-AIWEQELTDMRQRIQRMRQLFVNTLQEkG<br><br>LTGlAE-WHVPAAGMFLWIKVkgindVKELIEeKA-VKMGVLMLPGnafyvdssapspyLRASFSSASPEQMDVAFQVLAQLIkesl<br>ANR-DFsFIIKQNGMFSFSGL-----TKEQVL-RLrEEFGVYAVAS-------------GRVNVAGMTPDNMAPLCEAIVAVL----
647	2qlrA (425)<br>1aiaA (396)	N	13.89	TM-align: aliSize=332 (resi) RMSD=3.20 (Å)<br>mnyarfitaasaarnpspirtmtdilsrgpksmislagglpnpnmfpfktav---ITVENGKT-IQFGFeMMKRaLQYSPSAGIPELLSWLKQLQIKLhNPPTIHYPpsqgQMDLCVTSGSQQGLCKVFEMIIN--PGDNVLLDEPAYS<br>---mfenitaapadpilgladlfraderpgkinlgigvykdetgktpVLTSVKKAeQYLLE-NETT-KNYLGIDGIPEFGRCTQELLFGK-GSALINDK----RARTAQTPGGTGALRVAADFLAKntSVKRVWVSNPSWP<br><br>GTLQSLHP-LGCNIINVASD---ESGIVpDSLRDILSRWkpedaknpqkNTPKFLYTVPNGNNPTGNSLTSERKKEIYELARKYDFLIIEDDPYYFLQFNKFRV-PTFLSMD-VDGRVIRADSFS-II-SSGLRIGFLTGP------K-PLIERVILHIQVSTLHPSTFNQLMISQLLHEWG-EEGFMAH<br>NHKSVFNSaGLEVREYAYYDaenHTLDF-DALINSLNEA----------QAGDVVLFHGCCHNPTGIDPTLEQWQTLAQLSVEKGWLPLFDFAYQGFARGLEEDaEGLRAFAaMHKELIVASSYShNFgLYNERVGACTLVaadsetVdRAFSQMKAAIRANYSNPPAHGASVVATILSNDAIRAIWEQE<br><br>VDRVIDFYSNQKDAILAAADKWLTGLAEWH-VPAAGMFLWIKVkginDVkELIEEKAVKMGVLMLPGnafyvdssapspyLRASFSSASPEQMDVAFQVLAQLIkesl<br>LTDMRQRIQRMRQLFVNTLQEKGANRDFSFiIKQNGMFSFSGL----TK-EQVLRLREEFGVYAVAS------------GRVNVAGMTPDNMAPLCEAIVAVL----<br><br>SARST: aliSize=328 (resi) RMSD=11.64 (Å)<br>mnyarfitaasaarnpspirtmtdilsrgpk-------------------------SMISLAGGLPNPNMfpfktavitveNGKTIqfGFEMMKRA---------LQYSPSAGIPELLSWlkqLQIKLHNpptIHYPPS-QGQMDLCVTSGSQQGLCKVFF---MIINPGdNVLLDEPAYSGTLQSLHP<br>-------------------------------mfenitaapadpilgladlfraderpGKINLGIGVYKDET-----------GKTPV--LTSVKKAEqyllenettKNYLGIDGIPEFGRC---TQELLFG---KGSALInDKRARTAQTPGGTGALRVAADflaKNTSVK-RVWVSNPSWPNHKSVFNS<br><br>LGCNIINVASD---ESGIVPDSLrdilsrwkpEDAKNPqKNTPKFLYTVPNGNNPTGNSLTSERKKEIYELARKYDFLIIEDDPYYFLQFnkfrvpTFLSMDV-------DGRVIRADS---FSIISSGLRIGFLTGP-------KPLIERVILHIQVSTLHPSTFNQLMISQLLH-EWGEEGFMAHVDR<br>AGLEVREYAYYdaeNHTLDFDAL---------INSLNE-AQAGDVVLFHGCCHNPTGIDPTLEQWQTLAQLSVEKGWLPLFDFAYQGFAR------GLEEDAEglrafaaMHKELIVASsysHNFGLYNERVGACTLVaadsetvDRAFSQMKAAIRANYSNPPAHGASVVATILSnDALRAIWEQELTD<br><br>VIDFYSNQKDAILAAADKWltglaewhVPAAGM------FLWIKVKGinDVKELIEEKAVKMGVLMlpgnafyvDSSAPspylrASFSSASPEQMDVAFQVLAQ--likesl<br>MRQRIQRMRQLFVNTLQEK--------GANRDFsfiikqNGMFSFSG--LTKEQVLRLREEFGVYA--------VASGR-----VNVAGMTPDNMAPLCEAIVAvl------<br><br>BLAST: aliSize=31 (resi) iden=5.81% (23/396) simi=7.83% (31/396)<br>mnyarf----ITAASAarnpSPIRTMTDILSRGPK-SMISLAGGLPNPNmfPFKTAVIT-VENGKTIQFGEEMMKralQYSPSAGIPE--<br>------mfenITAAPA----DPILGLADLFRADERpGKINLGIGVYKDE--TGKTPVLTsVKKAEQYLLENETTK---NYLGIDGIPEfgrctqellfgkgsalindkrartaqtpggtgalrvaadflakntsvkrvwvsnpswpnhksvfnsaglevreyayydaenhtldfdalinslneaqagdvv<br><br>--<br>lfhgcchnptgidptleqwqtlaqlsvekgwlplfdfayqgfargleedaeglrafaamhkelivassyshnfglynervgactlvaadsetvdrafsqmkaairanysnppahgasvvatilsndalraiweqeltdmrqriqrmrqlfvntlqekganrdfsfiikqngmfsfsgltkeqvlrlreef<br><br>--------------------------------llswlkqlqiklhnpptihyppsqgqmdlcvtsgsqqglckvfemiinpgdnvlldepaysgtlqslhplgcniinvasdesgivpdslrdilsrwkpedaknpqkntpkflytvpngnnptgnsltserkkeiyelarkydfliieddpyyflqfnkf<br>gvyavasgrvnvagmtpdnmaplceaivavl---<br><br>rvptflsmdvdgrviradsfsiissglrigfltgpkpliervilhiqvstlhpstfnqlmisqllhewgeegfmahvdrvidfysnqkdailaaadkwltglaewhvpaagmflwikvkgindvkelieekavkmgvlmlpgnafyvdssapspylrasfssaspeqmdvafqvlaqlikesl<br>---<br><br>Proposed: aliSize=356 (resi) RMSD=2.71 (Å)<br>mNY-ArfItaasA-aRNPSPIRTMTDILSR-GPKSMISLaGGLPNPN-------mfpfKT-AV--ITvengktIQFGeeMMKRaLQYSPSAGIPELLSWLKQLQ-IKlhNPPTIHYPpsqgQMDLCVTSGSQQGLCKVFEMII----NpgDNVLLDEPAYSGTLQSLHP-LGCNIINVASD----EsGIV<br>-MFeN--I----Ta-APADPILGLADLFRAdERPGKINL-GIGVYKDetgktpv----LTsVKkaEQ------YLLE--NETT-KNYLGIDGIPEFGRCTQELLfGK--GSALINDK----RARTAQTPGGTGALRVAADFLAkntsV--KRVWVSNPSWPNHKSVFNSaGLEVREYAYYDaenhT-LDF<br><br>pDSLRDILSR-wkpedaknpqkNTPKFLYTVPNGNNPTGNSLTSERKKEIYELARKYDFLIIEDDPYYFLQfNK--fRV-PTFLSMD-VDGRVIRADSFSLII-SSGLRIGFLTGP------K-PLIERVILHIQVSTLHPSTFNQLMISQLLHEWG-EEGFMAHVDRVIDFYSNQKDAILAAADKW-LT<br>-DALINSLNEa-----------QAGDVVLFHGCCHNPTGIDPTLEQWQTLAQLSVEKGWLPLFDFAYQGFA-RGle-EDaEGLRAFAaMHKELIVASSYSHNFgLYNERVGACTLVaadsetVdRAFSQMKAAIRANYSNPPAHGASVVATILSNDAlRAIWEQELTDMRQRIQRMRQLFVNTLQEKgAN<br><br>GlAE-WHVPAAGMFLWIKVkgindVKELIEeKAVK-MGVLMLPGnafyvdssapspyLRASFSSASPEQMDVAFQVLAQLIkesl<br>R-DFsFIIKQNGMFSFSGL-----TKEQVL-RLREeFGVYAVAS-------------GRVNVAGMTPDNMAPLCEAIVAVL----

648	2qlrA (425)<br>1arhA (396)	N	12.63	TM-align: aliSize=331 (resi) RMSD=3.13 (Å)<br>[illegible]<br>SARST: aliSize=336 (resi) RMSD=12.82 (Å)<br>[illegible]<br>BLAST: aliSize=31 (resi) iden=5.81% (23/396) simi=7.83% (31/396)<br>[illegible]<br>Proposed: aliSize=355 (resi) RMSD=2.72 (Å)<br>[illegible]
649	2qlrA (425)<br>1ariA (396)	N	12.88	TM-align: aliSize=331 (resi) RMSD=3.05 (Å)<br>[illegible]<br>SARST: aliSize=346 (resi) RMSD=14.51 (Å)<br>[illegible]<br>BLAST: aliSize=31 (resi) iden=5.81% (23/396) simi=7.83% (31/396)<br>[illegible]<br>Proposed: aliSize=353 (resi) RMSD=2.61 (Å)<br>[illegible]
650	2qlrA (425)<br>1arsA (396)	N	13.38	TM-align: aliSize=331 (resi) RMSD=3.16 (Å)<br>[illegible]<br>SARST: aliSize=348 (resi) RMSD=14.77 (Å)<br>[illegible]<br>BLAST: aliSize=31 (resi) iden=5.81% (23/396) simi=7.83% (31/396)<br>[illegible]<br>Proposed: aliSize=354 (resi) RMSD=2.67 (Å)<br>[illegible]

651	2qlrA (425)<br>1asfA (396)	N	12.88	TM-align: aliSize=332 (resi) RMSD=3.22 (Å)<br>mnyarfitaasaarnpspirtmtdilsrgpksmislagglpnpnmfpfktav---ITVENGKT-IQFGEeMMKRaLQYSPSAGIPELLSWLKQLQIKLhNPPTIHYPpsqgQMDLCVTSGSQQGLCKVFFMIIN--PGDNVLLDEPAYS<br>---mfenitaapadpilgladlfraderpgkinlgigvykdetgktpVLTSVKKAeQYLLE-NETT-KNYLGIDGIPEFGRCTQELLFGK-GSALINDK----RARTAQTPGGTGALRVAADFLAKntSVKRVWVSNPSWP<br>GTLQSLHP-LGCNIINVASD---FSGIVpDSLRDILSRWkpedaknpqkNTPKFLYTVPNGNNPTGNSLTSFRKKEIYFLARKYDFLIIEDDPYYFLQFNKFRV-PTFLSMDV-DGRVIRADSFS-II-SSGLRIGFLTGP------KP-LIERVILHIQVSTLHPSTFNQLMISQLLHEWG-FEGFMAH<br>NHKSVFNSaGLEVREYAYYDaenHTLDF-DALINSLNEA-----------QAGDVVLFHGCCHNPTGIDPTLEQWQTLAQLSVEKGWLPLFDFAFQGFARGLEEDaEGLRAFAAmHKELIVASSYSkNFgLYNERVGACTLVaadsetVDrAFSQMKAAIRANYSNPPAHGASVVATILSNDAlRAIWEQE<br>VDRVIDFYSNQKDAILAAADKWLTGLAEWH-VPAAGMFLWIKVkgindVKFLIEFKAVKMGVLMLPGnafyvdssapspyLRASFSSASPFQMDVAFQVLAQlIKesl<br>LTDMRQRIQRMRQLFVNTLQEKGANRDFSFiIKQNGMFSFSGL-----TKEQVLRLREEFGVYAVAS-------------GRVNVAGMTPDNMAPLCEAIVA-VL---<br>SARST: aliSize=311 (resi) RMSD=5.67 (Å)<br>mnyarfitaasaarnpspirtmtdilsrgpksmislagglpnpnmfpfktavitvengktiqfgeemmkr------------------------------------AL---------------------QYSPSAGIPELLSWLKQlqiKLHNpptIHYPPS-QGQMDLCVTSGS--QQGLCKVFE<br>---mfenitaapadpilgladlfraderpgkinlgigvykdETgktpvltsvkkaeqyllenettkNYLGIDGIPEFGRCTQE---LLFG---KGSALInDKRARTAQTPGGtgALRVAADFL<br>MIINPGDNVLLDEPAYSGTLQSLHPLGCNIINVASD---FSGIVPDSLRDILSRWKPEdaknpqknTPkfLYTVPNGNNPTGNSLTSFRKKEIYFLARKYDFLIIEDDPYYFLQFNKfrvptFLSMDVDG------RVIRADS---FSIISSGLRIGFLTGP----KPLIERVILHIQVS---TLHPSTF<br>AKNTSVKRVWVSNPSWPNHKSVFNSAGLEVREYAYYdaeNHTLDFDALINSLNEAQAG--------DV--VLFHGCCHNPTGIDPTLEQWQTLAQLSVEKGWLPLFDFAFQGFARGL-----EEDAEGLRafaamhKELIVASsysKNFGLYNERVGACTLVaadsETVDRAFSQMKAAIranYSNPPAH<br>NQLMISQLLHEWGEFGFMAHVDRVIDFYSNQKDAILAAADKWlTGLAFWHVPAAGMFLWIKVKGINdvkFLIEEKAVKMGVLMLPGNAfyvdssapspylRASFSSASPFQMDVAFQVLAQ--likesl<br>GASVVATILSNDALRAIWEQELTDMRQRIQRMRQLFVNTLQE-KGANRDFSFIIKQNGMFSFSGLT---KEQVLRLREEFGVYAVASG------------RVNVAGMTPDNMAPLCEAIVAvl------<br>BLAST: aliSize=31 (resi) iden=5.81% (23/396) simi=7.83% (31/396)<br>mnyarf----ITAASAarnpSPIRTMTDILSRGPK-SMISLAGGLPNPNmfPFKTAVIT-VENGKTIQFGEEMMKralQYSPSAGIPE---<br>------mfenITAAPA----DPILGLADLFRADERpGKINLGIGVYKDE--TGKTPVLTsVKKAEQYLLENETTK---NYLGIDGIPEfgrctqellfgkgsalindkrartaqtpggtgalrvaadflakntsvkrvwvsnpswpnhksvfnsaglevreyayydaenhtldfdalinslneaqagdvv<br>--<br>lfhgcchnptgidptleqwqtlaqlsvekgwlplfdfafqgfargleedaeglrafaamhkelivassysknfglynervgactlvaadsetvdrafsqmkaairanysnppahgasvvatilsndalraiweqeltdmrqriqrmrqlfvntlqekganrdfsfiikqngmfsfsgltkeqvlrlreef<br>---------------------------------llswlkqlqiklhnpptihyppsqgqmdlcvtsgsqqglckvfemiinpgdnvlldepaysgtlqslhplgcniinvasdesgivpdslrdilsrwkpedaknpqkntpkflytvpngnnptgnsltserkkeiyelarkydfliieddpyyflqfnkf<br>gvyavasgrvnvagmtpdnmaplceaivavl---<br>rvptflsmdvdgrviradsfsiissglrigfltgpkplierv ilhiqvstlhpstfnqlmisqllhewgeegfmahvdrvidfysnqkdailaaadkwltglaewhvpaagmflwikvkgindvkelieekavkmgvlmlpgnafyvdssapspylrasfssaspeqmdvafqvlaqlikesl<br>--<br>Proposed: aliSize=358 (resi) RMSD=2.78 (Å)<br>mNY--Arfitaas AARNPSPIRTMTDILSRGPKSMISLAGGLPNPN------mfpFKT--AV-ITvengktIQFGeeMMKRaLQYSPSAGIPELLSWLKQLQ-IKlhNPPTIHYPpsqgQMDLCVTSGSQQGLCKVFEMI-----INpgDnVLLDEPAYSGTLQSLHP-LGCNIINVAS----DEsGIVp<br>-MFenI-------TAAPADPILGLADLFRADERPGKINLGIGVYKDetgktp---VLTsvKKaEQ------YLLE--NETT-KNYLGIDGIPEFGRCTQELLfGK--GSALINDK----RARTAQTPGGTGALRVAADFLakntsVK--R-VWVSNPSWPNHKSVFNSaGLEVREYAYYdaenHT-LDF-<br>DSLRDILSR-wkpedaknpqkNTPKFLYTVPNGNNPTGNSLTSFRKKEIYFLARKYDFLIIEDDPYYFLQfNK--fRV-PTFLSMDV-DGRVIRADSFSLII-SSGLRIGFLTGP------KP-LIERVILHIQVSTLHPSTFNQLMISQLLHEWG-FEGFMAHVDRVIDFYSNQKDAILAAADKWLTGL<br>DALINSLNEa-----------QAGDVVLFHGCCHNPTGIDPTLEQWQTLAQLSVEKGWLPLFDFAFQGFA-RGle-EDaEGLRAFAAmHKELIVASSYSKNFgLYNERVGACTLVaadsetVDrAFSQMKAAIRANYSNPPAHGASVVATILSNDAlRAIWEQELTDMRQRIQRMRQLFVNTLQEKGANR<br>AE-WHVPAAGMFLWIKVkgindVKFLIEeKAVKM-GVLMLPGnafyvdssapspyLRASFSSASPFQMDVAFQVLAQLIkesl<br>DFsFIIKQNGMFSFSGL-----TKEQVL-RLREEfGVYAVAS-------------GRVNVAGMTPDNMAPLCEAIVAVL----
652	2qlrA (425)<br>1b5oA (382)	N	20.68	TM-align: aliSize=331 (resi) RMSD=2.39 (Å)<br>mnyarfitaasaarnpspirtmtdilsrgpksmislagglpnpnmfpfktavi---TVENGKTI-QFGEeMMKrALQYSPSAGIPELLSWLKQLQIKLHNPpTIHyppsqgQMDLCVTSGSQQGLCKVFEMIINPGDNVLLDEPAYSGT<br>---mrglsrrvqamkpsatvavnakalelrrqgvdlvaltagepdfdTPEHVKEAaRRAL-AQG-KTKYAPPAGIPELREALAEKFRRENGL-SVT------PEETIVTVGGSQALFNLFQAILDPGDEVIVLSPYWVSY<br>LQSLHPLGCNIINVASD---ESGIVPDSLRDILsrWkpedaknpqkNTPKFLYTVPNgNNPTGNSLTSFRKKEIYFLARKYDFLIIEDDPYYFLQFNkFRVPTFLSMDVdGRVIRADSFS-II-SSGLRIGFLTGPKPLIERVILHIQVSTLHPSTFNQLMISQLLHeWgFEGFMAHVDRVIDFYSNQKD<br>PEMVRFAGGVVEVETLpeeGFVPDPERVRRAI--T----------PRTKALVVNSP-NNPTGAVYPKEVLEALARLAVEHDFYLVSDEIYEHLLYE-GEHFSPGRVAP-EHTLTVNGAAkAFaMTGWRIGYACGPKEVIKAMASVSSQSTTSPDTIAQWATLEALT-N-QEASRAFVEMAREAYRRRRD<br>AILAAADKWlTglAFWHVPAAGMFLWIKVKG-INDVKFLIFeKAVKMGVLMLPGNAFYvdSsapSPYLRASFSSASpFQMDVAFQVLAQLiKesl<br>LLLEGLTAL-G--LKAVRPSGAFYVLMDTSPiAPDEVRAAE-RLLEAGVAVVPGTDFA--A---FGHVRLSYATSE-ENLRKALERFARV-L---<br>SARST: aliSize=329 (resi) RMSD=6.24 (Å)<br>mnyarfitaasaarnpspirtmtdilsrgpksmislagglpnpnmfpfkt-------------------------------AVITVENGKTIQFGEEMMKRA--------LQYSPSAGIPELLSWLKQLQIKLHNPptiHYPPSQgqmdLCVTSGSQQGLCKVFEMIINPGDNVLLDEPAYSGTLQSLH<br>--mrglsrrvqamkpsatvavnakalelrrqgvdLVALTAGEPDFDTPEHVKEAArralaqgkTKYAPPAGIPELREALAEKFRRENGL---SVTPEE----TIVTVGGSQALFNLFQAILDPGDEVIVLSPYWVSYPEMVR<br>PLGCNIINVASD-ESG--IVPDSLRDilsRWKPEdaknpqkntPKFLYTVpNGNNPTGNSLTSFRKKEIYFLARKYDFLIIEDDPYYFLQFNkFRVPTFLSMDVDgRVIRADSFSIIS--SGLRIGFLTGPKPLIERVILHIQVSTLHPSTFNQLMISqlLHEWGFEGFMAHVDRVIDFYSNQKDAILAA<br>FAGGVVEVETLpEEGfvPDPERVRR---AITPR----------TKALVVN-SPNNPTGAVYPKEVLEALARLAVEHDFYLVSDEIYEHLLYE-GEHFSPGRVAPE-HTLTVNGAAKAFamTGWRIGYACGPKEVIKAMASVSSQSTTSPDTIAQWATL--EALTNQEASRAFVEMAREAYRRRRDLLLEG<br>ADKWltgLAFWHVPAAGMFLWIKvkgindVK------ELIEFKAVKMGVLMLPGNafyvdSSAPSPYLRASFsSASPFQMDVAFQVLAQ--likesl<br>LTAL---GLKAVRPSGAFYVLMD------TSpiapdeVRAAERLLEAGVAVVPGT-----DFAAFGHVRLSY-ATSEENLRKALERFARvl------<br>BLAST: aliSize=167 (resi) iden=25.92% (99/382) simi=43.72% (167/382)<br>mnyarfitaasaarnpspirtmtdilsrgpksmislagglpnpnmfpfktavitvengktiqfg---EEMMKRAL-----QYSPSAGIPELLSWLKQlqiKLHNPPTIHYPPSQgqmdLCVTSGSQQGLCKVFEMIINPGDNVL<br>---mrglsrrvqamkpsatvavnakalelrrqgvdlvaltagepdfdtpehvKEAARRALaqgktKYAPPAGIPELREALAE---KFRRENGLSVTPEE----TIVTVGGSQALFNLFQAILDPGDEVI<br>LDEPAYSGTLQSLHPLGCNIINVAS-DESGIVPDslrdilsrwkPEDAKnpQKNTPKFLYTVPNG-NNPTGNSLTSFRKKEIYFLARKYDFLIIEDDPYYFL-----QFNKFRVPTFLSMDVDGRvirADSFSIisSGLRIGFLTGPKPLIERVILHIQVSTLHPSTFNQLMISQLLHFwgFEGFMAHVD<br>VLSPYWVSYPEMVRFAGGVVEVETlPEEGFVPD-----------PERVR--RAITPRTKALVVNSpNNPTGAVYPKEVLEALARLAVEHDFYLVSDEIYEHLlyegeHFSPGRVAPEHTLTVNGA---AKAFAM--TGWRIGYACGPKEVIKAMASVSSQSTTSPDTIAQWATLEALTN--QEASRAFVE<br>RVIDFYSNQKDAILAAadkwLTGLAEWHV-PAAGMFLWIKVKGINDVKELIEFKAVKMGVLMLPGNAFyvdssAPSPYLRASFSSaSPFQMDVAFQVLAQLIkesl<br>MAREAYRRRRDLLLEG----LTALGLKAVrPSGAFYVLMDTSPIAPDEVRAAERLLEAGVAVVPGTDF-----AAFGHVRLSYAT-SEENLRKALERFARVL----<br>Proposed: aliSize=359 (resi) RMSD=2.10 (Å)<br>MNYArfITaASAARNPSPIRTMTDILSR--GPKSMI--slaggLPnP--nmFpFKTA-V--IT--V--FN--gktiqfgeemmkrALQYSPSAGIPELLSWLKQLQIKLHNPpTIhyppsqgqM--DLCVTSGSQQGLCKVFEMIINPGDNVLLDEPAYSGTLQSLHPLGCNIINVASD---ESGIVPDS<br>MRGL--SR-RVQAMKPSATVAVNAKALElrRQGVDLva-----LT-Age--P-DFDTpEhvKEaaRraLAqg-------------KTKYAPPAGIPELREALAEKFRRENGL-SV--------TpeETIVTVGGSQALFNLFQAILDPGDEVIVLSPYWVSYPEMVRFAGGVVEVETLpeeGFVPDPER<br>LRDILsrWkpedaknpqkNTPKFLYTVPNgNNPTGNSLTSFRKKEIYFLARKYDFLIIEDDPYYFLQFNkFRVPTFLSMDVdGRVIRADSFSLII-SSGLRIGFLTGPKPLIERVILHIQVSTLHPSTFNQLMISQLLHeWgFEGFMAHVDRVIDFYSNQKDAILAAADKWlTglAFWHVPAAGMFLWIK<br>VRRAI--T----------PRTKALVVNSP-NNPTGAVYPKEVLEALARLAVEHDFYLVSDEIYEHLLYE-GEHFSPGRVAP-EHTLTVNGAAKAFaMTGWRIGYACGPKEVIKAMASVSSQSTTSPDTIAQWATLEALT-N-QEASRAFVEMAREAYRRRRDLLLEGLTAL-G--LKAVRPSGAFYVLMD<br>VKG-INDVKFLIFeKAVKMGVLMLPGNAFY-vdssapSPYLRASFSSaSPFQMDVAFQVLAQLiKesl<br>TSPiAPDEVRAAE-RLLEAGVAVVPGTDFAa------FGHVRLSYAT-SEENLRKALERFARV-L---
653	2qlrA (425)<br>1bkgA (382)	N	20.42	TM-align: aliSize=331 (resi) RMSD=2.39 (Å)<br>mnyarfitaasaarnpspirtmtdilsrgpksmislagglpnpnmfpfktavi---TVENGKTI-QFGEeMMKrALQYSPSAGIPELLSWLKQLQIKLHNPpTIHyppsqgQMDLCVTSGSQQGLCKVFEMIINPGDNVLLDEPAYSGT<br>--mrglsrrvqamkpsatvavnakalelrrqgvdlvaltagepdfdTPEHVKEAaRRAL-AQG-KTKYAPPAGIPELREALAEKFRRENGL-SVT------PEETIVTVGGKQALFNLFQAILDPGDEVIVLSPYWVSY<br>LQSLHPLGCNIINVASD---ESGIVPDSLRDILsrWkpedaknpqkNTPKFLYTVPNgNNPTGNSLTSFRKKEIYFLARKYDFLIIEDDPYYFLQFNkFRVPTFLSMDVdGRVIRADSFS-II-SSGLRIGFLTGPKPLIERVILHIQVSTLHPSTFNQLMISQLLHeWgFEGFMAHVDRVIDFYSNQKD<br>PEMVRFAGGVVEVETLpeeGFVPDPERVRRAI--T----------PRTKALVVNSP-NNPTGAVYPKEVLEALARLAVEHDFYLVSDEIYEHLLYE-GEHFSPGRVAP-EHTLTVNGAAkAFaMTGWRIGYACGPKEVIKAMASVSSQSTTSPDTIAQWATLEALT-N-QEASRAFVEMAREAYRRRRD<br>AILAAADKWlTglAFWHVPAAGMFLWIKVKG-INDVKFLIFeKAVKMGVLMLPGNAFYvdssaPSPYLRASFSSASpFQMDVAFQVLAQLiKesl<br>LLLEGLTAL-G--LKAVRPSGAFYVLMDTSPiAPDEVRAAE-RLLEAGVAVVPGTDFA-----AFGHVRLSYATSE-ENLRKALERFARV-L---<br>SARST: aliSize=330 (resi) RMSD=4.92 (Å)<br>mnyarfitaasaarnpspirtmtdilsrgpksmislagglpnpnmfpfktavitv------------------------------------ENGKTIQFGEEMMKRA--------LQYSPSAGIPELLSWLKQLQIKLHNPptiHYPPSQgqmdLCVTSGSQQGLCKVFEMIINPGDNVLLDEPAYSGT<br>--mrglsrrvqamkpsatvavnakalelrrqgvdlvaltAGEPDFDTPEHVKEAArralaqgkTKYAPPAGIPELREALAEKFRRENGL---SVTPEE----TIVTVGGKQALFNLFQAILDPGDEVIVLSPYWVSY<br>LQSLHPLGCNIINVASDESG---IVPDSLRDilsRWKPEdaknpqkntPKFLYTVpNGNNPTGNSLTSFRKKEIYFLARKYDFLIIEDDPYYFLQFNkFRVPTFLSMDVDgRVIRADSFSI--ISSGLRIGFLTGPKPLIERVILHIQVSTLHPSTFNQLMISqlLHEWGFEGFMAHVDRVIDFYSNQKD<br>PEMVRFAGGVVEVETLPEEgfvPDPERVRR---AITPR---------TKALVVN-SPNNPTGAVYPKEVLEALARLAVEHDFYLVSDEIYEHLLYE-GEHFSPGRVAPE-HTLTVNGAAKafAMTGWRIGYACGPKEVIKAMASVSSQSTTSPDTIAQWATL--EALTNQEASRAFVEMAREAYRRRRD<br>AILAAADKWltgLAFWHVPAAGMFLWIKV-KGINDVKFLIFeKAVKMGVLMLPGNafyvdSSAPSPYLRASFsSASPFQMDVAFQVLAQL-ikesl<br>LLLEGLTAL---GLKAVRPSGAFYVLMDTsPIAPDEVRAAE-RLLEAGVAVVPGT-----DFAAFGHVRLSY-ATSEENLRKALERFARVl-----<br>BLAST: aliSize=168 (resi) iden=25.92% (99/382) simi=43.98% (168/382)<br>mnyarfitaasaarnpspirtmtdilsrgpksmislagglpnpnmfpfktavitvengktiqfg---EEMMKRAL-----QYSPSAGIPELLSWLKQlqiKLHNPPTIHYPPSQgqmdLCVTSGSQQGLCKVFEMIINPGDNVL<br>---mrglsrrvqamkpsatvavnakalelrrqgvdlvaltagepdfdtpehvKEAARRALaqgktKYAPPAGIPELREALAE---KFRRENGLSVTPEE----TIVTVGGKQALFNLFQAILDPGDEVI<br>LDEPAYSGTLQSLHPLGCNIINVAS-DESGIVPDslrdilsrwkPEDAKnpQKNTPKFLYTVPNG-NNPTGNSLTSFRKKEIYFLARKYDFLIIEDDPYYFL-----QFNKFRVPTFLSMDVDGRvirADSFSIisSGLRIGFLTGPKPLIERVILHIQVSTLHPSTFNQLMISQLLHFwgFEGFMAHVD<br>VLSPYWVSYPEMVRFAGGVVEVETlPEEGFVPD-----------PERVR--RAITPRTKALVVNSpNNPTGAVYPKEVLEALARLAVEHDFYLVSDEIYEHLlyegeHFSPGRVAPEHTLTVNGA---AKAFAM--TGWRIGYACGPKEVIKAMASVSSQSTTSPDTIAQWATLEALTN--QEASRAFVE<br>RVIDFYSNQKDAILAAadkwLTGLAEWHV-PAAGMFLWIKVKGINDVKELIEFKAVKMGVLMLPGNAFyvdssAPSPYLRASFSSaSPFQMDVAFQVLAQLIkesl<br>MAREAYRRRRDLLLEG----LTALGLKAVrPSGAFYVLMDTSPIAPDEVRAAERLLEAGVAVVPGTDF-----AAFGHVRLSYAT-SEENLRKALERFARVL----<br>Proposed: aliSize=361 (resi) RMSD=2.00 (Å)<br>MNYArfItAASAARNPSP-IRTMTDILS--RGPKSMISLaGGLPNPN-mfpFKT--AV--IT--VFN-gktiqfgeemmkraLQYSPSAGIPELLSWLKQLQIKLHNPpTIhyppsqgqM--DLCVTSGSQQGLCKVFEMIINPGDNVLLDEPAYSGTLQSLHPLGCNIINVASD---ESGIVPDSLRDI<br>MRGL--S-RRVQAMKPSAtVAVNAKALElrRQGVDLVAL-TAGEPDFd---TPEhvKEaaRRalAQGk-------------KTKYAPPAGIPELREALAEKFRRENGL-SV--------TpeETIVTVGGKQALFNLFQAILDPGDEVIVLSPYWVSYPEMVRFAGGVVEVETLpeeGFVPDPERVRRA<br>LsrWkpedaknpqkNTPKFLYTVPNgNNPTGNSLTSFRKKEIYFLARKYDFLIIEDDPYYFLQFNkFRVPTFLSMDVdGRVIRADSFSLII-SSGLRIGFLTGPKPLIERVILHIQVSTLHPSTFNQLMISQLLHeWgFEGFMAHVDRVIDFYSNQKDAILAAADKWlTglAFWHVPAAGMFLWIKVKG-<br>I--T----------PRTKALVVNSP-NNPTGAVYPKEVLEALARLAVEHDFYLVSDEIYEHLLYE-GEHFSPGRVAP-EHTLTVNGAAKAFaMTGWRIGYACGPKEVIKAMASVSSQSTTSPDTIAQWATLEALT-N-QEASRAFVEMAREAYRRRRDLLLEGLTAL-G--LKAVRPSGAFYVLMDTSPi<br>INDVKFLIFeKAVKMGVLMLPGNAFY-vdssapSPYLRASFSSAS--PFQMDVAFQVLaQlikesl<br>APDEVRAAE-RLLEAGVAVVPGTDFAa------FGHVRLSYATSEenLRKALERFARV-L------

<table>
<tr><td rowspan="2">654</td><td>2qlrA
(425)</td><td rowspan="2">N</td><td rowspan="2">12.63</td><td rowspan="2">TM-align: aliSize=331 (resi)   RMSD=3.18 (Å)
mnyarfitaasaarnpspirtmtdilsrgpksmislagglpnpnmfpfktavi--TVENG-KT-IQFGEeMMKRaLQYSPSAGIPFLLSWLKQLQIKLhNPPTIHYPpsqgQMDLCVTSGSQQGLCKVFEMIIN--PGDNVLLDEPAY
--mfenitaapadpilgladlfraderpgkinlgigvykdetgktpVLTSVkKAeQYLLE-NETT-KNYLGIDGIPEFGRCTQELLFGK-GSALINDK----RARTAQTPGGTGALRVAADFLAKntSVKRVWVSNPSW

SGTLQSLHP-LGCNIINVASD---ESGIVpDSLRDILSRWkpedaknpqkNTPKFLYTVPNGNNPTGNSLTSERKKEIYELARKYDFLIIEDDPYYFLQFNKFRV-PTFLSMD-VDGRVIRADSFS-II-SSGLRIGFLTGP------K-PLIERVILHIQVSTLHPSTFNQLMISQLLHEWG-EEGFMA
PNHKSVFNSaGLEVREYAYYDaenHTLDF-DALINSLNEA----------QAGDVVLFHGCCHAPTGIDPTLEQWQTLAQLSVEKGWLPLFDFAFQGFARGLEEDaEGLRAFAaMHKELIVASSYSkNFgLYNERVGACTLVaadsetVdRAFSQMKAAIRANYSNPPAHGASVVATILSNDAlRAIWEQ

HVDRVIDFYSNQKDAILAAADKWLTGLAEWH-VPAAGMFLWIKVkgindVKELIEEKAVKMGVLMLPGnafyvdssapspyLRASFSSASPEQMDVAFQVLAQLIkesl
ELTDMRQRIQRMRQLFVNTLQEKGANRDFSFiIKQNGMFSFSGL-----TKEQVLRLREEFGVYAVAS-------------GRVNVAGMTPDNMAPLCEAIVAVL----

SARST: aliSize=351 (resi)   RMSD=14.86 (Å)
mnyarfitaas--AARNPSPIRTMTD----ILSRGPKSMISLAGGLPNPNMfpfktavitveNGKTIqfGEEMMKRA---------LQYSPSAGIPFLLSWLKQLQiklhnpPTIHYPPS-QGQMDLCVTSGSQQGLCKVFE---MIINPGdNVLLDEPAYSGTLQSLHPLGCNIINVASD---ESGIVP
-----------mfENITAAPADPILGladlFRADERPGKINLGIGVYKDET-----------GKTPV--LTSVKKAEqyllenettKNYLGIDGIPEFGRCTQELL------FGKGSALInDKRARTAQTPGGTGALRVAADflaKNTSVK-RVWVSNPSWPNHKSVFNSAGLEVREYAYYdaeNHTLDF

DSLRDILSRWKpedaknpqKNTPkfLYTVPNGNNPTGNSLTSERKKEIYELARKYDFLIIEDDPYYFLQFnkfrvpTFLSMDV-------DGRVIRADS---FSIISSGLRIGFLTGP-------KPLIERVILHIQVSTLHPSTFNQLMISQLLHEWgEEGFMAHVDRVIDFYSNQKDAILAAADKWlT
DALINSLNEAQ--------AGDV--VLFHGCCHAPTGIDPTLEQWQTLAQLSVEKGWLPLFDFAFQGFAR------GLEEDAEglrafaaMHKELIVASsysKNFGLYNERVGACTLVaadsetvDRAFSQMKAAIRANYSNPPAHGASVVATILSND-ALRAIWEQELTDMRQRIQRMRQLFVNTLQ-E

GLAEWHVPA----AGMFLwikVKGINdvKELIEEKAVKMGVLMlpgnafyvDSSAPspylrASFSSASPEQMDVAFQVLAQ--likesl
KGANRDFSFiikqNGMFS---FSGLT--KEQVLRLREEFGVYA--------VASGR-----VNVAGMTPDNMAPLCEAIVAvl------

BLAST: aliSize=31 (resi)   iden=5.81% (23/396)   simi=7.83% (31/396)
mnyarf----ITAASAarnpSPIRTMTDILSRGPK-SMISLAGGLPNPNmfPFKTAVIT-VENGKTIQFGEEMMKralQYSPSAGIPF--
------mfenITAAPA----DPILGLADLFRADERpGKINLGIGVYKDE--TGKTPVLTsVKKAEQYLLENETTK---NYLGIDGIPEfgrctqellfgkgsalindkrartaqtpggtgalrvaadflakntsvkrvwvsnpswpnhksvfnsaglevreyayydaenhtldfdalinslneaqagdvv

----
lfhgcchaptgidptleqwqtlaqlsvekgwlplfdfafqgfargleedaeglrafaamhkelivassysknfglynervgactlvaadsetvdrafsqmkaairanysnppahgasvvatilsndalraiweqeltdmrqriqrmrqlfvntlqekganrdfsfiikqngmfsfsgltkeqvlrlreef

--------------------------------llswlkqlqiklhnpptihyppsqgqmdlcvtsgsqqglckvfemiinpgdnvlldepaysgtlqslhplgcniinvasdesgivpdslrdilsrwkpedaknpqkntpkflytvpngnnptgnsltserkkeiyelarkydfliieddpyyflqfnkf
gvyavasgrvnvagmtpdnmaplceaivavl--

rvptflsmdvdgrviradsfsiissglrigfltgpkplierviIhiqvstlhpstfnqlmisqllhewgeegfmahvdrvidfysnqkdailaaadkwltglaewhvpaagmflwikvkgindvkelieekavkmgvlmlpgnafyvdssapspylrasfssaspeqmdvafqvlaqlikesl
----

Proposed: aliSize=353 (resi)   RMSD=2.66 (Å)
mn--YArfItaasA-aRNPSPIRTMTDILSRG-PKSMISLAGGLPN-----PNMF--PF----ktavitvengktIQFGeeMMKRaLQYSPSAGIPFLLSWLKQLQIKLhNPPTI-HYppsqgQMDLCVTSGSQQGLCKVFEMII----NpgDNVLLDEPAYSGTLQSLHP-LGCNIINVASD----EsG
--mfEN--I----Ta-APADPILGLADLFRADeRPGKINLGIGVYKdetgkTPVLtsVKkaeq------------YLLE--NETT-KNYLGIDGIPEFGRCTQELLFGK-GSALInDK-----RARTAQTPGGTGALRVAADFLAkntsV--KRVWVSNPSWPNHKSVFNSaGLEVREYAYYDaenhT-L

IVpDSLRDI-LsR-wkpedaknpqkNTPKFLYTVPNGNNPTGNSLTSERKKEIYELARKYDFLIIEDDPYYFLQfNK--fRV-PTFLSMD-VDGRVIRADSFSLII-SSGLRIGFLTGP------K-PLIERVILHIQVSTLHPSTFNQLMISQLLHEWG-EEGFMAHVDRVIDFYSNQKDAILAADKW
DF-DALINSlN-Ea-----------QAGDVVLFHGCCHAPTGIDPTLEQWQTLAQLSVEKGWLPLFDFAFQGFA-RGle-EDaEGLRAFAaMHKELIVASSYSKNFgLYNERVGACTLVaadsetVdRAFSQMKAAIRANYSNPPAHGASVVATILSNDAlRAIWEQELTDMRQRIQRMRQLFVNTLQEK

LTGLaFW-H-VPAAGMFLWIKVkgindVKELIEeKAVKM-GVLMLPGnafyvdssapspyLRASFSSASPEQMDVAFQVLAQLIkesl
GANR-DFsFiIKQNGMFSFSGL-----TKEQVL-RLREEfGVYAVAS-------------GRVNVAGMTPDNMAPLCEAIVAVL----</td></tr>
<tr><td>1g4vA
(396)</td></tr>
<tr><td rowspan="2">655</td><td>2qlrA
(425)</td><td rowspan="2">N</td><td rowspan="2">13.13</td><td rowspan="2">TM-align: aliSize=332 (resi)   RMSD=3.29 (Å)
mnyarfitaasaarnpspirtmtdilsrgpksmislagglpnpnmfpfktav--ITVENGKT-IQFGEeMMKRaLQYSPSAGIPFLLSWLKQLQIKLhNPPTIHYPpsqgQMDLCVTSGSQQGLCKVFEMIIN--PGDNVLLDEPAYS
---mfenitaapadpilgladlfraderpgkinlgigvykdetgktpVLTSVKKAeQYLLE-NETT-KNYLGIDGIPEFGRCTQELLFGK-GSALINDK----RARTAQTPGGTGALRVAADFLAKntSVKRVWVSNPSWP

GTLQSLHP-LGCNIINVASD---ESGIVpDSLRDILSRWkpedaknpqkNTPKFLYTVPNGNNPTGNSLTSERKKEIYELARKYDFLIIEDDPYYFLQFNKFRV-PTFLSMD-VDGRVIRADSFS-II-SSGLRIGFLTGP------K-PLIERVILHIQVSTLHPSTFNQLMISQLLHEWG-EEGFMAH
NHKSVFNSaGLEVREYAYYDaenHTLDF-DALINSLNEA----------QAGDVVLFHGCCHAPTGIDPTLEQWQTLAQLSVEKGWLPLFDFAYQGFARGLEEDaEGLRAFAaMHKELIVASSYSkNFgLYNERVGACTLVaadsetVdRAFSQMKAAILANYSNPPAHGASVVATILSNDAlRAIWEQE

VDRVIDFYSNQKDAILAAADKWLTGLAEWHV-PAAGMFLWIKVkgindVKELIEEKAVKMGVLMLPGnafyvdssapspyLRASFSSASPEQMDVAFQVLAQLIkesl
LTDMRQRIQRMRQLFVNTLQEKGANRDFSFIiKQNGMFSFSGL-----TKEQVLRLREEFGVYAVAS-------------GRVNVAGMTPDNMAPLCEAIVAVL----

SARST: aliSize=335 (resi)   RMSD=14.07 (Å)
mnyarfitaasaarnpspirt-----------------MTDILSRgPKSMISLAGGLPNPNMfpfktavitveNGKTIqfGEEMMKRA---------LQYSPSAGIPFllsWLKQLQIKLHNpptIHY-PPSQGQMDLCVTSGSQQGLCKVFEM--IINPGDNVLLDEPAYSGTLQSLHPLGCNIINVAS
---------------------mfenitaapadpilglaDLFRADE-RPGKINLGIGVYKDET-----------GKTPV--LTSVKKAEqyllenettKNYLGIDGIPE---FGRCTQELLFG---KGSaLINDKRARTAQTPGGTGALRVAADFlaKNTSVKRVWVSNPSWPNHKSVFNSAGLEVREYAY

D---ESGIVPDSLRDILSRWKpedaknpqKNTPkfLYTVPNGNNPTGNSLTSERKKEIYELARKYDFLIIEDDPYYFLQFnkfrvpTFLSMDV-------DGRVIRADS---FSIISSGLRIGFLT----GPKPLIERV---ILHIQVSTLHPSTFNQLMISQLLH----EWGEEGFMAHVDRVIDFYSN
YdaeNHTLDFDALINSLNEAQ--------AGDV--VLFHGCCHAPTGIDPTLEQWQTLAQLSVEKGWLPLFDFAYQGFAR------GLEEDAEglrafaaMHKELIVASsysKNFGLYNERVGACTlvaaDSETVDRAFsqmKAAILANYSNPPAHGASVVATILSndalRAIWEQELTDMRQRIQRMRQ

QKDAILAAAdkwltglaEWHVPA------AGMFLWIKVKgindvKELIEEKAVKMGVLMlpgnafyvDSSAPspylrASFSSASPEQMDVAFQVLAQ--likesl
LFVNTLQEK--------GANRDFsfiikqNGMFSFSGLT-----KEQVLRLREEFGVYA--------VASGR-----VNVAGMTPDNMAPLCEAIVAvl------

BLAST: aliSize=31 (resi)   iden=5.81% (23/396)   simi=7.83% (31/396)
mnyarf----ITAASAarnpSPIRTMTDILSRGPK-SMISLAGGLPNPNmfPFKTAVIT-VENGKTIQFGEEMMKralQYSPSAGIPF--
------mfenITAAPA----DPILGLADLFRADERpGKINLGIGVYKDE--TGKTPVLTsVKKAEQYLLENETTK---NYLGIDGIPEfgrctqellfgkgsalindkrartaqtpggtgalrvaadflakntsvkrvwvsnpswpnhksvfnsaglevreyayydaenhtldfdalinslneaqagdvv

----
lfhgcchaptgidptleqwqtlaqlsvekgwlplfdfayqgfargleedaeglrafaamhkelivassysknfglynervgactlvaadsetvdrafsqmkaailanysnppahgasvvatilsndalraiweqeltdmrqriqrmrqlfvntlqekganrdfsfiikqngmfsfsgltkeqvlrlreef

--------------------------------llswlkqlqiklhnpptihyppsqgqmdlcvtsgsqqglckvfemiinpgdnvlldepaysgtlqslhplgcniinvasdesgivpdslrdilsrwkpedaknpqkntpkflytvpngnnptgnsltserkkeiyelarkydfliieddpyyflqfnkf
gvyavasgrvnvagmtpdnmaplceaivavl--

rvptflsmdvdgrviradsfsiissglrigfltgpkplierviIhiqvstlhpstfnqlmisqllhewgeegfmahvdrvidfysnqkdailaaadkwltglaewhvpaagmflwikvkgindvkelieekavkmgvlmlpgnafyvdssapspylrasfssaspeqmdvafqvlaqlikesl
----

Proposed: aliSize=352 (resi)   RMSD=2.68 (Å)
mn--YArfItaasA-aRNPSPIRTMTDILSRG-PKSMISLAGGLPN-----PNMF--PF----ktavitvengktIQFGeeMMKRaLQYSPSAGIPFLLSWLKQLQ-IKlhNPPTIHYPpsqgQMDLCVTSGSQQGLCKVFEMII----NpgDNVLLDEPAYSGTLQSLHP-LGCNIINVASD----EsG
--mfEN--I----Ta-APADPILGLADLFRADeRPGKINLGIGVYKdetgkTPVLtsVKkaeq------------YLLE--NETT-KNYLGIDGIPEFGRCTQELLfGK--GSALINDK----RARTAQTPGGTGALRVAADFLAkntsV--KRVWVSNPSWPNHKSVFNSaGLEVREYAYYDaenhT-L

IVpDSLRDI-LsR-wkpedaknpqkNTPKFLYTVPNGNNPTGNSLTSERKKEIYELARKYDFLIIEDDPYYFLQfNK--fRV-PTFLSMDV-DGRVIRADSFSLII-SSGLRIGFLTGP------K-PLIERVILHIQVSTLHPSTFNQLMISQLLHEWG-EEGFMAHVDRVIDFYSNQKDAILAAADKW
DF-DALINSlN-Ea-----------QAGDVVLFHGCCHAPTGIDPTLEQWQTLAQLSVEKGWLPLFDFAYQGFA-RGle-EDaEGLRAFAAmHKELIVASSYSKNFgLYNERVGACTLVaadsetVdRAFSQMKAAILANYSNPPAHGASVVATILSNDAlRAIWEQELTDMRQRIQRMRQLFVNTLQEK

-LTGlaFW-HV-PAAGMFLWIKVkgindVKELIEeKAVKM-GVLMLPGnafyvdssapspyLRASFSSASPEQMDVAFQVLAQLIkesl
gANR--DFsFIiKQNGMFSFSGL-----TKEQVL-RLREEfGVYAVAS-------------GRVNVAGMTPDNMAPLCEAIVAVL----</td></tr>
<tr><td>1g4xA
(396)</td></tr>
<tr><td rowspan="2">656</td><td>2qlrA
(425)</td><td rowspan="2">N</td><td rowspan="2">13.89</td><td rowspan="2">TM-align: aliSize=332 (resi)   RMSD=3.27 (Å)
mnyarfitaasaarnpspirtmtdilsrgpksmislagglpnpnmfpfktav--ITVENGKT-IQFGEeMMKRaLQYSPSAGIPFLLSWLKQLQIKLhNPPTIHYPpsqgQMDLCVTSGSQQGLCKVFEMIIN--PGDNVLLDEPAYS
---mfenitaapadpilgladlfraderpgkinlgigvykdetgktpVLTSVKKAeQYLLE-NETT-KNYLGIDGIPEFGRCTQELLFGK-GSALINDK----RARTAQTPGGTGALRVAADFLAKntSVKRVWVSNPSWP

GTLQSLHP-LGCNIINVASD---ESGIVpDSLRDILSRWkpedaknpqkNTPKFLYTVPNGNNPTGNSLTSERKKEIYELARKYDFLIIEDDPYYFLQFNKFRV-PTFLSMD-VDGRVIRADSFS-II-SSGLRIGFLTGP------K-PLIERVILHIQVSTLHPSTFNQLMISQLLHEWG-EEGFMAH
NHKSVFNSaGLEVREYAYYDaenHTLDF-DALINSLNEA----------QAGDVVLFHGCCHAPTGIDPTLEQWQTLAQLSVEKGWLPLFDFAYQGFARGLEEDaEGLRAFAaMHKELIVASSYSkNFgLYNERVGACTLVaadsetVdRAFSQMKAAIRANYSNPPAHGASVVATILSNDAlRAIWEQE

VDRVIDFYSNQKDAILAAADKWLTGLAEWH-VPAAGMFLWIKVkgindVKELIEEKAVKMGVLMLPGnafyvdssapspyLRASFSSASPEQMDVAFQVLAQLIkesl
LTDMRQRIQRMRQLFVNTLQEKGANRDFSFiIKQNGMFSFSGL-----TKEQVLRLREEFGVYAVAS-------------GLVNVAGMTPDNMAPLCEAIVAVL----

SARST: aliSize=330 (resi)   RMSD=12.17 (Å)
mnyarfitaasaarnpspirtmtdilsrg----------------------PKSMISLAGGLPNPNMfpfktavitveNGKTiqFGEEMMKRA---------LQYSPSAGIpelLSWLKQLQIKLHNpptIHYPP-SQGQMDLCVTSGSQQGLCKVFE---MIINPGdNVLLDEPAYSGTLQSLHPLG
----------------------------mfenitaapadpilgladlfradeRPGKINLGIGVYKDET-----------GKTP--VLTSVKKAEqyllenettKNYLGIDGI---PEFGRCTQELLFG---KGSALiNDKRARTAQTPGGTGALRVAADflaKNTSVK-RVWVSNPSWPNHKSVFNSAG

CNIINVASD---ESGIVPDSLRDILSRWKpedaknpqKNTPkfLYTVPNGNNPTGNSLTSERKKEIYELARKYDFLIIEDDPYYFLQFnkfrvpTFLSMD-------VDGRVIRADS---FSIISSGLRIGFLTGP-------KPLIERVILHIQVSTLHPSTFNQLMISQLLH-EWGEEGFMAHVDRVI
LEVREYAYYdaeNHTLDFDALINSLNEAQ--------AGDV--VLFHGCCHAPTGIDPTLEQWQTLAQLSVEKGWLPLFDFAYQGFAR------GLEEDAeglrafaAMHKELIVASsysKNFGLYNERVGACTLVaadsetvDRAFSQMKAAIRANYSNPPAHGASVVATILSnDALRAIWEQELTDMR

DFYSNQKDAILAAADKWLtglaeWHVPA------AGMFLWIKVKgindvKELIEEKAVKMGVLMlpgnafyvDSSAPspylrASFSSASPEQMDVAFQVLAQ--likesl
QRIQRMRQLFVNTLQEKG-----ANRDFsfiikqNGMFSFSGLT-----KEQVLRLREEFGVYA--------VASGL-----VNVAGMTPDNMAPLCEAIVAvl------

BLAST: aliSize=31 (resi)   iden=5.81% (23/396)   simi=7.83% (31/396)
mnyarf----ITAASAarnpSPIRTMTDILSRGPK-SMISLAGGLPNPNmfPFKTAVIT-VENGKTIQFGEEMMKralQYSPSAGIPF--
------mfenITAAPA----DPILGLADLFRADERpGKINLGIGVYKDE--TGKTPVLTsVKKAEQYLLENETTK---NYLGIDGIPEfgrctqellfgkgsalindkrartaqtpggtgalrvaadflakntsvkrvwvsnpswpnhksvfnsaglevreyayydaenhtldfdalinslneaqagdvv

----
lfhgcchaptgidptleqwqtlaqlsvekgwlplfdfayqgfargleedaeglrafaamhkelivassysknfglynervgactlvaadsetvdrafsqmkaairanysnppahgasvvatilsndalraiweqeltdmrqriqrmrqlfvntlqekganrdfsfiikqngmfsfsgltkeqvlrlreef

--------------------------------llswlkqlqiklhnpptihyppsqgqmdlcvtsgsqqglckvfemiinpgdnvlldepaysgtlqslhplgcniinvasdesgivpdslrdilsrwkpedaknpqkntpkflytvpngnnptgnsltserkkeiyelarkydfliieddpyyflqfnkf
gvyavasglvnvagmtpdnmaplceaivavl--

rvptflsmdvdgrviradsfsiissglrigfltgpkplierviIhiqvstlhpstfnqlmisqllhewgeegfmahvdrvidfysnqkdailaaadkwltglaewhvpaagmflwikvkgindvkelieekavkmgvlmlpgnafyvdssapspylrasfssaspeqmdvafqvlaqlikesl
----

Proposed: aliSize=350 (resi)   RMSD=2.77 (Å)
mn-YARfItaasAARNPSPIRTMTDILSR-GPKSMISLaGGLPNPN-------mfpfKT-AV--IT--VE-----ngktiqfgeemmkralQYSPSAGIPFLLSWLKQLQIKLhNPPTIHYPpsqgQMDLCVTSGSQQGLCKVFEMII----NpgDNVLLDEPAYSGTLQSLHP-LGCNIINVASD----
--mFEN-I----TAAPADPILGLADLFRAdERPGKINL-GIGVYKDetgktpv----LTsVKkaEQylLEnettk---------------NYLGIDGIPEFGRCTQELLFGK-GSALINDK----RARTAQTPGGTGALRVAADFLAkntsV--KRVWVSNPSWPNHKSVFNSaGLEVREYAYYDaenh

EsGIVpDSLRDILSR-WkpedAknpqknTpKFLYTVPNGNNPTGNSLTSERKKEIYELARKYDFLIIEDDPYYFLQfNK--fRV-PTFLSMD-VDGRVIRADSFSLII-SSGLRIGFLTGP------K-PLIERVILHIQVSTLHPSTFNQLMISQLLHEWG-EEGFMAHVDRVIDFYSNQKDAILAAAD
T-LDF-DALINSLNEaQ----A------G-DVVLFHGCCHAPTGIDPTLEQWQTLAQLSVEKGWLPLFDFAYQGFA-RGle-EDaEGLRAFAaMHKELIVASSYSKNFgLYNERVGACTLVaadsetVdRAFSQMKAAIRANYSNPPAHGASVVATILSNDAlRAIWEQELTDMRQRIQRMRQLFVNTLQ

KW-lTGLaFW-H-VPAAGMFLWIKVkgindVKELIEeKAVK-MGVLMLPGnafyvdssapspyLRASFSSASPEQMDVAFQVLAQLIkesl
EKg-ANR-DFsFiIKQNGMFSFSGL-----TKEQVL-RLREeFGVYAVAS-------------GLVNVAGMTPDNMAPLCEAIVAVL----</td></tr>
<tr><td>1g7wA
(396)</td></tr>
</table>

<table>
<tr><td>657</td><td>2qlrA (425)
1g7xA (396)</td><td>N</td><td>12.88</td><td>TM-align: aliSize=332 (resi) RMSD=3.26 (Å)
mnyarfitaasaarnpspirtmtdilsrgpksmislagglpnpnmfpfktav---ITVENGKT-IQFGEeMMKRaLQYSPSAGIPELLSWLKQLQIKLhNPPTIHYPpsqgQMDLCVTSGSQQGLCKVFEMIIN--PGDNVLLDEPAYS
--mfenitaapadpilgladlfraderpgkinlgigvykdetgktpVLTSVKKAeQYLLE-NETT-KNYLGIDGIPEFGRCTQELLFGK-GSALINDK----RARTAQTPGGTGALRVAADFLAKntSVKRVWVSNPSWP
GTLQSLHP-LGCNIINVASD---ESGIVpDSLRDILSRWkpedaknpqkNTPKFLYTVPNGNNPTGNSLTSERKKEIYELARKYDFLIIEDDPYYFLQFNKFRV-PTFLSMD-VDGRVIRADSFS-II-SSGLRIGFLTGP------K-PLIERVILHIQVSTLHPSTFNQLMISQLLHEWG-EEGFMAH
NHKSVFNSaGLEVREYAYYDaenHTLDF-DALINSLNEA----------QAGDVVLFHGCCHAPTGIDPTLEQWQTLAQLSVEKGWLPLFDFAYQGFARGLEEDaEGLRAFAaMHKELIVASSYSkNFgLYNERVGACTLVaadsetVdRAFSQMKAAIlANYSNPPAHGASVVATILSNDAIRAIWEQE
VDRVIDFYSNQKDAILAAADKWLTGLAE-WHVPAAGMFLWIKVkgindVKELIEEKAVKMGVLMLPGnafyvdssapspyLRASFSSASPEQMDVAFQVLAQLIkesl
LTDMRQRIQRMRQLFVNTLQEKGANRDFsFIIKQNGMFSFSGL-----TKEQVLRLREEFGVYAVAS-------------GLVNVAGMTPDNMAPLCEAIVAVL----

SARST: aliSize=284 (resi) RMSD=19.07 (Å)
mnyarfitaas--AARNPSPIRTMTD-----ILSRGpKSMISIAGGLPNPNMfpfktavitveNGKTIqfGEEMMKRA---------LQYSPSAGIPELlsWLKQLQIKLHNpptIHYPPS-QGQMDLCVTSGSQQGLCKVFE---MIINPGdNVLLDEPAYSGTLQSLHPLGCNIINVASD---ESGIV
-----------mfENITAAPADPILGladlfRADER-PGKINLGIGVYKDET----------GKTPV--LTSVKKAEqyllenettKNYLGIDGIPE---FGRCTQELLFG---KGSALInDKRARTAQTPGGTGALRVAADflaKNTSVK-RVWVSNPSWPNHKSVFNSAGLEVREYAYYdaeNHTLD
PDSLRDILSRWKpedaknpqKNTPkfLYTVPNGNNPTGNSLTSERKKEIYELARKYDFLIIEDDPYYFLQFnkfrvpTFLSMDV-----DGRV---IRADSFSIIS--SGLRIGFLTGP-------KPLIERVILHIQVSTLHPSTFNQlmisqllhewgeegfmahvdrvidfysnqkdaILAAADKWL
FDALINSLNEAQ--------AGDV--VLFHGCCHAPTGIDPTLEQWQTLAQLSVEKGWLPLFDFAYQGFAR------GLEEDAEglrafAAMHkelIVASSYSKNFglYNERVGACTLVaadsetvDRAFSQMKAAILANYSNPPAHGA---------------------------------SVVATILSN
tglaewhvpaagmflwikvkgindvKELIEEKAVkmgvlmlpgnafyvdssapspylrasfssaspEQMDVAFQVLAQLIK---esl
-------------------------DALRAIWEQ---------------------------------ELTDMRQRIQRMRQLfvntlqekganrdfsfiikqngmfsfsgltkeqvlrlreefgvyavasglvnvagmtpdnmaplceaivavl---

BLAST: aliSize=31 (resi) iden=5.81% (23/396) simi=7.83% (31/396)
mnyarf----ITAASAarnpSPIRTMTDILSRGPK-SMISIAGGLPNPNmfPFKTAVIT-VENGKTIQFGEEMMKralQYSPSAGIPE--
------mfenITAAPA----DPILGLADLFRADERpGKINLGIGVYKDE--TGKTPVLTsVKKAEQYLLENETTK---NYLGIDGIPEfgrctqellfgkgsalindkrartaqtpggtgalrvaadflakntsvkrvwvsnpswpnhksvfnsaglevreyayydaenhtldfdalinslneaqagdvv
----
lfhgcchaptgidptleqwqtlaqlsvekgwlplfdfayqgfargleedaeglrafaamhkelivassysknfglynervgactlvaadsetvdrafsqmkaailanysnppahgasvvatilsndalraiweqeltdmrqriqrmrqlfvntlqekganrdfsfiikqngmfsfsgltkeqvlrlreef
--------------------------------llswlkqlqiklhnpptihyppsqgqmdlcvtsgsqqglckvfemiinpgdnvlldepaysgtlqslhplgcniinvasdesgivpdslrdilsrwkpedaknpqkntpkflytvpngnnptgnsltserkkeiyelarkydfliieddpyyflqfnkf
gvyavasglvnvagmtpdnmaplceaivavl---
rvptflsmdvdgrviradsfsiissglrigfltgpkpliervilhiqvstlhpstfnqlmisqllhewgeegfmahvdrvidfysnqkdailaaadkwltglaewhvpaagmflwikvkgindvkelieekavkmgvlmlpgnafyvdssapspylrasfssaspeqmdvafqvlaqlikesl
----

Proposed: aliSize=354 (resi) RMSD=2.71 (Å)
mn--YArfItaasA-aRNPSPIRTMTDILSRG-PKSMISIAGGLPN-----PNMF--PF----ktavitvengktIQFGeeMMKRaLQYSPSAGIPELLSWLKQLQIKLhNPPTI-HYppsqgQMDLCVTSGSQQGLCKVFEMII----NpgDNVLLDEPAYSGTLQSLHP-LGCNIINVASD----EsG
--mfEN--I----Ta-APADPILGLADLFRADeRPGKINLGIGVYKdetgkTPVLtsVKkaeq------------YLLE--NETT-KNYLGIDGIPEFGRCTQELLFGK-GSALInDK-----RARTAQTPGGTGALRVAADFLAkntsV--KRVWVSNPSWPNHKSVFNSaGLEVREYAYYDaenhT-L
IVpDSLRDILSR-wkpedaknpqkNTPKFLYTVPNGNNPTGNSLTSERKKEIYELARKYDFLIIEDDPYYFLQfNK--fRV-PTFLSMD-VDGRVIRADSFSLII-SSGLRIGFLTGP------K--PlIERVILHIQVSTLHPSTFNQLMISQLLHEWG-EEGFMAHVDRVIDFYSNQKDAILAADKW
DF-DALINSLNEa-----------QAGDVVLFHGCCHAPTGIDPTLEQWQTLAQLSVEKGWLPLFDFAYQGFA-RGle-EDaEGLRAFAaMHKELIVASSYSKNFgLYNERVGACTLVaadsetVdrA-FSQMKAAILANYSNPPAHGASVVATILSNDAlRAIWEQELTDMRQRIQRMRQLFVNTLQEK
LTGLAE-WHVPAAGMFLWIKVkgindVKELIEeKAVK-MGVLMLPGnafyvdssapspyLRASFSSASPEQMDVAFQVLAQLIkesl
GANRDFsFIIKQNGMFSFSGL-----TKEQVL-RLREeFGVYAVAS------------GLVNVAGMTPDNMAPLCEAIVAVL----</td></tr>
<tr><td>658</td><td>2qlrA (425)
1gc3A (382)</td><td>N</td><td>19.90</td><td>TM-align: aliSize=331 (resi) RMSD=2.44 (Å)
mnyarfitaasaarnpspirtmtdilsrgpksmislagglpnpnmfpfktavi---------------------------------------TVENGKTI-QFGEeMMKrALQYSPSAGIPELLSWLKQLQIKLHNPpTIHyppsqgQMDLCVTSGSQQGLCKVFEMIINPGDNVLLDEPAYSGT
--mrglsrrvqamkpdavvavnakalelrrqgvdlvaltagepdfdTPEHVKEAaRRAL-AQG-KTKYAPPAGIPELREALAEKFRRENGL-SVT------PEETIVTVGGSQALFNLFQAILDPGDEVIVLSPYWVSY
LQSLHPLGCNIINVASD---ESGIVPDSLRDILsrWkpedaknpqkNTPKFLYTVPNgNNPTGNSLTSERKKEIYELARKYDFLIIEDDPYYFLQFNkFRVPTFLSMDVdGRVIRADSFS-II-SSGLRIGFLTGPKPLIERVILHIQVSTLHPSTFNQLMISQLLHeWgEEGFMAHVDRVIDFYSNQKD
PEMVRFAGGVVEVETLpeeGFVPDPERVRRAI--T----------PRTKALVVNSP-NNPTGAVYPKEVLEALARLAVEHDFYLVSDEIYEHLLYE-GEHFSPGRVAP-EHTLTVNGAAkAFaMTGWRIGYACGPKEVIKAMASVSRQSTTSPDTIAQWATLEALT-N-QEASRAFVEMAREAYRRRD
AILAAADKWlTglAEWHVPAAGMFLWIKVKG-INDVKELIEeKAVKMGVLMLPGNAFYvdssaPSPYLRASFSSASpEQMDVAFQVLAQLiKesl
LLLEGLTAL-G--LKAVRPSGAFYVLMDTSPiAPDEVRAAE-RLLEAGVAVVPGTDFA-----AFGHVRLSYATSE-ENLRKALERFARV-L---

SARST: aliSize=344 (resi) RMSD=12.64 (Å)
mnyarfitaasaarnpspir---------------------TMTDILSRGPksMISLAGG----LPNPNMfpfktavitvengktiqfgEEMMKRA-----LQYSPSAGIPELLSWLKQLQIKLHNPptiHYPPSQgqmdLCVTSGSQQGLCKVFEMIINPGDNVLLDEPAYSGTLQSLHPLGCNIINV
--------------------mrglsrrvqamkpdavvavnakALELRRQGVD--LVALTAGepdfdTPEHV--------------------KEAARRAlaqgkTKYAPPAGIPELREALAEKFRRENGL---SVTPEE----TIVTVGGSQALFNLFQAILDPGDEVIVLSPYWVSYPEMVRFAGGVVEV
ASDE-SG--IVPDslrDILSRWKPEdaknpqkntPKFLYTVpNGNNPTGNSLTSERKKEIYELARKYDFLIIEDDPYYFLQFNKFRVPTFLSMdvDGRVIRADSFSI--ISSGLRIGFLTGPKPLIERVILHIQVSTLHPSTFNQLMISQLLHEWGEEGFMAHVDRVIDFYSNQKDAILAAADkwltgLA
ETLPeEGfvPDPE---RVRRAITPR---------TKALVVN-SPNNPTGAVYPKEVLEALARLAVEHDFYLVSDEIYEHLLYEGEHFSPGRVA--PEHTLTVNGAAKafAMTGWRIGYACGPKEVIKAMASVSRQSTTSPDTIAQWATLEALTNQEASRAFVEMAREAYRRRRDLLLEGLTAL-----GL
EWHVPAAGMFLWIKV-KGINDVKElIEEKAVKMGVLMLPGNAFYVDSsapspyLRASFSSASPEQMDVAFQVLAQL-ikesl
KAVRPSGAFYVLMDTsPIAPDEVR-AAERLLEAGVAVVPGTDFAAFG------HVRLSYATSEENLRKALERFARVl-----

BLAST: aliSize=168 (resi) iden=25.92% (99/382) simi=43.98% (168/382)
mnyarfitaasaarnpspirtmtdilsrgpksmislagglpnpnmfpfktavitvengktiqfg-----------------------------EEMMKRAL-----QYSPSAGIPELLSWLKQlqiKLHNPPTIHYPPSQgqmdLCVTSGSQQGLCKVFEMIINPGDNVL
---mrglsrrvqamkpdavvavnakalelrrqgvdlvaltagepdfdtpehvKEAARRALaqgktKYAPPAGIPELREALAE---KFRRENGLSVTPEE----TIVTVGGSQALFNLFQAILDPGDEVI
LDEPAYSGTLQSLHPLGCNIINVAS-DESGIVPDslrdilsrwkPEDAKnpQKNTPKFLYTVPNG-NNPTGNSLTSERKKEIYELARKYDFLIIEDDPYYFL-----QFNKFRVPTFLSMDVDGRvirADSFSIisSGLRIGFLTGPKPLIERVILHIQVSTLHPSTFNQLMISQLLHFwgEEGFMAHVD
VLSPYWVSYPEMVRFAGGVVEVETlPEEGFVPD----------PERVR--RAITPRTKALVVNSpNNPTGAVYPKEVLEALARLAVEHDFYLVSDEIYEHLlyegeHFSPGRVAPEHTLTVNGA---AKAFAM--TGWRIGYACGPKEVIKAMASVSRQSTTSPDTIAQWATLEALTN--QEASRAFVE
RVIDFYSNQKDAILAAadkwLTGLAEWHV-PAAGMFLWIKVKGINDVKELIEEKAVKMGVLMLPGNAFyvdssAPSPYLRASFSSaSPEQMDVAFQVLAQLIkesl
MAREAYRRRRDLLLEG----LTALGLKAVrPSGAFYVLMDTSPIAPDEVRAAERLLEAGVAVVPGTDF-----AAFGHVRLSYAT-SEENLRKALERFARVL----

Proposed: aliSize=363 (resi) RMSD=2.06 (Å)
MNYArfITaASAARNPSPIRTMTDILSR--GPKSMISLaGGLPNPNmF-pFKT--AV--IT-V-ENgktiqfgeemmkrALQYSPSAGIPELLSWLKQLQIKLHNPpTIhyppsqgqM--DLCVTSGSQQGLCKVFEMIINPGDNVLLDEPAYSGTLQSLHPLGCNIINVASD---ESGIVPDSLRDILs
MRGL--SR-RVQAMKPDAVVAVNAKALElrRQGVDLVA-LTAGEPD-Fd-TPEhvKEaaRRaLaQG------------KTKYAPPAGIPELREALAEKFRRENGL-SV--------TpeETIVTVGGSQALFNLFQAILDPGDEVIVLSPYWVSYPEMVRFAGGVVEVETLpeeGFVPDPERVRRAI-
rWkpedaknpqkNTPKFLYTVPNgNNPTGNSLTSERKKEIYELARKYDFLIIEDDPYYFLQFNkFRVPTFLSMDVdGRVIRADSFSLII-SSGLRIGFLTGPKPLIERVILHIQVSTLHPSTFNQLMISQLLHeWgEEGFMAHVDRVIDFYSNQKDAILAAADKWlTglAEWHVPAAGMFLWIKVKG-IN
-T----------PRTKALVVNSP-NNPTGAVYPKEVLEALARLAVEHDFYLVSDEIYEHLLYE-GEHFSPGRVAP-EHTLTVNGAAKAFaMTGWRIGYACGPKEVIKAMASVSRQSTTSPDTIAQWATLEALT-N-QEASRAFVEMAREAYRRRRDLLLEGLTAL-G--LKAVRPSGAFYVLMDTSPiAP
DVKELIEeKAVKMGVLMLPGNAFY-vdssapSPYLRASFSSA-S-PEQMDVAFQVLaQlikesl
DEVRAAE-RLLEAGVAVVPGTDFAa------FGHVRLSYATSeEnLRKALERFARV-L------</td></tr>
<tr><td>659</td><td>2qlrA (425)
1gdeA (388)</td><td>N</td><td>20.62</td><td>TM-align: aliSize=340 (resi) RMSD=2.74 (Å)
mnyarfitaasaarnpspirtmtdilsrgpksmislagglpnpnmfpfktavi-------------------------------------TVENGKTI-QFGEEMMKrALQYSPSAGIPELLSWLKQLQIKLHNPpTIHypPsqgQMDLCVTSGSQQGLCKVFEMIINPGDNVLLDEPAYSGTLQSLHP
--alsdrlelvsaseirklfdiaagmkdvislgigepdfdTPQHIKEYaKEALDKG--LTHYGPNIGLLELREAIAEKLKKQNGI-EAD--P---KTEIMVLLGANQAFLMGLSAFLKDGEEVLIPTPAFVSYAPAVIL
LGCNIINVASD---ESGIVPDSLRDILsrWkpedaknpqKNtPKFLYTVPNgNNPTGNSLTSERKKEIYELARKYDFLIIEDDPYYFLQFNKFRVPTFLSMDVD-GRVIRADSFS-II-SSGLRIGFLTGPKPLIERVILHIQVSTLHPSTFNQLMISQLLHeWgeEGFMAHVDRVIDFYSNQKDAILAA
AGGKPVEVPTYeedEFRLNVDELKKYV--T---------DK-TRALIINSP-CNPTGAVLTKKDLEEIADFVVEHDLIVISDEVYEHFIYDDARHYSIASLDGMfERTITVNGFSkTFaMTGWRLGFVAAPSWIIERMVKFQMYNATCPVTFIQYAAAKALK-D--ERSWKAVEEMRKEYDRRRKLVWKR
ADKwLTglAEWHVPAAGMFLWIKV-KGINDVKELIEEKAVKMGVLMLPGNAFYVDSsapSPYLRASFSSASPeQMDVAFQVLAQLIK-ESL-
LNE-MG--LPTVKPKGAFYIFPRIrDTGLTSKKFSELMLKEARVAVVPGSAFGKAG---EGYVRISYATAYE-KLEEAMDRMERVLKeRKLv

SARST: aliSize=340 (resi) RMSD=5.23 (Å)
mnyarfitaasaarnpspirtmtdilsrgpksmislagglpnpnmfpfktavitv-----------------------------ENGKTIQFGEEMMKRA--------LQYSPSAGIPELLSWLKQLQIKLHNPptiHYPPSQGqmdLCVTSGSQQGLCKVFEMIINPGDNVLLDEPAYSGTLQSLHP
--alsdrlelvsaseirklfdiaagmkdvislgIGEPDFDTPQHIKEYAkealdkglTHYGPNIGLLELREAIAEKLKKQNGI---EADPKTE---IMVLLGANQAFLMGLSAFLKDGEEVLIPTPAFVSYAPAVIL
LGCNIINVASDE---SGIVPDSLrdiLSRWKPEdaknpqkntPKFLYTVpNGNNPTGNSLTSERKKEIYELARKYDFLIIEDDPYYFLQFNKFRVPTFLSM-DVDGRVIRADSFSI--ISSGLRIGFLTGPKPLIERVILHIQVSTLHPSTFNQLMISQLLHEWGEEGFMAHVDRVIDFYSNQKDAILAA
AGGKPVEVPTYEedeFRLNVDEL---KKYVTDK---------TRALIIN-SPCNPTGAVLTKKDLEEIADFVVEHDLIVISDEVYEHFIYDDARHYSIASLdGMFERTITVNGFSKtfAMTGWRLGFVAAPSWIIERMVKFQMYNATCPVTFIQYAAAKALKDERSWKAVEEMRKEYDRRRKLVWKRLNE
AdkwltgLAEWHVPAAGMFLWIKVKGINDVKE-LIEEKAVKMGVLMLPGNAFYVdSSApsPYLRASFSSASPEQMDVAFQVLAQLIKES--l
M------GLPTVKPKGAFYIFPRIRDTGLTSKkFSELMLKEARVAVVPGSAFGK-AGE--GYVRISYATAYEKLEEAMDRMERVLKERKlv-

BLAST: aliSize=184 (resi) iden=25.26% (98/388) simi=47.42% (184/388)
mnyarfitaasaarnp-----------SPIRTMTDIlSRGPKSMISLagGLPNPNmfpFKTAvitvENGKtiQFGEEMMKRAL-QYSPSAGIPELLSWLKQlQIKLHNppTIHYPPsqgQMDLCVTSGSQQGLCKVFEMIINPGDNVLLDEPAYSGTLQSLHPLGCNIINVASDEsgivpDSLRDILSRW
----------------alsdrlelvsaSEIRKLFDI-AAGMKDVISL--GIGEPD---FDTP----QHIK--EYAKEALDKGLtHYGPNIGLLELREAIAE-KLKKQN--GIEADP---KTEIMVLLGANQAFLMGLSAFLKDGEEVLIPTPAFVSYAPAVILAGGKPVEVPTYEE----DEFRLNVDEL
KpedaKNPQKNTPKFLYTVPngNNPTGNSLTSERKKEIYELARKYDFLIIEDDPYYFLQFNKFRVPTFLSMD-VDGRVIRADSFS--IISSGLRIGFLTGPKPLIERVILHIQVSTLHPSTFNQLMISQLLHFwgEEGFMAhVDRVIDFYSNQKDAILAAADKwlTGLAEWHvPAAGMFLWIKVKGINDV
K----KYVTDKTRALIINSP--CNPTGAVLTKKDLEEIADFVVEHDLIVISDEVYEHFIYDDARHYSIASLDgMFERTITVNGFSktFAMTGWRLGFVAAPSWIIERMVKFQMYNATCPVTFIQYAAAKALKD--ERSWKA-VEEMRKEYDRRRKLVWKRLNE--MGLPTVK-PKGAFYIFPRIRDTGLT
KELIEEKAVKMG-VLMLPGNAFyvdSSAPSPYLRASFSSASpEQMDVAFQVLAQLIKE----sl
SKKFSELMLKEArVAVVPGSAF---GKAGEGYVRISYATAY-EKLEEAMDRMERVLKErklv--

Proposed: aliSize=363 (resi) RMSD=2.21 (Å)
mnyarF-ITaASAARNPSPIRTMTDILSRGPkSMISLaGGLPNPN-mfpFKT--AV-IT--V--EngktiqfgeemmkrALQYSPSAGIPELLSWLKQLQIKLHNPptIhyppsqgqM----DLCVTSGSQQGLCKVFEMIINPGDNVLLDEPAYSGTLQSLHPLGCNIINVASD---ESGIVPDSLRDI
-----AlSD-RLELVSASEIRKLFDIAAGMK-DVISL-GIGEPDFd---TPQhiKEyAKeaLdkG-------------LTHYGPNIGLLELREAIAEKLKKQNGI--E--------AdpktEIMVLLGANQAFLMGLSAFLKDGEEVLIPTPAFVSYAPAVILAGGKPVEVPTYeedEFRLNVDELKKY
LsrWkpedaknpqkNTPKFLYTVPNgNNPTGNSLTSERKKEIYELARKYDFLIIEDDPYYFLQF-NkFRVPTFLSMDVD-GRVIRADSFSLII-SSGLRIGFLTGPKPLIERVILHIQVSTLHPSTFNQLMISQLLHeWgeEGFMAHVDRVIDFYSNQKDAILAAADKWLTglAEWHVPAAGMFLWIKV-
V--T----------DKTRALIINSP-CNPTGAVLTKKDLEEIADFVVEHDLIVISDEVYEHFIYdD-ARHYSIASLDGMfERTITVNGFSKTFaMTGWRLGFVAAPSWIIERMVKFQMYNATCPVTFIQYAAAKALK-D--ERSWKAVEEMRKEYDRRRKLVWKRLNE-MG--LPTVKPKGAFYIFPRIr
KGINDVKELIEeKAVKM-GVLMLPGNAFYvD--ssapSPYLRASFSSASPeQMDVAFQVLAQLIK-ESL---
DTGLTSKKFSE-LMLKEaRVAVVPGSAFG-Kag----EGYVRISYATAYE-KLEEAMDRMERVLKeRKLvee</td></tr>
</table>

660	2qlrA (425)<br>1ijiA (354)	N	13.84	TM-align: aliSize=309 (resi) RMSD=3.17 (Å)<br>[illegible]<br>SARST: aliSize=339 (resi) RMSD=12.41 (Å)<br>[illegible]<br>BLAST: aliSize=102 (resi) iden=14.69% (52/354) simi=28.81% (102/354)<br>[illegible]<br>Proposed: aliSize=334 (resi) RMSD=2.67 (Å)<br>[illegible]
661	2qlrA (425)<br>1ix6A (396)	N	13.38	TM-align: aliSize=332 (resi) RMSD=3.26 (Å)<br>[illegible]<br>SARST: aliSize=329 (resi) RMSD=12.51 (Å)<br>[illegible]<br>BLAST: aliSize=30 (resi) iden=5.81% (23/396) simi=7.58% (30/396)<br>[illegible]<br>Proposed: aliSize=352 (resi) RMSD=2.68 (Å)<br>[illegible]
662	2qlrA (425)<br>1ix8A (396)	N	13.13	TM-align: aliSize=332 (resi) RMSD=3.28 (Å)<br>[illegible]<br>SARST: aliSize=334 (resi) RMSD=12.27 (Å)<br>[illegible]<br>BLAST: aliSize=30 (resi) iden=5.81% (23/396) simi=7.58% (30/396)<br>[illegible]<br>Proposed: aliSize=350 (resi) RMSD=2.78 (Å)<br>[illegible]

663	2qlrA (425) / 1j32A (388)	N	16.49	TM-align: aliSize=336 (resi) RMSD=2.55 (Å) [sequence alignment] SARST: aliSize=361 (resi) RMSD=12.87 (Å) [sequence alignment] BLAST: aliSize=165 (resi) iden=24.74% (96/388) simi=42.53% (165/388) [sequence alignment] Proposed: aliSize=366 (resi) RMSD=2.07 (Å) [sequence alignment]
664	2qlrA (425) / 1o4sA (375)	N	18.67	TM-align: aliSize=327 (resi) RMSD=2.59 (Å) [sequence alignment] SARST: aliSize=351 (resi) RMSD=12.84 (Å) [sequence alignment] BLAST: aliSize=176 (resi) iden=27.20% (102/375) simi=46.93% (176/375) [sequence alignment] Proposed: aliSize=345 (resi) RMSD=2.07 (Å) [sequence alignment]
665	2qlrA (425) / 1qirA (396)	N	12.63	TM-align: aliSize=331 (resi) RMSD=3.09 (Å) [sequence alignment] SARST: aliSize=331 (resi) RMSD=12.50 (Å) [sequence alignment] BLAST: aliSize=31 (resi) iden=5.81% (23/396) simi=7.83% (31/396) [sequence alignment] Proposed: aliSize=355 (resi) RMSD=2.66 (Å) [sequence alignment]

666	2qlrA (425)	N	12.88	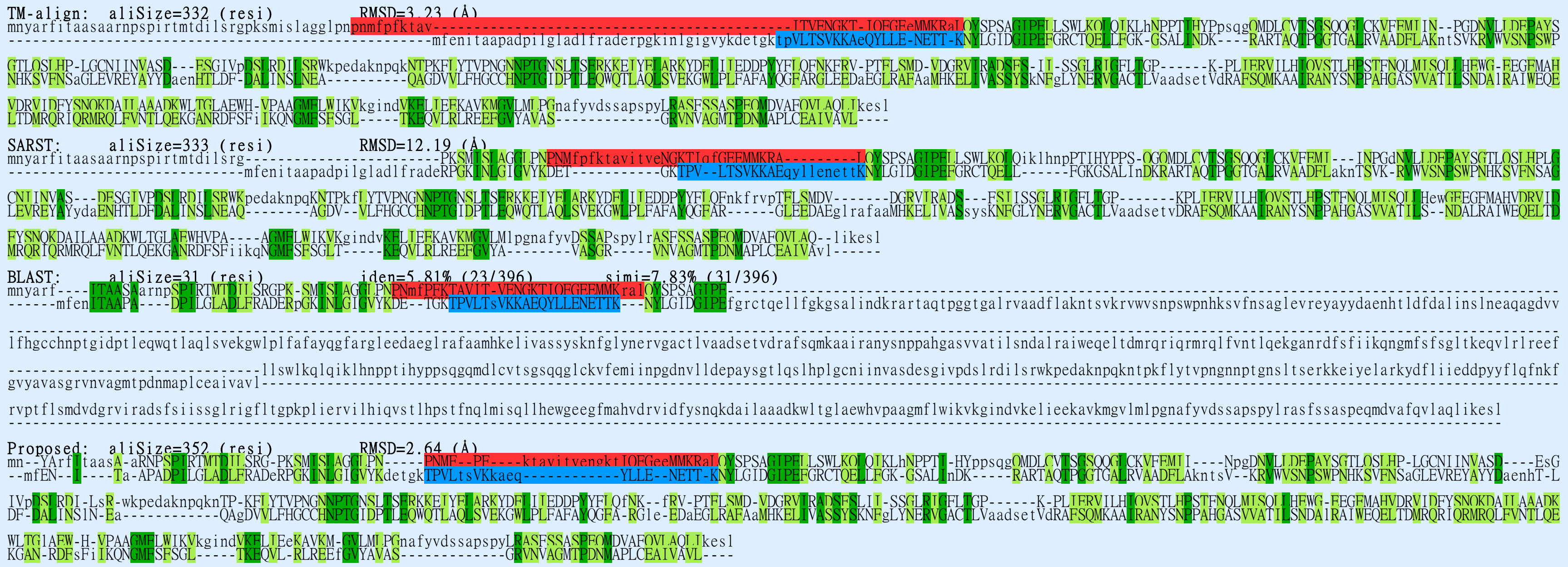
	1spaA (396)			
667	2qlrA (425)	N	11.64	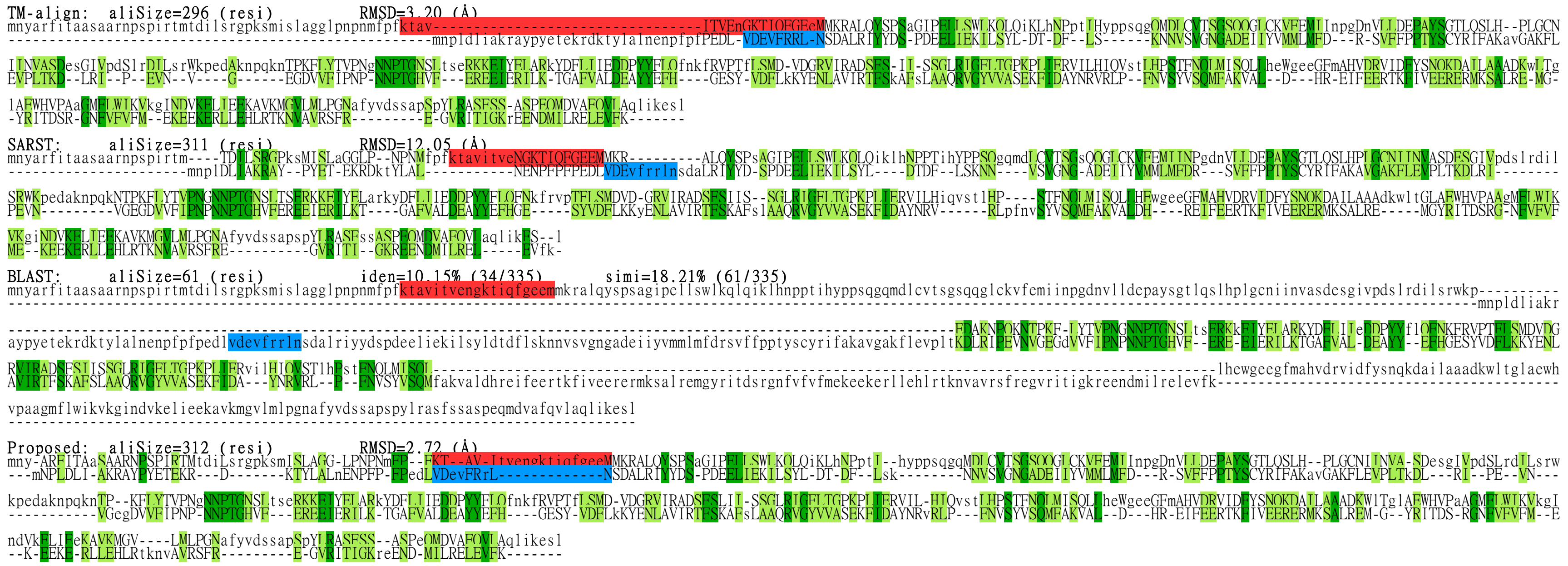
	1uu1C (335)			
668	2qlrA (425)	N	12.77	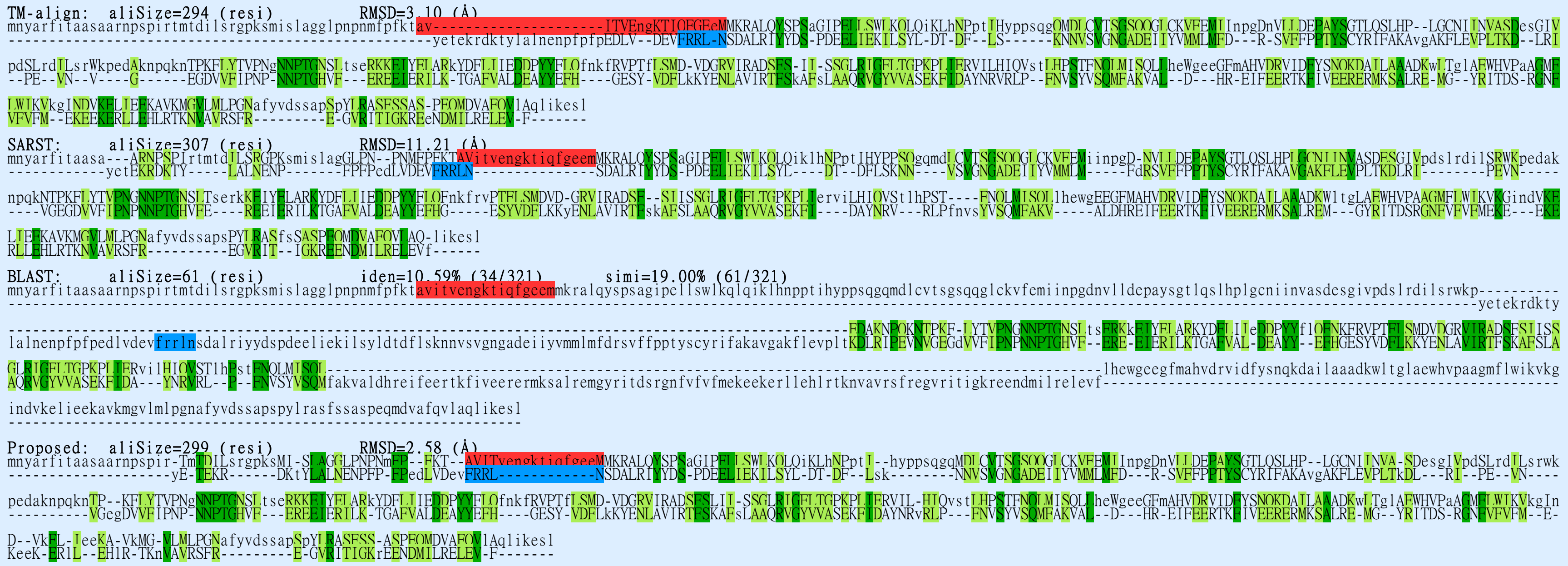
	1uu2A (321)			

669	2qlrA (425)<br>1v2fB (367)	N	20.44	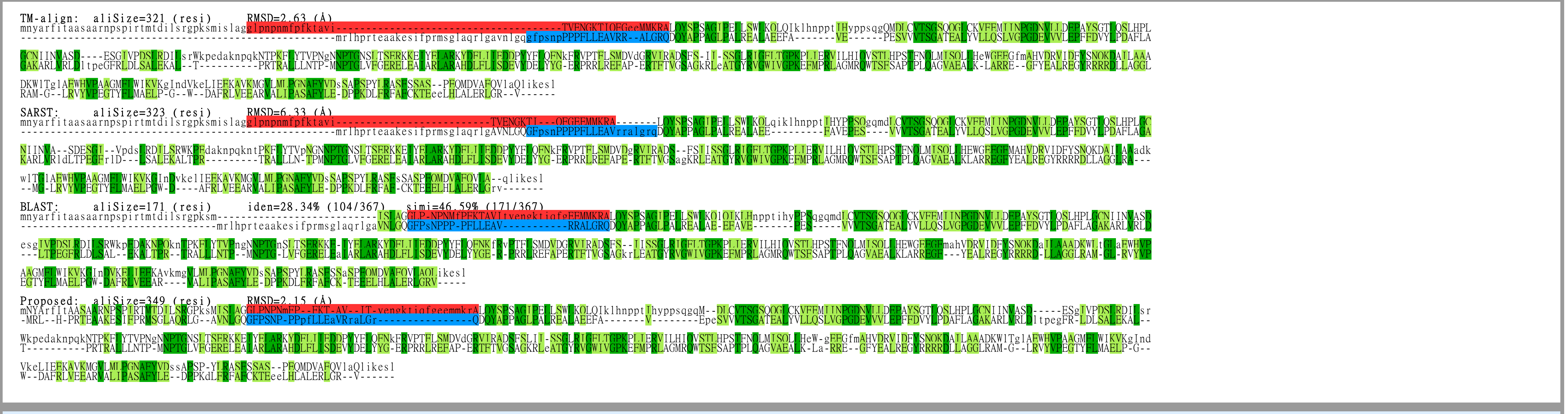
670	2qlrA (425)<br>1wstA (403)	N	29.28	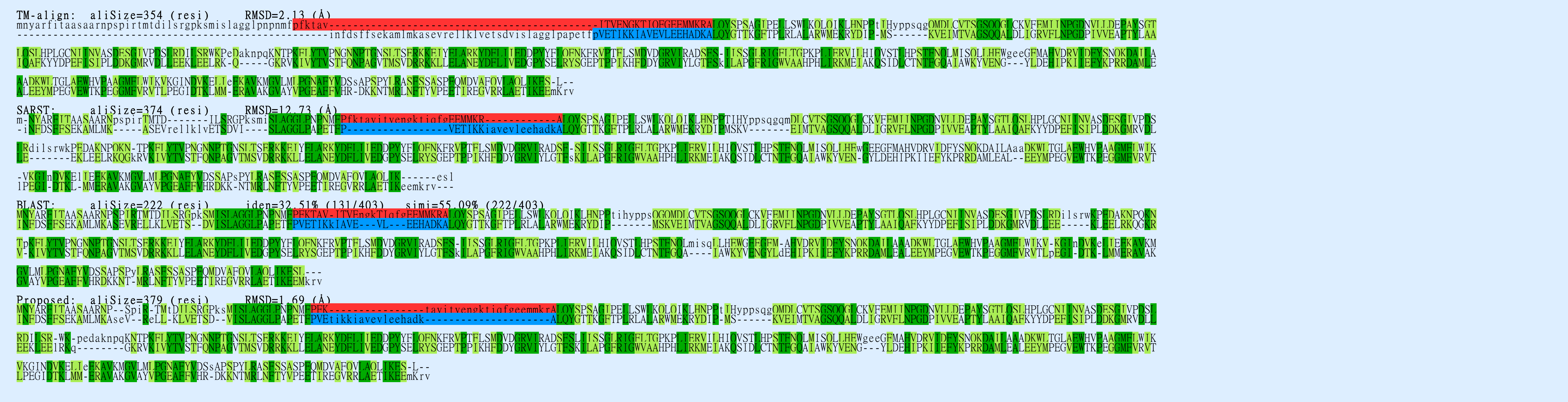
671	2qlrA (425)<br>1x0mA (403)	N	29.28	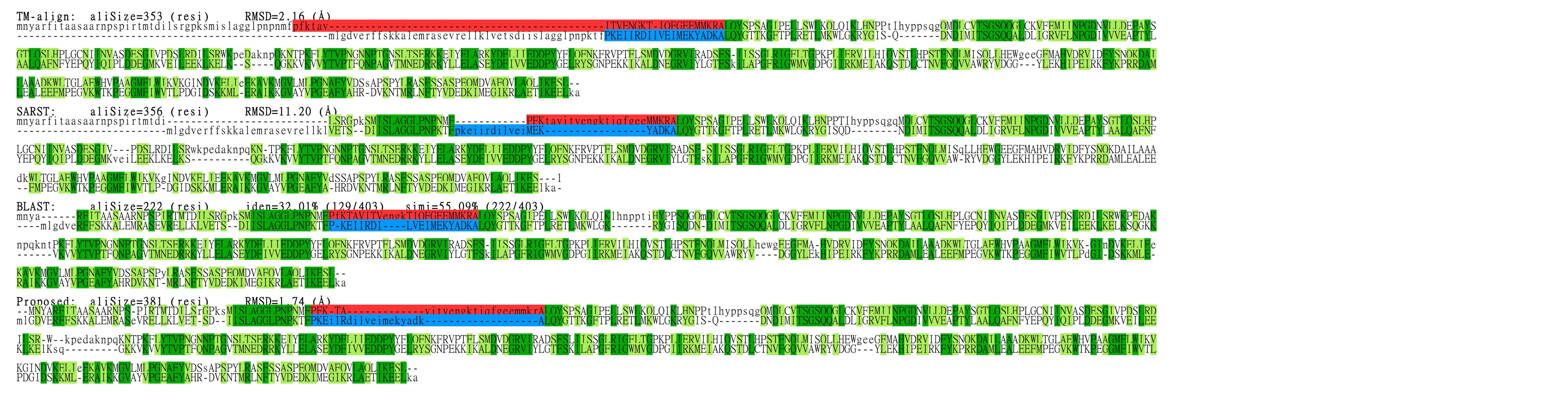

<table>
<tr><td rowspan="2">672</td><td>2qlrA
(425)</td><td rowspan="2">N</td><td rowspan="2">10.44</td><td rowspan="2">TM-align: aliSize=335 (resi) RMSD=2.96 (Å)
mnyarfitaasaarnpspirtmtdilsrgpksmislagglpnpnmfpfktavi-------------------------------TVEN--GK-TIQFGEEMMKraLQYSPSAGIPELLSWLKQLQIKLhNPPTIHYPpsqgQMDLCVTSGSQQGLCKVFEMII--NPGDNVLLD
--satlfnniellppdalfgikqrygqdqratkvdlgigayrddngkpwVLPSvkAAeKLIHNDSSYN--HEYLGITGLPSLTSNAAKIIFGT-QSDALQED----RVISVQSLSGTGALHISAKFFSkfFPDKLVYLS

EPAYSGTLQSLHP-LGCNIINVASD---ESGIVpDSLRDILSRWkpedaknpqkNTPKFLYTVPNGNNPTGNSLTSERKKEIYFLARKYDFLIIEDDPYYFLQFNK-FRV-PTFLSMDV--D--GRVIRADSFS-II-SSGLRIGFLTGPK---------P--LIERVILHIQVSTLHPSTFNQLMISQL
KPTWANHMAIFENqGLKTATYPYWAnetKSLDL-NGFLNAIQKA----------PEGSIFVLHSCAHNPTGLDPTSEQWVQIVDAIASKNHIALFDTAYQGFATGDlDKDaYAVRLGVEklStvSPVFVCQSFAkNAgMYGERVGCFHLALtkqaqnktiKpaVTSQLAKIIRSEVSNPPAYGAKIVAKL

IHEWG-EEGFMAHVDRVIDFYSNQKDAILAAADKWLTGLAEWH-VPAAGMFLWIKVkgindVKELIEEKAVKMGVLMLPGnafyvdssapspyLRASFSSASPEQMDVAFQVLAQLIKESL
LETPElTEQWHKDMVTMSSRITKMRHALRDHLVKLGTPGNWDHiVNQCGMFSFTGL-----TPQMVKRLEETHAVYLVAS-------------GRASIAGLNQGNVEYVAKAIDEVVRFYA

SARST: aliSize=334 (resi) RMSD=16.58 (Å)
mnyarfitaasaarnpspirt---------------------MTDILSRgPKSMISLAGGLPNPNMfpfktavitveNGKtiQFGEEMMKRA----------LQYSPSAGIPELLSWLKQLQIklhnppTIHYPP-SQGQMDLCVTSGSQQGLCKVFEMI--INPGDNVLLDEPAYSGTLQSLHPLGCNII
---------------------satlfnniellppdalfgikQRYGQDQ-RATKVDLGIGAYRDDN-----------GKP--WVLPSVKAAEklihndssynHEYLGITGLPSLTSNAAKIIF------GTQSDAlQEDRVISVQSLSGTGALHISAKFFskFFPDKLVYLSKPTWANHMAIFENQGLKTA

NVASD---ESGIVPDSLRDILSRWKpedaknpqKNTPkfLYTVPNGNNPTGNSLTSERKKEIYFLARKYDFLIIEDDPY---YFLqfnkfrvpTFLSMDV---------DGRVIRADS------FSIIssGLRIGFLTGP-----------KPLIERVILHIQVSTLHPSTFNQLMISQLIHewgeeGFM
TYPYWaneTKSLDLNGFLNAIQKAP---------EGSI--FVLHSCAHNPTGLDPTSEQWVQIVDAIASKNHIALFDTAYqgfATG---------DLDKDAYavrlgveklSTVSPVFVCqsfaknAGMY--GERVGCFHLAltkqaqnktikPAVTSQLAKIIRSEVSNPPAYGAKIVAKLLE-----TPE

AHVD------RVIDFYSNQKDAILAAADKWltgLAEWHVPA----AGMFLWIKVKgindvKELIEEKAVKMGVLMlpgnafyvDSSAPspylrASFSSASPEQMDVAFQVLAQLIK---esl
LTEQwhkdmvTMSSRITKMRHALRDHLVKL---GTPGNWDHivnqCGMFSFTGLT-----PQMVKRLEETHAVYL---------VASGR-----ASIAGLNQGNVEYVAKAIDEVVRfya---

BLAST: aliSize=85 (resi) iden=13.35% (55/412) simi=20.63% (85/412)
mnyarfitaasaarnpspirtmtdilsrgpksmislagglpnpnmfpfktavitvengktiqfgeemmkralqyspsagipellswlkqlqiklhnpptihyppsqgqmdlcvtsgsqqglckvfemiinpgdnvlldepaysgtlqslhplgcniinvasdesgivpdslrdilsrwkpedaknpqknt
----

pkflytvpng--
----------satlfnniellppdalfgikqrygqdqratkvdlgigayrddngkpwvlpsvkaaeklihndssynheylgitglpsltsnaakiifgtqsdalqedrvisvqslsgtgalhisakffskffpdklvylskptwanhmaifenqglktatypywanetksldlngflnaiqkapegsifv

-----NNPTGNSLTSERKKEIYFLARKYDFLIIEDDPYY-F----LQFNKFRVPTFLS-MDVDGRVIRADSFSIISS--GLRIG-F---LTGP------KPLIERVILHIQVSTL-HPSTFNQLMISQLIhEWGEEGFMAHVDRVIdfYSNQKDAILAAADKWLTGLA---FW-H-VPAAGMFLWIKVKG
lhscaHNPTGLDPTSEQWVQIVDAIASKNHIALFDTAYQgFatgdLDKDAYAVRLGVEkLSTVSPVFVCQSFAKNAGmyGERVGcFhlaLTKQaqnktiKPAVTSQLAKIIRSEVsNPPAYGAKIVAKLL-ETPELTEQWHKDMVT--MSSRITKMRHALRDHLVKLGtpgNWdHiVNQCGMFSFTGLTP

iNDVKELIEEKAV------KMGVLML-PGNAFYV------------dssapspylrasfssaspeqmdvafqvlaqlikesl
-QMVKRLEETHAVylvasgRASIAGLnQGNVEYVakaidevvrfya-----------------------------------

Proposed: aliSize=359 (resi) RMSD=2.59 (Å)
-MN-YARfItaasAARNPS---P-IRTMTDIlsrG-pksMISLAGGLP-NPN-------mfpFK-TA-VI--TvengkTiQFGeEMMkraLQYSPSAGIPELLSWLKQL-QIKlhNPPTIHYPpsqgQMDLCVTSGSQQGLCKVFEMII---NPgDNVLLDEPAYSGTLQSLHP-LGCNIINVASD----
sATlFNN-I----ELLPPDalfGiKQRYGQD---Qr---ATKVDLGIGaYRDdngkpwv---LPsVKaAEklI-----H-NDS-SYN---HEYLGITGLPSLTSNAAKIiFGT--QSDALQED----RVISVQSLSGTGALHISAKFFSkffPD-KLVYLSKPTWANHMAIFENqGLKTATYPYWAnetk

EsGiVPDSLRDILSRWkpedaknpqkNTPKFLYTVPNGNNPTGNSLTSERKKEIYFLARKYDFLIIEDDPYYFLQFNK--fRV-PTFLSMDV-DG---RVIRADSFSLII-SSGLRIGFLTGPK---------P--LiE-RVILHIQVSTLHPSTFNQLMISQLIHEWG-EEGFMAHVDRVIDFYSNQKD
S-L-DLNGFLNAIQKA----------PEGSIFVLHSCAHNPTGLDPTSEQWVQIVDAIASKNHIALFDTAYQGFATGDld-KDaYAVRLGVEkLStvsPVFVCQSFAKNAgMYGERVGCFHLALtkqaqnktiKpaV-TsQLAKIIRSEVSNPPAYGAKIVAKLLETPElTEQWHKDMVTMSSRITKMRH

AILAAADKW-LTGlAE-WH-VPAaGMFLWIKVkgindVKELIEeKAVKM-GVLMLPGnafyvdssapspyLRASFSSASPEQMDVAFQVLAQLIKESL
ALRDHLVKLgTPG-NWdHIvNQC-GMFSFTGL-----TPQMVK-RLEEThAVYLVAS-------------GRASIAGLNQGNVEYVAKAIDEVVRFYA</td></tr>
<tr><td>1yaaA
(412)</td></tr>
<tr><td rowspan="2">673</td><td>2qlrA
(425)</td><td rowspan="2">N</td><td rowspan="2">12.63</td><td rowspan="2">TM-align: aliSize=331 (resi) RMSD=3.09 (Å)
mnyarfitaasaarnpspirtmtdilsrgpksmislagglpnpnmfpfktavi-------------------------------TVENG-KT-IQFGEeMMKRaLQYSPSAGIPELLSWLKQLQIKLhNPPTIHYpPsqgQMDLCVTSGSQQGLCKVFEMIIN--PGDNVLLDEPAY
---mfenittapadpilgladllraderpgkidlgmgvyndetgktpVLTSVkKAeQYLLE-NETT-KNYLGIDGIPEFGRCTQELLFGK-GSALIND-K---RARTAQTPGGTGALRVAADFLAKntSVRRVWVSNPGW

SGTLQSLHP-LGCNIINVASD---ESGIVpDSLRDILSRWkpedaknpqkNTPKFLYTVPNGNNPTGNSLTSERKKEIYFLARKYDFLIIEDDPYYFLQFNKFRV-PTFLSMD-VDGRVIRADSFS-II-SSGLRIGFLTGP------KP-LIERVILHIQVSTLHPSTFNQLMISQLIHEWG-EEGFMA
PTHKSVFNSaGLEVREYAYYDaenHTLDF-DALINSLNEA----------QAGDVVLFHGCCHNPTGIDPTLEQWQTLAQLSVEKGWLPLFDFAYQGFARGLEEDaEGLRAFAaMHKELIVASSYSkNFgLYNERVGTCTLVaadsetVDrAFSQMKAAIRVNYSSPPAHGASVVATILGNDAlRAIWEQ

HVDRVIDFYSNQKDAILAAADKWLTGLAE-WHVPAAGMFLWIKVkgindVKELIEEKAVKMGVLMLPGnafyvdssapspyLRASFSSASPEQMDVAFQVLAQLIkesl
ELTDMRQRIQRMRQLFVNTLQEKGANRDFsFTIKQNGMFFFGGL-----TKEQVLRLREEFGVYAVAS-------------GRLNVAGMTPDNLAPLCEAIVAVL----

SARST: aliSize=330 (resi) RMSD=9.04 (Å)
mnyarfitaasaarnpspirtmtdilsrgpksmislagglpnp-------------------NMFPF-KTAVI--TVE----NGKTIqfGEEMMKRA---------LQYSPSAGIPELLSWLKQLqikLHNptIHY-PPSQGQMDLCVTSGSQQGLCKVFE---MIINPGdNVLLDEPAYSGTLQSLH
---------------------------------------mfenittapadpilgladlLRADERpGKIDLgmGVYndetGKTPV--LTSVKKAEqyllenettKNYLGIDGIPEFGRCTQEL---LFG---KGSaLINDKRARTAQTPGGTGALRVAADflaKNTSVR-RVWVSNPGWPTHKSVFN

PLGCNIINVA-SDESG--IVPDSLRDILSRWKpedaknpqKNTPkfLYTVPNGNNPTGNSLTSERKKEIYFLARKYDFLIIEDDPYYFLQFnkfrvpTFLSMDV-------DGRVIRADS---FSIISSGLRIGFLTGP-------KPLIERVILHIQVSTLHPSTFNQLMISQLIHEWGEEGFMAHVDR
SAGLEVREYAyYDAENhtLDFDALINSLNEAQ--------AGDV--VLFHGCCHNPTGIDPTLEQWQTLAQLSVEKGWLPLFDFAYQGFAR------GLEEDAEglrafaaMHKELIVASsysKNFGLYNERVGTCTLVaadsetvDRAFSQMKAAIRVNYSSPPAHGASVVATILGNDALRAIWEQELT

VIDFYSNQKDAILAAADKWlTGlaewhvPAAGM-----FLWIKVKGiNDVKELIEEKAVKMGVLMLpgNAFyvdssapspylRASFSSASPEQMDVAFQVLAqlikES-l
DMRQRIQRMRQLFVNTLQE-KG------ANRDFsftikQNGMFFFG-GLTKEQVLRLREEFGVYAV--ASG-----------RLNVAGMTPDNLAPLCEAIV----AVl-

BLAST: aliSize=42 (resi) iden=7.07% (28/396) simi=10.61% (42/396)
mnyarfitaasaarnpspirtmtdilsrgpksmislagglpnpnmfpfktavitvengktiqfgeemmkralqyspsagipellswlkqlqiklhnpptihyppsqgqmdlcvtsgsqqglckvfemiinpgdnvlldepaysgtlqslhplgcniinvasdesgivpdslrdilsrwkpedaknpqknt
----

pkflytvpng-----------------------------NNPTGNS--LTSERKKEIYFLARKY--DFLIIEDDPYY--FLQFNKFRVPTFLSMDVDGRVIRADSFSiisSGLRIG--FLTgPKPLIERVILHIQVSTLHPSTFN--
----------mfenittapadpilgladllraderpgkidlgmgvyNDETGKTpvLTSVKKAEQYLLENETtkNYLGIDGIPEFgrCTQELLFGKGSALINDKRARTAQTPGGT---GALRVAadFLA-KNTSVRRVWVSNPGWPTHKSVFNsaglevreyayydaenhtldfdalinslneaqagdvvl

----
fhgcchnptgidptleqwqtlaqlsvekgwlplfdfayqgfargleedaeglrafaamhkelivassysknfglynervgtctlvaadsetvdrafsqmkaairvnysspp ahgasvvatilgndalraiweqeltdmrqriqrmrqlfvntlqekganrdfsftikqngmfffggltkeqvlrlreefg

-----------------------------qlmisqllhewgeegfmahvdrvidfysnqkdailaaadkwltglaewhvpaagmflwikvkgindvkelieekavkmgvlmlpgnafyvdssapspylrasfssaspeqmdvafqvlaqlikesl
vyavasgrlnvagmtpdnlaplceaivavl--

Proposed: aliSize=355 (resi) RMSD=2.70 (Å)
mNYARfItaasAARNPS---PIR-TMTDILsRGPksMISlAGGLP-NPN-------mfpFK-TAV-ITvengktIQFGeeMMKRaLQYSPSAGIPELLSWLKQLQ-IKlhNPPTIHYpPsqgQMDLCVTSGSQQGLCKVFEMII----NpgDNVLLDEPAYSGTLQSLHP-LGCNIINVASD----ESgi
-MFEN-I----TTAPADpilGLAdLLRAD--ERP--GKI-DLGMGvYNDetgktpv---LTsVKKaEQ------YLLE--NETT-KNYLGIDGIPEFGRCTQELLfGK--GSALIND-K---RARTAQTPGGTGALRVAADFLAkntsV--RRVWVSNPGWPTHKSVFNSaGLEVREYAYYDaenhTL--

VPDSLRDILSR-wkpedaknpqkNTPKFLYTVPNGNNPTGNSLTSERKKEIYFLARKYDFLIIEDDPYYFLQfNK--fRV-PTFLSMD-VDGRVIRADSFSLII-SSGLRIGFLTGP------KP-LIERVILHIQVSTLHPSTFNQLMISQLIHEWG-EEGFMAHVDRVIDFYSNQKDAILAAADKW-L
DFDALINSLNEa-----------QAGDVVLFHGCCHNPTGIDPTLEQWQTLAQLSVEKGWLPLFDFAYQGFA-RGle-EDaEGLRAFAaMHKELIVASSYSKNFgLYNERVGTCTLVaadsetVDrAFSQMKAAIRVNYSSPPAHGASVVATILGNDAlRAIWEQELTDMRQRIQRMRQLFVNTLQEKgA

TGlAE-WHVPAAGMFLWIKVkgindVKELIEeKA-VKMGVLMLPGnafyvdssapspyLRASFSSASPEQMDVAFQVLAQLI-kesl
NR-DFsFTIKQNGMFFFGGL-----TKEQVL-RLrEEFGVYAVAS-------------GRLNVAGMTPDNLAPLCEAIVAVLi----</td></tr>
<tr><td>1yooA
(396)</td></tr>
<tr><td rowspan="2">674</td><td>2qlrA
(425)</td><td rowspan="2">N</td><td rowspan="2">12.63</td><td rowspan="2">TM-align: aliSize=331 (resi) RMSD=3.08 (Å)
mnyarfitaasaarnpspirtmtdilsrgpksmislagglpnpnmfpfktavi-------------------------------TVENG-KT-IQFGEeMMKRaLQYSPSAGIPELLSWLKQLQIKLhNPPTIHYPpsqgQMDLCVTSGSQQGLCKVFEMIIN--PGDNVLLDEPAY
---mfenittapadpilgladlfraderpgkinlgigvykdetgktpVLTSVkKAeQYLLE-NETT-KNYLGIDGIPEFGRCTQELLFGK-GSALINDK----RARTAQTPGGTGALRVAADFLAKntSVKRVWVSNPSW

SGTLQSLHP-LGCNIINVASD---ESGIVpDSLRDILSRWkpedaknpqkNTPKFLYTVPNGNNPTGNSLTSERKKEIYFLARKYDFLIIEDDPYYFLQFNKFRV-PTFLSMD-VDGRVIRADSFS-II-SSGLRIGFLTGP------KP-LIERVILHIQVSTLHPSTFNQLMISQLIHEWG-EEGFMA
PNHKSVFNSaGLEVREYAYYDaenHTLDF-DALINSLNEA----------QAGDVVLFHGCCHNPTGIDPTLEQWQTLAQLSVEKGWLPLFDFAYQGFARGLEEDaEGLRAFAaMHKELIVASSYSkNFgLYNERVGACTLVaadsetVDrAFSQMKAAIRVNYSSPPAHGASVVATILSNDAlRAIWEQ

HVDRVIDFYSNQKDAILAAADKW-LTGLAEWHVPAAGMFLWIKVkgindVKELIEEKAVKMGVLMLPGnafyvdssapspyLRASFSSASPEQMDVAFQVLAQLIkesl
ELTDMRQRIQRMRQLFVNTLQEKgANRDFSFTIKQNGMFFFGGL-----TKEQVLRLREEFGVYAVAS-------------GRLNVAGMTPDNLAPLCEAIVAVL----

SARST: aliSize=337 (resi) RMSD=12.15 (Å)
mnyarfitaasaarnpspirtmtdilsrg------------------------PKSMISLAGGLPNPNMfpfktavitveNGKTIqfGEEMMKRA---------LQYSPSAGIPELLSWLKQLQiklhnpPTIHYPPS-QGQMDLCVTSGSQQGLCKVFE---MIINPGdNVLLDEPAYSGTLQSLHPLG
----------------------------mfenittapadpilgladlfradeRPGKINLGIGVYKDET-----------GKTPV--LTSVKKAEqyllenettKNYLGIDGIPEFGRCTQELL------FGKGSALInDKRARTAQTPGGTGALRVAADflaKNTSVK-RVWVSNPSWPNHKSVFNSAG

CNIINVASD---ESGIVPDSLRDILSRWKpedaknpqKNTPkfLYTVPNGNNPTGNSLTSERKKEIYFLARKYDFLIIEDDPYYFLQFnkfrvpTFLSMDV-----DGRV---IRADSFSIIS--SGLRIGFLTGP-------KPLIERVILHIQVSTLHPSTFNQLMISQLIHEWGEEGFMAHVDRVID
LEVREYAYYdaeNHTLDFDALINSLNEAQ--------AGDV--VLFHGCCHNPTGIDPTLEQWQTLAQLSVEKGWLPLFDFAYQGFAR------GLEEDAEglrafAAMHkelIVASSYSKNFglYNERVGACTLVaadsetvDRAFSQMKAAIRVNYSSPPAHGASVVATILSNDALRAIWEQELTDMR

FYSNQKDAILAAADKWlTGlAEWHVPAAGMFLWIKVKGiNDVKELIEEKAVKMGVLMlpgnafyvDSSAPspylrASFSSASPEQMDVAFQVLAQ--likesl
QRIQRMRQLFVNTLQE-KG-ANRDFSFTIKQNGMFFFG-GLTKEQVLRLREEFGVYA--------VASGR-----LNVAGMTPDNLAPLCEAIVAvl------

BLAST: aliSize=42 (resi) iden=7.07% (28/396) simi=10.61% (42/396)
mnyarfitaasaarnpspirtmtdilsrgpksmislagglpnpnmfpfktavitvengktiqfgeemmkralqyspsagipellswlkqlqiklhnpptihyppsqgqmdlcvtsgsqqglckvfemiinpgdnvlldepaysgtlqslhplgcniinvasdesgivpdslrdilsrwkpedaknpqknt
----

pkf-----------------------------------LYTVPNGNNPTgnsLTSERKKEIYFLARKY--DFLIIEDDPYY--FLQFNKFRVPTFLSMDVDGRVIRADSFSiisSGLRIG--FLTgPKPLIERVILHIQVSTLHPSTFN--
---mfenittapadpilgladlfraderpgkinlgigVYKDETGKTPV---LTSVKKAEQYLLENETtkNYLGIDGIPEFgrCTQELLFGKGSALINDKRARTAQTPGGT---GALRVAadFLA-KNTSVKRVWVSNPSWPNHKSVFNsaglevreyayydaenhtldfdalinslneaqagdvvlfhgc

----
chnptgidptleqwqtlaqlsvekgwlplfdfayqgfargleedaeglrafaamhkelivassysknfglynervgactlvaadsetvdrafsqmkaairvnysspp ahgasvvatilsndalraiweqeltdmrqriqrmrqlfvntlqekganrdfsftikqngmfffggltkeqvlrlreefgvyav

--------------------------qlmisqllhewgeegfmahvdrvidfysnqkdailaaadkwltglaewhvpaagmflwikvkgindvkelieekavkmgvlmlpgnafyvdssapspylrasfssaspeqmdvafqvlaqlikesl
asgrlnvagmtpdnlaplceaivavl--

Proposed: aliSize=355 (resi) RMSD=2.67 (Å)
mNYARfItaasAARNPS---PI-RTMTDILsrGpksMISLAGGLP-NPN-------mfpfKT-A-VITvengkTIQFGeeMMKRaLQYSPSAGIPELLSWLKQLQ-IKlhNPPTIHYpPsqgQMDLCVTSGSQQGLCKVFEMII----NpgDNVLLDEPAYSGTLQSLHP-LGCNIINVASD----ESgi
-MFEN-I----TTAPADpilGLaDLFRADE--R---PGKINLGIGvYKDetgktpv----LTsVkKAE-----QYLLE--NETT-KNYLGIDGIPEFGRCTQELLfGK--GSALINDK----RARTAQTPGGTGALRVAADFLAkntsV--KRVWVSNPSWPNHKSVFNSaGLEVREYAYYDaenhTL--

VPDSLRDILSR-wkpedaknpqkNTPKFLYTVPNGNNPTGNSLTSERKKEIYFLARKYDFLIIEDDPYYFLQfNK--fRV-PTFLSMD-VDGRVIRADSFSLII-SSGLRIGFLTGP------KP-LIERVILHIQVSTLHPSTFNQLMISQLIHEWG-EEGFMAHVDRVIDFYSNQKDAILAAADKW-L
DFDALINSLNEa-----------QAGDVVLFHGCCHNPTGIDPTLEQWQTLAQLSVEKGWLPLFDFAYQGFA-RGle-EDaEGLRAFAaMHKELIVASSYSKNFgLYNERVGACTLVaadsetVDrAFSQMKAAIRVNYSSPPAHGASVVATILSNDAlRAIWEQELTDMRQRIQRMRQLFVNTLQEKgA

TGlAE-WHVPAAGMFLWIKVkgindVKELIEeKA-VKMGVLMLPGnafyvdssapspyLRASFSSASPEQMDVAFQVLAQLI-kesl
NR-DFsFTIKQNGMFFFGGL-----TKEQVL-RLrEEFGVYAVAS-------------GRLNVAGMTPDNLAPLCEAIVAVLi----</td></tr>
<tr><td>2d5yA
(396)</td></tr>
</table>

675	2qlrA (425)<br>2d61A (396)	N	14.14	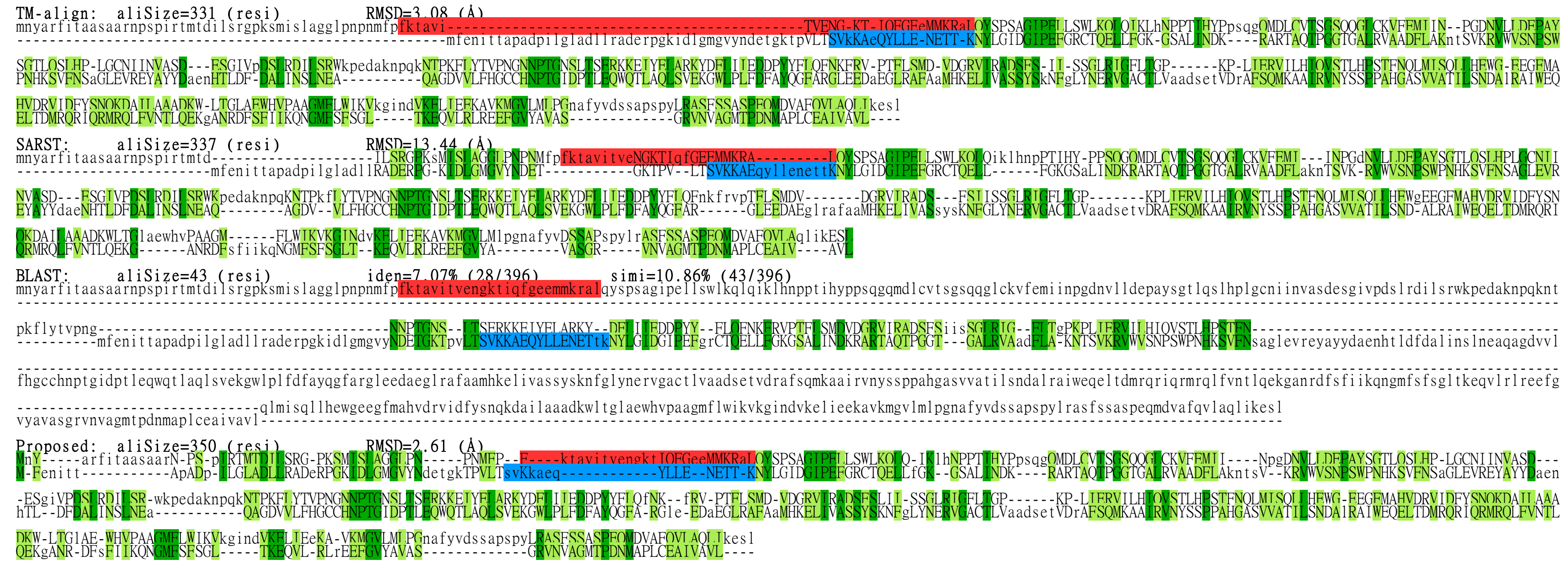
676	2qlrA (425)<br>2d64A (396)	N	13.89	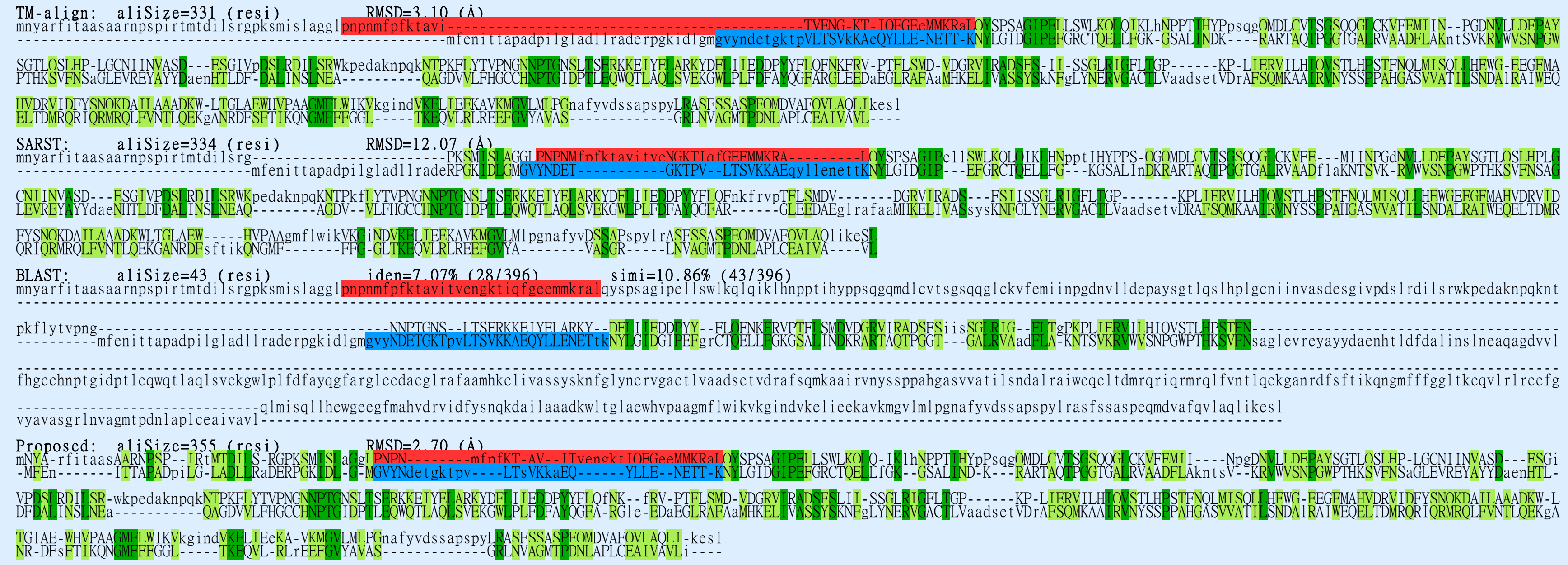
677	2qlrA (425)<br>2d66A (396)	N	12.63	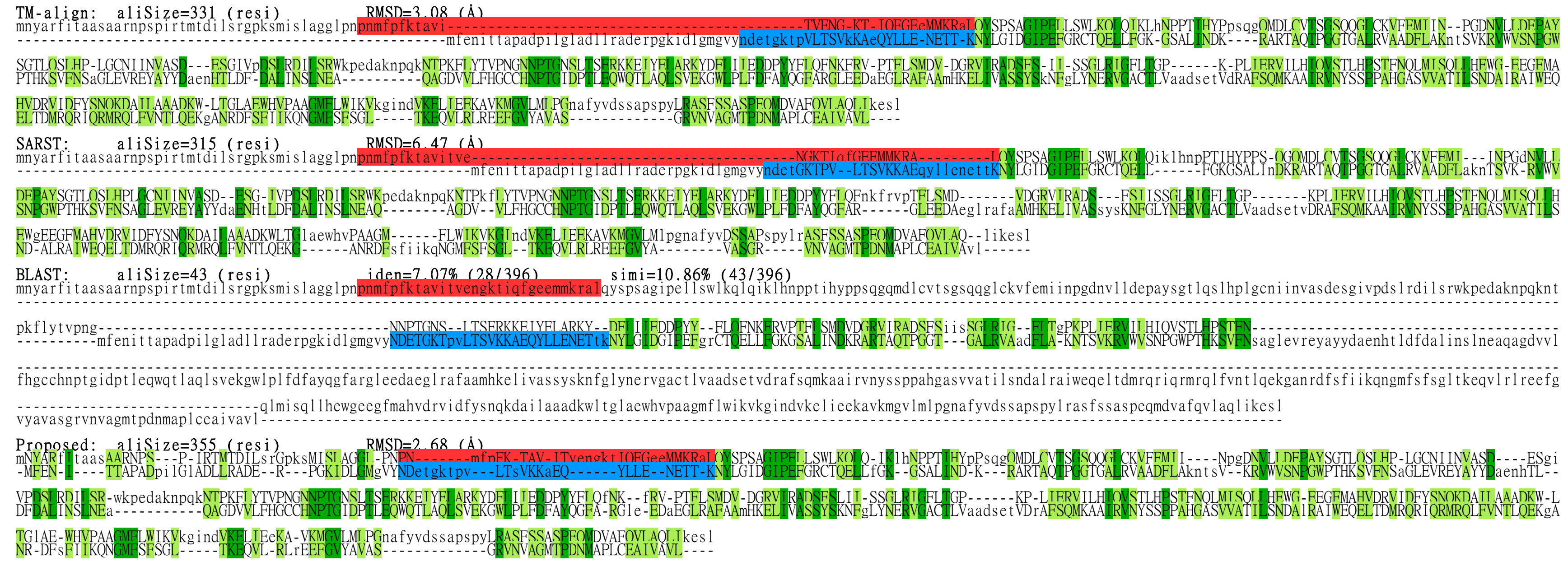

678	2qlrA<br>(425)<br>2douA<br>(372)	N	17.47	TM-align: aliSize=333 (resi) RMSD=2.76 (Å)<br>mnyarfitaasaarnpspirtmtdilsrgpksmislagglpnpnmfpfktavi-----------------------------------TVENGKTIQFGEEMMKRALQYSPSAGIPELLSWLKQLQIKLHNPPtIHypPsqgQMDLCVTSGSQQGLCKVFEMIINPGDNVLLDEPAYSGTLQSLHPLGCN<br>---vpepsvflvvdeakrkarergvglidlsigstdlpPPEAPLKALAEALNDPTTYGYCLKSCTLPFLEEAARWYEGRYGVG-LD--P---RREALALIGSQEGLAHLLLALTEPEDLLLLPEVAYPSYFGAARVASLR<br><br>IINVASD-FSGIVPDSLrdIlSRWkPeDaknpqknTPKFLYTVPNgNNPTGNSLTSERKKFIYELARKYDFLIIEDDPYYFLQFNKfRVPTFLSMDVD-GRVIRADSFS-II-SSGLRIGFLTGPKPLIERVILHIQVSTLHPSTFNQLMISQLIheWgeeGFMAHVDRVIDFYSNQKDAILAAADkwlT<br>TFLIPLReDGLADLKAV--P-EGV-W-R-------EAKVLLLNYP-NNPTGAVADWGYFEEALGLARKHGLWLIHDNPYVDQVYEG-EAPSPLALPGAkERVVELFSLSkSYnLAGFRLGFALGSEEALARLERVKGVIDFNQYAGVLRMGVEAL--K---TPKEVVRGYARVYRERALGMAEALK---G<br><br>gLAFWHVPAAGMFLWIKVKGINDVKFLIEeKAVKMGVLMLPGNAFYvDssAPSPYLRASFsSASPFQMDVAFQVLAQLIkesl<br>-VLSLLPPRATMYLWGRLPEGVDDLEFGL-RLVERGVALAPGRGFG-P--GGKGFVRIAL-VRPLEELLEAAKRIREAL----<br><br>SARST: aliSize=326 (resi) RMSD=5.38 (Å)<br>mnyarfitaasaarnpspirtmtdilsrgpksmislagglpnpnmfpfktavitv----------------------------ENGKTIQFGEEMMKR---------ALQYSPSAGIPELLSWLKQLQIKLHN---PPTIHyppsqgqmdLCVTSGSQQGLCKVFEMIINPGDNVLLDEPAYSGTLQSLH<br>---vpepsvflvvdeakrkarergvglidlsIGSTDLPPPEAPLKAlaealndptTYGYCLKSCTLPFLEEAARWYEGRYGvglDPRRE---------ALALIGSQEGLAHLLLALTEPEDLLLLPEVAYPSYFGAAR<br><br>PLGCNIINVASDFSG-IVpdslrdiLSRWKPEDAKnpqknTPKFLYTVpNGNNPTGNSLTSERKKFIYELARKYDFLIIEDDPYYFLQFNkFRVPTFLS-MDVDGRVIRADSF--SIISSGLRIGFLTGPKPLIERVILHIQVSTLHPSTFNQLMISQLIHewGEFGFMAHVDRVIDFYSNQKDAILaaa<br>VASLRTFLIPLREDGlAD-------LKAVPEGVWR-----EAKVLLLN-YPNNPTGAVADWGYFEEALGLARKHGLWLIHDNPYVDQVYE-GEAPSPLAlPGAKERVVELFSLskSYNLAGFRLGFALGSEEALARLERVKGVIDFNQYAGVLRMGVEALK--TPKEVVRGYARVYRERALGMAEAL---<br><br>dkwlTGLAFWHVPAAGMFLWIKVKgINDVKELIEEKAVKMGVLMLPGNAFYVDSsapSPYLRASFsSASPFQMDVAFQVLAQ--likesl<br>----KGVLSLLPPRATMYLWGRLP-EGVDDLEFGLRLVERGVALAPGRGFGPGG---KGFVRIAL-VRPLEELLEAAKRIREal------<br><br>BLAST: aliSize=123 (resi) iden=20.97% (78/372) simi=33.06% (123/372)<br>mnyarfitaasaarnpspirtmtdilsrgpksmislagglpnpnmfpfktavitvengktiqfgeemmkralqyspsagipellswlkqlqiklhnpptihyppsqgqmdlcvts---<br>---vpepsvflvvdeakrkarergvglidlsigstdlpppeaplkalaealndpttygyclksctlpfleeaarwyeg<br><br>----------------GSQQGLCKVFEMIINPGDNVLLDEPAYsgtlqslhPLGCNIINVASDFSGIVP---DSLRDILSrwKPEDAKNPQKntpKFLYTVPNgnNPTGNSLTSERKKFIYELARKYDFLIIEDDPYYFlQFNKFRVPTFLSM-DVDGRVIraDSFSIISS----GLRIGFLTGPKPLIE<br>rygvgldprrealaliGSQEGLAHLLLALTEPEDLLLLPEVAY--------PSYFGAARVASLRTFLIPlreDGLADLKA--VPEGVWREAK---VLLLNYPN--NPTGAVADWGYFEEALGLARKHGLWLIHDNPYVD-QVYEGEAPSPLALpGAKERVV--ELFSLSKSynlaGFRLGFALGSEEALA<br><br>RVilhIQVSTLhpSTFNQlmISQLIhEWGEFGFMAHVDRVIDFYSNQKDAILAAADKwLTGLAFWHVPAAGMFLWIKV-KGINDVKelIEEKAVKMGVLMLPGNAF-----------------------------yvdssapspylrasfssaspeqmdvafqvlaqlikesl<br>RL---ERVKGV--IDFNQ--YAGVL-RMGVEALKTPKEVVRGYARVYRERALGMAEA-LKGVLSLLPPRATMYLWGRLpEGVDDLE--FGLRLVERGVALAPGRGFgpggkgfvrialvrpleelleaakrireal-------------------------------------<br><br>Proposed: aliSize=349 (resi) RMSD=2.13 (Å)<br>mnyarfitaasaa--RNPSP--IRTMTDILSRGPksMISLaG-GLP-nPNmFPFKT--AV-IT--VEngktiqfgeemMKRALQYSPS-aGIPELLSWLKQLQIKLHNP-ptIhyppsqgqM---DLCVTSGSQQGLCKVFEMIINPGDNVLLDEPAYSGTLQSLHPLGCNIINVASD-FSGIVPDSLrd<br>-------------vpEPSVFlvVDEAKRKARERG--VGLI-DlSIGs-TD-LPPPEapLKaLAeaLN----------DPTTYGYCLKs-CTLPFLEEAARWYEGRYGVg--L--------DprrEALALIGSQEGLAHLLLALTEPEDLLLLPEVAYPSYFGAARVASLRTFLIPLReDGLADLKAV--<br><br>IlSR--WkpedaknpqknTPKFLYTVPNgNNPTGNSLTSERKKFIYELARKYDFLIIEDDPYYFLQFNKfRVPTFLSMDVD-GRVIRADSFSLII-SSGLRIGFLTGPKPLIERVILHIQVSTLHPSTFNQLMISQLIheWgeeGFMAHVDRVIDFYSNQKDAILAAADkwlTgLAFWHVPAAGMFLWIK<br>P-EGvwR----------EAKVLLLNYP-NNPTGAVADWGYFEEALGLARKHGLWLIHDNPYVDQVYEG-EAPSPLALPGAkERVVELFSLSKSYnLAGFRLGFALGSEEALARLERVKGVIDFNQYAGVLRMGVEAL--K---TPKEVVRGYARVYRERALGMAEALK---G-VLSLLPPRATMYLWGR<br><br>VKGINDVKFLIEeKAVKMGVLMLPGNAFYvD--ssapSPYLRASFsSASPFQMDVAFQVLAQLIkesl<br>LPEGVDDLEFGL-RLVERGVALAPGRGFG-Pgg----KGFVRIAL-VRPLEELLEAAKRIREAL----
679	2qlrA<br>(425)<br>2douB<br>(368)	N	17.66	TM-align: aliSize=333 (resi) RMSD=2.76 (Å)<br>mnyarfitaasaarnpspirtmtdilsrgpksmislagglpnpnmfpfktavi-----------------------------------TVENGKTIQFGEEMMKRALQYSPSAGIPELLSWLKQLQIKLHNPPtIHypPsqgQMDLCVTSGSQQGLCKVFEMIINPGDNVLLDEPAYSGTLQSLHPLGCNIINV<br>---svflvvdeakrkarergvglidlsigstdlpPPEAPLKALAEALNDPTTYGYCLKSCTLPFLEEAARWYEGRYGVG-LD--P---RREALALIGSQEGLAHLLLALTEPEDLLLLPEVAYPSYFGAARVASLRTFLI<br><br>ASD-FSGIVPDSLrdIlSRWkPedAknpqknTPKFLYTVPNgNNPTGNSLTSERKKFIYELARKYDFLIIEDDPYYFLQFNKfRVPTFLSMDVD-GRVIRADSFS-II-SSGLRIGFLTGPKPLIERVILHIQVSTLHPSTFNQLMISQLIheWgeeGFMAHVDRVIDFYSNQKDAILAAADkwlTgLAF<br>PLReDGLADLKAV--P-EGV-W--R------EAKVLLLNYP-NNPTGAVADWGYFEEALGLARKHGLWLIHDNPYVDQVYEG-EAPSPLALPGAkERVVELFSLSkSYnLAGFRLGFALGSEEALARLERVKGVIDFNQYAGVLRMGVEAL--K---TPKEVVRGYARVYRERALGMAEALK---G-VLS<br><br>WHVPAAGMFLWIKVKGINDVKFLIEeKAVKMGVLMLPGNAFYvDssAPSPYLRASFsSASPFQMDVAFQVLAQLIkesl<br>LLPPRATMYLWGRLPEGVDDLEFGL-RLVERGVALAPGRGFG-P--GGKGFVRIAL-VRPLEELLEAAKRIREAL----<br><br>SARST: aliSize=311 (resi) RMSD=4.04 (Å)<br>mnyarfitaasaarnpspirtmtdilsrgpksmislagglpnpnmfpfktavitvengktiqfgeemmkr--ALQYSPSAGIPELLSWLKQLQIKLHNPptiHYPPSQGqmdLCVTSGSQQGLCKVFEMIINPGDNVLLDEPAY<br>---svflvvdeakrkarergvglidlsigstdlpppeaplkalaealndptTYGYCLKSCTLPFLEEAARWYEGRYGV---GLDPRRE---ALALIGSQEGLAHLLLALTEPEDLLLLPEVAY<br><br>SGTLQSLHPLGCNIINVASDFSGIVpdslrdilsrwkpedakNPQKNT------PKFLYTVpNGNNPTGNSLTSERKKFIYELARKYDFLIIEDDPYYFLQFNKFRVPTFLSMDVDGRVIRADSFSIIS--SGLRIGFLTGPKPLIERVILHIQVSTLHPSTFNQLMISqLLHFWGFegfmahvDRVIDF<br>PSYFGAARVASLRTFLIPLREDGLA----------------DLKAVPegvwreAKVLLLN-YPNNPTGAVADWGYFEEALGLARKHGLWLIHDNPYVDQVYEGEAPSPLALPGAKERVVELFSLSKSYnlAGFRLGFALGSEEALARLERVKGVIDFNQYAGVLRMGV-EALKTPK-------EVVRGY<br><br>YSNQKDAILAAADKWLtGLAFWHVPAAGMFLWIKVKGINDvKELIEEKAVKMGVLMLPGNAFYVDSsapSPYLRASFSSASPFQMDVAFQVLAQ-likesl<br>ARVYRERALGMAEALK-GVLSLLPPRATMYLWGRLPEGVD-DLEFGLRLVERGVALAPGRGFGPGG---KGFVRIALVRPLEELLEAAKRIREAl------<br><br>BLAST: aliSize=123 (resi) iden=21.20% (78/368) simi=33.42% (123/368)<br>mnyarfitaasaarnpspirtmtdilsrgpksmislagglpnpnmfpfktavitvengktiqfgeemmkralqyspsagipellswlkqlqiklhnpptihyppsqgqmdlcvts---<br>---svflvvdeakrkarergvglidlsigstdlpppeaplkalaealndpttygyclksctlpfleeaarwyegrygv<br><br>------------GSQQGLCKVFEMIINPGDNVLLDEPAYsgtlqslhPLGCNIINVASDFSGIVP---DSLRDILSrwKPEDAKNPQKntpKFLYTVPNgnNPTGNSLTSERKKFIYELARKYDFLIIEDDPYYFlQFNKFRVPTFLSM-DVDGRVIraDSFSIISS----GLRIGFLTGPKPLIERVil<br>gldprrealaliGSQEGLAHLLLALTEPEDLLLLPEVAY--------PSYFGAARVASLRTFLIPlreDGLADLKA--VPEGVWREAK---VLLLNYPN--NPTGAVADWGYFEEALGLARKHGLWLIHDNPYVD-QVYEGEAPSPLALpGAKERVV--ELFSLSKSynlaGFRLGFALGSEEALARL--<br><br>hIQVSTLhpSTFNQlmISQLIhEWGEFGFMAHVDRVIDFYSNQKDAILAAADKwLTGLAFWHVPAAGMFLWIKV-KGINDVKelIEEKAVKMGVLMLPGNAF-----------------------------yvdssapspylrasfssaspeqmdvafqvlaqlikesl<br>-ERVKGV--IDFNQ--YAGVL-RMGVEALKTPKEVVRGYARVYRERALGMAEA-LKGVLSLLPPRATMYLWGRLpEGVDDLE--FGLRLVERGVALAPGRGFgpggkgfvrialvrpleelleaakrireal-------------------------------------<br><br>Proposed: aliSize=349 (resi) RMSD=2.18 (Å)<br>mnyarfitaasaarnPSpIRTMTDILSR-GP-kSMISLAGGLPNPNmFPFKT--AV-IT--VEngktiqfgeemMKRALQYSPS-aGIPELLSWLKQLQIKLHNP-ptI---hyppsqgqMDLCVTSGSQQGLCKVFEMIINPGDNVLLDEPAYSGTLQSLHPLGCNIINVASD-FSGIVPDSLrdIlSR<br>---------------SV-FLVVDEAKRKaREr-GVGLIDLSIGSTD-LPPPEapLKaLAeaLN----------DPTTYGYCLKs-CTLPFLEEAARWYEGRYGVg--Ldpr--------REALALIGSQEGLAHLLLALTEPEDLLLLPEVAYPSYFGAARVASLRTFLIPLReDGLADLKAV--P-EG<br><br>--WkpedaknpqknTPKFLYTVPNgNNPTGNSLTSERKKFIYELARKYDFLIIEDDPYYFLQFNKfRVPTFLSMDVD-GRVIRADSFSLII-SSGLRIGFLTGPKPLIERVILHIQVSTLHPSTFNQLMISQLIheWgeeGFMAHVDRVIDFYSNQKDAILAAADkwlTgLAFWHVPAAGMFLWIKVKGI<br>vwR-----------EAKVLLLNYP-NNPTGAVADWGYFEEALGLARKHGLWLIHDNPYVDQVYEG-EAPSPLALPGAkERVVELFSLSKSYnLAGFRLGFALGSEEALARLERVKGVIDFNQYAGVLRMGVEAL--K---TPKEVVRGYARVYRERALGMAEALK---G-VLSLLPPRATMYLWGRLPEG<br><br>NDVKFLIEeKAVKMGVLMLPGNAFYvD--ssapSPYLRASFsSASPFQMDVAFQVLAQLIkesl<br>VDDLEFGL-RLVERGVALAPGRGFG-Pgg----KGFVRIAL-VRPLEELLEAAKRIREAL----
680	2qlrA<br>(425)<br>2egyA<br>(392)	N	27.30	TM-align: aliSize=341 (resi) RMSD=2.07 (Å)<br>mnyarfitaasaarnpspirtmtdilsrgpksmislagglpnpnmfpfktav--ITVEnGKTI-QFGEEMMKRALQYSPSAGIPELLSWLKQLQIklhNpptIhyppsqgQMDLCVTSGSQQGLCKVFEMIINPGDNVLLDEPAYSGT<br>--swseafgkgagriqastirellkltqrpgilsfagglpapelfpKEEA-AEAAaRILREKGEVALQYSPTEGYAPLRAFVAEWIG---V---R-------PEEVLITTGSQQALDLVGKVFLDEGSPVLLEAPSYMGA<br><br>LQSLHPLGCNIINVASDFSGIVPDSLRDILSRWkpedaknpqknTPKFLYTVPNGNNPTGNSLTSERKKFIYELARKYDFLIIEDDPYYFLQFNKFRVPTFLSMD---VDGRVIRADSFS-IISSGLRIGFLTGPKPLIERVILHIQVSTLHPSTFNQLMISQLLHeWgeeGFMAHVDRVIDFYSNQKDA<br>IQAFRLQGPRFLTVPAGEEGPDLDALEEVLKRE----------RPRFLYLIPSFQNPTGGLTPLPARKRLLQMVMERGLVVVEDDAYRELYFGEARLPSLFELAreaGYPGVIYLGSFSkVLSPGLRVAFAVAHPEALQKLVQAKQGADLHTPMLNQMLVHELLK-E---GFSERLERVRRVYREKAQA<br><br>ILAAADKWLTGLAFWHVPAAGMFLWIKVKGINDVKELIeEKAVKMGVLMLPGNAFYVDSsAPSPYLRASFSSASPFQMDVAFQVLAQLIKESL--<br>MLHALDREVPKEVRYTRPKGGMFVWMELPKGLSAEGLF-RRALEENVAFVPGGPFFANG-GGENTLRLSYATLDREGIAEGVRRLGRALKGLLal<br><br>SARST: aliSize=370 (resi) RMSD=13.57 (Å)<br>mn-YARFITAASAARNPSPiRTMTDILSRGPKSMIsLAGGLPNPNMFpfktavitvengktiqfGEEMMKRA------------LQYSPSAGIPELLSWLKQLqiklhnpptIHYPPSQgqmdLCVTSGSQQGLCKVFEMIINPGDNVLLDEPAYSGTLQSLHPLGCNIINVASDFSGIVPDSLRDILSR<br>--sWSEAFGKGAGRIQAST-IRELLKLTQRPGILS-FAGGLPAPELF-----------------PKEEAAEAaarilrekgevaLQYSPTEGYAPLRAFVAEW---------IGVRPEE----VLITTGSQQALDLVGKVFLDEGSPVLLEAPSYMGAIQAFRLQGPRFLTVPAGEEGPDLDALEEVLKR<br><br>WKpedaknpqkntPKFLYTVPNGNNPTGNSLTSERKKFIYELARKYDFLIIEDDPYYFLQFNKFRVPTFLSMDVD---GRVIRADSFSI-ISSGLRIGFLTGPKPLIERVILHIQVSTLHPSTFNQLMISQLLHFwgeeGFMAHVDRVIDFYSNQKDAILAAADKWLTGLAFWHVPAAGMFlWIKVKGIN<br>ER-----------PRFLYLIPSFQNPTGGLTPLPARKRLLQMVMERGLVVVEDDAYRELYFGEARLPSLFELAREagyPGVIYLGSFSKvLSPGLRVAFAVAHPEALQKLVQAKQGADLHTPMLNQMLVHELLKE----GFSERLERVRRVYREKAQAMLHALDREVPKEVRYTRPKGGMF-VWMELPKG<br><br>DVKFLIEEKAVKMGVLMLPGNAFYVdSSAPSPYLRASFSSASPFQMDVAFQVLAQLIK-----esl<br>LSAEGLFRRALEENVAFVPGGPFFA-NGGGENTLRLSYATLDREGIAEGVRRLGRALKgllal---<br><br>BLAST: aliSize=203 (resi) iden=29.34% (115/392) simi=51.79% (203/392)<br>mnyarfitaasaarnp---------------SPIRTMTDILSRgPkSMISLAGGLPNPNMFPFKTAVitvENGKTIQfgEEMMKRALQYSPSAGIPELLSWLKQLqiklhnpptIHYPPSQgqmdLCVTSGSQQGLCKVFEMIINPGDNVLLDEPAYSGTLQSLHPLGCNIINVASDFSGIVPDSLRDIL<br>----------------swseafgkgagriqaSTIRELLKLTQR-P-GILSFAGGLPAPELFPKEEAA---EAAARIL--REKGEVALQYSPTEGYAPLRAFVAEW---------IGVRPEE----VLITTGSQQALDLVGKVFLDEGSPVLLEAPSYMGAIQAFRLQGPRFLTVPAGEEGPDLDALEEVL<br><br>SRWKPedaknpqkntpKFLYTVPNGNNPTGNSLTSERKKFIYELARKYDFLIIEDDPYYFLQFNKFRVPTFLSMDVDG---RVIRADSFS-IISSGLRIGFLTGPKPLIERVILHIQVSTLHPSTFNQLMISQLLhewgFEGFMAHVDRVIDFYSNQKDAILAAADKWLTGLAFWHVPAAGMFLWIKV-K<br>KRERP-----------RFLYLIPSFQNPTGGLTPLPARKRLLQMVMERGLVVVEDDAYRELYFGEARLPSLFELAREAgypGVIYLGSFSkVLSPGLRVAFAVAHPEALQKLVQAKQGADLHTPMLNQMLVHELL----KEGFSERLERVRRVYREKAQAMLHALDREVPKEVRYTRPKGGMFVWMELpK<br><br>GINdvKFLIEEKAVKMGVLMLPGNAFYVDSSAPSPyLRASFSSASPFQMDVAFQVLAQLIK-----esl<br>GLS--AEGLFRRALEENVAFVPGGPFFANGGGENT-LRLSYATLDREGIAEGVRRLGRALKgllal---<br><br>Proposed: aliSize=380 (resi) RMSD=1.82 (Å)<br>mNYARFITAASAARNPS-PiR-TMtDILSrGPKsMISLAGGLPNPNMFpFKT--AV--ItvengktiQFGEEMMKRALQYSPSAGIPELLSWLKQLQIklhNpptI-hyppsqgqMDLCVTSGSQQGLCKVFEMIINPGDNVLLDEPAYSGTLQSLHPLGCNIINVASDFSGIVPDSLRDILSRWkpeda<br>-SWSEAFGKGAGRIQAStI-ReLL-KLTQ-RPG-ILSFAGGLPAPELF-PKEeaAEaaA--------RILREKGEVALQYSPTEGYAPLRAFVAEWIG---V---Rp--------EEVLITTGSQQALDLVGKVFLDEGSPVLLEAPSYMGAIQAFRLQGPRFLTVPAGEEGPDLDALEEVLKRE-----<br><br>knpqknTPKFLYTVPNGNNPTGNSLTSERKKFIYELARKYDFLIIEDDPYYFLQFNKFRVPTFLSMD---VDGRVIRADSFSLIISSGLRIGFLTGPKPLIERVILHIQVSTLHPSTFNQLMISQLLHeWgeeGFMAHVDRVIDFYSNQKDAILAAADKWLTGLAFWHVPAAGMFLWIKVKGINDVKELI<br>------RPRFLYLIPSFQNPTGGLTPLPARKRLLQMVMERGLVVVEDDAYRELYFGEARLPSLFELAreaGYPGVIYLGSFSKVLSPGLRVAFAVAHPEALQKLVQAKQGADLHTPMLNQMLVHELLK-E---GFSERLERVRRVYREKAQAMLHALDREVPKEVRYTRPKGGMFVWMELPKGLSAEGLF<br><br>eEKAVKMGVLMLPGNAFYVDSsAPSPYLRASFSSASPFQMDVAFQVLAQLIKESL--<br>-RRALEENVAFVPGGPFFANG-GGENTLRLSYATLDREGIAEGVRRLGRALKGLLal

681	2qlrA<br>(425)<br><br>2egyB<br>(394)	N	26.65	TM-align: aliSize=341 (resi) RMSD=2.05 (Å)<br>mnyarfitaasaarnpspirtmtdilsrgpksmislagglpnpnmfpfktav---------------------------------------ITVEn-GKTIQFGEEMMKRALQYSPSAGIPELLSWLKQLQIklhnPptIhyppsqgQMDLCVTSGSQQGLCKVFFMIINPGDNVLLDEPAY<br>---kplswseafgkgagriqastirellkltqrpgilsfagglpapelfpKEEA-aEAAARILREKGEVALQYSPTEGYAPLRAFVAEWIG----V--R-------PEEVLITTGSQQALDLVGKVFLDEGSPVLLEAPSY<br><br>SGTLQSLHPLGCNIINVASDFSGIVPDSLRDILSRwkpedaknpqkNTPKFLYTVPNGNNPTGNSLTSERKKEIYELARKYDFLIIEDDPYYFLQFNKFRVPTFLSMD---VDGRVIRADSFS-IISSGLRIGFLTGPKPLIERVILHIQVSTLHPSTFNQLMISQLLHeWgeeGFMAHVDRVIDFYSNQ<br>MGAIQAFRLQGPRFLTVPAGEEGPDLDALEEVLKR-----------ERPRFLYLIPSFQNPTGGLTPLPARKRLLQMVMERGLVVVEDDAYRELYFGEARLPSLFELAreaGYPGVIYLGSFSkVLSPGLRVAFAVAHPEALQKLVQAKQGADLHTPMLNQMLVHELLK-E---GFSERLERVRRVYREK<br><br>KDAILAAADKWLTGLAEWHVPAAGMFLWIKVKGINDVKELIeFKAVKMGVLMLPGNAFYVDSsAPSPYLRASFSSASPEQMDVAFQVLAQLIKES-L<br>AQAMLHALDREVPKEVRYTRPKGGMFVWMELPKGLSAEGLF-RRALEENVAFVPGGPFFANG-GGENTLRLSYATLDREGIAEGVRRLGRALKGLlA<br><br>SARST: aliSize=375 (resi) RMSD=13.60 (Å)<br>m---NYARFITAASAARnpSPIRTMTDIL-SRGPKsMISLAGGLPNPNMFPFKtavitvengktiqfGEEMMKRA---------LQYSPSAGIPELLSWLKqlqiklhnpPTIHYPPSQgqmdLCVTSGSQQGLCKVFFMIINPGDNVLLDEPAYSGTLQSLHPLGCNIINVASDFSGIVPDSLRDILSR<br>-kplSWSEAFGKGAGRI--QASTIRELLKlTQRPG-ILSFAGGLPAPELFPKE-------------EAAEAAARilrekgevaLQYSPTEGYAPLRAFVA---------EWIGVRPEE----VLITTGSQQALDLVGKVFLDEGSPVLLEAPSYMGAIQAFRLQGPRFLTVPAGEEGPDLDALEEVLKR<br><br>WKpedaknpqkntPKFLYTVPNGNNPTGNSLTSERKKEIYELARKYDFLIIEDDPYYFLQFNKFRVPTFL---SMDVDGRVIRADS-FSIISSGLRIGFLTGPKPLIERVILHIQVSTLHPSTFNQLMISQLLhewgFEGFMAHVDRVIDFYSNQKDAILAAADKWLTGLAEWHVPAAGMFLWIKVKgIN<br>ER-----------PRFLYLIPSFQNPTGGLTPLPARKRLLQMVMERGLVVVEDDAYRELYFGEARLPSLFelaREAGYPGVIYLGSfSKVLSPGLRVAFAVAHPEALQKLVQAKQGADLHTPMLNQMLVHELL----KEGFSERLERVRRVYREKAQAMLHALDREVPKEVRYTRPKGGMFVWMELP-KG<br><br>DVKFLIEFKAVKMGVLMLPGNAFYVdSSAPSPYLRASFSSASPEQMDVAFQVLAQLIKES--l<br>LSAEGLFRRALEENVAFVPGGPFFA-NGGGENTLRLSYATLDREGIAEGVRRLGRALKGLla-<br><br>BLAST: aliSize=203 (resi) iden=29.19% (115/394) simi=51.52% (203/394)<br>mnyarfitaasaarnp------------------SPIRTMTDILSRgPkSMISLAGGLPNPNMFPFKTAVitvENGKTIQfgEEMMKRALQYSPSAGIPELLSWLKQLqiklhnpptIHYPPSQgqmdLCVTSGSQQGLCKVFFMIINPGDNVLLDEPAYSGTLQSLHPLGCNIINVASDFSGIVPDSLR<br>----------------kplswseafgkgagriqaSTIRELLKLTQR-P-GILSFAGGLPAPELFPKEEAA---EAAARIL--REKGEVALQYSPTEGYAPLRAFVAEW---------IGVRPEE----VLITTGSQQALDLVGKVFLDEGSPVLLEAPSYMGAIQAFRLQGPRFLTVPAGEEGPDLDALE<br><br>DILSRWKPedaknpqkntpKFLYTVPNGNNPTGNSLTSERKKEIYELARKYDFLIIEDDPYYFLQFNKFRVPTFLSMDVDG---RVIRADSFS-IISSGLRIGFLTGPKPLIERVILHIQVSTLHPSTFNQLMISQLLhewgFEGFMAHVDRVIDFYSNQKDAILAAADKWLTGLAEWHVPAAGMFLWIK<br>EVLKRERP-----------RFLYLIPSFQNPTGGLTPLPARKRLLQMVMERGLVVVEDDAYRELYFGEARLPSLFELAREAgypGVIYLGSFSkVLSPGLRVAFAVAHPEALQKLVQAKQGADLHTPMLNQMLVHELL----KEGFSERLERVRRVYREKAQAMLHALDREVPKEVRYTRPKGGMFVWME<br><br>V-KGINdvKFLIEFKAVKMGVLMLPGNAFYVDSSAPSPyLRASFSSASPEQMDVAFQVLAQLIK----esl<br>LpKGLS--AEGLFRRALEENVAFVPGGPFFANGGGENT-LRLSYATLDREGIAEGVRRLGRALKglla---<br><br>Proposed: aliSize=379 (resi) RMSD=1.78 (Å)<br>--MNYARFITAASAARNP--SpiR-TMtDILSrGPKsMISLAGGLPNPNMFpFKT--AV--ItvengktiQFGEEMMKRALQYSPSAGIPELLSWLKQLQIklhnpptIhyppsqgqM--DLCVTSGSQQGLCKVFFMIINPGDNVLLDEPAYSGTLQSLHPLGCNIINVASDFSGIVPDSLRDILSRwk<br>kpLSWSEAFGKGAGRIQAstI--ReLL-KLTQ-RPG-ILSFAGGLPAPELF-PKEeaAEaaA--------RILREKGEVALQYSPTEGYAPLRAFVAEWIG-------V--------RpeEVLITTGSQQALDLVGKVFLDEGSPVLLEAPSYMGAIQAFRLQGPRFLTVPAGEEGPDLDALEEVLKR--<br><br>pedaknpqkNTPKFLYTVPNGNNPTGNSLTSERKKEIYELARKYDFLIIEDDPYYFLQFNKFRVPTFLSMD---VDGRVIRADSFSLIISSGLRIGFLTGPKPLIERVILHIQVSTLHPSTFNQLMISQLLHeWgeeGFMAHVDRVIDFYSNQKDAILAAADKWLTGLAEWHVPAAGMFLWIKVKGINDV<br>---------ERPRFLYLIPSFQNPTGGLTPLPARKRLLQMVMERGLVVVEDDAYRELYFGEARLPSLFELAreaGYPGVIYLGSFSKVLSPGLRVAFAVAHPEALQKLVQAKQGADLHTPMLNQMLVHELLK-E---GFSERLERVRRVYREKAQAMLHALDREVPKEVRYTRPKGGMFVWMELPKGLSA<br><br>KELIeFKAVKMGVLMLPGNAFYVDSsAPSPYLRASFSSASPEQMDVAFQVLAQLIKES-L<br>EGLF-RRALEENVAFVPGGPFFANG-GGENTLRLSYATLDREGIAEGVRRLGRALKGLlA
682	2qlrA<br>(425)<br><br>2egyC<br>(396)	N	27.27	TM-align: aliSize=341 (resi) RMSD=2.09 (Å)<br>mnyarfitaasaarnpspirtmtdilsrgpksmislagglpnpnmfpfktav---------------------------------------ITVEn-GKTIQFGEEMMKRALQYSPSAGIPELLSWLKQLQIklhnPptIhyppsqgQMDLCVTSGSQQGLCKVFFMIINPGDNVLLDEPAY<br>---kplswseafgkgagriqastirellkltqrpgilsfagglpapelfpKEEA-aEAAARILREKGEVALQYSPTEGYAPLRAFVAEWIG----V--R-------PEEVLITTGSQQALDLVGKVFLDEGSPVLLEAPSY<br><br>SGTLQSLHPLGCNIINVASDFSGIVPDSLRDILSRWkpedaknpqkNTPKFLYTVPNGNNPTGNSLTSERKKEIYELARKYDFLIIEDDPYYFLQFNKFRVPTFLSMD---VDGRVIRADSFS-IISSGLRIGFLTGPKPLIERVILHIQVSTLHPSTFNQLMISQLLHeWgeeGFMAHVDRVIDFYSNQ<br>MGAIQAFRLQGPRFLTVPAGEEGPDLDALEEVLKRE----------RPRFLYLIPSFQNPTGGLTPLPARKRLLQMVMERGLVVVEDDAYRELYFGEARLPSLFELAreaGYPGVIYLGSFSkVLSPGLRVAFAVAHPEALQKLVQAKQGADLHTPMLNQMLVHELLK-E---GFSERLERVRRVYREK<br><br>KDAILAAADKWLTGLAEWHVPAAGMFLWIKVKGINDVKELIeFKAVKMGVLMLPGNAFYVDSsAPSPYLRASFSSASPEQMDVAFQVLAQLIKESL---<br>AQAMLHALDREVPKEVRYTRPKGGMFVWMELPKGLSAEGLF-RRALEENVAFVPGGPFFANG-GGENTLRLSYATLDREGIAEGVRRLGRALKGLLalv<br><br>SARST: aliSize=369 (resi) RMSD=12.83 (Å)<br>m---NYARFITAASAARNPSPirtMT----DILSRGPksmISLAGGLPNPNMFPFKtavitvengktiqfgEEMMKRA-----------LQYSPSAGIPELLSWLKQLqikLHNPPTIhyppsqgqmdLCVTSGSQQGLCKVFFMIINPGDNVLLDEPAYSGTLQSLHPLGCNIINVASDFSGIVPDSLRD<br>-kplSWSEAFGKGAGRIQAST---IRellkLTQRPGI---LSFAGGLPAPELFPKE---------------EAAEAAArilrekgevaLQYSPTEGYAPLRAFVAEW---IGVRPEE-----------VLITTGSQQALDLVGKVFLDEGSPVLLEAPSYMGAIQAFRLQGPRFLTVPAGEEGPDLDALEE<br><br>ILSRWKpedaknpqkntPKFLYTVPNGNNPTGNSLTSERKKEIYELARKYDFLIIEDDPYYFLQFNKFRVPTFL-SMDVDG--RVIRADSF-SIISSGLRIGFLTGPKPLIERVILHIQVSTLHPSTFNQLMIsqLLHEWGeeGFMAHVDRVIDFYSNQKDAILAAADKWLTGLAEWHVPAAGMFLWIKV<br>VLKRER------------PRFLYLIPSFQNPTGGLTPLPARKRLLQMVMERGLVVVEDDAYRELYFGEARLPSLFeLAREAGypGVIYLGSFsKVLSPGLRVAFAVAHPEALQKLVQAKQGADLHTPMLNQMLV--HELLKE--GFSERLERVRRVYREKAQAMLHALDREVPKEVRYTRPKGGMFVWMEL<br><br>KgINDVKFLIEFKAVKMGVLMLPGNAFYVdSSAPSPYLRASFSSASPEQMDVAFQVLAQLIK------esl<br>P-KGLSAEGLFRRALEENVAFVPGGPFFA-NGGGENTLRLSYATLDREGIAEGVRRLGRALKgllalv---<br><br>BLAST: aliSize=203 (resi) iden=29.04% (115/396) simi=51.26% (203/396)<br>mnyarfitaasaarnp------------------SPIRTMTDILSRgPkSMISLAGGLPNPNMFPFKTAVitvENGKTIQfgEEMMKRALQYSPSAGIPELLSWLKQLqiklhnpptIHYPPSQgqmdLCVTSGSQQGLCKVFFMIINPGDNVLLDEPAYSGTLQSLHPLGCNIINVASDFSGIVPDSLR<br>----------------kplswseafgkgagriqaSTIRELLKLTQR-P-GILSFAGGLPAPELFPKEEAA---EAAARIL--REKGEVALQYSPTEGYAPLRAFVAEW---------IGVRPEE----VLITTGSQQALDLVGKVFLDEGSPVLLEAPSYMGAIQAFRLQGPRFLTVPAGEEGPDLDALE<br><br>DILSRWKPedaknpqkntpKFLYTVPNGNNPTGNSLTSERKKEIYELARKYDFLIIEDDPYYFLQFNKFRVPTFLSMDVDG---RVIRADSFS-IISSGLRIGFLTGPKPLIERVILHIQVSTLHPSTFNQLMISQLLhewgFEGFMAHVDRVIDFYSNQKDAILAAADKWLTGLAEWHVPAAGMFLWIK<br>EVLKRERP-----------RFLYLIPSFQNPTGGLTPLPARKRLLQMVMERGLVVVEDDAYRELYFGEARLPSLFELAREAgypGVIYLGSFSkVLSPGLRVAFAVAHPEALQKLVQAKQGADLHTPMLNQMLVHELL----KEGFSERLERVRRVYREKAQAMLHALDREVPKEVRYTRPKGGMFVWME<br><br>V-KGINdvKFLIEFKAVKMGVLMLPGNAFYVDSSAPSPyLRASFSSASPEQMDVAFQVLAQLIK------esl<br>LpKGLS--AEGLFRRALEENVAFVPGGPFFANGGGENT-LRLSYATLDREGIAEGVRRLGRALKgllalv---<br><br>Proposed: aliSize=383 (resi) RMSD=1.78 (Å)<br>--MNYARFITAASAARNPSPIRTMTDILSrGPkSMISLAGGLPNPNMFpFKT--AV--ItvengktiQFGEEMMKRALQYSPSAGIPELLSWLKQLQIklhnPptI-hyppsqgqMDLCVTSGSQQGLCKVFFMIINPGDNVLLDEPAYSGTLQSLHPLGCNIINVASDFSGIVPDSLRDILSRWkpeda<br>kpLSWSEAFGKGAGRIQASTIRELLKLTQ-RP-GILSFAGGLPAPELF-PKEeaAEaaA--------RILREKGEVALQYSPTEGYAPLRAFVAEWIG----V--Rp--------EEVLITTGSQQALDLVGKVFLDEGSPVLLEAPSYMGAIQAFRLQGPRFLTVPAGEEGPDLDALEEVLKRE-----<br><br>knpqknTPKFLYTVPNGNNPTGNSLTSERKKEIYELARKYDFLIIEDDPYYFLQFNKFRVPTFLSMD---VDGRVIRADSFSLIISSGLRIGFLTGPKPLIERVILHIQVSTLHPSTFNQLMISQLLHeWgeeGFMAHVDRVIDFYSNQKDAILAAADKWLTGLAEWHVPAAGMFLWIKVKGINDVKELI<br>------RPRFLYLIPSFQNPTGGLTPLPARKRLLQMVMERGLVVVEDDAYRELYFGEARLPSLFELAreaGYPGVIYLGSFSKVLSPGLRVAFAVAHPEALQKLVQAKQGADLHTPMLNQMLVHELLK-E---GFSERLERVRRVYREKAQAMLHALDREVPKEVRYTRPKGGMFVWMELPKGLSAEGLF<br><br>eFKAVKMGVLMLPGNAFYVDSsAPSPYLRASFSSASPEQMDVAFQVLAQLIKESL---<br>-RRALEENVAFVPGGPFFANG-GGENTLRLSYATLDREGIAEGVRRLGRALKGLLalv
683	2qlrA<br>(425)<br><br>2egyD<br>(391)	N	27.37	TM-align: aliSize=341 (resi) RMSD=2.05 (Å)<br>mnyarfitaasaarnpspirtmtdilsrgpksmislagglpnpnmfpfktav--------------------------------------ITVEn-GKTIQFGEEMMKRALQYSPSAGIPELLSWLKQLQIklhnPptIhyppsqgQMDLCVTSGSQQGLCKVFFMIINPGDNVLLDEPAYSGT<br>--swseafgkgagriqastirellkltqrpgilsfagglpapelfpKEEA-aEAAARILREKGEVALQYSPTEGYAPLRAFVAEWIG----V--R-------PEEVLITTGSQQALDLVGKVFLDEGSPVLLEAPSYMGA<br><br>LQSLHPLGCNIINVASDFSGIVPDSLRDILSRWkpedaknpqkNTPKFLYTVPNGNNPTGNSLTSERKKEIYELARKYDFLIIEDDPYYFLQFNKFRVPTFLSMD---VDGRVIRADSFS-IISSGLRIGFLTGPKPLIERVILHIQVSTLHPSTFNQLMISQLLHeWgeeGFMAHVDRVIDFYSNQKDA<br>IQAFRLQGPRFLTVPAGEEGPDLDALEEVLKRE----------RPRFLYLIPSFQNPTGGLTPLPARKRLLQMVMERGLVVVEDDAYRELYFGEARLPSLFELAreaGYPGVIYLGSFSkVLSPGLRVAFAVAHPEALQKLVQAKQGADLHTPMLNQMLVHELLK-E---GFSERLERVRRVYREKAQA<br><br>ILAAADKWLTGLAEWHVPAAGMFLWIKVKGINDVKELIeFKAVKMGVLMLPGNAFYVDSsAPSPYLRASFSSASPEQMDVAFQVLAQLIKESL-<br>MLHALDREVPKEVRYTRPKGGMFVWMELPKGLSAEGLF-RRALEENVAFVPGGPFFANG-GGENTLRLSYATLDREGIAEGVRRLGRALKGLLa<br><br>SARST: aliSize=372 (resi) RMSD=13.61 (Å)<br>mn-YARFITAASAArnpSPIRTMTDILSRGPK-SMISLAGGLPNPNMEpFKtavitvengktiqfgEEMMKRA-----------LQYSPSAGIPELLSWLKQLQiklhnpptiHYPPSQgqmdLCVTSGSQQGLCKVFFMIINPGDNVLLDEPAYSGTLQSLHPLGCNIINVASDFSGIVPDSLRDILSR<br>--sWSEAFGKGAGR---IQASTIRELLKLTQRpGILSFAGGLPAPELF-PK---------------EEAAEAAarilrekgevaLQYSPTEGYAPLRAFVAEWI---------GVRPEE----VLITTGSQQALDLVGKVFLDEGSPVLLEAPSYMGAIQAFRLQGPRFLTVPAGEEGPDLDALEEVLKR<br><br>WKpedaknpqkntPKFLYTVPNGNNPTGNSLTSERKKEIYELARKYDFLIIEDDPYYFLQFNKFRVPTFLSMDVDGRV--IRADS--FSIISSGLRIGFLTGPKPLIERVILHIQVSTLHPSTFNQLMISQLLHFwgeeGFMAHVDRVIDFYSNQKDAILAAADKWLTGLAEWHVPAAGMFlWIKVKGIN<br>ER-----------PRFLYLIPSFQNPTGGLTPLPARKRLLQMVMERGLVVVEDDAYRELYFGEARLPSLFELAREAGYpgVIYLGsfSKVLSPGLRVAFAVAHPEALQKLVQAKQGADLHTPMLNQMLVHELLKE----GFSERLERVRRVYREKAQAMLHALDREVPKEVRYTRPKGGMF-VWMELPKG<br><br>DVKFLIEFKAVKMGVLMLPGNAFYVdSSAPSPYLRASFSSASPEQMDVAFQVLAQLIKES--l<br>LSAEGLFRRALEENVAFVPGGPFFA-NGGGENTLRLSYATLDREGIAEGVRRLGRALKGLla-<br><br>BLAST: aliSize=203 (resi) iden=29.41% (115/391) simi=51.92% (203/391)<br>mnyarfitaasaarnp--------------SPIRTMTDILSRgPkSMISLAGGLPNPNMFPFKTAVitvENGKTIQfgEEMMKRALQYSPSAGIPELLSWLKQLqiklhnpptIHYPPSQgqmdLCVTSGSQQGLCKVFFMIINPGDNVLLDEPAYSGTLQSLHPLGCNIINVASDFSGIVPDSLRDIL<br>----------------swseafgkgagriqaSTIRELLKLTQR-P-GILSFAGGLPAPELFPKEEAA---EAAARIL--REKGEVALQYSPTEGYAPLRAFVAEW---------IGVRPEE----VLITTGSQQALDLVGKVFLDEGSPVLLEAPSYMGAIQAFRLQGPRFLTVPAGEEGPDLDALEEVL<br><br>SRWKPedaknpqkntpKFLYTVPNGNNPTGNSLTSERKKEIYELARKYDFLIIEDDPYYFLQFNKFRVPTFLSMDVDG---RVIRADSFS-IISSGLRIGFLTGPKPLIERVILHIQVSTLHPSTFNQLMISQLLhewgFEGFMAHVDRVIDFYSNQKDAILAAADKWLTGLAEWHVPAAGMFLWIKV-K<br>KRERP-----------RFLYLIPSFQNPTGGLTPLPARKRLLQMVMERGLVVVEDDAYRELYFGEARLPSLFELAREAgypGVIYLGSFSkVLSPGLRVAFAVAHPEALQKLVQAKQGADLHTPMLNQMLVHELL----KEGFSERLERVRRVYREKAQAMLHALDREVPKEVRYTRPKGGMFVWMELpK<br><br>GINdvKFLIEFKAVKMGVLMLPGNAFYVDSSAPSPyLRASFSSASPEQMDVAFQVLAQLIK----esl<br>GLS--AEGLFRRALEENVAFVPGGPFFANGGGENT-LRLSYATLDREGIAEGVRRLGRALKglla---<br><br>Proposed: aliSize=376 (resi) RMSD=1.67 (Å)<br>mNYARFITAASAARNPSPIRTMTDILSrGPkSMISLAGGLPNPNMEpFKT--AV--I-----tvengktiqfgeeMMKRALQYSPSAGIPELLSWLKQLQIklhnpptIhyppsqgqM--DLCVTSGSQQGLCKVFFMIINPGDNVLLDEPAYSGTLQSLHPLGCNIINVASDFSGIVPDSLRDILSRWk<br>-SWSEAFGKGAGRIQASTIRELLKLTQ-RP-GILSFAGGLPAPELF-PKEeaAEaaArilre------------KGEVALQYSPTEGYAPLRAFVAEWIG-------V--------RpeEVLITTGSQQALDLVGKVFLDEGSPVLLEAPSYMGAIQAFRLQGPRFLTVPAGEEGPDLDALEEVLKRE-<br><br>pedaknpqknTPKFLYTVPNGNNPTGNSLTSERKKEIYELARKYDFLIIEDDPYYFLQFNKFRVPTFLSMD---VDGRVIRADSFSLIISSGLRIGFLTGPKPLIERVILHIQVSTLHPSTFNQLMISQLLHeWgeeGFMAHVDRVIDFYSNQKDAILAAADKWLTGLAEWHVPAAGMFLWIKVKGINDV<br>----------RPRFLYLIPSFQNPTGGLTPLPARKRLLQMVMERGLVVVEDDAYRELYFGEARLPSLFELAreaGYPGVIYLGSFSKVLSPGLRVAFAVAHPEALQKLVQAKQGADLHTPMLNQMLVHELLK-E---GFSERLERVRRVYREKAQAMLHALDREVPKEVRYTRPKGGMFVWMELPKGLSA<br><br>KELIeFKAVKMGVLMLPGNAFYVDSsAPSPYLRASFSSASPEQMDVAFQVLAQLIKESL-<br>EGLF-RRALEENVAFVPGGPFFANG-GGENTLRLSYATLDREGIAEGVRRLGRALKGLLa

<table>
<tr><td rowspan="2">684</td><td>2qlrA
(425)</td><td rowspan="2">N</td><td rowspan="2">11.64</td><td rowspan="2">TM-align: aliSize=295 (resi) RMSD=3.14 (Å)
mnyarfitaasaarnpspirtmtdilsrgpksmislagglpnpnmfpfktav------------------------------ITVEnGKTIQFGFEeMMKRALQYSPsAGIPELLSWLKQLQiKLhNPptIHyppsqgQMDLCVTSGSQQGLCKVFEMIInpgDnVLLDEPAYSGTLQSLH--PLGC
--hmnpldliakraypyetekrdktylalnenpfpfPEDL-VDEVFRRL-NSDALRIYYD-SPDEELIEKILSYL-DT-DF--LS------KNNVSVGNGADEIIYVMMLMFD---R-SVFFPPTYSCYRIFAKavGAKF

NIINVASDesGIVpdSLrdILsrWkpedAknpqknTPKFLYTVPNgNNPTGNSLtseRKKEIYFLARkYDFLIIEDDPYYFLQFnkfRVPTfLSMD-VDGRVIRADSFS-II-SSGLRIGFLTGPKPLIFRVILHIQVstLHPSTFNQLMISQLIheWgeeGFmAHVDRVIDFYSNQKDAILAAADKwLT
LEVPLTKD--LRI--PE--VN--V----G------EGDVVFIPNP-NNPTGHVF---EREEIERILK-TGAFVALDEAYYEFH----GESY-VDFLkKYENLAVIRTFSkAFsLAAQRVGYVVASEKFIDAYNRVRLP--FNVSYVSQMFAKVAL--D---HR-EIFEERTKFIVEERERMKSALRE-MG

glAFWHVPAaGMFLWIKVkgINDVKELIEFKAVKMGVLMLPGNafyvdssapSpYLRASFSSAS-PFQMDVAFQVlAqlikesl
--YRITDSR-GNFVFVFM--EKEEKERLLEHLRTKNVAVRSFR---------E-GVRITIGKREeNDMILRELEV-F-------

SARST: aliSize=293 (resi) RMSD=6.62 (Å)
mnyarfitaasaarnpspirtmtdilsrgpksmislagglpnpnmfpfktavitve---------------------------NGKTIQFGEEMMKRALqyspSAGIPELL--------SWLKQLQIKLHNPPTihYPPSQgqmdLCVTSGSQQGLCKVFEM-IINpgdnvLLDEPAYSGTLQSLHPLGC
--hmnpldliakraypyetekrdktylalNENPFPFPEDLVDEVF----RRLNSDALriyydspdEELIEKILSYLDTDF--LSKNN----VSVGNGADEIIYVMMLMfDRS-----VFFPPTYSCYRIFAKAVGA

NIINVASDFSGIVpdslrdilSRWKpedaknpqkNTPKFLYTVPNGNNPTGNSLTSERKKEIYFLarkyDFLIIEDDPYYFLQFNkfrvpTFLSMDVD-GRVIRADSFSIIS--SGLRIGFLTGPKplIERVILHIQVSTLHPSTFNQLMISQLlhewgEFGFMAHVDRVIDFYSNQKDAILAAADKWLt
KFLEVPLTKDLRI--------PEVN---------VGEGDVVFIPNPNNPTGHVFEREEIERILKT----GAFVALDEAYYEFHGE-----SYVDFLKKyENLAVIRTFSKAFslAAQRVGYVVASE--KFIDAYNRVRLPFNVSYVSQMFAKVA-----LDHREIFEERTKFIVEERERMKSALREMGY-

glaFWHVPAAgMFLWIKVKgiNDVKELIEFKAVKMGVLMLPGNAfyvdssapspYLRASFssASPFQMDVAFQVlaqlikE---sl
---RITDSRG-NFVFVFME--KEEKERLLEHLRTKNVAVRSFRE----------GVRITI--GKREENDMILRE------Levf--

BLAST: aliSize=61 (resi) iden=10.15% (34/335) simi=18.21% (61/335)
mnyarfitaasaarnpspirtmtdilsrgpksmislagglpnpnmfpfktavitvengktiqfgeemmkralqyspsagipellswlkqlqiklhnpptihyppsqgqmdlcvtsgsqqglckvfemiinpgdnvlldepaysgtlqslhplgcniinvasdesgivpdslrdilsrwkp----------
---hmnpldliak

---FDAKNPQKNTPKF-LYTVPNGNNPTGNSLtsERKKEIYFLARKYDFLIIeDDPYYflQFNKFRVPTFLSMDVD
raypyetekrdktylalnenpfpfpedlvdevfrrlnsdalriyydspdeeliekilsyldtdflsknnvsvgngadeiiyvmmlmfdrsvffpptyscyrifakavgakflevpltKDLRIPEVNVGEGdVVFIPNPNNPTGHVF--ERE-EIERILKTGAFVAL-DEAYY--EFHGESYVDFLKKYEN

GRVIRADSFSIISSGLRIGFLTGPKPLIFRvilHIQVSTlhPstFNQLMISQL---lhewgeegfmahvdrvidfysnqkdailaaadkwltglaewh
LAVIRTFSKAFSLAAQRVGYVVASEKFIDA---YNRVRL--P--FNVSYVSQMfakvaldhreifeertkfiveerermksalremgyritdsrgnfvfvfmekeekerllehlrtknvavrsfregvritigkreendmilrelevf--

vpaagmflwikvkgindvkelieekavkmgvlmlpgnafyvdssapspylrasfssaspeqmdvafqvlaqlikesl
----

Proposed: aliSize=312 (resi) RMSD=2.69 (Å)
mny-ARFITAaSA-ARNPSPiRTMTdiLsrgpksmISLAGG-LPNPNmFP--FKT-AV--ItvengktiqfgeemMKRALQYSPsAGIPELLSWLKQLQiKLhNPptI--hyppsqgqMDLCVTSGSQQGLCKVFEMIInpgDnVLLDEPAYSGTLQSLH--PLGCNIINVA-SDesgIVpdSLrdILsr
---hMNPLDL-IAkRAYPYE-TEKR--D-------KTYLALnENPFP-FPedLVDeVFrrL-------------NSDALRIYYD-SPDEELIEKILSYL-DT-DF--Lsk--------NNVSVGNGADEIIYVMMLMFD---R-SVFFPPTYSCYRIFAKavGAKFLEVPLTkDL---RI--PE--VN--

wkpedaknpqknTP--KFLYTVPNgNNPTGNSLtseRKKEIYFLARkYDFLIIEDDPYYFLQfnkfRVPTfLSMD-VDGRVIRADSFSLII-SSGLRIGFLTGPKPLIFRVIL-HIQvstLHPSTFNQLMISQLIhFWgeegFmAHVDRVIDFYSNQKDAILAAADKwLTglAFWHVPaAGMFLWIKVkg
-----------VGegDVVFIPNP-NNPTGHVF---EREEIERILK-TGAFVALDEAYYEFH----GESY-VDFLkKYENLAVIRTFSKAFsLAAQRVGYVVASEKFIDAYNRvRLP---FNVSYVSQMFAKVAL-DH----R-EIFEERTKFIVEERERMKSALRE-MG--YRITDS-RGNFVFVFM--

IndVkELIFeKAVKMGV----LMLPGNafyvdssapSpYLRASFSS-ASPFQMDVAFQVlAqlikesl
E--K-EEKE-RLLEHLRtknvAVRSFR---------E-GVRITIGKrEENDMILRELEV-F-------</td></tr>
<tr><td>2f8jA
(335)</td></tr>
<tr><td rowspan="2">685</td><td>2qlrA
(425)</td><td rowspan="2">N</td><td rowspan="2">25.63</td><td rowspan="2">TM-align: aliSize=341 (resi) RMSD=2.12 (Å)
mnyarfitaasaarnpspirtmtdilsrgpksmislagglpnpnmfpfktav---ITVEnGKTI-QFGFEEMMKRALQYSPSAGIPELLSWLKQLQIklhnPptIhyppsqgQMDLCVTSGSQQGLCKVFEMIINPGDNVLLDEPAYSG
--lswseafgkgagriqastirellkltqrpgilsfagglpapelfpKEEA-AEAAaRILREKGEVALQYSPTEGYAPLRAFVAEWIG----V--R-------PEEVLITTGSQQALDLVGKVFLDEGSPVLLEAPSYMG

TLQSLHPLGCNIINVASDFSGIVPDSLRDILSRWkpedaknpqknTPKFLYTVPNGNNPTGNSLTSERKKEIYFLARKYDFLIIEDDPYYFLQFNKFRVPTFLSMD---VDGRVIRADSFS-IISSGLRIGFLTGPKPLIERVILHIQVSTLHPSTFNQLMISQLIHeWgeeGFMAHVDRVIDFYSNQKD
AIQAFRLQGPRFLTVPAGEEGPDLDALEEVLKRE-----------RPRFLYLIPSFQNPTGGLTPLPARKRLLQMVMERGLVVVEDDAYRELYFGEARLPSLFELAreaGYPGVIYLGSFSkVLSPGLRVAFAVAHPEALQKLVQAKQGADLHTPMLNQMLVHELLK-E---GFSERLERVRRVYREKAQ

AILAAADKWLTGLAFWHVPAAGMFLWIKVKGINDVKELIFeKAVKMGVLMLPGNAFYVDSsAPSPYLRASFSSA---SPEQMDVAFQVLAQLIKESL
AMLHALDREVPKEVRYTRPKGGMFVWMELPKGLSAEGLFR-RALEENVAFVPGGPFFANG-GGENTLRLSYATLdreGIAEGVRRLGRALKGLLALV

SARST: aliSize=350 (resi) RMSD=11.60 (Å)
mnyarfitaasaarnpspirt----------------------MTDILSrgpKSMISLAGGLPNPNMFpfktavitvengktiqfGEEMMKR-----------ALQYSPSAGIPELLSWLKQLqiklhnpptIHYPPSQgqmdLCVTSGSQQGLCKVFEMIINPGDNVLLDEPAYSGTLQSLHPLGCNI
--------------------lswseafgkgagriqastirelLKLTQR---PGILSFAGGLPAPELF------------------PKEEAAEaaarilrekgevALQYSPTEGYAPLRAFVAEW---------IGVRPEE----VLITTGSQQALDLVGKVFLDEGSPVLLEAPSYMGAIQAFRLQGPRF

INVASDFSGIVPDSLRDILSRWKpedaknpqkntPKFLYTVPNGNNPTGNSLTSERKKEIYFLARKYDFLIIEDDPYYFLQFNKFRVPT-FLSMDVDG--RVIRADSF-SIISSGLRIGFLTGPKPLIERVILHIQVSTLHPSTFNQLmisqLIHFWGEFGFMAHVDRVIDFYSNQKDAILAAADKWLTG
LTVPAGEEGPDLDALEEVLKRER-----------PRFLYLIPSFQNPTGGLTPLPARKRLLQMVMERGLVVVEDDAYRELYFGEARLPSlFELAREAGypGVIYLGSFsKVLSPGLRVAFAVAHPEALQKLVQAKQGADLHTPMLNQM----LVHELLKEGFSERLERVRRVYREKAQAMLHALDREVPK

LAFWHVPAAGMFLWIKVKGINDvKELIEFKAVKMGVLMLPGNAFYVdSSAPSPYLRASFSSASPFQMDVAFQVLAQLIK------esl
EVRYTRPKGGMFVWMELPKGLS-AEGLFRRALEENVAFVPGGPFFA-NGGGENTLRLSYATLDREGIAEGVRRLGRALKgllalv---

BLAST: aliSize=203 (resi) iden=29.19% (115/394) simi=51.52% (203/394)
mnyarfitaasaarnp---------------SPIRTMTDILSRgPkSMISLAGGLPNPNMFPFKTAVitvENGKTIQfgEEMMKRALQYSPSAGIPELLSWLKQLqiklhnpptIHYPPSQgqmdLCVTSGSQQGLCKVFEMIINPGDNVLLDEPAYSGTLQSLHPLGCNIINVASDFSGIVPDSLRDI
---------------lswseafgkgagriqaSTIRELLKLTQR-P-GILSFAGGLPAPELFPKEEAA---EAAARIL--REKGEVALQYSPTEGYAPLRAFVAEW---------IGVRPEE----VLITTGSQQALDLVGKVFLDEGSPVLLEAPSYMGAIQAFRLQGPRFLTVPAGEEGPDLDALEEV

LSRWKPedaknpqkntpKFLYTVPNGNNPTGNSLTSERKKEIYFLARKYDFLIIEDDPYYFLQFNKFRVPTFLSMDVDG---RVIRADSFS-IISSGLRIGFLTGPKPLIERVILHIQVSTLHPSTFNQLMISQLIhewgEFGFMAHVDRVIDFYSNQKDAILAAADKWLTGLAFWHVPAAGMFLWIKV-
LKRERP-----------RFLYLIPSFQNPTGGLTPLPARKRLLQMVMERGLVVVEDDAYRELYFGEARLPSLFELAREAgypGVIYLGSFSkVLSPGLRVAFAVAHPEALQKLVQAKQGADLHTPMLNQMLVHELL----KEGFSERLERVRRVYREKAQAMLHALDREVPKEVRYTRPKGGMFVWMELp

KGINdvKELIEFKAVKMGVLMLPGNAFYVDSSAPSPyLRASFSSASPFQMDVAFQVLAQLIK------esl
KGLS--AEGLFRRALEENVAFVPGGPFFANGGGENT-LRLSYATLDREGIAEGVRRLGRALKgllalv---

Proposed: aliSize=381 (resi) RMSD=1.90 (Å)
MNYARFITAASAARN-PSPIRTMTDiLSRGPksMISLAGGLPNPNMFP--FKT--AVitvengktiQFGEEMMKRALQYSPSAGIPELLSWLKQLQIklhnpptIhyppsqgqM--DLCVTSGSQQGLCKVFEMIINPGDNVLLDEPAYSGTLQSLHPLGCNIINVASDFSGIVPDSLRDILSRWkpeda
LSWSEAFGKGAGRIQaSTIRELLKL-TQRPG--ILSFAGGLPAPELFPkeEAAeaAA---------RILREKGEVALQYSPTEGYAPLRAFVAEWIG-------V--------RpeEVLITTGSQQALDLVGKVFLDEGSPVLLEAPSYMGAIQAFRLQGPRFLTVPAGEEGPDLDALEEVLKRE-----

knpqknTPKFLYTVPNGNNPTGNSLTSERKKEIYFLARKYDFLIIEDDPYYFLQFNKFRVPTFLSMD---VDGRVIRADSFSLIISSGLRIGFLTGPKPLIERVILHIQVSTLHPSTFNQLMISQLIHeWgeeGFMAHVDRVIDFYSNQKDAILAAADKWLTGLAFWHVPAAGMFLWIKVKGINDVKELI
------RPRFLYLIPSFQNPTGGLTPLPARKRLLQMVMERGLVVVEDDAYRELYFGEARLPSLFELAreaGYPGVIYLGSFSKVLSPGLRVAFAVAHPEALQKLVQAKQGADLHTPMLNQMLVHELLK-E---GFSERLERVRRVYREKAQAMLHALDREVPKEVRYTRPKGGMFVWMELPKGLSAEGLF

FeKAVKMGVLMLPGNAFYVDSsAPSPYLRASFSSA---SPEQMDVAFQVLAQLIKESL-
R-RALEENVAFVPGGPFFANG-GGENTLRLSYATLdreGIAEGVRRLGRALKGLLALVl</td></tr>
<tr><td>2z1yB
(394)</td></tr>
<tr><td rowspan="2">686</td><td>2qlrA
(425)</td><td rowspan="2">N</td><td rowspan="2">28.15</td><td rowspan="2">TM-align: aliSize=352 (resi) RMSD=2.63 (Å)
mnyarfitaasaarnpspirtmtdilsrgpksmislagglpnpnmfpfktav---ITVENGKTIQFGEeMMKRALQYSPSAGIPELLSWLKQLQIK-LHNPpTIhyppsqgQMDLCVTSGSQQGLCKVFEMIINPGDNVLLDE
--mdytkylagranwikgsaladvmkkaselqkkgvklislaagdpdpeliPRAVLGEIAKEVLE-KEPKSVMYTPANGIPELREELAAFLKKyDHLE-VS-------PENIVITIGGTGALDLLGRVLIDPGDVVITEN

PAYSGTLQSLHPLGCNIINVASDESGIVPDSLRDILSRWkpeDaknpQKNTPKFLYTVPNGNNPTGNSLTSERKKEIYFLARKYDFLIIEDDPYYFLQFNKFRVPTFLSMDVDGRVIRADSFS-IISSGLRIGFLTGPKPLIERVILHIQVSTLHPSTFNQLMISQLIHEWgeeGFMAHVD-RVIDFYSN
PSYINTLLAFEQLGAKIEGVPVDNDGMRVDLLEEKIKELK--A----KGQKVKLIYTIPTGQNPMGVTMSMERRKALLEIASKYDLLIIEDTAYNFMRYEGGDIVPLKALDNEGRVIVAGTLSkVLGTGFRIGWIIAEGEILKKVLMQKQPIDFCAPAISQYIALEYLKRG---YFEKYHLeGALLGYKE

QKDAILAAADKWLTGlAFWHVPAAGMFLWIKVKGINDVKELIEFKAVKMGVLMLPGNAFYVDSsAPSPYLRASFSSASPFQMDVAFQVLAQLIKESL
KRDIMLKALENHLPN-AEFTKPIAGMFVMFFLPEGADGISFANELMEREGVVVVPGKPFYTDE-SGKNAIRLNFSRPSKEEIPIGIKKLAKLYKEKF

SARST: aliSize=355 (resi) RMSD=8.93 (Å)
mnyarfitaasaarnpspirtmtdilsrgpksm---------------------------------------ISLAGGLPNPNMFPfktavitvengktiqfgEEMMKR-----------ALQYSPSAGIPELLSWLKQLQIKLHNPptiHYPPSQgqmdLCVTSGSQQGLCKVFEMIINPGDNVLLDEPAYS
--------------------------------mdytkylagranwikgsaladvmkkaselqkkgvklISLAAGDPDPELIP-----------------RAVLGEiakevlekepkSVMYTPANGIPELREELAAFLKKYDHL---EVSPEN----IVITIGGTGALDLLGRVLIDPGDVVITENPSYI

GTLQSLHPLGCNIINVASDESGIVPDSLRDILSrwkPEDAKnpqkNTP-KFLYTVPNGNNPTGNSLTSERKKEIYFLARKYDFLIIEDDPYYFLQFNKFRVPTFLSMDVDGRVIRADSFSII-SSGLRIGFLTGPKPLIERVILHIQVSTLHPSTFNQLMISQLIHFwgEEGFMAHVDRVIDFYSNQKDA
NTLLAFEQLGAKIEGVPVDNDGMRVDLLEEKIK---ELKAK----GQKvKLIYTIPTGQNPMGVTMSMERRKALLEIASKYDLLIIEDTAYNFMRYEGGDIVPLKALDNEGRVIVAGTLSKVlGTGFRIGWIIAEGEILKKVLMQKQPIDFCAPAISQYIALEYLKR--GYFEKYHLEGALLGYKEKRDI

ILAAADKWlTGLAFWHVPAAGMFLWIKVKGINDVKELIEFKAVKMGVLMLPGNAFYVDsSAPSPYLRASFSSASPFQMDVAFQVLAQLIKES-l
MLKALENH-LPNAEFTKPIAGMFVMFFLPEGADGISFANELMEREGVVVVPGKPFYTD-ESGKNAIRLNFSRPSKEEIPIGIKKLAKLYKEKf-

BLAST: aliSize=227 (resi) iden=32.84% (133/405) simi=56.05% (227/405)
MNYARFITA-ASAARNPSPIRTM---TDILSRGPKsMISLAGGLPNPNMFPfkTAVITvENGKTIQfgeEMMKRALQYSPSAGIPELLSWLKQLqIKLHNPptIHYPPSqgqmDLCVTSGSQQGLCKVFEMIINPGDNVLLDEPAYSGTLQSLHPLGCNIINVASDESGIVPDSLRDILSRWkpedAKNp
MDYTKYLAGrANWIKGSALADVMkkaSELQKKGVK-LISLAAGDPDPELIP--RAVLG-EIAKEVL---EKEPKSVMYTPANGIPELREELAAF-LKKYDH--LEVSPE----NIVITIGGTGALDLLGRVLIDPGDVVITENPSYINTLLAFEQLGAKIEGVPVDNDGMRVDLLEEKIKELK---AKG-

QKntPKFLYTVPNGNNPTGNSLTSERKKEIYFLARKYDFLIIEDDPYYFLQFNKFRVPTFLSMDVDGRVIRADSFS-IISSGLRIGFLTGPKPLIERVILHIQvstlhPSTFNQLMISQLIH-FWGEEGFMA--HVDRVIDFYSNQKDAILAAADKWLTGlAFWHVPAAGMFLWIKVK----GINDVKEL
QK--VKLIYTIPTGQNPMGVTMSMERRKALLEIASKYDLLIIEDTAYNFMRYEGGDIVPLKALDNEGRVIVAGTLSkVLGTGFRIGWIIAEGEILKKVLMQKQ-----PIDFCAPAISQYIAlEYLKRGYFEkyHLEGALLGYKEKRDIMLKALENHLPN-AEFTKPIAGMFVMFFLPegadGISFANEL

IFFKavkmGVLMLPGNAFYVDSSAPSPyLRASFSSASPFQMDVAFQVLAQLIKF--sl
MERE----GVVVVPGKPFYTDESGKNA-IRLNFSRPSKEEIPIGIKKLAKLYKEkf--

Proposed: aliSize=385 (resi) RMSD=2.04 (Å)
MNYARFITAASAARNPS--P--IRTMTDILSRGPKsMISLAGGLPNPNMFP--FKT--AV--IT--vengktiqfgeemmKRALQYSPSAGIPELLSWLKQLQIK-LHNPptIhyppsqgqM--DLCVTSGSQQGLCKVFEMIINPGDNVLLDEPAYSGTLQSLHPLGCNIINVASDESGIVPDSLRDIL
MDYTKYLAGRANWIKGSalAdvMKKASELQKKGVK-LISLAAGDPDPELIPraVLGeiAKevLEke-------------PKSVMYTPANGIPELREELAAFLKKyDHLE--V--------SpeNIVITIGGTGALDLLGRVLIDPGDVVITENPSYINTLLAFEQLGAKIEGVPVDNDGMRVDLLEEKI

SR-W--kpedaknpqKNTPKFLYTVPNGNNPTGNSLTSERKKEIYFLARKYDFLIIEDDPYYFLQFNKFRVPTFLSMDVDGRVIRADSFSLIISSGLRIGFLTGPKPLIERVILHIQVSTLHPSTFNQLMISQLIHEWgeeGFMAHVD-RVIDFYSNQKDAILAAADKWLTGlAFWHVPAAGMFLWIKVK
KElKak---------GQKVKLIYTIPTGQNPMGVTMSMERRKALLEIASKYDLLIIEDTAYNFMRYEGGDIVPLKALDNEGRVIVAGTLSKVLGTGFRIGWIIAEGEILKKVLMQKQPIDFCAPAISQYIALEYLKRG---YFEKYHLeGALLGYKEKRDIMLKALENHLPN-AEFTKPIAGMFVMFFLP

GINDVKELIEFKAVKMGVLMLPGNAFYVDssA-PSPYLRASFSSASPFQMDVAFQVLAQLIKESL
EGADGISFANELMEREGVVVVPGKPFYTD--EsGKNAIRLNFSRPSKEEIPIGIKKLAKLYKEKF</td></tr>
<tr><td>2zc0A
(405)</td></tr>
</table>

687	2qlrA (425) / 3aatA (396)	N	12.37	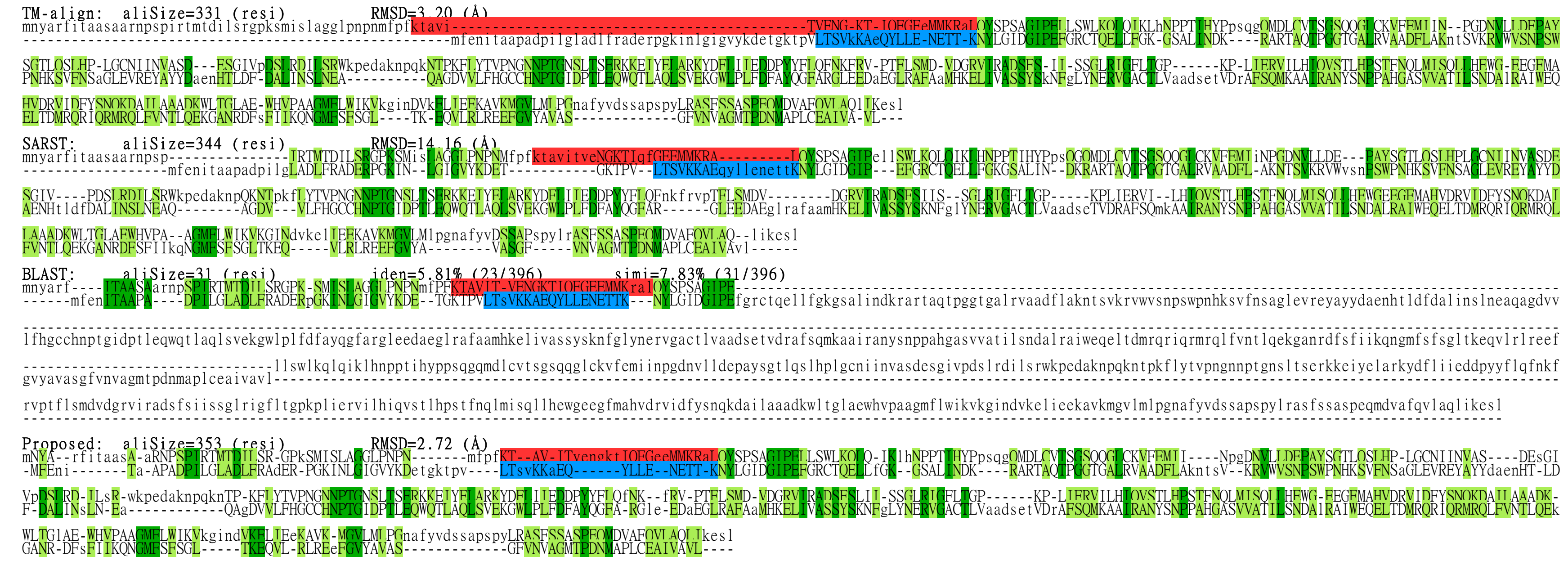
688	2qlrA (425) / 3tatA (397)	N	12.34	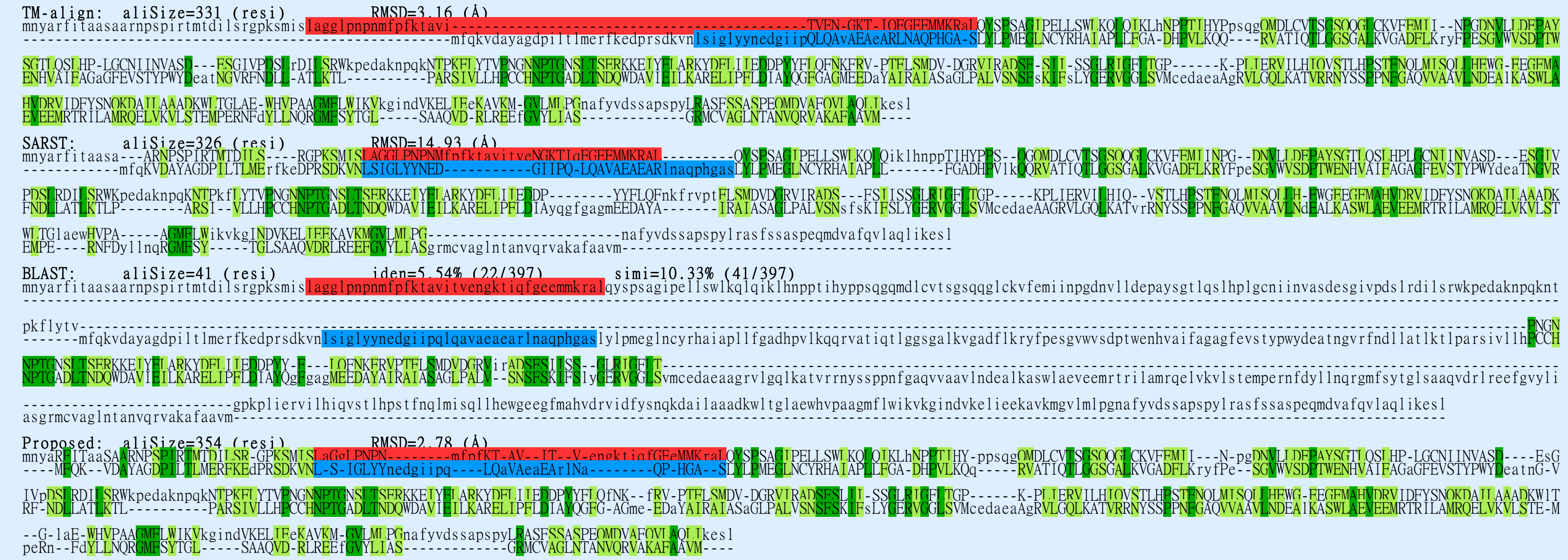
689	2qlrA (425) / 7aatA (401)	N	14.46	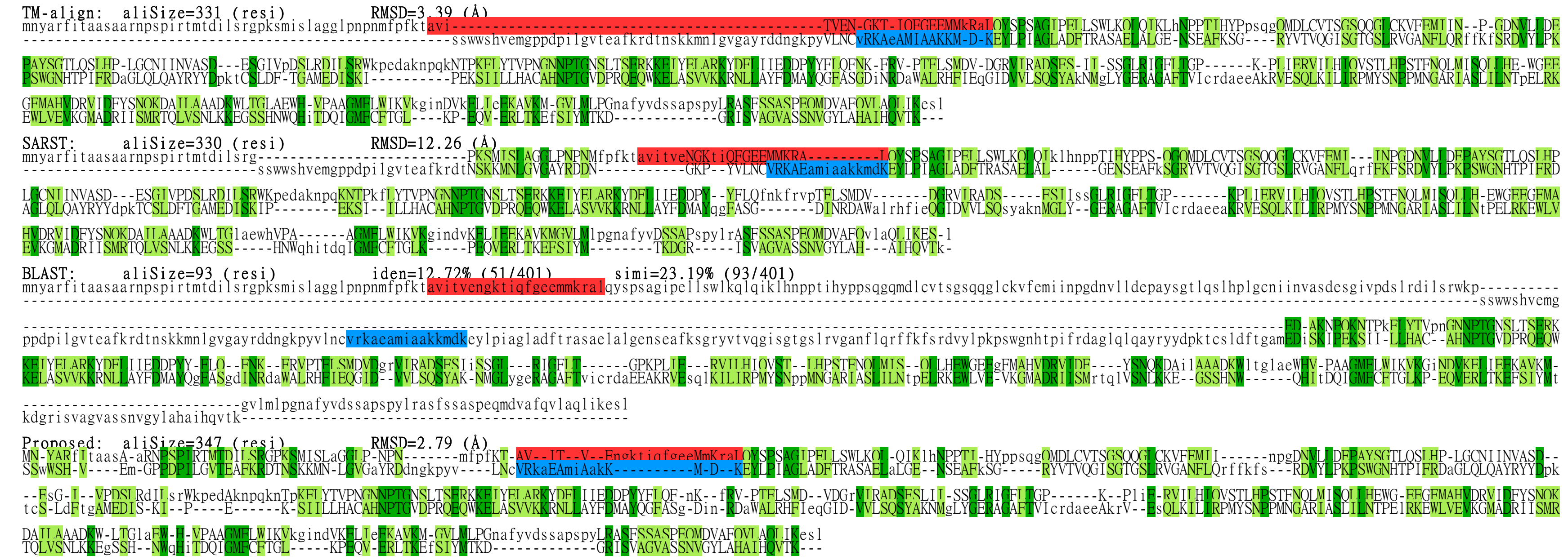

690	2r7hA (157)<br>1s3zB (145)	C	18.62	TM-align: aliSize=134 (resi) RMSD=2.41 (Å)<br>aVAFRRQVLpQDALLVRRVVEsTgFFTpEEAD-VAQELVDEHLHACGYHFVFATEDDD-AGYACYGPTPA------TEGTYDLYWIAVAPHRQHSGLGRALLAEVVHDVRLTGGRLLFAETSGIrkYAPTRRFYERAGFSAEAVLKaFYRagddkiiyrleva---<br>-MDIRQMNK-THLEHWRGLRK-Q-LWP-GHPDdAHLADGEEILQADHLASFIAMADGVaIGFADASIRHDyvngcdSSPVVFLEGIFVLPSFRQRGVAKQLIAAVQRWGTNKGCREMASDTSPE--NTISQKVHQALGFEETERVI-FYR------------krc<br><br>SARST: aliSize=130 (resi) RMSD=3.89 (Å)<br>ava-FRRQVLPQDALLVRRVVESTG-FFTPEEADVAQELVDEhlhACGYHFVFATEDDDAGYACYG-----PTPA--TEGTYDLYWIAVAPHRQHSGLGRALLAEVVHDVRLTGGRLLFAETSgiRKYAPTRRFYERAGFSAEAVLK------afyragddkiiyrleva<br>---mDIRQMNKTHLEHWRGLRKQLWpGHPDDAHLADGEEILQ---ADHLASFIAMADGVAIGFADAsirhdYVNGcdSSPVVFLEGIFVLPSFRQRGVAKQLIAAVQRWGTNKGCREMASDTS--PENTISQKVHQALGFEETERVIfyrkrc-----------------<br><br>BLAST: aliSize=54 (resi) iden=22.76% (33/145) simi=37.24% (54/145)<br>avafrrqvlpqdallvrrvvestgfft-------------------------PEEADVA--QELVD-EHL-----HACGYHFVFATEDDDAGY--AC-YGPTPATEGtydlywIAVAPHRQHSGLGRALLAEVVHDVRLTGGRLLFAETSgiRKYAPTRRFYERAGF-SAEAVLkaFYR---agddkiiy<br>--------------------------mdirqmnkthlehwrglrkqlwpghPDDAHLAdgEEILQaDHLasfiaMADGVAIGFADASIRHDYvnGCdSSPVVFLEG------IFVLPSFRQRGVAKQLIAAVQRWGTNKGCREMASDTS--PENTISQKVHQALGFeETERVI--FYRkrc--------<br><br>rleva<br>-----<br><br>Proposed: aliSize=138 (resi) RMSD=2.17 (Å)<br>aVAFRRQVLpQDALLVRRVVEsTgFFTpEEAD-VAQELVDEHLmHACGYHFVFATEDDDMAGYACYGPTPA------TEGTYDLYWIAVAPHRQHSGLGRALLAEVVHDVRLTGGRLLFAETSGiRkYAPTRRFYERAGFSAEAVLkafyragddkIIYRLEV-a<br>-MDIRQMNK-THLEHWRGLRK-Q-LWP-GHPDdAHLADGEEIL-QADHLASFIAMADGVAIGFADASIRHDyvngcdSSPVVFLEGIFVLPSFRQRGVAKQLIAAVQRWGTNKGCREMASDTSP-E-NTISQKVHQALGFEETERV----------IFYRKRCc-
691	2r7hA (157)<br>1s5kA (153)	C	15.69	TM-align: aliSize=136 (resi) RMSD=2.57 (Å)<br>-------AVAFRRQVLpQDALLVRRVVEsTgFFTPEEADVAQELVDEHLHACGYHFVFATEDDD-AGYACYGPTPA------TEGTYDLYWIAVAPHRQHSGLGRALLAEVVHDVRLTGGRLLFAETSGIrkYAPTRRFYERAGFSAEAVLKaFYRagddkiiyrleva---<br>glvprgsHMDIRQMNK-THLEHWRGLRK-Q-LWPGHPDDAHLADGEEILQADHLASFIAMADGVaIGFADASIRHDyvngcdSSPVVFLEGIFVLPSFRQRGVAKQLIAAVQRWGTNKGCREMASDTSPE--NTISQKVHQALGFEETERVI-FYR------------krc<br><br>SARST: aliSize=130 (resi) RMSD=3.81 (Å)<br>av--------AFRRQVLPQDALLVRRVVEST--GfFTPEEADVAQELVDEhlhACGYHFVFATEDDDA-GYACYG----PTPA--TEGTYDLYWIAVAPHRQHSGLGRALLAEVVHDVRLTGGRLLFAETSGirKYAPTRRFYERAGFSAEAVLK------afyragddkiiyrleva<br>--glvprgshMDIRQMNKTHLEHWRGLRKQLwpG-HPDDAHLADGEEILQ---ADHLASFIAMADGVAiGFADASirhdYVNGcdSSPVVFLEGIFVLPSFRQRGVAKQLIAAVQRWGTNKGCREMASDTSP--ENTISQKVHQALGFEETERVIfyrkrc-----------------<br><br>BLAST: aliSize=54 (resi) iden=21.57% (33/153) simi=35.29% (54/153)<br>avafrrqvlpqdallvrrvvestgfft---------------------------------PEEADVA--QELVD-EHL-----HACGYHFVFATEDDDAGY--AC-YGPTPATEGtydlywIAVAPHRQHSGLGRALLAEVVHDVRLTGGRLLFAETSgiRKYAPTRRFYERAGF-SAEAVLkaFYR---<br>--------------------------glvprgshmdirqmnkthlehwrglrkqlwpghPDDAHLAdgEEILQaDHLasfiaMADGVAIGFADASIRHDYvnGCdSSPVVFLEG------IFVLPSFRQRGVAKQLIAAVQRWGTNKGCREMASDTS--PENTISQKVHQALGFeETERVI--FYRkrc<br><br>agddkiiyrleva<br>-------------<br><br>Proposed: aliSize=138 (resi) RMSD=2.20 (Å)<br>-------AVAFRRQVLpQDALLVRRVVEsTgFFTPEeaD--VAQELVDEHLmHACGYHFVFATEDDDMAGYACYGPTPA------TEGTYDLYWIAVAPHRQHSGLGRALLAEVVHDVRLTGGRLLFAETSGiRkYAPTRRFYERAGFSAEAVLKAFyragdDKII-yrleva<br>glvprgsHMDIRQMNK-THLEHWRGLRK-Q-LWPGH--PddAHLADGEEIL-QADHLASFIAMADGVAIGFADASIRHDyvngcdSSPVVFLEGIFVLPSFRQRGVAKQLIAAVQRWGTNKGCREMASDTSP-E-NTISQKVHQALGFEETERVIFY-----RKRCc------
692	2r7hA (157)<br>1ufhA (155)	C	12.26	TM-align: aliSize=135 (resi) RMSD=3.49 (Å)<br>AVAFRRqVLPQDALLVRRVVEstgFF--T------------PE-EADVAQE-LVDEHLHACGYHFVFAT-E-DDDAGYACYGPT-PATEGTYDLYWIAVAPHRQHSGLGRALLAEVVHDVRLTGGRLLFAETSGirKYAPTRRFYERAGFSAEAVlKAfyrAGDdkiiyrleva-<br>TIMLTP-MQTEEFRSYLTYTT---KHyaEekvkagtwlpedAQlLSKQVFTdLLPRGLETPHHHLWSLKlNeKDIVGWLWIHAEpEHPQQEAFIYDFGLYEPYRGKGYAKQALAALDQAARSMGIRKLSLHVFA--HNQTARKLYEQTGFQETDV-VM---SKK---------l<br><br>SARST: aliSize=126 (resi) RMSD=5.82 (Å)<br>avafrrqvl-----------------PQDALLVRRVVESTGFFTPEEADVAQELVDEHLHAC-----GYHFVFATEDDDA--GYACYGPTPAT-EGTYDLYWIAVAPHRQHSGLGRALLAEVVHDVRLTGGRLLFAETsgIRKYAPTRRFYERAGFSAEAVL-----kafyragddkiiyrleva<br>---------timltpmqteefrsylTYTTKHYAEEKVKAGTWLPEDAQLLSKQVFTDLLPRgletpHHHLWSLKLNEKDivGWLWIHAEPEHpQQEAFIYDFGLYEPYRGKGYAKQALAALDQAARSMGIRKLSLHV--FAHNQTARKLYEQTGFQETDVVmskkl-----------------<br><br>BLAST: aliSize=50 (resi) iden=21.94% (34/155) simi=32.26% (50/155)<br>avafrrqvlpqdallvrrvvest----------------------------GFFTPEEADVAQELVDEHLHACG----YHFVFA---TEDDDAGYACYG--PT-PATEG-TYD--LYwiavAPHRQhSGLGRALLAEVVHDVRLTGGRLLFAETSGIRKYAptRRFYERAGFSAEAVL-----kafyr<br>-----------------------timltpmqteefrsyltyttkhyaeekvkaGTWLPEDAQLLSKQVFTDLLPRGletpHHHLWSlklNEKDIVGWLWIHaePEhPQQEAfIYDfgLY----EPYRG-KGYAKQALAALDQAARSMGIRKLSLHVFAHNQTA--RKLYEQTGFQETDVVmskkl-----<br><br>agddkiiyrleva<br>-------------<br><br>Proposed: aliSize=127 (resi) RMSD=2.52 (Å)<br>av-AFRRQVLPQDALLV-RRvV--estgfF--T-------------pE--EaD--VaqE--L-vdEHLMHaCGYHFVFAT-EDDDMAGYACYGPT-PATEGTYDLYWIAVAPHRQHSGLGRALLAEVVHDVRLTGGRLLFAETSGirKYAPTRRFYERAGFSAEAVlkafyragddkIIYRLEva<br>--tIMLTPMQTEEFRSYlTY-Ttk-----HyaEekvkagtwlpeda-QllS-KqvF--TdlLp--RGLET-PHHHLWSLKlNEKDIVGWLWIHAEpEHPQQEAFIYDFGLYEPYRGKGYAKQALAALDQAARSMGIRKLSLHVFA--HNQTARKLYEQTGFQETDV-----------VMSKKL--

693	2r7hA (157)<br>1ufhB (154)	C	12.99	TM-align: aliSize=135 (resi) RMSD=3.13 (Å)<br>aVAFRRqVLPQDALLVRRVVEstGFFTP-------------E-EADVAQE-LVDEHLHACGYHFVFATE--DDDAGYACYGPT-PATEGTYDLYWIAVAPHRQHSGLGRALLAEVVHDVRLTGGRLLFAETSGirKYAPTRRFYERAGFSAeAVLKAfyrAGDdkiiyrleva-<br>-IMLTP-MQTEEFRSYLTYTT--KHYAEekvkagtwlpedaQlLSKQVFTdLLPRGLETPHHHLWSLKLneKDIVGWLWIHAEpEHPQQEAFIYDFGLYEPYRGKGYAKQALAALDQAARSMGIRKLSLHVFA--HNQTARKLYEQTGFQE-TDVVM---SKK----------l<br><br>SARST: aliSize=126 (resi) RMSD=5.82 (Å)<br>avafrrqvl----------------PQDALLVRRVVESTGFFTPEEADVAQELVDEHLHAC-----GYHFVFATEDDDAG-YACYGPTPATE--GTYDLYWIAVAPHRQHSGLGRALLAEVVHDVRLTGGRLLFAETsgIRKYAPTRRFYERAGFSAEAVL-----kafyragddkiiyrleva<br>---------imltpmqteefrsylTYTTKHYAEEKVKAGTWLPEDAQLLSKQVFTDLLPRgletpHHHLWSLKLNEKDIvGWLWIHAEPEHpqQEAFIYDFGLYEPYRGKGYAKQALAALDQAARSMGIRKLSLHV--FAHNQTARKLYEQTGFQETDVVmskkl------------------<br><br>BLAST: aliSize=50 (resi) iden=22.08% (34/154) simi=32.47% (50/154)<br>avafrrqvlpqdallvrrvvest----------------------------GFFTPEEADVAQELVDEHLHACG----YHFVFA---TEDDDAGYACYG--PT-PATEG-TYD--LYwiavAPHRQhSGLGRALLAEVVHDVRLTGGRLLFAETSGIRKYAptRRFYERAGFSAEAVL-----kafyra<br>------------------------imltpmqteefrsyltyttkhyaeekvkaGTWLPEDAQLLSKQVFTDLLPRGletpHHHLWSlklNEKDIVGWLWIHaePEhPQQEAfIYDfgLY----EPYRG-KGYAKQALAALDQAARSMGIRKLSLHVFAHNQTA--RKLYEQTGFQETDVVmskkl------<br><br>gddkiiyrleva<br>------------<br><br>Proposed: aliSize=131 (resi) RMSD=2.45 (Å)<br>aVAFRRqVLPQDALLVRRVV-EstgFFT--------------PE-EaD-VAqE--L-vdEHLmHACGYHFVFATE--DdDMAGYACYGPT-PATEGTYDLYWIAVAPHRQHSGLGRALLAEVVHDVRLTGGRLLFAETSGirKYAPTRRFYERAGFSAeavlkAfyragdDKIIYRLEva<br>-IMLTP-MQTEEFRSYLTYTtK---HYAeekvkagtwlpedaQLlS-KqVF-TdlLp--RGL-ETPHHHLWSLKLneK-DIVGWLWIHAEpEHPQQEAFIYDFGLYEPYRGKGYAKQALAALDQAARSMGIRKLSLHVFA--HNQTARKLYEQTGFQE-----T------DVVMSKKL--
694	2r7hA (157)<br>2fe7B (166)	C	15.92	TM-align: aliSize=138 (resi) RMSD=2.80 (Å)<br>---------AVAFRRqVLPQDALLVRRVVEST-GFF--TPEEADVAQELVDEHLH-ACGYHFVFATEDDD-AGYACYGPTPAT---EGTYDLYWIAVAPHRQHSGLGRALLAEVVHDVRLTGGRLLFAETSGiRkYAPTRRFYERAGFSAEAVLKAFYragddkiiyrleva-----------<br>enlyfqghmTLEIRP-AVPADAEQILAFIIELaDYEraRHEVVTDVEGIRRSLFAeGSPTRALMCLSEGRpIGYAVFFYSYSTwlgRNGIYLEDLYVTPEYRGVGAGRRLLRELAREAVANDCGRLEWSVLD-W-NQPAIDFYRSIGALPQDEWVRYR--------------ldgealrkmae<br><br>SARST: aliSize=124 (resi) RMSD=4.48 (Å)<br>a---------VAFRRQVLPQDALLVRRVVE---------STGFFTPEEADVAQelvdeHLHACGYHFVFATEDDDAGYACYGPTP-----ATEgTYDLYWIAVAPHRQHSGLGRALLAEVVHDVRLTGGRLLFAETsgIRKYAPTRRFYERAGFSA-------------------eavlkafyragddki<br>-enlyfqghmTLEIRPAVPADAEQILAFIIeladyerarHEVVTDVEGIRRSL-----FAEGSPTRALMCLSEGRPIGYAVFFYSystwlGRN-GIYLEDLYVTPEYRGVGAGRRLLRELAREAVANDCGRLEWSV--LDWNQPAIDFYRSIGALPqdewvryrldgealrkmae--------------<br><br>iyrleva<br>-------<br><br>BLAST: aliSize=33 (resi) iden=18.06% (28/157) simi=21.29% (33/157)<br>avafrrqvlpqdallvrrvvestgfftpeeadvaqelvdehlhac--GYHFVFatedddagYAcYGPTPATEGTY--DLYwiaVAPHRQHSGLGRALLAEVVHD-VRLTGGRLlfaETSGI<br>---enlyfqghmtleirpavpadaeqilafiieladyerarhevvtdvegirrslfaegsptralmclsegrpiGYAVFF--------YS-YSTWLGRNGIYleDLY---VTPEYRGVGAGRRLLRELAREaVANDCGRL---EWSVL<br><br>RKYAPTRRFYERAG-------------------------fsaeavlkafyragddkiiyrleva<br>DWNQPAIDFYRSIGalpqdewvryrldgealrkmae-------------------------<br><br>Proposed: aliSize=142 (resi) RMSD=2.19 (Å)<br>---------AVAFRRqVLPQDALLVRRVVEST-GFF-TPEEADV-aQ-ELVDEHLMHACGYHFVFATEDDDMAGYACYGPTPaT----EGTYDLYWIAVAPHRQHSGLGRALLAEVVHDVRLTGGRLLFAETSGiRkYAPTRRFYERAGFSAeavLKafyragdDKIIYRLE---VA----<br>enlyfqghmTLEIRP-AVPADAEQILAFIIELaDYErARHEVVTd-VeGIRRSLFAEGSPTRALMCLSEGRPIGYAVFFYSY-StwlgRNGIYLEDLYVTPEYRGVGAGRRLLRELAREAVANDCGRLEWSVLD-W-NQPAIDFYRSIGALP---QD-------EWVRYRLDgeaLRkmae
695	2r7hA (157)<br>2vbqA (147)	C	16.33	TM-align: aliSize=136 (resi) RMSD=2.64 (Å)<br>-AVAFRRQVLPQDaLLVRRVVEStgFFTPEEADVAQELVDEHLHACGYHFVFATEDDD-AGYACYGPTPA------TEGTYDLYWIAVAPHRQHSGLGRALLAEVVHDVRLTGGRLLFAETSGIrkYAPTRRFYERAGFSAEAVLKaFYRagddkiiyrleva---<br>sHMDIRQMNKTHL-EHWRGLRKQ--LWPGHPDDAHLADGEEILQADHLASFIAMADGVaIGFADASIRHDyvngcdSSPVVFLEGIFVLPSFRQRGVAKQLIAAVQRWGTNKGCREMASDTSPE--NTISQKVHQALGFEETERVI-FYR-------------krc<br><br>SARST: aliSize=131 (resi) RMSD=3.70 (Å)<br>a-VAFRRQVLPQDALLVRRVVEST--GfFTPEEADVAQELVDEhlhACGYHFVFATEDDDAGYACYGPT-------PATEGTYDLYWIAVAPHRQHSGLGRALLAEVVHDVRLTGGRLLFAETSgiRKYAPTRRFYERAGFSAEAVLK------afyragddkiiyrleva<br>-sHMDIRQMNKTHLEHWRGLRKQLwpG-HPDDAHLADGEEILQ---ADHLASFIAMADGVAIGFADASIrhdyvngCDSSPVVFLEGIFVLPSFRQRGVAKQLIAAVQRWGTNKGCREMASDTS--PENTISQKVHQALGFEETERVIfyrkrc-----------------<br><br>BLAST: aliSize=54 (resi) iden=22.45% (33/147) simi=36.73% (54/147)<br>avafrrqvlpqdallvrrvvestgfft---------------------------PEEADVA--QELVD-EHL-----HACGYHFVFATEDDDAGY--AC-YGPTPATEGtydlywIAVAPHRQHSGLGRALLAEVVHDVRLTGGRLLFAETSgiRKYAPTRRFYERAGF-SAEAVLkaFYR---agddki<br>---------------------------shmdirqmnkthlehwrglrkqlwpghPDDAHLAdgEEILQaDHLasfiaMADGVAIGFADASIRHDYvnGCdSSPVVFLEG------IFVLPSFRQRGVAKQLIAAVQRWGTNKGCREMASDTS--PENTISQKVHQALGFeETERVI--FYRkrc------<br><br>iyrleva<br>-------<br><br>Proposed: aliSize=138 (resi) RMSD=2.27 (Å)<br>-AVAFRRQVLpQDALLVRRVVEStgFFTPEeaD--VAQELVDEHLmHACGYHFVFATEDDDMAGYACYGPTPA------TEGTYDLYWIAVAPHRQHSGLGRALLAEVVHDVRLTGGRLLFAETSGiRkYAPTRRFYERAGFSAEAVLKAFyragdDKIIyrleva<br>sHMDIRQMNK-THLEHWRGLRKQ--LWPGH--PddAHLADGEEIL-QADHLASFIAMADGVAIGFADASIRHDyvngcdSSPVVFLEGIFVLPSFRQRGVAKQLIAAVQRWGTNKGCREMASDTSP-E-NTISQKVHQALGFEETERVIFY-----RKRC------

696	2r7hA (157)	C	16.67	TM-align: aliSize=133 (resi) RMSD=2.43 (Å)<br>avAFRRQVLpQDALLVRRVVEsTgFFTpEEAD-VAQELVDEHLHACGYHFVFATEDDD-AGYACYGPTPA------TEGTYDLYWIAVAPHRQHSGLGRALLAEVVHDVRLTGGRLLFAETSGIrkYAPTRRFYERAGFSAEAVLKaFYRagddkiiyrleva---<br>--DIRQMNK-THLEHWRGLRK-Q-LWP-GHPDdAHLADGEEILQADHLASFIAMADGVaIGFADASIRHDyvngcdSSPVVFLEGIFVLPSFRQRGVAKQLIAAVQRWGTNKGCREMASDTSPE--NTISQKVHQALGFEETERVI-FYR-------------krc<br><br>SARST: aliSize=127 (resi) RMSD=3.80 (Å)<br>avafrr---QVLPQDALLVRRVVESTGF-FTPEEADVAQELVDEhlhACGYHFVFATEDDDAGYACYG-----PTPA--TEGTYDLYWIAVAPHRQHSGLGRALLAEVVHDVRLTGGRLLFAETSGirKYAPTRRFYERAGFSAEAVLK------afyragddkiiyrleva<br>------dirQMNKTHLEHWRGLRKQLWPgHPDDAHLADGEEILQ---ADHLASFIAMADGVAIGFADAsirhdYVNGcdSSPVVFLEGIFVLPSFRQRGVAKQLIAAVQRWGTNKGCREMASDTSP--ENTISQKVHQALGFEETERVIfyrkrc-----------------<br><br>BLAST: aliSize=54 (resi) iden=22.92% (33/144) simi=37.50% (54/144)<br>avafrrqvlpqdallvrrvvestgfft--------------------------PEEADVA--QELVD-EHL-----HACGYHFVFATEDDDAGY--AC-YGPTPATEGtydlywIAVAPHRQHSGLGRALLAEVVHDVRLTGGRLLFAETSgiRKYAPTRRFYERAGF-SAEAVLkaFYR---agddkiiyr<br>--------------------------dirqmnkthlehwrglrkqlwpghPDDAHLAdgEEILQaDHLasfiaMADGVAIGFADASIRHDYvnGCdSSPVVFLEG------IFVLPSFRQRGVAKQLIAAVQRWGTNKGCREMASDTS--PENTISQKVHQALGFeETERVI--FYRkrc---------<br><br>leva<br>----<br><br>Proposed: aliSize=137 (resi) RMSD=2.20 (Å)<br>avAFRRQVLpQDALLVRRVVEsTgFFTpEEAD-VAQELVDEHLmHACGYHFVFATEDDDMAGYACYGPTPA------TEGTYDLYWIAVAPHRQHSGLGRALLAEVVHDVRLTGGRLLFAETSGiRkYAPTRRFYERAGFSAEAVLkafyragddKIIYRLEva<br>--DIRQMNK-THLEHWRGLRK-Q-LWP-GHPDdAHLADGEEIL-QADHLASFIAMADGVAIGFADASIRHDyvngcdSSPVVFLEGIFVLPSFRQRGVAKQLIAAVQRWGTNKGCREMASDTSP-E-NTISQKVHQALGFEETERV----------IFYRKRC--
	2vbqB (144)			
697	2rbbA (129)	C	12.40	TM-align: aliSize=63 (resi) RMSD=2.95 (Å)<br>nadlsyvniftrdivasafyqQVFGFqeiesirspifrgldt--GKScigfnaheayelqlaqfsetsgi--------------------------KFLLNFDVDtkeAVDKLVPVAIAAGATLIK-APYETYYHWYQAVL<br>---------------------EDLFI----------------cidhvayacpdadeaskyyqetfgwhelhreenpeqgvveimmapaaklteHMT------------------------qvqvmaplndestvakwlakhngragLHHMAWRVD---DIDAVSATLRERGVQLLYdEPKLGTGGNRINFM<br><br>L--DPERNVFRINNVL--<br>HpkSGKGVLIELTQYPkn<br><br>SARST: aliSize=116 (resi) RMSD=12.78 (Å)<br>na-----DLSYVNIFTRD-IVASAFYQQVFGFQE-----IESIRSpIFRGLDT------GKSCIGFN------AHEAYELQLAQFSETSGikfLLNFDVDTKEAVdklVPVAIAAGATLIK-APYETYYHWYQAVLLDPE--RNVFRINNV---l<br>--edlfiCIDHVAYACPDaDEASKYYQETFGWHElhreeNPEQGV-VEIMMAPaaklteHMTQVQVMaplndeSTVAKWLAKHNGRAGLH---HMAWRVDDIDAV---SATLRERGVQLLYdEPKLGTGGNRINFMHPKSgkGVLIELTQYpkn-<br><br>BLAST: aliSize=13 (resi) iden=6.35% (8/129) simi=10.32% (13/129)<br>nadlsyvniftrdiv-------------------ASAFYQQVFGFQEIESIRSP---ifrgldtgkscigfnaheayelqlaqfset<br>---------------edlficidhvayacpdadeASKYYQETFGWHELHREENPeqgvveimmapaakltehmtqvqvmaplndestvakwlakhngraglhhmawrvddidavsatlrergvqllydepklgtggnrinfmhpksgkgvlieltqypkn------------------------------<br><br>sgikfllnfdvdtkeavdklvpvaiaagatlikapyetyyhwyqavlldpernvfrinnvl<br>---<br><br>Proposed: aliSize=107 (resi) RMSD=1.86 (Å)<br>-NMA-DLSYVNIFTRDIVAMSAFYQQVFGFQEIEsiRSP------IFRGLDTG------KSCIGFNAH------------------eayelmqlaqfsetsgiKFLLNFDVDtkeAVDKLVPVAIAAGATLIK-APYETYYHWYQAVLL--DPERNVFRINNVL--<br>eDLFiCIDHVAYACPDADEASKYYQETFGWHELH--REEnpeqgvVEIMMAPAakltehMTQVQVMAPlndestvakwlakhngrag-----------------LHHMAWRVD---DIDAVSATLRERGVQLLYdEPKLGTGGNRINFMHpkSGKGVLIELTQYPkn
	1jc5D (145)			
698	2vi7A (163)	C	17.11	TM-align: aliSize=140 (resi) RMSD=3.30 (Å)<br>---TIRLERYSERHVEGLTALYNDpaVARQVLQMPYQSVEQRRKRLHDSDDDRLLILVALH--QGDVIGSASLEQHpRIR-R-SHSGSIGmGVAVA--WQGKGVGSRLLGELLDIAdNWMNLRRVELTVYTDNAPALALYRKFGFETEGEMRDYavrdgrfvdvysmarlrr---<br>sedNITVRFVTENDKEGWQRLWKS--YQDFYEVSFPDDLDDFNFGRFLDPNIKMWAAVAVEssSEKIIGMINFFNH-MTTwDfKDKIYIN-DLYVDenSRVKGAGGKLIQFVYDEA-DKLGTPSVYWCTDESNHRAQLLYVKVGYKAPKILYKR-----------------kgy<br><br>SARST: aliSize=139 (resi) RMSD=4.93 (Å)<br>t----IRLERYSERHVEGLTALYNDPAVARQVLQMpyqsVEQRRKRL---HDSDDDrLLILVALH--QGDVIGSASLEQHPRIRRSHSGSIGMGVAV--AWQGKGVGSRLLGELLDIADNwMNLRRVELTVYTDNAPALALYRKFGFETEGemrdyavrdgrfvdvYSMARL--rr<br>-sednITVRFVTENDKEGWQRLWKSYQDFYEVSFP----DDLDDFNFgrfLDPNIK-MWAAVAVEssSEKIIGMINFFNHMTTWDFKDKIYINDLYVdeNSRVKGAGGKLIQFVYDEADK-LGTPSVYWCTDESNHRAQLLYVKVGYKAPK--------------ILYKRKgy--<br><br>BLAST: aliSize=24 (resi) iden=10.53% (16/152) simi=15.79% (24/152)<br>tirleryserhvegltalyndpavarqvlqmpyqsveqrrkrlhdsdddrllilvalhqgdvigsasleqhprirrshsgsigmgvavawqg---K<br>--sednitvrfvtendkegwqrlwksyqdfyevsfpddlddfnfgrfldpnikmwaavavesssekiigminffnhmttwdfkdkiyindlyvdensrvK<br><br>GVGSRLLGELLDIADN-------WM---NLRRVELtVYTD---NAPALALYRKfGFetegemrdyavrdgrfvdvysmarlrr<br>GAGGKLIQFVYDEADKlgtpsvyWCtdeSNHRAQL-LYVKvgyKAPKILYKRK-GY---------------------------<br><br>Proposed: aliSize=135 (resi) RMSD=2.40 (Å)<br>---TIRLERYSERHVEGLTALYNDpaVA-RQ-vlQMPyQSveQ-RRKRLHD--SDD-dRLLILVALH--QGDVIGSASLEQHpRiRR---SHSGSIGmGVAVAW--QGKGVGSRLLGELLDIADnWMNLRRVELTVYTDNAPALALYRKFGFETEGemrdyavrdgrFVDVYSM--arlrr<br>sedNITVRFVTENDKEGWQRLWKS--YQdFYe--VSF-PD--DlDDFNFGRflDPNi-KMWAAVAVEssSEKIIGMINFFNH-M-TTwdfKDKIYIN-DLYVDEnsRVKGAGGKLIQFVYDEAD-KLGTPSVYWCTDESNHRAQLLYVKVGYKAPK-----------ILYKRKGya-----
	1qsmD (152)			

699	2vi7A (163)<br>1s5kA (153)	C	15.03	TM-align: aliSize=135 (resi) RMSD=2.82 (Å)<br>-------TIRLERYSERHVEGLTALYNDpavArqvlQMPYQSvEQRRKRLHDS-DDDRLLILVALHQGDVIGSASLEQHPRIR----RSHSGSIGmGVAVA--WQGKGVGSRLLGELLDIADnWMNLRRVELTVYTDNAPALALYRKFGFETEGEMRDYavrdgrfvdvysmarlrr----<br>glvprgsHMDIRQMNKTHLEHWRGLRKQ---L----WPGHPD-DAHLADGEEIlQADHLASFIAMADGVAIGFADASIRHDYVngcdSSPVVFLE-GIFVLpsFRQRGVAKQLIAAVQRWGT-NKGCREMASDTSPENTISQKVHQALGFEETERVIFY------------------rkrc<br><br>SARST: aliSize=133 (resi) RMSD=3.36 (Å)<br>t--------IRLERYSERHVEGLTALYNDPavarqvlqMPYQSVEQRRKRLHDSD-DDRLLILVALHQGDVIGSASLEQHPRIRRS-HSG--SIGMGVAV--AWQGKGVGSRLLGELLDIADNWmNLRRVELTVYTDNAPALALYRKFGFETEGEMR------dyavrdgrfvdvysmarlrr<br>-glvprgshMDIRQMNKTHLEHWRGLRKQL--------WPGHPDDAHLADGEEILqADHLASFIAMADGVAIGFADASIRHDYVNGcDSSpvVFLEGIFVlpSFRQRGVAKQLIAAVQRWGTNK-GCREMASDTSPENTISQKVHQALGFEETERVIfyrkrc--------------------<br><br>BLAST: aliSize=48 (resi) iden=18.30% (28/153) simi=31.37% (48/153)<br>tirleryserhvegltalyndpavarqvlqmpyqsveqr--------------------------RKRLHDSD-DDRLL-----IL--------VALHQGDVIGSASLE-QHPRIRRSHSGSI----GMGVAVAWQGKGVGSRLLGELLDIADNwMNLRRVELTVYTDNAPALALYRKFGF-ETE------<br>---------------------------------------glvprgshmdirqmnkthlehwrglRKQLWPGHpDDAHLadgeeILqadhlasfIAMADGVAIGFADASiRHDYVNGCDSSPVvfleGIFVLPSFRQRGVAKQLIAAVQRWGTN-KGCREMASDTSPENTISQKVHQALGFeETErvifyr<br><br>---gemrdyavrdgrfvdvysmarlrr<br>krc------------------------<br><br>Proposed: aliSize=137 (resi) RMSD=2.27 (Å)<br>-------TIRLERYSERHVEGLTALYNDpavArqvlQMPYQSvEQRRKRLHDS-DDDRLLILVALHQGDVIGSASLEQHPRirR------SHSGSIGmGVAVA--WQGKGVGSRLLGELLDIADnWMNLRRVELTVYTDNAPALALYRKFGFETEGEmrdyavrdgrfVDVYSMAR-lrr<br>glvprgsHMDIRQMNKTHLEHWRGLRKQ---L----WPGHPD-DAHLADGEEIlQADHLASFIAMADGVAIGFADASIRHD--YvngcdsSPVVFLE-GIFVLpsFRQRGVAKQLIAAVQRWGT-NKGCREMASDTSPENTISQKVHQALGFEETER-----------VIFYRKRCc---
700	2vi7A (163)<br>2fe7B (166)	C	20.86	TM-align: aliSize=140 (resi) RMSD=3.08 (Å)<br>---------TIRLERYSERHVEGLTALYNDpaVAR-QVLQ-MPYQSVEQRRKRLHDSDDdRLLILVALHQGDVIGSASLEQHpRIR--RSHSGSIGmGVAVAW--QGKGVGSRLLGELLDIADnWMNLRRVELTVYTDNAPALALYRKFGFETEGEMRDYAvrdgrfvdvysmarlrr-----------<br>enlyfqghmTLEIRPAVPADAEQILAFIIE--LADyERARhEVVTDVEGIRRSLFAEGS-PTRALMCLSEGRPIGYAVFFYS-YSTwlGRNGIYLE-DLYVTPeyRGVGAGRRLLRELAREAV-ANDCGRLEWSVLDWNQPAIDFYRSIGALPQDEWVRYR------------------ldgealrkmae<br><br>SARST: aliSize=135 (resi) RMSD=3.20 (Å)<br>t----------IRLERYSERHVEGLTALYNDPAVARQVLQMPYQSVEQRRKRLHDSDDDrLLILVALHQGDVIGSASLEQHPRIRRSHSGSIGMGVAV--AWQGKGVGSRLLGELLDIADNWmNLRRVELTVYTDNAPALALYRKFGFET------------------egemrdyavrdgrfvdvysma<br>-enlyfqghmtLEIRPAVPADAEQILAFIIELADYERARHEVVTDVEGIRRSLFAEGSP-TRALMCLSEGRPIGYAVFFYSYSTWLGRNGIYLEDLYVtpEYRGVGAGRRLLRELAREAVAN-DCGRLEWSVLDWNQPAIDFYRSIGALPqdewvryrldgealrkmae--------------------<br><br>rlrr<br>----<br><br>BLAST: aliSize=32 (resi) iden=14.11% (23/163) simi=19.63% (32/163)<br>tirleryserhvegltalyndpavarqvlqmpyqsveqrrkrlhdsdddrllilvalhqgdvigsasleqhprirrshsgsigmg--VAVAWQGKGV<br>---enlyfqghmtleirpavpadaeqilafiieladyerarhevvtdvegirrslfaegsptralmclsegrpigyavffysystwlgrngiyledlyVTPEYRGVGA<br><br>GSRLLGELL-DIADNwmNLRRVELTVYTDNAPALALYRKFGFETEGEMRDYAVrDG--------rfvdvysmarlrr<br>GRRLLRELArEAVAN--DCGRLEWSVLDWNQPAIDFYRSIGALPQDEWVRYRL-DGealrkmae-------------<br><br>Proposed: aliSize=138 (resi) RMSD=2.29 (Å)<br>---------TIRLERYSERHVEGLTALYNDpaVARQV----lqMP-yQSVEQRRKRLHDSDDdRLLILVALHQGDVIGSASLEQHpRiRR---SHSGSIGmGVAVAW--QGKGVGSRLLGELLDIADnWMNLRRVELTVYTDNAPALALYRKFGFETEG--emrdyavrdGRFVDVYS-------marlrr<br>enlyfqghmTLEIRPAVPADAEQILAFIIE--LADYErarh--EVv-TDVEGIRRSLFAEGS-PTRALMCLSEGRPIGYAVFFYS-Y-STwlgRNGIYLE-DLYVTPeyRGVGAGRRLLRELAREAV-ANDCGRLEWSVLDWNQPAIDFYRSIGALPQDew---------VRYRLDGEalrkmae------
701	2vi7A (163)<br>2g3tA (169)	C	11.66	TM-align: aliSize=141 (resi) RMSD=3.26 (Å)<br>TIRLERYSERHVEGLTALYND--PAvaRQVLQMPYQSVEQRRKRLHDSDDdRLLILVALHQ--------GDVIGSASLEQHPRIRRSHSGSIGmGVAVAW--QGKGVGSRLLGELLDIADnWMNLRRVELTVYTDNAPALALYRKFGFETEGE---MRDYAvrdgrfvdvysmarlrr-------------<br>KFVIRPATAADCSDILRLIKElaKY--EYMEEQVILTEKDLLEDGFGEHP-FYHCLVAEVPkehwtpegHSIVGFAMYYFTYDPWIGKLLYLE-DFFVMSdyRGFGIGSEILKNLSQVAM-RCRCSSMHFLVAEWNEPSINFYKRRGASDLSSeegWRLFK------------------idkeyllkmatee<br><br>SARST: aliSize=128 (resi) RMSD=6.19 (Å)<br>ti--RLERYSERHVEGLTALYNDPAVARQVLQMPYQSVEQRRKRLHDSDDDRLLILVALH------QGD-VIGSASLEQhprirrshSGSIGMGVAVAW---------QGKGVGSRLLGELLDIADNWmNLRRVELTVYTDNAPALALYRKFGFETE-----------------------gemrdyavrd<br>--kfVIRPATAADCSDILRLIKELAKYEYMEEQVILTEKDLLEDGFGEHPFYHCLVAEVPkehwtpEGHsIVGFAMYYF--------TYDPWIGKLLYLedffvmsdyRGFGIGSEILKNLSQVAMRC-RCSSMHFLVAEWNEPSINFYKRRGASDLsseegwrlfkidkeyllkmatee----------<br><br>grfvdvysmarlrr<br>--------------<br><br>BLAST: aliSize=12 (resi) iden=3.68% (6/163) simi=7.36% (12/163)<br>tirleryserhvegltalyndpavarqvlqmpyqsveqrrkrlhdsdddrllilvalhqgdvigsasleqhprirrshsgsigmgvavawqgkgvgsrllgelldiadnwmnlrrvelt--<br>---kfvirpataadcsdilrlikelakyeymeeqviltekdlledgfgehpfyhclvaevpkehwtpeghsivg<br><br>---------------VYTDNAPALALYRKFGFETE---gemrdyavrdgrfvdvysmarlrr<br>famyyftydpwigklLYLEDFFVMSDYRGFGIGSEilknlsqvamrcrcssmhflvaewnepsinfykrrgasdlsseegwrlfkidkeyllkmatee-------------------------<br><br>Proposed: aliSize=143 (resi) RMSD=2.51 (Å)<br>TIRLERYSERHVEGLTALYND-PAVAR--qvlqMPY-QSVEQRRKRLHDSDD-drLLILVALHQ--------GDVIGSASLEQHpR-iR-RSHSGSIGmGVAVAW--QGKGVGSRLLGELLDIADnWMNLRRVELTVYTDNAPALALYRKFGFETEG--emrdyavrdGRFVDVYS---MA-R-LR--r<br>KFVIRPATAADCSDILRLIKElAKYEYme----EQViLTEKDLLEDGFGEHPf--YHCLVAEVPkehwtpegHSIVGFAMYYFT-Yd-PwIGKLLYLE-DFFVMSdyRGFGIGSEILKNLSQVAM-RCRCSSMHFLVAEWNEPSINFYKRRGASDLSse---------EGWRLFKIdkeYLlKmATee-

702	2vi7A<br>(163)<br>2vxkA<br>(165)	C	19.02	TM-align: aliSize=134 (resi) RMSD=2.25 (Å)<br>---------------------TIRLERYSERHVE-GLTALYNdpaVARqvlqMPYQSVE-QRRKRLHDS-DD-DRLLILVALH-QGDVIGSASLEQHPRIR--RSHSGSIGmGVAVA--WQGKGVGSRLLGELLDIADnWMNLRRVELTVYTDNapaLALYRKFGFETEGEMRdYAVrdgrfvdvysmar<br>entplfspslispdvlavlpaDYTIRPLCRSDYKrGYLDVLR---VLT----TVGDINEeQWNSRYEWIrARsDEYYLLVVCDgEGRIVGTGSLVVERKFIhsLGMVGHIE-DIAVEkgQQGKKLGLRIIQALDYVAE-KVGCYKTILDCSEAN---EGFYIKCGFKRAGLEM-AHY-------------<br>lrr-<br>---y<br>SARST: aliSize=133 (resi) RMSD=5.35 (Å)<br>t----------------------IRLERYSERHvegltaLYNDPAVARQVLQMPYQS-----VEQRRKRLHDSddDRLLILVALH-QGDVIGSASLEQHPRIRRSHSGS-IGMGVAV--AWQGKGVGSRLLGELLDIADNwMNLRRVELTVYTdnaPALALYRKFGFETEGemrdyavrdgrfvdvYSMA<br>-entplfspslispdvlavlpadYTIRPLCRSD------YKRGYLDVLRVLTTVGDIneeqwNSRYEWIRARS--DEYYLLVVCDgEGRIVGTGSLVVERKFIHSLGMVgHIEDIAVekGQQGKKLGLRIIQALDYVAEK-VGCYKTILDCSE---ANEGFYIKCGFKRAG---------------LEMA<br>RL-rr<br>HYy--<br>BLAST: aliSize=43 (resi) iden=15.95% (26/163) simi=26.38% (43/163)<br>tirleryserhvegltalyndpavarqvlqmpyqsveqrrkrlhdsd--DDRLLILVALHQGDVIGSASLEQHPRIrrSHS-GSIGMGVAVAWQgKGVGSRLLG-ELLDIADNWMNLRRVELTVYT<br>---entplfspslispdvlavlpadytirplcrsdykrgyldvlrvlttvgdineeqwnsryewirarsDEYYLLVVCDGEGRIVGTGSLVVERKF--IHSlGMVGHIEDIAVE-KGQQGKKLGlRIIQALDYVAEKVGCYKTILD<br>DNAPALALYRKFGFETEG-EMRDY-avrdgrfvdvysmarlrr<br>CSEANEGFYIKCGFKRAGlEMAHYy-------------------<br>Proposed: aliSize=134 (resi) RMSD=1.85 (Å)<br>---------------------TIRLERYSERHVE-GLTALYNdpaVARqvlqMPYQSVE-QRRKRLHDS-DD-DRLLILVALH-QGDVIGSASLEQHpRIRR---SHSGSIGmGVAVA--WQGKGVGSRLLGELLDIADnWMNLRRVELTVYTDNapaLALYRKFGFETEGemrdyavrdgrfvDVYSMA<br>entplfspslispdvlavlpaDYTIRPLCRSDYKrGYLDVLR---VLT----TVGDINEeQWNSRYEWIrARsDEYYLLVVCDgEGRIVGTGSLVVE-RKFIhslGMVGHIE-DIAVEkgQQGKKLGLRIIQALDYVAE-KVGCYKTILDCSEAN---EGFYIKCGFKRAG-------------LEMAHY<br>R-lrr<br>Yc---
703	5croA<br>(61)<br>1d1mB<br>(65)	C	91.80	TM-align: aliSize=59 (resi) RMSD=1.51 (Å)<br>MEQRITLKDYAMRFGQTKTAKDLGVYQSAINKAIHAGRKIFLTINADGSVYAEEVKPFPsn------<br>MEQRITLKDYAMRFGQTKTAKDLGVYQSAINKAIHAGRKIFLTINADGSVYAEEVKDGE--vkpwps<br>SARST: aliSize=55 (resi) RMSD=0.75 (Å)<br>m-EQRITLKDYAMRFGQTKTAKDLGVYQSAINKAIHAGRKIFLTINADGSVYAEEVK---------pfps<br>-mEQRITLKDYAMRFGQTKTAKDLGVYQSAINKAIHAGRKIFLTINADGSVYAEEVKdgevkpwps----<br>BLAST: aliSize=60 (resi) iden=98.33% (59/61) simi=100.00% (60/61)<br>MEQRITLKDYAMRFGQTKTAKDLGVYQSAINKAIHAGRKIFLTINADGSVYAE-----EVKPFPS<br>MEQRITLKDYAMRFGQTKTAKDLGVYQSAINKAIHAGRKIFLTINADGSVYAEevkdgEVKPWPS<br>Proposed: aliSize=61 (resi) RMSD=0.93 (Å)<br>MEQRITLKDYAMRFGQTKTAKDLGVYQSAINKAIHAGRKIFLTINADGSVYAEEVK----PFPSN<br>MEQRITLKDYAMRFGQTKTAKDLGVYQSAINKAIHAGRKIFLTINADGSVYAEEVKdgevKPWPS
704	5croA<br>(61)<br>2cw1A<br>(65)	C	19.67	TM-align: aliSize=52 (resi) RMSD=2.21 (Å)<br>meq--RIT-LKDYAMRFGQTKTAKD-LgVYQSAINKAIHAGRKIFLTINADGSVYAEEVKpfpsn---------<br>---mrKKLdLKKFVEDKNQEYAARAlG-LSQKLIEEVLKRGLPVYVETNKDGNIKVYITQ-----dgitqpfpp<br>SARST: aliSize=54 (resi) RMSD=2.48 (Å)<br>me--QRITLKDYAMRFGQTKTAKDLGVYQSAINKAIHAGRKIFLTINADGSVYAEEVK---------pfps<br>--mrKKLDLKKFVEDKNQEYAARALGLSQKLIEEVLKRGLPVYVETNKDGNIKVYITQdgitqpfpp----<br>BLAST: aliSize=32 (resi) iden=30.00% (18/61) simi=53.33% (32/61)<br>MEQRITLKDYAMRFGQTKTAKDLGVYQSAINKAIHAGRKIFLTINADGS--VYAEE---VKPFP-s<br>MRKKLDLKKFVEDKNQEYAARALGLSQKLIEEVLKRGLPVYVETNKDGNikVYITQdgiTQPFPp-<br>Proposed: aliSize=58 (resi) RMSD=2.03 (Å)<br>meq--RIT-LKDYAMRFGQTKTAKDLGVYQSAINKAIHAGRKIFLTINADGSVYAEE----VKPFPSN<br>---mrKKLdLKKFVEDKNQEYAARALGLSQKLIEEVLKRGLPVYVETNKDGNIKVYItqdgITQPFPP

705	5croA<br>(61)<br><br>2orcA<br>(71)	C	93.44	TM-align: aliSize=58 (resi) RMSD=1.68 (Å)<br>MEQRITLKDYAMRFGQTKTAKDLGVYQSAINKAIHAGRKIFLTINADGSVYAEEVKPFpsn-------------<br>MEQRITLKDYAMRFGQTKTAKDLGVYQSAINKAIHAGRKIFLTINADGSVYAEEVKDG---evkpfpsnkktta<br><br>SARST: aliSize=50 (resi) RMSD=1.19 (Å)<br>meqri-----TLKDYAMRFGQTKTAKDLGVYQSAINKAIHAGRKIFLTINADGSVYAEEV----------------kpfps<br>-----meqriTLKDYAMRFGQTKTAKDLGVYQSAINKAIHAGRKIFLTINADGSVYAEEVkdgevkpfpsnkktta-----<br><br>BLAST: aliSize=60 (resi) iden=100.00% (60/61) simi=100.00% (60/61)<br>MEQRITLKDYAMRFGQTKTAKDLGVYQSAINKAIHAGRKIFLTINADGSVYAEEVK-----PFPS------<br>MEQRITLKDYAMRFGQTKTAKDLGVYQSAINKAIHAGRKIFLTINADGSVYAEEVKdgevkPFPSnkktta<br><br>Proposed: aliSize=61 (resi) RMSD=1.49 (Å)<br>MEQRITLKDYAMRFGQTKTAKDLGVYQSAINKAIHAGRKIFLTINADGSVYAEEV-----KP-FPSN----<br>MEQRITLKDYAMRFGQTKTAKDLGVYQSAINKAIHAGRKIFLTINADGSVYAEEVkdgevKPfPSNKktta
706	1gheA<br>(170)<br><br>1qsmD<br>(152)	C	13.16	TM-align: aliSize=135 (resi) RMSD=2.63 (Å)<br>----AQLRRVtaeSFaHYRHGLAQLLFETVHgGASvgFadLDqQaYAWCDGLKADI-A-AGSLLLWVVA---EDdNVLASAQLSLCQKP-NGLNRAEVQKL-VLPSARGRGLGRQL-DEVEQVAVKHKRGLLHLDTEAGS-VAEAFYSALaYTRV-GELPGYcatpdgrlhptaiyfktl---<br>sednITVRFV---TE-NDKEGWQRLWKSYQD-FYE--V--SF-P-DDLDDFNFGRFlDpNIKMWAAVAVessSE-KIIGMINFFNHMTTwDFKDKIYINDLyVDENSRVKGAGGKLiQFVYDEADKLGTPSVYWCTDESNhRAQLLYVKV-GYKApKILYKR------------------kgy<br><br>SARST: aliSize=133 (resi) RMSD=4.63 (Å)<br>a-----QLRRVTAESFAHYRHGLAQLLFEtvhGGAsvgfadLDQQAYAWCDGLKADIAAGSLLLWVVAE--DDNVLASAQLSLCQKPNGLNRAEV--QKLVLPSARGRG-LGRQLDEVEQVAVKHKRGLLHLDTEAGSVAEAFYSALaytrVGELPG---------ycatpdgrlhptaiyfktl<br>-sedniTVRFVTENDKEGWQRLWKSYQDF---YEV------SFPDDLDDFNFGRFLDPNIKMWAAVAVEssSEKIIGMINFFNHMTTWDFKDKIYinDLYVDENSRVKGaGGKLIQFVYDEADKLGTPSVYWCTDESNHRAQLLYVK----VGYKAPkilykrkgy------------------<br><br>BLAST: aliSize=5 (resi) iden=3.29% (5/152) simi=3.29% (5/152)<br>aqlrrvtaesfahyrhglaqllfetvhggasvgfadldqqayawcdglkadiaagslllwvvaeddnvlasaqlslcqkpnglnra--<br>--sednitvrfvtendkegwqrlwksyqdfyevsfpddlddfnfgrfldpnikmwaavavesssekiigminffnhmttwdfkdkiyindlyvdensrvkgaggkl<br><br>------EVQKLVLPS---------------------------------argrglgrqldeveqvavkhkrgllhldteagsvaeafysalaytrvgelpgycatpdgrlhptaiyfktl<br>iqfvydEADKLGTPSvywctdesnhraqllyvkvgykapkilykrkgy---<br><br>Proposed: aliSize=137 (resi) RMSD=2.04 (Å)<br>----AQLRRVtaeSFaHYRHGLAQLLFETVHgGaSvgFMadL-dmqQaYAWCDGLKADI-A-AGSLLLWVVAE---DdNVLASAQLSLCqKpnG----LNRAEVQKLMVLPSARGRGLGRQLMDEVEQVAVKHKRGLLHLDTEAGS-VAEAFYSALAYTRVgELPGyCATPdG-rlhptaiyfktla<br>sednITVRFV---TE-NDKEGWQRLWKSYQD-F-Y--EV--Sf---P-DDLDDFNFGRFlDpNIKMWAAVAVEsssE-KIIGMINFFNH-M--TtwdfKDKIYINDLYVDENSRVKGAGGKLIQFVYDEADKLGTPSVYWCTDESNhRAQLLYVKVGYKAP-KILY-KRKG-Ya-------------
707	1k53A<br>(72)<br><br>2hj1A<br>(77)	C	8.33	TM-align: aliSize=49 (resi) RMSD=2.39 (Å)<br>mhhhhhhamEEVTIKANLIFaNASTQTAEFKGTF-EKaT-SeAYA--YADTLKKdNGE---WTVDVADKgytlnikfag--------------------<br>---------NQINIEIAYAF-PERYYLKSFQVDEgIT-VqT-AITqsGILSQFP-EIDlstNKIGIFSR-----------pikltdvlkegdrieiyrpll<br><br>SARST: aliSize=60 (resi) RMSD=9.10 (Å)<br>mhhhhhhame-EVTIKANLIFANASTQTAEFK---GTFEKATSEAYAYADTLK--KDNGEWTVD---------VADKGYTLNIKF--ag<br>----------nQINIEIAYAFPERYYLKSFQVdegITVQTAITQSGILSQFPEidLSTNKIGIFsrpikltdvLKEGDRIEIYRPll--<br><br>BLAST: aliSize=9 (resi) iden=6.94% (5/72) simi=12.50% (9/72)<br>mhhhhhhameevtikanlifanastqtaefkgtfekatse-------AYAYADTLKKDNgeWTVD--vadkgytlnikfag<br>--nqinieiAYAFPERYYLKS--FQVDegitvqtaitqsgilsqfpeidlstnkigifsrpikltdvlkegdrieiyrpll--------------<br><br>Proposed: aliSize=60 (resi) RMSD=2.36 (Å)<br>mhhhhhhamEEVTIKANLIFaNASTQTAEFKGTFEK-aTSEAYA--YADTLKKdNGE---WTVDVAD---------KGY-TLNIKFAG-<br>---------NQINIEIAYAF-PERYYLKSFQVDEGIt-VQTAITqsGILSQFP-EIDlstNKIGIFSrpikltdvlKEGdRIEIYRPLl

708	2c5jA (82)<br>1mi7R (103)	N	3.66	TM-align: aliSize=56 (resi) RMSD=2.53 (Å)<br>dpfqqvvkdtkeqln--------------------------------RInn--Yitrhntae--EEIQDILKDVEETIVDLDRSIIVMKRDENEDVSGREAQVKNIKQQLDALKlRF--D---------<br>---------------qqspysaamaeqrhqewlrfvdllknayqndlhLP--llN--------lmLTPDEREALGTRVRIVEELLRGEMSQRELKNELGAGIATITRGSNSLKAA-PVelRqwleevll<br><br>SARST: aliSize=80 (resi) RMSD=9.26 (Å)<br>d-----------PFQQVVKDTKEQLNRIN----NYITRHNTAEEEIQDILKDVEETIVDLDRSIIVMKRDENEDVSGREAQVKNIKQQL----DALKLRF----d<br>-qqspysaamaeQRHQEWLRFVDLLKNAYqndlHLPLLNLMLTPDEREALGTRVRIVEELLRGEMSQRELKNELGAGIATITRGSNSLKaapvELRQWLEevll-<br><br>BLAST: aliSize=19 (resi) iden=10.98% (9/82) simi=23.17% (19/82)<br>dpfqqvvkdtkeqlnrinnyitrhntaeeeiq---------------------DILKDVEETIVDLDRSIIVMKRDENEDVSGREAQVKNI--kqqldalklrfd<br>--------------------------------qqspysaamaeqrhqewlrfvDLLKNAYQNDLHLPLLNLMLTPDEREALGTRVRIVEELlrgemsqrelknelgagiatitrgsnslkaapvelrqwleevll------------<br><br>Proposed: aliSize=77 (resi) RMSD=1.64 (Å)<br>--DPFQQVVKDTKEQLNRINNYITRHNTA-----------eeeIQDILKDVEETIVDLDRSIIVMKRDENEDVSGREAQVKNIKQQLDALKL-rfD-----------<br>qqSPYSAAMAEQRHQEWLRFVDLLKNAYQndlhlpllnlml---TPDEREALGTRVRIVEELLRGEMSQRELKNELGAGIATITRGSNSLKAAp--Velrqwleevll
709	1pucA (101)<br>1qb3A (113)	C	71.29	TM-align: aliSize=86 (resi) RMSD=1.91 (Å)<br>sksgv----PRLLTASERERLEPFIDQIHYSPRYADDEYEYRHVMLPKAMLKAIPTDYFNPETGTLRILQEEEWRGLGITQSLGWEMYEVHVpephi-----LLFkrekd------------------<br>-----hafqGRKLTDQERARVLEFQDSIHYSPRYSDDNYEYRHVMLPKAMLKVIPSDYFNSEVGTLRILTEDEWRGLGITQSLGWEHYECHA-----pephiLLF-----krplnyeaelraataaaq<br><br>SARST: aliSize=96 (resi) RMSD=5.89 (Å)<br>sks--GVPRLLTASERERLEPFIDQIHYSPRYADDEYEYRHVMLPKAMLKAIPTDYFNPETGTLRILQEEEWRGLGITQSLGWEMYEVHVPEPHILLFKRE---------------kd<br>---haFQGRKLTDQERARVLEFQDSIHYSPRYSDDNYEYRHVMLPKAMLKVIPSDYFNSEVGTLRILTEDEWRGLGITQSLGWEHYECHAPEPHILLFKRPlnyeaelraataaaq--<br><br>BLAST: aliSize=76 (resi) iden=71.29% (72/101) simi=75.25% (76/101)<br>sksgvp------RLLTASERERLEPFIDQIHYSPRYADDEYEYRHVMLPKAMLKAIPTDYFNPETGTLRILQEEEWRGLGITQSLGWEMYEVHVPEPHILLFKR----------------ekd<br>------hafqggRKLTDQERARVLEFQDSIHYSPRYSDDNYEYRHVMLPKAMLKVIPSDYFNSEVGTLRILTEDEWRGLGITQSLGWEHYECHAPEPHILLFKRplnyeaelraataaaq---<br><br>Proposed: aliSize=96 (resi) RMSD=0.73 (Å)<br>sksgv----PRLLTASERERLEPFIDQIHYSPRYADDEYEYRHVMLPKAMLKAIPTDYFNPETGTLRILQEEEWRGLGITQSLGWEMYEVHVPEPHILLFKREKD-------------<br>-----hafqGRKLTDQERARVLEFQDSIHYSPRYSDDNYEYRHVMLPKAMLKVIPSDYFNSEVGTLRILTEDEWRGLGITQSLGWEHYECHAPEPHILLFKRPLNyeaelraataaaq
710	3bcoA (124)<br>3bcmA (119)	N	100.00	TM-align: aliSize=103 (resi) RMSD=0.35 (Å)<br>kesaaakferqhmdsgnsass---------------SSNYCNQMMCCRKMTQGKCKPVNTFVHESLADVKAVCSQKKVTCKDGQTNCYQSKSTMRITDCRETGSSKYPNCAYKTTQVEKHIIVACGGKPSVPVHFDASV<br>--------------------kesaaakferqhmdsgSSNYCNQMMCCRKMTQGKCKPVNTFVHESLADVKAVCSQKKVTCKDGQTNCYQSKSTMRITDCRETGSSKYPNCAYKTTQVEKHIIVACGGKPSVPVHFDASV<br><br>SARST: aliSize=117 (resi) RMSD=9.47 (Å)<br>k-ESAAAKFERQHMDSGNsassssNYCNQMMCCRKMTQGKCKPVNTFVHESLADVKAVCSQKKVTCKDGQTNCYQSKSTMRITDCRETGSSKYPNCAYKTTQVEKHIIVACGGKPSVPVHFDAS-v<br>-kESAAAKFERQHMDSGS-----SNYCNQMMCCRKMTQGKCKPVNTFVHESLADVKAVCSQKKVTCKDGQTNCYQSKSTMRITDCRETGSSKYPNCAYKTTQVEKHIIVACGGKPSVPVHFDASv-<br><br>BLAST: aliSize=119 (resi) iden=100.00% (119/119) simi=100.00% (119/119)<br>KESAAAKFERQHMDSGnsassSSNYCNQMMCCRKMTQGKCKPVNTFVHESLADVKAVCSQKKVTCKDGQTNCYQSKSTMRITDCRETGSSKYPNCAYKTTQVEKHIIVACGGKPSVPVHFDASV<br>KESAAAKFERQHMDSG-----SSNYCNQMMCCRKMTQGKCKPVNTFVHESLADVKAVCSQKKVTCKDGQTNCYQSKSTMRITDCRETGSSKYPNCAYKTTQVEKHIIVACGGKPSVPVHFDASV<br><br>Proposed: aliSize=119 (resi) RMSD=0.33 (Å)<br>KESAAAKFERQHMDSGnsassSSNYCNQMMCCRKMTQGKCKPVNTFVHESLADVKAVCSQKKVTCKDGQTNCYQSKSTMRITDCRETGSSKYPNCAYKTTQVEKHIIVACGGKPSVPVHFDASV<br>KESAAAKFERQHMDSG-----SSNYCNQMMCCRKMTQGKCKPVNTFVHESLADVKAVCSQKKVTCKDGQTNCYQSKSTMRITDCRETGSSKYPNCAYKTTQVEKHIIVACGGKPSVPVHFDASV

711	3bcoA (124)<br>1js0A (124)	N	74.19	TM-align: aliSize=95 (resi) RMSD=1.56 (Å)<br>kesaaakferqhmdsgnsas-------------------SSSNYCNQMMCCRKMTQGKCKPVNTFVHESLADVKAVCSQKKVTCKDGQTNCYQSKSTMRITDCRETGSSKYPNCAYKTTQVEKHIIVACGGKPSvpvhfdasv---------<br>-------------------ketaaakferqhmdsstsaaSSSNYCNQMMKSRNLTKDRCKPVNTFVHESLADVQAVCSQKNVACKNGQTNCYQSYSTMSITDCRETGSSKYPNCAYKTTQANKHIIVACEGNPY---------vpvhfdasv<br><br>SARST: aliSize=118 (resi) RMSD=11.07 (Å)<br>k-ESAAAKFERQHMDsgnsASSS----SNYCNQMMCCRKMTQGKCKPVNTFVHESLADVKAVCSQKKVTCKDGQTNCYQSKSTMRITDCRETGSSKYPNCAYKTTQVEKHIIVACGGKPSVPVHFDAS-v<br>-kETAAAKFERQHMD----SSTSaassSNYCNQMMKSRNLTKDRCKPVNTFVHESLADVQAVCSQKNVACKNGQTNCYQSYSTMSITDCRETGSSKYPNCAYKTTQANKHIIVACEGNPYVPVHFDASv-<br><br>BLAST: aliSize=109 (resi) iden=82.26% (102/124) simi=87.90% (109/124)<br>KESAAAKFERQHMDSGNSASSSSNYCNQMMCCRKMTQGKCKPVNTFVHESLADVKAVCSQKKVTCKDGQTNCYQSKSTMRITDCRETGSSKYPNCAYKTTQVEKHIIVACGGKPSVPVHFDASV<br>KETAAAKFERQHMDSSTSAASSSNYCNQMMKSRNLTKDRCKPVNTFVHESLADVQAVCSQKNVACKNGQTNCYQSYSTMSITDCRETGSSKYPNCAYKTTQANKHIIVACEGNPYVPVHFDASV<br><br>Proposed: aliSize=113 (resi) RMSD=0.93 (Å)<br>KESAAAKFERQHMDSGNSASSSSNYCNQMMCCRKMTQGKCKPVNTFVHESLADVKAVCSQKKVTCKDGQTNCYQSKSTMRITDCRETGSSKYPNCAYKTTQVEKHIIVACGGK-----------psvpvhfdasv<br>KETAAAKFERQHMDSSTSAASSSNYCNQMMKSRNLTKDRCKPVNTFVHESLADVQAVCSQKNVACKNGQTNCYQSYSTMSITDCRETGSSKYPNCAYKTTQANKHIIVACEGNpyvpvhfdasv-----------
712	3bcoA (124)<br>1js0A (124)	C	69.35	TM-align: aliSize=95 (resi) RMSD=1.56 (Å)<br>kesaaakferqhmdsgnsas-------------------SSSNYCNQMMCCRKMTQGKCKPVNTFVHESLADVKAVCSQKKVTCKDGQTNCYQSKSTMRITDCRETGSSKYPNCAYKTTQVEKHIIVACGGKPSvpvhfdasv---------<br>-------------------ketaaakferqhmdsstsaaSSSNYCNQMMKSRNLTKDRCKPVNTFVHESLADVQAVCSQKNVACKNGQTNCYQSYSTMSITDCRETGSSKYPNCAYKTTQANKHIIVACEGNPY---------vpvhfdasv<br><br>SARST: aliSize=118 (resi) RMSD=11.07 (Å)<br>k-ESAAAKFERQHMDsgnsASSS----SNYCNQMMCCRKMTQGKCKPVNTFVHESLADVKAVCSQKKVTCKDGQTNCYQSKSTMRITDCRETGSSKYPNCAYKTTQVEKHIIVACGGKPSVPVHFDAS-v<br>-kETAAAKFERQHMD----SSTSaassSNYCNQMMKSRNLTKDRCKPVNTFVHESLADVQAVCSQKNVACKNGQTNCYQSYSTMSITDCRETGSSKYPNCAYKTTQANKHIIVACEGNPYVPVHFDASv-<br><br>BLAST: aliSize=109 (resi) iden=82.26% (102/124) simi=87.90% (109/124)<br>KESAAAKFERQHMDSGNSASSSSNYCNQMMCCRKMTQGKCKPVNTFVHESLADVKAVCSQKKVTCKDGQTNCYQSKSTMRITDCRETGSSKYPNCAYKTTQVEKHIIVACGGKPSVPVHFDASV<br>KETAAAKFERQHMDSSTSAASSSNYCNQMMKSRNLTKDRCKPVNTFVHESLADVQAVCSQKNVACKNGQTNCYQSYSTMSITDCRETGSSKYPNCAYKTTQANKHIIVACEGNPYVPVHFDASV<br><br>Proposed: aliSize=104 (resi) RMSD=0.83 (Å)<br>kesaaakferqhmdsgnsas-------------------SSSNYCNQMMCCRKMTQGKCKPVNTFVHESLADVKAVCSQKKVTCKDGQTNCYQSKSTMRITDCRETGSSKYPNCAYKTTQVEKHIIVACGGKPSVPVHFDASV<br>-------------------ketaaakferqhmdsstsaaSSSNYCNQMMKSRNLTKDRCKPVNTFVHESLADVQAVCSQKNVACKNGQTNCYQSYSTMSITDCRETGSSKYPNCAYKTTQANKHIIVACEGNPYVPVHFDASV
713	3bcoA (124)<br>1r3mB (122)	N	96.72	TM-align: aliSize=104 (resi) RMSD=0.99 (Å)<br>kesaaakferqhmdsgnsas-----------------SSSNYCNQMMCCRKMTQGKCKPVNTFVHESLADVKAVCSQKKVTCKDGQTNCYQSKSTMRITDCRETGSSKYPNCAYKTTQVEKHIIVACGGKPSVPVHFDASV<br>-------------------kesaaakferqhmdsgnsPSSNYCNLMMCCRKMTQGKCKPVNTFVHESLADVKAVCSQKKVTCKNGQTNCYQSKSTMRITDCRETGSSKYPNCAYKTTQVEKHIIVACGGKPSVPVHFDASV<br><br>SARST: aliSize=120 (resi) RMSD=9.52 (Å)<br>k-ESAAAKFERQHMDSGnsASSSSNYCNQMMCCRKMTQGKCKPVNTFVHESLADVKAVCSQKKVTCKDGQTNCYQSKSTMRITDCRETGSSKYPNCAYKTTQVEKHIIVACGGKPSVPVHFDAS-v<br>-kESAAAKFERQHMDSG--NSPSSNYCNLMMCCRKMTQGKCKPVNTFVHESLADVKAVCSQKKVTCKNGQTNCYQSKSTMRITDCRETGSSKYPNCAYKTTQVEKHIIVACGGKPSVPVHFDASv-<br><br>BLAST: aliSize=120 (resi) iden=97.54% (119/122) simi=98.36% (120/122)<br>KESAAAKFERQHMDSGNSASSsNYCNQMMCCRKMTQGKCKPVNTFVHESLADVKAVCSQKKVTCKDGQTNCYQSKSTMRITDCRETGSSKYPNCAYKTTQVEKHIIVACGGKPSVPVHFDASV<br>KESAAAKFERQHMDSGNSPSS--NYCNLMMCCRKMTQGKCKPVNTFVHESLADVKAVCSQKKVTCKNGQTNCYQSKSTMRITDCRETGSSKYPNCAYKTTQVEKHIIVACGGKPSVPVHFDASV<br><br>Proposed: aliSize=122 (resi) RMSD=0.55 (Å)<br>KESAAAKFERQHMDSGNsASsSSNYCNQMMCCRKMTQGKCKPVNTFVHESLADVKAVCSQKKVTCKDGQTNCYQSKSTMRITDCRETGSSKYPNCAYKTTQVEKHIIVACGGKPSVPVHFDASV<br>KESAAAKFERQHMDSGN-SP-SSNYCNLMMCCRKMTQGKCKPVNTFVHESLADVKAVCSQKKVTCKNGQTNCYQSKSTMRITDCRETGSSKYPNCAYKTTQVEKHIIVACGGKPSVPVHFDASV

714	3bcoA<br>(124)<br>1a2wA<br>(124)	N	82.26	TM-align: aliSize=104 (resi) RMSD=0.72 (Å)<br>kesaaakferqhmdsgnsas--------------------SSSNYCNQMMCCRKMTQGKCKPVNTFVHESLADVKAVCSQKKVTCKDGQTNCYQSKSTMRITDCRETGSSKYPNCAYKTTQVEKHIIVACGGKPSVPVHFDASV<br>--------------------ketaaakferqhmdsstsaaSSSNYCNQMMKSRNLTKDRCKPVNTFVHESLADVQAVCSQKNVACKNGQTNCYQSYSTMSITDCRETGSSKYPNCAYKTTQANKHIIVACEGNPYVPVHFDASV<br><br>SARST: aliSize=120 (resi) RMSD=12.05 (Å)<br>k-ESAAAKFERQHMDsgNSASSS--SNYCNQMMCCRKMTQGKCKPVNTFVHESLADVKAVCSQKKVTCKDGQTNCYQSKSTMRITDCRETGSSKYPNCAYKTTQVEKHIIVACGGKPSVPVHFDAS-v<br>-kETAAAKFERQHMD--SSTSAAssSNYCNQMMKSRNLTKDRCKPVNTFVHESLADVQAVCSQKNVACKNGQTNCYQSYSTMSITDCRETGSSKYPNCAYKTTQANKHIIVACEGNPYVPVHFDASv-<br><br>BLAST: aliSize=109 (resi) iden=82.26% (102/124) simi=87.90% (109/124)<br>KESAAAKFERQHMDSGNSASSSSNYCNQMMCCRKMTQGKCKPVNTFVHESLADVKAVCSQKKVTCKDGQTNCYQSKSTMRITDCRETGSSKYPNCAYKTTQVEKHIIVACGGKPSVPVHFDASV<br>KETAAAKFERQHMDSSTSAASSSNYCNQMMKSRNLTKDRCKPVNTFVHESLADVQAVCSQKNVACKNGQTNCYQSYSTMSITDCRETGSSKYPNCAYKTTQANKHIIVACEGNPYVPVHFDASV<br><br>Proposed: aliSize=124 (resi) RMSD=1.01 (Å)<br>KESAAAKFERQHMDSGNSASSSSNYCNQMMCCRKMTQGKCKPVNTFVHESLADVKAVCSQKKVTCKDGQTNCYQSKSTMRITDCRETGSSKYPNCAYKTTQVEKHIIVACGGKPSVPVHFDASV<br>KETAAAKFERQHMDSSTSAASSSNYCNQMMKSRNLTKDRCKPVNTFVHESLADVQAVCSQKNVACKNGQTNCYQSYSTMSITDCRETGSSKYPNCAYKTTQANKHIIVACEGNPYVPVHFDASV
715	3bcoA<br>(124)<br>1h8xA<br>(125)	N	74.19	TM-align: aliSize=104 (resi) RMSD=1.40 (Å)<br>kesaaakferqhmdsgnsas--------------------SSSNYCNQMMCCRKMTQGKCKPVNTFVHESLADVKAVCSQKKVTCKDGQTNCYQSKSTMRITDCRETGSSKYPNCAYKTTQVEKHIIVACGGKPSVPVHFDASV-<br>--------------------kesaaakferqhmdsgnspsSSSTYCNQMMRRRNMTQGRCKPVNTFVHEPLVDVQNVCFQEKVTCKNGQGNCYKSNSSMHITDCRLTNGSRYPNCAYRTSQKERHIIVACEGSPYVPVHFDASVe<br><br>SARST: aliSize=120 (resi) RMSD=11.51 (Å)<br>k-ESAAAKFERQHMD--SGNSASSssNYCNQMMCCRKMTQGKCKPVNTFVHESLADVKAVCSQKKVTCKDGQTNCYQSKSTMRITDCRETGSSKYPNCAYKTTQVEKHIIVACGGKPSVPVHFDAS--v<br>-kESAAAKFERQHMDsgNSPSSSS--TYCNQMMRRRNMTQGRCKPVNTFVHEPLVDVQNVCFQEKVTCKNGQGNCYKSNSSMHITDCRLTNGSRYPNCAYRTSQKERHIIVACEGSPYVPVHFDASve-<br><br>BLAST: aliSize=105 (resi) iden=76.61% (95/124) simi=84.68% (105/124)<br>KESAAAKFERQHMDSGNSASSSSNYCNQMMCCRKMTQGKCKPVNTFVHESLADVKAVCSQKKVTCKDGQTNCYQSKSTMRITDCRETGSSKYPNCAYKTTQVEKHIIVACGGKPSVPVHFDASV-<br>KESAAAKFERQHMDSGNSPSSSSTYCNQMMRRRNMTQGRCKPVNTFVHEPLVDVQNVCFQEKVTCKNGQGNCYKSNSSMHITDCRLTNGSRYPNCAYRTSQKERHIIVACEGSPYVPVHFDASVe<br><br>Proposed: aliSize=123 (resi) RMSD=1.09 (Å)<br>KESAAAKFERQHMDSGnSAS-SSSNYCNQMMCCRKMTQGKCKPVNTFVHESLADVKAVCSQKKVTCKDGQTNCYQSKSTMRITDCRETGSSKYPNCAYKTTQVEKHIIVACGGKPSVPVHFDASV-<br>KESAAAKFERQHMDSG-NSPsSSSTYCNQMMRRRNMTQGRCKPVNTFVHEPLVDVQNVCFQEKVTCKNGQGNCYKSNSSMHITDCRLTNGSRYPNCAYRTSQKERHIIVACEGSPYVPVHFDASVe
716	1a2wA<br>(124)<br>1lbaA<br>(124)	N	80.65	TM-align: aliSize=104 (resi) RMSD=0.77 (Å)<br>ketaaakferqhmdsstsaa--------------------SSSNYCNQMMKSRNLTKDRCKPVNTFVHESLADVQAVCSQKNVACKNGQTNCYQSYSTMSITDCRETGSSKYPNCAYKTTQANKHIIVACEGNPYVPVHFDASV<br>--------------------kesaaakferqhmdsgnspsSSSNYCNLMMCCRKMTQGKCKPVNTFVHESLADVKAVCSQKKVTCKNGQTNCYQSKSTMRITDCRETGSSKYPNCAYKTTQVEKHIIVACGGKPSVPVHFDASV<br><br>SARST: aliSize=121 (resi) RMSD=12.23 (Å)<br>k-ETAAAKFERQHMDsSTSAASS-SNYCNQMMKSRNLTKDRCKPVNTFVHESLADVQAVCSQKNVACKNGQTNCYQSYSTMSITDCRETGSSKYPNCAYKTTQANKHIIVACEGNPYVPVHFDAS-v<br>-kESAAAKFERQHMD-SGNSPSSsSNYCNLMMCCRKMTQGKCKPVNTFVHESLADVKAVCSQKKVTCKNGQTNCYQSKSTMRITDCRETGSSKYPNCAYKTTQVEKHIIVACGGKPSVPVHFDASv-<br><br>BLAST: aliSize=107 (resi) iden=81.45% (101/124) simi=86.29% (107/124)<br>KETAAAKFERQHMDSSTSAASSSNYCNQMMKSRNLTKDRCKPVNTFVHESLADVQAVCSQKNVACKNGQTNCYQSYSTMSITDCRETGSSKYPNCAYKTTQANKHIIVACEGNPYVPVHFDASV<br>KESAAAKFERQHMDSGNSPSSSSNYCNLMMCCRKMTQGKCKPVNTFVHESLADVKAVCSQKKVTCKNGQTNCYQSKSTMRITDCRETGSSKYPNCAYKTTQVEKHIIVACGGKPSVPVHFDASV<br><br>Proposed: aliSize=123 (resi) RMSD=0.86 (Å)<br>KETAAAKFERQHMDSsTSAAS-SSNYCNQMMKSRNLTKDRCKPVNTFVHESLADVQAVCSQKNVACKNGQTNCYQSYSTMSITDCRETGSSKYPNCAYKTTQANKHIIVACEGNPYVPVHFDASV<br>KESAAAKFERQHMDS-GNSPSsSSNYCNLMMCCRKMTQGKCKPVNTFVHESLADVKAVCSQKKVTCKNGQTNCYQSKSTMRITDCRETGSSKYPNCAYKTTQVEKHIIVACGGKPSVPVHFDASV

717	3b8iA (283)<br>1oqfA (290)	C	26.15	TM-align: aliSize=249 (resi) RMSD=2.12 (Å)<br>ra-SHHeLRAMF-ALLDSSRCYHTASVFDPMSARIAADLGFECGILGGSVASLQVLAAPDFALITLSEFVEQATRIGRVARLPVIADADHGYGN-ALNVMRTVVELERAGIAALTIEDTLLPAQ-FGRKSTDLICVEEGVGKIRAALEARVDPALTIIARTN-AELIDVDAVIQRTLAYQEAGADGICLV<br>--sLHS-PGKAFrAALTKENPLQIVGTINANHALLAQRAGYQAIYLSGGVAAGSLGLPDLGISTLDDVLTDIRRITDVCSLPLLVDADIGFGSsAFNVARTVKSMIKAGAAGLHIEDQVGAKRcGHRPNKAIVSKEEMVDRIRAAVDAKTDPDFVIMARTDaLAVEGLDAAIERAQAYVEAGAEMLFPE<br><br>GVRDFAHLEAIAEHLHIPLMLVTYG---NPqLRDDARLARLGVRVVVNGHAAYFAAIKATYDCLREERGAVasdltaselskkytfpeeyqawardymevk--------------------------------<br>AITELAMYRQFADAVQVPILANITEfgaTP-LFTTDELRSAHVAMALYPLSAFRAMNRAAEHVYNVLRQEG------------------------------tqksvidtmqtrnelyesinyyqyeekldnlfa<br><br>SARST: aliSize=271 (resi) RMSD=11.39 (Å)<br>rashh----ELRAMFRALLDSSRCYHTAsVFDPMSARIAADLGFECGILGGSVASLQVLAAPDFALITLSEFVEQATRIGRVARLPVIADADHGYG-NALNVMRTVVELERAGIAALTIEDTLLP----AQFGrksTDLICVEEGVGKIRAALEARVDPALTIIARTNAEL-IDVDAVIQRTLAYQEAGA<br>-----slhsPGKAFRAALTKENPLQIVG-TINANHALLAQRAGYQAIYLSGGVAAGSLGLPDLGISTLDDVLTDIRRITDVCSLPLLVDADIGFGsSAFNVARTVKSMIKAGAAGLHIEDQVGAkrcgHRPN---KAIVSKEEMVDRIRAAVDAKTDPDFVIMARTDALAvEGLDAAIERAQAYVEAGA<br><br>DGICLVGVRDFAHLEAIAEHLHIPLML-VTYGNPQLR-DDARLARLGVRVVV--NGHAAYFAAIKATYDCLREERGA---VASDLTASELSKKYTFPEEYQAWARDY--mevk<br>EMLFPEAITELAMYRQFADAVQVPILAnITEFGATPLfTTDELRSAHVAMALypLSAFRAMNRAAEHVYNVLRQEGTqksVIDTMQTRNELYESINYYQYEEKLDNLfa----<br><br>BLAST: aliSize=144 (resi) iden=30.63% (87/283) simi=50.70% (144/283)<br>raSHHELRAMFRALLDSSRCYHTASVFDPMSARIAADLGFECGIL-GGSVASLQvLAAPDFALITLSEFVEQATRIGRVARLPVIADADHGYGN-ALNVMRTVVELERAGIAALTIEDTLLPAQFG-RKSTDLICVEEGVGKIRAALEARVDPALTIIARTNAELID-VDAVIQRTLAYQEAGADGICLV<br>--SLHSPGKAFRAALTKENPLQIVGTINANHALLAQRAGYQAIYLsGGGVAAGS-LGLPDLGISTLDDVLTDIRRITDVCSLPLLVDADIGFGSsAFNVARTVKSMIKAGAAGLHIEDQVGAKRCGhRPNKAIVSKEEMVDRIRAAVDAKTDPDFVIMARTDALAVEgLDAAIERAQAYVEAGAEMLFPE<br><br>GVRDFAHLEAIAEHLHIPLM--LVTYGNPQL--RDDARLARLGVRVV-VNGHAAYFAAIKATYDCLREE---RGAVASDLTASELSKKYTFpEEYQ--------awardymevk<br>AITELAMYRQFADAVQVPILanITEFGATPLftTDELRSAHVAMALYpLSAFRAMNRAAEHVYNVLRQEgtqKSVIDTMQTRNELYESINY-YQYEekldnlfa----------<br><br>Proposed: aliSize=268 (resi) RMSD=1.91 (Å)<br>ra--ShHeLRAMF-rALLDSSRCYHTASVFDPMSARIAADLGFECGILGGSVASLQVLAAPDFALITLSEFVEQATRIGRVARLPVIADADHGYGN-ALNVMRTVVELERAGIAALTIEDTLLpAQ---FGrKSTDLICVEEGVGKIRAALEARVDPALTIIARTN-AELIDVDAVIQRTLAYQEAGADG<br>--slH-S-PGKAFr-AALTKENPLQIVGTINANHALLAQRAGYQAIYLSGGVAAGSLGLPDLGISTLDDVLTDIRRITDVCSLPLLVDADIGFGSsAFNVARTVKSMIKAGAAGLHIEDQVG-AKrcgHR-PNKAIVSKEEMVDRIRAAVDAKTDPDFVIMARTDaLAVEGLDAAIERAQAYVEAGAEM<br><br>ICLVGVRDFAHLEAIAEHLHIPLMLVTYG---NPqLRDDARLARLGVRVVVNGHAAYFAAIKATYDCLREERG----avasdlTASELSKkyTFPEEYQA-WARDYME--VK----<br>LFPEAITELAMYRQFADAVQVPILANITEfgaTP-LFTTDELRSAHVAMALYPLSAFRAMNRAAEHVYNVLRQegtq------KSVIDTM--QTRNELYEsINYYQYEekLDnlfa
718	3b8iA (283)<br>1o5qA (271)	C	25.83	TM-align: aliSize=238 (resi) RMSD=1.99 (Å)<br>rashHeLRAMF-ALLDSSRCYHTASVFDPMSARIAADLGFECGILGGSVASLQVLAAPDFALITLSEFVEQATRIGRVARLPVIADADHGYGN-ALNVMRTVVELERAGIAALTIEDTLlpaqfgrkstDLICVEEGVGKIRAALEARVDPALTIIARTN-AELIDVDAVIQRTLAYQEAGADGICLVGV<br>----S-PGQAFrAALAKENPLQIVGAINANHALLAQRAGYQAIYLSGGVAAGSLGLPDLGISTLDDVLTDIRRITDVCPLPLLVDADIGFGSsAFNVARTVKSIAKAGAAALHIEDQV----------AIVSKEEMVDRIRAAVDARTDPNFVIMARTDaLAVEGLEAALDRAQAYVDAGADMLFPEAI<br><br>RDFAHLEAIAEHLHIPLMLVTYG--NPQLRDDARLARLGVRVVVNGHAAYFAAIKATYDCLREERGAVasdltaselskkytfpeeyqawardymevk----------------------------<br>TELSMYRRFADVAQVPILANITEfgATPLFTTDELRSAHVAMALYPLSAFRAMNRAAEKVYTVLRQEG-----------------------------tqknvidimqtrnelyesinyyqfeekl<br><br>SARST: aliSize=260 (resi) RMSD=9.31 (Å)<br>rashh-ELRAMFRALLDSSRCYHTAsVFDPMSARIAADLGFECGILGGSVASLQVLAAPDFALITLSEFVEQATRIGRVARLPVIADADHGYG-NALNVMRTVVELERAGIAALTIEDTllpaqfgrksTDLICVEEGVGKIRAALEARVDPALTIIARTNAE-LIDVDAVIQRTLAYQEAGADGICLVG<br>-----sPGQAFRAALAKENPLQIVG-AINANHALLAQRAGYQAIYLSGGVAAGSLGLPDLGISTLDDVLTDIRRITDVCPLPLLVDADIGFGsSAFNVARTVKSIAKAGAAALHIEDQ----------VAIVSKEEMVDRIRAAVDARTDPNFVIMARTDALaVEGLEAALDRAQAYVDAGADMLFPEA<br><br>VRDFAHLEAIAEHLHIPLMLVTY-GNPQLR-DDARLARLGVRVVVNGHAAYFAAIKATYDCLREER--GA---VASDLTASELSKKYTFPEEYQAW-ardymevk<br>ITELSMYRRFADVAQVPILANITeFGATPLfTTDELRSAHVAMALYPLSAFRAMNRAAEKVYTVLRqeGTqknVIDIMQTRNELYESINYYQFEEKl--------<br><br>BLAST: aliSize=135 (resi) iden=31.00% (84/271) simi=49.82% (135/271)<br>rashhelram-----FRALLDSSRCYHTASVFDPMSARIAADLGFECGIL-GGSVASLQvLAAPDFALITLSEFVEQATRIGRVARLPVIADADHGYGN-ALNVMRTVVELERAGIAALTIEDTLLpaqfgrkstdLICVEEGVGKIRAALEARVDPALTIIARTNAELID-VDAVIQRTLAYQEAGADG<br>----------spgqaFRAALAKENPLQIVGAINANHALLAQRAGYQAIYLsGGGVAAGS-LGLPDLGISTLDDVLTDIRRITDVCPLPLLVDADIGFGSsAFNVARTVKSIAKAGAAALHIEDQVA----------IVSKEEMVDRIRAAVDARTDPNFVIMARTDALAVEgLEAALDRAQAYVDAGADM<br><br>ICLVGVRDFAHLEAIAEHLHIPLM--LVTYGNPQL--RDDARLARLGVRVV-VNGHAAYFAAIKATYDCLREE---RGAVASDLTASELSKK---YTFPEE-yqawardymevk<br>LFPEAITELSMYRRFADVAQVPILanITEFGATPLftTDELRSAHVAMALYpLSAFRAMNRAAEKVYTVLRQEgtqKNVIDIMQTRNELYESinyYQFEEKl------------<br><br>Proposed: aliSize=255 (resi) RMSD=1.63 (Å)<br>rashHeLRAMF-rALLDS-sRCYHTASVFDPMSARIAADLGFECGILGGSVASLQVLAAPDFALITLSEFVEQATRIGRVARLPVIADADHGYGN-ALNVMRTVVELERAGIAALTIEDTLlpaqfgrkstDLICVEEGVGKIRAALEARVDPALTIIARTN-AELIDVDAVIQRTLAYQEAGADGICLV<br>----S-PGQAFr-AALAKe-NPLQIVGAINANHALLAQRAGYQAIYLSGGVAAGSLGLPDLGISTLDDVLTDIRRITDVCPLPLLVDADIGFGSsAFNVARTVKSIAKAGAAALHIEDQV----------AIVSKEEMVDRIRAAVDARTDPNFVIMARTDaLAVEGLEAALDRAQAYVDAGADMLFPE<br><br>GVRDFAHLEAIAEHLHIPLMLVTYG----NPqLR--DDARLarLgVRVVVNGHAAYFAAIKATYDCLREERG----avasdlTASELSKkyTFPEEYQA-WARDYMEVK-<br>AITELSMYRRFADVAQVPILANITEfgatPL-FTtdELRSA--H-VAMALYPLSAFRAMNRAAEKVYTVLRQegtq------KNVIDIM--QTRNELYEsINYYQFEEKl
719	3b8iA (283)<br>1zlpA (284)	C	23.67	TM-align: aliSize=244 (resi) RMSD=2.47 (Å)<br>rashHelRAMFALLDSSRCYHTASVFDPMSARIAADLGFECGILGGSVASLQVLAAPDFALITLSEFVEQATRIGRVA-RLPVIADADHGYGNALNVMRTVVELERAGIAALTIEDTLLPAQfgr----KSTDLICVEEGVGKIRAALEARVDPALTIIARTN-AELIDVDAVIQRTLAYQEAGADGICL<br>----K--TTMHRLIEEHGSVLMPGVQDALSAAVVEKTGFHAAFVSGYSVSAAMLGLPDFGLLTTTEVVEATRRITAAApNLCVVVDGDTGGGPLNVQRFIRELISAGAKGVFLEDQVWPKK---cghmRGKAVVPAEEHALKIAAAREAIGDSDFFLVARTDaRAPHGLEEGIRRANLYKEAGADATFV<br><br>VGVRDFAHLEAIAEHLHIPLMLVTYG--NPQLRDDARLARLGVRVVVNGHAAYFAAIKATYDCLREERGAVasdltaselskkytfpeeyqawardymevk--------------------------------<br>EAPANVDELKEVSAKTKGLRIANMIEggKTPLHTPEEFKEMGFHLIAHSLTAVYATARALVNIMKILKEKG-----------------------------ttrddldqmatfsefnelisleswyemeskfk<br><br>SARST: aliSize=271 (resi) RMSD=11.18 (Å)<br>rashhelr--AMFRALLDSSRCYHTAsVFDPMSARIAADLGFECGILGGSVASLQVLAAPDFALITLSEFVEQATRIGRVAR-LPVIADADHGYGNALNVMRTVVELERAGIAALTIEDTLLPAQFGRKSTDLICVEEGVGKIRAALEARVDPALTIIARTNA--ELIDVDAVIQRTLAYQEAGADGICL<br>--------ktTMHRLIEEHGSVLMPG-VQDALSAAVVEKTGFHAAFVSGYSVSAAMLGLPDFGLLTTTEVVEATRRITAAAPnLCVVVDGDTGGGPLNVQRFIRELISAGAKGVFLEDQVWPKKCGHMRGKAVVPAEEHALKIAAAREAIGDSDFFLVARTDarAPHGLEEGIRRANLYKEAGADATFV<br><br>VGVRDFAHLEAIAEHLHIPLMLVT-YGNP-QLRDDARLARLGVRVVV--NGHAAYFAAIKATYDCLREERGAVASD---LTASELSKKYTFPEEYQAWARDY-mevk<br>EAPANVDELKEVSAKTKGLRIANMiEGGKtPLHTPEEFKEMGFHLIAhsLTAVYATARALVNIMKILKEKGTTRDDldqMATFSEFNELISLESWYEMESKFk----<br><br>BLAST: aliSize=120 (resi) iden=27.46% (78/283) simi=42.25% (120/283)<br>rashhelramfralldssrcyhtas-----------------VFDPMSARIAADLGFECGILGGSVASLQVLAAPDFALITLSEFVEQATRIGRVA-RLPVIADADHGYGNALNVMRTVVELERAGIAALTIEDTLLPAQFGR-KSTDLICVEEGVGKIRAALEARVDPALTIIARTNAEL-IDVDAVI<br>-------------------------kttmhrlieehgsvlmpgVQDALSAAVVEKTGFHAAFVSGYSVSAAMLGLPDFGLLTTTEVVEATRRITAAApNLCVVVDGDTGGGPLNVQRFIRELISAGAKGVFLEDQVWPKKCGHmRGKAVVPAEEHALKIAAAREAIGDSDFFLVARTDARApHGLEEGI<br><br>QRTLAYQEAGADGICL---VGVRDFAHLEAIAEHLHIPLMlVTYGNPQLRDDARLARLGVRVVVNGHAAYFAAIKATYDCLR--EERGAVASDL----TASELSKKYTFPEEYQ------awardymevk<br>RRANLYKEAGADATFVeapANVDELKEVSAKTKGLRIANM-IEGGKTPLHTPEEFKEMGFHLIAHSLTAVYATARALVNIMKilKEKGTTRDDLdqmaTFSEFNELISLESWYEmeskfk----------<br><br>Proposed: aliSize=260 (resi) RMSD=1.95 (Å)<br>rashhel-RAmF-rALLDSSRCYHTASVFDPMSARIAADLGFECGILGGSVASLQVLAAPDFALITLSEFVEQATRIGRVA-RLPVIADADHGYGNALNVMRTVVELERAGIAALTIEDTLLPA------qfgrkSTDLICVEEGVGKIRAALEARVDPALTIIARTN-AELIDVDAVIQRTLAYQEAGA<br>-------kTT-Mh-RLIEEHGSVLMPGVQDALSAAVVEKTGFHAAFVSGYSVSAAMLGLPDFGLLTTTEVVEATRRITAAApNLCVVVDGDTGGGPLNVQRFIRELISAGAKGVFLEDQVWPKkcghmr-----GKAVVPAEEHALKIAAAREAIGDSDFFLVARTDaRAPHGLEEGIRRANLYKEAGA<br><br>DGICLVGVRDFAHLEAIAEHLHIPLMLVTYG---NPqLRDDARLARLGVRVVVNGHAAYFAAIKATYDCLREERG----------avasdlTASE-LSKKYTFPEEYQAWARDYmEvk<br>DATFVEAPANVDELKEVSAKTKGLRIANMIEggkTP-LHTPEEFKEMGFHLIAHSLTAVYATARALVNIMKILKEkgttrddldq------MATFsEFNELISLESWYEMESKF-K--

720	3b8iA (283)<br>1f61A (418)	C	19.43	TM-align: aliSize=251 (resi) RMSD=2.52 (Å)<br>r--ASHHFLRAMF-ALIDSSRCYHTASVFDPMSARIAADLGFFCGILGGSVASLQV----LAAPDFALITLSFFVFQATRIGRVA------------------RLPVIADADHGYGNALNVMRTVVELERAGIAALTIEDTLLPAQ<br>-asvvgtpksaeqiqqewdtnprwkdvtrtysaedvvalqgsvveehTLARRGAEVLwEQLHDLEWVNALGALTGNMAVQQVRAGLKAIYLSGWQVAGDAnlsgHTYPDQSLYPANSVPQVVRRINNALqradqiakiegdtsvenwLAPIVADGEAGFGGALNVYELQKALIAAGVAGSHWEDQLASEK<br>FG--RKSTDLICVFFGVGKIRAALEAR--VDPALTIIARTNAFL-----------------------------IDVDAVIQRTLAYQEaGADGICLVG-VRDFAHLFAIAF--H--L-HIPLMLVTYG---N---P---QL-RDDARLARLGVRVVVNGHAAYFAAIKATYDCLREERGAVasdltasel<br>KCghLGGKVLIPTQQHIRTLTSARLAAdvADVPTVVIARTDAEAatlitsdvderdqpfitgertregfyrtkNGIEPCIARAKAYAP-FADLIWMETgTPDLEAARQFSEavKaeYpDQMLAYNCSPsfnWkkhLddaTIaKFQKELAAMGFKFQFITLAGFHALNYSMFDLAYGYAQNQ---------<br>skkytfpeeyqawardymevk---<br>--------------------msayvelqerefaaeergytatkhqrevgagyfdriattvdpnssttal<br>SARST: aliSize=270 (resi) RMSD=14.13 (Å)<br>ras--HHELRAMFRALLDSSRCYHTaSVFDPMSARIAADLGFFCGILGGSVASLQ--VLA--APDFALIT------------LSFFVFQATRIGRVAR------LPVIADADHGYGNALNVMRTVVELERAGIAALTIEDTL-<br>---asvvgtpksaeqiqqewdtnprwkdvtrtysaedvvalqgsvveehtlaRRGAEVLWEQLHDLEWVNAL-GALTGNMAVQQVRAGLKAIYLSGWQVAGDanLSGhtYPDQSLYPansvpqvvrrinNALQRADQIAKIEGDTsvenwlAPIVADGEAGFGGALNVYELQKALIAAGVAGSHWEDQLa<br>--LPAQFGRKSTDLICVFFGVGKIRAALEA----RVDpalTIIARTNAFLI-------------------DV---------DAVIQRTLAYQEAGADGICL-VGVRDFAHLFAIAF-------HLHIPLMLVTYGNPQLRD---------DARLARLGVRVVV--------------------NGHAAYFA<br>seKKCGHLGGKVLIPTQQHIRTLTSARLAAdvadVPT---VVIARTDAEAAtlitsdvderdqpfitgerTRegfyrtkngIEPCIARAKAYAPFADLIWMeTGTPDLEAARQFSEavkaeypDQMLAYNCSPSFNWKKHLddatiakfQKELAAMGFKFQFitlagfhalnysmfdlaygyAQNQMSAY<br>AIKATYDCLREERGAVasdltaSELSKKYTFPEEYQAWARD--YMEV----k<br>VELQEREFAAEERGYT------ATKHQREVGAGYFDRIATTvdPNSSttal-<br>BLAST: aliSize=64 (resi) iden=15.49% (44/283) simi=22.54% (64/283)<br>rashhelramfralldssrcyhtasvfdpmsariaadlgfecgilggsv--ASLQVLAAPDFALITLSFFVFQATRIG-------RVARL----<br>---asvvgtpksaeqiqqewdtnprwkdvtrtysaedvvalqgsvveehtlarrgaevlweqlhdlewvnalgaltgnmavqqvraglkaiylsgwqvagdANLSGHTYPDQSLYPANSVPQVVRRINnalqradQIAKIegdt<br>-------PVIADADHGYGNALNVMRTVVELERAGIAALTIEDTLLP----AQFGRKStdLICVFFGVGKIRAA-LEARV-DPALTIIARTNAF---LI--DVD---<br>svenwlaPIVADGEAGFGGALNVYELQKALIAAGVAGSHWEDQLASekkcGHLGGKV--LIPTQQHIRTLTSArLAADVaDVPTVVIARTDAEaatLItsDVDerdqpfitgertregfyrtkngiepciarakayapfadliwmetgtpdleaarqfseavkaeypdqmlayncspsfnwkkhlddati<br>---aviqrtlayqeagadgiclvgvrdfahleaiaehlhiplmlvtygnpqlrddarlarlgvrvvvnghaayfaaikatydclreergavasdltaselskky<br>akfqkelaamgfkfqfitlagfhalnysmfdlaygyaqnqmsayvelqerefaaeergytatkhqrevgagyfdriattvdpnssttal---<br>tfpeeyqawardymevk<br>-----------------<br>Proposed: aliSize=262 (resi) RMSD=2.12 (Å)<br>ra---SH-HFLRAMF-rALIDSSRCYHTASVFDPMSARIAADLGFFCGILGGSVASLQV----LAAPDFALITLSFFVFQATRIGRVA------------------RLPVIADADHGYGNALNVMRTVVELERAGIAALTIEDTLLP<br>--asvvgtpksaeqiqqewdtnprwkdvtrtysaedvvalqgsvveehTLaRRGAEVLw-EQLHDLEWVNALGALTGNMAVQQVRAGLKAIYLSGWQVAGDAnlsgHTYPDQSLYPANSVPQVVRRINNALqradqiakiegdtsvenwLAPIVADGEAGFGGALNVYELQKALIAAGVAGSHWEDQLAS<br>AQfgR----KSTDLICVFFGVGKIRAALEAR--VDPALTIIARTNAFL-----------------------------IDVDAVIQRTLAYQEaGADGICLVG--VRDF--AHL-FAIAEHL-HIPLMLVTYG---N--------P--qlRDDaRLARLGVRVVVNGHAAYFAAIKATY-----------<br>EK--KcghlGGKVLIPTQQHIRTLTSARLAAdvADVPTVVIARTDAEAatlitsdvderdqpfitgertregfyrtkNGIEPCIARAKAYAP-FADLIWMETgtPDLEaaRQFsEAVKAEYpDQMLAYNCSPsfnWkkhlddatIak--FQK-ELAAMGFKFQFITLAGFHALNYSMFdlaygyaqnqms<br>-----------dclreergavASD---LtASELSKKYTFPEEYQAWARDYMEV------k<br>ayvelqerefa----------AEErgyT-ATK-HQREVGAGYFDRIATTVDPNssttal-
721	3b8iA (283)<br>1f8mA (427)	C	19.08	TM-align: aliSize=248 (resi) RMSD=2.50 (Å)<br>r--ASHHFLRAMF-ALIDSSRCYHTASVFDPMSARIAADLGFFCGILGGSVASLQ-V---LAAPDFALITLSFFVFQATRIGRVA------------------RLPVIADADHGYGNALNVMRTVVELERAGIAALTIEDTLLPAQ<br>-asvvgtpksaeqiqqewdtnprwkdvtrtysaedvvalqgsvveehTLARRGAEVLwEQLHDLEWVNALGALTGNMAVQQVRAGLKAIYLSGWQVAGDaNlsgHTYPDQSLYPANSVPQVVRRINNALqradqiakiegdtsvenwLAPIVADGEAGFGGALNVYELQKALIAAGVAGSHWEDQLASEK<br>fgr-----KSTDLICVFFGVGKIRAALEAR--VDPALTIIARTNAFL-----------------------------IDVDAVIQRTLAYQEaGADGICLVGVR-DFAHLFAIAF-HL----HIPLMLVTY--G-N---P---QL-RDDARLARLGVRVVVNGHAAYFAAIKATYDCLREERGAVasdlta<br>---kcghlGGKVLIPTQQHIRTLTSARLAAdvADVPTVVIARTDAEAatlitsdvderdqpfitgertregfyrtkNGIEPCIARAKAYAP-FADLIWMETGTpDLEAARQFSEaVKaeypDQMLAYNCSpsFnWkkhLddaTIaKFQKELAAMGFKFQFITLAGFHALNYSMFDLAYGYAQNQ------<br>selskkytfpeeyqawardymevk--<br>-----------------------msayvelqerefaaeergytatkhqrevgagyfdriattvdpnssttaltgsteegqf<br>SARST: aliSize=259 (resi) RMSD=9.77 (Å)<br>ras--HHELRAMFRALLDSSRCYhTASVFDPMSARIAADLGFFCGILGGSVASLQ--VLA--APDFALIT------------LSFFVFQATRIGRVAR------LPVIADADHGYGNALNVMRTVVELERAGIAALTIEDTL-<br>---asvvgtpksaeqiqqewdtnprwkdvtrtysaedvvalqgsvveehtlaRRGAEVLWEQLHDLEWVN-ALGALTGNMAVQQVRAGLKAIYLSGWQVAGDanLSGhtYPDQSLYPansvpqvvrrinNALQRADQIAKIEGDTsvenwlAPIVADGEAGFGGALNVYELQKALIAAGVAGSHWEDQLa<br>-LPAQFGRKSTDLICVFFGVGKIRAALEARVDP--ALTIIARTNAFL-------------------IDV---------DAVIQRTLAYQEAGADGICLVgVRD------FAHLFAIAEHL-HIPLMLV--TYGNPQLRD---------DARLARLGVRVVvnGHAAYFAAIKATYDClreergavasdltA<br>sEKKCGHLGGKVLIPTQQHIRTLTSARLAADVAdvPTVVIARTDAEAatlitsdvderdqpfitgeRTRegfyrtkngIEPCIARAKAYAPFADLIWME-TGTpdleaaRQFSEAVKAEYpDQMLAYNcsPSFNWKKHLddatiakfQKELAAMGFKFQ--FITLAGFHALNYSMF-------------D<br>SELSKKYTFPEEYQAWARD--ymevk<br>LAYGYAQNQMSAYVELQERefaaeergytatkhqrevgagyfdriattvdpnssttaltgsteegqf-----<br>BLAST: aliSize=64 (resi) iden=15.49% (44/283) simi=22.54% (64/283)<br>rashhelramfralldssrcyhtasvfdpmsariaadlgfecgilggsv--ASLQVLAAPDFALITLSFFVFQATRIG-------RVARL----<br>---asvvgtpksaeqiqqewdtnprwkdvtrtysaedvvalqgsvveehtlarrgaevlweqlhdlewvnalgaltgnmavqqvraglkaiylsgwqvagdANLSGHTYPDQSLYPANSVPQVVRRINnalqradQIAKIegdt<br>-------PVIADADHGYGNALNVMRTVVELERAGIAALTIEDTLLP----AQFGRKStdLICVFFGVGKIRAA-LEARV-DPALTIIARTNAF---LI--DVD---<br>svenwlaPIVADGEAGFGGALNVYELQKALIAAGVAGSHWEDQLASekkcGHLGGKV--LIPTQQHIRTLTSArLAADVaDVPTVVIARTDAEaatLItsDVDerdqpfitgertregfyrtkngiepciarakayapfadliwmetgtpdleaarqfseavkaeypdqmlayncspsfnwkkhlddati<br>--aviqrtlayqeagadgiclvgvrdfahleaiaehlhiplmlvtygnpqlrddarlarlgvrvvvnghaayfaaikatydclreergavasdl<br>akfqkelaamgfkfqfitlagfhalnysmfdlaygyaqnqmsayvelqerefaaeergytatkhqrevgagyfdriattvdpnssttaltgsteegqf--<br>taselskkytfpeeyqawardymevk<br>--------------------------<br>Proposed: aliSize=257 (resi) RMSD=2.05 (Å)<br>ra---SH-HFLRAMF-rALIDSSRCYHTASVFDPMSARIAADLGFFCGILGGSVASLQV----LAAPDFALITLSFFVFQATRIGRVA------------------RLPVIADADHGYGNALNVMRTVVELERAGIAALTIEDTLLP<br>--asvvgtpksaeqiqqewdtnprwkdvtrtysaedvvalqgsvveehTLaRRGAEVLw-EQLHDLEWVNALGALTGNMAVQQVRAGLKAIYLSGWQVAGDAnlsgHTYPDQSLYPANSVPQVVRRINNALqradqiakiegdtsvenwLAPIVADGEAGFGGALNVYELQKALIAAGVAGSHWEDQLAS<br>A-------qfgrkSTDLICVFFGVGKIRAALEAR--VDPALTIIARTNAFL-----------------------------IDVDAVIQRTLAYQEaGADGICLVG---vRDF--AHL-FAIAEHL-HIPLMLVTY----G---nP---Q---lRDDaRLARLGVRVVVNGHAAYFAAIKAT----------<br>Ekkcghlg-----GKVLIPTQQHIRTLTSARLAAdvADVPTVVIARTDAEAatlitsdvderdqpfitgertregfyrtkNGIEPCIARAKAYAP-FADLIWMETgtp-DLEaaRQFsEAVKAEYpDQMLAYNCSpsfnWkkh-LddaTiak-FQK-ELAAMGFKFQFITLAGFHALNYSMfdlaygyaq<br>-----------------ydclreergavASDLtASElSKKYTFPEEYQAWARDYMEV---------------k<br>nqmsayvelqerefaaee-----------RGYT-ATK-HQREVGAGYFDRIATTVDPNssttaltgsteegqf-
722	3b8iA (283)<br>1f8iA (427)	C	19.43	TM-align: aliSize=248 (resi) RMSD=2.48 (Å)<br>r--ASHHFLRAMF-ALIDSSRCYHTASVFDPMSARIAADLGFFCGILGGSVASLQ-V---LAAPDFALITLSFFVFQATRIGRVA------------------RLPVIADADHGYGNALNVMRTVVELERAGIAALTIEDTLLPAQ<br>-asvvgtpksaeqiqqewdtnprwkdvtrtysaedvvalqgsvveehTLARRGAEVLwEQLHDLEWVNALGALTGNMAVQQVRAGLKAIYLSGWQVAGDaNlsgHTYPDQSLYPANSVPQVVRRINNALqradqiakiegdtsvenwLAPIVADGEAGFGGALNVYELQKALIAAGVAGSHWEDQLASEK<br>fgr-----KSTDLICVFFGVGKIRAALEAR--VDPALTIIARTNAFL-----------------------------IDVDAVIQRTLAYQEaGADGICLVGVR-DFAHLFAIAF-HL----HIPLMLVTYG---N---P---QL-RDDARLARLGVRVVVNGHAAYFAAIKATYDCLREERGAVasdlta<br>---ksghlGGKVLIPTQQHIRTLTSARLAAdvADVPTVVIARTDAEAatlitsdvderdqpfitgertregfyrtkNGIEPCIARAKAYAP-FADLIWMETGTpDLEAARQFSEaVKaeYpDQMLAYNCSPsfnWkkhLddaTIaKFQKELAAMGFKFQFITLAGFHALNYSMFDLAYGYAQNQ------<br>selskkytfpeeyqawardymevk--<br>-----------------------msayvelqerefaaeergytatkhqrevgagyfdriattvdpnssttaltgsteegqf<br>SARST: aliSize=266 (resi) RMSD=10.74 (Å)<br>ras--HHELRAMFRALLDSSRCYHTAsVFDPMSARIAADLGFFCGILGGSVASLQ--VLA--APDFALIT------------LSFFVFQATRIGRVAR------LPVIADADHGYGNALNVMRTVVELERAGIAALTIEDTLL<br>---asvvgtpksaeqiqqewdtnprwkdvtrtysaedvvalqgsvveehtlaRRGAEVLWEQLHDLEWVNALG-ALTGNMAVQQVRAGLKAIYLSGWQVAGDanLSGhtYPDQSLYPansvpqvvrrinNALQRADQIAKIEGDTsvenwlAPIVADGEAGFGGALNVYELQKALIAAGVAGSHWEDQLA<br>PAQFGRK-STDLIC---VFFGVGKIRAALEARVDPALTIIARTNAFL-----------------------------IDVDAVIQRTLAYQEAgADGICL-VGVRDFAHLFAIA-------EHLHIPLMLVTYGNPQLRD---------DARLARLGVRVVVNGHAAYFAAIKATYDCLREergavasdltA<br>SEKKSGHlGGKVLIptqQHIRTLTSARLAADVADVPTVVIARTDAEAatlitsdvderdqpfitgertregfyrtkNGIEPCIARAKAYAPF-ADLIWMeTGTPDLEAARQFSeavkaeyPDQMLAYNCSPSFNWKKHLddatiakfQKELAAMGFKFQFITLAGFHALNYSMFDLAYG----------Y<br>SELSKKYTFPEEYQAWARDYM--evk<br>AQNQMSAYVELQEREFAAEERgytatkhqrevgagyfdriattvdpnssttaltgsteegqf---<br>BLAST: aliSize=64 (resi) iden=15.49% (44/283) simi=22.54% (64/283)<br>rashhelramfralldssrcyhtasvfdpmsariaadlgfecgilggsv--ASLQVLAAPDFALITLSFFVFQATRIG-------RVARL----<br>---asvvgtpksaeqiqqewdtnprwkdvtrtysaedvvalqgsvveehtlarrgaevlweqlhdlewvnalgaltgnmavqqvraglkaiylsgwqvagdANLSGHTYPDQSLYPANSVPQVVRRINnalqradQIAKIegdt<br>-------PVIADADHGYGNALNVMRTVVELERAGIAALTIEDTLL----PAQFGRKStdLICVFFGVGKIRAA-LEARV-DPALTIIARTNAF---LI--DVD---<br>svenwlaPIVADGEAGFGGALNVYELQKALIAAGVAGSHWEDQLAsekkSGHLGGKV--LIPTQQHIRTLTSArLAADVaDVPTVVIARTDAEaatLItsDVDerdqpfitgertregfyrtkngiepciarakayapfadliwmetgtpdleaarqfseavkaeypdqmlayncspsfnwkkhlddati<br>--aviqrtlayqeagadgiclvgvrdfahleaiaehlhiplmlvtygnpqlrddarlarlgvrvvvnghaayfaaikatydclreergavasdl<br>akfqkelaamgfkfqfitlagfhalnysmfdlaygyaqnqmsayvelqerefaaeergytatkhqrevgagyfdriattvdpnssttaltgsteegqf--<br>taselskkytfpeeyqawardymevk<br>--------------------------<br>Proposed: aliSize=257 (resi) RMSD=2.03 (Å)<br>ra---SH-HFLRAMF-rALIDSSRCYHTASVFDPMSARIAADLGFFCGILGGSVASLQ-V---LAAPDFALITLSFFVFQATRIGRVA------------------RLPVIADADHGYGNALNVMRTVVELERAGIAALTIEDTLLP<br>--asvvgtpksaeqiqqewdtnprwkdvtrtysaedvvalqgsvveehTLaRRGAEVLw-EQLHDLEWVNALGALTGNMAVQQVRAGLKAIYLSGWQVAGDaNlsgHTYPDQSLYPANSVPQVVRRINNALqradqiakiegdtsvenwLAPIVADGEAGFGGALNVYELQKALIAAGVAGSHWEDQLAS<br>A-------qfgrkSTDLICVFFGVGKIRAALEAR--VDPALTIIARTNAFL-----------------------------IDVDAVIQRTLAYQEaGADGICLVGVR-DFAHLFAIAF-HL----HIPLMLVTYG---N---P---Q--lRDDARLARLGVRVVVNGHAAYFAAL-kA------------<br>Ekksghlg-----GKVLIPTQQHIRTLTSARLAAdvADVPTVVIARTDAEAatlitsdvderdqpfitgertregfyrtkNGIEPCIARAKAYAP-FADLIWMETGTpDLEAARQFSEaVKaeYpDQMLAYNCSPsfnWkkhLddaTia-KFQKELAAMGFKFQFITLAGFHALNy-Smfdlaygyaqnq<br>-------------tydclreergavASD---L-TasELSKKYTFPEEYQAWARDYMEV---------------k<br>msayvelqerefa------------AEErgyTaT--K-HQREVGAGYFDRIATTVDPNssttaltgsteegqf-

723	1k51A (72)<br>2hj1A (77)	C	4.17	TM-align: aliSize=50 (resi) RMSD=2.45 (Å)<br>mhhhhhhaMeEVTIKANLIFaNGSTQTAEFKG-TFEKaT-SEaYA--YADT-LKkdNGE---WTVDVADKAYtlnikfag------------------<br>--------N-QINIEIAYAF-PERYYLKSFQVdEGIT-VqTA-ITqsGILSqFP--EIDlstNKIGIFSRPI--------kltdvlkegdrieiyrpll<br><br>SARST: aliSize=60 (resi) RMSD=9.14 (Å)<br>mhhhhhhame-EVTIKANLIFANGSTQTAEFK---GTFEKATSE--AYAYADTLKKDNGEWTVDVADKA--------YTLNIKF---ag<br>----------nQINIEIAYAFPERYYLKSFQVdegITVQTAITQsgILSQFPEIDLSTNKIGIFSRPIKltdvlkegDRIEIYRpll--<br><br>BLAST: aliSize=9 (resi) iden=6.94% (5/72) simi=12.50% (9/72)<br>mhhhhhhameevtikanlifangstqtaefkgtfekatse-------AYAYADTLKKDNgeWTVD---vadkaytlnikfag<br>--------------------------------------nqinieiAYAFPERYYLKS--FQVDegitvqtaitqsgilsqfpeidlstnkigifsrpikltdvlkegdrieiyrpll-------------<br><br>Proposed: aliSize=60 (resi) RMSD=2.30 (Å)<br>mhhhhhhaMeEVTIKANLIFaNGSTQTAEFKGTFEK-aT-SEAYA-YADTLKKdNGE---WTVDVADK---------AYTLNIKFAG--<br>--------N-QINIEIAYAF-PERYYLKSFQVDEGIt-VqTAITQsGILSQFP-EIDlstNKIGIFSRpikltdvlkEGDRIEIYRPll
724	1k52A (72)<br>2hj1A (77)	C	6.94	TM-align: aliSize=49 (resi) RMSD=2.38 (Å)<br>mhhhhhhamEEVTIKANLIFaNGSTQTAEFKGTF-EKaT-SEaYA--YADTLKKdNGE---WTVDVADGgytlnikfag--------------------<br>---------NQINIEIAYAF-PERYYLKSFQVDEgIT-VqTA-ITqsGILSQFP-EIDlstNKIGIFSR----------pikltdvlkegdrieiyrpll<br><br>SARST: aliSize=59 (resi) RMSD=8.76 (Å)<br>mhhhhhhame-EVTIKANLIFANgSTQTAEFKG----TFEKATSEAYAYADTLK--KDNGEWTVD--------VADGGYTLNIKF---ag<br>----------nQINIEIAYAFPE-RYYLKSFQVdegiTVQTAITQSGILSQFPEidLSTNKIGIFsrpikltdVLKEGDRIEIYRpll--<br><br>BLAST: aliSize=9 (resi) iden=6.94% (5/72) simi=12.50% (9/72)<br>mhhhhhhameevtikanlifangstqtaefkgtfekatse-------AYAYADTLKKDNgeWTVD---vadggytlnikfag<br>--------------------------------------nqinieiAYAFPERYYLKS--FQVDegitvqtaitqsgilsqfpeidlstnkigifsrpikltdvlkegdrieiyrpll-------------<br><br>Proposed: aliSize=59 (resi) RMSD=2.21 (Å)<br>mhhhhhhamEEVTIKANLIFaNGSTQTAEFKGTFEK-aTSEAYA--YADTLKKdNGE---WTVDVAD----------GGyTLNIKFAG--<br>---------NQINIEIAYAF-PERYYLKSFQVDEGIt-VQTAITqsGILSQFP-EIDlstNKIGIFSrpikltdvlkEG-DRIEIYRPll
725	1mo1A (87)<br>1y50A (87)	N	24.14	TM-align: aliSize=42 (resi) RMSD=2.38 (Å)<br>mvqqkvevrlkt-----------GLQARPAALFVQEANRFTSDVFLEKDGKKVNAKSI-GL-SLAVStgtevtliaqgedeqealeklaayvqeevlq-------------------------------<br>------------aektfkvvsdsGIHARPATILVQTASKWNSEIQLEYNGKTVNLKSImGVmSLGIP------------------------------kgatikitaegadaaeamaaltdtlakeglae<br><br>SARST: aliSize=80 (resi) RMSD=15.72 (Å)<br>mv-QQKVEVRLKTGLQARPAALFVQEANRFTSDVFLEKDGKKVNAKS--IGLSLAVSTGTEVTLIAQGED--EQEALEKLAAYVQEE--vlq<br>--aEKTFKVVSDSGIHARPATILVQTASKWNSEIQLEYNGKTVNLKSimGVMSLGIPKGATIKITAEGADaaEAMAALTDTLAKEGLae---<br><br>BLAST: aliSize=47 (resi) iden=35.29% (30/87) simi=55.29% (47/87)<br>mvqqkvevrlk----------TGLQARPAALFVQEANRFTSDVFLEKDGKKVNAKSI-G-LSLAVSTGTEVTLIAQGEDEQEALEKLAAYVQEEVL--q<br>-----------aektfkvvsdSGIHARPATILVQTASKWNSEIQLEYNGKTVNLKSImGvMSLGIPKGATIKITAEGADAAEAMAALTDTLAKEGLae-<br><br>Proposed: aliSize=52 (resi) RMSD=1.57 (Å)<br>mVQQKVEVRL-KT-glQARPAALFVQEANRFTSDVFLEKDGKKVNAKSIMGLMSLA-V--------------------------------stgtevtliaqgedeqealeklaayvqeevlq<br>-AEKTFKVVSdSGi--HARPATILVQTASKWNSEIQLEYNGKTVNLKSIMGVMSLGiPkgatikitaegadaaeamaaltdtlakeglae--------------------------------

726	1mo1A (87)<br>1y50A (87)	C	31.03	TM-align: aliSize=42 (resi) RMSD=2.38 (Å)<br>mvqqkvevrlkt-----------GLQARPAALFVQEANRFTSDVFLEKDGKKVNAKSI-GL-SLAVStgtevtliaqgedeqealeklaayvqeevlq--------------------------------<br>------------aektfkvvsdsGIHARPATILVQTASKWNSEIQLEYNGKTVNLKSImGVmSLGIP-------------------------------kgatikitaegadaaeamaaltdtlakeglae<br><br>SARST: aliSize=80 (resi) RMSD=15.72 (Å)<br>mv-QQKVEVRLKTGLQARPAALFVQEANRFTSDVFLEKDGKKVNAKS--IGLSLAVSTGTEVTLIAQGED--EQEALEKLAAYVQEE--vlq<br>--aEKTFKVVSDSGIHARPATILVQTASKWNSEIQLEYNGKTVNLKSimGVMSLGIPKGATIKITAEGADaaEAMAALTDTLAKEGLae---<br><br>BLAST: aliSize=47 (resi) iden=35.29% (30/87) simi=55.29% (47/87)<br>mvqqkvevrlk----------TGLQARPAALFVQEANRFTSDVFLEKDGKKVNAKSI-G-LSLAVSTGTEVTLIAQGEDEQEALEKLAAYVQEEVL--q<br>-----------aektfkvvsdSGIHARPATILVQTASKWNSEIQLEYNGKTVNLKSImGvMSLGIPKGATIKITAEGADAAEAMAALTDTLAKEGLae-<br><br>Proposed: aliSize=70 (resi) RMSD=1.63 (Å)<br>mvqqkvevrlktg------------LQARPAALFVQEANRFTSDVFLEKDGKKVNAKSIMGLMSL-aVSTGTEVTLIAQGEDEQEALEKLAAYVqEEV----lq<br>-------------aektfkvvsdsgIHARPATILVQTASKWNSEIQLEYNGKTVNLKSIMGVMSLg-IPKGATIKITAEGADAAEAMAALTDTL-AKEglae--
727	3bcmA (119)<br>1r5cA (124)	N	98.32	TM-align: aliSize=103 (resi) RMSD=0.41 (Å)<br>kesaaakferqhmdsg---------------------SSNYCNQMMCCRKMTQGKCKPVNTFVHESLADVKAVCSQKKVTCKDGQTNCYQSKSTMRITDCRETGSSKYPNCAYKTTQVEKHIIVACGGKPSVPVHFDASV<br>----------------kesaaakferqhmdsgnspssSSNYCNLMMCCRKMTQGKCKPVNTFVHESLADVKAVCSQKKVTCKNGQTNCYQSKSTMRITDCRETGSSKYPNCAYKTTQVEKHIIVACGGKPSVPVHFDASV<br><br>SARST: aliSize=117 (resi) RMSD=9.65 (Å)<br>k-ESAAAKFERQHMDSGS-----SNYCNQMMCCRKMTQGKCKPVNTFVHESLADVKAVCSQKKVTCKDGQTNCYQSKSTMRITDCRETGSSKYPNCAYKTTQVEKHIIVACGGKPSVPVHFDAS-v<br>-kESAAAKFERQHMDSGNspsssSNYCNLMMCCRKMTQGKCKPVNTFVHESLADVKAVCSQKKVTCKNGQTNCYQSKSTMRITDCRETGSSKYPNCAYKTTQVEKHIIVACGGKPSVPVHFDASv-<br><br>BLAST: aliSize=118 (resi) iden=98.32% (117/119) simi=99.16% (118/119)<br>KESAAAKFERQHMDSG-----SSNYCNQMMCCRKMTQGKCKPVNTFVHESLADVKAVCSQKKVTCKDGQTNCYQSKSTMRITDCRETGSSKYPNCAYKTTQVEKHIIVACGGKPSVPVHFDASV<br>KESAAAKFERQHMDSGnspssSSNYCNLMMCCRKMTQGKCKPVNTFVHESLADVKAVCSQKKVTCKNGQTNCYQSKSTMRITDCRETGSSKYPNCAYKTTQVEKHIIVACGGKPSVPVHFDASV<br><br>Proposed: aliSize=119 (resi) RMSD=0.39 (Å)<br>KESAAAKFERQHMDSG-----SSNYCNQMMCCRKMTQGKCKPVNTFVHESLADVKAVCSQKKVTCKDGQTNCYQSKSTMRITDCRETGSSKYPNCAYKTTQVEKHIIVACGGKPSVPVHFDASV<br>KESAAAKFERQHMDSGnspssSSNYCNLMMCCRKMTQGKCKPVNTFVHESLADVKAVCSQKKVTCKNGQTNCYQSKSTMRITDCRETGSSKYPNCAYKTTQVEKHIIVACGGKPSVPVHFDASV
728	3bcmA (119)<br>1js0A (124)	C	83.19	TM-align: aliSize=110 (resi) RMSD=1.41 (Å)<br>KESAAAKFERQHMDSG-----SSNYCNQMMCCRKMTQGKCKPVNTFVHESLADVKAVCSQKKVTCKDGQTNCYQSKSTMRITDCRETGSSKYPNCAYKTTQVEKHIIVACGGKPSvpvhfdasv---------<br>KETAAAKFERQHMDSStsaasSSNYCNQMMKSRNLTKDRCKPVNTFVHESLADVQAVCSQKNVACKNGQTNCYQSYSTMSITDCRETGSSKYPNCAYKTTQANKHIIVACEGNPY---------vpvhfdasv<br><br>SARST: aliSize=117 (resi) RMSD=6.59 (Å)<br>k-ESAAAKFERQHMD-----SGSSNYCNQMMCCRKMTQGKCKPVNTFVHESLADVKAVCSQKKVTCKDGQTNCYQSKSTMRITDCRETGSSKYPNCAYKTTQVEKHIIVACGGKPSVPVHFDAS-v<br>-kETAAAKFERQHMDsstsaASSSNYCNQMMKSRNLTKDRCKPVNTFVHESLADVQAVCSQKNVACKNGQTNCYQSYSTMSITDCRETGSSKYPNCAYKTTQANKHIIVACEGNPYVPVHFDASv-<br><br>BLAST: aliSize=105 (resi) iden=83.19% (99/119) simi=88.24% (105/119)<br>KESAAAKFERQHMDSG-----SSNYCNQMMCCRKMTQGKCKPVNTFVHESLADVKAVCSQKKVTCKDGQTNCYQSKSTMRITDCRETGSSKYPNCAYKTTQVEKHIIVACGGKPSVPVHFDASV<br>KETAAAKFERQHMDSStsaasSSNYCNQMMKSRNLTKDRCKPVNTFVHESLADVQAVCSQKNVACKNGQTNCYQSYSTMSITDCRETGSSKYPNCAYKTTQANKHIIVACEGNPYVPVHFDASV<br><br>Proposed: aliSize=119 (resi) RMSD=0.59 (Å)<br>KESAAAKFERQHMDS-G----SSNYCNQMMCCRKMTQGKCKPVNTFVHESLADVKAVCSQKKVTCKDGQTNCYQSKSTMRITDCRETGSSKYPNCAYKTTQVEKHIIVACGGKPSVPVHFDASV<br>KETAAAKFERQHMDSsTsaasSSNYCNQMMKSRNLTKDRCKPVNTFVHESLADVQAVCSQKNVACKNGQTNCYQSYSTMSITDCRETGSSKYPNCAYKTTQANKHIIVACEGNPYVPVHFDASV

729	3bcmA (119)<br>11bgA (124)	N	98.32	TM-align: aliSize=103 (resi) RMSD=0.42 (Å)<br>kesaaakferqhmdsg--------------------SSNYCNQMMCCRKMTQGKCKPVNTFVHESLADVKAVCSQKKVTCKDGQTNCYQSKSTMRITDCRETGSSKYPNCAYKTTQVEKHIIVACGGKPSVPVHFDASV<br>---------------kesaaakferqhmdsgnspssSSNYCNLMMCCRKMTQGKCKPVNTFVHESLADVKAVCSQKKVTCKNGQTNCYQSKSTMRITDCRETGSSKYPNCAYKTTQVEKHIIVACGGKPSVPVHFDASV<br><br>SARST: aliSize=117 (resi) RMSD=9.73 (Å)<br>k-ESAAAKFERQHMDSGS-----SNYCNQMMCCRKMTQGKCKPVNTFVHESLADVKAVCSQKKVTCKDGQTNCYQSKSTMRITDCRETGSSKYPNCAYKTTQVEKHIIVACGGKPSVPVHFDAS-v<br>-kESAAAKFERQHMDSGNspsssSNYCNLMMCCRKMTQGKCKPVNTFVHESLADVKAVCSQKKVTCKNGQTNCYQSKSTMRITDCRETGSSKYPNCAYKTTQVEKHIIVACGGKPSVPVHFDASv-<br><br>BLAST: aliSize=118 (resi) iden=98.32% (117/119) simi=99.16% (118/119)<br>KESAAAKFERQHMDSG-----SSNYCNQMMCCRKMTQGKCKPVNTFVHESLADVKAVCSQKKVTCKDGQTNCYQSKSTMRITDCRETGSSKYPNCAYKTTQVEKHIIVACGGKPSVPVHFDASV<br>KESAAAKFERQHMDSGnspssSSNYCNLMMCCRKMTQGKCKPVNTFVHESLADVKAVCSQKKVTCKNGQTNCYQSKSTMRITDCRETGSSKYPNCAYKTTQVEKHIIVACGGKPSVPVHFDASV<br><br>Proposed: aliSize=119 (resi) RMSD=0.42 (Å)<br>KESAAAKFERQHMDSG-----SSNYCNQMMCCRKMTQGKCKPVNTFVHESLADVKAVCSQKKVTCKDGQTNCYQSKSTMRITDCRETGSSKYPNCAYKTTQVEKHIIVACGGKPSVPVHFDASV<br>KESAAAKFERQHMDSGnspssSSNYCNLMMCCRKMTQGKCKPVNTFVHESLADVKAVCSQKKVTCKNGQTNCYQSKSTMRITDCRETGSSKYPNCAYKTTQVEKHIIVACGGKPSVPVHFDASV
730	3bcmA (119)<br>1tq9A (124)	N	96.64	TM-align: aliSize=101 (resi) RMSD=0.42 (Å)<br>kesaaakferqhmdsg--------------------SSNYCNQMMccRKMTQGKCKPVNTFVHESLADVKAVCSQKKVTCKDGQTNCYQSKSTMRITDCRETGSSKYPNCAYKTTQVEKHIIVACGGKPSVPVHFDASV<br>---------------kesaaakferqhmdsgnspssSSNYCNLMM--RKMTQGKCKPVNTFVHESLADVKAVCSQKKVTCKNGQTNCYQSKSTMRITDCRETGSSKYPNCAYKTTQVEKHIIVACGGKPSVPVHFDASV<br><br>SARST: aliSize=115 (resi) RMSD=11.28 (Å)<br>k-ESAAAKFERQHMDSGS-----SNYCNQMMCcrKMTQGKCKPVNTFVHESLADVKAVCSQKKVTCKDGQTNCYQSKSTMRITDCRETGSSKYPNCAYKTTQVEKHIIVACGGKPSVPVHFDAS-v<br>-kESAAAKFERQHMDSGNspsssSNYCNLMMR--KMTQGKCKPVNTFVHESLADVKAVCSQKKVTCKNGQTNCYQSKSTMRITDCRETGSSKYPNCAYKTTQVEKHIIVACGGKPSVPVHFDASv-<br><br>BLAST: aliSize=116 (resi) iden=96.64% (115/119) simi=97.48% (116/119)<br>KESAAAKFERQHMDSG-----SSNYCNQMMccRKMTQGKCKPVNTFVHESLADVKAVCSQKKVTCKDGQTNCYQSKSTMRITDCRETGSSKYPNCAYKTTQVEKHIIVACGGKPSVPVHFDASV<br>KESAAAKFERQHMDSGnspssSSNYCNLMM--RKMTQGKCKPVNTFVHESLADVKAVCSQKKVTCKNGQTNCYQSKSTMRITDCRETGSSKYPNCAYKTTQVEKHIIVACGGKPSVPVHFDASV<br><br>Proposed: aliSize=119 (resi) RMSD=0.41 (Å)<br>KESAAAKFERQHMDSG-----SSNYCNQMMCCRKMTQGKCKPVNTFVHESLADVKAVCSQKKVTCKDGQTNCYQSKSTMRITDCRETGSSKYPNCAYKTTQVEKHIIVACGGKPSVPVHFDASV<br>KESAAAKFERQHMDSGnspssSSNYCNLMMYYRKMTQGKCKPVNTFVHESLADVKAVCSQKKVTCKNGQTNCYQSKSTMRITDCRETGSSKYPNCAYKTTQVEKHIIVACGGKPSVPVHFDASV
731	3bcmA (119)<br>1r5dA (124)	N	98.32	TM-align: aliSize=103 (resi) RMSD=0.47 (Å)<br>kesaaakferqhmdsg--------------------SSNYCNQMMCCRKMTQGKCKPVNTFVHESLADVKAVCSQKKVTCKDGQTNCYQSKSTMRITDCRETGSSKYPNCAYKTTQVEKHIIVACGGKPSVPVHFDASV<br>---------------kesaaakferqhmdsgnspssSSNYCNLMMCCRKMTQGKCKPVNTFVHESLADVKAVCSQKKVTCKNGQTNCYQSKSTMRITDCRETGSSKYPNCAYKTTQVEKHIIVACGGKPSVPVHFDASV<br><br>SARST: aliSize=117 (resi) RMSD=9.67 (Å)<br>k-ESAAAKFERQHMDSGS-----SNYCNQMMCCRKMTQGKCKPVNTFVHESLADVKAVCSQKKVTCKDGQTNCYQSKSTMRITDCRETGSSKYPNCAYKTTQVEKHIIVACGGKPSVPVHFDAS-v<br>-kESAAAKFERQHMDSGNspsssSNYCNLMMCCRKMTQGKCKPVNTFVHESLADVKAVCSQKKVTCKNGQTNCYQSKSTMRITDCRETGSSKYPNCAYKTTQVEKHIIVACGGKPSVPVHFDASv-<br><br>BLAST: aliSize=118 (resi) iden=98.32% (117/119) simi=99.16% (118/119)<br>KESAAAKFERQHMDSG-----SSNYCNQMMCCRKMTQGKCKPVNTFVHESLADVKAVCSQKKVTCKDGQTNCYQSKSTMRITDCRETGSSKYPNCAYKTTQVEKHIIVACGGKPSVPVHFDASV<br>KESAAAKFERQHMDSGnspssSSNYCNLMMCCRKMTQGKCKPVNTFVHESLADVKAVCSQKKVTCKNGQTNCYQSKSTMRITDCRETGSSKYPNCAYKTTQVEKHIIVACGGKPSVPVHFDASV<br><br>Proposed: aliSize=119 (resi) RMSD=0.44 (Å)<br>KESAAAKFERQHMDSG-----SSNYCNQMMCCRKMTQGKCKPVNTFVHESLADVKAVCSQKKVTCKDGQTNCYQSKSTMRITDCRETGSSKYPNCAYKTTQVEKHIIVACGGKPSVPVHFDASV<br>KESAAAKFERQHMDSGnspssSSNYCNLMMCCRKMTQGKCKPVNTFVHESLADVKAVCSQKKVTCKNGQTNCYQSKSTMRITDCRETGSSKYPNCAYKTTQVEKHIIVACGGKPSVPVHFDASV

732	3bcmA (119)<br>1a2wA (124)	N	83.19	TM-align: aliSize=103 (resi) RMSD=0.51 (Å)<br>kesaaakferqhmdsg---------------------SSNYCNQMMCCRKMTQGKCKPVNTFVHESLADVKAVCSQKKVTCKDGQTNCYQSKSTMRITDCRETGSSKYPNCAYKTTQVEKHIIVACGGKPSVPVHFDASV<br>----------------ketaaakferqhmdsstsaasSSNYCNQMMKSRNLTKDRCKPVNTFVHESLADVQAVCSQKNVACKNGQTNCYQSYSTMSITDCRETGSSKYPNCAYKTTQANKHIIVACEGNPYVPVHFDASV<br><br>SARST: aliSize=117 (resi) RMSD=14.11 (Å)<br>k-ESAAAKFERQHMD-----SGSSNYCNQMMCCRKMTQGKCKPVNTFVHESLADVKAVCSQKKVTCKDGQTNCYQSKSTMRITDCRETGSSKYPNCAYKTTQVEKHIIVACGGKPSVPVHFDAS-v<br>-kETAAAKFERQHMDsstsaASSSNYCNQMMKSRNLTKDRCKPVNTFVHESLADVQAVCSQKNVACKNGQTNCYQSYSTMSITDCRETGSSKYPNCAYKTTQANKHIIVACEGNPYVPVHFDASv-<br><br>BLAST: aliSize=105 (resi) iden=83.19% (99/119) simi=88.24% (105/119)<br>KESAAAKFERQHMDSG-----SSNYCNQMMCCRKMTQGKCKPVNTFVHESLADVKAVCSQKKVTCKDGQTNCYQSKSTMRITDCRETGSSKYPNCAYKTTQVEKHIIVACGGKPSVPVHFDASV<br>KETAAAKFERQHMDSStsaasSSNYCNQMMKSRNLTKDRCKPVNTFVHESLADVQAVCSQKNVACKNGQTNCYQSYSTMSITDCRETGSSKYPNCAYKTTQANKHIIVACEGNPYVPVHFDASV<br><br>Proposed: aliSize=119 (resi) RMSD=0.58 (Å)<br>KESAAAKFERQHMDS-G----SSNYCNQMMCCRKMTQGKCKPVNTFVHESLADVKAVCSQKKVTCKDGQTNCYQSKSTMRITDCRETGSSKYPNCAYKTTQVEKHIIVACGGKPSVPVHFDASV<br>KETAAAKFERQHMDSsTsaasSSNYCNQMMKSRNLTKDRCKPVNTFVHESLADVQAVCSQKNVACKNGQTNCYQSYSTMSITDCRETGSSKYPNCAYKTTQANKHIIVACEGNPYVPVHFDASV
733	3bcmA (119)<br>1lbaA (124)	N	98.32	TM-align: aliSize=103 (resi) RMSD=0.38 (Å)<br>kesaaakferqhmdsg---------------------SSNYCNQMMCCRKMTQGKCKPVNTFVHESLADVKAVCSQKKVTCKDGQTNCYQSKSTMRITDCRETGSSKYPNCAYKTTQVEKHIIVACGGKPSVPVHFDASV<br>----------------kesaaakferqhmdsgnspssSSNYCNLMMCCRKMTQGKCKPVNTFVHESLADVKAVCSQKKVTCKNGQTNCYQSKSTMRITDCRETGSSKYPNCAYKTTQVEKHIIVACGGKPSVPVHFDASV<br><br>SARST: aliSize=117 (resi) RMSD=9.62 (Å)<br>k-ESAAAKFERQHMDSGS-----SNYCNQMMCCRKMTQGKCKPVNTFVHESLADVKAVCSQKKVTCKDGQTNCYQSKSTMRITDCRETGSSKYPNCAYKTTQVEKHIIVACGGKPSVPVHFDAS-v<br>-kESAAAKFERQHMDSGNspsssSNYCNLMMCCRKMTQGKCKPVNTFVHESLADVKAVCSQKKVTCKNGQTNCYQSKSTMRITDCRETGSSKYPNCAYKTTQVEKHIIVACGGKPSVPVHFDASv-<br><br>BLAST: aliSize=118 (resi) iden=98.32% (117/119) simi=99.16% (118/119)<br>KESAAAKFERQHMDSG-----SSNYCNQMMCCRKMTQGKCKPVNTFVHESLADVKAVCSQKKVTCKDGQTNCYQSKSTMRITDCRETGSSKYPNCAYKTTQVEKHIIVACGGKPSVPVHFDASV<br>KESAAAKFERQHMDSGnspssSSNYCNLMMCCRKMTQGKCKPVNTFVHESLADVKAVCSQKKVTCKNGQTNCYQSKSTMRITDCRETGSSKYPNCAYKTTQVEKHIIVACGGKPSVPVHFDASV<br><br>Proposed: aliSize=119 (resi) RMSD=0.36 (Å)<br>KESAAAKFERQHMDSG-----SSNYCNQMMCCRKMTQGKCKPVNTFVHESLADVKAVCSQKKVTCKDGQTNCYQSKSTMRITDCRETGSSKYPNCAYKTTQVEKHIIVACGGKPSVPVHFDASV<br>KESAAAKFERQHMDSGnspssSSNYCNLMMCCRKMTQGKCKPVNTFVHESLADVKAVCSQKKVTCKNGQTNCYQSKSTMRITDCRETGSSKYPNCAYKTTQVEKHIIVACGGKPSVPVHFDASV
734	3bcmA (119)<br>1h8xA (125)	N	75.63	TM-align: aliSize=103 (resi) RMSD=1.19 (Å)<br>kesaaakferqhmdsg---------------------SSNYCNQMMCCRKMTQGKCKPVNTFVHESLADVKAVCSQKKVTCKDGQTNCYQSKSTMRITDCRETGSSKYPNCAYKTTQVEKHIIVACGGKPSVPVHFDASV-<br>----------------kesaaakferqhmdsgnspssSSTYCNQMMRRRNMTQGRCKPVNTFVHEPLVDVQNVCFQEKVTCKNGQGNCYKSNSSMHITDCRLTNGSRYPNCAYRTSQKERHIIVACEGSPYVPVHFDASVe<br><br>SARST: aliSize=117 (resi) RMSD=14.08 (Å)<br>k-ESAAAKFERQHMD---SGSSN--YCNQMMCCRKMTQGKCKPVNTFVHESLADVKAVCSQKKVTCKDGQTNCYQSKSTMRITDCRETGSSKYPNCAYKTTQVEKHIIVACGGKPSVPVHFDAS--v<br>-kESAAAKFERQHMDsgnSPSSSstYCNQMMRRRNMTQGRCKPVNTFVHEPLVDVQNVCFQEKVTCKNGQGNCYKSNSSMHITDCRLTNGSRYPNCAYRTSQKERHIIVACEGSPYVPVHFDASve-<br><br>BLAST: aliSize=101 (resi) iden=76.47% (91/119) simi=84.87% (101/119)<br>KESAAAKFERQHMDSG-----SSNYCNQMMCCRKMTQGKCKPVNTFVHESLADVKAVCSQKKVTCKDGQTNCYQSKSTMRITDCRETGSSKYPNCAYKTTQVEKHIIVACGGKPSVPVHFDASV-<br>KESAAAKFERQHMDSGnspssSSTYCNQMMRRRNMTQGRCKPVNTFVHEPLVDVQNVCFQEKVTCKNGQGNCYKSNSSMHITDCRLTNGSRYPNCAYRTSQKERHIIVACEGSPYVPVHFDASVe<br><br>Proposed: aliSize=119 (resi) RMSD=1.04 (Å)<br>KESAAAKFERQHMDSG--S---SNYCNQMMCCRKMTQGKCKPVNTFVHESLADVKAVCSQKKVTCKDGQTNCYQSKSTMRITDCRETGSSKYPNCAYKTTQVEKHIIVACGGKPSVPVHFDASV-<br>KESAAAKFERQHMDSGnsPsssSTYCNQMMRRRNMTQGRCKPVNTFVHEPLVDVQNVCFQEKVTCKNGQGNCYKSNSSMHITDCRLTNGSRYPNCAYRTSQKERHIIVACEGSPYVPVHFDASVe

735	1tq9A<br>(124)<br>1r3mB<br>(122)	N	98.36	TM-align: aliSize=103 (resi) RMSD=1.21 (Å)<br>kesaaakferqhmdsgnsp-----------------SSSSNYCNLMM--RKMTQGKCKPVNTFVHESLADVKAVCSQKKVTCKNGQTNCYQSKSTMRITDCRETGSSKYPNCAYKTTQVEKHIIVACGGKPSVPVHFDASV<br>-------------------kesaaakferqhmdsgnSPSSNYCNLMMccRKMTQGKCKPVNTFVHESLADVKAVCSQKKVTCKNGQTNCYQSKSTMRITDCRETGSSKYPNCAYKTTQVEKHIIVACGGKPSVPVHFDASV<br><br>SARST: aliSize=120 (resi) RMSD=11.24 (Å)<br>k-ESAAAKFERQHMDSGNSPSSSSNYCNLMMRKMTQGKCKPVNTFVHESLADVKAVCSQKKVTCKNGQTNCYQSKSTMRITDCRETGSSKYPNCAYKTTQVEKHIIVACGGKPSVPVHFDAS-v<br>-kESAAAKFERQHMDSGNSPSSNYCNLMMCCRKMTQGKCKPVNTFVHESLADVKAVCSQKKVTCKNGQTNCYQSKSTMRITDCRETGSSKYPNCAYKTTQVEKHIIVACGGKPSVPVHFDASv-<br><br>BLAST: aliSize=120 (resi) iden=98.36% (120/122) simi=98.36% (120/122)<br>KESAAAKFERQHMDSGNSPSSsNYCNLMM--RKMTQGKCKPVNTFVHESLADVKAVCSQKKVTCKNGQTNCYQSKSTMRITDCRETGSSKYPNCAYKTTQVEKHIIVACGGKPSVPVHFDASV<br>KESAAAKFERQHMDSGNSPSS--NYCNLMMccRKMTQGKCKPVNTFVHESLADVKAVCSQKKVTCKNGQTNCYQSKSTMRITDCRETGSSKYPNCAYKTTQVEKHIIVACGGKPSVPVHFDASV<br><br>Proposed: aliSize=122 (resi) RMSD=0.62 (Å)<br>KESAAAKFERQHMDSGNSPssSSNYCNLMMYYRKMTQGKCKPVNTFVHESLADVKAVCSQKKVTCKNGQTNCYQSKSTMRITDCRETGSSKYPNCAYKTTQVEKHIIVACGGKPSVPVHFDASV<br>KESAAAKFERQHMDSGNSP--SSNYCNLMMCCRKMTQGKCKPVNTFVHESLADVKAVCSQKKVTCKNGQTNCYQSKSTMRITDCRETGSSKYPNCAYKTTQVEKHIIVACGGKPSVPVHFDASV
736	1k50A<br>(63)<br>1k51A<br>(72)	C	96.83	TM-align: aliSize=53 (resi) RMSD=1.02 (Å)<br>---------EEVTIKANLIFANGSTQTAEFKGTFEKATSEAYAYADTLKKDNGEWTADVADKgytlnikfag----------<br>mhhhhhhamEEVTIKANLIFANGSTQTAEFKGTFEKATSEAYAYADTLKKDNGEWTVDVADK----------aytlnikfag<br><br>SARST: aliSize=58 (resi) RMSD=8.41 (Å)<br>e----------EVTIKANLIFANGSTQTAEFKGTFEKATSEAYAYADTLKKDNGEWTADVAdkGYTLNIKF----ag<br>-mhhhhhhameEVTIKANLIFANGSTQTAEFKGTFEKATSEAYAYADTLKKDNGEWTVDVA--DKAYTLNIkfag--<br><br>BLAST: aliSize=61 (resi) iden=96.83% (61/63) simi=96.83% (61/63)<br>---------EEVTIKANLIFANGSTQTAEFKGTFEKATSEAYAYADTLKKDNGEWTADVADKGYTLNIKFAG<br>mhhhhhhamEEVTIKANLIFANGSTQTAEFKGTFEKATSEAYAYADTLKKDNGEWTVDVADKAYTLNIKFAG<br><br>Proposed: aliSize=63 (resi) RMSD=1.15 (Å)<br>---------EEVTIKANLIFANGSTQTAEFKGTFEKATSEAYAYADTLKKDNGEWTADVADKGYTLNIKFAG<br>mhhhhhhamEEVTIKANLIFANGSTQTAEFKGTFEKATSEAYAYADTLKKDNGEWTVDVADKAYTLNIKFAG
737	1k50A<br>(63)<br>1jmlA<br>(72)	C	93.65	TM-align: aliSize=53 (resi) RMSD=1.08 (Å)<br>---------EEVTIKANLIFANGSTQTAEFKGTFEKATSEAYAYADTLKKDNGEWTADVADKgytlnikfag----------<br>mhhhhhhgmEEVTIKANLIFANGSTQTAEFKGTFEKATSEAYAYADTLKKDNGEWTVDVVPK----------aytlnikfag<br><br>SARST: aliSize=58 (resi) RMSD=8.28 (Å)<br>e----------EVTIKANLIFANGSTQTAEFKGTFEKATSEAYAYADTLKKDNGEWTADVAdkGYTLNIKF----ag<br>-mhhhhhhgmeEVTIKANLIFANGSTQTAEFKGTFEKATSEAYAYADTLKKDNGEWTVDVV--PKAYTLNIkfag--<br><br>BLAST: aliSize=59 (resi) iden=93.65% (59/63) simi=93.65% (59/63)<br>---------EEVTIKANLIFANGSTQTAEFKGTFEKATSEAYAYADTLKKDNGEWTADVADKGYTLNIKFAG<br>mhhhhhhgmEEVTIKANLIFANGSTQTAEFKGTFEKATSEAYAYADTLKKDNGEWTVDVVPKAYTLNIKFAG<br><br>Proposed: aliSize=63 (resi) RMSD=1.19 (Å)<br>---------EEVTIKANLIFANGSTQTAEFKGTFEKATSEAYAYADTLKKDNGEWTADVADKGYTLNIKFAG<br>mhhhhhhgmEEVTIKANLIFANGSTQTAEFKGTFEKATSEAYAYADTLKKDNGEWTVDVVPKAYTLNIKFAG

738	1jmlA<br>(72)<br>2hj1A<br>(77)	C	4.17	TM-align: aliSize=50 (resi) RMSD=2.46 (Å)<br>mhhhhhhgMeEVTIKANLIFaNGSTQTAEFKG-TFEKaT-SEaYA--YADT-LkKdNGE---WTVDVVPKAYtlnikfag-------------------<br>--------N-QINIEIAYAF-PERYYLKSFQVdEGIT-VqTA-ITqsGILSqF-P-EIDlstNKIGIFSRPI--------kltdvlkegdrieiyrpll<br><br>SARST: aliSize=60 (resi) RMSD=8.92 (Å)<br>mhhhhhhgme-EVTIKANLIFANGSTQTAEFK---GTFEKATSEAYAYADTLK--KDNGEWTVDVVPKA--------YTLNIKF---ag<br>----------nQINIEIAYAFPERYYLKSFQVdegITVQTAITQSGILSQFPEidLSTNKIGIFSRPIKltdvlkegDRIEIYRpll--<br><br>BLAST: aliSize=9 (resi) iden=6.94% (5/72) simi=12.50% (9/72)<br>mhhhhhhgmeevtikanlifangstqtaefkgtfekatse-------AYAYADTLKKDNgeWTVD---vvpkaytlnikfag<br>--nqinieiAYAFPERYYLKS--FQVDegitvqtaitqsgilsqfpeidlstnkigifsrpikltdvlkegdrieiyrpll-------------<br><br>Proposed: aliSize=60 (resi) RMSD=2.36 (Å)<br>mhhhhhhgMeEVTIKANLIFaNGSTQTAEFKGTFEK-aT-SEAYA-YADTLKKdNGE---WTVDVVPK---------AYTLNIKFAG--<br>--------N-QINIEIAYAF-PERYYLKSFQVDEGIt-VqTAITQsGILSQFP-EIDlstNKIGIFSRpikltdvlkEGDRIEIYRPll
739	1mu4A<br>(86)<br>1y50A<br>(87)	N	26.74	TM-align: aliSize=44 (resi) RMSD=2.33 (Å)<br>mvqqkvevrlkt-----------GLQARPAALFVQEANRFTSDVFLEKDGKKVNAKSIMGLMSLAVStgtevtliaqgedeqealeklaayvqeevl--------------------------------<br>------------aektfkvvsdsGIHARPATILVQTASKWNSEIQLEYNGKTVNLKSIMGVMSLGIP-----------------------------kgatikitaegadaaeamaaltdtlakeglae<br><br>SARST: aliSize=81 (resi) RMSD=15.73 (Å)<br>mv-QQKVEVRLKTGLQARPAALFVQEANRFTSDVFLEKDGKKVNAKSIMGLMSLAVSTGTEVTLIAQGEDEQ--EALEKLAAYVQE---evl<br>--aEKTFKVVSDSGIHARPATILVQTASKWNSEIQLEYNGKTVNLKSIMGVMSLGIPKGATIKITAEGADAAeaMAALTDTLAKEGlae---<br><br>BLAST: aliSize=49 (resi) iden=37.21% (32/86) simi=56.98% (49/86)<br>mvqqkvevrlk----------TGLQARPAALFVQEANRFTSDVFLEKDGKKVNAKSIMGLMSLAVSTGTEVTLIAQGEDEQEALEKLAAYVQEEVL--<br>-----------aektfkvvsdSGIHARPATILVQTASKWNSEIQLEYNGKTVNLKSIMGVMSLGIPKGATIKITAEGADAAEAMAALTDTLAKEGLae<br><br>Proposed: aliSize=52 (resi) RMSD=1.55 (Å)<br>mVQQKVEVRL-KT-glQARPAALFVQEANRFTSDVFLEKDGKKVNAKSIMGLMSLA-V-------------------------------stgtevtliaqgedeqealeklaayvqeevl<br>-AEKTFKVVSdSGi--HARPATILVQTASKWNSEIQLEYNGKTVNLKSIMGVMSLGiPkgatikitaegadaaeamaaltdtlakeglae------------------------------
740	1mu4A<br>(86)<br>1y50A<br>(87)	C	34.88	TM-align: aliSize=44 (resi) RMSD=2.33 (Å)<br>mvqqkvevrlkt-----------GLQARPAALFVQEANRFTSDVFLEKDGKKVNAKSIMGLMSLAVStgtevtliaqgedeqealeklaayvqeevl--------------------------------<br>------------aektfkvvsdsGIHARPATILVQTASKWNSEIQLEYNGKTVNLKSIMGVMSLGIP-----------------------------kgatikitaegadaaeamaaltdtlakeglae<br><br>SARST: aliSize=81 (resi) RMSD=15.73 (Å)<br>mv-QQKVEVRLKTGLQARPAALFVQEANRFTSDVFLEKDGKKVNAKSIMGLMSLAVSTGTEVTLIAQGEDEQ--EALEKLAAYVQE---evl<br>--aEKTFKVVSDSGIHARPATILVQTASKWNSEIQLEYNGKTVNLKSIMGVMSLGIPKGATIKITAEGADAAeaMAALTDTLAKEGlae---<br><br>BLAST: aliSize=49 (resi) iden=37.21% (32/86) simi=56.98% (49/86)<br>mvqqkvevrlk----------TGLQARPAALFVQEANRFTSDVFLEKDGKKVNAKSIMGLMSLAVSTGTEVTLIAQGEDEQEALEKLAAYVQEEVL--<br>-----------aektfkvvsdSGIHARPATILVQTASKWNSEIQLEYNGKTVNLKSIMGVMSLGIPKGATIKITAEGADAAEAMAALTDTLAKEGLae<br><br>Proposed: aliSize=71 (resi) RMSD=1.75 (Å)<br>mvqqkvevrlktg------------LQARPAALFVQEANRFTSDVFLEKDGKKVNAKSIMGLMSLAVSTGTEVTLIAQGEDEQEALEKLAAYVQEE----vl<br>-------------aektfkvvsdsgIHARPATILVQTASKWNSEIQLEYNGKTVNLKSIMGVMSLGIPKGATIKITAEGADAAEAMAALTDTLAKEglae--

741	2ck2B (96)<br>1wwaX (105)	N	9.38	TM-align: aliSize=71 (resi) RMSD=3.60 (Å)<br>vsdvprdievva--------------VTptSALISWDAPaVT-IRYIRLTYGeTggnsPVQEI----TL-P--------GSKSTYTISGLKPGTDYTVTLYSVTGrgdspASSKPASINFRTEI------<br>------------vsfpasvqlhtaveMH--HWCIPFSVD-GQpAPSLRWLFN-G----SVLNEtsfiFTeFlepaanetVRHGCLRLNQPTHVNNGNYTLLAANP-----FGQASASIMAAFMDnpfefn<br><br>SARST: aliSize=82 (resi) RMSD=12.47 (Å)<br>vs---------DVPRDIEVvAVTptSALISWDAPavtiryIRLTYGETGGNSPV-----QEITLPGS-KSTYTISGLKPGT----DYTVTLYSvtGRGDSPASSKPASINFRTE----i<br>--vsfpasvqlHTAVEMHH-WCI--PFSVDGQPA------PSLRWLFNGSVLNEtsfifTEFLEPAAnETVRHGCLRLNQPthvnNGNYTLLA--ANPFGQASASIMAAFMDNPfefn-<br><br>BLAST: aliSize=23 (resi) iden=13.54% (13/96) simi=23.96% (23/96)<br>vsdvprdievvavtptsaliswdapavtir---YIRLTYGETGGNSPVQEITLPGSKSTYTISGlkpgtDYTvtLYSVTGRGDSPAS-------------skpasinfrtei<br>-----------------------------vsfpasvqlhtavemhhwcipfsvdgqpapslrwlfngsvlnetsFIFTEFLEPAANETVRHGCLRLNQPTHVNNG-----NYT--LLAANPFGQASASimaafmdnpfefn------------<br><br>Proposed: aliSize=78 (resi) RMSD=2.90 (Å)<br>-VSDVpRDIEVVAVTPT--saLISWDAPaVTIRYIRLTYGeT-ggnsPVQEI-----tL-----------pgSKSTYTISGLkPGT-DYTVTLYSVTGrgdspASSKPASINFRTEI------<br>vSFPA-SVQLHTAVEMHhw--CIPFSVD-GQPAPSLRWLF-Ng----SVLNEtsfif-Teflepaanetv--RHGCLRLNQP-THVnNGNYTLLAANP-----FGQASASIMAAFMDnpfefn
742	1f0vA (124)<br>3bcpA (124)	N	74.19	TM-align: aliSize=92 (resi) RMSD=1.15 (Å)<br>ketaaakferqhmdsstsaa--------------------SSSNYCNQMMksRNLTKDRCKPVNTFVHESLADVQAVCSQKNVACKNGQTNCYQSYSTMSITDCRETGSSKYPNCAYKTTQANKHIIVACEGNPyvpvhfdasv----------<br>--------------------kesaaakferqhmdsgnsasSSSNYCNQMM--RKMTQGKCKPVNTFVHESLADVKAVCSQKKVTCKDGQTNCYQSKSTMRITDCRETGSSKYPNCAYKTTQVEKHIIVACGGKP----------svpvhfdasv<br><br>SARST: aliSize=120 (resi) RMSD=16.64 (Å)<br>k-ETAAAKFERQHMDSSTsaASSSNYCNQMMKSRNLTKDRCKPVNTFVHESLADVQAVCSQKNVACKNGQTNCYQSYSTMSITDCRETGSSKYPNCAYKTTQANKHIIVACEGNPYVPVHFDAS-v<br>-kESAAAKFERQHMDSGN--SASSSSNYCNQMMRKMTQGKCKPVNTFVHESLADVKAVCSQKKVTCKDGQTNCYQSKSTMRITDCRETGSSKYPNCAYKTTQVEKHIIVACGGKPSVPVHFDASv-<br><br>BLAST: aliSize=109 (resi) iden=83.61% (102/124) simi=89.34% (109/124)<br>KETAAAKFERQHMDSSTSAASSSNYCNQMMksRNLTKDRCKPVNTFVHESLADVQAVCSQKNVACKNGQTNCYQSYSTMSITDCRETGSSKYPNCAYKTTQANKHIIVACEGNPYVPVHFDASV<br>KESAAAKFERQHMDSGNSASSSSNYCNQMM--RKMTQGKCKPVNTFVHESLADVKAVCSQKKVTCKDGQTNCYQSKSTMRITDCRETGSSKYPNCAYKTTQVEKHIIVACGGKPSVPVHFDASV<br><br>Proposed: aliSize=113 (resi) RMSD=1.04 (Å)<br>KETAAAKFERQHMDSSTSAASSSNYCNQMMKSRNLTKDRCKPVNTFVHESLADVQAVCSQKNVACKNGQTNCYQSYSTMSITDCRETGSSKYPNCAYKTTQANKHIIVACEGN----------pyvpvhfdasv<br>KESAAAKFERQHMDSGNSASSSSNYCNQMMYYRKMTQGKCKPVNTFVHESLADVKAVCSQKKVTCKDGQTNCYQSKSTMRITDCRETGSSKYPNCAYKTTQVEKHIIVACGGKpsvpvhfdasv----------
743	1f0vA (124)<br>3bcpA (124)	C	69.35	TM-align: aliSize=92 (resi) RMSD=1.15 (Å)<br>ketaaakferqhmdsstsaa--------------------SSSNYCNQMMksRNLTKDRCKPVNTFVHESLADVQAVCSQKNVACKNGQTNCYQSYSTMSITDCRETGSSKYPNCAYKTTQANKHIIVACEGNPyvpvhfdasv----------<br>--------------------kesaaakferqhmdsgnsasSSSNYCNQMM--RKMTQGKCKPVNTFVHESLADVKAVCSQKKVTCKDGQTNCYQSKSTMRITDCRETGSSKYPNCAYKTTQVEKHIIVACGGKP----------svpvhfdasv<br><br>SARST: aliSize=120 (resi) RMSD=16.64 (Å)<br>k-ETAAAKFERQHMDSSTsaASSSNYCNQMMKSRNLTKDRCKPVNTFVHESLADVQAVCSQKNVACKNGQTNCYQSYSTMSITDCRETGSSKYPNCAYKTTQANKHIIVACEGNPYVPVHFDAS-v<br>-kESAAAKFERQHMDSGN--SASSSSNYCNQMMRKMTQGKCKPVNTFVHESLADVKAVCSQKKVTCKDGQTNCYQSKSTMRITDCRETGSSKYPNCAYKTTQVEKHIIVACGGKPSVPVHFDASv-<br><br>BLAST: aliSize=109 (resi) iden=83.61% (102/124) simi=89.34% (109/124)<br>KETAAAKFERQHMDSSTSAASSSNYCNQMMksRNLTKDRCKPVNTFVHESLADVQAVCSQKNVACKNGQTNCYQSYSTMSITDCRETGSSKYPNCAYKTTQANKHIIVACEGNPYVPVHFDASV<br>KESAAAKFERQHMDSGNSASSSSNYCNQMM--RKMTQGKCKPVNTFVHESLADVKAVCSQKKVTCKDGQTNCYQSKSTMRITDCRETGSSKYPNCAYKTTQVEKHIIVACGGKPSVPVHFDASV<br><br>Proposed: aliSize=104 (resi) RMSD=0.83 (Å)<br>ketaaakferqhmdsstsaa--------------------SSSNYCNQMMKSRNLTKDRCKPVNTFVHESLADVQAVCSQKNVACKNGQTNCYQSYSTMSITDCRETGSSKYPNCAYKTTQANKHIIVACEGNPYVPVHFDASV<br>--------------------kesaaakferqhmdsgnsasSSSNYCNQMMYYRKMTQGKCKPVNTFVHESLADVKAVCSQKKVTCKDGQTNCYQSKSTMRITDCRETGSSKYPNCAYKTTQVEKHIIVACGGKPSVPVHFDASV

744	1f0vA (124)<br>3bcoA (124)	N	74.19	TM-align: aliSize=94 (resi) RMSD=1.16 (Å)<br>ketaaakferqhmdsstsaa--------------------SSSNYCNQMMKSRNLTKDRCKPVNTFVHESLADVQAVCSQKNVACKNGQTNCYQSYSTMSITDCRETGSSKYPNCAYKTTQANKHIIVACEGNPyvpvhfdasv----------<br>--------------------kesaaakferqhmdsgnsasSSSNYCNQMMCCRKMTQGKCKPVNTFVHESLADVKAVCSQKKVTCKDGQTNCYQSKSTMRITDCRETGSSKYPNCAYKTTQVEKHIIVACGGKP----------svpvhfdasv<br><br>SARST: aliSize=121 (resi) RMSD=12.72 (Å)<br>k-ETAAAKFERQHMDSsTSAASS-SNYCNQMMKSRNLTKDRCKPVNTFVHESLADVQAVCSQKNVACKNGQTNCYQSYSTMSITDCRETGSSKYPNCAYKTTQANKHIIVACEGNPYVPVHFDAS-v<br>-kESAAAKFERQHMDS-GNSASSsSNYCNQMMCCRKMTQGKCKPVNTFVHESLADVKAVCSQKKVTCKDGQTNCYQSKSTMRITDCRETGSSKYPNCAYKTTQVEKHIIVACGGKPSVPVHFDASv-<br><br>BLAST: aliSize=109 (resi) iden=82.26% (102/124) simi=87.90% (109/124)<br>KETAAAKFERQHMDSSTSAASSSNYCNQMMKSRNLTKDRCKPVNTFVHESLADVQAVCSQKNVACKNGQTNCYQSYSTMSITDCRETGSSKYPNCAYKTTQANKHIIVACEGNPYVPVHFDASV<br>KESAAAKFERQHMDSGNSASSSSNYCNQMMCCRKMTQGKCKPVNTFVHESLADVKAVCSQKKVTCKDGQTNCYQSKSTMRITDCRETGSSKYPNCAYKTTQVEKHIIVACGGKPSVPVHFDASV<br><br>Proposed: aliSize=113 (resi) RMSD=0.91 (Å)<br>KETAAAKFERQHMDSSTSAASSSNYCNQMMKSRNLTKDRCKPVNTFVHESLADVQAVCSQKNVACKNGQTNCYQSYSTMSITDCRETGSSKYPNCAYKTTQANKHIIVACEGN-----------pyvpvhfdasv<br>KESAAAKFERQHMDSGNSASSSSNYCNQMMCCRKMTQGKCKPVNTFVHESLADVKAVCSQKKVTCKDGQTNCYQSKSTMRITDCRETGSSKYPNCAYKTTQVEKHIIVACGGKpsvpvhfdasv-----------
745	1f0vA (124)<br>3bcoA (124)	C	69.35	TM-align: aliSize=94 (resi) RMSD=1.16 (Å)<br>ketaaakferqhmdsstsaa--------------------SSSNYCNQMMKSRNLTKDRCKPVNTFVHESLADVQAVCSQKNVACKNGQTNCYQSYSTMSITDCRETGSSKYPNCAYKTTQANKHIIVACEGNPyvpvhfdasv----------<br>--------------------kesaaakferqhmdsgnsasSSSNYCNQMMCCRKMTQGKCKPVNTFVHESLADVKAVCSQKKVTCKDGQTNCYQSKSTMRITDCRETGSSKYPNCAYKTTQVEKHIIVACGGKP----------svpvhfdasv<br><br>SARST: aliSize=121 (resi) RMSD=12.72 (Å)<br>k-ETAAAKFERQHMDSsTSAASS-SNYCNQMMKSRNLTKDRCKPVNTFVHESLADVQAVCSQKNVACKNGQTNCYQSYSTMSITDCRETGSSKYPNCAYKTTQANKHIIVACEGNPYVPVHFDAS-v<br>-kESAAAKFERQHMDS-GNSASSsSNYCNQMMCCRKMTQGKCKPVNTFVHESLADVKAVCSQKKVTCKDGQTNCYQSKSTMRITDCRETGSSKYPNCAYKTTQVEKHIIVACGGKPSVPVHFDASv-<br><br>BLAST: aliSize=109 (resi) iden=82.26% (102/124) simi=87.90% (109/124)<br>KETAAAKFERQHMDSSTSAASSSNYCNQMMKSRNLTKDRCKPVNTFVHESLADVQAVCSQKNVACKNGQTNCYQSYSTMSITDCRETGSSKYPNCAYKTTQANKHIIVACEGNPYVPVHFDASV<br>KESAAAKFERQHMDSGNSASSSSNYCNQMMCCRKMTQGKCKPVNTFVHESLADVKAVCSQKKVTCKDGQTNCYQSKSTMRITDCRETGSSKYPNCAYKTTQVEKHIIVACGGKPSVPVHFDASV<br><br>Proposed: aliSize=104 (resi) RMSD=0.83 (Å)<br>ketaaakferqhmdsstsaa--------------------SSSNYCNQMMKSRNLTKDRCKPVNTFVHESLADVQAVCSQKNVACKNGQTNCYQSYSTMSITDCRETGSSKYPNCAYKTTQANKHIIVACEGNPYVPVHFDASV<br>--------------------kesaaakferqhmdsgnsasSSSNYCNQMMCCRKMTQGKCKPVNTFVHESLADVKAVCSQKKVTCKDGQTNCYQSKSTMRITDCRETGSSKYPNCAYKTTQVEKHIIVACGGKPSVPVHFDASV
746	1f0vA (124)<br>3bcmA (119)	C	83.19	TM-align: aliSize=109 (resi) RMSD=0.96 (Å)<br>KETAAAKFERQHMDSsTsaasSSNYCNQMMKSRNLTKDRCKPVNTFVHESLADVQAVCSQKNVACKNGQTNCYQSYSTMSITDCRETGSSKYPNCAYKTTQANKHIIVACEGNPyvpvhfdasv----------<br>KESAAAKFERQHMDS-G----SSNYCNQMMCCRKMTQGKCKPVNTFVHESLADVKAVCSQKKVTCKDGQTNCYQSKSTMRITDCRETGSSKYPNCAYKTTQVEKHIIVACGGKP----------svpvhfdasv<br><br>SARST: aliSize=117 (resi) RMSD=9.20 (Å)<br>k-ETAAAKFERQHMDsstsaASSSNYCNQMMKSRNLTKDRCKPVNTFVHESLADVQAVCSQKNVACKNGQTNCYQSYSTMSITDCRETGSSKYPNCAYKTTQANKHIIVACEGNPYVPVHFDAS-v<br>-kESAAAKFERQHMD-----SGSSNYCNQMMCCRKMTQGKCKPVNTFVHESLADVKAVCSQKKVTCKDGQTNCYQSKSTMRITDCRETGSSKYPNCAYKTTQVEKHIIVACGGKPSVPVHFDASv-<br><br>BLAST: aliSize=105 (resi) iden=83.19% (99/119) simi=88.24% (105/119)<br>KETAAAKFERQHMDSStsaasSSNYCNQMMKSRNLTKDRCKPVNTFVHESLADVQAVCSQKNVACKNGQTNCYQSYSTMSITDCRETGSSKYPNCAYKTTQANKHIIVACEGNPYVPVHFDASV<br>KESAAAKFERQHMDSG-----SSNYCNQMMCCRKMTQGKCKPVNTFVHESLADVKAVCSQKKVTCKDGQTNCYQSKSTMRITDCRETGSSKYPNCAYKTTQVEKHIIVACGGKPSVPVHFDASV<br><br>Proposed: aliSize=119 (resi) RMSD=0.59 (Å)<br>KETAAAKFERQHMDSsTsaasSSNYCNQMMKSRNLTKDRCKPVNTFVHESLADVQAVCSQKNVACKNGQTNCYQSYSTMSITDCRETGSSKYPNCAYKTTQANKHIIVACEGNPYVPVHFDASV<br>KESAAAKFERQHMDS-G----SSNYCNQMMCCRKMTQGKCKPVNTFVHESLADVKAVCSQKKVTCKDGQTNCYQSKSTMRITDCRETGSSKYPNCAYKTTQVEKHIIVACGGKPSVPVHFDASV

747	1f0vA (124)<br>1r5cA (124)	N	72.58	TM-align: aliSize=95 (resi) RMSD=1.64 (Å)<br>ketaaakferqhmdsstsaa--------------------SSSNYCNQMMKSRNLTKDRCKPVNTFVHESLADVQAVCSQKNVACKNGQTNCYQSYSTMSITDCRETGSSKYPNCAYKTTQANKHIIVACEGNPYvpvhfdasv---------<br>--------------------kesaaakferqhmdsgnspsSSSNYCNLMMCCRKMTQGKCKPVNTFVHESLADVKAVCSQKKVTCKNGQTNCYQSKSTMRITDCRETGSSKYPNCAYKTTQVEKHIIVACGGKPS---------vpvhfdasv<br><br>SARST: aliSize=121 (resi) RMSD=12.83 (Å)<br>k-ETAAAKFERQHMDS-STSAASsSNYCNQMMKSRNLTKDRCKPVNTFVHESLADVQAVCSQKNVACKNGQTNCYQSYSTMSITDCRETGSSKYPNCAYKTTQANKHIIVACEGNPYVPVHFDAS-v<br>-kESAAAKFERQHMDSgNSPSSS-SNYCNLMMCCRKMTQGKCKPVNTFVHESLADVKAVCSQKKVTCKNGQTNCYQSKSTMRITDCRETGSSKYPNCAYKTTQVEKHIIVACGGKPSVPVHFDASv-<br><br>BLAST: aliSize=107 (resi) iden=81.45% (101/124) simi=86.29% (107/124)<br>KETAAAKFERQHMDSSTSAASSSNYCNQMMKSRNLTKDRCKPVNTFVHESLADVQAVCSQKNVACKNGQTNCYQSYSTMSITDCRETGSSKYPNCAYKTTQANKHIIVACEGNPYVPVHFDASV<br>KESAAAKFERQHMDSGNSPSSSSNYCNLMMCCRKMTQGKCKPVNTFVHESLADVKAVCSQKKVTCKNGQTNCYQSKSTMRITDCRETGSSKYPNCAYKTTQVEKHIIVACGGKPSVPVHFDASV<br><br>Proposed: aliSize=112 (resi) RMSD=0.84 (Å)<br>KETAAAKFERQHMDSSsTSA-ASSSNYCNQMMKSRNLTKDRCKPVNTFVHESLADVQAVCSQKNVACKNGQTNCYQSYSTMSITDCRETGSSKYPNCAYKTTQANKHIIVACEGN-----------pyvpvhfdasv<br>KESAAAKFERQHMDS-GNSpSSSSNYCNLMMCCRKMTQGKCKPVNTFVHESLADVKAVCSQKKVTCKNGQTNCYQSKSTMRITDCRETGSSKYPNCAYKTTQVEKHIIVACGGKpsvpvhfdasv-----------
748	1f0vA (124)<br>1r5cA (124)	C	68.55	TM-align: aliSize=95 (resi) RMSD=1.64 (Å)<br>ketaaakferqhmdsstsaa--------------------SSSNYCNQMMKSRNLTKDRCKPVNTFVHESLADVQAVCSQKNVACKNGQTNCYQSYSTMSITDCRETGSSKYPNCAYKTTQANKHIIVACEGNPYvpvhfdasv---------<br>--------------------kesaaakferqhmdsgnspsSSSNYCNLMMCCRKMTQGKCKPVNTFVHESLADVKAVCSQKKVTCKNGQTNCYQSKSTMRITDCRETGSSKYPNCAYKTTQVEKHIIVACGGKPS---------vpvhfdasv<br><br>SARST: aliSize=121 (resi) RMSD=12.83 (Å)<br>k-ETAAAKFERQHMDS-STSAASsSNYCNQMMKSRNLTKDRCKPVNTFVHESLADVQAVCSQKNVACKNGQTNCYQSYSTMSITDCRETGSSKYPNCAYKTTQANKHIIVACEGNPYVPVHFDAS-v<br>-kESAAAKFERQHMDSgNSPSSS-SNYCNLMMCCRKMTQGKCKPVNTFVHESLADVKAVCSQKKVTCKNGQTNCYQSKSTMRITDCRETGSSKYPNCAYKTTQVEKHIIVACGGKPSVPVHFDASv-<br><br>BLAST: aliSize=107 (resi) iden=81.45% (101/124) simi=86.29% (107/124)<br>KETAAAKFERQHMDSSTSAASSSNYCNQMMKSRNLTKDRCKPVNTFVHESLADVQAVCSQKNVACKNGQTNCYQSYSTMSITDCRETGSSKYPNCAYKTTQANKHIIVACEGNPYVPVHFDASV<br>KESAAAKFERQHMDSGNSPSSSSNYCNLMMCCRKMTQGKCKPVNTFVHESLADVKAVCSQKKVTCKNGQTNCYQSKSTMRITDCRETGSSKYPNCAYKTTQVEKHIIVACGGKPSVPVHFDASV<br><br>Proposed: aliSize=103 (resi) RMSD=0.65 (Å)<br>ketaaakferqhmdsstsaas---------------------SSNYCNQMMKSRNLTKDRCKPVNTFVHESLADVQAVCSQKNVACKNGQTNCYQSYSTMSITDCRETGSSKYPNCAYKTTQANKHIIVACEGNPYVPVHFDASV<br>---------------------kesaaakferqhmdsgnspssSSNYCNLMMCCRKMTQGKCKPVNTFVHESLADVKAVCSQKKVTCKNGQTNCYQSKSTMRITDCRETGSSKYPNCAYKTTQVEKHIIVACGGKPSVPVHFDASV
749	1f0vA (124)<br>1l1bgA (124)	N	73.39	TM-align: aliSize=94 (resi) RMSD=1.06 (Å)<br>ketaaakferqhmdsstsaa--------------------SSSNYCNQMMKSRNLTKDRCKPVNTFVHESLADVQAVCSQKNVACKNGQTNCYQSYSTMSITDCRETGSSKYPNCAYKTTQANKHIIVACEGNPyvpvhfdasv----------<br>--------------------kesaaakferqhmdsgnspsSSSNYCNLMMCCRKMTQGKCKPVNTFVHESLADVKAVCSQKKVTCKNGQTNCYQSKSTMRITDCRETGSSKYPNCAYKTTQVEKHIIVACGGKP----------svpvhfdasv<br><br>SARST: aliSize=121 (resi) RMSD=12.88 (Å)<br>k-ETAAAKFERQHMDS-STSAASsSNYCNQMMKSRNLTKDRCKPVNTFVHESLADVQAVCSQKNVACKNGQTNCYQSYSTMSITDCRETGSSKYPNCAYKTTQANKHIIVACEGNPYVPVHFDAS-v<br>-kESAAAKFERQHMDSgNSPSSS-SNYCNLMMCCRKMTQGKCKPVNTFVHESLADVKAVCSQKKVTCKNGQTNCYQSKSTMRITDCRETGSSKYPNCAYKTTQVEKHIIVACGGKPSVPVHFDASv-<br><br>BLAST: aliSize=107 (resi) iden=81.45% (101/124) simi=86.29% (107/124)<br>KETAAAKFERQHMDSSTSAASSSNYCNQMMKSRNLTKDRCKPVNTFVHESLADVQAVCSQKNVACKNGQTNCYQSYSTMSITDCRETGSSKYPNCAYKTTQANKHIIVACEGNPYVPVHFDASV<br>KESAAAKFERQHMDSGNSPSSSSNYCNLMMCCRKMTQGKCKPVNTFVHESLADVKAVCSQKKVTCKNGQTNCYQSKSTMRITDCRETGSSKYPNCAYKTTQVEKHIIVACGGKPSVPVHFDASV<br><br>Proposed: aliSize=113 (resi) RMSD=0.93 (Å)<br>KETAAAKFERQHMDSSsTSA-ASSSNYCNQMMKSRNLTKDRCKPVNTFVHESLADVQAVCSQKNVACKNGQTNCYQSYSTMSITDCRETGSSKYPNCAYKTTQANKHIIVACEGNP----------yvpvhfdasv<br>KESAAAKFERQHMDS-GNSpSSSSNYCNLMMCCRKMTQGKCKPVNTFVHESLADVKAVCSQKKVTCKNGQTNCYQSKSTMRITDCRETGSSKYPNCAYKTTQVEKHIIVACGGKPsvpvhfdasv----------

750	1f0vA (124)<br>11bgA (124)	C	69.35	TM-align: aliSize=94 (resi) RMSD=1.06 (Å)<br>ketaaakferqhmdsstsaa--------------------SSSNYCNQMMKSRNLTKDRCKPVNTFVHESLADVQAVCSQKNVACKNGQTNCYQSYSTMSITDCRETGSSKYPNCAYKTTQANKHIIVACEGNPyvpvhfdasv----------<br>--------------------kesaaakferqhmdsgnspsSSSNYCNLMMCCRKMTQGKCKPVNTFVHESLADVKAVCSQKKVTCKNGQTNCYQSKSTMRITDCRETGSSKYPNCAYKTTQVEKHIIVACGGKP----------svpvhfdasv<br><br>SARST: aliSize=121 (resi) RMSD=12.88 (Å)<br>k-ETAAAKFERQHMDS-STSAASsSNYCNQMMKSRNLTKDRCKPVNTFVHESLADVQAVCSQKNVACKNGQTNCYQSYSTMSITDCRETGSSKYPNCAYKTTQANKHIIVACEGNPYVPVHFDAS-v<br>-kESAAAKFERQHMDSgNSPSSS-SNYCNLMMCCRKMTQGKCKPVNTFVHESLADVKAVCSQKKVTCKNGQTNCYQSKSTMRITDCRETGSSKYPNCAYKTTQVEKHIIVACGGKPSVPVHFDASv-<br><br>BLAST: aliSize=107 (resi) iden=81.45% (101/124) simi=86.29% (107/124)<br>KETAAAKFERQHMDSSTSAASSSNYCNQMMKSRNLTKDRCKPVNTFVHESLADVQAVCSQKNVACKNGQTNCYQSYSTMSITDCRETGSSKYPNCAYKTTQANKHIIVACEGNPYVPVHFDASV<br>KESAAAKFERQHMDSGNSPSSSSNYCNLMMCCRKMTQGKCKPVNTFVHESLADVKAVCSQKKVTCKNGQTNCYQSKSTMRITDCRETGSSKYPNCAYKTTQVEKHIIVACGGKPSVPVHFDASV<br><br>Proposed: aliSize=104 (resi) RMSD=0.82 (Å)<br>ketaaakferqhmdsstsaa--------------------SSSNYCNQMMKSRNLTKDRCKPVNTFVHESLADVQAVCSQKNVACKNGQTNCYQSYSTMSITDCRETGSSKYPNCAYKTTQANKHIIVACEGNPYVPVHFDASV<br>--------------------kesaaakferqhmdsgnspsSSSNYCNLMMCCRKMTQGKCKPVNTFVHESLADVKAVCSQKKVTCKNGQTNCYQSKSTMRITDCRETGSSKYPNCAYKTTQVEKHIIVACGGKPSVPVHFDASV
751	1f0vA (124)<br>1tq9A (124)	N	73.39	TM-align: aliSize=93 (resi) RMSD=1.26 (Å)<br>ketaaakferqhmdsstsa-------------------ASSSNYCNQMMksRNLTKDRCKPVNTFVHESLADVQAVCSQKNVACKNGQTNCYQSYSTMSITDCRETGSSKYPNCAYKTTQANKHIIVACEGNPyvpvhfdasv----------<br>-------------------kesaaakferqhmdsgnspSSSSNYCNLMM--RKMTQGKCKPVNTFVHESLADVKAVCSQKKVTCKNGQTNCYQSKSTMRITDCRETGSSKYPNCAYKTTQVEKHIIVACGGKP----------svpvhfdasv<br><br>SARST: aliSize=119 (resi) RMSD=13.97 (Å)<br>k-ETAAAKFERQHMDSSTsAASS-SNYCNQMMKsrNLTKDRCKPVNTFVHESLADVQAVCSQKNVACKNGQTNCYQSYSTMSITDCRETGSSKYPNCAYKTTQANKHIIVACEGNPYVPVHFDAS-v<br>-kESAAAKFERQHMDSGN-SPSSsSNYCNLMMR--KMTQGKCKPVNTFVHESLADVKAVCSQKKVTCKNGQTNCYQSKSTMRITDCRETGSSKYPNCAYKTTQVEKHIIVACGGKPSVPVHFDASv-<br><br>BLAST: aliSize=107 (resi) iden=82.79% (101/124) simi=87.70% (107/124)<br>KETAAAKFERQHMDSSTSAASSSNYCNQMMksRNLTKDRCKPVNTFVHESLADVQAVCSQKNVACKNGQTNCYQSYSTMSITDCRETGSSKYPNCAYKTTQANKHIIVACEGNPYVPVHFDASV<br>KESAAAKFERQHMDSGNSPSSSSNYCNLMM--RKMTQGKCKPVNTFVHESLADVKAVCSQKKVTCKNGQTNCYQSKSTMRITDCRETGSSKYPNCAYKTTQVEKHIIVACGGKPSVPVHFDASV<br><br>Proposed: aliSize=113 (resi) RMSD=0.93 (Å)<br>KETAAAKFERQHMDSSTsAAS-SSNYCNQMMKSRNLTKDRCKPVNTFVHESLADVQAVCSQKNVACKNGQTNCYQSYSTMSITDCRETGSSKYPNCAYKTTQANKHIIVACEGNP----------yvpvhfdasv<br>KESAAAKFERQHMDSGN-SPSsSSNYCNLMMYYRKMTQGKCKPVNTFVHESLADVKAVCSQKKVTCKNGQTNCYQSKSTMRITDCRETGSSKYPNCAYKTTQVEKHIIVACGGKPsvpvhfdasv----------
752	1f0vA (124)<br>1tq9A (124)	C	69.35	TM-align: aliSize=93 (resi) RMSD=1.26 (Å)<br>ketaaakferqhmdsstsa-------------------ASSSNYCNQMMksRNLTKDRCKPVNTFVHESLADVQAVCSQKNVACKNGQTNCYQSYSTMSITDCRETGSSKYPNCAYKTTQANKHIIVACEGNPyvpvhfdasv----------<br>-------------------kesaaakferqhmdsgnspSSSSNYCNLMM--RKMTQGKCKPVNTFVHESLADVKAVCSQKKVTCKNGQTNCYQSKSTMRITDCRETGSSKYPNCAYKTTQVEKHIIVACGGKP----------svpvhfdasv<br><br>SARST: aliSize=119 (resi) RMSD=13.97 (Å)<br>k-ETAAAKFERQHMDSSTsAASS-SNYCNQMMKsrNLTKDRCKPVNTFVHESLADVQAVCSQKNVACKNGQTNCYQSYSTMSITDCRETGSSKYPNCAYKTTQANKHIIVACEGNPYVPVHFDAS-v<br>-kESAAAKFERQHMDSGN-SPSSsSNYCNLMMR--KMTQGKCKPVNTFVHESLADVKAVCSQKKVTCKNGQTNCYQSKSTMRITDCRETGSSKYPNCAYKTTQVEKHIIVACGGKPSVPVHFDASv-<br><br>BLAST: aliSize=107 (resi) iden=82.79% (101/124) simi=87.70% (107/124)<br>KETAAAKFERQHMDSSTSAASSSNYCNQMMksRNLTKDRCKPVNTFVHESLADVQAVCSQKNVACKNGQTNCYQSYSTMSITDCRETGSSKYPNCAYKTTQANKHIIVACEGNPYVPVHFDASV<br>KESAAAKFERQHMDSGNSPSSSSNYCNLMM--RKMTQGKCKPVNTFVHESLADVKAVCSQKKVTCKNGQTNCYQSKSTMRITDCRETGSSKYPNCAYKTTQVEKHIIVACGGKPSVPVHFDASV<br><br>Proposed: aliSize=104 (resi) RMSD=0.66 (Å)<br>ketaaakferqhmdsstsaa--------------------SSSNYCNQMMKSRNLTKDRCKPVNTFVHESLADVQAVCSQKNVACKNGQTNCYQSYSTMSITDCRETGSSKYPNCAYKTTQANKHIIVACEGNPYVPVHFDASV<br>--------------------kesaaakferqhmdsgnspsSSSNYCNLMMYYRKMTQGKCKPVNTFVHESLADVKAVCSQKKVTCKNGQTNCYQSKSTMRITDCRETGSSKYPNCAYKTTQVEKHIIVACGGKPSVPVHFDASV

753	1f0vA<br>(124)<br><br>1r3mB<br>(122)	C	81.15	TM-align: aliSize=112 (resi) RMSD=1.10 (Å)<br>KETAAAKFERQHMDSsTSAAsSSNYCNQMMKSRNLTKDRCKPVNTFVHESLADVQAVCSQKNVACKNGQTNCYQSYSTMSITDCRETGSSKYPNCAYKTTQANKHIIVACEGNPyvpvhfdasv----------<br>KESAAAKFERQHMDS-GNSP-SSNYCNLMMCCRKMTQGKCKPVNTFVHESLADVKAVCSQKKVTCKNGQTNCYQSKSTMRITDCRETGSSKYPNCAYKTTQVEKHIIVACGGKP----------svpvhfdasv<br><br>SARST: aliSize=120 (resi) RMSD=9.01 (Å)<br>k-ETAAAKFERQHMDssTSAASSSNYCNQMMKSRNLTKDRCKPVNTFVHESLADVQAVCSQKNVACKNGQTNCYQSYSTMSITDCRETGSSKYPNCAYKTTQANKHIIVACEGNPYVPVHFDAS-v<br>-kESAAAKFERQHMD--SGNSPSSNYCNLMMCCRKMTQGKCKPVNTFVHESLADVKAVCSQKKVTCKNGQTNCYQSKSTMRITDCRETGSSKYPNCAYKTTQVEKHIIVACGGKPSVPVHFDASv-<br><br>BLAST: aliSize=105 (resi) iden=81.97% (100/122) simi=86.07% (105/122)<br>KETAAAKFERQHMDSSTSAasSSNYCNQMMKSRNLTKDRCKPVNTFVHESLADVQAVCSQKNVACKNGQTNCYQSYSTMSITDCRETGSSKYPNCAYKTTQANKHIIVACEGNPYVPVHFDASV<br>KESAAAKFERQHMDSGNSP--SSNYCNLMMCCRKMTQGKCKPVNTFVHESLADVKAVCSQKKVTCKNGQTNCYQSKSTMRITDCRETGSSKYPNCAYKTTQVEKHIIVACGGKPSVPVHFDASV<br><br>Proposed: aliSize=122 (resi) RMSD=0.79 (Å)<br>KETAAAKFERQHMDSsTSAAsSSNYCNQMMKSRNLTKDRCKPVNTFVHESLADVQAVCSQKNVACKNGQTNCYQSYSTMSITDCRETGSSKYPNCAYKTTQANKHIIVACEGNPYVPVHFDASV<br>KESAAAKFERQHMDS-GNSP-SSNYCNLMMCCRKMTQGKCKPVNTFVHESLADVKAVCSQKKVTCKNGQTNCYQSKSTMRITDCRETGSSKYPNCAYKTTQVEKHIIVACGGKPSVPVHFDASV
754	1f0vA<br>(124)<br><br>1r5dA<br>(124)	N	72.58	TM-align: aliSize=95 (resi) RMSD=1.62 (Å)<br>ketaaakferqhmdsstsaa-------------------SSSNYCNQMMKSRNLTKDRCKPVNTFVHESLADVQAVCSQKNVACKNGQTNCYQSYSTMSITDCRETGSSKYPNCAYKTTQANKHIIVACEGNPYvpvhfdasv---------<br>-------------------kesaaakferqhmdsgnspsSSSNYCNLMMCCRKMTQGKCKPVNTFVHESLADVKAVCSQKKVTCKNGQTNCYQSKSTMRITDCRETGSSKYPNCAYKTTQVEKHIIVACGGKPS---------vpvhfdasv<br><br>SARST: aliSize=121 (resi) RMSD=12.84 (Å)<br>k-ETAAAKFERQHMDSsTSAASS-SNYCNQMMKSRNLTKDRCKPVNTFVHESLADVQAVCSQKNVACKNGQTNCYQSYSTMSITDCRETGSSKYPNCAYKTTQANKHIIVACEGNPYVPVHFDAS-v<br>-kESAAAKFERQHMDS-GNSPSSsSNYCNLMMCCRKMTQGKCKPVNTFVHESLADVKAVCSQKKVTCKNGQTNCYQSKSTMRITDCRETGSSKYPNCAYKTTQVEKHIIVACGGKPSVPVHFDASv-<br><br>BLAST: aliSize=107 (resi) iden=81.45% (101/124) simi=86.29% (107/124)<br>KETAAAKFERQHMDSSTSAASSSNYCNQMMKSRNLTKDRCKPVNTFVHESLADVQAVCSQKNVACKNGQTNCYQSYSTMSITDCRETGSSKYPNCAYKTTQANKHIIVACEGNPYVPVHFDASV<br>KESAAAKFERQHMDSGNSPSSSSNYCNLMMCCRKMTQGKCKPVNTFVHESLADVKAVCSQKKVTCKNGQTNCYQSKSTMRITDCRETGSSKYPNCAYKTTQVEKHIIVACGGKPSVPVHFDASV<br><br>Proposed: aliSize=112 (resi) RMSD=0.84 (Å)<br>KETAAAKFERQHMDSsTSA-ASSSNYCNQMMKSRNLTKDRCKPVNTFVHESLADVQAVCSQKNVACKNGQTNCYQSYSTMSITDCRETGSSKYPNCAYKTTQANKHIIVACEGN-----------pyvpvhfdasv<br>KESAAAKFERQHMDS-GNSpSSSSNYCNLMMCCRKMTQGKCKPVNTFVHESLADVKAVCSQKKVTCKNGQTNCYQSKSTMRITDCRETGSSKYPNCAYKTTQVEKHIIVACGGKpsvpvhfdasv-----------
755	1f0vA<br>(124)<br><br>1r5dA<br>(124)	C	68.55	TM-align: aliSize=95 (resi) RMSD=1.62 (Å)<br>ketaaakferqhmdsstsaa-------------------SSSNYCNQMMKSRNLTKDRCKPVNTFVHESLADVQAVCSQKNVACKNGQTNCYQSYSTMSITDCRETGSSKYPNCAYKTTQANKHIIVACEGNPYvpvhfdasv---------<br>-------------------kesaaakferqhmdsgnspsSSSNYCNLMMCCRKMTQGKCKPVNTFVHESLADVKAVCSQKKVTCKNGQTNCYQSKSTMRITDCRETGSSKYPNCAYKTTQVEKHIIVACGGKPS---------vpvhfdasv<br><br>SARST: aliSize=121 (resi) RMSD=12.84 (Å)<br>k-ETAAAKFERQHMDSsTSAASS-SNYCNQMMKSRNLTKDRCKPVNTFVHESLADVQAVCSQKNVACKNGQTNCYQSYSTMSITDCRETGSSKYPNCAYKTTQANKHIIVACEGNPYVPVHFDAS-v<br>-kESAAAKFERQHMDS-GNSPSSsSNYCNLMMCCRKMTQGKCKPVNTFVHESLADVKAVCSQKKVTCKNGQTNCYQSKSTMRITDCRETGSSKYPNCAYKTTQVEKHIIVACGGKPSVPVHFDASv-<br><br>BLAST: aliSize=107 (resi) iden=81.45% (101/124) simi=86.29% (107/124)<br>KETAAAKFERQHMDSSTSAASSSNYCNQMMKSRNLTKDRCKPVNTFVHESLADVQAVCSQKNVACKNGQTNCYQSYSTMSITDCRETGSSKYPNCAYKTTQANKHIIVACEGNPYVPVHFDASV<br>KESAAAKFERQHMDSGNSPSSSSNYCNLMMCCRKMTQGKCKPVNTFVHESLADVKAVCSQKKVTCKNGQTNCYQSKSTMRITDCRETGSSKYPNCAYKTTQVEKHIIVACGGKPSVPVHFDASV<br><br>Proposed: aliSize=103 (resi) RMSD=0.66 (Å)<br>ketaaakferqhmdsstsaas--------------------SSNYCNQMMKSRNLTKDRCKPVNTFVHESLADVQAVCSQKNVACKNGQTNCYQSYSTMSITDCRETGSSKYPNCAYKTTQANKHIIVACEGNPYVPVHFDASV<br>--------------------kesaaakferqhmdsgnspssSSNYCNLMMCCRKMTQGKCKPVNTFVHESLADVKAVCSQKKVTCKNGQTNCYQSKSTMRITDCRETGSSKYPNCAYKTTQVEKHIIVACGGKPSVPVHFDASV

756	1f0vA (124)<br>1lbaA (124)	N	72.58	TM-align: aliSize=94 (resi) RMSD=1.21 (Å)<br>ketaaakferqhmdsstsaa--------------------SSSNYCNQMMKSRNLTKDRCKPVNTFVHESLADVQAVCSQKNVACKNGQTNCYQSYSTMSITDCRETGSSKYPNCAYKTTQANKHIIVACEGNPyvpvhfdasv----------<br>--------------------kesaaakferqhmdsgnspsSSSNYCNLMMCCRKMTQGKCKPVNTFVHESLADVKAVCSQKKVTCKNGQTNCYQSKSTMRITDCRETGSSKYPNCAYKTTQVEKHIIVACGGKP----------svpvhfdasv<br><br>SARST: aliSize=121 (resi) RMSD=12.89 (Å)<br>k-ETAAAKFERQHMDSsTSAASS-SNYCNQMMKSRNLTKDRCKPVNTFVHESLADVQAVCSQKNVACKNGQTNCYQSYSTMSITDCRETGSSKYPNCAYKTTQANKHIIVACEGNPYVPVHFDAS-v<br>-kESAAAKFERQHMDS-GNSPSSsSNYCNLMMCCRKMTQGKCKPVNTFVHESLADVKAVCSQKKVTCKNGQTNCYQSKSTMRITDCRETGSSKYPNCAYKTTQVEKHIIVACGGKPSVPVHFDASv-<br><br>BLAST: aliSize=107 (resi) iden=81.45% (101/124) simi=86.29% (107/124)<br>KETAAAKFERQHMDSSTSAASSSNYCNQMMKSRNLTKDRCKPVNTFVHESLADVQAVCSQKNVACKNGQTNCYQSYSTMSITDCRETGSSKYPNCAYKTTQANKHIIVACEGNPYVPVHFDASV<br>KESAAAKFERQHMDSGNSPSSSSNYCNLMMCCRKMTQGKCKPVNTFVHESLADVKAVCSQKKVTCKNGQTNCYQSKSTMRITDCRETGSSKYPNCAYKTTQVEKHIIVACGGKPSVPVHFDASV<br><br>Proposed: aliSize=112 (resi) RMSD=0.75 (Å)<br>KETAAAKFERQHMDSsTSA-ASSSNYCNQMMKSRNLTKDRCKPVNTFVHESLADVQAVCSQKNVACKNGQTNCYQSYSTMSITDCRETGSSKYPNCAYKTTQANKHIIVACEGN-----------pyvpvhfdasv<br>KESAAAKFERQHMDS-GNSpSSSSNYCNLMMCCRKMTQGKCKPVNTFVHESLADVKAVCSQKKVTCKNGQTNCYQSKSTMRITDCRETGSSKYPNCAYKTTQVEKHIIVACGGKpsvpvhfdasv-----------
757	1f0vA (124)<br>1lbaA (124)	C	68.55	TM-align: aliSize=94 (resi) RMSD=1.21 (Å)<br>ketaaakferqhmdsstsaa--------------------SSSNYCNQMMKSRNLTKDRCKPVNTFVHESLADVQAVCSQKNVACKNGQTNCYQSYSTMSITDCRETGSSKYPNCAYKTTQANKHIIVACEGNPyvpvhfdasv----------<br>--------------------kesaaakferqhmdsgnspsSSSNYCNLMMCCRKMTQGKCKPVNTFVHESLADVKAVCSQKKVTCKNGQTNCYQSKSTMRITDCRETGSSKYPNCAYKTTQVEKHIIVACGGKP----------svpvhfdasv<br><br>SARST: aliSize=121 (resi) RMSD=12.89 (Å)<br>k-ETAAAKFERQHMDSsTSAASS-SNYCNQMMKSRNLTKDRCKPVNTFVHESLADVQAVCSQKNVACKNGQTNCYQSYSTMSITDCRETGSSKYPNCAYKTTQANKHIIVACEGNPYVPVHFDAS-v<br>-kESAAAKFERQHMDS-GNSPSSsSNYCNLMMCCRKMTQGKCKPVNTFVHESLADVKAVCSQKKVTCKNGQTNCYQSKSTMRITDCRETGSSKYPNCAYKTTQVEKHIIVACGGKPSVPVHFDASv-<br><br>BLAST: aliSize=107 (resi) iden=81.45% (101/124) simi=86.29% (107/124)<br>KETAAAKFERQHMDSSTSAASSSNYCNQMMKSRNLTKDRCKPVNTFVHESLADVQAVCSQKNVACKNGQTNCYQSYSTMSITDCRETGSSKYPNCAYKTTQANKHIIVACEGNPYVPVHFDASV<br>KESAAAKFERQHMDSGNSPSSSSNYCNLMMCCRKMTQGKCKPVNTFVHESLADVKAVCSQKKVTCKNGQTNCYQSKSTMRITDCRETGSSKYPNCAYKTTQVEKHIIVACGGKPSVPVHFDASV<br><br>Proposed: aliSize=103 (resi) RMSD=0.55 (Å)<br>ketaaakferqhmdsstsaas---------------------SSNYCNQMMKSRNLTKDRCKPVNTFVHESLADVQAVCSQKNVACKNGQTNCYQSYSTMSITDCRETGSSKYPNCAYKTTQANKHIIVACEGNPYVPVHFDASV<br>---------------------kesaaakferqhmdsgnspssSSNYCNLMMCCRKMTQGKCKPVNTFVHESLADVKAVCSQKKVTCKNGQTNCYQSKSTMRITDCRETGSSKYPNCAYKTTQVEKHIIVACGGKPSVPVHFDASV
758	1r5cA (124)<br>1js0A (124)	N	72.58	TM-align: aliSize=94 (resi) RMSD=1.43 (Å)<br>kesaaakferqhmdsgnsps--------------------SSSNYCNLMMCCRKMTQGKCKPVNTFVHESLADVKAVCSQKKVTCKNGQTNCYQSKSTMRITDCRETGSSKYPNCAYKTTQVEKHIIVACGGKPsvpvhfdasv----------<br>--------------------ketaaakferqhmdsstsaaSSSNYCNQMMKSRNLTKDRCKPVNTFVHESLADVQAVCSQKNVACKNGQTNCYQSYSTMSITDCRETGSSKYPNCAYKTTQANKHIIVACEGNP----------yvpvhfdasv<br><br>SARST: aliSize=121 (resi) RMSD=11.44 (Å)<br>k-ESAAAKFERQHMDSgNSPSSS-SNYCNLMMCCRKMTQGKCKPVNTFVHESLADVKAVCSQKKVTCKNGQTNCYQSKSTMRITDCRETGSSKYPNCAYKTTQVEKHIIVACGGKPSVPVHFDAS-v<br>-kETAAAKFERQHMDS-STSAASsSNYCNQMMKSRNLTKDRCKPVNTFVHESLADVQAVCSQKNVACKNGQTNCYQSYSTMSITDCRETGSSKYPNCAYKTTQANKHIIVACEGNPYVPVHFDASv-<br><br>BLAST: aliSize=107 (resi) iden=81.45% (101/124) simi=86.29% (107/124)<br>KESAAAKFERQHMDSGNSPSSSSNYCNLMMCCRKMTQGKCKPVNTFVHESLADVKAVCSQKKVTCKNGQTNCYQSKSTMRITDCRETGSSKYPNCAYKTTQVEKHIIVACGGKPSVPVHFDASV<br>KETAAAKFERQHMDSSTSAASSSNYCNQMMKSRNLTKDRCKPVNTFVHESLADVQAVCSQKNVACKNGQTNCYQSYSTMSITDCRETGSSKYPNCAYKTTQANKHIIVACEGNPYVPVHFDASV<br><br>Proposed: aliSize=112 (resi) RMSD=0.88 (Å)<br>KESAAAKFERQHMDS-GNSpSSSSNYCNLMMCCRKMTQGKCKPVNTFVHESLADVKAVCSQKKVTCKNGQTNCYQSKSTMRITDCRETGSSKYPNCAYKTTQVEKHIIVACGGK-----------psvpvhfdasv<br>KETAAAKFERQHMDSsTSA-ASSSNYCNQMMKSRNLTKDRCKPVNTFVHESLADVQAVCSQKNVACKNGQTNCYQSYSTMSITDCRETGSSKYPNCAYKTTQANKHIIVACEGNpyvpvhfdasv-----------

759	1r5cA (124)<br>1js0A (124)	C	68.55	TM-align: aliSize=94 (resi) RMSD=1.43 (Å)<br>kesaaakferqhmdsgnsps--------------------SSSNYCNLMMCCRKMTQGKCKPVNTFVHESLADVKAVCSQKKVTCKNGQTNCYQSKSTMRITDCRETGSSKYPNCAYKTTQVEKHIIVACGGKPsvpvhfdasv----------<br>--------------------ketaaakferqhmdsstsaaSSSNYCNQMMKSRNLTKDRCKPVNTFVHESLADVQAVCSQKNVACKNGQTNCYQSYSTMSITDCRETGSSKYPNCAYKTTQANKHIIVACEGNP----------yvpvhfdasv<br><br>SARST: aliSize=121 (resi) RMSD=11.44 (Å)<br>k-ESAAAKFERQHMDSgNSPSSS-SNYCNLMMCCRKMTQGKCKPVNTFVHESLADVKAVCSQKKVTCKNGQTNCYQSKSTMRITDCRETGSSKYPNCAYKTTQVEKHIIVACGGKPSVPVHFDAS-v<br>-kETAAAKFERQHMDS-STSAASsSNYCNQMMKSRNLTKDRCKPVNTFVHESLADVQAVCSQKNVACKNGQTNCYQSYSTMSITDCRETGSSKYPNCAYKTTQANKHIIVACEGNPYVPVHFDASv-<br><br>BLAST: aliSize=107 (resi) iden=81.45% (101/124) simi=86.29% (107/124)<br>KESAAAKFERQHMDSGNSPSSSSNYCNLMMCCRKMTQGKCKPVNTFVHESLADVKAVCSQKKVTCKNGQTNCYQSKSTMRITDCRETGSSKYPNCAYKTTQVEKHIIVACGGKPSVPVHFDASV<br>KETAAAKFERQHMDSSTSAASSSNYCNQMMKSRNLTKDRCKPVNTFVHESLADVQAVCSQKNVACKNGQTNCYQSYSTMSITDCRETGSSKYPNCAYKTTQANKHIIVACEGNPYVPVHFDASV<br><br>Proposed: aliSize=103 (resi) RMSD=0.67 (Å)<br>kesaaakferqhmdsgnspss---------------------SSNYCNLMMCCRKMTQGKCKPVNTFVHESLADVKAVCSQKKVTCKNGQTNCYQSKSTMRITDCRETGSSKYPNCAYKTTQVEKHIIVACGGKPSVPVHFDASV<br>---------------------ketaaakferqhmdsstsaasSSNYCNQMMKSRNLTKDRCKPVNTFVHESLADVQAVCSQKNVACKNGQTNCYQSYSTMSITDCRETGSSKYPNCAYKTTQANKHIIVACEGNPYVPVHFDASV
760	1r5cA (124)<br>1r3mB (122)	N	98.36	TM-align: aliSize=103 (resi) RMSD=0.35 (Å)<br>kesaaakferqhmdsgnspss-------------------SSNYCNLMMCCRKMTQGKCKPVNTFVHESLADVKAVCSQKKVTCKNGQTNCYQSKSTMRITDCRETGSSKYPNCAYKTTQVEKHIIVACGGKPSVPVHFDASV<br>---------------------kesaaakferqhmdsgnspSSNYCNLMMCCRKMTQGKCKPVNTFVHESLADVKAVCSQKKVTCKNGQTNCYQSKSTMRITDCRETGSSKYPNCAYKTTQVEKHIIVACGGKPSVPVHFDASV<br><br>SARST: aliSize=120 (resi) RMSD=9.87 (Å)<br>k-ESAAAKFERQHMDSGNSPSssSNYCNLMMCCRKMTQGKCKPVNTFVHESLADVKAVCSQKKVTCKNGQTNCYQSKSTMRITDCRETGSSKYPNCAYKTTQVEKHIIVACGGKPSVPVHFDAS-v<br>-kESAAAKFERQHMDSGNSPS--SNYCNLMMCCRKMTQGKCKPVNTFVHESLADVKAVCSQKKVTCKNGQTNCYQSKSTMRITDCRETGSSKYPNCAYKTTQVEKHIIVACGGKPSVPVHFDASv-<br><br>BLAST: aliSize=122 (resi) iden=100.00% (122/122) simi=100.00% (122/122)<br>KESAAAKFERQHMDSGNSPSSSsNYCNLMMCCRKMTQGKCKPVNTFVHESLADVKAVCSQKKVTCKNGQTNCYQSKSTMRITDCRETGSSKYPNCAYKTTQVEKHIIVACGGKPSVPVHFDASV<br>KESAAAKFERQHMDSGNSPSS--NYCNLMMCCRKMTQGKCKPVNTFVHESLADVKAVCSQKKVTCKNGQTNCYQSKSTMRITDCRETGSSKYPNCAYKTTQVEKHIIVACGGKPSVPVHFDASV<br><br>Proposed: aliSize=122 (resi) RMSD=0.51 (Å)<br>KESAAAKFERQHMDSGNsPSsSSNYCNLMMCCRKMTQGKCKPVNTFVHESLADVKAVCSQKKVTCKNGQTNCYQSKSTMRITDCRETGSSKYPNCAYKTTQVEKHIIVACGGKPSVPVHFDASV<br>KESAAAKFERQHMDSGN-SP-SSNYCNLMMCCRKMTQGKCKPVNTFVHESLADVKAVCSQKKVTCKNGQTNCYQSKSTMRITDCRETGSSKYPNCAYKTTQVEKHIIVACGGKPSVPVHFDASV
761	1r5cA (124)<br>1a2wA (124)	N	80.65	TM-align: aliSize=104 (resi) RMSD=0.89 (Å)<br>kesaaakferqhmdsgnsps--------------------SSSNYCNLMMCCRKMTQGKCKPVNTFVHESLADVKAVCSQKKVTCKNGQTNCYQSKSTMRITDCRETGSSKYPNCAYKTTQVEKHIIVACGGKPSVPVHFDASV<br>--------------------ketaaakferqhmdsstsaaSSSNYCNQMMKSRNLTKDRCKPVNTFVHESLADVQAVCSQKNVACKNGQTNCYQSYSTMSITDCRETGSSKYPNCAYKTTQANKHIIVACEGNPYVPVHFDASV<br><br>SARST: aliSize=118 (resi) RMSD=12.35 (Å)<br>k-ESAAAKFERQHMDsgnsPS--SS--SNYCNLMMCCRKMTQGKCKPVNTFVHESLADVKAVCSQKKVTCKNGQTNCYQSKSTMRITDCRETGSSKYPNCAYKTTQVEKHIIVACGGKPSVPVHFDAS-v<br>-kETAAAKFERQHMD----SStsAAssSNYCNQMMKSRNLTKDRCKPVNTFVHESLADVQAVCSQKNVACKNGQTNCYQSYSTMSITDCRETGSSKYPNCAYKTTQANKHIIVACEGNPYVPVHFDASv-<br><br>BLAST: aliSize=107 (resi) iden=81.45% (101/124) simi=86.29% (107/124)<br>KESAAAKFERQHMDSGNSPSSSSNYCNLMMCCRKMTQGKCKPVNTFVHESLADVKAVCSQKKVTCKNGQTNCYQSKSTMRITDCRETGSSKYPNCAYKTTQVEKHIIVACGGKPSVPVHFDASV<br>KETAAAKFERQHMDSSTSAASSSNYCNQMMKSRNLTKDRCKPVNTFVHESLADVQAVCSQKNVACKNGQTNCYQSYSTMSITDCRETGSSKYPNCAYKTTQANKHIIVACEGNPYVPVHFDASV<br><br>Proposed: aliSize=123 (resi) RMSD=0.92 (Å)<br>KESAAAKFERQHMDS-GNSpSSSSNYCNLMMCCRKMTQGKCKPVNTFVHESLADVKAVCSQKKVTCKNGQTNCYQSKSTMRITDCRETGSSKYPNCAYKTTQVEKHIIVACGGKPSVPVHFDASV<br>KETAAAKFERQHMDSsTSA-ASSSNYCNQMMKSRNLTKDRCKPVNTFVHESLADVQAVCSQKNVACKNGQTNCYQSYSTMSITDCRETGSSKYPNCAYKTTQANKHIIVACEGNPYVPVHFDASV

762	1r5cA (124)<br>1h8xA (125)	N	71.77	TM-align: aliSize=103 (resi) RMSD=1.36 (Å)<br>kesaaakferqhmdsgnsps--------------------SSSNYCNLMMCCRKMTQGKCKPVNTFVHESLADVKAVCSQKKVTC-KNgQTNCYQSKSTMRITDCRETGSSKYPNCAYKTTQVEKHIIVACGGKPSVPVHFDASV-<br>--------------------kesaaakferqhmdsgnspsSSSTYCNQMMRRRNMTQGRCKPVNTFVHEPLVDVQNVCFQEKVTCkNG-QGNCYKSNSSMHITDCRLTNGSRYPNCAYRTSQKERHIIVACEGSPYVPVHFDASVe<br><br>SARST: aliSize=120 (resi) RMSD=11.93 (Å)<br>k-ESAAAKFERQHMD--SGNSPSSSsnYCNLMMCCRKMTQGKCKPVNTFVHESLADVKAVCSQKKVTCKNGQTNCYQSKSTMRITDCRETGSSKYPNCAYKTTQVEKHIIVACGGKPSVPVHFDAS--v<br>-kESAAAKFERQHMDsgNSPSSSST--YCNQMMRRRNMTQGRCKPVNTFVHEPLVDVQNVCFQEKVTCKNGQGNCYKSNSSMHITDCRLTNGSRYPNCAYRTSQKERHIIVACEGSPYVPVHFDASve-<br><br>BLAST: aliSize=105 (resi) iden=77.42% (96/124) simi=84.68% (105/124)<br>KESAAAKFERQHMDSGNSPSSSSNYCNLMMCCRKMTQGKCKPVNTFVHESLADVKAVCSQKKVTCKNGQTNCYQSKSTMRITDCRETGSSKYPNCAYKTTQVEKHIIVACGGKPSVPVHFDASV-<br>KESAAAKFERQHMDSGNSPSSSSTYCNQMMRRRNMTQGRCKPVNTFVHEPLVDVQNVCFQEKVTCKNGQGNCYKSNSSMHITDCRLTNGSRYPNCAYRTSQKERHIIVACEGSPYVPVHFDASVe<br><br>Proposed: aliSize=122 (resi) RMSD=1.00 (Å)<br>KESAAAKFERQHMDSGnSPS-SSSNYCNLMMCCRKMTQGKCKPVNTFVHESLADVKAVCSQKKVTC-KNgQTNCYQSKSTMRITDCRETGSSKYPNCAYKTTQVEKHIIVACGGKPSVPVHFDASV-<br>KESAAAKFERQHMDSG-NSPsSSSTYCNQMMRRRNMTQGRCKPVNTFVHEPLVDVQNVCFQEKVTCkNG-QGNCYKSNSSMHITDCRLTNGSRYPNCAYRTSQKERHIIVACEGSPYVPVHFDASVe
763	1r3mB (122)<br>1r5dA (124)	N	99.18	TM-align: aliSize=103 (resi) RMSD=0.37 (Å)<br>kesaaakferqhmdsgnsp--------------------SSNYCNLMMCCRKMTQGKCKPVNTFVHESLADVKAVCSQKKVTCKNGQTNCYQSKSTMRITDCRETGSSKYPNCAYKTTQVEKHIIVACGGKPSVPVHFDASV<br>-------------------kesaaakferqhmdsgnspssSSNYCNLMMCCRKMTQGKCKPVNTFVHESLADVKAVCSQKKVTCKNGQTNCYQSKSTMRITDCRETGSSKYPNCAYKTTQVEKHIIVACGGKPSVPVHFDASV<br><br>SARST: aliSize=120 (resi) RMSD=9.88 (Å)<br>k-ESAAAKFERQHMDSGNSPS--SNYCNLMMCCRKMTQGKCKPVNTFVHESLADVKAVCSQKKVTCKNGQTNCYQSKSTMRITDCRETGSSKYPNCAYKTTQVEKHIIVACGGKPSVPVHFDAS-v<br>-kESAAAKFERQHMDSGNSPSssSNYCNLMMCCRKMTQGKCKPVNTFVHESLADVKAVCSQKKVTCKNGQTNCYQSKSTMRITDCRETGSSKYPNCAYKTTQVEKHIIVACGGKPSVPVHFDASv-<br><br>BLAST: aliSize=122 (resi) iden=100.00% (122/122) simi=100.00% (122/122)<br>KESAAAKFERQHMDSGNSPSS--NYCNLMMCCRKMTQGKCKPVNTFVHESLADVKAVCSQKKVTCKNGQTNCYQSKSTMRITDCRETGSSKYPNCAYKTTQVEKHIIVACGGKPSVPVHFDASV<br>KESAAAKFERQHMDSGNSPSSssNYCNLMMCCRKMTQGKCKPVNTFVHESLADVKAVCSQKKVTCKNGQTNCYQSKSTMRITDCRETGSSKYPNCAYKTTQVEKHIIVACGGKPSVPVHFDASV<br><br>Proposed: aliSize=122 (resi) RMSD=0.53 (Å)<br>KESAAAKFERQHMDSGNS-P-SSNYCNLMMCCRKMTQGKCKPVNTFVHESLADVKAVCSQKKVTCKNGQTNCYQSKSTMRITDCRETGSSKYPNCAYKTTQVEKHIIVACGGKPSVPVHFDASV<br>KESAAAKFERQHMDSGNSpSsSSNYCNLMMCCRKMTQGKCKPVNTFVHESLADVKAVCSQKKVTCKNGQTNCYQSKSTMRITDCRETGSSKYPNCAYKTTQVEKHIIVACGGKPSVPVHFDASV
764	1r3mB (122)<br>1a2wA (124)	N	81.15	TM-align: aliSize=105 (resi) RMSD=1.11 (Å)<br>kesaaakferqhmdsgn-------------------SPSSNYCNLMMCCRKMTQGKCKPVNTFVHESLADVKAVCSQKKVTCKNGQTNCYQSKSTMRITDCRETGSSKYPNCAYKTTQVEKHIIVACGGKPSVPVHFDASV<br>-----------------ketaaakferqhmdsstsaASSSNYCNQMMKSRNLTKDRCKPVNTFVHESLADVQAVCSQKNVACKNGQTNCYQSYSTMSITDCRETGSSKYPNCAYKTTQANKHIIVACEGNPYVPVHFDASV<br><br>SARST: aliSize=120 (resi) RMSD=14.21 (Å)<br>k-ESAAAKFERQHMDSGNSPS--SNYCNLMMCCRKMTQGKCKPVNTFVHESLADVKAVCSQKKVTCKNGQTNCYQSKSTMRITDCRETGSSKYPNCAYKTTQVEKHIIVACGGKPSVPVHFDAS-v<br>-kETAAAKFERQHMDSSTSAAssSNYCNQMMKSRNLTKDRCKPVNTFVHESLADVQAVCSQKNVACKNGQTNCYQSYSTMSITDCRETGSSKYPNCAYKTTQANKHIIVACEGNPYVPVHFDASv-<br><br>BLAST: aliSize=105 (resi) iden=81.97% (100/122) simi=86.07% (105/122)<br>KESAAAKFERQHMDSGNSP--SSNYCNLMMCCRKMTQGKCKPVNTFVHESLADVKAVCSQKKVTCKNGQTNCYQSKSTMRITDCRETGSSKYPNCAYKTTQVEKHIIVACGGKPSVPVHFDASV<br>KETAAAKFERQHMDSSTSAasSSNYCNQMMKSRNLTKDRCKPVNTFVHESLADVQAVCSQKNVACKNGQTNCYQSYSTMSITDCRETGSSKYPNCAYKTTQANKHIIVACEGNPYVPVHFDASV<br><br>Proposed: aliSize=122 (resi) RMSD=0.76 (Å)<br>KESAAAKFERQHMDS-GNSP-SSNYCNLMMCCRKMTQGKCKPVNTFVHESLADVKAVCSQKKVTCKNGQTNCYQSKSTMRITDCRETGSSKYPNCAYKTTQVEKHIIVACGGKPSVPVHFDASV<br>KETAAAKFERQHMDSsTSAAsSSNYCNQMMKSRNLTKDRCKPVNTFVHESLADVQAVCSQKNVACKNGQTNCYQSYSTMSITDCRETGSSKYPNCAYKTTQANKHIIVACEGNPYVPVHFDASV

765	1r3mB (122)<br>11baA (124)	N	99.18	TM-align: aliSize=103 (resi) RMSD=0.51 (Å)<br>kesaaakferqhmdsgnsp--------------------SSNYCNLMMCCRKMTQGKCKPVNTFVHESLADVKAVCSQKKVTCKNGQTNCYQSKSTMRITDCRETGSSKYPNCAYKTTQVEKHIIVACGGKPSVPVHFDASV<br>------------------kesaaakferqhmdsgnspssSSNYCNLMMCCRKMTQGKCKPVNTFVHESLADVKAVCSQKKVTCKNGQTNCYQSKSTMRITDCRETGSSKYPNCAYKTTQVEKHIIVACGGKPSVPVHFDASV<br><br>SARST: aliSize=120 (resi) RMSD=9.84 (Å)<br>k-ESAAAKFERQHMDSGNSPS--SNYCNLMMCCRKMTQGKCKPVNTFVHESLADVKAVCSQKKVTCKNGQTNCYQSKSTMRITDCRETGSSKYPNCAYKTTQVEKHIIVACGGKPSVPVHFDAS-v<br>-kESAAAKFERQHMDSGNSPSssSNYCNLMMCCRKMTQGKCKPVNTFVHESLADVKAVCSQKKVTCKNGQTNCYQSKSTMRITDCRETGSSKYPNCAYKTTQVEKHIIVACGGKPSVPVHFDASv-<br><br>BLAST: aliSize=122 (resi) iden=100.00% (122/122) simi=100.00% (122/122)<br>KESAAAKFERQHMDSGNSPSS--NYCNLMMCCRKMTQGKCKPVNTFVHESLADVKAVCSQKKVTCKNGQTNCYQSKSTMRITDCRETGSSKYPNCAYKTTQVEKHIIVACGGKPSVPVHFDASV<br>KESAAAKFERQHMDSGNSPSSSsNYCNLMMCCRKMTQGKCKPVNTFVHESLADVKAVCSQKKVTCKNGQTNCYQSKSTMRITDCRETGSSKYPNCAYKTTQVEKHIIVACGGKPSVPVHFDASV<br><br>Proposed: aliSize=122 (resi) RMSD=0.61 (Å)<br>KESAAAKFERQHMDSGNS-P-SSNYCNLMMCCRKMTQGKCKPVNTFVHESLADVKAVCSQKKVTCKNGQTNCYQSKSTMRITDCRETGSSKYPNCAYKTTQVEKHIIVACGGKPSVPVHFDASV<br>KESAAAKFERQHMDSGNSpSsSSNYCNLMMCCRKMTQGKCKPVNTFVHESLADVKAVCSQKKVTCKNGQTNCYQSKSTMRITDCRETGSSKYPNCAYKTTQVEKHIIVACGGKPSVPVHFDASV
766	1r3mB (122)<br>1h8xA (125)	N	73.77	TM-align: aliSize=103 (resi) RMSD=1.33 (Å)<br>kesaaakferqhmdsgns------------------P-SSNYCNLMMCCRKMTQGKCKPVNTFVHESLADVKAVCSQKKVTC-KNgQTNCYQSKSTMRITDCRETGSSKYPNCAYKTTQVEKHIIVACGGKPSVPVHFDASV-<br>-----------------kesaaakferqhmdsgnspSsSSTYCNQMMRRRNMTQGRCKPVNTFVHEPLVDVQNVCFQEKVTCkNG-QGNCYKSNSSMHITDCRLTNGSRYPNCAYRTSQKERHIIVACEGSPYVPVHFDASVe<br><br>SARST: aliSize=120 (resi) RMSD=14.10 (Å)<br>k-ESAAAKFERQHMDSGNSPSSN--YCNLMMCCRKMTQGKCKPVNTFVHESLADVKAVCSQKKVTCKNGQTNCYQSKSTMRITDCRETGSSKYPNCAYKTTQVEKHIIVACGGKPSVPVHFDAS--v<br>-kESAAAKFERQHMDSGNSPSSSstYCNQMMRRRNMTQGRCKPVNTFVHEPLVDVQNVCFQEKVTCKNGQGNCYKSNSSMHITDCRLTNGSRYPNCAYRTSQKERHIIVACEGSPYVPVHFDASve-<br><br>BLAST: aliSize=104 (resi) iden=77.05% (94/122) simi=85.25% (104/122)<br>KESAAAKFERQHMDSGNSPSSN--YCNLMMCCRKMTQGKCKPVNTFVHESLADVKAVCSQKKVTCKNGQTNCYQSKSTMRITDCRETGSSKYPNCAYKTTQVEKHIIVACGGKPSVPVHFDASV-<br>KESAAAKFERQHMDSGNSPSSSstYCNQMMRRRNMTQGRCKPVNTFVHEPLVDVQNVCFQEKVTCKNGQGNCYKSNSSMHITDCRLTNGSRYPNCAYRTSQKERHIIVACEGSPYVPVHFDASVe<br><br>Proposed: aliSize=120 (resi) RMSD=0.92 (Å)<br>KESAAAKFERQHMDSGNSP---sSNYCNLMMCCRKMTQGKCKPVNTFVHESLADVKAVCSQKKVTC-KNgQTNCYQSKSTMRITDCRETGSSKYPNCAYKTTQVEKHIIVACGGKPSVPVHFDASV-<br>KESAAAKFERQHMDSGNSPsss-STYCNQMMRRRNMTQGRCKPVNTFVHEPLVDVQNVCFQEKVTCkNG-QGNCYKSNSSMHITDCRLTNGSRYPNCAYRTSQKERHIIVACEGSPYVPVHFDASVe
767	1dz3A (123)<br>2ieyA (164)	C	8.94	TM-align: aliSize=90 (resi) RMSD=3.72 (Å)<br>-----SIKVCIAD-DNReLVSLLDEYISSqpdmev-----Igtayngq---------------------------Dcl-QMLEEKRPdILLLDI----IMPhLDGLAVLERIraG-FEH-QPNVIMLT--A-----F-GQEDVT-KKAVELGASYFILKPFDMEnlahhirqvygkt--------------<br>gdydyLIKLLALGdSGV-GKTTFLYRYTD------nkfnpK-------fittvgidfrekrvvydfkvhlqlwdtS--lTTAFFRDA-GFLLFDltsqQSF-LNVRNWSQLQ--AnAYCeNPDIVLIGnkAdlpdqReVNERQArELAEKYGIPYFETSAATGQ-------------nveksvetlldlikr<br><br>SARST: aliSize=114 (resi) RMSD=11.18 (Å)<br>si-------KVCIADDNRELVSLLDEYISSQPDMEVIGTA---------------------YNGQDClqMLEEKRPDILLLDIimphLDGLAVLER-----IRAGFEH-QPNVIMLTAFGQ---------EDVTKKAVELGASYFILKPFDMENLAHHIRQVYGK---t<br>--gdydyliKLLALGDSGVGKTTFLYRYTDNKFNPKFITTvgidfrekrvvydfkvhlqlWDTSLT--TAFFRDAGFLLFDL----TSQQSFLNVrnwsqLQANAYCeNPDIVLIGNKADlpdqrevneRQARELAEKYGIPYFETSAATGQNVEKSVETLLDLikr-<br><br>BLAST: aliSize=14 (resi) iden=4.88% (6/123) simi=11.38% (14/123)<br>sikvciaddnrelvslldeyissq---PDMEVIGTAYNGQDCLQMLEEKRPDI--------------------------------llldiim<br>------------------------gdydylikllalgdsgvgkttflyrytdnkfnpkfittvgidfrekrvvydfkvhlqlwdtslttaffrdagfllfdltsqqsflnvrnwsqlqanaycenPDIVLIGNKADLPDQREVNERQARELaekygipyfetsaatgqnveksvetlldlikr-------<br>phldglavleriragfehqpnvimltafgqedvtkkavelgasyfilkpfdmenlahhirqvygkt<br>--<br><br>Proposed: aliSize=97 (resi) RMSD=2.52 (Å)<br>-----SIKVCIAD-DN-reLVSLLDEYISS----------------------------------qpdmevigtayngqdclQMlEE--KRPDILLLDI-----IM--phlDGLAVLERIRaGFEHQ--PNVIMLTA-F-------GQEDVT-KKAVELGASYFIL--kpFDMENLAHHIRQVYGKT----<br>gdydyLIKLLALGdSGv--GKTTFLYRYTDnkfnpkfittvgidfrekrvvydfkvhlqlwdts-----------------LT-TAffRDAMGFLLMFdltsqQSfl---NVRNWMSQLQ-ANAYCenPDIVLIGNkAdlpdqreVNERQArELAEKYGIPYFETsa--ATGQNVEKSVETLLDLImkrm

768	1q10A<br>(56)<br><br>1k50A<br>(63)	C	5.36	TM-align: aliSize=36 (resi) RMSD=1.87 (Å)<br>--MQYKVILNGKT-LKGETTTEAVDaATAEKVV-KqFFNdNG-Vdgewtyddatktftvte----------------------<br>eeVTIKANLIFANgSTQTAEFKGTF-EKATSEAyA-YAD-TLkK-----------------dngewtadvadkgytlnikfag<br><br>SARST: aliSize=45 (resi) RMSD=8.15 (Å)<br>m---QYKVILNGKT-LKGETTTEAVDAATAEKVVKQFFNDNGVDGEWTYD--------------datktftvte<br>-eevTIKANLIFANgSTQTAEFKGTFEKATSEAYAYADTLKKDNGEWTADvadkgytlnikfag----------<br><br>BLAST: aliSize=22 (resi) iden=30.36% (17/56) simi=39.29% (22/56)<br>mqyk---------VILNGKTLKGETTTeAVDAATAEKVV--KQFFNDNGvdgEWTYDDATKTFTV------te<br>----eevtikanlIFANGSTQTAEFKG-TFEKATSEAYAyaDTLKKDNG---EWTADVADKGYTLnikfag--<br><br>Proposed: aliSize=55 (resi) RMSD=2.28 (Å)<br>-MQYKVILNG-K-TLKGETTTEAvDAATAEKVVKQFFNDNGV---DGEWTYDDATKTFTVTE--<br>eEVTIKANLIfAnGSTQTAEFKG-TFEKATSEAYAYADTLKKdngEWTADVADKGYTLNIKFag
769	1q10A<br>(56)<br><br>1k53A<br>(72)	C	5.36	TM-align: aliSize=36 (resi) RMSD=1.89 (Å)<br>-----------MQYKVILNGKT-LKGETTTEAVDaATAEKVV-KqFFNdNG-Vdgewtyddatktftvte----------------------<br>mhhhhhhameeVTIKANLIFANaSTQTAEFKGTF-EKATSEAyA-YAD-TLkK-----------------dngewtvdvadkgytlnikfag<br><br>SARST: aliSize=45 (resi) RMSD=8.08 (Å)<br>m------------QYKVILNGKT-LKGETTTEAVDAATAEKVVKQFFNDNGVDGEWTYD--------------datktftvte<br>-mhhhhhhameevTIKANLIFANaSTQTAEFKGTFEKATSEAYAYADTLKKDNGEWTVDvadkgytlnikfag----------<br><br>BLAST: aliSize=22 (resi) iden=28.57% (16/56) simi=39.29% (22/56)<br>mqykvilngktlkg-----------------------------------ETTTEAVdaATAEKVVKqffnDNGvdgEWTYDDATKTFTV------te<br>--------------mhhhhhhameevtikanlifanastqtaefkgtfeKATSEAY--AYADTLKK----DNG---EWTVDVADKGYTLnikfag--<br><br>Proposed: aliSize=55 (resi) RMSD=2.18 (Å)<br>----------MQYKVILNGK--TLKGETTTEAVDaATAEKVVKQFFNDNGV---DGEWTYDDATKTFTVTE--<br>mhhhhhhameEVTIKANLIFanASTQTAEFKGTF-EKATSEAYAYADTLKKdngEWTVDVADKGYTLNIKFag
770	1q10A<br>(56)<br><br>1k52A<br>(72)	C	16.07	TM-align: aliSize=36 (resi) RMSD=1.88 (Å)<br>-----------MQYKVILNGKT-LKGETTTEAVDaATAEKVV-KqFFNdNG-Vdgewtyddatktftvte----------------------<br>mhhhhhhameeVTIKANLIFANgSTQTAEFKGTF-EKATSEAyA-YAD-TLkK-----------------dngewtvdvadggytlnikfag<br><br>SARST: aliSize=45 (resi) RMSD=8.08 (Å)<br>m------------QYKVILNGKT-LKGETTTEAVDAATAEKVVKQFFNDNGVDGEWTYD--------------datktftvte<br>-mhhhhhhameevTIKANLIFANgSTQTAEFKGTFEKATSEAYAYADTLKKDNGEWTVDvadggytlnikfag----------<br><br>BLAST: aliSize=21 (resi) iden=28.57% (16/56) simi=37.50% (21/56)<br>mqyk------------------VILNGKTLKGETTTeAVDAATAEKVV--KQFFNDNGvdgEWTYDDATKTFTV------te<br>----mhhhhhhameevtikanlIFANGSTQTAEFKG-TFEKATSEAYAyaDTLKKDNG---EWTVDVADGGYTLnikfag--<br><br>Proposed: aliSize=55 (resi) RMSD=2.15 (Å)<br>----------MQYKVILNGK--TLKGETTTEAVDaATAEKVVKQFFNDNGVD-GEWTYDDA----TKTFTVTE<br>mhhhhhhameEVTIKANLIFanGSTQTAEFKGTF-EKATSEAYAYADTLKKDnGEWTVDVAdggyTLNIKFAG

771	1q10A (56)<br>2hj1A (77)	C	1.79	TM-align: aliSize=36 (resi) RMSD=2.21 (Å)<br>--MQYKVILNGKTLKGETTTEAV--DAA-TAEKVVkqFF-NdNGVdgewtyddatktftvte-----------------------------------<br>nqINIEIAYAFPERYYLKSFQVDegITVqTAITQS--GIlS-QFP-----------------eidlstnkigifsrpikltdvlkegdrieiyrpll<br><br>SARST: aliSize=45 (resi) RMSD=10.14 (Å)<br>m---QYKVILNGKTLKGETTTEA---VDAATAEKV---VKQFFNDNGVDGEWTYD-----------------------datktftvte<br>-nqiNIEIAYAFPERYYLKSFQVdegITVQTAITQsgiLSQFPEIDLSTNKIGIFsrpikltdvlkegdrieiyrpll----------<br><br>BLAST: aliSize=10 (resi) iden=12.50% (7/56) simi=17.86% (10/56)<br>mqykvilngkt----------------LKGETTTEAVDAATA---EKVVKQF------------------------------------fndngvdgewtyddatktftvte<br>-----------nqinieiayafperyyLKSFQVDEGITVQTAitqSGILSQFpeidlstnkigifsrpikltdvlkegdrieiyrpll-----------------------<br><br>Proposed: aliSize=49 (resi) RMSD=2.91 (Å)<br>-MQYKVILNGK--TLKGETTTEAVDAAtaEKVVKQ--FF-------NDNGVDGEWTY--D---dATKT--F---------tvte<br>nQINIEIAYAFpeRYYLKSFQVDEGIT--VQTAITqsGIlsqfpeiDLSTNKIGIFSrpIklt-DVLKegDrieiyrpll----
772	2rlzA (85)<br>1y50A (87)	N	27.06	TM-align: aliSize=44 (resi) RMSD=2.40 (Å)<br>mvqqkvevrlk----------TGLQARPAALFVQEANRFTSDVFLEKDGKKVnAK-SIMGLMSLAVStgtevtliaqgedeqealeklaayvqeev--------------------------------<br>-----------aektfkvvsdSGIHARPATILVQTASKWNSEIQLEYNGKTV-NLkSIMGVMSLGIP-----------------------------kgatikitaegadaaeamaaltdtlakeglae<br><br>SARST: aliSize=81 (resi) RMSD=16.69 (Å)<br>mv-QQKVEVRLKTGLQARPAALFVQEANRFTSDVFLEKDGKKVNAKSIMGLMSLAVSTGTEVTLIAQGED--EQEALEKLAAYVQE---ev<br>--aEKTFKVVSDSGIHARPATILVQTASKWNSEIQLEYNGKTVNLKSIMGVMSLGIPKGATIKITAEGADaaEAMAALTDTLAKEGlae--<br><br>BLAST: aliSize=48 (resi) iden=36.47% (31/85) simi=56.47% (48/85)<br>mvqqkvevrlk----------TGLQARPAALFVQEANRFTSDVFLEKDGKKVNAKSIMGLMSLAVSTGTEVTLIAQGEDEQEALEKLAAYVQEE----v<br>-----------aektfkvvsdSGIHARPATILVQTASKWNSEIQLEYNGKTVNLKSIMGVMSLGIPKGATIKITAEGADAAEAMAALTDTLAKEglae-<br><br>Proposed: aliSize=53 (resi) RMSD=1.92 (Å)<br>mVQQKVEVRLKT-gLQARPAALFVQEANRFTSDVFLEKDGKKVNAKSIMGLMSLA-V--------------------------------stgtevtliaqgedeqealeklaayvqeev<br>-AEKTFKVVSDSg-IHARPATILVQTASKWNSEIQLEYNGKTVNLKSIMGVMSLGiPkgatikitaegadaaeamaaltdtlakeglae------------------------------
773	2rlzA (85)<br>1y50A (87)	C	35.29	TM-align: aliSize=44 (resi) RMSD=2.40 (Å)<br>mvqqkvevrlk----------TGLQARPAALFVQEANRFTSDVFLEKDGKKVnAK-SIMGLMSLAVStgtevtliaqgedeqealeklaayvqeev--------------------------------<br>-----------aektfkvvsdSGIHARPATILVQTASKWNSEIQLEYNGKTV-NLkSIMGVMSLGIP-----------------------------kgatikitaegadaaeamaaltdtlakeglae<br><br>SARST: aliSize=81 (resi) RMSD=16.69 (Å)<br>mv-QQKVEVRLKTGLQARPAALFVQEANRFTSDVFLEKDGKKVNAKSIMGLMSLAVSTGTEVTLIAQGED--EQEALEKLAAYVQE---ev<br>--aEKTFKVVSDSGIHARPATILVQTASKWNSEIQLEYNGKTVNLKSIMGVMSLGIPKGATIKITAEGADaaEAMAALTDTLAKEGlae--<br><br>BLAST: aliSize=48 (resi) iden=36.47% (31/85) simi=56.47% (48/85)<br>mvqqkvevrlk----------TGLQARPAALFVQEANRFTSDVFLEKDGKKVNAKSIMGLMSLAVSTGTEVTLIAQGEDEQEALEKLAAYVQEE----v<br>-----------aektfkvvsdSGIHARPATILVQTASKWNSEIQLEYNGKTVNLKSIMGVMSLGIPKGATIKITAEGADAAEAMAALTDTLAKEglae-<br><br>Proposed: aliSize=73 (resi) RMSD=2.00 (Å)<br>mvqqkvevrlk----------TGLQARPAALFVQEANRFTSDVFLEKDGKKVNAKSIMGLMSLAVSTGTEVTLIAQGEDEQEALEKLAAYVQE-E---v<br>-----------aektfkvvsdSGIHARPATILVQTASKWNSEIQLEYNGKTVNLKSIMGVMSLGIPKGATIKITAEGADAAEAMAALTDTLAKeGlae-

774	1wwaX<br>(105)<br><br>1he7A<br>(107)	C	91.43	TM-align: aliSize=101 (resi) RMSD=1.41 (Å)<br>VSFPASVQLHTAVEMHHWCIPFSVDGQPAPSLRWLFNGSVLNETSFIFTEFLEPAANETVRHGCLRLNQPTHVNNGYTLLAANPFGQASASIMAAFMDNPfefn------<br>SHMPASVQLHTAVEMHHWCIPFSVDGQPAPSLRWLFNGSVLNETSFIFTEFLEPAANETVRHGCLRLNQPTHVNNGYTLLAANPFGQASASIMAAFMDNP----fefnpe<br><br>SARST: aliSize=98 (resi) RMSD=1.24 (Å)<br>vs--FPASVQLHTAVEMHHWCIPFSVDGQPAPSLRWLFNGSVLNETSFIFTEFLEPAANETVRHGCLRLNQPTHVNNGYTLLAANPFGQASASIMAAFMDN-------pfefn<br>--shMPASVQLHTAVEMHHWCIPFSVDGQPAPSLRWLFNGSVLNETSFIFTEFLEPAANETVRHGCLRLNQPTHVNNGYTLLAANPFGQASASIMAAFMDNpfefnpe-----<br><br>BLAST: aliSize=102 (resi) iden=97.14% (102/105) simi=97.14% (102/105)<br>vsf---PASVQLHTAVEMHHWCIPFSVDGQPAPSLRWLFNGSVLNETSFIFTEFLEPAANETVRHGCLRLNQPTHVNNGYTLLAANPFGQASASIMAAFMDNPFEFN--<br>---shmPASVQLHTAVEMHHWCIPFSVDGQPAPSLRWLFNGSVLNETSFIFTEFLEPAANETVRHGCLRLNQPTHVNNGYTLLAANPFGQASASIMAAFMDNPFEFNpe<br><br>Proposed: aliSize=105 (resi) RMSD=0.88 (Å)<br>VSFPASVQLHTAVEMHHWCIPFSVDGQPAPSLRWLFNGSVLNETSFIFTEFLEPAANETVRHGCLRLNQPTHVNNGYTLLAANPFGQASASIMAAFM-DN-PFEFN<br>SHMPASVQLHTAVEMHHWCIPFSVDGQPAPSLRWLFNGSVLNETSFIFTEFLEPAANETVRHGCLRLNQPTHVNNGYTLLAANPFGQASASIMAAFMdNPfEFNPE
775	1wwcA<br>(105)<br><br>1he7A<br>(107)	C	36.19	TM-align: aliSize=84 (resi) RMSD=1.93 (Å)<br>tvyypprvvsleep------------ELRLEHCIEFVVRGNPPPTLHWLHNGQPLRESKIIHVEYYQE---GEISEGCLLFNKPTHYNNGYTLIAKNPLGTANQTINGHFLKepfpvde--------<br>--------------shmpasvqlhtaVEMHHWCIPFSVDGQPAPSLRWLFNGSVLNETSFIFTEFLEPaanETVRHGCLRLNQPTHVNNGYTLLAANPFGQASASIMAAFMD-------npfefnpe<br><br>SARST: aliSize=96 (resi) RMSD=5.71 (Å)<br>tv-YYPPRVvSLEEPELRLEHCIEFVVRGNPPPTLHWLHNGQPLRESKIIHVEYYQEG---EISEGCLLFNKPTHYNNGYTLIAKNPLGTANQTINGHFLKE-------pfpvde<br>--sHMPASV-QLHTAVEMHHWCIPFSVDGQPAPSLRWLFNGSVLNETSFIFTEFLEPAaneTVRHGCLRLNQPTHVNNGYTLLAANPFGQASASIMAAFMDNpfefnpe------<br><br>BLAST: aliSize=55 (resi) iden=40.95% (43/105) simi=52.38% (55/105)<br>tvyyp---PRVVSLEEPeLRLEH-CIEFVVRGNPPPTLHWLHNGQPLRESKIIHVEYYQEGE---ISEGCLLFNKPTHYNNGYTLIAKNPLGTANQTINGHFLKEPF-----pvde<br>-----shmPASVQLHTA-VEMHHwCIPFSVDGQPAPSLRWLFNGSVLNETSFIFTEFLEPAAnetVRHGCLRLNQPTHVNNGYTLLAANPFGQASASIMAAFMDNPFefnpe----<br><br>Proposed: aliSize=89 (resi) RMSD=1.51 (Å)<br>tvyypprvvsleepe-------------LRLEHCIEFVVRGNPPPTLHWLHNGQPLRESKIIHVEYYQE----gEISEGCLLFNKPTHYNNGYTLIAKNPLGTANQTINGHFL-KEPFPVDE<br>---------------shmpasvqlhtavEMHHWCIPFSVDGQPAPSLRWLFNGSVLNETSFIFTEFLEPaane-TVRHGCLRLNQPTHVNNGYTLLAANPFGQASASIMAAFMdNPFEFNPE
776	3bcpA<br>(124)<br><br>3bcoA<br>(124)	N	98.39	TM-align: aliSize=103 (resi) RMSD=0.94 (Å)<br>kesaaakferqhmdsgnsa-------------------SSSSNYCNQMM--RKMTQGKCKPVNTFVHESLADVKAVCSQKKVTCKDGQTNCYQSKSTMRITDCRETGSSKYPNCAYKTTQVEKHIIVACGGKPSVPVHFDASV<br>-------------------kesaaakferqhmdsgnsaSSSSNYCNQMMccRKMTQGKCKPVNTFVHESLADVKAVCSQKKVTCKDGQTNCYQSKSTMRITDCRETGSSKYPNCAYKTTQVEKHIIVACGGKPSVPVHFDASV<br><br>SARST: aliSize=119 (resi) RMSD=11.47 (Å)<br>k-ESAAAKFERQHMDsGNSASSSSNYCNQMMR---KMTQGKCKPVNTFVHESLADVKAVCSQKKVTCKDGQTNCYQSKSTMRITDCRETGSSKYPNCAYKTTQVEKHIIVACGGKPSVPVHFDAS-v<br>-kESAAAKFERQHMD-SGNSASSSSNYCNQMMccrKMTQGKCKPVNTFVHESLADVKAVCSQKKVTCKDGQTNCYQSKSTMRITDCRETGSSKYPNCAYKTTQVEKHIIVACGGKPSVPVHFDASv-<br><br>BLAST: aliSize=122 (resi) iden=100.00% (122/124) simi=100.00% (122/124)<br>KESAAAKFERQHMDSGNSASSSSNYCNQMM--RKMTQGKCKPVNTFVHESLADVKAVCSQKKVTCKDGQTNCYQSKSTMRITDCRETGSSKYPNCAYKTTQVEKHIIVACGGKPSVPVHFDASV<br>KESAAAKFERQHMDSGNSASSSSNYCNQMMccRKMTQGKCKPVNTFVHESLADVKAVCSQKKVTCKDGQTNCYQSKSTMRITDCRETGSSKYPNCAYKTTQVEKHIIVACGGKPSVPVHFDASV<br><br>Proposed: aliSize=124 (resi) RMSD=0.97 (Å)<br>KESAAAKFERQHMDSGNSASSSSNYCNQMMYYRKMTQGKCKPVNTFVHESLADVKAVCSQKKVTCKDGQTNCYQSKSTMRITDCRETGSSKYPNCAYKTTQVEKHIIVACGGKPSVPVHFDASV<br>KESAAAKFERQHMDSGNSASSSSNYCNQMMCCRKMTQGKCKPVNTFVHESLADVKAVCSQKKVTCKDGQTNCYQSKSTMRITDCRETGSSKYPNCAYKTTQVEKHIIVACGGKPSVPVHFDASV

777	3bcpA<br>(124)<br><br>3bcmA<br>(119)	N	98.32	TM-align: aliSize=101 (resi) RMSD=0.75 (Å)<br>kesaaakferqhmdsgnsass---------------SSNYCNQMM--RKMTQGKCKPVNTFVHESLADVKAVCSQKKVTCKDGQTNCYQSKSTMRITDCRETGSSKYPNCAYKTTQVEKHIIVACGGKPSVPVHFDASV<br>--------------------kesaaakferqhmdsgSSNYCNQMMccRKMTQGKCKPVNTFVHESLADVKAVCSQKKVTCKDGQTNCYQSKSTMRITDCRETGSSKYPNCAYKTTQVEKHIIVACGGKPSVPVHFDASV<br><br>SARST: aliSize=117 (resi) RMSD=14.41 (Å)<br>k-ESAAAKFERQHMDsgnSASSSSNYCNQMMRKMTQGKCKPVNTFVHESLADVKAVCSQKKVTCKDGQTNCYQSKSTMRITDCRETGSSKYPNCAYKTTQVEKHIIVACGGKPSVPVHFDAS-v<br>-kESAAAKFERQHMD---SGSSNYCNQMMCCRKMTQGKCKPVNTFVHESLADVKAVCSQKKVTCKDGQTNCYQSKSTMRITDCRETGSSKYPNCAYKTTQVEKHIIVACGGKPSVPVHFDASv-<br><br>BLAST: aliSize=117 (resi) iden=98.32% (117/119) simi=98.32% (117/119)<br>KESAAAKFERQHMDSGnsassSSNYCNQMM--RKMTQGKCKPVNTFVHESLADVKAVCSQKKVTCKDGQTNCYQSKSTMRITDCRETGSSKYPNCAYKTTQVEKHIIVACGGKPSVPVHFDASV<br>KESAAAKFERQHMDSG-----SSNYCNQMMccRKMTQGKCKPVNTFVHESLADVKAVCSQKKVTCKDGQTNCYQSKSTMRITDCRETGSSKYPNCAYKTTQVEKHIIVACGGKPSVPVHFDASV<br><br>Proposed: aliSize=119 (resi) RMSD=0.72 (Å)<br>KESAAAKFERQHMDSGnsassSSNYCNQMMYYRKMTQGKCKPVNTFVHESLADVKAVCSQKKVTCKDGQTNCYQSKSTMRITDCRETGSSKYPNCAYKTTQVEKHIIVACGGKPSVPVHFDASV<br>KESAAAKFERQHMDSG-----SSNYCNQMMCCRKMTQGKCKPVNTFVHESLADVKAVCSQKKVTCKDGQTNCYQSKSTMRITDCRETGSSKYPNCAYKTTQVEKHIIVACGGKPSVPVHFDASV
778	3bcpA<br>(124)<br><br>1js0A<br>(124)	N	74.19	TM-align: aliSize=92 (resi) RMSD=1.49 (Å)<br>kesaaakferqhmdsgnsa-------------------SSSSNYCNQMM--RKMTQGKCKPVNTFVHESLADVKAVCSQKKVTCKDGQTNCYQSKSTMRITDCRETGSSKYPNCAYKTTQVEKHIIVACGGKpsvpvhfdasv-----------<br>-------------------ketaaakferqhmdsstsaASSSNYCNQMMksRNLTKDRCKPVNTFVHESLADVQAVCSQKNVACKNGQTNCYQSYSTMSITDCRETGSSKYPNCAYKTTQANKHIIVACEGN-----------pyvpvhfdasv<br><br>SARST: aliSize=120 (resi) RMSD=15.35 (Å)<br>k-ESAAAKFERQHMDSGNSASSSSNYCNQMMR--KMTQGKCKPVNTFVHESLADVKAVCSQKKVTCKDGQTNCYQSKSTMRITDCRETGSSKYPNCAYKTTQVEKHIIVACGGKPSVPVHFDAS-v<br>-kETAAAKFERQHMDSSTSAASSSNYCNQMMKsrNLTKDRCKPVNTFVHESLADVQAVCSQKNVACKNGQTNCYQSYSTMSITDCRETGSSKYPNCAYKTTQANKHIIVACEGNPYVPVHFDASv-<br><br>BLAST: aliSize=109 (resi) iden=83.61% (102/124) simi=89.34% (109/124)<br>KESAAAKFERQHMDSGNSASSSSNYCNQMM--RKMTQGKCKPVNTFVHESLADVKAVCSQKKVTCKDGQTNCYQSKSTMRITDCRETGSSKYPNCAYKTTQVEKHIIVACGGKPSVPVHFDASV<br>KETAAAKFERQHMDSSTSAASSSNYCNQMMksRNLTKDRCKPVNTFVHESLADVQAVCSQKNVACKNGQTNCYQSYSTMSITDCRETGSSKYPNCAYKTTQANKHIIVACEGNPYVPVHFDASV<br><br>Proposed: aliSize=112 (resi) RMSD=1.03 (Å)<br>KESAAAKFERQHMDSGNSA-sSSSNYCNQMMYYRKMTQGKCKPVNTFVHESLADVKAVCSQKKVTCKDGQTNCYQSKSTMRITDCRETGSSKYPNCAYKTTQVEKHIIVACGGK-----------psvpvhfdasv<br>KETAAAKFERQHMDSSTSAa-SSSNYCNQMMKSRNLTKDRCKPVNTFVHESLADVQAVCSQKNVACKNGQTNCYQSYSTMSITDCRETGSSKYPNCAYKTTQANKHIIVACEGNpyvpvhfdasv-----------
779	3bcpA<br>(124)<br><br>1js0A<br>(124)	C	69.35	TM-align: aliSize=92 (resi) RMSD=1.49 (Å)<br>kesaaakferqhmdsgnsa-------------------SSSSNYCNQMM--RKMTQGKCKPVNTFVHESLADVKAVCSQKKVTCKDGQTNCYQSKSTMRITDCRETGSSKYPNCAYKTTQVEKHIIVACGGKpsvpvhfdasv-----------<br>-------------------ketaaakferqhmdsstsaASSSNYCNQMMksRNLTKDRCKPVNTFVHESLADVQAVCSQKNVACKNGQTNCYQSYSTMSITDCRETGSSKYPNCAYKTTQANKHIIVACEGN-----------pyvpvhfdasv<br><br>SARST: aliSize=120 (resi) RMSD=15.35 (Å)<br>k-ESAAAKFERQHMDSGNSASSSSNYCNQMMR--KMTQGKCKPVNTFVHESLADVKAVCSQKKVTCKDGQTNCYQSKSTMRITDCRETGSSKYPNCAYKTTQVEKHIIVACGGKPSVPVHFDAS-v<br>-kETAAAKFERQHMDSSTSAASSSNYCNQMMKsrNLTKDRCKPVNTFVHESLADVQAVCSQKNVACKNGQTNCYQSYSTMSITDCRETGSSKYPNCAYKTTQANKHIIVACEGNPYVPVHFDASv-<br><br>BLAST: aliSize=109 (resi) iden=83.61% (102/124) simi=89.34% (109/124)<br>KESAAAKFERQHMDSGNSASSSSNYCNQMM--RKMTQGKCKPVNTFVHESLADVKAVCSQKKVTCKDGQTNCYQSKSTMRITDCRETGSSKYPNCAYKTTQVEKHIIVACGGKPSVPVHFDASV<br>KETAAAKFERQHMDSSTSAASSSNYCNQMMksRNLTKDRCKPVNTFVHESLADVQAVCSQKNVACKNGQTNCYQSYSTMSITDCRETGSSKYPNCAYKTTQANKHIIVACEGNPYVPVHFDASV<br><br>Proposed: aliSize=104 (resi) RMSD=0.91 (Å)<br>kesaaakferqhmdsgnsas--------------------SSSNYCNQMMYYRKMTQGKCKPVNTFVHESLADVKAVCSQKKVTCKDGQTNCYQSKSTMRITDCRETGSSKYPNCAYKTTQVEKHIIVACGGKPSVPVHFDASV<br>--------------------ketaaakferqhmdsstsaaSSSNYCNQMMKSRNLTKDRCKPVNTFVHESLADVQAVCSQKNVACKNGQTNCYQSYSTMSITDCRETGSSKYPNCAYKTTQANKHIIVACEGNPYVPVHFDASV

780	3bcpA (124)<br>1r3mB (122)	N	95.90	TM-align: aliSize=102 (resi) RMSD=1.13 (Å)<br>kesaaakferqhmdsgnsas------------------SSSNYCNQMM--RKMTQGKCKPVNTFVHESLADVKAVCSQKKVTCKDGQTNCYQSKSTMRITDCRETGSSKYPNCAYKTTQVEKHIIVACGGKPSVPVHFDASV<br>--------------------kesaaakferqhmdsgnsPSSNYCNLMMccRKMTQGKCKPVNTFVHESLADVKAVCSQKKVTCKNGQTNCYQSKSTMRITDCRETGSSKYPNCAYKTTQVEKHIIVACGGKPSVPVHFDASV<br><br>SARST: aliSize=120 (resi) RMSD=14.43 (Å)<br>k-ESAAAKFERQHMDSGNSASSSSNYCNQMMRKMTQGKCKPVNTFVHESLADVKAVCSQKKVTCKDGQTNCYQSKSTMRITDCRETGSSKYPNCAYKTTQVEKHIIVACGGKPSVPVHFDAS-v<br>-kESAAAKFERQHMDSGNSPSSNYCNLMMCCRKMTQGKCKPVNTFVHESLADVKAVCSQKKVTCKNGQTNCYQSKSTMRITDCRETGSSKYPNCAYKTTQVEKHIIVACGGKPSVPVHFDASv-<br><br>BLAST: aliSize=118 (resi) iden=95.90% (117/122) simi=96.72% (118/122)<br>KESAAAKFERQHMDSGNSASSsNYCNQMM--RKMTQGKCKPVNTFVHESLADVKAVCSQKKVTCKDGQTNCYQSKSTMRITDCRETGSSKYPNCAYKTTQVEKHIIVACGGKPSVPVHFDASV<br>KESAAAKFERQHMDSGNSPSS--NYCNLMMccRKMTQGKCKPVNTFVHESLADVKAVCSQKKVTCKNGQTNCYQSKSTMRITDCRETGSSKYPNCAYKTTQVEKHIIVACGGKPSVPVHFDASV<br><br>Proposed: aliSize=122 (resi) RMSD=0.85 (Å)<br>KESAAAKFERQHMDSGNSAssSSNYCNQMMYYRKMTQGKCKPVNTFVHESLADVKAVCSQKKVTCKDGQTNCYQSKSTMRITDCRETGSSKYPNCAYKTTQVEKHIIVACGGKPSVPVHFDASV<br>KESAAAKFERQHMDSGNSP--SSNYCNLMMCCRKMTQGKCKPVNTFVHESLADVKAVCSQKKVTCKNGQTNCYQSKSTMRITDCRETGSSKYPNCAYKTTQVEKHIIVACGGKPSVPVHFDASV
781	1js0A (124)<br>1lbgA (124)	N	72.58	TM-align: aliSize=94 (resi) RMSD=1.28 (Å)<br>ketaaakferqhmdsstsaa--------------------SSSNYCNQMMKSRNLTKDRCKPVNTFVHESLADVQAVCSQKNVACKNGQTNCYQSYSTMSITDCRETGSSKYPNCAYKTTQANKHIIVACEGNPyvpvhfdasv----------<br>--------------------kesaaakferqhmdsgnspsSSSNYCNLMMCCRKMTQGKCKPVNTFVHESLADVKAVCSQKKVTCKNGQTNCYQSKSTMRITDCRETGSSKYPNCAYKTTQVEKHIIVACGGKP----------svpvhfdasv<br><br>SARST: aliSize=121 (resi) RMSD=11.47 (Å)<br>k-ETAAAKFERQHMDS-STSAASsSNYCNQMMKSRNLTKDRCKPVNTFVHESLADVQAVCSQKNVACKNGQTNCYQSYSTMSITDCRETGSSKYPNCAYKTTQANKHIIVACEGNPYVPVHFDAS-v<br>-kESAAAKFERQHMDSgNSPSSS-SNYCNLMMCCRKMTQGKCKPVNTFVHESLADVKAVCSQKKVTCKNGQTNCYQSKSTMRITDCRETGSSKYPNCAYKTTQVEKHIIVACGGKPSVPVHFDASv-<br><br>BLAST: aliSize=107 (resi) iden=81.45% (101/124) simi=86.29% (107/124)<br>KETAAAKFERQHMDSSTSAASSSNYCNQMMKSRNLTKDRCKPVNTFVHESLADVQAVCSQKNVACKNGQTNCYQSYSTMSITDCRETGSSKYPNCAYKTTQANKHIIVACEGNPYVPVHFDASV<br>KESAAAKFERQHMDSGNSPSSSSNYCNLMMCCRKMTQGKCKPVNTFVHESLADVKAVCSQKKVTCKNGQTNCYQSKSTMRITDCRETGSSKYPNCAYKTTQVEKHIIVACGGKPSVPVHFDASV<br><br>Proposed: aliSize=112 (resi) RMSD=0.78 (Å)<br>KETAAAKFERQHMDSsTSA-ASSSNYCNQMMKSRNLTKDRCKPVNTFVHESLADVQAVCSQKNVACKNGQTNCYQSYSTMSITDCRETGSSKYPNCAYKTTQANKHIIVACEGN----------pyvpvhfdasv<br>KESAAAKFERQHMDS-GNSpSSSSNYCNLMMCCRKMTQGKCKPVNTFVHESLADVKAVCSQKKVTCKNGQTNCYQSKSTMRITDCRETGSSKYPNCAYKTTQVEKHIIVACGGKpsvpvhfdasv----------
782	1js0A (124)<br>1lbgA (124)	C	69.35	TM-align: aliSize=94 (resi) RMSD=1.28 (Å)<br>ketaaakferqhmdsstsaa--------------------SSSNYCNQMMKSRNLTKDRCKPVNTFVHESLADVQAVCSQKNVACKNGQTNCYQSYSTMSITDCRETGSSKYPNCAYKTTQANKHIIVACEGNPyvpvhfdasv----------<br>--------------------kesaaakferqhmdsgnspsSSSNYCNLMMCCRKMTQGKCKPVNTFVHESLADVKAVCSQKKVTCKNGQTNCYQSKSTMRITDCRETGSSKYPNCAYKTTQVEKHIIVACGGKP----------svpvhfdasv<br><br>SARST: aliSize=121 (resi) RMSD=11.47 (Å)<br>k-ETAAAKFERQHMDS-STSAASsSNYCNQMMKSRNLTKDRCKPVNTFVHESLADVQAVCSQKNVACKNGQTNCYQSYSTMSITDCRETGSSKYPNCAYKTTQANKHIIVACEGNPYVPVHFDAS-v<br>-kESAAAKFERQHMDSgNSPSSS-SNYCNLMMCCRKMTQGKCKPVNTFVHESLADVKAVCSQKKVTCKNGQTNCYQSKSTMRITDCRETGSSKYPNCAYKTTQVEKHIIVACGGKPSVPVHFDASv-<br><br>BLAST: aliSize=107 (resi) iden=81.45% (101/124) simi=86.29% (107/124)<br>KETAAAKFERQHMDSSTSAASSSNYCNQMMKSRNLTKDRCKPVNTFVHESLADVQAVCSQKNVACKNGQTNCYQSYSTMSITDCRETGSSKYPNCAYKTTQANKHIIVACEGNPYVPVHFDASV<br>KESAAAKFERQHMDSGNSPSSSSNYCNLMMCCRKMTQGKCKPVNTFVHESLADVKAVCSQKKVTCKNGQTNCYQSKSTMRITDCRETGSSKYPNCAYKTTQVEKHIIVACGGKPSVPVHFDASV<br><br>Proposed: aliSize=104 (resi) RMSD=0.74 (Å)<br>ketaaakferqhmdsstsaa--------------------SSSNYCNQMMKSRNLTKDRCKPVNTFVHESLADVQAVCSQKNVACKNGQTNCYQSYSTMSITDCRETGSSKYPNCAYKTTQANKHIIVACEGNPYVPVHFDASV<br>--------------------kesaaakferqhmdsgnspsSSSNYCNLMMCCRKMTQGKCKPVNTFVHESLADVKAVCSQKKVTCKNGQTNCYQSKSTMRITDCRETGSSKYPNCAYKTTQVEKHIIVACGGKPSVPVHFDASV

783	1js0A (124)<br>1tq9A (124)	N	72.58	TM-align: aliSize=94 (resi) RMSD=1.71 (Å)<br>ketaaakferqhmdsstsa-------------------ASSSNYCNQMMksRNLTKDRCKPVNTFVHESLADVQAVCSQKNVACKNGQTNCYQSYSTMSITDCRETGSSKYPNCAYKTTQANKHIIVACEGNPYvpvhfdasv---------<br>-------------------kesaaakferqhmdsgnspSSSSNYCNLMM--RKMTQGKCKPVNTFVHESLADVKAVCSQKKVTCKNGQTNCYQSKSTMRITDCRETGSSKYPNCAYKTTQVEKHIIVACGGKPS---------vpvhfdasv<br><br>SARST: aliSize=118 (resi) RMSD=12.73 (Å)<br>k-ETAAAKFERQHMDSSTsaasSSN--YCNQMMKSRNLTKDRCKPVNTFVHESLADVQAVCSQKNVACKNGQTNCYQSYSTMSITDCRETGSSKYPNCAYKTTQANKHIIVACEGNPYVPVHFDAS-v<br>-kESAAAKFERQHMDSGN----SPSssSNYCNLMMRKMTQGKCKPVNTFVHESLADVKAVCSQKKVTCKNGQTNCYQSKSTMRITDCRETGSSKYPNCAYKTTQVEKHIIVACGGKPSVPVHFDASv-<br><br>BLAST: aliSize=107 (resi) iden=82.79% (101/124) simi=87.70% (107/124)<br>KETAAAKFERQHMDSSTSAASSSNYCNQMMksRNLTKDRCKPVNTFVHESLADVQAVCSQKNVACKNGQTNCYQSYSTMSITDCRETGSSKYPNCAYKTTQANKHIIVACEGNPYVPVHFDASV<br>KESAAAKFERQHMDSGNSPSSSSNYCNLMM--RKMTQGKCKPVNTFVHESLADVKAVCSQKKVTCKNGQTNCYQSKSTMRITDCRETGSSKYPNCAYKTTQVEKHIIVACGGKPSVPVHFDASV<br><br>Proposed: aliSize=112 (resi) RMSD=0.78 (Å)<br>KETAAAKFERQHMDSSTsAAS-SSNYCNQMMKSRNLTKDRCKPVNTFVHESLADVQAVCSQKNVACKNGQTNCYQSYSTMSITDCRETGSSKYPNCAYKTTQANKHIIVACEGN-----------pyvpvhfdasv<br>KESAAAKFERQHMDSGN-SPSsSSNYCNLMMYYRKMTQGKCKPVNTFVHESLADVKAVCSQKKVTCKNGQTNCYQSKSTMRITDCRETGSSKYPNCAYKTTQVEKHIIVACGGKpsvpvhfdasv-----------
784	1js0A (124)<br>1tq9A (124)	C	69.35	TM-align: aliSize=94 (resi) RMSD=1.71 (Å)<br>ketaaakferqhmdsstsa-------------------ASSSNYCNQMMksRNLTKDRCKPVNTFVHESLADVQAVCSQKNVACKNGQTNCYQSYSTMSITDCRETGSSKYPNCAYKTTQANKHIIVACEGNPYvpvhfdasv---------<br>-------------------kesaaakferqhmdsgnspSSSSNYCNLMM--RKMTQGKCKPVNTFVHESLADVKAVCSQKKVTCKNGQTNCYQSKSTMRITDCRETGSSKYPNCAYKTTQVEKHIIVACGGKPS---------vpvhfdasv<br><br>SARST: aliSize=118 (resi) RMSD=12.73 (Å)<br>k-ETAAAKFERQHMDSSTsaasSSN--YCNQMMKSRNLTKDRCKPVNTFVHESLADVQAVCSQKNVACKNGQTNCYQSYSTMSITDCRETGSSKYPNCAYKTTQANKHIIVACEGNPYVPVHFDAS-v<br>-kESAAAKFERQHMDSGN----SPSssSNYCNLMMRKMTQGKCKPVNTFVHESLADVKAVCSQKKVTCKNGQTNCYQSKSTMRITDCRETGSSKYPNCAYKTTQVEKHIIVACGGKPSVPVHFDASv-<br><br>BLAST: aliSize=107 (resi) iden=82.79% (101/124) simi=87.70% (107/124)<br>KETAAAKFERQHMDSSTSAASSSNYCNQMMksRNLTKDRCKPVNTFVHESLADVQAVCSQKNVACKNGQTNCYQSYSTMSITDCRETGSSKYPNCAYKTTQANKHIIVACEGNPYVPVHFDASV<br>KESAAAKFERQHMDSGNSPSSSSNYCNLMM--RKMTQGKCKPVNTFVHESLADVKAVCSQKKVTCKNGQTNCYQSKSTMRITDCRETGSSKYPNCAYKTTQVEKHIIVACGGKPSVPVHFDASV<br><br>Proposed: aliSize=104 (resi) RMSD=0.66 (Å)<br>ketaaakferqhmdsstsaa--------------------SSSNYCNQMMKSRNLTKDRCKPVNTFVHESLADVQAVCSQKNVACKNGQTNCYQSYSTMSITDCRETGSSKYPNCAYKTTQANKHIIVACEGNPYVPVHFDASV<br>--------------------kesaaakferqhmdsgnspsSSSNYCNLMMYYRKMTQGKCKPVNTFVHESLADVKAVCSQKKVTCKNGQTNCYQSKSTMRITDCRETGSSKYPNCAYKTTQVEKHIIVACGGKPSVPVHFDASV
785	1js0A (124)<br>1r3mB (122)	C	81.15	TM-align: aliSize=112 (resi) RMSD=1.21 (Å)<br>KETAAAKFERQHMDSStSAAsSSNYCNQMMKSRNLTKDRCKPVNTFVHESLADVQAVCSQKNVACKNGQTNCYQSYSTMSITDCRETGSSKYPNCAYKTTQANKHIIVACEGNPyvpvhfdasv----------<br>KESAAAKFERQHMDSG-NSP-SSNYCNLMMCCRKMTQGKCKPVNTFVHESLADVKAVCSQKKVTCKNGQTNCYQSKSTMRITDCRETGSSKYPNCAYKTTQVEKHIIVACGGKP----------svpvhfdasv<br><br>SARST: aliSize=120 (resi) RMSD=6.44 (Å)<br>k-ETAAAKFERQHMDssTSAASSSNYCNQMMKSRNLTKDRCKPVNTFVHESLADVQAVCSQKNVACKNGQTNCYQSYSTMSITDCRETGSSKYPNCAYKTTQANKHIIVACEGNPYVPVHFDAS-v<br>-kESAAAKFERQHMD--SGNSPSSNYCNLMMCCRKMTQGKCKPVNTFVHESLADVKAVCSQKKVTCKNGQTNCYQSKSTMRITDCRETGSSKYPNCAYKTTQVEKHIIVACGGKPSVPVHFDASv-<br><br>BLAST: aliSize=105 (resi) iden=81.97% (100/122) simi=86.07% (105/122)<br>KETAAAKFERQHMDSSTSAasSSNYCNQMMKSRNLTKDRCKPVNTFVHESLADVQAVCSQKNVACKNGQTNCYQSYSTMSITDCRETGSSKYPNCAYKTTQANKHIIVACEGNPYVPVHFDASV<br>KESAAAKFERQHMDSGNSP--SSNYCNLMMCCRKMTQGKCKPVNTFVHESLADVKAVCSQKKVTCKNGQTNCYQSKSTMRITDCRETGSSKYPNCAYKTTQVEKHIIVACGGKPSVPVHFDASV<br><br>Proposed: aliSize=122 (resi) RMSD=0.79 (Å)<br>KETAAAKFERQHMDSStSAAsSSNYCNQMMKSRNLTKDRCKPVNTFVHESLADVQAVCSQKNVACKNGQTNCYQSYSTMSITDCRETGSSKYPNCAYKTTQANKHIIVACEGNPYVPVHFDASV<br>KESAAAKFERQHMDSG-NSP-SSNYCNLMMCCRKMTQGKCKPVNTFVHESLADVKAVCSQKKVTCKNGQTNCYQSKSTMRITDCRETGSSKYPNCAYKTTQVEKHIIVACGGKPSVPVHFDASV

786	1js0A<br>(124)<br><br>1r5dA<br>(124)	N	72.58	TM-align: aliSize=94 (resi) RMSD=1.44 (Å)<br>ketaaakferqhmdsstsaa--------------------SSSNYCNQMMKSRNLTKDRCKPVNTFVHESLADVQAVCSQKNVACKNGQTNCYQSYSTMSITDCRETGSSKYPNCAYKTTQANKHIIVACEGNPyvpvhfdasv----------<br>--------------------kesaaakferqhmdsgnspsSSSNYCNLMMCCRKMTQGKCKPVNTFVHESLADVKAVCSQKKVTCKNGQTNCYQSKSTMRITDCRETGSSKYPNCAYKTTQVEKHIIVACGGKP----------svpvhfdasv<br><br>SARST: aliSize=121 (resi) RMSD=11.44 (Å)<br>k-ETAAAKFERQHMDS-STSAASsSNYCNQMMKSRNLTKDRCKPVNTFVHESLADVQAVCSQKNVACKNGQTNCYQSYSTMSITDCRETGSSKYPNCAYKTTQANKHIIVACEGNPYVPVHFDAS-v<br>-kESAAAKFERQHMDSgNSPSSS-SNYCNLMMCCRKMTQGKCKPVNTFVHESLADVKAVCSQKKVTCKNGQTNCYQSKSTMRITDCRETGSSKYPNCAYKTTQVEKHIIVACGGKPSVPVHFDASv-<br><br>BLAST: aliSize=107 (resi) iden=81.45% (101/124) simi=86.29% (107/124)<br>KETAAAKFERQHMDSSTSAASSSNYCNQMMKSRNLTKDRCKPVNTFVHESLADVQAVCSQKNVACKNGQTNCYQSYSTMSITDCRETGSSKYPNCAYKTTQANKHIIVACEGNPYVPVHFDASV<br>KESAAAKFERQHMDSGNSPSSSSNYCNLMMCCRKMTQGKCKPVNTFVHESLADVKAVCSQKKVTCKNGQTNCYQSKSTMRITDCRETGSSKYPNCAYKTTQVEKHIIVACGGKPSVPVHFDASV<br><br>Proposed: aliSize=112 (resi) RMSD=0.91 (Å)<br>KETAAAKFERQHMDSsTSA-ASSSNYCNQMMKSRNLTKDRCKPVNTFVHESLADVQAVCSQKNVACKNGQTNCYQSYSTMSITDCRETGSSKYPNCAYKTTQANKHIIVACEGN----------pyvpvhfdasv<br>KESAAAKFERQHMDS-GNSpSSSSNYCNLMMCCRKMTQGKCKPVNTFVHESLADVKAVCSQKKVTCKNGQTNCYQSKSTMRITDCRETGSSKYPNCAYKTTQVEKHIIVACGGKpsvpvhfdasv----------
787	1js0A<br>(124)<br><br>1r5dA<br>(124)	C	68.55	TM-align: aliSize=94 (resi) RMSD=1.44 (Å)<br>ketaaakferqhmdsstsaa--------------------SSSNYCNQMMKSRNLTKDRCKPVNTFVHESLADVQAVCSQKNVACKNGQTNCYQSYSTMSITDCRETGSSKYPNCAYKTTQANKHIIVACEGNPyvpvhfdasv----------<br>--------------------kesaaakferqhmdsgnspsSSSNYCNLMMCCRKMTQGKCKPVNTFVHESLADVKAVCSQKKVTCKNGQTNCYQSKSTMRITDCRETGSSKYPNCAYKTTQVEKHIIVACGGKP----------svpvhfdasv<br><br>SARST: aliSize=121 (resi) RMSD=11.44 (Å)<br>k-ETAAAKFERQHMDS-STSAASsSNYCNQMMKSRNLTKDRCKPVNTFVHESLADVQAVCSQKNVACKNGQTNCYQSYSTMSITDCRETGSSKYPNCAYKTTQANKHIIVACEGNPYVPVHFDAS-v<br>-kESAAAKFERQHMDSgNSPSSS-SNYCNLMMCCRKMTQGKCKPVNTFVHESLADVKAVCSQKKVTCKNGQTNCYQSKSTMRITDCRETGSSKYPNCAYKTTQVEKHIIVACGGKPSVPVHFDASv-<br><br>BLAST: aliSize=107 (resi) iden=81.45% (101/124) simi=86.29% (107/124)<br>KETAAAKFERQHMDSSTSAASSSNYCNQMMKSRNLTKDRCKPVNTFVHESLADVQAVCSQKNVACKNGQTNCYQSYSTMSITDCRETGSSKYPNCAYKTTQANKHIIVACEGNPYVPVHFDASV<br>KESAAAKFERQHMDSGNSPSSSSNYCNLMMCCRKMTQGKCKPVNTFVHESLADVKAVCSQKKVTCKNGQTNCYQSKSTMRITDCRETGSSKYPNCAYKTTQVEKHIIVACGGKPSVPVHFDASV<br><br>Proposed: aliSize=103 (resi) RMSD=0.70 (Å)<br>ketaaakferqhmdsstsaas---------------------SSNYCNQMMKSRNLTKDRCKPVNTFVHESLADVQAVCSQKNVACKNGQTNCYQSYSTMSITDCRETGSSKYPNCAYKTTQANKHIIVACEGNPYVPVHFDASV<br>---------------------kesaaakferqhmdsgnspssSSNYCNLMMCCRKMTQGKCKPVNTFVHESLADVKAVCSQKKVTCKNGQTNCYQSKSTMRITDCRETGSSKYPNCAYKTTQVEKHIIVACGGKPSVPVHFDASV
788	1js0A<br>(124)<br><br>11baA<br>(124)	N	72.58	TM-align: aliSize=95 (resi) RMSD=1.58 (Å)<br>ketaaakferqhmdsstsaa--------------------SSSNYCNQMMKSRNLTKDRCKPVNTFVHESLADVQAVCSQKNVACKNGQTNCYQSYSTMSITDCRETGSSKYPNCAYKTTQANKHIIVACEGNPYvpvhfdasv---------<br>--------------------kesaaakferqhmdsgnspsSSSNYCNLMMCCRKMTQGKCKPVNTFVHESLADVKAVCSQKKVTCKNGQTNCYQSKSTMRITDCRETGSSKYPNCAYKTTQVEKHIIVACGGKPS---------vpvhfdasv<br><br>SARST: aliSize=121 (resi) RMSD=11.43 (Å)<br>k-ETAAAKFERQHMDS-STSAASsSNYCNQMMKSRNLTKDRCKPVNTFVHESLADVQAVCSQKNVACKNGQTNCYQSYSTMSITDCRETGSSKYPNCAYKTTQANKHIIVACEGNPYVPVHFDAS-v<br>-kESAAAKFERQHMDSgNSPSSS-SNYCNLMMCCRKMTQGKCKPVNTFVHESLADVKAVCSQKKVTCKNGQTNCYQSKSTMRITDCRETGSSKYPNCAYKTTQVEKHIIVACGGKPSVPVHFDASv-<br><br>BLAST: aliSize=107 (resi) iden=81.45% (101/124) simi=86.29% (107/124)<br>KETAAAKFERQHMDSSTSAASSSNYCNQMMKSRNLTKDRCKPVNTFVHESLADVQAVCSQKNVACKNGQTNCYQSYSTMSITDCRETGSSKYPNCAYKTTQANKHIIVACEGNPYVPVHFDASV<br>KESAAAKFERQHMDSGNSPSSSSNYCNLMMCCRKMTQGKCKPVNTFVHESLADVKAVCSQKKVTCKNGQTNCYQSKSTMRITDCRETGSSKYPNCAYKTTQVEKHIIVACGGKPSVPVHFDASV<br><br>Proposed: aliSize=112 (resi) RMSD=0.76 (Å)<br>KETAAAKFERQHMDSsTSA-ASSSNYCNQMMKSRNLTKDRCKPVNTFVHESLADVQAVCSQKNVACKNGQTNCYQSYSTMSITDCRETGSSKYPNCAYKTTQANKHIIVACEGN----------pyvpvhfdasv<br>KESAAAKFERQHMDS-GNSpSSSSNYCNLMMCCRKMTQGKCKPVNTFVHESLADVKAVCSQKKVTCKNGQTNCYQSKSTMRITDCRETGSSKYPNCAYKTTQVEKHIIVACGGKpsvpvhfdasv----------

789	1js0A (124)<br>1lbaA (124)	C	68.55	TM-align: aliSize=95 (resi) RMSD=1.58 (Å)<br>ketaaakferqhmdsstsaa--------------------SSSNYCNQMMKSRNLTKDRCKPVNTFVHESLADVQAVCSQKNVACKNGQTNCYQSYSTMSITDCRETGSSKYPNCAYKTTQANKHIIVACEGNPYvpvhfdasv---------<br>--------------------kesaaakferqhmdsgnspsSSSNYCNLMMCCRKMTQGKCKPVNTFVHESLADVKAVCSQKKVTCKNGQTNCYQSKSTMRITDCRETGSSKYPNCAYKTTQVEKHIIVACGGKPS---------vpvhfdasv<br><br>SARST: aliSize=121 (resi) RMSD=11.43 (Å)<br>k-ETAAAKFERQHMDS-STSAASsSNYCNQMMKSRNLTKDRCKPVNTFVHESLADVQAVCSQKNVACKNGQTNCYQSYSTMSITDCRETGSSKYPNCAYKTTQANKHIIVACEGNPYVPVHFDAS-v<br>-kESAAAKFERQHMDSgNSPSSS-SNYCNLMMCCRKMTQGKCKPVNTFVHESLADVKAVCSQKKVTCKNGQTNCYQSKSTMRITDCRETGSSKYPNCAYKTTQVEKHIIVACGGKPSVPVHFDASv-<br><br>BLAST: aliSize=107 (resi) iden=81.45% (101/124) simi=86.29% (107/124)<br>KETAAAKFERQHMDSSTSAASSSNYCNQMMKSRNLTKDRCKPVNTFVHESLADVQAVCSQKNVACKNGQTNCYQSYSTMSITDCRETGSSKYPNCAYKTTQANKHIIVACEGNPYVPVHFDASV<br>KESAAAKFERQHMDSGNSPSSSSNYCNLMMCCRKMTQGKCKPVNTFVHESLADVKAVCSQKKVTCKNGQTNCYQSKSTMRITDCRETGSSKYPNCAYKTTQVEKHIIVACGGKPSVPVHFDASV<br><br>Proposed: aliSize=103 (resi) RMSD=0.50 (Å)<br>ketaaakferqhmdsstsaas--------------------SSNYCNQMMKSRNLTKDRCKPVNTFVHESLADVQAVCSQKNVACKNGQTNCYQSYSTMSITDCRETGSSKYPNCAYKTTQANKHIIVACEGNPYVPVHFDASV<br>--------------------kesaaakferqhmdsgnspssSSNYCNLMMCCRKMTQGKCKPVNTFVHESLADVKAVCSQKKVTCKNGQTNCYQSKSTMRITDCRETGSSKYPNCAYKTTQVEKHIIVACGGKPSVPVHFDASV
790	1r5dA (124)<br>1a2wA (124)	N	80.65	TM-align: aliSize=104 (resi) RMSD=0.90 (Å)<br>kesaaakferqhmdsgnsps--------------------SSSNYCNLMMCCRKMTQGKCKPVNTFVHESLADVKAVCSQKKVTCKNGQTNCYQSKSTMRITDCRETGSSKYPNCAYKTTQVEKHIIVACGGKPSVPVHFDASV<br>--------------------ketaaakferqhmdsstsaaSSSNYCNQMMKSRNLTKDRCKPVNTFVHESLADVQAVCSQKNVACKNGQTNCYQSYSTMSITDCRETGSSKYPNCAYKTTQANKHIIVACEGNPYVPVHFDASV<br><br>SARST: aliSize=118 (resi) RMSD=12.33 (Å)<br>k-ESAAAKFERQHMDsgnsPS--SS--SNYCNLMMCCRKMTQGKCKPVNTFVHESLADVKAVCSQKKVTCKNGQTNCYQSKSTMRITDCRETGSSKYPNCAYKTTQVEKHIIVACGGKPSVPVHFDAS-v<br>-kETAAAKFERQHMD----SStsAAssSNYCNQMMKSRNLTKDRCKPVNTFVHESLADVQAVCSQKNVACKNGQTNCYQSYSTMSITDCRETGSSKYPNCAYKTTQANKHIIVACEGNPYVPVHFDASv-<br><br>BLAST: aliSize=107 (resi) iden=81.45% (101/124) simi=86.29% (107/124)<br>KESAAAKFERQHMDSGNSPSSSSNYCNLMMCCRKMTQGKCKPVNTFVHESLADVKAVCSQKKVTCKNGQTNCYQSKSTMRITDCRETGSSKYPNCAYKTTQVEKHIIVACGGKPSVPVHFDASV<br>KETAAAKFERQHMDSSTSAASSSNYCNQMMKSRNLTKDRCKPVNTFVHESLADVQAVCSQKNVACKNGQTNCYQSYSTMSITDCRETGSSKYPNCAYKTTQANKHIIVACEGNPYVPVHFDASV<br><br>Proposed: aliSize=123 (resi) RMSD=0.92 (Å)<br>KESAAAKFERQHMDS-GNSpSSSSNYCNLMMCCRKMTQGKCKPVNTFVHESLADVKAVCSQKKVTCKNGQTNCYQSKSTMRITDCRETGSSKYPNCAYKTTQVEKHIIVACGGKPSVPVHFDASV<br>KETAAAKFERQHMDSsTSA-ASSSNYCNQMMKSRNLTKDRCKPVNTFVHESLADVQAVCSQKNVACKNGQTNCYQSYSTMSITDCRETGSSKYPNCAYKTTQANKHIIVACEGNPYVPVHFDASV
791	1r5dA (124)<br>1h8xA (125)	N	71.77	TM-align: aliSize=103 (resi) RMSD=1.37 (Å)<br>kesaaakferqhmdsgnsps--------------------SSSNYCNLMMCCRKMTQGKCKPVNTFVHESLADVKAVCSQKKVTC-KNgQTNCYQSKSTMRITDCRETGSSKYPNCAYKTTQVEKHIIVACGGKPSVPVHFDASV-<br>--------------------kesaaakferqhmdsgnspsSSSTYCNQMMRRRNMTQGRCKPVNTFVHEPLVDVQNVCFQEKVTCkNG-QGNCYKSNSSMHITDCRLTNGSRYPNCAYRTSQKERHIIVACEGSPYVPVHFDASVe<br><br>SARST: aliSize=120 (resi) RMSD=11.86 (Å)<br>k-ESAAAKFERQHMD--SGNSPSSssNYCNLMMCCRKMTQGKCKPVNTFVHESLADVKAVCSQKKVTCKNGQTNCYQSKSTMRITDCRETGSSKYPNCAYKTTQVEKHIIVACGGKPSVPVHFDAS--v<br>-kESAAAKFERQHMDsgNSPSSSS--TYCNQMMRRRNMTQGRCKPVNTFVHEPLVDVQNVCFQEKVTCKNGQGNCYKSNSSMHITDCRLTNGSRYPNCAYRTSQKERHIIVACEGSPYVPVHFDASve-<br><br>BLAST: aliSize=105 (resi) iden=77.42% (96/124) simi=84.68% (105/124)<br>KESAAAKFERQHMDSGNSPSSSSNYCNLMMCCRKMTQGKCKPVNTFVHESLADVKAVCSQKKVTCKNGQTNCYQSKSTMRITDCRETGSSKYPNCAYKTTQVEKHIIVACGGKPSVPVHFDASV-<br>KESAAAKFERQHMDSGNSPSSSSTYCNQMMRRRNMTQGRCKPVNTFVHEPLVDVQNVCFQEKVTCKNGQGNCYKSNSSMHITDCRLTNGSRYPNCAYRTSQKERHIIVACEGSPYVPVHFDASVe<br><br>Proposed: aliSize=122 (resi) RMSD=1.01 (Å)<br>KESAAAKFERQHMDSGnSPS-SSSNYCNLMMCCRKMTQGKCKPVNTFVHESLADVKAVCSQKKVTC-KNgQTNCYQSKSTMRITDCRETGSSKYPNCAYKTTQVEKHIIVACGGKPSVPVHFDASV-<br>KESAAAKFERQHMDSG-NSPsSSSTYCNQMMRRRNMTQGRCKPVNTFVHEPLVDVQNVCFQEKVTCkNG-QGNCYKSNSSMHITDCRLTNGSRYPNCAYRTSQKERHIIVACEGSPYVPVHFDASVe

792	1g85A (159)<br>2hlvA (149)	C	92.62	TM-align: aliSize=116 (resi) RMSD=1.09 (Å)<br>aqeeeaeqnlSELSGPWRTVYIGSTNPEKIQENGPFRTYFRELVFDDEKGTVDFYFSVKRDGKWKNVHVKATKQDDGTYVADYEGQNVFKIVSLSRTHLVAHNINVDKHGQTTELTGLFVKLNvededlekfwkltedkgidkknvvnflenenhp----------------------------HPE----<br>----------SELSGPWRTVYIGSTNPEKIQENGPFRTYFRELVFDDEKGTVDFYFSVKRDGKCKNVHVKATKQDDGTYVADYEGQNVFKIVSLSRTHLVAHNINVDKHGQTTELTELFVKGL---------------------------------nvededlekfwkltedkgidkknvvnfleNEDcphp<br><br>SARST: aliSize=146 (resi) RMSD=13.39 (Å)<br>aqeeeaeqnls-ELSGPWRTVYIGSTNPEKIQENGPFRTYFRELVFDDEKGTVDFYFSVKRDGKWKNVHVKATKQDDGTYVADYEGQNVFKIVSLSRTHLVAHNINVDKHGQTTELTGLFVKL-NVEDEDLEKFWKLTEDKGIDKKNVVNFLENENHPH-pe<br>-----------sELSGPWRTVYIGSTNPEKIQENGPFRTYFRELVFDDEKGTVDFYFSVKRDGKCKNVHVKATKQDDGTYVADYEGQNVFKIVSLSRTHLVAHNINVDKHGQTTELTELFVKGlNVEDEDLEKFWKLTEDKGIDKKNVVNFLENEDCPHp--<br><br>BLAST: aliSize=145 (resi) iden=96.64% (144/149) simi=97.32% (145/149)<br>aqeeeaeqnlSELSGPWRTVYIGSTNPEKIQENGPFRTYFRELVFDDEKGTVDFYFSVKRDGKWKNVHVKATKQDDGTYVADYEGQNVFKIVSLSRTHLVAHNINVDKHGQTTELTGLFVK-LNVEDEDLEKFWKLTEDKGIDKKNVVNFLENENHPHPe<br>----------SELSGPWRTVYIGSTNPEKIQENGPFRTYFRELVFDDEKGTVDFYFSVKRDGKCKNVHVKATKQDDGTYVADYEGQNVFKIVSLSRTHLVAHNINVDKHGQTTELTELFVKgLNVEDEDLEKFWKLTEDKGIDKKNVVNFLENEDCPHP-<br><br>Proposed: aliSize=145 (resi) RMSD=0.88 (Å)<br>aqeeeaeqnlSELSGPWRTVYIGSTNPEKIQENGPFRTYFRELVFDDEKGTVDFYFSVKRDGKWKNVHVKATKQDDGTYVADYEGQNVFKIVSLSRTHLVAHNINVDKHGQTTELTGLFVK-LNVEDEDLEKFWKLTEDKGIDKKNVVNFLeNENHP---hpe<br>----------SELSGPWRTVYIGSTNPEKIQENGPFRTYFRELVFDDEKGTVDFYFSVKRDGKCKNVHVKATKQDDGTYVADYEGQNVFKIVSLSRTHLVAHNINVDKHGQTTELTELFVKgLNVEDEDLEKFWKLTEDKGIDKKNVVNFL-ENEDCphp---
793	1oy0A (248)<br>3b8iA (283)	C	15.32	TM-align: aliSize=213 (resi) RMSD=2.88 (Å)<br>-R---TKIRTHHLQRWkaDgHKWAMLTAYDYSTARIFDEAGIPVLLVGDSAANVV-YGYDTTVPISIDELIPLVRGVVRGAPhALVVADLPFGSYeAGpTAALAAATRFLKdGGAHAVKLEG-------------G--E-RVAEQIACLTA-A--G-IPVMAHIGFTPgDAAEQTIADAIAVAEAGAFAV<br>rAshhELRAMFALLDS--S-RCYHTASVFDPMSARIAADLGFECGILGGSVASLQvLAAPDFALITLSEFVEQATRIGRVAR-LPVIADADHGYG-NA-LNVMRTVVELER-AGIAALTIEDtllpaqfgrkstdLicVeEGVGKIRAALEaRvdPaLTIIARTNAEL-IDVDAVIQRTLAYQEAGADGI<br>VMEMVPA-ELATQITGKLTIPTVGIG--A--GP----N----CDGQVLVWqDmAgFSgaktarfvkryaDVGGELRRAAMQyAQEvaggvfpadeh---------------------------------<br>CLVGVRDfAHLEAIAEHLHIPLMLVTygNpqLRddarLarlgVRVVVNGH-A-A-YF------------AAIKATYDCLRE-ERG-----------avasdltaselskkytfpeeyqawardymevk<br>SARST: aliSize=210 (resi) RMSD=10.32 (Å)<br>r-------------------------TKIRTHHLQRWKADGHKWAMLTaydySTARIFDEAGipvllVGDsaanvvYGydttvPISIDELIPLVRGVVRGAPHALVVADLPFGsyeaGPTAALAAATRFLKDGGAHAVKL---EGG--------------ERVAEQIACLTAAGI--PVMAHIGFTPGDAA<br>-rashhelramfralldssrcyhtaSVFDPMSARIAADLGFECGILG----GSVASLQVLA-----APD------FA-----LITLSEFVEQATRIGRVARLPVIADADHGY----GNALNVMRTVVELERAGIAALTIedtLLPaqfgrkstdlicveEGVGKIRAALEARVDpaLTIIARTNAELIDV<br>EQTIADAIAVAEAGAFAVVMEMV-PAELATQITGKLTIPTVGIGAG---PNCDgqvlVWQDMAGFsgaktarFVKRYADVGGELRRAAMQYAQEVAGGVFPAD-----------------------------eh<br>DAVIQRTLAYQEAGADGICLVGVrDFAHLEAIAEHLHIPLMLVTYGnpqLRDD----ARLARLGV-------RVVVNGHAAYFAAIKATYDCLREERGAVASDltaselskkytfpeeyqawardymevk--<br>BLAST: aliSize=66 (resi) iden=16.53% (41/248) simi=26.61% (66/248)<br>rtkirthhlqrwkadghkwamltayd---------------------YSTARIFD--------EAGIPVLLVGDSAANV-VYGYDTTVPISIDELIPLVRGVVRGApHALVVADLPFGsYeaGPTAALAAATRFLKDGGAHAVKLEgGERVAEQIACLTAAGIPVMAHIGFTPGdAAEQTIADAIA-VAEA<br>---------------------------rashhelramfralldssrcYHTASVFDpmsariaaDLGFECGILGGSVASLqVLAAPDFALITLSEFVEQATRIGRVA-RLPVIADADHG-Y--GNALNVMRTVVELERAGIAALTIE-DTLLPAQFGRKSTDLICVEEGVGKIRA-ALEARVDPALTiIART<br>GAFAV-VMEMVPAELATQITGKLTIPTVGI--gagpncdgqvlvwqdmagfsgaktarfvkryadvggelrraamqyaqevaggvfpadeh<br>NAELIdVDAVIQRTLAYQEAGADGICLVGVrdfahleaiaehlhiplmlvtygnpqlrddarlarlgvrvvvnghaayfaaikatydclreergavasdltaselskkytfpeeyqawardymevk--<br>Proposed: aliSize=208 (resi) RMSD=2.26 (Å)<br>rt------K-IRT-hHLQRWkaDgHKWAMLTAYDYSTARIFDEAGIPVLLVGDSAANVV-YGYDTTVPISIDELIPLVRGVVRGAPhALVVADLPFGSYeAGpTAALAAATRFLKdGGAHAVKLEG---------------GERVAEQIACLTA-A--G-IPVMAHIGF-TPgdAAEQTIADAIAVAEA<br>--rashheLrAMFr-ALLDS--S-RCYHTASVFDPMSARIAADLGFECGILGGSVASLQvLAAPDFALITLSEFVEQATRIGRVAR-LPVIADADHGYG-NA-LNVMRTVVELER-AGIAALTIEDtllpaqfgrkstdlicVEEGVGKIRAALEaRvdPaLTIIARTNAeLI--DVDAVIQRTLAYQEA<br>GAFAVVMEMVPA-ELATQITGKLTIPTVGIG-----aGP----N----CDGQVLVWqDmagfsgaktarfvkryadVGGELRRAAMQYAQEVAG--------------------------------gvfpadeh<br>GADGICLVGVRDfAHLEAIAEHLHIPLMLVTygnpq-LRddarLarlgVRVVVNGH-A-----------------AYFAAIKATYDCLREERGavasdltaselskkytfpeeyqawardymevk--------
794	1dktB (71)<br>1cksB (78)	C	84.51	TM-align: aliSize=59 (resi) RMSD=1.06 (Å)<br>---QIYYSDKYDDEEFEYRHVMLPKDIAKLVPKTHLMSESEWRNLGVQQSQGWVHYMIHEPEphillfrrplpk----------------<br>ahkQIYYSDKYFDEHYEYRHVMLPRELSKQVPKTHLMSEEEWRRLGVQQSLGWVHYMIHEPE------------phillfrrplpkdqqk<br><br>SARST: aliSize=69 (resi) RMSD=14.00 (Å)<br>q----IYYSDKYDDEEFEYRHVMLPKDIAKLVPKTHLMSESEWRNLGVQQSQGWVHYMIHEPEPHILLFRRPLP-----k<br>-ahkqIYYSDKYFDEHYEYRHVMLPRELSKQVPKTHLMSEEEWRRLGVQQSLGWVHYMIHEPEPHILLFRRPLPkdqqk-<br><br>BLAST: aliSize=65 (resi) iden=84.51% (60/71) simi=91.55% (65/71)<br>---QIYYSDKYDDEEFEYRHVMLPKDIAKLVPKTHLMSESEWRNLGVQQSQGWVHYMIHEPEPHILLFRRPLPK----<br>ahkQIYYSDKYFDEHYEYRHVMLPRELSKQVPKTHLMSEEEWRRLGVQQSLGWVHYMIHEPEPHILLFRRPLPKdqqk<br><br>Proposed: aliSize=71 (resi) RMSD=1.18 (Å)<br>---QIYYSDKYDDEEFEYRHVMLPKDIAKLVPKTHLMSESEWRNLGVQQSQGWVHYMIHEPEPHILLFRRPLPK----<br>ahkQIYYSDKYFDEHYEYRHVMLPRELSKQVPKTHLMSEEEWRRLGVQQSLGWVHYMIHEPEPHILLFRRPLPKdqqk

795	1dktB<br>(71)<br>1qb3C<br>(109)	C	53.52	TM-align: aliSize=56 (resi) RMSD=1.93 (Å)<br>---------------------QIYYSDKYDDEEFEYRHVMLPKDIAK-L---VPKT-----HLMSESEWRNLGVQQSQGWVHYMIHepephillfrrplpk----------------------<br>hafqgrkltdqerarvlefqdSIHYSPRYSDDNYEYRHVMLPKAMLKvIpsdYFNSevgtlRILTEDEWRGLGITQSLGWEHYECH--------------apephillfkrplnyeaelraat<br><br>SARST: aliSize=66 (resi) RMSD=10.30 (Å)<br>q----------------------IYYSDKYDDEEFEYRHVMLPKDIAKLVPKT---------HLMSESEWRNLGVQQSQGWVH-YMIHePEPHILLFRRP-----------lpk<br>-hafqgrkltdqerarvlefqdsIHYSPRYSDDNYEYRHVMLPKAMLKVIPSDyfnsevgtlRILTEDEWRGLGITQSLGWEHyECHA-PEPHILLFKRPlnyeaelraat---<br><br>BLAST: aliSize=53 (resi) iden=56.34% (40/71) simi=74.65% (53/71)<br>q----------------------IYYSDKYDDEEFEYRHVMLPKDIAKLVPKTH---------LMSESEWRNLGVQQSQGWVHYMIHEPEPHILLFRRPL----------pk<br>-hafqgrkltdqerarvlefqdsIHYSPRYSDDNYEYRHVMLPKAMLKVIPSDYfnsevgtlrILTEDEWRGLGITQSLGWEHYECHAPEPHILLFKRPLnyeaelraat--<br><br>Proposed: aliSize=68 (resi) RMSD=1.31 (Å)<br>---------------------QIYYSDKYDDEEFEYRHVMLPKDIAK-L---vPKT------HLMSESEWRNLGVQQSQGWVHYM--iHePEPHILLFRRPLP--K------<br>hafqgrkltdqerarvlefqdSIHYSPRYSDDNYEYRHVMLPKAMLKvIpsd-YFNsevgtlRILTEDEWRGLGITQSLGWEHYEch-A-PEPHILLFKRPLNyeAelraat
796	1buhB<br>(70)<br>1cksB<br>(78)	C	84.29	TM-align: aliSize=59 (resi) RMSD=1.08 (Å)<br>---QIYYSDKYDDEEFEYRHVMLPKDIAKLVPKTHLMSESEWRNLGVQQSQGWVHYMIHEPEphillfrrplp----------------<br>ahkQIYYSDKYFDEHYEYRHVMLPRELSKQVPKTHLMSEEEWRRLGVQQSLGWVHYMIHEPE-----------phillfrrplpkdqqk<br><br>SARST: aliSize=67 (resi) RMSD=12.88 (Å)<br>qi-----YYSDKYDDEEFEYRHVMLPKDIAKLVPKTHLMSESEWRNLGVQQSQGWVHYMIHEPEPHILLFRRPL------p<br>--ahkqiYYSDKYFDEHYEYRHVMLPRELSKQVPKTHLMSEEEWRRLGVQQSLGWVHYMIHEPEPHILLFRRPLpkdqqk-<br><br>BLAST: aliSize=64 (resi) iden=84.29% (59/70) simi=91.43% (64/70)<br>---QIYYSDKYDDEEFEYRHVMLPKDIAKLVPKTHLMSESEWRNLGVQQSQGWVHYMIHEPEPHILLFRRPLP-----<br>ahkQIYYSDKYFDEHYEYRHVMLPRELSKQVPKTHLMSEEEWRRLGVQQSLGWVHYMIHEPEPHILLFRRPLPkdqqk<br><br>Proposed: aliSize=70 (resi) RMSD=1.11 (Å)<br>---QIYYSDKYDDEEFEYRHVMLPKDIAKLVPKTHLMSESEWRNLGVQQSQGWVHYMIHEPEPHILLFRRPLP-----<br>ahkQIYYSDKYFDEHYEYRHVMLPRELSKQVPKTHLMSEEEWRRLGVQQSLGWVHYMIHEPEPHILLFRRPLPkdqqk
797	1buhB<br>(70)<br>1qb3C<br>(109)	C	55.71	TM-align: aliSize=56 (resi) RMSD=1.75 (Å)<br>---------------------QIYYSDKYDDEEFEYRHVMLPKDIAK-L---VPK-----THLMSESEWRNLGVQQSQGWVHYMIHepephillfrrplp-----------------------<br>hafqgrkltdqerarvlefqdSIHYSPRYSDDNYEYRHVMLPKAMLKvIpsdYFNsevgtLRILTEDEWRGLGITQSLGWEHYECH--------------apephillfkrplnyeaelraat<br><br>SARST: aliSize=64 (resi) RMSD=10.62 (Å)<br>qiy------------------------YSDKYDDEEFEYRHVMLPKDIAKLVPK---------THLMSESEWRNLGVQQSQGWVH-YMIHePEPHILLfRRPL-----------p<br>---hafqgrkltdqerarvlefqdsihYSPRYSDDNYEYRHVMLPKAMLKVIPSdyfnsevgtLRILTEDEWRGLGITQSLGWEHyECHA-PEPHILL-FKRPlnyeaelraat-<br><br>BLAST: aliSize=53 (resi) iden=57.14% (40/70) simi=75.71% (53/70)<br>q----------------------IYYSDKYDDEEFEYRHVMLPKDIAKLVPKTH---------LMSESEWRNLGVQQSQGWVHYMIHEPEPHILLFRRPL----------p<br>-hafqgrkltdqerarvlefqdsIHYSPRYSDDNYEYRHVMLPKAMLKVIPSDYfnsevgtlrILTEDEWRGLGITQSLGWEHYECHAPEPHILLFKRPLnyeaelraat-<br><br>Proposed: aliSize=69 (resi) RMSD=1.59 (Å)<br>---------------------QIYYSDKYDDEEFEYRHVMLPKDIAK-L---VPK-----THLMSESEWRNLGVQQSQGWVHYM-iHEPEPHILLFRRPLP---------<br>hafqgrkltdqerarvlefqdSIHYSPRYSDDNYEYRHVMLPKAMLKvIpsdYFNsevgtLRILTEDEWRGLGITQSLGWEHYEc-HAPEPHILLFKRPLNyeaelraat

<table>
<tr><td rowspan="2">798</td><td>1dktA
(72)</td><td rowspan="2">C</td><td rowspan="2">83.33</td><td rowspan="2">TM-align: aliSize=59 (resi) RMSD=1.03 (Å)
---QIYYSDKYDDEEFEYRHVMLPKDIAKLVPKTHLMSESEWRNLGVQQSQGWVHYMIHEPEphillfrrplpkk----------------
ahkQIYYSDKYFDEHYEYRHVMLPRELSKQVPKTHLMSEEEWRRLGVQQSLGWVHYMIHEPE-------------phillfrrplpkdqqk

SARST: aliSize=69 (resi) RMSD=14.00 (Å)
q----IYYSDKYDDEEFEYRHVMLPKDIAKLVPKTHLMSESEWRNLGVQQSQGWVHYMIHEPEPHILLFRRPLP-----kk
-ahkqIYYSDKYFDEHYEYRHVMLPRELSKQVPKTHLMSEEEWRRLGVQQSLGWVHYMIHEPEPHILLFRRPLPkdqqk--

BLAST: aliSize=65 (resi) iden=83.33% (60/72) simi=90.28% (65/72)
---QIYYSDKYDDEEFEYRHVMLPKDIAKLVPKTHLMSESEWRNLGVQQSQGWVHYMIHEPEPHILLFRRPLPK----k
ahkQIYYSDKYFDEHYEYRHVMLPRELSKQVPKTHLMSEEEWRRLGVQQSLGWVHYMIHEPEPHILLFRRPLPKdqqk-

Proposed: aliSize=72 (resi) RMSD=1.09 (Å)
---QIYYSDKYDDEEFEYRHVMLPKDIAKLVPKTHLMSESEWRNLGVQQSQGWVHYMIHEPEPHILLFRRPLPKK---
ahkQIYYSDKYFDEHYEYRHVMLPRELSKQVPKTHLMSEEEWRRLGVQQSLGWVHYMIHEPEPHILLFRRPLPKDqqk</td></tr>
<tr><td>1cksB
(78)</td></tr>
<tr><td rowspan="2">799</td><td>1dktA
(72)</td><td rowspan="2">C</td><td rowspan="2">54.17</td><td rowspan="2">TM-align: aliSize=56 (resi) RMSD=1.87 (Å)
---------------------QIYYSDKYDDEEFEYRHVMLPKDIAK-LV--PKT------HLMSESEWRNLGVQQSQGWVHYMIHepephillfrrplpkk-----------------------
hafqgrkltdqerarvlefqdSIHYSPRYSDDNYEYRHVMLPKAMLKvIPsdYFNsevgtlRILTEDEWRGLGITQSLGWEHYECH----------------apephillfkrplnyeaelraat

SARST: aliSize=66 (resi) RMSD=10.24 (Å)
q----------------------IYYSDKYDDEEFEYRHVMLPKDIAKLVPKT---------HLMSESEWRNLGVQQSQGWVH-YMIHePEPHILLFRRP-----------lpkk
-hafqgrkltdqerarvlefqdsIHYSPRYSDDNYEYRHVMLPKAMLKVIPSDyfnsevgtlRILTEDEWRGLGITQSLGWEHyECHA-PEPHILLFKRPlnyeaelraat----

BLAST: aliSize=53 (resi) iden=55.56% (40/72) simi=73.61% (53/72)
q----------------------IYYSDKYDDEEFEYRHVMLPKDIAKLVPKTH---------LMSESEWRNLGVQQSQGWVHYMIHEPEPHILLFRRPL----------pkk
-hafqgrkltdqerarvlefqdsIHYSPRYSDDNYEYRHVMLPKAMLKVIPSDYfnsevgtlrILTEDEWRGLGITQSLGWEHYECHAPEPHILLFKRPLnyeaelraat---

Proposed: aliSize=69 (resi) RMSD=1.58 (Å)
---------------------QIYYSDKYDDEEFEYRHVMLPKDIAK-LV--PKT------HLMSESEWRNLGVQQSQGWVHYM-iHEPEPHILLFRRPLP---------kk
hafqgrkltdqerarvlefqdSIHYSPRYSDDNYEYRHVMLPKAMLKvIPsdYFNsevgtlRILTEDEWRGLGITQSLGWEHYEc-HAPEPHILLFKRPLNyeaelraat--</td></tr>
<tr><td>1qb3C
(109)</td></tr>
<tr><td rowspan="2">800</td><td>2astC
(69)</td><td rowspan="2">C</td><td rowspan="2">84.06</td><td rowspan="2">TM-align: aliSize=59 (resi) RMSD=0.95 (Å)
---QIYYSDKYDDEEFEYRHVMLPKDIAKLVPKTHLMSESEWRNLGVQQSQGWVHYMIHEPEphillfrrpl----------------
ahkQIYYSDKYFDEHYEYRHVMLPRELSKQVPKTHLMSEEEWRRLGVQQSLGWVHYMIHEPE----------phillfrrplpkdqqk

SARST: aliSize=67 (resi) RMSD=11.49 (Å)
q----IYYSDKYDDEEFEYRHVMLPKDIAKLVPKTHLMSESEWRNLGVQQSQGWVHYMIHEPEPHILLFRRP-------l
-ahkqIYYSDKYFDEHYEYRHVMLPRELSKQVPKTHLMSEEEWRRLGVQQSLGWVHYMIHEPEPHILLFRRPlpkdqqk-

BLAST: aliSize=63 (resi) iden=84.06% (58/69) simi=91.30% (63/69)
---QIYYSDKYDDEEFEYRHVMLPKDIAKLVPKTHLMSESEWRNLGVQQSQGWVHYMIHEPEPHILLFRRPL------
ahkQIYYSDKYFDEHYEYRHVMLPRELSKQVPKTHLMSEEEWRRLGVQQSLGWVHYMIHEPEPHILLFRRPLpkdqqk

Proposed: aliSize=69 (resi) RMSD=1.01 (Å)
---QIYYSDKYDDEEFEYRHVMLPKDIAKLVPKTHLMSESEWRNLGVQQSQGWVHYMIHEPEPHILLFRRPL------
ahkQIYYSDKYFDEHYEYRHVMLPRELSKQVPKTHLMSEEEWRRLGVQQSLGWVHYMIHEPEPHILLFRRPLpkdqqk</td></tr>
<tr><td>1cksB
(78)</td></tr>
</table>

801	2astC<br>(69)<br><br>1qb3C<br>(109)	C	56.52	TM-align: aliSize=56 (resi) RMSD=1.92 (Å)<br>---------------------QIYYSDKYDDEEFEYRHVMLPKDIAK-LV--PKT------HLMSESEWRNLGVQQSQGWVHYMIHepephillfrrpl-----------------------<br>hafqgrkltdqerarvlefqdSIHYSPRYSDDNYEYRHVMLPKAMLKvIPsdYFNsevgtlRILTEDEWRGLGITQSLGWEHYECH-------------apephillfkrplnyeaelraat<br><br>SARST: aliSize=66 (resi) RMSD=10.20 (Å)<br>q----------------------IYYSDKYDDEEFEYRHVMLPKDIAKLVPKT---------HLMSESEWRNLGVQQSQGWVH-YMIHePEPHILLFRRP-----------l<br>-hafqgrkltdqerarvlefqdsIHYSPRYSDDNYEYRHVMLPKAMLKVIPSDyfnsevgtlRILTEDEWRGLGITQSLGWEHyECHA-PEPHILLFKRPlnyeaelraat-<br><br>BLAST: aliSize=53 (resi) iden=57.97% (40/69) simi=76.81% (53/69)<br>q----------------------IYYSDKYDDEEFEYRHVMLPKDIAKLVPKTH---------LMSESEWRNLGVQQSQGWVHYMIHEPEPHILLFRRPL----------<br>-hafqgrkltdqerarvlefqdsIHYSPRYSDDNYEYRHVMLPKAMLKVIPSDYfnsevgtlrILTEDEWRGLGITQSLGWEHYECHAPEPHILLFKRPLnyeaelraat<br><br>Proposed: aliSize=68 (resi) RMSD=1.62 (Å)<br>---------------------QIYYSDKYDDEEFEYRHVMLPKDIAK-L---VPKT-----HLMSESEWRNLGVQQSQGWVHYM-iHEPEPHILLFRRPL----------<br>hafqgrkltdqerarvlefqdSIHYSPRYSDDNYEYRHVMLPKAMLKvIpsdYFNSevgtlRILTEDEWRGLGITQSLGWEHYEc-HAPEPHILLFKRPLnyeaelraat
802	2hdxA<br>(108)<br><br>2h46E<br>(98)	C	23.47	TM-align: aliSize=67 (resi) RMSD=2.36 (Å)<br>qpls--GYPWFHGMLSRLKAAQLVLEGgtGSHGVFLVRQSETRRGECVLTFNFQGKAKHLRLSLNAAGQCRVQHLhfqsifdmlehfrvhpiplesggssdvvlvsyvps-----------------------------<br>----mkPHPWFFGKIPRAKAEEMLSKQ--RHDGAFLIRESESAPGDFSLSVKFGNDVQHFKVLRDGAGKYFLWVV-----------------------------------kfnslnelvdyhrstsvsrnqqiflrdie<br><br>SARST: aliSize=82 (resi) RMSD=8.50 (Å)<br>qplsg---YPWFHGMLSRLKAAQLVLEGgtGSHGVFLVRQSETRRGECVLTFNFQGKAKHLRLSLNAAGQCRVQHLHFQSIFDMLEHFRVHP------------iplesggssdvvlvsyvps<br>-----mkpHPWFFGKIPRAKAEEMLSKQ--RHDGAFLIRESESAPGDFSLSVKFGNDVQHFKVLRDGAGKYFLWVVKFNSLNELVDYHRSTSvsrnqqiflrdie------------------<br><br>BLAST: aliSize=47 (resi) iden=23.47% (23/98) simi=47.96% (47/98)<br>qplsg---YPWFHGMLSRLKAAQLVLEGgtgSH-GVFLVRQSETRRGECVLTFNFQGKAKHLRLSLNAAGQCRVQHLHFQSIFDMLEHFR----------------vhpiplesggssdvvlvsyvps<br>-----mkpHPWFFGKIPRAKAEEMLSKQ---RHdGAFLIRESESAPGDFSLSVKFGNDVQHFKVLRDGAGKYFLWVVKFNSLNELVDYHRstsvsrnqqiflrdie----------------------<br><br>Proposed: aliSize=93 (resi) RMSD=2.00 (Å)<br>qpls--GYPWFHGMLSRLKAAQLVLEGgtGSHGVFLVRQSETRRGECVLTFNFQGKAKHLRLSLN-aAGQCRV--qhLHFQSIFDMLEHFRVHPIPLesgGsSDVVLVSYVps<br>----mkPHPWFFGKIPRAKAEEMLSKQ--RHDGAFLIRESESAPGDFSLSVKFGNDVQHFKVLRDg-AGKYFLwv--VKFNSLNELVDYHRSTSVSR---N-QQIFLRDIE--
803	2hdxA<br>(108)<br><br>2h5kB<br>(96)	C	21.88	TM-align: aliSize=66 (resi) RMSD=2.22 (Å)<br>qplsGYPWFHGMLSRLKAAQLVLEGgtGSHGVFLVRQSETRRGECVLTFNFQGKAKHLRLSLNa-AGQCRVQHLhfqsifdmlehfrvhpiplesggssdvvlvsyvps-----------------------------<br>----PHPWFFGKIPRAKAEEMLSKQ--RHDGAFLIRESESAPGDFSLSVKFGNDVQHFKVLRD-gAGKYFLWVV-----------------------------------kfnslnelvdyhrstsvsrnqqiflrdie<br><br>SARST: aliSize=92 (resi) RMSD=11.94 (Å)<br>qplsg-YPWFHGMLSRLKAA-QLVLEGGtgsHGVFLVRQSETRRGECVLTFNFQGKAKHLRLSLNAAGQCRVQHLHFQSIFDMLEHFRVHPIPleSGGSSDVVL-vsyvps<br>-----pHPWFFGKIPRAKAEeMLSKQRH---DGAFLIRESESAPGDFSLSVKFGNDVQHFKVLRDGAGKYFLWVVKFNSLNELVDYHRSTSVS--RNQQIFLRDi------<br><br>BLAST: aliSize=47 (resi) iden=24.21% (23/96) simi=49.47% (47/96)<br>qplsg-YPWFHGMLSRLKAAQLVLEGgtgSH-GVFLVRQSETRRGECVLTFNFQGKAKHLRLSLNAAGQCRVQHLHFQSIFDMLEHFR---------------vhpiplesggssdvvlvsyvps<br>-----pHPWFFGKIPRAKAEEMLSKQ---RHdGAFLIRESESAPGDFSLSVKFGNDVQHFKVLRDGAGKYFLWVVKFNSLNELVDYHRstsvsrnqqiflrdi----------------------<br><br>Proposed: aliSize=93 (resi) RMSD=1.82 (Å)<br>qplsGYPWFHGMLSRLKAAQLVLEGgtGSHGVFLVRQSETRRGECVLTFNFQGKAKHLRLSL---NaagQCRVQHLHFQSIFDMLEHFRVHPIPLesgGsSDVVLVSYVps<br>----PHPWFFGKIPRAKAEEMLSKQ--RHDGAFLIRESESAPGDFSLSVKFGNDVQHFKVLRdgaG---KYFLWVVKFNSLNELVDYHRSTSVSR---N-QQIFLRDIE--

804	2hdxA (108)<br>2aobB (93)	C	22.58	TM-align: aliSize=66 (resi) RMSD=2.52 (Å)<br>qplsgyPWFHGMLSRLKAAQLVLEGgtGSHGVFLVRQSETRRGECVLTFNFQGKAKHLRLSLNAAGQCRVQHLhfqsifdmlehfrv-------------Hpiplesggssdvvlsyvps------------<br>------PWFFGKIPRAKAEEMLSKQ--RHDGAFLIRESESAPGDFSLSVKFGNDVQHFKVLRDGAGKYFLWVV-------------kfnslnelvdyhrsT-------------------svsrnqqiflrdi<br><br>SARST: aliSize=79 (resi) RMSD=7.46 (Å)<br>qplsgyp-WFHGMLSRLKAAQLV-LEggtGSHGVFLVRQSETRRGECVLTFNFQGKAKHLRLSLNAAGQCRVQHLHFQSIFDMLEHFRVHP-----------iplesggssdvvlsyvps<br>-------pWFFGKIPRAKAEEMLsKQ---RHDGAFLIRESESAPGDFSLSVKFGNDVQHFKVLRDGAGKYFLWVVKFNSLNELVDYHRSTSvsrnqqiflrdi------------------<br><br>BLAST: aliSize=46 (resi) iden=24.73% (23/93) simi=49.46% (46/93)<br>qplsgyPWFHGMLSRLKAAQLVLEGgtgSH-GVFLVRQSETRRGECVLTFNFQGKAKHLRLSLNAAGQCRVQHLHFQSIFDMLEHFR--------------vhpiplesggssdvvlsyvps<br>------PWFFGKIPRAKAEEMLSKQ---RHdGAFLIRESESAPGDFSLSVKFGNDVQHFKVLRDGAGKYFLWVVKFNSLNELVDYHRstsvsrnqqiflrdi---------------------<br><br>Proposed: aliSize=90 (resi) RMSD=1.83 (Å)<br>qplsgyPWFHGMLSRLKAAQLVLEGgtGSHGVFLVRQSETRRGECVLTFNFQGKAKHLRLSL---NaagQCRVQHLHFQSIFDMLEHFRVHPIPLesgGsSDVVLVSYvps<br>------PWFFGKIPRAKAEEMLSKQ--RHDGAFLIRESESAPGDFSLSVKFGNDVQHFKVLRdgaG---KYFLWVVKFNSLNELVDYHRSTSVSR---N-QQIFLRDI---
805	2hdxA (108)<br>2aobA (96)	C	21.88	TM-align: aliSize=70 (resi) RMSD=2.67 (Å)<br>QplsGYPWFHGMLSRLKAAQLVLEGgtGSHGVFLVRQSETRRGECVLTFNFQGKAKHLRLSLNAAGQCRVQHLhfqsifdmlehfr------------VHpiplesggssdvvlsyvps------------<br>K---PHPWFFGKIPRAKAEEMLSKQ--RHDGAFLIRESESAPGDFSLSVKFGNDVQHFKVLRDGAGKYFLWVV------------kfnslnelvdyhrST-------------------svsrnqqiflrdi<br><br>SARST: aliSize=81 (resi) RMSD=7.35 (Å)<br>qplsg--YPWFHGMLS-RLKAAQLVLEggtGSHGVFLVRQSETRRGECVLTFNFQGKAKHLRLSLNAAGQCRVQHLHFQSIFDMLEHFRVHP-----------iplesggssdvvlsyvps<br>-----kpHPWFFGKIPrAKAEEMLSKQ---RHDGAFLIRESESAPGDFSLSVKFGNDVQHFKVLRDGAGKYFLWVVKFNSLNELVDYHRSTSvsrnqqiflrdi------------------<br><br>BLAST: aliSize=47 (resi) iden=23.96% (23/96) simi=48.96% (47/96)<br>qplsg--YPWFHGMLSRLKAAQLVLEGgtgSH-GVFLVRQSETRRGECVLTFNFQGKAKHLRLSLNAAGQCRVQHLHFQSIFDMLEHFR--------------vhpiplesggssdvvlsyvps<br>-----kpHPWFFGKIPRAKAEEMLSKQ---RHdGAFLIRESESAPGDFSLSVKFGNDVQHFKVLRDGAGKYFLWVVKFNSLNELVDYHRstsvsrnqqiflrdi---------------------<br><br>Proposed: aliSize=92 (resi) RMSD=1.68 (Å)<br>qpls-GYPWFHGMLSRLKAAQLVLEGgtGSHGVFLVRQSETRRGECVLTFNFQGKAKHLRLSL---NaagQCRVQHLHFQSIFDMLEHFRVHPIPLesgGsSDVVLVSYvps<br>----kPHPWFFGKIPRAKAEEMLSKQ--RHDGAFLIRESESAPGDFSLSVKFGNDVQHFKVLRdgaG---KYFLWVVKFNSLNELVDYHRSTSVSR---N-QQIFLRDI---
806	2hdxA (108)<br>2aoaA (93)	C	22.58	TM-align: aliSize=68 (resi) RMSD=2.52 (Å)<br>qplsgYPWFHGMLSRLKAAQLVLEGgtGSHGVFLVRQSETRRGECVLTFNFQGKAKHLRLSLNAAGQCRVQHLhfqsifdmlehfr------------VHpiplesggssdvvlsyvps------------<br>-----HPWFFGKIPRAKAEEMLSKQ--RHDGAFLIRESESAPGDFSLSVKFGNDVQHFKVLRDGAGKYFLWVV------------kfnslnelvdyhrST-------------------svsrnqqiflrd<br><br>SARST: aliSize=81 (resi) RMSD=7.28 (Å)<br>qplsgy-PWFHGMLSRLKAAQLVLEGGtgSHGVFLVRQSETRRGECVLTFNFQGKAKHLRLSLNAAGQCRVQHLHFQSIFDMLEHFRVHP----------iplesggssdvvlsyvps<br>------hPWFFGKIPRAKAEEMLSKQR--HDGAFLIRESESAPGDFSLSVKFGNDVQHFKVLRDGAGKYFLWVVKFNSLNELVDYHRSTSvsrnqqiflrd------------------<br><br>BLAST: aliSize=47 (resi) iden=24.73% (23/93) simi=50.54% (47/93)<br>qplsgYPWFHGMLSRLKAAQLVLEGgtgSH-GVFLVRQSETRRGECVLTFNFQGKAKHLRLSLNAAGQCRVQHLHFQSIFDMLEHFR-------------vhpiplesggssdvvlsyvps<br>-----HPWFFGKIPRAKAEEMLSKQ---RHdGAFLIRESESAPGDFSLSVKFGNDVQHFKVLRDGAGKYFLWVVKFNSLNELVDYHRstsvsrnqqiflrd---------------------<br><br>Proposed: aliSize=90 (resi) RMSD=1.62 (Å)<br>qplsgYPWFHGMLSRLKAAQLVLEGgtGSHGVFLVRQSETRRGECVLTFNFQGKAKHLRLSL---NaagQCRVQHLHFQSIFDMLEHFRVHPIPLesgGsSDVVLvSYvps<br>-----HPWFFGKIPRAKAEEMLSKQ--RHDGAFLIRESESAPGDFSLSVKFGNDVQHFKVLRdgaG---KYFLWVVKFNSLNELVDYHRSTSVSR---N-QQIFL-RD---

807	1p13A (102)<br>2h46E (98)	C	29.59	TM-align: aliSize=66 (resi) RMSD=1.32 (Å)<br>--AEEWYFGKITRRESERLLLNpENPRGTFLVRESE-TTKgAYCLSVSDFdnakgLNVKHYKIRKLDSGGFYITSRtqfsslqqlvayyskhadglchrltnvcp------------------------------<br>mkPHPWFFGKIPRAKAEEMLSK-QRHDGAFLIRESEsAPG-DFSLSVKFG-----NDVQHFKVLRDGAGKYFLWVV-----------------------------kfnslnelvdyhrstsvsrnqqiflrdie<br><br>SARST: aliSize=81 (resi) RMSD=8.80 (Å)<br>a---EEWYFGKITRRESERLLLNPENpRGTFLVRESETTKGAYCLSVSDfdnakGLNVKHYKIRKLDSGGFYITsRTQFSSLQQLVAYYSKH--------------adglchrltnvcp<br>-mkpHPWFFGKIPRAKAEEMLSKQRH-DGAFLIRESESAPGDFSLSVKF-----GNDVQHFKVLRDGAGKYFLW-VVKFNSLNELVDYHRSTsvsrnqqiflrdie-------------<br><br>BLAST: aliSize=51 (resi) iden=33.67% (33/98) simi=52.04% (51/98)<br>aee-----WYFGKITRRESERlLLNPENPRGTFLVRESETTKGAYCLSVSdFDNakglNVKHYKIRKlDSGGFYITSRTQFSSLQQLVAYY-----------------skhadglchrltnvcp<br>---mkphpWFFGKIPRAKAEE-MLSKQRHDGAFLIRESESAPGDFSLSVK-FGN----DVQHFKVLR-DGAGKYFLWVVKFNSLNELVDYHrstsvsrnqqiflrdie----------------<br><br>Proposed: aliSize=95 (resi) RMSD=1.74 (Å)<br>--AEEWYFGKITRRESERLLLNpENPRGTFLVRESETTKGAYCLSVSDFdnakgLNVKHYKIRKLDSGGFYITSrTQFSSLQQLVAYYSKHADGL-CHRLTNVCP<br>mkPHPWFFGKIPRAKAEEMLSK-QRHDGAFLIRESESAPGDFSLSVKFG-----NDVQHFKVLRDGAGKYFLWV-VKFNSLNELVDYHRSTSVSRnQQIFLRDIE
808	1p13A (102)<br>2h5kB (96)	C	29.17	TM-align: aliSize=66 (resi) RMSD=1.40 (Å)<br>AEEWYFGKITRRESERLLLNpENPRGTFLVRESE-TTKgAYCLSVSDFdnakgLNVKHYKIRKLDSGGFYITSRtqfsslqqlvayyskhadglchrltnvcp------------------------------<br>PHPWFFGKIPRAKAEEMLSK-QRHDGAFLIRESEsAPG-DFSLSVKFG-----NDVQHFKVLRDGAGKYFLWVV-----------------------------kfnslnelvdyhrstsvsrnqqiflrdie<br><br>SARST: aliSize=81 (resi) RMSD=8.95 (Å)<br>a-EEWYFGKITRRESERLLLnPENPRGTFLVRESETTKGAYCLSVSDfdnakGLNVKHYKIRKLDSGGFYITsRTQFSSLQQLVAYYSKH-------------adglchrltnvcp<br>-pHPWFFGKIPRAKAEEMLS-KQRHDGAFLIRESESAPGDFSLSVKF-----GNDVQHFKVLRDGAGKYFLW-VVKFNSLNELVDYHRSTsvsrnqqiflrdi-------------<br><br>BLAST: aliSize=51 (resi) iden=34.74% (33/96) simi=53.68% (51/96)<br>aee---WYFGKITRRESERlLLNPENPRGTFLVRESETTKGAYCLSVSdFDNakglNVKHYKIRKlDSGGFYITSRTQFSSLQQLVAYY----------------skhadglchrltnvcp<br>---phpWFFGKIPRAKAEE-MLSKQRHDGAFLIRESESAPGDFSLSVK-FGN----DVQHFKVLR-DGAGKYFLWVVKFNSLNELVDYHrstsvsrnqqiflrdi----------------<br><br>Proposed: aliSize=94 (resi) RMSD=1.65 (Å)<br>AEEWYFGKITRRESERLLLNpENPRGTFLVRESE-TTKgAYCLSVSDFdnakgLNVKHYKIRKLDSGGFYITSRtQFSSLQQLVAYYSKHADGL-CHRLTNVCP<br>PHPWFFGKIPRAKAEEMLSK-QRHDGAFLIRESEsAPG-DFSLSVKFG-----NDVQHFKVLRDGAGKYFLWVV-KFNSLNELVDYHRSTSVSRnQQIFLRDIE
809	1p13A (102)<br>2aobB (93)	C	32.26	TM-align: aliSize=68 (resi) RMSD=1.91 (Å)<br>aeEWYFGKITRRESERLLLNpENPRGTFLVRESETTKGAYCLSVSDFdnakgLNVKHYKIRKLDSGGFYITSRTqfsslqqlvayyskh------------ADglchrltnvcp-------------<br>--PWFFGKIPRAKAEEMLSK-QRHDGAFLIRESESAPGDFSLSVKFG-----NDVQHFKVLRDGAGKYFLWVVK---------------fnslnelvdyhrST-----------svsrnqqiflrdi<br><br>SARST: aliSize=78 (resi) RMSD=7.74 (Å)<br>aee-WYFGKIT-RRESERLLLNPenPRGTFLVRESETTKGAYCLSVSDfdnakGLNVKHYKIRKLDSGGFYITsRTQFSSLQQLVAYYSKH-------------adglchrltnvcp<br>---pWFFGKIPrAKAEEMLSKQR--HDGAFLIRESESAPGDFSLSVKF-----GNDVQHFKVLRDGAGKYFLW-VVKFNSLNELVDYHRSTsvsrnqqiflrdi-------------<br><br>BLAST: aliSize=51 (resi) iden=35.48% (33/93) simi=54.84% (51/93)<br>aee-WYFGKITRRESERlLLNPENPRGTFLVRESETTKGAYCLSVSdFDNakglNVKHYKIRKlDSGGFYITSRTQFSSLQQLVAYY----------------skhadglchrltnvcp<br>---pWFFGKIPRAKAEE-MLSKQRHDGAFLIRESESAPGDFSLSVK-FGN----DVQHFKVLR-DGAGKYFLWVVKFNSLNELVDYHrstsvsrnqqiflrdi----------------<br><br>Proposed: aliSize=91 (resi) RMSD=1.28 (Å)<br>aeEWYFGKITRRESERLLLNpENPRGTFLVRESETTKGAYCLSVSDFdnakgLNVKHYKIRKLDSGGFYITSRtQFSSLQQLVAYYSKHADGL-C-HRLTnVCp<br>--PWFFGKIPRAKAEEMLSK-QRHDGAFLIRESESAPGDFSLSVKFG-----NDVQHFKVLRDGAGKYFLWVV-KFNSLNELVDYHRSTSVSRnQqIFLR-DI-

810	1p13A (102)<br>2aobA (96)	C	30.21	TM-align: aliSize=69 (resi) RMSD=2.08 (Å)<br>-AEEWYFGKITRRESERLLLNpENPRGTFLVRESE-TTKgAYCLSVSDFdnakgLNVKHYKIRKLDSGGFYITSRTqfsslqqlvayyskh-----------ADglchrltnvcp------------<br>kPHPWFFGKIPRAKAEEMLSK-QRHDGAFLIRESEsAPG-DFSLSVKFG-----NDVQHFKVLRDGAGKYFLWVVK---------------fnslnelvdyhrST----------svsrnqqiflrdi<br><br>SARST: aliSize=80 (resi) RMSD=7.74 (Å)<br>a--EEWYFGKITRRESERLLlnPEN-PRGTFLVRESETTKGAYCLSVSDfdnakGLNVKHYKIRKLDSGGFYITsRTQFSSLQQLVAYYSKH------------adglchrltnvcp<br>-kpHPWFFGKIPRAKAEEML--SKQrHDGAFLIRESESAPGDFSLSVKF-----GNDVQHFKVLRDGAGKYFLW-VVKFNSLNELVDYHRSTsvsrnqqiflrdi------------<br><br>BLAST: aliSize=51 (resi) iden=34.38% (33/96) simi=53.12% (51/96)<br>aee----WYFGKITRRESERlLLNPENPRGTFLVRESETTKGAYCLSVSdFDNakglNVKHYKIRKlDSGGFYITSRTQFSSLQQLVAYY---------------skhadglchrltnvcp<br>---kphpWFFGKIPRAKAEE-MLSKQRHDGAFLIRESESAPGDFSLSVK-FGN----DVQHFKVLR-DGAGKYFLWVVKFNSLNELVDYHrstsvsrnqqiflrdi---------------<br><br>Proposed: aliSize=94 (resi) RMSD=1.79 (Å)<br>-AEEWYFGKITRRESERLLLNpENPRGTFLVRESETTKGAYCLSVSDFdnakgLNVKHYKIRKLDSGGFYITSRtQFSSLQQLVAYYSKHADGL-CHRLTNVCp<br>kPHPWFFGKIPRAKAEEMLSK-QRHDGAFLIRESESAPGDFSLSVKFG-----NDVQHFKVLRDGAGKYFLWVV-KFNSLNELVDYHRSTSVSRnQQIFLRDI-
811	1p13A (102)<br>2aoaA (93)	C	31.18	TM-align: aliSize=68 (resi) RMSD=2.04 (Å)<br>aEEWYFGKITRRESERLLLNpENPRGTFLVRESE-TTKgAYCLSVSDFdnakgLNVKHYKIRKLDSGGFYITSRTqfsslqqlvayyskh-----------ADglchrltnvcp-----------<br>-HPWFFGKIPRAKAEEMLSK-QRHDGAFLIRESEsAPG-DFSLSVKFG-----NDVQHFKVLRDGAGKYFLWVVK---------------fnslnelvdyhrST----------svsrnqqiflrd<br><br>SARST: aliSize=79 (resi) RMSD=7.76 (Å)<br>ae-EWYFGKITRRESERLLlnPEN-PRGTFLVRESETTKGAYCLSVSDfdnakGLNVKHYKIRKLDSGGFYITsRTQFSSLQQLVAYYSKH-----------adglchrltnvcp<br>--hPWFFGKIPRAKAEEML--SKQrHDGAFLIRESESAPGDFSLSVKF-----GNDVQHFKVLRDGAGKYFLW-VVKFNSLNELVDYHRSTsvsrnqqiflrd------------<br><br>BLAST: aliSize=51 (resi) iden=35.48% (33/93) simi=54.84% (51/93)<br>aee--WYFGKITRRESERlLLNPENPRGTFLVRESETTKGAYCLSVSdFDNakglNVKHYKIRKlDSGGFYITSRTQFSSLQQLVAYY--------------skhadglchrltnvcp<br>---hpWFFGKIPRAKAEE-MLSKQRHDGAFLIRESESAPGDFSLSVK-FGN----DVQHFKVLR-DGAGKYFLWVVKFNSLNELVDYHrstsvsrnqqiflrd---------------<br><br>Proposed: aliSize=92 (resi) RMSD=1.68 (Å)<br>aEEWYFGKITRRESERLLLNpENPRGTFLVRESETTKGAYCLSVSDFdnakgLNVKHYKIRKLDSGGFYITSRtQFSSLQQLVAYYSKHADGL-CHRLTnVCp<br>-HPWFFGKIPRAKAEEMLSK-QRHDGAFLIRESESAPGDFSLSVKFG-----NDVQHFKVLRDGAGKYFLWVV-KFNSLNELVDYHRSTSVSRnQQIFL-RD-
812	1rjaA (100)<br>2h46E (98)	C	38.78	TM-align: aliSize=66 (resi) RMSD=2.18 (Å)<br>s---EPWFFGCISRSEAVRRLQAeGNATGAFLIRVSEKPSADYVLSVRDTQAVRHYKIWRRAGGRLHLNeAVsflslpelvnyhraqslshglrlaapcrkhe-----------------------------<br>-mkpHPWFFGKIPRAKAEEMLSK-QRHDGAFLIRESESAPGDFSLSVKFGNDVQHFKVLRDGAGKYFLW-VV-------------------------------kfnslnelvdyhrstsvsrnqqiflrdie<br><br>SARST: aliSize=91 (resi) RMSD=12.53 (Å)<br>s---EPWFFGCISRSEAVRRLQAEGNaTGAFLIRVSEKPSADYVLSV-RDTQAVRHYKIWRRAGGRlhlNE-AVSFLSLPELVNYHRAQsLSHGLRLAAPCR--khe<br>-mkpHPWFFGKIPRAKAEEMLSKQRH-DGAFLIRESESAPGDFSLSVkFGNDVQHFKVLRDGAGKY---FLwVVKFNSLNELVDYHRST-SVSRNQQIFLRDie---<br><br>BLAST: aliSize=54 (resi) iden=39.80% (39/98) simi=55.10% (54/98)<br>se----PWFFGCISRSEAVRRLQAEGNaTGAFLIRVSEKPSADYVLSVRDTQAVRHYKIWRRAGGRLHLnEAVSFLSLPELVNYHRAQSLSHGLRL------aapcrkhe<br>--mkphPWFFGKIPRAKAEEMLSKQRH-DGAFLIRESESAPGDFSLSVKFGNDVQHFKVLRDGAGKYFL-WVVKFNSLNELVDYHRSTSVSRNQQIflrdie--------<br><br>Proposed: aliSize=91 (resi) RMSD=1.90 (Å)<br>s----EpWFFGCISRSEAVRRLQAeGNATGAFLIRVSEKPSADYVLSVRDTQAVRHYKIWRRAGGRLHLNeAVSFLSLPELVNYHRAQSlSH--G-LRLAAPcRkhe<br>-mkphP-WFFGKIPRAKAEEMLSK-QRHDGAFLIRESESAPGDFSLSVKFGNDVQHFKVLRDGAGKYFLW-VVKFNSLNELVDYHRSTS-VSrnQqIFLRDI-E---

813	1rjaA<br>(100)<br><br>2h5kB<br>(96)	C	39.58	TM-align: aliSize=66 (resi) RMSD=2.26 (Å)<br>s-EPWFFGCISRSEAVRRLQAeGNATGAFLIRVSEKPSADYVLSVRDTQAVRHYKIWRRAGGRLHLNeAVsflslpelvnyhraqslshglrlaapcrkhe-----------------------------<br>-pHPWFFGKIPRAKAEEMLSK-QRHDGAFLIRESESAPGDFSLSVKFGNDVQHFKVLRDGAGKYFLW-VV-------------------------------kfnslnelvdyhrstsvsrnqqiflrdie<br><br>SARST: aliSize=92 (resi) RMSD=12.86 (Å)<br>s-EPWFFGCISRSEAVRRLQAEGNaTGAFLIRVSEKPSADYVLSVRDTQAVRHYKIWRRAGGRLHLNeAVSFLSLPELVNYhraqSLSHGLRLAAPCRKH--e<br>-pHPWFFGKIPRAKAEEMLSKQRH-DGAFLIRESESAPGDFSLSVKFGNDVQHFKVLRDGAGKYFLW-VVKFNSLNELVDY----HRSTSVSRNQQIFLRdi-<br><br>BLAST: aliSize=54 (resi) iden=41.05% (39/96) simi=56.84% (54/96)<br>se--PWFFGCISRSEAVRRLQAEGNaTGAFLIRVSEKPSADYVLSVRDTQAVRHYKIWRRAGGRLHLnEAVSFLSLPELVNYHRAQSLSHGLRL-----aapcrkhe<br>--phPWFFGKIPRAKAEEMLSKQRH-DGAFLIRESESAPGDFSLSVKFGNDVQHFKVLRDGAGKYFL-WVVKFNSLNELVDYHRSTSVSRNQQIflrdi--------<br><br>Proposed: aliSize=91 (resi) RMSD=1.90 (Å)<br>s--EpWFFGCISRSEAVRRLQAeGNATGAFLIRVSEKPSADYVLSVRDTQAVRHYKIWRRAGGRLHLNeAVSFLSLPELVNYHRAQSlSH--G-LRLAAPcRkhe<br>-phP-WFFGKIPRAKAEEMLSK-QRHDGAFLIRESESAPGDFSLSVKFGNDVQHFKVLRDGAGKYFLW-VVKFNSLNELVDYHRSTS-VSrnQqIFLRDI-E---
814	1rjaA<br>(100)<br><br>2aobB<br>(93)	C	39.78	TM-align: aliSize=68 (resi) RMSD=2.83 (Å)<br>sEpWFFGCISRSEAVRRLQAeGNATGAFLIRVSEKPSADYVLSVRDTQAVRHYKIWRRAGGRLHLNEAVsflslpelvnyhr------------AQslshglrlaapcrkhe-------------<br>-P-WFFGKIPRAKAEEMLSK-QRHDGAFLIRESESAPGDFSLSVKFGNDVQHFKVLRDGAGKYFLWVVK-------------fnslnelvdyhrST----------------svsrnqqiflrdi<br><br>SARST: aliSize=76 (resi) RMSD=7.60 (Å)<br>sep-WFFGCISRSEAVRRLQAEGNaTGAFLIRVSEKPSADYVLSVR-DTQAVRHYKIWRRAGGrLHLNeAVSFLSLPELVNYHR---------------aqslshglrlaapcrkhe<br>---pWFFGKIPRAKAEEMLSKQRH-DGAFLIRESESAPGDFSLSVKfGNDVQHFKVLRDGAGK-YFLW-VVKFNSLNELVDYHRstsvsrnqqiflrdi------------------<br><br>BLAST: aliSize=54 (resi) iden=41.94% (39/93) simi=58.06% (54/93)<br>sePWFFGCISRSEAVRRLQAEGNaTGAFLIRVSEKPSADYVLSVRDTQAVRHYKIWRRAGGRLHLnEAVSFLSLPELVNYHRAQSLSHGLRL-----aapcrkhe<br>--PWFFGKIPRAKAEEMLSKQRH-DGAFLIRESESAPGDFSLSVKFGNDVQHFKVLRDGAGKYFL-WVVKFNSLNELVDYHRSTSVSRNQQIflrdi--------<br><br>Proposed: aliSize=88 (resi) RMSD=1.84 (Å)<br>sEpWFFGCISRSEAVRRLQAeGNATGAFLIRVSE-kpS-ADYVLSVRDTQAVRHYKIWRRAGGRLHLNEAvSFLSLPELVNYHRAQSlSH--G-LRLAAPcrkhe<br>-P-WFFGKIPRAKAEEMLSK-QRHDGAFLIRESEs--ApGDFSLSVKFGNDVQHFKVLRDGAGKYFLWVV-KFNSLNELVDYHRSTS-VSrnQqIFLRDI-----
815	1rjaA<br>(100)<br><br>2aobA<br>(96)	C	38.54	TM-align: aliSize=69 (resi) RMSD=2.60 (Å)<br>s--EPWFFGCISRSEAVRRLQAeGNATGAFLIRVSEKPSADYVLSVRDTQAVRHYKIWRRAGGRLHLNEAVsflslpelvnyhr------------AQslshglrlaapcrkhe-------------<br>-kpHPWFFGKIPRAKAEEMLSK-QRHDGAFLIRESESAPGDFSLSVKFGNDVQHFKVLRDGAGKYFLWVVK-------------fnslnelvdyhrST----------------svsrnqqiflrdi<br><br>SARST: aliSize=92 (resi) RMSD=10.76 (Å)<br>s--EPWFFGCISRSEAVRRLQAEGNaTGAFLIRVSEKPSADYVLSVR-DTQAVRHYKIWRRAGGrLHLNeAVSFLSLPELVNYHRAQsLSHGLRLAAPCR-khe<br>-kpHPWFFGKIPRAKAEEMLSKQRH-DGAFLIRESESAPGDFSLSVKfGNDVQHFKVLRDGAGK-YFLW-VVKFNSLNELVDYHRST-SVSRNQQIFLRDi---<br><br>BLAST: aliSize=54 (resi) iden=40.62% (39/96) simi=56.25% (54/96)<br>se---PWFFGCISRSEAVRRLQAEGNaTGAFLIRVSEKPSADYVLSVRDTQAVRHYKIWRRAGGRLHLnEAVSFLSLPELVNYHRAQSLSHGLRL-----aapcrkhe<br>--kphPWFFGKIPRAKAEEMLSKQRH-DGAFLIRESESAPGDFSLSVKFGNDVQHFKVLRDGAGKYFL-WVVKFNSLNELVDYHRSTSVSRNQQIflrdi--------<br><br>Proposed: aliSize=89 (resi) RMSD=1.62 (Å)<br>sep----WFFGCISRSEAVRRLQAeGNATGAFLIRVSEKPSADYVLSVRDTQAVRHYKIWRRAGGRLHLNEAvSFLSLPELVNYHRAQSlSH--G-LRLAAPcrkhe<br>---kphpWFFGKIPRAKAEEMLSK-QRHDGAFLIRESESAPGDFSLSVKFGNDVQHFKVLRDGAGKYFLWVV-KFNSLNELVDYHRSTS-VSrnQqIFLRDI-----

816	1rjaA<br>(100)<br>2aoaA<br>(93)	C	38.71	TM-align: aliSize=69 (resi) RMSD=2.62 (Å)<br>sEPWFFGCISRSEAVRRLQAeGNATGAFLIRVSEKPSADYVLSVRDTQAVRHYKIWRRAGGRLHLNEAVsflslpelvnyhr------------AQslshglrlaapcrkhe------------<br>-HPWFFGKIPRAKAEEMLSK-QRHDGAFLIRESESAPGDFSLSVKFGNDVQHFKVLRDGAGKYFLWVVK-------------fnslnelvdyhrST----------------svsrnqqiflrd<br><br>SARST: aliSize=85 (resi) RMSD=9.91 (Å)<br>sepw---FFGCISRSEAVRRLQaeGNAT-GAFLIRVSEKPSADYVLSVRDTQAVRHYKIWR--RAggRLHLNeAVSFLSLPELVNYHRAQSLSHGLRLAA--pcrkhe<br>----hpwFFGKIPRAKAEEMLS--KQRHdGAFLIRESESAPGDFSLSVKFGNDVQHFKVLRdgAG--KYFLW-VVKFNSLNELVDYHRSTSVSRNQQIFLrd------<br><br>BLAST: aliSize=54 (resi) iden=41.94% (39/93) simi=58.06% (54/93)<br>se-PWFFGCISRSEAVRRLQAEGNaTGAFLIRVSEKPSADYVLSVRDTQAVRHYKIWRRAGGRLHLnEAVSFLSLPELVNYHRAQSLSHGLRL----aapcrkhe<br>--hPWFFGKIPRAKAEEMLSKQRH-DGAFLIRESESAPGDFSLSVKFGNDVQHFKVLRDGAGKYFL-WVVKFNSLNELVDYHRSTSVSRNQQIflrd--------<br><br>Proposed: aliSize=89 (resi) RMSD=1.72 (Å)<br>sep--WFFGCISRSEAVRRLQAeGNATGAFLIRVSEKPSADYVLSVRDTQAVRHYKIWRRAGGRLHLNEAvSFLSLPELVNYHRAQSlSH--GLRLAAPcrkhe<br>---hpWFFGKIPRAKAEEMLSK-QRHDGAFLIRESESAPGDFSLSVKFGNDVQHFKVLRDGAGKYFLWVV-KFNSLNELVDYHRSTS-VSrnQQIFLRD-----
817	1r1pB<br>(100)<br>2h46E<br>(98)	C	52.04	TM-align: aliSize=68 (resi) RMSD=1.32 (Å)<br>gsfiDiEFPEWFHEGLSRHQAENLLMGK-DIGFFIIRASQSSPGDFSISVRHEDDVQHFKVMRDTKGNYFLWTEkfpslnklvdyyrttsiskqkqvflrd-----------------------------<br>----M-KPHPWFFGKIPRAKAEEMLSKQrHDGAFLIRESESAPGDFSLSVKFGNDVQHFKVLRDGAGKYFLWVV---------------------------kfnslnelvdyhrstsvsrnqqiflrdie<br><br>SARST: aliSize=93 (resi) RMSD=11.40 (Å)<br>gsfidi-EFPEWFHEGLSRHQAENLLMGK-DIGFFIIRASQSSPGDFSISVRHEDDVQHFKVMRDTKGNYFLWTEKFPSLNKLVDYYRTTSISKQKQVFLR---d<br>------mKPHPWFFGKIPRAKAEEMLSKQrHDGAFLIRESESAPGDFSLSVKFGNDVQHFKVLRDGAGKYFLWVVKFNSLNELVDYHRSTSVSRNQQIFLRdie-<br><br>BLAST: aliSize=69 (resi) iden=52.04% (51/98) simi=70.41% (69/98)<br>gsfidiefpe-----WFHEGLSRHQAENLLMG-KDIGFFIIRASQSSPGDFSISVRHEDDVQHFKVMRDTKGNYFLWTEKFPSLNKLVDYYRTTSISKQKQVFLRD--<br>----------mkphpWFFGKIPRAKAEEMLSKqRHDGAFLIRESESAPGDFSLSVKFGNDVQHFKVLRDGAGKYFLWVVKFNSLNELVDYHRSTSVSRNQQIFLRDie<br><br>Proposed: aliSize=95 (resi) RMSD=0.97 (Å)<br>gsfiDiEFPEWFHEGLSRHQAENLLMGK-DIGFFIIRASQSSPGDFSISVRHEDDVQHFKVMRDTKGNYFLWTEKFPSLNKLVDYYRTTSISKQKQVFLRD--<br>----M-KPHPWFFGKIPRAKAEEMLSKQrHDGAFLIRESESAPGDFSLSVKFGNDVQHFKVLRDGAGKYFLWVVKFNSLNELVDYHRSTSVSRNQQIFLRDie
818	1r1pB<br>(100)<br>2h5kB<br>(96)	C	53.13	TM-align: aliSize=66 (resi) RMSD=1.13 (Å)<br>gsfidieFPEWFHEGLSRHQAENLLMGK-DIGFFIIRASQSSPGDFSISVRHEDDVQHFKVMRDTKGNYFLWTEkfpslnklvdyyrttsiskqkqvflrd-----------------------------<br>-------PHPWFFGKIPRAKAEEMLSKQrHDGAFLIRESESAPGDFSLSVKFGNDVQHFKVLRDGAGKYFLWVV---------------------------kfnslnelvdyhrstsvsrnqqiflrdie<br><br>SARST: aliSize=90 (resi) RMSD=11.64 (Å)<br>gsfidiefp--EWFHEGLSRHQAENLLMGK-DIGFFIIRASQSSPGDFSISVRHEDDVQHFKVMRDTKGNYFLWTEKFPSLNKLVDYYRTTSISKQKQVFLR--d<br>---------phPWFFGKIPRAKAEEMLSKQrHDGAFLIRESESAPGDFSLSVKFGNDVQHFKVLRDGAGKYFLWVVKFNSLNELVDYHRSTSVSRNQQIFLRdi-<br><br>BLAST: aliSize=69 (resi) iden=53.68% (51/96) simi=72.63% (69/96)<br>gsfidiefpe---WFHEGLSRHQAENLLMG-KDIGFFIIRASQSSPGDFSISVRHEDDVQHFKVMRDTKGNYFLWTEKFPSLNKLVDYYRTTSISKQKQVFLRD-<br>----------phpWFFGKIPRAKAEEMLSKqRHDGAFLIRESESAPGDFSLSVKFGNDVQHFKVLRDGAGKYFLWVVKFNSLNELVDYHRSTSVSRNQQIFLRDi<br><br>Proposed: aliSize=93 (resi) RMSD=0.79 (Å)<br>gsfidieFPEWFHEGLSRHQAENLLMGK-DIGFFIIRASQSSPGDFSISVRHEDDVQHFKVMRDTKGNYFLWTEKFPSLNKLVDYYRTTSISKQKQVFLRD--<br>-------PHPWFFGKIPRAKAEEMLSKQrHDGAFLIRESESAPGDFSLSVKFGNDVQHFKVLRDGAGKYFLWVVKFNSLNELVDYHRSTSVSRNQQIFLRDie

<table>
<tr><td rowspan="2">819</td><td>1r1pB
(100)</td><td rowspan="2">C</td><td rowspan="2">53.76</td><td rowspan="2">TM-align: aliSize=64 (resi)   RMSD=1.68 (Å)
gsfidiefPeWFHEGLSRHQAENLLMGK-DIGFFIIRASQsSPG-DFSISVRHEDDVQHFKVMRDTKGNYFLWTEkfpslnklvdyyrt--------------Tsiskqkqvflrd-------------
--------P-WFFGKIPRAKAEEMLSKQrHDGAFLIRESE-SAPgDFSLSVKFGNDVQHFKVLRDGAGKYFLWVV--------------kfnslnelvdyhrsT------------svsrnqqiflrdi

SARST: aliSize=89 (resi)   RMSD=10.22 (Å)
gsfidiefpe-WFHEGLSRHQAENLLMGK-DIGFFIIRASQSSPGDFSISVRHEDDVQHFKVMRDTKGNYFLWTEKFPSLNKLVDYYRTTSISKQKQVFLR--d
----------pWFFGKIPRAKAEEMLSKQrHDGAFLIRESESAPGDFSLSVKFGNDVQHFKVLRDGAGKYFLWVVKFNSLNELVDYHRSTSVSRNQQIFLRdi-

BLAST: aliSize=69 (resi)   iden=54.84% (51/93)   simi=74.19% (69/93)
gsfidiefpe-WFHEGLSRHQAENLLMG-KDIGFFIIRASQSSPGDFSISVRHEDDVQHFKVMRDTKGNYFLWTEKFPSLNKLVDYYRTTSISKQKQVFLRD-
----------pWFFGKIPRAKAEEMLSKqRHDGAFLIRESESAPGDFSLSVKFGNDVQHFKVLRDGAGKYFLWVVKFNSLNELVDYHRSTSVSRNQQIFLRDi

Proposed: aliSize=90 (resi)   RMSD=1.22 (Å)
gsfidiefPeWFHEGLSRHQAENLLMGK-DIGFFIIRASQsSPG-DFSISVRHEDDVQHFKVMRDTKGNYFLWTEKFPSLNKLVDYYRTTSISKQKQVFLRD-
--------P-WFFGKIPRAKAEEMLSKQrHDGAFLIRESE-SAPgDFSLSVKFGNDVQHFKVLRDGAGKYFLWVVKFNSLNELVDYHRSTSVSRNQQIFLRDi</td></tr>
<tr><td>2aobB
(93)</td></tr>
<tr><td rowspan="2">820</td><td>1r1pB
(100)</td><td rowspan="2">C</td><td rowspan="2">53.13</td><td rowspan="2">TM-align: aliSize=69 (resi)   RMSD=1.80 (Å)
gsfidIEFPeWFHEGLSRHQAENLLMGK-DIGFFIIRASQSSPGDFSISVRHEDDVQHFKVMRDTKGNYFLWTEkfpslnklvdyyr-------------TTsiskqkqvflrd-------------
-----KPHP-WFFGKIPRAKAEEMLSKQrHDGAFLIRESESAPGDFSLSVKFGNDVQHFKVLRDGAGKYFLWVV-------------kfnslnelvdyhrST------------svsrnqqiflrdi

SARST: aliSize=90 (resi)   RMSD=10.06 (Å)
gsfidiefp---EWFHEGLSRHQAENLLMGK-DIGFFIIRASQSSPGDFSISVRHEDDVQHFKVMRDTKGNYFLWTEKFPSLNKLVDYYRTTSISKQKQVFLR--d
---------kphPWFFGKIPRAKAEEMLSKQrHDGAFLIRESESAPGDFSLSVKFGNDVQHFKVLRDGAGKYFLWVVKFNSLNELVDYHRSTSVSRNQQIFLRdi-

BLAST: aliSize=69 (resi)   iden=53.12% (51/96)   simi=71.88% (69/96)
gsfidiefpe----WFHEGLSRHQAENLLMG-KDIGFFIIRASQSSPGDFSISVRHEDDVQHFKVMRDTKGNYFLWTEKFPSLNKLVDYYRTTSISKQKQVFLRD-
----------kphpWFFGKIPRAKAEEMLSKqRHDGAFLIRESESAPGDFSLSVKFGNDVQHFKVLRDGAGKYFLWVVKFNSLNELVDYHRSTSVSRNQQIFLRDi

Proposed: aliSize=94 (resi)   RMSD=1.05 (Å)
gsfidiEFPEWFHEGLSRHQAENLLMGK-DIGFFIIRASQSSPGDFSISVRHEDDVQHFKVMRDTKGNYFLWTEKFPSLNKLVDYYRTTSISKQKQVFLRD-
------KPHPWFFGKIPRAKAEEMLSKQrHDGAFLIRESESAPGDFSLSVKFGNDVQHFKVLRDGAGKYFLWVVKFNSLNELVDYHRSTSVSRNQQIFLRDi</td></tr>
<tr><td>2aobA
(96)</td></tr>
<tr><td rowspan="2">821</td><td>1r1pB
(100)</td><td rowspan="2">C</td><td rowspan="2">55.91</td><td rowspan="2">TM-align: aliSize=67 (resi)   RMSD=1.63 (Å)
gsfidieFPeWFHEGLSRHQAENLLMGK-DIGFFIIRASQSSPGDFSISVRHEDDVQHFKVMRDTKGNYFLWTEkfpslnklvdyyr-------------TTsiskqkqvflrd------------
-------HP-WFFGKIPRAKAEEMLSKQrHDGAFLIRESESAPGDFSLSVKFGNDVQHFKVLRDGAGKYFLWVV-------------kfnslnelvdyhrST------------svsrnqqiflrd

SARST: aliSize=87 (resi)   RMSD=9.75 (Å)
gsfidiefp-EWFHEGLSRHQAENLLMGK-DIGFFIIRASQSSPGDFSISVRHEDDVQHFKVMRDTKGNYFLWTEKFPSLNKLVDYYRTTSISKQKQV----flrd
---------hPWFFGKIPRAKAEEMLSKQrHDGAFLIRESESAPGDFSLSVKFGNDVQHFKVLRDGAGKYFLWVVKFNSLNELVDYHRSTSVSRNQQIflrd----

BLAST: aliSize=69 (resi)   iden=54.84% (51/93)   simi=74.19% (69/93)
gsfidiefpe--WFHEGLSRHQAENLLMG-KDIGFFIIRASQSSPGDFSISVRHEDDVQHFKVMRDTKGNYFLWTEKFPSLNKLVDYYRTTSISKQKQVFLRD
----------hpWFFGKIPRAKAEEMLSKqRHDGAFLIRESESAPGDFSLSVKFGNDVQHFKVLRDGAGKYFLWVVKFNSLNELVDYHRSTSVSRNQQIFLRD

Proposed: aliSize=92 (resi)   RMSD=0.98 (Å)
gsfidieFPeWFHEGLSRHQAENLLMGK-DIGFFIIRASQSSPGDFSISVRHEDDVQHFKVMRDTKGNYFLWTEKFPSLNKLVDYYRTTSISKQKQVFLRD
-------HP-WFFGKIPRAKAEEMLSKQrHDGAFLIRESESAPGDFSLSVKFGNDVQHFKVLRDGAGKYFLWVVKFNSLNELVDYHRSTSVSRNQQIFLRD</td></tr>
<tr><td>2aoaA
(93)</td></tr>
</table>

822	1fhsA (112)<br>2h5kB (96)	C	90.63	TM-align: aliSize=66 (resi) RMSD=2.14 (Å)<br>giemkPHPWFFGKIPRAKAEEMLSKQRHDGAFLIRESE-SAPgDFSLSVKFGNDVQHFKVLRDGAGKYFLWVVkfnslnelvdyhrstsvsrnqqiflrdieqvpqqptyvqa-----------------------------<br>-----PHPWFFGKIPRAKAEEMLSKQRHDGAFLIRESEsAPG-DFSLSVKFGNDVQHFKVLRDGAGKYFLWVV--kfnslnelvdyhrstsvsrnqqiflrdie<br><br>SARST: aliSize=90 (resi) RMSD=12.11 (Å)<br>giemkphpw----FFGKIPRAKAEEMLSKQRHDGAFLIRESESAPGDFSLSVKFGNDVQHFKVLRDGAGKYFLWVVKFNSLNELVDYHRSTSVSRNQQIFLRD-ieqvpqqptyvqa<br>---------phpwFFGKIPRAKAEEMLSKQRHDGAFLIRESESAPGDFSLSVKFGNDVQHFKVLRDGAGKYFLWVVKFNSLNELVDYHRSTSVSRNQQIFLRDi-------------<br><br>BLAST: aliSize=95 (resi) iden=100.00% (95/96) simi=100.00% (95/96)<br>giemkPHPWFFGKIPRAKAEEMLSKQRHDGAFLIRESESAPGDFSLSVKFGNDVQHFKVLRDGAGKYFLWVVKFNSLNELVDYHRSTSVSRNQQIFLRDIeqvpqqptyvqa<br>-----PHPWFFGKIPRAKAEEMLSKQRHDGAFLIRESESAPGDFSLSVKFGNDVQHFKVLRDGAGKYFLWVVKFNSLNELVDYHRSTSVSRNQQIFLRDI------------<br><br>Proposed: aliSize=94 (resi) RMSD=1.73 (Å)<br>giemKpHPWFFGKIPRAKAEEMLSKQRHDGAFLIRESE-SAPgDFSLSVKFGNDVQHFKVLRDgAG-KYFLWVVKFNSLNELVDYHRSTSVSRNQQIFLRDIeQvpqqptyvqa<br>----P-HPWFFGKIPRAKAEEMLSKQRHDGAFLIRESEsAPG-DFSLSVKFGNDVQHFKVLRD-GAgKYFLWVVKFNSLNELVDYHRSTSVSRNQQIFLRDI-E----------
823	1fhsA (112)<br>2aobB (93)	C	95.70	TM-align: aliSize=65 (resi) RMSD=1.80 (Å)<br>giemkpHpWFFGKIPRAKAEEMLSKQRHDGAFLIRESESAPGDFSLSVKFGNDVQHFKVLRDgAG-KYFLWVVkfnslnelvdyhrs-------------Tsvsrnqqiflrdieqvpqqptyvqa------------<br>------P-WFFGKIPRAKAEEMLSKQRHDGAFLIRESESAPGDFSLSVKFGNDVQHFKVLRD-GAgKYFLWVV-------------kfnslnelvdyhrsT------------------------svsrnqqiflrdi<br><br>SARST: aliSize=85 (resi) RMSD=10.69 (Å)<br>giemkphpwffgki-------PRAKAEEMLSKQRHDGAFLIRESESAPGDFSLSVKFGNDVQHFKVLRDGAGKYFLWVVKFNSLNELVDYHRSTSVSRNQQIFLRD-ieqvpqqptyvqa<br>-------------pwffgkiPRAKAEEMLSKQRHDGAFLIRESESAPGDFSLSVKFGNDVQHFKVLRDGAGKYFLWVVKFNSLNELVDYHRSTSVSRNQQIFLRDi-------------<br><br>BLAST: aliSize=93 (resi) iden=100.00% (93/93) simi=100.00% (93/93)<br>giemkphPWFFGKIPRAKAEEMLSKQRHDGAFLIRESESAPGDFSLSVKFGNDVQHFKVLRDGAGKYFLWVVKFNSLNELVDYHRSTSVSRNQQIFLRDIeqvpqqptyvqa<br>-------PWFFGKIPRAKAEEMLSKQRHDGAFLIRESESAPGDFSLSVKFGNDVQHFKVLRDGAGKYFLWVVKFNSLNELVDYHRSTSVSRNQQIFLRDI------------<br><br>Proposed: aliSize=92 (resi) RMSD=1.50 (Å)<br>giemkpHpWFFGKIPRAKAEEMLSKQRHDGAFLIRESESAPGDFSLSVKFGNDVQHFKVLRDgAG-KYFLWVVKFNSLNELVDYHRSTSVSRNQQIFLRDIeqvpqqptyvqa<br>------P-WFFGKIPRAKAEEMLSKQRHDGAFLIRESESAPGDFSLSVKFGNDVQHFKVLRD-GAgKYFLWVVKFNSLNELVDYHRSTSVSRNQQIFLRDI------------
824	1fhsA (112)<br>2aobA (96)	C	91.67	TM-align: aliSize=68 (resi) RMSD=2.49 (Å)<br>giemKPHPWFFGKIPRAKAEEMLSKQRHDGAFLIRESE-SAPgDFSLSVKFGNDVQHFKVLRDgAG-KYFLWVVkfnslnelvdyhr-------------STsvsrnqqiflrdieqvpqqptyvqa------------<br>----KPHPWFFGKIPRAKAEEMLSKQRHDGAFLIRESEsAPG-DFSLSVKFGNDVQHFKVLRD-GAgKYFLWVV-------------kfnslnelvdyhrST------------------------svsrnqqiflrdi<br><br>SARST: aliSize=90 (resi) RMSD=10.42 (Å)<br>giemkphpw-----FFGKIPRAKAEEMLSKQRHDGAFLIRESESAPGDFSLSVKFGNDVQHFKVLRDGAGKYFLWVVKFNSLNELVDYHRSTSVSRNQQIFLRD-ieqvpqqptyvqa<br>---------kphpwFFGKIPRAKAEEMLSKQRHDGAFLIRESESAPGDFSLSVKFGNDVQHFKVLRDGAGKYFLWVVKFNSLNELVDYHRSTSVSRNQQIFLRDi-------------<br><br>BLAST: aliSize=96 (resi) iden=100.00% (96/96) simi=100.00% (96/96)<br>giemKPHPWFFGKIPRAKAEEMLSKQRHDGAFLIRESESAPGDFSLSVKFGNDVQHFKVLRDGAGKYFLWVVKFNSLNELVDYHRSTSVSRNQQIFLRDIeqvpqqptyvqa<br>----KPHPWFFGKIPRAKAEEMLSKQRHDGAFLIRESESAPGDFSLSVKFGNDVQHFKVLRDGAGKYFLWVVKFNSLNELVDYHRSTSVSRNQQIFLRDI------------<br><br>Proposed: aliSize=93 (resi) RMSD=1.70 (Å)<br>giemk-PHPWFFGKIPRAKAEEMLSKQRHDGAFLIRESE-SAPgDFSLSVKFGNDVQHFKVLRDgAG-KYFLWVVKFNSLNELVDYHRSTSVSRNQQIFLRDIeqvpqqptyvqa<br>-----kPHPWFFGKIPRAKAEEMLSKQRHDGAFLIRESEsAPG-DFSLSVKFGNDVQHFKVLRD-GAgKYFLWVVKFNSLNELVDYHRSTSVSRNQQIFLRDI------------

825	1fhsA (112)<br>2aoaA (93)	C	92.47	TM-align: aliSize=66 (resi) RMSD=2.15 (Å)<br>giemkpHPWFFGKIPRAKAEEMLSKQRHDGAFLIRESE-SAPgDFSLSVKFGNDVQHFKVLRDgAG-KYFLWVVkfnslnelvdyhr-------------STsvsrnqqiflrdieqvpqqptyvqa------------<br>------HPWFFGKIPRAKAEEMLSKQRHDGAFLIRESEsAPG-DFSLSVKFGNDVQHFKVLRD-GAgKYFLWVV-------------kfnslnelvdyhrST-------------------------svsrnqqiflrd<br><br>SARST: aliSize=86 (resi) RMSD=9.63 (Å)<br>giemkphpw---FFGKIPRAKAEEMLSKQRHDGAFLIRESESAPGDFSLSVKFGNDVQHFKVLRDGAGKYFLWVVKFNSLNELVDYHRSTSVSRNQQI----flrdieqvpqqptyvqa<br>---------hpwFFGKIPRAKAEEMLSKQRHDGAFLIRESESAPGDFSLSVKFGNDVQHFKVLRDGAGKYFLWVVKFNSLNELVDYHRSTSVSRNQQIflrd-----------------<br><br>BLAST: aliSize=93 (resi) iden=100.00% (93/93) simi=100.00% (93/93)<br>giemkpHPWFFGKIPRAKAEEMLSKQRHDGAFLIRESESAPGDFSLSVKFGNDVQHFKVLRDGAGKYFLWVVKFNSLNELVDYHRSTSVSRNQQIFLRDieqvpqqptyvqa<br>------HPWFFGKIPRAKAEEMLSKQRHDGAFLIRESESAPGDFSLSVKFGNDVQHFKVLRDGAGKYFLWVVKFNSLNELVDYHRSTSVSRNQQIFLRD-------------<br><br>Proposed: aliSize=91 (resi) RMSD=1.57 (Å)<br>giemkpHPWFFGKIPRAKAEEMLSKQRHDGAFLIRESE-SAPgDFSLSVKFGNDVQHFKVLRDgAG-KYFLWVVKFNSLNELVDYHRSTSVSRNQQIFLRDieqvpqqptyvqa<br>------HPWFFGKIPRAKAEEMLSKQRHDGAFLIRESEsAPG-DFSLSVKFGNDVQHFKVLRD-GAgKYFLWVVKFNSLNELVDYHRSTSVSRNQQIFLRD-------------
826	2jzjA (116)<br>1l5eA (101)	C	25.74	TM-align: aliSize=55 (resi) RMSD=2.35 (Å)<br>mQCNFANSCTGVELYGYILRGDCINEDGHPHATSINLNYYIGNDNGRLEYPGESfgsscvktalndghtltasckgadgqyhdssmdlnyvvgnsygymepcrasn---------------------Adhvlksss-------------E-----------<br>-LGKFSQTCYNSAIQGSVLTSTCERTNGGYNTSSIDLNSVIENVDGSLKWQPSN---fietcrntqlagsselaaecktR--------aqqfvstkinlddHianidgtlkye<br><br>SARST: aliSize=95 (resi) RMSD=15.55 (Å)<br>mq-CNFANSCTGVELYGYILRGDCINEDGHPHATSINLNYYIGNDNGRLEYPGESFGSSCVKTALNDGHTLTASCKGADGQYHDSSMDLNYVVGNSYGymepcrasn-----adhvlkssse<br>--lGKFSQTCYNSAIQGSVLTSTCERTNGGYNTSSIDLNSVIENVDGSLKWQPSNFIETCRNTQLAGSSELAAECKTRAQQFVSTKINLDDHIANIDG---------tlkye----------<br><br>BLAST: aliSize=47 (resi) iden=25.74% (26/101) simi=46.53% (47/101)<br>mqcn---FANSCTGVELYGYILRGDCINEDGHPHATSINLNYYIGNDNGRLEYPGESFGSSCVKTALNDGHTLTASCKGADGQYHDSSMDLNYVVGNSYGYME--pcrasnadhvlkssse<br>----lgkFSQTCYNSAIQGSVLTSTCERTNGGYNTSSIDLNSVIENVDGSLKWQPSNFIETCRNTQLAGSSELAAECKTRAQQFVSTKINLDDHIANIDGTLKye----------------<br><br>Proposed: aliSize=100 (resi) RMSD=1.27 (Å)<br>mQCNFANSCTGVELYGYILRGDCINEDGHPHATSINLNYYIGNDNGRLEYP-gESFGSSCVKTALNDGHTLTASCKGADGQYHDSSMDLNYVVGNSYGYMEPCrasnadhvlkssse<br>-LGKFSQTCYNSAIQGSVLTSTCERTNGGYNTSSIDLNSVIENVDGSLKWQp-SNFIETCRNTQLAGSSELAAECKTRAQQFVSTKINLDDHIANIDGTLKYE--------------
827	2z21A (102)<br>1l5eA (101)	N	94.06	TM-align: aliSize=52 (resi) RMSD=1.34 (Å)<br>LGNFSQACYNSAIQGSVLTSTCIRTNGGYNTSSIDLNSVIENVDGSLKWQGSnfietcrntqlagsselaaecktraqqfvstkinlddhiaaidgtlkyel---<br>LGKFSQTCYNSAIQGSVLTSTCERTNGGYNTSSIDLNSVIENVDGSLKWQPS---nfietcrntqlagsselaaecktraqqfvstkinlddhianidgtlkye<br><br>SARST: aliSize=99 (resi) RMSD=15.83 (Å)<br>l-GNFSQACYNSAIQGSVLTSTCIRTNGGYNTSSIDLNSVIENVDGSLKWQGSNFIETCRNTQLAGSSELAAECKTRAQQFVSTKINLDDHIAAIDGTLKY-el<br>-lGKFSQTCYNSAIQGSVLTSTCERTNGGYNTSSIDLNSVIENVDGSLKWQPSNFIETCRNTQLAGSSELAAECKTRAQQFVSTKINLDDHIANIDGTLKYe--<br><br>BLAST: aliSize=96 (resi) iden=95.05% (96/101) simi=95.05% (96/101)<br>LGNFSQACYNSAIQGSVLTSTCIRTNGGYNTSSIDLNSVIENVDGSLKWQGSNFIETCRNTQLAGSSELAAECKTRAQQFVSTKINLDDHIAAIDGTLKYEl<br>LGKFSQTCYNSAIQGSVLTSTCERTNGGYNTSSIDLNSVIENVDGSLKWQPSNFIETCRNTQLAGSSELAAECKTRAQQFVSTKINLDDHIANIDGTLKYE-<br><br>Proposed: aliSize=100 (resi) RMSD=0.79 (Å)<br>LGNFSQACYNSAIQGSVLTSTCIRTNGGYNTSSIDLNSVIENVDGSLKWQG-sNFIETCRNTQLAGSSELAAECKTRAQQFVSTKINLDDHIAAIDGTLKYEl<br>LGKFSQTCYNSAIQGSVLTSTCERTNGGYNTSSIDLNSVIENVDGSLKWQPs-NFIETCRNTQLAGSSELAAECKTRAQQFVSTKINLDDHIANIDGTLKYE-

828	2z21A (102)<br>1lomA (101)	N	94.06	TM-align: aliSize=53 (resi) RMSD=2.05 (Å)<br>lgnfsqacynsaiqgsvltstcirtnggyntssidln-------------------------------------Svienvdgslkw-----------QGSNFIETCRNTQLAGSSELAAECKTRAQQFVSTKINLDDHIAAIDGTLKYEl<br>-------------------------------------lgkfsqtcynsaiqgsvltstcertnggyntssidlnS-----------vienvdgslkwQSPNFIETCRNTQLAGSSELAAECKTRAQQFVSTKINLDDHIANIDGTLKYE-<br><br>SARST: aliSize=99 (resi) RMSD=16.41 (Å)<br>l-GNFSQACYNSAIQGSVLTSTCIRTNGGYNTSSIDLNSVIENVDGSLKWQGSNFIETCRNTQLAGSSELAAECKTRAQQFVSTKINLDDHIAAIDGTLKY-el<br>-lGKFSQTCYNSAIQGSVLTSTCERTNGGYNTSSIDLNSVIENVDGSLKWQSPNFIETCRNTQLAGSSELAAECKTRAQQFVSTKINLDDHIANIDGTLKYe--<br><br>BLAST: aliSize=95 (resi) iden=94.06% (95/101) simi=94.06% (95/101)<br>LGNFSQACYNSAIQGSVLTSTCIRTNGGYNTSSIDLNSVIENVDGSLKWQGSNFIETCRNTQLAGSSELAAECKTRAQQFVSTKINLDDHIAAIDGTLKYEl<br>LGKFSQTCYNSAIQGSVLTSTCERTNGGYNTSSIDLNSVIENVDGSLKWQSPNFIETCRNTQLAGSSELAAECKTRAQQFVSTKINLDDHIANIDGTLKYE-<br><br>Proposed: aliSize=100 (resi) RMSD=0.71 (Å)<br>LGNFSQACYNSAIQGSVLTSTCIRTNGGYNTSSIDLNSVIENVDGSLKWQG-sNFIETCRNTQLAGSSELAAECKTRAQQFVSTKINLDDHIAAIDGTLKYEl<br>LGKFSQTCYNSAIQGSVLTSTCERTNGGYNTSSIDLNSVIENVDGSLKWQSp-NFIETCRNTQLAGSSELAAECKTRAQQFVSTKINLDDHIANIDGTLKYE-
829	2rdkA (101)<br>1l5eA (101)	N	93.07	TM-align: aliSize=54 (resi) RMSD=2.03 (Å)<br>gnfsqacynsaiqgsvltstcirtnggyntssidl------------------------------------NSVienvdgslkwq----------GS-NFIETCRNTQLAGSSELAAECKTRAQQFVSTKINLDDHIAAIDGTLKYEl<br>-----------------------------------lgkfsqtcynsaiqgsvltstcertnggyntssidlNSV-----------ienvdgslkwQPsNFIETCRNTQLAGSSELAAECKTRAQQFVSTKINLDDHIANIDGTLKYE-<br><br>SARST: aliSize=98 (resi) RMSD=15.75 (Å)<br>g--NFSQACYNSAIQGSVLTSTCIRTNGGYNTSSIDLNSVIENVDGSLKWQGSNFIETCRNTQLAGSSELAAECKTRAQQFVSTKINLDDHIAAIDGTLKY-el<br>-lgKFSQTCYNSAIQGSVLTSTCERTNGGYNTSSIDLNSVIENVDGSLKWQPSNFIETCRNTQLAGSSELAAECKTRAQQFVSTKINLDDHIANIDGTLKYe--<br><br>BLAST: aliSize=95 (resi) iden=94.06% (95/101) simi=94.06% (95/101)<br>-GNFSQACYNSAIQGSVLTSTCIRTNGGYNTSSIDLNSVIENVDGSLKWQGSNFIETCRNTQLAGSSELAAECKTRAQQFVSTKINLDDHIAAIDGTLKYEl<br>lGKFSQTCYNSAIQGSVLTSTCERTNGGYNTSSIDLNSVIENVDGSLKWQPSNFIETCRNTQLAGSSELAAECKTRAQQFVSTKINLDDHIANIDGTLKYE-<br><br>Proposed: aliSize=99 (resi) RMSD=0.79 (Å)<br>-GNFSQACYNSAIQGSVLTSTCIRTNGGYNTSSIDLNSVIENVDGSLKWQG-sNFIETCRNTQLAGSSELAAECKTRAQQFVSTKINLDDHIAAIDGTLKYEl<br>lGKFSQTCYNSAIQGSVLTSTCERTNGGYNTSSIDLNSVIENVDGSLKWQPs-NFIETCRNTQLAGSSELAAECKTRAQQFVSTKINLDDHIANIDGTLKYE-
830	2rdkA (101)<br>1lomA (101)	N	93.07	TM-align: aliSize=53 (resi) RMSD=2.05 (Å)<br>gnfsqacynsaiqgsvltstcirtnggyntssidln-------------------------------------Svienvdgslkw-----------QGSNFIETCRNTQLAGSSELAAECKTRAQQFVSTKINLDDHIAAIDGTLKYEl<br>------------------------------------lgkfsqtcynsaiqgsvltstcertnggyntssidlnS-----------vienvdgslkwQSPNFIETCRNTQLAGSSELAAECKTRAQQFVSTKINLDDHIANIDGTLKYE-<br><br>SARST: aliSize=98 (resi) RMSD=16.32 (Å)<br>g--NFSQACYNSAIQGSVLTSTCIRTNGGYNTSSIDLNSVIENVDGSLKWQGSNFIETCRNTQLAGSSELAAECKTRAQQFVSTKINLDDHIAAIDGTLKY-el<br>-lgKFSQTCYNSAIQGSVLTSTCERTNGGYNTSSIDLNSVIENVDGSLKWQSPNFIETCRNTQLAGSSELAAECKTRAQQFVSTKINLDDHIANIDGTLKYe--<br><br>BLAST: aliSize=94 (resi) iden=93.07% (94/101) simi=93.07% (94/101)<br>-GNFSQACYNSAIQGSVLTSTCIRTNGGYNTSSIDLNSVIENVDGSLKWQGSNFIETCRNTQLAGSSELAAECKTRAQQFVSTKINLDDHIAAIDGTLKYEl<br>lGKFSQTCYNSAIQGSVLTSTCERTNGGYNTSSIDLNSVIENVDGSLKWQSPNFIETCRNTQLAGSSELAAECKTRAQQFVSTKINLDDHIANIDGTLKYE-<br><br>Proposed: aliSize=99 (resi) RMSD=0.69 (Å)<br>-GNFSQACYNSAIQGSVLTSTCIRTNGGYNTSSIDLNSVIENVDGSLKWQG-sNFIETCRNTQLAGSSELAAECKTRAQQFVSTKINLDDHIAAIDGTLKYEl<br>lGKFSQTCYNSAIQGSVLTSTCERTNGGYNTSSIDLNSVIENVDGSLKWQSp-NFIETCRNTQLAGSSELAAECKTRAQQFVSTKINLDDHIANIDGTLKYE-

<table>
<tr><td rowspan="2">831</td><td>2z21B
(102)</td><td rowspan="2">N</td><td rowspan="2">93.07</td><td rowspan="2">TM-align: aliSize=51 (resi) RMSD=1.27 (Å)
-GNFSQACYNSAIQGSVLTSTCIRTNGGYNTSSIDLNSVIENVDGSLKWQGSnfietcrntqlagsselaaecktraqqfvstkinlddhiaaidgtlkyele---
lGKFSQTCYNSAIQGSVLTSTCERTNGGYNTSSIDLNSVIENVDGSLKWQPS---nfietcrntqlagsselaaecktraqqfvstkinlddhianidgtlkye

SARST: aliSize=98 (resi) RMSD=15.81 (Å)
g--NFSQACYNSAIQGSVLTSTCIRTNGGYNTSSIDLNSVIENVDGSLKWQGSNFIETCRNTQLAGSSELAAECKTRAQQFVSTKINLDDHIAAIDGTLKY-ele
-lgKFSQTCYNSAIQGSVLTSTCERTNGGYNTSSIDLNSVIENVDGSLKWQPSNFIETCRNTQLAGSSELAAECKTRAQQFVSTKINLDDHIANIDGTLKYe---

BLAST: aliSize=95 (resi) iden=94.06% (95/101) simi=94.06% (95/101)
-GNFSQACYNSAIQGSVLTSTCIRTNGGYNTSSIDLNSVIENVDGSLKWQGSNFIETCRNTQLAGSSELAAECKTRAQQFVSTKINLDDHIAAIDGTLKYEle
lGKFSQTCYNSAIQGSVLTSTCERTNGGYNTSSIDLNSVIENVDGSLKWQPSNFIETCRNTQLAGSSELAAECKTRAQQFVSTKINLDDHIANIDGTLKYE--

Proposed: aliSize=99 (resi) RMSD=0.74 (Å)
-GNFSQACYNSAIQGSVLTSTCIRTNGGYNTSSIDLNSVIENVDGSLKWQG-sNFIETCRNTQLAGSSELAAECKTRAQQFVSTKINLDDHIAAIDGTLKYEle
lGKFSQTCYNSAIQGSVLTSTCERTNGGYNTSSIDLNSVIENVDGSLKWQPs-NFIETCRNTQLAGSSELAAECKTRAQQFVSTKINLDDHIANIDGTLKYE--</td></tr>
<tr><td>1l5eA
(101)</td></tr>
<tr><td rowspan="2">832</td><td>2z21B
(102)</td><td rowspan="2">N</td><td rowspan="2">93.07</td><td rowspan="2">TM-align: aliSize=53 (resi) RMSD=2.02 (Å)
gnfsqacynsaiqgsvltstcirtnggyntssidln-------------------------------------Svienvdgslkw-----------QGSNFIETCRNTQLAGSSELAAECKTRAQQFVSTKINLDDHIAAIDGTLKYEle
------------------------------------lgkfsqtcynsaiqgsvltstcertnggyntssidlnS-----------vienvdgslkwQSPNFIETCRNTQLAGSSELAAECKTRAQQFVSTKINLDDHIANIDGTLKYE--

SARST: aliSize=98 (resi) RMSD=16.37 (Å)
g--NFSQACYNSAIQGSVLTSTCIRTNGGYNTSSIDLNSVIENVDGSLKWQGSNFIETCRNTQLAGSSELAAECKTRAQQFVSTKINLDDHIAAIDGTLKY-ele
-lgKFSQTCYNSAIQGSVLTSTCERTNGGYNTSSIDLNSVIENVDGSLKWQSPNFIETCRNTQLAGSSELAAECKTRAQQFVSTKINLDDHIANIDGTLKYe---

BLAST: aliSize=94 (resi) iden=93.07% (94/101) simi=93.07% (94/101)
-GNFSQACYNSAIQGSVLTSTCIRTNGGYNTSSIDLNSVIENVDGSLKWQGSNFIETCRNTQLAGSSELAAECKTRAQQFVSTKINLDDHIAAIDGTLKYEle
lGKFSQTCYNSAIQGSVLTSTCERTNGGYNTSSIDLNSVIENVDGSLKWQSPNFIETCRNTQLAGSSELAAECKTRAQQFVSTKINLDDHIANIDGTLKYE--

Proposed: aliSize=99 (resi) RMSD=0.65 (Å)
-GNFSQACYNSAIQGSVLTSTCIRTNGGYNTSSIDLNSVIENVDGSLKWQG-sNFIETCRNTQLAGSSELAAECKTRAQQFVSTKINLDDHIAAIDGTLKYEle
lGKFSQTCYNSAIQGSVLTSTCERTNGGYNTSSIDLNSVIENVDGSLKWQSp-NFIETCRNTQLAGSSELAAECKTRAQQFVSTKINLDDHIANIDGTLKYE--</td></tr>
<tr><td>1lomA
(101)</td></tr>
<tr><td rowspan="2">833</td><td>2jzkA
(103)</td><td rowspan="2">N</td><td rowspan="2">25.74</td><td rowspan="2">TM-align: aliSize=50 (resi) RMSD=1.19 (Å)
---MSYADSSRNAVLTNGgRTLRAECRNADGNWVTSELDLDTCIGNPNGFLGWGmqnfshssedikleeggrkltcrpktvdggfrerqgidlnriqnvngrlvfq
lgkfsqtcynsaiqgsvltstcertnggyntssidlnsvienvdgslkwqpSNFIETCRNTQLAGS-SELAAECKTRAQQFVSTKINLDDHIANIDGTLKYE--

SARST: aliSize=96 (resi) RMSD=15.35 (Å)
ms---YADSSRNAVLTngGRTLRAECRNADGNWVTSELDLDTCIGNPNGFLGWGMQNFSHSSEDIKLeEGGRKLTCRpKTVDGGFRERQGIDLN-RIQNVNGRLVF-q
--lgkFSQTCYNSAIQ--GSVLTSTCERTNGGYNTSSIDLNSVIENVDGSLKWQPSNFIETCRNTQL-AGSSELAAE-CKTRAQQFVSTKINLDdHIANIDGTLKYe-

BLAST: aliSize=45 (resi) iden=26.73% (27/101) simi=44.55% (45/101)
msyadssrnavltng--------------GRTLRAECRNADGNWVTSELDLDTCIGNPNGFLGWGMQNFSHSSEDIKLeEGGRKLTCRPKTVDGGFRERQgIDL-NRIQNVNGRLVFQ
---------------lgkfsqtcynsaiqGSVLTSTCERTNGGYNTSSIDLNSVIENVDGSLKWQPSNFIETCRNTQL-AGSSELAAECKTRAQQFVSTK-INLdDHIANIDGTLKYE

Proposed: aliSize=95 (resi) RMSD=1.43 (Å)
-MSYADSSRNAVLTnGgRTLRAECRNADGNWVTSELDLDTCIGNPNGFLGWG--mqNFSHSSEDIKLEEGgRKLTCRPKT--vdGgFRERQGIDLN-RIQNVNGRLVFQ
lGKFSQTCYNSAIQ-G-SVLTSTCERTNGGYNTSSIDLNSVIENVDGSLKWQps--NFIETCRNTQLAGS-SELAAECKTra--Q-QFVSTKINLDdHIANIDGTLKYE</td></tr>
<tr><td>1l5eA
(101)</td></tr>
</table>

834	2rdkB<br>(99)<br><br>1l5eA<br>(101)	N	93.94	TM-align: aliSize=55 (resi) RMSD=2.26 (Å)<br>nfsqacynsaiqgsvltstcirtnggyntssidl------------------------------------NSVienvdgslkw----------QGSNFIETCRNTQLAGSSELAAECKTRAQQFVSTKINLDDHIAAIDGTLKYE<br>----------------------------------lgkfsqtcynsaiqgsvltstcertnggyntssidlNSV----------ienvdgslkwQPSNFIETCRNTQLAGSSELAAECKTRAQQFVSTKINLDDHIANIDGTLKYE<br><br>SARST: aliSize=97 (resi) RMSD=15.70 (Å)<br>n---FSQACYNSAIQGSVLTSTCIRTNGGYNTSSIDLNSVIENVDGSLKWQGSNFIETCRNTQLAGSSELAAECKTRAQQFVSTKINLDDHIAAIDGTLKY-e<br>-lgkFSQTCYNSAIQGSVLTSTCERTNGGYNTSSIDLNSVIENVDGSLKWQPSNFIETCRNTQLAGSSELAAECKTRAQQFVSTKINLDDHIANIDGTLKYe-<br><br>BLAST: aliSize=94 (resi) iden=94.95% (94/99) simi=94.95% (94/99)<br>n---FSQACYNSAIQGSVLTSTCIRTNGGYNTSSIDLNSVIENVDGSLKWQGSNFIETCRNTQLAGSSELAAECKTRAQQFVSTKINLDDHIAAIDGTLKYE<br>-lgkFSQTCYNSAIQGSVLTSTCERTNGGYNTSSIDLNSVIENVDGSLKWQPSNFIETCRNTQLAGSSELAAECKTRAQQFVSTKINLDDHIANIDGTLKYE<br><br>Proposed: aliSize=98 (resi) RMSD=0.73 (Å)<br>--NFSQACYNSAIQGSVLTSTCIRTNGGYNTSSIDLNSVIENVDGSLKWQG-sNFIETCRNTQLAGSSELAAECKTRAQQFVSTKINLDDHIAAIDGTLKYE<br>lgKFSQTCYNSAIQGSVLTSTCERTNGGYNTSSIDLNSVIENVDGSLKWQPs-NFIETCRNTQLAGSSELAAECKTRAQQFVSTKINLDDHIANIDGTLKYE
835	2rdkB<br>(99)<br><br>1lomA<br>(101)	N	93.94	TM-align: aliSize=53 (resi) RMSD=2.02 (Å)<br>nfsqacynsaiqgsvltstcirtnggyntssidln-------------------------------------Svienvdgslkw-----------QGSNFIETCRNTQLAGSSELAAECKTRAQQFVSTKINLDDHIAAIDGTLKYE<br>-----------------------------------lgkfsqtcynsaiqgsvltstcertnggyntssidlnS-----------vienvdgslkwQSPNFIETCRNTQLAGSSELAAECKTRAQQFVSTKINLDDHIANIDGTLKYE<br><br>SARST: aliSize=97 (resi) RMSD=16.28 (Å)<br>n---FSQACYNSAIQGSVLTSTCIRTNGGYNTSSIDLNSVIENVDGSLKWQGSNFIETCRNTQLAGSSELAAECKTRAQQFVSTKINLDDHIAAIDGTLKY-e<br>-lgkFSQTCYNSAIQGSVLTSTCERTNGGYNTSSIDLNSVIENVDGSLKWQSPNFIETCRNTQLAGSSELAAECKTRAQQFVSTKINLDDHIANIDGTLKYe-<br><br>BLAST: aliSize=93 (resi) iden=93.94% (93/99) simi=93.94% (93/99)<br>n---FSQACYNSAIQGSVLTSTCIRTNGGYNTSSIDLNSVIENVDGSLKWQGSNFIETCRNTQLAGSSELAAECKTRAQQFVSTKINLDDHIAAIDGTLKYE<br>-lgkFSQTCYNSAIQGSVLTSTCERTNGGYNTSSIDLNSVIENVDGSLKWQSPNFIETCRNTQLAGSSELAAECKTRAQQFVSTKINLDDHIANIDGTLKYE<br><br>Proposed: aliSize=98 (resi) RMSD=0.64 (Å)<br>--NFSQACYNSAIQGSVLTSTCIRTNGGYNTSSIDLNSVIENVDGSLKWQG-sNFIETCRNTQLAGSSELAAECKTRAQQFVSTKINLDDHIAAIDGTLKYE<br>lgKFSQTCYNSAIQGSVLTSTCERTNGGYNTSSIDLNSVIENVDGSLKWQSp-NFIETCRNTQLAGSSELAAECKTRAQQFVSTKINLDDHIANIDGTLKYE
836	2pysB<br>(103)<br><br>1l5eA<br>(101)	N	93.07	TM-align: aliSize=51 (resi) RMSD=1.29 (Å)<br>-GNFSQACYNSAIQGSVLTSTCIRTNGGYNTSSIDLNSVIENVDGSLKWQGSnfietcrntqlagsselaaecktraqqfvstkinlddhiaaidgtlkyeleh---<br>lGKFSQTCYNSAIQGSVLTSTCERTNGGYNTSSIDLNSVIENVDGSLKWQPS--nfietcrntqlagsselaaecktraqqfvstkinlddhianidgtlkye<br><br>SARST: aliSize=98 (resi) RMSD=15.82 (Å)<br>g--NFSQACYNSAIQGSVLTSTCIRTNGGYNTSSIDLNSVIENVDGSLKWQGSNFIETCRNTQLAGSSELAAECKTRAQQFVSTKINLDDHIAAIDGTLKY-eleh<br>-lgKFSQTCYNSAIQGSVLTSTCERTNGGYNTSSIDLNSVIENVDGSLKWQPSNFIETCRNTQLAGSSELAAECKTRAQQFVSTKINLDDHIANIDGTLKYe----<br><br>BLAST: aliSize=95 (resi) iden=94.06% (95/101) simi=94.06% (95/101)<br>-GNFSQACYNSAIQGSVLTSTCIRTNGGYNTSSIDLNSVIENVDGSLKWQGSNFIETCRNTQLAGSSELAAECKTRAQQFVSTKINLDDHIAAIDGTLKYEleh<br>lGKFSQTCYNSAIQGSVLTSTCERTNGGYNTSSIDLNSVIENVDGSLKWQPSNFIETCRNTQLAGSSELAAECKTRAQQFVSTKINLDDHIANIDGTLKYE---<br><br>Proposed: aliSize=99 (resi) RMSD=0.73 (Å)<br>-GNFSQACYNSAIQGSVLTSTCIRTNGGYNTSSIDLNSVIENVDGSLKWQG-sNFIETCRNTQLAGSSELAAECKTRAQQFVSTKINLDDHIAAIDGTLKYEleh<br>lGKFSQTCYNSAIQGSVLTSTCERTNGGYNTSSIDLNSVIENVDGSLKWQPs-NFIETCRNTQLAGSSELAAECKTRAQQFVSTKINLDDHIANIDGTLKYE---

837	2pysB (103)<br>1lomA (101)	N	93.07	TM-align: aliSize=53 (resi) RMSD=2.00 (Å)<br>gnfsqacynsaiqgsvltstcirtnggyntssidln-------------------------------------Svienvdgslkw-----------QGSNFIETCRNTQLAGSSELAAECKTRAQQFVSTKINLDDHIAAIDGTLKYEleh<br>------------------------------------lgkfsqtcynsaiqgsvltstcertnggyntssidlnS-----------vienvdgslkwQSPNFIETCRNTQLAGSSELAAECKTRAQQFVSTKINLDDHIANIDGTLKYE---<br><br>SARST: aliSize=98 (resi) RMSD=16.39 (Å)<br>g--NFSQACYNSAIQGSVLTSTCIRTNGGYNTSSIDLNSVIENVDGSLKWQGSNFIETCRNTQLAGSSELAAECKTRAQQFVSTKINLDDHIAAIDGTLKY-eleh<br>-lgKFSQTCYNSAIQGSVLTSTCERTNGGYNTSSIDLNSVIENVDGSLKWQSPNFIETCRNTQLAGSSELAAECKTRAQQFVSTKINLDDHIANIDGTLKYe----<br><br>BLAST: aliSize=94 (resi) iden=93.07% (94/101) simi=93.07% (94/101)<br>-GNFSQACYNSAIQGSVLTSTCIRTNGGYNTSSIDLNSVIENVDGSLKWQGSNFIETCRNTQLAGSSELAAECKTRAQQFVSTKINLDDHIAAIDGTLKYEleh<br>lGKFSQTCYNSAIQGSVLTSTCERTNGGYNTSSIDLNSVIENVDGSLKWQSPNFIETCRNTQLAGSSELAAECKTRAQQFVSTKINLDDHIANIDGTLKYE---<br><br>Proposed: aliSize=99 (resi) RMSD=0.63 (Å)<br>-GNFSQACYNSAIQGSVLTSTCIRTNGGYNTSSIDLNSVIENVDGSLKWQG-sNFIETCRNTQLAGSSELAAECKTRAQQFVSTKINLDDHIAAIDGTLKYEleh<br>lGKFSQTCYNSAIQGSVLTSTCERTNGGYNTSSIDLNSVIENVDGSLKWQSp-NFIETCRNTQLAGSSELAAECKTRAQQFVSTKINLDDHIANIDGTLKYE---
838	2vq8A (128)<br>1h8xA (125)	N	25.60	TM-align: aliSize=93 (resi) RMSD=1.91 (Å)<br>hmqpehvkeryknflnqhvgpdm------------------SV--Q-RCNSEIGpnNRKITlsgtDNGCKPVNTFILANKRLIKTVCGraGSPQG------NMVRSNQPFPVVKCVLN-NGERhPYCEYRGTRSTRYIVLKCEE--GWPVHYHEDevn--<br>-----------------------kesaaakferqhmdsgnsPSssStYCNQMMR--RRNMT----QGRCKPVNTFVHEPLVDVQNVCF--QEKVTckngqgNCYKSNSSMHITDCRLTnGSRY-PNCAYRTSQKERHIIVACEGspYVPVHFDAS---ve<br><br>SARST: aliSize=109 (resi) RMSD=13.18 (Å)<br>hmqpehvke---RYKNFLNQHVGPDMS--VQRCNSEIGPNNRKITLsGTdngCKPVNTFILANKRLIKTVCGRAGSPQGN----MVRSNQPFPVVKCVLNNGERHPYCEYRGTRSTRYIVLKCEEGWP--VHY-----hedevn<br>---------kesAAAKFERQHMDSGNSpsSSSTYCNQMMRRRNMTQ-GR---CKPVNTFVHEPLVDVQNVCFQEKVTCKNgqgnCYKSNSSMHITDCRLTNGSRYPNCAYRTSQKERHIIVACEGSPYvpVHFdasve------<br><br>BLAST: aliSize=55 (resi) iden=31.20% (39/125) simi=44.00% (55/125)<br>hmqpehvkerykn-------FLNQHV----GPDMSVQRCNSEIGPNNRkitlsgTDNGCKPVNTFILANKRLIKTVCGR----AGSPQGNMVRSNQPFPVVKCVLNNGERHPYCEYRGTRSTRYIVLKCEEG--WPVHY-----hedevn<br>-------------kesaaakFERQHMdsgnSPSSSSTYCNQMMRRRNM------TQGRCKPVNTFVHEPLVDVQNVCFQekvtCKNGQGNCYKSNSSMHITDCRLTNGSRYPNCAYRTSQKERHIIVACEGSpyVPVHFdasve------<br><br>Proposed: aliSize=109 (resi) RMSD=1.71 (Å)<br>hmqpehv-KERYKNFLNQHVGPDMSVQ----RCNSEIGpnNRKITlsgtDNGCKPVNTFILANKRLIKTVCGraGSPQG------NMVRSNQPFPVVKCVLN-NGERHpYCEYRGTRSTRYIVLKCEE--GWPVHYHED--evn<br>-------kESAAAKFERQHMDSGNSPSssstYCNQMMR--RRNMT----QGRCKPVNTFVHEPLVDVQNVCF--QEKVTckngqgNCYKSNSSMHITDCRLTnGSRYP-NCAYRTSQKERHIIVACEGspYVPVHFDASve---
839	1pu3A (105)<br>1h8xA (125)	N	21.90	TM-align: aliSize=87 (resi) RMSD=2.61 (Å)<br>mqdwltfqkkhitnt-------------------R--DVDCDNILSTNLFH---CKDKNTFIYSRPEPVKAICKG-Iia-------SKNVLTTSEFYLSDCNVTSRP----CKYKLKKSTNKFCVTCEN--QAPVHFVGVGSc<br>---------------kesaaakferqhmdsgnspSssSTYCNQMMRRRNMTqgrCKPVNTFVHEPLVDVQNVCFQeK--vtckngqGNCYKSNSSMHITDCRLTNGSrypnCAYRTSQKERHIIVACEGspYVPVHFDASVE-<br><br>SARST: aliSize=82 (resi) RMSD=3.46 (Å)<br>mqdwltfqkkhitntrdvd----------------------------CDNILSTNLFHCKDKNTFIYSRPEPVKAICKGIIAS------KNVLTTSEFYLSDCNVTsrPC------KYKLKKSTNKFCVTCEN--QAPVHFVGVG-sc<br>-------------------kesaaakferqhmdsgnspsssstycnqMMRRRNMTQGRCKPVNTFVHEPLVDVQNVCFQEKVTckngqgNCYKSNSSMHITDCRLT--NGsrypncAYRTSQKERHIIVACEGspYVPVHFDASVe--<br><br>BLAST: aliSize=38 (resi) iden=23.81% (25/105) simi=36.19% (38/105)<br>mqdwltfqkkhitntrdvdcdnilstnlfh---------------------------------------CKDKNTFIYSRPEPVKAIC-KGIIASKN-----VLTTSEFYLSDCNVT--SR-P-CKYKLKKSTNKFCVTCENQ--APVHF-----vgvgsc<br>------------------------------kesaaakferqhmdsgnspsssstycnqmmrrrnmtqgrCKPVNTFVHEPLVDVQNVCfQEKVTCKNgqgncYKSNSSMHITDCRLTngSRyPnCAYRTSQKERHIIVACEGSpyVPVHFdasve------<br><br>Proposed: aliSize=97 (resi) RMSD=1.58 (Å)<br>-MQDWLTFQKKHITN-TRD-----vDCDNILST-nLFH---CKDKNTFIYSRPEPVKAICK----------giiaSKNVLTTSEFYLSDCNVTSRP----CKYKLKKSTNKFCVTCEN--QAPVHFVGVG-sc<br>kESAAAKFERQHMDSgNSPsssst-YCNQMMRRr-NMTqgrCKPVNTFVHEPLVDVQNVCFqekvtckngq----GNCYKSNSSMHITDCRLTNGSrypnCAYRTSQKERHIIVACEGspYVPVHFDASVe--

840	1un4A (119)<br>1h8xA (125)	N	34.45	TM-align: aliSize=98 (resi) RMSD=1.99 (Å)<br>nsrythfltqhydakpq-----------------GR-DDRYCESIMRRRGLTS-PCKDINTFIHGNKRSIKAICEnknGNPHRE-----NLRISKSSFQVTACKLHGGSPWPPCQYRATAGFRNVVVACEN--GLPVHLDQSIFr<br>-----------------kesaaakferqhmdsgnsPSsSSTYCNQMMRRRNMTQgRCKPVNTFVHEPLVDVQNVCF---QEKVTCkngqgNCYKSNSSMHITDCRLTNGSRYPNCAYRTSQKERHIIVACEGspYVPVHFDASVE-<br><br>SARST: aliSize=105 (resi) RMSD=14.76 (Å)<br>ns---RYTHFLTQHYDAKP----QGRddrYCESIMRRRGLTS-PCKDINTFIHGNKRSIKAIC-----ENKNGNPHrenLRISKSSFQVTACKLHGGSPWPPCQYRATAGFRNVVVACEN--GLPVHL-----dqsifr<br>--kesAAAKFERQHMDSGNspssSST---YCNQMMRRRNMTQgRCKPVNTFVHEPLVDVQNVCfqekvTCKNGQGN---CYKSNSSMHITDCRLTNGSRYPNCAYRTSQKERHIIVACEGspYVPVHFdasve------<br><br>BLAST: aliSize=66 (resi) iden=37.82% (45/119) simi=55.46% (66/119)<br>nsryth-------FLTQHYDA--KPQGrDDRYCESIMRRRGLTSP-CKDINTFIHG---NKRSI----KAICENKNGNPHRENlriskSSFQVTACKLHGGSPWPPCQYRATAGFRNVVVACENG--LPVHLDQSI-fr<br>------kesaaakFERQHMDSgnSPSS-SSTYCNQMMRRRNMTQGrCKPVNTFVHEplvDVQNVcfqeKVTCKNGQGNCYKSN-----SSMHITDCRLTNGSRYPNCAYRTSQKERHIIVACEGSpyVPVHFDASVe--<br><br>Proposed: aliSize=110 (resi) RMSD=1.48 (Å)<br>n--SRYTHFLTQHYDAKPQGR---ddRYCESIMRRRGLTS-PCKDINTFIHGNKRSIKAICEnknGNPHRE-----NLRISKSSFQVTACKLHGGSPWPPCQYRATAGFRNVVVACEN--GLPVHLDQsI--fr<br>-keSAAAKFERQHMDSGNSPSsss--TYCNQMMRRRNMTQgRCKPVNTFVHEPLVDVQNVCF---QEKVTCkngqgNCYKSNSSMHITDCRLTNGSRYPNCAYRTSQKERHIIVACEGspYVPVHFDA-Sve--
841	1rnfA (120)<br>1h8xA (125)	N	41.67	TM-align: aliSize=100 (resi) RMSD=1.66 (Å)<br>mqdgmyqrflrqhvhpeet-----------------GG-SDRYCNLMMQRRKMTLYHCKRFNTFIHEDIWNIRSICSTTNIQCK-NGKmNCHEG--VVKVTDCRDTGSSRAPNCRYRAIASTRRVVIACEGNPQVPVHFDG---<br>-------------------kesaaakferqhmdsgnsPSsSSTYCNQMMRRRNMTQGRCKPVNTFVHEPLVDVQNVCFQEKVTCKnGQG-NCYKSnsSMHITDCRLTNGSRYPNCAYRTSQKERHIIVACEGSPYVPVHFDAsve<br><br>SARST: aliSize=112 (resi) RMSD=13.01 (Å)<br>mqdg---MYQRFLRQHVHPEEtggSDR----YCNLMMQRRKMTLYHCKRFNTFIHEDIWNIRSICSTTNIQCKNGKMNCHE--GVVKVTDCRDTGSSRAPNCRYRAIASTRRVVIACEGNPQVPVHFD----g<br>----kesAAAKFERQHMDSGN---SPSssstYCNQMMRRRNMTQGRCKPVNTFVHEPLVDVQNVCFQEKVTCKNGQGNCYKsnSSMHITDCRLTNGSRYPNCAYRTSQKERHIIVACEGSPYVPVHFDasve-<br><br>BLAST: aliSize=73 (resi) iden=43.33% (52/120) simi=60.83% (73/120)<br>mqdgmyq------RFLRQHVHPEET-GGSDRYCNLMMQRRKMTLYHCKRFNTFIHEDIWNIRSICSTTNIQCKNGKMNCHE--GVVKVTDCRDTGSSRAPNCRYRAIASTRRVVIACEGNPQVPVHFD----g<br>-------kesaaaKFERQHMDSGNSpSSSSTYCNQMMRRRNMTQGRCKPVNTFVHEPLVDVQNVCFQEKVTCKNGQGNCYKsnSSMHITDCRLTNGSRYPNCAYRTSQKERHIIVACEGSPYVPVHFDasve-<br><br>Proposed: aliSize=115 (resi) RMSD=1.37 (Å)<br>mqd--GMYQRFLRQHVHPEETGG--SdRYCNLMMQRRKMTLYHCKRFNTFIHEDIWNIRSICSTTNIQCK-NGKmNCHEG--VVKVTDCRDTGSSRAPNCRYRAIASTRRVVIACEGNPQVPVHFDG---<br>---keSAAAKFERQHMDSGNSPSssS-TYCNQMMRRRNMTQGRCKPVNTFVHEPLVDVQNVCFQEKVTCKnGQG-NCYKSnsSMHITDCRLTNGSRYPNCAYRTSQKERHIIVACEGSPYVPVHFDAsve
842	2j4tA (119)<br>1h8xA (125)	N	36.13	TM-align: aliSize=98 (resi) RMSD=1.75 (Å)<br>neryekflrqhydakpq-----------------GR-DDRYCESMMKERKLTS-PCKDVNTFIHGTKKNIRAICGkkGSPYG-----ENFRISNSPFQITTCTHSRGSPWPPCGYRAFKDFRYIVIACED--GWPVHFDEsFIsP<br>-----------------kesaaakferqhmdsgnsPSsSSTYCNQMMRRRNMTQgRCKPVNTFVHEPLVDVQNVCF--QEKVTckngqGNCYKSNSSMHITDCRLTNGSRYPNCAYRTSQKERHIIVACEGspYVPVHFDA-SV-E<br><br>SARST: aliSize=104 (resi) RMSD=13.70 (Å)<br>ne---RYEKFLRQHYDAKPQgrdDR----YCESMMKERKLTS-PCKDVNTFIHGTKKNIRAIC------GKKGSPYgenFRISNSPFQITTCTHSRGSPWPPCGYRAFKDFRYIVIACED--GWPVHF-----desfisp<br>--kesAAAKFERQHMDSGNS---PSssstYCNQMMRRRNMTQgRCKPVNTFVHEPLVDVQNVCfqekvtCKNGQGN---CYKSNSSMHITDCRLTNGSRYPNCAYRTSQKERHIIVACEGspYVPVHFdasve-------<br><br>BLAST: aliSize=62 (resi) iden=39.50% (47/119) simi=52.10% (62/119)<br>nerye------KFLRQHYDA--KPQGrDDRYCESMMKERKLTSP-CKDVNTFIHGTKKNIRAIC--GKKGSPYGE-NFRISNSPFQITTCTHSRGSPWPPCGYRAFKDFRYIVIACEDG--WPVHFDES--fisp<br>-----kesaaaKFERQHMDSgnSPSS-SSTYCNQMMRRRNMTQGrCKPVNTFVHEPLVDVQNVCfqEKVTCKNGQgNCYKSNSSMHITDCRLTNGSRYPNCAYRTSQKERHIIVACEGSpyVPVHFDASve----<br><br>Proposed: aliSize=113 (resi) RMSD=1.56 (Å)<br>n--ERYEKFLRQHYDAKPQ--GRDdRYCESMMKERKLTS-PCKDVNTFIHGTKKNIRAICGkkGSPYG-----ENFRISNSPFQITTCTHSRGSPWPPCGYRAFKDFRYIVIACED--GWPVHFDEsFIsP<br>-keSAAAKFERQHMDSGNSpsSSS-TYCNQMMRRRNMTQgRCKPVNTFVHEPLVDVQNVCF--QEKVTckngqGNCYKSNSSMHITDCRLTNGSRYPNCAYRTSQKERHIIVACEGspYVPVHFDA-SV-E

843	1b1eA (123)<br>1h8xA (125)	N	32.52	TM-align: aliSize=98 (resi) RMSD=1.99 (Å)<br>qdnsrythfltqhydakpq------------------GR-DDRYCESIMRRRGLTS-PCQDINTFIHGNKRSIKAICEnknGNPHRE-----NLRISKSSFQVTTCKLHGGSPWPPCQYRATAGFRNVVVACEN--GLPVHLDQsIFrRp<br>-------------------kesaaakferqhmdsgnsPSsSSTYCNQMMRRRNMTQgRCKPVNTFVHEPLVDVQNVCF---QEKVTCkngqgNCYKSNSSMHITDCRLTNGSRYPNCAYRTSQKERHIIVACEGspYVPVHFDA-SV-E-<br><br>SARST: aliSize=105 (resi) RMSD=13.80 (Å)<br>qdns---RYTHFLTQHYDAKP----QGRddrYCESIMRRRGLT-SPCQDINTFIHGNKRSIKAIC-----ENKNGNPHrenLRISKSSFQVTTCKLHGGSPWPPCQYRATAGFRNVVVACEN--GLPVHL-----dqsifrrp<br>----kesAAAKFERQHMDSGNspssSST---YCNQMMRRRNMTqGRCKPVNTFVHEPLVDVQNVCfqekvTCKNGQGN---CYKSNSSMHITDCRLTNGSRYPNCAYRTSQKERHIIVACEGspYVPVHFdasve--------<br><br>BLAST: aliSize=66 (resi) iden=35.77% (44/123) simi=53.66% (66/123)<br>qdnsryth-------FLTQHYDA--KPQGrDDRYCESIMRRRGLTSP-CQDINTFIHG---NKRSI----KAICENKNGNPHRENlriskSSFQVTTCKLHGGSPWPPCQYRATAGFRNVVVACENG--LPVHLDQSI-frrp<br>--------kesaaakFERQHMDSgnSPSS-SSTYCNQMMRRRNMTQGrCKPVNTFVHEplvDVQNVcfqeKVTCKNGQGNCYKSN-----SSMHITDCRLTNGSRYPNCAYRTSQKERHIIVACEGSpyVPVHFDASVe----<br><br>Proposed: aliSize=112 (resi) RMSD=1.63 (Å)<br>QdNSRYTHFLTQHYDAKPQGR---ddRYCESIMRRRGLTS-PCQDINTFIHGNKRSIKAICEnknGNPHRE-----NLRISKSSFQVTTCKLHGGSPWPPCQYRATAGFRNVVVACEN--GLPVHLDQsI--frrp<br>K-ESAAAKFERQHMDSGNSPSsss--TYCNQMMRRRNMTQgRCKPVNTFVHEPLVDVQNVCF---QEKVTCkngqgNCYKSNSSMHITDCRLTNGSRYPNCAYRTSQKERHIIVACEGspYVPVHFDA-Sve----
844	2hkyA (129)<br>1h8xA (125)	N	33.60	TM-align: aliSize=95 (resi) RMSD=1.94 (Å)<br>mkpkgmtssqwfkiqhmqpsp-----------------------QACNSAMK--NINKhtKRCKDLNTFLHEPFSSVAATCQTPKIAC---KngDkNCHQSHGPVSLTMCKLT-SGKYPNCRYKEKRQNKSYVVACKPPqkkdsqqfHLVPVHLDRVL-<br>---------------------kesaaakferqhmdsgnspssssTYCNQMMRrrNMTQ--GRCKPVNTFVHEPLVDVQNVCFQEKVTCkngQ--G-NCYKSNSSMHITDCRLTnGSRYPNCAYRTSQKERHIIVACEGS--------PYVPVHFDASVe<br><br>SARST: aliSize=114 (resi) RMSD=14.12 (Å)<br>mkpkg-MTSSQWFKIQHMQPsPQACNS---AMKNINKHTKRCKDL----NTFLHEPFSSVAATCQTPKIACKNGDKNCHQSHGPVSLTMCKLTSGKY-PNCRYKEKRQNKSYVVACKppqkkdsqQFHLVPVHLDRV--l<br>-----kESAAAKFERQHMDS-GNSPSSsstYCNQMMRRRNMTQGRckpvNTFVHEPLVDVQNVCFQEKVTCKNGQGNCYKSNSSMHITDCRLTNGSRyPNCAYRTSQKERHIIVACE--------GSPYVPVHFDASve-<br><br>BLAST: aliSize=68 (resi) iden=37.60% (47/125) simi=54.40% (68/125)<br>mkpkgmtssqw-------FKIQHMQP--SPQA----CNSAMKNINKHTKRCKDLNTFLHEPFSSVAATCQTPKIACKNGDKNCHQSHGPVSLTMCKLTSG-KYPNCRYKEKRQNKSYVVACkppqkkDSQQFhlVPVHLD----rvl<br>-----------kesaaakFERQHMDSgnSPSSsstyCNQMMRRRNMTQGRCKPVNTFVHEPLVDVQNVCFQEKVTCKNGQGNCYKSNSSMHITDCRLTNGsRYPNCAYRTSQKERHIIVAC------EGSPY--VPVHFDasve---<br><br>Proposed: aliSize=111 (resi) RMSD=1.78 (Å)<br>mkpKgMTSSQWFKIQHMQPSpQ-------ACNSAMK--NINKhtKRCKDLNTFLHEPFSSVAATCQTPKIAC---KngDkNCHQSHGPVSLTMCKLT-SGKYPNCRYKEKRQNKSYVVACKPPqkkdsqqfHLVPVHLDRVL-<br>---K-ESAAAKFERQHMDSG-NspsssstYCNQMMRrrNMTQ--GRCKPVNTFVHEPLVDVQNVCFQEKVTCkngQ--G-NCYKSNSSMHITDCRLTnGSRYPNCAYRTSQKERHIIVACEGS--------PYVPVHFDASVe
845	1m58A (106)<br>1h8xA (125)	N	23.58	TM-align: aliSize=86 (resi) RMSD=2.58 (Å)<br>mqnwetfqkkhltdtr----------------------DVKCDAEMKKALFD---CKQKNTFIYARPGRVQALCKN-Iiv-------SKNVLSTDEFYLSDCNRIK-LP---CHYKLKKSSNTICITCEN--KLPVHFVAVEEcp<br>----------------kesaaakferqhmdsgnspsssSTYCNQMMRRRNMTqgrCKPVNTFVHEPLVDVQNVCFQeK--vtckngqGNCYKSNSSMHITDCRLTNgSRypnCAYRTSQKERHIIVACEGspYVPVHFDASVE--<br><br>SARST: aliSize=90 (resi) RMSD=14.42 (Å)<br>mqn-----WETFQKK---HLTDTRDVKCDAEMKkALFDC-----KQKNTFIYARPGRVQALCK----------------NIIVSKNVLSTDEFYlsdcnriKLPCHYKLKK---SSNTICITCENKLPVHFVA---veecp<br>---kesaaAKFERQHmdsGNSPSSSSTYCNQMM-RRRNMtqgrcKPVNTFVHEPLVDVQNVCFqekvtckngqgncyksNSSMHITDCRLTNGS-------RYPNCAYRTSqkeRHIIVACEGSPYVPVHFDAsve-----<br><br>BLAST: aliSize=52 (resi) iden=28.30% (30/106) simi=49.06% (52/106)<br>mqnwet-------FQKKHLTD-----TRDVKCDAEMKKALFD---CKQKNTFIYARPGRVQALC-KNIIVSKN-----VLSTDEFYLSDC---NRIKLP-CHYKLKKSSNTICITCENK--LPVHF-VAVEecp<br>------kesaaakFERQHMDSgnspsSSSTYCNQMMRRRNMTqgrCKPVNTFVHEPLVDVQNVCfQEKVTCKNgqgncYKSNSSMHITDCrltNGSRYPnCAYRTSQKERHIIVACEGSpyVPVHFdASVE---<br><br>Proposed: aliSize=99 (resi) RMSD=2.07 (Å)<br>-MQNWETFQKKHLTDTRDV-----KCDAEMKK-ALFD--CKQKNTFIYARPGRVQALCkN----------iivSKNVLSTDEFYLSDCNRIK-LP---CHYKLKKSSNTICITCEN--KLPVHFVAVE-ecp<br>kESAAAKFERQHMDSGNSPssssstYCNQMMRRrNMTQgrCKPVNTFVHEPLVDVQNVC-Fqekvtckngq---GNCYKSNSSMHITDCRLTNgSRypnCAYRTSQKERHIIVACEGspYVPVHFDASVe---

846	2e0oB (125)<br>1h8xA (125)	N	86.40	TM-align: aliSize=105 (resi) RMSD=1.39 (Å)<br>mkesrakkfqrqhmdsdssp-------------------SS-SSTYCNQMMRRRNMTQGRCKPVNTFVHEPLLLVQLVCLQEKVTCKNGQGNCYKSNSSMHITDCRLTNGSRYPNCAYRTSPKERHIIVACEGSPYVPVHFDASV-<br>--------------------kesaaakferqhmdsgnsPSsSSTYCNQMMRRRNMTQGRCKPVNTFVHEPLVDVQNVCFQEKVTCKNGQGNCYKSNSSMHITDCRLTNGSRYPNCAYRTSQKERHIIVACEGSPYVPVHFDASVe<br><br>SARST: aliSize=122 (resi) RMSD=14.14 (Å)<br>mk-ESRAKKFQRQHMDSDSSPSSSSTYCNQMMRRRNMTQGRCKPVNTFVHEPLLLVQLVCLQEKVTCKNGQGNCYKSNSSMHITDCRLTNGSRYPNCAYRTSPKERHIIVACEGSPYVPVHFDAS--v<br>--kESAAAKFERQHMDSGNSPSSSSTYCNQMMRRRNMTQGRCKPVNTFVHEPLVDVQNVCFQEKVTCKNGQGNCYKSNSSMHITDCRLTNGSRYPNCAYRTSQKERHIIVACEGSPYVPVHFDASve-<br><br>BLAST: aliSize=117 (resi) iden=91.20% (114/125) simi=93.60% (117/125)<br>mKESRAKKFQRQHMDSDSSPSSSSTYCNQMMRRRNMTQGRCKPVNTFVHEPLLLVQLVCLQEKVTCKNGQGNCYKSNSSMHITDCRLTNGSRYPNCAYRTSPKERHIIVACEGSPYVPVHFDASV-<br>-KESAAAKFERQHMDSGNSPSSSSTYCNQMMRRRNMTQGRCKPVNTFVHEPLVDVQNVCFQEKVTCKNGQGNCYKSNSSMHITDCRLTNGSRYPNCAYRTSQKERHIIVACEGSPYVPVHFDASVe<br><br>Proposed: aliSize=121 (resi) RMSD=0.95 (Å)<br>mKESRAKKFQRQHMDSDSSPS--ssSTYCNQMMRRRNMTQGRCKPVNTFVHEPLLLVQLVCLQEKVTCK-NGQgNCYKSNSSMHITDCRLTNGSRYPNCAYRTSPKERHIIVACEGSPYVPVHFDASV-<br>-KESAAAKFERQHMDSGNSPSss--STYCNQMMRRRNMTQGRCKPVNTFVHEPLVDVQNVCFQEKVTCKnGQG-NCYKSNSSMHITDCRLTNGSRYPNCAYRTSQKERHIIVACEGSPYVPVHFDASVe
847	1ymnA (124)<br>1h8xA (125)	N	69.35	TM-align: aliSize=105 (resi) RMSD=1.80 (Å)<br>ketaaakferqhmdssts-----------------AASSSNYCNQMMKSRNLTKDRCKPVNTFVHESLADVQAVCSQKNVACK-NGQtNCYQSYSTMSITDCRETGSSKLPNCAYKTTQANKHIIVACEGNPYVPVHFDASV-<br>-----------------kesaaakferqhmdsgnsPSSSSTYCNQMMRRRNMTQGRCKPVNTFVHEPLVDVQNVCFQEKVTCKnGQG-NCYKSNSSMHITDCRLTNGSRYPNCAYRTSQKERHIIVACEGSPYVPVHFDASVe<br><br>SARST: aliSize=119 (resi) RMSD=14.46 (Å)<br>k-ETAAAKFERQHMDSST---SAASssnYCNQMMKSRNLTKDRCKPVNTFVHESLADVQAVCSQKNVACKNGQTNCYQSYSTMSITDCRETGSSKLPNCAYKTTQANKHIIVACEGNPYVPVHFDAS--v<br>-kESAAAKFERQHMDSGNspsSSST---YCNQMMRRRNMTQGRCKPVNTFVHEPLVDVQNVCFQEKVTCKNGQGNCYKSNSSMHITDCRLTNGSRYPNCAYRTSQKERHIIVACEGSPYVPVHFDASve-<br><br>BLAST: aliSize=103 (resi) iden=72.58% (90/124) simi=83.06% (103/124)<br>KETAAAKFERQHMDSSTSAASSSNYCNQMMKSRNLTKDRCKPVNTFVHESLADVQAVCSQKNVACKNGQTNCYQSYSTMSITDCRETGSSKLPNCAYKTTQANKHIIVACEGNPYVPVHFDASV-<br>KESAAAKFERQHMDSGNSPSSSSTYCNQMMRRRNMTQGRCKPVNTFVHEPLVDVQNVCFQEKVTCKNGQGNCYKSNSSMHITDCRLTNGSRYPNCAYRTSQKERHIIVACEGSPYVPVHFDASVe<br><br>Proposed: aliSize=122 (resi) RMSD=1.13 (Å)<br>KETAAAKFERQHMDSSTsAAS-SSNYCNQMMKSRNLTKDRCKPVNTFVHESLADVQAVCSQKNVACK-NGQtNCYQSYSTMSITDCRETGSSKLPNCAYKTTQANKHIIVACEGNPYVPVHFDASV-<br>KESAAAKFERQHMDSGN-SPSsSSTYCNQMMRRRNMTQGRCKPVNTFVHEPLVDVQNVCFQEKVTCKnGQG-NCYKSNSSMHITDCRLTNGSRYPNCAYRTSQKERHIIVACEGSPYVPVHFDASVe
848	1a5pA (124)<br>1h8xA (125)	N	68.55	TM-align: aliSize=105 (resi) RMSD=1.67 (Å)<br>ketaaakferqhmdssts-----------------AAs-SSNYCNQMMKSRNLTKDRAKPVNTFVHESLADVQAVCSQKNVACKNGQTNCYQSYSTMSITDCRETGSSKYPNAAYKTTQANKHIIVACEGNPYVPVHFDASV-<br>-----------------kesaaakferqhmdsgnsPS-sSSTYCNQMMRRRNMTQGRCKPVNTFVHEPLVDVQNVCFQEKVTCKNGQGNCYKSNSSMHITDCRLTNGSRYPNCAYRTSQKERHIIVACEGSPYVPVHFDASVe<br><br>SARST: aliSize=119 (resi) RMSD=14.37 (Å)<br>k-ETAAAKFERQHMDSST---SAAsssNYCNQMMKSRNLTKDRAKPVNTFVHESLADVQAVCSQKNVACKNGQTNCYQSYSTMSITDCRETGSSKYPNAAYKTTQANKHIIVACEGNPYVPVHFDAS--v<br>-kESAAAKFERQHMDSGNspsSSS---TYCNQMMRRRNMTQGRCKPVNTFVHEPLVDVQNVCFQEKVTCKNGQGNCYKSNSSMHITDCRLTNGSRYPNCAYRTSQKERHIIVACEGSPYVPVHFDASve-<br><br>BLAST: aliSize=102 (resi) iden=71.77% (89/124) simi=82.26% (102/124)<br>KETAAAKFERQHMDSSTSAASSSNYCNQMMKSRNLTKDRAKPVNTFVHESLADVQAVCSQKNVACKNGQTNCYQSYSTMSITDCRETGSSKYPNAAYKTTQANKHIIVACEGNPYVPVHFDASV-<br>KESAAAKFERQHMDSGNSPSSSSTYCNQMMRRRNMTQGRCKPVNTFVHEPLVDVQNVCFQEKVTCKNGQGNCYKSNSSMHITDCRLTNGSRYPNCAYRTSQKERHIIVACEGSPYVPVHFDASVe<br><br>Proposed: aliSize=122 (resi) RMSD=1.05 (Å)<br>KETAAAKFERQHMDSSTsAA-SSSNYCNQMMKSRNLTKDRAKPVNTFVHESLADVQAVCSQKNVACK-NGQtNCYQSYSTMSITDCRETGSSKYPNAAYKTTQANKHIIVACEGNPYVPVHFDASV-<br>KESAAAKFERQHMDSGN-SPsSSSTYCNQMMRRRNMTQGRCKPVNTFVHEPLVDVQNVCFQEKVTCKnGQG-NCYKSNSSMHITDCRLTNGSRYPNCAYRTSQKERHIIVACEGSPYVPVHFDASVe

849	2bwlA (118)<br>1h8xA (125)	N	37.29	TM-align: aliSize=97 (resi) RMSD=1.85 (Å)<br>ddsrytkfltqhhdakpkg-----------------RD--DRYCERMMKRRSLTS-PCKDVNTFIHGNKSNIKAICGanGSPYRE-----NLRMSKSPFQVTTCKHTGGSPRPPCQYRASAGFRHVVIACEN--GLPVHFDESFF<br>------------------kesaaakferqhmdsgnsPSssSTYCNQMMRRRNMTQgRCKPVNTFVHEPLVDVQNVCF--QEKVTCkngqgNCYKSNSSMHITDCRLTNGSRYPNCAYRTSQKERHIIVACEGspYVPVHFDASVE<br><br>SARST: aliSize=105 (resi) RMSD=14.53 (Å)<br>dd--SRYTKFLTQHHDAKPkgrDDR----YCERMMKRRSLTS-PCKDVNTFIHGNKSNIKAICG------ANGSPYrenLRMSKSPFQVTTCKHTGGSPRPPCQYRASAGFRHVVIACEN--GLPVHF-----desf<br>--keSAAAKFERQHMDSGN---SPSssstYCNQMMRRRNMTQgRCKPVNTFVHEPLVDVQNVCFqekvtcKNGQGN---CYKSNSSMHITDCRLTNGSRYPNCAYRTSQKERHIIVACEGspYVPVHFdasve----<br><br>BLAST: aliSize=64 (resi) iden=39.32% (46/118) simi=54.70% (64/118)<br>d-DSRYTKFLTQHHDA--KPKGrDDRYCERMMKRRSLTSP-CKDVNTFIHGNKSNIKAICGANGSPYRE---NLRMSKSPFQVTTCKHTGGSPRPPCQYRASAGFRHVVIACENG--LPVHFDES--f<br>-kESAAAKFERQHMDSgnSPSS-SSTYCNQMMRRRNMTQGrCKPVNTFVHEPLVDVQNVCFQEKVTCKNgqgNCYKSNSSMHITDCRLTNGSRYPNCAYRTSQKERHIIVACEGSpyVPVHFDASve-<br><br>Proposed: aliSize=113 (resi) RMSD=1.56 (Å)<br>DDSRYTKFLTQHHDAKPKGR---ddRYCERMMKRRSLTS-PCKDVNTFIHGNKSNIKAICGanGSPYRE-----NLRMSKSPFQVTTCKHTGGSPRPPCQYRASAGFRHVVIACEN--GLPVHFDEsF-F<br>KESAAAKFERQHMDSGNSPSsss--TYCNQMMRRRNMTQgRCKPVNTFVHEPLVDVQNVCF--QEKVTCkngqgNCYKSNSSMHITDCRLTNGSRYPNCAYRTSQKERHIIVACEGspYVPVHFDA-SvE
850	1gv7A (123)<br>1h8xA (125)	N	40.65	TM-align: aliSize=103 (resi) RMSD=2.17 (Å)<br>dnsrythfltqhydakpq-----------------GR-DDRYCESIMRRRGLTS-PCKDINTFIHGNKRSIKAICSQKNVACKNGQTNCYISKSSFQVTTCKLHGGSPWPPCQYRATAGFRNVVVACEN--GLPVHLDQsIFR<br>-----------------kesaaakferqhmdsgnsPSsSSTYCNQMMRRRNMTQgRCKPVNTFVHEPLVDVQNVCFQEKVTCKNGQGNCYKSNSSMHITDCRLTNGSRYPNCAYRTSQKERHIIVACEGspYVPVHFDA-SVE<br><br>SARST: aliSize=89 (resi) RMSD=15.31 (Å)<br>dns---RYTHFLTQHYDAKP----QGRddrYCESIMRRRGLTS-PCKDINTFIHGNKRSIKAICSQKNVACKN--------------------GQTncyiskssfqvttcklhggspWPPCQYRATAGFRNVVVACEN--GLPVHL-----dqsifr<br>---kesAAAKFERQHMDSGNspssSST---YCNQMMRRRNMTQgRCKPVNTFVHEPLVDVQNVCFQEKVTCKNgqgncyksnssmhitdcrltnGSR--------------------YPNCAYRTSQKERHIIVACEGspYVPVHFdasve------<br><br>BLAST: aliSize=69 (resi) iden=41.80% (51/123) simi=56.56% (69/123)<br>dnsryth-------FLTQHYDA--KPQGrDDRYCESIMRRRGLTSP-CKDINTFIHGNKRSIKAICSQKNVACKNGQTNCYISKSSFQVTTCKLHGGSPWPPCQYRATAGFRNVVVACENG--LPVHLDQSI-fr<br>-------kesaaakFERQHMDSgnSPSS-SSTYCNQMMRRRNMTQGrCKPVNTFVHEPLVDVQNVCFQEKVTCKNGQGNCYKSNSSMHITDCRLTNGSRYPNCAYRTSQKERHIIVACEGSpyVPVHFDASVe--<br><br>Proposed: aliSize=117 (resi) RMSD=1.67 (Å)<br>pDNSRYTHFLTQHYDAKPQGR---ddRYCESIMRRRGLTS-PCKDINTFIHGNKRSIKAICSQKNVACKNGQTNCYISKSSFQVTTCKLHGGSPWPPCQYRATAGFRNVVVACEN--GLPVHLDQsI--fr<br>-KESAAAKFERQHMDSGNSPSsss--TYCNQMMRRRNMTQgRCKPVNTFVHEPLVDVQNVCFQEKVTCKNGQGNCYKSNSSMHITDCRLTNGSRYPNCAYRTSQKERHIIVACEGspYVPVHFDA-Sve--
851	1rraA (124)<br>1h8xA (125)	N	62.10	TM-align: aliSize=104 (resi) RMSD=1.31 (Å)<br>aessadkfkrqhmdtegps-----------------KS-SPTYCNQMMKRQGMTKGSCKPVNTFVHEPLEDVQAICSQGQVTC-KNGRnNCHKSSSTLRITDCRLKGSSKYPNCDYTTTDSQKHIIIACDGNPYVPVHFDASV-<br>------------------kesaaakferqhmdsgnsPSsSSTYCNQMMRRRNMTQGRCKPVNTFVHEPLVDVQNVCFQEKVTCkNGQG-NCYKSNSSMHITDCRLTNGSRYPNCAYRTSQKERHIIVACEGSPYVPVHFDASVe<br><br>SARST: aliSize=120 (resi) RMSD=14.28 (Å)<br>a-ESSADKFKRQHMDTEGP--SKSspTYCNQMMKRQGMTKGSCKPVNTFVHEPLEDVQAICSQGQVTCKNGRNNCHKSSSTLRITDCRLKGSSKYPNCDYTTTDSQKHIIIACDGNPYVPVHFDAS--v<br>-kESAAAKFERQHMDSGNSpsSSS--TYCNQMMRRRNMTQGRCKPVNTFVHEPLVDVQNVCFQEKVTCKNGQGNCYKSNSSMHITDCRLTNGSRYPNCAYRTSQKERHIIVACEGSPYVPVHFDASve-<br><br>BLAST: aliSize=101 (resi) iden=65.32% (81/124) simi=81.45% (101/124)<br>a-ESSADKFKRQHMDTEGPSKSSPTYCNQMMKRQGMTKGSCKPVNTFVHEPLEDVQAICSQGQVTCKNGRNNCHKSSSTLRITDCRLKGSSKYPNCDYTTTDSQKHIIIACDGNPYVPVHFDASV-<br>-kESAAAKFERQHMDSGNSPSSSSTYCNQMMRRRNMTQGRCKPVNTFVHEPLVDVQNVCFQEKVTCKNGQGNCYKSNSSMHITDCRLTNGSRYPNCAYRTSQKERHIIVACEGSPYVPVHFDASVe<br><br>Proposed: aliSize=122 (resi) RMSD=1.14 (Å)<br>AESSADKFKRQHMDTEGPSK-SsPTYCNQMMKRQGMTKGSCKPVNTFVHEPLEDVQAICSQGQVTC-KNGRnNCHKSSSTLRITDCRLKGSSKYPNCDYTTTDSQKHIIIACDGNPYVPVHFDASV-<br>KESAAAKFERQHMDSGNSPSsS-STYCNQMMRRRNMTQGRCKPVNTFVHEPLVDVQNVCFQEKVTCkNGQG-NCYKSNSSMHITDCRLTNGSRYPNCAYRTSQKERHIIVACEGSPYVPVHFDASVe

<table>
<tr><td rowspan="2">852</td><td>2q4gX
(126)</td><td rowspan="2">N</td><td rowspan="2">89.60</td><td rowspan="2">TM-align: aliSize=106 (resi) RMSD=1.45 (Å)
esrakkfqrqhmdsdssp-----------------SS-SSTYCNQMMRRRNMTQGRCKPVNTFVHEPLVDVQNVCFQEKVTCKNGQGNCYKSNSSMHITDCRLTNGSRYPNCAYRTSPKERHIIVACEGSPYVPVHFDASVEds
-----------------kesaaakferqhmdsgnsPSsSSTYCNQMMRRRNMTQGRCKPVNTFVHEPLVDVQNVCFQEKVTCKNGQGNCYKSNSSMHITDCRLTNGSRYPNCAYRTSQKERHIIVACEGSPYVPVHFDASVE--

SARST: aliSize=118 (resi) RMSD=10.94 (Å)
esra-----KKFQRQHMDSDSSPSSSSTYCNQMMRRRNMTQGRCKPVNTFVHEPLVDVQNVCFQEKVTCKNGQGNCYKSNSSMHITDCRLTNGSRYPNCAYRTSPKERHIIVACEGSPYVPVHFDAS--veds
----kesaaAKFERQHMDSGNSPSSSSTYCNQMMRRRNMTQGRCKPVNTFVHEPLVDVQNVCFQEKVTCKNGQGNCYKSNSSMHITDCRLTNGSRYPNCAYRTSQKERHIIVACEGSPYVPVHFDASve----

BLAST: aliSize=120 (resi) iden=94.40% (118/125) simi=96.00% (120/125)
-ESRAKKFQRQHMDSDSSPSSSSTYCNQMMRRRNMTQGRCKPVNTFVHEPLVDVQNVCFQEKVTCKNGQGNCYKSNSSMHITDCRLTNGSRYPNCAYRTSPKERHIIVACEGSPYVPVHFDASVEds
kESAAAKFERQHMDSGNSPSSSSTYCNQMMRRRNMTQGRCKPVNTFVHEPLVDVQNVCFQEKVTCKNGQGNCYKSNSSMHITDCRLTNGSRYPNCAYRTSQKERHIIVACEGSPYVPVHFDASVE--

Proposed: aliSize=122 (resi) RMSD=1.11 (Å)
-ESRAKKFQRQHMDSDSSPS-SsSTYCNQMMRRRNMTQGRCKPVNTFVHEPLVDVQNVCFQEKVTC-KNGQgNCYKSNSSMHITDCRLTNGSRYPNCAYRTSPKERHIIVACEGSPYVPVHFDASVEds
kESAAAKFERQHMDSGNSPSsS-STYCNQMMRRRNMTQGRCKPVNTFVHEPLVDVQNVCFQEKVTCkNGQG-NCYKSNSSMHITDCRLTNGSRYPNCAYRTSQKERHIIVACEGSPYVPVHFDASVE--</td></tr>
<tr><td>1h8xA
(125)</td></tr>
<tr><td rowspan="2">853</td><td>2k11A
(127)</td><td rowspan="2">N</td><td rowspan="2">89.60</td><td rowspan="2">TM-align: aliSize=104 (resi) RMSD=1.73 (Å)
kesrakkfqrqhmdsdssp-----------------SSS--STYCNQMMRRRNMTQGRCKPVNTFVHEPLVDVQNVCFQEKVTC--KNgQgNCYKSNSSMHITDCRLTNGSRYPNCAYRTSPKERHIIVACEGSPYVPVHFDASVEds
-------------------kesaaakferqhmdsgnSPSssSTYCNQMMRRRNMTQGRCKPVNTFVHEPLVDVQNVCFQEKVTCknGQ-G-NCYKSNSSMHITDCRLTNGSRYPNCAYRTSQKERHIIVACEGSPYVPVHFDASVE--

SARST: aliSize=101 (resi) RMSD=2.03 (Å)
kesrakkfqrqhmdsdsspss--------------------SSTYCNQMMRRRNMTQGRCKPVNTFVHEPLVDVQNVCFQEKVTCKNGQGNCYKSNSSMHITDCRLTNGSRYPNCAYRTSPKERHIIVACEGSPYVPVHFDA---sveds
--------------------kesaaakferqhmdsgnspssSSTYCNQMMRRRNMTQGRCKPVNTFVHEPLVDVQNVCFQEKVTCKNGQGNCYKSNSSMHITDCRLTNGSRYPNCAYRTSQKERHIIVACEGSPYVPVHFDAsve-----

BLAST: aliSize=121 (resi) iden=95.20% (119/125) simi=96.80% (121/125)
KESRAKKFQRQHMDSDSSPSSSSTYCNQMMRRRNMTQGRCKPVNTFVHEPLVDVQNVCFQEKVTCKNGQGNCYKSNSSMHITDCRLTNGSRYPNCAYRTSPKERHIIVACEGSPYVPVHFDASVEds
KESAAAKFERQHMDSGNSPSSSSTYCNQMMRRRNMTQGRCKPVNTFVHEPLVDVQNVCFQEKVTCKNGQGNCYKSNSSMHITDCRLTNGSRYPNCAYRTSQKERHIIVACEGSPYVPVHFDASVE--

Proposed: aliSize=122 (resi) RMSD=1.58 (Å)
KESRAKKFQRQHMDSdSSP-SSSSTYCNQMMRRRNMTQGRCKPVNTFVHEPLVDVQNVCFQEKVTC--KNgQgNCYKSNSSMHITDCRLTNGSRYPNCAYRTSPKERHIIVACEGSPYVPVHFDASVEds
KESAAAKFERQHMDS-GNSpSSSSTYCNQMMRRRNMTQGRCKPVNTFVHEPLVDVQNVCFQEKVTCknGQ-G-NCYKSNSSMHITDCRLTNGSRYPNCAYRTSQKERHIIVACEGSPYVPVHFDASVE--</td></tr>
<tr><td>1h8xA
(125)</td></tr>
<tr><td rowspan="2">854</td><td>2aasA
(124)</td><td rowspan="2">N</td><td rowspan="2">70.16</td><td rowspan="2">TM-align: aliSize=105 (resi) RMSD=1.88 (Å)
ketaaakferqhmdsstsa------------------ASSSNYCNQMMKSRNLTKDRCKPVNTFVHESLADVQAVCSQKNVACKNGQTNCYQSYSTMSITDCRETGSSKYPNCAYKTTQANKHIIVACEGNPYVPVHFDASV-
------------------kesaaakferqhmdsgnspSSSSTYCNQMMRRRNMTQGRCKPVNTFVHEPLVDVQNVCFQEKVTCKNGQGNCYKSNSSMHITDCRLTNGSRYPNCAYRTSQKERHIIVACEGSPYVPVHFDASVe

SARST: aliSize=118 (resi) RMSD=14.39 (Å)
k-ETAAAKFERQHMDSSTsaasSS----NYCNQMMKSRNLTKDRCKPVNTFVHESLADVQAVCSQKNVACKNGQTNCYQSYSTMSITDCRETGSSKYPNCAYKTTQANKHIIVACEGNPYVPVHFDAS--v
-kESAAAKFERQHMDSGN----SPssssTYCNQMMRRRNMTQGRCKPVNTFVHEPLVDVQNVCFQEKVTCKNGQGNCYKSNSSMHITDCRLTNGSRYPNCAYRTSQKERHIIVACEGSPYVPVHFDASve-

BLAST: aliSize=104 (resi) iden=73.39% (91/124) simi=83.87% (104/124)
KETAAAKFERQHMDSSTSAASSSNYCNQMMKSRNLTKDRCKPVNTFVHESLADVQAVCSQKNVACKNGQTNCYQSYSTMSITDCRETGSSKYPNCAYKTTQANKHIIVACEGNPYVPVHFDASV-
KESAAAKFERQHMDSGNSPSSSSTYCNQMMRRRNMTQGRCKPVNTFVHEPLVDVQNVCFQEKVTCKNGQGNCYKSNSSMHITDCRLTNGSRYPNCAYRTSQKERHIIVACEGSPYVPVHFDASVe

Proposed: aliSize=120 (resi) RMSD=1.32 (Å)
KETAAAKFERQHMDSSTsAA--SS-snYCNQMMKSRNLTKDRCKPVNTFVHESLADVQAVCSQKNVACK-NgQTNCYQSYSTMSITDCRETGSSKYPNCAYKTTQANKHIIVACEGNPYVPVHFDASV-
KESAAAKFERQHMDSGN-SPssSSt--YCNQMMRRRNMTQGRCKPVNTFVHEPLVDVQNVCFQEKVTCKnG-QGNCYKSNSSMHITDCRLTNGSRYPNCAYRTSQKERHIIVACEGSPYVPVHFDASVe</td></tr>
<tr><td>1h8xA
(125)</td></tr>
</table>

855	2nuiA (124)<br>1h8xA (125)	N	68.55	TM-align: aliSize=106 (resi) RMSD=1.91 (Å)<br>ketaaakferqhmdssts-----------------AASSSNYCNQMMKSRNLTKDRCKPVNTFVHESLADVQAVCSQKNVACKNGQTNCYQSYSTMSITACRETGSSKYPNCAYKTTQANKHIIVACEGNPYVPVHFDASV-<br>-----------------kesaaakferqhmdsgnsPSSSSTYCNQMMRRRNMTQGRCKPVNTFVHEPLVDVQNVCFQEKVTCKNGQGNCYKSNSSMHITDCRLTNGSRYPNCAYRTSQKERHIIVACEGSPYVPVHFDASVe<br><br>SARST: aliSize=119 (resi) RMSD=14.38 (Å)<br>k-ETAAAKFERQHMDSST---SAASssnYCNQMMKSRNLTKDRCKPVNTFVHESLADVQAVCSQKNVACKNGQTNCYQSYSTMSITACRETGSSKYPNCAYKTTQANKHIIVACEGNPYVPVHFDAS--v<br>-kESAAAKFERQHMDSGNspsSSST---YCNQMMRRRNMTQGRCKPVNTFVHEPLVDVQNVCFQEKVTCKNGQGNCYKSNSSMHITDCRLTNGSRYPNCAYRTSQKERHIIVACEGSPYVPVHFDASve-<br><br>BLAST: aliSize=103 (resi) iden=72.58% (90/124) simi=83.06% (103/124)<br>KETAAAKFERQHMDSSTSAASSSNYCNQMMKSRNLTKDRCKPVNTFVHESLADVQAVCSQKNVACKNGQTNCYQSYSTMSITACRETGSSKYPNCAYKTTQANKHIIVACEGNPYVPVHFDASV-<br>KESAAAKFERQHMDSGNSPSSSSTYCNQMMRRRNMTQGRCKPVNTFVHEPLVDVQNVCFQEKVTCKNGQGNCYKSNSSMHITDCRLTNGSRYPNCAYRTSQKERHIIVACEGSPYVPVHFDASVe<br><br>Proposed: aliSize=122 (resi) RMSD=1.04 (Å)<br>KETAAAKFERQHMDSSTsAAS-SSNYCNQMMKSRNLTKDRCKPVNTFVHESLADVQAVCSQKNVAC-KNGQtNCYQSYSTMSITACRETGSSKYPNCAYKTTQANKHIIVACEGNPYVPVHFDASV-<br>KESAAAKFERQHMDSGN-SPSsSSTYCNQMMRRRNMTQGRCKPVNTFVHEPLVDVQNVCFQEKVTCkNGQG-NCYKSNSSMHITDCRLTNGSRYPNCAYRTSQKERHIIVACEGSPYVPVHFDASVe
856	1qwqA (124)<br>1h8xA (125)	N	70.97	TM-align: aliSize=104 (resi) RMSD=2.24 (Å)<br>kesaaakferqhmdsgns------------------PSSSSNYCNLMMCCRKMTQGKCKPVNTFVHESLADVKAVCSQKKVTC--KDgqTNCYQSKSTMRITDCRETGSSKYPNCAYKTTQVEKHIIVACGGKPSVPVHFDASV-<br>------------------kesaaakferqhmdsgnsPSSSSTYCNQMMRRRNMTQGRCKPVNTFVHEPLVDVQNVCFQEKVTCknGQ--GNCYKSNSSMHITDCRLTNGSRYPNCAYRTSQKERHIIVACEGSPYVPVHFDASVe<br><br>SARST: aliSize=99 (resi) RMSD=2.15 (Å)<br>kesaaakferqhmdsgnspssssn------------------------YCNLMMCCRKMTQGKCKPVNTFVHESLADVKAVCSQKKVTCKDGQTNCYQSKSTMRITDCRETGSSKYPNCAYKTTQVEKHIIVACGGKPSVPVHFDAS--v<br>------------------------kesaaakferqhmdsgnspsssstYCNQMMRRRNMTQGRCKPVNTFVHEPLVDVQNVCFQEKVTCKNGQGNCYKSNSSMHITDCRLTNGSRYPNCAYRTSQKERHIIVACEGSPYVPVHFDASve-<br><br>BLAST: aliSize=105 (resi) iden=76.61% (95/124) simi=84.68% (105/124)<br>KESAAAKFERQHMDSGNSPSSSSNYCNLMMCCRKMTQGKCKPVNTFVHESLADVKAVCSQKKVTCKDGQTNCYQSKSTMRITDCRETGSSKYPNCAYKTTQVEKHIIVACGGKPSVPVHFDASV-<br>KESAAAKFERQHMDSGNSPSSSSTYCNQMMRRRNMTQGRCKPVNTFVHEPLVDVQNVCFQEKVTCKNGQGNCYKSNSSMHITDCRLTNGSRYPNCAYRTSQKERHIIVACEGSPYVPVHFDASVe<br><br>Proposed: aliSize=118 (resi) RMSD=1.68 (Å)<br>KESAAAKFERQHMDSGN---sPSSssNYCNLMMCCRKMTQGKCKPVNTFVHESLADVKAVCSQKKVTC--KDgqTNCYQSKSTMRITDCRET-gSSKYPNCAYKTTQVEKHIIVACGGKPSVPVHFDASV-<br>KESAAAKFERQHMDSGNsps-SSS--TYCNQMMRRRNMTQGRCKPVNTFVHEPLVDVQNVCFQEKVTCknGQ--GNCYKSNSSMHITDCRLTn-GSRYPNCAYRTSQKERHIIVACEGSPYVPVHFDASVe
857	1izpA (124)<br>1h8xA (125)	N	68.55	TM-align: aliSize=105 (resi) RMSD=1.68 (Å)<br>ketaaakferqhmdssts-----------------AAs-SSNYCNQMMKSRNLTKDRCKPVNTLVHESLADVQAVCSQKNVACKNGQTNCYQSYSTMSITDCRETGSSKYPNCAYKTTQANKHIIVACEGNPYVPVHFDASV-<br>-----------------kesaaakferqhmdsgnsPS-sSSTYCNQMMRRRNMTQGRCKPVNTFVHEPLVDVQNVCFQEKVTCKNGQGNCYKSNSSMHITDCRLTNGSRYPNCAYRTSQKERHIIVACEGSPYVPVHFDASVe<br><br>SARST: aliSize=119 (resi) RMSD=14.37 (Å)<br>k-ETAAAKFERQHMDSST---SAAsssNYCNQMMKSRNLTKDRCKPVNTLVHESLADVQAVCSQKNVACKNGQTNCYQSYSTMSITDCRETGSSKYPNCAYKTTQANKHIIVACEGNPYVPVHFDAS--v<br>-kESAAAKFERQHMDSGNspsSSS---TYCNQMMRRRNMTQGRCKPVNTFVHEPLVDVQNVCFQEKVTCKNGQGNCYKSNSSMHITDCRLTNGSRYPNCAYRTSQKERHIIVACEGSPYVPVHFDASve-<br><br>BLAST: aliSize=103 (resi) iden=72.58% (90/124) simi=83.06% (103/124)<br>KETAAAKFERQHMDSSTSAASSSNYCNQMMKSRNLTKDRCKPVNTLVHESLADVQAVCSQKNVACKNGQTNCYQSYSTMSITDCRETGSSKYPNCAYKTTQANKHIIVACEGNPYVPVHFDASV-<br>KESAAAKFERQHMDSGNSPSSSSTYCNQMMRRRNMTQGRCKPVNTFVHEPLVDVQNVCFQEKVTCKNGQGNCYKSNSSMHITDCRLTNGSRYPNCAYRTSQKERHIIVACEGSPYVPVHFDASVe<br><br>Proposed: aliSize=121 (resi) RMSD=0.95 (Å)<br>KETAAAKFERQHMDSSTsAA--SsSNYCNQMMKSRNLTKDRCKPVNTLVHESLADVQAVCSQKNVACK-NGQtNCYQSYSTMSITDCRETGSSKYPNCAYKTTQANKHIIVACEGNPYVPVHFDASV-<br>KESAAAKFERQHMDSGN-SPssS-STYCNQMMRRRNMTQGRCKPVNTFVHEPLVDVQNVCFQEKVTCKnGQG-NCYKSNSSMHITDCRLTNGSRYPNCAYRTSQKERHIIVACEGSPYVPVHFDASVe

858	2vq8A (128)	11bgA (124)	N	22.58	TM-align: aliSize=93 (resi) RMSD=1.79 (Å)<br>hmqpehvkeryknflnqhvgpdm--------------------SVQRCNSEIGpnNRKITlsgtDNGCKPVNTFILANKRLIKTVCGraGSPQG-----N-MVRSNQPFPVVKCVLN-NGERHpYCEYRGTRSTRYIVLKCEE--GWPVHYHEDevn-<br>----------------------kesaaakferqhmdsgnspssSSNYCNLMMC--CRKMT----QGKCKPVNTFVHESLADVKAVCS--QKKVTckngqTnCYQSKSTMRITDCRETgSSKYP-NCAYKTTQVEKHIIVACGGkpSVPVHFDAS---v<br><br>SARST: aliSize=105 (resi) RMSD=9.62 (Å)<br>hmqpehvke---RYKNFLNQHV----GPDMsVQRCNSEIgPNNRKitlsGTDNGCKPVNTFILANKRLIKTVCGRAGSPQG----NMVRSNQPFPVVKCVLnnGE--RHPYCEYRGTRSTRYIVLKCEEGWP--VHY----hedevn<br>---------kesAAAKFERQHMdsgnSPSS-SSNYCNLM-MCCRK----MTQGKCKPVNTFVHESLADVKAVCSQKKVTCKngqtNCYQSKSTMRITDCRE--TGssKYPNCAYKTTQVEKHIIVACGGKPSvpVHFdasv------<br><br>BLAST: aliSize=54 (resi) iden=27.42% (34/124) simi=43.55% (54/124)<br>hmqpehvkerykn-------FLNQHV----GPDMSVQRCNSEIGPnnRKITlSGTdngCKPVNTFILANKRLIKTVCGR----AGSPQGNMVRSNQPFPVVKCVLNNGERHPYCEYRGTRSTRYIVLKC--EEGWPVHY----hedevn<br>-------------kesaaakFERQHMdsgnSPSSSSNYCNLMMCC--RKMT-QGK---CKPVNTFVHESLADVKAVCSQkkvtCKNGQTNCYQSKSTMRITDCRETGSSKYPNCAYKTTQVEKHIIVACggKPSVPVHFdasv------<br><br>Proposed: aliSize=108 (resi) RMSD=1.67 (Å)<br>hmqpehv-KERYKNFLNQHVGPD-MSVQ---RCNSEIGpnNRKITlsgtDNGCKPVNTFILANKRLIKTVCGraGSPQG-----N-MVRSNQPFPVVKCVLN-NGERHpYCEYRGTRSTRYIVLKCEE--GWPVHYHE--devn<br>-------kESAAAKFERQHMDSGnSPSSssnYCNLMMC--CRKMT----QGKCKPVNTFVHESLADVKAVCS--QKKVTckngqTnCYQSKSTMRITDCRETgSSKYP-NCAYKTTQVEKHIIVACGGkpSVPVHFDAsv----
859	2vq8A (128)	1y92B (124)	N	23.39	TM-align: aliSize=93 (resi) RMSD=1.84 (Å)<br>hmqpehvkeryknflnqhvgpdm-------------------SVQ-RCNSEIGpnNRKITlsgtDNGCKPVNTFILANKRLIKTVCGraGSPQG-----N-MVRSNQPFPVVKCVLN-NGERhPYCEYRGTRSTRYIVLKCEE--GWPVHYHEDevn-<br>----------------------kesaaakferqhmdsgnsasSSSnYCNLMMC--CRKMT----QGKCKPVNTFVHESLADVKAVCS--QKKVTckdgqTnCYQSKSTMRITDCRETgSSKY-PNCAYKTTQVEKHIIVACGGkpSVPVHFDAS---v<br><br>SARST: aliSize=105 (resi) RMSD=9.81 (Å)<br>hmqpehvke---RYKNFLNQHVGPDMS---VQRCNSEIgPNNRKitlsGTDNGCKPVNTFILANKRLIKTVCGRAGSP----QGNMVRSNQPFPVVKCVLNNGERHPYCEYRGTRSTRYIVLKCEEGWP---------vhyhedevn<br>---------kesAAAKFERQHMDSGNSassSSNYCNLM-MCCRK----MTQGKCKPVNTFVHESLADVKAVCSQKKVTckdgQTNCYQSKSTMRITDCRETGSSKYPNCAYKTTQVEKHIIVACGGKPSvpvhfdasv---------<br><br>BLAST: aliSize=40 (resi) iden=19.35% (24/124) simi=32.26% (40/124)<br>hmqpehvkeryknflnqhvgpdmsvqrcnseigpnnrkitlsg----------------------------------TDNGCKPVNTFILANKRLIKTVCGR----AGSPQGNMVRSNQPFPVVKCVLNNGERHPYCEYRGTRSTRYIVLKC--EEGWPVHY----hedevn<br>---kesaaakferqhmdsgnsassssnycnlmmccrkmTQGKCKPVNTFVHESLADVKAVCSQkkvtCKDGQTNCYQSKSTMRITDCRETGSSKYPNCAYKTTQVEKHIIVACggKPSVPVHFdasv------<br><br>Proposed: aliSize=109 (resi) RMSD=1.69 (Å)<br>hmqpehv-KERYKNFLNQHVGPDMSV-Q---RCNSEIGpnNRKITlsgtDNGCKPVNTFILANKRLIKTVCGraGSPQG-----N-MVRSNQPFPVVKCVLN-NGERHpYCEYRGTRSTRYIVLKCEE--GWPVHYHED-evn<br>-------kESAAAKFERQHMDSGNSAsSssnYCNLMMC--CRKMT----QGKCKPVNTFVHESLADVKAVCS--QKKVTckdgqTnCYQSKSTMRITDCRETgSSKYP-NCAYKTTQVEKHIIVACGGkpSVPVHFDASv---
860	2vq8A (128)	3bcoA (124)	N	22.58	TM-align: aliSize=93 (resi) RMSD=1.82 (Å)<br>hmqpehvkeryknflnqhvgpdm--------------------SVQRCNSEIGpnNRKITlsgtDNGCKPVNTFILANKRLIKTVCGraGSPQG-----N-MVRSNQPFPVVKCVLN-NGERHpYCEYRGTRSTRYIVLKCEE--GWPVHYHEDevn-<br>----------------------kesaaakferqhmdsgnsassSSNYCNQMMC--CRKMT----QGKCKPVNTFVHESLADVKAVCS--QKKVTckdgqTnCYQSKSTMRITDCRETgSSKYP-NCAYKTTQVEKHIIVACGGkpSVPVHFDAS---v<br><br>SARST: aliSize=102 (resi) RMSD=9.51 (Å)<br>hmqpehvke---RYKNFLNQHVG----PDMsVQRCNSEIgPNNRKitlsGTDNGCKPVNTFILANKRLIKTVCGRAGSP----QGNMVRSNQPFPVVKCVLnnGE--RHPYCEYRGTRSTRYIVLKCEEGWP---------vhyhedevn<br>---------kesAAAKFERQHMDsgnsASS-SSNYCNQM-MCCRK----MTQGKCKPVNTFVHESLADVKAVCSQKKVTckdgQTNCYQSKSTMRITDCRE--TGssKYPNCAYKTTQVEKHIIVACGGKPSvpvhfdasv---------<br><br>BLAST: aliSize=40 (resi) iden=19.35% (24/124) simi=32.26% (40/124)<br>hmqpehvkeryknflnqhvgpdmsvqrcnseigpnnrkitlsg----------------------------------TDNGCKPVNTFILANKRLIKTVCGR----AGSPQGNMVRSNQPFPVVKCVLNNGERHPYCEYRGTRSTRYIVLKC--EEGWPVHY----hedevn<br>---kesaaakferqhmdsgnsassssnycnqmmccrkmTQGKCKPVNTFVHESLADVKAVCSQkkvtCKDGQTNCYQSKSTMRITDCRETGSSKYPNCAYKTTQVEKHIIVACggKPSVPVHFdasv------<br><br>Proposed: aliSize=109 (resi) RMSD=1.78 (Å)<br>hmqpehv-KERYKNFLNQHVGPD-MSVQ---RCNSEIGpnNRKITlsgtDNGCKPVNTFILANKRLIKTVCGraGSPQG-----N-MVRSNQPFPVVKCVLN-NGERHpYCEYRGTRSTRYIVLKCE-E-GWPVHYHED-evn<br>-------kESAAAKFERQHMDSGnSASSssnYCNQMMC--CRKMT----QGKCKPVNTFVHESLADVKAVCS--QKKVTckdgqTnCYQSKSTMRITDCRETgSSKYP-NCAYKTTQVEKHIIVACGgKpSVPVHFDASv---

861	2vq8A (128)<br>1y94A (124)	N	22.58	TM-align: aliSize=92 (resi) RMSD=1.75 (Å)<br>hmqpehvkeryknflnqhvgpdm----------------------SVQRCNSEIGpnNRKITlsgtDNGCKPVNTFILANKRLIKTVCGraGSPQG-----N-MVRSNQPFPVVKCVLN--NGeRHpYCEYRGTRSTRYIVLKCEE--GWPVHYHEDevn-<br>------------------------kesaaakferqhmdsstsaasSSNYCNLMMC--CRKMT----QGKCKPVNTFVHESLADVKAVCS--QKKVTckdgqTnCYQSKSTMRITDCRETgsSK-YP-NCAYKTTQVEKHIIVACGGkpSVPVHFDAS---v<br><br>SARST: aliSize=104 (resi) RMSD=9.43 (Å)<br>hmqpehvke---RYKNFLNQHV----GPDMsVQRCNSEIgPNNRKitlsGTDNGCKPVNTFILANKRLIKTVCGRAGSPQG----NMVRSNQPFPVVKCVLNNGERHPYCEYRGTRSTRYIVLKCEEGWP---------vhyhedevn<br>---------kesAAAKFERQHMdsstSAAS-SSNYCNLM-MCCRK----MTQGKCKPVNTFVHESLADVKAVCSQKKVTCKdgqtNCYQSKSTMRITDCRETGSSKYPNCAYKTTQVEKHIIVACGGKPSvpvhfdasv---------<br><br>BLAST: aliSize=49 (resi) iden=25.00% (31/124) simi=39.52% (49/124)<br>hmqpehvkerykn-------FLNQHVgpDMSVQRCNSEIGPNNRKITLSGTDNGCKPVNTFILANKRLIKTVCGR----AGSPQGNMVRSNQPFPVVKCVLNNGERHPYCEYRGTRSTRYIVLKC--EEGWPVHY----hedevn<br>-------------kesaaakFERQHM--DSSTSAASSSNYCNLMMCCRKMTQGKCKPVNTFVHESLADVKAVCSQkkvtCKDGQTNCYQSKSTMRITDCRETGSSKYPNCAYKTTQVEKHIIVACggKPSVPVHFdasv------<br><br>Proposed: aliSize=109 (resi) RMSD=1.76 (Å)<br>hmqpehv-KERYKNFLNQHVGPD-MSVQ---RCNSEIGpnNRKITlsgtDNGCKPVNTFILANKRLIKTVCGraGSPQG-----N-MVRSNQPFPVVKCVLN-NGERHpYCEYRGTRSTRYIVLKCEE--GWPVHYHED-evn<br>-------kESAAAKFERQHMDSStSAASssnYCNLMMC--CRKMT----QGKCKPVNTFVHESLADVKAVCS--QKKVTckdgqTnCYQSKSTMRITDCRETgSSKYP-NCAYKTTQVEKHIIVACGGkpSVPVHFDASv---
862	1kvzA (107)<br>1lbgA (124)	N	24.30	TM-align: aliSize=85 (resi) RMSD=2.50 (Å)<br>mqdwatfkkkhltdtw---------------------D-VDCDNLMPTSLFD---CKDKNTFIYSLPGPVKALCRGvif---------SADVLSNSEFYLAECNVKPRK---PCKYKLKKSSNRICIRCEH--ELPVHFAGVGicp<br>----------------kesaaakferqhmdsgnspssSsNYCNLMMCCRKMTqgkCKPVNTFVHESLADVKAVCSQ---kkvtckngqTNCYQSKSTMRITDCRETGSSkypNCAYKTTQVEKHIIVACGGkpSVPVHFDASV---<br><br>SARST: aliSize=99 (resi) RMSD=10.44 (Å)<br>mqd----WATFKKKHLTDTWD-VDCDNLMPT--SLFDC-----KDKNTFIYSLPGPVKALCRGVIFSA------DVLSNSEFYLAECNVK----PRKPcKYKLKKSSNRICIRCEH--ELPVHFAGV-gicp<br>---kesaAAKFERQHMDSGNSpSSSSNYCNLmmCCRKMtqgkcKPVNTFVHESLADVKAVCSQKKVTCkngqtnCYQSKSTMRITDCRETgsskYPNC-AYKTTQVEKHIIVACGGkpSVPVHFDASv----<br><br>BLAST: aliSize=34 (resi) iden=21.50% (23/107) simi=31.78% (34/107)<br>mqdwatfkkkhltdtwdvdcdnlmptslfd---------------------------------------CKDKNTFIYSLPGPVKALC--RGVIFSADVL----SNSEFYLAECNV--KPRKP-CKYKLKKSSNRICIRC--EHELPVHF----agvgicp<br>------------------------------kesaaakferqhmdsgnspssssnycnlmmccrkmtqgkCKPVNTFVHESLADVKAVCsqKKVTCKNGQTncyqSKSTMRITDCREtgSSKYPnCAYKTTQVEKHIIVACggKPSVPVHFdasv-------<br><br>Proposed: aliSize=99 (resi) RMSD=2.11 (Å)<br>MQD-WATFKKKHLTDT-WDV---D-CDNLMPT-SLFD--CKDKNTFIYSLPGPVKALCR-----------gvifsADVLSNSEFYLAECNVKPRK---PCKYKLKKSSNRICIRCEH--ELPVHFAGVGicp<br>KESaAAKFERQHMDSGnSPSsssNyCNLMMCCrKMTQgkCKPVNTFVHESLADVKAVCSqkkvtckngqt-----NCYQSKSTMRITDCRETGSSkypNCAYKTTQVEKHIIVACGGkpSVPVHFDASV---
863	1kvzA (107)<br>1y92B (124)	N	23.36	TM-align: aliSize=85 (resi) RMSD=2.41 (Å)<br>mqdwatfkkkhltdtw---------------------D-VDCDNLMPTSLF-D--CKDKNTFIYSLPGPVKALCRGvif---------SADVLSNSEFYLAECNVKPRKP---CKYKLKKSSNRICIRCEH--ELPVHFAGVGicp<br>----------------kesaaakferqhmdsgnsassSsNYCNLMMCCRKMtQgkCKPVNTFVHESLADVKAVCSQ---kkvtckdgqTNCYQSKSTMRITDCRETGSSKypnCAYKTTQVEKHIIVACGGkpSVPVHFDASV---<br><br>SARST: aliSize=81 (resi) RMSD=3.45 (Å)<br>mqdwatfkkkhltdtwdvdcdn-------------------------------LMPTSLFD-CKDKNTFIYSLPGPVKALCRGVIFSA------DVLSNSEFYLAECNVKPRKPC---KYKLKKSSNRICIRCEH--ELPVHFAGV-gicp<br>-----------------------kesaaakferqhmdsgnsassssnycnlmmCCRKMTQGkCKPVNTFVHESLADVKAVCSQKKVTCkdgqtnCYQSKSTMRITDCRETGSSKYpncAYKTTQVEKHIIVACGGkpSVPVHFDASv----<br><br>BLAST: aliSize=36 (resi) iden=21.50% (23/107) simi=33.64% (36/107)<br>mqdwatfkkkhltdtwdvdcdnlmptslfd---------------------------------------CKDKNTFIYSLPGPVKALC-RGVIFSADVLSN-----SEFYLAECNV--KPRKP-CKYKLKKSSNRICIRC--EHELPVHF----agvgicp<br>------------------------------kesaaakferqhmdsgnsassssnycnlmmccrkmtqgkCKPVNTFVHESLADVKAVCsQKKVTCKDGQTNcyqskSTMRITDCREtgSSKYPnCAYKTTQVEKHIIVACggKPSVPVHFdasv-------<br><br>Proposed: aliSize=98 (resi) RMSD=2.04 (Å)<br>MQDWATFKKKHLTDTwdVD--------CDNLMPT-SLFD--CKDKNTFIYSLPGPVKALCR----------gvifSADVLSNSEFYLAECNVKPRKP---CKYKLKKSSNRICIRCEH--ELPVHFAGVGicp<br>KESAAAKFERQHMDS--GNsassssnyCNLMMCCrKMTQgkCKPVNTFVHESLADVKAVCSqkkvtckdgq----TNCYQSKSTMRITDCRETGSSKypnCAYKTTQVEKHIIVACGGkpSVPVHFDASV---

864	1kvzA (107)<br>3bcoA (124)	N	24.30	TM-align: aliSize=85 (resi) RMSD=2.50 (Å)<br>mqdwatfkkkhltdtw---------------------D-VDCDNLMPTSLF-D--CKDKNTFIYSLPGPVKALCRGvif---------SADVLSNSEFYLAECNVKPRKP---CKYKLKKSSNRICIRCEH--ELPVHFAGVGicp<br>----------------kesaaakferqhmdsgnsassSsNYCNQMMCCRKMtQgkCKPVNTFVHESLADVKAVCSQ---kkvtckdgqTNCYQSKSTMRITDCRETGSSKypnCAYKTTQVEKHIIVACGGkpSVPVHFDASV---<br><br>SARST: aliSize=94 (resi) RMSD=11.72 (Å)<br>mqd----WATFKKKHLTDTWD--VDCDNLMPTSLFDC------KDKNTFIYSLPGPVKALCRGVIFSA------DVLSNSEFYLAECNVK----PRKPcKYKLKKS-------SNRicIRCEHELPVHF-agvgicp<br>---kesaAAKFERQHMDSGNSasSSSNYCNQMMCCRKmtqgkcKPVNTFVHESLADVKAVCSQKKVTCkdgqtnCYQSKSTMRITDCRETgsskYPNC-AYKTTQVekhiivaCGG--KPSVPVHFDASv-------<br><br>BLAST: aliSize=36 (resi) iden=21.50% (23/107) simi=33.64% (36/107)<br>mqdwatfkkkhltdtwdvdcdnlmptslfd---------------------------------------CKDKNTFIYSLPGPVKALC-RGVIFSADVLSN-----SEFYLAECNV--KPRKP-CKYKLKKSSNRICIRC--EHELPVHF----agvgicp<br>------------------------------kesaaakferqhmdsgnsassssnycnqmmccrkmtqgkCKPVNTFVHESLADVKAVCsQKKVTCKDGQTNcyqskSTMRITDCREtgSSKYPnCAYKTTQVEKHIIVACggKPSVPVHFdasv-------<br><br>Proposed: aliSize=99 (resi) RMSD=2.10 (Å)<br>MQD-WATFKKKHLTDTWDV----D-CDNLMPT-SLFD--CKDKNTFIYSLPGPVKALCR-----------gvifsADVLSNSEFYLAECNVKPRK---PCKYKLKKSSNRICIRCE-H-ELPVHFAGVGicp<br>KESaAAKFERQHMDSGNSAssssNyCNQMMCCrKMTQgkCKPVNTFVHESLADVKAVCSqkkvtckdgqt-----NCYQSKSTMRITDCRETGSSkypNCAYKTTQVEKHIIVACGgKpSVPVHFDASV---
865	1kvzA (107)<br>1y94A (124)	N	24.30	TM-align: aliSize=85 (resi) RMSD=2.47 (Å)<br>mqdwatfkkkhltdtw---------------------D-VDCDNLMPTSLFD---CKDKNTFIYSLPGPVKALCRGvif---------SADVLSNSEFYLAECNVKPRKP---CKYKLKKSSNRICIRCEH--ELPVHFAGVGicp<br>----------------kesaaakferqhmdsstsaasSsNYCNLMMCCRKMTqgkCKPVNTFVHESLADVKAVCSQ---kkvtckdgqTNCYQSKSTMRITDCRETGSSKypnCAYKTTQVEKHIIVACGGkpSVPVHFDASV---<br><br>SARST: aliSize=99 (resi) RMSD=10.32 (Å)<br>mqd----WATFKKKHLTDTWD-VDCDNLM------PTSLFD-CKDKNTFIYSLPGPVKALCRGVIFSA------DVLSNSEFYLAECNVK----PRKPcKYKLKKSSNRICIRCEH--ELPVHFAGV-gicp<br>---kesaAAKFERQHMDSSTSaASSSNYCnlmmccRKMTQGkCKPVNTFVHESLADVKAVCSQKKVTCkdgqtnCYQSKSTMRITDCRETgsskYPNC-AYKTTQVEKHIIVACGGkpSVPVHFDASv----<br><br>BLAST: aliSize=36 (resi) iden=21.50% (23/107) simi=33.64% (36/107)<br>mqdwatfkkkhltdtwdvdcdnlmptslfd---------------------------------------CKDKNTFIYSLPGPVKALC-RGVIFSADVLSN-----SEFYLAECNV--KPRKP-CKYKLKKSSNRICIRC--EHELPVHF----agvgicp<br>------------------------------kesaaakferqhmdsstsaasssnycnlmmccrkmtqgkCKPVNTFVHESLADVKAVCsQKKVTCKDGQTNcyqskSTMRITDCREtgSSKYPnCAYKTTQVEKHIIVACggKPSVPVHFdasv-------<br><br>Proposed: aliSize=100 (resi) RMSD=2.15 (Å)<br>MQD-WATFKKKHLTDTWDV----D-CDNLMPT-SLFD--CKDKNTFIYSLPGPVKALCR----------gvifSADVLSNSEFYLAECNVKPRKP---CKYKLKKSSNRICIRCEH--ELPVHFAGVGicp<br>KESaAAKFERQHMDSSTSAasssNyCNLMMCCrKMTQgkCKPVNTFVHESLADVKAVCSqkkvtckdgq----TNCYQSKSTMRITDCRETGSSKypnCAYKTTQVEKHIIVACGGkpSVPVHFDASV---
866	1pu3A (105)<br>1lbgA (124)	N	23.81	TM-align: aliSize=84 (resi) RMSD=2.25 (Å)<br>mqdwltfqkkhitntr---------------------D-VDCDNILSTNLFH---CKDKNTFIYSRPEPVKAICKGiia---------SKNVLTTSEFYLSDCNVTSRP----CKYKLKKSTNKFCVTCE-N-QAPVHFVGVGsc<br>----------------kesaaakferqhmdsgnspssSsNYCNLMMCCRKMTqgkCKPVNTFVHESLADVKAVCSQ---kkvtckngqTNCYQSKSTMRITDCRETGSSkypnCAYKTTQVEKHIIVACGgKpSVPVHFDASV--<br><br>SARST: aliSize=99 (resi) RMSD=10.08 (Å)<br>mqd----WLTFQKKHITNTRDVD------CDNILSTNLFHCK--DKNTFIYSRPEPVKAICKGIIASK------NVLTTSEFYLSDCNVTS--RPCK--YKLKKSTNKFCVTCEN--QAPVHFVGV-gsc<br>---kesaAAKFERQHMDSGNSPSsssnycNLMMCCRKMTQGKckPVNTFVHESLADVKAVCSQKKVTCkngqtnCYQSKSTMRITDCRETGssKYPNcaYKTTQVEKHIIVACGGkpSVPVHFDASv---<br><br>BLAST: aliSize=37 (resi) iden=25.71% (27/105) simi=35.24% (37/105)<br>mqdwltfqkkhitntrdvdcdnilstnlfh---------------------------------------CKDKNTFIYSRPEPVKAIC-KGIIASKNVLTT-----SEFYLSDCNVT--SR-P-CKYKLKKSTNKFCVTCENQ--APVHF----vgvgsc<br>------------------------------kesaaakferqhmdsgnspssssnycnlmmccrkmtqgkCKPVNTFVHESLADVKAVCsQKKVTCKNGQTNcyqskSTMRITDCRETgsSKyPnCAYKTTQVEKHIIVACGGKpsVPVHFdasv------<br><br>Proposed: aliSize=98 (resi) RMSD=1.82 (Å)<br>-MQDWLTFQKKHITNT-RDV----DCDNILST-NLFH--CKDKNTFIYSRPEPVKAICK-----------giiasKNVLTTSEFYLSDCNVTSRP----CKYKLKKSTNKFCVTCE-N-QAPVHFVGVGsc<br>kESAAAKFERQHMDSGnSPSsssnYCNLMMCCrKMTQgkCKPVNTFVHESLADVKAVCSqkkvtckngqt-----NCYQSKSTMRITDCRETGSSkypnCAYKTTQVEKHIIVACGgKpSVPVHFDASV--

867	1pu3A<br>(105)<br><br>1y92B<br>(124)	N	23.81	TM-align: aliSize=85 (resi) RMSD=2.29 (Å)<br>mqdwltfqkkhitntr---------------------D-VDCDNILSTNLFH---CKDKNTFIYSRPEPVKAICKG-Iia-------SKNVLTTSEFYLSDCNVTSRP----CKYKLKKSTNKFCVTCE-N-QAPVHFVGVGsc<br>----------------kesaaakferqhmdsgnsassSsNYCNLMMCCRKMTqgkCKPVNTFVHESLADVKAVCSQkK--vtckdgqTNCYQSKSTMRITDCRETGSSkypnCAYKTTQVEKHIIVACGgKpSVPVHFDASV--<br><br>SARST: aliSize=83 (resi) RMSD=4.48 (Å)<br>mqdwltfqkkhitntrdvd----------------------------CDNILSTNLFHCKDKNTFIYSRPEPVKAIC------KGIIASKNVLTTSEFYLSDCNVTSRP----CKYKLKKSTNKFCVTCEN--QAPVHFVGV-gsc<br>-------------------kesaaakferqhmdsgnsassssnycnlMMCCRKMTQGKCKPVNTFVHESLADVKAVCsqkkvtCKDGQTNCYQSKSTMRITDCRETGSSkypnCAYKTTQVEKHIIVACGGkpSVPVHFDASv---<br><br>BLAST: aliSize=37 (resi) iden=24.76% (26/105) simi=35.24% (37/105)<br>mqdwltfqkkhitntrdvdcdnilstnlfh---------------------------------------CKDKNTFIYSRPEPVKAIC-KGIIASKNVLTT-----SEFYLSDCNVT--SR-P-CKYKLKKSTNKFCVTCENQ--APVHF----vgvgsc<br>------------------------------kesaaakferqhmdsgnsassssnycnlmmccrkmtqgkCKPVNTFVHESLADVKAVCsQKKVTCKDGQTNcyqskSTMRITDCRETgsSKyPnCAYKTTQVEKHIIVACGGKpsVPVHFdasv------<br><br>Proposed: aliSize=98 (resi) RMSD=1.77 (Å)<br>-MQDWLTFQKKHIT-NTRD----VDCDNILSTNLFH---CKDKNTFIYSRPEPVKAICK-----------giiasKNVLTTSEFYLSDCNVTSRP----CKYKLKKSTNKFCVTCE-N-QAPVHFVGVGsc<br>kESAAAKFERQHMDsGNSAssssNYCNLMMCCRKMTqgkCKPVNTFVHESLADVKAVCSqkkvtckdgqt-----NCYQSKSTMRITDCRETGSSkypnCAYKTTQVEKHIIVACGgKpSVPVHFDASV--
868	1pu3A<br>(105)<br><br>3bcoA<br>(124)	N	23.81	TM-align: aliSize=84 (resi) RMSD=2.21 (Å)<br>mqdwltfqkkhitntr---------------------D-VDCDNILSTNLFH---CKDKNTFIYSRPEPVKAICKGiia---------SKNVLTTSEFYLSDCNVTSRP----CKYKLKKSTNKFCVTCE--NQAPVHFVGVGsc<br>----------------kesaaakferqhmdsgnsassSsNYCNQMMCCRKMTqgkCKPVNTFVHESLADVKAVCSQ---kkvtckdgqTNCYQSKSTMRITDCRETGSSkypnCAYKTTQVEKHIIVACGgkPSVPVHFDASV--<br><br>SARST: aliSize=99 (resi) RMSD=9.93 (Å)<br>mqd----WLTFQKKHITNT---RDVDCDNILSTNLFHC-----KDKNTFIYSRPE-PVKAICKGIIAS-----KNVLTTSEFYLSDCNVTS--RPC--KYKLKKSTNKFCVTCEN--QAPVHFVGV-gsc<br>---kesaAAKFERQHMDSGnsaSSSSNYCNQMMCCRKMtqgkcKPVNTFVHESLAdVKAVCSQKKVTCkdgqtNCYQSKSTMRITDCRETGssKYPncAYKTTQVEKHIIVACGGkpSVPVHFDASv---<br><br>BLAST: aliSize=37 (resi) iden=24.76% (26/105) simi=35.24% (37/105)<br>mqdwltfqkkhitntrdvdcdnilstnlfh---------------------------------------CKDKNTFIYSRPEPVKAIC-KGIIASKNVLTT-----SEFYLSDCNVT--SR-P-CKYKLKKSTNKFCVTCENQ--APVHF----vgvgsc<br>------------------------------kesaaakferqhmdsgnsassssnycnqmmccrkmtqgkCKPVNTFVHESLADVKAVCsQKKVTCKDGQTNcyqskSTMRITDCRETgsSKyPnCAYKTTQVEKHIIVACGGKpsVPVHFdasv------<br><br>Proposed: aliSize=97 (resi) RMSD=1.68 (Å)<br>-MQDWLTFQKKHITNTRDV-----DCDNILST-nLFH---CKDKNTFIYSRPEPVKAICK-----------giiasKNVLTTSEFYLSDCNVTSRP----CKYKLKKSTNKFCVTCE--NQAPVHFVGVGsc<br>kESAAAKFERQHMDSGNSAssssnYCNQMMCCr-KMTqgkCKPVNTFVHESLADVKAVCSqkkvtckdgqt-----NCYQSKSTMRITDCRETGSSkypnCAYKTTQVEKHIIVACGgkPSVPVHFDASV--
869	1pu3A<br>(105)<br><br>1y94A<br>(124)	N	23.81	TM-align: aliSize=84 (resi) RMSD=2.18 (Å)<br>mqdwltfqkkhitntr---------------------D-VDCDNILSTNLFH---CKDKNTFIYSRPEPVKAICKGiia---------SKNVLTTSEFYLSDCNVTSRP----CKYKLKKSTNKFCVTCE-N-QAPVHFVGVGsc<br>----------------kesaaakferqhmdsstsaasSsNYCNLMMCCRKMTqgkCKPVNTFVHESLADVKAVCSQ---kkvtckdgqTNCYQSKSTMRITDCRETGSSkypnCAYKTTQVEKHIIVACGgKpSVPVHFDASV--<br><br>SARST: aliSize=99 (resi) RMSD=10.34 (Å)<br>mqd----WLTFQKKHITNTRDVD--------CDNILSTNLFHCKDKNTFIYSRPEPVKAIC------KGIIASKNVLTTSEFYLSDCNVTS--RPC--KYKLKKSTNKFCVTCEN--QAPVHFVGV-gsc<br>---kesaAAKFERQHMDSSTSAAsssnycnlMMCCRKMTQGKCKPVNTFVHESLADVKAVCsqkkvtCKDGQTNCYQSKSTMRITDCRETGssKYPncAYKTTQVEKHIIVACGGkpSVPVHFDASv---<br><br>BLAST: aliSize=37 (resi) iden=24.76% (26/105) simi=35.24% (37/105)<br>mqdwltfqkkhitntrdvdcdnilstnlfh---------------------------------------CKDKNTFIYSRPEPVKAIC-KGIIASKNVLTT-----SEFYLSDCNVT--SR-P-CKYKLKKSTNKFCVTCENQ--APVHF----vgvgsc<br>------------------------------kesaaakferqhmdsstsaasssnycnlmmccrkmtqgkCKPVNTFVHESLADVKAVCsQKKVTCKDGQTNcyqskSTMRITDCRETgsSKyPnCAYKTTQVEKHIIVACGGKpsVPVHFdasv------<br><br>Proposed: aliSize=97 (resi) RMSD=1.63 (Å)<br>-MQDWLTFQKKHITNT-RDV----DCDNILST-nLFH---CKDKNTFIYSRPEPVKAICK-----------giiasKNVLTTSEFYLSDCNVTSRP----CKYKLKKSTNKFCVTCE-N-QAPVHFVGVGsc<br>kESAAAKFERQHMDSStSAAsssnYCNLMMCCr-KMTqgkCKPVNTFVHESLADVKAVCSqkkvtckdgqt-----NCYQSKSTMRITDCRETGSSkypnCAYKTTQVEKHIIVACGgKpSVPVHFDASV--

<table>
<tr><td rowspan="2">870</td><td>1un4A
(119)</td><td rowspan="2">N</td><td rowspan="2">32.77</td><td rowspan="2">TM-align: aliSize=97 (resi) RMSD=1.87 (Å)
nsrythfltqhydakpq-------------------GRDDRYCESIMRRRGLT-SPCKDINTFIHGNKRSIKAICEnknGNPHR----EN-LRISKSSFQVTACKLHGGSPWPPCQYRATAGFRNVVVACE-N-GLPVHLDQsIFr
-----------------kesaaakferqhmdsgnspSSSSNYCNLMMCCRKMTqGKCKPVNTFVHESLADVKAVCS---QKKVTckngQTnCYQSKSTMRITDCRETGSSKYPNCAYKTTQVEKHIIVACGgKpSVPVHFDA-SV-

SARST: aliSize=104 (resi) RMSD=10.05 (Å)
ns---RYTHFLTQHYD----AKPQGRddrYCESIMRRRGLTS-PCKDINTFIHGNKRSIKAICenkNGNPH-----RENLRISKSSFQVTACKLhGGSPWP-PCQYRATAGFRNVVVACEN--GLPVHL----dqsifr
--kesAAAKFERQHMDsgnsPSSSSN---YCNLMMCCRKMTQgKCKPVNTFVHESLADVKAVC---SQKKVtckngQTNCYQSKSTMRITDCRE-TGSSKYpNCAYKTTQVEKHIIVACGGkpSVPVHFdasv------

BLAST: aliSize=65 (resi) iden=35.29% (42/119) simi=54.62% (65/119)
nsryth-------FLTQHYDA--KPQGRDDrYCESIMRRRGLTS-PCKDINTFIHGNKRSIKAICEN-----KNGnphRENLRISKSSFQVTACKLHGGSPWPPCQYRATAGFRNVVVAC--ENGLPVHLDQSIfr
------kesaaakFERQHMDSgnSPSSSSN-YCNLMMCCRKMTQgKCKPVNTFVHESLADVKAVCSQkkvtcKNG---QTNCYQSKSTMRITDCRETGSSKYPNCAYKTTQVEKHIIVACggKPSVPVHFDASV--

Proposed: aliSize=111 (resi) RMSD=1.37 (Å)
n--SRYTHFLTQHYDAK--PQGrDDRYCESIMRRRGLT-SPCKDINTFIHGNKRSIKAICEnknGNPHR-----ENLRISKSSFQVTACKLHGGSPWPPCQYRATAGFRNVVVACE-N-GLPVHLDQsI-fr
-keSAAAKFERQHMDSGnsPSS-SSNYCNLMMCCRKMTqGKCKPVNTFVHESLADVKAVCS---QKKVTckngqTNCYQSKSTMRITDCRETGSSKYPNCAYKTTQVEKHIIVACGgKpSVPVHFDA-Sv--</td></tr>
<tr><td>1lbgA
(124)</td></tr>
<tr><td rowspan="2">871</td><td>1un4A
(119)</td><td rowspan="2">N</td><td rowspan="2">30.25</td><td rowspan="2">TM-align: aliSize=97 (resi) RMSD=1.94 (Å)
nsrythfltqhydakpq-------------------GRDDRYCESIMRRRGLTS-PCKDINTFIHGNKRSIKAICEnknGNPHR---E-N-LRISKSSFQVTACKLHGGSPWPPCQYRATAGFRNVVVACEN--GLPVHLDQsIFr
-----------------kesaaakferqhmdsgnsaSSSSNYCNLMMCCRKMTQgKCKPVNTFVHESLADVKAVCS---QKKVTckdGqTnCYQSKSTMRITDCRETGSSKYPNCAYKTTQVEKHIIVACGGkpSVPVHFDA-SV-

SARST: aliSize=108 (resi) RMSD=10.00 (Å)
ns---RYTHFLTQHYD-AKPQGRDDRYCESIMRRRGLTS-PCKDINTFIHGNKRSIKAICenkNGNPHR-----ENLRISKSSFQVTACKLHGGSPWPPCQYRATAGFRNVVVACENG--LPVHL----dqsifr
--kesAAAKFERQHMDsGNSASSSSNYCNLMMCCRKMTQgKCKPVNTFVHESLADVKAVC---SQKKVTckdgqTNCYQSKSTMRITDCRETGSSKYPNCAYKTTQVEKHIIVACGGKpsVPVHFdasv------

BLAST: aliSize=61 (resi) iden=32.77% (39/119) simi=51.26% (61/119)
nsryth-------FLTQHYDA-KPQGRDDRYCESIMRRRGLTS-PCKDINTFIHGNKRSIKAICENK--NGNPHRENLRISKSSFQVTACKLHGGSPWPPCQYRATAGFRNVVVAC--ENGLPVHLDQSIfr
------kesaaakFERQHMDSgNSASSSSNYCNLMMCCRKMTQgKCKPVNTFVHESLADVKAVCSQKkvTCKDGQTNCYQSKSTMRITDCRETGSSKYPNCAYKTTQVEKHIIVACggKPSVPVHFDASV--

Proposed: aliSize=111 (resi) RMSD=1.35 (Å)
n--SRYTHFLTQHYDAKPQ-GRDDRYCESIMRRRGLTS-PCKDINTFIHGNKRSIKAICEnknGNPHR---E-N-LRISKSSFQVTACKLHG-GSpWPPCQYRATAGFRNVVVACEN--GLPVHLDQsI-fr
-keSAAAKFERQHMDSGNSaSSSSNYCNLMMCCRKMTQgKCKPVNTFVHESLADVKAVCS---QKKVTckdGqTnCYQSKSTMRITDCRETGsSK-YPNCAYKTTQVEKHIIVACGGkpSVPVHFDA-Sv--</td></tr>
<tr><td>1y92B
(124)</td></tr>
<tr><td rowspan="2">872</td><td>1un4A
(119)</td><td rowspan="2">N</td><td rowspan="2">31.93</td><td rowspan="2">TM-align: aliSize=97 (resi) RMSD=1.95 (Å)
nsrythfltqhydakpq-------------------GRDDRYCESIMRRRGLTS-PCKDINTFIHGNKRSIKAICEnknGNPHR----EN-LRISKSSFQVTACKLHGGSPWPPCQYRATAGFRNVVVACE-N-GLPVHLDQsIFr
-----------------kesaaakferqhmdsgnsaSSSSNYCNQMMCCRKMTQgKCKPVNTFVHESLADVKAVCS---QKKVTckdgQTnCYQSKSTMRITDCRETGSSKYPNCAYKTTQVEKHIIVACGgKpSVPVHFDA-SV-

SARST: aliSize=107 (resi) RMSD=10.25 (Å)
ns---RYTHFLTQHYDAKPQGRDDR-YCESIMRRRGLTS-PCKDINTFIHGNKRSIKAICenkNGNPH-----RENLRISKSSFQVTACKLHGGSPWPPCQYRATAGFRNVVVACEnGLPVHL-------dqsifr
--kesAAAKFERQHMDSGNSASSSSnYCNQMMCCRKMTQgKCKPVNTFVHESLADVKAVC---SQKKVtckdgQTNCYQSKSTMRITDCRETGSSKYPNCAYKTTQVEKHIIVACG-GKPSVPvhfdasv------

BLAST: aliSize=61 (resi) iden=32.77% (39/119) simi=51.26% (61/119)
nsryth-------FLTQHYDA-KPQGRDDRYCESIMRRRGLTS-PCKDINTFIHGNKRSIKAICENK--NGNPHRENLRISKSSFQVTACKLHGGSPWPPCQYRATAGFRNVVVAC--ENGLPVHLDQSIfr
------kesaaakFERQHMDSgNSASSSSNYCNQMMCCRKMTQgKCKPVNTFVHESLADVKAVCSQKkvTCKDGQTNCYQSKSTMRITDCRETGSSKYPNCAYKTTQVEKHIIVACggKPSVPVHFDASV--

Proposed: aliSize=112 (resi) RMSD=1.50 (Å)
n--SRYTHFLTQHYDAKPQGR-DDRYCESIMRRRGLTS-PCKDINTFIHGNKRSIKAICEnknGNPHR-----ENLRISKSSFQVTACKLHGGSPWPPCQYRATAGFRNVVVACE-N-GLPVHLDQsI-fr
-keSAAAKFERQHMDSGNSASsSSNYCNQMMCCRKMTQgKCKPVNTFVHESLADVKAVCS---QKKVTckdgqTNCYQSKSTMRITDCRETGSSKYPNCAYKTTQVEKHIIVACGgKpSVPVHFDA-Sv--</td></tr>
<tr><td>3bcoA
(124)</td></tr>
</table>

873	1un4A (119)<br>1y94A (124)	N	31.09	TM-align: aliSize=96 (resi) RMSD=1.91 (Å)<br>nsrythfltqhydakpq-------------------GRDDRYCESIMRRRGLTS-PCKDINTFIHGNKRSIKAICEnknGNPHR---E-N-LRISKSSFQVTACKLH-GGSpWPPCQYRATAGFRNVVVACE-N-GLPVHLDQsIFr<br>-----------------kesaaakferqhmdsstsaASSSNYCNLMMCCRKMTQgKCKPVNTFVHESLADVKAVCS---QKKVTckdGqTnCYQSKSTMRITDCRETgSSK-YPNCAYKTTQVEKHIIVACGgKpSVPVHFDA-SV-<br><br>SARST: aliSize=107 (resi) RMSD=10.02 (Å)<br>ns---RYTHFLTQHYD--AKPQGRDDRYCeSIMRRRGLTS-PCKDINTFIHGNKRSIKAICenkNGNPH-----RENLRISKSSFQVTACKLHGGSPWPPCQYRATAGFRNVVVACEN--GLPVHL----dqsifr<br>--kesAAAKFERQHMDssTSAASSSNYCN-LMMCCRKMTQgKCKPVNTFVHESLADVKAVC---SQKKVtckdgQTNCYQSKSTMRITDCRETGSSKYPNCAYKTTQVEKHIIVACGGkpSVPVHFdasv------<br><br>BLAST: aliSize=61 (resi) iden=32.77% (39/119) simi=51.26% (61/119)<br>nsryth-------FLTQHYDAKPQGRDD-RYCESIMRRRGLTS-PCKDINTFIHGNKRSIKAICENK--NGNPHRENLRISKSSFQVTACKLHGGSPWPPCQYRATAGFRNVVVAC--ENGLPVHLDQSIfr<br>------kesaaakFERQHMDSSTSAASSsNYCNLMMCCRKMTQgKCKPVNTFVHESLADVKAVCSQKkvTCKDGQTNCYQSKSTMRITDCRETGSSKYPNCAYKTTQVEKHIIVACggKPSVPVHFDASV--<br><br>Proposed: aliSize=111 (resi) RMSD=1.47 (Å)<br>n--SRYTHFLTQHYDAK--PQGrDDRYCESIMRRRGLTS-PCKDINTFIHGNKRSIKAICEnknGNPHR---E-N-LRISKSSFQVTACKLHGGSPWPPCQYRATAGFRNVVVACEN--GLPVHLDQsI-fr<br>-keSAAAKFERQHMDSStsAAS-SSNYCNLMMCCRKMTQgKCKPVNTFVHESLADVKAVCS---QKKVTckdGqTnCYQSKSTMRITDCRETGSSKYPNCAYKTTQVEKHIIVACGGkpSVPVHFDA-Sv--
874	1rnfA (120)<br>11bgA (124)	N	43.33	TM-align: aliSize=100 (resi) RMSD=1.52 (Å)<br>mqdgmyqrflrqhvhpeetg--------------------GSDRYCNLMMQRRKMTLYHCKRFNTFIHEDIWNIRSICSTTNIQCKNGKMNCHEG--VVKVTDCRDTGSSRAPNCRYRAIASTRRVVIACEGNPQVPVHFDG--<br>--------------------kesaaakferqhmdsgnspsSSSNYCNLMMCCRKMTQGKCKPVNTFVHESLADVKAVCSQKKVTCKNGQTNCYQSksTMRITDCRETGSSKYPNCAYKTTQVEKHIIVACGGKPSVPVHFDAsv<br><br>SARST: aliSize=112 (resi) RMSD=9.29 (Å)<br>mqdg---MYQRFLRQHVHpeeTGGSD----RYCNLMMQRRKMTLYHCKRFNTFIHEDIWNIRSICSTTNIQCKNGKMNCHE--GVVKVTDCRDTGSSRAPNCRYRAIASTRRVVIACEGNPQVPVHFD---g<br>----kesAAAKFERQHMD---SGNSPssssNYCNLMMCCRKMTQGKCKPVNTFVHESLADVKAVCSQKKVTCKNGQTNCYQskSTMRITDCRETGSSKYPNCAYKTTQVEKHIIVACGGKPSVPVHFDasv-<br><br>BLAST: aliSize=76 (resi) iden=43.33% (52/120) simi=63.33% (76/120)<br>mqdgmyq------RFLRQHVHPEET-GGSDRYCNLMMQRRKMTLYHCKRFNTFIHEDIWNIRSICSTTNIQCKNGKMNCHE--GVVKVTDCRDTGSSRAPNCRYRAIASTRRVVIACEGNPQVPVHFD---g<br>-------kesaaaKFERQHMDSGNSpSSSSNYCNLMMCCRKMTQGKCKPVNTFVHESLADVKAVCSQKKVTCKNGQTNCYQskSTMRITDCRETGSSKYPNCAYKTTQVEKHIIVACGGKPSVPVHFDasv-<br><br>Proposed: aliSize=117 (resi) RMSD=1.41 (Å)<br>mqd--GMYQRFLRQHVHPEETGGS-DRYCNLMMQRRKMTLYHCKRFNTFIHEDIWNIRSICSTTNIQCKNGKMNCHEG--VVKVTDCRDTGSSRAPNCRYRAIASTRRVVIACEGNPQVPVHFDG--<br>---keSAAAKFERQHMDSGNSPSSSsSNYCNLMMCCRKMTQGKCKPVNTFVHESLADVKAVCSQKKVTCKNGQTNCYQSksTMRITDCRETGSSKYPNCAYKTTQVEKHIIVACGGKPSVPVHFDAsv
875	1rnfA (120)<br>1y92B (124)	N	42.50	TM-align: aliSize=100 (resi) RMSD=1.34 (Å)<br>mqdgmyqrflrqhvhpeetg--------------------GSDRYCNLMMQRRKMTLYHCKRFNTFIHEDIWNIRSICSTTNIQCKNGKMNCHEG--VVKVTDCRDTGSSRAPNCRYRAIASTRRVVIACEGNPQVPVHFDG--<br>--------------------kesaaakferqhmdsgnsasSSSNYCNLMMCCRKMTQGKCKPVNTFVHESLADVKAVCSQKKVTCKDGQTNCYQSksTMRITDCRETGSSKYPNCAYKTTQVEKHIIVACGGKPSVPVHFDAsv<br><br>SARST: aliSize=114 (resi) RMSD=9.35 (Å)<br>mqdg---MYQRFLRQHVHPEEtGGSDR--YCNLMMQRRKMTLYHCKRFNTFIHEDIWNIRSICSTTNIQCKNGKMNCHE--GVVKVTDCRDTGSSRAPNCRYRAIASTRRVVIACEGNPQVPVHFD---g<br>----kesAAAKFERQHMDSGN-SASSSsnYCNLMMCCRKMTQGKCKPVNTFVHESLADVKAVCSQKKVTCKDGQTNCYQskSTMRITDCRETGSSKYPNCAYKTTQVEKHIIVACGGKPSVPVHFDasv-<br><br>BLAST: aliSize=75 (resi) iden=42.50% (51/120) simi=62.50% (75/120)<br>mqdgmyq------RFLRQHVHP-EETGGSDRYCNLMMQRRKMTLYHCKRFNTFIHEDIWNIRSICSTTNIQCKNGKMNCHE--GVVKVTDCRDTGSSRAPNCRYRAIASTRRVVIACEGNPQVPVHFD---g<br>-------kesaaaKFERQHMDSgNSASSSSNYCNLMMCCRKMTQGKCKPVNTFVHESLADVKAVCSQKKVTCKDGQTNCYQskSTMRITDCRETGSSKYPNCAYKTTQVEKHIIVACGGKPSVPVHFDasv-<br><br>Proposed: aliSize=116 (resi) RMSD=1.20 (Å)<br>mqd--GMYQRFLRQHVHPEETGGS--dRYCNLMMQRRKMTLYHCKRFNTFIHEDIWNIRSICSTTNIQCKNGKMNCHEG--VVKVTDCRDTGSSRAPNCRYRAIASTRRVVIACEGNPQVPVHFDG--<br>---keSAAAKFERQHMDSGNSASSSss-NYCNLMMCCRKMTQGKCKPVNTFVHESLADVKAVCSQKKVTCKDGQTNCYQSksTMRITDCRETGSSKYPNCAYKTTQVEKHIIVACGGKPSVPVHFDAsv

876	1rnfA (120)<br>3bcoA (124)	N	41.67	TM-align: aliSize=100 (resi) RMSD=1.55 (Å)<br>mqdgmyqrflrqhvhpeetg--------------------GSDRYCNLMMQRRKMTLYHCKRFNTFIHEDIWNIRSICSTTNIQCKNGKMNCHEG--VVKVTDCRDTGSSRAPNCRYRAIASTRRVVIACEGNPQVPVHFDG--<br>--------------------kesaaakferqhmdsgnsasSSSNYCNQMMCCRKMTQGKCKPVNTFVHESLADVKAVCSQKKVTCKDGQTNCYQSksTMRITDCRETGSSKYPNCAYKTTQVEKHIIVACGGKPSVPVHFDAsv<br><br>SARST: aliSize=113 (resi) RMSD=8.94 (Å)<br>mqdg---MYQRFLRQHVHpeE--TGGSDR-YCNLMMQRRKMTLYHCKRFNTFIHEDIWNIRSICSTTNIQCKNGKMNCHE--GVVKVTDCRDTGSSRAPNCRYRAIASTRRVVIACEGNPQVPVHFD---g<br>----kesAAAKFERQHMD--SgnSASSSSnYCNQMMCCRKMTQGKCKPVNTFVHESLADVKAVCSQKKVTCKDGQTNCYQskSTMRITDCRETGSSKYPNCAYKTTQVEKHIIVACGGKPSVPVHFDasv-<br><br>BLAST: aliSize=74 (resi) iden=41.67% (50/120) simi=61.67% (74/120)<br>mqdgmyq------RFLRQHVHP-EETGGSDRYCNLMMQRRKMTLYHCKRFNTFIHEDIWNIRSICSTTNIQCKNGKMNCHE--GVVKVTDCRDTGSSRAPNCRYRAIASTRRVVIACEGNPQVPVHFD---g<br>-------kesaaaKFERQHMDSgNSASSSSNYCNQMMCCRKMTQGKCKPVNTFVHESLADVKAVCSQKKVTCKDGQTNCYQskSTMRITDCRETGSSKYPNCAYKTTQVEKHIIVACGGKPSVPVHFDasv-<br><br>Proposed: aliSize=117 (resi) RMSD=1.40 (Å)<br>mqd--GMYQRFLRQHVHPEETGGS-DRYCNLMMQRRKMTLYHCKRFNTFIHEDIWNIRSICSTTNIQCKNGKMNCHEG--VVKVTDCRDTGSSRAPNCRYRAIASTRRVVIACEGNPQVPVHFDG--<br>---keSAAAKFERQHMDSGNSASSsSNYCNQMMCCRKMTQGKCKPVNTFVHESLADVKAVCSQKKVTCKDGQTNCYQSksTMRITDCRETGSSKYPNCAYKTTQVEKHIIVACGGKPSVPVHFDAsv
877	1rnfA (120)<br>1y94A (124)	N	42.50	TM-align: aliSize=100 (resi) RMSD=1.59 (Å)<br>mqdgmyqrflrqhvhpeetg--------------------GSDRYCNLMMQRRKMTLYHCKRFNTFIHEDIWNIRSICSTTNIQCKNGKMNCHEG--VVKVTDCRDTGSSRAPNCRYRAIASTRRVVIACEGNPQVPVHFDG--<br>--------------------kesaaakferqhmdsstsaaSSSNYCNLMMCCRKMTQGKCKPVNTFVHESLADVKAVCSQKKVTCKDGQTNCYQSksTMRITDCRETGSSKYPNCAYKTTQVEKHIIVACGGKPSVPVHFDAsv<br><br>SARST: aliSize=112 (resi) RMSD=8.88 (Å)<br>mqdg---MYQRFLRQHVH----PEETGgsdRYCNLMMQRRKMTLYHCKRFNTFIHEDIWNIRSICSTTNIQCKNGKMNCHE--GVVKVTDCRDTGSSRAPNCRYRAIASTRRVVIACEGNPQVPVHFD---g<br>----kesAAAKFERQHMDsstsAASSS---NYCNLMMCCRKMTQGKCKPVNTFVHESLADVKAVCSQKKVTCKDGQTNCYQskSTMRITDCRETGSSKYPNCAYKTTQVEKHIIVACGGKPSVPVHFDasv-<br><br>BLAST: aliSize=76 (resi) iden=42.50% (51/120) simi=63.33% (76/120)<br>mqdgmyq------RFLRQHVHPEETGGSD-RYCNLMMQRRKMTLYHCKRFNTFIHEDIWNIRSICSTTNIQCKNGKMNCHE--GVVKVTDCRDTGSSRAPNCRYRAIASTRRVVIACEGNPQVPVHFD---g<br>-------kesaaaKFERQHMDSSTSAASSsNYCNLMMCCRKMTQGKCKPVNTFVHESLADVKAVCSQKKVTCKDGQTNCYQskSTMRITDCRETGSSKYPNCAYKTTQVEKHIIVACGGKPSVPVHFDasv-<br><br>Proposed: aliSize=117 (resi) RMSD=1.41 (Å)<br>mqd--GMYQRFLRQHVHPEETGGS-DRYCNLMMQRRKMTLYHCKRFNTFIHEDIWNIRSICSTTNIQCKNGKMNCHEG--VVKVTDCRDTGSSRAPNCRYRAIASTRRVVIACEGNPQVPVHFDG--<br>---keSAAAKFERQHMDSSTSAASsSNYCNLMMCCRKMTQGKCKPVNTFVHESLADVKAVCSQKKVTCKDGQTNCYQSksTMRITDCRETGSSKYPNCAYKTTQVEKHIIVACGGKPSVPVHFDAsv
878	2j4tA (119)<br>1lbgA (124)	N	34.45	TM-align: aliSize=97 (resi) RMSD=1.62 (Å)<br>neryekflrqhydakpq------------------GRDDRYCESMMKERKLTS-PCKDVNTFIHGTKKNIRAICGkkGSPYG-----ENFRISNSPFQITTCTHSRGSPWPPCGYRAFKDFRYIVIACED--GWPVHFDEsFIsp<br>----------------kesaaakferqhmdsgnspSSSSNYCNLMMCCRKMTQgKCKPVNTFVHESLADVKAVCS--QKKVTcknggTNCYQSKSTMRITDCRETGSSKYPNCAYKTTQVEKHIIVACGGkpSVPVHFDA-SV--<br><br>SARST: aliSize=108 (resi) RMSD=9.51 (Å)<br>ne---RYEKFLRQHYDAKPQGRDDR-YCESMMKERKLTS-PCKDVNTFIHGTKKNIRAICgkKGSPY-----GENFRISNSPFQITTCTHSRGSPWPPCGYRAFKDFRYIVIACED--GWPVHF----desfisp<br>--kesAAAKFERQHMDSGNSPSSSSnYCNLMMCCRKMTQgKCKPVNTFVHESLADVKAVC--SQKKVtckngQTNCYQSKSTMRITDCRETGSSKYPNCAYKTTQVEKHIIVACGGkpSVPVHFdasv-------<br><br>BLAST: aliSize=65 (resi) iden=36.97% (44/119) simi=54.62% (65/119)<br>nerye------KFLRQHYDA--KPQGRDDrYCESMMKERKLTS-PCKDVNTFIHGTKKNIRAICGKKG--SPYGE-NFRISNSPFQITTCTHSRGSPWPPCGYRAFKDFRYIVIAC--EDGWPVHFDES-fisp<br>-----kesaaaKFERQHMDSgnSPSSSSN-YCNLMMCCRKMTQgKCKPVNTFVHESLADVKAVCSQKKvtCKNGQtNCYQSKSTMRITDCRETGSSKYPNCAYKTTQVEKHIIVACggKPSVPVHFDASv----<br><br>Proposed: aliSize=112 (resi) RMSD=1.29 (Å)<br>n--ERYEKFLRQHYDAK--PQGrDDRYCESMMKERKLTS-PCKDVNTFIHGTKKNIRAICGkkGSPYG-----ENFRISNSPFQITTCTHSRGSPWPPCGYRAFKDFRYIVIACED--GWPVHFDEsFIsp<br>-keSAAAKFERQHMDSGnsPSS-SSNYCNLMMCCRKMTQgKCKPVNTFVHESLADVKAVCS--QKKVTcknggTNCYQSKSTMRITDCRETGSSKYPNCAYKTTQVEKHIIVACGGkpSVPVHFDA-SV--

879	2j4tA (119)<br>1y92B (124)	N	33.61	TM-align: aliSize=97 (resi) RMSD=1.67 (Å)<br>neryekflrqhydakpq------------------GRDDRYCESMMKERRKLTS-PCKDVNTFIHGTKKNIRAICGkkGSPYG-----ENFRISNSPFQITTCTHSRGSPWPPCGYRAFKDFRYIVIACED--GWPVHFDEsFIsp<br>-----------------kesaaakferqhmdsgnsaSSSSNYCNLMMCCRKMTQgKCKPVNTFVHESLADVKAVCS--QKKVTckdgqTNCYQSKSTMRITDCRETGSSKYPNCAYKTTQVEKHIIVACGGkpSVPVHFDA-SV--<br><br>SARST: aliSize=108 (resi) RMSD=9.41 (Å)<br>ne---RYEKFLRQHYDAKPQGRDDR-YCESMMKERRKLTS-PCKDVNTFIHGTKKNIRAICgkKGSPY-----GENFRISNSPFQITTCTHSRGSPWPPCGYRAFKDFRYIVIACED--GWPVHF----desfisp<br>--kesAAAKFERQHMDSGNSASSSSnYCNLMMCCRKMTQgKCKPVNTFVHESLADVKAVC--SQKKVtckdgQTNCYQSKSTMRITDCRETGSSKYPNCAYKTTQVEKHIIVACGGkpSVPVHFdasv-------<br><br>BLAST: aliSize=63 (resi) iden=36.13% (43/119) simi=52.94% (63/119)<br>nerye------KFLRQHYDA-KPQGRDDRYCESMMKERRKLTS-PCKDVNTFIHGTKKNIRAICGKKGSPY--GE-NFRISNSPFQITTCTHSRGSPWPPCGYRAFKDFRYIVIAC--EDGWPVHFDES-fisp<br>-----kesaaaKFERQHMDSgNSASSSSNYCNLMMCCRKMTQgKCKPVNTFVHESLADVKAVCSQKKVTCkdGQtNCYQSKSTMRITDCRETGSSKYPNCAYKTTQVEKHIIVACggKPSVPVHFDASv----<br><br>Proposed: aliSize=113 (resi) RMSD=1.40 (Å)<br>n--ERYEKFLRQHYDAKPQ-GRDDRYCESMMKERRKLTS-PCKDVNTFIHGTKKNIRAICGkkGSPYG-----ENFRISNSPFQITTCTHSRGSPWPPCGYRAFKDFRYIVIACED--GWPVHFDEsFIsp<br>-keSAAAKFERQHMDSGNSaSSSSNYCNLMMCCRKMTQgKCKPVNTFVHESLADVKAVCS--QKKVTckdgqTNCYQSKSTMRITDCRETGSSKYPNCAYKTTQVEKHIIVACGGkpSVPVHFDA-SV--
880	2j4tA (119)<br>3bcoA (124)	N	33.61	TM-align: aliSize=97 (resi) RMSD=1.69 (Å)<br>neryekflrqhydakpq------------------GRDDRYCESMMKERRKLTS-PCKDVNTFIHGTKKNIRAICGkkGSPYG-----ENFRISNSPFQITTCTHSRGSPWPPCGYRAFKDFRYIVIACE-D-GWPVHFDEsFIsp<br>-----------------kesaaakferqhmdsgnsaSSSSNYCNQMMCCRKMTQgKCKPVNTFVHESLADVKAVCS--QKKVTckdgqTNCYQSKSTMRITDCRETGSSKYPNCAYKTTQVEKHIIVACGgKpSVPVHFDA-SV--<br><br>SARST: aliSize=109 (resi) RMSD=9.91 (Å)<br>ne---RYEKFLRQHYDAKPQGRDDR-YCESMMKERRKLTS-PCKDVNTFIHGTK-KNIRAICGKKGSP--YGENFRISNSPFQITTCTHSRGSPWPPCGYRAFKDFRYIVIACEdGWPVHF-------desfisp<br>--kesAAAKFERQHMDSGNSASSSSnYCNQMMCCRKMTQgKCKPVNTFVHESLaDVKAVCSQKKVTCkdGQTNCYQSKSTMRITDCRETGSSKYPNCAYKTTQVEKHIIVACG-GKPSVPvhfdasv-------<br><br>BLAST: aliSize=63 (resi) iden=36.13% (43/119) simi=52.94% (63/119)<br>nerye------KFLRQHYDA-KPQGRDDRYCESMMKERRKLTS-PCKDVNTFIHGTKKNIRAICGKKGSPY--GE-NFRISNSPFQITTCTHSRGSPWPPCGYRAFKDFRYIVIAC--EDGWPVHFDES-fisp<br>-----kesaaaKFERQHMDSgNSASSSSNYCNQMMCCRKMTQgKCKPVNTFVHESLADVKAVCSQKKVTCkdGQtNCYQSKSTMRITDCRETGSSKYPNCAYKTTQVEKHIIVACggKPSVPVHFDASv----<br><br>Proposed: aliSize=113 (resi) RMSD=1.43 (Å)<br>n--ERYEKFLRQHYDAKPQGR-DDRYCESMMKERRKLTS-PCKDVNTFIHGTKKNIRAICGkkGSPYG-----ENFRISNSPFQITTCTHSRGSPWPPCGYRAFKDFRYIVIACED--GWPVHFDEsFIsp<br>-keSAAAKFERQHMDSGNSASSsSNYCNQMMCCRKMTQgKCKPVNTFVHESLADVKAVCS--QKKVTckdgqTNCYQSKSTMRITDCRETGSSKYPNCAYKTTQVEKHIIVACGGkpSVPVHFDA-SV--
881	2j4tA (119)<br>1y94A (124)	N	33.61	TM-align: aliSize=97 (resi) RMSD=1.74 (Å)<br>neryekflrqhydakpq------------------GRDDRYCESMMKERRKLTS-PCKDVNTFIHGTKKNIRAICGkkGSPYG-----ENFRISNSPFQITTCTHSRGSPWPPCGYRAFKDFRYIVIACED--GWPVHFDEsFIsp<br>-----------------kesaaakferqhmdsstsaASSSNYCNLMMCCRKMTQgKCKPVNTFVHESLADVKAVCS--QKKVTckdgqTNCYQSKSTMRITDCRETGSSKYPNCAYKTTQVEKHIIVACGGkpSVPVHFDA-SV--<br><br>SARST: aliSize=108 (resi) RMSD=9.40 (Å)<br>ne---RYEKFLRQHYDAKPQGRDDR-YCESMMKERRKLTS-PCKDVNTFIHGTKKNIRAICgkKGSPY-----GENFRISNSPFQITTCTHSRGSPWPPCGYRAFKDFRYIVIACED--GWPVHF----desfisp<br>--kesAAAKFERQHMDSSTSAASSSnYCNLMMCCRKMTQgKCKPVNTFVHESLADVKAVC--SQKKVtckdgQTNCYQSKSTMRITDCRETGSSKYPNCAYKTTQVEKHIIVACGGkpSVPVHFdasv-------<br><br>BLAST: aliSize=63 (resi) iden=36.13% (43/119) simi=52.94% (63/119)<br>nerye------KFLRQHYDAKPQGRDD-RYCESMMKERRKLTS-PCKDVNTFIHGTKKNIRAICGKKGSPY--GE-NFRISNSPFQITTCTHSRGSPWPPCGYRAFKDFRYIVIAC--EDGWPVHFDES-fisp<br>-----kesaaaKFERQHMDSSTSAASSsNYCNLMMCCRKMTQgKCKPVNTFVHESLADVKAVCSQKKVTCkdGQtNCYQSKSTMRITDCRETGSSKYPNCAYKTTQVEKHIIVACggKPSVPVHFDASv----<br><br>Proposed: aliSize=112 (resi) RMSD=1.34 (Å)<br>n--ERYEKFLRQHYDAK--PQGrDDRYCESMMKERRKLTS-PCKDVNTFIHGTKKNIRAICGkkGSPYG-----ENFRISNSPFQITTCTHSRGSPWPPCGYRAFKDFRYIVIACED--GWPVHFDEsFIsp<br>-keSAAAKFERQHMDSStsAAS-SSNYCNLMMCCRKMTQgKCKPVNTFVHESLADVKAVCS--QKKVTckdgqTNCYQSKSTMRITDCRETGSSKYPNCAYKTTQVEKHIIVACGGkpSVPVHFDA-SV--

882	1b1eA (123)<br>11bgA (124)	N	30.89	TM-align: aliSize=97 (resi) RMSD=1.87 (Å)<br>qdnsrythfltqhydakpq------------------GRDDRYCESIMRRRGLTS-PCQDINTFIHGNKRSIKAICEnknGNPHR----EN-LRISKSSFQVTTCKLHGGSPWPPCQYRATAGFRNVVVACE-N-GLPVHLDQsIFrrp<br>-------------------kesaaakferqhmdsgnspSSSSNYCNLMMCCRKMTQgKCKPVNTFVHESLADVKAVCS---QKKVTckngQTnCYQSKSTMRITDCRETGSSKYPNCAYKTTQVEKHIIVACGgKpSVPVHFDA-SV---<br><br>SARST: aliSize=108 (resi) RMSD=9.99 (Å)<br>qdns---RYTHFLTQHYDAKPQGRDDR-YCESIMRRRGLT-SPCQDINTFIHGNKRSIKAICenkNGNPH-----RENLRISKSSFQVTTCKLHGGS--PWPPCQYRATAGFRNVVVACENGLPVHL----dqsifrrp<br>----kesAAAKFERQHMDSGNSPSSSSnYCNLMMCCRKMTqGKCKPVNTFVHESLADVKAVC---SQKKVtckngQTNCYQSKSTMRITDCRETGSSkyPNCAYKTTQVEKHIIVACGGKPSVPVHFdasv--------<br><br>BLAST: aliSize=65 (resi) iden=33.33% (41/123) simi=52.85% (65/123)<br>qdnsryth-------FLTQHYDA--KPQGRDDrYCESIMRRRGLTS-PCQDINTFIHGNKRSIKAICEN-----KNGnphRENLRISKSSFQVTTCKLHGGSPWPPCQYRATAGFRNVVVAC--ENGLPVHLDQSIfrrp<br>--------kesaaakFERQHMDSgnSPSSSSN-YCNLMMCCRKMTQgKCKPVNTFVHESLADVKAVCSQkkvtcKNG---QTNCYQSKSTMRITDCRETGSSKYPNCAYKTTQVEKHIIVACggKPSVPVHFDASV----<br><br>Proposed: aliSize=111 (resi) RMSD=1.34 (Å)<br>qdn--SRYTHFLTQHYDAK--PQGrDDRYCESIMRRRGLTS-PCQDINTFIHGNKRSIKAICEnknGNPHR-----ENLRISKSSFQVTTCKLHGGSPWPPCQYRATAGFRNVVVACE-N-GLPVHLDQsI-frrp<br>---keSAAAKFERQHMDSGnsPSS-SSNYCNLMMCCRKMTQgKCKPVNTFVHESLADVKAVCS---QKKVTckngqTNCYQSKSTMRITDCRETGSSKYPNCAYKTTQVEKHIIVACGgKpSVPVHFDA-Sv----
883	1b1eA (123)<br>1y92B (124)	N	28.46	TM-align: aliSize=97 (resi) RMSD=1.95 (Å)<br>qdnsrythfltqhydakpq------------------GRDDRYCESIMRRRGLTS-PCQDINTFIHGNKRSIKAICEnknGNPHR---E-N-LRISKSSFQVTTCKLHGGSPWPPCQYRATAGFRNVVVACEN--GLPVHLDQsIFrrp<br>-------------------kesaaakferqhmdsgnsaSSSSNYCNLMMCCRKMTQgKCKPVNTFVHESLADVKAVCS---QKKVTckdGqTnCYQSKSTMRITDCRETGSSKYPNCAYKTTQVEKHIIVACGGkpSVPVHFDA-SV---<br><br>SARST: aliSize=108 (resi) RMSD=9.31 (Å)<br>qdns---RYTHFLTQHYD-AKPQGRDDRYCESIMRRRGLT-SPCQDINTFIHGNKRSIKAICenkNGNPH-----RENLRISKSSFQVTTCKLHGGSPWPPCQYRATAGFRNVVVACEN--GLPVHL----dqsifrrp<br>----kesAAAKFERQHMDsGNSASSSSNYCNLMMCCRKMTqGKCKPVNTFVHESLADVKAVC---SQKKVtckdgQTNCYQSKSTMRITDCRETGSSKYPNCAYKTTQVEKHIIVACGGKPSVPVHFdasv--------<br><br>BLAST: aliSize=61 (resi) iden=30.89% (38/123) simi=49.59% (61/123)<br>qdnsryth-------FLTQHYDA-KPQGRDDRYCESIMRRRGLTS-PCQDINTFIHGNKRSIKAICENK--NGNPHRENLRISKSSFQVTTCKLHGGSPWPPCQYRATAGFRNVVVAC--ENGLPVHLDQSIfrrp<br>--------kesaaakFERQHMDSgNSASSSSNYCNLMMCCRKMTQgKCKPVNTFVHESLADVKAVCSQKkvTCKDGQTNCYQSKSTMRITDCRETGSSKYPNCAYKTTQVEKHIIVACggKPSVPVHFDASV----<br><br>Proposed: aliSize=112 (resi) RMSD=1.46 (Å)<br>Q-dnSRYTHFLTQHYDAKPQ-GRDDRYCESIMRRRGLTS-PCQDINTFIHGNKRSIKAICEnknGNPHR---E-N-LRISKSSFQVTTCKLHG-GSpWPPCQYRATAGFRNVVVACEN--GLPVHLDQsI-frrp<br>Ke--SAAAKFERQHMDSGNSaSSSSNYCNLMMCCRKMTQgKCKPVNTFVHESLADVKAVCS---QKKVTckdGqTnCYQSKSTMRITDCRETGsSK-YPNCAYKTTQVEKHIIVACGGkpSVPVHFDA-Sv----
884	1b1eA (123)<br>3bcoA (124)	N	28.46	TM-align: aliSize=97 (resi) RMSD=1.95 (Å)<br>qdnsrythfltqhydakpq------------------GRDDRYCESIMRRRGLTS-PCQDINTFIHGNKRSIKAICEnknGNPHR----EN-LRISKSSFQVTTCKLHGGSPWPPCQYRATAGFRNVVVACE-N-GLPVHLDQsIFrrp<br>-------------------kesaaakferqhmdsgnsaSSSSNYCNQMMCCRKMTQgKCKPVNTFVHESLADVKAVCS---QKKVTckdgQTnCYQSKSTMRITDCRETGSSKYPNCAYKTTQVEKHIIVACGgKpSVPVHFDA-SV---<br><br>SARST: aliSize=108 (resi) RMSD=9.41 (Å)<br>qdns---RYTHFLTQHYDAKPQGRDDR-YCESIMRRRGLT-SPCQDINTFIHGNKRSIKAICenkNGNPH-----RENLRISKSSFQVTTCKLHGGSPWPPCQYRATAGFRNVVVACEN--GLPVHL----dqsifrrp<br>----kesAAAKFERQHMDSGNSASSSSnYCNQMMCCRKMTqGKCKPVNTFVHESLADVKAVC---SQKKVtckdgQTNCYQSKSTMRITDCRETGSSKYPNCAYKTTQVEKHIIVACGGKPSVPVHFdasv--------<br><br>BLAST: aliSize=61 (resi) iden=30.89% (38/123) simi=49.59% (61/123)<br>qdnsryth-------FLTQHYDA-KPQGRDDRYCESIMRRRGLTS-PCQDINTFIHGNKRSIKAICENK--NGNPHRENLRISKSSFQVTTCKLHGGSPWPPCQYRATAGFRNVVVAC--ENGLPVHLDQSIfrrp<br>--------kesaaakFERQHMDSgNSASSSSNYCNQMMCCRKMTQgKCKPVNTFVHESLADVKAVCSQKkvTCKDGQTNCYQSKSTMRITDCRETGSSKYPNCAYKTTQVEKHIIVACggKPSVPVHFDASV----<br><br>Proposed: aliSize=111 (resi) RMSD=1.39 (Å)<br>Q-dnSRYTHFLTQHYDAK--PQGrDDRYCESIMRRRGLTS-PCQDINTFIHGNKRSIKAICEnknGNPHR-----ENLRISKSSFQVTTCKLH-GGSpWPPCQYRATAGFRNVVVACE-N-GLPVHLDQsI-frrp<br>Ke--SAAAKFERQHMDSGnsASS-SSNYCNQMMCCRKMTQgKCKPVNTFVHESLADVKAVCS---QKKVTckdgqTNCYQSKSTMRITDCRETgSSK-YPNCAYKTTQVEKHIIVACGgKpSVPVHFDA-Sv----

<table>
<tr><td rowspan="2">885</td><td>1b1eA
(123)</td><td rowspan="2">N</td><td rowspan="2">28.46</td><td rowspan="2">TM-align: aliSize=96 (resi) RMSD=1.89 (Å)
qdnsrythfltqhydakpq-------------------GRDDRYCESIMRRRGLTS-PCQDINTFIHGNKRSIKAICEnknGNPHR----EN-LRISKSSFQVTTCKLH-GGSpWPPCQYRATAGFRNVVVACE-N-GLPVHLDQsIFrrp
-------------------kesaaakferqhmdsstsaASSSNYCNLMMCCRKMTQgKCKPVNTFVHESLADVKAVCS---QKKVTckdgQTnCYQSKSTMRITDCRETgSSK-YPNCAYKTTQVEKHIIVACGgKpSVPVHFDA-SV---

SARST: aliSize=107 (resi) RMSD=9.23 (Å)
qdns---RYTHFLTQHYD--AKPQGRdDRYCESIMRRRGLT-SPCQDINTFIHGNKRSIKAICenkNGNPH-----RENLRISKSSFQVTTCKLHGGSPWPPCQYRATAGFRNVVVACEN--GLPVHL----dqsifrrp
----kesAAAKFERQHMDssTSAASS-SNYCNLMMCCRKMTqGKCKPVNTFVHESLADVKAVC---SQKKVtckdgQTNCYQSKSTMRITDCRETGSSKYPNCAYKTTQVEKHIIVACGGkpSVPVHFdasv--------

BLAST: aliSize=61 (resi) iden=30.89% (38/123) simi=49.59% (61/123)
qdnsryth-------FLTQHYDAKPQGRDD-RYCESIMRRRGLTS-PCQDINTFIHGNKRSIKAICENK--NGNPHRENLRISKSSFQVTTCKLHGGSPWPPCQYRATAGFRNVVVAC--ENGLPVHLDQSIfrrp
--------kesaaakFERQHMDSSTSAASSsNYCNLMMCCRKMTQgKCKPVNTFVHESLADVKAVCSQKkvTCKDGQTNCYQSKSTMRITDCRETGSSKYPNCAYKTTQVEKHIIVACggKPSVPVHFDASV----

Proposed: aliSize=110 (resi) RMSD=1.37 (Å)
Q-dnSRYTHFLTQHYDAK--PQG-rdDRYCESIMRRRGLTS-PCQDINTFIHGNKRSIKAICEnknGNPHR-----ENLRISKSSFQVTTCKLH-GGSpWPPCQYRATAGFRNVVVACE-N-GLPVHLDQsI-frrp
Ke--SAAAKFERQHMDSStsAASs--SNYCNLMMCCRKMTQgKCKPVNTFVHESLADVKAVCS---QKKVTckdgqTNCYQSKSTMRITDCRETgSSK-YPNCAYKTTQVEKHIIVACGgKpSVPVHFDA-Sv----</td></tr>
<tr><td>1y94A
(124)</td></tr>
<tr><td rowspan="2">886</td><td>2hkyA
(129)</td><td rowspan="2">N</td><td rowspan="2">35.48</td><td rowspan="2">TM-align: aliSize=97 (resi) RMSD=2.07 (Å)
mkpkgmtssqwfkiqhmqpsp-----------------------QACNSAMK--NINKhtKRCKDLNTFLHEPFSSVAATCQTPKIAC-KNgDKNCHQSHGPVSLTMCKLT-SGKYPNCRYKEKRQNKSYVVACKPPqkkdsqqfHLVPVHLDRVL
---------------------kesaaakferqhmdsgnspssssNYCNLMMCcrKMTQ--GKCKPVNTFVHESLADVKAVCSQKKVTCkNG-QTNCYQSKSTMRITDCRETgSSKYPNCAYKTTQVEKHIIVACGGK--------PSVPVHFDASV

SARST: aliSize=105 (resi) RMSD=10.27 (Å)
mkpkg-MTSSQWFKIQHMQ--PSPQACNSAMKNINKHTK----RCKDLNTFLHEPFSSVAATCQTPKIACKNGDKNCHQSHGPVSLTMCKL-TSGKYPNCRYKEKRQNKSYVVACKPP----------qkkdsqqfhlvpvhldrvl
-----kESAAAKFERQHMDsgNSPSSSSNYCNLMMCCRKmtqgKCKPVNTFVHESLADVKAVCSQKKVTCKNGQTNCYQSKSTMRITDCREtGSSKYPNCAYKTTQVEKHIIVACGGKpsvpvhfdasv------------------

BLAST: aliSize=66 (resi) iden=41.94% (52/124) simi=53.23% (66/124)
mkpkgmtssqw-------FKIQHMQP--SPQA----CNsAMKNINKHTK-RCKDLNTFLHEPFSSVAATCQTPKIACKNGDKNCHQSHGPVSLTMCKLT-SGKYPNCRYKEKRQNKSYVVACkppQKKDSqqfhlVPVHLD---rvl
-----------kesaaakFERQHMDSgnSPSSssnyCN-LMMCCRKMTQgKCKPVNTFVHESLADVKAVCSQKKVTCKNGQTNCYQSKSTMRITDCRETgSSKYPNCAYKTTQVEKHIIVAC---GGKPS-----VPVHFDasv---

Proposed: aliSize=113 (resi) RMSD=1.91 (Å)
mkpKgMTSSQWFKIQHMQPS--pQ-----ACNSAMK--NINKhtKRCKDLNTFLHEPFSSVAATCQTPKIAC-KNgDKNCHQSHGPVSLTMCKLT-SGKYPNCRYKEKRQNKSYVVACKPPqkkdsqqfHLVPVHLDRVL
---K-ESAAAKFERQHMDSGns-PssssnYCNLMMCcrKMTQ--GKCKPVNTFVHESLADVKAVCSQKKVTCkNG-QTNCYQSKSTMRITDCRETgSSKYPNCAYKTTQVEKHIIVACGGK--------PSVPVHFDASV</td></tr>
<tr><td>1lbgA
(124)</td></tr>
<tr><td rowspan="2">887</td><td>2hkyA
(129)</td><td rowspan="2">N</td><td rowspan="2">35.48</td><td rowspan="2">TM-align: aliSize=97 (resi) RMSD=1.89 (Å)
mkpkgmtssqwfkiqhmqpsp-----------------------QACNSAMK--NINKhtKRCKDLNTFLHEPFSSVAATCQTPKIAC-KNgDKNCHQSHGPVSLTMCKLT-SGKYPNCRYKEKRQNKSYVVACKPPqkkdsqqfHLVPVHLDRVL
---------------------kesaaakferqhmdsgnsassssNYCNLMMCcrKMTQ--GKCKPVNTFVHESLADVKAVCSQKKVTCkDG-QTNCYQSKSTMRITDCRETgSSKYPNCAYKTTQVEKHIIVACGGK--------PSVPVHFDASV

SARST: aliSize=105 (resi) RMSD=10.34 (Å)
mkpkg-MTSSQWFKIQHMQP--SPQACNSAMKNINKHTKRCKDL----NTFLHEPFSSVAATCQTPKIACKNGDKNCHQSHGPVSLTMCKL-TSGKYPNCRYKEKRQNKSYVVACKPP----------qkkdsqqfhlvpvhldrvl
-----kESAAAKFERQHMDSgnSASSSSNYCNLMMCCRKMTQGKckpvNTFVHESLADVKAVCSQKKVTCKDGQTNCYQSKSTMRITDCREtGSSKYPNCAYKTTQVEKHIIVACGGKpsvpvhfdasv------------------

BLAST: aliSize=65 (resi) iden=39.52% (49/124) simi=52.42% (65/124)
mkpkgmtssqw-------FKIQHMQPSPQACNSA-----MKNINKHTK-RCKDLNTFLHEPFSSVAATCQTPKIACKNGDKNCHQSHGPVSLTMCKLT-SGKYPNCRYKEKRQNKSYVVACkppQKKDSqqfhlVPVHLD---rvl
-----------kesaaakFERQHMDSGNSASSSSnycnlMMCCRKMTQgKCKPVNTFVHESLADVKAVCSQKKVTCKDGQTNCYQSKSTMRITDCRETgSSKYPNCAYKTTQVEKHIIVAC---GGKPS-----VPVHFDasv---

Proposed: aliSize=113 (resi) RMSD=1.68 (Å)
mkpKgMTSSQWFKIQHMQPSpQ-------ACNSAMK--NINKhtKRCKDLNTFLHEPFSSVAATCQTPKIAC-KNgDKNCHQSHGPVSLTMCKLT-SGKYPNCRYKEKRQNKSYVVACKPPqkkdsqqfHLVPVHLDRVL
---K-ESAAAKFERQHMDSG-NsassssnYCNLMMCcrKMTQ--GKCKPVNTFVHESLADVKAVCSQKKVTCkDG-QTNCYQSKSTMRITDCRETgSSKYPNCAYKTTQVEKHIIVACGGK--------PSVPVHFDASV</td></tr>
<tr><td>1y92B
(124)</td></tr>
</table>

888	2hkyA (129)<br>3bcoA (124)	N	35.48	TM-align: aliSize=97 (resi) RMSD=2.01 (Å)<br>mkpkgmtssqwfkiqhmqpsp-----------------------QACNSAMK--NINKhtKRCKDLNTFLHEPFSSVAATCQTPKIAC-KNgDKNCHQSHGPVSLTMCKLT-SGKYPNCRYKEKRQNKSYVVACKPPqkkdsqqfHLVPVHLDRVL<br>---------------------kesaaakferqhmdsgnsassssNYCNQMMCcrKMTQ--GKCKPVNTFVHESLADVKAVCSQKKVTCkDG-QTNCYQSKSTMRITDCRETgSSKYPNCAYKTTQVEKHIIVACGGK--------PSVPVHFDASV<br><br>SARST: aliSize=103 (resi) RMSD=10.12 (Å)<br>mkpkgm--TSSQWFKIQHMQPSPQ--ACNSAMKNINKHTK----RCKDLNTFLHEPFSSVAATCQTPKIACKNGDKNCHQSHGPVSLTMCKL--TSGKyPNCRYKEKRQNKSYVVACKPP-----------qkkdsqqfhlvpvhldrvl<br>------keSAAAKFERQHMDSGNSasSSSNYCNQMMCCRKmtqgKCKPVNTFVHESLADVKAVCSQKKVTCKDGQTNCYQSKSTMRITDCREtgSSKY-PNCAYKTTQVEKHIIVACGGKpsvpvhfdasv-------------------<br><br>BLAST: aliSize=65 (resi) iden=39.52% (49/124) simi=52.42% (65/124)<br>mkpkgmtssqw-------FKIQHMQPSPQACNSA-----MKNINKHTK-RCKDLNTFLHEPFSSVAATCQTPKIACKNGDKNCHQSHGPVSLTMCKLT-SGKYPNCRYKEKRQNKSYVVACkppQKKDSqqfhlVPVHLD---rvl<br>-----------kesaaakFERQHMDSGNSASSSSnycnqMMCCRKMTQgKCKPVNTFVHESLADVKAVCSQKKVTCKDGQTNCYQSKSTMRITDCRETgSSKYPNCAYKTTQVEKHIIVAC---GGKPS-----VPVHFDasv---<br><br>Proposed: aliSize=113 (resi) RMSD=1.86 (Å)<br>mkpKgMTSSQWFKIQHMQPS-------pQACNSAMK--NINKhtKRCKDLNTFLHEPFSSVAATCQTPKIAC-KNgDKNCHQSHGPVSLTMCKLT-SGKYPNCRYKEKRQNKSYVVACKPPqkkdsqqfHLVPVHLDRVL<br>---K-ESAAAKFERQHMDSGnsassss-NYCNQMMCcrKMTQ--GKCKPVNTFVHESLADVKAVCSQKKVTCkDG-QTNCYQSKSTMRITDCRETgSSKYPNCAYKTTQVEKHIIVACGGK--------PSVPVHFDASV
889	2hkyA (129)<br>1y94A (124)	N	36.29	TM-align: aliSize=97 (resi) RMSD=1.97 (Å)<br>mkpkgmtssqwfkiqhmqpsp-----------------------QACNSAMK--NINKhtKRCKDLNTFLHEPFSSVAATCQTPKIAC-KNgDKNCHQSHGPVSLTMCKLT-SGKYPNCRYKEKRQNKSYVVACKPPqkkdsqqfHLVPVHLDRVL<br>---------------------kesaaakferqhmdsstsaasssNYCNLMMCcrKMTQ--GKCKPVNTFVHESLADVKAVCSQKKVTCkDG-QTNCYQSKSTMRITDCRETgSSKYPNCAYKTTQVEKHIIVACGGK--------PSVPVHFDASV<br><br>SARST: aliSize=94 (resi) RMSD=2.96 (Å)<br>mkpkgmtssqwfkiqhmqpspqa-------------------------CNSAMKNINKHTKRCKDLNTFLHEPFSSVAATCQTPKIACKNGDKNCHQSHGPVSLTMCKLtsgKY----PNCRYKEKRQNKSYVVACKppqkkdsqQFHLVPVHLDRV-l<br>-----------------------kesaaakferqhmdsstsaasssnyCNLMMCCRKMTQGKCKPVNTFVHESLADVKAVCSQKKVTCKDGQTNCYQSKSTMRITDCRE---TGsskyPNCAYKTTQVEKHIIVACG--------GKPSVPVHFDASv-<br><br>BLAST: aliSize=66 (resi) iden=40.32% (50/124) simi=53.23% (66/124)<br>mkpkgmtssqw-------FKIQHMQPSPQACNSA-----MKNINKHTK-RCKDLNTFLHEPFSSVAATCQTPKIACKNGDKNCHQSHGPVSLTMCKLT-SGKYPNCRYKEKRQNKSYVVACkppQKKDSqqfhlVPVHLD---rvl<br>-----------kesaaakFERQHMDSSTSAASSSnycnlMMCCRKMTQgKCKPVNTFVHESLADVKAVCSQKKVTCKDGQTNCYQSKSTMRITDCRETgSSKYPNCAYKTTQVEKHIIVAC---GGKPS-----VPVHFDasv---<br><br>Proposed: aliSize=113 (resi) RMSD=1.82 (Å)<br>mkpKgMTSSQWFKIQHMQPS--pQ-----ACNSAMK--NINKhtKRCKDLNTFLHEPFSSVAATCQTPKIAC-KNgDKNCHQSHGPVSLTMCKLT-SGKYPNCRYKEKRQNKSYVVACKPPqkkdsqqfHLVPVHLDRVL<br>---K-ESAAAKFERQHMDSSts-AasssnYCNLMMCcrKMTQ--GKCKPVNTFVHESLADVKAVCSQKKVTCkDG-QTNCYQSKSTMRITDCRETgSSKYPNCAYKTTQVEKHIIVACGGK--------PSVPVHFDASV
890	1m58A (106)<br>1lbgA (124)	N	23.58	TM-align: aliSize=84 (resi) RMSD=2.47 (Å)<br>mqnwetfqkkhltdtr---------------------D-VKCDAEMKKALFD---CKQKNTFIYARPGRVQALCkNIiv---------SKNVLSTDEFYLSDCNRI-KL---PCHYKLKKSSNTICITCE-N-KLPVHFVAVEecp<br>----------------kesaaakferqhmdsgnspssSsNYCNLMMCCRKMTqgkCKPVNTFVHESLADVKAVC-SQ--kkvtckngqTNCYQSKSTMRITDCRETgSSkypNCAYKTTQVEKHIIVACGgKpSVPVHFDASV---<br><br>SARST: aliSize=98 (resi) RMSD=10.52 (Å)<br>mqn----WETFQKKHL--TDTRDV--KCDAEMKkALFDC-----KQKNTFIYARPGRVQALCKNIIVSK------NVLSTDEFYLSDCNR---IKLPCHYK---LKKSSNTICITCENKLPVHFVAV-eecp<br>---kesaAAKFERQHMdsGNSPSSssNYCNLMM-CCRKMtqgkcKPVNTFVHESLADVKAVCSQKKVTCkngqtnCYQSKSTMRITDCREtgsSKYPNCAYkttQVEKHIIVACGGKPSVPVHFDASv----<br><br>BLAST: aliSize=37 (resi) iden=24.53% (26/106) simi=34.91% (37/106)<br>mqnwetfqkkhltdtrdvkcdaemkkalfd---------------------------------------CKQKNTFIYARPGRVQALC-KNIIVSKN-----VLSTDEFYLSDCNRI---KLP-CHYKLKKSSNTICITCENK--LPVHFVA--veecp<br>------------------------------kesaaakferqhmdsgnspssssnycnlmmccrkmtqgkCKPVNTFVHESLADVKAVCsQKKVTCKNgqtncYQSKSTMRITDCRETgssKYPnCAYKTTQVEKHIIVACGGKpsVPVHFDAsv-----<br><br>Proposed: aliSize=97 (resi) RMSD=1.97 (Å)<br>m--QNWETFQKKHLTDTRDV-----KCDAEMKK-ALFD--CKQKNTFIYARPGRVQALCkN-----------iivsKNVLSTDEFYLSDCNRI-KLP---CHYKLKKSSNTICITCE-N-KLPVHFVAVEecp<br>-keSAAAKFERQHMDSGNSPssssnYCNLMMCCrKMTQgkCKPVNTFVHESLADVKAVC-Sqkkvtckngqt----NCYQSKSTMRITDCRETgSSKypnCAYKTTQVEKHIIVACGgKpSVPVHFDASV---

891	1m58A (106)<br>1y92B (124)	N	23.58	TM-align: aliSize=84 (resi) RMSD=2.31 (Å)<br>mqnwetfqkkhltdtr---------------------D-VKCDAEMKKALFD---CKQKNTFIYARPGRVQALCkNIiv---------SKNVLSTDEFYLSDCNRIK-LP---CHYKLKKSSNTICITCE-N-KLPVHFVAVEecp<br>----------------kesaaakferqhmdsgnsassSsNYCNLMMCCRKMTqgkCKPVNTFVHESLADVKAVC-SQ--kkvtckdgqTNCYQSKSTMRITDCRETGsSKypnCAYKTTQVEKHIIVACGgKpSVPVHFDASV---<br><br>SARST: aliSize=82 (resi) RMSD=4.57 (Å)<br>mqnwetfqkkhltdtrdvk---------------------------CDAEMKKALFD-CKQKNTFIYARPGRVQALCKNIIVSK------NVLSTDEFYLSDCNRI---KLPCHYKLKK---SSNTICITCENKLPVHFVA--veecp<br>-------------------kesaaakferqhmdsgnsassssnycnLMMCCRKMTQGkCKPVNTFVHESLADVKAVCSQKKVTCkdgqtnCYQSKSTMRITDCRETgssKYPNCAYKTTqveKHIIVACGGKPSVPVHFDAsv-----<br><br>BLAST: aliSize=36 (resi) iden=24.53% (26/106) simi=33.96% (36/106)<br>mqnwetfqkkhltdtrdvkcdaemkkalfd---------------------------------------CKQKNTFIYARPGRVQALC--KNIIVSK---NVL-STDEFYLSDCNRI---KLP-CHYKLKKSSNTICITCENK--LPVHFVA--veecp<br>-----------------------------kesaaakferqhmdsgnsassssnycnlmmccrkmtqgkCKPVNTFVHESLADVKAVCsqKKVTCKDgqtNCYqSKSTMRITDCRETgssKYPnCAYKTTQVEKHIIVACGGKpsVPVHFDAsv-----<br><br>Proposed: aliSize=99 (resi) RMSD=2.06 (Å)<br>-MQNWETFQKKHLT-DTRD----VKCDAEMKK-ALFD--CKQKNTFIYARPGRVQALCkN----------iivSKNVLSTDEFYLSDCNRIK-LP---CHYKLKKSSNTICITCEN--KLPVHFVAVEecp<br>kESAAAKFERQHMDsGNSAssssNYCNLMMCCrKMTQgkCKPVNTFVHESLADVKAVC-Sqkkvtckdgq---TNCYQSKSTMRITDCRETGsSKypnCAYKTTQVEKHIIVACGGkpSVPVHFDASV---
892	1m58A (106)<br>3bcoA (124)	N	23.58	TM-align: aliSize=84 (resi) RMSD=2.46 (Å)<br>mqnwetfqkkhltdtr---------------------D-VKCDAEMKKALFD---CKQKNTFIYARPGRVQALCkNIiv---------SKNVLSTDEFYLSDCNRI-KL---PCHYKLKKSSNTICITCE-N-KLPVHFVAVEecp<br>----------------kesaaakferqhmdsgnsassSsNYCNQMMCCRKMTqgkCKPVNTFVHESLADVKAVC-SQ--kkvtckdgqTNCYQSKSTMRITDCRETgSSkypNCAYKTTQVEKHIIVACGgKpSVPVHFDASV---<br><br>SARST: aliSize=99 (resi) RMSD=10.26 (Å)<br>mqn----WETFQKKHL--TDTRDVKCDAEMKKALFDC------KQKNTFIYARPGRVQALCKNIIVSK------NVLSTDEFYLSDCNRI---KLPCHYKLKK---SSNTICITCENKLPVHFVAV-eecp<br>---kesaAAKFERQHMdsGNSASSSSNYCNQMMCCRKmtqgkcKPVNTFVHESLADVKAVCSQKKVTCkdgqtnCYQSKSTMRITDCRETgssKYPNCAYKTTqveKHIIVACGGKPSVPVHFDASv----<br><br>BLAST: aliSize=36 (resi) iden=24.53% (26/106) simi=33.96% (36/106)<br>mqnwetfqkkhltdtrdvkcdaemkkalfd---------------------------------------CKQKNTFIYARPGRVQALC--KNIIVSK---NVL-STDEFYLSDCNRI---KLP-CHYKLKKSSNTICITCENK--LPVHFVA--veecp<br>-----------------------------kesaaakferqhmdsgnsassssnycnqmmccrkmtqgkCKPVNTFVHESLADVKAVCsqKKVTCKDgqtNCYqSKSTMRITDCRETgssKYPnCAYKTTQVEKHIIVACGGKpsVPVHFDAsv-----<br><br>Proposed: aliSize=98 (resi) RMSD=2.08 (Å)<br>-MQNWETFQKKHLTDTRDV-----KCDAEMKK-ALFD--CKQKNTFIYARPGRVQALCkN-----------iivsKNVLSTDEFYLSDCNRI-KLP---CHYKLKKSSNTICITCE--NKLPVHFVAVEecp<br>kESAAAKFERQHMDSGNSAssssnYCNQMMCCrKMTQgkCKPVNTFVHESLADVKAVC-Sqkkvtckdgqt----NCYQSKSTMRITDCRETgSSKypnCAYKTTQVEKHIIVACGgkPSVPVHFDASV---
893	1m58A (106)<br>1y94A (124)	N	23.58	TM-align: aliSize=84 (resi) RMSD=2.41 (Å)<br>mqnwetfqkkhltdtr---------------------D-VKCDAEMKKALFD---CKQKNTFIYARPGRVQALCkNIiv---------SKNVLSTDEFYLSDCNRI-KLP---CHYKLKKSSNTICITCE-N-KLPVHFVAVEecp<br>----------------kesaaakferqhmdsstsaasSsNYCNLMMCCRKMTqgkCKPVNTFVHESLADVKAVC-SQ--kkvtckdgqTNCYQSKSTMRITDCRETgSSKypnCAYKTTQVEKHIIVACGgKpSVPVHFDASV---<br><br>SARST: aliSize=93 (resi) RMSD=11.43 (Å)<br>mqn----WETFQKKHL--TDTRDVK--CDAEMKkALFDC-----KQKNTFIYARPGRVQALCKNIIVSK------NVLSTDEFYLSDCNRI---KLPCHYKLKK-------SSNTICITcenkLPVHFVA--veecp<br>---kesaAAKFERQHMdsSTSAASSsnYCNLMM-CCRKMtqgkcKPVNTFVHESLADVKAVCSQKKVTCkdgqtnCYQSKSTMRITDCRETgssKYPNCAYKTTqvekhiiVACGGKPS----VPVHFDAsv-----<br><br>BLAST: aliSize=47 (resi) iden=29.25% (31/106) simi=44.34% (47/106)<br>mqnwet-------FQKKHLTDTRDVK-----CDAEM---KKALFDCKQKNTFIYARPGRVQALC--KNIIVSK---NVL-STDEFYLSDCNRI---KLP-CHYKLKKSSNTICITCENK--LPVHFVA--veecp<br>------kesaaakFERQHMDSSTSAAsssnyCNLMMccrKMTQGKCKPVNTFVHESLADVKAVCsqKKVTCKDgqtNCYqSKSTMRITDCRETgssKYPnCAYKTTQVEKHIIVACGGKpsVPVHFDAsv-----<br><br>Proposed: aliSize=98 (resi) RMSD=2.02 (Å)<br>-MQNWETFQKKHLTDTRDV-----KCDAEMKK-ALFD--CKQKNTFIYARPGRVQALCkN-----------iivsKNVLSTDEFYLSDCNRI-KLP---CHYKLKKSSNTICITCE-N-KLPVHFVAVEecp<br>kESAAAKFERQHMDSSTSAasssnYCNLMMCCrKMTQgkCKPVNTFVHESLADVKAVC-Sqkkvtckdgqt----NCYQSKSTMRITDCRETgSSKypnCAYKTTQVEKHIIVACGgKpSVPVHFDASV---

894	2e0oB (125)<br>11bgA (124)	N	69.35	TM-align: aliSize=104 (resi) RMSD=1.10 (Å)<br>mkesrakkfqrqhmdsdssps-------------------SSSTYCNQMMRRRNMTQGRCKPVNTFVHEPLLLVQLVCLQEKVTCKNGQGNCYKSNSSMHITDCRLTNGSRYPNCAYRTSPKERHIIVACEGSPYVPVHFDASV<br>--------------------kesaaakferqhmdsgnspsSSSNYCNLMMCCRKMTQGKCKPVNTFVHESLADVKAVCSQKKVTCKNGQTNCYQSKSTMRITDCRETGSSKYPNCAYKTTQVEKHIIVACGGKPSVPVHFDASV<br><br>SARST: aliSize=122 (resi) RMSD=9.84 (Å)<br>mk-ESRAKKFQRQHMDSDSSPSSSSTYCNQMMRRRNMTQGRCKPVNTFVHEPLLLVQLVCLQEKVTCKNGQGNCYKSNSSMHITDCRLTNGSRYPNCAYRTSPKERHIIVACEGSPYVPVHFDAS-v<br>--kESAAAKFERQHMDSGNSPSSSSNYCNLMMCCRKMTQGKCKPVNTFVHESLADVKAVCSQKKVTCKNGQTNCYQSKSTMRITDCRETGSSKYPNCAYKTTQVEKHIIVACGGKPSVPVHFDASv-<br><br>BLAST: aliSize=100 (resi) iden=71.77% (89/124) simi=80.65% (100/124)<br>mKESRAKKFQRQHMDSDSSPSSSSTYCNQMMRRRNMTQGRCKPVNTFVHEPLLLVQLVCLQEKVTCKNGQGNCYKSNSSMHITDCRLTNGSRYPNCAYRTSPKERHIIVACEGSPYVPVHFDASV<br>-KESAAAKFERQHMDSGNSPSSSSNYCNLMMCCRKMTQGKCKPVNTFVHESLADVKAVCSQKKVTCKNGQTNCYQSKSTMRITDCRETGSSKYPNCAYKTTQVEKHIIVACGGKPSVPVHFDASV<br><br>Proposed: aliSize=123 (resi) RMSD=1.01 (Å)<br>mKESRAKKFQRQHMDSdSSPSS-SSTYCNQMMRRRNMTQGRCKPVNTFVHEPLLLVQLVCLQEKVTCKNGQGNCYKSNSSMHITDCRLTNGSRYPNCAYRTSPKERHIIVACEGSPYVPVHFDASV<br>-KESAAAKFERQHMDS-GNSPSsSSNYCNLMMCCRKMTQGKCKPVNTFVHESLADVKAVCSQKKVTCKNGQTNCYQSKSTMRITDCRETGSSKYPNCAYKTTQVEKHIIVACGGKPSVPVHFDASV
895	2e0oB (125)<br>1y92B (124)	N	70.16	TM-align: aliSize=104 (resi) RMSD=0.92 (Å)<br>mkesrakkfqrqhmdsdssps-------------------SSSTYCNQMMRRRNMTQGRCKPVNTFVHEPLLLVQLVCLQEKVTCKNGQGNCYKSNSSMHITDCRLTNGSRYPNCAYRTSPKERHIIVACEGSPYVPVHFDASV<br>--------------------kesaaakferqhmdsgnsasSSSNYCNLMMCCRKMTQGKCKPVNTFVHESLADVKAVCSQKKVTCKDGQTNCYQSKSTMRITDCRETGSSKYPNCAYKTTQVEKHIIVACGGKPSVPVHFDASV<br><br>SARST: aliSize=122 (resi) RMSD=9.65 (Å)<br>mk-ESRAKKFQRQHMDSDSSPSSSSTYCNQMMRRRNMTQGRCKPVNTFVHEPLLLVQLVCLQEKVTCKNGQGNCYKSNSSMHITDCRLTNGSRYPNCAYRTSPKERHIIVACEGSPYVPVHFDAS-v<br>--kESAAAKFERQHMDSGNSASSSSNYCNLMMCCRKMTQGKCKPVNTFVHESLADVKAVCSQKKVTCKDGQTNCYQSKSTMRITDCRETGSSKYPNCAYKTTQVEKHIIVACGGKPSVPVHFDASv-<br><br>BLAST: aliSize=99 (resi) iden=70.16% (87/124) simi=79.84% (99/124)<br>mKESRAKKFQRQHMDSDSSPSSSSTYCNQMMRRRNMTQGRCKPVNTFVHEPLLLVQLVCLQEKVTCKNGQGNCYKSNSSMHITDCRLTNGSRYPNCAYRTSPKERHIIVACEGSPYVPVHFDASV<br>-KESAAAKFERQHMDSGNSASSSSNYCNLMMCCRKMTQGKCKPVNTFVHESLADVKAVCSQKKVTCKDGQTNCYQSKSTMRITDCRETGSSKYPNCAYKTTQVEKHIIVACGGKPSVPVHFDASV<br><br>Proposed: aliSize=124 (resi) RMSD=0.89 (Å)<br>mKESRAKKFQRQHMDSDSSPSSSSTYCNQMMRRRNMTQGRCKPVNTFVHEPLLLVQLVCLQEKVTCKNGQGNCYKSNSSMHITDCRLTNGSRYPNCAYRTSPKERHIIVACEGSPYVPVHFDASV<br>-KESAAAKFERQHMDSGNSASSSSNYCNLMMCCRKMTQGKCKPVNTFVHESLADVKAVCSQKKVTCKDGQTNCYQSKSTMRITDCRETGSSKYPNCAYKTTQVEKHIIVACGGKPSVPVHFDASV
896	2e0oB (125)<br>3bcoA (124)	N	69.35	TM-align: aliSize=104 (resi) RMSD=1.07 (Å)<br>mkesrakkfqrqhmdsdssps-------------------SSSTYCNQMMRRRNMTQGRCKPVNTFVHEPLLLVQLVCLQEKVTCKNGQGNCYKSNSSMHITDCRLTNGSRYPNCAYRTSPKERHIIVACEGSPYVPVHFDASV<br>--------------------kesaaakferqhmdsgnsasSSSNYCNQMMCCRKMTQGKCKPVNTFVHESLADVKAVCSQKKVTCKDGQTNCYQSKSTMRITDCRETGSSKYPNCAYKTTQVEKHIIVACGGKPSVPVHFDASV<br><br>SARST: aliSize=120 (resi) RMSD=9.46 (Å)<br>mk-ESRAKKFQRQHMDsdSSPSSS--STYCNQMMRRRNMTQGRCKPVNTFVHEPLLLVQLVCLQEKVTCKNGQGNCYKSNSSMHITDCRLTNGSRYPNCAYRTSPKERHIIVACEGSPYVPVHFDAS-v<br>--kESAAAKFERQHMD--SGNSASsssSNYCNQMMCCRKMTQGKCKPVNTFVHESLADVKAVCSQKKVTCKDGQTNCYQSKSTMRITDCRETGSSKYPNCAYKTTQVEKHIIVACGGKPSVPVHFDASv-<br><br>BLAST: aliSize=100 (resi) iden=70.97% (88/124) simi=80.65% (100/124)<br>mKESRAKKFQRQHMDSDSSPSSSSTYCNQMMRRRNMTQGRCKPVNTFVHEPLLLVQLVCLQEKVTCKNGQGNCYKSNSSMHITDCRLTNGSRYPNCAYRTSPKERHIIVACEGSPYVPVHFDASV<br>-KESAAAKFERQHMDSGNSASSSSNYCNQMMCCRKMTQGKCKPVNTFVHESLADVKAVCSQKKVTCKDGQTNCYQSKSTMRITDCRETGSSKYPNCAYKTTQVEKHIIVACGGKPSVPVHFDASV<br><br>Proposed: aliSize=123 (resi) RMSD=0.93 (Å)<br>mKESRAKKFQRQHMDSdSSPSS-SSTYCNQMMRRRNMTQGRCKPVNTFVHEPLLLVQLVCLQEKVTCKNGQGNCYKSNSSMHITDCRLTNGSRYPNCAYRTSPKERHIIVACEGSPYVPVHFDASV<br>-KESAAAKFERQHMDS-GNSASsSSNYCNQMMCCRKMTQGKCKPVNTFVHESLADVKAVCSQKKVTCKDGQTNCYQSKSTMRITDCRETGSSKYPNCAYKTTQVEKHIIVACGGKPSVPVHFDASV

897	2e0oB<br>(125)<br><br>1y94A<br>(124)	N	68.55	TM-align: aliSize=104 (resi) RMSD=1.12 (Å)<br>mkesrakkfqrqhmdsdssps-------------------SSSTYCNQMMRRRNMTQGRCKPVNTFVHEPLLLVQLVCLQEKVTCKNGQGNCYKSNSSMHITDCRLTNGSRYPNCAYRTSPKERHIIVACEGSPYVPVHFDASV<br>--------------------kesaaakferqhmdsstsaaSSSNYCNLMMCCRKMTQGKCKPVNTFVHESLADVKAVCSQKKVTCKDGQTNCYQSKSTMRITDCRETGSSKYPNCAYKTTQVEKHIIVACGGKPSVPVHFDASV<br><br>SARST: aliSize=120 (resi) RMSD=9.46 (Å)<br>mk-ESRAKKFQRQHMDsdSSPS--SSSTYCNQMMRRRNMTQGRCKPVNTFVHEPLLLVQLVCLQEKVTCKNGQGNCYKSNSSMHITDCRLTNGSRYPNCAYRTSPKERHIIVACEGSPYVPVHFDAS-v<br>--kESAAAKFERQHMD--SSTSaaSSSNYCNLMMCCRKMTQGKCKPVNTFVHESLADVKAVCSQKKVTCKDGQTNCYQSKSTMRITDCRETGSSKYPNCAYKTTQVEKHIIVACGGKPSVPVHFDASv-<br><br>BLAST: aliSize=99 (resi) iden=69.35% (86/124) simi=79.84% (99/124)<br>mKESRAKKFQRQHMDSDSSPSSSSTYCNQMMRRRNMTQGRCKPVNTFVHEPLLLVQLVCLQEKVTCKNGQGNCYKSNSSMHITDCRLTNGSRYPNCAYRTSPKERHIIVACEGSPYVPVHFDASV<br>-KESAAAKFERQHMDSSTSAASSSNYCNLMMCCRKMTQGKCKPVNTFVHESLADVKAVCSQKKVTCKDGQTNCYQSKSTMRITDCRETGSSKYPNCAYKTTQVEKHIIVACGGKPSVPVHFDASV<br><br>Proposed: aliSize=123 (resi) RMSD=0.98 (Å)<br>mKESRAKKFQRQHMDSDSSPS-sSSTYCNQMMRRRNMTQGRCKPVNTFVHEPLLLVQLVCLQEKVTCKNGQGNCYKSNSSMHITDCRLTNGSRYPNCAYRTSPKERHIIVACEGSPYVPVHFDASV<br>-KESAAAKFERQHMDSSTSAAs-SSNYCNLMMCCRKMTQGKCKPVNTFVHESLADVKAVCSQKKVTCKDGQTNCYQSKSTMRITDCRETGSSKYPNCAYKTTQVEKHIIVACGGKPSVPVHFDASV
898	1ymnA<br>(124)<br><br>1lbgA<br>(124)	N	80.65	TM-align: aliSize=104 (resi) RMSD=1.07 (Å)<br>ketaaakferqhmdsstsaa-------------------SSSNYCNQMMKSRNLTKDRCKPVNTFVHESLADVQAVCSQKNVACKNGQTNCYQSYSTMSITDCRETGSSKLPNCAYKTTQANKHIIVACEGNPYVPVHFDASV<br>--------------------kesaaakferqhmdsgnspsSSSNYCNLMMCCRKMTQGKCKPVNTFVHESLADVKAVCSQKKVTCKNGQTNCYQSKSTMRITDCRETGSSKYPNCAYKTTQVEKHIIVACGGKPSVPVHFDASV<br><br>SARST: aliSize=121 (resi) RMSD=9.81 (Å)<br>k-ETAAAKFERQHMDS-STSAASsSNYCNQMMKSRNLTKDRCKPVNTFVHESLADVQAVCSQKNVACKNGQTNCYQSYSTMSITDCRETGSSKLPNCAYKTTQANKHIIVACEGNPYVPVHFDAS-v<br>-kESAAAKFERQHMDSgNSPSSS-SNYCNLMMCCRKMTQGKCKPVNTFVHESLADVKAVCSQKKVTCKNGQTNCYQSKSTMRITDCRETGSSKYPNCAYKTTQVEKHIIVACGGKPSVPVHFDASv-<br><br>BLAST: aliSize=106 (resi) iden=80.65% (100/124) simi=85.48% (106/124)<br>KETAAAKFERQHMDSSTSAASSSNYCNQMMKSRNLTKDRCKPVNTFVHESLADVQAVCSQKNVACKNGQTNCYQSYSTMSITDCRETGSSKLPNCAYKTTQANKHIIVACEGNPYVPVHFDASV<br>KESAAAKFERQHMDSGNSPSSSSNYCNLMMCCRKMTQGKCKPVNTFVHESLADVKAVCSQKKVTCKNGQTNCYQSKSTMRITDCRETGSSKYPNCAYKTTQVEKHIIVACGGKPSVPVHFDASV<br><br>Proposed: aliSize=124 (resi) RMSD=1.10 (Å)<br>KETAAAKFERQHMDSSTSAASSSNYCNQMMKSRNLTKDRCKPVNTFVHESLADVQAVCSQKNVACKNGQTNCYQSYSTMSITDCRETGSSKLPNCAYKTTQANKHIIVACEGNPYVPVHFDASV<br>KESAAAKFERQHMDSGNSPSSSSNYCNLMMCCRKMTQGKCKPVNTFVHESLADVKAVCSQKKVTCKNGQTNCYQSKSTMRITDCRETGSSKYPNCAYKTTQVEKHIIVACGGKPSVPVHFDASV
899	1ymnA<br>(124)<br><br>1y92B<br>(124)	N	80.65	TM-align: aliSize=104 (resi) RMSD=0.94 (Å)<br>ketaaakferqhmdsstsaa-------------------SSSNYCNQMMKSRNLTKDRCKPVNTFVHESLADVQAVCSQKNVACKNGQTNCYQSYSTMSITDCRETGSSKLPNCAYKTTQANKHIIVACEGNPYVPVHFDASV<br>--------------------kesaaakferqhmdsgnsasSSSNYCNLMMCCRKMTQGKCKPVNTFVHESLADVKAVCSQKKVTCKDGQTNCYQSKSTMRITDCRETGSSKYPNCAYKTTQVEKHIIVACGGKPSVPVHFDASV<br><br>SARST: aliSize=122 (resi) RMSD=9.71 (Å)<br>k-ETAAAKFERQHMDSSTSAASSSNYCNQMMKSRNLTKDRCKPVNTFVHESLADVQAVCSQKNVACKNGQTNCYQSYSTMSITDCRETGSSKLPNCAYKTTQANKHIIVACEGNPYVPVHFDAS-v<br>-kESAAAKFERQHMDSGNSASSSSNYCNLMMCCRKMTQGKCKPVNTFVHESLADVKAVCSQKKVTCKDGQTNCYQSKSTMRITDCRETGSSKYPNCAYKTTQVEKHIIVACGGKPSVPVHFDASv-<br><br>BLAST: aliSize=107 (resi) iden=80.65% (100/124) simi=86.29% (107/124)<br>KETAAAKFERQHMDSSTSAASSSNYCNQMMKSRNLTKDRCKPVNTFVHESLADVQAVCSQKNVACKNGQTNCYQSYSTMSITDCRETGSSKLPNCAYKTTQANKHIIVACEGNPYVPVHFDASV<br>KESAAAKFERQHMDSGNSASSSSNYCNLMMCCRKMTQGKCKPVNTFVHESLADVKAVCSQKKVTCKDGQTNCYQSKSTMRITDCRETGSSKYPNCAYKTTQVEKHIIVACGGKPSVPVHFDASV<br><br>Proposed: aliSize=124 (resi) RMSD=0.99 (Å)<br>KETAAAKFERQHMDSSTSAASSSNYCNQMMKSRNLTKDRCKPVNTFVHESLADVQAVCSQKNVACKNGQTNCYQSYSTMSITDCRETGSSKLPNCAYKTTQANKHIIVACEGNPYVPVHFDASV<br>KESAAAKFERQHMDSGNSASSSSNYCNLMMCCRKMTQGKCKPVNTFVHESLADVKAVCSQKKVTCKDGQTNCYQSKSTMRITDCRETGSSKYPNCAYKTTQVEKHIIVACGGKPSVPVHFDASV

900	1ymnA<br>(124)<br><br>3bcoA<br>(124)	N	81.45	TM-align: aliSize=104 (resi) RMSD=1.12 (Å)<br>ketaaakferqhmdsstsaa--------------------SSSNYCNQMMKSRNLTKDRCKPVNTFVHESLADVQAVCSQKNVACKNGQTNCYQSYSTMSITDCRETGSSKLPNCAYKTTQANKHIIVACEGNPYVPVHFDASV<br>--------------------kesaaakferqhmdsgnsasSSSNYCNQMMCCRKMTQGKCKPVNTFVHESLADVKAVCSQKKVTCKDGQTNCYQSKSTMRITDCRETGSSKYPNCAYKTTQVEKHIIVACGGKPSVPVHFDASV<br><br>SARST: aliSize=118 (resi) RMSD=9.26 (Å)<br>k-ETAAAKFERQHMD----SSTSaassSNYCNQMMKSRNLTKDRCKPVNTFVHESLADVQAVCSQKNVACKNGQTNCYQSYSTMSITDCRETGSSKLPNCAYKTTQANKHIIVACEGNPYVPVHFDAS-v<br>-kESAAAKFERQHMDsgnsASSS----SNYCNQMMCCRKMTQGKCKPVNTFVHESLADVKAVCSQKKVTCKDGQTNCYQSKSTMRITDCRETGSSKYPNCAYKTTQVEKHIIVACGGKPSVPVHFDASv-<br><br>BLAST: aliSize=108 (resi) iden=81.45% (101/124) simi=87.10% (108/124)<br>KETAAAKFERQHMDSSTSAASSSNYCNQMMKSRNLTKDRCKPVNTFVHESLADVQAVCSQKNVACKNGQTNCYQSYSTMSITDCRETGSSKLPNCAYKTTQANKHIIVACEGNPYVPVHFDASV<br>KESAAAKFERQHMDSGNSASSSSNYCNQMMCCRKMTQGKCKPVNTFVHESLADVKAVCSQKKVTCKDGQTNCYQSKSTMRITDCRETGSSKYPNCAYKTTQVEKHIIVACGGKPSVPVHFDASV<br><br>Proposed: aliSize=124 (resi) RMSD=1.09 (Å)<br>KETAAAKFERQHMDSSTSAASSSNYCNQMMKSRNLTKDRCKPVNTFVHESLADVQAVCSQKNVACKNGQTNCYQSYSTMSITDCRETGSSKLPNCAYKTTQANKHIIVACEGNPYVPVHFDASV<br>KESAAAKFERQHMDSGNSASSSSNYCNQMMCCRKMTQGKCKPVNTFVHESLADVKAVCSQKKVTCKDGQTNCYQSKSTMRITDCRETGSSKYPNCAYKTTQVEKHIIVACGGKPSVPVHFDASV
901	1ymnA<br>(124)<br><br>1y94A<br>(124)	N	83.06	TM-align: aliSize=104 (resi) RMSD=1.17 (Å)<br>ketaaakferqhmdsstsaa--------------------SSSNYCNQMMKSRNLTKDRCKPVNTFVHESLADVQAVCSQKNVACKNGQTNCYQSYSTMSITDCRETGSSKLPNCAYKTTQANKHIIVACEGNPYVPVHFDASV<br>--------------------kesaaakferqhmdsstsaaSSSNYCNLMMCCRKMTQGKCKPVNTFVHESLADVKAVCSQKKVTCKDGQTNCYQSKSTMRITDCRETGSSKYPNCAYKTTQVEKHIIVACGGKPSVPVHFDASV<br><br>SARST: aliSize=121 (resi) RMSD=9.62 (Å)<br>k-ETAAAKFERQHMDS-STSAASsSNYCNQMMKSRNLTKDRCKPVNTFVHESLADVQAVCSQKNVACKNGQTNCYQSYSTMSITDCRETGSSKLPNCAYKTTQANKHIIVACEGNPYVPVHFDAS-v<br>-kESAAAKFERQHMDSsTSAASS-SNYCNLMMCCRKMTQGKCKPVNTFVHESLADVKAVCSQKKVTCKDGQTNCYQSKSTMRITDCRETGSSKYPNCAYKTTQVEKHIIVACGGKPSVPVHFDASv-<br><br>BLAST: aliSize=109 (resi) iden=83.06% (103/124) simi=87.90% (109/124)<br>KETAAAKFERQHMDSSTSAASSSNYCNQMMKSRNLTKDRCKPVNTFVHESLADVQAVCSQKNVACKNGQTNCYQSYSTMSITDCRETGSSKLPNCAYKTTQANKHIIVACEGNPYVPVHFDASV<br>KESAAAKFERQHMDSSTSAASSSNYCNLMMCCRKMTQGKCKPVNTFVHESLADVKAVCSQKKVTCKDGQTNCYQSKSTMRITDCRETGSSKYPNCAYKTTQVEKHIIVACGGKPSVPVHFDASV<br><br>Proposed: aliSize=124 (resi) RMSD=1.15 (Å)<br>KETAAAKFERQHMDSSTSAASSSNYCNQMMKSRNLTKDRCKPVNTFVHESLADVQAVCSQKNVACKNGQTNCYQSYSTMSITDCRETGSSKLPNCAYKTTQANKHIIVACEGNPYVPVHFDASV<br>KESAAAKFERQHMDSSTSAASSSNYCNLMMCCRKMTQGKCKPVNTFVHESLADVKAVCSQKKVTCKDGQTNCYQSKSTMRITDCRETGSSKYPNCAYKTTQVEKHIIVACGGKPSVPVHFDASV
902	1a5pA<br>(124)<br><br>11bgA<br>(124)	N	79.84	TM-align: aliSize=104 (resi) RMSD=0.84 (Å)<br>ketaaakferqhmdsstsaa--------------------SSSNYCNQMMKSRNLTKDRAKPVNTFVHESLADVQAVCSQKNVACKNGQTNCYQSYSTMSITDCRETGSSKYPNAAYKTTQANKHIIVACEGNPYVPVHFDASV<br>--------------------kesaaakferqhmdsgnspsSSSNYCNLMMCCRKMTQGKCKPVNTFVHESLADVKAVCSQKKVTCKNGQTNCYQSKSTMRITDCRETGSSKYPNCAYKTTQVEKHIIVACGGKPSVPVHFDASV<br><br>SARST: aliSize=121 (resi) RMSD=9.75 (Å)<br>k-ETAAAKFERQHMDS-STSAASsSNYCNQMMKSRNLTKDRAKPVNTFVHESLADVQAVCSQKNVACKNGQTNCYQSYSTMSITDCRETGSSKYPNAAYKTTQANKHIIVACEGNPYVPVHFDAS-v<br>-kESAAAKFERQHMDSgNSPSSS-SNYCNLMMCCRKMTQGKCKPVNTFVHESLADVKAVCSQKKVTCKNGQTNCYQSKSTMRITDCRETGSSKYPNCAYKTTQVEKHIIVACGGKPSVPVHFDASv-<br><br>BLAST: aliSize=105 (resi) iden=79.84% (99/124) simi=84.68% (105/124)<br>KETAAAKFERQHMDSSTSAASSSNYCNQMMKSRNLTKDRAKPVNTFVHESLADVQAVCSQKNVACKNGQTNCYQSYSTMSITDCRETGSSKYPNAAYKTTQANKHIIVACEGNPYVPVHFDASV<br>KESAAAKFERQHMDSGNSPSSSSNYCNLMMCCRKMTQGKCKPVNTFVHESLADVKAVCSQKKVTCKNGQTNCYQSKSTMRITDCRETGSSKYPNCAYKTTQVEKHIIVACGGKPSVPVHFDASV<br><br>Proposed: aliSize=124 (resi) RMSD=0.91 (Å)<br>KETAAAKFERQHMDSSTSAASSSNYCNQMMKSRNLTKDRAKPVNTFVHESLADVQAVCSQKNVACKNGQTNCYQSYSTMSITDCRETGSSKYPNAAYKTTQANKHIIVACEGNPYVPVHFDASV<br>KESAAAKFERQHMDSGNSPSSSSNYCNLMMCCRKMTQGKCKPVNTFVHESLADVKAVCSQKKVTCKNGQTNCYQSKSTMRITDCRETGSSKYPNCAYKTTQVEKHIIVACGGKPSVPVHFDASV

903	1a5pA (124)<br>1y92B (124)	N	79.84	TM-align: aliSize=104 (resi) RMSD=0.81 (Å)<br>ketaaakferqhmdsstsaa--------------------SSSNYCNQMMKSRNLTKDRAKPVNTFVHESLADVQAVCSQKNVACKNGQTNCYQSYSTMSITDCRETGSSKYPNAAYKTTQANKHIIVACEGNPYVPVHFDASV<br>--------------------kesaaakferqhmdsgnsasSSSNYCNLMMCCRKMTQGKCKPVNTFVHESLADVKAVCSQKKVTCKDGQTNCYQSKSTMRITDCRETGSSKYPNCAYKTTQVEKHIIVACGGKPSVPVHFDASV<br><br>SARST: aliSize=122 (resi) RMSD=9.66 (Å)<br>k-ETAAAKFERQHMDSSTSAASSSNYCNQMMKSRNLTKDRAKPVNTFVHESLADVQAVCSQKNVACKNGQTNCYQSYSTMSITDCRETGSSKYPNAAYKTTQANKHIIVACEGNPYVPVHFDAS-v<br>-kESAAAKFERQHMDSGNSASSSSNYCNLMMCCRKMTQGKCKPVNTFVHESLADVKAVCSQKKVTCKDGQTNCYQSKSTMRITDCRETGSSKYPNCAYKTTQVEKHIIVACGGKPSVPVHFDASv-<br><br>BLAST: aliSize=106 (resi) iden=79.84% (99/124) simi=85.48% (106/124)<br>KETAAAKFERQHMDSSTSAASSSNYCNQMMKSRNLTKDRAKPVNTFVHESLADVQAVCSQKNVACKNGQTNCYQSYSTMSITDCRETGSSKYPNAAYKTTQANKHIIVACEGNPYVPVHFDASV<br>KESAAAKFERQHMDSGNSASSSSNYCNLMMCCRKMTQGKCKPVNTFVHESLADVKAVCSQKKVTCKDGQTNCYQSKSTMRITDCRETGSSKYPNCAYKTTQVEKHIIVACGGKPSVPVHFDASV<br><br>Proposed: aliSize=124 (resi) RMSD=0.89 (Å)<br>KETAAAKFERQHMDSSTSAASSSNYCNQMMKSRNLTKDRAKPVNTFVHESLADVQAVCSQKNVACKNGQTNCYQSYSTMSITDCRETGSSKYPNAAYKTTQANKHIIVACEGNPYVPVHFDASV<br>KESAAAKFERQHMDSGNSASSSSNYCNLMMCCRKMTQGKCKPVNTFVHESLADVKAVCSQKKVTCKDGQTNCYQSKSTMRITDCRETGSSKYPNCAYKTTQVEKHIIVACGGKPSVPVHFDASV
904	1a5pA (124)<br>3bcoA (124)	N	80.65	TM-align: aliSize=104 (resi) RMSD=0.88 (Å)<br>ketaaakferqhmdsstsaa--------------------SSSNYCNQMMKSRNLTKDRAKPVNTFVHESLADVQAVCSQKNVACKNGQTNCYQSYSTMSITDCRETGSSKYPNAAYKTTQANKHIIVACEGNPYVPVHFDASV<br>--------------------kesaaakferqhmdsgnsasSSSNYCNQMMCCRKMTQGKCKPVNTFVHESLADVKAVCSQKKVTCKDGQTNCYQSKSTMRITDCRETGSSKYPNCAYKTTQVEKHIIVACGGKPSVPVHFDASV<br><br>SARST: aliSize=121 (resi) RMSD=9.56 (Å)<br>k-ETAAAKFERQHMDSSsTSAASS-SNYCNQMMKSRNLTKDRAKPVNTFVHESLADVQAVCSQKNVACKNGQTNCYQSYSTMSITDCRETGSSKYPNAAYKTTQANKHIIVACEGNPYVPVHFDAS-v<br>-kESAAAKFERQHMDS-GNSASSsSNYCNQMMCCRKMTQGKCKPVNTFVHESLADVKAVCSQKKVTCKDGQTNCYQSKSTMRITDCRETGSSKYPNCAYKTTQVEKHIIVACGGKPSVPVHFDASv-<br><br>BLAST: aliSize=107 (resi) iden=80.65% (100/124) simi=86.29% (107/124)<br>KETAAAKFERQHMDSSTSAASSSNYCNQMMKSRNLTKDRAKPVNTFVHESLADVQAVCSQKNVACKNGQTNCYQSYSTMSITDCRETGSSKYPNAAYKTTQANKHIIVACEGNPYVPVHFDASV<br>KESAAAKFERQHMDSGNSASSSSNYCNQMMCCRKMTQGKCKPVNTFVHESLADVKAVCSQKKVTCKDGQTNCYQSKSTMRITDCRETGSSKYPNCAYKTTQVEKHIIVACGGKPSVPVHFDASV<br><br>Proposed: aliSize=124 (resi) RMSD=0.89 (Å)<br>KETAAAKFERQHMDSSTSAASSSNYCNQMMKSRNLTKDRAKPVNTFVHESLADVQAVCSQKNVACKNGQTNCYQSYSTMSITDCRETGSSKYPNAAYKTTQANKHIIVACEGNPYVPVHFDASV<br>KESAAAKFERQHMDSGNSASSSSNYCNQMMCCRKMTQGKCKPVNTFVHESLADVKAVCSQKKVTCKDGQTNCYQSKSTMRITDCRETGSSKYPNCAYKTTQVEKHIIVACGGKPSVPVHFDASV
905	1a5pA (124)<br>1y94A (124)	N	82.26	TM-align: aliSize=104 (resi) RMSD=0.97 (Å)<br>ketaaakferqhmdsstsaa--------------------SSSNYCNQMMKSRNLTKDRAKPVNTFVHESLADVQAVCSQKNVACKNGQTNCYQSYSTMSITDCRETGSSKYPNAAYKTTQANKHIIVACEGNPYVPVHFDASV<br>--------------------kesaaakferqhmdsstsaaSSSNYCNLMMCCRKMTQGKCKPVNTFVHESLADVKAVCSQKKVTCKDGQTNCYQSKSTMRITDCRETGSSKYPNCAYKTTQVEKHIIVACGGKPSVPVHFDASV<br><br>SARST: aliSize=121 (resi) RMSD=9.56 (Å)<br>k-ETAAAKFERQHMDS-STSAASsSNYCNQMMKSRNLTKDRAKPVNTFVHESLADVQAVCSQKNVACKNGQTNCYQSYSTMSITDCRETGSSKYPNAAYKTTQANKHIIVACEGNPYVPVHFDAS-v<br>-kESAAAKFERQHMDSsTSAASS-SNYCNLMMCCRKMTQGKCKPVNTFVHESLADVKAVCSQKKVTCKDGQTNCYQSKSTMRITDCRETGSSKYPNCAYKTTQVEKHIIVACGGKPSVPVHFDASv-<br><br>BLAST: aliSize=108 (resi) iden=82.26% (102/124) simi=87.10% (108/124)<br>KETAAAKFERQHMDSSTSAASSSNYCNQMMKSRNLTKDRAKPVNTFVHESLADVQAVCSQKNVACKNGQTNCYQSYSTMSITDCRETGSSKYPNAAYKTTQANKHIIVACEGNPYVPVHFDASV<br>KESAAAKFERQHMDSSTSAASSSNYCNLMMCCRKMTQGKCKPVNTFVHESLADVKAVCSQKKVTCKDGQTNCYQSKSTMRITDCRETGSSKYPNCAYKTTQVEKHIIVACGGKPSVPVHFDASV<br><br>Proposed: aliSize=124 (resi) RMSD=0.97 (Å)<br>KETAAAKFERQHMDSSTSAASSSNYCNQMMKSRNLTKDRAKPVNTFVHESLADVQAVCSQKNVACKNGQTNCYQSYSTMSITDCRETGSSKYPNAAYKTTQANKHIIVACEGNPYVPVHFDASV<br>KESAAAKFERQHMDSSTSAASSSNYCNLMMCCRKMTQGKCKPVNTFVHESLADVKAVCSQKKVTCKDGQTNCYQSKSTMRITDCRETGSSKYPNCAYKTTQVEKHIIVACGGKPSVPVHFDASV

906	1z5fA<br>(105)<br><br>11bgA<br>(124)	N	21.90	TM-align: aliSize=85 (resi) RMSD=3.09 (Å)<br>dwetfqkkhltdtk---------------------K-VKCDVEMAKALFD--CK-KTNTFIYALPGRVKALCKNIrd--------NTDVLSRDAFLLPQCD-RIKL---PCHYKLSSSTNTICITCV--NQLPIHFAGVGscp<br>--------------kesaaakferqhmdsgnspssSsNYCNLMMCCRKMTqgKCkPVNTFVHESLADVKAVCSQK--kvtckngqTNCYQSKSTMRITDCReTGSSkypNCAYKTTQVEKHIIVACGgkPSVPVHFDASV---<br><br>SARST: aliSize=84 (resi) RMSD=11.40 (Å)<br>d------WETFQKKHLTDTKKVkcDVE------MAKALFDCK--KTNTFIYAL-PGRVKALCKNIRDN-----TDVLSRDAFLlpqcdriklpchYKLSS----------------STNTICITCVnQLPIHFAGV----gscp<br>-kesaaaKFERQHMDSGNSPSS--SSNycnlmmCCRKMTQGKckPVNTFVHESlADVKAVCSQKKVTCkngqtNCYQSKSTMR------------ITDCRetgsskypncaykttqVEKHIIVACG-GKPSVPVHFdasv----<br><br>BLAST: aliSize=22 (resi) iden=14.42% (15/105) simi=21.15% (22/105)<br>dwetfqkkhltdtkkvkcdvemakalfd---------------------------------------CKKTNTFIYALPGRVKALCKNiRDNTdvlsrdafllpqCDRIKLPCHYKLSSSTNTIC--itcvnqlpihfagvgscp<br>----------------------------kesaaakferqhmdsgnspssssnycnlmmccrkmtqgkCKPVNTFVHESLADVKAVCSQ-KKVT------------CKNGQTNCYQSKSTMRITDCretgsskypncaykttqvekhiivacggkpsvpvhfdasv------------------<br><br>Proposed: aliSize=96 (resi) RMSD=2.32 (Å)<br>p---DWETFQKKHLTDTKKV-----KCDVEMAK-ALFD--CKKTNTFIYALPGRVKALCknI-----------rdnTDVLSRDAFLLPQCDRIKLP----CHYKLSSSTNTICITCV--NQLPIHFAGVGscp<br>-kesAAAKFERQHMDSGNSPssssnYCNLMMCCrKMTQgkCKPVNTFVHESLADVKAVC--Sqkkvtckngqt---NCYQSKSTMRITDCRETGSSkypnCAYKTTQVEKHIIVACGgkPSVPVHFDASV---
907	1z5fA<br>(105)<br><br>1y92B<br>(124)	N	20.95	TM-align: aliSize=85 (resi) RMSD=2.88 (Å)<br>dwetfqkkhltdtk---------------------K-VKCDVEMAKALFDC--K-KTNTFIYALPGRVKALCKNIrd--------NTDVLSRDAFLLPQCDRIKLP----CHYKLSSSTNTICITCV--NQLPIHFAGVGscp<br>--------------kesaaakferqhmdsgnsassSsNYCNLMMCCRKMTQgkCkPVNTFVHESLADVKAVCSQK--kvtckdgqTNCYQSKSTMRITDCRETGSSkypnCAYKTTQVEKHIIVACGgkPSVPVHFDASV---<br><br>SARST: aliSize=82 (resi) RMSD=6.67 (Å)<br>dwetfqkkhltdtkkvk--------------------------CDVEMAKALFD-CKKTNTFIYALPGRVKALCKNIRDNTDVLSRDAFL-----LPQCDRIKLP-----CHYKLSSSTNTICITCVnQLPIHFAGV----gscp<br>----------------kesaaakferqhmdsgnsassssnycnLMMCCRKMTQGkCKPVNTFVHESLADVKAVCSQKKVTCKDGQTNCYQskstmRITDCRETGSskypnCAYKTTQVEKHIIVACG-GKPSVPVHFdasv----<br><br>BLAST: aliSize=24 (resi) iden=15.38% (16/105) simi=23.08% (24/105)<br>dwetfqkkhltdtkkvkcdvemakalfd---------------------------------------CKKTNTFIYALPGRVKALCKNIRdntdVLSRDAfllpQCDriklpCHYKLSSSTNTIC--itcvnqlpihfagvgscp<br>----------------------------kesaaakferqhmdsgnsassssnycnlmmccrkmtqgkCKPVNTFVHESLADVKAVCSQKK----VTCKDG----QTN-----CYQSKSTMRITDCretgsskypncaykttqvekhiivacggkpsvpvhfdasv------------------<br><br>Proposed: aliSize=96 (resi) RMSD=2.27 (Å)<br>-PDWETFQKKHLTDT-kKV------KCDVEMAK-ALFD--CKKTNTFIYALPGRVKALCknI-----------rdnTDVLSRDAFLLPQCDRIKLP----CHYKLSSSTNTICITCV--NQLPIHFAGVGscp<br>kESAAAKFERQHMDSg-NSassssnYCNLMMCCrKMTQgkCKPVNTFVHESLADVKAVC--Sqkkvtckdgqt---NCYQSKSTMRITDCRETGSSkypnCAYKTTQVEKHIIVACGgkPSVPVHFDASV---
908	1z5fA<br>(105)<br><br>3bcoA<br>(124)	N	20.00	TM-align: aliSize=85 (resi) RMSD=3.04 (Å)<br>dwetfqkkhltdtk---------------------K-VKCDVEMAKALFD--CK-KTNTFIYALPGRVKALCKNIrd--------NTDVLSRDAFLLPQCDRIKLP----CHYKLSSSTNTICITCV--NQLPIHFAGVGscp<br>--------------kesaaakferqhmdsgnsassSsNYCNQMMCCRKMTqgKCkPVNTFVHESLADVKAVCSQK--kvtckdgqTNCYQSKSTMRITDCRETGSSkypnCAYKTTQVEKHIIVACGgkPSVPVHFDASV---<br><br>SARST: aliSize=82 (resi) RMSD=4.89 (Å)<br>dwetfqkkhltdtkkvk-------------------------CDVEMAKALFDCK--KTNTFIYAL-PGRVKALCKNIR-----DNTDVLSRDAFLLPQCDR---IKLPC-HYKLSSSTNTICITCVnQLPIHFAGV----gscp<br>----------------kesaaakferqhmdsgnsassssnycNQMMCCRKMTQGKckPVNTFVHESlADVKAVCSQKKVtckdgQTNCYQSKSTMRITDCREtgsSKYPNcAYKTTQVEKHIIVACG-GKPSVPVHFdasv----<br><br>BLAST: aliSize=24 (resi) iden=15.38% (16/105) simi=23.08% (24/105)<br>dwetfqkkhltdtkkvkcdvemakalfd---------------------------------------CKKTNTFIYALPGRVKALCKNIRdntdVLSRDAfllpQCDriklpCHYKLSSSTNTIC--itcvnqlpihfagvgscp<br>----------------------------kesaaakferqhmdsgnsassssnycnqmmccrkmtqgkCKPVNTFVHESLADVKAVCSQKK----VTCKDG----QTN-----CYQSKSTMRITDCretgsskypncaykttqvekhiivacggkpsvpvhfdasv------------------<br><br>Proposed: aliSize=97 (resi) RMSD=2.47 (Å)<br>-PDWETFQKKH-LTDT--KK---VKCDVEMAK-ALFD--CKKTNTFIYALPGRVKALCknI-----------rdnTDVLSRDAFLLPQCDRIKLP----CHYKLSSSTNTICITCV--NQLPIHFAGVGscp<br>kESAAAKFERQhMDSGnsASsssNYCNQMMCCrKMTQgkCKPVNTFVHESLADVKAVC--Sqkkvtckdgqt---NCYQSKSTMRITDCRETGSSkypnCAYKTTQVEKHIIVACGgkPSVPVHFDASV---

909	2bwlA (118)<br>11bgA (124)	N	35.59	TM-align: aliSize=96 (resi) RMSD=1.74 (Å)<br>ddsrytkfltqhhdakpk------------------GRDDRYCERMMKRRSLTS-PCKDVNTFIHGNKSNIKAICGanGSPYR-----ENLRMSKSPFQVTTCKHT-GGSpRPPCQYRASAGFRHVVIACE-N-GLPVHFDEsFF<br>-----------------kesaaakferqhmdsgnspSSSSNYCNLMMCCRKMTQgKCKPVNTFVHESLADVKAVCS--QKKVTckngqTNCYQSKSTMRITDCRETgSSK-YPNCAYKTTQVEKHIIVACGgKpSVPVHFDA-SV<br><br>SARST: aliSize=108 (resi) RMSD=9.84 (Å)<br>dd--SRYTKFLTQHHDakPkGRDD----RYCERMMKRRSLTS-PCKDVNTFIHGNKSNIKAICGANGSPYRE---NLRMSKSPFQVTTCKHTGGSPRPPCQYRASAGFRHVVIACEN--GLPVHF----desf<br>--keSAAAKFERQHMD--S-GNSPssssNYCNLMMCCRKMTQgKCKPVNTFVHESLADVKAVCSQKKVTCKNgqtNCYQSKSTMRITDCRETGSSKYPNCAYKTTQVEKHIIVACGGkpSVPVHFdasv----<br><br>BLAST: aliSize=66 (resi) iden=37.61% (44/118) simi=56.41% (66/118)<br>d-DSRYTKFLTQHHDA--KPKGRDDrYCERMMKRRSLTS-PCKDVNTFIHGNKSNIKAICGANGSPYRE---NLRMSKSPFQVTTCKHTGGSPRPPCQYRASAGFRHVVIAC--ENGLPVHFDES-f<br>-kESAAAKFERQHMDSgnSPSSSSN-YCNLMMCCRKMTQgKCKPVNTFVHESLADVKAVCSQKKVTCKNgqtNCYQSKSTMRITDCRETGSSKYPNCAYKTTQVEKHIIVACggKPSVPVHFDASv-<br><br>Proposed: aliSize=113 (resi) RMSD=1.46 (Å)<br>DDSRYTKFLTQHHDAK-PKG--rdDRYCERMMKRRSLTS-PCKDVNTFIHGNKSNIKAICGanGSPYR-----ENLRMSKSPFQVTTCKHTGGSPRPPCQYRASAGFRHVVIACEN--GLPVHFDEsFF<br>KESAAAKFERQHMDSGnSPSss--SNYCNLMMCCRKMTQgKCKPVNTFVHESLADVKAVCS--QKKVTckngqTNCYQSKSTMRITDCRETGSSKYPNCAYKTTQVEKHIIVACGGkpSVPVHFDA-SV
910	2bwlA (118)<br>1y92B (124)	N	34.75	TM-align: aliSize=96 (resi) RMSD=1.73 (Å)<br>ddsrytkfltqhhdakpk-------------------GrDDRYCERMMKRRSLTS-PCKDVNTFIHGNKSNIKAICGanGSPYR----EN-LRMSKSPFQVTTCKHTGGSPRPPCQYRASAGFRHVVIACEN--GLPVHFDEsFF<br>-----------------kesaaakferqhmdsgnsasS-SSNYCNLMMCCRKMTQgKCKPVNTFVHESLADVKAVCS--QKKVTckdgQTnCYQSKSTMRITDCRETGSSKYPNCAYKTTQVEKHIIVACGGkpSVPVHFDA-SV<br><br>SARST: aliSize=111 (resi) RMSD=9.65 (Å)<br>dd--SRYTKFLTQHHD-AKPKGRDDRYCERMMKRRSLTS-PCKDVNTFIHGNKSNIKAICGANGSPY---RENLRMSKSPFQVTTCKHTGGSPRPPCQYRASAGFRHVVIACEN--GLPVHF----desf<br>--keSAAAKFERQHMDsGNSASSSSNYCNLMMCCRKMTQgKCKPVNTFVHESLADVKAVCSQKKVTCkdgQTNCYQSKSTMRITDCRETGSSKYPNCAYKTTQVEKHIIVACGGkpSVPVHFdasv----<br><br>BLAST: aliSize=65 (resi) iden=36.75% (43/118) simi=55.56% (65/118)<br>d-DSRYTKFLTQHHDA-KPKGRDDRYCERMMKRRSLTS-PCKDVNTFIHGNKSNIKAICGANGSPYRE---NLRMSKSPFQVTTCKHTGGSPRPPCQYRASAGFRHVVIAC--ENGLPVHFDES-f<br>-kESAAAKFERQHMDSgNSASSSSNYCNLMMCCRKMTQgKCKPVNTFVHESLADVKAVCSQKKVTCKDgqtNCYQSKSTMRITDCRETGSSKYPNCAYKTTQVEKHIIVACggKPSVPVHFDASv-<br><br>Proposed: aliSize=114 (resi) RMSD=1.59 (Å)<br>DDSRYTKFLTQHHDAKPKGRD-DRYCERMMKRRSLTS-PCKDVNTFIHGNKSNIKAICGanGSPYR-----ENLRMSKSPFQVTTCKHTG-GSpRPPCQYRASAGFRHVVIACEN--GLPVHFDEsFF<br>KESAAAKFERQHMDSGNSASSsSNYCNLMMCCRKMTQgKCKPVNTFVHESLADVKAVCS--QKKVTckdgqTNCYQSKSTMRITDCRETGsSK-YPNCAYKTTQVEKHIIVACGGkpSVPVHFDA-SV
911	2bwlA (118)<br>3bcoA (124)	N	35.59	TM-align: aliSize=96 (resi) RMSD=1.82 (Å)<br>ddsrytkfltqhhdakpk------------------GRDDRYCERMMKRRSLTS-PCKDVNTFIHGNKSNIKAICGanGSPYR-----ENLRMSKSPFQVTTCKHT-GGSpRPPCQYRASAGFRHVVIACE-N-GLPVHFDEsFF<br>-----------------kesaaakferqhmdsgnsaSSSSNYCNQMMCCRKMTQgKCKPVNTFVHESLADVKAVCS--QKKVTckdgqTNCYQSKSTMRITDCRETgSSK-YPNCAYKTTQVEKHIIVACGgKpSVPVHFDA-SV<br><br>SARST: aliSize=111 (resi) RMSD=9.75 (Å)<br>dd--SRYTKFLTQHHDAKPKGRDDR-YCERMMKRRSLTS-PCKDVNTFIHGNKSNIKAICGANGSPY---RENLRMSKSPFQVTTCKHTGGSPRPPCQYRASAGFRHVVIACEN--GLPVHF----desf<br>--keSAAAKFERQHMDSGNSASSSSnYCNQMMCCRKMTQgKCKPVNTFVHESLADVKAVCSQKKVTCkdgQTNCYQSKSTMRITDCRETGSSKYPNCAYKTTQVEKHIIVACGGkpSVPVHFdasv----<br><br>BLAST: aliSize=66 (resi) iden=36.75% (43/118) simi=56.41% (66/118)<br>d-DSRYTKFLTQHHDA-KPKGRDDRYCERMMKRRSLTS-PCKDVNTFIHGNKSNIKAICGANGSPYRE---NLRMSKSPFQVTTCKHTGGSPRPPCQYRASAGFRHVVIAC--ENGLPVHFDES-f<br>-kESAAAKFERQHMDSgNSASSSSNYCNQMMCCRKMTQgKCKPVNTFVHESLADVKAVCSQKKVTCKDgqtNCYQSKSTMRITDCRETGSSKYPNCAYKTTQVEKHIIVACggKPSVPVHFDASv-<br><br>Proposed: aliSize=113 (resi) RMSD=1.54 (Å)<br>DDSRYTKFLTQHHDAK--PKG-rdDRYCERMMKRRSLTS-PCKDVNTFIHGNKSNIKAICGanGSPYR-----ENLRMSKSPFQVTTCKHTGGSPRPPCQYRASAGFRHVVIACE-N-GLPVHFDEsFF<br>KESAAAKFERQHMDSGnsASSs--SNYCNQMMCCRKMTQgKCKPVNTFVHESLADVKAVCS--QKKVTckdgqTNCYQSKSTMRITDCRETGSSKYPNCAYKTTQVEKHIIVACGgKpSVPVHFDA-SV

912	2bwlA (118)<br>1y94A (124)	N	33.90	TM-align: aliSize=96 (resi) RMSD=1.90 (Å)<br>ddsrytkfltqhhdakpk------------------GRDDRYCERMMKRRSLTS-PCKDVNTFIHGNKSNIKAICGanGSPYR----EN-LRMSKSPFQVTTCKHT-GGSpRPPCQYRASAGFRHVVIACEN--GLPVHFDEsFF<br>-----------------kesaaakferqhmdsstsaASSSNYCNLMMCCRKMTQgKCKPVNTFVHESLADVKAVCS--QKKVTckdgQTnCYQSKSTMRITDCRETgSSK-YPNCAYKTTQVEKHIIVACGGkpSVPVHFDA-SV<br><br>SARST: aliSize=110 (resi) RMSD=9.59 (Å)<br>dd--SRYTKFLTQHHD--AKPKGRDdRYCERMMKRRSLTS-PCKDVNTFIHGNKSNIKAICGANGSPYRE---NLRMSKSPFQVTTCKHTGGSPRPPCQYRASAGFRHVVIACEN--GLPVHF----desf<br>--keSAAAKFERQHMDssTSAASSS-NYCNLMMCCRKMTQgKCKPVNTFVHESLADVKAVCSQKKVTCKDgqtNCYQSKSTMRITDCRETGSSKYPNCAYKTTQVEKHIIVACGGkpSVPVHFdasv----<br><br>BLAST: aliSize=65 (resi) iden=36.75% (43/118) simi=55.56% (65/118)<br>d-DSRYTKFLTQHHDAKPKGRDD-RYCERMMKRRSLTS-PCKDVNTFIHGNKSNIKAICGANGSPYRE---NLRMSKSPFQVTTCKHTGGSPRPPCQYRASAGFRHVVIAC--ENGLPVHFDES-f<br>-kESAAAKFERQHMDSSTSAASSsNYCNLMMCCRKMTQgKCKPVNTFVHESLADVKAVCSQKKVTCKDgqtNCYQSKSTMRITDCRETGSSKYPNCAYKTTQVEKHIIVACggKPSVPVHFDASv-<br><br>Proposed: aliSize=112 (resi) RMSD=1.42 (Å)<br>DDSRYTKFLTQHHDAK-PKG--rdDRYCERMMKRRSLTS-PCKDVNTFIHGNKSNIKAICGanGSPYR-----ENLRMSKSPFQVTTCKHT-GGSpRPPCQYRASAGFRHVVIACEN--GLPVHFDEsFF<br>KESAAAKFERQHMDSStSAAss--SNYCNLMMCCRKMTQgKCKPVNTFVHESLADVKAVCS--QKKVTckdgqTNCYQSKSTMRITDCRETgSSK-YPNCAYKTTQVEKHIIVACGGkpSVPVHFDA-SV
913	1gv7A (123)<br>1lbgA (124)	N	40.65	TM-align: aliSize=102 (resi) RMSD=1.82 (Å)<br>dnsrythfltqhydakpq------------------GRDDRYCESIMRRRGLTS-PCKDINTFIHGNKRSIKAICSQKNVACKNGQTNCYISKSSFQVTTCKLHGGSPWPPCQYRATAGFRNVVVACE-N-GLPVHLDQsIFr<br>-----------------kesaaakferqhmdsgnspSSSSNYCNLMMCCRKMTQgKCKPVNTFVHESLADVKAVCSQKKVTCKNGQTNCYQSKSTMRITDCRETGSSKYPNCAYKTTQVEKHIIVACGgKpSVPVHFDA-SV-<br><br>SARST: aliSize=112 (resi) RMSD=9.32 (Å)<br>dns---RYTHFLTQHYDaKPQG-RDDR-YCESIMRRRGLTS-PCKDINTFIHGNKRSIKAICSQKNVACKNGQTNCYISKSSFQVTTCKLHGGSPWPPCQYRATAGFRNVVVACEN--GLPVHL----dqsifr<br>---kesAAAKFERQHMD-SGNSpSSSSnYCNLMMCCRKMTQgKCKPVNTFVHESLADVKAVCSQKKVTCKNGQTNCYQSKSTMRITDCRETGSSKYPNCAYKTTQVEKHIIVACGGkpSVPVHFdasv------<br><br>BLAST: aliSize=73 (resi) iden=41.80% (51/123) simi=59.84% (73/123)<br>dnsryth-------FLTQHYDA--KPQGRDDrYCESIMRRRGLTS-PCKDINTFIHGNKRSIKAICSQKNVACKNGQTNCYISKSSFQVTTCKLHGGSPWPPCQYRATAGFRNVVVAC--ENGLPVHLDQSIfr<br>-------kesaaakFERQHMDSgnSPSSSSN-YCNLMMCCRKMTQgKCKPVNTFVHESLADVKAVCSQKKVTCKNGQTNCYQSKSTMRITDCRETGSSKYPNCAYKTTQVEKHIIVACggKPSVPVHFDASV--<br><br>Proposed: aliSize=117 (resi) RMSD=1.42 (Å)<br>pdn--SRYTHFLTQHYDAKPQGR-DDRYCESIMRRRGLTS-PCKDINTFIHGNKRSIKAICSQKNVACKNGQTNCYISKSSFQVTTCKLHGGSPWPPCQYRATAGFRNVVVACE-N-GLPVHLDQsI-fr<br>---keSAAAKFERQHMDSGNSPSsSSNYCNLMMCCRKMTQgKCKPVNTFVHESLADVKAVCSQKKVTCKNGQTNCYQSKSTMRITDCRETGSSKYPNCAYKTTQVEKHIIVACGgKpSVPVHFDA-Sv--
914	1gv7A (123)<br>1y92B (124)	N	39.02	TM-align: aliSize=102 (resi) RMSD=1.89 (Å)<br>dnsrythfltqhydakpq-----------------GRDDRYCESIMRRRGLTS-PCKDINTFIHGNKRSIKAICSQKNVACKNGQTNCYISKSSFQVTTCKLHGGSPWPPCQYRATAGFRNVVVACEN--GLPVHLDQsIFr<br>-----------------kesaaakferqhmdsgnsaSSSSNYCNLMMCCRKMTQgKCKPVNTFVHESLADVKAVCSQKKVTCKDGQTNCYQSKSTMRITDCRETGSSKYPNCAYKTTQVEKHIIVACGGkpSVPVHFDA-SV-<br><br>SARST: aliSize=113 (resi) RMSD=9.17 (Å)<br>dns---RYTHFLTQHYD-AKPQGRDDRYCESIMRRRGLTS-PCKDINTFIHGNKRSIKAICSQKNVACKNGQTNCYISKSSFQVTTCKLHGGSPWPPCQYRATAGFRNVVVACEN--GLPVHL----dqsifr<br>---kesAAAKFERQHMDsGNSASSSSNYCNLMMCCRKMTQgKCKPVNTFVHESLADVKAVCSQKKVTCKDGQTNCYQSKSTMRITDCRETGSSKYPNCAYKTTQVEKHIIVACGGkpSVPVHFdasv------<br><br>BLAST: aliSize=71 (resi) iden=40.16% (49/123) simi=58.20% (71/123)<br>dnsryth-------FLTQHYDA-KPQGRDDRYCESIMRRRGLTS-PCKDINTFIHGNKRSIKAICSQKNVACKNGQTNCYISKSSFQVTTCKLHGGSPWPPCQYRATAGFRNVVVAC--ENGLPVHLDQSIfr<br>-------kesaaakFERQHMDSgNSASSSSNYCNLMMCCRKMTQgKCKPVNTFVHESLADVKAVCSQKKVTCKDGQTNCYQSKSTMRITDCRETGSSKYPNCAYKTTQVEKHIIVACggKPSVPVHFDASV--<br><br>Proposed: aliSize=115 (resi) RMSD=1.28 (Å)<br>pdn--SRYTHFLTQHYDAKPQGRD--dRYCESIMRRRGLTS-PCKDINTFIHGNKRSIKAICSQKNVACKNGQTNCYISKSSFQVTTCKLHG-GSpWPPCQYRATAGFRNVVVACEN--GLPVHLDQsI-fr<br>---keSAAAKFERQHMDSGNSASSss-NYCNLMMCCRKMTQgKCKPVNTFVHESLADVKAVCSQKKVTCKDGQTNCYQSKSTMRITDCRETGsSK-YPNCAYKTTQVEKHIIVACGGkpSVPVHFDA-Sv--

915	1gv7A (123)<br>3bcoA (124)	N	39.84	TM-align: aliSize=102 (resi) RMSD=1.88 (Å)<br>dnsrythfltqhydakpq------------------GRDDRYCESIMRRRGLTS-PCKDINTFIHGNKRSIKAICSQKNVACKNGQTNCYISKSSFQVTTCKLHGGSPWPPCQYRATAGFRNVVVACE-N-GLPVHLDQsIFr<br>-----------------kesaaakferqhmdsgnsaSSSSNYCNQMMCCRKMTQgKCKPVNTFVHESLADVKAVCSQKKVTCKDGQTNCYQSKSTMRITDCRETGSSKYPNCAYKTTQVEKHIIVACGgKpSVPVHFDA-SV-<br><br>SARST: aliSize=113 (resi) RMSD=9.26 (Å)<br>dns---RYTHFLTQHYDAKPQGRDDR-YCESIMRRRGLTS-PCKDINTFIHGNKRSIKAICSQKNVACKNGQTNCYISKSSFQVTTCKLHGGSPWPPCQYRATAGFRNVVVACEN--GLPVHL----dqsifr<br>---kesAAAKFERQHMDSGNSASSSSnYCNQMMCCRKMTQgKCKPVNTFVHESLADVKAVCSQKKVTCKDGQTNCYQSKSTMRITDCRETGSSKYPNCAYKTTQVEKHIIVACGGkpSVPVHFdasv------<br><br>BLAST: aliSize=71 (resi) iden=40.16% (49/123) simi=58.20% (71/123)<br>dnsryth-------FLTQHYDA-KPQGRDDRYCESIMRRRGLTS-PCKDINTFIHGNKRSIKAICSQKNVACKNGQTNCYISKSSFQVTTCKLHGGSPWPPCQYRATAGFRNVVVAC--ENGLPVHLDQSIfr<br>-------kesaaakFERQHMDSgNSASSSSNYCNQMMCCRKMTQgKCKPVNTFVHESLADVKAVCSQKKVTCKDGQTNCYQSKSTMRITDCRETGSSKYPNCAYKTTQVEKHIIVACggKPSVPVHFDASV--<br><br>Proposed: aliSize=115 (resi) RMSD=1.36 (Å)<br>pdn--SRYTHFLTQHYDAK--PQG-rdDRYCESIMRRRGLTS-PCKDINTFIHGNKRSIKAICSQKNVACKNGQTNCYISKSSFQVTTCKLHGGSPWPPCQYRATAGFRNVVVACE-N-GLPVHLDQsI-fr<br>---keSAAAKFERQHMDSGnsASSs--SNYCNQMMCCRKMTQgKCKPVNTFVHESLADVKAVCSQKKVTCKDGQTNCYQSKSTMRITDCRETGSSKYPNCAYKTTQVEKHIIVACGgKpSVPVHFDA-Sv--
916	1gv7A (123)<br>1y94A (124)	N	39.84	TM-align: aliSize=102 (resi) RMSD=1.92 (Å)<br>dnsrythfltqhydakpq------------------GRDDRYCESIMRRRGLTS-PCKDINTFIHGNKRSIKAICSQKNVACKNGQTNCYISKSSFQVTTCKLHGGSPWPPCQYRATAGFRNVVVACE-N-GLPVHLDQsIFr<br>-----------------kesaaakferqhmdsstsaASSSNYCNLMMCCRKMTQgKCKPVNTFVHESLADVKAVCSQKKVTCKDGQTNCYQSKSTMRITDCRETGSSKYPNCAYKTTQVEKHIIVACGgKpSVPVHFDA-SV-<br><br>SARST: aliSize=113 (resi) RMSD=9.17 (Å)<br>dns---RYTHFLTQHYD--AKPQGRDDRYCESIMRRRGLTSPCKDINTFIHGNKRSIKAICSQKNVACKNGQTNCYISKSSFQVTTCKLHGGSPWPPCQYRATAGFRNVVVACEN--GLPVHL----dqsifr<br>---kesAAAKFERQHMDssTSAASSSNYCNLMMCCRKMTQGKCKPVNTFVHESLADVKAVCSQKKVTCKDGQTNCYQSKSTMRITDCRETGSSKYPNCAYKTTQVEKHIIVACGGkpSVPVHFdasv------<br><br>BLAST: aliSize=71 (resi) iden=40.16% (49/123) simi=58.20% (71/123)<br>dnsryth-------FLTQHYDAKPQGRDD-RYCESIMRRRGLTS-PCKDINTFIHGNKRSIKAICSQKNVACKNGQTNCYISKSSFQVTTCKLHGGSPWPPCQYRATAGFRNVVVAC--ENGLPVHLDQSIfr<br>-------kesaaakFERQHMDSSTSAASSsNYCNLMMCCRKMTQgKCKPVNTFVHESLADVKAVCSQKKVTCKDGQTNCYQSKSTMRITDCRETGSSKYPNCAYKTTQVEKHIIVACggKPSVPVHFDASV--<br><br>Proposed: aliSize=115 (resi) RMSD=1.36 (Å)<br>pdn--SRYTHFLTQHYDAK--PQG-rdDRYCESIMRRRGLTS-PCKDINTFIHGNKRSIKAICSQKNVACKNGQTNCYISKSSFQVTTCKLHGGSPWPPCQYRATAGFRNVVVACEN--GLPVHLDQsI-fr<br>---keSAAAKFERQHMDSStsAASs--SNYCNLMMCCRKMTQgKCKPVNTFVHESLADVKAVCSQKKVTCKDGQTNCYQSKSTMRITDCRETGSSKYPNCAYKTTQVEKHIIVACGGkpSVPVHFDA-Sv--
917	1rraA (124)<br>1lbgA (124)	N	66.13	TM-align: aliSize=104 (resi) RMSD=1.03 (Å)<br>aessadkfkrqhmdtegpsk--------------------SSPTYCNQMMKRQGMTKGSCKPVNTFVHEPLEDVQAICSQGQVTCKNGRNNCHKSSSTLRITDCRLKGSSKYPNCDYTTTDSQKHIIIACDGNPYVPVHFDASV<br>-------------------kesaaakferqhmdsgnspsSSSNYCNLMMCCRKMTQGKCKPVNTFVHESLADVKAVCSQKKVTCKNGQTNCYQSKSTMRITDCRETGSSKYPNCAYKTTQVEKHIIVACGGKPSVPVHFDASV<br><br>SARST: aliSize=121 (resi) RMSD=9.62 (Å)<br>ae--SSADKFKRQHMDTEGPSKSSPTYCNQMMKRQGMTKGSCKPVNTFVHEPLEDVQAICSQGQVTCKNGRNNCHKSSSTLRITDCRLKGSSKYPNCDYTTTDSQKHIIIACDGNPYVPVHFDAS-v<br>--keSAAAKFERQHMDSGNSPSSSSNYCNLMMCCRKMTQGKCKPVNTFVHESLADVKAVCSQKKVTCKNGQTNCYQSKSTMRITDCRETGSSKYPNCAYKTTQVEKHIIVACGGKPSVPVHFDASv-<br><br>BLAST: aliSize=96 (resi) iden=66.13% (82/124) simi=77.42% (96/124)<br>a-ESSADKFKRQHMDTEGPSKSSPTYCNQMMKRQGMTKGSCKPVNTFVHEPLEDVQAICSQGQVTCKNGRNNCHKSSSTLRITDCRLKGSSKYPNCDYTTTDSQKHIIIACDGNPYVPVHFDASV<br>-kESAAAKFERQHMDSGNSPSSSSNYCNLMMCCRKMTQGKCKPVNTFVHESLADVKAVCSQKKVTCKNGQTNCYQSKSTMRITDCRETGSSKYPNCAYKTTQVEKHIIVACGGKPSVPVHFDASV<br><br>Proposed: aliSize=124 (resi) RMSD=1.18 (Å)<br>AESSADKFKRQHMDTEGPSKSSPTYCNQMMKRQGMTKGSCKPVNTFVHEPLEDVQAICSQGQVTCKNGRNNCHKSSSTLRITDCRLKGSSKYPNCDYTTTDSQKHIIIACDGNPYVPVHFDASV<br>KESAAAKFERQHMDSGNSPSSSSNYCNLMMCCRKMTQGKCKPVNTFVHESLADVKAVCSQKKVTCKNGQTNCYQSKSTMRITDCRETGSSKYPNCAYKTTQVEKHIIVACGGKPSVPVHFDASV

918	1rraA (124)<br>1y92B (124)	N	65.32	TM-align: aliSize=104 (resi) RMSD=0.82 (Å)<br>aessadkfkrqhmdtegpsk--------------------SSPTYCNQMMKRQGMTKGSCKPVNTFVHEPLEDVQAICSQGQVTCKNGRNNCHKSSSTLRITDCRLKGSSKYPNCDYTTTDSQKHIIACDGNPYVPVHFDASV<br>--------------------kesaaakferqhmdsgnsasSSSNYCNLMMCCRKMTQGKCKPVNTFVHESLADVKAVCSQKKVTCKDGQTNCYQSKSTMRITDCRETGSSKYPNCAYKTTQVEKHIIVACGGKPSVPVHFDASV<br><br>SARST: aliSize=122 (resi) RMSD=9.61 (Å)<br>a-ESSADKFKRQHMDTEGPSKSSPTYCNQMMKRQGMTKGSCKPVNTFVHEPLEDVQAICSQGQVTCKNGRNNCHKSSSTLRITDCRLKGSSKYPNCDYTTTDSQKHIIACDGNPYVPVHFDAS-v<br>-kESAAAKFERQHMDSGNSASSSSNYCNLMMCCRKMTQGKCKPVNTFVHESLADVKAVCSQKKVTCKDGQTNCYQSKSTMRITDCRETGSSKYPNCAYKTTQVEKHIIVACGGKPSVPVHFDASv-<br><br>BLAST: aliSize=97 (resi) iden=65.32% (81/124) simi=78.23% (97/124)<br>a-ESSADKFKRQHMDTEGPSKSSPTYCNQMMKRQGMTKGSCKPVNTFVHEPLEDVQAICSQGQVTCKNGRNNCHKSSSTLRITDCRLKGSSKYPNCDYTTTDSQKHIIACDGNPYVPVHFDASV<br>-kESAAAKFERQHMDSGNSASSSSNYCNLMMCCRKMTQGKCKPVNTFVHESLADVKAVCSQKKVTCKDGQTNCYQSKSTMRITDCRETGSSKYPNCAYKTTQVEKHIIVACGGKPSVPVHFDASV<br><br>Proposed: aliSize=124 (resi) RMSD=0.76 (Å)<br>AESSADKFKRQHMDTEGPSKSSPTYCNQMMKRQGMTKGSCKPVNTFVHEPLEDVQAICSQGQVTCKNGRNNCHKSSSTLRITDCRLKGSSKYPNCDYTTTDSQKHIIACDGNPYVPVHFDASV<br>KESAAAKFERQHMDSGNSASSSSNYCNLMMCCRKMTQGKCKPVNTFVHESLADVKAVCSQKKVTCKDGQTNCYQSKSTMRITDCRETGSSKYPNCAYKTTQVEKHIIVACGGKPSVPVHFDASV
919	1rraA (124)<br>3bcoA (124)	N	66.13	TM-align: aliSize=104 (resi) RMSD=1.06 (Å)<br>aessadkfkrqhmdtegpsk--------------------SSPTYCNQMMKRQGMTKGSCKPVNTFVHEPLEDVQAICSQGQVTCKNGRNNCHKSSSTLRITDCRLKGSSKYPNCDYTTTDSQKHIIACDGNPYVPVHFDASV<br>--------------------kesaaakferqhmdsgnsasSSSNYCNQMMCCRKMTQGKCKPVNTFVHESLADVKAVCSQKKVTCKDGQTNCYQSKSTMRITDCRETGSSKYPNCAYKTTQVEKHIIVACGGKPSVPVHFDASV<br><br>SARST: aliSize=120 (resi) RMSD=9.38 (Å)<br>a-ESSADKFKRQHMDteGPSK--SSPTYCNQMMKRQGMTKGSCKPVNTFVHEPLEDVQAICSQGQVTCKNGRNNCHKSSSTLRITDCRLKGSSKYPNCDYTTTDSQKHIIACDGNPYVPVHFDAS-v<br>-kESAAAKFERQHMD--SGNSasSSSNYCNQMMCCRKMTQGKCKPVNTFVHESLADVKAVCSQKKVTCKDGQTNCYQSKSTMRITDCRETGSSKYPNCAYKTTQVEKHIIVACGGKPSVPVHFDASv-<br><br>BLAST: aliSize=98 (resi) iden=66.13% (82/124) simi=79.03% (98/124)<br>a-ESSADKFKRQHMDTEGPSKSSPTYCNQMMKRQGMTKGSCKPVNTFVHEPLEDVQAICSQGQVTCKNGRNNCHKSSSTLRITDCRLKGSSKYPNCDYTTTDSQKHIIACDGNPYVPVHFDASV<br>-kESAAAKFERQHMDSGNSASSSSNYCNQMMCCRKMTQGKCKPVNTFVHESLADVKAVCSQKKVTCKDGQTNCYQSKSTMRITDCRETGSSKYPNCAYKTTQVEKHIIVACGGKPSVPVHFDASV<br><br>Proposed: aliSize=124 (resi) RMSD=1.13 (Å)<br>AESSADKFKRQHMDTEGPSKSSPTYCNQMMKRQGMTKGSCKPVNTFVHEPLEDVQAICSQGQVTCKNGRNNCHKSSSTLRITDCRLKGSSKYPNCDYTTTDSQKHIIACDGNPYVPVHFDASV<br>KESAAAKFERQHMDSGNSASSSSNYCNQMMCCRKMTQGKCKPVNTFVHESLADVKAVCSQKKVTCKDGQTNCYQSKSTMRITDCRETGSSKYPNCAYKTTQVEKHIIVACGGKPSVPVHFDASV
920	1rraA (124)<br>1y94A (124)	N	65.32	TM-align: aliSize=104 (resi) RMSD=1.05 (Å)<br>aessadkfkrqhmdtegpsk--------------------SSPTYCNQMMKRQGMTKGSCKPVNTFVHEPLEDVQAICSQGQVTCKNGRNNCHKSSSTLRITDCRLKGSSKYPNCDYTTTDSQKHIIACDGNPYVPVHFDASV<br>--------------------kesaaakferqhmdsstsaaSSSNYCNLMMCCRKMTQGKCKPVNTFVHESLADVKAVCSQKKVTCKDGQTNCYQSKSTMRITDCRETGSSKYPNCAYKTTQVEKHIIVACGGKPSVPVHFDASV<br><br>SARST: aliSize=122 (resi) RMSD=9.60 (Å)<br>a-ESSADKFKRQHMDTEGPSKSSPTYCNQMMKRQGMTKGSCKPVNTFVHEPLEDVQAICSQGQVTCKNGRNNCHKSSSTLRITDCRLKGSSKYPNCDYTTTDSQKHIIACDGNPYVPVHFDAS-v<br>-kESAAAKFERQHMDSSTSAASSSNYCNLMMCCRKMTQGKCKPVNTFVHESLADVKAVCSQKKVTCKDGQTNCYQSKSTMRITDCRETGSSKYPNCAYKTTQVEKHIIVACGGKPSVPVHFDASv-<br><br>BLAST: aliSize=97 (resi) iden=65.32% (81/124) simi=78.23% (97/124)<br>a-ESSADKFKRQHMDTEGPSKSSPTYCNQMMKRQGMTKGSCKPVNTFVHEPLEDVQAICSQGQVTCKNGRNNCHKSSSTLRITDCRLKGSSKYPNCDYTTTDSQKHIIACDGNPYVPVHFDASV<br>-kESAAAKFERQHMDSSTSAASSSNYCNLMMCCRKMTQGKCKPVNTFVHESLADVKAVCSQKKVTCKDGQTNCYQSKSTMRITDCRETGSSKYPNCAYKTTQVEKHIIVACGGKPSVPVHFDASV<br><br>Proposed: aliSize=124 (resi) RMSD=1.16 (Å)<br>AESSADKFKRQHMDTEGPSKSSPTYCNQMMKRQGMTKGSCKPVNTFVHEPLEDVQAICSQGQVTCKNGRNNCHKSSSTLRITDCRLKGSSKYPNCDYTTTDSQKHIIACDGNPYVPVHFDASV<br>KESAAAKFERQHMDSSTSAASSSNYCNLMMCCRKMTQGKCKPVNTFVHESLADVKAVCSQKKVTCKDGQTNCYQSKSTMRITDCRETGSSKYPNCAYKTTQVEKHIIVACGGKPSVPVHFDASV

921	2q4gX (126)<br>11bgA (124)	N	69.35	TM-align: aliSize=104 (resi) RMSD=0.92 (Å)<br>esrakkfqrqhmdsdssps-------------------SSSTYCNQMMRRRNMTQGRCKPVNTFVHEPLVDVQNVCFQEKVTCKNGQGNCYKSNSSMHITDCRLTNGSRYPNCAYRTSPKERHIIVACEGSPYVPVHFDASVeds<br>------------------kesaaakferqhmdsgnspsSSSNYCNLMMCCRKMTQGKCKPVNTFVHESLADVKAVCSQKKVTCKNGQTNCYQSKSTMRITDCRETGSSKYPNCAYKTTQVEKHIIVACGGKPSVPVHFDASV---<br><br>SARST: aliSize=121 (resi) RMSD=9.64 (Å)<br>e--SRAKKFQRQHMDSDSSPSSSSTYCNQMMRRRNMTQGRCKPVNTFVHEPLVDVQNVCFQEKVTCKNGQGNCYKSNSSMHITDCRLTNGSRYPNCAYRTSPKERHIIVACEGSPYVPVHFDAS-veds<br>-keSAAAKFERQHMDSGNSPSSSSNYCNLMMCCRKMTQGKCKPVNTFVHESLADVKAVCSQKKVTCKNGQTNCYQSKSTMRITDCRETGSSKYPNCAYKTTQVEKHIIVACGGKPSVPVHFDASv----<br><br>BLAST: aliSize=100 (resi) iden=71.77% (89/124) simi=80.65% (100/124)<br>-ESRAKKFQRQHMDSDSSPSSSSTYCNQMMRRRNMTQGRCKPVNTFVHEPLVDVQNVCFQEKVTCKNGQGNCYKSNSSMHITDCRLTNGSRYPNCAYRTSPKERHIIVACEGSPYVPVHFDASVeds<br>kESAAAKFERQHMDSGNSPSSSSNYCNLMMCCRKMTQGKCKPVNTFVHESLADVKAVCSQKKVTCKNGQTNCYQSKSTMRITDCRETGSSKYPNCAYKTTQVEKHIIVACGGKPSVPVHFDASV---<br><br>Proposed: aliSize=122 (resi) RMSD=0.85 (Å)<br>-ESRAKKFQRQHMDSdSSPSS-SSTYCNQMMRRRNMTQGRCKPVNTFVHEPLVDVQNVCFQEKVTCKNGQGNCYKSNSSMHITDCRLTNGSRYPNCAYRTSPKERHIIVACEGSPYVPVHFDASVeds<br>kESAAAKFERQHMDS-GNSPSsSSNYCNLMMCCRKMTQGKCKPVNTFVHESLADVKAVCSQKKVTCKNGQTNCYQSKSTMRITDCRETGSSKYPNCAYKTTQVEKHIIVACGGKPSVPVHFDASV---
922	2q4gX (126)<br>1y92B (124)	N	70.16	TM-align: aliSize=104 (resi) RMSD=0.89 (Å)<br>esrakkfqrqhmdsdssps-------------------SSSTYCNQMMRRRNMTQGRCKPVNTFVHEPLVDVQNVCFQEKVTCKNGQGNCYKSNSSMHITDCRLTNGSRYPNCAYRTSPKERHIIVACEGSPYVPVHFDASVeds<br>------------------kesaaakferqhmdsgnsasSSSNYCNLMMCCRKMTQGKCKPVNTFVHESLADVKAVCSQKKVTCKDGQTNCYQSKSTMRITDCRETGSSKYPNCAYKTTQVEKHIIVACGGKPSVPVHFDASV---<br><br>SARST: aliSize=118 (resi) RMSD=8.22 (Å)<br>esra-----KKFQRQHMDSDSSPSSSSTYCNQMMRRRNMTQGRCKPVNTFVHEPLVDVQNVCFQEKVTCKNGQGNCYKSNSSMHITDCRLTNGSRYPNCAYRTSPKERHIIVACEGSPYVPVHFDAS-veds<br>----kesaaAKFERQHMDSGNSASSSSNYCNLMMCCRKMTQGKCKPVNTFVHESLADVKAVCSQKKVTCKDGQTNCYQSKSTMRITDCRETGSSKYPNCAYKTTQVEKHIIVACGGKPSVPVHFDASv----<br><br>BLAST: aliSize=99 (resi) iden=70.16% (87/124) simi=79.84% (99/124)<br>-ESRAKKFQRQHMDSDSSPSSSSTYCNQMMRRRNMTQGRCKPVNTFVHEPLVDVQNVCFQEKVTCKNGQGNCYKSNSSMHITDCRLTNGSRYPNCAYRTSPKERHIIVACEGSPYVPVHFDASVeds<br>kESAAAKFERQHMDSGNSASSSSNYCNLMMCCRKMTQGKCKPVNTFVHESLADVKAVCSQKKVTCKDGQTNCYQSKSTMRITDCRETGSSKYPNCAYKTTQVEKHIIVACGGKPSVPVHFDASV---<br><br>Proposed: aliSize=123 (resi) RMSD=0.75 (Å)<br>-ESRAKKFQRQHMDSDSSPSSSSTYCNQMMRRRNMTQGRCKPVNTFVHEPLVDVQNVCFQEKVTCKNGQGNCYKSNSSMHITDCRLTNGSRYPNCAYRTSPKERHIIVACEGSPYVPVHFDASVeds<br>kESAAAKFERQHMDSGNSASSSSNYCNLMMCCRKMTQGKCKPVNTFVHESLADVKAVCSQKKVTCKDGQTNCYQSKSTMRITDCRETGSSKYPNCAYKTTQVEKHIIVACGGKPSVPVHFDASV---
923	2q4gX (126)<br>3bcoA (124)	N	70.97	TM-align: aliSize=104 (resi) RMSD=0.95 (Å)<br>esrakkfqrqhmdsdssps-------------------SSSTYCNQMMRRRNMTQGRCKPVNTFVHEPLVDVQNVCFQEKVTCKNGQGNCYKSNSSMHITDCRLTNGSRYPNCAYRTSPKERHIIVACEGSPYVPVHFDASVeds<br>------------------kesaaakferqhmdsgnsasSSSNYCNQMMCCRKMTQGKCKPVNTFVHESLADVKAVCSQKKVTCKDGQTNCYQSKSTMRITDCRETGSSKYPNCAYKTTQVEKHIIVACGGKPSVPVHFDASV---<br><br>SARST: aliSize=121 (resi) RMSD=9.43 (Å)<br>e--SRAKKFQRQHMDSDSSPSSSSTYCNQMMRRRNMTQGRCKPVNTFVHEPLVDVQNVCFQEKVTCKNGQGNCYKSNSSMHITDCRLTNGSRYPNCAYRTSPKERHIIVACEGSPYVPVHFDAS-veds<br>-keSAAAKFERQHMDSGNSASSSSNYCNQMMCCRKMTQGKCKPVNTFVHESLADVKAVCSQKKVTCKDGQTNCYQSKSTMRITDCRETGSSKYPNCAYKTTQVEKHIIVACGGKPSVPVHFDASv----<br><br>BLAST: aliSize=100 (resi) iden=70.97% (88/124) simi=80.65% (100/124)<br>-ESRAKKFQRQHMDSDSSPSSSSTYCNQMMRRRNMTQGRCKPVNTFVHEPLVDVQNVCFQEKVTCKNGQGNCYKSNSSMHITDCRLTNGSRYPNCAYRTSPKERHIIVACEGSPYVPVHFDASVeds<br>kESAAAKFERQHMDSGNSASSSSNYCNQMMCCRKMTQGKCKPVNTFVHESLADVKAVCSQKKVTCKDGQTNCYQSKSTMRITDCRETGSSKYPNCAYKTTQVEKHIIVACGGKPSVPVHFDASV---<br><br>Proposed: aliSize=123 (resi) RMSD=0.95 (Å)<br>-ESRAKKFQRQHMDSDSSPSSSSTYCNQMMRRRNMTQGRCKPVNTFVHEPLVDVQNVCFQEKVTCKNGQGNCYKSNSSMHITDCRLTNGSRYPNCAYRTSPKERHIIVACEGSPYVPVHFDASVeds<br>kESAAAKFERQHMDSGNSASSSSNYCNQMMCCRKMTQGKCKPVNTFVHESLADVKAVCSQKKVTCKDGQTNCYQSKSTMRITDCRETGSSKYPNCAYKTTQVEKHIIVACGGKPSVPVHFDASV---

924	2q4gX (126)<br>1y94A (124)	N	69.35	TM-align: aliSize=104 (resi) RMSD=1.01 (Å)<br>esrakkfqrqhmdsdssps--------------------SSSTYCNQMMRRRNMTQGRCKPVNTFVHEPLVDVQNVCFQEKVTCKNGQGNCYKSNSSMHITDCRLTNGSRYPNCAYRTSPKERHIIVACEGSPYVPVHFDASVeds<br>-------------------kesaaakferqhmdsstsaaSSSNYCNLMMCCRKMTQGKCKPVNTFVHESLADVKAVCSQKKVTCKDGQTNCYQSKSTMRITDCRETGSSKYPNCAYKTTQVEKHIIVACGGKPSVPVHFDASV---<br><br>SARST: aliSize=118 (resi) RMSD=8.23 (Å)<br>esra-----KKFQRQHMDSDSSPSSSSTYCNQMMRRRNMTQGRCKPVNTFVHEPLVDVQNVCFQEKVTCKNGQGNCYKSNSSMHITDCRLTNGSRYPNCAYRTSPKERHIIVACEGSPYVPVHFDAS-veds<br>----kesaaAKFERQHMDSSTSAASSSNYCNLMMCCRKMTQGKCKPVNTFVHESLADVKAVCSQKKVTCKDGQTNCYQSKSTMRITDCRETGSSKYPNCAYKTTQVEKHIIVACGGKPSVPVHFDASv----<br><br>BLAST: aliSize=99 (resi) iden=69.35% (86/124) simi=79.84% (99/124)<br>-ESRAKKFQRQHMDSDSSPSSSSTYCNQMMRRRNMTQGRCKPVNTFVHEPLVDVQNVCFQEKVTCKNGQGNCYKSNSSMHITDCRLTNGSRYPNCAYRTSPKERHIIVACEGSPYVPVHFDASVeds<br>kESAAAKFERQHMDSSTSAASSSNYCNLMMCCRKMTQGKCKPVNTFVHESLADVKAVCSQKKVTCKDGQTNCYQSKSTMRITDCRETGSSKYPNCAYKTTQVEKHIIVACGGKPSVPVHFDASV---<br><br>Proposed: aliSize=123 (resi) RMSD=1.03 (Å)<br>-ESRAKKFQRQHMDSDSSPSSSSTYCNQMMRRRNMTQGRCKPVNTFVHEPLVDVQNVCFQEKVTCKNGQGNCYKSNSSMHITDCRLTNGSRYPNCAYRTSPKERHIIVACEGSPYVPVHFDASVeds<br>kESAAAKFERQHMDSSTSAASSSNYCNLMMCCRKMTQGKCKPVNTFVHESLADVKAVCSQKKVTCKDGQTNCYQSKSTMRITDCRETGSSKYPNCAYKTTQVEKHIIVACGGKPSVPVHFDASV---
925	2k11A (127)<br>1lbgA (124)	N	67.74	TM-align: aliSize=103 (resi) RMSD=1.45 (Å)<br>kesrakkfqrqhmdsdssps--------------------SSSTYCNQMMRRRNMTQGRCKPVNTFVHEPLVDVQNVCFQEKVTC-KNGQgNCYKSNSSMHITDCRLTNGSRYPNCAYRTSPKERHIIVACEGSPYVPVHFDASVeds<br>--------------------kesaaakferqhmdsgnspsSSSNYCNLMMCCRKMTQGKCKPVNTFVHESLADVKAVCSQKKVTCkNGQT-NCYQSKSTMRITDCRETGSSKYPNCAYKTTQVEKHIIVACGGKPSVPVHFDASV---<br><br>SARST: aliSize=120 (resi) RMSD=9.69 (Å)<br>ke--SRAKKFQRQHMDSDSSPSSSSTYCNQMMRRRNMTQGRCKPVNTFVHEPLVDVQNVCFQEKVTCKNGQGNCYKSNSSMHITDCRLTNGSRYPNCAYRTSPKERHIIVACEGSPYVPVHFDA--sveds<br>--keSAAAKFERQHMDSGNSPSSSSNYCNLMMCCRKMTQGKCKPVNTFVHESLADVKAVCSQKKVTCKNGQTNCYQSKSTMRITDCRETGSSKYPNCAYKTTQVEKHIIVACGGKPSVPVHFDAsv-----<br><br>BLAST: aliSize=101 (resi) iden=72.58% (90/124) simi=81.45% (101/124)<br>KESRAKKFQRQHMDSDSSPSSSSTYCNQMMRRRNMTQGRCKPVNTFVHEPLVDVQNVCFQEKVTCKNGQGNCYKSNSSMHITDCRLTNGSRYPNCAYRTSPKERHIIVACEGSPYVPVHFDASVeds<br>KESAAAKFERQHMDSGNSPSSSSNYCNLMMCCRKMTQGKCKPVNTFVHESLADVKAVCSQKKVTCKNGQTNCYQSKSTMRITDCRETGSSKYPNCAYKTTQVEKHIIVACGGKPSVPVHFDASV---<br><br>Proposed: aliSize=121 (resi) RMSD=1.31 (Å)<br>KESRAKKFQRQHMDSdsSPS-S-SSTYCNQMMRRRNMTQGRCKPVNTFVHEPLVDVQNVCFQEKVTC-KNGQgNCYKSNSSMHITDCRLTNGSRYPNCAYRTSPKERHIIVACEGSPYVPVHFDASVeds<br>KESAAAKFERQHMDS--GNSpSsSSNYCNLMMCCRKMTQGKCKPVNTFVHESLADVKAVCSQKKVTCkNGQT-NCYQSKSTMRITDCRETGSSKYPNCAYKTTQVEKHIIVACGGKPSVPVHFDASV---
926	2k11A (127)<br>1y92B (124)	N	70.97	TM-align: aliSize=104 (resi) RMSD=1.45 (Å)<br>kesrakkfqrqhmdsdssps--------------------SSSTYCNQMMRRRNMTQGRCKPVNTFVHEPLVDVQNVCFQEKVTCKNGQGNCYKSNSSMHITDCRLTNGSRYPNCAYRTSPKERHIIVACEGSPYVPVHFDASVeds<br>--------------------kesaaakferqhmdsgnsasSSSNYCNLMMCCRKMTQGKCKPVNTFVHESLADVKAVCSQKKVTCKDGQTNCYQSKSTMRITDCRETGSSKYPNCAYKTTQVEKHIIVACGGKPSVPVHFDASV---<br><br>SARST: aliSize=121 (resi) RMSD=9.67 (Å)<br>k-ESRAKKFQRQHMDSDSSPSSSSTYCNQMMRRRNMTQGRCKPVNTFVHEPLVDVQNVCFQEKVTCKNGQGNCYKSNSSMHITDCRLTNGSRYPNCAYRTSPKERHIIVACEGSPYVPVHFDA--sveds<br>-kESAAAKFERQHMDSGNSASSSSNYCNLMMCCRKMTQGKCKPVNTFVHESLADVKAVCSQKKVTCKDGQTNCYQSKSTMRITDCRETGSSKYPNCAYKTTQVEKHIIVACGGKPSVPVHFDAsv-----<br><br>BLAST: aliSize=100 (resi) iden=70.97% (88/124) simi=80.65% (100/124)<br>KESRAKKFQRQHMDSDSSPSSSSTYCNQMMRRRNMTQGRCKPVNTFVHEPLVDVQNVCFQEKVTCKNGQGNCYKSNSSMHITDCRLTNGSRYPNCAYRTSPKERHIIVACEGSPYVPVHFDASVeds<br>KESAAAKFERQHMDSGNSASSSSNYCNLMMCCRKMTQGKCKPVNTFVHESLADVKAVCSQKKVTCKDGQTNCYQSKSTMRITDCRETGSSKYPNCAYKTTQVEKHIIVACGGKPSVPVHFDASV---<br><br>Proposed: aliSize=124 (resi) RMSD=1.43 (Å)<br>KESRAKKFQRQHMDSDSSPSSSSTYCNQMMRRRNMTQGRCKPVNTFVHEPLVDVQNVCFQEKVTCKNGQGNCYKSNSSMHITDCRLTNGSRYPNCAYRTSPKERHIIVACEGSPYVPVHFDASVeds<br>KESAAAKFERQHMDSGNSASSSSNYCNLMMCCRKMTQGKCKPVNTFVHESLADVKAVCSQKKVTCKDGQTNCYQSKSTMRITDCRETGSSKYPNCAYKTTQVEKHIIVACGGKPSVPVHFDASV---

927	2k11A (127)<br>3bcoA (124)	N	68.55	TM-align: aliSize=103 (resi) RMSD=1.45 (Å)<br>kesrakkfqrqhmdsdssps--------------------SSSTYCNQMMRRRNMTQGRCKPVNTFVHEPLVDVQNVCFQEKVTC-KNGQgNCYKSNSSMHITDCRLTNGSRYPNCAYRTSPKERHIIVACEGSPYVPVHFDASVeds<br>--------------------kesaaakferqhmdsgnsasSSSNYCNQMMCCRKMTQGKCKPVNTFVHESLADVKAVCSQKKVTCkDGQT-NCYQSKSTMRITDCRETGSSKYPNCAYKTTQVEKHIIVACGGKPSVPVHFDASV---<br><br>SARST: aliSize=117 (resi) RMSD=9.24 (Å)<br>k-ESRAKKFQRQHM----DSDSspssSSTYCNQMMRRRNMTQGRCKPVNTFVHEPLVDVQNVCFQEKVTCKNGQGNCYKSNSSMHITDCRLTNGSRYPNCAYRTSPKERHIIVACEGSPYVPVHFDA--sveds<br>-kESAAAKFERQHMdsgnSASS----SSNYCNQMMCCRKMTQGKCKPVNTFVHESLADVKAVCSQKKVTCKDGQTNCYQSKSTMRITDCRETGSSKYPNCAYKTTQVEKHIIVACGGKPSVPVHFDAsv-----<br><br>BLAST: aliSize=101 (resi) iden=71.77% (89/124) simi=81.45% (101/124)<br>KESRAKKFQRQHMDSDSSPSSSSTYCNQMMRRRNMTQGRCKPVNTFVHEPLVDVQNVCFQEKVTCKNGQGNCYKSNSSMHITDCRLTNGSRYPNCAYRTSPKERHIIVACEGSPYVPVHFDASVeds<br>KESAAAKFERQHMDSGNSASSSSNYCNQMMCCRKMTQGKCKPVNTFVHESLADVKAVCSQKKVTCKDGQTNCYQSKSTMRITDCRETGSSKYPNCAYKTTQVEKHIIVACGGKPSVPVHFDASV---<br><br>Proposed: aliSize=121 (resi) RMSD=1.31 (Å)<br>KESRAKKFQRQHMDSdsSPS-S-SSTYCNQMMRRRNMTQGRCKPVNTFVHEPLVDVQNVCFQEKVTC-KNGQgNCYKSNSSMHITDCRLTNGSRYPNCAYRTSPKERHIIVACEGSPYVPVHFDASVeds<br>KESAAAKFERQHMDS--GNSaSsSSNYCNQMMCCRKMTQGKCKPVNTFVHESLADVKAVCSQKKVTCkDGQT-NCYQSKSTMRITDCRETGSSKYPNCAYKTTQVEKHIIVACGGKPSVPVHFDASV---
928	2k11A (127)<br>1y94A (124)	N	67.74	TM-align: aliSize=103 (resi) RMSD=1.43 (Å)<br>kesrakkfqrqhmdsdssps--------------------SSSTYCNQMMRRRNMTQGRCKPVNTFVHEPLVDVQNVCFQEKVTC-KNGQgNCYKSNSSMHITDCRLTNGSRYPNCAYRTSPKERHIIVACEGSPYVPVHFDASVeds<br>--------------------kesaaakferqhmdsstsaaSSSNYCNLMMCCRKMTQGKCKPVNTFVHESLADVKAVCSQKKVTCkDGQT-NCYQSKSTMRITDCRETGSSKYPNCAYKTTQVEKHIIVACGGKPSVPVHFDASV---<br><br>SARST: aliSize=102 (resi) RMSD=1.59 (Å)<br>kesrakkfqrqhmdsdssps--------------------SSSTYCNQMMRRRNMTQGRCKPVNTFVHEPLVDVQNVCFQEKVTCKNGQGNCYKSNSSMHITDCRLTNGSRYPNCAYRTSPKERHIIVACEGSPYVPVHFDA--sveds<br>--------------------kesaaakferqhmdsstsaaSSSNYCNLMMCCRKMTQGKCKPVNTFVHESLADVKAVCSQKKVTCKDGQTNCYQSKSTMRITDCRETGSSKYPNCAYKTTQVEKHIIVACGGKPSVPVHFDAsv-----<br><br>BLAST: aliSize=100 (resi) iden=70.16% (87/124) simi=80.65% (100/124)<br>KESRAKKFQRQHMDSDSSPSSSSTYCNQMMRRRNMTQGRCKPVNTFVHEPLVDVQNVCFQEKVTCKNGQGNCYKSNSSMHITDCRLTNGSRYPNCAYRTSPKERHIIVACEGSPYVPVHFDASVeds<br>KESAAAKFERQHMDSSTSAASSSNYCNLMMCCRKMTQGKCKPVNTFVHESLADVKAVCSQKKVTCKDGQTNCYQSKSTMRITDCRETGSSKYPNCAYKTTQVEKHIIVACGGKPSVPVHFDASV---<br><br>Proposed: aliSize=121 (resi) RMSD=1.33 (Å)<br>KESRAKKFQRQHMDSdsSPS-SS-STYCNQMMRRRNMTQGRCKPVNTFVHEPLVDVQNVCFQEKVTC-KNGQgNCYKSNSSMHITDCRLTNGSRYPNCAYRTSPKERHIIVACEGSPYVPVHFDASVeds<br>KESAAAKFERQHMDS--STSaASsSNYCNLMMCCRKMTQGKCKPVNTFVHESLADVKAVCSQKKVTCkDGQT-NCYQSKSTMRITDCRETGSSKYPNCAYKTTQVEKHIIVACGGKPSVPVHFDASV---
929	2aasA (124)<br>1lbgA (124)	N	81.45	TM-align: aliSize=104 (resi) RMSD=1.43 (Å)<br>ketaaakferqhmdsstsaa--------------------SSSNYCNQMMKSRNLTKDRCKPVNTFVHESLADVQAVCSQKNVACKNGQTNCYQSYSTMSITDCRETGSSKYPNCAYKTTQANKHIIVACEGNPYVPVHFDASV<br>--------------------kesaaakferqhmdsgnspsSSSNYCNLMMCCRKMTQGKCKPVNTFVHESLADVKAVCSQKKVTCKNGQTNCYQSKSTMRITDCRETGSSKYPNCAYKTTQVEKHIIVACGGKPSVPVHFDASV<br><br>SARST: aliSize=118 (resi) RMSD=8.84 (Å)<br>keta----AAKFERQHMDS-STSAASsSNYCNQMMKSRNLTKDRCKPVNTFVHESLADVQAVCSQKNVACKNGQTNCYQSYSTMSITDCRETGSSKYPNCAYKTTQANKHIIVACEGNPYVPVHFDAS-v<br>----kesaAAKFERQHMDSgNSPSSS-SNYCNLMMCCRKMTQGKCKPVNTFVHESLADVKAVCSQKKVTCKNGQTNCYQSKSTMRITDCRETGSSKYPNCAYKTTQVEKHIIVACGGKPSVPVHFDASv-<br><br>BLAST: aliSize=107 (resi) iden=81.45% (101/124) simi=86.29% (107/124)<br>KETAAAKFERQHMDSSTSAASSSNYCNQMMKSRNLTKDRCKPVNTFVHESLADVQAVCSQKNVACKNGQTNCYQSYSTMSITDCRETGSSKYPNCAYKTTQANKHIIVACEGNPYVPVHFDASV<br>KESAAAKFERQHMDSGNSPSSSSNYCNLMMCCRKMTQGKCKPVNTFVHESLADVKAVCSQKKVTCKNGQTNCYQSKSTMRITDCRETGSSKYPNCAYKTTQVEKHIIVACGGKPSVPVHFDASV<br><br>Proposed: aliSize=124 (resi) RMSD=1.42 (Å)<br>KETAAAKFERQHMDSSTSAASSSNYCNQMMKSRNLTKDRCKPVNTFVHESLADVQAVCSQKNVACKNGQTNCYQSYSTMSITDCRETGSSKYPNCAYKTTQANKHIIVACEGNPYVPVHFDASV<br>KESAAAKFERQHMDSGNSPSSSSNYCNLMMCCRKMTQGKCKPVNTFVHESLADVKAVCSQKKVTCKNGQTNCYQSKSTMRITDCRETGSSKYPNCAYKTTQVEKHIIVACGGKPSVPVHFDASV

930	2aasA<br>(124)<br><br>1y92B<br>(124)	N	81.45	TM-align: aliSize=104 (resi) RMSD=1.37 (Å)<br>ketaaakferqhmdsstsaa--------------------SSSNYCNQMMKSRNLTKDRCKPVNTFVHESLADVQAVCSQKNVACKNGQTNCYQSYSTMSITDCRETGSSKYPNCAYKTTQANKHIIVACEGNPYVPVHFDASV<br>--------------------kesaaakferqhmdsgnsasSSSNYCNLMMCCRKMTQGKCKPVNTFVHESLADVKAVCSQKKVTCKDGQTNCYQSKSTMRITDCRETGSSKYPNCAYKTTQVEKHIIVACGGKPSVPVHFDASV<br><br>SARST: aliSize=122 (resi) RMSD=9.68 (Å)<br>k-ETAAAKFERQHMDSSTSAASSSNYCNQMMKSRNLTKDRCKPVNTFVHESLADVQAVCSQKNVACKNGQTNCYQSYSTMSITDCRETGSSKYPNCAYKTTQANKHIIVACEGNPYVPVHFDAS-v<br>-kESAAAKFERQHMDSGNSASSSSNYCNLMMCCRKMTQGKCKPVNTFVHESLADVKAVCSQKKVTCKDGQTNCYQSKSTMRITDCRETGSSKYPNCAYKTTQVEKHIIVACGGKPSVPVHFDASv-<br><br>BLAST: aliSize=108 (resi) iden=81.45% (101/124) simi=87.10% (108/124)<br>KETAAAKFERQHMDSSTSAASSSNYCNQMMKSRNLTKDRCKPVNTFVHESLADVQAVCSQKNVACKNGQTNCYQSYSTMSITDCRETGSSKYPNCAYKTTQANKHIIVACEGNPYVPVHFDASV<br>KESAAAKFERQHMDSGNSASSSSNYCNLMMCCRKMTQGKCKPVNTFVHESLADVKAVCSQKKVTCKDGQTNCYQSKSTMRITDCRETGSSKYPNCAYKTTQVEKHIIVACGGKPSVPVHFDASV<br><br>Proposed: aliSize=124 (resi) RMSD=1.38 (Å)<br>KETAAAKFERQHMDSSTSAASSSNYCNQMMKSRNLTKDRCKPVNTFVHESLADVQAVCSQKNVACKNGQTNCYQSYSTMSITDCRETGSSKYPNCAYKTTQANKHIIVACEGNPYVPVHFDASV<br>KESAAAKFERQHMDSGNSASSSSNYCNLMMCCRKMTQGKCKPVNTFVHESLADVKAVCSQKKVTCKDGQTNCYQSKSTMRITDCRETGSSKYPNCAYKTTQVEKHIIVACGGKPSVPVHFDASV
931	2aasA<br>(124)<br><br>3bcoA<br>(124)	N	82.26	TM-align: aliSize=104 (resi) RMSD=1.46 (Å)<br>ketaaakferqhmdsstsaa--------------------SSSNYCNQMMKSRNLTKDRCKPVNTFVHESLADVQAVCSQKNVACKNGQTNCYQSYSTMSITDCRETGSSKYPNCAYKTTQANKHIIVACEGNPYVPVHFDASV<br>--------------------kesaaakferqhmdsgnsasSSSNYCNQMMCCRKMTQGKCKPVNTFVHESLADVKAVCSQKKVTCKDGQTNCYQSKSTMRITDCRETGSSKYPNCAYKTTQVEKHIIVACGGKPSVPVHFDASV<br><br>SARST: aliSize=118 (resi) RMSD=9.22 (Å)<br>k-ETAAAKFERQHM----DSSTSaassSNYCNQMMKSRNLTKDRCKPVNTFVHESLADVQAVCSQKNVACKNGQTNCYQSYSTMSITDCRETGSSKYPNCAYKTTQANKHIIVACEGNPYVPVHFDAS-v<br>-kESAAAKFERQHMdsgnSASSS----SNYCNQMMCCRKMTQGKCKPVNTFVHESLADVKAVCSQKKVTCKDGQTNCYQSKSTMRITDCRETGSSKYPNCAYKTTQVEKHIIVACGGKPSVPVHFDASv-<br><br>BLAST: aliSize=109 (resi) iden=82.26% (102/124) simi=87.90% (109/124)<br>KETAAAKFERQHMDSSTSAASSSNYCNQMMKSRNLTKDRCKPVNTFVHESLADVQAVCSQKNVACKNGQTNCYQSYSTMSITDCRETGSSKYPNCAYKTTQANKHIIVACEGNPYVPVHFDASV<br>KESAAAKFERQHMDSGNSASSSSNYCNQMMCCRKMTQGKCKPVNTFVHESLADVKAVCSQKKVTCKDGQTNCYQSKSTMRITDCRETGSSKYPNCAYKTTQVEKHIIVACGGKPSVPVHFDASV<br><br>Proposed: aliSize=124 (resi) RMSD=1.41 (Å)<br>KETAAAKFERQHMDSSTSAASSSNYCNQMMKSRNLTKDRCKPVNTFVHESLADVQAVCSQKNVACKNGQTNCYQSYSTMSITDCRETGSSKYPNCAYKTTQANKHIIVACEGNPYVPVHFDASV<br>KESAAAKFERQHMDSGNSASSSSNYCNQMMCCRKMTQGKCKPVNTFVHESLADVKAVCSQKKVTCKDGQTNCYQSKSTMRITDCRETGSSKYPNCAYKTTQVEKHIIVACGGKPSVPVHFDASV
932	2aasA<br>(124)<br><br>1y94A<br>(124)	N	83.87	TM-align: aliSize=104 (resi) RMSD=1.52 (Å)<br>ketaaakferqhmdsstsaa--------------------SSSNYCNQMMKSRNLTKDRCKPVNTFVHESLADVQAVCSQKNVACKNGQTNCYQSYSTMSITDCRETGSSKYPNCAYKTTQANKHIIVACEGNPYVPVHFDASV<br>--------------------kesaaakferqhmdsstsaaSSSNYCNLMMCCRKMTQGKCKPVNTFVHESLADVKAVCSQKKVTCKDGQTNCYQSKSTMRITDCRETGSSKYPNCAYKTTQVEKHIIVACGGKPSVPVHFDASV<br><br>SARST: aliSize=118 (resi) RMSD=9.28 (Å)<br>k-ETAAAKFERQHMD----SSTSaassSNYCNQMMKSRNLTKDRCKPVNTFVHESLADVQAVCSQKNVACKNGQTNCYQSYSTMSITDCRETGSSKYPNCAYKTTQANKHIIVACEGNPYVPVHFDAS-v<br>-kESAAAKFERQHMDsstsAASS----SNYCNLMMCCRKMTQGKCKPVNTFVHESLADVKAVCSQKKVTCKDGQTNCYQSKSTMRITDCRETGSSKYPNCAYKTTQVEKHIIVACGGKPSVPVHFDASv-<br><br>BLAST: aliSize=110 (resi) iden=83.87% (104/124) simi=88.71% (110/124)<br>KETAAAKFERQHMDSSTSAASSSNYCNQMMKSRNLTKDRCKPVNTFVHESLADVQAVCSQKNVACKNGQTNCYQSYSTMSITDCRETGSSKYPNCAYKTTQANKHIIVACEGNPYVPVHFDASV<br>KESAAAKFERQHMDSSTSAASSSNYCNLMMCCRKMTQGKCKPVNTFVHESLADVKAVCSQKKVTCKDGQTNCYQSKSTMRITDCRETGSSKYPNCAYKTTQVEKHIIVACGGKPSVPVHFDASV<br><br>Proposed: aliSize=124 (resi) RMSD=1.47 (Å)<br>KETAAAKFERQHMDSSTSAASSSNYCNQMMKSRNLTKDRCKPVNTFVHESLADVQAVCSQKNVACKNGQTNCYQSYSTMSITDCRETGSSKYPNCAYKTTQANKHIIVACEGNPYVPVHFDASV<br>KESAAAKFERQHMDSSTSAASSSNYCNLMMCCRKMTQGKCKPVNTFVHESLADVKAVCSQKKVTCKDGQTNCYQSKSTMRITDCRETGSSKYPNCAYKTTQVEKHIIVACGGKPSVPVHFDASV

933	2nuiA<br>(124)<br><br>1lbgA<br>(124)	N	80.65	TM-align: aliSize=104 (resi) RMSD=0.79 (Å)<br>ketaaakferqhmdsstsaa--------------------SSSNYCNQMMKSRNLTKDRCKPVNTFVHESLADVQAVCSQKNVACKNGQTNCYQSYSTMSITACRETGSSKYPNCAYKTTQANKHIIVACEGNPYVPVHFDASV<br>--------------------kesaaakferqhmdsgnspsSSSNYCNLMMCCRKMTQGKCKPVNTFVHESLADVKAVCSQKKVTCKNGQTNCYQSKSTMRITDCRETGSSKYPNCAYKTTQVEKHIIVACGGKPSVPVHFDASV<br><br>SARST: aliSize=121 (resi) RMSD=9.85 (Å)<br>k-ETAAAKFERQHMDSsTSAASS-SNYCNQMMKSRNLTKDRCKPVNTFVHESLADVQAVCSQKNVACKNGQTNCYQSYSTMSITACRETGSSKYPNCAYKTTQANKHIIVACEGNPYVPVHFDAS-v<br>-kESAAAKFERQHMDS-GNSPSSsSNYCNLMMCCRKMTQGKCKPVNTFVHESLADVKAVCSQKKVTCKNGQTNCYQSKSTMRITDCRETGSSKYPNCAYKTTQVEKHIIVACGGKPSVPVHFDASv-<br><br>BLAST: aliSize=106 (resi) iden=80.65% (100/124) simi=85.48% (106/124)<br>KETAAAKFERQHMDSSTSAASSSNYCNQMMKSRNLTKDRCKPVNTFVHESLADVQAVCSQKNVACKNGQTNCYQSYSTMSITACRETGSSKYPNCAYKTTQANKHIIVACEGNPYVPVHFDASV<br>KESAAAKFERQHMDSGNSPSSSSNYCNLMMCCRKMTQGKCKPVNTFVHESLADVKAVCSQKKVTCKNGQTNCYQSKSTMRITDCRETGSSKYPNCAYKTTQVEKHIIVACGGKPSVPVHFDASV<br><br>Proposed: aliSize=124 (resi) RMSD=0.84 (Å)<br>KETAAAKFERQHMDSSTSAASSSNYCNQMMKSRNLTKDRCKPVNTFVHESLADVQAVCSQKNVACKNGQTNCYQSYSTMSITACRETGSSKYPNCAYKTTQANKHIIVACEGNPYVPVHFDASV<br>KESAAAKFERQHMDSGNSPSSSSNYCNLMMCCRKMTQGKCKPVNTFVHESLADVKAVCSQKKVTCKNGQTNCYQSKSTMRITDCRETGSSKYPNCAYKTTQVEKHIIVACGGKPSVPVHFDASV
934	2nuiA<br>(124)<br><br>1y92B<br>(124)	N	80.65	TM-align: aliSize=104 (resi) RMSD=0.83 (Å)<br>ketaaakferqhmdsstsaa--------------------SSSNYCNQMMKSRNLTKDRCKPVNTFVHESLADVQAVCSQKNVACKNGQTNCYQSYSTMSITACRETGSSKYPNCAYKTTQANKHIIVACEGNPYVPVHFDASV<br>--------------------kesaaakferqhmdsgnsasSSSNYCNLMMCCRKMTQGKCKPVNTFVHESLADVKAVCSQKKVTCKDGQTNCYQSKSTMRITDCRETGSSKYPNCAYKTTQVEKHIIVACGGKPSVPVHFDASV<br><br>SARST: aliSize=122 (resi) RMSD=9.72 (Å)<br>k-ETAAAKFERQHMDSSTSAASSSNYCNQMMKSRNLTKDRCKPVNTFVHESLADVQAVCSQKNVACKNGQTNCYQSYSTMSITACRETGSSKYPNCAYKTTQANKHIIVACEGNPYVPVHFDAS-v<br>-kESAAAKFERQHMDSGNSASSSSNYCNLMMCCRKMTQGKCKPVNTFVHESLADVKAVCSQKKVTCKDGQTNCYQSKSTMRITDCRETGSSKYPNCAYKTTQVEKHIIVACGGKPSVPVHFDASv-<br><br>BLAST: aliSize=107 (resi) iden=80.65% (100/124) simi=86.29% (107/124)<br>KETAAAKFERQHMDSSTSAASSSNYCNQMMKSRNLTKDRCKPVNTFVHESLADVQAVCSQKNVACKNGQTNCYQSYSTMSITACRETGSSKYPNCAYKTTQANKHIIVACEGNPYVPVHFDASV<br>KESAAAKFERQHMDSGNSASSSSNYCNLMMCCRKMTQGKCKPVNTFVHESLADVKAVCSQKKVTCKDGQTNCYQSKSTMRITDCRETGSSKYPNCAYKTTQVEKHIIVACGGKPSVPVHFDASV<br><br>Proposed: aliSize=124 (resi) RMSD=0.87 (Å)<br>KETAAAKFERQHMDSSTSAASSSNYCNQMMKSRNLTKDRCKPVNTFVHESLADVQAVCSQKNVACKNGQTNCYQSYSTMSITACRETGSSKYPNCAYKTTQANKHIIVACEGNPYVPVHFDASV<br>KESAAAKFERQHMDSGNSASSSSNYCNLMMCCRKMTQGKCKPVNTFVHESLADVKAVCSQKKVTCKDGQTNCYQSKSTMRITDCRETGSSKYPNCAYKTTQVEKHIIVACGGKPSVPVHFDASV
935	2nuiA<br>(124)<br><br>3bcoA<br>(124)	N	81.45	TM-align: aliSize=104 (resi) RMSD=0.86 (Å)<br>ketaaakferqhmdsstsaa--------------------SSSNYCNQMMKSRNLTKDRCKPVNTFVHESLADVQAVCSQKNVACKNGQTNCYQSYSTMSITACRETGSSKYPNCAYKTTQANKHIIVACEGNPYVPVHFDASV<br>--------------------kesaaakferqhmdsgnsasSSSNYCNQMMCCRKMTQGKCKPVNTFVHESLADVKAVCSQKKVTCKDGQTNCYQSKSTMRITDCRETGSSKYPNCAYKTTQVEKHIIVACGGKPSVPVHFDASV<br><br>SARST: aliSize=121 (resi) RMSD=9.62 (Å)<br>k-ETAAAKFERQHMDSsTSAASS-SNYCNQMMKSRNLTKDRCKPVNTFVHESLADVQAVCSQKNVACKNGQTNCYQSYSTMSITACRETGSSKYPNCAYKTTQANKHIIVACEGNPYVPVHFDAS-v<br>-kESAAAKFERQHMDS-GNSASSsSNYCNQMMCCRKMTQGKCKPVNTFVHESLADVKAVCSQKKVTCKDGQTNCYQSKSTMRITDCRETGSSKYPNCAYKTTQVEKHIIVACGGKPSVPVHFDASv-<br><br>BLAST: aliSize=108 (resi) iden=81.45% (101/124) simi=87.10% (108/124)<br>KETAAAKFERQHMDSSTSAASSSNYCNQMMKSRNLTKDRCKPVNTFVHESLADVQAVCSQKNVACKNGQTNCYQSYSTMSITACRETGSSKYPNCAYKTTQANKHIIVACEGNPYVPVHFDASV<br>KESAAAKFERQHMDSGNSASSSSNYCNQMMCCRKMTQGKCKPVNTFVHESLADVKAVCSQKKVTCKDGQTNCYQSKSTMRITDCRETGSSKYPNCAYKTTQVEKHIIVACGGKPSVPVHFDASV<br><br>Proposed: aliSize=124 (resi) RMSD=0.83 (Å)<br>KETAAAKFERQHMDSSTSAASSSNYCNQMMKSRNLTKDRCKPVNTFVHESLADVQAVCSQKNVACKNGQTNCYQSYSTMSITACRETGSSKYPNCAYKTTQANKHIIVACEGNPYVPVHFDASV<br>KESAAAKFERQHMDSGNSASSSSNYCNQMMCCRKMTQGKCKPVNTFVHESLADVKAVCSQKKVTCKDGQTNCYQSKSTMRITDCRETGSSKYPNCAYKTTQVEKHIIVACGGKPSVPVHFDASV

936	2nuiA<br>(124)<br><br>1y94A<br>(124)	N	83.06	TM-align: aliSize=104 (resi) RMSD=0.93 (Å)<br>ketaaakferqhmdsstsaa--------------------SSSNYCNQMMKSRNLTKDRCKPVNTFVHESLADVQAVCSQKNVACKNGQTNCYQSYSTMSITACRETGSSKYPNCAYKTTQANKHIIVACEGNPYVPVHFDASV<br>--------------------kesaaakferqhmdsstsaaSSSNYCNLMMCCRKMTQGKCKPVNTFVHESLADVKAVCSQKKVTCKDGQTNCYQSKSTMRITDCRETGSSKYPNCAYKTTQVEKHIIVACGGKPSVPVHFDASV<br><br>SARST: aliSize=121 (resi) RMSD=9.61 (Å)<br>k-ETAAAKFERQHMDS-STSAASsSNYCNQMMKSRNLTKDRCKPVNTFVHESLADVQAVCSQKNVACKNGQTNCYQSYSTMSITACRETGSSKYPNCAYKTTQANKHIIVACEGNPYVPVHFDAS-v<br>-kESAAAKFERQHMDSsTSAASS-SNYCNLMMCCRKMTQGKCKPVNTFVHESLADVKAVCSQKKVTCKDGQTNCYQSKSTMRITDCRETGSSKYPNCAYKTTQVEKHIIVACGGKPSVPVHFDASv-<br><br>BLAST: aliSize=109 (resi) iden=83.06% (103/124) simi=87.90% (109/124)<br>KETAAAKFERQHMDSSTSAASSSNYCNQMMKSRNLTKDRCKPVNTFVHESLADVQAVCSQKNVACKNGQTNCYQSYSTMSITACRETGSSKYPNCAYKTTQANKHIIVACEGNPYVPVHFDASV<br>KESAAAKFERQHMDSSTSAASSSNYCNLMMCCRKMTQGKCKPVNTFVHESLADVKAVCSQKKVTCKDGQTNCYQSKSTMRITDCRETGSSKYPNCAYKTTQVEKHIIVACGGKPSVPVHFDASV<br><br>Proposed: aliSize=124 (resi) RMSD=0.89 (Å)<br>KETAAAKFERQHMDSSTSAASSSNYCNQMMKSRNLTKDRCKPVNTFVHESLADVQAVCSQKNVACKNGQTNCYQSYSTMSITACRETGSSKYPNCAYKTTQANKHIIVACEGNPYVPVHFDASV<br>KESAAAKFERQHMDSSTSAASSSNYCNLMMCCRKMTQGKCKPVNTFVHESLADVKAVCSQKKVTCKDGQTNCYQSKSTMRITDCRETGSSKYPNCAYKTTQVEKHIIVACGGKPSVPVHFDASV
937	1qwqA<br>(124)<br><br>11bgA<br>(124)	N	92.74	TM-align: aliSize=105 (resi) RMSD=2.01 (Å)<br>kesaaakferqhmdsgnsp-------------------SSSSNYCNLMMCCRKMTQGKCKPVNTFVHESLADVKAVCSQKKVTCKDGQTNCYQSKSTMRITDCRETGSSKYPNCAYKTTQVEKHIIVACGGKPSVPVHFDASV<br>-------------------kesaaakferqhmdsgnspSSSSNYCNLMMCCRKMTQGKCKPVNTFVHESLADVKAVCSQKKVTCKNGQTNCYQSKSTMRITDCRETGSSKYPNCAYKTTQVEKHIIVACGGKPSVPVHFDASV<br><br>SARST: aliSize=118 (resi) RMSD=8.80 (Å)<br>kesaa---AKFERQHMDSGNSPS--SSSNYCNLMMCCRKMTQGKCKPVNTFVHESLADVKAVCSQKKVTCKDGQTNCYQSKSTMRITDCRETGSSKYPNCAYKTTQVEKHIIVACGGKPSVPVHFDAS-v<br>-----kesAAAKFERQHMDSGNSpsSSSNYCNLMMCCRKMTQGKCKPVNTFVHESLADVKAVCSQKKVTCKNGQTNCYQSKSTMRITDCRETGSSKYPNCAYKTTQVEKHIIVACGGKPSVPVHFDASv-<br><br>BLAST: aliSize=124 (resi) iden=99.19% (123/124) simi=100.00% (124/124)<br>KESAAAKFERQHMDSGNSPSSSSNYCNLMMCCRKMTQGKCKPVNTFVHESLADVKAVCSQKKVTCKDGQTNCYQSKSTMRITDCRETGSSKYPNCAYKTTQVEKHIIVACGGKPSVPVHFDASV<br>KESAAAKFERQHMDSGNSPSSSSNYCNLMMCCRKMTQGKCKPVNTFVHESLADVKAVCSQKKVTCKNGQTNCYQSKSTMRITDCRETGSSKYPNCAYKTTQVEKHIIVACGGKPSVPVHFDASV<br><br>Proposed: aliSize=121 (resi) RMSD=1.79 (Å)<br>KESAAAKFERQHMDSgNSP-SSSSNYCNLMMCCRKMTQGKCKPVNTFVHESLADVKAVCSQKKVTC-KDGqTNCYQSKSTMRITDCRET-gSSKYPNCAYKTTQVEKHIIVACGGKPSVPVHFDASV<br>KESAAAKFERQHMDS-GNSpSSSSNYCNLMMCCRKMTQGKCKPVNTFVHESLADVKAVCSQKKVTCkNGQ-TNCYQSKSTMRITDCRETg-SSKYPNCAYKTTQVEKHIIVACGGKPSVPVHFDASV
938	1qwqA<br>(124)<br><br>1y92B<br>(124)	N	98.39	TM-align: aliSize=105 (resi) RMSD=1.78 (Å)<br>kesaaakferqhmdsgnsp-------------------SSSSNYCNLMMCCRKMTQGKCKPVNTFVHESLADVKAVCSQKKVTCKDGQTNCYQSKSTMRITDCRETGSSKYPNCAYKTTQVEKHIIVACGGKPSVPVHFDASV<br>-------------------kesaaakferqhmdsgnsaSSSSNYCNLMMCCRKMTQGKCKPVNTFVHESLADVKAVCSQKKVTCKDGQTNCYQSKSTMRITDCRETGSSKYPNCAYKTTQVEKHIIVACGGKPSVPVHFDASV<br><br>SARST: aliSize=118 (resi) RMSD=8.53 (Å)<br>kesaa---AKFERQHMDSG--NSPSSSSNYCNLMMCCRKMTQGKCKPVNTFVHESLADVKAVCSQKKVTCKDGQTNCYQSKSTMRITDCRETGSSKYPNCAYKTTQVEKHIIVACGGKPSVPVHFDAS-v<br>-----kesAAAKFERQHMDsgNSASSSSNYCNLMMCCRKMTQGKCKPVNTFVHESLADVKAVCSQKKVTCKDGQTNCYQSKSTMRITDCRETGSSKYPNCAYKTTQVEKHIIVACGGKPSVPVHFDASv-<br><br>BLAST: aliSize=123 (resi) iden=99.19% (123/124) simi=99.19% (123/124)<br>KESAAAKFERQHMDSGNSPSSSSNYCNLMMCCRKMTQGKCKPVNTFVHESLADVKAVCSQKKVTCKDGQTNCYQSKSTMRITDCRETGSSKYPNCAYKTTQVEKHIIVACGGKPSVPVHFDASV<br>KESAAAKFERQHMDSGNSASSSSNYCNLMMCCRKMTQGKCKPVNTFVHESLADVKAVCSQKKVTCKDGQTNCYQSKSTMRITDCRETGSSKYPNCAYKTTQVEKHIIVACGGKPSVPVHFDASV<br><br>Proposed: aliSize=123 (resi) RMSD=1.59 (Å)<br>KESAAAKFERQHMDSGNSPSSSSNYCNLMMCCRKMTQGKCKPVNTFVHESLADVKAVCSQKKVTCKDGQTNCYQSKSTMRITDCRET-gSSKYPNCAYKTTQVEKHIIVACGGKPSVPVHFDASV<br>KESAAAKFERQHMDSGNSASSSSNYCNLMMCCRKMTQGKCKPVNTFVHESLADVKAVCSQKKVTCKDGQTNCYQSKSTMRITDCRETg-SSKYPNCAYKTTQVEKHIIVACGGKPSVPVHFDASV

939	1qwqA<br>(124)<br><br>3bcoA<br>(124)	N	92.74	TM-align: aliSize=104 (resi) RMSD=1.88 (Å)<br>kesaaakferqhmdsgnsp------------------SSSSNYCNLMMCCRKMTQGKCKPVNTFVHESLADVKAVCSQKKVTC-KDGqTNCYQSKSTMRITDCRETGSSKYPNCAYKTTQVEKHIIVACGGKPSVPVHFDASV<br>------------------kesaaakferqhmdsgnsaSSSSNYCNQMMCCRKMTQGKCKPVNTFVHESLADVKAVCSQKKVTCkDGQ-TNCYQSKSTMRITDCRETGSSKYPNCAYKTTQVEKHIIVACGGKPSVPVHFDASV<br><br>SARST: aliSize=122 (resi) RMSD=9.71 (Å)<br>k-ESAAAKFERQHMDSGNSPSSSSNYCNLMMCCRKMTQGKCKPVNTFVHESLADVKAVCSQKKVTCKDGQTNCYQSKSTMRITDCRETGSSKYPNCAYKTTQVEKHIIVACGGKPSVPVHFDAS-v<br>-kESAAAKFERQHMDSGNSASSSSNYCNQMMCCRKMTQGKCKPVNTFVHESLADVKAVCSQKKVTCKDGQTNCYQSKSTMRITDCRETGSSKYPNCAYKTTQVEKHIIVACGGKPSVPVHFDASv-<br><br>BLAST: aliSize=122 (resi) iden=98.39% (122/124) simi=98.39% (122/124)<br>KESAAAKFERQHMDSGNSPSSSSNYCNLMMCCRKMTQGKCKPVNTFVHESLADVKAVCSQKKVTCKDGQTNCYQSKSTMRITDCRETGSSKYPNCAYKTTQVEKHIIVACGGKPSVPVHFDASV<br>KESAAAKFERQHMDSGNSASSSSNYCNQMMCCRKMTQGKCKPVNTFVHESLADVKAVCSQKKVTCKDGQTNCYQSKSTMRITDCRETGSSKYPNCAYKTTQVEKHIIVACGGKPSVPVHFDASV<br><br>Proposed: aliSize=121 (resi) RMSD=1.78 (Å)<br>k-ESAAAKFERQHMDSGNSPSSSSNYCNLMMCCRKMTQGKCKPVNTFVHESLADVKAVCSQKKVTC-KDGQtNCYQSKSTMRITDCRET-gSSKYPNCAYKTTQVEKHIIVACGGKPSVPVHFDASV<br>-kESAAAKFERQHMDSGNSASSSSNYCNQMMCCRKMTQGKCKPVNTFVHESLADVKAVCSQKKVTCkDGQT-NCYQSKSTMRITDCRETg-SSKYPNCAYKTTQVEKHIIVACGGKPSVPVHFDASV
940	1qwqA<br>(124)<br><br>1y94A<br>(124)	N	95.97	TM-align: aliSize=105 (resi) RMSD=1.87 (Å)<br>kesaaakferqhmdsgnsp------------------SSSSNYCNLMMCCRKMTQGKCKPVNTFVHESLADVKAVCSQKKVTCKDGQTNCYQSKSTMRITDCRETGSSKYPNCAYKTTQVEKHIIVACGGKPSVPVHFDASV<br>------------------kesaaakferqhmdsstsaASSSNYCNLMMCCRKMTQGKCKPVNTFVHESLADVKAVCSQKKVTCKDGQTNCYQSKSTMRITDCRETGSSKYPNCAYKTTQVEKHIIVACGGKPSVPVHFDASV<br><br>SARST: aliSize=119 (resi) RMSD=8.77 (Å)<br>kesa----AAKFERQHMDSGNSPSSSSNYCNLMMCCRKMTQGKCKPVNTFVHESLADVKAVCSQKKVTCKDGQTNCYQSKSTMRITDCRETGSSKYPNCAYKTTQVEKHIIVACGGKPSVPVHFDAS-v<br>----kesaAAKFERQHMDSSTSAASSSNYCNLMMCCRKMTQGKCKPVNTFVHESLADVKAVCSQKKVTCKDGQTNCYQSKSTMRITDCRETGSSKYPNCAYKTTQVEKHIIVACGGKPSVPVHFDASv-<br><br>BLAST: aliSize=121 (resi) iden=96.77% (120/124) simi=97.58% (121/124)<br>KESAAAKFERQHMDSGNSPSSSSNYCNLMMCCRKMTQGKCKPVNTFVHESLADVKAVCSQKKVTCKDGQTNCYQSKSTMRITDCRETGSSKYPNCAYKTTQVEKHIIVACGGKPSVPVHFDASV<br>KESAAAKFERQHMDSSTSAASSSNYCNLMMCCRKMTQGKCKPVNTFVHESLADVKAVCSQKKVTCKDGQTNCYQSKSTMRITDCRETGSSKYPNCAYKTTQVEKHIIVACGGKPSVPVHFDASV<br><br>Proposed: aliSize=122 (resi) RMSD=1.79 (Å)<br>KESAAAKFERQHMDSG-nSPSSSSNYCNLMMCCRKMTQGKCKPVNTFVHESLADVKAVCSQKKVTCKDGQTNCYQSKSTMRITDCRET-gSSKYPNCAYKTTQVEKHIIVACGGKPSVPVHFDASV<br>KESAAAKFERQHMDSSt-SAASSSNYCNLMMCCRKMTQGKCKPVNTFVHESLADVKAVCSQKKVTCKDGQTNCYQSKSTMRITDCRETg-SSKYPNCAYKTTQVEKHIIVACGGKPSVPVHFDASV
941	1izpA<br>(124)<br><br>1lbgA<br>(124)	N	80.65	TM-align: aliSize=104 (resi) RMSD=0.85 (Å)<br>ketaaakferqhmdsstsaa--------------------SSSNYCNQMMKSRNLTKDRCKPVNTLVHESLADVQAVCSQKNVACKNGQTNCYQSYSTMSITDCRETGSSKYPNCAYKTTQANKHIIVACEGNPYVPVHFDASV<br>--------------------kesaaakferqhmdsgnspsSSSNYCNLMMCCRKMTQGKCKPVNTFVHESLADVKAVCSQKKVTCKNGQTNCYQSKSTMRITDCRETGSSKYPNCAYKTTQVEKHIIVACGGKPSVPVHFDASV<br><br>SARST: aliSize=121 (resi) RMSD=9.79 (Å)<br>k-ETAAAKFERQHMDS-STSAASsSNYCNQMMKSRNLTKDRCKPVNTLVHESLADVQAVCSQKNVACKNGQTNCYQSYSTMSITDCRETGSSKYPNCAYKTTQANKHIIVACEGNPYVPVHFDAS-v<br>-kESAAAKFERQHMDSgNSPSSS-SNYCNLMMCCRKMTQGKCKPVNTFVHESLADVKAVCSQKKVTCKNGQTNCYQSKSTMRITDCRETGSSKYPNCAYKTTQVEKHIIVACGGKPSVPVHFDASv-<br><br>BLAST: aliSize=106 (resi) iden=80.65% (100/124) simi=85.48% (106/124)<br>KETAAAKFERQHMDSSTSAASSSNYCNQMMKSRNLTKDRCKPVNTLVHESLADVQAVCSQKNVACKNGQTNCYQSYSTMSITDCRETGSSKYPNCAYKTTQANKHIIVACEGNPYVPVHFDASV<br>KESAAAKFERQHMDSGNSPSSSSNYCNLMMCCRKMTQGKCKPVNTFVHESLADVKAVCSQKKVTCKNGQTNCYQSKSTMRITDCRETGSSKYPNCAYKTTQVEKHIIVACGGKPSVPVHFDASV<br><br>Proposed: aliSize=124 (resi) RMSD=0.89 (Å)<br>KETAAAKFERQHMDSSTSAASSSNYCNQMMKSRNLTKDRCKPVNTLVHESLADVQAVCSQKNVACKNGQTNCYQSYSTMSITDCRETGSSKYPNCAYKTTQANKHIIVACEGNPYVPVHFDASV<br>KESAAAKFERQHMDSGNSPSSSSNYCNLMMCCRKMTQGKCKPVNTFVHESLADVKAVCSQKKVTCKNGQTNCYQSKSTMRITDCRETGSSKYPNCAYKTTQVEKHIIVACGGKPSVPVHFDASV

942	1izpA<br>(124)<br><br>1y92B<br>(124)	N	80.65	TM-align: aliSize=104 (resi) RMSD=0.77 (Å)<br>ketaaakferqhmdsstsaa--------------------SSSNYCNQMMKSRNLTKDRCKPVNTLVHESLADVQAVCSQKNVACKNGQTNCYQSYSTMSITDCRETGSSKYPNCAYKTTQANKHIIVACEGNPYVPVHFDASV<br>--------------------kesaaakferqhmdsgnsasSSSNYCNLMMCCRKMTQGKCKPVNTFVHESLADVKAVCSQKKVTCKDGQTNCYQSKSTMRITDCRETGSSKYPNCAYKTTQVEKHIIVACGGKPSVPVHFDASV<br><br>SARST: aliSize=122 (resi) RMSD=9.69 (Å)<br>k-ETAAAKFERQHMDSSTSAASSSNYCNQMMKSRNLTKDRCKPVNTLVHESLADVQAVCSQKNVACKNGQTNCYQSYSTMSITDCRETGSSKYPNCAYKTTQANKHIIVACEGNPYVPVHFDAS-v<br>-kESAAAKFERQHMDSGNSASSSSNYCNLMMCCRKMTQGKCKPVNTFVHESLADVKAVCSQKKVTCKDGQTNCYQSKSTMRITDCRETGSSKYPNCAYKTTQVEKHIIVACGGKPSVPVHFDASv-<br><br>BLAST: aliSize=107 (resi) iden=80.65% (100/124) simi=86.29% (107/124)<br>KETAAAKFERQHMDSSTSAASSSNYCNQMMKSRNLTKDRCKPVNTLVHESLADVQAVCSQKNVACKNGQTNCYQSYSTMSITDCRETGSSKYPNCAYKTTQANKHIIVACEGNPYVPVHFDASV<br>KESAAAKFERQHMDSGNSASSSSNYCNLMMCCRKMTQGKCKPVNTFVHESLADVKAVCSQKKVTCKDGQTNCYQSKSTMRITDCRETGSSKYPNCAYKTTQVEKHIIVACGGKPSVPVHFDASV<br><br>Proposed: aliSize=124 (resi) RMSD=0.86 (Å)<br>KETAAAKFERQHMDSSTSAASSSNYCNQMMKSRNLTKDRCKPVNTLVHESLADVQAVCSQKNVACKNGQTNCYQSYSTMSITDCRETGSSKYPNCAYKTTQANKHIIVACEGNPYVPVHFDASV<br>KESAAAKFERQHMDSGNSASSSSNYCNLMMCCRKMTQGKCKPVNTFVHESLADVKAVCSQKKVTCKDGQTNCYQSKSTMRITDCRETGSSKYPNCAYKTTQVEKHIIVACGGKPSVPVHFDASV
943	1izpA<br>(124)<br><br>3bcoA<br>(124)	N	81.45	TM-align: aliSize=104 (resi) RMSD=0.86 (Å)<br>ketaaakferqhmdsstsaa--------------------SSSNYCNQMMKSRNLTKDRCKPVNTLVHESLADVQAVCSQKNVACKNGQTNCYQSYSTMSITDCRETGSSKYPNCAYKTTQANKHIIVACEGNPYVPVHFDASV<br>--------------------kesaaakferqhmdsgnsasSSSNYCNQMMCCRKMTQGKCKPVNTFVHESLADVKAVCSQKKVTCKDGQTNCYQSKSTMRITDCRETGSSKYPNCAYKTTQVEKHIIVACGGKPSVPVHFDASV<br><br>SARST: aliSize=118 (resi) RMSD=9.22 (Å)<br>k-ETAAAKFERQHMD----SSTSaassSNYCNQMMKSRNLTKDRCKPVNTLVHESLADVQAVCSQKNVACKNGQTNCYQSYSTMSITDCRETGSSKYPNCAYKTTQANKHIIVACEGNPYVPVHFDAS-v<br>-kESAAAKFERQHMDsgnsASSS----SNYCNQMMCCRKMTQGKCKPVNTFVHESLADVKAVCSQKKVTCKDGQTNCYQSKSTMRITDCRETGSSKYPNCAYKTTQVEKHIIVACGGKPSVPVHFDASv-<br><br>BLAST: aliSize=108 (resi) iden=81.45% (101/124) simi=87.10% (108/124)<br>KETAAAKFERQHMDSSTSAASSSNYCNQMMKSRNLTKDRCKPVNTLVHESLADVQAVCSQKNVACKNGQTNCYQSYSTMSITDCRETGSSKYPNCAYKTTQANKHIIVACEGNPYVPVHFDASV<br>KESAAAKFERQHMDSGNSASSSSNYCNQMMCCRKMTQGKCKPVNTFVHESLADVKAVCSQKKVTCKDGQTNCYQSKSTMRITDCRETGSSKYPNCAYKTTQVEKHIIVACGGKPSVPVHFDASV<br><br>Proposed: aliSize=124 (resi) RMSD=0.85 (Å)<br>KETAAAKFERQHMDSSTSAASSSNYCNQMMKSRNLTKDRCKPVNTLVHESLADVQAVCSQKNVACKNGQTNCYQSYSTMSITDCRETGSSKYPNCAYKTTQANKHIIVACEGNPYVPVHFDASV<br>KESAAAKFERQHMDSGNSASSSSNYCNQMMCCRKMTQGKCKPVNTFVHESLADVKAVCSQKKVTCKDGQTNCYQSKSTMRITDCRETGSSKYPNCAYKTTQVEKHIIVACGGKPSVPVHFDASV
944	1izpA<br>(124)<br><br>1y94A<br>(124)	N	83.06	TM-align: aliSize=104 (resi) RMSD=0.98 (Å)<br>ketaaakferqhmdsstsaa--------------------SSSNYCNQMMKSRNLTKDRCKPVNTLVHESLADVQAVCSQKNVACKNGQTNCYQSYSTMSITDCRETGSSKYPNCAYKTTQANKHIIVACEGNPYVPVHFDASV<br>--------------------kesaaakferqhmdsstsaaSSSNYCNLMMCCRKMTQGKCKPVNTFVHESLADVKAVCSQKKVTCKDGQTNCYQSKSTMRITDCRETGSSKYPNCAYKTTQVEKHIIVACGGKPSVPVHFDASV<br><br>SARST: aliSize=118 (resi) RMSD=9.26 (Å)<br>k-ETAAAKFERQHMD----SSTSaassSNYCNQMMKSRNLTKDRCKPVNTLVHESLADVQAVCSQKNVACKNGQTNCYQSYSTMSITDCRETGSSKYPNCAYKTTQANKHIIVACEGNPYVPVHFDAS-v<br>-kESAAAKFERQHMDsstsAASS----SNYCNLMMCCRKMTQGKCKPVNTFVHESLADVKAVCSQKKVTCKDGQTNCYQSKSTMRITDCRETGSSKYPNCAYKTTQVEKHIIVACGGKPSVPVHFDASv-<br><br>BLAST: aliSize=109 (resi) iden=83.06% (103/124) simi=87.90% (109/124)<br>KETAAAKFERQHMDSSTSAASSSNYCNQMMKSRNLTKDRCKPVNTLVHESLADVQAVCSQKNVACKNGQTNCYQSYSTMSITDCRETGSSKYPNCAYKTTQANKHIIVACEGNPYVPVHFDASV<br>KESAAAKFERQHMDSSTSAASSSNYCNLMMCCRKMTQGKCKPVNTFVHESLADVKAVCSQKKVTCKDGQTNCYQSKSTMRITDCRETGSSKYPNCAYKTTQVEKHIIVACGGKPSVPVHFDASV<br><br>Proposed: aliSize=124 (resi) RMSD=0.95 (Å)<br>KETAAAKFERQHMDSSTSAASSSNYCNQMMKSRNLTKDRCKPVNTLVHESLADVQAVCSQKNVACKNGQTNCYQSYSTMSITDCRETGSSKYPNCAYKTTQANKHIIVACEGNPYVPVHFDASV<br>KESAAAKFERQHMDSSTSAASSSNYCNLMMCCRKMTQGKCKPVNTFVHESLADVKAVCSQKKVTCKDGQTNCYQSKSTMRITDCRETGSSKYPNCAYKTTQVEKHIIVACGGKPSVPVHFDASV

945	2v1qB (58)<br>1i07A (59)	C	31.03	TM-align: aliSize=36 (resi) RMSD=2.22 (Å)<br>-ERGIVQYDFMAESQDELTIKSGDKVYILD-DKKSkDWwmcqlvdsgksg---------Lvpaqfiepv------------<br>kKYAKSKYDFVARNSSELSVMKDDVLEILDdRRQW-WK------------vrnasgdsgF---------vpnnildimrtp<br><br>SARST: aliSize=52 (resi) RMSD=11.54 (Å)<br>er---GIVQYDFMAESQDELTIKSGDKVYILDDKKSkdWWMCQLvDSGKSGLVPAQFIEP----v<br>--kkyAKSKYDFVARNSSELSVMKDDVLEILDDRRQ--WWKVRN-ASGDSGFVPNNILDImrtp-<br><br>BLAST: aliSize=30 (resi) iden=32.76% (19/58) simi=51.72% (30/58)<br>ergiv------QYDFMAESQDELTIKSGDKVYILDDKksKDWWMCQLVdSGKSGLVPAQFIE-----pv<br>-----kkyaksKYDFVARNSSELSVMKDDVLEILDDR--RQWWKVRNA-SGDSGFVPNNILDimrtp--<br><br>Proposed: aliSize=53 (resi) RMSD=1.12 (Å)<br>-ERGIVQYDFMAESQDELTIKSGDKVYILD--dkksKDWWMCQLvDSGKSGLVPAQFIEPV---<br>kKYAKSKYDFVARNSSELSVMKDDVLEILDdr----RQWWKVRN-ASGDSGFVPNNILDIMrtp
946	1sshA (60)<br>1i07A (59)	C	27.12	TM-align: aliSize=36 (resi) RMSD=2.37 (Å)<br>gsSPKAVALYSFAGEESGDLPFRKGDVITILK-KSdSqnDWwtgrvngreg----------Ifpanyvelv------------<br>--KKYAKSKYDFVARNSSELSVMKDDVLEILDdRR-Q--WW----------kvrnasgdsgF---------vpnnildimrtp<br><br>SARST: aliSize=52 (resi) RMSD=10.91 (Å)<br>gssp--KAVALYSFAGEESGDLPFRKGDVITIlkkSDSQNDWWTGRV-NGREGIFPANYVEL----v<br>----kkYAKSKYDFVARNSSELSVMKDDVLEI---LDDRRQWWKVRNaSGDSGFVPNNILDImrtp-<br><br>BLAST: aliSize=27 (resi) iden=30.51% (18/59) simi=45.76% (27/59)<br>gsspk---AVALYSFAGEESGDLPFRKGDVITILkksDSQNDWWTGR-VNGREGIFPANYVELV---<br>-----kkyAKSKYDFVARNSSELSVMKDDVLEIL---DDRRQWWKVRnASGDSGFVPNNILDIMrtp<br><br>Proposed: aliSize=53 (resi) RMSD=1.41 (Å)<br>gsSPKAVALYSFAGEESGDLPFRKGDVITILK--ksdsqNDWWTGRVNG-REGIFPANYVELV---<br>--KKYAKSKYDFVARNSSELSVMKDDVLEILDdr-----RQWWKVRNASgDSGFVPNNILDIMrtp
947	1h3hA (60)<br>1i07A (59)	C	30.51	TM-align: aliSize=36 (resi) RMSD=2.64 (Å)<br>grv-RWARALYDFEALEEDELGFRSGEVVEVLD-SSNPSWwtgrlhnklg---------Lfpanyvapmmr------------<br>---kKYAKSKYDFVARNSSELSVMKDDVLEILDdRRQWWK----------vrnasgdsgF-----------vpnnildimrtp<br><br>SARST: aliSize=52 (resi) RMSD=11.14 (Å)<br>grvr--WARALYDFEALEEDELGFRSGEVVEVLDSSNpsWWTGRLH--NKLGLFPANYVAPM---mr<br>----kkYAKSKYDFVARNSSELSVMKDDVLEILDDRR--QWWKVRNasGDSGFVPNNILDIMrtp--<br><br>BLAST: aliSize=28 (resi) iden=32.20% (19/59) simi=47.46% (28/59)<br>grv-RWARALYDFEALEEDELGFRSGEVVEVLDSSNpSWWTGR-LHNKLGLFPANyVAPMMR--<br>---kKYAKSKYDFVARNSSELSVMKDDVLEILDDRR-QWWKVRnASGDSGFVPNN-ILDIMRtp<br><br>Proposed: aliSize=55 (resi) RMSD=1.48 (Å)<br>grv-RWARALYDFEALEEDELGFRSGEVVEVLD-SSNpsWWTGRLH-NKLGLFPANYVAPMMR-<br>---kKYAKSKYDFVARNSSELSVMKDDVLEILDdRRQ--WWKVRNAsGDSGFVPNNILDIMRTp

948	2semB<br>(59)<br><br>1i07A<br>(59)	C	22.03	TM-align: aliSize=37 (resi) RMSD=2.35 (Å)<br>TKFVQALFDFNPQESGELAFKRGDVITLIN-KDDPNWwegqlnnrrg---------Ifpsnyvcpynsn------------<br>KKYAKSKYDFVARNSSELSVMKDDVLEILDdRRQWWK----------vrnasgdsgF------------vpnnildimrtp<br><br>SARST: aliSize=52 (resi) RMSD=11.49 (Å)<br>tk--FVQALFDFNPQESGELAFKRGDVITLINKDDpnWWEGQLN--NRRGIFPSNYVCPY---nsn<br>--kkYAKSKYDFVARNSSELSVMKDDVLEILDDRR--QWWKVRNasGDSGFVPNNILDIMrtp---<br><br>BLAST: aliSize=28 (resi) iden=23.73% (14/59) simi=47.46% (28/59)<br>t-KFVQALFDFNPQESGELAFKRGDVITLINkDDPNWWE-GQLNNRRGIFPSN--------yvcpynsn<br>-kKYAKSKYDFVARNSSELSVMKDDVLEILD-DRRQWWKvRNASGDSGFVPNNildimrtp--------<br><br>Proposed: aliSize=56 (resi) RMSD=1.35 (Å)<br>TKFVQALFDFNPQESGELAFKRGDVITLIN--kddPNWWEGQLN-NRRGIFPSNYVCPYNSN<br>KKYAKSKYDFVARNSSELSVMKDDVLEILDdr---RQWWKVRNAsGDSGFVPNNILDIMRTP
949	1jo8A<br>(58)<br><br>1i07A<br>(59)	C	22.41	TM-align: aliSize=35 (resi) RMSD=2.41 (Å)<br>-PWATAEYDYDAAEDNELTFVENDKIInIE--FVDDDWwlgelekdgskg---------Lfpsnyvslgn------------<br>kKYAKSKYDFVARNSSELSVMKDDVLE-ILddRRQWWK------------vrnasgdsgF----------vpnnildimrtp<br><br>SARST: aliSize=49 (resi) RMSD=11.19 (Å)<br>p--WATAEYDYDAAEDNELTFVENDKII----NIEfvdddWWLGELEkDGSKGLFPSNYVSL----gn<br>-kkYAKSKYDFVARNSSELSVMKDDVLEilddRRQ-----WWKVRNA-SGDSGFVPNNILDImrtp--<br><br>BLAST: aliSize=30 (resi) iden=27.59% (16/58) simi=51.72% (30/58)<br>p--WATAEYDYDAAEDNELTFVENDKiinIEFVDD--DWWLGElEKDGSKGLFPSNYVSL----gn<br>-kkYAKSKYDFVARNSSELSVMKDDV---LEILDDrrQWWKVR-NASGDSGFVPNNILDImrtp--<br><br>Proposed: aliSize=54 (resi) RMSD=1.41 (Å)<br>-PWATAEYDYDAAEDNELTFVENDKIINIE--fvdDDWWLGELeKDGSKGLFPSNYVSLGN--<br>kKYAKSKYDFVARNSSELSVMKDDVLEILDdr---RQWWKVRN-ASGDSGFVPNNILDIMRtp
950	4hckA<br>(61)<br><br>1i07A<br>(59)	N	27.12	TM-align: aliSize=36 (resi) RMSD=2.58 (Å)<br>seDIIVVALYDYEAIHHEDLSFQKGDQMVVLE--ESGEWwkarslatrkeg---------Yipsnyvarvds------------<br>--KKYAKSKYDFVARNSSELSVMKDDVLEILDdrRQWWK------------vrnasgdsgF-----------vpnnildimrtp<br><br>SARST: aliSize=54 (resi) RMSD=12.28 (Å)<br>sedi--IVVALYDYEAIHHEDLSFQKGDQMVVlEESGEWWKARSLATRKEGYIPSNYVARV---ds<br>----kkYAKSKYDFVARNSSELSVMKDDVLEI-LDDRRQWWKVRNASGDSGFVPNNILDIMrtp--<br><br>BLAST: aliSize=29 (resi) iden=27.12% (16/59) simi=49.15% (29/59)<br>sediivval-------YDYEAIHHEDLSFQKGDQMVVLEESGEWWKARSlATRKEGYIPSN--------yvarvds<br>---------kkyakskYDFVARNSSELSVMKDDVLEILDDRRQWWKVRN-ASGDSGFVPNNildimrtp-------<br><br>Proposed: aliSize=57 (resi) RMSD=1.52 (Å)<br>seDIIVVALYDYEAIHHEDLSFQKGDQMVVLE-ESgEWWKARSlATRKEGYIPSNYVARVDS-<br>--KKYAKSKYDFVARNSSELSVMKDDVLEILDdRR-QWWKVRN-ASGDSGFVPNNILDIMRTp

951	1rn7A (112)<br>1r4cA (110)	N	52.73	TM-align: aliSize=64 (resi) RMSD=1.20 (Å)<br>aggihatdlndksvqraldfaiseynkvinkdeyysrplqvmaayqqi---VGGVNYYFNVKFGRTTCTKSQPNLDNCPFNDQPKLKEEEFCSFQINEVPWEDKISILNYKCRKV<br>---ggpmdasveeegvrraldfavgeynkasndmyhsralqvvrarkqiVAGVNYFLDVELGRTTCTKTQPNLDNCPFHDQPHLKRKAFCSFQIYAVPWQGTMTLSKSTCQDA<br><br>SARST: aliSize=108 (resi) RMSD=19.10 (Å)<br>agg--IHATDLNDKSVQRALDFAISEYNKViNKDEYYSRPLQVMAAYQQIVGGVNYYFNVKFGRTTCTKSQPNLDNCPFNDQPKLKEEEFCSFQINEVPWEDKISILNYKCRKV<br>---ggPMDASVEEEGVRRALDFAVGEYNKA-SNDMYHSRALQVVRARKQIVAGVNYFLDVELGRTTCTKTQPNLDNCPFHDQPHLKRKAFCSFQIYAVPWQGTMTLSKSTCQDA<br><br>BLAST: aliSize=80 (resi) iden=55.45% (61/110) simi=72.73% (80/110)<br>aGGIHATDLNDKSVQRALDFAISEYNKVINkDEYYSRPLQVMAAYQQIVGGVNYYFNVKFGRTTCTKSQPNLDNCPFNDQPKLKEEEFCSFQINEVPWEDKISILNYKCR--kv<br>-GGPMDASVEEEGVRRALDFAVGEYNKASN-DMYHSRALQVVRARKQIVAGVNYFLDVELGRTTCTKTQPNLDNCPFHDQPHLKRKAFCSFQIYAVPWQGTMTLSKSTCQda--<br><br>Proposed: aliSize=109 (resi) RMSD=0.92 (Å)<br>aGGIHATDLNDKSVQRALDFAISEYNKViNKDEYYSRPLQV-MAAYQQIvGGVNYYFNVKFGRTTCTKSQPNLDNCPFNDQPKLKEEEFCSFQINEVPWEDKISILNYKCRKV<br>-GGPMDASVEEEGVRRALDFAVGEYNKA-SNDMYHSRALQVvRARKQIV-AGVNYFLDVELGRTTCTKTQPNLDNCPFHDQPHLKRKAFCSFQIYAVPWQGTMTLSKSTCQDA
952	1yvbI (111)<br>1r4cA (110)	C	44.55	TM-align: aliSize=62 (resi) RMSD=1.82 (Å)<br>rllgapvpvdendeglqralqfamaeynrasndkyssrvvrvisakrql--VSGIKYILQVEIGRTTCPKSSGDLQSCEFHDEPEMAKYTTCTFVVYSIPWLNQIKLLESKCQ--<br>---ggpmdasveeegvrraldfavgeynkasndmyhsralqvvrarkqiVAGVNYFLDVELGRTTCTKTQPNLDNCPFHDQPHLKRKAFCSFQIYAVPWQGTMTLSKSTCQda<br><br>SARST: aliSize=101 (resi) RMSD=20.09 (Å)<br>rllga--PVPVDENDEGLQRALQFAMAEYNRASNDKYSSRVVRVISAKRQLVSGIKYILQVEIGRTTCP---KSSGDLQsceFHDePEMAK-YTTCTFVVYSIPWLNQIKLLESKC---q<br>-----ggPMDASVEEEGVRRALDFAVGEYNKASNDMYHSRALQVVRARKQIVAGVNYFLDVELGRTTCTktqPNLDNCP---FHD-QPHLKrKAFCSFQIYAVPWQGTMTLSKSTCqda-<br><br>BLAST: aliSize=76 (resi) iden=44.55% (49/110) simi=69.09% (76/110)<br>rllGAPVPVDENDEGLQRALQFAMAEYNRASNDKYSSRVVRVISAKRQLVSGIKYILQVEIGRTTCPKSSGDLQSCEFHDEPEMAKYTTCTFVVYSIPWLNQIKLLESKCQ--<br>---GGPMDASVEEEGVRRALDFAVGEYNKASNDMYHSRALQVVRARKQIVAGVNYFLDVELGRTTCTKTQPNLDNCPFHDQPHLKRKAFCSFQIYAVPWQGTMTLSKSTCQda<br><br>Proposed: aliSize=108 (resi) RMSD=1.24 (Å)<br>rllGAPVPVDENDEGLQRALQFAMAEYNRASNDKYSSRVVRVISAKRQLVSGIKYILQVEIGRTTCPKSSGDLQSCEFHDEPEMAKYTTCTFVVYSIPWLNQIKLLESKCQ--<br>---GGPMDASVEEEGVRRALDFAVGEYNKASNDMYHSRALQVVRARKQIVAGVNYFLDVELGRTTCTKTQPNLDNCPFHDQPHLKRKAFCSFQIYAVPWQGTMTLSKSTCQda
953	1yupE (154)<br>1g85A (159)	C	16.23	TM-align: aliSize=120 (resi) RMSD=2.79 (Å)<br>-----D-LDVQKVAGTWYSLAMAASDISLLDAqSAPLRVYVEELKPT-PGGDLEILLQKWENGKCAQKKIIAEKTEIPaVFKIDALNENKVLVLDTDYkKYLLFCMENSA-EPEQSLACQCLVRTPevddeamekfdkalkalpmhirlsfnptqle-------------------------------EQCRV<br>aqeeeAeQNLSELSGPWRTVYIGSTNPEKIQE-NGPFRTYFRELVFDdEKGTVDFYFSVKRDGKWKNVHVKATKQDDG-TYVADYEGQNVFKIVSLSR-THLVAHNINVDkHGQTTELTGLFVKLN-------------------------------vededlekfwkltedkgidkknvvnflenenHPHPE<br><br>SARST: aliSize=135 (resi) RMSD=13.52 (Å)<br>d-------LDVQKVAGTWYSLAMAASDISLLDAqSAPLRVYVEELKPTP-GGDLEILLQKWENGKCAQKKIIAEKTEiPAVFKIDALNENKVLVLDTdYKKYLLFCMENsaEPEQSL--ACQCLVRTPEVDDEAMEKFDKALKALPM---HIRL-----------sfnptqleeqcrv<br>-aqeeeaeQNLSELSGPWRTVYIGSTNPEKIQE-NGPFRTYFRELVFDDeKGTVDFYFSVKRDGKWKNVHVKATKQD-DGTYVADYEGQNVFKIVSL-SRTHLVAHNIN--VDKHGQttELTGLFVKLNVEDEDLEKFWKLTEDKGIdkkNVVNflenenhphpe-------------<br><br>BLAST: aliSize=55 (resi) iden=16.23% (25/154) simi=35.71% (55/154)<br>dl--------DVQKVAGTWYSLAMAASDISLLDaQSAPLRVYVEELK-PTPGGDLEILLQKWENGKCAQKKIIAEKTEiPAVFKIDALNENKVLVLDTDyKKYLLFCMENSAEPEQSLACQCLVRTPEVDDEAMEKFDK-------------------------alkalpmhirlsfnptqleeqcrv<br>--aqeeeaeqNLSELSGPWRTVYIGSTNPEKIQ-ENGPFRTYFRELVfDDEKGTVDFYFSVKRDGKWKNVHVKATKQD-DGTYVADYEGQNVFKIVSLS-RTHLVAHNINVDKHGQTTELTGLFVKLNVEDEDLEKFWKltedkgidkknvvnflenenhphpe------------------------<br><br>Proposed: aliSize=140 (resi) RMSD=1.94 (Å)<br>-----D-LDVQKVAGTWYSLAMAASDISLLDAqSAPLRVYVEELKPT-PGGDLEILLQKWENGKCAQKKIIAEKTEIPaVFKIDALNENKVLVLDTDYkKYLLFCMENsA----EpeQSLACQCLVRtPEVDDEAMEKFDKALKALPMH--IRLSFNptQ---leeqCR---v<br>aqeeeAeQNLSELSGPWRTVYIGSTNPEKIQE-NGPFRTYFRELVFDdEKGTVDFYFSVKRDGKWKNVHVKATKQDDG-TYVADYEGQNVFKIVSLSR-THLVAHNIN-VdkhgQ--TTELTGLFVK-LNVEDEDLEKFWKLTEDKGIDkkNVVNFL--Enen----HPhpe-

954	1yupE (154)<br>1pboB (151)	C	16.56	TM-align: aliSize=118 (resi) RMSD=2.77 (Å)<br>d1DVQKVAGTWYSLAMAASDISLLDAqSAPLRVYVEELKPT-PGGDLEILLQKWENGKCAQKKIIAEKTEIPaVFKIDALNENKVLVLDTDyKKYLLFCMENSA-EPEQSLACQCLVRTPevddeamekfdkalkalpmhirlsfnptqle-------------------------------EQCRV<br>--NLSELSGPWRTVYIGSTNPEKIQE-NGPFRTYFRELVFDdEKGTVDFYFSVKRDGKWKNVHVKATKQDDG-TYVADYEGQNVFKIVSLS-RTHLVAHNINVDkHGQKTELTGLFVKLN-------------------------------vededlekfwkltedkgidkknvvnflenedHPHPE<br><br>SARST: aliSize=132 (resi) RMSD=13.63 (Å)<br>d1dv--QKVAGTWYSLAMAASDISLLDAqSAPLRVYVEELKPT-PGGDLEILLQKWENGKCAQKKIIAEKTEiPAVFKIDALNENKVLVLDTdYKKYLLFCMENsaEPEQSL--ACQCLVRTPEVDDEAMEKFDKALKALPM---HIRL-----------sfnptqleeqcrv<br>----nlSELSGPWRTVYIGSTNPEKIQE-NGPFRTYFRELVFDdEKGTVDFYFSVKRDGKWKNVHVKATKQD-DGTYVADYEGQNVFKIVSL-SRTHLVAHNIN--VDKHGQktELTGLFVKLNVEDEDLEKFWKLTEDKGIdkkNVVNflenedhphpe-------------<br><br>BLAST: aliSize=54 (resi) iden=16.56% (25/151) simi=35.76% (54/151)<br>d1DVQKVAGTWYSLAMAASDISLLDaQSAPLRVYVEELK-PTPGGDLEILLQKWENGKCAQKKIIAEKTEiPAVFKIDALNENKVLVLDTDyKKYLLFCMENSAEPEQSLACQCLVRTPEVDDEAMEKFDK-------------------------alkalpmhirlsfnptqleeqcrv<br>--NLSELSGPWRTVYIGSTNPEKIQ-ENGPFRTYFRELVfDDEKGTVDFYFSVKRDGKWKNVHVKATKQD-DGTYVADYEGQNVFKIVSLS-RTHLVAHNINVDKHGQKTELTGLFVKLNVEDEDLEKFWKltedkgidkknvvnflenedhphpe------------------------<br><br>Proposed: aliSize=138 (resi) RMSD=1.89 (Å)<br>d1DVQKVAGTWYSLAMAASDISLLDAqSAPLRVYVEELKPT-PGGDLEILLQKWENGKCAQKKIIAEKTEIPaVFKIDALNENKVLVLDTDYkKYLLFCMENsA----EpeQSLACQCLVRtPEVDDEAMEKFDKALKALPMH--IRLSFNptQ---leeqCR---v<br>--NLSELSGPWRTVYIGSTNPEKIQE-NGPFRTYFRELVFDdEKGTVDFYFSVKRDGKWKNVHVKATKQDDG-TYVADYEGQNVFKIVSLSR-THLVAHNIN-VdkhgQ--KTELTGLFVK-LNVEDEDLEKFWKLTEDKGIDkkNVVNFL--Ened----HPhpe-
955	1yupE (154)<br>1gt3A (159)	C	16.23	TM-align: aliSize=120 (resi) RMSD=2.78 (Å)<br>-----D-LDVQKVAGTWYSLAMAASDISLLDAqSAPLRVYVEELKPT-PGGDLEILLQKWENGKCAQKKIIAEKTEIPaVFKIDALNENKVLVLDTDYkKYLLFCMENSA-EPEQSLACQCLVRTPevddeamekfdkalkalpmhirlsfnptqle-------------------------------EQCRV<br>aqeeeAeQNLSELSGPWRTVYIGSTNPEKIQE-NGPFRTYFRELVFDdEKGTVDFYFSVKRDGKWKNVHVKATKQDDG-TYVADYEGQNVFKIVSLSR-THLVAHNINVDkHGQTTELTELFVKLN-------------------------------vededlekfwkltedkgidkknvvnflenenHPHPE<br><br>SARST: aliSize=132 (resi) RMSD=13.66 (Å)<br>d-------LDVQKVAGTWYSLAMAASDISLLdaqsAPLR---VYVEELKPTP-GGDLEILLQKWENGKCAQKKIIAEKTEiPAVFKIDALNENKVLVLDTdYKKYLLFCMENsaEPEQSL--ACQCLVRTPEVDDEAMEKFDKALKALPM---HIRL-----------sfnptqleeqcrv<br>-aqeeeaeQNLSELSGPWRTVYIGSTNPEKI----QENGpfrTYFRELVFDDeKGTVDFYFSVKRDGKWKNVHVKATKQD-DGTYVADYEGQNVFKIVSL-SRTHLVAHNIN--VDKHGQttELTELFVKLNVEDEDLEKFWKLTEDKGIdkkNVVNflenenhphpe-------------<br><br>BLAST: aliSize=55 (resi) iden=16.23% (25/154) simi=35.71% (55/154)<br>d1--------DVQKVAGTWYSLAMAASDISLLDaQSAPLRVYVEELK-PTPGGDLEILLQKWENGKCAQKKIIAEKTEiPAVFKIDALNENKVLVLDTDyKKYLLFCMENSAEPEQSLACQCLVRTPEVDDEAMEKFDK-------------------------alkalpmhirlsfnptqleeqcrv<br>--aqeeeaeqNLSELSGPWRTVYIGSTNPEKIQ-ENGPFRTYFRELVfDDEKGTVDFYFSVKRDGKWKNVHVKATKQD-DGTYVADYEGQNVFKIVSLS-RTHLVAHNINVDKHGQTTELTELFVKLNVEDEDLEKFWKltedkgidkknvvnflenenhphpe------------------------<br><br>Proposed: aliSize=140 (resi) RMSD=1.94 (Å)<br>-----D-LDVQKVAGTWYSLAMAASDISLLDAqSAPLRVYVEELKPT-PGGDLEILLQKWENGKCAQKKIIAEKTEIPaVFKIDALNENKVLVLDTDyKKYLLFCMENsA----EpeQSLACQCLVRtPEVDDEAMEKFDKALKALPMH--IRLSFNptQ---leeqCR---v<br>aqeeeAeQNLSELSGPWRTVYIGSTNPEKIQE-NGPFRTYFRELVFDdEKGTVDFYFSVKRDGKWKNVHVKATKQDDG-TYVADYEGQNVFKIVSLS-RTHLVAHNIN-VdkhgQ--TTELTELFVK-LNVEDEDLEKFWKLTEDKGIDkkNVVNFL--Enen----HPhpe-
956	1yupE (154)<br>1gt1A (158)	C	16.23	TM-align: aliSize=120 (resi) RMSD=2.80 (Å)<br>----D-LDVQKVAGTWYSLAMAASDISLLDAqSAPLRVYVEELKPT-PGGDLEILLQKWENGKCAQKKIIAEKTEIPaVFKIDALNENKVLVLDTDYkKYLLFCMENSA-EPEQSLACQCLVRTPevddeamekfdkalkalpmhirlsfnptqle-------------------------------EQCRV<br>qeeeAeQNLSELSGPWRTVYIGSTNPEKIQE-NGPFRTYFRELVFDdEKGTVDFYFSVKRDGKWKNVHVKATKQDDG-TYVADYEGQNVFKIVSLSR-THLVAHNINVDkHGQTTELTELFVKLN-------------------------------vededlekfwkltedkgidkknvvnflenenHPHPE<br><br>SARST: aliSize=132 (resi) RMSD=14.12 (Å)<br>d------LDVQKVAGTWYSLAMAASDISLLdaqsAPLR---VYVEELKPTP-GGDLEILLQKWENGKCAQKKIIAEKTEiPAVFKIDALNENKVLVLDTdYKKYLLFCMENsaEPEQSLACQCLVRTPEVD--DEAMEKFDKALKALPM---HIRL-----------sfnptqleeqcrv<br>-qeeeaeQNLSELSGPWRTVYIGSTNPEKI----QENGpfrTYFRELVFDDeKGTVDFYFSVKRDGKWKNVHVKATKQD-DGTYVADYEGQNVFKIVSL-SRTHLVAHNIN--VDKHGQTTELTELFVKLNveDEDLEKFWKLTEDKGIdkkNVVNflenenhphpe-------------<br><br>BLAST: aliSize=55 (resi) iden=16.23% (25/154) simi=35.71% (55/154)<br>d1-------DVQKVAGTWYSLAMAASDISLLDaQSAPLRVYVEELK-PTPGGDLEILLQKWENGKCAQKKIIAEKTEiPAVFKIDALNENKVLVLDTDyKKYLLFCMENSAEPEQSLACQCLVRTPEVDDEAMEKFDK-------------------------alkalpmhirlsfnptqleeqcrv<br>--qeeeaeqNLSELSGPWRTVYIGSTNPEKIQ-ENGPFRTYFRELVfDDEKGTVDFYFSVKRDGKWKNVHVKATKQD-DGTYVADYEGQNVFKIVSLS-RTHLVAHNINVDKHGQTTELTELFVKLNVEDEDLEKFWKltedkgidkknvvnflenenhphpe------------------------<br><br>Proposed: aliSize=141 (resi) RMSD=2.03 (Å)<br>----D-LDVQKVAGTWYSLAMAASDISLLDAqSAPLRVYVEELKPT-PGGDLEILLQKWENGKCAQKKIIAEKTEIPaVFKIDALNENKVLVLDTDYkKYLLFCMENsA----EpeQSLACQCLVRtPEVDDEAMEKFDKALKALPMH--IRLSFNpTQ-leeqcRV----<br>qeeeAeQNLSELSGPWRTVYIGSTNPEKIQE-NGPFRTYFRELVFDdEKGTVDFYFSVKRDGKWKNVHVKATKQDDG-TYVADYEGQNVFKIVSLSR-THLVAHNIN-VdkhgQ--TTELTELFVK-LNVEDEDLEKFWKLTEDKGIDkkNVVNFL-ENe-----NHphpe

957	1qwdA<br>(167)<br>1g85A<br>(159)	C	8.18	TM-align: aliSize=107 (resi) RMSD=3.64 (Å)<br>hlestsl--Ykkssstpprgvtvv--NNFDAKRYLGTWYEIARFD-H-RFERGL---EKVTATYSLRDDG-GLNVINKGyNPDRgMWQQSEGKAYFTGAptRaALKVSFFgpFYGGYNVIALDreYRHALVCGPD-------RDYLWILSRTptisdevkqemlavatregfdvskfiwv----------<br>-------aqE--------------eeAEQNLSELSGPWRTVYIGStNpEKIQENgpfRTYFRELVFDDEKgTVDFYFSV-KRDG-KWKNVHVKATKQDD--G-TYVADYE--GQNVFKIVSLS--RTHLVAHNINvdkhgqtTELTGLFVKL--------------------------nvededlekf<br>-------------------------QQpg-<br>wkltedkgidkknvvnflenenhpHP--e<br>SARST: aliSize=140 (resi) RMSD=14.48 (Å)<br>hlestslykkssstppr--GVTVVnnFDAKRYLGTWYEIARF---DHRFERGLEKVTA--TYSLRD-DGGLNVINKGYNpdRGMWQQSEGKAYFtgAPTRAALKVSFfgpFYGGYNVIALDREYRHALVCGPDRD----YLWILSRTPTISDEVKQEMLAVATREGFDVSKFIWV----QQP---g<br>-----------------aqEEEAE--QNLSELSGPWRTVYIGStnPEKIQENGPFRTYfrELVFDDeKGTVDFYFSVKR--DGKWKNVHVKATK--QDDGTYVADYE---GQNVFKIVSLSRTHLVAHNINVDKHgqttELTGLFVKLNVEDEDLEKFWKLTEDKGIDKKNVVNFleneNHPhpe-<br>BLAST: aliSize=24 (resi) iden=9.43% (15/159) simi=15.09% (24/159)<br>hlestslykkssstpprgvtvvnnfdakrylgtwyeiarfdhrferglekvtatyslrddgglnvinkgynpdrgmwqqsegkayftgaptraal----------------------------KVSFFGPFYGGYNVIALDREYRHA-LVCGPDRDYLWilsRTPTISDEVKQEMLAVATREGFDVSKFI<br>---aqeeeaeqnlselsgpwrtvyigstnpeKIQENGPFRTYFRELVFDDEKGTVdFYFSVKRDGKW---KNVHVKATKQDDGTYVADYEGQNVFKIV<br>---wvqqpg<br>slsrthlvahninvdkhgqtteltglfvklnvededlekfwkltedkgidkknvvnflenenhphpe------<br>Proposed: aliSize=123 (resi) RMSD=2.34 (Å)<br>hlestslykkssstpprgvtvv-----NNFDAKRYLGTWYEIARF---D-H----RFERgLekVTATYS-L--rdDGGLNVINKGyN--pdRgMWQQSEGKAYFTG-aptRaALKVSFFgpFYGGYNVIALDreYRHALVCGP--------DrDYLWILSRtPTISDEVKQEMLAVATREGFDVSKFIWV<br>----------------------aqeeeAEQNLSELSGPWRTVYIGStnPeKiqenGPFR-T--YFRELVfDde--KGTVDFYFSV-Krd--G-KWKNVHVKATKQDd---G-TYVADYE--GQNVFKIVSLS--RTHLVAHNInvdkhgqtT-ELTGLFVK-LNVEDEDLEKFWKLTEDKGIDKKNVVNF<br>Q---------qpg<br>Lenenhphpe---
958	1qwdA<br>(167)<br>1gt3A<br>(159)	C	8.18	TM-align: aliSize=107 (resi) RMSD=3.63 (Å)<br>hlestsl--Ykkssstpprgvtvv--NNFDAKRYLGTWYEIARFD-H-RFERGL---EKVTATYSLRDDG-GLNVINKGyNPDRgMWQQSEGKAYFTGAptRaALKVSFFgpFYGGYNVIALDreYRHALVCGPD-------RDYLWILSRTptisdevkqemlavatregfdvskfiwv----------<br>-------aqE--------------eeAEQNLSELSGPWRTVYIGStNpEKIQENgpfRTYFRELVFDDEKgTVDFYFSV-KRDG-KWKNVHVKATKQDD--G-TYVADYE--GQNVFKIVSLS--RTHLVAHNINvdkhgqtTELTELFVKL--------------------------nvededlekf<br>-------------------------QQpg-<br>wkltedkgidkknvvnflenenhpHP--e<br>SARST: aliSize=140 (resi) RMSD=14.31 (Å)<br>hlestslykkssstppr--GVTVVnnFDAKRYLGTWYEIARF---DHRFERGL---EKVTATYSLRDDGGLNVINKGYNpdRGMWQQSEGKAYFtgAPTRAALKVSFfgpFYGGYNVIALDREYRHALVCGPDRD----YLWILSRTPTISDEVKQEMLAVATREGFDVSKFIWV----QQP---g<br>-----------------aqEEEAE--QNLSELSGPWRTVYIGStnPEKIQENGpfrTYFRELVFDDEKGTVDFYFSVKR--DGKWKNVHVKATK--QDDGTYVADYE---GQNVFKIVSLSRTHLVAHNINVDKHgqttELTELFVKLNVEDEDLEKFWKLTEDKGIDKKNVVNFleneNHPhpe-<br>BLAST: aliSize=30 (resi) iden=9.43% (15/159) simi=18.87% (30/159)<br>hl--ESTSLYKKSSSTppRGVTVVNNFDAKRYlGTWYEIARFDHRFERGLEKVTATYSLRDDGGL---NVIN-----------kgynpdrgmwqqsegkayftgaptra<br>--aqeeeaeqnlselsgpwrtvyigstnpekiqengpfrtyfrelvfddekgtvdfyfsvkrdgkwknvhvkatkqddgtyvadyEGQNVFKIVSLS--RTHLVAHNINVDKH-GQTTELTELFVKLNVEDEDLEKFWKLTEDKGIdkkNVVNflenenhphpe-------------------------<br>alkvsffgpfyggynvialdreyrhalvcgpdrdylwilsrtptisdevkqemlavatregfdvskfiwvqqpg<br>---<br>Proposed: aliSize=123 (resi) RMSD=2.33 (Å)<br>hlestslykkssstpprgvtvv-----NNFDAKRYLGTWYEIARF---D-H----RFERgLekVTATYS-L--rdDGGLNVINKGyN--pdRgMWQQSEGKAYFTG-aptRaALKVSFFgpFYGGYNVIALDreYRHALVCGP--------DrDYLWILSRtPTISDEVKQEMLAVATREGFDVSKFIWV<br>----------------------aqeeeAEQNLSELSGPWRTVYIGStnPeKiqenGPFR-T--YFRELVfDde--KGTVDFYFSV-Krd--G-KWKNVHVKATKQDd---G-TYVADYE--GQNVFKIVSLS--RTHLVAHNInvdkhgqtT-ELTELFVK-LNVEDEDLEKFWKLTEDKGIDKKNVVNF<br>Q---------qpg<br>Lenenhphpe---
959	1qwdA<br>(167)<br>1gt1A<br>(158)	C	8.23	TM-align: aliSize=107 (resi) RMSD=3.64 (Å)<br>hlestsl-Ykkssstpprgvtvv--NNFDAKRYLGTWYEIARFD-H-RFERGL---EKVTATYSLRDDG-GLNVINKGyNPDRgMWQQSEGKAYFTGAptRaALKVSFFgpFYGGYNVIALDreYRHALVCGPD-------RDYLWILSRTptisdevkqemlavatregfdvskfiwv----------<br>-------qE--------------eeAEQNLSELSGPWRTVYIGStNpEKIQENgpfRTYFRELVFDDEKgTVDFYFSV-KRDG-KWKNVHVKATKQDD--G-TYVADYE--GQNVFKIVSLS--RTHLVAHNINvdkhgqtTELTELFVKL--------------------------nvededlekfw<br>-------------------------QQpg-<br>kltedkgidkknvvnflenenhpHP--e<br>SARST: aliSize=133 (resi) RMSD=16.39 (Å)<br>hlestslykkssstppr-GVTVVnnFDAKRYLGTWYEIARF---DHRFERGL---EKVTATYSLRDDGGLNVINKGYNpdRGMWQQSEGKAYFT-------GAPTRAALKVSFFGPFYGGYNVIALDREYRHALVCGpdrdYLWILsrtpTISDEVKQEMLAVATREGFDVSKFIW-----------vqqpg<br>-----------------qEEEAE--QNLSELSGPWRTVYIGStnPEKIQENGpfrTYFRELVFDDEKGTVDFYFSVKR--DGKWKNVHVKATKQddgtyvaDYEGQNVFKIVSLSRTHLVAHNINVDKHGQTTELTE----LFVKL----NVEDEDLEKFWKLTEDKGIDKKNVVNflenenhphpe-----<br>BLAST: aliSize=30 (resi) iden=9.49% (15/158) simi=18.99% (30/158)<br>hl--ESTSLYKKSSSTppRGVTVVNNFDAKRYlGTWYEIARFDHRFERGLEKVTATYSLRDDGGL---NVIN-----------kgynpdrgmwqqsegkayftgaptraa<br>--qeeeaeqnlselsgpwrtvyigstnpekiqengpfrtyfrelvfddekgtvdfyfsvkrdgkwknvhvkatkqddgtyvadyEGQNVFKIVSLS--RTHLVAHNINVDKH-GQTTELTELFVKLNVEDEDLEKFWKLTEDKGIdkkNVVNflenenhphpe--------------------------<br>lkvsffgpfyggynvialdreyrhalvcgpdrdylwilsrtptisdevkqemlavatregfdvskfiwvqqpg<br>--<br>Proposed: aliSize=124 (resi) RMSD=2.40 (Å)<br>hlestslykkssstpprgvtvv----NNFDAKRYLGTWYEIARF---D-H----RFERgLekVTATYS-L--rdDGGLNVINKGyN--pdRgMWQQSEGKAYFTG-aptRaALKVSFFgpFYGGYNVIALDreYRHALVCGP--------DrDYLWILSRtPTISDEVKQEMLAVATREGFDVSKFIWVQ<br>----------------------qeeeAEQNLSELSGPWRTVYIGStnPeKiqenGPFR-T--YFRELVfDde--KGTVDFYFSV-Krd--G-KWKNVHVKATKQDd---G-TYVADYE--GQNVFKIVSLS--RTHLVAHNInvdkhgqtT-ELTELFVK-LNVEDEDLEKFWKLTEDKGIDKKNVVNFL<br>Q--------pg<br>Enenhphpe--

960	2q39B (151)<br>1pboB (151)	C	15.89	TM-align: aliSize=115 (resi) RMSD=2.37 (Å)<br>qtmkglDIQKVAGTWYSLAMAASDISLLDAqSAPLRVYVEELKPT-PEGDLEILLQKWENDECAQKKIIAEKTKIPaVFKIDALNENKVLVLDTDyKKYLLFCMENSA-EPEQSLVCQCLVRTPevddealekfdkalkalpmhirlsfnp---------------------------------TQ-<br>------NLSELSGPWRTVYIGSTNPEKIQE-NGPFRTYFRELVFDdEKGTVDFYFSVKRDGKWKNVHVKATKQDDG-TYVADYEGQNVFKIVSLS-RTHLVAHNINVDkHGQKTELTGLFVKLN---------------------------vededlekfwkltedkgidkknvvnflenedhpHPe<br><br>SARST: aliSize=135 (resi) RMSD=13.53 (Å)<br>qtmkgldi--QKVAGTWYSLAMAASDISLLDAqSAPLRVYVEELKPT-PEGDLEILLQKWENDECAQKKIIAEKTKiPAVFKIDALNENKVLVLDTDYKKYLLFCMENSAEPEQSLVCQCLVRTPEVDDEALEKFDKALKALPM--HIRL-----------sfnptq<br>--------nlSELSGPWRTVYIGSTNPEKIQE-NGPFRTYFRELVFDdEKGTVDFYFSVKRDGKWKNVHVKATKQD-DGTYVADYEGQNVFKIVSLSRTHLVAHNINVDKHGQKTELTGLFVKLNVEDEDLEKFWKLTEDKGIDkkNVVNflenedhphpe------<br><br>BLAST: aliSize=53 (resi) iden=16.56% (25/151) simi=35.10% (53/151)<br>qtmkglDIQKVAGTWYSLAMAASDISLLDaQSAPLRVYVEELKPTPE-GDLEILLQKWENDECAQKKIIAEKTKiPAVFKIDALNENKVLVLDTDyKKYLLFCMENSAEPEQSLVCQCLVRTPEVDDEALEKFDK-------------------------alkalpmhirlsfnptq<br>------NLSELSGPWRTVYIGSTNPEKIQ-ENGPFRTYFRELVFDDEkGTVDFYFSVKRDGKWKNVHVKATKQD-DGTYVADYEGQNVFKIVSLS-RTHLVAHNINVDKHGQKTELTGLFVKLNVEDEDLEKFWKltedkgidkknvvnflenedhphpe-----------------<br><br>Proposed: aliSize=139 (resi) RMSD=1.99 (Å)<br>qtmkglDIQKVAGTWYSLAMAASDISLLDAqSAPLRVYVEELKPT-PEGDLEILLQKWENDECAQKKIIAEKTKIPaVFKIDA-lNENKVLVLDTDyKKYLLFCMENSA--EPeQSLVCQCLVRtPEVDDEALEKFDKALKALPMH--IRLSFNPT--Q----<br>------NLSELSGPWRTVYIGSTNPEKIQE-NGPFRTYFRELVFDdEKGTVDFYFSVKRDGKWKNVHVKATKQDDG-TYVADYe-GQNVFKIVSLS-RTHLVAHNINVDkhGQ-KTELTGLFVK-LNVEDEDLEKFWKLTEDKGIDkkNVVNFLENedHphpe
961	1bj7A (150)<br>1g85A (159)	C	26.67	TM-align: aliSize=117 (resi) RMSD=2.11 (Å)<br>-------IDPSKIPGEWRIIYAAADNKDKIVEGGPLRNYYRRIECINDCESLSITFYLKDQGTCLLLTEVAKRQEGYVYVLEFYGTNTLEVIHVSENMLVTYVENYD-GERITKMTEGLAKGtsftpeelekyqqlnsergvpnenienlikt---------------------------------DNCpp-<br>aqeeeaeQNLSELSGPWRTVYIGSTNPEKIQENGPFRTYFRELVFDDEKGTVDFYFSVKRDGKWKNVHVKATKQDDGTYVADYEGQNVFKIVSLSRTHLVAHNINVDkHGQTTELTGLFVKL-------------------------------nvededlekfwkltedkgidkknvvnflenenhPHP--e<br><br>SARST: aliSize=137 (resi) RMSD=13.33 (Å)<br>idps-----------KIPGEWRIIYAAADNKDKIVEGGPLRNYYRRIECINDCESLSITFYLKDQGTCLLLTEVAKRQEGYVYVLEFYGTNTLEVIHVSENMLVTYVENY--DGERITKMTEGLAKGtsFTPEELEKYQQLNSERGVPNENIENLI---------ktdncpp<br>----aqeeeaeqnlsELSGPWRTVYIGSTNPEKIQENGPFRTYFRELVFDDEKGTVDFYFSVKRDGKWKNVHVKATKQDDGTYVADYEGQNVFKIVSLSRTHLVAHNINVdkHGQTTELTGLFVKLN--VEDEDLEKFWKLTEDKGIDKKNVVNFLenenhphpe-------<br><br>BLAST: aliSize=81 (resi) iden=30.67% (46/150) simi=54.00% (81/150)<br>idp----------SKIPGEWRIIYAAADNKDKIVEGGPLRNYYRriECINDCESLSITFY--LKDQGTCLLLTEVAKRQEGYVYVLEFYGTNTLEVIHVSENMLVTYVENYDGE-RITKMTeGLAKGTSFTPEELEKYQQLNSERGVPNENIENLIKTDNCP---p<br>---aqeeeaeqnlSELSGPWRTVYIGSTNPEKIQENGPFRTYFR--ELVFDDEKGTVDFYfsVKRDGKWKNVHVKATKQDDGTYVADYEGQNVFKIVSLSRTHLVAHNINVDKHgQTTELT-GLFVKLNVEDEDLEKFWKLTEDKGIDKKNVVNFLENENHPhpe-<br><br>Proposed: aliSize=147 (resi) RMSD=1.80 (Å)<br>i--------DPSKIPGEWRIIYAAADNKDKIVEGGPLRNYYRRIECINDCESLSITFYLKDQGTCLLLTEVAKRQEGYVYVLEFYGTNTLEVIHVSENMLVTYVENYD-GERITKMTEGLAKgTSFTPEELEKYQQLNSERGVPNENIENLIKT--DNCP-p<br>-aqeeeaeqNLSELSGPWRTVYIGSTNPEKIQENGPFRTYFRELVFDDEKGTVDFYFSVKRDGKWKNVHVKATKQDDGTYVADYEGQNVFKIVSLSRTHLVAHNINVDkHGQTTELTGLFVK-LNVEDEDLEKFWKLTEDKGIDKKNVVNFLENenHPHPe-
962	1bj7A (150)<br>1pboB (151)	C	26.67	TM-align: aliSize=117 (resi) RMSD=2.21 (Å)<br>iDPSKIPGEWRIIYAAADNKDKIVEGGPLRNYYRRIECINDCESLSITFYLKDQGTCLLLTEVAKRQEGYVYVLEFYGTNTLEVIHVSENMLVTYVENYD-GERITKMTEGLAKGtsftpeelekyqqlnsergvpnenienlik---------------------------------TDNCpp<br>-NLSELSGPWRTVYIGSTNPEKIQENGPFRTYFRELVFDDEKGTVDFYFSVKRDGKWKNVHVKATKQDDGTYVADYEGQNVFKIVSLSRTHLVAHNINVDkHGQKTELTGLFVKL------------------------------nvededlekfwkltedkgidkknvvnflenedhPHPE--<br><br>SARST: aliSize=134 (resi) RMSD=13.45 (Å)<br>idps---KIPGEWRIIYAAADNKDKIVEGGPLRNYYRRIECI--NDceSLSITFYLKDQGTCLLLTEVAKRqEGY-VYVLEFYGTNTLEVIHVSENMLVTYVENY--DGERITKMTEGLAKGtsFTPEELEKYQQLNSERGVPNENIENLI---------ktdncpp<br>----nlsELSGPWRTVYIGSTNPEKIQENGPFRTYFRELVFDdeKG--TVDFYFSVKRDGKWKNVHVKATK-QDDgTYVADYEGQNVFKIVSLSRTHLVAHNINVdkHGQKTELTGLFVKLN--VEDEDLEKFWKLTEDKGIDKKNVVNFLenedhphpe-------<br><br>BLAST: aliSize=83 (resi) iden=30.67% (46/150) simi=55.33% (83/150)<br>idp--SKIPGEWRIIYAAADNKDKIVEGGPLRNYYRriECINDCESLSITFY--LKDQGTCLLLTEVAKRQEGYVYVLEFYGTNTLEVIHVSENMLVTYVENYD--GERiTKMTeGLAKGTSFTPEELEKYQQLNSERGVPNENIENLIKTDNCP---p<br>---nlSELSGPWRTVYIGSTNPEKIQENGPFRTYFR--ELVFDDEKGTVDFYfsVKRDGKWKNVHVKATKQDDGTYVADYEGQNVFKIVSLSRTHLVAHNINVDkhGQK-TELT-GLFVKLNVEDEDLEKFWKLTEDKGIDKKNVVNFLENEDHPhpe-<br><br>Proposed: aliSize=147 (resi) RMSD=1.81 (Å)<br>iDPSKIPGEWRIIYAAADNKDKIVEGGPLRNYYRRIECINDCESLSITFYLKDQGTCLLLTEVAKRQEGYVYVLEFYGTNTLEVIHVSENMLVTYVENYD-GERITKMTEGLAKgTSFTPEELEKYQQLNSERGVPNENIENLIKT--DNCP-p<br>-NLSELSGPWRTVYIGSTNPEKIQENGPFRTYFRELVFDDEKGTVDFYFSVKRDGKWKNVHVKATKQDDGTYVADYEGQNVFKIVSLSRTHLVAHNINVDkHGQKTELTGLFVK-LNVEDEDLEKFWKLTEDKGIDKKNVVNFLENedHPHPe-

963	1bj7A (150)<br>1gt3A (159)	C	27.33	TM-align: aliSize=117 (resi) RMSD=2.10 (Å)<br>-------IDPSKIPGEWRIIYAAADNKDKIVEGGPLRNYYRRIECINDCESLSITFYLKDQGTCLLLTEVAKRQEGYVYVLEFYGTNTLEVIHVSENMLVTYVENYD-GERITKMTEGLAKGtsftpeelekyqqlnsergvpnenienlikt-------------------------------DNCpp-<br>aqeeeaeQNLSELSGPWRTVYIGSTNPEKIQENGPFRTYFRELVFDDEKGTVDFYFSVKRDGKWKNVHVKATKQDDGTYVADYEGQNVFKIVSLSRTHLVAHNINVDkHGQTTELTELFVKL-----------------------------nvededlekfwkltedkgidkknvvnflenenhPHP--e<br><br>SARST: aliSize=140 (resi) RMSD=13.19 (Å)<br>i--------DPSKIPGEWRIIYAAADNKDKIVEGGPLRNYYRRIECINDCESLSITFYLKDQGTCLLLTEVAKRQEGYVYVLEFYGTNTLEVIHVSENMLVTYVENY--DGERITKMTEGLAKGtsFTPEELEKYQQLNSERGVPNENIENLI---------ktdncpp<br>-aqeeeaeqNLSELSGPWRTVYIGSTNPEKIQENGPFRTYFRELVFDDEKGTVDFYFSVKRDGKWKNVHVKATKQDDGTYVADYEGQNVFKIVSLSRTHLVAHNINVdkHGQTTELTELFVKLN--VEDEDLEKFWKLTEDKGIDKKNVVNFLenenhphpe-------<br><br>BLAST: aliSize=81 (resi) iden=30.67% (46/150) simi=54.00% (81/150)<br>idp----------SKIPGEWRIIYAAADNKDKIVEGGPLRNYYRriECINDCESLSITFY--LKDQGTCLLLTEVAKRQEGYVYVLEFYGTNTLEVIHVSENMLVTYVENYDGE-RITKMTEGLAKgTSFTPEELEKYQQLNSERGVPNENIENLIKTDNCP---p<br>---aqeeeaeqnlSELSGPWRTVYIGSTNPEKIQENGPFRTYFR--ELVFDDEKGTVDFYfsVKRDGKWKNVHVKATKQDDGTYVADYEGQNVFKIVSLSRTHLVAHNINVDKHgQTTELTELFVK-LNVEDEDLEKFWKLTEDKGIDKKNVVNFLENENHPhpe-<br><br>Proposed: aliSize=147 (resi) RMSD=1.79 (Å)<br>i--------DPSKIPGEWRIIYAAADNKDKIVEGGPLRNYYRRIECINDCESLSITFYLKDQGTCLLLTEVAKRQEGYVYVLEFYGTNTLEVIHVSENMLVTYVENYD-GERITKMTEGLAKgTSFTPEELEKYQQLNSERGVPNENIENLIKT--DNCP-p<br>-aqeeeaeqNLSELSGPWRTVYIGSTNPEKIQENGPFRTYFRELVFDDEKGTVDFYFSVKRDGKWKNVHVKATKQDDGTYVADYEGQNVFKIVSLSRTHLVAHNINVDkHGQTTELTELFVK-LNVEDEDLEKFWKLTEDKGIDKKNVVNFLENenHPHPe-
964	1bj7A (150)<br>1gt1A (158)	C	27.33	TM-align: aliSize=117 (resi) RMSD=2.12 (Å)<br>------IDPSKIPGEWRIIYAAADNKDKIVEGGPLRNYYRRIECINDCESLSITFYLKDQGTCLLLTEVAKRQEGYVYVLEFYGTNTLEVIHVSENMLVTYVENYD-GERITKMTEGLAKGtsftpeelekyqqlnsergvpnenienlikt-------------------------------DNCpp-<br>qeeeaeQNLSELSGPWRTVYIGSTNPEKIQENGPFRTYFRELVFDDEKGTVDFYFSVKRDGKWKNVHVKATKQDDGTYVADYEGQNVFKIVSLSRTHLVAHNINVDkHGQTTELTELFVKL-----------------------------nvededlekfwkltedkgidkknvvnflenenhPHP--e<br><br>SARST: aliSize=135 (resi) RMSD=13.39 (Å)<br>idps----------KIPGEWRIIYAAADNKDKIVEGGPLRNYYRRIECI--NDceSLSITFYLKDQGTCLLLTEVAKRQEGYVYVLEFYGTNTLEVIHVSENMLVTYVENY--DGERITKMTEGLAkgTSFTPEELEKYQQLNSERGVPNENIENLI---------ktdncpp<br>----qeeeaeqnlsELSGPWRTVYIGSTNPEKIQENGPFRTYFRELVFDdeKG--TVDFYFSVKRDGKWKNVHVKATKQDDGTYVADYEGQNVFKIVSLSRTHLVAHNINVdkHGQTTELTELFVK--LNVEDEDLEKFWKLTEDKGIDKKNVVNFLenenhphpe-------<br><br>BLAST: aliSize=81 (resi) iden=30.67% (46/150) simi=54.00% (81/150)<br>idp---------SKIPGEWRIIYAAADNKDKIVEGGPLRNYYRriECINDCESLSITFY--LKDQGTCLLLTEVAKRQEGYVYVLEFYGTNTLEVIHVSENMLVTYVENYDGE-RITKMTEGLAKgTSFTPEELEKYQQLNSERGVPNENIENLIKTDNCP---p<br>---qeeeaeqnlSELSGPWRTVYIGSTNPEKIQENGPFRTYFR--ELVFDDEKGTVDFYfsVKRDGKWKNVHVKATKQDDGTYVADYEGQNVFKIVSLSRTHLVAHNINVDKHgQTTELTELFVK-LNVEDEDLEKFWKLTEDKGIDKKNVVNFLENENHPhpe-<br><br>Proposed: aliSize=147 (resi) RMSD=1.80 (Å)<br>i-------DPSKIPGEWRIIYAAADNKDKIVEGGPLRNYYRRIECINDCESLSITFYLKDQGTCLLLTEVAKRQEGYVYVLEFYGTNTLEVIHVSENMLVTYVENYD-GERITKMTEGLAKgTSFTPEELEKYQQLNSERGVPNENIENLIKT--DNCP-p<br>-qeeeaeqNLSELSGPWRTVYIGSTNPEKIQENGPFRTYFRELVFDDEKGTVDFYFSVKRDGKWKNVHVKATKQDDGTYVADYEGQNVFKIVSLSRTHLVAHNINVDkHGQTTELTELFVK-LNVEDEDLEKFWKLTEDKGIDKKNVVNFLENenHPHPe-
965	1dzkA (148)<br>1g85A (159)	C	42.57	TM-align: aliSize=115 (resi) RMSD=1.63 (Å)<br>---------F-ELSGKWITSYIGSSDLEKIGENAPFQVFMRSIEFDDKESKVYLNFFSKENGICEEFSLIGTKQEGNTYDVNYAGNNKFVVSYASETALIISNINVDEEGDKTIMTGLLGKGtdiedqdlekfkevtrengipeenivniier--------------------------------DDCpa<br>aqeeeaeqnLsELSGPWRTVYIGSTNPEKIQENGPFRTYFRELVFDDEKGTVDFYFSVKRDGKWKNVHVKATKQDDGTYVADYEGQNVFKIVSLSRTHLVAHNINVDKHGQTTELTGLFVKL-----------------------------nvededlekfwkltedkgidkknvvnflenenhpHPE--<br><br>SARST: aliSize=123 (resi) RMSD=14.09 (Å)<br>fe------------LSGKWITSYIGSSDLEKIGENAPFQVFMRSIEFDDKESKVYLNFFSKENGiceefsligtkqegn---------------TYDVNYAGNNKFVVSYASETALIISNINVDEEGDKTIMTGLLGKGTDIeDQDLEKFKEVTRENGIPEENIVNII---------erddcpa<br>--aqeeeaeqnlseLSGPWRTVYIGSTNPEKIQENGPFRTYFRELVFDDEKGTVDFYFSVKRDG---------------kwknvhvkatkqddgTYVADYEGQNVFKIVSLSRTHLVAHNINVDKHGQTTELTGLFVKLNVE-DEDLEKFWKLTEDKGIDKKNVVNFLenenhphpe-------<br><br>BLAST: aliSize=91 (resi) iden=42.57% (63/148) simi=61.49% (91/148)<br>f-----------ELSGKWITSYIGSSDLEKIGENAPFQVFMRSIEFDDKESKVYLNFFSKENGICEEFSLIGTKQEGNTYDVNYAGNNKFVVSYASETALIISNINVDEEGDKTIMTGLLGKgTDIEDQDLEKFKEVTRENGIPEENIVNIIERDDCP---a<br>-aqeeeaeqnlsELSGPWRTVYIGSTNPEKIQENGPFRTYFRELVFDDEKGTVDFYFSVKRDGKWKNVHVKATKQDDGTYVADYEGQNVFKIVSLSRTHLVAHNINVDKHGQTTELTGLFVK-LNVEDEDLEKFWKLTEDKGIDKKNVVNFLENENHPhpe-<br><br>Proposed: aliSize=146 (resi) RMSD=1.41 (Å)<br>---------F-ELSGKWITSYIGSSDLEKIGENAPFQVFMRSIEFDDKESKVYLNFFSKENGICEEFSLIGTKQEGNTYDVNYAGNNKFVVSYASETALIISNINVDEEGDKTIMTGLLGKgTDIEDQDLEKFKEVTRENGIPEENIVNIIER--DDCP-a<br>aqeeeaeqnLsELSGPWRTVYIGSTNPEKIQENGPFRTYFRELVFDDEKGTVDFYFSVKRDGKWKNVHVKATKQDDGTYVADYEGQNVFKIVSLSRTHLVAHNINVDKHGQTTELTGLFVK-LNVEDEDLEKFWKLTEDKGIDKKNVVNFLENenHPHPe-

966	1dzkA<br>(148)<br>1pboB<br>(151)	C	43.24	TM-align: aliSize=115 (resi) RMSD=1.67 (Å)<br>-F-ELSGKWITSYIGSSDLEKIGENAPFQVFMRSIEFDDKESKVYLNFFSKENGICEEFSLIGTKQEGNTYDVNYAGNNKFVVSYASETALIISNINVDEEGDKTIMTGLLGKGtdiedqdlekfkevtrengipeenivniier----------------------------------DDCpa<br>nLsELSGPWRTVYIGSTNPEKIQENGPFRTYFRELVFDDEKGTVDFYFSVKRDGKWKNVHVKATKQDDGTYVADYEGQNVFKIVSLSRTHLVAHNINVDKHGQKTELTGLFVKL-------------------------------nvededlekfwkltedkgidkknvvnflenedhpHPE--<br><br>SARST: aliSize=138 (resi) RMSD=13.43 (Å)<br>fe----LSGKWITSYIGSSDLEKIGENAPFQVFMRSIEFDDKESKVYLNFFSKENGICEEFSLIGTKQEGNTYDVNYAGNNKFVVSYASETALIISNINVDEEGDKTIMTGLLGkGTDIEDQDLEKFKEVTRENGIPEENIVNII---------erddcpa<br>--nlseLSGPWRTVYIGSTNPEKIQENGPFRTYFRELVFDDEKGTVDFYFSVKRDGKWKNVHVKATKQDDGTYVADYEGQNVFKIVSLSRTHLVAHNINVDKHGQKTELTGLFV-KLNVEDEDLEKFWKLTEDKGIDKKNVVNFLenedhphpe-------<br><br>BLAST: aliSize=92 (resi) iden=43.92% (65/148) simi=62.16% (92/148)<br>f---ELSGKWITSYIGSSDLEKIGENAPFQVFMRSIEFDDKESKVYLNFFSKENGICEEFSLIGTKQEGNTYDVNYAGNNKFVVSYASETALIISNINVDEEGDKTIMTGLLGKgTDIEDQDLEKFKEVTRENGIPEENIVNIIERDDCP---a<br>-nlsELSGPWRTVYIGSTNPEKIQENGPFRTYFRELVFDDEKGTVDFYFSVKRDGKWKNVHVKATKQDDGTYVADYEGQNVFKIVSLSRTHLVAHNINVDKHGQKTELTGLFVK-LNVEDEDLEKFWKLTEDKGIDKKNVVNFLENEDHPhpe-<br><br>Proposed: aliSize=146 (resi) RMSD=1.43 (Å)<br>-F-ELSGKWITSYIGSSDLEKIGENAPFQVFMRSIEFDDKESKVYLNFFSKENGICEEFSLIGTKQEGNTYDVNYAGNNKFVVSYASETALIISNINVDEEGDKTIMTGLLGKgTDIEDQDLEKFKEVTRENGIPEENIVNIIER--DDCP-a<br>nLsELSGPWRTVYIGSTNPEKIQENGPFRTYFRELVFDDEKGTVDFYFSVKRDGKWKNVHVKATKQDDGTYVADYEGQNVFKIVSLSRTHLVAHNINVDKHGQKTELTGLFVK-LNVEDEDLEKFWKLTEDKGIDKKNVVNFLENedHPHPe-
967	1dzkA<br>(148)<br>1gt3A<br>(159)	C	41.89	TM-align: aliSize=115 (resi) RMSD=1.62 (Å)<br>---------F-ELSGKWITSYIGSSDLEKIGENAPFQVFMRSIEFDDKESKVYLNFFSKENGICEEFSLIGTKQEGNTYDVNYAGNNKFVVSYASETALIISNINVDEEGDKTIMTGLLGKGtdiedqdlekfkevtrengipeenivniier----------------------------------DDCpa<br>aqeeeaeqnLsELSGPWRTVYIGSTNPEKIQENGPFRTYFRELVFDDEKGTVDFYFSVKRDGKWKNVHVKATKQDDGTYVADYEGQNVFKIVSLSRTHLVAHNINVDKHGQTTELTELFVKL-------------------------------nvededlekfwkltedkgidkknvvnflenenhpHPE--<br><br>SARST: aliSize=123 (resi) RMSD=14.07 (Å)<br>fe------------LSGKWITSYIGSSDLEKIGENAPFQVFMRSIEFDDKESKVYLNFFSKENGiceefsligtkqegn---------------TYDVNYAGNNKFVVSYASETALIISNINVDEEGDKTIMTGLLGKGTDIeDQDLEKFKEVTRENGIPEENIVNII---------erddcpa<br>--aqeeeaeqnlseLSGPWRTVYIGSTNPEKIQENGPFRTYFRELVFDDEKGTVDFYFSVKRDG---------------kwknvhvkatkqddgTYVADYEGQNVFKIVSLSRTHLVAHNINVDKHGQTTELTELFVKLNVE-DEDLEKFWKLTEDKGIDKKNVVNFLenenhphpe-------<br><br>BLAST: aliSize=90 (resi) iden=41.89% (62/148) simi=60.81% (90/148)<br>f-----------ELSGKWITSYIGSSDLEKIGENAPFQVFMRSIEFDDKESKVYLNFFSKENGICEEFSLIGTKQEGNTYDVNYAGNNKFVVSYASETALIISNINVDEEGDKTIMTGLLGKgTDIEDQDLEKFKEVTRENGIPEENIVNIIERDDCP---a<br>-aqeeeaeqnlsELSGPWRTVYIGSTNPEKIQENGPFRTYFRELVFDDEKGTVDFYFSVKRDGKWKNVHVKATKQDDGTYVADYEGQNVFKIVSLSRTHLVAHNINVDKHGQTTELTELFVK-LNVEDEDLEKFWKLTEDKGIDKKNVVNFLENENHPhpe-<br><br>Proposed: aliSize=146 (resi) RMSD=1.40 (Å)<br>---------F-ELSGKWITSYIGSSDLEKIGENAPFQVFMRSIEFDDKESKVYLNFFSKENGICEEFSLIGTKQEGNTYDVNYAGNNKFVVSYASETALIISNINVDEEGDKTIMTGLLGKgTDIEDQDLEKFKEVTRENGIPEENIVNIIER--DDCP-a<br>aqeeeaeqnLsELSGPWRTVYIGSTNPEKIQENGPFRTYFRELVFDDEKGTVDFYFSVKRDGKWKNVHVKATKQDDGTYVADYEGQNVFKIVSLSRTHLVAHNINVDKHGQTTELTELFVK-LNVEDEDLEKFWKLTEDKGIDKKNVVNFLENenHPHPe-
968	1dzkA<br>(148)<br>1gt1A<br>(158)	C	41.89	TM-align: aliSize=115 (resi) RMSD=1.64 (Å)<br>--------F-ELSGKWITSYIGSSDLEKIGENAPFQVFMRSIEFDDKESKVYLNFFSKENGICEEFSLIGTKQEGNTYDVNYAGNNKFVVSYASETALIISNINVDEEGDKTIMTGLLGKGtdiedqdlekfkevtrengipeenivniier----------------------------------DDCpa<br>qeeeaeqnLsELSGPWRTVYIGSTNPEKIQENGPFRTYFRELVFDDEKGTVDFYFSVKRDGKWKNVHVKATKQDDGTYVADYEGQNVFKIVSLSRTHLVAHNINVDKHGQTTELTELFVKL-------------------------------nvededlekfwkltedkgidkknvvnflenenhpHPE--<br><br>SARST: aliSize=123 (resi) RMSD=14.05 (Å)<br>fe-----------LSGKWITSYIGSSDLEKIGENAPFQVFMRSIEFDDKESKVYLNFFSKENGiceefsligtkqegn---------------TYDVNYAGNNKFVVSYASETALIISNINVDEEGDKTIMTGLLGkGTDIEDQDLEKFKEVTRENGIPEENIVNII---------erddcpa<br>--qeeeaeqnlseLSGPWRTVYIGSTNPEKIQENGPFRTYFRELVFDDEKGTVDFYFSVKRDG---------------kwknvhvkatkqddgTYVADYEGQNVFKIVSLSRTHLVAHNINVDKHGQTTELTELFV-KLNVEDEDLEKFWKLTEDKGIDKKNVVNFLenenhphpe-------<br><br>BLAST: aliSize=90 (resi) iden=41.89% (62/148) simi=60.81% (90/148)<br>f----------ELSGKWITSYIGSSDLEKIGENAPFQVFMRSIEFDDKESKVYLNFFSKENGICEEFSLIGTKQEGNTYDVNYAGNNKFVVSYASETALIISNINVDEEGDKTIMTGLLGKgTDIEDQDLEKFKEVTRENGIPEENIVNIIERDDCP---a<br>-qeeeaeqnlsELSGPWRTVYIGSTNPEKIQENGPFRTYFRELVFDDEKGTVDFYFSVKRDGKWKNVHVKATKQDDGTYVADYEGQNVFKIVSLSRTHLVAHNINVDKHGQTTELTELFVK-LNVEDEDLEKFWKLTEDKGIDKKNVVNFLENENHPhpe-<br><br>Proposed: aliSize=146 (resi) RMSD=1.42 (Å)<br>--------F-ELSGKWITSYIGSSDLEKIGENAPFQVFMRSIEFDDKESKVYLNFFSKENGICEEFSLIGTKQEGNTYDVNYAGNNKFVVSYASETALIISNINVDEEGDKTIMTGLLGKgTDIEDQDLEKFKEVTRENGIPEENIVNIIER--DDCP-a<br>qeeeaeqnLsELSGPWRTVYIGSTNPEKIQENGPFRTYFRELVFDDEKGTVDFYFSVKRDGKWKNVHVKATKQDDGTYVADYEGQNVFKIVSLSRTHLVAHNINVDKHGQTTELTELFVK-LNVEDEDLEKFWKLTEDKGIDKKNVVNFLENenHPHPe-

969	1ew3A (159)<br>1g85A (159)	N	16.35	TM-align: aliSize=119 (resi) RMSD=2.65 (Å)<br>vair-----N-FDISKISGEWYSIFLASDVKEKIEENGSMRVFVDVIRAL-DNSSLYAEYQTKVNGECTEFPMVFDKTEEDgVYSLNYDGYNVFRISEFeNDEHIILYLVNFD-KDRPFQLFEFYAREpdvspeikeefvkivqkrgivkeniidltkidr-----------------------------<br>----aqeeeAeQNLSELSGPWRTVYIGSTNPEKIQENGPFRTYFRELVFDdEKGTVDFYFSVKRDGKWKNVHVKATKQDDG-TYVADYEGQNVFKIVSL-SRTHLVAHNINVDkHGQTTELTGLFVKL---------------------------------nvededlekfwkltedkgidkknvvnfle<br>-CFQLRg--<br>nENHPH-pe<br><br>SARST: aliSize=148 (resi) RMSD=13.71 (Å)<br>v---AIRNFDISKISGEWYSIFLASDVKEKIEENGSMRVFVDVIRALD-NSSLYAEYQTKVNGECTEFPMVFDKTEEdGVYSLNYDGYNVFRISEFeNDEHIILYLVNFDKDRPFQLFEFYAREPDVSPEIKEEFVKIVQKRGIVKENIIDLTKI-------drcfqlrg<br>-aqeEEAEQNLSELSGPWRTVYIGSTNPEKIQENGPFRTYFRELVFDDeKGTVDFYFSVKRDGKWKNVHVKATKQDD-GTYVADYEGQNVFKIVSL-SRTHLVAHNINVDKHGQTTELTGLFVKLNVEDEDLEKFWKLTEDKGIDKKNVVNFLENenhphpe--------<br><br>BLAST: aliSize=80 (resi) iden=24.53% (39/159) simi=50.31% (80/159)<br>vairnf--------DISKISGEWYSIFLASDVKEKIEENGSMRV-FVDVIRALDNSSLYAEYQTKVNGECTEFPMVFDKtEEDGVYSLNYDGYNVFRISEFENdEHIILYLVNFDK-DRPFQLFEFYARePDVSPEIKEEFVKIVQKRGIVKENIID-----------ltkidrcfqlrg<br>------aqeeeaeqNLSELSGPWRTVYIGSTNPEKIQENGPFRTyFRELVFDDEKGTVDFYFSVKRDGKWKNVHVKATK-QDDGTYVADYEGQNVFKIVSLSR-THLVAHNINVDKhGQTTELTGLFVK-LNVEDEDLEKFWKLTEDKGIDKKNVVNflenenhphpe-----------<br><br>Proposed: aliSize=120 (resi) RMSD=1.83 (Å)<br>-VAIRNFD-ISKISGEWYSIFLASDVKEKIEENGSMRVFVDVIRAL-DNSSLYAEYQTKVNGECTEFPMVFDKTEEDgVYSLNYDGYNVFRISEFeNDEHIILYLVNFD-KDRPFQLFEFYARE---------------------------pdvspeikeefvkivqkrgivkeniidltkidrcfqlRG<br>aQEEEAEQnLSELSGPWRTVYIGSTNPEKIQENGPFRTYFRELVFDdEKGTVDFYFSVKRDGKWKNVHVKATKQDDG-TYVADYEGQNVFKIVSL-SRTHLVAHNINVDkHGQTTELTGLFVKLnvededlekfwkltedkgidkknvvnf-------------------------------------LE<br>--------<br>nenhphpe
970	1ew3A (159)<br>1g85A (159)	C	22.01	TM-align: aliSize=119 (resi) RMSD=2.65 (Å)<br>vair-----N-FDISKISGEWYSIFLASDVKEKIEENGSMRVFVDVIRAL-DNSSLYAEYQTKVNGECTEFPMVFDKTEEDgVYSLNYDGYNVFRISEFeNDEHIILYLVNFD-KDRPFQLFEFYAREpdvspeikeefvkivqkrgivkeniidltkidr-----------------------------<br>----aqeeeAeQNLSELSGPWRTVYIGSTNPEKIQENGPFRTYFRELVFDdEKGTVDFYFSVKRDGKWKNVHVKATKQDDG-TYVADYEGQNVFKIVSL-SRTHLVAHNINVDkHGQTTELTGLFVKL---------------------------------nvededlekfwkltedkgidkknvvnfle<br>-CFQLRg--<br>nENHPH-pe<br><br>SARST: aliSize=148 (resi) RMSD=13.71 (Å)<br>v---AIRNFDISKISGEWYSIFLASDVKEKIEENGSMRVFVDVIRALD-NSSLYAEYQTKVNGECTEFPMVFDKTEEdGVYSLNYDGYNVFRISEFeNDEHIILYLVNFDKDRPFQLFEFYAREPDVSPEIKEEFVKIVQKRGIVKENIIDLTKI-------drcfqlrg<br>-aqeEEAEQNLSELSGPWRTVYIGSTNPEKIQENGPFRTYFRELVFDDeKGTVDFYFSVKRDGKWKNVHVKATKQDD-GTYVADYEGQNVFKIVSL-SRTHLVAHNINVDKHGQTTELTGLFVKLNVEDEDLEKFWKLTEDKGIDKKNVVNFLENenhphpe--------<br><br>BLAST: aliSize=80 (resi) iden=24.53% (39/159) simi=50.31% (80/159)<br>vairnf--------DISKISGEWYSIFLASDVKEKIEENGSMRV-FVDVIRALDNSSLYAEYQTKVNGECTEFPMVFDKtEEDGVYSLNYDGYNVFRISEFENdEHIILYLVNFDK-DRPFQLFEFYARePDVSPEIKEEFVKIVQKRGIVKENIID-----------ltkidrcfqlrg<br>------aqeeeaeqNLSELSGPWRTVYIGSTNPEKIQENGPFRTyFRELVFDDEKGTVDFYFSVKRDGKWKNVHVKATK-QDDGTYVADYEGQNVFKIVSLSR-THLVAHNINVDKhGQTTELTGLFVK-LNVEDEDLEKFWKLTEDKGIDKKNVVNflenenhphpe-----------<br><br>Proposed: aliSize=148 (resi) RMSD=1.89 (Å)<br>vairnf--------DISKISGEWYSIFLASDVKEKIEENGSMRVFVDVIRAL-DNSSLYAEYQTKVNGECTEFPMVFDKTEEDgVYSLNYDGYNVFRISEFeNDEHIILYLVNFD-KDRPFQLFEFYARePDVSPEIKEEFVKIVQKRGIVKENIIDLTKID-RCFQLrg<br>------aqeeeaeqNLSELSGPWRTVYIGSTNPEKIQENGPFRTYFRELVFDdEKGTVDFYFSVKRDGKWKNVHVKATKQDDG-TYVADYEGQNVFKIVSL-SRTHLVAHNINVDkHGQTTELTGLFVK-LNVEDEDLEKFWKLTEDKGIDKKNVVNFLENEnHPHPE--
971	1ew3A (159)<br>1pboB (151)	C	23.18	TM-align: aliSize=117 (resi) RMSD=2.45 (Å)<br>vairnfDISKISGEWYSIFLASDVKEKIEENGSMRVFVDVIRAL-DNSSLYAEYQTKVNGECTEFPMVFDKTEEDgVYSLNYDGYNVFRISEFeNDEHIILYLVNFD-KDRPFQLFEFYAREpdvspeikeefvkivqkrgivkeniidltkidr------------------------------CFQLRg--<br>------NLSELSGPWRTVYIGSTNPEKIQENGPFRTYFRELVFDdEKGTVDFYFSVKRDGKWKNVHVKATKQDDG-TYVADYEGQNVFKIVSL-SRTHLVAHNINVDkHGQKTELTGLFVKL---------------------------------nvededlekfwkltedkgidkknvvnflenEDHPH-pe<br><br>SARST: aliSize=139 (resi) RMSD=13.73 (Å)<br>vairnfdisk----ISGEWYSIFLASDVKEKIEENGSMRVFVDVIRALD-NSSLYAEYQTKVNGECTEFPMVFDKTEEdGVYSLNYDGYNVFRISEFeNDEHIILYLVNFDKDRPFQLFEFYAREPDVSPEIKEEFVKIVQKRGIVKENIIDLTKI-------drcfqlrg<br>----------nlseLSGPWRTVYIGSTNPEKIQENGPFRTYFRELVFDDeKGTVDFYFSVKRDGKWKNVHVKATKQDD-GTYVADYEGQNVFKIVSL-SRTHLVAHNINVDKHGQKTELTGLFVKLNVEDEDLEKFWKLTEDKGIDKKNVVNFLENedhphpe--------<br><br>BLAST: aliSize=80 (resi) iden=25.83% (39/151) simi=52.98% (80/151)<br>vairnfDISKISGEWYSIFLASDVKEKIEENGSMRV-FVDVIRALDNSSLYAEYQTKVNGECTEFPMVFDKtEEDGVYSLNYDGYNVFRISEFENdEHIILYLVNFDK-DRPFQLFEFYARePDVSPEIKEEFVKIVQKRGIVKENIID-----------ltkidrcfqlrg<br>------NLSELSGPWRTVYIGSTNPEKIQENGPFRTyFRELVFDDEKGTVDFYFSVKRDGKWKNVHVKATK-QDDGTYVADYEGQNVFKIVSLSR-THLVAHNINVDKhGQKTELTGLFVK-LNVEDEDLEKFWKLTEDKGIDKKNVVNflenedhphpe-----------<br><br>Proposed: aliSize=148 (resi) RMSD=1.91 (Å)<br>vairnfDISKISGEWYSIFLASDVKEKIEENGSMRVFVDVIRAL-DNSSLYAEYQTKVNGECTEFPMVFDKTEEDgVYSLNYDGYNVFRISEFeNDEHIILYLVNFD-KDRPFQLFEFYARePDVSPEIKEEFVKIVQKRGIVKENIIDLTKID-RCFQLrg<br>------NLSELSGPWRTVYIGSTNPEKIQENGPFRTYFRELVFDdEKGTVDFYFSVKRDGKWKNVHVKATKQDDG-TYVADYEGQNVFKIVSL-SRTHLVAHNINVDkHGQKTELTGLFVK-LNVEDEDLEKFWKLTEDKGIDKKNVVNFLENEdHPHPE--

972	1ew3A<br>(159)<br>1gt3A<br>(159)	N	16.98	TM-align: aliSize=119 (resi) RMSD=2.65 (Å)<br>vair-----N-FDISKISGEWYSIFLASDVKEKIEENGSMRVFVDVIRAL-DNSSLYAEYQTKVNGECTEFPMVFDKTEEDgVYSLNYDGYNVFRISEFeNDEHIILYLVNFD-KDRPFQLFEFYAREpdvspeikeefvkivqkrgivkeniidltkidr-----------------------------<br>----aqeeeAeQNLSELSGPWRTVYIGSTNPEKIQENGPFRTYFRELVFDdEKGTVDFYFSVKRDGKWKNVHVKATKQDDG-TYVADYEGQNVFKIVSL-SRTHLVAHNINVDkHGQTTELTELFVKL---------------------------------nvededlekfwkltedkgidkknvvnfle<br>-CFQLRg--<br>nENHPH-pe<br><br>SARST: aliSize=148 (resi) RMSD=13.70 (Å)<br>v---AIRNFDISKISGEWYSIFLASDVKEKIEENGSMRVFVDVIRALD-NSSLYAEYQTKVNGECTEFPMVFDKTEEdGVYSLNYDGYNVFRISEFeNDEHIILYLVNFDKDRPFQLFEFYAREPDVSPEIKEEFVKIVQKRGIVKENIIDLTKI-------drcfqlrg<br>-aqeEEAEQNLSELSGPWRTVYIGSTNPEKIQENGPFRTYFRELVFDDeKGTVDFYFSVKRDGKWKNVHVKATKQDD-GTYVADYEGQNVFKIVSL-SRTHLVAHNINVDKHGQTTELTELFVKLNVEDEDLEKFWKLTEDKGIDKKNVVNFLENenhphpe--------<br><br>BLAST: aliSize=81 (resi) iden=25.16% (40/159) simi=50.94% (81/159)<br>vairnf--------DISKISGEWYSIFLASDVKEKIEENGSMRV-FVDVIRALDNSSLYAEYQTKVNGECTEFPMVFDKtEEDGVYSLNYDGYNVFRISEFENdEHIILYLVNFDK-DRPFQLFEFYARePDVSPEIKEEFVKIVQKRGIVKENIID-----------ltkidrcfqlrg<br>------aqeeeaeqNLSELSGPWRTVYIGSTNPEKIQENGPFRTyFRELVFDDEKGTVDFYFSVKRDGKWKNVHVKATK-QDDGTYVADYEGQNVFKIVSLSR-THLVAHNINVDKhGQTTELTELFVK-LNVEDEDLEKFWKLTEDKGIDKKNVVNflenenhphpe------------<br><br>Proposed: aliSize=120 (resi) RMSD=1.83 (Å)<br>-VAIRNFD-ISKISGEWYSIFLASDVKEKIEENGSMRVFVDVIRAL-DNSSLYAEYQTKVNGECTEFPMVFDKTEEDgVYSLNYDGYNVFRISEFeNDEHIILYLVNFD-KDRPFQLFEFYARE---------------------------pdvspeikeefvkivqkrgivkeniidltkidrcfqlRG<br>aQEEEAEQnLSELSGPWRTVYIGSTNPEKIQENGPFRTYFRELVFDdEKGTVDFYFSVKRDGKWKNVHVKATKQDDG-TYVADYEGQNVFKIVSL-SRTHLVAHNINVDkHGQTTELTELFVKLnvededlekfwkltedkgidkknvvnf-------------------------------------LE<br>--------<br>nenhphpe
973	1ew3A<br>(159)<br>1gt3A<br>(159)	C	22.64	TM-align: aliSize=119 (resi) RMSD=2.65 (Å)<br>vair-----N-FDISKISGEWYSIFLASDVKEKIEENGSMRVFVDVIRAL-DNSSLYAEYQTKVNGECTEFPMVFDKTEEDgVYSLNYDGYNVFRISEFeNDEHIILYLVNFD-KDRPFQLFEFYAREpdvspeikeefvkivqkrgivkeniidltkidr-----------------------------<br>----aqeeeAeQNLSELSGPWRTVYIGSTNPEKIQENGPFRTYFRELVFDdEKGTVDFYFSVKRDGKWKNVHVKATKQDDG-TYVADYEGQNVFKIVSL-SRTHLVAHNINVDkHGQTTELTELFVKL---------------------------------nvededlekfwkltedkgidkknvvnfle<br>-CFQLRg--<br>nENHPH-pe<br><br>SARST: aliSize=148 (resi) RMSD=13.70 (Å)<br>v---AIRNFDISKISGEWYSIFLASDVKEKIEENGSMRVFVDVIRALD-NSSLYAEYQTKVNGECTEFPMVFDKTEEdGVYSLNYDGYNVFRISEFeNDEHIILYLVNFDKDRPFQLFEFYAREPDVSPEIKEEFVKIVQKRGIVKENIIDLTKI-------drcfqlrg<br>-aqeEEAEQNLSELSGPWRTVYIGSTNPEKIQENGPFRTYFRELVFDDeKGTVDFYFSVKRDGKWKNVHVKATKQDD-GTYVADYEGQNVFKIVSL-SRTHLVAHNINVDKHGQTTELTELFVKLNVEDEDLEKFWKLTEDKGIDKKNVVNFLENenhphpe--------<br><br>BLAST: aliSize=81 (resi) iden=25.16% (40/159) simi=50.94% (81/159)<br>vairnf--------DISKISGEWYSIFLASDVKEKIEENGSMRV-FVDVIRALDNSSLYAEYQTKVNGECTEFPMVFDKtEEDGVYSLNYDGYNVFRISEFENdEHIILYLVNFDK-DRPFQLFEFYARePDVSPEIKEEFVKIVQKRGIVKENIID-----------ltkidrcfqlrg<br>------aqeeeaeqNLSELSGPWRTVYIGSTNPEKIQENGPFRTyFRELVFDDEKGTVDFYFSVKRDGKWKNVHVKATK-QDDGTYVADYEGQNVFKIVSLSR-THLVAHNINVDKhGQTTELTELFVK-LNVEDEDLEKFWKLTEDKGIDKKNVVNflenenhphpe------------<br><br>Proposed: aliSize=148 (resi) RMSD=1.89 (Å)<br>vairnf--------DISKISGEWYSIFLASDVKEKIEENGSMRVFVDVIRAL-DNSSLYAEYQTKVNGECTEFPMVFDKTEEDgVYSLNYDGYNVFRISEFeNDEHIILYLVNFD-KDRPFQLFEFYARePDVSPEIKEEFVKIVQKRGIVKENIIDLTKID-RCFQLrg<br>------aqeeeaeqNLSELSGPWRTVYIGSTNPEKIQENGPFRTYFRELVFDdEKGTVDFYFSVKRDGKWKNVHVKATKQDDG-TYVADYEGQNVFKIVSL-SRTHLVAHNINVDkHGQTTELTELFVK-LNVEDEDLEKFWKLTEDKGIDKKNVVNFLENEnHPHPE--
974	1ew3A<br>(159)<br>1gt1A<br>(158)	N	17.09	TM-align: aliSize=119 (resi) RMSD=2.66 (Å)<br>vair----N-FDISKISGEWYSIFLASDVKEKIEENGSMRVFVDVIRAL-DNSSLYAEYQTKVNGECTEFPMVFDKTEEDgVYSLNYDGYNVFRISEFeNDEHIILYLVNFD-KDRPFQLFEFYAREpdvspeikeefvkivqkrgivkeniidltkidr------------------------------<br>----qeeeAeQNLSELSGPWRTVYIGSTNPEKIQENGPFRTYFRELVFDdEKGTVDFYFSVKRDGKWKNVHVKATKQDDG-TYVADYEGQNVFKIVSL-SRTHLVAHNINVDkHGQTTELTELFVKL---------------------------------nvededlekfwkltedkgidkknvvnflen<br>CFQLRg--<br>ENHPH-pe<br><br>SARST: aliSize=146 (resi) RMSD=13.53 (Å)<br>vai----RNFDISKISGEWYSIFLASDVKEKIEENGSMRVFVDVIRALD-NSSLYAEYQTKVNGECTEFPMVFDKTEEdGVYSLNYDGYNVFRISEFeNDEHIILYLVNFDKDRPFQLFEFYAREPDVSPEIKEEFVKIVQKRGIVKENIIDLTKI-------drcfqlrg<br>---qeeeAEQNLSELSGPWRTVYIGSTNPEKIQENGPFRTYFRELVFDDeKGTVDFYFSVKRDGKWKNVHVKATKQDD-GTYVADYEGQNVFKIVSL-SRTHLVAHNINVDKHGQTTELTELFVKLNVEDEDLEKFWKLTEDKGIDKKNVVNFLENenhphpe--------<br><br>BLAST: aliSize=81 (resi) iden=25.32% (40/158) simi=51.27% (81/158)<br>vairnf-------DISKISGEWYSIFLASDVKEKIEENGSMRV-FVDVIRALDNSSLYAEYQTKVNGECTEFPMVFDKtEEDGVYSLNYDGYNVFRISEFENdEHIILYLVNFDK-DRPFQLFEFYARePDVSPEIKEEFVKIVQKRGIVKENIID-----------ltkidrcfqlrg<br>------qeeeaeqNLSELSGPWRTVYIGSTNPEKIQENGPFRTyFRELVFDDEKGTVDFYFSVKRDGKWKNVHVKATK-QDDGTYVADYEGQNVFKIVSLSR-THLVAHNINVDKhGQTTELTELFVK-LNVEDEDLEKFWKLTEDKGIDKKNVVNflenenhphpe------------<br><br>Proposed: aliSize=120 (resi) RMSD=1.83 (Å)<br>VAIRNFD-ISKISGEWYSIFLASDVKEKIEENGSMRVFVDVIRAL-DNSSLYAEYQTKVNGECTEFPMVFDKTEEDgVYSLNYDGYNVFRISEFeNDEHIILYLVNFD-KDRPFQLFEFYARE---------------------------pdvspeikeefvkivqkrgivkeniidltkidrcfqlRG-<br>QEEEAEQnLSELSGPWRTVYIGSTNPEKIQENGPFRTYFRELVFDdEKGTVDFYFSVKRDGKWKNVHVKATKQDDG-TYVADYEGQNVFKIVSL-SRTHLVAHNINVDkHGQTTELTELFVKLnvededlekfwkltedkgidkknvvnf-------------------------------------LEn<br>-------<br>enhphpe

975	1ew3A (159)<br>1gt1A (158)	C	22.78	TM-align: aliSize=119 (resi) RMSD=2.66 (Å)<br>vair----N-FDISKISGEWYSIFLASDVKEKIEENGSMRVFVDVIRAL-DNSSLYAEYQTKVNGECTEFPMVFDKTEEDgVYSLNYDGYNVFRISEFeNDEHIILYLVNFD-KDRPFQLFEFYAREpdvspeikeefvkivqkrgivkeniidltkidr-------------------------------<br>----qeeeAeQNLSELSGPWRTVYIGSTNPEKIQENGPFRTYFRELVFDdEKGTVDFYFSVKRDGKWKNVHVKATKQDDG-TYVADYEGQNVFKIVSL-SRTHLVAHNINVDkHGQTTELTELFVKL---------------------------------nvededlekfwkltedkgidkknvvnflen<br>CFQLRg--<br>ENHPH-pe<br>SARST: aliSize=146 (resi) RMSD=13.53 (Å)<br>vai----RNFDISKISGEWYSIFLASDVKEKIEENGSMRVFVDVIRALD-NSSLYAEYQTKVNGECTEFPMVFDKTEEdGVYSLNYDGYNVFRISEFeNDEHIILYLVNFDKDRPFQLFEFYAREPDVSPEIKEEFVKIVQKRGIVKENIIDLTKI-------drcfqlrg<br>---qeeeAEQNLSELSGPWRTVYIGSTNPEKIQENGPFRTYFRELVFDDeKGTVDFYFSVKRDGKWKNVHVKATKQDD-GTYVADYEGQNVFKIVSL-SRTHLVAHNINVDKHGQTTELTELFVKLNVEDEDLEKFWKLTEDKGIDKKNVVNFLENenhphpe--------<br>BLAST: aliSize=81 (resi) iden=25.32% (40/158) simi=51.27% (81/158)<br>vairnf-------DISKISGEWYSIFLASDVKEKIEENGSMRV-FVDVIRALDNSSLYAEYQTKVNGECTEFPMVFDKtEEDGVYSLNYDGYNVFRISEFENdEHIILYLVNFDK-DRPFQLFEFYARePDVSPEIKEEFVKIVQKRGIVKENIID-----------ltkidrcfqlrg<br>------qeeeaeqNLSELSGPWRTVYIGSTNPEKIQENGPFRTyFRELVFDDEKGTVDFYFSVKRDGKWKNVHVKATK-QDDGTYVADYEGQNVFKIVSLSR-THLVAHNINVDKhGQTTELTELFVK-LNVEDEDLEKFWKLTEDKGIDKKNVVNflenenhphpe-----------<br>Proposed: aliSize=148 (resi) RMSD=1.89 (Å)<br>vairnf-------DISKISGEWYSIFLASDVKEKIEENGSMRVFVDVIRAL-DNSSLYAEYQTKVNGECTEFPMVFDKTEEDgVYSLNYDGYNVFRISEFeNDEHIILYLVNFD-KDRPFQLFEFYARePDVSPEIKEEFVKIVQKRGIVKENIIDLTKID-RCFQLrg<br>------qeeeaeqNLSELSGPWRTVYIGSTNPEKIQENGPFRTYFRELVFDdEKGTVDFYFSVKRDGKWKNVHVKATKQDDG-TYVADYEGQNVFKIVSL-SRTHLVAHNINVDkHGQTTELTELFVK-LNVEDEDLEKFWKLTEDKGIDKKNVVNFLENEnHPHPE--
976	2a2uA (158)<br>1pboB (151)	C	26.49	TM-align: aliSize=114 (resi) RMSD=1.64 (Å)<br>eeasstrgnlDVAKLNGDWFSIVVASNKREKIEENGSMRVFMQHIDVL--ENSLGFKFRIKENGECRELYLVAYKTPEdGEYFVEYDGGNTFTILKTDYdRYVMFHLINFK-NGETFQLMVLYGRTkdlssdikekfaklceahgitrdniidltk--------------------------------<br>----------NLSELSGPWRTVYIGSTNPEKIQENGPFRTYFRELVFDdeKGTVDFYFSVKRDGKWKNVHVKATKQDD-GTYVADYEGQNVFKIVSLSR-THLVAHNINVDkHGQKTELTGLFVKL---------------------------------nvededlekfwkltedkgidkknvvnflenedhp<br>TDRcl<br>HPE--<br>SARST: aliSize=138 (resi) RMSD=13.67 (Å)<br>eeasstrgnldv--AKLNGDWFSIVVASNKREKIEENGSMRVFMQHIDVL--ENSLGFKFRIKENGECRELYLVAYKTPeDGEYFVEYDGGNTFTILKTDYDRYVMFHLINFKNGETFQLMVLYGrTKDLSSDIKEKFAKLCEAHGITRDNIIDLT---------ktdrcl<br>------------nlSELSGPWRTVYIGSTNPEKIQENGPFRTYFRELVFDdeKGTVDFYFSVKRDGKWKNVHVKATKQD-DGTYVADYEGQNVFKIVSLSRTHLVAHNINVDKHGQKTELTGLFV-KLNVEDEDLEKFWKLTEDKGIDKKNVVNFLenedhphpe------<br>BLAST: aliSize=82 (resi) iden=29.14% (44/151) simi=54.30% (82/151)<br>eeasstrgnlDVAKLNGDWFSIVVASNKREKIEENGSMRVFMQHI--DVLENSLGFKFRIKENGECRELYLVAYKTpEDGEYFVEYDGGNTFTILKTDYDRYVMfHLINF-KNGETFQLMVLYGRTKDLSSDIkEKFAKLCEAHGITRDNIIDLTKTD------rcl<br>----------NLSELSGPWRTVYIGSTNPEKIQENGPFRTYFRELvfDDEKGTVDFYFSVKRDGKWKNVHVKATKQ-DDGTYVADYEGQNVFKIVSLSRTHLVA-HNINVdKHGQKTELTGLFVKLNVEDEDL-EKFWKLTEDKGIDKKNVVNFLENEdhphpe---<br>Proposed: aliSize=144 (resi) RMSD=1.47 (Å)<br>eeasstrgnlDVAKLNGDWFSIVVASNKREKIEENGSMRVFMQHIDVL--ENSLGFKFRIKENGECRELYLVAYKTPEdGEYFVEYDGGNTFTILKTDYdRYVMFHLINFK-NGETFQLMVLYGRtkDLSSD-IKEKFAKLCEAHGITRDNIIDLTKTD-RCL--<br>----------NLSELSGPWRTVYIGSTNPEKIQENGPFRTYFRELVFDdeKGTVDFYFSVKRDGKWKNVHVKATKQDD-GTYVADYEGQNVFKIVSLSR-THLVAHNINVDkHGQKTELTGLFVK--LNVEDeDLEKFWKLTEDKGIDKKNVVNFLENEdHPHpe
977	1hcfX (101)<br>1he7A (107)	N	45.54	TM-align: aliSize=85 (resi) RMSD=2.04 (Å)<br>shmaptitflespts---------------DHHWCIPFTVKGNPKPALQWFYNGAILNESKYICTKIHVTN--HTEYHGCLQLDNPTHMNNGDYTLIAKNEYGKDEKQISAHFMGWPg------<br>---------------shmpasvqlhtaveMHHWCIPFSVDGQPAPSLRWLFNGSVLNETSFIFTEFLEPAanETVRHGCLRLNQPTHVNNGNYTLLAANPFGQASASIMAAFMDNP-fefnpe<br>SARST: aliSize=93 (resi) RMSD=15.54 (Å)<br>s-HMAPTItFLESPTSDHHWCIPFTVKGNPKPALQWFYNGAILNESKYICTKIHVTNH--TEYHGCLQLDNPTHMNNGDYTLIAKNEYGKDEKQISAH-----------fmgwpg<br>-sHMPASV-QLHTAVEMHHWCIPFSVDGQPAPSLRWLFNGSVLNETSFIFTEFLEPAAneTVRHGCLRLNQPTHVNNGNYTLLAANPFGQASASIMAAfmdnpfefnpe------<br>BLAST: aliSize=66 (resi) iden=48.51% (49/101) simi=65.35% (66/101)<br>SHMaPTITFLESPTSDHHWCIPFTVKGNPKPALQWFYNGAILNESKYICTKI--HVTNHTEYHGCLQLDNPTHMNNGDYTLIAKNEYGKDEKQISAHFMGWP------g<br>SHM-PASVQLHTAVEMHHWCIPFSVDGQPAPSLRWLFNGSVLNETSFIFTEFlePAANETVRHGCLRLNQPTHVNNGNYTLLAANPFGQASASIMAAFMDNPfefnpe-<br>Proposed: aliSize=97 (resi) RMSD=1.29 (Å)<br>SHMAPTItFLESPTSDHHWCIPFTVKGNPKPALQWFYNGAILNESKYICTKIHVTN--HTEYHGCLQLDNPTHMNNGDYTLIAKNEYGKDEKQISAHFMG--------wpg<br>SHMPASV-QLHTAVEMHHWCIPFSVDGQPAPSLRWLFNGSVLNETSFIFTEFLEPAanETVRHGCLRLNQPTHVNNGNYTLLAANPFGQASASIMAAFMDnpfefnpe---

978	1fhgA (102)<br>1he7A (107)	N	14.71	TM-align: aliSize=80 (resi) RMSD=1.88 (Å)<br>aeekphvkpyftktildmevve--------------GSAARFDCKVEGYPDPEVMWFKDDNPVKESRHFQIDY-DE-----EGNCSLTISEVCGDDDAKYTCKAVNSLGEATCTAELLVETM-------<br>----------------------shmpasvqlhtaveMHHWCIPFSVDGQPAPSLRWLFNGSVLNETSFIFTEFlEPaanetVRHGCLRLNQPTHVNNGNYTLLAANPFGQASASIMAAFMDNpfefnpe<br><br>SARST: aliSize=89 (resi) RMSD=14.98 (Å)<br>aeekph--VKPYFtkTILDMEVVEgsAARFDCKVEGYPDPEVMWFKDDNPVKESRHFQIDY--DEEGN----CSLTISEVCGDDDAKYTCKAVNSLGEATCTAELLV----------etm<br>------shMPASV--QLHTAVEMH--HWCIPFSVDGQPAPSLRWLFNGSVLNETSFIFTEFlePAANEtvrhGCLRLNQPTHVNNGNYTLLAANPFGQASASIMAAFmdnpfefnpe---<br><br>BLAST: aliSize=31 (resi) iden=14.71% (15/102) simi=30.39% (31/102)<br>aeekphvkpyftktildmevvegsaarfdck-----------------------VEGYPDPEVMWFKDDNPVKESRHFQIDYDEEG-------NCsLTISEVCGDDDAKYTCKAVNSLGEATCT---------------aellvetm<br>-------------------------------shmpasvqlhtavemhhwcipfsVDGQPAPSLRWLFNGSVLNETSFIFTEFLEPAanetvrhGC-LRLNQPTHVNNGNYTLLAANPFGQASASimaafmdnpfefnpe--------<br><br>Proposed: aliSize=94 (resi) RMSD=1.99 (Å)<br>aeEKPHVkPYFTKTILDMevvegSAARFDCKVEGYPDPEVMWFKDDNPVKESRHFQIDY-DE-----EGNCSLTISEVCGDDDAKYTCKAVNSLGEATCTAELLVETM-------<br>--SHMPA-SVQLHTAVEM-----HHWCIPFSVDGQPAPSLRWLFNGSVLNETSFIFTEFlEPaanetVRHGCLRLNQPTHVNNGNYTLLAANPFGQASASIMAAFMDNpfefnpe
979	2dm3A (110)<br>1he7A (107)	N	18.69	TM-align: aliSize=82 (resi) RMSD=2.05 (Å)<br>gssgssgfrphflqapgdltvqe-------------GKLCRMDCKVSGLPTPDLSWQLDGKPVRPDSAHKMLV-RE-----NGVHSLIIEPVTSRDAGIYTCIATNRAGQNSFSLELVVAAKESgpssg-----<br>----------------------shmpasvqlhtaveMHHWCIPFSVDGQPAPSLRWLFNGSVLNETSFIFTEFlEPaanetVRHGCLRLNQPTHVNNGNYTLLAANPFGQASASIMAAFMDNPF-----efnpe<br><br>SARST: aliSize=91 (resi) RMSD=13.07 (Å)<br>gssgssgfrphfl-QAPGDLTVQEGKLCRMDC---KVSGLPTPDLSWQLDGKPVRPDSA--HKMLVRENGV----HSLIIEPVTSRDAGIYTCIATNRAGQNSFSLELVVAAKE------sgpssg<br>-------------sHMPASVQLHTAVEMHHWCipfSVDGQPAPSLRWLFNGSVLNETSFifTEFLEPAANEtvrhGCLRLNQPTHVNNGNYTLLAANPFGQASASIMAAFMDNPfefnpe------<br><br>BLAST: aliSize=29 (resi) iden=19.63% (21/107) simi=27.10% (29/107)<br>gssgssgfrphflqapgdltvqegklcrmdck----------------------VSGLPTPDLSWQLDGKPVRPDSA--HKML---VRENGVHSLI-IEPVTSRDAGIYTCIATNRAGQNSFSL--------------elvvaakesgpssg<br>-------------------------------shmpasvqlhtavemhhwcipfsVDGQPAPSLRWLFNGSVLNETSFifTEFLepaANETVRHGCLrLNQPTHVNNGNYTLLAANPFGQASASImaafmdnpfefnpe--------------<br><br>Proposed: aliSize=95 (resi) RMSD=1.90 (Å)<br>gssgsSGFRPHFlqAPGDLTVQegkLCRMDCKVSGLPTPDLSWQLDGKPVRPDSAHKMLV-RE-----NGVHSLIIEPVTSRDAGIYTCIATNRAGQNSFSLELVVAAK-eS-----gpssg<br>-----SHMPASV--QLHTAVEM--HHWCIPFSVDGQPAPSLRWLFNGSVLNETSFIFTEFlEPaanetVRHGCLRLNQPTHVNNGNYTLLAANPFGQASASIMAAFMDNp-Fefnpe-----
980	1g1cA (98)<br>1he7A (107)	N	17.35	TM-align: aliSize=80 (resi) RMSD=1.63 (Å)<br>smeapkiferiqsqtvgq-------------GSDAHFRVRVVGKPDPECEWYKNGVKIERSDRIYWYWPED------NVCELVIRDVTGEDSASIMVKAINIAGETSSHAFLLVQAK-------<br>-----------------shmpasvqlhtaveMHHWCIPFSVDGQPAPSLRWLFNGSVLNETSFIFTEFLEPaanetvRHGCLRLNQPTHVNNGNYTLLAANPFGQASASIMAAFMDNpfefnpe<br><br>SARST: aliSize=87 (resi) RMSD=12.43 (Å)<br>smeapkif--ERIQSQTVGQ--GSDAHFRVRVVGKPDPECEWYKNGVKIERSDRIYWYW-----PEDNVC-ELVIRDVTGEDSASIMVKAINIAGETSSHAFLLV----------qak<br>--------shMPASVQLHTAveMHHWCIPFSVDGQPAPSLRWLFNGSVLNETSFIFTEFlepaaNETVRHgCLRLNQPTHVNNGNYTLLAANPFGQASASIMAAFmdnpfefnpe---<br><br>BLAST: aliSize=16 (resi) iden=11.22% (11/98) simi=16.33% (16/98)<br>smeapkiferiqsqtvgqgsdahfrvr-----------------------VVGKPDPECEWYKNGVKIERSDRIYWYWPEDNVCELV---irdvtgedsasimvkainiagetsshafllvqak<br>--------------------------shmpasvqlhtavemhhwcipfsVDGQPAPSLRWLFNGSVLNETSFIFTEFLEPAANETVrhgclrlnqpthvnngnytllaanpfgqasasimaafmdnpfefnpe----------------------------------<br><br>Proposed: aliSize=94 (resi) RMSD=1.75 (Å)<br>smEAPKIfeRIQSQTVGQGSDAHFRVRVVGKPDPECEWYKNGVKIERSDRIYWYWPED------NVCELVIRDVTGEDSASIMVKAINIAGETSSHAFLLVQAK-------<br>--SHMPA--SVQLHTAVEMHHWCIPFSVDGQPAPSLRWLFNGSVLNETSFIFTEFLEPaanetvRHGCLRLNQPTHVNNGNYTLLAANPFGQASASIMAAFMDNpfefnpe

981	1u2hA (96)<br>1he7A (107)	N	19.79	TM-align: aliSize=79 (resi) RMSD=1.40 (Å)<br>kapptfkvslmdqsvre--------------GQDVIMSIRVQGEPKPVVSWLRNRQPVRPDQRRFAEEA-E-----GGLCRLRILAAERGDAGFYTCKAVNEYGARQCEARLEVR-G-------<br>-----------------shmpasvqlhtaveMHHWCIPFSVDGQPAPSLRWLFNGSVLNETSFIFTEFLePaanetVRHGCLRLNQPTHVNNGNYTLLAANPFGQASASIMAAFMdNpfefnpe<br><br>SARST: aliSize=79 (resi) RMSD=15.38 (Å)<br>kapptfk--VSLMDQSVREGQDVIMS--IRVQGEPKPVVSWLRNRQPVRPDQRRFAEEAE--GGL----CRLRILAAERGDAGFYTCKAVN----------------EYGAR--qcearlevrg<br>-------shMPASVQLHTAVEMHHWCipFSVDGQPAPSLRWLFNGSVLNETSFIFTEFLEpaANEtvrhGCLRLNQPTHVNNGNYTLLAANpfgqasasimaafmdnPFEFNpe----------<br><br>BLAST: aliSize=25 (resi) iden=19.79% (19/96) simi=26.04% (25/96)<br>kapptfkvslmdqsvregqdvimsir----------------------VQGEPKPVVSWLRNRQPVRPDQ---RRFAEEAEGGLCR---LRILAAERGDAGFYTCKAVNEYG-------------------arqcearlevrg<br>-------------------------shmpasvqlhtavemhhwcipfsVDGQPAPSLRWLFNGSVLNETSfifTEFLEPAANETVRhgcLRLNQPTHVNNGNYTLLAANPFGqasasimaafmdnpfefnpe-----------<br><br>Proposed: aliSize=92 (resi) RMSD=1.46 (Å)<br>kApPTF-kvSLMDQSVREGQDVIMSIRVQGEPKPVVSWLRNRQPVRPDQRRFAEEA-EG-----GLCRLRILAAERGDAGFYTCKAVNEYGARQCEARLEVR-G-------<br>-S-HMPa--SVQLHTAVEMHHWCIPFSVDGQPAPSLRWLFNGSVLNETSFIFTEFLePAanetvRHGCLRLNQPTHVNNGNYTLLAANPFGQASASIMAAFMdNpfefnpe
982	2a5mA (177)<br>1ytqA (181)	C	33.90	TM-align: aliSize=90 (resi) RMSD=1.66 (Å)<br>skTGGKISFYEDRNFQGRRYDCDCDCADFRSY-LSRCNSIRVEGGTWAVYERPNFSGHMYILPQGEYPEYQRWMG-L-NDRLGSCRAVHLSSGGQakiqvfekgdfngqmyettedcpsimeqfhlreihsckvvegtwifyelpnyrgrqylldkkeyrkpvdwgaaspaiqsfrrive----------<br>--LNPKIIIFEQENFQGHSHELNGPCPNLKETgVEKAGSVLVQAGPWVGYEQANCKGEQFVFEKGEYPRWDSWTSsRrTDSLSSLRPIKVDSQEH---kiilyenpnf<br><br>--<br>tgkkmeiidddvpsfhahgyqekvssvrvqsgtwvgyqypgyrglqyllekgdykdssdfgaphpqvqsvrrirdmqw<br><br>SARST: aliSize=170 (resi) RMSD=15.72 (Å)<br>skt-GGKISFYEDRNFQGRRYDCDCDCADFRSY-LSRCNSIRVEGGTWAVYERPNFSGHMYILPQGEYPEYQRW--MGLNDRLGSCRAVHLSSGgQAKIQVFEKGDFNGQM-YETTEDCPSIMEqFHLRE-IHSCKVVEGTWIFYELPNYRGRQYLLDKKEYRKPVDWGAASPAIQSFRRI-----ve<br>---lNPKIIIFEQENFQGHSHELNGPCPNLKETgVEKAGSVLVQAGPWVGYEQANCKGEQFVFEKGEYPRWDSWtsSRRTDSLSSLRPIKVDSQ-EHKIILYENPNFTGKKmEIIDDDVPSFHA-HGYQEkVSSVRVQSGTWVGYQYPGYRGLQYLLEKGDYKDSSDFGAPHPQVQSVRRIrdmqw--<br><br>BLAST: aliSize=94 (resi) iden=34.46% (61/177) simi=53.11% (94/177)<br>sktgg---KISFYEDRNFQGRRYDCDCDCADFRSY-LSRCNSIRVEGGTWAVYERPNFSGHMYILPQGEYPeyqRW-----MGLNDRLGSCRAVHLSSgGQAKIQVFEKGDFNGQMYE-TTEDCPSIMEQFHLREIHSCKVVEGTWIFYELPNYRGRQYLLDKKEYRKPVDWGAASPAIQSFRRI-----ve<br>-----lnpKIIIFEQENFQGHSHELNGPCPNLKETgVEKAGSVLVQAGPWVGYEQANCKGEQFVFEKGEYP---RWdswtsSRRTDSLSSLRPIKVDS-QEHKIILYENPNFTGKKMEiIDDDVPSFHAHGYQEKVSSVRVQSGTWVGYQYPGYRGLQYLLEKGDYKDSSDFGAPHPQVQSVRRIrdmqw--<br><br>Proposed: aliSize=174 (resi) RMSD=1.42 (Å)<br>skTGGKISFYEDRNFQGRRYDCDCDCADFRSY-LSRCNSIRVEGGTWAVYERPNFSGHMYILPQGEYPEYQRWMG-L-NDRLGSCRAVHLSSGgQAKIQVFEKGDFNGQMYETT-EDCPSIMEQFHLREIHSCKVVEGTWIFYELPNYRGRQYLLDKKEYRKPVDWGAASPAIQSFRRIVE---<br>--LNPKIIIFEQENFQGHSHELNGPCPNLKETgVEKAGSVLVQAGPWVGYEQANCKGEQFVFEKGEYPRWDSWTSsRrTDSLSSLRPIKVDSQ-EHKIILYENPNFTGKKMEIIdDDVPSFHAHGYQEKVSSVRVQSGTWVGYQYPGYRGLQYLLEKGDYKDSSDFGAPHPQVQSVRRIRDmqw
983	2a5mA (177)<br>2bb2A (181)	C	33.90	TM-align: aliSize=89 (resi) RMSD=1.56 (Å)<br>s---KTGGKISFYEDRNFQGRRYDCD-CDCADFR--SYLSRCNSIRVEGGTWAVYERPNFSGHMYILPQGEYPEYQRWMGLNDRLGSCRAVHLSsgggakiqvf<br>-lnpkiiifeqenfqghshelngpcpnlketgvekagsvlvqagpwvgyeqanckgeqfvfekgeyprwdswtssrrtdslsslrpikvdSQEHKITLYENPNFTGKKMEVIdDDVPSFHahGYQEKVSSVRVQSGTWVGYQYPGYRGLQYLLEKGDYKDSGDFGAPQPQVQSVRRIRDM----------<br><br>ekgdfngqmyettedcpsimeqfhlreihsckvvEGtwifyelpnyrgrqylldkkeyrkpvdwgaaspaiqsfrrive<br>----------------------------------QW---<br><br>SARST: aliSize=169 (resi) RMSD=16.17 (Å)<br>skt-GGKISFYEDRNFQGRRYDCDCDCADFRSY-LSRCNSIRVEGGTWAVYERPNFSGHMYILPQGEYPEYQRW---MgLNDRLGSCRAVHLSSGGQaKIQVFEKGDFNGQMYET-TEDCPSIMeQFHLRE-IHSCKVVEGTWIFYELPNYRGRQYLLDKKEYRKPVDWGAASPAIQSFRRI-----ve<br>---lNPKIIIFEQENFQGHSHELNGPCPNLKETgVEKAGSVLVQAGPWVGYEQANCKGEQFVFEKGEYPRWDSWtssR-RTDSLSSLRPIKVDSQEH-KITLYENPNFTGKKMEViDDDVPSFH-AHGYQEkVSSVRVQSGTWVGYQYPGYRGLQYLLEKGDYKDSGDFGAPQPQVQSVRRIrdmqw--<br><br>BLAST: aliSize=94 (resi) iden=34.46% (61/177) simi=53.11% (94/177)<br>sktgg---KISFYEDRNFQGRRYDCDCDCADFRSY-LSRCNSIRVEGGTWAVYERPNFSGHMYILPQGEYPeyqRW-----MGLNDRLGSCRAVHLSSgGQAKIQVFEKGDFNGQMYET-TEDCPSIMEQFHLREIHSCKVVEGTWIFYELPNYRGRQYLLDKKEYRKPVDWGAASPAIQSFRRI-----ve<br>-----lnpKIIIFEQENFQGHSHELNGPCPNLKETgVEKAGSVLVQAGPWVGYEQANCKGEQFVFEKGEYP---RWdswtsSRRTDSLSSLRPIKVDS-QEHKITLYENPNFTGKKMEViDDDVPSFHAHGYQEKVSSVRVQSGTWVGYQYPGYRGLQYLLEKGDYKDSGDFGAPQPQVQSVRRIrdmqw--<br><br>Proposed: aliSize=174 (resi) RMSD=1.43 (Å)<br>skTGGKISFYEDRNFQGRRYDCDCDCADFRSY-LSRCNSIRVEGGTWAVYERPNFSGHMYILPQGEYPEYQRWMG-L-NDRLGSCRAVHLSSGGgAKIQVFEKGDFNGQMYETT-EDCPSIMEQFHLREIHSCKVVEGTWIFYELPNYRGRQYLLDKKEYRKPVDWGAASPAIQSFRRIVE---<br>--LNPKIIIFEQENFQGHSHELNGPCPNLKETgVEKAGSVLVQAGPWVGYEQANCKGEQFVFEKGEYPRWDSWTSsRrTDSLSSLRPIKVDSQE-HKITLYENPNFTGKKMEVIdDDVPSFHAHGYQEKVSSVRVQSGTWVGYQYPGYRGLQYLLEKGDYKDSGDFGAPQPQVQSVRRIRDmqw

984	1a45A (173)<br>1ytqA (181)	C	35.26	TM-align: aliSize=87 (resi) RMSD=1.72 (Å)<br>--GKITFYEDRGFQGRHYECSSDHSNLQPY-FSRCNSIRVDSGCWMLYEQPNFQGPQYFLRRGDYPDYQQWMG-L-NDSIRSCRLIPHTGSHrlriveredyrgqmveitedcsslhdrfhfseihsfnvlegwwvlyemtnyrgrqyllrpgdyrryhdwgatnarvgslrravdfy-----------<br>lnPKIIIFEQENFQGHSHELNGPCPNLKETgVEKAGSVLVQAGPWVGYEQANCKGEQFVFEKGEYPRWDSWTSsRrTDSLSSLRPIKVDSQE--hkiilyenpnft<br>---<br>gkkmeiidddvpsfhahgyqekvssvrvqsgtwvgyqypgyrglqyllekgdykdssdfgaphpqvqsvrrirdmqw<br>SARST: aliSize=167 (resi) RMSD=15.88 (Å)<br>g---KITFYEDRGFQGRHYECSSDHSNLQPY-FSRCNSIRVDSGCWMLYEQPNFQGPQYFLRRGDYPDYQQW--MGLNDSIRSCRLIPHTGSHRLR-IYEREDYRGQMVEI-TEDCSSL-HDRFHFsEIHSFNVLEGWWVLYEMTNYRGRQYLLRPGDYRRYHDWGATNARVGSLRRA-----vdfy<br>-lnpKIIIFEQENFQGHSHELNGPCPNLKETgVEKAGSVLVQAGPWVGYEQANCKGEQFVFEKGEYPRWDSWtsSRRTDSLSSLRPIKVDSQEHKIiLYENPNFTGKKMEIiDDDVPSFhAHGYQE-KVSSVRVQSGTWVGYQYPGYRGLQYLLEKGDYKDSSDFGAPHPQVQSVRRIrdmqw----<br>BLAST: aliSize=93 (resi) iden=35.26% (61/173) simi=53.76% (93/173)<br>g---KITFYEDRGFQGRHYECSSDHSNLQPY-FSRCNSIRVDSGCWMLYEQPNFQGPQYFLRRGDYPDYQQWMG--LNDSIRSCRLIP-HTGSHRLRIYEREDYRGQMVE-ITEDCSSLHDRFHFSEIHSFNVLEGWWVLYEMTNYRGRQYLLRPGDYRRYHDWGATNARVGSLRRAVD---fy<br>-lnpKIIIFEQENFQGHSHELNGPCPNLKETgVEKAGSVLVQAGPWVGYEQANCKGEQFVFEKGEYPRWDSWTSsrRTDSLSSLRPIKvDSQEHKIILYENPNFTGKKMEiIDDDVPSFHAHGYQEKVSSVRVQSGTWVGYQYPGYRGLQYLLEKGDYKDSSDFGAPHPQVQSVRRIRDmqw--<br>Proposed: aliSize=170 (resi) RMSD=1.51 (Å)<br>--GKITFYEDRGFQGRHYECSSDHSNLQPY-FSRCNSIRVDSGCWMLYEQPNFQGPQYFLRRGDYPDYQQWMG-L-NDSIRSCRLIPHT-GSHRLRIYEREDYRGQMVEIT-EDCSSLhDRFHFSE-IHSFNVLEGWWVLYEMTNYRGRQYLLRPGDYRRYHDWGATNARVGSLRRAVD---fy<br>lnPKIIIFEQENFQGHSHELNGPCPNLKETgVEKAGSVLVQAGPWVGYEQANCKGEQFVFEKGEYPRWDSWTSsRrTDSLSSLRPIKVDsQEHKIILYENPNFTGKKMEIIdDDVPSF-HAHGYQEkVSSVRVQSGTWVGYQYPGYRGLQYLLEKGDYKDSSDFGAPHPQVQSVRRIRDmqw--
985	2v2uA (173)<br>2bb2A (181)	N	35.84	TM-align: aliSize=86 (resi) RMSD=1.00 (Å)<br>--GKITFFEDRSFQGRCYECS-SDCPNLQ--TYFSRCNSVRVDSGCWMLYERPNYQGHQYFLRRGEYPDYQQWMGFSDSIRSCRLIPHAGShrmrlyeke<br>lnpkiiifeqenfqghshelngpcpnlketgvekagsvlvqagpwvgyeqanckgeqfvfekgeyprwdswtssrrtdslsslrpikvdsqeHKITLYENPNFTGKKMEVIdDDVPSFHahGYQEKVSSVRVQSGTWVGYQYPGYRGLQYLLEKGDYKDSGDFGAPQPQVQSVRRIRDMQW---------<br>dhkgvmmelsedcsciqdrfhlsevrslqvlegcwvlyempnyrgrqyllrpqeyrrfqdwgsvdakagslrrvvdly<br>---<br>SARST: aliSize=167 (resi) RMSD=16.38 (Å)<br>g---KITFFEDRSFQGRCYECSSDCPNLQTY-FSRCNSVRVDSGCWMLYERPNYQGHQYFLRRGEYPDYQQW---MgFSDSIRSCRLIPHAG-SHRMRLYEKEDHKGVMMEL-SEDCSCIQDRFHLSEVRSLQVLEGCWVLYEMPNYRGRQYLLRPQEYRRFQDWGSVDAKAGSLRRV-----vdly<br>-lnpKIIIFEQENFQGHSHELNGPCPNLKETgVEKAGSVLVQAGPWVGYEQANCKGEQFVFEKGEYPRWDSWtssR-RTDSLSSLRPIKVDSqEHKITLYENPNFTGKKMEViDDDVPSFHAHGYQEKVSSVRVQSGTWVGYQYPGYRGLQYLLEKGDYKDSGDFGAPQPQVQSVRRIrdmqw----<br>BLAST: aliSize=96 (resi) iden=36.42% (63/173) simi=55.49% (96/173)<br>g---KITFFEDRSFQGRCYECSSDCPNL-QTYFSRCNSVRVDSGCWMLYERPNYQGHQYFLRRGEYPDYQQWMGF--SDSIRSCRLIP-HAGSHRMRLYEKEDHKGVMME-LSEDCSCIQDRFHLSEVRSLQVLEGCWVLYEMPNYRGRQYLLRPQEYRRFQDWGSVDAKAGSLRRVVDL--y<br>-lnpKIIIFEQENFQGHSHELNGPCPNLkETGVEKAGSVLVQAGPWVGYEQANCKGEQFVFEKGEYPRWDSWTSSrrTDSLSSLRPIKvDSQEHKITLYENPNFTGKKMEvIDDDVPSFHAHGYQEKVSSVRVQSGTWVGYQYPGYRGLQYLLEKGDYKDSGDFGAPQPQVQSVRRIRDMqw-<br>Proposed: aliSize=171 (resi) RMSD=1.22 (Å)<br>--GKITFFEDRSFQGRCYECSSDCPNLQTY-FSRCNSVRVDSGCWMLYERPNYQGHQYFLRRGEYPDYQQWM--GFSDSIRSCRLIPHAGS-HRMRLYEKEDHKGVMMELS-EDCSCIQDRFHLSEVRSLQVLEGCWVLYEMPNYRGRQYLLRPQEYRRFQDWGSVDAKAGSLRRVVD---ly<br>lnPKIIIFEQENFQGHSHELNGPCPNLKETgVEKAGSVLVQAGPWVGYEQANCKGEQFVFEKGEYPRWDSWTssRRTDSLSSLRPIKVDSQeHKITLYENPNFTGKKMEVIdDDVPSFHAHGYQEKVSSVRVQSGTWVGYQYPGYRGLQYLLEKGDYKDSGDFGAPQPQVQSVRRIRDmqw--
986	1ammA (174)<br>2bb2A (181)	C	36.78	TM-align: aliSize=86 (resi) RMSD=1.10 (Å)<br>--GKITFYEDRGFQGHCYECS-SDCPNLQ--PYFSRCNSIRVDSGCWMLYERPNYQGHQYFLRRGDYPDYQQWMGFNDSIRSCRLIPQHtGTfrmriyer<br>lnpkiiifeqenfqghshelngpcpnlketgvekagsvlvqagpwvgyeqanckgeqfvfekgeyprwdswtssrrtdslsslrpikvdsqeHKITLYENPNFTGKKMEVIdDDVPSFHahGYQEKVSSVRVQSGTWVGYQYPGYRGLQYLLEKGDYKDSGDFGAPQPQVQSVRRIRDM-QW--------<br>ddfrgqmseitddcpslqdrfhltevhslnvlegswvlyempsyrgrqyllrpgeyrryldwgamnakvgslrrvmdfy<br>---<br>SARST: aliSize=167 (resi) RMSD=16.50 (Å)<br>g---KITFYEDRGFQGHCYECSSDCPNLQPY-FSRCNSIRVDSGCWMLYERPNYQGHQYFLRRGDYPDYQQW---MgFNDSIRSCRLIPQHTGTFRMRIYERDDFRGQMSEI-TDDCPSlQDRFHLTE-VHSLNVLEGSWVLYEMPSYRGRQYLLRPGEYRRYLDWGAMNAKVGSLRRV-----mdfy<br>-lnpKIIIFEQENFQGHSHELNGPCPNLKETgVEKAGSVLVQAGPWVGYEQANCKGEQFVFEKGEYPRWDSWtssR-RTDSLSSLRPIKVDSQEHKITLYENPNFTGKKMEViDDDVPS-FHAHGYQEkVSSVRVQSGTWVGYQYPGYRGLQYLLEKGDYKDSGDFGAPQPQVQSVRRIrdmqw----<br>BLAST: aliSize=97 (resi) iden=37.36% (65/174) simi=55.75% (97/174)<br>g---KITFYEDRGFQGHCYECSSDCPNL-QPYFSRCNSIRVDSGCWMLYERPNYQGHQYFLRRGDYPDYQQWMGF--NDSIRSCRLIPQHTGTFRMRIYERDDFRG-QMSEITDDCPSLQDRFHLTEVHSLNVLEGSWVLYEMPSYRGRQYLLRPGEYRRYLDWGAMNAKVGSLRRVMD---fy<br>-lnpKIIIFEQENFQGHSHELNGPCPNLkETGVEKAGSVLVQAGPWVGYEQANCKGEQFVFEKGEYPRWDSWTSSrrTDSLSSLRPIKVDSQEHKITLYENPNFTGkKMEVIDDDVPSFHAHGYQEKVSSVRVQSGTWVGYQYPGYRGLQYLLEKGDYKDSGDFGAPQPQVQSVRRIRDmqw--<br>Proposed: aliSize=172 (resi) RMSD=1.23 (Å)<br>--GKITFYEDRGFQGHCYECSSDCPNLQPY-FSRCNSIRVDSGCWMLYERPNYQGHQYFLRRGDYPDYQQWM--GFNDSIRSCRLIPQHTGTFRMRIYERDDFRGQMSEIT-DDCPSLQDRFHLTEVHSLNVLEGSWVLYEMPSYRGRQYLLRPGEYRRYLDWGAMNAKVGSLRRVMD---fy<br>lnPKIIIFEQENFQGHSHELNGPCPNLKETgVEKAGSVLVQAGPWVGYEQANCKGEQFVFEKGEYPRWDSWTssRRTDSLSSLRPIKVDSQEHKITLYENPNFTGKKMEVIdDDVPSFHAHGYQEKVSSVRVQSGTWVGYQYPGYRGLQYLLEKGDYKDSGDFGAPQPQVQSVRRIRDmqw--

987	1zwmA (177)<br>1ytqA (181)	C	33.90	TM-align: aliSize=90 (resi) RMSD=1.59 (Å)<br>--SKTGGKISFYEDRNFQGRRYDCD-CDCADFR--SYLSRCNSIRVEGGTWAVYERPNFSGHMYILPQGEYPEYQRWMGLNDRLGSCRAVHLSSGgqakiqvfe<br>lnpkiiifeqenfqghshelngpcpnlketgvekagsvlvqagpwvgyeqanckgeqfvfekgeyprwdswtssrrtdslsslrpikvDSQEHKIILYENPNFTGKKMEIIdDDVPSFHahGYQEKVSSVRVQSGTWVGYQYPGYRGLQYLLEKGDYKDSSDFGAPHPQVQSVRRIRDMQW---------<br>kgdfngqmyettedcpsimeqfhlreihsckvvegtwifyelpnyrgrqylldkkeyrkpvdwgaaspaiqsfrrive<br>--<br>SARST: aliSize=170 (resi) RMSD=15.34 (Å)<br>skt-GGKISFYEDRNFQGRRYDCDCDCADFRSY-LSRCNSIRVEGGTWAVYERPNFSGHMYILPQGEYPEYQRW--MGLNDRLGSCRAVHLSSgGQAKIQVFEKGDFNGQM-YETTEDCPSIMEqFHLRE-IHSCKVVEGTWIFYELPNYRGRQYLLDKKEYRKPVDWGAASPAIQSFRRI-----v<br>---lNPKIIIFEQENFQGHSHELNGPCPNLKETgVEKAGSVLVQAGPWVGYEQANCKGEQFVFEKGEYPRWDSWtsSRRTDSLSSLRPIKVDS-QEHKIILYENPNFTGKKmEIIDDDVPSFHA-HGYQEkVSSVRVQSGTWVGYQYPGYRGLQYLLEKGDYKDSSDFGAPHPQVQSVRRIrdmqw-<br>BLAST: aliSize=94 (resi) iden=34.66% (61/177) simi=53.41% (94/177)<br>sktgg---KISFYEDRNFQGRRYDCDCDCADFRSY-LSRCNSIRVEGGTWAVYERPNFSGHMYILPQGEYPeyqRW-----MGLNDRLGSCRAVHLSSgGQAKIQVFEKGDFNGQMYE-TTEDCPSIMEQFHLREIHSCKVVEGTWIFYELPNYRGRQYLLDKKEYRKPVDWGAASPAIQSFRRI-----v<br>-----lnpKIIIFEQENFQGHSHELNGPCPNLKETgVEKAGSVLVQAGPWVGYEQANCKGEQFVFEKGEYP---RWdswtsSRRTDSLSSLRPIKVDS-QEHKIILYENPNFTGKKMEiIDDDVPSFHAHGYQEKVSSVRVQSGTWVGYQYPGYRGLQYLLEKGDYKDSSDFGAPHPQVQSVRRIrdmqw-<br>Proposed: aliSize=174 (resi) RMSD=1.45 (Å)<br>skTGGKISFYEDRNFQGRRYDCDCDCADFRSY-LSRCNSIRVEGGTWAVYERPNFSGHMYILPQGEYPEYQRWMG-L-NDRLGSCRAVHLSSGgQAKIQVFEKGDFNGQMYETT-EDCPSIMEQFHLREIHSCKVVEGTWIFYELPNYRGRQYLLDKKEYRKPVDWGAASPAIQSFRRIVE---<br>--LNPKIIIFEQENFQGHSHELNGPCPNLKETgVEKAGSVLVQAGPWVGYEQANCKGEQFVFEKGEYPRWDSWTSsRrTDSLSSLRPIKVDSQ-EHKIILYENPNFTGKKMEIIdDDVPSFHAHGYQEKVSSVRVQSGTWVGYQYPGYRGLQYLLEKGDYKDSSDFGAPHPQVQSVRRIRDmqw
988	2jdfA (175)<br>1ytqA (181)	C	34.86	TM-align: aliSize=89 (resi) RMSD=1.84 (Å)<br>gkitfyedrafqgrsyecttdcpnlqpyfsrcnsirvesgcwmiyerpnyqghqyflrrgeypdyqqwmglsdsirsccclipph--SGAYRMKIYDRDELRGQ<br>---lnpkiiifeqenfqghshelngpcpnlketgvekagsvlvqagpwvgyeqanckgeqfvfekgeyprwdswtssrrtdslsslrpikvdSQEHKIILYENPNFTGK<br>MSELT-DDCLSVQDRFHLTEIHSLNVLEGSWILYEMPNYRGRQYLLRPGEYRRFLDWGAPNAKVGSLRRVMDLyl--<br>KMEIIdDDVPSFHAHGYQEKVSSVRVQSGTWVGYQYPGYRGLQYLLEKGDYKDSSDFGAPHPQVQSVRRIRDM--qw<br>SARST: aliSize=168 (resi) RMSD=15.88 (Å)<br>g---KITFYEDRAFQGRSYECTTDCPNLQPY-FSRCNSIRVESGCWMIYERPNYQGHQYFLRRGEYPDYQQW--MGLSDSIRSCCLIPPHSGAYRMKIYDRDELRGQMSEL-TDDCLSvQDRFHLTE-IHSLNVLEGSWILYEMPNYRGRQYLLRPGEYRRFLDWGAPNAKVGSLRRV-----mdlyl<br>-lnpKIIIFEQENFQGHSHELNGPCPNLKETgVEKAGSVLVQAGPWVGYEQANCKGEQFVFEKGEYPRWDSWtsSRRTDSLSSLRPIKVDSQEHKIILYENPNFTGKKMEIiDDDVPS-FHAHGYQEkVSSVRVQSGTWVGYQYPGYRGLQYLLEKGDYKDSSDFGAPHPQVQSVRRIrdmqw-----<br>BLAST: aliSize=99 (resi) iden=34.86% (61/175) simi=56.57% (99/175)<br>g---KITFYEDRAFQGRSYECTTDCPNL-QPYFSRCNSIRVESGCWMIYERPNYQGHQYFLRRGEYPDYQQWMG--LSDSIRSCCLIPPHSGAYRMKIYDRDELRGQMSELTDDCL-SVQDRFHLTEIHSLNVLEGSWILYEMPNYRGRQYLLRPGEYRRFLDWGAPNAKVGSLRRVMDL--yl<br>-lnpKIIIFEQENFQGHSHELNGPCPNLkETGVEKAGSVLVQAGPWVGYEQANCKGEQFVFEKGEYPRWDSWTSsrRTDSLSSLRPIKVDSQEHKIILYENPNFTGKKMEIIDDDVpSFHAHGYQEKVSSVRVQSGTWVGYQYPGYRGLQYLLEKGDYKDSSDFGAPHPQVQSVRRIRDMqw--<br>Proposed: aliSize=172 (resi) RMSD=1.30 (Å)<br>--GKITFYEDRAFQGRSYECTTDCPNLQPY-FSRCNSIRVESGCWMIYERPNYQGHQYFLRRGEYPDYQQWMG-L-SDSIRSCCLIPPHSGAYRMKIYDRDELRGQMSELT-DDCLSVQDRFHLTEIHSLNVLEGSWILYEMPNYRGRQYLLRPGEYRRFLDWGAPNAKVGSLRRVMD---lyl<br>lnPKIIIFEQENFQGHSHELNGPCPNLKETgVEKAGSVLVQAGPWVGYEQANCKGEQFVFEKGEYPRWDSWTSsRrTDSLSSLRPIKVDSQEHKIILYENPNFTGKKMEIIdDDVPSFHAHGYQEKVSSVRVQSGTWVGYQYPGYRGLQYLLEKGDYKDSSDFGAPHPQVQSVRRIRDmqw---
989	2jdfA (175)<br>2bb2A (181)	C	34.86	TM-align: aliSize=86 (resi) RMSD=1.15 (Å)<br>--GKITFYEDRAFQGRSYECT-TDCPNLQ--PYFSRCNSIRVESGCWMIYERPNYQGHQYFLRRGEYPDYQQWMGLSDSIRSCCLIPPHsGAyrmkiydr<br>lnpkiiifeqenfqghshelngpcpnlketgvekagsvlvqagpwvgyeqanckgeqfvfekgeyprwdswtssrrtdslsslrpikvdsqeHKITLYENPNFTGKKMEVIdDDVPSFHahGYQEKVSSVRVQSGTWVGYQYPGYRGLQYLLEKGDYKDSGDFGAPQPQVQSVRRIRDM-QW--------<br>delrgqmseltddclsvqdrfhlteihslnvlegswilyempnyrgrqyllrpgeyrrfldwgapnakvgslrrvmdlyl<br>--<br>SARST: aliSize=167 (resi) RMSD=16.38 (Å)<br>g---KITFYEDRAFQGRSYECTTDCPNLQPY-FSRCNSIRVESGCWMIYERPNYQGHQYFLRRGEYPDYQQW---MgLSDSIRSCCLIPPHSGAYRMKIYDRDELRGQMSEL-TDDCLSvQDRFHLTE-IHSLNVLEGSWILYEMPNYRGRQYLLRPGEYRRFLDWGAPNAKVGSLRRV-----mdlyl<br>-lnpKIIIFEQENFQGHSHELNGPCPNLKETgVEKAGSVLVQAGPWVGYEQANCKGEQFVFEKGEYPRWDSWtssR-RTDSLSSLRPIKVDSQEHKITLYENPNFTGKKMEViDDDVPS-FHAHGYQEkVSSVRVQSGTWVGYQYPGYRGLQYLLEKGDYKDSGDFGAPQPQVQSVRRIrdmqw-----<br>BLAST: aliSize=98 (resi) iden=34.86% (61/175) simi=56.00% (98/175)<br>g---KITFYEDRAFQGRSYECTTDCPNL-QPYFSRCNSIRVESGCWMIYERPNYQGHQYFLRRGEYPDYQQWMG--LSDSIRSCCLIPPHSGAYRMKIYDRDELRGQMSELTDDCL-SVQDRFHLTEIHSLNVLEGSWILYEMPNYRGRQYLLRPGEYRRFLDWGAPNAKVGSLRRVMDL--yl<br>-lnpKIIIFEQENFQGHSHELNGPCPNLkETGVEKAGSVLVQAGPWVGYEQANCKGEQFVFEKGEYPRWDSWTSsrRTDSLSSLRPIKVDSQEHKITLYENPNFTGKKMEVIDDDVpSFHAHGYQEKVSSVRVQSGTWVGYQYPGYRGLQYLLEKGDYKDSGDFGAPQPQVQSVRRIRDMqw--<br>Proposed: aliSize=172 (resi) RMSD=1.31 (Å)<br>--GKITFYEDRAFQGRSYECTTDCPNLQPY-FSRCNSIRVESGCWMIYERPNYQGHQYFLRRGEYPDYQQWMG-L-SDSIRSCCLIPPHSGAYRMKIYDRDELRGQMSELT-DDCLSVQDRFHLTEIHSLNVLEGSWILYEMPNYRGRQYLLRPGEYRRFLDWGAPNAKVGSLRRVMD---lyl<br>lnPKIIIFEQENFQGHSHELNGPCPNLKETgVEKAGSVLVQAGPWVGYEQANCKGEQFVFEKGEYPRWDSWTSsRrTDSLSSLRPIKVDSQEHKITLYENPNFTGKKMEVIdDDVPSFHAHGYQEKVSSVRVQSGTWVGYQYPGYRGLQYLLEKGDYKDSGDFGAPQPQVQSVRRIRDmqw---

990	1h4aX (173)<br>2bb2A (181)	N	34.68	TM-align: aliSize=89 (resi) RMSD=1.79 (Å)<br>gkitlyedrgfqgrhyecssdhpnlqpylsrcnsarvdsgcwmlyeqpnysglqyflhrgdyadhqqwmglsdsvrscrliphs---GSHRIRLYEREDYRGQ<br>---lnpkiiifeqenfqghshelngpcpnlketgvekagsvlvqagpwvgyeqanckgeqfvfekgeyprwdswtssrrtdslsslrpikvdsQEHKITLYENPNFTGK<br>MIEFT-EDCSCLQDRFRFNEIHSLNVLEGSWVLYELSNYRGRQYLLMPGDYRRYQDWGATNARVGSLRRVIDFS-<br>KMEVIdDDVPSFHAHGYQEKVSSVRVQSGTWVGYQYPGYRGLQYLLEKGDYKDSGDFGAPQPQVQSVRRIRDMQw<br><br>SARST: aliSize=166 (resi) RMSD=16.42 (Å)<br>g---KITLYEDRGFQGRHYECSSDHPNLQPY-LSRCNSARVDSGCWMLYEQPNYSGLQYFLHRGDYADHQQW---MgLSDSVRSCRLIPHSG-SHRIRLYEREDYRGQM-IEFTEDCSCLQDR-FRFnEIHSLNVLEGSWVLYELSNYRGRQYLLMPGDYRRYQDWGATNARVGSLRRV-----idfs<br>-lnpKIIIFEQENFQGHSHELNGPCPNLKETgVEKAGSVLVQAGPWVGYEQANCKGEQFVFEKGEYPRWDSWtssR-RTDSLSSLRPIKVDSqEHKITLYENPNFTGKKmEVIDDDVPSFHAHgYQE-KVSSVRVQSGTWVGYQYPGYRGLQYLLEKGDYKDSGDFGAPQPQVQSVRRIrdmqw----<br><br>BLAST: aliSize=92 (resi) iden=35.84% (62/173) simi=53.18% (92/173)<br>g---KITLYEDRGFQGRHYECSSDHPNL-QPYLSRCNSARVDSGCWMLYEQPNYSGLQYFLHRGDYADHQQWMG--LSDSVRSCRLIP-HSGSHRIRLYEREDYRGQMIEFTEDCSCLQDRFRFNE-IHSLNVLEGSWVLYELSNYRGRQYLLMPGDYRRYQDWGATNARVGSLRRVID---fs<br>-lnpKIIIFEQENFQGHSHELNGPCPNLkETGVEKAGSVLVQAGPWVGYEQANCKGEQFVFEKGEYPRWDSWTSsrRTDSLSSLRPIKvDSQEHKITLYENPNFTGKKMEVIDDDVPSFHAHGYQEkVSSVRVQSGTWVGYQYPGYRGLQYLLEKGDYKDSGDFGAPQPQVQSVRRIRDmqw--<br><br>Proposed: aliSize=171 (resi) RMSD=1.23 (Å)<br>--GKITLYEDRGFQGRHYECSSDHPNLQPY-LSRCNSARVDSGCWMLYEQPNYSGLQYFLHRGDYADHQQWM--GLSDSVRSCRLIPHSGS-HRIRLYEREDYRGQMIEFT-EDCSCLQDRFRFNEIHSLNVLEGSWVLYELSNYRGRQYLLMPGDYRRYQDWGATNARVGSLRRVID---fs<br>lnPKIIIFEQENFQGHSHELNGPCPNLKETgVEKAGSVLVQAGPWVGYEQANCKGEQFVFEKGEYPRWDSWTssRRTDSLSSLRPIKVDSQeHKITLYENPNFTGKKMEVIdDDVPSFHAHGYQEKVSSVRVQSGTWVGYQYPGYRGLQYLLEKGDYKDSGDFGAPQPQVQSVRRIRDmqw--
991	2jdgA (171)<br>1ytqA (181)	C	34.50	TM-align: aliSize=87 (resi) RMSD=1.49 (Å)<br>--GSIIFLEDRAFQGRIYGCTTDCPNLQPY-FSRCNSIVVQSGCWMIYERPNYQGHQYFLRRGEYPDYQQWMG-L-SDSIRSCCLIPPHSGAvrmkiydrdelrgqmseltddclsvqdrfhlteihslnvlegswilyempnyrgrqyllrpgeyrrfldwgapnakvgslrrvm--------------<br>lnPKIIIFEQENFQGHSHELNGPCPNLKETgVEKAGSVLVQAGPWVGYEQANCKGEQFVFEKGEYPRWDSWTSsRrTDSLSSLRPIKVDSQE--hkiilyenpnftgk<br>---<br>kmeiidddvpsfhahgyqekvssvrvqsgtwvgyqypgyrglqyllekgdykdssdfgaphpqvqsvrrirdmqw<br><br>SARST: aliSize=168 (resi) RMSD=16.07 (Å)<br>g---SIIFLEDRAFQGRIYGCTTDCPNLQPY-FSRCNSIVVQSGCWMIYERPNYQGHQYFLRRGEYPDYQQW--MGLSDSIRSCCLIPPHSGAYRMKIYDRDELRGQMSEL-TDDCLSVQDrFHLTE-IHSLNVLEGSWILYEMPNYRGRQYLLRPGEYRRFLDWGAPNAKVGSLRRV-----m<br>-lnpKIIIFEQENFQGHSHELNGPCPNLKETgVEKAGSVLVQAGPWVGYEQANCKGEQFVFEKGEYPRWDSWtsSRRTDSLSSLRPIKVDSQEHKIILYENPNFTGKKMEIiDDDVPSFHA-HGYQEkVSSVRVQSGTWVGYQYPGYRGLQYLLEKGDYKDSSDFGAPHPQVQSVRRIrdmqw-<br><br>BLAST: aliSize=95 (resi) iden=34.50% (59/171) simi=55.56% (95/171)<br>gs----IIFLEDRAFQGRIYGCTTDCPNL-QPYFSRCNSIVVQSGCWMIYERPNYQGHQYFLRRGEYPDYQQWMG--LSDSIRSCCLIPPHSGAYRMKIYDRDELRGQMSELTDDCL-SVQDRFHLTEIHSLNVLEGSWILYEMPNYRGRQYLLRPGEYRRFLDWGAPNAKVGSLRRV-----m<br>--lnpkIIIFEQENFQGHSHELNGPCPNLkETGVEKAGSVLVQAGPWVGYEQANCKGEQFVFEKGEYPRWDSWTSsrRTDSLSSLRPIKVDSQEHKIILYENPNFTGKKMEIIDDDVpSFHAHGYQEKVSSVRVQSGTWVGYQYPGYRGLQYLLEKGDYKDSSDFGAPHPQVQSVRRIrdmqw-<br><br>Proposed: aliSize=171 (resi) RMSD=1.29 (Å)<br>--GSIIFLEDRAFQGRIYGCTTDCPNLQPY-FSRCNSIVVQSGCWMIYERPNYQGHQYFLRRGEYPDYQQWMG-L-SDSIRSCCLIPPHSGAYRMKIYDRDELRGQMSELT-DDCLSVQDRFHLTEIHSLNVLEGSWILYEMPNYRGRQYLLRPGEYRRFLDWGAPNAKVGSLRRVM----<br>lnPKIIIFEQENFQGHSHELNGPCPNLKETgVEKAGSVLVQAGPWVGYEQANCKGEQFVFEKGEYPRWDSWTSsRrTDSLSSLRPIKVDSQEHKIILYENPNFTGKKMEIIdDDVPSFHAHGYQEKVSSVRVQSGTWVGYQYPGYRGLQYLLEKGDYKDSSDFGAPHPQVQSVRRIRdmqw
992	1ez3B (124)<br>2c5jB (89)	C	12.36	TM-align: aliSize=68 (resi) RMSD=2.49 (Å)<br>drfmdeffeqvEEIRGFIDKIAENVEEVKRKHSAILaSpNPDEKTKEELEELMSDIKKTANKVRSKLKSIEQSIEQEEgLnr-SSadlrirktqhstlsrkfvevmseynatqsdyrerckgriq-------------------<br>-----------DPFQQVVKDTKEQLNRINNYITRHN-T-AGDDDQEEEIQDILKDVEETIVDLDRSIIVMKRDENEDV-S--gRE--aqvknikqqldalklrfdrr<br><br>SARST: aliSize=86 (resi) RMSD=20.39 (Å)<br>drfmdeffeqveeir-GFIDKIAENVEEVKRKHSAILASPNPDEKTKEELEELMSDIKKTANKVRSKLKSIEQSIeqeeglnrssadlRIRKTQHSTLSRKFVEVMSEYNATQSD--yrerckgriq<br>---------------dPFQQVVKDTKEQLNRINNYITRHNTAGDDDQEEEIQDILKDVEETIVDLDRSIIVMKRD-------------ENEDVSGREAQVKNIKQQLDALKLRFDrr----------<br><br>BLAST: aliSize=30 (resi) iden=14.61% (13/89) simi=33.71% (30/89)<br>drfmdeff------------------------------EQVEEIRGFIDKIAENVEEVKRkhsAILASPNpDEktKEELEELMSDIKKTANKVRS-KLK----sieqsieqeeglnrssadlrirktqhstlsrkfvevmseynatqsdyrerckgriq<br>--------dpfqqvvkdtkeqlnrinnyitrhntagddDQEEEIQDILKDVEETIVDLDR---SIIVMKR-DE--NEDVSGREAQVKNIKQQLDAlKLRfdrr--<br><br>Proposed: aliSize=76 (resi) RMSD=1.59 (Å)<br>drfmdeffeqveeirgfIdKIAENVEEVKRKHSAILASP--N-----------pDEKTKEELEELMSDIKKTANKVRSKLKSIEQSIEQEEglnrssadlRIRKTQHSTLSRKFVEVmseynatqsdyrerckgriq<br>-----------------D-PFQQVVKDTKEQLNRINNYItrHntagdddqeee-IQDILKDVEETIVDLDRSIIVMKRDENEDVSGREAQV---------KNIKQQLDALKLRFDRR--------------------

<table>
<tr><td rowspan="2">993</td><td>1lvfA
(106)</td><td rowspan="2">C</td><td rowspan="2">22.47</td><td rowspan="2">TM-align: aliSize=67 (resi) RMSD=2.99 (Å)
edpffvvkGEVQKAVNTAQGLFQRWTELLQ--G-PSAA-TREEIDWTTNELRNNLRSIEWDLEDLDETISIVEaNPRKFNldatelsirkafitstrqivrdmkdqmsas------------------
--------DPFQQVVKDTKEQLNRINNYITrhNtAGDDdQEEEIQDILKDVEETIVDLDRSIIVMKRDENEDV-SGREAQ------------------------------vknikqqldalklrfdrr

SARST: aliSize=86 (resi) RMSD=17.51 (Å)
edpffvvk-GEVQKAVNTAQGLFQRWTELLQGPSAATREEIDWTTNELRNNLRSIEWDLEDLDETISIVEANPRKfnldATELSIRKAFITSTRQIVRD--mkdqmsas
--------dPFQQVVKDTKEQLNRINNYITRHNTAGDDDQEEEIQDILKDVEETIVDLDRSIIVMKRDENEDVSG----REAQVKNIKQQLDALKLRFDrr--------

BLAST: aliSize=33 (resi) iden=24.72% (22/89) simi=37.08% (33/89)
eDPFFVVKGEVQKAVNTAQGLFQRWTELLQGPSAATREEIDWTTNELRNNL-RSIEWDLEDLDETISIVEANPR--KFNLDATEL-----sirkafitstrqivrdmkdqmsas
-DPFQQVVKDTKEQLNRINNYITRHNTAGDDDQEEEIQDILKDVEETIVDLdRSIIVMKRDENEDVSGREAQVKniKQQLDALKLrfdrr-----------------------

Proposed: aliSize=88 (resi) RMSD=1.90 (Å)
eDPFFVVKGEVQKAVNTAQGLFQRWtELLqgpsaatreEIDWTTNELRNNLRSIEWDLEDLDETISIveanpRK-fnLDATELSIRKAFITSTRQIVRDMKDQMSAS
-DPFQQVVKDTKEQLNRINNYITRH-NTA---------GDDDQEEEIQDILKDVEETIVDLDRSIIV-----MKr--DENEDVSGREAQVKNIKQQLDALKLRFDRR</td></tr>
<tr><td>2c5jB
(89)</td></tr>
<tr><td rowspan="2">994</td><td>2iggA
(64)</td><td rowspan="2">C</td><td rowspan="2">7.81</td><td rowspan="2">TM-align: aliSize=52 (resi) RMSD=2.16 (Å)
------LTPAVTTYKLVINGKT-LKGETTTEAVDaATAEKVFKQYANDNG---VDGEWTYDDatKtftvtekpe----------
mhhhhhHAMEEVTIKANLIFANgSTQTAEFKGTF-EKATSEAYAYADTLKkdnGEWTVDVAD--K----------aytlnikfag

SARST: aliSize=59 (resi) RMSD=10.22 (Å)
lt--------PAVTTYKLVINGKTLKGETTTEAVDAATAEKVFKQYAN--DNGVDGEWTYDdATKTFTVTEK---pe
--mhhhhhhaMEEVTIKANLIFANGSTQTAEFKGTFEKATSEAYAYADtlKKDNGEWTVDV-ADKAYTLNIKfag--

BLAST: aliSize=24 (resi) iden=29.69% (19/64) simi=37.50% (24/64)
ltpavttyklvi---------------------NGKTLKGETTTeAVDAATAEKVfkQYANDNGVD-GEWTYDDATKTFTVTEK---pe
------------mhhhhhhameevtikanlifaNGSTQTAEFKG-TFEKATSEAY--AYADTLKKDnGEWTVDVADKAYTLNIKfag--

Proposed: aliSize=59 (resi) RMSD=2.10 (Å)
------LTPAVTTYKLVINGKT-LKGETTTEAVDaATAEKVFKQYANDNG---VDGEWTYD-daTKTFTVTEK--pe
mhhhhhHAMEEVTIKANLIFANgSTQTAEFKGTF-EKATSEAYAYADTLKkdnGEWTVDVAd--KAYTLNIKFag--</td></tr>
<tr><td>1k51A
(72)</td></tr>
<tr><td rowspan="2">995</td><td>2igdA
(61)</td><td rowspan="2">C</td><td rowspan="2">14.75</td><td rowspan="2">TM-align: aliSize=51 (resi) RMSD=2.17 (Å)
mt--------PAVTTYKLVINGKT-LKGETTTKAVdAETAEKAFKQYANDNG--V-DGVWTYDDaTKtftvte---------
--mhhhhhhaMEEVTIKANLIFANgSTQTAEFKGT-FEKATSEAYAYADTLKkdNgEWTVDVAD-KA------ytlnikfag

SARST: aliSize=57 (resi) RMSD=8.22 (Å)
m-------TPAVTTYKLVINGKTLKGETTTKAVDAETAEKAFKQY----ANDNGVDGVWTYDdaTKTFTVT----e
-mhhhhhhAMEEVTIKANLIFANGSTQTAEFKGTFEKATSEAYAYadtlKKDNGEWTVDVAD--KAYTLNIkfag-

BLAST: aliSize=16 (resi) iden=22.95% (14/61) simi=26.23% (16/61)
mtpavttyklvingktlk---------------------GETTTKAVDAeTAEKAFKQ---YAN----DNG---VD--------------gvwtyddatktftvte
------------------mhhhhhhameevtikanlifanGSTQTAEFKG-TFEKATSEayaYADtlkkDNGewtVDvadkaytlnikfag----------------

Proposed: aliSize=58 (resi) RMSD=2.02 (Å)
mt--------PAVTTYKLVINGKT-LKGETTTKAVDaETAEKAFKQYANDNG--V-DGVWTYDD--ATKTFTVTE
--mhhhhhhaMEEVTIKANLIFANgSTQTAEFKGTF-EKATSEAYAYADTLKkdNgEWTVDVADkaYTLNIKFAG</td></tr>
<tr><td>1k51A
(72)</td></tr>
</table>

<table>
<tr><td rowspan="2">996</td><td>1mhhE
(63)</td><td rowspan="2">C</td><td rowspan="2">58.73</td><td rowspan="2">TM-align: aliSize=53 (resi) RMSD=1.43 (Å)
----------EVTIKVNLIFADGKIQTAEFKGTFEEATAEAYRYAALLAKVNGEWTADLEDGgNhmnikfagk---------
mhhhhhhameEVTIKANLIFANGSTQTAEFKGTFEKATSEAYAYADTLKKDNGEWTVDVADK-A---------ytlnikfag

SARST: aliSize=57 (resi) RMSD=8.50 (Å)
e-----------VTIKVNLIFADGKIQTAEFKGTFEEATAEAYRYAALLAKVNGEWTADLEdgGNHMNIKF----agk
-mhhhhhhameeVTIKANLIFANGSTQTAEFKGTFEKATSEAYAYADTLKKDNGEWTVDVA--DKAYTLNIkfag---

BLAST: aliSize=48 (resi) iden=68.25% (43/63) simi=76.19% (48/63)
----------EVTIKVNLIFADGKIQTAEFKGTFEEATAEAYRYAALLAKVNGEWTADLEDGGNHMNIKFAGk
mhhhhhhameEVTIKANLIFANGSTQTAEFKGTFEKATSEAYAYADTLKKDNGEWTVDVADKAYTLNIKFAG-

Proposed: aliSize=62 (resi) RMSD=0.93 (Å)
----------EVTIKVNLIFADGKIQTAEFKGTFEEATAEAYRYAALLAKVNGEWTADLEDGGnHMNIKFAGK
mhhhhhhameEVTIKANLIFANGSTQTAEFKGTFEKATSEAYAYADTLKKDNGEWTVDVADKA-YTLNIKFAG</td></tr>
<tr><td>1k51A
(72)</td></tr>
<tr><td rowspan="2">997</td><td>1kh0A
(65)</td><td rowspan="2">C</td><td rowspan="2">78.46</td><td rowspan="2">TM-align: aliSize=55 (resi) RMSD=1.34 (Å)
---------EEVTIKANLIFANGSTQTAEFKGTKEKALSEVLAYADTLKKDNGEWTIDKRVTnGViilnikfag--------
mhhhhhhamEEVTIKANLIFANGSTQTAEFKGTFEKATSEAYAYADTLKKDNGEWTVDVADK-AY---------tlnikfag

SARST: aliSize=61 (resi) RMSD=12.21 (Å)
e----------EVTIKANLIFANGSTQTAEFKGTKEKALSEVLAYADTLKKDNGEwtIDKRVTNGVIILNIKFA-g
-mhhhhhhameEVTIKANLIFANGSTQTAEFKGTFEKATSEAYAYADTLKKDNGE--WTVDVADKAYTLNIKFAg-

BLAST: aliSize=54 (resi) iden=80.00% (52/65) simi=83.08% (54/65)
---------EEVTIKANLIFANGSTQTAEFKGTKEKALSEVLAYADTLKKDNGEWTIDkrVTNGVIILNIKFAG
mhhhhhhamEEVTIKANLIFANGSTQTAEFKGTFEKATSEAYAYADTLKKDNGEWTVD--VADKAYTLNIKFAG

Proposed: aliSize=63 (resi) RMSD=0.86 (Å)
---------EEVTIKANLIFANGSTQTAEFKGTKEKALSEVLAYADTLKKDNGEWTIDKRVTNgvIILNIKFAG
mhhhhhhamEEVTIKANLIFANGSTQTAEFKGTFEKATSEAYAYADTLKKDNGEWTVDVADKA--YTLNIKFAG</td></tr>
<tr><td>1k51A
(72)</td></tr>
<tr><td rowspan="2">998</td><td>1hz6B
(63)</td><td rowspan="2">C</td><td rowspan="2">98.41</td><td rowspan="2">TM-align: aliSize=53 (resi) RMSD=1.21 (Å)
---------EEVTIKANLIFANGSTQTAEFKGTFEKATSEAYAYADTLKKDNGEWTVDVADKgytlnikfag----------
mhhhhhhamEEVTIKANLIFANGSTQTAEFKGTFEKATSEAYAYADTLKKDNGEWTVDVADK----------aytlnikfag

SARST: aliSize=60 (resi) RMSD=11.74 (Å)
ee-----------VTIKANLIFANGSTQTAEFKGTFEKATSEAYAYADTLKKDNGEWTVDVADKGYTLNIKFA-g
--mhhhhhhameeVTIKANLIFANGSTQTAEFKGTFEKATSEAYAYADTLKKDNGEWTVDVADKAYTLNIKFAg-

BLAST: aliSize=62 (resi) iden=98.41% (62/63) simi=98.41% (62/63)
---------EEVTIKANLIFANGSTQTAEFKGTFEKATSEAYAYADTLKKDNGEWTVDVADKGYTLNIKFAG
mhhhhhhamEEVTIKANLIFANGSTQTAEFKGTFEKATSEAYAYADTLKKDNGEWTVDVADKAYTLNIKFAG

Proposed: aliSize=63 (resi) RMSD=1.29 (Å)
---------EEVTIKANLIFANGSTQTAEFKGTFEKATSEAYAYADTLKKDNGEWTVDVADKGYTLNIKFAG
mhhhhhhamEEVTIKANLIFANGSTQTAEFKGTFEKATSEAYAYADTLKKDNGEWTVDVADKAYTLNIKFAG</td></tr>
<tr><td>1k51A
(72)</td></tr>
</table>

999	1le4A (139)<br>1ow6A (134)	N	11.94	TM-align: aliSize=97 (resi) RMSD=3.02 (Å)<br>qrwelalgrfw-------------------------DYl-RWVqtlseqVqeeLlssqVtqELRALMDETMKELKAYKSELEEQL-TPVaEETRARLSKELQAAQARLGADMEDVRGRLVqYRgeVqamlgqsTeeLrvRLASHLRKLRKRLLRDADDLQKRLAVY-----------<br>-----------nldrsndkvyenvtglvkaviemsSK-iQPA------P---P----E--EYVPMVKEVGLALRTLLATVDETIpLLP-ASTHREIEMAQKLLNSDLGELINKMKLAQQ-YV--M-------T--S--LQQEYKKQMLTAAHALAVDAKNLLDVIdqarlkmlgqt<br><br>SARST: aliSize=118 (resi) RMSD=11.75 (Å)<br>q---------RWELALGRFWDYLRWVQTLSeqvqeellSSQVTQELRALMDETMKELKAYKSELEEQ-LTPVAEETRARLSKELQAAQARLGADMEDVRGR---LVQYRGEVQAMlgqsteelRVRLASHLRKLRKRLLRDADDLQKR---lavy<br>-nldrsndkvYENVTGLVKAVIEMSSKIQP--------APPEEYVPMVKEVGLALRTLLATVDETIPlLPASTHREIEMAQKLLNSDLGELINKMKLAQQYvmtSLQQEYKKQML--------TAAHALAVDAKNLLDVIDQARLKMLgqt----<br><br>BLAST: aliSize=50 (resi) iden=20.15% (27/134) simi=37.31% (50/134)<br>qrwelalgrfwdylrw---VQTLSEQVQEE--LLSSQVTQEL---RALMDETMKELkAYKSELEEQ--LTPVAEETRARLskeLQAAQArLGADMEDVRGRLVQYRGEvqaMLGQSteelrvrlashlrklrkrllrdaddlqkrlavy<br>---------------nldrsndkvyenvtglvkaviemsskiqpappeeyvpmvkevglaLRTLLATVDETipLLPASTHREIemaQKLLNSDLGEL-INKMKLAQQyvMTSLQQEYKKQM---LTAAHA-LAVDAKNLLDVIDQARLK---MLGQT---------------------------------<br><br>Proposed: aliSize=111 (resi) RMSD=2.31 (Å)<br>----------QRWELALGRFWDYLRWVQT-----lseqvqeellssqVTQELRALMDETMKELKAYKSELEEqLtPvaeetRaRLSKELQAAQARLGADMEDVRGRLVQYRGEVqamlgqSTeELRVRLASHLRKLRKRLLRDADDLQKRLAVY--------<br>nldrsndkvyENVTGLVKAVIEMSSKIQPappee-------------YVPMVKEVGLALRTLLATVDETIPL-L-P-----A-STHREIEMAQKLLNSDLGELINKMKLAQQYV------MT-SLQQEYKKQMLTAAHALAVDAKNLLDVIDQArlkmlgqt
1000	1k40A (126)<br>1ow6A (134)	N	96.83	TM-align: aliSize=107 (resi) RMSD=1.74 (Å)<br>ndkvyenvtglvkavi---------------------Ems--SKi-QPAPPEEYVPMVKEVGLALRTLLATVDETIPALPASTHREIEMAQKLLNSDLGELISKMKLAQQYVMTSLQQEYKKQMLTAAHALAVDAKNLLDVIDQARLKM-L--<br>---------------nldrsndkvyenvtglvkaviE--msSK-iQPAPPEEYVPMVKEVGLALRTLLATVDETIPLLPASTHREIEMAQKLLNSDLGELINKMKLAQQYVMTSLQQEYKKQMLTAAHALAVDAKNLLDVIDQARLKMlGqt<br><br>SARST: aliSize=115 (resi) RMSD=10.06 (Å)<br>ndkv-------YENVTGLVKAVIEMSSKIQP--APPEEYVPMVKEVGLALRTLLATVDETIPALPASTHREIEMAQKLLNSDLGELISKMKLAQQY-------VMTSLQQEYKKQMLTAAHALAVDAKNLLDVID---qarlkml<br>----nldrsndKVYENVTGLVKAVIEMSSKIqpAPPEEYVPMVKEVGLALRTLLATVDETIPLLPASTHREIEMAQKLLNSDLGELINKMKLAQQYvmtslqqEYKKQMLTAAHALAVDAKNLLDVIDQARLKMLgqt-------<br><br>BLAST: aliSize=125 (resi) iden=98.41% (124/126) simi=99.21% (125/126)<br>-----NDKVYENVTGLVKAVIEMSSKIQPAPPEEYVPMVKEVGLALRTLLATVDETIPALPASTHREIEMAQKLLNSDLGELISKMKLAQQYVMTSLQQEYKKQMLTAAHALAVDAKNLLDVIDQARLKML---<br>nldrsNDKVYENVTGLVKAVIEMSSKIQPAPPEEYVPMVKEVGLALRTLLATVDETIPLLPASTHREIEMAQKLLNSDLGELINKMKLAQQYVMTSLQQEYKKQMLTAAHALAVDAKNLLDVIDQARLKMLgqt<br><br>Proposed: aliSize=126 (resi) RMSD=1.05 (Å)<br>---N--DKVYENVTGLVKAVIEMSSKIQPAPPEEYVPMVKEVGLALRTLLATVDETIPALPASTHREIEMAQKLLNSDLGELISKMKLAQQYVMTSLQQEYKKQMLTAAHALAVDAKNLLDVIDQARLKM-L--<br>nldRsnDKVYENVTGLVKAVIEMSSKIQPAPPEEYVPMVKEVGLALRTLLATVDETIPLLPASTHREIEMAQKLLNSDLGELINKMKLAQQYVMTSLQQEYKKQMLTAAHALAVDAKNLLDVIDQARLKMlGqt
1001	1e87A (117)<br>1oz7B (123)	M	21.37	TM-align: aliSize=97 (resi) RMSD=1.85 (Å)<br>sSCSEDWVGYQRKCYFISTVKRSWTSAQNACSEH-G-ATLAVIDSEKDMNFLKR-YA---GREEHWVGLKKEPGHPwkwsngkefnnwfnvtgs------------------DKCVFLKN--TEVSSMECEKNLYWICNKPYk<br>-NCLPDWSVYEGYCYKVFKERMNWADAEKFCTKQhKdGHLVSFRNSKEVDFVISlAFpmlKNDLVWIGLTDYWRDC------------------nwewsdgaqldykawdneRHCFIYKNtdNQWTRRDCTWTFSFVCKCPA-<br><br>SARST: aliSize=92 (resi) RMSD=6.55 (Å)<br>ss-CSEDWVGYQRKCYFISTVKRSWTSAQNACSEH--GATLAVIDSEKDMNFLKRYAGREE----HWVGL--------KKE---------------PGHPWKW-SNGkefnnwfnvtgsdkcvflkntEVSSMECEKNLYWICNKP-yk<br>--nCLPDWSVYEGYCYKVFKERMNWADAEKFCTKQhkDGHLVSFRNSKEVDFVISLAFPMLkndlVWIGLtdywrdcnWEWsdgaqldykawdneRHCFIYKnTDN---------------------QWTRRDCTWTFSFVCKCPa--<br><br>BLAST: aliSize=54 (resi) iden=25.64% (30/117) simi=46.15% (54/117)<br>sSCSEDWVGYQRKCYFISTVKRSWTSAQNACS-EH-GATLAVIDSEKDMNFLKRYA----GREEHWVGLKKE-PGHPWKWSNGK--EFNNWFNvtgSDKCVFLKNT--EVSSMECEKNLYWICNKP-yk<br>-NCLPDWSVYEGYCYKVFKERMNWADAEKFCTkQHkDGHLVSFRNSKEVDFVISLAfpmlKNDLVWIGLTDYwRDCNWEWSDGAqlDYKAWDN---ERHCFIYKNTdnQWTRRDCTWTFSFVCKCPa--<br><br>Proposed: aliSize=112 (resi) RMSD=1.65 (Å)<br>sSCSEDWVGYQRKCYFISTVKRSWTSAQNACSEH-G-ATLAVIDSEKDMNFLKR-YA---GREEHWVGLKKEPG-HPWKWSNGKEFNnWFNVT--gsDKCVFLKN--TEVSSMECEKNLYWICNKPYk<br>-NCLPDWSVYEGYCYKVFKERMNWADAEKFCTKQhKdGHLVSFRNSKEVDFVISlAFpmlKNDLVWIGLTDYWRdCNWEWSDGAQLD-YKAWDne--RHCFIYKNtdNQWTRRDCTWTFSFVCKCPA-

1002	1e87A<br>(117)<br><br>1ukmB<br>(124)	M	22.22	TM-align: aliSize=96 (resi) RMSD=1.85 (Å)<br>ssCSEDWVGYQRKCYFISTVKRSWTSAQNACSEH-G-ATLAVIDSEKDMNFLKRYAG----REEHWVGLKKEPGHPwkwsngkefnnwfnvtgs-----------------DKCVFLKNT----EVSSMECEKNLYWICNKPYk<br>--CPLGWSSFDQHCYKVFEPVKNWTEAEEICMQQhKgSRLASIHSSEEEAFVSKLASkalkFTSMWIGLNNPWKDC-----------------kwewsdnarfdykawkrrPYCTVMVVKpdriFWFTRGCEKSVSFVCKFLT-<br><br>SARST: aliSize=112 (resi) RMSD=9.68 (Å)<br>ssc-SEDWVGYQRKCYFISTVKRSWTSAQNACSEH--GATLAVIDSEKDMNFLKRYAGR----EEHWVGLKK-EPGHPWKWSNGKEFNN-WFNVTGSDKCVFLKNTE--VSSMECEKNLYWICNKP-yk<br>---cPLGWSSFDQHCYKVFEPVKNWTEAEEICMQQhkGSRLASIHSSEEEAFVSKLASKalkfTSMWIGLNNpWKDCKWEWSDNARFDYkAWKRRPYCTVMVVKPDRifWFTRGCEKSVSFVCKFLt--<br><br>BLAST: aliSize=57 (resi) iden=27.35% (32/117) simi=48.72% (57/117)<br>ssCSEDWVGYQRKCYFISTVKRSWTSAQNAC-SEH-GATLAVIDSEKDMNFLKRYAGRE----EHWVGLKkepgHPWK-----WS-NGK-EFNNWFNVTGSDKCVfLKNTEV--SSMECEKNLYWIC----nkpyk<br>--CPLGWSSFDQHCYKVFEPVKNWTEAEEICmQQHkGSRLASIHSSEEEAFVSKLASKAlkftSMWIGLN----NPWKdckweWSdNARfDYKAWKRRPYCTVMV-VKPDRIfwFTRGCEKSVSFVCkflt-----<br><br>Proposed: aliSize=111 (resi) RMSD=1.67 (Å)<br>ssCSEDWVGYQRKCYFISTVKRSWTSAQNACSEH-G-ATLAVIDSEKDMNFLKRYAG----REEHWVGLKK-eP-GHPWKWSNGKEFNNwFNVTGS-dKCVFLKNT----EVSSMECEKNLYWICNKPYk<br>--CPLGWSSFDQHCYKVFEPVKNWTEAEEICMQQhKgSRLASIHSSEEEAFVSKLASkalkFTSMWIGLNNp-WkDCKWEWSDNARFDY-KAWKRRp-YCTVMVVKpdriFWFTRGCEKSVSFVCKFLT-
1003	1e87A<br>(117)<br><br>1ukmA<br>(131)	M	24.79	TM-align: aliSize=98 (resi) RMSD=2.07 (Å)<br>sSCSEDWVGYQRKCYFISTVKRSWTSAQNACSE-HG-ATLAVIDSEKDMNFLKRYAG--R---E-EHWVGLKKEP-G-HPwkwsngkefnnwfnvtg------------------SDKCVFLKN-T---EVSSMECEKNLYWICNKPYk<br>-DCPSDWTAYDQHCYLAIGEPQNWYEAERFCTEqAKdGHLVSIQSREEGNFVAQLVSgfMhrsEiYVWIGLRDRReEqQC----------------npewndgskiiyvnwkegeSKMCQGLTKwTnfhDWNNINCEDLYPFVCKFSA-<br><br>SARST: aliSize=113 (resi) RMSD=9.22 (Å)<br>ss-CSEDWVGYQRKCYFISTVKRSWTSAQNACSEH--GATLAVIDSEKDMNFL-----KRYAGRE-EHWVGLKKE--PGHPW-KWSNGKEFNNWFNVTGS-DKCVFLKN----TEVSSMECEKNLYWICNKP-yk<br>--dCPSDWTAYDQHCYLAIGEPQNWYEAERFCTEQakDGHLVSIQSREEGNFVaqlvsGFMHRSEiYVWIGLRDRreEQQCNpEWNDGSKIIYVNWKEGEsKMCQGLTKwtnfHDWNNINCEDLYPFVCKFSa--<br><br>BLAST: aliSize=64 (resi) iden=31.62% (37/117) simi=54.70% (64/117)<br>ss-CSEDWVGYQRKCYFISTVKRSWTSAQNACSEHGAT--LAVIDSEKDMNFLKRY-AG---REEH--WVGLK----KEPGHPwKWSNGKE--FNNWfnVTGSDK-CVFL-KNT---EVSSMECEkNLY-WIC----nkpyk<br>--dCPSDWTAYDQHCYLAIGEPQNWYEAERFCTEQAKDghLVSIQSREEGNFVAQLvSGfmhRSEIyvWIGLRdrreEQQCNP-EWNDGSKiiYVNW--KEGESKmCQGLtKWTnfhDWNNINCE-DLYpFVCkfsa-----<br><br>Proposed: aliSize=113 (resi) RMSD=1.60 (Å)<br>sSCSEDWVGYQRKCYFISTVKRSWTSAQNACSE-HG-ATLAVIDSEKDMNFLKRYAG--R---E-EHWVGLKKEP----gHPWKWSNGKEFNNwFNVT--GSDKCVFLK---NT-EVSSMECEKNLYWICNKPYk<br>-DCPSDWTAYDQHCYLAIGEPQNWYEAERFCTEqAKdGHLVSIQSREEGNFVAQLVSgfMhrsEiYVWIGLRDRReeqq-CNPEWNDGSKIIY-VNWKegESKMCQGLTkwtNFhDWNNINCEDLYPFVCKFSA-
1004	1e87A<br>(117)<br><br>1uexA<br>(125)	M	26.50	TM-align: aliSize=95 (resi) RMSD=1.76 (Å)<br>sSCSEDWVGYQRKCYFISTVKRSWTSAQNACSEHGATLAVIDSEKDMNFLKRYAG-----R-EEHWVGLKKEPGHpwkwsngkefnnwfnvtg-------------------SDKCVFL-K--N-TEVSSMECEKNLYWICNKpyk<br>-GCLPDWSSYKGHCYKVFKKVGTWEDAEKFCVENSGHLASIDSKEEADFVTKLASqtltkFvYDAWIGLRDESKT-----------------qqcspqwtdgssvvyenvdePTKCFGLdVhtEyRTWTDLPCGEKNPFICKS---<br><br>SARST: aliSize=110 (resi) RMSD=8.79 (Å)<br>ss-CSEDWVGYQRKCYFISTVKRSWTSAQNACSEHGATLAVIDSEKDMNFLKRYA------GREEHWVGLKKEPGHPW---KWSNGKEFNNwFNVTGSDKCVFLKN----TEVSSMECEKNLYWICN-kpyk<br>--gCLPDWSSYKGHCYKVFKKVGTWEDAEKFCVENSGHLASIDSKEEADFVTKLAsqtltkFVYDAWIGLRDESKTQQcspQWTDGSSVVY-ENVDEPTKCFGLDVhteyRTWTDLPCGEKNPFICKs----<br><br>BLAST: aliSize=60 (resi) iden=31.62% (37/117) simi=51.28% (60/117)<br>ss-CSEDWVGYQRKCYFISTVKRSWTSAQNACSEHGATLAVIDSEKDMNFLKRYAGRE------EHWVGLKKEPG----HPwKWSNGKEFnNWFNVTGSDKCVFLK-NTEV---SSMEC-EKNLYwIC--nkpyk<br>--gCLPDWSSYKGHCYKVFKKVGTWEDAEKFCVENSGHLASIDSKEEADFVTKLASQTltkfvyDAWIGLRDESKtqqcSP-QWTDGSSV-VYENVDEPTKCFGLDvHTEYrtwTDLPCgEKNPF-ICks-----<br><br>Proposed: aliSize=112 (resi) RMSD=1.64 (Å)<br>sSCSEDWVGYQRKCYFISTVKRSWTSAQNACSEHGATLAVIDSEKDMNFLKRYAG-----R-EEHWVGLKKEPG---HPWKWSNGKEFNNwFNVTGSDKCVFL-K--N-TEVSSMECEKNLYWICNKpyk<br>-GCLPDWSSYKGHCYKVFKKVGTWEDAEKFCVENSGHLASIDSKEEADFVTKLASqtltkFvYDAWIGLRDESKtqqCSPQWTDGSSVVY-ENVDEPTKCFGLdVhtEyRTWTDLPCGEKNPFICKS---

1005	1e87A (117)<br>1j34B (123)	M	23.08	TM-align: aliSize=97 (resi) RMSD=1.80 (Å)<br>sSCSEDWVGYQRKCYFISTVKRSWTSAQNACSEH-G-ATLAVIDSEKDMNFLKRYAG----REEHWVGLKKE-PGHPwkwsngkefnnwfnvtgs-----------------DKCVFLK-NT-EVSSMECEKNLYWICNKPYk<br>-DCPSDWSSYEGHCYKPFSEPKNWADAENFCTQQhAgGHLVSFQSSEEADFVVKLAFqtfgHSIFWMGLSNVwNQCN-----------------wqwsnaamlrykawaeeSYCVYFKsTNnKWRSRACRMMAQFVCEFQA-<br><br>SARST: aliSize=104 (resi) RMSD=9.02 (Å)<br>ssc--SEDWVGYQRKCYFISTVKRSWTSAQNACSEH--GATLAVIDSEKDMNFLKRYA----GREEHWVGLKkepG----HPWKWSNGKEFNnwfnvTGSDKC----VFLKNTE--VSSMECEKNLYWICNKP-yk<br>---dcPSDWSSYEGHCYKPFSEPKNWADAENFCTQQhaGGHLVSFQSSEEADFVVKLAfqtfGHSIFWMGLS---NvwnqCNWQWSNAAMLR-----YKAWAEesycVYFKSTNnkWRSRACRMMAQFVCEFQa--<br><br>BLAST: aliSize=51 (resi) iden=27.35% (32/117) simi=43.59% (51/117)<br>ss-CSEDWVGYQRKCYFISTVKRSWTSAQNACS-EH-GATLAVIDSEKDMNFLKRYA----GREEHWVGLKKEPGH-PWKWSNGK--EFNNWfnvTGSDKCVFLK--NTEVSSMECEKNLYWIC----nkpyk<br>--dCPSDWSSYEGHCYKPFSEPKNWADAENFCTqQHaGGHLVSFQSSEEADFVVKLAfqtfGHSIFWMGLSNVWNQcNWQWSNAAmlRYKAW---AEESYCVYFKstNNKWRSRACRMMAQFVCefqa-----<br><br>Proposed: aliSize=113 (resi) RMSD=1.62 (Å)<br>sSCSEDWVGYQRKCYFISTVKRSWTSAQNACSEH-G-ATLAVIDSEKDMNFLKRYAG----REEHWVGLKKEP-GHPWKWSNGKEFNnWFNVTG-SdKCVFL-KNT-EVSSMECEKNLYWICNKPYk<br>-DCPSDWSSYEGHCYKPFSEPKNWADAENFCTQQhAgGHLVSFQSSEEADFVVKLAFqtfgHSIFWMGLSNVWnQCNWQWSNAAMLR-YKAWAEeS-YCVYFkSTNnKWRSRACRMMAQFVCEFQA-
1006	1e87A (117)<br>1jwiB (123)	M	19.66	TM-align: aliSize=97 (resi) RMSD=1.54 (Å)<br>sSCSEDWVGYQRKCYFISTVKRSWTSAQNACSEH-G-ATLAVIDSEKDMNFLKRYAG----REEHWVGLKKEPGHPwkwsngkefnnwfnvtgs-----------------DKCVFLKNT--EVSSMECEKNLYWICNKPYk<br>-GCLPDWSSYKGHCYKVFKVEKTWADAEKFCKELvNgGHLMSVNSREEGEFISKLALekmrIVLVWIGLSHFWRIC-----------------plrwtdgarldyralsdePICFVAESFhnKWIQWTCNRKKSFVCKYRV-<br><br>SARST: aliSize=111 (resi) RMSD=8.93 (Å)<br>ss-CSEDWVGYQRKCYFISTVKRSWTSAQNACSEH--GATLAVIDSEKDMNFLKRYAGR----EEHWVGLKKE-PGHPWKWSNGKEFNnwFNVTGSDKCVFLK---NTEVSSMECEKNLYWICNKP-yk<br>--gCLPDWSSYKGHCYKVFKVEKTWADAEKFCKELvnGGHLMSVNSREEGEFISKLALEkmriVLVWIGLSHFwRICPLRWTDGARLD--YRALSDEPICFVAesfHNKWIQWTCNRKKSFVCKYRv--<br><br>BLAST: aliSize=53 (resi) iden=24.79% (29/117) simi=45.30% (53/117)<br>ss-CSEDWVGYQRKCYFISTVKRSWTSAQNACSE--HGATLAVIDSEKDMNFLKRYAGREEH----WVGLkkepGHPW-----KWSNGKEFNnwFNVTGSDKCVFLknteVSSMEcEKNLYWICNK----------pyk<br>--gCLPDWSSYKGHCYKVFKVEKTWADAEKFCKElvNGGHLMSVNSREEGEFISKLALEKMRivlvWIGL----SHFWricplRWTDGARLD--YRALSDEPICFV----AESFH-NKWIQWTCNRkksfvckyrv---<br><br>Proposed: aliSize=114 (resi) RMSD=1.67 (Å)<br>sSCSEDWVGYQRKCYFISTVKRSWTSAQNACSEH-G-ATLAVIDSEKDMNFLKRYAG----REEHWVGLKKEPGHP-WKWSNGKEFNNWFNVTGSdKCVFLKNT--EVSSMECEKNLYWICNKPYk<br>-GCLPDWSSYKGHCYKVFKVEKTWADAEKFCKELvNgGHLMSVNSREEGEFISKLALekmrIVLVWIGLSHFWRICpLRWTDGARLDYRALSDEP-ICFVAESFhnKWIQWTCNRKKSFVCKYRV-
1007	1e87A (117)<br>1v4lB (125)	M	22.22	TM-align: aliSize=98 (resi) RMSD=1.72 (Å)<br>-SSCSEDWVGYQRKCYFISTVKRSWTSAQNACSEH-G-ATLAVIDSEKDMNFLKRYAG----REEHWVGLKKEPGHPwkwsngkefnnwfnvtgs-----------------DKCVFLK-NT-EVSSMECEKNLYWICNKPYk<br>gFCCPLGWSSYDEHCYQVFQQKMNWEDAEKFCTQQhRgSHLVSFHSSEEVDFVVSKTSpilkHDFVWMGLSNVWNEC-----------------akewsdgtkldykawsgqSDCITSKtTDnQWLSMDCSSKRYVVCKFQA-<br><br>SARST: aliSize=104 (resi) RMSD=9.61 (Å)<br>sscsed-------WVGYQRKCYFISTVKRSWTSAQNACSEH--GATLAVIDSEKDMNFLKR----YAGREEHWVGLKkepG----HPWKWSNGKEFNnwFNVTGSDKC-VFLKNTE--VSSMECEKNLYWICNkPY-k<br>------gfccplgWSSYDEHCYQVFQQKMNWEDAEKFCTQQhrGSHLVSFHSSEEVDFVVSktspILKHDFVWMGLS---NvwneCAKEWSDGTKLD--YKAWSGQSDcITSKTTDnqWLSMDCSSKRYVVCK-FQa-<br><br>BLAST: aliSize=55 (resi) iden=27.35% (32/117) simi=47.01% (55/117)<br>ss---CSEDWVGYQRKCYFISTVKRSWTSAQNACSE-H-GATLAVIDSEKDMNFL-KRYAGREEH---WVGLKKEPGHPWK-WSNGK--EFNNWfnvTGSDKCVFLKNTEVS--SMECEKNLYWIC----nkpyk<br>--gfcCPLGWSSYDEHCYQVFQQKMNWEDAEKFCTQqHrGSHLVSFHSSEEVDFVvSKTSPILKHdfvWMGLSNVWNECAKeWSDGTklDYKAW---SGQSDCITSKTTDNQwlSMDCSSKRYVVCkfqa-----<br><br>Proposed: aliSize=115 (resi) RMSD=1.81 (Å)<br>-SSCSEDWVGYQRKCYFISTVKRSWTSAQNACSE-HG-ATLAVIDSEKDMNFLKRYAG----REEHWVGLKKEPGHP-WKWSNGKEFNNwFNVTGSDKCVFL-KNT-EVSSMECEKNLYWICNKPYk<br>gFCCPLGWSSYDEHCYQVFQQKMNWEDAEKFCTQqHRgSHLVSFHSSEEVDFVVSKTSpilkHDFVWMGLSNVWNECaKEWSDGTKLDY-KAWSGQSDCITSkTTDnQWLSMDCSSKRYVVCKFQA-

1008	1e87A (117)<br>1iodA (129)	M	23.08	TM-align: aliSize=95 (resi) RMSD=1.80 (Å)<br>sSCSEDWVGYQRKCYFISTVKRSWTSAQNACSEH-G-ATLAVIDSEKDMNFLKRYAG---RE---EHWVGLKKEP----GHpwkwsngkefnnwfnvtg-----------------SDKCVFLKN-T---EVSSMECEKNLYWICNKpyk<br>-DCSSGWSSYEGHCYKVFKQSKTWADAESFCTKQvNgGHLVSIESSGEADFVGQLIAqkiKSakiHVWIGLRAQNkekqCS-----------------iewsdgssisyenwieeeSKKCLGVHIeTgfhKWENFYCEQQDPFVCEA---<br><br>SARST: aliSize=106 (resi) RMSD=10.35 (Å)<br>ss-CSEDWVGYQRKCYFISTVKRSWTSAQNACSEH--GATLAVIDSEKDMNFLKRYAGREEHWVGLKKEPGHPW---------KWSNGKEFNnwfnvTGSD------KCVFLKNT----EVSSMECEKNLYWICN-kpyk<br>--dCSSGWSSYEGHCYKVFKQSKTWADAESFCTKQvnGGHLVSIESSGEADFVGQLIAQKIKSAKIHVWIGLRAqnkekqcsiEWSDGSSIS-----YENWieeeskKCLGVHIEtgfhKWENFYCEQQDPFVCEa----<br><br>BLAST: aliSize=59 (resi) iden=28.21% (33/117) simi=50.43% (59/117)<br>ss-CSEDWVGYQRKCYFISTVKRSWTSAQNACSE--HGATLAVIDSEKDMNFL-----KRYAGREEH-WVGLK---KEPGHPWKWSNGKE--FNNWFNvTGSDKCVFLK-NTEVSSME---CEKNLYWIC--nkpyk<br>--dCSSGWSSYEGHCYKVFKQSKTWADAESFCTKqvNGGHLVSIESSGEADFVgqliaQKIKSAKIHvWIGLRaqnKEKQCSIEWSDGSSisYENWIE-EESKKCLGVHiETGFHKWEnfyCEQQDPFVCea-----<br><br>Proposed: aliSize=111 (resi) RMSD=1.50 (Å)<br>sSCSEDWVGYQRKCYFISTVKRSWTSAQNACSEH-G-ATLAVIDSEKDMNFLKRYAG---RE---EHWVGLKKEP----GHPwKWSNGKEFNnWFNVT--GSDKCVFLKN-T---EVSSMECEKNLYWICNKpyk<br>-DCSSGWSSYEGHCYKVFKQSKTWADAESFCTKQvNgGHLVSIESSGEADFVGQLIAqkiKSakiHVWIGLRAQNkekqCSI-EWSDGSSIS-YENWIeeESKKCLGVHIeTgfhKWENFYCEQQDPFVCEA---
1009	1e87A (117)<br>1uosA (133)	M	26.50	TM-align: aliSize=98 (resi) RMSD=1.92 (Å)<br>sSCSEDWVGYQRKCYFISTVKRSWTSAQNACSEHG--ATLAVIDSEKDMNFLKRYAG----R--EEHWVGLKKEPGHpwkwsngkefnnwfnvtg---------------------SDKCVFLKN-T---EVSSMECEKNLYWICNKPYK-<br>-HCPSDWYYYDQHCYRIFNEEMNWEDAEWFCTKQAkgAHLVSIKSAKEADFVAWMVTqnieEsfSHVSIGLRVQNKE-----------------kqcstkwsdgssvsydnlldlyITKCSLLKKeTgfrKWFVASCIGKIPFVCKFPPQc<br><br>SARST: aliSize=110 (resi) RMSD=9.32 (Å)<br>ss-CSEDWVGYQRKCYFISTVKRSWTSAQNACSEH--GATLAVIDSEKDMNFLKRYAGREE------HWVGLKKEPGH---PWKWSNGKEFNnwFNVTG-----SDkcVFLKNT----EVSSMECEKNLYWICNKPY--k<br>--hCPSDWYYYDQHCYRIFNEEMNWEDAEWFCTKQakGAHLVSIKSAKEADFVAWMVTQNIeesfshVSIGLRVQNKEkqcSTKWSDGSSVS--YDNLLdlyitKC--SLLKKEtgfrKWFVASCIGKIPFVCKFPPqc-<br><br>BLAST: aliSize=50 (resi) iden=27.35% (32/117) simi=42.74% (50/117)<br>ss-CSEDWVGYQRKCYFISTVKRSWTSAQNACSEH--GATLAVIDSEKDMNFLKRYAGR--EEHW----VGLK---KEPGHPWKWSNGK--EFNNWFNVTgSDKCVFLK--------------------------ntevssmeceknlywicnkpyk<br>--hCPSDWYYYDQHCYRIFNEEMNWEDAEWFCTKQakGAHLVSIKSAKEADFVAWMVTQniEESFshvsIGLRvqnKEKQCSTKWSDGSsvSYDNLLDLY-ITKCSLLKketgfrkwfvascigkipfvckfppqc---------------------<br><br>Proposed: aliSize=114 (resi) RMSD=1.58 (Å)<br>sSCSEDWVGYQRKCYFISTVKRSWTSAQNACSEHG--ATLAVIDSEKDMNFLKRYAG-------rEEHWVGLKKEPG---HPWKWSNGKEFNNwFNVT--GSDKCVFLKN-T---EVSSMECEKNLYWICNKPYK-<br>-HCPSDWYYYDQHCYRIFNEEMNWEDAEWFCTKQAkgAHLVSIKSAKEADFVAWMVTqnieesf-SHVSIGLRVQNKekqCSTKWSDGSSVSY-DNLLdlYITKCSLLKKeTgfrKWFVASCIGKIPFVCKFPPQc
1010	1e87A (117)<br>1j34A (129)	M	21.37	TM-align: aliSize=95 (resi) RMSD=1.74 (Å)<br>sSCSEDWVGYQRKCYFISTVKRSWTSAQNACSEH-G-ATLAVIDSEKDMNFLKRYAG-----R-EEHWVGLKK-E-P--Ghpwkwsngkefnnwfnvt-----------------GSDKCVFLKN-T---EVSSMECEKNLYWICNKpyk<br>-DCPSGWSSYEGHCYKPFKLYKTWDDAERFCTEQaKgGHLVSIESAGEADFVAQLVTeniqnTkSYVWIGLRVqGkEkqC-----------------ssewsdgssvsyenwieaESKTCLGLEKeTgfrKWVNIYCGQQNPFVCEA---<br><br>SARST: aliSize=109 (resi) RMSD=8.85 (Å)<br>ss-CSEDWVGYQRKCYFISTVKRSWTSAQNACSEH--GATLAVIDSEKDMNFLKRYAG------REEHWVGLKkePGHPWKWSNGKEFNNWFNVTGSD------KCVFLKN----TEVSSMECEKNLYWICN-kpyk<br>--dCPSGWSSYEGHCYKPFKLYKTWDDAERFCTEQakGGHLVSIESAGEADFVAQLVTeniqntKSYVWIGLR--VQGKEKQCSSEWSDGSSVSYENWieaeskTCLGLEKetgfRKWVNIYCGQQNPFVCEa----<br><br>BLAST: aliSize=48 (resi) iden=24.79% (29/117) simi=41.03% (48/117)<br>ss-CSEDWVGYQRKCYFISTVKRSWTSAQNACSEH--GATLAVIDSEKDMNFLKRYA-----GREEH-WVGLK---KEPGHPWKWSNGKE--FNNWFNVTgSDKCVFL-KNT-------------------evssmeceknlywicnkpyk<br>--dCPSGWSSYEGHCYKPFKLYKTWDDAERFCTEQakGGHLVSIESAGEADFVAQLVteniqNTKSYvWIGLRvqgKEKQCSSEWSDGSSvsYENWIEAE-SKTCLGLeKETgfrkwvniycgqqnpfvcea-------------------<br><br>Proposed: aliSize=109 (resi) RMSD=1.50 (Å)<br>sSCSEDWVGYQRKCYFISTVKRSWTSAQNACSEH-G-ATLAVIDSEKDMNFLKRYAG-----R-EEHWVGLKK------epgHPWKWSNGKEFNnWFNVT--GSDKCVFLKNTE----VSSMECEKNLYWICNKpyk<br>-DCPSGWSSYEGHCYKPFKLYKTWDDAERFCTEQaKgGHLVSIESAGEADFVAQLVTeniqnTkSYVWIGLRVqgkekq---CSSEWSDGSSVS-YENWIeaESKTCLGLEKETgfrkWVNIYCGQQNPFVCEA---

1011	1p4lD<br>(122)<br><br>1ukmA<br>(131)	M	19.67	TM-align: aliSize=97 (resi) RMSD=2.12 (Å)<br>rgvk----YWFCYSTKCYYFIMNKTTWSGCKANCQHYG--VPILKIEDEDELKFLQRHV-I-P---G-NYWIGLSYDKKkKewawidngpskldmkikk-MNFK-----------------SRGCVFLS---K-ARIEDIDCNIPYYCICGKKLdk<br>----dcpsDWTAYDQHCYLAIGEPQNWYEAERFCTEQAkdGHLVSIQSREEGNFVAQLVsGfMhrsEiYVWIGLRDRRE-E------------------qQCNPewndgskiiyvnwkegeSKMCQGLTkwtNfHDWNNINCEDLYPFVCKFSA--<br><br>SARST: aliSize=89 (resi) RMSD=8.04 (Å)<br>rgvkyw------FCYSTKCYYFIMNKTTWSGCKANCQHY--GVPILKIEDEDELKFL------QRHVIPGNYWIGLSYDKKKKE--WAWIDNG-------------------------PSkldmkikkmnfksrgcvflskariEDIDCNIPYYCICGKK-ldk<br>------dcpsdwTAYDQHCYLAIGEPQNWYEAERFCTEQakDGHLVSIQSREEGNFVaqlvsgFMHRSEIYVWIGLRDRREEQQcnPEWNDGSkiiyvnwkegeskmcqgltkwtnfhDW------------------------NNINCEDLYPFVCKFSa---<br><br>BLAST: aliSize=53 (resi) iden=25.41% (31/122) simi=43.44% (53/122)<br>rgvky-----WFCYSTKCYYFIMNKTTWSGCKANC--QHYGVPILKIEDEDELKFLQRhVIPG------NY-WIGLSyDKKKKEWA---WidNGPSKLdMKIKKMNFKSRGCVFLSK-ARIED---IDCNIPYYCIC----gkkldk<br>-----dcpsdWTAYDQHCYLAIGEPQNWYEAERFCteQAKDGHLVSIQSREEGNFVAQ-LVSGfmhrseIYvWIGLR-DRREEQQCnpeW--NDGSKI-IYVNWKEGESKMCQGLTKwTNFHDwnnINCEDLYPFVCkfsa------<br><br>Proposed: aliSize=110 (resi) RMSD=2.10 (Å)<br>rgvk----YWFCYSTKCYYFIMNKTTWSGCKANCQHYG--VPILKIEDEDELKFLQRHV-I-P---G-NYWIGLSYDKK---kkEW-AWIDNGPSKLDMKI-KKmnfkSRGCVFLS---K-ARIEDIDCNIPYYCICGKKLdk<br>----dcpsDWTAYDQHCYLAIGEPQNWYEAERFCTEQAkdGHLVSIQSREEGNFVAQLVsGfMhrsEiYVWIGLRDRREeqq--CNpEWNDGSKIIYVNWKeGE----SKMCQGLTkwtNfHDWNNINCEDLYPFVCKFSA--
1012	1p4lD<br>(122)<br><br>1j34B<br>(123)	M	15.57	TM-align: aliSize=96 (resi) RMSD=1.94 (Å)<br>rgvk----YWFCYSTKCYYFIMNKTTWSGCKANCQHYG--VPILKIEDEDELKFLQRHVI----PGNYWIGLS-YDKkkKEwawidngpskldmkikkmNF------------K--SRGCVFLS-KA-RIEDIDCNIPYYCICGKKLdk<br>----dcpsDWSSYEGHCYKPFSEPKNWADAENFCTQQHagGHLVSFQSSEEADFVVKLAFqtfgHSIFWMGLSnVWN--QC------------------NWqwsnaamlrykaWaeESYCVYFKsTNnKWRSRACRMMAQFVCEFQA--<br><br>SARST: aliSize=109 (resi) RMSD=9.13 (Å)<br>rgvky-----WFCYSTKCYYFIMNKTTWSGCKANCQHY--GVPILKIEDEDELKFLQR----HVIPGNYWIGLSYDKKKKEWAWIDNGPSKLdmkikKMNFKSRGCVFLSKAR--IEDIDCNIPYYCICGKK-ldk<br>-----dcpsdWSSYEGHCYKPFSEPKNWADAENFCTQQhaGGHLVSFQSSEEADFVVKlafqTFGHSIFWMGLSNVWNQCNWQWSNAAMLRY-----KAWAEESYCVYFKSTNnkWRSRACRMMAQFVCEFQa---<br><br>BLAST: aliSize=30 (resi) iden=14.75% (18/122) simi=24.59% (30/122)<br>rgvky-----WFCYSTKCYYFIMNKTTWSGCKANC--QHYGVPILKIEDEDELKFLQRHVIP--GN--YWIGLSYDKKKKEWAW--idngpskldmkikkmnfksrgcvflskariedidcnipyycicgkkldk<br>-----dcpsdWSSYEGHCYKPFSEPKNWADAENFCtqQHAGGHLVSFQSSEEADFVVKLAFQtfGHsiFWMGLSNVWNQCNWQWsnaamlrykawaeesycvyfkstnnkwrsracrmmaqfvcefqa---<br><br>Proposed: aliSize=109 (resi) RMSD=2.06 (Å)<br>rgvk----YWFCYSTKCYYFIMNKTTWSGCKANCQHYG--VPILKIEDEDELKFLQRHVI----PGNYWIGLSYDKKKKEWAWI-dngpSkLDMKIKKM-nfKSRGCVFLS-KA-RIEDIDCNIPYYCICGKKLdk<br>----dcpsDWSSYEGHCYKPFSEPKNWADAENFCTQQHagGHLVSFQSSEEADFVVKLAFqtfgHSIFWMGLSNVWNQCNWQWSn----A-AMLRYKAWa--EESYCVYFKsTNnKWRSRACRMMAQFVCEFQA--
1013	1p4lD<br>(122)<br><br>1jwiB<br>(123)	M	16.39	TM-align: aliSize=97 (resi) RMSD=1.86 (Å)<br>rgvk----YWFCYSTKCYYFIMNKTTWSGCKANCQHYG--VPILKIEDEDELKFLQRHVI----PGNYWIGLSYDKKKKEwawidngpskldmkikkmn------------FKS--RGCVFLSKA--RIEDIDCNIPYYCICGKKLdk<br>----gclpDWSSYKGHCYKVFKVEKTWADAEKFCKELVngGHLMSVNSREEGEFISKLALekmrIVLVWIGLSHFWRICP-------------------lrwtdgarldyrALSdePICFVAESFhnKWIQWTCNRKKSFVCKYRV--<br><br>SARST: aliSize=111 (resi) RMSD=8.97 (Å)<br>r-GVKYWFCYSTKCYYFIMNKTTWSGCKANCQHY--GVPILKIEDEDE----LKFLQRHVIPGNYWIGLSYDKKKKEWAWIDNGPSKLdmkikkmNFKSRGCVFLS----KARIEDIDCNIPYYCICGKK-ldk<br>-gCLPDWSSYKGHCYKVFKVEKTWADAEKFCKELvnGGHLMSVNSREEgefiSKLALEKMRIVLVWIGLSHFWRICPLRWTDGARLDY-------RALSDEPICFVaesfHNKWIQWTCNRKKSFVCKYRv---<br><br>BLAST: aliSize=36 (resi) iden=15.57% (19/122) simi=29.51% (36/122)<br>rgvky-----WFCYSTKCYYFIMNKTTWSGCKANCQHY--GVPILKIEDEDELKFLQRHVIPGN----YWIGLSYDKKKKEWAWIDNgpSKLDMK-----------------------------------ikkmnfksrgcvflskariedidcnipyycicgkkldk<br>-----gclpdWSSYKGHCYKVFKVEKTWADAEKFCKELvnGGHLMSVNSREEGEFISKLALEKMrivlVWIGLSHFWRICPLRWTDG--ARLDYRalsdepicfvaesfhnkwiqwtcnrkksfvckyrv--------------------------------------<br><br>Proposed: aliSize=110 (resi) RMSD=1.70 (Å)<br>rgvk----YWFCYSTKCYYFIMNKTTWSGCKANCQHYG--VPILKIEDEDELKFLQRHVI----PGNYWIGLSYDKKKKEWAWIDNGPSKLDMKIK-kmnfksRGCVFLSKA--RIEDIDCNIPYYCICGKKLdk<br>----gclpDWSSYKGHCYKVFKVEKTWADAEKFCKELVngGHLMSVNSREEGEFISKLALekmrIVLVWIGLSHFWRICPLRWTDGARLDYRALSDe------PICFVAESFhnKWIQWTCNRKKSFVCKYRV--

1014	1p4lD (122)<br>1v4lB (125)	M	17.21	TM-align: aliSize=95 (resi) RMSD=1.89 (Å)<br>rgvk------YWFCYSTKCYYFIMNKTTWSGCKANCQHYG--VPILKIEDEDELKFLQRHV--I-P-GNYWIGLS-YDKkKKEwawidngpskldmkikkmnf-------------K--SRGCVFLS-KA-RIEDIDCNIPYYCICGKKLdk<br>----gfccplGWSSYDEHCYQVFQQKMNWEDAEKFCTQQHrgSHLVSFHSSEEVDFVVSKTspIlKhDFVWMGLSnVWN-ECA-------------------kewsdgtkldykaWsgQSDCITSKtTDnQWLSMDCSSKRYVVCKFQA--<br><br>SARST: aliSize=109 (resi) RMSD=11.16 (Å)<br>rgvkyw--------FCYSTKCYYFIMNKTTWSGCKANCQHY--GVPILKIEDEDELKFLQR---HVIPGNYWIGLSYDKKKKEWAWIDNGPSKL-DMKIKKMNFKSRGCVflskARIEDIDCNIPYYCICGKK-ldk<br>------gfccplgwSSYDEHCYQVFQQKMNWEDAEKFCTQQhrGSHLVSFHSSEEVDFVVSktspILKHDFVWMGLSNVWNECAKEWSDGTKLDYkAWSGQSDCITSKTTD----NQWLSMDCSSKRYVVCKFQa---<br><br>BLAST: aliSize=54 (resi) iden=27.05% (33/122) simi=44.26% (54/122)<br>rgvky-------WFCYSTKCYYFIMNKTTWSGCKANC--QHYCVPILKIEDEDELKFLQRHVIP---GNY-WIGLS--YDKKKKEWAwidNGpSKLDMKIkkMNFKSrGCVfLSKARIED---IDCNIPYYCIC----gkkldk<br>-----gfccplgWSSYDEHCYQVFQQKMNWEDAEKFCtqQHRGSHLVSFHSSEEVDFVVSKTSPilkHDFvWMGLSnvWNECAKEWS---DG-TKLDYKA--WSGQS-DCI-TSKTTDNQwlsMDCSSKRYVVCkfqa------<br><br>Proposed: aliSize=109 (resi) RMSD=2.05 (Å)<br>rgvk------YWFCYSTKCYYFIMNKTTWSGCKANCQHYG--VPILKIEDEDELKFLQRHV--I-P-GNYWIGLSYDKKKKEWAWI-dngpSkLDMKIKKM-nfKSRGCVFLS-KA-RIEDIDCNIPYYCICGKKLdk<br>----gfccplGWSSYDEHCYQVFQQKMNWEDAEKFCTQQHrgSHLVSFHSSEEVDFVVSKTspIlKhDFVWMGLSNVWNECAKEWSd----G-TKLDYKAWs--GQSDCITSKtTDnQWLSMDCSSKRYVVCKFQA--
1015	1p4lD (122)<br>1iodA (129)	M	19.67	TM-align: aliSize=95 (resi) RMSD=2.32 (Å)<br>rgvk----YWFCYSTKCYYFIMNKTTWSGCKANCQHYG--VPILKIEDEDELKFLQRHV---IPG---NYWIGLSYDKKkKewawidngpskldmkikk-MN-F-------K---------SRGCVFLSK-A---RIEDIDCNIPYYCICGKkldk<br>----dcssGWSSYEGHCYKVFKQSKTWADAESFCTKQVngGHLVSIESSGEADFVGQLIaqkIKSakiHVWIGLRAQNK-E-----------------kQCsIewsdgssIsyenwieeeSKKCLGVHIeTgfhKWENFYCEQQDPFVCEA----<br><br>SARST: aliSize=105 (resi) RMSD=9.99 (Å)<br>rgvky-----WFCYSTKCYYFIMNKTTWSGCKANCQHY--GVPILKIEDEDELKFLQRH------VIPGNYWIGLSYDKKK---KEwAWIDNGPSKL-DM--KIKKMNfksrgcVFLS-KA---RIEDIDCNIPYYCICG-kkldk<br>-----dcssgWSSYEGHCYKVFKQSKTWADAESFCTKQvnGGHLVSIESSGEADFVGQLiaqkikSAKIHVWIGLRAQNKEkqcSI-EWSDGSSISYeNWieEESKKC------LGVHiETgfhKWENFYCEQQDPFVCEa-----<br><br>BLAST: aliSize=50 (resi) iden=24.59% (30/122) simi=40.98% (50/122)<br>rgvky-----WFCYSTKCYYFIMNKTTWSGCKANC--QHYGVPILKIEDEDELKFLQRHV---IPG---NYWIGLSYDKKKKEWA--WIDNGPSKLDMKIKKMNFKSRGcVFLSKA--RIEDIDC--NIPYYC--icgkkldk<br>-----dcssgWSSYEGHCYKVFKQSKTWADAESFCtkQVNGGHLVSIESSGEADFVGQLIaqkIKSakiHVWIGLRAQNKEKQCSieWSDGSSISYENWIEEESKKCLG-VHIETGfhKWENFYCeqQDPFVCea--------<br><br>Proposed: aliSize=109 (resi) RMSD=2.09 (Å)<br>rgvk----YWFCYSTKCYYFIMNKTTWSGCKANCQHYG--VPILKIEDEDELKFLQRHVI---PG---NYWIGLSYDKK---kKEWAWIDNGPSKLDMKIK-KmnfkSRGCVFLS---KA-RIEDIDCNIPYYCICGKkldk<br>----dcssGWSSYEGHCYKVFKQSKTWADAESFCTKQVngGHLVSIESSGEADFVGQLIAqkiKSakiHVWIGLRAQNKekq-CSIEWSDGSSISYENWIEeE----SKKCLGVHietGFhKWENFYCEQQDPFVCEA----
1016	1r13A (145)<br>1oz7B (123)	M	17.07	TM-align: aliSize=100 (resi) RMSD=2.74 (Å)<br>deelqtelyeikhqIlqtMgVLslqgSMLSVGDKVFSTNGQSVNFDTIKEMCTRA-G-GNIAVPRTPEENEAIAS--IAKKYNNYVYLGMIEDQTPg-Dfhyldgasvsytnwypgeprgq--------------GK-EKCVEMYTD-GTWNDRGCLQYRLAVCEF--<br>-------------N---C-LP----DWSVYEGYCYKVFKERMNWADAEKFCTKQhKdGHLVSFRNSKEVDFVISlaFPMLKNDLVWIGLTDYWRD-cN---------------------wewsdgaqldykawDNeRHCFIYKNTdNQWTRRDCTWTFSFVCKCpa<br><br>SARST: aliSize=109 (resi) RMSD=9.27 (Å)<br>deelqtelyeikhqilqtmgvlslqgs-----MLSVGDKVFSTNGQSVNFDTIKEMCTRA--GGNIAVPRTPEENEAIASIAKKY--NNYVYLGMIEDQTPGDFHYLDGASVSYTNWY--PGEPrgqgkekcVEMYTDGTWNDRGCLQYRLAV-CE--f<br>--------------------------nclpdWSVYEGYCYKVFKERMNWADAEKFCTKQhkDGHLVSFRNSKEVDFVISLAFPMlkNDLVWIGLTDYWRDCNWEWSDGAQLDYKAWDneRHCF--------IYKNTDNQWTRRDCTWTFSFVcKCpa-<br><br>BLAST: aliSize=51 (resi) iden=21.95% (27/123) simi=41.46% (51/123)<br>deelqtelyeikhqilqtmgvlslqgsmlsvgd--------------KVFStngQSVNFDTIKEMCTRA--GGNIAVPRTPEENEAIASIAKKY--NNYVYLGMIEDQTPGDFHYLDGASVSYTNWypgeprgQGKEKC-VEMYTDGTWNDRGCLQYRLAVCE---f<br>---------------------------------nclpdwsvyegycyKVFK---ERMNWADAEKFCTKQhkDGHLVSFRNSKEVDFVISLAFPMlkNDLVWIGLTDYWRDCNWEWSDGAQLDYKAW-------DNERHCfIYKNTDNQWTRRDCTWTFSFVCKcpa-<br><br>Proposed: aliSize=114 (resi) RMSD=2.03 (Å)<br>deelqtelyeikhqIlqtM-gvLslqgSMLSVGDKVFSTNGQSVNFDTIKEMCTRA-G-GNIAVPRTPEENEAIAS-IAK-KYNNYVYLGMIEDQTpGD-FHYLDGASVSYTNWYPGeprgqgkEKCVEMYTD-GTWNDRGCLQYRLAVCEF--<br>-------------N---Cl--P----DWSVYEGYCYKVFKERMNWADAEKFCTKQhKdGHLVSFRNSKEVDFVISlAFPmLKNDLVWIGLTDYWR-DCnWEWSDGAQLDYKAWDNE-------RHCFIYKNTdNQWTRRDCTWTFSFVCKCpa

1017	1r13A (145)<br>1ukmB (124)	M	19.35	TM-align: aliSize=98 (resi) RMSD=2.23 (Å)<br>deelqtelyeikhqilqtMgVLslqgSMLSVGDKVFSTNGQSVNFDTIKEMCTRAG--GNIAVPRTPEENEAIASIAKK-Y-NNYVYLGMI-E-DQTPgdfhyldgasvsytnwypgeprgq---------------GKEKCVEMYTD---GTWNDRGCLQYRLAVCEF--<br>------------------C-PL----GWSSFDQHCYKVFEPVKNWTEAEEICMQQHkgSRLASIHSSEEEAFVSKLASKaLkFTSMWIGLNnPwKDCK-------------------------wewsdnarfdykawkRRPYCTVMVVKpdrIFWFTRGCEKSVSFVCKFlt<br><br>SARST: aliSize=109 (resi) RMSD=11.26 (Å)<br>deelqtelyeikhqilqtmgvlslqgs----MLSVGDKVFSTNGQSVNFDTIKEMCTRA--GGNIAVPRTPEENEAIASIAKKYnnyVYLGMIEDQTPG-----DFHYLDGASVSYTNWYPGEPRGqgkekCVEMYTDGTWN-DRGCLQYRLAVCE---f<br>---------------------------cplgWSSFDQHCYKVFEPVKNWTEAEEICMQQhkGSRLASIHSSEEEAFVSKLASKA---LKFTSMWIGLNNpwkdcKWEWSDNARFDYKAWKRRPYCT-----VMVVKPDRIFWfTRGCEKSVSFVCKflt-<br><br>BLAST: aliSize=44 (resi) iden=19.35% (24/124) simi=35.48% (44/124)<br>deelqtelyeikhqilqtmgvlslqgsmlsvgdkvfstngqsv--------------------NFDTIKEMCTRA--GGNIAVPRTPEENEAIASIAKKYNNY--VYLGMIEDQTPGDFHYLDGASVSYTNWypgeprgQGKEKCVEMYT--DGT-WNDRGCLQYRLAVCEF--<br>---cplgwssfdqhcykvfepvkNWTEAEEICMQQhkGSRLASIHSSEEEAFVSKLASKALKFtsMWIGLNNPWKDCKWEWSDNARFDYKAW-------KRRPYCTVMVVkpDRIfWFTRGCEKSVSFVCKFlt<br><br>Proposed: aliSize=110 (resi) RMSD=1.93 (Å)<br>deelqtelyeikhqilqtM-gvLslqgSMLSVGDKVFSTNGQSVNFDTIKEMCTRAG--GNIAVPRTPEENEAIASIAKK-Y-NNYVYLGMI----edqtPGDFHYLDGASVSYTNWYpgeprgqGKEKCVEMYTD---GTWNDRGCLQYRLAVCEF--<br>------------------Cp--L----GWSSFDQHCYKVFEPVKNWTEAEEICMQQHkgSRLASIHSSEEEAFVSKLASKaLkFTSMWIGLNnpwk----DCKWEWSDNARFDYKAWK-------RRPYCTVMVVKpdrIFWFTRGCEKSVSFVCKFlt
1018	1r13A (145)<br>1ukmA (131)	M	18.32	TM-align: aliSize=101 (resi) RMSD=2.36 (Å)<br>deelqtelyeikhqiLqtMgVLslqgSMLSVGDKVFSTNGQSVNFDTIKEMCTRA-G-GNIAVPRTPEENEAIASIAKKYN---N-YVYLGMIEDQ---TPGdfhyldgasvsytnwypgeprg----------------QGKEKCVEMYT--D-GTWNDRGCLQYRLAVCEF--<br>---------------D--C-PS----DWTAYDQHCYLAIGEPQNWYEAERFCTEQaKdGHLVSIQSREEGNFVAQLVSGFMhrsEiYVWIGLRDRReeqQCN----------------------pewndgskiiyvnwkeGESKMCQGLTKwtNfHDWNNINCEDLYPFVCKFsa<br><br>SARST: aliSize=113 (resi) RMSD=10.13 (Å)<br>deelqtelyeikhqilqtmgvlslq---GSMLSVGDKVFSTNGQSVNFDTIKEMCTRA--GGNIAVPRTPEENEAIASIAKKYNNYVYlGMIEDQTP-------GDFHYLDGASVSYTNWYPGEPRgqgkeKCVEMYTDG---TWNDRGCLQYRLAVCE---f<br>-------------------------dcpSDWTAYDQHCYLAIGEPQNWYEAERFCTEQakDGHLVSIQSREEGNFVAQLVSGFMHRSE-IYVWIGLRdrreeqqCNPEWNDGSKIIYVNWKEGESK-----MCQGLTKWTnfhDWNNINCEDLYPFVCKfsa-<br><br>BLAST: aliSize=53 (resi) iden=21.37% (28/131) simi=40.46% (53/131)<br>deelqtelyeikhqilqtmgvlslqgsmlsvgdkvfstn-----------------GQSVNFDTIKEMCTRAG--GNIAVPRTPEENEAIASIAKKYNN----YVYLGMIE--DQTPGDFHYLDGASVSYTNWYPGEPR-GQGKEKCVEMYtDgtWNDRGCLQYRLAVCEF--<br>---------------------------------------dcpsdwtaydqhcylaiGEPQNWYEAERFCTEQAkdGHLVSIQSREEGNFVAQLVSGFMHrseiYVWIGLRDrrEEQQCNPEWNDGSKIIYVNWKEGESKmCQGLTKWTNFH-D--WNNINCEDLYPFVCKFsa<br><br>Proposed: aliSize=115 (resi) RMSD=1.86 (Å)<br>deelqtelyeikhqiLqtM-gvLslqgSMLSVGDKVFSTNGQSVNFDTIKEMCTRA-G-GNIAVPRTPEENEAIASIAKKYN---N-YVYLGMIEDQ---tpGDF-HYLDGASVSYTNWYPGeprgqGKEKCVEMYT--D-GTWNDRGCLQYRLAVCEF--<br>---------------D--Cp--S----DWTAYDQHCYLAIGEPQNWYEAERFCTEQaKdGHLVSIQSREEGNFVAQLVSGFMhrsEiYVWIGLRDRReeq--QCNpEWNDGSKIIYVNWKEG-----ESKMCQGLTKwtNfHDWNNINCEDLYPFVCKFsa
1019	1r13A (145)<br>1uexA (125)	M	16.80	TM-align: aliSize=102 (resi) RMSD=2.42 (Å)<br>deelqtelyeikhqilqtMGVLslqgSMLSVGDKVFSTNGQSVNFDTIKEMCTRAGGNIAVPRTPEENEAIASIAK-KY--N-NYVYLGMIEDQ-T--PGdfhyldgasvsytnwypgepr-------------GQGKEKCVEMYT--D-GTWNDRGCLQYRLAVCEF<br>------------------GCLP----DWSSYKGHCYKVFKKVGTWEDAEKFCVENSGHLASIDSKEEADFVTKLASqTLtkFvYDAWIGLRDESkTqqCS---------------------pqwtdgssvvyenVDEPTKCFGLDVhtEyRTWTDLPCGEKNPFICKS<br><br>SARST: aliSize=106 (resi) RMSD=11.43 (Å)<br>deelqtelyeikhqilqtmgvlslqg----SMLSVGDKVFSTNGQSVNFDTIKEMCTRAGGNIAVPRTPEENEAIASIAKKYN----NYVYLGMIE---DQtPGDFHYLDGASVSYTNW--YPGEPRG--QGKEKCVEMYTDGTWNDR----gclqyrlavcef<br>--------------------------gclpDWSSYKGHCYKVFKKVGTWEDAEKFCVENSGHLASIDSKEEADFVTKLASQTLtkfvYDAWIGLRDeskTQ-QCSPQWTDGSSVVYENVdePTKCFGLdvHTEYRTWTDLPCGEKNPFicks------------<br><br>BLAST: aliSize=55 (resi) iden=23.20% (29/125) simi=44.00% (55/125)<br>deelqtelyeikhqilqtmgvlslqgsmlsvgd--------------KVFSTNGqsvNFDTIKEMCTRAGGNIAVPRTPEENEAIASIAKK-YNNYVY---LGMIEDQTPGDF--HYLDGASVSYTNwyPGEPRgqgkeKC--VEMYTD-GTWNDRGCLQYRLAVCE-f<br>---------------------------------gclpdwssykghcyKVFKKVG---TWEDAEKFCVENSGHLASIDSKEEADFVTKLASQtLTKFVYdawIGLRDESKTQQCspQWTDGSSVVYEN--VDEPT-----KCfgLDVHTEyRTWTDLPCGEKNPFICKs-<br><br>Proposed: aliSize=113 (resi) RMSD=1.79 (Å)<br>deelqtelyeikhqilqTM-gvLslqgSMLSVGDKVFSTNGQSVNFDTIKEMCTRAGGNIAVPRTPEENEAIASIAK-KY--N-NYVYLGMIEDQ----tpGDFHYLDGASVSYTNWYpgeprgqGKEKCVEMYT--D-GTWNDRGCLQYRLAVCEF<br>-----------------GCl--P----DWSSYKGHCYKVFKKVGTWEDAEKFCVENSGHLASIDSKEEADFVTKLASqTLtkFvYDAWIGLRDESktqq--CSPQWTDGSSVVYENVD-------EPTKCFGLDVhtEyRTWTDLPCGEKNPFICKS

1020	1r13A (145)<br>1j34B (123)	M	17.07	TM-align: aliSize=101 (resi) RMSD=2.43 (Å)<br>deelqtelyeikhqiLqtMgVLslqgSMLSVGDKVFSTNGQSVNFDTIKEMCTRA-G-GNIAVPRTPEENEAIASIAKK-Y-NNYVYLGMI-ED-QTPGdfhyldgasvsytnwypgeprg-------------QGKEKCVEMYT-DGTWNDRGCLQYRLAVCEF--<br>---------------D--C-PS----DWSSYEGHCYKPFSEPKNWADAENFCTQQhAgGHLVSFQSSEEADFVVKLAFQtFgHSIFWMGLSnVWnQCNW----------------------qwsnaamlrykawAEESYCVYFKStNNKWRSRACRMMAQFVCEFqa<br><br>SARST: aliSize=109 (resi) RMSD=10.71 (Å)<br>deelqtelyeikhqilqtmgvlslqg----SMLSVGDKVFSTNGQSVNFDTIKEMCTRA--GGNIAVPRTPEENEAIASIAKKYNNYVylgMIEDQTPG-----DFHYLDGASVSYTNWYPGEPRGqgkekcVEMYTDGTWNDRGCLQYRLAVCE---f<br>--------------------------dcpsDWSSYEGHCYKPFSEPKNWADAENFCTQQhaGGHLVSFQSSEEADFVVKLAFQTFGHS---IFWMGLSNvwnqcNWQWSNAAMLRYKAWAEESYCV------YFKSTNNKWRSRACRMMAQFVCEfqa-<br><br>BLAST: aliSize=48 (resi) iden=19.51% (24/123) simi=39.02% (48/123)<br>deelqtelyeikhqilqtmgvlslqgsmlsvgdkvfstngqsv---------------------NFDTIKEMCTR--AGGNIAVPRTPEENEAIASIA-KKYNNYVY-LGMIEDQTPGDFHYLDGASVSYTNWypGEprgqgKEKCVEMY-TDGTWNDRGCLQYRLAVCEF--<br>---dcpsdwssyeghcykpfsepkNWADAENFCTQqhAGGHLVSFQSSEEADFVVKLAfQTFGHSIFwMGLSNVWNQCNWQWSNAAMLRYKAW--AE-----ESYCVYFKsTNNKWRSRACRMMAQFVCEFqa<br><br>Proposed: aliSize=114 (resi) RMSD=1.88 (Å)<br>deelqtelyeikhqIlqtM-gvLslqgSMLSVGDKVFSTNGQSVNFDTIKEMCTRA-G-GNIAVPRTPEENEAIASIAKK-Y-NNYVYLGMI-EDQTPgDFHYLDGASVSYTNWYPGeprgqgkEKCVEMYT-DGTWNDRGCLQYRLAVCEF--<br>-------------D---Cp--S----DWSSYEGHCYKPFSEPKNWADAENFCTQQhAgGHLVSFQSSEEADFVVKLAFQtFgHSIFWMGLSnVWNQC-NWQWSNAAMLRYKAWAEE-------SYCVYFKStNNKWRSRACRMMAQFVCEFqa
1021	1r13A (145)<br>1jwiB (123)	M	15.45	TM-align: aliSize=100 (resi) RMSD=2.52 (Å)<br>deelqtelyeikhqIlqtMgVLslqgSMLSVGDKVFSTNGQSVNFDTIKEMCTRAG--GNIAVPRTPEENEAIASIAK--KYNNYVYLGMIED--QTPGdfhyldgasvsytnwypgeprgq-------------G-KEKCVEMYTD-GTWNDRGCLQYRLAVCEF--<br>-------------G---C-LP----DWSSYKGHCYKVFKVEKTWADAEKFCKELVngGHLMSVNSREEGEFISKLALekMRIVLVWIGLSHFwrICPL-----------------------rwtdgarldyralSdEPICFVAESFhNKWIQWTCNRKKSFVCKYrv<br><br>SARST: aliSize=111 (resi) RMSD=9.40 (Å)<br>deelqtelyeikhqilqtmgvlslqgs-----MLSVGDKVFSTNGQSVNFDTIKEMCTRA--GGNIAVPRTPEENEAIASIAKK-YNN-YVYLGMIEDQTPGDFHYLDGASVSYTNWYpGEPRGqgkekCVEMYTDGTWNDRGCLQYRLAVCE---f<br>---------------------------gclpdWSSYKGHCYKVFKVEKTWADAEKFCKELvnGGHLMSVNSREEGEFISKLALEkMRIvLVWIGLSHFWRICPLRWTDGARLDYRALS-DEPIC-----FVAESFHNKWIQWTCNRKKSFVCKyrv-<br><br>BLAST: aliSize=32 (resi) iden=16.26% (20/123) simi=26.02% (32/123)<br>deelqtelyeikhqilqtmgvlslqgsmlsvgd-----------------KVFSTNGQSVNFdtIKEMCTraGGNIAVPRTPEENEAIASIA--KKYNNYVYLGMIEDQTPGDFHYLDGASVSY------------------------------------tnwypgeprgqgkekcvemytdgtwndrgc<br>---------------------------------gclpdwssykghcykvfKVEKTWADAEKF--CKELVN--GGHLMSVNSREEGEFISKLAleKMRIVLVWIGLSHFWRICPLRWTDGARLDYralsdepicfvaesfhnkwiqwtcnrkksfvckyrv------------------------------<br>lqyrlavcef<br>----------<br><br>Proposed: aliSize=114 (resi) RMSD=1.85 (Å)<br>deelqtelyeikhqIlqtM-gvLslqgSMLSVGDKVFSTNGQSVNFDTIKEMCTRAG--GNIAVPRTPEENEAIASIAK--KYNNYVYLGMIEDQ-tPGDFHYLDGASVSYTNWYPGepRgqgkeKCVEMYTD-GTWNDRGCLQYRLAVCEF--<br>-------------G---Cl--P----DWSSYKGHCYKVFKVEKTWADAEKFCKELVngGHLMSVNSREEGEFISKLALekMRIVLVWIGLSHFWr-ICPLRWTDGARLDYRALSDE--P-----ICFVAESFhNKWIQWTCNRKKSFVCKYrv
1022	1r13A (145)<br>1v4lB (125)	M	19.20	TM-align: aliSize=102 (resi) RMSD=2.54 (Å)<br>deelqtelyeiKhqILqtMgVLslqgSMLSVGDKVFSTNGQSVNFDTIKEMCTRA-G-GNIAVPRTPEENEAIASIAK-KYN-NYVYLGMI-E-DQTPGdfhyldgasvsytnwypgeprgq--------------GKEKCVEMYT-DGTWNDRGCLQYRLAVCEF--<br>-----------G--FC--C-PL----GWSSYDEHCYQVFQQKMNWEDAEKFCTQQhRgSHLVSFHSSEEVDFVVSKTSpILKhDFVWMGLSnVwNECAK-----------------------ewsdgtkldykawsGQSDCITSKTtDNQWLSMDCSSKRYVVCKFqa<br><br>SARST: aliSize=110 (resi) RMSD=12.54 (Å)<br>deelqtelyeikhqilqtmgvlslqgsm--------LSVGDKVFSTNGQSVNFDTIKEMCTRA--GGNIAVPRTPEENEAIASIAKKY--NNYVYLGMIEDQTPGDFHYLDGASVSYTNW--YPGEPRGQGKEKCVEMYTDGTWNDRGclQYRL-avcef<br>----------------------------gfccplgwSSYDEHCYQVFQQKMNWEDAEKFCTQQhrGSHLVSFHSSEEVDFVVSKTSPIlkHDFVWMGLSNVWNECAKEWSDGTKLDYKAWsgQSDCITSKTTDNQWLSMDCSSKRYVV--CKFQa-----<br><br>BLAST: aliSize=46 (resi) iden=19.20% (24/125) simi=36.80% (46/125)<br>deelqtelyeikhqilqtmgvlslqgsmlsvgdkvfstng--------------------QSVNFDTIKEMCTRA--GGNIAVPRTPEENEAIASIAKKY--NNYVYLGMIEDQTPGDFHYLDGASVSYTNWypgeprgQGKEKCV-EMYTDGTWNDRGCLQYRLAVCEF--<br>--gfccplgwssydehcyqvfqQKMNWEDAEKFCTQQhrGSHLVSFHSSEEVDFVVSKTSPIlkHDFVWMGLSNVWNECAKEWSDGTKLDYKAW-------SGQSDCItSKTTDNQWLSMDCSSKRYVVCKFqa<br><br>Proposed: aliSize=113 (resi) RMSD=2.01 (Å)<br>deelqtelyeiKhqILqtM-gvLslqgSMLSVGDKVFSTNGQSVNFDTIKEMCTRA-G-GNIAVPRTPEENEAIASIAK-KYN-NYVYLGMI----edqtPGDFHYLDGASVSYTNWYPgeprgqgKEKCVEMYT-DGTWNDRGCLQYRLAVCEF--<br>-----------G--FC--Cp--L----GWSSYDEHCYQVFQQKMNWEDAEKFCTQQhRgSHLVSFHSSEEVDFVVSKTSpILKhDFVWMGLSnvwn----ECAKEWSDGTKLDYKAWSG-------QSDCITSKTtDNQWLSMDCSSKRYVVCKFqa

1023	1r13A (145)<br>1iodA (129)	M	18.60	TM-align: aliSize=100 (resi) RMSD=2.39 (Å)<br>deelqtelyeikhqilqtMGVLslqgSMLSVGDKVFSTNGQSVNFDTIKEMCTRA-G-GNIAVPRTPEENEAIASIAKK-YNN---YVYLGMIEDQ----TPgdfhyldgasvsytnwypgeprg----------------QGKEKCVEMYT--D-GTWNDRGCLQYRLAVCEF<br>------------------DCSS----GWSSYEGHCYKVFKQSKTWADAESFCTKQvNgGHLVSIESSGEADFVGQLIAQkIKSakiHVWIGLRAQNkekqCS----------------------iewsdgssisyenwieEESKKCLGVHIetGfHKWENFYCEQQDPFVCEA<br><br>SARST: aliSize=110 (resi) RMSD=9.27 (Å)<br>deelqtelyeikhqilqtmgvlslqgs-----MLSVGDKVFSTNGQSVNFDTIKEMCTRA--GGNIAVPRTPEENEAIASIAKK----YNNYVYLGMIE---DQTPGdFHYLDGASVSYTNWypgeprGQGKEKCVEMY--TDG--TWNDRGCLQYRLAVCE-f<br>---------------------------dcssgWSSYEGHCYKVFKQSKTWADAESFCTKQvnGGHLVSIESSGEADFVGQLIAQkiksAKIHVWIGLRAqnkEKQCS-IEWSDGSSISYENW------IEEESKKCLGVhiETGfhKWENFYCEQQDPFVCEa-<br><br>BLAST: aliSize=56 (resi) iden=24.81% (32/129) simi=43.41% (56/129)<br>deelqtelyeikhqilqtmgvlslqgsmlsvgd--------------KVFStngQSVNFDTIKEMCTRA--GGNIAVPRTPEENEAIAS-IAKKYNN---YVYLGMIED--QTPGDFHYLDGASVSYTNWYPGEprgqgKEKCVEMYTD-G--TWNDRGCLQYRLAVCE-f<br>---------------------------------dcssgwssyeghcyKVFK---QSKTWADAESFCTKQvnGGHLVSIESSGEADFVGQlIAQKIKSakiHVWIGLRAQnkEKQCSIEWSDGSSISYENWIEEE-----SKKCLGVHIEtGfhKWENFYCEQQDPFVCEa-<br><br>Proposed: aliSize=116 (resi) RMSD=1.94 (Å)<br>deelqtelyeikhqiLqtM-gvLslqgSMLSVGDKVFSTNGQSVNFDTIKEMCTRAG--GNIAVPRTPEENEAIASIAKK-YNN---YVYLGMIEDQ---tPGDFHYLDGASVSYTNWYPGeprgqGKEKCVEMYT--D-GTWNDRGCLQYRLAVCEF<br>---------------D--Cs--S----GWSSYEGHCYKVFKQSKTWADAESFCTKQVngGHLVSIESSGEADFVGQLIAQkIKSakiHVWIGLRAQNkek-QCSIEWSDGSSISYENWIEE-----ESKKCLGVHIetGfHKWENFYCEQQDPFVCEA
1024	1r13A (145)<br>1j34A (129)	M	19.38	TM-align: aliSize=100 (resi) RMSD=2.16 (Å)<br>deelqtelyeikhqIlqtMgVLslqgSMLSVGDKVFSTNGQSVNFDTIKEMCTRA-G-GNIAVPRTPEENEAIASIAKK-Y--N-NYVYLGMIED--Q--TPgdfhyldgasvsytnwypgeprg----------------QGKEKCVEMYT--D-GTWNDRGCLQYRLAVCEF<br>--------------D---C-PS----GWSSYEGHCYKPFKLYKTWDDAERFCTEQaKgGHLVSIESAGEADFVAQLVTEnIqnTkSYVWIGLRVQgkEkqCS----------------------sewsdgssvsyenwieAESKTCLGLEKetGfRKWVNIYCGQQNPFVCEA<br><br>SARST: aliSize=111 (resi) RMSD=9.03 (Å)<br>deelqtelyeikhqilqtmgvlslqgs-----MLSVGDKVFSTNGQSVNFDTIKEMCTRA--GGNIAVPRTPEENEAIASIAKKY----NNYVYLGMIEDQTPGDF--HYLDGASVSYTNWypgeprGQGKE-KCVEMY-TDG--TWNDRGCLQYRLAVCE-f<br>---------------------------dcpsgWSSYEGHCYKPFKLYKTWDDAERFCTEQakGGHLVSIESAGEADFVAQLVTENiqntKSYVWIGLRVQGKEKQCssEWSDGSSVSYENW------IEAESkTCLGLEkETGfrKWVNIYCGQQNPFVCEa-<br><br>BLAST: aliSize=48 (resi) iden=22.48% (29/129) simi=37.21% (48/129)<br>deelqtelyeikhqilqtmgvlslqgsmlsvgdkvfstngqsvn----------------------FDTIKEMCTRA--GGNIAVPRTPEENEAIAS-IAKKYNN---YVYLGM-IEDQTPG-DFHYLDGASVSYTNWYPGEPRG-QGKEKCVEMytdGTWNDRGCLQYRLAVCE-f<br>--dcpsgwssyeghcykpfklyktWDDAERFCTEQakGGHLVSIESAGEADFVAQlVTENIQNtksYVWIGLrVQGKEKQcSSEWSDGSSVSYENWIEAESKTcLGLEKETGF---RKWVNIYCGQQNPFVCEa-<br><br>Proposed: aliSize=115 (resi) RMSD=1.84 (Å)<br>deelqtelyeikhqIlqtM-gvLslqgSMLSVGDKVFSTNGQSVNFDTIKEMCTRA-G-GNIAVPRTPEENEAIASIAKK-YN---NYVYLGMIED----qtPGDFHYLDGASVSYTNWYPGeprgqGKEKCVEMYT--D-GTWNDRGCLQYRLAVCEF<br>--------------D---Cp--S----GWSSYEGHCYKPFKLYKTWDDAERFCTEQaKgGHLVSIESAGEADFVAQLVTEnIQntkSYVWIGLRVQgkek--QCSSEWSDGSSVSYENWIEA-----ESKTCLGLEKetGfRKWVNIYCGQQNPFVCEA
1025	1tdqB (126)<br>1oz7B (123)	M	29.27	TM-align: aliSize=102 (resi) RMSD=2.17 (Å)<br>eQCEEGWTKFQGHCYRHFPDRETWVDAERRCREQ-Q-SHLSSIVTPEEQEFVNK-NA----QDYQWIGLNDRTIEGDfrwsdghslqfekwrpnqpdnf-------------FATGEDCVVMIWHERgEWNDVPCNYQLPFTCKKG-<br>-NCLPDWSVYEGYCYKVFKERMNWADAEKFCTKQhKdGHLVSFRNSKEVDFVISlAFpmlkNDLVWIGLTDYWRDCN----------------------wewsdgaqldykaWDNERHCFIYKNTDN-QWTRRDCTWTFSFVCKCPa<br><br>SARST: aliSize=110 (resi) RMSD=9.62 (Å)<br>eqc--EEGWTKFQGHCYRHFPDRETWVDAERRCRE--QQSHLSSIVTPEEQEFVNKN-----AQDYQWIGLNDRTIEGDFRWSDGHSLQFEKWR--PNQPdnffatgedcvVMIWHeRGEWNDVPCNYQLPFTCKK--g<br>---ncLPDWSVYEGYCYKVFKERMNWADAEKFCTKqhKDGHLVSFRNSKEVDFVISLafpmlKNDLVWIGLTDYWRDCNWEWSDGAQLDYKAWDneRHCF-----------IYKNT-DNQWTRRDCTWTFSFVCKCpa-<br><br>BLAST: aliSize=58 (resi) iden=32.52% (40/123) simi=47.15% (58/123)<br>eq-CEEGWTKFQGHCYRHFPDRETWVDAERRCREQQS--HLSSIVTPEEQEFVNKNA-----QDYQWIGLNDRTIEGDFRWSDGHSLQFEKWrPNQPDNFFATGEDcvvmiwherGEWNDVPCNYQLPFTCK---kg<br>--nCLPDWSVYEGYCYKVFKERMNWADAEKFCTKQHKdgHLVSFRNSKEVDFVISLAfpmlkNDLVWIGLTDYWRDCNWEWSDGAQLDYKAW-DNERHCFIYKNTD---------NQWTRRDCTWTFSFVCKcpa--<br><br>Proposed: aliSize=112 (resi) RMSD=1.36 (Å)<br>eQCEEGWTKFQGHCYRHFPDRETWVDAERRCRE-QQ-SHLSSIVTPEEQEFVNK-NA----QDYQWIGLNDR-tieG-D-FRWSDGHSLQFEKWrpnqpdnffATGEDCVVMIWHERgEWNDVPCNYQLPFTCKKG-<br>-NCLPDWSVYEGYCYKVFKERMNWADAEKFCTKqHKdGHLVSFRNSKEVDFVISlAFpmlkNDLVWIGLTDYw---RdCnWEWSDGAQLDYKAW---------DNERHCFIYKNTDN-QWTRRDCTWTFSFVCKCPa

1026	1tdqB (126)<br>1ukmB (124)	M	32.26	TM-align: aliSize=101 (resi) RMSD=2.07 (Å)<br>eqCEEGWTKFQGHCYRHFPDRETWVDAERRCREQ-Q-SHLSSIVTPEEQEFVNKNAQ-----DYQWIGLNDRTIEGDfrwsdghslqfekwrpnqpdnf-------------FATGeDCVVMIWH-ERGEWNDVPCNYQLPFTCKKG-<br>--CPLGWSSFDQHCYKVFEPVKNWTEAEEICMQQhKgSRLASIHSSEEEAFVSKLASkalkfTSMWIGLNNPWKDCK---------------------wewsdnarfdykawKRRP-YCTVMVVKpDRIFWFTRGCEKSVSFVCKFLt<br><br>SARST: aliSize=114 (resi) RMSD=9.67 (Å)<br>eqc-EEGWTKFQGHCYRHFPDRETWVDAERRCREQ--QSHLSSIVTPEEQE--FVNKNAQD---YQWIGLNDRTIEGDFRWSDGHSLQFEkwrpnqpDNFFATGEDCVVMIWHErGEWNDVPCNYQLPFTCKK--g<br>---cPLGWSSFDQHCYKVFEPVKNWTEAEEICMQQhkGSRLASIHSSEEEAfvSKLASKALkftSMWIGLNNPWKDCKWEWSDNARFDYK-------AWKRRPYCTVMVVKPDR-IFWFTRGCEKSVSFVCKFlt-<br><br>BLAST: aliSize=63 (resi) iden=33.06% (41/124) simi=50.81% (63/124)<br>eqCEEGWTKFQGHCYRHFPDRETWVDAERRCREQQ--SHLSSIVTPEEQEFVNKNAQ-----DYQWIGLNDRTIEGDFRWSDGHSLQFEKWRpNQPdnfFATgedcVVMIWHERGEWNDVPCNYQLPFTCK---kg<br>--CPLGWSSFDQHCYKVFEPVKNWTEAEEICMQQHkgSRLASIHSSEEEAFVSKLASkalkfTSMWIGLNNPWKDCKWEWSDNARFDYKAWK-RRP---YCT----VMVVKPDRIFWFTRGCEKSVSFVCKflt--<br><br>Proposed: aliSize=111 (resi) RMSD=1.54 (Å)<br>eqCEEGWTKFQGHCYRHFPDRETWVDAERRCREQ-Q-SHLSSIVTPEEQEFVNKNAQ-----DYQWIGLND-rtiE--GDFRWSDGHSLqFEkWrpnQPDNF-fatgeDCVVMIWH-ERGEWNDVPCNYQLPFTCKKG-<br>--CPLGWSSFDQHCYKVFEPVKNWTEAEEICMQQhKgSRLASIHSSEEEAFVSKLASkalkfTSMWIGLNNp---WkdCKWEWSDNARF-DY-K---AWKRRp-----YCTVMVVKpDRIFWFTRGCEKSVSFVCKFLt
1027	1tdqB (126)<br>1ukmA (131)	M	30.95	TM-align: aliSize=102 (resi) RMSD=1.90 (Å)<br>eQCEEGWTKFQGHCYRHFPDRETWVDAERRCRE-QQ-SHLSSIVTPEEQEFVNKNAQ-D------YQWIGLNDRTI--EGDfrwsdghslqfekwrpnqpdn----------------FFaTgEDCVVMIWHER-GEWNDVPCNYQLPFTCKKG-<br>-DCPSDWTAYDQHCYLAIGEPQNWYEAERFCTEqAKdGHLVSIQSREEGNFVAQLVSgFmhrseiYVWIGLRDRREeqQCN---------------------pewndgskiiyvnwkeGE-S-KMCQGLTKWTNfHDWNNINCEDLYPFVCKFSa<br><br>SARST: aliSize=115 (resi) RMSD=9.44 (Å)<br>eqc--EEGWTKFQGHCYRHFPDRETWVDAERRCREQ--QSHLSSIVTPEEQEFVNK----NAQD---YQWIGLNDRTIEGD--FRWSDGHSLQFEKWRPNQPdnffatgEDCVVMIWHERG-EWNDVPCNYQLPFTCKK--g<br>---dcPSDWTAYDQHCYLAIGEPQNWYEAERFCTEQakDGHLVSIQSREEGNFVAQlvsgFMHRseiYVWIGLRDRREEQQcnPEWNDGSKIIYVNWKEGES-------KMCQGLTKWTNFhDWNNINCEDLYPFVCKFsa-<br><br>BLAST: aliSize=61 (resi) iden=33.33% (42/126) simi=48.41% (61/126)<br>eq-CEEGWTKFQGHCYRHFPDRETWVDAERRCREQ--QSHLSSIVTPEEQEFVNK-------NAQDYQWIGLNDRTIEGDF--RWSDGHSLQFEKWRpnQPDNFFATGedcvVMIWHERGEWNDVPCNYQLPFTCK---kg<br>--dCPSDWTAYDQHCYLAIGEPQNWYEAERFCTEQakDGHLVSIQSREEGNFVAQlvsgfmhRSEIYVWIGLRDRREEQQCnpEWNDGSKIIYVNWK--EGESKMCQG----LTKWTNFHDWNNINCEDLYPFVCKfsa--<br><br>Proposed: aliSize=117 (resi) RMSD=1.38 (Å)<br>eQCEEGWTKFQGHCYRHFPDRETWVDAERRCRE-QQ-SHLSSIVTPEEQEFVNKNAQ-D------YQWIGLNDRTI--eGDF-RWSDGHSLQFEKWRPNQPdnffatgEDCVVMIWHER-GEWNDVPCNYQLPFTCKKG-<br>-DCPSDWTAYDQHCYLAIGEPQNWYEAERFCTEqAKdGHLVSIQSREEGNFVAQLVSgFmhrseiYVWIGLRDRREeq-QCNpEWNDGSKIIYVNWKEGES-------KMCQGLTKWTNfHDWNNINCEDLYPFVCKFSa
1028	1tdqB (126)<br>1uexA (125)	M	35.20	TM-align: aliSize=101 (resi) RMSD=1.91 (Å)<br>eQCEEGWTKFQGHCYRHFPDRETWVDAERRCREQQSHLSSIVTPEEQEFVNKNAQ--D-----YQWIGLNDrTI-E--GDfrwsdghslqfekwrpnqpdnf-------------FATGEDCVVMIWHER-GEWNDVPCNYQLPFTCKKg<br>-GCLPDWSSYKGHCYKVFKKVGTWEDAEKFCVENSGHLASIDSKEEADFVTKLASqtLtkfvyDAWIGLRD-ESkTqqCS----------------------pqwtdgssvvyenVDEPTKCFGLDVHTEyRTWTDLPCGEKNPFICKS-<br><br>SARST: aliSize=109 (resi) RMSD=8.93 (Å)<br>eqceeg-----WTKFQGHCYRHFPDRETWVDAERRCREQQSHLSSIVTPEEQEFVNKNA-------QDYQWIGLN--DRTIEGDFRWSDGHSLQFEKwrpnqpdnfFATGEDCVVMIWHERG-EWNDVPCNYQLPFTCK-kg<br>------gclpdWSSYKGHCYKVFKKVGTWEDAEKFCVENSGHLASIDSKEEADFVTKLAsqtltkfVYDAWIGLRdeSKTQQCSPQWTDGSSVVYEN---------VDEPTKCFGLDVHTEYrTWTDLPCGEKNPFICKs--<br><br>BLAST: aliSize=68 (resi) iden=39.20% (49/125) simi=54.40% (68/125)<br>eq-CEEGWTKFQGHCYRHFPDRETWVDAERRCREQQSHLSSIVTPEEQEFVNKNAQD------YQ-WIGLND--RTIEGDFRWSDGHSLQFEKwrPNQPDNFFatGEDcvvmIWHERGEWNDVPCNYQLPFTCK-kg<br>--gCLPDWSSYKGHCYKVFKKVGTWEDAEKFCVENSGHLASIDSKEEADFVTKLASQtltkfvYDaWIGLRDesKTQQCSPQWTDGSSVVYEN--VDEPTKCF--GLD----VHTEYRTWTDLPCGEKNPFICKs--<br><br>Proposed: aliSize=115 (resi) RMSD=1.77 (Å)<br>eQCEEGWTKFQGHCYRHFPDRETWVDAERRCREQQSHLSSIVTPEEQEFVNKNAQ--D-----YQWIGLNDRTIE-GDF-RWSDGHSLQFEkWrpnqpdnfFATGEDCVVMIWHER-GEWNDVPCNYQLPFTCKKg<br>-GCLPDWSSYKGHCYKVFKKVGTWEDAEKFCVENSGHLASIDSKEEADFVTKLASqtLtkfvyDAWIGLRDESKTqQCSpQWTDGSSVVYE-N---------VDEPTKCFGLDVHTEyRTWTDLPCGEKNPFICKS-

1029	1tdqB (126)<br>1jwiB (123)	M	32.52	TM-align: aliSize=102 (resi) RMSD=2.01 (Å)<br>eQCEEGWTKFQGHCYRHFPDRETWVDAERRCREQ-Q-SHLSSIVTPEEQEFVNKNAQ-----DYQWIGLNDR-TIEGDfrwsdghslqfekwrpnqpdnf------------FATGEDCVVMIWHeRGEWNDVPCNYQLPFTCKKG-<br>-GCLPDWSSYKGHCYKVFKVEKTWADAEKFCKELvNgGHLMSVNSREEGEFISKLALekmriVLVWIGLSHFwRICPL---------------------rwtdgarldyraLSDEPICFVAESF-HNKWIQWTCNRKKSFVCKYRv<br><br>SARST: aliSize=111 (resi) RMSD=9.24 (Å)<br>eq-CEEGWTKFQGHCYRHFPDRETWVDAERRCREQ--QSHLSSIVTPEEQEFVN----KNAQD-YQWIGL--NDRTIEgdFRWSDGHSLQFEkwrpNQPDnffatGEDCVVMIWHeRGEWNDVPCNYQLPFTCKK--g<br>--gCLPDWSSYKGHCYKVFKVEKTWADAEKFCKELvnGGHLMSVNSREEGEFISklalEKMRIvLVWIGLshFWRICP--LRWTDGARLDYR----ALSD-----EPICFVAESF-HNKWIQWTCNRKKSFVCKYrv-<br><br>BLAST: aliSize=63 (resi) iden=34.15% (42/123) simi=51.22% (63/123)<br>eq-CEEGWTKFQGHCYRHFPDRETWVDAERRCRE--QQSHLSSIVTPEEQEFVNKNAQDYQ-----WIGLNDRTIEGDFRWSDGHSLQFeKWRPNQPDNFFATGedcvvmiWHERgeWNDVPCNYQLPFTCK---kg<br>--gCLPDWSSYKGHCYKVFKVEKTWADAEKFCKElvNGGHLMSVNSREEGEFISKLALEKMrivlvWIGLSHFWRICPLRWTDGARLDY-RALSDEPICFVAES-------FHNK--WIQWTCNRKKSFVCKyrv--<br><br>Proposed: aliSize=113 (resi) RMSD=1.36 (Å)<br>eQCEEGWTKFQGHCYRHFPDRETWVDAERRCREQ-Q-SHLSSIVTPEEQEFVNKNAQ-----DYQWIGLNDR--tiEGDFRWSDGHSLQFEKWrpnqpdnffATGEDCVVMIWHeRGEWNDVPCNYQLPFTCKKG-<br>-GCLPDWSSYKGHCYKVFKVEKTWADAEKFCKELvNgGHLMSVNSREEGEFISKLALekmriVLVWIGLSHFwr--ICPLRWTDGARLDYRAL---------SDEPICFVAESF-HNKWIQWTCNRKKSFVCKYRv
1030	1tdqB (126)<br>1v4lB (125)	M	28.80	TM-align: aliSize=103 (resi) RMSD=2.16 (Å)<br>-EQCEEGWTKFQGHCYRHFPDRETWVDAERRCREQ-Q-SHLSSIVTPEEQEFVNKNAQ-----DYQWIGLNDRTIEGDfrwsdghslqfekwrpnqpdnf-------------FATGEDCVVMIWHeRGEWNDVPCNYQLPFTCKKG-<br>gFCCPLGWSSYDEHCYQVFQQKMNWEDAEKFCTQQhRgSHLVSFHSSEEVDFVVSKTSpilkhDFVWMGLSNVWNECA----------------------kewsdgtkldykaWSGQSDCITSKTT-DNQWLSMDCSSKRYVVCKFQa<br><br>SARST: aliSize=112 (resi) RMSD=9.85 (Å)<br>eqc----EEGWTKFQGHCYRHFPDRETWVDAERRCREQ--QSHLSSIVTPEEQEFVNKNA-----QDYQWIGLNDRTIEGDFRWSDGHSLQFEKWRpnqpdnffatGEDCVVMIWHERGEWNDVPCNYQLPFTCKK--g<br>---gfccPLGWSSYDEHCYQVFQQKMNWEDAEKFCTQQhrGSHLVSFHSSEEVDFVVSKTspilkHDFVWMGLSNVWNECAKEWSDGTKLDYKAWS----------GQSDCITSKTTDNQWLSMDCSSKRYVVCKFqa-<br><br>BLAST: aliSize=64 (resi) iden=32.00% (40/125) simi=51.20% (64/125)<br>eq---CEEGWTKFQGHCYRHFPDRETWVDAERRCREQQ--SHLSSIVTPEEQEF-VNKNA----QDYQWIGLNDRTIEGDFRWSDGHSLQFEKWrpnqpdnffaTGE-DCVVMIWHErGEWNDVPCNYQLPFTCK---kg<br>--gfcCPLGWSSYDEHCYQVFQQKMNWEDAEKFCTQQHrgSHLVSFHSSEEVDFvVSKTSpilkHDFVWMGLSNVWNECAKEWSDGTKLDYKAW----------SGQsDCITSKTTD-NQWLSMDCSSKRYVVCKfqa--<br><br>Proposed: aliSize=114 (resi) RMSD=1.59 (Å)<br>-EQCEEGWTKFQGHCYRHFPDRETWVDAERRCRE-QQ-SHLSSIVTPEEQEFVNKNAQ-----DYQWIGLNDRtiE--GDFRWSDGHSLqFEKWrpnQpdnffATGEDCVVMIWHeRGEWNDVPCNYQLPFTCKKG-<br>gFCCPLGWSSYDEHCYQVFQQKMNWEDAEKFCTQqHRgSHLVSFHSSEEVDFVVSKTSpilkhDFVWMGLSNV--WneCAKEWSDGTKL-DYKA---W-----SGQSDCITSKTT-DNQWLSMDCSSKRYVVCKFQa
1031	1tdqB (126)<br>1iodA (129)	M	31.75	TM-align: aliSize=102 (resi) RMSD=1.97 (Å)<br>eQCEEGWTKFQGHCYRHFPDRETWVDAERRCREQ-Q-SHLSSIVTPEEQEFVNKNAQ--D-----YQWIGLNDRTI--EGDfrwsdghslqfekwrpnqpdnf-------------FAT--GEDCVVMIWHER-GEWNDVPCNYQLPFTCKKg<br>-DCSSGWSSYEGHCYKVFKQSKTWADAESFCTKQvNgGHLVSIESSGEADFVGQLIAqkIksakiHVWIGLRAQNKekQCS----------------------iewsdgssisyenWIEeeSKKCLGVHIETGfHKWENFYCEQQDPFVCEA-<br><br>SARST: aliSize=113 (resi) RMSD=9.89 (Å)<br>eqc--EEGWTKFQGHCYRHFPDRETWVDAERRCREQ--QSHLSSIVTPEEQEFVNKNA-------QDYQWIGLNDR---TIEGDfRWSDGHSLQFEKWRpnqpdnFFATgEDCVVMIWHERG-EWNDVPCNYQLPFTCK-kg<br>---dcSSGWSSYEGHCYKVFKQSKTWADAESFCTKQvnGGHLVSIESSGEADFVGQLIaqkiksaKIHVWIGLRAQnkeKQCSI-EWSDGSSISYENWI------EEES-KKCLGVHIETGFhKWENFYCEQQDPFVCEa--<br><br>BLAST: aliSize=68 (resi) iden=37.30% (47/126) simi=53.97% (68/126)<br>eq-CEEGWTKFQGHCYRHFPDRETWVDAERRCREQQS--HLSSIVTPEEQEFVN-------KNAQDYQWIGLNDRTIEG--DFRWSDGHSLQFEKWRPNQpdnffatGEDCV-VMIwhERG--EWNDVPCNYQLPFTCK-kg<br>--dCSSGWSSYEGHCYKVFKQSKTWADAESFCTKQVNggHLVSIESSGEADFVGqliaqkiKSAKIHVWIGLRAQNKEKqcSIEWSDGSSISYENWIEEE-------SKKCLgVHI--ETGfhKWENFYCEQQDPFVCEa--<br><br>Proposed: aliSize=116 (resi) RMSD=1.43 (Å)<br>eQCEEGWTKFQGHCYRHFPDRETWVDAERRCREQ-Q-SHLSSIVTPEEQEFVNKNAQ--D-----YQWIGLNDRTI--eGD-FRWSDGHSLQFEKWRPNQpdnffatGEDCVVMIWHER-GEWNDVPCNYQLPFTCKKg<br>-DCSSGWSSYEGHCYKVFKQSKTWADAESFCTKQvNgGHLVSIESSGEADFVGQLIAqkIksakiHVWIGLRAQNKek-QCsIEWSDGSSISYENWIEEE-------SKKCLGVHIETGfHKWENFYCEQQDPFVCEA-

1032	1tdqB (126)<br>1uosA (133)	M	28.57	TM-align: aliSize=101 (resi) RMSD=1.57 (Å)<br>eQCEEGWTKFQGHCYRHFPDRETWVDAERRCREQQ--SHLSSIVTPEEQEFVNKNAQ-------DYQWIGLNDrTI---EGdfrwsdghslqfekwrpnqpdnf-------------FAT--GEDCVVMIWHER-GEWNDVPCNYQLPFTCKKG---<br>-HCPSDWYYYDQHCYRIFNEEMNWEDAEWFCTKQAkgAHLVSIKSAKEADFVAWMVTqnieesfSHVSIGLRV-QNkekQC----------------------stkwsdgssvsydnLLDlyITKCSLLKKETGfRKWFVASCIGKIPFVCKFPpqc<br><br>SARST: aliSize=115 (resi) RMSD=9.74 (Å)<br>eqc--EEGWTKFQGHCYRHFPDRETWVDAERRCREQ--QSHLSSIVTPEEQEFVNK-------NAQDYQWIGLNDRTIE--GDFRWSDGHSLQFEKWRPnqpdnffATGEDCVVMIWHERG-EWNDVPCNYQLPFTCKK----g<br>---hcPSDWYYYDQHCYRIFNEEMNWEDAEWFCTKQakGAHLVSIKSAKEADFVAWmvtqnieESFSHVSIGLRVQNKEkqCSTKWSDGSSVSYDNLLD-------LYITKCSLLKKETGFrKWFVASCIGKIPFVCKFppqc-<br><br>BLAST: aliSize=64 (resi) iden=33.33% (42/126) simi=50.79% (64/126)<br>eq-CEEGWTKFQGHCYRHFPDRETWVDAERRCREQQ--SHLSSIVTPEEQEFV----NKNAQD---YQWIGLNDRTIEGDF--RWSDGHSLQFEkwrpNQPDnFFATgeDCVvMIWHERG--EWNDVPCNYQLPFTCK-----kg<br>--hCPSDWYYYDQHCYRIFNEEMNWEDAEWFCTKQAkgAHLVSIKSAKEADFVawmvTQNIEEsfsHVSIGLRVQNKEKQCstKWSDGSSVSYD----NLLD-LYIT--KCS-LLKKETGfrKWFVASCIGKIPFVCKfppqc--<br><br>Proposed: aliSize=114 (resi) RMSD=1.55 (Å)<br>eQCEEGWTKFQGHCYRHFPDRETWVDAERRCREQQ--SHLSSIVTPEEQEFVNKNAQ-------DYQWIGLND-----rtieGDF-RWSDGHSLQFEkWrpnQPDNFfatGEDCVVMIWHER-GEWNDVPCNYQLPFTCKKG---<br>-HCPSDWYYYDQHCYRIFNEEMNWEDAEWFCTKQAkgAHLVSIKSAKEADFVAWMVTqnieesfSHVSIGLRVqnkek----QCStKWSDGSSVSYD-N---LLDLY---ITKCSLLKKETGfRKWFVASCIGKIPFVCKFPpqc
1033	1tdqB (126)<br>1j34A (129)	M	34.92	TM-align: aliSize=102 (resi) RMSD=1.86 (Å)<br>eQCEEGWTKFQGHCYRHFPDRETWVDAERRCREQ-Q-SHLSSIVTPEEQEFVNKNAQ--D-----YQWIGLNDRTIE-GDfrwsdghslqfekwrpnqpdnf-------------FAT--GEDCVVMIWHER-GEWNDVPCNYQLPFTCKKg<br>-DCPSGWSSYEGHCYKPFKLYKTWDDAERFCTEQaKgGHLVSIESAGEADFVAQLVTenIqntksYVWIGLRVQGKEkQC---------------------ssewsdgssvsyenWIEaeSKTCLGLEKETGfRKWVNIYCGQQNPFVCEA-<br><br>SARST: aliSize=114 (resi) RMSD=9.66 (Å)<br>eqc--EEGWTKFQGHCYRHFPDRETWVDAERRCREQ--QSHLSSIVTPEEQEFVNKN-------AQDYQWIGLNDRTIEGD--FRWSDGHSLQFEKWRpnqpdnFFATgEDCVVMIWHERG-EWNDVPCNYQLPFTCK-kg<br>---dcPSGWSSYEGHCYKPFKLYKTWDDAERFCTEQakGGHLVSIESAGEADFVAQLvteniqnTKSYVWIGLRVQGKEKQcsSEWSDGSSVSYENWI------EAES-KTCLGLEKETGFrKWVNIYCGQQNPFVCEa--<br><br>BLAST: aliSize=68 (resi) iden=38.10% (48/126) simi=53.97% (68/126)<br>eq-CEEGWTKFQGHCYRHFPDRETWVDAERRCREQQ--SHLSSIVTPEEQEFV------N-KNAQDYQWIGL--NDRTIEGDFRWSDGHSLQFEKWrpnqpdnFFATGEDCvVMIWHERG--EWNDVPCNYQLPFTCK-kg<br>--dCPSGWSSYEGHCYKPFKLYKTWDDAERFCTEQAkgGHLVSIESAGEADFVaqlvteNiQNTKSYVWIGLrvQGKEKQCSSEWSDGSSVSYENW-------IEAESKTC-LGLEKETGfrKWVNIYCGQQNPFVCEa--<br><br>Proposed: aliSize=117 (resi) RMSD=1.51 (Å)<br>eQCEEGWTKFQGHCYRHFPDRETWVDAERRCREQ-Q-SHLSSIVTPEEQEFVNKNAQ--D-----YQWIGLNDRTIE-GDF-RWSDGHSLQFEKWRPNQpdnffatGEDCVVMIWHERG-EWNDVPCNYQLPFTCKKg<br>-DCPSGWSSYEGHCYKPFKLYKTWDDAERFCTEQaKgGHLVSIESAGEADFVAQLVTenIqntksYVWIGLRVQGKEkQCSsEWSDGSSVSYENWIEAE-------SKTCLGLEKETGFrKWVNIYCGQQNPFVCEA-
1034	3bc7C (128)<br>1oz7B (123)	M	26.02	TM-align: aliSize=97 (resi) RMSD=2.09 (Å)<br>----GWKYFKGNFYYFSLIPKTWYSAEQFCVSR-N-SHLTSVTSESEQEFLYKT-AG---GLIYWIGLTKA-GM-EGdwswvddtpfnkvqsvrfwipgepnnag------------N-NEHCGNIKAPSlQAWNDAPCDKTFLFICKRPYvp<br>nclpDWSVYEGYCYKVFKERMNWADAEKFCTKQhKdGHLVSFRNSKEVDFVISLaFPmlkNDLVWIGLTDYwRDcNW--------------------------ewsdgaqldykawDnERHCFIYKNTD-NQWTRRDCTWTFSFVCKCPA--<br><br>SARST: aliSize=106 (resi) RMSD=9.76 (Å)<br>gw------KYFKGNFYYFSLIPKTWYSAEQFCVS--RNSHLTSVTSESEQEFLYKTAG----GLIYWIGLT--KAGMEgdWSWVDDTPFnkvqsvrfWIPGEPNNAGnnehcgnIKAPSLQA--WNDAPCDKTFLFICKRP-yvp<br>--nclpdwSVYEGYCYKVFKERMNWADAEKFCTKqhKDGHLVSFRNSKEVDFVISLAFpmlkNDLVWIGLTdyWRDCN--WEWSDGAQL--------DYKAWDNERH-------CFIYKNTDnqWTRRDCTWTFSFVCKCPa---<br><br>BLAST: aliSize=51 (resi) iden=29.27% (36/123) simi=41.46% (51/123)<br>g-----WKYFKGNFYYFSLIPKTWYSAEQFCVS--RNSHLTSVTSESEQEFLYKTAGGLI----YWIGLTKAGMEGDWSWVDDTPFNkvqsVRFWipgepnnaGNNEHCGNIKAPSLQaWNDAPCDKTFLFICKRP-yvp<br>-nclpdWSVYEGYCYKVFKERMNWADAEKFCTKqhKDGHLVSFRNSKEVDFVISLAFPMLkndlVWIGLTDYWRDCNWEWSDGAQLD----YKAW--------DNERHCFIYKNTDNQ-WTRRDCTWTFSFVCKCPa---<br><br>Proposed: aliSize=109 (resi) RMSD=1.75 (Å)<br>----GWKYFKGNFYYFSLIPKTWYSAEQFCVSR-N-SHLTSVTSESEQEFLYKT-AG---GLIYWIGLTKA---gmeGDWSWvdDTPFNKVQsVrFWIpGepnnagNEHCGNIKAP-SlqAWNDAPCDKTFLFICKRPYvp<br>nclpDWSVYEGYCYKVFKERMNWADAEKFCTKQhKdGHLVSFRNSKEVDFVISLaFPmlkNDLVWIGLTDYwrd---CNWEW--SDGAQLDY-K-AWD-N-------ERHCFIYKNTdN--QWTRRDCTWTFSFVCKCPA--

1035	3bc7C (128)<br>1ukmB (124)	M	25.81	TM-align: aliSize=98 (resi) RMSD=2.06 (Å)<br>---GWKYFKGNFYYFSLIPKTWYSAEQFCVSR-N-SHLTSVTSESEQEFLYKTAG----GLIYWIGLTK-AGMEGdwswvddtpfnkvqsvrfwipgepnna--------------GNNEHCGNIKAP--SLqAWNDAPCDKTFLFICKRPYvp<br>cplGWSSFDQHCYKVFEPVKNWTEAEEICMQQhKgSRLASIHSSEEEAFVSKLASkalkFTSMWIGLNNpWKDCK---------------------------wewsdnarfdykawKRRPYCTVMVVKpdRI-FWFTRGCEKSVSFVCKFLT--<br><br>SARST: aliSize=108 (resi) RMSD=9.28 (Å)<br>g----WKYFKGNFYYFSLIPKTWYSAEQFCVSR--NSHLTSVTSESEQEFLYKTAG----GLIYWIGLtkAGME-GD-WSWVDDTPfnkvqsvrfwipgePNNAGNNEHCGNIKAPSLQAW---NDAPCDKTFLFICKRP-yvp<br>-cplgWSSFDQHCYKVFEPVKNWTEAEEICMQQhkGSRLASIHSSEEEAFVSKLASkalkFTSMWIGL--NNPWkDCkWEWSDNAR-------------FDYKAWKRRPYCTVMVVKPDRifwFTRGCEKSVSFVCKFLt---<br><br>BLAST: aliSize=55 (resi) iden=29.03% (36/124) simi=44.35% (55/124)<br>---GWKYFKGNFY-YFSLIpKTWYSAEQFCVSRN--SHLTSVTSESEQEFLYKTAGGLI----YWIGLTKAGMEGDWSWVDDTPFNkvqsVRFWiPGEPnnagnneHCG-NIKAPSLQAWNDAPCDKTFLFICK---rpyvp<br>cplGWSSFDQHCYkVFEPV-KNWTEAEEICMQQHkgSRLASIHSSEEEAFVSKLASKALkftsMWIGLNNPWKDCKWEWSDNARFD----YKAW-KRRP-------YCTvMVVKPDRIFWFTRGCEKSVSFVCKflt-----<br><br>Proposed: aliSize=110 (resi) RMSD=1.60 (Å)<br>---GWKYFKGNFYYFSLIPKTWYSAEQFCVSR-N-SHLTSVTSESEQEFLYKTAG----GLIYWIGLTK---agmEGDWSWVDDTPFnKvqSVrfWipgePNnagNNEHCGNIKAP--SLqAWNDAPCDKTFLFICKRPYvp<br>cplGWSSFDQHCYKVFEPVKNWTEAEEICMQQhKgSRLASIHSSEEEAFVSKLASkalkFTSMWIGLNNpwk---DCKWEWSDNARF-D--YK--A----WK---RRPYCTVMVVKpdRI-FWFTRGCEKSVSFVCKFLT--
1036	3bc7C (128)<br>1ukmA (131)	M	22.66	TM-align: aliSize=99 (resi) RMSD=2.15 (Å)<br>----GWKYFKGNFYYFSLIPKTWYSAEQFCVS-RN-SHLTSVTSESEQEFLYKTAG--G---L-IYWIGLTKAG-M-EGDwswvddtpfnkvqsvrfwipgepnn---------------AGnNEHCGNIK--APSLqAWNDAPCDKTFLFICKRPYvp<br>dcpsDWTAYDQHCYLAIGEPQNWYEAERFCTEqAKdGHLVSIQSREEGNFVAQLVSgfMhrsEiYVWIGLRDRReEqQCN------------------------pewndgskiiyvnwkeGE-SKMCQGLTkwTNFH-DWNNINCEDLYPFVCKFSA--<br><br>SARST: aliSize=111 (resi) RMSD=9.56 (Å)<br>g-----WKYFKGNFYYFSLIPKTWYSAEQFCVSR--NSHLTSVTSESEQEFLYKTAG------GLIYWIGLTKAGMEG--DWSWVDDTPFnkvqSVRFWIPGEPnnagnnEHCGNikaPSLQA----WNDAPCDKTFLFICKRP-yvp<br>-dcpsdWTAYDQHCYLAIGEPQNWYEAERFCTEQakDGHLVSIQSREEGNFVAQLVSgfmhrsEIYVWIGLRDRREEQqcNPEWNDGSKI----IYVNWKEGES------KMCQG---LTKWTnfhdWNNINCEDLYPFVCKFSa---<br><br>BLAST: aliSize=51 (resi) iden=24.22% (31/128) simi=39.84% (51/128)<br>g-----WKYFKGNFYYFSLIPKTWYSAEQFCV--SRNSHLTSVTSESEQEFLYKTAGGLIYWigltkagmegdwswvddtpfnkvQSVRFWI------------P----GEP----N-NAGNNEHC-GNIKAPSLQAWNDAPCDKTFLFICK---rpyvp<br>-dcpsdWTAYDQHCYLAIGEPQNWYEAERFCTeqAKDGHLVSIQSREEGNFVAQLVSGFMHR----------------------SEIYVWIglrdrreeqqcnPewndGSKiiyvNwKEGESKMCqGLTKWTNFHDWNNINCEDLYPFVCKfsa-----<br><br>Proposed: aliSize=114 (resi) RMSD=1.89 (Å)<br>----GWKYFKGNFYYFSLIPKTWYSAEQFCVSR-N-SHLTSVTSESEQEFLYKTAG--G-L---IYWIGLTKAG---mEGDWSWVDDTPFnKvqSVrfWipgEPNnAgNNEHCGNIK--APSLqAWNDAPCDKTFLFICKRPYvp<br>dcpsDWTAYDQHCYLAIGEPQNWYEAERFCTEQaKdGHLVSIQSREEGNFVAQLVSgfMhRseiYVWIGLRDRReeq-QCNPEWNDGSKI-I--YV--N---WKE-G-ESKMCQGLTkwTNFH-DWNNINCEDLYPFVCKFSA--
1037	3bc7C (128)<br>1j34B (123)	M	24.39	TM-align: aliSize=98 (resi) RMSD=1.91 (Å)<br>----GWKYFKGNFYYFSLIPKTWYSAEQFCVSR-N-SHLTSVTSESEQEFLYKTAG----GLIYWIGLTKA-GMEGdwswvddtpfnkvqsvrfwipgepnna-------------GNNEHCGNIKAPSLqAWNDAPCDKTFLFICKRPYvp<br>dcpsDWSSYEGHCYKPFSEPKNWADAENFCTQQhAgGHLVSFQSSEEADFVVKLAFqtfgHSIFWMGLSNVwNQCN--------------------------wqwsnaamlrykawAEESYCVYFKSTNN-KWRSRACRMMAQFVCEFQA--<br><br>SARST: aliSize=109 (resi) RMSD=8.73 (Å)<br>gw------KYFKGNFYYFSLIPKTWYSAEQFCVSR--NSHLTSVTSESEQEFLYKTA----GGLIYWIGLTKAGMEGDWSWVDDTPfnkvqsvrfwipgePNNAGNNEHCGNIKAPSLQAW-NDAPCDKTFLFICKRP-yvp<br>--dcpsdwSSYEGHCYKPFSEPKNWADAENFCTQQhaGGHLVSFQSSEEADFVVKLAfqtfGHSIFWMGLSNVWNQCNWQWSNAAM-------------LRYKAWAEESYCVYFKSTNNKwRSRACRMMAQFVCEFQa---<br><br>BLAST: aliSize=54 (resi) iden=27.64% (34/123) simi=43.90% (54/123)<br>g-----WKYFKGNFYY-FSLiPKTWYSAEQFCVSRNS--HLTSVTSESEQEFLYKTA----GGLIYWIGLTKAGMEGDWSWVDDTPFNkvqsVRFWipgepnnaGNNEHCGNIKAPSlQAWNDAPCDKTFLFICK---rpyvp<br>-dcpsdWSSYEGHCYKpFSE-PKNWADAENFCTQQHAggHLVSFQSSEEADFVVKLAfqtfGHSIFWMGLSNVWNQCNWQWSNAAMLR----YKAW--------AEESYCVYFKSTN-NKWRSRACRMMAQFVCEfqa-----<br><br>Proposed: aliSize=111 (resi) RMSD=1.72 (Å)<br>----GWKYFKGNFYYFSLIPKTWYSAEQFCVSR-N-SHLTSVTSESEQEFLYKTAG----GLIYWIGLTKA--gmEGDWSWVDDTPFNkvqSVrfWipgEpnnaGNNEHCGNIKAPSLqAWNDAPCDKTFLFICKRPYvp<br>dcpsDWSSYEGHCYKPFSEPKNWADAENFCTQQhAgGHLVSFQSSEEADFVVKLAFqtfgHSIFWMGLSNVwn--QCNWQWSNAAMLR---YK--A---W----AEESYCVYFKSTNN-KWRSRACRMMAQFVCEFQA--

1038	3bc7C (128)<br>1jwiB (123)	M	27.64	TM-align: aliSize=99 (resi) RMSD=2.26 (Å)<br>----GWKYFKGNFYYFSLIPKTWYSAEQFCVSRN--SHLTSVTSESEQEFLYKTAG----GLIYWIGLTKA-GMEGDwswvddtpfnkvqsvrfwipgepnna------------GN-NEHCGNIKAPSLqAWNDAPCDKTFLFICKRPYvp<br>gclpDWSSYKGHCYKVFKVEKTWADAEKFCKELVngGHLMSVNSREEGEFISKLALekmrIVLVWIGLSHFwRICPL-------------------------rwtdgarldyraLSdEPICFVAESFHN-KWIQWTCNRKKSFVCKYRV--<br><br>SARST: aliSize=90 (resi) RMSD=11.12 (Å)<br>g-----WKYFKGNFYYFSLIPKTWYSAEQFCVS-------------------------RNshltsVTSeseqeflyktaggliYWIGLTKAGMEGDWSWVDDTPFNkvqsvrfwIPGEPNNAgnnehcGNIKAPSLQAW-NDAPCDKTFLFICKRP-yvp<br>-gclpdWSSYKGHCYKVFKVEKTWADAEKFCKElvngghlmsvnsreegefisklalekMR-----IVL---------------VWIGLSHFWRICPLRWTDGARLD--------YRALSDEP------ICFVAESFHNKwIQWTCNRKKSFVCKYRv---<br><br>BLAST: aliSize=36 (resi) iden=21.95% (27/123) simi=29.27% (36/123)<br>g-----WKYFKGNFYYFSLIPKTWYSAEQFC--VSRNSHLTSVTSESEQEFLYKTA----GGLIYWIGLTKAGMEGDWSWVD--dtpfnkvqsvrfwipgepnnagnnehcgnikapslqawndapcdktflfickrpyvp<br>-gclpdWSSYKGHCYKVFKVEKTWADAEKFCkeLVNGGHLMSVNSREEGEFISKLAlekmRIVLVWIGLSHFWRICPLRWTDgarldyralsdepicfvaesfhnkwiqwtcnrkksfvckyrv--<br><br>Proposed: aliSize=111 (resi) RMSD=1.80 (Å)<br>----GWKYFKGNFYYFSLIPKTWYSAEQFCVSR-N-SHLTSVTSESEQEFLYKTAG----GLIYWIGLTKA--gmEGDWSWVDdTpFNKvqSVrfWipgEPNNagnNEHCGNIKAPSLqAWNDAPCDKTFLFICKRPYvp<br>gclpDWSSYKGHCYKVFKVEKTWADAEKFCKELvNgGHLMSVNSREEGEFISKLALekmrIVLVWIGLSHFwr--ICPLRWTD-G-ARL--DY--R---ALSD---EPICFVAESFHN-KWIQWTCNRKKSFVCKYRV--
1039	3bc7C (128)<br>1v4lB (125)	M	22.40	TM-align: aliSize=98 (resi) RMSD=2.16 (Å)<br>------GWKYFKGNFYYFSLIPKTWYSAEQFCVSR-N-SHLTSVTSESEQEFLYKTAG----GLIYWIGLT-KAGMEGdwswvddtpfnkvqsvrfwipgepnna--------------GNNEHCGNIKAPSLqAWNDAPCDKTFLFICKRPYvp<br>gfccplGWSSYDEHCYQVFQQKMNWEDAEKFCTQQhRgSHLVSFHSSEEVDFVVSKTSpilkHDFVWMGLSnVWNECA---------------------------kewsdgtkldykawSGQSDCITSKTTDN-QWLSMDCSSKRYVVCKFQA--<br><br>SARST: aliSize=110 (resi) RMSD=9.79 (Å)<br>g-------WKYFKGNFYYFSLIPKTWYSAEQFCVSR--NSHLTSVTSESEQEFLY----KTAGGLIYWIGLTKAGMEGDWSWVDDTpfnkvqsvrfwIPGEPNNAGNNEHCGNIKapsLQAW-NDAPCDKTFLFICKRP-yvp<br>-gfccplgWSSYDEHCYQVFQQKMNWEDAEKFCTQQhrGSHLVSFHSSEEVDFVVsktsPILKHDFVWMGLSNVWNECAKEWSDGT-----------KLDYKAWSGQSDCITSKT---TDNQwLSMDCSSKRYVVCKFQa---<br><br>BLAST: aliSize=42 (resi) iden=21.60% (27/125) simi=33.60% (42/125)<br>------GWKYFKGNFYYFSLIPKTWYSAEQFCVS--RNSHLTSVTSESEQEFLyktaggliywIGLTKAGMEGDWSWVDdtpfnkVQSVrfWipgepnNAGNNEHCGNIKApSLQAWN-DAPC---------------------------dktflfickrpyvp<br>gfccplGWSSYDEHCYQVFQQKMNWEDAEKFCTQqhRGSHLVSFHSSEEVDFV----------VSKTSPILKHDFVWMG------LSNV--W------NECAKEWSDGTKL-DYKAWSgQSDCitskttdnqwlsmdcsskryvvckfqa-------------<br><br>Proposed: aliSize=109 (resi) RMSD=1.62 (Å)<br>------GWKYFKGNFYYFSLIPKTWYSAEQFCVSR-N-SHLTSVTSESEQEFLYKTAG----GLIYWIGLT----kagmEGDWSWVDDTPFNKvqSVrfWipgEPNnagnNEHCGNIKAPSLqAWNDAPCDKTFLFICKRPYvp<br>gfccplGWSSYDEHCYQVFQQKMNWEDAEKFCTQQhRgSHLVSFHSSEEVDFVVSKTSpilkHDFVWMGLSnvwn----ECAKEWSDGTKLD---YK--A---WSG----QSDCITSKTTDN-QWLSMDCSSKRYVVCKFQA--
1040	3bc7C (128)<br>1iodA (129)	M	22.66	TM-align: aliSize=97 (resi) RMSD=2.10 (Å)<br>----GWKYFKGNFYYFSLIPKTWYSAEQFCVSR-N-SHLTSVTSESEQEFLYKTAG---GL---IYWIGLTKAG-M-EGDwswvddtpfnkvqsvrfwipgepnna----------------GNNEHCGNIK--APSLqAWNDAPCDKTFLFICKRpyvp<br>dcssGWSSYEGHCYKVFKQSKTWADAESFCTKQvNgGHLVSIESSGEADFVGQLIAqkiKSakiHVWIGLRAQNkEkQCS--------------------------iewsdgssisyenwieEESKKCLGVHieTGFH-KWENFYCEQQDPFVCEA----<br><br>SARST: aliSize=110 (resi) RMSD=8.96 (Å)<br>g-----WKYFKGNFYYFSLIPKTWYSAEQFCVSR--NSHLTSVTSESEQEFLYKTA------GGLIYWIGLTKagMEGDWSWvdDTPFnkvqsVRFWIPGEPNNA-GNNEHCGNIkapSLQA----WNDAPCDKTFLFICK-rpyvp<br>-dcssgWSSYEGHCYKVFKQSKTWADAESFCTKQvnGGHLVSIESSGEADFVGQLIaqkiksAKIHVWIGLRA--QNKEKQC--SIEW-----SDGSSISYENWIeEESKKCLGV---HIETgfhkWENFYCEQQDPFVCEa-----<br><br>BLAST: aliSize=54 (resi) iden=28.12% (36/128) simi=42.19% (54/128)<br>----GWKYFKGNFYYFSLIPKTWYSAEQFCVSR--NSHLTSVTSESEQEFL-----YKTAGGLIY-WIGLTKAGMEGDWS--WVDDTPFnkvqSVRFWIPGEpnnagnNEHCGNIKAPS-LQAWNDAPCDKTFLFICK-rpyvp<br>dcssGWSSYEGHCYKVFKQSKTWADAESFCTKQvnGGHLVSIESSGEADFVgqliaQKIKSAKIHvWIGLRAQNKEKQCSieWSDGSSI----SYENWIEEE------SKKCLGVHIETgFHKWENFYCEQQDPFVCEa-----<br><br>Proposed: aliSize=112 (resi) RMSD=1.85 (Å)<br>----GWKYFKGNFYYFSLIPKTWYSAEQFCVSR-N-SHLTSVTSESEQEFLYKTAG---GL---IYWIGLTKAGmEGDW---SWVDDTPFNkvqSVrfWipgEPNnaGNNEHCGNIK--APSLqAWNDAPCDKTFLFICKRpyvp<br>dcssGWSSYEGHCYKVFKQSKTWADAESFCTKQvNgGHLVSIESSGEADFVGQLIAqkiKSakiHVWIGLRAQN-KEKQcsiEWSDGSSIS---YE--N---WIE--EESKKCLGVHieTGFH-KWENFYCEQQDPFVCEA----

1041	3bc7C (128)<br>1uosA (133)	M	20.31	TM-align: aliSize=102 (resi) RMSD=2.19 (Å)<br>----GWKYFKGNFYYFSLIPKTWYSAEQFCVSRN--SHLTSVTSESEQEFLYKTAG--G----LIYWIGLTKAGME-GDwswvddtpfnkvqsvrfwipgepnn---------------AGNNEHCGNIK--APSLqAWNDAPCDKTFLFICKRPYVP<br>hcpsDWYYYDQHCYRIFNEEMNWEDAEWFCTKQAkgAHLVSIKSAKEADFVAWMVTqnIeesfSHVSIGLRVQNKEkQC------------------------stkwsdgssvsydnlLDLYITKCSLLKkeTGFR-KWFVASCIGKIPFVCKFPPQC<br><br>SARST: aliSize=110 (resi) RMSD=9.30 (Å)<br>g-----WKYFKGNFYYFSLIPKTWYSAEQFCVS--RNSHLTSVTS-ESEQEFLYKTAGGLI-----YWIGLT--KAGMEGDWSWVDDtpfnkvqsvrfWIPGEPNNAGNN-EHCGNI------KAPslqawNDAPCDKTFLFICKRPYV-p<br>-hcpsdWYYYDQHCYRIFNEEMNWEDAEWFCTKqaKGAHLVSIKSaKEADFVAWMVTQNIEesfshVSIGLRvqNKEKQCSTKWSDG-----------SSVSYDNLLDLYiTKCSLLkketgfRKW-----FVASCIGKIPFVCKFPPQc-<br><br>BLAST: aliSize=52 (resi) iden=26.56% (34/128) simi=40.62% (52/128)<br>g-----WKYFKGNFYYFSLIPKTWYSAEQFCV--SRNSHLTSVTSESEQEFlyktagglIYWIgLTKaGMEGDWSWVDD--TPFNK-VQSVRFWIPGEPNNAGN-----NEHCGNIKAPS-LQAWNDAPCDKTFLFICKRP---yvp<br>-hcpsdWYYYDQHCYRIFNEEMNWEDAEWFCTkqAKGAHLVSIKSAKEADF--------VAWM-VTQ-NIEESFSHVSIglRVQNKeKQCSTKWSDGSSVSYDNlldlyITKCSLLKKETgFRKWFVASCIGKIPFVCKFPpqc---<br><br>Proposed: aliSize=113 (resi) RMSD=1.77 (Å)<br>----GWKYFKGNFYYFSLIPKTWYSAEQFCVSRN--SHLTSVTSESEQEFLYKTAG-------gLIYWIGLTKAGMeGDWSWvddtPF-----NKVqSVrfwipgEPNNaGNNEHCGNIK--APSLqAWNDAPCDKTFLFICKRPYVP<br>hcpsDWYYYDQHCYRIFNEEMNWEDAEWFCTKQAkgAHLVSIKSAKEADFVAWMVTqnieesf-SHVSIGLRVQNK-EKQCS----TKwsdgsSVS-YD------NLLD-LYITKCSLLKkeTGFR-KWFVASCIGKIPFVCKFPPQC
1042	3bc7C (128)<br>1j34A (129)	M	21.88	TM-align: aliSize=97 (resi) RMSD=2.07 (Å)<br>----GWKYFKGNFYYFSLIPKTWYSAEQFCVSR-N-SHLTSVTSESEQEFLYKTAG-----G-LIYWIGLTKAG-M-EGdwswvddtpfnkvqsvrfwipgepnn---------------AGNNEHCGNIK--APSLqAWNDAPCDKTFLFICKRpyvp<br>dcpsGWSSYEGHCYKPFKLYKTWDDAERFCTEQaKgGHLVSIESAGEADFVAQLVTeniqnTkSYVWIGLRVQGkEkQC-------------------------ssewsdgssvsyenwiEAESKTCLGLEkeTGFR-KWVNIYCGQQNPFVCEA----<br><br>SARST: aliSize=109 (resi) RMSD=9.17 (Å)<br>gw------KYFKGNFYYFSLIPKTWYSAEQFCVSR--NSHLTSVTSESEQEFLYKTA------GGLIYWIGLTKAGmegDWSWVDDTPfnkvqsVRFWIPGEPNNA-GNNEHCGNIkapSLQA----WNDAPCDKTFLFICK-rpyvp<br>--dcpsgwSSYEGHCYKPFKLYKTWDDAERFCTEQakGGHLVSIESAGEADFVAQLVteniqnTKSYVWIGLRVQG---KEKQCSSEW------SDGSSVSYENWIeAESKTCLGL---EKETgfrkWVNIYCGQQNPFVCEa-----<br><br>BLAST: aliSize=59 (resi) iden=32.03% (41/128) simi=46.09% (59/128)<br>----GWKYFKGNFYY-FSLIpKTWYSAEQFCV--SRNSHLTSVTSESEQEFLYKTAGGLI-----Y-WIGLTKAGMEGDWS--WVDDTPFnkvqSVRFWIPGEpnnagnNEHC-GNIKAPSLQAWNDAPCDKTFLFICK-rpyvp<br>dcpsGWSSYEGHCYKpFKLY-KTWDDAERFCTeqAKGGHLVSIESAGEADFVAQLVTENIqntksYvWIGLRVQGKEKQCSseWSDGSSV----SYENWIEAE------SKTClGLEKETGFRKWVNIYCGQQNPFVCEa-----<br><br>Proposed: aliSize=110 (resi) RMSD=1.79 (Å)<br>----GWKYFKGNFYYFSLIPKTWYSAEQFCVSR-N-SHLTSVTSESEQEFLYKTAG-----G-LIYWIGLTKAGmEGDWSWvddtPF-----NKVqsVrfWipgEPNNAgNNEHCGNIK--APSLqAWNDAPCDKTFLFICKRpyvp<br>dcpsGWSSYEGHCYKPFKLYKTWDDAERFCTEQaKgGHLVSIESAGEADFVAQLVTeniqnTkSYVWIGLRVQG-KEKQCS----SEwsdgsSVS--Y--E---NWIEA-ESKTCLGLEkeTGFR-KWVNIYCGQQNPFVCEA----
1043	1byfA (123)<br>1oz7B (123)	M	21.14	TM-align: aliSize=95 (resi) RMSD=2.70 (Å)<br>---------DYEILFSDETMNYADAGTYCQSRG--MALVSsaMRDSTMVKAILAF-TE-VKGHDYWVGADNLQDGAYNflwndgvslptdsdlwspnepsnpq-----------SWQ--LCVQIWSKYNLLDDVGCGGARRVICEKELd<br>nclpdwsvyeGYCYKVFKERMNWADAEKFCTKQHkdGHLVS--FRNSKEVDFVISLaFPmLKNDLVWIGLTDYWRDCNW------------------------ewsdgaqldykaWDNerHCFIYKNTDNQWTRRDCTWTFSFVCKCPA-<br><br>SARST: aliSize=109 (resi) RMSD=9.05 (Å)<br>d----------YEILFSDETMNYADAGTYCQSR--GMALVSSAMRDSTMVKAILAFTEVKGHDYWVGADNLQDGAYNfLWNDGVSLptdsdlwspNEPSNPQSWQLCvQIWSKYNLLDDVGCGGARRVICEKE-ld<br>-nclpdwsvyegYCYKVFKERMNWADAEKFCTKQhkDGHLVSFRNSKEVDFVISLAFPMLKNDLVWIGLTDYWRDCNW-EWSDGAQL---------DYKAWDNERHCF-IYKNTDNQWTRRDCTWTFSFVCKCPa--<br><br>BLAST: aliSize=45 (resi) iden=26.83% (33/123) simi=36.59% (45/123)<br>d------------YEIlFSdETMNYADAGTYC--QSRGMALVSsaMRDSTMVKAI--LAFTEVKGHDYWVG-ADNLQDgaYNFLWNDGVSLptDSDLWSpNE------------------------------psnpqswqlcvqiwskynllddvgcggarrvicekeld<br>-nclpdwsvyegycYKV-FK-ERMNWADAEKFCtkQHKDGHLVS--FRNSKEVDFVisLAFPMLKNDLVWIGlTDYWRD--CNWEWSDGAQL--DYKAWD-NErhcfiykntdnqwtrrdctwtfsfvckcpa-------------------------------------<br><br>Proposed: aliSize=105 (resi) RMSD=1.81 (Å)<br>---------DYEILFSDETMNYADAGTYCQSR-G-MALVSsaMRDSTMVKAILAF-TeV--KGHDYWVGADNLqdgAYN--FLWNDGVSLPtdSdlWspnEPSN-PqswqLCVQIWSKYNLLDDVGCGGARRVICEKELd<br>nclpdwsvyeGYCYKVFKERMNWADAEKFCTKQhKdGHLVS--FRNSKEVDFVISLaF-PmlKNDLVWIGLTDY---WRDcnWEWSDGAQLD--Y--K---AWDNeR----HCFIYKNTDNQWTRRDCTWTFSFVCKCPA-

1044	1byfA (123)<br>1ukmB (124)	M	12.20	TM-align: aliSize=96 (resi) RMSD=2.74 (Å)<br>---------DYEILFSDETMNYADAGTYCQSRG--MALVSsaMRDSTMVKAILAFTEV-K-GHDYWVGADNLQDGAYnflwndgvslptdsdlwspnepsn-------------PQSWQLCVQIWSK--YNLLDDVGCGGARRVICEKELd<br>cplgwssfdQHCYKVFEPVKNWTEAEEICMQQHkgSRLAS--IHSSEEEAFVSKLASKaLkFTSMWIGLNNPWKDCK------------------------wewsdnarfdykaWKRRPYCTVMVVKpdRIFWFTRGCEKSVSFVCKFLT-<br><br>SARST: aliSize=108 (resi) RMSD=9.94 (Å)<br>d----------YEILFSDETMNYADAGTYCQSR--GMALVSSAMrdsTMVKAILAFTEVKGH---DYWVGADNLqDGAYNFLWNDGVSLptdsdLWSPnePSNPQsWQLCVQIWSKYNLLDDVGCGGARRVICEKE-ld<br>-cplgwssfdqHCYKVFEPVKNWTEAEEICMQQhkGSRLASIHS---SEEEAFVSKLASKALkftSMWIGLNNP-WKDCKWEWSDNARF-----DYKA--WKRRP-YCTVMVVKPDRIFWFTRGCEKSVSFVCKFLt--<br><br>BLAST: aliSize=33 (resi) iden=15.45% (19/123) simi=26.83% (33/123)<br>d------------YEILfsDETMNYADAGTYC--QSRGMALVSSAMRDSTMVKAILAFTEVKGHDYWVGADN-LQDGAYNflWNDGVSLptDSDLW-----------------------------------spnepsnpqswqlcvqiwskynllddvgcggarrvicekeld<br>-cplgwssfdqhcYKVF--EPVKNWTEAEEICmqQHKGSRLASIHSSEEEAFVSKLASKALKFTSMWIGLNNpWKDCKWE--WSDNARF--DYKAWkrrpyctvmvvkpdrifwftrgceksvsfvckflt--<br><br>Proposed: aliSize=105 (resi) RMSD=1.96 (Å)<br>---------DYEILFSDETMNYADAGTYCQSR-G-MALVSsaMRDSTMVKAILAFTEV-K-GHDYWVGADN---lqdGAyNFLWNDGVSLPtdSdlWspnEPSnpqSWQLCVQIWSkY---NLLDDVGCGGARRVICEKELd<br>cplgwssfdQHCYKVFEPVKNWTEAEEICMQQhKgSRLAS--IHSSEEEAFVSKLASKaLkFTSMWIGLNNpwk---DC-KWEWSDNARFD--Y--K---AWK---RRPYCTVMVV-KpdrIFWFTRGCEKSVSFVCKFLT-
1045	1byfA (123)<br>1ukmA (131)	M	16.26	TM-align: aliSize=94 (resi) RMSD=2.20 (Å)<br>----------DYEILFSDETMNYADAGTYCQSR-G-MALVSsaMRDSTMVKAILAFTEVKG---H-DYWVGADNLQD--GAYnflwndgvslptdsdlwspnepsnpq-----------------SWQLCVQIWSKY-N-LLDDVGCGGARRVICEKELd<br>dcpsdwtaydQHCYLAIGEPQNWYEAERFCTEQaKdGHLVS--IQSREEGNFVAQLVSGFMhrsEiYVWIGLRDRREeqQCN--------------------------pewndgskiiyvnwkegESKMCQGLTKWTnFhDWNNINCEDLYPFVCKFSA-<br><br>SARST: aliSize=110 (resi) RMSD=9.08 (Å)<br>d----------YEILFSDETMNYADAGTYCQSR--GMALVSsaMRDSTMVKAILAFTEVKGHD----YWVGAD-NLQDGAYNFLWNDGVSLptDSDLWSPNepsnpqSWQLCVQIWSKYN--LLDDVGCGGARRVICEKE-ld<br>-dcpsdwtaydqHCYLAIGEPQNWYEAERFCTEQakDGHLVS--IQSREEGNFVAQLVSGFMHRseiyVWIGLRdRREEQQCNPEWNDGSKI--IYVNWKEG------ESKMCQGLTKWTNfhDWNNINCEDLYPFVCKFSa--<br><br>BLAST: aliSize=49 (resi) iden=20.33% (25/123) simi=39.84% (49/123)<br>dyeilfsd------------------ETMNYADAGTYC--QSRGMALVSSAMRDS-TMVKAILAFTEVKGHDY-WVGA-DNLQDGAYNFLWNDGVSLPTDSdlWSPNEPSNPQSwqlcVQIWSKYNLLDDVGCGGARRVICE---keld<br>--------dcpsdwtaydqhcylaigEPQNWYEAERFCteQAKDGHLVSIQSREEgNFVAQLVSGFMHRSEIYvWIGLrDRREEQQCNPEWNDGSKIIYVN--WKEGESKMCQG----LTKWTNFHDWNNINCEDLYPFVCKfsa----<br><br>Proposed: aliSize=110 (resi) RMSD=1.88 (Å)<br>----------DYEILFSDETMNYADAGTYCQSR-G-MALVSsaMRDSTMVKAILAFTEVKG---H-DYWVGADNLQ---dgAYNFLWNDGVSLPtDSDlWSPNEpsnpqsWQLCVQIWSKY-N-LLDDVGCGGARRVICEKELd<br>dcpsdwtaydQHCYLAIGEPQNWYEAERFCTEQaKdGHLVS--IQSREEGNFVAQLVSGFMhrsEiYVWIGLRDRReeq--QCNPEWNDGSKII-YVN-WKEGE------SKMCQGLTKWTnFhDWNNINCEDLYPFVCKFSA-
1046	1byfA (123)<br>1uexA (125)	M	14.63	TM-align: aliSize=94 (resi) RMSD=2.22 (Å)<br>---------D-YEILFSDETMNYADAGTYCQSRGMALVSsaMRDSTMVKAILAFTEV-K--G-HDYWVGADNLQDG--AYNflwndgvslptdsdlwspnepsn------------Pq-SWQLCVQIWSKY-N-LLDDVGCGGARRVICEKeld<br>gclpdwssyKgHCYKVFKKVGTWEDAEKFCVENSGHLAS--IDSKEEADFVTKLASQtLtkFvYDAWIGLRDESKTqqCSP-----------------------qwtdgssvvyenV-dEPTKCFGLDVHTeYrTWTDLPCGEKNPFICKS---<br><br>SARST: aliSize=104 (resi) RMSD=8.32 (Å)<br>d----------YEILFSDETMNYADAGTYCQSRGMALV--SsamrDSTMVKAILAFTEVK----GHDYWVGADNLQDGA-YNFLWNDGVSLptdsdLWSpnepsNPQSWQLCVQIWSKYN--LLDDVGCGGARRVICE-keld<br>-gclpdwssykgHCYKVFKKVGTWEDAEKFCVENSGHLAsiD----SKEEADFVTKLASQTltkfVYDAWIGLRDESKTQqCSPQWTDGSSV-----VYE-----NVDEPTKCFGLDVHTEyrTWTDLPCGEKNPFICKs----<br><br>BLAST: aliSize=52 (resi) iden=21.95% (27/123) simi=42.28% (52/123)<br>dyeilfsdetmn----------------------YADAGTYC-QSRG-MALVSSAmRDSTMVKAILAFTEVKG-HDYWVGA-DNLQDGAYNFLWNDGVSLPTDSdlwsPNEPSnpQSWQLCVQiwSKYNLLDDVGCGGARRVICE-keld<br>------------gclpdwssykghcykvfkkvgtWEDAEKFCvENSGhLASIDSK-EEADFVTKLASQTLTKFvYDAWIGLrDESKTQQCSPQWTDGSSVVYEN----VDEPT--KCFGLDVH--TEYRTWTDLPCGEKNPFICKs----<br><br>Proposed: aliSize=108 (resi) RMSD=1.95 (Å)<br>---------D-YEILFSDETMNYADAGTYCQSRGMALVSsaMRDSTMVKAILAFTEV-K--G-HDYWVGADNLQDG-AYNFLWNDgVSLPtdSdlWspnEPSNPqsWQLCVQIWSKY-N-LLDDVGCGGARRVICEKeld<br>gclpdwssyKgHCYKVFKKVGTWEDAEKFCVENSGHLAS--IDSKEEADFVTKLASQtLtkFvYDAWIGLRDESKTqQCSPQWTD-GSSV--V--Y---ENVDE--PTKCFGLDVHTeYrTWTDLPCGEKNPFICKS---

1047	1byfA (123)<br>1j34B (123)	M	18.70	TM-align: aliSize=95 (resi) RMSD=2.58 (Å)<br>----------DYEILFSDETMNYADAGTYCQSR-G-MALVSsaMRDSTMVKAILAFTEV--KGHDYWVGADNLQDGAYnflwndgvslptdsdlwspnepsn-------------Pq-SWQLCVQIWSKYNLLDDVGCGGARRVICEKELd<br>dcpsdwssyeGHCYKPFSEPKNWADAENFCTQQhAgGHLVS--FQSSEEADFVVKLAFQtfGHSIFWMGLSNVWNQCN----------------------wqwsnaamlrykaW-aEESYCVYFKSTNNKWRSRACRMMAQFVCEFQA-<br><br>SARST: aliSize=104 (resi) RMSD=8.38 (Å)<br>d-----------YEILFSDETMNYADAGTYCQSR--GMALVSsaMRDSTMVKAILAFTEVKGHDY--WVGADNLQDGAyNFLWNDGVSlptdsdlwspnePSNPqSW---QLCVQIWSKYNLLDDVGCGGARRVICEKE-ld<br>-dcpsdwssyegHCYKPFSEPKNWADAENFCTQQhaGGHLVS--FQSSEEADFVVKLAFQTFGHSifWMGLSNVWNQC-NWQWSNAAM------------LRYK-AWaeeSYCVYFKSTNNKWRSRACRMMAQFVCEFQa--<br><br>BLAST: aliSize=54 (resi) iden=29.27% (36/123) simi=43.90% (54/123)<br>dyeil----------------FSdETMNYADAGTYC--QSRGMALVS--SAMRDSTMVKaiLAFtEVKGHD-YWVGADNLQDGAyNFLWNDGVSLPTDSdlWSPnepsnpQSWqlCVQIWSKYNLLDDVGCGGARRVICE---keld<br>-----dcpsdwssyeghcykpFS-EPKNWADAENFCtqQHAGGHLVSfqSSEEADFVVK--LAF-QTFGHSiFWMGLSNVWNQC-NWQWSNAAMLRYKA--WAE------ESY--CVYFKSTNNKWRSRACRMMAQFVCEfqa----<br><br>Proposed: aliSize=107 (resi) RMSD=2.10 (Å)<br>----------DYEILFSDETMNYADAGTYCQSR-G-MALVSsaMRDSTMVKAILAFTEV--KGHDYWVGADNLqdGAY-NFLWNDGVSLPtdSdlWspnEPSNpqsWQLCVQIWSkYN-LLDDVGCGGARRVICEKELd<br>dcpsdwssyeGHCYKPFSEPKNWADAENFCTQQhAgGHLVS--FQSSEEADFVVKLAFQtfGHSIFWMGLSNV--WNQcNWQWSNAAMLR--Y--K---AWAE---ESYCVYFKS-TNnKWRSRACRMMAQFVCEFQA-
1048	1byfA (123)<br>1jwiB (123)	M	15.45	TM-align: aliSize=95 (resi) RMSD=2.44 (Å)<br>----------DYEILFSDETMNYADAGTYCQSRG--MALVSsaMRDSTMVKAILAFTEV--KGHDYWVGADNL-QDGAYnflwndgvslptdsdlwspnepsnp------------QS-WQLCVQIWSKYNLLDDVGCGGARRVICEKELd<br>gclpdwssykGHCYKVFKVEKTWADAEKFCKELVngGHLMS--VNSREEGEFISKLALEkmRIVLVWIGLSHFwRICPL---------------------rwtdgarldyraLSdEPICFVAESFHNKWIQWTCNRKKSFVCKYRV-<br><br>SARST: aliSize=106 (resi) RMSD=9.03 (Å)<br>d-----------YEILFSDETMNYADAGTYCQSR--GMALVSsaMRDSTMVKAILAFTEVK-GH-DYWVG-ADNLQDGayNFLWNDGVSLPtdsdlwSPNEPSNPqswqLCVQIWSKYNLLDDVGCGGARRVICEKE-ld<br>-gclpdwssykgHCYKVFKVEKTWADAEKFCKELvnGGHLMS--VNSREEGEFISKLALEKmRIvLVWIGlSHFWRIC--PLRWTDGARLD------YRALSDEP----ICFVAESFHNKWIQWTCNRKKSFVCKYRv--<br><br>BLAST: aliSize=33 (resi) iden=14.63% (18/123) simi=26.83% (33/123)<br>d-------------YEILFSDETmnYADAGTYCQS--RGMALVSSAMRDSTMVKAILAFTEVKGHDYWVGADNLQdGAYNFLWNDGVSL--------------------------------------ptdsdlwspnepsnpqswqlcvqiwskynllddvgcggarrvicekeld<br>-gclpdwssykghcYKVFKVEKT--WADAEKFCKElvNGGHLMSVNSREEGEFISKLALEKMRIVLVWIGLSHFW-RICPLRWTDGARLdyralsdepicfvaesfhnkwiqwtcnrkksfvckyrv---<br><br>Proposed: aliSize=106 (resi) RMSD=1.95 (Å)<br>----------DYEILFSDETMNYADAGTYCQSRG--MALVSsaMRDSTMVKAILAFTEV--KGHDYWVGADNLqdgAY--NFLWNDGVSLPtdSdlWspnEPSNpqsWQLCVQIWS-kYNLLDDVGCGGARRVICEKELd<br>gclpdwssykGHCYKVFKVEKTWADAEKFCKELVngGHLMS--VNSREEGEFISKLALEkmRIVLVWIGLSHF---WRicPLRWTDGARLD--Y--R---ALSD---EPICFVAESf-HNKWIQWTCNRKKSFVCKYRV-
1049	1byfA (123)<br>1v4lB (125)	M	17.07	TM-align: aliSize=95 (resi) RMSD=2.71 (Å)<br>------------DYEILFSDETMNYADAGTYCQSR-G-MALVSsaMRDSTMVKAILAFTEV--KGHDYWVGAD-NLQDGAYnflwndgvslptdsdlwspnepsnp-------------QSWQLCVQIWSKYNLLDDVGCGGARRVICEKELd<br>gfccplgwssydEHCYQVFQQKMNWEDAEKFCTQQhRgSHLVS--FHSSEEVDFVVSKTSPilKHDFVWMGLSnVWNECAK----------------------ewsdgtkldykawSGQSDCITSKTTDNQWLSMDCSSKRYVVCKFQA-<br><br>SARST: aliSize=104 (resi) RMSD=9.09 (Å)<br>d-------------YEILFSDETMNYADAGTYCQSR--GMALVS---SAmrdsTMVKAILAFTEVKG-HDYWVGADNLQDGAyNFLWNDGVSL-PTDSdlwspnepsnpQSWQLCVQIWSKYNLLDDVGCGGARRVICEKE-ld<br>-gfccplgwssydeHCYQVFQQKMNWEDAEKFCTQQhrGSHLVSfhsSE----EVDFVVSKTSPILKhDFVWMGLSNVWNEC-AKEWSDGTKLdYKAW-----------SGQSDCITSKTTDNQWLSMDCSSKRYVVCKFQa--<br><br>BLAST: aliSize=52 (resi) iden=26.83% (33/123) simi=42.28% (52/123)<br>d---------------YEILfsDETMNYADAGTYC--QSRGMALVSsaMRDSTMVKAILAFTE-VKGHDY-WVGADNLQDGAYNFlWNDGVSLptDSDLWSPNEPsnpqswqlCVQIWSKYNLLDDVGCGGARRVICE---keld<br>-gfccplgwssydehcYQVF--QQKMNWEDAEKFCtqQHRGSHLVS--FHSSEEVDFVVSKTSpILKHDFvWMGLSNVWNECAKE-WSDGTKL--DYKAWSGQSD--------CITSKTTDNQWLSMDCSSKRYVVCKfqa----<br><br>Proposed: aliSize=108 (resi) RMSD=2.46 (Å)<br>------------DYEILFSDETMNYADAGTYCQSRG--MALVSsaMRDSTMVKAILAFTEV--KGHDYWVGADNLqdGAYNflwndgvslPTDSDLWSPNEPSNPQSWQLCVQIWS-kYNLLDDVGCGGARRVICEKELd<br>gfccplgwssydEHCYQVFQQKMNWEDAEKFCTQQHrgSHLVS--FHSSEEVDFVVSKTSPilKHDFVWMGLSNV--WNEC---------AKEWSDGTKLDYKAWSGQSDCITSKTt-DNQWLSMDCSSKRYVVCKFQA-

1050	1byfA (123)<br>1iodA (129)	M	16.26	TM-align: aliSize=94 (resi) RMSD=2.54 (Å)<br>---------D-YEILFSDETMNYADAGTYCQSR-G-MALVSsaMRDSTMVKAILAFTEV-KGH---DYWVGADNLQDG-AYNflwndgvslptdsdlwspnepsn---------------Pq-SWQLCVQIWSKY-N-LLDDVGCGGARRVICEKeld<br>dcssgwssyEgHCYKVFKQSKTWADAESFCTKQvNgGHLVS--IESSGEADFVGQLIAQkIKSakiHVWIGLRAQNKEkQCS----------------------iewsdgssisyenwiE-eESKKCLGVHIETgFhKWENFYCEQQDPFVCEA---<br><br>SARST: aliSize=107 (resi) RMSD=8.97 (Å)<br>d-----------YEILFSDETMNYADAGTYCQSR--GMALVSsaMRDSTMVKAILAFTEVK----GHDYWVGADNLQDGAYN-FLWNDGVSLptdsdLWSpnepSNP-QSWQLCVQIWSKYN--LLDDVGCGGARRVICE-keld<br>-dcssgwssyegHCYKVFKQSKTWADAESFCTKQvnGGHLVS--IESSGEADFVGQLIAQKiksaKIHVWIGLRAQNKEKQCsIEWSDGSSI-----SYE----NWIeEESKKCLGVHIETGfhKWENFYCEQQDPFVCEa----<br><br>BLAST: aliSize=56 (resi) iden=25.20% (31/123) simi=45.53% (56/123)<br>d-------------YEILFSDETmnYADAGTYC--QSRGMALVS-SAMRDSTMVKAILA--FTEVKGHdYWVG--ADNlQDGAYNFLWNDGVSLPTDSdlWSPNEPSNpqswQLCVQIWSKYNLLDDVGCGGARRVICE-keld<br>-dcssgwssyeghcYKVFKQSKT--WADAESFCtkQVNGGHLVSiESSGEADFVGQLIAqkIKSAKIH-VWIGlrAQN-KEKQCSIEWSDGSSISYEN--WIEEESKK----CLGVHIETGFHKWENFYCEQQDPFVCEa----<br><br>Proposed: aliSize=108 (resi) RMSD=1.89 (Å)<br>---------D-YEILFSDETMNYADAGTYCQSR-G-MALVSsaMRDSTMVKAILAFTEV-KGH---DYWVGADNLQ---dgAYNFLWNDGVSLPtdSDLWSPNEpsnpqsWQLCVQIWSKY-N-LLDDVGCGGARRVICEKeld<br>dcssgwssyEgHCYKVFKQSKTWADAESFCTKQvNgGHLVS--IESSGEADFVGQLIAQkIKSakiHVWIGLRAQNkek--QCSIEWSDGSSIS--YENWIEEE------SKKCLGVHIETgFhKWENFYCEQQDPFVCEA---
1051	1byfA (123)<br>1uosA (133)	M	14.63	TM-align: aliSize=96 (resi) RMSD=2.46 (Å)<br>---------DYEILFSDETMNYADAGTYCQSRG--MALVSsaMRDSTMVKAILAFTEV-K-G--HDYWVGADNlQDG--AYNflwndgvslptdsdlwspneps---------------NPqsWQLCVQIWSKY-N-LLDDVGCGGARRVICEKELD-<br>hcpsdwyyydQHCYRIFNEEMNWEDAEWFCTKQAkgAHLVS--IKSAKEADFVAWMVTQnIeEsfSHVSIGLRV-QNKekQCS---------------------tkwsdgssvsydnlldLY--ITKCSLLKKETgFrKWFVASCIGKIPFVCKFPPQc<br><br>SARST: aliSize=110 (resi) RMSD=8.52 (Å)<br>d-----------YEILFSDETMNYADAGTYCQSR--GMALVSsaMRDSTMVKAILAFTE----VKGHDYWVGADNLQDGA-YNFLWNDGVSLptdsdlwspNEPSNPQSWQL-CVQIWSKYN--LLDDVGCGGARRVI-CEKEL-d<br>-hcpsdwyyydqHCYRIFNEEMNWEDAEWFCTKQakGAHLVS--IKSAKEADFVAWMVTqnieESFSHVSIGLRVQNKEKqCSTKWSDGSSV---------SYDNLLDLYITkCSLLKKETGfrKWFVASCIGKIPFVcKFPPQc-<br><br>BLAST: aliSize=52 (resi) iden=21.95% (27/123) simi=42.28% (52/123)<br>d-------------YEIlFSDEtMNYADAGTYC--QSRGMALVS-SAMRDSTMVKAILA--FTEVKGH-DYWVGADNlQDGAYNFLWNDGVSLPTDS--DLWSPN-----EPSNPQSWQL--CV-------------qiwskynllddvgcggarrvicekeld<br>-hcpsdwyyydqhcYRI-FNEE-MNWEDAEWFCtkQAKGAHLVSiKSAKEADFVAWMVTqnIEESFSHvSIGLRVQN-KEKQCSTKWSDGSSVSYDNllDLYITKcsllkKETGFRKWFVasCIgkipfvckfppqc--------------------------<br><br>Proposed: aliSize=109 (resi) RMSD=2.04 (Å)<br>---------DYEILFSDETMNYADAGTYCQSRG--MALVSsaMRDSTMVKAILAFTEV-K-G--HDYWVGADN-lqdgA----YNFLWNDGVSLPtDSD1WSPNEpsnpqsWQLCVQIWSKY-N-LLDDVGCGGARRVICEKELD-<br>hcpsdwyyydQHCYRIFNEEMNWEDAEWFCTKQAkgAHLVS--IKSAKEADFVAWMVTQnIeEsfSHVSIGLRVq----NkekqCSTKWSDGSSVS-YDN-LLDLY------ITKCSLLKKETgFrKWFVASCIGKIPFVCKFPPQc
1052	1byfA (123)<br>1j34A (129)	M	13.82	TM-align: aliSize=94 (resi) RMSD=2.50 (Å)<br>----------DYEILFSDETMNYADAGTYCQSR-G-MALVSsaMRDSTMVKAILAFTEV-K--G-HDYWVGADNLQDG-AYNflwndgvslptdsdlwspnepsn---------------Pq-SWQLCVQIWSKY-N-LLDDVGCGGARRVICEKeld<br>dcpsgwssyeGHCYKPFKLYKTWDDAERFCTEQaKgGHLVS--IESAGEADFVAQLVTEnIqnTkSYVWIGLRVQGKEkQCS----------------------sewsdgssvsyenwiE-aESKTCLGLEKETgFrKWVNIYCGQQNPFVCEA---<br><br>SARST: aliSize=105 (resi) RMSD=9.00 (Å)<br>d-----------YEILFSDETMNYADAGTYCQSR--GMALVSsaMRDSTMVKAILAFTEV----KGHDYWVGA---DNLQdgAYNFLWNDGvslptdsdlWSPNEPSNP-QSWQLCVQIWSKYN--LLDDVGCGGARRVICE-keld<br>-dcpsgwssyegHCYKPFKLYKTWDDAERFCTEQakGGHLVS--IESAGEADFVAQLVTEniqnTKSYVWIGLrvqGKEK--QCSSEWSDG---------SSVSYENWIeAESKTCLGLEKETGfrKWVNIYCGQQNPFVCEa----<br><br>BLAST: aliSize=47 (resi) iden=19.51% (24/123) simi=38.21% (47/123)<br>dyeilfsdetmnya------------------------DAGTYC--QSRGMALVS-SAMRDSTMVKAILAFTEVKGHDY-WVGAdNLQ--DGAYNFLWNDGVSLPTDSdlWSPNEPSNpqswQLCVQIWSKYNLLDDVGCGGARRVICE-keld<br>--------------dcpsgwssyeghcykpfklyktwdDAERFCteQAKGGHLVSiESAGEADFVAQLVTENIQNTKSYvWIGL-RVQgkEKQCSSEWSDGSSVSYEN--WIEAESKT----CLGLEKETGFRKWVNIYCGQQNPFVCEa----<br><br>Proposed: aliSize=108 (resi) RMSD=2.01 (Å)<br>----------DYEILFSDETMNYADAGTYCQSR-G-MALVSsaMRDSTMVKAILAFTEV-K--G-HDYWVGADNLQ--dgAYN-FLWNDGVSLPtDSdlWspnEPSNPqsWQLCVQIWSKY-N-LLDDVGCGGARRVICEKeld<br>dcpsgwssyeGHCYKPFKLYKTWDDAERFCTEQaKgGHLVS--IESAGEADFVAQLVTEnIqnTkSYVWIGLRVQGke--KQCsSEWSDGSSVS-YE--N---WIEAE--SKTCLGLEKETgFrKWVNIYCGQQNPFVCEA---

1053	1kza1 (115)<br>1oz7B (123)	M	19.13	TM-align: aliSize=91 (resi) RMSD=2.05 (Å)<br>-------NVGKKYFMSSVRRMPLNRAKALCSE-LQ-GTVATPRNAEENRAIQN-VA----KDVAFLGITDQRTEn-Vfedltgnrvrytnwnegepnnvg--------------S-GENCVVLLTN-GKWNDVPCSDSFLVVCEFS-<br>nclpdwsVYEGYCYKVFKERMNWADAEKFCTKqHKdGHLVSFRNSKEVDFVISlAFpmlkNDLVWIGLTDYWRD-cN-----------------------wewsdgaqldykawDnERHCFIYKNTdNQWTRRDCTWTFSFVCKCPa<br><br>SARST: aliSize=84 (resi) RMSD=5.85 (Å)<br>n--------VGKKYFMSSVRRMPLNRAKALCSEL--QGTVATPRNAEENRAIQNVAK-----DVAFLGITD----QRT----------------ENVFED--LTGnrvrytnwnegepnnvgsgencvvlltngKWNDVPCSDSFLVVCEF--s<br>-nclpdwsvYEGYCYKVFKERMNWADAEKFCTKQhkDGHLVSFRNSKEVDFVISLAFpmlknDLVWIGLTDywrdCNWewsdgaqldykawdneRHCFIYknTDN-----------------------------QWTRRDCTWTFSFVCKCpa-<br><br>BLAST: aliSize=50 (resi) iden=21.74% (25/115) simi=43.48% (50/115)<br>nvgkkyfmssvr-------------------RMPLNRAKALCSELQ--GTVATPRNAEE-----NRAIQNVAKDVAFLGITDQRTENVFEDLTGNRVRYTNWNegepNNvgsgENCVVLL-TNGKWNDVPCSDSFLVVCE---fs<br>------------nclpdwsvyegycykvfkeRMNWADAEKFCTKQHkdGHLVSFRNSKEvdfviSLAFPMLKNDLVWIGLTDYWRDCNWEWSDGAQLDYKAWD----NE----RHCFIYKnTDNQWTRRDCTWTFSFVCKcpa--<br><br>Proposed: aliSize=106 (resi) RMSD=1.68 (Å)<br>-------NVGKKYFMSSVRRMPLNRAKALCSE-LQ-GTVATPRNAEENRAIQN-VA----KDVAFLGITDQRTeNV-FEDLTGNRVRYTNWNEgepnnvgsGENCVVLLTN-GKWNDVPCSDSFLVVCEFS-<br>nclpdwsVYEGYCYKVFKERMNWADAEKFCTKqHKdGHLVSFRNSKEVDFVISlAFpmlkNDLVWIGLTDYWR-DCnWEWSDGAQLDYKAWDN--------ERHCFIYKNTdNQWTRRDCTWTFSFVCKCPa
1054	1kza1 (115)<br>1ukmB (124)	M	16.52	TM-align: aliSize=93 (resi) RMSD=2.28 (Å)<br>------NVGKKYFMSSVRRMPLNRAKALCSEL-Q-GTVATPRNAEENRAIQNVA---K--DVAFLGITDQRTENVfedltgnrvrytnwnegepnnv--------------GSGENCVVLLTN---GKWNDVPCSDSFLVVCEFS-<br>cplgwsSFDQHCYKVFEPVKNWTEAEEICMQQhKgSRLASIHSSEEEAFVSKLAskaLkfTSMWIGLNNPWKDCK----------------------wewsdnarfdykawKRRPYCTVMVVKpdrIFWFTRGCEKSVSFVCKFLt<br><br>SARST: aliSize=106 (resi) RMSD=9.81 (Å)<br>n-------VGKKYFMSSVRRMPLNRAKALCSEL--QGTVATPRNAEENRAIQN----VAKD-VAFLGITDQRtEN-VFEDLTGNRVRYTNwnegePNNVGsGENCVVLLTNG-KWNDVPCSDSFLVVCEF--s<br>-cplgwssFDQHCYKVFEPVKNWTEAEEICMQQhkGSRLASIHSSEEEAFVSKlaskALKFtSMWIGLNNPW-KDcKWEWSDNARFDYKA-----WKRRP-YCTVMVVKPDRiFWFTRGCEKSVSFVCKFlt-<br><br>BLAST: aliSize=43 (resi) iden=20.00% (23/115) simi=37.39% (43/115)<br>nvgkkyfmssvrrmplnr------------------------AKALCSEL-QGT-VATPRNAEENRAIQNVA-KDVAF----LGITDQRTENVFEDLTGNRVRYTNWNEgEPNnvgsgenCVVLLTNGK---WNDVPCSDSFLVVCEF--s<br>------------------cplgwssfdqhcykvfepvknwteAEEICMQQhKGSrLASIHSSEEEAFVSKLAsKALKFtsmwIGLNNPWKDCKWEWSDNARFDYKAWKR-RPY-------CTVMVVKPDrifWFTRGCEKSVSFVCKFlt-<br><br>Proposed: aliSize=106 (resi) RMSD=1.64 (Å)<br>------NVGKKYFMSSVRRMPLNRAKALCSE-LQ-GTVATPRNAEENRAIQNVA---K--DVAFLGIT-DQRTEnVFEDLTGNRVRYTNWNegepnnvgSGENCVVLLTN---GKWNDVPCSDSFLVVCEFS-<br>cplgwsSFDQHCYKVFEPVKNWTEAEEICMQqHKgSRLASIHSSEEEAFVSKLAskaLkfTSMWIGLNnPWKDC-KWEWSDNARFDYKAWK--------RRPYCTVMVVKpdrIFWFTRGCEKSVSFVCKFLt
1055	1kza1 (115)<br>1ukmA (131)	M	22.61	TM-align: aliSize=93 (resi) RMSD=1.88 (Å)<br>-------NVGKKYFMSSVRRMPLNRAKALCSE-LQ-GTVATPRNAEENRAIQNVA-KD------VAFLGITDQR-T--ENvfedltgnrvrytnwnegepnn-------------VG--SGENCVVLLT-N--GKWNDVPCSDSFLVVCEFS-<br>dcpsdwtAYDQHCYLAIGEPQNWYEAERFCTEqAKdGHLVSIQSREEGNFVAQLVsGFmhrseiYVWIGLRDRReEqqCN----------------------pewndgskiiyvnWKegESKMCQGLTKwTnfHDWNNINCEDLYPFVCKFSa<br><br>SARST: aliSize=106 (resi) RMSD=9.51 (Å)<br>n--------VGKKYFMSSVRRMPLNRAKALCSEL--QGTVATPRNAEENRAIQN--------VAKDvAFLGITDQRTENV--FEDLTGNRVRYTNWNEGepnnvgSGENCVVLL-TNG--KWNDVPCSDSFLVVCEF--s<br>-dcpsdwtaYDQHCYLAIGEPQNWYEAERFCTEQakDGHLVSIQSREEGNFVAQlvsgfmhrSEIY-VWIGLRDRREEQQcnPEWNDGSKIIYVNWKEG------ESKMCQGLTkWTNfhDWNNINCEDLYPFVCKFsa-<br><br>BLAST: aliSize=57 (resi) iden=29.57% (34/115) simi=49.57% (57/115)<br>nvgkkyfmssvrrm-------------------PLN--RAKALCSEL--QGTVATPRNAEE-NRAIQNVA-----KDV-AFLGITDQRTENVF--EDLTGNRVRYTNWNEGEpNNVGSGencVVLLTN-GKWNDVPCSDSFLVVCEFS-<br>--------------dcpsdwtaydqhcylaigePQNwyEAERFCTEQakDGHLVSIQSREEgNFVAQLVSgfmhrSEIyVWIGLRDRREEQQCnpEWNDGSKIIYVNWKEGE-SKMCQG---LTKWTNfHDWNNINCEDLYPFVCKFSa<br><br>Proposed: aliSize=107 (resi) RMSD=1.34 (Å)<br>-------NVGKKYFMSSVRRMPLNRAKALCSE-LQ-GTVATPRNAEENRAIQNVA-KD------VAFLGITDQR----teNVFEDLTGNRVRYTNWNEGepnnvgSGENCVVLLT-N--GKWNDVPCSDSFLVVCEFS-<br>dcpsdwtAYDQHCYLAIGEPQNWYEAERFCTEqAKdGHLVSIQSREEGNFVAQLVsGFmhrseiYVWIGLRDRReeqq--CNPEWNDGSKIIYVNWKEG------ESKMCQGLTKwTnfHDWNNINCEDLYPFVCKFSa

1056	1kza1<br>(115)<br><br>1uexA<br>(125)	M	19.13	TM-align: aliSize=92 (resi) RMSD=1.80 (Å)<br>-------NVGKKYFMSSVRRMPLNRAKALCSELQGTVATPRNAEENRAIQNVA---K----DVAFLGITDQRT---ENvfedltgnrvrytnwnegepnn------------VGSGENCVVLLT--N-GKWNDVPCSDSFLVVCEFs<br>gclpdwsSYKGHCYKVFKKVGTWEDAEKFCVENSGHLASIDSKEEADFVTKLAsqtLtkfvYDAWIGLRDESKtqqCS---------------------pqwtdgssvvyenVDEPTKCFGLDVhtEyRTWTDLPCGEKNPFICKS-<br><br>SARST: aliSize=101 (resi) RMSD=8.48 (Å)<br>n--------VGKKYFMSSVRRMPLNRAKALCSELQGTVATPRNAEENRAIQNV-------AKDVAFLGITdqrTENVFEDL-----TGNRVRYTNWnegepnnvGSGENCVVLLTNG---KWNDVPCSDSFLVVCE-fs<br>-gclpdwssYKGHCYKVFKKVGTWEDAEKFCVENSGHLASIDSKEEADFVTKLasqtltkFVYDAWIGLR---DESKTQQCspqwtDGSSVVYENV--------DEPTKCFGLDVHTeyrTWTDLPCGEKNPFICKs--<br><br>BLAST: aliSize=47 (resi) iden=25.22% (29/115) simi=40.87% (47/115)<br>nvgkkyfmssvrrmplnr------------------------AKALCSELQGTVATPRNAEE-----NRAIQNVAKDV--AFLGITDQ-RTENVFEDLT-GNRVRYTNWNEgePNNVgSGENcvVLLTNGKWNDVPCSDSFLVVCE-fs<br>-----------------gclpdwssykghcykvfkkvgtwedAEKFCVENSGHLASIDSKEEadfvtKLASQTLTKFVydAWIGLRDEsKTQQCSPQWTdGSSVVYENVDE--PTKC-FGLD--VHTEYRTWTDLPCGEKNPFICKs--<br><br>Proposed: aliSize=105 (resi) RMSD=1.53 (Å)<br>-------NVGKKYFMSSVRRMPLNRAKALCSELQGTVATPRNAEENRAIQNVA---K----DVAFLGITDQRT---eNVFEDLTGNRVRYTNWnegepnnvGSGENCVVLLT--N-GKWNDVPCSDSFLVVCEFs<br>gclpdwsSYKGHCYKVFKKVGTWEDAEKFCVENSGHLASIDSKEEADFVTKLAsqtLtkfvYDAWIGLRDESKtqq-CSPQWTDGSSVVYENV--------DEPTKCFGLDVhtEyRTWTDLPCGEKNPFICKS-
1057	1kza1<br>(115)<br><br>1j34B<br>(123)	M	17.39	TM-align: aliSize=93 (resi) RMSD=2.08 (Å)<br>-------NVGKKYFMSSVRRMPLNRAKALCSE-LQ-GTVATPRNAEENRAIQNVA---K--DVAFLGIT-DQRTENvfedltgnrvrytnwnegepnn------------VGSGENCVVLLT-NGKWNDVPCSDSFLVVCEFS-<br>dcpsdwsSYEGHCYKPFSEPKNWADAENFCTQqHAgGHLVSFQSSEEADFVVKLAfqtFghSIFWMGLSnVWNQCN---------------------wqwsnaamlrykaWAEESYCVYFKStNNKWRSRACRMMAQFVCEFQa<br><br>SARST: aliSize=106 (resi) RMSD=10.16 (Å)<br>n--------VGKKYFMSSVRRMPLNRAKALCSEL--QGTVATPRNA-----EENRAIQNVAKDVAFLGITDQRTENVFEDLTGNRVRYTNWNEgepnnvgSGENCVVLLTNGKWNDVPCSDSFLVVCEF--s<br>-dcpsdwssYEGHCYKPFSEPKNWADAENFCTQQhaGGHLVSFQSSeeadfVVKLAFQTFGHSIFWMGLSNVWNQCNWQWSNAAMLRYKAWAE-------ESYCVYFKSTNNKWRSRACRMMAQFVCEFqa-<br><br>BLAST: aliSize=44 (resi) iden=23.48% (27/115) simi=38.26% (44/115)<br>nvgkkyfmssvrrm------------------PLNRAKA--LCSELQ--GTVATPRNAEE-----NRAIQNVAKDVAFLGITDQRTENVFEDLTGNRVRYTNWNEgEpnnvgsgENCVVLL-TNGKWNDVPCSDSFLVVCEF--s<br>-------------dcpsdwssyeghcykpfsePKNWADAenFCTQQHagGHLVSFQSSEEadfvvKLAFQTFGHSIFWMGLSNVWNQCNWQWSNAAMLRYKAWAE-E-------SYCVYFKsTNNKWRSRACRMMAQFVCEFqa-<br><br>Proposed: aliSize=105 (resi) RMSD=1.68 (Å)<br>-------NVGKKYFMSSVRRMPLNRAKALCSE-LQ-GTVATPRNAEENRAIQNVA---K--DVAFLGITDQ--rtENVFEDLTGNRVRYTNWnegepnnvGSGENCVVLLT-NGKWNDVPCSDSFLVVCEFS-<br>dcpsdwsSYEGHCYKPFSEPKNWADAENFCTQqHAgGHLVSFQSSEEADFVVKLAfqtFghSIFWMGLSNVwn--QCNWQWSNAAMLRYKAW--------AEESYCVYFKStNNKWRSRACRMMAQFVCEFQa
1058	1kza1<br>(115)<br><br>1jwiB<br>(123)	M	16.52	TM-align: aliSize=93 (resi) RMSD=2.32 (Å)<br>-------NVGKKYFMSSVRRMPLNRAKALCSEL-Q-GTVATPRNAEENRAIQNVA-----KDVAFLGITDQ-RTENVfedltgnrvrytnwnegepnnv------------GS-GENCVVLLTN-GKWNDVPCSDSFLVVCEFS-<br>gclpdwsSYKGHCYKVFKVEKTWADAEKFCKELvNgGHLMSVNSREEGEFISKLAlekmrIVLVWIGLSHFwRICPL----------------------rwtdgarldyraLSdEPICFVAESFhNKWIQWTCNRKKSFVCKYRv<br><br>SARST: aliSize=83 (resi) RMSD=6.92 (Å)<br>n--------VGKKYFMSSVRRMPLNRAKALCSEL--QGTVATPRNAEENRAIQN------VAKDvAFLGIT-----------DQ------RTENVFED-----LTGnrvrytnwnegepnnvgsgencvvlltngKWNDVPCSDSFLVVCEF--s<br>-gclpdwssYKGHCYKVFKVEKTWADAEKFCKELvnGGHLMSVNSREEGEFISKlalekmRIVL-VWIGLShfwricplrwtDGarldyrALSDEPICfvaesFHN-----------------------------KWIQWTCNRKKSFVCKYrv-<br><br>BLAST: aliSize=40 (resi) iden=20.87% (24/115) simi=34.78% (40/115)<br>nvgkkyfmssvrrmplnr------------------------AKALCSELQ--GTVATPRNAEENRAIQNVAkdvaflgITDQRTENVFEDLTGN-RVRYTNWNEGEPNNVG--SGEN-CVVLLT-NGKWNDVPCSDSFLVVCEF--s<br>-----------------gclpdwssykghcykvfkvektwadAEKFCKELVngGHLMSVNSREEGEFISKLA-------LEKMRIVLVWIGLSHFwRICPLRWTDGARLDYRalSDEPiCFVAESfHNKWIQWTCNRKKSFVCKYrv-<br><br>Proposed: aliSize=105 (resi) RMSD=1.69 (Å)<br>-------NVGKKYFMSSVRRMPLNRAKALCSE-LQ-GTVATPRNAEENRAIQNVA-----KDVAFLGITDQ--rtENVFEDLTGNRVRYTNWNegEpnnvgsGENCVVLLTN-GKWNDVPCSDSFLVVCEFS-<br>gclpdwsSYKGHCYKVFKVEKTWADAEKFCKElVNgGHLMSVNSREEGEFISKLAlekmrIVLVWIGLSHFwr--ICPLRWTDGARLDYRALS--D------EPICFVAESFhNKWIQWTCNRKKSFVCKYRv

1059	1kza1 (115)<br>1v4lB (125)	M	17.39	TM-align: aliSize=92 (resi) RMSD=2.03 (Å)<br>---------NVGKKYFMSSVRRMPLNRAKALCSE-LQ-GTVATPRNAEENRAIQNVA---K--DVAFLGIT-DQRTENvfedltgnrvrytnwnegepnnv--------------GSGENCVVLL-TNGKWNDVPCSDSFLVVCEFS-<br>gfccplgwsSYDEHCYQVFQQKMNWEDAEKFCTQqHRgSHLVSFHSSEEVDFVVSKTspiLkhDFVWMGLSnVWNECA-----------------------kewsdgtkldykawSGQSDCITSKtTDNQWLSMDCSSKRYVVCKFQa<br><br>SARST: aliSize=105 (resi) RMSD=9.80 (Å)<br>n----------VGKKYFMSSVRRMPLNRAKALCSEL--QGTVATPRNAEENRA-----IQNVAKDVAFLGITDQRTENVFEDLTGNRVRYTNWnegepNNVgsgENCVVLLTN-GKWNDVPCSDSFLVVCEF--s<br>-gfccplgwssYDEHCYQVFQQKMNWEDAEKFCTQQhrGSHLVSFHSSEEVDFvvsktSPILKHDFVWMGLSNVWNECAKEWSDGTKLDYKAW-----SGQ---SDCITSKTTdNQWLSMDCSSKRYVVCKFqa-<br><br>BLAST: aliSize=32 (resi) iden=14.78% (17/115) simi=27.83% (32/115)<br>nvgkkyfmssvrrmplnrakalcselqgtvatprnaeenraiqnvak---DVAFLGITDQRTENVFEDLTGNRVRYTNWNeGEpnnvgsgENCVVL-LTNGKWNDVPCSDSFLVVCEF--s<br>---gfccplgwssydehcyqvfqqkmnwedaekfctqqhrgshlvsfhsseevdfvvsktspilkhDFVWMGLSNVWNECAKEWSDGTKLDYKAWS-GQ-------SDCITSkTTDNQWLSMDCSSKRYVVCKFqa-<br><br>Proposed: aliSize=106 (resi) RMSD=1.56 (Å)<br>---------NVGKKYFMSSVRRMPLNRAKALCSE-LQ-GTVATPRNAEENRAIQNVA---K--DVAFLGIT-DQRTEnVFEDLTGNRVRYTNWNEgepnnvgsGENCVVLL-TNGKWNDVPCSDSFLVVCEFS-<br>gfccplgwsSYDEHCYQVFQQKMNWEDAEKFCTQqHRgSHLVSFHSSEEVDFVVSKTspiLkhDFVWMGLSnVWNEC-AKEWSDGTKLDYKAWSG--------QSDCITSKtTDNQWLSMDCSSKRYVVCKFQa
1060	1kza1 (115)<br>1iodA (129)	M	17.39	TM-align: aliSize=92 (resi) RMSD=2.04 (Å)<br>-------NVGKKYFMSSVRRMPLNRAKALCSEL-Q-GTVATPRNAEENRAIQNVA---KD----VAFLGITDQRT--ENvfedltgnrvrytnwnegepnn----------------VGSGENCVVLLT--N-GKWNDVPCSDSFLVVCEFs<br>dcssgwsSYEGHCYKVFKQSKTWADAESFCTKQvNgGHLVSIESSGEADFVGQLIaqkIKsakiHVWIGLRAQNKekQC----------------------siewsdgssisyenwiEEESKKCLGVHIetGfHKWENFYCEQQDPFVCEA-<br><br>SARST: aliSize=85 (resi) RMSD=9.51 (Å)<br>n--------VGKKYFMSSVRRMPLNRAKALCSEL--QGTVATPRNAEENRAIQN------VA-KDVAFLGITDQrtenvfedltgnrvrytnWNEGEPNnvgsgencvVLLTNGKW--------------------------NDVPCSDSFLVVCE-fs<br>-dcssgwssYEGHCYKVFKQSKTWADAESFCTKQvnGGHLVSIESSGEADFVGQliaqkiKSaKIHVWIGLRAQ------------------NKEKQCS---------IEWSDGSSisyenwieeeskkclgvhietgfhkwENFYCEQQDPFVCEa--<br><br>BLAST: aliSize=30 (resi) iden=18.26% (21/115) simi=26.09% (30/115)<br>nvgkkyfmssvrrmplnrakalcselqgtvatprnaeenraiqnv---AKDVAFLGITDQRTENV--FEDLTGNRVRYTNWNEGEpnnvgsGENCV-VLLTNG--KWNDVPCSDSFLVVCE-fs<br>---dcssgwssyeghcykvfkqsktwadaesfctkqvngghlvsiessgeadfvgqliaqkiksAKIHVWIGLRAQNKEKQcsIEWSDGSSISYENWIEEE------SKKCLgVHIETGfhKWENFYCEQQDPFVCEa--<br><br>Proposed: aliSize=107 (resi) RMSD=1.53 (Å)<br>-------NVGKKYFMSSVRRMPLNRAKALCSE-LQ-GTVATPRNAEENRAIQNVA--KD-----VAFLGITDQR---tENVFEDLTGNRVRYTNWNEgepnnvGSGENCVVLLT--N-GKWNDVPCSDSFLVVCEFs<br>dcssgwsSYEGHCYKVFKQSKTWADAESFCTKqVNgGHLVSIESSGEADFVGQLIaqKIksakiHVWIGLRAQNkek-QCSIEWSDGSSISYENWIE------EESKKCLGVHIetGfHKWENFYCEQQDPFVCEA-
1061	1kza1 (115)<br>1uosA (133)	M	15.65	TM-align: aliSize=93 (resi) RMSD=1.99 (Å)<br>-------NVGKKYFMSSVRRMPLNRAKALCSELQ--GTVATPRNAEENRAIQNVA---K----DVAFLGITDQRT---ENvfedltgnrvrytnwnegepnn---------------VGSGENCVVLLT--N-GKWNDVPCSDSFLVVCEFS---<br>hcpsdwyYYDQHCYRIFNEEMNWEDAEWFCTKQAkgAHLVSIKSAKEADFVAWMVtqnIeesfSHVSIGLRVQNKekqCS----------------------tkwsdgssvsydnllDLYITKCSLLKKetGfRKWFVASCIGKIPFVCKFPpqc<br><br>SARST: aliSize=98 (resi) RMSD=9.83 (Å)<br>n--------VGKKYFMSSVRRMPLNRAKALCSEL--QGTVATPRNAEENRAIQNV--------AKDvAFLGITDQRTE--NVFEDLTGNRVRYTNWNEgepnnvGSGENCvvlltngkW-----------NDVPCSDSFLVVCEF----s<br>-hcpsdwyyYDQHCYRIFNEEMNWEDAEWFCTKQakGAHLVSIKSAKEADFVAWMvtqnieesFSH-VSIGLRVQNKEkqCSTKWSDGSSVSYDNLLD------LYITKC--------SllkketgfrkwFVASCIGKIPFVCKFppqc-<br><br>BLAST: aliSize=34 (resi) iden=19.13% (22/115) simi=29.57% (34/115)<br>nvgkkyfmssvrrmplnrakalcselqgtvatprnaeenraiqnv--AKDVAFLG-ITDQRTENVFEDLT-GNRVRY------TNWNEGEP---NNVGSG--ENCVVLLTN-G--KWNDVPCSDSFLVVCEF----s<br>---hcpsdwyyydqhcyrifneemnwedaewfctkqakgahlvsiksAKEADFVAwMVTQNIEESFSHVSiGLRVQNkekqcsTKWSDGSSvsyDNLLDLyiTKCSLLKKEtGfrKWFVASCIGKIPFVCKFppqc-<br><br>Proposed: aliSize=105 (resi) RMSD=1.36 (Å)<br>-------NVGKKYFMSSVRRMPLNRAKALCSELQ--GTVATPRNAEENRAIQNVA---K----DVAFLGITD------qrteNVFEDLTGNRVRYTNWNEgepnnvGSGENCVVLLT--N-GKWNDVPCSDSFLVVCEFS---<br>hcpsdwyYYDQHCYRIFNEEMNWEDAEWFCTKQAkgAHLVSIKSAKEADFVAWMVtqnIeesfSHVSIGLRVqnkekq----CSTKWSDGSSVSYDNLLD------LYITKCSLLKKetGfRKWFVASCIGKIPFVCKFPpqc

1062	1kza1 (115)<br>1j34A (129)	M	20.87	TM-align: aliSize=92 (resi) RMSD=1.93 (Å)<br>-------NVGKKYFMSSVRRMPLNRAKALCSEL-Q-GTVATPRNAEENRAIQNVA---K----DVAFLGITDQR-T-ENvfedltgnrvrytnwnegepnn----------------VGSGENCVVLLT--N-GKWNDVPCSDSFLVVCEFs<br>dcpsgwsSYEGHCYKPFKLYKTWDDAERFCTEQaKgGHLVSIESAGEADFVAQLVtenIqntkSYVWIGLRVQGkEkQC----------------------ssewsdgssvsyenwiEAESKTCLGLEKetGfRKWVNIYCGQQNPFVCEA-<br><br>SARST: aliSize=85 (resi) RMSD=11.67 (Å)<br>n--------VGKKYFMSSVRRMPLNRAKALCSEL--QGTVATPRNAEENRAIQN---------------------VAKD----------VAFLGITDQRTENVFED--LTGnrvrytnwnegepnnvgsgencvvlltNGKWNDVPCSDSFLVVCE-fs<br>-dcpsgwssYEGHCYKPFKLYKTWDDAERFCTEQakGGHLVSIESAGEADFVAQlvteniqntksyvwiglrvqgKEKQcssewsdgssVSYENWIEAESKTCLGLekETG---------------------------FRKWVNIYCGQQNPFVCEa--<br><br>BLAST: aliSize=34 (resi) iden=22.61% (26/115) simi=29.57% (34/115)<br>nvgkkyfmssvrrmplnrakalcselqgtvatprnae--ENraIQNVaKDVAFLGITDQRTENVF--EDLTGNRVRYTNWNEGEpnnvgsGENCVVL-LTNG--KWNDVPCSDSFLVVCE-fs<br>-------------------------------------dcpsgwssyeghcykpfklyktwddaerfcteqakgghlvsiesageadfvaqlvtEN--IQNT-KSYVWIGLRVQGKEKQCssEWSDGSSVSYENWIEAE------SKTCLGLeKETGfrKWVNIYCGQQNPFVCEa--<br><br>Proposed: aliSize=107 (resi) RMSD=1.53 (Å)<br>-------NVGKKYFMSSVRRMPLNRAKALCSEL-Q-GTVATPRNAEENRAIQNVA---K----DVAFLGITDQR---tENVFEDLTGNRVRYTNWNEGEpnnvgsGENCVVLLT--N-GKWNDVPCSDSFLVVCEFs<br>dcpsgwsSYEGHCYKPFKLYKTWDDAERFCTEQaKgGHLVSIESAGEADFVAQLVtenIqntkSYVWIGLRVQGkek-QCSSEWSDGSSVSYENWIEAE------SKTCLGLEKetGfRKWVNIYCGQQNPFVCEA-
1063	1yttA (115)<br>1oz7B (123)	M	19.13	TM-align: aliSize=91 (resi) RMSD=2.18 (Å)<br>--------SGKKFFVTNHERMPFSKVKALCSEL-R-GTVAIPRNAEENKAIQE-VA----KTSAFLGITDEVTEg-Qfmyvtggrltysnwkkdepndhg--------------S-GEDCVTIV-DNGLWNDISCQASHTAVCEFPA<br>nclpdwsvYEGYCYKVFKERMNWADAEKFCTKQhKdGHLVSFRNSKEVDFVISlAFpmlkNDLVWIGLTDYWRD-cN-----------------------wewsdgaqldykawDnERHCFIYKnTDNQWTRRDCTWTFSFVCKCPA<br><br>SARST: aliSize=104 (resi) RMSD=9.43 (Å)<br>s---------GKKFFVTNHERMPFSKVKALCSEL--RGTVAIPRNAEENKAIQEV-----AKTSAFLGITDEVTEGQFMYVTGGRLTYSNWK--KDEPndhgsgedcVTIVDNGLWNDISCQASHTAVCEFP-a<br>-nclpdwsvyEGYCYKVFKERMNWADAEKFCTKQhkDGHLVSFRNSKEVDFVISLafpmlKNDLVWIGLTDYWRDCNWEWSDGAQLDYKAWDneRHCF---------IYKNTDNQWTRRDCTWTFSFVCKCPa-<br><br>BLAST: aliSize=48 (resi) iden=23.48% (27/115) simi=41.74% (48/115)<br>sgkkffvtnh------------------ERMPFSKVKALCSELR--GTVAIPRNAEENKAIQEVA----KTS-AFLGITDEVTEGQFMYVTGGRLTYSNWKKDEpndHgsgedCVTI--VDNGlWNDISCQASHTAVCEFPA<br>----------nclpdwsvyegycykvfkERMNWADAEKFCTKQHkdGHLVSFRNSKEVDFVISLAfpmlKNDlVWIGLTDYWRDCNWEWSDGAQLDYKAWDNER---H-----CFIYknTDNQ-WTRRDCTWTFSFVCKCPA<br><br>Proposed: aliSize=106 (resi) RMSD=1.75 (Å)<br>--------SGKKFFVTNHERMPFSKVKALCSEL-R-GTVAIPRNAEENKAIQE-VA----KTSAFLGITDEVT-eGQFMYVTGGRLTYSNWKKDepndhgsgEDCVTIV-DNGLWNDISCQASHTAVCEFPA<br>nclpdwsvYEGYCYKVFKERMNWADAEKFCTKQhKdGHLVSFRNSKEVDFVISlAFpmlkNDLVWIGLTDYWRd-CNWEWSDGAQLDYKAWDNE--------RHCFIYKnTDNQWTRRDCTWTFSFVCKCPA
1064	1yttA (115)<br>1ukmB (124)	M	17.39	TM-align: aliSize=93 (resi) RMSD=2.44 (Å)<br>-------SGKKFFVTNHERMPFSKVKALCSELR--GTVAIPRNAEENKAIQEVAK-----TSAFLGITDEVTEGQfmyvtggrltysnwkkdepndh--------------GSGEDCVTIVD---NGLWNDISCQASHTAVCEFPA<br>cplgwssFDQHCYKVFEPVKNWTEAEEICMQQHkgSRLASIHSSEEEAFVSKLASkalkfTSMWIGLNNPWKDCK----------------------wewsdnarfdykawKRRPYCTVMVVkpdRIFWFTRGCEKSVSFVCKFLT<br><br>SARST: aliSize=107 (resi) RMSD=9.73 (Å)<br>s--------GKKFFVTNHERMPFSKVKALCSEL--RGTVAIPRNAEENKAIQEVA-----KTSAFLGITDEVTEGQFMYVTGGRLTYSNWkkdepnDHGSGEDCVTIVDNG-LWNDISCQASHTAVCEFP-a<br>-cplgwssfDQHCYKVFEPVKNWTEAEEICMQQhkGSRLASIHSSEEEAFVSKLAskalkFTSMWIGLNNPWKDCKWEWSDNARFDYKAW------KRRPYCTVMVVKPDRiFWFTRGCEKSVSFVCKFLt-<br><br>BLAST: aliSize=50 (resi) iden=20.00% (23/115) simi=43.48% (50/115)<br>sgkkffvtnhermp---------------------FSKVKALCSEL-RGT-VAIPRNAEENKAIQEVAK-----TSAFLGITDEVTEGQFMYVTGGRLTYSNWKKdEPNdhgsgedCVTIV---DNGLWNDISCQASHTAVCEF--pa<br>--------------cplgwssfdqhcykvfepvknWTEAEEICMQQhKGSrLASIHSSEEEAFVSKLASkalkfTSMWIGLNNPWKDCKWEWSDNARFDYKAWKR-RPY-------CTVMVvkpDRIFWFTRGCEKSVSFVCKFlt--<br><br>Proposed: aliSize=104 (resi) RMSD=1.80 (Å)<br>-------SGKKFFVTNHERMPFSKVKALCSELR--GTVAIPRNAEENKAIQEVAK-----TSAFLGITD---evtEGQFMYVTGGRLTYSNWKkdepndhgSGEDCVTIVD---NGLWNDISCQASHTAVCEFPA<br>cplgwssFDQHCYKVFEPVKNWTEAEEICMQQHkgSRLASIHSSEEEAFVSKLASkalkfTSMWIGLNNpwk---DCKWEWSDNARFDYKAWK--------RRPYCTVMVVkpdRIFWFTRGCEKSVSFVCKFLT

<table>
<tr><td>1065</td><td>1yttA
(115)

1ukmA
(131)</td><td>M</td><td>19.13</td><td>TM-align: aliSize=93 (resi)   RMSD=2.10 (Å)
--------SGKKFFVTNHERMPFSKVKALCSEL-R-GTVAIPRNAEENKAIQEVAK-T------SAFLGITDEV-T--EGqfmyvtggrltysnwkkdepnd-------------HG--SGEDCVTIV-DN--GLWNDISCQASHTAVCEFPA
dcpsdwtaYDQHCYLAIGEPQNWYEAERFCTEQaKdGHLVSIQSREEGNFVAQLVSgFmhrseiYVWIGLRDRReEqqCN---------------------pewndgskiiyvnWKegESKMCQGLTkWTnfHDWNNINCEDLYPFVCKFSA

SARST: aliSize=107 (resi)   RMSD=9.29 (Å)
s---------GKKFFVTNHERMPFSKVKALCSEL--RGTVAIPRNAEENKAIQEV----AKT---SAFLGITDEVTEGQ--FMYVTGGRLTYSNWKKDepndhgSGEDCVTIV--DNG-LWNDISCQASHTAVCEFP-a
-dcpsdwtayDQHCYLAIGEPQNWYEAERFCTEQakDGHLVSIQSREEGNFVAQLvsgfMHRseiYVWIGLRDRREEQQcnPEWNDGSKIIYVNWKEG------ESKMCQGLTkwTNFhDWNNINCEDLYPFVCKFSa-

BLAST: aliSize=31 (resi)   iden=16.52% (19/115)   simi=26.96% (31/115)
sgkkffvtnhermpfskvkalcselrgtvaiprnaeenkaiqevaktsa--FLGITDEVTEGQFM--YVTGGRLTYSNWKKDEPNDHGSGEDCVTIVDnglWNDISCQASHTAVCEFPA
--dcpsdwtaydqhcylaigepqnwyeaerfcteqakdghlvsiqsreegnfvaqlvsgfmhrseiyvWIGLRDRREEQQCNpeWNDGSKIIYVNWKEGESKMCQGLTKWTNFHD---WNNINCEDLYPFVCKFSA

Proposed: aliSize=107 (resi)   RMSD=1.61 (Å)
--------SGKKFFVTNHERMPFSKVKALCSEL-R-GTVAIPRNAEENKAIQEVAK-T------SAFLGITDEV---teGQF-MYVTGGRLTYSNWKKDepndhgSGEDCVTIVD-N--GLWNDISCQASHTAVCEFPA
dcpsdwtaYDQHCYLAIGEPQNWYEAERFCTEQaKdGHLVSIQSREEGNFVAQLVSgFmhrseiYVWIGLRDRReeq--QCNpEWNDGSKIIYVNWKEG------ESKMCQGLTKwTnfHDWNNINCEDLYPFVCKFSA</td></tr>
<tr><td>1066</td><td>1yttA
(115)

1j34B
(123)</td><td>M</td><td>17.39</td><td>TM-align: aliSize=92 (resi)   RMSD=2.06 (Å)
--------SGKKFFVTNHERMPFSKVKALCSEL-R-GTVAIPRNAEENKAIQEVAK-----TSAFLGIT-DEVTEGqfmyvtggrltysnwkkdepndh-------------GSG-EDCVTIV-DNGLWNDISCQASHTAVCEFPA
dcpsdwssYEGHCYKPFSEPKNWADAENFCTQQhAgGHLVSFQSSEEADFVVKLAFqtfghSIFWMGLSnVWNQCN----------------------wqwsnaamlrykaWAEeSYCVYFKsTNNKWRSRACRMMAQFVCEFQA

SARST: aliSize=106 (resi)   RMSD=9.74 (Å)
s---------GKKFFVTNHERMPFSKVKALCSEL--RGTVAIPRNAEENKAIQEVA-----KTSAFLGITDEVTEGQFMYVTGGRLTYSNWKKdepndhgSGEDCVTIVDNGLWNDISCQASHTAVCEFP-a
-dcpsdwssyEGHCYKPFSEPKNWADAENFCTQQhaGGHLVSFQSSEEADFVVKLAfqtfgHSIFWMGLSNVWNQCNWQWSNAAMLRYKAWAE-------ESYCVYFKSTNNKWRSRACRMMAQFVCEFQa-

BLAST: aliSize=29 (resi)   iden=13.91% (16/115)   simi=25.22% (29/115)
sgkkffvtnhermpfskvkalcselrgtvaiprnaeenk--AIQEVAKTSAFLGITDEVTEGQFMYVTGGRLTYSNWKKDEPndhgsgedCVTIVD-NGLWNDISCQASHTAVCEFPA
---------------------------------------dcpsdwssyeghcykpfsepknwadaenfctqqhagghlvsfqsseeadfvvklAFQTFGHSIFWMGLSNVWNQCNWQWSNAAMLRYKAWAEESY--------CVYFKStNNKWRSRACRMMAQFVCEFQA

Proposed: aliSize=106 (resi)   RMSD=1.74 (Å)
--------SGKKFFVTNHERMPFSKVKALCSEL-R-GTVAIPRNAEENKAIQEVAK-----TSAFLGIT-DEVTEgQFMYVTGGRLTYSNWKKDepndhgsgEDCVTIV-DNGLWNDISCQASHTAVCEFPA
dcpsdwssYEGHCYKPFSEPKNWADAENFCTQQhAgGHLVSFQSSEEADFVVKLAFqtfghSIFWMGLSnVWNQC-NWQWSNAAMLRYKAWAEE--------SYCVYFKsTNNKWRSRACRMMAQFVCEFQA</td></tr>
<tr><td>1067</td><td>1yttA
(115)

1jwiB
(123)</td><td>M</td><td>15.65</td><td>TM-align: aliSize=93 (resi)   RMSD=2.36 (Å)
--------SGKKFFVTNHERMPFSKVKALCSELR--GTVAIPRNAEENKAIQEVAK-----TSAFLGITDE-VTEGQfmyvtggrltysnwkkdepndh------------GS-GEDCVTIV-DNGLWNDISCQASHTAVCEFPA
gclpdwssYKGHCYKVFKVEKTWADAEKFCKELVngGHLMSVNSREEGEFISKLALekmriVLVWIGLSHFwRICPL----------------------rwtdgarldyraLSdEPICFVAEsFHNKWIQWTCNRKKSFVCKYRV

SARST: aliSize=104 (resi)   RMSD=9.10 (Å)
s---------GKKFFVTNHERMPFSKVKALCSEL--RGTVAIPRNAEENKAIQEVA----KT-SAFLGIT--DEVTEgqFMYVTGGRLTYSnwkkDEPNDHgsgEDCVTIVDNGLWNDISCQASHTAVCEFP-a
-gclpdwssyKGHCYKVFKVEKTWADAEKFCKELvnGGHLMSVNSREEGEFISKLAlekmRIvLVWIGLShfWRICP--LRWTDGARLDYR----ALSDEP---ICFVAESFHNKWIQWTCNRKKSFVCKYRv-

BLAST: aliSize=5 (resi)   iden=3.48% (4/115)   simi=4.35% (5/115)
sg---KKFFVTNH--ermpfskvkalcselrgtvaiprnaeenkaiqevaktsaflgitdevtegqfmyvtggrltysnw
--gclpdwssykghcykvfkvektwadaekfckelvngghlmsvnsreegefisklalekmrivlvwiglshfwricplrwtdgarldyralsdepicfvaesfhnkwiqwtcnrKKSFVCKYrv---
kkdepndhgsgedcvtivdnglwndiscqashtavcefpa

Proposed: aliSize=105 (resi)   RMSD=2.05 (Å)
--------SGKKFFVTNHERMPFSKVKALCSELR--GTVAIPRNAEENKAIQEVAK-----TSAFLGITDEvtEG--QFMYVTGGRLTySnWkkdEPNDhgsGEDCVTIV-DNGLWNDISCQASHTAVCEFPA
gclpdwssYKGHCYKVFKVEKTWADAEKFCKELVngGHLMSVNSREEGEFISKLALekmriVLVWIGLSHF--WRicPLRWTDGARLD-Y-R---ALSD---EPICFVAEsFHNKWIQWTCNRKKSFVCKYRV</td></tr>
</table>

1068	1yttA<br>(115)<br>1v4lB<br>(125)	M	16.52	TM-align: aliSize=92 (resi) RMSD=2.19 (Å)<br>----------SGKKFFVTNHERMPFSKVKALCSEL-R-GTVAIPRNAEENKAIQEVAK--T---SAFLGIT-DEVTEGqfmyvtggrltysnwkkdepndh--------------GSGEDCVTIV-DNGLWNDISCQASHTAVCEFPA<br>gfccplgwssYDEHCYQVFQQKMNWEDAEKFCTQQhRgSHLVSFHSSEEVDFVVSKTSpiLkhdFVWMGLSnVWNECA-----------------------kewsdgtkldykawSGQSDCITSKtTDNQWLSMDCSSKRYVVCKFQA<br><br>SARST: aliSize=103 (resi) RMSD=9.54 (Å)<br>sg------------KKFFVTNHERMPFSKVKALCSEL--RGTVAIPRNAEENKAIQEVA-----KTSAFLGITDEVTEGQFMYVTGGRLTYSNWkkdepndhgSGEDCVTIVDNGL--WNDISCQASHTAVCEFP-a<br>--gfccplgwssydEHCYQVFQQKMNWEDAEKFCTQQhrGSHLVSFHSSEEVDFVVSKTspilkHDFVWMGLSNVWNECAKEWSDGTKLDYKAW---------SGQSDCITSKTTDnqWLSMDCSSKRYVVCKFQa-<br><br>BLAST: aliSize=48 (resi) iden=25.22% (29/115) simi=41.74% (48/115)<br>sgkkffvtnh--------------------ERMPFSKVKALCSEL-RGTVAIP-RNAEENKAIqeVAKTSAFLGiTDEVTEGqfMYVTGGRLTySNWKKDEPNDH--GSGE-DCVT--IVDNgLWNDISCQASHTAVCEFPA<br>----------gfccplgwssydehcyqvfqQKMNWEDAEKFCTQQhRGSHLVSfHSSEEVDFV--VSKTSPILK-HDFVWMG--LSNVWNECA-KEWSDGTKLDYkaWSGQsDCITskTTDN-QWLSMDCSSKRYVVCKFQA<br><br>Proposed: aliSize=106 (resi) RMSD=1.72 (Å)<br>----------SGKKFFVTNHERMPFSKVKALCSEL-R-GTVAIPRNAEENKAIQEVAK-----TSAFLGIT-DEVTEgQFMYVTGGRLTYSNWKKdepndhgsGEDCVTIV-DNGLWNDISCQASHTAVCEFPA<br>gfccplgwssYDEHCYQVFQQKMNWEDAEKFCTQQhRgSHLVSFHSSEEVDFVVSKTSpilkhDFVWMGLSnVWNEC-AKEWSDGTKLDYKAWSG--------QSDCITSKtTDNQWLSMDCSSKRYVVCKFQA
1069	1yttA<br>(115)<br>1iodA<br>(129)	M	12.17	TM-align: aliSize=91 (resi) RMSD=2.19 (Å)<br>--------SGKKFFVTNHERMPFSKVKALCSEL-R-GTVAIPRNAEENKAIQEVAK--T-----SAFLGITDEVT--EGQfmyvtggrltysnwkkdepndh----------------GSGEDCVTIV--DN-GLWNDISCQASHTAVCEFpa<br>dcssgwssYEGHCYKVFKQSKTWADAESFCTKQvNgGHLVSIESSGEADFVGQLIAqkIksakiHVWIGLRAQNKekQCS----------------------iewsdgssisyenwieEESKKCLGVHieTGfHKWENFYCEQQDPFVCEA--<br><br>SARST: aliSize=104 (resi) RMSD=9.93 (Å)<br>s---------GKKFFVTNHERMPFSKVKALCSEL--RGTVAIPRNAEENKAIQEVA-------KTSAFLGITDE---VTEGQfMYVTGGRLTYSNWKKDepndhgSGEDCVTIV--DNG-LWNDISCQASHTAVCE-fpa<br>-dcssgwssyEGHCYKVFKQSKTWADAESFCTKQvnGGHLVSIESSGEADFVGQLIaqkiksaKIHVWIGLRAQnkeKQCSI-EWSDGSSISYENWIEE------ESKKCLGVHieTGFhKWENFYCEQQDPFVCEa---<br><br>BLAST: aliSize=33 (resi) iden=14.78% (17/115) simi=28.70% (33/115)<br>sgkkffvtnhermpfskvkalcselrgtvaiprnaeenkaiqev---AKTSAFLGITDEVTEGQ--FMYVTGGRLTYSNWKKDEpndhgsGEDCVTI-VDNGL--WNDISCQASHTAVCE-fpa<br>--dcssgwssyeghcykvfkqsktwadaesfctkqvngghlvsiessgeadfvgqliaqkiksAKIHVWIGLRAQNKEKQcsIEWSDGSSISYENWIEEE------SKKCLGVhIETGFhkWENFYCEQQDPFVCEa---<br><br>Proposed: aliSize=106 (resi) RMSD=1.69 (Å)<br>--------SGKKFFVTNHERMPFSKVKALCSELR--GTVAIPRNAEENKAIQEVAK--T-----SAFLGITDEV---tEGQFMYVTGGRLTYSNWKKDepndhgSGEDCVTIV--DN-GLWNDISCQASHTAVCEFpa<br>dcssgwssYEGHCYKVFKQSKTWADAESFCTKQVngGHLVSIESSGEADFVGQLIAqkIksakiHVWIGLRAQNkek-QCSIEWSDGSSISYENWIEE------ESKKCLGVHieTGfHKWENFYCEQQDPFVCEA--
1070	1yttA<br>(115)<br>1uosA<br>(133)	M	14.78	TM-align: aliSize=92 (resi) RMSD=2.00 (Å)<br>--------SGKKFFVTNHERMPFSKVKALCSELR--GTVAIPRNAEENKAIQEVAK-------TSAFLGITDEVT---EGqfmyvtggrltysnwkkdepndh---------------GSGEDCVTIV--DN-GLWNDISCQASHTAVCEFPA--<br>hcpsdwyyYDQHCYRIFNEEMNWEDAEWFCTKQAkgAHLVSIKSAKEADFVAWMVTqnieesfSHVSIGLRVQNKekqCS----------------------tkwsdgssvsydnlldLYITKCSLLKkeTGfRKWFVASCIGKIPFVCKFPPqc<br><br>SARST: aliSize=98 (resi) RMSD=9.76 (Å)<br>sg----------KKFFVTNHERMPFSKVKALCSEL--RGTVAIPRNAEENKAIQEVA--KTS-----AFLGITDEVTE--GQFMYVTGGRLTYSNWKKdepndhGSGEDCvtivdnglW-----------NDISCQASHTAVCEFP---a<br>--hcpsdwyyydQHCYRIFNEEMNWEDAEWFCTKQakGAHLVSIKSAKEADFVAWMVtqNIEesfshVSIGLRVQNKEkqCSTKWSDGSSVSYDNLLD------LYITKC--------SllkketgfrkwFVASCIGKIPFVCKFPpqc-<br><br>BLAST: aliSize=8 (resi) iden=6.09% (7/115) simi=6.96% (8/115)<br>sgkkffvtnhermpfskvkalcselrgtvaiprnaeenkaiqevaktsaflgitdevtegqfmyvtggrltysnwkkdepndhgsgedcvtivdngl---<br>---hcpsdwyyydqhcyrifneemnwedaewfctkqakgahlvsiksakeadfvawmvtqnieesfshvsiglrvqnkekqcstkwsdgssvsydn<br>--------------------WNDISCQASHTAVCEFP---a<br>lldlyitkcsllkketgfrkWFVASCIGKIPFVCKFPpqc-<br><br>Proposed: aliSize=108 (resi) RMSD=1.80 (Å)<br>--------SGKKFFVTNHERMPFSKVKALCSELR--GTVAIPRNAEENKAIQEVAK-------TSAFLGITDEVT---eGQFMYVTGGRLTYSNWKKdepndhGSGEDCVTIV--DN-GLWNDISCQASHTAVCEFPA--<br>hcpsdwyyYDQHCYRIFNEEMNWEDAEWFCTKQAkgAHLVSIKSAKEADFVAWMVTqnieesfSHVSIGLRVQNKekq-CSTKWSDGSSVSYDNLLD------LYITKCSLLKkeTGfRKWFVASCIGKIPFVCKFPPqc

<table>
<tr><td rowspan="2">1071</td><td>1yttA
(115)</td><td rowspan="2">M</td><td rowspan="2">14.78</td><td rowspan="2">TM-align: aliSize=91 (resi)   RMSD=2.10 (Å)
--------SGKKFFVTNHERMPFSKVKALCSEL-R-GTVAIPRNAEENKAIQEVAK--T-----SAFLGITDEV-T-EGqfmyvtggrltysnwkkdepnd----------------HGSGEDCVTIVD--N-GLWNDISCQASHTAVCEFpa
dcpsgwssYEGHCYKPFKLYKTWDDAERFCTEQaKgGHLVSIESAGEADFVAQLVTenIqntksYVWIGLRVQGkEkQC----------------------ssewsdgssvsyenwiEAESKTCLGLEKetGfRKWVNIYCGQQNPFVCEA--

SARST: aliSize=103 (resi)   RMSD=9.85 (Å)
s---------GKKFFVTNHERMPFSKVKALCSEL--RGTVAIPRNAEENKAIQEVA-------KTSAFLGITDevTEGQ----FMYVTGGRLTYSNWKKDepndhgSGEDCVTIV---DNGLWNDISCQASHTAVCE-fpa
-dcpsgwssyEGHCYKPFKLYKTWDDAERFCTEQakGGHLVSIESAGEADFVAQLVteniqntKSYVWIGLRV--QGKEkqcsSEWSDGSSVSYENWIEA------ESKTCLGLEketGFRKWVNIYCGQQNPFVCEa---

BLAST: aliSize=36 (resi)   iden=18.26% (21/115)   simi=31.30% (36/115)
sgkkffvtnhermpfskvkalcselrgtvaiprnae--ENkaIQEvAKTSAFLGITDEVTEGQFM--YVTGGRLTYSNWKKDEpndhgsGEDCVTI-VDNGL--WNDISCQASHTAVCE-fpa
------------------------------------dcpsgwssyeghcykpfklyktwddaerfcteqakgghlvsiesageadfvaqlvtEN--IQN-TKSYVWIGLRVQGKEKQCSSeWSDGSSVSYENWIEAE------SKTCLGLeKETGFrkWVNIYCGQQNPFVCEa---

Proposed: aliSize=105 (resi)   RMSD=1.59 (Å)
--------SGKKFFVTNHERMPFSKVKALCSEL-R-GTVAIPRNAEENKAIQEVAK--T-----SAFLGITDEV----teGQFMYVTGGRLTYSNWKKDepndhgSGEDCVTIVD--N-GLWNDISCQASHTAVCEFpa
dcpsgwssYEGHCYKPFKLYKTWDDAERFCTEQaKgGHLVSIESAGEADFVAQLVTenIqntksYVWIGLRVQGkekq--CSSEWSDGSSVSYENWIEA------ESKTCLGLEKetGfRKWVNIYCGQQNPFVCEA--</td></tr>
<tr><td>1j34A
(129)</td></tr>
<tr><td rowspan="2">1072</td><td>2cw1A
(65)</td><td rowspan="2">C</td><td rowspan="2">19.67</td><td rowspan="2">TM-align: aliSize=53 (resi)   RMSD=2.30 (Å)
mr---KKLdLKKFVEDKNQEYAARALGLSQKLIEEVLKRGLPVYVETNKDGNIKVYITQdgitqpfpp-----
--meqRIT-LKDYAMRFGQTKTAKDLGVYQSAINKAIHAGRKIFLTINADGSVYAEEVK---------pwpsn

SARST: aliSize=54 (resi)   RMSD=2.51 (Å)
mr--KKLDLKKFVEDKNQEYAARALGLSQKLIEEVLKRGLPVYVETNKDGNIKVYITQ-----dgitqpfpp
--meQRITLKDYAMRFGQTKTAKDLGVYQSAINKAIHAGRKIFLTINADGSVYAEEVKpwpsn---------

BLAST: aliSize=32 (resi)   iden=27.87% (17/61)   simi=52.46% (32/61)
MRKKLDLKKFVEDKNQEYAARALGLSQKLIEEVLKRGLPVYVETNKDGNikVYITQdgiTQPFP--p
MEQRITLKDYAMRFGQTKTAKDLGVYQSAINKAIHAGRKIFLTINADGS--VYAEE---VKPWPsn-

Proposed: aliSize=58 (resi)   RMSD=2.11 (Å)
mr---KKLdLKKFVEDKNQEYAARALGLSQKLIEEVLKRGLPVYVETNKDGNIKVYItqdgITQPFPP
--meqRIT-LKDYAMRFGQTKTAKDLGVYQSAINKAIHAGRKIFLTINADGSVYAEE----VKPWPSN</td></tr>
<tr><td>1d1lA
(61)</td></tr>
<tr><td rowspan="2">1073</td><td>2cw1A
(65)</td><td rowspan="2">C</td><td rowspan="2">20.00</td><td rowspan="2">TM-align: aliSize=54 (resi)   RMSD=2.38 (Å)
mr---KKLdLKKFVEDKNQEYAARaLG-LSQKLIEEVLKRGLPVYVETNKDGNIKVYITQdGItqpfpp--
--meqRIT-LKDYAMRFGQTKTAK-DLgVYPSSINQAIHAGRKIFLTINADGSVYAEEVK-PF------ps

SARST: aliSize=55 (resi)   RMSD=2.86 (Å)
m-RKKLDLKKFVEDKNQEYAARALGLSQKLIEEVLKRGLPVYVETNKDGNIKVYITQ----dgitqpfpp
-mEQRITLKDYAMRFGQTKTAKDLGVYPSSINQAIHAGRKIFLTINADGSVYAEEVKpfps---------

BLAST: aliSize=31 (resi)   iden=28.33% (17/60)   simi=51.67% (31/60)
MRKKLDLKKFVEDKNQEYAARALGLSQKLIEEVLKRGLPVYVETNKDGNikVYITQdgiTQPFP-p
MEQRITLKDYAMRFGQTKTAKDLGVYPSSINQAIHAGRKIFLTINADGS--VYAEE---VKPFPs-

Proposed: aliSize=57 (resi)   RMSD=2.14 (Å)
mr---KkLDLKKFVEDKNQEYAARALGLSQKLIEEVLKRGLPVYVETNKDGNIKVYITQdgiTQPFpp
--meqR-ITLKDYAMRFGQTKTAKDLGVYPSSINQAIHAGRKIFLTINADGSVYAEEVK---PFPS--</td></tr>
<tr><td>2ecsA
(60)</td></tr>
</table>

1074	2orcA<br>(71)<br>2a63A<br>(66)	C	81.82	TM-align: aliSize=58 (resi) RMSD=1.84 (Å)<br>-MeQRITLKDYAMRFGQTKTAKDLGVYQSAINKAIHAGRKIFLTINADGSVYAEEVKDGEvkpfpsnkktta-------<br>mE-QRITLKDYAMRFGQTKTAKDLGVQQSAINKWIHAGRKIFLTINADGSVYAEEVKPDP------------snkktta<br><br>SARST: aliSize=63 (resi) RMSD=5.51 (Å)<br>me--QRITLKDYAMRFGQTKTAKDLGVYQSAINKAIHAGRKIFLTINADGSVYAEEVKdgEVKPFPSnkKT-ta<br>--meQRITLKDYAMRFGQTKTAKDLGVQQSAINKWIHAGRKIFLTINADGSVYAEEVK--PDPSNKK--TTa--<br><br>BLAST: aliSize=63 (resi) iden=95.45% (63/66) simi=95.45% (63/66)<br>MEQRITLKDYAMRFGQTKTAKDLGVYQSAINKAIHAGRKIFLTINADGSVYAEevkdgEVKPFPSNKKTTA<br>MEQRITLKDYAMRFGQTKTAKDLGVQQSAINKWIHAGRKIFLTINADGSVYAE-----EVKPDPSNKKTTA<br><br>Proposed: aliSize=66 (resi) RMSD=1.71 (Å)<br>MEQRITLKDYAMRFGQTKTAKDLGVYQSAINKAIHAGRKIFLTINADGSVYAEEVKDgEVKPfpSNKKTta<br>MEQRITLKDYAMRFGQTKTAKDLGVQQSAINKWIHAGRKIFLTINADGSVYAEEVKP-DPSN--KKTTA--
1075	1d1mB<br>(65)<br>1d1lA<br>(61)	C	91.80	TM-align: aliSize=59 (resi) RMSD=1.55 (Å)<br>MEQRITLKDYAMRFGQTKTAKDLGVYQSAINKAIHAGRKIFLTINADGSVYAEEVKDGEvkpwps--<br>MEQRITLKDYAMRFGQTKTAKDLGVYQSAINKAIHAGRKIFLTINADGSVYAEEVKPWP------sn<br><br>SARST: aliSize=55 (resi) RMSD=0.74 (Å)<br>m-EQRITLKDYAMRFGQTKTAKDLGVYQSAINKAIHAGRKIFLTINADGSVYAEEVK-----dgevkpwps<br>-mEQRITLKDYAMRFGQTKTAKDLGVYQSAINKAIHAGRKIFLTINADGSVYAEEVKpwpsn---------<br><br>BLAST: aliSize=60 (resi) iden=98.36% (60/61) simi=98.36% (60/61)<br>MEQRITLKDYAMRFGQTKTAKDLGVYQSAINKAIHAGRKIFLTINADGSVYAEEVKdgevkPWPS-<br>MEQRITLKDYAMRFGQTKTAKDLGVYQSAINKAIHAGRKIFLTINADGSVYAEEVK-----PWPSn<br><br>Proposed: aliSize=61 (resi) RMSD=0.91 (Å)<br>MEQRITLKDYAMRFGQTKTAKDLGVYQSAINKAIHAGRKIFLTINADGSVYAEEVKdgevKPWPS<br>MEQRITLKDYAMRFGQTKTAKDLGVYQSAINKAIHAGRKIFLTINADGSVYAEEVK----PWPSN
1076	1d1mB<br>(65)<br>2a63A<br>(66)	C	81.54	TM-align: aliSize=59 (resi) RMSD=1.99 (Å)<br>MEQRITLKDYAMRFGQTKTAKDLGVYQSAINKAIHAGRKIFLTINADGSVYAEEVKDGEvkpwps-------<br>MEQRITLKDYAMRFGQTKTAKDLGVQQSAINKWIHAGRKIFLTINADGSVYAEEVKPDP------snkktta<br><br>SARST: aliSize=55 (resi) RMSD=1.20 (Å)<br>m-EQRITLKDYAMRFGQTKTAKDLGVYQSAINKAIHAGRKIFLTINADGSVYAEEVK----------dgevkpwps<br>-mEQRITLKDYAMRFGQTKTAKDLGVQQSAINKWIHAGRKIFLTINADGSVYAEEVKpdpsnkktta---------<br><br>BLAST: aliSize=54 (resi) iden=83.08% (54/65) simi=83.08% (54/65)<br>MEQRITLKDYAMRFGQTKTAKDLGVYQSAINKAIHAGRKIFLTINADGSVYAEEVK----------dgevkpwps<br>MEQRITLKDYAMRFGQTKTAKDLGVQQSAINKWIHAGRKIFLTINADGSVYAEEVKpdpsnkktta---------<br><br>Proposed: aliSize=64 (resi) RMSD=1.37 (Å)<br>m-EQRITLKDYAMRFGQTKTAKDLGVYQSAINKAIHAGRKIFLTINADGSVYAEEVKDGEVKPWPS-<br>-mEQRITLKDYAMRFGQTKTAKDLGVQQSAINKWIHAGRKIFLTINADGSVYAEEVKPDPSNKKTTa

1077	1gcpA<br>(65)<br><br>1i07A<br>(59)	N	18.64	TM-align: aliSize=34 (resi) RMSD=2.60 (Å)<br>--KMEVFQEYYGIpPppgafGPFLRLNPGDIVELTK-AEaeHNWwegrntatnevg----------Wfpcnrvhpyvh------------<br>kkYAKSKYDFVAR-N-----SSELSVMKDDVLEILDdRR--QWW------------kvrnasgdsgF-----------vpnnildimrtp<br><br>SARST: aliSize=54 (resi) RMSD=14.34 (Å)<br>k---MEVFQEYygipppPGAFGPFLRLNPGDIVELtkaEAEHNWWEGRNTATNEVGWFPCNRVHPYV--h<br>-kkyAKSKYDF------VARNSSELSVMKDDVLEI---LDDRRQWWKVRNASGDSGFVPNNILDIMRtp-<br><br>BLAST: aliSize=18 (resi) iden=16.95% (10/59) simi=30.51% (18/59)<br>kmevfqeyygippppgafgpflrlnpg----------------------DIVELTkaEAEHNWWEGRNtATNEVGWFPCN--------rvhpyvh<br>--------------------------kkyakskydfvarnsselsvmkdDVLEIL--DDRRQWWKVRN-ASGDSGFVPNNildimrtp-------<br><br>Proposed: aliSize=54 (resi) RMSD=1.46 (Å)<br>--KMEVFQEYYGIpP-ppgafgPFLRLNPGDIVELTK-AEaehNWWEGRNtATNEVGWFPCNRVHPYVH-<br>kkYAKSKYDFVAR-Ns------SELSVMKDDVLEILDdRR---QWWKVRN-ASGDSGFVPNNILDIMRTp
1078	1ckaA<br>(57)<br><br>1i07A<br>(59)	C	26.32	TM-align: aliSize=36 (resi) RMSD=2.39 (Å)<br>AEYVRALFDFNGNDEEDLPFKKGDILRIRD--KPEeQWwnaedsegkrg---------Mipvpyveky------------<br>KKYAKSKYDFVARNSSELSVMKDDVLEILDdrRQW-WK-----------vrnasgdsgF---------vpnnildimrtp<br><br>SARST: aliSize=53 (resi) RMSD=11.24 (Å)<br>a-EYVRALFDFNGNDEEDLPFKKGDILRIRDKPEeQWWNAEDSEGKRGMIPVPYVE-----k<br>-kKYAKSKYDFVARNSSELSVMKDDVLEILDDRR-QWWKVRNASGDSGFVPNNILDimrtp-<br><br>BLAST: aliSize=25 (resi) iden=26.79% (15/57) simi=44.64% (25/57)<br>a-EYVRALFDFNGNDEEDLPFKKGDILRIRDKpEEQWWNAEDSEGKRGMIP----------vpyvek<br>-kKYAKSKYDFVARNSSELSVMKDDVLEILDD-RRQWWKVRNASGDSGFVPnnildimrtp------<br><br>Proposed: aliSize=53 (resi) RMSD=0.82 (Å)<br>AEYVRALFDFNGNDEEDLPFKKGDILRIRD---kpeeQWWNAEDSEGKRGMIPVPYVEKY---<br>KKYAKSKYDFVARNSSELSVMKDDVLEILDdrr----QWWKVRNASGDSGFVPNNILDIMrtp
1079	1m3aA<br>(57)<br><br>1i07A<br>(59)	C	26.32	TM-align: aliSize=34 (resi) RMSD=2.36 (Å)<br>-CYVRALFDFNGNDEEDLPFKKGDILRIRD-KPEEQwwnaedsegkrgmipvpyvekyg-----------------------<br>kKYAKSKYDFVARNSSELSVMKDDVLEILDdRRQWW-----------------------kvrnasgdsgfvpnnildimrtp<br><br>SARST: aliSize=50 (resi) RMSD=11.67 (Å)<br>c--YVRALFDFNGNDEEDLPFKKGDILRIrdkpEEQ---WWNAEDSEGKRGMIPVPYVEK----yg<br>-kkYAKSKYDFVARNSSELSVMKDDVLEI----LDDrrqWWKVRNASGDSGFVPNNILDImrtp--<br><br>BLAST: aliSize=24 (resi) iden=26.32% (15/57) simi=42.11% (24/57)<br>c--YVRALFDFNGNDEEDLPFKKGDILRIRDKpEEQWWNAEDSEGKRGMIP----------vpyvekyg<br>-kkYAKSKYDFVARNSSELSVMKDDVLEILDD-RRQWWKVRNASGDSGFVPnnildimrtp--------<br><br>Proposed: aliSize=53 (resi) RMSD=1.59 (Å)<br>-CYVRALFDFNGNDEEDLPFKKGDILRIRD---kpeeQWWNAEDSEGKRGMIPVPYVEKYG--<br>kKYAKSKYDFVARNSSELSVMKDDVLEILDdrr----QWWKVRNASGDSGFVPNNILDIMRtp

1080	1zuyA (58)<br>1i07A (59)	C	24.14	TM-align: aliSize=35 (resi) RMSD=2.28 (Å)<br>-PMFEAAYDFPGSgSPSELPLKKGDVIYITR--EEPSGwslgklldgskeg---------Wvptaymkph------------<br>kKYAKSKYDFVAR-NSSELSVMKDDVLEILDdrRQWWK-------------vrnasgdsgF---------vpnnildimrtp<br><br>SARST: aliSize=50 (resi) RMSD=12.25 (Å)<br>pm---FEAAYDFPGSgSPSELPLKKGDVIYItreEPSG--WSLGKLLDGsKEGWVPTAYMKP----h<br>--kkyAKSKYDFVAR-NSSELSVMKDDVLEI---LDDRrqWWKVRNASG-DSGFVPNNILDImrtp-<br><br>BLAST: aliSize=24 (resi) iden=27.59% (16/58) simi=41.38% (24/58)<br>pmf----EAAYDFPGSGSpSELPLKKGDVIYITREEPSGWSLgKLLDGSkEGWVP----------taymkph<br>---kkyaKSKYDFVARNS-SELSVMKDDVLEILDDRRQWWKV-RNASGD-SGFVPnnildimrtp-------<br><br>Proposed: aliSize=54 (resi) RMSD=1.53 (Å)<br>-PMFEAAYDFPGSgSPSELPLKKGDVIYITR-eePSGWSLGKLlDGSKEGWVPTAYMKPH---<br>kKYAKSKYDFVAR-NSSELSVMKDDVLEILDd--RRQWWKVRN-ASGDSGFVPNNILDIMrtp
1081	1bu1C (56)<br>1i07A (59)	C	26.79	TM-align: aliSize=35 (resi) RMSD=2.06 (Å)<br>-IIVVALYDYEAIHHEDLSFQKGDQMVVLE-ESGEWwkarslatrkeg----------Yipsnyvarv------------<br>kKYAKSKYDFVARNSSELSVMKDDVLEILDdRRQWW------------kvrnasgdsgF---------vpnnildimrtp<br><br>SARST: aliSize=53 (resi) RMSD=11.51 (Å)<br>i--IVVALYDYEAIHHEDLSFQKGDQMVVLEESGEWWKARSlATRKEGYIPSNYVAR----v<br>-kkYAKSKYDFVARNSSELSVMKDDVLEILDDRRQWWKVRN-ASGDSGFVPNNILDImrtp-<br><br>BLAST: aliSize=29 (resi) iden=28.57% (16/56) simi=51.79% (29/56)<br>iivval-------YDYEAIHHEDLSFQKGDQMVVLEESGEWWKARSlATRKEGYIPSN--------yvarv<br>------kkyakskYDFVARNSSELSVMKDDVLEILDDRRQWWKVRN-ASGDSGFVPNNildimrtp-----<br><br>Proposed: aliSize=52 (resi) RMSD=0.72 (Å)<br>-IIVVALYDYEAIHHEDLSFQKGDQMVVLE---esgEWWKARSLaTRKEGYIPSNYVARV---<br>kKYAKSKYDFVARNSSELSVMKDDVLEILDdrr---QWWKVRNA-SGDSGFVPNNILDIMrtp
1082	1avzC (57)<br>1i07A (59)	C	28.07	TM-align: aliSize=36 (resi) RMSD=2.29 (Å)<br>-TLFVALYDYEARTEDDLSFHKGEKFQILN-SSEGDWwearslttgetg---------Yipsnyvapv------------<br>kKYAKSKYDFVARNSSELSVMKDDVLEILDdRRQWWK------------vrnasgdsgF---------vpnnildimrtp<br><br>SARST: aliSize=53 (resi) RMSD=11.64 (Å)<br>t--LFVALYDYEARTEDDLSFHKGEKFQILNsSEGDWWEARSLTtGETGYIPSNYVAP----v<br>-kkYAKSKYDFVARNSSELSVMKDDVLEILD-DRRQWWKVRNAS-GDSGFVPNNILDImrtp-<br><br>BLAST: aliSize=29 (resi) iden=28.07% (16/57) simi=50.88% (29/57)<br>tlfval-------YDYEARTEDDLSFHKGEKFQILNSSEgDWWEARSlTTGETGYIPSN--------yvapv<br>------kkyakskYDFVARNSSELSVMKDDVLEILDDRR-QWWKVRN-ASGDSGFVPNNildimrtp-----<br><br>Proposed: aliSize=53 (resi) RMSD=1.13 (Å)<br>-TLFVALYDYEARTEDDLSFHKGEKFQILN--sseGDWWEARSlTTGETGYIPSNYVAPV---<br>kKYAKSKYDFVARNSSELSVMKDDVLEILDdr---RQWWKVRN-ASGDSGFVPNNILDIMrtp

1083	2ebpA (73)<br>1i07A (59)	C	16.95	TM-align: aliSize=35 (resi) RMSD=1.80 (Å)<br>gssgssgFcGRARVHTDFTPSpyDTDSLKLKKGDIIDIIS-KPpmGTwmgllnnkvg----------Tfkfiyvdvlssgpssg-----------<br>-------K-KYAKSKYDFVAR--NSSELSVMKDDVLEILDdRR--QW----------wkvrnasgdsgF---------------vpnnildimrtp<br><br>SARST: aliSize=55 (resi) RMSD=12.07 (Å)<br>gssgssgfcg--RARVHTDFTPspYDTDSLKLKKGDIIDIISKPPMGTWMGLLNNKVGTFKFIYVDVLS--sgpssg<br>----------kkYAKSKYDFVA--RNSSELSVMKDDVLEILDDRRQWWKVRNASGDSGFVPNNILDIMRtp------<br><br>BLAST: aliSize=9 (resi) iden=11.86% (7/59) simi=15.25% (9/59)<br>g---------------------------------------SSGSSGFCGRARVHTDFTPspydtdslklkkgdiidiiskppmgtwmgllnnkvgtfkfiyvdvlssgpssg<br>-kkyakskydfvarnsselsvmkddvleilddrrqwwkvrnASGDSGFVPNNILDIMRTP---<br><br>Proposed: aliSize=55 (resi) RMSD=1.55 (Å)<br>gssgssgFcGRARVHTDFTPSpyDTDSLKLKKGDIIDIIS---kppmGTWMGLL-NNKVGTFKFIYVDVLSSgpsSg<br>-------K-KYAKSKYDFVAR--NSSELSVMKDDVLEILDdrr----QWWKVRNaSGDSGFVPNNILDIMRT---P-
1084	2eyxA (67)<br>1i07A (59)	N	13.56	TM-align: aliSize=36 (resi) RMSD=2.41 (Å)<br>nlqngPIYARVIQKRVPNayDKTALALEVGELVKVTK-I-NVSgQwegecngkrg---------Hfpfthvrlldqqn-----------<br>-----KKYAKSKYDFVAR--NSSELSVMKDDVLEILDdRrQWW-K----------vrnasgdsgF------------vpnnildimrtp<br><br>SARST: aliSize=43 (resi) RMSD=10.12 (Å)<br>nlqngp-IYARVIQKRVPnaYDKTALALEVGELVKvtkinvsgqwegECNG------------KRGHFPFTHVRLL---dqqn<br>------kKYAKSKYDFVA--RNSSELSVMKDDVLE------------ILDDrrqwwkvrnasgDSGFVPNNILDIMrtp----<br><br>BLAST: aliSize=5 (resi) iden=8.47% (5/59) simi=8.47% (5/59)<br>nlqngpiyarviqkrvpnaydktalalevgelv-----------------------------------KVTkiNVSG---------------qwegecngkrghfpfthvrlldqqn<br>--------------------------------kkyakskydfvarnsselsvmkddvleilddrrqwwKVR--NASGdsgfvpnnildimrtp------------------------<br><br>Proposed: aliSize=57 (resi) RMSD=1.54 (Å)<br>nlqngPIYARVIQKRVPNayDKTALALEVGELVKVTK-INVsgQWEGECN-GKRGHFPFTHVRLLDQQn<br>-----KKYAKSKYDFVAR--NSSELSVMKDDVLEILDdRRQ--WWKVRNAsGDSGFVPNNILDIMRTP-
1085	2vq9A (123)<br>1h8xA (125)	N	30.89	TM-align: aliSize=97 (resi) RMSD=1.92 (Å)<br>irrryehfltqhvyggi-----------------TE--Q-TCDRVMRQRRITrfptGNDCKEVNTFIQANGNHVRTVCtGgGTRQTD---NRDLYMSNDQFTVITCTLR--SGERhpNCRYRGKESSRKIVVACEG--EWPAHYERGVIv<br>-----------------kesaaakferqhmdsgnsPSsssStYCNQMMRRRNMT----QGRCKPVNTFVHEPLVDVQNVC-F-QEKVTCkngQGNCYKSNSSMHITDCRLTngSRYP--NCAYRTSQKERHIIVACEGspYVPVHFDASVE-<br><br>SARST: aliSize=113 (resi) RMSD=13.05 (Å)<br>irr---RYEHFLTQHVYGGITE----QTCDRVMRQRRitrfPTGNDCKEVNTFIQANGNHVRTVCT-GGGTRQTDNRDLYMSNDQFTVITCTLRSGERHPNCRYRGKESSRKIVVACEG--EWPAHYERG--viv<br>---kesAAAKFERQHMDSGNSPssssTYCNQMMRRRN----MTQGRCKPVNTFVHEPLVDVQNVCFqEKVTCKNGQGNCYKSNSSMHITDCRLTNGSRYPNCAYRTSQKERHIIVACEGspYVPVHFDASve---<br><br>BLAST: aliSize=62 (resi) iden=34.15% (42/123) simi=50.41% (62/123)<br>irrryeh-------FLTQHVYGGITEQT----CDRVMRQRRITRfptGNdCKEVNTFIQANGNHVRTVCTGGG-TRQTDNRDLYMSNDQFTVITCTLRSGERHPNCRYRGKESSRKIVVACEGE--WPAHYERGV-iv<br>-------kesaaakFERQHMDSGNSPSSsstyCNQMMRRRNMTQ---GR-CKPVNTFVHEPLVDVQNVCFQEKvTCKNGQGNCYKSNSSMHITDCRLTNGSRYPNCAYRTSQKERHIIVACEGSpyVPVHFDASVe--<br><br>Proposed: aliSize=112 (resi) RMSD=1.66 (Å)<br>i-RRRYEHFLTQHVYGGITE---Q-TCDRVMRQRRITrfptGNDCKEVNTFIQANGNHVRTVCtGgGTRQtDN----RDLYMSNDQFTVITCTLR--SGERhpNCRYRGKESSRKIVVACEG--EWPAHYERGVIv<br>-kESAAAKFERQHMDSGNSPssssStYCNQMMRRRNMT----QGRCKPVNTFVHEPLVDVQNVC-F-QEKV-TCkngqGNCYKSNSSMHITDCRLTngSRYP--NCAYRTSQKERHIIVACEGspYVPVHFDASVE-

1086	1yv7A (102)<br>1h8xA (125)	N	24.51	TM-align: aliSize=86 (resi) RMSD=2.50 (Å)<br>dwltfqkkhitnt-------------------R--DVDCDNIMSTNLFH---CKDKNTFIYSRPEPVKAICKG-Iia-------SKNVLTTSEFYLSDCNVTSRP----CKYKLKKSTNKFSVTCEN--QAPVHFVGVG-<br>-------------kesaaakferqhmdsgnspSssSTYCNQMMRRRNMTqgrCKPVNTFVHEPLVDVQNVCFQeK--vtckngqGNCYKSNSSMHITDCRLTNGSrypnCAYRTSQKERHIIVACEGspYVPVHFDASVe<br><br>SARST: aliSize=96 (resi) RMSD=12.82 (Å)<br>d----WLTFQKKHIT-NT---RDVDCDNIMStNLFHC-----KDKNTFIYSRPEPVKAICKGIIAS------KNVLTTSEFYLSDCNVT-----SRPcKYKLKKSTNKFSVTCEN--QAPVHFVG---vg<br>-kesaAAKFERQHMDsGNspsSSSTYCNQMM-RRRNMtqgrcKPVNTFVHEPLVDVQNVCFQEKVTckngqgNCYKSNSSMHITDCRLTngsryPNC-AYRTSQKERHIIVACEGspYVPVHFDAsve--<br><br>BLAST: aliSize=52 (resi) iden=29.70% (30/102) simi=51.49% (52/102)<br>dwlt-------FQKKHITN-----TRDVDCDNIMST-NLFH--CKDKNTFIYSRPEPVKAIC-KGIIASKN-----VLTTSEFYLSDCNVT--SR-P-CKYKLKKSTNKFSVTCENQ--APVHF-----vgvg<br>----kesaaakFERQHMDSgnspsSSSTYCNQMMRRrNMTQgrCKPVNTFVHEPLVDVQNVCfQEKVTCKNgqgncYKSNSSMHITDCRLTngSRyPnCAYRTSQKERHIIVACEGSpyVPVHFdasve----<br><br>Proposed: aliSize=97 (resi) RMSD=1.67 (Å)<br>--PDWLTFQKKHITN-TRD-----vDCDNIMST-NLFH--CKDKNTFIYSRPEPVKAICK----------giiaSKNVLTTSEFYLSDCNVTSRP----CKYKLKKSTNKFSVTCEN--QAPVHFVGVG-<br>keSAAAKFERQHMDSgNSPsssst-YCNQMMRRrNMTQgrCKPVNTFVHEPLVDVQNVCFqekvtckngq----GNCYKSNSSMHITDCRLTNGSrypnCAYRTSQKERHIIVACEGspYVPVHFDASVe
1087	1k5aA (118)<br>1h8xA (125)	N	34.75	TM-align: aliSize=98 (resi) RMSD=2.00 (Å)<br>nsrythfltqhydakpq-----------------GR-DDRYCESIMRRRGLTS-PCKDINTFIHGNKRSIKAICEnknGNPHRE-----NLRISKSSFQVTTCKLHGGSPWPPCQYRATAGFRNVVVACEN--GLPVHLDQSAA<br>-----------------kesaaakferqhmdsgnsPSsSSTYCNQMMRRRNMTQgRCKPVNTFVHEPLVDVQNVCF---QEKVTCkngqgNCYKSNSSMHITDCRLTNGSRYPNCAYRTSQKERHIIVACEGspYVPVHFDASVE<br><br>SARST: aliSize=107 (resi) RMSD=13.33 (Å)<br>ns---RYTHFLTQHYDAKPQ--GRDDRYCESIMRrRGLTS-PCKDINTFIHGNKRSIKAICenkNGNPH-REN----LRISKSSFQVTTCKLHGGSPWPPCQYRATAGFRNVVVACEN--GLPVHL-----dqsaa<br>--kesAAAKFERQHMDSGNSpsSSSTYCNQMMRR-RNMTQgRCKPVNTFVHEPLVDVQNVC---FQEKVtCKNgqgnCYKSNSSMHITDCRLTNGSRYPNCAYRTSQKERHIIVACEGspYVPVHFdasve-----<br><br>BLAST: aliSize=65 (resi) iden=38.14% (45/118) simi=55.08% (65/118)<br>nsryth-------FLTQHYDA--KPQGrDDRYCESIMRRRGLTSP-CKDINTFIHG---NKRSI----KAICENKNGNPHRENlriskSSFQVTTCKLHGGSPWPPCQYRATAGFRNVVVACENG--LPVHLDQS--aa<br>------kesaaakFERQHMDSgnSPSS-SSTYCNQMMRRRNMTQGrCKPVNTFVHEplvDVQNVcfqeKVTCKNGQGNCYKSN-----SSMHITDCRLTNGSRYPNCAYRTSQKERHIIVACEGSpyVPVHFDASve--<br><br>Proposed: aliSize=111 (resi) RMSD=1.50 (Å)<br>-NSRYTHFLTQHYDAKPQGR---ddRYCESIMRRRGLTS-PCKDINTFIHGNKRSIKAICEnknGNPHRE-----NLRISKSSFQVTTCKLHGGSPWPPCQYRATAGFRNVVVACEN--GLPVHLDQsA--a<br>kESAAAKFERQHMDSGNSPSsss--TYCNQMMRRRNMTQgRCKPVNTFVHEPLVDVQNVCF---QEKVTCkngqgNCYKSNSSMHITDCRLTNGSRYPNCAYRTSQKERHIIVACEGspYVPVHFDA-Sve-
1088	1un5A (121)<br>1h8xA (125)	N	35.54	TM-align: aliSize=99 (resi) RMSD=2.03 (Å)<br>nsrythfltqhydakpq-----------------GR-DDRYCESIMRRRGLTSDRCKPINTFIHGNKRSIKAICEnknGNPHRE-----NLRISKSSFQVTTCKLHGGSPWPPCQYRATAGFRNVVVACEN--GLPVHLDQsIFR<br>-----------------kesaaakferqhmdsgnsPSsSSTYCNQMMRRRNMTQGRCKPVNTFVHEPLVDVQNVCF---QEKVTCkngqgNCYKSNSSMHITDCRLTNGSRYPNCAYRTSQKERHIIVACEGspYVPVHFDA-SVE<br><br>SARST: aliSize=106 (resi) RMSD=14.68 (Å)<br>s---RYTHFLTQHYDAKPQgrdDR----YCESIMRRRGLTSDRCKPINTFIHGNKRSIKAIC-----ENKNGNPHrenLRISKSSFQVTTCKLHGGSPWPPCQYRATAGFRNVVVACEN--GLPVHL-----dqsifr<br>-kesAAAKFERQHMDSGNS---PSssstYCNQMMRRRNMTQGRCKPVNTFVHEPLVDVQNVCfqekvTCKNGQGN---CYKSNSSMHITDCRLTNGSRYPNCAYRTSQKERHIIVACEGspYVPVHFdasve------<br><br>BLAST: aliSize=68 (resi) iden=39.50% (47/121) simi=57.14% (68/121)<br>sryth-------FLTQHYDA--KPQGrDDRYCESIMRRRGLTSDRCKPINTFIHG---NKRSI----KAICENKNGNPHRENlriskSSFQVTTCKLHGGSPWPPCQYRATAGFRNVVVACENG--LPVHLDQSI-fr<br>-----kesaaakFERQHMDSgnSPSS-SSTYCNQMMRRRNMTQGRCKPVNTFVHEplvDVQNVcfqeKVTCKNGQGNCYKSN-----SSMHITDCRLTNGSRYPNCAYRTSQKERHIIVACEGSpyVPVHFDASVe--<br><br>Proposed: aliSize=112 (resi) RMSD=1.52 (Å)<br>n--SRYTHFLTQHYDAKPQGR--DdRYCESIMRRRGLTSDRCKPINTFIHGNKRSIKAICEnknGNPHRE-----NLRISKSSFQVTTCKLHGGSPWPPCQYRATAGFRNVVVACEN--GLPVHLDQsI--frr<br>-keSAAAKFERQHMDSGNSPSssS-TYCNQMMRRRNMTQGRCKPVNTFVHEPLVDVQNVCF---QEKVTCkngqgNCYKSNSSMHITDCRLTNGSRYPNCAYRTSQKERHIIVACEGspYVPVHFDA-Sve---

1089	1a5qA<br>(124)<br><br>1h8xA<br>(125)	N	69.35	TM-align: aliSize=105 (resi) RMSD=1.66 (Å)<br>ketaaakferqhmdssts------------------AAs-SSNYCNQMMKSRNLTKDRCKPVNTFVHESLADVQAVCSQKNVACKNGQTNCYQSYSTMSITDCRETGSSKYANCAYKTTQANKHIIVACEGNPYVPVHFDASV-<br>------------------kesaaakferqhmdsgnsPS-sSSTYCNQMMRRRNMTQGRCKPVNTFVHEPLVDVQNVCFQEKVTCKNGQGNCYKSNSSMHITDCRLTNGSRYPNCAYRTSQKERHIIVACEGSPYVPVHFDASVe<br><br>SARST: aliSize=98 (resi) RMSD=16.11 (Å)<br>k-ETAAAKFERQHMDSST---SAAsssNYCNQMMKSRNLTKDRCKPVNTFVHESLADVQAVCSQKNVACKN---------------------GQtncyqsystmsitdcretgssKYANCAYKTTQANKHIIVACEGNPYVPVHFDAS--v<br>-kESAAAKFERQHMDSGNspsSSS---TYCNQMMRRRNMTQGRCKPVNTFVHEPLVDVQNVCFQEKVTCKNgqgncyksnssmhitdcrltnGS---------------------RYPNCAYRTSQKERHIIVACEGSPYVPVHFDASve-<br><br>BLAST: aliSize=103 (resi) iden=72.58% (90/124) simi=83.06% (103/124)<br>KETAAAKFERQHMDSSTSAASSSNYCNQMMKSRNLTKDRCKPVNTFVHESLADVQAVCSQKNVACKNGQTNCYQSYSTMSITDCRETGSSKYANCAYKTTQANKHIIVACEGNPYVPVHFDASV-<br>KESAAAKFERQHMDSGNSPSSSSTYCNQMMRRRNMTQGRCKPVNTFVHEPLVDVQNVCFQEKVTCKNGQGNCYKSNSSMHITDCRLTNGSRYPNCAYRTSQKERHIIVACEGSPYVPVHFDASVe<br><br>Proposed: aliSize=122 (resi) RMSD=1.03 (Å)<br>KETAAAKFERQHMDSSTsAA-SSSNYCNQMMKSRNLTKDRCKPVNTFVHESLADVQAVCSQKNVACK-NGQtNCYQSYSTMSITDCRETGSSKYANCAYKTTQANKHIIVACEGNPYVPVHFDASV-<br>KESAAAKFERQHMDSGN-SPsSSSTYCNQMMRRRNMTQGRCKPVNTFVHEPLVDVQNVCFQEKVTCKnGQG-NCYKSNSSMHITDCRLTNGSRYPNCAYRTSQKERHIIVACEGSPYVPVHFDASVe
1090	1lbgA<br>(124)<br><br>1r3mB<br>(122)	N	99.18	TM-align: aliSize=104 (resi) RMSD=1.02 (Å)<br>kesaaakferqhmdsgnsps------------------SSSNYCNLMMCCRKMTQGKCKPVNTFVHESLADVKAVCSQKKVTCKNGQTNCYQSKSTMRITDCRETGSSKYPNCAYKTTQVEKHIIVACGGKPSVPVHFDASV<br>--------------------kesaaakferqhmdsgnsPSSNYCNLMMCCRKMTQGKCKPVNTFVHESLADVKAVCSQKKVTCKNGQTNCYQSKSTMRITDCRETGSSKYPNCAYKTTQVEKHIIVACGGKPSVPVHFDASV<br><br>SARST: aliSize=120 (resi) RMSD=9.91 (Å)<br>k-ESAAAKFERQHMDSGNSPSssSNYCNLMMCCRKMTQGKCKPVNTFVHESLADVKAVCSQKKVTCKNGQTNCYQSKSTMRITDCRETGSSKYPNCAYKTTQVEKHIIVACGGKPSVPVHFDAS-v<br>-kESAAAKFERQHMDSGNSPS--SNYCNLMMCCRKMTQGKCKPVNTFVHESLADVKAVCSQKKVTCKNGQTNCYQSKSTMRITDCRETGSSKYPNCAYKTTQVEKHIIVACGGKPSVPVHFDASv-<br><br>BLAST: aliSize=122 (resi) iden=100.00% (122/122) simi=100.00% (122/122)<br>KESAAAKFERQHMDSGNSPSSsNYCNLMMCCRKMTQGKCKPVNTFVHESLADVKAVCSQKKVTCKNGQTNCYQSKSTMRITDCRETGSSKYPNCAYKTTQVEKHIIVACGGKPSVPVHFDASV<br>KESAAAKFERQHMDSGNSPSS--NYCNLMMCCRKMTQGKCKPVNTFVHESLADVKAVCSQKKVTCKNGQTNCYQSKSTMRITDCRETGSSKYPNCAYKTTQVEKHIIVACGGKPSVPVHFDASV<br><br>Proposed: aliSize=122 (resi) RMSD=0.70 (Å)<br>KESAAAKFERQHMDSGNSpSsSSNYCNLMMCCRKMTQGKCKPVNTFVHESLADVKAVCSQKKVTCKNGQTNCYQSKSTMRITDCRETGSSKYPNCAYKTTQVEKHIIVACGGKPSVPVHFDASV<br>KESAAAKFERQHMDSGNS-P-SSNYCNLMMCCRKMTQGKCKPVNTFVHESLADVKAVCSQKKVTCKNGQTNCYQSKSTMRITDCRETGSSKYPNCAYKTTQVEKHIIVACGGKPSVPVHFDASV
1091	1lbgA<br>(124)<br><br>1a2wA<br>(124)	N	80.65	TM-align: aliSize=104 (resi) RMSD=0.70 (Å)<br>kesaaakferqhmdsgnsps--------------------SSSNYCNLMMCCRKMTQGKCKPVNTFVHESLADVKAVCSQKKVTCKNGQTNCYQSKSTMRITDCRETGSSKYPNCAYKTTQVEKHIIVACGGKPSVPVHFDASV<br>--------------------ketaaakferqhmdsstsaaSSSNYCNQMMKSRNLTKDRCKPVNTFVHESLADVQAVCSQKNVACKNGQTNCYQSYSTMSITDCRETGSSKYPNCAYKTTQANKHIIVACEGNPYVPVHFDASV<br><br>SARST: aliSize=121 (resi) RMSD=11.86 (Å)<br>k-ESAAAKFERQHMD-SGNSPSSsSNYCNLMMCCRKMTQGKCKPVNTFVHESLADVKAVCSQKKVTCKNGQTNCYQSKSTMRITDCRETGSSKYPNCAYKTTQVEKHIIVACGGKPSVPVHFDAS-v<br>-kETAAAKFERQHMDsSTSAASS-SNYCNQMMKSRNLTKDRCKPVNTFVHESLADVQAVCSQKNVACKNGQTNCYQSYSTMSITDCRETGSSKYPNCAYKTTQANKHIIVACEGNPYVPVHFDASv-<br><br>BLAST: aliSize=107 (resi) iden=81.45% (101/124) simi=86.29% (107/124)<br>KESAAAKFERQHMDSGNSPSSSSNYCNLMMCCRKMTQGKCKPVNTFVHESLADVKAVCSQKKVTCKNGQTNCYQSKSTMRITDCRETGSSKYPNCAYKTTQVEKHIIVACGGKPSVPVHFDASV<br>KETAAAKFERQHMDSSTSAASSSNYCNQMMKSRNLTKDRCKPVNTFVHESLADVQAVCSQKNVACKNGQTNCYQSYSTMSITDCRETGSSKYPNCAYKTTQANKHIIVACEGNPYVPVHFDASV<br><br>Proposed: aliSize=123 (resi) RMSD=0.90 (Å)<br>KESAAAKFERQHMDSGNSPS-sSSNYCNLMMCCRKMTQGKCKPVNTFVHESLADVKAVCSQKKVTCKNGQTNCYQSKSTMRITDCRETGSSKYPNCAYKTTQVEKHIIVACGGKPSVPVHFDASV<br>KETAAAKFERQHMDSSTSAAs-SSNYCNQMMKSRNLTKDRCKPVNTFVHESLADVQAVCSQKNVACKNGQTNCYQSYSTMSITDCRETGSSKYPNCAYKTTQANKHIIVACEGNPYVPVHFDASV

1092	11bgA (124)<br>1h8xA (125)	N	71.77	TM-align: aliSize=104 (resi) RMSD=1.47 (Å)<br>kesaaakferqhmdsgnsps--------------------SSSNYCNLMMCCRKMTQGKCKPVNTFVHESLADVKAVCSQKKVTCKNGQTNCYQSKSTMRITDCRETGSSKYPNCAYKTTQVEKHIIVACGGKPSVPVHFDASV-<br>--------------------kesaaakferqhmdsgnspsSSSTYCNQMMRRRNMTQGRCKPVNTFVHEPLVDVQNVCFQEKVTCKNGQGNCYKSNSSMHITDCRLTNGSRYPNCAYRTSQKERHIIVACEGSPYVPVHFDASVe<br><br>SARST: aliSize=120 (resi) RMSD=11.31 (Å)<br>k-ESAAAKFERQHMD--SGNSPSSssNYCNLMMCCRKMTQGKCKPVNTFVHESLADVKAVCSQKKVTCKNGQTNCYQSKSTMRITDCRETGSSKYPNCAYKTTQVEKHIIVACGGKPSVPVHFDAS--v<br>-kESAAAKFERQHMDsgNSPSSSS--TYCNQMMRRRNMTQGRCKPVNTFVHEPLVDVQNVCFQEKVTCKNGQGNCYKSNSSMHITDCRLTNGSRYPNCAYRTSQKERHIIVACEGSPYVPVHFDASve-<br><br>BLAST: aliSize=105 (resi) iden=77.42% (96/124) simi=84.68% (105/124)<br>KESAAAKFERQHMDSGNSPSSSSNYCNLMMCCRKMTQGKCKPVNTFVHESLADVKAVCSQKKVTCKNGQTNCYQSKSTMRITDCRETGSSKYPNCAYKTTQVEKHIIVACGGKPSVPVHFDASV-<br>KESAAAKFERQHMDSGNSPSSSSTYCNQMMRRRNMTQGRCKPVNTFVHEPLVDVQNVCFQEKVTCKNGQGNCYKSNSSMHITDCRLTNGSRYPNCAYRTSQKERHIIVACEGSPYVPVHFDASVe<br><br>Proposed: aliSize=122 (resi) RMSD=1.05 (Å)<br>KESAAAKFERQHMDSGnSPS-SSSNYCNLMMCCRKMTQGKCKPVNTFVHESLADVKAVCSQKKVTC-KNgQTNCYQSKSTMRITDCRETGSSKYPNCAYKTTQVEKHIIVACGGKPSVPVHFDASV-<br>KESAAAKFERQHMDSG-NSPsSSSTYCNQMMRRRNMTQGRCKPVNTFVHEPLVDVQNVCFQEKVTCkNG-QGNCYKSNSSMHITDCRLTNGSRYPNCAYRTSQKERHIIVACEGSPYVPVHFDASVe
1093	11baA (124)<br>1h8xA (125)	N	74.19	TM-align: aliSize=104 (resi) RMSD=1.43 (Å)<br>kesaaakferqhmdsgnsps--------------------SSSNYCNLMMCCRKMTQGKCKPVNTFVHESLADVKAVCSQKKVTCKNGQTNCYQSKSTMRITDCRETGSSKYPNCAYKTTQVEKHIIVACGGKPSVPVHFDASV-<br>--------------------kesaaakferqhmdsgnspsSSSTYCNQMMRRRNMTQGRCKPVNTFVHEPLVDVQNVCFQEKVTCKNGQGNCYKSNSSMHITDCRLTNGSRYPNCAYRTSQKERHIIVACEGSPYVPVHFDASVe<br><br>SARST: aliSize=120 (resi) RMSD=11.65 (Å)<br>k-ESAAAKFERQHMD--SGNSPSSssNYCNLMMCCRKMTQGKCKPVNTFVHESLADVKAVCSQKKVTCKNGQTNCYQSKSTMRITDCRETGSSKYPNCAYKTTQVEKHIIVACGGKPSVPVHFDAS--v<br>-kESAAAKFERQHMDsgNSPSSSS--TYCNQMMRRRNMTQGRCKPVNTFVHEPLVDVQNVCFQEKVTCKNGQGNCYKSNSSMHITDCRLTNGSRYPNCAYRTSQKERHIIVACEGSPYVPVHFDASve-<br><br>BLAST: aliSize=105 (resi) iden=77.42% (96/124) simi=84.68% (105/124)<br>KESAAAKFERQHMDSGNSPSSSSNYCNLMMCCRKMTQGKCKPVNTFVHESLADVKAVCSQKKVTCKNGQTNCYQSKSTMRITDCRETGSSKYPNCAYKTTQVEKHIIVACGGKPSVPVHFDASV-<br>KESAAAKFERQHMDSGNSPSSSSTYCNQMMRRRNMTQGRCKPVNTFVHEPLVDVQNVCFQEKVTCKNGQGNCYKSNSSMHITDCRLTNGSRYPNCAYRTSQKERHIIVACEGSPYVPVHFDASVe<br><br>Proposed: aliSize=123 (resi) RMSD=1.16 (Å)<br>KESAAAKFERQHMDSGnSPS-SSSNYCNLMMCCRKMTQGKCKPVNTFVHESLADVKAVCSQKKVTCKNGQTNCYQSKSTMRITDCRETGSSKYPNCAYKTTQVEKHIIVACGGKPSVPVHFDASV-<br>KESAAAKFERQHMDSG-NSPsSSSTYCNQMMRRRNMTQGRCKPVNTFVHEPLVDVQNVCFQEKVTCKNGQGNCYKSNSSMHITDCRLTNGSRYPNCAYRTSQKERHIIVACEGSPYVPVHFDASVe
