# Supplementary material for: Detection and Alignment of 3D Domain Swapping Proteins Using Angle-Distance Image-Based Secondary Structural Matching Techniques
Source: PLoS One. 2010 Oct 14;5(10):e13361. doi: 10.1371/journal.pone.0013361 (PMC2955075; doi:10.1371/journal.pone.0013361)
Supplement: Table S6 — Number of SSEs in the swapped domains. Here an SSE means an α-helix or a β-strand. The number of SSEs that a swapped domain contains roughly reflects the size of the domain. The ranges of SSEs were extracted from the PDB files according to the HELIX and SHEET records. (0.07 MB PDF) [file pone.0013361.s010.pdf]

Table S6. Number of SSEs in the swapped domains

Number of SSEs	Number of swapped domains possessing specific numbers of SSEs		
	N-terminal-swapped domains	C-terminal-swapped domains	Middle-swapped domains
0	26	21	12
1	118	253	16
2	58	89	16
3	25	46	1
≥4	35	39	0

Here an SSE means an α-helix or a β-strand. The number of SSEs that a swapped domain contains roughly reflects the size of the domain. The ranges of SSEs were extracted from the PDB files according to the HELIX and SHEET records.
